# Supplementary material for: A common East-Asian ALDH2 mutation causes metabolic disorders and the therapeutic effect of ALDH2 activators
Source: Nat Commun. 2023 Sep 25;14:5971. doi: 10.1038/s41467-023-41570-6 (PMC10520061; doi:10.1038/s41467-023-41570-6)

Supplementary Materials for

A common East-Asian *ALDH2* mutation causes metabolic disorders and the therapeutic effect of ALDH2 activators

Yi-Cheng Chang, Hsiao-Lin Lee, Wenjin Yang, Meng-Lun Hsieh, Cai-Cin Liu, Tung-Yuan Lee, Jing-Yong Huang, Jiun-Yi Nong, Fu-An Li, Hsiao-Li Chuang, Zhi-Zhong Ding, Wei-Lun Su, Li-Yun Chueh, Yi-Ting Tsai, Wei-Shun Yang, Che-Hong Chen, Daria Mochly-Rosen* and Lee-Ming Chuang*

*Co-corresponding author, e-mail: leeming@ntu.edu.tw

*Co-corresponding author, e-mail: mochly@stanford.edu

LIST OF SUPPLEMENTARY MATERIALS

Materials and Methods

Table S1. Primary sequences for real-time-quantitative PCR

Table S2. (a) SI table of identified 4-hydroxynonenal (4-HNE)-adducted proteins of brown adipose tissue isolated from *Aldh2* wild-type (WT) and knock-in (KI) mice by liquid chromatography tandem mass spectrometry (LC-MS/MS). SI table of identified 4-hydroxynonenal (4-HNE)-adducted proteins of brown adipose tissue isolated from (b) *Aldh2* knock-in (KI) mice and (c) *Aldh2* knock-in (KI) mice treated or not treated with AD9308 (60mg/kg/day) by LC-MS/MS.

Table S3. Pharmacokinetic study of AD-9308 after single intravenous injection or single oral gavage in mice and dogs.

Table S4. Pathological examination of liver and kidney from *Aldh2* knock-in and wild-type mice treated with 0, 20 or 60 mg/kg/day of AD-9308 for 20 weeks scored by H&E stain.

Figure S1. Respiratory exchange rate (RER) of *Aldh2* homozygous knock-in (KI) and heterozygous knock-in (HE) and wild-type (WT) mice.

Figure S2. Energy expenditure measured by indirect calorimetry after high-fat high-sucrose (HFHSD) feeding of *Aldh2* homozygous knock-in (KI) and heterozygous knick-in (HE) mice compared with wild-type (WT) mice.

Figure S3. (a) Rectal temperature and (b) energy expenditure in 4-hr acute cold tolerance tests of *Aldh2* homozygous knock-in (KI) and heterozygous knock-in (HE) mice compared with wild-type (WT) mice.

Figure S4. Serum norepinephrine levels of *Aldh2* homozygous knick-in (KI) and heterozygous knock-in (HE) mice compared with wild-type (WT) mice.

Figure S5. (a) Serum total cholesterol and (b) triglycerides of *Aldh2* homozygous knock-in (KI) and heterozygous knock-in (HE) mice compared with wild-type (WT) mice.

Figure S6. Expression levels of genes involved in thermogenesis including (**a**) *Ucp1*, (**b**) *Pgc1α*, (**c**) *Prdm16,* (**d**) *Cidea,* and (**e**) *Dio2* among BAT of *Aldh2* homozygous knock-in (KI), heterozygous knock-in (HE) and wild-type (WT) mice using and real-time quantitative PCR (RT-qPCR).

Figure S7. Expression of *Ucp1* relative to *Ppia* in white fat including inguinal and perigonadal fat between the *Aldh2* knock-in (KI) and wild-type (WT) mice measured by real-time quantitative PCR (RT-qPCR).

Figure S8. (a) Morphology showing the differentiation and **(b)** expression (**c**) and densitometric histogram of *Ucp1* of primary brown adipocytes isolated from *Aldh2* knock-in (KI) and wild-type (WT) mice

Figure S9. Immunoblots of the components of mitochondrial respiratory complex I (Ndufb8), II (Sdhb), III (Uqcrc2), IV (Mtco1), and V (Atpa5) in the brown adipose tissues of the *Aldh2* knock-in (KI) and wild-type (WT) mice.

Figure S10. Microscopic appearance of brown adipose tissue from the *Aldh2* knock-in (KI) and wild-type (WT) mice using H&E stain

Figure S11. Fatty acid oxidation of skeletal muscle and liver from *Aldh2* knock-in (KI) and wild-type (WT) mice

Figure S12. (a) Ribbon diagram showing the binding pocket of **Alda-1** within human ALDH2. **(b)** LigPlot showing the bonds between **Alda-1** and human ALDH2. **(c)** Ribbon diagram showing the binding of **Alda-1** and NAD^+ with human ALDH2.

Figure S13. (a) Immunoblots and **(b)** densitometry histogram showing the Aldh2 expression in the brown

adipose tissue (BAT) from chow-fed and high-fat high-sucrose (HFHSD)-fed C57BL6/J mice

Figure S14. Serum alanine aminotransferase (ALT) and creatinine levels of *Aldh2* KI and WT mice treated with 0, 20 or 60 mg/kg/day of AD-9308 for 20 weeks

Figure S15. (a) Number and (**b**) list of 4-HNE-adducted mitochondrial proteins of the BAT from the *Aldh2* WT mice receiving or not receiving AD9308 identified by liquid-chromatography tandem mass spectrometry (LC-MS/MS)

Figure S16. (a) Number and (**b**) list of 4-HNE-adducted mitochondrial proteins of the BAT from the *Aldh2* KI mice receiving or not receiving AD9308 identified by liquid-chromatography tandem mass spectrometry (LC-MS/MS)

Figure S17. (a) Schematic graphic of the generation of *Aldh2* mutant allele mimicking human Glu504Lys mutation (b) Sanger sequencing of Aldh2 wild-type and heterozygous knock-in mice

Uncropped images of all immunoblots in Supplementary Materials

Materials and Methods

Pharmacokinetic study of AD-9308 after single intravenous injection or single oral gavage in mice and dogs.

The pharmacokinetics study of AD-9308/AD-9551 was conducted in CD-1 mice and Beagle dogs were performed according to previous methods (37). Briefly, a single intravenous or a single oral gavage of AD-9308 was administered, and the plasma concentrations of AD-5591 were measured by LC-MS/MS. The $T_{1/2}$ (half-life), Cl (clearance), Vss (steady-state volume of distribution), AUC (area under curve), C_{max} (maximum plasma concentration), T_{max} (time taken to reach C_{max}), and F(%): bioavailability were calculated according to published formula (*1*).

Pathological examinations

Pathological examination was performed by the contract pathological core service of the Animal Centers of Medical College, National Taiwan University. Pathological changes of liver and kidney from *Aldh2* KI and WT mice treated with 0, 20 or 60 mg/kg/day of AD-9308 for 20 weeks was examined and scored by H&E stain. Serum alanine aminotransferase (ALT) and creatinine levels of *Aldh2* KI and WT mice treated with 0, 20 or 60 mg/kg/day of AD-9308 for 20 weeks were assayed using the FUJI DRI-CHEM clinical chemistry analyzer. The report was summarized in **Supplementary Table S4**.

Glucose and insulin tolerance test

Oral and intraperitoneal glucose tolerance tests were evaluated after 6 hours of fasting. Tail blood glucose was collected at 0, 15, 30, 45, 60, 90, and 120 min after oral gavage or intraperitoneal injection of glucose water (1 g/kg) and measured by a glucometer (ACCU-CHECK Performa, Roche). For the insulin tolerance test, mice were intraperitoneally injected 1 U/kg insulin (Humulin R, Eli Lilly) after 4 hours of fasting. Tail blood glucose was collected at 0, 15, 30, 45, 60, 90, 120, and 180 min after injection.

Immunoblots

Samples prepared with Laemmli sample buffer with or without 1mM DTT were separated by 10%

SDS-PAGE gel and transferred to PVDF membrane. The membrane was blocked with 10% skim milk in PBST and incubated at 4°C overnight with following primary antibodies: rabbit anti-UCP1 (1:1000; cat. no. GTX10983, GeneTex), HSP70 (1:5000; cat. no. ab78434, Abcam) and OXPHOS (1:1000; cat. no. MS604, MitoSciences). The membrane was then incubated with HRP conjugated rabbit IgG (1:10000; cat. no. GTX26721, GeneTex) or mouse IgG (1:10000; cat. no. GTX213111, GeneTex) and detected chemiluminescence signals.

Generation of Aldh2 knock-in mice

Aldh2 KI mice carrying the human ALDH2 Glu504Lys mutation were generated by introducing the Glu504Lys mutation within the mouse gene [2]. The details of generation were described previously[2]. Briefly, *Aldh2* knock-in mice were generated by homologous recombination with an 8.0-kb genomic fragment encompassing the mouse ALDH2 locus carrying a site-directed mutagenesis within exon 12 of the *Aldh2* genomic fragment corresponding to the position of human E487K mutation. The schematic graphic was shown in **Supplementary Figure S17**. The sequence of specific primers EG475, EG460, and EG399 were used for the amplification of a 1.3-kb fragment from exon 13 to the 3´ untranslated region (UTR) of neomycin marker and a 3.0-kb fragment from exon 13 to downstream of the neomycin marker, respectively, for the mutated allele. In contrast, for the wild-type allele, a 1.4-kb fragment devoid of the neomycin marker was amplified using EG475 and EG399 primers[29]. The founder mice were backcrossed to the C57BL/6J background for at least nine generations to achieve a homogeneous genetic background. Both *Aldh*2 WT controls and *Aldh2* KI mice were littermates from mated heterozygous mice

Table S1. Primary sequences for real-time quantitative PCR

**Gene**	**Forward primer (5'-3')**	**Reverse primer (5'-3')**
Ucp1	CGT ACC AAG CTG TGC GAT GT	TAG AAG CCC AAT GAT GTT CAG T
Dio2	CGC CCC AGT GTC AAG TTGT	CCC GTA AGC TAC GTT GGC ATT
Cidea	TGCTCTTCTGTATCGCCCAGT	GCC GTG TTA AGG AAT CTG CTG
Prdm16	CAG CAC GGT GAA GCCATTC	GCG TGC ATC CGC TTG TG
PGC1α	TGC GGG ATG ATG GAG ACA	GCG AAA GCG TCA CAG GTG TA
Ppia	GCA TAC GGG TCC TGG CAT CTT GTC C	ATG GTG ATC TTC TTG CTG GTC TTG C

Table S2. (a) SI table of identified 4-hydroxynonenal (4-HNE)-adducted proteins of brown adipose tissue isolated from *Aldh2* wild-type (WT) and knock-in (KI) mice by liquid chromatography tandem mass spectrometry (LC-MS/MS). SI table of identified 4-hydroxynonenal (4-HNE)-adducted proteins of brown adipose tissue isolated from (b) *Aldh2* knock-in (KI) mice and (c) *Aldh2* knock-in (KI) mice treated or not treated with AD9308 (60mg/kg/day) by LC-MS/MS.

(a)

***Aldh2* knock-in mice**							
**Uniprot ID**	**Protein name**	**Gene name**	**Mascot score**	**Peptide Num.**	**emPAI**	**Sequence**	**Adducted residue**
Q8BWT1	3-ketoacyl-CoA thiolase, mitochondrial	*Acca2*	3226	31	29.48	R.TPFGAYGGLLK.D + HNE (K)	K25
Q91ZA3	Propionyl-CoA carboxylase alpha chain, mitochondrial	*Pcca*	715	15	1.28	R.HIEIQVLGDKHGNALWLNERECSIQR.R +HNE (H)	H275
Q9CQ54	NADH dehydrogenase [ubiquinone] 1 subunit C2	*Ndufc2*	351	4	2.14	M.MNGRPGHEPLKFLPDEAR.S + HNE (H)	H8
Q9CQC7	NADH dehydrogenase [ubiquinone] 1 beta subcomplex subunit 4	*Ndufb4*	220	4	2.85	R.VSHIEDPALIR.W + HNE (H)	H59

Q9CQA3	Succinate dehydrogenase [ubiquinone] iron-sulfur subunit, mitochondrial	*Sdhb*	1366	16	15.7	R.CHTIMNCTQTCPK.G + HNE (H)	H246
P35486	Pyruvate dehydrogenase E1 component subunit alpha, somatic form, mitochondrial	*Pdha1*	747	18	5.58	R.AHGFTFTR.G + HNE (H)	H121
Q99KI0	Aconitate hydratase, mitochondrial	*Aco2*	5325	47	16.12	R.AKDINQEVYNFLATAGAK.Y + HNE (K)	K144
Q504P4	Heat shock cognate 71 kDa protein (627aa)	*Hspa8*	211	6	0.43	R.IINEPTAAAIAYGLDK.K + HNE (K)	K168
A2AQR0	Glycerol-3-phosphate dehydrogenase	*Gpd2*	3813	40	7.92	R.FHKFDEDEKGFITIVDVQR.V + HNE (K)	K652
						R.FHKFDEDEKGFITIVDVQR.V + HNE (K)	K658
G5E8R3	Pyruvate carboxylase	*Pcx*	2028	33	2.15	R.DAHQSLLATR.V + HNE (H)	H574
Q5SX39	Myosin-4	*Myh4*	601	16	0.37	K.NLQQEISDLTEQIAEGGK.H + HNE (K)	K1525
***Aldh2* knock-in &wild-type mice**							
**Uniprot ID**	**Protein name**	**Gene**	**Mascot**	**Peptide**	**emPAI**	**Sequence**	**Adducted**

		name	score	Num.			residue
Q8BWT1	3-ketoacyl-CoA thiolase, mitochondrial	*Acaa2*	2275	30	21.75	R.GGKYAVGSACIGGGQGIALIIQNTA.- + HNE (K)	K375
Q9CQB4	Cytochrome b-c1 complex subunit 7	*Uqcrb*	967	7	13.84	R.DDTLHETEDVKEAIR.R + HNE (H)	H39
A0A0A6YVZ0	Cytochrome b-c1 complex subunit 1, mitochondrial	*Uqcrc1*	133	2	9.36	-.DEKNNGAGYFLEHLAFK.L + HNE (K)	K3
Q9CR68	Cytochrome b-c1 complex subunit Rieske, mitochondrial	*Uqcrfs1*	466	9	2.49	R.AEVLDSTKSSKESSEAR.K + HNE (K)	K101
Q8CAQ8	MICOS complex subunit Mic60	*Immt*	1829	36	5.49	R.KAVDEAADALLK.A + HNE (K)	K296
E9Q800	MICOS complex subunit Mic60	*Immt*	1826	35	6.54	R.KAVDEAADALLK.A + HNE (K)	K218
Q03265	ATP synthase subunit alpha, mitochondrial	*Atp5f1a* (*Atp5a1*)	1118	22	3.58	R.RVGLKAPGIIPR.I + HNE (K)	K175
D3Z6F5	ATP synthase subunit alpha	*Atp5a1*	1067	22	4.28	R.RVGLKAPGIIPR.I + HNE (K)	K125
P63017	Heat shock cognate 71 kDa protein	*Hspa8*	281	7	0.5	R.IINEPTAAAIAYGLDK.K + HNE (K)	K187
***Aldh2* wild-type mice**							

Uniprot ID	Protein name	Gene name	Mascot score	Peptide Num.	emPAI	Sequence	Adducted residue
D3Z0L4	MICOS complex subunit	*Chchd3*	555	11	7.88	R.VTFEADENENITVVK.G + HNE (K)	K24

(b)

Aldh2 wild-type mice +AD9308							
**Uniprot ID**	**Protein name**	**Gene name**	**Mascot score**	**Peptide Num.**	**emPAI**	**Sequence**	**Adducted residue (residue site)**
Q9DCS9	NADH dehydrogenase [ubiquinone] 1 beta subcomplex subunit 10	*Ndufb10*	1750	11	13.72	R.DFKVDQEIMNIIQER.L+HNE(K)	K97
***Aldh2* wild-type mice & *Aldh2* wild-type mice +AD9308**							
**Uniprot ID**	**Protein name**	**Gene name**	**Mascot score**	**Peptide Num.**	**emPAI**	**Sequence**	**Adducted residue (residue site)**
Q99KI0	Aconitate hydratase, mitochondrial	*Aco2*	4878	46	16.91	K.GGTGAIVEYHGPGVDSISCTGMATICNMGAEIGATTSVFPYNHR.M+HNE (C)	C277
						K.GGTGAIVEYHGPGVDSISCTGMATICNMGAEIGATTSVFPYNHR.M+ HNE (H)	H268
						R.VGLIGSCTNSSYEDMGR.S+ HNE (C)	C385
			5971	49	16.91	K.GGTGAIVEYHGPGVDSISCTGMATICNMGAEIGATTSVFPYNHR.M+ HNE (C)	C284
						K.CKSQFTITPGSEQIR.A+ HNE (C)	C410
			7751	49	17.80	R.AKDINQEVYNFLATAGAK.Y + HNE (K)	K144
Q9D0M3	Cytochrome c1, heme protein, mitochondrial	*Cyc1*	3496	11	4.07	R.GLLSSLDHTSIR.R+ HNE (H)	H107
P58281-2	Dynamin-like 120 kDa protein, mitochondrial	*Opa1*	1470	32	2.25	R.SIVTDLVSQMDPHGR.R+ HNE (H)	H495
			1879	48	5.65	R.GVEVDPSLIKDTWHQVYR.R+ HNE (H)	H875

***Aldh2* wild-type mice**							
**Uniprot ID**	**Protein name**	**Gene name**	**Mascot score**	**Peptide Num.**	**emPAI**	**Sequence**	**Adducted residue (residue site)**
Q64521	Glycerol-3-phosphate dehydrogenase, mitochondrial	Gpd2	20156	72	82.82	R.FHKFDEDEKGFITIVDVQR.V+ HNE (H)	H633
						R.FHKFDEDEKGFITIVDVQR.V+ HNE (K)	K634
						R.KMDDKNVVPICQPSAGVHIVMPGYYSPENMGLLDPATSDGR.V+ HNE (C)	C309
P12242	Mitochondrial brown fat uncoupling protein 1	*Ucp1*	17843	30	81.02	MVNPTTSEVQPTMGVKIFSAGVSACLADIITFPLDTAK.V+ HNE (C)	C25
Q8BMS1	Trifunctional enzyme subunit alpha, mitochondrial	*Hadha*	14659	66	61.16	K.VNTLNKEVQSEFIEVMNEIWANDQIR.S+HNE(K)	K66
						K.SLNSEMDNILANLRLPAKPEVSSDEDVQYR.V+HNE(K)	K664
			21868	73	143.81	R.SAVLISSKPGCFVAGADINMLSSCTTPQEATR.I+ HNE (C)	C97
			16307	66	71.16	R.SAVLISSKPGCFVAGADINMLSSCTTPQEATR.I + HNE (K)	K94
Q8K2B3	Succinate dehydrogenase [ubiquinone] flavoprotein subunit, mitochondrial	*Sdha*	11698	39	16.62	K.HVNGQDQIVPGLYACGEAACASVHGANR.L+ HNE (C)	C438
						R.GMVWNTDLVETLELQNLMLCALQTIYGAEAR.K+ HNE (C)	C574
Q9CZ13	Cytochrome b-c1 complex subunit 1, mitochondrial	*Uqcrc1*	11361	37	81.43	R.EMQENDASMQNVVFDYLHATAFQGTPLAQAVEGPSENVR.R +HNE(H)	H188
			13397	37	95.23	R.PGNALEKEVESIGAHLNAYSTR.E + HNE (K)	K111
D3Z041	Arachidonate--CoA ligase	*Acsl1*	9567	54	26.14	R.VKPKPPEPEDLAIICFTSGTTGNPK.G + HNE (K)	K264
			13678	59	40.28	R.GFKPCSEQFIGLFSQNRPEWVIVEQGCFSYSMVVVPLYDTLGADAITYIVNK.A+HNE(C)	C168
			10512	50	23.44	R.NKDINKAILDDLLK.L + HNE (K)	K633
Q99JY0	Trifunctional enzyme subunit beta, mitochondrial	*Hadhb*	5485	32	30.25	R.EAALGAGFSDKTPAHTVTMACISSNQAMTTAVGLIASGQCDVVVAGGVELMSDVPIR.H	H133

			8324	37	53.73	K.DGGQYALVAACAAGGQGHAMIVEAYPK.-+ HNE (C)	C459
			8796	37	120.86	R.EAALGAGFSDKTPAHTVTMACISSNQAMTTAVGLIASGQCDVVVAGGVELMSDVPIR.H+ HNE (C)	C139, C158
Q8BWT1	3-ketoacyl-CoA thiolase, mitochondrial	*Acaa2*	7996	38	53.59	R.TPFGAYGGLLK.D+HNE (K)	K25
			6773	35	43.91	R.LCGSGFQSIVSGCQEICSKDAEVVLCGGTESMSQSPYCVR.N + HNE (C)	C103
						R.LCGSGFQSIVSGCQEICSKDAEVVLCGGTESMSQSPYCVR.N+ HNE (C)	C107
Q924L1	LETM1 domain-containing protein 1	*Letmd1*	2304	16	5.44	K.QQIDFLDVYHGLR.R + HNE (H)	H184
			2406	15	4.84	R.SHSEVITHLR.R + HNE (H)	H197
Q9CQB4	Cytochrome b-c1 complex subunit 7	*Uqcrb*	1736	11	35.40	R.DDTLHETEDVKEAIR.R + HNE (H)	H39
Q9CPQ1	Cytochrome c oxidase subunit 6C		1084	10	182.34	R.LRVHIAGAFIVALGVAAAYKFGVAEPR.K+ HNE (H)	H23

(C)

Aldh2 knock-in type mice +AD9308							
**Uniprot ID**	**Protein name**	**Gene name**	**Mascot score**	**Peptide Num.**	**emPAI**	**Sequence**	**Adducted residue (residue site)**
P19096	Fatty acid synthase	*Fasn*	19141	139	1 1.1	R.LQEMSSKTDSATDTTAPK.S+ HNE (K)	K2187
			11711	110	5.92	R.ISSCMEVLDLFLNQPHAVLSSFVLAEK.K+ HNE (C)	C2084
			5520	76	2.41	R.DHKDNLEFFLTNLGK.V+ HNE (H)	H789
Q9D0M3	Cytochrome c1, heme protein, mitochondrial	*Cyc1*	3837	11	4.07	R.GLLSSLDHTSIR.R+ HNE (H)	H107
Q9CR68	Cytochrome b-c1 complex subunit	*Uqcrfs1*	5148	23	41.34	R.AEVLDSTKSSKESSEAR.K+ HNE (K)	K101

	Rieske, mitochondrial						
						R.KGFSYLVTATTTVGVAYAAKNVVSQFVSSMSASADVLAMSK.I + HNE (K)	K151
			3304	20	31.08	R.KGFSYLVTATTTVGVAYAAKNVVSQFVSSMSASADVLAMSK.I+ HNE (K)	K130
***Aldh2* knock-in mice & *Aldh2* knock-in type mice +AD9308**							
Uniprot ID	Protein name	Gene name	Mascot score	Peptide Num.	emPAI	Sequence	Adducted residue (residue site)
Q64521	Glycerol-3-phosphate dehydrogenase, mitochondrial	*Gpd2*	15457	66	46.88	R.EAQLMTLKNTPEFDILVIGGGATGCGCALDAVTR.G+ HNE (K)	K65
						R.YLQKAIMNLDVEQYR.M+ HNE (K)	K124
						R.FHKFDEDEKGFITIVDVQR.V+ HNE (H)	H633
						R.FHKFDEDEKGFITIVDVQR.V+ HNE (K)	K634

Q8BWT1	3-ketoacyl-CoA thiolase, mitochondrial	*Acaa2*	6075	32	39.73	R.TPFGAYGGLLK.D+ HNE (K)	K25
						R.LCGSGFQSIVSGCQEICSKDAEVVLCGGTESMSQSPYCVR.N+ HNE (K)	K109
						K.AGLSLKDMDLIDVNEAFAPQFLSVQK.A+ HNE (K)	K312
			7944	38	143.8	R.LCGSGFQSIVSGCQEICSKDAEVVLCGGTESMSQSPYCVR.N HNE (C)	C103
						R.ITAHLVHELR.R+ HNE (H)	H367
Q9DCS9	NADH dehydrogenase [ubiquinone] 1 beta subcomplex subunit 10	*Ndufb10*	1874	12	13.72	R.DFKVDQEIMNIIQER.L+ HNE (K)	K97
P15105	Glutamine synthetase	*Glul*	212	7	1.16	R.LTGFHETSNINDFSAGVANR.G HNE (H)	H304
***Aldh2* knock-in mice**							

Uniprot ID	Protein name	Gene name	Mascot score	Peptide Num.	emPAI	Sequence	Adducted residue (residue site)
Q8BMS1	Trifunctional enzyme subunit alpha, mitochondrial	*Hadha*	12862	67	45.11	K.VNTLNKEVQSEFIEVMNEIWANDQIR.S+ HNE (K)	K66
						K.SLNSEMDNILANLRLPAKPEVSSDEDVQYR.V+ HNE (K)	K664
			17298	65	95.11	R.SAVLISSKPGCFVAGADINMLSSCTTPQEATR.I HNE (C)	C97
						R.KYESAYGTQFTPCQLLLDHANNSSK.K HNE (C)	C747
P12242	Mitochondrial brown fat uncoupling protein 1	*Ucp1*	18845	31	169.95	M.VNPTTSEVQPTMGVKIFSAGVSACLADIITFPLDTAK.V+ HNE (K)	K16
						M.VNPTTSEVQPTMGVKIFSAGVSACLADIITFPLDTAK.V HNE (C)	C25

						R.NVIINCTELVTYDLMKGALVNNKILADDVPCHLLSALVAGFCTTLLASPVDVVK.T HNE (C)	C189
						R.NVIINCTELVTYDLMKGALVNNKILADDVPCHLLSALVAGFCTTLLASPVDVVK.T+ HNE (H)	H215
Q8K2B3	Succinate dehydrogenase [ubiquinone] flavoprotein subunit, mitochondrial	*Sdha*	11131	44	18.72	K.HVNGQDQIVPGLYACGEAACASVHGANR.L HNE (C)	C438
						K.TFDRGMVWNTDLVETLELQNLMLCALQTIYGAEAR.K HNE (C)	C574
Q9CZ13	Cytochrome b-c1 complex subunit 1, mitochondrial	*Uqcrc1*	10563	39	81.43	R.EMQENDASMQNVVFDYLHATAFQGTPLAQAVEGPSENVR.R+ HNE (H)	H188
						R.IQEVDAQMLRDICSKYFYDQCPAVAGYGPIEQLPDYNR.I+ HNE (C)	C453

						R.IQEVDAQMLRDICSKYFYDQCPAVAGYGPIEQLPDYNR.I+ HNE (K)	K447
			11859	35	75.29	K.NRPGNALEKEVESIGAHLNAYSTR.E+ HNE (K)	K111
Q9DB77	Cytochrome b-c1 complex subunit 2, mitochondrial	*Uqcrc2*	77992	30	50.13	R.IIENLHDVAYKNALANPLYCPDYR.M+ HNE (K)	K183
Q99JY0	Trifunctional enzyme subunit beta, mitochondrial	*Hadhb*	4458	30	27.85	R.EAALGAGFSDKTPAHTVTMACISSNQAMTTAVGLIASGQCDVVVAGGVELMSDVPIR.H+ HNE (H)	H133
						R.EAALGAGFSDKTPAHTVTMACISSNQAMTTAVGLIASGQCDVVVAGGVELMSDVPIR.H HNE (C)	C139,C158
						K.DGGQYALVAACAAGGQGHAMIVEAYPK.- HNE (C)	C459
			6831	34	42.05	K.LKPAFIKPYGTVTAANSSFLTDGASAMLIMSEDR.A+ HNE (K)	K299
						K.AYLRDFIYVSQDPKDQLLLGPTYATPK.V+ HNE (K)	K349
Q62425	Cytochrome c	*Ndufa4*	2894	15	5457.11	R.QILGQAKKHPSLIPLFVFIGAGGTGAALYVMR.L+ HNE (H)	H12

	oxidase subunit NDUFA4						
						K.NNPEPWNKLGPNEQYKFYSVNVDYSK.L+ HNE (K)	K56
						K.NNPEPWNKLGPNEQYKFYSVNVDYSK.L+ HNE (K)	K64
Q9Z2Z6	Mitochondrial carnitine/acylcarnitine carrier protein	*Slc25a20*	2677	25	58.13	M.ADEPKPISPFKNLLAGGFGGMCLVFVGHPLDTVK.V+ HNE (K)	K12
						R.IKCLLQIQASSGENKYSGTLDCAK.K+HNE (C)	C136
			2527	27	74.71	K.LQQKSPEDELSYPQLFTAGMLSGVFTTGIMTPGER.I+ HNE (K)	K102
Q9CQB4	Cytochrome b-c1 complex subunit 7	*Uqcrb*	2116	12	88.41	R.DDTLHETEDVKEAIR.R+ HNE (H)	H39
						K.DQWTKYEEDKFYLEPYLK.E+ HNE (K)	K88
Q924L1	LETM1 domain-containing	*Letmd1*	1942	15	4.3	K.QQIDFLDVYHGLR.R+ HNE (H)	H184

	protein 1						
						R.SHSEVITHLR.R+ HNE (H)	H197
Q99LC3	NADH dehydrogenase [ubiquinone] 1 alpha subcomplex subunit 10	*Ndufa10*	2934	24	14.28	R.LTLPEYLPPHAVIYIDVPVPEVQSR.I+ HNE (H)	H202
P58281-2	Dynamin-like 120 kDa protein, mitochondrial	*Opa1*	1448	36	2.89	R.SIVTDLVSQMDPHGR.R+ HNE (H)	H495
P52503	NADH dehydrogenase [ubiquinone] iron-sulfur protein 6,	*Ndufs6*	1478	8	38.76	R.FVDRQKEVNENFAIDLIAQQPVNEVEHR.I+ HNE (K)	K53

	mitochondrial						
Q9CPQ1	Cytochrome c oxidase subunit 6C	*Cox6c*	676	7	26.56	R.LRVHIAGAFIVALGVAAAYKFGVAEPR.K+ HNE (H)	H23

Table S3. Pharmacokinetic study of AD-9308 after single intravenous injection or single oral gavage in mice and dogs.

Compound	Mouse (CD-1) (dose: IV, 5 mg/kg)				Mouse (CD-1) (dose: PO, 10 mg/kg)				
	$T_{1/2}$ (hr)	CL (ml/min/kg)	V_{ss} (L/kg)	AUC_{INF} (hr*ng/mL)	$T_{1/2}$ (hr)	C_{max} (ng/mL)	T_{max} (hr)	AUC_{INF} (hr*ng/mL)	F (%)
AD-5591 After AD-9308 Dosing	7.26	55.8	4.89	1155	4.31	455	0.25	778	33.7

Compound	Dog (Beagle) (dose: IV, 5 mg/kg)				Dog (Beagle) (dose: PO, 10 mg/kg)				
	$T_{1/2}$ (hr)	CL (ml/min/kg)	V_{ss} (L/kg)	AUC_{INF} (hr*ng/mL)	$T_{1/2}$ (hr)	C_{max} (ng/mL)	T_{max} (hr)	AUC_{INF} (hr*ng/mL)	F (%)
AD-5591 After AD-9308 Dosing	4.47	14.35	2.94	5002	7.69	1125	1.25	7500	63.2

$T_{1/2}$: half-life, Cl: clearance, Vss: steady-state volume of distribution, AUC: area under curve, C_{max}: maximum serum concentration, T_{max}: time taken to reach C_{max}, F(%): bioavailability.

Table S4. Pathological examination of liver and kidney from *Aldh2* KI and WT mice treated with 0, 20 or 60 mg/kg/day of AD-9308 for 20 weeks scored by H&E stain.

Organ	Histopathological finding	Pathological number																							
		WT												KI											
		0 mg/kg				20 mg/kg				60 mg/kg				0 mg/kg				20 mg/kg				60 mg/kg			
		#1	#2	#3	#4	#1	#2	#3	#4	#1	#2	#3	#4	#1	#2	#3	#4	#1	#2	#3	#4	#1	#2	#3	#4
Liver	Fatty change, hepatocyte	3	1	3	3	2	1	1	2	3	2	2	-	1	1	1	2	1	1	2	1	1	1	2	1
	Accumulation, glycogen, hepatocyte	4	2	2	2	2	1	1	4	3	3	2	2	2	1	1	3	2	2	4	3	3	2	1	2
	Extramedullary hematopoiesis	1	1	1	1	1	1	1	1	1	1	1	1	1	1	1	1	1	1	1	1	1	1	1	1
Kidney	Vacuolation, cytoplasmic, renal tubule	2	2	2	2	2	2	2	2	2	2	N	2	2	2	2	2	2	1	2	1	2	1	2	2
	Infiltration, mononuclear cell, interstitium	1	1	1	1	1	1	1	1	1	1	N	1	2	1	1	1	1	1	1	2	2	1	1	1

N: No available tissue. -: No significant lesions. Degree of lesions stained with HE was graded from one to five depending on severity: 1 = minimal (< 1%); 2: slight (1-25%); 3 = moderate (26-50%); 4 = moderate/severe (51-75%); 5 = severe/high (76-100%).

*Contract pathological examination by the Pathology core of the National Animal Research Laboratory, Taiwan

Figure S1

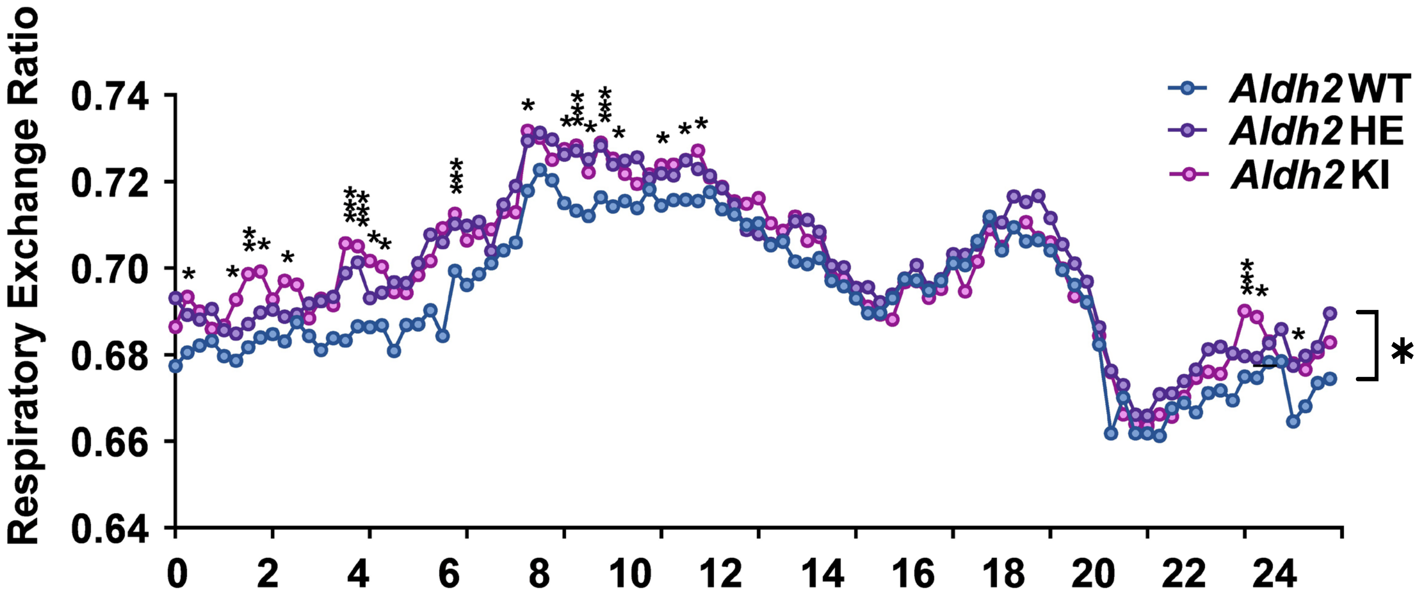

Figure S1. Respiratory exchange rate (RER) of *Aldh2* homozygous knock-in (KI) and heterozygous knock-in (HE) and wild-type (WT) mice at the age of 8-10 weeks (n=19:27:13; repeated measures analysis of variance [ANOVA] P=0.021). Dara was analyzed using tests for linear trends and further analyzed using repeated measures ANOVA. All data are presented as mean and standard error (S.E.M.) and all reported sample size are independent biological repeats. The asterisks indicate two-sided * P < 0.05.

Figure S2

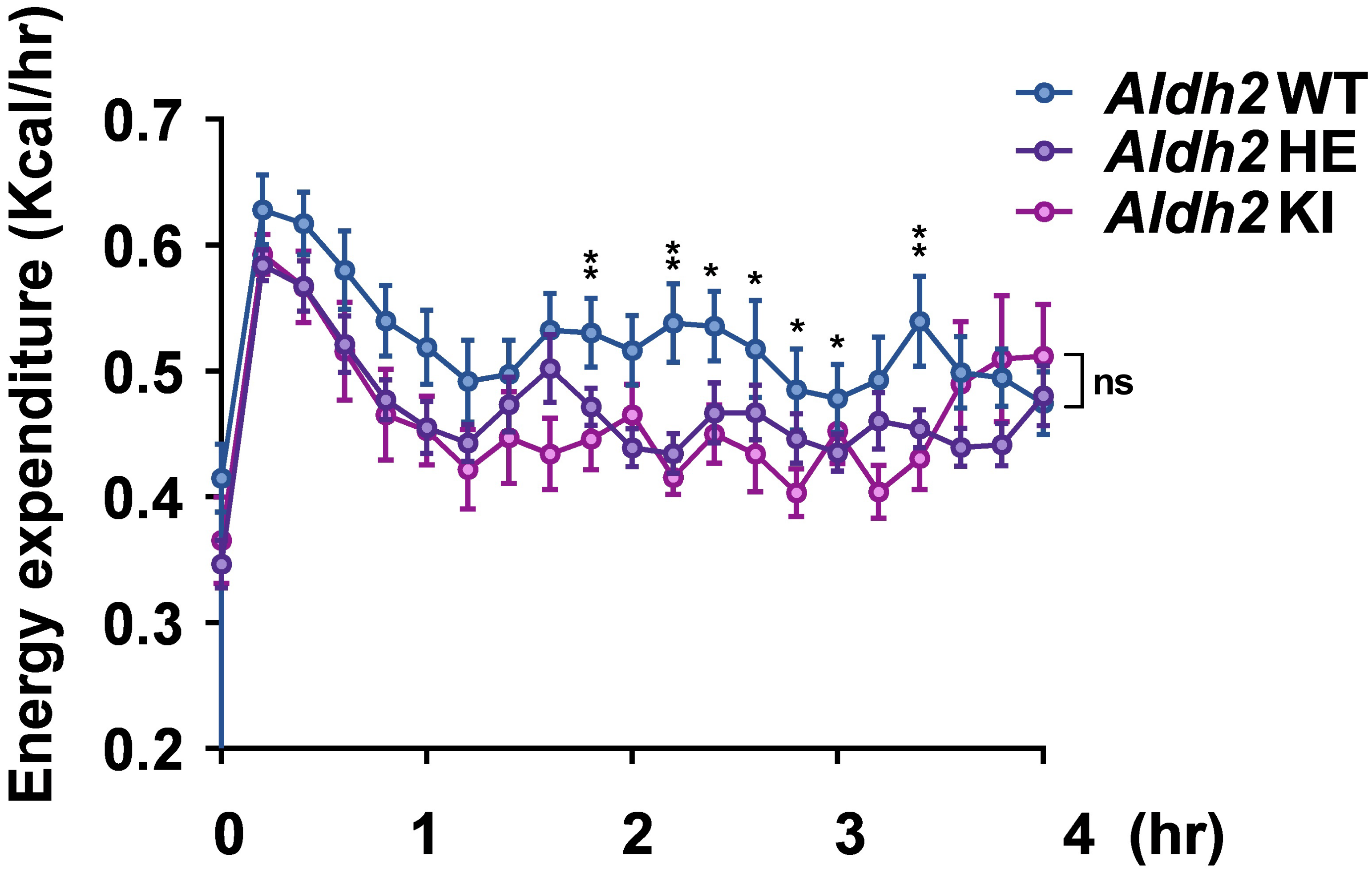

Figure S2. Diet-induced increased energy expenditure of *Aldh2* wild-type (WT), heterozygous knock-in (HE), and homozygous knock-in mice (KI) (n=10:14:8; repeated measures analysis of variance [ANOVA] P=0.052). Energy expenditure measured by indirect calorimetry after high-fat high-sucrose feeding for 4 hours

after overnight fasting at the age of 8-10 weeks. Dara was analyzed using tests for linear trends and further analyzed using repeated measures ANOVA. All data are presented as mean and standard error (S.E.M.) and all reported sample size are independent biological repeats.

Figure S3.

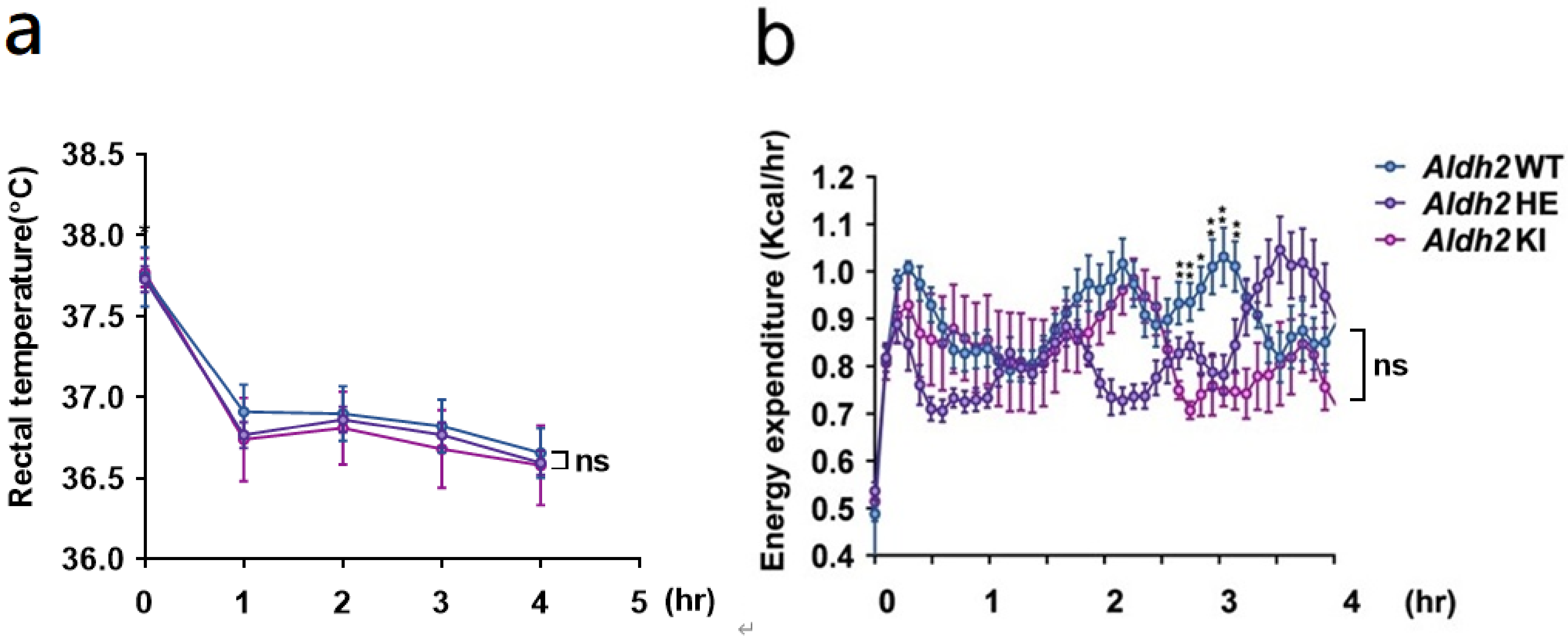

Figure S3. (a) Rectal temperature (n=9:24:10; repeated measures analysis of variance [ANOVA] P=0.72) and (**b**) energy expenditure in 4-hr acute cold tolerance tests of *Aldh2* homozygous knock-in (KI) and heterozygous knock-in (HE) mice compared with wild-type (WT) mice (n=6:6:4; repeated measures ANOVA P=0.17) at the age of 8-10 weeks. Energy expenditure measured by indirect calorimetry after high-fat high-sucrose feeding for 4 hours after overnight fasting at the age of 8-10 weeks. Dara was analyzed using tests for linear trends and further analyzed using repeated measures ANOVA. All data are presented as mean and

standard error (S.E.M.) and all reported sample size are independent biological repeats.

Figure S4

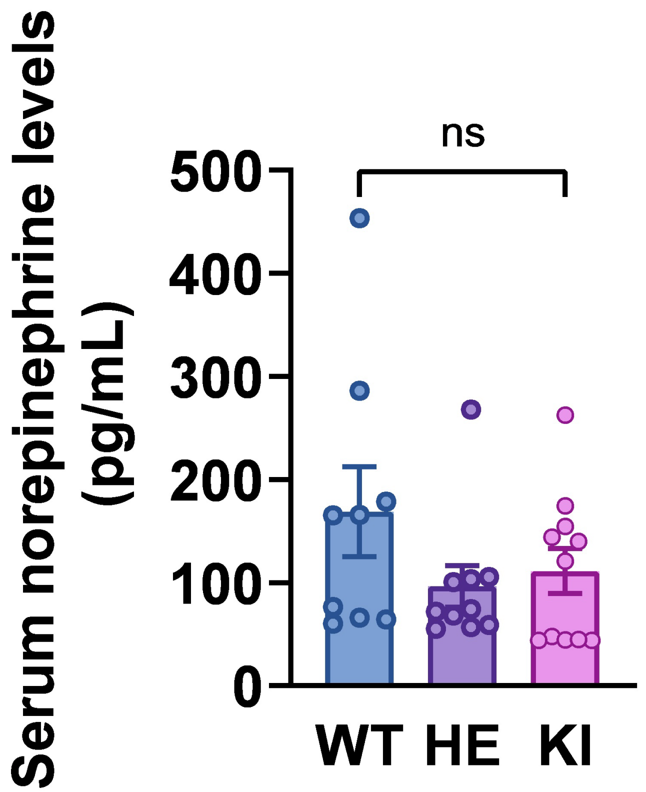

Figure S4. Serum norepinephrine levels of *Aldh2* homozygous knock-in (KI) and heterozygous knock-in (HE) mice compared with wild-type (WT) mice (n=9:10:11 in duplicates; P-for-trend=0.19) at the age of 24 weeks. Dara was analyzed using tests for linear trends. All data are presented as mean and standard error (S.E.M.) and all reported sample size are independent biological repeats. The asterisks indicate two-sided * P < 0.05.

Figure S5

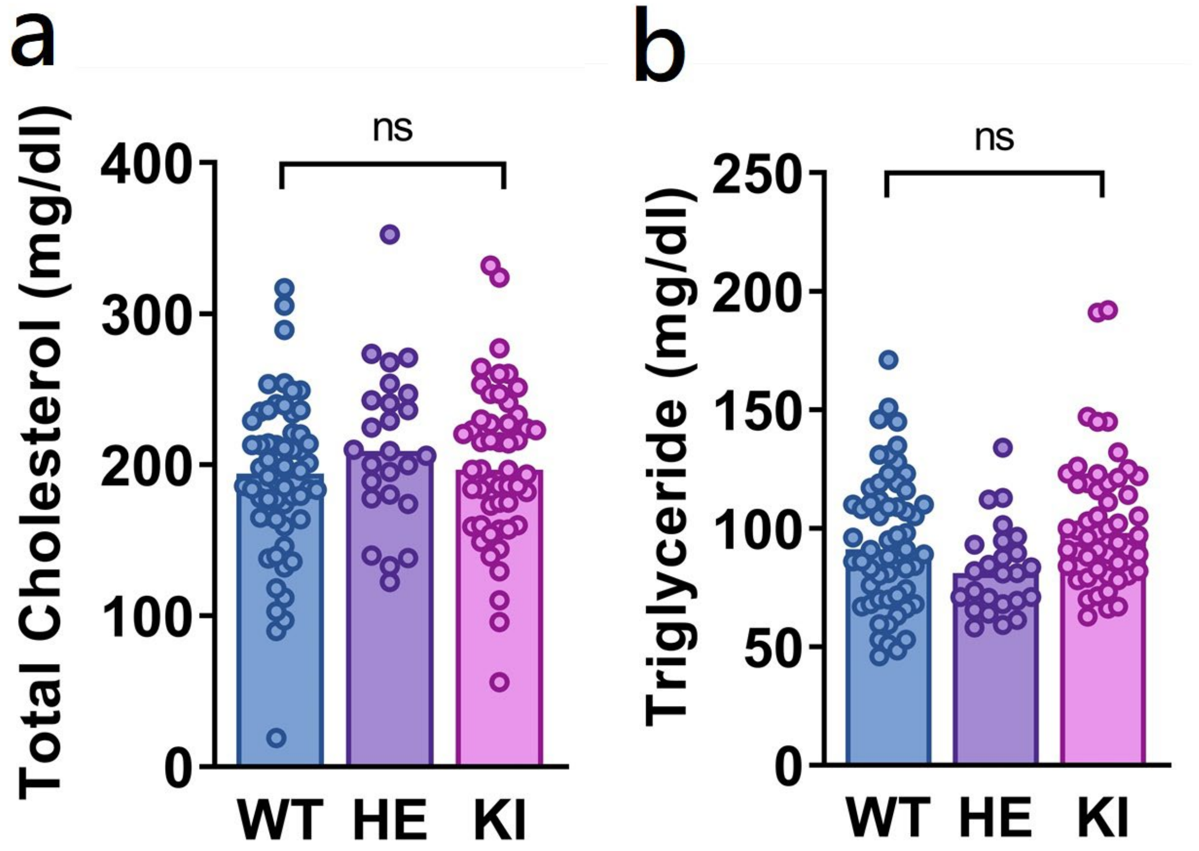

Figure S5. (a) Serum total cholesterol and (**b**) triglycerides (n=58:25:51 in duplicates; P-for-trend=0.38 and 0.13) of *Aldh2* homozygous knock-in (KI) and heterozygous knock-in (HE) mice compared with wild-type (WT) mice at the age of 24 weeks. Dara was analyzed using tests for linear trends. All data are presented as mean and standard error (S.E.M.). The n values represent biological repeats and the number of technical repeats was expressed as duplicates or triplicates.

Figure S6.

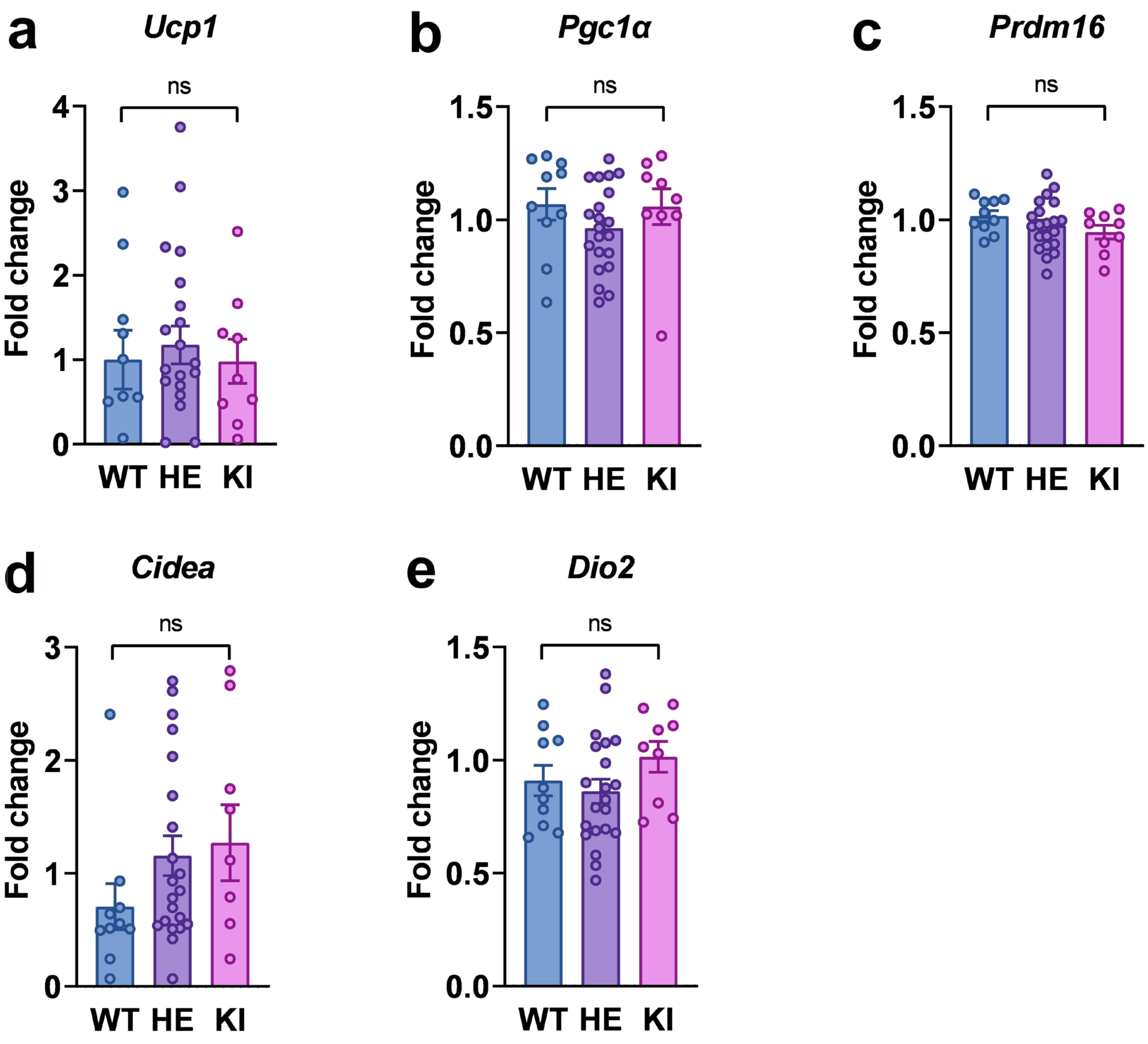
a
Ucp1
ns
Fold change
0
1
2
3
4
WT HE KI
b
Pgc1α
ns
Fold change
0.0
0.5
1.0
1.5
WT HE KI
c
Prdm16
ns
Fold change
0.0
0.5
1.0
1.5
WT HE KI
d
Cidea
ns
Fold change
0
1
2
3
WT HE KI
e
Dio2
ns
Fold change
0.0
0.5
1.0
1.5
WT HE KI

Figure S6. Expression levels of genes involved in thermogenesis including (**a**) *Ucp1*, (**b**) *Pgc1α*, (**c**) *Prdm16,* (**d**) *Cidea,* and (**e**) *Dio2* among BAT of *Aldh2* homozygous knock-in (KI), heterozygous knock-in (HE) and wild-type (WT) mice using real-time quantitative PCR (RT-qPCR) at the age of 24 weeks. Dara was analyzed using tests for linear trends (P-for-trend=0.98, 0.87, 0.11, 0.14 in duplicates, and 0.35 respectively). All data are presented as mean and standard error (S.E.M.). The n values represent biological repeats and the number of technical repeats was expressed as duplicates or triplicates.

Figure S7.

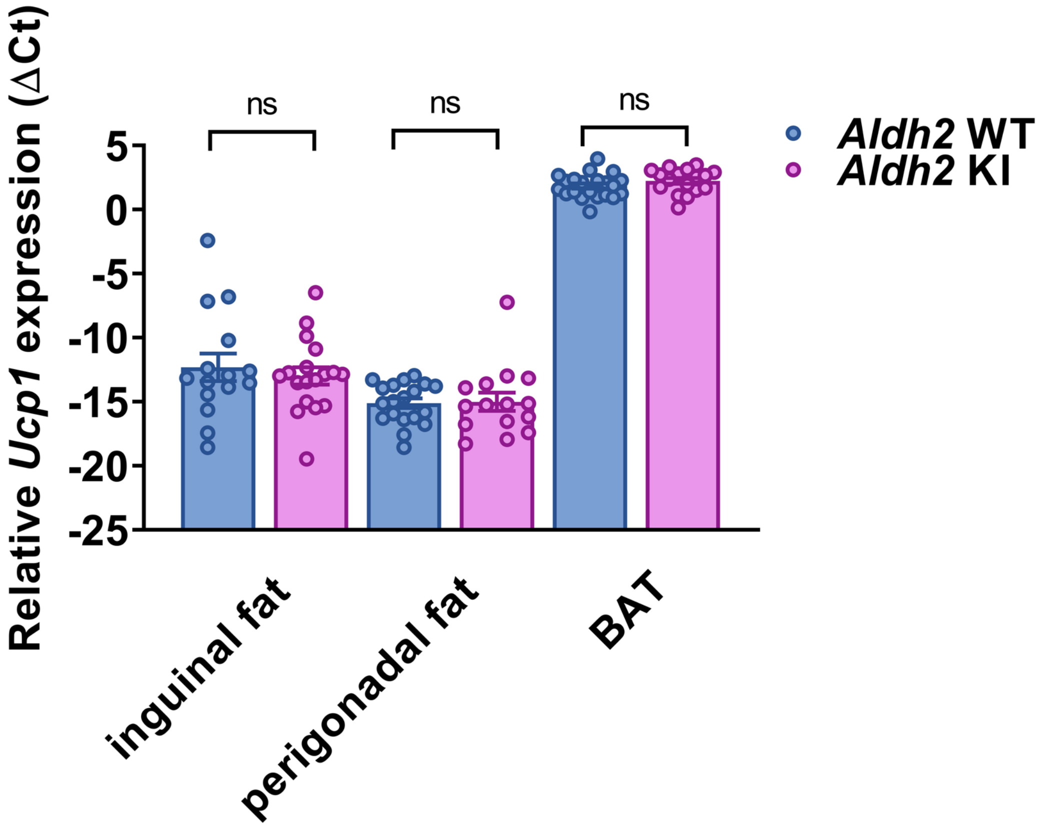

Figure S7. Expression of *Ucp1* relative to *Ppia* in white fat including inguinal and perigonadal fat between the *Aldh2* knock-in (KI) and wild-type (WT) mice measured by real-time quantitative PCR (RT-qPCR) at the age of 24 weeks (n=15: 16 in duplicates). Data was analyzed using two-sample independent *t*-tests (P=0.59, 0.88, and 0.18 respectively). All data are presented as mean and standard error (S.E.M.). The n values represent biological repeats and the number of technical repeats was expressed as duplicates or triplicates.

Figure S8.

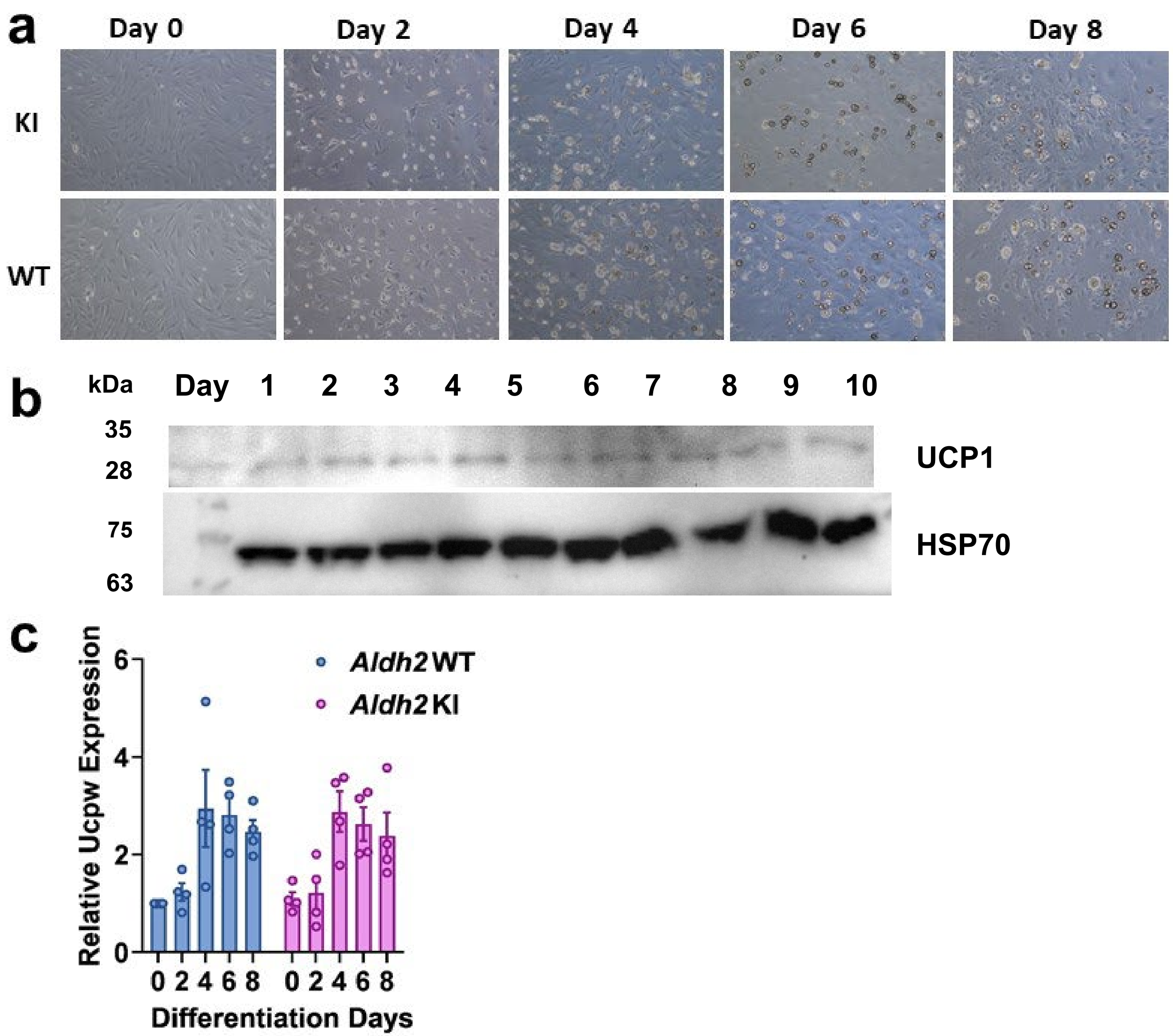

Figure S8. (a) Morphology showing the differentiation and **(b)** expression (c) and densitometric histogram of *Ucp1* of primary brown adipocytes isolated from *Aldh2* knock-in (KI) and wild-type (WT) mice. All data are presented as mean and standard error (S.E.M.).

Figure S9.

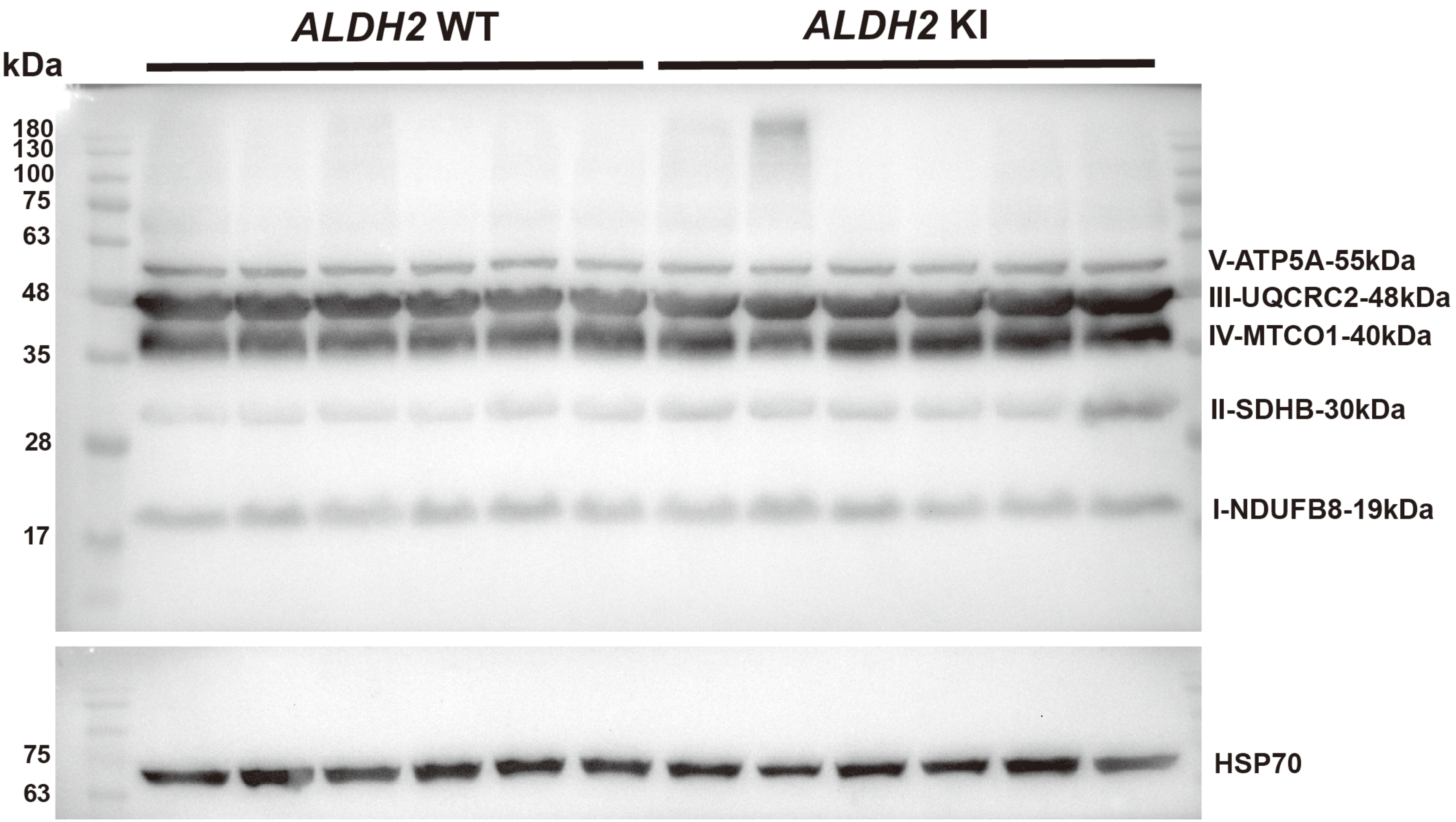

Figure S9. Immunoblots of the components of mitochondrial respiratory complex I (NDUFB8), II (SDHB), III (UQCRC2), IV (MTCO1), and V (ATPA5) in the brown adipose tissues of the *Aldh2* KI and WT mice at the age of 24 weeks.

Figure S10.

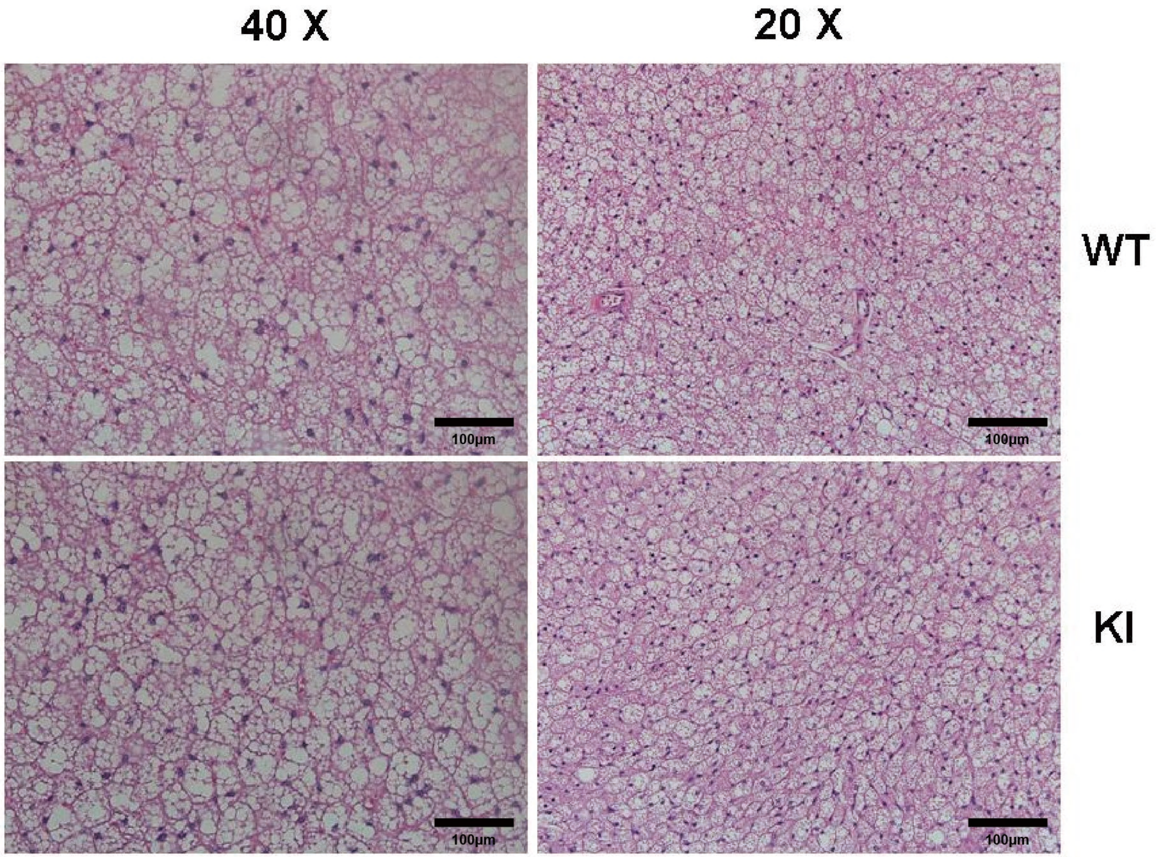

Figure S10. Microscopic appearance of brown adipose tissue from the *Aldh2* KI and WT mice using H&E stain at the age of 24 weeks. The scale bar is 100 μm.

Figure S11

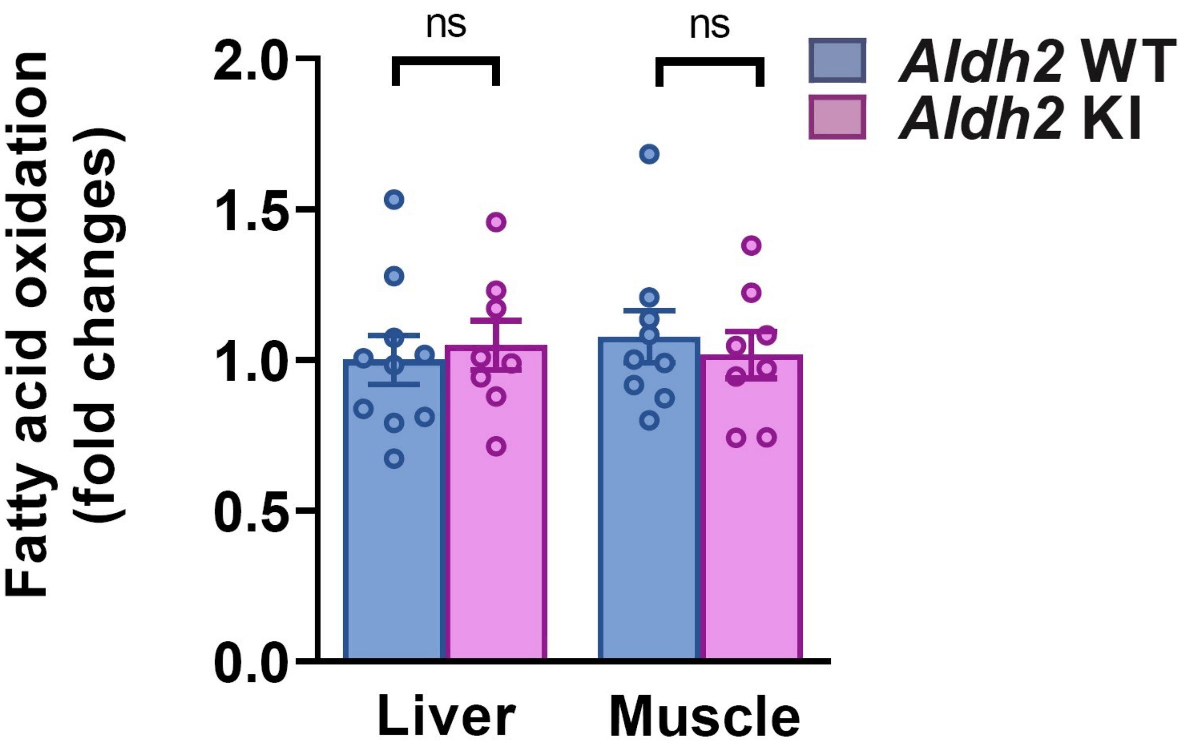

Figure S11. Fatty acid oxidation of skeletal muscle (quadriceps) (n=9:8) and liver (n=10:8) from *Aldh2* knock-in (KI) and wild-type (WT) mice at the age of 24 weeks. Data was analyzed using two-sample independent *t*-tests (P=0.67 and 0.81). All data are presented as mean and standard error (S.E.M.). The n values represent biological repeats.

Figure S12

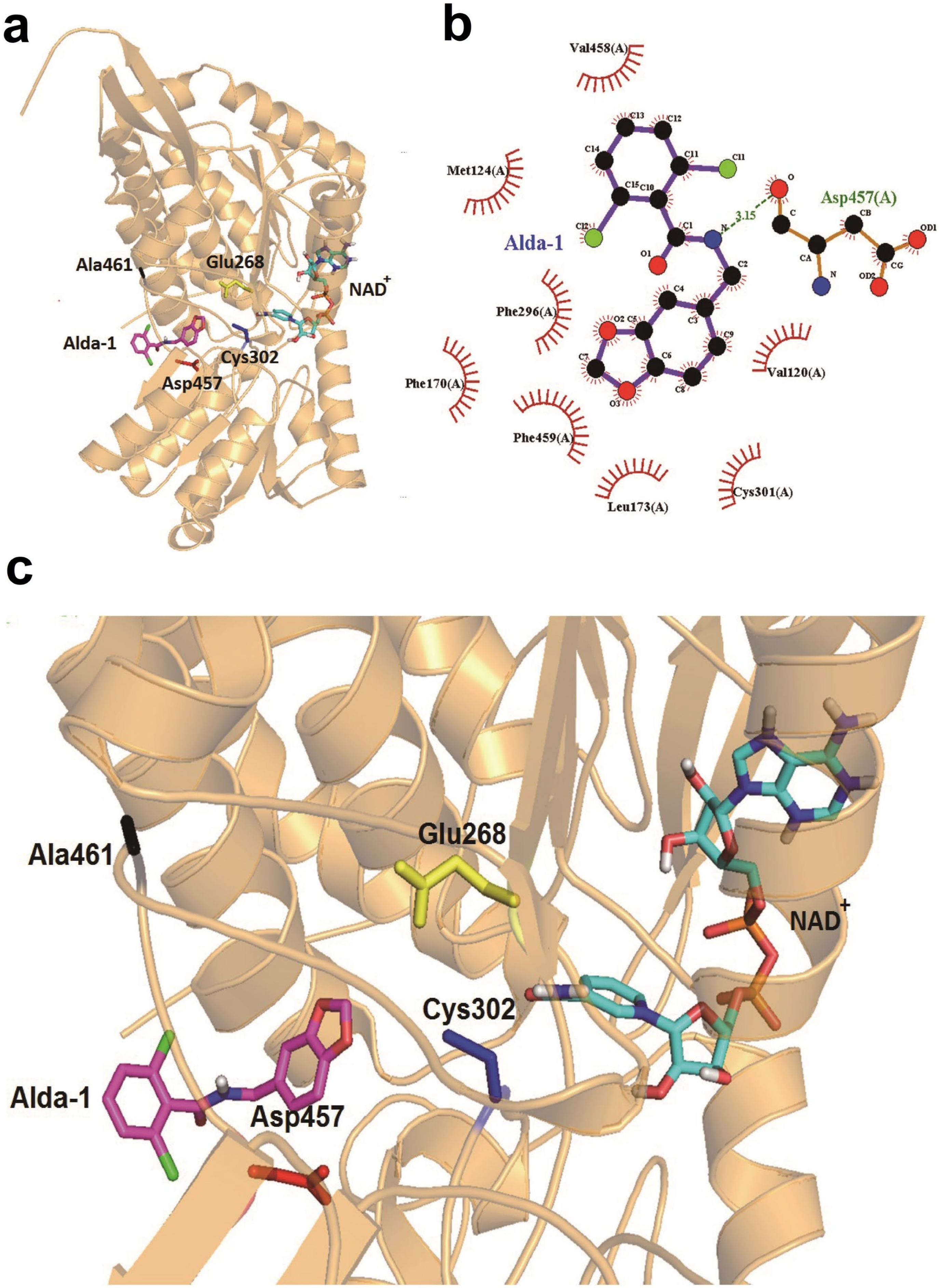
a
Ala461
Glu268
NAD+
Alda-1
Cys302
Asp457
b
Val458(A)
Met124(A)
Alda-1
Asp457(A)
3.15
Phe296(A)
Phe170(A)
Val120(A)
Phe459(A)
Leu173(A)
Cys301(A)
c
Ala461
Glu268
NAD+
Cys302
Alda-1
Asp457

Figure S12. (a) Ribbon diagram showing the binding pocket of **Alda-1** within human ALDH2. **(b)** LigPlot showing the bonds between Alda-1 and human ALDH2. **(c)** Ribbon diagram showing the binding of **Alda-1** and NAD^+ with human ALDH2.

Figure S13.

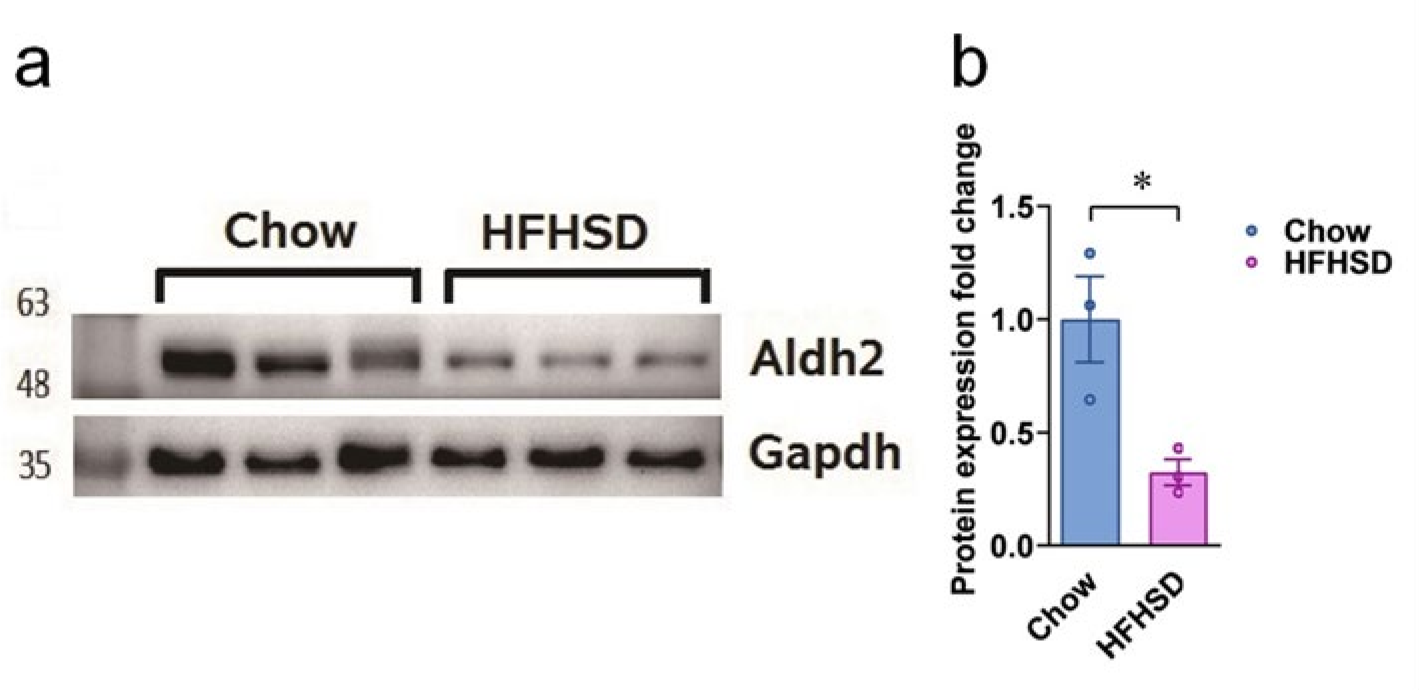

Figure S13. (a) Immunoblots and **(b)** densitometry histogram showing the Aldh2 expression in the brown adipose tissue (BAT) from chow-fed and high-fat high-sucrose (HFHSD)-fed C57BL6/J mice at the age of 24 weeks (n=3:3). Data was analyzed using two-sample independent *t*-tests (P=0.026). All data are presented as mean and standard error (S.E.M.) and all reported sample size are independent biological repeats. The asterisks indicate two-sided * P < 0.05.

Figure S14.

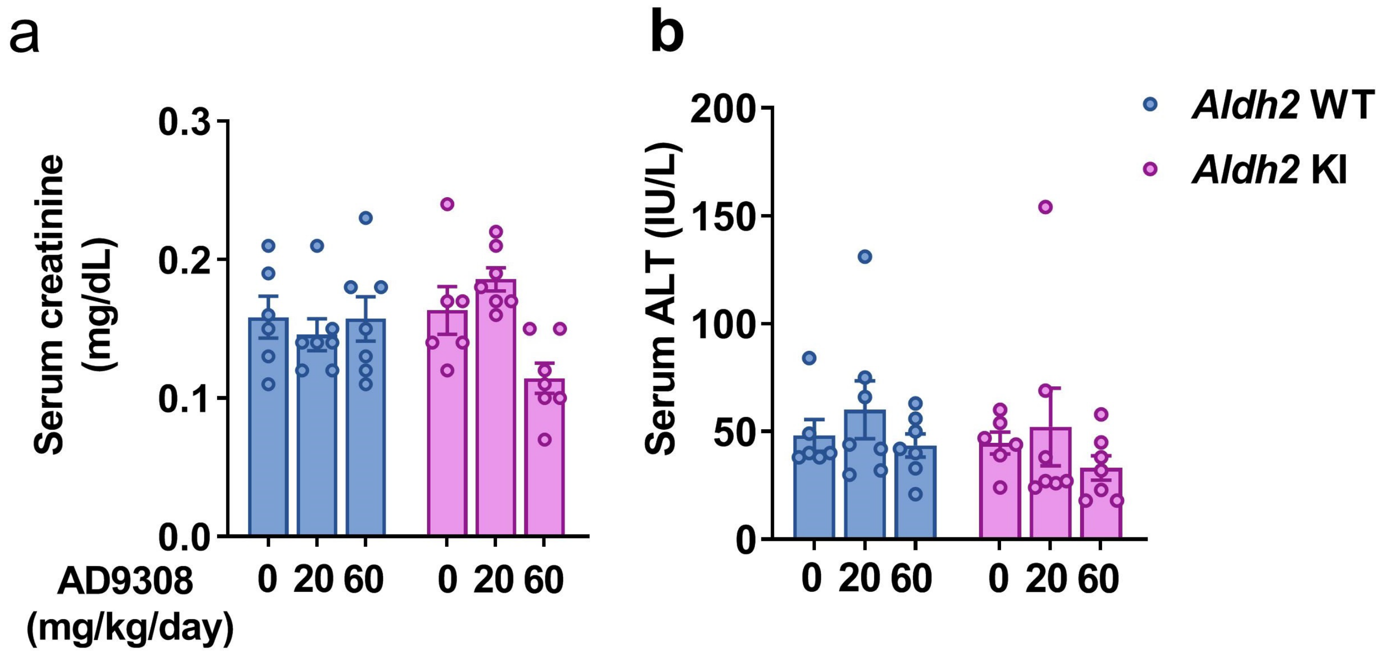

Figure S14. Serum (**a**) creatinine and (**b**) alanine aminotransferase (ALT) levels of *Aldh2* KI and WT mice treated with 0, 20 or 60 mg/kg/day of AD-9308 for 20 weeks (n=6:6:7) at the age of 24 weeks. All data are presented as mean and standard error (S.E.M.) and all reported sample size are independent biological repeats.

Figure S15.

a

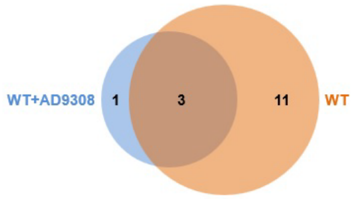

b

Gene name	protein name	biological function
***Aldh2* wild-type mice + AD9308**		
Ndufb10	NADH dehydrogenase [ubiquinone] 1 beta subcomplex subunit 10	Mitochondrial electron transfer
***Aldh2* wild-type mice & wild-type mice + AD9308**		
Aco2	Aconitate hydratase, mitochondrial	Tricarboxylic acid cycle
Cyc1	Cytochrome c1, heme protein, mitochondrial	Mitochondrial electron transfer
Opa1	Dynamin-like 120 kDa protein, mitochondrial	Mitochondrial fisssion and fusion
***Aldh2* wild-type mice**		
Gpd2	Glycerol-3-phosphate dehydrogenase, mitochondrial	Glycerol-3-phosphate metabolic process
Ucp1	Mitochondrial brown fat uncoupling protein 1	Thermogenesis
Hadha	Trifunctional enzyme subunit alpha, mitochondrial	Fatty acid beta-oxidation
Sdha	Succinate dehydrogenase [ubiquinone] flavoprotein subunit, mitochondrial	Mitochondrial electron transfer
Uqcrc1	Cytochrome b-c1 complex subunit 1, mitochondrial	Mitochondrial electron transfer
Acsl1	Long-chain-fatty-acid--CoA ligase	Fatty acid beta-oxidation and lipid synthesis
Hadhb	Trifunctional enzyme subunit beta, mitochondrial	Fatty acid beta-oxidation
Acaa2	3-ketoacyl-CoA thiolase, mitochondrial	Fatty acid beta-oxidation
Letmd1	LETM1 domain-containing protein 1	Thermogenesis
Uqcrb	Cytochrome b-c1 complex subunit 7	Mitochondrial electron transfer
Cox6c	Cytochrome c oxidase subunit 6C	Mitochondrial electron transfer

Figure S15. (a) Number and (**b**) list of 4-HNE-adducted mitochondrial proteins of the BAT from the *Aldh2* WT mice receiving or not receiving AD9308 identified by liquid-chromatography tandem mass spectrometry (LC-MS/MS) (n=3:3) at the age of 24 weeks.

Figure S16.

a

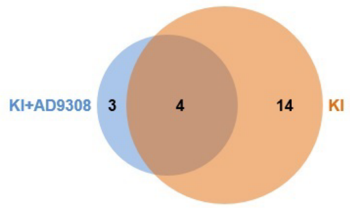

b

Gene name	protein name	biological function
***Aldh2* knock-in mice + AD9308**		
Fasn	Fatty acid synthase	Fatty acid synthesis
Cyc1	Cytochrome c1, heme protein, mitochondrial	Mitochondrial electron transfer
Uqcrfs1	Cytochrome b-c1 complex subunit Rieske, mitochondrial	Mitochondrial electron transfer
***Aldh2* knock-in mice & knock-in mice + AD9308**		
Gpd2	Glycerol-3-phosphate dehydrogenase, mitochondrial	Glycerol-3-phosphate metabolic process
Acaa2	3-ketoacyl-CoA thiolase, mitochondrial	Fatty acid beta-oxidation
Ndufb10	Mitochondrial intermembrane space import and assembly	Mitochondrial electron transfer
Glul	Glutamine synthetase	Glutamine synthesis
***Aldh2* knock-in mice**		
Hadha	Trifunctional enzyme subunit alpha, mitochondrial	Fatty acid beta-oxidation
Ucp1	Mitochondrial brown fat uncoupling protein 1	Thermogenesis
Sdha	Succinate dehydrogenase [ubiquinone] flavoprotein subunit, mitochondrial	Mitochondrial electron transfer
Uqcrc1	Cytochrome b-c1 complex subunit 1, mitochondrial	Mitochondrial electron transfer
Uqcrc2	Cytochrome b-c1 complex subunit 2, mitochondrial	Mitochondrial electron transfer
Hadhb	Trifunctional enzyme subunit beta, mitochondrial	Fatty acid beta-oxidation
Ndufa4	Cytochrome c oxidase subunit NDUFA4	Mitochondrial electron transfer
Slc25a20	Mitochondrial carnitine/acylcarnitine carrier protein	Fatty acid beta-oxidation
Uqcrb	Cytochrome b-c1 complex subunit 7	Mitochondrial electron transfer
Letmd1	LETM1 domain-containing protein 1	Thermogenesis
Ndufa10	NADH dehydrogenase [ubiquinone] 1 beta subcomplex subunit 10	Mitochondrial electron transfer
Opa1	Dynamin-like 120 kDa protein, mitochondrial	Mitochondrial fisssion and fusion
Ndufs6	NADH dehydrogenase [ubiquinone] iron-sulfur protein 6, mitochondrial	Mitochondrial electron transfer
Cox6c	Cytochrome c oxidase subunit 6C	Mitochondrial electron transfer

Figure S16. (a) Number and (**b**) list of 4-HNE-adducted mitochondrial proteins of the BAT from the *Aldh2* KI mice receiving or not receiving AD9308 identified by liquid-chromatography tandem mass spectrometry (LC-MS/MS) (n=3:3) at the age of 30 weeks.

Figure S17.

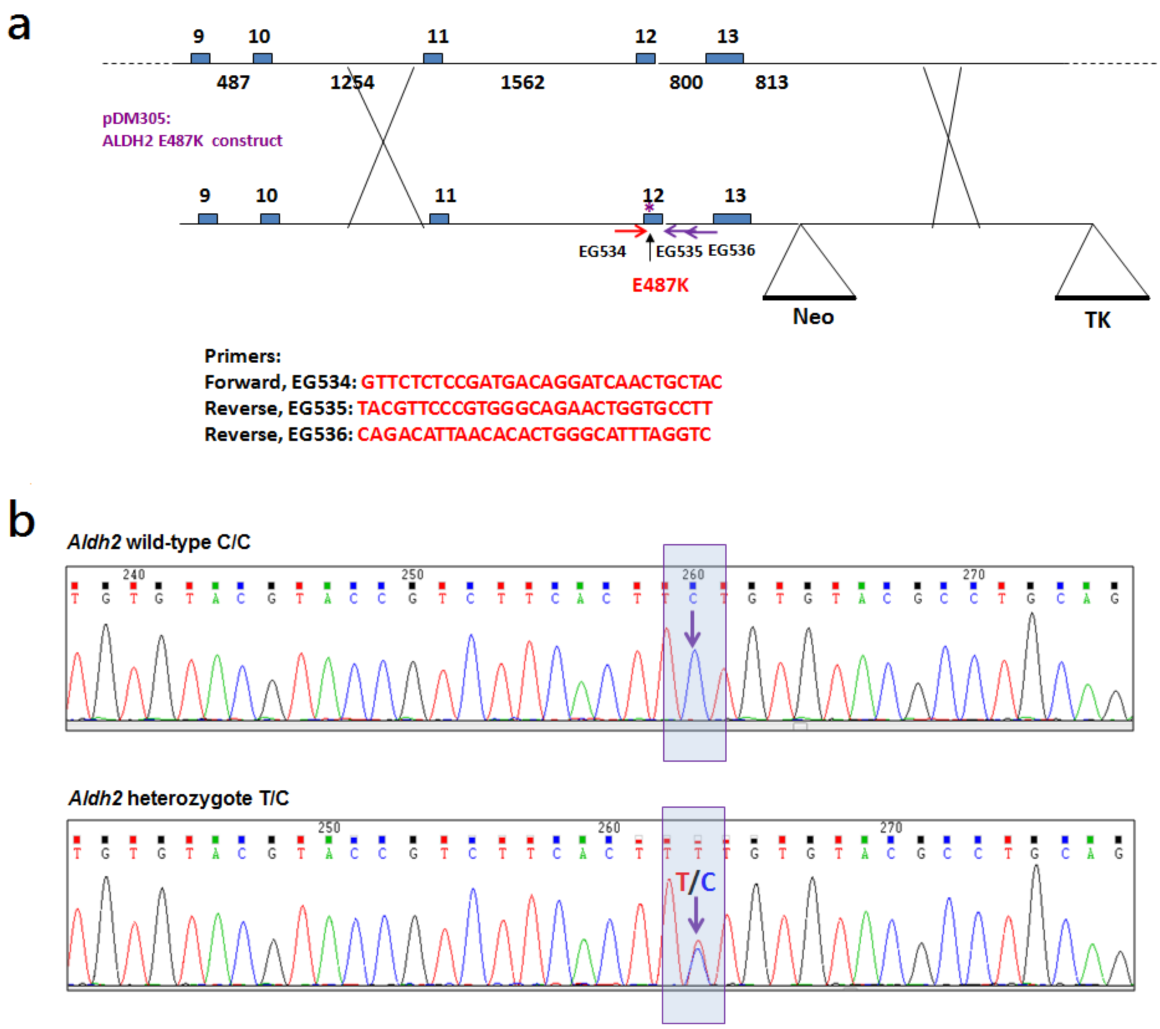

Figure S17. (a) Schematic graphic of the generation of *Aldh2* mutant allele mimicking human Glu504Lys mutation (b) Sanger sequencing of Aldh2 wild-type and heterozygous knock-in mice

REFERENCES

1. R. Urso, P. Blardi, G. Giorgi, A short introduction to pharmacokinetics. *Eur Rev Med Pharmacol Sci* **6**, 33-44 (2002).

2. Zambelli, V. O., Gross, E. R., Chen, C.H., Gutierrez, V. P., Cury Y. & Daria, M-R. Aldehyde dehydrogenase-2 regulates nociception in rodent models of acute inflammatory pain. *Sci. Transl. Med.* **6***:251ra118* (2014).

Uncropped image of all immunoblots in Supplementary Materials

Supplementary Fig S3 OXOPHOS

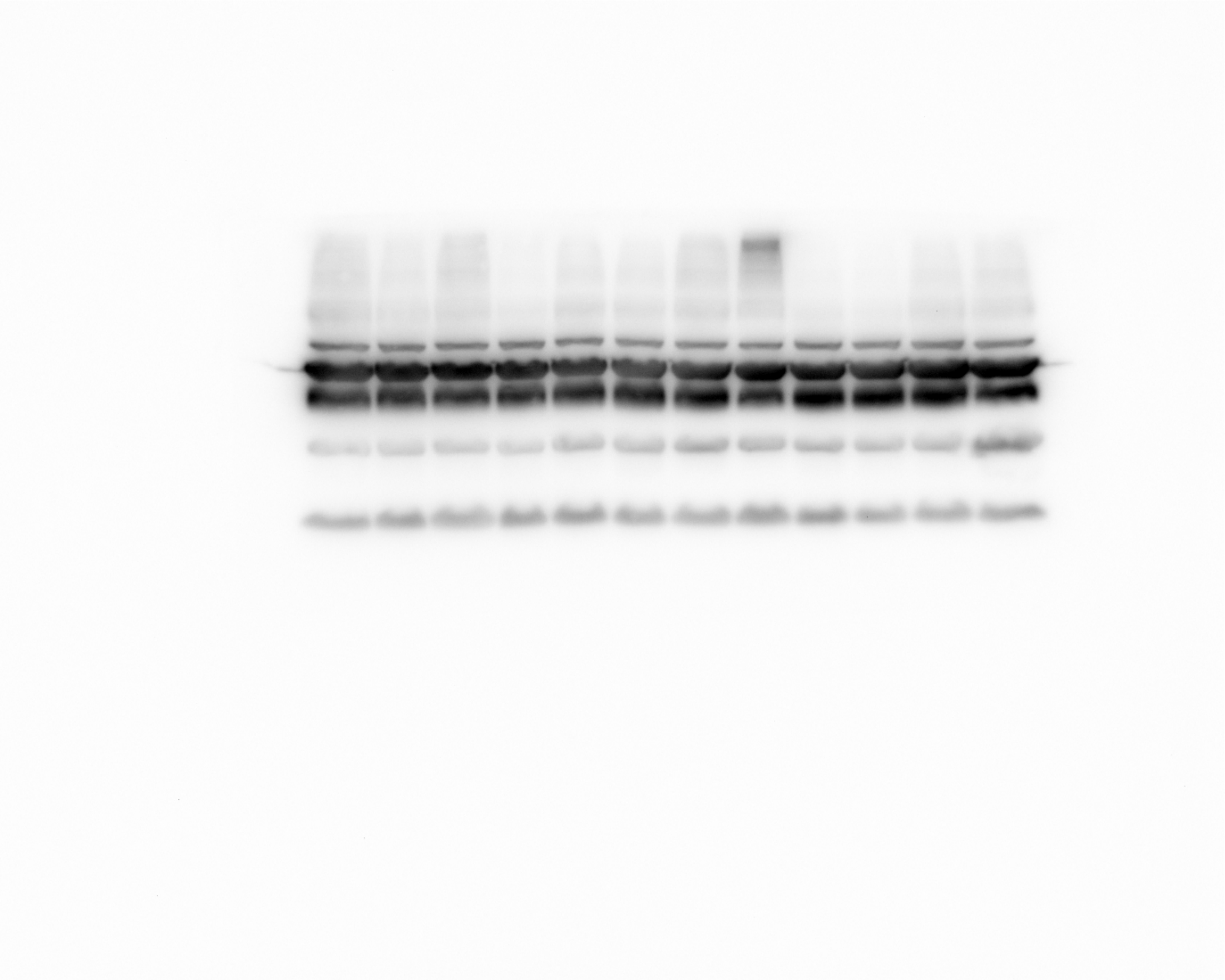

Supplementary Fig S3 OXOPHOS Bright Field

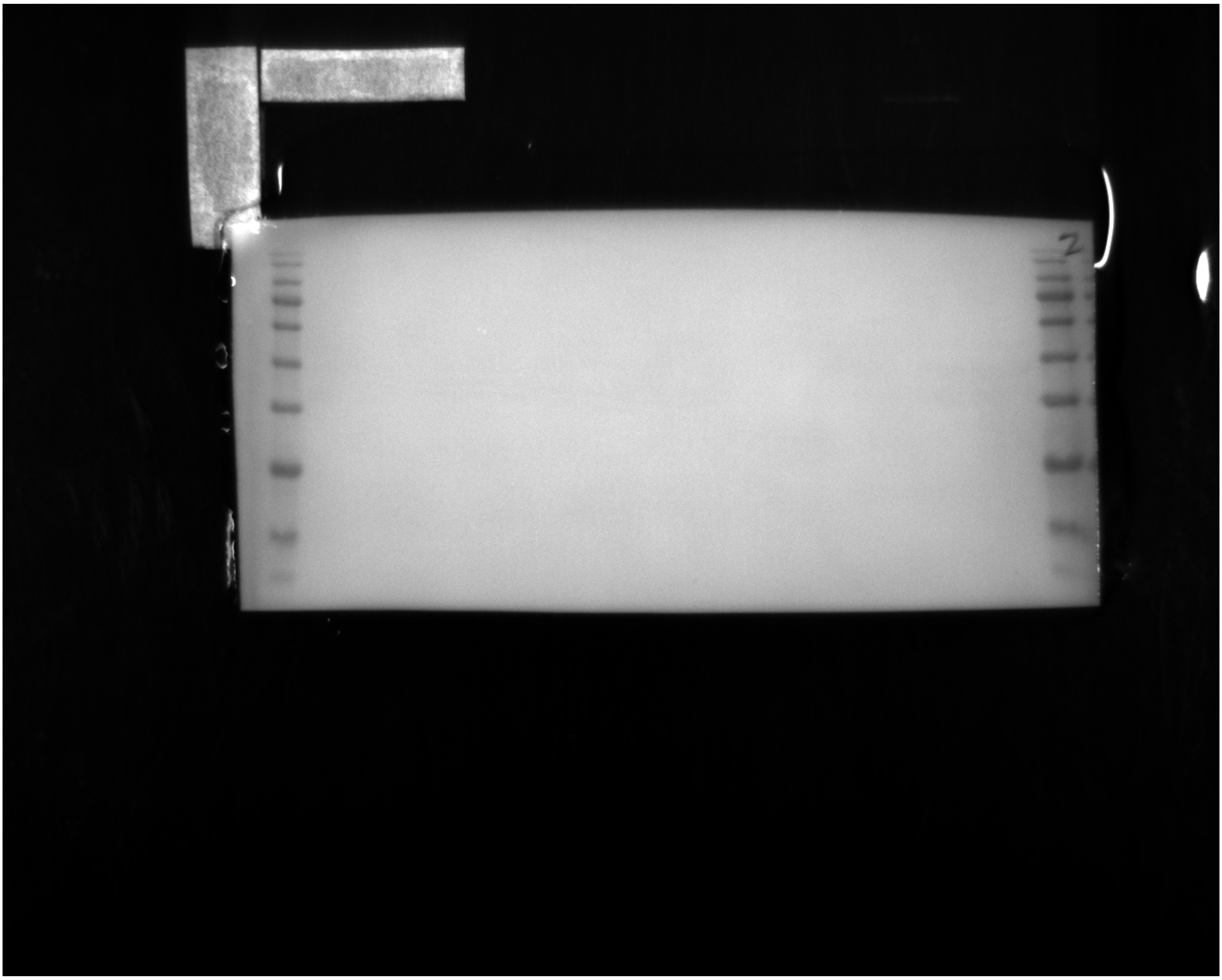

Supplementary Fig S6 Aldh2

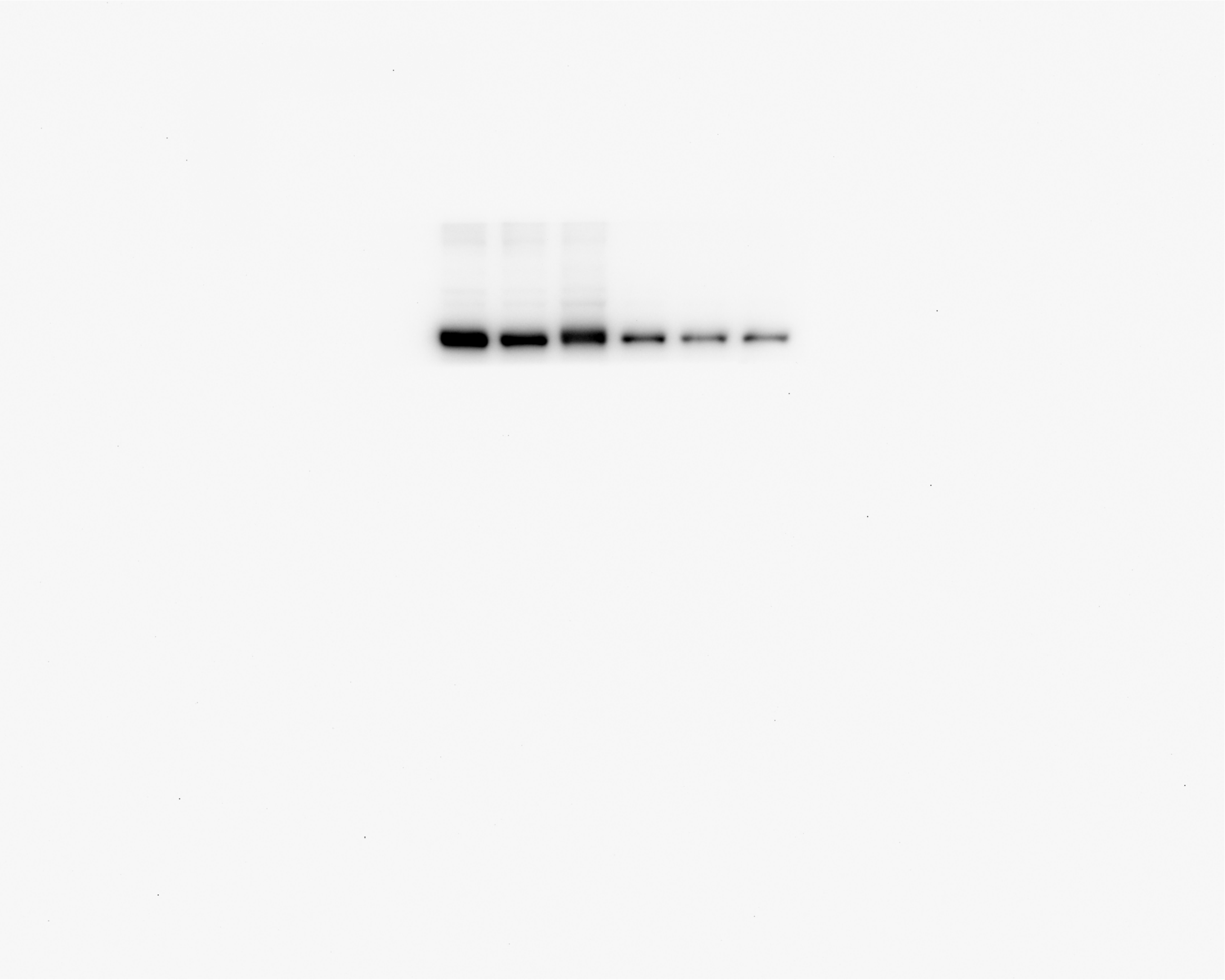

Supplementary Fig S6 Aldh2 Bright Field

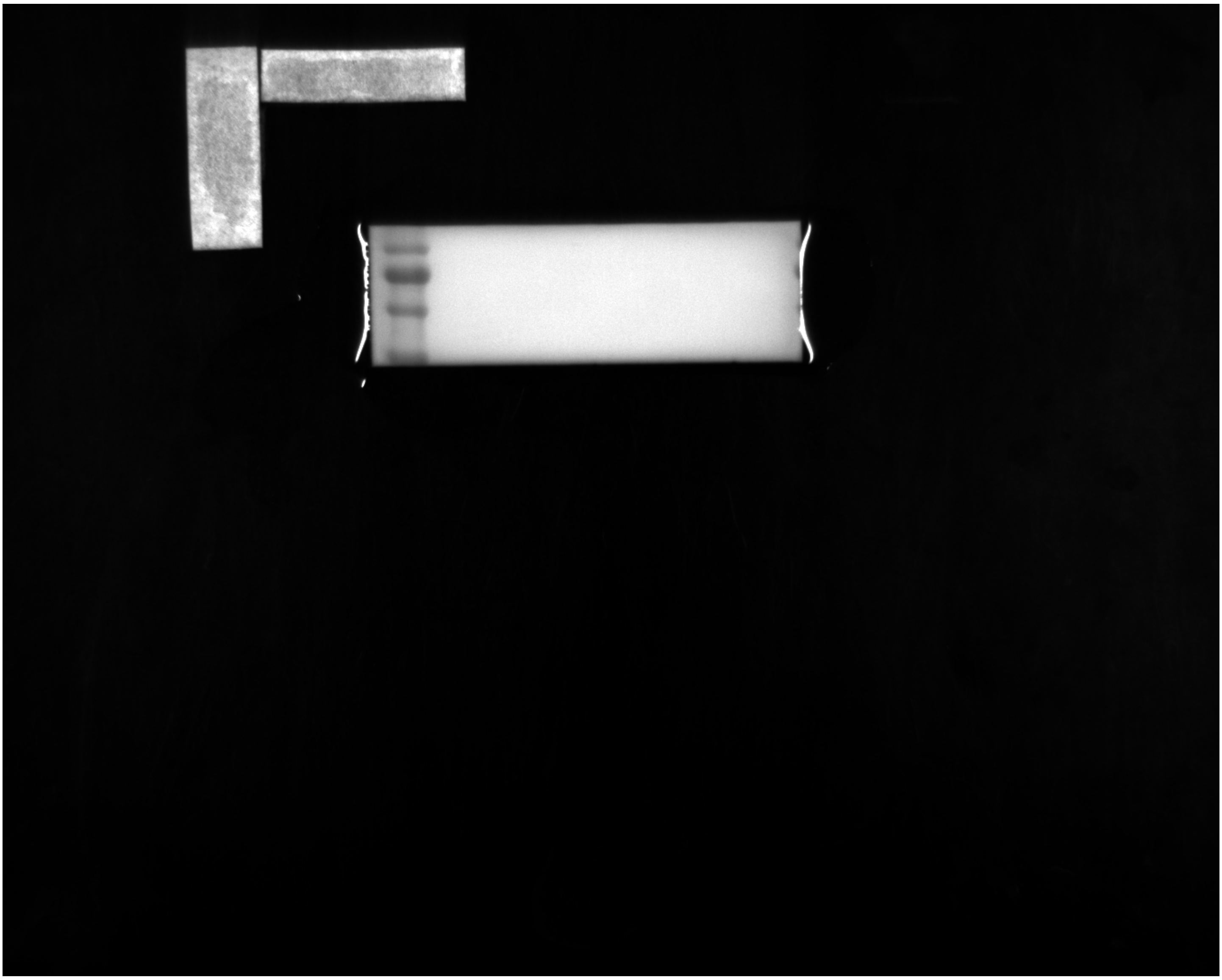

Supplementary Fig S6 Gapdh

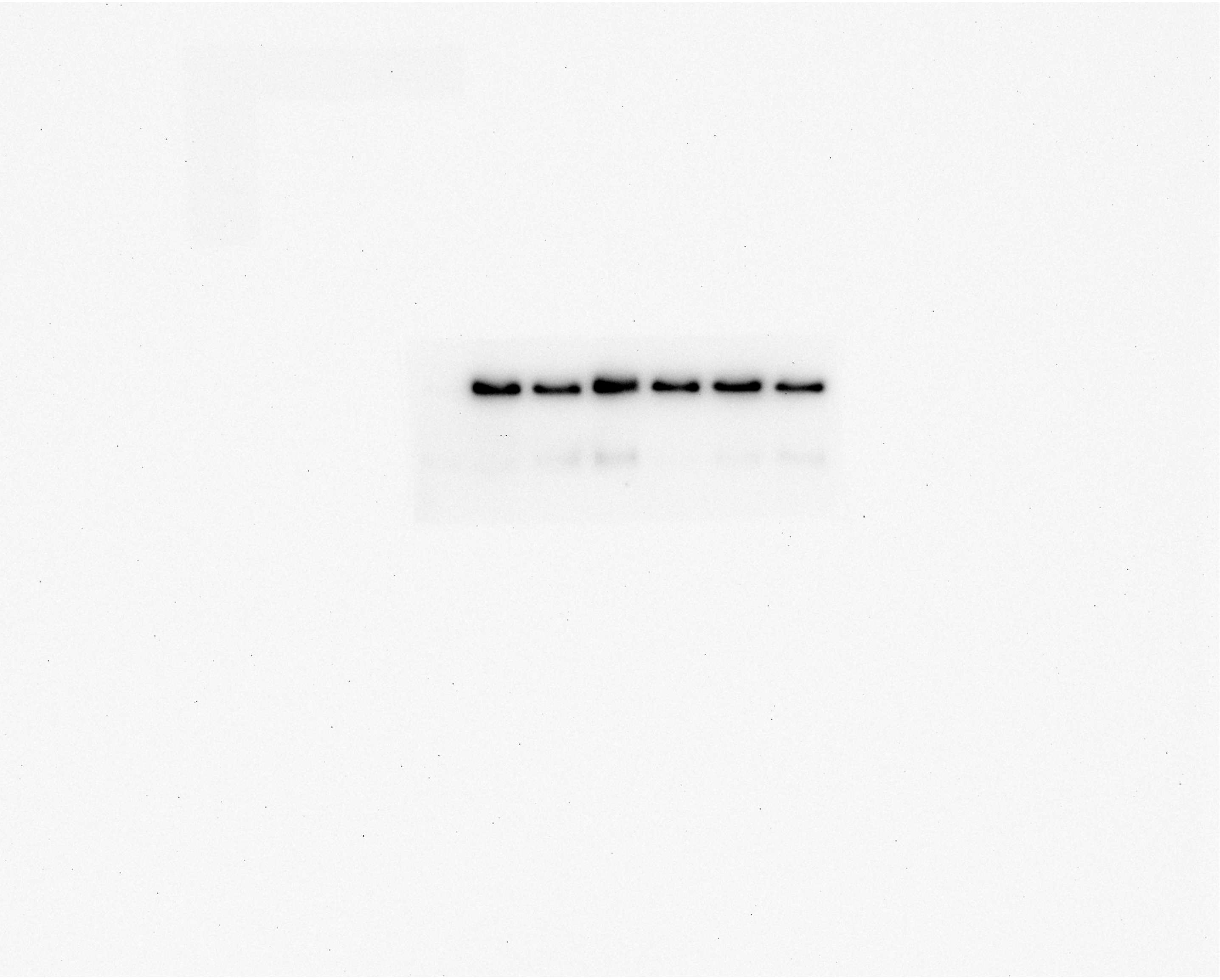

Supplementary Fig S6 Gapdh Bright Field

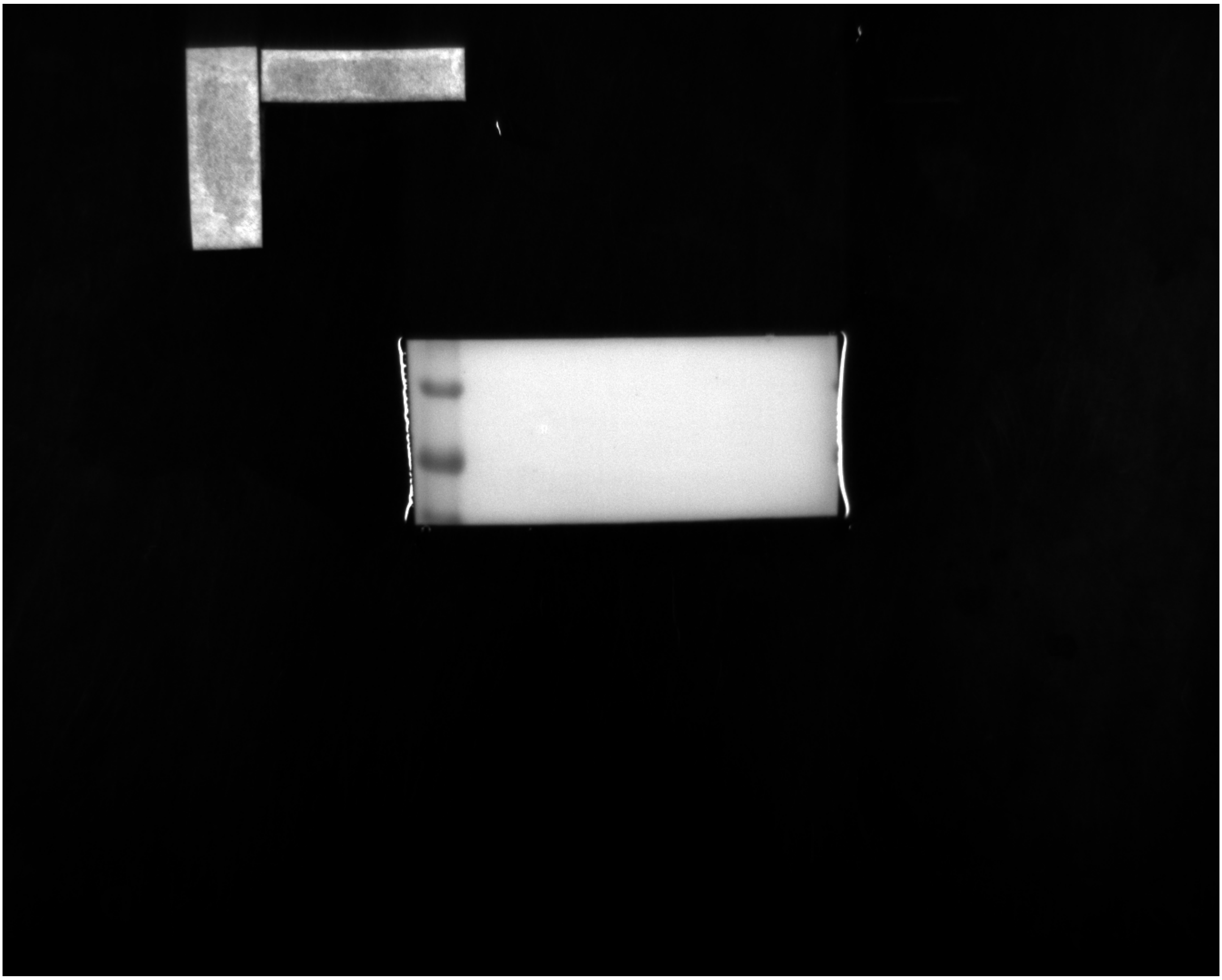

Supplement: Supplementary file 1 — Supplementary Information [file 41467_2023_41570_MOESM1_ESM.pdf]
