# Supplementary material for: A common East-Asian ALDH2 mutation causes metabolic disorders and the therapeutic effect of ALDH2 activators
Source: Nat Commun. 2023 Sep 25;14:5971. doi: 10.1038/s41467-023-41570-6 (PMC10520061; doi:10.1038/s41467-023-41570-6)
Supplement: Supplementary file 3 — Description of Additional Supplementary Files [file 41467_2023_41570_MOESM3_ESM.pdf]

Description of Additional Supplementary Files

Title: **Supplementary Data 1**

Description: MASCOT mass spectrum analyses for identified 4-HNE adducted proteins by liquid chromatography tandem mass spectrometry (LC-MS/MS)
