# Supplementary material for: A common East-Asian ALDH2 mutation causes metabolic disorders and the therapeutic effect of ALDH2 activators
Source: Nat Commun. 2023 Sep 25;14:5971. doi: 10.1038/s41467-023-41570-6 (PMC10520061; doi:10.1038/s41467-023-41570-6)
Supplement: Supplementary file 4 — Supplementary Data 1 [file 41467_2023_41570_MOESM4_ESM.zip › Table S5b/D3Z041/D3Z041_WTO-1_K264.html]

Mascot Search Results: D3Z041
 

# MASCOT Search Results

## Protein View: D3Z041

### Arachidonate--CoA ligase OS=Mus musculus OX=10090 GN=Acsl1 PE=1 SV=1

|  |  |
| --- | --- |
| Database: | Mouse\_UniProt\_proteomes |
| Score: | 9567 |
| Monoisotopic mass (Mr): | 79011 |
| Calculated pI: | 7.43 |

Sequence similarity is available as an NCBI BLAST search of D3Z041 against nr.

### Search parameters

|  |  |
| --- | --- |
| MS data file: | `D:\LCMSMS\2023 Users' data\230529-1\230529-1-WTO-1.raw` |
| Enzyme: | Trypsin/P: cuts C-term side of KR. |
| Fixed modifications: | Carbamidomethyl (C) |
| Variable modifications: | Deamidated (NQ), HNE (C), HNE (H), HNE (K), Oxidation (M) |

### Protein sequence coverage: 66%

Matched peptides shown in ***bold red***.

|  |  |  |  |  |  |
| --- | --- | --- | --- | --- | --- |
| `1` | `MEVHELFRYF` | `RMPELIDIRQ` | `YVRTLPTNTL` | `MGFGAFAALT` | `TFWYATRPKA` |
| `51` | `LKPPCDLSMQ` | `SVEIAGTTDG` | `IRRSAVLEDD` | `KLLVYYYDDV` | `RTMYDGFQRG` |
| `101` | `IQVSNNGPCL` | `GSRKPNQPYE` | `WISYKEVAEL` | `AECIGSGLIQ` | `KGFKPCSEQF` |
| `151` | `IGLFSQNRPE` | `WVIVEQGCFS` | `YSMVVVPLYD` | `TLGADAITYI` | `VNKAELSVIF` |
| `201` | `ADKPEKAKLL` | `LEGVENKLTP` | `CLKIIVIMDS` | `YGSDLVERGK` | `KCGVEIISLK` |
| `251` | `ALEDLGRVNR` | `VKPKPPEPED` | `LAIICFTSGT` | `TGNPKGAMIT` | `HQNIINDCSG` |
| `301` | `FIKATESALT` | `LNASDTQISY` | `LPLAHMYEQQ` | `LQCVMLCHGA` | `KIGFFQGDIR` |
| `351` | `LLMDDLKVLQ` | `PTIFPVVPRL` | `LNRMFDRIFG` | `QANTSLKRWL` | `LDFASKRKEA` |
| `401` | `ELRSGIVRNN` | `SLWDKLIFHK` | `IQSSLGGKVR` | `LMITGAAPVS` | `ATVLTFLRTA` |
| `451` | `LGCQFYEGYG` | `QTECTAGCCL` | `SLPGDWTAGH` | `VGAPMPCNYV` | `KLVDVEEMNY` |
| `501` | `LASKGEGEVC` | `VKGANVFKGY` | `LKDPARTAEA` | `LDKDGWLHTG` | `DIGKWLPNGT` |
| `551` | `LKIIDRKKHI` | `FKLAQGEYIA` | `PEKIENIYLR` | `SEAVAQVFVH` | `GESLQAFLIA` |
| `601` | `VVVPDVESLP` | `SWAQKRGLQG` | `SFEELCRNKD` | `INKAILDDLL` | `KLGKEAGLKP` |
| `651` | `FEQVKGIAVH` | `PELFSIDNGL` | `LTPTLKAKRP` | `ELRNYFRSQI` | `DELYATIKI` |

Unformatted sequence string: 699 residues (for pasting into other applications).

|  |  |  |  |
| --- | --- | --- | --- |
| Sort by | residue number | increasing mass | decreasing mass |
| Show | matched peptides only | predicted peptides also |  |

| Query | Start | – | End | Observed | Mr(expt) | Mr(calc) | ppm | M | Score | Expect | Rank | U | Peptide |
| --- | --- | --- | --- | --- | --- | --- | --- | --- | --- | --- | --- | --- | --- |
| 59637 | 9 | – | 19 | 484.9264 | 1451.7573 | 1451.7595 | -1.53 | 1 | 24 | 0.0064 | 1Score **> 34** indicates **identity** Score **> 15** indicates **homology** | U | R.YFRMPELIDIR.Q |
| 59640 | 9 | – | 19 | 484.9268 | 1451.7585 | 1451.7595 | -0.67 | 1 | 41 | 0.00026 | 1Score **> 34** indicates **identity** Score **> 18** indicates **homology** | U | R.YFRMPELIDIR.Q |
| 59642 | 9 | – | 19 | 484.9270 | 1451.7591 | 1451.7595 | -0.28 | 1 | 39 | 0.00032 | 1Score **> 34** indicates **identity** Score **> 17** indicates **homology** | U | R.YFRMPELIDIR.Q |
| 59643 | 9 | – | 19 | 484.9272 | 1451.7596 | 1451.7595 | 0.10 | 1 | 41 | 0.00024 | 1Score **> 34** indicates **identity** Score **> 17** indicates **homology** | U | R.YFRMPELIDIR.Q |
| 59644 | 9 | – | 19 | 484.9273 | 1451.7602 | 1451.7595 | 0.47 | 1 | 32 | 0.0012 | 1Score **> 34** indicates **identity** Score **> 15** indicates **homology** | U | R.YFRMPELIDIR.Q |
| 59645 | 9 | – | 19 | 484.9280 | 1451.7622 | 1451.7595 | 1.91 | 1 | 24 | 0.01 | 1Score **> 34** indicates **identity** Score **> 17** indicates **homology** | U | R.YFRMPELIDIR.Q |
| 59648 | 9 | – | 19 | 484.9285 | 1451.7636 | 1451.7595 | 2.87 | 1 | 25 | 0.0056 | 1Score **> 34** indicates **identity** Score **> 15** indicates **homology** | U | R.YFRMPELIDIR.Q |
| 13245 | 12 | – | 19 | 493.7711 | 985.5277 | 985.5266 | 1.12 | 0 | 47 | 0.00016 | 1Score **> 31** indicates **identity** Score **> 21** indicates **homology** | U | R.MPELIDIR.Q |
| 13247 | 12 | – | 19 | 493.7713 | 985.5280 | 985.5266 | 1.35 | 0 | 41 | 0.00048 | 1Score **> 31** indicates **identity** Score **> 20** indicates **homology** | U | R.MPELIDIR.Q |
| 13248 | 12 | – | 19 | 493.7715 | 985.5285 | 985.5266 | 1.88 | 0 | 47 | 0.00035 | 1Score **> 31** indicates **identity** Score **> 25** indicates **homology** | U | R.MPELIDIR.Q |
| 13250 | 12 | – | 19 | 493.7717 | 985.5288 | 985.5266 | 2.23 | 0 | 43 | 0.00074 | 1Score **> 31** indicates **identity** Score **> 24** indicates **homology** | U | R.MPELIDIR.Q |
| 14296 | 12 | – | 19 | 501.7675 | 1001.5204 | 1001.5215 | -1.12 | 0 | 35 | 0.0015 | 1Score **> 33** indicates **identity** Score **> 20** indicates **homology** | U | R.MPELIDIR.Q  + Oxidation (M) |
| 14297 | 12 | – | 19 | 501.7676 | 1001.5206 | 1001.5215 | -0.97 | 0 | 38 | 0.00091 | 1Score **> 33** indicates **identity** Score **> 20** indicates **homology** | U | R.MPELIDIR.Q  + Oxidation (M) |
| 14298 | 12 | – | 19 | 501.7677 | 1001.5208 | 1001.5215 | -0.72 | 0 | 34 | 0.00066 | 1Score **> 33** indicates **identity** Score **> 15** indicates **homology** | U | R.MPELIDIR.Q  + Oxidation (M) |
| 14299 | 12 | – | 19 | 501.7685 | 1001.5225 | 1001.5215 | 0.99 | 0 | 14 | 0.047 | 1Score **> 33** indicates **identity** Score **> 13** indicates **homology** | U | R.MPELIDIR.Q  + Oxidation (M) |
| 14300 | 12 | – | 19 | 501.7687 | 1001.5229 | 1001.5215 | 1.31 | 0 | 23 | 0.019 | 1Score **> 33** indicates **identity** Score **> 19** indicates **homology** | U | R.MPELIDIR.Q  + Oxidation (M) |
| 14301 | 12 | – | 19 | 501.7693 | 1001.5240 | 1001.5215 | 2.47 | 0 | 37 | 0.012 | 1Score **> 33** indicates **identity** Score **> 30** indicates **homology** | U | R.MPELIDIR.Q  + Oxidation (M) |
| 14303 | 12 | – | 19 | 501.7696 | 1001.5247 | 1001.5215 | 3.14 | 0 | 37 | 0.016 | 1Score **> 31** indicates **identity** Score **> 31** indicates **homology** | U | R.MPELIDIR.Q  + Oxidation (M) |
| 169723 | 24 | – | 49 | 959.8351 | 2876.4836 | 2876.4571 | 9.19 | 1 | 19 | 0.017 | 1Score **> 37** indicates **identity** Score **> 14** indicates **homology** | U | R.TLPTNTLMGFGAFAALTTFWYATRPK.A  + Deamidated (NQ) |
| 160312 | 50 | – | 73 | 654.5882 | 2614.3237 | 2614.3207 | 1.14 | 2 | 42 | 0.00011 | 1Score **> 37** indicates **identity** Score **> 15** indicates **homology** | U | K.ALKPPCDLSMQSVEIAGTTDGIRR.S |
| 160313 | 50 | – | 73 | 654.5883 | 2614.3240 | 2614.3207 | 1.25 | 2 | 32 | 0.001 | 1Score **> 37** indicates **identity** Score **> 15** indicates **homology** | U | K.ALKPPCDLSMQSVEIAGTTDGIRR.S |
| 160315 | 50 | – | 73 | 654.5887 | 2614.3256 | 2614.3207 | 1.89 | 2 | 54 | 7.9e-06 | 1Score **> 37** indicates **identity** Score **> 16** indicates **homology** | U | K.ALKPPCDLSMQSVEIAGTTDGIRR.S |
| 145834 | 73 | – | 91 | 583.8018 | 2331.1781 | 2331.1747 | 1.47 | 2 | 26 | 0.0034 | 1Score **> 37** indicates **identity** Score **> 14** indicates **homology** | U | R.RSAVLEDDKLLVYYYDDVR.T |
| 145837 | 73 | – | 91 | 583.8037 | 2331.1857 | 2331.1747 | 4.75 | 2 | 34 | 0.00067 | 1Score **> 37** indicates **identity** Score **> 15** indicates **homology** | U | R.RSAVLEDDKLLVYYYDDVR.T |
| 145838 | 73 | – | 91 | 583.8049 | 2331.1904 | 2331.1747 | 6.75 | 2 | 24 | 0.0058 | 1Score **> 37** indicates **identity** Score **> 14** indicates **homology** | U | R.RSAVLEDDKLLVYYYDDVR.T |
| 134669 | 74 | – | 91 | 726.0267 | 2175.0582 | 2175.0735 | -7.06 | 1 | 49 | 2.3e-05 | 1Score **> 36** indicates **identity** Score **> 16** indicates **homology** | U | R.SAVLEDDKLLVYYYDDVR.T |
| 134674 | 74 | – | 91 | 726.0279 | 2175.0618 | 2175.0735 | -5.41 | 1 | 24 | 0.0059 | 1Score **> 36** indicates **identity** Score **> 14** indicates **homology** | U | R.SAVLEDDKLLVYYYDDVR.T |
| 134677 | 74 | – | 91 | 726.0285 | 2175.0636 | 2175.0735 | -4.57 | 1 | 64 | 9.3e-07 | 1Score **> 36** indicates **identity** Score **> 17** indicates **homology** | U | R.SAVLEDDKLLVYYYDDVR.T |
| 134681 | 74 | – | 91 | 726.0290 | 2175.0652 | 2175.0735 | -3.82 | 1 | 69 | 3.5e-07 | 1Score **> 36** indicates **identity** Score **> 17** indicates **homology** | U | R.SAVLEDDKLLVYYYDDVR.T |
| 134682 | 74 | – | 91 | 726.0292 | 2175.0657 | 2175.0735 | -3.62 | 1 | 68 | 4.1e-07 | 1Score **> 36** indicates **identity** Score **> 17** indicates **homology** | U | R.SAVLEDDKLLVYYYDDVR.T |
| 134685 | 74 | – | 91 | 1088.5404 | 2175.0662 | 2175.0735 | -3.37 | 1 | 20 | 0.015 | 1Score **> 36** indicates **identity** Score **> 14** indicates **homology** | U | R.SAVLEDDKLLVYYYDDVR.T |
| 134686 | 74 | – | 91 | 726.0294 | 2175.0665 | 2175.0735 | -3.24 | 1 | 54 | 8.5e-06 | 1Score **> 36** indicates **identity** Score **> 16** indicates **homology** | U | R.SAVLEDDKLLVYYYDDVR.T |
| 134688 | 74 | – | 91 | 726.0295 | 2175.0668 | 2175.0735 | -3.12 | 1 | 51 | 1.8e-05 | 1Score **> 36** indicates **identity** Score **> 16** indicates **homology** | U | R.SAVLEDDKLLVYYYDDVR.T |
| 134693 | 74 | – | 91 | 726.0300 | 2175.0680 | 2175.0735 | -2.53 | 1 | 70 | 2.7e-07 | 1Score **> 36** indicates **identity** Score **> 17** indicates **homology** | U | R.SAVLEDDKLLVYYYDDVR.T |
| 134694 | 74 | – | 91 | 726.0300 | 2175.0683 | 2175.0735 | -2.41 | 1 | 51 | 1.6e-05 | 1Score **> 36** indicates **identity** Score **> 16** indicates **homology** | U | R.SAVLEDDKLLVYYYDDVR.T |
| 134695 | 74 | – | 91 | 1088.5415 | 2175.0684 | 2175.0735 | -2.38 | 1 | 98 | 6.4e-10 | 1Score **> 36** indicates **identity** Score **> 19** indicates **homology** | U | R.SAVLEDDKLLVYYYDDVR.T |
| 134698 | 74 | – | 91 | 726.0302 | 2175.0688 | 2175.0735 | -2.19 | 1 | 39 | 0.00023 | 1Score **> 36** indicates **identity** Score **> 15** indicates **homology** | U | R.SAVLEDDKLLVYYYDDVR.T |
| 134700 | 74 | – | 91 | 726.0303 | 2175.0689 | 2175.0735 | -2.12 | 1 | 82 | 2e-08 | 1Score **> 36** indicates **identity** Score **> 18** indicates **homology** | U | R.SAVLEDDKLLVYYYDDVR.T |
| 134705 | 74 | – | 91 | 1088.5420 | 2175.0695 | 2175.0735 | -1.86 | 1 | 47 | 4.3e-05 | 1Score **> 36** indicates **identity** Score **> 15** indicates **homology** | U | R.SAVLEDDKLLVYYYDDVR.T |
| 134706 | 74 | – | 91 | 726.0306 | 2175.0700 | 2175.0735 | -1.63 | 1 | 69 | 3.1e-07 | 1Score **> 36** indicates **identity** Score **> 17** indicates **homology** | U | R.SAVLEDDKLLVYYYDDVR.T |
| 134707 | 74 | – | 91 | 1088.5424 | 2175.0703 | 2175.0735 | -1.50 | 1 | 90 | 4e-09 | 1Score **> 36** indicates **identity** Score **> 18** indicates **homology** | U | R.SAVLEDDKLLVYYYDDVR.T |
| 134708 | 74 | – | 91 | 726.0307 | 2175.0704 | 2175.0735 | -1.47 | 1 | 66 | 6.3e-07 | 1Score **> 36** indicates **identity** Score **> 17** indicates **homology** | U | R.SAVLEDDKLLVYYYDDVR.T |
| 134709 | 74 | – | 91 | 1088.5425 | 2175.0705 | 2175.0735 | -1.42 | 1 | 97 | 7.3e-10 | 1Score **> 36** indicates **identity** Score **> 19** indicates **homology** | U | R.SAVLEDDKLLVYYYDDVR.T |
| 134710 | 74 | – | 91 | 726.0308 | 2175.0705 | 2175.0735 | -1.39 | 1 | 57 | 4.4e-06 | 1Score **> 36** indicates **identity** Score **> 16** indicates **homology** | U | R.SAVLEDDKLLVYYYDDVR.T |
| 134711 | 74 | – | 91 | 1088.5427 | 2175.0708 | 2175.0735 | -1.26 | 1 | 103 | 2.3e-10 | 1Score **> 36** indicates **identity** Score **> 19** indicates **homology** | U | R.SAVLEDDKLLVYYYDDVR.T |
| 134714 | 74 | – | 91 | 1088.5429 | 2175.0712 | 2175.0735 | -1.09 | 1 | 15 | 0.04 | 1Score **> 36** indicates **identity** Score **> 13** indicates **homology** | U | R.SAVLEDDKLLVYYYDDVR.T |
| 134717 | 74 | – | 91 | 726.0313 | 2175.0720 | 2175.0735 | -0.73 | 1 | 70 | 2.7e-07 | 1Score **> 36** indicates **identity** Score **> 17** indicates **homology** | U | R.SAVLEDDKLLVYYYDDVR.T |
| 134718 | 74 | – | 91 | 726.0313 | 2175.0721 | 2175.0735 | -0.66 | 1 | 66 | 6.5e-07 | 1Score **> 36** indicates **identity** Score **> 17** indicates **homology** | U | R.SAVLEDDKLLVYYYDDVR.T |
| 134720 | 74 | – | 91 | 726.0314 | 2175.0725 | 2175.0735 | -0.48 | 1 | 78 | 4.6e-08 | 1Score **> 36** indicates **identity** Score **> 17** indicates **homology** | U | R.SAVLEDDKLLVYYYDDVR.T |
| 134721 | 74 | – | 91 | 726.0315 | 2175.0726 | 2175.0735 | -0.42 | 1 | 54 | 8.3e-06 | 1Score **> 36** indicates **identity** Score **> 16** indicates **homology** | U | R.SAVLEDDKLLVYYYDDVR.T |
| 134723 | 74 | – | 91 | 726.0315 | 2175.0728 | 2175.0735 | -0.34 | 1 | 54 | 8.4e-06 | 1Score **> 36** indicates **identity** Score **> 16** indicates **homology** | U | R.SAVLEDDKLLVYYYDDVR.T |
| 134724 | 74 | – | 91 | 1088.5437 | 2175.0728 | 2175.0735 | -0.33 | 1 | 94 | 1.7e-09 | 1Score **> 36** indicates **identity** Score **> 18** indicates **homology** | U | R.SAVLEDDKLLVYYYDDVR.T |
| 134725 | 74 | – | 91 | 726.0317 | 2175.0732 | 2175.0735 | -0.18 | 1 | 68 | 4.1e-07 | 1Score **> 36** indicates **identity** Score **> 17** indicates **homology** | U | R.SAVLEDDKLLVYYYDDVR.T |
| 134727 | 74 | – | 91 | 726.0318 | 2175.0735 | 2175.0735 | -0.015 | 1 | 18 | 0.02 | 1Score **> 36** indicates **identity** Score **> 14** indicates **homology** | U | R.SAVLEDDKLLVYYYDDVR.T |
| 134729 | 74 | – | 91 | 726.0322 | 2175.0746 | 2175.0735 | 0.50 | 1 | 41 | 0.00014 | 1Score **> 36** indicates **identity** Score **> 15** indicates **homology** | U | R.SAVLEDDKLLVYYYDDVR.T |
| 134730 | 74 | – | 91 | 726.0323 | 2175.0751 | 2175.0735 | 0.73 | 1 | 66 | 6.2e-07 | 1Score **> 36** indicates **identity** Score **> 17** indicates **homology** | U | R.SAVLEDDKLLVYYYDDVR.T |
| 134734 | 74 | – | 91 | 726.0329 | 2175.0768 | 2175.0735 | 1.51 | 1 | 24 | 0.0059 | 1Score **> 36** indicates **identity** Score **> 14** indicates **homology** | U | R.SAVLEDDKLLVYYYDDVR.T |
| 134738 | 74 | – | 91 | 726.0337 | 2175.0793 | 2175.0735 | 2.64 | 1 | 29 | 0.0021 | 1Score **> 36** indicates **identity** Score **> 14** indicates **homology** | U | R.SAVLEDDKLLVYYYDDVR.T |
| 134740 | 74 | – | 91 | 726.0339 | 2175.0798 | 2175.0735 | 2.90 | 1 | 22 | 0.0078 | 1Score **> 36** indicates **identity** Score **> 14** indicates **homology** | U | R.SAVLEDDKLLVYYYDDVR.T |
| 134741 | 74 | – | 91 | 726.0339 | 2175.0800 | 2175.0735 | 2.95 | 1 | 18 | 0.018 | 1Score **> 36** indicates **identity** Score **> 14** indicates **homology** | U | R.SAVLEDDKLLVYYYDDVR.T |
| 134744 | 74 | – | 91 | 726.0357 | 2175.0852 | 2175.0735 | 5.37 | 1 | 14 | 0.045 | 1Score **> 36** indicates **identity** Score **> 13** indicates **homology** | U | R.SAVLEDDKLLVYYYDDVR.T |
| 134746 | 74 | – | 91 | 726.0369 | 2175.0889 | 2175.0735 | 7.04 | 1 | 43 | 8.9e-05 | 1Score **> 37** indicates **identity** Score **> 15** indicates **homology** | U | R.SAVLEDDKLLVYYYDDVR.T |
| 134747 | 74 | – | 91 | 726.0374 | 2175.0904 | 2175.0735 | 7.73 | 1 | 52 | 1.3e-05 | 1Score **> 37** indicates **identity** Score **> 16** indicates **homology** | U | R.SAVLEDDKLLVYYYDDVR.T |
| 60240 | 100 | – | 113 | 729.8536 | 1457.6926 | 1457.7045 | -8.14 | 0 | 69 | 3.7e-07 | 1Score **> 32** indicates **identity** Score **> 17** indicates **homology** | U | R.GIQVSNNGPCLGSR.K |
| 60242 | 100 | – | 113 | 729.8552 | 1457.6959 | 1457.7045 | -5.94 | 0 | 85 | 1.2e-08 | 1Score **> 32** indicates **identity** Score **> 18** indicates **homology** | U | R.GIQVSNNGPCLGSR.K |
| 60243 | 100 | – | 113 | 729.8567 | 1457.6989 | 1457.7045 | -3.85 | 0 | 35 | 0.0005 | 1Score **> 32** indicates **identity** Score **> 15** indicates **homology** | U | R.GIQVSNNGPCLGSR.K |
| 60245 | 100 | – | 113 | 729.8590 | 1457.7034 | 1457.7045 | -0.74 | 0 | 105 | 1.3e-10 | 1Score **> 32** indicates **identity** Score **> 19** indicates **homology** | U | R.GIQVSNNGPCLGSR.K |
| 60246 | 100 | – | 113 | 729.8592 | 1457.7038 | 1457.7045 | -0.51 | 0 | 89 | 4.2e-09 | 1Score **> 32** indicates **identity** Score **> 18** indicates **homology** | U | R.GIQVSNNGPCLGSR.K |
| 60247 | 100 | – | 113 | 729.8592 | 1457.7039 | 1457.7045 | -0.43 | 0 | 81 | 2.7e-08 | 1Score **> 32** indicates **identity** Score **> 18** indicates **homology** | U | R.GIQVSNNGPCLGSR.K |
| 60345 | 100 | – | 113 | 730.3509 | 1458.6872 | 1458.6885 | -0.90 | 0 | 54 | 9.6e-06 | 1Score **> 31** indicates **identity** Score **> 16** indicates **homology** | U | R.GIQVSNNGPCLGSR.K  + Deamidated (NQ) |
| 60346 | 100 | – | 113 | 730.3510 | 1458.6875 | 1458.6885 | -0.73 | 0 | 86 | 9.3e-09 | 1Score **> 31** indicates **identity** Score **> 18** indicates **homology** | U | R.GIQVSNNGPCLGSR.K  + Deamidated (NQ) |
| 60349 | 100 | – | 113 | 730.3523 | 1458.6900 | 1458.6885 | 1.03 | 0 | 87 | 7e-09 | 1Score **> 31** indicates **identity** Score **> 18** indicates **homology** | U | R.GIQVSNNGPCLGSR.K  + Deamidated (NQ) |
| 60350 | 100 | – | 113 | 730.3537 | 1458.6928 | 1458.6885 | 2.93 | 0 | 71 | 2.2e-07 | 1Score **> 31** indicates **identity** Score **> 17** indicates **homology** | U | R.GIQVSNNGPCLGSR.K  + Deamidated (NQ) |
| 60353 | 100 | – | 113 | 730.3574 | 1458.7003 | 1458.6885 | 8.05 | 0 | 51 | 1.7e-05 | 1Score **> 32** indicates **identity** Score **> 16** indicates **homology** | U | R.GIQVSNNGPCLGSR.K  + Deamidated (NQ) |
| 71654 | 114 | – | 125 | 776.8929 | 1551.7712 | 1551.7721 | -0.58 | 1 | 86 | 8.9e-09 | 1Score **> 34** indicates **identity** Score **> 18** indicates **homology** | U | R.KPNQPYEWISYK.E |
| 178153 | 114 | – | 141 | 1084.2217 | 3249.6432 | 3249.6379 | 1.63 | 2 | 70 | 3e-07 | 1Score **> 38** indicates **identity** Score **> 17** indicates **homology** | U | R.KPNQPYEWISYKEVAELAECIGSGLIQK.G |
| 178154 | 114 | – | 141 | 813.4182 | 3249.6437 | 3249.6379 | 1.77 | 2 | 58 | 3.8e-06 | 1Score **> 38** indicates **identity** Score **> 16** indicates **homology** | U | R.KPNQPYEWISYKEVAELAECIGSGLIQK.G |
| 178155 | 114 | – | 141 | 813.4193 | 3249.6482 | 3249.6379 | 3.16 | 2 | 49 | 2.6e-05 | 1Score **> 37** indicates **identity** Score **> 16** indicates **homology** | U | R.KPNQPYEWISYKEVAELAECIGSGLIQK.G |
| 92397 | 126 | – | 141 | 572.9662 | 1715.8768 | 1715.8764 | 0.25 | 0 | 23 | 0.0071 | 1Score **> 35** indicates **identity** Score **> 14** indicates **homology** | U | K.EVAELAECIGSGLIQK.G |
| 92401 | 126 | – | 141 | 572.9664 | 1715.8773 | 1715.8764 | 0.54 | 0 | 51 | 1.6e-05 | 1Score **> 35** indicates **identity** Score **> 16** indicates **homology** | U | K.EVAELAECIGSGLIQK.G |
| 92402 | 126 | – | 141 | 572.9664 | 1715.8774 | 1715.8764 | 0.63 | 0 | 32 | 0.00093 | 1Score **> 35** indicates **identity** Score **> 15** indicates **homology** | U | K.EVAELAECIGSGLIQK.G |
| 92405 | 126 | – | 141 | 572.9669 | 1715.8789 | 1715.8764 | 1.46 | 0 | 39 | 0.00021 | 1Score **> 35** indicates **identity** Score **> 15** indicates **homology** | U | K.EVAELAECIGSGLIQK.G |
| 58868 | 194 | – | 206 | 723.8948 | 1445.7750 | 1445.7766 | -1.11 | 1 | 66 | 6.2e-07 | 1Score **> 35** indicates **identity** Score **> 17** indicates **homology** | U | K.AELSVIFADKPEK.A |
| 58871 | 194 | – | 206 | 482.9327 | 1445.7762 | 1445.7766 | -0.26 | 1 | 54 | 1.7e-05 | 1Score **> 35** indicates **identity** Score **> 19** indicates **homology** | U | K.AELSVIFADKPEK.A |
| 58873 | 194 | – | 206 | 482.9329 | 1445.7767 | 1445.7766 | 0.12 | 1 | 53 | 9.8e-06 | 1Score **> 35** indicates **identity** Score **> 16** indicates **homology** | U | K.AELSVIFADKPEK.A |
| 58874 | 194 | – | 206 | 723.8956 | 1445.7767 | 1445.7766 | 0.12 | 1 | 61 | 1.8e-06 | 1Score **> 35** indicates **identity** Score **> 16** indicates **homology** | U | K.AELSVIFADKPEK.A |
| 58876 | 194 | – | 206 | 482.9329 | 1445.7768 | 1445.7766 | 0.18 | 1 | 45 | 5.6e-05 | 1Score **> 35** indicates **identity** Score **> 15** indicates **homology** | U | K.AELSVIFADKPEK.A |
| 58878 | 194 | – | 206 | 723.8959 | 1445.7772 | 1445.7766 | 0.47 | 1 | 62 | 1.4e-06 | 1Score **> 35** indicates **identity** Score **> 16** indicates **homology** | U | K.AELSVIFADKPEK.A |
| 58879 | 194 | – | 206 | 482.9331 | 1445.7775 | 1445.7766 | 0.65 | 1 | 53 | 1.1e-05 | 1Score **> 35** indicates **identity** Score **> 16** indicates **homology** | U | K.AELSVIFADKPEK.A |
| 58880 | 194 | – | 206 | 723.8961 | 1445.7777 | 1445.7766 | 0.78 | 1 | 64 | 9.9e-07 | 1Score **> 35** indicates **identity** Score **> 17** indicates **homology** | U | K.AELSVIFADKPEK.A |
| 58883 | 194 | – | 206 | 482.9335 | 1445.7787 | 1445.7766 | 1.46 | 1 | 48 | 3.1e-05 | 1Score **> 35** indicates **identity** Score **> 16** indicates **homology** | U | K.AELSVIFADKPEK.A |
| 58884 | 194 | – | 206 | 482.9335 | 1445.7787 | 1445.7766 | 1.50 | 1 | 23 | 0.0064 | 1Score **> 35** indicates **identity** Score **> 14** indicates **homology** | U | K.AELSVIFADKPEK.A |
| 58886 | 194 | – | 206 | 482.9340 | 1445.7803 | 1445.7766 | 2.59 | 1 | 30 | 0.0015 | 1Score **> 35** indicates **identity** Score **> 14** indicates **homology** | U | K.AELSVIFADKPEK.A |
| 112997 | 207 | – | 223 | 482.2838 | 1925.1061 | 1925.1019 | 2.17 | 2 | 16 | 0.031 | 1Score **> 32** indicates **identity** Score **> 14** indicates **homology** | U | K.AKLLLEGVENKLTPCLK.I |
| 15039 | 209 | – | 217 | 507.7952 | 1013.5759 | 1013.5757 | 0.22 | 0 | 58 | 9.2e-05 | 1Score **> 31** indicates **identity** | U | K.LLLEGVENK.L |
| 15040 | 209 | – | 217 | 507.7953 | 1013.5761 | 1013.5757 | 0.44 | 0 | 53 | 0.0003 | 1Score **> 30** indicates **identity** | U | K.LLLEGVENK.L |
| 93591 | 209 | – | 223 | 576.3299 | 1725.9678 | 1725.9699 | -1.21 | 1 | 54 | 2.7e-05 | 1Score **> 34** indicates **identity** Score **> 21** indicates **homology** | U | K.LLLEGVENKLTPCLK.I |
| 93593 | 209 | – | 223 | 576.3309 | 1725.9709 | 1725.9699 | 0.61 | 1 | 60 | 4.2e-06 | 1Score **> 34** indicates **identity** Score **> 18** indicates **homology** | U | K.LLLEGVENKLTPCLK.I |
| 93594 | 209 | – | 223 | 576.3310 | 1725.9710 | 1725.9699 | 0.69 | 1 | 54 | 8.6e-06 | 1Score **> 34** indicates **identity** Score **> 16** indicates **homology** | U | K.LLLEGVENKLTPCLK.I |
| 91579 | 224 | – | 238 | 855.4397 | 1708.8648 | 1708.8706 | -3.35 | 0 | 61 | 1.8e-06 | 1Score **> 35** indicates **identity** Score **> 16** indicates **homology** | U | K.IIVIMDSYGSDLVER.G |
| 91582 | 224 | – | 238 | 855.4405 | 1708.8664 | 1708.8706 | -2.44 | 0 | 62 | 1.5e-06 | 1Score **> 35** indicates **identity** Score **> 16** indicates **homology** | U | K.IIVIMDSYGSDLVER.G |
| 91583 | 224 | – | 238 | 855.4413 | 1708.8680 | 1708.8706 | -1.51 | 0 | 61 | 1.8e-06 | 1Score **> 35** indicates **identity** Score **> 16** indicates **homology** | U | K.IIVIMDSYGSDLVER.G |
| 91585 | 224 | – | 238 | 855.4430 | 1708.8715 | 1708.8706 | 0.56 | 0 | 61 | 1.9e-06 | 1Score **> 35** indicates **identity** Score **> 16** indicates **homology** | U | K.IIVIMDSYGSDLVER.G |
| 91587 | 224 | – | 238 | 855.4440 | 1708.8734 | 1708.8706 | 1.67 | 0 | 60 | 2.3e-06 | 1Score **> 35** indicates **identity** Score **> 16** indicates **homology** | U | K.IIVIMDSYGSDLVER.G |
| 26786 | 241 | – | 250 | 382.8898 | 1145.6477 | 1145.6478 | -0.081 | 1 | 30 | 0.018 | 1Score **> 34** indicates **identity** Score **> 25** indicates **homology** | U | K.KCGVEIISLK.A |
| 26787 | 241 | – | 250 | 573.8317 | 1145.6488 | 1145.6478 | 0.84 | 1 | 46 | 0.0019 | 1Score **> 33** indicates **identity** Score **> 32** indicates **homology** | U | K.KCGVEIISLK.A |
| 15337 | 242 | – | 250 | 509.7835 | 1017.5525 | 1017.5529 | -0.34 | 0 | 50 | 2.3e-05 | 1Score **> 33** indicates **identity** Score **> 16** indicates **homology** | U | K.CGVEIISLK.A |
| 15339 | 242 | – | 250 | 509.7837 | 1017.5529 | 1017.5529 | 0.077 | 0 | 51 | 2.3e-05 | 1Score **> 33** indicates **identity** Score **> 17** indicates **homology** | U | K.CGVEIISLK.A |
| 15340 | 242 | – | 250 | 509.7838 | 1017.5531 | 1017.5529 | 0.25 | 0 | 56 | 1.6e-05 | 1Score **> 33** indicates **identity** Score **> 21** indicates **homology** | U | K.CGVEIISLK.A |
| 15341 | 242 | – | 250 | 509.7839 | 1017.5532 | 1017.5529 | 0.37 | 0 | 62 | 5.8e-06 | 1Score **> 33** indicates **identity** Score **> 23** indicates **homology** | U | K.CGVEIISLK.A |
| 15342 | 242 | – | 250 | 509.7839 | 1017.5533 | 1017.5529 | 0.43 | 0 | 56 | 1.5e-05 | 1Score **> 33** indicates **identity** Score **> 21** indicates **homology** | U | K.CGVEIISLK.A |
| 15343 | 242 | – | 250 | 509.7840 | 1017.5534 | 1017.5529 | 0.51 | 0 | 64 | 4.5e-06 | 1Score **> 33** indicates **identity** Score **> 23** indicates **homology** | U | K.CGVEIISLK.A |
| 15344 | 242 | – | 250 | 509.7840 | 1017.5534 | 1017.5529 | 0.57 | 0 | 67 | 3.4e-06 | 1Score **> 33** indicates **identity** Score **> 25** indicates **homology** | U | K.CGVEIISLK.A |
| 15345 | 242 | – | 250 | 509.7841 | 1017.5537 | 1017.5529 | 0.86 | 0 | 51 | 2.3e-05 | 1Score **> 33** indicates **identity** Score **> 17** indicates **homology** | U | K.CGVEIISLK.A |
| 98655 | 242 | – | 257 | 591.6576 | 1771.9510 | 1771.9502 | 0.43 | 1 | 36 | 0.00041 | 1Score **> 36** indicates **identity** Score **> 15** indicates **homology** | U | K.CGVEIISLKALEDLGR.V |
| 2505 | 251 | – | 257 | 387.2110 | 772.4074 | 772.4079 | -0.65 | 0 | 35 | 0.0084 | 1Score **> 26** indicates **identity** | U | K.ALEDLGR.V |
| 2506 | 251 | – | 257 | 387.2110 | 772.4074 | 772.4079 | -0.64 | 0 | 45 | 0.00074 | 1Score **> 26** indicates **identity** | U | K.ALEDLGR.V |
| 2507 | 251 | – | 257 | 387.2112 | 772.4079 | 772.4079 | 0.049 | 0 | 49 | 0.00034 | 1Score **> 27** indicates **identity** | U | K.ALEDLGR.V |
| 2508 | 251 | – | 257 | 387.2113 | 772.4081 | 772.4079 | 0.26 | 0 | 34 | 0.011 | 1Score **> 27** indicates **identity** | U | K.ALEDLGR.V |
| 26322 | 251 | – | 260 | 381.5472 | 1141.6198 | 1141.6203 | -0.51 | 1 | 28 | 0.022 | 1Score **> 32** indicates **identity** Score **> 25** indicates **homology** | U | K.ALEDLGRVNR.V |
| 163495 | 261 | – | 285 | 899.4702 | 2695.3887 | 2695.3891 | -0.16 | 2 | 21 | 0.011 | 1Score **> 37** indicates **identity** Score **> 14** indicates **homology** | U | R.VKPKPPEPEDLAIICFTSGTTGNPK.G |
| 163496 | 261 | – | 285 | 674.8546 | 2695.3892 | 2695.3891 | 0.030 | 2 | 39 | 0.00024 | 1Score **> 37** indicates **identity** Score **> 15** indicates **homology** | U | R.VKPKPPEPEDLAIICFTSGTTGNPK.G |
| 163497 | 261 | – | 285 | 674.8549 | 2695.3904 | 2695.3891 | 0.49 | 2 | 34 | 0.00064 | 1Score **> 37** indicates **identity** Score **> 15** indicates **homology** | U | R.VKPKPPEPEDLAIICFTSGTTGNPK.G |
| 163498 | 261 | – | 285 | 899.4708 | 2695.3905 | 2695.3891 | 0.53 | 2 | 56 | 5.4e-06 | 1Score **> 37** indicates **identity** Score **> 16** indicates **homology** | U | R.VKPKPPEPEDLAIICFTSGTTGNPK.G |
| 163499 | 261 | – | 285 | 674.8552 | 2695.3915 | 2695.3891 | 0.90 | 2 | 32 | 0.0011 | 1Score **> 37** indicates **identity** Score **> 15** indicates **homology** | U | R.VKPKPPEPEDLAIICFTSGTTGNPK.G |
| 163500 | 261 | – | 285 | 899.4711 | 2695.3915 | 2695.3891 | 0.90 | 2 | 46 | 4.8e-05 | 1Score **> 37** indicates **identity** Score **> 15** indicates **homology** | U | R.VKPKPPEPEDLAIICFTSGTTGNPK.G |
| 163501 | 261 | – | 285 | 899.4712 | 2695.3917 | 2695.3891 | 0.95 | 2 | 55 | 6.4e-06 | 1Score **> 37** indicates **identity** Score **> 16** indicates **homology** | U | R.VKPKPPEPEDLAIICFTSGTTGNPK.G |
| 163502 | 261 | – | 285 | 674.8554 | 2695.3924 | 2695.3891 | 1.24 | 2 | 34 | 0.0006 | 1Score **> 37** indicates **identity** Score **> 15** indicates **homology** | U | R.VKPKPPEPEDLAIICFTSGTTGNPK.G |
| 163503 | 261 | – | 285 | 899.4716 | 2695.3929 | 2695.3891 | 1.39 | 2 | 19 | 0.016 | 1Score **> 37** indicates **identity** Score **> 14** indicates **homology** | U | R.VKPKPPEPEDLAIICFTSGTTGNPK.G |
| 163504 | 261 | – | 285 | 674.8566 | 2695.3972 | 2695.3891 | 3.01 | 2 | 16 | 0.032 | 1Score **> 37** indicates **identity** Score **> 14** indicates **homology** | U | R.VKPKPPEPEDLAIICFTSGTTGNPK.G |
| 163505 | 261 | – | 285 | 674.8570 | 2695.3991 | 2695.3891 | 3.71 | 2 | 24 | 0.0063 | 1Score **> 37** indicates **identity** Score **> 14** indicates **homology** | U | R.VKPKPPEPEDLAIICFTSGTTGNPK.G |
| 163507 | 261 | – | 285 | 899.4767 | 2695.4084 | 2695.3891 | 7.15 | 2 | 27 | 0.0032 | 1Score **> 37** indicates **identity** Score **> 14** indicates **homology** | U | R.VKPKPPEPEDLAIICFTSGTTGNPK.G |
| 169031 | 261 | – | 285 | 951.5053 | 2851.4940 | 2851.5041 | -3.57 | 2 | 49 | 2.5e-05 | 1Score **> 37** indicates **identity** Score **> 16** indicates **homology** | U | R.VKPKPPEPEDLAIICFTSGTTGNPK.G  + HNE (K) |
| 121215 | 286 | – | 303 | 673.6635 | 2017.9686 | 2017.9714 | -1.36 | 0 | 35 | 0.00057 | 1Score **> 34** indicates **identity** Score **> 15** indicates **homology** | U | K.GAMITHQNIINDCSGFIK.A |
| 121216 | 286 | – | 303 | 1009.9919 | 2017.9693 | 2017.9714 | -1.03 | 0 | 64 | 9.4e-07 | 1Score **> 35** indicates **identity** Score **> 17** indicates **homology** | U | K.GAMITHQNIINDCSGFIK.A |
| 121218 | 286 | – | 303 | 673.6643 | 2017.9711 | 2017.9714 | -0.15 | 0 | 63 | 1.2e-06 | 1Score **> 35** indicates **identity** Score **> 16** indicates **homology** | U | K.GAMITHQNIINDCSGFIK.A |
| 121219 | 286 | – | 303 | 673.6644 | 2017.9713 | 2017.9714 | -0.016 | 0 | 23 | 0.0065 | 1Score **> 35** indicates **identity** Score **> 14** indicates **homology** | U | K.GAMITHQNIINDCSGFIK.A |
| 121221 | 286 | – | 303 | 673.6648 | 2017.9727 | 2017.9714 | 0.65 | 0 | 19 | 0.015 | 1Score **> 35** indicates **identity** Score **> 14** indicates **homology** | U | K.GAMITHQNIINDCSGFIK.A |
| 17646 | 342 | – | 350 | 526.7784 | 1051.5422 | 1051.5451 | -2.73 | 0 | 56 | 6.5e-05 | 1Score **> 31** indicates **identity** Score **> 26** indicates **homology** |  | K.IGFFQGDIR.L |
| 17648 | 342 | – | 350 | 526.7792 | 1051.5438 | 1051.5451 | -1.23 | 0 | 26 | 0.0035 | 1Score **> 31** indicates **identity** Score **> 14** indicates **homology** |  | K.IGFFQGDIR.L |
| 17649 | 342 | – | 350 | 526.7793 | 1051.5441 | 1051.5451 | -0.91 | 0 | 47 | 0.00018 | 1Score **> 31** indicates **identity** Score **> 22** indicates **homology** |  | K.IGFFQGDIR.L |
| 17650 | 342 | – | 350 | 526.7794 | 1051.5442 | 1051.5451 | -0.81 | 0 | 47 | 0.00018 | 1Score **> 31** indicates **identity** Score **> 22** indicates **homology** |  | K.IGFFQGDIR.L |
| 17651 | 342 | – | 350 | 526.7795 | 1051.5445 | 1051.5451 | -0.54 | 0 | 47 | 0.00018 | 1Score **> 31** indicates **identity** Score **> 22** indicates **homology** |  | K.IGFFQGDIR.L |
| 17652 | 342 | – | 350 | 526.7796 | 1051.5446 | 1051.5451 | -0.45 | 0 | 33 | 0.0027 | 1Score **> 31** indicates **identity** Score **> 20** indicates **homology** |  | K.IGFFQGDIR.L |
| 17653 | 342 | – | 350 | 526.7797 | 1051.5449 | 1051.5451 | -0.23 | 0 | 53 | 0.00012 | 1Score **> 31** indicates **identity** Score **> 27** indicates **homology** |  | K.IGFFQGDIR.L |
| 17654 | 342 | – | 350 | 526.7797 | 1051.5449 | 1051.5451 | -0.20 | 0 | 47 | 0.00017 | 1Score **> 31** indicates **identity** Score **> 22** indicates **homology** |  | K.IGFFQGDIR.L |
| 17655 | 342 | – | 350 | 526.7798 | 1051.5450 | 1051.5451 | -0.12 | 0 | 48 | 0.00017 | 1Score **> 31** indicates **identity** Score **> 23** indicates **homology** |  | K.IGFFQGDIR.L |
| 17656 | 342 | – | 350 | 526.7798 | 1051.5451 | 1051.5451 | 0.048 | 0 | 56 | 7.9e-05 | 1Score **> 31** indicates **identity** Score **> 27** indicates **homology** |  | K.IGFFQGDIR.L |
| 17657 | 342 | – | 350 | 526.7800 | 1051.5454 | 1051.5451 | 0.26 | 0 | 47 | 0.00017 | 1Score **> 31** indicates **identity** Score **> 22** indicates **homology** |  | K.IGFFQGDIR.L |
| 17658 | 342 | – | 350 | 526.7800 | 1051.5455 | 1051.5451 | 0.39 | 0 | 54 | 0.00012 | 1Score **> 31** indicates **identity** Score **> 27** indicates **homology** |  | K.IGFFQGDIR.L |
| 17659 | 342 | – | 350 | 526.7802 | 1051.5458 | 1051.5451 | 0.72 | 0 | 51 | 9.5e-05 | 1Score **> 31** indicates **identity** Score **> 23** indicates **homology** |  | K.IGFFQGDIR.L |
| 17660 | 342 | – | 350 | 526.7802 | 1051.5459 | 1051.5451 | 0.74 | 0 | 53 | 0.00012 | 1Score **> 31** indicates **identity** Score **> 27** indicates **homology** |  | K.IGFFQGDIR.L |
| 17661 | 342 | – | 350 | 526.7803 | 1051.5461 | 1051.5451 | 0.96 | 0 | 51 | 0.00011 | 1Score **> 31** indicates **identity** Score **> 24** indicates **homology** |  | K.IGFFQGDIR.L |
| 17662 | 342 | – | 350 | 526.7805 | 1051.5464 | 1051.5451 | 1.28 | 0 | 48 | 0.00019 | 1Score **> 31** indicates **identity** Score **> 24** indicates **homology** |  | K.IGFFQGDIR.L |
| 17663 | 342 | – | 350 | 526.7807 | 1051.5468 | 1051.5451 | 1.63 | 0 | 50 | 0.0001 | 1Score **> 31** indicates **identity** Score **> 22** indicates **homology** |  | K.IGFFQGDIR.L |
| 17664 | 342 | – | 350 | 526.7808 | 1051.5471 | 1051.5451 | 1.91 | 0 | 34 | 0.0013 | 1Score **> 31** indicates **identity** Score **> 18** indicates **homology** |  | K.IGFFQGDIR.L |
| 17666 | 342 | – | 350 | 526.7810 | 1051.5474 | 1051.5451 | 2.22 | 0 | 29 | 0.0057 | 1Score **> 31** indicates **identity** Score **> 19** indicates **homology** |  | K.IGFFQGDIR.L |
| 17667 | 342 | – | 350 | 526.7813 | 1051.5481 | 1051.5451 | 2.90 | 0 | 45 | 5.6e-05 | 1Score **> 31** indicates **identity** Score **> 15** indicates **homology** |  | K.IGFFQGDIR.L |
| 5713 | 351 | – | 357 | 424.2322 | 846.4499 | 846.4521 | -2.54 | 0 | 16 | 0.03 | 1Score **> 30** indicates **identity** Score **> 14** indicates **homology** | U | R.LLMDDLK.V |
| 5715 | 351 | – | 357 | 424.2332 | 846.4517 | 846.4521 | -0.38 | 0 | 31 | 0.0035 | 1Score **> 31** indicates **identity** Score **> 19** indicates **homology** | U | R.LLMDDLK.V |
| 5717 | 351 | – | 357 | 424.2333 | 846.4520 | 846.4521 | -0.035 | 0 | 28 | 0.0047 | 1Score **> 31** indicates **identity** Score **> 17** indicates **homology** | U | R.LLMDDLK.V |
| 5718 | 351 | – | 357 | 424.2333 | 846.4521 | 846.4521 | 0.059 | 0 | 44 | 0.00097 | 1Score **> 31** indicates **identity** Score **> 26** indicates **homology** | U | R.LLMDDLK.V |
| 6477 | 351 | – | 357 | 432.2300 | 862.4454 | 862.4470 | -1.85 | 0 | 15 | 0.047 | 1Score **> 29** indicates **identity** Score **> 15** indicates **homology** | U | R.LLMDDLK.V  + Oxidation (M) |
| 6479 | 351 | – | 357 | 432.2307 | 862.4469 | 862.4470 | -0.092 | 0 | 21 | 0.025 | 1Score **> 30** indicates **identity** Score **> 17** indicates **homology** | U | R.LLMDDLK.V  + Oxidation (M) |
| 50341 | 358 | – | 369 | 455.9464 | 1364.8172 | 1364.8180 | -0.58 | 0 | 41 | 0.00014 | 1Score **> 30** indicates **identity** Score **> 15** indicates **homology** | U | K.VLQPTIFPVVPR.L |
| 50342 | 358 | – | 369 | 683.4161 | 1364.8177 | 1364.8180 | -0.22 | 0 | 57 | 9.7e-05 | 1Score **> 30** indicates **identity** | U | K.VLQPTIFPVVPR.L |
| 50343 | 358 | – | 369 | 683.4162 | 1364.8179 | 1364.8180 | -0.12 | 0 | 50 | 0.00052 | 1Score **> 30** indicates **identity** | U | K.VLQPTIFPVVPR.L |
| 50346 | 358 | – | 369 | 455.9468 | 1364.8185 | 1364.8180 | 0.31 | 0 | 40 | 0.00017 | 1Score **> 30** indicates **identity** Score **> 15** indicates **homology** | U | K.VLQPTIFPVVPR.L |
| 50349 | 358 | – | 369 | 683.4172 | 1364.8198 | 1364.8180 | 1.29 | 0 | 49 | 0.00069 | 1Score **> 30** indicates **identity** | U | K.VLQPTIFPVVPR.L |
| 50352 | 358 | – | 369 | 683.4186 | 1364.8227 | 1364.8180 | 3.43 | 0 | 42 | 0.0033 | 1Score **> 29** indicates **identity** | U | K.VLQPTIFPVVPR.L |
| 99699 | 374 | – | 388 | 595.3134 | 1782.9183 | 1782.9199 | -0.90 | 2 | 36 | 0.05 | 1Score **> 35** indicates **identity** | U | R.MFDRIFGQANTSLKR.W |
| 20023 | 378 | – | 387 | 539.7982 | 1077.5818 | 1077.5818 | -0.013 | 0 | 24 | 0.0095 | 1Score **> 31** indicates **identity** Score **> 16** indicates **homology** | U | R.IFGQANTSLK.R |
| 20025 | 378 | – | 387 | 539.7996 | 1077.5847 | 1077.5818 | 2.62 | 0 | 52 | 0.00021 | 1Score **> 32** indicates **identity** Score **> 28** indicates **homology** | U | R.IFGQANTSLK.R |
| 36440 | 378 | – | 388 | 412.2340 | 1233.6803 | 1233.6830 | -2.17 | 1 | 21 | 0.01 | 1Score **> 33** indicates **identity** Score **> 14** indicates **homology** | U | R.IFGQANTSLKR.W |
| 36444 | 378 | – | 388 | 617.8484 | 1233.6823 | 1233.6830 | -0.53 | 1 | 35 | 0.0028 | 1Score **> 33** indicates **identity** Score **> 21** indicates **homology** | U | R.IFGQANTSLKR.W |
| 36445 | 378 | – | 388 | 412.2347 | 1233.6824 | 1233.6830 | -0.49 | 1 | 69 | 1.4e-06 | 1Score **> 33** indicates **identity** Score **> 23** indicates **homology** | U | R.IFGQANTSLKR.W |
| 36446 | 378 | – | 388 | 617.8485 | 1233.6824 | 1233.6830 | -0.42 | 1 | 54 | 4.8e-05 | 1Score **> 33** indicates **identity** Score **> 23** indicates **homology** | U | R.IFGQANTSLKR.W |
| 36449 | 378 | – | 388 | 617.8488 | 1233.6831 | 1233.6830 | 0.12 | 1 | 41 | 0.00072 | 1Score **> 33** indicates **identity** Score **> 22** indicates **homology** | U | R.IFGQANTSLKR.W |
| 36450 | 378 | – | 388 | 412.2350 | 1233.6831 | 1233.6830 | 0.15 | 1 | 34 | 0.00072 | 1Score **> 33** indicates **identity** Score **> 15** indicates **homology** | U | R.IFGQANTSLKR.W |
| 36452 | 378 | – | 388 | 412.2351 | 1233.6835 | 1233.6830 | 0.41 | 1 | 63 | 1.9e-06 | 1Score **> 33** indicates **identity** Score **> 19** indicates **homology** | U | R.IFGQANTSLKR.W |
| 12888 | 389 | – | 396 | 490.2657 | 978.5168 | 978.5175 | -0.72 | 0 | 17 | 0.023 | 1Score **> 30** indicates **identity** Score **> 14** indicates **homology** | U | R.WLLDFASK.R |
| 12889 | 389 | – | 396 | 490.2659 | 978.5173 | 978.5175 | -0.16 | 0 | 24 | 0.006 | 1Score **> 30** indicates **identity** Score **> 14** indicates **homology** | U | R.WLLDFASK.R |
| 12890 | 389 | – | 396 | 490.2662 | 978.5179 | 978.5175 | 0.47 | 0 | 19 | 0.015 | 1Score **> 31** indicates **identity** Score **> 14** indicates **homology** | U | R.WLLDFASK.R |
| 12891 | 389 | – | 396 | 490.2663 | 978.5181 | 978.5175 | 0.69 | 0 | 19 | 0.015 | 1Score **> 31** indicates **identity** Score **> 14** indicates **homology** | U | R.WLLDFASK.R |
| 12892 | 389 | – | 396 | 490.2664 | 978.5182 | 978.5175 | 0.72 | 0 | 19 | 0.016 | 1Score **> 31** indicates **identity** Score **> 14** indicates **homology** | U | R.WLLDFASK.R |
| 12893 | 389 | – | 396 | 490.2664 | 978.5182 | 978.5175 | 0.76 | 0 | 18 | 0.019 | 1Score **> 31** indicates **identity** Score **> 14** indicates **homology** | U | R.WLLDFASK.R |
| 12894 | 389 | – | 396 | 490.2667 | 978.5188 | 978.5175 | 1.41 | 0 | 21 | 0.011 | 1Score **> 31** indicates **identity** Score **> 14** indicates **homology** | U | R.WLLDFASK.R |
| 12895 | 389 | – | 396 | 490.2667 | 978.5189 | 978.5175 | 1.52 | 0 | 21 | 0.012 | 1Score **> 31** indicates **identity** Score **> 14** indicates **homology** | U | R.WLLDFASK.R |
| 12896 | 389 | – | 396 | 490.2668 | 978.5191 | 978.5175 | 1.71 | 0 | 18 | 0.022 | 1Score **> 31** indicates **identity** Score **> 14** indicates **homology** | U | R.WLLDFASK.R |
| 12897 | 389 | – | 396 | 490.2675 | 978.5205 | 978.5175 | 3.08 | 0 | 17 | 0.024 | 1Score **> 31** indicates **identity** Score **> 14** indicates **homology** | U | R.WLLDFASK.R |
| 25634 | 389 | – | 397 | 379.2137 | 1134.6191 | 1134.6186 | 0.50 | 1 | 33 | 0.0042 | 1Score **> 32** indicates **identity** Score **> 21** indicates **homology** | U | R.WLLDFASKR.K |
| 25636 | 389 | – | 397 | 379.2139 | 1134.6197 | 1134.6186 | 1.04 | 1 | 33 | 0.0011 | 1Score **> 32** indicates **identity** Score **> 16** indicates **homology** | U | R.WLLDFASKR.K |
| 25637 | 389 | – | 397 | 379.2139 | 1134.6199 | 1134.6186 | 1.20 | 1 | 24 | 0.005 | 1Score **> 32** indicates **identity** Score **> 14** indicates **homology** | U | R.WLLDFASKR.K |
| 7112 | 409 | – | 415 | 438.7135 | 875.4124 | 875.4137 | -1.46 | 0 | 23 | 0.019 | 1Score **> 27** indicates **identity** Score **> 18** indicates **homology** | U | R.NNSLWDK.L |
| 7113 | 409 | – | 415 | 438.7141 | 875.4136 | 875.4137 | -0.14 | 0 | 26 | 0.011 | 1Score **> 27** indicates **identity** Score **> 18** indicates **homology** | U | R.NNSLWDK.L |
| 7114 | 409 | – | 415 | 438.7142 | 875.4139 | 875.4137 | 0.22 | 0 | 18 | 0.022 | 1Score **> 27** indicates **identity** Score **> 14** indicates **homology** | U | R.NNSLWDK.L |
| 67004 | 409 | – | 420 | 505.6074 | 1513.8002 | 1513.8041 | -2.57 | 1 | 52 | 1.4e-05 | 1Score **> 35** indicates **identity** Score **> 16** indicates **homology** | U | R.NNSLWDKLIFHK.I |
| 67007 | 409 | – | 420 | 505.6076 | 1513.8009 | 1513.8041 | -2.13 | 1 | 48 | 3.3e-05 | 1Score **> 34** indicates **identity** Score **> 15** indicates **homology** | U | R.NNSLWDKLIFHK.I |
| 67008 | 409 | – | 420 | 505.6080 | 1513.8021 | 1513.8041 | -1.33 | 1 | 49 | 2.7e-05 | 1Score **> 34** indicates **identity** Score **> 16** indicates **homology** | U | R.NNSLWDKLIFHK.I |
| 67010 | 409 | – | 420 | 505.6083 | 1513.8030 | 1513.8041 | -0.76 | 1 | 46 | 5.1e-05 | 1Score **> 35** indicates **identity** Score **> 15** indicates **homology** | U | R.NNSLWDKLIFHK.I |
| 67012 | 409 | – | 420 | 505.6083 | 1513.8030 | 1513.8041 | -0.75 | 1 | 32 | 0.00096 | 1Score **> 35** indicates **identity** Score **> 15** indicates **homology** | U | R.NNSLWDKLIFHK.I |
| 67014 | 409 | – | 420 | 505.6084 | 1513.8034 | 1513.8041 | -0.51 | 1 | 48 | 3.1e-05 | 1Score **> 35** indicates **identity** Score **> 16** indicates **homology** | U | R.NNSLWDKLIFHK.I |
| 67015 | 409 | – | 420 | 505.6084 | 1513.8034 | 1513.8041 | -0.46 | 1 | 45 | 6.3e-05 | 1Score **> 35** indicates **identity** Score **> 15** indicates **homology** | U | R.NNSLWDKLIFHK.I |
| 67016 | 409 | – | 420 | 505.6085 | 1513.8036 | 1513.8041 | -0.35 | 1 | 49 | 2.7e-05 | 1Score **> 35** indicates **identity** Score **> 16** indicates **homology** | U | R.NNSLWDKLIFHK.I |
| 67017 | 409 | – | 420 | 505.6086 | 1513.8039 | 1513.8041 | -0.16 | 1 | 39 | 0.00024 | 1Score **> 35** indicates **identity** Score **> 15** indicates **homology** | U | R.NNSLWDKLIFHK.I |
| 67018 | 409 | – | 420 | 505.6086 | 1513.8040 | 1513.8041 | -0.086 | 1 | 14 | 0.044 | 1Score **> 35** indicates **identity** Score **> 13** indicates **homology** | U | R.NNSLWDKLIFHK.I |
| 67019 | 409 | – | 420 | 505.6086 | 1513.8041 | 1513.8041 | -0.032 | 1 | 49 | 2.6e-05 | 1Score **> 34** indicates **identity** Score **> 16** indicates **homology** | U | R.NNSLWDKLIFHK.I |
| 67022 | 409 | – | 420 | 505.6087 | 1513.8042 | 1513.8041 | 0.049 | 1 | 49 | 2.8e-05 | 1Score **> 34** indicates **identity** Score **> 16** indicates **homology** | U | R.NNSLWDKLIFHK.I |
| 67023 | 409 | – | 420 | 505.6087 | 1513.8043 | 1513.8041 | 0.085 | 1 | 49 | 2.6e-05 | 1Score **> 34** indicates **identity** Score **> 16** indicates **homology** | U | R.NNSLWDKLIFHK.I |
| 67024 | 409 | – | 420 | 505.6087 | 1513.8043 | 1513.8041 | 0.14 | 1 | 31 | 0.0012 | 1Score **> 34** indicates **identity** Score **> 14** indicates **homology** | U | R.NNSLWDKLIFHK.I |
| 67025 | 409 | – | 420 | 505.6088 | 1513.8045 | 1513.8041 | 0.26 | 1 | 40 | 0.00018 | 1Score **> 34** indicates **identity** Score **> 15** indicates **homology** | U | R.NNSLWDKLIFHK.I |
| 67026 | 409 | – | 420 | 505.6088 | 1513.8045 | 1513.8041 | 0.26 | 1 | 37 | 0.00036 | 1Score **> 34** indicates **identity** Score **> 15** indicates **homology** | U | R.NNSLWDKLIFHK.I |
| 67028 | 409 | – | 420 | 505.6090 | 1513.8051 | 1513.8041 | 0.67 | 1 | 53 | 1.1e-05 | 1Score **> 34** indicates **identity** Score **> 16** indicates **homology** | U | R.NNSLWDKLIFHK.I |
| 67029 | 409 | – | 420 | 505.6091 | 1513.8054 | 1513.8041 | 0.85 | 1 | 51 | 1.5e-05 | 1Score **> 34** indicates **identity** Score **> 16** indicates **homology** | U | R.NNSLWDKLIFHK.I |
| 67030 | 409 | – | 420 | 505.6094 | 1513.8063 | 1513.8041 | 1.42 | 1 | 16 | 0.034 | 1Score **> 35** indicates **identity** Score **> 13** indicates **homology** | U | R.NNSLWDKLIFHK.I |
| 67032 | 409 | – | 420 | 505.6096 | 1513.8070 | 1513.8041 | 1.90 | 1 | 18 | 0.019 | 1Score **> 35** indicates **identity** Score **> 14** indicates **homology** | U | R.NNSLWDKLIFHK.I |
| 67040 | 409 | – | 420 | 505.6136 | 1513.8188 | 1513.8041 | 9.71 | 1 | 23 | 0.016 | 1Score **> 35** indicates **identity** Score **> 17** indicates **homology** | U | R.NNSLWDKLIFHK.I |
| 67165 | 409 | – | 420 | 505.9363 | 1514.7871 | 1514.7881 | -0.68 | 1 | 37 | 0.00035 | 1Score **> 35** indicates **identity** Score **> 15** indicates **homology** | U | R.NNSLWDKLIFHK.I  + Deamidated (NQ) |
| 67172 | 409 | – | 420 | 505.9373 | 1514.7901 | 1514.7881 | 1.31 | 1 | 38 | 0.00026 | 1Score **> 35** indicates **identity** Score **> 15** indicates **homology** | U | R.NNSLWDKLIFHK.I  + Deamidated (NQ) |
| 67185 | 409 | – | 420 | 505.9401 | 1514.7984 | 1514.7881 | 6.81 | 1 | 17 | 0.028 | 1Score **> 35** indicates **identity** Score **> 14** indicates **homology** | U | R.NNSLWDKLIFHK.I  + Deamidated (NQ) |
| 107312 | 431 | – | 448 | 621.0251 | 1860.0534 | 1860.0543 | -0.47 | 0 | 36 | 0.00046 | 1Score **> 34** indicates **identity** Score **> 15** indicates **homology** | U | R.LMITGAAPVSATVLTFLR.T |
| 107313 | 431 | – | 448 | 931.0354 | 1860.0562 | 1860.0543 | 1.03 | 0 | 71 | 2.2e-07 | 1Score **> 33** indicates **identity** Score **> 17** indicates **homology** | U | R.LMITGAAPVSATVLTFLR.T |
| 107314 | 431 | – | 448 | 931.0354 | 1860.0563 | 1860.0543 | 1.09 | 0 | 55 | 7.6e-06 | 1Score **> 33** indicates **identity** Score **> 16** indicates **homology** | U | R.LMITGAAPVSATVLTFLR.T |
| 107315 | 431 | – | 448 | 621.0261 | 1860.0565 | 1860.0543 | 1.20 | 0 | 79 | 3.9e-08 | 1Score **> 33** indicates **identity** Score **> 17** indicates **homology** | U | R.LMITGAAPVSATVLTFLR.T |
| 107316 | 431 | – | 448 | 621.0273 | 1860.0600 | 1860.0543 | 3.06 | 0 | 31 | 0.0013 | 1Score **> 33** indicates **identity** Score **> 14** indicates **homology** | U | R.LMITGAAPVSATVLTFLR.T |
| 108875 | 431 | – | 448 | 626.3564 | 1876.0473 | 1876.0492 | -1.01 | 0 | 54 | 8.1e-06 | 1Score **> 34** indicates **identity** Score **> 16** indicates **homology** | U | R.LMITGAAPVSATVLTFLR.T  + Oxidation (M) |
| 66515 | 492 | – | 504 | 755.8765 | 1509.7385 | 1509.7385 | 0.025 | 0 | 34 | 0.00059 | 1Score **> 34** indicates **identity** Score **> 15** indicates **homology** | U | K.LVDVEEMNYLASK.G |
| 66516 | 492 | – | 504 | 755.8769 | 1509.7393 | 1509.7385 | 0.56 | 0 | 84 | 1.4e-08 | 1Score **> 34** indicates **identity** Score **> 18** indicates **homology** | U | K.LVDVEEMNYLASK.G |
| 66517 | 492 | – | 504 | 755.8769 | 1509.7393 | 1509.7385 | 0.57 | 0 | 53 | 9.8e-06 | 1Score **> 34** indicates **identity** Score **> 16** indicates **homology** | U | K.LVDVEEMNYLASK.G |
| 66518 | 492 | – | 504 | 755.8790 | 1509.7434 | 1509.7385 | 3.25 | 0 | 53 | 1.1e-05 | 1Score **> 35** indicates **identity** Score **> 16** indicates **homology** | U | K.LVDVEEMNYLASK.G |
| 147962 | 492 | – | 512 | 790.3835 | 2368.1287 | 2368.1290 | -0.12 | 1 | 17 | 0.023 | 1Score **> 35** indicates **identity** Score **> 14** indicates **homology** | U | K.LVDVEEMNYLASKGEGEVCVK.G |
| 69656 | 513 | – | 526 | 512.6145 | 1534.8216 | 1534.8256 | -2.61 | 2 | 16 | 0.03 | 1Score **> 35** indicates **identity** Score **> 14** indicates **homology** | U | K.GANVFKGYLKDPAR.T |
| 69658 | 513 | – | 526 | 384.7134 | 1534.8246 | 1534.8256 | -0.66 | 2 | 18 | 0.019 | 1Score **> 35** indicates **identity** Score **> 14** indicates **homology** | U | K.GANVFKGYLKDPAR.T |
| 69659 | 513 | – | 526 | 512.6156 | 1534.8249 | 1534.8256 | -0.44 | 2 | 46 | 0.00023 | 1Score **> 35** indicates **identity** Score **> 22** indicates **homology** | U | K.GANVFKGYLKDPAR.T |
| 69660 | 513 | – | 526 | 384.7138 | 1534.8261 | 1534.8256 | 0.31 | 2 | 28 | 0.0026 | 1Score **> 34** indicates **identity** Score **> 14** indicates **homology** | U | K.GANVFKGYLKDPAR.T |
| 69661 | 513 | – | 526 | 512.6165 | 1534.8277 | 1534.8256 | 1.36 | 2 | 29 | 0.0021 | 1Score **> 35** indicates **identity** Score **> 14** indicates **homology** | U | K.GANVFKGYLKDPAR.T |
| 69663 | 513 | – | 526 | 384.7145 | 1534.8288 | 1534.8256 | 2.06 | 2 | 14 | 0.048 | 1Score **> 35** indicates **identity** Score **> 13** indicates **homology** | U | K.GANVFKGYLKDPAR.T |
| 9431 | 519 | – | 526 | 460.2532 | 918.4918 | 918.4923 | -0.49 | 1 | 26 | 0.0084 | 1Score **> 31** indicates **identity** Score **> 18** indicates **homology** | U | K.GYLKDPAR.T |
| 9435 | 519 | – | 526 | 460.2536 | 918.4927 | 918.4923 | 0.44 | 1 | 42 | 0.00068 | 1Score **> 31** indicates **identity** Score **> 22** indicates **homology** | U | K.GYLKDPAR.T |
| 9436 | 519 | – | 526 | 460.2539 | 918.4932 | 918.4923 | 1.03 | 1 | 22 | 0.0093 | 1Score **> 31** indicates **identity** Score **> 14** indicates **homology** | U | K.GYLKDPAR.T |
| 9437 | 519 | – | 526 | 460.2551 | 918.4956 | 918.4923 | 3.64 | 1 | 15 | 0.039 | 1Score **> 31** indicates **identity** Score **> 13** indicates **homology** | U | K.GYLKDPAR.T |
| 113026 | 527 | – | 544 | 642.9879 | 1925.9420 | 1925.9483 | -3.26 | 1 | 36 | 0.00038 | 1Score **> 35** indicates **identity** Score **> 15** indicates **homology** | U | R.TAEALDKDGWLHTGDIGK.W |
| 113030 | 527 | – | 544 | 963.9800 | 1925.9455 | 1925.9483 | -1.45 | 1 | 55 | 6.6e-06 | 1Score **> 35** indicates **identity** Score **> 16** indicates **homology** | U | R.TAEALDKDGWLHTGDIGK.W |
| 113031 | 527 | – | 544 | 642.9892 | 1925.9458 | 1925.9483 | -1.32 | 1 | 50 | 2.1e-05 | 1Score **> 35** indicates **identity** Score **> 16** indicates **homology** | U | R.TAEALDKDGWLHTGDIGK.W |
| 113033 | 527 | – | 544 | 642.9894 | 1925.9465 | 1925.9483 | -0.95 | 1 | 35 | 0.00053 | 1Score **> 35** indicates **identity** Score **> 15** indicates **homology** | U | R.TAEALDKDGWLHTGDIGK.W |
| 113036 | 527 | – | 544 | 482.4941 | 1925.9471 | 1925.9483 | -0.62 | 1 | 35 | 0.00047 | 1Score **> 35** indicates **identity** Score **> 15** indicates **homology** | U | R.TAEALDKDGWLHTGDIGK.W |
| 113039 | 527 | – | 544 | 482.4941 | 1925.9475 | 1925.9483 | -0.43 | 1 | 37 | 0.00036 | 1Score **> 35** indicates **identity** Score **> 15** indicates **homology** | U | R.TAEALDKDGWLHTGDIGK.W |
| 113040 | 527 | – | 544 | 963.9811 | 1925.9477 | 1925.9483 | -0.30 | 1 | 77 | 5.5e-08 | 1Score **> 35** indicates **identity** Score **> 17** indicates **homology** | U | R.TAEALDKDGWLHTGDIGK.W |
| 113041 | 527 | – | 544 | 642.9899 | 1925.9479 | 1925.9483 | -0.22 | 1 | 32 | 0.00095 | 1Score **> 35** indicates **identity** Score **> 15** indicates **homology** | U | R.TAEALDKDGWLHTGDIGK.W |
| 113042 | 527 | – | 544 | 482.4943 | 1925.9482 | 1925.9483 | -0.029 | 1 | 40 | 0.00019 | 1Score **> 35** indicates **identity** Score **> 15** indicates **homology** | U | R.TAEALDKDGWLHTGDIGK.W |
| 113043 | 527 | – | 544 | 963.9815 | 1925.9485 | 1925.9483 | 0.10 | 1 | 78 | 5.1e-08 | 1Score **> 35** indicates **identity** Score **> 17** indicates **homology** | U | R.TAEALDKDGWLHTGDIGK.W |
| 113044 | 527 | – | 544 | 642.9901 | 1925.9486 | 1925.9483 | 0.15 | 1 | 64 | 9.8e-07 | 1Score **> 35** indicates **identity** Score **> 17** indicates **homology** | U | R.TAEALDKDGWLHTGDIGK.W |
| 113045 | 527 | – | 544 | 642.9902 | 1925.9487 | 1925.9483 | 0.22 | 1 | 61 | 2.1e-06 | 1Score **> 35** indicates **identity** Score **> 16** indicates **homology** | U | R.TAEALDKDGWLHTGDIGK.W |
| 113046 | 527 | – | 544 | 482.4946 | 1925.9494 | 1925.9483 | 0.55 | 1 | 28 | 0.0026 | 1Score **> 35** indicates **identity** Score **> 14** indicates **homology** | U | R.TAEALDKDGWLHTGDIGK.W |
| 113048 | 527 | – | 544 | 642.9906 | 1925.9499 | 1925.9483 | 0.83 | 1 | 53 | 1e-05 | 1Score **> 35** indicates **identity** Score **> 16** indicates **homology** | U | R.TAEALDKDGWLHTGDIGK.W |
| 113051 | 527 | – | 544 | 642.9912 | 1925.9517 | 1925.9483 | 1.76 | 1 | 64 | 1.1e-06 | 1Score **> 35** indicates **identity** Score **> 16** indicates **homology** | U | R.TAEALDKDGWLHTGDIGK.W |
| 113054 | 527 | – | 544 | 642.9915 | 1925.9527 | 1925.9483 | 2.29 | 1 | 16 | 0.031 | 1Score **> 35** indicates **identity** Score **> 14** indicates **homology** | U | R.TAEALDKDGWLHTGDIGK.W |
| 113055 | 527 | – | 544 | 642.9915 | 1925.9528 | 1925.9483 | 2.31 | 1 | 74 | 1.1e-07 | 1Score **> 35** indicates **identity** Score **> 17** indicates **homology** | U | R.TAEALDKDGWLHTGDIGK.W |
| 113057 | 527 | – | 544 | 642.9917 | 1925.9532 | 1925.9483 | 2.54 | 1 | 27 | 0.0029 | 1Score **> 35** indicates **identity** Score **> 14** indicates **homology** | U | R.TAEALDKDGWLHTGDIGK.W |
| 113058 | 527 | – | 544 | 642.9917 | 1925.9533 | 1925.9483 | 2.58 | 1 | 17 | 0.026 | 1Score **> 35** indicates **identity** Score **> 14** indicates **homology** | U | R.TAEALDKDGWLHTGDIGK.W |
| 168561 | 527 | – | 552 | 709.8689 | 2835.4466 | 2835.4555 | -3.16 | 2 | 35 | 0.00047 | 1Score **> 37** indicates **identity** Score **> 15** indicates **homology** | U | R.TAEALDKDGWLHTGDIGKWLPNGTLK.I |
| 168566 | 527 | – | 552 | 709.8712 | 2835.4559 | 2835.4555 | 0.12 | 2 | 27 | 0.0028 | 1Score **> 37** indicates **identity** Score **> 14** indicates **homology** | U | R.TAEALDKDGWLHTGDIGKWLPNGTLK.I |
| 168582 | 527 | – | 552 | 710.1174 | 2836.4406 | 2836.4395 | 0.36 | 2 | 22 | 0.0096 | 1Score **> 37** indicates **identity** Score **> 14** indicates **homology** | U | R.TAEALDKDGWLHTGDIGKWLPNGTLK.I  + Deamidated (NQ) |
| 9834 | 545 | – | 552 | 465.2584 | 928.5022 | 928.5018 | 0.46 | 0 | 21 | 0.015 | 1Score **> 31** indicates **identity** Score **> 15** indicates **homology** |  | K.WLPNGTLK.I  + Deamidated (NQ) |
| 9835 | 545 | – | 552 | 465.2585 | 928.5025 | 928.5018 | 0.71 | 0 | 33 | 0.0024 | 1Score **> 31** indicates **identity** Score **> 20** indicates **homology** |  | K.WLPNGTLK.I  + Deamidated (NQ) |
| 34569 | 563 | – | 573 | 609.8211 | 1217.6276 | 1217.6292 | -1.25 | 0 | 63 | 1.3e-06 | 1Score **> 34** indicates **identity** Score **> 17** indicates **homology** |  | K.LAQGEYIAPEK.I |
| 34570 | 563 | – | 573 | 609.8213 | 1217.6280 | 1217.6292 | -0.94 | 0 | 34 | 0.00064 | 1Score **> 34** indicates **identity** Score **> 15** indicates **homology** |  | K.LAQGEYIAPEK.I |
| 34571 | 563 | – | 573 | 609.8214 | 1217.6282 | 1217.6292 | -0.78 | 0 | 23 | 0.0073 | 1Score **> 34** indicates **identity** Score **> 14** indicates **homology** |  | K.LAQGEYIAPEK.I |
| 130099 | 563 | – | 580 | 707.3830 | 2119.1273 | 2119.1313 | -1.88 | 1 | 19 | 0.016 | 1Score **> 36** indicates **identity** Score **> 14** indicates **homology** | U | K.LAQGEYIAPEKIENIYLR.S |
| 130101 | 563 | – | 580 | 707.3839 | 2119.1298 | 2119.1313 | -0.70 | 1 | 56 | 5.1e-06 | 1Score **> 36** indicates **identity** Score **> 16** indicates **homology** | U | K.LAQGEYIAPEKIENIYLR.S |
| 130102 | 563 | – | 580 | 707.3841 | 2119.1306 | 2119.1313 | -0.34 | 1 | 64 | 1e-06 | 1Score **> 36** indicates **identity** Score **> 16** indicates **homology** | U | K.LAQGEYIAPEKIENIYLR.S |
| 130103 | 563 | – | 580 | 707.3842 | 2119.1308 | 2119.1313 | -0.21 | 1 | 63 | 1.3e-06 | 1Score **> 36** indicates **identity** Score **> 16** indicates **homology** | U | K.LAQGEYIAPEKIENIYLR.S |
| 130104 | 563 | – | 580 | 707.3843 | 2119.1311 | 2119.1313 | -0.077 | 1 | 56 | 5.9e-06 | 1Score **> 36** indicates **identity** Score **> 16** indicates **homology** | U | K.LAQGEYIAPEKIENIYLR.S |
| 130105 | 563 | – | 580 | 1060.5729 | 2119.1313 | 2119.1313 | 0.0071 | 1 | 71 | 2.3e-07 | 1Score **> 36** indicates **identity** Score **> 17** indicates **homology** | U | K.LAQGEYIAPEKIENIYLR.S |
| 130107 | 563 | – | 580 | 707.3845 | 2119.1315 | 2119.1313 | 0.12 | 1 | 52 | 1.4e-05 | 1Score **> 36** indicates **identity** Score **> 16** indicates **homology** | U | K.LAQGEYIAPEKIENIYLR.S |
| 130108 | 563 | – | 580 | 1060.5731 | 2119.1317 | 2119.1313 | 0.19 | 1 | 80 | 3.3e-08 | 1Score **> 36** indicates **identity** Score **> 17** indicates **homology** | U | K.LAQGEYIAPEKIENIYLR.S |
| 130110 | 563 | – | 580 | 707.3848 | 2119.1327 | 2119.1313 | 0.66 | 1 | 22 | 0.0078 | 1Score **> 36** indicates **identity** Score **> 14** indicates **homology** | U | K.LAQGEYIAPEKIENIYLR.S |
| 130112 | 563 | – | 580 | 707.3850 | 2119.1331 | 2119.1313 | 0.86 | 1 | 64 | 9.9e-07 | 1Score **> 36** indicates **identity** Score **> 17** indicates **homology** | U | K.LAQGEYIAPEKIENIYLR.S |
| 130113 | 563 | – | 580 | 707.3851 | 2119.1335 | 2119.1313 | 1.04 | 1 | 18 | 0.018 | 1Score **> 36** indicates **identity** Score **> 14** indicates **homology** | U | K.LAQGEYIAPEKIENIYLR.S |
| 130114 | 563 | – | 580 | 1060.5746 | 2119.1346 | 2119.1313 | 1.56 | 1 | 41 | 0.00015 | 1Score **> 36** indicates **identity** Score **> 15** indicates **homology** | U | K.LAQGEYIAPEKIENIYLR.S |
| 9488 | 574 | – | 580 | 460.7633 | 919.5121 | 919.5127 | -0.64 | 0 | 42 | 0.0026 | 1Score **> 28** indicates **identity** | U | K.IENIYLR.S |
| 9490 | 574 | – | 580 | 460.7637 | 919.5128 | 919.5127 | 0.17 | 0 | 42 | 0.0026 | 1Score **> 28** indicates **identity** | U | K.IENIYLR.S |
| 59501 | 616 | – | 627 | 484.5736 | 1450.6989 | 1450.6987 | 0.13 | 1 | 58 | 2e-05 | 1Score **> 33** indicates **identity** Score **> 23** indicates **homology** | U | K.RGLQGSFEELCR.N |
| 59502 | 616 | – | 627 | 484.5736 | 1450.6991 | 1450.6987 | 0.26 | 1 | 43 | 0.00028 | 1Score **> 33** indicates **identity** Score **> 20** indicates **homology** | U | K.RGLQGSFEELCR.N |
| 59503 | 616 | – | 627 | 484.5738 | 1450.6995 | 1450.6987 | 0.57 | 1 | 58 | 2e-05 | 1Score **> 33** indicates **identity** Score **> 24** indicates **homology** | U | K.RGLQGSFEELCR.N |
| 59504 | 616 | – | 627 | 484.5738 | 1450.6997 | 1450.6987 | 0.70 | 1 | 41 | 0.00019 | 1Score **> 33** indicates **identity** Score **> 16** indicates **homology** | U | K.RGLQGSFEELCR.N |
| 59505 | 616 | – | 627 | 726.3571 | 1450.6997 | 1450.6987 | 0.71 | 1 | 48 | 0.00026 | 1Score **> 33** indicates **identity** Score **> 25** indicates **homology** | U | K.RGLQGSFEELCR.N |
| 59506 | 616 | – | 627 | 726.3572 | 1450.6998 | 1450.6987 | 0.80 | 1 | 42 | 0.00087 | 1Score **> 33** indicates **identity** Score **> 24** indicates **homology** | U | K.RGLQGSFEELCR.N |
| 59507 | 616 | – | 627 | 484.5739 | 1450.6999 | 1450.6987 | 0.85 | 1 | 62 | 9e-06 | 1Score **> 33** indicates **identity** Score **> 24** indicates **homology** | U | K.RGLQGSFEELCR.N |
| 59509 | 616 | – | 627 | 726.3576 | 1450.7006 | 1450.6987 | 1.31 | 1 | 17 | 0.025 | 1Score **> 33** indicates **identity** Score **> 14** indicates **homology** | U | K.RGLQGSFEELCR.N |
| 59510 | 616 | – | 627 | 484.5742 | 1450.7008 | 1450.6987 | 1.47 | 1 | 47 | 4.3e-05 | 1Score **> 33** indicates **identity** Score **> 15** indicates **homology** | U | K.RGLQGSFEELCR.N |
| 59511 | 616 | – | 627 | 484.5742 | 1450.7009 | 1450.6987 | 1.52 | 1 | 62 | 9.7e-06 | 1Score **> 33** indicates **identity** Score **> 24** indicates **homology** | U | K.RGLQGSFEELCR.N |
| 59512 | 616 | – | 627 | 484.5744 | 1450.7014 | 1450.6987 | 1.86 | 1 | 36 | 0.00038 | 1Score **> 33** indicates **identity** Score **> 15** indicates **homology** | U | K.RGLQGSFEELCR.N |
| 59513 | 616 | – | 627 | 484.5746 | 1450.7018 | 1450.6987 | 2.16 | 1 | 36 | 0.00041 | 1Score **> 33** indicates **identity** Score **> 15** indicates **homology** | U | K.RGLQGSFEELCR.N |
| 42923 | 617 | – | 627 | 648.3040 | 1294.5935 | 1294.5976 | -3.12 | 0 | 17 | 0.024 | 1Score **> 30** indicates **identity** Score **> 14** indicates **homology** | U | R.GLQGSFEELCR.N |
| 42924 | 617 | – | 627 | 648.3044 | 1294.5943 | 1294.5976 | -2.51 | 0 | 27 | 0.003 | 1Score **> 30** indicates **identity** Score **> 14** indicates **homology** | U | R.GLQGSFEELCR.N |
| 42925 | 617 | – | 627 | 648.3062 | 1294.5978 | 1294.5976 | 0.20 | 0 | 51 | 1.5e-05 | 1Score **> 31** indicates **identity** Score **> 16** indicates **homology** | U | R.GLQGSFEELCR.N |
| 42926 | 617 | – | 627 | 648.3062 | 1294.5979 | 1294.5976 | 0.21 | 0 | 61 | 2.2e-06 | 1Score **> 31** indicates **identity** Score **> 17** indicates **homology** | U | R.GLQGSFEELCR.N |
| 42927 | 617 | – | 627 | 648.3063 | 1294.5980 | 1294.5976 | 0.29 | 0 | 51 | 1.9e-05 | 1Score **> 31** indicates **identity** Score **> 16** indicates **homology** | U | R.GLQGSFEELCR.N |
| 42928 | 617 | – | 627 | 648.3063 | 1294.5981 | 1294.5976 | 0.37 | 0 | 59 | 3.7e-06 | 1Score **> 31** indicates **identity** Score **> 18** indicates **homology** | U | R.GLQGSFEELCR.N |
| 42929 | 617 | – | 627 | 648.3070 | 1294.5994 | 1294.5976 | 1.42 | 0 | 26 | 0.0037 | 1Score **> 31** indicates **identity** Score **> 14** indicates **homology** | U | R.GLQGSFEELCR.N |
| 42930 | 617 | – | 627 | 648.3074 | 1294.6003 | 1294.5976 | 2.09 | 0 | 33 | 0.00078 | 1Score **> 31** indicates **identity** Score **> 15** indicates **homology** | U | R.GLQGSFEELCR.N |
| 43036 | 617 | – | 627 | 648.8042 | 1295.5938 | 1295.5816 | 9.43 | 0 | 15 | 0.037 | 1Score **> 31** indicates **identity** Score **> 13** indicates **homology** | U | R.GLQGSFEELCR.N  + Deamidated (NQ) |
| 79508 | 628 | – | 641 | 538.3136 | 1611.9189 | 1611.9195 | -0.40 | 2 | 33 | 0.00085 | 1Score **> 33** indicates **identity** Score **> 15** indicates **homology** | U | R.NKDINKAILDDLLK.L |
| 79509 | 628 | – | 641 | 538.3138 | 1611.9195 | 1611.9195 | -0.036 | 2 | 31 | 0.0014 | 1Score **> 33** indicates **identity** Score **> 14** indicates **homology** | U | R.NKDINKAILDDLLK.L |
| 79510 | 628 | – | 641 | 403.9872 | 1611.9197 | 1611.9195 | 0.14 | 2 | 34 | 0.0029 | 1Score **> 33** indicates **identity** Score **> 21** indicates **homology** | U | R.NKDINKAILDDLLK.L |
| 79512 | 628 | – | 641 | 538.3139 | 1611.9200 | 1611.9195 | 0.28 | 2 | 57 | 4.8e-06 | 1Score **> 33** indicates **identity** Score **> 16** indicates **homology** | U | R.NKDINKAILDDLLK.L |
| 79515 | 628 | – | 641 | 403.9874 | 1611.9206 | 1611.9195 | 0.67 | 2 | 18 | 0.018 | 1Score **> 33** indicates **identity** Score **> 14** indicates **homology** | U | R.NKDINKAILDDLLK.L |
| 79516 | 628 | – | 641 | 538.3142 | 1611.9209 | 1611.9195 | 0.85 | 2 | 14 | 0.047 | 1Score **> 33** indicates **identity** Score **> 13** indicates **homology** | U | R.NKDINKAILDDLLK.L |
| 79517 | 628 | – | 641 | 538.3144 | 1611.9212 | 1611.9195 | 1.07 | 2 | 59 | 4.7e-06 | 1Score **> 33** indicates **identity** Score **> 18** indicates **homology** | U | R.NKDINKAILDDLLK.L |
| 79518 | 628 | – | 641 | 538.3147 | 1611.9222 | 1611.9195 | 1.69 | 2 | 48 | 3.2e-05 | 1Score **> 33** indicates **identity** Score **> 16** indicates **homology** | U | R.NKDINKAILDDLLK.L |
| 50748 | 630 | – | 641 | 457.6010 | 1369.7810 | 1369.7816 | -0.45 | 1 | 52 | 0.00096 | 1Score **> 34** indicates **identity** | U | K.DINKAILDDLLK.L |
| 50749 | 630 | – | 641 | 457.6014 | 1369.7824 | 1369.7816 | 0.56 | 1 | 46 | 0.0017 | 1Score **> 34** indicates **identity** Score **> 30** indicates **homology** | U | K.DINKAILDDLLK.L |
| 8236 | 634 | – | 641 | 450.7734 | 899.5323 | 899.5328 | -0.55 | 0 | 37 | 0.0039 | 1Score **> 28** indicates **identity** Score **> 26** indicates **homology** | U | K.AILDDLLK.L |
| 8237 | 634 | – | 641 | 450.7735 | 899.5324 | 899.5328 | -0.36 | 0 | 46 | 0.00083 | 1Score **> 28** indicates **identity** Score **> 28** indicates **homology** | U | K.AILDDLLK.L |
| 8238 | 634 | – | 641 | 450.7735 | 899.5325 | 899.5328 | -0.25 | 0 | 32 | 0.013 | 1Score **> 28** indicates **identity** Score **> 26** indicates **homology** | U | K.AILDDLLK.L |
| 8240 | 634 | – | 641 | 450.7736 | 899.5327 | 899.5328 | -0.11 | 0 | 37 | 0.004 | 1Score **> 28** indicates **identity** Score **> 26** indicates **homology** | U | K.AILDDLLK.L |
| 8243 | 634 | – | 641 | 450.7737 | 899.5328 | 899.5328 | 0.043 | 0 | 44 | 0.0011 | 1Score **> 28** indicates **identity** Score **> 27** indicates **homology** | U | K.AILDDLLK.L |
| 8244 | 634 | – | 641 | 450.7737 | 899.5328 | 899.5328 | 0.092 | 0 | 41 | 0.0015 | 1Score **> 28** indicates **identity** Score **> 26** indicates **homology** | U | K.AILDDLLK.L |
| 8246 | 634 | – | 641 | 450.7737 | 899.5329 | 899.5328 | 0.18 | 0 | 22 | 0.024 | 1Score **> 28** indicates **identity** Score **> 18** indicates **homology** | U | K.AILDDLLK.L |
| 8248 | 634 | – | 641 | 450.7737 | 899.5329 | 899.5328 | 0.19 | 0 | 44 | 0.0012 | 1Score **> 28** indicates **identity** Score **> 27** indicates **homology** | U | K.AILDDLLK.L |
| 8249 | 634 | – | 641 | 450.7738 | 899.5331 | 899.5328 | 0.42 | 0 | 46 | 0.00084 | 1Score **> 28** indicates **identity** Score **> 28** indicates **homology** | U | K.AILDDLLK.L |
| 8250 | 634 | – | 641 | 450.7741 | 899.5336 | 899.5328 | 0.98 | 0 | 33 | 0.0056 | 1Score **> 28** indicates **identity** Score **> 23** indicates **homology** | U | K.AILDDLLK.L |
| 8252 | 634 | – | 641 | 450.7743 | 899.5340 | 899.5328 | 1.32 | 0 | 34 | 0.006 | 1Score **> 28** indicates **identity** Score **> 24** indicates **homology** | U | K.AILDDLLK.L |
| 8253 | 634 | – | 641 | 450.7744 | 899.5342 | 899.5328 | 1.55 | 0 | 40 | 0.0023 | 1Score **> 28** indicates **identity** Score **> 26** indicates **homology** | U | K.AILDDLLK.L |
| 8254 | 634 | – | 641 | 450.7747 | 899.5348 | 899.5328 | 2.22 | 0 | 28 | 0.012 | 1Score **> 28** indicates **identity** Score **> 21** indicates **homology** | U | K.AILDDLLK.L |
| 32595 | 634 | – | 644 | 599.8730 | 1197.7315 | 1197.7332 | -1.43 | 1 | 25 | 0.016 | 1Score **> 29** indicates **identity** Score **> 19** indicates **homology** | U | K.AILDDLLKLGK.E |
| 32597 | 634 | – | 644 | 599.8734 | 1197.7323 | 1197.7332 | -0.82 | 1 | 21 | 0.011 | 1Score **> 29** indicates **identity** Score **> 14** indicates **homology** | U | K.AILDDLLKLGK.E |
| 32600 | 634 | – | 644 | 400.2517 | 1197.7333 | 1197.7332 | 0.012 | 1 | 37 | 0.00032 | 1Score **> 30** indicates **identity** Score **> 15** indicates **homology** | U | K.AILDDLLKLGK.E |
| 32601 | 634 | – | 644 | 400.2518 | 1197.7335 | 1197.7332 | 0.19 | 1 | 42 | 0.00011 | 1Score **> 30** indicates **identity** Score **> 15** indicates **homology** | U | K.AILDDLLKLGK.E |
| 32603 | 634 | – | 644 | 400.2519 | 1197.7338 | 1197.7332 | 0.49 | 1 | 45 | 6.3e-05 | 1Score **> 30** indicates **identity** Score **> 15** indicates **homology** | U | K.AILDDLLKLGK.E |
| 70633 | 642 | – | 655 | 386.7259 | 1542.8747 | 1542.8770 | -1.47 | 2 | 18 | 0.025 | 1Score **> 34** indicates **identity** Score **> 15** indicates **homology** | U | K.LGKEAGLKPFEQVK.G |
| 70634 | 642 | – | 655 | 515.2992 | 1542.8757 | 1542.8770 | -0.78 | 2 | 42 | 0.00012 | 1Score **> 34** indicates **identity** Score **> 15** indicates **homology** | U | K.LGKEAGLKPFEQVK.G |
| 70636 | 642 | – | 655 | 515.2992 | 1542.8758 | 1542.8770 | -0.73 | 2 | 39 | 0.00026 | 1Score **> 34** indicates **identity** Score **> 16** indicates **homology** | U | K.LGKEAGLKPFEQVK.G |
| 70637 | 642 | – | 655 | 386.7263 | 1542.8759 | 1542.8770 | -0.67 | 2 | 27 | 0.0027 | 1Score **> 34** indicates **identity** Score **> 14** indicates **homology** | U | K.LGKEAGLKPFEQVK.G |
| 70638 | 642 | – | 655 | 386.7263 | 1542.8762 | 1542.8770 | -0.46 | 2 | 23 | 0.0075 | 1Score **> 34** indicates **identity** Score **> 14** indicates **homology** | U | K.LGKEAGLKPFEQVK.G |
| 70639 | 642 | – | 655 | 515.2997 | 1542.8774 | 1542.8770 | 0.26 | 2 | 23 | 0.0069 | 1Score **> 34** indicates **identity** Score **> 14** indicates **homology** | U | K.LGKEAGLKPFEQVK.G |
| 37516 | 645 | – | 655 | 415.8988 | 1244.6746 | 1244.6765 | -1.47 | 1 | 17 | 0.03 | 1Score **> 34** indicates **identity** Score **> 14** indicates **homology** | U | K.EAGLKPFEQVK.G |
| 37517 | 645 | – | 655 | 415.8990 | 1244.6753 | 1244.6765 | -0.95 | 1 | 27 | 0.0036 | 1Score **> 34** indicates **identity** Score **> 16** indicates **homology** | U | K.EAGLKPFEQVK.G |
| 37518 | 645 | – | 655 | 623.3449 | 1244.6753 | 1244.6765 | -0.91 | 1 | 27 | 0.012 | 1Score **> 34** indicates **identity** Score **> 20** indicates **homology** | U | K.EAGLKPFEQVK.G |
| 37519 | 645 | – | 655 | 415.8992 | 1244.6758 | 1244.6765 | -0.53 | 1 | 28 | 0.0035 | 1Score **> 34** indicates **identity** Score **> 16** indicates **homology** | U | K.EAGLKPFEQVK.G |
| 37520 | 645 | – | 655 | 623.3453 | 1244.6761 | 1244.6765 | -0.33 | 1 | 50 | 9e-05 | 1Score **> 34** indicates **identity** Score **> 22** indicates **homology** | U | K.EAGLKPFEQVK.G |
| 37523 | 645 | – | 655 | 415.8994 | 1244.6763 | 1244.6765 | -0.13 | 1 | 28 | 0.0024 | 1Score **> 34** indicates **identity** Score **> 15** indicates **homology** | U | K.EAGLKPFEQVK.G |
| 37524 | 645 | – | 655 | 623.3455 | 1244.6765 | 1244.6765 | 0.043 | 1 | 52 | 0.00044 | 1Score **> 34** indicates **identity** Score **> 31** indicates **homology** | U | K.EAGLKPFEQVK.G |
| 37526 | 645 | – | 655 | 415.8995 | 1244.6766 | 1244.6765 | 0.089 | 1 | 24 | 0.0057 | 1Score **> 34** indicates **identity** Score **> 14** indicates **homology** | U | K.EAGLKPFEQVK.G |
| 37527 | 645 | – | 655 | 623.3456 | 1244.6767 | 1244.6765 | 0.17 | 1 | 65 | 1.4e-05 | 1Score **> 34** indicates **identity** Score **> 29** indicates **homology** | U | K.EAGLKPFEQVK.G |
| 37528 | 645 | – | 655 | 415.8995 | 1244.6767 | 1244.6765 | 0.22 | 1 | 28 | 0.0033 | 1Score **> 34** indicates **identity** Score **> 16** indicates **homology** | U | K.EAGLKPFEQVK.G |
| 139248 | 656 | – | 676 | 745.7511 | 2234.2313 | 2234.2311 | 0.12 | 0 | 65 | 8.8e-07 | 1Score **> 35** indicates **identity** Score **> 17** indicates **homology** | U | K.GIAVHPELFSIDNGLLTPTLK.A |
| 139249 | 656 | – | 676 | 745.7513 | 2234.2320 | 2234.2311 | 0.40 | 0 | 30 | 0.0015 | 1Score **> 34** indicates **identity** Score **> 14** indicates **homology** | U | K.GIAVHPELFSIDNGLLTPTLK.A |
| 139250 | 656 | – | 676 | 1118.1236 | 2234.2326 | 2234.2311 | 0.69 | 0 | 95 | 1.2e-09 | 1Score **> 34** indicates **identity** Score **> 18** indicates **homology** | U | K.GIAVHPELFSIDNGLLTPTLK.A |
| 139251 | 656 | – | 676 | 1118.1244 | 2234.2342 | 2234.2311 | 1.39 | 0 | 27 | 0.0031 | 1Score **> 34** indicates **identity** Score **> 14** indicates **homology** | U | K.GIAVHPELFSIDNGLLTPTLK.A |
| 139361 | 656 | – | 676 | 746.0804 | 2235.2195 | 2235.2151 | 1.96 | 0 | 69 | 3.7e-07 | 1Score **> 35** indicates **identity** Score **> 17** indicates **homology** | U | K.GIAVHPELFSIDNGLLTPTLK.A  + Deamidated (NQ) |
| 139362 | 656 | – | 676 | 746.0816 | 2235.2229 | 2235.2151 | 3.49 | 0 | 63 | 1.1e-06 | 1Score **> 35** indicates **identity** Score **> 16** indicates **homology** | U | K.GIAVHPELFSIDNGLLTPTLK.A  + Deamidated (NQ) |
| 139363 | 656 | – | 676 | 1118.6197 | 2235.2248 | 2235.2151 | 4.35 | 0 | 116 | 1.4e-11 | 1Score **> 35** indicates **identity** Score **> 20** indicates **homology** | U | K.GIAVHPELFSIDNGLLTPTLK.A  + Deamidated (NQ) |
| 139365 | 656 | – | 676 | 746.0849 | 2235.2330 | 2235.2151 | 8.01 | 0 | 34 | 0.00066 | 1Score **> 35** indicates **identity** Score **> 15** indicates **homology** | U | K.GIAVHPELFSIDNGLLTPTLK.A  + Deamidated (NQ) |
| 41457 | 688 | – | 698 | 640.8402 | 1279.6659 | 1279.6660 | -0.012 | 0 | 28 | 0.0024 | 1Score **> 33** indicates **identity** Score **> 14** indicates **homology** | U | R.SQIDELYATIK.I |
| 41460 | 688 | – | 698 | 640.8407 | 1279.6669 | 1279.6660 | 0.77 | 0 | 48 | 3.5e-05 | 1Score **> 33** indicates **identity** Score **> 15** indicates **homology** | U | R.SQIDELYATIK.I |
| 41466 | 688 | – | 698 | 640.8423 | 1279.6701 | 1279.6660 | 3.26 | 0 | 28 | 0.0026 | 1Score **> 33** indicates **identity** Score **> 14** indicates **homology** | U | R.SQIDELYATIK.I |
| 52938 | 688 | – | 699 | 697.3811 | 1392.7476 | 1392.7500 | -1.70 | 1 | 48 | 3.3e-05 | 1Score **> 34** indicates **identity** Score **> 15** indicates **homology** | U | R.SQIDELYATIKI.- |
| 52939 | 688 | – | 699 | 697.3821 | 1392.7497 | 1392.7500 | -0.24 | 1 | 45 | 5.5e-05 | 1Score **> 34** indicates **identity** Score **> 15** indicates **homology** | U | R.SQIDELYATIKI.- |
| 52940 | 688 | – | 699 | 697.3824 | 1392.7502 | 1392.7500 | 0.16 | 1 | 57 | 4.2e-06 | 1Score **> 34** indicates **identity** Score **> 16** indicates **homology** | U | R.SQIDELYATIKI.- |
| 52941 | 688 | – | 699 | 697.3825 | 1392.7505 | 1392.7500 | 0.38 | 1 | 57 | 4.2e-06 | 1Score **> 34** indicates **identity** Score **> 16** indicates **homology** | U | R.SQIDELYATIKI.- |
| 52943 | 688 | – | 699 | 697.3828 | 1392.7510 | 1392.7500 | 0.74 | 1 | 46 | 4.6e-05 | 1Score **> 34** indicates **identity** Score **> 15** indicates **homology** | U | R.SQIDELYATIKI.- |
| 52944 | 688 | – | 699 | 465.2577 | 1392.7512 | 1392.7500 | 0.88 | 1 | 31 | 0.0014 | 1Score **> 34** indicates **identity** Score **> 14** indicates **homology** | U | R.SQIDELYATIKI.- |
| 52945 | 688 | – | 699 | 697.3829 | 1392.7513 | 1392.7500 | 0.93 | 1 | 57 | 4.4e-06 | 1Score **> 34** indicates **identity** Score **> 16** indicates **homology** | U | R.SQIDELYATIKI.- |
| 52946 | 688 | – | 699 | 697.3830 | 1392.7514 | 1392.7500 | 0.99 | 1 | 57 | 4.4e-06 | 1Score **> 34** indicates **identity** Score **> 16** indicates **homology** | U | R.SQIDELYATIKI.- |
| 52949 | 688 | – | 699 | 697.3834 | 1392.7523 | 1392.7500 | 1.62 | 1 | 61 | 1.8e-06 | 1Score **> 34** indicates **identity** Score **> 16** indicates **homology** | U | R.SQIDELYATIKI.- |
| 52950 | 688 | – | 699 | 697.3838 | 1392.7530 | 1392.7500 | 2.15 | 1 | 48 | 3.3e-05 | 1Score **> 34** indicates **identity** Score **> 15** indicates **homology** | U | R.SQIDELYATIKI.- |
| 52951 | 688 | – | 699 | 697.3843 | 1392.7541 | 1392.7500 | 2.94 | 1 | 22 | 0.0087 | 1Score **> 33** indicates **identity** Score **> 14** indicates **homology** | U | R.SQIDELYATIKI.- |
| 52954 | 688 | – | 699 | 697.3861 | 1392.7576 | 1392.7500 | 5.46 | 1 | 31 | 0.0014 | 1Score **> 33** indicates **identity** Score **> 14** indicates **homology** | U | R.SQIDELYATIKI.- |

---

```
ID   D3Z041_MOUSE            Unreviewed;       699 AA.
AC   D3Z041;
DT   20-APR-2010, integrated into UniProtKB/TrEMBL.
DT   20-APR-2010, sequence version 1.
DT   28-JUN-2023, entry version 79.
DE   RecName: Full=Long-chain-fatty-acid--CoA ligase {ECO:0000256|RuleBase:RU369030};
DE            EC=6.2.1.15 {ECO:0000256|RuleBase:RU369030};
DE            EC=6.2.1.3 {ECO:0000256|RuleBase:RU369030};
DE   AltName: Full=Acyl-CoA synthetase {ECO:0000256|RuleBase:RU369030};
DE   AltName: Full=Long-chain acyl-CoA synthetase {ECO:0000256|RuleBase:RU369030};
GN   Name=Acsl1 {ECO:0000313|Ensembl:ENSMUSP00000106000.2,
GN   ECO:0000313|MGI:MGI:102797};
OS   Mus musculus (Mouse).
OC   Eukaryota; Metazoa; Chordata; Craniata; Vertebrata; Euteleostomi; Mammalia;
OC   Eutheria; Euarchontoglires; Glires; Rodentia; Myomorpha; Muroidea; Muridae;
OC   Murinae; Mus; Mus.
OX   NCBI_TaxID=10090 {ECO:0000313|Ensembl:ENSMUSP00000106000.2, ECO:0000313|Proteomes:UP000000589};
RN   [1] {ECO:0007829|PubMed:17242355}
RP   IDENTIFICATION BY MASS SPECTROMETRY [LARGE SCALE ANALYSIS].
RX   PubMed=17242355; DOI=10.1073/pnas.0609836104;
RA   Villen J., Beausoleil S.A., Gerber S.A., Gygi S.P.;
RT   "Large-scale phosphorylation analysis of mouse liver.";
RL   Proc. Natl. Acad. Sci. U.S.A. 104:1488-1493(2007).
RN   [2] {ECO:0000313|Ensembl:ENSMUSP00000106000.2, ECO:0000313|Proteomes:UP000000589}
RP   NUCLEOTIDE SEQUENCE [LARGE SCALE GENOMIC DNA].
RC   STRAIN=C57BL/6J {ECO:0000313|Ensembl:ENSMUSP00000106000.2,
RC   ECO:0000313|Proteomes:UP000000589};
RX   PubMed=19468303; DOI=10.1371/journal.pbio.1000112;
RA   Church D.M., Goodstadt L., Hillier L.W., Zody M.C., Goldstein S., She X.,
RA   Bult C.J., Agarwala R., Cherry J.L., DiCuccio M., Hlavina W., Kapustin Y.,
RA   Meric P., Maglott D., Birtle Z., Marques A.C., Graves T., Zhou S.,
RA   Teague B., Potamousis K., Churas C., Place M., Herschleb J., Runnheim R.,
RA   Forrest D., Amos-Landgraf J., Schwartz D.C., Cheng Z., Lindblad-Toh K.,
RA   Eichler E.E., Ponting C.P.;
RT   "Lineage-specific biology revealed by a finished genome assembly of the
RT   mouse.";
RL   PLoS Biol. 7:E1000112-E1000112(2009).
RN   [3] {ECO:0007829|PubMed:21183079}
RP   IDENTIFICATION BY MASS SPECTROMETRY [LARGE SCALE ANALYSIS].
RX   PubMed=21183079; DOI=10.1016/j.cell.2010.12.001;
RA   Huttlin E.L., Jedrychowski M.P., Elias J.E., Goswami T., Rad R.,
RA   Beausoleil S.A., Villen J., Haas W., Sowa M.E., Gygi S.P.;
RT   "A tissue-specific atlas of mouse protein phosphorylation and expression.";
RL   Cell 143:1174-1189(2010).
RN   [4] {ECO:0007829|PubMed:23576753}
RP   IDENTIFICATION BY MASS SPECTROMETRY [LARGE SCALE ANALYSIS].
RX   PubMed=23576753; DOI=10.1073/pnas.1302961110;
RA   Rardin M.J., Newman J.C., Held J.M., Cusack M.P., Sorensen D.J., Li B.,
RA   Schilling B., Mooney S.D., Kahn C.R., Verdin E., Gibson B.W.;
RT   "Label-free quantitative proteomics of the lysine acetylome in mitochondria
RT   identifies substrates of SIRT3 in metabolic pathways.";
RL   Proc. Natl. Acad. Sci. U.S.A. 110:6601-6606(2013).
RN   [5] {ECO:0000313|Ensembl:ENSMUSP00000106000.2}
RP   IDENTIFICATION.
RC   STRAIN=C57BL/6J {ECO:0000313|Ensembl:ENSMUSP00000106000.2};
RG   Ensembl;
RL   Submitted (MAR-2023) to UniProtKB.
CC   -!- FUNCTION: Catalyzes the conversion of long-chain fatty acids to their
CC       active form acyl-CoAs for both synthesis of cellular lipids, and
CC       degradation via beta-oxidation. {ECO:0000256|RuleBase:RU369030}.
CC   -!- CATALYTIC ACTIVITY:
CC       Reaction=(5Z,8Z,11Z,14Z)-eicosatetraenoate + ATP + CoA =
CC         (5Z,8Z,11Z,14Z)-eicosatetraenoyl-CoA + AMP + diphosphate;
CC         Xref=Rhea:RHEA:19713, ChEBI:CHEBI:30616, ChEBI:CHEBI:32395,
CC         ChEBI:CHEBI:33019, ChEBI:CHEBI:57287, ChEBI:CHEBI:57368,
CC         ChEBI:CHEBI:456215; EC=6.2.1.15;
CC         Evidence={ECO:0000256|ARBA:ARBA00024548,
CC         ECO:0000256|RuleBase:RU369030};
CC       PhysiologicalDirection=left-to-right; Xref=Rhea:RHEA:19714;
CC         Evidence={ECO:0000256|ARBA:ARBA00024548,
CC         ECO:0000256|RuleBase:RU369030};
CC   -!- CATALYTIC ACTIVITY:
CC       Reaction=(E)-hexadec-2-enoate + ATP + CoA = (2E)-hexadecenoyl-CoA + AMP
CC         + diphosphate; Xref=Rhea:RHEA:36139, ChEBI:CHEBI:30616,
CC         ChEBI:CHEBI:33019, ChEBI:CHEBI:57287, ChEBI:CHEBI:61526,
CC         ChEBI:CHEBI:72745, ChEBI:CHEBI:456215;
CC         Evidence={ECO:0000256|ARBA:ARBA00024565,
CC         ECO:0000256|RuleBase:RU369030};
CC       PhysiologicalDirection=left-to-right; Xref=Rhea:RHEA:36140;
CC         Evidence={ECO:0000256|ARBA:ARBA00024565,
CC         ECO:0000256|RuleBase:RU369030};
CC   -!- CATALYTIC ACTIVITY:
CC       Reaction=12-hydroxy-(5Z,8Z,10E,14Z)-eicosatetraenoate + ATP + CoA = 12-
CC         hydroxy-(5Z,8Z,10E,14Z)-eicosatetraenoyl-CoA + AMP + diphosphate;
CC         Xref=Rhea:RHEA:52112, ChEBI:CHEBI:30616, ChEBI:CHEBI:33019,
CC         ChEBI:CHEBI:57287, ChEBI:CHEBI:90718, ChEBI:CHEBI:136408,
CC         ChEBI:CHEBI:456215; Evidence={ECO:0000256|ARBA:ARBA00024495,
CC         ECO:0000256|RuleBase:RU369030};
CC       PhysiologicalDirection=left-to-right; Xref=Rhea:RHEA:52113;
CC         Evidence={ECO:0000256|ARBA:ARBA00024495,
CC         ECO:0000256|RuleBase:RU369030};
CC   -!- CATALYTIC ACTIVITY:
CC       Reaction=15-hydroxy-(5Z,8Z,11Z,13E)-eicosatetraenoate + ATP + CoA = 15-
CC         hydroxy-(5Z,8Z,11Z,13E)-eicosatetraenoyl-CoA + AMP + diphosphate;
CC         Xref=Rhea:RHEA:52116, ChEBI:CHEBI:30616, ChEBI:CHEBI:33019,
CC         ChEBI:CHEBI:57287, ChEBI:CHEBI:78832, ChEBI:CHEBI:136409,
CC         ChEBI:CHEBI:456215; Evidence={ECO:0000256|ARBA:ARBA00024532,
CC         ECO:0000256|RuleBase:RU369030};
CC       PhysiologicalDirection=left-to-right; Xref=Rhea:RHEA:52117;
CC         Evidence={ECO:0000256|ARBA:ARBA00024532,
CC         ECO:0000256|RuleBase:RU369030};
CC   -!- CATALYTIC ACTIVITY:
CC       Reaction=5-hydroxy-(6E,8Z,11Z,14Z)-eicosatetraenoate + ATP + CoA = 5-
CC         hydroxy-(6E,8Z,11Z,14Z)-eicosatetraenoyl-CoA + AMP + diphosphate;
CC         Xref=Rhea:RHEA:52108, ChEBI:CHEBI:30616, ChEBI:CHEBI:33019,
CC         ChEBI:CHEBI:57287, ChEBI:CHEBI:65341, ChEBI:CHEBI:136407,
CC         ChEBI:CHEBI:456215; Evidence={ECO:0000256|ARBA:ARBA00024469,
CC         ECO:0000256|RuleBase:RU369030};
CC       PhysiologicalDirection=left-to-right; Xref=Rhea:RHEA:52109;
CC         Evidence={ECO:0000256|ARBA:ARBA00024469,
CC         ECO:0000256|RuleBase:RU369030};
CC   -!- CATALYTIC ACTIVITY:
CC       Reaction=ATP + CoA + hexadecanoate = AMP + diphosphate + hexadecanoyl-
CC         CoA; Xref=Rhea:RHEA:30751, ChEBI:CHEBI:7896, ChEBI:CHEBI:30616,
CC         ChEBI:CHEBI:33019, ChEBI:CHEBI:57287, ChEBI:CHEBI:57379,
CC         ChEBI:CHEBI:456215; Evidence={ECO:0000256|ARBA:ARBA00024497,
CC         ECO:0000256|RuleBase:RU369030};
CC       PhysiologicalDirection=left-to-right; Xref=Rhea:RHEA:30752;
CC         Evidence={ECO:0000256|ARBA:ARBA00024497,
CC         ECO:0000256|RuleBase:RU369030};
CC   -!- CATALYTIC ACTIVITY:
CC       Reaction=a long-chain fatty acid + ATP + CoA = a long-chain fatty acyl-
CC         CoA + AMP + diphosphate; Xref=Rhea:RHEA:15421, ChEBI:CHEBI:30616,
CC         ChEBI:CHEBI:33019, ChEBI:CHEBI:57287, ChEBI:CHEBI:57560,
CC         ChEBI:CHEBI:83139, ChEBI:CHEBI:456215; EC=6.2.1.3;
CC         Evidence={ECO:0000256|ARBA:ARBA00024484};
CC       PhysiologicalDirection=left-to-right; Xref=Rhea:RHEA:15422;
CC         Evidence={ECO:0000256|ARBA:ARBA00024484};
CC   -!- SUBCELLULAR LOCATION: Endoplasmic reticulum membrane
CC       {ECO:0000256|ARBA:ARBA00004643}; Single-pass type III membrane protein
CC       {ECO:0000256|ARBA:ARBA00004643}. Membrane
CC       {ECO:0000256|ARBA:ARBA00004183}; Single-pass type III membrane protein
CC       {ECO:0000256|ARBA:ARBA00004183}. Mitochondrion outer membrane
CC       {ECO:0000256|RuleBase:RU369030}; Single-pass membrane protein
CC       {ECO:0000256|RuleBase:RU369030}. Endoplasmic reticulum membrane
CC       {ECO:0000256|RuleBase:RU369030}; Single-pass membrane protein
CC       {ECO:0000256|RuleBase:RU369030}. Mitochondrion outer membrane
CC       {ECO:0000256|ARBA:ARBA00025703}; Single-pass type III membrane protein
CC       {ECO:0000256|ARBA:ARBA00025703}.
CC   -!- SIMILARITY: Belongs to the ATP-dependent AMP-binding enzyme family.
CC       {ECO:0000256|ARBA:ARBA00006432, ECO:0000256|RuleBase:RU369030}.
CC   ---------------------------------------------------------------------------
CC   Copyrighted by the UniProt Consortium, see https://www.uniprot.org/terms
CC   Distributed under the Creative Commons Attribution (CC BY 4.0) License
CC   ---------------------------------------------------------------------------
DR   AlphaFoldDB; D3Z041; -.
DR   SMR; D3Z041; -.
DR   SwissPalm; D3Z041; -.
DR   EPD; D3Z041; -.
DR   jPOST; D3Z041; -.
DR   MaxQB; D3Z041; -.
DR   PeptideAtlas; D3Z041; -.
DR   ProteomicsDB; 310638; -.
DR   Antibodypedia; 1946; 328 antibodies from 35 providers.
DR   Ensembl; ENSMUST00000110371.8; ENSMUSP00000106000.2; ENSMUSG00000018796.14.
DR   AGR; MGI:102797; -.
DR   MGI; MGI:102797; Acsl1.
DR   VEuPathDB; HostDB:ENSMUSG00000018796; -.
DR   GeneTree; ENSGT00940000154508; -.
DR   OMA; WTIGAQV; -.
DR   ChiTaRS; Acsl1; mouse.
DR   Proteomes; UP000000589; Chromosome 8.
DR   Bgee; ENSMUSG00000018796; Expressed in brown adipose tissue and 285 other tissues.
DR   ExpressionAtlas; D3Z041; baseline and differential.
DR   GO; GO:0005789; C:endoplasmic reticulum membrane; IEA:UniProtKB-SubCell.
DR   GO; GO:0005741; C:mitochondrial outer membrane; IEA:UniProtKB-SubCell.
DR   GO; GO:0005524; F:ATP binding; IEA:UniProtKB-KW.
DR   GO; GO:0004467; F:long-chain fatty acid-CoA ligase activity; IEA:InterPro.
DR   CDD; cd05927; LC-FACS_euk; 1.
DR   Gene3D; 3.40.50.12780; N-terminal domain of ligase-like; 1.
DR   InterPro; IPR025110; AMP-bd_C.
DR   InterPro; IPR020845; AMP-binding_CS.
DR   InterPro; IPR000873; AMP-dep_Synth/Lig_com.
DR   InterPro; IPR042099; ANL_N_sf.
DR   InterPro; IPR045311; LC-FACS_euk.
DR   PANTHER; PTHR43272; LONG-CHAIN-FATTY-ACID--COA LIGASE; 1.
DR   PANTHER; PTHR43272:SF28; LONG-CHAIN-FATTY-ACID--COA LIGASE 1; 1.
DR   Pfam; PF00501; AMP-binding; 1.
DR   Pfam; PF13193; AMP-binding_C; 1.
DR   SUPFAM; SSF56801; Acetyl-CoA synthetase-like; 1.
DR   PROSITE; PS00455; AMP_BINDING; 1.
PE   1: Evidence at protein level;
KW   ATP-binding {ECO:0000256|RuleBase:RU369030};
KW   Fatty acid metabolism {ECO:0000256|ARBA:ARBA00022832,
KW   ECO:0000256|RuleBase:RU369030}; Ligase {ECO:0000256|RuleBase:RU369030};
KW   Lipid metabolism {ECO:0000256|ARBA:ARBA00023098,
KW   ECO:0000256|RuleBase:RU369030}; Membrane {ECO:0000256|RuleBase:RU369030};
KW   Nucleotide-binding {ECO:0000256|RuleBase:RU369030};
KW   Proteomics identification {ECO:0007829|EPD:D3Z041,
KW   ECO:0007829|MaxQB:D3Z041};
KW   Reference proteome {ECO:0000313|Proteomes:UP000000589};
KW   Transmembrane {ECO:0000256|RuleBase:RU369030};
KW   Transmembrane helix {ECO:0000256|RuleBase:RU369030}.
FT   TRANSMEM        21..45
FT                   /note="Helical"
FT                   /evidence="ECO:0000256|RuleBase:RU369030"
FT   DOMAIN          116..563
FT                   /note="AMP-dependent synthetase/ligase"
FT                   /evidence="ECO:0000259|Pfam:PF00501"
FT   DOMAIN          573..629
FT                   /note="AMP-binding enzyme C-terminal"
FT                   /evidence="ECO:0000259|Pfam:PF13193"
SQ   SEQUENCE   699 AA;  78034 MW;  D10B6FE1982D3474 CRC64;
     MEVHELFRYF RMPELIDIRQ YVRTLPTNTL MGFGAFAALT TFWYATRPKA LKPPCDLSMQ
     SVEIAGTTDG IRRSAVLEDD KLLVYYYDDV RTMYDGFQRG IQVSNNGPCL GSRKPNQPYE
     WISYKEVAEL AECIGSGLIQ KGFKPCSEQF IGLFSQNRPE WVIVEQGCFS YSMVVVPLYD
     TLGADAITYI VNKAELSVIF ADKPEKAKLL LEGVENKLTP CLKIIVIMDS YGSDLVERGK
     KCGVEIISLK ALEDLGRVNR VKPKPPEPED LAIICFTSGT TGNPKGAMIT HQNIINDCSG
     FIKATESALT LNASDTQISY LPLAHMYEQQ LQCVMLCHGA KIGFFQGDIR LLMDDLKVLQ
     PTIFPVVPRL LNRMFDRIFG QANTSLKRWL LDFASKRKEA ELRSGIVRNN SLWDKLIFHK
     IQSSLGGKVR LMITGAAPVS ATVLTFLRTA LGCQFYEGYG QTECTAGCCL SLPGDWTAGH
     VGAPMPCNYV KLVDVEEMNY LASKGEGEVC VKGANVFKGY LKDPARTAEA LDKDGWLHTG
     DIGKWLPNGT LKIIDRKKHI FKLAQGEYIA PEKIENIYLR SEAVAQVFVH GESLQAFLIA
     VVVPDVESLP SWAQKRGLQG SFEELCRNKD INKAILDDLL KLGKEAGLKP FEQVKGIAVH
     PELFSIDNGL LTPTLKAKRP ELRNYFRSQI DELYATIKI
//
```

|  |
| --- |
| **Mascot:** http://www.matrixscience.com/ |

HNE (K) (+156.1150)
