# Supplementary material for: A common East-Asian ALDH2 mutation causes metabolic disorders and the therapeutic effect of ALDH2 activators
Source: Nat Commun. 2023 Sep 25;14:5971. doi: 10.1038/s41467-023-41570-6 (PMC10520061; doi:10.1038/s41467-023-41570-6)
Supplement: Supplementary file 4 — Supplementary Data 1 [file 41467_2023_41570_MOESM4_ESM.zip › Table S5b/D3Z041/D3Z041_WTO-2_C168.html]

Mascot Search Results: D3Z041
 

# MASCOT Search Results

## Protein View: D3Z041

### Arachidonate--CoA ligase OS=Mus musculus OX=10090 GN=Acsl1 PE=1 SV=1

|  |  |
| --- | --- |
| Database: | Mouse\_UniProt\_proteomes |
| Score: | 13678 |
| Monoisotopic mass (Mr): | 79011 |
| Calculated pI: | 7.43 |

Sequence similarity is available as an NCBI BLAST search of D3Z041 against nr.

### Search parameters

|  |  |
| --- | --- |
| MS data file: | `D:\LCMSMS\2023 Users' data\230529-1\230529-1-WTO-2.raw` |
| Enzyme: | Trypsin/P: cuts C-term side of KR. |
| Fixed modifications: | Carbamidomethyl (C) |
| Variable modifications: | Deamidated (NQ), HNE (C), HNE (H), HNE (K), Oxidation (M) |

### Protein sequence coverage: 79%

Matched peptides shown in ***bold red***.

|  |  |  |  |  |  |
| --- | --- | --- | --- | --- | --- |
| `1` | `MEVHELFRYF` | `RMPELIDIRQ` | `YVRTLPTNTL` | `MGFGAFAALT` | `TFWYATRPKA` |
| `51` | `LKPPCDLSMQ` | `SVEIAGTTDG` | `IRRSAVLEDD` | `KLLVYYYDDV` | `RTMYDGFQRG` |
| `101` | `IQVSNNGPCL` | `GSRKPNQPYE` | `WISYKEVAEL` | `AECIGSGLIQ` | `KGFKPCSEQF` |
| `151` | `IGLFSQNRPE` | `WVIVEQGCFS` | `YSMVVVPLYD` | `TLGADAITYI` | `VNKAELSVIF` |
| `201` | `ADKPEKAKLL` | `LEGVENKLTP` | `CLKIIVIMDS` | `YGSDLVERGK` | `KCGVEIISLK` |
| `251` | `ALEDLGRVNR` | `VKPKPPEPED` | `LAIICFTSGT` | `TGNPKGAMIT` | `HQNIINDCSG` |
| `301` | `FIKATESALT` | `LNASDTQISY` | `LPLAHMYEQQ` | `LQCVMLCHGA` | `KIGFFQGDIR` |
| `351` | `LLMDDLKVLQ` | `PTIFPVVPRL` | `LNRMFDRIFG` | `QANTSLKRWL` | `LDFASKRKEA` |
| `401` | `ELRSGIVRNN` | `SLWDKLIFHK` | `IQSSLGGKVR` | `LMITGAAPVS` | `ATVLTFLRTA` |
| `451` | `LGCQFYEGYG` | `QTECTAGCCL` | `SLPGDWTAGH` | `VGAPMPCNYV` | `KLVDVEEMNY` |
| `501` | `LASKGEGEVC` | `VKGANVFKGY` | `LKDPARTAEA` | `LDKDGWLHTG` | `DIGKWLPNGT` |
| `551` | `LKIIDRKKHI` | `FKLAQGEYIA` | `PEKIENIYLR` | `SEAVAQVFVH` | `GESLQAFLIA` |
| `601` | `VVVPDVESLP` | `SWAQKRGLQG` | `SFEELCRNKD` | `INKAILDDLL` | `KLGKEAGLKP` |
| `651` | `FEQVKGIAVH` | `PELFSIDNGL` | `LTPTLKAKRP` | `ELRNYFRSQI` | `DELYATIKI` |

Unformatted sequence string: 699 residues (for pasting into other applications).

|  |  |  |  |
| --- | --- | --- | --- |
| Sort by | residue number | increasing mass | decreasing mass |
| Show | matched peptides only | predicted peptides also |  |

| Query | Start | – | End | Observed | Mr(expt) | Mr(calc) | ppm | M | Score | Expect | Rank | U | Peptide |
| --- | --- | --- | --- | --- | --- | --- | --- | --- | --- | --- | --- | --- | --- |
| 61260 | 9 | – | 19 | 484.9264 | 1451.7575 | 1451.7595 | -1.37 | 1 | 33 | 0.0013 | 1Score **> 34** indicates **identity** Score **> 17** indicates **homology** | U | R.YFRMPELIDIR.Q |
| 61261 | 9 | – | 19 | 484.9267 | 1451.7584 | 1451.7595 | -0.74 | 1 | 33 | 0.00092 | 1Score **> 34** indicates **identity** Score **> 15** indicates **homology** | U | R.YFRMPELIDIR.Q |
| 61262 | 9 | – | 19 | 484.9268 | 1451.7585 | 1451.7595 | -0.68 | 1 | 32 | 0.0012 | 1Score **> 34** indicates **identity** Score **> 15** indicates **homology** | U | R.YFRMPELIDIR.Q |
| 61263 | 9 | – | 19 | 484.9268 | 1451.7586 | 1451.7595 | -0.60 | 1 | 41 | 0.00021 | 1Score **> 34** indicates **identity** Score **> 17** indicates **homology** | U | R.YFRMPELIDIR.Q |
| 61264 | 9 | – | 19 | 484.9268 | 1451.7586 | 1451.7595 | -0.59 | 1 | 43 | 0.00014 | 1Score **> 34** indicates **identity** Score **> 17** indicates **homology** | U | R.YFRMPELIDIR.Q |
| 61267 | 9 | – | 19 | 484.9272 | 1451.7596 | 1451.7595 | 0.12 | 1 | 23 | 0.0076 | 1Score **> 34** indicates **identity** Score **> 14** indicates **homology** | U | R.YFRMPELIDIR.Q |
| 61275 | 9 | – | 19 | 484.9279 | 1451.7619 | 1451.7595 | 1.68 | 1 | 34 | 0.00079 | 1Score **> 34** indicates **identity** Score **> 15** indicates **homology** | U | R.YFRMPELIDIR.Q |
| 61277 | 9 | – | 19 | 484.9280 | 1451.7621 | 1451.7595 | 1.78 | 1 | 20 | 0.013 | 1Score **> 34** indicates **identity** Score **> 14** indicates **homology** | U | R.YFRMPELIDIR.Q |
| 13914 | 12 | – | 19 | 493.7699 | 985.5253 | 985.5266 | -1.31 | 0 | 33 | 0.00085 | 1Score **> 31** indicates **identity** Score **> 15** indicates **homology** | U | R.MPELIDIR.Q |
| 13918 | 12 | – | 19 | 493.7709 | 985.5273 | 985.5266 | 0.64 | 0 | 48 | 0.00013 | 1Score **> 31** indicates **identity** Score **> 22** indicates **homology** | U | R.MPELIDIR.Q |
| 13920 | 12 | – | 19 | 493.7710 | 985.5275 | 985.5266 | 0.88 | 0 | 49 | 0.00013 | 1Score **> 31** indicates **identity** Score **> 23** indicates **homology** | U | R.MPELIDIR.Q |
| 13921 | 12 | – | 19 | 493.7710 | 985.5275 | 985.5266 | 0.89 | 0 | 49 | 0.00013 | 1Score **> 31** indicates **identity** Score **> 23** indicates **homology** | U | R.MPELIDIR.Q |
| 13922 | 12 | – | 19 | 493.7712 | 985.5278 | 985.5266 | 1.17 | 0 | 42 | 0.00022 | 1Score **> 31** indicates **identity** Score **> 18** indicates **homology** | U | R.MPELIDIR.Q |
| 13923 | 12 | – | 19 | 493.7712 | 985.5278 | 985.5266 | 1.19 | 0 | 40 | 0.00019 | 1Score **> 31** indicates **identity** Score **> 15** indicates **homology** | U | R.MPELIDIR.Q |
| 13924 | 12 | – | 19 | 493.7714 | 985.5283 | 985.5266 | 1.73 | 0 | 35 | 0.0021 | 1Score **> 31** indicates **identity** Score **> 21** indicates **homology** | U | R.MPELIDIR.Q |
| 15046 | 12 | – | 19 | 501.7661 | 1001.5176 | 1001.5215 | -3.93 | 0 | 26 | 0.007 | 1Score **> 32** indicates **identity** Score **> 17** indicates **homology** | U | R.MPELIDIR.Q  + Oxidation (M) |
| 15050 | 12 | – | 19 | 501.7671 | 1001.5197 | 1001.5215 | -1.80 | 0 | 35 | 0.00053 | 1Score **> 33** indicates **identity** Score **> 15** indicates **homology** | U | R.MPELIDIR.Q  + Oxidation (M) |
| 15051 | 12 | – | 19 | 501.7671 | 1001.5197 | 1001.5215 | -1.80 | 0 | 37 | 0.00031 | 1Score **> 33** indicates **identity** Score **> 15** indicates **homology** | U | R.MPELIDIR.Q  + Oxidation (M) |
| 15052 | 12 | – | 19 | 501.7673 | 1001.5201 | 1001.5215 | -1.44 | 0 | 27 | 0.0032 | 1Score **> 33** indicates **identity** Score **> 14** indicates **homology** | U | R.MPELIDIR.Q  + Oxidation (M) |
| 15055 | 12 | – | 19 | 501.7681 | 1001.5217 | 1001.5215 | 0.15 | 0 | 35 | 0.00052 | 1Score **> 33** indicates **identity** Score **> 15** indicates **homology** | U | R.MPELIDIR.Q  + Oxidation (M) |
| 15056 | 12 | – | 19 | 501.7684 | 1001.5222 | 1001.5215 | 0.70 | 0 | 26 | 0.011 | 1Score **> 33** indicates **identity** Score **> 19** indicates **homology** | U | R.MPELIDIR.Q  + Oxidation (M) |
| 175655 | 24 | – | 49 | 959.5065 | 2875.4978 | 2875.4731 | 8.57 | 1 | 80 | 3.4e-08 | 1Score **> 37** indicates **identity** Score **> 17** indicates **homology** | U | R.TLPTNTLMGFGAFAALTTFWYATRPK.A |
| 158352 | 50 | – | 72 | 820.4146 | 2458.2218 | 2458.2196 | 0.91 | 1 | 22 | 0.0091 | 1Score **> 37** indicates **identity** Score **> 14** indicates **homology** | U | K.ALKPPCDLSMQSVEIAGTTDGIR.R |
| 158491 | 50 | – | 72 | 820.7426 | 2459.2060 | 2459.2036 | 1.00 | 1 | 27 | 0.0026 | 1Score **> 37** indicates **identity** Score **> 14** indicates **homology** | U | K.ALKPPCDLSMQSVEIAGTTDGIR.R  + Deamidated (NQ) |
| 165772 | 50 | – | 73 | 654.5868 | 2614.3183 | 2614.3207 | -0.92 | 2 | 31 | 0.0014 | 1Score **> 37** indicates **identity** Score **> 14** indicates **homology** | U | K.ALKPPCDLSMQSVEIAGTTDGIRR.S |
| 165773 | 50 | – | 73 | 872.4473 | 2614.3201 | 2614.3207 | -0.24 | 2 | 18 | 0.02 | 1Score **> 37** indicates **identity** Score **> 14** indicates **homology** | U | K.ALKPPCDLSMQSVEIAGTTDGIRR.S |
| 165774 | 50 | – | 73 | 654.5876 | 2614.3213 | 2614.3207 | 0.23 | 2 | 60 | 2.4e-06 | 1Score **> 37** indicates **identity** Score **> 16** indicates **homology** | U | K.ALKPPCDLSMQSVEIAGTTDGIRR.S |
| 165777 | 50 | – | 73 | 654.5881 | 2614.3234 | 2614.3207 | 1.04 | 2 | 47 | 3.7e-05 | 1Score **> 37** indicates **identity** Score **> 15** indicates **homology** | U | K.ALKPPCDLSMQSVEIAGTTDGIRR.S |
| 165779 | 50 | – | 73 | 654.5882 | 2614.3238 | 2614.3207 | 1.20 | 2 | 62 | 1.6e-06 | 1Score **> 37** indicates **identity** Score **> 16** indicates **homology** | U | K.ALKPPCDLSMQSVEIAGTTDGIRR.S |
| 165780 | 50 | – | 73 | 654.5885 | 2614.3250 | 2614.3207 | 1.63 | 2 | 53 | 9.8e-06 | 1Score **> 37** indicates **identity** Score **> 16** indicates **homology** | U | K.ALKPPCDLSMQSVEIAGTTDGIRR.S |
| 150722 | 73 | – | 91 | 583.8013 | 2331.1760 | 2331.1747 | 0.58 | 2 | 29 | 0.002 | 1Score **> 37** indicates **identity** Score **> 14** indicates **homology** | U | R.RSAVLEDDKLLVYYYDDVR.T |
| 139040 | 74 | – | 91 | 726.0257 | 2175.0553 | 2175.0735 | -8.37 | 1 | 65 | 7.7e-07 | 1Score **> 36** indicates **identity** Score **> 17** indicates **homology** | U | R.SAVLEDDKLLVYYYDDVR.T |
| 139045 | 74 | – | 91 | 1088.5357 | 2175.0569 | 2175.0735 | -7.63 | 1 | 18 | 0.026 | 1Score **> 36** indicates **identity** Score **> 15** indicates **homology** | U | R.SAVLEDDKLLVYYYDDVR.T |
| 139054 | 74 | – | 91 | 726.0278 | 2175.0614 | 2175.0735 | -5.56 | 1 | 65 | 8.3e-07 | 1Score **> 36** indicates **identity** Score **> 17** indicates **homology** | U | R.SAVLEDDKLLVYYYDDVR.T |
| 139057 | 74 | – | 91 | 726.0282 | 2175.0627 | 2175.0735 | -4.98 | 1 | 21 | 0.011 | 1Score **> 36** indicates **identity** Score **> 14** indicates **homology** | U | R.SAVLEDDKLLVYYYDDVR.T |
| 139058 | 74 | – | 91 | 726.0283 | 2175.0632 | 2175.0735 | -4.76 | 1 | 27 | 0.0032 | 1Score **> 36** indicates **identity** Score **> 14** indicates **homology** | U | R.SAVLEDDKLLVYYYDDVR.T |
| 139060 | 74 | – | 91 | 726.0286 | 2175.0638 | 2175.0735 | -4.47 | 1 | 44 | 6.7e-05 | 1Score **> 36** indicates **identity** Score **> 15** indicates **homology** | U | R.SAVLEDDKLLVYYYDDVR.T |
| 139061 | 74 | – | 91 | 1088.5393 | 2175.0640 | 2175.0735 | -4.40 | 1 | 38 | 0.00027 | 1Score **> 36** indicates **identity** Score **> 15** indicates **homology** | U | R.SAVLEDDKLLVYYYDDVR.T |
| 139064 | 74 | – | 91 | 726.0292 | 2175.0656 | 2175.0735 | -3.64 | 1 | 32 | 0.00098 | 1Score **> 36** indicates **identity** Score **> 15** indicates **homology** | U | R.SAVLEDDKLLVYYYDDVR.T |
| 139068 | 74 | – | 91 | 726.0296 | 2175.0669 | 2175.0735 | -3.08 | 1 | 26 | 0.0036 | 1Score **> 36** indicates **identity** Score **> 14** indicates **homology** | U | R.SAVLEDDKLLVYYYDDVR.T |
| 139069 | 74 | – | 91 | 726.0296 | 2175.0669 | 2175.0735 | -3.06 | 1 | 86 | 8.6e-09 | 1Score **> 36** indicates **identity** Score **> 18** indicates **homology** | U | R.SAVLEDDKLLVYYYDDVR.T |
| 139070 | 74 | – | 91 | 726.0296 | 2175.0669 | 2175.0735 | -3.05 | 1 | 62 | 1.5e-06 | 1Score **> 36** indicates **identity** Score **> 16** indicates **homology** | U | R.SAVLEDDKLLVYYYDDVR.T |
| 139071 | 74 | – | 91 | 726.0296 | 2175.0671 | 2175.0735 | -2.97 | 1 | 50 | 2.1e-05 | 1Score **> 36** indicates **identity** Score **> 16** indicates **homology** | U | R.SAVLEDDKLLVYYYDDVR.T |
| 139073 | 74 | – | 91 | 726.0299 | 2175.0678 | 2175.0735 | -2.63 | 1 | 18 | 0.021 | 1Score **> 36** indicates **identity** Score **> 14** indicates **homology** | U | R.SAVLEDDKLLVYYYDDVR.T |
| 139074 | 74 | – | 91 | 726.0299 | 2175.0680 | 2175.0735 | -2.55 | 1 | 40 | 0.0002 | 1Score **> 36** indicates **identity** Score **> 15** indicates **homology** | U | R.SAVLEDDKLLVYYYDDVR.T |
| 139075 | 74 | – | 91 | 1088.5413 | 2175.0681 | 2175.0735 | -2.49 | 1 | 66 | 7.1e-07 | 1Score **> 36** indicates **identity** Score **> 17** indicates **homology** | U | R.SAVLEDDKLLVYYYDDVR.T |
| 139077 | 74 | – | 91 | 726.0301 | 2175.0684 | 2175.0735 | -2.38 | 1 | 54 | 8.8e-06 | 1Score **> 36** indicates **identity** Score **> 16** indicates **homology** | U | R.SAVLEDDKLLVYYYDDVR.T |
| 139078 | 74 | – | 91 | 726.0301 | 2175.0684 | 2175.0735 | -2.37 | 1 | 50 | 2e-05 | 1Score **> 36** indicates **identity** Score **> 16** indicates **homology** | U | R.SAVLEDDKLLVYYYDDVR.T |
| 139080 | 74 | – | 91 | 726.0301 | 2175.0686 | 2175.0735 | -2.30 | 1 | 78 | 4.8e-08 | 1Score **> 36** indicates **identity** Score **> 17** indicates **homology** | U | R.SAVLEDDKLLVYYYDDVR.T |
| 139081 | 74 | – | 91 | 726.0302 | 2175.0687 | 2175.0735 | -2.21 | 1 | 29 | 0.0019 | 1Score **> 36** indicates **identity** Score **> 14** indicates **homology** | U | R.SAVLEDDKLLVYYYDDVR.T |
| 139082 | 74 | – | 91 | 726.0302 | 2175.0688 | 2175.0735 | -2.18 | 1 | 47 | 3.7e-05 | 1Score **> 36** indicates **identity** Score **> 15** indicates **homology** | U | R.SAVLEDDKLLVYYYDDVR.T |
| 139083 | 74 | – | 91 | 726.0302 | 2175.0689 | 2175.0735 | -2.15 | 1 | 74 | 1.1e-07 | 1Score **> 36** indicates **identity** Score **> 17** indicates **homology** | U | R.SAVLEDDKLLVYYYDDVR.T |
| 139084 | 74 | – | 91 | 726.0302 | 2175.0689 | 2175.0735 | -2.15 | 1 | 51 | 1.7e-05 | 1Score **> 36** indicates **identity** Score **> 16** indicates **homology** | U | R.SAVLEDDKLLVYYYDDVR.T |
| 139085 | 74 | – | 91 | 726.0302 | 2175.0689 | 2175.0735 | -2.13 | 1 | 77 | 5.9e-08 | 1Score **> 36** indicates **identity** Score **> 17** indicates **homology** | U | R.SAVLEDDKLLVYYYDDVR.T |
| 139086 | 74 | – | 91 | 1088.5421 | 2175.0696 | 2175.0735 | -1.81 | 1 | 84 | 1.2e-08 | 1Score **> 36** indicates **identity** Score **> 18** indicates **homology** | U | R.SAVLEDDKLLVYYYDDVR.T |
| 139087 | 74 | – | 91 | 726.0305 | 2175.0696 | 2175.0735 | -1.80 | 1 | 39 | 0.0002 | 1Score **> 36** indicates **identity** Score **> 15** indicates **homology** | U | R.SAVLEDDKLLVYYYDDVR.T |
| 139089 | 74 | – | 91 | 726.0306 | 2175.0699 | 2175.0735 | -1.66 | 1 | 50 | 2.3e-05 | 1Score **> 36** indicates **identity** Score **> 16** indicates **homology** | U | R.SAVLEDDKLLVYYYDDVR.T |
| 139090 | 74 | – | 91 | 726.0306 | 2175.0701 | 2175.0735 | -1.59 | 1 | 35 | 0.00052 | 1Score **> 36** indicates **identity** Score **> 15** indicates **homology** | U | R.SAVLEDDKLLVYYYDDVR.T |
| 139091 | 74 | – | 91 | 726.0307 | 2175.0702 | 2175.0735 | -1.54 | 1 | 36 | 0.00046 | 1Score **> 36** indicates **identity** Score **> 15** indicates **homology** | U | R.SAVLEDDKLLVYYYDDVR.T |
| 139092 | 74 | – | 91 | 1088.5425 | 2175.0704 | 2175.0735 | -1.47 | 1 | 49 | 2.3e-05 | 1Score **> 36** indicates **identity** Score **> 16** indicates **homology** | U | R.SAVLEDDKLLVYYYDDVR.T |
| 139094 | 74 | – | 91 | 726.0308 | 2175.0707 | 2175.0735 | -1.31 | 1 | 66 | 6.1e-07 | 1Score **> 36** indicates **identity** Score **> 17** indicates **homology** | U | R.SAVLEDDKLLVYYYDDVR.T |
| 139095 | 74 | – | 91 | 726.0309 | 2175.0708 | 2175.0735 | -1.28 | 1 | 66 | 6.4e-07 | 1Score **> 36** indicates **identity** Score **> 17** indicates **homology** | U | R.SAVLEDDKLLVYYYDDVR.T |
| 139097 | 74 | – | 91 | 726.0309 | 2175.0709 | 2175.0735 | -1.23 | 1 | 63 | 1.3e-06 | 1Score **> 36** indicates **identity** Score **> 16** indicates **homology** | U | R.SAVLEDDKLLVYYYDDVR.T |
| 139099 | 74 | – | 91 | 1088.5428 | 2175.0710 | 2175.0735 | -1.19 | 1 | 64 | 1e-06 | 1Score **> 36** indicates **identity** Score **> 16** indicates **homology** | U | R.SAVLEDDKLLVYYYDDVR.T |
| 139102 | 74 | – | 91 | 726.0312 | 2175.0717 | 2175.0735 | -0.84 | 1 | 66 | 7.1e-07 | 1Score **> 36** indicates **identity** Score **> 17** indicates **homology** | U | R.SAVLEDDKLLVYYYDDVR.T |
| 139103 | 74 | – | 91 | 726.0313 | 2175.0720 | 2175.0735 | -0.69 | 1 | 90 | 3.5e-09 | 1Score **> 36** indicates **identity** Score **> 18** indicates **homology** | U | R.SAVLEDDKLLVYYYDDVR.T |
| 139104 | 74 | – | 91 | 726.0314 | 2175.0723 | 2175.0735 | -0.59 | 1 | 60 | 2.5e-06 | 1Score **> 36** indicates **identity** Score **> 16** indicates **homology** | U | R.SAVLEDDKLLVYYYDDVR.T |
| 139105 | 74 | – | 91 | 1088.5436 | 2175.0727 | 2175.0735 | -0.39 | 1 | 103 | 2.4e-10 | 1Score **> 36** indicates **identity** Score **> 19** indicates **homology** | U | R.SAVLEDDKLLVYYYDDVR.T |
| 139106 | 74 | – | 91 | 726.0315 | 2175.0728 | 2175.0735 | -0.34 | 1 | 53 | 1e-05 | 1Score **> 36** indicates **identity** Score **> 16** indicates **homology** | U | R.SAVLEDDKLLVYYYDDVR.T |
| 139108 | 74 | – | 91 | 726.0316 | 2175.0730 | 2175.0735 | -0.26 | 1 | 66 | 6.2e-07 | 1Score **> 36** indicates **identity** Score **> 17** indicates **homology** | U | R.SAVLEDDKLLVYYYDDVR.T |
| 139109 | 74 | – | 91 | 726.0316 | 2175.0730 | 2175.0735 | -0.25 | 1 | 24 | 0.0055 | 1Score **> 36** indicates **identity** Score **> 14** indicates **homology** | U | R.SAVLEDDKLLVYYYDDVR.T |
| 139111 | 74 | – | 91 | 726.0317 | 2175.0731 | 2175.0735 | -0.19 | 1 | 35 | 0.00055 | 1Score **> 36** indicates **identity** Score **> 15** indicates **homology** | U | R.SAVLEDDKLLVYYYDDVR.T |
| 139112 | 74 | – | 91 | 726.0317 | 2175.0731 | 2175.0735 | -0.18 | 1 | 37 | 0.00033 | 1Score **> 36** indicates **identity** Score **> 15** indicates **homology** | U | R.SAVLEDDKLLVYYYDDVR.T |
| 139114 | 74 | – | 91 | 1088.5439 | 2175.0732 | 2175.0735 | -0.14 | 1 | 105 | 1.5e-10 | 1Score **> 36** indicates **identity** Score **> 19** indicates **homology** | U | R.SAVLEDDKLLVYYYDDVR.T |
| 139115 | 74 | – | 91 | 726.0317 | 2175.0734 | 2175.0735 | -0.078 | 1 | 20 | 0.013 | 1Score **> 36** indicates **identity** Score **> 14** indicates **homology** | U | R.SAVLEDDKLLVYYYDDVR.T |
| 139116 | 74 | – | 91 | 1088.5440 | 2175.0735 | 2175.0735 | -0.024 | 1 | 105 | 1.5e-10 | 1Score **> 36** indicates **identity** Score **> 19** indicates **homology** | U | R.SAVLEDDKLLVYYYDDVR.T |
| 139117 | 74 | – | 91 | 726.0318 | 2175.0735 | 2175.0735 | -0.013 | 1 | 52 | 1.2e-05 | 1Score **> 36** indicates **identity** Score **> 16** indicates **homology** | U | R.SAVLEDDKLLVYYYDDVR.T |
| 139118 | 74 | – | 91 | 1088.5440 | 2175.0735 | 2175.0735 | -0.0032 | 1 | 32 | 0.0011 | 1Score **> 36** indicates **identity** Score **> 15** indicates **homology** | U | R.SAVLEDDKLLVYYYDDVR.T |
| 139119 | 74 | – | 91 | 726.0318 | 2175.0737 | 2175.0735 | 0.075 | 1 | 54 | 9.2e-06 | 1Score **> 36** indicates **identity** Score **> 16** indicates **homology** | U | R.SAVLEDDKLLVYYYDDVR.T |
| 139120 | 74 | – | 91 | 726.0318 | 2175.0737 | 2175.0735 | 0.076 | 1 | 24 | 0.0055 | 1Score **> 36** indicates **identity** Score **> 14** indicates **homology** | U | R.SAVLEDDKLLVYYYDDVR.T |
| 139121 | 74 | – | 91 | 1088.5442 | 2175.0738 | 2175.0735 | 0.13 | 1 | 103 | 2.4e-10 | 1Score **> 36** indicates **identity** Score **> 19** indicates **homology** | U | R.SAVLEDDKLLVYYYDDVR.T |
| 139122 | 74 | – | 91 | 726.0319 | 2175.0740 | 2175.0735 | 0.19 | 1 | 16 | 0.035 | 1Score **> 36** indicates **identity** Score **> 13** indicates **homology** | U | R.SAVLEDDKLLVYYYDDVR.T |
| 139123 | 74 | – | 91 | 726.0319 | 2175.0740 | 2175.0735 | 0.22 | 1 | 19 | 0.016 | 1Score **> 36** indicates **identity** Score **> 14** indicates **homology** | U | R.SAVLEDDKLLVYYYDDVR.T |
| 139124 | 74 | – | 91 | 1088.5444 | 2175.0742 | 2175.0735 | 0.29 | 1 | 96 | 9.6e-10 | 1Score **> 36** indicates **identity** Score **> 19** indicates **homology** | U | R.SAVLEDDKLLVYYYDDVR.T |
| 139127 | 74 | – | 91 | 726.0321 | 2175.0744 | 2175.0735 | 0.38 | 1 | 31 | 0.0013 | 1Score **> 36** indicates **identity** Score **> 14** indicates **homology** | U | R.SAVLEDDKLLVYYYDDVR.T |
| 139130 | 74 | – | 91 | 726.0321 | 2175.0746 | 2175.0735 | 0.49 | 1 | 32 | 0.00092 | 1Score **> 36** indicates **identity** Score **> 15** indicates **homology** | U | R.SAVLEDDKLLVYYYDDVR.T |
| 139132 | 74 | – | 91 | 1088.5447 | 2175.0748 | 2175.0735 | 0.60 | 1 | 15 | 0.036 | 1Score **> 36** indicates **identity** Score **> 13** indicates **homology** | U | R.SAVLEDDKLLVYYYDDVR.T |
| 139134 | 74 | – | 91 | 726.0323 | 2175.0750 | 2175.0735 | 0.67 | 1 | 30 | 0.0017 | 1Score **> 36** indicates **identity** Score **> 14** indicates **homology** | U | R.SAVLEDDKLLVYYYDDVR.T |
| 139135 | 74 | – | 91 | 726.0324 | 2175.0753 | 2175.0735 | 0.80 | 1 | 55 | 7.7e-06 | 1Score **> 36** indicates **identity** Score **> 16** indicates **homology** | U | R.SAVLEDDKLLVYYYDDVR.T |
| 139136 | 74 | – | 91 | 726.0324 | 2175.0754 | 2175.0735 | 0.85 | 1 | 66 | 6.3e-07 | 1Score **> 36** indicates **identity** Score **> 17** indicates **homology** | U | R.SAVLEDDKLLVYYYDDVR.T |
| 139137 | 74 | – | 91 | 726.0325 | 2175.0757 | 2175.0735 | 0.97 | 1 | 24 | 0.0053 | 1Score **> 36** indicates **identity** Score **> 14** indicates **homology** | U | R.SAVLEDDKLLVYYYDDVR.T |
| 139138 | 74 | – | 91 | 726.0326 | 2175.0759 | 2175.0735 | 1.10 | 1 | 91 | 3.2e-09 | 1Score **> 36** indicates **identity** Score **> 18** indicates **homology** | U | R.SAVLEDDKLLVYYYDDVR.T |
| 139139 | 74 | – | 91 | 726.0326 | 2175.0761 | 2175.0735 | 1.16 | 1 | 33 | 0.00074 | 1Score **> 36** indicates **identity** Score **> 15** indicates **homology** | U | R.SAVLEDDKLLVYYYDDVR.T |
| 139140 | 74 | – | 91 | 1088.5453 | 2175.0761 | 2175.0735 | 1.19 | 1 | 98 | 6.5e-10 | 1Score **> 36** indicates **identity** Score **> 19** indicates **homology** | U | R.SAVLEDDKLLVYYYDDVR.T |
| 139141 | 74 | – | 91 | 726.0327 | 2175.0764 | 2175.0735 | 1.30 | 1 | 19 | 0.016 | 1Score **> 36** indicates **identity** Score **> 14** indicates **homology** | U | R.SAVLEDDKLLVYYYDDVR.T |
| 139142 | 74 | – | 91 | 726.0328 | 2175.0766 | 2175.0735 | 1.42 | 1 | 44 | 7.2e-05 | 1Score **> 36** indicates **identity** Score **> 15** indicates **homology** | U | R.SAVLEDDKLLVYYYDDVR.T |
| 139143 | 74 | – | 91 | 726.0330 | 2175.0772 | 2175.0735 | 1.68 | 1 | 31 | 0.0013 | 1Score **> 36** indicates **identity** Score **> 14** indicates **homology** | U | R.SAVLEDDKLLVYYYDDVR.T |
| 139144 | 74 | – | 91 | 726.0331 | 2175.0776 | 2175.0735 | 1.86 | 1 | 23 | 0.0069 | 1Score **> 36** indicates **identity** Score **> 14** indicates **homology** | U | R.SAVLEDDKLLVYYYDDVR.T |
| 139145 | 74 | – | 91 | 1088.5461 | 2175.0777 | 2175.0735 | 1.93 | 1 | 84 | 1.2e-08 | 1Score **> 36** indicates **identity** Score **> 18** indicates **homology** | U | R.SAVLEDDKLLVYYYDDVR.T |
| 139146 | 74 | – | 91 | 726.0332 | 2175.0778 | 2175.0735 | 1.96 | 1 | 27 | 0.0027 | 1Score **> 36** indicates **identity** Score **> 14** indicates **homology** | U | R.SAVLEDDKLLVYYYDDVR.T |
| 139147 | 74 | – | 91 | 726.0332 | 2175.0779 | 2175.0735 | 2.00 | 1 | 43 | 9.8e-05 | 1Score **> 36** indicates **identity** Score **> 15** indicates **homology** | U | R.SAVLEDDKLLVYYYDDVR.T |
| 139148 | 74 | – | 91 | 726.0333 | 2175.0782 | 2175.0735 | 2.15 | 1 | 34 | 0.0007 | 1Score **> 36** indicates **identity** Score **> 15** indicates **homology** | U | R.SAVLEDDKLLVYYYDDVR.T |
| 139153 | 74 | – | 91 | 726.0362 | 2175.0869 | 2175.0735 | 6.13 | 1 | 40 | 0.00018 | 1Score **> 37** indicates **identity** Score **> 15** indicates **homology** | U | R.SAVLEDDKLLVYYYDDVR.T |
| 139154 | 74 | – | 91 | 726.0364 | 2175.0872 | 2175.0735 | 6.29 | 1 | 19 | 0.017 | 1Score **> 37** indicates **identity** Score **> 14** indicates **homology** | U | R.SAVLEDDKLLVYYYDDVR.T |
| 46694 | 82 | – | 91 | 659.8394 | 1317.6643 | 1317.6605 | 2.86 | 0 | 24 | 0.0059 | 1Score **> 33** indicates **identity** Score **> 14** indicates **homology** | U | K.LLVYYYDDVR.T |
| 15951 | 92 | – | 99 | 509.2257 | 1016.4368 | 1016.4386 | -1.74 | 0 | 17 | 0.023 | 1Score **> 24** indicates **identity** Score **> 14** indicates **homology** | U | R.TMYDGFQR.G |
| 61861 | 100 | – | 113 | 729.8579 | 1457.7013 | 1457.7045 | -2.20 | 0 | 82 | 1.9e-08 | 1Score **> 32** indicates **identity** Score **> 18** indicates **homology** | U | R.GIQVSNNGPCLGSR.K |
| 61862 | 100 | – | 113 | 729.8582 | 1457.7018 | 1457.7045 | -1.84 | 0 | 71 | 2.3e-07 | 1Score **> 32** indicates **identity** Score **> 17** indicates **homology** | U | R.GIQVSNNGPCLGSR.K |
| 61864 | 100 | – | 113 | 729.8587 | 1457.7028 | 1457.7045 | -1.16 | 0 | 78 | 5.4e-08 | 1Score **> 32** indicates **identity** Score **> 17** indicates **homology** | U | R.GIQVSNNGPCLGSR.K |
| 61865 | 100 | – | 113 | 729.8588 | 1457.7030 | 1457.7045 | -1.02 | 0 | 70 | 2.5e-07 | 1Score **> 32** indicates **identity** Score **> 17** indicates **homology** | U | R.GIQVSNNGPCLGSR.K |
| 61867 | 100 | – | 113 | 729.8593 | 1457.7040 | 1457.7045 | -0.34 | 0 | 77 | 6.6e-08 | 1Score **> 32** indicates **identity** Score **> 17** indicates **homology** | U | R.GIQVSNNGPCLGSR.K |
| 61868 | 100 | – | 113 | 729.8593 | 1457.7040 | 1457.7045 | -0.33 | 0 | 106 | 1.3e-10 | 1Score **> 32** indicates **identity** Score **> 19** indicates **homology** | U | R.GIQVSNNGPCLGSR.K |
| 61869 | 100 | – | 113 | 729.8594 | 1457.7042 | 1457.7045 | -0.23 | 0 | 86 | 8.5e-09 | 1Score **> 32** indicates **identity** Score **> 18** indicates **homology** | U | R.GIQVSNNGPCLGSR.K |
| 61872 | 100 | – | 113 | 729.8599 | 1457.7052 | 1457.7045 | 0.50 | 0 | 50 | 2.1e-05 | 1Score **> 32** indicates **identity** Score **> 16** indicates **homology** | U | R.GIQVSNNGPCLGSR.K |
| 61956 | 100 | – | 113 | 730.3493 | 1458.6841 | 1458.6885 | -3.00 | 0 | 34 | 0.00085 | 1Score **> 31** indicates **identity** Score **> 16** indicates **homology** | U | R.GIQVSNNGPCLGSR.K  + Deamidated (NQ) |
| 61957 | 100 | – | 113 | 730.3507 | 1458.6868 | 1458.6885 | -1.18 | 0 | 82 | 2.1e-08 | 1Score **> 31** indicates **identity** Score **> 18** indicates **homology** | U | R.GIQVSNNGPCLGSR.K  + Deamidated (NQ) |
| 61959 | 100 | – | 113 | 730.3512 | 1458.6879 | 1458.6885 | -0.40 | 0 | 87 | 7.2e-09 | 1Score **> 31** indicates **identity** Score **> 18** indicates **homology** | U | R.GIQVSNNGPCLGSR.K  + Deamidated (NQ) |
| 61962 | 100 | – | 113 | 730.3528 | 1458.6910 | 1458.6885 | 1.70 | 0 | 75 | 8.8e-08 | 1Score **> 31** indicates **identity** Score **> 17** indicates **homology** | U | R.GIQVSNNGPCLGSR.K  + Deamidated (NQ) |
| 61967 | 100 | – | 113 | 730.3564 | 1458.6982 | 1458.6885 | 6.62 | 0 | 44 | 7e-05 | 1Score **> 32** indicates **identity** Score **> 15** indicates **homology** | U | R.GIQVSNNGPCLGSR.K  + Deamidated (NQ) |
| 73491 | 114 | – | 125 | 776.8919 | 1551.7692 | 1551.7721 | -1.86 | 1 | 69 | 3.1e-07 | 1Score **> 34** indicates **identity** Score **> 17** indicates **homology** | U | R.KPNQPYEWISYK.E |
| 73492 | 114 | – | 125 | 776.8932 | 1551.7718 | 1551.7721 | -0.24 | 1 | 86 | 2.1e-08 | 1Score **> 34** indicates **identity** Score **> 22** indicates **homology** | U | R.KPNQPYEWISYK.E |
| 73493 | 114 | – | 125 | 518.2646 | 1551.7720 | 1551.7721 | -0.12 | 1 | 35 | 0.0005 | 1Score **> 34** indicates **identity** Score **> 15** indicates **homology** | U | R.KPNQPYEWISYK.E |
| 73494 | 114 | – | 125 | 776.8933 | 1551.7721 | 1551.7721 | -0.035 | 1 | 88 | 5.4e-09 | 1Score **> 34** indicates **identity** Score **> 18** indicates **homology** | U | R.KPNQPYEWISYK.E |
| 73495 | 114 | – | 125 | 518.2648 | 1551.7727 | 1551.7721 | 0.34 | 1 | 36 | 0.0004 | 1Score **> 34** indicates **identity** Score **> 15** indicates **homology** | U | R.KPNQPYEWISYK.E |
| 73497 | 114 | – | 125 | 776.8939 | 1551.7732 | 1551.7721 | 0.71 | 1 | 39 | 0.00023 | 1Score **> 34** indicates **identity** Score **> 15** indicates **homology** | U | R.KPNQPYEWISYK.E |
| 184203 | 114 | – | 141 | 813.4176 | 3249.6411 | 3249.6379 | 0.98 | 2 | 62 | 1.4e-06 | 1Score **> 38** indicates **identity** Score **> 16** indicates **homology** | U | R.KPNQPYEWISYKEVAELAECIGSGLIQK.G |
| 184204 | 114 | – | 141 | 813.4178 | 3249.6422 | 3249.6379 | 1.32 | 2 | 59 | 2.7e-06 | 1Score **> 38** indicates **identity** Score **> 16** indicates **homology** | U | R.KPNQPYEWISYKEVAELAECIGSGLIQK.G |
| 184205 | 114 | – | 141 | 1084.2214 | 3249.6424 | 3249.6379 | 1.36 | 2 | 112 | 2.9e-11 | 1Score **> 38** indicates **identity** Score **> 20** indicates **homology** | U | R.KPNQPYEWISYKEVAELAECIGSGLIQK.G |
| 184216 | 114 | – | 141 | 1084.5577 | 3250.6512 | 3250.6219 | 9.01 | 2 | 57 | 4.9e-06 | 1Score **> 38** indicates **identity** Score **> 16** indicates **homology** | U | R.KPNQPYEWISYKEVAELAECIGSGLIQK.G  + Deamidated (NQ) |
| 94980 | 126 | – | 141 | 858.9447 | 1715.8749 | 1715.8764 | -0.84 | 0 | 54 | 8.8e-06 | 1Score **> 35** indicates **identity** Score **> 16** indicates **homology** | U | K.EVAELAECIGSGLIQK.G |
| 94982 | 126 | – | 141 | 572.9662 | 1715.8767 | 1715.8764 | 0.19 | 0 | 47 | 3.5e-05 | 1Score **> 35** indicates **identity** Score **> 15** indicates **homology** | U | K.EVAELAECIGSGLIQK.G |
| 94983 | 126 | – | 141 | 572.9664 | 1715.8775 | 1715.8764 | 0.65 | 0 | 39 | 0.00022 | 1Score **> 35** indicates **identity** Score **> 15** indicates **homology** | U | K.EVAELAECIGSGLIQK.G |
| 94984 | 126 | – | 141 | 572.9666 | 1715.8780 | 1715.8764 | 0.98 | 0 | 21 | 0.012 | 1Score **> 35** indicates **identity** Score **> 14** indicates **homology** | U | K.EVAELAECIGSGLIQK.G |
| 94986 | 126 | – | 141 | 858.9466 | 1715.8787 | 1715.8764 | 1.38 | 0 | 74 | 2.2e-07 | 1Score **> 35** indicates **identity** Score **> 20** indicates **homology** | U | K.EVAELAECIGSGLIQK.G |
| 195929 | 142 | – | 193 | 1218.6110 | 6088.0188 | 6088.0029 | 2.61 | 2 | 24 | 0.0051 | 1Score **> 35** indicates **identity** Score **> 14** indicates **homology** | U | K.GFKPCSEQFIGLFSQNRPEWVIVEQGCFSYSMVVVPLYDTLGADAITYIVNK.A  + Deamidated (NQ); HNE (C); Oxidation (M) |
| 60448 | 194 | – | 206 | 482.9327 | 1445.7763 | 1445.7766 | -0.20 | 1 | 45 | 6.1e-05 | 1Score **> 35** indicates **identity** Score **> 15** indicates **homology** | U | K.AELSVIFADKPEK.A |
| 60449 | 194 | – | 206 | 482.9328 | 1445.7765 | 1445.7766 | -0.058 | 1 | 48 | 3e-05 | 1Score **> 35** indicates **identity** Score **> 16** indicates **homology** | U | K.AELSVIFADKPEK.A |
| 60450 | 194 | – | 206 | 723.8956 | 1445.7767 | 1445.7766 | 0.071 | 1 | 68 | 4.2e-07 | 1Score **> 35** indicates **identity** Score **> 17** indicates **homology** | U | K.AELSVIFADKPEK.A |
| 60451 | 194 | – | 206 | 723.8957 | 1445.7769 | 1445.7766 | 0.21 | 1 | 50 | 2e-05 | 1Score **> 35** indicates **identity** Score **> 16** indicates **homology** | U | K.AELSVIFADKPEK.A |
| 60452 | 194 | – | 206 | 482.9330 | 1445.7771 | 1445.7766 | 0.34 | 1 | 53 | 1e-05 | 1Score **> 35** indicates **identity** Score **> 16** indicates **homology** | U | K.AELSVIFADKPEK.A |
| 60453 | 194 | – | 206 | 723.8958 | 1445.7771 | 1445.7766 | 0.34 | 1 | 68 | 4.4e-07 | 1Score **> 35** indicates **identity** Score **> 17** indicates **homology** | U | K.AELSVIFADKPEK.A |
| 60454 | 194 | – | 206 | 482.9332 | 1445.7777 | 1445.7766 | 0.79 | 1 | 49 | 2.4e-05 | 1Score **> 35** indicates **identity** Score **> 16** indicates **homology** | U | K.AELSVIFADKPEK.A |
| 60455 | 194 | – | 206 | 723.8962 | 1445.7778 | 1445.7766 | 0.85 | 1 | 44 | 7.6e-05 | 1Score **> 35** indicates **identity** Score **> 15** indicates **homology** | U | K.AELSVIFADKPEK.A |
| 60456 | 194 | – | 206 | 482.9332 | 1445.7778 | 1445.7766 | 0.87 | 1 | 48 | 3.1e-05 | 1Score **> 35** indicates **identity** Score **> 16** indicates **homology** | U | K.AELSVIFADKPEK.A |
| 60457 | 194 | – | 206 | 723.8962 | 1445.7778 | 1445.7766 | 0.89 | 1 | 44 | 7.7e-05 | 1Score **> 35** indicates **identity** Score **> 15** indicates **homology** | U | K.AELSVIFADKPEK.A |
| 60458 | 194 | – | 206 | 482.9333 | 1445.7781 | 1445.7766 | 1.06 | 1 | 34 | 0.00061 | 1Score **> 35** indicates **identity** Score **> 15** indicates **homology** | U | K.AELSVIFADKPEK.A |
| 34797 | 207 | – | 217 | 405.2434 | 1212.7085 | 1212.7077 | 0.60 | 1 | 21 | 0.012 | 1Score **> 32** indicates **identity** Score **> 14** indicates **homology** | U | K.AKLLLEGVENK.L |
| 116293 | 207 | – | 223 | 642.7092 | 1925.1057 | 1925.1019 | 1.96 | 2 | 21 | 0.011 | 1Score **> 32** indicates **identity** Score **> 14** indicates **homology** | U | K.AKLLLEGVENKLTPCLK.I |
| 15788 | 209 | – | 217 | 507.7952 | 1013.5759 | 1013.5757 | 0.17 | 0 | 57 | 0.00012 | 1Score **> 31** indicates **identity** | U | K.LLLEGVENK.L |
| 15789 | 209 | – | 217 | 507.7952 | 1013.5759 | 1013.5757 | 0.22 | 0 | 55 | 0.00019 | 1Score **> 31** indicates **identity** | U | K.LLLEGVENK.L |
| 15791 | 209 | – | 217 | 507.7960 | 1013.5774 | 1013.5757 | 1.69 | 0 | 34 | 0.036 | 1Score **> 32** indicates **identity** | U | K.LLLEGVENK.L |
| 96247 | 209 | – | 223 | 863.9918 | 1725.9690 | 1725.9699 | -0.53 | 1 | 70 | 4.5e-07 | 1Score **> 33** indicates **identity** Score **> 19** indicates **homology** | U | K.LLLEGVENKLTPCLK.I |
| 96248 | 209 | – | 223 | 576.3303 | 1725.9691 | 1725.9699 | -0.45 | 1 | 59 | 2.8e-06 | 1Score **> 33** indicates **identity** Score **> 16** indicates **homology** | U | K.LLLEGVENKLTPCLK.I |
| 96249 | 209 | – | 223 | 863.9920 | 1725.9694 | 1725.9699 | -0.25 | 1 | 65 | 1e-06 | 1Score **> 33** indicates **identity** Score **> 17** indicates **homology** | U | K.LLLEGVENKLTPCLK.I |
| 96250 | 209 | – | 223 | 576.3309 | 1725.9708 | 1725.9699 | 0.53 | 1 | 65 | 2.2e-06 | 1Score **> 34** indicates **identity** Score **> 21** indicates **homology** | U | K.LLLEGVENKLTPCLK.I |
| 96253 | 209 | – | 223 | 576.3315 | 1725.9725 | 1725.9699 | 1.54 | 1 | 35 | 0.0005 | 1Score **> 34** indicates **identity** Score **> 15** indicates **homology** | U | K.LLLEGVENKLTPCLK.I |
| 96254 | 209 | – | 223 | 576.3316 | 1725.9729 | 1725.9699 | 1.76 | 1 | 17 | 0.048 | 1Score **> 34** indicates **identity** Score **> 16** indicates **homology** | U | K.LLLEGVENKLTPCLK.I |
| 96256 | 209 | – | 223 | 576.3323 | 1725.9749 | 1725.9699 | 2.94 | 1 | 51 | 1.8e-05 | 1Score **> 34** indicates **identity** Score **> 16** indicates **homology** | U | K.LLLEGVENKLTPCLK.I |
| 94109 | 224 | – | 238 | 855.4395 | 1708.8644 | 1708.8706 | -3.63 | 0 | 82 | 1.9e-08 | 1Score **> 35** indicates **identity** Score **> 18** indicates **homology** | U | K.IIVIMDSYGSDLVER.G |
| 94117 | 224 | – | 238 | 855.4411 | 1708.8676 | 1708.8706 | -1.74 | 0 | 44 | 8e-05 | 1Score **> 35** indicates **identity** Score **> 15** indicates **homology** | U | K.IIVIMDSYGSDLVER.G |
| 94122 | 224 | – | 238 | 855.4434 | 1708.8723 | 1708.8706 | 0.99 | 0 | 73 | 1.5e-07 | 1Score **> 35** indicates **identity** Score **> 17** indicates **homology** | U | K.IIVIMDSYGSDLVER.G |
| 94124 | 224 | – | 238 | 855.4443 | 1708.8741 | 1708.8706 | 2.06 | 0 | 61 | 2e-06 | 1Score **> 35** indicates **identity** Score **> 16** indicates **homology** | U | K.IIVIMDSYGSDLVER.G |
| 94125 | 224 | – | 238 | 855.4446 | 1708.8746 | 1708.8706 | 2.34 | 0 | 97 | 8.5e-10 | 1Score **> 35** indicates **identity** Score **> 19** indicates **homology** | U | K.IIVIMDSYGSDLVER.G |
| 94126 | 224 | – | 238 | 855.4453 | 1708.8760 | 1708.8706 | 3.18 | 0 | 59 | 2.7e-06 | 1Score **> 35** indicates **identity** Score **> 16** indicates **homology** | U | K.IIVIMDSYGSDLVER.G |
| 94128 | 224 | – | 238 | 855.4457 | 1708.8769 | 1708.8706 | 3.69 | 0 | 66 | 6.5e-07 | 1Score **> 35** indicates **identity** Score **> 17** indicates **homology** | U | K.IIVIMDSYGSDLVER.G |
| 94129 | 224 | – | 238 | 855.4461 | 1708.8777 | 1708.8706 | 4.19 | 0 | 65 | 7.4e-07 | 1Score **> 35** indicates **identity** Score **> 17** indicates **homology** | U | K.IIVIMDSYGSDLVER.G |
| 94130 | 224 | – | 238 | 855.4467 | 1708.8788 | 1708.8706 | 4.80 | 0 | 59 | 2.7e-06 | 1Score **> 35** indicates **identity** Score **> 16** indicates **homology** | U | K.IIVIMDSYGSDLVER.G |
| 94135 | 224 | – | 238 | 855.4487 | 1708.8828 | 1708.8706 | 7.14 | 0 | 83 | 1.6e-08 | 1Score **> 35** indicates **identity** Score **> 18** indicates **homology** | U | K.IIVIMDSYGSDLVER.G |
| 16051 | 242 | – | 250 | 509.7837 | 1017.5528 | 1017.5529 | -0.088 | 0 | 58 | 1.2e-05 | 1Score **> 33** indicates **identity** Score **> 21** indicates **homology** | U | K.CGVEIISLK.A |
| 16052 | 242 | – | 250 | 509.7838 | 1017.5531 | 1017.5529 | 0.25 | 0 | 51 | 2.3e-05 | 1Score **> 33** indicates **identity** Score **> 18** indicates **homology** | U | K.CGVEIISLK.A |
| 16053 | 242 | – | 250 | 509.7839 | 1017.5533 | 1017.5529 | 0.42 | 0 | 59 | 9.6e-06 | 1Score **> 33** indicates **identity** Score **> 21** indicates **homology** | U | K.CGVEIISLK.A |
| 16054 | 242 | – | 250 | 509.7842 | 1017.5538 | 1017.5529 | 0.88 | 0 | 65 | 4.7e-06 | 1Score **> 33** indicates **identity** Score **> 24** indicates **homology** | U | K.CGVEIISLK.A |
| 16055 | 242 | – | 250 | 509.7843 | 1017.5540 | 1017.5529 | 1.16 | 0 | 67 | 3.5e-06 | 1Score **> 34** indicates **identity** Score **> 25** indicates **homology** | U | K.CGVEIISLK.A |
| 16056 | 242 | – | 250 | 509.7843 | 1017.5541 | 1017.5529 | 1.26 | 0 | 67 | 3.4e-06 | 1Score **> 34** indicates **identity** Score **> 25** indicates **homology** | U | K.CGVEIISLK.A |
| 16057 | 242 | – | 250 | 509.7845 | 1017.5544 | 1017.5529 | 1.53 | 0 | 51 | 4.6e-05 | 1Score **> 34** indicates **identity** Score **> 20** indicates **homology** | U | K.CGVEIISLK.A |
| 101465 | 242 | – | 257 | 591.6607 | 1771.9602 | 1771.9502 | 5.66 | 1 | 42 | 0.00011 | 1Score **> 35** indicates **identity** Score **> 15** indicates **homology** | U | K.CGVEIISLKALEDLGR.V |
| 2651 | 251 | – | 257 | 387.2110 | 772.4075 | 772.4079 | -0.51 | 0 | 30 | 0.025 | 1Score **> 26** indicates **identity** | U | K.ALEDLGR.V |
| 2652 | 251 | – | 257 | 387.2111 | 772.4076 | 772.4079 | -0.37 | 0 | 30 | 0.026 | 1Score **> 27** indicates **identity** | U | K.ALEDLGR.V |
| 2653 | 251 | – | 257 | 387.2111 | 772.4076 | 772.4079 | -0.34 | 0 | 33 | 0.012 | 1Score **> 27** indicates **identity** | U | K.ALEDLGR.V |
| 2654 | 251 | – | 257 | 387.2111 | 772.4077 | 772.4079 | -0.31 | 0 | 46 | 0.00069 | 1Score **> 27** indicates **identity** | U | K.ALEDLGR.V |
| 2655 | 251 | – | 257 | 387.2111 | 772.4077 | 772.4079 | -0.29 | 0 | 49 | 0.00033 | 1Score **> 27** indicates **identity** | U | K.ALEDLGR.V |
| 27225 | 251 | – | 260 | 381.5474 | 1141.6202 | 1141.6203 | -0.088 | 1 | 28 | 0.025 | 1Score **> 32** indicates **identity** Score **> 24** indicates **homology** | U | K.ALEDLGRVNR.V |
| 169153 | 261 | – | 285 | 674.8547 | 2695.3896 | 2695.3891 | 0.18 | 2 | 35 | 0.00058 | 1Score **> 37** indicates **identity** Score **> 15** indicates **homology** | U | R.VKPKPPEPEDLAIICFTSGTTGNPK.G |
| 169154 | 261 | – | 285 | 674.8548 | 2695.3901 | 2695.3891 | 0.36 | 2 | 38 | 0.00029 | 1Score **> 37** indicates **identity** Score **> 15** indicates **homology** | U | R.VKPKPPEPEDLAIICFTSGTTGNPK.G |
| 169156 | 261 | – | 285 | 674.8550 | 2695.3907 | 2695.3891 | 0.60 | 2 | 37 | 0.00034 | 1Score **> 37** indicates **identity** Score **> 15** indicates **homology** | U | R.VKPKPPEPEDLAIICFTSGTTGNPK.G |
| 169157 | 261 | – | 285 | 674.8550 | 2695.3908 | 2695.3891 | 0.65 | 2 | 31 | 0.0013 | 1Score **> 37** indicates **identity** Score **> 14** indicates **homology** | U | R.VKPKPPEPEDLAIICFTSGTTGNPK.G |
| 169158 | 261 | – | 285 | 899.4709 | 2695.3909 | 2695.3891 | 0.68 | 2 | 54 | 8.4e-06 | 1Score **> 37** indicates **identity** Score **> 16** indicates **homology** | U | R.VKPKPPEPEDLAIICFTSGTTGNPK.G |
| 169159 | 261 | – | 285 | 674.8551 | 2695.3911 | 2695.3891 | 0.75 | 2 | 40 | 0.00017 | 1Score **> 37** indicates **identity** Score **> 15** indicates **homology** | U | R.VKPKPPEPEDLAIICFTSGTTGNPK.G |
| 169160 | 261 | – | 285 | 674.8551 | 2695.3912 | 2695.3891 | 0.78 | 2 | 28 | 0.0026 | 1Score **> 37** indicates **identity** Score **> 15** indicates **homology** | U | R.VKPKPPEPEDLAIICFTSGTTGNPK.G |
| 169161 | 261 | – | 285 | 899.4711 | 2695.3914 | 2695.3891 | 0.84 | 2 | 55 | 6.8e-06 | 1Score **> 37** indicates **identity** Score **> 16** indicates **homology** | U | R.VKPKPPEPEDLAIICFTSGTTGNPK.G |
| 169162 | 261 | – | 285 | 674.8552 | 2695.3915 | 2695.3891 | 0.90 | 2 | 31 | 0.0012 | 1Score **> 37** indicates **identity** Score **> 15** indicates **homology** | U | R.VKPKPPEPEDLAIICFTSGTTGNPK.G |
| 169164 | 261 | – | 285 | 899.4711 | 2695.3916 | 2695.3891 | 0.93 | 2 | 46 | 4.8e-05 | 1Score **> 37** indicates **identity** Score **> 15** indicates **homology** | U | R.VKPKPPEPEDLAIICFTSGTTGNPK.G |
| 169165 | 261 | – | 285 | 1348.7031 | 2695.3916 | 2695.3891 | 0.93 | 2 | 19 | 0.017 | 1Score **> 37** indicates **identity** Score **> 14** indicates **homology** | U | R.VKPKPPEPEDLAIICFTSGTTGNPK.G |
| 169166 | 261 | – | 285 | 899.4714 | 2695.3924 | 2695.3891 | 1.21 | 2 | 53 | 1.7e-05 | 1Score **> 37** indicates **identity** Score **> 18** indicates **homology** | U | R.VKPKPPEPEDLAIICFTSGTTGNPK.G |
| 169167 | 261 | – | 285 | 899.4716 | 2695.3929 | 2695.3891 | 1.41 | 2 | 46 | 4.7e-05 | 1Score **> 37** indicates **identity** Score **> 15** indicates **homology** | U | R.VKPKPPEPEDLAIICFTSGTTGNPK.G |
| 169168 | 261 | – | 285 | 674.8556 | 2695.3933 | 2695.3891 | 1.57 | 2 | 15 | 0.042 | 1Score **> 37** indicates **identity** Score **> 13** indicates **homology** | U | R.VKPKPPEPEDLAIICFTSGTTGNPK.G |
| 169173 | 261 | – | 285 | 674.8582 | 2695.4038 | 2695.3891 | 5.47 | 2 | 23 | 0.0098 | 1Score **> 37** indicates **identity** Score **> 15** indicates **homology** | U | R.VKPKPPEPEDLAIICFTSGTTGNPK.G |
| 124762 | 286 | – | 303 | 673.6634 | 2017.9682 | 2017.9714 | -1.55 | 0 | 25 | 0.0049 | 1Score **> 34** indicates **identity** Score **> 14** indicates **homology** | U | K.GAMITHQNIINDCSGFIK.A |
| 124764 | 286 | – | 303 | 673.6642 | 2017.9707 | 2017.9714 | -0.33 | 0 | 41 | 0.00013 | 1Score **> 35** indicates **identity** Score **> 15** indicates **homology** | U | K.GAMITHQNIINDCSGFIK.A |
| 124765 | 286 | – | 303 | 1009.9927 | 2017.9709 | 2017.9714 | -0.22 | 0 | 89 | 5.9e-09 | 1Score **> 35** indicates **identity** Score **> 20** indicates **homology** | U | K.GAMITHQNIINDCSGFIK.A |
| 124766 | 286 | – | 303 | 673.6644 | 2017.9714 | 2017.9714 | 0.023 | 0 | 78 | 4.5e-08 | 1Score **> 35** indicates **identity** Score **> 17** indicates **homology** | U | K.GAMITHQNIINDCSGFIK.A |
| 124767 | 286 | – | 303 | 1009.9932 | 2017.9718 | 2017.9714 | 0.24 | 0 | 37 | 0.00032 | 1Score **> 35** indicates **identity** Score **> 15** indicates **homology** | U | K.GAMITHQNIINDCSGFIK.A |
| 124768 | 286 | – | 303 | 673.6647 | 2017.9723 | 2017.9714 | 0.46 | 0 | 77 | 5.7e-08 | 1Score **> 35** indicates **identity** Score **> 17** indicates **homology** | U | K.GAMITHQNIINDCSGFIK.A |
| 124769 | 286 | – | 303 | 1009.9943 | 2017.9741 | 2017.9714 | 1.35 | 0 | 71 | 2.1e-07 | 1Score **> 35** indicates **identity** Score **> 17** indicates **homology** | U | K.GAMITHQNIINDCSGFIK.A |
| 18419 | 342 | – | 350 | 526.7788 | 1051.5430 | 1051.5451 | -1.96 | 0 | 45 | 0.00017 | 1Score **> 31** indicates **identity** Score **> 20** indicates **homology** |  | K.IGFFQGDIR.L |
| 18420 | 342 | – | 350 | 526.7788 | 1051.5431 | 1051.5451 | -1.85 | 0 | 29 | 0.0052 | 1Score **> 31** indicates **identity** Score **> 19** indicates **homology** |  | K.IGFFQGDIR.L |
| 18421 | 342 | – | 350 | 526.7790 | 1051.5434 | 1051.5451 | -1.58 | 0 | 46 | 0.00017 | 1Score **> 31** indicates **identity** Score **> 21** indicates **homology** |  | K.IGFFQGDIR.L |
| 18422 | 342 | – | 350 | 526.7797 | 1051.5449 | 1051.5451 | -0.19 | 0 | 42 | 0.00034 | 1Score **> 31** indicates **identity** Score **> 20** indicates **homology** |  | K.IGFFQGDIR.L |
| 18423 | 342 | – | 350 | 526.7798 | 1051.5451 | 1051.5451 | 0.017 | 0 | 54 | 0.00012 | 1Score **> 31** indicates **identity** Score **> 27** indicates **homology** |  | K.IGFFQGDIR.L |
| 18424 | 342 | – | 350 | 526.7798 | 1051.5451 | 1051.5451 | 0.048 | 0 | 45 | 0.00017 | 1Score **> 31** indicates **identity** Score **> 20** indicates **homology** |  | K.IGFFQGDIR.L |
| 18425 | 342 | – | 350 | 526.7801 | 1051.5456 | 1051.5451 | 0.49 | 0 | 51 | 9.9e-05 | 1Score **> 31** indicates **identity** Score **> 23** indicates **homology** |  | K.IGFFQGDIR.L |
| 18426 | 342 | – | 350 | 526.7802 | 1051.5459 | 1051.5451 | 0.80 | 0 | 53 | 0.00012 | 1Score **> 31** indicates **identity** Score **> 26** indicates **homology** |  | K.IGFFQGDIR.L |
| 18427 | 342 | – | 350 | 526.7803 | 1051.5460 | 1051.5451 | 0.82 | 0 | 53 | 0.00012 | 1Score **> 31** indicates **identity** Score **> 27** indicates **homology** |  | K.IGFFQGDIR.L |
| 18428 | 342 | – | 350 | 526.7803 | 1051.5460 | 1051.5451 | 0.86 | 0 | 53 | 0.00012 | 1Score **> 31** indicates **identity** Score **> 26** indicates **homology** |  | K.IGFFQGDIR.L |
| 18429 | 342 | – | 350 | 526.7804 | 1051.5462 | 1051.5451 | 1.09 | 0 | 58 | 7.2e-05 | 1Score **> 31** indicates **identity** Score **> 29** indicates **homology** |  | K.IGFFQGDIR.L |
| 18430 | 342 | – | 350 | 526.7806 | 1051.5465 | 1051.5451 | 1.39 | 0 | 62 | 2.4e-05 | 1Score **> 31** indicates **identity** Score **> 28** indicates **homology** |  | K.IGFFQGDIR.L |
| 18431 | 342 | – | 350 | 526.7806 | 1051.5466 | 1051.5451 | 1.39 | 0 | 52 | 0.00012 | 1Score **> 31** indicates **identity** Score **> 26** indicates **homology** |  | K.IGFFQGDIR.L |
| 18432 | 342 | – | 350 | 526.7806 | 1051.5466 | 1051.5451 | 1.41 | 0 | 44 | 0.00027 | 1Score **> 31** indicates **identity** Score **> 21** indicates **homology** |  | K.IGFFQGDIR.L |
| 18433 | 342 | – | 350 | 526.7808 | 1051.5470 | 1051.5451 | 1.78 | 0 | 53 | 0.00012 | 1Score **> 31** indicates **identity** Score **> 27** indicates **homology** |  | K.IGFFQGDIR.L |
| 18434 | 342 | – | 350 | 526.7809 | 1051.5472 | 1051.5451 | 1.97 | 0 | 52 | 0.00012 | 1Score **> 31** indicates **identity** Score **> 25** indicates **homology** |  | K.IGFFQGDIR.L |
| 18435 | 342 | – | 350 | 526.7809 | 1051.5473 | 1051.5451 | 2.09 | 0 | 27 | 0.003 | 1Score **> 31** indicates **identity** Score **> 14** indicates **homology** |  | K.IGFFQGDIR.L |
| 6027 | 351 | – | 357 | 424.2330 | 846.4515 | 846.4521 | -0.62 | 0 | 27 | 0.0045 | 1Score **> 31** indicates **identity** Score **> 16** indicates **homology** | U | R.LLMDDLK.V |
| 6028 | 351 | – | 357 | 424.2332 | 846.4519 | 846.4521 | -0.23 | 0 | 14 | 0.048 | 1Score **> 31** indicates **identity** Score **> 13** indicates **homology** | U | R.LLMDDLK.V |
| 6029 | 351 | – | 357 | 424.2335 | 846.4524 | 846.4521 | 0.34 | 0 | 46 | 0.00076 | 1Score **> 31** indicates **identity** Score **> 27** indicates **homology** | U | R.LLMDDLK.V |
| 6030 | 351 | – | 357 | 424.2335 | 846.4525 | 846.4521 | 0.48 | 0 | 23 | 0.0072 | 1Score **> 31** indicates **identity** Score **> 14** indicates **homology** | U | R.LLMDDLK.V |
| 6031 | 351 | – | 357 | 424.2336 | 846.4527 | 846.4521 | 0.79 | 0 | 46 | 0.0013 | 1Score **> 31** indicates **identity** Score **> 30** indicates **homology** | U | R.LLMDDLK.V |
| 6032 | 351 | – | 357 | 424.2337 | 846.4528 | 846.4521 | 0.91 | 0 | 21 | 0.016 | 1Score **> 31** indicates **identity** Score **> 16** indicates **homology** | U | R.LLMDDLK.V |
| 6033 | 351 | – | 357 | 424.2339 | 846.4533 | 846.4521 | 1.42 | 0 | 46 | 0.00075 | 1Score **> 29** indicates **identity** Score **> 27** indicates **homology** | U | R.LLMDDLK.V |
| 6034 | 351 | – | 357 | 424.2341 | 846.4536 | 846.4521 | 1.84 | 0 | 28 | 0.0051 | 1Score **> 29** indicates **identity** Score **> 18** indicates **homology** | U | R.LLMDDLK.V |
| 6950 | 351 | – | 357 | 432.2303 | 862.4461 | 862.4470 | -1.01 | 0 | 25 | 0.0043 | 1Score **> 28** indicates **identity** Score **> 14** indicates **homology** | U | R.LLMDDLK.V  + Oxidation (M) |
| 141030 | 351 | – | 369 | 549.3214 | 2193.2564 | 2193.2595 | -1.45 | 1 | 14 | 0.045 | 1Score **> 32** indicates **identity** Score **> 13** indicates **homology** | U | R.LLMDDLKVLQPTIFPVVPR.L |
| 51617 | 358 | – | 369 | 683.4160 | 1364.8175 | 1364.8180 | -0.41 | 0 | 43 | 0.0029 | 1Score **> 30** indicates **identity** | U | K.VLQPTIFPVVPR.L |
| 51618 | 358 | – | 369 | 455.9465 | 1364.8176 | 1364.8180 | -0.32 | 0 | 41 | 0.0039 | 2Score **> 30** indicates **identity** | U | K.VLQPTIFPVVPR.L |
| 51619 | 358 | – | 369 | 683.4161 | 1364.8176 | 1364.8180 | -0.31 | 0 | 45 | 0.0018 | 1Score **> 30** indicates **identity** | U | K.VLQPTIFPVVPR.L |
| 51621 | 358 | – | 369 | 455.9465 | 1364.8177 | 1364.8180 | -0.23 | 0 | 36 | 0.00044 | 1Score **> 30** indicates **identity** Score **> 15** indicates **homology** | U | K.VLQPTIFPVVPR.L |
| 51623 | 358 | – | 369 | 683.4167 | 1364.8188 | 1364.8180 | 0.57 | 0 | 50 | 0.00056 | 1Score **> 30** indicates **identity** | U | K.VLQPTIFPVVPR.L |
| 51624 | 358 | – | 369 | 455.9469 | 1364.8188 | 1364.8180 | 0.57 | 0 | 44 | 6.8e-05 | 1Score **> 30** indicates **identity** Score **> 15** indicates **homology** | U | K.VLQPTIFPVVPR.L |
| 51625 | 358 | – | 369 | 683.4167 | 1364.8188 | 1364.8180 | 0.58 | 0 | 50 | 0.00056 | 1Score **> 30** indicates **identity** | U | K.VLQPTIFPVVPR.L |
| 51633 | 358 | – | 369 | 455.9491 | 1364.8256 | 1364.8180 | 5.54 | 0 | 44 | 7.9e-05 | 1Score **> 29** indicates **identity** Score **> 15** indicates **homology** | U | K.VLQPTIFPVVPR.L |
| 20889 | 378 | – | 387 | 539.7990 | 1077.5834 | 1077.5818 | 1.48 | 0 | 67 | 8.1e-06 | 1Score **> 32** indicates **identity** Score **> 29** indicates **homology** | U | R.IFGQANTSLK.R |
| 20890 | 378 | – | 387 | 539.8016 | 1077.5887 | 1077.5818 | 6.33 | 0 | 36 | 0.0039 | 1Score **> 32** indicates **identity** Score **> 25** indicates **homology** | U | R.IFGQANTSLK.R |
| 37223 | 378 | – | 388 | 412.2349 | 1233.6829 | 1233.6830 | -0.066 | 1 | 38 | 0.0003 | 1Score **> 33** indicates **identity** Score **> 15** indicates **homology** | U | R.IFGQANTSLKR.W |
| 37224 | 378 | – | 388 | 412.2349 | 1233.6829 | 1233.6830 | -0.024 | 1 | 68 | 9.2e-07 | 1Score **> 33** indicates **identity** Score **> 20** indicates **homology** | U | R.IFGQANTSLKR.W |
| 37225 | 378 | – | 388 | 617.8488 | 1233.6830 | 1233.6830 | -0.0024 | 1 | 58 | 2.8e-05 | 1Score **> 33** indicates **identity** Score **> 25** indicates **homology** | U | R.IFGQANTSLKR.W |
| 37226 | 378 | – | 388 | 617.8488 | 1233.6831 | 1233.6830 | 0.10 | 1 | 41 | 0.00084 | 1Score **> 33** indicates **identity** Score **> 23** indicates **homology** | U | R.IFGQANTSLKR.W |
| 37227 | 378 | – | 388 | 412.2350 | 1233.6831 | 1233.6830 | 0.14 | 1 | 34 | 0.00069 | 1Score **> 33** indicates **identity** Score **> 15** indicates **homology** | U | R.IFGQANTSLKR.W |
| 37228 | 378 | – | 388 | 412.2350 | 1233.6831 | 1233.6830 | 0.16 | 1 | 63 | 2.3e-06 | 1Score **> 33** indicates **identity** Score **> 20** indicates **homology** | U | R.IFGQANTSLKR.W |
| 37229 | 378 | – | 388 | 412.2350 | 1233.6832 | 1233.6830 | 0.18 | 1 | 33 | 0.00084 | 1Score **> 33** indicates **identity** Score **> 15** indicates **homology** | U | R.IFGQANTSLKR.W |
| 37230 | 378 | – | 388 | 412.2350 | 1233.6832 | 1233.6830 | 0.18 | 1 | 29 | 0.002 | 1Score **> 33** indicates **identity** Score **> 14** indicates **homology** | U | R.IFGQANTSLKR.W |
| 37231 | 378 | – | 388 | 617.8489 | 1233.6833 | 1233.6830 | 0.28 | 1 | 31 | 0.004 | 1Score **> 33** indicates **identity** Score **> 19** indicates **homology** | U | R.IFGQANTSLKR.W |
| 37233 | 378 | – | 388 | 412.2353 | 1233.6842 | 1233.6830 | 1.00 | 1 | 30 | 0.0016 | 1Score **> 32** indicates **identity** Score **> 14** indicates **homology** | U | R.IFGQANTSLKR.W |
| 37234 | 378 | – | 388 | 412.2354 | 1233.6844 | 1233.6830 | 1.16 | 1 | 33 | 0.00088 | 1Score **> 32** indicates **identity** Score **> 15** indicates **homology** | U | R.IFGQANTSLKR.W |
| 13546 | 389 | – | 396 | 490.2645 | 978.5145 | 978.5175 | -3.02 | 0 | 18 | 0.023 | 1Score **> 30** indicates **identity** Score **> 14** indicates **homology** | U | R.WLLDFASK.R |
| 13548 | 389 | – | 396 | 490.2652 | 978.5159 | 978.5175 | -1.61 | 0 | 26 | 0.0034 | 1Score **> 30** indicates **identity** Score **> 14** indicates **homology** | U | R.WLLDFASK.R |
| 13549 | 389 | – | 396 | 490.2660 | 978.5174 | 978.5175 | -0.10 | 0 | 19 | 0.015 | 1Score **> 30** indicates **identity** Score **> 14** indicates **homology** | U | R.WLLDFASK.R |
| 13550 | 389 | – | 396 | 490.2660 | 978.5175 | 978.5175 | 0.086 | 0 | 24 | 0.0057 | 1Score **> 30** indicates **identity** Score **> 14** indicates **homology** | U | R.WLLDFASK.R |
| 13551 | 389 | – | 396 | 490.2663 | 978.5180 | 978.5175 | 0.57 | 0 | 20 | 0.014 | 1Score **> 31** indicates **identity** Score **> 14** indicates **homology** | U | R.WLLDFASK.R |
| 13552 | 389 | – | 396 | 490.2663 | 978.5181 | 978.5175 | 0.69 | 0 | 19 | 0.017 | 1Score **> 31** indicates **identity** Score **> 14** indicates **homology** | U | R.WLLDFASK.R |
| 13553 | 389 | – | 396 | 490.2663 | 978.5181 | 978.5175 | 0.70 | 0 | 22 | 0.0085 | 1Score **> 31** indicates **identity** Score **> 14** indicates **homology** | U | R.WLLDFASK.R |
| 13554 | 389 | – | 396 | 490.2665 | 978.5185 | 978.5175 | 1.02 | 0 | 22 | 0.0087 | 1Score **> 31** indicates **identity** Score **> 14** indicates **homology** | U | R.WLLDFASK.R |
| 13555 | 389 | – | 396 | 490.2666 | 978.5187 | 978.5175 | 1.28 | 0 | 19 | 0.017 | 1Score **> 31** indicates **identity** Score **> 14** indicates **homology** | U | R.WLLDFASK.R |
| 13556 | 389 | – | 396 | 490.2667 | 978.5189 | 978.5175 | 1.45 | 0 | 17 | 0.028 | 1Score **> 31** indicates **identity** Score **> 14** indicates **homology** | U | R.WLLDFASK.R |
| 13557 | 389 | – | 396 | 490.2667 | 978.5189 | 978.5175 | 1.45 | 0 | 18 | 0.022 | 1Score **> 31** indicates **identity** Score **> 14** indicates **homology** | U | R.WLLDFASK.R |
| 13558 | 389 | – | 396 | 490.2668 | 978.5189 | 978.5175 | 1.52 | 0 | 16 | 0.028 | 1Score **> 31** indicates **identity** Score **> 14** indicates **homology** | U | R.WLLDFASK.R |
| 13560 | 389 | – | 396 | 490.2674 | 978.5202 | 978.5175 | 2.83 | 0 | 21 | 0.0099 | 1Score **> 31** indicates **identity** Score **> 14** indicates **homology** | U | R.WLLDFASK.R |
| 26567 | 389 | – | 397 | 379.2130 | 1134.6173 | 1134.6186 | -1.11 | 1 | 29 | 0.0019 | 1Score **> 33** indicates **identity** Score **> 14** indicates **homology** | U | R.WLLDFASKR.K |
| 26569 | 389 | – | 397 | 379.2133 | 1134.6180 | 1134.6186 | -0.48 | 1 | 31 | 0.0092 | 1Score **> 33** indicates **identity** Score **> 24** indicates **homology** | U | R.WLLDFASKR.K |
| 26570 | 389 | – | 397 | 379.2135 | 1134.6186 | 1134.6186 | -0.0053 | 1 | 24 | 0.0084 | 1Score **> 33** indicates **identity** Score **> 16** indicates **homology** | U | R.WLLDFASKR.K |
| 26571 | 389 | – | 397 | 379.2135 | 1134.6187 | 1134.6186 | 0.093 | 1 | 34 | 0.0049 | 1Score **> 33** indicates **identity** Score **> 24** indicates **homology** | U | R.WLLDFASKR.K |
| 26574 | 389 | – | 397 | 379.2140 | 1134.6200 | 1134.6186 | 1.31 | 1 | 37 | 0.00034 | 1Score **> 32** indicates **identity** Score **> 15** indicates **homology** | U | R.WLLDFASKR.K |
| 7599 | 409 | – | 415 | 438.7143 | 875.4141 | 875.4137 | 0.48 | 0 | 29 | 0.0051 | 1Score **> 27** indicates **identity** Score **> 18** indicates **homology** | U | R.NNSLWDK.L |
| 7600 | 409 | – | 415 | 438.7144 | 875.4142 | 875.4137 | 0.58 | 0 | 39 | 0.0017 | 1Score **> 27** indicates **identity** Score **> 23** indicates **homology** | U | R.NNSLWDK.L |
| 68631 | 409 | – | 420 | 505.6078 | 1513.8015 | 1513.8041 | -1.73 | 1 | 48 | 3e-05 | 1Score **> 34** indicates **identity** Score **> 16** indicates **homology** | U | R.NNSLWDKLIFHK.I |
| 68632 | 409 | – | 420 | 505.6078 | 1513.8015 | 1513.8041 | -1.71 | 1 | 38 | 0.00026 | 1Score **> 34** indicates **identity** Score **> 15** indicates **homology** | U | R.NNSLWDKLIFHK.I |
| 68634 | 409 | – | 420 | 505.6081 | 1513.8026 | 1513.8041 | -1.01 | 1 | 52 | 1.2e-05 | 1Score **> 35** indicates **identity** Score **> 16** indicates **homology** | U | R.NNSLWDKLIFHK.I |
| 68637 | 409 | – | 420 | 505.6084 | 1513.8033 | 1513.8041 | -0.52 | 1 | 34 | 0.00062 | 1Score **> 35** indicates **identity** Score **> 15** indicates **homology** | U | R.NNSLWDKLIFHK.I |
| 68638 | 409 | – | 420 | 757.9090 | 1513.8034 | 1513.8041 | -0.48 | 1 | 24 | 0.0051 | 1Score **> 35** indicates **identity** Score **> 14** indicates **homology** | U | R.NNSLWDKLIFHK.I |
| 68639 | 409 | – | 420 | 505.6084 | 1513.8034 | 1513.8041 | -0.46 | 1 | 39 | 0.0002 | 1Score **> 35** indicates **identity** Score **> 15** indicates **homology** | U | R.NNSLWDKLIFHK.I |
| 68640 | 409 | – | 420 | 505.6085 | 1513.8035 | 1513.8041 | -0.39 | 1 | 49 | 2.8e-05 | 1Score **> 35** indicates **identity** Score **> 16** indicates **homology** | U | R.NNSLWDKLIFHK.I |
| 68642 | 409 | – | 420 | 505.6085 | 1513.8037 | 1513.8041 | -0.31 | 1 | 36 | 0.00046 | 1Score **> 35** indicates **identity** Score **> 15** indicates **homology** | U | R.NNSLWDKLIFHK.I |
| 68644 | 409 | – | 420 | 505.6085 | 1513.8038 | 1513.8041 | -0.20 | 1 | 52 | 1.3e-05 | 1Score **> 35** indicates **identity** Score **> 16** indicates **homology** | U | R.NNSLWDKLIFHK.I |
| 68645 | 409 | – | 420 | 505.6086 | 1513.8039 | 1513.8041 | -0.14 | 1 | 53 | 1.1e-05 | 1Score **> 35** indicates **identity** Score **> 16** indicates **homology** | U | R.NNSLWDKLIFHK.I |
| 68646 | 409 | – | 420 | 505.6086 | 1513.8040 | 1513.8041 | -0.082 | 1 | 36 | 0.00043 | 1Score **> 35** indicates **identity** Score **> 15** indicates **homology** | U | R.NNSLWDKLIFHK.I |
| 68647 | 409 | – | 420 | 505.6086 | 1513.8041 | 1513.8041 | -0.038 | 1 | 37 | 0.00031 | 1Score **> 34** indicates **identity** Score **> 15** indicates **homology** | U | R.NNSLWDKLIFHK.I |
| 68648 | 409 | – | 420 | 505.6086 | 1513.8041 | 1513.8041 | -0.032 | 1 | 51 | 1.6e-05 | 1Score **> 34** indicates **identity** Score **> 16** indicates **homology** | U | R.NNSLWDKLIFHK.I |
| 68649 | 409 | – | 420 | 505.6087 | 1513.8042 | 1513.8041 | 0.039 | 1 | 18 | 0.02 | 1Score **> 34** indicates **identity** Score **> 14** indicates **homology** | U | R.NNSLWDKLIFHK.I |
| 68650 | 409 | – | 420 | 505.6087 | 1513.8043 | 1513.8041 | 0.10 | 1 | 32 | 0.0011 | 1Score **> 34** indicates **identity** Score **> 14** indicates **homology** | U | R.NNSLWDKLIFHK.I |
| 68651 | 409 | – | 420 | 757.9094 | 1513.8043 | 1513.8041 | 0.11 | 1 | 19 | 0.016 | 1Score **> 34** indicates **identity** Score **> 14** indicates **homology** | U | R.NNSLWDKLIFHK.I |
| 68653 | 409 | – | 420 | 505.6087 | 1513.8044 | 1513.8041 | 0.16 | 1 | 34 | 0.00069 | 1Score **> 34** indicates **identity** Score **> 15** indicates **homology** | U | R.NNSLWDKLIFHK.I |
| 68654 | 409 | – | 420 | 505.6087 | 1513.8044 | 1513.8041 | 0.17 | 1 | 49 | 2.7e-05 | 1Score **> 34** indicates **identity** Score **> 16** indicates **homology** | U | R.NNSLWDKLIFHK.I |
| 68655 | 409 | – | 420 | 505.6088 | 1513.8045 | 1513.8041 | 0.22 | 1 | 51 | 1.5e-05 | 1Score **> 34** indicates **identity** Score **> 16** indicates **homology** | U | R.NNSLWDKLIFHK.I |
| 68656 | 409 | – | 420 | 505.6088 | 1513.8045 | 1513.8041 | 0.23 | 1 | 34 | 0.00062 | 1Score **> 34** indicates **identity** Score **> 15** indicates **homology** | U | R.NNSLWDKLIFHK.I |
| 68657 | 409 | – | 420 | 505.6088 | 1513.8045 | 1513.8041 | 0.23 | 1 | 48 | 3.1e-05 | 1Score **> 34** indicates **identity** Score **> 16** indicates **homology** | U | R.NNSLWDKLIFHK.I |
| 68660 | 409 | – | 420 | 505.6088 | 1513.8045 | 1513.8041 | 0.26 | 1 | 46 | 4.9e-05 | 1Score **> 34** indicates **identity** Score **> 15** indicates **homology** | U | R.NNSLWDKLIFHK.I |
| 68661 | 409 | – | 420 | 505.6088 | 1513.8046 | 1513.8041 | 0.30 | 1 | 33 | 0.00084 | 1Score **> 34** indicates **identity** Score **> 15** indicates **homology** | U | R.NNSLWDKLIFHK.I |
| 68662 | 409 | – | 420 | 505.6089 | 1513.8047 | 1513.8041 | 0.40 | 1 | 52 | 1.3e-05 | 1Score **> 34** indicates **identity** Score **> 16** indicates **homology** | U | R.NNSLWDKLIFHK.I |
| 68664 | 409 | – | 420 | 505.6089 | 1513.8049 | 1513.8041 | 0.49 | 1 | 52 | 1.2e-05 | 1Score **> 34** indicates **identity** Score **> 16** indicates **homology** | U | R.NNSLWDKLIFHK.I |
| 68666 | 409 | – | 420 | 505.6089 | 1513.8050 | 1513.8041 | 0.55 | 1 | 48 | 3e-05 | 1Score **> 34** indicates **identity** Score **> 16** indicates **homology** | U | R.NNSLWDKLIFHK.I |
| 68668 | 409 | – | 420 | 757.9098 | 1513.8051 | 1513.8041 | 0.65 | 1 | 56 | 5.1e-06 | 1Score **> 34** indicates **identity** Score **> 16** indicates **homology** | U | R.NNSLWDKLIFHK.I |
| 68669 | 409 | – | 420 | 757.9099 | 1513.8052 | 1513.8041 | 0.72 | 1 | 51 | 1.8e-05 | 1Score **> 34** indicates **identity** Score **> 16** indicates **homology** | U | R.NNSLWDKLIFHK.I |
| 68670 | 409 | – | 420 | 757.9100 | 1513.8054 | 1513.8041 | 0.87 | 1 | 19 | 0.017 | 1Score **> 34** indicates **identity** Score **> 14** indicates **homology** | U | R.NNSLWDKLIFHK.I |
| 68671 | 409 | – | 420 | 505.6091 | 1513.8054 | 1513.8041 | 0.87 | 1 | 35 | 0.00055 | 1Score **> 34** indicates **identity** Score **> 15** indicates **homology** | U | R.NNSLWDKLIFHK.I |
| 68672 | 409 | – | 420 | 505.6091 | 1513.8055 | 1513.8041 | 0.89 | 1 | 19 | 0.017 | 1Score **> 34** indicates **identity** Score **> 14** indicates **homology** | U | R.NNSLWDKLIFHK.I |
| 68673 | 409 | – | 420 | 505.6091 | 1513.8055 | 1513.8041 | 0.93 | 1 | 49 | 2.8e-05 | 1Score **> 34** indicates **identity** Score **> 16** indicates **homology** | U | R.NNSLWDKLIFHK.I |
| 68674 | 409 | – | 420 | 505.6094 | 1513.8064 | 1513.8041 | 1.53 | 1 | 38 | 0.0003 | 1Score **> 35** indicates **identity** Score **> 15** indicates **homology** | U | R.NNSLWDKLIFHK.I |
| 68677 | 409 | – | 420 | 505.6098 | 1513.8076 | 1513.8041 | 2.28 | 1 | 26 | 0.0036 | 1Score **> 34** indicates **identity** Score **> 14** indicates **homology** | U | R.NNSLWDKLIFHK.I |
| 68820 | 409 | – | 420 | 505.9359 | 1514.7858 | 1514.7881 | -1.53 | 1 | 22 | 0.0085 | 1Score **> 34** indicates **identity** Score **> 14** indicates **homology** | U | R.NNSLWDKLIFHK.I  + Deamidated (NQ) |
| 68822 | 409 | – | 420 | 505.9360 | 1514.7863 | 1514.7881 | -1.22 | 1 | 25 | 0.0045 | 1Score **> 35** indicates **identity** Score **> 14** indicates **homology** | U | R.NNSLWDKLIFHK.I  + Deamidated (NQ) |
| 68823 | 409 | – | 420 | 505.9361 | 1514.7865 | 1514.7881 | -1.11 | 1 | 42 | 0.00012 | 1Score **> 35** indicates **identity** Score **> 15** indicates **homology** | U | R.NNSLWDKLIFHK.I  + Deamidated (NQ) |
| 68824 | 409 | – | 420 | 505.9361 | 1514.7865 | 1514.7881 | -1.11 | 1 | 40 | 0.00019 | 1Score **> 35** indicates **identity** Score **> 15** indicates **homology** | U | R.NNSLWDKLIFHK.I  + Deamidated (NQ) |
| 68827 | 409 | – | 420 | 505.9368 | 1514.7885 | 1514.7881 | 0.27 | 1 | 19 | 0.016 | 1Score **> 34** indicates **identity** Score **> 14** indicates **homology** | U | R.NNSLWDKLIFHK.I  + Deamidated (NQ) |
| 68830 | 409 | – | 420 | 505.9371 | 1514.7894 | 1514.7881 | 0.83 | 1 | 38 | 0.00025 | 1Score **> 34** indicates **identity** Score **> 15** indicates **homology** | U | R.NNSLWDKLIFHK.I  + Deamidated (NQ) |
| 68831 | 409 | – | 420 | 505.9371 | 1514.7895 | 1514.7881 | 0.90 | 1 | 40 | 0.00018 | 1Score **> 34** indicates **identity** Score **> 15** indicates **homology** | U | R.NNSLWDKLIFHK.I  + Deamidated (NQ) |
| 68832 | 409 | – | 420 | 505.9371 | 1514.7895 | 1514.7881 | 0.93 | 1 | 42 | 0.00013 | 1Score **> 34** indicates **identity** Score **> 15** indicates **homology** | U | R.NNSLWDKLIFHK.I  + Deamidated (NQ) |
| 68839 | 409 | – | 420 | 505.9390 | 1514.7952 | 1514.7881 | 4.69 | 1 | 42 | 0.00012 | 1Score **> 35** indicates **identity** Score **> 15** indicates **homology** | U | R.NNSLWDKLIFHK.I  + Deamidated (NQ) |
| 68844 | 409 | – | 420 | 505.9402 | 1514.7986 | 1514.7881 | 6.93 | 1 | 18 | 0.019 | 1Score **> 35** indicates **identity** Score **> 14** indicates **homology** | U | R.NNSLWDKLIFHK.I  + Deamidated (NQ) |
| 68845 | 409 | – | 420 | 505.9414 | 1514.8024 | 1514.7881 | 9.41 | 1 | 18 | 0.022 | 1Score **> 35** indicates **identity** Score **> 14** indicates **homology** | U | R.NNSLWDKLIFHK.I  + Deamidated (NQ) |
| 110345 | 431 | – | 448 | 931.0343 | 1860.0540 | 1860.0543 | -0.18 | 0 | 24 | 0.006 | 1Score **> 34** indicates **identity** Score **> 14** indicates **homology** | U | R.LMITGAAPVSATVLTFLR.T |
| 110346 | 431 | – | 448 | 621.0256 | 1860.0549 | 1860.0543 | 0.32 | 0 | 76 | 7.4e-08 | 1Score **> 34** indicates **identity** Score **> 17** indicates **homology** | U | R.LMITGAAPVSATVLTFLR.T |
| 110348 | 431 | – | 448 | 621.0262 | 1860.0569 | 1860.0543 | 1.39 | 0 | 61 | 2e-06 | 1Score **> 34** indicates **identity** Score **> 16** indicates **homology** | U | R.LMITGAAPVSATVLTFLR.T |
| 110349 | 431 | – | 448 | 931.0357 | 1860.0569 | 1860.0543 | 1.39 | 0 | 67 | 1.2e-06 | 1Score **> 34** indicates **identity** Score **> 21** indicates **homology** | U | R.LMITGAAPVSATVLTFLR.T |
| 110350 | 431 | – | 448 | 621.0267 | 1860.0581 | 1860.0543 | 2.07 | 0 | 53 | 1e-05 | 1Score **> 33** indicates **identity** Score **> 16** indicates **homology** | U | R.LMITGAAPVSATVLTFLR.T |
| 110351 | 431 | – | 448 | 931.0373 | 1860.0601 | 1860.0543 | 3.14 | 0 | 56 | 5.7e-06 | 1Score **> 33** indicates **identity** Score **> 16** indicates **homology** | U | R.LMITGAAPVSATVLTFLR.T |
| 111968 | 431 | – | 448 | 626.3588 | 1876.0545 | 1876.0492 | 2.80 | 0 | 51 | 1.6e-05 | 1Score **> 33** indicates **identity** Score **> 16** indicates **homology** | U | R.LMITGAAPVSATVLTFLR.T  + Oxidation (M) |
| 68143 | 492 | – | 504 | 755.8724 | 1509.7302 | 1509.7385 | -5.49 | 0 | 23 | 0.0066 | 1Score **> 34** indicates **identity** Score **> 14** indicates **homology** | U | K.LVDVEEMNYLASK.G |
| 68144 | 492 | – | 504 | 755.8729 | 1509.7312 | 1509.7385 | -4.78 | 0 | 66 | 6.6e-07 | 1Score **> 34** indicates **identity** Score **> 17** indicates **homology** | U | K.LVDVEEMNYLASK.G |
| 68146 | 492 | – | 504 | 755.8758 | 1509.7370 | 1509.7385 | -0.96 | 0 | 85 | 1.1e-08 | 1Score **> 34** indicates **identity** Score **> 18** indicates **homology** | U | K.LVDVEEMNYLASK.G |
| 68148 | 492 | – | 504 | 755.8775 | 1509.7404 | 1509.7385 | 1.30 | 0 | 85 | 1.4e-08 | 1Score **> 34** indicates **identity** Score **> 18** indicates **homology** | U | K.LVDVEEMNYLASK.G |
| 152907 | 492 | – | 512 | 790.3811 | 2368.1214 | 2368.1290 | -3.22 | 1 | 61 | 2e-06 | 1Score **> 35** indicates **identity** Score **> 16** indicates **homology** | U | K.LVDVEEMNYLASKGEGEVCVK.G |
| 152916 | 492 | – | 512 | 790.3814 | 2368.1223 | 2368.1290 | -2.83 | 1 | 27 | 0.0031 | 1Score **> 35** indicates **identity** Score **> 14** indicates **homology** | U | K.LVDVEEMNYLASKGEGEVCVK.G |
| 152922 | 492 | – | 512 | 790.3815 | 2368.1227 | 2368.1290 | -2.66 | 1 | 52 | 1.3e-05 | 1Score **> 35** indicates **identity** Score **> 16** indicates **homology** | U | K.LVDVEEMNYLASKGEGEVCVK.G |
| 152923 | 492 | – | 512 | 790.3816 | 2368.1229 | 2368.1290 | -2.60 | 1 | 15 | 0.042 | 1Score **> 34** indicates **identity** Score **> 13** indicates **homology** | U | K.LVDVEEMNYLASKGEGEVCVK.G |
| 152930 | 492 | – | 512 | 790.3824 | 2368.1254 | 2368.1290 | -1.52 | 1 | 48 | 3.2e-05 | 1Score **> 35** indicates **identity** Score **> 15** indicates **homology** | U | K.LVDVEEMNYLASKGEGEVCVK.G |
| 152931 | 492 | – | 512 | 790.3826 | 2368.1259 | 2368.1290 | -1.34 | 1 | 57 | 4.7e-06 | 1Score **> 35** indicates **identity** Score **> 16** indicates **homology** | U | K.LVDVEEMNYLASKGEGEVCVK.G |
| 152936 | 492 | – | 512 | 790.3838 | 2368.1297 | 2368.1290 | 0.29 | 1 | 33 | 0.00078 | 1Score **> 35** indicates **identity** Score **> 15** indicates **homology** | U | K.LVDVEEMNYLASKGEGEVCVK.G |
| 152937 | 492 | – | 512 | 790.3841 | 2368.1306 | 2368.1290 | 0.64 | 1 | 17 | 0.024 | 1Score **> 35** indicates **identity** Score **> 14** indicates **homology** | U | K.LVDVEEMNYLASKGEGEVCVK.G |
| 152945 | 492 | – | 512 | 790.3863 | 2368.1372 | 2368.1290 | 3.44 | 1 | 52 | 1.4e-05 | 1Score **> 35** indicates **identity** Score **> 16** indicates **homology** | U | K.LVDVEEMNYLASKGEGEVCVK.G |
| 152990 | 492 | – | 512 | 790.7170 | 2369.1292 | 2369.1130 | 6.83 | 1 | 23 | 0.0069 | 1Score **> 35** indicates **identity** Score **> 14** indicates **homology** | U | K.LVDVEEMNYLASKGEGEVCVK.G  + Deamidated (NQ) |
| 71377 | 513 | – | 526 | 512.6109 | 1534.8110 | 1534.8256 | -9.50 | 2 | 29 | 0.0018 | 1Score **> 35** indicates **identity** Score **> 14** indicates **homology** | U | K.GANVFKGYLKDPAR.T |
| 71379 | 513 | – | 526 | 384.7137 | 1534.8256 | 1534.8256 | 0.029 | 2 | 42 | 0.00012 | 1Score **> 35** indicates **identity** Score **> 15** indicates **homology** | U | K.GANVFKGYLKDPAR.T |
| 71380 | 513 | – | 526 | 384.7137 | 1534.8258 | 1534.8256 | 0.16 | 2 | 22 | 0.0086 | 1Score **> 34** indicates **identity** Score **> 14** indicates **homology** | U | K.GANVFKGYLKDPAR.T |
| 71381 | 513 | – | 526 | 512.6163 | 1534.8269 | 1534.8256 | 0.88 | 2 | 40 | 0.00076 | 1Score **> 34** indicates **identity** Score **> 22** indicates **homology** | U | K.GANVFKGYLKDPAR.T |
| 9993 | 519 | – | 526 | 460.2516 | 918.4887 | 918.4923 | -3.88 | 1 | 19 | 0.017 | 1Score **> 31** indicates **identity** Score **> 14** indicates **homology** | U | K.GYLKDPAR.T |
| 9995 | 519 | – | 526 | 460.2532 | 918.4919 | 918.4923 | -0.44 | 1 | 30 | 0.0085 | 1Score **> 31** indicates **identity** Score **> 22** indicates **homology** | U | K.GYLKDPAR.T |
| 9997 | 519 | – | 526 | 460.2534 | 918.4923 | 918.4923 | 0.035 | 1 | 38 | 0.001 | 1Score **> 31** indicates **identity** Score **> 21** indicates **homology** | U | K.GYLKDPAR.T |
| 116327 | 527 | – | 544 | 642.9883 | 1925.9432 | 1925.9483 | -2.67 | 1 | 47 | 3.9e-05 | 1Score **> 35** indicates **identity** Score **> 15** indicates **homology** | U | R.TAEALDKDGWLHTGDIGK.W |
| 116331 | 527 | – | 544 | 482.4939 | 1925.9463 | 1925.9483 | -1.04 | 1 | 21 | 0.011 | 1Score **> 35** indicates **identity** Score **> 14** indicates **homology** | U | R.TAEALDKDGWLHTGDIGK.W |
| 116334 | 527 | – | 544 | 482.4940 | 1925.9471 | 1925.9483 | -0.63 | 1 | 15 | 0.039 | 1Score **> 35** indicates **identity** Score **> 13** indicates **homology** | U | R.TAEALDKDGWLHTGDIGK.W |
| 116335 | 527 | – | 544 | 482.4941 | 1925.9474 | 1925.9483 | -0.48 | 1 | 22 | 0.0083 | 1Score **> 35** indicates **identity** Score **> 14** indicates **homology** | U | R.TAEALDKDGWLHTGDIGK.W |
| 116336 | 527 | – | 544 | 642.9898 | 1925.9477 | 1925.9483 | -0.33 | 1 | 65 | 7.3e-07 | 1Score **> 35** indicates **identity** Score **> 17** indicates **homology** | U | R.TAEALDKDGWLHTGDIGK.W |
| 116337 | 527 | – | 544 | 642.9899 | 1925.9479 | 1925.9483 | -0.20 | 1 | 43 | 8.5e-05 | 1Score **> 35** indicates **identity** Score **> 15** indicates **homology** | U | R.TAEALDKDGWLHTGDIGK.W |
| 116338 | 527 | – | 544 | 482.4943 | 1925.9480 | 1925.9483 | -0.15 | 1 | 21 | 0.011 | 1Score **> 35** indicates **identity** Score **> 14** indicates **homology** | U | R.TAEALDKDGWLHTGDIGK.W |
| 116339 | 527 | – | 544 | 482.4943 | 1925.9480 | 1925.9483 | -0.14 | 1 | 24 | 0.0057 | 1Score **> 35** indicates **identity** Score **> 14** indicates **homology** | U | R.TAEALDKDGWLHTGDIGK.W |
| 116340 | 527 | – | 544 | 642.9900 | 1925.9483 | 1925.9483 | -0.023 | 1 | 47 | 4.2e-05 | 1Score **> 35** indicates **identity** Score **> 15** indicates **homology** | U | R.TAEALDKDGWLHTGDIGK.W |
| 116342 | 527 | – | 544 | 963.9815 | 1925.9485 | 1925.9483 | 0.088 | 1 | 78 | 4.6e-08 | 1Score **> 35** indicates **identity** Score **> 17** indicates **homology** | U | R.TAEALDKDGWLHTGDIGK.W |
| 116343 | 527 | – | 544 | 482.4944 | 1925.9485 | 1925.9483 | 0.11 | 1 | 34 | 0.00071 | 1Score **> 35** indicates **identity** Score **> 15** indicates **homology** | U | R.TAEALDKDGWLHTGDIGK.W |
| 116344 | 527 | – | 544 | 963.9815 | 1925.9485 | 1925.9483 | 0.12 | 1 | 64 | 1.1e-06 | 1Score **> 35** indicates **identity** Score **> 16** indicates **homology** | U | R.TAEALDKDGWLHTGDIGK.W |
| 116345 | 527 | – | 544 | 482.4944 | 1925.9487 | 1925.9483 | 0.19 | 1 | 37 | 0.00031 | 1Score **> 35** indicates **identity** Score **> 15** indicates **homology** | U | R.TAEALDKDGWLHTGDIGK.W |
| 116346 | 527 | – | 544 | 482.4944 | 1925.9487 | 1925.9483 | 0.20 | 1 | 35 | 0.0005 | 1Score **> 35** indicates **identity** Score **> 15** indicates **homology** | U | R.TAEALDKDGWLHTGDIGK.W |
| 116348 | 527 | – | 544 | 642.9902 | 1925.9489 | 1925.9483 | 0.30 | 1 | 45 | 6e-05 | 1Score **> 35** indicates **identity** Score **> 15** indicates **homology** | U | R.TAEALDKDGWLHTGDIGK.W |
| 116349 | 527 | – | 544 | 642.9902 | 1925.9489 | 1925.9483 | 0.31 | 1 | 68 | 4.7e-07 | 1Score **> 35** indicates **identity** Score **> 17** indicates **homology** | U | R.TAEALDKDGWLHTGDIGK.W |
| 116350 | 527 | – | 544 | 642.9904 | 1925.9492 | 1925.9483 | 0.49 | 1 | 66 | 5.8e-07 | 1Score **> 35** indicates **identity** Score **> 17** indicates **homology** | U | R.TAEALDKDGWLHTGDIGK.W |
| 116351 | 527 | – | 544 | 642.9906 | 1925.9501 | 1925.9483 | 0.93 | 1 | 72 | 1.8e-07 | 1Score **> 35** indicates **identity** Score **> 17** indicates **homology** | U | R.TAEALDKDGWLHTGDIGK.W |
| 116352 | 527 | – | 544 | 963.9824 | 1925.9503 | 1925.9483 | 1.02 | 1 | 83 | 1.6e-08 | 1Score **> 35** indicates **identity** Score **> 18** indicates **homology** | U | R.TAEALDKDGWLHTGDIGK.W |
| 116353 | 527 | – | 544 | 963.9824 | 1925.9503 | 1925.9483 | 1.03 | 1 | 94 | 1.8e-09 | 1Score **> 35** indicates **identity** Score **> 19** indicates **homology** | U | R.TAEALDKDGWLHTGDIGK.W |
| 116354 | 527 | – | 544 | 642.9909 | 1925.9508 | 1925.9483 | 1.30 | 1 | 55 | 6.8e-06 | 1Score **> 35** indicates **identity** Score **> 16** indicates **homology** | U | R.TAEALDKDGWLHTGDIGK.W |
| 116355 | 527 | – | 544 | 963.9830 | 1925.9515 | 1925.9483 | 1.68 | 1 | 83 | 1.6e-08 | 1Score **> 35** indicates **identity** Score **> 18** indicates **homology** | U | R.TAEALDKDGWLHTGDIGK.W |
| 116356 | 527 | – | 544 | 482.4952 | 1925.9518 | 1925.9483 | 1.81 | 1 | 18 | 0.022 | 1Score **> 35** indicates **identity** Score **> 14** indicates **homology** | U | R.TAEALDKDGWLHTGDIGK.W |
| 116357 | 527 | – | 544 | 642.9912 | 1925.9518 | 1925.9483 | 1.81 | 1 | 63 | 1.3e-06 | 1Score **> 35** indicates **identity** Score **> 16** indicates **homology** | U | R.TAEALDKDGWLHTGDIGK.W |
| 116360 | 527 | – | 544 | 963.9837 | 1925.9528 | 1925.9483 | 2.34 | 1 | 15 | 0.035 | 1Score **> 35** indicates **identity** Score **> 13** indicates **homology** | U | R.TAEALDKDGWLHTGDIGK.W |
| 116361 | 527 | – | 544 | 642.9916 | 1925.9530 | 1925.9483 | 2.46 | 1 | 46 | 5e-05 | 1Score **> 35** indicates **identity** Score **> 15** indicates **homology** | U | R.TAEALDKDGWLHTGDIGK.W |
| 116362 | 527 | – | 544 | 642.9917 | 1925.9531 | 1925.9483 | 2.50 | 1 | 29 | 0.0021 | 1Score **> 35** indicates **identity** Score **> 14** indicates **homology** | U | R.TAEALDKDGWLHTGDIGK.W |
| 116363 | 527 | – | 544 | 642.9918 | 1925.9536 | 1925.9483 | 2.75 | 1 | 27 | 0.0031 | 1Score **> 35** indicates **identity** Score **> 14** indicates **homology** | U | R.TAEALDKDGWLHTGDIGK.W |
| 116368 | 527 | – | 544 | 482.4961 | 1925.9555 | 1925.9483 | 3.72 | 1 | 26 | 0.0036 | 1Score **> 35** indicates **identity** Score **> 14** indicates **homology** | U | R.TAEALDKDGWLHTGDIGK.W |
| 116370 | 527 | – | 544 | 642.9925 | 1925.9558 | 1925.9483 | 3.90 | 1 | 47 | 3.6e-05 | 1Score **> 35** indicates **identity** Score **> 15** indicates **homology** | U | R.TAEALDKDGWLHTGDIGK.W |
| 174481 | 527 | – | 552 | 709.8727 | 2835.4618 | 2835.4555 | 2.20 | 2 | 20 | 0.014 | 1Score **> 37** indicates **identity** Score **> 14** indicates **homology** | U | R.TAEALDKDGWLHTGDIGKWLPNGTLK.I |
| 174482 | 527 | – | 552 | 709.8735 | 2835.4649 | 2835.4555 | 3.31 | 2 | 18 | 0.019 | 1Score **> 37** indicates **identity** Score **> 14** indicates **homology** | U | R.TAEALDKDGWLHTGDIGKWLPNGTLK.I |
| 174483 | 527 | – | 552 | 709.8743 | 2835.4682 | 2835.4555 | 4.48 | 2 | 43 | 9.5e-05 | 1Score **> 37** indicates **identity** Score **> 15** indicates **homology** | U | R.TAEALDKDGWLHTGDIGKWLPNGTLK.I |
| 174495 | 527 | – | 552 | 710.1149 | 2836.4306 | 2836.4395 | -3.16 | 2 | 37 | 0.00036 | 1Score **> 38** indicates **identity** Score **> 15** indicates **homology** | U | R.TAEALDKDGWLHTGDIGKWLPNGTLK.I  + Deamidated (NQ) |
| 174496 | 527 | – | 552 | 710.1150 | 2836.4309 | 2836.4395 | -3.04 | 2 | 18 | 0.021 | 1Score **> 38** indicates **identity** Score **> 14** indicates **homology** | U | R.TAEALDKDGWLHTGDIGKWLPNGTLK.I  + Deamidated (NQ) |
| 33256 | 534 | – | 544 | 400.2003 | 1197.5790 | 1197.5778 | 0.99 | 0 | 17 | 0.025 | 1Score **> 30** indicates **identity** Score **> 14** indicates **homology** | U | K.DGWLHTGDIGK.W |
| 33258 | 534 | – | 544 | 400.2006 | 1197.5801 | 1197.5778 | 1.89 | 0 | 15 | 0.038 | 1Score **> 30** indicates **identity** Score **> 13** indicates **homology** | U | K.DGWLHTGDIGK.W |
| 35344 | 563 | – | 573 | 609.8213 | 1217.6281 | 1217.6292 | -0.86 | 0 | 23 | 0.0077 | 1Score **> 34** indicates **identity** Score **> 14** indicates **homology** |  | K.LAQGEYIAPEK.I |
| 35345 | 563 | – | 573 | 609.8217 | 1217.6288 | 1217.6292 | -0.34 | 0 | 70 | 6.7e-07 | 1Score **> 34** indicates **identity** Score **> 21** indicates **homology** |  | K.LAQGEYIAPEK.I |
| 35346 | 563 | – | 573 | 609.8220 | 1217.6295 | 1217.6292 | 0.27 | 0 | 47 | 4.4e-05 | 1Score **> 34** indicates **identity** Score **> 15** indicates **homology** |  | K.LAQGEYIAPEK.I |
| 134188 | 563 | – | 580 | 707.3811 | 2119.1214 | 2119.1313 | -4.68 | 1 | 18 | 0.019 | 1Score **> 36** indicates **identity** Score **> 14** indicates **homology** | U | K.LAQGEYIAPEKIENIYLR.S |
| 134195 | 563 | – | 580 | 1060.5728 | 2119.1311 | 2119.1313 | -0.11 | 1 | 50 | 1.9e-05 | 1Score **> 36** indicates **identity** Score **> 16** indicates **homology** | U | K.LAQGEYIAPEKIENIYLR.S |
| 134197 | 563 | – | 580 | 707.3845 | 2119.1316 | 2119.1313 | 0.15 | 1 | 45 | 6.2e-05 | 1Score **> 36** indicates **identity** Score **> 15** indicates **homology** | U | K.LAQGEYIAPEKIENIYLR.S |
| 134199 | 563 | – | 580 | 1060.5732 | 2119.1318 | 2119.1313 | 0.26 | 1 | 77 | 5.4e-08 | 1Score **> 36** indicates **identity** Score **> 17** indicates **homology** | U | K.LAQGEYIAPEKIENIYLR.S |
| 134200 | 563 | – | 580 | 707.3846 | 2119.1321 | 2119.1313 | 0.39 | 1 | 48 | 2.9e-05 | 1Score **> 36** indicates **identity** Score **> 16** indicates **homology** | U | K.LAQGEYIAPEKIENIYLR.S |
| 134201 | 563 | – | 580 | 707.3847 | 2119.1324 | 2119.1313 | 0.52 | 1 | 52 | 1.4e-05 | 1Score **> 36** indicates **identity** Score **> 16** indicates **homology** | U | K.LAQGEYIAPEKIENIYLR.S |
| 134202 | 563 | – | 580 | 1060.5736 | 2119.1327 | 2119.1313 | 0.67 | 1 | 83 | 1.7e-08 | 1Score **> 36** indicates **identity** Score **> 18** indicates **homology** | U | K.LAQGEYIAPEKIENIYLR.S |
| 134203 | 563 | – | 580 | 707.3850 | 2119.1332 | 2119.1313 | 0.89 | 1 | 60 | 2.5e-06 | 1Score **> 36** indicates **identity** Score **> 16** indicates **homology** | U | K.LAQGEYIAPEKIENIYLR.S |
| 134204 | 563 | – | 580 | 707.3850 | 2119.1333 | 2119.1313 | 0.94 | 1 | 46 | 4.7e-05 | 1Score **> 36** indicates **identity** Score **> 15** indicates **homology** | U | K.LAQGEYIAPEKIENIYLR.S |
| 134205 | 563 | – | 580 | 707.3852 | 2119.1337 | 2119.1313 | 1.16 | 1 | 56 | 5.4e-06 | 1Score **> 36** indicates **identity** Score **> 16** indicates **homology** | U | K.LAQGEYIAPEKIENIYLR.S |
| 134207 | 563 | – | 580 | 707.3858 | 2119.1356 | 2119.1313 | 2.04 | 1 | 66 | 6.2e-07 | 1Score **> 36** indicates **identity** Score **> 17** indicates **homology** | U | K.LAQGEYIAPEKIENIYLR.S |
| 134210 | 563 | – | 580 | 1060.5763 | 2119.1381 | 2119.1313 | 3.20 | 1 | 71 | 2.4e-07 | 1Score **> 35** indicates **identity** Score **> 17** indicates **homology** | U | K.LAQGEYIAPEKIENIYLR.S |
| 134211 | 563 | – | 580 | 707.3867 | 2119.1381 | 2119.1313 | 3.24 | 1 | 16 | 0.028 | 1Score **> 35** indicates **identity** Score **> 14** indicates **homology** | U | K.LAQGEYIAPEKIENIYLR.S |
| 134315 | 563 | – | 580 | 1061.0735 | 2120.1324 | 2120.1153 | 8.07 | 1 | 52 | 1.4e-05 | 1Score **> 36** indicates **identity** Score **> 16** indicates **homology** | U | K.LAQGEYIAPEKIENIYLR.S  + Deamidated (NQ) |
| 10040 | 574 | – | 580 | 460.7634 | 919.5123 | 919.5127 | -0.37 | 0 | 41 | 0.0032 | 1Score **> 28** indicates **identity** | U | K.IENIYLR.S |
| 10041 | 574 | – | 580 | 460.7638 | 919.5130 | 919.5127 | 0.37 | 0 | 42 | 0.0026 | 1Score **> 28** indicates **identity** | U | K.IENIYLR.S |
| 190437 | 581 | – | 615 | 938.5040 | 3749.9870 | 3749.9669 | 5.38 | 0 | 20 | 0.013 | 1Score **> 36** indicates **identity** Score **> 14** indicates **homology** | U | R.SEAVAQVFVHGESLQAFLIAVVVPDVESLPSWAQK.R |
| 190445 | 581 | – | 615 | 1251.3361 | 3750.9863 | 3750.9509 | 9.45 | 0 | 41 | 0.00014 | 1Score **> 36** indicates **identity** Score **> 15** indicates **homology** | U | R.SEAVAQVFVHGESLQAFLIAVVVPDVESLPSWAQK.R  + Deamidated (NQ) |
| 61135 | 616 | – | 627 | 484.5736 | 1450.6990 | 1450.6987 | 0.20 | 1 | 45 | 6.7e-05 | 1Score **> 33** indicates **identity** Score **> 15** indicates **homology** | U | K.RGLQGSFEELCR.N |
| 61137 | 616 | – | 627 | 484.5737 | 1450.6991 | 1450.6987 | 0.32 | 1 | 58 | 3e-05 | 1Score **> 33** indicates **identity** Score **> 25** indicates **homology** | U | K.RGLQGSFEELCR.N |
| 61138 | 616 | – | 627 | 484.5737 | 1450.6992 | 1450.6987 | 0.34 | 1 | 33 | 0.00079 | 1Score **> 33** indicates **identity** Score **> 15** indicates **homology** | U | K.RGLQGSFEELCR.N |
| 61139 | 616 | – | 627 | 726.3569 | 1450.6992 | 1450.6987 | 0.36 | 1 | 41 | 0.0011 | 1Score **> 33** indicates **identity** Score **> 24** indicates **homology** | U | K.RGLQGSFEELCR.N |
| 61140 | 616 | – | 627 | 484.5737 | 1450.6994 | 1450.6987 | 0.47 | 1 | 52 | 2.1e-05 | 1Score **> 33** indicates **identity** Score **> 18** indicates **homology** | U | K.RGLQGSFEELCR.N |
| 61141 | 616 | – | 627 | 726.3570 | 1450.6995 | 1450.6987 | 0.55 | 1 | 43 | 0.00046 | 1Score **> 33** indicates **identity** Score **> 22** indicates **homology** | U | K.RGLQGSFEELCR.N |
| 61142 | 616 | – | 627 | 484.5738 | 1450.6997 | 1450.6987 | 0.70 | 1 | 62 | 1.4e-05 | 1Score **> 33** indicates **identity** Score **> 26** indicates **homology** | U | K.RGLQGSFEELCR.N |
| 61143 | 616 | – | 627 | 726.3572 | 1450.6998 | 1450.6987 | 0.77 | 1 | 22 | 0.0095 | 1Score **> 33** indicates **identity** Score **> 14** indicates **homology** | U | K.RGLQGSFEELCR.N |
| 61144 | 616 | – | 627 | 484.5739 | 1450.6999 | 1450.6987 | 0.82 | 1 | 55 | 2.2e-05 | 1Score **> 33** indicates **identity** Score **> 21** indicates **homology** | U | K.RGLQGSFEELCR.N |
| 61145 | 616 | – | 627 | 484.5740 | 1450.7000 | 1450.6987 | 0.93 | 1 | 61 | 1.3e-05 | 1Score **> 33** indicates **identity** Score **> 25** indicates **homology** | U | K.RGLQGSFEELCR.N |
| 61146 | 616 | – | 627 | 484.5743 | 1450.7012 | 1450.6987 | 1.71 | 1 | 26 | 0.0034 | 1Score **> 33** indicates **identity** Score **> 14** indicates **homology** | U | K.RGLQGSFEELCR.N |
| 61147 | 616 | – | 627 | 484.5743 | 1450.7012 | 1450.6987 | 1.72 | 1 | 17 | 0.027 | 1Score **> 33** indicates **identity** Score **> 14** indicates **homology** | U | K.RGLQGSFEELCR.N |
| 61148 | 616 | – | 627 | 726.3584 | 1450.7022 | 1450.6987 | 2.39 | 1 | 15 | 0.037 | 1Score **> 33** indicates **identity** Score **> 13** indicates **homology** | U | K.RGLQGSFEELCR.N |
| 43958 | 617 | – | 627 | 648.3047 | 1294.5948 | 1294.5976 | -2.14 | 0 | 43 | 9.1e-05 | 1Score **> 31** indicates **identity** Score **> 15** indicates **homology** | U | R.GLQGSFEELCR.N |
| 43959 | 617 | – | 627 | 648.3049 | 1294.5953 | 1294.5976 | -1.75 | 0 | 37 | 0.00033 | 1Score **> 31** indicates **identity** Score **> 15** indicates **homology** | U | R.GLQGSFEELCR.N |
| 43960 | 617 | – | 627 | 648.3057 | 1294.5969 | 1294.5976 | -0.53 | 0 | 58 | 3.9e-06 | 1Score **> 31** indicates **identity** Score **> 17** indicates **homology** | U | R.GLQGSFEELCR.N |
| 43961 | 617 | – | 627 | 648.3064 | 1294.5982 | 1294.5976 | 0.50 | 0 | 58 | 1.1e-05 | 1Score **> 31** indicates **identity** Score **> 21** indicates **homology** | U | R.GLQGSFEELCR.N |
| 43962 | 617 | – | 627 | 648.3065 | 1294.5985 | 1294.5976 | 0.68 | 0 | 40 | 0.0002 | 1Score **> 31** indicates **identity** Score **> 15** indicates **homology** | U | R.GLQGSFEELCR.N |
| 43963 | 617 | – | 627 | 648.3069 | 1294.5992 | 1294.5976 | 1.27 | 0 | 37 | 0.00033 | 1Score **> 31** indicates **identity** Score **> 15** indicates **homology** | U | R.GLQGSFEELCR.N |
| 43964 | 617 | – | 627 | 648.3070 | 1294.5994 | 1294.5976 | 1.44 | 0 | 66 | 9.7e-07 | 1Score **> 31** indicates **identity** Score **> 18** indicates **homology** | U | R.GLQGSFEELCR.N |
| 43965 | 617 | – | 627 | 648.3071 | 1294.5996 | 1294.5976 | 1.54 | 0 | 66 | 2.2e-06 | 1Score **> 31** indicates **identity** Score **> 22** indicates **homology** | U | R.GLQGSFEELCR.N |
| 81633 | 628 | – | 641 | 403.9869 | 1611.9185 | 1611.9195 | -0.65 | 2 | 19 | 0.036 | 1Score **> 33** indicates **identity** Score **> 17** indicates **homology** | U | R.NKDINKAILDDLLK.L |
| 81634 | 628 | – | 641 | 403.9870 | 1611.9191 | 1611.9195 | -0.27 | 2 | 34 | 0.0019 | 1Score **> 33** indicates **identity** Score **> 20** indicates **homology** | U | R.NKDINKAILDDLLK.L |
| 81636 | 628 | – | 641 | 538.3139 | 1611.9200 | 1611.9195 | 0.28 | 2 | 29 | 0.0019 | 1Score **> 33** indicates **identity** Score **> 14** indicates **homology** | U | R.NKDINKAILDDLLK.L |
| 81638 | 628 | – | 641 | 538.3144 | 1611.9213 | 1611.9195 | 1.10 | 2 | 63 | 2e-06 | 1Score **> 33** indicates **identity** Score **> 18** indicates **homology** | U | R.NKDINKAILDDLLK.L |
| 81639 | 628 | – | 641 | 538.3145 | 1611.9217 | 1611.9195 | 1.34 | 2 | 56 | 7.4e-06 | 1Score **> 33** indicates **identity** Score **> 18** indicates **homology** | U | R.NKDINKAILDDLLK.L |
| 81640 | 628 | – | 641 | 538.3146 | 1611.9220 | 1611.9195 | 1.56 | 2 | 41 | 0.00021 | 1Score **> 33** indicates **identity** Score **> 17** indicates **homology** | U | R.NKDINKAILDDLLK.L |
| 81642 | 628 | – | 641 | 538.3148 | 1611.9225 | 1611.9195 | 1.88 | 2 | 21 | 0.011 | 1Score **> 33** indicates **identity** Score **> 14** indicates **homology** | U | R.NKDINKAILDDLLK.L |
| 81643 | 628 | – | 641 | 403.9880 | 1611.9229 | 1611.9195 | 2.12 | 2 | 35 | 0.005 | 1Score **> 33** indicates **identity** Score **> 25** indicates **homology** | U | R.NKDINKAILDDLLK.L |
| 52024 | 630 | – | 641 | 457.6011 | 1369.7814 | 1369.7816 | -0.19 | 1 | 55 | 0.00045 | 1Score **> 34** indicates **identity** | U | K.DINKAILDDLLK.L |
| 52025 | 630 | – | 641 | 457.6013 | 1369.7820 | 1369.7816 | 0.24 | 1 | 28 | 0.01 | 1Score **> 34** indicates **identity** Score **> 20** indicates **homology** | U | K.DINKAILDDLLK.L |
| 52026 | 630 | – | 641 | 457.6016 | 1369.7829 | 1369.7816 | 0.92 | 1 | 52 | 0.00076 | 1Score **> 34** indicates **identity** | U | K.DINKAILDDLLK.L |
| 52027 | 630 | – | 641 | 457.6016 | 1369.7831 | 1369.7816 | 1.06 | 1 | 40 | 0.0052 | 1Score **> 34** indicates **identity** Score **> 30** indicates **homology** | U | K.DINKAILDDLLK.L |
| 8761 | 634 | – | 641 | 450.7730 | 899.5314 | 899.5328 | -1.51 | 0 | 19 | 0.022 | 1Score **> 29** indicates **identity** Score **> 15** indicates **homology** | U | K.AILDDLLK.L |
| 8762 | 634 | – | 641 | 450.7730 | 899.5315 | 899.5328 | -1.39 | 0 | 40 | 0.0029 | 1Score **> 29** indicates **identity** Score **> 27** indicates **homology** | U | K.AILDDLLK.L |
| 8763 | 634 | – | 641 | 450.7732 | 899.5318 | 899.5328 | -1.03 | 0 | 45 | 0.0013 | 1Score **> 29** indicates **identity** Score **> 28** indicates **homology** | U | K.AILDDLLK.L |
| 8764 | 634 | – | 641 | 450.7733 | 899.5321 | 899.5328 | -0.69 | 0 | 41 | 0.0017 | 1Score **> 28** indicates **identity** Score **> 26** indicates **homology** | U | K.AILDDLLK.L |
| 8765 | 634 | – | 641 | 450.7734 | 899.5322 | 899.5328 | -0.66 | 0 | 41 | 0.0022 | 1Score **> 28** indicates **identity** Score **> 27** indicates **homology** | U | K.AILDDLLK.L |
| 8770 | 634 | – | 641 | 450.7737 | 899.5328 | 899.5328 | 0.032 | 0 | 36 | 0.0062 | 1Score **> 28** indicates **identity** Score **> 27** indicates **homology** | U | K.AILDDLLK.L |
| 8773 | 634 | – | 641 | 450.7738 | 899.5330 | 899.5328 | 0.21 | 0 | 39 | 0.0017 | 1Score **> 28** indicates **identity** Score **> 24** indicates **homology** | U | K.AILDDLLK.L |
| 8774 | 634 | – | 641 | 450.7738 | 899.5331 | 899.5328 | 0.40 | 0 | 45 | 0.00093 | 1Score **> 28** indicates **identity** Score **> 27** indicates **homology** | U | K.AILDDLLK.L |
| 8775 | 634 | – | 641 | 450.7740 | 899.5335 | 899.5328 | 0.81 | 0 | 46 | 0.00083 | 1Score **> 28** indicates **identity** Score **> 28** indicates **homology** | U | K.AILDDLLK.L |
| 8776 | 634 | – | 641 | 450.7741 | 899.5336 | 899.5328 | 0.93 | 0 | 27 | 0.021 | 1Score **> 28** indicates **identity** Score **> 23** indicates **homology** | U | K.AILDDLLK.L |
| 8777 | 634 | – | 641 | 450.7741 | 899.5336 | 899.5328 | 0.98 | 0 | 46 | 0.00083 | 1Score **> 28** indicates **identity** Score **> 28** indicates **homology** | U | K.AILDDLLK.L |
| 8778 | 634 | – | 641 | 450.7741 | 899.5336 | 899.5328 | 0.99 | 0 | 43 | 0.0016 | 1Score **> 28** indicates **identity** Score **> 27** indicates **homology** | U | K.AILDDLLK.L |
| 8779 | 634 | – | 641 | 450.7741 | 899.5337 | 899.5328 | 1.00 | 0 | 22 | 0.0087 | 1Score **> 28** indicates **identity** Score **> 14** indicates **homology** | U | K.AILDDLLK.L |
| 8781 | 634 | – | 641 | 450.7743 | 899.5340 | 899.5328 | 1.40 | 0 | 46 | 0.00087 | 1Score **> 28** indicates **identity** Score **> 28** indicates **homology** | U | K.AILDDLLK.L |
| 8782 | 634 | – | 641 | 450.7744 | 899.5343 | 899.5328 | 1.68 | 0 | 44 | 0.0012 | 1Score **> 28** indicates **identity** Score **> 28** indicates **homology** | U | K.AILDDLLK.L |
| 33348 | 634 | – | 644 | 400.2515 | 1197.7326 | 1197.7332 | -0.55 | 1 | 32 | 0.00097 | 1Score **> 30** indicates **identity** Score **> 15** indicates **homology** | U | K.AILDDLLKLGK.E |
| 33349 | 634 | – | 644 | 400.2516 | 1197.7330 | 1197.7332 | -0.18 | 1 | 32 | 0.001 | 1Score **> 30** indicates **identity** Score **> 14** indicates **homology** | U | K.AILDDLLKLGK.E |
| 33350 | 634 | – | 644 | 400.2516 | 1197.7331 | 1197.7332 | -0.14 | 1 | 34 | 0.00069 | 1Score **> 30** indicates **identity** Score **> 15** indicates **homology** | U | K.AILDDLLKLGK.E |
| 33351 | 634 | – | 644 | 599.8739 | 1197.7333 | 1197.7332 | 0.068 | 1 | 16 | 0.033 | 1Score **> 30** indicates **identity** Score **> 13** indicates **homology** | U | K.AILDDLLKLGK.E |
| 33352 | 634 | – | 644 | 400.2518 | 1197.7335 | 1197.7332 | 0.21 | 1 | 35 | 0.00054 | 1Score **> 30** indicates **identity** Score **> 15** indicates **homology** | U | K.AILDDLLKLGK.E |
| 33353 | 634 | – | 644 | 400.2519 | 1197.7338 | 1197.7332 | 0.48 | 1 | 33 | 0.00089 | 1Score **> 30** indicates **identity** Score **> 15** indicates **homology** | U | K.AILDDLLKLGK.E |
| 72379 | 642 | – | 655 | 386.7262 | 1542.8757 | 1542.8770 | -0.84 | 2 | 23 | 0.0063 | 1Score **> 34** indicates **identity** Score **> 14** indicates **homology** | U | K.LGKEAGLKPFEQVK.G |
| 72380 | 642 | – | 655 | 515.2992 | 1542.8759 | 1542.8770 | -0.70 | 2 | 50 | 2.1e-05 | 1Score **> 34** indicates **identity** Score **> 16** indicates **homology** | U | K.LGKEAGLKPFEQVK.G |
| 72381 | 642 | – | 655 | 386.7263 | 1542.8760 | 1542.8770 | -0.59 | 2 | 28 | 0.0023 | 1Score **> 34** indicates **identity** Score **> 14** indicates **homology** | U | K.LGKEAGLKPFEQVK.G |
| 72382 | 642 | – | 655 | 515.2995 | 1542.8766 | 1542.8770 | -0.23 | 2 | 38 | 0.0003 | 1Score **> 34** indicates **identity** Score **> 15** indicates **homology** | U | K.LGKEAGLKPFEQVK.G |
| 38339 | 645 | – | 655 | 415.8992 | 1244.6757 | 1244.6765 | -0.60 | 1 | 28 | 0.0035 | 1Score **> 34** indicates **identity** Score **> 16** indicates **homology** | U | K.EAGLKPFEQVK.G |
| 38340 | 645 | – | 655 | 415.8992 | 1244.6757 | 1244.6765 | -0.60 | 1 | 22 | 0.0091 | 1Score **> 34** indicates **identity** Score **> 14** indicates **homology** | U | K.EAGLKPFEQVK.G |
| 38341 | 645 | – | 655 | 415.8992 | 1244.6758 | 1244.6765 | -0.51 | 1 | 23 | 0.0077 | 1Score **> 34** indicates **identity** Score **> 14** indicates **homology** | U | K.EAGLKPFEQVK.G |
| 38343 | 645 | – | 655 | 415.8993 | 1244.6762 | 1244.6765 | -0.21 | 1 | 26 | 0.0048 | 1Score **> 34** indicates **identity** Score **> 16** indicates **homology** | U | K.EAGLKPFEQVK.G |
| 38344 | 645 | – | 655 | 623.3454 | 1244.6763 | 1244.6765 | -0.17 | 1 | 48 | 0.00018 | 1Score **> 34** indicates **identity** Score **> 23** indicates **homology** | U | K.EAGLKPFEQVK.G |
| 38345 | 645 | – | 655 | 415.8994 | 1244.6764 | 1244.6765 | -0.048 | 1 | 29 | 0.002 | 1Score **> 34** indicates **identity** Score **> 15** indicates **homology** | U | K.EAGLKPFEQVK.G |
| 38346 | 645 | – | 655 | 623.3455 | 1244.6765 | 1244.6765 | -0.013 | 1 | 52 | 0.001 | 1Score **> 34** indicates **identity** | U | K.EAGLKPFEQVK.G |
| 38347 | 645 | – | 655 | 415.8994 | 1244.6765 | 1244.6765 | -0.0096 | 1 | 26 | 0.0039 | 1Score **> 34** indicates **identity** Score **> 14** indicates **homology** | U | K.EAGLKPFEQVK.G |
| 38348 | 645 | – | 655 | 623.3456 | 1244.6767 | 1244.6765 | 0.16 | 1 | 38 | 0.00083 | 1Score **> 34** indicates **identity** Score **> 20** indicates **homology** | U | K.EAGLKPFEQVK.G |
| 38349 | 645 | – | 655 | 415.8995 | 1244.6767 | 1244.6765 | 0.21 | 1 | 28 | 0.0033 | 1Score **> 34** indicates **identity** Score **> 16** indicates **homology** | U | K.EAGLKPFEQVK.G |
| 38350 | 645 | – | 655 | 623.3458 | 1244.6770 | 1244.6765 | 0.39 | 1 | 57 | 2.2e-05 | 1Score **> 34** indicates **identity** Score **> 23** indicates **homology** | U | K.EAGLKPFEQVK.G |
| 38351 | 645 | – | 655 | 623.3458 | 1244.6771 | 1244.6765 | 0.54 | 1 | 60 | 4.3e-05 | 1Score **> 34** indicates **identity** Score **> 29** indicates **homology** | U | K.EAGLKPFEQVK.G |
| 143848 | 656 | – | 676 | 1118.1221 | 2234.2297 | 2234.2311 | -0.60 | 0 | 61 | 1.8e-06 | 1Score **> 34** indicates **identity** Score **> 16** indicates **homology** | U | K.GIAVHPELFSIDNGLLTPTLK.A |
| 143850 | 656 | – | 676 | 745.7511 | 2234.2316 | 2234.2311 | 0.23 | 0 | 31 | 0.0014 | 1Score **> 35** indicates **identity** Score **> 14** indicates **homology** | U | K.GIAVHPELFSIDNGLLTPTLK.A |
| 143851 | 656 | – | 676 | 745.7514 | 2234.2325 | 2234.2311 | 0.65 | 0 | 70 | 2.6e-07 | 1Score **> 34** indicates **identity** Score **> 17** indicates **homology** | U | K.GIAVHPELFSIDNGLLTPTLK.A |
| 143852 | 656 | – | 676 | 1118.1239 | 2234.2332 | 2234.2311 | 0.93 | 0 | 67 | 4.8e-07 | 1Score **> 34** indicates **identity** Score **> 17** indicates **homology** | U | K.GIAVHPELFSIDNGLLTPTLK.A |
| 143853 | 656 | – | 676 | 745.7540 | 2234.2401 | 2234.2311 | 4.03 | 0 | 56 | 5.7e-06 | 1Score **> 34** indicates **identity** Score **> 16** indicates **homology** | U | K.GIAVHPELFSIDNGLLTPTLK.A |
| 143854 | 656 | – | 676 | 745.7560 | 2234.2462 | 2234.2311 | 6.78 | 0 | 41 | 0.00015 | 1Score **> 34** indicates **identity** Score **> 15** indicates **homology** | U | K.GIAVHPELFSIDNGLLTPTLK.A |
| 144001 | 656 | – | 676 | 1118.6163 | 2235.2180 | 2235.2151 | 1.32 | 0 | 93 | 1.8e-09 | 1Score **> 35** indicates **identity** Score **> 18** indicates **homology** | U | K.GIAVHPELFSIDNGLLTPTLK.A  + Deamidated (NQ) |
| 144002 | 656 | – | 676 | 1118.6166 | 2235.2187 | 2235.2151 | 1.64 | 0 | 51 | 1.6e-05 | 1Score **> 35** indicates **identity** Score **> 16** indicates **homology** | U | K.GIAVHPELFSIDNGLLTPTLK.A  + Deamidated (NQ) |
| 144003 | 656 | – | 676 | 746.0802 | 2235.2188 | 2235.2151 | 1.65 | 0 | 42 | 0.00012 | 1Score **> 35** indicates **identity** Score **> 15** indicates **homology** | U | K.GIAVHPELFSIDNGLLTPTLK.A  + Deamidated (NQ) |
| 144004 | 656 | – | 676 | 746.0805 | 2235.2198 | 2235.2151 | 2.09 | 0 | 71 | 2.1e-07 | 1Score **> 35** indicates **identity** Score **> 17** indicates **homology** | U | K.GIAVHPELFSIDNGLLTPTLK.A  + Deamidated (NQ) |
| 144005 | 656 | – | 676 | 746.0819 | 2235.2240 | 2235.2151 | 3.98 | 0 | 45 | 6.3e-05 | 1Score **> 35** indicates **identity** Score **> 15** indicates **homology** | U | K.GIAVHPELFSIDNGLLTPTLK.A  + Deamidated (NQ) |
| 42404 | 688 | – | 698 | 640.8401 | 1279.6657 | 1279.6660 | -0.18 | 0 | 34 | 0.00063 | 1Score **> 33** indicates **identity** Score **> 15** indicates **homology** | U | R.SQIDELYATIK.I |
| 42406 | 688 | – | 698 | 640.8412 | 1279.6678 | 1279.6660 | 1.42 | 0 | 63 | 1.3e-06 | 1Score **> 33** indicates **identity** Score **> 16** indicates **homology** | U | R.SQIDELYATIK.I |
| 42407 | 688 | – | 698 | 640.8414 | 1279.6682 | 1279.6660 | 1.73 | 0 | 54 | 8.9e-06 | 1Score **> 33** indicates **identity** Score **> 16** indicates **homology** | U | R.SQIDELYATIK.I |
| 54361 | 688 | – | 699 | 697.3821 | 1392.7497 | 1392.7500 | -0.25 | 1 | 57 | 4.3e-06 | 1Score **> 34** indicates **identity** Score **> 16** indicates **homology** | U | R.SQIDELYATIKI.- |
| 54363 | 688 | – | 699 | 697.3824 | 1392.7503 | 1392.7500 | 0.20 | 1 | 74 | 1.1e-07 | 1Score **> 34** indicates **identity** Score **> 17** indicates **homology** | U | R.SQIDELYATIKI.- |
| 54364 | 688 | – | 699 | 697.3825 | 1392.7505 | 1392.7500 | 0.37 | 1 | 57 | 4.1e-06 | 1Score **> 34** indicates **identity** Score **> 16** indicates **homology** | U | R.SQIDELYATIKI.- |
| 54365 | 688 | – | 699 | 465.2575 | 1392.7505 | 1392.7500 | 0.38 | 1 | 33 | 0.00077 | 1Score **> 34** indicates **identity** Score **> 15** indicates **homology** | U | R.SQIDELYATIKI.- |
| 54366 | 688 | – | 699 | 465.2575 | 1392.7508 | 1392.7500 | 0.54 | 1 | 30 | 0.0014 | 1Score **> 34** indicates **identity** Score **> 14** indicates **homology** | U | R.SQIDELYATIKI.- |
| 54367 | 688 | – | 699 | 697.3829 | 1392.7512 | 1392.7500 | 0.84 | 1 | 47 | 4.3e-05 | 1Score **> 34** indicates **identity** Score **> 15** indicates **homology** | U | R.SQIDELYATIKI.- |
| 54369 | 688 | – | 699 | 697.3830 | 1392.7515 | 1392.7500 | 1.08 | 1 | 46 | 5.4e-05 | 1Score **> 34** indicates **identity** Score **> 15** indicates **homology** | U | R.SQIDELYATIKI.- |
| 54371 | 688 | – | 699 | 697.3834 | 1392.7522 | 1392.7500 | 1.59 | 1 | 57 | 4.5e-06 | 1Score **> 34** indicates **identity** Score **> 16** indicates **homology** | U | R.SQIDELYATIKI.- |
| 54372 | 688 | – | 699 | 697.3834 | 1392.7522 | 1392.7500 | 1.60 | 1 | 42 | 0.00011 | 1Score **> 34** indicates **identity** Score **> 15** indicates **homology** | U | R.SQIDELYATIKI.- |
| 54373 | 688 | – | 699 | 697.3841 | 1392.7537 | 1392.7500 | 2.65 | 1 | 38 | 0.0003 | 1Score **> 34** indicates **identity** Score **> 15** indicates **homology** | U | R.SQIDELYATIKI.- |
| 54374 | 688 | – | 699 | 697.3847 | 1392.7548 | 1392.7500 | 3.44 | 1 | 56 | 5.6e-06 | 1Score **> 34** indicates **identity** Score **> 16** indicates **homology** | U | R.SQIDELYATIKI.- |

---

```
ID   D3Z041_MOUSE            Unreviewed;       699 AA.
AC   D3Z041;
DT   20-APR-2010, integrated into UniProtKB/TrEMBL.
DT   20-APR-2010, sequence version 1.
DT   28-JUN-2023, entry version 79.
DE   RecName: Full=Long-chain-fatty-acid--CoA ligase {ECO:0000256|RuleBase:RU369030};
DE            EC=6.2.1.15 {ECO:0000256|RuleBase:RU369030};
DE            EC=6.2.1.3 {ECO:0000256|RuleBase:RU369030};
DE   AltName: Full=Acyl-CoA synthetase {ECO:0000256|RuleBase:RU369030};
DE   AltName: Full=Long-chain acyl-CoA synthetase {ECO:0000256|RuleBase:RU369030};
GN   Name=Acsl1 {ECO:0000313|Ensembl:ENSMUSP00000106000.2,
GN   ECO:0000313|MGI:MGI:102797};
OS   Mus musculus (Mouse).
OC   Eukaryota; Metazoa; Chordata; Craniata; Vertebrata; Euteleostomi; Mammalia;
OC   Eutheria; Euarchontoglires; Glires; Rodentia; Myomorpha; Muroidea; Muridae;
OC   Murinae; Mus; Mus.
OX   NCBI_TaxID=10090 {ECO:0000313|Ensembl:ENSMUSP00000106000.2, ECO:0000313|Proteomes:UP000000589};
RN   [1] {ECO:0007829|PubMed:17242355}
RP   IDENTIFICATION BY MASS SPECTROMETRY [LARGE SCALE ANALYSIS].
RX   PubMed=17242355; DOI=10.1073/pnas.0609836104;
RA   Villen J., Beausoleil S.A., Gerber S.A., Gygi S.P.;
RT   "Large-scale phosphorylation analysis of mouse liver.";
RL   Proc. Natl. Acad. Sci. U.S.A. 104:1488-1493(2007).
RN   [2] {ECO:0000313|Ensembl:ENSMUSP00000106000.2, ECO:0000313|Proteomes:UP000000589}
RP   NUCLEOTIDE SEQUENCE [LARGE SCALE GENOMIC DNA].
RC   STRAIN=C57BL/6J {ECO:0000313|Ensembl:ENSMUSP00000106000.2,
RC   ECO:0000313|Proteomes:UP000000589};
RX   PubMed=19468303; DOI=10.1371/journal.pbio.1000112;
RA   Church D.M., Goodstadt L., Hillier L.W., Zody M.C., Goldstein S., She X.,
RA   Bult C.J., Agarwala R., Cherry J.L., DiCuccio M., Hlavina W., Kapustin Y.,
RA   Meric P., Maglott D., Birtle Z., Marques A.C., Graves T., Zhou S.,
RA   Teague B., Potamousis K., Churas C., Place M., Herschleb J., Runnheim R.,
RA   Forrest D., Amos-Landgraf J., Schwartz D.C., Cheng Z., Lindblad-Toh K.,
RA   Eichler E.E., Ponting C.P.;
RT   "Lineage-specific biology revealed by a finished genome assembly of the
RT   mouse.";
RL   PLoS Biol. 7:E1000112-E1000112(2009).
RN   [3] {ECO:0007829|PubMed:21183079}
RP   IDENTIFICATION BY MASS SPECTROMETRY [LARGE SCALE ANALYSIS].
RX   PubMed=21183079; DOI=10.1016/j.cell.2010.12.001;
RA   Huttlin E.L., Jedrychowski M.P., Elias J.E., Goswami T., Rad R.,
RA   Beausoleil S.A., Villen J., Haas W., Sowa M.E., Gygi S.P.;
RT   "A tissue-specific atlas of mouse protein phosphorylation and expression.";
RL   Cell 143:1174-1189(2010).
RN   [4] {ECO:0007829|PubMed:23576753}
RP   IDENTIFICATION BY MASS SPECTROMETRY [LARGE SCALE ANALYSIS].
RX   PubMed=23576753; DOI=10.1073/pnas.1302961110;
RA   Rardin M.J., Newman J.C., Held J.M., Cusack M.P., Sorensen D.J., Li B.,
RA   Schilling B., Mooney S.D., Kahn C.R., Verdin E., Gibson B.W.;
RT   "Label-free quantitative proteomics of the lysine acetylome in mitochondria
RT   identifies substrates of SIRT3 in metabolic pathways.";
RL   Proc. Natl. Acad. Sci. U.S.A. 110:6601-6606(2013).
RN   [5] {ECO:0000313|Ensembl:ENSMUSP00000106000.2}
RP   IDENTIFICATION.
RC   STRAIN=C57BL/6J {ECO:0000313|Ensembl:ENSMUSP00000106000.2};
RG   Ensembl;
RL   Submitted (MAR-2023) to UniProtKB.
CC   -!- FUNCTION: Catalyzes the conversion of long-chain fatty acids to their
CC       active form acyl-CoAs for both synthesis of cellular lipids, and
CC       degradation via beta-oxidation. {ECO:0000256|RuleBase:RU369030}.
CC   -!- CATALYTIC ACTIVITY:
CC       Reaction=(5Z,8Z,11Z,14Z)-eicosatetraenoate + ATP + CoA =
CC         (5Z,8Z,11Z,14Z)-eicosatetraenoyl-CoA + AMP + diphosphate;
CC         Xref=Rhea:RHEA:19713, ChEBI:CHEBI:30616, ChEBI:CHEBI:32395,
CC         ChEBI:CHEBI:33019, ChEBI:CHEBI:57287, ChEBI:CHEBI:57368,
CC         ChEBI:CHEBI:456215; EC=6.2.1.15;
CC         Evidence={ECO:0000256|ARBA:ARBA00024548,
CC         ECO:0000256|RuleBase:RU369030};
CC       PhysiologicalDirection=left-to-right; Xref=Rhea:RHEA:19714;
CC         Evidence={ECO:0000256|ARBA:ARBA00024548,
CC         ECO:0000256|RuleBase:RU369030};
CC   -!- CATALYTIC ACTIVITY:
CC       Reaction=(E)-hexadec-2-enoate + ATP + CoA = (2E)-hexadecenoyl-CoA + AMP
CC         + diphosphate; Xref=Rhea:RHEA:36139, ChEBI:CHEBI:30616,
CC         ChEBI:CHEBI:33019, ChEBI:CHEBI:57287, ChEBI:CHEBI:61526,
CC         ChEBI:CHEBI:72745, ChEBI:CHEBI:456215;
CC         Evidence={ECO:0000256|ARBA:ARBA00024565,
CC         ECO:0000256|RuleBase:RU369030};
CC       PhysiologicalDirection=left-to-right; Xref=Rhea:RHEA:36140;
CC         Evidence={ECO:0000256|ARBA:ARBA00024565,
CC         ECO:0000256|RuleBase:RU369030};
CC   -!- CATALYTIC ACTIVITY:
CC       Reaction=12-hydroxy-(5Z,8Z,10E,14Z)-eicosatetraenoate + ATP + CoA = 12-
CC         hydroxy-(5Z,8Z,10E,14Z)-eicosatetraenoyl-CoA + AMP + diphosphate;
CC         Xref=Rhea:RHEA:52112, ChEBI:CHEBI:30616, ChEBI:CHEBI:33019,
CC         ChEBI:CHEBI:57287, ChEBI:CHEBI:90718, ChEBI:CHEBI:136408,
CC         ChEBI:CHEBI:456215; Evidence={ECO:0000256|ARBA:ARBA00024495,
CC         ECO:0000256|RuleBase:RU369030};
CC       PhysiologicalDirection=left-to-right; Xref=Rhea:RHEA:52113;
CC         Evidence={ECO:0000256|ARBA:ARBA00024495,
CC         ECO:0000256|RuleBase:RU369030};
CC   -!- CATALYTIC ACTIVITY:
CC       Reaction=15-hydroxy-(5Z,8Z,11Z,13E)-eicosatetraenoate + ATP + CoA = 15-
CC         hydroxy-(5Z,8Z,11Z,13E)-eicosatetraenoyl-CoA + AMP + diphosphate;
CC         Xref=Rhea:RHEA:52116, ChEBI:CHEBI:30616, ChEBI:CHEBI:33019,
CC         ChEBI:CHEBI:57287, ChEBI:CHEBI:78832, ChEBI:CHEBI:136409,
CC         ChEBI:CHEBI:456215; Evidence={ECO:0000256|ARBA:ARBA00024532,
CC         ECO:0000256|RuleBase:RU369030};
CC       PhysiologicalDirection=left-to-right; Xref=Rhea:RHEA:52117;
CC         Evidence={ECO:0000256|ARBA:ARBA00024532,
CC         ECO:0000256|RuleBase:RU369030};
CC   -!- CATALYTIC ACTIVITY:
CC       Reaction=5-hydroxy-(6E,8Z,11Z,14Z)-eicosatetraenoate + ATP + CoA = 5-
CC         hydroxy-(6E,8Z,11Z,14Z)-eicosatetraenoyl-CoA + AMP + diphosphate;
CC         Xref=Rhea:RHEA:52108, ChEBI:CHEBI:30616, ChEBI:CHEBI:33019,
CC         ChEBI:CHEBI:57287, ChEBI:CHEBI:65341, ChEBI:CHEBI:136407,
CC         ChEBI:CHEBI:456215; Evidence={ECO:0000256|ARBA:ARBA00024469,
CC         ECO:0000256|RuleBase:RU369030};
CC       PhysiologicalDirection=left-to-right; Xref=Rhea:RHEA:52109;
CC         Evidence={ECO:0000256|ARBA:ARBA00024469,
CC         ECO:0000256|RuleBase:RU369030};
CC   -!- CATALYTIC ACTIVITY:
CC       Reaction=ATP + CoA + hexadecanoate = AMP + diphosphate + hexadecanoyl-
CC         CoA; Xref=Rhea:RHEA:30751, ChEBI:CHEBI:7896, ChEBI:CHEBI:30616,
CC         ChEBI:CHEBI:33019, ChEBI:CHEBI:57287, ChEBI:CHEBI:57379,
CC         ChEBI:CHEBI:456215; Evidence={ECO:0000256|ARBA:ARBA00024497,
CC         ECO:0000256|RuleBase:RU369030};
CC       PhysiologicalDirection=left-to-right; Xref=Rhea:RHEA:30752;
CC         Evidence={ECO:0000256|ARBA:ARBA00024497,
CC         ECO:0000256|RuleBase:RU369030};
CC   -!- CATALYTIC ACTIVITY:
CC       Reaction=a long-chain fatty acid + ATP + CoA = a long-chain fatty acyl-
CC         CoA + AMP + diphosphate; Xref=Rhea:RHEA:15421, ChEBI:CHEBI:30616,
CC         ChEBI:CHEBI:33019, ChEBI:CHEBI:57287, ChEBI:CHEBI:57560,
CC         ChEBI:CHEBI:83139, ChEBI:CHEBI:456215; EC=6.2.1.3;
CC         Evidence={ECO:0000256|ARBA:ARBA00024484};
CC       PhysiologicalDirection=left-to-right; Xref=Rhea:RHEA:15422;
CC         Evidence={ECO:0000256|ARBA:ARBA00024484};
CC   -!- SUBCELLULAR LOCATION: Endoplasmic reticulum membrane
CC       {ECO:0000256|ARBA:ARBA00004643}; Single-pass type III membrane protein
CC       {ECO:0000256|ARBA:ARBA00004643}. Membrane
CC       {ECO:0000256|ARBA:ARBA00004183}; Single-pass type III membrane protein
CC       {ECO:0000256|ARBA:ARBA00004183}. Mitochondrion outer membrane
CC       {ECO:0000256|RuleBase:RU369030}; Single-pass membrane protein
CC       {ECO:0000256|RuleBase:RU369030}. Endoplasmic reticulum membrane
CC       {ECO:0000256|RuleBase:RU369030}; Single-pass membrane protein
CC       {ECO:0000256|RuleBase:RU369030}. Mitochondrion outer membrane
CC       {ECO:0000256|ARBA:ARBA00025703}; Single-pass type III membrane protein
CC       {ECO:0000256|ARBA:ARBA00025703}.
CC   -!- SIMILARITY: Belongs to the ATP-dependent AMP-binding enzyme family.
CC       {ECO:0000256|ARBA:ARBA00006432, ECO:0000256|RuleBase:RU369030}.
CC   ---------------------------------------------------------------------------
CC   Copyrighted by the UniProt Consortium, see https://www.uniprot.org/terms
CC   Distributed under the Creative Commons Attribution (CC BY 4.0) License
CC   ---------------------------------------------------------------------------
DR   AlphaFoldDB; D3Z041; -.
DR   SMR; D3Z041; -.
DR   SwissPalm; D3Z041; -.
DR   EPD; D3Z041; -.
DR   jPOST; D3Z041; -.
DR   MaxQB; D3Z041; -.
DR   PeptideAtlas; D3Z041; -.
DR   ProteomicsDB; 310638; -.
DR   Antibodypedia; 1946; 328 antibodies from 35 providers.
DR   Ensembl; ENSMUST00000110371.8; ENSMUSP00000106000.2; ENSMUSG00000018796.14.
DR   AGR; MGI:102797; -.
DR   MGI; MGI:102797; Acsl1.
DR   VEuPathDB; HostDB:ENSMUSG00000018796; -.
DR   GeneTree; ENSGT00940000154508; -.
DR   OMA; WTIGAQV; -.
DR   ChiTaRS; Acsl1; mouse.
DR   Proteomes; UP000000589; Chromosome 8.
DR   Bgee; ENSMUSG00000018796; Expressed in brown adipose tissue and 285 other tissues.
DR   ExpressionAtlas; D3Z041; baseline and differential.
DR   GO; GO:0005789; C:endoplasmic reticulum membrane; IEA:UniProtKB-SubCell.
DR   GO; GO:0005741; C:mitochondrial outer membrane; IEA:UniProtKB-SubCell.
DR   GO; GO:0005524; F:ATP binding; IEA:UniProtKB-KW.
DR   GO; GO:0004467; F:long-chain fatty acid-CoA ligase activity; IEA:InterPro.
DR   CDD; cd05927; LC-FACS_euk; 1.
DR   Gene3D; 3.40.50.12780; N-terminal domain of ligase-like; 1.
DR   InterPro; IPR025110; AMP-bd_C.
DR   InterPro; IPR020845; AMP-binding_CS.
DR   InterPro; IPR000873; AMP-dep_Synth/Lig_com.
DR   InterPro; IPR042099; ANL_N_sf.
DR   InterPro; IPR045311; LC-FACS_euk.
DR   PANTHER; PTHR43272; LONG-CHAIN-FATTY-ACID--COA LIGASE; 1.
DR   PANTHER; PTHR43272:SF28; LONG-CHAIN-FATTY-ACID--COA LIGASE 1; 1.
DR   Pfam; PF00501; AMP-binding; 1.
DR   Pfam; PF13193; AMP-binding_C; 1.
DR   SUPFAM; SSF56801; Acetyl-CoA synthetase-like; 1.
DR   PROSITE; PS00455; AMP_BINDING; 1.
PE   1: Evidence at protein level;
KW   ATP-binding {ECO:0000256|RuleBase:RU369030};
KW   Fatty acid metabolism {ECO:0000256|ARBA:ARBA00022832,
KW   ECO:0000256|RuleBase:RU369030}; Ligase {ECO:0000256|RuleBase:RU369030};
KW   Lipid metabolism {ECO:0000256|ARBA:ARBA00023098,
KW   ECO:0000256|RuleBase:RU369030}; Membrane {ECO:0000256|RuleBase:RU369030};
KW   Nucleotide-binding {ECO:0000256|RuleBase:RU369030};
KW   Proteomics identification {ECO:0007829|EPD:D3Z041,
KW   ECO:0007829|MaxQB:D3Z041};
KW   Reference proteome {ECO:0000313|Proteomes:UP000000589};
KW   Transmembrane {ECO:0000256|RuleBase:RU369030};
KW   Transmembrane helix {ECO:0000256|RuleBase:RU369030}.
FT   TRANSMEM        21..45
FT                   /note="Helical"
FT                   /evidence="ECO:0000256|RuleBase:RU369030"
FT   DOMAIN          116..563
FT                   /note="AMP-dependent synthetase/ligase"
FT                   /evidence="ECO:0000259|Pfam:PF00501"
FT   DOMAIN          573..629
FT                   /note="AMP-binding enzyme C-terminal"
FT                   /evidence="ECO:0000259|Pfam:PF13193"
SQ   SEQUENCE   699 AA;  78034 MW;  D10B6FE1982D3474 CRC64;
     MEVHELFRYF RMPELIDIRQ YVRTLPTNTL MGFGAFAALT TFWYATRPKA LKPPCDLSMQ
     SVEIAGTTDG IRRSAVLEDD KLLVYYYDDV RTMYDGFQRG IQVSNNGPCL GSRKPNQPYE
     WISYKEVAEL AECIGSGLIQ KGFKPCSEQF IGLFSQNRPE WVIVEQGCFS YSMVVVPLYD
     TLGADAITYI VNKAELSVIF ADKPEKAKLL LEGVENKLTP CLKIIVIMDS YGSDLVERGK
     KCGVEIISLK ALEDLGRVNR VKPKPPEPED LAIICFTSGT TGNPKGAMIT HQNIINDCSG
     FIKATESALT LNASDTQISY LPLAHMYEQQ LQCVMLCHGA KIGFFQGDIR LLMDDLKVLQ
     PTIFPVVPRL LNRMFDRIFG QANTSLKRWL LDFASKRKEA ELRSGIVRNN SLWDKLIFHK
     IQSSLGGKVR LMITGAAPVS ATVLTFLRTA LGCQFYEGYG QTECTAGCCL SLPGDWTAGH
     VGAPMPCNYV KLVDVEEMNY LASKGEGEVC VKGANVFKGY LKDPARTAEA LDKDGWLHTG
     DIGKWLPNGT LKIIDRKKHI FKLAQGEYIA PEKIENIYLR SEAVAQVFVH GESLQAFLIA
     VVVPDVESLP SWAQKRGLQG SFEELCRNKD INKAILDDLL KLGKEAGLKP FEQVKGIAVH
     PELFSIDNGL LTPTLKAKRP ELRNYFRSQI DELYATIKI
//
```

|  |
| --- |
| **Mascot:** http://www.matrixscience.com/ |

Oxidation (M) (+15.9949)
