# Supplementary material for: A common East-Asian ALDH2 mutation causes metabolic disorders and the therapeutic effect of ALDH2 activators
Source: Nat Commun. 2023 Sep 25;14:5971. doi: 10.1038/s41467-023-41570-6 (PMC10520061; doi:10.1038/s41467-023-41570-6)
Supplement: Supplementary file 4 — Supplementary Data 1 [file 41467_2023_41570_MOESM4_ESM.zip › Table S5b/D3Z041/D3Z041_WTO-3_K633.html]

Mascot Search Results: D3Z041
 

# MASCOT Search Results

## Protein View: D3Z041

### Arachidonate--CoA ligase OS=Mus musculus OX=10090 GN=Acsl1 PE=1 SV=1

|  |  |
| --- | --- |
| Database: | Mouse\_UniProt\_proteomes |
| Score: | 10512 |
| Monoisotopic mass (Mr): | 79011 |
| Calculated pI: | 7.43 |

Sequence similarity is available as an NCBI BLAST search of D3Z041 against nr.

### Search parameters

|  |  |
| --- | --- |
| MS data file: | `D:\LCMSMS\2023 Users' data\230529-1\230529-1-WTO-3_20230601175054.raw` |
| Enzyme: | Trypsin/P: cuts C-term side of KR. |
| Fixed modifications: | Carbamidomethyl (C) |
| Variable modifications: | Deamidated (NQ), HNE (C), HNE (H), HNE (K), Oxidation (M) |

### Protein sequence coverage: 61%

Matched peptides shown in ***bold red***.

|  |  |  |  |  |  |
| --- | --- | --- | --- | --- | --- |
| `1` | `MEVHELFRYF` | `RMPELIDIRQ` | `YVRTLPTNTL` | `MGFGAFAALT` | `TFWYATRPKA` |
| `51` | `LKPPCDLSMQ` | `SVEIAGTTDG` | `IRRSAVLEDD` | `KLLVYYYDDV` | `RTMYDGFQRG` |
| `101` | `IQVSNNGPCL` | `GSRKPNQPYE` | `WISYKEVAEL` | `AECIGSGLIQ` | `KGFKPCSEQF` |
| `151` | `IGLFSQNRPE` | `WVIVEQGCFS` | `YSMVVVPLYD` | `TLGADAITYI` | `VNKAELSVIF` |
| `201` | `ADKPEKAKLL` | `LEGVENKLTP` | `CLKIIVIMDS` | `YGSDLVERGK` | `KCGVEIISLK` |
| `251` | `ALEDLGRVNR` | `VKPKPPEPED` | `LAIICFTSGT` | `TGNPKGAMIT` | `HQNIINDCSG` |
| `301` | `FIKATESALT` | `LNASDTQISY` | `LPLAHMYEQQ` | `LQCVMLCHGA` | `KIGFFQGDIR` |
| `351` | `LLMDDLKVLQ` | `PTIFPVVPRL` | `LNRMFDRIFG` | `QANTSLKRWL` | `LDFASKRKEA` |
| `401` | `ELRSGIVRNN` | `SLWDKLIFHK` | `IQSSLGGKVR` | `LMITGAAPVS` | `ATVLTFLRTA` |
| `451` | `LGCQFYEGYG` | `QTECTAGCCL` | `SLPGDWTAGH` | `VGAPMPCNYV` | `KLVDVEEMNY` |
| `501` | `LASKGEGEVC` | `VKGANVFKGY` | `LKDPARTAEA` | `LDKDGWLHTG` | `DIGKWLPNGT` |
| `551` | `LKIIDRKKHI` | `FKLAQGEYIA` | `PEKIENIYLR` | `SEAVAQVFVH` | `GESLQAFLIA` |
| `601` | `VVVPDVESLP` | `SWAQKRGLQG` | `SFEELCRNKD` | `INKAILDDLL` | `KLGKEAGLKP` |
| `651` | `FEQVKGIAVH` | `PELFSIDNGL` | `LTPTLKAKRP` | `ELRNYFRSQI` | `DELYATIKI` |

Unformatted sequence string: 699 residues (for pasting into other applications).

|  |  |  |  |
| --- | --- | --- | --- |
| Sort by | residue number | increasing mass | decreasing mass |
| Show | matched peptides only | predicted peptides also |  |

| Query | Start | – | End | Observed | Mr(expt) | Mr(calc) | ppm | M | Score | Expect | Rank | U | Peptide |
| --- | --- | --- | --- | --- | --- | --- | --- | --- | --- | --- | --- | --- | --- |
| 60767 | 9 | – | 19 | 484.9260 | 1451.7561 | 1451.7595 | -2.31 | 1 | 33 | 0.001 | 1Score **> 34** indicates **identity** Score **> 15** indicates **homology** | U | R.YFRMPELIDIR.Q |
| 60768 | 9 | – | 19 | 484.9260 | 1451.7562 | 1451.7595 | -2.26 | 1 | 16 | 0.033 | 1Score **> 34** indicates **identity** Score **> 14** indicates **homology** | U | R.YFRMPELIDIR.Q |
| 60770 | 9 | – | 19 | 484.9264 | 1451.7573 | 1451.7595 | -1.53 | 1 | 32 | 0.0012 | 1Score **> 34** indicates **identity** Score **> 15** indicates **homology** | U | R.YFRMPELIDIR.Q |
| 60772 | 9 | – | 19 | 484.9267 | 1451.7581 | 1451.7595 | -0.92 | 1 | 34 | 0.0016 | 1Score **> 34** indicates **identity** Score **> 19** indicates **homology** | U | R.YFRMPELIDIR.Q |
| 60773 | 9 | – | 19 | 484.9267 | 1451.7582 | 1451.7595 | -0.87 | 1 | 43 | 0.00022 | 1Score **> 34** indicates **identity** Score **> 19** indicates **homology** | U | R.YFRMPELIDIR.Q |
| 60775 | 9 | – | 19 | 484.9273 | 1451.7601 | 1451.7595 | 0.40 | 1 | 43 | 0.00014 | 1Score **> 34** indicates **identity** Score **> 18** indicates **homology** | U | R.YFRMPELIDIR.Q |
| 60776 | 9 | – | 19 | 484.9273 | 1451.7601 | 1451.7595 | 0.40 | 1 | 22 | 0.0096 | 1Score **> 34** indicates **identity** Score **> 15** indicates **homology** | U | R.YFRMPELIDIR.Q |
| 60778 | 9 | – | 19 | 484.9274 | 1451.7602 | 1451.7595 | 0.53 | 1 | 21 | 0.016 | 1Score **> 34** indicates **identity** Score **> 15** indicates **homology** | U | R.YFRMPELIDIR.Q |
| 60781 | 9 | – | 19 | 484.9282 | 1451.7626 | 1451.7595 | 2.18 | 1 | 25 | 0.006 | 1Score **> 34** indicates **identity** Score **> 15** indicates **homology** | U | R.YFRMPELIDIR.Q |
| 13756 | 12 | – | 19 | 493.7697 | 985.5248 | 985.5266 | -1.90 | 0 | 22 | 0.022 | 1Score **> 31** indicates **identity** Score **> 18** indicates **homology** | U | R.MPELIDIR.Q |
| 13758 | 12 | – | 19 | 493.7701 | 985.5256 | 985.5266 | -1.05 | 0 | 31 | 0.0013 | 1Score **> 31** indicates **identity** Score **> 14** indicates **homology** | U | R.MPELIDIR.Q |
| 13761 | 12 | – | 19 | 493.7707 | 985.5268 | 985.5266 | 0.14 | 0 | 49 | 0.00011 | 1Score **> 31** indicates **identity** Score **> 22** indicates **homology** | U | R.MPELIDIR.Q |
| 13763 | 12 | – | 19 | 493.7709 | 985.5272 | 985.5266 | 0.61 | 0 | 51 | 8.6e-05 | 1Score **> 31** indicates **identity** Score **> 23** indicates **homology** | U | R.MPELIDIR.Q |
| 13764 | 12 | – | 19 | 493.7709 | 985.5273 | 985.5266 | 0.70 | 0 | 47 | 0.00016 | 1Score **> 31** indicates **identity** Score **> 22** indicates **homology** | U | R.MPELIDIR.Q |
| 13765 | 12 | – | 19 | 493.7711 | 985.5276 | 985.5266 | 1.01 | 0 | 28 | 0.003 | 1Score **> 31** indicates **identity** Score **> 15** indicates **homology** | U | R.MPELIDIR.Q |
| 13766 | 12 | – | 19 | 493.7712 | 985.5279 | 985.5266 | 1.25 | 0 | 31 | 0.0012 | 1Score **> 31** indicates **identity** Score **> 14** indicates **homology** | U | R.MPELIDIR.Q |
| 14876 | 12 | – | 19 | 501.7693 | 1001.5240 | 1001.5215 | 2.41 | 0 | 20 | 0.027 | 1Score **> 33** indicates **identity** Score **> 17** indicates **homology** | U | R.MPELIDIR.Q  + Oxidation (M) |
| 14882 | 12 | – | 19 | 501.7706 | 1001.5267 | 1001.5215 | 5.16 | 0 | 15 | 0.041 | 1Score **> 32** indicates **identity** Score **> 13** indicates **homology** | U | R.MPELIDIR.Q  + Oxidation (M) |
| 166104 | 50 | – | 73 | 654.5867 | 2614.3179 | 2614.3207 | -1.08 | 2 | 49 | 2.8e-05 | 1Score **> 37** indicates **identity** Score **> 16** indicates **homology** | U | K.ALKPPCDLSMQSVEIAGTTDGIRR.S |
| 166108 | 50 | – | 73 | 654.5876 | 2614.3215 | 2614.3207 | 0.31 | 2 | 48 | 3e-05 | 1Score **> 37** indicates **identity** Score **> 16** indicates **homology** | U | K.ALKPPCDLSMQSVEIAGTTDGIRR.S |
| 166109 | 50 | – | 73 | 654.5877 | 2614.3218 | 2614.3207 | 0.41 | 2 | 54 | 9.4e-06 | 1Score **> 37** indicates **identity** Score **> 16** indicates **homology** | U | K.ALKPPCDLSMQSVEIAGTTDGIRR.S |
| 166175 | 50 | – | 73 | 654.8373 | 2615.3200 | 2615.3047 | 5.86 | 2 | 27 | 0.0027 | 1Score **> 37** indicates **identity** Score **> 14** indicates **homology** | U | K.ALKPPCDLSMQSVEIAGTTDGIRR.S  + Deamidated (NQ) |
| 150748 | 73 | – | 91 | 583.8016 | 2331.1774 | 2331.1747 | 1.19 | 2 | 21 | 0.011 | 1Score **> 37** indicates **identity** Score **> 14** indicates **homology** | U | R.RSAVLEDDKLLVYYYDDVR.T |
| 150749 | 73 | – | 91 | 583.8020 | 2331.1787 | 2331.1747 | 1.74 | 2 | 27 | 0.0031 | 1Score **> 37** indicates **identity** Score **> 14** indicates **homology** | U | R.RSAVLEDDKLLVYYYDDVR.T |
| 150750 | 73 | – | 91 | 583.8023 | 2331.1801 | 2331.1747 | 2.34 | 2 | 32 | 0.001 | 1Score **> 37** indicates **identity** Score **> 14** indicates **homology** | U | R.RSAVLEDDKLLVYYYDDVR.T |
| 138922 | 74 | – | 91 | 726.0249 | 2175.0528 | 2175.0735 | -9.52 | 1 | 30 | 0.0017 | 1Score **> 36** indicates **identity** Score **> 14** indicates **homology** | U | R.SAVLEDDKLLVYYYDDVR.T |
| 138925 | 74 | – | 91 | 1088.5355 | 2175.0564 | 2175.0735 | -7.89 | 1 | 43 | 9.6e-05 | 1Score **> 36** indicates **identity** Score **> 15** indicates **homology** | U | R.SAVLEDDKLLVYYYDDVR.T |
| 138932 | 74 | – | 91 | 726.0282 | 2175.0628 | 2175.0735 | -4.92 | 1 | 19 | 0.015 | 1Score **> 36** indicates **identity** Score **> 14** indicates **homology** | U | R.SAVLEDDKLLVYYYDDVR.T |
| 138933 | 74 | – | 91 | 726.0283 | 2175.0631 | 2175.0735 | -4.79 | 1 | 27 | 0.0034 | 1Score **> 36** indicates **identity** Score **> 15** indicates **homology** | U | R.SAVLEDDKLLVYYYDDVR.T |
| 138936 | 74 | – | 91 | 726.0286 | 2175.0640 | 2175.0735 | -4.39 | 1 | 49 | 2.4e-05 | 1Score **> 36** indicates **identity** Score **> 16** indicates **homology** | U | R.SAVLEDDKLLVYYYDDVR.T |
| 138937 | 74 | – | 91 | 726.0287 | 2175.0643 | 2175.0735 | -4.23 | 1 | 65 | 7.8e-07 | 1Score **> 36** indicates **identity** Score **> 17** indicates **homology** | U | R.SAVLEDDKLLVYYYDDVR.T |
| 138938 | 74 | – | 91 | 726.0287 | 2175.0644 | 2175.0735 | -4.22 | 1 | 65 | 8.1e-07 | 1Score **> 36** indicates **identity** Score **> 17** indicates **homology** | U | R.SAVLEDDKLLVYYYDDVR.T |
| 138943 | 74 | – | 91 | 726.0292 | 2175.0656 | 2175.0735 | -3.64 | 1 | 65 | 7.3e-07 | 1Score **> 36** indicates **identity** Score **> 17** indicates **homology** | U | R.SAVLEDDKLLVYYYDDVR.T |
| 138944 | 74 | – | 91 | 726.0292 | 2175.0656 | 2175.0735 | -3.63 | 1 | 70 | 2.8e-07 | 1Score **> 36** indicates **identity** Score **> 17** indicates **homology** | U | R.SAVLEDDKLLVYYYDDVR.T |
| 138946 | 74 | – | 91 | 726.0292 | 2175.0658 | 2175.0735 | -3.54 | 1 | 78 | 5.2e-08 | 1Score **> 36** indicates **identity** Score **> 17** indicates **homology** | U | R.SAVLEDDKLLVYYYDDVR.T |
| 138948 | 74 | – | 91 | 1088.5403 | 2175.0660 | 2175.0735 | -3.45 | 1 | 62 | 1.6e-06 | 1Score **> 36** indicates **identity** Score **> 16** indicates **homology** | U | R.SAVLEDDKLLVYYYDDVR.T |
| 138950 | 74 | – | 91 | 726.0293 | 2175.0662 | 2175.0735 | -3.40 | 1 | 30 | 0.0016 | 1Score **> 36** indicates **identity** Score **> 14** indicates **homology** | U | R.SAVLEDDKLLVYYYDDVR.T |
| 138952 | 74 | – | 91 | 726.0294 | 2175.0665 | 2175.0735 | -3.24 | 1 | 66 | 6.9e-07 | 1Score **> 36** indicates **identity** Score **> 17** indicates **homology** | U | R.SAVLEDDKLLVYYYDDVR.T |
| 138957 | 74 | – | 91 | 726.0297 | 2175.0672 | 2175.0735 | -2.93 | 1 | 27 | 0.0028 | 1Score **> 36** indicates **identity** Score **> 14** indicates **homology** | U | R.SAVLEDDKLLVYYYDDVR.T |
| 138959 | 74 | – | 91 | 726.0297 | 2175.0673 | 2175.0735 | -2.89 | 1 | 61 | 2e-06 | 1Score **> 36** indicates **identity** Score **> 16** indicates **homology** | U | R.SAVLEDDKLLVYYYDDVR.T |
| 138960 | 74 | – | 91 | 1088.5409 | 2175.0673 | 2175.0735 | -2.89 | 1 | 135 | 2.4e-13 | 1Score **> 36** indicates **identity** Score **> 21** indicates **homology** | U | R.SAVLEDDKLLVYYYDDVR.T |
| 138961 | 74 | – | 91 | 726.0298 | 2175.0675 | 2175.0735 | -2.80 | 1 | 63 | 1.3e-06 | 1Score **> 36** indicates **identity** Score **> 16** indicates **homology** | U | R.SAVLEDDKLLVYYYDDVR.T |
| 138962 | 74 | – | 91 | 1088.5410 | 2175.0675 | 2175.0735 | -2.79 | 1 | 86 | 8.5e-09 | 1Score **> 36** indicates **identity** Score **> 18** indicates **homology** | U | R.SAVLEDDKLLVYYYDDVR.T |
| 138964 | 74 | – | 91 | 726.0299 | 2175.0679 | 2175.0735 | -2.60 | 1 | 25 | 0.0047 | 1Score **> 36** indicates **identity** Score **> 14** indicates **homology** | U | R.SAVLEDDKLLVYYYDDVR.T |
| 138966 | 74 | – | 91 | 726.0300 | 2175.0682 | 2175.0735 | -2.48 | 1 | 70 | 2.7e-07 | 1Score **> 36** indicates **identity** Score **> 17** indicates **homology** | U | R.SAVLEDDKLLVYYYDDVR.T |
| 138971 | 74 | – | 91 | 1088.5419 | 2175.0693 | 2175.0735 | -1.97 | 1 | 98 | 6.4e-10 | 1Score **> 36** indicates **identity** Score **> 19** indicates **homology** | U | R.SAVLEDDKLLVYYYDDVR.T |
| 138972 | 74 | – | 91 | 1088.5420 | 2175.0695 | 2175.0735 | -1.88 | 1 | 118 | 9.5e-12 | 1Score **> 36** indicates **identity** Score **> 20** indicates **homology** | U | R.SAVLEDDKLLVYYYDDVR.T |
| 138973 | 74 | – | 91 | 726.0305 | 2175.0696 | 2175.0735 | -1.81 | 1 | 66 | 6.3e-07 | 1Score **> 36** indicates **identity** Score **> 17** indicates **homology** | U | R.SAVLEDDKLLVYYYDDVR.T |
| 138974 | 74 | – | 91 | 726.0306 | 2175.0700 | 2175.0735 | -1.61 | 1 | 40 | 0.00017 | 1Score **> 36** indicates **identity** Score **> 15** indicates **homology** | U | R.SAVLEDDKLLVYYYDDVR.T |
| 138976 | 74 | – | 91 | 1088.5426 | 2175.0706 | 2175.0735 | -1.35 | 1 | 17 | 0.024 | 1Score **> 36** indicates **identity** Score **> 14** indicates **homology** | U | R.SAVLEDDKLLVYYYDDVR.T |
| 138977 | 74 | – | 91 | 726.0308 | 2175.0707 | 2175.0735 | -1.33 | 1 | 65 | 8e-07 | 1Score **> 36** indicates **identity** Score **> 17** indicates **homology** | U | R.SAVLEDDKLLVYYYDDVR.T |
| 138979 | 74 | – | 91 | 726.0312 | 2175.0717 | 2175.0735 | -0.87 | 1 | 50 | 2.3e-05 | 1Score **> 36** indicates **identity** Score **> 16** indicates **homology** | U | R.SAVLEDDKLLVYYYDDVR.T |
| 138980 | 74 | – | 91 | 726.0312 | 2175.0718 | 2175.0735 | -0.82 | 1 | 78 | 5e-08 | 1Score **> 36** indicates **identity** Score **> 17** indicates **homology** | U | R.SAVLEDDKLLVYYYDDVR.T |
| 138982 | 74 | – | 91 | 726.0313 | 2175.0720 | 2175.0735 | -0.70 | 1 | 49 | 2.6e-05 | 1Score **> 36** indicates **identity** Score **> 16** indicates **homology** | U | R.SAVLEDDKLLVYYYDDVR.T |
| 138983 | 74 | – | 91 | 726.0313 | 2175.0722 | 2175.0735 | -0.64 | 1 | 29 | 0.0021 | 1Score **> 36** indicates **identity** Score **> 14** indicates **homology** | U | R.SAVLEDDKLLVYYYDDVR.T |
| 138984 | 74 | – | 91 | 726.0314 | 2175.0723 | 2175.0735 | -0.57 | 1 | 78 | 4.9e-08 | 1Score **> 36** indicates **identity** Score **> 17** indicates **homology** | U | R.SAVLEDDKLLVYYYDDVR.T |
| 138986 | 74 | – | 91 | 1088.5436 | 2175.0727 | 2175.0735 | -0.38 | 1 | 96 | 9.7e-10 | 1Score **> 36** indicates **identity** Score **> 19** indicates **homology** | U | R.SAVLEDDKLLVYYYDDVR.T |
| 138987 | 74 | – | 91 | 726.0315 | 2175.0728 | 2175.0735 | -0.36 | 1 | 65 | 7.7e-07 | 1Score **> 36** indicates **identity** Score **> 17** indicates **homology** | U | R.SAVLEDDKLLVYYYDDVR.T |
| 138988 | 74 | – | 91 | 726.0316 | 2175.0730 | 2175.0735 | -0.27 | 1 | 49 | 2.6e-05 | 1Score **> 36** indicates **identity** Score **> 16** indicates **homology** | U | R.SAVLEDDKLLVYYYDDVR.T |
| 138989 | 74 | – | 91 | 726.0317 | 2175.0732 | 2175.0735 | -0.14 | 1 | 33 | 0.00079 | 1Score **> 36** indicates **identity** Score **> 15** indicates **homology** | U | R.SAVLEDDKLLVYYYDDVR.T |
| 138990 | 74 | – | 91 | 726.0319 | 2175.0740 | 2175.0735 | 0.21 | 1 | 68 | 3.8e-07 | 1Score **> 36** indicates **identity** Score **> 17** indicates **homology** | U | R.SAVLEDDKLLVYYYDDVR.T |
| 138993 | 74 | – | 91 | 726.0320 | 2175.0743 | 2175.0735 | 0.33 | 1 | 78 | 4.9e-08 | 1Score **> 36** indicates **identity** Score **> 17** indicates **homology** | U | R.SAVLEDDKLLVYYYDDVR.T |
| 138994 | 74 | – | 91 | 726.0322 | 2175.0748 | 2175.0735 | 0.56 | 1 | 15 | 0.041 | 1Score **> 36** indicates **identity** Score **> 13** indicates **homology** | U | R.SAVLEDDKLLVYYYDDVR.T |
| 138995 | 74 | – | 91 | 726.0323 | 2175.0751 | 2175.0735 | 0.73 | 1 | 66 | 6.6e-07 | 1Score **> 36** indicates **identity** Score **> 17** indicates **homology** | U | R.SAVLEDDKLLVYYYDDVR.T |
| 138996 | 74 | – | 91 | 726.0323 | 2175.0752 | 2175.0735 | 0.77 | 1 | 69 | 3.7e-07 | 1Score **> 36** indicates **identity** Score **> 17** indicates **homology** | U | R.SAVLEDDKLLVYYYDDVR.T |
| 138997 | 74 | – | 91 | 1088.5452 | 2175.0759 | 2175.0735 | 1.09 | 1 | 74 | 1.1e-07 | 1Score **> 36** indicates **identity** Score **> 17** indicates **homology** | U | R.SAVLEDDKLLVYYYDDVR.T |
| 138998 | 74 | – | 91 | 726.0326 | 2175.0760 | 2175.0735 | 1.14 | 1 | 14 | 0.048 | 1Score **> 36** indicates **identity** Score **> 13** indicates **homology** | U | R.SAVLEDDKLLVYYYDDVR.T |
| 138999 | 74 | – | 91 | 726.0327 | 2175.0762 | 2175.0735 | 1.24 | 1 | 16 | 0.035 | 1Score **> 36** indicates **identity** Score **> 13** indicates **homology** | U | R.SAVLEDDKLLVYYYDDVR.T |
| 139001 | 74 | – | 91 | 726.0327 | 2175.0763 | 2175.0735 | 1.25 | 1 | 30 | 0.0015 | 1Score **> 36** indicates **identity** Score **> 14** indicates **homology** | U | R.SAVLEDDKLLVYYYDDVR.T |
| 139002 | 74 | – | 91 | 726.0329 | 2175.0768 | 2175.0735 | 1.49 | 1 | 68 | 4.1e-07 | 1Score **> 36** indicates **identity** Score **> 17** indicates **homology** | U | R.SAVLEDDKLLVYYYDDVR.T |
| 139006 | 74 | – | 91 | 726.0342 | 2175.0808 | 2175.0735 | 3.33 | 1 | 53 | 1.2e-05 | 1Score **> 37** indicates **identity** Score **> 16** indicates **homology** | U | R.SAVLEDDKLLVYYYDDVR.T |
| 139010 | 74 | – | 91 | 726.0349 | 2175.0829 | 2175.0735 | 4.28 | 1 | 21 | 0.011 | 1Score **> 37** indicates **identity** Score **> 14** indicates **homology** | U | R.SAVLEDDKLLVYYYDDVR.T |
| 139012 | 74 | – | 91 | 726.0351 | 2175.0834 | 2175.0735 | 4.51 | 1 | 20 | 0.013 | 1Score **> 37** indicates **identity** Score **> 14** indicates **homology** | U | R.SAVLEDDKLLVYYYDDVR.T |
| 139013 | 74 | – | 91 | 1088.5493 | 2175.0840 | 2175.0735 | 4.81 | 1 | 79 | 3.8e-08 | 1Score **> 36** indicates **identity** Score **> 17** indicates **homology** | U | R.SAVLEDDKLLVYYYDDVR.T |
| 139016 | 74 | – | 91 | 1088.5499 | 2175.0853 | 2175.0735 | 5.38 | 1 | 132 | 4.1e-13 | 1Score **> 36** indicates **identity** Score **> 21** indicates **homology** | U | R.SAVLEDDKLLVYYYDDVR.T |
| 139017 | 74 | – | 91 | 726.0358 | 2175.0855 | 2175.0735 | 5.48 | 1 | 32 | 0.001 | 1Score **> 36** indicates **identity** Score **> 14** indicates **homology** | U | R.SAVLEDDKLLVYYYDDVR.T |
| 139018 | 74 | – | 91 | 726.0361 | 2175.0864 | 2175.0735 | 5.92 | 1 | 46 | 4.8e-05 | 1Score **> 37** indicates **identity** Score **> 15** indicates **homology** | U | R.SAVLEDDKLLVYYYDDVR.T |
| 139021 | 74 | – | 91 | 726.0383 | 2175.0929 | 2175.0735 | 8.91 | 1 | 69 | 3.2e-07 | 1Score **> 37** indicates **identity** Score **> 17** indicates **homology** | U | R.SAVLEDDKLLVYYYDDVR.T |
| 61381 | 100 | – | 113 | 729.8566 | 1457.6986 | 1457.7045 | -4.08 | 0 | 24 | 0.0062 | 1Score **> 32** indicates **identity** Score **> 14** indicates **homology** | U | R.GIQVSNNGPCLGSR.K |
| 61382 | 100 | – | 113 | 729.8580 | 1457.7014 | 1457.7045 | -2.14 | 0 | 65 | 8e-07 | 1Score **> 32** indicates **identity** Score **> 17** indicates **homology** | U | R.GIQVSNNGPCLGSR.K |
| 61383 | 100 | – | 113 | 729.8584 | 1457.7023 | 1457.7045 | -1.50 | 0 | 74 | 1.2e-07 | 1Score **> 32** indicates **identity** Score **> 17** indicates **homology** | U | R.GIQVSNNGPCLGSR.K |
| 61384 | 100 | – | 113 | 729.8590 | 1457.7034 | 1457.7045 | -0.76 | 0 | 43 | 8.5e-05 | 1Score **> 32** indicates **identity** Score **> 15** indicates **homology** | U | R.GIQVSNNGPCLGSR.K |
| 61385 | 100 | – | 113 | 729.8591 | 1457.7036 | 1457.7045 | -0.64 | 0 | 74 | 1e-07 | 1Score **> 32** indicates **identity** Score **> 17** indicates **homology** | U | R.GIQVSNNGPCLGSR.K |
| 61386 | 100 | – | 113 | 729.8593 | 1457.7041 | 1457.7045 | -0.26 | 0 | 89 | 4.2e-09 | 1Score **> 32** indicates **identity** Score **> 18** indicates **homology** | U | R.GIQVSNNGPCLGSR.K |
| 61387 | 100 | – | 113 | 729.8599 | 1457.7053 | 1457.7045 | 0.52 | 0 | 70 | 2.5e-07 | 1Score **> 32** indicates **identity** Score **> 17** indicates **homology** | U | R.GIQVSNNGPCLGSR.K |
| 61484 | 100 | – | 113 | 730.3511 | 1458.6877 | 1458.6885 | -0.56 | 0 | 55 | 6.4e-06 | 1Score **> 31** indicates **identity** Score **> 16** indicates **homology** | U | R.GIQVSNNGPCLGSR.K  + Deamidated (NQ) |
| 61485 | 100 | – | 113 | 730.3514 | 1458.6883 | 1458.6885 | -0.13 | 0 | 43 | 9.9e-05 | 1Score **> 31** indicates **identity** Score **> 15** indicates **homology** | U | R.GIQVSNNGPCLGSR.K  + Deamidated (NQ) |
| 61486 | 100 | – | 113 | 730.3515 | 1458.6884 | 1458.6885 | -0.062 | 0 | 91 | 2.8e-09 | 1Score **> 31** indicates **identity** Score **> 18** indicates **homology** | U | R.GIQVSNNGPCLGSR.K  + Deamidated (NQ) |
| 61487 | 100 | – | 113 | 730.3525 | 1458.6904 | 1458.6885 | 1.27 | 0 | 43 | 9.4e-05 | 1Score **> 31** indicates **identity** Score **> 15** indicates **homology** | U | R.GIQVSNNGPCLGSR.K  + Deamidated (NQ) |
| 61490 | 100 | – | 113 | 730.3541 | 1458.6937 | 1458.6885 | 3.53 | 0 | 65 | 7.5e-07 | 1Score **> 31** indicates **identity** Score **> 17** indicates **homology** | U | R.GIQVSNNGPCLGSR.K  + Deamidated (NQ) |
| 61566 | 100 | – | 113 | 730.8503 | 1459.6861 | 1459.6725 | 9.28 | 0 | 50 | 2.2e-05 | 1Score **> 31** indicates **identity** Score **> 16** indicates **homology** | U | R.GIQVSNNGPCLGSR.K  + 2 Deamidated (NQ) |
| 73018 | 114 | – | 125 | 776.8927 | 1551.7708 | 1551.7721 | -0.89 | 1 | 86 | 8.7e-09 | 1Score **> 34** indicates **identity** Score **> 18** indicates **homology** | U | R.KPNQPYEWISYK.E |
| 73021 | 114 | – | 125 | 518.2646 | 1551.7720 | 1551.7721 | -0.076 | 1 | 38 | 0.00029 | 1Score **> 34** indicates **identity** Score **> 15** indicates **homology** | U | R.KPNQPYEWISYK.E |
| 73022 | 114 | – | 125 | 776.8937 | 1551.7728 | 1551.7721 | 0.42 | 1 | 86 | 8.4e-09 | 1Score **> 34** indicates **identity** Score **> 18** indicates **homology** | U | R.KPNQPYEWISYK.E |
| 184797 | 114 | – | 141 | 1084.2209 | 3249.6409 | 3249.6379 | 0.90 | 2 | 79 | 4.1e-08 | 1Score **> 38** indicates **identity** Score **> 17** indicates **homology** | U | R.KPNQPYEWISYKEVAELAECIGSGLIQK.G |
| 184798 | 114 | – | 141 | 813.4175 | 3249.6411 | 3249.6379 | 0.96 | 2 | 47 | 3.6e-05 | 1Score **> 38** indicates **identity** Score **> 15** indicates **homology** | U | R.KPNQPYEWISYKEVAELAECIGSGLIQK.G |
| 184799 | 114 | – | 141 | 813.4186 | 3249.6453 | 3249.6379 | 2.28 | 2 | 68 | 4.4e-07 | 1Score **> 38** indicates **identity** Score **> 17** indicates **homology** | U | R.KPNQPYEWISYKEVAELAECIGSGLIQK.G |
| 184800 | 114 | – | 141 | 1084.2227 | 3249.6462 | 3249.6379 | 2.53 | 2 | 86 | 9.6e-09 | 1Score **> 38** indicates **identity** Score **> 18** indicates **homology** | U | R.KPNQPYEWISYKEVAELAECIGSGLIQK.G |
| 94758 | 126 | – | 141 | 858.9451 | 1715.8757 | 1715.8764 | -0.39 | 0 | 54 | 8.3e-06 | 1Score **> 35** indicates **identity** Score **> 16** indicates **homology** | U | K.EVAELAECIGSGLIQK.G |
| 94760 | 126 | – | 141 | 572.9663 | 1715.8771 | 1715.8764 | 0.43 | 0 | 54 | 8.7e-06 | 1Score **> 35** indicates **identity** Score **> 16** indicates **homology** | U | K.EVAELAECIGSGLIQK.G |
| 94761 | 126 | – | 141 | 572.9664 | 1715.8775 | 1715.8764 | 0.64 | 0 | 42 | 0.00014 | 1Score **> 35** indicates **identity** Score **> 16** indicates **homology** | U | K.EVAELAECIGSGLIQK.G |
| 94762 | 126 | – | 141 | 572.9667 | 1715.8782 | 1715.8764 | 1.10 | 0 | 18 | 0.019 | 1Score **> 35** indicates **identity** Score **> 14** indicates **homology** | U | K.EVAELAECIGSGLIQK.G |
| 59939 | 194 | – | 206 | 482.9317 | 1445.7733 | 1445.7766 | -2.29 | 1 | 36 | 0.00039 | 1Score **> 35** indicates **identity** Score **> 15** indicates **homology** | U | K.AELSVIFADKPEK.A |
| 59942 | 194 | – | 206 | 482.9324 | 1445.7754 | 1445.7766 | -0.77 | 1 | 45 | 5.7e-05 | 1Score **> 35** indicates **identity** Score **> 15** indicates **homology** | U | K.AELSVIFADKPEK.A |
| 59943 | 194 | – | 206 | 723.8950 | 1445.7755 | 1445.7766 | -0.71 | 1 | 40 | 0.00017 | 1Score **> 35** indicates **identity** Score **> 15** indicates **homology** | U | K.AELSVIFADKPEK.A |
| 59945 | 194 | – | 206 | 482.9326 | 1445.7758 | 1445.7766 | -0.51 | 1 | 43 | 8.9e-05 | 1Score **> 35** indicates **identity** Score **> 15** indicates **homology** | U | K.AELSVIFADKPEK.A |
| 59946 | 194 | – | 206 | 482.9326 | 1445.7759 | 1445.7766 | -0.45 | 1 | 53 | 1e-05 | 1Score **> 35** indicates **identity** Score **> 16** indicates **homology** | U | K.AELSVIFADKPEK.A |
| 59947 | 194 | – | 206 | 723.8953 | 1445.7760 | 1445.7766 | -0.41 | 1 | 68 | 4.2e-07 | 1Score **> 35** indicates **identity** Score **> 17** indicates **homology** | U | K.AELSVIFADKPEK.A |
| 59948 | 194 | – | 206 | 723.8953 | 1445.7761 | 1445.7766 | -0.30 | 1 | 67 | 5e-07 | 1Score **> 35** indicates **identity** Score **> 17** indicates **homology** | U | K.AELSVIFADKPEK.A |
| 59949 | 194 | – | 206 | 723.8954 | 1445.7762 | 1445.7766 | -0.27 | 1 | 62 | 1.6e-06 | 1Score **> 35** indicates **identity** Score **> 16** indicates **homology** | U | K.AELSVIFADKPEK.A |
| 59950 | 194 | – | 206 | 482.9327 | 1445.7763 | 1445.7766 | -0.17 | 1 | 54 | 9.5e-06 | 1Score **> 35** indicates **identity** Score **> 16** indicates **homology** | U | K.AELSVIFADKPEK.A |
| 59951 | 194 | – | 206 | 482.9328 | 1445.7766 | 1445.7766 | 0.042 | 1 | 53 | 9.7e-06 | 1Score **> 35** indicates **identity** Score **> 16** indicates **homology** | U | K.AELSVIFADKPEK.A |
| 59952 | 194 | – | 206 | 723.8956 | 1445.7766 | 1445.7766 | 0.051 | 1 | 59 | 2.7e-06 | 1Score **> 35** indicates **identity** Score **> 16** indicates **homology** | U | K.AELSVIFADKPEK.A |
| 59953 | 194 | – | 206 | 482.9329 | 1445.7767 | 1445.7766 | 0.12 | 1 | 48 | 3e-05 | 1Score **> 35** indicates **identity** Score **> 16** indicates **homology** | U | K.AELSVIFADKPEK.A |
| 15579 | 209 | – | 217 | 507.7955 | 1013.5764 | 1013.5757 | 0.73 | 0 | 57 | 0.00011 | 1Score **> 30** indicates **identity** | U | K.LLLEGVENK.L |
| 15580 | 209 | – | 217 | 507.7955 | 1013.5765 | 1013.5757 | 0.81 | 0 | 55 | 0.00017 | 1Score **> 30** indicates **identity** | U | K.LLLEGVENK.L |
| 15583 | 209 | – | 217 | 507.7963 | 1013.5781 | 1013.5757 | 2.43 | 0 | 35 | 0.033 | 1Score **> 32** indicates **identity** | U | K.LLLEGVENK.L |
| 96004 | 209 | – | 223 | 576.3306 | 1725.9700 | 1725.9699 | 0.076 | 1 | 60 | 2.6e-06 | 1Score **> 33** indicates **identity** Score **> 16** indicates **homology** | U | K.LLLEGVENKLTPCLK.I |
| 96005 | 209 | – | 223 | 576.3310 | 1725.9711 | 1725.9699 | 0.70 | 1 | 42 | 0.00012 | 1Score **> 34** indicates **identity** Score **> 15** indicates **homology** | U | K.LLLEGVENKLTPCLK.I |
| 96006 | 209 | – | 223 | 576.3313 | 1725.9721 | 1725.9699 | 1.29 | 1 | 56 | 1.3e-05 | 1Score **> 34** indicates **identity** Score **> 20** indicates **homology** | U | K.LLLEGVENKLTPCLK.I |
| 96007 | 209 | – | 223 | 576.3314 | 1725.9724 | 1725.9699 | 1.45 | 1 | 47 | 4.2e-05 | 1Score **> 34** indicates **identity** Score **> 15** indicates **homology** | U | K.LLLEGVENKLTPCLK.I |
| 93873 | 224 | – | 238 | 855.4392 | 1708.8639 | 1708.8706 | -3.91 | 0 | 45 | 6e-05 | 1Score **> 35** indicates **identity** Score **> 15** indicates **homology** | U | K.IIVIMDSYGSDLVER.G |
| 93880 | 224 | – | 238 | 855.4402 | 1708.8659 | 1708.8706 | -2.71 | 0 | 60 | 2.2e-06 | 1Score **> 35** indicates **identity** Score **> 16** indicates **homology** | U | K.IIVIMDSYGSDLVER.G |
| 93885 | 224 | – | 238 | 855.4418 | 1708.8691 | 1708.8706 | -0.86 | 0 | 65 | 7.3e-07 | 1Score **> 35** indicates **identity** Score **> 17** indicates **homology** | U | K.IIVIMDSYGSDLVER.G |
| 93888 | 224 | – | 238 | 855.4430 | 1708.8715 | 1708.8706 | 0.55 | 0 | 36 | 0.00047 | 1Score **> 35** indicates **identity** Score **> 15** indicates **homology** | U | K.IIVIMDSYGSDLVER.G |
| 93889 | 224 | – | 238 | 855.4438 | 1708.8731 | 1708.8706 | 1.51 | 0 | 53 | 1e-05 | 1Score **> 35** indicates **identity** Score **> 16** indicates **homology** | U | K.IIVIMDSYGSDLVER.G |
| 93891 | 224 | – | 238 | 855.4440 | 1708.8734 | 1708.8706 | 1.64 | 0 | 62 | 1.5e-06 | 1Score **> 35** indicates **identity** Score **> 16** indicates **homology** | U | K.IIVIMDSYGSDLVER.G |
| 93899 | 224 | – | 238 | 855.4479 | 1708.8812 | 1708.8706 | 6.20 | 0 | 50 | 1.9e-05 | 1Score **> 35** indicates **identity** Score **> 16** indicates **homology** | U | K.IIVIMDSYGSDLVER.G |
| 15853 | 242 | – | 250 | 509.7836 | 1017.5527 | 1017.5529 | -0.15 | 0 | 51 | 1.6e-05 | 1Score **> 33** indicates **identity** Score **> 16** indicates **homology** | U | K.CGVEIISLK.A |
| 15854 | 242 | – | 250 | 509.7837 | 1017.5528 | 1017.5529 | -0.067 | 0 | 64 | 4.2e-06 | 1Score **> 33** indicates **identity** Score **> 23** indicates **homology** | U | K.CGVEIISLK.A |
| 15855 | 242 | – | 250 | 509.7838 | 1017.5531 | 1017.5529 | 0.20 | 0 | 51 | 1.6e-05 | 1Score **> 33** indicates **identity** Score **> 16** indicates **homology** | U | K.CGVEIISLK.A |
| 15856 | 242 | – | 250 | 509.7838 | 1017.5531 | 1017.5529 | 0.25 | 0 | 50 | 2.3e-05 | 1Score **> 33** indicates **identity** Score **> 16** indicates **homology** | U | K.CGVEIISLK.A |
| 15857 | 242 | – | 250 | 509.7839 | 1017.5533 | 1017.5529 | 0.47 | 0 | 64 | 4.4e-06 | 1Score **> 33** indicates **identity** Score **> 23** indicates **homology** | U | K.CGVEIISLK.A |
| 15858 | 242 | – | 250 | 509.7842 | 1017.5538 | 1017.5529 | 0.91 | 0 | 56 | 1.5e-05 | 1Score **> 33** indicates **identity** Score **> 20** indicates **homology** | U | K.CGVEIISLK.A |
| 15859 | 242 | – | 250 | 509.7842 | 1017.5538 | 1017.5529 | 0.91 | 0 | 65 | 3.5e-06 | 1Score **> 33** indicates **identity** Score **> 23** indicates **homology** | U | K.CGVEIISLK.A |
| 2623 | 251 | – | 257 | 387.2108 | 772.4071 | 772.4079 | -1.02 | 0 | 33 | 0.012 | 1Score **> 26** indicates **identity** | U | K.ALEDLGR.V |
| 2624 | 251 | – | 257 | 387.2112 | 772.4078 | 772.4079 | -0.18 | 0 | 49 | 0.00033 | 1Score **> 27** indicates **identity** | U | K.ALEDLGR.V |
| 2625 | 251 | – | 257 | 387.2112 | 772.4078 | 772.4079 | -0.083 | 0 | 49 | 0.00031 | 1Score **> 27** indicates **identity** | U | K.ALEDLGR.V |
| 2626 | 251 | – | 257 | 387.2113 | 772.4080 | 772.4079 | 0.20 | 0 | 34 | 0.01 | 1Score **> 27** indicates **identity** | U | K.ALEDLGR.V |
| 26906 | 251 | – | 260 | 381.5475 | 1141.6206 | 1141.6203 | 0.18 | 1 | 26 | 0.039 | 1Score **> 32** indicates **identity** Score **> 24** indicates **homology** | U | K.ALEDLGRVNR.V |
| 169485 | 261 | – | 285 | 899.4699 | 2695.3879 | 2695.3891 | -0.43 | 2 | 24 | 0.0062 | 1Score **> 37** indicates **identity** Score **> 14** indicates **homology** | U | R.VKPKPPEPEDLAIICFTSGTTGNPK.G |
| 169486 | 261 | – | 285 | 899.4702 | 2695.3888 | 2695.3891 | -0.12 | 2 | 43 | 9e-05 | 1Score **> 37** indicates **identity** Score **> 15** indicates **homology** | U | R.VKPKPPEPEDLAIICFTSGTTGNPK.G |
| 169487 | 261 | – | 285 | 674.8548 | 2695.3902 | 2695.3891 | 0.41 | 2 | 37 | 0.00042 | 1Score **> 37** indicates **identity** Score **> 16** indicates **homology** | U | R.VKPKPPEPEDLAIICFTSGTTGNPK.G |
| 169488 | 261 | – | 285 | 674.8549 | 2695.3905 | 2695.3891 | 0.53 | 2 | 35 | 0.00059 | 1Score **> 37** indicates **identity** Score **> 15** indicates **homology** | U | R.VKPKPPEPEDLAIICFTSGTTGNPK.G |
| 169489 | 261 | – | 285 | 674.8550 | 2695.3909 | 2695.3891 | 0.68 | 2 | 33 | 0.00087 | 1Score **> 37** indicates **identity** Score **> 15** indicates **homology** | U | R.VKPKPPEPEDLAIICFTSGTTGNPK.G |
| 169490 | 261 | – | 285 | 899.4710 | 2695.3911 | 2695.3891 | 0.75 | 2 | 49 | 2.8e-05 | 1Score **> 37** indicates **identity** Score **> 16** indicates **homology** | U | R.VKPKPPEPEDLAIICFTSGTTGNPK.G |
| 169491 | 261 | – | 285 | 899.4710 | 2695.3912 | 2695.3891 | 0.78 | 2 | 49 | 2.5e-05 | 1Score **> 37** indicates **identity** Score **> 16** indicates **homology** | U | R.VKPKPPEPEDLAIICFTSGTTGNPK.G |
| 169492 | 261 | – | 285 | 674.8553 | 2695.3921 | 2695.3891 | 1.12 | 2 | 33 | 0.00074 | 1Score **> 37** indicates **identity** Score **> 15** indicates **homology** | U | R.VKPKPPEPEDLAIICFTSGTTGNPK.G |
| 169493 | 261 | – | 285 | 674.8554 | 2695.3924 | 2695.3891 | 1.22 | 2 | 32 | 0.001 | 1Score **> 37** indicates **identity** Score **> 15** indicates **homology** | U | R.VKPKPPEPEDLAIICFTSGTTGNPK.G |
| 169497 | 261 | – | 285 | 674.8581 | 2695.4034 | 2695.3891 | 5.31 | 2 | 49 | 2.9e-05 | 1Score **> 37** indicates **identity** Score **> 16** indicates **homology** | U | R.VKPKPPEPEDLAIICFTSGTTGNPK.G |
| 169498 | 261 | – | 285 | 899.4765 | 2695.4076 | 2695.3891 | 6.88 | 2 | 22 | 0.0087 | 1Score **> 37** indicates **identity** Score **> 14** indicates **homology** | U | R.VKPKPPEPEDLAIICFTSGTTGNPK.G |
| 124782 | 286 | – | 303 | 673.6631 | 2017.9675 | 2017.9714 | -1.90 | 0 | 31 | 0.0011 | 1Score **> 34** indicates **identity** Score **> 14** indicates **homology** | U | K.GAMITHQNIINDCSGFIK.A |
| 124783 | 286 | – | 303 | 673.6639 | 2017.9699 | 2017.9714 | -0.73 | 0 | 47 | 4.1e-05 | 1Score **> 35** indicates **identity** Score **> 15** indicates **homology** | U | K.GAMITHQNIINDCSGFIK.A |
| 124784 | 286 | – | 303 | 673.6644 | 2017.9714 | 2017.9714 | 0.014 | 0 | 28 | 0.0023 | 1Score **> 35** indicates **identity** Score **> 14** indicates **homology** | U | K.GAMITHQNIINDCSGFIK.A |
| 18206 | 342 | – | 350 | 526.7785 | 1051.5424 | 1051.5451 | -2.55 | 0 | 46 | 0.00015 | 1Score **> 31** indicates **identity** Score **> 20** indicates **homology** |  | K.IGFFQGDIR.L |
| 18207 | 342 | – | 350 | 526.7786 | 1051.5427 | 1051.5451 | -2.27 | 0 | 19 | 0.016 | 1Score **> 31** indicates **identity** Score **> 14** indicates **homology** |  | K.IGFFQGDIR.L |
| 18209 | 342 | – | 350 | 526.7789 | 1051.5432 | 1051.5451 | -1.82 | 0 | 33 | 0.0028 | 1Score **> 31** indicates **identity** Score **> 20** indicates **homology** |  | K.IGFFQGDIR.L |
| 18210 | 342 | – | 350 | 526.7791 | 1051.5437 | 1051.5451 | -1.35 | 0 | 44 | 0.00027 | 1Score **> 31** indicates **identity** Score **> 21** indicates **homology** |  | K.IGFFQGDIR.L |
| 18211 | 342 | – | 350 | 526.7792 | 1051.5438 | 1051.5451 | -1.26 | 0 | 31 | 0.0013 | 1Score **> 31** indicates **identity** Score **> 14** indicates **homology** |  | K.IGFFQGDIR.L |
| 18212 | 342 | – | 350 | 526.7792 | 1051.5439 | 1051.5451 | -1.12 | 0 | 52 | 0.00012 | 1Score **> 31** indicates **identity** Score **> 25** indicates **homology** |  | K.IGFFQGDIR.L |
| 18213 | 342 | – | 350 | 526.7793 | 1051.5440 | 1051.5451 | -1.05 | 0 | 46 | 0.00017 | 1Score **> 31** indicates **identity** Score **> 21** indicates **homology** |  | K.IGFFQGDIR.L |
| 18214 | 342 | – | 350 | 526.7796 | 1051.5447 | 1051.5451 | -0.42 | 0 | 47 | 0.00018 | 1Score **> 31** indicates **identity** Score **> 22** indicates **homology** |  | K.IGFFQGDIR.L |
| 18215 | 342 | – | 350 | 526.7797 | 1051.5449 | 1051.5451 | -0.22 | 0 | 52 | 0.00012 | 1Score **> 31** indicates **identity** Score **> 26** indicates **homology** |  | K.IGFFQGDIR.L |
| 18217 | 342 | – | 350 | 526.7798 | 1051.5450 | 1051.5451 | -0.068 | 0 | 62 | 1.9e-05 | 1Score **> 31** indicates **identity** Score **> 27** indicates **homology** |  | K.IGFFQGDIR.L |
| 18218 | 342 | – | 350 | 526.7800 | 1051.5455 | 1051.5451 | 0.34 | 0 | 48 | 0.00018 | 1Score **> 31** indicates **identity** Score **> 23** indicates **homology** |  | K.IGFFQGDIR.L |
| 18219 | 342 | – | 350 | 526.7800 | 1051.5455 | 1051.5451 | 0.36 | 0 | 54 | 0.00011 | 1Score **> 31** indicates **identity** Score **> 27** indicates **homology** |  | K.IGFFQGDIR.L |
| 18220 | 342 | – | 350 | 526.7801 | 1051.5457 | 1051.5451 | 0.62 | 0 | 48 | 0.00018 | 1Score **> 31** indicates **identity** Score **> 23** indicates **homology** |  | K.IGFFQGDIR.L |
| 18221 | 342 | – | 350 | 526.7803 | 1051.5460 | 1051.5451 | 0.83 | 0 | 54 | 9.1e-05 | 1Score **> 31** indicates **identity** Score **> 26** indicates **homology** |  | K.IGFFQGDIR.L |
| 18222 | 342 | – | 350 | 526.7803 | 1051.5461 | 1051.5451 | 0.97 | 0 | 61 | 3.8e-05 | 1Score **> 31** indicates **identity** Score **> 30** indicates **homology** |  | K.IGFFQGDIR.L |
| 18223 | 342 | – | 350 | 526.7806 | 1051.5467 | 1051.5451 | 1.54 | 0 | 45 | 0.00014 | 1Score **> 31** indicates **identity** Score **> 19** indicates **homology** |  | K.IGFFQGDIR.L |
| 18224 | 342 | – | 350 | 526.7806 | 1051.5467 | 1051.5451 | 1.55 | 0 | 33 | 0.001 | 1Score **> 31** indicates **identity** Score **> 15** indicates **homology** |  | K.IGFFQGDIR.L |
| 18225 | 342 | – | 350 | 526.7809 | 1051.5473 | 1051.5451 | 2.11 | 0 | 51 | 0.00012 | 1Score **> 31** indicates **identity** Score **> 24** indicates **homology** |  | K.IGFFQGDIR.L |
| 5859 | 351 | – | 357 | 424.2319 | 846.4492 | 846.4521 | -3.45 | 0 | 18 | 0.023 | 1Score **> 30** indicates **identity** Score **> 14** indicates **homology** | U | R.LLMDDLK.V |
| 5860 | 351 | – | 357 | 424.2320 | 846.4495 | 846.4521 | -3.04 | 0 | 16 | 0.034 | 1Score **> 30** indicates **identity** Score **> 13** indicates **homology** | U | R.LLMDDLK.V |
| 5862 | 351 | – | 357 | 424.2332 | 846.4519 | 846.4521 | -0.26 | 0 | 46 | 0.00075 | 1Score **> 31** indicates **identity** Score **> 28** indicates **homology** | U | R.LLMDDLK.V |
| 5863 | 351 | – | 357 | 424.2332 | 846.4519 | 846.4521 | -0.23 | 0 | 34 | 0.0026 | 1Score **> 31** indicates **identity** Score **> 21** indicates **homology** | U | R.LLMDDLK.V |
| 5864 | 351 | – | 357 | 424.2336 | 846.4526 | 846.4521 | 0.61 | 0 | 46 | 0.00074 | 1Score **> 31** indicates **identity** Score **> 27** indicates **homology** | U | R.LLMDDLK.V |
| 51234 | 358 | – | 369 | 683.4144 | 1364.8142 | 1364.8180 | -2.82 | 0 | 33 | 0.00081 | 1Score **> 30** indicates **identity** Score **> 15** indicates **homology** | U | K.VLQPTIFPVVPR.L |
| 51239 | 358 | – | 369 | 455.9461 | 1364.8166 | 1364.8180 | -1.03 | 0 | 29 | 0.0021 | 1Score **> 30** indicates **identity** Score **> 14** indicates **homology** | U | K.VLQPTIFPVVPR.L |
| 51240 | 358 | – | 369 | 683.4158 | 1364.8170 | 1364.8180 | -0.72 | 0 | 42 | 0.0031 | 1Score **> 30** indicates **identity** | U | K.VLQPTIFPVVPR.L |
| 51243 | 358 | – | 369 | 683.4162 | 1364.8179 | 1364.8180 | -0.086 | 0 | 62 | 3.4e-05 | 1Score **> 30** indicates **identity** | U | K.VLQPTIFPVVPR.L |
| 51244 | 358 | – | 369 | 455.9466 | 1364.8180 | 1364.8180 | -0.025 | 0 | 56 | 5.2e-06 | 1Score **> 30** indicates **identity** Score **> 16** indicates **homology** | U | K.VLQPTIFPVVPR.L |
| 51245 | 358 | – | 369 | 455.9467 | 1364.8181 | 1364.8180 | 0.074 | 0 | 59 | 2.9e-06 | 1Score **> 30** indicates **identity** Score **> 16** indicates **homology** | U | K.VLQPTIFPVVPR.L |
| 51246 | 358 | – | 369 | 683.4166 | 1364.8187 | 1364.8180 | 0.49 | 0 | 45 | 0.0016 | 1Score **> 30** indicates **identity** | U | K.VLQPTIFPVVPR.L |
| 51247 | 358 | – | 369 | 683.4168 | 1364.8191 | 1364.8180 | 0.79 | 0 | 44 | 0.0023 | 1Score **> 30** indicates **identity** | U | K.VLQPTIFPVVPR.L |
| 51248 | 358 | – | 369 | 455.9470 | 1364.8191 | 1364.8180 | 0.79 | 0 | 18 | 0.018 | 1Score **> 30** indicates **identity** Score **> 14** indicates **homology** | U | K.VLQPTIFPVVPR.L |
| 20585 | 378 | – | 387 | 539.7998 | 1077.5850 | 1077.5818 | 2.95 | 0 | 51 | 0.00035 | 1Score **> 32** indicates **identity** Score **> 29** indicates **homology** | U | R.IFGQANTSLK.R |
| 20586 | 378 | – | 387 | 539.7998 | 1077.5850 | 1077.5818 | 2.95 | 0 | 38 | 0.0011 | 1Score **> 32** indicates **identity** Score **> 21** indicates **homology** | U | R.IFGQANTSLK.R |
| 36972 | 378 | – | 388 | 412.2346 | 1233.6821 | 1233.6830 | -0.73 | 1 | 39 | 0.00023 | 1Score **> 33** indicates **identity** Score **> 15** indicates **homology** | U | R.IFGQANTSLKR.W |
| 36974 | 378 | – | 388 | 617.8484 | 1233.6823 | 1233.6830 | -0.50 | 1 | 36 | 0.0018 | 1Score **> 33** indicates **identity** Score **> 21** indicates **homology** | U | R.IFGQANTSLKR.W |
| 36976 | 378 | – | 388 | 617.8487 | 1233.6828 | 1233.6830 | -0.11 | 1 | 59 | 2.2e-05 | 1Score **> 33** indicates **identity** Score **> 25** indicates **homology** | U | R.IFGQANTSLKR.W |
| 36977 | 378 | – | 388 | 412.2349 | 1233.6829 | 1233.6830 | -0.034 | 1 | 22 | 0.0089 | 1Score **> 33** indicates **identity** Score **> 14** indicates **homology** | U | R.IFGQANTSLKR.W |
| 36979 | 378 | – | 388 | 412.2350 | 1233.6831 | 1233.6830 | 0.12 | 1 | 55 | 1.4e-05 | 1Score **> 33** indicates **identity** Score **> 18** indicates **homology** | U | R.IFGQANTSLKR.W |
| 36981 | 378 | – | 388 | 412.2350 | 1233.6833 | 1233.6830 | 0.28 | 1 | 53 | 2e-05 | 1Score **> 33** indicates **identity** Score **> 18** indicates **homology** | U | R.IFGQANTSLKR.W |
| 36982 | 378 | – | 388 | 617.8490 | 1233.6834 | 1233.6830 | 0.34 | 1 | 34 | 0.0018 | 1Score **> 33** indicates **identity** Score **> 20** indicates **homology** | U | R.IFGQANTSLKR.W |
| 36985 | 378 | – | 388 | 412.2354 | 1233.6844 | 1233.6830 | 1.21 | 1 | 32 | 0.00091 | 1Score **> 32** indicates **identity** Score **> 15** indicates **homology** | U | R.IFGQANTSLKR.W |
| 13354 | 389 | – | 396 | 490.2656 | 978.5167 | 978.5175 | -0.78 | 0 | 19 | 0.018 | 1Score **> 30** indicates **identity** Score **> 14** indicates **homology** | U | R.WLLDFASK.R |
| 13355 | 389 | – | 396 | 490.2660 | 978.5174 | 978.5175 | -0.031 | 0 | 18 | 0.022 | 1Score **> 30** indicates **identity** Score **> 14** indicates **homology** | U | R.WLLDFASK.R |
| 13357 | 389 | – | 396 | 490.2662 | 978.5178 | 978.5175 | 0.31 | 0 | 22 | 0.0087 | 1Score **> 31** indicates **identity** Score **> 14** indicates **homology** | U | R.WLLDFASK.R |
| 13358 | 389 | – | 396 | 490.2663 | 978.5180 | 978.5175 | 0.53 | 0 | 18 | 0.02 | 1Score **> 31** indicates **identity** Score **> 14** indicates **homology** | U | R.WLLDFASK.R |
| 13359 | 389 | – | 396 | 490.2663 | 978.5180 | 978.5175 | 0.53 | 0 | 24 | 0.0062 | 1Score **> 31** indicates **identity** Score **> 14** indicates **homology** | U | R.WLLDFASK.R |
| 13360 | 389 | – | 396 | 490.2663 | 978.5180 | 978.5175 | 0.54 | 0 | 20 | 0.013 | 1Score **> 31** indicates **identity** Score **> 14** indicates **homology** | U | R.WLLDFASK.R |
| 13361 | 389 | – | 396 | 490.2663 | 978.5180 | 978.5175 | 0.58 | 0 | 22 | 0.0082 | 1Score **> 31** indicates **identity** Score **> 14** indicates **homology** | U | R.WLLDFASK.R |
| 13362 | 389 | – | 396 | 490.2663 | 978.5180 | 978.5175 | 0.59 | 0 | 20 | 0.012 | 1Score **> 31** indicates **identity** Score **> 14** indicates **homology** | U | R.WLLDFASK.R |
| 13363 | 389 | – | 396 | 490.2663 | 978.5181 | 978.5175 | 0.65 | 0 | 22 | 0.0085 | 1Score **> 31** indicates **identity** Score **> 14** indicates **homology** | U | R.WLLDFASK.R |
| 13364 | 389 | – | 396 | 490.2664 | 978.5183 | 978.5175 | 0.83 | 0 | 21 | 0.011 | 1Score **> 31** indicates **identity** Score **> 14** indicates **homology** | U | R.WLLDFASK.R |
| 13365 | 389 | – | 396 | 490.2666 | 978.5186 | 978.5175 | 1.22 | 0 | 29 | 0.0019 | 1Score **> 31** indicates **identity** Score **> 14** indicates **homology** | U | R.WLLDFASK.R |
| 13366 | 389 | – | 396 | 490.2666 | 978.5187 | 978.5175 | 1.31 | 0 | 18 | 0.021 | 1Score **> 31** indicates **identity** Score **> 14** indicates **homology** | U | R.WLLDFASK.R |
| 13367 | 389 | – | 396 | 490.2668 | 978.5191 | 978.5175 | 1.63 | 0 | 19 | 0.015 | 1Score **> 31** indicates **identity** Score **> 14** indicates **homology** | U | R.WLLDFASK.R |
| 26258 | 389 | – | 396 | 379.2146 | 1134.6220 | 1134.6325 | -9.27 | 0 | 25 | 0.0047 | 1Score **> 33** indicates **identity** Score **> 14** indicates **homology** | U | R.WLLDFASK.R  + HNE (K) |
| 26245 | 389 | – | 397 | 379.2129 | 1134.6168 | 1134.6186 | -1.54 | 1 | 27 | 0.022 | 1Score **> 32** indicates **identity** Score **> 23** indicates **homology** | U | R.WLLDFASKR.K |
| 26247 | 389 | – | 397 | 379.2131 | 1134.6174 | 1134.6186 | -1.05 | 1 | 26 | 0.0044 | 1Score **> 33** indicates **identity** Score **> 15** indicates **homology** | U | R.WLLDFASKR.K |
| 26250 | 389 | – | 397 | 379.2133 | 1134.6180 | 1134.6186 | -0.48 | 1 | 42 | 0.0022 | 1Score **> 33** indicates **identity** Score **> 28** indicates **homology** | U | R.WLLDFASKR.K |
| 26252 | 389 | – | 397 | 379.2134 | 1134.6184 | 1134.6186 | -0.17 | 1 | 37 | 0.00033 | 1Score **> 32** indicates **identity** Score **> 15** indicates **homology** | U | R.WLLDFASKR.K |
| 26254 | 389 | – | 397 | 379.2136 | 1134.6190 | 1134.6186 | 0.35 | 1 | 37 | 0.0035 | 1Score **> 32** indicates **identity** Score **> 25** indicates **homology** | U | R.WLLDFASKR.K |
| 26255 | 389 | – | 397 | 379.2137 | 1134.6193 | 1134.6186 | 0.63 | 1 | 40 | 0.00016 | 1Score **> 32** indicates **identity** Score **> 15** indicates **homology** | U | R.WLLDFASKR.K |
| 7353 | 409 | – | 415 | 438.7138 | 875.4131 | 875.4137 | -0.75 | 0 | 41 | 0.00038 | 1Score **> 27** indicates **identity** Score **> 19** indicates **homology** | U | R.NNSLWDK.L |
| 7354 | 409 | – | 415 | 438.7139 | 875.4132 | 875.4137 | -0.61 | 0 | 33 | 0.003 | 1Score **> 27** indicates **identity** Score **> 20** indicates **homology** | U | R.NNSLWDK.L |
| 7355 | 409 | – | 415 | 438.7140 | 875.4135 | 875.4137 | -0.29 | 0 | 30 | 0.031 | 1Score **> 27** indicates **identity** | U | R.NNSLWDK.L |
| 68216 | 409 | – | 420 | 505.6081 | 1513.8023 | 1513.8041 | -1.18 | 1 | 21 | 0.0098 | 1Score **> 35** indicates **identity** Score **> 14** indicates **homology** | U | R.NNSLWDKLIFHK.I |
| 68218 | 409 | – | 420 | 505.6083 | 1513.8030 | 1513.8041 | -0.75 | 1 | 32 | 0.00091 | 1Score **> 35** indicates **identity** Score **> 15** indicates **homology** | U | R.NNSLWDKLIFHK.I |
| 68220 | 409 | – | 420 | 505.6084 | 1513.8032 | 1513.8041 | -0.59 | 1 | 48 | 2.9e-05 | 1Score **> 35** indicates **identity** Score **> 16** indicates **homology** | U | R.NNSLWDKLIFHK.I |
| 68223 | 409 | – | 420 | 505.6085 | 1513.8036 | 1513.8041 | -0.34 | 1 | 34 | 0.00065 | 1Score **> 35** indicates **identity** Score **> 15** indicates **homology** | U | R.NNSLWDKLIFHK.I |
| 68224 | 409 | – | 420 | 505.6085 | 1513.8037 | 1513.8041 | -0.30 | 1 | 34 | 0.00071 | 1Score **> 35** indicates **identity** Score **> 15** indicates **homology** | U | R.NNSLWDKLIFHK.I |
| 68226 | 409 | – | 420 | 505.6085 | 1513.8038 | 1513.8041 | -0.20 | 1 | 27 | 0.0027 | 1Score **> 35** indicates **identity** Score **> 14** indicates **homology** | U | R.NNSLWDKLIFHK.I |
| 68227 | 409 | – | 420 | 505.6086 | 1513.8039 | 1513.8041 | -0.16 | 1 | 34 | 0.00067 | 1Score **> 35** indicates **identity** Score **> 15** indicates **homology** | U | R.NNSLWDKLIFHK.I |
| 68229 | 409 | – | 420 | 505.6086 | 1513.8040 | 1513.8041 | -0.100 | 1 | 53 | 1.1e-05 | 1Score **> 35** indicates **identity** Score **> 16** indicates **homology** | U | R.NNSLWDKLIFHK.I |
| 68231 | 409 | – | 420 | 505.6086 | 1513.8041 | 1513.8041 | -0.024 | 1 | 48 | 3.1e-05 | 1Score **> 34** indicates **identity** Score **> 16** indicates **homology** | U | R.NNSLWDKLIFHK.I |
| 68232 | 409 | – | 420 | 505.6087 | 1513.8041 | 1513.8041 | 0.0073 | 1 | 53 | 1.1e-05 | 1Score **> 34** indicates **identity** Score **> 16** indicates **homology** | U | R.NNSLWDKLIFHK.I |
| 68233 | 409 | – | 420 | 379.4583 | 1513.8042 | 1513.8041 | 0.024 | 1 | 20 | 0.013 | 1Score **> 34** indicates **identity** Score **> 14** indicates **homology** | U | R.NNSLWDKLIFHK.I |
| 68234 | 409 | – | 420 | 757.9094 | 1513.8042 | 1513.8041 | 0.075 | 1 | 50 | 2.1e-05 | 1Score **> 34** indicates **identity** Score **> 16** indicates **homology** | U | R.NNSLWDKLIFHK.I |
| 68236 | 409 | – | 420 | 505.6088 | 1513.8045 | 1513.8041 | 0.26 | 1 | 53 | 1.1e-05 | 1Score **> 34** indicates **identity** Score **> 16** indicates **homology** | U | R.NNSLWDKLIFHK.I |
| 68237 | 409 | – | 420 | 757.9096 | 1513.8046 | 1513.8041 | 0.31 | 1 | 21 | 0.01 | 1Score **> 34** indicates **identity** Score **> 14** indicates **homology** | U | R.NNSLWDKLIFHK.I |
| 68238 | 409 | – | 420 | 505.6088 | 1513.8046 | 1513.8041 | 0.34 | 1 | 37 | 0.00038 | 1Score **> 34** indicates **identity** Score **> 15** indicates **homology** | U | R.NNSLWDKLIFHK.I |
| 68239 | 409 | – | 420 | 505.6089 | 1513.8048 | 1513.8041 | 0.43 | 1 | 48 | 2.9e-05 | 1Score **> 34** indicates **identity** Score **> 16** indicates **homology** | U | R.NNSLWDKLIFHK.I |
| 68240 | 409 | – | 420 | 505.6089 | 1513.8048 | 1513.8041 | 0.45 | 1 | 46 | 5.1e-05 | 1Score **> 34** indicates **identity** Score **> 15** indicates **homology** | U | R.NNSLWDKLIFHK.I |
| 68244 | 409 | – | 420 | 505.6090 | 1513.8051 | 1513.8041 | 0.67 | 1 | 48 | 2.9e-05 | 1Score **> 34** indicates **identity** Score **> 16** indicates **homology** | U | R.NNSLWDKLIFHK.I |
| 68246 | 409 | – | 420 | 505.6091 | 1513.8053 | 1513.8041 | 0.81 | 1 | 52 | 1.4e-05 | 1Score **> 34** indicates **identity** Score **> 16** indicates **homology** | U | R.NNSLWDKLIFHK.I |
| 68247 | 409 | – | 420 | 505.6091 | 1513.8055 | 1513.8041 | 0.92 | 1 | 29 | 0.0021 | 1Score **> 34** indicates **identity** Score **> 14** indicates **homology** | U | R.NNSLWDKLIFHK.I |
| 68249 | 409 | – | 420 | 505.6092 | 1513.8056 | 1513.8041 | 1.00 | 1 | 22 | 0.009 | 1Score **> 34** indicates **identity** Score **> 14** indicates **homology** | U | R.NNSLWDKLIFHK.I |
| 68251 | 409 | – | 420 | 505.6092 | 1513.8057 | 1513.8041 | 1.04 | 1 | 51 | 1.6e-05 | 1Score **> 34** indicates **identity** Score **> 16** indicates **homology** | U | R.NNSLWDKLIFHK.I |
| 68252 | 409 | – | 420 | 505.6092 | 1513.8057 | 1513.8041 | 1.04 | 1 | 48 | 2.9e-05 | 1Score **> 34** indicates **identity** Score **> 16** indicates **homology** | U | R.NNSLWDKLIFHK.I |
| 68254 | 409 | – | 420 | 505.6093 | 1513.8062 | 1513.8041 | 1.35 | 1 | 35 | 0.00057 | 1Score **> 35** indicates **identity** Score **> 15** indicates **homology** | U | R.NNSLWDKLIFHK.I |
| 68259 | 409 | – | 420 | 757.9128 | 1513.8110 | 1513.8041 | 4.54 | 1 | 18 | 0.021 | 1Score **> 35** indicates **identity** Score **> 14** indicates **homology** | U | R.NNSLWDKLIFHK.I |
| 68389 | 409 | – | 420 | 505.9346 | 1514.7819 | 1514.7881 | -4.13 | 1 | 24 | 0.0055 | 1Score **> 35** indicates **identity** Score **> 14** indicates **homology** | U | R.NNSLWDKLIFHK.I  + Deamidated (NQ) |
| 68392 | 409 | – | 420 | 505.9362 | 1514.7868 | 1514.7881 | -0.88 | 1 | 42 | 0.00011 | 1Score **> 35** indicates **identity** Score **> 15** indicates **homology** | U | R.NNSLWDKLIFHK.I  + Deamidated (NQ) |
| 68395 | 409 | – | 420 | 505.9367 | 1514.7884 | 1514.7881 | 0.19 | 1 | 27 | 0.0032 | 1Score **> 34** indicates **identity** Score **> 14** indicates **homology** | U | R.NNSLWDKLIFHK.I  + Deamidated (NQ) |
| 68396 | 409 | – | 420 | 505.9371 | 1514.7895 | 1514.7881 | 0.89 | 1 | 24 | 0.0051 | 1Score **> 34** indicates **identity** Score **> 14** indicates **homology** | U | R.NNSLWDKLIFHK.I  + Deamidated (NQ) |
| 68401 | 409 | – | 420 | 505.9386 | 1514.7939 | 1514.7881 | 3.79 | 1 | 29 | 0.0021 | 1Score **> 35** indicates **identity** Score **> 14** indicates **homology** | U | R.NNSLWDKLIFHK.I  + Deamidated (NQ) |
| 110185 | 431 | – | 448 | 621.0263 | 1860.0572 | 1860.0543 | 1.57 | 0 | 61 | 1.7e-06 | 1Score **> 33** indicates **identity** Score **> 16** indicates **homology** | U | R.LMITGAAPVSATVLTFLR.T |
| 110186 | 431 | – | 448 | 931.0363 | 1860.0580 | 1860.0543 | 1.97 | 0 | 63 | 1.2e-06 | 1Score **> 33** indicates **identity** Score **> 16** indicates **homology** | U | R.LMITGAAPVSATVLTFLR.T |
| 110187 | 431 | – | 448 | 621.0267 | 1860.0584 | 1860.0543 | 2.19 | 0 | 65 | 8.5e-07 | 1Score **> 33** indicates **identity** Score **> 17** indicates **homology** | U | R.LMITGAAPVSATVLTFLR.T |
| 110188 | 431 | – | 448 | 931.0387 | 1860.0628 | 1860.0543 | 4.55 | 0 | 62 | 3.7e-06 | 1Score **> 33** indicates **identity** Score **> 20** indicates **homology** | U | R.LMITGAAPVSATVLTFLR.T |
| 67694 | 492 | – | 504 | 755.8768 | 1509.7391 | 1509.7385 | 0.43 | 0 | 53 | 1.1e-05 | 1Score **> 34** indicates **identity** Score **> 16** indicates **homology** | U | K.LVDVEEMNYLASK.G |
| 67695 | 492 | – | 504 | 755.8769 | 1509.7393 | 1509.7385 | 0.56 | 0 | 67 | 5.6e-07 | 1Score **> 34** indicates **identity** Score **> 17** indicates **homology** | U | K.LVDVEEMNYLASK.G |
| 67696 | 492 | – | 504 | 755.8775 | 1509.7404 | 1509.7385 | 1.29 | 0 | 40 | 0.0002 | 1Score **> 34** indicates **identity** Score **> 15** indicates **homology** | U | K.LVDVEEMNYLASK.G |
| 152931 | 492 | – | 512 | 790.3805 | 2368.1196 | 2368.1290 | -3.98 | 1 | 31 | 0.0013 | 1Score **> 34** indicates **identity** Score **> 14** indicates **homology** | U | K.LVDVEEMNYLASKGEGEVCVK.G |
| 152949 | 492 | – | 512 | 790.3819 | 2368.1240 | 2368.1290 | -2.13 | 1 | 36 | 0.00038 | 1Score **> 35** indicates **identity** Score **> 15** indicates **homology** | U | K.LVDVEEMNYLASKGEGEVCVK.G |
| 152955 | 492 | – | 512 | 790.3846 | 2368.1320 | 2368.1290 | 1.26 | 1 | 16 | 0.034 | 1Score **> 35** indicates **identity** Score **> 13** indicates **homology** | U | K.LVDVEEMNYLASKGEGEVCVK.G |
| 70844 | 513 | – | 526 | 512.6135 | 1534.8187 | 1534.8256 | -4.50 | 2 | 26 | 0.0037 | 1Score **> 34** indicates **identity** Score **> 14** indicates **homology** | U | K.GANVFKGYLKDPAR.T |
| 70846 | 513 | – | 526 | 384.7137 | 1534.8259 | 1534.8256 | 0.17 | 2 | 20 | 0.014 | 1Score **> 34** indicates **identity** Score **> 14** indicates **homology** | U | K.GANVFKGYLKDPAR.T |
| 70847 | 513 | – | 526 | 384.7138 | 1534.8261 | 1534.8256 | 0.30 | 2 | 33 | 0.0014 | 1Score **> 34** indicates **identity** Score **> 17** indicates **homology** | U | K.GANVFKGYLKDPAR.T |
| 70849 | 513 | – | 526 | 512.6161 | 1534.8264 | 1534.8256 | 0.53 | 2 | 35 | 0.0021 | 1Score **> 34** indicates **identity** Score **> 21** indicates **homology** | U | K.GANVFKGYLKDPAR.T |
| 9788 | 519 | – | 526 | 460.2538 | 918.4930 | 918.4923 | 0.73 | 1 | 40 | 0.00071 | 1Score **> 31** indicates **identity** Score **> 21** indicates **homology** | U | K.GYLKDPAR.T |
| 9789 | 519 | – | 526 | 460.2539 | 918.4932 | 918.4923 | 1.02 | 1 | 29 | 0.0039 | 1Score **> 31** indicates **identity** Score **> 18** indicates **homology** | U | K.GYLKDPAR.T |
| 9790 | 519 | – | 526 | 460.2558 | 918.4970 | 918.4923 | 5.08 | 1 | 17 | 0.023 | 1Score **> 31** indicates **identity** Score **> 14** indicates **homology** | U | K.GYLKDPAR.T |
| 116225 | 527 | – | 544 | 642.9872 | 1925.9397 | 1925.9483 | -4.47 | 1 | 30 | 0.0016 | 1Score **> 35** indicates **identity** Score **> 14** indicates **homology** | U | R.TAEALDKDGWLHTGDIGK.W |
| 116226 | 527 | – | 544 | 642.9874 | 1925.9405 | 1925.9483 | -4.08 | 1 | 26 | 0.0035 | 1Score **> 35** indicates **identity** Score **> 14** indicates **homology** | U | R.TAEALDKDGWLHTGDIGK.W |
| 116230 | 527 | – | 544 | 642.9885 | 1925.9435 | 1925.9483 | -2.48 | 1 | 23 | 0.0072 | 1Score **> 35** indicates **identity** Score **> 14** indicates **homology** | U | R.TAEALDKDGWLHTGDIGK.W |
| 116241 | 527 | – | 544 | 482.4940 | 1925.9471 | 1925.9483 | -0.64 | 1 | 35 | 0.00049 | 1Score **> 35** indicates **identity** Score **> 15** indicates **homology** | U | R.TAEALDKDGWLHTGDIGK.W |
| 116243 | 527 | – | 544 | 482.4941 | 1925.9473 | 1925.9483 | -0.53 | 1 | 27 | 0.0027 | 1Score **> 35** indicates **identity** Score **> 14** indicates **homology** | U | R.TAEALDKDGWLHTGDIGK.W |
| 116245 | 527 | – | 544 | 963.9812 | 1925.9479 | 1925.9483 | -0.19 | 1 | 91 | 3.1e-09 | 1Score **> 35** indicates **identity** Score **> 18** indicates **homology** | U | R.TAEALDKDGWLHTGDIGK.W |
| 116246 | 527 | – | 544 | 642.9899 | 1925.9480 | 1925.9483 | -0.18 | 1 | 47 | 4e-05 | 1Score **> 35** indicates **identity** Score **> 15** indicates **homology** | U | R.TAEALDKDGWLHTGDIGK.W |
| 116248 | 527 | – | 544 | 482.4943 | 1925.9482 | 1925.9483 | -0.064 | 1 | 39 | 0.00023 | 1Score **> 35** indicates **identity** Score **> 15** indicates **homology** | U | R.TAEALDKDGWLHTGDIGK.W |
| 116249 | 527 | – | 544 | 642.9901 | 1925.9484 | 1925.9483 | 0.061 | 1 | 61 | 1.8e-06 | 1Score **> 35** indicates **identity** Score **> 16** indicates **homology** | U | R.TAEALDKDGWLHTGDIGK.W |
| 116250 | 527 | – | 544 | 963.9815 | 1925.9485 | 1925.9483 | 0.11 | 1 | 38 | 0.00025 | 1Score **> 35** indicates **identity** Score **> 15** indicates **homology** | U | R.TAEALDKDGWLHTGDIGK.W |
| 116252 | 527 | – | 544 | 642.9902 | 1925.9486 | 1925.9483 | 0.17 | 1 | 69 | 3.6e-07 | 1Score **> 35** indicates **identity** Score **> 17** indicates **homology** | U | R.TAEALDKDGWLHTGDIGK.W |
| 116253 | 527 | – | 544 | 482.4945 | 1925.9487 | 1925.9483 | 0.23 | 1 | 34 | 0.00067 | 1Score **> 35** indicates **identity** Score **> 15** indicates **homology** | U | R.TAEALDKDGWLHTGDIGK.W |
| 116254 | 527 | – | 544 | 642.9902 | 1925.9489 | 1925.9483 | 0.31 | 1 | 64 | 1e-06 | 1Score **> 35** indicates **identity** Score **> 16** indicates **homology** | U | R.TAEALDKDGWLHTGDIGK.W |
| 116255 | 527 | – | 544 | 642.9903 | 1925.9490 | 1925.9483 | 0.37 | 1 | 65 | 8.3e-07 | 1Score **> 35** indicates **identity** Score **> 17** indicates **homology** | U | R.TAEALDKDGWLHTGDIGK.W |
| 116257 | 527 | – | 544 | 642.9904 | 1925.9495 | 1925.9483 | 0.63 | 1 | 54 | 8.5e-06 | 1Score **> 35** indicates **identity** Score **> 16** indicates **homology** | U | R.TAEALDKDGWLHTGDIGK.W |
| 116258 | 527 | – | 544 | 642.9905 | 1925.9496 | 1925.9483 | 0.68 | 1 | 46 | 4.8e-05 | 1Score **> 35** indicates **identity** Score **> 15** indicates **homology** | U | R.TAEALDKDGWLHTGDIGK.W |
| 116260 | 527 | – | 544 | 482.4949 | 1925.9505 | 1925.9483 | 1.15 | 1 | 14 | 0.046 | 1Score **> 35** indicates **identity** Score **> 13** indicates **homology** | U | R.TAEALDKDGWLHTGDIGK.W |
| 116262 | 527 | – | 544 | 642.9914 | 1925.9524 | 1925.9483 | 2.15 | 1 | 28 | 0.0024 | 1Score **> 35** indicates **identity** Score **> 14** indicates **homology** | U | R.TAEALDKDGWLHTGDIGK.W |
| 116267 | 527 | – | 544 | 642.9930 | 1925.9573 | 1925.9483 | 4.66 | 1 | 35 | 0.00056 | 1Score **> 35** indicates **identity** Score **> 15** indicates **homology** | U | R.TAEALDKDGWLHTGDIGK.W |
| 174753 | 527 | – | 552 | 709.8708 | 2835.4543 | 2835.4555 | -0.45 | 2 | 19 | 0.016 | 1Score **> 37** indicates **identity** Score **> 14** indicates **homology** | U | R.TAEALDKDGWLHTGDIGKWLPNGTLK.I |
| 174754 | 527 | – | 552 | 709.8711 | 2835.4551 | 2835.4555 | -0.15 | 2 | 30 | 0.0017 | 1Score **> 37** indicates **identity** Score **> 14** indicates **homology** | U | R.TAEALDKDGWLHTGDIGKWLPNGTLK.I |
| 174771 | 527 | – | 552 | 710.1153 | 2836.4322 | 2836.4395 | -2.58 | 2 | 22 | 0.0086 | 1Score **> 37** indicates **identity** Score **> 14** indicates **homology** | U | R.TAEALDKDGWLHTGDIGKWLPNGTLK.I  + Deamidated (NQ) |
| 174776 | 527 | – | 552 | 710.1179 | 2836.4424 | 2836.4395 | 1.02 | 2 | 29 | 0.002 | 1Score **> 37** indicates **identity** Score **> 14** indicates **homology** | U | R.TAEALDKDGWLHTGDIGKWLPNGTLK.I  + Deamidated (NQ) |
| 33002 | 534 | – | 544 | 400.2007 | 1197.5804 | 1197.5778 | 2.14 | 0 | 16 | 0.03 | 1Score **> 30** indicates **identity** Score **> 14** indicates **homology** | U | K.DGWLHTGDIGK.W |
| 10241 | 545 | – | 552 | 465.2588 | 928.5031 | 928.5018 | 1.44 | 0 | 27 | 0.0095 | 1Score **> 31** indicates **identity** Score **> 19** indicates **homology** |  | K.WLPNGTLK.I  + Deamidated (NQ) |
| 35089 | 563 | – | 573 | 609.8214 | 1217.6283 | 1217.6292 | -0.74 | 0 | 68 | 1e-06 | 1Score **> 34** indicates **identity** Score **> 20** indicates **homology** |  | K.LAQGEYIAPEK.I |
| 35090 | 563 | – | 573 | 609.8215 | 1217.6284 | 1217.6292 | -0.64 | 0 | 26 | 0.0035 | 1Score **> 34** indicates **identity** Score **> 14** indicates **homology** |  | K.LAQGEYIAPEK.I |
| 35091 | 563 | – | 573 | 609.8222 | 1217.6298 | 1217.6292 | 0.53 | 0 | 34 | 0.00062 | 1Score **> 34** indicates **identity** Score **> 15** indicates **homology** |  | K.LAQGEYIAPEK.I |
| 133966 | 563 | – | 580 | 1060.5728 | 2119.1311 | 2119.1313 | -0.099 | 1 | 62 | 1.6e-06 | 1Score **> 36** indicates **identity** Score **> 16** indicates **homology** | U | K.LAQGEYIAPEKIENIYLR.S |
| 133967 | 563 | – | 580 | 1060.5730 | 2119.1315 | 2119.1313 | 0.082 | 1 | 71 | 2.3e-07 | 1Score **> 36** indicates **identity** Score **> 17** indicates **homology** | U | K.LAQGEYIAPEKIENIYLR.S |
| 133968 | 563 | – | 580 | 707.3844 | 2119.1315 | 2119.1313 | 0.094 | 1 | 46 | 5e-05 | 1Score **> 36** indicates **identity** Score **> 15** indicates **homology** | U | K.LAQGEYIAPEKIENIYLR.S |
| 133969 | 563 | – | 580 | 707.3844 | 2119.1315 | 2119.1313 | 0.100 | 1 | 49 | 2.8e-05 | 1Score **> 36** indicates **identity** Score **> 16** indicates **homology** | U | K.LAQGEYIAPEKIENIYLR.S |
| 133970 | 563 | – | 580 | 1060.5731 | 2119.1317 | 2119.1313 | 0.20 | 1 | 86 | 8.9e-09 | 1Score **> 36** indicates **identity** Score **> 18** indicates **homology** | U | K.LAQGEYIAPEKIENIYLR.S |
| 133971 | 563 | – | 580 | 707.3846 | 2119.1321 | 2119.1313 | 0.36 | 1 | 64 | 1.1e-06 | 1Score **> 36** indicates **identity** Score **> 16** indicates **homology** | U | K.LAQGEYIAPEKIENIYLR.S |
| 133972 | 563 | – | 580 | 707.3847 | 2119.1322 | 2119.1313 | 0.45 | 1 | 58 | 3.5e-06 | 1Score **> 36** indicates **identity** Score **> 16** indicates **homology** | U | K.LAQGEYIAPEKIENIYLR.S |
| 133973 | 563 | – | 580 | 707.3847 | 2119.1323 | 2119.1313 | 0.47 | 1 | 43 | 9.8e-05 | 1Score **> 36** indicates **identity** Score **> 15** indicates **homology** | U | K.LAQGEYIAPEKIENIYLR.S |
| 133974 | 563 | – | 580 | 1060.5740 | 2119.1335 | 2119.1313 | 1.04 | 1 | 72 | 1.9e-07 | 1Score **> 36** indicates **identity** Score **> 17** indicates **homology** | U | K.LAQGEYIAPEKIENIYLR.S |
| 133976 | 563 | – | 580 | 707.3858 | 2119.1355 | 2119.1313 | 1.97 | 1 | 30 | 0.0016 | 1Score **> 36** indicates **identity** Score **> 14** indicates **homology** | U | K.LAQGEYIAPEKIENIYLR.S |
| 133978 | 563 | – | 580 | 707.3869 | 2119.1388 | 2119.1313 | 3.53 | 1 | 56 | 5.9e-06 | 1Score **> 35** indicates **identity** Score **> 16** indicates **homology** | U | K.LAQGEYIAPEKIENIYLR.S |
| 134108 | 563 | – | 580 | 1061.0674 | 2120.1202 | 2120.1153 | 2.31 | 1 | 29 | 0.0051 | 1Score **> 36** indicates **identity** Score **> 19** indicates **homology** | U | K.LAQGEYIAPEKIENIYLR.S  + Deamidated (NQ) |
| 134109 | 563 | – | 580 | 707.7142 | 2120.1207 | 2120.1153 | 2.54 | 1 | 26 | 0.0036 | 1Score **> 36** indicates **identity** Score **> 14** indicates **homology** | U | K.LAQGEYIAPEKIENIYLR.S  + Deamidated (NQ) |
| 9824 | 574 | – | 580 | 460.7634 | 919.5122 | 919.5127 | -0.56 | 0 | 41 | 0.0029 | 1Score **> 28** indicates **identity** | U | K.IENIYLR.S |
| 9827 | 574 | – | 580 | 460.7636 | 919.5126 | 919.5127 | -0.035 | 0 | 42 | 0.0027 | 1Score **> 28** indicates **identity** | U | K.IENIYLR.S |
| 60616 | 616 | – | 627 | 484.5732 | 1450.6978 | 1450.6987 | -0.62 | 1 | 41 | 0.00021 | 1Score **> 32** indicates **identity** Score **> 17** indicates **homology** | U | K.RGLQGSFEELCR.N |
| 60617 | 616 | – | 627 | 484.5733 | 1450.6980 | 1450.6987 | -0.49 | 1 | 44 | 8.2e-05 | 1Score **> 32** indicates **identity** Score **> 15** indicates **homology** | U | K.RGLQGSFEELCR.N |
| 60618 | 616 | – | 627 | 484.5734 | 1450.6985 | 1450.6987 | -0.12 | 1 | 55 | 2.9e-05 | 1Score **> 33** indicates **identity** Score **> 22** indicates **homology** | U | K.RGLQGSFEELCR.N |
| 60619 | 616 | – | 627 | 726.3565 | 1450.6985 | 1450.6987 | -0.12 | 1 | 26 | 0.024 | 1Score **> 33** indicates **identity** Score **> 22** indicates **homology** | U | K.RGLQGSFEELCR.N |
| 60620 | 616 | – | 627 | 484.5735 | 1450.6986 | 1450.6987 | -0.046 | 1 | 36 | 0.00058 | 1Score **> 33** indicates **identity** Score **> 16** indicates **homology** | U | K.RGLQGSFEELCR.N |
| 60621 | 616 | – | 627 | 726.3570 | 1450.6994 | 1450.6987 | 0.48 | 1 | 46 | 0.00034 | 1Score **> 33** indicates **identity** Score **> 24** indicates **homology** | U | K.RGLQGSFEELCR.N |
| 60622 | 616 | – | 627 | 484.5737 | 1450.6994 | 1450.6987 | 0.49 | 1 | 58 | 1.6e-05 | 1Score **> 33** indicates **identity** Score **> 23** indicates **homology** | U | K.RGLQGSFEELCR.N |
| 60623 | 616 | – | 627 | 484.5738 | 1450.6995 | 1450.6987 | 0.56 | 1 | 58 | 3e-05 | 1Score **> 33** indicates **identity** Score **> 25** indicates **homology** | U | K.RGLQGSFEELCR.N |
| 60625 | 616 | – | 627 | 484.5739 | 1450.6998 | 1450.6987 | 0.75 | 1 | 40 | 0.00016 | 1Score **> 33** indicates **identity** Score **> 15** indicates **homology** | U | K.RGLQGSFEELCR.N |
| 60626 | 616 | – | 627 | 726.3572 | 1450.6998 | 1450.6987 | 0.76 | 1 | 45 | 0.00054 | 1Score **> 33** indicates **identity** Score **> 24** indicates **homology** | U | K.RGLQGSFEELCR.N |
| 60627 | 616 | – | 627 | 484.5739 | 1450.6999 | 1450.6987 | 0.86 | 1 | 64 | 5.5e-06 | 1Score **> 33** indicates **identity** Score **> 24** indicates **homology** | U | K.RGLQGSFEELCR.N |
| 43581 | 617 | – | 627 | 648.3037 | 1294.5929 | 1294.5976 | -3.64 | 0 | 20 | 0.012 | 1Score **> 30** indicates **identity** Score **> 14** indicates **homology** | U | R.GLQGSFEELCR.N |
| 43582 | 617 | – | 627 | 648.3055 | 1294.5964 | 1294.5976 | -0.94 | 0 | 27 | 0.0028 | 1Score **> 31** indicates **identity** Score **> 14** indicates **homology** | U | R.GLQGSFEELCR.N |
| 43583 | 617 | – | 627 | 648.3055 | 1294.5965 | 1294.5976 | -0.84 | 0 | 54 | 1.1e-05 | 1Score **> 31** indicates **identity** Score **> 16** indicates **homology** | U | R.GLQGSFEELCR.N |
| 43584 | 617 | – | 627 | 648.3063 | 1294.5980 | 1294.5976 | 0.29 | 0 | 52 | 4e-05 | 1Score **> 31** indicates **identity** Score **> 20** indicates **homology** | U | R.GLQGSFEELCR.N |
| 43585 | 617 | – | 627 | 648.3064 | 1294.5982 | 1294.5976 | 0.48 | 0 | 61 | 2.2e-06 | 1Score **> 31** indicates **identity** Score **> 17** indicates **homology** | U | R.GLQGSFEELCR.N |
| 43586 | 617 | – | 627 | 648.3064 | 1294.5982 | 1294.5976 | 0.50 | 0 | 45 | 5.5e-05 | 1Score **> 31** indicates **identity** Score **> 15** indicates **homology** | U | R.GLQGSFEELCR.N |
| 43684 | 617 | – | 627 | 648.8039 | 1295.5933 | 1295.5816 | 9.06 | 0 | 14 | 0.045 | 1Score **> 30** indicates **identity** Score **> 13** indicates **homology** | U | R.GLQGSFEELCR.N  + Deamidated (NQ) |
| 81275 | 628 | – | 641 | 538.3109 | 1611.9109 | 1611.9195 | -5.37 | 2 | 14 | 0.046 | 1Score **> 33** indicates **identity** Score **> 13** indicates **homology** | U | R.NKDINKAILDDLLK.L |
| 81276 | 628 | – | 641 | 538.3123 | 1611.9150 | 1611.9195 | -2.82 | 2 | 32 | 0.00092 | 1Score **> 33** indicates **identity** Score **> 15** indicates **homology** | U | R.NKDINKAILDDLLK.L |
| 81278 | 628 | – | 641 | 403.9869 | 1611.9184 | 1611.9195 | -0.71 | 2 | 33 | 0.00078 | 1Score **> 33** indicates **identity** Score **> 15** indicates **homology** | U | R.NKDINKAILDDLLK.L |
| 81279 | 628 | – | 641 | 403.9869 | 1611.9186 | 1611.9195 | -0.59 | 2 | 43 | 0.00062 | 1Score **> 33** indicates **identity** Score **> 23** indicates **homology** | U | R.NKDINKAILDDLLK.L |
| 81281 | 628 | – | 641 | 538.3136 | 1611.9189 | 1611.9195 | -0.41 | 2 | 24 | 0.0061 | 1Score **> 33** indicates **identity** Score **> 14** indicates **homology** | U | R.NKDINKAILDDLLK.L |
| 81282 | 628 | – | 641 | 538.3140 | 1611.9202 | 1611.9195 | 0.40 | 2 | 35 | 0.00057 | 1Score **> 33** indicates **identity** Score **> 15** indicates **homology** | U | R.NKDINKAILDDLLK.L |
| 81285 | 628 | – | 641 | 538.3144 | 1611.9215 | 1611.9195 | 1.21 | 2 | 55 | 6.7e-06 | 1Score **> 33** indicates **identity** Score **> 16** indicates **homology** | U | R.NKDINKAILDDLLK.L |
| 81286 | 628 | – | 641 | 538.3144 | 1611.9215 | 1611.9195 | 1.22 | 2 | 21 | 0.01 | 1Score **> 33** indicates **identity** Score **> 14** indicates **homology** | U | R.NKDINKAILDDLLK.L |
| 81287 | 628 | – | 641 | 538.3149 | 1611.9230 | 1611.9195 | 2.14 | 2 | 39 | 0.00022 | 1Score **> 33** indicates **identity** Score **> 15** indicates **homology** | U | R.NKDINKAILDDLLK.L |
| 100996 | 628 | – | 641 | 591.0027 | 1769.9864 | 1770.0026 | -9.13 | 2 | 14 | 0.045 | 1Score **> 34** indicates **identity** Score **> 13** indicates **homology** | U | R.NKDINKAILDDLLK.L  + 2 Deamidated (NQ); HNE (K) |
| 51677 | 630 | – | 641 | 457.5993 | 1369.7761 | 1369.7816 | -4.05 | 1 | 53 | 2.5e-05 | 1Score **> 34** indicates **identity** Score **> 20** indicates **homology** | U | K.DINKAILDDLLK.L |
| 51680 | 630 | – | 641 | 457.6010 | 1369.7812 | 1369.7816 | -0.32 | 1 | 42 | 0.0025 | 1Score **> 34** indicates **identity** Score **> 28** indicates **homology** | U | K.DINKAILDDLLK.L |
| 51682 | 630 | – | 641 | 457.6014 | 1369.7825 | 1369.7816 | 0.59 | 1 | 52 | 0.0009 | 1Score **> 34** indicates **identity** | U | K.DINKAILDDLLK.L |
| 8557 | 634 | – | 641 | 450.7732 | 899.5319 | 899.5328 | -0.96 | 0 | 35 | 0.0065 | 1Score **> 28** indicates **identity** Score **> 25** indicates **homology** | U | K.AILDDLLK.L |
| 8558 | 634 | – | 641 | 450.7733 | 899.5321 | 899.5328 | -0.76 | 0 | 40 | 0.0016 | 1Score **> 28** indicates **identity** Score **> 24** indicates **homology** | U | K.AILDDLLK.L |
| 8559 | 634 | – | 641 | 450.7733 | 899.5321 | 899.5328 | -0.71 | 0 | 44 | 0.00097 | 1Score **> 28** indicates **identity** Score **> 27** indicates **homology** | U | K.AILDDLLK.L |
| 8561 | 634 | – | 641 | 450.7734 | 899.5322 | 899.5328 | -0.65 | 0 | 46 | 0.00084 | 1Score **> 28** indicates **identity** Score **> 27** indicates **homology** | U | K.AILDDLLK.L |
| 8562 | 634 | – | 641 | 450.7734 | 899.5323 | 899.5328 | -0.51 | 0 | 38 | 0.0025 | 1Score **> 28** indicates **identity** Score **> 24** indicates **homology** | U | K.AILDDLLK.L |
| 8564 | 634 | – | 641 | 450.7735 | 899.5325 | 899.5328 | -0.32 | 0 | 46 | 0.00083 | 1Score **> 28** indicates **identity** Score **> 28** indicates **homology** | U | K.AILDDLLK.L |
| 8566 | 634 | – | 641 | 450.7736 | 899.5327 | 899.5328 | -0.077 | 0 | 44 | 0.0011 | 1Score **> 28** indicates **identity** Score **> 27** indicates **homology** | U | K.AILDDLLK.L |
| 8567 | 634 | – | 641 | 450.7736 | 899.5327 | 899.5328 | -0.014 | 0 | 46 | 0.00085 | 1Score **> 28** indicates **identity** Score **> 28** indicates **homology** | U | K.AILDDLLK.L |
| 8571 | 634 | – | 641 | 450.7738 | 899.5330 | 899.5328 | 0.29 | 0 | 17 | 0.026 | 1Score **> 28** indicates **identity** Score **> 14** indicates **homology** | U | K.AILDDLLK.L |
| 8572 | 634 | – | 641 | 450.7738 | 899.5330 | 899.5328 | 0.32 | 0 | 46 | 0.00083 | 1Score **> 28** indicates **identity** Score **> 28** indicates **homology** | U | K.AILDDLLK.L |
| 8573 | 634 | – | 641 | 450.7739 | 899.5332 | 899.5328 | 0.51 | 0 | 35 | 0.0044 | 1Score **> 28** indicates **identity** Score **> 24** indicates **homology** | U | K.AILDDLLK.L |
| 8574 | 634 | – | 641 | 450.7739 | 899.5333 | 899.5328 | 0.57 | 0 | 32 | 0.0091 | 1Score **> 28** indicates **identity** Score **> 24** indicates **homology** | U | K.AILDDLLK.L |
| 8576 | 634 | – | 641 | 450.7741 | 899.5337 | 899.5328 | 1.07 | 0 | 24 | 0.04 | 1Score **> 28** indicates **identity** Score **> 23** indicates **homology** | U | K.AILDDLLK.L |
| 8577 | 634 | – | 641 | 450.7744 | 899.5342 | 899.5328 | 1.59 | 0 | 36 | 0.0037 | 1Score **> 28** indicates **identity** Score **> 24** indicates **homology** | U | K.AILDDLLK.L |
| 33085 | 634 | – | 644 | 400.2508 | 1197.7305 | 1197.7332 | -2.27 | 1 | 41 | 0.00016 | 1Score **> 30** indicates **identity** Score **> 15** indicates **homology** | U | K.AILDDLLKLGK.E |
| 33086 | 634 | – | 644 | 599.8727 | 1197.7308 | 1197.7332 | -2.05 | 1 | 15 | 0.036 | 1Score **> 30** indicates **identity** Score **> 13** indicates **homology** | U | K.AILDDLLKLGK.E |
| 33089 | 634 | – | 644 | 400.2513 | 1197.7322 | 1197.7332 | -0.91 | 1 | 39 | 0.00021 | 1Score **> 29** indicates **identity** Score **> 15** indicates **homology** | U | K.AILDDLLKLGK.E |
| 33090 | 634 | – | 644 | 400.2516 | 1197.7328 | 1197.7332 | -0.35 | 1 | 30 | 0.0016 | 1Score **> 30** indicates **identity** Score **> 14** indicates **homology** | U | K.AILDDLLKLGK.E |
| 33091 | 634 | – | 644 | 400.2524 | 1197.7353 | 1197.7332 | 1.70 | 1 | 31 | 0.0012 | 1Score **> 30** indicates **identity** Score **> 14** indicates **homology** | U | K.AILDDLLKLGK.E |
| 71858 | 642 | – | 655 | 515.2992 | 1542.8756 | 1542.8770 | -0.85 | 2 | 22 | 0.0095 | 1Score **> 34** indicates **identity** Score **> 14** indicates **homology** | U | K.LGKEAGLKPFEQVK.G |
| 71861 | 642 | – | 655 | 515.2994 | 1542.8763 | 1542.8770 | -0.39 | 2 | 48 | 6.9e-05 | 1Score **> 34** indicates **identity** Score **> 19** indicates **homology** | U | K.LGKEAGLKPFEQVK.G |
| 71862 | 642 | – | 655 | 515.2995 | 1542.8766 | 1542.8770 | -0.26 | 2 | 17 | 0.027 | 1Score **> 34** indicates **identity** Score **> 14** indicates **homology** | U | K.LGKEAGLKPFEQVK.G |
| 71863 | 642 | – | 655 | 386.7264 | 1542.8766 | 1542.8770 | -0.22 | 2 | 27 | 0.0045 | 1Score **> 34** indicates **identity** Score **> 16** indicates **homology** | U | K.LGKEAGLKPFEQVK.G |
| 71865 | 642 | – | 655 | 386.7265 | 1542.8770 | 1542.8770 | 0.017 | 2 | 22 | 0.012 | 1Score **> 34** indicates **identity** Score **> 15** indicates **homology** | U | K.LGKEAGLKPFEQVK.G |
| 71867 | 642 | – | 655 | 386.7266 | 1542.8773 | 1542.8770 | 0.24 | 2 | 23 | 0.0071 | 1Score **> 34** indicates **identity** Score **> 14** indicates **homology** | U | K.LGKEAGLKPFEQVK.G |
| 71868 | 642 | – | 655 | 515.2998 | 1542.8775 | 1542.8770 | 0.38 | 2 | 17 | 0.023 | 1Score **> 34** indicates **identity** Score **> 14** indicates **homology** | U | K.LGKEAGLKPFEQVK.G |
| 38025 | 645 | – | 655 | 623.3453 | 1244.6760 | 1244.6765 | -0.34 | 1 | 20 | 0.022 | 1Score **> 34** indicates **identity** Score **> 16** indicates **homology** | U | K.EAGLKPFEQVK.G |
| 38027 | 645 | – | 655 | 415.8994 | 1244.6763 | 1244.6765 | -0.16 | 1 | 26 | 0.0038 | 1Score **> 34** indicates **identity** Score **> 14** indicates **homology** | U | K.EAGLKPFEQVK.G |
| 38028 | 645 | – | 655 | 415.8994 | 1244.6763 | 1244.6765 | -0.10 | 1 | 28 | 0.0036 | 1Score **> 34** indicates **identity** Score **> 16** indicates **homology** | U | K.EAGLKPFEQVK.G |
| 38029 | 645 | – | 655 | 623.3455 | 1244.6765 | 1244.6765 | 0.0016 | 1 | 53 | 7.8e-05 | 1Score **> 34** indicates **identity** Score **> 24** indicates **homology** | U | K.EAGLKPFEQVK.G |
| 38031 | 645 | – | 655 | 623.3456 | 1244.6766 | 1244.6765 | 0.098 | 1 | 64 | 2.5e-05 | 1Score **> 34** indicates **identity** Score **> 31** indicates **homology** | U | K.EAGLKPFEQVK.G |
| 38032 | 645 | – | 655 | 415.8995 | 1244.6767 | 1244.6765 | 0.20 | 1 | 31 | 0.0019 | 1Score **> 34** indicates **identity** Score **> 16** indicates **homology** | U | K.EAGLKPFEQVK.G |
| 38033 | 645 | – | 655 | 623.3457 | 1244.6769 | 1244.6765 | 0.31 | 1 | 39 | 0.0052 | 1Score **> 34** indicates **identity** Score **> 29** indicates **homology** | U | K.EAGLKPFEQVK.G |
| 38034 | 645 | – | 655 | 415.8996 | 1244.6770 | 1244.6765 | 0.40 | 1 | 15 | 0.036 | 1Score **> 34** indicates **identity** Score **> 13** indicates **homology** | U | K.EAGLKPFEQVK.G |
| 38035 | 645 | – | 655 | 415.8996 | 1244.6771 | 1244.6765 | 0.47 | 1 | 28 | 0.0025 | 1Score **> 34** indicates **identity** Score **> 15** indicates **homology** | U | K.EAGLKPFEQVK.G |
| 38036 | 645 | – | 655 | 623.3459 | 1244.6772 | 1244.6765 | 0.56 | 1 | 48 | 0.00083 | 1Score **> 34** indicates **identity** Score **> 29** indicates **homology** | U | K.EAGLKPFEQVK.G |
| 38037 | 645 | – | 655 | 415.8997 | 1244.6772 | 1244.6765 | 0.60 | 1 | 29 | 0.0025 | 1Score **> 34** indicates **identity** Score **> 15** indicates **homology** | U | K.EAGLKPFEQVK.G |
| 143829 | 656 | – | 676 | 1118.1223 | 2234.2301 | 2234.2311 | -0.45 | 0 | 59 | 3e-06 | 1Score **> 35** indicates **identity** Score **> 16** indicates **homology** | U | K.GIAVHPELFSIDNGLLTPTLK.A |
| 143830 | 656 | – | 676 | 1118.1229 | 2234.2313 | 2234.2311 | 0.11 | 0 | 59 | 2.8e-06 | 1Score **> 35** indicates **identity** Score **> 16** indicates **homology** | U | K.GIAVHPELFSIDNGLLTPTLK.A |
| 143831 | 656 | – | 676 | 745.7511 | 2234.2316 | 2234.2311 | 0.23 | 0 | 52 | 1.3e-05 | 1Score **> 35** indicates **identity** Score **> 16** indicates **homology** | U | K.GIAVHPELFSIDNGLLTPTLK.A |
| 143832 | 656 | – | 676 | 745.7521 | 2234.2343 | 2234.2311 | 1.47 | 0 | 42 | 0.00011 | 1Score **> 34** indicates **identity** Score **> 15** indicates **homology** | U | K.GIAVHPELFSIDNGLLTPTLK.A |
| 143956 | 656 | – | 676 | 746.0787 | 2235.2144 | 2235.2151 | -0.31 | 0 | 51 | 1.7e-05 | 1Score **> 35** indicates **identity** Score **> 16** indicates **homology** | U | K.GIAVHPELFSIDNGLLTPTLK.A  + Deamidated (NQ) |
| 143957 | 656 | – | 676 | 746.0799 | 2235.2179 | 2235.2151 | 1.25 | 0 | 46 | 4.5e-05 | 1Score **> 35** indicates **identity** Score **> 15** indicates **homology** | U | K.GIAVHPELFSIDNGLLTPTLK.A  + Deamidated (NQ) |
| 143958 | 656 | – | 676 | 1118.6162 | 2235.2179 | 2235.2151 | 1.27 | 0 | 99 | 5.6e-10 | 1Score **> 35** indicates **identity** Score **> 19** indicates **homology** | U | K.GIAVHPELFSIDNGLLTPTLK.A  + Deamidated (NQ) |
| 143959 | 656 | – | 676 | 746.0831 | 2235.2273 | 2235.2151 | 5.49 | 0 | 35 | 0.00053 | 1Score **> 35** indicates **identity** Score **> 15** indicates **homology** | U | K.GIAVHPELFSIDNGLLTPTLK.A  + Deamidated (NQ) |
| 143960 | 656 | – | 676 | 746.0841 | 2235.2305 | 2235.2151 | 6.90 | 0 | 14 | 0.046 | 1Score **> 35** indicates **identity** Score **> 13** indicates **homology** | U | K.GIAVHPELFSIDNGLLTPTLK.A  + Deamidated (NQ) |
| 143961 | 656 | – | 676 | 746.0858 | 2235.2356 | 2235.2151 | 9.20 | 0 | 22 | 0.0083 | 1Score **> 35** indicates **identity** Score **> 14** indicates **homology** | U | K.GIAVHPELFSIDNGLLTPTLK.A  + Deamidated (NQ) |
| 42036 | 688 | – | 698 | 640.8404 | 1279.6662 | 1279.6660 | 0.20 | 0 | 42 | 0.00011 | 1Score **> 33** indicates **identity** Score **> 15** indicates **homology** | U | R.SQIDELYATIK.I |
| 42039 | 688 | – | 698 | 640.8408 | 1279.6671 | 1279.6660 | 0.87 | 0 | 47 | 3.9e-05 | 1Score **> 33** indicates **identity** Score **> 15** indicates **homology** | U | R.SQIDELYATIK.I |
| 42040 | 688 | – | 698 | 640.8413 | 1279.6680 | 1279.6660 | 1.61 | 0 | 62 | 1.4e-06 | 1Score **> 33** indicates **identity** Score **> 16** indicates **homology** | U | R.SQIDELYATIK.I |
| 53959 | 688 | – | 699 | 697.3820 | 1392.7495 | 1392.7500 | -0.40 | 1 | 57 | 4.2e-06 | 1Score **> 34** indicates **identity** Score **> 16** indicates **homology** | U | R.SQIDELYATIKI.- |
| 53960 | 688 | – | 699 | 697.3821 | 1392.7496 | 1392.7500 | -0.31 | 1 | 57 | 4.2e-06 | 1Score **> 34** indicates **identity** Score **> 16** indicates **homology** | U | R.SQIDELYATIKI.- |
| 53961 | 688 | – | 699 | 465.2571 | 1392.7496 | 1392.7500 | -0.29 | 1 | 32 | 0.001 | 1Score **> 34** indicates **identity** Score **> 14** indicates **homology** | U | R.SQIDELYATIKI.- |
| 53962 | 688 | – | 699 | 465.2572 | 1392.7497 | 1392.7500 | -0.19 | 1 | 30 | 0.0017 | 1Score **> 34** indicates **identity** Score **> 14** indicates **homology** | U | R.SQIDELYATIKI.- |
| 53963 | 688 | – | 699 | 697.3823 | 1392.7500 | 1392.7500 | 0.024 | 1 | 74 | 1e-07 | 1Score **> 34** indicates **identity** Score **> 17** indicates **homology** | U | R.SQIDELYATIKI.- |
| 53964 | 688 | – | 699 | 697.3823 | 1392.7501 | 1392.7500 | 0.035 | 1 | 57 | 4.8e-06 | 1Score **> 34** indicates **identity** Score **> 16** indicates **homology** | U | R.SQIDELYATIKI.- |
| 53965 | 688 | – | 699 | 697.3825 | 1392.7504 | 1392.7500 | 0.31 | 1 | 31 | 0.0011 | 1Score **> 34** indicates **identity** Score **> 14** indicates **homology** | U | R.SQIDELYATIKI.- |
| 53967 | 688 | – | 699 | 697.3829 | 1392.7513 | 1392.7500 | 0.90 | 1 | 42 | 0.00013 | 1Score **> 34** indicates **identity** Score **> 15** indicates **homology** | U | R.SQIDELYATIKI.- |
| 53969 | 688 | – | 699 | 697.3830 | 1392.7515 | 1392.7500 | 1.10 | 1 | 57 | 4.3e-06 | 1Score **> 34** indicates **identity** Score **> 16** indicates **homology** | U | R.SQIDELYATIKI.- |
| 53973 | 688 | – | 699 | 697.3838 | 1392.7530 | 1392.7500 | 2.15 | 1 | 45 | 6.3e-05 | 1Score **> 34** indicates **identity** Score **> 15** indicates **homology** | U | R.SQIDELYATIKI.- |
| 53975 | 688 | – | 699 | 697.3845 | 1392.7545 | 1392.7500 | 3.21 | 1 | 47 | 3.6e-05 | 1Score **> 33** indicates **identity** Score **> 15** indicates **homology** | U | R.SQIDELYATIKI.- |

---

```
ID   D3Z041_MOUSE            Unreviewed;       699 AA.
AC   D3Z041;
DT   20-APR-2010, integrated into UniProtKB/TrEMBL.
DT   20-APR-2010, sequence version 1.
DT   28-JUN-2023, entry version 79.
DE   RecName: Full=Long-chain-fatty-acid--CoA ligase {ECO:0000256|RuleBase:RU369030};
DE            EC=6.2.1.15 {ECO:0000256|RuleBase:RU369030};
DE            EC=6.2.1.3 {ECO:0000256|RuleBase:RU369030};
DE   AltName: Full=Acyl-CoA synthetase {ECO:0000256|RuleBase:RU369030};
DE   AltName: Full=Long-chain acyl-CoA synthetase {ECO:0000256|RuleBase:RU369030};
GN   Name=Acsl1 {ECO:0000313|Ensembl:ENSMUSP00000106000.2,
GN   ECO:0000313|MGI:MGI:102797};
OS   Mus musculus (Mouse).
OC   Eukaryota; Metazoa; Chordata; Craniata; Vertebrata; Euteleostomi; Mammalia;
OC   Eutheria; Euarchontoglires; Glires; Rodentia; Myomorpha; Muroidea; Muridae;
OC   Murinae; Mus; Mus.
OX   NCBI_TaxID=10090 {ECO:0000313|Ensembl:ENSMUSP00000106000.2, ECO:0000313|Proteomes:UP000000589};
RN   [1] {ECO:0007829|PubMed:17242355}
RP   IDENTIFICATION BY MASS SPECTROMETRY [LARGE SCALE ANALYSIS].
RX   PubMed=17242355; DOI=10.1073/pnas.0609836104;
RA   Villen J., Beausoleil S.A., Gerber S.A., Gygi S.P.;
RT   "Large-scale phosphorylation analysis of mouse liver.";
RL   Proc. Natl. Acad. Sci. U.S.A. 104:1488-1493(2007).
RN   [2] {ECO:0000313|Ensembl:ENSMUSP00000106000.2, ECO:0000313|Proteomes:UP000000589}
RP   NUCLEOTIDE SEQUENCE [LARGE SCALE GENOMIC DNA].
RC   STRAIN=C57BL/6J {ECO:0000313|Ensembl:ENSMUSP00000106000.2,
RC   ECO:0000313|Proteomes:UP000000589};
RX   PubMed=19468303; DOI=10.1371/journal.pbio.1000112;
RA   Church D.M., Goodstadt L., Hillier L.W., Zody M.C., Goldstein S., She X.,
RA   Bult C.J., Agarwala R., Cherry J.L., DiCuccio M., Hlavina W., Kapustin Y.,
RA   Meric P., Maglott D., Birtle Z., Marques A.C., Graves T., Zhou S.,
RA   Teague B., Potamousis K., Churas C., Place M., Herschleb J., Runnheim R.,
RA   Forrest D., Amos-Landgraf J., Schwartz D.C., Cheng Z., Lindblad-Toh K.,
RA   Eichler E.E., Ponting C.P.;
RT   "Lineage-specific biology revealed by a finished genome assembly of the
RT   mouse.";
RL   PLoS Biol. 7:E1000112-E1000112(2009).
RN   [3] {ECO:0007829|PubMed:21183079}
RP   IDENTIFICATION BY MASS SPECTROMETRY [LARGE SCALE ANALYSIS].
RX   PubMed=21183079; DOI=10.1016/j.cell.2010.12.001;
RA   Huttlin E.L., Jedrychowski M.P., Elias J.E., Goswami T., Rad R.,
RA   Beausoleil S.A., Villen J., Haas W., Sowa M.E., Gygi S.P.;
RT   "A tissue-specific atlas of mouse protein phosphorylation and expression.";
RL   Cell 143:1174-1189(2010).
RN   [4] {ECO:0007829|PubMed:23576753}
RP   IDENTIFICATION BY MASS SPECTROMETRY [LARGE SCALE ANALYSIS].
RX   PubMed=23576753; DOI=10.1073/pnas.1302961110;
RA   Rardin M.J., Newman J.C., Held J.M., Cusack M.P., Sorensen D.J., Li B.,
RA   Schilling B., Mooney S.D., Kahn C.R., Verdin E., Gibson B.W.;
RT   "Label-free quantitative proteomics of the lysine acetylome in mitochondria
RT   identifies substrates of SIRT3 in metabolic pathways.";
RL   Proc. Natl. Acad. Sci. U.S.A. 110:6601-6606(2013).
RN   [5] {ECO:0000313|Ensembl:ENSMUSP00000106000.2}
RP   IDENTIFICATION.
RC   STRAIN=C57BL/6J {ECO:0000313|Ensembl:ENSMUSP00000106000.2};
RG   Ensembl;
RL   Submitted (MAR-2023) to UniProtKB.
CC   -!- FUNCTION: Catalyzes the conversion of long-chain fatty acids to their
CC       active form acyl-CoAs for both synthesis of cellular lipids, and
CC       degradation via beta-oxidation. {ECO:0000256|RuleBase:RU369030}.
CC   -!- CATALYTIC ACTIVITY:
CC       Reaction=(5Z,8Z,11Z,14Z)-eicosatetraenoate + ATP + CoA =
CC         (5Z,8Z,11Z,14Z)-eicosatetraenoyl-CoA + AMP + diphosphate;
CC         Xref=Rhea:RHEA:19713, ChEBI:CHEBI:30616, ChEBI:CHEBI:32395,
CC         ChEBI:CHEBI:33019, ChEBI:CHEBI:57287, ChEBI:CHEBI:57368,
CC         ChEBI:CHEBI:456215; EC=6.2.1.15;
CC         Evidence={ECO:0000256|ARBA:ARBA00024548,
CC         ECO:0000256|RuleBase:RU369030};
CC       PhysiologicalDirection=left-to-right; Xref=Rhea:RHEA:19714;
CC         Evidence={ECO:0000256|ARBA:ARBA00024548,
CC         ECO:0000256|RuleBase:RU369030};
CC   -!- CATALYTIC ACTIVITY:
CC       Reaction=(E)-hexadec-2-enoate + ATP + CoA = (2E)-hexadecenoyl-CoA + AMP
CC         + diphosphate; Xref=Rhea:RHEA:36139, ChEBI:CHEBI:30616,
CC         ChEBI:CHEBI:33019, ChEBI:CHEBI:57287, ChEBI:CHEBI:61526,
CC         ChEBI:CHEBI:72745, ChEBI:CHEBI:456215;
CC         Evidence={ECO:0000256|ARBA:ARBA00024565,
CC         ECO:0000256|RuleBase:RU369030};
CC       PhysiologicalDirection=left-to-right; Xref=Rhea:RHEA:36140;
CC         Evidence={ECO:0000256|ARBA:ARBA00024565,
CC         ECO:0000256|RuleBase:RU369030};
CC   -!- CATALYTIC ACTIVITY:
CC       Reaction=12-hydroxy-(5Z,8Z,10E,14Z)-eicosatetraenoate + ATP + CoA = 12-
CC         hydroxy-(5Z,8Z,10E,14Z)-eicosatetraenoyl-CoA + AMP + diphosphate;
CC         Xref=Rhea:RHEA:52112, ChEBI:CHEBI:30616, ChEBI:CHEBI:33019,
CC         ChEBI:CHEBI:57287, ChEBI:CHEBI:90718, ChEBI:CHEBI:136408,
CC         ChEBI:CHEBI:456215; Evidence={ECO:0000256|ARBA:ARBA00024495,
CC         ECO:0000256|RuleBase:RU369030};
CC       PhysiologicalDirection=left-to-right; Xref=Rhea:RHEA:52113;
CC         Evidence={ECO:0000256|ARBA:ARBA00024495,
CC         ECO:0000256|RuleBase:RU369030};
CC   -!- CATALYTIC ACTIVITY:
CC       Reaction=15-hydroxy-(5Z,8Z,11Z,13E)-eicosatetraenoate + ATP + CoA = 15-
CC         hydroxy-(5Z,8Z,11Z,13E)-eicosatetraenoyl-CoA + AMP + diphosphate;
CC         Xref=Rhea:RHEA:52116, ChEBI:CHEBI:30616, ChEBI:CHEBI:33019,
CC         ChEBI:CHEBI:57287, ChEBI:CHEBI:78832, ChEBI:CHEBI:136409,
CC         ChEBI:CHEBI:456215; Evidence={ECO:0000256|ARBA:ARBA00024532,
CC         ECO:0000256|RuleBase:RU369030};
CC       PhysiologicalDirection=left-to-right; Xref=Rhea:RHEA:52117;
CC         Evidence={ECO:0000256|ARBA:ARBA00024532,
CC         ECO:0000256|RuleBase:RU369030};
CC   -!- CATALYTIC ACTIVITY:
CC       Reaction=5-hydroxy-(6E,8Z,11Z,14Z)-eicosatetraenoate + ATP + CoA = 5-
CC         hydroxy-(6E,8Z,11Z,14Z)-eicosatetraenoyl-CoA + AMP + diphosphate;
CC         Xref=Rhea:RHEA:52108, ChEBI:CHEBI:30616, ChEBI:CHEBI:33019,
CC         ChEBI:CHEBI:57287, ChEBI:CHEBI:65341, ChEBI:CHEBI:136407,
CC         ChEBI:CHEBI:456215; Evidence={ECO:0000256|ARBA:ARBA00024469,
CC         ECO:0000256|RuleBase:RU369030};
CC       PhysiologicalDirection=left-to-right; Xref=Rhea:RHEA:52109;
CC         Evidence={ECO:0000256|ARBA:ARBA00024469,
CC         ECO:0000256|RuleBase:RU369030};
CC   -!- CATALYTIC ACTIVITY:
CC       Reaction=ATP + CoA + hexadecanoate = AMP + diphosphate + hexadecanoyl-
CC         CoA; Xref=Rhea:RHEA:30751, ChEBI:CHEBI:7896, ChEBI:CHEBI:30616,
CC         ChEBI:CHEBI:33019, ChEBI:CHEBI:57287, ChEBI:CHEBI:57379,
CC         ChEBI:CHEBI:456215; Evidence={ECO:0000256|ARBA:ARBA00024497,
CC         ECO:0000256|RuleBase:RU369030};
CC       PhysiologicalDirection=left-to-right; Xref=Rhea:RHEA:30752;
CC         Evidence={ECO:0000256|ARBA:ARBA00024497,
CC         ECO:0000256|RuleBase:RU369030};
CC   -!- CATALYTIC ACTIVITY:
CC       Reaction=a long-chain fatty acid + ATP + CoA = a long-chain fatty acyl-
CC         CoA + AMP + diphosphate; Xref=Rhea:RHEA:15421, ChEBI:CHEBI:30616,
CC         ChEBI:CHEBI:33019, ChEBI:CHEBI:57287, ChEBI:CHEBI:57560,
CC         ChEBI:CHEBI:83139, ChEBI:CHEBI:456215; EC=6.2.1.3;
CC         Evidence={ECO:0000256|ARBA:ARBA00024484};
CC       PhysiologicalDirection=left-to-right; Xref=Rhea:RHEA:15422;
CC         Evidence={ECO:0000256|ARBA:ARBA00024484};
CC   -!- SUBCELLULAR LOCATION: Endoplasmic reticulum membrane
CC       {ECO:0000256|ARBA:ARBA00004643}; Single-pass type III membrane protein
CC       {ECO:0000256|ARBA:ARBA00004643}. Membrane
CC       {ECO:0000256|ARBA:ARBA00004183}; Single-pass type III membrane protein
CC       {ECO:0000256|ARBA:ARBA00004183}. Mitochondrion outer membrane
CC       {ECO:0000256|RuleBase:RU369030}; Single-pass membrane protein
CC       {ECO:0000256|RuleBase:RU369030}. Endoplasmic reticulum membrane
CC       {ECO:0000256|RuleBase:RU369030}; Single-pass membrane protein
CC       {ECO:0000256|RuleBase:RU369030}. Mitochondrion outer membrane
CC       {ECO:0000256|ARBA:ARBA00025703}; Single-pass type III membrane protein
CC       {ECO:0000256|ARBA:ARBA00025703}.
CC   -!- SIMILARITY: Belongs to the ATP-dependent AMP-binding enzyme family.
CC       {ECO:0000256|ARBA:ARBA00006432, ECO:0000256|RuleBase:RU369030}.
CC   ---------------------------------------------------------------------------
CC   Copyrighted by the UniProt Consortium, see https://www.uniprot.org/terms
CC   Distributed under the Creative Commons Attribution (CC BY 4.0) License
CC   ---------------------------------------------------------------------------
DR   AlphaFoldDB; D3Z041; -.
DR   SMR; D3Z041; -.
DR   SwissPalm; D3Z041; -.
DR   EPD; D3Z041; -.
DR   jPOST; D3Z041; -.
DR   MaxQB; D3Z041; -.
DR   PeptideAtlas; D3Z041; -.
DR   ProteomicsDB; 310638; -.
DR   Antibodypedia; 1946; 328 antibodies from 35 providers.
DR   Ensembl; ENSMUST00000110371.8; ENSMUSP00000106000.2; ENSMUSG00000018796.14.
DR   AGR; MGI:102797; -.
DR   MGI; MGI:102797; Acsl1.
DR   VEuPathDB; HostDB:ENSMUSG00000018796; -.
DR   GeneTree; ENSGT00940000154508; -.
DR   OMA; WTIGAQV; -.
DR   ChiTaRS; Acsl1; mouse.
DR   Proteomes; UP000000589; Chromosome 8.
DR   Bgee; ENSMUSG00000018796; Expressed in brown adipose tissue and 285 other tissues.
DR   ExpressionAtlas; D3Z041; baseline and differential.
DR   GO; GO:0005789; C:endoplasmic reticulum membrane; IEA:UniProtKB-SubCell.
DR   GO; GO:0005741; C:mitochondrial outer membrane; IEA:UniProtKB-SubCell.
DR   GO; GO:0005524; F:ATP binding; IEA:UniProtKB-KW.
DR   GO; GO:0004467; F:long-chain fatty acid-CoA ligase activity; IEA:InterPro.
DR   CDD; cd05927; LC-FACS_euk; 1.
DR   Gene3D; 3.40.50.12780; N-terminal domain of ligase-like; 1.
DR   InterPro; IPR025110; AMP-bd_C.
DR   InterPro; IPR020845; AMP-binding_CS.
DR   InterPro; IPR000873; AMP-dep_Synth/Lig_com.
DR   InterPro; IPR042099; ANL_N_sf.
DR   InterPro; IPR045311; LC-FACS_euk.
DR   PANTHER; PTHR43272; LONG-CHAIN-FATTY-ACID--COA LIGASE; 1.
DR   PANTHER; PTHR43272:SF28; LONG-CHAIN-FATTY-ACID--COA LIGASE 1; 1.
DR   Pfam; PF00501; AMP-binding; 1.
DR   Pfam; PF13193; AMP-binding_C; 1.
DR   SUPFAM; SSF56801; Acetyl-CoA synthetase-like; 1.
DR   PROSITE; PS00455; AMP_BINDING; 1.
PE   1: Evidence at protein level;
KW   ATP-binding {ECO:0000256|RuleBase:RU369030};
KW   Fatty acid metabolism {ECO:0000256|ARBA:ARBA00022832,
KW   ECO:0000256|RuleBase:RU369030}; Ligase {ECO:0000256|RuleBase:RU369030};
KW   Lipid metabolism {ECO:0000256|ARBA:ARBA00023098,
KW   ECO:0000256|RuleBase:RU369030}; Membrane {ECO:0000256|RuleBase:RU369030};
KW   Nucleotide-binding {ECO:0000256|RuleBase:RU369030};
KW   Proteomics identification {ECO:0007829|EPD:D3Z041,
KW   ECO:0007829|MaxQB:D3Z041};
KW   Reference proteome {ECO:0000313|Proteomes:UP000000589};
KW   Transmembrane {ECO:0000256|RuleBase:RU369030};
KW   Transmembrane helix {ECO:0000256|RuleBase:RU369030}.
FT   TRANSMEM        21..45
FT                   /note="Helical"
FT                   /evidence="ECO:0000256|RuleBase:RU369030"
FT   DOMAIN          116..563
FT                   /note="AMP-dependent synthetase/ligase"
FT                   /evidence="ECO:0000259|Pfam:PF00501"
FT   DOMAIN          573..629
FT                   /note="AMP-binding enzyme C-terminal"
FT                   /evidence="ECO:0000259|Pfam:PF13193"
SQ   SEQUENCE   699 AA;  78034 MW;  D10B6FE1982D3474 CRC64;
     MEVHELFRYF RMPELIDIRQ YVRTLPTNTL MGFGAFAALT TFWYATRPKA LKPPCDLSMQ
     SVEIAGTTDG IRRSAVLEDD KLLVYYYDDV RTMYDGFQRG IQVSNNGPCL GSRKPNQPYE
     WISYKEVAEL AECIGSGLIQ KGFKPCSEQF IGLFSQNRPE WVIVEQGCFS YSMVVVPLYD
     TLGADAITYI VNKAELSVIF ADKPEKAKLL LEGVENKLTP CLKIIVIMDS YGSDLVERGK
     KCGVEIISLK ALEDLGRVNR VKPKPPEPED LAIICFTSGT TGNPKGAMIT HQNIINDCSG
     FIKATESALT LNASDTQISY LPLAHMYEQQ LQCVMLCHGA KIGFFQGDIR LLMDDLKVLQ
     PTIFPVVPRL LNRMFDRIFG QANTSLKRWL LDFASKRKEA ELRSGIVRNN SLWDKLIFHK
     IQSSLGGKVR LMITGAAPVS ATVLTFLRTA LGCQFYEGYG QTECTAGCCL SLPGDWTAGH
     VGAPMPCNYV KLVDVEEMNY LASKGEGEVC VKGANVFKGY LKDPARTAEA LDKDGWLHTG
     DIGKWLPNGT LKIIDRKKHI FKLAQGEYIA PEKIENIYLR SEAVAQVFVH GESLQAFLIA
     VVVPDVESLP SWAQKRGLQG SFEELCRNKD INKAILDDLL KLGKEAGLKP FEQVKGIAVH
     PELFSIDNGL LTPTLKAKRP ELRNYFRSQI DELYATIKI
//
```

|  |
| --- |
| **Mascot:** http://www.matrixscience.com/ |

Deamidated (NQ) (+0.9840)
