# Supplementary material for: A common East-Asian ALDH2 mutation causes metabolic disorders and the therapeutic effect of ALDH2 activators
Source: Nat Commun. 2023 Sep 25;14:5971. doi: 10.1038/s41467-023-41570-6 (PMC10520061; doi:10.1038/s41467-023-41570-6)
Supplement: Supplementary file 4 — Supplementary Data 1 [file 41467_2023_41570_MOESM4_ESM.zip › Table S5b/P12242/P12242_WTO-1_C25.html]

Mascot Search Results: P12242
 

# MASCOT Search Results

## Protein View: P12242

### Mitochondrial brown fat uncoupling protein 1 OS=Mus musculus OX=10090 GN=Ucp1 PE=1 SV=2

|  |  |
| --- | --- |
| Database: | Mouse\_UniProt\_proteomes |
| Score: | 17843 |
| Monoisotopic mass (Mr): | 33625 |
| Calculated pI: | 9.29 |

Sequence similarity is available as an NCBI BLAST search of P12242 against nr.

### Search parameters

|  |  |
| --- | --- |
| MS data file: | `D:\LCMSMS\2023 Users' data\230529-1\230529-1-WTO-1.raw` |
| Enzyme: | Trypsin/P: cuts C-term side of KR. |
| Fixed modifications: | Carbamidomethyl (C) |
| Variable modifications: | Deamidated (NQ), HNE (C), HNE (H), HNE (K), Oxidation (M) |

### Protein sequence coverage: 95%

Matched peptides shown in ***bold red***.

|  |  |  |  |  |  |
| --- | --- | --- | --- | --- | --- |
| `1` | `MVNPTTSEVQ` | `PTMGVKIFSA` | `GVSACLADII` | `TFPLDTAKVR` | `LQIQGEGQAS` |
| `51` | `STIRYKGVLG` | `TITTLAKTEG` | `LPKLYSGLPA` | `GIQRQISFAS` | `LRIGLYDSVQ` |
| `101` | `EYFSSGRETP` | `ASLGNKISAG` | `LMTGGVAVFI` | `GQPTEVVKVR` | `MQAQSHLHGI` |
| `151` | `KPRYTGTYNA` | `YRVIATTESL` | `STLWKGTTPN` | `LMRNVIINCT` | `ELVTYDLMKG` |
| `201` | `ALVNNKILAD` | `DVPCHLLSAL` | `VAGFCTTLLA` | `SPVDVVKTRF` | `INSLPGQYPS` |
| `251` | `VPSCAMSMYT` | `KEGPTAFFKG` | `FVASFLRLGS` | `WNVIMFVCFE` | `QLKKELMKSR` |
| `301` | `QTVDCTT` |  |  |  |  |

Unformatted sequence string: 307 residues (for pasting into other applications).

|  |  |  |  |
| --- | --- | --- | --- |
| Sort by | residue number | increasing mass | decreasing mass |
| Show | matched peptides only | predicted peptides also |  |

| Query | Start | – | End | Observed | Mr(expt) | Mr(calc) | ppm | M | Score | Expect | Rank | U | Peptide |
| --- | --- | --- | --- | --- | --- | --- | --- | --- | --- | --- | --- | --- | --- |
| 185657 | 1 | – | 38 | 1028.0286 | 4108.0852 | 4108.1186 | -8.15 | 1 | 26 | 0.0035 | 1Score **> 37** indicates **identity** Score **> 14** indicates **homology** | U | -.MVNPTTSEVQPTMGVKIFSAGVSACLADIITFPLDTAK.V  + HNE (C) |
| 76266 | 2 | – | 16 | 794.4040 | 1586.7935 | 1586.7974 | -2.47 | 0 | 36 | 0.00039 | 1Score **> 34** indicates **identity** Score **> 15** indicates **homology** | U | M.VNPTTSEVQPTMGVK.I |
| 76272 | 2 | – | 16 | 794.4056 | 1586.7967 | 1586.7974 | -0.48 | 0 | 75 | 1e-07 | 1Score **> 34** indicates **identity** Score **> 17** indicates **homology** | U | M.VNPTTSEVQPTMGVK.I |
| 76273 | 2 | – | 16 | 794.4058 | 1586.7971 | 1586.7974 | -0.21 | 0 | 70 | 2.6e-07 | 1Score **> 34** indicates **identity** Score **> 17** indicates **homology** | U | M.VNPTTSEVQPTMGVK.I |
| 76277 | 2 | – | 16 | 794.4065 | 1586.7985 | 1586.7974 | 0.68 | 0 | 76 | 1.3e-07 | 1Score **> 34** indicates **identity** Score **> 19** indicates **homology** | U | M.VNPTTSEVQPTMGVK.I |
| 76280 | 2 | – | 16 | 794.4067 | 1586.7989 | 1586.7974 | 0.94 | 0 | 50 | 2.3e-05 | 1Score **> 34** indicates **identity** Score **> 16** indicates **homology** | U | M.VNPTTSEVQPTMGVK.I |
| 78369 | 2 | – | 16 | 802.3998 | 1602.7851 | 1602.7923 | -4.52 | 0 | 50 | 2e-05 | 1Score **> 34** indicates **identity** Score **> 16** indicates **homology** | U | M.VNPTTSEVQPTMGVK.I  + Oxidation (M) |
| 78370 | 2 | – | 16 | 802.4003 | 1602.7860 | 1602.7923 | -3.94 | 0 | 37 | 0.00035 | 1Score **> 34** indicates **identity** Score **> 15** indicates **homology** | U | M.VNPTTSEVQPTMGVK.I  + Oxidation (M) |
| 78372 | 2 | – | 16 | 802.4004 | 1602.7863 | 1602.7923 | -3.75 | 0 | 64 | 9.2e-07 | 1Score **> 34** indicates **identity** Score **> 17** indicates **homology** | U | M.VNPTTSEVQPTMGVK.I  + Oxidation (M) |
| 78376 | 2 | – | 16 | 802.4011 | 1602.7877 | 1602.7923 | -2.91 | 0 | 60 | 2.1e-06 | 1Score **> 34** indicates **identity** Score **> 16** indicates **homology** | U | M.VNPTTSEVQPTMGVK.I  + Oxidation (M) |
| 78377 | 2 | – | 16 | 802.4012 | 1602.7878 | 1602.7923 | -2.85 | 0 | 51 | 3.7e-05 | 1Score **> 34** indicates **identity** Score **> 19** indicates **homology** | U | M.VNPTTSEVQPTMGVK.I  + Oxidation (M) |
| 78378 | 2 | – | 16 | 802.4012 | 1602.7878 | 1602.7923 | -2.82 | 0 | 45 | 6.6e-05 | 1Score **> 34** indicates **identity** Score **> 15** indicates **homology** | U | M.VNPTTSEVQPTMGVK.I  + Oxidation (M) |
| 78382 | 2 | – | 16 | 802.4017 | 1602.7889 | 1602.7923 | -2.13 | 0 | 54 | 9.3e-06 | 1Score **> 34** indicates **identity** Score **> 16** indicates **homology** | U | M.VNPTTSEVQPTMGVK.I  + Oxidation (M) |
| 78383 | 2 | – | 16 | 802.4018 | 1602.7891 | 1602.7923 | -2.02 | 0 | 45 | 5.7e-05 | 1Score **> 34** indicates **identity** Score **> 15** indicates **homology** | U | M.VNPTTSEVQPTMGVK.I  + Oxidation (M) |
| 78384 | 2 | – | 16 | 802.4023 | 1602.7900 | 1602.7923 | -1.49 | 0 | 60 | 2.2e-06 | 1Score **> 34** indicates **identity** Score **> 16** indicates **homology** | U | M.VNPTTSEVQPTMGVK.I  + Oxidation (M) |
| 78386 | 2 | – | 16 | 802.4024 | 1602.7902 | 1602.7923 | -1.31 | 0 | 69 | 3.2e-07 | 1Score **> 34** indicates **identity** Score **> 17** indicates **homology** | U | M.VNPTTSEVQPTMGVK.I  + Oxidation (M) |
| 78388 | 2 | – | 16 | 802.4025 | 1602.7904 | 1602.7923 | -1.24 | 0 | 65 | 7.5e-07 | 1Score **> 34** indicates **identity** Score **> 17** indicates **homology** | U | M.VNPTTSEVQPTMGVK.I  + Oxidation (M) |
| 78389 | 2 | – | 16 | 802.4025 | 1602.7904 | 1602.7923 | -1.23 | 0 | 54 | 9.5e-06 | 1Score **> 34** indicates **identity** Score **> 16** indicates **homology** | U | M.VNPTTSEVQPTMGVK.I  + Oxidation (M) |
| 78391 | 2 | – | 16 | 802.4026 | 1602.7907 | 1602.7923 | -1.03 | 0 | 53 | 1.8e-05 | 1Score **> 34** indicates **identity** Score **> 19** indicates **homology** | U | M.VNPTTSEVQPTMGVK.I  + Oxidation (M) |
| 78392 | 2 | – | 16 | 802.4028 | 1602.7911 | 1602.7923 | -0.80 | 0 | 49 | 2.6e-05 | 1Score **> 34** indicates **identity** Score **> 16** indicates **homology** | U | M.VNPTTSEVQPTMGVK.I  + Oxidation (M) |
| 78393 | 2 | – | 16 | 802.4029 | 1602.7912 | 1602.7923 | -0.69 | 0 | 24 | 0.0053 | 1Score **> 34** indicates **identity** Score **> 14** indicates **homology** | U | M.VNPTTSEVQPTMGVK.I  + Oxidation (M) |
| 78394 | 2 | – | 16 | 802.4029 | 1602.7913 | 1602.7923 | -0.66 | 0 | 49 | 2.3e-05 | 1Score **> 34** indicates **identity** Score **> 16** indicates **homology** | U | M.VNPTTSEVQPTMGVK.I  + Oxidation (M) |
| 78396 | 2 | – | 16 | 802.4031 | 1602.7916 | 1602.7923 | -0.48 | 0 | 57 | 5.6e-06 | 1Score **> 34** indicates **identity** Score **> 17** indicates **homology** | U | M.VNPTTSEVQPTMGVK.I  + Oxidation (M) |
| 78397 | 2 | – | 16 | 802.4032 | 1602.7918 | 1602.7923 | -0.33 | 0 | 41 | 0.00015 | 1Score **> 34** indicates **identity** Score **> 15** indicates **homology** | U | M.VNPTTSEVQPTMGVK.I  + Oxidation (M) |
| 78398 | 2 | – | 16 | 802.4033 | 1602.7921 | 1602.7923 | -0.17 | 0 | 51 | 1.5e-05 | 1Score **> 34** indicates **identity** Score **> 16** indicates **homology** | U | M.VNPTTSEVQPTMGVK.I  + Oxidation (M) |
| 78399 | 2 | – | 16 | 802.4033 | 1602.7921 | 1602.7923 | -0.13 | 0 | 45 | 6.5e-05 | 1Score **> 34** indicates **identity** Score **> 15** indicates **homology** | U | M.VNPTTSEVQPTMGVK.I  + Oxidation (M) |
| 78400 | 2 | – | 16 | 802.4034 | 1602.7922 | 1602.7923 | -0.089 | 0 | 45 | 0.00023 | 1Score **> 34** indicates **identity** Score **> 21** indicates **homology** | U | M.VNPTTSEVQPTMGVK.I  + Oxidation (M) |
| 78401 | 2 | – | 16 | 802.4034 | 1602.7923 | 1602.7923 | -0.054 | 0 | 42 | 0.00012 | 1Score **> 34** indicates **identity** Score **> 15** indicates **homology** | U | M.VNPTTSEVQPTMGVK.I  + Oxidation (M) |
| 78403 | 2 | – | 16 | 802.4036 | 1602.7926 | 1602.7923 | 0.14 | 0 | 67 | 1.3e-06 | 1Score **> 34** indicates **identity** Score **> 21** indicates **homology** | U | M.VNPTTSEVQPTMGVK.I  + Oxidation (M) |
| 78405 | 2 | – | 16 | 802.4038 | 1602.7931 | 1602.7923 | 0.44 | 0 | 52 | 1.3e-05 | 1Score **> 34** indicates **identity** Score **> 16** indicates **homology** | U | M.VNPTTSEVQPTMGVK.I  + Oxidation (M) |
| 78407 | 2 | – | 16 | 802.4040 | 1602.7933 | 1602.7923 | 0.63 | 0 | 63 | 1.3e-06 | 1Score **> 34** indicates **identity** Score **> 16** indicates **homology** | U | M.VNPTTSEVQPTMGVK.I  + Oxidation (M) |
| 78408 | 2 | – | 16 | 802.4040 | 1602.7934 | 1602.7923 | 0.65 | 0 | 59 | 2.8e-06 | 1Score **> 34** indicates **identity** Score **> 16** indicates **homology** | U | M.VNPTTSEVQPTMGVK.I  + Oxidation (M) |
| 78410 | 2 | – | 16 | 802.4046 | 1602.7946 | 1602.7923 | 1.41 | 0 | 30 | 0.0019 | 1Score **> 34** indicates **identity** Score **> 15** indicates **homology** | U | M.VNPTTSEVQPTMGVK.I  + Oxidation (M) |
| 78412 | 2 | – | 16 | 802.4047 | 1602.7948 | 1602.7923 | 1.54 | 0 | 46 | 5.3e-05 | 1Score **> 34** indicates **identity** Score **> 15** indicates **homology** | U | M.VNPTTSEVQPTMGVK.I  + Oxidation (M) |
| 78413 | 2 | – | 16 | 802.4048 | 1602.7951 | 1602.7923 | 1.71 | 0 | 60 | 2.3e-06 | 1Score **> 34** indicates **identity** Score **> 16** indicates **homology** | U | M.VNPTTSEVQPTMGVK.I  + Oxidation (M) |
| 78415 | 2 | – | 16 | 802.4053 | 1602.7961 | 1602.7923 | 2.34 | 0 | 54 | 1.3e-05 | 1Score **> 34** indicates **identity** Score **> 18** indicates **homology** | U | M.VNPTTSEVQPTMGVK.I  + Oxidation (M) |
| 78416 | 2 | – | 16 | 802.4057 | 1602.7969 | 1602.7923 | 2.84 | 0 | 39 | 0.00021 | 1Score **> 34** indicates **identity** Score **> 15** indicates **homology** | U | M.VNPTTSEVQPTMGVK.I  + Oxidation (M) |
| 78419 | 2 | – | 16 | 802.4069 | 1602.7992 | 1602.7923 | 4.30 | 0 | 40 | 0.00018 | 1Score **> 34** indicates **identity** Score **> 15** indicates **homology** | U | M.VNPTTSEVQPTMGVK.I  + Oxidation (M) |
| 78420 | 2 | – | 16 | 802.4070 | 1602.7995 | 1602.7923 | 4.48 | 0 | 38 | 0.00029 | 1Score **> 34** indicates **identity** Score **> 15** indicates **homology** | U | M.VNPTTSEVQPTMGVK.I  + Oxidation (M) |
| 78568 | 2 | – | 16 | 802.8993 | 1603.7840 | 1603.7764 | 4.77 | 0 | 30 | 0.0015 | 1Score **> 34** indicates **identity** Score **> 14** indicates **homology** | U | M.VNPTTSEVQPTMGVK.I  + Deamidated (NQ); Oxidation (M) |
| 78584 | 2 | – | 16 | 802.9012 | 1603.7878 | 1603.7764 | 7.16 | 0 | 20 | 0.013 | 1Score **> 34** indicates **identity** Score **> 14** indicates **homology** | U | M.VNPTTSEVQPTMGVK.I  + Deamidated (NQ); Oxidation (M) |
| 144262 | 17 | – | 38 | 770.7366 | 2309.1881 | 2309.1977 | -4.17 | 0 | 15 | 0.04 | 1Score **> 37** indicates **identity** Score **> 13** indicates **homology** | U | K.IFSAGVSACLADIITFPLDTAK.V |
| 144264 | 17 | – | 38 | 1155.6026 | 2309.1907 | 2309.1977 | -3.05 | 0 | 27 | 0.003 | 1Score **> 37** indicates **identity** Score **> 14** indicates **homology** | U | K.IFSAGVSACLADIITFPLDTAK.V |
| 144267 | 17 | – | 38 | 1155.6035 | 2309.1925 | 2309.1977 | -2.28 | 0 | 71 | 2.4e-07 | 1Score **> 37** indicates **identity** Score **> 17** indicates **homology** | U | K.IFSAGVSACLADIITFPLDTAK.V |
| 144273 | 17 | – | 38 | 770.7392 | 2309.1958 | 2309.1977 | -0.85 | 0 | 19 | 0.017 | 1Score **> 37** indicates **identity** Score **> 14** indicates **homology** | U | K.IFSAGVSACLADIITFPLDTAK.V |
| 144275 | 17 | – | 38 | 1155.6055 | 2309.1964 | 2309.1977 | -0.59 | 0 | 63 | 1.3e-06 | 1Score **> 37** indicates **identity** Score **> 16** indicates **homology** | U | K.IFSAGVSACLADIITFPLDTAK.V |
| 144278 | 17 | – | 38 | 770.7397 | 2309.1973 | 2309.1977 | -0.20 | 0 | 21 | 0.011 | 1Score **> 37** indicates **identity** Score **> 14** indicates **homology** | U | K.IFSAGVSACLADIITFPLDTAK.V |
| 144282 | 17 | – | 38 | 1155.6063 | 2309.1980 | 2309.1977 | 0.11 | 0 | 18 | 0.02 | 1Score **> 37** indicates **identity** Score **> 14** indicates **homology** | U | K.IFSAGVSACLADIITFPLDTAK.V |
| 144283 | 17 | – | 38 | 1155.6064 | 2309.1982 | 2309.1977 | 0.20 | 0 | 84 | 1.4e-08 | 1Score **> 37** indicates **identity** Score **> 18** indicates **homology** | U | K.IFSAGVSACLADIITFPLDTAK.V |
| 144284 | 17 | – | 38 | 770.7401 | 2309.1986 | 2309.1977 | 0.37 | 0 | 20 | 0.012 | 1Score **> 37** indicates **identity** Score **> 14** indicates **homology** | U | K.IFSAGVSACLADIITFPLDTAK.V |
| 144285 | 17 | – | 38 | 1155.6067 | 2309.1989 | 2309.1977 | 0.50 | 0 | 138 | 1.2e-13 | 1Score **> 37** indicates **identity** Score **> 21** indicates **homology** | U | K.IFSAGVSACLADIITFPLDTAK.V |
| 144290 | 17 | – | 38 | 1155.6070 | 2309.1994 | 2309.1977 | 0.74 | 0 | 78 | 5.2e-08 | 1Score **> 37** indicates **identity** Score **> 17** indicates **homology** | U | K.IFSAGVSACLADIITFPLDTAK.V |
| 144293 | 17 | – | 38 | 1155.6071 | 2309.1997 | 2309.1977 | 0.87 | 0 | 109 | 5.4e-11 | 1Score **> 37** indicates **identity** Score **> 19** indicates **homology** | U | K.IFSAGVSACLADIITFPLDTAK.V |
| 144294 | 17 | – | 38 | 770.7405 | 2309.1998 | 2309.1977 | 0.91 | 0 | 48 | 3.4e-05 | 1Score **> 37** indicates **identity** Score **> 15** indicates **homology** | U | K.IFSAGVSACLADIITFPLDTAK.V |
| 144297 | 17 | – | 38 | 770.7409 | 2309.2008 | 2309.1977 | 1.34 | 0 | 59 | 2.7e-06 | 1Score **> 37** indicates **identity** Score **> 16** indicates **homology** | U | K.IFSAGVSACLADIITFPLDTAK.V |
| 144298 | 17 | – | 38 | 770.7409 | 2309.2009 | 2309.1977 | 1.37 | 0 | 47 | 3.6e-05 | 1Score **> 37** indicates **identity** Score **> 15** indicates **homology** | U | K.IFSAGVSACLADIITFPLDTAK.V |
| 144300 | 17 | – | 38 | 770.7410 | 2309.2012 | 2309.1977 | 1.51 | 0 | 45 | 5.7e-05 | 1Score **> 37** indicates **identity** Score **> 15** indicates **homology** | U | K.IFSAGVSACLADIITFPLDTAK.V |
| 144303 | 17 | – | 38 | 1155.6081 | 2309.2016 | 2309.1977 | 1.69 | 0 | 125 | 2e-12 | 1Score **> 37** indicates **identity** Score **> 20** indicates **homology** | U | K.IFSAGVSACLADIITFPLDTAK.V |
| 144306 | 17 | – | 38 | 1155.6088 | 2309.2030 | 2309.1977 | 2.29 | 0 | 79 | 4.4e-08 | 1Score **> 37** indicates **identity** Score **> 18** indicates **homology** | U | K.IFSAGVSACLADIITFPLDTAK.V |
| 144308 | 17 | – | 38 | 770.7418 | 2309.2035 | 2309.1977 | 2.52 | 0 | 27 | 0.0032 | 1Score **> 36** indicates **identity** Score **> 14** indicates **homology** | U | K.IFSAGVSACLADIITFPLDTAK.V |
| 144311 | 17 | – | 38 | 770.7421 | 2309.2044 | 2309.1977 | 2.88 | 0 | 15 | 0.038 | 1Score **> 37** indicates **identity** Score **> 13** indicates **homology** | U | K.IFSAGVSACLADIITFPLDTAK.V |
| 144312 | 17 | – | 38 | 770.7422 | 2309.2048 | 2309.1977 | 3.07 | 0 | 36 | 0.00043 | 1Score **> 37** indicates **identity** Score **> 15** indicates **homology** | U | K.IFSAGVSACLADIITFPLDTAK.V |
| 144315 | 17 | – | 38 | 770.7424 | 2309.2052 | 2309.1977 | 3.25 | 0 | 24 | 0.0069 | 1Score **> 37** indicates **identity** Score **> 15** indicates **homology** | U | K.IFSAGVSACLADIITFPLDTAK.V |
| 144316 | 17 | – | 38 | 1155.6100 | 2309.2054 | 2309.1977 | 3.33 | 0 | 21 | 0.021 | 1Score **> 37** indicates **identity** Score **> 17** indicates **homology** | U | K.IFSAGVSACLADIITFPLDTAK.V |
| 144321 | 17 | – | 38 | 1155.6106 | 2309.2067 | 2309.1977 | 3.87 | 0 | 85 | 1.1e-08 | 1Score **> 37** indicates **identity** Score **> 18** indicates **homology** | U | K.IFSAGVSACLADIITFPLDTAK.V |
| 144326 | 17 | – | 38 | 770.7433 | 2309.2081 | 2309.1977 | 4.51 | 0 | 46 | 5.1e-05 | 1Score **> 37** indicates **identity** Score **> 15** indicates **homology** | U | K.IFSAGVSACLADIITFPLDTAK.V |
| 144328 | 17 | – | 38 | 1155.6116 | 2309.2087 | 2309.1977 | 4.77 | 0 | 81 | 2.4e-08 | 1Score **> 37** indicates **identity** Score **> 18** indicates **homology** | U | K.IFSAGVSACLADIITFPLDTAK.V |
| 144329 | 17 | – | 38 | 770.7436 | 2309.2089 | 2309.1977 | 4.85 | 0 | 19 | 0.017 | 1Score **> 37** indicates **identity** Score **> 14** indicates **homology** | U | K.IFSAGVSACLADIITFPLDTAK.V |
| 144330 | 17 | – | 38 | 1155.6119 | 2309.2093 | 2309.1977 | 5.00 | 0 | 72 | 1.7e-07 | 1Score **> 37** indicates **identity** Score **> 17** indicates **homology** | U | K.IFSAGVSACLADIITFPLDTAK.V |
| 144331 | 17 | – | 38 | 1155.6121 | 2309.2096 | 2309.1977 | 5.14 | 0 | 23 | 0.0071 | 1Score **> 37** indicates **identity** Score **> 14** indicates **homology** | U | K.IFSAGVSACLADIITFPLDTAK.V |
| 144332 | 17 | – | 38 | 770.7438 | 2309.2096 | 2309.1977 | 5.15 | 0 | 38 | 0.00025 | 1Score **> 37** indicates **identity** Score **> 15** indicates **homology** | U | K.IFSAGVSACLADIITFPLDTAK.V |
| 144334 | 17 | – | 38 | 1155.6135 | 2309.2124 | 2309.1977 | 6.35 | 0 | 75 | 9.4e-08 | 1Score **> 37** indicates **identity** Score **> 17** indicates **homology** | U | K.IFSAGVSACLADIITFPLDTAK.V |
| 144335 | 17 | – | 38 | 1155.6135 | 2309.2124 | 2309.1977 | 6.36 | 0 | 65 | 7.6e-07 | 1Score **> 37** indicates **identity** Score **> 17** indicates **homology** | U | K.IFSAGVSACLADIITFPLDTAK.V |
| 144336 | 17 | – | 38 | 770.7448 | 2309.2124 | 2309.1977 | 6.37 | 0 | 35 | 0.00049 | 1Score **> 37** indicates **identity** Score **> 15** indicates **homology** | U | K.IFSAGVSACLADIITFPLDTAK.V |
| 144337 | 17 | – | 38 | 770.7449 | 2309.2129 | 2309.1977 | 6.55 | 0 | 26 | 0.0035 | 1Score **> 37** indicates **identity** Score **> 14** indicates **homology** | U | K.IFSAGVSACLADIITFPLDTAK.V |
| 144338 | 17 | – | 38 | 1155.6140 | 2309.2135 | 2309.1977 | 6.84 | 0 | 16 | 0.033 | 1Score **> 37** indicates **identity** Score **> 14** indicates **homology** | U | K.IFSAGVSACLADIITFPLDTAK.V |
| 144339 | 17 | – | 38 | 770.7451 | 2309.2136 | 2309.1977 | 6.87 | 0 | 23 | 0.0066 | 1Score **> 36** indicates **identity** Score **> 14** indicates **homology** | U | K.IFSAGVSACLADIITFPLDTAK.V |
| 144340 | 17 | – | 38 | 770.7454 | 2309.2144 | 2309.1977 | 7.22 | 0 | 39 | 0.0002 | 1Score **> 36** indicates **identity** Score **> 15** indicates **homology** | U | K.IFSAGVSACLADIITFPLDTAK.V |
| 144341 | 17 | – | 38 | 1155.6145 | 2309.2145 | 2309.1977 | 7.28 | 0 | 60 | 2.6e-06 | 1Score **> 36** indicates **identity** Score **> 16** indicates **homology** | U | K.IFSAGVSACLADIITFPLDTAK.V |
| 144343 | 17 | – | 38 | 1155.6150 | 2309.2155 | 2309.1977 | 7.72 | 0 | 63 | 1.3e-06 | 1Score **> 37** indicates **identity** Score **> 16** indicates **homology** | U | K.IFSAGVSACLADIITFPLDTAK.V |
| 144344 | 17 | – | 38 | 770.7459 | 2309.2159 | 2309.1977 | 7.87 | 0 | 25 | 0.0047 | 1Score **> 37** indicates **identity** Score **> 14** indicates **homology** | U | K.IFSAGVSACLADIITFPLDTAK.V |
| 144346 | 17 | – | 38 | 770.7469 | 2309.2190 | 2309.1977 | 9.20 | 0 | 16 | 0.029 | 1Score **> 37** indicates **identity** Score **> 14** indicates **homology** | U | K.IFSAGVSACLADIITFPLDTAK.V |
| 144347 | 17 | – | 38 | 770.7472 | 2309.2197 | 2309.1977 | 9.50 | 0 | 33 | 0.00083 | 1Score **> 37** indicates **identity** Score **> 15** indicates **homology** | U | K.IFSAGVSACLADIITFPLDTAK.V |
| 144348 | 17 | – | 38 | 770.7475 | 2309.2208 | 2309.1977 | 9.98 | 0 | 25 | 0.0051 | 1Score **> 37** indicates **identity** Score **> 14** indicates **homology** | U | K.IFSAGVSACLADIITFPLDTAK.V |
| 95351 | 39 | – | 54 | 581.6542 | 1741.9408 | 1741.9435 | -1.51 | 1 | 15 | 0.038 | 1Score **> 35** indicates **identity** Score **> 13** indicates **homology** | U | K.VRLQIQGEGQASSTIR.Y |
| 95355 | 39 | – | 54 | 581.6546 | 1741.9420 | 1741.9435 | -0.86 | 1 | 43 | 8.7e-05 | 1Score **> 35** indicates **identity** Score **> 15** indicates **homology** | U | K.VRLQIQGEGQASSTIR.Y |
| 95356 | 39 | – | 54 | 581.6548 | 1741.9426 | 1741.9435 | -0.48 | 1 | 27 | 0.0034 | 1Score **> 35** indicates **identity** Score **> 15** indicates **homology** | U | K.VRLQIQGEGQASSTIR.Y |
| 95357 | 39 | – | 54 | 581.6550 | 1741.9432 | 1741.9435 | -0.17 | 1 | 58 | 4.8e-06 | 1Score **> 35** indicates **identity** Score **> 18** indicates **homology** | U | K.VRLQIQGEGQASSTIR.Y |
| 95358 | 39 | – | 54 | 581.6551 | 1741.9434 | 1741.9435 | -0.028 | 1 | 20 | 0.013 | 1Score **> 35** indicates **identity** Score **> 14** indicates **homology** | U | K.VRLQIQGEGQASSTIR.Y |
| 95365 | 39 | – | 54 | 581.6554 | 1741.9444 | 1741.9435 | 0.54 | 1 | 20 | 0.013 | 1Score **> 35** indicates **identity** Score **> 14** indicates **homology** | U | K.VRLQIQGEGQASSTIR.Y |
| 63589 | 41 | – | 54 | 744.3868 | 1486.7591 | 1486.7740 | -10.00 | 0 | 22 | 0.0089 | 1Score **> 34** indicates **identity** Score **> 14** indicates **homology** | U | R.LQIQGEGQASSTIR.Y |
| 63591 | 41 | – | 54 | 744.3873 | 1486.7601 | 1486.7740 | -9.34 | 0 | 16 | 0.034 | 1Score **> 34** indicates **identity** Score **> 13** indicates **homology** | U | R.LQIQGEGQASSTIR.Y |
| 63593 | 41 | – | 54 | 744.3879 | 1486.7612 | 1486.7740 | -8.60 | 0 | 25 | 0.011 | 1Score **> 34** indicates **identity** Score **> 18** indicates **homology** | U | R.LQIQGEGQASSTIR.Y |
| 63596 | 41 | – | 54 | 744.3885 | 1486.7624 | 1486.7740 | -7.76 | 0 | 20 | 0.014 | 1Score **> 34** indicates **identity** Score **> 14** indicates **homology** | U | R.LQIQGEGQASSTIR.Y |
| 63598 | 41 | – | 54 | 744.3888 | 1486.7630 | 1486.7740 | -7.36 | 0 | 22 | 0.0087 | 1Score **> 34** indicates **identity** Score **> 14** indicates **homology** | U | R.LQIQGEGQASSTIR.Y |
| 63601 | 41 | – | 54 | 744.3896 | 1486.7646 | 1486.7740 | -6.30 | 0 | 18 | 0.02 | 1Score **> 34** indicates **identity** Score **> 14** indicates **homology** | U | R.LQIQGEGQASSTIR.Y |
| 63602 | 41 | – | 54 | 744.3897 | 1486.7648 | 1486.7740 | -6.15 | 0 | 47 | 0.00031 | 1Score **> 34** indicates **identity** Score **> 24** indicates **homology** | U | R.LQIQGEGQASSTIR.Y |
| 63603 | 41 | – | 54 | 744.3898 | 1486.7649 | 1486.7740 | -6.06 | 0 | 30 | 0.002 | 1Score **> 34** indicates **identity** Score **> 15** indicates **homology** | U | R.LQIQGEGQASSTIR.Y |
| 63604 | 41 | – | 54 | 744.3905 | 1486.7664 | 1486.7740 | -5.08 | 0 | 30 | 0.0016 | 1Score **> 34** indicates **identity** Score **> 14** indicates **homology** | U | R.LQIQGEGQASSTIR.Y |
| 63605 | 41 | – | 54 | 744.3907 | 1486.7668 | 1486.7740 | -4.82 | 0 | 49 | 8e-05 | 1Score **> 34** indicates **identity** Score **> 21** indicates **homology** | U | R.LQIQGEGQASSTIR.Y |
| 63606 | 41 | – | 54 | 744.3907 | 1486.7669 | 1486.7740 | -4.74 | 0 | 19 | 0.016 | 1Score **> 34** indicates **identity** Score **> 14** indicates **homology** | U | R.LQIQGEGQASSTIR.Y |
| 63607 | 41 | – | 54 | 744.3912 | 1486.7678 | 1486.7740 | -4.17 | 0 | 21 | 0.035 | 1Score **> 34** indicates **identity** Score **> 18** indicates **homology** | U | R.LQIQGEGQASSTIR.Y |
| 63608 | 41 | – | 54 | 744.3912 | 1486.7679 | 1486.7740 | -4.06 | 0 | 76 | 7.4e-07 | 1Score **> 34** indicates **identity** Score **> 27** indicates **homology** | U | R.LQIQGEGQASSTIR.Y |
| 63609 | 41 | – | 54 | 744.3915 | 1486.7684 | 1486.7740 | -3.73 | 0 | 78 | 4.1e-07 | 1Score **> 34** indicates **identity** Score **> 27** indicates **homology** | U | R.LQIQGEGQASSTIR.Y |
| 63610 | 41 | – | 54 | 744.3916 | 1486.7686 | 1486.7740 | -3.59 | 0 | 37 | 0.00052 | 1Score **> 34** indicates **identity** Score **> 16** indicates **homology** | U | R.LQIQGEGQASSTIR.Y |
| 63613 | 41 | – | 54 | 744.3916 | 1486.7687 | 1486.7740 | -3.51 | 0 | 32 | 0.00092 | 1Score **> 34** indicates **identity** Score **> 15** indicates **homology** | U | R.LQIQGEGQASSTIR.Y |
| 63614 | 41 | – | 54 | 744.3917 | 1486.7688 | 1486.7740 | -3.50 | 0 | 56 | 3.5e-05 | 1Score **> 34** indicates **identity** Score **> 24** indicates **homology** | U | R.LQIQGEGQASSTIR.Y |
| 63615 | 41 | – | 54 | 744.3918 | 1486.7690 | 1486.7740 | -3.35 | 0 | 47 | 5.5e-05 | 1Score **> 34** indicates **identity** Score **> 17** indicates **homology** | U | R.LQIQGEGQASSTIR.Y |
| 63616 | 41 | – | 54 | 744.3918 | 1486.7691 | 1486.7740 | -3.27 | 0 | 55 | 4.4e-05 | 1Score **> 34** indicates **identity** Score **> 24** indicates **homology** | U | R.LQIQGEGQASSTIR.Y |
| 63618 | 41 | – | 54 | 744.3922 | 1486.7698 | 1486.7740 | -2.76 | 0 | 53 | 2.1e-05 | 1Score **> 34** indicates **identity** Score **> 19** indicates **homology** | U | R.LQIQGEGQASSTIR.Y |
| 63619 | 41 | – | 54 | 744.3922 | 1486.7699 | 1486.7740 | -2.75 | 0 | 41 | 0.00063 | 1Score **> 34** indicates **identity** Score **> 21** indicates **homology** | U | R.LQIQGEGQASSTIR.Y |
| 63620 | 41 | – | 54 | 744.3923 | 1486.7700 | 1486.7740 | -2.68 | 0 | 52 | 9.8e-05 | 1Score **> 34** indicates **identity** Score **> 24** indicates **homology** | U | R.LQIQGEGQASSTIR.Y |
| 63622 | 41 | – | 54 | 744.3923 | 1486.7701 | 1486.7740 | -2.61 | 0 | 53 | 9.4e-05 | 1Score **> 34** indicates **identity** Score **> 25** indicates **homology** | U | R.LQIQGEGQASSTIR.Y |
| 63623 | 41 | – | 54 | 744.3927 | 1486.7709 | 1486.7740 | -2.04 | 0 | 61 | 1.8e-05 | 1Score **> 34** indicates **identity** Score **> 26** indicates **homology** | U | R.LQIQGEGQASSTIR.Y |
| 63624 | 41 | – | 54 | 744.3928 | 1486.7710 | 1486.7740 | -1.96 | 0 | 22 | 0.0081 | 1Score **> 34** indicates **identity** Score **> 14** indicates **homology** | U | R.LQIQGEGQASSTIR.Y |
| 63625 | 41 | – | 54 | 744.3928 | 1486.7711 | 1486.7740 | -1.93 | 0 | 58 | 1.2e-05 | 1Score **> 34** indicates **identity** Score **> 21** indicates **homology** | U | R.LQIQGEGQASSTIR.Y |
| 63626 | 41 | – | 54 | 744.3929 | 1486.7712 | 1486.7740 | -1.88 | 0 | 41 | 0.00032 | 1Score **> 34** indicates **identity** Score **> 19** indicates **homology** | U | R.LQIQGEGQASSTIR.Y |
| 63628 | 41 | – | 54 | 744.3929 | 1486.7712 | 1486.7740 | -1.86 | 0 | 32 | 0.0017 | 1Score **> 34** indicates **identity** Score **> 17** indicates **homology** | U | R.LQIQGEGQASSTIR.Y |
| 63629 | 41 | – | 54 | 744.3929 | 1486.7713 | 1486.7740 | -1.79 | 0 | 28 | 0.0035 | 1Score **> 34** indicates **identity** Score **> 16** indicates **homology** | U | R.LQIQGEGQASSTIR.Y |
| 63630 | 41 | – | 54 | 744.3930 | 1486.7714 | 1486.7740 | -1.71 | 0 | 55 | 7.2e-05 | 1Score **> 34** indicates **identity** Score **> 26** indicates **homology** | U | R.LQIQGEGQASSTIR.Y |
| 63631 | 41 | – | 54 | 744.3930 | 1486.7714 | 1486.7740 | -1.70 | 0 | 36 | 0.0004 | 1Score **> 34** indicates **identity** Score **> 15** indicates **homology** | U | R.LQIQGEGQASSTIR.Y |
| 63632 | 41 | – | 54 | 744.3930 | 1486.7715 | 1486.7740 | -1.62 | 0 | 26 | 0.0035 | 1Score **> 34** indicates **identity** Score **> 14** indicates **homology** | U | R.LQIQGEGQASSTIR.Y |
| 63633 | 41 | – | 54 | 744.3931 | 1486.7716 | 1486.7740 | -1.61 | 0 | 27 | 0.0054 | 1Score **> 34** indicates **identity** Score **> 17** indicates **homology** | U | R.LQIQGEGQASSTIR.Y |
| 63634 | 41 | – | 54 | 744.3931 | 1486.7717 | 1486.7740 | -1.55 | 0 | 55 | 0.00042 | 1Score **> 34** indicates **identity** Score **> 33** indicates **homology** | U | R.LQIQGEGQASSTIR.Y |
| 63635 | 41 | – | 54 | 744.3931 | 1486.7717 | 1486.7740 | -1.54 | 0 | 55 | 3.5e-05 | 1Score **> 34** indicates **identity** Score **> 23** indicates **homology** | U | R.LQIQGEGQASSTIR.Y |
| 63636 | 41 | – | 54 | 744.3932 | 1486.7718 | 1486.7740 | -1.46 | 0 | 60 | 1.7e-05 | 1Score **> 34** indicates **identity** Score **> 25** indicates **homology** | U | R.LQIQGEGQASSTIR.Y |
| 63638 | 41 | – | 54 | 744.3932 | 1486.7719 | 1486.7740 | -1.38 | 0 | 61 | 1.8e-05 | 1Score **> 34** indicates **identity** Score **> 26** indicates **homology** | U | R.LQIQGEGQASSTIR.Y |
| 63639 | 41 | – | 54 | 744.3932 | 1486.7719 | 1486.7740 | -1.36 | 0 | 44 | 0.00026 | 1Score **> 34** indicates **identity** Score **> 21** indicates **homology** | U | R.LQIQGEGQASSTIR.Y |
| 63640 | 41 | – | 54 | 744.3933 | 1486.7721 | 1486.7740 | -1.27 | 0 | 45 | 0.00013 | 1Score **> 34** indicates **identity** Score **> 18** indicates **homology** | U | R.LQIQGEGQASSTIR.Y |
| 63641 | 41 | – | 54 | 744.3933 | 1486.7721 | 1486.7740 | -1.22 | 0 | 33 | 0.00077 | 1Score **> 34** indicates **identity** Score **> 15** indicates **homology** | U | R.LQIQGEGQASSTIR.Y |
| 63642 | 41 | – | 54 | 744.3934 | 1486.7721 | 1486.7740 | -1.22 | 0 | 55 | 4.8e-05 | 1Score **> 34** indicates **identity** Score **> 24** indicates **homology** | U | R.LQIQGEGQASSTIR.Y |
| 63643 | 41 | – | 54 | 744.3934 | 1486.7722 | 1486.7740 | -1.21 | 0 | 55 | 3.3e-05 | 1Score **> 34** indicates **identity** Score **> 23** indicates **homology** | U | R.LQIQGEGQASSTIR.Y |
| 63644 | 41 | – | 54 | 744.3934 | 1486.7722 | 1486.7740 | -1.21 | 0 | 68 | 3.7e-06 | 1Score **> 34** indicates **identity** Score **> 26** indicates **homology** | U | R.LQIQGEGQASSTIR.Y |
| 63645 | 41 | – | 54 | 744.3934 | 1486.7723 | 1486.7740 | -1.14 | 0 | 76 | 1.1e-06 | 1Score **> 34** indicates **identity** Score **> 29** indicates **homology** | U | R.LQIQGEGQASSTIR.Y |
| 63647 | 41 | – | 54 | 744.3935 | 1486.7724 | 1486.7740 | -1.05 | 0 | 36 | 0.0014 | 1Score **> 34** indicates **identity** Score **> 20** indicates **homology** | U | R.LQIQGEGQASSTIR.Y |
| 63649 | 41 | – | 54 | 744.3935 | 1486.7725 | 1486.7740 | -0.98 | 0 | 81 | 7.8e-07 | 1Score **> 34** indicates **identity** Score **> 33** indicates **homology** | U | R.LQIQGEGQASSTIR.Y |
| 63650 | 41 | – | 54 | 744.3935 | 1486.7725 | 1486.7740 | -0.97 | 0 | 80 | 4.7e-07 | 1Score **> 34** indicates **identity** Score **> 29** indicates **homology** | U | R.LQIQGEGQASSTIR.Y |
| 63651 | 41 | – | 54 | 744.3935 | 1486.7725 | 1486.7740 | -0.96 | 0 | 75 | 1.1e-06 | 1Score **> 34** indicates **identity** Score **> 28** indicates **homology** | U | R.LQIQGEGQASSTIR.Y |
| 63652 | 41 | – | 54 | 744.3936 | 1486.7726 | 1486.7740 | -0.95 | 0 | 56 | 3.6e-05 | 1Score **> 34** indicates **identity** Score **> 24** indicates **homology** | U | R.LQIQGEGQASSTIR.Y |
| 63653 | 41 | – | 54 | 744.3936 | 1486.7726 | 1486.7740 | -0.90 | 0 | 74 | 1.5e-06 | 1Score **> 34** indicates **identity** Score **> 28** indicates **homology** | U | R.LQIQGEGQASSTIR.Y |
| 63655 | 41 | – | 54 | 744.3936 | 1486.7727 | 1486.7740 | -0.82 | 0 | 61 | 1.2e-05 | 1Score **> 34** indicates **identity** Score **> 24** indicates **homology** | U | R.LQIQGEGQASSTIR.Y |
| 63657 | 41 | – | 54 | 744.3937 | 1486.7728 | 1486.7740 | -0.80 | 0 | 62 | 3.7e-06 | 1Score **> 34** indicates **identity** Score **> 21** indicates **homology** | U | R.LQIQGEGQASSTIR.Y |
| 63659 | 41 | – | 54 | 744.3937 | 1486.7728 | 1486.7740 | -0.78 | 0 | 66 | 8.2e-06 | 1Score **> 34** indicates **identity** Score **> 27** indicates **homology** | U | R.LQIQGEGQASSTIR.Y |
| 63660 | 41 | – | 54 | 744.3937 | 1486.7729 | 1486.7740 | -0.73 | 0 | 72 | 2.3e-06 | 1Score **> 34** indicates **identity** Score **> 28** indicates **homology** | U | R.LQIQGEGQASSTIR.Y |
| 63661 | 41 | – | 54 | 744.3937 | 1486.7729 | 1486.7740 | -0.73 | 0 | 58 | 2.3e-05 | 1Score **> 34** indicates **identity** Score **> 24** indicates **homology** | U | R.LQIQGEGQASSTIR.Y |
| 63662 | 41 | – | 54 | 744.3937 | 1486.7729 | 1486.7740 | -0.72 | 0 | 33 | 0.0034 | 1Score **> 34** indicates **identity** Score **> 21** indicates **homology** | U | R.LQIQGEGQASSTIR.Y |
| 63663 | 41 | – | 54 | 744.3937 | 1486.7729 | 1486.7740 | -0.70 | 0 | 62 | 5.5e-06 | 1Score **> 34** indicates **identity** Score **> 22** indicates **homology** | U | R.LQIQGEGQASSTIR.Y |
| 63664 | 41 | – | 54 | 744.3938 | 1486.7730 | 1486.7740 | -0.63 | 0 | 46 | 0.00018 | 1Score **> 34** indicates **identity** Score **> 21** indicates **homology** | U | R.LQIQGEGQASSTIR.Y |
| 63665 | 41 | – | 54 | 744.3938 | 1486.7730 | 1486.7740 | -0.63 | 0 | 83 | 4.5e-07 | 1Score **> 34** indicates **identity** Score **> 32** indicates **homology** | U | R.LQIQGEGQASSTIR.Y |
| 63666 | 41 | – | 54 | 744.3938 | 1486.7730 | 1486.7740 | -0.62 | 0 | 75 | 3.3e-06 | 1Score **> 34** indicates **identity** Score **> 33** indicates **homology** | U | R.LQIQGEGQASSTIR.Y |
| 63668 | 41 | – | 54 | 744.3938 | 1486.7731 | 1486.7740 | -0.56 | 0 | 68 | 4.9e-06 | 1Score **> 34** indicates **identity** Score **> 28** indicates **homology** | U | R.LQIQGEGQASSTIR.Y |
| 63669 | 41 | – | 54 | 744.3939 | 1486.7732 | 1486.7740 | -0.54 | 0 | 73 | 1.6e-06 | 1Score **> 34** indicates **identity** Score **> 28** indicates **homology** | U | R.LQIQGEGQASSTIR.Y |
| 63670 | 41 | – | 54 | 744.3939 | 1486.7732 | 1486.7740 | -0.54 | 0 | 74 | 1.6e-06 | 1Score **> 34** indicates **identity** Score **> 28** indicates **homology** | U | R.LQIQGEGQASSTIR.Y |
| 63671 | 41 | – | 54 | 744.3939 | 1486.7732 | 1486.7740 | -0.54 | 0 | 32 | 0.0031 | 1Score **> 34** indicates **identity** Score **> 20** indicates **homology** | U | R.LQIQGEGQASSTIR.Y |
| 63672 | 41 | – | 54 | 744.3939 | 1486.7732 | 1486.7740 | -0.52 | 0 | 55 | 0.00016 | 1Score **> 34** indicates **identity** Score **> 30** indicates **homology** | U | R.LQIQGEGQASSTIR.Y |
| 63673 | 41 | – | 54 | 744.3939 | 1486.7732 | 1486.7740 | -0.49 | 0 | 52 | 2.5e-05 | 1Score **> 34** indicates **identity** Score **> 19** indicates **homology** | U | R.LQIQGEGQASSTIR.Y |
| 63674 | 41 | – | 54 | 744.3939 | 1486.7732 | 1486.7740 | -0.49 | 0 | 55 | 3.1e-05 | 1Score **> 34** indicates **identity** Score **> 22** indicates **homology** | U | R.LQIQGEGQASSTIR.Y |
| 63675 | 41 | – | 54 | 744.3939 | 1486.7732 | 1486.7740 | -0.48 | 0 | 60 | 1.3e-05 | 1Score **> 34** indicates **identity** Score **> 24** indicates **homology** | U | R.LQIQGEGQASSTIR.Y |
| 63676 | 41 | – | 54 | 744.3939 | 1486.7732 | 1486.7740 | -0.47 | 0 | 97 | 1.5e-08 | 1Score **> 34** indicates **identity** Score **> 32** indicates **homology** | U | R.LQIQGEGQASSTIR.Y |
| 63677 | 41 | – | 54 | 744.3939 | 1486.7733 | 1486.7740 | -0.47 | 0 | 72 | 1.9e-06 | 1Score **> 34** indicates **identity** Score **> 28** indicates **homology** | U | R.LQIQGEGQASSTIR.Y |
| 63678 | 41 | – | 54 | 744.3939 | 1486.7733 | 1486.7740 | -0.46 | 0 | 75 | 1.9e-06 | 1Score **> 34** indicates **identity** Score **> 30** indicates **homology** | U | R.LQIQGEGQASSTIR.Y |
| 63679 | 41 | – | 54 | 744.3940 | 1486.7734 | 1486.7740 | -0.39 | 0 | 58 | 1.9e-05 | 1Score **> 34** indicates **identity** Score **> 23** indicates **homology** | U | R.LQIQGEGQASSTIR.Y |
| 63680 | 41 | – | 54 | 744.3940 | 1486.7734 | 1486.7740 | -0.38 | 0 | 56 | 3.7e-05 | 1Score **> 34** indicates **identity** Score **> 24** indicates **homology** | U | R.LQIQGEGQASSTIR.Y |
| 63681 | 41 | – | 54 | 744.3940 | 1486.7734 | 1486.7740 | -0.37 | 0 | 70 | 2.2e-06 | 1Score **> 34** indicates **identity** Score **> 26** indicates **homology** | U | R.LQIQGEGQASSTIR.Y |
| 63682 | 41 | – | 54 | 744.3940 | 1486.7734 | 1486.7740 | -0.37 | 0 | 67 | 1.3e-05 | 1Score **> 34** indicates **identity** Score **> 31** indicates **homology** | U | R.LQIQGEGQASSTIR.Y |
| 63683 | 41 | – | 54 | 744.3940 | 1486.7734 | 1486.7740 | -0.37 | 0 | 80 | 9.4e-07 | 1Score **> 34** indicates **identity** Score **> 32** indicates **homology** | U | R.LQIQGEGQASSTIR.Y |
| 63684 | 41 | – | 54 | 744.3940 | 1486.7735 | 1486.7740 | -0.32 | 0 | 31 | 0.0025 | 1Score **> 34** indicates **identity** Score **> 18** indicates **homology** | U | R.LQIQGEGQASSTIR.Y |
| 63685 | 41 | – | 54 | 744.3940 | 1486.7735 | 1486.7740 | -0.31 | 0 | 69 | 5.4e-06 | 1Score **> 34** indicates **identity** Score **> 29** indicates **homology** | U | R.LQIQGEGQASSTIR.Y |
| 63686 | 41 | – | 54 | 744.3941 | 1486.7736 | 1486.7740 | -0.24 | 0 | 73 | 1.3e-06 | 1Score **> 34** indicates **identity** Score **> 27** indicates **homology** | U | R.LQIQGEGQASSTIR.Y |
| 63687 | 41 | – | 54 | 744.3941 | 1486.7736 | 1486.7740 | -0.23 | 0 | 76 | 1.7e-06 | 1Score **> 34** indicates **identity** Score **> 31** indicates **homology** | U | R.LQIQGEGQASSTIR.Y |
| 63688 | 41 | – | 54 | 744.3941 | 1486.7736 | 1486.7740 | -0.23 | 0 | 49 | 7.8e-05 | 1Score **> 34** indicates **identity** Score **> 21** indicates **homology** | U | R.LQIQGEGQASSTIR.Y |
| 63689 | 41 | – | 54 | 744.3941 | 1486.7736 | 1486.7740 | -0.22 | 0 | 55 | 4e-05 | 1Score **> 34** indicates **identity** Score **> 23** indicates **homology** | U | R.LQIQGEGQASSTIR.Y |
| 63690 | 41 | – | 54 | 744.3941 | 1486.7736 | 1486.7740 | -0.21 | 0 | 66 | 4.9e-06 | 1Score **> 34** indicates **identity** Score **> 26** indicates **homology** | U | R.LQIQGEGQASSTIR.Y |
| 63691 | 41 | – | 54 | 744.3941 | 1486.7737 | 1486.7740 | -0.15 | 0 | 64 | 9.3e-06 | 1Score **> 34** indicates **identity** Score **> 27** indicates **homology** | U | R.LQIQGEGQASSTIR.Y |
| 63692 | 41 | – | 54 | 744.3942 | 1486.7737 | 1486.7740 | -0.14 | 0 | 68 | 6.4e-06 | 1Score **> 34** indicates **identity** Score **> 28** indicates **homology** | U | R.LQIQGEGQASSTIR.Y |
| 63693 | 41 | – | 54 | 744.3942 | 1486.7737 | 1486.7740 | -0.14 | 0 | 83 | 7.2e-07 | 1Score **> 34** indicates **identity** | U | R.LQIQGEGQASSTIR.Y |
| 63694 | 41 | – | 54 | 744.3942 | 1486.7738 | 1486.7740 | -0.14 | 0 | 67 | 6.6e-06 | 1Score **> 34** indicates **identity** Score **> 27** indicates **homology** | U | R.LQIQGEGQASSTIR.Y |
| 63695 | 41 | – | 54 | 744.3942 | 1486.7738 | 1486.7740 | -0.14 | 0 | 46 | 0.00047 | 1Score **> 34** indicates **identity** Score **> 25** indicates **homology** | U | R.LQIQGEGQASSTIR.Y |
| 63696 | 41 | – | 54 | 744.3942 | 1486.7738 | 1486.7740 | -0.12 | 0 | 79 | 6e-07 | 1Score **> 34** indicates **identity** Score **> 29** indicates **homology** | U | R.LQIQGEGQASSTIR.Y |
| 63697 | 41 | – | 54 | 744.3942 | 1486.7738 | 1486.7740 | -0.097 | 0 | 79 | 3.8e-07 | 1Score **> 34** indicates **identity** Score **> 27** indicates **homology** | U | R.LQIQGEGQASSTIR.Y |
| 63698 | 41 | – | 54 | 744.3942 | 1486.7738 | 1486.7740 | -0.071 | 0 | 29 | 0.005 | 1Score **> 34** indicates **identity** Score **> 19** indicates **homology** | U | R.LQIQGEGQASSTIR.Y |
| 63699 | 41 | – | 54 | 744.3942 | 1486.7739 | 1486.7740 | -0.050 | 0 | 78 | 6.4e-07 | 1Score **> 34** indicates **identity** Score **> 29** indicates **homology** | U | R.LQIQGEGQASSTIR.Y |
| 63700 | 41 | – | 54 | 744.3942 | 1486.7739 | 1486.7740 | -0.047 | 0 | 77 | 2.8e-06 | 1Score **> 34** indicates **identity** Score **> 34** indicates **homology** | U | R.LQIQGEGQASSTIR.Y |
| 63701 | 41 | – | 54 | 744.3942 | 1486.7739 | 1486.7740 | -0.047 | 0 | 77 | 1.4e-06 | 1Score **> 34** indicates **identity** Score **> 31** indicates **homology** | U | R.LQIQGEGQASSTIR.Y |
| 63702 | 41 | – | 54 | 744.3942 | 1486.7739 | 1486.7740 | -0.043 | 0 | 72 | 2.3e-06 | 1Score **> 34** indicates **identity** Score **> 28** indicates **homology** | U | R.LQIQGEGQASSTIR.Y |
| 63703 | 41 | – | 54 | 744.3942 | 1486.7739 | 1486.7740 | -0.0067 | 0 | 45 | 0.00029 | 1Score **> 34** indicates **identity** Score **> 22** indicates **homology** | U | R.LQIQGEGQASSTIR.Y |
| 63704 | 41 | – | 54 | 744.3943 | 1486.7740 | 1486.7740 | 0.0040 | 0 | 72 | 2.2e-06 | 1Score **> 34** indicates **identity** Score **> 28** indicates **homology** | U | R.LQIQGEGQASSTIR.Y |
| 63705 | 41 | – | 54 | 744.3943 | 1486.7740 | 1486.7740 | 0.0067 | 0 | 49 | 8.8e-05 | 1Score **> 34** indicates **identity** Score **> 21** indicates **homology** | U | R.LQIQGEGQASSTIR.Y |
| 63707 | 41 | – | 54 | 744.3943 | 1486.7741 | 1486.7740 | 0.096 | 0 | 57 | 9.3e-05 | 1Score **> 34** indicates **identity** Score **> 29** indicates **homology** | U | R.LQIQGEGQASSTIR.Y |
| 63708 | 41 | – | 54 | 744.3943 | 1486.7741 | 1486.7740 | 0.10 | 0 | 29 | 0.0026 | 1Score **> 34** indicates **identity** Score **> 15** indicates **homology** | U | R.LQIQGEGQASSTIR.Y |
| 63709 | 41 | – | 54 | 744.3943 | 1486.7741 | 1486.7740 | 0.11 | 0 | 75 | 9.2e-07 | 1Score **> 34** indicates **identity** Score **> 28** indicates **homology** | U | R.LQIQGEGQASSTIR.Y |
| 63710 | 41 | – | 54 | 744.3943 | 1486.7741 | 1486.7740 | 0.11 | 0 | 73 | 2.5e-06 | 1Score **> 34** indicates **identity** Score **> 29** indicates **homology** | U | R.LQIQGEGQASSTIR.Y |
| 63711 | 41 | – | 54 | 744.3943 | 1486.7741 | 1486.7740 | 0.12 | 0 | 73 | 2e-06 | 1Score **> 34** indicates **identity** Score **> 28** indicates **homology** | U | R.LQIQGEGQASSTIR.Y |
| 63712 | 41 | – | 54 | 744.3943 | 1486.7741 | 1486.7740 | 0.12 | 0 | 39 | 0.00074 | 1Score **> 34** indicates **identity** Score **> 20** indicates **homology** | U | R.LQIQGEGQASSTIR.Y |
| 63713 | 41 | – | 54 | 744.3943 | 1486.7741 | 1486.7740 | 0.12 | 0 | 67 | 7.1e-06 | 1Score **> 34** indicates **identity** Score **> 28** indicates **homology** | U | R.LQIQGEGQASSTIR.Y |
| 63714 | 41 | – | 54 | 744.3943 | 1486.7741 | 1486.7740 | 0.12 | 0 | 49 | 0.00018 | 1Score **> 34** indicates **identity** Score **> 24** indicates **homology** | U | R.LQIQGEGQASSTIR.Y |
| 63716 | 41 | – | 54 | 744.3943 | 1486.7741 | 1486.7740 | 0.13 | 0 | 21 | 0.014 | 1Score **> 34** indicates **identity** Score **> 15** indicates **homology** | U | R.LQIQGEGQASSTIR.Y |
| 63719 | 41 | – | 54 | 744.3944 | 1486.7742 | 1486.7740 | 0.17 | 0 | 48 | 8.5e-05 | 1Score **> 34** indicates **identity** Score **> 20** indicates **homology** | U | R.LQIQGEGQASSTIR.Y |
| 63720 | 41 | – | 54 | 744.3944 | 1486.7742 | 1486.7740 | 0.19 | 0 | 54 | 0.00012 | 1Score **> 34** indicates **identity** Score **> 28** indicates **homology** | U | R.LQIQGEGQASSTIR.Y |
| 63721 | 41 | – | 54 | 744.3944 | 1486.7742 | 1486.7740 | 0.19 | 0 | 75 | 1e-06 | 1Score **> 34** indicates **identity** Score **> 28** indicates **homology** | U | R.LQIQGEGQASSTIR.Y |
| 63722 | 41 | – | 54 | 744.3944 | 1486.7743 | 1486.7740 | 0.21 | 0 | 37 | 0.00034 | 1Score **> 34** indicates **identity** Score **> 15** indicates **homology** | U | R.LQIQGEGQASSTIR.Y |
| 63723 | 41 | – | 54 | 744.3944 | 1486.7743 | 1486.7740 | 0.24 | 0 | 67 | 5.8e-06 | 1Score **> 34** indicates **identity** Score **> 27** indicates **homology** | U | R.LQIQGEGQASSTIR.Y |
| 63724 | 41 | – | 54 | 744.3944 | 1486.7743 | 1486.7740 | 0.25 | 0 | 77 | 7.1e-07 | 1Score **> 35** indicates **identity** Score **> 28** indicates **homology** | U | R.LQIQGEGQASSTIR.Y |
| 63726 | 41 | – | 54 | 744.3945 | 1486.7744 | 1486.7740 | 0.28 | 0 | 66 | 4.6e-06 | 1Score **> 35** indicates **identity** Score **> 25** indicates **homology** | U | R.LQIQGEGQASSTIR.Y |
| 63728 | 41 | – | 54 | 744.3945 | 1486.7745 | 1486.7740 | 0.35 | 0 | 37 | 0.0003 | 1Score **> 35** indicates **identity** Score **> 15** indicates **homology** | U | R.LQIQGEGQASSTIR.Y |
| 63729 | 41 | – | 54 | 744.3945 | 1486.7745 | 1486.7740 | 0.35 | 0 | 64 | 8.8e-06 | 1Score **> 35** indicates **identity** Score **> 26** indicates **homology** | U | R.LQIQGEGQASSTIR.Y |
| 63730 | 41 | – | 54 | 744.3945 | 1486.7745 | 1486.7740 | 0.36 | 0 | 65 | 3.5e-06 | 1Score **> 35** indicates **identity** Score **> 23** indicates **homology** | U | R.LQIQGEGQASSTIR.Y |
| 63731 | 41 | – | 54 | 744.3946 | 1486.7746 | 1486.7740 | 0.42 | 0 | 81 | 3.5e-07 | 1Score **> 35** indicates **identity** Score **> 29** indicates **homology** | U | R.LQIQGEGQASSTIR.Y |
| 63732 | 41 | – | 54 | 744.3946 | 1486.7746 | 1486.7740 | 0.43 | 0 | 81 | 3.6e-07 | 1Score **> 35** indicates **identity** Score **> 29** indicates **homology** | U | R.LQIQGEGQASSTIR.Y |
| 63733 | 41 | – | 54 | 744.3946 | 1486.7746 | 1486.7740 | 0.44 | 0 | 68 | 6.1e-06 | 1Score **> 35** indicates **identity** Score **> 28** indicates **homology** | U | R.LQIQGEGQASSTIR.Y |
| 63734 | 41 | – | 54 | 744.3946 | 1486.7746 | 1486.7740 | 0.44 | 0 | 70 | 6.5e-07 | 1Score **> 35** indicates **identity** Score **> 20** indicates **homology** | U | R.LQIQGEGQASSTIR.Y |
| 63735 | 41 | – | 54 | 744.3946 | 1486.7747 | 1486.7740 | 0.51 | 0 | 87 | 1.8e-07 | 1Score **> 35** indicates **identity** Score **> 32** indicates **homology** | U | R.LQIQGEGQASSTIR.Y |
| 63736 | 41 | – | 54 | 744.3946 | 1486.7747 | 1486.7740 | 0.51 | 0 | 47 | 0.00015 | 1Score **> 35** indicates **identity** Score **> 21** indicates **homology** | U | R.LQIQGEGQASSTIR.Y |
| 63737 | 41 | – | 54 | 744.3946 | 1486.7747 | 1486.7740 | 0.52 | 0 | 68 | 6e-06 | 1Score **> 35** indicates **identity** Score **> 28** indicates **homology** | U | R.LQIQGEGQASSTIR.Y |
| 63738 | 41 | – | 54 | 744.3946 | 1486.7747 | 1486.7740 | 0.52 | 0 | 68 | 2.5e-05 | 1Score **> 35** indicates **identity** Score **> 34** indicates **homology** | U | R.LQIQGEGQASSTIR.Y |
| 63739 | 41 | – | 54 | 744.3947 | 1486.7748 | 1486.7740 | 0.58 | 0 | 65 | 4.9e-06 | 1Score **> 35** indicates **identity** Score **> 24** indicates **homology** | U | R.LQIQGEGQASSTIR.Y |
| 63741 | 41 | – | 54 | 744.3947 | 1486.7749 | 1486.7740 | 0.61 | 0 | 56 | 3e-05 | 1Score **> 35** indicates **identity** Score **> 23** indicates **homology** | U | R.LQIQGEGQASSTIR.Y |
| 63743 | 41 | – | 54 | 744.3948 | 1486.7750 | 1486.7740 | 0.68 | 0 | 56 | 5e-05 | 1Score **> 35** indicates **identity** Score **> 26** indicates **homology** | U | R.LQIQGEGQASSTIR.Y |
| 63744 | 41 | – | 54 | 744.3948 | 1486.7750 | 1486.7740 | 0.68 | 0 | 50 | 9.2e-05 | 1Score **> 35** indicates **identity** Score **> 22** indicates **homology** | U | R.LQIQGEGQASSTIR.Y |
| 63745 | 41 | – | 54 | 744.3948 | 1486.7750 | 1486.7740 | 0.68 | 0 | 78 | 6.5e-07 | 1Score **> 35** indicates **identity** Score **> 28** indicates **homology** | U | R.LQIQGEGQASSTIR.Y |
| 63746 | 41 | – | 54 | 744.3948 | 1486.7750 | 1486.7740 | 0.69 | 0 | 49 | 6e-05 | 1Score **> 35** indicates **identity** Score **> 20** indicates **homology** | U | R.LQIQGEGQASSTIR.Y |
| 63747 | 41 | – | 54 | 744.3948 | 1486.7750 | 1486.7740 | 0.69 | 0 | 76 | 1.4e-06 | 1Score **> 35** indicates **identity** Score **> 30** indicates **homology** | U | R.LQIQGEGQASSTIR.Y |
| 63748 | 41 | – | 54 | 744.3948 | 1486.7751 | 1486.7740 | 0.75 | 0 | 72 | 2.5e-06 | 1Score **> 35** indicates **identity** Score **> 28** indicates **homology** | U | R.LQIQGEGQASSTIR.Y |
| 63749 | 41 | – | 54 | 744.3948 | 1486.7751 | 1486.7740 | 0.75 | 0 | 91 | 1.1e-07 | 1Score **> 35** indicates **identity** Score **> 34** indicates **homology** | U | R.LQIQGEGQASSTIR.Y |
| 63751 | 41 | – | 54 | 744.3948 | 1486.7751 | 1486.7740 | 0.75 | 0 | 56 | 3.7e-05 | 1Score **> 35** indicates **identity** Score **> 25** indicates **homology** | U | R.LQIQGEGQASSTIR.Y |
| 63752 | 41 | – | 54 | 744.3948 | 1486.7751 | 1486.7740 | 0.76 | 0 | 73 | 2.4e-06 | 1Score **> 35** indicates **identity** Score **> 29** indicates **homology** | U | R.LQIQGEGQASSTIR.Y |
| 63754 | 41 | – | 54 | 744.3948 | 1486.7751 | 1486.7740 | 0.76 | 0 | 38 | 0.00063 | 1Score **> 35** indicates **identity** Score **> 19** indicates **homology** | U | R.LQIQGEGQASSTIR.Y |
| 63756 | 41 | – | 54 | 744.3949 | 1486.7752 | 1486.7740 | 0.84 | 0 | 17 | 0.025 | 1Score **> 35** indicates **identity** Score **> 14** indicates **homology** | U | R.LQIQGEGQASSTIR.Y |
| 63757 | 41 | – | 54 | 744.3949 | 1486.7752 | 1486.7740 | 0.85 | 0 | 67 | 4.3e-06 | 1Score **> 35** indicates **identity** Score **> 26** indicates **homology** | U | R.LQIQGEGQASSTIR.Y |
| 63758 | 41 | – | 54 | 744.3949 | 1486.7752 | 1486.7740 | 0.85 | 0 | 35 | 0.0013 | 1Score **> 35** indicates **identity** Score **> 18** indicates **homology** | U | R.LQIQGEGQASSTIR.Y |
| 63759 | 41 | – | 54 | 744.3949 | 1486.7752 | 1486.7740 | 0.86 | 0 | 44 | 0.00018 | 1Score **> 35** indicates **identity** Score **> 19** indicates **homology** | U | R.LQIQGEGQASSTIR.Y |
| 63760 | 41 | – | 54 | 744.3949 | 1486.7753 | 1486.7740 | 0.92 | 0 | 58 | 1.7e-05 | 1Score **> 35** indicates **identity** Score **> 23** indicates **homology** | U | R.LQIQGEGQASSTIR.Y |
| 63761 | 41 | – | 54 | 744.3949 | 1486.7753 | 1486.7740 | 0.92 | 0 | 73 | 1.9e-06 | 1Score **> 35** indicates **identity** Score **> 28** indicates **homology** | U | R.LQIQGEGQASSTIR.Y |
| 63764 | 41 | – | 54 | 744.3951 | 1486.7756 | 1486.7740 | 1.07 | 0 | 41 | 0.00015 | 1Score **> 35** indicates **identity** Score **> 15** indicates **homology** | U | R.LQIQGEGQASSTIR.Y |
| 63765 | 41 | – | 54 | 744.3951 | 1486.7756 | 1486.7740 | 1.09 | 0 | 57 | 3e-05 | 1Score **> 35** indicates **identity** Score **> 25** indicates **homology** | U | R.LQIQGEGQASSTIR.Y |
| 63766 | 41 | – | 54 | 744.3951 | 1486.7756 | 1486.7740 | 1.10 | 0 | 75 | 9.1e-07 | 1Score **> 35** indicates **identity** Score **> 27** indicates **homology** | U | R.LQIQGEGQASSTIR.Y |
| 63767 | 41 | – | 54 | 744.3952 | 1486.7758 | 1486.7740 | 1.23 | 0 | 56 | 5.2e-05 | 1Score **> 35** indicates **identity** Score **> 26** indicates **homology** | U | R.LQIQGEGQASSTIR.Y |
| 63769 | 41 | – | 54 | 744.3952 | 1486.7758 | 1486.7740 | 1.24 | 0 | 55 | 4.9e-05 | 1Score **> 35** indicates **identity** Score **> 24** indicates **homology** | U | R.LQIQGEGQASSTIR.Y |
| 63770 | 41 | – | 54 | 744.3952 | 1486.7759 | 1486.7740 | 1.32 | 0 | 20 | 0.014 | 1Score **> 35** indicates **identity** Score **> 14** indicates **homology** | U | R.LQIQGEGQASSTIR.Y |
| 63771 | 41 | – | 54 | 744.3952 | 1486.7759 | 1486.7740 | 1.33 | 0 | 54 | 6.7e-05 | 1Score **> 35** indicates **identity** Score **> 25** indicates **homology** | U | R.LQIQGEGQASSTIR.Y |
| 63772 | 41 | – | 54 | 744.3953 | 1486.7760 | 1486.7740 | 1.40 | 0 | 78 | 4.9e-07 | 1Score **> 35** indicates **identity** Score **> 28** indicates **homology** | U | R.LQIQGEGQASSTIR.Y |
| 63774 | 41 | – | 54 | 744.3953 | 1486.7760 | 1486.7740 | 1.41 | 0 | 43 | 0.00045 | 1Score **> 35** indicates **identity** Score **> 22** indicates **homology** | U | R.LQIQGEGQASSTIR.Y |
| 63775 | 41 | – | 54 | 744.3953 | 1486.7761 | 1486.7740 | 1.41 | 0 | 79 | 4.1e-07 | 1Score **> 35** indicates **identity** Score **> 28** indicates **homology** | U | R.LQIQGEGQASSTIR.Y |
| 63776 | 41 | – | 54 | 744.3953 | 1486.7761 | 1486.7740 | 1.41 | 0 | 28 | 0.0059 | 1Score **> 35** indicates **identity** Score **> 18** indicates **homology** | U | R.LQIQGEGQASSTIR.Y |
| 63777 | 41 | – | 54 | 744.3953 | 1486.7761 | 1486.7740 | 1.42 | 0 | 28 | 0.0031 | 1Score **> 35** indicates **identity** Score **> 15** indicates **homology** | U | R.LQIQGEGQASSTIR.Y |
| 63778 | 41 | – | 54 | 744.3953 | 1486.7761 | 1486.7740 | 1.44 | 0 | 57 | 2.7e-05 | 1Score **> 35** indicates **identity** Score **> 23** indicates **homology** | U | R.LQIQGEGQASSTIR.Y |
| 63779 | 41 | – | 54 | 744.3954 | 1486.7762 | 1486.7740 | 1.52 | 0 | 34 | 0.0048 | 1Score **> 35** indicates **identity** Score **> 23** indicates **homology** | U | R.LQIQGEGQASSTIR.Y |
| 63781 | 41 | – | 54 | 744.3954 | 1486.7763 | 1486.7740 | 1.57 | 0 | 71 | 2e-06 | 1Score **> 35** indicates **identity** Score **> 27** indicates **homology** | U | R.LQIQGEGQASSTIR.Y |
| 63782 | 41 | – | 54 | 744.3954 | 1486.7763 | 1486.7740 | 1.57 | 0 | 27 | 0.012 | 1Score **> 35** indicates **identity** Score **> 21** indicates **homology** | U | R.LQIQGEGQASSTIR.Y |
| 63783 | 41 | – | 54 | 744.3954 | 1486.7763 | 1486.7740 | 1.58 | 0 | 69 | 4.1e-06 | 1Score **> 35** indicates **identity** Score **> 28** indicates **homology** | U | R.LQIQGEGQASSTIR.Y |
| 63784 | 41 | – | 54 | 744.3954 | 1486.7763 | 1486.7740 | 1.59 | 0 | 23 | 0.0068 | 1Score **> 35** indicates **identity** Score **> 14** indicates **homology** | U | R.LQIQGEGQASSTIR.Y |
| 63785 | 41 | – | 54 | 744.3955 | 1486.7764 | 1486.7740 | 1.66 | 0 | 43 | 0.00051 | 1Score **> 35** indicates **identity** Score **> 22** indicates **homology** | U | R.LQIQGEGQASSTIR.Y |
| 63786 | 41 | – | 54 | 744.3956 | 1486.7767 | 1486.7740 | 1.82 | 0 | 38 | 0.00025 | 1Score **> 35** indicates **identity** Score **> 15** indicates **homology** | U | R.LQIQGEGQASSTIR.Y |
| 63787 | 41 | – | 54 | 744.3957 | 1486.7768 | 1486.7740 | 1.91 | 0 | 55 | 3.3e-05 | 1Score **> 35** indicates **identity** Score **> 23** indicates **homology** | U | R.LQIQGEGQASSTIR.Y |
| 63788 | 41 | – | 54 | 744.3957 | 1486.7768 | 1486.7740 | 1.92 | 0 | 30 | 0.0016 | 1Score **> 35** indicates **identity** Score **> 14** indicates **homology** | U | R.LQIQGEGQASSTIR.Y |
| 63789 | 41 | – | 54 | 744.3957 | 1486.7768 | 1486.7740 | 1.93 | 0 | 47 | 0.00014 | 1Score **> 35** indicates **identity** Score **> 21** indicates **homology** | U | R.LQIQGEGQASSTIR.Y |
| 63790 | 41 | – | 54 | 744.3957 | 1486.7768 | 1486.7740 | 1.93 | 0 | 38 | 0.0098 | 1Score **> 35** indicates **identity** Score **> 30** indicates **homology** | U | R.LQIQGEGQASSTIR.Y |
| 63791 | 41 | – | 54 | 744.3957 | 1486.7769 | 1486.7740 | 1.99 | 0 | 56 | 0.00027 | 1Score **> 35** indicates **identity** Score **> 32** indicates **homology** | U | R.LQIQGEGQASSTIR.Y |
| 63792 | 41 | – | 54 | 744.3958 | 1486.7770 | 1486.7740 | 2.06 | 0 | 72 | 2.4e-06 | 1Score **> 35** indicates **identity** Score **> 28** indicates **homology** | U | R.LQIQGEGQASSTIR.Y |
| 63793 | 41 | – | 54 | 744.3958 | 1486.7770 | 1486.7740 | 2.07 | 0 | 63 | 5.8e-06 | 1Score **> 35** indicates **identity** Score **> 23** indicates **homology** | U | R.LQIQGEGQASSTIR.Y |
| 63794 | 41 | – | 54 | 744.3958 | 1486.7770 | 1486.7740 | 2.08 | 0 | 49 | 3.7e-05 | 1Score **> 35** indicates **identity** Score **> 17** indicates **homology** | U | R.LQIQGEGQASSTIR.Y |
| 63795 | 41 | – | 54 | 744.3958 | 1486.7771 | 1486.7740 | 2.08 | 0 | 60 | 3.7e-05 | 1Score **> 35** indicates **identity** Score **> 28** indicates **homology** | U | R.LQIQGEGQASSTIR.Y |
| 63796 | 41 | – | 54 | 744.3959 | 1486.7773 | 1486.7740 | 2.23 | 0 | 61 | 2.3e-05 | 1Score **> 35** indicates **identity** Score **> 27** indicates **homology** | U | R.LQIQGEGQASSTIR.Y |
| 63797 | 41 | – | 54 | 744.3959 | 1486.7773 | 1486.7740 | 2.24 | 0 | 73 | 7.8e-07 | 1Score **> 35** indicates **identity** Score **> 25** indicates **homology** | U | R.LQIQGEGQASSTIR.Y |
| 63798 | 41 | – | 54 | 744.3959 | 1486.7773 | 1486.7740 | 2.24 | 0 | 82 | 2.2e-07 | 1Score **> 35** indicates **identity** Score **> 28** indicates **homology** | U | R.LQIQGEGQASSTIR.Y |
| 63799 | 41 | – | 54 | 744.3959 | 1486.7773 | 1486.7740 | 2.25 | 0 | 41 | 0.00037 | 1Score **> 35** indicates **identity** Score **> 19** indicates **homology** | U | R.LQIQGEGQASSTIR.Y |
| 63800 | 41 | – | 54 | 744.3959 | 1486.7773 | 1486.7740 | 2.25 | 0 | 44 | 0.00034 | 1Score **> 35** indicates **identity** Score **> 22** indicates **homology** | U | R.LQIQGEGQASSTIR.Y |
| 63801 | 41 | – | 54 | 744.3960 | 1486.7774 | 1486.7740 | 2.30 | 0 | 72 | 3e-06 | 1Score **> 35** indicates **identity** Score **> 29** indicates **homology** | U | R.LQIQGEGQASSTIR.Y |
| 63804 | 41 | – | 54 | 744.3962 | 1486.7778 | 1486.7740 | 2.58 | 0 | 73 | 3.8e-06 | 1Score **> 35** indicates **identity** Score **> 31** indicates **homology** | U | R.LQIQGEGQASSTIR.Y |
| 63805 | 41 | – | 54 | 744.3962 | 1486.7779 | 1486.7740 | 2.67 | 0 | 61 | 2.1e-05 | 1Score **> 35** indicates **identity** Score **> 27** indicates **homology** | U | R.LQIQGEGQASSTIR.Y |
| 63806 | 41 | – | 54 | 744.3963 | 1486.7781 | 1486.7740 | 2.77 | 0 | 30 | 0.011 | 1Score **> 35** indicates **identity** Score **> 23** indicates **homology** | U | R.LQIQGEGQASSTIR.Y |
| 63807 | 41 | – | 54 | 744.3964 | 1486.7783 | 1486.7740 | 2.90 | 0 | 56 | 2.3e-05 | 1Score **> 35** indicates **identity** Score **> 22** indicates **homology** | U | R.LQIQGEGQASSTIR.Y |
| 63808 | 41 | – | 54 | 744.3964 | 1486.7783 | 1486.7740 | 2.90 | 0 | 44 | 0.00039 | 1Score **> 35** indicates **identity** Score **> 22** indicates **homology** | U | R.LQIQGEGQASSTIR.Y |
| 63809 | 41 | – | 54 | 744.3964 | 1486.7783 | 1486.7740 | 2.91 | 0 | 56 | 5.3e-05 | 1Score **> 35** indicates **identity** Score **> 26** indicates **homology** | U | R.LQIQGEGQASSTIR.Y |
| 63810 | 41 | – | 54 | 744.3965 | 1486.7784 | 1486.7740 | 2.98 | 0 | 61 | 1.2e-05 | 1Score **> 35** indicates **identity** Score **> 25** indicates **homology** | U | R.LQIQGEGQASSTIR.Y |
| 63811 | 41 | – | 54 | 744.3965 | 1486.7785 | 1486.7740 | 3.06 | 0 | 44 | 0.00026 | 1Score **> 35** indicates **identity** Score **> 21** indicates **homology** | U | R.LQIQGEGQASSTIR.Y |
| 63812 | 41 | – | 54 | 744.3965 | 1486.7785 | 1486.7740 | 3.08 | 0 | 22 | 0.0084 | 1Score **> 35** indicates **identity** Score **> 14** indicates **homology** | U | R.LQIQGEGQASSTIR.Y |
| 63813 | 41 | – | 54 | 744.3966 | 1486.7787 | 1486.7740 | 3.21 | 0 | 53 | 5.8e-05 | 1Score **> 35** indicates **identity** Score **> 24** indicates **homology** | U | R.LQIQGEGQASSTIR.Y |
| 63814 | 41 | – | 54 | 744.3967 | 1486.7788 | 1486.7740 | 3.24 | 0 | 61 | 1.2e-05 | 1Score **> 35** indicates **identity** Score **> 24** indicates **homology** | U | R.LQIQGEGQASSTIR.Y |
| 63815 | 41 | – | 54 | 744.3969 | 1486.7793 | 1486.7740 | 3.61 | 0 | 56 | 3.7e-05 | 1Score **> 35** indicates **identity** Score **> 24** indicates **homology** | U | R.LQIQGEGQASSTIR.Y |
| 63816 | 41 | – | 54 | 744.3969 | 1486.7793 | 1486.7740 | 3.62 | 0 | 71 | 3.8e-06 | 1Score **> 35** indicates **identity** Score **> 29** indicates **homology** | U | R.LQIQGEGQASSTIR.Y |
| 63817 | 41 | – | 54 | 744.3971 | 1486.7797 | 1486.7740 | 3.88 | 0 | 56 | 3.5e-05 | 1Score **> 35** indicates **identity** Score **> 24** indicates **homology** | U | R.LQIQGEGQASSTIR.Y |
| 63819 | 41 | – | 54 | 744.3972 | 1486.7798 | 1486.7740 | 3.95 | 0 | 63 | 2e-05 | 1Score **> 35** indicates **identity** Score **> 29** indicates **homology** | U | R.LQIQGEGQASSTIR.Y |
| 63821 | 41 | – | 54 | 744.3974 | 1486.7802 | 1486.7740 | 4.22 | 0 | 70 | 2.2e-06 | 1Score **> 35** indicates **identity** Score **> 26** indicates **homology** | U | R.LQIQGEGQASSTIR.Y |
| 63822 | 41 | – | 54 | 744.3975 | 1486.7804 | 1486.7740 | 4.31 | 0 | 68 | 6.6e-06 | 1Score **> 35** indicates **identity** Score **> 28** indicates **homology** | U | R.LQIQGEGQASSTIR.Y |
| 63823 | 41 | – | 54 | 744.3976 | 1486.7806 | 1486.7740 | 4.48 | 0 | 55 | 3.6e-05 | 1Score **> 35** indicates **identity** Score **> 23** indicates **homology** | U | R.LQIQGEGQASSTIR.Y |
| 63824 | 41 | – | 54 | 744.3979 | 1486.7812 | 1486.7740 | 4.88 | 0 | 29 | 0.0019 | 1Score **> 35** indicates **identity** Score **> 14** indicates **homology** | U | R.LQIQGEGQASSTIR.Y |
| 63827 | 41 | – | 54 | 744.3992 | 1486.7839 | 1486.7740 | 6.67 | 0 | 48 | 0.00011 | 1Score **> 34** indicates **identity** Score **> 21** indicates **homology** | U | R.LQIQGEGQASSTIR.Y |
| 63828 | 41 | – | 54 | 744.3993 | 1486.7840 | 1486.7740 | 6.78 | 0 | 41 | 0.0012 | 1Score **> 34** indicates **identity** Score **> 24** indicates **homology** | U | R.LQIQGEGQASSTIR.Y |
| 63829 | 41 | – | 54 | 744.3998 | 1486.7851 | 1486.7740 | 7.51 | 0 | 42 | 0.00071 | 1Score **> 35** indicates **identity** Score **> 23** indicates **homology** | U | R.LQIQGEGQASSTIR.Y |
| 63832 | 41 | – | 54 | 744.4003 | 1486.7861 | 1486.7740 | 8.15 | 0 | 16 | 0.035 | 1Score **> 35** indicates **identity** Score **> 14** indicates **homology** | U | R.LQIQGEGQASSTIR.Y |
| 63837 | 41 | – | 54 | 744.4011 | 1486.7876 | 1486.7740 | 9.14 | 0 | 64 | 5.7e-06 | 1Score **> 35** indicates **identity** Score **> 24** indicates **homology** | U | R.LQIQGEGQASSTIR.Y |
| 50172 | 55 | – | 67 | 682.9091 | 1363.8036 | 1363.8075 | -2.89 | 1 | 26 | 0.022 | 1Score **> 30** indicates **identity** Score **> 22** indicates **homology** | U | R.YKGVLGTITTLAK.T |
| 50176 | 55 | – | 67 | 455.6093 | 1363.8061 | 1363.8075 | -1.03 | 1 | 53 | 0.00033 | 1Score **> 31** indicates **identity** | U | R.YKGVLGTITTLAK.T |
| 50181 | 55 | – | 67 | 455.6096 | 1363.8070 | 1363.8075 | -0.37 | 1 | 43 | 0.0035 | 1Score **> 31** indicates **identity** Score **> 30** indicates **homology** | U | R.YKGVLGTITTLAK.T |
| 50183 | 55 | – | 67 | 455.6098 | 1363.8074 | 1363.8075 | -0.043 | 1 | 47 | 0.0011 | 1Score **> 30** indicates **identity** | U | R.YKGVLGTITTLAK.T |
| 50184 | 55 | – | 67 | 455.6098 | 1363.8075 | 1363.8075 | 0.026 | 1 | 45 | 0.001 | 1Score **> 30** indicates **identity** Score **> 27** indicates **homology** | U | R.YKGVLGTITTLAK.T |
| 50186 | 55 | – | 67 | 682.9110 | 1363.8075 | 1363.8075 | 0.030 | 1 | 34 | 0.0037 | 1Score **> 30** indicates **identity** Score **> 22** indicates **homology** | U | R.YKGVLGTITTLAK.T |
| 50187 | 55 | – | 67 | 682.9111 | 1363.8076 | 1363.8075 | 0.054 | 1 | 48 | 0.00024 | 1Score **> 30** indicates **identity** Score **> 25** indicates **homology** | U | R.YKGVLGTITTLAK.T |
| 50191 | 55 | – | 67 | 455.6099 | 1363.8078 | 1363.8075 | 0.19 | 1 | 55 | 0.00014 | 1Score **> 30** indicates **identity** Score **> 29** indicates **homology** | U | R.YKGVLGTITTLAK.T |
| 50192 | 55 | – | 67 | 455.6099 | 1363.8079 | 1363.8075 | 0.30 | 1 | 50 | 0.00061 | 1Score **> 30** indicates **identity** | U | R.YKGVLGTITTLAK.T |
| 50193 | 55 | – | 67 | 682.9112 | 1363.8079 | 1363.8075 | 0.31 | 1 | 102 | 3.2e-09 | 1Score **> 30** indicates **identity** | U | R.YKGVLGTITTLAK.T |
| 50194 | 55 | – | 67 | 682.9112 | 1363.8079 | 1363.8075 | 0.31 | 1 | 14 | 0.049 | 1Score **> 30** indicates **identity** Score **> 13** indicates **homology** | U | R.YKGVLGTITTLAK.T |
| 50195 | 55 | – | 67 | 455.6100 | 1363.8081 | 1363.8075 | 0.41 | 1 | 43 | 0.0031 | 1Score **> 30** indicates **identity** | U | R.YKGVLGTITTLAK.T |
| 50196 | 55 | – | 67 | 455.6100 | 1363.8082 | 1363.8075 | 0.50 | 1 | 46 | 0.00084 | 1Score **> 30** indicates **identity** Score **> 27** indicates **homology** | U | R.YKGVLGTITTLAK.T |
| 50197 | 55 | – | 67 | 682.9114 | 1363.8082 | 1363.8075 | 0.52 | 1 | 95 | 1.6e-08 | 1Score **> 30** indicates **identity** | U | R.YKGVLGTITTLAK.T |
| 50199 | 55 | – | 67 | 455.6100 | 1363.8083 | 1363.8075 | 0.61 | 1 | 36 | 0.0058 | 1Score **> 30** indicates **identity** Score **> 26** indicates **homology** | U | R.YKGVLGTITTLAK.T |
| 50200 | 55 | – | 67 | 682.9114 | 1363.8083 | 1363.8075 | 0.62 | 1 | 39 | 0.0017 | 1Score **> 30** indicates **identity** Score **> 24** indicates **homology** | U | R.YKGVLGTITTLAK.T |
| 50201 | 55 | – | 67 | 455.6101 | 1363.8084 | 1363.8075 | 0.63 | 1 | 38 | 0.0029 | 1Score **> 30** indicates **identity** Score **> 25** indicates **homology** | U | R.YKGVLGTITTLAK.T |
| 50202 | 55 | – | 67 | 455.6101 | 1363.8084 | 1363.8075 | 0.65 | 1 | 20 | 0.016 | 1Score **> 30** indicates **identity** Score **> 15** indicates **homology** | U | R.YKGVLGTITTLAK.T |
| 50203 | 55 | – | 67 | 682.9115 | 1363.8085 | 1363.8075 | 0.73 | 1 | 97 | 9.7e-09 | 1Score **> 30** indicates **identity** Score **> 30** indicates **homology** | U | R.YKGVLGTITTLAK.T |
| 50204 | 55 | – | 67 | 682.9115 | 1363.8085 | 1363.8075 | 0.75 | 1 | 107 | 1e-09 | 1Score **> 30** indicates **identity** | U | R.YKGVLGTITTLAK.T |
| 50208 | 55 | – | 67 | 455.6102 | 1363.8088 | 1363.8075 | 0.98 | 1 | 45 | 0.0012 | 1Score **> 30** indicates **identity** Score **> 28** indicates **homology** | U | R.YKGVLGTITTLAK.T |
| 50210 | 55 | – | 67 | 682.9117 | 1363.8089 | 1363.8075 | 1.02 | 1 | 102 | 3.7e-09 | 1Score **> 30** indicates **identity** | U | R.YKGVLGTITTLAK.T |
| 50212 | 55 | – | 67 | 682.9118 | 1363.8090 | 1363.8075 | 1.07 | 1 | 37 | 0.0026 | 1Score **> 30** indicates **identity** Score **> 24** indicates **homology** | U | R.YKGVLGTITTLAK.T |
| 50214 | 55 | – | 67 | 682.9121 | 1363.8096 | 1363.8075 | 1.55 | 1 | 60 | 2.4e-05 | 1Score **> 30** indicates **identity** Score **> 27** indicates **homology** | U | R.YKGVLGTITTLAK.T |
| 50215 | 55 | – | 67 | 455.6105 | 1363.8097 | 1363.8075 | 1.61 | 1 | 40 | 0.0031 | 1Score **> 30** indicates **identity** Score **> 27** indicates **homology** | U | R.YKGVLGTITTLAK.T |
| 50219 | 55 | – | 67 | 682.9124 | 1363.8103 | 1363.8075 | 2.07 | 1 | 43 | 0.00062 | 1Score **> 30** indicates **identity** Score **> 23** indicates **homology** | U | R.YKGVLGTITTLAK.T |
| 50220 | 55 | – | 67 | 455.6108 | 1363.8105 | 1363.8075 | 2.21 | 1 | 18 | 0.024 | 1Score **> 30** indicates **identity** Score **> 14** indicates **homology** | U | R.YKGVLGTITTLAK.T |
| 50222 | 55 | – | 67 | 455.6110 | 1363.8110 | 1363.8075 | 2.59 | 1 | 39 | 0.0029 | 1Score **> 30** indicates **identity** Score **> 26** indicates **homology** | U | R.YKGVLGTITTLAK.T |
| 118842 | 55 | – | 73 | 498.2962 | 1989.1559 | 1989.1510 | 2.44 | 2 | 26 | 0.0036 | 1Score **> 31** indicates **identity** Score **> 14** indicates **homology** | U | R.YKGVLGTITTLAKTEGLPK.L |
| 19519 | 57 | – | 67 | 537.3329 | 1072.6512 | 1072.6492 | 1.82 | 0 | 51 | 0.00018 | 1Score **> 29** indicates **identity** Score **> 26** indicates **homology** | U | K.GVLGTITTLAK.T |
| 19520 | 57 | – | 67 | 537.3330 | 1072.6514 | 1072.6492 | 2.06 | 0 | 53 | 0.00011 | 1Score **> 29** indicates **identity** Score **> 26** indicates **homology** | U | K.GVLGTITTLAK.T |
| 19521 | 57 | – | 67 | 537.3331 | 1072.6517 | 1072.6492 | 2.28 | 0 | 40 | 0.0033 | 1Score **> 29** indicates **identity** Score **> 27** indicates **homology** | U | K.GVLGTITTLAK.T |
| 19522 | 57 | – | 67 | 537.3331 | 1072.6517 | 1072.6492 | 2.30 | 0 | 56 | 0.00011 | 1Score **> 29** indicates **identity** Score **> 29** indicates **homology** | U | K.GVLGTITTLAK.T |
| 19523 | 57 | – | 67 | 537.3331 | 1072.6517 | 1072.6492 | 2.32 | 0 | 40 | 0.0007 | 1Score **> 29** indicates **identity** Score **> 21** indicates **homology** | U | K.GVLGTITTLAK.T |
| 29785 | 74 | – | 84 | 587.8322 | 1173.6499 | 1173.6506 | -0.55 | 0 | 27 | 0.0032 | 1Score **> 34** indicates **identity** Score **> 14** indicates **homology** | U | K.LYSGLPAGIQR.Q |
| 29787 | 74 | – | 84 | 587.8324 | 1173.6502 | 1173.6506 | -0.31 | 0 | 19 | 0.018 | 1Score **> 35** indicates **identity** Score **> 14** indicates **homology** | U | K.LYSGLPAGIQR.Q |
| 29788 | 74 | – | 84 | 587.8324 | 1173.6503 | 1173.6506 | -0.21 | 0 | 54 | 1.9e-05 | 1Score **> 35** indicates **identity** Score **> 20** indicates **homology** | U | K.LYSGLPAGIQR.Q |
| 29790 | 74 | – | 84 | 587.8326 | 1173.6506 | 1173.6506 | -0.0077 | 0 | 23 | 0.0065 | 1Score **> 35** indicates **identity** Score **> 14** indicates **homology** | U | K.LYSGLPAGIQR.Q |
| 29791 | 74 | – | 84 | 587.8326 | 1173.6507 | 1173.6506 | 0.12 | 0 | 56 | 5.9e-06 | 1Score **> 35** indicates **identity** Score **> 17** indicates **homology** | U | K.LYSGLPAGIQR.Q |
| 29792 | 74 | – | 84 | 587.8327 | 1173.6508 | 1173.6506 | 0.18 | 0 | 55 | 8.3e-06 | 1Score **> 35** indicates **identity** Score **> 17** indicates **homology** | U | K.LYSGLPAGIQR.Q |
| 29793 | 74 | – | 84 | 587.8328 | 1173.6511 | 1173.6506 | 0.40 | 0 | 36 | 0.0004 | 1Score **> 35** indicates **identity** Score **> 15** indicates **homology** | U | K.LYSGLPAGIQR.Q |
| 29794 | 74 | – | 84 | 587.8328 | 1173.6511 | 1173.6506 | 0.41 | 0 | 59 | 3.7e-06 | 1Score **> 35** indicates **identity** Score **> 17** indicates **homology** | U | K.LYSGLPAGIQR.Q |
| 29795 | 74 | – | 84 | 587.8329 | 1173.6512 | 1173.6506 | 0.52 | 0 | 18 | 0.023 | 1Score **> 34** indicates **identity** Score **> 14** indicates **homology** | U | K.LYSGLPAGIQR.Q |
| 29797 | 74 | – | 84 | 587.8330 | 1173.6515 | 1173.6506 | 0.81 | 0 | 32 | 0.00099 | 1Score **> 34** indicates **identity** Score **> 15** indicates **homology** | U | K.LYSGLPAGIQR.Q |
| 29798 | 74 | – | 84 | 587.8330 | 1173.6515 | 1173.6506 | 0.81 | 0 | 51 | 1.6e-05 | 1Score **> 34** indicates **identity** Score **> 16** indicates **homology** | U | K.LYSGLPAGIQR.Q |
| 29799 | 74 | – | 84 | 587.8332 | 1173.6518 | 1173.6506 | 1.02 | 0 | 50 | 2e-05 | 1Score **> 34** indicates **identity** Score **> 16** indicates **homology** | U | K.LYSGLPAGIQR.Q |
| 29801 | 74 | – | 84 | 587.8334 | 1173.6522 | 1173.6506 | 1.34 | 0 | 46 | 4.7e-05 | 1Score **> 34** indicates **identity** Score **> 15** indicates **homology** | U | K.LYSGLPAGIQR.Q |
| 29802 | 74 | – | 84 | 587.8334 | 1173.6523 | 1173.6506 | 1.44 | 0 | 51 | 1.8e-05 | 1Score **> 34** indicates **identity** Score **> 16** indicates **homology** | U | K.LYSGLPAGIQR.Q |
| 29803 | 74 | – | 84 | 587.8334 | 1173.6523 | 1173.6506 | 1.45 | 0 | 36 | 0.0004 | 1Score **> 34** indicates **identity** Score **> 15** indicates **homology** | U | K.LYSGLPAGIQR.Q |
| 29805 | 74 | – | 84 | 587.8336 | 1173.6526 | 1173.6506 | 1.74 | 0 | 37 | 0.00032 | 1Score **> 34** indicates **identity** Score **> 15** indicates **homology** | U | K.LYSGLPAGIQR.Q |
| 29809 | 74 | – | 84 | 587.8341 | 1173.6536 | 1173.6506 | 2.59 | 0 | 30 | 0.0016 | 1Score **> 34** indicates **identity** Score **> 14** indicates **homology** | U | K.LYSGLPAGIQR.Q |
| 29813 | 74 | – | 84 | 587.8354 | 1173.6563 | 1173.6506 | 4.88 | 0 | 18 | 0.021 | 1Score **> 34** indicates **identity** Score **> 14** indicates **homology** | U | K.LYSGLPAGIQR.Q |
| 29817 | 74 | – | 84 | 587.8367 | 1173.6588 | 1173.6506 | 6.95 | 0 | 20 | 0.013 | 1Score **> 34** indicates **identity** Score **> 14** indicates **homology** | U | K.LYSGLPAGIQR.Q |
| 9549 | 85 | – | 92 | 461.2603 | 920.5061 | 920.5080 | -2.00 | 0 | 37 | 0.0045 | 1Score **> 28** indicates **identity** Score **> 26** indicates **homology** | U | R.QISFASLR.I |
| 9551 | 85 | – | 92 | 461.2605 | 920.5064 | 920.5080 | -1.64 | 0 | 29 | 0.045 | 1Score **> 29** indicates **identity** Score **> 28** indicates **homology** | U | R.QISFASLR.I |
| 9552 | 85 | – | 92 | 461.2605 | 920.5065 | 920.5080 | -1.59 | 0 | 25 | 0.017 | 1Score **> 29** indicates **identity** Score **> 20** indicates **homology** | U | R.QISFASLR.I |
| 9553 | 85 | – | 92 | 461.2608 | 920.5070 | 920.5080 | -1.00 | 0 | 28 | 0.019 | 1Score **> 29** indicates **identity** Score **> 23** indicates **homology** | U | R.QISFASLR.I |
| 9554 | 85 | – | 92 | 461.2608 | 920.5071 | 920.5080 | -0.92 | 0 | 32 | 0.011 | 1Score **> 29** indicates **identity** Score **> 25** indicates **homology** | U | R.QISFASLR.I |
| 9555 | 85 | – | 92 | 461.2609 | 920.5073 | 920.5080 | -0.71 | 0 | 32 | 0.012 | 1Score **> 29** indicates **identity** Score **> 25** indicates **homology** | U | R.QISFASLR.I |
| 9556 | 85 | – | 92 | 461.2609 | 920.5073 | 920.5080 | -0.71 | 0 | 26 | 0.0093 | 1Score **> 29** indicates **identity** Score **> 18** indicates **homology** | U | R.QISFASLR.I |
| 9557 | 85 | – | 92 | 461.2610 | 920.5075 | 920.5080 | -0.51 | 0 | 37 | 0.0042 | 1Score **> 29** indicates **identity** Score **> 25** indicates **homology** | U | R.QISFASLR.I |
| 9558 | 85 | – | 92 | 461.2610 | 920.5075 | 920.5080 | -0.48 | 0 | 41 | 0.0015 | 1Score **> 29** indicates **identity** Score **> 25** indicates **homology** | U | R.QISFASLR.I |
| 9559 | 85 | – | 92 | 461.2610 | 920.5075 | 920.5080 | -0.48 | 0 | 38 | 0.0057 | 1Score **> 29** indicates **identity** | U | R.QISFASLR.I |
| 9561 | 85 | – | 92 | 461.2611 | 920.5076 | 920.5080 | -0.38 | 0 | 39 | 0.0036 | 1Score **> 29** indicates **identity** Score **> 27** indicates **homology** | U | R.QISFASLR.I |
| 9562 | 85 | – | 92 | 461.2611 | 920.5076 | 920.5080 | -0.34 | 0 | 44 | 0.0013 | 1Score **> 29** indicates **identity** Score **> 28** indicates **homology** | U | R.QISFASLR.I |
| 9563 | 85 | – | 92 | 461.2611 | 920.5076 | 920.5080 | -0.33 | 0 | 45 | 0.0014 | 1Score **> 29** indicates **identity** | U | R.QISFASLR.I |
| 9564 | 85 | – | 92 | 461.2611 | 920.5076 | 920.5080 | -0.33 | 0 | 29 | 0.0047 | 1Score **> 29** indicates **identity** Score **> 18** indicates **homology** | U | R.QISFASLR.I |
| 9565 | 85 | – | 92 | 461.2611 | 920.5077 | 920.5080 | -0.32 | 0 | 21 | 0.037 | 1Score **> 29** indicates **identity** Score **> 19** indicates **homology** | U | R.QISFASLR.I |
| 9566 | 85 | – | 92 | 461.2611 | 920.5077 | 920.5080 | -0.32 | 0 | 30 | 0.0072 | 1Score **> 29** indicates **identity** Score **> 21** indicates **homology** | U | R.QISFASLR.I |
| 9567 | 85 | – | 92 | 461.2612 | 920.5079 | 920.5080 | -0.059 | 0 | 45 | 0.00092 | 1Score **> 29** indicates **identity** Score **> 27** indicates **homology** | U | R.QISFASLR.I |
| 9569 | 85 | – | 92 | 461.2614 | 920.5082 | 920.5080 | 0.32 | 0 | 23 | 0.012 | 1Score **> 29** indicates **identity** Score **> 17** indicates **homology** | U | R.QISFASLR.I |
| 9570 | 85 | – | 92 | 461.2614 | 920.5083 | 920.5080 | 0.38 | 0 | 38 | 0.0037 | 1Score **> 29** indicates **identity** Score **> 26** indicates **homology** | U | R.QISFASLR.I |
| 9571 | 85 | – | 92 | 461.2616 | 920.5086 | 920.5080 | 0.68 | 0 | 33 | 0.008 | 1Score **> 29** indicates **identity** Score **> 24** indicates **homology** | U | R.QISFASLR.I |
| 9572 | 85 | – | 92 | 461.2617 | 920.5088 | 920.5080 | 0.88 | 0 | 37 | 0.0042 | 1Score **> 29** indicates **identity** Score **> 25** indicates **homology** | U | R.QISFASLR.I |
| 9573 | 85 | – | 92 | 461.2620 | 920.5094 | 920.5080 | 1.53 | 0 | 31 | 0.0035 | 1Score **> 27** indicates **identity** Score **> 19** indicates **homology** | U | R.QISFASLR.I |
| 9574 | 85 | – | 92 | 461.2621 | 920.5097 | 920.5080 | 1.92 | 0 | 18 | 0.021 | 1Score **> 27** indicates **identity** Score **> 14** indicates **homology** | U | R.QISFASLR.I |
| 92780 | 93 | – | 107 | 574.2746 | 1719.8021 | 1719.8104 | -4.83 | 0 | 20 | 0.014 | 1Score **> 33** indicates **identity** Score **> 14** indicates **homology** | U | R.IGLYDSVQEYFSSGR.E |
| 92781 | 93 | – | 107 | 860.9088 | 1719.8030 | 1719.8104 | -4.30 | 0 | 39 | 0.00023 | 1Score **> 33** indicates **identity** Score **> 15** indicates **homology** | U | R.IGLYDSVQEYFSSGR.E |
| 92783 | 93 | – | 107 | 860.9098 | 1719.8051 | 1719.8104 | -3.10 | 0 | 76 | 6.8e-08 | 1Score **> 33** indicates **identity** Score **> 17** indicates **homology** | U | R.IGLYDSVQEYFSSGR.E |
| 92785 | 93 | – | 107 | 860.9101 | 1719.8056 | 1719.8104 | -2.79 | 0 | 68 | 3.8e-07 | 1Score **> 33** indicates **identity** Score **> 17** indicates **homology** | U | R.IGLYDSVQEYFSSGR.E |
| 92787 | 93 | – | 107 | 860.9106 | 1719.8066 | 1719.8104 | -2.22 | 0 | 73 | 1.6e-07 | 1Score **> 33** indicates **identity** Score **> 17** indicates **homology** | U | R.IGLYDSVQEYFSSGR.E |
| 92788 | 93 | – | 107 | 860.9107 | 1719.8068 | 1719.8104 | -2.11 | 0 | 68 | 4.1e-07 | 1Score **> 33** indicates **identity** Score **> 17** indicates **homology** | U | R.IGLYDSVQEYFSSGR.E |
| 92789 | 93 | – | 107 | 860.9108 | 1719.8071 | 1719.8104 | -1.93 | 0 | 86 | 7.9e-09 | 1Score **> 33** indicates **identity** Score **> 18** indicates **homology** | U | R.IGLYDSVQEYFSSGR.E |
| 92791 | 93 | – | 107 | 860.9112 | 1719.8078 | 1719.8104 | -1.51 | 0 | 52 | 1.2e-05 | 1Score **> 33** indicates **identity** Score **> 16** indicates **homology** | U | R.IGLYDSVQEYFSSGR.E |
| 92792 | 93 | – | 107 | 860.9115 | 1719.8085 | 1719.8104 | -1.11 | 0 | 71 | 2.2e-07 | 1Score **> 33** indicates **identity** Score **> 17** indicates **homology** | U | R.IGLYDSVQEYFSSGR.E |
| 92795 | 93 | – | 107 | 860.9118 | 1719.8091 | 1719.8104 | -0.76 | 0 | 90 | 3.8e-09 | 1Score **> 33** indicates **identity** Score **> 18** indicates **homology** | U | R.IGLYDSVQEYFSSGR.E |
| 92796 | 93 | – | 107 | 860.9120 | 1719.8094 | 1719.8104 | -0.58 | 0 | 96 | 9.6e-10 | 1Score **> 33** indicates **identity** Score **> 19** indicates **homology** | U | R.IGLYDSVQEYFSSGR.E |
| 92797 | 93 | – | 107 | 574.2771 | 1719.8094 | 1719.8104 | -0.56 | 0 | 32 | 0.00096 | 1Score **> 33** indicates **identity** Score **> 15** indicates **homology** | U | R.IGLYDSVQEYFSSGR.E |
| 92798 | 93 | – | 107 | 860.9120 | 1719.8095 | 1719.8104 | -0.52 | 0 | 87 | 7.6e-09 | 1Score **> 33** indicates **identity** Score **> 18** indicates **homology** | U | R.IGLYDSVQEYFSSGR.E |
| 92800 | 93 | – | 107 | 860.9121 | 1719.8096 | 1719.8104 | -0.47 | 0 | 57 | 5e-06 | 1Score **> 33** indicates **identity** Score **> 16** indicates **homology** | U | R.IGLYDSVQEYFSSGR.E |
| 92801 | 93 | – | 107 | 860.9121 | 1719.8096 | 1719.8104 | -0.46 | 0 | 91 | 3.1e-09 | 1Score **> 33** indicates **identity** Score **> 18** indicates **homology** | U | R.IGLYDSVQEYFSSGR.E |
| 92803 | 93 | – | 107 | 860.9123 | 1719.8100 | 1719.8104 | -0.23 | 0 | 91 | 2.7e-09 | 1Score **> 33** indicates **identity** Score **> 18** indicates **homology** | U | R.IGLYDSVQEYFSSGR.E |
| 92804 | 93 | – | 107 | 860.9123 | 1719.8100 | 1719.8104 | -0.22 | 0 | 77 | 5.9e-08 | 1Score **> 33** indicates **identity** Score **> 17** indicates **homology** | U | R.IGLYDSVQEYFSSGR.E |
| 92805 | 93 | – | 107 | 860.9123 | 1719.8101 | 1719.8104 | -0.18 | 0 | 74 | 1.3e-07 | 1Score **> 33** indicates **identity** Score **> 17** indicates **homology** | U | R.IGLYDSVQEYFSSGR.E |
| 92806 | 93 | – | 107 | 860.9124 | 1719.8103 | 1719.8104 | -0.080 | 0 | 79 | 4e-08 | 1Score **> 33** indicates **identity** Score **> 17** indicates **homology** | U | R.IGLYDSVQEYFSSGR.E |
| 92807 | 93 | – | 107 | 860.9125 | 1719.8105 | 1719.8104 | 0.049 | 0 | 91 | 2.8e-09 | 1Score **> 33** indicates **identity** Score **> 18** indicates **homology** | U | R.IGLYDSVQEYFSSGR.E |
| 92808 | 93 | – | 107 | 860.9125 | 1719.8105 | 1719.8104 | 0.058 | 0 | 92 | 2.2e-09 | 1Score **> 33** indicates **identity** Score **> 18** indicates **homology** | U | R.IGLYDSVQEYFSSGR.E |
| 92810 | 93 | – | 107 | 860.9128 | 1719.8110 | 1719.8104 | 0.34 | 0 | 89 | 5e-09 | 1Score **> 33** indicates **identity** Score **> 18** indicates **homology** | U | R.IGLYDSVQEYFSSGR.E |
| 92811 | 93 | – | 107 | 860.9129 | 1719.8112 | 1719.8104 | 0.46 | 0 | 82 | 1.9e-08 | 1Score **> 33** indicates **identity** Score **> 18** indicates **homology** | U | R.IGLYDSVQEYFSSGR.E |
| 92812 | 93 | – | 107 | 574.2777 | 1719.8112 | 1719.8104 | 0.46 | 0 | 26 | 0.0035 | 1Score **> 33** indicates **identity** Score **> 14** indicates **homology** | U | R.IGLYDSVQEYFSSGR.E |
| 92815 | 93 | – | 107 | 860.9131 | 1719.8116 | 1719.8104 | 0.69 | 0 | 91 | 2.9e-09 | 1Score **> 33** indicates **identity** Score **> 18** indicates **homology** | U | R.IGLYDSVQEYFSSGR.E |
| 92818 | 93 | – | 107 | 860.9136 | 1719.8126 | 1719.8104 | 1.27 | 0 | 73 | 1.5e-07 | 1Score **> 33** indicates **identity** Score **> 17** indicates **homology** | U | R.IGLYDSVQEYFSSGR.E |
| 92819 | 93 | – | 107 | 860.9137 | 1719.8129 | 1719.8104 | 1.42 | 0 | 31 | 0.0012 | 1Score **> 33** indicates **identity** Score **> 14** indicates **homology** | U | R.IGLYDSVQEYFSSGR.E |
| 92821 | 93 | – | 107 | 860.9140 | 1719.8134 | 1719.8104 | 1.75 | 0 | 53 | 1.1e-05 | 1Score **> 33** indicates **identity** Score **> 16** indicates **homology** | U | R.IGLYDSVQEYFSSGR.E |
| 92822 | 93 | – | 107 | 574.2784 | 1719.8134 | 1719.8104 | 1.75 | 0 | 25 | 0.0048 | 1Score **> 33** indicates **identity** Score **> 14** indicates **homology** | U | R.IGLYDSVQEYFSSGR.E |
| 92824 | 93 | – | 107 | 860.9143 | 1719.8140 | 1719.8104 | 2.09 | 0 | 39 | 0.00024 | 1Score **> 33** indicates **identity** Score **> 15** indicates **homology** | U | R.IGLYDSVQEYFSSGR.E |
| 92826 | 93 | – | 107 | 860.9144 | 1719.8142 | 1719.8104 | 2.23 | 0 | 53 | 1.1e-05 | 1Score **> 33** indicates **identity** Score **> 16** indicates **homology** | U | R.IGLYDSVQEYFSSGR.E |
| 92827 | 93 | – | 107 | 860.9148 | 1719.8150 | 1719.8104 | 2.65 | 0 | 56 | 5.1e-06 | 1Score **> 33** indicates **identity** Score **> 16** indicates **homology** | U | R.IGLYDSVQEYFSSGR.E |
| 92829 | 93 | – | 107 | 860.9154 | 1719.8162 | 1719.8104 | 3.36 | 0 | 58 | 3.4e-06 | 1Score **> 33** indicates **identity** Score **> 16** indicates **homology** | U | R.IGLYDSVQEYFSSGR.E |
| 160497 | 93 | – | 116 | 873.4258 | 2617.2557 | 2617.2660 | -3.94 | 1 | 23 | 0.0076 | 1Score **> 36** indicates **identity** Score **> 14** indicates **homology** | U | R.IGLYDSVQEYFSSGRETPASLGNK.I |
| 160498 | 93 | – | 116 | 873.4273 | 2617.2602 | 2617.2660 | -2.21 | 1 | 38 | 0.00026 | 1Score **> 36** indicates **identity** Score **> 15** indicates **homology** | U | R.IGLYDSVQEYFSSGRETPASLGNK.I |
| 160499 | 93 | – | 116 | 873.4275 | 2617.2607 | 2617.2660 | -2.03 | 1 | 47 | 3.8e-05 | 1Score **> 36** indicates **identity** Score **> 15** indicates **homology** | U | R.IGLYDSVQEYFSSGRETPASLGNK.I |
| 160500 | 93 | – | 116 | 873.4285 | 2617.2637 | 2617.2660 | -0.87 | 1 | 41 | 0.00014 | 1Score **> 36** indicates **identity** Score **> 15** indicates **homology** | U | R.IGLYDSVQEYFSSGRETPASLGNK.I |
| 160501 | 93 | – | 116 | 873.4287 | 2617.2642 | 2617.2660 | -0.70 | 1 | 47 | 4.1e-05 | 1Score **> 36** indicates **identity** Score **> 15** indicates **homology** | U | R.IGLYDSVQEYFSSGRETPASLGNK.I |
| 160504 | 93 | – | 116 | 873.4292 | 2617.2659 | 2617.2660 | -0.044 | 1 | 44 | 8e-05 | 1Score **> 36** indicates **identity** Score **> 15** indicates **homology** | U | R.IGLYDSVQEYFSSGRETPASLGNK.I |
| 160505 | 93 | – | 116 | 873.4293 | 2617.2660 | 2617.2660 | -0.0057 | 1 | 57 | 4.5e-06 | 1Score **> 36** indicates **identity** Score **> 16** indicates **homology** | U | R.IGLYDSVQEYFSSGRETPASLGNK.I |
| 160506 | 93 | – | 116 | 873.4295 | 2617.2665 | 2617.2660 | 0.21 | 1 | 57 | 4.4e-06 | 1Score **> 36** indicates **identity** Score **> 16** indicates **homology** | U | R.IGLYDSVQEYFSSGRETPASLGNK.I |
| 160507 | 93 | – | 116 | 1309.6409 | 2617.2672 | 2617.2660 | 0.45 | 1 | 37 | 0.00032 | 1Score **> 36** indicates **identity** Score **> 15** indicates **homology** | U | R.IGLYDSVQEYFSSGRETPASLGNK.I |
| 160508 | 93 | – | 116 | 873.4298 | 2617.2674 | 2617.2660 | 0.55 | 1 | 64 | 9.3e-07 | 1Score **> 36** indicates **identity** Score **> 17** indicates **homology** | U | R.IGLYDSVQEYFSSGRETPASLGNK.I |
| 160509 | 93 | – | 116 | 1309.6410 | 2617.2675 | 2617.2660 | 0.59 | 1 | 54 | 7.9e-06 | 1Score **> 36** indicates **identity** Score **> 16** indicates **homology** | U | R.IGLYDSVQEYFSSGRETPASLGNK.I |
| 160510 | 93 | – | 116 | 873.4306 | 2617.2701 | 2617.2660 | 1.58 | 1 | 32 | 0.0011 | 1Score **> 36** indicates **identity** Score **> 14** indicates **homology** | U | R.IGLYDSVQEYFSSGRETPASLGNK.I |
| 160511 | 93 | – | 116 | 873.4311 | 2617.2714 | 2617.2660 | 2.05 | 1 | 55 | 6.8e-06 | 1Score **> 36** indicates **identity** Score **> 16** indicates **homology** | U | R.IGLYDSVQEYFSSGRETPASLGNK.I |
| 160517 | 93 | – | 116 | 873.4364 | 2617.2875 | 2617.2660 | 8.21 | 1 | 55 | 6.5e-06 | 1Score **> 37** indicates **identity** Score **> 16** indicates **homology** | U | R.IGLYDSVQEYFSSGRETPASLGNK.I |
| 9224 | 108 | – | 116 | 458.7396 | 915.4647 | 915.4661 | -1.60 | 0 | 36 | 0.00039 | 1Score **> 29** indicates **identity** Score **> 15** indicates **homology** | U | R.ETPASLGNK.I |
| 9225 | 108 | – | 116 | 458.7406 | 915.4667 | 915.4661 | 0.61 | 0 | 23 | 0.0076 | 1Score **> 29** indicates **identity** Score **> 14** indicates **homology** | U | R.ETPASLGNK.I |
| 9226 | 108 | – | 116 | 458.7409 | 915.4673 | 915.4661 | 1.29 | 0 | 26 | 0.004 | 1Score **> 29** indicates **identity** Score **> 14** indicates **homology** | U | R.ETPASLGNK.I |
| 174950 | 108 | – | 138 | 1024.5546 | 3070.6419 | 3070.6373 | 1.50 | 1 | 47 | 4e-05 | 1Score **> 36** indicates **identity** Score **> 15** indicates **homology** | U | R.ETPASLGNKISAGLMTGGVAVFIGQPTEVVK.V |
| 179465 | 108 | – | 140 | 832.4621 | 3325.8192 | 3325.8068 | 3.73 | 2 | 18 | 0.022 | 1Score **> 34** indicates **identity** Score **> 14** indicates **homology** | U | R.ETPASLGNKISAGLMTGGVAVFIGQPTEVVKVR.M |
| 134539 | 117 | – | 138 | 1087.5909 | 2173.1672 | 2173.1817 | -6.67 | 0 | 48 | 2.9e-05 | 1Score **> 35** indicates **identity** Score **> 16** indicates **homology** | U | K.ISAGLMTGGVAVFIGQPTEVVK.V |
| 134545 | 117 | – | 138 | 1087.5971 | 2173.1797 | 2173.1817 | -0.94 | 0 | 55 | 7.2e-06 | 1Score **> 35** indicates **identity** Score **> 16** indicates **homology** | U | K.ISAGLMTGGVAVFIGQPTEVVK.V |
| 134550 | 117 | – | 138 | 725.4019 | 2173.1839 | 2173.1817 | 1.02 | 0 | 64 | 9.7e-07 | 1Score **> 35** indicates **identity** Score **> 17** indicates **homology** | U | K.ISAGLMTGGVAVFIGQPTEVVK.V |
| 134551 | 117 | – | 138 | 1087.5994 | 2173.1842 | 2173.1817 | 1.13 | 0 | 70 | 2.5e-07 | 1Score **> 35** indicates **identity** Score **> 17** indicates **homology** | U | K.ISAGLMTGGVAVFIGQPTEVVK.V |
| 134553 | 117 | – | 138 | 1087.6002 | 2173.1858 | 2173.1817 | 1.87 | 0 | 94 | 1.4e-09 | 1Score **> 35** indicates **identity** Score **> 18** indicates **homology** | U | K.ISAGLMTGGVAVFIGQPTEVVK.V |
| 134555 | 117 | – | 138 | 725.4026 | 2173.1859 | 2173.1817 | 1.93 | 0 | 30 | 0.0015 | 1Score **> 35** indicates **identity** Score **> 14** indicates **homology** | U | K.ISAGLMTGGVAVFIGQPTEVVK.V |
| 134556 | 117 | – | 138 | 725.4028 | 2173.1866 | 2173.1817 | 2.25 | 0 | 46 | 4.5e-05 | 1Score **> 35** indicates **identity** Score **> 15** indicates **homology** | U | K.ISAGLMTGGVAVFIGQPTEVVK.V |
| 134561 | 117 | – | 138 | 1087.6049 | 2173.1953 | 2173.1817 | 6.26 | 0 | 48 | 3.3e-05 | 1Score **> 35** indicates **identity** Score **> 15** indicates **homology** | U | K.ISAGLMTGGVAVFIGQPTEVVK.V |
| 136190 | 117 | – | 138 | 1095.6027 | 2189.1909 | 2189.1766 | 6.53 | 0 | 36 | 0.00043 | 1Score **> 35** indicates **identity** Score **> 15** indicates **homology** | U | K.ISAGLMTGGVAVFIGQPTEVVK.V  + Oxidation (M) |
| 151653 | 117 | – | 140 | 810.4591 | 2428.3555 | 2428.3512 | 1.76 | 1 | 15 | 0.04 | 1Score **> 34** indicates **identity** Score **> 13** indicates **homology** | U | K.ISAGLMTGGVAVFIGQPTEVVKVR.M |
| 65628 | 141 | – | 153 | 376.4557 | 1501.7938 | 1501.7936 | 0.13 | 1 | 35 | 0.00055 | 1Score **> 35** indicates **identity** Score **> 15** indicates **homology** | U | R.MQAQSHLHGIKPR.Y |
| 65629 | 141 | – | 153 | 501.6052 | 1501.7938 | 1501.7936 | 0.15 | 1 | 34 | 0.00066 | 1Score **> 35** indicates **identity** Score **> 15** indicates **homology** | U | R.MQAQSHLHGIKPR.Y |
| 65630 | 141 | – | 153 | 376.4557 | 1501.7938 | 1501.7936 | 0.17 | 1 | 37 | 0.00038 | 1Score **> 35** indicates **identity** Score **> 15** indicates **homology** | U | R.MQAQSHLHGIKPR.Y |
| 65631 | 141 | – | 153 | 751.9043 | 1501.7940 | 1501.7936 | 0.29 | 1 | 95 | 1.3e-09 | 1Score **> 35** indicates **identity** Score **> 19** indicates **homology** | U | R.MQAQSHLHGIKPR.Y |
| 65632 | 141 | – | 153 | 501.6053 | 1501.7942 | 1501.7936 | 0.40 | 1 | 43 | 0.0001 | 1Score **> 35** indicates **identity** Score **> 15** indicates **homology** | U | R.MQAQSHLHGIKPR.Y |
| 65635 | 141 | – | 153 | 376.4561 | 1501.7952 | 1501.7936 | 1.07 | 1 | 23 | 0.007 | 1Score **> 35** indicates **identity** Score **> 14** indicates **homology** | U | R.MQAQSHLHGIKPR.Y |
| 67512 | 141 | – | 153 | 380.4538 | 1517.7862 | 1517.7885 | -1.54 | 1 | 21 | 0.0098 | 1Score **> 35** indicates **identity** Score **> 14** indicates **homology** | U | R.MQAQSHLHGIKPR.Y  + Oxidation (M) |
| 67513 | 141 | – | 153 | 380.4538 | 1517.7863 | 1517.7885 | -1.47 | 1 | 29 | 0.002 | 1Score **> 35** indicates **identity** Score **> 14** indicates **homology** | U | R.MQAQSHLHGIKPR.Y  + Oxidation (M) |
| 67514 | 141 | – | 153 | 380.4540 | 1517.7869 | 1517.7885 | -1.05 | 1 | 21 | 0.011 | 1Score **> 35** indicates **identity** Score **> 14** indicates **homology** | U | R.MQAQSHLHGIKPR.Y  + Oxidation (M) |
| 67515 | 141 | – | 153 | 380.4540 | 1517.7870 | 1517.7885 | -1.00 | 1 | 32 | 0.0011 | 1Score **> 35** indicates **identity** Score **> 14** indicates **homology** | U | R.MQAQSHLHGIKPR.Y  + Oxidation (M) |
| 67517 | 141 | – | 153 | 380.4542 | 1517.7875 | 1517.7885 | -0.64 | 1 | 26 | 0.0033 | 1Score **> 35** indicates **identity** Score **> 14** indicates **homology** | U | R.MQAQSHLHGIKPR.Y  + Oxidation (M) |
| 67518 | 141 | – | 153 | 380.4542 | 1517.7877 | 1517.7885 | -0.50 | 1 | 35 | 0.00052 | 1Score **> 35** indicates **identity** Score **> 15** indicates **homology** | U | R.MQAQSHLHGIKPR.Y  + Oxidation (M) |
| 67519 | 141 | – | 153 | 380.4543 | 1517.7880 | 1517.7885 | -0.33 | 1 | 24 | 0.0052 | 1Score **> 35** indicates **identity** Score **> 14** indicates **homology** | U | R.MQAQSHLHGIKPR.Y  + Oxidation (M) |
| 67520 | 141 | – | 153 | 380.4543 | 1517.7881 | 1517.7885 | -0.25 | 1 | 15 | 0.038 | 1Score **> 35** indicates **identity** Score **> 13** indicates **homology** | U | R.MQAQSHLHGIKPR.Y  + Oxidation (M) |
| 67521 | 141 | – | 153 | 506.9367 | 1517.7881 | 1517.7885 | -0.25 | 1 | 30 | 0.0015 | 1Score **> 35** indicates **identity** Score **> 14** indicates **homology** | U | R.MQAQSHLHGIKPR.Y  + Oxidation (M) |
| 67522 | 141 | – | 153 | 506.9367 | 1517.7882 | 1517.7885 | -0.21 | 1 | 22 | 0.0096 | 1Score **> 35** indicates **identity** Score **> 14** indicates **homology** | U | R.MQAQSHLHGIKPR.Y  + Oxidation (M) |
| 67523 | 141 | – | 153 | 380.4543 | 1517.7883 | 1517.7885 | -0.15 | 1 | 28 | 0.0026 | 1Score **> 35** indicates **identity** Score **> 14** indicates **homology** | U | R.MQAQSHLHGIKPR.Y  + Oxidation (M) |
| 67525 | 141 | – | 153 | 506.9367 | 1517.7884 | 1517.7885 | -0.072 | 1 | 28 | 0.0023 | 1Score **> 35** indicates **identity** Score **> 14** indicates **homology** | U | R.MQAQSHLHGIKPR.Y  + Oxidation (M) |
| 67526 | 141 | – | 153 | 380.4544 | 1517.7884 | 1517.7885 | -0.065 | 1 | 23 | 0.0067 | 1Score **> 35** indicates **identity** Score **> 14** indicates **homology** | U | R.MQAQSHLHGIKPR.Y  + Oxidation (M) |
| 67527 | 141 | – | 153 | 380.4544 | 1517.7884 | 1517.7885 | -0.059 | 1 | 24 | 0.0062 | 1Score **> 35** indicates **identity** Score **> 14** indicates **homology** | U | R.MQAQSHLHGIKPR.Y  + Oxidation (M) |
| 67528 | 141 | – | 153 | 506.9368 | 1517.7885 | 1517.7885 | -0.026 | 1 | 37 | 0.00035 | 1Score **> 35** indicates **identity** Score **> 15** indicates **homology** | U | R.MQAQSHLHGIKPR.Y  + Oxidation (M) |
| 67529 | 141 | – | 153 | 380.4544 | 1517.7886 | 1517.7885 | 0.094 | 1 | 28 | 0.0025 | 1Score **> 35** indicates **identity** Score **> 14** indicates **homology** | U | R.MQAQSHLHGIKPR.Y  + Oxidation (M) |
| 67530 | 141 | – | 153 | 380.4545 | 1517.7888 | 1517.7885 | 0.22 | 1 | 20 | 0.012 | 1Score **> 35** indicates **identity** Score **> 14** indicates **homology** | U | R.MQAQSHLHGIKPR.Y  + Oxidation (M) |
| 67531 | 141 | – | 153 | 380.4545 | 1517.7889 | 1517.7885 | 0.27 | 1 | 14 | 0.046 | 1Score **> 35** indicates **identity** Score **> 13** indicates **homology** | U | R.MQAQSHLHGIKPR.Y  + Oxidation (M) |
| 67532 | 141 | – | 153 | 380.4546 | 1517.7891 | 1517.7885 | 0.43 | 1 | 25 | 0.0043 | 1Score **> 35** indicates **identity** Score **> 14** indicates **homology** | U | R.MQAQSHLHGIKPR.Y  + Oxidation (M) |
| 67533 | 141 | – | 153 | 506.9371 | 1517.7896 | 1517.7885 | 0.73 | 1 | 20 | 0.014 | 1Score **> 35** indicates **identity** Score **> 14** indicates **homology** | U | R.MQAQSHLHGIKPR.Y  + Oxidation (M) |
| 67534 | 141 | – | 153 | 506.9372 | 1517.7899 | 1517.7885 | 0.91 | 1 | 26 | 0.0039 | 1Score **> 35** indicates **identity** Score **> 14** indicates **homology** | U | R.MQAQSHLHGIKPR.Y  + Oxidation (M) |
| 67536 | 141 | – | 153 | 506.9374 | 1517.7905 | 1517.7885 | 1.33 | 1 | 17 | 0.023 | 1Score **> 35** indicates **identity** Score **> 14** indicates **homology** | U | R.MQAQSHLHGIKPR.Y  + Oxidation (M) |
| 67538 | 141 | – | 153 | 506.9377 | 1517.7912 | 1517.7885 | 1.78 | 1 | 15 | 0.036 | 1Score **> 35** indicates **identity** Score **> 13** indicates **homology** | U | R.MQAQSHLHGIKPR.Y  + Oxidation (M) |
| 22834 | 154 | – | 162 | 554.7541 | 1107.4937 | 1107.4985 | -4.30 | 0 | 37 | 0.00033 | 1Score **> 28** indicates **identity** Score **> 15** indicates **homology** | U | R.YTGTYNAYR.V |
| 22835 | 154 | – | 162 | 554.7554 | 1107.4962 | 1107.4985 | -2.12 | 0 | 30 | 0.0014 | 1Score **> 29** indicates **identity** Score **> 14** indicates **homology** | U | R.YTGTYNAYR.V |
| 22837 | 154 | – | 162 | 554.7557 | 1107.4968 | 1107.4985 | -1.55 | 0 | 41 | 0.00013 | 1Score **> 29** indicates **identity** Score **> 15** indicates **homology** | U | R.YTGTYNAYR.V |
| 22838 | 154 | – | 162 | 554.7558 | 1107.4970 | 1107.4985 | -1.33 | 0 | 48 | 2.9e-05 | 1Score **> 29** indicates **identity** Score **> 16** indicates **homology** | U | R.YTGTYNAYR.V |
| 22839 | 154 | – | 162 | 554.7558 | 1107.4971 | 1107.4985 | -1.24 | 0 | 47 | 6.9e-05 | 1Score **> 29** indicates **identity** Score **> 18** indicates **homology** | U | R.YTGTYNAYR.V |
| 22840 | 154 | – | 162 | 554.7561 | 1107.4976 | 1107.4985 | -0.82 | 0 | 56 | 1.7e-05 | 1Score **> 29** indicates **identity** Score **> 21** indicates **homology** | U | R.YTGTYNAYR.V |
| 22841 | 154 | – | 162 | 554.7562 | 1107.4978 | 1107.4985 | -0.68 | 0 | 67 | 5.5e-07 | 1Score **> 28** indicates **identity** Score **> 17** indicates **homology** | U | R.YTGTYNAYR.V |
| 22842 | 154 | – | 162 | 554.7563 | 1107.4981 | 1107.4985 | -0.38 | 0 | 49 | 2.7e-05 | 1Score **> 28** indicates **identity** Score **> 16** indicates **homology** | U | R.YTGTYNAYR.V |
| 22843 | 154 | – | 162 | 554.7565 | 1107.4985 | 1107.4985 | -0.046 | 0 | 42 | 0.00012 | 1Score **> 28** indicates **identity** Score **> 15** indicates **homology** | U | R.YTGTYNAYR.V |
| 22844 | 154 | – | 162 | 554.7565 | 1107.4985 | 1107.4985 | -0.035 | 0 | 43 | 8.9e-05 | 1Score **> 28** indicates **identity** Score **> 15** indicates **homology** | U | R.YTGTYNAYR.V |
| 22845 | 154 | – | 162 | 554.7565 | 1107.4985 | 1107.4985 | -0.012 | 0 | 28 | 0.0022 | 1Score **> 28** indicates **identity** Score **> 14** indicates **homology** | U | R.YTGTYNAYR.V |
| 22847 | 154 | – | 162 | 554.7566 | 1107.4986 | 1107.4985 | 0.098 | 0 | 55 | 7.3e-06 | 1Score **> 28** indicates **identity** Score **> 16** indicates **homology** | U | R.YTGTYNAYR.V |
| 22848 | 154 | – | 162 | 554.7569 | 1107.4993 | 1107.4985 | 0.75 | 0 | 37 | 0.00037 | 1Score **> 28** indicates **identity** Score **> 15** indicates **homology** | U | R.YTGTYNAYR.V |
| 22849 | 154 | – | 162 | 554.7574 | 1107.5002 | 1107.4985 | 1.50 | 0 | 20 | 0.015 | 1Score **> 28** indicates **identity** Score **> 14** indicates **homology** | U | R.YTGTYNAYR.V |
| 22850 | 154 | – | 162 | 554.7583 | 1107.5021 | 1107.4985 | 3.28 | 0 | 24 | 0.006 | 1Score **> 30** indicates **identity** Score **> 14** indicates **homology** | U | R.YTGTYNAYR.V |
| 22927 | 154 | – | 162 | 555.2473 | 1108.4800 | 1108.4825 | -2.23 | 0 | 30 | 0.0017 | 1Score **> 27** indicates **identity** Score **> 14** indicates **homology** | U | R.YTGTYNAYR.V  + Deamidated (NQ) |
| 22928 | 154 | – | 162 | 555.2476 | 1108.4807 | 1108.4825 | -1.65 | 0 | 35 | 0.00057 | 1Score **> 27** indicates **identity** Score **> 15** indicates **homology** | U | R.YTGTYNAYR.V  + Deamidated (NQ) |
| 22930 | 154 | – | 162 | 555.2483 | 1108.4820 | 1108.4825 | -0.48 | 0 | 28 | 0.0025 | 1Score **> 27** indicates **identity** Score **> 14** indicates **homology** | U | R.YTGTYNAYR.V  + Deamidated (NQ) |
| 157124 | 154 | – | 175 | 846.7697 | 2537.2873 | 2537.2802 | 2.79 | 1 | 47 | 4.3e-05 | 1Score **> 37** indicates **identity** Score **> 15** indicates **homology** | U | R.YTGTYNAYRVIATTESLSTLWK.G |
| 59126 | 163 | – | 175 | 724.9012 | 1447.7879 | 1447.7922 | -2.97 | 0 | 65 | 8.4e-07 | 1Score **> 34** indicates **identity** Score **> 17** indicates **homology** | U | R.VIATTESLSTLWK.G |
| 59128 | 163 | – | 175 | 724.9014 | 1447.7883 | 1447.7922 | -2.74 | 0 | 83 | 1.8e-08 | 1Score **> 34** indicates **identity** Score **> 18** indicates **homology** | U | R.VIATTESLSTLWK.G |
| 59129 | 163 | – | 175 | 724.9016 | 1447.7887 | 1447.7922 | -2.47 | 0 | 37 | 0.00037 | 1Score **> 34** indicates **identity** Score **> 15** indicates **homology** | U | R.VIATTESLSTLWK.G |
| 59130 | 163 | – | 175 | 724.9017 | 1447.7888 | 1447.7922 | -2.38 | 0 | 83 | 1.5e-08 | 1Score **> 34** indicates **identity** Score **> 18** indicates **homology** | U | R.VIATTESLSTLWK.G |
| 59131 | 163 | – | 175 | 724.9020 | 1447.7895 | 1447.7922 | -1.89 | 0 | 44 | 7.8e-05 | 1Score **> 34** indicates **identity** Score **> 15** indicates **homology** | U | R.VIATTESLSTLWK.G |
| 59135 | 163 | – | 175 | 724.9023 | 1447.7901 | 1447.7922 | -1.46 | 0 | 79 | 3.7e-08 | 1Score **> 34** indicates **identity** Score **> 17** indicates **homology** | U | R.VIATTESLSTLWK.G |
| 59136 | 163 | – | 175 | 724.9024 | 1447.7902 | 1447.7922 | -1.39 | 0 | 31 | 0.0012 | 1Score **> 34** indicates **identity** Score **> 14** indicates **homology** | U | R.VIATTESLSTLWK.G |
| 59138 | 163 | – | 175 | 724.9027 | 1447.7908 | 1447.7922 | -0.97 | 0 | 70 | 3e-07 | 1Score **> 34** indicates **identity** Score **> 17** indicates **homology** | U | R.VIATTESLSTLWK.G |
| 59140 | 163 | – | 175 | 724.9027 | 1447.7908 | 1447.7922 | -0.97 | 0 | 98 | 6e-10 | 1Score **> 34** indicates **identity** Score **> 19** indicates **homology** | U | R.VIATTESLSTLWK.G |
| 59141 | 163 | – | 175 | 724.9028 | 1447.7911 | 1447.7922 | -0.80 | 0 | 64 | 1.1e-06 | 1Score **> 34** indicates **identity** Score **> 16** indicates **homology** | U | R.VIATTESLSTLWK.G |
| 59143 | 163 | – | 175 | 724.9029 | 1447.7913 | 1447.7922 | -0.63 | 0 | 65 | 7.5e-07 | 1Score **> 34** indicates **identity** Score **> 17** indicates **homology** | U | R.VIATTESLSTLWK.G |
| 59144 | 163 | – | 175 | 724.9029 | 1447.7913 | 1447.7922 | -0.62 | 0 | 83 | 1.6e-08 | 1Score **> 34** indicates **identity** Score **> 18** indicates **homology** | U | R.VIATTESLSTLWK.G |
| 59145 | 163 | – | 175 | 724.9030 | 1447.7915 | 1447.7922 | -0.54 | 0 | 80 | 3.5e-08 | 1Score **> 34** indicates **identity** Score **> 17** indicates **homology** | U | R.VIATTESLSTLWK.G |
| 59146 | 163 | – | 175 | 724.9031 | 1447.7917 | 1447.7922 | -0.38 | 0 | 65 | 7.3e-07 | 1Score **> 34** indicates **identity** Score **> 17** indicates **homology** | U | R.VIATTESLSTLWK.G |
| 59147 | 163 | – | 175 | 724.9032 | 1447.7918 | 1447.7922 | -0.27 | 0 | 34 | 0.00065 | 1Score **> 34** indicates **identity** Score **> 15** indicates **homology** | U | R.VIATTESLSTLWK.G |
| 59149 | 163 | – | 175 | 724.9032 | 1447.7919 | 1447.7922 | -0.21 | 0 | 61 | 1.9e-06 | 1Score **> 34** indicates **identity** Score **> 16** indicates **homology** | U | R.VIATTESLSTLWK.G |
| 59150 | 163 | – | 175 | 724.9034 | 1447.7923 | 1447.7922 | 0.048 | 0 | 119 | 8.2e-12 | 1Score **> 34** indicates **identity** Score **> 20** indicates **homology** | U | R.VIATTESLSTLWK.G |
| 59151 | 163 | – | 175 | 483.6047 | 1447.7923 | 1447.7922 | 0.054 | 0 | 15 | 0.042 | 1Score **> 34** indicates **identity** Score **> 13** indicates **homology** | U | R.VIATTESLSTLWK.G |
| 59152 | 163 | – | 175 | 724.9034 | 1447.7923 | 1447.7922 | 0.055 | 0 | 99 | 5.6e-10 | 1Score **> 34** indicates **identity** Score **> 19** indicates **homology** | U | R.VIATTESLSTLWK.G |
| 59153 | 163 | – | 175 | 724.9035 | 1447.7924 | 1447.7922 | 0.12 | 0 | 83 | 1.6e-08 | 1Score **> 34** indicates **identity** Score **> 18** indicates **homology** | U | R.VIATTESLSTLWK.G |
| 59154 | 163 | – | 175 | 724.9035 | 1447.7924 | 1447.7922 | 0.13 | 0 | 119 | 8.4e-12 | 1Score **> 34** indicates **identity** Score **> 21** indicates **homology** | U | R.VIATTESLSTLWK.G |
| 59155 | 163 | – | 175 | 483.6048 | 1447.7924 | 1447.7922 | 0.14 | 0 | 53 | 1e-05 | 1Score **> 34** indicates **identity** Score **> 16** indicates **homology** | U | R.VIATTESLSTLWK.G |
| 59156 | 163 | – | 175 | 724.9035 | 1447.7924 | 1447.7922 | 0.15 | 0 | 119 | 7.3e-12 | 1Score **> 34** indicates **identity** Score **> 20** indicates **homology** | U | R.VIATTESLSTLWK.G |
| 59158 | 163 | – | 175 | 724.9036 | 1447.7927 | 1447.7922 | 0.30 | 0 | 40 | 0.00019 | 1Score **> 34** indicates **identity** Score **> 15** indicates **homology** | U | R.VIATTESLSTLWK.G |
| 59159 | 163 | – | 175 | 724.9036 | 1447.7927 | 1447.7922 | 0.31 | 0 | 83 | 1.5e-08 | 1Score **> 34** indicates **identity** Score **> 18** indicates **homology** | U | R.VIATTESLSTLWK.G |
| 59160 | 163 | – | 175 | 724.9037 | 1447.7928 | 1447.7922 | 0.38 | 0 | 99 | 7.9e-10 | 1Score **> 34** indicates **identity** Score **> 21** indicates **homology** | U | R.VIATTESLSTLWK.G |
| 59161 | 163 | – | 175 | 724.9038 | 1447.7930 | 1447.7922 | 0.56 | 0 | 119 | 8.3e-12 | 1Score **> 34** indicates **identity** Score **> 20** indicates **homology** | U | R.VIATTESLSTLWK.G |
| 59162 | 163 | – | 175 | 724.9038 | 1447.7931 | 1447.7922 | 0.62 | 0 | 98 | 6.6e-10 | 1Score **> 34** indicates **identity** Score **> 19** indicates **homology** | U | R.VIATTESLSTLWK.G |
| 59163 | 163 | – | 175 | 724.9039 | 1447.7933 | 1447.7922 | 0.71 | 0 | 36 | 0.00041 | 1Score **> 34** indicates **identity** Score **> 15** indicates **homology** | U | R.VIATTESLSTLWK.G |
| 59164 | 163 | – | 175 | 724.9039 | 1447.7933 | 1447.7922 | 0.72 | 0 | 91 | 3e-09 | 1Score **> 34** indicates **identity** Score **> 18** indicates **homology** | U | R.VIATTESLSTLWK.G |
| 59166 | 163 | – | 175 | 724.9040 | 1447.7934 | 1447.7922 | 0.79 | 0 | 79 | 4.3e-08 | 1Score **> 34** indicates **identity** Score **> 17** indicates **homology** | U | R.VIATTESLSTLWK.G |
| 59167 | 163 | – | 175 | 724.9041 | 1447.7937 | 1447.7922 | 0.99 | 0 | 119 | 8.3e-12 | 1Score **> 34** indicates **identity** Score **> 20** indicates **homology** | U | R.VIATTESLSTLWK.G |
| 59168 | 163 | – | 175 | 724.9041 | 1447.7937 | 1447.7922 | 0.99 | 0 | 48 | 3.1e-05 | 1Score **> 34** indicates **identity** Score **> 16** indicates **homology** | U | R.VIATTESLSTLWK.G |
| 59169 | 163 | – | 175 | 724.9042 | 1447.7939 | 1447.7922 | 1.14 | 0 | 78 | 5e-08 | 1Score **> 34** indicates **identity** Score **> 17** indicates **homology** | U | R.VIATTESLSTLWK.G |
| 59170 | 163 | – | 175 | 724.9046 | 1447.7946 | 1447.7922 | 1.63 | 0 | 106 | 2e-10 | 1Score **> 34** indicates **identity** Score **> 21** indicates **homology** | U | R.VIATTESLSTLWK.G |
| 59171 | 163 | – | 175 | 724.9046 | 1447.7946 | 1447.7922 | 1.63 | 0 | 50 | 2.2e-05 | 1Score **> 34** indicates **identity** Score **> 16** indicates **homology** | U | R.VIATTESLSTLWK.G |
| 59173 | 163 | – | 175 | 724.9046 | 1447.7947 | 1447.7922 | 1.72 | 0 | 27 | 0.0032 | 1Score **> 34** indicates **identity** Score **> 14** indicates **homology** | U | R.VIATTESLSTLWK.G |
| 59174 | 163 | – | 175 | 724.9047 | 1447.7948 | 1447.7922 | 1.75 | 0 | 99 | 8.7e-10 | 1Score **> 34** indicates **identity** Score **> 20** indicates **homology** | U | R.VIATTESLSTLWK.G |
| 59175 | 163 | – | 175 | 724.9048 | 1447.7951 | 1447.7922 | 1.98 | 0 | 99 | 8.5e-10 | 1Score **> 34** indicates **identity** Score **> 21** indicates **homology** | U | R.VIATTESLSTLWK.G |
| 59176 | 163 | – | 175 | 724.9050 | 1447.7954 | 1447.7922 | 2.17 | 0 | 62 | 1.6e-06 | 1Score **> 34** indicates **identity** Score **> 16** indicates **homology** | U | R.VIATTESLSTLWK.G |
| 59177 | 163 | – | 175 | 724.9055 | 1447.7964 | 1447.7922 | 2.91 | 0 | 64 | 9.9e-07 | 1Score **> 33** indicates **identity** Score **> 17** indicates **homology** | U | R.VIATTESLSTLWK.G |
| 59180 | 163 | – | 175 | 724.9056 | 1447.7967 | 1447.7922 | 3.10 | 0 | 99 | 8.5e-10 | 1Score **> 33** indicates **identity** Score **> 20** indicates **homology** | U | R.VIATTESLSTLWK.G |
| 59181 | 163 | – | 175 | 724.9057 | 1447.7969 | 1447.7922 | 3.25 | 0 | 98 | 5.8e-10 | 1Score **> 33** indicates **identity** Score **> 19** indicates **homology** | U | R.VIATTESLSTLWK.G |
| 59183 | 163 | – | 175 | 724.9060 | 1447.7974 | 1447.7922 | 3.58 | 0 | 65 | 7.9e-07 | 1Score **> 33** indicates **identity** Score **> 17** indicates **homology** | U | R.VIATTESLSTLWK.G |
| 59187 | 163 | – | 175 | 724.9062 | 1447.7979 | 1447.7922 | 3.93 | 0 | 78 | 5.1e-08 | 1Score **> 33** indicates **identity** Score **> 17** indicates **homology** | U | R.VIATTESLSTLWK.G |
| 59188 | 163 | – | 175 | 724.9063 | 1447.7981 | 1447.7922 | 4.02 | 0 | 38 | 0.00026 | 1Score **> 33** indicates **identity** Score **> 15** indicates **homology** | U | R.VIATTESLSTLWK.G |
| 59189 | 163 | – | 175 | 724.9065 | 1447.7985 | 1447.7922 | 4.33 | 0 | 77 | 5.9e-08 | 1Score **> 33** indicates **identity** Score **> 17** indicates **homology** | U | R.VIATTESLSTLWK.G |
| 59190 | 163 | – | 175 | 724.9066 | 1447.7986 | 1447.7922 | 4.42 | 0 | 50 | 2.2e-05 | 1Score **> 33** indicates **identity** Score **> 16** indicates **homology** | U | R.VIATTESLSTLWK.G |
| 59191 | 163 | – | 175 | 724.9070 | 1447.7994 | 1447.7922 | 4.92 | 0 | 40 | 0.00017 | 1Score **> 33** indicates **identity** Score **> 15** indicates **homology** | U | R.VIATTESLSTLWK.G |
| 59192 | 163 | – | 175 | 724.9072 | 1447.7999 | 1447.7922 | 5.27 | 0 | 46 | 5.4e-05 | 1Score **> 33** indicates **identity** Score **> 15** indicates **homology** | U | R.VIATTESLSTLWK.G |
| 59193 | 163 | – | 175 | 724.9072 | 1447.7999 | 1447.7922 | 5.27 | 0 | 82 | 1.9e-08 | 1Score **> 33** indicates **identity** Score **> 18** indicates **homology** | U | R.VIATTESLSTLWK.G |
| 59194 | 163 | – | 175 | 724.9074 | 1447.8002 | 1447.7922 | 5.52 | 0 | 48 | 2.9e-05 | 1Score **> 33** indicates **identity** Score **> 16** indicates **homology** | U | R.VIATTESLSTLWK.G |
| 8497 | 176 | – | 183 | 453.2270 | 904.4395 | 904.4436 | -4.62 | 0 | 19 | 0.016 | 1Score **> 30** indicates **identity** Score **> 14** indicates **homology** | U | K.GTTPNLMR.N  + Oxidation (M) |
| 8500 | 176 | – | 183 | 453.2281 | 904.4417 | 904.4436 | -2.17 | 0 | 20 | 0.037 | 1Score **> 31** indicates **identity** Score **> 19** indicates **homology** | U | K.GTTPNLMR.N  + Oxidation (M) |
| 8501 | 176 | – | 183 | 453.2282 | 904.4419 | 904.4436 | -1.91 | 0 | 22 | 0.009 | 1Score **> 31** indicates **identity** Score **> 14** indicates **homology** | U | K.GTTPNLMR.N  + Oxidation (M) |
| 8505 | 176 | – | 183 | 453.2286 | 904.4426 | 904.4436 | -1.11 | 0 | 29 | 0.0025 | 1Score **> 31** indicates **identity** Score **> 16** indicates **homology** | U | K.GTTPNLMR.N  + Oxidation (M) |
| 8506 | 176 | – | 183 | 453.2287 | 904.4429 | 904.4436 | -0.85 | 0 | 21 | 0.011 | 1Score **> 31** indicates **identity** Score **> 14** indicates **homology** | U | K.GTTPNLMR.N  + Oxidation (M) |
| 8507 | 176 | – | 183 | 453.2288 | 904.4430 | 904.4436 | -0.70 | 0 | 27 | 0.021 | 1Score **> 31** indicates **identity** Score **> 22** indicates **homology** | U | K.GTTPNLMR.N  + Oxidation (M) |
| 8509 | 176 | – | 183 | 453.2290 | 904.4435 | 904.4436 | -0.16 | 0 | 29 | 0.0023 | 1Score **> 31** indicates **identity** Score **> 15** indicates **homology** | U | K.GTTPNLMR.N  + Oxidation (M) |
| 8510 | 176 | – | 183 | 453.2293 | 904.4440 | 904.4436 | 0.37 | 0 | 18 | 0.021 | 1Score **> 31** indicates **identity** Score **> 14** indicates **homology** | U | K.GTTPNLMR.N  + Oxidation (M) |
| 8511 | 176 | – | 183 | 453.2295 | 904.4444 | 904.4436 | 0.82 | 0 | 23 | 0.007 | 1Score **> 31** indicates **identity** Score **> 14** indicates **homology** | U | K.GTTPNLMR.N  + Oxidation (M) |
| 8513 | 176 | – | 183 | 453.2297 | 904.4448 | 904.4436 | 1.31 | 0 | 16 | 0.034 | 1Score **> 31** indicates **identity** Score **> 13** indicates **homology** | U | K.GTTPNLMR.N  + Oxidation (M) |
| 112969 | 184 | – | 199 | 963.4889 | 1924.9632 | 1924.9638 | -0.31 | 0 | 99 | 4.9e-10 | 1Score **> 35** indicates **identity** Score **> 19** indicates **homology** | U | R.NVIINCTELVTYDLMK.G |
| 112970 | 184 | – | 199 | 642.6618 | 1924.9637 | 1924.9638 | -0.064 | 0 | 82 | 1.9e-08 | 1Score **> 36** indicates **identity** Score **> 18** indicates **homology** | U | R.NVIINCTELVTYDLMK.G |
| 112972 | 184 | – | 199 | 642.6623 | 1924.9651 | 1924.9638 | 0.68 | 0 | 40 | 0.00017 | 1Score **> 35** indicates **identity** Score **> 15** indicates **homology** | U | R.NVIINCTELVTYDLMK.G |
| 112974 | 184 | – | 199 | 963.4901 | 1924.9657 | 1924.9638 | 0.99 | 0 | 71 | 2.2e-07 | 1Score **> 35** indicates **identity** Score **> 17** indicates **homology** | U | R.NVIINCTELVTYDLMK.G |
| 112976 | 184 | – | 199 | 963.4908 | 1924.9671 | 1924.9638 | 1.70 | 0 | 79 | 4.2e-08 | 1Score **> 35** indicates **identity** Score **> 18** indicates **homology** | U | R.NVIINCTELVTYDLMK.G |
| 112977 | 184 | – | 199 | 963.4911 | 1924.9677 | 1924.9638 | 2.01 | 0 | 87 | 6.7e-09 | 1Score **> 36** indicates **identity** Score **> 18** indicates **homology** | U | R.NVIINCTELVTYDLMK.G |
| 112981 | 184 | – | 199 | 642.6642 | 1924.9708 | 1924.9638 | 3.65 | 0 | 53 | 1.1e-05 | 1Score **> 36** indicates **identity** Score **> 16** indicates **homology** | U | R.NVIINCTELVTYDLMK.G |
| 112986 | 184 | – | 199 | 963.4956 | 1924.9767 | 1924.9638 | 6.71 | 0 | 86 | 8.1e-09 | 1Score **> 35** indicates **identity** Score **> 18** indicates **homology** | U | R.NVIINCTELVTYDLMK.G |
| 114429 | 184 | – | 199 | 971.4821 | 1940.9497 | 1940.9587 | -4.66 | 0 | 15 | 0.042 | 1Score **> 35** indicates **identity** Score **> 13** indicates **homology** | U | R.NVIINCTELVTYDLMK.G  + Oxidation (M) |
| 114437 | 184 | – | 199 | 647.9926 | 1940.9561 | 1940.9587 | -1.35 | 0 | 23 | 0.0071 | 1Score **> 35** indicates **identity** Score **> 14** indicates **homology** | U | R.NVIINCTELVTYDLMK.G  + Oxidation (M) |
| 114442 | 184 | – | 199 | 647.9932 | 1940.9577 | 1940.9587 | -0.53 | 0 | 36 | 0.00041 | 1Score **> 35** indicates **identity** Score **> 15** indicates **homology** | U | R.NVIINCTELVTYDLMK.G  + Oxidation (M) |
| 114443 | 184 | – | 199 | 647.9933 | 1940.9582 | 1940.9587 | -0.27 | 0 | 26 | 0.0039 | 1Score **> 35** indicates **identity** Score **> 14** indicates **homology** | U | R.NVIINCTELVTYDLMK.G  + Oxidation (M) |
| 114450 | 184 | – | 199 | 647.9943 | 1940.9611 | 1940.9587 | 1.21 | 0 | 18 | 0.02 | 1Score **> 35** indicates **identity** Score **> 14** indicates **homology** | U | R.NVIINCTELVTYDLMK.G  + Oxidation (M) |
| 114452 | 184 | – | 199 | 971.4883 | 1940.9620 | 1940.9587 | 1.67 | 0 | 20 | 0.014 | 1Score **> 35** indicates **identity** Score **> 14** indicates **homology** | U | R.NVIINCTELVTYDLMK.G  + Oxidation (M) |
| 114459 | 184 | – | 199 | 971.4912 | 1940.9679 | 1940.9587 | 4.73 | 0 | 29 | 0.0018 | 1Score **> 35** indicates **identity** Score **> 14** indicates **homology** | U | R.NVIINCTELVTYDLMK.G  + Oxidation (M) |
| 114461 | 184 | – | 199 | 647.9966 | 1940.9681 | 1940.9587 | 4.82 | 0 | 20 | 0.014 | 1Score **> 35** indicates **identity** Score **> 14** indicates **homology** | U | R.NVIINCTELVTYDLMK.G  + Oxidation (M) |
| 114462 | 184 | – | 199 | 647.9969 | 1940.9688 | 1940.9587 | 5.18 | 0 | 22 | 0.0093 | 1Score **> 35** indicates **identity** Score **> 14** indicates **homology** | U | R.NVIINCTELVTYDLMK.G  + Oxidation (M) |
| 160724 | 184 | – | 206 | 874.7938 | 2621.3597 | 2621.3557 | 1.54 | 1 | 22 | 0.009 | 1Score **> 37** indicates **identity** Score **> 14** indicates **homology** | U | R.NVIINCTELVTYDLMKGALVNNK.I |
| 178861 | 207 | – | 237 | 1098.9207 | 3293.7402 | 3293.7404 | -0.050 | 0 | 59 | 2.9e-06 | 1Score **> 37** indicates **identity** Score **> 16** indicates **homology** | U | K.ILADDVPCHLLSALVAGFCTTLLASPVDVVK.T |
| 178865 | 207 | – | 237 | 824.4444 | 3293.7487 | 3293.7404 | 2.52 | 0 | 54 | 7.8e-06 | 1Score **> 36** indicates **identity** Score **> 16** indicates **homology** | U | K.ILADDVPCHLLSALVAGFCTTLLASPVDVVK.T |
| 154094 | 240 | – | 261 | 826.7258 | 2477.1554 | 2477.1429 | 5.05 | 0 | 36 | 0.00042 | 1Score **> 34** indicates **identity** Score **> 15** indicates **homology** | U | R.FINSLPGQYPSVPSCAMSMYTK.E |
| 154099 | 240 | – | 261 | 826.7267 | 2477.1582 | 2477.1429 | 6.17 | 0 | 30 | 0.0017 | 1Score **> 34** indicates **identity** Score **> 14** indicates **homology** | U | R.FINSLPGQYPSVPSCAMSMYTK.E |
| 7961 | 262 | – | 269 | 448.7288 | 895.4431 | 895.4440 | -1.00 | 0 | 39 | 0.0012 | 1Score **> 27** indicates **identity** Score **> 23** indicates **homology** | U | K.EGPTAFFK.G |
| 7962 | 262 | – | 269 | 448.7292 | 895.4438 | 895.4440 | -0.18 | 0 | 45 | 0.00087 | 1Score **> 27** indicates **identity** | U | K.EGPTAFFK.G |
| 7964 | 262 | – | 269 | 448.7296 | 895.4447 | 895.4440 | 0.84 | 0 | 43 | 0.0015 | 1Score **> 27** indicates **identity** Score **> 27** indicates **homology** | U | K.EGPTAFFK.G |
| 98775 | 262 | – | 277 | 591.9831 | 1772.9276 | 1772.9250 | 1.45 | 1 | 30 | 0.0017 | 1Score **> 35** indicates **identity** Score **> 14** indicates **homology** | U | K.EGPTAFFKGFVASFLR.L |
| 7976 | 270 | – | 277 | 448.7526 | 895.4906 | 895.4916 | -1.09 | 0 | 25 | 0.0042 | 1Score **> 27** indicates **identity** Score **> 14** indicates **homology** | U | K.GFVASFLR.L |
| 7977 | 270 | – | 277 | 448.7531 | 895.4917 | 895.4916 | 0.16 | 0 | 31 | 0.0012 | 1Score **> 27** indicates **identity** Score **> 14** indicates **homology** | U | K.GFVASFLR.L |
| 7978 | 270 | – | 277 | 448.7533 | 895.4920 | 895.4916 | 0.43 | 0 | 27 | 0.0045 | 1Score **> 27** indicates **identity** Score **> 16** indicates **homology** | U | K.GFVASFLR.L |
| 7979 | 270 | – | 277 | 448.7533 | 895.4921 | 895.4916 | 0.56 | 0 | 37 | 0.0012 | 1Score **> 27** indicates **identity** Score **> 20** indicates **homology** | U | K.GFVASFLR.L |
| 7980 | 270 | – | 277 | 448.7534 | 895.4922 | 895.4916 | 0.70 | 0 | 38 | 0.00086 | 1Score **> 27** indicates **identity** Score **> 20** indicates **homology** | U | K.GFVASFLR.L |
| 7981 | 270 | – | 277 | 448.7534 | 895.4922 | 895.4916 | 0.70 | 0 | 35 | 0.002 | 1Score **> 27** indicates **identity** Score **> 20** indicates **homology** | U | K.GFVASFLR.L |
| 7982 | 270 | – | 277 | 448.7537 | 895.4929 | 895.4916 | 1.42 | 0 | 16 | 0.034 | 1Score **> 27** indicates **identity** Score **> 13** indicates **homology** | U | K.GFVASFLR.L |
| 7984 | 270 | – | 277 | 448.7540 | 895.4933 | 895.4916 | 1.96 | 0 | 35 | 0.0021 | 1Score **> 27** indicates **identity** Score **> 20** indicates **homology** | U | K.GFVASFLR.L |
| 128204 | 278 | – | 294 | 700.3638 | 2098.0696 | 2098.0744 | -2.27 | 1 | 21 | 0.011 | 1Score **> 36** indicates **identity** Score **> 14** indicates **homology** | U | R.LGSWNVIMFVCFEQLKK.E |

---

```
ID   UCP1_MOUSE              Reviewed;         307 AA.
AC   P12242;
DT   01-OCT-1989, integrated into UniProtKB/Swiss-Prot.
DT   23-JAN-2007, sequence version 2.
DT   28-JUN-2023, entry version 182.
DE   RecName: Full=Mitochondrial brown fat uncoupling protein 1 {ECO:0000305};
DE            Short=UCP 1 {ECO:0000305};
DE   AltName: Full=Solute carrier family 25 member 7 {ECO:0000250|UniProtKB:P25874};
DE   AltName: Full=Thermogenin {ECO:0000250|UniProtKB:P04575};
GN   Name=Ucp1 {ECO:0000312|MGI:MGI:98894};
GN   Synonyms=Slc25a7 {ECO:0000250|UniProtKB:P25874}, Ucp
GN   {ECO:0000303|PubMed:3410843};
OS   Mus musculus (Mouse).
OC   Eukaryota; Metazoa; Chordata; Craniata; Vertebrata; Euteleostomi; Mammalia;
OC   Eutheria; Euarchontoglires; Glires; Rodentia; Myomorpha; Muroidea; Muridae;
OC   Murinae; Mus; Mus.
OX   NCBI_TaxID=10090;
RN   [1]
RP   NUCLEOTIDE SEQUENCE [GENOMIC DNA].
RX   PubMed=3410843; DOI=10.1016/s0021-9258(18)37751-2;
RA   Kozak L.P., Britton J.H., Kozak U.C., Wells J.M.;
RT   "The mitochondrial uncoupling protein gene. Correlation of exon structure
RT   to transmembrane domains.";
RL   J. Biol. Chem. 263:12274-12277(1988).
RN   [2]
RP   NUCLEOTIDE SEQUENCE, AND TISSUE SPECIFICITY.
RC   STRAIN=129/SvJ;
RX   PubMed=8264627; DOI=10.1128/mcb.14.1.59-67.1994;
RA   Kozak U.C., Kopecky J., Teisinger J., Enerbaeck S., Boyer B., Kozak L.P.;
RT   "An upstream enhancer regulating brown-fat-specific expression of the
RT   mitochondrial uncoupling protein gene.";
RL   Mol. Cell. Biol. 14:59-67(1994).
RN   [3]
RP   NUCLEOTIDE SEQUENCE [LARGE SCALE MRNA].
RC   STRAIN=FVB/N; TISSUE=Salivary gland;
RX   PubMed=15489334; DOI=10.1101/gr.2596504;
RG   The MGC Project Team;
RT   "The status, quality, and expansion of the NIH full-length cDNA project:
RT   the Mammalian Gene Collection (MGC).";
RL   Genome Res. 14:2121-2127(2004).
RN   [4]
RP   FUNCTION, AND DISRUPTION PHENOTYPE.
RX   PubMed=9139827; DOI=10.1038/387090a0;
RA   Enerbaeck S., Jacobsson A., Simpson E.M., Guerra C., Yamashita H.,
RA   Harper M.E., Kozak L.P.;
RT   "Mice lacking mitochondrial uncoupling protein are cold-sensitive but not
RT   obese.";
RL   Nature 387:90-94(1997).
RN   [5]
RP   FUNCTION, INDUCTION BY HIGH-FAT DIET, AND DISRUPTION PHENOTYPE.
RX   PubMed=19187776; DOI=10.1016/j.cmet.2008.12.014;
RA   Feldmann H.M., Golozoubova V., Cannon B., Nedergaard J.;
RT   "UCP1 ablation induces obesity and abolishes diet-induced thermogenesis in
RT   mice exempt from thermal stress by living at thermoneutrality.";
RL   Cell Metab. 9:203-209(2009).
RN   [6]
RP   FUNCTION, AND DISRUPTION PHENOTYPE.
RX   PubMed=20416274; DOI=10.1016/j.bbabio.2010.04.008;
RA   Dlaskova A., Clarke K.J., Porter R.K.;
RT   "The role of UCP 1 in production of reactive oxygen species by mitochondria
RT   isolated from brown adipose tissue.";
RL   Biochim. Biophys. Acta 1797:1470-1476(2010).
RN   [7]
RP   IDENTIFICATION BY MASS SPECTROMETRY [LARGE SCALE ANALYSIS].
RC   TISSUE=Brown adipose tissue, Heart, Kidney, Liver, Lung, and Spleen;
RX   PubMed=21183079; DOI=10.1016/j.cell.2010.12.001;
RA   Huttlin E.L., Jedrychowski M.P., Elias J.E., Goswami T., Rad R.,
RA   Beausoleil S.A., Villen J., Haas W., Sowa M.E., Gygi S.P.;
RT   "A tissue-specific atlas of mouse protein phosphorylation and expression.";
RL   Cell 143:1174-1189(2010).
RN   [8]
RP   FUNCTION, DISRUPTION PHENOTYPE, AND INDUCTION BY COLD.
RX   PubMed=20466728; DOI=10.1074/jbc.m110.122861;
RA   Oelkrug R., Kutschke M., Meyer C.W., Heldmaier G., Jastroch M.;
RT   "Uncoupling protein 1 decreases superoxide production in brown adipose
RT   tissue mitochondria.";
RL   J. Biol. Chem. 285:21961-21968(2010).
RN   [9]
RP   FUNCTION, TRANSPORTER ACTIVITY, ACTIVITY REGULATION, AND SUBCELLULAR
RP   LOCATION.
RX   PubMed=23063128; DOI=10.1016/j.cell.2012.09.010;
RA   Fedorenko A., Lishko P.V., Kirichok Y.;
RT   "Mechanism of fatty-acid-dependent UCP1 uncoupling in brown fat
RT   mitochondria.";
RL   Cell 151:400-413(2012).
RN   [10]
RP   INDUCTION BY COLD.
RX   PubMed=25578880; DOI=10.1016/j.molcel.2014.12.005;
RA   Dempersmier J., Sambeat A., Gulyaeva O., Paul S.M., Hudak C.S.,
RA   Raposo H.F., Kwan H.Y., Kang C., Wong R.H., Sul H.S.;
RT   "Cold-inducible Zfp516 activates UCP1 transcription to promote browning of
RT   white fat and development of brown fat.";
RL   Mol. Cell 57:235-246(2015).
RN   [11]
RP   FUNCTION, ACTIVITY REGULATION, OXIDATION AT CYS-254, AND MUTAGENESIS OF
RP   CYS-25; CYS-189; CYS-214; CYS-225; CYS-254; CYS-288 AND CYS-305.
RX   PubMed=27027295; DOI=10.1038/nature17399;
RA   Chouchani E.T., Kazak L., Jedrychowski M.P., Lu G.Z., Erickson B.K.,
RA   Szpyt J., Pierce K.A., Laznik-Bogoslavski D., Vetrivelan R., Clish C.B.,
RA   Robinson A.J., Gygi S.P., Spiegelman B.M.;
RT   "Mitochondrial ROS regulate thermogenic energy expenditure and
RT   sulfenylation of UCP1.";
RL   Nature 532:112-116(2016).
RN   [12]
RP   ACTIVITY REGULATION.
RX   PubMed=32358195; DOI=10.1073/pnas.2001387117;
RA   Jedrychowski M.P., Lu G.Z., Szpyt J., Mariotti M., Garrity R., Paulo J.A.,
RA   Schweppe D.K., Laznik-Bogoslavski D., Kazak L., Murphy M.P.,
RA   Gladyshev V.N., Gygi S.P., Chouchani E.T., Spiegelman B.M.;
RT   "Facultative protein selenation regulates redox sensitivity, adipose tissue
RT   thermogenesis, and obesity.";
RL   Proc. Natl. Acad. Sci. U.S.A. 117:10789-10796(2020).
CC   -!- FUNCTION: Mitochondrial transporter that functions as a long-chain
CC       fatty acid/LCFA and proton symporter, simultaneously transporting one
CC       LCFA and one proton through the inner mitochondrial membrane. However,
CC       LCFAs remaining associated with the transporter via their hydrophobic
CC       tails, it results in an apparent transport of protons activated by
CC       LCFAs. Thereby, dissipates the mitochondrial proton gradient and
CC       converts the energy of substrate oxydation into heat instead of ATP
CC       (PubMed:23063128). Responsible for thermogenic respiration, a
CC       specialized capacity of brown adipose tissue and beige fat that
CC       participates in non-shivering adaptive thermogenesis to temperature and
CC       diet variations and more generally to the regulation of energy balance
CC       (PubMed:9139827, PubMed:19187776, PubMed:23063128, PubMed:27027295).
CC       Regulates the production of reactive oxygen species/ROS by mitochondria
CC       (PubMed:20416274, PubMed:20466728). {ECO:0000269|PubMed:19187776,
CC       ECO:0000269|PubMed:20416274, ECO:0000269|PubMed:20466728,
CC       ECO:0000269|PubMed:23063128, ECO:0000269|PubMed:27027295,
CC       ECO:0000269|PubMed:9139827}.
CC   -!- CATALYTIC ACTIVITY:
CC       Reaction=H(+)(in) = H(+)(out); Xref=Rhea:RHEA:34979, ChEBI:CHEBI:15378;
CC         Evidence={ECO:0000269|PubMed:23063128};
CC   -!- ACTIVITY REGULATION: Has no constitutive proton transporter activity
CC       and has to be activated by long-chain fatty acids/LCFAs. Inhibited by
CC       purine nucleotides. Both purine nucleotides and LCFAs bind the
CC       cytosolic side of the transporter and directly compete to activate or
CC       inhibit it (PubMed:23063128). Activated by noradrenaline and reactive
CC       oxygen species (PubMed:27027295). Despite lacking canonical
CC       translational encoding for selenocysteine, a small pool of the protein
CC       has been observed to selectively incorporate selenocysteine at 'Cys-
CC       254' (PubMed:32358195). Selenocysteine-modified protein is highly
CC       sensitive to redox modification and may constitute a pool of protein
CC       highly sensitive to activation by elevated levels of reactive oxygen
CC       species (ROS) (PubMed:32358195). {ECO:0000269|PubMed:23063128,
CC       ECO:0000269|PubMed:27027295, ECO:0000269|PubMed:32358195}.
CC   -!- SUBUNIT: Most probably functions as a monomer. Binds one purine
CC       nucleotide per monomer. However, has also been suggested to function as
CC       a homodimer or a homotetramer. Tightly associates with cardiolipin in
CC       the mitochondrion inner membrane; may stabilize and regulate its
CC       activity. {ECO:0000250|UniProtKB:P25874, ECO:0000250|UniProtKB:W5PSH7}.
CC   -!- SUBCELLULAR LOCATION: Mitochondrion inner membrane
CC       {ECO:0000269|PubMed:23063128}; Multi-pass membrane protein
CC       {ECO:0000250|UniProtKB:P04633}.
CC   -!- TISSUE SPECIFICITY: Expressed in brown adipose tissue.
CC       {ECO:0000269|PubMed:8264627}.
CC   -!- INDUCTION: Up-regulated in response to cold in brown adipose tissue
CC       where it may regulate non-shivering thermogenesis (at protein level)
CC       (PubMed:20466728, PubMed:25578880). Up-regulated by high-fat diet (at
CC       protein level) (PubMed:19187776). {ECO:0000269|PubMed:19187776,
CC       ECO:0000269|PubMed:20466728, ECO:0000269|PubMed:25578880}.
CC   -!- PTM: Sulfenylation at Cys-254 is increased upon cold exposure. It
CC       increases the sensitivity of UCP1 thermogenic function to the
CC       activation by noradrenaline probably through structural effects.
CC       {ECO:0000269|PubMed:27027295}.
CC   -!- PTM: May undergo ubiquitin-mediated proteasomal degradation.
CC       {ECO:0000250|UniProtKB:P04633}.
CC   -!- DISRUPTION PHENOTYPE: Mice lacking Ucp1 display an absence of adaptive
CC       thermogenesis in response to cold. Compared to wild-type mice, they are
CC       sensitive to cold and consume less oxygen upon treatment with beta-3-
CC       adrenergic-receptor agonists that normally activate thermogenesis
CC       (PubMed:9139827, PubMed:19187776). They also display impaired adaptive
CC       thermogenesis in response to diet variation (PubMed:19187776). If they
CC       display lipid accumulation in adipocytes of brown adipose tissues, no
CC       overt obesity is observed when mice are housed under classical
CC       conditions, i.e. 18 to 20 degrees Celsius (PubMed:9139827). However,
CC       when mice are housed at thermoneutrality, i.e. at 30 degrees Celsius,
CC       obesity is clearly observed and exacerbated by high fat diet
CC       (PubMed:19187776). The brown adipose tissue of mice lacking Ucp1
CC       produce higher levels of reactive oxygen species (PubMed:20416274,
CC       PubMed:20466728). {ECO:0000269|PubMed:19187776,
CC       ECO:0000269|PubMed:20416274, ECO:0000269|PubMed:20466728,
CC       ECO:0000269|PubMed:9139827}.
CC   -!- SIMILARITY: Belongs to the mitochondrial carrier (TC 2.A.29) family.
CC       {ECO:0000305}.
CC   ---------------------------------------------------------------------------
CC   Copyrighted by the UniProt Consortium, see https://www.uniprot.org/terms
CC   Distributed under the Creative Commons Attribution (CC BY 4.0) License
CC   ---------------------------------------------------------------------------
DR   EMBL; M21247; AAA40521.1; -; Genomic_DNA.
DR   EMBL; M21222; AAA40521.1; JOINED; Genomic_DNA.
DR   EMBL; M21244; AAA40521.1; JOINED; Genomic_DNA.
DR   EMBL; M21245; AAA40521.1; JOINED; Genomic_DNA.
DR   EMBL; M21246; AAA40521.1; JOINED; Genomic_DNA.
DR   EMBL; U63419; AAB05870.1; -; mRNA.
DR   EMBL; U63418; AAB07367.1; -; Genomic_DNA.
DR   EMBL; BC012701; AAH12701.1; -; mRNA.
DR   CCDS; CCDS22449.1; -.
DR   PIR; A31106; A31106.
DR   RefSeq; NP_033489.1; NM_009463.3.
DR   AlphaFoldDB; P12242; -.
DR   SMR; P12242; -.
DR   STRING; 10090.ENSMUSP00000034146; -.
DR   iPTMnet; P12242; -.
DR   PhosphoSitePlus; P12242; -.
DR   MaxQB; P12242; -.
DR   PaxDb; P12242; -.
DR   ProteomicsDB; 297718; -.
DR   Antibodypedia; 16250; 477 antibodies from 38 providers.
DR   DNASU; 22227; -.
DR   Ensembl; ENSMUST00000034146; ENSMUSP00000034146; ENSMUSG00000031710.
DR   GeneID; 22227; -.
DR   KEGG; mmu:22227; -.
DR   UCSC; uc009mjx.2; mouse.
DR   AGR; MGI:98894; -.
DR   CTD; 7350; -.
DR   MGI; MGI:98894; Ucp1.
DR   VEuPathDB; HostDB:ENSMUSG00000031710; -.
DR   eggNOG; KOG0753; Eukaryota.
DR   GeneTree; ENSGT00940000160382; -.
DR   HOGENOM; CLU_015166_14_2_1; -.
DR   InParanoid; P12242; -.
DR   OMA; NCAMKMF; -.
DR   OrthoDB; 1832865at2759; -.
DR   PhylomeDB; P12242; -.
DR   TreeFam; TF323211; -.
DR   Reactome; R-MMU-167826; The fatty acid cycling model.
DR   Reactome; R-MMU-167827; The proton buffering model.
DR   BioGRID-ORCS; 22227; 2 hits in 76 CRISPR screens.
DR   ChiTaRS; Ucp1; mouse.
DR   PRO; PR:P12242; -.
DR   Proteomes; UP000000589; Chromosome 8.
DR   RNAct; P12242; protein.
DR   Bgee; ENSMUSG00000031710; Expressed in intercostal muscle and 56 other tissues.
DR   Genevisible; P12242; MM.
DR   GO; GO:0005740; C:mitochondrial envelope; IDA:MGI.
DR   GO; GO:0005743; C:mitochondrial inner membrane; IDA:UniProtKB.
DR   GO; GO:0005739; C:mitochondrion; IDA:UniProtKB.
DR   GO; GO:1901612; F:cardiolipin binding; ISS:UniProtKB.
DR   GO; GO:0019003; F:GDP binding; ISO:MGI.
DR   GO; GO:0005525; F:GTP binding; ISO:MGI.
DR   GO; GO:0036041; F:long-chain fatty acid binding; IDA:UniProtKB.
DR   GO; GO:0017077; F:oxidative phosphorylation uncoupler activity; IDA:UniProtKB.
DR   GO; GO:0032555; F:purine ribonucleotide binding; IDA:UniProtKB.
DR   GO; GO:0022857; F:transmembrane transporter activity; ISO:MGI.
DR   GO; GO:1990845; P:adaptive thermogenesis; IMP:UniProtKB.
DR   GO; GO:0050873; P:brown fat cell differentiation; IDA:MGI.
DR   GO; GO:0070417; P:cellular response to cold; IDA:MGI.
DR   GO; GO:1903495; P:cellular response to dehydroepiandrosterone; IEA:Ensembl.
DR   GO; GO:0071398; P:cellular response to fatty acid; IDA:UniProtKB.
DR   GO; GO:0032870; P:cellular response to hormone stimulus; IMP:UniProtKB.
DR   GO; GO:0034614; P:cellular response to reactive oxygen species; IMP:UniProtKB.
DR   GO; GO:0002024; P:diet induced thermogenesis; IMP:UniProtKB.
DR   GO; GO:1990542; P:mitochondrial transmembrane transport; IDA:UniProtKB.
DR   GO; GO:0006839; P:mitochondrial transport; ISO:MGI.
DR   GO; GO:0120162; P:positive regulation of cold-induced thermogenesis; IMP:YuBioLab.
DR   GO; GO:1902600; P:proton transmembrane transport; IDA:UniProtKB.
DR   GO; GO:1903426; P:regulation of reactive oxygen species biosynthetic process; IMP:UniProtKB.
DR   GO; GO:0006357; P:regulation of transcription by RNA polymerase II; IDA:MGI.
DR   GO; GO:0009409; P:response to cold; IBA:GO_Central.
DR   GO; GO:0031667; P:response to nutrient levels; IMP:UniProtKB.
DR   GO; GO:0009266; P:response to temperature stimulus; IMP:UniProtKB.
DR   Gene3D; 1.50.40.10; Mitochondrial carrier domain; 1.
DR   InterPro; IPR002030; Mit_uncoupling_UCP-like.
DR   InterPro; IPR018108; Mitochondrial_sb/sol_carrier.
DR   InterPro; IPR023395; Mt_carrier_dom_sf.
DR   PANTHER; PTHR45618:SF19; MITOCHONDRIAL BROWN FAT UNCOUPLING PROTEIN 1; 1.
DR   PANTHER; PTHR45618; MITOCHONDRIAL DICARBOXYLATE CARRIER-RELATED; 1.
DR   Pfam; PF00153; Mito_carr; 3.
DR   PRINTS; PR00784; MTUNCOUPLING.
DR   SUPFAM; SSF103506; Mitochondrial carrier; 1.
DR   PROSITE; PS50920; SOLCAR; 3.
PE   1: Evidence at protein level;
KW   Ion channel; Ion transport; Membrane; Mitochondrion;
KW   Mitochondrion inner membrane; Oxidation; Reference proteome; Repeat;
KW   Selenium; Selenocysteine; Transmembrane; Transmembrane helix; Transport.
FT   CHAIN           1..307
FT                   /note="Mitochondrial brown fat uncoupling protein 1"
FT                   /id="PRO_0000090659"
FT   TOPO_DOM        1..10
FT                   /note="Mitochondrial intermembrane"
FT                   /evidence="ECO:0000250|UniProtKB:P04633"
FT   TRANSMEM        11..32
FT                   /note="Helical; Name=1"
FT                   /evidence="ECO:0000255"
FT   TOPO_DOM        33..73
FT                   /note="Mitochondrial matrix"
FT                   /evidence="ECO:0000250|UniProtKB:P04633"
FT   TRANSMEM        74..96
FT                   /note="Helical; Name=2"
FT                   /evidence="ECO:0000255"
FT   TOPO_DOM        97..116
FT                   /note="Mitochondrial intermembrane"
FT                   /evidence="ECO:0000250|UniProtKB:P04633"
FT   TRANSMEM        117..133
FT                   /note="Helical; Name=3"
FT                   /evidence="ECO:0000255"
FT   TOPO_DOM        134..178
FT                   /note="Mitochondrial matrix"
FT                   /evidence="ECO:0000250|UniProtKB:P04633"
FT   TRANSMEM        179..195
FT                   /note="Helical; Name=4"
FT                   /evidence="ECO:0000255"
FT   TOPO_DOM        196..212
FT                   /note="Mitochondrial intermembrane"
FT                   /evidence="ECO:0000250|UniProtKB:P04633"
FT   TRANSMEM        213..232
FT                   /note="Helical; Name=5"
FT                   /evidence="ECO:0000255"
FT   TOPO_DOM        233..266
FT                   /note="Mitochondrial matrix"
FT                   /evidence="ECO:0000250|UniProtKB:P04633"
FT   TRANSMEM        267..289
FT                   /note="Helical; Name=6"
FT                   /evidence="ECO:0000255"
FT   TOPO_DOM        290..307
FT                   /note="Mitochondrial intermembrane"
FT                   /evidence="ECO:0000250|UniProtKB:P04633"
FT   REPEAT          11..102
FT                   /note="Solcar 1"
FT   REPEAT          111..201
FT                   /note="Solcar 2"
FT   REPEAT          210..295
FT                   /note="Solcar 3"
FT   BINDING         56
FT                   /ligand="fatty acid 16:0"
FT                   /ligand_id="ChEBI:CHEBI:78123"
FT                   /evidence="ECO:0000250|UniProtKB:P25874"
FT   BINDING         269
FT                   /ligand="fatty acid 16:0"
FT                   /ligand_id="ChEBI:CHEBI:78123"
FT                   /evidence="ECO:0000250|UniProtKB:P25874"
FT   MOD_RES         254
FT                   /note="Cysteine sulfenic acid (-SOH)"
FT                   /evidence="ECO:0000269|PubMed:27027295"
FT   MUTAGEN         25
FT                   /note="C->A: No effect on UCP1 activity in thermogenic
FT                   respiration."
FT                   /evidence="ECO:0000269|PubMed:27027295"
FT   MUTAGEN         189
FT                   /note="C->A: No effect on UCP1 activity in thermogenic
FT                   respiration."
FT                   /evidence="ECO:0000269|PubMed:27027295"
FT   MUTAGEN         214
FT                   /note="C->A: No effect on UCP1 activity in thermogenic
FT                   respiration."
FT                   /evidence="ECO:0000269|PubMed:27027295"
FT   MUTAGEN         225
FT                   /note="C->A: Decreased UCP1 activity in thermogenic
FT                   respiration."
FT                   /evidence="ECO:0000269|PubMed:27027295"
FT   MUTAGEN         254
FT                   /note="C->A: Decreased sensitivity to activation by
FT                   noradrenaline in thermogenic respiration."
FT                   /evidence="ECO:0000269|PubMed:27027295"
FT   MUTAGEN         288
FT                   /note="C->A: No effect on UCP1 activity in thermogenic
FT                   respiration."
FT                   /evidence="ECO:0000269|PubMed:27027295"
FT   MUTAGEN         305
FT                   /note="C->A: No effect on UCP1 activity in thermogenic
FT                   respiration."
FT                   /evidence="ECO:0000269|PubMed:27027295"
SQ   SEQUENCE   307 AA;  33248 MW;  33D107EDF04BD1AF CRC64;
     MVNPTTSEVQ PTMGVKIFSA GVSACLADII TFPLDTAKVR LQIQGEGQAS STIRYKGVLG
     TITTLAKTEG LPKLYSGLPA GIQRQISFAS LRIGLYDSVQ EYFSSGRETP ASLGNKISAG
     LMTGGVAVFI GQPTEVVKVR MQAQSHLHGI KPRYTGTYNA YRVIATTESL STLWKGTTPN
     LMRNVIINCT ELVTYDLMKG ALVNNKILAD DVPCHLLSAL VAGFCTTLLA SPVDVVKTRF
     INSLPGQYPS VPSCAMSMYT KEGPTAFFKG FVASFLRLGS WNVIMFVCFE QLKKELMKSR
     QTVDCTT
//
```

|  |
| --- |
| **Mascot:** http://www.matrixscience.com/ |

HNE (C) (+156.1150)
