# Supplementary material for: A common East-Asian ALDH2 mutation causes metabolic disorders and the therapeutic effect of ALDH2 activators
Source: Nat Commun. 2023 Sep 25;14:5971. doi: 10.1038/s41467-023-41570-6 (PMC10520061; doi:10.1038/s41467-023-41570-6)
Supplement: Supplementary file 4 — Supplementary Data 1 [file 41467_2023_41570_MOESM4_ESM.zip › Table S5b/P58281-2/P58281-2_WTO-1_H495.html]

Mascot Search Results: P58281-2
 

# MASCOT Search Results

## Protein View: P58281-2

### Isoform 2 of Dynamin-like 120 kDa protein, mitochondrial OS=Mus musculus OX=10090 GN=Opa1

|  |  |
| --- | --- |
| Database: | Mouse\_UniProt\_proteomes |
| Score: | 1470 |
| Monoisotopic mass (Mr): | 116034 |
| Calculated pI: | 7.19 |

Sequence similarity is available as an NCBI BLAST search of P58281-2 against nr.

### Search parameters

|  |  |
| --- | --- |
| MS data file: | `D:\LCMSMS\2023 Users' data\230529-1\230529-1-WTO-1.raw` |
| Enzyme: | Trypsin/P: cuts C-term side of KR. |
| Fixed modifications: | Carbamidomethyl (C) |
| Variable modifications: | Deamidated (NQ), HNE (C), HNE (H), HNE (K), Oxidation (M) |

### Protein sequence coverage: 37%

Matched peptides shown in ***bold red***.

|  |  |  |  |  |  |
| --- | --- | --- | --- | --- | --- |
| `1` | `MWRAGRAAVA` | `CEVCQSLVKH` | `SSGIQRNVPL` | `QKLHLVSRSI` | `YRSHHPALKL` |
| `51` | `QRPQLRTPFQ` | `QFSSLTHLSL` | `HKLKLSPIKY` | `GYQPRRNFWP` | `ARLAARLLKL` |
| `101` | `RYIILGSAVG` | `GGYTAKKTFD` | `EWKDMIPDLS` | `DYKWIVPDFI` | `WEIDEYIDLE` |
| `151` | `KIRKALPSSE` | `DLASLAPDLD` | `KITESLSLLK` | `DFFTAGSPGE` | `TAFRATDHGS` |
| `201` | `ESDKHYRKGL` | `LGELILLQQQ` | `IQEHEEEARR` | `AAGQYSTSYA` | `QQKRKVSDKE` |
| `251` | `KIDQLQEELL` | `HTQLKYQRIL` | `ERLEKENKEL` | `RKLVLQKDDK` | `GIHHRKLKKS` |
| `301` | `LIDMYSEVLD` | `VLSDYDASYN` | `TQDHLPRVVV` | `VGDQSAGKTS` | `VLEMIAQARI` |
| `351` | `FPRGSGEMMT` | `RSPVKVTLSE` | `GPHHVALFKD` | `SSREFDLTKE` | `EDLAALRHEI` |
| `401` | `ELRMRKNVKE` | `GCTVSPETIS` | `LNVKGPGLQR` | `MVLVDLPGVI` | `NTVTSGMAPD` |
| `451` | `TKETIFSISK` | `AYMQNPNAII` | `LCIQDGSVDA` | `ERSIVTDLVS` | `QMDPHGRRTI` |
| `501` | `FVLTKVDLAE` | `KNVASPSRIQ` | `QIIEGKLFPM` | `KALGYFAVVT` | `GKGNSSESIE` |
| `551` | `AIREYEEEFF` | `QNSKLLKTSM` | `LKAHQVTTRN` | `LSLAVSDCFW` | `KMVRESVEQQ` |
| `601` | `ADSFKATRFN` | `LETEWKNNYP` | `RLRELDRNEL` | `FEKAKNEILD` | `EVISLSQVTP` |
| `651` | `KHWEEILQQS` | `LWERVSTHVI` | `ENIYLPAAQT` | `MNSGTFNTTV` | `DIKLKQWTDK` |
| `701` | `QLPNKAVEVA` | `WETLQEEFSR` | `FMTEPKGKEH` | `DDIFDKLKEA` | `VKEESIKRHK` |
| `751` | `WNDFAEDSLR` | `VIQHNALEDR` | `SISDKQQWDA` | `AIYFMEEALQ` | `GRLKDTENAI` |
| `801` | `ENMIGPDWKK` | `RWMYWKNRTQ` | `EQCVHNETKN` | `ELEKMLKVND` | `EHPAYLASDE` |
| `851` | `ITTVRKNLES` | `RGVEVDPSLI` | `KDTWHQVYRR` | `HFLKTALNHC` | `NLCRRGFYYY` |
| `901` | `QRHFIDSELE` | `CNDVVLFWRI` | `QRMLAITANT` | `LRQQLTNTEV` | `RRLEKNVKEV` |
| `951` | `LEDFAEDGEK` | `KVKLLTGKRV` | `QLAEDLKKVR` | `EIQEKLDAFI` | `EALHQEK` |

Unformatted sequence string: 997 residues (for pasting into other applications).

|  |  |  |  |
| --- | --- | --- | --- |
| Sort by | residue number | increasing mass | decreasing mass |
| Show | matched peptides only | predicted peptides also |  |

| Query | Start | – | End | Observed | Mr(expt) | Mr(calc) | ppm | M | Score | Expect | Rank | U | Peptide |
| --- | --- | --- | --- | --- | --- | --- | --- | --- | --- | --- | --- | --- | --- |
| 53788 | 231 | – | 243 | 701.8317 | 1401.6488 | 1401.6524 | -2.56 | 0 | 53 | 1.2e-05 | 1Score **> 30** indicates **identity** Score **> 16** indicates **homology** | U | R.AAGQYSTSYAQQK.R |
| 53791 | 231 | – | 243 | 701.8340 | 1401.6535 | 1401.6524 | 0.75 | 0 | 35 | 0.00053 | 1Score **> 31** indicates **identity** Score **> 15** indicates **homology** | U | R.AAGQYSTSYAQQK.R |
| 91337 | 252 | – | 265 | 569.9803 | 1706.9191 | 1706.9203 | -0.67 | 0 | 15 | 0.036 | 1Score **> 35** indicates **identity** Score **> 13** indicates **homology** | U | K.IDQLQEELLHTQLK.Y |
| 18245 | 328 | – | 338 | 529.7961 | 1057.5777 | 1057.5768 | 0.86 | 0 | 50 | 0.00027 | 1Score **> 33** indicates **identity** Score **> 26** indicates **homology** | U | R.VVVVGDQSAGK.T |
| 34580 | 339 | – | 349 | 609.8303 | 1217.6460 | 1217.6438 | 1.85 | 0 | 39 | 0.00022 | 1Score **> 34** indicates **identity** Score **> 15** indicates **homology** | U | K.TSVLEMIAQAR.I |
| 69553 | 366 | – | 379 | 384.4652 | 1533.8318 | 1533.8304 | 0.95 | 0 | 32 | 0.0011 | 1Score **> 34** indicates **identity** Score **> 14** indicates **homology** | U | K.VTLSEGPHHVALFK.D |
| 69554 | 366 | – | 379 | 384.4654 | 1533.8326 | 1533.8304 | 1.49 | 0 | 23 | 0.007 | 1Score **> 34** indicates **identity** Score **> 14** indicates **homology** | U | K.VTLSEGPHHVALFK.D |
| 84199 | 384 | – | 397 | 550.6183 | 1648.8332 | 1648.8308 | 1.44 | 1 | 31 | 0.0011 | 1Score **> 35** indicates **identity** Score **> 14** indicates **homology** | U | R.EFDLTKEEDLAALR.H |
| 151546 | 384 | – | 403 | 607.5682 | 2426.2436 | 2426.2441 | -0.22 | 2 | 18 | 0.022 | 1Score **> 37** indicates **identity** Score **> 14** indicates **homology** | U | R.EFDLTKEEDLAALRHEIELR.M |
| 151547 | 384 | – | 403 | 486.2561 | 2426.2442 | 2426.2441 | 0.029 | 2 | 23 | 0.0086 | 1Score **> 37** indicates **identity** Score **> 15** indicates **homology** | U | R.EFDLTKEEDLAALRHEIELR.M |
| 151549 | 384 | – | 403 | 486.2562 | 2426.2448 | 2426.2441 | 0.29 | 2 | 24 | 0.0067 | 1Score **> 37** indicates **identity** Score **> 15** indicates **homology** | U | R.EFDLTKEEDLAALRHEIELR.M |
| 151550 | 384 | – | 403 | 607.5687 | 2426.2455 | 2426.2441 | 0.58 | 2 | 40 | 0.00018 | 1Score **> 37** indicates **identity** Score **> 15** indicates **homology** | U | R.EFDLTKEEDLAALRHEIELR.M |
| 151552 | 384 | – | 403 | 607.5692 | 2426.2477 | 2426.2441 | 1.50 | 2 | 27 | 0.0027 | 1Score **> 37** indicates **identity** Score **> 14** indicates **homology** | U | R.EFDLTKEEDLAALRHEIELR.M |
| 82229 | 410 | – | 424 | 817.4080 | 1632.8014 | 1632.8029 | -0.94 | 0 | 26 | 0.0034 | 1Score **> 34** indicates **identity** Score **> 14** indicates **homology** | U | K.EGCTVSPETISLNVK.G |
| 82231 | 410 | – | 424 | 817.4086 | 1632.8027 | 1632.8029 | -0.13 | 0 | 40 | 0.00019 | 1Score **> 34** indicates **identity** Score **> 15** indicates **homology** | U | K.EGCTVSPETISLNVK.G |
| 82234 | 410 | – | 424 | 817.4094 | 1632.8042 | 1632.8029 | 0.77 | 0 | 53 | 1.1e-05 | 1Score **> 34** indicates **identity** Score **> 16** indicates **homology** | U | K.EGCTVSPETISLNVK.G |
| 139983 | 410 | – | 430 | 748.0540 | 2241.1401 | 2241.1423 | -0.98 | 1 | 41 | 0.00013 | 1Score **> 37** indicates **identity** Score **> 15** indicates **homology** | U | K.EGCTVSPETISLNVKGPGLQR.M |
| 139987 | 410 | – | 430 | 748.0555 | 2241.1448 | 2241.1423 | 1.10 | 1 | 20 | 0.012 | 1Score **> 37** indicates **identity** Score **> 14** indicates **homology** | U | K.EGCTVSPETISLNVKGPGLQR.M |
| 139988 | 410 | – | 430 | 748.0569 | 2241.1490 | 2241.1423 | 2.98 | 1 | 36 | 0.00045 | 1Score **> 37** indicates **identity** Score **> 15** indicates **homology** | U | K.EGCTVSPETISLNVKGPGLQR.M |
| 102433 | 483 | – | 497 | 453.4863 | 1809.9163 | 1809.9295 | -7.31 | 0 | 17 | 0.035 | 1Score **> 35** indicates **identity** Score **> 15** indicates **homology** | U | R.SIVTDLVSQMDPHGR.R  + HNE (H) |
| 52125 | 506 | – | 518 | 462.5844 | 1384.7315 | 1384.7310 | 0.32 | 1 | 57 | 6.8e-06 | 1Score **> 34** indicates **identity** Score **> 18** indicates **homology** | U | K.VDLAEKNVASPSR.I |
| 52126 | 506 | – | 518 | 693.3735 | 1384.7324 | 1384.7310 | 1.01 | 1 | 45 | 5.8e-05 | 1Score **> 34** indicates **identity** Score **> 15** indicates **homology** | U | K.VDLAEKNVASPSR.I |
| 52127 | 506 | – | 518 | 693.3735 | 1384.7325 | 1384.7310 | 1.06 | 1 | 60 | 2.4e-06 | 1Score **> 34** indicates **identity** Score **> 16** indicates **homology** | U | K.VDLAEKNVASPSR.I |
| 9797 | 519 | – | 526 | 464.7773 | 927.5401 | 927.5389 | 1.24 | 0 | 31 | 0.0034 | 1Score **> 28** indicates **identity** Score **> 18** indicates **homology** | U | R.IQQIIEGK.L |
| 9798 | 519 | – | 526 | 464.7773 | 927.5401 | 927.5389 | 1.24 | 0 | 32 | 0.0019 | 1Score **> 28** indicates **identity** Score **> 18** indicates **homology** | U | R.IQQIIEGK.L |
| 24516 | 532 | – | 542 | 563.3186 | 1124.6226 | 1124.6230 | -0.35 | 0 | 24 | 0.0059 | 1Score **> 31** indicates **identity** Score **> 14** indicates **homology** | U | K.ALGYFAVVTGK.G |
| 24522 | 532 | – | 542 | 563.3199 | 1124.6253 | 1124.6230 | 2.06 | 0 | 21 | 0.01 | 1Score **> 31** indicates **identity** Score **> 14** indicates **homology** | U | K.ALGYFAVVTGK.G |
| 24524 | 532 | – | 542 | 563.3210 | 1124.6274 | 1124.6230 | 3.89 | 0 | 41 | 0.00016 | 1Score **> 31** indicates **identity** Score **> 15** indicates **homology** | U | K.ALGYFAVVTGK.G |
| 159449 | 543 | – | 564 | 865.3940 | 2593.1601 | 2593.1456 | 5.61 | 1 | 16 | 0.03 | 1Score **> 32** indicates **identity** Score **> 14** indicates **homology** | U | K.GNSSESIEAIREYEEEFFQNSK.L  + Deamidated (NQ) |
| 57937 | 580 | – | 591 | 720.3560 | 1438.6974 | 1438.6915 | 4.13 | 0 | 63 | 1.2e-06 | 1Score **> 33** indicates **identity** Score **> 16** indicates **homology** | U | R.NLSLAVSDCFWK.M |
| 57940 | 580 | – | 591 | 720.3575 | 1438.7004 | 1438.6915 | 6.19 | 0 | 62 | 1.5e-06 | 1Score **> 33** indicates **identity** Score **> 16** indicates **homology** | U | R.NLSLAVSDCFWK.M |
| 39974 | 595 | – | 605 | 634.2925 | 1266.5704 | 1266.5728 | -1.91 | 0 | 75 | 8.7e-08 | 1Score **> 29** indicates **identity** Score **> 17** indicates **homology** | U | R.ESVEQQADSFK.A |
| 39975 | 595 | – | 605 | 634.2928 | 1266.5710 | 1266.5728 | -1.41 | 0 | 33 | 0.00083 | 1Score **> 29** indicates **identity** Score **> 15** indicates **homology** | U | R.ESVEQQADSFK.A |
| 39976 | 595 | – | 605 | 634.2938 | 1266.5731 | 1266.5728 | 0.21 | 0 | 70 | 2.6e-07 | 1Score **> 29** indicates **identity** Score **> 17** indicates **homology** | U | R.ESVEQQADSFK.A |
| 99805 | 636 | – | 651 | 595.6596 | 1783.9571 | 1783.9567 | 0.20 | 0 | 36 | 0.00041 | 1Score **> 35** indicates **identity** Score **> 15** indicates **homology** | U | K.NEILDEVISLSQVTPK.H |
| 99808 | 636 | – | 651 | 892.9864 | 1783.9583 | 1783.9567 | 0.89 | 0 | 88 | 5.1e-09 | 1Score **> 35** indicates **identity** Score **> 18** indicates **homology** | U | K.NEILDEVISLSQVTPK.H |
| 99810 | 636 | – | 651 | 892.9886 | 1783.9626 | 1783.9567 | 3.28 | 0 | 57 | 4.9e-06 | 1Score **> 35** indicates **identity** Score **> 16** indicates **homology** | U | K.NEILDEVISLSQVTPK.H |
| 100590 | 706 | – | 720 | 598.6277 | 1792.8613 | 1792.8632 | -1.03 | 0 | 32 | 0.001 | 1Score **> 34** indicates **identity** Score **> 14** indicates **homology** | U | K.AVEVAWETLQEEFSR.F |
| 100593 | 706 | – | 720 | 897.4384 | 1792.8622 | 1792.8632 | -0.52 | 0 | 55 | 7.1e-06 | 1Score **> 34** indicates **identity** Score **> 16** indicates **homology** | U | K.AVEVAWETLQEEFSR.F |
| 100594 | 706 | – | 720 | 897.4389 | 1792.8633 | 1792.8632 | 0.092 | 0 | 54 | 8.6e-06 | 1Score **> 34** indicates **identity** Score **> 16** indicates **homology** | U | K.AVEVAWETLQEEFSR.F |
| 31404 | 739 | – | 748 | 396.8910 | 1187.6512 | 1187.6510 | 0.20 | 2 | 32 | 0.0014 | 1Score **> 34** indicates **identity** Score **> 16** indicates **homology** | U | K.EAVKEESIKR.H |
| 67325 | 749 | – | 760 | 506.5748 | 1516.7026 | 1516.7059 | -2.18 | 1 | 24 | 0.0056 | 1Score **> 30** indicates **identity** Score **> 14** indicates **homology** | U | R.HKWNDFAEDSLR.V |
| 32084 | 761 | – | 770 | 597.8146 | 1193.6146 | 1193.6153 | -0.54 | 0 | 43 | 0.0001 | 1Score **> 33** indicates **identity** Score **> 15** indicates **homology** | U | R.VIQHNALEDR.S |
| 32085 | 761 | – | 770 | 398.8789 | 1193.6149 | 1193.6153 | -0.29 | 0 | 35 | 0.00076 | 1Score **> 33** indicates **identity** Score **> 17** indicates **homology** | U | R.VIQHNALEDR.S |
| 32088 | 761 | – | 770 | 597.8153 | 1193.6160 | 1193.6153 | 0.60 | 0 | 44 | 0.00012 | 1Score **> 32** indicates **identity** Score **> 18** indicates **homology** | U | R.VIQHNALEDR.S |
| 32091 | 761 | – | 770 | 398.8797 | 1193.6173 | 1193.6153 | 1.68 | 0 | 24 | 0.0062 | 1Score **> 33** indicates **identity** Score **> 14** indicates **homology** | U | R.VIQHNALEDR.S |
| 140844 | 817 | – | 834 | 565.0207 | 2256.0536 | 2256.0553 | -0.76 | 2 | 55 | 7e-06 | 1Score **> 34** indicates **identity** Score **> 16** indicates **homology** | U | K.NRTQEQCVHNETKNELEK.M |
| 118508 | 819 | – | 834 | 662.9776 | 1985.9109 | 1985.9112 | -0.18 | 1 | 14 | 0.045 | 1Score **> 33** indicates **identity** Score **> 13** indicates **homology** | U | R.TQEQCVHNETKNELEK.M |
| 118509 | 819 | – | 834 | 662.9780 | 1985.9123 | 1985.9112 | 0.54 | 1 | 18 | 0.019 | 1Score **> 33** indicates **identity** Score **> 14** indicates **homology** | U | R.TQEQCVHNETKNELEK.M |
| 118510 | 819 | – | 834 | 497.4856 | 1985.9134 | 1985.9112 | 1.07 | 1 | 14 | 0.048 | 1Score **> 33** indicates **identity** Score **> 13** indicates **homology** | U | R.TQEQCVHNETKNELEK.M |
| 122181 | 838 | – | 855 | 677.3336 | 2028.9791 | 2028.9752 | 1.90 | 0 | 21 | 0.012 | 1Score **> 35** indicates **identity** Score **> 14** indicates **homology** | U | K.VNDEHPAYLASDEITTVR.K |
| 122182 | 838 | – | 855 | 677.3337 | 2028.9794 | 2028.9752 | 2.05 | 0 | 55 | 6.9e-06 | 1Score **> 35** indicates **identity** Score **> 16** indicates **homology** | U | K.VNDEHPAYLASDEITTVR.K |
| 122184 | 838 | – | 855 | 677.3345 | 2028.9816 | 2028.9752 | 3.14 | 0 | 14 | 0.044 | 1Score **> 35** indicates **identity** Score **> 13** indicates **homology** | U | K.VNDEHPAYLASDEITTVR.K |
| 122258 | 838 | – | 855 | 677.6663 | 2029.9771 | 2029.9592 | 8.78 | 0 | 30 | 0.0015 | 1Score **> 35** indicates **identity** Score **> 14** indicates **homology** | U | K.VNDEHPAYLASDEITTVR.K  + Deamidated (NQ) |
| 132023 | 862 | – | 879 | 536.2815 | 2141.0969 | 2141.0906 | 2.96 | 1 | 20 | 0.013 | 1Score **> 36** indicates **identity** Score **> 14** indicates **homology** | U | R.GVEVDPSLIKDTWHQVYR.R |
| 38983 | 885 | – | 894 | 420.1964 | 1257.5674 | 1257.5706 | -2.59 | 0 | 19 | 0.015 | 1Score **> 28** indicates **identity** Score **> 14** indicates **homology** | U | K.TALNHCNLCR.R |
| 38984 | 885 | – | 894 | 629.7910 | 1257.5674 | 1257.5706 | -2.56 | 0 | 33 | 0.00083 | 1Score **> 28** indicates **identity** Score **> 15** indicates **homology** | U | K.TALNHCNLCR.R |
| 38985 | 885 | – | 894 | 420.1977 | 1257.5711 | 1257.5706 | 0.40 | 0 | 23 | 0.0073 | 1Score **> 29** indicates **identity** Score **> 14** indicates **homology** | U | K.TALNHCNLCR.R |
| 20951 | 933 | – | 941 | 544.7859 | 1087.5573 | 1087.5622 | -4.45 | 0 | 33 | 0.005 | 1Score **> 33** indicates **identity** Score **> 22** indicates **homology** | U | R.QQLTNTEVR.R |
| 20952 | 933 | – | 941 | 544.7866 | 1087.5587 | 1087.5622 | -3.24 | 0 | 61 | 1.8e-05 | 1Score **> 33** indicates **identity** Score **> 26** indicates **homology** | U | R.QQLTNTEVR.R |
| 106222 | 946 | – | 961 | 617.3110 | 1848.9112 | 1848.9105 | 0.41 | 2 | 15 | 0.043 | 1Score **> 35** indicates **identity** Score **> 13** indicates **homology** | U | K.NVKEVLEDFAEDGEKK.V |
| 51604 | 949 | – | 960 | 690.8120 | 1379.6095 | 1379.6092 | 0.22 | 0 | 53 | 9.6e-06 | 1Score **> 28** indicates **identity** Score **> 16** indicates **homology** | U | K.EVLEDFAEDGEK.K |
| 51605 | 949 | – | 960 | 690.8125 | 1379.6104 | 1379.6092 | 0.84 | 0 | 33 | 0.00089 | 1Score **> 29** indicates **identity** Score **> 15** indicates **homology** | U | K.EVLEDFAEDGEK.K |
| 32694 | 969 | – | 978 | 400.5749 | 1198.7028 | 1198.7033 | -0.41 | 2 | 22 | 0.0079 | 1Score **> 32** indicates **identity** Score **> 14** indicates **homology** | U | K.RVQLAEDLKK.V |
| 123207 | 981 | – | 997 | 511.0202 | 2040.0517 | 2040.0527 | -0.52 | 1 | 25 | 0.0045 | 1Score **> 36** indicates **identity** Score **> 14** indicates **homology** | U | R.EIQEKLDAFIEALHQEK.- |
| 123208 | 981 | – | 997 | 681.0247 | 2040.0522 | 2040.0527 | -0.25 | 1 | 48 | 3.5e-05 | 1Score **> 36** indicates **identity** Score **> 15** indicates **homology** | U | R.EIQEKLDAFIEALHQEK.- |
| 123209 | 981 | – | 997 | 511.0205 | 2040.0528 | 2040.0527 | 0.042 | 1 | 28 | 0.0024 | 1Score **> 36** indicates **identity** Score **> 14** indicates **homology** | U | R.EIQEKLDAFIEALHQEK.- |
| 123212 | 981 | – | 997 | 511.0214 | 2040.0566 | 2040.0527 | 1.88 | 1 | 27 | 0.0031 | 1Score **> 36** indicates **identity** Score **> 14** indicates **homology** | U | R.EIQEKLDAFIEALHQEK.- |
| 55150 | 986 | – | 997 | 471.9164 | 1412.7274 | 1412.7300 | -1.80 | 0 | 36 | 0.00041 | 1Score **> 33** indicates **identity** Score **> 15** indicates **homology** | U | K.LDAFIEALHQEK.- |
| 55151 | 986 | – | 997 | 471.9173 | 1412.7300 | 1412.7300 | 0.050 | 0 | 39 | 0.00023 | 1Score **> 33** indicates **identity** Score **> 15** indicates **homology** | U | K.LDAFIEALHQEK.- |

---

```
ID   OPA1_MOUSE              Reviewed;         997 AA.
AC   P58281-2;
DT   26-SEP-2001, integrated into UniProtKB/Swiss-Prot.
DT   26-SEP-2001, sequence version 1.
DT   28-JUN-2023, entry version 174.
DE   RecName: Full=Isoform 2 of Dynamin-like 120 kDa protein, mitochondrial;
DE            EC=3.6.5.5;
DE   AltName: Full=Large GTP-binding protein;
DE            Short=LargeG;
DE   AltName: Full=Optic atrophy protein 1 homolog;
DE   Contains:
DE     RecName: Full=Dynamin-like 120 kDa protein, form S1;
DE   Flags: Precursor;
GN   Name=Opa1 {ECO:0000303|PubMed:28746876, ECO:0000312|MGI:MGI:1921393};
OS   Mus musculus (Mouse).
OC   Eukaryota; Metazoa; Chordata; Craniata; Vertebrata; Euteleostomi; Mammalia;
OC   Eutheria; Euarchontoglires; Glires; Rodentia; Myomorpha; Muroidea; Muridae;
OC   Murinae; Mus; Mus.
OX   NCBI_TaxID=10090;
RN   [1]
RP   NUCLEOTIDE SEQUENCE [MRNA] (ISOFORM 1), FUNCTION, SUBCELLULAR LOCATION, AND
RP   TISSUE SPECIFICITY.
RC   TISSUE=Brain;
RX   PubMed=11847212; DOI=10.1074/jbc.m109260200;
RA   Misaka T., Miyashita T., Kubo Y.;
RT   "Primary structure of a dynamin-related mouse mitochondrial GTPase and its
RT   distribution in brain, subcellular localization, and effect on
RT   mitochondrial morphology.";
RL   J. Biol. Chem. 277:15834-15842(2002).
RN   [2]
RP   NUCLEOTIDE SEQUENCE [LARGE SCALE MRNA] (ISOFORM 1), AND NUCLEOTIDE SEQUENCE
RP   [LARGE SCALE MRNA] OF 1-365 (ISOFORM 2).
RC   STRAIN=C57BL/6J;
RC   TISSUE=Blastocyst, Hypothalamus, Liver, Retina, and Skin;
RX   PubMed=16141072; DOI=10.1126/science.1112014;
RA   Carninci P., Kasukawa T., Katayama S., Gough J., Frith M.C., Maeda N.,
RA   Oyama R., Ravasi T., Lenhard B., Wells C., Kodzius R., Shimokawa K.,
RA   Bajic V.B., Brenner S.E., Batalov S., Forrest A.R., Zavolan M., Davis M.J.,
RA   Wilming L.G., Aidinis V., Allen J.E., Ambesi-Impiombato A., Apweiler R.,
RA   Aturaliya R.N., Bailey T.L., Bansal M., Baxter L., Beisel K.W., Bersano T.,
RA   Bono H., Chalk A.M., Chiu K.P., Choudhary V., Christoffels A.,
RA   Clutterbuck D.R., Crowe M.L., Dalla E., Dalrymple B.P., de Bono B.,
RA   Della Gatta G., di Bernardo D., Down T., Engstrom P., Fagiolini M.,
RA   Faulkner G., Fletcher C.F., Fukushima T., Furuno M., Futaki S.,
RA   Gariboldi M., Georgii-Hemming P., Gingeras T.R., Gojobori T., Green R.E.,
RA   Gustincich S., Harbers M., Hayashi Y., Hensch T.K., Hirokawa N., Hill D.,
RA   Huminiecki L., Iacono M., Ikeo K., Iwama A., Ishikawa T., Jakt M.,
RA   Kanapin A., Katoh M., Kawasawa Y., Kelso J., Kitamura H., Kitano H.,
RA   Kollias G., Krishnan S.P., Kruger A., Kummerfeld S.K., Kurochkin I.V.,
RA   Lareau L.F., Lazarevic D., Lipovich L., Liu J., Liuni S., McWilliam S.,
RA   Madan Babu M., Madera M., Marchionni L., Matsuda H., Matsuzawa S., Miki H.,
RA   Mignone F., Miyake S., Morris K., Mottagui-Tabar S., Mulder N., Nakano N.,
RA   Nakauchi H., Ng P., Nilsson R., Nishiguchi S., Nishikawa S., Nori F.,
RA   Ohara O., Okazaki Y., Orlando V., Pang K.C., Pavan W.J., Pavesi G.,
RA   Pesole G., Petrovsky N., Piazza S., Reed J., Reid J.F., Ring B.Z.,
RA   Ringwald M., Rost B., Ruan Y., Salzberg S.L., Sandelin A., Schneider C.,
RA   Schoenbach C., Sekiguchi K., Semple C.A., Seno S., Sessa L., Sheng Y.,
RA   Shibata Y., Shimada H., Shimada K., Silva D., Sinclair B., Sperling S.,
RA   Stupka E., Sugiura K., Sultana R., Takenaka Y., Taki K., Tammoja K.,
RA   Tan S.L., Tang S., Taylor M.S., Tegner J., Teichmann S.A., Ueda H.R.,
RA   van Nimwegen E., Verardo R., Wei C.L., Yagi K., Yamanishi H.,
RA   Zabarovsky E., Zhu S., Zimmer A., Hide W., Bult C., Grimmond S.M.,
RA   Teasdale R.D., Liu E.T., Brusic V., Quackenbush J., Wahlestedt C.,
RA   Mattick J.S., Hume D.A., Kai C., Sasaki D., Tomaru Y., Fukuda S.,
RA   Kanamori-Katayama M., Suzuki M., Aoki J., Arakawa T., Iida J., Imamura K.,
RA   Itoh M., Kato T., Kawaji H., Kawagashira N., Kawashima T., Kojima M.,
RA   Kondo S., Konno H., Nakano K., Ninomiya N., Nishio T., Okada M., Plessy C.,
RA   Shibata K., Shiraki T., Suzuki S., Tagami M., Waki K., Watahiki A.,
RA   Okamura-Oho Y., Suzuki H., Kawai J., Hayashizaki Y.;
RT   "The transcriptional landscape of the mammalian genome.";
RL   Science 309:1559-1563(2005).
RN   [3]
RP   NUCLEOTIDE SEQUENCE [LARGE SCALE MRNA] (ISOFORM 1).
RC   STRAIN=Czech II; TISSUE=Brain, and Mammary gland;
RX   PubMed=15489334; DOI=10.1101/gr.2596504;
RG   The MGC Project Team;
RT   "The status, quality, and expansion of the NIH full-length cDNA project:
RT   the Mammalian Gene Collection (MGC).";
RL   Genome Res. 14:2121-2127(2004).
RN   [4]
RP   FUNCTION, SUBCELLULAR LOCATION, AND INTERACTION WITH PARL.
RX   PubMed=16839884; DOI=10.1016/j.cell.2006.06.021;
RA   Cipolat S., Rudka T., Hartmann D., Costa V., Serneels L., Craessaerts K.,
RA   Metzger K., Frezza C., Annaert W., D'Adamio L., Derks C., Dejaegere T.,
RA   Pellegrini L., D'Hooge R., Scorrano L., De Strooper B.;
RT   "Mitochondrial rhomboid PARL regulates cytochrome c release during
RT   apoptosis via OPA1-dependent cristae remodeling.";
RL   Cell 126:163-175(2006).
RN   [5]
RP   FUNCTION, AND SUBUNIT.
RX   PubMed=16839885; DOI=10.1016/j.cell.2006.06.025;
RA   Frezza C., Cipolat S., Martins de Brito O., Micaroni M., Beznoussenko G.V.,
RA   Rudka T., Bartoli D., Polishuck R.S., Danial N.N., De Strooper B.,
RA   Scorrano L.;
RT   "OPA1 controls apoptotic cristae remodeling independently from
RT   mitochondrial fusion.";
RL   Cell 126:177-189(2006).
RN   [6]
RP   FUNCTION (DYNAMIN-LIKE 120 KDA PROTEIN; FORM S1), AND PROTEOLYTIC
RP   PROCESSING.
RX   PubMed=20038678; DOI=10.1083/jcb.200906084;
RA   Ehses S., Raschke I., Mancuso G., Bernacchia A., Geimer S., Tondera D.,
RA   Martinou J.C., Westermann B., Rugarli E.I., Langer T.;
RT   "Regulation of OPA1 processing and mitochondrial fusion by m-AAA protease
RT   isoenzymes and OMA1.";
RL   J. Cell Biol. 187:1023-1036(2009).
RN   [7]
RP   IDENTIFICATION BY MASS SPECTROMETRY [LARGE SCALE ANALYSIS].
RC   TISSUE=Brain, Brown adipose tissue, Heart, Kidney, Liver, Lung,
RC   Pancreas, Spleen, and Testis;
RX   PubMed=21183079; DOI=10.1016/j.cell.2010.12.001;
RA   Huttlin E.L., Jedrychowski M.P., Elias J.E., Goswami T., Rad R.,
RA   Beausoleil S.A., Villen J., Haas W., Sowa M.E., Gygi S.P.;
RT   "A tissue-specific atlas of mouse protein phosphorylation and expression.";
RL   Cell 143:1174-1189(2010).
RN   [8]
RP   INTERACTION WITH CHCHD3 AND IMMT, AND SUBCELLULAR LOCATION.
RX   PubMed=21081504; DOI=10.1074/jbc.m110.171975;
RA   Darshi M., Mendiola V.L., Mackey M.R., Murphy A.N., Koller A.,
RA   Perkins G.A., Ellisman M.H., Taylor S.S.;
RT   "ChChd3, an inner mitochondrial membrane protein, is essential for
RT   maintaining crista integrity and mitochondrial function.";
RL   J. Biol. Chem. 286:2918-2932(2011).
RN   [9]
RP   FUNCTION (DYNAMIN-LIKE 120 KDA PROTEIN; FORM S1), AND PROTEOLYTIC
RP   PROCESSING.
RX   PubMed=22433842; DOI=10.1038/emboj.2012.70;
RA   Quiros P.M., Ramsay A.J., Sala D., Fernandez-Vizarra E., Rodriguez F.,
RA   Peinado J.R., Fernandez-Garcia M.S., Vega J.A., Enriquez J.A., Zorzano A.,
RA   Lopez-Otin C.;
RT   "Loss of mitochondrial protease OMA1 alters processing of the GTPase OPA1
RT   and causes obesity and defective thermogenesis in mice.";
RL   EMBO J. 31:2117-2133(2012).
RN   [10]
RP   PROTEOLYTIC PROCESSING.
RX   PubMed=24550258; DOI=10.1002/embj.201386474;
RA   Baker M.J., Lampe P.A., Stojanovski D., Korwitz A., Anand R., Tatsuta T.,
RA   Langer T.;
RT   "Stress-induced OMA1 activation and autocatalytic turnover regulate OPA1-
RT   dependent mitochondrial dynamics.";
RL   EMBO J. 33:578-593(2014).
RN   [11]
RP   FUNCTION, AND PROTEOLYTIC CLEAVAGE.
RX   PubMed=24616225; DOI=10.1083/jcb.201308006;
RA   Anand R., Wai T., Baker M.J., Kladt N., Schauss A.C., Rugarli E.,
RA   Langer T.;
RT   "The i-AAA protease YME1L and OMA1 cleave OPA1 to balance mitochondrial
RT   fusion and fission.";
RL   J. Cell Biol. 204:919-929(2014).
RN   [12]
RP   FUNCTION, AND PROTEOLYTIC CLEAVAGE.
RX   PubMed=26785494; DOI=10.1126/science.aad0116;
RA   Wai T., Garcia-Prieto J., Baker M.J., Merkwirth C., Benit P., Rustin P.,
RA   Ruperez F.J., Barbas C., Ibanez B., Langer T.;
RT   "Imbalanced OPA1 processing and mitochondrial fragmentation cause heart
RT   failure in mice.";
RL   Science 350:116-116(2015).
RN   [13]
RP   SUBUNIT, FUNCTION, AND SUBCELLULAR LOCATION.
RX   PubMed=28746876; DOI=10.1016/j.celrep.2017.06.090;
RA   Huang G., Massoudi D., Muir A.M., Joshi D.C., Zhang C.L., Chiu S.Y.,
RA   Greenspan D.S.;
RT   "WBSCR16 Is a Guanine Nucleotide Exchange Factor Important for
RT   Mitochondrial Fusion.";
RL   Cell Rep. 20:923-934(2017).
CC   -!- ALTERNATIVE PRODUCTS:
CC       Event=Alternative splicing; Named isoforms=2;
CC       Name=1;
CC         IsoId=P58281-1; Sequence=Displayed;
CC       Name=2;
CC         IsoId=P58281-2; Sequence=VSP_021037;
CC   -!- PTM: Cleavage at position S2 is mediated by YME1L (PubMed:24616225,
CC       PubMed:26785494). Cleavage may occur in the sequence motif Leu-Gln-Gln-
CC       Gln-Ile-Gln (LQQQIQ) (By similarity). {ECO:0000250|UniProtKB:Q2TA68,
CC       ECO:0000269|PubMed:24616225, ECO:0000269|PubMed:26785494}.
CC   ---------------------------------------------------------------------------
CC   Copyrighted by the UniProt Consortium, see https://www.uniprot.org/terms
CC   Distributed under the Creative Commons Attribution (CC BY 4.0) License
CC   ---------------------------------------------------------------------------
DR   ProteomicsDB; 294078; -.
DR   Ensembl; ENSMUST00000161186; ENSMUSP00000123880; ENSMUSG00000038084.
DR   Proteomes; UP000000589; Chromosome 16.
PE   1: Evidence at protein level;
FT   MOTIF           217..222
FT                   /note="LQQQIQ motif"
FT                   /evidence="ECO:0000250|UniProtKB:Q2TA68"
SQ   SEQUENCE   997 AA;  115593 MW;  FADCB1430E585522 CRC64;
     MWRAGRAAVA CEVCQSLVKH SSGIQRNVPL QKLHLVSRSI YRSHHPALKL QRPQLRTPFQ
     QFSSLTHLSL HKLKLSPIKY GYQPRRNFWP ARLAARLLKL RYIILGSAVG GGYTAKKTFD
     EWKDMIPDLS DYKWIVPDFI WEIDEYIDLE KIRKALPSSE DLASLAPDLD KITESLSLLK
     DFFTAGSPGE TAFRATDHGS ESDKHYRKGL LGELILLQQQ IQEHEEEARR AAGQYSTSYA
     QQKRKVSDKE KIDQLQEELL HTQLKYQRIL ERLEKENKEL RKLVLQKDDK GIHHRKLKKS
     LIDMYSEVLD VLSDYDASYN TQDHLPRVVV VGDQSAGKTS VLEMIAQARI FPRGSGEMMT
     RSPVKVTLSE GPHHVALFKD SSREFDLTKE EDLAALRHEI ELRMRKNVKE GCTVSPETIS
     LNVKGPGLQR MVLVDLPGVI NTVTSGMAPD TKETIFSISK AYMQNPNAII LCIQDGSVDA
     ERSIVTDLVS QMDPHGRRTI FVLTKVDLAE KNVASPSRIQ QIIEGKLFPM KALGYFAVVT
     GKGNSSESIE AIREYEEEFF QNSKLLKTSM LKAHQVTTRN LSLAVSDCFW KMVRESVEQQ
     ADSFKATRFN LETEWKNNYP RLRELDRNEL FEKAKNEILD EVISLSQVTP KHWEEILQQS
     LWERVSTHVI ENIYLPAAQT MNSGTFNTTV DIKLKQWTDK QLPNKAVEVA WETLQEEFSR
     FMTEPKGKEH DDIFDKLKEA VKEESIKRHK WNDFAEDSLR VIQHNALEDR SISDKQQWDA
     AIYFMEEALQ GRLKDTENAI ENMIGPDWKK RWMYWKNRTQ EQCVHNETKN ELEKMLKVND
     EHPAYLASDE ITTVRKNLES RGVEVDPSLI KDTWHQVYRR HFLKTALNHC NLCRRGFYYY
     QRHFIDSELE CNDVVLFWRI QRMLAITANT LRQQLTNTEV RRLEKNVKEV LEDFAEDGEK
     KVKLLTGKRV QLAEDLKKVR EIQEKLDAFI EALHQEK
//
```

|  |
| --- |
| **Mascot:** http://www.matrixscience.com/ |

HNE (H) (+156.1150)
