# Supplementary material for: A common East-Asian ALDH2 mutation causes metabolic disorders and the therapeutic effect of ALDH2 activators
Source: Nat Commun. 2023 Sep 25;14:5971. doi: 10.1038/s41467-023-41570-6 (PMC10520061; doi:10.1038/s41467-023-41570-6)
Supplement: Supplementary file 4 — Supplementary Data 1 [file 41467_2023_41570_MOESM4_ESM.zip › Table S5b/Q64521/Q64521_WTO-1_C309_H633_K634.html]

Mascot Search Results: Q64521
 

# MASCOT Search Results

## Protein View: Q64521

### Glycerol-3-phosphate dehydrogenase, mitochondrial OS=Mus musculus OX=10090 GN=Gpd2 PE=1 SV=2

|  |  |
| --- | --- |
| Database: | Mouse\_UniProt\_proteomes |
| Score: | 20156 |
| Monoisotopic mass (Mr): | 81416 |
| Calculated pI: | 6.17 |

Sequence similarity is available as an NCBI BLAST search of Q64521 against nr.

### Search parameters

|  |  |
| --- | --- |
| MS data file: | `D:\LCMSMS\2023 Users' data\230529-1\230529-1-WTO-1.raw` |
| Enzyme: | Trypsin/P: cuts C-term side of KR. |
| Fixed modifications: | Carbamidomethyl (C) |
| Variable modifications: | Deamidated (NQ), HNE (C), HNE (H), HNE (K), Oxidation (M) |

### Protein sequence coverage: 85%

Matched peptides shown in ***bold red***.

|  |  |  |  |  |  |
| --- | --- | --- | --- | --- | --- |
| `1` | `MAFQKAVKGT` | `ILVGGGALAT` | `VLGLSPFAHY` | `RRKQVSLAYV` | `EAAGYLTEPV` |
| `51` | `NREPPSREAQ` | `LMTLKNTPEF` | `DILVIGGGAT` | `GCGCALDAVT` | `RGLKTALVER` |
| `101` | `DDFSSGTSSR` | `STKLIHGGVR` | `YLQKAIMNLD` | `VEQYRMVKEA` | `LHERANLLEI` |
| `151` | `APHLSAPLPI` | `MLPLYKWWQL` | `PYYWVGIKMY` | `DLVAGSQCLK` | `SSYVLSKSRA` |
| `201` | `LEHFPMLQKD` | `KLVGAIVYYD` | `GQHNDARMNL` | `AIALTAARYG` | `AATANYMEVV` |
| `251` | `SLLKKTDPET` | `GKERVSGARC` | `KDVLTGQEFD` | `VRAKCVINAS` | `GPFTDSVRKM` |
| `301` | `DDKNVVPICQ` | `PSAGVHIVMP` | `GYYSPENMGL` | `LDPATSDGRV` | `IFFLPWEKMT` |
| `351` | `IAGTTDTPTD` | `VTHHPIPSEE` | `DINFILNEVR` | `NYLSSDVEVR` | `RGDVLAAWSG` |
| `401` | `IRPLVTDPKS` | `ADTQSISRNH` | `VVDISDSGLI` | `TIAGGKWTTY` | `RSMAEDTVDA` |
| `451` | `AVKFHNLNAG` | `PSRTVGLFLQ` | `GGKDWSPTLY` | `IRLVQDYGLE` | `SEVAQHLAKT` |
| `501` | `YGDKAFEVAK` | `MASVTGKRWP` | `VVGVRLVSEF` | `PYIEAEVKYG` | `IKEYACTAVD` |
| `551` | `MISRRTRLAF` | `LNVQAAEEAL` | `PRIVELMGRE` | `LNWSELRKQE` | `ELETATRFLY` |
| `601` | `YEMGYKSRTE` | `QLTDSTEISL` | `LPSDIDRYKK` | `RFHKFDEDEK` | `GFITIVDVQR` |
| `651` | `VLESINVQMD` | `ENTLHEILCE` | `VDLNKNGQVE` | `LHEFLQLMSA` | `VQKGRVSGSR` |
| `701` | `LAILMKTAEE` | `NLDRRVPIPV` | `DRSCGGL` |  |  |

Unformatted sequence string: 727 residues (for pasting into other applications).

|  |  |  |  |
| --- | --- | --- | --- |
| Sort by | residue number | increasing mass | decreasing mass |
| Show | matched peptides only | predicted peptides also |  |

| Query | Start | – | End | Observed | Mr(expt) | Mr(calc) | ppm | M | Score | Expect | Rank | U | Peptide |
| --- | --- | --- | --- | --- | --- | --- | --- | --- | --- | --- | --- | --- | --- |
| 141832 | 9 | – | 31 | 757.4265 | 2269.2575 | 2269.2583 | -0.34 | 0 | 37 | 0.00033 | 1Score **> 35** indicates **identity** Score **> 15** indicates **homology** | U | K.GTILVGGGALATVLGLSPFAHYR.R |
| 10077 | 58 | – | 65 | 467.2581 | 932.5016 | 932.5001 | 1.58 | 0 | 36 | 0.00087 | 1Score **> 32** indicates **identity** Score **> 18** indicates **homology** | U | R.EAQLMTLK.N |
| 10078 | 58 | – | 65 | 467.2581 | 932.5017 | 932.5001 | 1.77 | 0 | 37 | 0.00066 | 1Score **> 32** indicates **identity** Score **> 18** indicates **homology** | U | R.EAQLMTLK.N |
| 10079 | 58 | – | 65 | 467.2583 | 932.5020 | 932.5001 | 2.10 | 0 | 37 | 0.00073 | 1Score **> 32** indicates **identity** Score **> 18** indicates **homology** | U | R.EAQLMTLK.N |
| 11063 | 58 | – | 65 | 475.2539 | 948.4932 | 948.4950 | -1.85 | 0 | 19 | 0.017 | 1Score **> 31** indicates **identity** Score **> 14** indicates **homology** | U | R.EAQLMTLK.N  + Oxidation (M) |
| 11069 | 58 | – | 65 | 475.2545 | 948.4945 | 948.4950 | -0.51 | 0 | 20 | 0.013 | 1Score **> 31** indicates **identity** Score **> 14** indicates **homology** | U | R.EAQLMTLK.N  + Oxidation (M) |
| 11070 | 58 | – | 65 | 475.2547 | 948.4949 | 948.4950 | -0.11 | 0 | 29 | 0.002 | 1Score **> 31** indicates **identity** Score **> 15** indicates **homology** | U | R.EAQLMTLK.N  + Oxidation (M) |
| 11074 | 58 | – | 65 | 475.2549 | 948.4952 | 948.4950 | 0.20 | 0 | 17 | 0.025 | 1Score **> 31** indicates **identity** Score **> 14** indicates **homology** | U | R.EAQLMTLK.N  + Oxidation (M) |
| 11075 | 58 | – | 65 | 475.2549 | 948.4952 | 948.4950 | 0.23 | 0 | 23 | 0.0065 | 1Score **> 31** indicates **identity** Score **> 14** indicates **homology** | U | R.EAQLMTLK.N  + Oxidation (M) |
| 11076 | 58 | – | 65 | 475.2550 | 948.4955 | 948.4950 | 0.49 | 0 | 17 | 0.027 | 1Score **> 31** indicates **identity** Score **> 14** indicates **homology** | U | R.EAQLMTLK.N  + Oxidation (M) |
| 162115 | 66 | – | 91 | 888.7561 | 2663.2464 | 2663.2684 | -8.25 | 0 | 42 | 0.0001 | 1Score **> 35** indicates **identity** Score **> 15** indicates **homology** | U | K.NTPEFDILVIGGGATGCGCALDAVTR.G |
| 162116 | 66 | – | 91 | 1332.6346 | 2663.2547 | 2663.2684 | -5.14 | 0 | 92 | 2.5e-09 | 1Score **> 35** indicates **identity** Score **> 18** indicates **homology** | U | K.NTPEFDILVIGGGATGCGCALDAVTR.G |
| 162117 | 66 | – | 91 | 888.7608 | 2663.2607 | 2663.2684 | -2.89 | 0 | 82 | 2.1e-08 | 1Score **> 36** indicates **identity** Score **> 18** indicates **homology** | U | K.NTPEFDILVIGGGATGCGCALDAVTR.G |
| 162118 | 66 | – | 91 | 888.7609 | 2663.2608 | 2663.2684 | -2.86 | 0 | 16 | 0.029 | 1Score **> 36** indicates **identity** Score **> 14** indicates **homology** | U | K.NTPEFDILVIGGGATGCGCALDAVTR.G |
| 162121 | 66 | – | 91 | 888.7615 | 2663.2627 | 2663.2684 | -2.13 | 0 | 29 | 0.0019 | 1Score **> 36** indicates **identity** Score **> 14** indicates **homology** | U | K.NTPEFDILVIGGGATGCGCALDAVTR.G |
| 162122 | 66 | – | 91 | 888.7618 | 2663.2635 | 2663.2684 | -1.85 | 0 | 38 | 0.00027 | 1Score **> 36** indicates **identity** Score **> 15** indicates **homology** | U | K.NTPEFDILVIGGGATGCGCALDAVTR.G |
| 162123 | 66 | – | 91 | 1332.6390 | 2663.2635 | 2663.2684 | -1.82 | 0 | 103 | 2.5e-10 | 1Score **> 36** indicates **identity** Score **> 20** indicates **homology** | U | K.NTPEFDILVIGGGATGCGCALDAVTR.G |
| 162126 | 66 | – | 91 | 888.7625 | 2663.2656 | 2663.2684 | -1.03 | 0 | 101 | 3.3e-10 | 1Score **> 36** indicates **identity** Score **> 19** indicates **homology** | U | K.NTPEFDILVIGGGATGCGCALDAVTR.G |
| 162127 | 66 | – | 91 | 888.7626 | 2663.2660 | 2663.2684 | -0.90 | 0 | 43 | 8.9e-05 | 1Score **> 36** indicates **identity** Score **> 15** indicates **homology** | U | K.NTPEFDILVIGGGATGCGCALDAVTR.G |
| 162128 | 66 | – | 91 | 888.7629 | 2663.2668 | 2663.2684 | -0.61 | 0 | 35 | 0.00058 | 1Score **> 36** indicates **identity** Score **> 15** indicates **homology** | U | K.NTPEFDILVIGGGATGCGCALDAVTR.G |
| 162130 | 66 | – | 91 | 888.7630 | 2663.2670 | 2663.2684 | -0.50 | 0 | 59 | 3e-06 | 1Score **> 36** indicates **identity** Score **> 16** indicates **homology** | U | K.NTPEFDILVIGGGATGCGCALDAVTR.G |
| 162131 | 66 | – | 91 | 888.7630 | 2663.2671 | 2663.2684 | -0.46 | 0 | 95 | 1.3e-09 | 1Score **> 36** indicates **identity** Score **> 18** indicates **homology** | U | K.NTPEFDILVIGGGATGCGCALDAVTR.G |
| 162132 | 66 | – | 91 | 888.7630 | 2663.2671 | 2663.2684 | -0.46 | 0 | 54 | 7.9e-06 | 1Score **> 36** indicates **identity** Score **> 16** indicates **homology** | U | K.NTPEFDILVIGGGATGCGCALDAVTR.G |
| 162133 | 66 | – | 91 | 888.7631 | 2663.2673 | 2663.2684 | -0.39 | 0 | 120 | 5.4e-12 | 1Score **> 36** indicates **identity** Score **> 20** indicates **homology** | U | K.NTPEFDILVIGGGATGCGCALDAVTR.G |
| 162135 | 66 | – | 91 | 888.7631 | 2663.2675 | 2663.2684 | -0.31 | 0 | 75 | 1.3e-07 | 1Score **> 36** indicates **identity** Score **> 18** indicates **homology** | U | K.NTPEFDILVIGGGATGCGCALDAVTR.G |
| 162139 | 66 | – | 91 | 888.7636 | 2663.2690 | 2663.2684 | 0.23 | 0 | 30 | 0.0017 | 1Score **> 36** indicates **identity** Score **> 14** indicates **homology** | U | K.NTPEFDILVIGGGATGCGCALDAVTR.G |
| 162140 | 66 | – | 91 | 888.7637 | 2663.2693 | 2663.2684 | 0.34 | 0 | 28 | 0.0024 | 1Score **> 36** indicates **identity** Score **> 14** indicates **homology** | U | K.NTPEFDILVIGGGATGCGCALDAVTR.G |
| 162143 | 66 | – | 91 | 888.7640 | 2663.2701 | 2663.2684 | 0.65 | 0 | 52 | 1.3e-05 | 1Score **> 36** indicates **identity** Score **> 16** indicates **homology** | U | K.NTPEFDILVIGGGATGCGCALDAVTR.G |
| 162145 | 66 | – | 91 | 888.7640 | 2663.2702 | 2663.2684 | 0.67 | 0 | 20 | 0.016 | 1Score **> 36** indicates **identity** Score **> 14** indicates **homology** | U | K.NTPEFDILVIGGGATGCGCALDAVTR.G |
| 162147 | 66 | – | 91 | 888.7641 | 2663.2705 | 2663.2684 | 0.81 | 0 | 39 | 0.00022 | 1Score **> 36** indicates **identity** Score **> 15** indicates **homology** | U | K.NTPEFDILVIGGGATGCGCALDAVTR.G |
| 162148 | 66 | – | 91 | 888.7642 | 2663.2707 | 2663.2684 | 0.86 | 0 | 73 | 1.4e-07 | 1Score **> 36** indicates **identity** Score **> 17** indicates **homology** | U | K.NTPEFDILVIGGGATGCGCALDAVTR.G |
| 162149 | 66 | – | 91 | 1332.6427 | 2663.2708 | 2663.2684 | 0.90 | 0 | 96 | 1.3e-09 | 1Score **> 36** indicates **identity** Score **> 19** indicates **homology** | U | K.NTPEFDILVIGGGATGCGCALDAVTR.G |
| 162150 | 66 | – | 91 | 1332.6428 | 2663.2711 | 2663.2684 | 1.04 | 0 | 89 | 4.1e-09 | 1Score **> 36** indicates **identity** Score **> 18** indicates **homology** | U | K.NTPEFDILVIGGGATGCGCALDAVTR.G |
| 162151 | 66 | – | 91 | 888.7643 | 2663.2712 | 2663.2684 | 1.06 | 0 | 35 | 0.00047 | 1Score **> 36** indicates **identity** Score **> 15** indicates **homology** | U | K.NTPEFDILVIGGGATGCGCALDAVTR.G |
| 162152 | 66 | – | 91 | 888.7646 | 2663.2720 | 2663.2684 | 1.36 | 0 | 149 | 1e-14 | 1Score **> 36** indicates **identity** Score **> 22** indicates **homology** | U | K.NTPEFDILVIGGGATGCGCALDAVTR.G |
| 162154 | 66 | – | 91 | 888.7649 | 2663.2729 | 2663.2684 | 1.71 | 0 | 33 | 0.00078 | 1Score **> 36** indicates **identity** Score **> 15** indicates **homology** | U | K.NTPEFDILVIGGGATGCGCALDAVTR.G |
| 162156 | 66 | – | 91 | 1332.6441 | 2663.2737 | 2663.2684 | 2.01 | 0 | 99 | 6.8e-10 | 1Score **> 36** indicates **identity** Score **> 20** indicates **homology** | U | K.NTPEFDILVIGGGATGCGCALDAVTR.G |
| 162157 | 66 | – | 91 | 888.7657 | 2663.2751 | 2663.2684 | 2.54 | 0 | 20 | 0.012 | 1Score **> 36** indicates **identity** Score **> 14** indicates **homology** | U | K.NTPEFDILVIGGGATGCGCALDAVTR.G |
| 162159 | 66 | – | 91 | 1332.6449 | 2663.2752 | 2663.2684 | 2.57 | 0 | 54 | 8.6e-06 | 1Score **> 36** indicates **identity** Score **> 16** indicates **homology** | U | K.NTPEFDILVIGGGATGCGCALDAVTR.G |
| 162160 | 66 | – | 91 | 888.7657 | 2663.2753 | 2663.2684 | 2.61 | 0 | 18 | 0.02 | 1Score **> 36** indicates **identity** Score **> 14** indicates **homology** | U | K.NTPEFDILVIGGGATGCGCALDAVTR.G |
| 162161 | 66 | – | 91 | 888.7659 | 2663.2760 | 2663.2684 | 2.85 | 0 | 57 | 4.2e-06 | 1Score **> 36** indicates **identity** Score **> 16** indicates **homology** | U | K.NTPEFDILVIGGGATGCGCALDAVTR.G |
| 162163 | 66 | – | 91 | 888.7662 | 2663.2767 | 2663.2684 | 3.13 | 0 | 21 | 0.014 | 1Score **> 36** indicates **identity** Score **> 15** indicates **homology** | U | K.NTPEFDILVIGGGATGCGCALDAVTR.G |
| 162166 | 66 | – | 91 | 888.7664 | 2663.2773 | 2663.2684 | 3.34 | 0 | 55 | 7.1e-06 | 1Score **> 36** indicates **identity** Score **> 16** indicates **homology** | U | K.NTPEFDILVIGGGATGCGCALDAVTR.G |
| 162168 | 66 | – | 91 | 888.7672 | 2663.2796 | 2663.2684 | 4.23 | 0 | 32 | 0.00091 | 1Score **> 36** indicates **identity** Score **> 15** indicates **homology** | U | K.NTPEFDILVIGGGATGCGCALDAVTR.G |
| 162169 | 66 | – | 91 | 888.7675 | 2663.2807 | 2663.2684 | 4.63 | 0 | 37 | 0.00037 | 1Score **> 36** indicates **identity** Score **> 15** indicates **homology** | U | K.NTPEFDILVIGGGATGCGCALDAVTR.G |
| 162171 | 66 | – | 91 | 888.7680 | 2663.2820 | 2663.2684 | 5.14 | 0 | 30 | 0.0014 | 1Score **> 36** indicates **identity** Score **> 14** indicates **homology** | U | K.NTPEFDILVIGGGATGCGCALDAVTR.G |
| 162172 | 66 | – | 91 | 888.7682 | 2663.2827 | 2663.2684 | 5.38 | 0 | 53 | 1.1e-05 | 1Score **> 36** indicates **identity** Score **> 16** indicates **homology** | U | K.NTPEFDILVIGGGATGCGCALDAVTR.G |
| 162173 | 66 | – | 91 | 888.7684 | 2663.2834 | 2663.2684 | 5.63 | 0 | 43 | 9.3e-05 | 1Score **> 36** indicates **identity** Score **> 15** indicates **homology** | U | K.NTPEFDILVIGGGATGCGCALDAVTR.G |
| 162174 | 66 | – | 91 | 888.7688 | 2663.2845 | 2663.2684 | 6.06 | 0 | 56 | 5.8e-06 | 1Score **> 36** indicates **identity** Score **> 16** indicates **homology** | U | K.NTPEFDILVIGGGATGCGCALDAVTR.G |
| 162175 | 66 | – | 91 | 888.7689 | 2663.2850 | 2663.2684 | 6.24 | 0 | 28 | 0.0025 | 1Score **> 36** indicates **identity** Score **> 14** indicates **homology** | U | K.NTPEFDILVIGGGATGCGCALDAVTR.G |
| 162177 | 66 | – | 91 | 888.7695 | 2663.2866 | 2663.2684 | 6.83 | 0 | 34 | 0.00063 | 1Score **> 36** indicates **identity** Score **> 15** indicates **homology** | U | K.NTPEFDILVIGGGATGCGCALDAVTR.G |
| 162179 | 66 | – | 91 | 888.7702 | 2663.2889 | 2663.2684 | 7.69 | 0 | 43 | 9.6e-05 | 1Score **> 37** indicates **identity** Score **> 15** indicates **homology** | U | K.NTPEFDILVIGGGATGCGCALDAVTR.G |
| 162180 | 66 | – | 91 | 888.7712 | 2663.2919 | 2663.2684 | 8.83 | 0 | 21 | 0.0099 | 1Score **> 36** indicates **identity** Score **> 14** indicates **homology** | U | K.NTPEFDILVIGGGATGCGCALDAVTR.G |
| 162181 | 66 | – | 91 | 888.7717 | 2663.2933 | 2663.2684 | 9.35 | 0 | 39 | 0.00023 | 1Score **> 37** indicates **identity** Score **> 15** indicates **homology** | U | K.NTPEFDILVIGGGATGCGCALDAVTR.G |
| 162182 | 66 | – | 91 | 888.7721 | 2663.2946 | 2663.2684 | 9.84 | 0 | 47 | 4.3e-05 | 1Score **> 37** indicates **identity** Score **> 15** indicates **homology** | U | K.NTPEFDILVIGGGATGCGCALDAVTR.G |
| 162210 | 66 | – | 91 | 889.0930 | 2664.2570 | 2664.2524 | 1.75 | 0 | 18 | 0.022 | 1Score **> 35** indicates **identity** Score **> 14** indicates **homology** | U | K.NTPEFDILVIGGGATGCGCALDAVTR.G  + Deamidated (NQ) |
| 162215 | 66 | – | 91 | 889.0967 | 2664.2681 | 2664.2524 | 5.92 | 0 | 28 | 0.0023 | 1Score **> 36** indicates **identity** Score **> 14** indicates **homology** | U | K.NTPEFDILVIGGGATGCGCALDAVTR.G  + Deamidated (NQ) |
| 162217 | 66 | – | 91 | 889.0971 | 2664.2694 | 2664.2524 | 6.39 | 0 | 42 | 0.00011 | 1Score **> 36** indicates **identity** Score **> 15** indicates **homology** | U | K.NTPEFDILVIGGGATGCGCALDAVTR.G  + Deamidated (NQ) |
| 162218 | 66 | – | 91 | 889.0971 | 2664.2695 | 2664.2524 | 6.41 | 0 | 67 | 5.5e-07 | 1Score **> 36** indicates **identity** Score **> 17** indicates **homology** | U | K.NTPEFDILVIGGGATGCGCALDAVTR.G  + Deamidated (NQ) |
| 162225 | 66 | – | 91 | 889.0993 | 2664.2760 | 2664.2524 | 8.87 | 0 | 30 | 0.0014 | 1Score **> 36** indicates **identity** Score **> 14** indicates **homology** | U | K.NTPEFDILVIGGGATGCGCALDAVTR.G  + Deamidated (NQ) |
| 162226 | 66 | – | 91 | 889.0995 | 2664.2766 | 2664.2524 | 9.10 | 0 | 21 | 0.012 | 1Score **> 36** indicates **identity** Score **> 14** indicates **homology** | U | K.NTPEFDILVIGGGATGCGCALDAVTR.G  + Deamidated (NQ) |
| 162227 | 66 | – | 91 | 889.0997 | 2664.2772 | 2664.2524 | 9.31 | 0 | 38 | 0.00029 | 1Score **> 36** indicates **identity** Score **> 15** indicates **homology** | U | K.NTPEFDILVIGGGATGCGCALDAVTR.G  + Deamidated (NQ) |
| 162228 | 66 | – | 91 | 889.0998 | 2664.2775 | 2664.2524 | 9.43 | 0 | 48 | 3.4e-05 | 1Score **> 36** indicates **identity** Score **> 15** indicates **homology** | U | K.NTPEFDILVIGGGATGCGCALDAVTR.G  + Deamidated (NQ) |
| 162229 | 66 | – | 91 | 889.0999 | 2664.2780 | 2664.2524 | 9.62 | 0 | 62 | 1.4e-06 | 1Score **> 36** indicates **identity** Score **> 16** indicates **homology** | U | K.NTPEFDILVIGGGATGCGCALDAVTR.G  + Deamidated (NQ) |
| 162230 | 66 | – | 91 | 889.1002 | 2664.2788 | 2664.2524 | 9.93 | 0 | 38 | 0.00027 | 1Score **> 36** indicates **identity** Score **> 15** indicates **homology** | U | K.NTPEFDILVIGGGATGCGCALDAVTR.G  + Deamidated (NQ) |
| 93623 | 95 | – | 110 | 864.4110 | 1726.8074 | 1726.8122 | -2.76 | 1 | 44 | 7.2e-05 | 1Score **> 33** indicates **identity** Score **> 15** indicates **homology** | U | K.TALVERDDFSSGTSSR.S |
| 93624 | 95 | – | 110 | 576.6100 | 1726.8083 | 1726.8122 | -2.29 | 1 | 56 | 5.9e-06 | 1Score **> 32** indicates **identity** Score **> 16** indicates **homology** | U | K.TALVERDDFSSGTSSR.S |
| 93627 | 95 | – | 110 | 576.6103 | 1726.8091 | 1726.8122 | -1.78 | 1 | 54 | 8.8e-06 | 1Score **> 32** indicates **identity** Score **> 16** indicates **homology** | U | K.TALVERDDFSSGTSSR.S |
| 93628 | 95 | – | 110 | 864.4118 | 1726.8091 | 1726.8122 | -1.78 | 1 | 23 | 0.0073 | 1Score **> 32** indicates **identity** Score **> 14** indicates **homology** | U | K.TALVERDDFSSGTSSR.S |
| 93629 | 95 | – | 110 | 864.4118 | 1726.8091 | 1726.8122 | -1.78 | 1 | 94 | 1.4e-09 | 1Score **> 32** indicates **identity** Score **> 18** indicates **homology** | U | K.TALVERDDFSSGTSSR.S |
| 93630 | 95 | – | 110 | 576.6103 | 1726.8092 | 1726.8122 | -1.77 | 1 | 58 | 3.9e-06 | 1Score **> 32** indicates **identity** Score **> 16** indicates **homology** | U | K.TALVERDDFSSGTSSR.S |
| 93631 | 95 | – | 110 | 576.6105 | 1726.8096 | 1726.8122 | -1.52 | 1 | 66 | 6.1e-07 | 1Score **> 32** indicates **identity** Score **> 17** indicates **homology** | U | K.TALVERDDFSSGTSSR.S |
| 93632 | 95 | – | 110 | 864.4123 | 1726.8100 | 1726.8122 | -1.27 | 1 | 109 | 6.7e-11 | 1Score **> 33** indicates **identity** Score **> 20** indicates **homology** | U | K.TALVERDDFSSGTSSR.S |
| 93633 | 95 | – | 110 | 576.6106 | 1726.8101 | 1726.8122 | -1.24 | 1 | 51 | 1.8e-05 | 1Score **> 33** indicates **identity** Score **> 16** indicates **homology** | U | K.TALVERDDFSSGTSSR.S |
| 93634 | 95 | – | 110 | 576.6107 | 1726.8102 | 1726.8122 | -1.14 | 1 | 61 | 2.1e-06 | 1Score **> 33** indicates **identity** Score **> 16** indicates **homology** | U | K.TALVERDDFSSGTSSR.S |
| 93635 | 95 | – | 110 | 864.4124 | 1726.8103 | 1726.8122 | -1.12 | 1 | 46 | 0.00012 | 1Score **> 33** indicates **identity** Score **> 19** indicates **homology** | U | K.TALVERDDFSSGTSSR.S |
| 93636 | 95 | – | 110 | 864.4125 | 1726.8105 | 1726.8122 | -0.98 | 1 | 100 | 4e-10 | 1Score **> 33** indicates **identity** Score **> 19** indicates **homology** | U | K.TALVERDDFSSGTSSR.S |
| 93637 | 95 | – | 110 | 576.6108 | 1726.8106 | 1726.8122 | -0.92 | 1 | 62 | 1.6e-06 | 1Score **> 33** indicates **identity** Score **> 16** indicates **homology** | U | K.TALVERDDFSSGTSSR.S |
| 93638 | 95 | – | 110 | 864.4126 | 1726.8106 | 1726.8122 | -0.92 | 1 | 85 | 9.9e-09 | 1Score **> 33** indicates **identity** Score **> 18** indicates **homology** | U | K.TALVERDDFSSGTSSR.S |
| 93639 | 95 | – | 110 | 576.6108 | 1726.8106 | 1726.8122 | -0.90 | 1 | 62 | 1.6e-06 | 1Score **> 33** indicates **identity** Score **> 16** indicates **homology** | U | K.TALVERDDFSSGTSSR.S |
| 93641 | 95 | – | 110 | 576.6110 | 1726.8112 | 1726.8122 | -0.59 | 1 | 64 | 9.7e-07 | 1Score **> 33** indicates **identity** Score **> 17** indicates **homology** | U | K.TALVERDDFSSGTSSR.S |
| 93642 | 95 | – | 110 | 576.6110 | 1726.8113 | 1726.8122 | -0.52 | 1 | 57 | 4.8e-06 | 1Score **> 33** indicates **identity** Score **> 16** indicates **homology** | U | K.TALVERDDFSSGTSSR.S |
| 93643 | 95 | – | 110 | 864.4130 | 1726.8115 | 1726.8122 | -0.44 | 1 | 89 | 4.7e-09 | 1Score **> 33** indicates **identity** Score **> 18** indicates **homology** | U | K.TALVERDDFSSGTSSR.S |
| 93644 | 95 | – | 110 | 576.6112 | 1726.8118 | 1726.8122 | -0.26 | 1 | 61 | 1.7e-06 | 1Score **> 33** indicates **identity** Score **> 16** indicates **homology** | U | K.TALVERDDFSSGTSSR.S |
| 93645 | 95 | – | 110 | 576.6112 | 1726.8118 | 1726.8122 | -0.26 | 1 | 14 | 0.05 | 1Score **> 33** indicates **identity** Score **> 13** indicates **homology** | U | K.TALVERDDFSSGTSSR.S |
| 93646 | 95 | – | 110 | 576.6114 | 1726.8123 | 1726.8122 | 0.063 | 1 | 53 | 1e-05 | 1Score **> 33** indicates **identity** Score **> 16** indicates **homology** | U | K.TALVERDDFSSGTSSR.S |
| 93648 | 95 | – | 110 | 576.6115 | 1726.8126 | 1726.8122 | 0.24 | 1 | 35 | 0.00053 | 1Score **> 32** indicates **identity** Score **> 15** indicates **homology** | U | K.TALVERDDFSSGTSSR.S |
| 93650 | 95 | – | 110 | 576.6117 | 1726.8133 | 1726.8122 | 0.66 | 1 | 16 | 0.029 | 1Score **> 33** indicates **identity** Score **> 14** indicates **homology** | U | K.TALVERDDFSSGTSSR.S |
| 93651 | 95 | – | 110 | 864.4141 | 1726.8137 | 1726.8122 | 0.85 | 1 | 83 | 4.8e-08 | 1Score **> 33** indicates **identity** Score **> 22** indicates **homology** | U | K.TALVERDDFSSGTSSR.S |
| 93655 | 95 | – | 110 | 576.6123 | 1726.8150 | 1726.8122 | 1.63 | 1 | 18 | 0.019 | 1Score **> 33** indicates **identity** Score **> 14** indicates **homology** | U | K.TALVERDDFSSGTSSR.S |
| 18186 | 101 | – | 110 | 529.7221 | 1057.4297 | 1057.4312 | -1.43 | 0 | 37 | 0.00034 | 1Score **> 22** indicates **identity** Score **> 15** indicates **homology** | U | R.DDFSSGTSSR.S |
| 18187 | 101 | – | 110 | 529.7226 | 1057.4307 | 1057.4312 | -0.48 | 0 | 43 | 0.0001 | 1Score **> 22** indicates **identity** Score **> 15** indicates **homology** | U | R.DDFSSGTSSR.S |
| 18188 | 101 | – | 110 | 529.7230 | 1057.4314 | 1057.4312 | 0.12 | 0 | 21 | 0.011 | 1Score **> 22** indicates **identity** Score **> 14** indicates **homology** | U | R.DDFSSGTSSR.S |
| 19023 | 111 | – | 120 | 356.5493 | 1066.6261 | 1066.6247 | 1.30 | 1 | 18 | 0.02 | 1Score **> 28** indicates **identity** Score **> 14** indicates **homology** | U | R.STKLIHGGVR.Y |
| 1786 | 114 | – | 120 | 376.2312 | 750.4478 | 750.4501 | -3.02 | 0 | 21 | 0.0073 | 1Score **> 13** indicates **identity** | U | K.LIHGGVR.Y |
| 1788 | 114 | – | 120 | 376.2325 | 750.4504 | 750.4501 | 0.45 | 0 | 24 | 0.0036 | 1Score **> 13** indicates **identity** | U | K.LIHGGVR.Y |
| 48873 | 125 | – | 135 | 676.3370 | 1350.6595 | 1350.6602 | -0.51 | 0 | 24 | 0.0054 | 1Score **> 33** indicates **identity** Score **> 14** indicates **homology** | U | K.AIMNLDVEQYR.M |
| 48874 | 125 | – | 135 | 676.3371 | 1350.6596 | 1350.6602 | -0.42 | 0 | 25 | 0.0043 | 1Score **> 33** indicates **identity** Score **> 14** indicates **homology** | U | K.AIMNLDVEQYR.M |
| 50447 | 125 | – | 135 | 684.3326 | 1366.6506 | 1366.6551 | -3.28 | 0 | 49 | 2.8e-05 | 1Score **> 32** indicates **identity** Score **> 16** indicates **homology** | U | K.AIMNLDVEQYR.M  + Oxidation (M) |
| 50453 | 125 | – | 135 | 684.3352 | 1366.6558 | 1366.6551 | 0.55 | 0 | 50 | 2e-05 | 1Score **> 32** indicates **identity** Score **> 16** indicates **homology** | U | K.AIMNLDVEQYR.M  + Oxidation (M) |
| 50454 | 125 | – | 135 | 684.3355 | 1366.6565 | 1366.6551 | 1.07 | 0 | 51 | 1.6e-05 | 1Score **> 32** indicates **identity** Score **> 16** indicates **homology** | U | K.AIMNLDVEQYR.M  + Oxidation (M) |
| 24807 | 136 | – | 144 | 376.8656 | 1127.5749 | 1127.5757 | -0.73 | 1 | 21 | 0.029 | 1Score **> 30** indicates **identity** Score **> 18** indicates **homology** | U | R.MVKEALHER.A  + Oxidation (M) |
| 150853 | 145 | – | 166 | 805.4707 | 2413.3902 | 2413.3806 | 3.95 | 0 | 74 | 1.2e-07 | 1Score **> 31** indicates **identity** Score **> 17** indicates **homology** | U | R.ANLLEIAPHLSAPLPIMLPLYK.W |
| 82775 | 167 | – | 178 | 819.9273 | 1637.8401 | 1637.8395 | 0.42 | 0 | 35 | 0.00054 | 1Score **> 35** indicates **identity** Score **> 15** indicates **homology** | U | K.WWQLPYYWVGIK.M |
| 82781 | 167 | – | 178 | 819.9291 | 1637.8436 | 1637.8395 | 2.51 | 0 | 27 | 0.0031 | 1Score **> 35** indicates **identity** Score **> 14** indicates **homology** | U | K.WWQLPYYWVGIK.M |
| 52021 | 179 | – | 190 | 692.8331 | 1383.6516 | 1383.6526 | -0.79 | 0 | 58 | 4e-06 | 1Score **> 31** indicates **identity** Score **> 16** indicates **homology** | U | K.MYDLVAGSQCLK.S |
| 52022 | 179 | – | 190 | 692.8337 | 1383.6529 | 1383.6526 | 0.17 | 0 | 61 | 1.9e-06 | 1Score **> 32** indicates **identity** Score **> 16** indicates **homology** | U | K.MYDLVAGSQCLK.S |
| 52024 | 179 | – | 190 | 692.8343 | 1383.6541 | 1383.6526 | 1.07 | 0 | 35 | 0.00054 | 1Score **> 32** indicates **identity** Score **> 15** indicates **homology** | U | K.MYDLVAGSQCLK.S |
| 53607 | 179 | – | 190 | 700.8333 | 1399.6520 | 1399.6476 | 3.20 | 0 | 15 | 0.043 | 1Score **> 31** indicates **identity** Score **> 13** indicates **homology** | U | K.MYDLVAGSQCLK.S  + Oxidation (M) |
| 2832 | 191 | – | 197 | 392.2158 | 782.4170 | 782.4174 | -0.54 | 0 | 46 | 7.1e-05 | 1Score **> 17** indicates **identity** | U | K.SSYVLSK.S |
| 2833 | 191 | – | 197 | 392.2159 | 782.4173 | 782.4174 | -0.17 | 0 | 46 | 6.4e-05 | 1Score **> 17** indicates **identity** | U | K.SSYVLSK.S |
| 2834 | 191 | – | 197 | 392.2161 | 782.4176 | 782.4174 | 0.19 | 0 | 40 | 0.00027 | 1Score **> 17** indicates **identity** | U | K.SSYVLSK.S |
| 34025 | 200 | – | 209 | 405.2154 | 1212.6243 | 1212.6325 | -6.72 | 0 | 19 | 0.016 | 1Score **> 32** indicates **identity** Score **> 14** indicates **homology** | U | R.ALEHFPMLQK.D |
| 34027 | 200 | – | 209 | 405.2170 | 1212.6291 | 1212.6325 | -2.76 | 0 | 24 | 0.0061 | 1Score **> 32** indicates **identity** Score **> 14** indicates **homology** | U | R.ALEHFPMLQK.D |
| 34029 | 200 | – | 209 | 405.2174 | 1212.6302 | 1212.6325 | -1.85 | 0 | 25 | 0.005 | 1Score **> 32** indicates **identity** Score **> 14** indicates **homology** | U | R.ALEHFPMLQK.D |
| 34031 | 200 | – | 209 | 405.2177 | 1212.6313 | 1212.6325 | -1.01 | 0 | 21 | 0.01 | 1Score **> 32** indicates **identity** Score **> 14** indicates **homology** | U | R.ALEHFPMLQK.D |
| 34032 | 200 | – | 209 | 405.2179 | 1212.6320 | 1212.6325 | -0.40 | 0 | 21 | 0.011 | 1Score **> 32** indicates **identity** Score **> 14** indicates **homology** | U | R.ALEHFPMLQK.D |
| 122526 | 210 | – | 227 | 678.6679 | 2032.9818 | 2032.9966 | -7.31 | 1 | 21 | 0.011 | 1Score **> 35** indicates **identity** Score **> 14** indicates **homology** | U | K.DKLVGAIVYYDGQHNDAR.M |
| 122529 | 210 | – | 227 | 678.6688 | 2032.9846 | 2032.9966 | -5.94 | 1 | 27 | 0.0032 | 1Score **> 35** indicates **identity** Score **> 14** indicates **homology** | U | K.DKLVGAIVYYDGQHNDAR.M |
| 122531 | 210 | – | 227 | 678.6705 | 2032.9897 | 2032.9966 | -3.40 | 1 | 31 | 0.0012 | 1Score **> 36** indicates **identity** Score **> 14** indicates **homology** | U | K.DKLVGAIVYYDGQHNDAR.M |
| 122533 | 210 | – | 227 | 678.6723 | 2032.9949 | 2032.9966 | -0.84 | 1 | 37 | 0.00031 | 1Score **> 36** indicates **identity** Score **> 15** indicates **homology** | U | K.DKLVGAIVYYDGQHNDAR.M |
| 122534 | 210 | – | 227 | 678.6724 | 2032.9954 | 2032.9966 | -0.60 | 1 | 17 | 0.025 | 1Score **> 36** indicates **identity** Score **> 14** indicates **homology** | U | K.DKLVGAIVYYDGQHNDAR.M |
| 122536 | 210 | – | 227 | 678.6726 | 2032.9959 | 2032.9966 | -0.35 | 1 | 64 | 1.1e-06 | 1Score **> 36** indicates **identity** Score **> 16** indicates **homology** | U | K.DKLVGAIVYYDGQHNDAR.M |
| 122540 | 210 | – | 227 | 678.6730 | 2032.9973 | 2032.9966 | 0.32 | 1 | 56 | 5.2e-06 | 1Score **> 36** indicates **identity** Score **> 16** indicates **homology** | U | K.DKLVGAIVYYDGQHNDAR.M |
| 122541 | 210 | – | 227 | 678.6731 | 2032.9975 | 2032.9966 | 0.42 | 1 | 60 | 2.1e-06 | 1Score **> 36** indicates **identity** Score **> 16** indicates **homology** | U | K.DKLVGAIVYYDGQHNDAR.M |
| 122542 | 210 | – | 227 | 509.2567 | 2032.9976 | 2032.9966 | 0.49 | 1 | 24 | 0.0053 | 1Score **> 36** indicates **identity** Score **> 14** indicates **homology** | U | K.DKLVGAIVYYDGQHNDAR.M |
| 122543 | 210 | – | 227 | 678.6732 | 2032.9976 | 2032.9966 | 0.49 | 1 | 46 | 4.7e-05 | 1Score **> 36** indicates **identity** Score **> 15** indicates **homology** | U | K.DKLVGAIVYYDGQHNDAR.M |
| 122544 | 210 | – | 227 | 678.6734 | 2032.9985 | 2032.9966 | 0.92 | 1 | 77 | 6.3e-08 | 1Score **> 36** indicates **identity** Score **> 17** indicates **homology** | U | K.DKLVGAIVYYDGQHNDAR.M |
| 122545 | 210 | – | 227 | 509.2569 | 2032.9986 | 2032.9966 | 0.95 | 1 | 39 | 0.00021 | 1Score **> 36** indicates **identity** Score **> 15** indicates **homology** | U | K.DKLVGAIVYYDGQHNDAR.M |
| 122547 | 210 | – | 227 | 509.2570 | 2032.9990 | 2032.9966 | 1.14 | 1 | 33 | 0.00089 | 1Score **> 36** indicates **identity** Score **> 15** indicates **homology** | U | K.DKLVGAIVYYDGQHNDAR.M |
| 122548 | 210 | – | 227 | 509.2570 | 2032.9990 | 2032.9966 | 1.17 | 1 | 44 | 8.3e-05 | 1Score **> 36** indicates **identity** Score **> 15** indicates **homology** | U | K.DKLVGAIVYYDGQHNDAR.M |
| 122550 | 210 | – | 227 | 509.2571 | 2032.9992 | 2032.9966 | 1.27 | 1 | 41 | 0.00014 | 1Score **> 36** indicates **identity** Score **> 15** indicates **homology** | U | K.DKLVGAIVYYDGQHNDAR.M |
| 122552 | 210 | – | 227 | 1017.5069 | 2032.9993 | 2032.9966 | 1.30 | 1 | 80 | 3.2e-08 | 1Score **> 36** indicates **identity** Score **> 17** indicates **homology** | U | K.DKLVGAIVYYDGQHNDAR.M |
| 122555 | 210 | – | 227 | 1017.5073 | 2033.0000 | 2032.9966 | 1.66 | 1 | 79 | 4e-08 | 1Score **> 36** indicates **identity** Score **> 17** indicates **homology** | U | K.DKLVGAIVYYDGQHNDAR.M |
| 122560 | 210 | – | 227 | 678.6750 | 2033.0032 | 2032.9966 | 3.23 | 1 | 24 | 0.006 | 1Score **> 36** indicates **identity** Score **> 14** indicates **homology** | U | K.DKLVGAIVYYDGQHNDAR.M |
| 122564 | 210 | – | 227 | 509.2584 | 2033.0045 | 2032.9966 | 3.88 | 1 | 36 | 0.00046 | 1Score **> 36** indicates **identity** Score **> 15** indicates **homology** | U | K.DKLVGAIVYYDGQHNDAR.M |
| 122570 | 210 | – | 227 | 509.2594 | 2033.0084 | 2032.9966 | 5.77 | 1 | 14 | 0.047 | 1Score **> 36** indicates **identity** Score **> 13** indicates **homology** | U | K.DKLVGAIVYYDGQHNDAR.M |
| 122571 | 210 | – | 227 | 678.6768 | 2033.0084 | 2032.9966 | 5.79 | 1 | 21 | 0.01 | 1Score **> 36** indicates **identity** Score **> 14** indicates **homology** | U | K.DKLVGAIVYYDGQHNDAR.M |
| 122575 | 210 | – | 227 | 678.6784 | 2033.0134 | 2032.9966 | 8.26 | 1 | 16 | 0.034 | 1Score **> 36** indicates **identity** Score **> 13** indicates **homology** | U | K.DKLVGAIVYYDGQHNDAR.M |
| 122658 | 210 | – | 227 | 1018.0076 | 2034.0006 | 2033.9807 | 9.80 | 1 | 31 | 0.0012 | 1Score **> 36** indicates **identity** Score **> 14** indicates **homology** | U | K.DKLVGAIVYYDGQHNDAR.M  + Deamidated (NQ) |
| 100286 | 212 | – | 227 | 597.6304 | 1789.8693 | 1789.8747 | -3.05 | 0 | 18 | 0.019 | 1Score **> 35** indicates **identity** Score **> 14** indicates **homology** | U | K.LVGAIVYYDGQHNDAR.M |
| 100287 | 212 | – | 227 | 597.6306 | 1789.8699 | 1789.8747 | -2.69 | 0 | 33 | 0.00075 | 1Score **> 35** indicates **identity** Score **> 15** indicates **homology** | U | K.LVGAIVYYDGQHNDAR.M |
| 100288 | 212 | – | 227 | 597.6308 | 1789.8705 | 1789.8747 | -2.36 | 0 | 29 | 0.0019 | 1Score **> 35** indicates **identity** Score **> 14** indicates **homology** | U | K.LVGAIVYYDGQHNDAR.M |
| 100290 | 212 | – | 227 | 895.9432 | 1789.8719 | 1789.8747 | -1.61 | 0 | 54 | 7.9e-06 | 1Score **> 35** indicates **identity** Score **> 16** indicates **homology** | U | K.LVGAIVYYDGQHNDAR.M |
| 100291 | 212 | – | 227 | 597.6313 | 1789.8720 | 1789.8747 | -1.51 | 0 | 33 | 0.00085 | 1Score **> 35** indicates **identity** Score **> 15** indicates **homology** | U | K.LVGAIVYYDGQHNDAR.M |
| 100292 | 212 | – | 227 | 597.6315 | 1789.8725 | 1789.8747 | -1.23 | 0 | 24 | 0.0051 | 1Score **> 35** indicates **identity** Score **> 14** indicates **homology** | U | K.LVGAIVYYDGQHNDAR.M |
| 100293 | 212 | – | 227 | 895.9444 | 1789.8743 | 1789.8747 | -0.25 | 0 | 63 | 1.3e-06 | 1Score **> 35** indicates **identity** Score **> 16** indicates **homology** | U | K.LVGAIVYYDGQHNDAR.M |
| 100294 | 212 | – | 227 | 597.6321 | 1789.8746 | 1789.8747 | -0.10 | 0 | 49 | 2.7e-05 | 1Score **> 35** indicates **identity** Score **> 16** indicates **homology** | U | K.LVGAIVYYDGQHNDAR.M |
| 100295 | 212 | – | 227 | 597.6323 | 1789.8750 | 1789.8747 | 0.17 | 0 | 34 | 0.0007 | 1Score **> 35** indicates **identity** Score **> 15** indicates **homology** | U | K.LVGAIVYYDGQHNDAR.M |
| 100296 | 212 | – | 227 | 895.9449 | 1789.8753 | 1789.8747 | 0.31 | 0 | 77 | 5.6e-08 | 1Score **> 35** indicates **identity** Score **> 17** indicates **homology** | U | K.LVGAIVYYDGQHNDAR.M |
| 100297 | 212 | – | 227 | 597.6324 | 1789.8754 | 1789.8747 | 0.37 | 0 | 32 | 0.001 | 1Score **> 35** indicates **identity** Score **> 14** indicates **homology** | U | K.LVGAIVYYDGQHNDAR.M |
| 100298 | 212 | – | 227 | 597.6324 | 1789.8755 | 1789.8747 | 0.41 | 0 | 39 | 0.00021 | 1Score **> 35** indicates **identity** Score **> 15** indicates **homology** | U | K.LVGAIVYYDGQHNDAR.M |
| 100299 | 212 | – | 227 | 597.6324 | 1789.8755 | 1789.8747 | 0.42 | 0 | 36 | 0.0004 | 1Score **> 35** indicates **identity** Score **> 15** indicates **homology** | U | K.LVGAIVYYDGQHNDAR.M |
| 100300 | 212 | – | 227 | 895.9451 | 1789.8757 | 1789.8747 | 0.52 | 0 | 49 | 2.7e-05 | 1Score **> 35** indicates **identity** Score **> 16** indicates **homology** | U | K.LVGAIVYYDGQHNDAR.M |
| 100301 | 212 | – | 227 | 895.9452 | 1789.8758 | 1789.8747 | 0.58 | 0 | 67 | 5.1e-07 | 1Score **> 35** indicates **identity** Score **> 17** indicates **homology** | U | K.LVGAIVYYDGQHNDAR.M |
| 100302 | 212 | – | 227 | 895.9453 | 1789.8760 | 1789.8747 | 0.70 | 0 | 60 | 2.3e-06 | 1Score **> 35** indicates **identity** Score **> 16** indicates **homology** | U | K.LVGAIVYYDGQHNDAR.M |
| 100303 | 212 | – | 227 | 895.9454 | 1789.8762 | 1789.8747 | 0.81 | 0 | 67 | 5.4e-07 | 1Score **> 35** indicates **identity** Score **> 17** indicates **homology** | U | K.LVGAIVYYDGQHNDAR.M |
| 100304 | 212 | – | 227 | 597.6329 | 1789.8767 | 1789.8747 | 1.11 | 0 | 32 | 0.00094 | 1Score **> 35** indicates **identity** Score **> 15** indicates **homology** | U | K.LVGAIVYYDGQHNDAR.M |
| 100306 | 212 | – | 227 | 895.9458 | 1789.8771 | 1789.8747 | 1.29 | 0 | 45 | 5.6e-05 | 1Score **> 35** indicates **identity** Score **> 15** indicates **homology** | U | K.LVGAIVYYDGQHNDAR.M |
| 100307 | 212 | – | 227 | 597.6330 | 1789.8771 | 1789.8747 | 1.33 | 0 | 29 | 0.0018 | 1Score **> 35** indicates **identity** Score **> 14** indicates **homology** | U | K.LVGAIVYYDGQHNDAR.M |
| 100308 | 212 | – | 227 | 597.6330 | 1789.8772 | 1789.8747 | 1.36 | 0 | 14 | 0.046 | 1Score **> 35** indicates **identity** Score **> 13** indicates **homology** | U | K.LVGAIVYYDGQHNDAR.M |
| 100310 | 212 | – | 227 | 895.9475 | 1789.8804 | 1789.8747 | 3.18 | 0 | 42 | 0.00013 | 1Score **> 35** indicates **identity** Score **> 15** indicates **homology** | U | K.LVGAIVYYDGQHNDAR.M |
| 26599 | 228 | – | 238 | 572.8283 | 1143.6420 | 1143.6434 | -1.16 | 0 | 52 | 0.00023 | 1Score **> 34** indicates **identity** Score **> 28** indicates **homology** | U | R.MNLAIALTAAR.Y |
| 26602 | 228 | – | 238 | 572.8287 | 1143.6429 | 1143.6434 | -0.43 | 0 | 27 | 0.006 | 1Score **> 34** indicates **identity** Score **> 18** indicates **homology** | U | R.MNLAIALTAAR.Y |
| 26603 | 228 | – | 238 | 572.8291 | 1143.6436 | 1143.6434 | 0.22 | 0 | 71 | 6.4e-06 | 1Score **> 34** indicates **identity** Score **> 31** indicates **homology** | U | R.MNLAIALTAAR.Y |
| 26604 | 228 | – | 238 | 572.8291 | 1143.6437 | 1143.6434 | 0.32 | 0 | 66 | 1.7e-05 | 1Score **> 34** indicates **identity** Score **> 31** indicates **homology** | U | R.MNLAIALTAAR.Y |
| 26606 | 228 | – | 238 | 572.8293 | 1143.6441 | 1143.6434 | 0.63 | 0 | 63 | 6.9e-05 | 1Score **> 34** indicates **identity** | U | R.MNLAIALTAAR.Y |
| 26608 | 228 | – | 238 | 572.8295 | 1143.6445 | 1143.6434 | 0.95 | 0 | 71 | 6.8e-06 | 1Score **> 34** indicates **identity** Score **> 32** indicates **homology** | U | R.MNLAIALTAAR.Y |
| 26609 | 228 | – | 238 | 572.8296 | 1143.6446 | 1143.6434 | 1.07 | 0 | 15 | 0.043 | 1Score **> 34** indicates **identity** Score **> 13** indicates **homology** | U | R.MNLAIALTAAR.Y |
| 26610 | 228 | – | 238 | 572.8296 | 1143.6447 | 1143.6434 | 1.18 | 0 | 35 | 0.0017 | 1Score **> 34** indicates **identity** Score **> 19** indicates **homology** | U | R.MNLAIALTAAR.Y |
| 26611 | 228 | – | 238 | 572.8298 | 1143.6450 | 1143.6434 | 1.39 | 0 | 44 | 0.00015 | 1Score **> 34** indicates **identity** Score **> 19** indicates **homology** | U | R.MNLAIALTAAR.Y |
| 26612 | 228 | – | 238 | 572.8299 | 1143.6452 | 1143.6434 | 1.63 | 0 | 33 | 0.0034 | 1Score **> 34** indicates **identity** Score **> 21** indicates **homology** | U | R.MNLAIALTAAR.Y |
| 28321 | 228 | – | 238 | 580.8279 | 1159.6412 | 1159.6383 | 2.49 | 0 | 41 | 0.00082 | 1Score **> 34** indicates **identity** Score **> 23** indicates **homology** | U | R.MNLAIALTAAR.Y  + Oxidation (M) |
| 93913 | 239 | – | 254 | 865.4367 | 1728.8589 | 1728.8756 | -9.70 | 0 | 58 | 3.5e-06 | 1Score **> 34** indicates **identity** Score **> 16** indicates **homology** | U | R.YGAATANYMEVVSLLK.K |
| 93934 | 239 | – | 254 | 865.4457 | 1728.8768 | 1728.8756 | 0.68 | 0 | 107 | 8.7e-11 | 1Score **> 35** indicates **identity** Score **> 19** indicates **homology** | U | R.YGAATANYMEVVSLLK.K |
| 93939 | 239 | – | 254 | 865.4474 | 1728.8803 | 1728.8756 | 2.70 | 0 | 88 | 6e-09 | 1Score **> 35** indicates **identity** Score **> 18** indicates **homology** | U | R.YGAATANYMEVVSLLK.K |
| 93946 | 239 | – | 254 | 865.4494 | 1728.8842 | 1728.8756 | 4.96 | 0 | 58 | 3.3e-06 | 1Score **> 35** indicates **identity** Score **> 16** indicates **homology** | U | R.YGAATANYMEVVSLLK.K |
| 107050 | 239 | – | 255 | 619.9975 | 1856.9706 | 1856.9706 | 0.012 | 1 | 78 | 4.5e-08 | 1Score **> 36** indicates **identity** Score **> 17** indicates **homology** | U | R.YGAATANYMEVVSLLKK.T |
| 107052 | 239 | – | 255 | 619.9979 | 1856.9717 | 1856.9706 | 0.63 | 1 | 62 | 1.5e-06 | 1Score **> 36** indicates **identity** Score **> 16** indicates **homology** | U | R.YGAATANYMEVVSLLKK.T |
| 107053 | 239 | – | 255 | 619.9979 | 1856.9718 | 1856.9706 | 0.67 | 1 | 45 | 6.3e-05 | 1Score **> 36** indicates **identity** Score **> 15** indicates **homology** | U | R.YGAATANYMEVVSLLKK.T |
| 107054 | 239 | – | 255 | 619.9979 | 1856.9720 | 1856.9706 | 0.76 | 1 | 74 | 1.1e-07 | 1Score **> 35** indicates **identity** Score **> 17** indicates **homology** | U | R.YGAATANYMEVVSLLKK.T |
| 107055 | 239 | – | 255 | 619.9980 | 1856.9722 | 1856.9706 | 0.89 | 1 | 34 | 0.00059 | 1Score **> 35** indicates **identity** Score **> 15** indicates **homology** | U | R.YGAATANYMEVVSLLKK.T |
| 73430 | 270 | – | 282 | 783.8798 | 1565.7450 | 1565.7508 | -3.74 | 1 | 64 | 1.2e-06 | 1Score **> 33** indicates **identity** Score **> 17** indicates **homology** | U | R.CKDVLTGQEFDVR.A |
| 73431 | 270 | – | 282 | 783.8809 | 1565.7472 | 1565.7508 | -2.28 | 1 | 54 | 9.7e-06 | 1Score **> 33** indicates **identity** Score **> 16** indicates **homology** | U | R.CKDVLTGQEFDVR.A |
| 73432 | 270 | – | 282 | 783.8809 | 1565.7472 | 1565.7508 | -2.27 | 1 | 76 | 7.7e-08 | 1Score **> 33** indicates **identity** Score **> 18** indicates **homology** | U | R.CKDVLTGQEFDVR.A |
| 73433 | 270 | – | 282 | 522.9234 | 1565.7485 | 1565.7508 | -1.46 | 1 | 36 | 0.00047 | 1Score **> 33** indicates **identity** Score **> 15** indicates **homology** | U | R.CKDVLTGQEFDVR.A |
| 73434 | 270 | – | 282 | 522.9235 | 1565.7487 | 1565.7508 | -1.35 | 1 | 49 | 2.4e-05 | 1Score **> 33** indicates **identity** Score **> 16** indicates **homology** | U | R.CKDVLTGQEFDVR.A |
| 73435 | 270 | – | 282 | 522.9237 | 1565.7493 | 1565.7508 | -0.99 | 1 | 41 | 0.00016 | 1Score **> 33** indicates **identity** Score **> 15** indicates **homology** | U | R.CKDVLTGQEFDVR.A |
| 73436 | 270 | – | 282 | 522.9239 | 1565.7497 | 1565.7508 | -0.68 | 1 | 58 | 4e-06 | 1Score **> 33** indicates **identity** Score **> 16** indicates **homology** | U | R.CKDVLTGQEFDVR.A |
| 73437 | 270 | – | 282 | 783.8822 | 1565.7499 | 1565.7508 | -0.55 | 1 | 63 | 1.2e-06 | 1Score **> 33** indicates **identity** Score **> 17** indicates **homology** | U | R.CKDVLTGQEFDVR.A |
| 73438 | 270 | – | 282 | 783.8824 | 1565.7503 | 1565.7508 | -0.31 | 1 | 70 | 3.6e-07 | 1Score **> 33** indicates **identity** Score **> 18** indicates **homology** | U | R.CKDVLTGQEFDVR.A |
| 73440 | 270 | – | 282 | 522.9243 | 1565.7511 | 1565.7508 | 0.19 | 1 | 31 | 0.0012 | 1Score **> 33** indicates **identity** Score **> 14** indicates **homology** | U | R.CKDVLTGQEFDVR.A |
| 73441 | 270 | – | 282 | 522.9244 | 1565.7513 | 1565.7508 | 0.30 | 1 | 37 | 0.00036 | 1Score **> 33** indicates **identity** Score **> 15** indicates **homology** | U | R.CKDVLTGQEFDVR.A |
| 73442 | 270 | – | 282 | 783.8830 | 1565.7515 | 1565.7508 | 0.46 | 1 | 75 | 1.1e-07 | 1Score **> 33** indicates **identity** Score **> 18** indicates **homology** | U | R.CKDVLTGQEFDVR.A |
| 73443 | 270 | – | 282 | 783.8831 | 1565.7516 | 1565.7508 | 0.53 | 1 | 73 | 1.4e-07 | 1Score **> 33** indicates **identity** Score **> 17** indicates **homology** | U | R.CKDVLTGQEFDVR.A |
| 73444 | 270 | – | 282 | 522.9245 | 1565.7517 | 1565.7508 | 0.54 | 1 | 76 | 7.7e-08 | 1Score **> 33** indicates **identity** Score **> 17** indicates **homology** | U | R.CKDVLTGQEFDVR.A |
| 73445 | 270 | – | 282 | 783.8832 | 1565.7519 | 1565.7508 | 0.67 | 1 | 63 | 1.2e-06 | 1Score **> 33** indicates **identity** Score **> 16** indicates **homology** | U | R.CKDVLTGQEFDVR.A |
| 73446 | 270 | – | 282 | 522.9246 | 1565.7520 | 1565.7508 | 0.75 | 1 | 64 | 1.1e-06 | 1Score **> 33** indicates **identity** Score **> 16** indicates **homology** | U | R.CKDVLTGQEFDVR.A |
| 73447 | 270 | – | 282 | 522.9246 | 1565.7520 | 1565.7508 | 0.75 | 1 | 59 | 3.1e-06 | 1Score **> 33** indicates **identity** Score **> 16** indicates **homology** | U | R.CKDVLTGQEFDVR.A |
| 73448 | 270 | – | 282 | 783.8835 | 1565.7524 | 1565.7508 | 1.03 | 1 | 65 | 8e-07 | 1Score **> 33** indicates **identity** Score **> 17** indicates **homology** | U | R.CKDVLTGQEFDVR.A |
| 73450 | 270 | – | 282 | 522.9252 | 1565.7537 | 1565.7508 | 1.84 | 1 | 29 | 0.002 | 1Score **> 33** indicates **identity** Score **> 14** indicates **homology** | U | R.CKDVLTGQEFDVR.A |
| 73453 | 270 | – | 282 | 783.8847 | 1565.7548 | 1565.7508 | 2.57 | 1 | 64 | 2.4e-06 | 1Score **> 33** indicates **identity** Score **> 20** indicates **homology** | U | R.CKDVLTGQEFDVR.A |
| 73454 | 270 | – | 282 | 522.9256 | 1565.7551 | 1565.7508 | 2.71 | 1 | 37 | 0.00036 | 1Score **> 33** indicates **identity** Score **> 15** indicates **homology** | U | R.CKDVLTGQEFDVR.A |
| 73455 | 270 | – | 282 | 522.9257 | 1565.7554 | 1565.7508 | 2.94 | 1 | 44 | 7.6e-05 | 1Score **> 33** indicates **identity** Score **> 15** indicates **homology** | U | R.CKDVLTGQEFDVR.A |
| 73456 | 270 | – | 282 | 522.9262 | 1565.7567 | 1565.7508 | 3.75 | 1 | 33 | 0.00074 | 1Score **> 33** indicates **identity** Score **> 15** indicates **homology** | U | R.CKDVLTGQEFDVR.A |
| 73459 | 270 | – | 282 | 783.8897 | 1565.7649 | 1565.7508 | 8.97 | 1 | 38 | 0.00026 | 1Score **> 34** indicates **identity** Score **> 15** indicates **homology** | U | R.CKDVLTGQEFDVR.A |
| 41199 | 272 | – | 282 | 639.8190 | 1277.6235 | 1277.6252 | -1.35 | 0 | 40 | 0.00018 | 1Score **> 33** indicates **identity** Score **> 16** indicates **homology** | U | K.DVLTGQEFDVR.A |
| 41200 | 272 | – | 282 | 639.8193 | 1277.6241 | 1277.6252 | -0.85 | 0 | 40 | 0.00018 | 1Score **> 33** indicates **identity** Score **> 15** indicates **homology** | U | K.DVLTGQEFDVR.A |
| 41201 | 272 | – | 282 | 639.8199 | 1277.6253 | 1277.6252 | 0.069 | 0 | 16 | 0.033 | 1Score **> 33** indicates **identity** Score **> 13** indicates **homology** | U | K.DVLTGQEFDVR.A |
| 41202 | 272 | – | 282 | 639.8201 | 1277.6257 | 1277.6252 | 0.39 | 0 | 76 | 2.1e-07 | 1Score **> 33** indicates **identity** Score **> 21** indicates **homology** | U | K.DVLTGQEFDVR.A |
| 41203 | 272 | – | 282 | 639.8201 | 1277.6257 | 1277.6252 | 0.40 | 0 | 68 | 1.2e-06 | 1Score **> 33** indicates **identity** Score **> 21** indicates **homology** | U | K.DVLTGQEFDVR.A |
| 92935 | 283 | – | 298 | 861.4364 | 1720.8582 | 1720.8567 | 0.91 | 1 | 56 | 6.1e-06 | 1Score **> 35** indicates **identity** Score **> 16** indicates **homology** | U | R.AKCVINASGPFTDSVR.K |
| 92936 | 283 | – | 298 | 574.6268 | 1720.8586 | 1720.8567 | 1.11 | 1 | 50 | 2e-05 | 1Score **> 35** indicates **identity** Score **> 16** indicates **homology** | U | R.AKCVINASGPFTDSVR.K |
| 92937 | 283 | – | 298 | 574.6269 | 1720.8590 | 1720.8567 | 1.34 | 1 | 44 | 7.5e-05 | 1Score **> 35** indicates **identity** Score **> 15** indicates **homology** | U | R.AKCVINASGPFTDSVR.K |
| 92941 | 283 | – | 298 | 574.6275 | 1720.8608 | 1720.8567 | 2.39 | 1 | 75 | 1e-07 | 1Score **> 35** indicates **identity** Score **> 17** indicates **homology** | U | R.AKCVINASGPFTDSVR.K |
| 92942 | 283 | – | 298 | 861.4378 | 1720.8611 | 1720.8567 | 2.60 | 1 | 43 | 0.0001 | 1Score **> 35** indicates **identity** Score **> 15** indicates **homology** | U | R.AKCVINASGPFTDSVR.K |
| 92944 | 283 | – | 298 | 574.6286 | 1720.8640 | 1720.8567 | 4.25 | 1 | 15 | 0.042 | 1Score **> 35** indicates **identity** Score **> 13** indicates **homology** | U | R.AKCVINASGPFTDSVR.K |
| 92945 | 283 | – | 298 | 574.6288 | 1720.8645 | 1720.8567 | 4.53 | 1 | 61 | 2.1e-06 | 1Score **> 35** indicates **identity** Score **> 16** indicates **homology** | U | R.AKCVINASGPFTDSVR.K |
| 92946 | 283 | – | 298 | 861.4399 | 1720.8652 | 1720.8567 | 4.94 | 1 | 57 | 5.3e-06 | 1Score **> 35** indicates **identity** Score **> 17** indicates **homology** | U | R.AKCVINASGPFTDSVR.K |
| 67996 | 285 | – | 298 | 761.8656 | 1521.7166 | 1521.7246 | -5.23 | 0 | 50 | 2.1e-05 | 1Score **> 32** indicates **identity** Score **> 16** indicates **homology** | U | K.CVINASGPFTDSVR.K |
| 67998 | 285 | – | 298 | 761.8675 | 1521.7204 | 1521.7246 | -2.76 | 0 | 80 | 2.9e-08 | 1Score **> 32** indicates **identity** Score **> 18** indicates **homology** | U | K.CVINASGPFTDSVR.K |
| 67999 | 285 | – | 298 | 761.8676 | 1521.7206 | 1521.7246 | -2.61 | 0 | 74 | 1.6e-07 | 1Score **> 33** indicates **identity** Score **> 18** indicates **homology** | U | K.CVINASGPFTDSVR.K |
| 68000 | 285 | – | 298 | 761.8678 | 1521.7211 | 1521.7246 | -2.29 | 0 | 73 | 1.6e-07 | 1Score **> 32** indicates **identity** Score **> 18** indicates **homology** | U | K.CVINASGPFTDSVR.K |
| 68001 | 285 | – | 298 | 761.8684 | 1521.7223 | 1521.7246 | -1.48 | 0 | 50 | 1.9e-05 | 1Score **> 32** indicates **identity** Score **> 16** indicates **homology** | U | K.CVINASGPFTDSVR.K |
| 68002 | 285 | – | 298 | 761.8687 | 1521.7228 | 1521.7246 | -1.17 | 0 | 43 | 0.00012 | 1Score **> 32** indicates **identity** Score **> 17** indicates **homology** | U | K.CVINASGPFTDSVR.K |
| 68003 | 285 | – | 298 | 761.8687 | 1521.7228 | 1521.7246 | -1.16 | 0 | 62 | 1.5e-06 | 1Score **> 32** indicates **identity** Score **> 16** indicates **homology** | U | K.CVINASGPFTDSVR.K |
| 68005 | 285 | – | 298 | 761.8688 | 1521.7230 | 1521.7246 | -1.05 | 0 | 76 | 1.2e-07 | 1Score **> 32** indicates **identity** Score **> 20** indicates **homology** | U | K.CVINASGPFTDSVR.K |
| 68006 | 285 | – | 298 | 761.8688 | 1521.7231 | 1521.7246 | -1.00 | 0 | 59 | 2.8e-06 | 1Score **> 32** indicates **identity** Score **> 16** indicates **homology** | U | K.CVINASGPFTDSVR.K |
| 68007 | 285 | – | 298 | 761.8688 | 1521.7231 | 1521.7246 | -0.97 | 0 | 44 | 6.8e-05 | 1Score **> 32** indicates **identity** Score **> 15** indicates **homology** | U | K.CVINASGPFTDSVR.K |
| 68008 | 285 | – | 298 | 761.8689 | 1521.7233 | 1521.7246 | -0.83 | 0 | 76 | 9.1e-08 | 1Score **> 32** indicates **identity** Score **> 18** indicates **homology** | U | K.CVINASGPFTDSVR.K |
| 68009 | 285 | – | 298 | 761.8691 | 1521.7237 | 1521.7246 | -0.59 | 0 | 89 | 2e-08 | 1Score **> 32** indicates **identity** Score **> 25** indicates **homology** | U | K.CVINASGPFTDSVR.K |
| 68010 | 285 | – | 298 | 761.8693 | 1521.7240 | 1521.7246 | -0.36 | 0 | 77 | 9e-08 | 1Score **> 32** indicates **identity** Score **> 19** indicates **homology** | U | K.CVINASGPFTDSVR.K |
| 68011 | 285 | – | 298 | 761.8693 | 1521.7241 | 1521.7246 | -0.35 | 0 | 44 | 0.00013 | 1Score **> 32** indicates **identity** Score **> 17** indicates **homology** | U | K.CVINASGPFTDSVR.K |
| 68012 | 285 | – | 298 | 761.8694 | 1521.7242 | 1521.7246 | -0.29 | 0 | 97 | 3.7e-09 | 1Score **> 33** indicates **identity** Score **> 25** indicates **homology** | U | K.CVINASGPFTDSVR.K |
| 68013 | 285 | – | 298 | 761.8694 | 1521.7242 | 1521.7246 | -0.27 | 0 | 40 | 0.0002 | 1Score **> 33** indicates **identity** Score **> 15** indicates **homology** | U | K.CVINASGPFTDSVR.K |
| 68014 | 285 | – | 298 | 761.8694 | 1521.7242 | 1521.7246 | -0.26 | 0 | 100 | 1.9e-09 | 1Score **> 33** indicates **identity** Score **> 26** indicates **homology** | U | K.CVINASGPFTDSVR.K |
| 68015 | 285 | – | 298 | 761.8694 | 1521.7243 | 1521.7246 | -0.20 | 0 | 87 | 3e-08 | 1Score **> 33** indicates **identity** Score **> 25** indicates **homology** | U | K.CVINASGPFTDSVR.K |
| 68017 | 285 | – | 298 | 761.8698 | 1521.7250 | 1521.7246 | 0.23 | 0 | 96 | 7.2e-09 | 1Score **> 32** indicates **identity** Score **> 27** indicates **homology** | U | K.CVINASGPFTDSVR.K |
| 68018 | 285 | – | 298 | 761.8699 | 1521.7253 | 1521.7246 | 0.45 | 0 | 47 | 0.00015 | 1Score **> 32** indicates **identity** Score **> 22** indicates **homology** | U | K.CVINASGPFTDSVR.K |
| 68020 | 285 | – | 298 | 761.8708 | 1521.7271 | 1521.7246 | 1.68 | 0 | 62 | 1.6e-06 | 1Score **> 33** indicates **identity** Score **> 16** indicates **homology** | U | K.CVINASGPFTDSVR.K |
| 68021 | 285 | – | 298 | 761.8709 | 1521.7273 | 1521.7246 | 1.76 | 0 | 27 | 0.0028 | 1Score **> 33** indicates **identity** Score **> 14** indicates **homology** | U | K.CVINASGPFTDSVR.K |
| 68022 | 285 | – | 298 | 761.8723 | 1521.7300 | 1521.7246 | 3.56 | 0 | 78 | 1.5e-07 | 1Score **> 33** indicates **identity** Score **> 22** indicates **homology** | U | K.CVINASGPFTDSVR.K |
| 68145 | 285 | – | 298 | 762.3680 | 1522.7215 | 1522.7086 | 8.46 | 0 | 44 | 7.9e-05 | 1Score **> 32** indicates **identity** Score **> 15** indicates **homology** | U | K.CVINASGPFTDSVR.K  + Deamidated (NQ) |
| 68147 | 285 | – | 298 | 762.3688 | 1522.7230 | 1522.7086 | 9.43 | 0 | 31 | 0.0012 | 1Score **> 33** indicates **identity** Score **> 14** indicates **homology** | U | K.CVINASGPFTDSVR.K  + Deamidated (NQ) |
| 84291 | 285 | – | 299 | 550.9459 | 1649.8158 | 1649.8196 | -2.30 | 1 | 42 | 0.00011 | 1Score **> 34** indicates **identity** Score **> 15** indicates **homology** | U | K.CVINASGPFTDSVRK.M |
| 84292 | 285 | – | 299 | 550.9459 | 1649.8160 | 1649.8196 | -2.18 | 1 | 20 | 0.015 | 1Score **> 34** indicates **identity** Score **> 14** indicates **homology** | U | K.CVINASGPFTDSVRK.M |
| 84293 | 285 | – | 299 | 550.9468 | 1649.8187 | 1649.8196 | -0.52 | 1 | 57 | 4.7e-06 | 1Score **> 34** indicates **identity** Score **> 16** indicates **homology** | U | K.CVINASGPFTDSVRK.M |
| 84294 | 285 | – | 299 | 550.9470 | 1649.8191 | 1649.8196 | -0.30 | 1 | 51 | 1.6e-05 | 1Score **> 34** indicates **identity** Score **> 16** indicates **homology** | U | K.CVINASGPFTDSVRK.M |
| 84412 | 285 | – | 299 | 551.2805 | 1650.8197 | 1650.8036 | 9.78 | 1 | 37 | 0.00034 | 1Score **> 35** indicates **identity** Score **> 15** indicates **homology** | U | K.CVINASGPFTDSVRK.M  + Deamidated (NQ) |
| 187509 | 299 | – | 339 | 915.8433 | 4574.1799 | 4574.1841 | -0.92 | 2 | 29 | 0.002 | 1Score **> 36** indicates **identity** Score **> 14** indicates **homology** | U | R.KMDDKNVVPICQPSAGVHIVMPGYYSPENMGLLDPATSDGR.V  + Deamidated (NQ); HNE (C); Oxidation (M) |
| 30311 | 340 | – | 348 | 589.8333 | 1177.6521 | 1177.6536 | -1.27 | 0 | 25 | 0.0053 | 1Score **> 32** indicates **identity** Score **> 14** indicates **homology** | U | R.VIFFLPWEK.M |
| 30312 | 340 | – | 348 | 589.8334 | 1177.6522 | 1177.6536 | -1.18 | 0 | 29 | 0.0026 | 1Score **> 32** indicates **identity** Score **> 16** indicates **homology** | U | R.VIFFLPWEK.M |
| 30313 | 340 | – | 348 | 589.8335 | 1177.6524 | 1177.6536 | -0.98 | 0 | 33 | 0.0047 | 1Score **> 32** indicates **identity** Score **> 23** indicates **homology** | U | R.VIFFLPWEK.M |
| 30314 | 340 | – | 348 | 589.8337 | 1177.6528 | 1177.6536 | -0.69 | 0 | 16 | 0.039 | 1Score **> 32** indicates **identity** Score **> 15** indicates **homology** | U | R.VIFFLPWEK.M |
| 30316 | 340 | – | 348 | 589.8338 | 1177.6530 | 1177.6536 | -0.44 | 0 | 31 | 0.0044 | 1Score **> 32** indicates **identity** Score **> 20** indicates **homology** | U | R.VIFFLPWEK.M |
| 30318 | 340 | – | 348 | 589.8338 | 1177.6531 | 1177.6536 | -0.37 | 0 | 28 | 0.0026 | 1Score **> 32** indicates **identity** Score **> 14** indicates **homology** | U | R.VIFFLPWEK.M |
| 30322 | 340 | – | 348 | 589.8342 | 1177.6539 | 1177.6536 | 0.29 | 0 | 38 | 0.0022 | 1Score **> 32** indicates **identity** Score **> 24** indicates **homology** | U | R.VIFFLPWEK.M |
| 30323 | 340 | – | 348 | 589.8343 | 1177.6540 | 1177.6536 | 0.38 | 0 | 33 | 0.0045 | 1Score **> 32** indicates **identity** Score **> 22** indicates **homology** | U | R.VIFFLPWEK.M |
| 30326 | 340 | – | 348 | 589.8343 | 1177.6541 | 1177.6536 | 0.49 | 0 | 40 | 0.00032 | 1Score **> 32** indicates **identity** Score **> 18** indicates **homology** | U | R.VIFFLPWEK.M |
| 30328 | 340 | – | 348 | 589.8344 | 1177.6543 | 1177.6536 | 0.59 | 0 | 22 | 0.0087 | 1Score **> 32** indicates **identity** Score **> 14** indicates **homology** | U | R.VIFFLPWEK.M |
| 30329 | 340 | – | 348 | 589.8344 | 1177.6543 | 1177.6536 | 0.59 | 0 | 32 | 0.0047 | 1Score **> 32** indicates **identity** Score **> 22** indicates **homology** | U | R.VIFFLPWEK.M |
| 30331 | 340 | – | 348 | 589.8345 | 1177.6544 | 1177.6536 | 0.68 | 0 | 21 | 0.0098 | 1Score **> 32** indicates **identity** Score **> 14** indicates **homology** | U | R.VIFFLPWEK.M |
| 30333 | 340 | – | 348 | 589.8346 | 1177.6546 | 1177.6536 | 0.86 | 0 | 28 | 0.0026 | 1Score **> 32** indicates **identity** Score **> 14** indicates **homology** | U | R.VIFFLPWEK.M |
| 30335 | 340 | – | 348 | 589.8346 | 1177.6546 | 1177.6536 | 0.89 | 0 | 33 | 0.0046 | 1Score **> 32** indicates **identity** Score **> 22** indicates **homology** | U | R.VIFFLPWEK.M |
| 30339 | 340 | – | 348 | 589.8347 | 1177.6549 | 1177.6536 | 1.10 | 0 | 21 | 0.011 | 1Score **> 32** indicates **identity** Score **> 14** indicates **homology** | U | R.VIFFLPWEK.M |
| 30340 | 340 | – | 348 | 589.8348 | 1177.6550 | 1177.6536 | 1.21 | 0 | 20 | 0.013 | 1Score **> 32** indicates **identity** Score **> 14** indicates **homology** | U | R.VIFFLPWEK.M |
| 30342 | 340 | – | 348 | 589.8349 | 1177.6552 | 1177.6536 | 1.40 | 0 | 30 | 0.0058 | 1Score **> 32** indicates **identity** Score **> 20** indicates **homology** | U | R.VIFFLPWEK.M |
| 30343 | 340 | – | 348 | 589.8349 | 1177.6552 | 1177.6536 | 1.41 | 0 | 23 | 0.0065 | 1Score **> 32** indicates **identity** Score **> 14** indicates **homology** | U | R.VIFFLPWEK.M |
| 30344 | 340 | – | 348 | 589.8349 | 1177.6552 | 1177.6536 | 1.42 | 0 | 15 | 0.038 | 1Score **> 32** indicates **identity** Score **> 13** indicates **homology** | U | R.VIFFLPWEK.M |
| 30346 | 340 | – | 348 | 589.8351 | 1177.6556 | 1177.6536 | 1.69 | 0 | 22 | 0.014 | 1Score **> 32** indicates **identity** Score **> 16** indicates **homology** | U | R.VIFFLPWEK.M |
| 30350 | 340 | – | 348 | 589.8354 | 1177.6562 | 1177.6536 | 2.24 | 0 | 25 | 0.0064 | 1Score **> 32** indicates **identity** Score **> 16** indicates **homology** | U | R.VIFFLPWEK.M |
| 30352 | 340 | – | 348 | 589.8354 | 1177.6562 | 1177.6536 | 2.26 | 0 | 33 | 0.0046 | 1Score **> 32** indicates **identity** Score **> 22** indicates **homology** | U | R.VIFFLPWEK.M |
| 30354 | 340 | – | 348 | 589.8356 | 1177.6566 | 1177.6536 | 2.56 | 0 | 18 | 0.022 | 1Score **> 32** indicates **identity** Score **> 14** indicates **homology** | U | R.VIFFLPWEK.M |
| 30355 | 340 | – | 348 | 589.8356 | 1177.6567 | 1177.6536 | 2.66 | 0 | 17 | 0.024 | 1Score **> 32** indicates **identity** Score **> 14** indicates **homology** | U | R.VIFFLPWEK.M |
| 30356 | 340 | – | 348 | 589.8357 | 1177.6569 | 1177.6536 | 2.84 | 0 | 15 | 0.037 | 1Score **> 32** indicates **identity** Score **> 13** indicates **homology** | U | R.VIFFLPWEK.M |
| 30358 | 340 | – | 348 | 589.8359 | 1177.6573 | 1177.6536 | 3.15 | 0 | 18 | 0.02 | 1Score **> 32** indicates **identity** Score **> 14** indicates **homology** | U | R.VIFFLPWEK.M |
| 30359 | 340 | – | 348 | 589.8359 | 1177.6573 | 1177.6536 | 3.20 | 0 | 17 | 0.023 | 1Score **> 31** indicates **identity** Score **> 14** indicates **homology** | U | R.VIFFLPWEK.M |
| 30361 | 340 | – | 348 | 589.8361 | 1177.6576 | 1177.6536 | 3.44 | 0 | 22 | 0.009 | 1Score **> 31** indicates **identity** Score **> 14** indicates **homology** | U | R.VIFFLPWEK.M |
| 30363 | 340 | – | 348 | 589.8378 | 1177.6611 | 1177.6536 | 6.39 | 0 | 29 | 0.0087 | 1Score **> 32** indicates **identity** Score **> 21** indicates **homology** | U | R.VIFFLPWEK.M |
| 30364 | 340 | – | 348 | 589.8380 | 1177.6614 | 1177.6536 | 6.61 | 0 | 21 | 0.0098 | 1Score **> 32** indicates **identity** Score **> 14** indicates **homology** | U | R.VIFFLPWEK.M |
| 30365 | 340 | – | 348 | 589.8385 | 1177.6625 | 1177.6536 | 7.62 | 0 | 23 | 0.0072 | 1Score **> 32** indicates **identity** Score **> 14** indicates **homology** | U | R.VIFFLPWEK.M |
| 182724 | 349 | – | 380 | 891.6885 | 3562.7248 | 3562.7250 | -0.054 | 0 | 15 | 0.036 | 1Score **> 37** indicates **identity** Score **> 13** indicates **homology** | U | K.MTIAGTTDTPTDVTHHPIPSEEDINFILNEVR.N |
| 182725 | 349 | – | 380 | 1188.5831 | 3562.7276 | 3562.7250 | 0.72 | 0 | 60 | 2.2e-06 | 1Score **> 37** indicates **identity** Score **> 16** indicates **homology** | U | K.MTIAGTTDTPTDVTHHPIPSEEDINFILNEVR.N |
| 182726 | 349 | – | 380 | 891.6900 | 3562.7308 | 3562.7250 | 1.63 | 0 | 25 | 0.0048 | 1Score **> 37** indicates **identity** Score **> 14** indicates **homology** | U | K.MTIAGTTDTPTDVTHHPIPSEEDINFILNEVR.N |
| 30579 | 381 | – | 390 | 591.2906 | 1180.5666 | 1180.5724 | -4.91 | 0 | 16 | 0.035 | 1Score **> 31** indicates **identity** Score **> 13** indicates **homology** | U | R.NYLSSDVEVR.R |
| 30580 | 381 | – | 390 | 591.2907 | 1180.5669 | 1180.5724 | -4.69 | 0 | 31 | 0.0013 | 1Score **> 31** indicates **identity** Score **> 14** indicates **homology** | U | R.NYLSSDVEVR.R |
| 30585 | 381 | – | 390 | 591.2924 | 1180.5702 | 1180.5724 | -1.90 | 0 | 32 | 0.0009 | 1Score **> 31** indicates **identity** Score **> 15** indicates **homology** | U | R.NYLSSDVEVR.R |
| 30586 | 381 | – | 390 | 591.2925 | 1180.5704 | 1180.5724 | -1.70 | 0 | 35 | 0.00058 | 1Score **> 31** indicates **identity** Score **> 15** indicates **homology** | U | R.NYLSSDVEVR.R |
| 30587 | 381 | – | 390 | 591.2928 | 1180.5711 | 1180.5724 | -1.08 | 0 | 48 | 2.9e-05 | 1Score **> 31** indicates **identity** Score **> 16** indicates **homology** | U | R.NYLSSDVEVR.R |
| 30588 | 381 | – | 390 | 591.2930 | 1180.5714 | 1180.5724 | -0.87 | 0 | 55 | 6.9e-06 | 1Score **> 32** indicates **identity** Score **> 16** indicates **homology** | U | R.NYLSSDVEVR.R |
| 30589 | 381 | – | 390 | 591.2930 | 1180.5715 | 1180.5724 | -0.77 | 0 | 42 | 0.0002 | 1Score **> 32** indicates **identity** Score **> 18** indicates **homology** | U | R.NYLSSDVEVR.R |
| 30590 | 381 | – | 390 | 591.2930 | 1180.5715 | 1180.5724 | -0.77 | 0 | 29 | 0.0017 | 1Score **> 32** indicates **identity** Score **> 14** indicates **homology** | U | R.NYLSSDVEVR.R |
| 30591 | 381 | – | 390 | 591.2933 | 1180.5720 | 1180.5724 | -0.37 | 0 | 53 | 1.1e-05 | 1Score **> 32** indicates **identity** Score **> 16** indicates **homology** | U | R.NYLSSDVEVR.R |
| 30592 | 381 | – | 390 | 591.2933 | 1180.5721 | 1180.5724 | -0.24 | 0 | 58 | 3.7e-06 | 1Score **> 32** indicates **identity** Score **> 16** indicates **homology** | U | R.NYLSSDVEVR.R |
| 30593 | 381 | – | 390 | 591.2934 | 1180.5722 | 1180.5724 | -0.14 | 0 | 55 | 6.9e-06 | 1Score **> 32** indicates **identity** Score **> 16** indicates **homology** | U | R.NYLSSDVEVR.R |
| 30594 | 381 | – | 390 | 591.2934 | 1180.5723 | 1180.5724 | -0.072 | 0 | 49 | 2.3e-05 | 1Score **> 32** indicates **identity** Score **> 16** indicates **homology** | U | R.NYLSSDVEVR.R |
| 30595 | 381 | – | 390 | 591.2934 | 1180.5723 | 1180.5724 | -0.052 | 0 | 60 | 3e-06 | 1Score **> 32** indicates **identity** Score **> 17** indicates **homology** | U | R.NYLSSDVEVR.R |
| 30596 | 381 | – | 390 | 591.2936 | 1180.5727 | 1180.5724 | 0.28 | 0 | 43 | 8.4e-05 | 1Score **> 32** indicates **identity** Score **> 15** indicates **homology** | U | R.NYLSSDVEVR.R |
| 30597 | 381 | – | 390 | 591.2937 | 1180.5728 | 1180.5724 | 0.35 | 0 | 52 | 1.3e-05 | 1Score **> 32** indicates **identity** Score **> 16** indicates **homology** | U | R.NYLSSDVEVR.R |
| 30599 | 381 | – | 390 | 591.2943 | 1180.5740 | 1180.5724 | 1.39 | 0 | 62 | 1.7e-06 | 1Score **> 31** indicates **identity** Score **> 16** indicates **homology** | U | R.NYLSSDVEVR.R |
| 30603 | 381 | – | 390 | 591.2950 | 1180.5755 | 1180.5724 | 2.65 | 0 | 18 | 0.02 | 1Score **> 32** indicates **identity** Score **> 14** indicates **homology** | U | R.NYLSSDVEVR.R |
| 30652 | 381 | – | 390 | 591.2991 | 1180.5836 | 1180.5724 | 9.49 | 0 | 14 | 0.046 | 1Score **> 32** indicates **identity** Score **> 13** indicates **homology** | U | R.NYLSSDVEVR.R |
| 30729 | 381 | – | 390 | 591.7833 | 1181.5521 | 1181.5564 | -3.67 | 0 | 23 | 0.0071 | 1Score **> 30** indicates **identity** Score **> 14** indicates **homology** | U | R.NYLSSDVEVR.R  + Deamidated (NQ) |
| 30731 | 381 | – | 390 | 591.7870 | 1181.5595 | 1181.5564 | 2.62 | 0 | 29 | 0.0041 | 1Score **> 30** indicates **identity** Score **> 18** indicates **homology** | U | R.NYLSSDVEVR.R  + Deamidated (NQ) |
| 47476 | 381 | – | 391 | 669.3427 | 1336.6709 | 1336.6735 | -1.95 | 1 | 28 | 0.0064 | 1Score **> 33** indicates **identity** Score **> 18** indicates **homology** | U | R.NYLSSDVEVRR.G |
| 47477 | 381 | – | 391 | 446.5644 | 1336.6715 | 1336.6735 | -1.49 | 1 | 23 | 0.0068 | 1Score **> 33** indicates **identity** Score **> 14** indicates **homology** | U | R.NYLSSDVEVRR.G |
| 124327 | 391 | – | 409 | 684.3843 | 2050.1311 | 2050.1324 | -0.60 | 2 | 24 | 0.0072 | 1Score **> 35** indicates **identity** Score **> 15** indicates **homology** | U | R.RGDVLAAWSGIRPLVTDPK.S |
| 124328 | 391 | – | 409 | 513.5401 | 2050.1314 | 2050.1324 | -0.45 | 2 | 27 | 0.018 | 1Score **> 35** indicates **identity** Score **> 22** indicates **homology** | U | R.RGDVLAAWSGIRPLVTDPK.S |
| 124329 | 391 | – | 409 | 513.5403 | 2050.1320 | 2050.1324 | -0.19 | 2 | 19 | 0.032 | 1Score **> 35** indicates **identity** Score **> 17** indicates **homology** | U | R.RGDVLAAWSGIRPLVTDPK.S |
| 124330 | 391 | – | 409 | 684.3846 | 2050.1321 | 2050.1324 | -0.12 | 2 | 42 | 0.00015 | 1Score **> 35** indicates **identity** Score **> 17** indicates **homology** | U | R.RGDVLAAWSGIRPLVTDPK.S |
| 124331 | 391 | – | 409 | 1026.0733 | 2050.1321 | 2050.1324 | -0.12 | 2 | 15 | 0.042 | 1Score **> 35** indicates **identity** Score **> 13** indicates **homology** | U | R.RGDVLAAWSGIRPLVTDPK.S |
| 124332 | 391 | – | 409 | 513.5403 | 2050.1323 | 2050.1324 | -0.042 | 2 | 28 | 0.012 | 1Score **> 35** indicates **identity** Score **> 21** indicates **homology** | U | R.RGDVLAAWSGIRPLVTDPK.S |
| 124333 | 391 | – | 409 | 513.5404 | 2050.1323 | 2050.1324 | -0.0073 | 2 | 23 | 0.0096 | 1Score **> 35** indicates **identity** Score **> 15** indicates **homology** | U | R.RGDVLAAWSGIRPLVTDPK.S |
| 124335 | 391 | – | 409 | 684.3849 | 2050.1328 | 2050.1324 | 0.20 | 2 | 47 | 6e-05 | 1Score **> 35** indicates **identity** Score **> 17** indicates **homology** | U | R.RGDVLAAWSGIRPLVTDPK.S |
| 124338 | 391 | – | 409 | 513.5406 | 2050.1331 | 2050.1324 | 0.38 | 2 | 18 | 0.042 | 1Score **> 35** indicates **identity** Score **> 16** indicates **homology** | U | R.RGDVLAAWSGIRPLVTDPK.S |
| 124339 | 391 | – | 409 | 684.3851 | 2050.1334 | 2050.1324 | 0.49 | 2 | 43 | 0.00014 | 1Score **> 35** indicates **identity** Score **> 17** indicates **homology** | U | R.RGDVLAAWSGIRPLVTDPK.S |
| 124343 | 391 | – | 409 | 684.3852 | 2050.1339 | 2050.1324 | 0.75 | 2 | 41 | 0.00026 | 1Score **> 34** indicates **identity** Score **> 18** indicates **homology** | U | R.RGDVLAAWSGIRPLVTDPK.S |
| 124345 | 391 | – | 409 | 513.5408 | 2050.1341 | 2050.1324 | 0.85 | 2 | 18 | 0.03 | 1Score **> 34** indicates **identity** Score **> 16** indicates **homology** | U | R.RGDVLAAWSGIRPLVTDPK.S |
| 124347 | 391 | – | 409 | 513.5408 | 2050.1341 | 2050.1324 | 0.87 | 2 | 26 | 0.025 | 1Score **> 34** indicates **identity** Score **> 22** indicates **homology** | U | R.RGDVLAAWSGIRPLVTDPK.S |
| 124348 | 391 | – | 409 | 513.5408 | 2050.1342 | 2050.1324 | 0.88 | 2 | 19 | 0.025 | 1Score **> 34** indicates **identity** Score **> 16** indicates **homology** | U | R.RGDVLAAWSGIRPLVTDPK.S |
| 124349 | 391 | – | 409 | 513.5408 | 2050.1342 | 2050.1324 | 0.91 | 2 | 21 | 0.013 | 1Score **> 34** indicates **identity** Score **> 14** indicates **homology** | U | R.RGDVLAAWSGIRPLVTDPK.S |
| 124350 | 391 | – | 409 | 513.5408 | 2050.1342 | 2050.1324 | 0.91 | 2 | 20 | 0.019 | 1Score **> 34** indicates **identity** Score **> 15** indicates **homology** | U | R.RGDVLAAWSGIRPLVTDPK.S |
| 124351 | 391 | – | 409 | 513.5408 | 2050.1343 | 2050.1324 | 0.94 | 2 | 27 | 0.017 | 1Score **> 34** indicates **identity** Score **> 22** indicates **homology** | U | R.RGDVLAAWSGIRPLVTDPK.S |
| 124352 | 391 | – | 409 | 684.3854 | 2050.1344 | 2050.1324 | 0.99 | 2 | 17 | 0.024 | 1Score **> 34** indicates **identity** Score **> 14** indicates **homology** | U | R.RGDVLAAWSGIRPLVTDPK.S |
| 124354 | 391 | – | 409 | 684.3856 | 2050.1349 | 2050.1324 | 1.25 | 2 | 29 | 0.0027 | 1Score **> 34** indicates **identity** Score **> 16** indicates **homology** | U | R.RGDVLAAWSGIRPLVTDPK.S |
| 124361 | 391 | – | 409 | 513.5420 | 2050.1388 | 2050.1324 | 3.17 | 2 | 23 | 0.019 | 1Score **> 34** indicates **identity** Score **> 18** indicates **homology** | U | R.RGDVLAAWSGIRPLVTDPK.S |
| 124362 | 391 | – | 409 | 513.5420 | 2050.1389 | 2050.1324 | 3.21 | 2 | 16 | 0.031 | 1Score **> 34** indicates **identity** Score **> 14** indicates **homology** | U | R.RGDVLAAWSGIRPLVTDPK.S |
| 124368 | 391 | – | 409 | 513.5433 | 2050.1442 | 2050.1324 | 5.77 | 2 | 17 | 0.029 | 1Score **> 34** indicates **identity** Score **> 14** indicates **homology** | U | R.RGDVLAAWSGIRPLVTDPK.S |
| 110396 | 392 | – | 409 | 632.3504 | 1894.0294 | 1894.0312 | -1.00 | 1 | 21 | 0.012 | 1Score **> 35** indicates **identity** Score **> 14** indicates **homology** | U | R.GDVLAAWSGIRPLVTDPK.S |
| 110397 | 392 | – | 409 | 632.3508 | 1894.0306 | 1894.0312 | -0.35 | 1 | 25 | 0.0048 | 1Score **> 35** indicates **identity** Score **> 14** indicates **homology** | U | R.GDVLAAWSGIRPLVTDPK.S |
| 11907 | 410 | – | 418 | 482.7381 | 963.4617 | 963.4621 | -0.46 | 0 | 62 | 4.8e-05 | 1Score **> 31** indicates **identity** | U | K.SADTQSISR.N |
| 11908 | 410 | – | 418 | 482.7382 | 963.4617 | 963.4621 | -0.39 | 0 | 32 | 0.0043 | 1Score **> 31** indicates **identity** Score **> 21** indicates **homology** | U | K.SADTQSISR.N |
| 165178 | 410 | – | 436 | 686.3530 | 2741.3829 | 2741.3832 | -0.093 | 1 | 25 | 0.005 | 1Score **> 37** indicates **identity** Score **> 14** indicates **homology** | U | K.SADTQSISRNHVVDISDSGLITIAGGK.W  + Deamidated (NQ) |
| 100834 | 419 | – | 436 | 599.3209 | 1794.9408 | 1794.9476 | -3.80 | 0 | 51 | 1.6e-05 | 1Score **> 35** indicates **identity** Score **> 16** indicates **homology** | U | R.NHVVDISDSGLITIAGGK.W |
| 100837 | 419 | – | 436 | 898.4782 | 1794.9419 | 1794.9476 | -3.17 | 0 | 71 | 2.3e-07 | 1Score **> 36** indicates **identity** Score **> 17** indicates **homology** | U | R.NHVVDISDSGLITIAGGK.W |
| 100841 | 419 | – | 436 | 599.3220 | 1794.9442 | 1794.9476 | -1.88 | 0 | 58 | 4e-06 | 1Score **> 36** indicates **identity** Score **> 16** indicates **homology** | U | R.NHVVDISDSGLITIAGGK.W |
| 100843 | 419 | – | 436 | 898.4796 | 1794.9447 | 1794.9476 | -1.62 | 0 | 90 | 3.8e-09 | 1Score **> 35** indicates **identity** Score **> 18** indicates **homology** | U | R.NHVVDISDSGLITIAGGK.W |
| 100848 | 419 | – | 436 | 599.3226 | 1794.9458 | 1794.9476 | -0.98 | 0 | 75 | 9.8e-08 | 1Score **> 36** indicates **identity** Score **> 17** indicates **homology** | U | R.NHVVDISDSGLITIAGGK.W |
| 100853 | 419 | – | 436 | 898.4805 | 1794.9465 | 1794.9476 | -0.61 | 0 | 24 | 0.0055 | 1Score **> 36** indicates **identity** Score **> 14** indicates **homology** | U | R.NHVVDISDSGLITIAGGK.W |
| 100856 | 419 | – | 436 | 599.3229 | 1794.9467 | 1794.9476 | -0.47 | 0 | 78 | 5.3e-08 | 1Score **> 36** indicates **identity** Score **> 17** indicates **homology** | U | R.NHVVDISDSGLITIAGGK.W |
| 100857 | 419 | – | 436 | 599.3229 | 1794.9468 | 1794.9476 | -0.44 | 0 | 28 | 0.0023 | 1Score **> 36** indicates **identity** Score **> 14** indicates **homology** | U | R.NHVVDISDSGLITIAGGK.W |
| 100858 | 419 | – | 436 | 898.4808 | 1794.9470 | 1794.9476 | -0.33 | 0 | 36 | 0.00042 | 1Score **> 36** indicates **identity** Score **> 15** indicates **homology** | U | R.NHVVDISDSGLITIAGGK.W |
| 100859 | 419 | – | 436 | 599.3230 | 1794.9471 | 1794.9476 | -0.27 | 0 | 45 | 6.2e-05 | 1Score **> 36** indicates **identity** Score **> 15** indicates **homology** | U | R.NHVVDISDSGLITIAGGK.W |
| 100863 | 419 | – | 436 | 599.3233 | 1794.9481 | 1794.9476 | 0.26 | 0 | 113 | 3.4e-11 | 1Score **> 35** indicates **identity** Score **> 21** indicates **homology** | U | R.NHVVDISDSGLITIAGGK.W |
| 100866 | 419 | – | 436 | 599.3234 | 1794.9484 | 1794.9476 | 0.43 | 0 | 74 | 1.2e-07 | 1Score **> 36** indicates **identity** Score **> 17** indicates **homology** | U | R.NHVVDISDSGLITIAGGK.W |
| 100867 | 419 | – | 436 | 599.3235 | 1794.9486 | 1794.9476 | 0.57 | 0 | 74 | 1.1e-07 | 1Score **> 36** indicates **identity** Score **> 17** indicates **homology** | U | R.NHVVDISDSGLITIAGGK.W |
| 100868 | 419 | – | 436 | 599.3235 | 1794.9486 | 1794.9476 | 0.58 | 0 | 92 | 2.6e-09 | 1Score **> 36** indicates **identity** Score **> 19** indicates **homology** | U | R.NHVVDISDSGLITIAGGK.W |
| 100873 | 419 | – | 436 | 599.3236 | 1794.9489 | 1794.9476 | 0.75 | 0 | 81 | 2.5e-08 | 1Score **> 36** indicates **identity** Score **> 18** indicates **homology** | U | R.NHVVDISDSGLITIAGGK.W |
| 100875 | 419 | – | 436 | 898.4818 | 1794.9490 | 1794.9476 | 0.80 | 0 | 78 | 5e-08 | 1Score **> 36** indicates **identity** Score **> 17** indicates **homology** | U | R.NHVVDISDSGLITIAGGK.W |
| 100876 | 419 | – | 436 | 599.3236 | 1794.9490 | 1794.9476 | 0.80 | 0 | 78 | 5.1e-08 | 1Score **> 36** indicates **identity** Score **> 18** indicates **homology** | U | R.NHVVDISDSGLITIAGGK.W |
| 100877 | 419 | – | 436 | 599.3236 | 1794.9491 | 1794.9476 | 0.84 | 0 | 56 | 5.8e-06 | 1Score **> 36** indicates **identity** Score **> 16** indicates **homology** | U | R.NHVVDISDSGLITIAGGK.W |
| 100880 | 419 | – | 436 | 599.3237 | 1794.9493 | 1794.9476 | 0.95 | 0 | 90 | 5.3e-09 | 1Score **> 36** indicates **identity** Score **> 19** indicates **homology** | U | R.NHVVDISDSGLITIAGGK.W |
| 100881 | 419 | – | 436 | 599.3237 | 1794.9494 | 1794.9476 | 1.00 | 0 | 67 | 4.7e-07 | 1Score **> 36** indicates **identity** Score **> 17** indicates **homology** | U | R.NHVVDISDSGLITIAGGK.W |
| 100882 | 419 | – | 436 | 898.4820 | 1794.9494 | 1794.9476 | 1.01 | 0 | 113 | 2.3e-11 | 1Score **> 36** indicates **identity** Score **> 20** indicates **homology** | U | R.NHVVDISDSGLITIAGGK.W |
| 100883 | 419 | – | 436 | 599.3238 | 1794.9495 | 1794.9476 | 1.07 | 0 | 113 | 3.5e-11 | 1Score **> 36** indicates **identity** Score **> 21** indicates **homology** | U | R.NHVVDISDSGLITIAGGK.W |
| 100886 | 419 | – | 436 | 599.3239 | 1794.9498 | 1794.9476 | 1.25 | 0 | 99 | 7e-10 | 1Score **> 36** indicates **identity** Score **> 20** indicates **homology** | U | R.NHVVDISDSGLITIAGGK.W |
| 100887 | 419 | – | 436 | 599.3239 | 1794.9498 | 1794.9476 | 1.25 | 0 | 24 | 0.0057 | 1Score **> 36** indicates **identity** Score **> 14** indicates **homology** | U | R.NHVVDISDSGLITIAGGK.W |
| 100889 | 419 | – | 436 | 898.4825 | 1794.9504 | 1794.9476 | 1.55 | 0 | 71 | 2.2e-07 | 1Score **> 36** indicates **identity** Score **> 17** indicates **homology** | U | R.NHVVDISDSGLITIAGGK.W |
| 100890 | 419 | – | 436 | 898.4825 | 1794.9505 | 1794.9476 | 1.62 | 0 | 84 | 1.3e-08 | 1Score **> 36** indicates **identity** Score **> 18** indicates **homology** | U | R.NHVVDISDSGLITIAGGK.W |
| 100891 | 419 | – | 436 | 898.4825 | 1794.9505 | 1794.9476 | 1.62 | 0 | 90 | 3.7e-09 | 1Score **> 36** indicates **identity** Score **> 18** indicates **homology** | U | R.NHVVDISDSGLITIAGGK.W |
| 100892 | 419 | – | 436 | 599.3242 | 1794.9507 | 1794.9476 | 1.75 | 0 | 44 | 7.8e-05 | 1Score **> 36** indicates **identity** Score **> 15** indicates **homology** | U | R.NHVVDISDSGLITIAGGK.W |
| 100894 | 419 | – | 436 | 599.3242 | 1794.9508 | 1794.9476 | 1.80 | 0 | 81 | 2.6e-08 | 1Score **> 36** indicates **identity** Score **> 18** indicates **homology** | U | R.NHVVDISDSGLITIAGGK.W |
| 100897 | 419 | – | 436 | 599.3243 | 1794.9512 | 1794.9476 | 1.99 | 0 | 68 | 4.5e-07 | 1Score **> 36** indicates **identity** Score **> 17** indicates **homology** | U | R.NHVVDISDSGLITIAGGK.W |
| 100899 | 419 | – | 436 | 599.3244 | 1794.9515 | 1794.9476 | 2.16 | 0 | 73 | 1.4e-07 | 1Score **> 36** indicates **identity** Score **> 17** indicates **homology** | U | R.NHVVDISDSGLITIAGGK.W |
| 100900 | 419 | – | 436 | 599.3244 | 1794.9515 | 1794.9476 | 2.19 | 0 | 62 | 1.6e-06 | 1Score **> 36** indicates **identity** Score **> 16** indicates **homology** | U | R.NHVVDISDSGLITIAGGK.W |
| 100904 | 419 | – | 436 | 599.3250 | 1794.9532 | 1794.9476 | 3.13 | 0 | 40 | 0.00017 | 1Score **> 36** indicates **identity** Score **> 15** indicates **homology** | U | R.NHVVDISDSGLITIAGGK.W |
| 100905 | 419 | – | 436 | 599.3251 | 1794.9533 | 1794.9476 | 3.21 | 0 | 81 | 2.5e-08 | 1Score **> 36** indicates **identity** Score **> 18** indicates **homology** | U | R.NHVVDISDSGLITIAGGK.W |
| 100906 | 419 | – | 436 | 599.3251 | 1794.9534 | 1794.9476 | 3.22 | 0 | 42 | 0.00012 | 1Score **> 36** indicates **identity** Score **> 15** indicates **homology** | U | R.NHVVDISDSGLITIAGGK.W |
| 100910 | 419 | – | 436 | 599.3254 | 1794.9545 | 1794.9476 | 3.85 | 0 | 70 | 2.6e-07 | 1Score **> 35** indicates **identity** Score **> 17** indicates **homology** | U | R.NHVVDISDSGLITIAGGK.W |
| 100911 | 419 | – | 436 | 599.3255 | 1794.9546 | 1794.9476 | 3.90 | 0 | 69 | 3.2e-07 | 1Score **> 35** indicates **identity** Score **> 17** indicates **homology** | U | R.NHVVDISDSGLITIAGGK.W |
| 100912 | 419 | – | 436 | 599.3255 | 1794.9548 | 1794.9476 | 4.02 | 0 | 28 | 0.0023 | 1Score **> 36** indicates **identity** Score **> 14** indicates **homology** | U | R.NHVVDISDSGLITIAGGK.W |
| 100914 | 419 | – | 436 | 599.3258 | 1794.9555 | 1794.9476 | 4.42 | 0 | 81 | 2.4e-08 | 1Score **> 35** indicates **identity** Score **> 18** indicates **homology** | U | R.NHVVDISDSGLITIAGGK.W |
| 100915 | 419 | – | 436 | 599.3258 | 1794.9557 | 1794.9476 | 4.51 | 0 | 77 | 5.5e-08 | 1Score **> 35** indicates **identity** Score **> 17** indicates **homology** | U | R.NHVVDISDSGLITIAGGK.W |
| 100918 | 419 | – | 436 | 599.3261 | 1794.9565 | 1794.9476 | 4.94 | 0 | 63 | 1.2e-06 | 1Score **> 36** indicates **identity** Score **> 16** indicates **homology** | U | R.NHVVDISDSGLITIAGGK.W |
| 100922 | 419 | – | 436 | 599.3268 | 1794.9585 | 1794.9476 | 6.09 | 0 | 25 | 0.0061 | 1Score **> 35** indicates **identity** Score **> 15** indicates **homology** | U | R.NHVVDISDSGLITIAGGK.W |
| 100923 | 419 | – | 436 | 599.3268 | 1794.9587 | 1794.9476 | 6.19 | 0 | 80 | 3e-08 | 1Score **> 35** indicates **identity** Score **> 18** indicates **homology** | U | R.NHVVDISDSGLITIAGGK.W |
| 100924 | 419 | – | 436 | 599.3270 | 1794.9591 | 1794.9476 | 6.42 | 0 | 71 | 2e-07 | 1Score **> 35** indicates **identity** Score **> 17** indicates **homology** | U | R.NHVVDISDSGLITIAGGK.W |
| 100927 | 419 | – | 436 | 599.3278 | 1794.9616 | 1794.9476 | 7.78 | 0 | 62 | 1.5e-06 | 1Score **> 35** indicates **identity** Score **> 16** indicates **homology** | U | R.NHVVDISDSGLITIAGGK.W |
| 100928 | 419 | – | 436 | 599.3283 | 1794.9630 | 1794.9476 | 8.59 | 0 | 81 | 2.8e-08 | 1Score **> 35** indicates **identity** Score **> 18** indicates **homology** | U | R.NHVVDISDSGLITIAGGK.W |
| 100930 | 419 | – | 436 | 599.3286 | 1794.9641 | 1794.9476 | 9.21 | 0 | 23 | 0.0073 | 1Score **> 35** indicates **identity** Score **> 14** indicates **homology** | U | R.NHVVDISDSGLITIAGGK.W |
| 100979 | 419 | – | 436 | 599.6498 | 1795.9275 | 1795.9316 | -2.29 | 0 | 32 | 0.00095 | 1Score **> 35** indicates **identity** Score **> 15** indicates **homology** | U | R.NHVVDISDSGLITIAGGK.W  + Deamidated (NQ) |
| 100989 | 419 | – | 436 | 599.6568 | 1795.9486 | 1795.9316 | 9.44 | 0 | 30 | 0.0015 | 1Score **> 36** indicates **identity** Score **> 14** indicates **homology** | U | R.NHVVDISDSGLITIAGGK.W  + Deamidated (NQ) |
| 100991 | 419 | – | 436 | 599.6571 | 1795.9495 | 1795.9316 | 9.96 | 0 | 31 | 0.0013 | 1Score **> 35** indicates **identity** Score **> 14** indicates **homology** | U | R.NHVVDISDSGLITIAGGK.W  + Deamidated (NQ) |
| 100992 | 419 | – | 436 | 599.6571 | 1795.9495 | 1795.9316 | 10.00 | 0 | 49 | 2.6e-05 | 1Score **> 35** indicates **identity** Score **> 16** indicates **homology** | U | R.NHVVDISDSGLITIAGGK.W  + Deamidated (NQ) |
| 155530 | 419 | – | 441 | 835.1040 | 2502.2902 | 2502.2867 | 1.40 | 1 | 68 | 4e-07 | 1Score **> 37** indicates **identity** Score **> 17** indicates **homology** | U | R.NHVVDISDSGLITIAGGKWTTYR.S |
| 155531 | 419 | – | 441 | 626.5799 | 2502.2903 | 2502.2867 | 1.45 | 1 | 62 | 1.4e-06 | 1Score **> 37** indicates **identity** Score **> 16** indicates **homology** | U | R.NHVVDISDSGLITIAGGKWTTYR.S |
| 155532 | 419 | – | 441 | 626.5810 | 2502.2950 | 2502.2867 | 3.31 | 1 | 45 | 5.5e-05 | 1Score **> 37** indicates **identity** Score **> 15** indicates **homology** | U | R.NHVVDISDSGLITIAGGKWTTYR.S |
| 155533 | 419 | – | 441 | 835.1064 | 2502.2974 | 2502.2867 | 4.26 | 1 | 63 | 1.3e-06 | 1Score **> 37** indicates **identity** Score **> 16** indicates **homology** | U | R.NHVVDISDSGLITIAGGKWTTYR.S |
| 155536 | 419 | – | 441 | 835.1079 | 2502.3019 | 2502.2867 | 6.09 | 1 | 60 | 2.4e-06 | 1Score **> 37** indicates **identity** Score **> 16** indicates **homology** | U | R.NHVVDISDSGLITIAGGKWTTYR.S |
| 36589 | 442 | – | 453 | 618.7921 | 1235.5697 | 1235.5704 | -0.56 | 0 | 27 | 0.0032 | 1Score **> 30** indicates **identity** Score **> 14** indicates **homology** | U | R.SMAEDTVDAAVK.F |
| 36590 | 442 | – | 453 | 618.7923 | 1235.5701 | 1235.5704 | -0.20 | 0 | 42 | 0.00013 | 1Score **> 30** indicates **identity** Score **> 15** indicates **homology** | U | R.SMAEDTVDAAVK.F |
| 38261 | 442 | – | 453 | 626.7896 | 1251.5646 | 1251.5653 | -0.50 | 0 | 20 | 0.012 | 1Score **> 29** indicates **identity** Score **> 14** indicates **homology** | U | R.SMAEDTVDAAVK.F  + Oxidation (M) |
| 145677 | 442 | – | 463 | 583.2862 | 2329.1158 | 2329.1121 | 1.59 | 1 | 42 | 0.00011 | 1Score **> 35** indicates **identity** Score **> 15** indicates **homology** | U | R.SMAEDTVDAAVKFHNLNAGPSR.T |
| 145679 | 442 | – | 463 | 583.2870 | 2329.1190 | 2329.1121 | 2.98 | 1 | 21 | 0.012 | 1Score **> 35** indicates **identity** Score **> 14** indicates **homology** | U | R.SMAEDTVDAAVKFHNLNAGPSR.T |
| 23185 | 454 | – | 463 | 556.7817 | 1111.5489 | 1111.5523 | -3.04 | 0 | 16 | 0.046 | 1Score **> 30** indicates **identity** Score **> 15** indicates **homology** | U | K.FHNLNAGPSR.T |
| 23188 | 454 | – | 463 | 371.5246 | 1111.5518 | 1111.5523 | -0.40 | 0 | 37 | 0.00038 | 1Score **> 31** indicates **identity** Score **> 15** indicates **homology** | U | K.FHNLNAGPSR.T |
| 23189 | 454 | – | 463 | 556.7832 | 1111.5519 | 1111.5523 | -0.30 | 0 | 37 | 0.00074 | 1Score **> 31** indicates **identity** Score **> 18** indicates **homology** | U | K.FHNLNAGPSR.T |
| 23190 | 454 | – | 463 | 371.5246 | 1111.5521 | 1111.5523 | -0.18 | 0 | 36 | 0.0004 | 1Score **> 31** indicates **identity** Score **> 15** indicates **homology** | U | K.FHNLNAGPSR.T |
| 23191 | 454 | – | 463 | 556.7834 | 1111.5522 | 1111.5523 | -0.075 | 0 | 46 | 0.00011 | 1Score **> 31** indicates **identity** Score **> 19** indicates **homology** | U | K.FHNLNAGPSR.T |
| 23192 | 454 | – | 463 | 556.7834 | 1111.5523 | 1111.5523 | 0.024 | 0 | 25 | 0.0068 | 1Score **> 31** indicates **identity** Score **> 16** indicates **homology** | U | K.FHNLNAGPSR.T |
| 23194 | 454 | – | 463 | 371.5248 | 1111.5524 | 1111.5523 | 0.13 | 0 | 54 | 8.9e-06 | 1Score **> 31** indicates **identity** Score **> 16** indicates **homology** | U | K.FHNLNAGPSR.T |
| 23195 | 454 | – | 463 | 371.5248 | 1111.5526 | 1111.5523 | 0.26 | 0 | 26 | 0.0035 | 1Score **> 31** indicates **identity** Score **> 14** indicates **homology** | U | K.FHNLNAGPSR.T |
| 23197 | 454 | – | 463 | 556.7836 | 1111.5527 | 1111.5523 | 0.37 | 0 | 49 | 0.00015 | 1Score **> 31** indicates **identity** Score **> 23** indicates **homology** | U | K.FHNLNAGPSR.T |
| 23198 | 454 | – | 463 | 371.5249 | 1111.5529 | 1111.5523 | 0.52 | 0 | 29 | 0.0019 | 1Score **> 31** indicates **identity** Score **> 14** indicates **homology** | U | K.FHNLNAGPSR.T |
| 23199 | 454 | – | 463 | 556.7837 | 1111.5529 | 1111.5523 | 0.58 | 0 | 38 | 0.0015 | 1Score **> 31** indicates **identity** Score **> 22** indicates **homology** | U | K.FHNLNAGPSR.T |
| 23200 | 454 | – | 463 | 371.5249 | 1111.5530 | 1111.5523 | 0.61 | 0 | 31 | 0.0011 | 1Score **> 31** indicates **identity** Score **> 14** indicates **homology** | U | K.FHNLNAGPSR.T |
| 23201 | 454 | – | 463 | 371.5251 | 1111.5534 | 1111.5523 | 0.97 | 0 | 51 | 1.8e-05 | 1Score **> 31** indicates **identity** Score **> 16** indicates **homology** | U | K.FHNLNAGPSR.T |
| 23202 | 454 | – | 463 | 556.7840 | 1111.5535 | 1111.5523 | 1.07 | 0 | 40 | 0.00059 | 1Score **> 31** indicates **identity** Score **> 20** indicates **homology** | U | K.FHNLNAGPSR.T |
| 15431 | 464 | – | 473 | 510.2975 | 1018.5804 | 1018.5811 | -0.74 | 0 | 44 | 7.3e-05 | 1Score **> 31** indicates **identity** Score **> 15** indicates **homology** | U | R.TVGLFLQGGK.D |
| 15432 | 464 | – | 473 | 510.2980 | 1018.5815 | 1018.5811 | 0.35 | 0 | 49 | 2.4e-05 | 1Score **> 31** indicates **identity** Score **> 16** indicates **homology** | U | R.TVGLFLQGGK.D |
| 15433 | 464 | – | 473 | 510.2986 | 1018.5825 | 1018.5811 | 1.38 | 0 | 51 | 1.5e-05 | 1Score **> 32** indicates **identity** Score **> 16** indicates **homology** | U | R.TVGLFLQGGK.D |
| 15434 | 464 | – | 473 | 510.2986 | 1018.5825 | 1018.5811 | 1.38 | 0 | 45 | 6e-05 | 1Score **> 32** indicates **identity** Score **> 15** indicates **homology** | U | R.TVGLFLQGGK.D |
| 15435 | 464 | – | 473 | 510.2990 | 1018.5835 | 1018.5811 | 2.31 | 0 | 45 | 5.6e-05 | 1Score **> 30** indicates **identity** Score **> 15** indicates **homology** | U | R.TVGLFLQGGK.D |
| 132876 | 464 | – | 482 | 717.7179 | 2150.1319 | 2150.1524 | -9.57 | 1 | 18 | 0.019 | 1Score **> 36** indicates **identity** Score **> 14** indicates **homology** | U | R.TVGLFLQGGKDWSPTLYIR.L |
| 132892 | 464 | – | 482 | 717.7234 | 2150.1483 | 2150.1524 | -1.91 | 1 | 24 | 0.006 | 1Score **> 36** indicates **identity** Score **> 14** indicates **homology** | U | R.TVGLFLQGGKDWSPTLYIR.L |
| 132903 | 464 | – | 482 | 1076.0840 | 2150.1534 | 2150.1524 | 0.44 | 1 | 102 | 3e-10 | 1Score **> 36** indicates **identity** Score **> 19** indicates **homology** | U | R.TVGLFLQGGKDWSPTLYIR.L |
| 132904 | 464 | – | 482 | 717.7251 | 2150.1534 | 2150.1524 | 0.45 | 1 | 19 | 0.016 | 1Score **> 36** indicates **identity** Score **> 14** indicates **homology** | U | R.TVGLFLQGGKDWSPTLYIR.L |
| 132905 | 464 | – | 482 | 1076.0840 | 2150.1535 | 2150.1524 | 0.52 | 1 | 42 | 0.00011 | 1Score **> 36** indicates **identity** Score **> 15** indicates **homology** | U | R.TVGLFLQGGKDWSPTLYIR.L |
| 132906 | 464 | – | 482 | 717.7252 | 2150.1539 | 2150.1524 | 0.68 | 1 | 61 | 1.7e-06 | 1Score **> 36** indicates **identity** Score **> 16** indicates **homology** | U | R.TVGLFLQGGKDWSPTLYIR.L |
| 132907 | 464 | – | 482 | 717.7253 | 2150.1540 | 2150.1524 | 0.73 | 1 | 65 | 8.7e-07 | 1Score **> 36** indicates **identity** Score **> 17** indicates **homology** | U | R.TVGLFLQGGKDWSPTLYIR.L |
| 132908 | 464 | – | 482 | 717.7253 | 2150.1542 | 2150.1524 | 0.81 | 1 | 74 | 1.2e-07 | 1Score **> 36** indicates **identity** Score **> 17** indicates **homology** | U | R.TVGLFLQGGKDWSPTLYIR.L |
| 132910 | 464 | – | 482 | 717.7255 | 2150.1547 | 2150.1524 | 1.03 | 1 | 43 | 0.0001 | 1Score **> 36** indicates **identity** Score **> 15** indicates **homology** | U | R.TVGLFLQGGKDWSPTLYIR.L |
| 132911 | 464 | – | 482 | 1076.0847 | 2150.1548 | 2150.1524 | 1.10 | 1 | 88 | 5.8e-09 | 1Score **> 36** indicates **identity** Score **> 18** indicates **homology** | U | R.TVGLFLQGGKDWSPTLYIR.L |
| 132912 | 464 | – | 482 | 717.7259 | 2150.1558 | 2150.1524 | 1.57 | 1 | 72 | 1.6e-07 | 1Score **> 36** indicates **identity** Score **> 17** indicates **homology** | U | R.TVGLFLQGGKDWSPTLYIR.L |
| 132914 | 464 | – | 482 | 717.7266 | 2150.1578 | 2150.1524 | 2.51 | 1 | 31 | 0.0012 | 1Score **> 36** indicates **identity** Score **> 14** indicates **homology** | U | R.TVGLFLQGGKDWSPTLYIR.L |
| 132917 | 464 | – | 482 | 717.7268 | 2150.1586 | 2150.1524 | 2.84 | 1 | 37 | 0.00036 | 1Score **> 36** indicates **identity** Score **> 15** indicates **homology** | U | R.TVGLFLQGGKDWSPTLYIR.L |
| 132918 | 464 | – | 482 | 1076.0867 | 2150.1588 | 2150.1524 | 2.94 | 1 | 113 | 2.6e-11 | 1Score **> 36** indicates **identity** Score **> 20** indicates **homology** | U | R.TVGLFLQGGKDWSPTLYIR.L |
| 132923 | 464 | – | 482 | 717.7295 | 2150.1665 | 2150.1524 | 6.55 | 1 | 57 | 4.7e-06 | 1Score **> 36** indicates **identity** Score **> 16** indicates **homology** | U | R.TVGLFLQGGKDWSPTLYIR.L |
| 132997 | 464 | – | 482 | 1076.5731 | 2151.1317 | 2151.1364 | -2.21 | 1 | 27 | 0.0031 | 1Score **> 36** indicates **identity** Score **> 14** indicates **homology** | U | R.TVGLFLQGGKDWSPTLYIR.L  + Deamidated (NQ) |
| 133011 | 464 | – | 482 | 718.0591 | 2151.1555 | 2151.1364 | 8.87 | 1 | 40 | 0.00017 | 1Score **> 36** indicates **identity** Score **> 15** indicates **homology** | U | R.TVGLFLQGGKDWSPTLYIR.L  + Deamidated (NQ) |
| 27243 | 474 | – | 482 | 575.7972 | 1149.5798 | 1149.5818 | -1.78 | 0 | 24 | 0.0054 | 1Score **> 32** indicates **identity** Score **> 14** indicates **homology** | U | K.DWSPTLYIR.L |
| 27244 | 474 | – | 482 | 575.7973 | 1149.5800 | 1149.5818 | -1.60 | 0 | 18 | 0.021 | 1Score **> 32** indicates **identity** Score **> 14** indicates **homology** | U | K.DWSPTLYIR.L |
| 27248 | 474 | – | 482 | 575.7984 | 1149.5822 | 1149.5818 | 0.35 | 0 | 33 | 0.00087 | 1Score **> 32** indicates **identity** Score **> 15** indicates **homology** | U | K.DWSPTLYIR.L |
| 27250 | 474 | – | 482 | 575.7984 | 1149.5823 | 1149.5818 | 0.43 | 0 | 33 | 0.00076 | 1Score **> 32** indicates **identity** Score **> 15** indicates **homology** | U | K.DWSPTLYIR.L |
| 27251 | 474 | – | 482 | 575.7985 | 1149.5825 | 1149.5818 | 0.56 | 0 | 33 | 0.00087 | 1Score **> 32** indicates **identity** Score **> 15** indicates **homology** | U | K.DWSPTLYIR.L |
| 27252 | 474 | – | 482 | 575.7988 | 1149.5831 | 1149.5818 | 1.06 | 0 | 32 | 0.00097 | 1Score **> 32** indicates **identity** Score **> 15** indicates **homology** | U | K.DWSPTLYIR.L |
| 110735 | 483 | – | 499 | 633.9964 | 1898.9673 | 1898.9738 | -3.41 | 0 | 17 | 0.025 | 1Score **> 35** indicates **identity** Score **> 14** indicates **homology** | U | R.LVQDYGLESEVAQHLAK.T |
| 110737 | 483 | – | 499 | 633.9974 | 1898.9703 | 1898.9738 | -1.85 | 0 | 60 | 2.3e-06 | 1Score **> 36** indicates **identity** Score **> 16** indicates **homology** | U | R.LVQDYGLESEVAQHLAK.T |
| 110738 | 483 | – | 499 | 950.4940 | 1898.9735 | 1898.9738 | -0.16 | 0 | 89 | 4.8e-09 | 1Score **> 36** indicates **identity** Score **> 18** indicates **homology** | U | R.LVQDYGLESEVAQHLAK.T |
| 110739 | 483 | – | 499 | 633.9985 | 1898.9738 | 1898.9738 | -0.0042 | 0 | 62 | 1.4e-06 | 1Score **> 36** indicates **identity** Score **> 16** indicates **homology** | U | R.LVQDYGLESEVAQHLAK.T |
| 110740 | 483 | – | 499 | 950.4942 | 1898.9738 | 1898.9738 | 0.032 | 0 | 91 | 3e-09 | 1Score **> 36** indicates **identity** Score **> 18** indicates **homology** | U | R.LVQDYGLESEVAQHLAK.T |
| 110741 | 483 | – | 499 | 633.9986 | 1898.9739 | 1898.9738 | 0.061 | 0 | 34 | 0.00058 | 1Score **> 36** indicates **identity** Score **> 15** indicates **homology** | U | R.LVQDYGLESEVAQHLAK.T |
| 110742 | 483 | – | 499 | 633.9986 | 1898.9740 | 1898.9738 | 0.12 | 0 | 99 | 5.5e-10 | 1Score **> 36** indicates **identity** Score **> 19** indicates **homology** | U | R.LVQDYGLESEVAQHLAK.T |
| 110743 | 483 | – | 499 | 950.4943 | 1898.9740 | 1898.9738 | 0.14 | 0 | 90 | 3.6e-09 | 1Score **> 36** indicates **identity** Score **> 18** indicates **homology** | U | R.LVQDYGLESEVAQHLAK.T |
| 110744 | 483 | – | 499 | 633.9987 | 1898.9742 | 1898.9738 | 0.23 | 0 | 84 | 1.4e-08 | 1Score **> 36** indicates **identity** Score **> 18** indicates **homology** | U | R.LVQDYGLESEVAQHLAK.T |
| 110745 | 483 | – | 499 | 633.9987 | 1898.9743 | 1898.9738 | 0.29 | 0 | 84 | 1.2e-08 | 1Score **> 36** indicates **identity** Score **> 18** indicates **homology** | U | R.LVQDYGLESEVAQHLAK.T |
| 110747 | 483 | – | 499 | 633.9988 | 1898.9744 | 1898.9738 | 0.35 | 0 | 94 | 1.4e-09 | 1Score **> 36** indicates **identity** Score **> 18** indicates **homology** | U | R.LVQDYGLESEVAQHLAK.T |
| 110748 | 483 | – | 499 | 950.4946 | 1898.9746 | 1898.9738 | 0.46 | 0 | 56 | 1.4e-05 | 1Score **> 36** indicates **identity** Score **> 20** indicates **homology** | U | R.LVQDYGLESEVAQHLAK.T |
| 110749 | 483 | – | 499 | 633.9989 | 1898.9748 | 1898.9738 | 0.57 | 0 | 32 | 0.00095 | 1Score **> 36** indicates **identity** Score **> 15** indicates **homology** | U | R.LVQDYGLESEVAQHLAK.T |
| 110750 | 483 | – | 499 | 633.9989 | 1898.9750 | 1898.9738 | 0.64 | 0 | 15 | 0.042 | 1Score **> 36** indicates **identity** Score **> 13** indicates **homology** | U | R.LVQDYGLESEVAQHLAK.T |
| 110751 | 483 | – | 499 | 633.9990 | 1898.9751 | 1898.9738 | 0.68 | 0 | 85 | 1.1e-08 | 1Score **> 35** indicates **identity** Score **> 18** indicates **homology** | U | R.LVQDYGLESEVAQHLAK.T |
| 110753 | 483 | – | 499 | 633.9993 | 1898.9760 | 1898.9738 | 1.20 | 0 | 59 | 2.7e-06 | 1Score **> 36** indicates **identity** Score **> 16** indicates **homology** | U | R.LVQDYGLESEVAQHLAK.T |
| 110757 | 483 | – | 499 | 634.0000 | 1898.9781 | 1898.9738 | 2.26 | 0 | 82 | 1.9e-08 | 1Score **> 36** indicates **identity** Score **> 18** indicates **homology** | U | R.LVQDYGLESEVAQHLAK.T |
| 110758 | 483 | – | 499 | 634.0003 | 1898.9790 | 1898.9738 | 2.75 | 0 | 24 | 0.0057 | 1Score **> 36** indicates **identity** Score **> 14** indicates **homology** | U | R.LVQDYGLESEVAQHLAK.T |
| 110760 | 483 | – | 499 | 950.4971 | 1898.9796 | 1898.9738 | 3.06 | 0 | 58 | 3.6e-06 | 1Score **> 35** indicates **identity** Score **> 16** indicates **homology** | U | R.LVQDYGLESEVAQHLAK.T |
| 110761 | 483 | – | 499 | 634.0005 | 1898.9798 | 1898.9738 | 3.16 | 0 | 79 | 4.3e-08 | 1Score **> 35** indicates **identity** Score **> 17** indicates **homology** | U | R.LVQDYGLESEVAQHLAK.T |
| 110762 | 483 | – | 499 | 634.0008 | 1898.9804 | 1898.9738 | 3.51 | 0 | 53 | 1.1e-05 | 1Score **> 36** indicates **identity** Score **> 16** indicates **homology** | U | R.LVQDYGLESEVAQHLAK.T |
| 110763 | 483 | – | 499 | 634.0010 | 1898.9811 | 1898.9738 | 3.87 | 0 | 88 | 5.8e-09 | 1Score **> 36** indicates **identity** Score **> 18** indicates **homology** | U | R.LVQDYGLESEVAQHLAK.T |
| 110766 | 483 | – | 499 | 634.0014 | 1898.9823 | 1898.9738 | 4.51 | 0 | 57 | 4.1e-06 | 1Score **> 36** indicates **identity** Score **> 16** indicates **homology** | U | R.LVQDYGLESEVAQHLAK.T |
| 110840 | 483 | – | 499 | 634.3288 | 1899.9646 | 1899.9578 | 3.57 | 0 | 25 | 0.0044 | 2Score **> 35** indicates **identity** Score **> 14** indicates **homology** | U | R.LVQDYGLESEVAQHLAK.T  + Deamidated (NQ) |
| 110843 | 483 | – | 499 | 634.3299 | 1899.9679 | 1899.9578 | 5.30 | 0 | 33 | 0.0014 | 1Score **> 35** indicates **identity** Score **> 17** indicates **homology** | U | R.LVQDYGLESEVAQHLAK.T  + Deamidated (NQ) |
| 35655 | 500 | – | 510 | 614.8127 | 1227.6108 | 1227.6135 | -2.27 | 1 | 18 | 0.02 | 1Score **> 31** indicates **identity** Score **> 14** indicates **homology** | U | K.TYGDKAFEVAK.M |
| 35656 | 500 | – | 510 | 410.2110 | 1227.6111 | 1227.6135 | -1.97 | 1 | 34 | 0.00061 | 1Score **> 31** indicates **identity** Score **> 15** indicates **homology** | U | K.TYGDKAFEVAK.M |
| 35660 | 500 | – | 510 | 410.2112 | 1227.6116 | 1227.6135 | -1.55 | 1 | 50 | 2.2e-05 | 1Score **> 31** indicates **identity** Score **> 16** indicates **homology** | U | K.TYGDKAFEVAK.M |
| 35661 | 500 | – | 510 | 410.2112 | 1227.6118 | 1227.6135 | -1.43 | 1 | 33 | 0.00072 | 1Score **> 31** indicates **identity** Score **> 15** indicates **homology** | U | K.TYGDKAFEVAK.M |
| 35662 | 500 | – | 510 | 614.8132 | 1227.6119 | 1227.6135 | -1.34 | 1 | 51 | 1.6e-05 | 1Score **> 31** indicates **identity** Score **> 16** indicates **homology** | U | K.TYGDKAFEVAK.M |
| 35663 | 500 | – | 510 | 614.8133 | 1227.6120 | 1227.6135 | -1.25 | 1 | 17 | 0.028 | 1Score **> 31** indicates **identity** Score **> 14** indicates **homology** | U | K.TYGDKAFEVAK.M |
| 35664 | 500 | – | 510 | 410.2113 | 1227.6122 | 1227.6135 | -1.08 | 1 | 45 | 6.4e-05 | 1Score **> 31** indicates **identity** Score **> 15** indicates **homology** | U | K.TYGDKAFEVAK.M |
| 35665 | 500 | – | 510 | 614.8134 | 1227.6123 | 1227.6135 | -1.05 | 1 | 52 | 2.3e-05 | 1Score **> 31** indicates **identity** Score **> 18** indicates **homology** | U | K.TYGDKAFEVAK.M |
| 35666 | 500 | – | 510 | 614.8135 | 1227.6124 | 1227.6135 | -0.94 | 1 | 23 | 0.0072 | 1Score **> 31** indicates **identity** Score **> 14** indicates **homology** | U | K.TYGDKAFEVAK.M |
| 35668 | 500 | – | 510 | 410.2115 | 1227.6126 | 1227.6135 | -0.78 | 1 | 27 | 0.003 | 1Score **> 31** indicates **identity** Score **> 14** indicates **homology** | U | K.TYGDKAFEVAK.M |
| 35669 | 500 | – | 510 | 410.2115 | 1227.6126 | 1227.6135 | -0.75 | 1 | 21 | 0.0098 | 1Score **> 31** indicates **identity** Score **> 14** indicates **homology** | U | K.TYGDKAFEVAK.M |
| 35670 | 500 | – | 510 | 614.8137 | 1227.6129 | 1227.6135 | -0.55 | 1 | 76 | 2.4e-07 | 1Score **> 31** indicates **identity** Score **> 22** indicates **homology** | U | K.TYGDKAFEVAK.M |
| 35671 | 500 | – | 510 | 410.2116 | 1227.6129 | 1227.6135 | -0.52 | 1 | 51 | 1.6e-05 | 1Score **> 31** indicates **identity** Score **> 16** indicates **homology** | U | K.TYGDKAFEVAK.M |
| 35672 | 500 | – | 510 | 410.2116 | 1227.6130 | 1227.6135 | -0.47 | 1 | 30 | 0.0015 | 1Score **> 31** indicates **identity** Score **> 14** indicates **homology** | U | K.TYGDKAFEVAK.M |
| 35673 | 500 | – | 510 | 614.8139 | 1227.6133 | 1227.6135 | -0.17 | 1 | 80 | 1.1e-07 | 1Score **> 31** indicates **identity** Score **> 23** indicates **homology** | U | K.TYGDKAFEVAK.M |
| 35675 | 500 | – | 510 | 410.2120 | 1227.6141 | 1227.6135 | 0.46 | 1 | 34 | 0.00063 | 1Score **> 31** indicates **identity** Score **> 15** indicates **homology** | U | K.TYGDKAFEVAK.M |
| 35676 | 500 | – | 510 | 614.8144 | 1227.6142 | 1227.6135 | 0.50 | 1 | 84 | 2.3e-08 | 1Score **> 31** indicates **identity** Score **> 20** indicates **homology** | U | K.TYGDKAFEVAK.M |
| 12140 | 518 | – | 525 | 484.7925 | 967.5704 | 967.5716 | -1.22 | 1 | 41 | 0.0017 | 1Score **> 26** indicates **identity** Score **> 26** indicates **homology** | U | K.RWPVVGVR.L |
| 12142 | 518 | – | 525 | 484.7927 | 967.5708 | 967.5716 | -0.79 | 1 | 42 | 0.00086 | 1Score **> 26** indicates **identity** Score **> 23** indicates **homology** | U | K.RWPVVGVR.L |
| 12143 | 518 | – | 525 | 484.7927 | 967.5708 | 967.5716 | -0.78 | 1 | 32 | 0.006 | 1Score **> 26** indicates **identity** Score **> 22** indicates **homology** | U | K.RWPVVGVR.L |
| 12145 | 518 | – | 525 | 484.7928 | 967.5711 | 967.5716 | -0.47 | 1 | 40 | 0.0025 | 1Score **> 26** indicates **identity** Score **> 26** indicates **homology** | U | K.RWPVVGVR.L |
| 12147 | 518 | – | 525 | 484.7929 | 967.5712 | 967.5716 | -0.35 | 1 | 39 | 0.0017 | 1Score **> 26** indicates **identity** Score **> 24** indicates **homology** | U | K.RWPVVGVR.L |
| 12148 | 518 | – | 525 | 484.7929 | 967.5713 | 967.5716 | -0.34 | 1 | 20 | 0.042 | 1Score **> 26** indicates **identity** Score **> 19** indicates **homology** | U | K.RWPVVGVR.L |
| 12149 | 518 | – | 525 | 484.7929 | 967.5713 | 967.5716 | -0.33 | 1 | 37 | 0.0024 | 1Score **> 26** indicates **identity** Score **> 23** indicates **homology** | U | K.RWPVVGVR.L |
| 12152 | 518 | – | 525 | 484.7931 | 967.5716 | 967.5716 | 0.022 | 1 | 35 | 0.0034 | 1Score **> 26** indicates **identity** Score **> 23** indicates **homology** | U | K.RWPVVGVR.L |
| 4027 | 519 | – | 525 | 406.7418 | 811.4690 | 811.4705 | -1.79 | 0 | 23 | 0.0072 | 1Score **> 21** indicates **identity** Score **> 14** indicates **homology** | U | R.WPVVGVR.L |
| 4028 | 519 | – | 525 | 406.7422 | 811.4697 | 811.4705 | -0.89 | 0 | 23 | 0.0072 | 1Score **> 21** indicates **identity** Score **> 14** indicates **homology** | U | R.WPVVGVR.L |
| 4029 | 519 | – | 525 | 406.7424 | 811.4702 | 811.4705 | -0.36 | 0 | 20 | 0.012 | 1Score **> 21** indicates **identity** Score **> 14** indicates **homology** | U | R.WPVVGVR.L |
| 4030 | 519 | – | 525 | 406.7424 | 811.4702 | 811.4705 | -0.35 | 0 | 21 | 0.012 | 1Score **> 21** indicates **identity** Score **> 14** indicates **homology** | U | R.WPVVGVR.L |
| 4032 | 519 | – | 525 | 406.7424 | 811.4703 | 811.4705 | -0.23 | 0 | 20 | 0.013 | 1Score **> 21** indicates **identity** Score **> 14** indicates **homology** | U | R.WPVVGVR.L |
| 4033 | 519 | – | 525 | 406.7425 | 811.4704 | 811.4705 | -0.15 | 0 | 34 | 0.0006 | 1Score **> 21** indicates **identity** Score **> 15** indicates **homology** | U | R.WPVVGVR.L |
| 4034 | 519 | – | 525 | 406.7425 | 811.4704 | 811.4705 | -0.13 | 0 | 19 | 0.018 | 1Score **> 21** indicates **identity** Score **> 14** indicates **homology** | U | R.WPVVGVR.L |
| 4035 | 519 | – | 525 | 406.7425 | 811.4704 | 811.4705 | -0.064 | 0 | 20 | 0.013 | 1Score **> 21** indicates **identity** Score **> 14** indicates **homology** | U | R.WPVVGVR.L |
| 4036 | 519 | – | 525 | 406.7425 | 811.4705 | 811.4705 | -0.0074 | 0 | 16 | 0.034 | 1Score **> 21** indicates **identity** Score **> 13** indicates **homology** | U | R.WPVVGVR.L |
| 4037 | 519 | – | 525 | 406.7425 | 811.4705 | 811.4705 | 0.0074 | 0 | 23 | 0.0074 | 1Score **> 21** indicates **identity** Score **> 14** indicates **homology** | U | R.WPVVGVR.L |
| 4038 | 519 | – | 525 | 406.7426 | 811.4706 | 811.4705 | 0.15 | 0 | 19 | 0.015 | 1Score **> 21** indicates **identity** Score **> 14** indicates **homology** | U | R.WPVVGVR.L |
| 4039 | 519 | – | 525 | 406.7426 | 811.4707 | 811.4705 | 0.30 | 0 | 23 | 0.007 | 1Score **> 21** indicates **identity** Score **> 14** indicates **homology** | U | R.WPVVGVR.L |
| 4040 | 519 | – | 525 | 406.7426 | 811.4707 | 811.4705 | 0.31 | 0 | 25 | 0.0044 | 1Score **> 21** indicates **identity** Score **> 14** indicates **homology** | U | R.WPVVGVR.L |
| 4041 | 519 | – | 525 | 406.7427 | 811.4708 | 811.4705 | 0.39 | 0 | 16 | 0.029 | 1Score **> 21** indicates **identity** Score **> 14** indicates **homology** | U | R.WPVVGVR.L |
| 4042 | 519 | – | 525 | 406.7427 | 811.4709 | 811.4705 | 0.52 | 0 | 33 | 0.00088 | 1Score **> 21** indicates **identity** Score **> 15** indicates **homology** | U | R.WPVVGVR.L |
| 4043 | 519 | – | 525 | 406.7427 | 811.4709 | 811.4705 | 0.55 | 0 | 19 | 0.018 | 1Score **> 21** indicates **identity** Score **> 14** indicates **homology** | U | R.WPVVGVR.L |
| 4044 | 519 | – | 525 | 406.7429 | 811.4712 | 811.4705 | 0.89 | 0 | 20 | 0.015 | 1Score **> 21** indicates **identity** Score **> 14** indicates **homology** | U | R.WPVVGVR.L |
| 4045 | 519 | – | 525 | 406.7430 | 811.4714 | 811.4705 | 1.15 | 0 | 22 | 0.009 | 1Score **> 21** indicates **identity** Score **> 14** indicates **homology** | U | R.WPVVGVR.L |
| 4046 | 519 | – | 525 | 406.7430 | 811.4715 | 811.4705 | 1.30 | 0 | 16 | 0.034 | 1Score **> 21** indicates **identity** Score **> 13** indicates **homology** | U | R.WPVVGVR.L |
| 4047 | 519 | – | 525 | 406.7431 | 811.4716 | 811.4705 | 1.35 | 0 | 25 | 0.0046 | 1Score **> 21** indicates **identity** Score **> 14** indicates **homology** | U | R.WPVVGVR.L |
| 68190 | 526 | – | 538 | 762.4024 | 1522.7902 | 1522.7919 | -1.07 | 0 | 21 | 0.011 | 1Score **> 35** indicates **identity** Score **> 14** indicates **homology** | U | R.LVSEFPYIEAEVK.Y |
| 68193 | 526 | – | 538 | 762.4032 | 1522.7919 | 1522.7919 | 0.015 | 0 | 40 | 0.00016 | 1Score **> 35** indicates **identity** Score **> 15** indicates **homology** | U | R.LVSEFPYIEAEVK.Y |
| 68194 | 526 | – | 538 | 508.6046 | 1522.7920 | 1522.7919 | 0.10 | 0 | 32 | 0.0011 | 1Score **> 35** indicates **identity** Score **> 14** indicates **homology** | U | R.LVSEFPYIEAEVK.Y |
| 68195 | 526 | – | 538 | 762.4034 | 1522.7923 | 1522.7919 | 0.27 | 0 | 66 | 2.4e-06 | 1Score **> 35** indicates **identity** Score **> 22** indicates **homology** | U | R.LVSEFPYIEAEVK.Y |
| 68197 | 526 | – | 538 | 508.6048 | 1522.7926 | 1522.7919 | 0.50 | 0 | 18 | 0.019 | 1Score **> 35** indicates **identity** Score **> 14** indicates **homology** | U | R.LVSEFPYIEAEVK.Y |
| 68198 | 526 | – | 538 | 762.4037 | 1522.7928 | 1522.7919 | 0.59 | 0 | 61 | 5.1e-06 | 1Score **> 35** indicates **identity** Score **> 21** indicates **homology** | U | R.LVSEFPYIEAEVK.Y |
| 68199 | 526 | – | 538 | 762.4037 | 1522.7928 | 1522.7919 | 0.59 | 0 | 71 | 6.8e-07 | 1Score **> 35** indicates **identity** Score **> 22** indicates **homology** | U | R.LVSEFPYIEAEVK.Y |
| 68202 | 526 | – | 538 | 762.4041 | 1522.7936 | 1522.7919 | 1.16 | 0 | 55 | 6.5e-06 | 1Score **> 35** indicates **identity** Score **> 16** indicates **homology** | U | R.LVSEFPYIEAEVK.Y |
| 68205 | 526 | – | 538 | 762.4052 | 1522.7958 | 1522.7919 | 2.58 | 0 | 53 | 2.4e-05 | 1Score **> 35** indicates **identity** Score **> 20** indicates **homology** | U | R.LVSEFPYIEAEVK.Y |
| 68206 | 526 | – | 538 | 762.4056 | 1522.7966 | 1522.7919 | 3.10 | 0 | 39 | 0.00021 | 1Score **> 35** indicates **identity** Score **> 15** indicates **homology** | U | R.LVSEFPYIEAEVK.Y |
| 68207 | 526 | – | 538 | 762.4057 | 1522.7969 | 1522.7919 | 3.31 | 0 | 29 | 0.0019 | 1Score **> 35** indicates **identity** Score **> 14** indicates **homology** | U | R.LVSEFPYIEAEVK.Y |
| 68208 | 526 | – | 538 | 762.4064 | 1522.7983 | 1522.7919 | 4.24 | 0 | 28 | 0.0022 | 1Score **> 35** indicates **identity** Score **> 14** indicates **homology** | U | R.LVSEFPYIEAEVK.Y |
| 68209 | 526 | – | 538 | 762.4076 | 1522.8006 | 1522.7919 | 5.74 | 0 | 21 | 0.012 | 1Score **> 35** indicates **identity** Score **> 14** indicates **homology** | U | R.LVSEFPYIEAEVK.Y |
| 108753 | 539 | – | 554 | 626.3031 | 1875.8876 | 1875.8859 | 0.93 | 1 | 25 | 0.0047 | 1Score **> 34** indicates **identity** Score **> 14** indicates **homology** | U | K.YGIKEYACTAVDMISR.R |
| 108754 | 539 | – | 554 | 938.9512 | 1875.8879 | 1875.8859 | 1.07 | 1 | 21 | 0.0096 | 1Score **> 34** indicates **identity** Score **> 14** indicates **homology** | U | K.YGIKEYACTAVDMISR.R |
| 108772 | 539 | – | 554 | 626.3039 | 1875.8900 | 1875.8859 | 2.20 | 1 | 43 | 9.7e-05 | 1Score **> 34** indicates **identity** Score **> 15** indicates **homology** | U | K.YGIKEYACTAVDMISR.R |
| 108790 | 539 | – | 554 | 626.3047 | 1875.8922 | 1875.8859 | 3.36 | 1 | 43 | 9.2e-05 | 1Score **> 34** indicates **identity** Score **> 15** indicates **homology** | U | K.YGIKEYACTAVDMISR.R |
| 122411 | 539 | – | 555 | 678.3362 | 2031.9867 | 2031.9870 | -0.13 | 2 | 18 | 0.022 | 1Score **> 35** indicates **identity** Score **> 14** indicates **homology** | U | K.YGIKEYACTAVDMISRR.T |
| 122412 | 539 | – | 555 | 509.0043 | 2031.9882 | 2031.9870 | 0.61 | 2 | 51 | 9.9e-05 | 1Score **> 35** indicates **identity** Score **> 23** indicates **homology** | U | K.YGIKEYACTAVDMISRR.T |
| 122413 | 539 | – | 555 | 509.0044 | 2031.9884 | 2031.9870 | 0.72 | 2 | 61 | 2e-06 | 1Score **> 35** indicates **identity** Score **> 16** indicates **homology** | U | K.YGIKEYACTAVDMISRR.T |
| 122415 | 539 | – | 555 | 678.3374 | 2031.9903 | 2031.9870 | 1.64 | 2 | 37 | 0.00037 | 1Score **> 35** indicates **identity** Score **> 15** indicates **homology** | U | K.YGIKEYACTAVDMISRR.T |
| 122416 | 539 | – | 555 | 509.0049 | 2031.9905 | 2031.9870 | 1.73 | 2 | 36 | 0.00045 | 1Score **> 35** indicates **identity** Score **> 15** indicates **homology** | U | K.YGIKEYACTAVDMISRR.T |
| 122418 | 539 | – | 555 | 509.0053 | 2031.9922 | 2031.9870 | 2.59 | 2 | 74 | 1.3e-07 | 1Score **> 35** indicates **identity** Score **> 17** indicates **homology** | U | K.YGIKEYACTAVDMISRR.T |
| 55320 | 543 | – | 554 | 708.3166 | 1414.6187 | 1414.6221 | -2.36 | 0 | 29 | 0.0018 | 1Score **> 27** indicates **identity** Score **> 14** indicates **homology** | U | K.EYACTAVDMISR.R |
| 55321 | 543 | – | 554 | 708.3177 | 1414.6208 | 1414.6221 | -0.92 | 0 | 56 | 5.3e-06 | 1Score **> 27** indicates **identity** Score **> 16** indicates **homology** | U | K.EYACTAVDMISR.R |
| 55322 | 543 | – | 554 | 708.3187 | 1414.6229 | 1414.6221 | 0.56 | 0 | 52 | 1.2e-05 | 1Score **> 28** indicates **identity** Score **> 16** indicates **homology** | U | K.EYACTAVDMISR.R |
| 73992 | 543 | – | 555 | 786.3677 | 1570.7209 | 1570.7232 | -1.47 | 1 | 15 | 0.04 | 1Score **> 31** indicates **identity** Score **> 13** indicates **homology** | U | K.EYACTAVDMISRR.T |
| 73993 | 543 | – | 555 | 524.5812 | 1570.7216 | 1570.7232 | -0.98 | 1 | 21 | 0.012 | 1Score **> 31** indicates **identity** Score **> 14** indicates **homology** | U | K.EYACTAVDMISRR.T |
| 73995 | 543 | – | 555 | 524.5818 | 1570.7234 | 1570.7232 | 0.16 | 1 | 35 | 0.00048 | 1Score **> 31** indicates **identity** Score **> 15** indicates **homology** | U | K.EYACTAVDMISRR.T |
| 73996 | 543 | – | 555 | 524.5823 | 1570.7251 | 1570.7232 | 1.22 | 1 | 31 | 0.0012 | 1Score **> 31** indicates **identity** Score **> 14** indicates **homology** | U | K.EYACTAVDMISRR.T |
| 73998 | 543 | – | 555 | 786.3703 | 1570.7261 | 1570.7232 | 1.88 | 1 | 24 | 0.008 | 1Score **> 32** indicates **identity** Score **> 15** indicates **homology** | U | K.EYACTAVDMISRR.T |
| 110658 | 556 | – | 572 | 950.0210 | 1898.0274 | 1898.0374 | -5.26 | 1 | 33 | 0.00086 | 1Score **> 35** indicates **identity** Score **> 15** indicates **homology** | U | R.TRLAFLNVQAAEEALPR.I |
| 110664 | 556 | – | 572 | 633.6865 | 1898.0378 | 1898.0374 | 0.24 | 1 | 50 | 2.3e-05 | 1Score **> 35** indicates **identity** Score **> 16** indicates **homology** | U | R.TRLAFLNVQAAEEALPR.I |
| 110665 | 556 | – | 572 | 633.6866 | 1898.0381 | 1898.0374 | 0.39 | 1 | 27 | 0.003 | 1Score **> 35** indicates **identity** Score **> 14** indicates **homology** | U | R.TRLAFLNVQAAEEALPR.I |
| 110666 | 556 | – | 572 | 633.6867 | 1898.0384 | 1898.0374 | 0.56 | 1 | 25 | 0.0049 | 1Score **> 35** indicates **identity** Score **> 14** indicates **homology** | U | R.TRLAFLNVQAAEEALPR.I |
| 110667 | 556 | – | 572 | 633.6869 | 1898.0390 | 1898.0374 | 0.85 | 1 | 67 | 5.8e-07 | 1Score **> 35** indicates **identity** Score **> 17** indicates **homology** | U | R.TRLAFLNVQAAEEALPR.I |
| 110669 | 556 | – | 572 | 633.6870 | 1898.0392 | 1898.0374 | 0.99 | 1 | 72 | 2.5e-07 | 1Score **> 35** indicates **identity** Score **> 19** indicates **homology** | U | R.TRLAFLNVQAAEEALPR.I |
| 110670 | 556 | – | 572 | 633.6871 | 1898.0394 | 1898.0374 | 1.05 | 1 | 60 | 2.4e-06 | 1Score **> 35** indicates **identity** Score **> 16** indicates **homology** | U | R.TRLAFLNVQAAEEALPR.I |
| 110671 | 556 | – | 572 | 950.0271 | 1898.0396 | 1898.0374 | 1.17 | 1 | 60 | 2.2e-06 | 1Score **> 35** indicates **identity** Score **> 16** indicates **homology** | U | R.TRLAFLNVQAAEEALPR.I |
| 110672 | 556 | – | 572 | 633.6872 | 1898.0396 | 1898.0374 | 1.20 | 1 | 65 | 8.5e-07 | 1Score **> 35** indicates **identity** Score **> 17** indicates **homology** | U | R.TRLAFLNVQAAEEALPR.I |
| 110674 | 556 | – | 572 | 950.0282 | 1898.0418 | 1898.0374 | 2.35 | 1 | 45 | 9.5e-05 | 1Score **> 35** indicates **identity** Score **> 17** indicates **homology** | U | R.TRLAFLNVQAAEEALPR.I |
| 110688 | 556 | – | 572 | 633.6903 | 1898.0491 | 1898.0374 | 6.18 | 1 | 83 | 1.8e-08 | 1Score **> 35** indicates **identity** Score **> 18** indicates **homology** | U | R.TRLAFLNVQAAEEALPR.I |
| 83147 | 558 | – | 572 | 821.4492 | 1640.8838 | 1640.8886 | -2.92 | 0 | 62 | 1.6e-06 | 1Score **> 35** indicates **identity** Score **> 16** indicates **homology** | U | R.LAFLNVQAAEEALPR.I |
| 83148 | 558 | – | 572 | 547.9695 | 1640.8867 | 1640.8886 | -1.12 | 0 | 56 | 5.7e-06 | 1Score **> 35** indicates **identity** Score **> 16** indicates **homology** | U | R.LAFLNVQAAEEALPR.I |
| 83151 | 558 | – | 572 | 547.9701 | 1640.8884 | 1640.8886 | -0.095 | 0 | 73 | 1.5e-07 | 1Score **> 35** indicates **identity** Score **> 18** indicates **homology** | U | R.LAFLNVQAAEEALPR.I |
| 83152 | 558 | – | 572 | 547.9701 | 1640.8884 | 1640.8886 | -0.080 | 0 | 88 | 6.1e-09 | 1Score **> 35** indicates **identity** Score **> 18** indicates **homology** | U | R.LAFLNVQAAEEALPR.I |
| 83154 | 558 | – | 572 | 547.9701 | 1640.8885 | 1640.8886 | -0.029 | 0 | 85 | 1.1e-08 | 1Score **> 35** indicates **identity** Score **> 18** indicates **homology** | U | R.LAFLNVQAAEEALPR.I |
| 83155 | 558 | – | 572 | 547.9701 | 1640.8886 | 1640.8886 | 0.011 | 0 | 88 | 6.5e-09 | 1Score **> 35** indicates **identity** Score **> 19** indicates **homology** | U | R.LAFLNVQAAEEALPR.I |
| 83157 | 558 | – | 572 | 821.4517 | 1640.8888 | 1640.8886 | 0.17 | 0 | 98 | 6.8e-10 | 1Score **> 35** indicates **identity** Score **> 19** indicates **homology** | U | R.LAFLNVQAAEEALPR.I |
| 83158 | 558 | – | 572 | 821.4517 | 1640.8889 | 1640.8886 | 0.19 | 0 | 112 | 3.2e-11 | 1Score **> 35** indicates **identity** Score **> 19** indicates **homology** | U | R.LAFLNVQAAEEALPR.I |
| 83159 | 558 | – | 572 | 821.4518 | 1640.8890 | 1640.8886 | 0.27 | 0 | 127 | 1.4e-12 | 1Score **> 35** indicates **identity** Score **> 20** indicates **homology** | U | R.LAFLNVQAAEEALPR.I |
| 83161 | 558 | – | 572 | 547.9703 | 1640.8892 | 1640.8886 | 0.36 | 0 | 88 | 6.4e-09 | 1Score **> 35** indicates **identity** Score **> 19** indicates **homology** | U | R.LAFLNVQAAEEALPR.I |
| 83162 | 558 | – | 572 | 547.9705 | 1640.8897 | 1640.8886 | 0.71 | 0 | 47 | 4.2e-05 | 1Score **> 35** indicates **identity** Score **> 15** indicates **homology** | U | R.LAFLNVQAAEEALPR.I |
| 83163 | 558 | – | 572 | 547.9708 | 1640.8904 | 1640.8886 | 1.13 | 0 | 27 | 0.0028 | 1Score **> 35** indicates **identity** Score **> 14** indicates **homology** | U | R.LAFLNVQAAEEALPR.I |
| 83164 | 558 | – | 572 | 547.9708 | 1640.8906 | 1640.8886 | 1.25 | 0 | 36 | 0.00046 | 1Score **> 35** indicates **identity** Score **> 15** indicates **homology** | U | R.LAFLNVQAAEEALPR.I |
| 83165 | 558 | – | 572 | 821.4527 | 1640.8909 | 1640.8886 | 1.43 | 0 | 98 | 6.9e-10 | 1Score **> 35** indicates **identity** Score **> 19** indicates **homology** | U | R.LAFLNVQAAEEALPR.I |
| 83169 | 558 | – | 572 | 547.9716 | 1640.8931 | 1640.8886 | 2.76 | 0 | 46 | 4.9e-05 | 1Score **> 35** indicates **identity** Score **> 15** indicates **homology** | U | R.LAFLNVQAAEEALPR.I |
| 5029 | 573 | – | 579 | 417.2303 | 832.4461 | 832.4477 | -1.83 | 0 | 30 | 0.0024 | 1Score **> 29** indicates **identity** Score **> 16** indicates **homology** | U | R.IVELMGR.E  + Oxidation (M) |
| 5030 | 573 | – | 579 | 417.2308 | 832.4470 | 832.4477 | -0.80 | 0 | 20 | 0.012 | 1Score **> 29** indicates **identity** Score **> 14** indicates **homology** | U | R.IVELMGR.E  + Oxidation (M) |
| 5032 | 573 | – | 579 | 417.2309 | 832.4473 | 832.4477 | -0.41 | 0 | 19 | 0.022 | 1Score **> 30** indicates **identity** Score **> 15** indicates **homology** | U | R.IVELMGR.E  + Oxidation (M) |
| 5034 | 573 | – | 579 | 417.2313 | 832.4481 | 832.4477 | 0.49 | 0 | 21 | 0.022 | 1Score **> 30** indicates **identity** Score **> 17** indicates **homology** | U | R.IVELMGR.E  + Oxidation (M) |
| 5035 | 573 | – | 579 | 417.2313 | 832.4481 | 832.4477 | 0.56 | 0 | 22 | 0.015 | 1Score **> 30** indicates **identity** Score **> 16** indicates **homology** | U | R.IVELMGR.E  + Oxidation (M) |
| 5036 | 573 | – | 579 | 417.2314 | 832.4482 | 832.4477 | 0.59 | 0 | 18 | 0.032 | 1Score **> 30** indicates **identity** Score **> 15** indicates **homology** | U | R.IVELMGR.E  + Oxidation (M) |
| 5039 | 573 | – | 579 | 417.2321 | 832.4497 | 832.4477 | 2.40 | 0 | 29 | 0.0019 | 1Score **> 28** indicates **identity** Score **> 14** indicates **homology** | U | R.IVELMGR.E  + Oxidation (M) |
| 17039 | 580 | – | 587 | 523.7660 | 1045.5174 | 1045.5192 | -1.74 | 0 | 27 | 0.01 | 1Score **> 32** indicates **identity** Score **> 20** indicates **homology** | U | R.ELNWSELR.K |
| 17040 | 580 | – | 587 | 523.7663 | 1045.5180 | 1045.5192 | -1.15 | 0 | 36 | 0.0088 | 1Score **> 32** indicates **identity** Score **> 28** indicates **homology** | U | R.ELNWSELR.K |
| 17041 | 580 | – | 587 | 523.7667 | 1045.5189 | 1045.5192 | -0.34 | 0 | 44 | 0.00058 | 1Score **> 33** indicates **identity** Score **> 24** indicates **homology** | U | R.ELNWSELR.K |
| 17042 | 580 | – | 587 | 523.7669 | 1045.5193 | 1045.5192 | 0.027 | 0 | 44 | 0.00071 | 1Score **> 33** indicates **identity** Score **> 25** indicates **homology** | U | R.ELNWSELR.K |
| 17044 | 580 | – | 587 | 523.7670 | 1045.5194 | 1045.5192 | 0.12 | 0 | 43 | 0.00083 | 1Score **> 33** indicates **identity** Score **> 24** indicates **homology** | U | R.ELNWSELR.K |
| 17045 | 580 | – | 587 | 523.7670 | 1045.5195 | 1045.5192 | 0.25 | 0 | 42 | 0.00086 | 1Score **> 33** indicates **identity** Score **> 24** indicates **homology** | U | R.ELNWSELR.K |
| 17046 | 580 | – | 587 | 523.7677 | 1045.5208 | 1045.5192 | 1.51 | 0 | 35 | 0.0024 | 1Score **> 33** indicates **identity** Score **> 21** indicates **homology** | U | R.ELNWSELR.K |
| 17047 | 580 | – | 587 | 523.7681 | 1045.5217 | 1045.5192 | 2.37 | 0 | 36 | 0.0088 | 1Score **> 33** indicates **identity** Score **> 28** indicates **homology** | U | R.ELNWSELR.K |
| 29743 | 580 | – | 588 | 392.2122 | 1173.6147 | 1173.6142 | 0.42 | 1 | 22 | 0.012 | 1Score **> 34** indicates **identity** Score **> 15** indicates **homology** | U | R.ELNWSELRK.Q |
| 29747 | 580 | – | 588 | 392.2126 | 1173.6158 | 1173.6142 | 1.40 | 1 | 15 | 0.041 | 1Score **> 33** indicates **identity** Score **> 13** indicates **homology** | U | R.ELNWSELRK.Q |
| 139030 | 580 | – | 597 | 558.7865 | 2231.1170 | 2231.1182 | -0.53 | 2 | 30 | 0.0016 | 1Score **> 37** indicates **identity** Score **> 15** indicates **homology** | U | R.ELNWSELRKQEELETATR.F |
| 139031 | 580 | – | 597 | 744.7134 | 2231.1183 | 2231.1182 | 0.053 | 2 | 40 | 0.00019 | 1Score **> 37** indicates **identity** Score **> 15** indicates **homology** | U | R.ELNWSELRKQEELETATR.F |
| 139032 | 580 | – | 597 | 558.7869 | 2231.1185 | 2231.1182 | 0.17 | 2 | 32 | 0.00094 | 1Score **> 37** indicates **identity** Score **> 15** indicates **homology** | U | R.ELNWSELRKQEELETATR.F |
| 139034 | 580 | – | 597 | 744.7140 | 2231.1203 | 2231.1182 | 0.97 | 2 | 25 | 0.0042 | 1Score **> 37** indicates **identity** Score **> 14** indicates **homology** | U | R.ELNWSELRKQEELETATR.F |
| 139037 | 580 | – | 597 | 558.7876 | 2231.1213 | 2231.1182 | 1.40 | 2 | 17 | 0.026 | 1Score **> 37** indicates **identity** Score **> 14** indicates **homology** | U | R.ELNWSELRKQEELETATR.F |
| 33161 | 588 | – | 597 | 402.2088 | 1203.6047 | 1203.6095 | -4.01 | 1 | 39 | 0.013 | 1Score **> 33** indicates **identity** Score **> 33** indicates **homology** | U | R.KQEELETATR.F |
| 33164 | 588 | – | 597 | 602.8100 | 1203.6055 | 1203.6095 | -3.35 | 1 | 68 | 1.9e-05 | 1Score **> 34** indicates **identity** | U | R.KQEELETATR.F |
| 33165 | 588 | – | 597 | 602.8109 | 1203.6073 | 1203.6095 | -1.86 | 1 | 43 | 0.0074 | 1Score **> 34** indicates **identity** | U | R.KQEELETATR.F |
| 33166 | 588 | – | 597 | 402.2099 | 1203.6080 | 1203.6095 | -1.26 | 1 | 30 | 0.026 | 1Score **> 34** indicates **identity** Score **> 27** indicates **homology** | U | R.KQEELETATR.F |
| 33169 | 588 | – | 597 | 602.8119 | 1203.6093 | 1203.6095 | -0.14 | 1 | 35 | 0.033 | 1Score **> 34** indicates **identity** Score **> 33** indicates **homology** | U | R.KQEELETATR.F |
| 33175 | 588 | – | 597 | 602.8133 | 1203.6120 | 1203.6095 | 2.07 | 1 | 44 | 0.00049 | 1Score **> 34** indicates **identity** Score **> 24** indicates **homology** | U | R.KQEELETATR.F |
| 33176 | 588 | – | 597 | 402.2120 | 1203.6142 | 1203.6095 | 3.88 | 1 | 22 | 0.023 | 1Score **> 34** indicates **identity** Score **> 18** indicates **homology** | U | R.KQEELETATR.F |
| 150891 | 588 | – | 606 | 604.7849 | 2415.1105 | 2415.1304 | -8.22 | 2 | 16 | 0.03 | 1Score **> 34** indicates **identity** Score **> 14** indicates **homology** | U | R.KQEELETATRFLYYEMGYK.S  + Deamidated (NQ); Oxidation (M) |
| 19749 | 589 | – | 597 | 538.7645 | 1075.5145 | 1075.5145 | -0.082 | 0 | 39 | 0.004 | 1Score **> 31** indicates **identity** Score **> 27** indicates **homology** | U | K.QEELETATR.F |
| 19751 | 589 | – | 597 | 538.7661 | 1075.5176 | 1075.5145 | 2.87 | 0 | 43 | 0.00066 | 1Score **> 31** indicates **identity** Score **> 24** indicates **homology** | U | K.QEELETATR.F |
| 33982 | 598 | – | 606 | 607.2829 | 1212.5512 | 1212.5525 | -1.04 | 0 | 24 | 0.0054 | 1Score **> 28** indicates **identity** Score **> 14** indicates **homology** | U | R.FLYYEMGYK.S |
| 33985 | 598 | – | 606 | 607.2841 | 1212.5536 | 1212.5525 | 0.87 | 0 | 24 | 0.0058 | 1Score **> 28** indicates **identity** Score **> 14** indicates **homology** | U | R.FLYYEMGYK.S |
| 148444 | 607 | – | 627 | 792.7328 | 2375.1767 | 2375.1816 | -2.05 | 1 | 75 | 1e-07 | 1Score **> 37** indicates **identity** Score **> 17** indicates **homology** | U | K.SRTEQLTDSTEISLLPSDIDR.Y |
| 148451 | 607 | – | 627 | 1188.5982 | 2375.1819 | 2375.1816 | 0.14 | 1 | 35 | 0.006 | 1Score **> 37** indicates **identity** Score **> 25** indicates **homology** | U | K.SRTEQLTDSTEISLLPSDIDR.Y |
| 148455 | 607 | – | 627 | 792.7350 | 2375.1830 | 2375.1816 | 0.61 | 1 | 69 | 3.4e-07 | 1Score **> 37** indicates **identity** Score **> 17** indicates **homology** | U | K.SRTEQLTDSTEISLLPSDIDR.Y |
| 148457 | 607 | – | 627 | 792.7351 | 2375.1836 | 2375.1816 | 0.84 | 1 | 35 | 0.00049 | 1Score **> 37** indicates **identity** Score **> 15** indicates **homology** | U | K.SRTEQLTDSTEISLLPSDIDR.Y |
| 148458 | 607 | – | 627 | 792.7352 | 2375.1839 | 2375.1816 | 0.95 | 1 | 65 | 1.1e-06 | 1Score **> 37** indicates **identity** Score **> 18** indicates **homology** | U | K.SRTEQLTDSTEISLLPSDIDR.Y |
| 148459 | 607 | – | 627 | 1188.5992 | 2375.1839 | 2375.1816 | 0.99 | 1 | 47 | 5.4e-05 | 1Score **> 37** indicates **identity** Score **> 17** indicates **homology** | U | K.SRTEQLTDSTEISLLPSDIDR.Y |
| 148461 | 607 | – | 627 | 1188.5993 | 2375.1841 | 2375.1816 | 1.06 | 1 | 63 | 2.7e-06 | 1Score **> 37** indicates **identity** Score **> 20** indicates **homology** | U | K.SRTEQLTDSTEISLLPSDIDR.Y |
| 148462 | 607 | – | 627 | 792.7356 | 2375.1849 | 2375.1816 | 1.40 | 1 | 61 | 1.8e-06 | 1Score **> 37** indicates **identity** Score **> 16** indicates **homology** | U | K.SRTEQLTDSTEISLLPSDIDR.Y |
| 148463 | 607 | – | 627 | 1188.5998 | 2375.1851 | 2375.1816 | 1.47 | 1 | 16 | 0.03 | 1Score **> 37** indicates **identity** Score **> 14** indicates **homology** | U | K.SRTEQLTDSTEISLLPSDIDR.Y |
| 148512 | 607 | – | 627 | 793.0682 | 2376.1827 | 2376.1656 | 7.19 | 1 | 29 | 0.0021 | 1Score **> 37** indicates **identity** Score **> 14** indicates **homology** | U | K.SRTEQLTDSTEISLLPSDIDR.Y  + Deamidated (NQ) |
| 148516 | 607 | – | 627 | 793.0689 | 2376.1850 | 2376.1656 | 8.15 | 1 | 24 | 0.0058 | 1Score **> 37** indicates **identity** Score **> 14** indicates **homology** | U | K.SRTEQLTDSTEISLLPSDIDR.Y  + Deamidated (NQ) |
| 162363 | 607 | – | 629 | 889.7827 | 2666.3264 | 2666.3399 | -5.06 | 2 | 30 | 0.0016 | 1Score **> 37** indicates **identity** Score **> 14** indicates **homology** | U | K.SRTEQLTDSTEISLLPSDIDRYK.K |
| 162365 | 607 | – | 629 | 667.5907 | 2666.3337 | 2666.3399 | -2.31 | 2 | 17 | 0.039 | 1Score **> 37** indicates **identity** Score **> 15** indicates **homology** | U | K.SRTEQLTDSTEISLLPSDIDRYK.K |
| 162369 | 607 | – | 629 | 889.7868 | 2666.3384 | 2666.3399 | -0.54 | 2 | 55 | 2.8e-05 | 1Score **> 37** indicates **identity** Score **> 22** indicates **homology** | U | K.SRTEQLTDSTEISLLPSDIDRYK.K |
| 162370 | 607 | – | 629 | 889.7871 | 2666.3396 | 2666.3399 | -0.11 | 2 | 39 | 0.00023 | 1Score **> 37** indicates **identity** Score **> 16** indicates **homology** | U | K.SRTEQLTDSTEISLLPSDIDRYK.K |
| 162371 | 607 | – | 629 | 667.5923 | 2666.3399 | 2666.3399 | 0.026 | 2 | 50 | 5.1e-05 | 1Score **> 37** indicates **identity** Score **> 20** indicates **homology** | U | K.SRTEQLTDSTEISLLPSDIDRYK.K |
| 162373 | 607 | – | 629 | 667.5925 | 2666.3410 | 2666.3399 | 0.41 | 2 | 51 | 4.6e-05 | 1Score **> 37** indicates **identity** Score **> 20** indicates **homology** | U | K.SRTEQLTDSTEISLLPSDIDRYK.K |
| 162374 | 607 | – | 629 | 667.5926 | 2666.3411 | 2666.3399 | 0.47 | 2 | 23 | 0.0065 | 1Score **> 37** indicates **identity** Score **> 14** indicates **homology** | U | K.SRTEQLTDSTEISLLPSDIDRYK.K |
| 162375 | 607 | – | 629 | 667.5927 | 2666.3417 | 2666.3399 | 0.68 | 2 | 26 | 0.015 | 1Score **> 37** indicates **identity** Score **> 20** indicates **homology** | U | K.SRTEQLTDSTEISLLPSDIDRYK.K |
| 162376 | 607 | – | 629 | 889.7879 | 2666.3420 | 2666.3399 | 0.80 | 2 | 58 | 8.7e-06 | 1Score **> 37** indicates **identity** Score **> 19** indicates **homology** | U | K.SRTEQLTDSTEISLLPSDIDRYK.K |
| 162377 | 607 | – | 629 | 889.7880 | 2666.3421 | 2666.3399 | 0.82 | 2 | 65 | 1.5e-06 | 1Score **> 37** indicates **identity** Score **> 19** indicates **homology** | U | K.SRTEQLTDSTEISLLPSDIDRYK.K |
| 162378 | 607 | – | 629 | 667.5933 | 2666.3441 | 2666.3399 | 1.58 | 2 | 54 | 5.9e-05 | 1Score **> 37** indicates **identity** Score **> 24** indicates **homology** | U | K.SRTEQLTDSTEISLLPSDIDRYK.K |
| 131078 | 609 | – | 627 | 1067.0277 | 2132.0408 | 2132.0485 | -3.58 | 0 | 51 | 2.7e-05 | 1Score **> 36** indicates **identity** Score **> 18** indicates **homology** | U | R.TEQLTDSTEISLLPSDIDR.Y |
| 131082 | 609 | – | 627 | 1067.0320 | 2132.0494 | 2132.0485 | 0.44 | 0 | 108 | 1.8e-09 | 1Score **> 36** indicates **identity** Score **> 33** indicates **homology** | U | R.TEQLTDSTEISLLPSDIDR.Y |
| 131083 | 609 | – | 627 | 1067.0323 | 2132.0501 | 2132.0485 | 0.77 | 0 | 99 | 9.4e-09 | 1Score **> 36** indicates **identity** Score **> 32** indicates **homology** | U | R.TEQLTDSTEISLLPSDIDR.Y |
| 131084 | 609 | – | 627 | 1067.0325 | 2132.0504 | 2132.0485 | 0.93 | 0 | 90 | 8.9e-08 | 1Score **> 36** indicates **identity** Score **> 32** indicates **homology** | U | R.TEQLTDSTEISLLPSDIDR.Y |
| 131085 | 609 | – | 627 | 1067.0327 | 2132.0508 | 2132.0485 | 1.09 | 0 | 59 | 3.3e-06 | 1Score **> 36** indicates **identity** Score **> 16** indicates **homology** | U | R.TEQLTDSTEISLLPSDIDR.Y |
| 131086 | 609 | – | 627 | 711.6909 | 2132.0510 | 2132.0485 | 1.18 | 0 | 27 | 0.0032 | 1Score **> 36** indicates **identity** Score **> 14** indicates **homology** | U | R.TEQLTDSTEISLLPSDIDR.Y |
| 131087 | 609 | – | 627 | 1067.0328 | 2132.0510 | 2132.0485 | 1.20 | 0 | 104 | 3.3e-09 | 1Score **> 36** indicates **identity** Score **> 32** indicates **homology** | U | R.TEQLTDSTEISLLPSDIDR.Y |
| 131088 | 609 | – | 627 | 711.6910 | 2132.0511 | 2132.0485 | 1.25 | 0 | 24 | 0.0052 | 1Score **> 36** indicates **identity** Score **> 14** indicates **homology** | U | R.TEQLTDSTEISLLPSDIDR.Y |
| 131089 | 609 | – | 627 | 1067.0329 | 2132.0512 | 2132.0485 | 1.28 | 0 | 108 | 1.8e-09 | 1Score **> 36** indicates **identity** Score **> 33** indicates **homology** | U | R.TEQLTDSTEISLLPSDIDR.Y |
| 131090 | 609 | – | 627 | 1067.0330 | 2132.0514 | 2132.0485 | 1.37 | 0 | 86 | 2.4e-07 | 1Score **> 36** indicates **identity** Score **> 32** indicates **homology** | U | R.TEQLTDSTEISLLPSDIDR.Y |
| 131092 | 609 | – | 627 | 711.6912 | 2132.0516 | 2132.0485 | 1.49 | 0 | 19 | 0.016 | 1Score **> 36** indicates **identity** Score **> 14** indicates **homology** | U | R.TEQLTDSTEISLLPSDIDR.Y |
| 131093 | 609 | – | 627 | 1067.0331 | 2132.0517 | 2132.0485 | 1.51 | 0 | 108 | 1.8e-09 | 1Score **> 36** indicates **identity** Score **> 33** indicates **homology** | U | R.TEQLTDSTEISLLPSDIDR.Y |
| 131095 | 609 | – | 627 | 1067.0333 | 2132.0520 | 2132.0485 | 1.65 | 0 | 108 | 1.8e-09 | 1Score **> 36** indicates **identity** Score **> 33** indicates **homology** | U | R.TEQLTDSTEISLLPSDIDR.Y |
| 131097 | 609 | – | 627 | 1067.0359 | 2132.0573 | 2132.0485 | 4.13 | 0 | 89 | 1.2e-07 | 1Score **> 36** indicates **identity** Score **> 32** indicates **homology** | U | R.TEQLTDSTEISLLPSDIDR.Y |
| 131099 | 609 | – | 627 | 1067.0371 | 2132.0596 | 2132.0485 | 5.22 | 0 | 94 | 5.7e-08 | 1Score **> 36** indicates **identity** Score **> 34** indicates **homology** | U | R.TEQLTDSTEISLLPSDIDR.Y |
| 151340 | 609 | – | 629 | 808.7421 | 2423.2046 | 2423.2067 | -0.90 | 1 | 32 | 0.0011 | 1Score **> 36** indicates **identity** Score **> 14** indicates **homology** | U | R.TEQLTDSTEISLLPSDIDRYK.K |
| 151345 | 609 | – | 629 | 808.7435 | 2423.2086 | 2423.2067 | 0.76 | 1 | 41 | 0.00017 | 1Score **> 37** indicates **identity** Score **> 16** indicates **homology** | U | R.TEQLTDSTEISLLPSDIDRYK.K |
| 151348 | 609 | – | 629 | 808.7437 | 2423.2092 | 2423.2067 | 1.02 | 1 | 41 | 0.00015 | 1Score **> 37** indicates **identity** Score **> 15** indicates **homology** | U | R.TEQLTDSTEISLLPSDIDRYK.K |
| 151350 | 609 | – | 629 | 808.7439 | 2423.2098 | 2423.2067 | 1.26 | 1 | 44 | 8.2e-05 | 1Score **> 37** indicates **identity** Score **> 15** indicates **homology** | U | R.TEQLTDSTEISLLPSDIDRYK.K |
| 151351 | 609 | – | 629 | 1212.6124 | 2423.2103 | 2423.2067 | 1.45 | 1 | 80 | 2.9e-08 | 1Score **> 37** indicates **identity** Score **> 18** indicates **homology** | U | R.TEQLTDSTEISLLPSDIDRYK.K |
| 151352 | 609 | – | 629 | 1212.6124 | 2423.2103 | 2423.2067 | 1.46 | 1 | 91 | 2.9e-09 | 1Score **> 37** indicates **identity** Score **> 18** indicates **homology** | U | R.TEQLTDSTEISLLPSDIDRYK.K |
| 151353 | 609 | – | 629 | 1212.6127 | 2423.2108 | 2423.2067 | 1.67 | 1 | 75 | 8.6e-08 | 1Score **> 37** indicates **identity** Score **> 17** indicates **homology** | U | R.TEQLTDSTEISLLPSDIDRYK.K |
| 151356 | 609 | – | 629 | 808.7450 | 2423.2133 | 2423.2067 | 2.69 | 1 | 49 | 2.4e-05 | 1Score **> 37** indicates **identity** Score **> 16** indicates **homology** | U | R.TEQLTDSTEISLLPSDIDRYK.K |
| 151358 | 609 | – | 629 | 808.7455 | 2423.2147 | 2423.2067 | 3.30 | 1 | 26 | 0.0036 | 1Score **> 37** indicates **identity** Score **> 14** indicates **homology** | U | R.TEQLTDSTEISLLPSDIDRYK.K |
| 145205 | 632 | – | 650 | 775.0600 | 2322.1581 | 2322.1645 | -2.72 | 2 | 18 | 0.021 | 1Score **> 37** indicates **identity** Score **> 14** indicates **homology** | U | R.FHKFDEDEKGFITIVDVQR.V |
| 145208 | 632 | – | 650 | 581.5474 | 2322.1604 | 2322.1645 | -1.73 | 2 | 23 | 0.0069 | 1Score **> 37** indicates **identity** Score **> 14** indicates **homology** | U | R.FHKFDEDEKGFITIVDVQR.V |
| 145209 | 632 | – | 650 | 581.5475 | 2322.1608 | 2322.1645 | -1.57 | 2 | 14 | 0.046 | 1Score **> 37** indicates **identity** Score **> 13** indicates **homology** | U | R.FHKFDEDEKGFITIVDVQR.V |
| 145211 | 632 | – | 650 | 581.5476 | 2322.1615 | 2322.1645 | -1.29 | 2 | 46 | 5e-05 | 1Score **> 37** indicates **identity** Score **> 15** indicates **homology** | U | R.FHKFDEDEKGFITIVDVQR.V |
| 145215 | 632 | – | 650 | 581.5482 | 2322.1637 | 2322.1645 | -0.31 | 2 | 50 | 2.1e-05 | 1Score **> 37** indicates **identity** Score **> 16** indicates **homology** | U | R.FHKFDEDEKGFITIVDVQR.V |
| 145216 | 632 | – | 650 | 775.0619 | 2322.1639 | 2322.1645 | -0.22 | 2 | 29 | 0.002 | 1Score **> 37** indicates **identity** Score **> 14** indicates **homology** | U | R.FHKFDEDEKGFITIVDVQR.V |
| 145217 | 632 | – | 650 | 775.0620 | 2322.1640 | 2322.1645 | -0.18 | 2 | 30 | 0.0014 | 1Score **> 37** indicates **identity** Score **> 14** indicates **homology** | U | R.FHKFDEDEKGFITIVDVQR.V |
| 145220 | 632 | – | 650 | 581.5484 | 2322.1645 | 2322.1645 | -0.0013 | 2 | 34 | 0.00064 | 1Score **> 37** indicates **identity** Score **> 15** indicates **homology** | U | R.FHKFDEDEKGFITIVDVQR.V |
| 145221 | 632 | – | 650 | 775.0622 | 2322.1648 | 2322.1645 | 0.13 | 2 | 37 | 0.00031 | 1Score **> 37** indicates **identity** Score **> 15** indicates **homology** | U | R.FHKFDEDEKGFITIVDVQR.V |
| 145222 | 632 | – | 650 | 581.5485 | 2322.1648 | 2322.1645 | 0.13 | 2 | 44 | 8.1e-05 | 1Score **> 37** indicates **identity** Score **> 16** indicates **homology** | U | R.FHKFDEDEKGFITIVDVQR.V |
| 145223 | 632 | – | 650 | 581.5485 | 2322.1649 | 2322.1645 | 0.18 | 2 | 39 | 0.0002 | 1Score **> 37** indicates **identity** Score **> 15** indicates **homology** | U | R.FHKFDEDEKGFITIVDVQR.V |
| 145224 | 632 | – | 650 | 581.5485 | 2322.1649 | 2322.1645 | 0.21 | 2 | 44 | 8.1e-05 | 1Score **> 37** indicates **identity** Score **> 15** indicates **homology** | U | R.FHKFDEDEKGFITIVDVQR.V |
| 145225 | 632 | – | 650 | 581.5486 | 2322.1653 | 2322.1645 | 0.35 | 2 | 34 | 0.00066 | 1Score **> 37** indicates **identity** Score **> 15** indicates **homology** | U | R.FHKFDEDEKGFITIVDVQR.V |
| 145226 | 632 | – | 650 | 581.5486 | 2322.1654 | 2322.1645 | 0.39 | 2 | 44 | 8.8e-05 | 1Score **> 37** indicates **identity** Score **> 16** indicates **homology** | U | R.FHKFDEDEKGFITIVDVQR.V |
| 145227 | 632 | – | 650 | 581.5486 | 2322.1654 | 2322.1645 | 0.42 | 2 | 42 | 0.00011 | 1Score **> 37** indicates **identity** Score **> 15** indicates **homology** | U | R.FHKFDEDEKGFITIVDVQR.V |
| 145228 | 632 | – | 650 | 775.0624 | 2322.1654 | 2322.1645 | 0.42 | 2 | 36 | 0.00044 | 1Score **> 37** indicates **identity** Score **> 15** indicates **homology** | U | R.FHKFDEDEKGFITIVDVQR.V |
| 145231 | 632 | – | 650 | 775.0625 | 2322.1657 | 2322.1645 | 0.55 | 2 | 47 | 3.6e-05 | 1Score **> 37** indicates **identity** Score **> 15** indicates **homology** | U | R.FHKFDEDEKGFITIVDVQR.V |
| 145233 | 632 | – | 650 | 581.5488 | 2322.1660 | 2322.1645 | 0.67 | 2 | 29 | 0.0021 | 1Score **> 37** indicates **identity** Score **> 14** indicates **homology** | U | R.FHKFDEDEKGFITIVDVQR.V |
| 145234 | 632 | – | 650 | 465.4405 | 2322.1660 | 2322.1645 | 0.67 | 2 | 21 | 0.024 | 1Score **> 37** indicates **identity** Score **> 17** indicates **homology** | U | R.FHKFDEDEKGFITIVDVQR.V |
| 145235 | 632 | – | 650 | 1162.0903 | 2322.1660 | 2322.1645 | 0.68 | 2 | 40 | 0.00019 | 1Score **> 37** indicates **identity** Score **> 15** indicates **homology** | U | R.FHKFDEDEKGFITIVDVQR.V |
| 145236 | 632 | – | 650 | 581.5488 | 2322.1663 | 2322.1645 | 0.78 | 2 | 47 | 4e-05 | 1Score **> 37** indicates **identity** Score **> 15** indicates **homology** | U | R.FHKFDEDEKGFITIVDVQR.V |
| 145237 | 632 | – | 650 | 775.0627 | 2322.1663 | 2322.1645 | 0.78 | 2 | 67 | 5.8e-07 | 1Score **> 37** indicates **identity** Score **> 17** indicates **homology** | U | R.FHKFDEDEKGFITIVDVQR.V |
| 145239 | 632 | – | 650 | 581.5489 | 2322.1663 | 2322.1645 | 0.79 | 2 | 42 | 0.00012 | 1Score **> 37** indicates **identity** Score **> 15** indicates **homology** | U | R.FHKFDEDEKGFITIVDVQR.V |
| 145240 | 632 | – | 650 | 775.0627 | 2322.1663 | 2322.1645 | 0.79 | 2 | 44 | 7.2e-05 | 1Score **> 37** indicates **identity** Score **> 15** indicates **homology** | U | R.FHKFDEDEKGFITIVDVQR.V |
| 145241 | 632 | – | 650 | 775.0627 | 2322.1664 | 2322.1645 | 0.81 | 2 | 52 | 1.2e-05 | 1Score **> 37** indicates **identity** Score **> 16** indicates **homology** | U | R.FHKFDEDEKGFITIVDVQR.V |
| 145242 | 632 | – | 650 | 581.5489 | 2322.1664 | 2322.1645 | 0.82 | 2 | 39 | 0.00024 | 1Score **> 37** indicates **identity** Score **> 15** indicates **homology** | U | R.FHKFDEDEKGFITIVDVQR.V |
| 145243 | 632 | – | 650 | 581.5489 | 2322.1664 | 2322.1645 | 0.82 | 2 | 37 | 0.00039 | 1Score **> 37** indicates **identity** Score **> 15** indicates **homology** | U | R.FHKFDEDEKGFITIVDVQR.V |
| 145245 | 632 | – | 650 | 581.5489 | 2322.1665 | 2322.1645 | 0.87 | 2 | 37 | 0.00035 | 1Score **> 37** indicates **identity** Score **> 15** indicates **homology** | U | R.FHKFDEDEKGFITIVDVQR.V |
| 145248 | 632 | – | 650 | 581.5490 | 2322.1667 | 2322.1645 | 0.97 | 2 | 34 | 0.00059 | 1Score **> 37** indicates **identity** Score **> 15** indicates **homology** | U | R.FHKFDEDEKGFITIVDVQR.V |
| 145249 | 632 | – | 650 | 581.5490 | 2322.1669 | 2322.1645 | 1.06 | 2 | 29 | 0.002 | 1Score **> 37** indicates **identity** Score **> 14** indicates **homology** | U | R.FHKFDEDEKGFITIVDVQR.V |
| 145252 | 632 | – | 650 | 581.5494 | 2322.1683 | 2322.1645 | 1.67 | 2 | 16 | 0.033 | 1Score **> 37** indicates **identity** Score **> 13** indicates **homology** | U | R.FHKFDEDEKGFITIVDVQR.V |
| 145253 | 632 | – | 650 | 581.5494 | 2322.1685 | 2322.1645 | 1.72 | 2 | 16 | 0.034 | 1Score **> 37** indicates **identity** Score **> 13** indicates **homology** | U | R.FHKFDEDEKGFITIVDVQR.V |
| 145254 | 632 | – | 650 | 581.5494 | 2322.1685 | 2322.1645 | 1.73 | 2 | 39 | 0.00023 | 1Score **> 37** indicates **identity** Score **> 16** indicates **homology** | U | R.FHKFDEDEKGFITIVDVQR.V |
| 145255 | 632 | – | 650 | 581.5495 | 2322.1687 | 2322.1645 | 1.83 | 2 | 42 | 0.00011 | 1Score **> 37** indicates **identity** Score **> 15** indicates **homology** | U | R.FHKFDEDEKGFITIVDVQR.V |
| 145258 | 632 | – | 650 | 581.5496 | 2322.1691 | 2322.1645 | 2.01 | 2 | 16 | 0.036 | 1Score **> 37** indicates **identity** Score **> 14** indicates **homology** | U | R.FHKFDEDEKGFITIVDVQR.V |
| 145260 | 632 | – | 650 | 581.5496 | 2322.1693 | 2322.1645 | 2.09 | 2 | 23 | 0.0074 | 1Score **> 37** indicates **identity** Score **> 14** indicates **homology** | U | R.FHKFDEDEKGFITIVDVQR.V |
| 145263 | 632 | – | 650 | 581.5501 | 2322.1715 | 2322.1645 | 3.02 | 2 | 18 | 0.023 | 1Score **> 37** indicates **identity** Score **> 14** indicates **homology** | U | R.FHKFDEDEKGFITIVDVQR.V |
| 145266 | 632 | – | 650 | 581.5515 | 2322.1767 | 2322.1645 | 5.28 | 2 | 42 | 0.00012 | 1Score **> 37** indicates **identity** Score **> 15** indicates **homology** | U | R.FHKFDEDEKGFITIVDVQR.V |
| 154176 | 632 | – | 650 | 496.6602 | 2478.2647 | 2478.2795 | -5.97 | 2 | 32 | 0.0011 | 1Score **> 37** indicates **identity** Score **> 14** indicates **homology** | U | R.FHKFDEDEKGFITIVDVQR.V  + HNE (H) |
| 154178 | 632 | – | 650 | 620.5736 | 2478.2654 | 2478.2795 | -5.68 | 2 | 25 | 0.034 | 1Score **> 37** indicates **identity** Score **> 23** indicates **homology** | U | R.FHKFDEDEKGFITIVDVQR.V  + HNE (H) |
| 154180 | 632 | – | 650 | 620.5737 | 2478.2657 | 2478.2795 | -5.58 | 2 | 34 | 0.00061 | 1Score **> 37** indicates **identity** Score **> 15** indicates **homology** | U | R.FHKFDEDEKGFITIVDVQR.V  + HNE (H) |
| 154183 | 632 | – | 650 | 496.6605 | 2478.2663 | 2478.2795 | -5.34 | 2 | 33 | 0.00072 | 1Score **> 37** indicates **identity** Score **> 15** indicates **homology** | U | R.FHKFDEDEKGFITIVDVQR.V  + HNE (H) |
| 154184 | 632 | – | 650 | 496.6606 | 2478.2668 | 2478.2795 | -5.12 | 2 | 23 | 0.0067 | 1Score **> 37** indicates **identity** Score **> 14** indicates **homology** | U | R.FHKFDEDEKGFITIVDVQR.V  + HNE (H) |
| 154185 | 632 | – | 650 | 496.6607 | 2478.2670 | 2478.2795 | -5.03 | 2 | 33 | 0.0008 | 1Score **> 37** indicates **identity** Score **> 15** indicates **homology** | U | R.FHKFDEDEKGFITIVDVQR.V  + HNE (H) |
| 154188 | 632 | – | 650 | 620.5742 | 2478.2676 | 2478.2795 | -4.79 | 2 | 31 | 0.0011 | 1Score **> 37** indicates **identity** Score **> 14** indicates **homology** | U | R.FHKFDEDEKGFITIVDVQR.V  + HNE (H) |
| 154193 | 632 | – | 650 | 620.5746 | 2478.2694 | 2478.2795 | -4.08 | 2 | 26 | 0.019 | 1Score **> 37** indicates **identity** Score **> 21** indicates **homology** | U | R.FHKFDEDEKGFITIVDVQR.V  + HNE (H) |
| 154194 | 632 | – | 650 | 496.6612 | 2478.2697 | 2478.2795 | -3.95 | 2 | 15 | 0.038 | 1Score **> 37** indicates **identity** Score **> 13** indicates **homology** | U | R.FHKFDEDEKGFITIVDVQR.V  + HNE (K) |
| 154199 | 632 | – | 650 | 620.5759 | 2478.2747 | 2478.2795 | -1.95 | 2 | 15 | 0.038 | 1Score **> 37** indicates **identity** Score **> 13** indicates **homology** | U | R.FHKFDEDEKGFITIVDVQR.V  + HNE (H) |
| 154200 | 632 | – | 650 | 620.5761 | 2478.2754 | 2478.2795 | -1.67 | 2 | 21 | 0.011 | 1Score **> 37** indicates **identity** Score **> 14** indicates **homology** | U | R.FHKFDEDEKGFITIVDVQR.V  + HNE (K) |
| 111595 | 635 | – | 650 | 637.6534 | 1909.9383 | 1909.9422 | -2.03 | 1 | 42 | 0.00011 | 1Score **> 35** indicates **identity** Score **> 15** indicates **homology** | U | K.FDEDEKGFITIVDVQR.V |
| 111597 | 635 | – | 650 | 637.6548 | 1909.9426 | 1909.9422 | 0.24 | 1 | 39 | 0.00022 | 1Score **> 35** indicates **identity** Score **> 15** indicates **homology** | U | K.FDEDEKGFITIVDVQR.V |
| 125839 | 676 | – | 693 | 691.0276 | 2070.0608 | 2070.0568 | 1.96 | 0 | 69 | 3.1e-07 | 1Score **> 36** indicates **identity** Score **> 17** indicates **homology** | U | K.NGQVELHEFLQLMSAVQK.G |
| 10824 | 707 | – | 714 | 474.2235 | 946.4325 | 946.4356 | -3.27 | 0 | 28 | 0.0022 | 1Score **> 28** indicates **identity** Score **> 14** indicates **homology** | U | K.TAEENLDR.R |
| 22367 | 707 | – | 715 | 368.5198 | 1102.5374 | 1102.5367 | 0.70 | 1 | 17 | 0.025 | 1Score **> 31** indicates **identity** Score **> 14** indicates **homology** | U | K.TAEENLDRR.V |
| 109168 | 707 | – | 722 | 627.3388 | 1878.9947 | 1878.9912 | 1.89 | 2 | 16 | 0.032 | 1Score **> 36** indicates **identity** Score **> 13** indicates **homology** | U | K.TAEENLDRRVPIPVDR.S |
| 11199 | 715 | – | 722 | 476.2901 | 950.5657 | 950.5661 | -0.47 | 1 | 15 | 0.038 | 1Score **> 23** indicates **identity** Score **> 13** indicates **homology** | U | R.RVPIPVDR.S |
| 11201 | 715 | – | 722 | 476.2907 | 950.5668 | 950.5661 | 0.67 | 1 | 15 | 0.046 | 1Score **> 23** indicates **identity** Score **> 14** indicates **homology** | U | R.RVPIPVDR.S |
| 11202 | 715 | – | 722 | 476.2907 | 950.5668 | 950.5661 | 0.74 | 1 | 25 | 0.028 | 1Score **> 23** indicates **identity** Score **> 22** indicates **homology** | U | R.RVPIPVDR.S |
| 11203 | 715 | – | 722 | 476.2907 | 950.5669 | 950.5661 | 0.81 | 1 | 31 | 0.009 | 1Score **> 23** indicates **identity** | U | R.RVPIPVDR.S |
| 11204 | 715 | – | 722 | 476.2907 | 950.5669 | 950.5661 | 0.82 | 1 | 33 | 0.0056 | 1Score **> 23** indicates **identity** Score **> 23** indicates **homology** | U | R.RVPIPVDR.S |
| 11205 | 715 | – | 722 | 476.2909 | 950.5672 | 950.5661 | 1.13 | 1 | 39 | 0.0014 | 1Score **> 23** indicates **identity** | U | R.RVPIPVDR.S |
| 3353 | 716 | – | 722 | 398.2399 | 794.4653 | 794.4650 | 0.32 | 0 | 30 | 0.012 | 1Score **> 23** indicates **identity** | U | R.VPIPVDR.S |
| 3355 | 716 | – | 722 | 398.2403 | 794.4660 | 794.4650 | 1.17 | 0 | 34 | 0.0038 | 1Score **> 23** indicates **identity** | U | R.VPIPVDR.S |

---

```
ID   GPDM_MOUSE              Reviewed;         727 AA.
AC   Q64521; Q3TK51; Q3UDY8; Q61507; Q8CBX6; Q8K4U5; Q8VDT0; Q9ERP0;
DT   01-NOV-1997, integrated into UniProtKB/Swiss-Prot.
DT   01-MAY-2007, sequence version 2.
DT   28-JUN-2023, entry version 186.
DE   RecName: Full=Glycerol-3-phosphate dehydrogenase, mitochondrial;
DE            Short=GPD-M;
DE            Short=GPDH-M;
DE            EC=1.1.5.3 {ECO:0000250|UniProtKB:P43304};
DE   AltName: Full=Protein TISP38;
DE   Flags: Precursor;
GN   Name=Gpd2 {ECO:0000312|MGI:MGI:99778}; Synonyms=Gdm1;
OS   Mus musculus (Mouse).
OC   Eukaryota; Metazoa; Chordata; Craniata; Vertebrata; Euteleostomi; Mammalia;
OC   Eutheria; Euarchontoglires; Glires; Rodentia; Myomorpha; Muroidea; Muridae;
OC   Murinae; Mus; Mus.
OX   NCBI_TaxID=10090;
RN   [1]
RP   NUCLEOTIDE SEQUENCE [MRNA].
RC   STRAIN=C57BL/6J; TISSUE=Adipocyte;
RX   PubMed=8951039; DOI=10.1006/abbi.1996.0536;
RA   Koza R.A., Kozak U.C., Brown L.J., Leiter E.H., Macdonald M.J., Kozak L.P.;
RT   "Sequence and tissue-dependent RNA expression of mouse FAD-linked glycerol-
RT   3-phosphate dehydrogenase.";
RL   Arch. Biochem. Biophys. 336:97-104(1996).
RN   [2]
RP   NUCLEOTIDE SEQUENCE [MRNA].
RC   STRAIN=C57BL/6J;
RX   PubMed=8772729; DOI=10.2337/diab.45.9.1238;
RA   Ishihara H., Nakazaki M., Kanegae Y., Inukai K., Asano T., Katagiri H.,
RA   Yazaki Y., Kikuchi M., Miyazaki J., Saito I., Oka Y.;
RT   "Effect of mitochondrial and/or cytosolic glycerol 3-phosphate
RT   dehydrogenase overexpression on glucose-stimulated insulin secretion from
RT   MIN6 and HIT cells.";
RL   Diabetes 45:1238-1244(1996).
RN   [3]
RP   NUCLEOTIDE SEQUENCE [LARGE SCALE MRNA].
RC   STRAIN=C57BL/6J; TISSUE=Bone marrow, Diencephalon, and Lung;
RX   PubMed=16141072; DOI=10.1126/science.1112014;
RA   Carninci P., Kasukawa T., Katayama S., Gough J., Frith M.C., Maeda N.,
RA   Oyama R., Ravasi T., Lenhard B., Wells C., Kodzius R., Shimokawa K.,
RA   Bajic V.B., Brenner S.E., Batalov S., Forrest A.R., Zavolan M., Davis M.J.,
RA   Wilming L.G., Aidinis V., Allen J.E., Ambesi-Impiombato A., Apweiler R.,
RA   Aturaliya R.N., Bailey T.L., Bansal M., Baxter L., Beisel K.W., Bersano T.,
RA   Bono H., Chalk A.M., Chiu K.P., Choudhary V., Christoffels A.,
RA   Clutterbuck D.R., Crowe M.L., Dalla E., Dalrymple B.P., de Bono B.,
RA   Della Gatta G., di Bernardo D., Down T., Engstrom P., Fagiolini M.,
RA   Faulkner G., Fletcher C.F., Fukushima T., Furuno M., Futaki S.,
RA   Gariboldi M., Georgii-Hemming P., Gingeras T.R., Gojobori T., Green R.E.,
RA   Gustincich S., Harbers M., Hayashi Y., Hensch T.K., Hirokawa N., Hill D.,
RA   Huminiecki L., Iacono M., Ikeo K., Iwama A., Ishikawa T., Jakt M.,
RA   Kanapin A., Katoh M., Kawasawa Y., Kelso J., Kitamura H., Kitano H.,
RA   Kollias G., Krishnan S.P., Kruger A., Kummerfeld S.K., Kurochkin I.V.,
RA   Lareau L.F., Lazarevic D., Lipovich L., Liu J., Liuni S., McWilliam S.,
RA   Madan Babu M., Madera M., Marchionni L., Matsuda H., Matsuzawa S., Miki H.,
RA   Mignone F., Miyake S., Morris K., Mottagui-Tabar S., Mulder N., Nakano N.,
RA   Nakauchi H., Ng P., Nilsson R., Nishiguchi S., Nishikawa S., Nori F.,
RA   Ohara O., Okazaki Y., Orlando V., Pang K.C., Pavan W.J., Pavesi G.,
RA   Pesole G., Petrovsky N., Piazza S., Reed J., Reid J.F., Ring B.Z.,
RA   Ringwald M., Rost B., Ruan Y., Salzberg S.L., Sandelin A., Schneider C.,
RA   Schoenbach C., Sekiguchi K., Semple C.A., Seno S., Sessa L., Sheng Y.,
RA   Shibata Y., Shimada H., Shimada K., Silva D., Sinclair B., Sperling S.,
RA   Stupka E., Sugiura K., Sultana R., Takenaka Y., Taki K., Tammoja K.,
RA   Tan S.L., Tang S., Taylor M.S., Tegner J., Teichmann S.A., Ueda H.R.,
RA   van Nimwegen E., Verardo R., Wei C.L., Yagi K., Yamanishi H.,
RA   Zabarovsky E., Zhu S., Zimmer A., Hide W., Bult C., Grimmond S.M.,
RA   Teasdale R.D., Liu E.T., Brusic V., Quackenbush J., Wahlestedt C.,
RA   Mattick J.S., Hume D.A., Kai C., Sasaki D., Tomaru Y., Fukuda S.,
RA   Kanamori-Katayama M., Suzuki M., Aoki J., Arakawa T., Iida J., Imamura K.,
RA   Itoh M., Kato T., Kawaji H., Kawagashira N., Kawashima T., Kojima M.,
RA   Kondo S., Konno H., Nakano K., Ninomiya N., Nishio T., Okada M., Plessy C.,
RA   Shibata K., Shiraki T., Suzuki S., Tagami M., Waki K., Watahiki A.,
RA   Okamura-Oho Y., Suzuki H., Kawai J., Hayashizaki Y.;
RT   "The transcriptional landscape of the mammalian genome.";
RL   Science 309:1559-1563(2005).
RN   [4]
RP   NUCLEOTIDE SEQUENCE [LARGE SCALE MRNA].
RC   STRAIN=Czech II; TISSUE=Mammary tumor;
RX   PubMed=15489334; DOI=10.1101/gr.2596504;
RG   The MGC Project Team;
RT   "The status, quality, and expansion of the NIH full-length cDNA project:
RT   the Mammalian Gene Collection (MGC).";
RL   Genome Res. 14:2121-2127(2004).
RN   [5]
RP   PROTEIN SEQUENCE OF 58-91; 95-110; 125-135; 167-178; 200-209; 212-254;
RP   285-298; 340-348; 381-390; 392-409; 442-453; 464-473; 483-499; 519-538;
RP   558-579; 598-606; 609-627 AND 635-693.
RC   STRAIN=C57BL/6J; TISSUE=Brain, and Hippocampus;
RA   Lubec G., Klug S., Kang S.U.;
RL   Submitted (APR-2007) to UniProtKB.
RN   [6]
RP   NUCLEOTIDE SEQUENCE [GENOMIC DNA] OF 390-686.
RC   STRAIN=129/Sv; TISSUE=Liver;
RA   Weitzel J.M.;
RT   "Genomic sequence of mouse mitochondrial glycerol-3-phosphate dehydrogenase
RT   (mGPDH).";
RL   Submitted (AUG-2000) to the EMBL/GenBank/DDBJ databases.
RN   [7]
RP   NUCLEOTIDE SEQUENCE [MRNA] OF 611-727.
RC   TISSUE=Testis;
RA   Tamura K., Nishimune Y., Nojima H.;
RT   "Mus musculus TISP38 mRNA.";
RL   Submitted (JUL-2000) to the EMBL/GenBank/DDBJ databases.
RN   [8]
RP   PHOSPHORYLATION [LARGE SCALE ANALYSIS] AT TYR-601, AND IDENTIFICATION BY
RP   MASS SPECTROMETRY [LARGE SCALE ANALYSIS].
RC   TISSUE=Brain;
RX   PubMed=18034455; DOI=10.1021/pr0701254;
RA   Ballif B.A., Carey G.R., Sunyaev S.R., Gygi S.P.;
RT   "Large-scale identification and evolution indexing of tyrosine
RT   phosphorylation sites from murine brain.";
RL   J. Proteome Res. 7:311-318(2008).
RN   [9]
RP   IDENTIFICATION BY MASS SPECTROMETRY [LARGE SCALE ANALYSIS].
RC   TISSUE=Brain, Brown adipose tissue, Heart, Kidney, Liver, Lung,
RC   Pancreas, Spleen, and Testis;
RX   PubMed=21183079; DOI=10.1016/j.cell.2010.12.001;
RA   Huttlin E.L., Jedrychowski M.P., Elias J.E., Goswami T., Rad R.,
RA   Beausoleil S.A., Villen J., Haas W., Sowa M.E., Gygi S.P.;
RT   "A tissue-specific atlas of mouse protein phosphorylation and expression.";
RL   Cell 143:1174-1189(2010).
CC   -!- FUNCTION: Calcium-responsive mitochondrial glycerol-3-phosphate
CC       dehydrogenase which seems to be a key component of the pancreatic beta-
CC       cell glucose-sensing device. {ECO:0000250|UniProtKB:P43304}.
CC   -!- CATALYTIC ACTIVITY:
CC       Reaction=a quinone + sn-glycerol 3-phosphate = a quinol +
CC         dihydroxyacetone phosphate; Xref=Rhea:RHEA:18977, ChEBI:CHEBI:24646,
CC         ChEBI:CHEBI:57597, ChEBI:CHEBI:57642, ChEBI:CHEBI:132124; EC=1.1.5.3;
CC         Evidence={ECO:0000250|UniProtKB:P43304};
CC       PhysiologicalDirection=left-to-right; Xref=Rhea:RHEA:18978;
CC         Evidence={ECO:0000250|UniProtKB:P43304};
CC   -!- COFACTOR:
CC       Name=FAD; Xref=ChEBI:CHEBI:57692;
CC   -!- ACTIVITY REGULATION: Calcium-binding enhance the activity of the
CC       enzyme. {ECO:0000250|UniProtKB:P43304}.
CC   -!- PATHWAY: Polyol metabolism; glycerol degradation via glycerol kinase
CC       pathway; glycerone phosphate from sn-glycerol 3-phosphate (anaerobic
CC       route): step 1/1.
CC   -!- SUBCELLULAR LOCATION: Mitochondrion inner membrane.
CC   -!- SIMILARITY: Belongs to the FAD-dependent glycerol-3-phosphate
CC       dehydrogenase family. {ECO:0000305}.
CC   ---------------------------------------------------------------------------
CC   Copyrighted by the UniProt Consortium, see https://www.uniprot.org/terms
CC   Distributed under the Creative Commons Attribution (CC BY 4.0) License
CC   ---------------------------------------------------------------------------
DR   EMBL; U60987; AAB50545.1; -; mRNA.
DR   EMBL; D50430; BAA08926.1; -; mRNA.
DR   EMBL; AK034353; BAC28685.1; -; mRNA.
DR   EMBL; AK144716; BAE26028.1; -; mRNA.
DR   EMBL; AK149851; BAE29123.1; -; mRNA.
DR   EMBL; AK167152; BAE39294.1; -; mRNA.
DR   EMBL; BC021359; AAH21359.1; -; mRNA.
DR   EMBL; AH009802; AAG12342.1; -; Genomic_DNA.
DR   EMBL; AB045714; BAB97201.1; -; mRNA.
DR   CCDS; CCDS16045.1; -.
DR   RefSeq; NP_001139292.1; NM_001145820.1.
DR   RefSeq; NP_034404.3; NM_010274.3.
DR   AlphaFoldDB; Q64521; -.
DR   SMR; Q64521; -.
DR   BioGRID; 199894; 21.
DR   IntAct; Q64521; 2.
DR   STRING; 10090.ENSMUSP00000108237; -.
DR   GlyGen; Q64521; 1 site, 1 O-linked glycan (1 site).
DR   iPTMnet; Q64521; -.
DR   PhosphoSitePlus; Q64521; -.
DR   SwissPalm; Q64521; -.
DR   REPRODUCTION-2DPAGE; Q64521; -.
DR   EPD; Q64521; -.
DR   jPOST; Q64521; -.
DR   MaxQB; Q64521; -.
DR   PaxDb; Q64521; -.
DR   PeptideAtlas; Q64521; -.
DR   ProteomicsDB; 271263; -.
DR   Antibodypedia; 2370; 316 antibodies from 30 providers.
DR   DNASU; 14571; -.
DR   Ensembl; ENSMUST00000028167; ENSMUSP00000028167; ENSMUSG00000026827.
DR   Ensembl; ENSMUST00000169687; ENSMUSP00000130992; ENSMUSG00000026827.
DR   GeneID; 14571; -.
DR   KEGG; mmu:14571; -.
DR   UCSC; uc008jsc.2; mouse.
DR   AGR; MGI:99778; -.
DR   CTD; 2820; -.
DR   MGI; MGI:99778; Gpd2.
DR   VEuPathDB; HostDB:ENSMUSG00000026827; -.
DR   eggNOG; KOG0042; Eukaryota.
DR   GeneTree; ENSGT00390000001718; -.
DR   InParanoid; Q64521; -.
DR   OMA; WAMAPHI; -.
DR   OrthoDB; 989271at2759; -.
DR   BRENDA; 1.1.5.3; 3474.
DR   Reactome; R-MMU-1483166; Synthesis of PA.
DR   Reactome; R-MMU-163560; Triglyceride catabolism.
DR   UniPathway; UPA00618; UER00673.
DR   BioGRID-ORCS; 14571; 2 hits in 78 CRISPR screens.
DR   ChiTaRS; Gpd2; mouse.
DR   PRO; PR:Q64521; -.
DR   Proteomes; UP000000589; Chromosome 2.
DR   RNAct; Q64521; protein.
DR   Bgee; ENSMUSG00000026827; Expressed in seminiferous tubule of testis and 269 other tissues.
DR   ExpressionAtlas; Q64521; baseline and differential.
DR   Genevisible; Q64521; MM.
DR   GO; GO:0009331; C:glycerol-3-phosphate dehydrogenase complex; TAS:MGI.
DR   GO; GO:0005743; C:mitochondrial inner membrane; HDA:MGI.
DR   GO; GO:0005739; C:mitochondrion; HDA:MGI.
DR   GO; GO:0005509; F:calcium ion binding; IEA:InterPro.
DR   GO; GO:0004368; F:glycerol-3-phosphate dehydrogenase (quinone) activity; ISO:MGI.
DR   GO; GO:0047952; F:glycerol-3-phosphate dehydrogenase [NAD(P)+] activity; ISO:MGI.
DR   GO; GO:0052591; F:sn-glycerol-3-phosphate:ubiquinone-8 oxidoreductase activity; IEA:UniProtKB-EC.
DR   GO; GO:0043010; P:camera-type eye development; IGI:MGI.
DR   GO; GO:0006094; P:gluconeogenesis; IMP:MGI.
DR   GO; GO:0019563; P:glycerol catabolic process; IEA:UniProtKB-UniPathway.
DR   GO; GO:0006072; P:glycerol-3-phosphate metabolic process; IMP:MGI.
DR   GO; GO:0035264; P:multicellular organism growth; IGI:MGI.
DR   GO; GO:0006734; P:NADH metabolic process; ISO:MGI.
DR   CDD; cd00051; EFh; 1.
DR   Gene3D; 1.10.8.870; Alpha-glycerophosphate oxidase, cap domain; 1.
DR   Gene3D; 3.30.9.10; D-Amino Acid Oxidase, subunit A, domain 2; 1.
DR   Gene3D; 1.10.238.10; EF-hand; 1.
DR   Gene3D; 3.50.50.60; FAD/NAD(P)-binding domain; 1.
DR   InterPro; IPR031656; DAO_C.
DR   InterPro; IPR038299; DAO_C_sf.
DR   InterPro; IPR011992; EF-hand-dom_pair.
DR   InterPro; IPR018247; EF_Hand_1_Ca_BS.
DR   InterPro; IPR002048; EF_hand_dom.
DR   InterPro; IPR006076; FAD-dep_OxRdtase.
DR   InterPro; IPR036188; FAD/NAD-bd_sf.
DR   InterPro; IPR000447; G3P_DH_FAD-dep.
DR   PANTHER; PTHR11985; GLYCEROL-3-PHOSPHATE DEHYDROGENASE; 1.
DR   PANTHER; PTHR11985:SF15; GLYCEROL-3-PHOSPHATE DEHYDROGENASE, MITOCHONDRIAL; 1.
DR   Pfam; PF01266; DAO; 1.
DR   Pfam; PF16901; DAO_C; 1.
DR   Pfam; PF13499; EF-hand_7; 1.
DR   PRINTS; PR01001; FADG3PDH.
DR   SMART; SM00054; EFh; 2.
DR   SUPFAM; SSF47473; EF-hand; 1.
DR   SUPFAM; SSF54373; FAD-linked reductases, C-terminal domain; 1.
DR   SUPFAM; SSF51905; FAD/NAD(P)-binding domain; 1.
DR   PROSITE; PS00018; EF_HAND_1; 1.
DR   PROSITE; PS50222; EF_HAND_2; 2.
DR   PROSITE; PS00977; FAD_G3PDH_1; 1.
DR   PROSITE; PS00978; FAD_G3PDH_2; 1.
PE   1: Evidence at protein level;
KW   Calcium; Direct protein sequencing; FAD; Flavoprotein; Membrane;
KW   Metal-binding; Mitochondrion; Mitochondrion inner membrane; Oxidoreductase;
KW   Phosphoprotein; Reference proteome; Repeat; Transit peptide.
FT   TRANSIT         1..42
FT                   /note="Mitochondrion"
FT                   /evidence="ECO:0000250"
FT   CHAIN           43..727
FT                   /note="Glycerol-3-phosphate dehydrogenase, mitochondrial"
FT                   /id="PRO_0000010430"
FT   DOMAIN          623..658
FT                   /note="EF-hand 1"
FT                   /evidence="ECO:0000255|PROSITE-ProRule:PRU00448"
FT   DOMAIN          659..694
FT                   /note="EF-hand 2"
FT                   /evidence="ECO:0000255|PROSITE-ProRule:PRU00448"
FT   BINDING         71..99
FT                   /ligand="FAD"
FT                   /ligand_id="ChEBI:CHEBI:57692"
FT                   /evidence="ECO:0000255"
FT   BINDING         672
FT                   /ligand="Ca(2+)"
FT                   /ligand_id="ChEBI:CHEBI:29108"
FT                   /evidence="ECO:0000255|PROSITE-ProRule:PRU00448"
FT   BINDING         674
FT                   /ligand="Ca(2+)"
FT                   /ligand_id="ChEBI:CHEBI:29108"
FT                   /evidence="ECO:0000255|PROSITE-ProRule:PRU00448"
FT   BINDING         676
FT                   /ligand="Ca(2+)"
FT                   /ligand_id="ChEBI:CHEBI:29108"
FT                   /evidence="ECO:0000255|PROSITE-ProRule:PRU00448"
FT   BINDING         678
FT                   /ligand="Ca(2+)"
FT                   /ligand_id="ChEBI:CHEBI:29108"
FT                   /evidence="ECO:0000255|PROSITE-ProRule:PRU00448"
FT   BINDING         683
FT                   /ligand="Ca(2+)"
FT                   /ligand_id="ChEBI:CHEBI:29108"
FT                   /evidence="ECO:0000255|PROSITE-ProRule:PRU00448"
FT   MOD_RES         601
FT                   /note="Phosphotyrosine"
FT                   /evidence="ECO:0007744|PubMed:18034455"
FT   CONFLICT        26
FT                   /note="P -> Q (in Ref. 1; AAB50545 and 4; AAH21359)"
FT                   /evidence="ECO:0000305"
FT   CONFLICT        42
FT                   /note="Missing (in Ref. 3; BAC28685)"
FT                   /evidence="ECO:0000305"
FT   CONFLICT        158
FT                   /note="L -> V (in Ref. 1; AAB50545)"
FT                   /evidence="ECO:0000305"
FT   CONFLICT        295
FT                   /note="D -> E (in Ref. 2; BAA08926)"
FT                   /evidence="ECO:0000305"
FT   CONFLICT        449
FT                   /note="D -> N (in Ref. 4; AAH21359)"
FT                   /evidence="ECO:0000305"
FT   CONFLICT        498
FT                   /note="A -> P (in Ref. 2; BAA08926)"
FT                   /evidence="ECO:0000305"
FT   CONFLICT        582
FT                   /note="N -> D (in Ref. 1; AAB50545)"
FT                   /evidence="ECO:0000305"
FT   CONFLICT        593
FT                   /note="E -> G (in Ref. 1; AAB50545)"
FT                   /evidence="ECO:0000305"
SQ   SEQUENCE   727 AA;  80954 MW;  319F52E31CFDFC44 CRC64;
     MAFQKAVKGT ILVGGGALAT VLGLSPFAHY RRKQVSLAYV EAAGYLTEPV NREPPSREAQ
     LMTLKNTPEF DILVIGGGAT GCGCALDAVT RGLKTALVER DDFSSGTSSR STKLIHGGVR
     YLQKAIMNLD VEQYRMVKEA LHERANLLEI APHLSAPLPI MLPLYKWWQL PYYWVGIKMY
     DLVAGSQCLK SSYVLSKSRA LEHFPMLQKD KLVGAIVYYD GQHNDARMNL AIALTAARYG
     AATANYMEVV SLLKKTDPET GKERVSGARC KDVLTGQEFD VRAKCVINAS GPFTDSVRKM
     DDKNVVPICQ PSAGVHIVMP GYYSPENMGL LDPATSDGRV IFFLPWEKMT IAGTTDTPTD
     VTHHPIPSEE DINFILNEVR NYLSSDVEVR RGDVLAAWSG IRPLVTDPKS ADTQSISRNH
     VVDISDSGLI TIAGGKWTTY RSMAEDTVDA AVKFHNLNAG PSRTVGLFLQ GGKDWSPTLY
     IRLVQDYGLE SEVAQHLAKT YGDKAFEVAK MASVTGKRWP VVGVRLVSEF PYIEAEVKYG
     IKEYACTAVD MISRRTRLAF LNVQAAEEAL PRIVELMGRE LNWSELRKQE ELETATRFLY
     YEMGYKSRTE QLTDSTEISL LPSDIDRYKK RFHKFDEDEK GFITIVDVQR VLESINVQMD
     ENTLHEILCE VDLNKNGQVE LHEFLQLMSA VQKGRVSGSR LAILMKTAEE NLDRRVPIPV
     DRSCGGL
//
```

|  |
| --- |
| **Mascot:** http://www.matrixscience.com/ |

HNE (H) (+156.1150)
