# Supplementary material for: A common East-Asian ALDH2 mutation causes metabolic disorders and the therapeutic effect of ALDH2 activators
Source: Nat Commun. 2023 Sep 25;14:5971. doi: 10.1038/s41467-023-41570-6 (PMC10520061; doi:10.1038/s41467-023-41570-6)
Supplement: Supplementary file 4 — Supplementary Data 1 [file 41467_2023_41570_MOESM4_ESM.zip › Table S5b/Q8BMS1/Q8BMS1_WTO-1_K66_K664.html]

Mascot Search Results: Q8BMS1
 

# MASCOT Search Results

## Protein View: Q8BMS1

### Trifunctional enzyme subunit alpha, mitochondrial OS=Mus musculus OX=10090 GN=Hadha PE=1 SV=1

|  |  |
| --- | --- |
| Database: | Mouse\_UniProt\_proteomes |
| Score: | 14659 |
| Monoisotopic mass (Mr): | 83302 |
| Calculated pI: | 9.24 |

Sequence similarity is available as an NCBI BLAST search of Q8BMS1 against nr.

### Search parameters

|  |  |
| --- | --- |
| MS data file: | `D:\LCMSMS\2023 Users' data\230529-1\230529-1-WTO-1.raw` |
| Enzyme: | Trypsin/P: cuts C-term side of KR. |
| Fixed modifications: | Carbamidomethyl (C) |
| Variable modifications: | Deamidated (NQ), HNE (C), HNE (H), HNE (K), Oxidation (M) |

### Protein sequence coverage: 69%

Matched peptides shown in ***bold red***.

|  |  |  |  |  |  |
| --- | --- | --- | --- | --- | --- |
| `1` | `MVASRAIGSL` | `SRFSAFRILR` | `SRGCICRSFT` | `TSSALLTRTH` | `INYGVKGDVA` |
| `51` | `VIRINSPNSK` | `VNTLNKEVQS` | `EFIEVMNEIW` | `ANDQIRSAVL` | `ISSKPGCFVA` |
| `101` | `GADINMLSSC` | `TTPQEATRIS` | `QEGQRMFEKL` | `EKSPKPVVAA` | `ISGSCLGGGL` |
| `151` | `ELAIACQYRI` | `ATKDRKTVLG` | `VPEVLLGILP` | `GAGGTQRLPK` | `MVGVPAAFDM` |
| `201` | `MLTGRNIRAD` | `RAKKMGLVDQ` | `LVEPLGPGIK` | `SPEERTIEYL` | `EEVAVNFAKG` |
| `251` | `LADRKVSAKQ` | `SKGLVEKLTT` | `YAMTVPFVRQ` | `QVYKTVEEKV` | `KKQTKGLYPA` |
| `301` | `PLKIIDAVKA` | `GLEQGSDAGY` | `LAESQKFGEL` | `ALTKESKALM` | `GLYNGQVLCK` |
| `351` | `KNKFGAPQKN` | `VQQLAILGAG` | `LMGAGIAQVS` | `VDKGLKTLLK` | `DTTVTGLGRG` |
| `401` | `QQQVFKGLND` | `KVKKKALTSF` | `ERDSIFSNLI` | `GQLDYKGFEK` | `ADMVIEAVFE` |
| `451` | `DLGVKHKVLK` | `EVESVTPEHC` | `IFASNTSALP` | `INQIAAVSKR` | `PEKVIGMHYF` |
| `501` | `SPVDKMQLLE` | `IITTDKTSKD` | `TTASAVAVGL` | `RQGKVIIVVK` | `DGPGFYTTRC` |
| `551` | `LAPMMSEVMR` | `ILQEGVDPKK` | `LDALTTGFGF` | `PVGAATLADE` | `VGVDVAQHVA` |
| `601` | `EDLGKAFGER` | `FGGGSVELLK` | `QMVSKGFLGR` | `KSGKGFYIYQ` | `EGSKNKSLNS` |
| `651` | `EMDNILANLR` | `LPAKPEVSSD` | `EDVQYRVITR` | `FVNEAVLCLQ` | `EGILATPAEG` |
| `701` | `DIGAVFGLGF` | `PPCLGGPFRF` | `VDLYGAQKVV` | `DRLRKYESAY` | `GTQFTPCQLL` |
| `751` | `LDHANNSSKK` | `FYQ` |  |  |  |

Unformatted sequence string: 763 residues (for pasting into other applications).

|  |  |  |  |
| --- | --- | --- | --- |
| Sort by | residue number | increasing mass | decreasing mass |
| Show | matched peptides only | predicted peptides also |  |

| Query | Start | – | End | Observed | Mr(expt) | Mr(calc) | ppm | M | Score | Expect | Rank | U | Peptide |
| --- | --- | --- | --- | --- | --- | --- | --- | --- | --- | --- | --- | --- | --- |
| 9946 | 39 | – | 46 | 466.2533 | 930.4920 | 930.4923 | -0.35 | 0 | 24 | 0.025 | 1Score **> 31** indicates **identity** Score **> 20** indicates **homology** | U | R.THINYGVK.G |
| 9948 | 39 | – | 46 | 466.2536 | 930.4926 | 930.4923 | 0.27 | 0 | 38 | 0.0013 | 1Score **> 31** indicates **identity** Score **> 22** indicates **homology** | U | R.THINYGVK.G |
| 83168 | 39 | – | 53 | 411.2305 | 1640.8930 | 1640.8998 | -4.17 | 1 | 27 | 0.0031 | 1Score **> 35** indicates **identity** Score **> 14** indicates **homology** | U | R.THINYGVKGDVAVIR.I |
| 83173 | 39 | – | 53 | 547.9733 | 1640.8981 | 1640.8998 | -1.08 | 1 | 70 | 2.7e-07 | 1Score **> 35** indicates **identity** Score **> 17** indicates **homology** | U | R.THINYGVKGDVAVIR.I |
| 83175 | 39 | – | 53 | 411.2320 | 1640.8990 | 1640.8998 | -0.48 | 1 | 23 | 0.0065 | 1Score **> 35** indicates **identity** Score **> 14** indicates **homology** | U | R.THINYGVKGDVAVIR.I |
| 83178 | 39 | – | 53 | 547.9738 | 1640.8994 | 1640.8998 | -0.26 | 1 | 20 | 0.015 | 1Score **> 35** indicates **identity** Score **> 14** indicates **homology** | U | R.THINYGVKGDVAVIR.I |
| 83179 | 39 | – | 53 | 547.9738 | 1640.8996 | 1640.8998 | -0.16 | 1 | 45 | 5.8e-05 | 1Score **> 35** indicates **identity** Score **> 15** indicates **homology** | U | R.THINYGVKGDVAVIR.I |
| 83181 | 39 | – | 53 | 547.9740 | 1640.9001 | 1640.8998 | 0.15 | 1 | 44 | 7.6e-05 | 1Score **> 35** indicates **identity** Score **> 15** indicates **homology** | U | R.THINYGVKGDVAVIR.I |
| 83183 | 39 | – | 53 | 547.9740 | 1640.9003 | 1640.8998 | 0.28 | 1 | 31 | 0.0012 | 1Score **> 35** indicates **identity** Score **> 14** indicates **homology** | U | R.THINYGVKGDVAVIR.I |
| 83185 | 39 | – | 53 | 547.9741 | 1640.9005 | 1640.8998 | 0.38 | 1 | 50 | 1.9e-05 | 1Score **> 35** indicates **identity** Score **> 16** indicates **homology** | U | R.THINYGVKGDVAVIR.I |
| 83186 | 39 | – | 53 | 547.9741 | 1640.9005 | 1640.8998 | 0.42 | 1 | 44 | 7.4e-05 | 1Score **> 35** indicates **identity** Score **> 15** indicates **homology** | U | R.THINYGVKGDVAVIR.I |
| 83187 | 39 | – | 53 | 547.9741 | 1640.9005 | 1640.8998 | 0.42 | 1 | 39 | 0.0002 | 1Score **> 35** indicates **identity** Score **> 15** indicates **homology** | U | R.THINYGVKGDVAVIR.I |
| 83189 | 39 | – | 53 | 547.9742 | 1640.9009 | 1640.8998 | 0.63 | 1 | 37 | 0.00032 | 1Score **> 35** indicates **identity** Score **> 15** indicates **homology** | U | R.THINYGVKGDVAVIR.I |
| 1066 | 47 | – | 53 | 365.2162 | 728.4179 | 728.4181 | -0.29 | 0 | 24 | 0.048 | 1Score **> 23** indicates **identity** | U | K.GDVAVIR.I |
| 1067 | 47 | – | 53 | 365.2162 | 728.4179 | 728.4181 | -0.20 | 0 | 50 | 0.0001 | 1Score **> 23** indicates **identity** | U | K.GDVAVIR.I |
| 1068 | 47 | – | 53 | 365.2163 | 728.4180 | 728.4181 | -0.11 | 0 | 50 | 0.0001 | 1Score **> 23** indicates **identity** | U | K.GDVAVIR.I |
| 1069 | 47 | – | 53 | 365.2165 | 728.4185 | 728.4181 | 0.55 | 0 | 35 | 0.0036 | 1Score **> 23** indicates **identity** | U | K.GDVAVIR.I |
| 175819 | 61 | – | 86 | 1040.5235 | 3118.5486 | 3118.5393 | 2.97 | 1 | 47 | 4e-05 | 1Score **> 37** indicates **identity** Score **> 15** indicates **homology** | U | K.VNTLNKEVQSEFIEVMNEIWANDQIR.S |
| 175820 | 61 | – | 86 | 1040.5292 | 3118.5656 | 3118.5393 | 8.44 | 1 | 37 | 0.00036 | 1Score **> 37** indicates **identity** Score **> 15** indicates **homology** | U | K.VNTLNKEVQSEFIEVMNEIWANDQIR.S |
| 178822 | 61 | – | 86 | 823.9099 | 3291.6105 | 3291.6333 | -6.90 | 1 | 18 | 0.021 | 1Score **> 37** indicates **identity** Score **> 14** indicates **homology** | U | K.VNTLNKEVQSEFIEVMNEIWANDQIR.S  + Deamidated (NQ); HNE (K); Oxidation (M) |
| 152760 | 67 | – | 86 | 817.7286 | 2450.1640 | 2450.1423 | 8.85 | 0 | 24 | 0.0053 | 1Score **> 35** indicates **identity** Score **> 14** indicates **homology** | U | K.EVQSEFIEVMNEIWANDQIR.S  + Deamidated (NQ) |
| 180024 | 87 | – | 118 | 1123.5531 | 3367.6374 | 3367.6210 | 4.85 | 1 | 74 | 1.3e-07 | 1Score **> 37** indicates **identity** Score **> 17** indicates **homology** | U | R.SAVLISSKPGCFVAGADINMLSSCTTPQEATR.I |
| 180025 | 87 | – | 118 | 1123.5542 | 3367.6408 | 3367.6210 | 5.86 | 1 | 95 | 1.1e-09 | 1Score **> 37** indicates **identity** Score **> 18** indicates **homology** | U | R.SAVLISSKPGCFVAGADINMLSSCTTPQEATR.I |
| 180027 | 87 | – | 118 | 1123.5550 | 3367.6431 | 3367.6210 | 6.54 | 1 | 86 | 8.6e-09 | 1Score **> 37** indicates **identity** Score **> 18** indicates **homology** | U | R.SAVLISSKPGCFVAGADINMLSSCTTPQEATR.I |
| 180028 | 87 | – | 118 | 842.9181 | 3367.6435 | 3367.6210 | 6.66 | 1 | 32 | 0.001 | 1Score **> 37** indicates **identity** Score **> 14** indicates **homology** | U | R.SAVLISSKPGCFVAGADINMLSSCTTPQEATR.I |
| 180029 | 87 | – | 118 | 1123.5557 | 3367.6454 | 3367.6210 | 7.22 | 1 | 103 | 2e-10 | 1Score **> 37** indicates **identity** Score **> 19** indicates **homology** | U | R.SAVLISSKPGCFVAGADINMLSSCTTPQEATR.I |
| 180030 | 87 | – | 118 | 1123.5561 | 3367.6464 | 3367.6210 | 7.54 | 1 | 83 | 1.8e-08 | 1Score **> 37** indicates **identity** Score **> 18** indicates **homology** | U | R.SAVLISSKPGCFVAGADINMLSSCTTPQEATR.I |
| 180040 | 87 | – | 118 | 1123.8816 | 3368.6229 | 3368.6051 | 5.31 | 1 | 31 | 0.0012 | 1Score **> 37** indicates **identity** Score **> 14** indicates **homology** | U | R.SAVLISSKPGCFVAGADINMLSSCTTPQEATR.I  + Deamidated (NQ) |
| 180041 | 87 | – | 118 | 1123.8837 | 3368.6291 | 3368.6051 | 7.15 | 1 | 59 | 3.2e-06 | 1Score **> 37** indicates **identity** Score **> 16** indicates **homology** | U | R.SAVLISSKPGCFVAGADINMLSSCTTPQEATR.I  + Deamidated (NQ) |
| 180343 | 87 | – | 118 | 1128.8787 | 3383.6142 | 3383.6160 | -0.53 | 1 | 52 | 1.4e-05 | 1Score **> 36** indicates **identity** Score **> 16** indicates **homology** | U | R.SAVLISSKPGCFVAGADINMLSSCTTPQEATR.I  + Oxidation (M) |
| 180350 | 87 | – | 118 | 1129.2097 | 3384.6073 | 3384.6000 | 2.16 | 1 | 36 | 0.00044 | 1Score **> 36** indicates **identity** Score **> 15** indicates **homology** | U | R.SAVLISSKPGCFVAGADINMLSSCTTPQEATR.I  + Deamidated (NQ); Oxidation (M) |
| 180353 | 87 | – | 118 | 1129.2151 | 3384.6233 | 3384.6000 | 6.91 | 1 | 57 | 4.3e-06 | 1Score **> 37** indicates **identity** Score **> 16** indicates **homology** | U | R.SAVLISSKPGCFVAGADINMLSSCTTPQEATR.I  + Deamidated (NQ); Oxidation (M) |
| 180361 | 87 | – | 118 | 1129.5462 | 3385.6169 | 3385.5840 | 9.73 | 1 | 23 | 0.0076 | 1Score **> 36** indicates **identity** Score **> 14** indicates **homology** | U | R.SAVLISSKPGCFVAGADINMLSSCTTPQEATR.I  + 2 Deamidated (NQ); Oxidation (M) |
| 176134 | 130 | – | 159 | 786.9197 | 3143.6499 | 3143.6471 | 0.88 | 2 | 30 | 0.0014 | 1Score **> 37** indicates **identity** Score **> 14** indicates **homology** | U | K.LEKSPKPVVAAISGSCLGGGLELAIACQYR.I |
| 176135 | 130 | – | 159 | 786.9211 | 3143.6554 | 3143.6471 | 2.64 | 2 | 49 | 2.4e-05 | 1Score **> 37** indicates **identity** Score **> 16** indicates **homology** | U | K.LEKSPKPVVAAISGSCLGGGLELAIACQYR.I |
| 166176 | 133 | – | 159 | 925.4802 | 2773.4188 | 2773.4255 | -2.41 | 1 | 72 | 1.9e-07 | 1Score **> 37** indicates **identity** Score **> 17** indicates **homology** | U | K.SPKPVVAAISGSCLGGGLELAIACQYR.I |
| 166177 | 133 | – | 159 | 925.4805 | 2773.4197 | 2773.4255 | -2.09 | 1 | 54 | 8.5e-06 | 1Score **> 37** indicates **identity** Score **> 16** indicates **homology** | U | K.SPKPVVAAISGSCLGGGLELAIACQYR.I |
| 166178 | 133 | – | 159 | 925.4809 | 2773.4210 | 2773.4255 | -1.61 | 1 | 24 | 0.0082 | 1Score **> 37** indicates **identity** Score **> 16** indicates **homology** | U | K.SPKPVVAAISGSCLGGGLELAIACQYR.I |
| 166179 | 133 | – | 159 | 925.4809 | 2773.4210 | 2773.4255 | -1.61 | 1 | 25 | 0.0045 | 1Score **> 37** indicates **identity** Score **> 14** indicates **homology** | U | K.SPKPVVAAISGSCLGGGLELAIACQYR.I |
| 166181 | 133 | – | 159 | 925.4813 | 2773.4221 | 2773.4255 | -1.22 | 1 | 27 | 0.0028 | 1Score **> 37** indicates **identity** Score **> 14** indicates **homology** | U | K.SPKPVVAAISGSCLGGGLELAIACQYR.I |
| 166183 | 133 | – | 159 | 925.4815 | 2773.4227 | 2773.4255 | -1.01 | 1 | 50 | 1.9e-05 | 1Score **> 37** indicates **identity** Score **> 16** indicates **homology** | U | K.SPKPVVAAISGSCLGGGLELAIACQYR.I |
| 166184 | 133 | – | 159 | 925.4815 | 2773.4228 | 2773.4255 | -0.97 | 1 | 19 | 0.018 | 1Score **> 37** indicates **identity** Score **> 14** indicates **homology** | U | K.SPKPVVAAISGSCLGGGLELAIACQYR.I |
| 166185 | 133 | – | 159 | 925.4816 | 2773.4231 | 2773.4255 | -0.88 | 1 | 33 | 0.00074 | 1Score **> 37** indicates **identity** Score **> 15** indicates **homology** | U | K.SPKPVVAAISGSCLGGGLELAIACQYR.I |
| 166186 | 133 | – | 159 | 925.4818 | 2773.4234 | 2773.4255 | -0.75 | 1 | 36 | 0.00038 | 1Score **> 37** indicates **identity** Score **> 15** indicates **homology** | U | K.SPKPVVAAISGSCLGGGLELAIACQYR.I |
| 166187 | 133 | – | 159 | 694.3633 | 2773.4242 | 2773.4255 | -0.48 | 1 | 90 | 3.7e-09 | 1Score **> 37** indicates **identity** Score **> 18** indicates **homology** | U | K.SPKPVVAAISGSCLGGGLELAIACQYR.I |
| 166189 | 133 | – | 159 | 925.4822 | 2773.4249 | 2773.4255 | -0.23 | 1 | 17 | 0.023 | 1Score **> 37** indicates **identity** Score **> 14** indicates **homology** | U | K.SPKPVVAAISGSCLGGGLELAIACQYR.I |
| 166190 | 133 | – | 159 | 925.4824 | 2773.4253 | 2773.4255 | -0.078 | 1 | 109 | 5.6e-11 | 1Score **> 37** indicates **identity** Score **> 19** indicates **homology** | U | K.SPKPVVAAISGSCLGGGLELAIACQYR.I |
| 166191 | 133 | – | 159 | 925.4824 | 2773.4254 | 2773.4255 | -0.041 | 1 | 34 | 0.00059 | 1Score **> 37** indicates **identity** Score **> 15** indicates **homology** | U | K.SPKPVVAAISGSCLGGGLELAIACQYR.I |
| 166192 | 133 | – | 159 | 925.4824 | 2773.4254 | 2773.4255 | -0.037 | 1 | 72 | 1.8e-07 | 1Score **> 37** indicates **identity** Score **> 17** indicates **homology** | U | K.SPKPVVAAISGSCLGGGLELAIACQYR.I |
| 166193 | 133 | – | 159 | 694.3638 | 2773.4259 | 2773.4255 | 0.16 | 1 | 21 | 0.011 | 1Score **> 37** indicates **identity** Score **> 14** indicates **homology** | U | K.SPKPVVAAISGSCLGGGLELAIACQYR.I |
| 166194 | 133 | – | 159 | 694.3638 | 2773.4259 | 2773.4255 | 0.16 | 1 | 33 | 0.00086 | 1Score **> 37** indicates **identity** Score **> 15** indicates **homology** | U | K.SPKPVVAAISGSCLGGGLELAIACQYR.I |
| 166195 | 133 | – | 159 | 925.4826 | 2773.4261 | 2773.4255 | 0.22 | 1 | 16 | 0.034 | 1Score **> 37** indicates **identity** Score **> 13** indicates **homology** | U | K.SPKPVVAAISGSCLGGGLELAIACQYR.I |
| 166196 | 133 | – | 159 | 925.4829 | 2773.4269 | 2773.4255 | 0.52 | 1 | 31 | 0.0013 | 1Score **> 37** indicates **identity** Score **> 14** indicates **homology** | U | K.SPKPVVAAISGSCLGGGLELAIACQYR.I |
| 166197 | 133 | – | 159 | 925.4829 | 2773.4270 | 2773.4255 | 0.53 | 1 | 124 | 2.4e-12 | 1Score **> 37** indicates **identity** Score **> 20** indicates **homology** | U | K.SPKPVVAAISGSCLGGGLELAIACQYR.I |
| 166198 | 133 | – | 159 | 925.4829 | 2773.4270 | 2773.4255 | 0.53 | 1 | 103 | 2.2e-10 | 1Score **> 37** indicates **identity** Score **> 19** indicates **homology** | U | K.SPKPVVAAISGSCLGGGLELAIACQYR.I |
| 166199 | 133 | – | 159 | 925.4830 | 2773.4272 | 2773.4255 | 0.61 | 1 | 121 | 4.4e-12 | 1Score **> 37** indicates **identity** Score **> 20** indicates **homology** | U | K.SPKPVVAAISGSCLGGGLELAIACQYR.I |
| 166200 | 133 | – | 159 | 925.4831 | 2773.4274 | 2773.4255 | 0.67 | 1 | 43 | 0.00014 | 1Score **> 37** indicates **identity** Score **> 17** indicates **homology** | U | K.SPKPVVAAISGSCLGGGLELAIACQYR.I |
| 166201 | 133 | – | 159 | 694.3641 | 2773.4275 | 2773.4255 | 0.71 | 1 | 59 | 2.9e-06 | 1Score **> 37** indicates **identity** Score **> 16** indicates **homology** | U | K.SPKPVVAAISGSCLGGGLELAIACQYR.I |
| 166204 | 133 | – | 159 | 925.4832 | 2773.4277 | 2773.4255 | 0.81 | 1 | 20 | 0.013 | 1Score **> 37** indicates **identity** Score **> 14** indicates **homology** | U | K.SPKPVVAAISGSCLGGGLELAIACQYR.I |
| 166205 | 133 | – | 159 | 925.4832 | 2773.4277 | 2773.4255 | 0.81 | 1 | 55 | 6.6e-06 | 1Score **> 37** indicates **identity** Score **> 16** indicates **homology** | U | K.SPKPVVAAISGSCLGGGLELAIACQYR.I |
| 166208 | 133 | – | 159 | 925.4837 | 2773.4291 | 2773.4255 | 1.32 | 1 | 101 | 3.1e-10 | 1Score **> 37** indicates **identity** Score **> 19** indicates **homology** | U | K.SPKPVVAAISGSCLGGGLELAIACQYR.I |
| 166209 | 133 | – | 159 | 925.4837 | 2773.4292 | 2773.4255 | 1.34 | 1 | 107 | 9.2e-11 | 1Score **> 37** indicates **identity** Score **> 19** indicates **homology** | U | K.SPKPVVAAISGSCLGGGLELAIACQYR.I |
| 166211 | 133 | – | 159 | 925.4842 | 2773.4306 | 2773.4255 | 1.85 | 1 | 35 | 0.00056 | 1Score **> 37** indicates **identity** Score **> 15** indicates **homology** | U | K.SPKPVVAAISGSCLGGGLELAIACQYR.I |
| 166212 | 133 | – | 159 | 925.4842 | 2773.4307 | 2773.4255 | 1.89 | 1 | 33 | 0.00085 | 1Score **> 37** indicates **identity** Score **> 15** indicates **homology** | U | K.SPKPVVAAISGSCLGGGLELAIACQYR.I |
| 166214 | 133 | – | 159 | 925.4844 | 2773.4313 | 2773.4255 | 2.08 | 1 | 28 | 0.0023 | 1Score **> 37** indicates **identity** Score **> 14** indicates **homology** | U | K.SPKPVVAAISGSCLGGGLELAIACQYR.I |
| 166215 | 133 | – | 159 | 925.4846 | 2773.4318 | 2773.4255 | 2.29 | 1 | 16 | 0.029 | 1Score **> 37** indicates **identity** Score **> 14** indicates **homology** | U | K.SPKPVVAAISGSCLGGGLELAIACQYR.I |
| 166216 | 133 | – | 159 | 925.4849 | 2773.4328 | 2773.4255 | 2.63 | 1 | 31 | 0.0011 | 1Score **> 37** indicates **identity** Score **> 14** indicates **homology** | U | K.SPKPVVAAISGSCLGGGLELAIACQYR.I |
| 166218 | 133 | – | 159 | 925.4849 | 2773.4330 | 2773.4255 | 2.70 | 1 | 24 | 0.0052 | 1Score **> 37** indicates **identity** Score **> 14** indicates **homology** | U | K.SPKPVVAAISGSCLGGGLELAIACQYR.I |
| 166220 | 133 | – | 159 | 925.4850 | 2773.4330 | 2773.4255 | 2.72 | 1 | 79 | 4.3e-08 | 1Score **> 37** indicates **identity** Score **> 17** indicates **homology** | U | K.SPKPVVAAISGSCLGGGLELAIACQYR.I |
| 166222 | 133 | – | 159 | 925.4850 | 2773.4333 | 2773.4255 | 2.80 | 1 | 56 | 7.3e-06 | 1Score **> 37** indicates **identity** Score **> 17** indicates **homology** | U | K.SPKPVVAAISGSCLGGGLELAIACQYR.I |
| 166223 | 133 | – | 159 | 925.4851 | 2773.4334 | 2773.4255 | 2.84 | 1 | 15 | 0.042 | 1Score **> 37** indicates **identity** Score **> 13** indicates **homology** | U | K.SPKPVVAAISGSCLGGGLELAIACQYR.I |
| 166225 | 133 | – | 159 | 925.4854 | 2773.4344 | 2773.4255 | 3.20 | 1 | 62 | 1.6e-06 | 1Score **> 37** indicates **identity** Score **> 16** indicates **homology** | U | K.SPKPVVAAISGSCLGGGLELAIACQYR.I |
| 166226 | 133 | – | 159 | 925.4854 | 2773.4344 | 2773.4255 | 3.20 | 1 | 46 | 4.4e-05 | 1Score **> 37** indicates **identity** Score **> 15** indicates **homology** | U | K.SPKPVVAAISGSCLGGGLELAIACQYR.I |
| 166229 | 133 | – | 159 | 925.4857 | 2773.4354 | 2773.4255 | 3.58 | 1 | 45 | 0.0001 | 1Score **> 37** indicates **identity** Score **> 18** indicates **homology** | U | K.SPKPVVAAISGSCLGGGLELAIACQYR.I |
| 166230 | 133 | – | 159 | 925.4859 | 2773.4358 | 2773.4255 | 3.71 | 1 | 67 | 5.3e-07 | 1Score **> 37** indicates **identity** Score **> 17** indicates **homology** | U | K.SPKPVVAAISGSCLGGGLELAIACQYR.I |
| 166231 | 133 | – | 159 | 925.4861 | 2773.4364 | 2773.4255 | 3.93 | 1 | 40 | 0.0006 | 1Score **> 37** indicates **identity** Score **> 21** indicates **homology** | U | K.SPKPVVAAISGSCLGGGLELAIACQYR.I |
| 166234 | 133 | – | 159 | 925.4870 | 2773.4392 | 2773.4255 | 4.94 | 1 | 17 | 0.028 | 1Score **> 37** indicates **identity** Score **> 14** indicates **homology** | U | K.SPKPVVAAISGSCLGGGLELAIACQYR.I |
| 166238 | 133 | – | 159 | 925.4875 | 2773.4406 | 2773.4255 | 5.43 | 1 | 20 | 0.013 | 1Score **> 37** indicates **identity** Score **> 14** indicates **homology** | U | K.SPKPVVAAISGSCLGGGLELAIACQYR.I |
| 166239 | 133 | – | 159 | 925.4879 | 2773.4418 | 2773.4255 | 5.88 | 1 | 35 | 0.00048 | 1Score **> 37** indicates **identity** Score **> 15** indicates **homology** | U | K.SPKPVVAAISGSCLGGGLELAIACQYR.I |
| 166242 | 133 | – | 159 | 925.4892 | 2773.4457 | 2773.4255 | 7.29 | 1 | 18 | 0.021 | 1Score **> 37** indicates **identity** Score **> 14** indicates **homology** | U | K.SPKPVVAAISGSCLGGGLELAIACQYR.I |
| 166243 | 133 | – | 159 | 925.4896 | 2773.4469 | 2773.4255 | 7.74 | 1 | 21 | 0.012 | 1Score **> 37** indicates **identity** Score **> 14** indicates **homology** | U | K.SPKPVVAAISGSCLGGGLELAIACQYR.I |
| 166244 | 133 | – | 159 | 925.4911 | 2773.4514 | 2773.4255 | 9.33 | 1 | 19 | 0.017 | 1Score **> 37** indicates **identity** Score **> 14** indicates **homology** | U | K.SPKPVVAAISGSCLGGGLELAIACQYR.I |
| 166245 | 133 | – | 159 | 925.4914 | 2773.4523 | 2773.4255 | 9.66 | 1 | 41 | 0.00016 | 1Score **> 37** indicates **identity** Score **> 15** indicates **homology** | U | K.SPKPVVAAISGSCLGGGLELAIACQYR.I |
| 166246 | 133 | – | 159 | 925.4914 | 2773.4523 | 2773.4255 | 9.68 | 1 | 23 | 0.0077 | 1Score **> 37** indicates **identity** Score **> 14** indicates **homology** | U | K.SPKPVVAAISGSCLGGGLELAIACQYR.I |
| 166248 | 133 | – | 159 | 925.4917 | 2773.4532 | 2773.4255 | 10.0 | 1 | 74 | 1.1e-07 | 1Score **> 37** indicates **identity** Score **> 17** indicates **homology** | U | K.SPKPVVAAISGSCLGGGLELAIACQYR.I |
| 166274 | 133 | – | 159 | 925.8127 | 2774.4163 | 2774.4095 | 2.44 | 1 | 24 | 0.0056 | 1Score **> 37** indicates **identity** Score **> 14** indicates **homology** | U | K.SPKPVVAAISGSCLGGGLELAIACQYR.I  + Deamidated (NQ) |
| 166275 | 133 | – | 159 | 925.8136 | 2774.4189 | 2774.4095 | 3.40 | 1 | 17 | 0.026 | 1Score **> 37** indicates **identity** Score **> 14** indicates **homology** | U | K.SPKPVVAAISGSCLGGGLELAIACQYR.I  + Deamidated (NQ) |
| 166285 | 133 | – | 159 | 925.8174 | 2774.4303 | 2774.4095 | 7.49 | 1 | 40 | 0.00019 | 1Score **> 37** indicates **identity** Score **> 15** indicates **homology** | U | K.SPKPVVAAISGSCLGGGLELAIACQYR.I  + Deamidated (NQ) |
| 166288 | 133 | – | 159 | 925.8181 | 2774.4324 | 2774.4095 | 8.27 | 1 | 16 | 0.034 | 1Score **> 37** indicates **identity** Score **> 13** indicates **homology** | U | K.SPKPVVAAISGSCLGGGLELAIACQYR.I  + Deamidated (NQ) |
| 166293 | 133 | – | 159 | 925.8187 | 2774.4344 | 2774.4095 | 8.96 | 1 | 15 | 0.036 | 1Score **> 37** indicates **identity** Score **> 13** indicates **homology** | U | K.SPKPVVAAISGSCLGGGLELAIACQYR.I  + Deamidated (NQ) |
| 166294 | 133 | – | 159 | 925.8190 | 2774.4353 | 2774.4095 | 9.29 | 1 | 47 | 4e-05 | 1Score **> 37** indicates **identity** Score **> 15** indicates **homology** | U | K.SPKPVVAAISGSCLGGGLELAIACQYR.I  + Deamidated (NQ) |
| 134626 | 166 | – | 187 | 725.7655 | 2174.2748 | 2174.2787 | -1.80 | 1 | 23 | 0.0065 | 1Score **> 30** indicates **identity** Score **> 14** indicates **homology** | U | R.KTVLGVPEVLLGILPGAGGTQR.L |
| 134627 | 166 | – | 187 | 725.7674 | 2174.2803 | 2174.2787 | 0.72 | 1 | 29 | 0.0019 | 1Score **> 29** indicates **identity** Score **> 14** indicates **homology** | U | R.KTVLGVPEVLLGILPGAGGTQR.L |
| 134628 | 166 | – | 187 | 1088.1536 | 2174.2927 | 2174.2787 | 6.43 | 1 | 56 | 5.4e-06 | 1Score **> 28** indicates **identity** Score **> 16** indicates **homology** | U | R.KTVLGVPEVLLGILPGAGGTQR.L |
| 155992 | 166 | – | 190 | 629.1355 | 2512.5128 | 2512.5105 | 0.92 | 2 | 20 | 0.014 | 1Score **> 24** indicates **identity** Score **> 14** indicates **homology** | U | R.KTVLGVPEVLLGILPGAGGTQRLPK.M |
| 155993 | 166 | – | 190 | 629.1356 | 2512.5132 | 2512.5105 | 1.09 | 2 | 30 | 0.0015 | 1Score **> 24** indicates **identity** Score **> 14** indicates **homology** | U | R.KTVLGVPEVLLGILPGAGGTQRLPK.M |
| 155994 | 166 | – | 190 | 838.5129 | 2512.5168 | 2512.5105 | 2.51 | 2 | 60 | 2.6e-06 | 1Score **> 24** indicates **identity** Score **> 16** indicates **homology** | U | R.KTVLGVPEVLLGILPGAGGTQRLPK.M |
| 123903 | 167 | – | 187 | 1024.0983 | 2046.1821 | 2046.1837 | -0.79 | 0 | 57 | 5.2e-06 | 1Score **> 31** indicates **identity** Score **> 17** indicates **homology** | U | K.TVLGVPEVLLGILPGAGGTQR.L |
| 123905 | 167 | – | 187 | 683.0681 | 2046.1825 | 2046.1837 | -0.62 | 0 | 67 | 5.3e-07 | 1Score **> 31** indicates **identity** Score **> 17** indicates **homology** | U | K.TVLGVPEVLLGILPGAGGTQR.L |
| 123909 | 167 | – | 187 | 1024.0990 | 2046.1833 | 2046.1837 | -0.19 | 0 | 72 | 1.6e-07 | 1Score **> 31** indicates **identity** Score **> 17** indicates **homology** | U | K.TVLGVPEVLLGILPGAGGTQR.L |
| 123913 | 167 | – | 187 | 683.0697 | 2046.1872 | 2046.1837 | 1.70 | 0 | 52 | 1.2e-05 | 1Score **> 31** indicates **identity** Score **> 16** indicates **homology** | U | K.TVLGVPEVLLGILPGAGGTQR.L |
| 123915 | 167 | – | 187 | 683.0701 | 2046.1885 | 2046.1837 | 2.34 | 0 | 35 | 0.00049 | 1Score **> 31** indicates **identity** Score **> 15** indicates **homology** | U | K.TVLGVPEVLLGILPGAGGTQR.L |
| 123916 | 167 | – | 187 | 1024.1018 | 2046.1890 | 2046.1837 | 2.55 | 0 | 61 | 1.8e-06 | 1Score **> 31** indicates **identity** Score **> 16** indicates **homology** | U | K.TVLGVPEVLLGILPGAGGTQR.L |
| 123917 | 167 | – | 187 | 683.0704 | 2046.1893 | 2046.1837 | 2.70 | 0 | 37 | 0.00032 | 1Score **> 31** indicates **identity** Score **> 15** indicates **homology** | U | K.TVLGVPEVLLGILPGAGGTQR.L |
| 123919 | 167 | – | 187 | 1024.1026 | 2046.1907 | 2046.1837 | 3.40 | 0 | 25 | 0.0045 | 1Score **> 31** indicates **identity** Score **> 14** indicates **homology** | U | K.TVLGVPEVLLGILPGAGGTQR.L |
| 123920 | 167 | – | 187 | 1024.1028 | 2046.1910 | 2046.1837 | 3.53 | 0 | 19 | 0.018 | 1Score **> 31** indicates **identity** Score **> 14** indicates **homology** | U | K.TVLGVPEVLLGILPGAGGTQR.L |
| 123921 | 167 | – | 187 | 1024.1042 | 2046.1939 | 2046.1837 | 4.98 | 0 | 68 | 8.7e-07 | 1Score **> 30** indicates **identity** Score **> 20** indicates **homology** | U | K.TVLGVPEVLLGILPGAGGTQR.L |
| 123922 | 167 | – | 187 | 683.0731 | 2046.1974 | 2046.1837 | 6.67 | 0 | 38 | 0.00026 | 1Score **> 30** indicates **identity** Score **> 15** indicates **homology** | U | K.TVLGVPEVLLGILPGAGGTQR.L |
| 123923 | 167 | – | 187 | 683.0748 | 2046.2026 | 2046.1837 | 9.21 | 0 | 22 | 0.0092 | 1Score **> 30** indicates **identity** Score **> 14** indicates **homology** | U | K.TVLGVPEVLLGILPGAGGTQR.L |
| 149032 | 167 | – | 190 | 795.8106 | 2384.4099 | 2384.4155 | -2.36 | 1 | 18 | 0.02 | 1Score **> 28** indicates **identity** Score **> 14** indicates **homology** | U | K.TVLGVPEVLLGILPGAGGTQRLPK.M |
| 149034 | 167 | – | 190 | 795.8122 | 2384.4148 | 2384.4155 | -0.30 | 1 | 35 | 0.00054 | 1Score **> 27** indicates **identity** Score **> 15** indicates **homology** | U | K.TVLGVPEVLLGILPGAGGTQRLPK.M |
| 149035 | 167 | – | 190 | 795.8125 | 2384.4157 | 2384.4155 | 0.092 | 1 | 28 | 0.0026 | 1Score **> 27** indicates **identity** Score **> 14** indicates **homology** | U | K.TVLGVPEVLLGILPGAGGTQRLPK.M |
| 149036 | 167 | – | 190 | 597.1114 | 2384.4164 | 2384.4155 | 0.35 | 1 | 25 | 0.005 | 1Score **> 27** indicates **identity** Score **> 14** indicates **homology** | U | K.TVLGVPEVLLGILPGAGGTQRLPK.M |
| 149037 | 167 | – | 190 | 795.8130 | 2384.4171 | 2384.4155 | 0.65 | 1 | 38 | 0.00029 | 1Score **> 27** indicates **identity** Score **> 15** indicates **homology** | U | K.TVLGVPEVLLGILPGAGGTQRLPK.M |
| 149038 | 167 | – | 190 | 1193.2164 | 2384.4183 | 2384.4155 | 1.15 | 1 | 15 | 0.038 | 1Score **> 27** indicates **identity** Score **> 13** indicates **homology** | U | K.TVLGVPEVLLGILPGAGGTQRLPK.M |
| 149039 | 167 | – | 190 | 1193.2193 | 2384.4241 | 2384.4155 | 3.61 | 1 | 60 | 2.5e-06 | 1Score **> 26** indicates **identity** Score **> 16** indicates **homology** | U | K.TVLGVPEVLLGILPGAGGTQRLPK.M |
| 100664 | 214 | – | 230 | 598.6781 | 1793.0126 | 1793.0121 | 0.27 | 1 | 28 | 0.0023 | 1Score **> 33** indicates **identity** Score **> 14** indicates **homology** | U | K.KMGLVDQLVEPLGPGIK.S |
| 100665 | 214 | – | 230 | 598.6782 | 1793.0127 | 1793.0121 | 0.37 | 1 | 36 | 0.00042 | 1Score **> 33** indicates **identity** Score **> 15** indicates **homology** | U | K.KMGLVDQLVEPLGPGIK.S |
| 100667 | 214 | – | 230 | 598.6792 | 1793.0159 | 1793.0121 | 2.12 | 1 | 54 | 9.4e-06 | 1Score **> 33** indicates **identity** Score **> 16** indicates **homology** | U | K.KMGLVDQLVEPLGPGIK.S |
| 149519 | 214 | – | 235 | 798.0981 | 2391.2724 | 2391.2832 | -4.49 | 2 | 53 | 1.2e-05 | 1Score **> 36** indicates **identity** Score **> 16** indicates **homology** | U | K.KMGLVDQLVEPLGPGIKSPEER.T |
| 149523 | 214 | – | 235 | 598.8271 | 2391.2794 | 2391.2832 | -1.58 | 2 | 26 | 0.0037 | 1Score **> 36** indicates **identity** Score **> 14** indicates **homology** | U | K.KMGLVDQLVEPLGPGIKSPEER.T |
| 149526 | 214 | – | 235 | 598.8275 | 2391.2809 | 2391.2832 | -0.95 | 2 | 42 | 0.00013 | 1Score **> 36** indicates **identity** Score **> 15** indicates **homology** | U | K.KMGLVDQLVEPLGPGIKSPEER.T |
| 149528 | 214 | – | 235 | 598.8290 | 2391.2871 | 2391.2832 | 1.64 | 2 | 48 | 3e-05 | 1Score **> 36** indicates **identity** Score **> 16** indicates **homology** | U | K.KMGLVDQLVEPLGPGIKSPEER.T |
| 149531 | 214 | – | 235 | 798.1060 | 2391.2962 | 2391.2832 | 5.47 | 2 | 50 | 2.1e-05 | 1Score **> 35** indicates **identity** Score **> 16** indicates **homology** | U | K.KMGLVDQLVEPLGPGIKSPEER.T |
| 149589 | 214 | – | 235 | 798.4355 | 2392.2848 | 2392.2672 | 7.36 | 2 | 47 | 3.7e-05 | 1Score **> 36** indicates **identity** Score **> 15** indicates **homology** | U | K.KMGLVDQLVEPLGPGIKSPEER.T  + Deamidated (NQ) |
| 86214 | 215 | – | 230 | 833.4636 | 1664.9126 | 1664.9171 | -2.71 | 0 | 41 | 0.00016 | 1Score **> 34** indicates **identity** Score **> 15** indicates **homology** | U | K.MGLVDQLVEPLGPGIK.S |
| 86216 | 215 | – | 230 | 555.9791 | 1664.9153 | 1664.9171 | -1.08 | 0 | 36 | 0.00044 | 1Score **> 34** indicates **identity** Score **> 15** indicates **homology** | U | K.MGLVDQLVEPLGPGIK.S |
| 86220 | 215 | – | 230 | 555.9800 | 1664.9180 | 1664.9171 | 0.55 | 0 | 19 | 0.017 | 1Score **> 34** indicates **identity** Score **> 14** indicates **homology** | U | K.MGLVDQLVEPLGPGIK.S |
| 86221 | 215 | – | 230 | 555.9804 | 1664.9193 | 1664.9171 | 1.31 | 0 | 35 | 0.00047 | 1Score **> 34** indicates **identity** Score **> 15** indicates **homology** | U | K.MGLVDQLVEPLGPGIK.S |
| 86222 | 215 | – | 230 | 833.4675 | 1664.9204 | 1664.9171 | 1.94 | 0 | 60 | 2.6e-06 | 1Score **> 34** indicates **identity** Score **> 16** indicates **homology** | U | K.MGLVDQLVEPLGPGIK.S |
| 86223 | 215 | – | 230 | 833.4683 | 1664.9220 | 1664.9171 | 2.95 | 0 | 44 | 7.6e-05 | 1Score **> 34** indicates **identity** Score **> 15** indicates **homology** | U | K.MGLVDQLVEPLGPGIK.S |
| 141367 | 215 | – | 235 | 755.4043 | 2263.1912 | 2263.1882 | 1.33 | 1 | 60 | 2.6e-06 | 1Score **> 37** indicates **identity** Score **> 16** indicates **homology** | U | K.MGLVDQLVEPLGPGIKSPEER.T |
| 141370 | 215 | – | 235 | 755.4083 | 2263.2031 | 2263.1882 | 6.58 | 1 | 59 | 3.2e-06 | 1Score **> 36** indicates **identity** Score **> 16** indicates **homology** | U | K.MGLVDQLVEPLGPGIKSPEER.T |
| 141371 | 215 | – | 235 | 755.4089 | 2263.2047 | 2263.1882 | 7.31 | 1 | 62 | 1.6e-06 | 1Score **> 36** indicates **identity** Score **> 16** indicates **homology** | U | K.MGLVDQLVEPLGPGIKSPEER.T |
| 81163 | 236 | – | 249 | 813.4219 | 1624.8292 | 1624.8348 | -3.47 | 0 | 33 | 0.00088 | 1Score **> 35** indicates **identity** Score **> 15** indicates **homology** | U | R.TIEYLEEVAVNFAK.G |
| 81164 | 236 | – | 249 | 813.4221 | 1624.8297 | 1624.8348 | -3.17 | 0 | 63 | 1.2e-06 | 1Score **> 34** indicates **identity** Score **> 16** indicates **homology** | U | R.TIEYLEEVAVNFAK.G |
| 81167 | 236 | – | 249 | 813.4223 | 1624.8301 | 1624.8348 | -2.87 | 0 | 51 | 1.5e-05 | 1Score **> 34** indicates **identity** Score **> 16** indicates **homology** | U | R.TIEYLEEVAVNFAK.G |
| 81170 | 236 | – | 249 | 813.4226 | 1624.8307 | 1624.8348 | -2.55 | 0 | 60 | 2.2e-06 | 1Score **> 34** indicates **identity** Score **> 16** indicates **homology** | U | R.TIEYLEEVAVNFAK.G |
| 81172 | 236 | – | 249 | 813.4227 | 1624.8309 | 1624.8348 | -2.43 | 0 | 32 | 0.00098 | 1Score **> 34** indicates **identity** Score **> 15** indicates **homology** | U | R.TIEYLEEVAVNFAK.G |
| 81173 | 236 | – | 249 | 813.4228 | 1624.8310 | 1624.8348 | -2.35 | 0 | 64 | 9.6e-07 | 1Score **> 34** indicates **identity** Score **> 17** indicates **homology** | U | R.TIEYLEEVAVNFAK.G |
| 81183 | 236 | – | 249 | 813.4239 | 1624.8332 | 1624.8348 | -1.00 | 0 | 24 | 0.0058 | 1Score **> 35** indicates **identity** Score **> 14** indicates **homology** | U | R.TIEYLEEVAVNFAK.G |
| 81185 | 236 | – | 249 | 813.4240 | 1624.8334 | 1624.8348 | -0.84 | 0 | 54 | 9.3e-06 | 1Score **> 34** indicates **identity** Score **> 16** indicates **homology** | U | R.TIEYLEEVAVNFAK.G |
| 81187 | 236 | – | 249 | 542.6186 | 1624.8341 | 1624.8348 | -0.45 | 0 | 29 | 0.0021 | 1Score **> 34** indicates **identity** Score **> 14** indicates **homology** | U | R.TIEYLEEVAVNFAK.G |
| 81189 | 236 | – | 249 | 813.4243 | 1624.8341 | 1624.8348 | -0.42 | 0 | 19 | 0.018 | 1Score **> 34** indicates **identity** Score **> 14** indicates **homology** | U | R.TIEYLEEVAVNFAK.G |
| 81191 | 236 | – | 249 | 542.6188 | 1624.8345 | 1624.8348 | -0.19 | 0 | 52 | 1.4e-05 | 1Score **> 34** indicates **identity** Score **> 16** indicates **homology** | U | R.TIEYLEEVAVNFAK.G |
| 81192 | 236 | – | 249 | 542.6189 | 1624.8348 | 1624.8348 | 0.0062 | 0 | 24 | 0.0057 | 1Score **> 34** indicates **identity** Score **> 14** indicates **homology** | U | R.TIEYLEEVAVNFAK.G |
| 81194 | 236 | – | 249 | 542.6189 | 1624.8350 | 1624.8348 | 0.10 | 0 | 62 | 1.7e-06 | 1Score **> 34** indicates **identity** Score **> 16** indicates **homology** | U | R.TIEYLEEVAVNFAK.G |
| 81195 | 236 | – | 249 | 813.4249 | 1624.8352 | 1624.8348 | 0.22 | 0 | 41 | 0.00015 | 1Score **> 34** indicates **identity** Score **> 15** indicates **homology** | U | R.TIEYLEEVAVNFAK.G |
| 81197 | 236 | – | 249 | 813.4249 | 1624.8352 | 1624.8348 | 0.25 | 0 | 23 | 0.0071 | 1Score **> 34** indicates **identity** Score **> 14** indicates **homology** | U | R.TIEYLEEVAVNFAK.G |
| 81199 | 236 | – | 249 | 813.4250 | 1624.8354 | 1624.8348 | 0.34 | 0 | 31 | 0.0012 | 1Score **> 34** indicates **identity** Score **> 14** indicates **homology** | U | R.TIEYLEEVAVNFAK.G |
| 81201 | 236 | – | 249 | 813.4250 | 1624.8354 | 1624.8348 | 0.35 | 0 | 35 | 0.00052 | 1Score **> 34** indicates **identity** Score **> 15** indicates **homology** | U | R.TIEYLEEVAVNFAK.G |
| 81206 | 236 | – | 249 | 542.6192 | 1624.8357 | 1624.8348 | 0.52 | 0 | 45 | 5.9e-05 | 1Score **> 34** indicates **identity** Score **> 15** indicates **homology** | U | R.TIEYLEEVAVNFAK.G |
| 81208 | 236 | – | 249 | 813.4251 | 1624.8357 | 1624.8348 | 0.57 | 0 | 84 | 1.4e-08 | 1Score **> 34** indicates **identity** Score **> 18** indicates **homology** | U | R.TIEYLEEVAVNFAK.G |
| 81209 | 236 | – | 249 | 542.6193 | 1624.8359 | 1624.8348 | 0.70 | 0 | 38 | 0.0003 | 1Score **> 34** indicates **identity** Score **> 15** indicates **homology** | U | R.TIEYLEEVAVNFAK.G |
| 81212 | 236 | – | 249 | 542.6193 | 1624.8361 | 1624.8348 | 0.78 | 0 | 36 | 0.00039 | 1Score **> 34** indicates **identity** Score **> 15** indicates **homology** | U | R.TIEYLEEVAVNFAK.G |
| 81213 | 236 | – | 249 | 542.6193 | 1624.8362 | 1624.8348 | 0.83 | 0 | 51 | 1.7e-05 | 1Score **> 34** indicates **identity** Score **> 16** indicates **homology** | U | R.TIEYLEEVAVNFAK.G |
| 81214 | 236 | – | 249 | 813.4254 | 1624.8362 | 1624.8348 | 0.86 | 0 | 61 | 1.8e-06 | 1Score **> 34** indicates **identity** Score **> 16** indicates **homology** | U | R.TIEYLEEVAVNFAK.G |
| 81215 | 236 | – | 249 | 542.6194 | 1624.8363 | 1624.8348 | 0.91 | 0 | 49 | 2.5e-05 | 1Score **> 34** indicates **identity** Score **> 16** indicates **homology** | U | R.TIEYLEEVAVNFAK.G |
| 81217 | 236 | – | 249 | 542.6194 | 1624.8363 | 1624.8348 | 0.92 | 0 | 60 | 2.4e-06 | 1Score **> 34** indicates **identity** Score **> 16** indicates **homology** | U | R.TIEYLEEVAVNFAK.G |
| 81218 | 236 | – | 249 | 813.4254 | 1624.8363 | 1624.8348 | 0.95 | 0 | 20 | 0.012 | 1Score **> 34** indicates **identity** Score **> 14** indicates **homology** | U | R.TIEYLEEVAVNFAK.G |
| 81219 | 236 | – | 249 | 813.4255 | 1624.8364 | 1624.8348 | 0.96 | 0 | 40 | 0.00016 | 1Score **> 34** indicates **identity** Score **> 15** indicates **homology** | U | R.TIEYLEEVAVNFAK.G |
| 81221 | 236 | – | 249 | 813.4256 | 1624.8366 | 1624.8348 | 1.13 | 0 | 86 | 8.1e-09 | 1Score **> 34** indicates **identity** Score **> 18** indicates **homology** | U | R.TIEYLEEVAVNFAK.G |
| 81222 | 236 | – | 249 | 542.6195 | 1624.8367 | 1624.8348 | 1.15 | 0 | 56 | 5.1e-06 | 1Score **> 34** indicates **identity** Score **> 16** indicates **homology** | U | R.TIEYLEEVAVNFAK.G |
| 81224 | 236 | – | 249 | 542.6195 | 1624.8368 | 1624.8348 | 1.22 | 0 | 35 | 0.00055 | 1Score **> 34** indicates **identity** Score **> 15** indicates **homology** | U | R.TIEYLEEVAVNFAK.G |
| 81226 | 236 | – | 249 | 813.4258 | 1624.8370 | 1624.8348 | 1.35 | 0 | 74 | 1.1e-07 | 1Score **> 34** indicates **identity** Score **> 17** indicates **homology** | U | R.TIEYLEEVAVNFAK.G |
| 81227 | 236 | – | 249 | 813.4258 | 1624.8371 | 1624.8348 | 1.42 | 0 | 48 | 3.3e-05 | 1Score **> 34** indicates **identity** Score **> 15** indicates **homology** | U | R.TIEYLEEVAVNFAK.G |
| 81231 | 236 | – | 249 | 542.6197 | 1624.8374 | 1624.8348 | 1.58 | 0 | 25 | 0.0042 | 1Score **> 34** indicates **identity** Score **> 14** indicates **homology** | U | R.TIEYLEEVAVNFAK.G |
| 81233 | 236 | – | 249 | 813.4260 | 1624.8375 | 1624.8348 | 1.63 | 0 | 22 | 0.008 | 1Score **> 34** indicates **identity** Score **> 14** indicates **homology** | U | R.TIEYLEEVAVNFAK.G |
| 81235 | 236 | – | 249 | 813.4260 | 1624.8375 | 1624.8348 | 1.64 | 0 | 60 | 2.2e-06 | 1Score **> 34** indicates **identity** Score **> 16** indicates **homology** | U | R.TIEYLEEVAVNFAK.G |
| 81236 | 236 | – | 249 | 813.4260 | 1624.8375 | 1624.8348 | 1.64 | 0 | 23 | 0.0066 | 1Score **> 34** indicates **identity** Score **> 14** indicates **homology** | U | R.TIEYLEEVAVNFAK.G |
| 81240 | 236 | – | 249 | 813.4262 | 1624.8378 | 1624.8348 | 1.85 | 0 | 68 | 4.4e-07 | 1Score **> 34** indicates **identity** Score **> 17** indicates **homology** | U | R.TIEYLEEVAVNFAK.G |
| 81241 | 236 | – | 249 | 813.4262 | 1624.8378 | 1624.8348 | 1.85 | 0 | 79 | 3.9e-08 | 1Score **> 34** indicates **identity** Score **> 17** indicates **homology** | U | R.TIEYLEEVAVNFAK.G |
| 81243 | 236 | – | 249 | 813.4263 | 1624.8380 | 1624.8348 | 1.95 | 0 | 89 | 4.4e-09 | 1Score **> 34** indicates **identity** Score **> 18** indicates **homology** | U | R.TIEYLEEVAVNFAK.G |
| 81247 | 236 | – | 249 | 813.4264 | 1624.8382 | 1624.8348 | 2.08 | 0 | 60 | 2.4e-06 | 1Score **> 34** indicates **identity** Score **> 16** indicates **homology** | U | R.TIEYLEEVAVNFAK.G |
| 81249 | 236 | – | 249 | 542.6200 | 1624.8382 | 1624.8348 | 2.10 | 0 | 51 | 1.8e-05 | 1Score **> 34** indicates **identity** Score **> 16** indicates **homology** | U | R.TIEYLEEVAVNFAK.G |
| 81251 | 236 | – | 249 | 813.4265 | 1624.8384 | 1624.8348 | 2.18 | 0 | 64 | 9.9e-07 | 1Score **> 34** indicates **identity** Score **> 17** indicates **homology** | U | R.TIEYLEEVAVNFAK.G |
| 81254 | 236 | – | 249 | 813.4266 | 1624.8386 | 1624.8348 | 2.32 | 0 | 19 | 0.016 | 1Score **> 34** indicates **identity** Score **> 14** indicates **homology** | U | R.TIEYLEEVAVNFAK.G |
| 81255 | 236 | – | 249 | 813.4266 | 1624.8386 | 1624.8348 | 2.34 | 0 | 68 | 4.4e-07 | 1Score **> 34** indicates **identity** Score **> 17** indicates **homology** | U | R.TIEYLEEVAVNFAK.G |
| 81258 | 236 | – | 249 | 542.6202 | 1624.8387 | 1624.8348 | 2.38 | 0 | 24 | 0.0054 | 1Score **> 34** indicates **identity** Score **> 14** indicates **homology** | U | R.TIEYLEEVAVNFAK.G |
| 81260 | 236 | – | 249 | 813.4268 | 1624.8390 | 1624.8348 | 2.56 | 0 | 79 | 3.7e-08 | 1Score **> 34** indicates **identity** Score **> 17** indicates **homology** | U | R.TIEYLEEVAVNFAK.G |
| 81262 | 236 | – | 249 | 813.4272 | 1624.8398 | 1624.8348 | 3.05 | 0 | 63 | 1.2e-06 | 1Score **> 35** indicates **identity** Score **> 16** indicates **homology** | U | R.TIEYLEEVAVNFAK.G |
| 81265 | 236 | – | 249 | 813.4278 | 1624.8410 | 1624.8348 | 3.81 | 0 | 74 | 1.3e-07 | 1Score **> 34** indicates **identity** Score **> 17** indicates **homology** | U | R.TIEYLEEVAVNFAK.G |
| 81267 | 236 | – | 249 | 813.4282 | 1624.8418 | 1624.8348 | 4.28 | 0 | 22 | 0.0088 | 1Score **> 34** indicates **identity** Score **> 14** indicates **homology** | U | R.TIEYLEEVAVNFAK.G |
| 81268 | 236 | – | 249 | 542.6212 | 1624.8418 | 1624.8348 | 4.33 | 0 | 44 | 7.2e-05 | 1Score **> 34** indicates **identity** Score **> 15** indicates **homology** | U | R.TIEYLEEVAVNFAK.G |
| 81269 | 236 | – | 249 | 813.4283 | 1624.8420 | 1624.8348 | 4.44 | 0 | 37 | 0.00031 | 1Score **> 34** indicates **identity** Score **> 15** indicates **homology** | U | R.TIEYLEEVAVNFAK.G |
| 81270 | 236 | – | 249 | 813.4283 | 1624.8421 | 1624.8348 | 4.47 | 0 | 15 | 0.039 | 1Score **> 34** indicates **identity** Score **> 13** indicates **homology** | U | R.TIEYLEEVAVNFAK.G |
| 81271 | 236 | – | 249 | 813.4284 | 1624.8423 | 1624.8348 | 4.58 | 0 | 46 | 6.5e-05 | 1Score **> 34** indicates **identity** Score **> 16** indicates **homology** | U | R.TIEYLEEVAVNFAK.G |
| 81272 | 236 | – | 249 | 813.4284 | 1624.8423 | 1624.8348 | 4.63 | 0 | 64 | 1.1e-06 | 1Score **> 34** indicates **identity** Score **> 16** indicates **homology** | U | R.TIEYLEEVAVNFAK.G |
| 81274 | 236 | – | 249 | 813.4287 | 1624.8428 | 1624.8348 | 4.91 | 0 | 21 | 0.012 | 1Score **> 34** indicates **identity** Score **> 14** indicates **homology** | U | R.TIEYLEEVAVNFAK.G |
| 81275 | 236 | – | 249 | 542.6217 | 1624.8431 | 1624.8348 | 5.13 | 0 | 45 | 5.7e-05 | 1Score **> 34** indicates **identity** Score **> 15** indicates **homology** | U | R.TIEYLEEVAVNFAK.G |
| 81276 | 236 | – | 249 | 813.4290 | 1624.8434 | 1624.8348 | 5.31 | 0 | 61 | 2.1e-06 | 1Score **> 34** indicates **identity** Score **> 16** indicates **homology** | U | R.TIEYLEEVAVNFAK.G |
| 81278 | 236 | – | 249 | 813.4295 | 1624.8445 | 1624.8348 | 5.98 | 0 | 50 | 2.1e-05 | 1Score **> 35** indicates **identity** Score **> 16** indicates **homology** | U | R.TIEYLEEVAVNFAK.G |
| 81279 | 236 | – | 249 | 813.4297 | 1624.8448 | 1624.8348 | 6.13 | 0 | 45 | 6.1e-05 | 1Score **> 34** indicates **identity** Score **> 15** indicates **homology** | U | R.TIEYLEEVAVNFAK.G |
| 81280 | 236 | – | 249 | 813.4299 | 1624.8452 | 1624.8348 | 6.43 | 0 | 64 | 9.8e-07 | 1Score **> 34** indicates **identity** Score **> 17** indicates **homology** | U | R.TIEYLEEVAVNFAK.G |
| 81282 | 236 | – | 249 | 813.4304 | 1624.8462 | 1624.8348 | 7.03 | 0 | 57 | 4.8e-06 | 1Score **> 34** indicates **identity** Score **> 16** indicates **homology** | U | R.TIEYLEEVAVNFAK.G |
| 81283 | 236 | – | 249 | 813.4306 | 1624.8466 | 1624.8348 | 7.25 | 0 | 54 | 8.2e-06 | 1Score **> 34** indicates **identity** Score **> 16** indicates **homology** | U | R.TIEYLEEVAVNFAK.G |
| 81284 | 236 | – | 249 | 813.4308 | 1624.8471 | 1624.8348 | 7.57 | 0 | 61 | 2e-06 | 1Score **> 34** indicates **identity** Score **> 16** indicates **homology** | U | R.TIEYLEEVAVNFAK.G |
| 81285 | 236 | – | 249 | 813.4309 | 1624.8473 | 1624.8348 | 7.66 | 0 | 47 | 4.2e-05 | 1Score **> 34** indicates **identity** Score **> 15** indicates **homology** | U | R.TIEYLEEVAVNFAK.G |
| 81286 | 236 | – | 249 | 813.4309 | 1624.8473 | 1624.8348 | 7.67 | 0 | 35 | 0.00053 | 1Score **> 34** indicates **identity** Score **> 15** indicates **homology** | U | R.TIEYLEEVAVNFAK.G |
| 81293 | 236 | – | 249 | 813.4327 | 1624.8509 | 1624.8348 | 9.89 | 0 | 73 | 1.3e-07 | 1Score **> 34** indicates **identity** Score **> 17** indicates **homology** | U | R.TIEYLEEVAVNFAK.G |
| 81396 | 236 | – | 249 | 813.9244 | 1625.8343 | 1625.8188 | 9.54 | 0 | 20 | 0.013 | 1Score **> 34** indicates **identity** Score **> 14** indicates **homology** | U | R.TIEYLEEVAVNFAK.G  + Deamidated (NQ) |
| 81397 | 236 | – | 249 | 542.9522 | 1625.8349 | 1625.8188 | 9.91 | 0 | 24 | 0.0056 | 1Score **> 34** indicates **identity** Score **> 14** indicates **homology** | U | R.TIEYLEEVAVNFAK.G  + Deamidated (NQ) |
| 53411 | 268 | – | 279 | 699.8763 | 1397.7380 | 1397.7377 | 0.18 | 0 | 71 | 1e-06 | 1Score **> 34** indicates **identity** Score **> 23** indicates **homology** | U | K.LTTYAMTVPFVR.Q |
| 53412 | 268 | – | 279 | 699.8765 | 1397.7385 | 1397.7377 | 0.54 | 0 | 71 | 1e-06 | 1Score **> 34** indicates **identity** Score **> 23** indicates **homology** | U | K.LTTYAMTVPFVR.Q |
| 53414 | 268 | – | 279 | 699.8767 | 1397.7389 | 1397.7377 | 0.87 | 0 | 51 | 1.8e-05 | 1Score **> 34** indicates **identity** Score **> 16** indicates **homology** | U | K.LTTYAMTVPFVR.Q |
| 53416 | 268 | – | 279 | 699.8775 | 1397.7404 | 1397.7377 | 1.93 | 0 | 70 | 1.1e-06 | 1Score **> 33** indicates **identity** Score **> 22** indicates **homology** | U | K.LTTYAMTVPFVR.Q |
| 55260 | 268 | – | 279 | 707.8724 | 1413.7303 | 1413.7326 | -1.67 | 0 | 37 | 0.00037 | 1Score **> 34** indicates **identity** Score **> 15** indicates **homology** | U | K.LTTYAMTVPFVR.Q  + Oxidation (M) |
| 55263 | 268 | – | 279 | 707.8728 | 1413.7310 | 1413.7326 | -1.15 | 0 | 27 | 0.0028 | 1Score **> 34** indicates **identity** Score **> 14** indicates **homology** | U | K.LTTYAMTVPFVR.Q  + Oxidation (M) |
| 6282 | 296 | – | 303 | 429.7577 | 857.5009 | 857.5011 | -0.16 | 0 | 31 | 0.0024 | 1Score **> 32** indicates **identity** Score **> 18** indicates **homology** | U | K.GLYPAPLK.I |
| 6283 | 296 | – | 303 | 429.7577 | 857.5009 | 857.5011 | -0.16 | 0 | 15 | 0.041 | 1Score **> 32** indicates **identity** Score **> 13** indicates **homology** | U | K.GLYPAPLK.I |
| 6285 | 296 | – | 303 | 429.7579 | 857.5013 | 857.5011 | 0.25 | 0 | 35 | 0.0014 | 1Score **> 32** indicates **identity** Score **> 19** indicates **homology** | U | K.GLYPAPLK.I |
| 6286 | 296 | – | 303 | 429.7580 | 857.5015 | 857.5011 | 0.55 | 0 | 33 | 0.0021 | 1Score **> 32** indicates **identity** Score **> 19** indicates **homology** | U | K.GLYPAPLK.I |
| 6287 | 296 | – | 303 | 429.7582 | 857.5018 | 857.5011 | 0.83 | 0 | 35 | 0.0014 | 1Score **> 32** indicates **identity** Score **> 19** indicates **homology** | U | K.GLYPAPLK.I |
| 6288 | 296 | – | 303 | 429.7583 | 857.5021 | 857.5011 | 1.18 | 0 | 19 | 0.021 | 1Score **> 32** indicates **identity** Score **> 15** indicates **homology** | U | K.GLYPAPLK.I |
| 65064 | 296 | – | 309 | 499.9716 | 1496.8930 | 1496.8966 | -2.45 | 1 | 17 | 0.024 | 1Score **> 32** indicates **identity** Score **> 14** indicates **homology** | U | K.GLYPAPLKIIDAVK.A |
| 65067 | 296 | – | 309 | 499.9726 | 1496.8960 | 1496.8966 | -0.38 | 1 | 51 | 1.8e-05 | 1Score **> 32** indicates **identity** Score **> 16** indicates **homology** | U | K.GLYPAPLKIIDAVK.A |
| 65068 | 296 | – | 309 | 499.9727 | 1496.8963 | 1496.8966 | -0.22 | 1 | 25 | 0.0044 | 1Score **> 32** indicates **identity** Score **> 14** indicates **homology** | U | K.GLYPAPLKIIDAVK.A |
| 65069 | 296 | – | 309 | 499.9728 | 1496.8965 | 1496.8966 | -0.092 | 1 | 37 | 0.00038 | 1Score **> 31** indicates **identity** Score **> 15** indicates **homology** | U | K.GLYPAPLKIIDAVK.A |
| 65072 | 296 | – | 309 | 749.4557 | 1496.8969 | 1496.8966 | 0.16 | 1 | 50 | 2.1e-05 | 1Score **> 31** indicates **identity** Score **> 16** indicates **homology** | U | K.GLYPAPLKIIDAVK.A |
| 65073 | 296 | – | 309 | 749.4558 | 1496.8970 | 1496.8966 | 0.27 | 1 | 32 | 0.001 | 1Score **> 31** indicates **identity** Score **> 14** indicates **homology** | U | K.GLYPAPLKIIDAVK.A |
| 65075 | 296 | – | 309 | 499.9731 | 1496.8973 | 1496.8966 | 0.47 | 1 | 61 | 1.9e-06 | 1Score **> 31** indicates **identity** Score **> 16** indicates **homology** | U | K.GLYPAPLKIIDAVK.A |
| 65076 | 296 | – | 309 | 499.9733 | 1496.8981 | 1496.8966 | 1.01 | 1 | 44 | 9.3e-05 | 1Score **> 31** indicates **identity** Score **> 16** indicates **homology** | U | K.GLYPAPLKIIDAVK.A |
| 93148 | 310 | – | 326 | 862.4082 | 1722.8018 | 1722.8060 | -2.45 | 0 | 56 | 5.8e-06 | 1Score **> 32** indicates **identity** Score **> 16** indicates **homology** | U | K.AGLEQGSDAGYLAESQK.F |
| 93149 | 310 | – | 326 | 862.4085 | 1722.8024 | 1722.8060 | -2.10 | 0 | 73 | 1.4e-07 | 1Score **> 32** indicates **identity** Score **> 17** indicates **homology** | U | K.AGLEQGSDAGYLAESQK.F |
| 93151 | 310 | – | 326 | 862.4095 | 1722.8044 | 1722.8060 | -0.95 | 0 | 71 | 2.4e-07 | 1Score **> 32** indicates **identity** Score **> 17** indicates **homology** | U | K.AGLEQGSDAGYLAESQK.F |
| 93153 | 310 | – | 326 | 862.4098 | 1722.8051 | 1722.8060 | -0.51 | 0 | 113 | 4.9e-11 | 1Score **> 32** indicates **identity** Score **> 22** indicates **homology** | U | K.AGLEQGSDAGYLAESQK.F |
| 93154 | 310 | – | 326 | 862.4100 | 1722.8054 | 1722.8060 | -0.34 | 0 | 76 | 7.9e-08 | 1Score **> 32** indicates **identity** Score **> 17** indicates **homology** | U | K.AGLEQGSDAGYLAESQK.F |
| 93155 | 310 | – | 326 | 862.4101 | 1722.8056 | 1722.8060 | -0.24 | 0 | 113 | 1e-10 | 1Score **> 32** indicates **identity** Score **> 25** indicates **homology** | U | K.AGLEQGSDAGYLAESQK.F |
| 93156 | 310 | – | 326 | 862.4103 | 1722.8060 | 1722.8060 | -0.023 | 0 | 113 | 1.1e-10 | 1Score **> 32** indicates **identity** Score **> 26** indicates **homology** | U | K.AGLEQGSDAGYLAESQK.F |
| 93157 | 310 | – | 326 | 575.2760 | 1722.8061 | 1722.8060 | 0.053 | 0 | 20 | 0.014 | 1Score **> 32** indicates **identity** Score **> 14** indicates **homology** | U | K.AGLEQGSDAGYLAESQK.F |
| 93158 | 310 | – | 326 | 862.4105 | 1722.8064 | 1722.8060 | 0.24 | 0 | 91 | 3e-09 | 1Score **> 32** indicates **identity** Score **> 18** indicates **homology** | U | K.AGLEQGSDAGYLAESQK.F |
| 93159 | 310 | – | 326 | 575.2761 | 1722.8065 | 1722.8060 | 0.26 | 0 | 49 | 2.6e-05 | 1Score **> 32** indicates **identity** Score **> 16** indicates **homology** | U | K.AGLEQGSDAGYLAESQK.F |
| 93160 | 310 | – | 326 | 575.2762 | 1722.8069 | 1722.8060 | 0.49 | 0 | 58 | 3.7e-06 | 1Score **> 32** indicates **identity** Score **> 16** indicates **homology** | U | K.AGLEQGSDAGYLAESQK.F |
| 93162 | 310 | – | 326 | 862.4111 | 1722.8076 | 1722.8060 | 0.89 | 0 | 112 | 6.5e-11 | 1Score **> 33** indicates **identity** Score **> 23** indicates **homology** | U | K.AGLEQGSDAGYLAESQK.F |
| 93164 | 310 | – | 326 | 862.4115 | 1722.8084 | 1722.8060 | 1.38 | 0 | 75 | 2.1e-07 | 1Score **> 33** indicates **identity** Score **> 21** indicates **homology** | U | K.AGLEQGSDAGYLAESQK.F |
| 93166 | 310 | – | 326 | 862.4119 | 1722.8092 | 1722.8060 | 1.81 | 0 | 93 | 1.8e-09 | 1Score **> 33** indicates **identity** Score **> 18** indicates **homology** | U | K.AGLEQGSDAGYLAESQK.F |
| 93167 | 310 | – | 326 | 575.2770 | 1722.8092 | 1722.8060 | 1.82 | 0 | 24 | 0.0058 | 1Score **> 33** indicates **identity** Score **> 14** indicates **homology** | U | K.AGLEQGSDAGYLAESQK.F |
| 158988 | 310 | – | 334 | 861.7693 | 2582.2860 | 2582.2864 | -0.14 | 1 | 14 | 0.049 | 1Score **> 37** indicates **identity** Score **> 13** indicates **homology** | U | K.AGLEQGSDAGYLAESQKFGELALTK.E |
| 158989 | 310 | – | 334 | 1292.1511 | 2582.2877 | 2582.2864 | 0.51 | 1 | 145 | 2.5e-14 | 1Score **> 37** indicates **identity** Score **> 22** indicates **homology** | U | K.AGLEQGSDAGYLAESQKFGELALTK.E |
| 158990 | 310 | – | 334 | 1292.1513 | 2582.2880 | 2582.2864 | 0.62 | 1 | 110 | 4.6e-11 | 1Score **> 37** indicates **identity** Score **> 19** indicates **homology** | U | K.AGLEQGSDAGYLAESQKFGELALTK.E |
| 158991 | 310 | – | 334 | 861.7699 | 2582.2880 | 2582.2864 | 0.64 | 1 | 69 | 3.4e-07 | 1Score **> 37** indicates **identity** Score **> 17** indicates **homology** | U | K.AGLEQGSDAGYLAESQKFGELALTK.E |
| 158992 | 310 | – | 334 | 861.7702 | 2582.2888 | 2582.2864 | 0.92 | 1 | 81 | 2.4e-08 | 1Score **> 37** indicates **identity** Score **> 18** indicates **homology** | U | K.AGLEQGSDAGYLAESQKFGELALTK.E |
| 158993 | 310 | – | 334 | 861.7703 | 2582.2892 | 2582.2864 | 1.08 | 1 | 69 | 3.3e-07 | 1Score **> 37** indicates **identity** Score **> 17** indicates **homology** | U | K.AGLEQGSDAGYLAESQKFGELALTK.E |
| 158994 | 310 | – | 334 | 861.7712 | 2582.2917 | 2582.2864 | 2.05 | 1 | 85 | 9.8e-09 | 1Score **> 37** indicates **identity** Score **> 18** indicates **homology** | U | K.AGLEQGSDAGYLAESQKFGELALTK.E |
| 158996 | 310 | – | 334 | 861.7722 | 2582.2949 | 2582.2864 | 3.31 | 1 | 60 | 2.5e-06 | 1Score **> 37** indicates **identity** Score **> 16** indicates **homology** | U | K.AGLEQGSDAGYLAESQKFGELALTK.E |
| 158998 | 310 | – | 334 | 861.7736 | 2582.2990 | 2582.2864 | 4.88 | 1 | 24 | 0.0052 | 1Score **> 37** indicates **identity** Score **> 14** indicates **homology** | U | K.AGLEQGSDAGYLAESQKFGELALTK.E |
| 158999 | 310 | – | 334 | 861.7745 | 2582.3018 | 2582.2864 | 5.98 | 1 | 50 | 2.2e-05 | 1Score **> 37** indicates **identity** Score **> 16** indicates **homology** | U | K.AGLEQGSDAGYLAESQKFGELALTK.E |
| 159000 | 310 | – | 334 | 861.7758 | 2582.3056 | 2582.2864 | 7.44 | 1 | 52 | 1.3e-05 | 1Score **> 37** indicates **identity** Score **> 16** indicates **homology** | U | K.AGLEQGSDAGYLAESQKFGELALTK.E |
| 159046 | 310 | – | 334 | 1292.6521 | 2583.2896 | 2583.2704 | 7.43 | 1 | 103 | 2e-10 | 1Score **> 37** indicates **identity** Score **> 19** indicates **homology** | U | K.AGLEQGSDAGYLAESQKFGELALTK.E  + Deamidated (NQ) |
| 159047 | 310 | – | 334 | 862.1039 | 2583.2900 | 2583.2704 | 7.58 | 1 | 37 | 0.00036 | 1Score **> 37** indicates **identity** Score **> 15** indicates **homology** | U | K.AGLEQGSDAGYLAESQKFGELALTK.E  + Deamidated (NQ) |
| 170996 | 310 | – | 337 | 732.6236 | 2926.4653 | 2926.4559 | 3.20 | 2 | 56 | 5.6e-06 | 1Score **> 38** indicates **identity** Score **> 16** indicates **homology** | U | K.AGLEQGSDAGYLAESQKFGELALTKESK.A |
| 7248 | 327 | – | 334 | 439.7518 | 877.4891 | 877.4909 | -2.08 | 0 | 31 | 0.0022 | 1Score **> 26** indicates **identity** Score **> 16** indicates **homology** | U | K.FGELALTK.E |
| 7249 | 327 | – | 334 | 439.7519 | 877.4892 | 877.4909 | -1.91 | 0 | 29 | 0.0021 | 1Score **> 26** indicates **identity** Score **> 14** indicates **homology** | U | K.FGELALTK.E |
| 7250 | 327 | – | 334 | 439.7522 | 877.4898 | 877.4909 | -1.24 | 0 | 20 | 0.013 | 1Score **> 25** indicates **identity** Score **> 14** indicates **homology** | U | K.FGELALTK.E |
| 7251 | 327 | – | 334 | 439.7523 | 877.4900 | 877.4909 | -1.03 | 0 | 40 | 0.00022 | 1Score **> 25** indicates **identity** Score **> 16** indicates **homology** | U | K.FGELALTK.E |
| 7252 | 327 | – | 334 | 439.7523 | 877.4901 | 877.4909 | -0.95 | 0 | 16 | 0.029 | 1Score **> 25** indicates **identity** Score **> 14** indicates **homology** | U | K.FGELALTK.E |
| 7253 | 327 | – | 334 | 439.7525 | 877.4904 | 877.4909 | -0.53 | 0 | 32 | 0.0018 | 1Score **> 25** indicates **identity** Score **> 17** indicates **homology** | U | K.FGELALTK.E |
| 7254 | 327 | – | 334 | 439.7527 | 877.4909 | 877.4909 | -0.034 | 0 | 46 | 7.8e-05 | 1Score **> 26** indicates **identity** Score **> 18** indicates **homology** | U | K.FGELALTK.E |
| 7255 | 327 | – | 334 | 439.7529 | 877.4913 | 877.4909 | 0.41 | 0 | 46 | 7.9e-05 | 1Score **> 26** indicates **identity** Score **> 18** indicates **homology** | U | K.FGELALTK.E |
| 7256 | 327 | – | 334 | 439.7531 | 877.4917 | 877.4909 | 0.92 | 0 | 46 | 0.0001 | 1Score **> 26** indicates **identity** Score **> 19** indicates **homology** | U | K.FGELALTK.E |
| 7257 | 327 | – | 334 | 439.7532 | 877.4918 | 877.4909 | 0.98 | 0 | 20 | 0.028 | 1Score **> 26** indicates **identity** Score **> 17** indicates **homology** | U | K.FGELALTK.E |
| 7258 | 327 | – | 334 | 439.7533 | 877.4920 | 877.4909 | 1.28 | 0 | 29 | 0.0055 | 1Score **> 26** indicates **identity** Score **> 19** indicates **homology** | U | K.FGELALTK.E |
| 7259 | 327 | – | 334 | 439.7534 | 877.4923 | 877.4909 | 1.62 | 0 | 46 | 0.00012 | 1Score **> 26** indicates **identity** Score **> 20** indicates **homology** | U | K.FGELALTK.E |
| 7260 | 327 | – | 334 | 439.7534 | 877.4923 | 877.4909 | 1.64 | 0 | 34 | 0.00064 | 1Score **> 26** indicates **identity** Score **> 15** indicates **homology** | U | K.FGELALTK.E |
| 7261 | 327 | – | 334 | 439.7535 | 877.4924 | 877.4909 | 1.68 | 0 | 44 | 0.00023 | 1Score **> 26** indicates **identity** Score **> 20** indicates **homology** | U | K.FGELALTK.E |
| 7262 | 327 | – | 334 | 439.7537 | 877.4928 | 877.4909 | 2.11 | 0 | 20 | 0.027 | 1Score **> 26** indicates **identity** Score **> 17** indicates **homology** | U | K.FGELALTK.E |
| 7263 | 327 | – | 334 | 439.7537 | 877.4928 | 877.4909 | 2.19 | 0 | 27 | 0.0073 | 1Score **> 26** indicates **identity** Score **> 18** indicates **homology** | U | K.FGELALTK.E |
| 34996 | 327 | – | 337 | 611.8342 | 1221.6539 | 1221.6605 | -5.37 | 1 | 28 | 0.0026 | 1Score **> 33** indicates **identity** Score **> 14** indicates **homology** | U | K.FGELALTKESK.A |
| 61149 | 338 | – | 350 | 733.8781 | 1465.7416 | 1465.7421 | -0.33 | 0 | 66 | 6.8e-07 | 1Score **> 34** indicates **identity** Score **> 17** indicates **homology** | U | K.ALMGLYNGQVLCK.K |
| 61150 | 338 | – | 350 | 733.8792 | 1465.7438 | 1465.7421 | 1.17 | 0 | 49 | 2.5e-05 | 1Score **> 34** indicates **identity** Score **> 16** indicates **homology** | U | K.ALMGLYNGQVLCK.K |
| 77211 | 338 | – | 351 | 797.9257 | 1593.8369 | 1593.8371 | -0.10 | 1 | 23 | 0.0071 | 1Score **> 35** indicates **identity** Score **> 14** indicates **homology** | U | K.ALMGLYNGQVLCKK.N |
| 177714 | 352 | – | 383 | 807.1957 | 3224.7535 | 3224.7227 | 9.55 | 2 | 14 | 0.047 | 1Score **> 35** indicates **identity** Score **> 13** indicates **homology** | U | K.NKFGAPQKNVQQLAILGAGLMGAGIAQVSVDK.G  + 2 Deamidated (NQ) |
| 147051 | 360 | – | 383 | 785.1039 | 2352.2898 | 2352.2835 | 2.67 | 0 | 102 | 2.9e-10 | 1Score **> 35** indicates **identity** Score **> 19** indicates **homology** | U | K.NVQQLAILGAGLMGAGIAQVSVDK.G |
| 147052 | 360 | – | 383 | 785.1040 | 2352.2903 | 2352.2835 | 2.88 | 0 | 17 | 0.027 | 1Score **> 35** indicates **identity** Score **> 14** indicates **homology** | U | K.NVQQLAILGAGLMGAGIAQVSVDK.G |
| 147053 | 360 | – | 383 | 1177.1533 | 2352.2920 | 2352.2835 | 3.62 | 0 | 114 | 1.9e-11 | 1Score **> 35** indicates **identity** Score **> 20** indicates **homology** | U | K.NVQQLAILGAGLMGAGIAQVSVDK.G |
| 161694 | 360 | – | 386 | 884.5039 | 2650.4897 | 2650.4840 | 2.17 | 1 | 65 | 8.7e-07 | 1Score **> 33** indicates **identity** Score **> 17** indicates **homology** | U | K.NVQQLAILGAGLMGAGIAQVSVDKGLK.T |
| 51099 | 387 | – | 399 | 458.9362 | 1373.7868 | 1373.7878 | -0.79 | 1 | 43 | 0.00012 | 1Score **> 33** indicates **identity** Score **> 16** indicates **homology** | U | K.TLLKDTTVTGLGR.G |
| 51100 | 387 | – | 399 | 458.9363 | 1373.7869 | 1373.7878 | -0.65 | 1 | 42 | 0.0001 | 1Score **> 33** indicates **identity** Score **> 15** indicates **homology** | U | K.TLLKDTTVTGLGR.G |
| 51101 | 387 | – | 399 | 458.9363 | 1373.7871 | 1373.7878 | -0.51 | 1 | 49 | 2.7e-05 | 1Score **> 33** indicates **identity** Score **> 16** indicates **homology** | U | K.TLLKDTTVTGLGR.G |
| 51102 | 387 | – | 399 | 687.9010 | 1373.7874 | 1373.7878 | -0.33 | 1 | 40 | 0.00018 | 1Score **> 33** indicates **identity** Score **> 15** indicates **homology** | U | K.TLLKDTTVTGLGR.G |
| 51104 | 387 | – | 399 | 458.9364 | 1373.7874 | 1373.7878 | -0.29 | 1 | 28 | 0.0025 | 1Score **> 33** indicates **identity** Score **> 14** indicates **homology** | U | K.TLLKDTTVTGLGR.G |
| 51105 | 387 | – | 399 | 458.9365 | 1373.7875 | 1373.7878 | -0.22 | 1 | 35 | 0.00056 | 1Score **> 33** indicates **identity** Score **> 15** indicates **homology** | U | K.TLLKDTTVTGLGR.G |
| 51106 | 387 | – | 399 | 687.9011 | 1373.7876 | 1373.7878 | -0.14 | 1 | 26 | 0.0039 | 1Score **> 33** indicates **identity** Score **> 14** indicates **homology** | U | K.TLLKDTTVTGLGR.G |
| 51107 | 387 | – | 399 | 687.9011 | 1373.7877 | 1373.7878 | -0.13 | 1 | 17 | 0.025 | 1Score **> 33** indicates **identity** Score **> 14** indicates **homology** | U | K.TLLKDTTVTGLGR.G |
| 51108 | 387 | – | 399 | 458.9365 | 1373.7877 | 1373.7878 | -0.076 | 1 | 24 | 0.0051 | 1Score **> 33** indicates **identity** Score **> 14** indicates **homology** | U | K.TLLKDTTVTGLGR.G |
| 51109 | 387 | – | 399 | 458.9366 | 1373.7879 | 1373.7878 | 0.036 | 1 | 47 | 4.2e-05 | 1Score **> 33** indicates **identity** Score **> 15** indicates **homology** | U | K.TLLKDTTVTGLGR.G |
| 51110 | 387 | – | 399 | 458.9368 | 1373.7884 | 1373.7878 | 0.44 | 1 | 38 | 0.00075 | 1Score **> 33** indicates **identity** Score **> 19** indicates **homology** | U | K.TLLKDTTVTGLGR.G |
| 51111 | 387 | – | 399 | 458.9368 | 1373.7885 | 1373.7878 | 0.51 | 1 | 45 | 0.00011 | 1Score **> 33** indicates **identity** Score **> 18** indicates **homology** | U | K.TLLKDTTVTGLGR.G |
| 51112 | 387 | – | 399 | 458.9369 | 1373.7888 | 1373.7878 | 0.71 | 1 | 34 | 0.001 | 1Score **> 33** indicates **identity** Score **> 17** indicates **homology** | U | K.TLLKDTTVTGLGR.G |
| 51113 | 387 | – | 399 | 458.9369 | 1373.7890 | 1373.7878 | 0.83 | 1 | 27 | 0.0027 | 1Score **> 33** indicates **identity** Score **> 14** indicates **homology** | U | K.TLLKDTTVTGLGR.G |
| 51114 | 387 | – | 399 | 458.9370 | 1373.7892 | 1373.7878 | 0.97 | 1 | 27 | 0.003 | 1Score **> 33** indicates **identity** Score **> 14** indicates **homology** | U | K.TLLKDTTVTGLGR.G |
| 9405 | 391 | – | 399 | 460.2460 | 918.4774 | 918.4771 | 0.31 | 0 | 31 | 0.036 | 1Score **> 31** indicates **identity** Score **> 29** indicates **homology** | U | K.DTTVTGLGR.G |
| 49863 | 400 | – | 411 | 454.5771 | 1360.7094 | 1360.7099 | -0.36 | 1 | 22 | 0.0079 | 1Score **> 35** indicates **identity** Score **> 14** indicates **homology** | U | R.GQQQVFKGLNDK.V |
| 20121 | 414 | – | 422 | 360.5452 | 1078.6137 | 1078.6135 | 0.19 | 2 | 57 | 4.7e-06 | 1Score **> 29** indicates **identity** Score **> 16** indicates **homology** | U | K.KKALTSFER.D |
| 11184 | 415 | – | 422 | 476.2667 | 950.5189 | 950.5185 | 0.39 | 1 | 27 | 0.012 | 1Score **> 28** indicates **identity** Score **> 20** indicates **homology** | U | K.KALTSFER.D |
| 11185 | 415 | – | 422 | 476.2668 | 950.5191 | 950.5185 | 0.59 | 1 | 31 | 0.0053 | 1Score **> 28** indicates **identity** Score **> 21** indicates **homology** | U | K.KALTSFER.D |
| 157455 | 415 | – | 436 | 637.0870 | 2544.3191 | 2544.3224 | -1.30 | 2 | 29 | 0.0021 | 1Score **> 37** indicates **identity** Score **> 14** indicates **homology** | U | K.KALTSFERDSIFSNLIGQLDYK.G |
| 4580 | 416 | – | 422 | 412.2192 | 822.4238 | 822.4236 | 0.30 | 0 | 43 | 0.00095 | 1Score **> 26** indicates **identity** | U | K.ALTSFER.D |
| 4581 | 416 | – | 422 | 412.2194 | 822.4243 | 822.4236 | 0.86 | 0 | 36 | 0.0051 | 1Score **> 25** indicates **identity** | U | K.ALTSFER.D |
| 150952 | 416 | – | 436 | 1209.1216 | 2416.2286 | 2416.2274 | 0.48 | 1 | 42 | 0.00012 | 1Score **> 37** indicates **identity** Score **> 15** indicates **homology** | U | K.ALTSFERDSIFSNLIGQLDYK.G |
| 169754 | 416 | – | 440 | 960.1609 | 2877.4610 | 2877.4548 | 2.14 | 2 | 45 | 5.6e-05 | 1Score **> 37** indicates **identity** Score **> 15** indicates **homology** | U | K.ALTSFERDSIFSNLIGQLDYKGFEK.A |
| 79472 | 423 | – | 436 | 806.9163 | 1611.8180 | 1611.8144 | 2.23 | 0 | 91 | 3.1e-09 | 1Score **> 34** indicates **identity** Score **> 18** indicates **homology** | U | R.DSIFSNLIGQLDYK.G |
| 79473 | 423 | – | 436 | 806.9164 | 1611.8183 | 1611.8144 | 2.41 | 0 | 90 | 4.7e-09 | 1Score **> 34** indicates **identity** Score **> 20** indicates **homology** | U | R.DSIFSNLIGQLDYK.G |
| 126031 | 423 | – | 440 | 1037.5211 | 2073.0277 | 2073.0419 | -6.82 | 1 | 69 | 4.1e-07 | 1Score **> 36** indicates **identity** Score **> 18** indicates **homology** | U | R.DSIFSNLIGQLDYKGFEK.A |
| 126033 | 423 | – | 440 | 692.0213 | 2073.0422 | 2073.0419 | 0.17 | 1 | 32 | 0.00092 | 1Score **> 36** indicates **identity** Score **> 15** indicates **homology** | U | R.DSIFSNLIGQLDYKGFEK.A |
| 126034 | 423 | – | 440 | 1037.5285 | 2073.0424 | 2073.0419 | 0.29 | 1 | 94 | 3.8e-09 | 1Score **> 36** indicates **identity** Score **> 22** indicates **homology** | U | R.DSIFSNLIGQLDYKGFEK.A |
| 82420 | 441 | – | 455 | 545.9482 | 1634.8228 | 1634.8226 | 0.16 | 0 | 33 | 0.0008 | 1Score **> 35** indicates **identity** Score **> 15** indicates **homology** | U | K.ADMVIEAVFEDLGVK.H |
| 82426 | 441 | – | 455 | 545.9489 | 1634.8249 | 1634.8226 | 1.44 | 0 | 74 | 1.1e-07 | 1Score **> 35** indicates **identity** Score **> 17** indicates **homology** | U | K.ADMVIEAVFEDLGVK.H |
| 82432 | 441 | – | 455 | 818.4204 | 1634.8263 | 1634.8226 | 2.31 | 0 | 97 | 7.4e-10 | 1Score **> 35** indicates **identity** Score **> 19** indicates **homology** | U | K.ADMVIEAVFEDLGVK.H |
| 84403 | 441 | – | 455 | 551.2751 | 1650.8035 | 1650.8175 | -8.44 | 0 | 20 | 0.014 | 1Score **> 34** indicates **identity** Score **> 14** indicates **homology** | U | K.ADMVIEAVFEDLGVK.H  + Oxidation (M) |
| 84406 | 441 | – | 455 | 826.4133 | 1650.8121 | 1650.8175 | -3.23 | 0 | 30 | 0.0015 | 1Score **> 34** indicates **identity** Score **> 14** indicates **homology** | U | K.ADMVIEAVFEDLGVK.H  + Oxidation (M) |
| 84409 | 441 | – | 455 | 826.4140 | 1650.8135 | 1650.8175 | -2.39 | 0 | 35 | 0.00056 | 1Score **> 34** indicates **identity** Score **> 15** indicates **homology** | U | K.ADMVIEAVFEDLGVK.H  + Oxidation (M) |
| 84413 | 441 | – | 455 | 551.2809 | 1650.8210 | 1650.8175 | 2.13 | 0 | 44 | 7.3e-05 | 1Score **> 34** indicates **identity** Score **> 15** indicates **homology** | U | K.ADMVIEAVFEDLGVK.H  + Oxidation (M) |
| 181428 | 458 | – | 489 | 863.9573 | 3451.8000 | 3451.8021 | -0.60 | 1 | 33 | 0.00089 | 1Score **> 37** indicates **identity** Score **> 15** indicates **homology** | U | K.VLKEVESVTPEHCIFASNTSALPINQIAAVSK.R |
| 181429 | 458 | – | 489 | 863.9575 | 3451.8010 | 3451.8021 | -0.32 | 1 | 43 | 8.5e-05 | 1Score **> 37** indicates **identity** Score **> 15** indicates **homology** | U | K.VLKEVESVTPEHCIFASNTSALPINQIAAVSK.R |
| 181430 | 458 | – | 489 | 1151.6077 | 3451.8014 | 3451.8021 | -0.20 | 1 | 82 | 1.9e-08 | 1Score **> 37** indicates **identity** Score **> 18** indicates **homology** | U | K.VLKEVESVTPEHCIFASNTSALPINQIAAVSK.R |
| 181431 | 458 | – | 489 | 1151.6088 | 3451.8047 | 3451.8021 | 0.75 | 1 | 85 | 1.1e-08 | 1Score **> 37** indicates **identity** Score **> 18** indicates **homology** | U | K.VLKEVESVTPEHCIFASNTSALPINQIAAVSK.R |
| 181432 | 458 | – | 489 | 1151.6104 | 3451.8093 | 3451.8021 | 2.10 | 1 | 91 | 2.8e-09 | 1Score **> 37** indicates **identity** Score **> 18** indicates **homology** | U | K.VLKEVESVTPEHCIFASNTSALPINQIAAVSK.R |
| 181433 | 458 | – | 489 | 863.9597 | 3451.8096 | 3451.8021 | 2.18 | 1 | 42 | 0.0001 | 1Score **> 37** indicates **identity** Score **> 15** indicates **homology** | U | K.VLKEVESVTPEHCIFASNTSALPINQIAAVSK.R |
| 181434 | 458 | – | 489 | 1151.6161 | 3451.8266 | 3451.8021 | 7.09 | 1 | 27 | 0.003 | 1Score **> 36** indicates **identity** Score **> 14** indicates **homology** | U | K.VLKEVESVTPEHCIFASNTSALPINQIAAVSK.R |
| 175682 | 461 | – | 489 | 1038.1921 | 3111.5545 | 3111.5546 | -0.055 | 0 | 40 | 0.00017 | 1Score **> 38** indicates **identity** Score **> 15** indicates **homology** | U | K.EVESVTPEHCIFASNTSALPINQIAAVSK.R |
| 175683 | 461 | – | 489 | 1038.1923 | 3111.5549 | 3111.5546 | 0.091 | 0 | 68 | 4.5e-07 | 1Score **> 38** indicates **identity** Score **> 17** indicates **homology** | U | K.EVESVTPEHCIFASNTSALPINQIAAVSK.R |
| 175684 | 461 | – | 489 | 1038.1925 | 3111.5556 | 3111.5546 | 0.32 | 0 | 63 | 1.4e-06 | 1Score **> 38** indicates **identity** Score **> 16** indicates **homology** | U | K.EVESVTPEHCIFASNTSALPINQIAAVSK.R |
| 175686 | 461 | – | 489 | 1038.1931 | 3111.5574 | 3111.5546 | 0.88 | 0 | 65 | 8.4e-07 | 1Score **> 38** indicates **identity** Score **> 17** indicates **homology** | U | K.EVESVTPEHCIFASNTSALPINQIAAVSK.R |
| 175689 | 461 | – | 489 | 1038.1935 | 3111.5588 | 3111.5546 | 1.33 | 0 | 14 | 0.049 | 1Score **> 38** indicates **identity** Score **> 13** indicates **homology** | U | K.EVESVTPEHCIFASNTSALPINQIAAVSK.R |
| 175693 | 461 | – | 489 | 1038.1951 | 3111.5634 | 3111.5546 | 2.81 | 0 | 74 | 1e-07 | 1Score **> 38** indicates **identity** Score **> 17** indicates **homology** | U | K.EVESVTPEHCIFASNTSALPINQIAAVSK.R |
| 175716 | 461 | – | 489 | 1557.2856 | 3112.5566 | 3112.5387 | 5.77 | 0 | 20 | 0.021 | 1Score **> 38** indicates **identity** Score **> 16** indicates **homology** | U | K.EVESVTPEHCIFASNTSALPINQIAAVSK.R  + Deamidated (NQ) |
| 175720 | 461 | – | 489 | 1038.5288 | 3112.5645 | 3112.5387 | 8.29 | 0 | 18 | 0.019 | 1Score **> 38** indicates **identity** Score **> 14** indicates **homology** | U | K.EVESVTPEHCIFASNTSALPINQIAAVSK.R  + Deamidated (NQ) |
| 175721 | 461 | – | 489 | 1038.5299 | 3112.5679 | 3112.5387 | 9.38 | 0 | 30 | 0.0015 | 1Score **> 38** indicates **identity** Score **> 14** indicates **homology** | U | K.EVESVTPEHCIFASNTSALPINQIAAVSK.R  + Deamidated (NQ) |
| 175722 | 461 | – | 489 | 1038.5300 | 3112.5681 | 3112.5387 | 9.46 | 0 | 22 | 0.0091 | 1Score **> 38** indicates **identity** Score **> 14** indicates **homology** | U | K.EVESVTPEHCIFASNTSALPINQIAAVSK.R  + Deamidated (NQ) |
| 183246 | 461 | – | 493 | 906.4680 | 3621.8429 | 3621.8461 | -0.88 | 2 | 24 | 0.006 | 1Score **> 37** indicates **identity** Score **> 14** indicates **homology** | U | K.EVESVTPEHCIFASNTSALPINQIAAVSKRPEK.V |
| 183248 | 461 | – | 493 | 1208.2898 | 3621.8475 | 3621.8461 | 0.41 | 2 | 16 | 0.033 | 1Score **> 37** indicates **identity** Score **> 13** indicates **homology** | U | K.EVESVTPEHCIFASNTSALPINQIAAVSKRPEK.V |
| 183249 | 461 | – | 493 | 906.4692 | 3621.8477 | 3621.8461 | 0.47 | 2 | 33 | 0.00079 | 1Score **> 37** indicates **identity** Score **> 15** indicates **homology** | U | K.EVESVTPEHCIFASNTSALPINQIAAVSKRPEK.V |
| 183250 | 461 | – | 493 | 1208.2899 | 3621.8480 | 3621.8461 | 0.54 | 2 | 17 | 0.024 | 1Score **> 37** indicates **identity** Score **> 14** indicates **homology** | U | K.EVESVTPEHCIFASNTSALPINQIAAVSKRPEK.V |
| 183251 | 461 | – | 493 | 725.3769 | 3621.8481 | 3621.8461 | 0.58 | 2 | 27 | 0.0029 | 1Score **> 37** indicates **identity** Score **> 14** indicates **homology** | U | K.EVESVTPEHCIFASNTSALPINQIAAVSKRPEK.V |
| 183252 | 461 | – | 493 | 725.3774 | 3621.8505 | 3621.8461 | 1.23 | 2 | 20 | 0.014 | 1Score **> 37** indicates **identity** Score **> 14** indicates **homology** | U | K.EVESVTPEHCIFASNTSALPINQIAAVSKRPEK.V |
| 183253 | 461 | – | 493 | 906.4700 | 3621.8507 | 3621.8461 | 1.29 | 2 | 31 | 0.0012 | 1Score **> 37** indicates **identity** Score **> 14** indicates **homology** | U | K.EVESVTPEHCIFASNTSALPINQIAAVSKRPEK.V |
| 183254 | 461 | – | 493 | 906.4707 | 3621.8536 | 3621.8461 | 2.09 | 2 | 18 | 0.02 | 1Score **> 37** indicates **identity** Score **> 14** indicates **homology** | U | K.EVESVTPEHCIFASNTSALPINQIAAVSKRPEK.V |
| 52779 | 494 | – | 505 | 464.9044 | 1391.6912 | 1391.6908 | 0.33 | 0 | 19 | 0.017 | 1Score **> 33** indicates **identity** Score **> 14** indicates **homology** | U | K.VIGMHYFSPVDK.M |
| 80587 | 506 | – | 519 | 540.9676 | 1619.8809 | 1619.8804 | 0.34 | 1 | 30 | 0.0016 | 1Score **> 34** indicates **identity** Score **> 14** indicates **homology** | U | K.MQLLEIITTDKTSK.D |
| 80588 | 506 | – | 519 | 540.9677 | 1619.8812 | 1619.8804 | 0.53 | 1 | 43 | 8.7e-05 | 1Score **> 34** indicates **identity** Score **> 15** indicates **homology** | U | K.MQLLEIITTDKTSK.D |
| 80589 | 506 | – | 519 | 540.9678 | 1619.8816 | 1619.8804 | 0.75 | 1 | 64 | 9.1e-07 | 1Score **> 34** indicates **identity** Score **> 17** indicates **homology** | U | K.MQLLEIITTDKTSK.D |
| 80590 | 506 | – | 519 | 540.9678 | 1619.8817 | 1619.8804 | 0.80 | 1 | 35 | 0.00047 | 1Score **> 34** indicates **identity** Score **> 15** indicates **homology** | U | K.MQLLEIITTDKTSK.D |
| 80591 | 506 | – | 519 | 540.9687 | 1619.8843 | 1619.8804 | 2.42 | 1 | 24 | 0.0056 | 1Score **> 34** indicates **identity** Score **> 14** indicates **homology** | U | K.MQLLEIITTDKTSK.D |
| 165813 | 506 | – | 531 | 691.3779 | 2761.4825 | 2761.4895 | -2.54 | 2 | 29 | 0.0019 | 1Score **> 36** indicates **identity** Score **> 14** indicates **homology** | U | K.MQLLEIITTDKTSKDTTASAVAVGLR.Q |
| 165814 | 506 | – | 531 | 921.5020 | 2761.4841 | 2761.4895 | -1.95 | 2 | 45 | 5.9e-05 | 1Score **> 36** indicates **identity** Score **> 15** indicates **homology** | U | K.MQLLEIITTDKTSKDTTASAVAVGLR.Q |
| 165815 | 506 | – | 531 | 691.3800 | 2761.4909 | 2761.4895 | 0.49 | 2 | 55 | 6.8e-06 | 1Score **> 36** indicates **identity** Score **> 16** indicates **homology** | U | K.MQLLEIITTDKTSKDTTASAVAVGLR.Q |
| 165816 | 506 | – | 531 | 691.3855 | 2761.5131 | 2761.4895 | 8.53 | 2 | 31 | 0.0011 | 1Score **> 35** indicates **identity** Score **> 14** indicates **homology** | U | K.MQLLEIITTDKTSKDTTASAVAVGLR.Q |
| 62368 | 517 | – | 531 | 738.9017 | 1475.7889 | 1475.7944 | -3.68 | 1 | 57 | 4.8e-06 | 1Score **> 35** indicates **identity** Score **> 16** indicates **homology** | U | K.TSKDTTASAVAVGLR.Q |
| 62371 | 517 | – | 531 | 738.9028 | 1475.7911 | 1475.7944 | -2.25 | 1 | 94 | 1.4e-09 | 1Score **> 35** indicates **identity** Score **> 18** indicates **homology** | U | K.TSKDTTASAVAVGLR.Q |
| 62372 | 517 | – | 531 | 492.9376 | 1475.7911 | 1475.7944 | -2.25 | 1 | 60 | 2.5e-06 | 1Score **> 35** indicates **identity** Score **> 17** indicates **homology** | U | K.TSKDTTASAVAVGLR.Q |
| 62374 | 517 | – | 531 | 738.9036 | 1475.7927 | 1475.7944 | -1.11 | 1 | 68 | 4.3e-07 | 1Score **> 35** indicates **identity** Score **> 17** indicates **homology** | U | K.TSKDTTASAVAVGLR.Q |
| 62375 | 517 | – | 531 | 492.9385 | 1475.7938 | 1475.7944 | -0.42 | 1 | 19 | 0.025 | 1Score **> 35** indicates **identity** Score **> 16** indicates **homology** | U | K.TSKDTTASAVAVGLR.Q |
| 62376 | 517 | – | 531 | 492.9386 | 1475.7938 | 1475.7944 | -0.36 | 1 | 31 | 0.0014 | 1Score **> 35** indicates **identity** Score **> 14** indicates **homology** | U | K.TSKDTTASAVAVGLR.Q |
| 62377 | 517 | – | 531 | 738.9043 | 1475.7940 | 1475.7944 | -0.23 | 1 | 38 | 0.00029 | 1Score **> 35** indicates **identity** Score **> 15** indicates **homology** | U | K.TSKDTTASAVAVGLR.Q |
| 28301 | 520 | – | 531 | 580.8158 | 1159.6170 | 1159.6197 | -2.37 | 0 | 34 | 0.00071 | 1Score **> 34** indicates **identity** Score **> 15** indicates **homology** | U | K.DTTASAVAVGLR.Q |
| 28302 | 520 | – | 531 | 580.8163 | 1159.6180 | 1159.6197 | -1.49 | 0 | 21 | 0.01 | 1Score **> 34** indicates **identity** Score **> 14** indicates **homology** | U | K.DTTASAVAVGLR.Q |
| 28303 | 520 | – | 531 | 580.8166 | 1159.6187 | 1159.6197 | -0.87 | 0 | 67 | 5.8e-07 | 1Score **> 34** indicates **identity** Score **> 17** indicates **homology** | U | K.DTTASAVAVGLR.Q |
| 28304 | 520 | – | 531 | 580.8166 | 1159.6187 | 1159.6197 | -0.87 | 0 | 54 | 7.8e-06 | 1Score **> 34** indicates **identity** Score **> 16** indicates **homology** | U | K.DTTASAVAVGLR.Q |
| 28306 | 520 | – | 531 | 580.8169 | 1159.6193 | 1159.6197 | -0.34 | 0 | 59 | 1.9e-05 | 1Score **> 34** indicates **identity** Score **> 25** indicates **homology** | U | K.DTTASAVAVGLR.Q |
| 28307 | 520 | – | 531 | 580.8171 | 1159.6197 | 1159.6197 | -0.027 | 0 | 63 | 1.1e-05 | 1Score **> 34** indicates **identity** Score **> 26** indicates **homology** | U | K.DTTASAVAVGLR.Q |
| 28308 | 520 | – | 531 | 580.8171 | 1159.6197 | 1159.6197 | 0.015 | 0 | 74 | 1.2e-06 | 1Score **> 34** indicates **identity** Score **> 27** indicates **homology** | U | K.DTTASAVAVGLR.Q |
| 28310 | 520 | – | 531 | 580.8174 | 1159.6202 | 1159.6197 | 0.43 | 0 | 62 | 2.6e-06 | 1Score **> 34** indicates **identity** Score **> 19** indicates **homology** | U | K.DTTASAVAVGLR.Q |
| 28311 | 520 | – | 531 | 580.8178 | 1159.6210 | 1159.6197 | 1.12 | 0 | 20 | 0.02 | 1Score **> 34** indicates **identity** Score **> 15** indicates **homology** | U | K.DTTASAVAVGLR.Q |
| 28313 | 520 | – | 531 | 580.8185 | 1159.6224 | 1159.6197 | 2.28 | 0 | 67 | 2.5e-06 | 1Score **> 34** indicates **identity** Score **> 23** indicates **homology** | U | K.DTTASAVAVGLR.Q |
| 86079 | 535 | – | 549 | 555.6502 | 1663.9287 | 1663.9298 | -0.65 | 1 | 26 | 0.0037 | 1Score **> 34** indicates **identity** Score **> 14** indicates **homology** | U | K.VIIVVKDGPGFYTTR.C |
| 86080 | 535 | – | 549 | 555.6509 | 1663.9309 | 1663.9298 | 0.71 | 1 | 58 | 3.3e-06 | 1Score **> 34** indicates **identity** Score **> 16** indicates **homology** | U | K.VIIVVKDGPGFYTTR.C |
| 86081 | 535 | – | 549 | 555.6510 | 1663.9311 | 1663.9298 | 0.81 | 1 | 19 | 0.016 | 1Score **> 33** indicates **identity** Score **> 14** indicates **homology** | U | K.VIIVVKDGPGFYTTR.C |
| 86082 | 535 | – | 549 | 555.6510 | 1663.9312 | 1663.9298 | 0.87 | 1 | 43 | 9.1e-05 | 1Score **> 33** indicates **identity** Score **> 15** indicates **homology** | U | K.VIIVVKDGPGFYTTR.C |
| 86083 | 535 | – | 549 | 555.6512 | 1663.9318 | 1663.9298 | 1.22 | 1 | 64 | 1e-06 | 1Score **> 33** indicates **identity** Score **> 16** indicates **homology** | U | K.VIIVVKDGPGFYTTR.C |
| 86084 | 535 | – | 549 | 555.6512 | 1663.9318 | 1663.9298 | 1.24 | 1 | 46 | 5.4e-05 | 1Score **> 33** indicates **identity** Score **> 15** indicates **homology** | U | K.VIIVVKDGPGFYTTR.C |
| 86085 | 535 | – | 549 | 555.6513 | 1663.9322 | 1663.9298 | 1.46 | 1 | 37 | 0.00037 | 1Score **> 33** indicates **identity** Score **> 15** indicates **homology** | U | K.VIIVVKDGPGFYTTR.C |
| 86086 | 535 | – | 549 | 555.6518 | 1663.9337 | 1663.9298 | 2.37 | 1 | 29 | 0.002 | 1Score **> 33** indicates **identity** Score **> 14** indicates **homology** | U | K.VIIVVKDGPGFYTTR.C |
| 86087 | 535 | – | 549 | 555.6523 | 1663.9352 | 1663.9298 | 3.25 | 1 | 38 | 0.00029 | 1Score **> 33** indicates **identity** Score **> 15** indicates **homology** | U | K.VIIVVKDGPGFYTTR.C |
| 14939 | 541 | – | 549 | 507.2357 | 1012.4569 | 1012.4614 | -4.44 | 0 | 20 | 0.013 | 1Score **> 27** indicates **identity** Score **> 14** indicates **homology** | U | K.DGPGFYTTR.C |
| 14940 | 541 | – | 549 | 507.2358 | 1012.4571 | 1012.4614 | -4.25 | 0 | 34 | 0.00066 | 1Score **> 27** indicates **identity** Score **> 15** indicates **homology** | U | K.DGPGFYTTR.C |
| 14941 | 541 | – | 549 | 507.2359 | 1012.4573 | 1012.4614 | -4.08 | 0 | 45 | 5.8e-05 | 1Score **> 26** indicates **identity** Score **> 15** indicates **homology** | U | K.DGPGFYTTR.C |
| 14942 | 541 | – | 549 | 507.2367 | 1012.4588 | 1012.4614 | -2.63 | 0 | 36 | 0.00043 | 1Score **> 27** indicates **identity** Score **> 15** indicates **homology** | U | K.DGPGFYTTR.C |
| 14943 | 541 | – | 549 | 507.2377 | 1012.4609 | 1012.4614 | -0.48 | 0 | 45 | 6.6e-05 | 1Score **> 27** indicates **identity** Score **> 15** indicates **homology** | U | K.DGPGFYTTR.C |
| 14944 | 541 | – | 549 | 507.2379 | 1012.4612 | 1012.4614 | -0.21 | 0 | 48 | 2.9e-05 | 1Score **> 27** indicates **identity** Score **> 16** indicates **homology** | U | K.DGPGFYTTR.C |
| 14945 | 541 | – | 549 | 507.2380 | 1012.4614 | 1012.4614 | 0.0099 | 0 | 35 | 0.00047 | 1Score **> 27** indicates **identity** Score **> 15** indicates **homology** | U | K.DGPGFYTTR.C |
| 14049 | 561 | – | 569 | 499.7788 | 997.5430 | 997.5444 | -1.41 | 0 | 44 | 0.00012 | 1Score **> 29** indicates **identity** Score **> 17** indicates **homology** | U | R.ILQEGVDPK.K |
| 14050 | 561 | – | 569 | 499.7792 | 997.5438 | 997.5444 | -0.61 | 0 | 46 | 7.5e-05 | 1Score **> 29** indicates **identity** Score **> 17** indicates **homology** | U | R.ILQEGVDPK.K |
| 14051 | 561 | – | 569 | 499.7795 | 997.5444 | 997.5444 | -0.0050 | 0 | 39 | 0.00024 | 1Score **> 29** indicates **identity** Score **> 15** indicates **homology** | U | R.ILQEGVDPK.K |
| 14052 | 561 | – | 569 | 499.7796 | 997.5446 | 997.5444 | 0.16 | 0 | 34 | 0.0007 | 1Score **> 28** indicates **identity** Score **> 15** indicates **homology** | U | R.ILQEGVDPK.K |
| 14575 | 611 | – | 620 | 503.7817 | 1005.5488 | 1005.5495 | -0.72 | 0 | 53 | 2.3e-05 | 1Score **> 31** indicates **identity** Score **> 19** indicates **homology** | U | R.FGGGSVELLK.Q |
| 14578 | 611 | – | 620 | 503.7819 | 1005.5492 | 1005.5495 | -0.29 | 0 | 60 | 4.6e-06 | 1Score **> 31** indicates **identity** Score **> 19** indicates **homology** | U | R.FGGGSVELLK.Q |
| 14579 | 611 | – | 620 | 503.7819 | 1005.5493 | 1005.5495 | -0.16 | 0 | 59 | 6.1e-06 | 1Score **> 31** indicates **identity** Score **> 19** indicates **homology** | U | R.FGGGSVELLK.Q |
| 14582 | 611 | – | 620 | 503.7822 | 1005.5499 | 1005.5495 | 0.37 | 0 | 46 | 6.1e-05 | 1Score **> 31** indicates **identity** Score **> 17** indicates **homology** | U | R.FGGGSVELLK.Q |
| 14583 | 611 | – | 620 | 503.7823 | 1005.5501 | 1005.5495 | 0.55 | 0 | 43 | 9.5e-05 | 1Score **> 31** indicates **identity** Score **> 15** indicates **homology** | U | R.FGGGSVELLK.Q |
| 14584 | 611 | – | 620 | 503.7823 | 1005.5501 | 1005.5495 | 0.56 | 0 | 46 | 0.00012 | 1Score **> 31** indicates **identity** Score **> 19** indicates **homology** | U | R.FGGGSVELLK.Q |
| 14585 | 611 | – | 620 | 503.7823 | 1005.5501 | 1005.5495 | 0.58 | 0 | 60 | 4.7e-06 | 1Score **> 31** indicates **identity** Score **> 19** indicates **homology** | U | R.FGGGSVELLK.Q |
| 14586 | 611 | – | 620 | 503.7823 | 1005.5501 | 1005.5495 | 0.62 | 0 | 48 | 3.9e-05 | 1Score **> 31** indicates **identity** Score **> 16** indicates **homology** | U | R.FGGGSVELLK.Q |
| 14587 | 611 | – | 620 | 503.7823 | 1005.5501 | 1005.5495 | 0.64 | 0 | 37 | 0.00037 | 1Score **> 31** indicates **identity** Score **> 15** indicates **homology** | U | R.FGGGSVELLK.Q |
| 14589 | 611 | – | 620 | 503.7825 | 1005.5504 | 1005.5495 | 0.92 | 0 | 60 | 4.6e-06 | 1Score **> 31** indicates **identity** Score **> 19** indicates **homology** | U | R.FGGGSVELLK.Q |
| 14590 | 611 | – | 620 | 503.7826 | 1005.5506 | 1005.5495 | 1.05 | 0 | 32 | 0.0011 | 1Score **> 31** indicates **identity** Score **> 14** indicates **homology** | U | R.FGGGSVELLK.Q |
| 14591 | 611 | – | 620 | 503.7827 | 1005.5508 | 1005.5495 | 1.24 | 0 | 50 | 2.6e-05 | 1Score **> 32** indicates **identity** Score **> 17** indicates **homology** | U | R.FGGGSVELLK.Q |
| 14592 | 611 | – | 620 | 503.7827 | 1005.5508 | 1005.5495 | 1.30 | 0 | 29 | 0.0021 | 1Score **> 32** indicates **identity** Score **> 14** indicates **homology** | U | R.FGGGSVELLK.Q |
| 14593 | 611 | – | 620 | 503.7827 | 1005.5508 | 1005.5495 | 1.30 | 0 | 33 | 0.00086 | 1Score **> 32** indicates **identity** Score **> 15** indicates **homology** | U | R.FGGGSVELLK.Q |
| 60755 | 632 | – | 644 | 732.3612 | 1462.7078 | 1462.7092 | -0.95 | 1 | 61 | 2.5e-06 | 1Score **> 33** indicates **identity** Score **> 18** indicates **homology** | U | K.SGKGFYIYQEGSK.N |
| 60756 | 632 | – | 644 | 488.5766 | 1462.7081 | 1462.7092 | -0.76 | 1 | 30 | 0.0016 | 1Score **> 33** indicates **identity** Score **> 14** indicates **homology** | U | K.SGKGFYIYQEGSK.N |
| 60758 | 632 | – | 644 | 732.3615 | 1462.7085 | 1462.7092 | -0.48 | 1 | 70 | 2.6e-07 | 1Score **> 33** indicates **identity** Score **> 17** indicates **homology** | U | K.SGKGFYIYQEGSK.N |
| 60760 | 632 | – | 644 | 732.3618 | 1462.7091 | 1462.7092 | -0.092 | 1 | 64 | 9.6e-07 | 1Score **> 33** indicates **identity** Score **> 17** indicates **homology** | U | K.SGKGFYIYQEGSK.N |
| 60761 | 632 | – | 644 | 488.5772 | 1462.7099 | 1462.7092 | 0.43 | 1 | 30 | 0.0017 | 1Score **> 33** indicates **identity** Score **> 14** indicates **homology** | U | K.SGKGFYIYQEGSK.N |
| 60762 | 632 | – | 644 | 488.5774 | 1462.7104 | 1462.7092 | 0.80 | 1 | 15 | 0.036 | 1Score **> 33** indicates **identity** Score **> 13** indicates **homology** | U | K.SGKGFYIYQEGSK.N |
| 60763 | 632 | – | 644 | 732.3635 | 1462.7124 | 1462.7092 | 2.17 | 1 | 54 | 8.9e-06 | 1Score **> 33** indicates **identity** Score **> 16** indicates **homology** | U | K.SGKGFYIYQEGSK.N |
| 31677 | 635 | – | 644 | 596.2837 | 1190.5528 | 1190.5608 | -6.69 | 0 | 30 | 0.0016 | 1Score **> 31** indicates **identity** Score **> 14** indicates **homology** | U | K.GFYIYQEGSK.N |
| 31683 | 635 | – | 644 | 596.2851 | 1190.5557 | 1190.5608 | -4.29 | 0 | 30 | 0.0015 | 1Score **> 31** indicates **identity** Score **> 14** indicates **homology** | U | K.GFYIYQEGSK.N |
| 31686 | 635 | – | 644 | 596.2861 | 1190.5577 | 1190.5608 | -2.62 | 0 | 17 | 0.028 | 1Score **> 31** indicates **identity** Score **> 14** indicates **homology** | U | K.GFYIYQEGSK.N |
| 31689 | 635 | – | 644 | 596.2869 | 1190.5593 | 1190.5608 | -1.25 | 0 | 41 | 0.00015 | 1Score **> 31** indicates **identity** Score **> 15** indicates **homology** | U | K.GFYIYQEGSK.N |
| 31692 | 635 | – | 644 | 596.2872 | 1190.5598 | 1190.5608 | -0.86 | 0 | 35 | 0.00051 | 1Score **> 31** indicates **identity** Score **> 15** indicates **homology** | U | K.GFYIYQEGSK.N |
| 31693 | 635 | – | 644 | 596.2872 | 1190.5598 | 1190.5608 | -0.86 | 0 | 21 | 0.01 | 1Score **> 31** indicates **identity** Score **> 14** indicates **homology** | U | K.GFYIYQEGSK.N |
| 31695 | 635 | – | 644 | 596.2874 | 1190.5601 | 1190.5608 | -0.53 | 0 | 31 | 0.0014 | 1Score **> 32** indicates **identity** Score **> 14** indicates **homology** | U | K.GFYIYQEGSK.N |
| 31697 | 635 | – | 644 | 596.2874 | 1190.5602 | 1190.5608 | -0.51 | 0 | 33 | 0.00085 | 1Score **> 32** indicates **identity** Score **> 15** indicates **homology** | U | K.GFYIYQEGSK.N |
| 57318 | 635 | – | 646 | 478.5735 | 1432.6988 | 1432.6987 | 0.11 | 1 | 20 | 0.012 | 1Score **> 33** indicates **identity** Score **> 14** indicates **homology** | U | K.GFYIYQEGSKNK.S |
| 57320 | 635 | – | 646 | 717.3575 | 1432.7005 | 1432.6987 | 1.30 | 1 | 44 | 7.6e-05 | 1Score **> 33** indicates **identity** Score **> 15** indicates **homology** | U | K.GFYIYQEGSKNK.S |
| 76565 | 647 | – | 660 | 795.3994 | 1588.7843 | 1588.7879 | -2.21 | 0 | 91 | 2.7e-09 | 1Score **> 34** indicates **identity** Score **> 18** indicates **homology** | U | K.SLNSEMDNILANLR.L |
| 76584 | 647 | – | 660 | 795.4049 | 1588.7952 | 1588.7879 | 4.64 | 0 | 73 | 1.3e-07 | 1Score **> 35** indicates **identity** Score **> 17** indicates **homology** | U | K.SLNSEMDNILANLR.L |
| 180583 | 647 | – | 676 | 1135.2333 | 3402.6782 | 3402.6725 | 1.67 | 2 | 39 | 0.00022 | 1Score **> 37** indicates **identity** Score **> 15** indicates **homology** | U | K.SLNSEMDNILANLRLPAKPEVSSDEDVQYR.V |
| 182703 | 647 | – | 676 | 890.9443 | 3559.7480 | 3559.7715 | -6.62 | 2 | 20 | 0.012 | 1Score **> 37** indicates **identity** Score **> 14** indicates **homology** | U | K.SLNSEMDNILANLRLPAKPEVSSDEDVQYR.V  + Deamidated (NQ); HNE (K) |
| 104670 | 661 | – | 676 | 611.6386 | 1831.8941 | 1831.8952 | -0.62 | 1 | 84 | 1.2e-08 | 1Score **> 35** indicates **identity** Score **> 18** indicates **homology** | U | R.LPAKPEVSSDEDVQYR.V |
| 104671 | 661 | – | 676 | 611.6387 | 1831.8943 | 1831.8952 | -0.47 | 1 | 80 | 3.3e-08 | 1Score **> 35** indicates **identity** Score **> 17** indicates **homology** | U | R.LPAKPEVSSDEDVQYR.V |
| 104672 | 661 | – | 676 | 611.6389 | 1831.8947 | 1831.8952 | -0.25 | 1 | 69 | 3.4e-07 | 1Score **> 35** indicates **identity** Score **> 17** indicates **homology** | U | R.LPAKPEVSSDEDVQYR.V |
| 104673 | 661 | – | 676 | 611.6389 | 1831.8948 | 1831.8952 | -0.21 | 1 | 67 | 5.5e-07 | 1Score **> 35** indicates **identity** Score **> 17** indicates **homology** | U | R.LPAKPEVSSDEDVQYR.V |
| 104674 | 661 | – | 676 | 611.6390 | 1831.8951 | 1831.8952 | -0.059 | 1 | 92 | 2.4e-09 | 1Score **> 35** indicates **identity** Score **> 18** indicates **homology** | U | R.LPAKPEVSSDEDVQYR.V |
| 104676 | 661 | – | 676 | 611.6391 | 1831.8956 | 1831.8952 | 0.23 | 1 | 92 | 2.3e-09 | 1Score **> 35** indicates **identity** Score **> 18** indicates **homology** | U | R.LPAKPEVSSDEDVQYR.V |
| 104679 | 661 | – | 676 | 611.6394 | 1831.8965 | 1831.8952 | 0.72 | 1 | 60 | 2.4e-06 | 1Score **> 35** indicates **identity** Score **> 16** indicates **homology** | U | R.LPAKPEVSSDEDVQYR.V |
| 104680 | 661 | – | 676 | 611.6395 | 1831.8966 | 1831.8952 | 0.75 | 1 | 89 | 4.4e-09 | 1Score **> 35** indicates **identity** Score **> 18** indicates **homology** | U | R.LPAKPEVSSDEDVQYR.V |
| 104681 | 661 | – | 676 | 611.6396 | 1831.8971 | 1831.8952 | 1.04 | 1 | 35 | 0.00047 | 1Score **> 35** indicates **identity** Score **> 15** indicates **homology** | U | R.LPAKPEVSSDEDVQYR.V |
| 104682 | 661 | – | 676 | 916.9559 | 1831.8973 | 1831.8952 | 1.14 | 1 | 87 | 7.2e-09 | 1Score **> 35** indicates **identity** Score **> 18** indicates **homology** | U | R.LPAKPEVSSDEDVQYR.V |
| 104683 | 661 | – | 676 | 611.6399 | 1831.8980 | 1831.8952 | 1.53 | 1 | 80 | 2.9e-08 | 1Score **> 35** indicates **identity** Score **> 18** indicates **homology** | U | R.LPAKPEVSSDEDVQYR.V |
| 104685 | 661 | – | 676 | 916.9589 | 1831.9033 | 1831.8952 | 4.42 | 1 | 57 | 4.8e-06 | 1Score **> 35** indicates **identity** Score **> 16** indicates **homology** | U | R.LPAKPEVSSDEDVQYR.V |
| 143892 | 661 | – | 680 | 768.0728 | 2301.1967 | 2301.1965 | 0.11 | 2 | 20 | 0.013 | 1Score **> 37** indicates **identity** Score **> 14** indicates **homology** | U | R.LPAKPEVSSDEDVQYRVITR.F |
| 16668 | 720 | – | 728 | 520.7734 | 1039.5322 | 1039.5338 | -1.60 | 0 | 22 | 0.0089 | 1Score **> 31** indicates **identity** Score **> 14** indicates **homology** | U | R.FVDLYGAQK.V |
| 16670 | 720 | – | 728 | 520.7735 | 1039.5324 | 1039.5338 | -1.35 | 0 | 26 | 0.0038 | 1Score **> 31** indicates **identity** Score **> 14** indicates **homology** | U | R.FVDLYGAQK.V |
| 16671 | 720 | – | 728 | 520.7735 | 1039.5325 | 1039.5338 | -1.28 | 0 | 18 | 0.02 | 1Score **> 31** indicates **identity** Score **> 14** indicates **homology** | U | R.FVDLYGAQK.V |
| 16672 | 720 | – | 728 | 520.7736 | 1039.5326 | 1039.5338 | -1.24 | 0 | 30 | 0.0016 | 1Score **> 31** indicates **identity** Score **> 14** indicates **homology** | U | R.FVDLYGAQK.V |
| 16674 | 720 | – | 728 | 520.7739 | 1039.5332 | 1039.5338 | -0.58 | 0 | 16 | 0.031 | 1Score **> 31** indicates **identity** Score **> 14** indicates **homology** | U | R.FVDLYGAQK.V |
| 16675 | 720 | – | 728 | 520.7740 | 1039.5334 | 1039.5338 | -0.46 | 0 | 26 | 0.0034 | 1Score **> 31** indicates **identity** Score **> 14** indicates **homology** | U | R.FVDLYGAQK.V |
| 16676 | 720 | – | 728 | 520.7741 | 1039.5336 | 1039.5338 | -0.25 | 0 | 43 | 8.7e-05 | 1Score **> 31** indicates **identity** Score **> 15** indicates **homology** | U | R.FVDLYGAQK.V |
| 16678 | 720 | – | 728 | 520.7741 | 1039.5337 | 1039.5338 | -0.098 | 0 | 45 | 6.6e-05 | 1Score **> 30** indicates **identity** Score **> 15** indicates **homology** | U | R.FVDLYGAQK.V |
| 16680 | 720 | – | 728 | 520.7744 | 1039.5343 | 1039.5338 | 0.39 | 0 | 40 | 0.00019 | 1Score **> 30** indicates **identity** Score **> 15** indicates **homology** | U | R.FVDLYGAQK.V |
| 16681 | 720 | – | 728 | 520.7745 | 1039.5345 | 1039.5338 | 0.65 | 0 | 38 | 0.00027 | 1Score **> 30** indicates **identity** Score **> 15** indicates **homology** | U | R.FVDLYGAQK.V |
| 16682 | 720 | – | 728 | 520.7746 | 1039.5346 | 1039.5338 | 0.77 | 0 | 35 | 0.0005 | 1Score **> 30** indicates **identity** Score **> 15** indicates **homology** | U | R.FVDLYGAQK.V |
| 16683 | 720 | – | 728 | 520.7746 | 1039.5347 | 1039.5338 | 0.83 | 0 | 41 | 0.00013 | 1Score **> 31** indicates **identity** Score **> 15** indicates **homology** | U | R.FVDLYGAQK.V |
| 176106 | 733 | – | 759 | 786.1437 | 3140.5457 | 3140.5349 | 3.46 | 2 | 31 | 0.0012 | 1Score **> 37** indicates **identity** Score **> 14** indicates **homology** | U | R.LRKYESAYGTQFTPCQLLLDHANNSSK.K |
| 169576 | 735 | – | 759 | 958.1231 | 2871.3474 | 2871.3497 | -0.80 | 1 | 55 | 7.4e-06 | 1Score **> 35** indicates **identity** Score **> 16** indicates **homology** | U | R.KYESAYGTQFTPCQLLLDHANNSSK.K |
| 169577 | 735 | – | 759 | 718.8441 | 2871.3474 | 2871.3497 | -0.80 | 1 | 49 | 2.7e-05 | 1Score **> 35** indicates **identity** Score **> 16** indicates **homology** | U | R.KYESAYGTQFTPCQLLLDHANNSSK.K |
| 169578 | 735 | – | 759 | 718.8443 | 2871.3481 | 2871.3497 | -0.56 | 1 | 14 | 0.044 | 1Score **> 35** indicates **identity** Score **> 13** indicates **homology** | U | R.KYESAYGTQFTPCQLLLDHANNSSK.K |
| 169580 | 735 | – | 759 | 718.8450 | 2871.3511 | 2871.3497 | 0.47 | 1 | 66 | 7.2e-07 | 1Score **> 36** indicates **identity** Score **> 17** indicates **homology** | U | R.KYESAYGTQFTPCQLLLDHANNSSK.K |
| 169581 | 735 | – | 759 | 958.1244 | 2871.3513 | 2871.3497 | 0.56 | 1 | 97 | 8.7e-10 | 1Score **> 36** indicates **identity** Score **> 19** indicates **homology** | U | R.KYESAYGTQFTPCQLLLDHANNSSK.K |
| 169582 | 735 | – | 759 | 958.1245 | 2871.3517 | 2871.3497 | 0.68 | 1 | 103 | 2.4e-10 | 1Score **> 36** indicates **identity** Score **> 19** indicates **homology** | U | R.KYESAYGTQFTPCQLLLDHANNSSK.K |
| 169583 | 735 | – | 759 | 718.8453 | 2871.3520 | 2871.3497 | 0.81 | 1 | 71 | 2.1e-07 | 1Score **> 36** indicates **identity** Score **> 17** indicates **homology** | U | R.KYESAYGTQFTPCQLLLDHANNSSK.K |
| 169584 | 735 | – | 759 | 958.1247 | 2871.3521 | 2871.3497 | 0.84 | 1 | 101 | 3.2e-10 | 1Score **> 36** indicates **identity** Score **> 19** indicates **homology** | U | R.KYESAYGTQFTPCQLLLDHANNSSK.K |
| 169585 | 735 | – | 759 | 718.8454 | 2871.3524 | 2871.3497 | 0.92 | 1 | 16 | 0.033 | 1Score **> 36** indicates **identity** Score **> 13** indicates **homology** | U | R.KYESAYGTQFTPCQLLLDHANNSSK.K |
| 169586 | 735 | – | 759 | 718.8454 | 2871.3526 | 2871.3497 | 1.01 | 1 | 70 | 2.5e-07 | 1Score **> 36** indicates **identity** Score **> 17** indicates **homology** | U | R.KYESAYGTQFTPCQLLLDHANNSSK.K |
| 169607 | 735 | – | 759 | 719.0907 | 2872.3336 | 2872.3337 | -0.054 | 1 | 73 | 1.5e-07 | 1Score **> 35** indicates **identity** Score **> 17** indicates **homology** | U | R.KYESAYGTQFTPCQLLLDHANNSSK.K  + Deamidated (NQ) |
| 169608 | 735 | – | 759 | 719.0909 | 2872.3345 | 2872.3337 | 0.27 | 1 | 59 | 2.7e-06 | 1Score **> 35** indicates **identity** Score **> 16** indicates **homology** | U | R.KYESAYGTQFTPCQLLLDHANNSSK.K  + Deamidated (NQ) |
| 169609 | 735 | – | 759 | 958.4529 | 2872.3368 | 2872.3337 | 1.07 | 1 | 43 | 8.5e-05 | 1Score **> 35** indicates **identity** Score **> 15** indicates **homology** | U | R.KYESAYGTQFTPCQLLLDHANNSSK.K  + Deamidated (NQ) |
| 169610 | 735 | – | 759 | 958.4533 | 2872.3381 | 2872.3337 | 1.51 | 1 | 74 | 1.2e-07 | 1Score **> 35** indicates **identity** Score **> 17** indicates **homology** | U | R.KYESAYGTQFTPCQLLLDHANNSSK.K  + Deamidated (NQ) |
| 169611 | 735 | – | 759 | 719.0921 | 2872.3394 | 2872.3337 | 1.96 | 1 | 56 | 5.5e-06 | 1Score **> 35** indicates **identity** Score **> 16** indicates **homology** | U | R.KYESAYGTQFTPCQLLLDHANNSSK.K  + Deamidated (NQ) |
| 169612 | 735 | – | 759 | 958.4539 | 2872.3399 | 2872.3337 | 2.15 | 1 | 66 | 6.1e-07 | 1Score **> 35** indicates **identity** Score **> 17** indicates **homology** | U | R.KYESAYGTQFTPCQLLLDHANNSSK.K  + Deamidated (NQ) |
| 169613 | 735 | – | 759 | 958.4543 | 2872.3410 | 2872.3337 | 2.53 | 1 | 94 | 1.5e-09 | 1Score **> 35** indicates **identity** Score **> 18** indicates **homology** | U | R.KYESAYGTQFTPCQLLLDHANNSSK.K  + Deamidated (NQ) |
| 169616 | 735 | – | 759 | 958.4575 | 2872.3508 | 2872.3337 | 5.93 | 1 | 76 | 8.1e-08 | 1Score **> 35** indicates **identity** Score **> 17** indicates **homology** | U | R.KYESAYGTQFTPCQLLLDHANNSSK.K  + Deamidated (NQ) |
| 169617 | 735 | – | 759 | 958.4577 | 2872.3514 | 2872.3337 | 6.15 | 1 | 18 | 0.022 | 1Score **> 35** indicates **identity** Score **> 14** indicates **homology** | U | R.KYESAYGTQFTPCQLLLDHANNSSK.K  + Deamidated (NQ) |
| 169618 | 735 | – | 759 | 719.0959 | 2872.3544 | 2872.3337 | 7.21 | 1 | 41 | 0.00014 | 1Score **> 35** indicates **identity** Score **> 15** indicates **homology** | U | R.KYESAYGTQFTPCQLLLDHANNSSK.K  + Deamidated (NQ) |
| 174009 | 735 | – | 759 | 1010.8085 | 3029.4038 | 3029.4328 | -9.57 | 1 | 14 | 0.049 | 1Score **> 35** indicates **identity** Score **> 13** indicates **homology** | U | R.KYESAYGTQFTPCQLLLDHANNSSK.K  + 2 Deamidated (NQ); HNE (K) |
| 173358 | 735 | – | 760 | 750.8684 | 2999.4446 | 2999.4447 | -0.030 | 2 | 16 | 0.03 | 1Score **> 37** indicates **identity** Score **> 14** indicates **homology** | U | R.KYESAYGTQFTPCQLLLDHANNSSKK.F |
| 173359 | 735 | – | 760 | 750.8685 | 2999.4449 | 2999.4447 | 0.058 | 2 | 19 | 0.017 | 1Score **> 37** indicates **identity** Score **> 14** indicates **homology** | U | R.KYESAYGTQFTPCQLLLDHANNSSKK.F |
| 173360 | 735 | – | 760 | 600.8963 | 2999.4452 | 2999.4447 | 0.18 | 2 | 34 | 0.0006 | 1Score **> 37** indicates **identity** Score **> 15** indicates **homology** | U | R.KYESAYGTQFTPCQLLLDHANNSSKK.F |
| 173361 | 735 | – | 760 | 600.8966 | 2999.4465 | 2999.4447 | 0.61 | 2 | 38 | 0.00026 | 1Score **> 37** indicates **identity** Score **> 15** indicates **homology** | U | R.KYESAYGTQFTPCQLLLDHANNSSKK.F |
| 173362 | 735 | – | 760 | 750.8690 | 2999.4468 | 2999.4447 | 0.72 | 2 | 58 | 3.6e-06 | 1Score **> 37** indicates **identity** Score **> 16** indicates **homology** | U | R.KYESAYGTQFTPCQLLLDHANNSSKK.F |
| 173363 | 735 | – | 760 | 750.8691 | 2999.4471 | 2999.4447 | 0.81 | 2 | 64 | 1e-06 | 1Score **> 37** indicates **identity** Score **> 16** indicates **homology** | U | R.KYESAYGTQFTPCQLLLDHANNSSKK.F |
| 173364 | 735 | – | 760 | 1000.8232 | 2999.4476 | 2999.4447 | 0.99 | 2 | 52 | 1.4e-05 | 1Score **> 37** indicates **identity** Score **> 16** indicates **homology** | U | R.KYESAYGTQFTPCQLLLDHANNSSKK.F |
| 173365 | 735 | – | 760 | 600.8969 | 2999.4479 | 2999.4447 | 1.08 | 2 | 40 | 0.0002 | 1Score **> 37** indicates **identity** Score **> 15** indicates **homology** | U | R.KYESAYGTQFTPCQLLLDHANNSSKK.F |
| 173367 | 735 | – | 760 | 1000.8246 | 2999.4520 | 2999.4447 | 2.42 | 2 | 36 | 0.00042 | 1Score **> 37** indicates **identity** Score **> 15** indicates **homology** | U | R.KYESAYGTQFTPCQLLLDHANNSSKK.F |
| 173404 | 735 | – | 760 | 1001.1582 | 3000.4527 | 3000.4287 | 8.01 | 2 | 78 | 4.7e-08 | 1Score **> 37** indicates **identity** Score **> 17** indicates **homology** | U | R.KYESAYGTQFTPCQLLLDHANNSSKK.F  + Deamidated (NQ) |
| 165214 | 736 | – | 759 | 915.4259 | 2743.2558 | 2743.2548 | 0.36 | 0 | 49 | 2.6e-05 | 1Score **> 34** indicates **identity** Score **> 16** indicates **homology** | U | K.YESAYGTQFTPCQLLLDHANNSSK.K |
| 165215 | 736 | – | 759 | 915.4262 | 2743.2566 | 2743.2548 | 0.68 | 0 | 57 | 4.6e-06 | 1Score **> 34** indicates **identity** Score **> 16** indicates **homology** | U | K.YESAYGTQFTPCQLLLDHANNSSK.K |
| 165216 | 736 | – | 759 | 915.4273 | 2743.2601 | 2743.2548 | 1.94 | 0 | 47 | 3.5e-05 | 1Score **> 34** indicates **identity** Score **> 15** indicates **homology** | U | K.YESAYGTQFTPCQLLLDHANNSSK.K |
| 165261 | 736 | – | 759 | 916.0898 | 2745.2476 | 2745.2228 | 9.04 | 0 | 18 | 0.022 | 1Score **> 33** indicates **identity** Score **> 14** indicates **homology** | U | K.YESAYGTQFTPCQLLLDHANNSSK.K  + 2 Deamidated (NQ) |

---

```
ID   ECHA_MOUSE              Reviewed;         763 AA.
AC   Q8BMS1; Q3TCY3; Q5U5Y5; Q8QZU4;
DT   18-MAR-2008, integrated into UniProtKB/Swiss-Prot.
DT   01-MAR-2003, sequence version 1.
DT   28-JUN-2023, entry version 183.
DE   RecName: Full=Trifunctional enzyme subunit alpha, mitochondrial;
DE   AltName: Full=Monolysocardiolipin acyltransferase {ECO:0000250|UniProtKB:P40939};
DE            EC=2.3.1.- {ECO:0000250|UniProtKB:P40939};
DE   AltName: Full=TP-alpha;
DE   Includes:
DE     RecName: Full=Long-chain enoyl-CoA hydratase;
DE              EC=4.2.1.17 {ECO:0000250|UniProtKB:P40939};
DE   Includes:
DE     RecName: Full=Long chain 3-hydroxyacyl-CoA dehydrogenase;
DE              EC=1.1.1.211 {ECO:0000250|UniProtKB:P40939};
DE   Flags: Precursor;
GN   Name=Hadha;
OS   Mus musculus (Mouse).
OC   Eukaryota; Metazoa; Chordata; Craniata; Vertebrata; Euteleostomi; Mammalia;
OC   Eutheria; Euarchontoglires; Glires; Rodentia; Myomorpha; Muroidea; Muridae;
OC   Murinae; Mus; Mus.
OX   NCBI_TaxID=10090;
RN   [1]
RP   NUCLEOTIDE SEQUENCE [LARGE SCALE MRNA].
RC   STRAIN=C57BL/6J, and NOD; TISSUE=Skin;
RX   PubMed=16141072; DOI=10.1126/science.1112014;
RA   Carninci P., Kasukawa T., Katayama S., Gough J., Frith M.C., Maeda N.,
RA   Oyama R., Ravasi T., Lenhard B., Wells C., Kodzius R., Shimokawa K.,
RA   Bajic V.B., Brenner S.E., Batalov S., Forrest A.R., Zavolan M., Davis M.J.,
RA   Wilming L.G., Aidinis V., Allen J.E., Ambesi-Impiombato A., Apweiler R.,
RA   Aturaliya R.N., Bailey T.L., Bansal M., Baxter L., Beisel K.W., Bersano T.,
RA   Bono H., Chalk A.M., Chiu K.P., Choudhary V., Christoffels A.,
RA   Clutterbuck D.R., Crowe M.L., Dalla E., Dalrymple B.P., de Bono B.,
RA   Della Gatta G., di Bernardo D., Down T., Engstrom P., Fagiolini M.,
RA   Faulkner G., Fletcher C.F., Fukushima T., Furuno M., Futaki S.,
RA   Gariboldi M., Georgii-Hemming P., Gingeras T.R., Gojobori T., Green R.E.,
RA   Gustincich S., Harbers M., Hayashi Y., Hensch T.K., Hirokawa N., Hill D.,
RA   Huminiecki L., Iacono M., Ikeo K., Iwama A., Ishikawa T., Jakt M.,
RA   Kanapin A., Katoh M., Kawasawa Y., Kelso J., Kitamura H., Kitano H.,
RA   Kollias G., Krishnan S.P., Kruger A., Kummerfeld S.K., Kurochkin I.V.,
RA   Lareau L.F., Lazarevic D., Lipovich L., Liu J., Liuni S., McWilliam S.,
RA   Madan Babu M., Madera M., Marchionni L., Matsuda H., Matsuzawa S., Miki H.,
RA   Mignone F., Miyake S., Morris K., Mottagui-Tabar S., Mulder N., Nakano N.,
RA   Nakauchi H., Ng P., Nilsson R., Nishiguchi S., Nishikawa S., Nori F.,
RA   Ohara O., Okazaki Y., Orlando V., Pang K.C., Pavan W.J., Pavesi G.,
RA   Pesole G., Petrovsky N., Piazza S., Reed J., Reid J.F., Ring B.Z.,
RA   Ringwald M., Rost B., Ruan Y., Salzberg S.L., Sandelin A., Schneider C.,
RA   Schoenbach C., Sekiguchi K., Semple C.A., Seno S., Sessa L., Sheng Y.,
RA   Shibata Y., Shimada H., Shimada K., Silva D., Sinclair B., Sperling S.,
RA   Stupka E., Sugiura K., Sultana R., Takenaka Y., Taki K., Tammoja K.,
RA   Tan S.L., Tang S., Taylor M.S., Tegner J., Teichmann S.A., Ueda H.R.,
RA   van Nimwegen E., Verardo R., Wei C.L., Yagi K., Yamanishi H.,
RA   Zabarovsky E., Zhu S., Zimmer A., Hide W., Bult C., Grimmond S.M.,
RA   Teasdale R.D., Liu E.T., Brusic V., Quackenbush J., Wahlestedt C.,
RA   Mattick J.S., Hume D.A., Kai C., Sasaki D., Tomaru Y., Fukuda S.,
RA   Kanamori-Katayama M., Suzuki M., Aoki J., Arakawa T., Iida J., Imamura K.,
RA   Itoh M., Kato T., Kawaji H., Kawagashira N., Kawashima T., Kojima M.,
RA   Kondo S., Konno H., Nakano K., Ninomiya N., Nishio T., Okada M., Plessy C.,
RA   Shibata K., Shiraki T., Suzuki S., Tagami M., Waki K., Watahiki A.,
RA   Okamura-Oho Y., Suzuki H., Kawai J., Hayashizaki Y.;
RT   "The transcriptional landscape of the mammalian genome.";
RL   Science 309:1559-1563(2005).
RN   [2]
RP   NUCLEOTIDE SEQUENCE [LARGE SCALE MRNA].
RC   STRAIN=FVB/N; TISSUE=Eye, Liver, and Olfactory epithelium;
RX   PubMed=15489334; DOI=10.1101/gr.2596504;
RG   The MGC Project Team;
RT   "The status, quality, and expansion of the NIH full-length cDNA project:
RT   the Mammalian Gene Collection (MGC).";
RL   Genome Res. 14:2121-2127(2004).
RN   [3]
RP   ACETYLATION [LARGE SCALE ANALYSIS] AT LYS-129, AND IDENTIFICATION BY MASS
RP   SPECTROMETRY [LARGE SCALE ANALYSIS].
RC   TISSUE=Liver;
RX   PubMed=16916647; DOI=10.1016/j.molcel.2006.06.026;
RA   Kim S.C., Sprung R., Chen Y., Xu Y., Ball H., Pei J., Cheng T., Kho Y.,
RA   Xiao H., Xiao L., Grishin N.V., White M., Yang X.-J., Zhao Y.;
RT   "Substrate and functional diversity of lysine acetylation revealed by a
RT   proteomics survey.";
RL   Mol. Cell 23:607-618(2006).
RN   [4]
RP   PHOSPHORYLATION [LARGE SCALE ANALYSIS] AT SER-231; SER-316; THR-395 AND
RP   SER-647, AND IDENTIFICATION BY MASS SPECTROMETRY [LARGE SCALE ANALYSIS].
RC   TISSUE=Brain, Brown adipose tissue, Heart, Kidney, Liver, Lung,
RC   Pancreas, Spleen, and Testis;
RX   PubMed=21183079; DOI=10.1016/j.cell.2010.12.001;
RA   Huttlin E.L., Jedrychowski M.P., Elias J.E., Goswami T., Rad R.,
RA   Beausoleil S.A., Villen J., Haas W., Sowa M.E., Gygi S.P.;
RT   "A tissue-specific atlas of mouse protein phosphorylation and expression.";
RL   Cell 143:1174-1189(2010).
RN   [5]
RP   ACETYLATION [LARGE SCALE ANALYSIS] AT LYS-60 AND LYS-406, SUCCINYLATION
RP   [LARGE SCALE ANALYSIS] AT LYS-46; LYS-60; LYS-166; LYS-213; LYS-214;
RP   LYS-230; LYS-249; LYS-303; LYS-326; LYS-334; LYS-350; LYS-406; LYS-411;
RP   LYS-415; LYS-436; LYS-440; LYS-460; LYS-505; LYS-519; LYS-569; LYS-620;
RP   LYS-634; LYS-644; LYS-646; LYS-664; LYS-728 AND LYS-759, AND IDENTIFICATION
RP   BY MASS SPECTROMETRY [LARGE SCALE ANALYSIS].
RC   TISSUE=Embryonic fibroblast, and Liver;
RX   PubMed=23806337; DOI=10.1016/j.molcel.2013.06.001;
RA   Park J., Chen Y., Tishkoff D.X., Peng C., Tan M., Dai L., Xie Z., Zhang Y.,
RA   Zwaans B.M., Skinner M.E., Lombard D.B., Zhao Y.;
RT   "SIRT5-mediated lysine desuccinylation impacts diverse metabolic
RT   pathways.";
RL   Mol. Cell 50:919-930(2013).
RN   [6]
RP   ACETYLATION [LARGE SCALE ANALYSIS] AT LYS-46; LYS-60; LYS-129; LYS-166;
RP   LYS-214; LYS-249; LYS-289; LYS-303; LYS-326; LYS-334; LYS-350; LYS-353;
RP   LYS-406; LYS-411; LYS-436; LYS-460; LYS-505; LYS-519; LYS-540; LYS-569;
RP   LYS-644; LYS-664; LYS-728; LYS-735 AND LYS-759, AND IDENTIFICATION BY MASS
RP   SPECTROMETRY [LARGE SCALE ANALYSIS].
RC   TISSUE=Liver;
RX   PubMed=23576753; DOI=10.1073/pnas.1302961110;
RA   Rardin M.J., Newman J.C., Held J.M., Cusack M.P., Sorensen D.J., Li B.,
RA   Schilling B., Mooney S.D., Kahn C.R., Verdin E., Gibson B.W.;
RT   "Label-free quantitative proteomics of the lysine acetylome in mitochondria
RT   identifies substrates of SIRT3 in metabolic pathways.";
RL   Proc. Natl. Acad. Sci. U.S.A. 110:6601-6606(2013).
RN   [7]
RP   METHYLATION [LARGE SCALE ANALYSIS] AT ARG-399, AND IDENTIFICATION BY MASS
RP   SPECTROMETRY [LARGE SCALE ANALYSIS].
RC   TISSUE=Brain;
RX   PubMed=24129315; DOI=10.1074/mcp.o113.027870;
RA   Guo A., Gu H., Zhou J., Mulhern D., Wang Y., Lee K.A., Yang V., Aguiar M.,
RA   Kornhauser J., Jia X., Ren J., Beausoleil S.A., Silva J.C., Vemulapalli V.,
RA   Bedford M.T., Comb M.J.;
RT   "Immunoaffinity enrichment and mass spectrometry analysis of protein
RT   methylation.";
RL   Mol. Cell. Proteomics 13:372-387(2014).
RN   [8]
RP   INTERACTION WITH MTLN, AND IDENTIFICATION BY MASS SPECTROMETRY.
RX   PubMed=29949755; DOI=10.1016/j.celrep.2018.05.058;
RA   Makarewich C.A., Baskin K.K., Munir A.Z., Bezprozvannaya S., Sharma G.,
RA   Khemtong C., Shah A.M., McAnally J.R., Malloy C.R., Szweda L.I.,
RA   Bassel-Duby R., Olson E.N.;
RT   "MOXI Is a Mitochondrial Micropeptide That Enhances Fatty Acid beta-
RT   Oxidation.";
RL   Cell Rep. 23:3701-3709(2018).
CC   -!- FUNCTION: Mitochondrial trifunctional enzyme catalyzes the last three
CC       of the four reactions of the mitochondrial beta-oxidation pathway. The
CC       mitochondrial beta-oxidation pathway is the major energy-producing
CC       process in tissues and is performed through four consecutive reactions
CC       breaking down fatty acids into acetyl-CoA. Among the enzymes involved
CC       in this pathway, the trifunctional enzyme exhibits specificity for
CC       long-chain fatty acids. Mitochondrial trifunctional enzyme is a
CC       heterotetrameric complex composed of two proteins, the trifunctional
CC       enzyme subunit alpha/HADHA described here carries the 2,3-enoyl-CoA
CC       hydratase and the 3-hydroxyacyl-CoA dehydrogenase activities while the
CC       trifunctional enzyme subunit beta/HADHB bears the 3-ketoacyl-CoA
CC       thiolase activity. Independently of the subunit beta, the trifunctional
CC       enzyme subunit alpha/HADHA also has a monolysocardiolipin
CC       acyltransferase activity. It acylates monolysocardiolipin into
CC       cardiolipin, a major mitochondrial membrane phospholipid which plays a
CC       key role in apoptosis and supports mitochondrial respiratory chain
CC       complexes in the generation of ATP. Allows the acylation of
CC       monolysocardiolipin with different acyl-CoA substrates including
CC       oleoyl-CoA for which it displays the highest activity.
CC       {ECO:0000250|UniProtKB:P40939}.
CC   -!- CATALYTIC ACTIVITY:
CC       Reaction=a (3S)-3-hydroxyacyl-CoA = a (2E)-enoyl-CoA + H2O;
CC         Xref=Rhea:RHEA:16105, ChEBI:CHEBI:15377, ChEBI:CHEBI:57318,
CC         ChEBI:CHEBI:58856; EC=4.2.1.17;
CC         Evidence={ECO:0000250|UniProtKB:P40939};
CC       PhysiologicalDirection=right-to-left; Xref=Rhea:RHEA:16107;
CC         Evidence={ECO:0000250|UniProtKB:P40939};
CC   -!- CATALYTIC ACTIVITY:
CC       Reaction=a 4-saturated-(3S)-3-hydroxyacyl-CoA = a (3E)-enoyl-CoA + H2O;
CC         Xref=Rhea:RHEA:20724, ChEBI:CHEBI:15377, ChEBI:CHEBI:58521,
CC         ChEBI:CHEBI:137480; EC=4.2.1.17;
CC         Evidence={ECO:0000250|UniProtKB:P40939};
CC       PhysiologicalDirection=right-to-left; Xref=Rhea:RHEA:20726;
CC         Evidence={ECO:0000250|UniProtKB:P40939};
CC   -!- CATALYTIC ACTIVITY:
CC       Reaction=(3S)-hydroxyoctanoyl-CoA = (2E)-octenoyl-CoA + H2O;
CC         Xref=Rhea:RHEA:31199, ChEBI:CHEBI:15377, ChEBI:CHEBI:62242,
CC         ChEBI:CHEBI:62617; Evidence={ECO:0000250|UniProtKB:P40939};
CC       PhysiologicalDirection=right-to-left; Xref=Rhea:RHEA:31201;
CC         Evidence={ECO:0000250|UniProtKB:P40939};
CC   -!- CATALYTIC ACTIVITY:
CC       Reaction=(3S)-3-hydroxydodecanoyl-CoA = (2E)-dodecenoyl-CoA + H2O;
CC         Xref=Rhea:RHEA:31075, ChEBI:CHEBI:15377, ChEBI:CHEBI:57330,
CC         ChEBI:CHEBI:62558; Evidence={ECO:0000250|UniProtKB:P40939};
CC       PhysiologicalDirection=right-to-left; Xref=Rhea:RHEA:31077;
CC         Evidence={ECO:0000250|UniProtKB:P40939};
CC   -!- CATALYTIC ACTIVITY:
CC       Reaction=(3S)-hydroxyhexadecanoyl-CoA = (2E)-hexadecenoyl-CoA + H2O;
CC         Xref=Rhea:RHEA:31163, ChEBI:CHEBI:15377, ChEBI:CHEBI:61526,
CC         ChEBI:CHEBI:62613; Evidence={ECO:0000250|UniProtKB:P40939};
CC       PhysiologicalDirection=right-to-left; Xref=Rhea:RHEA:31165;
CC         Evidence={ECO:0000250|UniProtKB:P40939};
CC   -!- CATALYTIC ACTIVITY:
CC       Reaction=a long-chain (3S)-3-hydroxy fatty acyl-CoA + NAD(+) = a long-
CC         chain 3-oxo-fatty acyl-CoA + H(+) + NADH; Xref=Rhea:RHEA:52656,
CC         ChEBI:CHEBI:15378, ChEBI:CHEBI:57540, ChEBI:CHEBI:57945,
CC         ChEBI:CHEBI:136757, ChEBI:CHEBI:136758; EC=1.1.1.211;
CC         Evidence={ECO:0000250|UniProtKB:P40939};
CC       PhysiologicalDirection=left-to-right; Xref=Rhea:RHEA:52657;
CC         Evidence={ECO:0000250|UniProtKB:P40939};
CC   -!- CATALYTIC ACTIVITY:
CC       Reaction=(3S)-hydroxyoctanoyl-CoA + NAD(+) = 3-oxooctanoyl-CoA + H(+) +
CC         NADH; Xref=Rhea:RHEA:31195, ChEBI:CHEBI:15378, ChEBI:CHEBI:57540,
CC         ChEBI:CHEBI:57945, ChEBI:CHEBI:62617, ChEBI:CHEBI:62619;
CC         Evidence={ECO:0000250|UniProtKB:P40939};
CC       PhysiologicalDirection=left-to-right; Xref=Rhea:RHEA:31196;
CC         Evidence={ECO:0000250|UniProtKB:P40939};
CC   -!- CATALYTIC ACTIVITY:
CC       Reaction=(3S)-hydroxydecanoyl-CoA + NAD(+) = 3-oxodecanoyl-CoA + H(+) +
CC         NADH; Xref=Rhea:RHEA:31187, ChEBI:CHEBI:15378, ChEBI:CHEBI:57540,
CC         ChEBI:CHEBI:57945, ChEBI:CHEBI:62548, ChEBI:CHEBI:62616;
CC         Evidence={ECO:0000250|UniProtKB:P40939};
CC       PhysiologicalDirection=left-to-right; Xref=Rhea:RHEA:31188;
CC         Evidence={ECO:0000250|UniProtKB:P40939};
CC   -!- CATALYTIC ACTIVITY:
CC       Reaction=(3S)-3-hydroxydodecanoyl-CoA + NAD(+) = 3-oxododecanoyl-CoA +
CC         H(+) + NADH; Xref=Rhea:RHEA:31179, ChEBI:CHEBI:15378,
CC         ChEBI:CHEBI:57540, ChEBI:CHEBI:57945, ChEBI:CHEBI:62558,
CC         ChEBI:CHEBI:62615; Evidence={ECO:0000250|UniProtKB:P40939};
CC       PhysiologicalDirection=left-to-right; Xref=Rhea:RHEA:31180;
CC         Evidence={ECO:0000250|UniProtKB:P40939};
CC   -!- CATALYTIC ACTIVITY:
CC       Reaction=(3S)-hydroxytetradecanoyl-CoA + NAD(+) = 3-oxotetradecanoyl-
CC         CoA + H(+) + NADH; Xref=Rhea:RHEA:31167, ChEBI:CHEBI:15378,
CC         ChEBI:CHEBI:57540, ChEBI:CHEBI:57945, ChEBI:CHEBI:62543,
CC         ChEBI:CHEBI:62614; Evidence={ECO:0000250|UniProtKB:P40939};
CC       PhysiologicalDirection=left-to-right; Xref=Rhea:RHEA:31168;
CC         Evidence={ECO:0000250|UniProtKB:P40939};
CC   -!- CATALYTIC ACTIVITY:
CC       Reaction=(3S)-hydroxyhexadecanoyl-CoA + NAD(+) = 3-oxohexadecanoyl-CoA
CC         + H(+) + NADH; Xref=Rhea:RHEA:31159, ChEBI:CHEBI:15378,
CC         ChEBI:CHEBI:57349, ChEBI:CHEBI:57540, ChEBI:CHEBI:57945,
CC         ChEBI:CHEBI:62613; Evidence={ECO:0000250|UniProtKB:P40939};
CC       PhysiologicalDirection=left-to-right; Xref=Rhea:RHEA:31160;
CC         Evidence={ECO:0000250|UniProtKB:P40939};
CC   -!- CATALYTIC ACTIVITY:
CC       Reaction=1'-[1,2-di-(9Z,12Z-octadecadienoyl)-sn-glycero-3-phospho]-3'-
CC         [1-(9Z,12Z-octadecadienoyl)-sn-glycero-3-phospho]-glycerol +
CC         hexadecanoyl-CoA = 1'-[1,2-di-(9Z,12Z-octadecadienoyl)-sn-glycero-3-
CC         phospho]-3'-[1-(9Z,12Z-octadecadienoyl)-2-hexadecanoyl-sn-glycero-3-
CC         phospho]-glycerol + CoA; Xref=Rhea:RHEA:43680, ChEBI:CHEBI:57287,
CC         ChEBI:CHEBI:57379, ChEBI:CHEBI:83580, ChEBI:CHEBI:83583;
CC         Evidence={ECO:0000250|UniProtKB:P40939};
CC       PhysiologicalDirection=left-to-right; Xref=Rhea:RHEA:43681;
CC         Evidence={ECO:0000250|UniProtKB:P40939};
CC   -!- CATALYTIC ACTIVITY:
CC       Reaction=(9Z)-octadecenoyl-CoA + 1'-[1,2-di-(9Z,12Z-octadecadienoyl)-
CC         sn-glycero-3-phospho]-3'-[1-(9Z,12Z-octadecadienoyl)-sn-glycero-3-
CC         phospho]-glycerol = 1'-[1,2-di-(9Z,12Z-octadecadienoyl)-sn-glycero-3-
CC         phospho]-3'-[1-(9Z,12Z-octadecadienoyl)-2-(9Z-octadecenoyl)-sn-
CC         glycero-3-phospho]-glycerol + CoA; Xref=Rhea:RHEA:43676,
CC         ChEBI:CHEBI:57287, ChEBI:CHEBI:57387, ChEBI:CHEBI:83580,
CC         ChEBI:CHEBI:83582; Evidence={ECO:0000250|UniProtKB:P40939};
CC       PhysiologicalDirection=left-to-right; Xref=Rhea:RHEA:43677;
CC         Evidence={ECO:0000250|UniProtKB:P40939};
CC   -!- CATALYTIC ACTIVITY:
CC       Reaction=(9Z,12Z)-octadecadienoyl-CoA + 1'-[1,2-di-(9Z,12Z-
CC         octadecadienoyl)-sn-glycero-3-phospho]-3'-[1-(9Z,12Z-
CC         octadecadienoyl)-sn-glycero-3-phospho]-glycerol = 1',3'-bis-[1,2-di-
CC         (9Z,12Z-octadecadienoyl)-sn-glycero-3-phospho]-glycerol + CoA;
CC         Xref=Rhea:RHEA:43672, ChEBI:CHEBI:57287, ChEBI:CHEBI:57383,
CC         ChEBI:CHEBI:83580, ChEBI:CHEBI:83581;
CC         Evidence={ECO:0000250|UniProtKB:P40939};
CC       PhysiologicalDirection=left-to-right; Xref=Rhea:RHEA:43673;
CC         Evidence={ECO:0000250|UniProtKB:P40939};
CC   -!- PATHWAY: Lipid metabolism; fatty acid beta-oxidation.
CC       {ECO:0000250|UniProtKB:P40939}.
CC   -!- SUBUNIT: Heterotetramer of 2 alpha/HADHA and 2 beta/HADHB subunits;
CC       forms the mitochondrial trifunctional enzyme (By similarity). Also
CC       purified as higher order heterooligomers including a 4 alpha/HADHA and
CC       4 beta/HADHB heterooligomer which physiological significance remains
CC       unclear (By similarity). The mitochondrial trifunctional enzyme
CC       interacts with MTLN (PubMed:29949755). {ECO:0000250|UniProtKB:P40939,
CC       ECO:0000269|PubMed:29949755}.
CC   -!- SUBCELLULAR LOCATION: Mitochondrion {ECO:0000250|UniProtKB:P40939}.
CC       Mitochondrion inner membrane {ECO:0000250|UniProtKB:P40939}.
CC       Note=Protein stability and association with mitochondrion inner
CC       membrane do not require HADHB. {ECO:0000250|UniProtKB:P40939}.
CC   -!- PTM: Acetylation of Lys-569 and Lys-728 is observed in liver
CC       mitochondria from fasted mice but not from fed mice.
CC   -!- SIMILARITY: In the N-terminal section; belongs to the enoyl-CoA
CC       hydratase/isomerase family. {ECO:0000305}.
CC   -!- SIMILARITY: In the central section; belongs to the 3-hydroxyacyl-CoA
CC       dehydrogenase family. {ECO:0000305}.
CC   ---------------------------------------------------------------------------
CC   Copyrighted by the UniProt Consortium, see https://www.uniprot.org/terms
CC   Distributed under the Creative Commons Attribution (CC BY 4.0) License
CC   ---------------------------------------------------------------------------
DR   EMBL; AK029017; BAC26245.1; -; mRNA.
DR   EMBL; AK170478; BAE41822.1; -; mRNA.
DR   EMBL; AK170683; BAE41956.1; -; mRNA.
DR   EMBL; BC027156; AAH27156.1; -; mRNA.
DR   EMBL; BC037009; AAH37009.1; -; mRNA.
DR   EMBL; BC046978; AAH46978.1; -; mRNA.
DR   EMBL; BC058569; AAH58569.1; -; mRNA.
DR   CCDS; CCDS19155.1; -.
DR   RefSeq; NP_849209.1; NM_178878.2.
DR   AlphaFoldDB; Q8BMS1; -.
DR   SMR; Q8BMS1; -.
DR   BioGRID; 220648; 43.
DR   IntAct; Q8BMS1; 8.
DR   MINT; Q8BMS1; -.
DR   STRING; 10090.ENSMUSP00000120976; -.
DR   GlyGen; Q8BMS1; 1 site, 1 O-linked glycan (1 site).
DR   iPTMnet; Q8BMS1; -.
DR   PhosphoSitePlus; Q8BMS1; -.
DR   SwissPalm; Q8BMS1; -.
DR   REPRODUCTION-2DPAGE; IPI00223092; -.
DR   EPD; Q8BMS1; -.
DR   jPOST; Q8BMS1; -.
DR   MaxQB; Q8BMS1; -.
DR   PaxDb; Q8BMS1; -.
DR   PeptideAtlas; Q8BMS1; -.
DR   ProteomicsDB; 277753; -.
DR   Antibodypedia; 3074; 238 antibodies from 32 providers.
DR   DNASU; 97212; -.
DR   Ensembl; ENSMUST00000156859; ENSMUSP00000120976; ENSMUSG00000025745.
DR   GeneID; 97212; -.
DR   KEGG; mmu:97212; -.
DR   UCSC; uc008wvc.1; mouse.
DR   AGR; MGI:2135593; -.
DR   CTD; 3030; -.
DR   MGI; MGI:2135593; Hadha.
DR   VEuPathDB; HostDB:ENSMUSG00000025745; -.
DR   eggNOG; KOG1683; Eukaryota.
DR   GeneTree; ENSGT00940000154677; -.
DR   HOGENOM; CLU_009834_16_1_1; -.
DR   InParanoid; Q8BMS1; -.
DR   OMA; PFRYMDT; -.
DR   OrthoDB; 622692at2759; -.
DR   PhylomeDB; Q8BMS1; -.
DR   TreeFam; TF352288; -.
DR   Reactome; R-MMU-1482798; Acyl chain remodeling of CL.
DR   Reactome; R-MMU-77285; Beta oxidation of myristoyl-CoA to lauroyl-CoA.
DR   Reactome; R-MMU-77305; Beta oxidation of palmitoyl-CoA to myristoyl-CoA.
DR   Reactome; R-MMU-77310; Beta oxidation of lauroyl-CoA to decanoyl-CoA-CoA.
DR   Reactome; R-MMU-77346; Beta oxidation of decanoyl-CoA to octanoyl-CoA-CoA.
DR   Reactome; R-MMU-77348; Beta oxidation of octanoyl-CoA to hexanoyl-CoA.
DR   Reactome; R-MMU-77350; Beta oxidation of hexanoyl-CoA to butanoyl-CoA.
DR   UniPathway; UPA00659; -.
DR   BioGRID-ORCS; 97212; 4 hits in 79 CRISPR screens.
DR   ChiTaRS; Hadha; mouse.
DR   PRO; PR:Q8BMS1; -.
DR   Proteomes; UP000000589; Chromosome 5.
DR   RNAct; Q8BMS1; protein.
DR   Bgee; ENSMUSG00000025745; Expressed in myocardium of ventricle and 247 other tissues.
DR   Genevisible; Q8BMS1; MM.
DR   GO; GO:0016507; C:mitochondrial fatty acid beta-oxidation multienzyme complex; ISO:MGI.
DR   GO; GO:0005743; C:mitochondrial inner membrane; HDA:MGI.
DR   GO; GO:0042645; C:mitochondrial nucleoid; ISO:MGI.
DR   GO; GO:0005739; C:mitochondrion; HDA:MGI.
DR   GO; GO:0003857; F:3-hydroxyacyl-CoA dehydrogenase activity; ISO:MGI.
DR   GO; GO:0003988; F:acetyl-CoA C-acyltransferase activity; ISO:MGI.
DR   GO; GO:0004300; F:enoyl-CoA hydratase activity; ISO:MGI.
DR   GO; GO:0000062; F:fatty-acyl-CoA binding; ISO:MGI.
DR   GO; GO:0016509; F:long-chain-3-hydroxyacyl-CoA dehydrogenase activity; IDA:MGI.
DR   GO; GO:0051287; F:NAD binding; ISO:MGI.
DR   GO; GO:0070403; F:NAD+ binding; IEA:InterPro.
DR   GO; GO:0044877; F:protein-containing complex binding; ISO:MGI.
DR   GO; GO:0035965; P:cardiolipin acyl-chain remodeling; ISS:UniProtKB.
DR   GO; GO:0006635; P:fatty acid beta-oxidation; IMP:MGI.
DR   GO; GO:0032868; P:response to insulin; IMP:MGI.
DR   GO; GO:0009410; P:response to xenobiotic stimulus; ISO:MGI.
DR   CDD; cd06558; crotonase-like; 1.
DR   Gene3D; 1.10.1040.50; -; 1.
DR   Gene3D; 3.40.50.720; NAD(P)-binding Rossmann-like Domain; 1.
DR   InterPro; IPR006180; 3-OHacyl-CoA_DH_CS.
DR   InterPro; IPR006176; 3-OHacyl-CoA_DH_NAD-bd.
DR   InterPro; IPR006108; 3HC_DH_C.
DR   InterPro; IPR008927; 6-PGluconate_DH-like_C_sf.
DR   InterPro; IPR029045; ClpP/crotonase-like_dom_sf.
DR   InterPro; IPR018376; Enoyl-CoA_hyd/isom_CS.
DR   InterPro; IPR001753; Enoyl-CoA_hydra/iso.
DR   InterPro; IPR012803; Fa_ox_alpha_mit.
DR   InterPro; IPR036291; NAD(P)-bd_dom_sf.
DR   PANTHER; PTHR43612; TRIFUNCTIONAL ENZYME SUBUNIT ALPHA; 1.
DR   PANTHER; PTHR43612:SF3; TRIFUNCTIONAL ENZYME SUBUNIT ALPHA, MITOCHONDRIAL; 1.
DR   Pfam; PF00725; 3HCDH; 2.
DR   Pfam; PF02737; 3HCDH_N; 1.
DR   Pfam; PF00378; ECH_1; 1.
DR   SUPFAM; SSF48179; 6-phosphogluconate dehydrogenase C-terminal domain-like; 2.
DR   SUPFAM; SSF52096; ClpP/crotonase; 1.
DR   SUPFAM; SSF51735; NAD(P)-binding Rossmann-fold domains; 1.
DR   PROSITE; PS00067; 3HCDH; 1.
DR   PROSITE; PS00166; ENOYL_COA_HYDRATASE; 1.
DR   TIGRFAMs; TIGR02441; fa_ox_alpha_mit; 1.
PE   1: Evidence at protein level;
KW   Acetylation; Fatty acid metabolism; Lipid metabolism; Lyase; Membrane;
KW   Methylation; Mitochondrion; Mitochondrion inner membrane;
KW   Multifunctional enzyme; NAD; Oxidoreductase; Phosphoprotein;
KW   Reference proteome; Transferase; Transit peptide.
FT   TRANSIT         1..36
FT                   /note="Mitochondrion"
FT                   /evidence="ECO:0000255"
FT   CHAIN           37..763
FT                   /note="Trifunctional enzyme subunit alpha, mitochondrial"
FT                   /id="PRO_0000322639"
FT   ACT_SITE        510
FT                   /note="For hydroxyacyl-coenzyme A dehydrogenase activity"
FT                   /evidence="ECO:0000250|UniProtKB:P40939"
FT   SITE            151
FT                   /note="Important for long-chain enoyl-CoA hydratase
FT                   activity"
FT                   /evidence="ECO:0000250|UniProtKB:P40939"
FT   SITE            173
FT                   /note="Important for long-chain enoyl-CoA hydratase
FT                   activity"
FT                   /evidence="ECO:0000250|UniProtKB:P40939"
FT   SITE            498
FT                   /note="Important for hydroxyacyl-coenzyme A dehydrogenase
FT                   activity"
FT                   /evidence="ECO:0000250|UniProtKB:P40939"
FT   MOD_RES         46
FT                   /note="N6-acetyllysine; alternate"
FT                   /evidence="ECO:0007744|PubMed:23576753"
FT   MOD_RES         46
FT                   /note="N6-succinyllysine; alternate"
FT                   /evidence="ECO:0007744|PubMed:23806337"
FT   MOD_RES         60
FT                   /note="N6-acetyllysine; alternate"
FT                   /evidence="ECO:0007744|PubMed:23576753,
FT                   ECO:0007744|PubMed:23806337"
FT   MOD_RES         60
FT                   /note="N6-succinyllysine; alternate"
FT                   /evidence="ECO:0007744|PubMed:23806337"
FT   MOD_RES         129
FT                   /note="N6-acetyllysine"
FT                   /evidence="ECO:0007744|PubMed:16916647,
FT                   ECO:0007744|PubMed:23576753"
FT   MOD_RES         166
FT                   /note="N6-acetyllysine; alternate"
FT                   /evidence="ECO:0007744|PubMed:23576753"
FT   MOD_RES         166
FT                   /note="N6-succinyllysine; alternate"
FT                   /evidence="ECO:0007744|PubMed:23806337"
FT   MOD_RES         213
FT                   /note="N6-succinyllysine"
FT                   /evidence="ECO:0007744|PubMed:23806337"
FT   MOD_RES         214
FT                   /note="N6-acetyllysine; alternate"
FT                   /evidence="ECO:0007744|PubMed:23576753"
FT   MOD_RES         214
FT                   /note="N6-succinyllysine; alternate"
FT                   /evidence="ECO:0007744|PubMed:23806337"
FT   MOD_RES         230
FT                   /note="N6-succinyllysine"
FT                   /evidence="ECO:0007744|PubMed:23806337"
FT   MOD_RES         231
FT                   /note="Phosphoserine"
FT                   /evidence="ECO:0007744|PubMed:21183079"
FT   MOD_RES         249
FT                   /note="N6-acetyllysine; alternate"
FT                   /evidence="ECO:0007744|PubMed:23576753"
FT   MOD_RES         249
FT                   /note="N6-succinyllysine; alternate"
FT                   /evidence="ECO:0007744|PubMed:23806337"
FT   MOD_RES         289
FT                   /note="N6-acetyllysine"
FT                   /evidence="ECO:0007744|PubMed:23576753"
FT   MOD_RES         295
FT                   /note="N6-acetyllysine"
FT                   /evidence="ECO:0000250|UniProtKB:P40939"
FT   MOD_RES         303
FT                   /note="N6-acetyllysine; alternate"
FT                   /evidence="ECO:0007744|PubMed:23576753"
FT   MOD_RES         303
FT                   /note="N6-succinyllysine; alternate"
FT                   /evidence="ECO:0007744|PubMed:23806337"
FT   MOD_RES         316
FT                   /note="Phosphoserine"
FT                   /evidence="ECO:0007744|PubMed:21183079"
FT   MOD_RES         326
FT                   /note="N6-acetyllysine; alternate"
FT                   /evidence="ECO:0007744|PubMed:23576753"
FT   MOD_RES         326
FT                   /note="N6-succinyllysine; alternate"
FT                   /evidence="ECO:0007744|PubMed:23806337"
FT   MOD_RES         334
FT                   /note="N6-acetyllysine; alternate"
FT                   /evidence="ECO:0007744|PubMed:23576753"
FT   MOD_RES         334
FT                   /note="N6-succinyllysine; alternate"
FT                   /evidence="ECO:0007744|PubMed:23806337"
FT   MOD_RES         350
FT                   /note="N6-acetyllysine; alternate"
FT                   /evidence="ECO:0007744|PubMed:23576753"
FT   MOD_RES         350
FT                   /note="N6-succinyllysine; alternate"
FT                   /evidence="ECO:0007744|PubMed:23806337"
FT   MOD_RES         353
FT                   /note="N6-acetyllysine"
FT                   /evidence="ECO:0007744|PubMed:23576753"
FT   MOD_RES         395
FT                   /note="Phosphothreonine"
FT                   /evidence="ECO:0007744|PubMed:21183079"
FT   MOD_RES         399
FT                   /note="Omega-N-methylarginine"
FT                   /evidence="ECO:0007744|PubMed:24129315"
FT   MOD_RES         406
FT                   /note="N6-acetyllysine; alternate"
FT                   /evidence="ECO:0007744|PubMed:23576753,
FT                   ECO:0007744|PubMed:23806337"
FT   MOD_RES         406
FT                   /note="N6-succinyllysine; alternate"
FT                   /evidence="ECO:0007744|PubMed:23806337"
FT   MOD_RES         411
FT                   /note="N6-acetyllysine; alternate"
FT                   /evidence="ECO:0007744|PubMed:23576753"
FT   MOD_RES         411
FT                   /note="N6-succinyllysine; alternate"
FT                   /evidence="ECO:0007744|PubMed:23806337"
FT   MOD_RES         415
FT                   /note="N6-succinyllysine"
FT                   /evidence="ECO:0007744|PubMed:23806337"
FT   MOD_RES         419
FT                   /note="Phosphoserine"
FT                   /evidence="ECO:0000250|UniProtKB:Q64428"
FT   MOD_RES         436
FT                   /note="N6-acetyllysine; alternate"
FT                   /evidence="ECO:0007744|PubMed:23576753"
FT   MOD_RES         436
FT                   /note="N6-succinyllysine; alternate"
FT                   /evidence="ECO:0007744|PubMed:23806337"
FT   MOD_RES         440
FT                   /note="N6-succinyllysine"
FT                   /evidence="ECO:0007744|PubMed:23806337"
FT   MOD_RES         460
FT                   /note="N6-acetyllysine; alternate"
FT                   /evidence="ECO:0007744|PubMed:23576753"
FT   MOD_RES         460
FT                   /note="N6-succinyllysine; alternate"
FT                   /evidence="ECO:0007744|PubMed:23806337"
FT   MOD_RES         505
FT                   /note="N6-acetyllysine; alternate"
FT                   /evidence="ECO:0007744|PubMed:23576753"
FT   MOD_RES         505
FT                   /note="N6-succinyllysine; alternate"
FT                   /evidence="ECO:0007744|PubMed:23806337"
FT   MOD_RES         519
FT                   /note="N6-acetyllysine; alternate"
FT                   /evidence="ECO:0007744|PubMed:23576753"
FT   MOD_RES         519
FT                   /note="N6-succinyllysine; alternate"
FT                   /evidence="ECO:0007744|PubMed:23806337"
FT   MOD_RES         540
FT                   /note="N6-acetyllysine"
FT                   /evidence="ECO:0007744|PubMed:23576753"
FT   MOD_RES         569
FT                   /note="N6-acetyllysine; alternate"
FT                   /evidence="ECO:0007744|PubMed:23576753"
FT   MOD_RES         569
FT                   /note="N6-succinyllysine; alternate"
FT                   /evidence="ECO:0007744|PubMed:23806337"
FT   MOD_RES         620
FT                   /note="N6-succinyllysine"
FT                   /evidence="ECO:0007744|PubMed:23806337"
FT   MOD_RES         634
FT                   /note="N6-succinyllysine"
FT                   /evidence="ECO:0007744|PubMed:23806337"
FT   MOD_RES         644
FT                   /note="N6-acetyllysine; alternate"
FT                   /evidence="ECO:0007744|PubMed:23576753"
FT   MOD_RES         644
FT                   /note="N6-succinyllysine; alternate"
FT                   /evidence="ECO:0007744|PubMed:23806337"
FT   MOD_RES         646
FT                   /note="N6-succinyllysine"
FT                   /evidence="ECO:0007744|PubMed:23806337"
FT   MOD_RES         647
FT                   /note="Phosphoserine"
FT                   /evidence="ECO:0007744|PubMed:21183079"
FT   MOD_RES         650
FT                   /note="Phosphoserine"
FT                   /evidence="ECO:0000250|UniProtKB:Q64428"
FT   MOD_RES         664
FT                   /note="N6-acetyllysine; alternate"
FT                   /evidence="ECO:0007744|PubMed:23576753"
FT   MOD_RES         664
FT                   /note="N6-succinyllysine; alternate"
FT                   /evidence="ECO:0007744|PubMed:23806337"
FT   MOD_RES         728
FT                   /note="N6-acetyllysine; alternate"
FT                   /evidence="ECO:0007744|PubMed:23576753"
FT   MOD_RES         728
FT                   /note="N6-succinyllysine; alternate"
FT                   /evidence="ECO:0007744|PubMed:23806337"
FT   MOD_RES         735
FT                   /note="N6-acetyllysine"
FT                   /evidence="ECO:0007744|PubMed:23576753"
FT   MOD_RES         759
FT                   /note="N6-acetyllysine; alternate"
FT                   /evidence="ECO:0007744|PubMed:23576753"
FT   MOD_RES         759
FT                   /note="N6-succinyllysine; alternate"
FT                   /evidence="ECO:0007744|PubMed:23806337"
FT   CONFLICT        196
FT                   /note="A -> D (in Ref. 1; BAE41822)"
FT                   /evidence="ECO:0000305"
FT   CONFLICT        459
FT                   /note="L -> S (in Ref. 2; AAH37009)"
FT                   /evidence="ECO:0000305"
SQ   SEQUENCE   763 AA;  82670 MW;  73D203795D5C1141 CRC64;
     MVASRAIGSL SRFSAFRILR SRGCICRSFT TSSALLTRTH INYGVKGDVA VIRINSPNSK
     VNTLNKEVQS EFIEVMNEIW ANDQIRSAVL ISSKPGCFVA GADINMLSSC TTPQEATRIS
     QEGQRMFEKL EKSPKPVVAA ISGSCLGGGL ELAIACQYRI ATKDRKTVLG VPEVLLGILP
     GAGGTQRLPK MVGVPAAFDM MLTGRNIRAD RAKKMGLVDQ LVEPLGPGIK SPEERTIEYL
     EEVAVNFAKG LADRKVSAKQ SKGLVEKLTT YAMTVPFVRQ QVYKTVEEKV KKQTKGLYPA
     PLKIIDAVKA GLEQGSDAGY LAESQKFGEL ALTKESKALM GLYNGQVLCK KNKFGAPQKN
     VQQLAILGAG LMGAGIAQVS VDKGLKTLLK DTTVTGLGRG QQQVFKGLND KVKKKALTSF
     ERDSIFSNLI GQLDYKGFEK ADMVIEAVFE DLGVKHKVLK EVESVTPEHC IFASNTSALP
     INQIAAVSKR PEKVIGMHYF SPVDKMQLLE IITTDKTSKD TTASAVAVGL RQGKVIIVVK
     DGPGFYTTRC LAPMMSEVMR ILQEGVDPKK LDALTTGFGF PVGAATLADE VGVDVAQHVA
     EDLGKAFGER FGGGSVELLK QMVSKGFLGR KSGKGFYIYQ EGSKNKSLNS EMDNILANLR
     LPAKPEVSSD EDVQYRVITR FVNEAVLCLQ EGILATPAEG DIGAVFGLGF PPCLGGPFRF
     VDLYGAQKVV DRLRKYESAY GTQFTPCQLL LDHANNSSKK FYQ
//
```

|  |
| --- |
| **Mascot:** http://www.matrixscience.com/ |

HNE (K) (+156.1150)
