# Supplementary material for: A common East-Asian ALDH2 mutation causes metabolic disorders and the therapeutic effect of ALDH2 activators
Source: Nat Commun. 2023 Sep 25;14:5971. doi: 10.1038/s41467-023-41570-6 (PMC10520061; doi:10.1038/s41467-023-41570-6)
Supplement: Supplementary file 4 — Supplementary Data 1 [file 41467_2023_41570_MOESM4_ESM.zip › Table S5b/Q8BMS1/Q8BMS1_WTO-2_C97.html]

Mascot Search Results: Q8BMS1
  

# MASCOT Search Results

## Protein View: Q8BMS1

### Trifunctional enzyme subunit alpha, mitochondrial OS=Mus musculus OX=10090 GN=Hadha PE=1 SV=1

|  |  |
| --- | --- |
| Database: | Mouse\_UniProt\_proteomes |
| Score: | 21868 |
| Monoisotopic mass (Mr): | 83302 |
| Calculated pI: | 9.24 |

Sequence similarity is available as an NCBI BLAST search of Q8BMS1 against nr.

### Search parameters

|  |  |
| --- | --- |
| MS data file: | `D:\LCMSMS\2023 Users' data\230529-1\230529-1-WTO-2.raw` |
| Enzyme: | Trypsin/P: cuts C-term side of KR. |
| Fixed modifications: | Carbamidomethyl (C) |
| Variable modifications: | Deamidated (NQ), HNE (C), HNE (H), HNE (K), Oxidation (M) |

### Protein sequence coverage: 72%

Matched peptides shown in ***bold red***.

|  |  |  |  |  |  |
| --- | --- | --- | --- | --- | --- |
| `1` | `MVASRAIGSL` | `SRFSAFRILR` | `SRGCICRSFT` | `TSSALLTRTH` | `INYGVKGDVA` |
| `51` | `VIRINSPNSK` | `VNTLNKEVQS` | `EFIEVMNEIW` | `ANDQIRSAVL` | `ISSKPGCFVA` |
| `101` | `GADINMLSSC` | `TTPQEATRIS` | `QEGQRMFEKL` | `EKSPKPVVAA` | `ISGSCLGGGL` |
| `151` | `ELAIACQYRI` | `ATKDRKTVLG` | `VPEVLLGILP` | `GAGGTQRLPK` | `MVGVPAAFDM` |
| `201` | `MLTGRNIRAD` | `RAKKMGLVDQ` | `LVEPLGPGIK` | `SPEERTIEYL` | `EEVAVNFAKG` |
| `251` | `LADRKVSAKQ` | `SKGLVEKLTT` | `YAMTVPFVRQ` | `QVYKTVEEKV` | `KKQTKGLYPA` |
| `301` | `PLKIIDAVKA` | `GLEQGSDAGY` | `LAESQKFGEL` | `ALTKESKALM` | `GLYNGQVLCK` |
| `351` | `KNKFGAPQKN` | `VQQLAILGAG` | `LMGAGIAQVS` | `VDKGLKTLLK` | `DTTVTGLGRG` |
| `401` | `QQQVFKGLND` | `KVKKKALTSF` | `ERDSIFSNLI` | `GQLDYKGFEK` | `ADMVIEAVFE` |
| `451` | `DLGVKHKVLK` | `EVESVTPEHC` | `IFASNTSALP` | `INQIAAVSKR` | `PEKVIGMHYF` |
| `501` | `SPVDKMQLLE` | `IITTDKTSKD` | `TTASAVAVGL` | `RQGKVIIVVK` | `DGPGFYTTRC` |
| `551` | `LAPMMSEVMR` | `ILQEGVDPKK` | `LDALTTGFGF` | `PVGAATLADE` | `VGVDVAQHVA` |
| `601` | `EDLGKAFGER` | `FGGGSVELLK` | `QMVSKGFLGR` | `KSGKGFYIYQ` | `EGSKNKSLNS` |
| `651` | `EMDNILANLR` | `LPAKPEVSSD` | `EDVQYRVITR` | `FVNEAVLCLQ` | `EGILATPAEG` |
| `701` | `DIGAVFGLGF` | `PPCLGGPFRF` | `VDLYGAQKVV` | `DRLRKYESAY` | `GTQFTPCQLL` |
| `751` | `LDHANNSSKK` | `FYQ` |  |  |  |

Unformatted sequence string: 763 residues (for pasting into other applications).

|  |  |  |  |
| --- | --- | --- | --- |
| Sort by | residue number | increasing mass | decreasing mass |
| Show | matched peptides only | predicted peptides also |  |

| Query | Start | – | End | Observed | Mr(expt) | Mr(calc) | ppm | M | Score | Expect | Rank | U | Peptide |
| --- | --- | --- | --- | --- | --- | --- | --- | --- | --- | --- | --- | --- | --- |
| 10579 | 39 | – | 46 | 466.2535 | 930.4924 | 930.4923 | 0.077 | 0 | 38 | 0.0014 | 1Score **> 31** indicates **identity** Score **> 22** indicates **homology** | U | R.THINYGVK.G |
| 85353 | 39 | – | 53 | 411.2292 | 1640.8876 | 1640.8998 | -7.46 | 1 | 27 | 0.0028 | 1Score **> 35** indicates **identity** Score **> 14** indicates **homology** | U | R.THINYGVKGDVAVIR.I |
| 85380 | 39 | – | 53 | 411.2305 | 1640.8930 | 1640.8998 | -4.20 | 1 | 33 | 0.00075 | 1Score **> 35** indicates **identity** Score **> 15** indicates **homology** | U | R.THINYGVKGDVAVIR.I |
| 85391 | 39 | – | 53 | 547.9728 | 1640.8966 | 1640.8998 | -1.98 | 1 | 17 | 0.026 | 1Score **> 35** indicates **identity** Score **> 14** indicates **homology** | U | R.THINYGVKGDVAVIR.I |
| 85402 | 39 | – | 53 | 547.9737 | 1640.8994 | 1640.8998 | -0.28 | 1 | 33 | 0.00074 | 1Score **> 35** indicates **identity** Score **> 15** indicates **homology** | U | R.THINYGVKGDVAVIR.I |
| 85403 | 39 | – | 53 | 547.9739 | 1640.8999 | 1640.8998 | 0.061 | 1 | 50 | 2e-05 | 1Score **> 35** indicates **identity** Score **> 16** indicates **homology** | U | R.THINYGVKGDVAVIR.I |
| 85405 | 39 | – | 53 | 547.9740 | 1640.9001 | 1640.8998 | 0.17 | 1 | 50 | 2.3e-05 | 1Score **> 35** indicates **identity** Score **> 16** indicates **homology** | U | R.THINYGVKGDVAVIR.I |
| 85406 | 39 | – | 53 | 547.9740 | 1640.9002 | 1640.8998 | 0.19 | 1 | 75 | 9.5e-08 | 1Score **> 35** indicates **identity** Score **> 17** indicates **homology** | U | R.THINYGVKGDVAVIR.I |
| 85407 | 39 | – | 53 | 547.9740 | 1640.9002 | 1640.8998 | 0.20 | 1 | 18 | 0.019 | 1Score **> 35** indicates **identity** Score **> 14** indicates **homology** | U | R.THINYGVKGDVAVIR.I |
| 85410 | 39 | – | 53 | 547.9741 | 1640.9005 | 1640.8998 | 0.37 | 1 | 63 | 1.3e-06 | 1Score **> 35** indicates **identity** Score **> 16** indicates **homology** | U | R.THINYGVKGDVAVIR.I |
| 85411 | 39 | – | 53 | 411.2324 | 1640.9006 | 1640.8998 | 0.47 | 1 | 22 | 0.0083 | 1Score **> 35** indicates **identity** Score **> 14** indicates **homology** | U | R.THINYGVKGDVAVIR.I |
| 85412 | 39 | – | 53 | 411.2324 | 1640.9006 | 1640.8998 | 0.49 | 1 | 17 | 0.023 | 1Score **> 35** indicates **identity** Score **> 14** indicates **homology** | U | R.THINYGVKGDVAVIR.I |
| 85414 | 39 | – | 53 | 411.2325 | 1640.9007 | 1640.8998 | 0.55 | 1 | 16 | 0.031 | 1Score **> 35** indicates **identity** Score **> 14** indicates **homology** | U | R.THINYGVKGDVAVIR.I |
| 85416 | 39 | – | 53 | 547.9743 | 1640.9010 | 1640.8998 | 0.72 | 1 | 50 | 2.1e-05 | 1Score **> 35** indicates **identity** Score **> 16** indicates **homology** | U | R.THINYGVKGDVAVIR.I |
| 85417 | 39 | – | 53 | 547.9744 | 1640.9014 | 1640.8998 | 0.95 | 1 | 25 | 0.0046 | 1Score **> 34** indicates **identity** Score **> 14** indicates **homology** | U | R.THINYGVKGDVAVIR.I |
| 85418 | 39 | – | 53 | 547.9745 | 1640.9016 | 1640.8998 | 1.09 | 1 | 51 | 1.6e-05 | 1Score **> 34** indicates **identity** Score **> 16** indicates **homology** | U | R.THINYGVKGDVAVIR.I |
| 85419 | 39 | – | 53 | 547.9746 | 1640.9020 | 1640.8998 | 1.30 | 1 | 19 | 0.017 | 1Score **> 34** indicates **identity** Score **> 14** indicates **homology** | U | R.THINYGVKGDVAVIR.I |
| 85420 | 39 | – | 53 | 411.2329 | 1640.9026 | 1640.8998 | 1.69 | 1 | 24 | 0.0062 | 1Score **> 34** indicates **identity** Score **> 14** indicates **homology** | U | R.THINYGVKGDVAVIR.I |
| 85422 | 39 | – | 53 | 547.9749 | 1640.9027 | 1640.8998 | 1.76 | 1 | 23 | 0.0068 | 1Score **> 34** indicates **identity** Score **> 14** indicates **homology** | U | R.THINYGVKGDVAVIR.I |
| 85423 | 39 | – | 53 | 547.9749 | 1640.9029 | 1640.8998 | 1.86 | 1 | 22 | 0.0087 | 1Score **> 34** indicates **identity** Score **> 14** indicates **homology** | U | R.THINYGVKGDVAVIR.I |
| 85424 | 39 | – | 53 | 411.2331 | 1640.9031 | 1640.8998 | 2.02 | 1 | 28 | 0.0022 | 1Score **> 34** indicates **identity** Score **> 14** indicates **homology** | U | R.THINYGVKGDVAVIR.I |
| 85427 | 39 | – | 53 | 547.9753 | 1640.9040 | 1640.8998 | 2.52 | 1 | 37 | 0.00032 | 1Score **> 34** indicates **identity** Score **> 15** indicates **homology** | U | R.THINYGVKGDVAVIR.I |
| 85430 | 39 | – | 53 | 547.9757 | 1640.9052 | 1640.8998 | 3.24 | 1 | 31 | 0.0096 | 1Score **> 34** indicates **identity** Score **> 23** indicates **homology** | U | R.THINYGVKGDVAVIR.I |
| 85437 | 39 | – | 53 | 547.9765 | 1640.9077 | 1640.8998 | 4.82 | 1 | 59 | 3.2e-06 | 1Score **> 34** indicates **identity** Score **> 16** indicates **homology** | U | R.THINYGVKGDVAVIR.I |
| 1114 | 47 | – | 53 | 365.2158 | 728.4170 | 728.4181 | -1.47 | 0 | 37 | 0.0022 | 1Score **> 23** indicates **identity** | U | K.GDVAVIR.I |
| 1115 | 47 | – | 53 | 365.2163 | 728.4180 | 728.4181 | -0.19 | 0 | 50 | 9.9e-05 | 1Score **> 23** indicates **identity** | U | K.GDVAVIR.I |
| 1116 | 47 | – | 53 | 365.2163 | 728.4181 | 728.4181 | -0.048 | 0 | 50 | 0.0001 | 1Score **> 23** indicates **identity** | U | K.GDVAVIR.I |
| 157898 | 67 | – | 86 | 1225.5947 | 2449.1749 | 2449.1583 | 6.76 | 0 | 73 | 1.4e-07 | 1Score **> 36** indicates **identity** Score **> 17** indicates **homology** | U | K.EVQSEFIEVMNEIWANDQIR.S |
| 186158 | 87 | – | 118 | 1123.5498 | 3367.6277 | 3367.6210 | 1.97 | 1 | 85 | 1.2e-08 | 1Score **> 37** indicates **identity** Score **> 18** indicates **homology** | U | R.SAVLISSKPGCFVAGADINMLSSCTTPQEATR.I |
| 186159 | 87 | – | 118 | 1123.5499 | 3367.6280 | 3367.6210 | 2.05 | 1 | 74 | 1.3e-07 | 1Score **> 37** indicates **identity** Score **> 17** indicates **homology** | U | R.SAVLISSKPGCFVAGADINMLSSCTTPQEATR.I |
| 186160 | 87 | – | 118 | 1123.5512 | 3367.6317 | 3367.6210 | 3.18 | 1 | 101 | 3.1e-10 | 1Score **> 37** indicates **identity** Score **> 19** indicates **homology** | U | R.SAVLISSKPGCFVAGADINMLSSCTTPQEATR.I |
| 186161 | 87 | – | 118 | 842.9159 | 3367.6345 | 3367.6210 | 4.00 | 1 | 36 | 0.00042 | 1Score **> 37** indicates **identity** Score **> 15** indicates **homology** | U | R.SAVLISSKPGCFVAGADINMLSSCTTPQEATR.I |
| 186162 | 87 | – | 118 | 1123.5531 | 3367.6374 | 3367.6210 | 4.86 | 1 | 106 | 1.1e-10 | 1Score **> 37** indicates **identity** Score **> 19** indicates **homology** | U | R.SAVLISSKPGCFVAGADINMLSSCTTPQEATR.I |
| 186163 | 87 | – | 118 | 842.9168 | 3367.6381 | 3367.6210 | 5.08 | 1 | 22 | 0.0096 | 1Score **> 37** indicates **identity** Score **> 15** indicates **homology** | U | R.SAVLISSKPGCFVAGADINMLSSCTTPQEATR.I |
| 186164 | 87 | – | 118 | 1123.5536 | 3367.6391 | 3367.6210 | 5.36 | 1 | 85 | 1.1e-08 | 1Score **> 37** indicates **identity** Score **> 18** indicates **homology** | U | R.SAVLISSKPGCFVAGADINMLSSCTTPQEATR.I |
| 186165 | 87 | – | 118 | 1123.5537 | 3367.6393 | 3367.6210 | 5.41 | 1 | 64 | 1e-06 | 1Score **> 37** indicates **identity** Score **> 16** indicates **homology** | U | R.SAVLISSKPGCFVAGADINMLSSCTTPQEATR.I |
| 186166 | 87 | – | 118 | 1123.5537 | 3367.6394 | 3367.6210 | 5.45 | 1 | 88 | 5.2e-09 | 1Score **> 37** indicates **identity** Score **> 18** indicates **homology** | U | R.SAVLISSKPGCFVAGADINMLSSCTTPQEATR.I |
| 186167 | 87 | – | 118 | 842.9174 | 3367.6405 | 3367.6210 | 5.77 | 1 | 59 | 3e-06 | 1Score **> 37** indicates **identity** Score **> 16** indicates **homology** | U | R.SAVLISSKPGCFVAGADINMLSSCTTPQEATR.I |
| 186168 | 87 | – | 118 | 1123.5545 | 3367.6416 | 3367.6210 | 6.09 | 1 | 56 | 5.6e-06 | 1Score **> 37** indicates **identity** Score **> 16** indicates **homology** | U | R.SAVLISSKPGCFVAGADINMLSSCTTPQEATR.I |
| 186169 | 87 | – | 118 | 1123.5556 | 3367.6449 | 3367.6210 | 7.07 | 1 | 73 | 1.3e-07 | 1Score **> 37** indicates **identity** Score **> 17** indicates **homology** | U | R.SAVLISSKPGCFVAGADINMLSSCTTPQEATR.I |
| 186173 | 87 | – | 118 | 1123.8810 | 3368.6213 | 3368.6051 | 4.81 | 1 | 48 | 2.9e-05 | 1Score **> 37** indicates **identity** Score **> 16** indicates **homology** | U | R.SAVLISSKPGCFVAGADINMLSSCTTPQEATR.I  + Deamidated (NQ) |
| 186174 | 87 | – | 118 | 1123.8823 | 3368.6250 | 3368.6051 | 5.92 | 1 | 40 | 0.00016 | 1Score **> 37** indicates **identity** Score **> 15** indicates **homology** | U | R.SAVLISSKPGCFVAGADINMLSSCTTPQEATR.I  + Deamidated (NQ) |
| 186175 | 87 | – | 118 | 1123.8843 | 3368.6310 | 3368.6051 | 7.70 | 1 | 42 | 0.00011 | 1Score **> 37** indicates **identity** Score **> 15** indicates **homology** | U | R.SAVLISSKPGCFVAGADINMLSSCTTPQEATR.I  + Deamidated (NQ) |
| 186495 | 87 | – | 118 | 1128.8768 | 3383.6087 | 3383.6160 | -2.14 | 1 | 61 | 1.8e-06 | 1Score **> 36** indicates **identity** Score **> 16** indicates **homology** | U | R.SAVLISSKPGCFVAGADINMLSSCTTPQEATR.I  + Oxidation (M) |
| 186503 | 87 | – | 118 | 1129.2126 | 3384.6161 | 3384.6000 | 4.77 | 1 | 62 | 1.4e-06 | 1Score **> 36** indicates **identity** Score **> 16** indicates **homology** | U | R.SAVLISSKPGCFVAGADINMLSSCTTPQEATR.I  + Deamidated (NQ); Oxidation (M) |
| 188030 | 87 | – | 118 | 871.9247 | 3483.6696 | 3483.6935 | -6.86 | 1 | 22 | 0.0095 | 1Score **> 36** indicates **identity** Score **> 14** indicates **homology** | U | R.SAVLISSKPGCFVAGADINMLSSCTTPQEATR.I  + Deamidated (NQ); HNE (C); Oxidation (M) |
| 10281 | 126 | – | 132 | 462.7464 | 923.4783 | 923.4786 | -0.34 | 1 | 25 | 0.019 | 1Score **> 29** indicates **identity** Score **> 21** indicates **homology** | U | R.MFEKLEK.S |
| 10282 | 126 | – | 132 | 462.7469 | 923.4793 | 923.4786 | 0.79 | 1 | 21 | 0.021 | 1Score **> 29** indicates **identity** Score **> 17** indicates **homology** | U | R.MFEKLEK.S |
| 182228 | 130 | – | 159 | 786.9181 | 3143.6431 | 3143.6471 | -1.26 | 2 | 37 | 0.00034 | 1Score **> 37** indicates **identity** Score **> 15** indicates **homology** | U | K.LEKSPKPVVAAISGSCLGGGLELAIACQYR.I |
| 182229 | 130 | – | 159 | 1048.8902 | 3143.6488 | 3143.6471 | 0.56 | 2 | 51 | 1.5e-05 | 1Score **> 37** indicates **identity** Score **> 16** indicates **homology** | U | K.LEKSPKPVVAAISGSCLGGGLELAIACQYR.I |
| 182230 | 130 | – | 159 | 786.9215 | 3143.6567 | 3143.6471 | 3.06 | 2 | 45 | 5.9e-05 | 1Score **> 36** indicates **identity** Score **> 15** indicates **homology** | U | K.LEKSPKPVVAAISGSCLGGGLELAIACQYR.I |
| 182231 | 130 | – | 159 | 786.9222 | 3143.6597 | 3143.6471 | 4.02 | 2 | 49 | 2.5e-05 | 1Score **> 36** indicates **identity** Score **> 16** indicates **homology** | U | K.LEKSPKPVVAAISGSCLGGGLELAIACQYR.I |
| 182235 | 130 | – | 159 | 1049.2251 | 3144.6536 | 3144.6311 | 7.16 | 2 | 15 | 0.036 | 1Score **> 36** indicates **identity** Score **> 13** indicates **homology** | U | K.LEKSPKPVVAAISGSCLGGGLELAIACQYR.I  + Deamidated (NQ) |
| 171958 | 133 | – | 159 | 925.4737 | 2773.3992 | 2773.4255 | -9.49 | 1 | 17 | 0.024 | 1Score **> 37** indicates **identity** Score **> 14** indicates **homology** | U | K.SPKPVVAAISGSCLGGGLELAIACQYR.I |
| 171959 | 133 | – | 159 | 925.4743 | 2773.4011 | 2773.4255 | -8.81 | 1 | 72 | 1.9e-07 | 1Score **> 37** indicates **identity** Score **> 17** indicates **homology** | U | K.SPKPVVAAISGSCLGGGLELAIACQYR.I |
| 171960 | 133 | – | 159 | 925.4749 | 2773.4029 | 2773.4255 | -8.14 | 1 | 30 | 0.0017 | 1Score **> 37** indicates **identity** Score **> 14** indicates **homology** | U | K.SPKPVVAAISGSCLGGGLELAIACQYR.I |
| 171964 | 133 | – | 159 | 925.4771 | 2773.4094 | 2773.4255 | -5.79 | 1 | 30 | 0.0016 | 1Score **> 37** indicates **identity** Score **> 15** indicates **homology** | U | K.SPKPVVAAISGSCLGGGLELAIACQYR.I |
| 171966 | 133 | – | 159 | 925.4791 | 2773.4155 | 2773.4255 | -3.60 | 1 | 30 | 0.0014 | 1Score **> 37** indicates **identity** Score **> 14** indicates **homology** | U | K.SPKPVVAAISGSCLGGGLELAIACQYR.I |
| 171967 | 133 | – | 159 | 925.4792 | 2773.4159 | 2773.4255 | -3.46 | 1 | 54 | 1.3e-05 | 1Score **> 37** indicates **identity** Score **> 18** indicates **homology** | U | K.SPKPVVAAISGSCLGGGLELAIACQYR.I |
| 171969 | 133 | – | 159 | 925.4800 | 2773.4181 | 2773.4255 | -2.68 | 1 | 69 | 6.4e-07 | 1Score **> 37** indicates **identity** Score **> 19** indicates **homology** | U | K.SPKPVVAAISGSCLGGGLELAIACQYR.I |
| 171971 | 133 | – | 159 | 925.4801 | 2773.4186 | 2773.4255 | -2.48 | 1 | 21 | 0.011 | 1Score **> 37** indicates **identity** Score **> 14** indicates **homology** | U | K.SPKPVVAAISGSCLGGGLELAIACQYR.I |
| 171973 | 133 | – | 159 | 925.4803 | 2773.4192 | 2773.4255 | -2.28 | 1 | 32 | 0.00091 | 1Score **> 37** indicates **identity** Score **> 15** indicates **homology** | U | K.SPKPVVAAISGSCLGGGLELAIACQYR.I |
| 171975 | 133 | – | 159 | 925.4807 | 2773.4204 | 2773.4255 | -1.84 | 1 | 57 | 5e-06 | 1Score **> 37** indicates **identity** Score **> 17** indicates **homology** | U | K.SPKPVVAAISGSCLGGGLELAIACQYR.I |
| 171977 | 133 | – | 159 | 925.4808 | 2773.4206 | 2773.4255 | -1.76 | 1 | 43 | 0.0001 | 1Score **> 37** indicates **identity** Score **> 15** indicates **homology** | U | K.SPKPVVAAISGSCLGGGLELAIACQYR.I |
| 171978 | 133 | – | 159 | 925.4808 | 2773.4207 | 2773.4255 | -1.74 | 1 | 60 | 2.3e-06 | 1Score **> 37** indicates **identity** Score **> 16** indicates **homology** | U | K.SPKPVVAAISGSCLGGGLELAIACQYR.I |
| 171979 | 133 | – | 159 | 925.4810 | 2773.4213 | 2773.4255 | -1.51 | 1 | 51 | 1.5e-05 | 1Score **> 37** indicates **identity** Score **> 16** indicates **homology** | U | K.SPKPVVAAISGSCLGGGLELAIACQYR.I |
| 171981 | 133 | – | 159 | 925.4814 | 2773.4224 | 2773.4255 | -1.11 | 1 | 41 | 0.00013 | 1Score **> 37** indicates **identity** Score **> 15** indicates **homology** | U | K.SPKPVVAAISGSCLGGGLELAIACQYR.I |
| 171982 | 133 | – | 159 | 694.3635 | 2773.4250 | 2773.4255 | -0.19 | 1 | 75 | 1e-07 | 1Score **> 37** indicates **identity** Score **> 17** indicates **homology** | U | K.SPKPVVAAISGSCLGGGLELAIACQYR.I |
| 171983 | 133 | – | 159 | 694.3636 | 2773.4253 | 2773.4255 | -0.052 | 1 | 34 | 0.00061 | 1Score **> 37** indicates **identity** Score **> 15** indicates **homology** | U | K.SPKPVVAAISGSCLGGGLELAIACQYR.I |
| 171984 | 133 | – | 159 | 925.4824 | 2773.4255 | 2773.4255 | 0.011 | 1 | 115 | 1.7e-11 | 1Score **> 37** indicates **identity** Score **> 20** indicates **homology** | U | K.SPKPVVAAISGSCLGGGLELAIACQYR.I |
| 171985 | 133 | – | 159 | 925.4825 | 2773.4255 | 2773.4255 | 0.021 | 1 | 107 | 1e-10 | 1Score **> 37** indicates **identity** Score **> 19** indicates **homology** | U | K.SPKPVVAAISGSCLGGGLELAIACQYR.I |
| 171986 | 133 | – | 159 | 925.4825 | 2773.4256 | 2773.4255 | 0.033 | 1 | 45 | 5.7e-05 | 1Score **> 37** indicates **identity** Score **> 15** indicates **homology** | U | K.SPKPVVAAISGSCLGGGLELAIACQYR.I |
| 171987 | 133 | – | 159 | 925.4826 | 2773.4259 | 2773.4255 | 0.16 | 1 | 76 | 6.8e-08 | 1Score **> 37** indicates **identity** Score **> 17** indicates **homology** | U | K.SPKPVVAAISGSCLGGGLELAIACQYR.I |
| 171988 | 133 | – | 159 | 694.3638 | 2773.4262 | 2773.4255 | 0.24 | 1 | 61 | 1.8e-06 | 1Score **> 37** indicates **identity** Score **> 16** indicates **homology** | U | K.SPKPVVAAISGSCLGGGLELAIACQYR.I |
| 171989 | 133 | – | 159 | 925.4827 | 2773.4264 | 2773.4255 | 0.32 | 1 | 95 | 1.1e-09 | 1Score **> 37** indicates **identity** Score **> 18** indicates **homology** | U | K.SPKPVVAAISGSCLGGGLELAIACQYR.I |
| 171990 | 133 | – | 159 | 925.4828 | 2773.4264 | 2773.4255 | 0.34 | 1 | 114 | 2e-11 | 1Score **> 37** indicates **identity** Score **> 20** indicates **homology** | U | K.SPKPVVAAISGSCLGGGLELAIACQYR.I |
| 171991 | 133 | – | 159 | 925.4829 | 2773.4267 | 2773.4255 | 0.44 | 1 | 104 | 1.9e-10 | 1Score **> 37** indicates **identity** Score **> 19** indicates **homology** | U | K.SPKPVVAAISGSCLGGGLELAIACQYR.I |
| 171993 | 133 | – | 159 | 925.4829 | 2773.4268 | 2773.4255 | 0.49 | 1 | 102 | 2.8e-10 | 1Score **> 37** indicates **identity** Score **> 19** indicates **homology** | U | K.SPKPVVAAISGSCLGGGLELAIACQYR.I |
| 171995 | 133 | – | 159 | 925.4830 | 2773.4272 | 2773.4255 | 0.61 | 1 | 102 | 2.5e-10 | 1Score **> 37** indicates **identity** Score **> 19** indicates **homology** | U | K.SPKPVVAAISGSCLGGGLELAIACQYR.I |
| 171996 | 133 | – | 159 | 694.3642 | 2773.4276 | 2773.4255 | 0.74 | 1 | 64 | 9e-07 | 1Score **> 37** indicates **identity** Score **> 17** indicates **homology** | U | K.SPKPVVAAISGSCLGGGLELAIACQYR.I |
| 171997 | 133 | – | 159 | 925.4832 | 2773.4277 | 2773.4255 | 0.78 | 1 | 109 | 5.9e-11 | 1Score **> 37** indicates **identity** Score **> 19** indicates **homology** | U | K.SPKPVVAAISGSCLGGGLELAIACQYR.I |
| 171998 | 133 | – | 159 | 925.4832 | 2773.4279 | 2773.4255 | 0.86 | 1 | 64 | 9.5e-07 | 1Score **> 37** indicates **identity** Score **> 17** indicates **homology** | U | K.SPKPVVAAISGSCLGGGLELAIACQYR.I |
| 172001 | 133 | – | 159 | 925.4833 | 2773.4281 | 2773.4255 | 0.95 | 1 | 58 | 3.6e-06 | 1Score **> 37** indicates **identity** Score **> 16** indicates **homology** | U | K.SPKPVVAAISGSCLGGGLELAIACQYR.I |
| 172002 | 133 | – | 159 | 694.3643 | 2773.4283 | 2773.4255 | 1.01 | 1 | 50 | 2e-05 | 1Score **> 37** indicates **identity** Score **> 16** indicates **homology** | U | K.SPKPVVAAISGSCLGGGLELAIACQYR.I |
| 172003 | 133 | – | 159 | 925.4835 | 2773.4286 | 2773.4255 | 1.11 | 1 | 123 | 3.1e-12 | 1Score **> 37** indicates **identity** Score **> 20** indicates **homology** | U | K.SPKPVVAAISGSCLGGGLELAIACQYR.I |
| 172006 | 133 | – | 159 | 925.4836 | 2773.4289 | 2773.4255 | 1.22 | 1 | 37 | 0.00033 | 1Score **> 37** indicates **identity** Score **> 15** indicates **homology** | U | K.SPKPVVAAISGSCLGGGLELAIACQYR.I |
| 172007 | 133 | – | 159 | 925.4837 | 2773.4291 | 2773.4255 | 1.31 | 1 | 21 | 0.011 | 1Score **> 37** indicates **identity** Score **> 14** indicates **homology** | U | K.SPKPVVAAISGSCLGGGLELAIACQYR.I |
| 172009 | 133 | – | 159 | 925.4837 | 2773.4293 | 2773.4255 | 1.37 | 1 | 53 | 1.4e-05 | 1Score **> 37** indicates **identity** Score **> 17** indicates **homology** | U | K.SPKPVVAAISGSCLGGGLELAIACQYR.I |
| 172010 | 133 | – | 159 | 694.3646 | 2773.4293 | 2773.4255 | 1.37 | 1 | 23 | 0.0066 | 1Score **> 37** indicates **identity** Score **> 14** indicates **homology** | U | K.SPKPVVAAISGSCLGGGLELAIACQYR.I |
| 172011 | 133 | – | 159 | 925.4837 | 2773.4294 | 2773.4255 | 1.39 | 1 | 75 | 9.8e-08 | 1Score **> 37** indicates **identity** Score **> 17** indicates **homology** | U | K.SPKPVVAAISGSCLGGGLELAIACQYR.I |
| 172013 | 133 | – | 159 | 925.4838 | 2773.4295 | 2773.4255 | 1.46 | 1 | 82 | 2e-08 | 1Score **> 37** indicates **identity** Score **> 18** indicates **homology** | U | K.SPKPVVAAISGSCLGGGLELAIACQYR.I |
| 172015 | 133 | – | 159 | 925.4839 | 2773.4298 | 2773.4255 | 1.56 | 1 | 51 | 1.7e-05 | 1Score **> 37** indicates **identity** Score **> 16** indicates **homology** | U | K.SPKPVVAAISGSCLGGGLELAIACQYR.I |
| 172016 | 133 | – | 159 | 925.4839 | 2773.4299 | 2773.4255 | 1.60 | 1 | 114 | 1.9e-11 | 1Score **> 37** indicates **identity** Score **> 20** indicates **homology** | U | K.SPKPVVAAISGSCLGGGLELAIACQYR.I |
| 172017 | 133 | – | 159 | 925.4840 | 2773.4301 | 2773.4255 | 1.67 | 1 | 48 | 3.4e-05 | 1Score **> 37** indicates **identity** Score **> 15** indicates **homology** | U | K.SPKPVVAAISGSCLGGGLELAIACQYR.I |
| 172018 | 133 | – | 159 | 925.4840 | 2773.4301 | 2773.4255 | 1.67 | 1 | 57 | 5.1e-06 | 1Score **> 37** indicates **identity** Score **> 16** indicates **homology** | U | K.SPKPVVAAISGSCLGGGLELAIACQYR.I |
| 172019 | 133 | – | 159 | 925.4840 | 2773.4303 | 2773.4255 | 1.74 | 1 | 56 | 9e-06 | 1Score **> 37** indicates **identity** Score **> 19** indicates **homology** | U | K.SPKPVVAAISGSCLGGGLELAIACQYR.I |
| 172020 | 133 | – | 159 | 925.4841 | 2773.4303 | 2773.4255 | 1.75 | 1 | 56 | 5.5e-06 | 1Score **> 37** indicates **identity** Score **> 16** indicates **homology** | U | K.SPKPVVAAISGSCLGGGLELAIACQYR.I |
| 172021 | 133 | – | 159 | 925.4841 | 2773.4306 | 2773.4255 | 1.85 | 1 | 42 | 0.00012 | 1Score **> 37** indicates **identity** Score **> 15** indicates **homology** | U | K.SPKPVVAAISGSCLGGGLELAIACQYR.I |
| 172023 | 133 | – | 159 | 925.4842 | 2773.4307 | 2773.4255 | 1.89 | 1 | 29 | 0.0021 | 1Score **> 37** indicates **identity** Score **> 15** indicates **homology** | U | K.SPKPVVAAISGSCLGGGLELAIACQYR.I |
| 172026 | 133 | – | 159 | 925.4842 | 2773.4309 | 2773.4255 | 1.95 | 1 | 69 | 3.3e-07 | 1Score **> 37** indicates **identity** Score **> 17** indicates **homology** | U | K.SPKPVVAAISGSCLGGGLELAIACQYR.I |
| 172027 | 133 | – | 159 | 925.4844 | 2773.4313 | 2773.4255 | 2.10 | 1 | 42 | 0.00011 | 1Score **> 37** indicates **identity** Score **> 15** indicates **homology** | U | K.SPKPVVAAISGSCLGGGLELAIACQYR.I |
| 172028 | 133 | – | 159 | 925.4847 | 2773.4323 | 2773.4255 | 2.44 | 1 | 61 | 1.7e-06 | 1Score **> 37** indicates **identity** Score **> 16** indicates **homology** | U | K.SPKPVVAAISGSCLGGGLELAIACQYR.I |
| 172030 | 133 | – | 159 | 925.4849 | 2773.4329 | 2773.4255 | 2.69 | 1 | 33 | 0.00088 | 1Score **> 37** indicates **identity** Score **> 15** indicates **homology** | U | K.SPKPVVAAISGSCLGGGLELAIACQYR.I |
| 172031 | 133 | – | 159 | 925.4849 | 2773.4329 | 2773.4255 | 2.69 | 1 | 50 | 2e-05 | 1Score **> 37** indicates **identity** Score **> 16** indicates **homology** | U | K.SPKPVVAAISGSCLGGGLELAIACQYR.I |
| 172032 | 133 | – | 159 | 925.4849 | 2773.4330 | 2773.4255 | 2.71 | 1 | 60 | 2.6e-06 | 1Score **> 37** indicates **identity** Score **> 16** indicates **homology** | U | K.SPKPVVAAISGSCLGGGLELAIACQYR.I |
| 172033 | 133 | – | 159 | 925.4850 | 2773.4332 | 2773.4255 | 2.77 | 1 | 46 | 0.0001 | 1Score **> 37** indicates **identity** Score **> 18** indicates **homology** | U | K.SPKPVVAAISGSCLGGGLELAIACQYR.I |
| 172034 | 133 | – | 159 | 925.4850 | 2773.4333 | 2773.4255 | 2.82 | 1 | 18 | 0.02 | 1Score **> 37** indicates **identity** Score **> 14** indicates **homology** | U | K.SPKPVVAAISGSCLGGGLELAIACQYR.I |
| 172035 | 133 | – | 159 | 925.4852 | 2773.4338 | 2773.4255 | 3.01 | 1 | 46 | 5.4e-05 | 1Score **> 37** indicates **identity** Score **> 15** indicates **homology** | U | K.SPKPVVAAISGSCLGGGLELAIACQYR.I |
| 172037 | 133 | – | 159 | 925.4856 | 2773.4349 | 2773.4255 | 3.38 | 1 | 20 | 0.013 | 1Score **> 37** indicates **identity** Score **> 14** indicates **homology** | U | K.SPKPVVAAISGSCLGGGLELAIACQYR.I |
| 172038 | 133 | – | 159 | 925.4857 | 2773.4353 | 2773.4255 | 3.53 | 1 | 35 | 0.00047 | 1Score **> 37** indicates **identity** Score **> 15** indicates **homology** | U | K.SPKPVVAAISGSCLGGGLELAIACQYR.I |
| 172042 | 133 | – | 159 | 925.4864 | 2773.4375 | 2773.4255 | 4.32 | 1 | 29 | 0.0018 | 1Score **> 37** indicates **identity** Score **> 14** indicates **homology** | U | K.SPKPVVAAISGSCLGGGLELAIACQYR.I |
| 172043 | 133 | – | 159 | 925.4870 | 2773.4391 | 2773.4255 | 4.92 | 1 | 40 | 0.00021 | 1Score **> 37** indicates **identity** Score **> 16** indicates **homology** | U | K.SPKPVVAAISGSCLGGGLELAIACQYR.I |
| 172045 | 133 | – | 159 | 925.4875 | 2773.4406 | 2773.4255 | 5.45 | 1 | 86 | 9.6e-09 | 1Score **> 37** indicates **identity** Score **> 18** indicates **homology** | U | K.SPKPVVAAISGSCLGGGLELAIACQYR.I |
| 172046 | 133 | – | 159 | 925.4877 | 2773.4414 | 2773.4255 | 5.72 | 1 | 27 | 0.0029 | 1Score **> 37** indicates **identity** Score **> 14** indicates **homology** | U | K.SPKPVVAAISGSCLGGGLELAIACQYR.I |
| 172048 | 133 | – | 159 | 925.4879 | 2773.4418 | 2773.4255 | 5.89 | 1 | 37 | 0.00037 | 1Score **> 37** indicates **identity** Score **> 15** indicates **homology** | U | K.SPKPVVAAISGSCLGGGLELAIACQYR.I |
| 172049 | 133 | – | 159 | 925.4881 | 2773.4425 | 2773.4255 | 6.12 | 1 | 66 | 6.9e-07 | 1Score **> 37** indicates **identity** Score **> 17** indicates **homology** | U | K.SPKPVVAAISGSCLGGGLELAIACQYR.I |
| 172051 | 133 | – | 159 | 925.4883 | 2773.4432 | 2773.4255 | 6.38 | 1 | 14 | 0.048 | 1Score **> 37** indicates **identity** Score **> 13** indicates **homology** | U | K.SPKPVVAAISGSCLGGGLELAIACQYR.I |
| 172052 | 133 | – | 159 | 925.4889 | 2773.4448 | 2773.4255 | 6.96 | 1 | 64 | 9e-07 | 1Score **> 37** indicates **identity** Score **> 17** indicates **homology** | U | K.SPKPVVAAISGSCLGGGLELAIACQYR.I |
| 172053 | 133 | – | 159 | 925.4896 | 2773.4470 | 2773.4255 | 7.75 | 1 | 32 | 0.0011 | 1Score **> 37** indicates **identity** Score **> 14** indicates **homology** | U | K.SPKPVVAAISGSCLGGGLELAIACQYR.I |
| 172054 | 133 | – | 159 | 925.4903 | 2773.4492 | 2773.4255 | 8.54 | 1 | 54 | 9.5e-06 | 1Score **> 37** indicates **identity** Score **> 16** indicates **homology** | U | K.SPKPVVAAISGSCLGGGLELAIACQYR.I |
| 172055 | 133 | – | 159 | 925.4917 | 2773.4531 | 2773.4255 | 9.97 | 1 | 24 | 0.0059 | 1Score **> 37** indicates **identity** Score **> 14** indicates **homology** | U | K.SPKPVVAAISGSCLGGGLELAIACQYR.I |
| 172083 | 133 | – | 159 | 925.8167 | 2774.4281 | 2774.4095 | 6.72 | 1 | 40 | 0.00017 | 1Score **> 37** indicates **identity** Score **> 15** indicates **homology** | U | K.SPKPVVAAISGSCLGGGLELAIACQYR.I  + Deamidated (NQ) |
| 172086 | 133 | – | 159 | 925.8181 | 2774.4326 | 2774.4095 | 8.33 | 1 | 33 | 0.00085 | 1Score **> 37** indicates **identity** Score **> 15** indicates **homology** | U | K.SPKPVVAAISGSCLGGGLELAIACQYR.I  + Deamidated (NQ) |
| 172088 | 133 | – | 159 | 925.8183 | 2774.4331 | 2774.4095 | 8.49 | 1 | 38 | 0.00026 | 1Score **> 37** indicates **identity** Score **> 15** indicates **homology** | U | K.SPKPVVAAISGSCLGGGLELAIACQYR.I  + Deamidated (NQ) |
| 138986 | 166 | – | 187 | 725.7645 | 2174.2718 | 2174.2787 | -3.18 | 1 | 33 | 0.00087 | 1Score **> 30** indicates **identity** Score **> 15** indicates **homology** | U | R.KTVLGVPEVLLGILPGAGGTQR.L |
| 138987 | 166 | – | 187 | 1088.1483 | 2174.2821 | 2174.2787 | 1.55 | 1 | 60 | 2.2e-06 | 1Score **> 29** indicates **identity** Score **> 16** indicates **homology** | U | R.KTVLGVPEVLLGILPGAGGTQR.L |
| 161224 | 166 | – | 190 | 629.1335 | 2512.5047 | 2512.5105 | -2.29 | 2 | 28 | 0.0025 | 1Score **> 25** indicates **identity** Score **> 14** indicates **homology** | U | R.KTVLGVPEVLLGILPGAGGTQRLPK.M |
| 161225 | 166 | – | 190 | 629.1354 | 2512.5124 | 2512.5105 | 0.76 | 2 | 33 | 0.00089 | 1Score **> 24** indicates **identity** Score **> 15** indicates **homology** | U | R.KTVLGVPEVLLGILPGAGGTQRLPK.M |
| 161226 | 166 | – | 190 | 838.5115 | 2512.5127 | 2512.5105 | 0.87 | 2 | 44 | 7e-05 | 1Score **> 24** indicates **identity** Score **> 15** indicates **homology** | U | R.KTVLGVPEVLLGILPGAGGTQRLPK.M |
| 127614 | 167 | – | 187 | 683.0674 | 2046.1803 | 2046.1837 | -1.69 | 0 | 39 | 0.0002 | 1Score **> 31** indicates **identity** Score **> 15** indicates **homology** | U | K.TVLGVPEVLLGILPGAGGTQR.L |
| 127617 | 167 | – | 187 | 683.0683 | 2046.1832 | 2046.1837 | -0.29 | 0 | 56 | 5.8e-06 | 1Score **> 31** indicates **identity** Score **> 16** indicates **homology** | U | K.TVLGVPEVLLGILPGAGGTQR.L |
| 127619 | 167 | – | 187 | 1024.0995 | 2046.1845 | 2046.1837 | 0.36 | 0 | 53 | 1.1e-05 | 1Score **> 31** indicates **identity** Score **> 16** indicates **homology** | U | K.TVLGVPEVLLGILPGAGGTQR.L |
| 127623 | 167 | – | 187 | 683.0698 | 2046.1877 | 2046.1837 | 1.94 | 0 | 26 | 0.0033 | 1Score **> 31** indicates **identity** Score **> 14** indicates **homology** | U | K.TVLGVPEVLLGILPGAGGTQR.L |
| 127624 | 167 | – | 187 | 1024.1016 | 2046.1887 | 2046.1837 | 2.41 | 0 | 78 | 4.9e-08 | 1Score **> 31** indicates **identity** Score **> 17** indicates **homology** | U | K.TVLGVPEVLLGILPGAGGTQR.L |
| 127625 | 167 | – | 187 | 683.0702 | 2046.1887 | 2046.1837 | 2.42 | 0 | 56 | 6.2e-06 | 1Score **> 31** indicates **identity** Score **> 16** indicates **homology** | U | K.TVLGVPEVLLGILPGAGGTQR.L |
| 127626 | 167 | – | 187 | 1024.1020 | 2046.1894 | 2046.1837 | 2.79 | 0 | 27 | 0.0027 | 1Score **> 31** indicates **identity** Score **> 14** indicates **homology** | U | K.TVLGVPEVLLGILPGAGGTQR.L |
| 127627 | 167 | – | 187 | 1024.1020 | 2046.1895 | 2046.1837 | 2.80 | 0 | 83 | 1.6e-08 | 1Score **> 31** indicates **identity** Score **> 18** indicates **homology** | U | K.TVLGVPEVLLGILPGAGGTQR.L |
| 127628 | 167 | – | 187 | 1024.1021 | 2046.1897 | 2046.1837 | 2.91 | 0 | 70 | 2.8e-07 | 1Score **> 31** indicates **identity** Score **> 17** indicates **homology** | U | K.TVLGVPEVLLGILPGAGGTQR.L |
| 127629 | 167 | – | 187 | 1024.1021 | 2046.1897 | 2046.1837 | 2.91 | 0 | 43 | 8.5e-05 | 1Score **> 31** indicates **identity** Score **> 15** indicates **homology** | U | K.TVLGVPEVLLGILPGAGGTQR.L |
| 127630 | 167 | – | 187 | 1024.1045 | 2046.1945 | 2046.1837 | 5.24 | 0 | 64 | 1.1e-06 | 1Score **> 30** indicates **identity** Score **> 16** indicates **homology** | U | K.TVLGVPEVLLGILPGAGGTQR.L |
| 127631 | 167 | – | 187 | 683.0724 | 2046.1952 | 2046.1837 | 5.62 | 0 | 40 | 0.00017 | 1Score **> 30** indicates **identity** Score **> 15** indicates **homology** | U | K.TVLGVPEVLLGILPGAGGTQR.L |
| 127632 | 167 | – | 187 | 1024.1054 | 2046.1962 | 2046.1837 | 6.10 | 0 | 64 | 1.8e-06 | 1Score **> 30** indicates **identity** Score **> 19** indicates **homology** | U | K.TVLGVPEVLLGILPGAGGTQR.L |
| 127633 | 167 | – | 187 | 683.0727 | 2046.1962 | 2046.1837 | 6.10 | 0 | 55 | 6.4e-06 | 1Score **> 30** indicates **identity** Score **> 16** indicates **homology** | U | K.TVLGVPEVLLGILPGAGGTQR.L |
| 127636 | 167 | – | 187 | 683.0733 | 2046.1980 | 2046.1837 | 6.98 | 0 | 42 | 0.00011 | 1Score **> 30** indicates **identity** Score **> 15** indicates **homology** | U | K.TVLGVPEVLLGILPGAGGTQR.L |
| 154118 | 167 | – | 190 | 795.8092 | 2384.4057 | 2384.4155 | -4.14 | 1 | 20 | 0.013 | 1Score **> 29** indicates **identity** Score **> 14** indicates **homology** | U | K.TVLGVPEVLLGILPGAGGTQRLPK.M |
| 154119 | 167 | – | 190 | 795.8102 | 2384.4089 | 2384.4155 | -2.80 | 1 | 15 | 0.038 | 1Score **> 28** indicates **identity** Score **> 13** indicates **homology** | U | K.TVLGVPEVLLGILPGAGGTQRLPK.M |
| 154120 | 167 | – | 190 | 597.1112 | 2384.4155 | 2384.4155 | 0.00042 | 1 | 30 | 0.0014 | 1Score **> 27** indicates **identity** Score **> 14** indicates **homology** | U | K.TVLGVPEVLLGILPGAGGTQRLPK.M |
| 154121 | 167 | – | 190 | 795.8125 | 2384.4156 | 2384.4155 | 0.032 | 1 | 34 | 0.00066 | 1Score **> 27** indicates **identity** Score **> 15** indicates **homology** | U | K.TVLGVPEVLLGILPGAGGTQRLPK.M |
| 154122 | 167 | – | 190 | 795.8129 | 2384.4168 | 2384.4155 | 0.55 | 1 | 33 | 0.00079 | 1Score **> 27** indicates **identity** Score **> 15** indicates **homology** | U | K.TVLGVPEVLLGILPGAGGTQRLPK.M |
| 154124 | 167 | – | 190 | 795.8145 | 2384.4216 | 2384.4155 | 2.56 | 1 | 28 | 0.0025 | 1Score **> 27** indicates **identity** Score **> 14** indicates **homology** | U | K.TVLGVPEVLLGILPGAGGTQRLPK.M |
| 154125 | 167 | – | 190 | 1193.2191 | 2384.4236 | 2384.4155 | 3.38 | 1 | 47 | 4e-05 | 1Score **> 27** indicates **identity** Score **> 15** indicates **homology** | U | K.TVLGVPEVLLGILPGAGGTQRLPK.M |
| 154127 | 167 | – | 190 | 795.8157 | 2384.4252 | 2384.4155 | 4.06 | 1 | 16 | 0.031 | 1Score **> 26** indicates **identity** Score **> 14** indicates **homology** | U | K.TVLGVPEVLLGILPGAGGTQRLPK.M |
| 154174 | 167 | – | 190 | 1193.7162 | 2385.4178 | 2385.3995 | 7.66 | 1 | 20 | 0.015 | 1Score **> 28** indicates **identity** Score **> 14** indicates **homology** | U | K.TVLGVPEVLLGILPGAGGTQRLPK.M  + Deamidated (NQ) |
| 79307 | 191 | – | 205 | 798.3894 | 1594.7642 | 1594.7670 | -1.73 | 0 | 35 | 0.00053 | 1Score **> 33** indicates **identity** Score **> 15** indicates **homology** | U | K.MVGVPAAFDMMLTGR.N |
| 103518 | 214 | – | 230 | 897.5133 | 1793.0120 | 1793.0121 | -0.053 | 1 | 50 | 2e-05 | 1Score **> 33** indicates **identity** Score **> 16** indicates **homology** | U | K.KMGLVDQLVEPLGPGIK.S |
| 103520 | 214 | – | 230 | 598.6785 | 1793.0135 | 1793.0121 | 0.81 | 1 | 20 | 0.015 | 1Score **> 33** indicates **identity** Score **> 14** indicates **homology** | U | K.KMGLVDQLVEPLGPGIK.S |
| 103521 | 214 | – | 230 | 598.6786 | 1793.0139 | 1793.0121 | 1.01 | 1 | 44 | 7.8e-05 | 1Score **> 33** indicates **identity** Score **> 15** indicates **homology** | U | K.KMGLVDQLVEPLGPGIK.S |
| 103522 | 214 | – | 230 | 598.6786 | 1793.0140 | 1793.0121 | 1.05 | 1 | 49 | 2.3e-05 | 1Score **> 33** indicates **identity** Score **> 16** indicates **homology** | U | K.KMGLVDQLVEPLGPGIK.S |
| 103523 | 214 | – | 230 | 598.6788 | 1793.0146 | 1793.0121 | 1.42 | 1 | 40 | 0.00017 | 1Score **> 33** indicates **identity** Score **> 15** indicates **homology** | U | K.KMGLVDQLVEPLGPGIK.S |
| 103524 | 214 | – | 230 | 897.5154 | 1793.0163 | 1793.0121 | 2.34 | 1 | 53 | 1.1e-05 | 1Score **> 33** indicates **identity** Score **> 16** indicates **homology** | U | K.KMGLVDQLVEPLGPGIK.S |
| 154596 | 214 | – | 235 | 798.1000 | 2391.2781 | 2391.2832 | -2.13 | 2 | 36 | 0.00043 | 1Score **> 36** indicates **identity** Score **> 15** indicates **homology** | U | K.KMGLVDQLVEPLGPGIKSPEER.T |
| 154600 | 214 | – | 235 | 798.1019 | 2391.2838 | 2391.2832 | 0.26 | 2 | 49 | 2.6e-05 | 1Score **> 36** indicates **identity** Score **> 16** indicates **homology** | U | K.KMGLVDQLVEPLGPGIKSPEER.T |
| 154601 | 214 | – | 235 | 798.1025 | 2391.2856 | 2391.2832 | 1.03 | 2 | 78 | 5.4e-08 | 1Score **> 36** indicates **identity** Score **> 17** indicates **homology** | U | K.KMGLVDQLVEPLGPGIKSPEER.T |
| 154602 | 214 | – | 235 | 598.8287 | 2391.2858 | 2391.2832 | 1.09 | 2 | 48 | 3.2e-05 | 1Score **> 36** indicates **identity** Score **> 15** indicates **homology** | U | K.KMGLVDQLVEPLGPGIKSPEER.T |
| 154605 | 214 | – | 235 | 598.8289 | 2391.2864 | 2391.2832 | 1.36 | 2 | 39 | 0.00024 | 1Score **> 36** indicates **identity** Score **> 15** indicates **homology** | U | K.KMGLVDQLVEPLGPGIKSPEER.T |
| 154606 | 214 | – | 235 | 598.8289 | 2391.2867 | 2391.2832 | 1.47 | 2 | 59 | 2.9e-06 | 1Score **> 36** indicates **identity** Score **> 16** indicates **homology** | U | K.KMGLVDQLVEPLGPGIKSPEER.T |
| 154607 | 214 | – | 235 | 598.8290 | 2391.2871 | 2391.2832 | 1.65 | 2 | 34 | 0.0007 | 1Score **> 36** indicates **identity** Score **> 15** indicates **homology** | U | K.KMGLVDQLVEPLGPGIKSPEER.T |
| 154609 | 214 | – | 235 | 798.1032 | 2391.2877 | 2391.2832 | 1.90 | 2 | 72 | 2e-07 | 1Score **> 36** indicates **identity** Score **> 17** indicates **homology** | U | K.KMGLVDQLVEPLGPGIKSPEER.T |
| 88573 | 215 | – | 230 | 555.9776 | 1664.9109 | 1664.9171 | -3.75 | 0 | 25 | 0.0043 | 1Score **> 34** indicates **identity** Score **> 14** indicates **homology** | U | K.MGLVDQLVEPLGPGIK.S |
| 88576 | 215 | – | 230 | 833.4644 | 1664.9143 | 1664.9171 | -1.72 | 0 | 56 | 5.4e-06 | 1Score **> 34** indicates **identity** Score **> 16** indicates **homology** | U | K.MGLVDQLVEPLGPGIK.S |
| 88580 | 215 | – | 230 | 555.9800 | 1664.9181 | 1664.9171 | 0.57 | 0 | 40 | 0.00018 | 1Score **> 34** indicates **identity** Score **> 15** indicates **homology** | U | K.MGLVDQLVEPLGPGIK.S |
| 88581 | 215 | – | 230 | 555.9800 | 1664.9183 | 1664.9171 | 0.68 | 0 | 52 | 1.2e-05 | 1Score **> 34** indicates **identity** Score **> 16** indicates **homology** | U | K.MGLVDQLVEPLGPGIK.S |
| 88582 | 215 | – | 230 | 833.4669 | 1664.9193 | 1664.9171 | 1.30 | 0 | 57 | 4.4e-06 | 1Score **> 34** indicates **identity** Score **> 16** indicates **homology** | U | K.MGLVDQLVEPLGPGIK.S |
| 88583 | 215 | – | 230 | 833.4670 | 1664.9195 | 1664.9171 | 1.39 | 0 | 59 | 2.7e-06 | 1Score **> 34** indicates **identity** Score **> 16** indicates **homology** | U | K.MGLVDQLVEPLGPGIK.S |
| 88584 | 215 | – | 230 | 833.4671 | 1664.9196 | 1664.9171 | 1.48 | 0 | 56 | 6e-06 | 1Score **> 34** indicates **identity** Score **> 16** indicates **homology** | U | K.MGLVDQLVEPLGPGIK.S |
| 88585 | 215 | – | 230 | 555.9807 | 1664.9201 | 1664.9171 | 1.80 | 0 | 49 | 2.4e-05 | 1Score **> 34** indicates **identity** Score **> 16** indicates **homology** | U | K.MGLVDQLVEPLGPGIK.S |
| 146109 | 215 | – | 235 | 755.4047 | 2263.1922 | 2263.1882 | 1.77 | 1 | 65 | 8.6e-07 | 1Score **> 37** indicates **identity** Score **> 17** indicates **homology** | U | K.MGLVDQLVEPLGPGIKSPEER.T |
| 146110 | 215 | – | 235 | 755.4051 | 2263.1935 | 2263.1882 | 2.36 | 1 | 58 | 3.6e-06 | 1Score **> 37** indicates **identity** Score **> 16** indicates **homology** | U | K.MGLVDQLVEPLGPGIKSPEER.T |
| 146111 | 215 | – | 235 | 1132.6049 | 2263.1953 | 2263.1882 | 3.12 | 1 | 78 | 5.3e-08 | 1Score **> 37** indicates **identity** Score **> 17** indicates **homology** | U | K.MGLVDQLVEPLGPGIKSPEER.T |
| 146113 | 215 | – | 235 | 755.4065 | 2263.1978 | 2263.1882 | 4.24 | 1 | 18 | 0.022 | 1Score **> 36** indicates **identity** Score **> 14** indicates **homology** | U | K.MGLVDQLVEPLGPGIKSPEER.T |
| 146114 | 215 | – | 235 | 755.4067 | 2263.1982 | 2263.1882 | 4.41 | 1 | 58 | 3.3e-06 | 1Score **> 37** indicates **identity** Score **> 16** indicates **homology** | U | K.MGLVDQLVEPLGPGIKSPEER.T |
| 147184 | 215 | – | 235 | 760.7363 | 2279.1871 | 2279.1831 | 1.73 | 1 | 31 | 0.0015 | 1Score **> 37** indicates **identity** Score **> 15** indicates **homology** | U | K.MGLVDQLVEPLGPGIKSPEER.T  + Oxidation (M) |
| 83388 | 236 | – | 249 | 542.6152 | 1624.8239 | 1624.8348 | -6.73 | 0 | 21 | 0.011 | 1Score **> 34** indicates **identity** Score **> 14** indicates **homology** | U | R.TIEYLEEVAVNFAK.G |
| 83392 | 236 | – | 249 | 813.4210 | 1624.8275 | 1624.8348 | -4.51 | 0 | 61 | 2e-06 | 1Score **> 35** indicates **identity** Score **> 16** indicates **homology** | U | R.TIEYLEEVAVNFAK.G |
| 83394 | 236 | – | 249 | 813.4218 | 1624.8290 | 1624.8348 | -3.61 | 0 | 64 | 9.2e-07 | 1Score **> 35** indicates **identity** Score **> 17** indicates **homology** | U | R.TIEYLEEVAVNFAK.G |
| 83395 | 236 | – | 249 | 813.4220 | 1624.8294 | 1624.8348 | -3.33 | 0 | 33 | 0.00073 | 1Score **> 34** indicates **identity** Score **> 15** indicates **homology** | U | R.TIEYLEEVAVNFAK.G |
| 83397 | 236 | – | 249 | 542.6172 | 1624.8297 | 1624.8348 | -3.16 | 0 | 27 | 0.0031 | 1Score **> 34** indicates **identity** Score **> 14** indicates **homology** | U | R.TIEYLEEVAVNFAK.G |
| 83399 | 236 | – | 249 | 813.4230 | 1624.8314 | 1624.8348 | -2.10 | 0 | 62 | 1.5e-06 | 1Score **> 35** indicates **identity** Score **> 16** indicates **homology** | U | R.TIEYLEEVAVNFAK.G |
| 83400 | 236 | – | 249 | 542.6178 | 1624.8317 | 1624.8348 | -1.94 | 0 | 38 | 0.0003 | 1Score **> 35** indicates **identity** Score **> 15** indicates **homology** | U | R.TIEYLEEVAVNFAK.G |
| 83403 | 236 | – | 249 | 542.6179 | 1624.8319 | 1624.8348 | -1.77 | 0 | 23 | 0.007 | 1Score **> 35** indicates **identity** Score **> 14** indicates **homology** | U | R.TIEYLEEVAVNFAK.G |
| 83405 | 236 | – | 249 | 542.6181 | 1624.8324 | 1624.8348 | -1.45 | 0 | 15 | 0.043 | 1Score **> 35** indicates **identity** Score **> 13** indicates **homology** | U | R.TIEYLEEVAVNFAK.G |
| 83406 | 236 | – | 249 | 542.6181 | 1624.8326 | 1624.8348 | -1.37 | 0 | 31 | 0.0012 | 1Score **> 35** indicates **identity** Score **> 14** indicates **homology** | U | R.TIEYLEEVAVNFAK.G |
| 83407 | 236 | – | 249 | 813.4236 | 1624.8326 | 1624.8348 | -1.36 | 0 | 64 | 1.1e-06 | 1Score **> 35** indicates **identity** Score **> 16** indicates **homology** | U | R.TIEYLEEVAVNFAK.G |
| 83408 | 236 | – | 249 | 813.4236 | 1624.8327 | 1624.8348 | -1.28 | 0 | 63 | 1.3e-06 | 1Score **> 35** indicates **identity** Score **> 16** indicates **homology** | U | R.TIEYLEEVAVNFAK.G |
| 83409 | 236 | – | 249 | 813.4237 | 1624.8328 | 1624.8348 | -1.23 | 0 | 65 | 7.6e-07 | 1Score **> 35** indicates **identity** Score **> 17** indicates **homology** | U | R.TIEYLEEVAVNFAK.G |
| 83410 | 236 | – | 249 | 813.4237 | 1624.8329 | 1624.8348 | -1.15 | 0 | 83 | 1.7e-08 | 1Score **> 35** indicates **identity** Score **> 18** indicates **homology** | U | R.TIEYLEEVAVNFAK.G |
| 83411 | 236 | – | 249 | 542.6184 | 1624.8333 | 1624.8348 | -0.94 | 0 | 41 | 0.00016 | 1Score **> 35** indicates **identity** Score **> 15** indicates **homology** | U | R.TIEYLEEVAVNFAK.G |
| 83412 | 236 | – | 249 | 542.6184 | 1624.8335 | 1624.8348 | -0.79 | 0 | 58 | 4e-06 | 1Score **> 34** indicates **identity** Score **> 16** indicates **homology** | U | R.TIEYLEEVAVNFAK.G |
| 83413 | 236 | – | 249 | 813.4242 | 1624.8338 | 1624.8348 | -0.61 | 0 | 61 | 1.8e-06 | 1Score **> 34** indicates **identity** Score **> 16** indicates **homology** | U | R.TIEYLEEVAVNFAK.G |
| 83414 | 236 | – | 249 | 542.6186 | 1624.8339 | 1624.8348 | -0.55 | 0 | 41 | 0.00013 | 1Score **> 34** indicates **identity** Score **> 15** indicates **homology** | U | R.TIEYLEEVAVNFAK.G |
| 83415 | 236 | – | 249 | 542.6186 | 1624.8339 | 1624.8348 | -0.53 | 0 | 62 | 1.5e-06 | 1Score **> 34** indicates **identity** Score **> 16** indicates **homology** | U | R.TIEYLEEVAVNFAK.G |
| 83416 | 236 | – | 249 | 542.6186 | 1624.8341 | 1624.8348 | -0.44 | 0 | 25 | 0.0045 | 1Score **> 34** indicates **identity** Score **> 14** indicates **homology** | U | R.TIEYLEEVAVNFAK.G |
| 83417 | 236 | – | 249 | 813.4244 | 1624.8342 | 1624.8348 | -0.36 | 0 | 64 | 9.7e-07 | 1Score **> 34** indicates **identity** Score **> 17** indicates **homology** | U | R.TIEYLEEVAVNFAK.G |
| 83419 | 236 | – | 249 | 542.6187 | 1624.8344 | 1624.8348 | -0.27 | 0 | 39 | 0.00022 | 1Score **> 34** indicates **identity** Score **> 15** indicates **homology** | U | R.TIEYLEEVAVNFAK.G |
| 83420 | 236 | – | 249 | 813.4245 | 1624.8344 | 1624.8348 | -0.24 | 0 | 84 | 1.3e-08 | 1Score **> 34** indicates **identity** Score **> 18** indicates **homology** | U | R.TIEYLEEVAVNFAK.G |
| 83421 | 236 | – | 249 | 813.4245 | 1624.8344 | 1624.8348 | -0.22 | 0 | 65 | 8.7e-07 | 1Score **> 34** indicates **identity** Score **> 17** indicates **homology** | U | R.TIEYLEEVAVNFAK.G |
| 83422 | 236 | – | 249 | 542.6188 | 1624.8346 | 1624.8348 | -0.14 | 0 | 64 | 9.6e-07 | 1Score **> 34** indicates **identity** Score **> 17** indicates **homology** | U | R.TIEYLEEVAVNFAK.G |
| 83426 | 236 | – | 249 | 813.4247 | 1624.8349 | 1624.8348 | 0.079 | 0 | 68 | 4.4e-07 | 1Score **> 34** indicates **identity** Score **> 17** indicates **homology** | U | R.TIEYLEEVAVNFAK.G |
| 83429 | 236 | – | 249 | 813.4248 | 1624.8350 | 1624.8348 | 0.14 | 0 | 63 | 1.2e-06 | 1Score **> 34** indicates **identity** Score **> 16** indicates **homology** | U | R.TIEYLEEVAVNFAK.G |
| 83430 | 236 | – | 249 | 813.4248 | 1624.8350 | 1624.8348 | 0.15 | 0 | 33 | 0.00076 | 1Score **> 34** indicates **identity** Score **> 15** indicates **homology** | U | R.TIEYLEEVAVNFAK.G |
| 83431 | 236 | – | 249 | 813.4248 | 1624.8351 | 1624.8348 | 0.21 | 0 | 42 | 0.00011 | 1Score **> 34** indicates **identity** Score **> 15** indicates **homology** | U | R.TIEYLEEVAVNFAK.G |
| 83434 | 236 | – | 249 | 542.6190 | 1624.8352 | 1624.8348 | 0.22 | 0 | 21 | 0.01 | 1Score **> 34** indicates **identity** Score **> 14** indicates **homology** | U | R.TIEYLEEVAVNFAK.G |
| 83437 | 236 | – | 249 | 813.4250 | 1624.8354 | 1624.8348 | 0.35 | 0 | 83 | 1.5e-08 | 1Score **> 34** indicates **identity** Score **> 18** indicates **homology** | U | R.TIEYLEEVAVNFAK.G |
| 83439 | 236 | – | 249 | 813.4250 | 1624.8354 | 1624.8348 | 0.37 | 0 | 83 | 1.8e-08 | 1Score **> 34** indicates **identity** Score **> 18** indicates **homology** | U | R.TIEYLEEVAVNFAK.G |
| 83441 | 236 | – | 249 | 542.6191 | 1624.8355 | 1624.8348 | 0.43 | 0 | 64 | 9.4e-07 | 1Score **> 34** indicates **identity** Score **> 17** indicates **homology** | U | R.TIEYLEEVAVNFAK.G |
| 83442 | 236 | – | 249 | 542.6191 | 1624.8356 | 1624.8348 | 0.47 | 0 | 62 | 1.6e-06 | 1Score **> 34** indicates **identity** Score **> 16** indicates **homology** | U | R.TIEYLEEVAVNFAK.G |
| 83446 | 236 | – | 249 | 542.6192 | 1624.8358 | 1624.8348 | 0.59 | 0 | 42 | 0.00013 | 1Score **> 34** indicates **identity** Score **> 15** indicates **homology** | U | R.TIEYLEEVAVNFAK.G |
| 83447 | 236 | – | 249 | 542.6193 | 1624.8361 | 1624.8348 | 0.78 | 0 | 46 | 4.9e-05 | 1Score **> 34** indicates **identity** Score **> 15** indicates **homology** | U | R.TIEYLEEVAVNFAK.G |
| 83448 | 236 | – | 249 | 542.6193 | 1624.8361 | 1624.8348 | 0.81 | 0 | 29 | 0.0019 | 1Score **> 34** indicates **identity** Score **> 14** indicates **homology** | U | R.TIEYLEEVAVNFAK.G |
| 83450 | 236 | – | 249 | 813.4254 | 1624.8362 | 1624.8348 | 0.87 | 0 | 45 | 5.8e-05 | 1Score **> 34** indicates **identity** Score **> 15** indicates **homology** | U | R.TIEYLEEVAVNFAK.G |
| 83451 | 236 | – | 249 | 542.6194 | 1624.8363 | 1624.8348 | 0.93 | 0 | 16 | 0.028 | 1Score **> 34** indicates **identity** Score **> 14** indicates **homology** | U | R.TIEYLEEVAVNFAK.G |
| 83452 | 236 | – | 249 | 542.6194 | 1624.8364 | 1624.8348 | 0.98 | 0 | 65 | 7.9e-07 | 1Score **> 34** indicates **identity** Score **> 17** indicates **homology** | U | R.TIEYLEEVAVNFAK.G |
| 83454 | 236 | – | 249 | 542.6194 | 1624.8365 | 1624.8348 | 1.03 | 0 | 24 | 0.0062 | 1Score **> 34** indicates **identity** Score **> 14** indicates **homology** | U | R.TIEYLEEVAVNFAK.G |
| 83455 | 236 | – | 249 | 813.4255 | 1624.8365 | 1624.8348 | 1.04 | 0 | 89 | 4.6e-09 | 1Score **> 34** indicates **identity** Score **> 18** indicates **homology** | U | R.TIEYLEEVAVNFAK.G |
| 83458 | 236 | – | 249 | 813.4256 | 1624.8366 | 1624.8348 | 1.12 | 0 | 48 | 3e-05 | 1Score **> 34** indicates **identity** Score **> 16** indicates **homology** | U | R.TIEYLEEVAVNFAK.G |
| 83462 | 236 | – | 249 | 542.6195 | 1624.8368 | 1624.8348 | 1.24 | 0 | 56 | 5.2e-06 | 1Score **> 34** indicates **identity** Score **> 16** indicates **homology** | U | R.TIEYLEEVAVNFAK.G |
| 83463 | 236 | – | 249 | 813.4258 | 1624.8370 | 1624.8348 | 1.35 | 0 | 81 | 2.3e-08 | 1Score **> 34** indicates **identity** Score **> 18** indicates **homology** | U | R.TIEYLEEVAVNFAK.G |
| 83464 | 236 | – | 249 | 813.4258 | 1624.8371 | 1624.8348 | 1.39 | 0 | 66 | 6.3e-07 | 1Score **> 34** indicates **identity** Score **> 17** indicates **homology** | U | R.TIEYLEEVAVNFAK.G |
| 83466 | 236 | – | 249 | 813.4258 | 1624.8371 | 1624.8348 | 1.42 | 0 | 88 | 5.8e-09 | 1Score **> 34** indicates **identity** Score **> 18** indicates **homology** | U | R.TIEYLEEVAVNFAK.G |
| 83469 | 236 | – | 249 | 542.6197 | 1624.8372 | 1624.8348 | 1.46 | 0 | 22 | 0.0079 | 1Score **> 34** indicates **identity** Score **> 14** indicates **homology** | U | R.TIEYLEEVAVNFAK.G |
| 83470 | 236 | – | 249 | 813.4260 | 1624.8374 | 1624.8348 | 1.58 | 0 | 60 | 2.4e-06 | 1Score **> 34** indicates **identity** Score **> 16** indicates **homology** | U | R.TIEYLEEVAVNFAK.G |
| 83471 | 236 | – | 249 | 813.4260 | 1624.8375 | 1624.8348 | 1.63 | 0 | 79 | 4e-08 | 1Score **> 34** indicates **identity** Score **> 17** indicates **homology** | U | R.TIEYLEEVAVNFAK.G |
| 83472 | 236 | – | 249 | 542.6198 | 1624.8375 | 1624.8348 | 1.68 | 0 | 17 | 0.028 | 1Score **> 34** indicates **identity** Score **> 14** indicates **homology** | U | R.TIEYLEEVAVNFAK.G |
| 83473 | 236 | – | 249 | 542.6198 | 1624.8376 | 1624.8348 | 1.72 | 0 | 52 | 1.4e-05 | 1Score **> 34** indicates **identity** Score **> 16** indicates **homology** | U | R.TIEYLEEVAVNFAK.G |
| 83474 | 236 | – | 249 | 542.6198 | 1624.8376 | 1624.8348 | 1.72 | 0 | 62 | 1.5e-06 | 1Score **> 34** indicates **identity** Score **> 16** indicates **homology** | U | R.TIEYLEEVAVNFAK.G |
| 83476 | 236 | – | 249 | 813.4261 | 1624.8377 | 1624.8348 | 1.79 | 0 | 65 | 8.4e-07 | 1Score **> 34** indicates **identity** Score **> 17** indicates **homology** | U | R.TIEYLEEVAVNFAK.G |
| 83477 | 236 | – | 249 | 813.4262 | 1624.8379 | 1624.8348 | 1.89 | 0 | 61 | 2e-06 | 1Score **> 34** indicates **identity** Score **> 16** indicates **homology** | U | R.TIEYLEEVAVNFAK.G |
| 83478 | 236 | – | 249 | 542.6199 | 1624.8380 | 1624.8348 | 1.96 | 0 | 61 | 1.8e-06 | 1Score **> 34** indicates **identity** Score **> 16** indicates **homology** | U | R.TIEYLEEVAVNFAK.G |
| 83479 | 236 | – | 249 | 813.4263 | 1624.8380 | 1624.8348 | 1.99 | 0 | 65 | 8.6e-07 | 1Score **> 34** indicates **identity** Score **> 17** indicates **homology** | U | R.TIEYLEEVAVNFAK.G |
| 83482 | 236 | – | 249 | 542.6200 | 1624.8381 | 1624.8348 | 2.03 | 0 | 22 | 0.0095 | 1Score **> 34** indicates **identity** Score **> 14** indicates **homology** | U | R.TIEYLEEVAVNFAK.G |
| 83483 | 236 | – | 249 | 813.4265 | 1624.8385 | 1624.8348 | 2.25 | 0 | 26 | 0.0069 | 1Score **> 34** indicates **identity** Score **> 17** indicates **homology** | U | R.TIEYLEEVAVNFAK.G |
| 83485 | 236 | – | 249 | 813.4266 | 1624.8386 | 1624.8348 | 2.33 | 0 | 49 | 2.7e-05 | 1Score **> 34** indicates **identity** Score **> 16** indicates **homology** | U | R.TIEYLEEVAVNFAK.G |
| 83486 | 236 | – | 249 | 542.6202 | 1624.8387 | 1624.8348 | 2.37 | 0 | 35 | 0.00049 | 1Score **> 34** indicates **identity** Score **> 15** indicates **homology** | U | R.TIEYLEEVAVNFAK.G |
| 83487 | 236 | – | 249 | 542.6202 | 1624.8387 | 1624.8348 | 2.37 | 0 | 25 | 0.0049 | 1Score **> 34** indicates **identity** Score **> 14** indicates **homology** | U | R.TIEYLEEVAVNFAK.G |
| 83488 | 236 | – | 249 | 813.4266 | 1624.8387 | 1624.8348 | 2.39 | 0 | 66 | 7e-07 | 1Score **> 34** indicates **identity** Score **> 17** indicates **homology** | U | R.TIEYLEEVAVNFAK.G |
| 83489 | 236 | – | 249 | 813.4267 | 1624.8388 | 1624.8348 | 2.43 | 0 | 83 | 1.6e-08 | 1Score **> 34** indicates **identity** Score **> 18** indicates **homology** | U | R.TIEYLEEVAVNFAK.G |
| 83490 | 236 | – | 249 | 542.6202 | 1624.8388 | 1624.8348 | 2.45 | 0 | 21 | 0.011 | 1Score **> 34** indicates **identity** Score **> 14** indicates **homology** | U | R.TIEYLEEVAVNFAK.G |
| 83491 | 236 | – | 249 | 542.6202 | 1624.8389 | 1624.8348 | 2.50 | 0 | 43 | 9.5e-05 | 1Score **> 34** indicates **identity** Score **> 15** indicates **homology** | U | R.TIEYLEEVAVNFAK.G |
| 83492 | 236 | – | 249 | 813.4268 | 1624.8390 | 1624.8348 | 2.56 | 0 | 64 | 1e-06 | 1Score **> 34** indicates **identity** Score **> 16** indicates **homology** | U | R.TIEYLEEVAVNFAK.G |
| 83493 | 236 | – | 249 | 813.4269 | 1624.8393 | 1624.8348 | 2.77 | 0 | 79 | 4.3e-08 | 1Score **> 34** indicates **identity** Score **> 17** indicates **homology** | U | R.TIEYLEEVAVNFAK.G |
| 83497 | 236 | – | 249 | 813.4270 | 1624.8395 | 1624.8348 | 2.91 | 0 | 62 | 1.7e-06 | 1Score **> 34** indicates **identity** Score **> 16** indicates **homology** | U | R.TIEYLEEVAVNFAK.G |
| 83498 | 236 | – | 249 | 813.4271 | 1624.8397 | 1624.8348 | 3.04 | 0 | 61 | 2.1e-06 | 1Score **> 35** indicates **identity** Score **> 16** indicates **homology** | U | R.TIEYLEEVAVNFAK.G |
| 83499 | 236 | – | 249 | 813.4274 | 1624.8403 | 1624.8348 | 3.36 | 0 | 42 | 0.00012 | 1Score **> 34** indicates **identity** Score **> 15** indicates **homology** | U | R.TIEYLEEVAVNFAK.G |
| 83500 | 236 | – | 249 | 813.4274 | 1624.8403 | 1624.8348 | 3.37 | 0 | 83 | 1.6e-08 | 1Score **> 34** indicates **identity** Score **> 18** indicates **homology** | U | R.TIEYLEEVAVNFAK.G |
| 83501 | 236 | – | 249 | 813.4274 | 1624.8403 | 1624.8348 | 3.38 | 0 | 70 | 2.6e-07 | 1Score **> 34** indicates **identity** Score **> 17** indicates **homology** | U | R.TIEYLEEVAVNFAK.G |
| 83502 | 236 | – | 249 | 813.4276 | 1624.8406 | 1624.8348 | 3.58 | 0 | 65 | 8.4e-07 | 1Score **> 34** indicates **identity** Score **> 17** indicates **homology** | U | R.TIEYLEEVAVNFAK.G |
| 83503 | 236 | – | 249 | 813.4277 | 1624.8408 | 1624.8348 | 3.67 | 0 | 70 | 2.8e-07 | 1Score **> 34** indicates **identity** Score **> 17** indicates **homology** | U | R.TIEYLEEVAVNFAK.G |
| 83504 | 236 | – | 249 | 813.4277 | 1624.8408 | 1624.8348 | 3.68 | 0 | 84 | 1.3e-08 | 1Score **> 34** indicates **identity** Score **> 18** indicates **homology** | U | R.TIEYLEEVAVNFAK.G |
| 83505 | 236 | – | 249 | 813.4277 | 1624.8409 | 1624.8348 | 3.72 | 0 | 30 | 0.0017 | 1Score **> 34** indicates **identity** Score **> 14** indicates **homology** | U | R.TIEYLEEVAVNFAK.G |
| 83506 | 236 | – | 249 | 813.4277 | 1624.8409 | 1624.8348 | 3.76 | 0 | 61 | 2e-06 | 1Score **> 34** indicates **identity** Score **> 16** indicates **homology** | U | R.TIEYLEEVAVNFAK.G |
| 83507 | 236 | – | 249 | 542.6209 | 1624.8410 | 1624.8348 | 3.82 | 0 | 46 | 5.2e-05 | 1Score **> 34** indicates **identity** Score **> 15** indicates **homology** | U | R.TIEYLEEVAVNFAK.G |
| 83510 | 236 | – | 249 | 813.4279 | 1624.8413 | 1624.8348 | 3.98 | 0 | 35 | 0.00057 | 1Score **> 34** indicates **identity** Score **> 15** indicates **homology** | U | R.TIEYLEEVAVNFAK.G |
| 83512 | 236 | – | 249 | 542.6211 | 1624.8414 | 1624.8348 | 4.05 | 0 | 34 | 0.00063 | 1Score **> 34** indicates **identity** Score **> 15** indicates **homology** | U | R.TIEYLEEVAVNFAK.G |
| 83513 | 236 | – | 249 | 813.4280 | 1624.8415 | 1624.8348 | 4.12 | 0 | 83 | 1.6e-08 | 1Score **> 34** indicates **identity** Score **> 18** indicates **homology** | U | R.TIEYLEEVAVNFAK.G |
| 83514 | 236 | – | 249 | 813.4281 | 1624.8416 | 1624.8348 | 4.18 | 0 | 87 | 6.6e-09 | 1Score **> 34** indicates **identity** Score **> 18** indicates **homology** | U | R.TIEYLEEVAVNFAK.G |
| 83517 | 236 | – | 249 | 542.6212 | 1624.8419 | 1624.8348 | 4.37 | 0 | 17 | 0.027 | 1Score **> 34** indicates **identity** Score **> 14** indicates **homology** | U | R.TIEYLEEVAVNFAK.G |
| 83522 | 236 | – | 249 | 813.4291 | 1624.8437 | 1624.8348 | 5.48 | 0 | 82 | 2.2e-08 | 1Score **> 34** indicates **identity** Score **> 18** indicates **homology** | U | R.TIEYLEEVAVNFAK.G |
| 83523 | 236 | – | 249 | 813.4292 | 1624.8439 | 1624.8348 | 5.63 | 0 | 82 | 2.2e-08 | 1Score **> 34** indicates **identity** Score **> 18** indicates **homology** | U | R.TIEYLEEVAVNFAK.G |
| 83524 | 236 | – | 249 | 813.4293 | 1624.8441 | 1624.8348 | 5.69 | 0 | 33 | 0.00087 | 1Score **> 34** indicates **identity** Score **> 15** indicates **homology** | U | R.TIEYLEEVAVNFAK.G |
| 83525 | 236 | – | 249 | 813.4294 | 1624.8442 | 1624.8348 | 5.76 | 0 | 50 | 2e-05 | 1Score **> 34** indicates **identity** Score **> 16** indicates **homology** | U | R.TIEYLEEVAVNFAK.G |
| 83528 | 236 | – | 249 | 813.4299 | 1624.8453 | 1624.8348 | 6.43 | 0 | 64 | 9.1e-07 | 1Score **> 34** indicates **identity** Score **> 17** indicates **homology** | U | R.TIEYLEEVAVNFAK.G |
| 83529 | 236 | – | 249 | 813.4300 | 1624.8454 | 1624.8348 | 6.49 | 0 | 70 | 2.8e-07 | 1Score **> 34** indicates **identity** Score **> 17** indicates **homology** | U | R.TIEYLEEVAVNFAK.G |
| 83530 | 236 | – | 249 | 813.4300 | 1624.8455 | 1624.8348 | 6.61 | 0 | 50 | 1.9e-05 | 1Score **> 34** indicates **identity** Score **> 16** indicates **homology** | U | R.TIEYLEEVAVNFAK.G |
| 83532 | 236 | – | 249 | 542.6227 | 1624.8462 | 1624.8348 | 7.02 | 0 | 16 | 0.033 | 1Score **> 34** indicates **identity** Score **> 13** indicates **homology** | U | R.TIEYLEEVAVNFAK.G |
| 83533 | 236 | – | 249 | 542.6228 | 1624.8466 | 1624.8348 | 7.23 | 0 | 16 | 0.031 | 1Score **> 34** indicates **identity** Score **> 14** indicates **homology** | U | R.TIEYLEEVAVNFAK.G |
| 83534 | 236 | – | 249 | 813.4310 | 1624.8475 | 1624.8348 | 7.83 | 0 | 64 | 1e-06 | 1Score **> 34** indicates **identity** Score **> 16** indicates **homology** | U | R.TIEYLEEVAVNFAK.G |
| 83535 | 236 | – | 249 | 813.4311 | 1624.8477 | 1624.8348 | 7.93 | 0 | 88 | 5.2e-09 | 1Score **> 34** indicates **identity** Score **> 18** indicates **homology** | U | R.TIEYLEEVAVNFAK.G |
| 83536 | 236 | – | 249 | 813.4312 | 1624.8478 | 1624.8348 | 8.02 | 0 | 77 | 5.6e-08 | 1Score **> 34** indicates **identity** Score **> 17** indicates **homology** | U | R.TIEYLEEVAVNFAK.G |
| 83538 | 236 | – | 249 | 813.4314 | 1624.8482 | 1624.8348 | 8.23 | 0 | 22 | 0.0085 | 1Score **> 34** indicates **identity** Score **> 14** indicates **homology** | U | R.TIEYLEEVAVNFAK.G |
| 54821 | 268 | – | 279 | 699.8764 | 1397.7383 | 1397.7377 | 0.41 | 0 | 71 | 1e-06 | 1Score **> 34** indicates **identity** Score **> 24** indicates **homology** | U | K.LTTYAMTVPFVR.Q |
| 54822 | 268 | – | 279 | 699.8767 | 1397.7388 | 1397.7377 | 0.81 | 0 | 71 | 1e-06 | 1Score **> 34** indicates **identity** Score **> 24** indicates **homology** | U | K.LTTYAMTVPFVR.Q |
| 54823 | 268 | – | 279 | 699.8767 | 1397.7389 | 1397.7377 | 0.87 | 0 | 71 | 1e-06 | 1Score **> 34** indicates **identity** Score **> 23** indicates **homology** | U | K.LTTYAMTVPFVR.Q |
| 54824 | 268 | – | 279 | 466.9203 | 1397.7390 | 1397.7377 | 0.91 | 0 | 15 | 0.041 | 1Score **> 34** indicates **identity** Score **> 13** indicates **homology** | U | K.LTTYAMTVPFVR.Q |
| 54826 | 268 | – | 279 | 466.9203 | 1397.7391 | 1397.7377 | 1.01 | 0 | 18 | 0.02 | 1Score **> 34** indicates **identity** Score **> 14** indicates **homology** | U | K.LTTYAMTVPFVR.Q |
| 54827 | 268 | – | 279 | 699.8770 | 1397.7395 | 1397.7377 | 1.26 | 0 | 70 | 1e-06 | 1Score **> 34** indicates **identity** Score **> 22** indicates **homology** | U | K.LTTYAMTVPFVR.Q |
| 54828 | 268 | – | 279 | 699.8770 | 1397.7395 | 1397.7377 | 1.26 | 0 | 66 | 8.5e-07 | 1Score **> 34** indicates **identity** Score **> 18** indicates **homology** | U | K.LTTYAMTVPFVR.Q |
| 56681 | 268 | – | 279 | 707.8739 | 1413.7332 | 1413.7326 | 0.42 | 0 | 19 | 0.017 | 1Score **> 34** indicates **identity** Score **> 14** indicates **homology** | U | K.LTTYAMTVPFVR.Q  + Oxidation (M) |
| 56689 | 268 | – | 279 | 707.8785 | 1413.7425 | 1413.7326 | 6.97 | 0 | 15 | 0.038 | 1Score **> 34** indicates **identity** Score **> 13** indicates **homology** | U | K.LTTYAMTVPFVR.Q  + Oxidation (M) |
| 6702 | 296 | – | 303 | 429.7573 | 857.5001 | 857.5011 | -1.08 | 0 | 17 | 0.027 | 1Score **> 32** indicates **identity** Score **> 14** indicates **homology** | U | K.GLYPAPLK.I |
| 6703 | 296 | – | 303 | 429.7574 | 857.5002 | 857.5011 | -1.02 | 0 | 16 | 0.031 | 1Score **> 32** indicates **identity** Score **> 14** indicates **homology** | U | K.GLYPAPLK.I |
| 6704 | 296 | – | 303 | 429.7577 | 857.5009 | 857.5011 | -0.16 | 0 | 25 | 0.0045 | 1Score **> 32** indicates **identity** Score **> 14** indicates **homology** | U | K.GLYPAPLK.I |
| 6706 | 296 | – | 303 | 429.7579 | 857.5012 | 857.5011 | 0.13 | 0 | 34 | 0.0021 | 1Score **> 32** indicates **identity** Score **> 19** indicates **homology** | U | K.GLYPAPLK.I |
| 6707 | 296 | – | 303 | 429.7579 | 857.5012 | 857.5011 | 0.17 | 0 | 34 | 0.0021 | 1Score **> 32** indicates **identity** Score **> 19** indicates **homology** | U | K.GLYPAPLK.I |
| 6708 | 296 | – | 303 | 429.7580 | 857.5015 | 857.5011 | 0.53 | 0 | 34 | 0.002 | 1Score **> 32** indicates **identity** Score **> 19** indicates **homology** | U | K.GLYPAPLK.I |
| 6709 | 296 | – | 303 | 429.7583 | 857.5020 | 857.5011 | 1.06 | 0 | 31 | 0.0024 | 1Score **> 32** indicates **identity** Score **> 17** indicates **homology** | U | K.GLYPAPLK.I |
| 6710 | 296 | – | 303 | 429.7583 | 857.5021 | 857.5011 | 1.19 | 0 | 29 | 0.0032 | 1Score **> 32** indicates **identity** Score **> 16** indicates **homology** | U | K.GLYPAPLK.I |
| 6711 | 296 | – | 303 | 429.7585 | 857.5024 | 857.5011 | 1.50 | 0 | 15 | 0.047 | 1Score **> 32** indicates **identity** Score **> 15** indicates **homology** | U | K.GLYPAPLK.I |
| 66679 | 296 | – | 309 | 749.4548 | 1496.8951 | 1496.8966 | -1.05 | 1 | 36 | 0.00041 | 1Score **> 31** indicates **identity** Score **> 15** indicates **homology** | U | K.GLYPAPLKIIDAVK.A |
| 66680 | 296 | – | 309 | 499.9724 | 1496.8954 | 1496.8966 | -0.78 | 1 | 25 | 0.0047 | 1Score **> 31** indicates **identity** Score **> 14** indicates **homology** | U | K.GLYPAPLKIIDAVK.A |
| 66681 | 296 | – | 309 | 499.9726 | 1496.8960 | 1496.8966 | -0.41 | 1 | 31 | 0.0026 | 1Score **> 32** indicates **identity** Score **> 17** indicates **homology** | U | K.GLYPAPLKIIDAVK.A |
| 66682 | 296 | – | 309 | 499.9726 | 1496.8960 | 1496.8966 | -0.40 | 1 | 50 | 2.1e-05 | 1Score **> 32** indicates **identity** Score **> 16** indicates **homology** | U | K.GLYPAPLKIIDAVK.A |
| 66684 | 296 | – | 309 | 499.9728 | 1496.8965 | 1496.8966 | -0.063 | 1 | 48 | 3.1e-05 | 1Score **> 31** indicates **identity** Score **> 16** indicates **homology** | U | K.GLYPAPLKIIDAVK.A |
| 66685 | 296 | – | 309 | 499.9730 | 1496.8970 | 1496.8966 | 0.28 | 1 | 42 | 0.00012 | 1Score **> 31** indicates **identity** Score **> 15** indicates **homology** | U | K.GLYPAPLKIIDAVK.A |
| 66686 | 296 | – | 309 | 749.4560 | 1496.8974 | 1496.8966 | 0.53 | 1 | 39 | 0.00023 | 1Score **> 31** indicates **identity** Score **> 15** indicates **homology** | U | K.GLYPAPLKIIDAVK.A |
| 66688 | 296 | – | 309 | 499.9732 | 1496.8978 | 1496.8966 | 0.78 | 1 | 62 | 1.7e-06 | 1Score **> 31** indicates **identity** Score **> 17** indicates **homology** | U | K.GLYPAPLKIIDAVK.A |
| 66690 | 296 | – | 309 | 499.9736 | 1496.8989 | 1496.8966 | 1.50 | 1 | 26 | 0.0037 | 1Score **> 31** indicates **identity** Score **> 14** indicates **homology** | U | K.GLYPAPLKIIDAVK.A |
| 95789 | 310 | – | 326 | 862.4098 | 1722.8051 | 1722.8060 | -0.53 | 0 | 111 | 6e-11 | 1Score **> 32** indicates **identity** Score **> 21** indicates **homology** | U | K.AGLEQGSDAGYLAESQK.F |
| 95790 | 310 | – | 326 | 575.2757 | 1722.8052 | 1722.8060 | -0.49 | 0 | 24 | 0.0052 | 1Score **> 32** indicates **identity** Score **> 14** indicates **homology** | U | K.AGLEQGSDAGYLAESQK.F |
| 95791 | 310 | – | 326 | 862.4101 | 1722.8057 | 1722.8060 | -0.18 | 0 | 95 | 1.2e-09 | 1Score **> 32** indicates **identity** Score **> 18** indicates **homology** | U | K.AGLEQGSDAGYLAESQK.F |
| 95793 | 310 | – | 326 | 862.4103 | 1722.8060 | 1722.8060 | -0.043 | 0 | 113 | 1.1e-10 | 1Score **> 32** indicates **identity** Score **> 26** indicates **homology** | U | K.AGLEQGSDAGYLAESQK.F |
| 95794 | 310 | – | 326 | 862.4105 | 1722.8064 | 1722.8060 | 0.24 | 0 | 95 | 1e-08 | 1Score **> 32** indicates **identity** Score **> 27** indicates **homology** | U | K.AGLEQGSDAGYLAESQK.F |
| 95795 | 310 | – | 326 | 862.4105 | 1722.8064 | 1722.8060 | 0.24 | 0 | 69 | 3.3e-07 | 1Score **> 32** indicates **identity** Score **> 17** indicates **homology** | U | K.AGLEQGSDAGYLAESQK.F |
| 95796 | 310 | – | 326 | 575.2761 | 1722.8065 | 1722.8060 | 0.26 | 0 | 58 | 3.9e-06 | 1Score **> 32** indicates **identity** Score **> 16** indicates **homology** | U | K.AGLEQGSDAGYLAESQK.F |
| 95797 | 310 | – | 326 | 575.2761 | 1722.8066 | 1722.8060 | 0.31 | 0 | 24 | 0.0057 | 1Score **> 32** indicates **identity** Score **> 14** indicates **homology** | U | K.AGLEQGSDAGYLAESQK.F |
| 95798 | 310 | – | 326 | 575.2762 | 1722.8066 | 1722.8060 | 0.35 | 0 | 21 | 0.011 | 1Score **> 32** indicates **identity** Score **> 14** indicates **homology** | U | K.AGLEQGSDAGYLAESQK.F |
| 95799 | 310 | – | 326 | 862.4106 | 1722.8066 | 1722.8060 | 0.36 | 0 | 69 | 3.3e-07 | 1Score **> 32** indicates **identity** Score **> 17** indicates **homology** | U | K.AGLEQGSDAGYLAESQK.F |
| 95801 | 310 | – | 326 | 862.4107 | 1722.8068 | 1722.8060 | 0.44 | 0 | 98 | 6e-10 | 1Score **> 32** indicates **identity** Score **> 19** indicates **homology** | U | K.AGLEQGSDAGYLAESQK.F |
| 95802 | 310 | – | 326 | 575.2762 | 1722.8068 | 1722.8060 | 0.46 | 0 | 47 | 4.2e-05 | 1Score **> 32** indicates **identity** Score **> 15** indicates **homology** | U | K.AGLEQGSDAGYLAESQK.F |
| 95803 | 310 | – | 326 | 862.4108 | 1722.8071 | 1722.8060 | 0.62 | 0 | 90 | 1.6e-08 | 1Score **> 32** indicates **identity** Score **> 25** indicates **homology** | U | K.AGLEQGSDAGYLAESQK.F |
| 95804 | 310 | – | 326 | 862.4108 | 1722.8071 | 1722.8060 | 0.62 | 0 | 113 | 5.7e-11 | 1Score **> 32** indicates **identity** Score **> 23** indicates **homology** | U | K.AGLEQGSDAGYLAESQK.F |
| 95806 | 310 | – | 326 | 862.4109 | 1722.8073 | 1722.8060 | 0.75 | 0 | 70 | 2.5e-07 | 1Score **> 32** indicates **identity** Score **> 17** indicates **homology** | U | K.AGLEQGSDAGYLAESQK.F |
| 95807 | 310 | – | 326 | 575.2765 | 1722.8076 | 1722.8060 | 0.93 | 0 | 25 | 0.0043 | 1Score **> 33** indicates **identity** Score **> 14** indicates **homology** | U | K.AGLEQGSDAGYLAESQK.F |
| 95809 | 310 | – | 326 | 862.4112 | 1722.8078 | 1722.8060 | 1.04 | 0 | 113 | 4.6e-11 | 1Score **> 33** indicates **identity** Score **> 22** indicates **homology** | U | K.AGLEQGSDAGYLAESQK.F |
| 95810 | 310 | – | 326 | 862.4116 | 1722.8086 | 1722.8060 | 1.46 | 0 | 59 | 2.7e-06 | 1Score **> 33** indicates **identity** Score **> 16** indicates **homology** | U | K.AGLEQGSDAGYLAESQK.F |
| 95935 | 310 | – | 326 | 862.9105 | 1723.8065 | 1723.7900 | 9.54 | 0 | 62 | 1.6e-06 | 1Score **> 32** indicates **identity** Score **> 16** indicates **homology** | U | K.AGLEQGSDAGYLAESQK.F  + Deamidated (NQ) |
| 164375 | 310 | – | 334 | 861.7701 | 2582.2884 | 2582.2864 | 0.79 | 1 | 37 | 0.00036 | 1Score **> 37** indicates **identity** Score **> 15** indicates **homology** | U | K.AGLEQGSDAGYLAESQKFGELALTK.E |
| 164377 | 310 | – | 334 | 1292.1517 | 2582.2889 | 2582.2864 | 0.97 | 1 | 95 | 1.1e-09 | 1Score **> 37** indicates **identity** Score **> 18** indicates **homology** | U | K.AGLEQGSDAGYLAESQKFGELALTK.E |
| 164378 | 310 | – | 334 | 861.7704 | 2582.2893 | 2582.2864 | 1.14 | 1 | 69 | 3.3e-07 | 1Score **> 37** indicates **identity** Score **> 17** indicates **homology** | U | K.AGLEQGSDAGYLAESQKFGELALTK.E |
| 164379 | 310 | – | 334 | 1292.1520 | 2582.2895 | 2582.2864 | 1.20 | 1 | 116 | 1.3e-11 | 1Score **> 37** indicates **identity** Score **> 20** indicates **homology** | U | K.AGLEQGSDAGYLAESQKFGELALTK.E |
| 164380 | 310 | – | 334 | 861.7706 | 2582.2898 | 2582.2864 | 1.34 | 1 | 69 | 3.4e-07 | 1Score **> 37** indicates **identity** Score **> 17** indicates **homology** | U | K.AGLEQGSDAGYLAESQKFGELALTK.E |
| 164381 | 310 | – | 334 | 861.7709 | 2582.2907 | 2582.2864 | 1.69 | 1 | 33 | 0.0008 | 1Score **> 37** indicates **identity** Score **> 15** indicates **homology** | U | K.AGLEQGSDAGYLAESQKFGELALTK.E |
| 164382 | 310 | – | 334 | 1292.1528 | 2582.2910 | 2582.2864 | 1.77 | 1 | 144 | 2.9e-14 | 1Score **> 37** indicates **identity** Score **> 22** indicates **homology** | U | K.AGLEQGSDAGYLAESQKFGELALTK.E |
| 164383 | 310 | – | 334 | 861.7714 | 2582.2924 | 2582.2864 | 2.34 | 1 | 71 | 2e-07 | 1Score **> 37** indicates **identity** Score **> 17** indicates **homology** | U | K.AGLEQGSDAGYLAESQKFGELALTK.E |
| 164384 | 310 | – | 334 | 861.7718 | 2582.2936 | 2582.2864 | 2.80 | 1 | 81 | 2.4e-08 | 1Score **> 37** indicates **identity** Score **> 18** indicates **homology** | U | K.AGLEQGSDAGYLAESQKFGELALTK.E |
| 164385 | 310 | – | 334 | 861.7721 | 2582.2944 | 2582.2864 | 3.10 | 1 | 44 | 7.6e-05 | 1Score **> 37** indicates **identity** Score **> 15** indicates **homology** | U | K.AGLEQGSDAGYLAESQKFGELALTK.E |
| 164386 | 310 | – | 334 | 861.7724 | 2582.2953 | 2582.2864 | 3.45 | 1 | 69 | 3.3e-07 | 1Score **> 37** indicates **identity** Score **> 17** indicates **homology** | U | K.AGLEQGSDAGYLAESQKFGELALTK.E |
| 164387 | 310 | – | 334 | 861.7725 | 2582.2957 | 2582.2864 | 3.62 | 1 | 47 | 4.2e-05 | 1Score **> 37** indicates **identity** Score **> 15** indicates **homology** | U | K.AGLEQGSDAGYLAESQKFGELALTK.E |
| 164388 | 310 | – | 334 | 861.7748 | 2582.3026 | 2582.2864 | 6.28 | 1 | 36 | 0.00042 | 1Score **> 37** indicates **identity** Score **> 15** indicates **homology** | U | K.AGLEQGSDAGYLAESQKFGELALTK.E |
| 164445 | 310 | – | 334 | 1292.6521 | 2583.2896 | 2583.2704 | 7.42 | 1 | 73 | 1.4e-07 | 1Score **> 37** indicates **identity** Score **> 17** indicates **homology** | U | K.AGLEQGSDAGYLAESQKFGELALTK.E  + Deamidated (NQ) |
| 164446 | 310 | – | 334 | 862.1051 | 2583.2933 | 2583.2704 | 8.88 | 1 | 18 | 0.02 | 1Score **> 37** indicates **identity** Score **> 14** indicates **homology** | U | K.AGLEQGSDAGYLAESQKFGELALTK.E  + Deamidated (NQ) |
| 164447 | 310 | – | 334 | 862.1052 | 2583.2939 | 2583.2704 | 9.09 | 1 | 16 | 0.031 | 1Score **> 37** indicates **identity** Score **> 14** indicates **homology** | U | K.AGLEQGSDAGYLAESQKFGELALTK.E  + Deamidated (NQ) |
| 164448 | 310 | – | 334 | 862.1056 | 2583.2949 | 2583.2704 | 9.49 | 1 | 32 | 0.0011 | 1Score **> 37** indicates **identity** Score **> 14** indicates **homology** | U | K.AGLEQGSDAGYLAESQKFGELALTK.E  + Deamidated (NQ) |
| 177032 | 310 | – | 337 | 732.6213 | 2926.4561 | 2926.4559 | 0.056 | 2 | 37 | 0.00034 | 1Score **> 38** indicates **identity** Score **> 15** indicates **homology** | U | K.AGLEQGSDAGYLAESQKFGELALTKESK.A |
| 177033 | 310 | – | 337 | 732.6228 | 2926.4621 | 2926.4559 | 2.10 | 2 | 35 | 0.00052 | 1Score **> 38** indicates **identity** Score **> 15** indicates **homology** | U | K.AGLEQGSDAGYLAESQKFGELALTKESK.A |
| 7725 | 327 | – | 334 | 439.7526 | 877.4907 | 877.4909 | -0.18 | 0 | 41 | 0.00022 | 1Score **> 26** indicates **identity** Score **> 17** indicates **homology** | U | K.FGELALTK.E |
| 7726 | 327 | – | 334 | 439.7527 | 877.4909 | 877.4909 | 0.018 | 0 | 36 | 0.00065 | 1Score **> 26** indicates **identity** Score **> 17** indicates **homology** | U | K.FGELALTK.E |
| 7727 | 327 | – | 334 | 439.7528 | 877.4910 | 877.4909 | 0.11 | 0 | 53 | 9.9e-06 | 1Score **> 26** indicates **identity** Score **> 16** indicates **homology** | U | K.FGELALTK.E |
| 7728 | 327 | – | 334 | 439.7528 | 877.4910 | 877.4909 | 0.11 | 0 | 44 | 0.00029 | 1Score **> 26** indicates **identity** Score **> 21** indicates **homology** | U | K.FGELALTK.E |
| 7729 | 327 | – | 334 | 439.7530 | 877.4914 | 877.4909 | 0.52 | 0 | 44 | 0.00014 | 1Score **> 26** indicates **identity** Score **> 18** indicates **homology** | U | K.FGELALTK.E |
| 7730 | 327 | – | 334 | 439.7530 | 877.4914 | 877.4909 | 0.52 | 0 | 46 | 0.00014 | 1Score **> 26** indicates **identity** Score **> 20** indicates **homology** | U | K.FGELALTK.E |
| 7731 | 327 | – | 334 | 439.7530 | 877.4914 | 877.4909 | 0.54 | 0 | 47 | 7.5e-05 | 1Score **> 26** indicates **identity** Score **> 18** indicates **homology** | U | K.FGELALTK.E |
| 7732 | 327 | – | 334 | 439.7530 | 877.4914 | 877.4909 | 0.55 | 0 | 59 | 7.3e-06 | 1Score **> 26** indicates **identity** Score **> 20** indicates **homology** | U | K.FGELALTK.E |
| 7733 | 327 | – | 334 | 439.7530 | 877.4914 | 877.4909 | 0.58 | 0 | 22 | 0.0083 | 1Score **> 26** indicates **identity** Score **> 14** indicates **homology** | U | K.FGELALTK.E |
| 7734 | 327 | – | 334 | 439.7530 | 877.4914 | 877.4909 | 0.58 | 0 | 38 | 0.0003 | 1Score **> 26** indicates **identity** Score **> 15** indicates **homology** | U | K.FGELALTK.E |
| 7735 | 327 | – | 334 | 439.7530 | 877.4914 | 877.4909 | 0.58 | 0 | 31 | 0.002 | 1Score **> 26** indicates **identity** Score **> 17** indicates **homology** | U | K.FGELALTK.E |
| 7736 | 327 | – | 334 | 439.7530 | 877.4915 | 877.4909 | 0.73 | 0 | 25 | 0.0056 | 1Score **> 26** indicates **identity** Score **> 15** indicates **homology** | U | K.FGELALTK.E |
| 7737 | 327 | – | 334 | 439.7531 | 877.4916 | 877.4909 | 0.77 | 0 | 45 | 0.00011 | 1Score **> 26** indicates **identity** Score **> 18** indicates **homology** | U | K.FGELALTK.E |
| 7738 | 327 | – | 334 | 439.7532 | 877.4918 | 877.4909 | 0.99 | 0 | 34 | 0.00062 | 1Score **> 26** indicates **identity** Score **> 15** indicates **homology** | U | K.FGELALTK.E |
| 7739 | 327 | – | 334 | 439.7534 | 877.4923 | 877.4909 | 1.56 | 0 | 18 | 0.031 | 1Score **> 26** indicates **identity** Score **> 15** indicates **homology** | U | K.FGELALTK.E |
| 7740 | 327 | – | 334 | 439.7536 | 877.4926 | 877.4909 | 1.89 | 0 | 18 | 0.021 | 1Score **> 26** indicates **identity** Score **> 14** indicates **homology** | U | K.FGELALTK.E |
| 7741 | 327 | – | 334 | 439.7539 | 877.4932 | 877.4909 | 2.66 | 0 | 29 | 0.0088 | 1Score **> 25** indicates **identity** Score **> 21** indicates **homology** | U | K.FGELALTK.E |
| 7742 | 327 | – | 334 | 439.7539 | 877.4933 | 877.4909 | 2.72 | 0 | 32 | 0.0011 | 1Score **> 25** indicates **identity** Score **> 15** indicates **homology** | U | K.FGELALTK.E |
| 35779 | 327 | – | 337 | 408.2255 | 1221.6546 | 1221.6605 | -4.79 | 1 | 24 | 0.005 | 1Score **> 33** indicates **identity** Score **> 14** indicates **homology** | U | K.FGELALTKESK.A |
| 35780 | 327 | – | 337 | 408.2255 | 1221.6547 | 1221.6605 | -4.71 | 1 | 15 | 0.036 | 1Score **> 33** indicates **identity** Score **> 13** indicates **homology** | U | K.FGELALTKESK.A |
| 62768 | 338 | – | 350 | 733.8778 | 1465.7410 | 1465.7421 | -0.75 | 0 | 84 | 1.4e-08 | 1Score **> 34** indicates **identity** Score **> 18** indicates **homology** | U | K.ALMGLYNGQVLCK.K |
| 62771 | 338 | – | 350 | 733.8793 | 1465.7440 | 1465.7421 | 1.27 | 0 | 85 | 1.1e-08 | 1Score **> 34** indicates **identity** Score **> 18** indicates **homology** | U | K.ALMGLYNGQVLCK.K |
| 62772 | 338 | – | 350 | 733.8797 | 1465.7448 | 1465.7421 | 1.82 | 0 | 42 | 0.00011 | 1Score **> 34** indicates **identity** Score **> 15** indicates **homology** | U | K.ALMGLYNGQVLCK.K |
| 62861 | 338 | – | 350 | 734.3700 | 1466.7254 | 1466.7261 | -0.51 | 0 | 39 | 0.00021 | 1Score **> 34** indicates **identity** Score **> 15** indicates **homology** | U | K.ALMGLYNGQVLCK.K  + Deamidated (NQ) |
| 62864 | 338 | – | 350 | 734.3727 | 1466.7309 | 1466.7261 | 3.23 | 0 | 35 | 0.00056 | 1Score **> 34** indicates **identity** Score **> 15** indicates **homology** | U | K.ALMGLYNGQVLCK.K  + Deamidated (NQ) |
| 79230 | 338 | – | 351 | 797.9244 | 1593.8342 | 1593.8371 | -1.84 | 1 | 34 | 0.00065 | 1Score **> 35** indicates **identity** Score **> 15** indicates **homology** | U | K.ALMGLYNGQVLCKK.N |
| 79231 | 338 | – | 351 | 797.9244 | 1593.8342 | 1593.8371 | -1.81 | 1 | 34 | 0.00094 | 1Score **> 35** indicates **identity** Score **> 16** indicates **homology** | U | K.ALMGLYNGQVLCKK.N |
| 79232 | 338 | – | 351 | 797.9266 | 1593.8387 | 1593.8371 | 1.01 | 1 | 50 | 1.9e-05 | 1Score **> 34** indicates **identity** Score **> 16** indicates **homology** | U | K.ALMGLYNGQVLCKK.N |
| 79332 | 338 | – | 351 | 798.4171 | 1594.8196 | 1594.8211 | -0.95 | 1 | 33 | 0.00079 | 1Score **> 35** indicates **identity** Score **> 15** indicates **homology** | U | K.ALMGLYNGQVLCKK.N  + Deamidated (NQ) |
| 79333 | 338 | – | 351 | 798.4171 | 1594.8196 | 1594.8211 | -0.92 | 1 | 29 | 0.0017 | 1Score **> 35** indicates **identity** Score **> 14** indicates **homology** | U | K.ALMGLYNGQVLCKK.N  + Deamidated (NQ) |
| 79336 | 338 | – | 351 | 532.6148 | 1594.8227 | 1594.8211 | 1.02 | 1 | 25 | 0.0041 | 1Score **> 34** indicates **identity** Score **> 14** indicates **homology** | U | K.ALMGLYNGQVLCKK.N  + Deamidated (NQ) |
| 81378 | 338 | – | 351 | 537.6192 | 1609.8357 | 1609.8320 | 2.33 | 1 | 19 | 0.018 | 1Score **> 34** indicates **identity** Score **> 14** indicates **homology** | U | K.ALMGLYNGQVLCKK.N  + Oxidation (M) |
| 8223 | 352 | – | 359 | 445.2485 | 888.4824 | 888.4817 | 0.73 | 1 | 15 | 0.039 | 1Score **> 31** indicates **identity** Score **> 13** indicates **homology** | U | K.NKFGAPQK.N |
| 183751 | 352 | – | 383 | 806.7006 | 3222.7735 | 3222.7547 | 5.82 | 2 | 23 | 0.0069 | 1Score **> 34** indicates **identity** Score **> 14** indicates **homology** | U | K.NKFGAPQKNVQQLAILGAGLMGAGIAQVSVDK.G |
| 151973 | 360 | – | 383 | 785.1006 | 2352.2799 | 2352.2835 | -1.52 | 0 | 39 | 0.00023 | 1Score **> 35** indicates **identity** Score **> 15** indicates **homology** | U | K.NVQQLAILGAGLMGAGIAQVSVDK.G |
| 151975 | 360 | – | 383 | 785.1013 | 2352.2821 | 2352.2835 | -0.61 | 0 | 19 | 0.017 | 1Score **> 35** indicates **identity** Score **> 14** indicates **homology** | U | K.NVQQLAILGAGLMGAGIAQVSVDK.G |
| 151980 | 360 | – | 383 | 785.1037 | 2352.2893 | 2352.2835 | 2.48 | 0 | 41 | 0.00016 | 1Score **> 35** indicates **identity** Score **> 15** indicates **homology** | U | K.NVQQLAILGAGLMGAGIAQVSVDK.G |
| 151983 | 360 | – | 383 | 1177.1523 | 2352.2901 | 2352.2835 | 2.80 | 0 | 125 | 2.1e-12 | 1Score **> 35** indicates **identity** Score **> 20** indicates **homology** | U | K.NVQQLAILGAGLMGAGIAQVSVDK.G |
| 151984 | 360 | – | 383 | 785.1042 | 2352.2909 | 2352.2835 | 3.14 | 0 | 20 | 0.014 | 1Score **> 35** indicates **identity** Score **> 14** indicates **homology** | U | K.NVQQLAILGAGLMGAGIAQVSVDK.G |
| 151990 | 360 | – | 383 | 785.1056 | 2352.2949 | 2352.2835 | 4.86 | 0 | 124 | 2.4e-12 | 1Score **> 35** indicates **identity** Score **> 20** indicates **homology** | U | K.NVQQLAILGAGLMGAGIAQVSVDK.G |
| 151991 | 360 | – | 383 | 785.1057 | 2352.2953 | 2352.2835 | 5.03 | 0 | 17 | 0.027 | 1Score **> 35** indicates **identity** Score **> 14** indicates **homology** | U | K.NVQQLAILGAGLMGAGIAQVSVDK.G |
| 151994 | 360 | – | 383 | 785.1059 | 2352.2960 | 2352.2835 | 5.31 | 0 | 22 | 0.009 | 1Score **> 35** indicates **identity** Score **> 14** indicates **homology** | U | K.NVQQLAILGAGLMGAGIAQVSVDK.G |
| 151996 | 360 | – | 383 | 785.1061 | 2352.2965 | 2352.2835 | 5.54 | 0 | 38 | 0.00028 | 1Score **> 35** indicates **identity** Score **> 15** indicates **homology** | U | K.NVQQLAILGAGLMGAGIAQVSVDK.G |
| 151998 | 360 | – | 383 | 785.1064 | 2352.2974 | 2352.2835 | 5.92 | 0 | 39 | 0.00024 | 1Score **> 35** indicates **identity** Score **> 15** indicates **homology** | U | K.NVQQLAILGAGLMGAGIAQVSVDK.G |
| 152006 | 360 | – | 383 | 785.1073 | 2352.3002 | 2352.2835 | 7.10 | 0 | 21 | 0.011 | 1Score **> 35** indicates **identity** Score **> 14** indicates **homology** | U | K.NVQQLAILGAGLMGAGIAQVSVDK.G |
| 152014 | 360 | – | 383 | 785.1095 | 2352.3068 | 2352.2835 | 9.90 | 0 | 16 | 0.029 | 1Score **> 34** indicates **identity** Score **> 14** indicates **homology** | U | K.NVQQLAILGAGLMGAGIAQVSVDK.G |
| 152080 | 360 | – | 383 | 1177.6517 | 2353.2888 | 2353.2675 | 9.05 | 0 | 94 | 1.6e-09 | 1Score **> 35** indicates **identity** Score **> 18** indicates **homology** | U | K.NVQQLAILGAGLMGAGIAQVSVDK.G  + Deamidated (NQ) |
| 167243 | 360 | – | 386 | 663.6290 | 2650.4868 | 2650.4840 | 1.05 | 1 | 32 | 0.0011 | 1Score **> 33** indicates **identity** Score **> 14** indicates **homology** | U | K.NVQQLAILGAGLMGAGIAQVSVDKGLK.T |
| 167244 | 360 | – | 386 | 884.5045 | 2650.4918 | 2650.4840 | 2.93 | 1 | 69 | 3.4e-07 | 1Score **> 33** indicates **identity** Score **> 17** indicates **homology** | U | K.NVQQLAILGAGLMGAGIAQVSVDKGLK.T |
| 167307 | 360 | – | 386 | 884.8386 | 2651.4940 | 2651.4680 | 9.82 | 1 | 54 | 8.7e-06 | 1Score **> 33** indicates **identity** Score **> 16** indicates **homology** | U | K.NVQQLAILGAGLMGAGIAQVSVDKGLK.T  + Deamidated (NQ) |
| 52397 | 387 | – | 399 | 458.9363 | 1373.7871 | 1373.7878 | -0.56 | 1 | 41 | 0.00015 | 1Score **> 33** indicates **identity** Score **> 15** indicates **homology** | U | K.TLLKDTTVTGLGR.G |
| 52399 | 387 | – | 399 | 458.9365 | 1373.7877 | 1373.7878 | -0.12 | 1 | 41 | 0.00026 | 1Score **> 33** indicates **identity** Score **> 18** indicates **homology** | U | K.TLLKDTTVTGLGR.G |
| 52400 | 387 | – | 399 | 687.9011 | 1373.7877 | 1373.7878 | -0.087 | 1 | 15 | 0.04 | 1Score **> 33** indicates **identity** Score **> 13** indicates **homology** | U | K.TLLKDTTVTGLGR.G |
| 52401 | 387 | – | 399 | 687.9014 | 1373.7882 | 1373.7878 | 0.24 | 1 | 30 | 0.0014 | 1Score **> 33** indicates **identity** Score **> 14** indicates **homology** | U | K.TLLKDTTVTGLGR.G |
| 52402 | 387 | – | 399 | 458.9367 | 1373.7884 | 1373.7878 | 0.39 | 1 | 37 | 0.00036 | 1Score **> 33** indicates **identity** Score **> 15** indicates **homology** | U | K.TLLKDTTVTGLGR.G |
| 52403 | 387 | – | 399 | 687.9015 | 1373.7884 | 1373.7878 | 0.44 | 1 | 17 | 0.024 | 1Score **> 33** indicates **identity** Score **> 14** indicates **homology** | U | K.TLLKDTTVTGLGR.G |
| 52404 | 387 | – | 399 | 458.9368 | 1373.7885 | 1373.7878 | 0.48 | 1 | 26 | 0.0035 | 1Score **> 33** indicates **identity** Score **> 14** indicates **homology** | U | K.TLLKDTTVTGLGR.G |
| 52405 | 387 | – | 399 | 458.9368 | 1373.7885 | 1373.7878 | 0.51 | 1 | 38 | 0.00027 | 1Score **> 33** indicates **identity** Score **> 15** indicates **homology** | U | K.TLLKDTTVTGLGR.G |
| 52406 | 387 | – | 399 | 458.9368 | 1373.7886 | 1373.7878 | 0.58 | 1 | 53 | 1e-05 | 1Score **> 33** indicates **identity** Score **> 16** indicates **homology** | U | K.TLLKDTTVTGLGR.G |
| 52407 | 387 | – | 399 | 458.9368 | 1373.7887 | 1373.7878 | 0.61 | 1 | 30 | 0.0016 | 1Score **> 33** indicates **identity** Score **> 14** indicates **homology** | U | K.TLLKDTTVTGLGR.G |
| 52408 | 387 | – | 399 | 458.9368 | 1373.7887 | 1373.7878 | 0.62 | 1 | 37 | 0.00035 | 1Score **> 33** indicates **identity** Score **> 15** indicates **homology** | U | K.TLLKDTTVTGLGR.G |
| 52409 | 387 | – | 399 | 687.9016 | 1373.7887 | 1373.7878 | 0.65 | 1 | 20 | 0.012 | 1Score **> 33** indicates **identity** Score **> 14** indicates **homology** | U | K.TLLKDTTVTGLGR.G |
| 52410 | 387 | – | 399 | 458.9369 | 1373.7888 | 1373.7878 | 0.71 | 1 | 45 | 0.00011 | 1Score **> 33** indicates **identity** Score **> 18** indicates **homology** | U | K.TLLKDTTVTGLGR.G |
| 52411 | 387 | – | 399 | 687.9017 | 1373.7889 | 1373.7878 | 0.79 | 1 | 43 | 9.7e-05 | 1Score **> 33** indicates **identity** Score **> 15** indicates **homology** | U | K.TLLKDTTVTGLGR.G |
| 52412 | 387 | – | 399 | 687.9017 | 1373.7889 | 1373.7878 | 0.80 | 1 | 16 | 0.029 | 1Score **> 33** indicates **identity** Score **> 14** indicates **homology** | U | K.TLLKDTTVTGLGR.G |
| 52413 | 387 | – | 399 | 458.9369 | 1373.7890 | 1373.7878 | 0.85 | 1 | 36 | 0.00072 | 1Score **> 33** indicates **identity** Score **> 17** indicates **homology** | U | K.TLLKDTTVTGLGR.G |
| 52414 | 387 | – | 399 | 687.9018 | 1373.7890 | 1373.7878 | 0.86 | 1 | 24 | 0.0053 | 1Score **> 33** indicates **identity** Score **> 14** indicates **homology** | U | K.TLLKDTTVTGLGR.G |
| 52416 | 387 | – | 399 | 458.9370 | 1373.7892 | 1373.7878 | 0.97 | 1 | 46 | 5.7e-05 | 1Score **> 33** indicates **identity** Score **> 16** indicates **homology** | U | K.TLLKDTTVTGLGR.G |
| 52417 | 387 | – | 399 | 687.9019 | 1373.7892 | 1373.7878 | 1.02 | 1 | 18 | 0.02 | 1Score **> 33** indicates **identity** Score **> 14** indicates **homology** | U | K.TLLKDTTVTGLGR.G |
| 52418 | 387 | – | 399 | 458.9371 | 1373.7895 | 1373.7878 | 1.23 | 1 | 32 | 0.0009 | 1Score **> 34** indicates **identity** Score **> 15** indicates **homology** | U | K.TLLKDTTVTGLGR.G |
| 52419 | 387 | – | 399 | 458.9372 | 1373.7897 | 1373.7878 | 1.36 | 1 | 35 | 0.00057 | 1Score **> 34** indicates **identity** Score **> 15** indicates **homology** | U | K.TLLKDTTVTGLGR.G |
| 52420 | 387 | – | 399 | 687.9023 | 1373.7900 | 1373.7878 | 1.57 | 1 | 18 | 0.02 | 1Score **> 34** indicates **identity** Score **> 14** indicates **homology** | U | K.TLLKDTTVTGLGR.G |
| 52421 | 387 | – | 399 | 687.9023 | 1373.7900 | 1373.7878 | 1.58 | 1 | 20 | 0.013 | 1Score **> 34** indicates **identity** Score **> 14** indicates **homology** | U | K.TLLKDTTVTGLGR.G |
| 9966 | 391 | – | 399 | 460.2463 | 918.4781 | 918.4771 | 1.13 | 0 | 32 | 0.045 | 1Score **> 31** indicates **identity** | U | K.DTTVTGLGR.G |
| 51139 | 400 | – | 411 | 454.5768 | 1360.7085 | 1360.7099 | -1.04 | 1 | 17 | 0.025 | 1Score **> 35** indicates **identity** Score **> 14** indicates **homology** | U | R.GQQQVFKGLNDK.V |
| 20998 | 414 | – | 422 | 360.5453 | 1078.6142 | 1078.6135 | 0.67 | 2 | 34 | 0.00063 | 1Score **> 30** indicates **identity** Score **> 15** indicates **homology** | U | K.KKALTSFER.D |
| 20999 | 414 | – | 422 | 360.5454 | 1078.6145 | 1078.6135 | 0.94 | 2 | 44 | 0.00016 | 1Score **> 30** indicates **identity** Score **> 18** indicates **homology** | U | K.KKALTSFER.D |
| 11831 | 415 | – | 422 | 476.2655 | 950.5164 | 950.5185 | -2.17 | 1 | 31 | 0.01 | 1Score **> 30** indicates **identity** Score **> 23** indicates **homology** | U | K.KALTSFER.D |
| 11834 | 415 | – | 422 | 476.2670 | 950.5195 | 950.5185 | 1.02 | 1 | 35 | 0.002 | 1Score **> 28** indicates **identity** Score **> 20** indicates **homology** | U | K.KALTSFER.D |
| 162641 | 415 | – | 436 | 637.0881 | 2544.3232 | 2544.3224 | 0.33 | 2 | 25 | 0.005 | 1Score **> 37** indicates **identity** Score **> 14** indicates **homology** | U | K.KALTSFERDSIFSNLIGQLDYK.G |
| 162646 | 415 | – | 436 | 849.1183 | 2544.3330 | 2544.3224 | 4.17 | 2 | 24 | 0.0057 | 1Score **> 37** indicates **identity** Score **> 14** indicates **homology** | U | K.KALTSFERDSIFSNLIGQLDYK.G |
| 4876 | 416 | – | 422 | 412.2188 | 822.4231 | 822.4236 | -0.60 | 0 | 43 | 0.001 | 1Score **> 26** indicates **identity** | U | K.ALTSFER.D |
| 4877 | 416 | – | 422 | 412.2190 | 822.4235 | 822.4236 | -0.11 | 0 | 36 | 0.0052 | 1Score **> 26** indicates **identity** | U | K.ALTSFER.D |
| 156043 | 416 | – | 436 | 806.4165 | 2416.2276 | 2416.2274 | 0.094 | 1 | 45 | 5.5e-05 | 1Score **> 37** indicates **identity** Score **> 15** indicates **homology** | U | K.ALTSFERDSIFSNLIGQLDYK.G |
| 156044 | 416 | – | 436 | 1209.1212 | 2416.2279 | 2416.2274 | 0.19 | 1 | 28 | 0.0024 | 1Score **> 37** indicates **identity** Score **> 14** indicates **homology** | U | K.ALTSFERDSIFSNLIGQLDYK.G |
| 156045 | 416 | – | 436 | 1209.1229 | 2416.2312 | 2416.2274 | 1.56 | 1 | 33 | 0.00077 | 1Score **> 37** indicates **identity** Score **> 15** indicates **homology** | U | K.ALTSFERDSIFSNLIGQLDYK.G |
| 175735 | 416 | – | 440 | 720.3712 | 2877.4557 | 2877.4548 | 0.29 | 2 | 14 | 0.047 | 1Score **> 37** indicates **identity** Score **> 13** indicates **homology** | U | K.ALTSFERDSIFSNLIGQLDYKGFEK.A |
| 175736 | 416 | – | 440 | 960.1602 | 2877.4587 | 2877.4548 | 1.33 | 2 | 49 | 2.4e-05 | 1Score **> 37** indicates **identity** Score **> 16** indicates **homology** | U | K.ALTSFERDSIFSNLIGQLDYKGFEK.A |
| 81589 | 423 | – | 436 | 806.9156 | 1611.8166 | 1611.8144 | 1.36 | 0 | 91 | 2.8e-09 | 1Score **> 34** indicates **identity** Score **> 18** indicates **homology** | U | R.DSIFSNLIGQLDYK.G |
| 81591 | 423 | – | 436 | 538.2800 | 1611.8182 | 1611.8144 | 2.34 | 0 | 22 | 0.01 | 1Score **> 34** indicates **identity** Score **> 15** indicates **homology** | U | R.DSIFSNLIGQLDYK.G |
| 81593 | 423 | – | 436 | 806.9184 | 1611.8222 | 1611.8144 | 4.82 | 0 | 91 | 3.5e-09 | 1Score **> 34** indicates **identity** Score **> 19** indicates **homology** | U | R.DSIFSNLIGQLDYK.G |
| 129875 | 423 | – | 440 | 1037.5188 | 2073.0230 | 2073.0419 | -9.11 | 1 | 92 | 2.4e-09 | 1Score **> 36** indicates **identity** Score **> 18** indicates **homology** | U | R.DSIFSNLIGQLDYKGFEK.A |
| 129877 | 423 | – | 440 | 692.0220 | 2073.0440 | 2073.0419 | 1.06 | 1 | 32 | 0.0011 | 1Score **> 36** indicates **identity** Score **> 14** indicates **homology** | U | R.DSIFSNLIGQLDYKGFEK.A |
| 129878 | 423 | – | 440 | 1037.5297 | 2073.0449 | 2073.0419 | 1.45 | 1 | 73 | 1.6e-07 | 1Score **> 36** indicates **identity** Score **> 17** indicates **homology** | U | R.DSIFSNLIGQLDYKGFEK.A |
| 129879 | 423 | – | 440 | 692.0229 | 2073.0468 | 2073.0419 | 2.41 | 1 | 21 | 0.0096 | 1Score **> 36** indicates **identity** Score **> 14** indicates **homology** | U | R.DSIFSNLIGQLDYKGFEK.A |
| 129880 | 423 | – | 440 | 692.0238 | 2073.0497 | 2073.0419 | 3.76 | 1 | 27 | 0.0028 | 1Score **> 36** indicates **identity** Score **> 14** indicates **homology** | U | R.DSIFSNLIGQLDYKGFEK.A |
| 132053 | 437 | – | 455 | 699.6938 | 2096.0594 | 2096.0500 | 4.50 | 1 | 35 | 0.0005 | 1Score **> 36** indicates **identity** Score **> 15** indicates **homology** | U | K.GFEKADMVIEAVFEDLGVK.H |
| 84633 | 441 | – | 455 | 545.9468 | 1634.8187 | 1634.8226 | -2.38 | 0 | 58 | 4e-06 | 1Score **> 35** indicates **identity** Score **> 16** indicates **homology** | U | K.ADMVIEAVFEDLGVK.H |
| 84639 | 441 | – | 455 | 818.4194 | 1634.8243 | 1634.8226 | 1.05 | 0 | 46 | 4.4e-05 | 1Score **> 35** indicates **identity** Score **> 15** indicates **homology** | U | K.ADMVIEAVFEDLGVK.H |
| 84642 | 441 | – | 455 | 545.9492 | 1634.8257 | 1634.8226 | 1.91 | 0 | 17 | 0.027 | 1Score **> 35** indicates **identity** Score **> 14** indicates **homology** | U | K.ADMVIEAVFEDLGVK.H |
| 84643 | 441 | – | 455 | 545.9494 | 1634.8262 | 1634.8226 | 2.24 | 0 | 82 | 1.9e-08 | 1Score **> 35** indicates **identity** Score **> 18** indicates **homology** | U | K.ADMVIEAVFEDLGVK.H |
| 84648 | 441 | – | 455 | 818.4209 | 1634.8272 | 1634.8226 | 2.85 | 0 | 84 | 1.3e-08 | 1Score **> 35** indicates **identity** Score **> 18** indicates **homology** | U | K.ADMVIEAVFEDLGVK.H |
| 86783 | 441 | – | 455 | 826.4134 | 1650.8123 | 1650.8175 | -3.15 | 0 | 21 | 0.0099 | 1Score **> 34** indicates **identity** Score **> 14** indicates **homology** | U | K.ADMVIEAVFEDLGVK.H  + Oxidation (M) |
| 86789 | 441 | – | 455 | 551.2787 | 1650.8143 | 1650.8175 | -1.90 | 0 | 52 | 1.2e-05 | 1Score **> 34** indicates **identity** Score **> 16** indicates **homology** | U | K.ADMVIEAVFEDLGVK.H  + Oxidation (M) |
| 86791 | 441 | – | 455 | 826.4162 | 1650.8178 | 1650.8175 | 0.19 | 0 | 51 | 1.8e-05 | 1Score **> 34** indicates **identity** Score **> 16** indicates **homology** | U | K.ADMVIEAVFEDLGVK.H  + Oxidation (M) |
| 86797 | 441 | – | 455 | 551.2850 | 1650.8332 | 1650.8175 | 9.52 | 0 | 17 | 0.023 | 1Score **> 35** indicates **identity** Score **> 14** indicates **homology** | U | K.ADMVIEAVFEDLGVK.H  + Oxidation (M) |
| 187580 | 458 | – | 489 | 863.9570 | 3451.7990 | 3451.8021 | -0.89 | 1 | 34 | 0.00066 | 1Score **> 37** indicates **identity** Score **> 15** indicates **homology** | U | K.VLKEVESVTPEHCIFASNTSALPINQIAAVSK.R |
| 187583 | 458 | – | 489 | 1151.6086 | 3451.8040 | 3451.8021 | 0.56 | 1 | 75 | 8.8e-08 | 1Score **> 37** indicates **identity** Score **> 17** indicates **homology** | U | K.VLKEVESVTPEHCIFASNTSALPINQIAAVSK.R |
| 187584 | 458 | – | 489 | 863.9583 | 3451.8041 | 3451.8021 | 0.58 | 1 | 43 | 8.9e-05 | 1Score **> 37** indicates **identity** Score **> 15** indicates **homology** | U | K.VLKEVESVTPEHCIFASNTSALPINQIAAVSK.R |
| 187585 | 458 | – | 489 | 1151.6088 | 3451.8044 | 3451.8021 | 0.68 | 1 | 80 | 3.2e-08 | 1Score **> 37** indicates **identity** Score **> 18** indicates **homology** | U | K.VLKEVESVTPEHCIFASNTSALPINQIAAVSK.R |
| 187586 | 458 | – | 489 | 1151.6090 | 3451.8051 | 3451.8021 | 0.87 | 1 | 67 | 4.8e-07 | 1Score **> 37** indicates **identity** Score **> 17** indicates **homology** | U | K.VLKEVESVTPEHCIFASNTSALPINQIAAVSK.R |
| 187587 | 458 | – | 489 | 863.9587 | 3451.8056 | 3451.8021 | 1.02 | 1 | 60 | 2.2e-06 | 1Score **> 37** indicates **identity** Score **> 16** indicates **homology** | U | K.VLKEVESVTPEHCIFASNTSALPINQIAAVSK.R |
| 187588 | 458 | – | 489 | 1151.6099 | 3451.8078 | 3451.8021 | 1.65 | 1 | 47 | 4.2e-05 | 1Score **> 37** indicates **identity** Score **> 15** indicates **homology** | U | K.VLKEVESVTPEHCIFASNTSALPINQIAAVSK.R |
| 187589 | 458 | – | 489 | 863.9593 | 3451.8082 | 3451.8021 | 1.77 | 1 | 42 | 0.00013 | 1Score **> 37** indicates **identity** Score **> 15** indicates **homology** | U | K.VLKEVESVTPEHCIFASNTSALPINQIAAVSK.R |
| 187591 | 458 | – | 489 | 1151.6102 | 3451.8087 | 3451.8021 | 1.93 | 1 | 41 | 0.00014 | 1Score **> 37** indicates **identity** Score **> 15** indicates **homology** | U | K.VLKEVESVTPEHCIFASNTSALPINQIAAVSK.R |
| 187593 | 458 | – | 489 | 1151.6126 | 3451.8161 | 3451.8021 | 4.05 | 1 | 56 | 6e-06 | 1Score **> 37** indicates **identity** Score **> 16** indicates **homology** | U | K.VLKEVESVTPEHCIFASNTSALPINQIAAVSK.R |
| 187608 | 458 | – | 489 | 864.2077 | 3452.8016 | 3452.7861 | 4.51 | 1 | 18 | 0.02 | 1Score **> 37** indicates **identity** Score **> 14** indicates **homology** | U | K.VLKEVESVTPEHCIFASNTSALPINQIAAVSK.R  + Deamidated (NQ) |
| 181714 | 461 | – | 489 | 1038.1911 | 3111.5515 | 3111.5546 | -1.02 | 0 | 25 | 0.0043 | 1Score **> 38** indicates **identity** Score **> 14** indicates **homology** | U | K.EVESVTPEHCIFASNTSALPINQIAAVSK.R |
| 181717 | 461 | – | 489 | 1038.1925 | 3111.5557 | 3111.5546 | 0.35 | 0 | 71 | 2.1e-07 | 1Score **> 38** indicates **identity** Score **> 17** indicates **homology** | U | K.EVESVTPEHCIFASNTSALPINQIAAVSK.R |
| 181718 | 461 | – | 489 | 778.8963 | 3111.5560 | 3111.5546 | 0.43 | 0 | 19 | 0.018 | 1Score **> 38** indicates **identity** Score **> 14** indicates **homology** | U | K.EVESVTPEHCIFASNTSALPINQIAAVSK.R |
| 181720 | 461 | – | 489 | 1038.1928 | 3111.5565 | 3111.5546 | 0.60 | 0 | 66 | 6.8e-07 | 1Score **> 38** indicates **identity** Score **> 17** indicates **homology** | U | K.EVESVTPEHCIFASNTSALPINQIAAVSK.R |
| 181721 | 461 | – | 489 | 778.8966 | 3111.5571 | 3111.5546 | 0.79 | 0 | 17 | 0.023 | 1Score **> 38** indicates **identity** Score **> 14** indicates **homology** | U | K.EVESVTPEHCIFASNTSALPINQIAAVSK.R |
| 181722 | 461 | – | 489 | 1556.7859 | 3111.5572 | 3111.5546 | 0.81 | 0 | 66 | 5.9e-07 | 1Score **> 38** indicates **identity** Score **> 17** indicates **homology** | U | K.EVESVTPEHCIFASNTSALPINQIAAVSK.R |
| 181723 | 461 | – | 489 | 1038.1931 | 3111.5574 | 3111.5546 | 0.87 | 0 | 57 | 4.6e-06 | 1Score **> 38** indicates **identity** Score **> 16** indicates **homology** | U | K.EVESVTPEHCIFASNTSALPINQIAAVSK.R |
| 181724 | 461 | – | 489 | 1038.1931 | 3111.5576 | 3111.5546 | 0.96 | 0 | 72 | 1.8e-07 | 1Score **> 38** indicates **identity** Score **> 17** indicates **homology** | U | K.EVESVTPEHCIFASNTSALPINQIAAVSK.R |
| 181725 | 461 | – | 489 | 1038.1932 | 3111.5578 | 3111.5546 | 1.02 | 0 | 68 | 3.8e-07 | 1Score **> 38** indicates **identity** Score **> 17** indicates **homology** | U | K.EVESVTPEHCIFASNTSALPINQIAAVSK.R |
| 181726 | 461 | – | 489 | 1556.7864 | 3111.5582 | 3111.5546 | 1.13 | 0 | 44 | 8.3e-05 | 1Score **> 38** indicates **identity** Score **> 15** indicates **homology** | U | K.EVESVTPEHCIFASNTSALPINQIAAVSK.R |
| 181729 | 461 | – | 489 | 1038.1957 | 3111.5653 | 3111.5546 | 3.43 | 0 | 46 | 4.6e-05 | 1Score **> 38** indicates **identity** Score **> 15** indicates **homology** | U | K.EVESVTPEHCIFASNTSALPINQIAAVSK.R |
| 181730 | 461 | – | 489 | 1038.1961 | 3111.5663 | 3111.5546 | 3.76 | 0 | 43 | 9.2e-05 | 1Score **> 38** indicates **identity** Score **> 15** indicates **homology** | U | K.EVESVTPEHCIFASNTSALPINQIAAVSK.R |
| 181731 | 461 | – | 489 | 1038.1976 | 3111.5709 | 3111.5546 | 5.22 | 0 | 46 | 5.2e-05 | 1Score **> 38** indicates **identity** Score **> 15** indicates **homology** | U | K.EVESVTPEHCIFASNTSALPINQIAAVSK.R |
| 181732 | 461 | – | 489 | 1038.1980 | 3111.5722 | 3111.5546 | 5.65 | 0 | 53 | 9.7e-06 | 1Score **> 38** indicates **identity** Score **> 16** indicates **homology** | U | K.EVESVTPEHCIFASNTSALPINQIAAVSK.R |
| 181733 | 461 | – | 489 | 1038.1992 | 3111.5759 | 3111.5546 | 6.82 | 0 | 31 | 0.0013 | 1Score **> 38** indicates **identity** Score **> 14** indicates **homology** | U | K.EVESVTPEHCIFASNTSALPINQIAAVSK.R |
| 181757 | 461 | – | 489 | 1038.5197 | 3112.5371 | 3112.5387 | -0.49 | 0 | 28 | 0.0025 | 1Score **> 38** indicates **identity** Score **> 14** indicates **homology** | U | K.EVESVTPEHCIFASNTSALPINQIAAVSK.R  + Deamidated (NQ) |
| 181760 | 461 | – | 489 | 1038.5239 | 3112.5499 | 3112.5387 | 3.62 | 0 | 26 | 0.0038 | 1Score **> 38** indicates **identity** Score **> 14** indicates **homology** | U | K.EVESVTPEHCIFASNTSALPINQIAAVSK.R  + Deamidated (NQ) |
| 181761 | 461 | – | 489 | 1038.5242 | 3112.5509 | 3112.5387 | 3.93 | 0 | 54 | 9e-06 | 1Score **> 38** indicates **identity** Score **> 16** indicates **homology** | U | K.EVESVTPEHCIFASNTSALPINQIAAVSK.R  + Deamidated (NQ) |
| 181762 | 461 | – | 489 | 1038.5249 | 3112.5528 | 3112.5387 | 4.54 | 0 | 40 | 0.00017 | 1Score **> 38** indicates **identity** Score **> 15** indicates **homology** | U | K.EVESVTPEHCIFASNTSALPINQIAAVSK.R  + Deamidated (NQ) |
| 181763 | 461 | – | 489 | 1038.5258 | 3112.5557 | 3112.5387 | 5.48 | 0 | 31 | 0.0013 | 1Score **> 38** indicates **identity** Score **> 14** indicates **homology** | U | K.EVESVTPEHCIFASNTSALPINQIAAVSK.R  + Deamidated (NQ) |
| 181764 | 461 | – | 489 | 1038.5266 | 3112.5578 | 3112.5387 | 6.16 | 0 | 20 | 0.012 | 1Score **> 38** indicates **identity** Score **> 14** indicates **homology** | U | K.EVESVTPEHCIFASNTSALPINQIAAVSK.R  + Deamidated (NQ) |
| 181765 | 461 | – | 489 | 1038.5281 | 3112.5624 | 3112.5387 | 7.64 | 0 | 30 | 0.0014 | 1Score **> 38** indicates **identity** Score **> 14** indicates **homology** | U | K.EVESVTPEHCIFASNTSALPINQIAAVSK.R  + Deamidated (NQ) |
| 181766 | 461 | – | 489 | 1038.5289 | 3112.5648 | 3112.5387 | 8.42 | 0 | 45 | 6.3e-05 | 1Score **> 38** indicates **identity** Score **> 15** indicates **homology** | U | K.EVESVTPEHCIFASNTSALPINQIAAVSK.R  + Deamidated (NQ) |
| 181767 | 461 | – | 489 | 1038.5292 | 3112.5659 | 3112.5387 | 8.74 | 0 | 48 | 2.8e-05 | 1Score **> 38** indicates **identity** Score **> 16** indicates **homology** | U | K.EVESVTPEHCIFASNTSALPINQIAAVSK.R  + Deamidated (NQ) |
| 189440 | 461 | – | 493 | 906.4678 | 3621.8421 | 3621.8461 | -1.10 | 2 | 17 | 0.028 | 1Score **> 37** indicates **identity** Score **> 14** indicates **homology** | U | K.EVESVTPEHCIFASNTSALPINQIAAVSKRPEK.V |
| 189441 | 461 | – | 493 | 906.4693 | 3621.8481 | 3621.8461 | 0.56 | 2 | 28 | 0.0025 | 1Score **> 37** indicates **identity** Score **> 14** indicates **homology** | U | K.EVESVTPEHCIFASNTSALPINQIAAVSKRPEK.V |
| 189443 | 461 | – | 493 | 1208.2902 | 3621.8486 | 3621.8461 | 0.71 | 2 | 34 | 0.00069 | 1Score **> 37** indicates **identity** Score **> 15** indicates **homology** | U | K.EVESVTPEHCIFASNTSALPINQIAAVSKRPEK.V |
| 189444 | 461 | – | 493 | 906.4695 | 3621.8488 | 3621.8461 | 0.74 | 2 | 32 | 0.0011 | 1Score **> 37** indicates **identity** Score **> 14** indicates **homology** | U | K.EVESVTPEHCIFASNTSALPINQIAAVSKRPEK.V |
| 189445 | 461 | – | 493 | 725.3771 | 3621.8490 | 3621.8461 | 0.82 | 2 | 18 | 0.019 | 1Score **> 37** indicates **identity** Score **> 14** indicates **homology** | U | K.EVESVTPEHCIFASNTSALPINQIAAVSKRPEK.V |
| 189446 | 461 | – | 493 | 1208.2904 | 3621.8495 | 3621.8461 | 0.95 | 2 | 17 | 0.024 | 1Score **> 37** indicates **identity** Score **> 14** indicates **homology** | U | K.EVESVTPEHCIFASNTSALPINQIAAVSKRPEK.V |
| 189447 | 461 | – | 493 | 1208.2906 | 3621.8501 | 3621.8461 | 1.11 | 2 | 37 | 0.00034 | 1Score **> 37** indicates **identity** Score **> 15** indicates **homology** | U | K.EVESVTPEHCIFASNTSALPINQIAAVSKRPEK.V |
| 189448 | 461 | – | 493 | 906.4699 | 3621.8504 | 3621.8461 | 1.21 | 2 | 32 | 0.0011 | 1Score **> 37** indicates **identity** Score **> 14** indicates **homology** | U | K.EVESVTPEHCIFASNTSALPINQIAAVSKRPEK.V |
| 189449 | 461 | – | 493 | 725.3775 | 3621.8513 | 3621.8461 | 1.44 | 2 | 21 | 0.0098 | 1Score **> 37** indicates **identity** Score **> 14** indicates **homology** | U | K.EVESVTPEHCIFASNTSALPINQIAAVSKRPEK.V |
| 189451 | 461 | – | 493 | 906.4702 | 3621.8516 | 3621.8461 | 1.52 | 2 | 35 | 0.00048 | 1Score **> 37** indicates **identity** Score **> 15** indicates **homology** | U | K.EVESVTPEHCIFASNTSALPINQIAAVSKRPEK.V |
| 54196 | 494 | – | 505 | 464.9040 | 1391.6901 | 1391.6908 | -0.46 | 0 | 16 | 0.032 | 1Score **> 33** indicates **identity** Score **> 13** indicates **homology** | U | K.VIGMHYFSPVDK.M |
| 54197 | 494 | – | 505 | 464.9042 | 1391.6909 | 1391.6908 | 0.065 | 0 | 29 | 0.0019 | 1Score **> 33** indicates **identity** Score **> 14** indicates **homology** | U | K.VIGMHYFSPVDK.M |
| 54198 | 494 | – | 505 | 464.9044 | 1391.6913 | 1391.6908 | 0.38 | 0 | 29 | 0.0019 | 1Score **> 33** indicates **identity** Score **> 14** indicates **homology** | U | K.VIGMHYFSPVDK.M |
| 54199 | 494 | – | 505 | 696.8530 | 1391.6914 | 1391.6908 | 0.46 | 0 | 27 | 0.003 | 1Score **> 33** indicates **identity** Score **> 14** indicates **homology** | U | K.VIGMHYFSPVDK.M |
| 54201 | 494 | – | 505 | 464.9046 | 1391.6921 | 1391.6908 | 0.94 | 0 | 38 | 0.00027 | 1Score **> 33** indicates **identity** Score **> 15** indicates **homology** | U | K.VIGMHYFSPVDK.M |
| 54205 | 494 | – | 505 | 696.8542 | 1391.6939 | 1391.6908 | 2.28 | 0 | 16 | 0.032 | 1Score **> 33** indicates **identity** Score **> 13** indicates **homology** | U | K.VIGMHYFSPVDK.M |
| 168426 | 494 | – | 516 | 670.3554 | 2677.3925 | 2677.3859 | 2.46 | 1 | 48 | 3.1e-05 | 1Score **> 37** indicates **identity** Score **> 16** indicates **homology** | U | K.VIGMHYFSPVDKMQLLEIITTDK.T |
| 45072 | 506 | – | 516 | 652.8601 | 1303.7056 | 1303.7057 | -0.076 | 0 | 42 | 0.00012 | 1Score **> 34** indicates **identity** Score **> 15** indicates **homology** | U | K.MQLLEIITTDK.T |
| 45073 | 506 | – | 516 | 652.8602 | 1303.7059 | 1303.7057 | 0.13 | 0 | 51 | 2.6e-05 | 1Score **> 34** indicates **identity** Score **> 17** indicates **homology** | U | K.MQLLEIITTDK.T |
| 45074 | 506 | – | 516 | 652.8603 | 1303.7060 | 1303.7057 | 0.21 | 0 | 34 | 0.00071 | 1Score **> 34** indicates **identity** Score **> 15** indicates **homology** | U | K.MQLLEIITTDK.T |
| 45075 | 506 | – | 516 | 652.8605 | 1303.7064 | 1303.7057 | 0.48 | 0 | 54 | 1.2e-05 | 1Score **> 34** indicates **identity** Score **> 18** indicates **homology** | U | K.MQLLEIITTDK.T |
| 45077 | 506 | – | 516 | 652.8606 | 1303.7066 | 1303.7057 | 0.67 | 0 | 55 | 1.2e-05 | 1Score **> 34** indicates **identity** Score **> 19** indicates **homology** | U | K.MQLLEIITTDK.T |
| 45080 | 506 | – | 516 | 652.8621 | 1303.7097 | 1303.7057 | 3.02 | 0 | 33 | 0.00079 | 1Score **> 33** indicates **identity** Score **> 15** indicates **homology** | U | K.MQLLEIITTDK.T |
| 82785 | 506 | – | 519 | 540.9673 | 1619.8802 | 1619.8804 | -0.14 | 1 | 27 | 0.0027 | 1Score **> 34** indicates **identity** Score **> 14** indicates **homology** | U | K.MQLLEIITTDKTSK.D |
| 82786 | 506 | – | 519 | 540.9674 | 1619.8804 | 1619.8804 | 0.0043 | 1 | 45 | 5.9e-05 | 1Score **> 34** indicates **identity** Score **> 15** indicates **homology** | U | K.MQLLEIITTDKTSK.D |
| 82787 | 506 | – | 519 | 540.9676 | 1619.8809 | 1619.8804 | 0.33 | 1 | 37 | 0.00035 | 1Score **> 34** indicates **identity** Score **> 15** indicates **homology** | U | K.MQLLEIITTDKTSK.D |
| 82788 | 506 | – | 519 | 540.9678 | 1619.8816 | 1619.8804 | 0.76 | 1 | 60 | 2.5e-06 | 1Score **> 34** indicates **identity** Score **> 16** indicates **homology** | U | K.MQLLEIITTDKTSK.D |
| 82789 | 506 | – | 519 | 540.9679 | 1619.8818 | 1619.8804 | 0.88 | 1 | 64 | 1e-06 | 1Score **> 34** indicates **identity** Score **> 17** indicates **homology** | U | K.MQLLEIITTDKTSK.D |
| 82790 | 506 | – | 519 | 540.9681 | 1619.8826 | 1619.8804 | 1.35 | 1 | 64 | 9.1e-07 | 1Score **> 34** indicates **identity** Score **> 17** indicates **homology** | U | K.MQLLEIITTDKTSK.D |
| 82791 | 506 | – | 519 | 540.9685 | 1619.8837 | 1619.8804 | 2.04 | 1 | 46 | 5e-05 | 1Score **> 34** indicates **identity** Score **> 15** indicates **homology** | U | K.MQLLEIITTDKTSK.D |
| 82792 | 506 | – | 519 | 540.9696 | 1619.8869 | 1619.8804 | 4.02 | 1 | 45 | 6.1e-05 | 1Score **> 34** indicates **identity** Score **> 15** indicates **homology** | U | K.MQLLEIITTDKTSK.D |
| 82793 | 506 | – | 519 | 540.9707 | 1619.8904 | 1619.8804 | 6.16 | 1 | 53 | 1.1e-05 | 1Score **> 34** indicates **identity** Score **> 16** indicates **homology** | U | K.MQLLEIITTDKTSK.D |
| 82795 | 506 | – | 519 | 810.9534 | 1619.8922 | 1619.8804 | 7.27 | 1 | 43 | 8.6e-05 | 1Score **> 34** indicates **identity** Score **> 15** indicates **homology** | U | K.MQLLEIITTDKTSK.D |
| 171571 | 506 | – | 531 | 691.3795 | 2761.4889 | 2761.4895 | -0.23 | 2 | 51 | 1.5e-05 | 1Score **> 36** indicates **identity** Score **> 16** indicates **homology** | U | K.MQLLEIITTDKTSKDTTASAVAVGLR.Q |
| 171572 | 506 | – | 531 | 921.5038 | 2761.4897 | 2761.4895 | 0.057 | 2 | 72 | 1.9e-07 | 1Score **> 36** indicates **identity** Score **> 17** indicates **homology** | U | K.MQLLEIITTDKTSKDTTASAVAVGLR.Q |
| 171573 | 506 | – | 531 | 691.3801 | 2761.4912 | 2761.4895 | 0.59 | 2 | 44 | 7e-05 | 1Score **> 36** indicates **identity** Score **> 15** indicates **homology** | U | K.MQLLEIITTDKTSKDTTASAVAVGLR.Q |
| 171574 | 506 | – | 531 | 691.3807 | 2761.4938 | 2761.4895 | 1.54 | 2 | 65 | 7.9e-07 | 1Score **> 36** indicates **identity** Score **> 17** indicates **homology** | U | K.MQLLEIITTDKTSKDTTASAVAVGLR.Q |
| 171575 | 506 | – | 531 | 921.5057 | 2761.4951 | 2761.4895 | 2.03 | 2 | 40 | 0.00018 | 1Score **> 36** indicates **identity** Score **> 15** indicates **homology** | U | K.MQLLEIITTDKTSKDTTASAVAVGLR.Q |
| 171576 | 506 | – | 531 | 921.5080 | 2761.5022 | 2761.4895 | 4.59 | 2 | 38 | 0.00025 | 1Score **> 35** indicates **identity** Score **> 15** indicates **homology** | U | K.MQLLEIITTDKTSKDTTASAVAVGLR.Q |
| 171618 | 506 | – | 531 | 921.8389 | 2762.4950 | 2762.4735 | 7.75 | 2 | 49 | 2.5e-05 | 1Score **> 35** indicates **identity** Score **> 16** indicates **homology** | U | K.MQLLEIITTDKTSKDTTASAVAVGLR.Q  + Deamidated (NQ) |
| 63951 | 517 | – | 531 | 492.9381 | 1475.7924 | 1475.7944 | -1.36 | 1 | 25 | 0.0041 | 1Score **> 35** indicates **identity** Score **> 14** indicates **homology** | U | K.TSKDTTASAVAVGLR.Q |
| 63953 | 517 | – | 531 | 492.9382 | 1475.7928 | 1475.7944 | -1.04 | 1 | 33 | 0.00085 | 1Score **> 35** indicates **identity** Score **> 15** indicates **homology** | U | K.TSKDTTASAVAVGLR.Q |
| 63954 | 517 | – | 531 | 738.9037 | 1475.7929 | 1475.7944 | -1.03 | 1 | 94 | 1.4e-09 | 1Score **> 35** indicates **identity** Score **> 18** indicates **homology** | U | K.TSKDTTASAVAVGLR.Q |
| 63955 | 517 | – | 531 | 738.9037 | 1475.7929 | 1475.7944 | -1.00 | 1 | 78 | 4.6e-08 | 1Score **> 35** indicates **identity** Score **> 17** indicates **homology** | U | K.TSKDTTASAVAVGLR.Q |
| 63956 | 517 | – | 531 | 492.9383 | 1475.7931 | 1475.7944 | -0.89 | 1 | 57 | 5.4e-06 | 1Score **> 35** indicates **identity** Score **> 16** indicates **homology** | U | K.TSKDTTASAVAVGLR.Q |
| 63957 | 517 | – | 531 | 492.9384 | 1475.7935 | 1475.7944 | -0.61 | 1 | 28 | 0.0024 | 1Score **> 35** indicates **identity** Score **> 14** indicates **homology** | U | K.TSKDTTASAVAVGLR.Q |
| 63958 | 517 | – | 531 | 492.9384 | 1475.7935 | 1475.7944 | -0.61 | 1 | 23 | 0.0076 | 1Score **> 35** indicates **identity** Score **> 14** indicates **homology** | U | K.TSKDTTASAVAVGLR.Q |
| 63961 | 517 | – | 531 | 492.9390 | 1475.7951 | 1475.7944 | 0.51 | 1 | 25 | 0.005 | 1Score **> 35** indicates **identity** Score **> 14** indicates **homology** | U | K.TSKDTTASAVAVGLR.Q |
| 63962 | 517 | – | 531 | 738.9054 | 1475.7963 | 1475.7944 | 1.32 | 1 | 52 | 1.2e-05 | 1Score **> 35** indicates **identity** Score **> 16** indicates **homology** | U | K.TSKDTTASAVAVGLR.Q |
| 29085 | 520 | – | 531 | 580.8158 | 1159.6170 | 1159.6197 | -2.33 | 0 | 18 | 0.022 | 1Score **> 34** indicates **identity** Score **> 14** indicates **homology** | U | K.DTTASAVAVGLR.Q |
| 29086 | 520 | – | 531 | 580.8165 | 1159.6185 | 1159.6197 | -1.04 | 0 | 46 | 5.3e-05 | 1Score **> 34** indicates **identity** Score **> 15** indicates **homology** | U | K.DTTASAVAVGLR.Q |
| 29087 | 520 | – | 531 | 580.8168 | 1159.6190 | 1159.6197 | -0.64 | 0 | 51 | 1.6e-05 | 1Score **> 34** indicates **identity** Score **> 16** indicates **homology** | U | K.DTTASAVAVGLR.Q |
| 29088 | 520 | – | 531 | 580.8170 | 1159.6195 | 1159.6197 | -0.16 | 0 | 67 | 4.5e-06 | 1Score **> 34** indicates **identity** Score **> 26** indicates **homology** | U | K.DTTASAVAVGLR.Q |
| 29089 | 520 | – | 531 | 580.8171 | 1159.6196 | 1159.6197 | -0.099 | 0 | 64 | 9.8e-06 | 1Score **> 34** indicates **identity** Score **> 26** indicates **homology** | U | K.DTTASAVAVGLR.Q |
| 29090 | 520 | – | 531 | 580.8171 | 1159.6196 | 1159.6197 | -0.096 | 0 | 71 | 1.4e-06 | 1Score **> 34** indicates **identity** Score **> 25** indicates **homology** | U | K.DTTASAVAVGLR.Q |
| 29091 | 520 | – | 531 | 580.8173 | 1159.6201 | 1159.6197 | 0.33 | 0 | 63 | 1.1e-05 | 1Score **> 34** indicates **identity** Score **> 26** indicates **homology** | U | K.DTTASAVAVGLR.Q |
| 29092 | 520 | – | 531 | 580.8175 | 1159.6205 | 1159.6197 | 0.65 | 0 | 68 | 2.6e-06 | 1Score **> 34** indicates **identity** Score **> 25** indicates **homology** | U | K.DTTASAVAVGLR.Q |
| 29093 | 520 | – | 531 | 580.8177 | 1159.6209 | 1159.6197 | 1.05 | 0 | 61 | 7.6e-06 | 1Score **> 34** indicates **identity** Score **> 22** indicates **homology** | U | K.DTTASAVAVGLR.Q |
| 29094 | 520 | – | 531 | 580.8180 | 1159.6215 | 1159.6197 | 1.57 | 0 | 70 | 3.5e-06 | 1Score **> 33** indicates **identity** Score **> 28** indicates **homology** | U | K.DTTASAVAVGLR.Q |
| 29095 | 520 | – | 531 | 580.8182 | 1159.6219 | 1159.6197 | 1.87 | 0 | 60 | 1.1e-05 | 1Score **> 33** indicates **identity** Score **> 23** indicates **homology** | U | K.DTTASAVAVGLR.Q |
| 29096 | 520 | – | 531 | 580.8200 | 1159.6254 | 1159.6197 | 4.95 | 0 | 47 | 0.00011 | 1Score **> 34** indicates **identity** Score **> 20** indicates **homology** | U | K.DTTASAVAVGLR.Q |
| 121195 | 532 | – | 549 | 495.5343 | 1978.1082 | 1978.0888 | 9.83 | 2 | 16 | 0.03 | 1Score **> 34** indicates **identity** Score **> 14** indicates **homology** | U | R.QGKVIIVVKDGPGFYTTR.C  + Deamidated (NQ) |
| 88448 | 535 | – | 549 | 555.6500 | 1663.9282 | 1663.9298 | -0.94 | 1 | 17 | 0.024 | 1Score **> 34** indicates **identity** Score **> 14** indicates **homology** | U | K.VIIVVKDGPGFYTTR.C |
| 88449 | 535 | – | 549 | 832.9723 | 1663.9301 | 1663.9298 | 0.23 | 1 | 37 | 0.00034 | 1Score **> 34** indicates **identity** Score **> 15** indicates **homology** | U | K.VIIVVKDGPGFYTTR.C |
| 88450 | 535 | – | 549 | 555.6509 | 1663.9308 | 1663.9298 | 0.60 | 1 | 48 | 3.2e-05 | 1Score **> 34** indicates **identity** Score **> 15** indicates **homology** | U | K.VIIVVKDGPGFYTTR.C |
| 88451 | 535 | – | 549 | 555.6509 | 1663.9310 | 1663.9298 | 0.73 | 1 | 43 | 9.9e-05 | 1Score **> 34** indicates **identity** Score **> 15** indicates **homology** | U | K.VIIVVKDGPGFYTTR.C |
| 88452 | 535 | – | 549 | 555.6510 | 1663.9312 | 1663.9298 | 0.84 | 1 | 56 | 5.2e-06 | 1Score **> 33** indicates **identity** Score **> 16** indicates **homology** | U | K.VIIVVKDGPGFYTTR.C |
| 88453 | 535 | – | 549 | 555.6511 | 1663.9314 | 1663.9298 | 0.98 | 1 | 20 | 0.012 | 1Score **> 33** indicates **identity** Score **> 14** indicates **homology** | U | K.VIIVVKDGPGFYTTR.C |
| 88454 | 535 | – | 549 | 555.6511 | 1663.9315 | 1663.9298 | 1.02 | 1 | 25 | 0.0042 | 1Score **> 33** indicates **identity** Score **> 14** indicates **homology** | U | K.VIIVVKDGPGFYTTR.C |
| 88455 | 535 | – | 549 | 555.6511 | 1663.9315 | 1663.9298 | 1.03 | 1 | 26 | 0.0034 | 1Score **> 33** indicates **identity** Score **> 14** indicates **homology** | U | K.VIIVVKDGPGFYTTR.C |
| 88456 | 535 | – | 549 | 555.6512 | 1663.9317 | 1663.9298 | 1.16 | 1 | 35 | 0.00054 | 1Score **> 33** indicates **identity** Score **> 15** indicates **homology** | U | K.VIIVVKDGPGFYTTR.C |
| 88457 | 535 | – | 549 | 555.6512 | 1663.9319 | 1663.9298 | 1.27 | 1 | 56 | 6e-06 | 1Score **> 33** indicates **identity** Score **> 16** indicates **homology** | U | K.VIIVVKDGPGFYTTR.C |
| 88458 | 535 | – | 549 | 555.6517 | 1663.9332 | 1663.9298 | 2.04 | 1 | 26 | 0.0039 | 1Score **> 33** indicates **identity** Score **> 14** indicates **homology** | U | K.VIIVVKDGPGFYTTR.C |
| 88459 | 535 | – | 549 | 832.9739 | 1663.9333 | 1663.9298 | 2.14 | 1 | 31 | 0.0013 | 1Score **> 33** indicates **identity** Score **> 14** indicates **homology** | U | K.VIIVVKDGPGFYTTR.C |
| 88460 | 535 | – | 549 | 555.6521 | 1663.9344 | 1663.9298 | 2.81 | 1 | 36 | 0.00044 | 1Score **> 33** indicates **identity** Score **> 15** indicates **homology** | U | K.VIIVVKDGPGFYTTR.C |
| 15686 | 541 | – | 549 | 507.2356 | 1012.4567 | 1012.4614 | -4.62 | 0 | 35 | 0.0005 | 1Score **> 27** indicates **identity** Score **> 15** indicates **homology** | U | K.DGPGFYTTR.C |
| 15687 | 541 | – | 549 | 507.2371 | 1012.4596 | 1012.4614 | -1.78 | 0 | 36 | 0.0004 | 1Score **> 27** indicates **identity** Score **> 15** indicates **homology** | U | K.DGPGFYTTR.C |
| 15688 | 541 | – | 549 | 507.2378 | 1012.4610 | 1012.4614 | -0.39 | 0 | 42 | 0.00011 | 1Score **> 27** indicates **identity** Score **> 15** indicates **homology** | U | K.DGPGFYTTR.C |
| 15689 | 541 | – | 549 | 507.2381 | 1012.4617 | 1012.4614 | 0.30 | 0 | 48 | 2.9e-05 | 1Score **> 27** indicates **identity** Score **> 16** indicates **homology** | U | K.DGPGFYTTR.C |
| 15690 | 541 | – | 549 | 507.2382 | 1012.4618 | 1012.4614 | 0.41 | 0 | 42 | 0.00012 | 1Score **> 27** indicates **identity** Score **> 15** indicates **homology** | U | K.DGPGFYTTR.C |
| 15691 | 541 | – | 549 | 507.2383 | 1012.4620 | 1012.4614 | 0.61 | 0 | 28 | 0.0024 | 1Score **> 27** indicates **identity** Score **> 14** indicates **homology** | U | K.DGPGFYTTR.C |
| 47265 | 550 | – | 560 | 662.7981 | 1323.5817 | 1323.5807 | 0.75 | 0 | 31 | 0.0012 | 1Score **> 28** indicates **identity** Score **> 14** indicates **homology** | U | R.CLAPMMSEVMR.I |
| 14757 | 561 | – | 569 | 499.7795 | 997.5444 | 997.5444 | -0.047 | 0 | 57 | 4.4e-06 | 1Score **> 29** indicates **identity** Score **> 16** indicates **homology** | U | R.ILQEGVDPK.K |
| 14758 | 561 | – | 569 | 499.7795 | 997.5444 | 997.5444 | -0.0090 | 0 | 41 | 0.00026 | 1Score **> 29** indicates **identity** Score **> 18** indicates **homology** | U | R.ILQEGVDPK.K |
| 14759 | 561 | – | 569 | 499.7798 | 997.5450 | 997.5444 | 0.56 | 0 | 39 | 0.00024 | 1Score **> 28** indicates **identity** Score **> 15** indicates **homology** | U | R.ILQEGVDPK.K |
| 14760 | 561 | – | 569 | 499.7799 | 997.5452 | 997.5444 | 0.82 | 0 | 48 | 4.7e-05 | 1Score **> 28** indicates **identity** Score **> 17** indicates **homology** | U | R.ILQEGVDPK.K |
| 15332 | 611 | – | 620 | 503.7815 | 1005.5485 | 1005.5495 | -1.03 | 0 | 20 | 0.013 | 1Score **> 31** indicates **identity** Score **> 14** indicates **homology** | U | R.FGGGSVELLK.Q |
| 15341 | 611 | – | 620 | 503.7820 | 1005.5494 | 1005.5495 | -0.14 | 0 | 70 | 6.6e-07 | 1Score **> 31** indicates **identity** Score **> 21** indicates **homology** | U | R.FGGGSVELLK.Q |
| 15344 | 611 | – | 620 | 503.7821 | 1005.5496 | 1005.5495 | 0.13 | 0 | 58 | 7.6e-06 | 1Score **> 31** indicates **identity** Score **> 19** indicates **homology** | U | R.FGGGSVELLK.Q |
| 15346 | 611 | – | 620 | 503.7821 | 1005.5497 | 1005.5495 | 0.20 | 0 | 55 | 6.5e-06 | 1Score **> 31** indicates **identity** Score **> 16** indicates **homology** | U | R.FGGGSVELLK.Q |
| 15347 | 611 | – | 620 | 503.7822 | 1005.5498 | 1005.5495 | 0.27 | 0 | 62 | 3.8e-06 | 1Score **> 31** indicates **identity** Score **> 20** indicates **homology** | U | R.FGGGSVELLK.Q |
| 15348 | 611 | – | 620 | 503.7822 | 1005.5498 | 1005.5495 | 0.32 | 0 | 48 | 5.7e-05 | 1Score **> 31** indicates **identity** Score **> 18** indicates **homology** | U | R.FGGGSVELLK.Q |
| 15349 | 611 | – | 620 | 503.7822 | 1005.5498 | 1005.5495 | 0.33 | 0 | 58 | 7.4e-06 | 1Score **> 31** indicates **identity** Score **> 19** indicates **homology** | U | R.FGGGSVELLK.Q |
| 15351 | 611 | – | 620 | 503.7823 | 1005.5501 | 1005.5495 | 0.63 | 0 | 59 | 6e-06 | 1Score **> 31** indicates **identity** Score **> 19** indicates **homology** | U | R.FGGGSVELLK.Q |
| 15352 | 611 | – | 620 | 503.7824 | 1005.5502 | 1005.5495 | 0.69 | 0 | 58 | 7.5e-06 | 1Score **> 31** indicates **identity** Score **> 19** indicates **homology** | U | R.FGGGSVELLK.Q |
| 15353 | 611 | – | 620 | 503.7824 | 1005.5502 | 1005.5495 | 0.70 | 0 | 60 | 5.8e-06 | 1Score **> 31** indicates **identity** Score **> 20** indicates **homology** | U | R.FGGGSVELLK.Q |
| 15354 | 611 | – | 620 | 503.7824 | 1005.5502 | 1005.5495 | 0.70 | 0 | 54 | 1.9e-05 | 1Score **> 31** indicates **identity** Score **> 19** indicates **homology** | U | R.FGGGSVELLK.Q |
| 15355 | 611 | – | 620 | 503.7825 | 1005.5504 | 1005.5495 | 0.93 | 0 | 58 | 7.5e-06 | 1Score **> 31** indicates **identity** Score **> 19** indicates **homology** | U | R.FGGGSVELLK.Q |
| 15358 | 611 | – | 620 | 503.7828 | 1005.5510 | 1005.5495 | 1.48 | 0 | 45 | 5.4e-05 | 1Score **> 32** indicates **identity** Score **> 15** indicates **homology** | U | R.FGGGSVELLK.Q |
| 15359 | 611 | – | 620 | 503.7828 | 1005.5510 | 1005.5495 | 1.53 | 0 | 34 | 0.00064 | 1Score **> 32** indicates **identity** Score **> 15** indicates **homology** | U | R.FGGGSVELLK.Q |
| 15364 | 611 | – | 620 | 503.7831 | 1005.5516 | 1005.5495 | 2.09 | 0 | 16 | 0.03 | 1Score **> 32** indicates **identity** Score **> 14** indicates **homology** | U | R.FGGGSVELLK.Q |
| 15365 | 611 | – | 620 | 503.7837 | 1005.5529 | 1005.5495 | 3.36 | 0 | 47 | 3.9e-05 | 1Score **> 31** indicates **identity** Score **> 15** indicates **homology** | U | R.FGGGSVELLK.Q |
| 15366 | 611 | – | 620 | 503.7838 | 1005.5531 | 1005.5495 | 3.55 | 0 | 44 | 7.2e-05 | 1Score **> 31** indicates **identity** Score **> 15** indicates **homology** | U | R.FGGGSVELLK.Q |
| 62373 | 632 | – | 644 | 732.3620 | 1462.7095 | 1462.7092 | 0.19 | 1 | 73 | 1.6e-07 | 1Score **> 33** indicates **identity** Score **> 17** indicates **homology** | U | K.SGKGFYIYQEGSK.N |
| 62374 | 632 | – | 644 | 732.3622 | 1462.7098 | 1462.7092 | 0.36 | 1 | 88 | 5.4e-09 | 1Score **> 33** indicates **identity** Score **> 18** indicates **homology** | U | K.SGKGFYIYQEGSK.N |
| 62375 | 632 | – | 644 | 488.5772 | 1462.7098 | 1462.7092 | 0.41 | 1 | 33 | 0.0008 | 1Score **> 33** indicates **identity** Score **> 15** indicates **homology** | U | K.SGKGFYIYQEGSK.N |
| 62376 | 632 | – | 644 | 488.5772 | 1462.7099 | 1462.7092 | 0.45 | 1 | 29 | 0.0019 | 1Score **> 33** indicates **identity** Score **> 14** indicates **homology** | U | K.SGKGFYIYQEGSK.N |
| 62377 | 632 | – | 644 | 732.3622 | 1462.7099 | 1462.7092 | 0.46 | 1 | 54 | 8.7e-06 | 1Score **> 33** indicates **identity** Score **> 16** indicates **homology** | U | K.SGKGFYIYQEGSK.N |
| 32461 | 635 | – | 644 | 596.2839 | 1190.5532 | 1190.5608 | -6.40 | 0 | 28 | 0.0026 | 1Score **> 31** indicates **identity** Score **> 14** indicates **homology** | U | K.GFYIYQEGSK.N |
| 32465 | 635 | – | 644 | 596.2846 | 1190.5547 | 1190.5608 | -5.08 | 0 | 15 | 0.038 | 1Score **> 31** indicates **identity** Score **> 13** indicates **homology** | U | K.GFYIYQEGSK.N |
| 32475 | 635 | – | 644 | 596.2872 | 1190.5598 | 1190.5608 | -0.86 | 0 | 38 | 0.00026 | 1Score **> 31** indicates **identity** Score **> 15** indicates **homology** | U | K.GFYIYQEGSK.N |
| 32476 | 635 | – | 644 | 596.2874 | 1190.5602 | 1190.5608 | -0.44 | 0 | 38 | 0.00029 | 1Score **> 32** indicates **identity** Score **> 15** indicates **homology** | U | K.GFYIYQEGSK.N |
| 32477 | 635 | – | 644 | 596.2880 | 1190.5614 | 1190.5608 | 0.49 | 0 | 33 | 0.00075 | 1Score **> 31** indicates **identity** Score **> 15** indicates **homology** | U | K.GFYIYQEGSK.N |
| 32478 | 635 | – | 644 | 596.2880 | 1190.5614 | 1190.5608 | 0.49 | 0 | 35 | 0.00052 | 1Score **> 31** indicates **identity** Score **> 15** indicates **homology** | U | K.GFYIYQEGSK.N |
| 32479 | 635 | – | 644 | 596.2880 | 1190.5615 | 1190.5608 | 0.63 | 0 | 39 | 0.00022 | 1Score **> 31** indicates **identity** Score **> 15** indicates **homology** | U | K.GFYIYQEGSK.N |
| 32480 | 635 | – | 644 | 596.2883 | 1190.5621 | 1190.5608 | 1.08 | 0 | 31 | 0.0014 | 1Score **> 31** indicates **identity** Score **> 14** indicates **homology** | U | K.GFYIYQEGSK.N |
| 32481 | 635 | – | 644 | 596.2883 | 1190.5621 | 1190.5608 | 1.09 | 0 | 38 | 0.0003 | 1Score **> 31** indicates **identity** Score **> 15** indicates **homology** | U | K.GFYIYQEGSK.N |
| 58824 | 635 | – | 646 | 717.3571 | 1432.6997 | 1432.6987 | 0.72 | 1 | 16 | 0.031 | 1Score **> 33** indicates **identity** Score **> 14** indicates **homology** | U | K.GFYIYQEGSKNK.S |
| 78619 | 647 | – | 660 | 530.6026 | 1588.7859 | 1588.7879 | -1.21 | 0 | 44 | 7e-05 | 1Score **> 34** indicates **identity** Score **> 15** indicates **homology** | U | K.SLNSEMDNILANLR.L |
| 78622 | 647 | – | 660 | 795.4014 | 1588.7882 | 1588.7879 | 0.20 | 0 | 111 | 3.8e-11 | 1Score **> 35** indicates **identity** Score **> 19** indicates **homology** | U | K.SLNSEMDNILANLR.L |
| 78626 | 647 | – | 660 | 530.6041 | 1588.7903 | 1588.7879 | 1.56 | 0 | 53 | 1.5e-05 | 1Score **> 34** indicates **identity** Score **> 17** indicates **homology** | U | K.SLNSEMDNILANLR.L |
| 186720 | 647 | – | 676 | 1135.5697 | 3403.6871 | 3403.6565 | 9.00 | 2 | 49 | 2.8e-05 | 1Score **> 37** indicates **identity** Score **> 16** indicates **homology** | U | K.SLNSEMDNILANLRLPAKPEVSSDEDVQYR.V  + Deamidated (NQ) |
| 188892 | 647 | – | 676 | 890.9439 | 3559.7465 | 3559.7715 | -7.04 | 2 | 25 | 0.0049 | 1Score **> 37** indicates **identity** Score **> 14** indicates **homology** | U | K.SLNSEMDNILANLRLPAKPEVSSDEDVQYR.V  + Deamidated (NQ); HNE (K) |
| 107633 | 661 | – | 676 | 611.6375 | 1831.8908 | 1831.8952 | -2.39 | 1 | 51 | 1.8e-05 | 1Score **> 34** indicates **identity** Score **> 16** indicates **homology** | U | R.LPAKPEVSSDEDVQYR.V |
| 107635 | 661 | – | 676 | 916.9536 | 1831.8927 | 1831.8952 | -1.35 | 1 | 44 | 7.2e-05 | 1Score **> 35** indicates **identity** Score **> 15** indicates **homology** | U | R.LPAKPEVSSDEDVQYR.V |
| 107636 | 661 | – | 676 | 611.6388 | 1831.8944 | 1831.8952 | -0.41 | 1 | 34 | 0.00064 | 1Score **> 35** indicates **identity** Score **> 15** indicates **homology** | U | R.LPAKPEVSSDEDVQYR.V |
| 107637 | 661 | – | 676 | 611.6389 | 1831.8948 | 1831.8952 | -0.19 | 1 | 67 | 4.7e-07 | 1Score **> 35** indicates **identity** Score **> 17** indicates **homology** | U | R.LPAKPEVSSDEDVQYR.V |
| 107638 | 661 | – | 676 | 916.9547 | 1831.8949 | 1831.8952 | -0.13 | 1 | 67 | 5.7e-07 | 1Score **> 35** indicates **identity** Score **> 17** indicates **homology** | U | R.LPAKPEVSSDEDVQYR.V |
| 107639 | 661 | – | 676 | 611.6390 | 1831.8951 | 1831.8952 | -0.046 | 1 | 32 | 0.0013 | 1Score **> 35** indicates **identity** Score **> 16** indicates **homology** | U | R.LPAKPEVSSDEDVQYR.V |
| 107640 | 661 | – | 676 | 611.6390 | 1831.8953 | 1831.8952 | 0.036 | 1 | 42 | 0.00018 | 1Score **> 35** indicates **identity** Score **> 17** indicates **homology** | U | R.LPAKPEVSSDEDVQYR.V |
| 107641 | 661 | – | 676 | 916.9551 | 1831.8956 | 1831.8952 | 0.23 | 1 | 82 | 2.1e-08 | 1Score **> 35** indicates **identity** Score **> 18** indicates **homology** | U | R.LPAKPEVSSDEDVQYR.V |
| 107642 | 661 | – | 676 | 611.6392 | 1831.8958 | 1831.8952 | 0.31 | 1 | 88 | 5.8e-09 | 1Score **> 35** indicates **identity** Score **> 18** indicates **homology** | U | R.LPAKPEVSSDEDVQYR.V |
| 107643 | 661 | – | 676 | 611.6393 | 1831.8960 | 1831.8952 | 0.42 | 1 | 80 | 3.3e-08 | 1Score **> 35** indicates **identity** Score **> 17** indicates **homology** | U | R.LPAKPEVSSDEDVQYR.V |
| 107644 | 661 | – | 676 | 611.6393 | 1831.8961 | 1831.8952 | 0.47 | 1 | 67 | 4.9e-07 | 1Score **> 35** indicates **identity** Score **> 17** indicates **homology** | U | R.LPAKPEVSSDEDVQYR.V |
| 107645 | 661 | – | 676 | 611.6394 | 1831.8963 | 1831.8952 | 0.60 | 1 | 30 | 0.0014 | 1Score **> 35** indicates **identity** Score **> 14** indicates **homology** | U | R.LPAKPEVSSDEDVQYR.V |
| 107646 | 661 | – | 676 | 611.6394 | 1831.8963 | 1831.8952 | 0.62 | 1 | 60 | 2.3e-06 | 1Score **> 35** indicates **identity** Score **> 16** indicates **homology** | U | R.LPAKPEVSSDEDVQYR.V |
| 107647 | 661 | – | 676 | 611.6394 | 1831.8965 | 1831.8952 | 0.71 | 1 | 31 | 0.0012 | 1Score **> 35** indicates **identity** Score **> 14** indicates **homology** | U | R.LPAKPEVSSDEDVQYR.V |
| 107648 | 661 | – | 676 | 611.6395 | 1831.8966 | 1831.8952 | 0.79 | 1 | 24 | 0.0056 | 1Score **> 35** indicates **identity** Score **> 14** indicates **homology** | U | R.LPAKPEVSSDEDVQYR.V |
| 107649 | 661 | – | 676 | 611.6395 | 1831.8967 | 1831.8952 | 0.82 | 1 | 58 | 3.3e-06 | 1Score **> 35** indicates **identity** Score **> 16** indicates **homology** | U | R.LPAKPEVSSDEDVQYR.V |
| 107651 | 661 | – | 676 | 611.6405 | 1831.8997 | 1831.8952 | 2.47 | 1 | 91 | 3e-09 | 1Score **> 35** indicates **identity** Score **> 18** indicates **homology** | U | R.LPAKPEVSSDEDVQYR.V |
| 107652 | 661 | – | 676 | 916.9574 | 1831.9002 | 1831.8952 | 2.73 | 1 | 66 | 6.5e-07 | 1Score **> 35** indicates **identity** Score **> 17** indicates **homology** | U | R.LPAKPEVSSDEDVQYR.V |
| 148622 | 661 | – | 680 | 768.0703 | 2301.1889 | 2301.1965 | -3.27 | 2 | 16 | 0.034 | 1Score **> 37** indicates **identity** Score **> 13** indicates **homology** | U | R.LPAKPEVSSDEDVQYRVITR.F |
| 148624 | 661 | – | 680 | 768.0728 | 2301.1966 | 2301.1965 | 0.053 | 2 | 20 | 0.014 | 1Score **> 37** indicates **identity** Score **> 14** indicates **homology** | U | R.LPAKPEVSSDEDVQYRVITR.F |
| 17454 | 720 | – | 728 | 520.7742 | 1039.5337 | 1039.5338 | -0.092 | 0 | 36 | 0.00038 | 1Score **> 30** indicates **identity** Score **> 15** indicates **homology** | U | R.FVDLYGAQK.V |
| 17455 | 720 | – | 728 | 520.7742 | 1039.5338 | 1039.5338 | -0.085 | 0 | 26 | 0.0033 | 1Score **> 30** indicates **identity** Score **> 14** indicates **homology** | U | R.FVDLYGAQK.V |
| 17457 | 720 | – | 728 | 520.7743 | 1039.5340 | 1039.5338 | 0.17 | 0 | 43 | 9.1e-05 | 1Score **> 30** indicates **identity** Score **> 15** indicates **homology** | U | R.FVDLYGAQK.V |
| 17458 | 720 | – | 728 | 520.7743 | 1039.5341 | 1039.5338 | 0.25 | 0 | 18 | 0.019 | 1Score **> 30** indicates **identity** Score **> 14** indicates **homology** | U | R.FVDLYGAQK.V |
| 17459 | 720 | – | 728 | 520.7744 | 1039.5342 | 1039.5338 | 0.29 | 0 | 42 | 0.00012 | 1Score **> 30** indicates **identity** Score **> 15** indicates **homology** | U | R.FVDLYGAQK.V |
| 17460 | 720 | – | 728 | 520.7744 | 1039.5342 | 1039.5338 | 0.32 | 0 | 37 | 0.00032 | 1Score **> 30** indicates **identity** Score **> 15** indicates **homology** | U | R.FVDLYGAQK.V |
| 17461 | 720 | – | 728 | 520.7744 | 1039.5342 | 1039.5338 | 0.32 | 0 | 42 | 0.00011 | 1Score **> 30** indicates **identity** Score **> 15** indicates **homology** | U | R.FVDLYGAQK.V |
| 17462 | 720 | – | 728 | 520.7745 | 1039.5344 | 1039.5338 | 0.49 | 0 | 24 | 0.006 | 1Score **> 30** indicates **identity** Score **> 14** indicates **homology** | U | R.FVDLYGAQK.V |
| 17463 | 720 | – | 728 | 520.7745 | 1039.5345 | 1039.5338 | 0.62 | 0 | 39 | 0.00024 | 1Score **> 30** indicates **identity** Score **> 15** indicates **homology** | U | R.FVDLYGAQK.V |
| 17468 | 720 | – | 728 | 520.7747 | 1039.5348 | 1039.5338 | 0.88 | 0 | 30 | 0.0016 | 1Score **> 31** indicates **identity** Score **> 14** indicates **homology** | U | R.FVDLYGAQK.V |
| 17469 | 720 | – | 728 | 520.7747 | 1039.5349 | 1039.5338 | 0.98 | 0 | 25 | 0.0042 | 1Score **> 31** indicates **identity** Score **> 14** indicates **homology** | U | R.FVDLYGAQK.V |
| 17471 | 720 | – | 728 | 520.7749 | 1039.5352 | 1039.5338 | 1.30 | 0 | 26 | 0.0038 | 1Score **> 31** indicates **identity** Score **> 14** indicates **homology** | U | R.FVDLYGAQK.V |
| 17472 | 720 | – | 728 | 520.7750 | 1039.5355 | 1039.5338 | 1.56 | 0 | 25 | 0.0048 | 1Score **> 31** indicates **identity** Score **> 14** indicates **homology** | U | R.FVDLYGAQK.V |
| 17473 | 720 | – | 728 | 520.7751 | 1039.5356 | 1039.5338 | 1.71 | 0 | 14 | 0.049 | 1Score **> 31** indicates **identity** Score **> 13** indicates **homology** | U | R.FVDLYGAQK.V |
| 182194 | 733 | – | 759 | 786.1445 | 3140.5490 | 3140.5349 | 4.49 | 2 | 43 | 8.4e-05 | 1Score **> 37** indicates **identity** Score **> 15** indicates **homology** | U | R.LRKYESAYGTQFTPCQLLLDHANNSSK.K |
| 182208 | 733 | – | 759 | 786.3922 | 3141.5395 | 3141.5189 | 6.56 | 2 | 49 | 2.4e-05 | 1Score **> 37** indicates **identity** Score **> 16** indicates **homology** | U | R.LRKYESAYGTQFTPCQLLLDHANNSSK.K  + Deamidated (NQ) |
| 175514 | 735 | – | 759 | 718.8446 | 2871.3495 | 2871.3497 | -0.086 | 1 | 20 | 0.014 | 1Score **> 35** indicates **identity** Score **> 14** indicates **homology** | U | R.KYESAYGTQFTPCQLLLDHANNSSK.K |
| 175515 | 735 | – | 759 | 958.1238 | 2871.3497 | 2871.3497 | -0.0094 | 1 | 28 | 0.0024 | 1Score **> 35** indicates **identity** Score **> 14** indicates **homology** | U | R.KYESAYGTQFTPCQLLLDHANNSSK.K |
| 175516 | 735 | – | 759 | 718.8447 | 2871.3497 | 2871.3497 | -0.0024 | 1 | 26 | 0.0034 | 1Score **> 35** indicates **identity** Score **> 14** indicates **homology** | U | R.KYESAYGTQFTPCQLLLDHANNSSK.K |
| 175517 | 735 | – | 759 | 718.8449 | 2871.3503 | 2871.3497 | 0.20 | 1 | 64 | 1e-06 | 1Score **> 35** indicates **identity** Score **> 16** indicates **homology** | U | R.KYESAYGTQFTPCQLLLDHANNSSK.K |
| 175518 | 735 | – | 759 | 718.8449 | 2871.3505 | 2871.3497 | 0.28 | 1 | 31 | 0.0013 | 1Score **> 36** indicates **identity** Score **> 14** indicates **homology** | U | R.KYESAYGTQFTPCQLLLDHANNSSK.K |
| 175519 | 735 | – | 759 | 1436.6826 | 2871.3506 | 2871.3497 | 0.31 | 1 | 64 | 1.1e-06 | 1Score **> 35** indicates **identity** Score **> 16** indicates **homology** | U | R.KYESAYGTQFTPCQLLLDHANNSSK.K |
| 175521 | 735 | – | 759 | 958.1243 | 2871.3512 | 2871.3497 | 0.51 | 1 | 21 | 0.012 | 1Score **> 36** indicates **identity** Score **> 14** indicates **homology** | U | R.KYESAYGTQFTPCQLLLDHANNSSK.K |
| 175522 | 735 | – | 759 | 718.8452 | 2871.3515 | 2871.3497 | 0.63 | 1 | 24 | 0.0057 | 1Score **> 36** indicates **identity** Score **> 14** indicates **homology** | U | R.KYESAYGTQFTPCQLLLDHANNSSK.K |
| 175523 | 735 | – | 759 | 718.8452 | 2871.3517 | 2871.3497 | 0.68 | 1 | 74 | 1.2e-07 | 1Score **> 36** indicates **identity** Score **> 17** indicates **homology** | U | R.KYESAYGTQFTPCQLLLDHANNSSK.K |
| 175524 | 735 | – | 759 | 958.1246 | 2871.3520 | 2871.3497 | 0.78 | 1 | 103 | 2.2e-10 | 1Score **> 36** indicates **identity** Score **> 19** indicates **homology** | U | R.KYESAYGTQFTPCQLLLDHANNSSK.K |
| 175525 | 735 | – | 759 | 958.1246 | 2871.3520 | 2871.3497 | 0.78 | 1 | 63 | 1.2e-06 | 1Score **> 36** indicates **identity** Score **> 16** indicates **homology** | U | R.KYESAYGTQFTPCQLLLDHANNSSK.K |
| 175526 | 735 | – | 759 | 958.1246 | 2871.3521 | 2871.3497 | 0.83 | 1 | 109 | 6.4e-11 | 1Score **> 36** indicates **identity** Score **> 19** indicates **homology** | U | R.KYESAYGTQFTPCQLLLDHANNSSK.K |
| 175527 | 735 | – | 759 | 718.8453 | 2871.3521 | 2871.3497 | 0.83 | 1 | 44 | 7.4e-05 | 1Score **> 36** indicates **identity** Score **> 15** indicates **homology** | U | R.KYESAYGTQFTPCQLLLDHANNSSK.K |
| 175529 | 735 | – | 759 | 958.1247 | 2871.3522 | 2871.3497 | 0.85 | 1 | 52 | 1.2e-05 | 1Score **> 36** indicates **identity** Score **> 16** indicates **homology** | U | R.KYESAYGTQFTPCQLLLDHANNSSK.K |
| 175530 | 735 | – | 759 | 718.8454 | 2871.3524 | 2871.3497 | 0.92 | 1 | 68 | 3.9e-07 | 1Score **> 36** indicates **identity** Score **> 17** indicates **homology** | U | R.KYESAYGTQFTPCQLLLDHANNSSK.K |
| 175531 | 735 | – | 759 | 1436.6835 | 2871.3524 | 2871.3497 | 0.94 | 1 | 55 | 7.5e-06 | 1Score **> 36** indicates **identity** Score **> 16** indicates **homology** | U | R.KYESAYGTQFTPCQLLLDHANNSSK.K |
| 175532 | 735 | – | 759 | 718.8455 | 2871.3527 | 2871.3497 | 1.04 | 1 | 31 | 0.0012 | 1Score **> 36** indicates **identity** Score **> 14** indicates **homology** | U | R.KYESAYGTQFTPCQLLLDHANNSSK.K |
| 175533 | 735 | – | 759 | 718.8455 | 2871.3527 | 2871.3497 | 1.05 | 1 | 65 | 7.8e-07 | 1Score **> 36** indicates **identity** Score **> 17** indicates **homology** | U | R.KYESAYGTQFTPCQLLLDHANNSSK.K |
| 175534 | 735 | – | 759 | 958.1249 | 2871.3528 | 2871.3497 | 1.06 | 1 | 87 | 7.5e-09 | 1Score **> 36** indicates **identity** Score **> 18** indicates **homology** | U | R.KYESAYGTQFTPCQLLLDHANNSSK.K |
| 175535 | 735 | – | 759 | 958.1249 | 2871.3528 | 2871.3497 | 1.06 | 1 | 109 | 6.3e-11 | 1Score **> 36** indicates **identity** Score **> 19** indicates **homology** | U | R.KYESAYGTQFTPCQLLLDHANNSSK.K |
| 175568 | 735 | – | 759 | 719.0913 | 2872.3360 | 2872.3337 | 0.80 | 1 | 70 | 2.7e-07 | 1Score **> 35** indicates **identity** Score **> 17** indicates **homology** | U | R.KYESAYGTQFTPCQLLLDHANNSSK.K  + Deamidated (NQ) |
| 175569 | 735 | – | 759 | 958.4529 | 2872.3368 | 2872.3337 | 1.08 | 1 | 106 | 1.1e-10 | 1Score **> 35** indicates **identity** Score **> 19** indicates **homology** | U | R.KYESAYGTQFTPCQLLLDHANNSSK.K  + Deamidated (NQ) |
| 175570 | 735 | – | 759 | 958.4535 | 2872.3386 | 2872.3337 | 1.70 | 1 | 91 | 3e-09 | 1Score **> 35** indicates **identity** Score **> 18** indicates **homology** | U | R.KYESAYGTQFTPCQLLLDHANNSSK.K  + Deamidated (NQ) |
| 175574 | 735 | – | 759 | 719.0932 | 2872.3435 | 2872.3337 | 3.41 | 1 | 74 | 1.3e-07 | 1Score **> 35** indicates **identity** Score **> 17** indicates **homology** | U | R.KYESAYGTQFTPCQLLLDHANNSSK.K  + Deamidated (NQ) |
| 175575 | 735 | – | 759 | 719.0932 | 2872.3438 | 2872.3337 | 3.51 | 1 | 59 | 3.2e-06 | 1Score **> 35** indicates **identity** Score **> 16** indicates **homology** | U | R.KYESAYGTQFTPCQLLLDHANNSSK.K  + Deamidated (NQ) |
| 175577 | 735 | – | 759 | 719.0942 | 2872.3475 | 2872.3337 | 4.80 | 1 | 65 | 8.6e-07 | 1Score **> 35** indicates **identity** Score **> 17** indicates **homology** | U | R.KYESAYGTQFTPCQLLLDHANNSSK.K  + Deamidated (NQ) |
| 175578 | 735 | – | 759 | 958.4575 | 2872.3507 | 2872.3337 | 5.92 | 1 | 81 | 2.4e-08 | 1Score **> 35** indicates **identity** Score **> 18** indicates **homology** | U | R.KYESAYGTQFTPCQLLLDHANNSSK.K  + Deamidated (NQ) |
| 179563 | 735 | – | 760 | 600.8935 | 2999.4311 | 2999.4447 | -4.52 | 2 | 32 | 0.001 | 1Score **> 36** indicates **identity** Score **> 14** indicates **homology** | U | R.KYESAYGTQFTPCQLLLDHANNSSKK.F |
| 179565 | 735 | – | 760 | 1000.8201 | 2999.4383 | 2999.4447 | -2.12 | 2 | 61 | 1.8e-06 | 1Score **> 37** indicates **identity** Score **> 16** indicates **homology** | U | R.KYESAYGTQFTPCQLLLDHANNSSKK.F |
| 179566 | 735 | – | 760 | 750.8683 | 2999.4442 | 2999.4447 | -0.17 | 2 | 47 | 4.1e-05 | 1Score **> 37** indicates **identity** Score **> 15** indicates **homology** | U | R.KYESAYGTQFTPCQLLLDHANNSSKK.F |
| 179567 | 735 | – | 760 | 600.8962 | 2999.4446 | 2999.4447 | -0.027 | 2 | 34 | 0.00062 | 1Score **> 37** indicates **identity** Score **> 15** indicates **homology** | U | R.KYESAYGTQFTPCQLLLDHANNSSKK.F |
| 179568 | 735 | – | 760 | 750.8686 | 2999.4452 | 2999.4447 | 0.17 | 2 | 64 | 9.3e-07 | 1Score **> 37** indicates **identity** Score **> 17** indicates **homology** | U | R.KYESAYGTQFTPCQLLLDHANNSSKK.F |
| 179569 | 735 | – | 760 | 750.8686 | 2999.4455 | 2999.4447 | 0.27 | 2 | 17 | 0.026 | 1Score **> 37** indicates **identity** Score **> 14** indicates **homology** | U | R.KYESAYGTQFTPCQLLLDHANNSSKK.F |
| 179571 | 735 | – | 760 | 1000.8225 | 2999.4457 | 2999.4447 | 0.32 | 2 | 78 | 5.2e-08 | 1Score **> 37** indicates **identity** Score **> 17** indicates **homology** | U | R.KYESAYGTQFTPCQLLLDHANNSSKK.F |
| 179572 | 735 | – | 760 | 600.8965 | 2999.4463 | 2999.4447 | 0.53 | 2 | 43 | 0.0001 | 1Score **> 37** indicates **identity** Score **> 15** indicates **homology** | U | R.KYESAYGTQFTPCQLLLDHANNSSKK.F |
| 179574 | 735 | – | 760 | 600.8966 | 2999.4466 | 2999.4447 | 0.65 | 2 | 43 | 8.7e-05 | 1Score **> 37** indicates **identity** Score **> 15** indicates **homology** | U | R.KYESAYGTQFTPCQLLLDHANNSSKK.F |
| 179575 | 735 | – | 760 | 750.8691 | 2999.4473 | 2999.4447 | 0.87 | 2 | 43 | 9.3e-05 | 1Score **> 37** indicates **identity** Score **> 15** indicates **homology** | U | R.KYESAYGTQFTPCQLLLDHANNSSKK.F |
| 179576 | 735 | – | 760 | 1000.8234 | 2999.4483 | 2999.4447 | 1.20 | 2 | 86 | 8.8e-09 | 1Score **> 37** indicates **identity** Score **> 18** indicates **homology** | U | R.KYESAYGTQFTPCQLLLDHANNSSKK.F |
| 179584 | 735 | – | 760 | 751.1105 | 3000.4128 | 3000.4287 | -5.28 | 2 | 34 | 0.0007 | 1Score **> 36** indicates **identity** Score **> 15** indicates **homology** | U | R.KYESAYGTQFTPCQLLLDHANNSSKK.F  + Deamidated (NQ) |
| 179593 | 735 | – | 760 | 751.1114 | 3000.4167 | 3000.4287 | -4.00 | 2 | 15 | 0.037 | 1Score **> 36** indicates **identity** Score **> 13** indicates **homology** | U | R.KYESAYGTQFTPCQLLLDHANNSSKK.F  + Deamidated (NQ) |
| 179595 | 735 | – | 760 | 751.1116 | 3000.4173 | 3000.4287 | -3.80 | 2 | 32 | 0.001 | 1Score **> 36** indicates **identity** Score **> 14** indicates **homology** | U | R.KYESAYGTQFTPCQLLLDHANNSSKK.F  + Deamidated (NQ) |
| 179599 | 735 | – | 760 | 1001.1478 | 3000.4216 | 3000.4287 | -2.37 | 2 | 28 | 0.0023 | 1Score **> 36** indicates **identity** Score **> 14** indicates **homology** | U | R.KYESAYGTQFTPCQLLLDHANNSSKK.F  + Deamidated (NQ) |
| 179602 | 735 | – | 760 | 751.1142 | 3000.4278 | 3000.4287 | -0.29 | 2 | 44 | 7.8e-05 | 1Score **> 36** indicates **identity** Score **> 15** indicates **homology** | U | R.KYESAYGTQFTPCQLLLDHANNSSKK.F  + Deamidated (NQ) |
| 179603 | 735 | – | 760 | 751.1143 | 3000.4281 | 3000.4287 | -0.20 | 2 | 38 | 0.00029 | 1Score **> 36** indicates **identity** Score **> 15** indicates **homology** | U | R.KYESAYGTQFTPCQLLLDHANNSSKK.F  + Deamidated (NQ) |
| 179604 | 735 | – | 760 | 751.1146 | 3000.4292 | 3000.4287 | 0.15 | 2 | 44 | 6.8e-05 | 1Score **> 36** indicates **identity** Score **> 15** indicates **homology** | U | R.KYESAYGTQFTPCQLLLDHANNSSKK.F  + Deamidated (NQ) |
| 179639 | 735 | – | 760 | 1001.4878 | 3001.4415 | 3001.4127 | 9.59 | 2 | 27 | 0.0027 | 1Score **> 37** indicates **identity** Score **> 14** indicates **homology** | U | R.KYESAYGTQFTPCQLLLDHANNSSKK.F  + 2 Deamidated (NQ) |
| 170896 | 736 | – | 759 | 915.4244 | 2743.2514 | 2743.2548 | -1.22 | 0 | 26 | 0.0034 | 1Score **> 33** indicates **identity** Score **> 14** indicates **homology** | U | K.YESAYGTQFTPCQLLLDHANNSSK.K |
| 170897 | 736 | – | 759 | 915.4266 | 2743.2580 | 2743.2548 | 1.17 | 0 | 56 | 5.5e-06 | 1Score **> 34** indicates **identity** Score **> 16** indicates **homology** | U | K.YESAYGTQFTPCQLLLDHANNSSK.K |
| 170898 | 736 | – | 759 | 915.4270 | 2743.2591 | 2743.2548 | 1.59 | 0 | 63 | 1.3e-06 | 1Score **> 34** indicates **identity** Score **> 16** indicates **homology** | U | K.YESAYGTQFTPCQLLLDHANNSSK.K |
| 170911 | 736 | – | 759 | 915.7535 | 2744.2387 | 2744.2388 | -0.029 | 0 | 28 | 0.0023 | 1Score **> 32** indicates **identity** Score **> 14** indicates **homology** | U | K.YESAYGTQFTPCQLLLDHANNSSK.K  + Deamidated (NQ) |
| 170914 | 736 | – | 759 | 915.7586 | 2744.2539 | 2744.2388 | 5.51 | 0 | 20 | 0.013 | 1Score **> 34** indicates **identity** Score **> 14** indicates **homology** | U | K.YESAYGTQFTPCQLLLDHANNSSK.K  + Deamidated (NQ) |
| 170918 | 736 | – | 759 | 915.7612 | 2744.2619 | 2744.2388 | 8.41 | 0 | 57 | 4.7e-06 | 1Score **> 34** indicates **identity** Score **> 16** indicates **homology** | U | K.YESAYGTQFTPCQLLLDHANNSSK.K  + Deamidated (NQ) |
| 170937 | 736 | – | 759 | 916.0888 | 2745.2447 | 2745.2228 | 7.98 | 0 | 18 | 0.019 | 1Score **> 33** indicates **identity** Score **> 14** indicates **homology** | U | K.YESAYGTQFTPCQLLLDHANNSSK.K  + 2 Deamidated (NQ) |

---

```
ID   ECHA_MOUSE              Reviewed;         763 AA.
AC   Q8BMS1; Q3TCY3; Q5U5Y5; Q8QZU4;
DT   18-MAR-2008, integrated into UniProtKB/Swiss-Prot.
DT   01-MAR-2003, sequence version 1.
DT   28-JUN-2023, entry version 183.
DE   RecName: Full=Trifunctional enzyme subunit alpha, mitochondrial;
DE   AltName: Full=Monolysocardiolipin acyltransferase {ECO:0000250|UniProtKB:P40939};
DE            EC=2.3.1.- {ECO:0000250|UniProtKB:P40939};
DE   AltName: Full=TP-alpha;
DE   Includes:
DE     RecName: Full=Long-chain enoyl-CoA hydratase;
DE              EC=4.2.1.17 {ECO:0000250|UniProtKB:P40939};
DE   Includes:
DE     RecName: Full=Long chain 3-hydroxyacyl-CoA dehydrogenase;
DE              EC=1.1.1.211 {ECO:0000250|UniProtKB:P40939};
DE   Flags: Precursor;
GN   Name=Hadha;
OS   Mus musculus (Mouse).
OC   Eukaryota; Metazoa; Chordata; Craniata; Vertebrata; Euteleostomi; Mammalia;
OC   Eutheria; Euarchontoglires; Glires; Rodentia; Myomorpha; Muroidea; Muridae;
OC   Murinae; Mus; Mus.
OX   NCBI_TaxID=10090;
RN   [1]
RP   NUCLEOTIDE SEQUENCE [LARGE SCALE MRNA].
RC   STRAIN=C57BL/6J, and NOD; TISSUE=Skin;
RX   PubMed=16141072; DOI=10.1126/science.1112014;
RA   Carninci P., Kasukawa T., Katayama S., Gough J., Frith M.C., Maeda N.,
RA   Oyama R., Ravasi T., Lenhard B., Wells C., Kodzius R., Shimokawa K.,
RA   Bajic V.B., Brenner S.E., Batalov S., Forrest A.R., Zavolan M., Davis M.J.,
RA   Wilming L.G., Aidinis V., Allen J.E., Ambesi-Impiombato A., Apweiler R.,
RA   Aturaliya R.N., Bailey T.L., Bansal M., Baxter L., Beisel K.W., Bersano T.,
RA   Bono H., Chalk A.M., Chiu K.P., Choudhary V., Christoffels A.,
RA   Clutterbuck D.R., Crowe M.L., Dalla E., Dalrymple B.P., de Bono B.,
RA   Della Gatta G., di Bernardo D., Down T., Engstrom P., Fagiolini M.,
RA   Faulkner G., Fletcher C.F., Fukushima T., Furuno M., Futaki S.,
RA   Gariboldi M., Georgii-Hemming P., Gingeras T.R., Gojobori T., Green R.E.,
RA   Gustincich S., Harbers M., Hayashi Y., Hensch T.K., Hirokawa N., Hill D.,
RA   Huminiecki L., Iacono M., Ikeo K., Iwama A., Ishikawa T., Jakt M.,
RA   Kanapin A., Katoh M., Kawasawa Y., Kelso J., Kitamura H., Kitano H.,
RA   Kollias G., Krishnan S.P., Kruger A., Kummerfeld S.K., Kurochkin I.V.,
RA   Lareau L.F., Lazarevic D., Lipovich L., Liu J., Liuni S., McWilliam S.,
RA   Madan Babu M., Madera M., Marchionni L., Matsuda H., Matsuzawa S., Miki H.,
RA   Mignone F., Miyake S., Morris K., Mottagui-Tabar S., Mulder N., Nakano N.,
RA   Nakauchi H., Ng P., Nilsson R., Nishiguchi S., Nishikawa S., Nori F.,
RA   Ohara O., Okazaki Y., Orlando V., Pang K.C., Pavan W.J., Pavesi G.,
RA   Pesole G., Petrovsky N., Piazza S., Reed J., Reid J.F., Ring B.Z.,
RA   Ringwald M., Rost B., Ruan Y., Salzberg S.L., Sandelin A., Schneider C.,
RA   Schoenbach C., Sekiguchi K., Semple C.A., Seno S., Sessa L., Sheng Y.,
RA   Shibata Y., Shimada H., Shimada K., Silva D., Sinclair B., Sperling S.,
RA   Stupka E., Sugiura K., Sultana R., Takenaka Y., Taki K., Tammoja K.,
RA   Tan S.L., Tang S., Taylor M.S., Tegner J., Teichmann S.A., Ueda H.R.,
RA   van Nimwegen E., Verardo R., Wei C.L., Yagi K., Yamanishi H.,
RA   Zabarovsky E., Zhu S., Zimmer A., Hide W., Bult C., Grimmond S.M.,
RA   Teasdale R.D., Liu E.T., Brusic V., Quackenbush J., Wahlestedt C.,
RA   Mattick J.S., Hume D.A., Kai C., Sasaki D., Tomaru Y., Fukuda S.,
RA   Kanamori-Katayama M., Suzuki M., Aoki J., Arakawa T., Iida J., Imamura K.,
RA   Itoh M., Kato T., Kawaji H., Kawagashira N., Kawashima T., Kojima M.,
RA   Kondo S., Konno H., Nakano K., Ninomiya N., Nishio T., Okada M., Plessy C.,
RA   Shibata K., Shiraki T., Suzuki S., Tagami M., Waki K., Watahiki A.,
RA   Okamura-Oho Y., Suzuki H., Kawai J., Hayashizaki Y.;
RT   "The transcriptional landscape of the mammalian genome.";
RL   Science 309:1559-1563(2005).
RN   [2]
RP   NUCLEOTIDE SEQUENCE [LARGE SCALE MRNA].
RC   STRAIN=FVB/N; TISSUE=Eye, Liver, and Olfactory epithelium;
RX   PubMed=15489334; DOI=10.1101/gr.2596504;
RG   The MGC Project Team;
RT   "The status, quality, and expansion of the NIH full-length cDNA project:
RT   the Mammalian Gene Collection (MGC).";
RL   Genome Res. 14:2121-2127(2004).
RN   [3]
RP   ACETYLATION [LARGE SCALE ANALYSIS] AT LYS-129, AND IDENTIFICATION BY MASS
RP   SPECTROMETRY [LARGE SCALE ANALYSIS].
RC   TISSUE=Liver;
RX   PubMed=16916647; DOI=10.1016/j.molcel.2006.06.026;
RA   Kim S.C., Sprung R., Chen Y., Xu Y., Ball H., Pei J., Cheng T., Kho Y.,
RA   Xiao H., Xiao L., Grishin N.V., White M., Yang X.-J., Zhao Y.;
RT   "Substrate and functional diversity of lysine acetylation revealed by a
RT   proteomics survey.";
RL   Mol. Cell 23:607-618(2006).
RN   [4]
RP   PHOSPHORYLATION [LARGE SCALE ANALYSIS] AT SER-231; SER-316; THR-395 AND
RP   SER-647, AND IDENTIFICATION BY MASS SPECTROMETRY [LARGE SCALE ANALYSIS].
RC   TISSUE=Brain, Brown adipose tissue, Heart, Kidney, Liver, Lung,
RC   Pancreas, Spleen, and Testis;
RX   PubMed=21183079; DOI=10.1016/j.cell.2010.12.001;
RA   Huttlin E.L., Jedrychowski M.P., Elias J.E., Goswami T., Rad R.,
RA   Beausoleil S.A., Villen J., Haas W., Sowa M.E., Gygi S.P.;
RT   "A tissue-specific atlas of mouse protein phosphorylation and expression.";
RL   Cell 143:1174-1189(2010).
RN   [5]
RP   ACETYLATION [LARGE SCALE ANALYSIS] AT LYS-60 AND LYS-406, SUCCINYLATION
RP   [LARGE SCALE ANALYSIS] AT LYS-46; LYS-60; LYS-166; LYS-213; LYS-214;
RP   LYS-230; LYS-249; LYS-303; LYS-326; LYS-334; LYS-350; LYS-406; LYS-411;
RP   LYS-415; LYS-436; LYS-440; LYS-460; LYS-505; LYS-519; LYS-569; LYS-620;
RP   LYS-634; LYS-644; LYS-646; LYS-664; LYS-728 AND LYS-759, AND IDENTIFICATION
RP   BY MASS SPECTROMETRY [LARGE SCALE ANALYSIS].
RC   TISSUE=Embryonic fibroblast, and Liver;
RX   PubMed=23806337; DOI=10.1016/j.molcel.2013.06.001;
RA   Park J., Chen Y., Tishkoff D.X., Peng C., Tan M., Dai L., Xie Z., Zhang Y.,
RA   Zwaans B.M., Skinner M.E., Lombard D.B., Zhao Y.;
RT   "SIRT5-mediated lysine desuccinylation impacts diverse metabolic
RT   pathways.";
RL   Mol. Cell 50:919-930(2013).
RN   [6]
RP   ACETYLATION [LARGE SCALE ANALYSIS] AT LYS-46; LYS-60; LYS-129; LYS-166;
RP   LYS-214; LYS-249; LYS-289; LYS-303; LYS-326; LYS-334; LYS-350; LYS-353;
RP   LYS-406; LYS-411; LYS-436; LYS-460; LYS-505; LYS-519; LYS-540; LYS-569;
RP   LYS-644; LYS-664; LYS-728; LYS-735 AND LYS-759, AND IDENTIFICATION BY MASS
RP   SPECTROMETRY [LARGE SCALE ANALYSIS].
RC   TISSUE=Liver;
RX   PubMed=23576753; DOI=10.1073/pnas.1302961110;
RA   Rardin M.J., Newman J.C., Held J.M., Cusack M.P., Sorensen D.J., Li B.,
RA   Schilling B., Mooney S.D., Kahn C.R., Verdin E., Gibson B.W.;
RT   "Label-free quantitative proteomics of the lysine acetylome in mitochondria
RT   identifies substrates of SIRT3 in metabolic pathways.";
RL   Proc. Natl. Acad. Sci. U.S.A. 110:6601-6606(2013).
RN   [7]
RP   METHYLATION [LARGE SCALE ANALYSIS] AT ARG-399, AND IDENTIFICATION BY MASS
RP   SPECTROMETRY [LARGE SCALE ANALYSIS].
RC   TISSUE=Brain;
RX   PubMed=24129315; DOI=10.1074/mcp.o113.027870;
RA   Guo A., Gu H., Zhou J., Mulhern D., Wang Y., Lee K.A., Yang V., Aguiar M.,
RA   Kornhauser J., Jia X., Ren J., Beausoleil S.A., Silva J.C., Vemulapalli V.,
RA   Bedford M.T., Comb M.J.;
RT   "Immunoaffinity enrichment and mass spectrometry analysis of protein
RT   methylation.";
RL   Mol. Cell. Proteomics 13:372-387(2014).
RN   [8]
RP   INTERACTION WITH MTLN, AND IDENTIFICATION BY MASS SPECTROMETRY.
RX   PubMed=29949755; DOI=10.1016/j.celrep.2018.05.058;
RA   Makarewich C.A., Baskin K.K., Munir A.Z., Bezprozvannaya S., Sharma G.,
RA   Khemtong C., Shah A.M., McAnally J.R., Malloy C.R., Szweda L.I.,
RA   Bassel-Duby R., Olson E.N.;
RT   "MOXI Is a Mitochondrial Micropeptide That Enhances Fatty Acid beta-
RT   Oxidation.";
RL   Cell Rep. 23:3701-3709(2018).
CC   -!- FUNCTION: Mitochondrial trifunctional enzyme catalyzes the last three
CC       of the four reactions of the mitochondrial beta-oxidation pathway. The
CC       mitochondrial beta-oxidation pathway is the major energy-producing
CC       process in tissues and is performed through four consecutive reactions
CC       breaking down fatty acids into acetyl-CoA. Among the enzymes involved
CC       in this pathway, the trifunctional enzyme exhibits specificity for
CC       long-chain fatty acids. Mitochondrial trifunctional enzyme is a
CC       heterotetrameric complex composed of two proteins, the trifunctional
CC       enzyme subunit alpha/HADHA described here carries the 2,3-enoyl-CoA
CC       hydratase and the 3-hydroxyacyl-CoA dehydrogenase activities while the
CC       trifunctional enzyme subunit beta/HADHB bears the 3-ketoacyl-CoA
CC       thiolase activity. Independently of the subunit beta, the trifunctional
CC       enzyme subunit alpha/HADHA also has a monolysocardiolipin
CC       acyltransferase activity. It acylates monolysocardiolipin into
CC       cardiolipin, a major mitochondrial membrane phospholipid which plays a
CC       key role in apoptosis and supports mitochondrial respiratory chain
CC       complexes in the generation of ATP. Allows the acylation of
CC       monolysocardiolipin with different acyl-CoA substrates including
CC       oleoyl-CoA for which it displays the highest activity.
CC       {ECO:0000250|UniProtKB:P40939}.
CC   -!- CATALYTIC ACTIVITY:
CC       Reaction=a (3S)-3-hydroxyacyl-CoA = a (2E)-enoyl-CoA + H2O;
CC         Xref=Rhea:RHEA:16105, ChEBI:CHEBI:15377, ChEBI:CHEBI:57318,
CC         ChEBI:CHEBI:58856; EC=4.2.1.17;
CC         Evidence={ECO:0000250|UniProtKB:P40939};
CC       PhysiologicalDirection=right-to-left; Xref=Rhea:RHEA:16107;
CC         Evidence={ECO:0000250|UniProtKB:P40939};
CC   -!- CATALYTIC ACTIVITY:
CC       Reaction=a 4-saturated-(3S)-3-hydroxyacyl-CoA = a (3E)-enoyl-CoA + H2O;
CC         Xref=Rhea:RHEA:20724, ChEBI:CHEBI:15377, ChEBI:CHEBI:58521,
CC         ChEBI:CHEBI:137480; EC=4.2.1.17;
CC         Evidence={ECO:0000250|UniProtKB:P40939};
CC       PhysiologicalDirection=right-to-left; Xref=Rhea:RHEA:20726;
CC         Evidence={ECO:0000250|UniProtKB:P40939};
CC   -!- CATALYTIC ACTIVITY:
CC       Reaction=(3S)-hydroxyoctanoyl-CoA = (2E)-octenoyl-CoA + H2O;
CC         Xref=Rhea:RHEA:31199, ChEBI:CHEBI:15377, ChEBI:CHEBI:62242,
CC         ChEBI:CHEBI:62617; Evidence={ECO:0000250|UniProtKB:P40939};
CC       PhysiologicalDirection=right-to-left; Xref=Rhea:RHEA:31201;
CC         Evidence={ECO:0000250|UniProtKB:P40939};
CC   -!- CATALYTIC ACTIVITY:
CC       Reaction=(3S)-3-hydroxydodecanoyl-CoA = (2E)-dodecenoyl-CoA + H2O;
CC         Xref=Rhea:RHEA:31075, ChEBI:CHEBI:15377, ChEBI:CHEBI:57330,
CC         ChEBI:CHEBI:62558; Evidence={ECO:0000250|UniProtKB:P40939};
CC       PhysiologicalDirection=right-to-left; Xref=Rhea:RHEA:31077;
CC         Evidence={ECO:0000250|UniProtKB:P40939};
CC   -!- CATALYTIC ACTIVITY:
CC       Reaction=(3S)-hydroxyhexadecanoyl-CoA = (2E)-hexadecenoyl-CoA + H2O;
CC         Xref=Rhea:RHEA:31163, ChEBI:CHEBI:15377, ChEBI:CHEBI:61526,
CC         ChEBI:CHEBI:62613; Evidence={ECO:0000250|UniProtKB:P40939};
CC       PhysiologicalDirection=right-to-left; Xref=Rhea:RHEA:31165;
CC         Evidence={ECO:0000250|UniProtKB:P40939};
CC   -!- CATALYTIC ACTIVITY:
CC       Reaction=a long-chain (3S)-3-hydroxy fatty acyl-CoA + NAD(+) = a long-
CC         chain 3-oxo-fatty acyl-CoA + H(+) + NADH; Xref=Rhea:RHEA:52656,
CC         ChEBI:CHEBI:15378, ChEBI:CHEBI:57540, ChEBI:CHEBI:57945,
CC         ChEBI:CHEBI:136757, ChEBI:CHEBI:136758; EC=1.1.1.211;
CC         Evidence={ECO:0000250|UniProtKB:P40939};
CC       PhysiologicalDirection=left-to-right; Xref=Rhea:RHEA:52657;
CC         Evidence={ECO:0000250|UniProtKB:P40939};
CC   -!- CATALYTIC ACTIVITY:
CC       Reaction=(3S)-hydroxyoctanoyl-CoA + NAD(+) = 3-oxooctanoyl-CoA + H(+) +
CC         NADH; Xref=Rhea:RHEA:31195, ChEBI:CHEBI:15378, ChEBI:CHEBI:57540,
CC         ChEBI:CHEBI:57945, ChEBI:CHEBI:62617, ChEBI:CHEBI:62619;
CC         Evidence={ECO:0000250|UniProtKB:P40939};
CC       PhysiologicalDirection=left-to-right; Xref=Rhea:RHEA:31196;
CC         Evidence={ECO:0000250|UniProtKB:P40939};
CC   -!- CATALYTIC ACTIVITY:
CC       Reaction=(3S)-hydroxydecanoyl-CoA + NAD(+) = 3-oxodecanoyl-CoA + H(+) +
CC         NADH; Xref=Rhea:RHEA:31187, ChEBI:CHEBI:15378, ChEBI:CHEBI:57540,
CC         ChEBI:CHEBI:57945, ChEBI:CHEBI:62548, ChEBI:CHEBI:62616;
CC         Evidence={ECO:0000250|UniProtKB:P40939};
CC       PhysiologicalDirection=left-to-right; Xref=Rhea:RHEA:31188;
CC         Evidence={ECO:0000250|UniProtKB:P40939};
CC   -!- CATALYTIC ACTIVITY:
CC       Reaction=(3S)-3-hydroxydodecanoyl-CoA + NAD(+) = 3-oxododecanoyl-CoA +
CC         H(+) + NADH; Xref=Rhea:RHEA:31179, ChEBI:CHEBI:15378,
CC         ChEBI:CHEBI:57540, ChEBI:CHEBI:57945, ChEBI:CHEBI:62558,
CC         ChEBI:CHEBI:62615; Evidence={ECO:0000250|UniProtKB:P40939};
CC       PhysiologicalDirection=left-to-right; Xref=Rhea:RHEA:31180;
CC         Evidence={ECO:0000250|UniProtKB:P40939};
CC   -!- CATALYTIC ACTIVITY:
CC       Reaction=(3S)-hydroxytetradecanoyl-CoA + NAD(+) = 3-oxotetradecanoyl-
CC         CoA + H(+) + NADH; Xref=Rhea:RHEA:31167, ChEBI:CHEBI:15378,
CC         ChEBI:CHEBI:57540, ChEBI:CHEBI:57945, ChEBI:CHEBI:62543,
CC         ChEBI:CHEBI:62614; Evidence={ECO:0000250|UniProtKB:P40939};
CC       PhysiologicalDirection=left-to-right; Xref=Rhea:RHEA:31168;
CC         Evidence={ECO:0000250|UniProtKB:P40939};
CC   -!- CATALYTIC ACTIVITY:
CC       Reaction=(3S)-hydroxyhexadecanoyl-CoA + NAD(+) = 3-oxohexadecanoyl-CoA
CC         + H(+) + NADH; Xref=Rhea:RHEA:31159, ChEBI:CHEBI:15378,
CC         ChEBI:CHEBI:57349, ChEBI:CHEBI:57540, ChEBI:CHEBI:57945,
CC         ChEBI:CHEBI:62613; Evidence={ECO:0000250|UniProtKB:P40939};
CC       PhysiologicalDirection=left-to-right; Xref=Rhea:RHEA:31160;
CC         Evidence={ECO:0000250|UniProtKB:P40939};
CC   -!- CATALYTIC ACTIVITY:
CC       Reaction=1'-[1,2-di-(9Z,12Z-octadecadienoyl)-sn-glycero-3-phospho]-3'-
CC         [1-(9Z,12Z-octadecadienoyl)-sn-glycero-3-phospho]-glycerol +
CC         hexadecanoyl-CoA = 1'-[1,2-di-(9Z,12Z-octadecadienoyl)-sn-glycero-3-
CC         phospho]-3'-[1-(9Z,12Z-octadecadienoyl)-2-hexadecanoyl-sn-glycero-3-
CC         phospho]-glycerol + CoA; Xref=Rhea:RHEA:43680, ChEBI:CHEBI:57287,
CC         ChEBI:CHEBI:57379, ChEBI:CHEBI:83580, ChEBI:CHEBI:83583;
CC         Evidence={ECO:0000250|UniProtKB:P40939};
CC       PhysiologicalDirection=left-to-right; Xref=Rhea:RHEA:43681;
CC         Evidence={ECO:0000250|UniProtKB:P40939};
CC   -!- CATALYTIC ACTIVITY:
CC       Reaction=(9Z)-octadecenoyl-CoA + 1'-[1,2-di-(9Z,12Z-octadecadienoyl)-
CC         sn-glycero-3-phospho]-3'-[1-(9Z,12Z-octadecadienoyl)-sn-glycero-3-
CC         phospho]-glycerol = 1'-[1,2-di-(9Z,12Z-octadecadienoyl)-sn-glycero-3-
CC         phospho]-3'-[1-(9Z,12Z-octadecadienoyl)-2-(9Z-octadecenoyl)-sn-
CC         glycero-3-phospho]-glycerol + CoA; Xref=Rhea:RHEA:43676,
CC         ChEBI:CHEBI:57287, ChEBI:CHEBI:57387, ChEBI:CHEBI:83580,
CC         ChEBI:CHEBI:83582; Evidence={ECO:0000250|UniProtKB:P40939};
CC       PhysiologicalDirection=left-to-right; Xref=Rhea:RHEA:43677;
CC         Evidence={ECO:0000250|UniProtKB:P40939};
CC   -!- CATALYTIC ACTIVITY:
CC       Reaction=(9Z,12Z)-octadecadienoyl-CoA + 1'-[1,2-di-(9Z,12Z-
CC         octadecadienoyl)-sn-glycero-3-phospho]-3'-[1-(9Z,12Z-
CC         octadecadienoyl)-sn-glycero-3-phospho]-glycerol = 1',3'-bis-[1,2-di-
CC         (9Z,12Z-octadecadienoyl)-sn-glycero-3-phospho]-glycerol + CoA;
CC         Xref=Rhea:RHEA:43672, ChEBI:CHEBI:57287, ChEBI:CHEBI:57383,
CC         ChEBI:CHEBI:83580, ChEBI:CHEBI:83581;
CC         Evidence={ECO:0000250|UniProtKB:P40939};
CC       PhysiologicalDirection=left-to-right; Xref=Rhea:RHEA:43673;
CC         Evidence={ECO:0000250|UniProtKB:P40939};
CC   -!- PATHWAY: Lipid metabolism; fatty acid beta-oxidation.
CC       {ECO:0000250|UniProtKB:P40939}.
CC   -!- SUBUNIT: Heterotetramer of 2 alpha/HADHA and 2 beta/HADHB subunits;
CC       forms the mitochondrial trifunctional enzyme (By similarity). Also
CC       purified as higher order heterooligomers including a 4 alpha/HADHA and
CC       4 beta/HADHB heterooligomer which physiological significance remains
CC       unclear (By similarity). The mitochondrial trifunctional enzyme
CC       interacts with MTLN (PubMed:29949755). {ECO:0000250|UniProtKB:P40939,
CC       ECO:0000269|PubMed:29949755}.
CC   -!- SUBCELLULAR LOCATION: Mitochondrion {ECO:0000250|UniProtKB:P40939}.
CC       Mitochondrion inner membrane {ECO:0000250|UniProtKB:P40939}.
CC       Note=Protein stability and association with mitochondrion inner
CC       membrane do not require HADHB. {ECO:0000250|UniProtKB:P40939}.
CC   -!- PTM: Acetylation of Lys-569 and Lys-728 is observed in liver
CC       mitochondria from fasted mice but not from fed mice.
CC   -!- SIMILARITY: In the N-terminal section; belongs to the enoyl-CoA
CC       hydratase/isomerase family. {ECO:0000305}.
CC   -!- SIMILARITY: In the central section; belongs to the 3-hydroxyacyl-CoA
CC       dehydrogenase family. {ECO:0000305}.
CC   ---------------------------------------------------------------------------
CC   Copyrighted by the UniProt Consortium, see https://www.uniprot.org/terms
CC   Distributed under the Creative Commons Attribution (CC BY 4.0) License
CC   ---------------------------------------------------------------------------
DR   EMBL; AK029017; BAC26245.1; -; mRNA.
DR   EMBL; AK170478; BAE41822.1; -; mRNA.
DR   EMBL; AK170683; BAE41956.1; -; mRNA.
DR   EMBL; BC027156; AAH27156.1; -; mRNA.
DR   EMBL; BC037009; AAH37009.1; -; mRNA.
DR   EMBL; BC046978; AAH46978.1; -; mRNA.
DR   EMBL; BC058569; AAH58569.1; -; mRNA.
DR   CCDS; CCDS19155.1; -.
DR   RefSeq; NP_849209.1; NM_178878.2.
DR   AlphaFoldDB; Q8BMS1; -.
DR   SMR; Q8BMS1; -.
DR   BioGRID; 220648; 43.
DR   IntAct; Q8BMS1; 8.
DR   MINT; Q8BMS1; -.
DR   STRING; 10090.ENSMUSP00000120976; -.
DR   GlyGen; Q8BMS1; 1 site, 1 O-linked glycan (1 site).
DR   iPTMnet; Q8BMS1; -.
DR   PhosphoSitePlus; Q8BMS1; -.
DR   SwissPalm; Q8BMS1; -.
DR   REPRODUCTION-2DPAGE; IPI00223092; -.
DR   EPD; Q8BMS1; -.
DR   jPOST; Q8BMS1; -.
DR   MaxQB; Q8BMS1; -.
DR   PaxDb; Q8BMS1; -.
DR   PeptideAtlas; Q8BMS1; -.
DR   ProteomicsDB; 277753; -.
DR   Antibodypedia; 3074; 238 antibodies from 32 providers.
DR   DNASU; 97212; -.
DR   Ensembl; ENSMUST00000156859; ENSMUSP00000120976; ENSMUSG00000025745.
DR   GeneID; 97212; -.
DR   KEGG; mmu:97212; -.
DR   UCSC; uc008wvc.1; mouse.
DR   AGR; MGI:2135593; -.
DR   CTD; 3030; -.
DR   MGI; MGI:2135593; Hadha.
DR   VEuPathDB; HostDB:ENSMUSG00000025745; -.
DR   eggNOG; KOG1683; Eukaryota.
DR   GeneTree; ENSGT00940000154677; -.
DR   HOGENOM; CLU_009834_16_1_1; -.
DR   InParanoid; Q8BMS1; -.
DR   OMA; PFRYMDT; -.
DR   OrthoDB; 622692at2759; -.
DR   PhylomeDB; Q8BMS1; -.
DR   TreeFam; TF352288; -.
DR   Reactome; R-MMU-1482798; Acyl chain remodeling of CL.
DR   Reactome; R-MMU-77285; Beta oxidation of myristoyl-CoA to lauroyl-CoA.
DR   Reactome; R-MMU-77305; Beta oxidation of palmitoyl-CoA to myristoyl-CoA.
DR   Reactome; R-MMU-77310; Beta oxidation of lauroyl-CoA to decanoyl-CoA-CoA.
DR   Reactome; R-MMU-77346; Beta oxidation of decanoyl-CoA to octanoyl-CoA-CoA.
DR   Reactome; R-MMU-77348; Beta oxidation of octanoyl-CoA to hexanoyl-CoA.
DR   Reactome; R-MMU-77350; Beta oxidation of hexanoyl-CoA to butanoyl-CoA.
DR   UniPathway; UPA00659; -.
DR   BioGRID-ORCS; 97212; 4 hits in 79 CRISPR screens.
DR   ChiTaRS; Hadha; mouse.
DR   PRO; PR:Q8BMS1; -.
DR   Proteomes; UP000000589; Chromosome 5.
DR   RNAct; Q8BMS1; protein.
DR   Bgee; ENSMUSG00000025745; Expressed in myocardium of ventricle and 247 other tissues.
DR   Genevisible; Q8BMS1; MM.
DR   GO; GO:0016507; C:mitochondrial fatty acid beta-oxidation multienzyme complex; ISO:MGI.
DR   GO; GO:0005743; C:mitochondrial inner membrane; HDA:MGI.
DR   GO; GO:0042645; C:mitochondrial nucleoid; ISO:MGI.
DR   GO; GO:0005739; C:mitochondrion; HDA:MGI.
DR   GO; GO:0003857; F:3-hydroxyacyl-CoA dehydrogenase activity; ISO:MGI.
DR   GO; GO:0003988; F:acetyl-CoA C-acyltransferase activity; ISO:MGI.
DR   GO; GO:0004300; F:enoyl-CoA hydratase activity; ISO:MGI.
DR   GO; GO:0000062; F:fatty-acyl-CoA binding; ISO:MGI.
DR   GO; GO:0016509; F:long-chain-3-hydroxyacyl-CoA dehydrogenase activity; IDA:MGI.
DR   GO; GO:0051287; F:NAD binding; ISO:MGI.
DR   GO; GO:0070403; F:NAD+ binding; IEA:InterPro.
DR   GO; GO:0044877; F:protein-containing complex binding; ISO:MGI.
DR   GO; GO:0035965; P:cardiolipin acyl-chain remodeling; ISS:UniProtKB.
DR   GO; GO:0006635; P:fatty acid beta-oxidation; IMP:MGI.
DR   GO; GO:0032868; P:response to insulin; IMP:MGI.
DR   GO; GO:0009410; P:response to xenobiotic stimulus; ISO:MGI.
DR   CDD; cd06558; crotonase-like; 1.
DR   Gene3D; 1.10.1040.50; -; 1.
DR   Gene3D; 3.40.50.720; NAD(P)-binding Rossmann-like Domain; 1.
DR   InterPro; IPR006180; 3-OHacyl-CoA_DH_CS.
DR   InterPro; IPR006176; 3-OHacyl-CoA_DH_NAD-bd.
DR   InterPro; IPR006108; 3HC_DH_C.
DR   InterPro; IPR008927; 6-PGluconate_DH-like_C_sf.
DR   InterPro; IPR029045; ClpP/crotonase-like_dom_sf.
DR   InterPro; IPR018376; Enoyl-CoA_hyd/isom_CS.
DR   InterPro; IPR001753; Enoyl-CoA_hydra/iso.
DR   InterPro; IPR012803; Fa_ox_alpha_mit.
DR   InterPro; IPR036291; NAD(P)-bd_dom_sf.
DR   PANTHER; PTHR43612; TRIFUNCTIONAL ENZYME SUBUNIT ALPHA; 1.
DR   PANTHER; PTHR43612:SF3; TRIFUNCTIONAL ENZYME SUBUNIT ALPHA, MITOCHONDRIAL; 1.
DR   Pfam; PF00725; 3HCDH; 2.
DR   Pfam; PF02737; 3HCDH_N; 1.
DR   Pfam; PF00378; ECH_1; 1.
DR   SUPFAM; SSF48179; 6-phosphogluconate dehydrogenase C-terminal domain-like; 2.
DR   SUPFAM; SSF52096; ClpP/crotonase; 1.
DR   SUPFAM; SSF51735; NAD(P)-binding Rossmann-fold domains; 1.
DR   PROSITE; PS00067; 3HCDH; 1.
DR   PROSITE; PS00166; ENOYL_COA_HYDRATASE; 1.
DR   TIGRFAMs; TIGR02441; fa_ox_alpha_mit; 1.
PE   1: Evidence at protein level;
KW   Acetylation; Fatty acid metabolism; Lipid metabolism; Lyase; Membrane;
KW   Methylation; Mitochondrion; Mitochondrion inner membrane;
KW   Multifunctional enzyme; NAD; Oxidoreductase; Phosphoprotein;
KW   Reference proteome; Transferase; Transit peptide.
FT   TRANSIT         1..36
FT                   /note="Mitochondrion"
FT                   /evidence="ECO:0000255"
FT   CHAIN           37..763
FT                   /note="Trifunctional enzyme subunit alpha, mitochondrial"
FT                   /id="PRO_0000322639"
FT   ACT_SITE        510
FT                   /note="For hydroxyacyl-coenzyme A dehydrogenase activity"
FT                   /evidence="ECO:0000250|UniProtKB:P40939"
FT   SITE            151
FT                   /note="Important for long-chain enoyl-CoA hydratase
FT                   activity"
FT                   /evidence="ECO:0000250|UniProtKB:P40939"
FT   SITE            173
FT                   /note="Important for long-chain enoyl-CoA hydratase
FT                   activity"
FT                   /evidence="ECO:0000250|UniProtKB:P40939"
FT   SITE            498
FT                   /note="Important for hydroxyacyl-coenzyme A dehydrogenase
FT                   activity"
FT                   /evidence="ECO:0000250|UniProtKB:P40939"
FT   MOD_RES         46
FT                   /note="N6-acetyllysine; alternate"
FT                   /evidence="ECO:0007744|PubMed:23576753"
FT   MOD_RES         46
FT                   /note="N6-succinyllysine; alternate"
FT                   /evidence="ECO:0007744|PubMed:23806337"
FT   MOD_RES         60
FT                   /note="N6-acetyllysine; alternate"
FT                   /evidence="ECO:0007744|PubMed:23576753,
FT                   ECO:0007744|PubMed:23806337"
FT   MOD_RES         60
FT                   /note="N6-succinyllysine; alternate"
FT                   /evidence="ECO:0007744|PubMed:23806337"
FT   MOD_RES         129
FT                   /note="N6-acetyllysine"
FT                   /evidence="ECO:0007744|PubMed:16916647,
FT                   ECO:0007744|PubMed:23576753"
FT   MOD_RES         166
FT                   /note="N6-acetyllysine; alternate"
FT                   /evidence="ECO:0007744|PubMed:23576753"
FT   MOD_RES         166
FT                   /note="N6-succinyllysine; alternate"
FT                   /evidence="ECO:0007744|PubMed:23806337"
FT   MOD_RES         213
FT                   /note="N6-succinyllysine"
FT                   /evidence="ECO:0007744|PubMed:23806337"
FT   MOD_RES         214
FT                   /note="N6-acetyllysine; alternate"
FT                   /evidence="ECO:0007744|PubMed:23576753"
FT   MOD_RES         214
FT                   /note="N6-succinyllysine; alternate"
FT                   /evidence="ECO:0007744|PubMed:23806337"
FT   MOD_RES         230
FT                   /note="N6-succinyllysine"
FT                   /evidence="ECO:0007744|PubMed:23806337"
FT   MOD_RES         231
FT                   /note="Phosphoserine"
FT                   /evidence="ECO:0007744|PubMed:21183079"
FT   MOD_RES         249
FT                   /note="N6-acetyllysine; alternate"
FT                   /evidence="ECO:0007744|PubMed:23576753"
FT   MOD_RES         249
FT                   /note="N6-succinyllysine; alternate"
FT                   /evidence="ECO:0007744|PubMed:23806337"
FT   MOD_RES         289
FT                   /note="N6-acetyllysine"
FT                   /evidence="ECO:0007744|PubMed:23576753"
FT   MOD_RES         295
FT                   /note="N6-acetyllysine"
FT                   /evidence="ECO:0000250|UniProtKB:P40939"
FT   MOD_RES         303
FT                   /note="N6-acetyllysine; alternate"
FT                   /evidence="ECO:0007744|PubMed:23576753"
FT   MOD_RES         303
FT                   /note="N6-succinyllysine; alternate"
FT                   /evidence="ECO:0007744|PubMed:23806337"
FT   MOD_RES         316
FT                   /note="Phosphoserine"
FT                   /evidence="ECO:0007744|PubMed:21183079"
FT   MOD_RES         326
FT                   /note="N6-acetyllysine; alternate"
FT                   /evidence="ECO:0007744|PubMed:23576753"
FT   MOD_RES         326
FT                   /note="N6-succinyllysine; alternate"
FT                   /evidence="ECO:0007744|PubMed:23806337"
FT   MOD_RES         334
FT                   /note="N6-acetyllysine; alternate"
FT                   /evidence="ECO:0007744|PubMed:23576753"
FT   MOD_RES         334
FT                   /note="N6-succinyllysine; alternate"
FT                   /evidence="ECO:0007744|PubMed:23806337"
FT   MOD_RES         350
FT                   /note="N6-acetyllysine; alternate"
FT                   /evidence="ECO:0007744|PubMed:23576753"
FT   MOD_RES         350
FT                   /note="N6-succinyllysine; alternate"
FT                   /evidence="ECO:0007744|PubMed:23806337"
FT   MOD_RES         353
FT                   /note="N6-acetyllysine"
FT                   /evidence="ECO:0007744|PubMed:23576753"
FT   MOD_RES         395
FT                   /note="Phosphothreonine"
FT                   /evidence="ECO:0007744|PubMed:21183079"
FT   MOD_RES         399
FT                   /note="Omega-N-methylarginine"
FT                   /evidence="ECO:0007744|PubMed:24129315"
FT   MOD_RES         406
FT                   /note="N6-acetyllysine; alternate"
FT                   /evidence="ECO:0007744|PubMed:23576753,
FT                   ECO:0007744|PubMed:23806337"
FT   MOD_RES         406
FT                   /note="N6-succinyllysine; alternate"
FT                   /evidence="ECO:0007744|PubMed:23806337"
FT   MOD_RES         411
FT                   /note="N6-acetyllysine; alternate"
FT                   /evidence="ECO:0007744|PubMed:23576753"
FT   MOD_RES         411
FT                   /note="N6-succinyllysine; alternate"
FT                   /evidence="ECO:0007744|PubMed:23806337"
FT   MOD_RES         415
FT                   /note="N6-succinyllysine"
FT                   /evidence="ECO:0007744|PubMed:23806337"
FT   MOD_RES         419
FT                   /note="Phosphoserine"
FT                   /evidence="ECO:0000250|UniProtKB:Q64428"
FT   MOD_RES         436
FT                   /note="N6-acetyllysine; alternate"
FT                   /evidence="ECO:0007744|PubMed:23576753"
FT   MOD_RES         436
FT                   /note="N6-succinyllysine; alternate"
FT                   /evidence="ECO:0007744|PubMed:23806337"
FT   MOD_RES         440
FT                   /note="N6-succinyllysine"
FT                   /evidence="ECO:0007744|PubMed:23806337"
FT   MOD_RES         460
FT                   /note="N6-acetyllysine; alternate"
FT                   /evidence="ECO:0007744|PubMed:23576753"
FT   MOD_RES         460
FT                   /note="N6-succinyllysine; alternate"
FT                   /evidence="ECO:0007744|PubMed:23806337"
FT   MOD_RES         505
FT                   /note="N6-acetyllysine; alternate"
FT                   /evidence="ECO:0007744|PubMed:23576753"
FT   MOD_RES         505
FT                   /note="N6-succinyllysine; alternate"
FT                   /evidence="ECO:0007744|PubMed:23806337"
FT   MOD_RES         519
FT                   /note="N6-acetyllysine; alternate"
FT                   /evidence="ECO:0007744|PubMed:23576753"
FT   MOD_RES         519
FT                   /note="N6-succinyllysine; alternate"
FT                   /evidence="ECO:0007744|PubMed:23806337"
FT   MOD_RES         540
FT                   /note="N6-acetyllysine"
FT                   /evidence="ECO:0007744|PubMed:23576753"
FT   MOD_RES         569
FT                   /note="N6-acetyllysine; alternate"
FT                   /evidence="ECO:0007744|PubMed:23576753"
FT   MOD_RES         569
FT                   /note="N6-succinyllysine; alternate"
FT                   /evidence="ECO:0007744|PubMed:23806337"
FT   MOD_RES         620
FT                   /note="N6-succinyllysine"
FT                   /evidence="ECO:0007744|PubMed:23806337"
FT   MOD_RES         634
FT                   /note="N6-succinyllysine"
FT                   /evidence="ECO:0007744|PubMed:23806337"
FT   MOD_RES         644
FT                   /note="N6-acetyllysine; alternate"
FT                   /evidence="ECO:0007744|PubMed:23576753"
FT   MOD_RES         644
FT                   /note="N6-succinyllysine; alternate"
FT                   /evidence="ECO:0007744|PubMed:23806337"
FT   MOD_RES         646
FT                   /note="N6-succinyllysine"
FT                   /evidence="ECO:0007744|PubMed:23806337"
FT   MOD_RES         647
FT                   /note="Phosphoserine"
FT                   /evidence="ECO:0007744|PubMed:21183079"
FT   MOD_RES         650
FT                   /note="Phosphoserine"
FT                   /evidence="ECO:0000250|UniProtKB:Q64428"
FT   MOD_RES         664
FT                   /note="N6-acetyllysine; alternate"
FT                   /evidence="ECO:0007744|PubMed:23576753"
FT   MOD_RES         664
FT                   /note="N6-succinyllysine; alternate"
FT                   /evidence="ECO:0007744|PubMed:23806337"
FT   MOD_RES         728
FT                   /note="N6-acetyllysine; alternate"
FT                   /evidence="ECO:0007744|PubMed:23576753"
FT   MOD_RES         728
FT                   /note="N6-succinyllysine; alternate"
FT                   /evidence="ECO:0007744|PubMed:23806337"
FT   MOD_RES         735
FT                   /note="N6-acetyllysine"
FT                   /evidence="ECO:0007744|PubMed:23576753"
FT   MOD_RES         759
FT                   /note="N6-acetyllysine; alternate"
FT                   /evidence="ECO:0007744|PubMed:23576753"
FT   MOD_RES         759
FT                   /note="N6-succinyllysine; alternate"
FT                   /evidence="ECO:0007744|PubMed:23806337"
FT   CONFLICT        196
FT                   /note="A -> D (in Ref. 1; BAE41822)"
FT                   /evidence="ECO:0000305"
FT   CONFLICT        459
FT                   /note="L -> S (in Ref. 2; AAH37009)"
FT                   /evidence="ECO:0000305"
SQ   SEQUENCE   763 AA;  82670 MW;  73D203795D5C1141 CRC64;
     MVASRAIGSL SRFSAFRILR SRGCICRSFT TSSALLTRTH INYGVKGDVA VIRINSPNSK
     VNTLNKEVQS EFIEVMNEIW ANDQIRSAVL ISSKPGCFVA GADINMLSSC TTPQEATRIS
     QEGQRMFEKL EKSPKPVVAA ISGSCLGGGL ELAIACQYRI ATKDRKTVLG VPEVLLGILP
     GAGGTQRLPK MVGVPAAFDM MLTGRNIRAD RAKKMGLVDQ LVEPLGPGIK SPEERTIEYL
     EEVAVNFAKG LADRKVSAKQ SKGLVEKLTT YAMTVPFVRQ QVYKTVEEKV KKQTKGLYPA
     PLKIIDAVKA GLEQGSDAGY LAESQKFGEL ALTKESKALM GLYNGQVLCK KNKFGAPQKN
     VQQLAILGAG LMGAGIAQVS VDKGLKTLLK DTTVTGLGRG QQQVFKGLND KVKKKALTSF
     ERDSIFSNLI GQLDYKGFEK ADMVIEAVFE DLGVKHKVLK EVESVTPEHC IFASNTSALP
     INQIAAVSKR PEKVIGMHYF SPVDKMQLLE IITTDKTSKD TTASAVAVGL RQGKVIIVVK
     DGPGFYTTRC LAPMMSEVMR ILQEGVDPKK LDALTTGFGF PVGAATLADE VGVDVAQHVA
     EDLGKAFGER FGGGSVELLK QMVSKGFLGR KSGKGFYIYQ EGSKNKSLNS EMDNILANLR
     LPAKPEVSSD EDVQYRVITR FVNEAVLCLQ EGILATPAEG DIGAVFGLGF PPCLGGPFRF
     VDLYGAQKVV DRLRKYESAY GTQFTPCQLL LDHANNSSKK FYQ
//
```

|  |
| --- |
| **Mascot:** http://www.matrixscience.com/ |

HNE (C) (+156.1150)
