# Supplementary material for: A common East-Asian ALDH2 mutation causes metabolic disorders and the therapeutic effect of ALDH2 activators
Source: Nat Commun. 2023 Sep 25;14:5971. doi: 10.1038/s41467-023-41570-6 (PMC10520061; doi:10.1038/s41467-023-41570-6)
Supplement: Supplementary file 4 — Supplementary Data 1 [file 41467_2023_41570_MOESM4_ESM.zip › Table S5b/Q8BMS1/Q8BMS1_WTO-3_K94.html]

Mascot Search Results: Q8BMS1


# MASCOT Search Results

## Protein View: Q8BMS1

### Trifunctional enzyme subunit alpha, mitochondrial OS=Mus musculus OX=10090 GN=Hadha PE=1 SV=1

|  |  |
| --- | --- |
| Database: | Mouse\_UniProt\_proteomes |
| Score: | 16307 |
| Monoisotopic mass (Mr): | 83302 |
| Calculated pI: | 9.24 |

Sequence similarity is available as an NCBI BLAST search of Q8BMS1 against nr.

### Search parameters

|  |  |
| --- | --- |
| MS data file: | `D:\LCMSMS\2023 Users' data\230529-1\230529-1-WTO-3_20230601175054.raw` |
| Enzyme: | Trypsin/P: cuts C-term side of KR. |
| Fixed modifications: | Carbamidomethyl (C) |
| Variable modifications: | Deamidated (NQ), HNE (C), HNE (H), HNE (K), Oxidation (M) |

### Protein sequence coverage: 67%

Matched peptides shown in ***bold red***.

|  |  |  |  |  |  |
| --- | --- | --- | --- | --- | --- |
| `1` | `MVASRAIGSL` | `SRFSAFRILR` | `SRGCICRSFT` | `TSSALLTRTH` | `INYGVKGDVA` |
| `51` | `VIRINSPNSK` | `VNTLNKEVQS` | `EFIEVMNEIW` | `ANDQIRSAVL` | `ISSKPGCFVA` |
| `101` | `GADINMLSSC` | `TTPQEATRIS` | `QEGQRMFEKL` | `EKSPKPVVAA` | `ISGSCLGGGL` |
| `151` | `ELAIACQYRI` | `ATKDRKTVLG` | `VPEVLLGILP` | `GAGGTQRLPK` | `MVGVPAAFDM` |
| `201` | `MLTGRNIRAD` | `RAKKMGLVDQ` | `LVEPLGPGIK` | `SPEERTIEYL` | `EEVAVNFAKG` |
| `251` | `LADRKVSAKQ` | `SKGLVEKLTT` | `YAMTVPFVRQ` | `QVYKTVEEKV` | `KKQTKGLYPA` |
| `301` | `PLKIIDAVKA` | `GLEQGSDAGY` | `LAESQKFGEL` | `ALTKESKALM` | `GLYNGQVLCK` |
| `351` | `KNKFGAPQKN` | `VQQLAILGAG` | `LMGAGIAQVS` | `VDKGLKTLLK` | `DTTVTGLGRG` |
| `401` | `QQQVFKGLND` | `KVKKKALTSF` | `ERDSIFSNLI` | `GQLDYKGFEK` | `ADMVIEAVFE` |
| `451` | `DLGVKHKVLK` | `EVESVTPEHC` | `IFASNTSALP` | `INQIAAVSKR` | `PEKVIGMHYF` |
| `501` | `SPVDKMQLLE` | `IITTDKTSKD` | `TTASAVAVGL` | `RQGKVIIVVK` | `DGPGFYTTRC` |
| `551` | `LAPMMSEVMR` | `ILQEGVDPKK` | `LDALTTGFGF` | `PVGAATLADE` | `VGVDVAQHVA` |
| `601` | `EDLGKAFGER` | `FGGGSVELLK` | `QMVSKGFLGR` | `KSGKGFYIYQ` | `EGSKNKSLNS` |
| `651` | `EMDNILANLR` | `LPAKPEVSSD` | `EDVQYRVITR` | `FVNEAVLCLQ` | `EGILATPAEG` |
| `701` | `DIGAVFGLGF` | `PPCLGGPFRF` | `VDLYGAQKVV` | `DRLRKYESAY` | `GTQFTPCQLL` |
| `751` | `LDHANNSSKK` | `FYQ` |  |  |  |

Unformatted sequence string: 763 residues (for pasting into other applications).

|  |  |  |  |
| --- | --- | --- | --- |
| Sort by | residue number | increasing mass | decreasing mass |
| Show | matched peptides only | predicted peptides also |  |

| Query | Start | – | End | Observed | Mr(expt) | Mr(calc) | ppm | M | Score | Expect | Rank | U | Peptide |
| --- | --- | --- | --- | --- | --- | --- | --- | --- | --- | --- | --- | --- | --- |
| 10347 | 39 | – | 46 | 466.2539 | 930.4932 | 930.4923 | 0.95 | 0 | 38 | 0.0015 | 1Score **> 33** indicates **identity** Score **> 23** indicates **homology** | U | R.THINYGVK.G |
| 10350 | 39 | – | 46 | 466.2545 | 930.4944 | 930.4923 | 2.27 | 0 | 20 | 0.019 | 1Score **> 32** indicates **identity** Score **> 15** indicates **homology** | U | R.THINYGVK.G |
| 85075 | 39 | – | 53 | 411.2297 | 1640.8897 | 1640.8998 | -6.20 | 1 | 22 | 0.0087 | 1Score **> 35** indicates **identity** Score **> 14** indicates **homology** | U | R.THINYGVKGDVAVIR.I |
| 85082 | 39 | – | 53 | 411.2313 | 1640.8962 | 1640.8998 | -2.21 | 1 | 28 | 0.0026 | 1Score **> 35** indicates **identity** Score **> 14** indicates **homology** | U | R.THINYGVKGDVAVIR.I |
| 85086 | 39 | – | 53 | 547.9733 | 1640.8979 | 1640.8998 | -1.15 | 1 | 43 | 0.0001 | 1Score **> 35** indicates **identity** Score **> 15** indicates **homology** | U | R.THINYGVKGDVAVIR.I |
| 85087 | 39 | – | 53 | 547.9733 | 1640.8981 | 1640.8998 | -1.05 | 1 | 23 | 0.0065 | 1Score **> 35** indicates **identity** Score **> 14** indicates **homology** | U | R.THINYGVKGDVAVIR.I |
| 85093 | 39 | – | 53 | 547.9741 | 1640.9005 | 1640.8998 | 0.42 | 1 | 60 | 2.1e-06 | 1Score **> 35** indicates **identity** Score **> 16** indicates **homology** | U | R.THINYGVKGDVAVIR.I |
| 85095 | 39 | – | 53 | 547.9742 | 1640.9009 | 1640.8998 | 0.62 | 1 | 52 | 1.3e-05 | 1Score **> 35** indicates **identity** Score **> 16** indicates **homology** | U | R.THINYGVKGDVAVIR.I |
| 85096 | 39 | – | 53 | 547.9742 | 1640.9009 | 1640.8998 | 0.63 | 1 | 71 | 2e-07 | 1Score **> 35** indicates **identity** Score **> 17** indicates **homology** | U | R.THINYGVKGDVAVIR.I |
| 85098 | 39 | – | 53 | 547.9743 | 1640.9012 | 1640.8998 | 0.82 | 1 | 37 | 0.00037 | 1Score **> 34** indicates **identity** Score **> 15** indicates **homology** | U | R.THINYGVKGDVAVIR.I |
| 85100 | 39 | – | 53 | 547.9745 | 1640.9015 | 1640.8998 | 1.04 | 1 | 17 | 0.025 | 1Score **> 34** indicates **identity** Score **> 14** indicates **homology** | U | R.THINYGVKGDVAVIR.I |
| 85102 | 39 | – | 53 | 411.2328 | 1640.9020 | 1640.8998 | 1.33 | 1 | 17 | 0.026 | 1Score **> 34** indicates **identity** Score **> 14** indicates **homology** | U | R.THINYGVKGDVAVIR.I |
| 85103 | 39 | – | 53 | 547.9746 | 1640.9021 | 1640.8998 | 1.39 | 1 | 27 | 0.003 | 1Score **> 34** indicates **identity** Score **> 14** indicates **homology** | U | R.THINYGVKGDVAVIR.I |
| 85105 | 39 | – | 53 | 547.9750 | 1640.9031 | 1640.8998 | 1.96 | 1 | 48 | 3.4e-05 | 1Score **> 34** indicates **identity** Score **> 15** indicates **homology** | U | R.THINYGVKGDVAVIR.I |
| 85106 | 39 | – | 53 | 547.9750 | 1640.9031 | 1640.8998 | 1.96 | 1 | 40 | 0.00018 | 1Score **> 34** indicates **identity** Score **> 15** indicates **homology** | U | R.THINYGVKGDVAVIR.I |
| 85107 | 39 | – | 53 | 411.2331 | 1640.9034 | 1640.8998 | 2.15 | 1 | 22 | 0.0086 | 1Score **> 34** indicates **identity** Score **> 14** indicates **homology** | U | R.THINYGVKGDVAVIR.I |
| 85109 | 39 | – | 53 | 411.2334 | 1640.9045 | 1640.8998 | 2.87 | 1 | 25 | 0.0044 | 1Score **> 34** indicates **identity** Score **> 14** indicates **homology** | U | R.THINYGVKGDVAVIR.I |
| 1065 | 47 | – | 53 | 365.2155 | 728.4165 | 728.4181 | -2.24 | 0 | 29 | 0.015 | 1Score **> 23** indicates **identity** | U | K.GDVAVIR.I |
| 1066 | 47 | – | 53 | 365.2159 | 728.4172 | 728.4181 | -1.24 | 0 | 50 | 0.0001 | 1Score **> 23** indicates **identity** | U | K.GDVAVIR.I |
| 1068 | 47 | – | 53 | 365.2160 | 728.4175 | 728.4181 | -0.81 | 0 | 50 | 0.0001 | 1Score **> 23** indicates **identity** | U | K.GDVAVIR.I |
| 182438 | 61 | – | 86 | 1040.5248 | 3118.5526 | 3118.5393 | 4.25 | 1 | 38 | 0.00039 | 1Score **> 37** indicates **identity** Score **> 16** indicates **homology** | U | K.VNTLNKEVQSEFIEVMNEIWANDQIR.S |
| 157994 | 67 | – | 86 | 817.3929 | 2449.1568 | 2449.1583 | -0.62 | 0 | 42 | 0.00011 | 1Score **> 35** indicates **identity** Score **> 15** indicates **homology** | U | K.EVQSEFIEVMNEIWANDQIR.S |
| 158064 | 67 | – | 86 | 1226.0873 | 2450.1600 | 2450.1423 | 7.19 | 0 | 27 | 0.0027 | 1Score **> 35** indicates **identity** Score **> 14** indicates **homology** | U | K.EVQSEFIEVMNEIWANDQIR.S  + Deamidated (NQ) |
| 186715 | 87 | – | 118 | 1123.5499 | 3367.6278 | 3367.6210 | 2.02 | 1 | 61 | 2.1e-06 | 1Score **> 37** indicates **identity** Score **> 16** indicates **homology** | U | R.SAVLISSKPGCFVAGADINMLSSCTTPQEATR.I |
| 186717 | 87 | – | 118 | 1123.5541 | 3367.6406 | 3367.6210 | 5.81 | 1 | 68 | 4.3e-07 | 1Score **> 37** indicates **identity** Score **> 17** indicates **homology** | U | R.SAVLISSKPGCFVAGADINMLSSCTTPQEATR.I |
| 186718 | 87 | – | 118 | 842.9186 | 3367.6453 | 3367.6210 | 7.19 | 1 | 43 | 8.7e-05 | 1Score **> 37** indicates **identity** Score **> 15** indicates **homology** | U | R.SAVLISSKPGCFVAGADINMLSSCTTPQEATR.I |
| 186719 | 87 | – | 118 | 1123.5557 | 3367.6453 | 3367.6210 | 7.20 | 1 | 93 | 2.1e-09 | 1Score **> 37** indicates **identity** Score **> 18** indicates **homology** | U | R.SAVLISSKPGCFVAGADINMLSSCTTPQEATR.I |
| 186721 | 87 | – | 118 | 1123.5565 | 3367.6476 | 3367.6210 | 7.87 | 1 | 87 | 7.2e-09 | 1Score **> 37** indicates **identity** Score **> 18** indicates **homology** | U | R.SAVLISSKPGCFVAGADINMLSSCTTPQEATR.I |
| 186722 | 87 | – | 118 | 1123.5565 | 3367.6478 | 3367.6210 | 7.94 | 1 | 84 | 1.3e-08 | 1Score **> 37** indicates **identity** Score **> 18** indicates **homology** | U | R.SAVLISSKPGCFVAGADINMLSSCTTPQEATR.I |
| 186723 | 87 | – | 118 | 1123.5574 | 3367.6505 | 3367.6210 | 8.74 | 1 | 66 | 5.9e-07 | 1Score **> 37** indicates **identity** Score **> 17** indicates **homology** | U | R.SAVLISSKPGCFVAGADINMLSSCTTPQEATR.I |
| 188596 | 87 | – | 118 | 1161.9032 | 3482.6878 | 3482.7095 | -6.25 | 1 | 40 | 0.00017 | 1Score **> 37** indicates **identity** Score **> 15** indicates **homology** | U | R.SAVLISSKPGCFVAGADINMLSSCTTPQEATR.I  + HNE (C); Oxidation (M) |
| 188620 | 87 | – | 118 | 1162.2429 | 3483.7068 | 3483.6935 | 3.81 | 1 | 37 | 0.00036 | 1Score **> 37** indicates **identity** Score **> 15** indicates **homology** | U | R.SAVLISSKPGCFVAGADINMLSSCTTPQEATR.I  + Deamidated (NQ); HNE (C); Oxidation (M) |
| 189385 | 87 | – | 118 | 1181.2381 | 3540.6925 | 3540.7150 | -6.36 | 1 | 14 | 0.046 | 1Score **> 37** indicates **identity** Score **> 13** indicates **homology** | U | R.SAVLISSKPGCFVAGADINMLSSCTTPQEATR.I  + Deamidated (NQ); HNE (K); Oxidation (M) |
| 10040 | 126 | – | 132 | 462.7471 | 923.4796 | 923.4786 | 1.10 | 1 | 16 | 0.032 | 1Score **> 29** indicates **identity** Score **> 13** indicates **homology** | U | R.MFEKLEK.S |
| 182762 | 130 | – | 159 | 786.9218 | 3143.6580 | 3143.6471 | 3.48 | 2 | 21 | 0.012 | 1Score **> 36** indicates **identity** Score **> 14** indicates **homology** | U | K.LEKSPKPVVAAISGSCLGGGLELAIACQYR.I |
| 182763 | 130 | – | 159 | 786.9230 | 3143.6630 | 3143.6471 | 5.05 | 2 | 31 | 0.0013 | 1Score **> 36** indicates **identity** Score **> 14** indicates **homology** | U | K.LEKSPKPVVAAISGSCLGGGLELAIACQYR.I |
| 172283 | 133 | – | 159 | 925.4760 | 2773.4061 | 2773.4255 | -6.98 | 1 | 22 | 0.0083 | 1Score **> 37** indicates **identity** Score **> 14** indicates **homology** | U | K.SPKPVVAAISGSCLGGGLELAIACQYR.I |
| 172287 | 133 | – | 159 | 925.4792 | 2773.4158 | 2773.4255 | -3.48 | 1 | 56 | 5.4e-06 | 1Score **> 37** indicates **identity** Score **> 16** indicates **homology** | U | K.SPKPVVAAISGSCLGGGLELAIACQYR.I |
| 172289 | 133 | – | 159 | 925.4800 | 2773.4183 | 2773.4255 | -2.60 | 1 | 48 | 3.3e-05 | 1Score **> 37** indicates **identity** Score **> 15** indicates **homology** | U | K.SPKPVVAAISGSCLGGGLELAIACQYR.I |
| 172290 | 133 | – | 159 | 925.4803 | 2773.4191 | 2773.4255 | -2.29 | 1 | 86 | 8e-09 | 1Score **> 37** indicates **identity** Score **> 18** indicates **homology** | U | K.SPKPVVAAISGSCLGGGLELAIACQYR.I |
| 172291 | 133 | – | 159 | 925.4807 | 2773.4204 | 2773.4255 | -1.84 | 1 | 45 | 6.3e-05 | 1Score **> 37** indicates **identity** Score **> 15** indicates **homology** | U | K.SPKPVVAAISGSCLGGGLELAIACQYR.I |
| 172292 | 133 | – | 159 | 925.4808 | 2773.4204 | 2773.4255 | -1.83 | 1 | 25 | 0.005 | 1Score **> 37** indicates **identity** Score **> 14** indicates **homology** | U | K.SPKPVVAAISGSCLGGGLELAIACQYR.I |
| 172293 | 133 | – | 159 | 925.4809 | 2773.4208 | 2773.4255 | -1.70 | 1 | 45 | 5.7e-05 | 1Score **> 37** indicates **identity** Score **> 15** indicates **homology** | U | K.SPKPVVAAISGSCLGGGLELAIACQYR.I |
| 172294 | 133 | – | 159 | 925.4810 | 2773.4213 | 2773.4255 | -1.52 | 1 | 27 | 0.0027 | 1Score **> 37** indicates **identity** Score **> 14** indicates **homology** | U | K.SPKPVVAAISGSCLGGGLELAIACQYR.I |
| 172295 | 133 | – | 159 | 925.4817 | 2773.4232 | 2773.4255 | -0.83 | 1 | 27 | 0.0031 | 1Score **> 37** indicates **identity** Score **> 14** indicates **homology** | U | K.SPKPVVAAISGSCLGGGLELAIACQYR.I |
| 172297 | 133 | – | 159 | 925.4819 | 2773.4240 | 2773.4255 | -0.55 | 1 | 41 | 0.00015 | 1Score **> 37** indicates **identity** Score **> 15** indicates **homology** | U | K.SPKPVVAAISGSCLGGGLELAIACQYR.I |
| 172299 | 133 | – | 159 | 925.4824 | 2773.4254 | 2773.4255 | -0.044 | 1 | 71 | 2.2e-07 | 1Score **> 37** indicates **identity** Score **> 17** indicates **homology** | U | K.SPKPVVAAISGSCLGGGLELAIACQYR.I |
| 172300 | 133 | – | 159 | 694.3638 | 2773.4260 | 2773.4255 | 0.17 | 1 | 64 | 1.1e-06 | 1Score **> 37** indicates **identity** Score **> 16** indicates **homology** | U | K.SPKPVVAAISGSCLGGGLELAIACQYR.I |
| 172301 | 133 | – | 159 | 925.4826 | 2773.4260 | 2773.4255 | 0.17 | 1 | 105 | 1.4e-10 | 1Score **> 37** indicates **identity** Score **> 19** indicates **homology** | U | K.SPKPVVAAISGSCLGGGLELAIACQYR.I |
| 172302 | 133 | – | 159 | 694.3638 | 2773.4260 | 2773.4255 | 0.20 | 1 | 74 | 1.2e-07 | 1Score **> 37** indicates **identity** Score **> 17** indicates **homology** | U | K.SPKPVVAAISGSCLGGGLELAIACQYR.I |
| 172303 | 133 | – | 159 | 925.4828 | 2773.4266 | 2773.4255 | 0.40 | 1 | 36 | 0.00045 | 1Score **> 37** indicates **identity** Score **> 15** indicates **homology** | U | K.SPKPVVAAISGSCLGGGLELAIACQYR.I |
| 172304 | 133 | – | 159 | 925.4830 | 2773.4270 | 2773.4255 | 0.55 | 1 | 62 | 1.5e-06 | 1Score **> 37** indicates **identity** Score **> 16** indicates **homology** | U | K.SPKPVVAAISGSCLGGGLELAIACQYR.I |
| 172305 | 133 | – | 159 | 925.4830 | 2773.4271 | 2773.4255 | 0.56 | 1 | 110 | 5.3e-11 | 1Score **> 37** indicates **identity** Score **> 19** indicates **homology** | U | K.SPKPVVAAISGSCLGGGLELAIACQYR.I |
| 172306 | 133 | – | 159 | 925.4830 | 2773.4272 | 2773.4255 | 0.62 | 1 | 100 | 4.5e-10 | 1Score **> 37** indicates **identity** Score **> 19** indicates **homology** | U | K.SPKPVVAAISGSCLGGGLELAIACQYR.I |
| 172307 | 133 | – | 159 | 694.3641 | 2773.4274 | 2773.4255 | 0.68 | 1 | 45 | 6.6e-05 | 1Score **> 37** indicates **identity** Score **> 15** indicates **homology** | U | K.SPKPVVAAISGSCLGGGLELAIACQYR.I |
| 172308 | 133 | – | 159 | 925.4831 | 2773.4274 | 2773.4255 | 0.70 | 1 | 46 | 4.5e-05 | 1Score **> 37** indicates **identity** Score **> 15** indicates **homology** | U | K.SPKPVVAAISGSCLGGGLELAIACQYR.I |
| 172309 | 133 | – | 159 | 925.4831 | 2773.4275 | 2773.4255 | 0.73 | 1 | 54 | 9.1e-06 | 1Score **> 37** indicates **identity** Score **> 16** indicates **homology** | U | K.SPKPVVAAISGSCLGGGLELAIACQYR.I |
| 172311 | 133 | – | 159 | 925.4831 | 2773.4276 | 2773.4255 | 0.76 | 1 | 99 | 4.7e-10 | 1Score **> 37** indicates **identity** Score **> 19** indicates **homology** | U | K.SPKPVVAAISGSCLGGGLELAIACQYR.I |
| 172312 | 133 | – | 159 | 925.4832 | 2773.4278 | 2773.4255 | 0.82 | 1 | 90 | 3.7e-09 | 1Score **> 37** indicates **identity** Score **> 18** indicates **homology** | U | K.SPKPVVAAISGSCLGGGLELAIACQYR.I |
| 172313 | 133 | – | 159 | 925.4833 | 2773.4279 | 2773.4255 | 0.88 | 1 | 40 | 0.00019 | 1Score **> 37** indicates **identity** Score **> 16** indicates **homology** | U | K.SPKPVVAAISGSCLGGGLELAIACQYR.I |
| 172314 | 133 | – | 159 | 925.4833 | 2773.4280 | 2773.4255 | 0.89 | 1 | 74 | 1.1e-07 | 1Score **> 37** indicates **identity** Score **> 17** indicates **homology** | U | K.SPKPVVAAISGSCLGGGLELAIACQYR.I |
| 172316 | 133 | – | 159 | 925.4835 | 2773.4286 | 2773.4255 | 1.13 | 1 | 67 | 4.7e-07 | 1Score **> 37** indicates **identity** Score **> 17** indicates **homology** | U | K.SPKPVVAAISGSCLGGGLELAIACQYR.I |
| 172319 | 133 | – | 159 | 925.4837 | 2773.4293 | 2773.4255 | 1.37 | 1 | 50 | 2.2e-05 | 1Score **> 37** indicates **identity** Score **> 16** indicates **homology** | U | K.SPKPVVAAISGSCLGGGLELAIACQYR.I |
| 172321 | 133 | – | 159 | 925.4838 | 2773.4295 | 2773.4255 | 1.45 | 1 | 99 | 4.9e-10 | 1Score **> 37** indicates **identity** Score **> 19** indicates **homology** | U | K.SPKPVVAAISGSCLGGGLELAIACQYR.I |
| 172322 | 133 | – | 159 | 925.4838 | 2773.4296 | 2773.4255 | 1.50 | 1 | 50 | 2.3e-05 | 1Score **> 37** indicates **identity** Score **> 16** indicates **homology** | U | K.SPKPVVAAISGSCLGGGLELAIACQYR.I |
| 172323 | 133 | – | 159 | 925.4839 | 2773.4298 | 2773.4255 | 1.55 | 1 | 31 | 0.0012 | 1Score **> 37** indicates **identity** Score **> 14** indicates **homology** | U | K.SPKPVVAAISGSCLGGGLELAIACQYR.I |
| 172324 | 133 | – | 159 | 925.4839 | 2773.4299 | 2773.4255 | 1.60 | 1 | 50 | 2e-05 | 1Score **> 37** indicates **identity** Score **> 16** indicates **homology** | U | K.SPKPVVAAISGSCLGGGLELAIACQYR.I |
| 172326 | 133 | – | 159 | 925.4841 | 2773.4303 | 2773.4255 | 1.75 | 1 | 39 | 0.0002 | 1Score **> 37** indicates **identity** Score **> 15** indicates **homology** | U | K.SPKPVVAAISGSCLGGGLELAIACQYR.I |
| 172327 | 133 | – | 159 | 925.4842 | 2773.4309 | 2773.4255 | 1.95 | 1 | 98 | 7.2e-10 | 1Score **> 37** indicates **identity** Score **> 19** indicates **homology** | U | K.SPKPVVAAISGSCLGGGLELAIACQYR.I |
| 172328 | 133 | – | 159 | 925.4843 | 2773.4312 | 2773.4255 | 2.05 | 1 | 38 | 0.00025 | 1Score **> 37** indicates **identity** Score **> 15** indicates **homology** | U | K.SPKPVVAAISGSCLGGGLELAIACQYR.I |
| 172329 | 133 | – | 159 | 925.4845 | 2773.4317 | 2773.4255 | 2.23 | 1 | 50 | 3e-05 | 1Score **> 37** indicates **identity** Score **> 18** indicates **homology** | U | K.SPKPVVAAISGSCLGGGLELAIACQYR.I |
| 172331 | 133 | – | 159 | 925.4847 | 2773.4323 | 2773.4255 | 2.45 | 1 | 51 | 1.8e-05 | 1Score **> 37** indicates **identity** Score **> 16** indicates **homology** | U | K.SPKPVVAAISGSCLGGGLELAIACQYR.I |
| 172336 | 133 | – | 159 | 925.4848 | 2773.4327 | 2773.4255 | 2.60 | 1 | 42 | 0.00011 | 1Score **> 37** indicates **identity** Score **> 15** indicates **homology** | U | K.SPKPVVAAISGSCLGGGLELAIACQYR.I |
| 172338 | 133 | – | 159 | 925.4850 | 2773.4331 | 2773.4255 | 2.74 | 1 | 48 | 2.9e-05 | 1Score **> 37** indicates **identity** Score **> 16** indicates **homology** | U | K.SPKPVVAAISGSCLGGGLELAIACQYR.I |
| 172339 | 133 | – | 159 | 925.4850 | 2773.4331 | 2773.4255 | 2.74 | 1 | 18 | 0.022 | 1Score **> 37** indicates **identity** Score **> 14** indicates **homology** | U | K.SPKPVVAAISGSCLGGGLELAIACQYR.I |
| 172340 | 133 | – | 159 | 925.4853 | 2773.4341 | 2773.4255 | 3.10 | 1 | 31 | 0.0012 | 1Score **> 37** indicates **identity** Score **> 14** indicates **homology** | U | K.SPKPVVAAISGSCLGGGLELAIACQYR.I |
| 172341 | 133 | – | 159 | 925.4854 | 2773.4344 | 2773.4255 | 3.21 | 1 | 46 | 8.6e-05 | 1Score **> 37** indicates **identity** Score **> 18** indicates **homology** | U | K.SPKPVVAAISGSCLGGGLELAIACQYR.I |
| 172342 | 133 | – | 159 | 925.4855 | 2773.4346 | 2773.4255 | 3.28 | 1 | 23 | 0.0066 | 1Score **> 37** indicates **identity** Score **> 14** indicates **homology** | U | K.SPKPVVAAISGSCLGGGLELAIACQYR.I |
| 172343 | 133 | – | 159 | 925.4861 | 2773.4366 | 2773.4255 | 4.01 | 1 | 39 | 0.00024 | 1Score **> 37** indicates **identity** Score **> 15** indicates **homology** | U | K.SPKPVVAAISGSCLGGGLELAIACQYR.I |
| 172346 | 133 | – | 159 | 925.4864 | 2773.4375 | 2773.4255 | 4.33 | 1 | 21 | 0.012 | 1Score **> 37** indicates **identity** Score **> 14** indicates **homology** | U | K.SPKPVVAAISGSCLGGGLELAIACQYR.I |
| 172348 | 133 | – | 159 | 1387.7266 | 2773.4387 | 2773.4255 | 4.78 | 1 | 40 | 0.00016 | 1Score **> 37** indicates **identity** Score **> 15** indicates **homology** | U | K.SPKPVVAAISGSCLGGGLELAIACQYR.I |
| 172349 | 133 | – | 159 | 925.4870 | 2773.4391 | 2773.4255 | 4.90 | 1 | 50 | 2e-05 | 1Score **> 37** indicates **identity** Score **> 16** indicates **homology** | U | K.SPKPVVAAISGSCLGGGLELAIACQYR.I |
| 172350 | 133 | – | 159 | 925.4871 | 2773.4395 | 2773.4255 | 5.06 | 1 | 72 | 1.9e-07 | 1Score **> 37** indicates **identity** Score **> 17** indicates **homology** | U | K.SPKPVVAAISGSCLGGGLELAIACQYR.I |
| 172351 | 133 | – | 159 | 925.4877 | 2773.4412 | 2773.4255 | 5.68 | 1 | 55 | 6.5e-06 | 1Score **> 37** indicates **identity** Score **> 16** indicates **homology** | U | K.SPKPVVAAISGSCLGGGLELAIACQYR.I |
| 172352 | 133 | – | 159 | 925.4880 | 2773.4423 | 2773.4255 | 6.05 | 1 | 31 | 0.0011 | 1Score **> 37** indicates **identity** Score **> 14** indicates **homology** | U | K.SPKPVVAAISGSCLGGGLELAIACQYR.I |
| 172354 | 133 | – | 159 | 925.4899 | 2773.4478 | 2773.4255 | 8.05 | 1 | 57 | 4.6e-06 | 1Score **> 37** indicates **identity** Score **> 16** indicates **homology** | U | K.SPKPVVAAISGSCLGGGLELAIACQYR.I |
| 172355 | 133 | – | 159 | 925.4908 | 2773.4505 | 2773.4255 | 9.02 | 1 | 54 | 1e-05 | 1Score **> 37** indicates **identity** Score **> 16** indicates **homology** | U | K.SPKPVVAAISGSCLGGGLELAIACQYR.I |
| 172356 | 133 | – | 159 | 925.4909 | 2773.4508 | 2773.4255 | 9.14 | 1 | 54 | 9.5e-06 | 1Score **> 37** indicates **identity** Score **> 16** indicates **homology** | U | K.SPKPVVAAISGSCLGGGLELAIACQYR.I |
| 172357 | 133 | – | 159 | 925.4911 | 2773.4514 | 2773.4255 | 9.34 | 1 | 21 | 0.011 | 1Score **> 37** indicates **identity** Score **> 14** indicates **homology** | U | K.SPKPVVAAISGSCLGGGLELAIACQYR.I |
| 172402 | 133 | – | 159 | 1388.2182 | 2774.4219 | 2774.4095 | 4.48 | 1 | 27 | 0.0028 | 1Score **> 37** indicates **identity** Score **> 14** indicates **homology** | U | K.SPKPVVAAISGSCLGGGLELAIACQYR.I  + Deamidated (NQ) |
| 172412 | 133 | – | 159 | 925.8184 | 2774.4332 | 2774.4095 | 8.56 | 1 | 14 | 0.046 | 1Score **> 37** indicates **identity** Score **> 13** indicates **homology** | U | K.SPKPVVAAISGSCLGGGLELAIACQYR.I  + Deamidated (NQ) |
| 138876 | 166 | – | 187 | 725.7658 | 2174.2757 | 2174.2787 | -1.39 | 1 | 30 | 0.0015 | 1Score **> 30** indicates **identity** Score **> 14** indicates **homology** | U | R.KTVLGVPEVLLGILPGAGGTQR.L |
| 138877 | 166 | – | 187 | 1088.1482 | 2174.2819 | 2174.2787 | 1.45 | 1 | 21 | 0.012 | 1Score **> 29** indicates **identity** Score **> 14** indicates **homology** | U | R.KTVLGVPEVLLGILPGAGGTQR.L |
| 161491 | 166 | – | 190 | 629.1356 | 2512.5134 | 2512.5105 | 1.17 | 2 | 14 | 0.046 | 1Score **> 24** indicates **identity** Score **> 13** indicates **homology** | U | R.KTVLGVPEVLLGILPGAGGTQRLPK.M |
| 161492 | 166 | – | 190 | 629.1359 | 2512.5146 | 2512.5105 | 1.63 | 2 | 33 | 0.00084 | 1Score **> 24** indicates **identity** Score **> 15** indicates **homology** | U | R.KTVLGVPEVLLGILPGAGGTQRLPK.M |
| 161532 | 166 | – | 190 | 838.8458 | 2513.5154 | 2513.4945 | 8.33 | 2 | 27 | 0.0027 | 1Score **> 23** indicates **identity** Score **> 14** indicates **homology** | U | R.KTVLGVPEVLLGILPGAGGTQRLPK.M  + Deamidated (NQ) |
| 127556 | 167 | – | 187 | 683.0681 | 2046.1824 | 2046.1837 | -0.65 | 0 | 56 | 5.9e-06 | 1Score **> 31** indicates **identity** Score **> 16** indicates **homology** | U | K.TVLGVPEVLLGILPGAGGTQR.L |
| 127558 | 167 | – | 187 | 1024.0986 | 2046.1826 | 2046.1837 | -0.55 | 0 | 72 | 1.6e-07 | 1Score **> 31** indicates **identity** Score **> 17** indicates **homology** | U | K.TVLGVPEVLLGILPGAGGTQR.L |
| 127561 | 167 | – | 187 | 683.0694 | 2046.1865 | 2046.1837 | 1.32 | 0 | 45 | 6.1e-05 | 1Score **> 31** indicates **identity** Score **> 15** indicates **homology** | U | K.TVLGVPEVLLGILPGAGGTQR.L |
| 127562 | 167 | – | 187 | 1024.1010 | 2046.1875 | 2046.1837 | 1.85 | 0 | 48 | 3.2e-05 | 1Score **> 31** indicates **identity** Score **> 15** indicates **homology** | U | K.TVLGVPEVLLGILPGAGGTQR.L |
| 127563 | 167 | – | 187 | 1024.1011 | 2046.1877 | 2046.1837 | 1.95 | 0 | 64 | 2.4e-06 | 1Score **> 31** indicates **identity** Score **> 20** indicates **homology** | U | K.TVLGVPEVLLGILPGAGGTQR.L |
| 127564 | 167 | – | 187 | 1024.1012 | 2046.1879 | 2046.1837 | 2.02 | 0 | 76 | 1.1e-07 | 1Score **> 31** indicates **identity** Score **> 19** indicates **homology** | U | K.TVLGVPEVLLGILPGAGGTQR.L |
| 127565 | 167 | – | 187 | 683.0701 | 2046.1884 | 2046.1837 | 2.28 | 0 | 56 | 6.2e-06 | 1Score **> 31** indicates **identity** Score **> 16** indicates **homology** | U | K.TVLGVPEVLLGILPGAGGTQR.L |
| 127568 | 167 | – | 187 | 1024.1030 | 2046.1914 | 2046.1837 | 3.75 | 0 | 29 | 0.0018 | 1Score **> 31** indicates **identity** Score **> 14** indicates **homology** | U | K.TVLGVPEVLLGILPGAGGTQR.L |
| 127569 | 167 | – | 187 | 683.0711 | 2046.1916 | 2046.1837 | 3.82 | 0 | 49 | 2.7e-05 | 1Score **> 31** indicates **identity** Score **> 16** indicates **homology** | U | K.TVLGVPEVLLGILPGAGGTQR.L |
| 127570 | 167 | – | 187 | 1024.1032 | 2046.1919 | 2046.1837 | 3.98 | 0 | 44 | 7.8e-05 | 1Score **> 31** indicates **identity** Score **> 15** indicates **homology** | U | K.TVLGVPEVLLGILPGAGGTQR.L |
| 127571 | 167 | – | 187 | 683.0713 | 2046.1922 | 2046.1837 | 4.12 | 0 | 40 | 0.00017 | 1Score **> 31** indicates **identity** Score **> 15** indicates **homology** | U | K.TVLGVPEVLLGILPGAGGTQR.L |
| 127572 | 167 | – | 187 | 1024.1035 | 2046.1925 | 2046.1837 | 4.26 | 0 | 40 | 0.00017 | 1Score **> 31** indicates **identity** Score **> 15** indicates **homology** | U | K.TVLGVPEVLLGILPGAGGTQR.L |
| 127573 | 167 | – | 187 | 1024.1039 | 2046.1932 | 2046.1837 | 4.62 | 0 | 26 | 0.0037 | 1Score **> 30** indicates **identity** Score **> 14** indicates **homology** | U | K.TVLGVPEVLLGILPGAGGTQR.L |
| 127574 | 167 | – | 187 | 683.0738 | 2046.1995 | 2046.1837 | 7.69 | 0 | 44 | 6.8e-05 | 1Score **> 30** indicates **identity** Score **> 15** indicates **homology** | U | K.TVLGVPEVLLGILPGAGGTQR.L |
| 154134 | 167 | – | 190 | 795.8101 | 2384.4085 | 2384.4155 | -2.93 | 1 | 31 | 0.0012 | 1Score **> 28** indicates **identity** Score **> 14** indicates **homology** | U | K.TVLGVPEVLLGILPGAGGTQRLPK.M |
| 154135 | 167 | – | 190 | 795.8119 | 2384.4139 | 2384.4155 | -0.69 | 1 | 28 | 0.0022 | 1Score **> 28** indicates **identity** Score **> 14** indicates **homology** | U | K.TVLGVPEVLLGILPGAGGTQRLPK.M |
| 154136 | 167 | – | 190 | 795.8122 | 2384.4149 | 2384.4155 | -0.25 | 1 | 27 | 0.0031 | 1Score **> 27** indicates **identity** Score **> 14** indicates **homology** | U | K.TVLGVPEVLLGILPGAGGTQRLPK.M |
| 154137 | 167 | – | 190 | 597.1113 | 2384.4161 | 2384.4155 | 0.23 | 1 | 25 | 0.0043 | 1Score **> 27** indicates **identity** Score **> 14** indicates **homology** | U | K.TVLGVPEVLLGILPGAGGTQRLPK.M |
| 154138 | 167 | – | 190 | 1193.2163 | 2384.4181 | 2384.4155 | 1.07 | 1 | 33 | 0.00083 | 1Score **> 27** indicates **identity** Score **> 15** indicates **homology** | U | K.TVLGVPEVLLGILPGAGGTQRLPK.M |
| 154139 | 167 | – | 190 | 795.8140 | 2384.4200 | 2384.4155 | 1.89 | 1 | 24 | 0.0054 | 1Score **> 27** indicates **identity** Score **> 14** indicates **homology** | U | K.TVLGVPEVLLGILPGAGGTQRLPK.M |
| 103314 | 214 | – | 230 | 598.6787 | 1793.0143 | 1793.0121 | 1.21 | 1 | 49 | 2.3e-05 | 1Score **> 33** indicates **identity** Score **> 16** indicates **homology** | U | K.KMGLVDQLVEPLGPGIK.S |
| 103315 | 214 | – | 230 | 598.6789 | 1793.0150 | 1793.0121 | 1.62 | 1 | 37 | 0.00034 | 1Score **> 33** indicates **identity** Score **> 15** indicates **homology** | U | K.KMGLVDQLVEPLGPGIK.S |
| 154633 | 214 | – | 235 | 598.8277 | 2391.2816 | 2391.2832 | -0.65 | 2 | 29 | 0.0018 | 1Score **> 36** indicates **identity** Score **> 14** indicates **homology** | U | K.KMGLVDQLVEPLGPGIKSPEER.T |
| 154638 | 214 | – | 235 | 598.8282 | 2391.2839 | 2391.2832 | 0.30 | 2 | 51 | 1.7e-05 | 1Score **> 36** indicates **identity** Score **> 16** indicates **homology** | U | K.KMGLVDQLVEPLGPGIKSPEER.T |
| 154639 | 214 | – | 235 | 598.8285 | 2391.2849 | 2391.2832 | 0.71 | 2 | 43 | 8.8e-05 | 1Score **> 36** indicates **identity** Score **> 15** indicates **homology** | U | K.KMGLVDQLVEPLGPGIKSPEER.T |
| 154641 | 214 | – | 235 | 798.1039 | 2391.2898 | 2391.2832 | 2.78 | 2 | 71 | 2.4e-07 | 1Score **> 36** indicates **identity** Score **> 17** indicates **homology** | U | K.KMGLVDQLVEPLGPGIKSPEER.T |
| 154703 | 214 | – | 235 | 798.4364 | 2392.2875 | 2392.2672 | 8.51 | 2 | 31 | 0.0011 | 1Score **> 36** indicates **identity** Score **> 14** indicates **homology** | U | K.KMGLVDQLVEPLGPGIKSPEER.T  + Deamidated (NQ) |
| 154704 | 214 | – | 235 | 798.4376 | 2392.2910 | 2392.2672 | 9.95 | 2 | 32 | 0.00093 | 1Score **> 36** indicates **identity** Score **> 15** indicates **homology** | U | K.KMGLVDQLVEPLGPGIKSPEER.T  + Deamidated (NQ) |
| 88263 | 215 | – | 230 | 833.4650 | 1664.9155 | 1664.9171 | -0.98 | 0 | 62 | 1.5e-06 | 1Score **> 34** indicates **identity** Score **> 16** indicates **homology** | U | K.MGLVDQLVEPLGPGIK.S |
| 88264 | 215 | – | 230 | 555.9795 | 1664.9168 | 1664.9171 | -0.19 | 0 | 14 | 0.047 | 1Score **> 34** indicates **identity** Score **> 13** indicates **homology** | U | K.MGLVDQLVEPLGPGIK.S |
| 88265 | 215 | – | 230 | 555.9796 | 1664.9169 | 1664.9171 | -0.12 | 0 | 42 | 0.00011 | 1Score **> 34** indicates **identity** Score **> 15** indicates **homology** | U | K.MGLVDQLVEPLGPGIK.S |
| 88266 | 215 | – | 230 | 833.4664 | 1664.9183 | 1664.9171 | 0.70 | 0 | 55 | 7e-06 | 1Score **> 34** indicates **identity** Score **> 16** indicates **homology** | U | K.MGLVDQLVEPLGPGIK.S |
| 88267 | 215 | – | 230 | 555.9803 | 1664.9190 | 1664.9171 | 1.12 | 0 | 34 | 0.0006 | 1Score **> 34** indicates **identity** Score **> 15** indicates **homology** | U | K.MGLVDQLVEPLGPGIK.S |
| 88268 | 215 | – | 230 | 833.4678 | 1664.9211 | 1664.9171 | 2.39 | 0 | 59 | 3.1e-06 | 1Score **> 34** indicates **identity** Score **> 16** indicates **homology** | U | K.MGLVDQLVEPLGPGIK.S |
| 146081 | 215 | – | 235 | 755.4057 | 2263.1952 | 2263.1882 | 3.10 | 1 | 69 | 3.7e-07 | 1Score **> 37** indicates **identity** Score **> 17** indicates **homology** | U | K.MGLVDQLVEPLGPGIKSPEER.T |
| 146082 | 215 | – | 235 | 755.4058 | 2263.1956 | 2263.1882 | 3.25 | 1 | 41 | 0.00014 | 1Score **> 37** indicates **identity** Score **> 15** indicates **homology** | U | K.MGLVDQLVEPLGPGIKSPEER.T |
| 146083 | 215 | – | 235 | 755.4062 | 2263.1969 | 2263.1882 | 3.83 | 1 | 60 | 2.4e-06 | 1Score **> 37** indicates **identity** Score **> 16** indicates **homology** | U | K.MGLVDQLVEPLGPGIKSPEER.T |
| 146085 | 215 | – | 235 | 755.4096 | 2263.2071 | 2263.1882 | 8.33 | 1 | 44 | 8.2e-05 | 1Score **> 36** indicates **identity** Score **> 15** indicates **homology** | U | K.MGLVDQLVEPLGPGIKSPEER.T |
| 82993 | 236 | – | 249 | 813.4212 | 1624.8279 | 1624.8348 | -4.25 | 0 | 39 | 0.00021 | 1Score **> 35** indicates **identity** Score **> 15** indicates **homology** | U | R.TIEYLEEVAVNFAK.G |
| 82995 | 236 | – | 249 | 542.6171 | 1624.8295 | 1624.8348 | -3.28 | 0 | 20 | 0.015 | 1Score **> 34** indicates **identity** Score **> 14** indicates **homology** | U | R.TIEYLEEVAVNFAK.G |
| 82996 | 236 | – | 249 | 542.6176 | 1624.8310 | 1624.8348 | -2.36 | 0 | 33 | 0.00086 | 1Score **> 34** indicates **identity** Score **> 15** indicates **homology** | U | R.TIEYLEEVAVNFAK.G |
| 83001 | 236 | – | 249 | 542.6182 | 1624.8328 | 1624.8348 | -1.26 | 0 | 18 | 0.019 | 1Score **> 35** indicates **identity** Score **> 14** indicates **homology** | U | R.TIEYLEEVAVNFAK.G |
| 83002 | 236 | – | 249 | 813.4237 | 1624.8329 | 1624.8348 | -1.16 | 0 | 70 | 2.8e-07 | 1Score **> 35** indicates **identity** Score **> 17** indicates **homology** | U | R.TIEYLEEVAVNFAK.G |
| 83003 | 236 | – | 249 | 542.6183 | 1624.8330 | 1624.8348 | -1.14 | 0 | 27 | 0.0028 | 1Score **> 35** indicates **identity** Score **> 14** indicates **homology** | U | R.TIEYLEEVAVNFAK.G |
| 83004 | 236 | – | 249 | 813.4239 | 1624.8332 | 1624.8348 | -0.99 | 0 | 49 | 2.8e-05 | 1Score **> 35** indicates **identity** Score **> 16** indicates **homology** | U | R.TIEYLEEVAVNFAK.G |
| 83005 | 236 | – | 249 | 813.4239 | 1624.8333 | 1624.8348 | -0.93 | 0 | 62 | 1.5e-06 | 1Score **> 35** indicates **identity** Score **> 16** indicates **homology** | U | R.TIEYLEEVAVNFAK.G |
| 83006 | 236 | – | 249 | 813.4240 | 1624.8334 | 1624.8348 | -0.86 | 0 | 82 | 1.9e-08 | 1Score **> 34** indicates **identity** Score **> 18** indicates **homology** | U | R.TIEYLEEVAVNFAK.G |
| 83007 | 236 | – | 249 | 542.6185 | 1624.8335 | 1624.8348 | -0.78 | 0 | 32 | 0.001 | 1Score **> 34** indicates **identity** Score **> 14** indicates **homology** | U | R.TIEYLEEVAVNFAK.G |
| 83008 | 236 | – | 249 | 542.6186 | 1624.8338 | 1624.8348 | -0.60 | 0 | 41 | 0.00014 | 1Score **> 34** indicates **identity** Score **> 15** indicates **homology** | U | R.TIEYLEEVAVNFAK.G |
| 83009 | 236 | – | 249 | 813.4243 | 1624.8340 | 1624.8348 | -0.50 | 0 | 51 | 1.5e-05 | 1Score **> 34** indicates **identity** Score **> 16** indicates **homology** | U | R.TIEYLEEVAVNFAK.G |
| 83014 | 236 | – | 249 | 813.4244 | 1624.8342 | 1624.8348 | -0.39 | 0 | 75 | 9.6e-08 | 1Score **> 34** indicates **identity** Score **> 17** indicates **homology** | U | R.TIEYLEEVAVNFAK.G |
| 83015 | 236 | – | 249 | 542.6187 | 1624.8343 | 1624.8348 | -0.31 | 0 | 62 | 1.6e-06 | 1Score **> 34** indicates **identity** Score **> 16** indicates **homology** | U | R.TIEYLEEVAVNFAK.G |
| 83020 | 236 | – | 249 | 542.6188 | 1624.8347 | 1624.8348 | -0.073 | 0 | 14 | 0.047 | 1Score **> 34** indicates **identity** Score **> 13** indicates **homology** | U | R.TIEYLEEVAVNFAK.G |
| 83021 | 236 | – | 249 | 813.4247 | 1624.8348 | 1624.8348 | -0.030 | 0 | 79 | 3.8e-08 | 1Score **> 34** indicates **identity** Score **> 17** indicates **homology** | U | R.TIEYLEEVAVNFAK.G |
| 83022 | 236 | – | 249 | 542.6189 | 1624.8348 | 1624.8348 | -0.022 | 0 | 58 | 3.9e-06 | 1Score **> 34** indicates **identity** Score **> 16** indicates **homology** | U | R.TIEYLEEVAVNFAK.G |
| 83024 | 236 | – | 249 | 542.6189 | 1624.8350 | 1624.8348 | 0.095 | 0 | 39 | 0.00024 | 1Score **> 34** indicates **identity** Score **> 15** indicates **homology** | U | R.TIEYLEEVAVNFAK.G |
| 83025 | 236 | – | 249 | 542.6189 | 1624.8350 | 1624.8348 | 0.097 | 0 | 62 | 1.5e-06 | 1Score **> 34** indicates **identity** Score **> 16** indicates **homology** | U | R.TIEYLEEVAVNFAK.G |
| 83026 | 236 | – | 249 | 542.6189 | 1624.8350 | 1624.8348 | 0.098 | 0 | 58 | 4e-06 | 1Score **> 34** indicates **identity** Score **> 16** indicates **homology** | U | R.TIEYLEEVAVNFAK.G |
| 83032 | 236 | – | 249 | 813.4248 | 1624.8351 | 1624.8348 | 0.15 | 0 | 64 | 1e-06 | 1Score **> 34** indicates **identity** Score **> 16** indicates **homology** | U | R.TIEYLEEVAVNFAK.G |
| 83034 | 236 | – | 249 | 542.6191 | 1624.8355 | 1624.8348 | 0.44 | 0 | 57 | 5e-06 | 1Score **> 34** indicates **identity** Score **> 16** indicates **homology** | U | R.TIEYLEEVAVNFAK.G |
| 83035 | 236 | – | 249 | 542.6192 | 1624.8357 | 1624.8348 | 0.54 | 0 | 45 | 5.6e-05 | 1Score **> 34** indicates **identity** Score **> 15** indicates **homology** | U | R.TIEYLEEVAVNFAK.G |
| 83037 | 236 | – | 249 | 542.6192 | 1624.8357 | 1624.8348 | 0.55 | 0 | 17 | 0.026 | 1Score **> 34** indicates **identity** Score **> 14** indicates **homology** | U | R.TIEYLEEVAVNFAK.G |
| 83038 | 236 | – | 249 | 542.6192 | 1624.8357 | 1624.8348 | 0.56 | 0 | 46 | 5.4e-05 | 1Score **> 34** indicates **identity** Score **> 15** indicates **homology** | U | R.TIEYLEEVAVNFAK.G |
| 83043 | 236 | – | 249 | 813.4252 | 1624.8359 | 1624.8348 | 0.65 | 0 | 55 | 6.7e-06 | 1Score **> 34** indicates **identity** Score **> 16** indicates **homology** | U | R.TIEYLEEVAVNFAK.G |
| 83044 | 236 | – | 249 | 813.4252 | 1624.8359 | 1624.8348 | 0.66 | 0 | 84 | 1.3e-08 | 1Score **> 34** indicates **identity** Score **> 18** indicates **homology** | U | R.TIEYLEEVAVNFAK.G |
| 83045 | 236 | – | 249 | 813.4252 | 1624.8359 | 1624.8348 | 0.67 | 0 | 57 | 4.2e-06 | 1Score **> 34** indicates **identity** Score **> 16** indicates **homology** | U | R.TIEYLEEVAVNFAK.G |
| 83046 | 236 | – | 249 | 813.4252 | 1624.8359 | 1624.8348 | 0.70 | 0 | 49 | 2.7e-05 | 1Score **> 34** indicates **identity** Score **> 16** indicates **homology** | U | R.TIEYLEEVAVNFAK.G |
| 83047 | 236 | – | 249 | 813.4253 | 1624.8360 | 1624.8348 | 0.70 | 0 | 64 | 9.9e-07 | 1Score **> 34** indicates **identity** Score **> 17** indicates **homology** | U | R.TIEYLEEVAVNFAK.G |
| 83050 | 236 | – | 249 | 813.4253 | 1624.8360 | 1624.8348 | 0.74 | 0 | 59 | 2.7e-06 | 1Score **> 34** indicates **identity** Score **> 16** indicates **homology** | U | R.TIEYLEEVAVNFAK.G |
| 83051 | 236 | – | 249 | 542.6193 | 1624.8361 | 1624.8348 | 0.78 | 0 | 47 | 3.9e-05 | 1Score **> 34** indicates **identity** Score **> 15** indicates **homology** | U | R.TIEYLEEVAVNFAK.G |
| 83053 | 236 | – | 249 | 813.4253 | 1624.8361 | 1624.8348 | 0.81 | 0 | 78 | 5.1e-08 | 1Score **> 34** indicates **identity** Score **> 17** indicates **homology** | U | R.TIEYLEEVAVNFAK.G |
| 83057 | 236 | – | 249 | 542.6194 | 1624.8363 | 1624.8348 | 0.90 | 0 | 32 | 0.001 | 1Score **> 34** indicates **identity** Score **> 14** indicates **homology** | U | R.TIEYLEEVAVNFAK.G |
| 83060 | 236 | – | 249 | 813.4255 | 1624.8365 | 1624.8348 | 1.03 | 0 | 55 | 7.1e-06 | 1Score **> 34** indicates **identity** Score **> 16** indicates **homology** | U | R.TIEYLEEVAVNFAK.G |
| 83068 | 236 | – | 249 | 542.6196 | 1624.8370 | 1624.8348 | 1.34 | 0 | 19 | 0.018 | 1Score **> 34** indicates **identity** Score **> 14** indicates **homology** | U | R.TIEYLEEVAVNFAK.G |
| 83071 | 236 | – | 249 | 542.6197 | 1624.8372 | 1624.8348 | 1.44 | 0 | 52 | 1.2e-05 | 1Score **> 34** indicates **identity** Score **> 16** indicates **homology** | U | R.TIEYLEEVAVNFAK.G |
| 83072 | 236 | – | 249 | 542.6197 | 1624.8372 | 1624.8348 | 1.50 | 0 | 58 | 3.8e-06 | 1Score **> 34** indicates **identity** Score **> 16** indicates **homology** | U | R.TIEYLEEVAVNFAK.G |
| 83073 | 236 | – | 249 | 813.4259 | 1624.8373 | 1624.8348 | 1.55 | 0 | 52 | 1.3e-05 | 1Score **> 34** indicates **identity** Score **> 16** indicates **homology** | U | R.TIEYLEEVAVNFAK.G |
| 83075 | 236 | – | 249 | 813.4260 | 1624.8374 | 1624.8348 | 1.57 | 0 | 84 | 1.4e-08 | 1Score **> 34** indicates **identity** Score **> 18** indicates **homology** | U | R.TIEYLEEVAVNFAK.G |
| 83076 | 236 | – | 249 | 813.4260 | 1624.8374 | 1624.8348 | 1.58 | 0 | 77 | 5.7e-08 | 1Score **> 34** indicates **identity** Score **> 17** indicates **homology** | U | R.TIEYLEEVAVNFAK.G |
| 83078 | 236 | – | 249 | 542.6198 | 1624.8374 | 1624.8348 | 1.61 | 0 | 68 | 4.2e-07 | 1Score **> 34** indicates **identity** Score **> 17** indicates **homology** | U | R.TIEYLEEVAVNFAK.G |
| 83080 | 236 | – | 249 | 813.4261 | 1624.8377 | 1624.8348 | 1.77 | 0 | 80 | 3e-08 | 1Score **> 34** indicates **identity** Score **> 18** indicates **homology** | U | R.TIEYLEEVAVNFAK.G |
| 83084 | 236 | – | 249 | 542.6200 | 1624.8382 | 1624.8348 | 2.06 | 0 | 19 | 0.017 | 1Score **> 34** indicates **identity** Score **> 14** indicates **homology** | U | R.TIEYLEEVAVNFAK.G |
| 83085 | 236 | – | 249 | 542.6200 | 1624.8383 | 1624.8348 | 2.12 | 0 | 36 | 0.00045 | 1Score **> 34** indicates **identity** Score **> 15** indicates **homology** | U | R.TIEYLEEVAVNFAK.G |
| 83086 | 236 | – | 249 | 813.4264 | 1624.8383 | 1624.8348 | 2.15 | 0 | 70 | 2.7e-07 | 1Score **> 34** indicates **identity** Score **> 17** indicates **homology** | U | R.TIEYLEEVAVNFAK.G |
| 83087 | 236 | – | 249 | 542.6201 | 1624.8385 | 1624.8348 | 2.25 | 0 | 15 | 0.042 | 1Score **> 34** indicates **identity** Score **> 13** indicates **homology** | U | R.TIEYLEEVAVNFAK.G |
| 83089 | 236 | – | 249 | 813.4266 | 1624.8387 | 1624.8348 | 2.37 | 0 | 88 | 5.5e-09 | 1Score **> 34** indicates **identity** Score **> 18** indicates **homology** | U | R.TIEYLEEVAVNFAK.G |
| 83090 | 236 | – | 249 | 813.4267 | 1624.8389 | 1624.8348 | 2.54 | 0 | 69 | 3.1e-07 | 1Score **> 34** indicates **identity** Score **> 17** indicates **homology** | U | R.TIEYLEEVAVNFAK.G |
| 83091 | 236 | – | 249 | 813.4268 | 1624.8390 | 1624.8348 | 2.57 | 0 | 67 | 5.3e-07 | 1Score **> 34** indicates **identity** Score **> 17** indicates **homology** | U | R.TIEYLEEVAVNFAK.G |
| 83092 | 236 | – | 249 | 813.4268 | 1624.8390 | 1624.8348 | 2.58 | 0 | 45 | 5.5e-05 | 1Score **> 34** indicates **identity** Score **> 15** indicates **homology** | U | R.TIEYLEEVAVNFAK.G |
| 83093 | 236 | – | 249 | 542.6203 | 1624.8390 | 1624.8348 | 2.59 | 0 | 55 | 6.3e-06 | 1Score **> 34** indicates **identity** Score **> 16** indicates **homology** | U | R.TIEYLEEVAVNFAK.G |
| 83095 | 236 | – | 249 | 813.4270 | 1624.8394 | 1624.8348 | 2.80 | 0 | 39 | 0.00022 | 1Score **> 34** indicates **identity** Score **> 15** indicates **homology** | U | R.TIEYLEEVAVNFAK.G |
| 83096 | 236 | – | 249 | 813.4270 | 1624.8395 | 1624.8348 | 2.91 | 0 | 74 | 1.1e-07 | 1Score **> 34** indicates **identity** Score **> 17** indicates **homology** | U | R.TIEYLEEVAVNFAK.G |
| 83097 | 236 | – | 249 | 813.4270 | 1624.8395 | 1624.8348 | 2.91 | 0 | 88 | 5.4e-09 | 1Score **> 34** indicates **identity** Score **> 18** indicates **homology** | U | R.TIEYLEEVAVNFAK.G |
| 83098 | 236 | – | 249 | 813.4271 | 1624.8396 | 1624.8348 | 2.95 | 0 | 58 | 3.4e-06 | 1Score **> 34** indicates **identity** Score **> 16** indicates **homology** | U | R.TIEYLEEVAVNFAK.G |
| 83101 | 236 | – | 249 | 813.4274 | 1624.8402 | 1624.8348 | 3.31 | 0 | 34 | 0.00066 | 1Score **> 34** indicates **identity** Score **> 15** indicates **homology** | U | R.TIEYLEEVAVNFAK.G |
| 83103 | 236 | – | 249 | 813.4275 | 1624.8404 | 1624.8348 | 3.46 | 0 | 60 | 2.3e-06 | 1Score **> 34** indicates **identity** Score **> 16** indicates **homology** | U | R.TIEYLEEVAVNFAK.G |
| 83105 | 236 | – | 249 | 813.4275 | 1624.8405 | 1624.8348 | 3.52 | 0 | 74 | 1.1e-07 | 1Score **> 34** indicates **identity** Score **> 17** indicates **homology** | U | R.TIEYLEEVAVNFAK.G |
| 83106 | 236 | – | 249 | 813.4275 | 1624.8405 | 1624.8348 | 3.53 | 0 | 40 | 0.00018 | 1Score **> 34** indicates **identity** Score **> 15** indicates **homology** | U | R.TIEYLEEVAVNFAK.G |
| 83108 | 236 | – | 249 | 813.4277 | 1624.8408 | 1624.8348 | 3.68 | 0 | 22 | 0.0091 | 1Score **> 34** indicates **identity** Score **> 14** indicates **homology** | U | R.TIEYLEEVAVNFAK.G |
| 83109 | 236 | – | 249 | 813.4277 | 1624.8408 | 1624.8348 | 3.68 | 0 | 77 | 5.5e-08 | 1Score **> 34** indicates **identity** Score **> 17** indicates **homology** | U | R.TIEYLEEVAVNFAK.G |
| 83110 | 236 | – | 249 | 813.4277 | 1624.8409 | 1624.8348 | 3.74 | 0 | 61 | 1.8e-06 | 1Score **> 34** indicates **identity** Score **> 16** indicates **homology** | U | R.TIEYLEEVAVNFAK.G |
| 83111 | 236 | – | 249 | 813.4278 | 1624.8411 | 1624.8348 | 3.90 | 0 | 58 | 3.4e-06 | 1Score **> 34** indicates **identity** Score **> 16** indicates **homology** | U | R.TIEYLEEVAVNFAK.G |
| 83112 | 236 | – | 249 | 813.4280 | 1624.8415 | 1624.8348 | 4.10 | 0 | 42 | 0.00011 | 1Score **> 34** indicates **identity** Score **> 15** indicates **homology** | U | R.TIEYLEEVAVNFAK.G |
| 83114 | 236 | – | 249 | 813.4282 | 1624.8419 | 1624.8348 | 4.38 | 0 | 63 | 1.2e-06 | 1Score **> 34** indicates **identity** Score **> 16** indicates **homology** | U | R.TIEYLEEVAVNFAK.G |
| 83115 | 236 | – | 249 | 813.4283 | 1624.8420 | 1624.8348 | 4.44 | 0 | 35 | 0.00052 | 1Score **> 34** indicates **identity** Score **> 15** indicates **homology** | U | R.TIEYLEEVAVNFAK.G |
| 83117 | 236 | – | 249 | 813.4284 | 1624.8423 | 1624.8348 | 4.58 | 0 | 45 | 5.7e-05 | 1Score **> 34** indicates **identity** Score **> 15** indicates **homology** | U | R.TIEYLEEVAVNFAK.G |
| 83118 | 236 | – | 249 | 813.4284 | 1624.8423 | 1624.8348 | 4.64 | 0 | 49 | 2.4e-05 | 1Score **> 34** indicates **identity** Score **> 16** indicates **homology** | U | R.TIEYLEEVAVNFAK.G |
| 83119 | 236 | – | 249 | 813.4286 | 1624.8427 | 1624.8348 | 4.86 | 0 | 74 | 1.3e-07 | 1Score **> 34** indicates **identity** Score **> 17** indicates **homology** | U | R.TIEYLEEVAVNFAK.G |
| 83120 | 236 | – | 249 | 813.4286 | 1624.8427 | 1624.8348 | 4.89 | 0 | 48 | 3e-05 | 1Score **> 34** indicates **identity** Score **> 16** indicates **homology** | U | R.TIEYLEEVAVNFAK.G |
| 83121 | 236 | – | 249 | 813.4289 | 1624.8433 | 1624.8348 | 5.24 | 0 | 32 | 0.00093 | 1Score **> 34** indicates **identity** Score **> 15** indicates **homology** | U | R.TIEYLEEVAVNFAK.G |
| 83122 | 236 | – | 249 | 813.4290 | 1624.8435 | 1624.8348 | 5.34 | 0 | 61 | 2e-06 | 1Score **> 34** indicates **identity** Score **> 16** indicates **homology** | U | R.TIEYLEEVAVNFAK.G |
| 83125 | 236 | – | 249 | 813.4294 | 1624.8442 | 1624.8348 | 5.75 | 0 | 64 | 9.2e-07 | 1Score **> 34** indicates **identity** Score **> 17** indicates **homology** | U | R.TIEYLEEVAVNFAK.G |
| 83126 | 236 | – | 249 | 813.4297 | 1624.8448 | 1624.8348 | 6.16 | 0 | 48 | 2.9e-05 | 1Score **> 34** indicates **identity** Score **> 16** indicates **homology** | U | R.TIEYLEEVAVNFAK.G |
| 83127 | 236 | – | 249 | 813.4298 | 1624.8451 | 1624.8348 | 6.35 | 0 | 34 | 0.00069 | 1Score **> 34** indicates **identity** Score **> 15** indicates **homology** | U | R.TIEYLEEVAVNFAK.G |
| 83130 | 236 | – | 249 | 813.4308 | 1624.8469 | 1624.8348 | 7.47 | 0 | 66 | 6.1e-07 | 1Score **> 34** indicates **identity** Score **> 17** indicates **homology** | U | R.TIEYLEEVAVNFAK.G |
| 83132 | 236 | – | 249 | 813.4312 | 1624.8478 | 1624.8348 | 8.02 | 0 | 77 | 6.7e-08 | 1Score **> 34** indicates **identity** Score **> 17** indicates **homology** | U | R.TIEYLEEVAVNFAK.G |
| 83133 | 236 | – | 249 | 813.4314 | 1624.8482 | 1624.8348 | 8.21 | 0 | 46 | 4.5e-05 | 1Score **> 34** indicates **identity** Score **> 15** indicates **homology** | U | R.TIEYLEEVAVNFAK.G |
| 83134 | 236 | – | 249 | 813.4314 | 1624.8483 | 1624.8348 | 8.29 | 0 | 65 | 8.9e-07 | 1Score **> 34** indicates **identity** Score **> 17** indicates **homology** | U | R.TIEYLEEVAVNFAK.G |
| 83138 | 236 | – | 249 | 813.4319 | 1624.8493 | 1624.8348 | 8.92 | 0 | 36 | 0.00041 | 1Score **> 34** indicates **identity** Score **> 15** indicates **homology** | U | R.TIEYLEEVAVNFAK.G |
| 83139 | 236 | – | 249 | 813.4322 | 1624.8499 | 1624.8348 | 9.30 | 0 | 82 | 2.1e-08 | 1Score **> 34** indicates **identity** Score **> 18** indicates **homology** | U | R.TIEYLEEVAVNFAK.G |
| 83140 | 236 | – | 249 | 813.4323 | 1624.8500 | 1624.8348 | 9.32 | 0 | 54 | 7.9e-06 | 1Score **> 34** indicates **identity** Score **> 16** indicates **homology** | U | R.TIEYLEEVAVNFAK.G |
| 54418 | 268 | – | 279 | 699.8759 | 1397.7372 | 1397.7377 | -0.40 | 0 | 64 | 9.4e-07 | 1Score **> 33** indicates **identity** Score **> 17** indicates **homology** | U | K.LTTYAMTVPFVR.Q |
| 54419 | 268 | – | 279 | 699.8760 | 1397.7375 | 1397.7377 | -0.17 | 0 | 71 | 1e-06 | 1Score **> 34** indicates **identity** Score **> 23** indicates **homology** | U | K.LTTYAMTVPFVR.Q |
| 54422 | 268 | – | 279 | 699.8766 | 1397.7386 | 1397.7377 | 0.63 | 0 | 67 | 9.3e-07 | 1Score **> 34** indicates **identity** Score **> 19** indicates **homology** | U | K.LTTYAMTVPFVR.Q |
| 54423 | 268 | – | 279 | 699.8768 | 1397.7390 | 1397.7377 | 0.89 | 0 | 67 | 8.7e-07 | 1Score **> 34** indicates **identity** Score **> 19** indicates **homology** | U | K.LTTYAMTVPFVR.Q |
| 54424 | 268 | – | 279 | 699.8781 | 1397.7417 | 1397.7377 | 2.88 | 0 | 57 | 1.6e-05 | 1Score **> 34** indicates **identity** Score **> 21** indicates **homology** | U | K.LTTYAMTVPFVR.Q |
| 6502 | 296 | – | 303 | 429.7580 | 857.5015 | 857.5011 | 0.46 | 0 | 36 | 0.00082 | 1Score **> 32** indicates **identity** Score **> 18** indicates **homology** | U | K.GLYPAPLK.I |
| 6505 | 296 | – | 303 | 429.7581 | 857.5017 | 857.5011 | 0.76 | 0 | 34 | 0.0014 | 1Score **> 32** indicates **identity** Score **> 18** indicates **homology** | U | K.GLYPAPLK.I |
| 6506 | 296 | – | 303 | 429.7583 | 857.5020 | 857.5011 | 1.04 | 0 | 21 | 0.021 | 1Score **> 32** indicates **identity** Score **> 17** indicates **homology** | U | K.GLYPAPLK.I |
| 6507 | 296 | – | 303 | 429.7583 | 857.5020 | 857.5011 | 1.04 | 0 | 28 | 0.0033 | 1Score **> 32** indicates **identity** Score **> 16** indicates **homology** | U | K.GLYPAPLK.I |
| 6508 | 296 | – | 303 | 429.7584 | 857.5022 | 857.5011 | 1.33 | 0 | 30 | 0.0034 | 1Score **> 32** indicates **identity** Score **> 18** indicates **homology** | U | K.GLYPAPLK.I |
| 6509 | 296 | – | 303 | 429.7585 | 857.5024 | 857.5011 | 1.56 | 0 | 15 | 0.038 | 1Score **> 32** indicates **identity** Score **> 13** indicates **homology** | U | K.GLYPAPLK.I |
| 6510 | 296 | – | 303 | 429.7585 | 857.5025 | 857.5011 | 1.61 | 0 | 34 | 0.0007 | 1Score **> 32** indicates **identity** Score **> 15** indicates **homology** | U | K.GLYPAPLK.I |
| 66209 | 296 | – | 309 | 499.9720 | 1496.8943 | 1496.8966 | -1.54 | 1 | 42 | 0.00012 | 1Score **> 31** indicates **identity** Score **> 16** indicates **homology** | U | K.GLYPAPLKIIDAVK.A |
| 66211 | 296 | – | 309 | 499.9724 | 1496.8953 | 1496.8966 | -0.88 | 1 | 62 | 1.5e-06 | 1Score **> 31** indicates **identity** Score **> 16** indicates **homology** | U | K.GLYPAPLKIIDAVK.A |
| 66212 | 296 | – | 309 | 499.9725 | 1496.8957 | 1496.8966 | -0.63 | 1 | 41 | 0.00014 | 1Score **> 32** indicates **identity** Score **> 15** indicates **homology** | U | K.GLYPAPLKIIDAVK.A |
| 66213 | 296 | – | 309 | 499.9726 | 1496.8960 | 1496.8966 | -0.43 | 1 | 36 | 0.00044 | 1Score **> 32** indicates **identity** Score **> 15** indicates **homology** | U | K.GLYPAPLKIIDAVK.A |
| 66216 | 296 | – | 309 | 749.4559 | 1496.8972 | 1496.8966 | 0.39 | 1 | 15 | 0.037 | 1Score **> 31** indicates **identity** Score **> 13** indicates **homology** | U | K.GLYPAPLKIIDAVK.A |
| 66217 | 296 | – | 309 | 499.9732 | 1496.8977 | 1496.8966 | 0.69 | 1 | 48 | 3.5e-05 | 1Score **> 31** indicates **identity** Score **> 16** indicates **homology** | U | K.GLYPAPLKIIDAVK.A |
| 95598 | 310 | – | 326 | 575.2749 | 1722.8028 | 1722.8060 | -1.87 | 0 | 18 | 0.02 | 1Score **> 32** indicates **identity** Score **> 14** indicates **homology** | U | K.AGLEQGSDAGYLAESQK.F |
| 95599 | 310 | – | 326 | 862.4088 | 1722.8030 | 1722.8060 | -1.73 | 0 | 94 | 1.7e-09 | 1Score **> 32** indicates **identity** Score **> 18** indicates **homology** | U | K.AGLEQGSDAGYLAESQK.F |
| 95601 | 310 | – | 326 | 862.4103 | 1722.8061 | 1722.8060 | 0.041 | 0 | 40 | 0.00016 | 1Score **> 32** indicates **identity** Score **> 15** indicates **homology** | U | K.AGLEQGSDAGYLAESQK.F |
| 95602 | 310 | – | 326 | 862.4104 | 1722.8062 | 1722.8060 | 0.10 | 0 | 32 | 0.00098 | 1Score **> 32** indicates **identity** Score **> 15** indicates **homology** | U | K.AGLEQGSDAGYLAESQK.F |
| 95603 | 310 | – | 326 | 862.4104 | 1722.8062 | 1722.8060 | 0.11 | 0 | 65 | 8.8e-07 | 1Score **> 32** indicates **identity** Score **> 17** indicates **homology** | U | K.AGLEQGSDAGYLAESQK.F |
| 95604 | 310 | – | 326 | 575.2761 | 1722.8065 | 1722.8060 | 0.25 | 0 | 44 | 8e-05 | 1Score **> 32** indicates **identity** Score **> 16** indicates **homology** | U | K.AGLEQGSDAGYLAESQK.F |
| 95605 | 310 | – | 326 | 862.4106 | 1722.8067 | 1722.8060 | 0.37 | 0 | 113 | 2.7e-11 | 1Score **> 32** indicates **identity** Score **> 20** indicates **homology** | U | K.AGLEQGSDAGYLAESQK.F |
| 95606 | 310 | – | 326 | 862.4106 | 1722.8067 | 1722.8060 | 0.38 | 0 | 113 | 9.5e-11 | 1Score **> 32** indicates **identity** Score **> 25** indicates **homology** | U | K.AGLEQGSDAGYLAESQK.F |
| 95607 | 310 | – | 326 | 862.4107 | 1722.8068 | 1722.8060 | 0.44 | 0 | 93 | 9.1e-09 | 1Score **> 32** indicates **identity** Score **> 25** indicates **homology** | U | K.AGLEQGSDAGYLAESQK.F |
| 95608 | 310 | – | 326 | 862.4107 | 1722.8069 | 1722.8060 | 0.53 | 0 | 112 | 7.2e-11 | 1Score **> 32** indicates **identity** Score **> 23** indicates **homology** | U | K.AGLEQGSDAGYLAESQK.F |
| 95609 | 310 | – | 326 | 575.2763 | 1722.8070 | 1722.8060 | 0.57 | 0 | 54 | 7.8e-06 | 1Score **> 32** indicates **identity** Score **> 16** indicates **homology** | U | K.AGLEQGSDAGYLAESQK.F |
| 95611 | 310 | – | 326 | 862.4108 | 1722.8071 | 1722.8060 | 0.61 | 0 | 99 | 1.6e-09 | 1Score **> 32** indicates **identity** Score **> 24** indicates **homology** | U | K.AGLEQGSDAGYLAESQK.F |
| 95612 | 310 | – | 326 | 862.4110 | 1722.8074 | 1722.8060 | 0.80 | 0 | 77 | 5.7e-08 | 1Score **> 32** indicates **identity** Score **> 17** indicates **homology** | U | K.AGLEQGSDAGYLAESQK.F |
| 95613 | 310 | – | 326 | 862.4111 | 1722.8076 | 1722.8060 | 0.91 | 0 | 67 | 5.4e-07 | 1Score **> 33** indicates **identity** Score **> 17** indicates **homology** | U | K.AGLEQGSDAGYLAESQK.F |
| 95614 | 310 | – | 326 | 862.4113 | 1722.8080 | 1722.8060 | 1.12 | 0 | 112 | 3e-11 | 1Score **> 33** indicates **identity** Score **> 20** indicates **homology** | U | K.AGLEQGSDAGYLAESQK.F |
| 95615 | 310 | – | 326 | 575.2768 | 1722.8085 | 1722.8060 | 1.42 | 0 | 25 | 0.0048 | 1Score **> 33** indicates **identity** Score **> 14** indicates **homology** | U | K.AGLEQGSDAGYLAESQK.F |
| 95718 | 310 | – | 326 | 862.9095 | 1723.8045 | 1723.7900 | 8.39 | 0 | 20 | 0.012 | 1Score **> 32** indicates **identity** Score **> 14** indicates **homology** | U | K.AGLEQGSDAGYLAESQK.F  + Deamidated (NQ) |
| 164709 | 310 | – | 334 | 861.7699 | 2582.2878 | 2582.2864 | 0.54 | 1 | 59 | 2.9e-06 | 1Score **> 37** indicates **identity** Score **> 16** indicates **homology** | U | K.AGLEQGSDAGYLAESQKFGELALTK.E |
| 164710 | 310 | – | 334 | 1292.1515 | 2582.2885 | 2582.2864 | 0.82 | 1 | 128 | 1e-12 | 1Score **> 37** indicates **identity** Score **> 20** indicates **homology** | U | K.AGLEQGSDAGYLAESQKFGELALTK.E |
| 164711 | 310 | – | 334 | 861.7701 | 2582.2885 | 2582.2864 | 0.83 | 1 | 80 | 3.1e-08 | 1Score **> 37** indicates **identity** Score **> 18** indicates **homology** | U | K.AGLEQGSDAGYLAESQKFGELALTK.E |
| 164712 | 310 | – | 334 | 861.7702 | 2582.2889 | 2582.2864 | 0.98 | 1 | 68 | 4.7e-07 | 1Score **> 37** indicates **identity** Score **> 17** indicates **homology** | U | K.AGLEQGSDAGYLAESQKFGELALTK.E |
| 164713 | 310 | – | 334 | 1292.1518 | 2582.2890 | 2582.2864 | 1.03 | 1 | 79 | 4.2e-08 | 1Score **> 37** indicates **identity** Score **> 17** indicates **homology** | U | K.AGLEQGSDAGYLAESQKFGELALTK.E |
| 164714 | 310 | – | 334 | 861.7714 | 2582.2922 | 2582.2864 | 2.27 | 1 | 71 | 2e-07 | 1Score **> 37** indicates **identity** Score **> 17** indicates **homology** | U | K.AGLEQGSDAGYLAESQKFGELALTK.E |
| 164715 | 310 | – | 334 | 861.7716 | 2582.2928 | 2582.2864 | 2.51 | 1 | 67 | 4.8e-07 | 1Score **> 37** indicates **identity** Score **> 17** indicates **homology** | U | K.AGLEQGSDAGYLAESQKFGELALTK.E |
| 164717 | 310 | – | 334 | 861.7727 | 2582.2963 | 2582.2864 | 3.83 | 1 | 58 | 3.7e-06 | 1Score **> 37** indicates **identity** Score **> 16** indicates **homology** | U | K.AGLEQGSDAGYLAESQKFGELALTK.E |
| 164719 | 310 | – | 334 | 861.7737 | 2582.2993 | 2582.2864 | 5.01 | 1 | 62 | 1.4e-06 | 1Score **> 37** indicates **identity** Score **> 16** indicates **homology** | U | K.AGLEQGSDAGYLAESQKFGELALTK.E |
| 164790 | 310 | – | 334 | 1292.6507 | 2583.2869 | 2583.2704 | 6.38 | 1 | 89 | 4.3e-09 | 1Score **> 37** indicates **identity** Score **> 18** indicates **homology** | U | K.AGLEQGSDAGYLAESQKFGELALTK.E  + Deamidated (NQ) |
| 177395 | 310 | – | 337 | 732.6163 | 2926.4360 | 2926.4559 | -6.83 | 2 | 23 | 0.0067 | 1Score **> 37** indicates **identity** Score **> 14** indicates **homology** | U | K.AGLEQGSDAGYLAESQKFGELALTKESK.A |
| 177399 | 310 | – | 337 | 732.6224 | 2926.4605 | 2926.4559 | 1.57 | 2 | 30 | 0.0014 | 1Score **> 38** indicates **identity** Score **> 14** indicates **homology** | U | K.AGLEQGSDAGYLAESQKFGELALTKESK.A |
| 7507 | 327 | – | 334 | 439.7520 | 877.4895 | 877.4909 | -1.57 | 0 | 26 | 0.0074 | 1Score **> 26** indicates **identity** Score **> 17** indicates **homology** | U | K.FGELALTK.E |
| 7508 | 327 | – | 334 | 439.7522 | 877.4899 | 877.4909 | -1.10 | 0 | 30 | 0.0033 | 1Score **> 25** indicates **identity** Score **> 17** indicates **homology** | U | K.FGELALTK.E |
| 7509 | 327 | – | 334 | 439.7523 | 877.4900 | 877.4909 | -1.07 | 0 | 36 | 0.00047 | 1Score **> 25** indicates **identity** Score **> 15** indicates **homology** | U | K.FGELALTK.E |
| 7510 | 327 | – | 334 | 439.7524 | 877.4903 | 877.4909 | -0.66 | 0 | 21 | 0.0096 | 1Score **> 25** indicates **identity** Score **> 14** indicates **homology** | U | K.FGELALTK.E |
| 7511 | 327 | – | 334 | 439.7525 | 877.4905 | 877.4909 | -0.50 | 0 | 58 | 4.5e-06 | 1Score **> 25** indicates **identity** Score **> 17** indicates **homology** | U | K.FGELALTK.E |
| 7513 | 327 | – | 334 | 439.7528 | 877.4910 | 877.4909 | 0.055 | 0 | 59 | 5e-06 | 1Score **> 26** indicates **identity** Score **> 19** indicates **homology** | U | K.FGELALTK.E |
| 7514 | 327 | – | 334 | 439.7528 | 877.4910 | 877.4909 | 0.10 | 0 | 46 | 8.6e-05 | 1Score **> 26** indicates **identity** Score **> 18** indicates **homology** | U | K.FGELALTK.E |
| 7515 | 327 | – | 334 | 439.7529 | 877.4912 | 877.4909 | 0.36 | 0 | 28 | 0.0033 | 1Score **> 26** indicates **identity** Score **> 15** indicates **homology** | U | K.FGELALTK.E |
| 7517 | 327 | – | 334 | 439.7530 | 877.4915 | 877.4909 | 0.70 | 0 | 39 | 0.00029 | 1Score **> 26** indicates **identity** Score **> 16** indicates **homology** | U | K.FGELALTK.E |
| 7518 | 327 | – | 334 | 439.7531 | 877.4916 | 877.4909 | 0.78 | 0 | 28 | 0.0035 | 1Score **> 26** indicates **identity** Score **> 16** indicates **homology** | U | K.FGELALTK.E |
| 7519 | 327 | – | 334 | 439.7531 | 877.4917 | 877.4909 | 0.92 | 0 | 35 | 0.0013 | 1Score **> 26** indicates **identity** Score **> 19** indicates **homology** | U | K.FGELALTK.E |
| 7520 | 327 | – | 334 | 439.7531 | 877.4917 | 877.4909 | 0.95 | 0 | 46 | 9.5e-05 | 1Score **> 26** indicates **identity** Score **> 18** indicates **homology** | U | K.FGELALTK.E |
| 7521 | 327 | – | 334 | 439.7532 | 877.4919 | 877.4909 | 1.15 | 0 | 20 | 0.013 | 1Score **> 26** indicates **identity** Score **> 14** indicates **homology** | U | K.FGELALTK.E |
| 7522 | 327 | – | 334 | 439.7534 | 877.4923 | 877.4909 | 1.55 | 0 | 43 | 0.00019 | 1Score **> 26** indicates **identity** Score **> 18** indicates **homology** | U | K.FGELALTK.E |
| 7523 | 327 | – | 334 | 439.7541 | 877.4936 | 877.4909 | 3.08 | 0 | 36 | 0.0025 | 1Score **> 28** indicates **identity** Score **> 23** indicates **homology** | U | K.FGELALTK.E |
| 35532 | 327 | – | 337 | 611.8341 | 1221.6537 | 1221.6605 | -5.51 | 1 | 26 | 0.0033 | 1Score **> 33** indicates **identity** Score **> 14** indicates **homology** | U | K.FGELALTKESK.A |
| 35533 | 327 | – | 337 | 408.2252 | 1221.6538 | 1221.6605 | -5.47 | 1 | 15 | 0.037 | 1Score **> 33** indicates **identity** Score **> 13** indicates **homology** | U | K.FGELALTKESK.A |
| 62285 | 338 | – | 350 | 733.8777 | 1465.7408 | 1465.7421 | -0.92 | 0 | 40 | 0.00018 | 1Score **> 34** indicates **identity** Score **> 15** indicates **homology** | U | K.ALMGLYNGQVLCK.K |
| 62286 | 338 | – | 350 | 733.8791 | 1465.7436 | 1465.7421 | 1.01 | 0 | 84 | 1.3e-08 | 1Score **> 34** indicates **identity** Score **> 18** indicates **homology** | U | K.ALMGLYNGQVLCK.K |
| 62287 | 338 | – | 350 | 733.8794 | 1465.7443 | 1465.7421 | 1.48 | 0 | 49 | 2.6e-05 | 1Score **> 34** indicates **identity** Score **> 16** indicates **homology** | U | K.ALMGLYNGQVLCK.K |
| 78782 | 338 | – | 351 | 797.9241 | 1593.8337 | 1593.8371 | -2.14 | 1 | 33 | 0.00073 | 1Score **> 35** indicates **identity** Score **> 15** indicates **homology** | U | K.ALMGLYNGQVLCKK.N |
| 152004 | 360 | – | 383 | 785.1053 | 2352.2940 | 2352.2835 | 4.45 | 0 | 100 | 4.1e-10 | 1Score **> 35** indicates **identity** Score **> 19** indicates **homology** | U | K.NVQQLAILGAGLMGAGIAQVSVDK.G |
| 152005 | 360 | – | 383 | 1177.1543 | 2352.2940 | 2352.2835 | 4.46 | 0 | 69 | 3.3e-07 | 1Score **> 35** indicates **identity** Score **> 17** indicates **homology** | U | K.NVQQLAILGAGLMGAGIAQVSVDK.G |
| 167550 | 360 | – | 386 | 884.5022 | 2650.4847 | 2650.4840 | 0.26 | 1 | 92 | 2.5e-09 | 1Score **> 33** indicates **identity** Score **> 18** indicates **homology** | U | K.NVQQLAILGAGLMGAGIAQVSVDKGLK.T |
| 167551 | 360 | – | 386 | 884.5022 | 2650.4847 | 2650.4840 | 0.26 | 1 | 44 | 7.9e-05 | 1Score **> 33** indicates **identity** Score **> 15** indicates **homology** | U | K.NVQQLAILGAGLMGAGIAQVSVDKGLK.T |
| 52034 | 387 | – | 399 | 458.9364 | 1373.7874 | 1373.7878 | -0.36 | 1 | 27 | 0.004 | 1Score **> 33** indicates **identity** Score **> 16** indicates **homology** | U | K.TLLKDTTVTGLGR.G |
| 52035 | 387 | – | 399 | 458.9364 | 1373.7875 | 1373.7878 | -0.27 | 1 | 44 | 9.1e-05 | 1Score **> 33** indicates **identity** Score **> 16** indicates **homology** | U | K.TLLKDTTVTGLGR.G |
| 52036 | 387 | – | 399 | 458.9365 | 1373.7875 | 1373.7878 | -0.21 | 1 | 50 | 2e-05 | 1Score **> 33** indicates **identity** Score **> 16** indicates **homology** | U | K.TLLKDTTVTGLGR.G |
| 52037 | 387 | – | 399 | 687.9011 | 1373.7876 | 1373.7878 | -0.21 | 1 | 18 | 0.021 | 1Score **> 33** indicates **identity** Score **> 14** indicates **homology** | U | K.TLLKDTTVTGLGR.G |
| 52038 | 387 | – | 399 | 458.9365 | 1373.7878 | 1373.7878 | -0.039 | 1 | 29 | 0.0021 | 1Score **> 33** indicates **identity** Score **> 14** indicates **homology** | U | K.TLLKDTTVTGLGR.G |
| 52039 | 387 | – | 399 | 458.9365 | 1373.7878 | 1373.7878 | -0.019 | 1 | 48 | 3e-05 | 1Score **> 33** indicates **identity** Score **> 16** indicates **homology** | U | K.TLLKDTTVTGLGR.G |
| 52040 | 387 | – | 399 | 458.9366 | 1373.7878 | 1373.7878 | -0.0058 | 1 | 40 | 0.00022 | 1Score **> 33** indicates **identity** Score **> 16** indicates **homology** | U | K.TLLKDTTVTGLGR.G |
| 52041 | 387 | – | 399 | 458.9366 | 1373.7881 | 1373.7878 | 0.18 | 1 | 44 | 8.2e-05 | 1Score **> 33** indicates **identity** Score **> 16** indicates **homology** | U | K.TLLKDTTVTGLGR.G |
| 52042 | 387 | – | 399 | 458.9366 | 1373.7881 | 1373.7878 | 0.19 | 1 | 47 | 0.00014 | 1Score **> 33** indicates **identity** Score **> 21** indicates **homology** | U | K.TLLKDTTVTGLGR.G |
| 52044 | 387 | – | 399 | 687.9014 | 1373.7883 | 1373.7878 | 0.31 | 1 | 36 | 0.00039 | 1Score **> 33** indicates **identity** Score **> 15** indicates **homology** | U | K.TLLKDTTVTGLGR.G |
| 52046 | 387 | – | 399 | 458.9368 | 1373.7885 | 1373.7878 | 0.48 | 1 | 32 | 0.0011 | 1Score **> 33** indicates **identity** Score **> 14** indicates **homology** | U | K.TLLKDTTVTGLGR.G |
| 52047 | 387 | – | 399 | 458.9368 | 1373.7885 | 1373.7878 | 0.50 | 1 | 29 | 0.002 | 1Score **> 33** indicates **identity** Score **> 14** indicates **homology** | U | K.TLLKDTTVTGLGR.G |
| 52048 | 387 | – | 399 | 687.9016 | 1373.7886 | 1373.7878 | 0.52 | 1 | 35 | 0.00054 | 1Score **> 33** indicates **identity** Score **> 15** indicates **homology** | U | K.TLLKDTTVTGLGR.G |
| 52049 | 387 | – | 399 | 458.9369 | 1373.7888 | 1373.7878 | 0.70 | 1 | 52 | 1.5e-05 | 1Score **> 33** indicates **identity** Score **> 16** indicates **homology** | U | K.TLLKDTTVTGLGR.G |
| 52050 | 387 | – | 399 | 458.9369 | 1373.7889 | 1373.7878 | 0.78 | 1 | 26 | 0.0038 | 1Score **> 33** indicates **identity** Score **> 14** indicates **homology** | U | K.TLLKDTTVTGLGR.G |
| 9764 | 391 | – | 399 | 460.2462 | 918.4779 | 918.4771 | 0.92 | 0 | 37 | 0.013 | 1Score **> 31** indicates **identity** | U | K.DTTVTGLGR.G |
| 9765 | 391 | – | 399 | 460.2463 | 918.4780 | 918.4771 | 0.97 | 0 | 33 | 0.0075 | 1Score **> 31** indicates **identity** Score **> 25** indicates **homology** | U | K.DTTVTGLGR.G |
| 20694 | 414 | – | 422 | 360.5452 | 1078.6139 | 1078.6135 | 0.36 | 2 | 43 | 0.0002 | 1Score **> 29** indicates **identity** Score **> 19** indicates **homology** | U | K.KKALTSFER.D |
| 11644 | 415 | – | 422 | 476.2664 | 950.5183 | 950.5185 | -0.26 | 1 | 31 | 0.0012 | 1Score **> 28** indicates **identity** Score **> 14** indicates **homology** | U | K.KALTSFER.D |
| 163063 | 415 | – | 436 | 637.3383 | 2545.3239 | 2545.3064 | 6.89 | 2 | 22 | 0.0086 | 1Score **> 37** indicates **identity** Score **> 14** indicates **homology** | U | K.KALTSFERDSIFSNLIGQLDYK.G  + Deamidated (NQ) |
| 4766 | 416 | – | 422 | 412.2192 | 822.4239 | 822.4236 | 0.42 | 0 | 43 | 0.00099 | 1Score **> 26** indicates **identity** | U | K.ALTSFER.D |
| 4769 | 416 | – | 422 | 412.2195 | 822.4244 | 822.4236 | 1.00 | 0 | 40 | 0.0018 | 1Score **> 25** indicates **identity** | U | K.ALTSFER.D |
| 156044 | 416 | – | 436 | 1209.1208 | 2416.2271 | 2416.2274 | -0.12 | 1 | 56 | 5.6e-06 | 1Score **> 37** indicates **identity** Score **> 16** indicates **homology** | U | K.ALTSFERDSIFSNLIGQLDYK.G |
| 156045 | 416 | – | 436 | 806.4182 | 2416.2327 | 2416.2274 | 2.20 | 1 | 33 | 0.00082 | 1Score **> 37** indicates **identity** Score **> 15** indicates **homology** | U | K.ALTSFERDSIFSNLIGQLDYK.G |
| 176029 | 416 | – | 440 | 960.1600 | 2877.4583 | 2877.4548 | 1.20 | 2 | 64 | 9.5e-07 | 1Score **> 37** indicates **identity** Score **> 17** indicates **homology** | U | K.ALTSFERDSIFSNLIGQLDYKGFEK.A |
| 176030 | 416 | – | 440 | 720.3735 | 2877.4649 | 2877.4548 | 3.50 | 2 | 27 | 0.0027 | 1Score **> 37** indicates **identity** Score **> 14** indicates **homology** | U | K.ALTSFERDSIFSNLIGQLDYKGFEK.A |
| 81237 | 423 | – | 436 | 806.9113 | 1611.8081 | 1611.8144 | -3.90 | 0 | 37 | 0.00031 | 1Score **> 34** indicates **identity** Score **> 15** indicates **homology** | U | R.DSIFSNLIGQLDYK.G |
| 81240 | 423 | – | 436 | 538.2773 | 1611.8100 | 1611.8144 | -2.76 | 0 | 28 | 0.003 | 1Score **> 34** indicates **identity** Score **> 16** indicates **homology** | U | R.DSIFSNLIGQLDYK.G |
| 81243 | 423 | – | 436 | 806.9159 | 1611.8172 | 1611.8144 | 1.75 | 0 | 87 | 7.1e-09 | 1Score **> 34** indicates **identity** Score **> 18** indicates **homology** | U | R.DSIFSNLIGQLDYK.G |
| 129748 | 423 | – | 440 | 1037.5238 | 2073.0330 | 2073.0419 | -4.27 | 1 | 87 | 8.1e-09 | 1Score **> 36** indicates **identity** Score **> 19** indicates **homology** | U | R.DSIFSNLIGQLDYKGFEK.A |
| 129750 | 423 | – | 440 | 1037.5277 | 2073.0409 | 2073.0419 | -0.44 | 1 | 29 | 0.0021 | 1Score **> 36** indicates **identity** Score **> 14** indicates **homology** | U | R.DSIFSNLIGQLDYKGFEK.A |
| 129752 | 423 | – | 440 | 692.0214 | 2073.0424 | 2073.0419 | 0.25 | 1 | 33 | 0.00086 | 1Score **> 36** indicates **identity** Score **> 15** indicates **homology** | U | R.DSIFSNLIGQLDYKGFEK.A |
| 129757 | 423 | – | 440 | 692.0243 | 2073.0510 | 2073.0419 | 4.39 | 1 | 29 | 0.0019 | 1Score **> 36** indicates **identity** Score **> 14** indicates **homology** | U | R.DSIFSNLIGQLDYKGFEK.A |
| 129759 | 423 | – | 440 | 692.0256 | 2073.0550 | 2073.0419 | 6.33 | 1 | 22 | 0.009 | 1Score **> 36** indicates **identity** Score **> 14** indicates **homology** | U | R.DSIFSNLIGQLDYKGFEK.A |
| 84271 | 441 | – | 455 | 545.9479 | 1634.8220 | 1634.8226 | -0.33 | 0 | 69 | 3.3e-07 | 1Score **> 35** indicates **identity** Score **> 17** indicates **homology** | U | K.ADMVIEAVFEDLGVK.H |
| 84272 | 441 | – | 455 | 545.9482 | 1634.8227 | 1634.8226 | 0.083 | 0 | 82 | 1.9e-08 | 1Score **> 35** indicates **identity** Score **> 18** indicates **homology** | U | K.ADMVIEAVFEDLGVK.H |
| 84276 | 441 | – | 455 | 818.4208 | 1634.8271 | 1634.8226 | 2.76 | 0 | 97 | 7.5e-10 | 1Score **> 35** indicates **identity** Score **> 19** indicates **homology** | U | K.ADMVIEAVFEDLGVK.H |
| 86398 | 441 | – | 455 | 826.4128 | 1650.8111 | 1650.8175 | -3.88 | 0 | 33 | 0.00082 | 1Score **> 34** indicates **identity** Score **> 15** indicates **homology** | U | K.ADMVIEAVFEDLGVK.H  + Oxidation (M) |
| 86401 | 441 | – | 455 | 551.2782 | 1650.8127 | 1650.8175 | -2.89 | 0 | 36 | 0.00045 | 1Score **> 34** indicates **identity** Score **> 15** indicates **homology** | U | K.ADMVIEAVFEDLGVK.H  + Oxidation (M) |
| 86408 | 441 | – | 455 | 826.4195 | 1650.8245 | 1650.8175 | 4.27 | 0 | 24 | 0.0053 | 1Score **> 35** indicates **identity** Score **> 14** indicates **homology** | U | K.ADMVIEAVFEDLGVK.H  + Oxidation (M) |
| 188139 | 458 | – | 489 | 863.9577 | 3451.8018 | 3451.8021 | -0.092 | 1 | 29 | 0.002 | 1Score **> 37** indicates **identity** Score **> 14** indicates **homology** | U | K.VLKEVESVTPEHCIFASNTSALPINQIAAVSK.R |
| 188140 | 458 | – | 489 | 863.9580 | 3451.8030 | 3451.8021 | 0.26 | 1 | 44 | 7.9e-05 | 1Score **> 37** indicates **identity** Score **> 15** indicates **homology** | U | K.VLKEVESVTPEHCIFASNTSALPINQIAAVSK.R |
| 188141 | 458 | – | 489 | 1151.6088 | 3451.8046 | 3451.8021 | 0.74 | 1 | 79 | 3.9e-08 | 1Score **> 37** indicates **identity** Score **> 17** indicates **homology** | U | K.VLKEVESVTPEHCIFASNTSALPINQIAAVSK.R |
| 188142 | 458 | – | 489 | 1151.6088 | 3451.8046 | 3451.8021 | 0.74 | 1 | 73 | 1.5e-07 | 1Score **> 37** indicates **identity** Score **> 17** indicates **homology** | U | K.VLKEVESVTPEHCIFASNTSALPINQIAAVSK.R |
| 188143 | 458 | – | 489 | 863.9588 | 3451.8062 | 3451.8021 | 1.19 | 1 | 32 | 0.00091 | 1Score **> 37** indicates **identity** Score **> 15** indicates **homology** | U | K.VLKEVESVTPEHCIFASNTSALPINQIAAVSK.R |
| 188144 | 458 | – | 489 | 863.9590 | 3451.8070 | 3451.8021 | 1.42 | 1 | 39 | 0.00023 | 1Score **> 37** indicates **identity** Score **> 15** indicates **homology** | U | K.VLKEVESVTPEHCIFASNTSALPINQIAAVSK.R |
| 188145 | 458 | – | 489 | 1151.6097 | 3451.8072 | 3451.8021 | 1.50 | 1 | 66 | 6.9e-07 | 1Score **> 37** indicates **identity** Score **> 17** indicates **homology** | U | K.VLKEVESVTPEHCIFASNTSALPINQIAAVSK.R |
| 182297 | 461 | – | 489 | 1038.1922 | 3111.5548 | 3111.5546 | 0.050 | 0 | 71 | 2.3e-07 | 1Score **> 38** indicates **identity** Score **> 17** indicates **homology** | U | K.EVESVTPEHCIFASNTSALPINQIAAVSK.R |
| 182299 | 461 | – | 489 | 1556.7852 | 3111.5559 | 3111.5546 | 0.40 | 0 | 23 | 0.0074 | 1Score **> 38** indicates **identity** Score **> 14** indicates **homology** | U | K.EVESVTPEHCIFASNTSALPINQIAAVSK.R |
| 182300 | 461 | – | 489 | 1556.7853 | 3111.5560 | 3111.5546 | 0.45 | 0 | 84 | 1.5e-08 | 1Score **> 38** indicates **identity** Score **> 19** indicates **homology** | U | K.EVESVTPEHCIFASNTSALPINQIAAVSK.R |
| 182301 | 461 | – | 489 | 1038.1929 | 3111.5569 | 3111.5546 | 0.72 | 0 | 22 | 0.0078 | 1Score **> 38** indicates **identity** Score **> 14** indicates **homology** | U | K.EVESVTPEHCIFASNTSALPINQIAAVSK.R |
| 182303 | 461 | – | 489 | 1556.7861 | 3111.5577 | 3111.5546 | 1.00 | 0 | 54 | 1.5e-05 | 1Score **> 38** indicates **identity** Score **> 18** indicates **homology** | U | K.EVESVTPEHCIFASNTSALPINQIAAVSK.R |
| 182304 | 461 | – | 489 | 1038.1933 | 3111.5581 | 3111.5546 | 1.11 | 0 | 62 | 1.5e-06 | 1Score **> 38** indicates **identity** Score **> 16** indicates **homology** | U | K.EVESVTPEHCIFASNTSALPINQIAAVSK.R |
| 182306 | 461 | – | 489 | 1038.1934 | 3111.5582 | 3111.5546 | 1.15 | 0 | 69 | 3.4e-07 | 1Score **> 38** indicates **identity** Score **> 17** indicates **homology** | U | K.EVESVTPEHCIFASNTSALPINQIAAVSK.R |
| 182307 | 461 | – | 489 | 1038.1934 | 3111.5585 | 3111.5546 | 1.24 | 0 | 65 | 7.4e-07 | 1Score **> 38** indicates **identity** Score **> 17** indicates **homology** | U | K.EVESVTPEHCIFASNTSALPINQIAAVSK.R |
| 182308 | 461 | – | 489 | 1556.7868 | 3111.5590 | 3111.5546 | 1.41 | 0 | 32 | 0.00093 | 1Score **> 38** indicates **identity** Score **> 15** indicates **homology** | U | K.EVESVTPEHCIFASNTSALPINQIAAVSK.R |
| 182310 | 461 | – | 489 | 1038.1938 | 3111.5596 | 3111.5546 | 1.60 | 0 | 24 | 0.0062 | 1Score **> 38** indicates **identity** Score **> 14** indicates **homology** | U | K.EVESVTPEHCIFASNTSALPINQIAAVSK.R |
| 182311 | 461 | – | 489 | 1038.1940 | 3111.5601 | 3111.5546 | 1.77 | 0 | 78 | 4.8e-08 | 1Score **> 38** indicates **identity** Score **> 17** indicates **homology** | U | K.EVESVTPEHCIFASNTSALPINQIAAVSK.R |
| 182312 | 461 | – | 489 | 1038.1955 | 3111.5648 | 3111.5546 | 3.27 | 0 | 56 | 6.2e-06 | 1Score **> 38** indicates **identity** Score **> 16** indicates **homology** | U | K.EVESVTPEHCIFASNTSALPINQIAAVSK.R |
| 182314 | 461 | – | 489 | 1038.1983 | 3111.5731 | 3111.5546 | 5.94 | 0 | 19 | 0.015 | 1Score **> 38** indicates **identity** Score **> 14** indicates **homology** | U | K.EVESVTPEHCIFASNTSALPINQIAAVSK.R |
| 182316 | 461 | – | 489 | 1038.1994 | 3111.5765 | 3111.5546 | 7.02 | 0 | 25 | 0.0046 | 1Score **> 38** indicates **identity** Score **> 14** indicates **homology** | U | K.EVESVTPEHCIFASNTSALPINQIAAVSK.R |
| 182343 | 461 | – | 489 | 1557.2815 | 3112.5484 | 3112.5387 | 3.13 | 0 | 46 | 5e-05 | 1Score **> 38** indicates **identity** Score **> 15** indicates **homology** | U | K.EVESVTPEHCIFASNTSALPINQIAAVSK.R  + Deamidated (NQ) |
| 182346 | 461 | – | 489 | 1038.5270 | 3112.5593 | 3112.5387 | 6.63 | 0 | 31 | 0.0013 | 1Score **> 38** indicates **identity** Score **> 14** indicates **homology** | U | K.EVESVTPEHCIFASNTSALPINQIAAVSK.R  + Deamidated (NQ) |
| 182347 | 461 | – | 489 | 1038.5275 | 3112.5607 | 3112.5387 | 7.10 | 0 | 15 | 0.039 | 1Score **> 38** indicates **identity** Score **> 13** indicates **homology** | U | K.EVESVTPEHCIFASNTSALPINQIAAVSK.R  + Deamidated (NQ) |
| 182385 | 461 | – | 489 | 1038.8582 | 3113.5527 | 3113.5227 | 9.66 | 0 | 19 | 0.018 | 1Score **> 38** indicates **identity** Score **> 14** indicates **homology** | U | K.EVESVTPEHCIFASNTSALPINQIAAVSK.R  + 2 Deamidated (NQ) |
| 190123 | 461 | – | 493 | 1208.2895 | 3621.8468 | 3621.8461 | 0.19 | 2 | 34 | 0.00067 | 1Score **> 37** indicates **identity** Score **> 15** indicates **homology** | U | K.EVESVTPEHCIFASNTSALPINQIAAVSKRPEK.V |
| 190124 | 461 | – | 493 | 906.4691 | 3621.8475 | 3621.8461 | 0.40 | 2 | 28 | 0.0025 | 1Score **> 37** indicates **identity** Score **> 14** indicates **homology** | U | K.EVESVTPEHCIFASNTSALPINQIAAVSKRPEK.V |
| 190126 | 461 | – | 493 | 1208.2901 | 3621.8484 | 3621.8461 | 0.65 | 2 | 22 | 0.0092 | 1Score **> 37** indicates **identity** Score **> 14** indicates **homology** | U | K.EVESVTPEHCIFASNTSALPINQIAAVSKRPEK.V |
| 190127 | 461 | – | 493 | 725.3771 | 3621.8491 | 3621.8461 | 0.83 | 2 | 28 | 0.0026 | 1Score **> 37** indicates **identity** Score **> 14** indicates **homology** | U | K.EVESVTPEHCIFASNTSALPINQIAAVSKRPEK.V |
| 190129 | 461 | – | 493 | 906.4702 | 3621.8519 | 3621.8461 | 1.61 | 2 | 34 | 0.00061 | 1Score **> 37** indicates **identity** Score **> 15** indicates **homology** | U | K.EVESVTPEHCIFASNTSALPINQIAAVSKRPEK.V |
| 190131 | 461 | – | 493 | 1208.2921 | 3621.8546 | 3621.8461 | 2.36 | 2 | 14 | 0.044 | 1Score **> 37** indicates **identity** Score **> 13** indicates **homology** | U | K.EVESVTPEHCIFASNTSALPINQIAAVSKRPEK.V |
| 190132 | 461 | – | 493 | 906.4709 | 3621.8546 | 3621.8461 | 2.36 | 2 | 31 | 0.0011 | 1Score **> 37** indicates **identity** Score **> 14** indicates **homology** | U | K.EVESVTPEHCIFASNTSALPINQIAAVSKRPEK.V |
| 53800 | 494 | – | 505 | 464.9030 | 1391.6873 | 1391.6908 | -2.50 | 0 | 21 | 0.0097 | 1Score **> 33** indicates **identity** Score **> 14** indicates **homology** | U | K.VIGMHYFSPVDK.M |
| 53802 | 494 | – | 505 | 464.9046 | 1391.6919 | 1391.6908 | 0.84 | 0 | 24 | 0.0056 | 1Score **> 33** indicates **identity** Score **> 14** indicates **homology** | U | K.VIGMHYFSPVDK.M |
| 44661 | 506 | – | 516 | 652.8595 | 1303.7045 | 1303.7057 | -0.92 | 0 | 40 | 0.00019 | 1Score **> 34** indicates **identity** Score **> 15** indicates **homology** | U | K.MQLLEIITTDK.T |
| 44662 | 506 | – | 516 | 652.8597 | 1303.7049 | 1303.7057 | -0.65 | 0 | 47 | 4e-05 | 1Score **> 34** indicates **identity** Score **> 16** indicates **homology** | U | K.MQLLEIITTDK.T |
| 82414 | 506 | – | 519 | 540.9675 | 1619.8807 | 1619.8804 | 0.19 | 1 | 23 | 0.0072 | 1Score **> 34** indicates **identity** Score **> 14** indicates **homology** | U | K.MQLLEIITTDKTSK.D |
| 82415 | 506 | – | 519 | 540.9679 | 1619.8818 | 1619.8804 | 0.87 | 1 | 39 | 0.0002 | 1Score **> 34** indicates **identity** Score **> 15** indicates **homology** | U | K.MQLLEIITTDKTSK.D |
| 82416 | 506 | – | 519 | 540.9679 | 1619.8818 | 1619.8804 | 0.88 | 1 | 57 | 4.7e-06 | 1Score **> 34** indicates **identity** Score **> 16** indicates **homology** | U | K.MQLLEIITTDKTSK.D |
| 171906 | 506 | – | 531 | 691.3772 | 2761.4797 | 2761.4895 | -3.57 | 2 | 47 | 3.7e-05 | 1Score **> 36** indicates **identity** Score **> 15** indicates **homology** | U | K.MQLLEIITTDKTSKDTTASAVAVGLR.Q |
| 171907 | 506 | – | 531 | 691.3794 | 2761.4884 | 2761.4895 | -0.40 | 2 | 45 | 5.7e-05 | 1Score **> 36** indicates **identity** Score **> 15** indicates **homology** | U | K.MQLLEIITTDKTSKDTTASAVAVGLR.Q |
| 171908 | 506 | – | 531 | 691.3795 | 2761.4890 | 2761.4895 | -0.20 | 2 | 46 | 5.1e-05 | 1Score **> 36** indicates **identity** Score **> 15** indicates **homology** | U | K.MQLLEIITTDKTSKDTTASAVAVGLR.Q |
| 171910 | 506 | – | 531 | 691.3841 | 2761.5073 | 2761.4895 | 6.44 | 2 | 51 | 1.7e-05 | 1Score **> 35** indicates **identity** Score **> 16** indicates **homology** | U | K.MQLLEIITTDKTSKDTTASAVAVGLR.Q |
| 171939 | 506 | – | 531 | 921.8367 | 2762.4883 | 2762.4735 | 5.35 | 2 | 39 | 0.00023 | 1Score **> 36** indicates **identity** Score **> 15** indicates **homology** | U | K.MQLLEIITTDKTSKDTTASAVAVGLR.Q  + Deamidated (NQ) |
| 171940 | 506 | – | 531 | 921.8378 | 2762.4917 | 2762.4735 | 6.57 | 2 | 37 | 0.00037 | 1Score **> 36** indicates **identity** Score **> 15** indicates **homology** | U | K.MQLLEIITTDKTSKDTTASAVAVGLR.Q  + Deamidated (NQ) |
| 63455 | 517 | – | 531 | 492.9380 | 1475.7923 | 1475.7944 | -1.43 | 1 | 58 | 5.3e-06 | 1Score **> 35** indicates **identity** Score **> 18** indicates **homology** | U | K.TSKDTTASAVAVGLR.Q |
| 63456 | 517 | – | 531 | 738.9035 | 1475.7925 | 1475.7944 | -1.28 | 1 | 85 | 1.1e-08 | 1Score **> 35** indicates **identity** Score **> 18** indicates **homology** | U | K.TSKDTTASAVAVGLR.Q |
| 63457 | 517 | – | 531 | 738.9036 | 1475.7927 | 1475.7944 | -1.14 | 1 | 64 | 1.1e-06 | 1Score **> 35** indicates **identity** Score **> 16** indicates **homology** | U | K.TSKDTTASAVAVGLR.Q |
| 63458 | 517 | – | 531 | 738.9037 | 1475.7929 | 1475.7944 | -0.99 | 1 | 49 | 2.5e-05 | 1Score **> 35** indicates **identity** Score **> 16** indicates **homology** | U | K.TSKDTTASAVAVGLR.Q |
| 63459 | 517 | – | 531 | 492.9382 | 1475.7929 | 1475.7944 | -0.99 | 1 | 27 | 0.0029 | 1Score **> 35** indicates **identity** Score **> 14** indicates **homology** | U | K.TSKDTTASAVAVGLR.Q |
| 63462 | 517 | – | 531 | 492.9385 | 1475.7937 | 1475.7944 | -0.47 | 1 | 22 | 0.0084 | 1Score **> 35** indicates **identity** Score **> 14** indicates **homology** | U | K.TSKDTTASAVAVGLR.Q |
| 63463 | 517 | – | 531 | 492.9391 | 1475.7953 | 1475.7944 | 0.65 | 1 | 20 | 0.014 | 1Score **> 35** indicates **identity** Score **> 14** indicates **homology** | U | K.TSKDTTASAVAVGLR.Q |
| 28852 | 520 | – | 531 | 580.8168 | 1159.6191 | 1159.6197 | -0.55 | 0 | 71 | 2.2e-07 | 1Score **> 34** indicates **identity** Score **> 17** indicates **homology** | U | K.DTTASAVAVGLR.Q |
| 28853 | 520 | – | 531 | 580.8168 | 1159.6191 | 1159.6197 | -0.52 | 0 | 58 | 3.4e-06 | 1Score **> 34** indicates **identity** Score **> 16** indicates **homology** | U | K.DTTASAVAVGLR.Q |
| 28854 | 520 | – | 531 | 580.8171 | 1159.6196 | 1159.6197 | -0.12 | 0 | 70 | 3.5e-06 | 1Score **> 34** indicates **identity** Score **> 28** indicates **homology** | U | K.DTTASAVAVGLR.Q |
| 28855 | 520 | – | 531 | 580.8171 | 1159.6197 | 1159.6197 | -0.030 | 0 | 63 | 1.1e-05 | 1Score **> 34** indicates **identity** Score **> 26** indicates **homology** | U | K.DTTASAVAVGLR.Q |
| 28856 | 520 | – | 531 | 580.8173 | 1159.6201 | 1159.6197 | 0.31 | 0 | 76 | 7.2e-07 | 1Score **> 34** indicates **identity** Score **> 27** indicates **homology** | U | K.DTTASAVAVGLR.Q |
| 28857 | 520 | – | 531 | 580.8174 | 1159.6202 | 1159.6197 | 0.43 | 0 | 45 | 0.00016 | 1Score **> 34** indicates **identity** Score **> 20** indicates **homology** | U | K.DTTASAVAVGLR.Q |
| 28858 | 520 | – | 531 | 580.8176 | 1159.6207 | 1159.6197 | 0.86 | 0 | 72 | 1.6e-06 | 1Score **> 34** indicates **identity** Score **> 26** indicates **homology** | U | K.DTTASAVAVGLR.Q |
| 28859 | 520 | – | 531 | 580.8177 | 1159.6209 | 1159.6197 | 1.02 | 0 | 58 | 1.6e-05 | 1Score **> 34** indicates **identity** Score **> 22** indicates **homology** | U | K.DTTASAVAVGLR.Q |
| 28861 | 520 | – | 531 | 580.8182 | 1159.6219 | 1159.6197 | 1.89 | 0 | 21 | 0.02 | 1Score **> 33** indicates **identity** Score **> 17** indicates **homology** | U | K.DTTASAVAVGLR.Q |
| 88133 | 535 | – | 549 | 555.6501 | 1663.9284 | 1663.9298 | -0.82 | 1 | 42 | 0.00011 | 1Score **> 34** indicates **identity** Score **> 15** indicates **homology** | U | K.VIIVVKDGPGFYTTR.C |
| 88134 | 535 | – | 549 | 555.6507 | 1663.9304 | 1663.9298 | 0.39 | 1 | 20 | 0.013 | 1Score **> 34** indicates **identity** Score **> 14** indicates **homology** | U | K.VIIVVKDGPGFYTTR.C |
| 88135 | 535 | – | 549 | 555.6508 | 1663.9306 | 1663.9298 | 0.49 | 1 | 54 | 8.2e-06 | 1Score **> 34** indicates **identity** Score **> 16** indicates **homology** | U | K.VIIVVKDGPGFYTTR.C |
| 88136 | 535 | – | 549 | 555.6509 | 1663.9309 | 1663.9298 | 0.70 | 1 | 48 | 3.1e-05 | 1Score **> 34** indicates **identity** Score **> 16** indicates **homology** | U | K.VIIVVKDGPGFYTTR.C |
| 88137 | 535 | – | 549 | 555.6511 | 1663.9314 | 1663.9298 | 1.01 | 1 | 57 | 4.1e-06 | 1Score **> 33** indicates **identity** Score **> 16** indicates **homology** | U | K.VIIVVKDGPGFYTTR.C |
| 88138 | 535 | – | 549 | 555.6514 | 1663.9323 | 1663.9298 | 1.50 | 1 | 33 | 0.00085 | 1Score **> 33** indicates **identity** Score **> 15** indicates **homology** | U | K.VIIVVKDGPGFYTTR.C |
| 88140 | 535 | – | 549 | 555.6522 | 1663.9349 | 1663.9298 | 3.07 | 1 | 32 | 0.0011 | 1Score **> 33** indicates **identity** Score **> 14** indicates **homology** | U | K.VIIVVKDGPGFYTTR.C |
| 15486 | 541 | – | 549 | 507.2370 | 1012.4595 | 1012.4614 | -1.92 | 0 | 34 | 0.00071 | 1Score **> 27** indicates **identity** Score **> 15** indicates **homology** | U | K.DGPGFYTTR.C |
| 15487 | 541 | – | 549 | 507.2371 | 1012.4596 | 1012.4614 | -1.85 | 0 | 35 | 0.00053 | 1Score **> 27** indicates **identity** Score **> 15** indicates **homology** | U | K.DGPGFYTTR.C |
| 15488 | 541 | – | 549 | 507.2372 | 1012.4599 | 1012.4614 | -1.54 | 0 | 36 | 0.00043 | 1Score **> 27** indicates **identity** Score **> 15** indicates **homology** | U | K.DGPGFYTTR.C |
| 15489 | 541 | – | 549 | 507.2373 | 1012.4601 | 1012.4614 | -1.32 | 0 | 19 | 0.015 | 1Score **> 27** indicates **identity** Score **> 14** indicates **homology** | U | K.DGPGFYTTR.C |
| 15490 | 541 | – | 549 | 507.2374 | 1012.4602 | 1012.4614 | -1.20 | 0 | 36 | 0.00046 | 1Score **> 27** indicates **identity** Score **> 15** indicates **homology** | U | K.DGPGFYTTR.C |
| 15491 | 541 | – | 549 | 507.2376 | 1012.4607 | 1012.4614 | -0.75 | 0 | 42 | 0.00011 | 1Score **> 27** indicates **identity** Score **> 15** indicates **homology** | U | K.DGPGFYTTR.C |
| 15492 | 541 | – | 549 | 507.2379 | 1012.4612 | 1012.4614 | -0.22 | 0 | 51 | 1.6e-05 | 1Score **> 27** indicates **identity** Score **> 16** indicates **homology** | U | K.DGPGFYTTR.C |
| 15493 | 541 | – | 549 | 507.2380 | 1012.4614 | 1012.4614 | -0.018 | 0 | 51 | 1.7e-05 | 1Score **> 27** indicates **identity** Score **> 16** indicates **homology** | U | K.DGPGFYTTR.C |
| 15494 | 541 | – | 549 | 507.2385 | 1012.4625 | 1012.4614 | 1.04 | 0 | 31 | 0.0012 | 1Score **> 27** indicates **identity** Score **> 14** indicates **homology** | U | K.DGPGFYTTR.C |
| 14603 | 561 | – | 569 | 499.7795 | 997.5444 | 997.5444 | -0.0050 | 0 | 38 | 0.00028 | 1Score **> 29** indicates **identity** Score **> 15** indicates **homology** | U | R.ILQEGVDPK.K |
| 14604 | 561 | – | 569 | 499.7796 | 997.5446 | 997.5444 | 0.17 | 0 | 39 | 0.00024 | 1Score **> 28** indicates **identity** Score **> 15** indicates **homology** | U | R.ILQEGVDPK.K |
| 14605 | 561 | – | 569 | 499.7797 | 997.5448 | 997.5444 | 0.42 | 0 | 43 | 0.00013 | 1Score **> 28** indicates **identity** Score **> 17** indicates **homology** | U | R.ILQEGVDPK.K |
| 14606 | 561 | – | 569 | 499.7797 | 997.5448 | 997.5444 | 0.43 | 0 | 44 | 9.4e-05 | 1Score **> 28** indicates **identity** Score **> 16** indicates **homology** | U | R.ILQEGVDPK.K |
| 15145 | 611 | – | 620 | 503.7819 | 1005.5492 | 1005.5495 | -0.34 | 0 | 58 | 7.3e-06 | 1Score **> 31** indicates **identity** Score **> 19** indicates **homology** | U | R.FGGGSVELLK.Q |
| 15146 | 611 | – | 620 | 503.7819 | 1005.5493 | 1005.5495 | -0.21 | 0 | 58 | 7.3e-06 | 1Score **> 31** indicates **identity** Score **> 19** indicates **homology** | U | R.FGGGSVELLK.Q |
| 15147 | 611 | – | 620 | 503.7820 | 1005.5494 | 1005.5495 | -0.11 | 0 | 50 | 1.9e-05 | 1Score **> 31** indicates **identity** Score **> 16** indicates **homology** | U | R.FGGGSVELLK.Q |
| 15148 | 611 | – | 620 | 503.7820 | 1005.5495 | 1005.5495 | -0.038 | 0 | 60 | 5.7e-06 | 1Score **> 31** indicates **identity** Score **> 20** indicates **homology** | U | R.FGGGSVELLK.Q |
| 15149 | 611 | – | 620 | 503.7821 | 1005.5496 | 1005.5495 | 0.058 | 0 | 60 | 4.7e-06 | 1Score **> 31** indicates **identity** Score **> 19** indicates **homology** | U | R.FGGGSVELLK.Q |
| 15150 | 611 | – | 620 | 503.7821 | 1005.5496 | 1005.5495 | 0.14 | 0 | 59 | 6.2e-06 | 1Score **> 31** indicates **identity** Score **> 19** indicates **homology** | U | R.FGGGSVELLK.Q |
| 15153 | 611 | – | 620 | 503.7822 | 1005.5498 | 1005.5495 | 0.27 | 0 | 56 | 9.1e-06 | 1Score **> 31** indicates **identity** Score **> 18** indicates **homology** | U | R.FGGGSVELLK.Q |
| 15154 | 611 | – | 620 | 503.7822 | 1005.5498 | 1005.5495 | 0.30 | 0 | 70 | 7.7e-07 | 1Score **> 31** indicates **identity** Score **> 21** indicates **homology** | U | R.FGGGSVELLK.Q |
| 15155 | 611 | – | 620 | 503.7822 | 1005.5498 | 1005.5495 | 0.32 | 0 | 49 | 4.5e-05 | 1Score **> 31** indicates **identity** Score **> 18** indicates **homology** | U | R.FGGGSVELLK.Q |
| 15156 | 611 | – | 620 | 503.7822 | 1005.5499 | 1005.5495 | 0.37 | 0 | 41 | 0.00016 | 1Score **> 31** indicates **identity** Score **> 15** indicates **homology** | U | R.FGGGSVELLK.Q |
| 15158 | 611 | – | 620 | 503.7823 | 1005.5501 | 1005.5495 | 0.61 | 0 | 31 | 0.0012 | 1Score **> 31** indicates **identity** Score **> 14** indicates **homology** | U | R.FGGGSVELLK.Q |
| 15159 | 611 | – | 620 | 503.7824 | 1005.5502 | 1005.5495 | 0.74 | 0 | 39 | 0.00022 | 1Score **> 31** indicates **identity** Score **> 15** indicates **homology** | U | R.FGGGSVELLK.Q |
| 15161 | 611 | – | 620 | 503.7826 | 1005.5506 | 1005.5495 | 1.10 | 0 | 38 | 0.00028 | 1Score **> 31** indicates **identity** Score **> 15** indicates **homology** | U | R.FGGGSVELLK.Q |
| 15162 | 611 | – | 620 | 503.7827 | 1005.5509 | 1005.5495 | 1.43 | 0 | 39 | 0.00023 | 1Score **> 32** indicates **identity** Score **> 15** indicates **homology** | U | R.FGGGSVELLK.Q |
| 15166 | 611 | – | 620 | 503.7833 | 1005.5520 | 1005.5495 | 2.51 | 0 | 43 | 8.8e-05 | 1Score **> 31** indicates **identity** Score **> 15** indicates **homology** | U | R.FGGGSVELLK.Q |
| 61849 | 632 | – | 644 | 732.3578 | 1462.7010 | 1462.7092 | -5.59 | 1 | 59 | 2.8e-06 | 1Score **> 32** indicates **identity** Score **> 16** indicates **homology** | U | K.SGKGFYIYQEGSK.N |
| 61854 | 632 | – | 644 | 732.3619 | 1462.7092 | 1462.7092 | -0.036 | 1 | 75 | 9.6e-08 | 1Score **> 33** indicates **identity** Score **> 17** indicates **homology** | U | K.SGKGFYIYQEGSK.N |
| 61855 | 632 | – | 644 | 488.5771 | 1462.7094 | 1462.7092 | 0.13 | 1 | 31 | 0.0012 | 1Score **> 34** indicates **identity** Score **> 14** indicates **homology** | U | K.SGKGFYIYQEGSK.N |
| 61856 | 632 | – | 644 | 732.3622 | 1462.7098 | 1462.7092 | 0.39 | 1 | 56 | 6.3e-06 | 1Score **> 33** indicates **identity** Score **> 16** indicates **homology** | U | K.SGKGFYIYQEGSK.N |
| 61858 | 632 | – | 644 | 488.5773 | 1462.7102 | 1462.7092 | 0.65 | 1 | 31 | 0.0012 | 1Score **> 33** indicates **identity** Score **> 14** indicates **homology** | U | K.SGKGFYIYQEGSK.N |
| 32229 | 635 | – | 644 | 596.2842 | 1190.5538 | 1190.5608 | -5.84 | 0 | 22 | 0.0083 | 1Score **> 30** indicates **identity** Score **> 14** indicates **homology** | U | K.GFYIYQEGSK.N |
| 32232 | 635 | – | 644 | 596.2848 | 1190.5550 | 1190.5608 | -4.85 | 0 | 25 | 0.0046 | 1Score **> 31** indicates **identity** Score **> 14** indicates **homology** | U | K.GFYIYQEGSK.N |
| 32233 | 635 | – | 644 | 596.2850 | 1190.5555 | 1190.5608 | -4.40 | 0 | 17 | 0.023 | 1Score **> 31** indicates **identity** Score **> 14** indicates **homology** | U | K.GFYIYQEGSK.N |
| 32241 | 635 | – | 644 | 596.2875 | 1190.5605 | 1190.5608 | -0.27 | 0 | 33 | 0.00079 | 1Score **> 32** indicates **identity** Score **> 15** indicates **homology** | U | K.GFYIYQEGSK.N |
| 32242 | 635 | – | 644 | 596.2877 | 1190.5608 | 1190.5608 | -0.018 | 0 | 40 | 0.00018 | 1Score **> 32** indicates **identity** Score **> 15** indicates **homology** | U | K.GFYIYQEGSK.N |
| 32243 | 635 | – | 644 | 596.2877 | 1190.5608 | 1190.5608 | 0.020 | 0 | 35 | 0.00051 | 1Score **> 31** indicates **identity** Score **> 15** indicates **homology** | U | K.GFYIYQEGSK.N |
| 32244 | 635 | – | 644 | 596.2878 | 1190.5611 | 1190.5608 | 0.26 | 0 | 32 | 0.001 | 1Score **> 31** indicates **identity** Score **> 14** indicates **homology** | U | K.GFYIYQEGSK.N |
| 32246 | 635 | – | 644 | 596.2882 | 1190.5619 | 1190.5608 | 0.98 | 0 | 24 | 0.0053 | 1Score **> 31** indicates **identity** Score **> 14** indicates **homology** | U | K.GFYIYQEGSK.N |
| 58398 | 635 | – | 646 | 478.5726 | 1432.6961 | 1432.6987 | -1.81 | 1 | 18 | 0.019 | 1Score **> 33** indicates **identity** Score **> 14** indicates **homology** | U | K.GFYIYQEGSKNK.S |
| 58400 | 635 | – | 646 | 717.3559 | 1432.6972 | 1432.6987 | -1.01 | 1 | 30 | 0.0023 | 1Score **> 33** indicates **identity** Score **> 16** indicates **homology** | U | K.GFYIYQEGSKNK.S |
| 58406 | 635 | – | 646 | 717.3570 | 1432.6995 | 1432.6987 | 0.59 | 1 | 22 | 0.0092 | 1Score **> 33** indicates **identity** Score **> 14** indicates **homology** | U | K.GFYIYQEGSKNK.S |
| 78113 | 647 | – | 660 | 795.3985 | 1588.7824 | 1588.7879 | -3.45 | 0 | 77 | 5.9e-08 | 1Score **> 34** indicates **identity** Score **> 17** indicates **homology** | U | K.SLNSEMDNILANLR.L |
| 78117 | 647 | – | 660 | 795.4007 | 1588.7868 | 1588.7879 | -0.64 | 0 | 101 | 3.2e-10 | 1Score **> 34** indicates **identity** Score **> 19** indicates **homology** | U | K.SLNSEMDNILANLR.L |
| 78119 | 647 | – | 660 | 530.6037 | 1588.7892 | 1588.7879 | 0.86 | 0 | 24 | 0.0053 | 1Score **> 34** indicates **identity** Score **> 14** indicates **homology** | U | K.SLNSEMDNILANLR.L |
| 187271 | 647 | – | 676 | 1135.2331 | 3402.6776 | 3402.6725 | 1.51 | 2 | 38 | 0.00025 | 1Score **> 37** indicates **identity** Score **> 15** indicates **homology** | U | K.SLNSEMDNILANLRLPAKPEVSSDEDVQYR.V |
| 189582 | 647 | – | 676 | 890.9433 | 3559.7442 | 3559.7715 | -7.67 | 2 | 20 | 0.012 | 1Score **> 37** indicates **identity** Score **> 14** indicates **homology** | U | K.SLNSEMDNILANLRLPAKPEVSSDEDVQYR.V  + Deamidated (NQ); HNE (K) |
| 189583 | 647 | – | 676 | 890.9434 | 3559.7447 | 3559.7715 | -7.55 | 2 | 17 | 0.025 | 1Score **> 37** indicates **identity** Score **> 14** indicates **homology** | U | K.SLNSEMDNILANLRLPAKPEVSSDEDVQYR.V  + Deamidated (NQ); HNE (K) |
| 107526 | 661 | – | 676 | 611.6388 | 1831.8945 | 1831.8952 | -0.35 | 1 | 85 | 9.9e-09 | 1Score **> 35** indicates **identity** Score **> 18** indicates **homology** | U | R.LPAKPEVSSDEDVQYR.V |
| 107527 | 661 | – | 676 | 611.6388 | 1831.8946 | 1831.8952 | -0.32 | 1 | 30 | 0.002 | 1Score **> 35** indicates **identity** Score **> 15** indicates **homology** | U | R.LPAKPEVSSDEDVQYR.V |
| 107528 | 661 | – | 676 | 611.6389 | 1831.8950 | 1831.8952 | -0.098 | 1 | 86 | 8.2e-09 | 1Score **> 35** indicates **identity** Score **> 18** indicates **homology** | U | R.LPAKPEVSSDEDVQYR.V |
| 107530 | 661 | – | 676 | 611.6391 | 1831.8954 | 1831.8952 | 0.11 | 1 | 73 | 1.5e-07 | 1Score **> 35** indicates **identity** Score **> 17** indicates **homology** | U | R.LPAKPEVSSDEDVQYR.V |
| 107531 | 661 | – | 676 | 611.6391 | 1831.8956 | 1831.8952 | 0.21 | 1 | 17 | 0.024 | 1Score **> 35** indicates **identity** Score **> 14** indicates **homology** | U | R.LPAKPEVSSDEDVQYR.V |
| 107532 | 661 | – | 676 | 611.6392 | 1831.8958 | 1831.8952 | 0.34 | 1 | 92 | 2.3e-09 | 1Score **> 35** indicates **identity** Score **> 18** indicates **homology** | U | R.LPAKPEVSSDEDVQYR.V |
| 107533 | 661 | – | 676 | 611.6396 | 1831.8968 | 1831.8952 | 0.89 | 1 | 70 | 2.5e-07 | 1Score **> 35** indicates **identity** Score **> 17** indicates **homology** | U | R.LPAKPEVSSDEDVQYR.V |
| 107534 | 661 | – | 676 | 611.6396 | 1831.8969 | 1831.8952 | 0.91 | 1 | 70 | 2.6e-07 | 1Score **> 35** indicates **identity** Score **> 17** indicates **homology** | U | R.LPAKPEVSSDEDVQYR.V |
| 107537 | 661 | – | 676 | 611.6398 | 1831.8976 | 1831.8952 | 1.31 | 1 | 86 | 8.5e-09 | 1Score **> 35** indicates **identity** Score **> 18** indicates **homology** | U | R.LPAKPEVSSDEDVQYR.V |
| 107538 | 661 | – | 676 | 611.6400 | 1831.8980 | 1831.8952 | 1.55 | 1 | 56 | 5.6e-06 | 1Score **> 35** indicates **identity** Score **> 16** indicates **homology** | U | R.LPAKPEVSSDEDVQYR.V |
| 107539 | 661 | – | 676 | 916.9571 | 1831.8997 | 1831.8952 | 2.48 | 1 | 77 | 6.5e-08 | 1Score **> 35** indicates **identity** Score **> 17** indicates **homology** | U | R.LPAKPEVSSDEDVQYR.V |
| 107540 | 661 | – | 676 | 611.6406 | 1831.8999 | 1831.8952 | 2.59 | 1 | 40 | 0.00016 | 1Score **> 35** indicates **identity** Score **> 15** indicates **homology** | U | R.LPAKPEVSSDEDVQYR.V |
| 107541 | 661 | – | 676 | 916.9574 | 1831.9002 | 1831.8952 | 2.76 | 1 | 43 | 9.2e-05 | 1Score **> 35** indicates **identity** Score **> 15** indicates **homology** | U | R.LPAKPEVSSDEDVQYR.V |
| 107543 | 661 | – | 676 | 916.9579 | 1831.9013 | 1831.8952 | 3.32 | 1 | 54 | 8.9e-06 | 1Score **> 35** indicates **identity** Score **> 16** indicates **homology** | U | R.LPAKPEVSSDEDVQYR.V |
| 148671 | 661 | – | 680 | 768.0743 | 2301.2012 | 2301.1965 | 2.05 | 2 | 23 | 0.0063 | 1Score **> 37** indicates **identity** Score **> 14** indicates **homology** | U | R.LPAKPEVSSDEDVQYRVITR.F |
| 17201 | 720 | – | 728 | 520.7736 | 1039.5326 | 1039.5338 | -1.15 | 0 | 26 | 0.0038 | 1Score **> 31** indicates **identity** Score **> 14** indicates **homology** | U | R.FVDLYGAQK.V |
| 17202 | 720 | – | 728 | 520.7737 | 1039.5328 | 1039.5338 | -1.04 | 0 | 26 | 0.0033 | 1Score **> 31** indicates **identity** Score **> 14** indicates **homology** | U | R.FVDLYGAQK.V |
| 17203 | 720 | – | 728 | 520.7739 | 1039.5333 | 1039.5338 | -0.53 | 0 | 30 | 0.0016 | 1Score **> 31** indicates **identity** Score **> 14** indicates **homology** | U | R.FVDLYGAQK.V |
| 17205 | 720 | – | 728 | 520.7740 | 1039.5335 | 1039.5338 | -0.30 | 0 | 35 | 0.00056 | 1Score **> 31** indicates **identity** Score **> 15** indicates **homology** | U | R.FVDLYGAQK.V |
| 17207 | 720 | – | 728 | 520.7742 | 1039.5338 | 1039.5338 | -0.060 | 0 | 42 | 0.00012 | 1Score **> 30** indicates **identity** Score **> 15** indicates **homology** | U | R.FVDLYGAQK.V |
| 17208 | 720 | – | 728 | 520.7743 | 1039.5340 | 1039.5338 | 0.13 | 0 | 36 | 0.00043 | 1Score **> 30** indicates **identity** Score **> 15** indicates **homology** | U | R.FVDLYGAQK.V |
| 17211 | 720 | – | 728 | 520.7744 | 1039.5342 | 1039.5338 | 0.37 | 0 | 28 | 0.0026 | 1Score **> 30** indicates **identity** Score **> 14** indicates **homology** | U | R.FVDLYGAQK.V |
| 17212 | 720 | – | 728 | 520.7744 | 1039.5343 | 1039.5338 | 0.40 | 0 | 45 | 6.6e-05 | 1Score **> 30** indicates **identity** Score **> 15** indicates **homology** | U | R.FVDLYGAQK.V |
| 17215 | 720 | – | 728 | 520.7745 | 1039.5344 | 1039.5338 | 0.53 | 0 | 14 | 0.049 | 1Score **> 30** indicates **identity** Score **> 13** indicates **homology** | U | R.FVDLYGAQK.V |
| 17216 | 720 | – | 728 | 520.7745 | 1039.5345 | 1039.5338 | 0.61 | 0 | 47 | 3.5e-05 | 1Score **> 30** indicates **identity** Score **> 15** indicates **homology** | U | R.FVDLYGAQK.V |
| 17217 | 720 | – | 728 | 520.7745 | 1039.5345 | 1039.5338 | 0.63 | 0 | 45 | 6.1e-05 | 1Score **> 30** indicates **identity** Score **> 15** indicates **homology** | U | R.FVDLYGAQK.V |
| 17218 | 720 | – | 728 | 520.7745 | 1039.5345 | 1039.5338 | 0.65 | 0 | 27 | 0.0031 | 1Score **> 30** indicates **identity** Score **> 14** indicates **homology** | U | R.FVDLYGAQK.V |
| 17220 | 720 | – | 728 | 520.7753 | 1039.5361 | 1039.5338 | 2.13 | 0 | 16 | 0.03 | 1Score **> 31** indicates **identity** Score **> 14** indicates **homology** | U | R.FVDLYGAQK.V |
| 17221 | 720 | – | 728 | 520.7755 | 1039.5365 | 1039.5338 | 2.60 | 0 | 27 | 0.0028 | 1Score **> 30** indicates **identity** Score **> 14** indicates **homology** | U | R.FVDLYGAQK.V |
| 182750 | 733 | – | 759 | 786.3926 | 3141.5414 | 3141.5189 | 7.16 | 2 | 38 | 0.00025 | 1Score **> 37** indicates **identity** Score **> 15** indicates **homology** | U | R.LRKYESAYGTQFTPCQLLLDHANNSSK.K  + Deamidated (NQ) |
| 175819 | 735 | – | 759 | 958.1221 | 2871.3446 | 2871.3497 | -1.80 | 1 | 15 | 0.039 | 1Score **> 35** indicates **identity** Score **> 13** indicates **homology** | U | R.KYESAYGTQFTPCQLLLDHANNSSK.K |
| 175821 | 735 | – | 759 | 718.8438 | 2871.3463 | 2871.3497 | -1.21 | 1 | 49 | 2.7e-05 | 1Score **> 35** indicates **identity** Score **> 16** indicates **homology** | U | R.KYESAYGTQFTPCQLLLDHANNSSK.K |
| 175823 | 735 | – | 759 | 718.8443 | 2871.3483 | 2871.3497 | -0.50 | 1 | 51 | 1.6e-05 | 1Score **> 35** indicates **identity** Score **> 16** indicates **homology** | U | R.KYESAYGTQFTPCQLLLDHANNSSK.K |
| 175824 | 735 | – | 759 | 718.8444 | 2871.3483 | 2871.3497 | -0.49 | 1 | 69 | 3.3e-07 | 1Score **> 35** indicates **identity** Score **> 17** indicates **homology** | U | R.KYESAYGTQFTPCQLLLDHANNSSK.K |
| 175826 | 735 | – | 759 | 718.8444 | 2871.3487 | 2871.3497 | -0.37 | 1 | 71 | 2.3e-07 | 1Score **> 35** indicates **identity** Score **> 17** indicates **homology** | U | R.KYESAYGTQFTPCQLLLDHANNSSK.K |
| 175827 | 735 | – | 759 | 958.1236 | 2871.3490 | 2871.3497 | -0.24 | 1 | 31 | 0.0011 | 1Score **> 35** indicates **identity** Score **> 14** indicates **homology** | U | R.KYESAYGTQFTPCQLLLDHANNSSK.K |
| 175828 | 735 | – | 759 | 718.8446 | 2871.3492 | 2871.3497 | -0.17 | 1 | 24 | 0.0055 | 1Score **> 35** indicates **identity** Score **> 14** indicates **homology** | U | R.KYESAYGTQFTPCQLLLDHANNSSK.K |
| 175829 | 735 | – | 759 | 958.1237 | 2871.3493 | 2871.3497 | -0.14 | 1 | 99 | 5e-10 | 1Score **> 35** indicates **identity** Score **> 19** indicates **homology** | U | R.KYESAYGTQFTPCQLLLDHANNSSK.K |
| 175830 | 735 | – | 759 | 958.1238 | 2871.3495 | 2871.3497 | -0.072 | 1 | 79 | 3.6e-08 | 1Score **> 35** indicates **identity** Score **> 17** indicates **homology** | U | R.KYESAYGTQFTPCQLLLDHANNSSK.K |
| 175831 | 735 | – | 759 | 958.1239 | 2871.3499 | 2871.3497 | 0.056 | 1 | 109 | 6.3e-11 | 1Score **> 35** indicates **identity** Score **> 19** indicates **homology** | U | R.KYESAYGTQFTPCQLLLDHANNSSK.K |
| 175833 | 735 | – | 759 | 958.1243 | 2871.3511 | 2871.3497 | 0.49 | 1 | 96 | 9.7e-10 | 1Score **> 36** indicates **identity** Score **> 19** indicates **homology** | U | R.KYESAYGTQFTPCQLLLDHANNSSK.K |
| 175834 | 735 | – | 759 | 718.8453 | 2871.3522 | 2871.3497 | 0.85 | 1 | 73 | 1.3e-07 | 1Score **> 36** indicates **identity** Score **> 17** indicates **homology** | U | R.KYESAYGTQFTPCQLLLDHANNSSK.K |
| 175872 | 735 | – | 759 | 719.0881 | 2872.3232 | 2872.3337 | -3.67 | 1 | 22 | 0.0079 | 1Score **> 34** indicates **identity** Score **> 14** indicates **homology** | U | R.KYESAYGTQFTPCQLLLDHANNSSK.K  + Deamidated (NQ) |
| 175874 | 735 | – | 759 | 958.4513 | 2872.3322 | 2872.3337 | -0.55 | 1 | 63 | 1.2e-06 | 1Score **> 35** indicates **identity** Score **> 16** indicates **homology** | U | R.KYESAYGTQFTPCQLLLDHANNSSK.K  + Deamidated (NQ) |
| 175875 | 735 | – | 759 | 958.4518 | 2872.3335 | 2872.3337 | -0.089 | 1 | 93 | 1.8e-09 | 1Score **> 35** indicates **identity** Score **> 18** indicates **homology** | U | R.KYESAYGTQFTPCQLLLDHANNSSK.K  + Deamidated (NQ) |
| 175877 | 735 | – | 759 | 719.0908 | 2872.3341 | 2872.3337 | 0.13 | 1 | 52 | 1.4e-05 | 1Score **> 35** indicates **identity** Score **> 16** indicates **homology** | U | R.KYESAYGTQFTPCQLLLDHANNSSK.K  + Deamidated (NQ) |
| 175878 | 735 | – | 759 | 719.0912 | 2872.3355 | 2872.3337 | 0.63 | 1 | 78 | 4.7e-08 | 1Score **> 35** indicates **identity** Score **> 17** indicates **homology** | U | R.KYESAYGTQFTPCQLLLDHANNSSK.K  + Deamidated (NQ) |
| 175879 | 735 | – | 759 | 958.4528 | 2872.3366 | 2872.3337 | 0.99 | 1 | 23 | 0.0076 | 1Score **> 35** indicates **identity** Score **> 14** indicates **homology** | U | R.KYESAYGTQFTPCQLLLDHANNSSK.K  + Deamidated (NQ) |
| 175880 | 735 | – | 759 | 958.4536 | 2872.3390 | 2872.3337 | 1.81 | 1 | 101 | 3.3e-10 | 1Score **> 35** indicates **identity** Score **> 19** indicates **homology** | U | R.KYESAYGTQFTPCQLLLDHANNSSK.K  + Deamidated (NQ) |
| 175882 | 735 | – | 759 | 719.0936 | 2872.3454 | 2872.3337 | 4.06 | 1 | 32 | 0.0011 | 1Score **> 35** indicates **identity** Score **> 14** indicates **homology** | U | R.KYESAYGTQFTPCQLLLDHANNSSK.K  + Deamidated (NQ) |
| 175887 | 735 | – | 759 | 958.4578 | 2872.3514 | 2872.3337 | 6.17 | 1 | 18 | 0.019 | 1Score **> 35** indicates **identity** Score **> 14** indicates **homology** | U | R.KYESAYGTQFTPCQLLLDHANNSSK.K  + Deamidated (NQ) |
| 175915 | 735 | – | 759 | 958.7872 | 2873.3396 | 2873.3177 | 7.62 | 1 | 59 | 3.1e-06 | 1Score **> 35** indicates **identity** Score **> 16** indicates **homology** | U | R.KYESAYGTQFTPCQLLLDHANNSSK.K  + 2 Deamidated (NQ) |
| 180536 | 735 | – | 759 | 1010.8104 | 3029.4092 | 3029.4328 | -7.77 | 1 | 25 | 0.0045 | 1Score **> 35** indicates **identity** Score **> 14** indicates **homology** | U | R.KYESAYGTQFTPCQLLLDHANNSSK.K  + 2 Deamidated (NQ); HNE (K) |
| 179857 | 735 | – | 760 | 600.8944 | 2999.4358 | 2999.4447 | -2.97 | 2 | 39 | 0.00023 | 1Score **> 36** indicates **identity** Score **> 15** indicates **homology** | U | R.KYESAYGTQFTPCQLLLDHANNSSKK.F |
| 179862 | 735 | – | 760 | 600.8963 | 2999.4450 | 2999.4447 | 0.10 | 2 | 25 | 0.0044 | 1Score **> 37** indicates **identity** Score **> 14** indicates **homology** | U | R.KYESAYGTQFTPCQLLLDHANNSSKK.F |
| 179864 | 735 | – | 760 | 750.8688 | 2999.4463 | 2999.4447 | 0.53 | 2 | 56 | 5.4e-06 | 1Score **> 37** indicates **identity** Score **> 16** indicates **homology** | U | R.KYESAYGTQFTPCQLLLDHANNSSKK.F |
| 179866 | 735 | – | 760 | 750.8691 | 2999.4472 | 2999.4447 | 0.85 | 2 | 18 | 0.02 | 1Score **> 37** indicates **identity** Score **> 14** indicates **homology** | U | R.KYESAYGTQFTPCQLLLDHANNSSKK.F |
| 179867 | 735 | – | 760 | 600.8968 | 2999.4478 | 2999.4447 | 1.03 | 2 | 44 | 7.1e-05 | 1Score **> 37** indicates **identity** Score **> 15** indicates **homology** | U | R.KYESAYGTQFTPCQLLLDHANNSSKK.F |
| 179868 | 735 | – | 760 | 1000.8233 | 2999.4480 | 2999.4447 | 1.11 | 2 | 83 | 1.8e-08 | 1Score **> 37** indicates **identity** Score **> 18** indicates **homology** | U | R.KYESAYGTQFTPCQLLLDHANNSSKK.F |
| 179869 | 735 | – | 760 | 1000.8239 | 2999.4499 | 2999.4447 | 1.74 | 2 | 33 | 0.00078 | 1Score **> 37** indicates **identity** Score **> 15** indicates **homology** | U | R.KYESAYGTQFTPCQLLLDHANNSSKK.F |
| 179893 | 735 | – | 760 | 751.1109 | 3000.4145 | 3000.4287 | -4.74 | 2 | 18 | 0.022 | 1Score **> 36** indicates **identity** Score **> 14** indicates **homology** | U | R.KYESAYGTQFTPCQLLLDHANNSSKK.F  + Deamidated (NQ) |
| 179914 | 735 | – | 760 | 751.1192 | 3000.4477 | 3000.4287 | 6.34 | 2 | 54 | 8.5e-06 | 1Score **> 37** indicates **identity** Score **> 16** indicates **homology** | U | R.KYESAYGTQFTPCQLLLDHANNSSKK.F  + Deamidated (NQ) |
| 171232 | 736 | – | 759 | 915.4261 | 2743.2564 | 2743.2548 | 0.59 | 0 | 47 | 4.2e-05 | 1Score **> 34** indicates **identity** Score **> 15** indicates **homology** | U | K.YESAYGTQFTPCQLLLDHANNSSK.K |
| 171233 | 736 | – | 759 | 915.4269 | 2743.2590 | 2743.2548 | 1.53 | 0 | 50 | 1.9e-05 | 1Score **> 34** indicates **identity** Score **> 16** indicates **homology** | U | K.YESAYGTQFTPCQLLLDHANNSSK.K |
| 171234 | 736 | – | 759 | 915.4271 | 2743.2595 | 2743.2548 | 1.72 | 0 | 46 | 4.9e-05 | 1Score **> 34** indicates **identity** Score **> 15** indicates **homology** | U | K.YESAYGTQFTPCQLLLDHANNSSK.K |
| 171236 | 736 | – | 759 | 915.4305 | 2743.2697 | 2743.2548 | 5.43 | 0 | 28 | 0.0024 | 1Score **> 35** indicates **identity** Score **> 14** indicates **homology** | U | K.YESAYGTQFTPCQLLLDHANNSSK.K |
| 171257 | 736 | – | 759 | 915.7559 | 2744.2458 | 2744.2388 | 2.54 | 0 | 18 | 0.02 | 1Score **> 33** indicates **identity** Score **> 14** indicates **homology** | U | K.YESAYGTQFTPCQLLLDHANNSSK.K  + Deamidated (NQ) |
| 171260 | 736 | – | 759 | 915.7619 | 2744.2638 | 2744.2388 | 9.11 | 0 | 42 | 0.00012 | 1Score **> 34** indicates **identity** Score **> 15** indicates **homology** | U | K.YESAYGTQFTPCQLLLDHANNSSK.K  + Deamidated (NQ) |
| 171279 | 736 | – | 759 | 916.0892 | 2745.2458 | 2745.2228 | 8.37 | 0 | 14 | 0.048 | 1Score **> 33** indicates **identity** Score **> 13** indicates **homology** | U | K.YESAYGTQFTPCQLLLDHANNSSK.K  + 2 Deamidated (NQ) |

---

```
ID   ECHA_MOUSE              Reviewed;         763 AA.
AC   Q8BMS1; Q3TCY3; Q5U5Y5; Q8QZU4;
DT   18-MAR-2008, integrated into UniProtKB/Swiss-Prot.
DT   01-MAR-2003, sequence version 1.
DT   28-JUN-2023, entry version 183.
DE   RecName: Full=Trifunctional enzyme subunit alpha, mitochondrial;
DE   AltName: Full=Monolysocardiolipin acyltransferase {ECO:0000250|UniProtKB:P40939};
DE            EC=2.3.1.- {ECO:0000250|UniProtKB:P40939};
DE   AltName: Full=TP-alpha;
DE   Includes:
DE     RecName: Full=Long-chain enoyl-CoA hydratase;
DE              EC=4.2.1.17 {ECO:0000250|UniProtKB:P40939};
DE   Includes:
DE     RecName: Full=Long chain 3-hydroxyacyl-CoA dehydrogenase;
DE              EC=1.1.1.211 {ECO:0000250|UniProtKB:P40939};
DE   Flags: Precursor;
GN   Name=Hadha;
OS   Mus musculus (Mouse).
OC   Eukaryota; Metazoa; Chordata; Craniata; Vertebrata; Euteleostomi; Mammalia;
OC   Eutheria; Euarchontoglires; Glires; Rodentia; Myomorpha; Muroidea; Muridae;
OC   Murinae; Mus; Mus.
OX   NCBI_TaxID=10090;
RN   [1]
RP   NUCLEOTIDE SEQUENCE [LARGE SCALE MRNA].
RC   STRAIN=C57BL/6J, and NOD; TISSUE=Skin;
RX   PubMed=16141072; DOI=10.1126/science.1112014;
RA   Carninci P., Kasukawa T., Katayama S., Gough J., Frith M.C., Maeda N.,
RA   Oyama R., Ravasi T., Lenhard B., Wells C., Kodzius R., Shimokawa K.,
RA   Bajic V.B., Brenner S.E., Batalov S., Forrest A.R., Zavolan M., Davis M.J.,
RA   Wilming L.G., Aidinis V., Allen J.E., Ambesi-Impiombato A., Apweiler R.,
RA   Aturaliya R.N., Bailey T.L., Bansal M., Baxter L., Beisel K.W., Bersano T.,
RA   Bono H., Chalk A.M., Chiu K.P., Choudhary V., Christoffels A.,
RA   Clutterbuck D.R., Crowe M.L., Dalla E., Dalrymple B.P., de Bono B.,
RA   Della Gatta G., di Bernardo D., Down T., Engstrom P., Fagiolini M.,
RA   Faulkner G., Fletcher C.F., Fukushima T., Furuno M., Futaki S.,
RA   Gariboldi M., Georgii-Hemming P., Gingeras T.R., Gojobori T., Green R.E.,
RA   Gustincich S., Harbers M., Hayashi Y., Hensch T.K., Hirokawa N., Hill D.,
RA   Huminiecki L., Iacono M., Ikeo K., Iwama A., Ishikawa T., Jakt M.,
RA   Kanapin A., Katoh M., Kawasawa Y., Kelso J., Kitamura H., Kitano H.,
RA   Kollias G., Krishnan S.P., Kruger A., Kummerfeld S.K., Kurochkin I.V.,
RA   Lareau L.F., Lazarevic D., Lipovich L., Liu J., Liuni S., McWilliam S.,
RA   Madan Babu M., Madera M., Marchionni L., Matsuda H., Matsuzawa S., Miki H.,
RA   Mignone F., Miyake S., Morris K., Mottagui-Tabar S., Mulder N., Nakano N.,
RA   Nakauchi H., Ng P., Nilsson R., Nishiguchi S., Nishikawa S., Nori F.,
RA   Ohara O., Okazaki Y., Orlando V., Pang K.C., Pavan W.J., Pavesi G.,
RA   Pesole G., Petrovsky N., Piazza S., Reed J., Reid J.F., Ring B.Z.,
RA   Ringwald M., Rost B., Ruan Y., Salzberg S.L., Sandelin A., Schneider C.,
RA   Schoenbach C., Sekiguchi K., Semple C.A., Seno S., Sessa L., Sheng Y.,
RA   Shibata Y., Shimada H., Shimada K., Silva D., Sinclair B., Sperling S.,
RA   Stupka E., Sugiura K., Sultana R., Takenaka Y., Taki K., Tammoja K.,
RA   Tan S.L., Tang S., Taylor M.S., Tegner J., Teichmann S.A., Ueda H.R.,
RA   van Nimwegen E., Verardo R., Wei C.L., Yagi K., Yamanishi H.,
RA   Zabarovsky E., Zhu S., Zimmer A., Hide W., Bult C., Grimmond S.M.,
RA   Teasdale R.D., Liu E.T., Brusic V., Quackenbush J., Wahlestedt C.,
RA   Mattick J.S., Hume D.A., Kai C., Sasaki D., Tomaru Y., Fukuda S.,
RA   Kanamori-Katayama M., Suzuki M., Aoki J., Arakawa T., Iida J., Imamura K.,
RA   Itoh M., Kato T., Kawaji H., Kawagashira N., Kawashima T., Kojima M.,
RA   Kondo S., Konno H., Nakano K., Ninomiya N., Nishio T., Okada M., Plessy C.,
RA   Shibata K., Shiraki T., Suzuki S., Tagami M., Waki K., Watahiki A.,
RA   Okamura-Oho Y., Suzuki H., Kawai J., Hayashizaki Y.;
RT   "The transcriptional landscape of the mammalian genome.";
RL   Science 309:1559-1563(2005).
RN   [2]
RP   NUCLEOTIDE SEQUENCE [LARGE SCALE MRNA].
RC   STRAIN=FVB/N; TISSUE=Eye, Liver, and Olfactory epithelium;
RX   PubMed=15489334; DOI=10.1101/gr.2596504;
RG   The MGC Project Team;
RT   "The status, quality, and expansion of the NIH full-length cDNA project:
RT   the Mammalian Gene Collection (MGC).";
RL   Genome Res. 14:2121-2127(2004).
RN   [3]
RP   ACETYLATION [LARGE SCALE ANALYSIS] AT LYS-129, AND IDENTIFICATION BY MASS
RP   SPECTROMETRY [LARGE SCALE ANALYSIS].
RC   TISSUE=Liver;
RX   PubMed=16916647; DOI=10.1016/j.molcel.2006.06.026;
RA   Kim S.C., Sprung R., Chen Y., Xu Y., Ball H., Pei J., Cheng T., Kho Y.,
RA   Xiao H., Xiao L., Grishin N.V., White M., Yang X.-J., Zhao Y.;
RT   "Substrate and functional diversity of lysine acetylation revealed by a
RT   proteomics survey.";
RL   Mol. Cell 23:607-618(2006).
RN   [4]
RP   PHOSPHORYLATION [LARGE SCALE ANALYSIS] AT SER-231; SER-316; THR-395 AND
RP   SER-647, AND IDENTIFICATION BY MASS SPECTROMETRY [LARGE SCALE ANALYSIS].
RC   TISSUE=Brain, Brown adipose tissue, Heart, Kidney, Liver, Lung,
RC   Pancreas, Spleen, and Testis;
RX   PubMed=21183079; DOI=10.1016/j.cell.2010.12.001;
RA   Huttlin E.L., Jedrychowski M.P., Elias J.E., Goswami T., Rad R.,
RA   Beausoleil S.A., Villen J., Haas W., Sowa M.E., Gygi S.P.;
RT   "A tissue-specific atlas of mouse protein phosphorylation and expression.";
RL   Cell 143:1174-1189(2010).
RN   [5]
RP   ACETYLATION [LARGE SCALE ANALYSIS] AT LYS-60 AND LYS-406, SUCCINYLATION
RP   [LARGE SCALE ANALYSIS] AT LYS-46; LYS-60; LYS-166; LYS-213; LYS-214;
RP   LYS-230; LYS-249; LYS-303; LYS-326; LYS-334; LYS-350; LYS-406; LYS-411;
RP   LYS-415; LYS-436; LYS-440; LYS-460; LYS-505; LYS-519; LYS-569; LYS-620;
RP   LYS-634; LYS-644; LYS-646; LYS-664; LYS-728 AND LYS-759, AND IDENTIFICATION
RP   BY MASS SPECTROMETRY [LARGE SCALE ANALYSIS].
RC   TISSUE=Embryonic fibroblast, and Liver;
RX   PubMed=23806337; DOI=10.1016/j.molcel.2013.06.001;
RA   Park J., Chen Y., Tishkoff D.X., Peng C., Tan M., Dai L., Xie Z., Zhang Y.,
RA   Zwaans B.M., Skinner M.E., Lombard D.B., Zhao Y.;
RT   "SIRT5-mediated lysine desuccinylation impacts diverse metabolic
RT   pathways.";
RL   Mol. Cell 50:919-930(2013).
RN   [6]
RP   ACETYLATION [LARGE SCALE ANALYSIS] AT LYS-46; LYS-60; LYS-129; LYS-166;
RP   LYS-214; LYS-249; LYS-289; LYS-303; LYS-326; LYS-334; LYS-350; LYS-353;
RP   LYS-406; LYS-411; LYS-436; LYS-460; LYS-505; LYS-519; LYS-540; LYS-569;
RP   LYS-644; LYS-664; LYS-728; LYS-735 AND LYS-759, AND IDENTIFICATION BY MASS
RP   SPECTROMETRY [LARGE SCALE ANALYSIS].
RC   TISSUE=Liver;
RX   PubMed=23576753; DOI=10.1073/pnas.1302961110;
RA   Rardin M.J., Newman J.C., Held J.M., Cusack M.P., Sorensen D.J., Li B.,
RA   Schilling B., Mooney S.D., Kahn C.R., Verdin E., Gibson B.W.;
RT   "Label-free quantitative proteomics of the lysine acetylome in mitochondria
RT   identifies substrates of SIRT3 in metabolic pathways.";
RL   Proc. Natl. Acad. Sci. U.S.A. 110:6601-6606(2013).
RN   [7]
RP   METHYLATION [LARGE SCALE ANALYSIS] AT ARG-399, AND IDENTIFICATION BY MASS
RP   SPECTROMETRY [LARGE SCALE ANALYSIS].
RC   TISSUE=Brain;
RX   PubMed=24129315; DOI=10.1074/mcp.o113.027870;
RA   Guo A., Gu H., Zhou J., Mulhern D., Wang Y., Lee K.A., Yang V., Aguiar M.,
RA   Kornhauser J., Jia X., Ren J., Beausoleil S.A., Silva J.C., Vemulapalli V.,
RA   Bedford M.T., Comb M.J.;
RT   "Immunoaffinity enrichment and mass spectrometry analysis of protein
RT   methylation.";
RL   Mol. Cell. Proteomics 13:372-387(2014).
RN   [8]
RP   INTERACTION WITH MTLN, AND IDENTIFICATION BY MASS SPECTROMETRY.
RX   PubMed=29949755; DOI=10.1016/j.celrep.2018.05.058;
RA   Makarewich C.A., Baskin K.K., Munir A.Z., Bezprozvannaya S., Sharma G.,
RA   Khemtong C., Shah A.M., McAnally J.R., Malloy C.R., Szweda L.I.,
RA   Bassel-Duby R., Olson E.N.;
RT   "MOXI Is a Mitochondrial Micropeptide That Enhances Fatty Acid beta-
RT   Oxidation.";
RL   Cell Rep. 23:3701-3709(2018).
CC   -!- FUNCTION: Mitochondrial trifunctional enzyme catalyzes the last three
CC       of the four reactions of the mitochondrial beta-oxidation pathway. The
CC       mitochondrial beta-oxidation pathway is the major energy-producing
CC       process in tissues and is performed through four consecutive reactions
CC       breaking down fatty acids into acetyl-CoA. Among the enzymes involved
CC       in this pathway, the trifunctional enzyme exhibits specificity for
CC       long-chain fatty acids. Mitochondrial trifunctional enzyme is a
CC       heterotetrameric complex composed of two proteins, the trifunctional
CC       enzyme subunit alpha/HADHA described here carries the 2,3-enoyl-CoA
CC       hydratase and the 3-hydroxyacyl-CoA dehydrogenase activities while the
CC       trifunctional enzyme subunit beta/HADHB bears the 3-ketoacyl-CoA
CC       thiolase activity. Independently of the subunit beta, the trifunctional
CC       enzyme subunit alpha/HADHA also has a monolysocardiolipin
CC       acyltransferase activity. It acylates monolysocardiolipin into
CC       cardiolipin, a major mitochondrial membrane phospholipid which plays a
CC       key role in apoptosis and supports mitochondrial respiratory chain
CC       complexes in the generation of ATP. Allows the acylation of
CC       monolysocardiolipin with different acyl-CoA substrates including
CC       oleoyl-CoA for which it displays the highest activity.
CC       {ECO:0000250|UniProtKB:P40939}.
CC   -!- CATALYTIC ACTIVITY:
CC       Reaction=a (3S)-3-hydroxyacyl-CoA = a (2E)-enoyl-CoA + H2O;
CC         Xref=Rhea:RHEA:16105, ChEBI:CHEBI:15377, ChEBI:CHEBI:57318,
CC         ChEBI:CHEBI:58856; EC=4.2.1.17;
CC         Evidence={ECO:0000250|UniProtKB:P40939};
CC       PhysiologicalDirection=right-to-left; Xref=Rhea:RHEA:16107;
CC         Evidence={ECO:0000250|UniProtKB:P40939};
CC   -!- CATALYTIC ACTIVITY:
CC       Reaction=a 4-saturated-(3S)-3-hydroxyacyl-CoA = a (3E)-enoyl-CoA + H2O;
CC         Xref=Rhea:RHEA:20724, ChEBI:CHEBI:15377, ChEBI:CHEBI:58521,
CC         ChEBI:CHEBI:137480; EC=4.2.1.17;
CC         Evidence={ECO:0000250|UniProtKB:P40939};
CC       PhysiologicalDirection=right-to-left; Xref=Rhea:RHEA:20726;
CC         Evidence={ECO:0000250|UniProtKB:P40939};
CC   -!- CATALYTIC ACTIVITY:
CC       Reaction=(3S)-hydroxyoctanoyl-CoA = (2E)-octenoyl-CoA + H2O;
CC         Xref=Rhea:RHEA:31199, ChEBI:CHEBI:15377, ChEBI:CHEBI:62242,
CC         ChEBI:CHEBI:62617; Evidence={ECO:0000250|UniProtKB:P40939};
CC       PhysiologicalDirection=right-to-left; Xref=Rhea:RHEA:31201;
CC         Evidence={ECO:0000250|UniProtKB:P40939};
CC   -!- CATALYTIC ACTIVITY:
CC       Reaction=(3S)-3-hydroxydodecanoyl-CoA = (2E)-dodecenoyl-CoA + H2O;
CC         Xref=Rhea:RHEA:31075, ChEBI:CHEBI:15377, ChEBI:CHEBI:57330,
CC         ChEBI:CHEBI:62558; Evidence={ECO:0000250|UniProtKB:P40939};
CC       PhysiologicalDirection=right-to-left; Xref=Rhea:RHEA:31077;
CC         Evidence={ECO:0000250|UniProtKB:P40939};
CC   -!- CATALYTIC ACTIVITY:
CC       Reaction=(3S)-hydroxyhexadecanoyl-CoA = (2E)-hexadecenoyl-CoA + H2O;
CC         Xref=Rhea:RHEA:31163, ChEBI:CHEBI:15377, ChEBI:CHEBI:61526,
CC         ChEBI:CHEBI:62613; Evidence={ECO:0000250|UniProtKB:P40939};
CC       PhysiologicalDirection=right-to-left; Xref=Rhea:RHEA:31165;
CC         Evidence={ECO:0000250|UniProtKB:P40939};
CC   -!- CATALYTIC ACTIVITY:
CC       Reaction=a long-chain (3S)-3-hydroxy fatty acyl-CoA + NAD(+) = a long-
CC         chain 3-oxo-fatty acyl-CoA + H(+) + NADH; Xref=Rhea:RHEA:52656,
CC         ChEBI:CHEBI:15378, ChEBI:CHEBI:57540, ChEBI:CHEBI:57945,
CC         ChEBI:CHEBI:136757, ChEBI:CHEBI:136758; EC=1.1.1.211;
CC         Evidence={ECO:0000250|UniProtKB:P40939};
CC       PhysiologicalDirection=left-to-right; Xref=Rhea:RHEA:52657;
CC         Evidence={ECO:0000250|UniProtKB:P40939};
CC   -!- CATALYTIC ACTIVITY:
CC       Reaction=(3S)-hydroxyoctanoyl-CoA + NAD(+) = 3-oxooctanoyl-CoA + H(+) +
CC         NADH; Xref=Rhea:RHEA:31195, ChEBI:CHEBI:15378, ChEBI:CHEBI:57540,
CC         ChEBI:CHEBI:57945, ChEBI:CHEBI:62617, ChEBI:CHEBI:62619;
CC         Evidence={ECO:0000250|UniProtKB:P40939};
CC       PhysiologicalDirection=left-to-right; Xref=Rhea:RHEA:31196;
CC         Evidence={ECO:0000250|UniProtKB:P40939};
CC   -!- CATALYTIC ACTIVITY:
CC       Reaction=(3S)-hydroxydecanoyl-CoA + NAD(+) = 3-oxodecanoyl-CoA + H(+) +
CC         NADH; Xref=Rhea:RHEA:31187, ChEBI:CHEBI:15378, ChEBI:CHEBI:57540,
CC         ChEBI:CHEBI:57945, ChEBI:CHEBI:62548, ChEBI:CHEBI:62616;
CC         Evidence={ECO:0000250|UniProtKB:P40939};
CC       PhysiologicalDirection=left-to-right; Xref=Rhea:RHEA:31188;
CC         Evidence={ECO:0000250|UniProtKB:P40939};
CC   -!- CATALYTIC ACTIVITY:
CC       Reaction=(3S)-3-hydroxydodecanoyl-CoA + NAD(+) = 3-oxododecanoyl-CoA +
CC         H(+) + NADH; Xref=Rhea:RHEA:31179, ChEBI:CHEBI:15378,
CC         ChEBI:CHEBI:57540, ChEBI:CHEBI:57945, ChEBI:CHEBI:62558,
CC         ChEBI:CHEBI:62615; Evidence={ECO:0000250|UniProtKB:P40939};
CC       PhysiologicalDirection=left-to-right; Xref=Rhea:RHEA:31180;
CC         Evidence={ECO:0000250|UniProtKB:P40939};
CC   -!- CATALYTIC ACTIVITY:
CC       Reaction=(3S)-hydroxytetradecanoyl-CoA + NAD(+) = 3-oxotetradecanoyl-
CC         CoA + H(+) + NADH; Xref=Rhea:RHEA:31167, ChEBI:CHEBI:15378,
CC         ChEBI:CHEBI:57540, ChEBI:CHEBI:57945, ChEBI:CHEBI:62543,
CC         ChEBI:CHEBI:62614; Evidence={ECO:0000250|UniProtKB:P40939};
CC       PhysiologicalDirection=left-to-right; Xref=Rhea:RHEA:31168;
CC         Evidence={ECO:0000250|UniProtKB:P40939};
CC   -!- CATALYTIC ACTIVITY:
CC       Reaction=(3S)-hydroxyhexadecanoyl-CoA + NAD(+) = 3-oxohexadecanoyl-CoA
CC         + H(+) + NADH; Xref=Rhea:RHEA:31159, ChEBI:CHEBI:15378,
CC         ChEBI:CHEBI:57349, ChEBI:CHEBI:57540, ChEBI:CHEBI:57945,
CC         ChEBI:CHEBI:62613; Evidence={ECO:0000250|UniProtKB:P40939};
CC       PhysiologicalDirection=left-to-right; Xref=Rhea:RHEA:31160;
CC         Evidence={ECO:0000250|UniProtKB:P40939};
CC   -!- CATALYTIC ACTIVITY:
CC       Reaction=1'-[1,2-di-(9Z,12Z-octadecadienoyl)-sn-glycero-3-phospho]-3'-
CC         [1-(9Z,12Z-octadecadienoyl)-sn-glycero-3-phospho]-glycerol +
CC         hexadecanoyl-CoA = 1'-[1,2-di-(9Z,12Z-octadecadienoyl)-sn-glycero-3-
CC         phospho]-3'-[1-(9Z,12Z-octadecadienoyl)-2-hexadecanoyl-sn-glycero-3-
CC         phospho]-glycerol + CoA; Xref=Rhea:RHEA:43680, ChEBI:CHEBI:57287,
CC         ChEBI:CHEBI:57379, ChEBI:CHEBI:83580, ChEBI:CHEBI:83583;
CC         Evidence={ECO:0000250|UniProtKB:P40939};
CC       PhysiologicalDirection=left-to-right; Xref=Rhea:RHEA:43681;
CC         Evidence={ECO:0000250|UniProtKB:P40939};
CC   -!- CATALYTIC ACTIVITY:
CC       Reaction=(9Z)-octadecenoyl-CoA + 1'-[1,2-di-(9Z,12Z-octadecadienoyl)-
CC         sn-glycero-3-phospho]-3'-[1-(9Z,12Z-octadecadienoyl)-sn-glycero-3-
CC         phospho]-glycerol = 1'-[1,2-di-(9Z,12Z-octadecadienoyl)-sn-glycero-3-
CC         phospho]-3'-[1-(9Z,12Z-octadecadienoyl)-2-(9Z-octadecenoyl)-sn-
CC         glycero-3-phospho]-glycerol + CoA; Xref=Rhea:RHEA:43676,
CC         ChEBI:CHEBI:57287, ChEBI:CHEBI:57387, ChEBI:CHEBI:83580,
CC         ChEBI:CHEBI:83582; Evidence={ECO:0000250|UniProtKB:P40939};
CC       PhysiologicalDirection=left-to-right; Xref=Rhea:RHEA:43677;
CC         Evidence={ECO:0000250|UniProtKB:P40939};
CC   -!- CATALYTIC ACTIVITY:
CC       Reaction=(9Z,12Z)-octadecadienoyl-CoA + 1'-[1,2-di-(9Z,12Z-
CC         octadecadienoyl)-sn-glycero-3-phospho]-3'-[1-(9Z,12Z-
CC         octadecadienoyl)-sn-glycero-3-phospho]-glycerol = 1',3'-bis-[1,2-di-
CC         (9Z,12Z-octadecadienoyl)-sn-glycero-3-phospho]-glycerol + CoA;
CC         Xref=Rhea:RHEA:43672, ChEBI:CHEBI:57287, ChEBI:CHEBI:57383,
CC         ChEBI:CHEBI:83580, ChEBI:CHEBI:83581;
CC         Evidence={ECO:0000250|UniProtKB:P40939};
CC       PhysiologicalDirection=left-to-right; Xref=Rhea:RHEA:43673;
CC         Evidence={ECO:0000250|UniProtKB:P40939};
CC   -!- PATHWAY: Lipid metabolism; fatty acid beta-oxidation.
CC       {ECO:0000250|UniProtKB:P40939}.
CC   -!- SUBUNIT: Heterotetramer of 2 alpha/HADHA and 2 beta/HADHB subunits;
CC       forms the mitochondrial trifunctional enzyme (By similarity). Also
CC       purified as higher order heterooligomers including a 4 alpha/HADHA and
CC       4 beta/HADHB heterooligomer which physiological significance remains
CC       unclear (By similarity). The mitochondrial trifunctional enzyme
CC       interacts with MTLN (PubMed:29949755). {ECO:0000250|UniProtKB:P40939,
CC       ECO:0000269|PubMed:29949755}.
CC   -!- SUBCELLULAR LOCATION: Mitochondrion {ECO:0000250|UniProtKB:P40939}.
CC       Mitochondrion inner membrane {ECO:0000250|UniProtKB:P40939}.
CC       Note=Protein stability and association with mitochondrion inner
CC       membrane do not require HADHB. {ECO:0000250|UniProtKB:P40939}.
CC   -!- PTM: Acetylation of Lys-569 and Lys-728 is observed in liver
CC       mitochondria from fasted mice but not from fed mice.
CC   -!- SIMILARITY: In the N-terminal section; belongs to the enoyl-CoA
CC       hydratase/isomerase family. {ECO:0000305}.
CC   -!- SIMILARITY: In the central section; belongs to the 3-hydroxyacyl-CoA
CC       dehydrogenase family. {ECO:0000305}.
CC   ---------------------------------------------------------------------------
CC   Copyrighted by the UniProt Consortium, see https://www.uniprot.org/terms
CC   Distributed under the Creative Commons Attribution (CC BY 4.0) License
CC   ---------------------------------------------------------------------------
DR   EMBL; AK029017; BAC26245.1; -; mRNA.
DR   EMBL; AK170478; BAE41822.1; -; mRNA.
DR   EMBL; AK170683; BAE41956.1; -; mRNA.
DR   EMBL; BC027156; AAH27156.1; -; mRNA.
DR   EMBL; BC037009; AAH37009.1; -; mRNA.
DR   EMBL; BC046978; AAH46978.1; -; mRNA.
DR   EMBL; BC058569; AAH58569.1; -; mRNA.
DR   CCDS; CCDS19155.1; -.
DR   RefSeq; NP_849209.1; NM_178878.2.
DR   AlphaFoldDB; Q8BMS1; -.
DR   SMR; Q8BMS1; -.
DR   BioGRID; 220648; 43.
DR   IntAct; Q8BMS1; 8.
DR   MINT; Q8BMS1; -.
DR   STRING; 10090.ENSMUSP00000120976; -.
DR   GlyGen; Q8BMS1; 1 site, 1 O-linked glycan (1 site).
DR   iPTMnet; Q8BMS1; -.
DR   PhosphoSitePlus; Q8BMS1; -.
DR   SwissPalm; Q8BMS1; -.
DR   REPRODUCTION-2DPAGE; IPI00223092; -.
DR   EPD; Q8BMS1; -.
DR   jPOST; Q8BMS1; -.
DR   MaxQB; Q8BMS1; -.
DR   PaxDb; Q8BMS1; -.
DR   PeptideAtlas; Q8BMS1; -.
DR   ProteomicsDB; 277753; -.
DR   Antibodypedia; 3074; 238 antibodies from 32 providers.
DR   DNASU; 97212; -.
DR   Ensembl; ENSMUST00000156859; ENSMUSP00000120976; ENSMUSG00000025745.
DR   GeneID; 97212; -.
DR   KEGG; mmu:97212; -.
DR   UCSC; uc008wvc.1; mouse.
DR   AGR; MGI:2135593; -.
DR   CTD; 3030; -.
DR   MGI; MGI:2135593; Hadha.
DR   VEuPathDB; HostDB:ENSMUSG00000025745; -.
DR   eggNOG; KOG1683; Eukaryota.
DR   GeneTree; ENSGT00940000154677; -.
DR   HOGENOM; CLU_009834_16_1_1; -.
DR   InParanoid; Q8BMS1; -.
DR   OMA; PFRYMDT; -.
DR   OrthoDB; 622692at2759; -.
DR   PhylomeDB; Q8BMS1; -.
DR   TreeFam; TF352288; -.
DR   Reactome; R-MMU-1482798; Acyl chain remodeling of CL.
DR   Reactome; R-MMU-77285; Beta oxidation of myristoyl-CoA to lauroyl-CoA.
DR   Reactome; R-MMU-77305; Beta oxidation of palmitoyl-CoA to myristoyl-CoA.
DR   Reactome; R-MMU-77310; Beta oxidation of lauroyl-CoA to decanoyl-CoA-CoA.
DR   Reactome; R-MMU-77346; Beta oxidation of decanoyl-CoA to octanoyl-CoA-CoA.
DR   Reactome; R-MMU-77348; Beta oxidation of octanoyl-CoA to hexanoyl-CoA.
DR   Reactome; R-MMU-77350; Beta oxidation of hexanoyl-CoA to butanoyl-CoA.
DR   UniPathway; UPA00659; -.
DR   BioGRID-ORCS; 97212; 4 hits in 79 CRISPR screens.
DR   ChiTaRS; Hadha; mouse.
DR   PRO; PR:Q8BMS1; -.
DR   Proteomes; UP000000589; Chromosome 5.
DR   RNAct; Q8BMS1; protein.
DR   Bgee; ENSMUSG00000025745; Expressed in myocardium of ventricle and 247 other tissues.
DR   Genevisible; Q8BMS1; MM.
DR   GO; GO:0016507; C:mitochondrial fatty acid beta-oxidation multienzyme complex; ISO:MGI.
DR   GO; GO:0005743; C:mitochondrial inner membrane; HDA:MGI.
DR   GO; GO:0042645; C:mitochondrial nucleoid; ISO:MGI.
DR   GO; GO:0005739; C:mitochondrion; HDA:MGI.
DR   GO; GO:0003857; F:3-hydroxyacyl-CoA dehydrogenase activity; ISO:MGI.
DR   GO; GO:0003988; F:acetyl-CoA C-acyltransferase activity; ISO:MGI.
DR   GO; GO:0004300; F:enoyl-CoA hydratase activity; ISO:MGI.
DR   GO; GO:0000062; F:fatty-acyl-CoA binding; ISO:MGI.
DR   GO; GO:0016509; F:long-chain-3-hydroxyacyl-CoA dehydrogenase activity; IDA:MGI.
DR   GO; GO:0051287; F:NAD binding; ISO:MGI.
DR   GO; GO:0070403; F:NAD+ binding; IEA:InterPro.
DR   GO; GO:0044877; F:protein-containing complex binding; ISO:MGI.
DR   GO; GO:0035965; P:cardiolipin acyl-chain remodeling; ISS:UniProtKB.
DR   GO; GO:0006635; P:fatty acid beta-oxidation; IMP:MGI.
DR   GO; GO:0032868; P:response to insulin; IMP:MGI.
DR   GO; GO:0009410; P:response to xenobiotic stimulus; ISO:MGI.
DR   CDD; cd06558; crotonase-like; 1.
DR   Gene3D; 1.10.1040.50; -; 1.
DR   Gene3D; 3.40.50.720; NAD(P)-binding Rossmann-like Domain; 1.
DR   InterPro; IPR006180; 3-OHacyl-CoA_DH_CS.
DR   InterPro; IPR006176; 3-OHacyl-CoA_DH_NAD-bd.
DR   InterPro; IPR006108; 3HC_DH_C.
DR   InterPro; IPR008927; 6-PGluconate_DH-like_C_sf.
DR   InterPro; IPR029045; ClpP/crotonase-like_dom_sf.
DR   InterPro; IPR018376; Enoyl-CoA_hyd/isom_CS.
DR   InterPro; IPR001753; Enoyl-CoA_hydra/iso.
DR   InterPro; IPR012803; Fa_ox_alpha_mit.
DR   InterPro; IPR036291; NAD(P)-bd_dom_sf.
DR   PANTHER; PTHR43612; TRIFUNCTIONAL ENZYME SUBUNIT ALPHA; 1.
DR   PANTHER; PTHR43612:SF3; TRIFUNCTIONAL ENZYME SUBUNIT ALPHA, MITOCHONDRIAL; 1.
DR   Pfam; PF00725; 3HCDH; 2.
DR   Pfam; PF02737; 3HCDH_N; 1.
DR   Pfam; PF00378; ECH_1; 1.
DR   SUPFAM; SSF48179; 6-phosphogluconate dehydrogenase C-terminal domain-like; 2.
DR   SUPFAM; SSF52096; ClpP/crotonase; 1.
DR   SUPFAM; SSF51735; NAD(P)-binding Rossmann-fold domains; 1.
DR   PROSITE; PS00067; 3HCDH; 1.
DR   PROSITE; PS00166; ENOYL_COA_HYDRATASE; 1.
DR   TIGRFAMs; TIGR02441; fa_ox_alpha_mit; 1.
PE   1: Evidence at protein level;
KW   Acetylation; Fatty acid metabolism; Lipid metabolism; Lyase; Membrane;
KW   Methylation; Mitochondrion; Mitochondrion inner membrane;
KW   Multifunctional enzyme; NAD; Oxidoreductase; Phosphoprotein;
KW   Reference proteome; Transferase; Transit peptide.
FT   TRANSIT         1..36
FT                   /note="Mitochondrion"
FT                   /evidence="ECO:0000255"
FT   CHAIN           37..763
FT                   /note="Trifunctional enzyme subunit alpha, mitochondrial"
FT                   /id="PRO_0000322639"
FT   ACT_SITE        510
FT                   /note="For hydroxyacyl-coenzyme A dehydrogenase activity"
FT                   /evidence="ECO:0000250|UniProtKB:P40939"
FT   SITE            151
FT                   /note="Important for long-chain enoyl-CoA hydratase
FT                   activity"
FT                   /evidence="ECO:0000250|UniProtKB:P40939"
FT   SITE            173
FT                   /note="Important for long-chain enoyl-CoA hydratase
FT                   activity"
FT                   /evidence="ECO:0000250|UniProtKB:P40939"
FT   SITE            498
FT                   /note="Important for hydroxyacyl-coenzyme A dehydrogenase
FT                   activity"
FT                   /evidence="ECO:0000250|UniProtKB:P40939"
FT   MOD_RES         46
FT                   /note="N6-acetyllysine; alternate"
FT                   /evidence="ECO:0007744|PubMed:23576753"
FT   MOD_RES         46
FT                   /note="N6-succinyllysine; alternate"
FT                   /evidence="ECO:0007744|PubMed:23806337"
FT   MOD_RES         60
FT                   /note="N6-acetyllysine; alternate"
FT                   /evidence="ECO:0007744|PubMed:23576753,
FT                   ECO:0007744|PubMed:23806337"
FT   MOD_RES         60
FT                   /note="N6-succinyllysine; alternate"
FT                   /evidence="ECO:0007744|PubMed:23806337"
FT   MOD_RES         129
FT                   /note="N6-acetyllysine"
FT                   /evidence="ECO:0007744|PubMed:16916647,
FT                   ECO:0007744|PubMed:23576753"
FT   MOD_RES         166
FT                   /note="N6-acetyllysine; alternate"
FT                   /evidence="ECO:0007744|PubMed:23576753"
FT   MOD_RES         166
FT                   /note="N6-succinyllysine; alternate"
FT                   /evidence="ECO:0007744|PubMed:23806337"
FT   MOD_RES         213
FT                   /note="N6-succinyllysine"
FT                   /evidence="ECO:0007744|PubMed:23806337"
FT   MOD_RES         214
FT                   /note="N6-acetyllysine; alternate"
FT                   /evidence="ECO:0007744|PubMed:23576753"
FT   MOD_RES         214
FT                   /note="N6-succinyllysine; alternate"
FT                   /evidence="ECO:0007744|PubMed:23806337"
FT   MOD_RES         230
FT                   /note="N6-succinyllysine"
FT                   /evidence="ECO:0007744|PubMed:23806337"
FT   MOD_RES         231
FT                   /note="Phosphoserine"
FT                   /evidence="ECO:0007744|PubMed:21183079"
FT   MOD_RES         249
FT                   /note="N6-acetyllysine; alternate"
FT                   /evidence="ECO:0007744|PubMed:23576753"
FT   MOD_RES         249
FT                   /note="N6-succinyllysine; alternate"
FT                   /evidence="ECO:0007744|PubMed:23806337"
FT   MOD_RES         289
FT                   /note="N6-acetyllysine"
FT                   /evidence="ECO:0007744|PubMed:23576753"
FT   MOD_RES         295
FT                   /note="N6-acetyllysine"
FT                   /evidence="ECO:0000250|UniProtKB:P40939"
FT   MOD_RES         303
FT                   /note="N6-acetyllysine; alternate"
FT                   /evidence="ECO:0007744|PubMed:23576753"
FT   MOD_RES         303
FT                   /note="N6-succinyllysine; alternate"
FT                   /evidence="ECO:0007744|PubMed:23806337"
FT   MOD_RES         316
FT                   /note="Phosphoserine"
FT                   /evidence="ECO:0007744|PubMed:21183079"
FT   MOD_RES         326
FT                   /note="N6-acetyllysine; alternate"
FT                   /evidence="ECO:0007744|PubMed:23576753"
FT   MOD_RES         326
FT                   /note="N6-succinyllysine; alternate"
FT                   /evidence="ECO:0007744|PubMed:23806337"
FT   MOD_RES         334
FT                   /note="N6-acetyllysine; alternate"
FT                   /evidence="ECO:0007744|PubMed:23576753"
FT   MOD_RES         334
FT                   /note="N6-succinyllysine; alternate"
FT                   /evidence="ECO:0007744|PubMed:23806337"
FT   MOD_RES         350
FT                   /note="N6-acetyllysine; alternate"
FT                   /evidence="ECO:0007744|PubMed:23576753"
FT   MOD_RES         350
FT                   /note="N6-succinyllysine; alternate"
FT                   /evidence="ECO:0007744|PubMed:23806337"
FT   MOD_RES         353
FT                   /note="N6-acetyllysine"
FT                   /evidence="ECO:0007744|PubMed:23576753"
FT   MOD_RES         395
FT                   /note="Phosphothreonine"
FT                   /evidence="ECO:0007744|PubMed:21183079"
FT   MOD_RES         399
FT                   /note="Omega-N-methylarginine"
FT                   /evidence="ECO:0007744|PubMed:24129315"
FT   MOD_RES         406
FT                   /note="N6-acetyllysine; alternate"
FT                   /evidence="ECO:0007744|PubMed:23576753,
FT                   ECO:0007744|PubMed:23806337"
FT   MOD_RES         406
FT                   /note="N6-succinyllysine; alternate"
FT                   /evidence="ECO:0007744|PubMed:23806337"
FT   MOD_RES         411
FT                   /note="N6-acetyllysine; alternate"
FT                   /evidence="ECO:0007744|PubMed:23576753"
FT   MOD_RES         411
FT                   /note="N6-succinyllysine; alternate"
FT                   /evidence="ECO:0007744|PubMed:23806337"
FT   MOD_RES         415
FT                   /note="N6-succinyllysine"
FT                   /evidence="ECO:0007744|PubMed:23806337"
FT   MOD_RES         419
FT                   /note="Phosphoserine"
FT                   /evidence="ECO:0000250|UniProtKB:Q64428"
FT   MOD_RES         436
FT                   /note="N6-acetyllysine; alternate"
FT                   /evidence="ECO:0007744|PubMed:23576753"
FT   MOD_RES         436
FT                   /note="N6-succinyllysine; alternate"
FT                   /evidence="ECO:0007744|PubMed:23806337"
FT   MOD_RES         440
FT                   /note="N6-succinyllysine"
FT                   /evidence="ECO:0007744|PubMed:23806337"
FT   MOD_RES         460
FT                   /note="N6-acetyllysine; alternate"
FT                   /evidence="ECO:0007744|PubMed:23576753"
FT   MOD_RES         460
FT                   /note="N6-succinyllysine; alternate"
FT                   /evidence="ECO:0007744|PubMed:23806337"
FT   MOD_RES         505
FT                   /note="N6-acetyllysine; alternate"
FT                   /evidence="ECO:0007744|PubMed:23576753"
FT   MOD_RES         505
FT                   /note="N6-succinyllysine; alternate"
FT                   /evidence="ECO:0007744|PubMed:23806337"
FT   MOD_RES         519
FT                   /note="N6-acetyllysine; alternate"
FT                   /evidence="ECO:0007744|PubMed:23576753"
FT   MOD_RES         519
FT                   /note="N6-succinyllysine; alternate"
FT                   /evidence="ECO:0007744|PubMed:23806337"
FT   MOD_RES         540
FT                   /note="N6-acetyllysine"
FT                   /evidence="ECO:0007744|PubMed:23576753"
FT   MOD_RES         569
FT                   /note="N6-acetyllysine; alternate"
FT                   /evidence="ECO:0007744|PubMed:23576753"
FT   MOD_RES         569
FT                   /note="N6-succinyllysine; alternate"
FT                   /evidence="ECO:0007744|PubMed:23806337"
FT   MOD_RES         620
FT                   /note="N6-succinyllysine"
FT                   /evidence="ECO:0007744|PubMed:23806337"
FT   MOD_RES         634
FT                   /note="N6-succinyllysine"
FT                   /evidence="ECO:0007744|PubMed:23806337"
FT   MOD_RES         644
FT                   /note="N6-acetyllysine; alternate"
FT                   /evidence="ECO:0007744|PubMed:23576753"
FT   MOD_RES         644
FT                   /note="N6-succinyllysine; alternate"
FT                   /evidence="ECO:0007744|PubMed:23806337"
FT   MOD_RES         646
FT                   /note="N6-succinyllysine"
FT                   /evidence="ECO:0007744|PubMed:23806337"
FT   MOD_RES         647
FT                   /note="Phosphoserine"
FT                   /evidence="ECO:0007744|PubMed:21183079"
FT   MOD_RES         650
FT                   /note="Phosphoserine"
FT                   /evidence="ECO:0000250|UniProtKB:Q64428"
FT   MOD_RES         664
FT                   /note="N6-acetyllysine; alternate"
FT                   /evidence="ECO:0007744|PubMed:23576753"
FT   MOD_RES         664
FT                   /note="N6-succinyllysine; alternate"
FT                   /evidence="ECO:0007744|PubMed:23806337"
FT   MOD_RES         728
FT                   /note="N6-acetyllysine; alternate"
FT                   /evidence="ECO:0007744|PubMed:23576753"
FT   MOD_RES         728
FT                   /note="N6-succinyllysine; alternate"
FT                   /evidence="ECO:0007744|PubMed:23806337"
FT   MOD_RES         735
FT                   /note="N6-acetyllysine"
FT                   /evidence="ECO:0007744|PubMed:23576753"
FT   MOD_RES         759
FT                   /note="N6-acetyllysine; alternate"
FT                   /evidence="ECO:0007744|PubMed:23576753"
FT   MOD_RES         759
FT                   /note="N6-succinyllysine; alternate"
FT                   /evidence="ECO:0007744|PubMed:23806337"
FT   CONFLICT        196
FT                   /note="A -> D (in Ref. 1; BAE41822)"
FT                   /evidence="ECO:0000305"
FT   CONFLICT        459
FT                   /note="L -> S (in Ref. 2; AAH37009)"
FT                   /evidence="ECO:0000305"
SQ   SEQUENCE   763 AA;  82670 MW;  73D203795D5C1141 CRC64;
     MVASRAIGSL SRFSAFRILR SRGCICRSFT TSSALLTRTH INYGVKGDVA VIRINSPNSK
     VNTLNKEVQS EFIEVMNEIW ANDQIRSAVL ISSKPGCFVA GADINMLSSC TTPQEATRIS
     QEGQRMFEKL EKSPKPVVAA ISGSCLGGGL ELAIACQYRI ATKDRKTVLG VPEVLLGILP
     GAGGTQRLPK MVGVPAAFDM MLTGRNIRAD RAKKMGLVDQ LVEPLGPGIK SPEERTIEYL
     EEVAVNFAKG LADRKVSAKQ SKGLVEKLTT YAMTVPFVRQ QVYKTVEEKV KKQTKGLYPA
     PLKIIDAVKA GLEQGSDAGY LAESQKFGEL ALTKESKALM GLYNGQVLCK KNKFGAPQKN
     VQQLAILGAG LMGAGIAQVS VDKGLKTLLK DTTVTGLGRG QQQVFKGLND KVKKKALTSF
     ERDSIFSNLI GQLDYKGFEK ADMVIEAVFE DLGVKHKVLK EVESVTPEHC IFASNTSALP
     INQIAAVSKR PEKVIGMHYF SPVDKMQLLE IITTDKTSKD TTASAVAVGL RQGKVIIVVK
     DGPGFYTTRC LAPMMSEVMR ILQEGVDPKK LDALTTGFGF PVGAATLADE VGVDVAQHVA
     EDLGKAFGER FGGGSVELLK QMVSKGFLGR KSGKGFYIYQ EGSKNKSLNS EMDNILANLR
     LPAKPEVSSD EDVQYRVITR FVNEAVLCLQ EGILATPAEG DIGAVFGLGF PPCLGGPFRF
     VDLYGAQKVV DRLRKYESAY GTQFTPCQLL LDHANNSSKK FYQ
//
```

|  |
| --- |
| **Mascot:** http://www.matrixscience.com/ |
