# Supplementary material for: A common East-Asian ALDH2 mutation causes metabolic disorders and the therapeutic effect of ALDH2 activators
Source: Nat Commun. 2023 Sep 25;14:5971. doi: 10.1038/s41467-023-41570-6 (PMC10520061; doi:10.1038/s41467-023-41570-6)
Supplement: Supplementary file 4 — Supplementary Data 1 [file 41467_2023_41570_MOESM4_ESM.zip › Table S5b/Q8BWT1/Q8BWT1_WTO-1-K25.html]

Mascot Search Results: Q8BWT1
 

# MASCOT Search Results

## Protein View: Q8BWT1

### 3-ketoacyl-CoA thiolase, mitochondrial OS=Mus musculus OX=10090 GN=Acaa2 PE=1 SV=3

|  |  |
| --- | --- |
| Database: | Mouse\_UniProt\_proteomes |
| Score: | 7996 |
| Monoisotopic mass (Mr): | 42260 |
| Calculated pI: | 8.33 |

Sequence similarity is available as an NCBI BLAST search of Q8BWT1 against nr.

### Search parameters

|  |  |
| --- | --- |
| MS data file: | `D:\LCMSMS\2023 Users' data\230529-1\230529-1-WTO-1.raw` |
| Enzyme: | Trypsin/P: cuts C-term side of KR. |
| Fixed modifications: | Carbamidomethyl (C) |
| Variable modifications: | Deamidated (NQ), HNE (C), HNE (H), HNE (K), Oxidation (M) |

### Protein sequence coverage: 85%

Matched peptides shown in ***bold red***.

|  |  |  |  |  |  |
| --- | --- | --- | --- | --- | --- |
| `1` | `MALLRGVFIV` | `AAKRTPFGAY` | `GGLLKDFSAT` | `DLTEFAARAA` | `LSAGKVPPET` |
| `51` | `IDSVIVGNVM` | `QSSSDAAYLA` | `RHVGLRVGVP` | `TETGALTLNR` | `LCGSGFQSIV` |
| `101` | `SGCQEICSKD` | `AEVVLCGGTE` | `SMSQSPYCVR` | `NVRFGTKFGL` | `DLKLEDTLWA` |
| `151` | `GLTDQHVKLP` | `MGMTAENLAA` | `KYNISREDCD` | `RYALQSQQRW` | `KAANEAGYFN` |
| `201` | `EEMAPIEVKT` | `KKGKQTMQVD` | `EHARPQTTLE` | `QLQKLPSVFK` | `KDGTVTAGNA` |
| `251` | `SGVSDGAGAV` | `IIASEDAVKK` | `HNFTPLARVV` | `GYFVSGCDPT` | `IMGIGPVPAI` |
| `301` | `NGALKKAGLS` | `LKDMDLIDVN` | `EAFAPQFLSV` | `QKALDLDPSK` | `TNVSGGAIAL` |
| `351` | `GHPLGGSGSR` | `ITAHLVHELR` | `RRGGKYAVGS` | `ACIGGGQGIA` | `LIIQNTA` |

Unformatted sequence string: 397 residues (for pasting into other applications).

|  |  |  |  |
| --- | --- | --- | --- |
| Sort by | residue number | increasing mass | decreasing mass |
| Show | matched peptides only | predicted peptides also |  |

| Query | Start | – | End | Observed | Mr(expt) | Mr(calc) | ppm | M | Score | Expect | Rank | U | Peptide |
| --- | --- | --- | --- | --- | --- | --- | --- | --- | --- | --- | --- | --- | --- |
| 3685 | 6 | – | 13 | 402.7521 | 803.4897 | 803.4905 | -1.02 | 0 | 27 | 0.0038 | 1Score **> 22** indicates **identity** Score **> 15** indicates **homology** | U | R.GVFIVAAK.R |
| 3686 | 6 | – | 13 | 402.7523 | 803.4900 | 803.4905 | -0.65 | 0 | 23 | 0.0063 | 1Score **> 22** indicates **identity** Score **> 14** indicates **homology** | U | R.GVFIVAAK.R |
| 3687 | 6 | – | 13 | 402.7523 | 803.4900 | 803.4905 | -0.63 | 0 | 30 | 0.0018 | 1Score **> 22** indicates **identity** Score **> 16** indicates **homology** | U | R.GVFIVAAK.R |
| 3688 | 6 | – | 13 | 402.7524 | 803.4902 | 803.4905 | -0.42 | 0 | 29 | 0.0019 | 1Score **> 22** indicates **identity** Score **> 14** indicates **homology** | U | R.GVFIVAAK.R |
| 3689 | 6 | – | 13 | 402.7524 | 803.4903 | 803.4905 | -0.31 | 0 | 33 | 0.0013 | 1Score **> 22** indicates **identity** Score **> 16** indicates **homology** | U | R.GVFIVAAK.R |
| 11718 | 6 | – | 14 | 480.8020 | 959.5894 | 959.5916 | -2.28 | 1 | 29 | 0.0021 | 1Score **> 25** indicates **identity** Score **> 14** indicates **homology** | U | R.GVFIVAAKR.T |
| 41384 | 14 | – | 25 | 427.2434 | 1278.7083 | 1278.7084 | -0.082 | 1 | 52 | 4.5e-05 | 1Score **> 32** indicates **identity** Score **> 21** indicates **homology** | U | K.RTPFGAYGGLLK.D |
| 41385 | 14 | – | 25 | 640.3616 | 1278.7086 | 1278.7084 | 0.12 | 1 | 33 | 0.0042 | 1Score **> 32** indicates **identity** Score **> 22** indicates **homology** | U | K.RTPFGAYGGLLK.D |
| 41386 | 14 | – | 25 | 427.2435 | 1278.7086 | 1278.7084 | 0.12 | 1 | 29 | 0.0029 | 1Score **> 32** indicates **identity** Score **> 17** indicates **homology** | U | K.RTPFGAYGGLLK.D |
| 41388 | 14 | – | 25 | 640.3616 | 1278.7087 | 1278.7084 | 0.20 | 1 | 64 | 4.1e-05 | 1Score **> 32** indicates **identity** | U | K.RTPFGAYGGLLK.D |
| 41389 | 14 | – | 25 | 640.3616 | 1278.7087 | 1278.7084 | 0.23 | 1 | 55 | 0.00029 | 1Score **> 32** indicates **identity** | U | K.RTPFGAYGGLLK.D |
| 41390 | 14 | – | 25 | 427.2436 | 1278.7090 | 1278.7084 | 0.41 | 1 | 39 | 0.00044 | 1Score **> 32** indicates **identity** Score **> 18** indicates **homology** | U | K.RTPFGAYGGLLK.D |
| 41392 | 14 | – | 25 | 427.2436 | 1278.7091 | 1278.7084 | 0.52 | 1 | 44 | 0.00011 | 1Score **> 32** indicates **identity** Score **> 17** indicates **homology** | U | K.RTPFGAYGGLLK.D |
| 41394 | 14 | – | 25 | 640.3620 | 1278.7095 | 1278.7084 | 0.80 | 1 | 54 | 0.00041 | 1Score **> 32** indicates **identity** | U | K.RTPFGAYGGLLK.D |
| 163726 | 14 | – | 38 | 676.8487 | 2703.3657 | 2703.3657 | 0.026 | 2 | 75 | 9.6e-08 | 1Score **> 37** indicates **identity** Score **> 17** indicates **homology** | U | K.RTPFGAYGGLLKDFSATDLTEFAAR.A |
| 163728 | 14 | – | 38 | 1352.6909 | 2703.3672 | 2703.3657 | 0.55 | 2 | 16 | 0.031 | 1Score **> 37** indicates **identity** Score **> 14** indicates **homology** | U | K.RTPFGAYGGLLKDFSATDLTEFAAR.A |
| 163729 | 14 | – | 38 | 902.1306 | 2703.3699 | 2703.3657 | 1.56 | 2 | 94 | 1.7e-09 | 1Score **> 37** indicates **identity** Score **> 18** indicates **homology** | U | K.RTPFGAYGGLLKDFSATDLTEFAAR.A |
| 163730 | 14 | – | 38 | 676.8500 | 2703.3709 | 2703.3657 | 1.94 | 2 | 75 | 1e-07 | 1Score **> 37** indicates **identity** Score **> 17** indicates **homology** | U | K.RTPFGAYGGLLKDFSATDLTEFAAR.A |
| 24320 | 15 | – | 25 | 562.3061 | 1122.5977 | 1122.6073 | -8.59 | 0 | 30 | 0.0043 | 1Score **> 31** indicates **identity** Score **> 19** indicates **homology** | U | R.TPFGAYGGLLK.D |
| 24323 | 15 | – | 25 | 562.3085 | 1122.6024 | 1122.6073 | -4.42 | 0 | 20 | 0.014 | 1Score **> 32** indicates **identity** Score **> 14** indicates **homology** | U | R.TPFGAYGGLLK.D |
| 24326 | 15 | – | 25 | 562.3103 | 1122.6060 | 1122.6073 | -1.17 | 0 | 15 | 0.04 | 1Score **> 32** indicates **identity** Score **> 13** indicates **homology** | U | R.TPFGAYGGLLK.D |
| 24328 | 15 | – | 25 | 562.3115 | 1122.6085 | 1122.6073 | 1.01 | 0 | 66 | 7.1e-07 | 1Score **> 32** indicates **identity** Score **> 17** indicates **homology** | U | R.TPFGAYGGLLK.D |
| 24329 | 15 | – | 25 | 562.3119 | 1122.6092 | 1122.6073 | 1.63 | 0 | 67 | 8.6e-07 | 1Score **> 32** indicates **identity** Score **> 19** indicates **homology** | U | R.TPFGAYGGLLK.D |
| 24330 | 15 | – | 25 | 562.3122 | 1122.6099 | 1122.6073 | 2.28 | 0 | 43 | 0.0001 | 1Score **> 32** indicates **identity** Score **> 15** indicates **homology** | U | R.TPFGAYGGLLK.D |
| 24331 | 15 | – | 25 | 562.3141 | 1122.6137 | 1122.6073 | 5.67 | 0 | 38 | 0.00026 | 1Score **> 32** indicates **identity** Score **> 15** indicates **homology** | U | R.TPFGAYGGLLK.D |
| 41396 | 15 | – | 25 | 427.2444 | 1278.7112 | 1278.7224 | -8.71 | 0 | 32 | 0.001 | 1Score **> 32** indicates **identity** Score **> 14** indicates **homology** | U | R.TPFGAYGGLLK.D  + HNE (K) |
| 41398 | 15 | – | 25 | 427.2445 | 1278.7117 | 1278.7224 | -8.38 | 0 | 44 | 7.1e-05 | 1Score **> 32** indicates **identity** Score **> 15** indicates **homology** | U | R.TPFGAYGGLLK.D  + HNE (K) |
| 157541 | 15 | – | 38 | 850.0984 | 2547.2732 | 2547.2646 | 3.41 | 1 | 82 | 2.2e-08 | 1Score **> 37** indicates **identity** Score **> 18** indicates **homology** | U | R.TPFGAYGGLLKDFSATDLTEFAAR.A |
| 58434 | 26 | – | 38 | 722.3379 | 1442.6613 | 1442.6678 | -4.49 | 0 | 46 | 4.6e-05 | 1Score **> 30** indicates **identity** Score **> 15** indicates **homology** | U | K.DFSATDLTEFAAR.A |
| 58435 | 26 | – | 38 | 722.3380 | 1442.6614 | 1442.6678 | -4.39 | 0 | 47 | 4.1e-05 | 1Score **> 30** indicates **identity** Score **> 15** indicates **homology** | U | K.DFSATDLTEFAAR.A |
| 58436 | 26 | – | 38 | 722.3382 | 1442.6619 | 1442.6678 | -4.08 | 0 | 53 | 1e-05 | 1Score **> 30** indicates **identity** Score **> 16** indicates **homology** | U | K.DFSATDLTEFAAR.A |
| 58437 | 26 | – | 38 | 722.3387 | 1442.6628 | 1442.6678 | -3.47 | 0 | 59 | 3e-06 | 1Score **> 30** indicates **identity** Score **> 16** indicates **homology** | U | K.DFSATDLTEFAAR.A |
| 58438 | 26 | – | 38 | 722.3387 | 1442.6629 | 1442.6678 | -3.35 | 0 | 51 | 1.7e-05 | 1Score **> 30** indicates **identity** Score **> 16** indicates **homology** | U | K.DFSATDLTEFAAR.A |
| 58439 | 26 | – | 38 | 722.3388 | 1442.6631 | 1442.6678 | -3.22 | 0 | 62 | 1.7e-06 | 1Score **> 30** indicates **identity** Score **> 16** indicates **homology** | U | K.DFSATDLTEFAAR.A |
| 58440 | 26 | – | 38 | 722.3390 | 1442.6634 | 1442.6678 | -3.04 | 0 | 74 | 1.1e-07 | 1Score **> 30** indicates **identity** Score **> 17** indicates **homology** | U | K.DFSATDLTEFAAR.A |
| 58441 | 26 | – | 38 | 722.3390 | 1442.6634 | 1442.6678 | -3.02 | 0 | 49 | 2.4e-05 | 1Score **> 30** indicates **identity** Score **> 16** indicates **homology** | U | K.DFSATDLTEFAAR.A |
| 58442 | 26 | – | 38 | 722.3393 | 1442.6640 | 1442.6678 | -2.59 | 0 | 50 | 2.1e-05 | 1Score **> 30** indicates **identity** Score **> 16** indicates **homology** | U | K.DFSATDLTEFAAR.A |
| 58443 | 26 | – | 38 | 722.3398 | 1442.6650 | 1442.6678 | -1.95 | 0 | 79 | 3.9e-08 | 1Score **> 30** indicates **identity** Score **> 17** indicates **homology** | U | K.DFSATDLTEFAAR.A |
| 58444 | 26 | – | 38 | 722.3398 | 1442.6650 | 1442.6678 | -1.93 | 0 | 55 | 6.3e-06 | 1Score **> 30** indicates **identity** Score **> 16** indicates **homology** | U | K.DFSATDLTEFAAR.A |
| 58445 | 26 | – | 38 | 722.3401 | 1442.6656 | 1442.6678 | -1.52 | 0 | 40 | 0.00019 | 1Score **> 31** indicates **identity** Score **> 15** indicates **homology** | U | K.DFSATDLTEFAAR.A |
| 58447 | 26 | – | 38 | 722.3402 | 1442.6658 | 1442.6678 | -1.34 | 0 | 72 | 1.7e-07 | 1Score **> 30** indicates **identity** Score **> 17** indicates **homology** | U | K.DFSATDLTEFAAR.A |
| 58449 | 26 | – | 38 | 722.3406 | 1442.6666 | 1442.6678 | -0.79 | 0 | 36 | 0.00041 | 1Score **> 30** indicates **identity** Score **> 15** indicates **homology** | U | K.DFSATDLTEFAAR.A |
| 58450 | 26 | – | 38 | 722.3407 | 1442.6668 | 1442.6678 | -0.65 | 0 | 85 | 1.2e-08 | 1Score **> 30** indicates **identity** Score **> 18** indicates **homology** | U | K.DFSATDLTEFAAR.A |
| 58451 | 26 | – | 38 | 722.3407 | 1442.6669 | 1442.6678 | -0.59 | 0 | 78 | 4.8e-08 | 1Score **> 30** indicates **identity** Score **> 17** indicates **homology** | U | K.DFSATDLTEFAAR.A |
| 58453 | 26 | – | 38 | 722.3409 | 1442.6672 | 1442.6678 | -0.42 | 0 | 80 | 3.3e-08 | 1Score **> 30** indicates **identity** Score **> 17** indicates **homology** | U | K.DFSATDLTEFAAR.A |
| 58454 | 26 | – | 38 | 722.3411 | 1442.6676 | 1442.6678 | -0.090 | 0 | 83 | 1.6e-08 | 1Score **> 30** indicates **identity** Score **> 18** indicates **homology** | U | K.DFSATDLTEFAAR.A |
| 58456 | 26 | – | 38 | 722.3412 | 1442.6679 | 1442.6678 | 0.12 | 0 | 85 | 1.1e-08 | 1Score **> 31** indicates **identity** Score **> 18** indicates **homology** | U | K.DFSATDLTEFAAR.A |
| 58457 | 26 | – | 38 | 722.3417 | 1442.6688 | 1442.6678 | 0.71 | 0 | 38 | 0.00029 | 1Score **> 31** indicates **identity** Score **> 15** indicates **homology** | U | K.DFSATDLTEFAAR.A |
| 58459 | 26 | – | 38 | 722.3417 | 1442.6689 | 1442.6678 | 0.76 | 0 | 35 | 0.00053 | 1Score **> 31** indicates **identity** Score **> 15** indicates **homology** | U | K.DFSATDLTEFAAR.A |
| 58462 | 26 | – | 38 | 722.3437 | 1442.6728 | 1442.6678 | 3.49 | 0 | 51 | 1.7e-05 | 1Score **> 31** indicates **identity** Score **> 16** indicates **homology** | U | K.DFSATDLTEFAAR.A |
| 58463 | 26 | – | 38 | 722.3444 | 1442.6743 | 1442.6678 | 4.56 | 0 | 52 | 1.4e-05 | 1Score **> 31** indicates **identity** Score **> 16** indicates **homology** | U | K.DFSATDLTEFAAR.A |
| 179317 | 39 | – | 71 | 1106.5676 | 3316.6808 | 3316.6973 | -4.96 | 1 | 91 | 3.8e-09 | 1Score **> 38** indicates **identity** Score **> 19** indicates **homology** | U | R.AALSAGKVPPETIDSVIVGNVMQSSSDAAYLAR.H |
| 179318 | 39 | – | 71 | 830.1825 | 3316.7009 | 3316.6973 | 1.10 | 1 | 55 | 7.6e-06 | 1Score **> 37** indicates **identity** Score **> 16** indicates **homology** | U | R.AALSAGKVPPETIDSVIVGNVMQSSSDAAYLAR.H |
| 179319 | 39 | – | 71 | 1106.5746 | 3316.7020 | 3316.6973 | 1.43 | 1 | 104 | 1.7e-10 | 1Score **> 37** indicates **identity** Score **> 19** indicates **homology** | U | R.AALSAGKVPPETIDSVIVGNVMQSSSDAAYLAR.H |
| 179320 | 39 | – | 71 | 830.1834 | 3316.7045 | 3316.6973 | 2.19 | 1 | 37 | 0.00033 | 1Score **> 37** indicates **identity** Score **> 15** indicates **homology** | U | R.AALSAGKVPPETIDSVIVGNVMQSSSDAAYLAR.H |
| 164218 | 46 | – | 71 | 907.1258 | 2718.3556 | 2718.3534 | 0.79 | 0 | 65 | 7.9e-07 | 1Score **> 37** indicates **identity** Score **> 17** indicates **homology** | U | K.VPPETIDSVIVGNVMQSSSDAAYLAR.H |
| 164220 | 46 | – | 71 | 680.5964 | 2718.3565 | 2718.3534 | 1.11 | 0 | 27 | 0.0032 | 1Score **> 37** indicates **identity** Score **> 14** indicates **homology** | U | K.VPPETIDSVIVGNVMQSSSDAAYLAR.H |
| 164221 | 46 | – | 71 | 907.1267 | 2718.3582 | 2718.3534 | 1.75 | 0 | 19 | 0.017 | 1Score **> 37** indicates **identity** Score **> 14** indicates **homology** | U | K.VPPETIDSVIVGNVMQSSSDAAYLAR.H |
| 164223 | 46 | – | 71 | 907.1314 | 2718.3722 | 2718.3534 | 6.92 | 0 | 87 | 6.9e-09 | 1Score **> 37** indicates **identity** Score **> 18** indicates **homology** | U | K.VPPETIDSVIVGNVMQSSSDAAYLAR.H |
| 56668 | 77 | – | 90 | 714.3960 | 1426.7774 | 1426.7780 | -0.40 | 0 | 55 | 7.6e-06 | 1Score **> 34** indicates **identity** Score **> 16** indicates **homology** | U | R.VGVPTETGALTLNR.L |
| 56669 | 77 | – | 90 | 714.3962 | 1426.7778 | 1426.7780 | -0.17 | 0 | 66 | 6.3e-07 | 1Score **> 34** indicates **identity** Score **> 17** indicates **homology** | U | R.VGVPTETGALTLNR.L |
| 56670 | 77 | – | 90 | 714.3962 | 1426.7778 | 1426.7780 | -0.14 | 0 | 75 | 1.2e-07 | 1Score **> 34** indicates **identity** Score **> 19** indicates **homology** | U | R.VGVPTETGALTLNR.L |
| 56671 | 77 | – | 90 | 714.3963 | 1426.7780 | 1426.7780 | 0.0042 | 0 | 82 | 2.6e-08 | 1Score **> 34** indicates **identity** Score **> 19** indicates **homology** | U | R.VGVPTETGALTLNR.L |
| 56672 | 77 | – | 90 | 714.3963 | 1426.7780 | 1426.7780 | 0.014 | 0 | 78 | 7.3e-08 | 1Score **> 34** indicates **identity** Score **> 19** indicates **homology** | U | R.VGVPTETGALTLNR.L |
| 56673 | 77 | – | 90 | 714.3963 | 1426.7780 | 1426.7780 | 0.021 | 0 | 80 | 4.1e-08 | 1Score **> 34** indicates **identity** Score **> 19** indicates **homology** | U | R.VGVPTETGALTLNR.L |
| 56674 | 77 | – | 90 | 714.3963 | 1426.7781 | 1426.7780 | 0.088 | 0 | 78 | 6e-08 | 1Score **> 34** indicates **identity** Score **> 19** indicates **homology** | U | R.VGVPTETGALTLNR.L |
| 56675 | 77 | – | 90 | 714.3964 | 1426.7781 | 1426.7780 | 0.10 | 0 | 80 | 4e-08 | 1Score **> 34** indicates **identity** Score **> 18** indicates **homology** | U | R.VGVPTETGALTLNR.L |
| 56676 | 77 | – | 90 | 476.6003 | 1426.7791 | 1426.7780 | 0.74 | 0 | 83 | 1.4e-07 | 1Score **> 34** indicates **identity** Score **> 27** indicates **homology** | U | R.VGVPTETGALTLNR.L |
| 56677 | 77 | – | 90 | 714.3972 | 1426.7799 | 1426.7780 | 1.36 | 0 | 64 | 1.1e-06 | 1Score **> 34** indicates **identity** Score **> 17** indicates **homology** | U | R.VGVPTETGALTLNR.L |
| 56679 | 77 | – | 90 | 714.3974 | 1426.7803 | 1426.7780 | 1.62 | 0 | 28 | 0.0024 | 1Score **> 34** indicates **identity** Score **> 14** indicates **homology** | U | R.VGVPTETGALTLNR.L |
| 129774 | 91 | – | 109 | 1058.9771 | 2115.9396 | 2115.9388 | 0.38 | 0 | 86 | 8.3e-09 | 1Score **> 30** indicates **identity** Score **> 18** indicates **homology** | U | R.LCGSGFQSIVSGCQEICSK.D |
| 129775 | 91 | – | 109 | 706.3205 | 2115.9396 | 2115.9388 | 0.38 | 0 | 72 | 1.9e-07 | 1Score **> 30** indicates **identity** Score **> 17** indicates **homology** | U | R.LCGSGFQSIVSGCQEICSK.D |
| 129776 | 91 | – | 109 | 706.3208 | 2115.9407 | 2115.9388 | 0.92 | 0 | 72 | 1.7e-07 | 1Score **> 30** indicates **identity** Score **> 17** indicates **homology** | U | R.LCGSGFQSIVSGCQEICSK.D |
| 129777 | 91 | – | 109 | 1058.9777 | 2115.9408 | 2115.9388 | 0.94 | 0 | 83 | 1.6e-08 | 1Score **> 30** indicates **identity** Score **> 18** indicates **homology** | U | R.LCGSGFQSIVSGCQEICSK.D |
| 129778 | 91 | – | 109 | 706.3209 | 2115.9408 | 2115.9388 | 0.94 | 0 | 15 | 0.038 | 1Score **> 30** indicates **identity** Score **> 13** indicates **homology** | U | R.LCGSGFQSIVSGCQEICSK.D |
| 129779 | 91 | – | 109 | 1058.9777 | 2115.9408 | 2115.9388 | 0.95 | 0 | 103 | 2e-10 | 1Score **> 30** indicates **identity** Score **> 19** indicates **homology** | U | R.LCGSGFQSIVSGCQEICSK.D |
| 129780 | 91 | – | 109 | 1058.9777 | 2115.9409 | 2115.9388 | 1.03 | 0 | 116 | 1.4e-11 | 1Score **> 30** indicates **identity** Score **> 20** indicates **homology** | U | R.LCGSGFQSIVSGCQEICSK.D |
| 129781 | 91 | – | 109 | 1058.9781 | 2115.9416 | 2115.9388 | 1.32 | 0 | 96 | 9.4e-10 | 1Score **> 30** indicates **identity** Score **> 19** indicates **homology** | U | R.LCGSGFQSIVSGCQEICSK.D |
| 129783 | 91 | – | 109 | 706.3214 | 2115.9424 | 2115.9388 | 1.72 | 0 | 31 | 0.0012 | 1Score **> 30** indicates **identity** Score **> 14** indicates **homology** | U | R.LCGSGFQSIVSGCQEICSK.D |
| 129784 | 91 | – | 109 | 706.3214 | 2115.9425 | 2115.9388 | 1.77 | 0 | 73 | 1.6e-07 | 1Score **> 30** indicates **identity** Score **> 17** indicates **homology** | U | R.LCGSGFQSIVSGCQEICSK.D |
| 129785 | 91 | – | 109 | 1058.9788 | 2115.9430 | 2115.9388 | 1.98 | 0 | 52 | 1.4e-05 | 1Score **> 30** indicates **identity** Score **> 16** indicates **homology** | U | R.LCGSGFQSIVSGCQEICSK.D |
| 129786 | 91 | – | 109 | 1058.9788 | 2115.9430 | 2115.9388 | 1.98 | 0 | 77 | 5.9e-08 | 1Score **> 30** indicates **identity** Score **> 17** indicates **homology** | U | R.LCGSGFQSIVSGCQEICSK.D |
| 129787 | 91 | – | 109 | 1058.9790 | 2115.9435 | 2115.9388 | 2.23 | 0 | 102 | 2.7e-10 | 1Score **> 30** indicates **identity** Score **> 19** indicates **homology** | U | R.LCGSGFQSIVSGCQEICSK.D |
| 129788 | 91 | – | 109 | 1058.9800 | 2115.9454 | 2115.9388 | 3.11 | 0 | 100 | 4.2e-10 | 1Score **> 30** indicates **identity** Score **> 19** indicates **homology** | U | R.LCGSGFQSIVSGCQEICSK.D |
| 129789 | 91 | – | 109 | 1058.9801 | 2115.9456 | 2115.9388 | 3.21 | 0 | 77 | 5.9e-08 | 1Score **> 30** indicates **identity** Score **> 17** indicates **homology** | U | R.LCGSGFQSIVSGCQEICSK.D |
| 129790 | 91 | – | 109 | 1058.9813 | 2115.9481 | 2115.9388 | 4.41 | 0 | 61 | 1.9e-06 | 1Score **> 31** indicates **identity** Score **> 16** indicates **homology** | U | R.LCGSGFQSIVSGCQEICSK.D |
| 129883 | 91 | – | 109 | 1059.4777 | 2116.9409 | 2116.9228 | 8.58 | 0 | 25 | 0.0047 | 1Score **> 30** indicates **identity** Score **> 14** indicates **homology** | U | R.LCGSGFQSIVSGCQEICSK.D  + Deamidated (NQ) |
| 129884 | 91 | – | 109 | 706.6543 | 2116.9411 | 2116.9228 | 8.64 | 0 | 41 | 0.00015 | 1Score **> 30** indicates **identity** Score **> 15** indicates **homology** | U | R.LCGSGFQSIVSGCQEICSK.D  + Deamidated (NQ) |
| 146514 | 110 | – | 130 | 1173.0140 | 2344.0134 | 2344.0134 | 0.0026 | 0 | 17 | 0.023 | 1Score **> 29** indicates **identity** Score **> 14** indicates **homology** | U | K.DAEVVLCGGTESMSQSPYCVR.N |
| 146517 | 110 | – | 130 | 1173.0172 | 2344.0199 | 2344.0134 | 2.77 | 0 | 74 | 1.2e-07 | 1Score **> 29** indicates **identity** Score **> 17** indicates **homology** | U | K.DAEVVLCGGTESMSQSPYCVR.N |
| 146518 | 110 | – | 130 | 1173.0184 | 2344.0222 | 2344.0134 | 3.77 | 0 | 68 | 4.1e-07 | 1Score **> 29** indicates **identity** Score **> 17** indicates **homology** | U | K.DAEVVLCGGTESMSQSPYCVR.N |
| 24513 | 134 | – | 143 | 375.8813 | 1124.6221 | 1124.6230 | -0.78 | 1 | 36 | 0.0024 | 1Score **> 31** indicates **identity** Score **> 22** indicates **homology** | U | R.FGTKFGLDLK.L |
| 24514 | 134 | – | 143 | 375.8813 | 1124.6222 | 1124.6230 | -0.71 | 1 | 29 | 0.008 | 1Score **> 31** indicates **identity** Score **> 20** indicates **homology** | U | R.FGTKFGLDLK.L |
| 24518 | 134 | – | 143 | 375.8815 | 1124.6227 | 1124.6230 | -0.31 | 1 | 36 | 0.0024 | 1Score **> 31** indicates **identity** Score **> 22** indicates **homology** | U | R.FGTKFGLDLK.L |
| 24520 | 134 | – | 143 | 375.8818 | 1124.6235 | 1124.6230 | 0.42 | 1 | 28 | 0.013 | 1Score **> 31** indicates **identity** Score **> 21** indicates **homology** | U | R.FGTKFGLDLK.L |
| 168378 | 134 | – | 158 | 708.8785 | 2831.4849 | 2831.4858 | -0.33 | 2 | 30 | 0.0016 | 1Score **> 37** indicates **identity** Score **> 14** indicates **homology** | U | R.FGTKFGLDLKLEDTLWAGLTDQHVK.L |
| 168379 | 134 | – | 158 | 567.3045 | 2831.4859 | 2831.4858 | 0.034 | 2 | 26 | 0.0037 | 1Score **> 37** indicates **identity** Score **> 14** indicates **homology** | U | R.FGTKFGLDLKLEDTLWAGLTDQHVK.L |
| 168380 | 134 | – | 158 | 708.8801 | 2831.4912 | 2831.4858 | 1.92 | 2 | 46 | 5.1e-05 | 1Score **> 37** indicates **identity** Score **> 15** indicates **homology** | U | R.FGTKFGLDLKLEDTLWAGLTDQHVK.L |
| 149998 | 138 | – | 158 | 600.5701 | 2398.2515 | 2398.2533 | -0.75 | 1 | 20 | 0.013 | 1Score **> 37** indicates **identity** Score **> 14** indicates **homology** | U | K.FGLDLKLEDTLWAGLTDQHVK.L |
| 150000 | 138 | – | 158 | 600.5704 | 2398.2524 | 2398.2533 | -0.38 | 1 | 23 | 0.0063 | 1Score **> 37** indicates **identity** Score **> 14** indicates **homology** | U | K.FGLDLKLEDTLWAGLTDQHVK.L |
| 150002 | 138 | – | 158 | 800.4248 | 2398.2525 | 2398.2533 | -0.31 | 1 | 15 | 0.036 | 1Score **> 37** indicates **identity** Score **> 13** indicates **homology** | U | K.FGLDLKLEDTLWAGLTDQHVK.L |
| 150005 | 138 | – | 158 | 600.5709 | 2398.2543 | 2398.2533 | 0.44 | 1 | 32 | 0.00095 | 1Score **> 37** indicates **identity** Score **> 15** indicates **homology** | U | K.FGLDLKLEDTLWAGLTDQHVK.L |
| 150007 | 138 | – | 158 | 800.4256 | 2398.2550 | 2398.2533 | 0.73 | 1 | 33 | 0.00072 | 1Score **> 37** indicates **identity** Score **> 15** indicates **homology** | U | K.FGLDLKLEDTLWAGLTDQHVK.L |
| 150009 | 138 | – | 158 | 600.5711 | 2398.2552 | 2398.2533 | 0.79 | 1 | 47 | 4e-05 | 1Score **> 37** indicates **identity** Score **> 15** indicates **homology** | U | K.FGLDLKLEDTLWAGLTDQHVK.L |
| 150012 | 138 | – | 158 | 800.4257 | 2398.2552 | 2398.2533 | 0.82 | 1 | 35 | 0.00056 | 1Score **> 37** indicates **identity** Score **> 15** indicates **homology** | U | K.FGLDLKLEDTLWAGLTDQHVK.L |
| 150014 | 138 | – | 158 | 600.5712 | 2398.2558 | 2398.2533 | 1.08 | 1 | 33 | 0.00074 | 1Score **> 37** indicates **identity** Score **> 15** indicates **homology** | U | K.FGLDLKLEDTLWAGLTDQHVK.L |
| 150015 | 138 | – | 158 | 800.4259 | 2398.2559 | 2398.2533 | 1.09 | 1 | 54 | 8.6e-06 | 1Score **> 37** indicates **identity** Score **> 16** indicates **homology** | U | K.FGLDLKLEDTLWAGLTDQHVK.L |
| 150017 | 138 | – | 158 | 800.4262 | 2398.2566 | 2398.2533 | 1.40 | 1 | 22 | 0.0087 | 1Score **> 37** indicates **identity** Score **> 14** indicates **homology** | U | K.FGLDLKLEDTLWAGLTDQHVK.L |
| 150020 | 138 | – | 158 | 800.4264 | 2398.2573 | 2398.2533 | 1.68 | 1 | 29 | 0.0021 | 1Score **> 37** indicates **identity** Score **> 14** indicates **homology** | U | K.FGLDLKLEDTLWAGLTDQHVK.L |
| 150022 | 138 | – | 158 | 600.5717 | 2398.2577 | 2398.2533 | 1.86 | 1 | 39 | 0.00024 | 1Score **> 37** indicates **identity** Score **> 15** indicates **homology** | U | K.FGLDLKLEDTLWAGLTDQHVK.L |
| 150027 | 138 | – | 158 | 600.5727 | 2398.2618 | 2398.2533 | 3.54 | 1 | 38 | 0.00026 | 1Score **> 37** indicates **identity** Score **> 15** indicates **homology** | U | K.FGLDLKLEDTLWAGLTDQHVK.L |
| 93458 | 144 | – | 158 | 863.4425 | 1724.8704 | 1724.8734 | -1.70 | 0 | 66 | 5.9e-07 | 1Score **> 35** indicates **identity** Score **> 17** indicates **homology** | U | K.LEDTLWAGLTDQHVK.L |
| 93460 | 144 | – | 158 | 575.9650 | 1724.8732 | 1724.8734 | -0.092 | 0 | 36 | 0.00047 | 1Score **> 35** indicates **identity** Score **> 15** indicates **homology** | U | K.LEDTLWAGLTDQHVK.L |
| 93461 | 144 | – | 158 | 575.9651 | 1724.8734 | 1724.8734 | 0.045 | 0 | 33 | 0.00081 | 1Score **> 35** indicates **identity** Score **> 15** indicates **homology** | U | K.LEDTLWAGLTDQHVK.L |
| 93463 | 144 | – | 158 | 863.4448 | 1724.8750 | 1724.8734 | 0.97 | 0 | 29 | 0.0019 | 1Score **> 35** indicates **identity** Score **> 14** indicates **homology** | U | K.LEDTLWAGLTDQHVK.L |
| 93464 | 144 | – | 158 | 575.9660 | 1724.8763 | 1724.8734 | 1.72 | 0 | 32 | 0.0009 | 1Score **> 35** indicates **identity** Score **> 15** indicates **homology** | U | K.LEDTLWAGLTDQHVK.L |
| 93465 | 144 | – | 158 | 575.9662 | 1724.8768 | 1724.8734 | 2.03 | 0 | 28 | 0.0023 | 1Score **> 35** indicates **identity** Score **> 14** indicates **homology** | U | K.LEDTLWAGLTDQHVK.L |
| 46445 | 172 | – | 181 | 664.2888 | 1326.5631 | 1326.5622 | 0.68 | 1 | 19 | 0.019 | 1Score **> 26** indicates **identity** Score **> 14** indicates **homology** | U | K.YNISREDCDR.Y |
| 143856 | 172 | – | 189 | 768.0257 | 2301.0552 | 2301.0556 | -0.16 | 2 | 29 | 0.002 | 1Score **> 33** indicates **identity** Score **> 14** indicates **homology** | U | K.YNISREDCDRYALQSQQR.W |
| 143858 | 172 | – | 189 | 768.0257 | 2301.0553 | 2301.0556 | -0.15 | 2 | 17 | 0.024 | 1Score **> 33** indicates **identity** Score **> 14** indicates **homology** | U | K.YNISREDCDRYALQSQQR.W |
| 143859 | 172 | – | 189 | 768.0259 | 2301.0559 | 2301.0556 | 0.12 | 2 | 33 | 0.00087 | 1Score **> 33** indicates **identity** Score **> 15** indicates **homology** | U | K.YNISREDCDRYALQSQQR.W |
| 86475 | 177 | – | 189 | 556.9175 | 1667.7306 | 1667.7322 | -0.95 | 1 | 19 | 0.015 | 1Score **> 28** indicates **identity** Score **> 14** indicates **homology** | U | R.EDCDRYALQSQQR.W |
| 86476 | 177 | – | 189 | 834.8727 | 1667.7308 | 1667.7322 | -0.84 | 1 | 40 | 0.00017 | 1Score **> 28** indicates **identity** Score **> 15** indicates **homology** | U | R.EDCDRYALQSQQR.W |
| 86477 | 177 | – | 189 | 556.9176 | 1667.7311 | 1667.7322 | -0.65 | 1 | 35 | 0.00055 | 1Score **> 28** indicates **identity** Score **> 15** indicates **homology** | U | R.EDCDRYALQSQQR.W |
| 86478 | 177 | – | 189 | 556.9177 | 1667.7313 | 1667.7322 | -0.51 | 1 | 39 | 0.00021 | 1Score **> 28** indicates **identity** Score **> 15** indicates **homology** | U | R.EDCDRYALQSQQR.W |
| 86479 | 177 | – | 189 | 834.8733 | 1667.7320 | 1667.7322 | -0.10 | 1 | 51 | 6.5e-05 | 1Score **> 28** indicates **identity** Score **> 21** indicates **homology** | U | R.EDCDRYALQSQQR.W |
| 86480 | 177 | – | 189 | 556.9180 | 1667.7322 | 1667.7322 | 0.037 | 1 | 34 | 0.00061 | 1Score **> 28** indicates **identity** Score **> 15** indicates **homology** | U | R.EDCDRYALQSQQR.W |
| 86481 | 177 | – | 189 | 556.9181 | 1667.7326 | 1667.7322 | 0.23 | 1 | 15 | 0.037 | 1Score **> 28** indicates **identity** Score **> 13** indicates **homology** | U | R.EDCDRYALQSQQR.W |
| 86483 | 177 | – | 189 | 556.9181 | 1667.7326 | 1667.7322 | 0.26 | 1 | 34 | 0.0006 | 1Score **> 28** indicates **identity** Score **> 15** indicates **homology** | U | R.EDCDRYALQSQQR.W |
| 86484 | 177 | – | 189 | 834.8736 | 1667.7327 | 1667.7322 | 0.33 | 1 | 26 | 0.0075 | 1Score **> 28** indicates **identity** Score **> 17** indicates **homology** | U | R.EDCDRYALQSQQR.W |
| 86485 | 177 | – | 189 | 556.9184 | 1667.7335 | 1667.7322 | 0.82 | 1 | 17 | 0.025 | 1Score **> 29** indicates **identity** Score **> 14** indicates **homology** | U | R.EDCDRYALQSQQR.W |
| 13710 | 182 | – | 189 | 497.2582 | 992.5018 | 992.5039 | -2.14 | 0 | 25 | 0.0042 | 1Score **> 32** indicates **identity** Score **> 14** indicates **homology** | U | R.YALQSQQR.W |
| 13711 | 182 | – | 189 | 497.2582 | 992.5018 | 992.5039 | -2.10 | 0 | 41 | 0.00025 | 1Score **> 32** indicates **identity** Score **> 18** indicates **homology** | U | R.YALQSQQR.W |
| 13713 | 182 | – | 189 | 497.2587 | 992.5028 | 992.5039 | -1.16 | 0 | 42 | 0.00011 | 1Score **> 31** indicates **identity** Score **> 15** indicates **homology** | U | R.YALQSQQR.W |
| 143528 | 190 | – | 209 | 766.3639 | 2296.0699 | 2296.0834 | -5.86 | 1 | 15 | 0.037 | 1Score **> 34** indicates **identity** Score **> 13** indicates **homology** | U | R.WKAANEAGYFNEEMAPIEVK.T |
| 143530 | 190 | – | 209 | 766.3680 | 2296.0823 | 2296.0834 | -0.46 | 1 | 25 | 0.0045 | 1Score **> 35** indicates **identity** Score **> 14** indicates **homology** | U | R.WKAANEAGYFNEEMAPIEVK.T |
| 143532 | 190 | – | 209 | 766.3698 | 2296.0874 | 2296.0834 | 1.78 | 1 | 28 | 0.0023 | 1Score **> 35** indicates **identity** Score **> 14** indicates **homology** | U | R.WKAANEAGYFNEEMAPIEVK.T |
| 118043 | 192 | – | 209 | 991.9612 | 1981.9078 | 1981.9091 | -0.66 | 0 | 59 | 2.9e-06 | 1Score **> 33** indicates **identity** Score **> 16** indicates **homology** | U | K.AANEAGYFNEEMAPIEVK.T |
| 118044 | 192 | – | 209 | 991.9620 | 1981.9094 | 1981.9091 | 0.15 | 0 | 81 | 2.5e-08 | 1Score **> 33** indicates **identity** Score **> 18** indicates **homology** | U | K.AANEAGYFNEEMAPIEVK.T |
| 118045 | 192 | – | 209 | 991.9628 | 1981.9111 | 1981.9091 | 0.99 | 0 | 69 | 3.2e-07 | 1Score **> 33** indicates **identity** Score **> 17** indicates **homology** | U | K.AANEAGYFNEEMAPIEVK.T |
| 118047 | 192 | – | 209 | 991.9645 | 1981.9145 | 1981.9091 | 2.73 | 0 | 57 | 4.8e-06 | 1Score **> 33** indicates **identity** Score **> 16** indicates **homology** | U | K.AANEAGYFNEEMAPIEVK.T |
| 148737 | 215 | – | 234 | 596.0510 | 2380.1748 | 2380.1805 | -2.38 | 1 | 23 | 0.0072 | 1Score **> 36** indicates **identity** Score **> 14** indicates **homology** | U | K.QTMQVDEHARPQTTLEQLQK.L |
| 148742 | 215 | – | 234 | 794.4014 | 2380.1824 | 2380.1805 | 0.81 | 1 | 34 | 0.0007 | 1Score **> 37** indicates **identity** Score **> 15** indicates **homology** | U | K.QTMQVDEHARPQTTLEQLQK.L |
| 148745 | 215 | – | 234 | 794.4025 | 2380.1858 | 2380.1805 | 2.23 | 1 | 37 | 0.00034 | 1Score **> 37** indicates **identity** Score **> 15** indicates **homology** | U | K.QTMQVDEHARPQTTLEQLQK.L |
| 148752 | 215 | – | 234 | 596.0553 | 2380.1921 | 2380.1805 | 4.89 | 1 | 21 | 0.012 | 1Score **> 37** indicates **identity** Score **> 14** indicates **homology** | U | K.QTMQVDEHARPQTTLEQLQK.L |
| 4350 | 235 | – | 241 | 409.7600 | 817.5055 | 817.5062 | -0.78 | 1 | 25 | 0.026 | 1Score **> 22** indicates **identity** Score **> 22** indicates **homology** | U | K.LPSVFKK.D |
| 4351 | 235 | – | 241 | 409.7601 | 817.5057 | 817.5062 | -0.51 | 1 | 36 | 0.002 | 1Score **> 22** indicates **identity** | U | K.LPSVFKK.D |
| 4352 | 235 | – | 241 | 409.7604 | 817.5062 | 817.5062 | 0.10 | 1 | 34 | 0.0032 | 1Score **> 22** indicates **identity** | U | K.LPSVFKK.D |
| 162008 | 241 | – | 269 | 887.4564 | 2659.3473 | 2659.3301 | 6.46 | 1 | 50 | 2e-05 | 1Score **> 37** indicates **identity** Score **> 16** indicates **homology** | U | K.KDGTVTAGNASGVSDGAGAVIIASEDAVK.K |
| 166799 | 241 | – | 270 | 697.8645 | 2787.4291 | 2787.4250 | 1.45 | 2 | 30 | 0.0014 | 1Score **> 37** indicates **identity** Score **> 14** indicates **homology** | U | K.KDGTVTAGNASGVSDGAGAVIIASEDAVKK.H |
| 156806 | 242 | – | 269 | 1266.6235 | 2531.2325 | 2531.2351 | -1.05 | 0 | 114 | 2.1e-11 | 1Score **> 36** indicates **identity** Score **> 20** indicates **homology** | U | K.DGTVTAGNASGVSDGAGAVIIASEDAVK.K |
| 156809 | 242 | – | 269 | 844.7516 | 2531.2331 | 2531.2351 | -0.80 | 0 | 41 | 0.00014 | 1Score **> 36** indicates **identity** Score **> 15** indicates **homology** | U | K.DGTVTAGNASGVSDGAGAVIIASEDAVK.K |
| 156810 | 242 | – | 269 | 844.7519 | 2531.2340 | 2531.2351 | -0.44 | 0 | 33 | 0.00083 | 1Score **> 36** indicates **identity** Score **> 15** indicates **homology** | U | K.DGTVTAGNASGVSDGAGAVIIASEDAVK.K |
| 156811 | 242 | – | 269 | 844.7524 | 2531.2353 | 2531.2351 | 0.051 | 0 | 83 | 1.6e-08 | 1Score **> 36** indicates **identity** Score **> 18** indicates **homology** | U | K.DGTVTAGNASGVSDGAGAVIIASEDAVK.K |
| 156812 | 242 | – | 269 | 844.7525 | 2531.2357 | 2531.2351 | 0.23 | 0 | 111 | 3.9e-11 | 1Score **> 36** indicates **identity** Score **> 20** indicates **homology** | U | K.DGTVTAGNASGVSDGAGAVIIASEDAVK.K |
| 156813 | 242 | – | 269 | 844.7526 | 2531.2360 | 2531.2351 | 0.37 | 0 | 48 | 3.1e-05 | 1Score **> 36** indicates **identity** Score **> 16** indicates **homology** | U | K.DGTVTAGNASGVSDGAGAVIIASEDAVK.K |
| 156815 | 242 | – | 269 | 844.7528 | 2531.2365 | 2531.2351 | 0.56 | 0 | 35 | 0.00056 | 1Score **> 37** indicates **identity** Score **> 15** indicates **homology** | U | K.DGTVTAGNASGVSDGAGAVIIASEDAVK.K |
| 156817 | 242 | – | 269 | 1266.6259 | 2531.2372 | 2531.2351 | 0.83 | 0 | 153 | 5.5e-15 | 1Score **> 37** indicates **identity** Score **> 23** indicates **homology** | U | K.DGTVTAGNASGVSDGAGAVIIASEDAVK.K |
| 156819 | 242 | – | 269 | 1266.6263 | 2531.2381 | 2531.2351 | 1.19 | 0 | 113 | 2.7e-11 | 1Score **> 37** indicates **identity** Score **> 20** indicates **homology** | U | K.DGTVTAGNASGVSDGAGAVIIASEDAVK.K |
| 156820 | 242 | – | 269 | 844.7534 | 2531.2384 | 2531.2351 | 1.30 | 0 | 50 | 2e-05 | 1Score **> 37** indicates **identity** Score **> 16** indicates **homology** | U | K.DGTVTAGNASGVSDGAGAVIIASEDAVK.K |
| 156821 | 242 | – | 269 | 844.7535 | 2531.2385 | 2531.2351 | 1.34 | 0 | 90 | 3.5e-09 | 1Score **> 37** indicates **identity** Score **> 18** indicates **homology** | U | K.DGTVTAGNASGVSDGAGAVIIASEDAVK.K |
| 156825 | 242 | – | 269 | 1266.6303 | 2531.2461 | 2531.2351 | 4.35 | 0 | 142 | 4.6e-14 | 1Score **> 37** indicates **identity** Score **> 21** indicates **homology** | U | K.DGTVTAGNASGVSDGAGAVIIASEDAVK.K |
| 156849 | 242 | – | 269 | 845.0854 | 2532.2345 | 2532.2191 | 6.06 | 0 | 16 | 0.032 | 1Score **> 36** indicates **identity** Score **> 13** indicates **homology** | U | K.DGTVTAGNASGVSDGAGAVIIASEDAVK.K  + Deamidated (NQ) |
| 156850 | 242 | – | 269 | 1267.1253 | 2532.2361 | 2532.2191 | 6.72 | 0 | 32 | 0.0011 | 1Score **> 36** indicates **identity** Score **> 15** indicates **homology** | U | K.DGTVTAGNASGVSDGAGAVIIASEDAVK.K  + Deamidated (NQ) |
| 156851 | 242 | – | 269 | 1267.1258 | 2532.2370 | 2532.2191 | 7.08 | 0 | 76 | 7.9e-08 | 1Score **> 36** indicates **identity** Score **> 17** indicates **homology** | U | K.DGTVTAGNASGVSDGAGAVIIASEDAVK.K  + Deamidated (NQ) |
| 156853 | 242 | – | 269 | 1267.1265 | 2532.2384 | 2532.2191 | 7.60 | 0 | 95 | 1.2e-09 | 1Score **> 37** indicates **identity** Score **> 18** indicates **homology** | U | K.DGTVTAGNASGVSDGAGAVIIASEDAVK.K  + Deamidated (NQ) |
| 156854 | 242 | – | 269 | 1267.1273 | 2532.2401 | 2532.2191 | 8.29 | 0 | 45 | 6.5e-05 | 1Score **> 37** indicates **identity** Score **> 15** indicates **homology** | U | K.DGTVTAGNASGVSDGAGAVIIASEDAVK.K  + Deamidated (NQ) |
| 161991 | 242 | – | 270 | 887.4447 | 2659.3122 | 2659.3301 | -6.71 | 1 | 31 | 0.0012 | 1Score **> 37** indicates **identity** Score **> 14** indicates **homology** | U | K.DGTVTAGNASGVSDGAGAVIIASEDAVKK.H |
| 161993 | 242 | – | 270 | 887.4462 | 2659.3169 | 2659.3301 | -4.97 | 1 | 18 | 0.02 | 1Score **> 37** indicates **identity** Score **> 14** indicates **homology** | U | K.DGTVTAGNASGVSDGAGAVIIASEDAVKK.H |
| 161995 | 242 | – | 270 | 887.4479 | 2659.3219 | 2659.3301 | -3.06 | 1 | 52 | 1.3e-05 | 1Score **> 37** indicates **identity** Score **> 16** indicates **homology** | U | K.DGTVTAGNASGVSDGAGAVIIASEDAVKK.H |
| 161997 | 242 | – | 270 | 887.4497 | 2659.3274 | 2659.3301 | -1.01 | 1 | 33 | 0.00089 | 1Score **> 37** indicates **identity** Score **> 15** indicates **homology** | U | K.DGTVTAGNASGVSDGAGAVIIASEDAVKK.H |
| 161998 | 242 | – | 270 | 887.4508 | 2659.3305 | 2659.3301 | 0.16 | 1 | 84 | 1.2e-08 | 1Score **> 37** indicates **identity** Score **> 18** indicates **homology** | U | K.DGTVTAGNASGVSDGAGAVIIASEDAVKK.H |
| 161999 | 242 | – | 270 | 887.4513 | 2659.3322 | 2659.3301 | 0.79 | 1 | 60 | 2.4e-06 | 1Score **> 37** indicates **identity** Score **> 16** indicates **homology** | U | K.DGTVTAGNASGVSDGAGAVIIASEDAVKK.H |
| 162000 | 242 | – | 270 | 887.4514 | 2659.3324 | 2659.3301 | 0.87 | 1 | 102 | 2.9e-10 | 1Score **> 37** indicates **identity** Score **> 19** indicates **homology** | U | K.DGTVTAGNASGVSDGAGAVIIASEDAVKK.H |
| 162001 | 242 | – | 270 | 1330.6736 | 2659.3327 | 2659.3301 | 1.00 | 1 | 76 | 7.8e-08 | 1Score **> 37** indicates **identity** Score **> 17** indicates **homology** | U | K.DGTVTAGNASGVSDGAGAVIIASEDAVKK.H |
| 162002 | 242 | – | 270 | 887.4515 | 2659.3328 | 2659.3301 | 1.01 | 1 | 95 | 1.3e-09 | 1Score **> 37** indicates **identity** Score **> 18** indicates **homology** | U | K.DGTVTAGNASGVSDGAGAVIIASEDAVKK.H |
| 162003 | 242 | – | 270 | 887.4516 | 2659.3330 | 2659.3301 | 1.08 | 1 | 114 | 1.9e-11 | 1Score **> 37** indicates **identity** Score **> 20** indicates **homology** | U | K.DGTVTAGNASGVSDGAGAVIIASEDAVKK.H |
| 162004 | 242 | – | 270 | 887.4518 | 2659.3335 | 2659.3301 | 1.27 | 1 | 29 | 0.0021 | 1Score **> 37** indicates **identity** Score **> 14** indicates **homology** | U | K.DGTVTAGNASGVSDGAGAVIIASEDAVKK.H |
| 162005 | 242 | – | 270 | 1330.6741 | 2659.3337 | 2659.3301 | 1.37 | 1 | 60 | 2.3e-06 | 1Score **> 37** indicates **identity** Score **> 16** indicates **homology** | U | K.DGTVTAGNASGVSDGAGAVIIASEDAVKK.H |
| 162006 | 242 | – | 270 | 887.4520 | 2659.3340 | 2659.3301 | 1.49 | 1 | 68 | 4e-07 | 1Score **> 37** indicates **identity** Score **> 17** indicates **homology** | U | K.DGTVTAGNASGVSDGAGAVIIASEDAVKK.H |
| 162007 | 242 | – | 270 | 887.4525 | 2659.3356 | 2659.3301 | 2.08 | 1 | 112 | 3.4e-11 | 1Score **> 37** indicates **identity** Score **> 19** indicates **homology** | U | K.DGTVTAGNASGVSDGAGAVIIASEDAVKK.H |
| 162010 | 242 | – | 270 | 887.4568 | 2659.3487 | 2659.3301 | 6.99 | 1 | 59 | 2.9e-06 | 1Score **> 37** indicates **identity** Score **> 16** indicates **homology** | U | K.DGTVTAGNASGVSDGAGAVIIASEDAVKK.H |
| 162033 | 242 | – | 270 | 887.7797 | 2660.3173 | 2660.3141 | 1.19 | 1 | 24 | 0.0057 | 1Score **> 37** indicates **identity** Score **> 14** indicates **homology** | U | K.DGTVTAGNASGVSDGAGAVIIASEDAVKK.H  + Deamidated (NQ) |
| 162037 | 242 | – | 270 | 887.7824 | 2660.3253 | 2660.3141 | 4.20 | 1 | 23 | 0.007 | 1Score **> 37** indicates **identity** Score **> 14** indicates **homology** | U | K.DGTVTAGNASGVSDGAGAVIIASEDAVKK.H  + Deamidated (NQ) |
| 162038 | 242 | – | 270 | 887.7829 | 2660.3268 | 2660.3141 | 4.78 | 1 | 30 | 0.0014 | 1Score **> 37** indicates **identity** Score **> 14** indicates **homology** | U | K.DGTVTAGNASGVSDGAGAVIIASEDAVKK.H  + Deamidated (NQ) |
| 162039 | 242 | – | 270 | 887.7834 | 2660.3283 | 2660.3141 | 5.33 | 1 | 43 | 9.1e-05 | 1Score **> 37** indicates **identity** Score **> 15** indicates **homology** | U | K.DGTVTAGNASGVSDGAGAVIIASEDAVKK.H  + Deamidated (NQ) |
| 162040 | 242 | – | 270 | 887.7838 | 2660.3296 | 2660.3141 | 5.82 | 1 | 57 | 4.7e-06 | 1Score **> 37** indicates **identity** Score **> 16** indicates **homology** | U | K.DGTVTAGNASGVSDGAGAVIIASEDAVKK.H  + Deamidated (NQ) |
| 162043 | 242 | – | 270 | 887.7855 | 2660.3347 | 2660.3141 | 7.75 | 1 | 59 | 2.9e-06 | 1Score **> 37** indicates **identity** Score **> 16** indicates **homology** | U | K.DGTVTAGNASGVSDGAGAVIIASEDAVKK.H  + Deamidated (NQ) |
| 162044 | 242 | – | 270 | 887.7856 | 2660.3350 | 2660.3141 | 7.87 | 1 | 27 | 0.0032 | 1Score **> 37** indicates **identity** Score **> 14** indicates **homology** | U | K.DGTVTAGNASGVSDGAGAVIIASEDAVKK.H  + Deamidated (NQ) |
| 162045 | 242 | – | 270 | 887.7856 | 2660.3350 | 2660.3141 | 7.87 | 1 | 14 | 0.046 | 1Score **> 37** indicates **identity** Score **> 13** indicates **homology** | U | K.DGTVTAGNASGVSDGAGAVIIASEDAVKK.H  + Deamidated (NQ) |
| 20458 | 270 | – | 278 | 361.8731 | 1082.5976 | 1082.5985 | -0.86 | 1 | 27 | 0.0028 | 1Score **> 31** indicates **identity** Score **> 14** indicates **homology** | U | K.KHNFTPLAR.V |
| 20461 | 270 | – | 278 | 542.3063 | 1082.5981 | 1082.5985 | -0.39 | 1 | 24 | 0.0058 | 1Score **> 31** indicates **identity** Score **> 14** indicates **homology** | U | K.KHNFTPLAR.V |
| 20462 | 270 | – | 278 | 361.8734 | 1082.5982 | 1082.5985 | -0.24 | 1 | 38 | 0.00037 | 1Score **> 30** indicates **identity** Score **> 16** indicates **homology** | U | K.KHNFTPLAR.V |
| 20463 | 270 | – | 278 | 542.3065 | 1082.5985 | 1082.5985 | -0.023 | 1 | 33 | 0.0012 | 1Score **> 30** indicates **identity** Score **> 16** indicates **homology** | U | K.KHNFTPLAR.V |
| 20465 | 270 | – | 278 | 361.8737 | 1082.5992 | 1082.5985 | 0.65 | 1 | 35 | 0.00049 | 1Score **> 30** indicates **identity** Score **> 15** indicates **homology** | U | K.KHNFTPLAR.V |
| 20467 | 270 | – | 278 | 361.8739 | 1082.5998 | 1082.5985 | 1.24 | 1 | 21 | 0.01 | 1Score **> 30** indicates **identity** Score **> 14** indicates **homology** | U | K.KHNFTPLAR.V |
| 11404 | 271 | – | 278 | 478.2591 | 954.5036 | 954.5035 | 0.034 | 0 | 34 | 0.0027 | 1Score **> 28** indicates **identity** Score **> 20** indicates **homology** | U | K.HNFTPLAR.V |
| 11405 | 271 | – | 278 | 478.2593 | 954.5040 | 954.5035 | 0.46 | 0 | 29 | 0.0068 | 1Score **> 30** indicates **identity** Score **> 20** indicates **homology** | U | K.HNFTPLAR.V |
| 11406 | 271 | – | 278 | 478.2593 | 954.5040 | 954.5035 | 0.53 | 0 | 33 | 0.0027 | 1Score **> 30** indicates **identity** Score **> 20** indicates **homology** | U | K.HNFTPLAR.V |
| 11407 | 271 | – | 278 | 478.2594 | 954.5042 | 954.5035 | 0.72 | 0 | 22 | 0.023 | 1Score **> 30** indicates **identity** Score **> 18** indicates **homology** | U | K.HNFTPLAR.V |
| 11410 | 271 | – | 278 | 478.2599 | 954.5052 | 954.5035 | 1.74 | 0 | 33 | 0.0026 | 1Score **> 30** indicates **identity** Score **> 20** indicates **homology** | U | K.HNFTPLAR.V |
| 11411 | 271 | – | 278 | 478.2601 | 954.5056 | 954.5035 | 2.14 | 0 | 31 | 0.0046 | 1Score **> 29** indicates **identity** Score **> 20** indicates **homology** | U | K.HNFTPLAR.V |
| 11412 | 271 | – | 278 | 478.2601 | 954.5056 | 954.5035 | 2.18 | 0 | 27 | 0.0029 | 1Score **> 29** indicates **identity** Score **> 15** indicates **homology** | U | K.HNFTPLAR.V |
| 11413 | 271 | – | 278 | 478.2603 | 954.5060 | 954.5035 | 2.57 | 0 | 28 | 0.0063 | 1Score **> 29** indicates **identity** Score **> 18** indicates **homology** | U | K.HNFTPLAR.V |
| 11416 | 271 | – | 278 | 478.2607 | 954.5068 | 954.5035 | 3.43 | 0 | 23 | 0.0087 | 1Score **> 29** indicates **identity** Score **> 15** indicates **homology** | U | K.HNFTPLAR.V |
| 169255 | 279 | – | 306 | 954.4991 | 2860.4756 | 2860.4867 | -3.89 | 1 | 20 | 0.013 | 1Score **> 37** indicates **identity** Score **> 14** indicates **homology** | U | R.VVGYFVSGCDPTIMGIGPVPAINGALKK.A  + Deamidated (NQ) |
| 169256 | 279 | – | 306 | 954.5017 | 2860.4834 | 2860.4867 | -1.16 | 1 | 51 | 1.5e-05 | 1Score **> 37** indicates **identity** Score **> 16** indicates **homology** | U | R.VVGYFVSGCDPTIMGIGPVPAINGALKK.A  + Deamidated (NQ) |
| 168937 | 307 | – | 332 | 950.4991 | 2848.4754 | 2848.4681 | 2.57 | 1 | 71 | 2.3e-07 | 1Score **> 37** indicates **identity** Score **> 17** indicates **homology** | U | K.AGLSLKDMDLIDVNEAFAPQFLSVQK.A |
| 142440 | 313 | – | 332 | 1140.5666 | 2279.1187 | 2279.1144 | 1.89 | 0 | 66 | 3.9e-06 | 1Score **> 36** indicates **identity** Score **> 24** indicates **homology** | U | K.DMDLIDVNEAFAPQFLSVQK.A |
| 142442 | 313 | – | 332 | 1140.5719 | 2279.1292 | 2279.1144 | 6.52 | 0 | 31 | 0.0014 | 1Score **> 36** indicates **identity** Score **> 14** indicates **homology** | U | K.DMDLIDVNEAFAPQFLSVQK.A |
| 6252 | 333 | – | 340 | 429.7306 | 857.4466 | 857.4494 | -3.28 | 0 | 25 | 0.0091 | 1Score **> 28** indicates **identity** Score **> 17** indicates **homology** | U | K.ALDLDPSK.T |
| 6255 | 333 | – | 340 | 429.7316 | 857.4486 | 857.4494 | -0.94 | 0 | 43 | 0.00058 | 1Score **> 27** indicates **identity** Score **> 23** indicates **homology** | U | K.ALDLDPSK.T |
| 6256 | 333 | – | 340 | 429.7317 | 857.4489 | 857.4494 | -0.61 | 0 | 42 | 0.0011 | 1Score **> 27** indicates **identity** Score **> 25** indicates **homology** | U | K.ALDLDPSK.T |
| 161568 | 333 | – | 360 | 883.1321 | 2646.3746 | 2646.3725 | 0.77 | 1 | 48 | 3.4e-05 | 1Score **> 37** indicates **identity** Score **> 15** indicates **homology** | U | K.ALDLDPSKTNVSGGAIALGHPLGGSGSR.I |
| 161569 | 333 | – | 360 | 883.1324 | 2646.3755 | 2646.3725 | 1.10 | 1 | 75 | 8.6e-08 | 1Score **> 37** indicates **identity** Score **> 17** indicates **homology** | U | K.ALDLDPSKTNVSGGAIALGHPLGGSGSR.I |
| 161570 | 333 | – | 360 | 662.6012 | 2646.3757 | 2646.3725 | 1.21 | 1 | 32 | 0.00098 | 1Score **> 37** indicates **identity** Score **> 15** indicates **homology** | U | K.ALDLDPSKTNVSGGAIALGHPLGGSGSR.I |
| 161571 | 333 | – | 360 | 883.1327 | 2646.3762 | 2646.3725 | 1.37 | 1 | 35 | 0.00049 | 1Score **> 37** indicates **identity** Score **> 15** indicates **homology** | U | K.ALDLDPSKTNVSGGAIALGHPLGGSGSR.I |
| 161572 | 333 | – | 360 | 662.6013 | 2646.3762 | 2646.3725 | 1.38 | 1 | 29 | 0.0019 | 1Score **> 37** indicates **identity** Score **> 14** indicates **homology** | U | K.ALDLDPSKTNVSGGAIALGHPLGGSGSR.I |
| 161573 | 333 | – | 360 | 662.6014 | 2646.3763 | 2646.3725 | 1.43 | 1 | 37 | 0.00033 | 1Score **> 37** indicates **identity** Score **> 15** indicates **homology** | U | K.ALDLDPSKTNVSGGAIALGHPLGGSGSR.I |
| 161574 | 333 | – | 360 | 883.1328 | 2646.3767 | 2646.3725 | 1.56 | 1 | 50 | 1.9e-05 | 1Score **> 37** indicates **identity** Score **> 16** indicates **homology** | U | K.ALDLDPSKTNVSGGAIALGHPLGGSGSR.I |
| 161575 | 333 | – | 360 | 662.6015 | 2646.3767 | 2646.3725 | 1.57 | 1 | 49 | 2.5e-05 | 1Score **> 37** indicates **identity** Score **> 16** indicates **homology** | U | K.ALDLDPSKTNVSGGAIALGHPLGGSGSR.I |
| 161576 | 333 | – | 360 | 662.6015 | 2646.3769 | 2646.3725 | 1.65 | 1 | 51 | 1.6e-05 | 1Score **> 37** indicates **identity** Score **> 16** indicates **homology** | U | K.ALDLDPSKTNVSGGAIALGHPLGGSGSR.I |
| 161577 | 333 | – | 360 | 883.1330 | 2646.3771 | 2646.3725 | 1.71 | 1 | 26 | 0.0038 | 1Score **> 37** indicates **identity** Score **> 14** indicates **homology** | U | K.ALDLDPSKTNVSGGAIALGHPLGGSGSR.I |
| 161578 | 333 | – | 360 | 662.6018 | 2646.3779 | 2646.3725 | 2.04 | 1 | 43 | 9.4e-05 | 1Score **> 37** indicates **identity** Score **> 15** indicates **homology** | U | K.ALDLDPSKTNVSGGAIALGHPLGGSGSR.I |
| 161579 | 333 | – | 360 | 883.1333 | 2646.3779 | 2646.3725 | 2.04 | 1 | 70 | 2.8e-07 | 1Score **> 37** indicates **identity** Score **> 17** indicates **homology** | U | K.ALDLDPSKTNVSGGAIALGHPLGGSGSR.I |
| 161580 | 333 | – | 360 | 662.6018 | 2646.3780 | 2646.3725 | 2.06 | 1 | 41 | 0.00014 | 1Score **> 37** indicates **identity** Score **> 15** indicates **homology** | U | K.ALDLDPSKTNVSGGAIALGHPLGGSGSR.I |
| 102088 | 341 | – | 360 | 904.4724 | 1806.9302 | 1806.9337 | -1.91 | 0 | 69 | 3.2e-07 | 1Score **> 35** indicates **identity** Score **> 17** indicates **homology** | U | K.TNVSGGAIALGHPLGGSGSR.I |
| 102089 | 341 | – | 360 | 904.4728 | 1806.9310 | 1806.9337 | -1.48 | 0 | 81 | 2.3e-08 | 1Score **> 35** indicates **identity** Score **> 18** indicates **homology** | U | K.TNVSGGAIALGHPLGGSGSR.I |
| 102090 | 341 | – | 360 | 603.3182 | 1806.9327 | 1806.9337 | -0.53 | 0 | 21 | 0.015 | 1Score **> 35** indicates **identity** Score **> 15** indicates **homology** | U | K.TNVSGGAIALGHPLGGSGSR.I |
| 102091 | 341 | – | 360 | 603.3182 | 1806.9329 | 1806.9337 | -0.45 | 0 | 46 | 0.0008 | 1Score **> 35** indicates **identity** Score **> 27** indicates **homology** | U | K.TNVSGGAIALGHPLGGSGSR.I |
| 102092 | 341 | – | 360 | 904.4739 | 1806.9333 | 1806.9337 | -0.18 | 0 | 96 | 1e-09 | 1Score **> 35** indicates **identity** Score **> 18** indicates **homology** | U | K.TNVSGGAIALGHPLGGSGSR.I |
| 102093 | 341 | – | 360 | 603.3185 | 1806.9336 | 1806.9337 | -0.030 | 0 | 32 | 0.00095 | 1Score **> 36** indicates **identity** Score **> 15** indicates **homology** | U | K.TNVSGGAIALGHPLGGSGSR.I |
| 102094 | 341 | – | 360 | 603.3187 | 1806.9342 | 1806.9337 | 0.27 | 0 | 31 | 0.0012 | 1Score **> 36** indicates **identity** Score **> 14** indicates **homology** | U | K.TNVSGGAIALGHPLGGSGSR.I |
| 102095 | 341 | – | 360 | 603.3188 | 1806.9345 | 1806.9337 | 0.44 | 0 | 60 | 2.4e-05 | 1Score **> 36** indicates **identity** Score **> 27** indicates **homology** | U | K.TNVSGGAIALGHPLGGSGSR.I |
| 102097 | 341 | – | 360 | 603.3191 | 1806.9356 | 1806.9337 | 1.05 | 0 | 28 | 0.0021 | 1Score **> 36** indicates **identity** Score **> 14** indicates **homology** | U | K.TNVSGGAIALGHPLGGSGSR.I |
| 102099 | 341 | – | 360 | 904.4752 | 1806.9358 | 1806.9337 | 1.20 | 0 | 116 | 2e-11 | 1Score **> 36** indicates **identity** Score **> 21** indicates **homology** | U | K.TNVSGGAIALGHPLGGSGSR.I |
| 102100 | 341 | – | 360 | 603.3193 | 1806.9360 | 1806.9337 | 1.27 | 0 | 46 | 0.00072 | 1Score **> 36** indicates **identity** Score **> 27** indicates **homology** | U | K.TNVSGGAIALGHPLGGSGSR.I |
| 102102 | 341 | – | 360 | 603.3199 | 1806.9380 | 1806.9337 | 2.38 | 0 | 33 | 0.0015 | 1Score **> 36** indicates **identity** Score **> 17** indicates **homology** | U | K.TNVSGGAIALGHPLGGSGSR.I |
| 31419 | 361 | – | 370 | 396.8997 | 1187.6771 | 1187.6775 | -0.29 | 0 | 32 | 0.01 | 1Score **> 33** indicates **identity** Score **> 25** indicates **homology** | U | R.ITAHLVHELR.R |
| 31420 | 361 | – | 370 | 396.8997 | 1187.6771 | 1187.6775 | -0.28 | 0 | 30 | 0.0078 | 1Score **> 33** indicates **identity** Score **> 21** indicates **homology** | U | R.ITAHLVHELR.R |
| 31421 | 361 | – | 370 | 396.8997 | 1187.6773 | 1187.6775 | -0.15 | 0 | 37 | 0.0077 | 1Score **> 33** indicates **identity** Score **> 29** indicates **homology** | U | R.ITAHLVHELR.R |
| 31423 | 361 | – | 370 | 396.9005 | 1187.6798 | 1187.6775 | 1.95 | 0 | 35 | 0.006 | 1Score **> 33** indicates **identity** Score **> 25** indicates **homology** | U | R.ITAHLVHELR.R |
| 31424 | 361 | – | 370 | 396.9012 | 1187.6818 | 1187.6775 | 3.63 | 0 | 28 | 0.013 | 1Score **> 33** indicates **identity** Score **> 21** indicates **homology** | U | R.ITAHLVHELR.R |
| 48232 | 361 | – | 371 | 448.9335 | 1343.7786 | 1343.7786 | -0.00074 | 1 | 34 | 0.00064 | 1Score **> 34** indicates **identity** Score **> 15** indicates **homology** | U | R.ITAHLVHELRR.R |
| 48233 | 361 | – | 371 | 448.9336 | 1343.7790 | 1343.7786 | 0.31 | 1 | 30 | 0.0028 | 1Score **> 34** indicates **identity** Score **> 17** indicates **homology** | U | R.ITAHLVHELRR.R |
| 48236 | 361 | – | 371 | 448.9338 | 1343.7796 | 1343.7786 | 0.73 | 1 | 35 | 0.039 | 1Score **> 33** indicates **identity** | U | R.ITAHLVHELRR.R |
| 48238 | 361 | – | 371 | 448.9341 | 1343.7806 | 1343.7786 | 1.48 | 1 | 35 | 0.038 | 1Score **> 33** indicates **identity** | U | R.ITAHLVHELRR.R |

---

```
ID   THIM_MOUSE              Reviewed;         397 AA.
AC   Q8BWT1; Q3TIT9; Q8JZR8;
DT   30-AUG-2005, integrated into UniProtKB/Swiss-Prot.
DT   27-JUL-2011, sequence version 3.
DT   28-JUN-2023, entry version 149.
DE   RecName: Full=3-ketoacyl-CoA thiolase, mitochondrial {ECO:0000305};
DE            EC=2.3.1.16 {ECO:0000250|UniProtKB:P42765};
DE   AltName: Full=Acetyl-CoA acetyltransferase {ECO:0000305};
DE            EC=2.3.1.9 {ECO:0000255|PROSITE-ProRule:PRU10020};
DE   AltName: Full=Acetyl-CoA acyltransferase;
DE   AltName: Full=Acyl-CoA hydrolase, mitochondrial {ECO:0000305};
DE            EC=3.1.2.- {ECO:0000250|UniProtKB:P42765};
DE            EC=3.1.2.1 {ECO:0000250|UniProtKB:P42765};
DE            EC=3.1.2.2 {ECO:0000250|UniProtKB:P13437};
DE   AltName: Full=Beta-ketothiolase;
DE   AltName: Full=Mitochondrial 3-oxoacyl-CoA thiolase;
GN   Name=Acaa2;
OS   Mus musculus (Mouse).
OC   Eukaryota; Metazoa; Chordata; Craniata; Vertebrata; Euteleostomi; Mammalia;
OC   Eutheria; Euarchontoglires; Glires; Rodentia; Myomorpha; Muroidea; Muridae;
OC   Murinae; Mus; Mus.
OX   NCBI_TaxID=10090;
RN   [1]
RP   NUCLEOTIDE SEQUENCE [LARGE SCALE MRNA].
RC   STRAIN=C57BL/6J; TISSUE=Amnion, Heart, and Liver;
RX   PubMed=16141072; DOI=10.1126/science.1112014;
RA   Carninci P., Kasukawa T., Katayama S., Gough J., Frith M.C., Maeda N.,
RA   Oyama R., Ravasi T., Lenhard B., Wells C., Kodzius R., Shimokawa K.,
RA   Bajic V.B., Brenner S.E., Batalov S., Forrest A.R., Zavolan M., Davis M.J.,
RA   Wilming L.G., Aidinis V., Allen J.E., Ambesi-Impiombato A., Apweiler R.,
RA   Aturaliya R.N., Bailey T.L., Bansal M., Baxter L., Beisel K.W., Bersano T.,
RA   Bono H., Chalk A.M., Chiu K.P., Choudhary V., Christoffels A.,
RA   Clutterbuck D.R., Crowe M.L., Dalla E., Dalrymple B.P., de Bono B.,
RA   Della Gatta G., di Bernardo D., Down T., Engstrom P., Fagiolini M.,
RA   Faulkner G., Fletcher C.F., Fukushima T., Furuno M., Futaki S.,
RA   Gariboldi M., Georgii-Hemming P., Gingeras T.R., Gojobori T., Green R.E.,
RA   Gustincich S., Harbers M., Hayashi Y., Hensch T.K., Hirokawa N., Hill D.,
RA   Huminiecki L., Iacono M., Ikeo K., Iwama A., Ishikawa T., Jakt M.,
RA   Kanapin A., Katoh M., Kawasawa Y., Kelso J., Kitamura H., Kitano H.,
RA   Kollias G., Krishnan S.P., Kruger A., Kummerfeld S.K., Kurochkin I.V.,
RA   Lareau L.F., Lazarevic D., Lipovich L., Liu J., Liuni S., McWilliam S.,
RA   Madan Babu M., Madera M., Marchionni L., Matsuda H., Matsuzawa S., Miki H.,
RA   Mignone F., Miyake S., Morris K., Mottagui-Tabar S., Mulder N., Nakano N.,
RA   Nakauchi H., Ng P., Nilsson R., Nishiguchi S., Nishikawa S., Nori F.,
RA   Ohara O., Okazaki Y., Orlando V., Pang K.C., Pavan W.J., Pavesi G.,
RA   Pesole G., Petrovsky N., Piazza S., Reed J., Reid J.F., Ring B.Z.,
RA   Ringwald M., Rost B., Ruan Y., Salzberg S.L., Sandelin A., Schneider C.,
RA   Schoenbach C., Sekiguchi K., Semple C.A., Seno S., Sessa L., Sheng Y.,
RA   Shibata Y., Shimada H., Shimada K., Silva D., Sinclair B., Sperling S.,
RA   Stupka E., Sugiura K., Sultana R., Takenaka Y., Taki K., Tammoja K.,
RA   Tan S.L., Tang S., Taylor M.S., Tegner J., Teichmann S.A., Ueda H.R.,
RA   van Nimwegen E., Verardo R., Wei C.L., Yagi K., Yamanishi H.,
RA   Zabarovsky E., Zhu S., Zimmer A., Hide W., Bult C., Grimmond S.M.,
RA   Teasdale R.D., Liu E.T., Brusic V., Quackenbush J., Wahlestedt C.,
RA   Mattick J.S., Hume D.A., Kai C., Sasaki D., Tomaru Y., Fukuda S.,
RA   Kanamori-Katayama M., Suzuki M., Aoki J., Arakawa T., Iida J., Imamura K.,
RA   Itoh M., Kato T., Kawaji H., Kawagashira N., Kawashima T., Kojima M.,
RA   Kondo S., Konno H., Nakano K., Ninomiya N., Nishio T., Okada M., Plessy C.,
RA   Shibata K., Shiraki T., Suzuki S., Tagami M., Waki K., Watahiki A.,
RA   Okamura-Oho Y., Suzuki H., Kawai J., Hayashizaki Y.;
RT   "The transcriptional landscape of the mammalian genome.";
RL   Science 309:1559-1563(2005).
RN   [2]
RP   NUCLEOTIDE SEQUENCE [LARGE SCALE GENOMIC DNA].
RA   Mural R.J., Adams M.D., Myers E.W., Smith H.O., Venter J.C.;
RL   Submitted (SEP-2005) to the EMBL/GenBank/DDBJ databases.
RN   [3]
RP   NUCLEOTIDE SEQUENCE [LARGE SCALE MRNA].
RC   STRAIN=FVB/N; TISSUE=Liver;
RX   PubMed=15489334; DOI=10.1101/gr.2596504;
RG   The MGC Project Team;
RT   "The status, quality, and expansion of the NIH full-length cDNA project:
RT   the Mammalian Gene Collection (MGC).";
RL   Genome Res. 14:2121-2127(2004).
RN   [4]
RP   PHOSPHORYLATION [LARGE SCALE ANALYSIS] AT SER-344, AND IDENTIFICATION BY
RP   MASS SPECTROMETRY [LARGE SCALE ANALYSIS].
RC   TISSUE=Liver;
RX   PubMed=17208939; DOI=10.1074/mcp.m600218-mcp200;
RA   Lee J., Xu Y., Chen Y., Sprung R., Kim S.C., Xie S., Zhao Y.;
RT   "Mitochondrial phosphoproteome revealed by an improved IMAC method and
RT   MS/MS/MS.";
RL   Mol. Cell. Proteomics 6:669-676(2007).
RN   [5]
RP   PHOSPHORYLATION [LARGE SCALE ANALYSIS] AT SER-28, AND IDENTIFICATION BY
RP   MASS SPECTROMETRY [LARGE SCALE ANALYSIS].
RC   TISSUE=Liver;
RX   PubMed=17242355; DOI=10.1073/pnas.0609836104;
RA   Villen J., Beausoleil S.A., Gerber S.A., Gygi S.P.;
RT   "Large-scale phosphorylation analysis of mouse liver.";
RL   Proc. Natl. Acad. Sci. U.S.A. 104:1488-1493(2007).
RN   [6]
RP   PHOSPHORYLATION [LARGE SCALE ANALYSIS] AT SER-28; THR-136 AND SER-310, AND
RP   IDENTIFICATION BY MASS SPECTROMETRY [LARGE SCALE ANALYSIS].
RC   TISSUE=Brain, Brown adipose tissue, Heart, Kidney, Liver, Lung,
RC   Pancreas, Spleen, and Testis;
RX   PubMed=21183079; DOI=10.1016/j.cell.2010.12.001;
RA   Huttlin E.L., Jedrychowski M.P., Elias J.E., Goswami T., Rad R.,
RA   Beausoleil S.A., Villen J., Haas W., Sowa M.E., Gygi S.P.;
RT   "A tissue-specific atlas of mouse protein phosphorylation and expression.";
RL   Cell 143:1174-1189(2010).
RN   [7]
RP   ACETYLATION [LARGE SCALE ANALYSIS] AT LYS-191, SUCCINYLATION [LARGE SCALE
RP   ANALYSIS] AT LYS-25; LYS-45; LYS-137; LYS-143; LYS-158; LYS-171; LYS-191;
RP   LYS-209; LYS-211; LYS-212; LYS-214; LYS-234; LYS-240; LYS-305 AND LYS-312,
RP   AND IDENTIFICATION BY MASS SPECTROMETRY [LARGE SCALE ANALYSIS].
RC   TISSUE=Liver;
RX   PubMed=23806337; DOI=10.1016/j.molcel.2013.06.001;
RA   Park J., Chen Y., Tishkoff D.X., Peng C., Tan M., Dai L., Xie Z., Zhang Y.,
RA   Zwaans B.M., Skinner M.E., Lombard D.B., Zhao Y.;
RT   "SIRT5-mediated lysine desuccinylation impacts diverse metabolic
RT   pathways.";
RL   Mol. Cell 50:919-930(2013).
RN   [8]
RP   ACETYLATION [LARGE SCALE ANALYSIS] AT LYS-25; LYS-137; LYS-143; LYS-158;
RP   LYS-171; LYS-191; LYS-209; LYS-234; LYS-241; LYS-269; LYS-270; LYS-305;
RP   LYS-312; LYS-340 AND LYS-375, AND IDENTIFICATION BY MASS SPECTROMETRY
RP   [LARGE SCALE ANALYSIS].
RC   TISSUE=Liver;
RX   PubMed=23576753; DOI=10.1073/pnas.1302961110;
RA   Rardin M.J., Newman J.C., Held J.M., Cusack M.P., Sorensen D.J., Li B.,
RA   Schilling B., Mooney S.D., Kahn C.R., Verdin E., Gibson B.W.;
RT   "Label-free quantitative proteomics of the lysine acetylome in mitochondria
RT   identifies substrates of SIRT3 in metabolic pathways.";
RL   Proc. Natl. Acad. Sci. U.S.A. 110:6601-6606(2013).
CC   -!- FUNCTION: In the production of energy from fats, this is one of the
CC       enzymes that catalyzes the last step of the mitochondrial beta-
CC       oxidation pathway, an aerobic process breaking down fatty acids into
CC       acetyl-CoA. Using free coenzyme A/CoA, catalyzes the thiolytic cleavage
CC       of medium- to long-chain unbranched 3-oxoacyl-CoAs into acetyl-CoA and
CC       a fatty acyl-CoA shortened by two carbon atoms. Also catalyzes the
CC       condensation of two acetyl-CoA molecules into acetoacetyl-CoA and could
CC       be involved in the production of ketone bodies. Also displays hydrolase
CC       activity on various fatty acyl-CoAs (By similarity). Thereby, could be
CC       responsible for the production of acetate in a side reaction to beta-
CC       oxidation (By similarity). Abolishes BNIP3-mediated apoptosis and
CC       mitochondrial damage (By similarity). {ECO:0000250|UniProtKB:P13437,
CC       ECO:0000250|UniProtKB:P42765}.
CC   -!- CATALYTIC ACTIVITY:
CC       Reaction=acetyl-CoA + an acyl-CoA = a 3-oxoacyl-CoA + CoA;
CC         Xref=Rhea:RHEA:21564, ChEBI:CHEBI:57287, ChEBI:CHEBI:57288,
CC         ChEBI:CHEBI:58342, ChEBI:CHEBI:90726; EC=2.3.1.16;
CC         Evidence={ECO:0000250|UniProtKB:P42765};
CC       PhysiologicalDirection=left-to-right; Xref=Rhea:RHEA:21565;
CC         Evidence={ECO:0000250|UniProtKB:P42765};
CC       PhysiologicalDirection=right-to-left; Xref=Rhea:RHEA:21566;
CC         Evidence={ECO:0000250|UniProtKB:P42765};
CC   -!- CATALYTIC ACTIVITY:
CC       Reaction=2 acetyl-CoA = acetoacetyl-CoA + CoA; Xref=Rhea:RHEA:21036,
CC         ChEBI:CHEBI:57286, ChEBI:CHEBI:57287, ChEBI:CHEBI:57288; EC=2.3.1.9;
CC         Evidence={ECO:0000255|PROSITE-ProRule:PRU10020};
CC       PhysiologicalDirection=left-to-right; Xref=Rhea:RHEA:21037;
CC         Evidence={ECO:0000250|UniProtKB:P42765};
CC       PhysiologicalDirection=right-to-left; Xref=Rhea:RHEA:21038;
CC         Evidence={ECO:0000250|UniProtKB:P42765};
CC   -!- CATALYTIC ACTIVITY:
CC       Reaction=acetyl-CoA + H2O = acetate + CoA + H(+); Xref=Rhea:RHEA:20289,
CC         ChEBI:CHEBI:15377, ChEBI:CHEBI:15378, ChEBI:CHEBI:30089,
CC         ChEBI:CHEBI:57287, ChEBI:CHEBI:57288; EC=3.1.2.1;
CC         Evidence={ECO:0000250|UniProtKB:P42765};
CC       PhysiologicalDirection=left-to-right; Xref=Rhea:RHEA:20290;
CC         Evidence={ECO:0000250|UniProtKB:P42765};
CC   -!- CATALYTIC ACTIVITY:
CC       Reaction=H2O + propanoyl-CoA = CoA + H(+) + propanoate;
CC         Xref=Rhea:RHEA:40103, ChEBI:CHEBI:15377, ChEBI:CHEBI:15378,
CC         ChEBI:CHEBI:17272, ChEBI:CHEBI:57287, ChEBI:CHEBI:57392;
CC         Evidence={ECO:0000250|UniProtKB:P42765};
CC       PhysiologicalDirection=left-to-right; Xref=Rhea:RHEA:40104;
CC         Evidence={ECO:0000250|UniProtKB:P42765};
CC   -!- CATALYTIC ACTIVITY:
CC       Reaction=butanoyl-CoA + H2O = butanoate + CoA + H(+);
CC         Xref=Rhea:RHEA:40111, ChEBI:CHEBI:15377, ChEBI:CHEBI:15378,
CC         ChEBI:CHEBI:17968, ChEBI:CHEBI:57287, ChEBI:CHEBI:57371;
CC         Evidence={ECO:0000250|UniProtKB:P42765};
CC       PhysiologicalDirection=left-to-right; Xref=Rhea:RHEA:40112;
CC         Evidence={ECO:0000250|UniProtKB:P42765};
CC   -!- CATALYTIC ACTIVITY:
CC       Reaction=H2O + hexanoyl-CoA = CoA + H(+) + hexanoate;
CC         Xref=Rhea:RHEA:40115, ChEBI:CHEBI:15377, ChEBI:CHEBI:15378,
CC         ChEBI:CHEBI:17120, ChEBI:CHEBI:57287, ChEBI:CHEBI:62620;
CC         Evidence={ECO:0000250|UniProtKB:P42765};
CC       PhysiologicalDirection=left-to-right; Xref=Rhea:RHEA:40116;
CC         Evidence={ECO:0000250|UniProtKB:P42765};
CC   -!- CATALYTIC ACTIVITY:
CC       Reaction=H2O + octanoyl-CoA = CoA + H(+) + octanoate;
CC         Xref=Rhea:RHEA:30143, ChEBI:CHEBI:15377, ChEBI:CHEBI:15378,
CC         ChEBI:CHEBI:25646, ChEBI:CHEBI:57287, ChEBI:CHEBI:57386;
CC         Evidence={ECO:0000250|UniProtKB:P42765};
CC       PhysiologicalDirection=left-to-right; Xref=Rhea:RHEA:30144;
CC         Evidence={ECO:0000250|UniProtKB:P42765};
CC   -!- CATALYTIC ACTIVITY:
CC       Reaction=decanoyl-CoA + H2O = CoA + decanoate + H(+);
CC         Xref=Rhea:RHEA:40059, ChEBI:CHEBI:15377, ChEBI:CHEBI:15378,
CC         ChEBI:CHEBI:27689, ChEBI:CHEBI:57287, ChEBI:CHEBI:61430;
CC         Evidence={ECO:0000250|UniProtKB:P42765};
CC       PhysiologicalDirection=left-to-right; Xref=Rhea:RHEA:40060;
CC         Evidence={ECO:0000250|UniProtKB:P42765};
CC   -!- CATALYTIC ACTIVITY:
CC       Reaction=dodecanoyl-CoA + H2O = CoA + dodecanoate + H(+);
CC         Xref=Rhea:RHEA:30135, ChEBI:CHEBI:15377, ChEBI:CHEBI:15378,
CC         ChEBI:CHEBI:18262, ChEBI:CHEBI:57287, ChEBI:CHEBI:57375;
CC         Evidence={ECO:0000250|UniProtKB:P42765};
CC       PhysiologicalDirection=left-to-right; Xref=Rhea:RHEA:30136;
CC         Evidence={ECO:0000250|UniProtKB:P42765};
CC   -!- CATALYTIC ACTIVITY:
CC       Reaction=H2O + tetradecanoyl-CoA = CoA + H(+) + tetradecanoate;
CC         Xref=Rhea:RHEA:40119, ChEBI:CHEBI:15377, ChEBI:CHEBI:15378,
CC         ChEBI:CHEBI:30807, ChEBI:CHEBI:57287, ChEBI:CHEBI:57385;
CC         Evidence={ECO:0000250|UniProtKB:P42765};
CC       PhysiologicalDirection=left-to-right; Xref=Rhea:RHEA:40120;
CC         Evidence={ECO:0000250|UniProtKB:P42765};
CC   -!- CATALYTIC ACTIVITY:
CC       Reaction=H2O + hexadecanoyl-CoA = CoA + H(+) + hexadecanoate;
CC         Xref=Rhea:RHEA:16645, ChEBI:CHEBI:7896, ChEBI:CHEBI:15377,
CC         ChEBI:CHEBI:15378, ChEBI:CHEBI:57287, ChEBI:CHEBI:57379; EC=3.1.2.2;
CC         Evidence={ECO:0000250|UniProtKB:P13437};
CC       PhysiologicalDirection=left-to-right; Xref=Rhea:RHEA:16646;
CC         Evidence={ECO:0000250|UniProtKB:P42765};
CC   -!- PATHWAY: Lipid metabolism; fatty acid beta-oxidation.
CC       {ECO:0000250|UniProtKB:P42765}.
CC   -!- SUBUNIT: Homotetramer. Interacts with BNIP3.
CC       {ECO:0000250|UniProtKB:P42765}.
CC   -!- SUBCELLULAR LOCATION: Mitochondrion {ECO:0000250|UniProtKB:P42765}.
CC   -!- SIMILARITY: Belongs to the thiolase-like superfamily. Thiolase family.
CC       {ECO:0000305}.
CC   ---------------------------------------------------------------------------
CC   Copyrighted by the UniProt Consortium, see https://www.uniprot.org/terms
CC   Distributed under the Creative Commons Attribution (CC BY 4.0) License
CC   ---------------------------------------------------------------------------
DR   EMBL; AK050101; BAC34067.1; -; mRNA.
DR   EMBL; AK167567; BAE39630.1; -; mRNA.
DR   EMBL; AK167715; BAE39757.1; -; mRNA.
DR   EMBL; AK169359; BAE41108.1; -; mRNA.
DR   EMBL; CH466528; EDL09521.1; -; Genomic_DNA.
DR   EMBL; BC028901; AAH28901.1; -; mRNA.
DR   CCDS; CCDS29342.1; -.
DR   RefSeq; NP_803421.1; NM_177470.3.
DR   AlphaFoldDB; Q8BWT1; -.
DR   SMR; Q8BWT1; -.
DR   BioGRID; 206649; 30.
DR   IntAct; Q8BWT1; 9.
DR   MINT; Q8BWT1; -.
DR   STRING; 10090.ENSMUSP00000037348; -.
DR   GlyGen; Q8BWT1; 1 site, 1 O-linked glycan (1 site).
DR   iPTMnet; Q8BWT1; -.
DR   PhosphoSitePlus; Q8BWT1; -.
DR   SwissPalm; Q8BWT1; -.
DR   REPRODUCTION-2DPAGE; Q8BWT1; -.
DR   CPTAC; non-CPTAC-3618; -.
DR   CPTAC; non-CPTAC-3883; -.
DR   EPD; Q8BWT1; -.
DR   jPOST; Q8BWT1; -.
DR   MaxQB; Q8BWT1; -.
DR   PaxDb; Q8BWT1; -.
DR   PeptideAtlas; Q8BWT1; -.
DR   ProteomicsDB; 258872; -.
DR   Antibodypedia; 22617; 409 antibodies from 30 providers.
DR   Ensembl; ENSMUST00000041053; ENSMUSP00000037348; ENSMUSG00000036880.
DR   GeneID; 52538; -.
DR   KEGG; mmu:52538; -.
DR   UCSC; uc008fpt.1; mouse.
DR   AGR; MGI:1098623; -.
DR   CTD; 10449; -.
DR   MGI; MGI:1098623; Acaa2.
DR   VEuPathDB; HostDB:ENSMUSG00000036880; -.
DR   eggNOG; KOG1391; Eukaryota.
DR   GeneTree; ENSGT01030000234626; -.
DR   HOGENOM; CLU_031026_0_0_1; -.
DR   InParanoid; Q8BWT1; -.
DR   OMA; DYYWGMG; -.
DR   OrthoDB; 5481312at2759; -.
DR   PhylomeDB; Q8BWT1; -.
DR   TreeFam; TF105696; -.
DR   BRENDA; 2.3.1.16; 3474.
DR   Reactome; R-MMU-77289; Mitochondrial Fatty Acid Beta-Oxidation.
DR   UniPathway; UPA00659; -.
DR   BioGRID-ORCS; 52538; 1 hit in 80 CRISPR screens.
DR   ChiTaRS; Acaa2; mouse.
DR   PRO; PR:Q8BWT1; -.
DR   Proteomes; UP000000589; Chromosome 18.
DR   RNAct; Q8BWT1; protein.
DR   Bgee; ENSMUSG00000036880; Expressed in gall bladder and 285 other tissues.
DR   ExpressionAtlas; Q8BWT1; baseline and differential.
DR   Genevisible; Q8BWT1; MM.
DR   GO; GO:0005743; C:mitochondrial inner membrane; HDA:MGI.
DR   GO; GO:0005759; C:mitochondrial matrix; ISO:MGI.
DR   GO; GO:0005739; C:mitochondrion; HDA:MGI.
DR   GO; GO:0003985; F:acetyl-CoA C-acetyltransferase activity; ISS:UniProtKB.
DR   GO; GO:0003988; F:acetyl-CoA C-acyltransferase activity; ISS:UniProtKB.
DR   GO; GO:0003986; F:acetyl-CoA hydrolase activity; IEA:UniProtKB-EC.
DR   GO; GO:0047617; F:acyl-CoA hydrolase activity; ISS:UniProtKB.
DR   GO; GO:0102991; F:myristoyl-CoA hydrolase activity; IEA:UniProtKB-EC.
DR   GO; GO:0016290; F:palmitoyl-CoA hydrolase activity; IEA:UniProtKB-EC.
DR   GO; GO:0006084; P:acetyl-CoA metabolic process; ISO:MGI.
DR   GO; GO:0071456; P:cellular response to hypoxia; ISO:MGI.
DR   GO; GO:0006635; P:fatty acid beta-oxidation; ISO:MGI.
DR   GO; GO:1902109; P:negative regulation of mitochondrial membrane permeability involved in apoptotic process; ISO:MGI.
DR   GO; GO:1901029; P:negative regulation of mitochondrial outer membrane permeabilization involved in apoptotic signaling pathway; ISS:UniProtKB.
DR   CDD; cd00751; thiolase; 1.
DR   Gene3D; 3.40.47.10; -; 2.
DR   InterPro; IPR002155; Thiolase.
DR   InterPro; IPR016039; Thiolase-like.
DR   InterPro; IPR020615; Thiolase_acyl_enz_int_AS.
DR   InterPro; IPR020610; Thiolase_AS.
DR   InterPro; IPR020617; Thiolase_C.
DR   InterPro; IPR020613; Thiolase_CS.
DR   InterPro; IPR020616; Thiolase_N.
DR   PANTHER; PTHR18919:SF107; 3-KETOACYL-COA THIOLASE, MITOCHONDRIAL; 1.
DR   PANTHER; PTHR18919; ACETYL-COA C-ACYLTRANSFERASE; 1.
DR   Pfam; PF02803; Thiolase_C; 1.
DR   Pfam; PF00108; Thiolase_N; 1.
DR   PIRSF; PIRSF000429; Ac-CoA_Ac_transf; 1.
DR   SUPFAM; SSF53901; Thiolase-like; 2.
DR   PROSITE; PS00098; THIOLASE_1; 1.
DR   PROSITE; PS00737; THIOLASE_2; 1.
DR   PROSITE; PS00099; THIOLASE_3; 1.
DR   TIGRFAMs; TIGR01930; AcCoA-C-Actrans; 1.
PE   1: Evidence at protein level;
KW   Acetylation; Acyltransferase; Fatty acid metabolism; Hydrolase;
KW   Lipid metabolism; Mitochondrion; Phosphoprotein; Reference proteome;
KW   Transferase; Transit peptide.
FT   CHAIN           1..397
FT                   /note="3-ketoacyl-CoA thiolase, mitochondrial"
FT                   /id="PRO_0000223300"
FT   TRANSIT         1..16
FT                   /note="Mitochondrion; not cleaved"
FT                   /evidence="ECO:0000250"
FT   ACT_SITE        92
FT                   /note="Acyl-thioester intermediate"
FT                   /evidence="ECO:0000250|UniProtKB:P42765"
FT   ACT_SITE        382
FT                   /note="Proton donor/acceptor"
FT                   /evidence="ECO:0000250|UniProtKB:P42765"
FT   BINDING         224
FT                   /ligand="CoA"
FT                   /ligand_id="ChEBI:CHEBI:57287"
FT                   /evidence="ECO:0000250|UniProtKB:P42765"
FT   BINDING         227
FT                   /ligand="CoA"
FT                   /ligand_id="ChEBI:CHEBI:57287"
FT                   /evidence="ECO:0000250|UniProtKB:P42765"
FT   BINDING         251
FT                   /ligand="CoA"
FT                   /ligand_id="ChEBI:CHEBI:57287"
FT                   /evidence="ECO:0000250|UniProtKB:P42765"
FT   SITE            352
FT                   /note="Increases nucleophilicity of active site Cys"
FT                   /evidence="ECO:0000250|UniProtKB:P42765"
FT   MOD_RES         25
FT                   /note="N6-acetyllysine; alternate"
FT                   /evidence="ECO:0007744|PubMed:23576753"
FT   MOD_RES         25
FT                   /note="N6-succinyllysine; alternate"
FT                   /evidence="ECO:0007744|PubMed:23806337"
FT   MOD_RES         28
FT                   /note="Phosphoserine"
FT                   /evidence="ECO:0007744|PubMed:17242355,
FT                   ECO:0007744|PubMed:21183079"
FT   MOD_RES         45
FT                   /note="N6-succinyllysine"
FT                   /evidence="ECO:0007744|PubMed:23806337"
FT   MOD_RES         119
FT                   /note="Phosphothreonine"
FT                   /evidence="ECO:0000250|UniProtKB:P42765"
FT   MOD_RES         121
FT                   /note="Phosphoserine"
FT                   /evidence="ECO:0000250|UniProtKB:P42765"
FT   MOD_RES         127
FT                   /note="Phosphotyrosine"
FT                   /evidence="ECO:0000250|UniProtKB:P42765"
FT   MOD_RES         136
FT                   /note="Phosphothreonine"
FT                   /evidence="ECO:0007744|PubMed:21183079"
FT   MOD_RES         137
FT                   /note="N6-acetyllysine; alternate"
FT                   /evidence="ECO:0007744|PubMed:23576753"
FT   MOD_RES         137
FT                   /note="N6-succinyllysine; alternate"
FT                   /evidence="ECO:0007744|PubMed:23806337"
FT   MOD_RES         143
FT                   /note="N6-acetyllysine; alternate"
FT                   /evidence="ECO:0007744|PubMed:23576753"
FT   MOD_RES         143
FT                   /note="N6-succinyllysine; alternate"
FT                   /evidence="ECO:0007744|PubMed:23806337"
FT   MOD_RES         158
FT                   /note="N6-acetyllysine; alternate"
FT                   /evidence="ECO:0007744|PubMed:23576753"
FT   MOD_RES         158
FT                   /note="N6-succinyllysine; alternate"
FT                   /evidence="ECO:0007744|PubMed:23806337"
FT   MOD_RES         171
FT                   /note="N6-acetyllysine; alternate"
FT                   /evidence="ECO:0007744|PubMed:23576753"
FT   MOD_RES         171
FT                   /note="N6-succinyllysine; alternate"
FT                   /evidence="ECO:0007744|PubMed:23806337"
FT   MOD_RES         191
FT                   /note="N6-acetyllysine; alternate"
FT                   /evidence="ECO:0007744|PubMed:23576753,
FT                   ECO:0007744|PubMed:23806337"
FT   MOD_RES         191
FT                   /note="N6-succinyllysine; alternate"
FT                   /evidence="ECO:0007744|PubMed:23806337"
FT   MOD_RES         209
FT                   /note="N6-acetyllysine; alternate"
FT                   /evidence="ECO:0007744|PubMed:23576753"
FT   MOD_RES         209
FT                   /note="N6-succinyllysine; alternate"
FT                   /evidence="ECO:0007744|PubMed:23806337"
FT   MOD_RES         211
FT                   /note="N6-succinyllysine"
FT                   /evidence="ECO:0007744|PubMed:23806337"
FT   MOD_RES         212
FT                   /note="N6-succinyllysine"
FT                   /evidence="ECO:0007744|PubMed:23806337"
FT   MOD_RES         214
FT                   /note="N6-succinyllysine"
FT                   /evidence="ECO:0007744|PubMed:23806337"
FT   MOD_RES         234
FT                   /note="N6-acetyllysine; alternate"
FT                   /evidence="ECO:0007744|PubMed:23576753"
FT   MOD_RES         234
FT                   /note="N6-succinyllysine; alternate"
FT                   /evidence="ECO:0007744|PubMed:23806337"
FT   MOD_RES         240
FT                   /note="N6-succinyllysine"
FT                   /evidence="ECO:0007744|PubMed:23806337"
FT   MOD_RES         241
FT                   /note="N6-acetyllysine"
FT                   /evidence="ECO:0007744|PubMed:23576753"
FT   MOD_RES         269
FT                   /note="N6-acetyllysine"
FT                   /evidence="ECO:0007744|PubMed:23576753"
FT   MOD_RES         270
FT                   /note="N6-acetyllysine"
FT                   /evidence="ECO:0007744|PubMed:23576753"
FT   MOD_RES         305
FT                   /note="N6-acetyllysine; alternate"
FT                   /evidence="ECO:0007744|PubMed:23576753"
FT   MOD_RES         305
FT                   /note="N6-succinyllysine; alternate"
FT                   /evidence="ECO:0007744|PubMed:23806337"
FT   MOD_RES         310
FT                   /note="Phosphoserine"
FT                   /evidence="ECO:0007744|PubMed:21183079"
FT   MOD_RES         312
FT                   /note="N6-acetyllysine; alternate"
FT                   /evidence="ECO:0007744|PubMed:23576753"
FT   MOD_RES         312
FT                   /note="N6-succinyllysine; alternate"
FT                   /evidence="ECO:0007744|PubMed:23806337"
FT   MOD_RES         340
FT                   /note="N6-acetyllysine"
FT                   /evidence="ECO:0007744|PubMed:23576753"
FT   MOD_RES         344
FT                   /note="Phosphoserine"
FT                   /evidence="ECO:0007744|PubMed:17208939"
FT   MOD_RES         375
FT                   /note="N6-acetyllysine"
FT                   /evidence="ECO:0007744|PubMed:23576753"
FT   CONFLICT        397
FT                   /note="A -> V (in Ref. 3; AAH28901)"
FT                   /evidence="ECO:0000305"
SQ   SEQUENCE   397 AA;  41830 MW;  856840546F5CB8DA CRC64;
     MALLRGVFIV AAKRTPFGAY GGLLKDFSAT DLTEFAARAA LSAGKVPPET IDSVIVGNVM
     QSSSDAAYLA RHVGLRVGVP TETGALTLNR LCGSGFQSIV SGCQEICSKD AEVVLCGGTE
     SMSQSPYCVR NVRFGTKFGL DLKLEDTLWA GLTDQHVKLP MGMTAENLAA KYNISREDCD
     RYALQSQQRW KAANEAGYFN EEMAPIEVKT KKGKQTMQVD EHARPQTTLE QLQKLPSVFK
     KDGTVTAGNA SGVSDGAGAV IIASEDAVKK HNFTPLARVV GYFVSGCDPT IMGIGPVPAI
     NGALKKAGLS LKDMDLIDVN EAFAPQFLSV QKALDLDPSK TNVSGGAIAL GHPLGGSGSR
     ITAHLVHELR RRGGKYAVGS ACIGGGQGIA LIIQNTA
//
```

|  |
| --- |
| **Mascot:** http://www.matrixscience.com/ |
