# Supplementary material for: A common East-Asian ALDH2 mutation causes metabolic disorders and the therapeutic effect of ALDH2 activators
Source: Nat Commun. 2023 Sep 25;14:5971. doi: 10.1038/s41467-023-41570-6 (PMC10520061; doi:10.1038/s41467-023-41570-6)
Supplement: Supplementary file 4 — Supplementary Data 1 [file 41467_2023_41570_MOESM4_ESM.zip › Table S5b/Q8BWT1/Q8BWT1_WTO-2_C103_C107.html]

Mascot Search Results: Q8BWT1
 

# MASCOT Search Results

## Protein View: Q8BWT1

### 3-ketoacyl-CoA thiolase, mitochondrial OS=Mus musculus OX=10090 GN=Acaa2 PE=1 SV=3

|  |  |
| --- | --- |
| Database: | Mouse\_UniProt\_proteomes |
| Score: | 6773 |
| Monoisotopic mass (Mr): | 42260 |
| Calculated pI: | 8.33 |

Sequence similarity is available as an NCBI BLAST search of Q8BWT1 against nr.

### Search parameters

|  |  |
| --- | --- |
| MS data file: | `D:\LCMSMS\2023 Users' data\230529-1\230529-1-WTO-2.raw` |
| Enzyme: | Trypsin/P: cuts C-term side of KR. |
| Fixed modifications: | Carbamidomethyl (C) |
| Variable modifications: | Deamidated (NQ), HNE (C), HNE (H), HNE (K), Oxidation (M) |

### Protein sequence coverage: 85%

Matched peptides shown in ***bold red***.

|  |  |  |  |  |  |
| --- | --- | --- | --- | --- | --- |
| `1` | `MALLRGVFIV` | `AAKRTPFGAY` | `GGLLKDFSAT` | `DLTEFAARAA` | `LSAGKVPPET` |
| `51` | `IDSVIVGNVM` | `QSSSDAAYLA` | `RHVGLRVGVP` | `TETGALTLNR` | `LCGSGFQSIV` |
| `101` | `SGCQEICSKD` | `AEVVLCGGTE` | `SMSQSPYCVR` | `NVRFGTKFGL` | `DLKLEDTLWA` |
| `151` | `GLTDQHVKLP` | `MGMTAENLAA` | `KYNISREDCD` | `RYALQSQQRW` | `KAANEAGYFN` |
| `201` | `EEMAPIEVKT` | `KKGKQTMQVD` | `EHARPQTTLE` | `QLQKLPSVFK` | `KDGTVTAGNA` |
| `251` | `SGVSDGAGAV` | `IIASEDAVKK` | `HNFTPLARVV` | `GYFVSGCDPT` | `IMGIGPVPAI` |
| `301` | `NGALKKAGLS` | `LKDMDLIDVN` | `EAFAPQFLSV` | `QKALDLDPSK` | `TNVSGGAIAL` |
| `351` | `GHPLGGSGSR` | `ITAHLVHELR` | `RRGGKYAVGS` | `ACIGGGQGIA` | `LIIQNTA` |

Unformatted sequence string: 397 residues (for pasting into other applications).

|  |  |  |  |
| --- | --- | --- | --- |
| Sort by | residue number | increasing mass | decreasing mass |
| Show | matched peptides only | predicted peptides also |  |

| Query | Start | – | End | Observed | Mr(expt) | Mr(calc) | ppm | M | Score | Expect | Rank | U | Peptide |
| --- | --- | --- | --- | --- | --- | --- | --- | --- | --- | --- | --- | --- | --- |
| 3950 | 6 | – | 13 | 402.7524 | 803.4902 | 803.4905 | -0.36 | 0 | 33 | 0.00087 | 1Score **> 22** indicates **identity** Score **> 15** indicates **homology** | U | R.GVFIVAAK.R |
| 3951 | 6 | – | 13 | 402.7526 | 803.4906 | 803.4905 | 0.050 | 0 | 23 | 0.0066 | 1Score **> 22** indicates **identity** Score **> 14** indicates **homology** | U | R.GVFIVAAK.R |
| 3952 | 6 | – | 13 | 402.7526 | 803.4906 | 803.4905 | 0.14 | 0 | 29 | 0.0019 | 1Score **> 22** indicates **identity** Score **> 14** indicates **homology** | U | R.GVFIVAAK.R |
| 3953 | 6 | – | 13 | 402.7530 | 803.4914 | 803.4905 | 1.13 | 0 | 20 | 0.013 | 1Score **> 22** indicates **identity** Score **> 14** indicates **homology** | U | R.GVFIVAAK.R |
| 42339 | 14 | – | 25 | 427.2434 | 1278.7083 | 1278.7084 | -0.15 | 1 | 40 | 0.00029 | 1Score **> 32** indicates **identity** Score **> 18** indicates **homology** | U | K.RTPFGAYGGLLK.D |
| 42343 | 14 | – | 25 | 640.3617 | 1278.7088 | 1278.7084 | 0.28 | 1 | 66 | 2.6e-05 | 1Score **> 32** indicates **identity** | U | K.RTPFGAYGGLLK.D |
| 42344 | 14 | – | 25 | 427.2436 | 1278.7089 | 1278.7084 | 0.31 | 1 | 21 | 0.01 | 1Score **> 32** indicates **identity** Score **> 14** indicates **homology** | U | K.RTPFGAYGGLLK.D |
| 42345 | 14 | – | 25 | 427.2436 | 1278.7090 | 1278.7084 | 0.45 | 1 | 41 | 0.00036 | 1Score **> 32** indicates **identity** Score **> 19** indicates **homology** | U | K.RTPFGAYGGLLK.D |
| 42346 | 14 | – | 25 | 640.3619 | 1278.7092 | 1278.7084 | 0.55 | 1 | 57 | 0.0002 | 1Score **> 32** indicates **identity** Score **> 32** indicates **homology** | U | K.RTPFGAYGGLLK.D |
| 42348 | 14 | – | 25 | 640.3620 | 1278.7095 | 1278.7084 | 0.81 | 1 | 53 | 0.00045 | 1Score **> 32** indicates **identity** | U | K.RTPFGAYGGLLK.D |
| 42349 | 14 | – | 25 | 427.2438 | 1278.7096 | 1278.7084 | 0.94 | 1 | 35 | 0.00098 | 1Score **> 32** indicates **identity** Score **> 17** indicates **homology** | U | K.RTPFGAYGGLLK.D |
| 169366 | 14 | – | 38 | 676.8486 | 2703.3655 | 2703.3657 | -0.064 | 2 | 59 | 2.7e-06 | 1Score **> 37** indicates **identity** Score **> 16** indicates **homology** | U | K.RTPFGAYGGLLKDFSATDLTEFAAR.A |
| 169367 | 14 | – | 38 | 676.8491 | 2703.3674 | 2703.3657 | 0.66 | 2 | 72 | 1.9e-07 | 1Score **> 37** indicates **identity** Score **> 17** indicates **homology** | U | K.RTPFGAYGGLLKDFSATDLTEFAAR.A |
| 169368 | 14 | – | 38 | 902.1307 | 2703.3703 | 2703.3657 | 1.73 | 2 | 95 | 1.1e-09 | 1Score **> 37** indicates **identity** Score **> 18** indicates **homology** | U | K.RTPFGAYGGLLKDFSATDLTEFAAR.A |
| 25259 | 15 | – | 25 | 562.3098 | 1122.6050 | 1122.6073 | -2.05 | 0 | 29 | 0.0019 | 1Score **> 31** indicates **identity** Score **> 14** indicates **homology** | U | R.TPFGAYGGLLK.D |
| 25260 | 15 | – | 25 | 562.3116 | 1122.6086 | 1122.6073 | 1.13 | 0 | 59 | 3.8e-06 | 1Score **> 32** indicates **identity** Score **> 17** indicates **homology** | U | R.TPFGAYGGLLK.D |
| 25261 | 15 | – | 25 | 562.3120 | 1122.6095 | 1122.6073 | 1.89 | 0 | 55 | 6.9e-06 | 1Score **> 32** indicates **identity** Score **> 16** indicates **homology** | U | R.TPFGAYGGLLK.D |
| 25263 | 15 | – | 25 | 562.3134 | 1122.6122 | 1122.6073 | 4.28 | 0 | 56 | 5.5e-06 | 1Score **> 31** indicates **identity** Score **> 16** indicates **homology** | U | R.TPFGAYGGLLK.D |
| 42350 | 15 | – | 25 | 427.2440 | 1278.7103 | 1278.7224 | -9.44 | 0 | 43 | 8.5e-05 | 1Score **> 32** indicates **identity** Score **> 15** indicates **homology** | U | R.TPFGAYGGLLK.D  + HNE (K) |
| 42351 | 15 | – | 25 | 427.2442 | 1278.7108 | 1278.7224 | -9.09 | 0 | 51 | 1.8e-05 | 1Score **> 32** indicates **identity** Score **> 16** indicates **homology** | U | R.TPFGAYGGLLK.D  + HNE (K) |
| 42352 | 15 | – | 25 | 427.2445 | 1278.7117 | 1278.7224 | -8.33 | 0 | 37 | 0.00034 | 1Score **> 32** indicates **identity** Score **> 15** indicates **homology** | U | R.TPFGAYGGLLK.D  + HNE (K) |
| 162723 | 15 | – | 38 | 850.0945 | 2547.2616 | 2547.2646 | -1.18 | 1 | 83 | 1.5e-08 | 1Score **> 37** indicates **identity** Score **> 18** indicates **homology** | U | R.TPFGAYGGLLKDFSATDLTEFAAR.A |
| 59976 | 26 | – | 38 | 722.3385 | 1442.6625 | 1442.6678 | -3.62 | 0 | 49 | 2.3e-05 | 1Score **> 30** indicates **identity** Score **> 16** indicates **homology** | U | K.DFSATDLTEFAAR.A |
| 59977 | 26 | – | 38 | 722.3389 | 1442.6633 | 1442.6678 | -3.10 | 0 | 52 | 1.3e-05 | 1Score **> 30** indicates **identity** Score **> 16** indicates **homology** | U | K.DFSATDLTEFAAR.A |
| 59979 | 26 | – | 38 | 722.3393 | 1442.6640 | 1442.6678 | -2.62 | 0 | 48 | 3e-05 | 1Score **> 30** indicates **identity** Score **> 16** indicates **homology** | U | K.DFSATDLTEFAAR.A |
| 59980 | 26 | – | 38 | 722.3394 | 1442.6642 | 1442.6678 | -2.45 | 0 | 74 | 1.2e-07 | 1Score **> 30** indicates **identity** Score **> 17** indicates **homology** | U | K.DFSATDLTEFAAR.A |
| 59981 | 26 | – | 38 | 722.3397 | 1442.6648 | 1442.6678 | -2.08 | 0 | 68 | 3.9e-07 | 1Score **> 30** indicates **identity** Score **> 17** indicates **homology** | U | K.DFSATDLTEFAAR.A |
| 59982 | 26 | – | 38 | 722.3404 | 1442.6662 | 1442.6678 | -1.11 | 0 | 46 | 5.1e-05 | 1Score **> 31** indicates **identity** Score **> 15** indicates **homology** | U | K.DFSATDLTEFAAR.A |
| 59983 | 26 | – | 38 | 722.3405 | 1442.6664 | 1442.6678 | -0.98 | 0 | 36 | 0.00043 | 1Score **> 31** indicates **identity** Score **> 15** indicates **homology** | U | K.DFSATDLTEFAAR.A |
| 59984 | 26 | – | 38 | 722.3406 | 1442.6666 | 1442.6678 | -0.84 | 0 | 43 | 9.8e-05 | 1Score **> 30** indicates **identity** Score **> 15** indicates **homology** | U | K.DFSATDLTEFAAR.A |
| 59985 | 26 | – | 38 | 722.3406 | 1442.6667 | 1442.6678 | -0.76 | 0 | 73 | 1.4e-07 | 1Score **> 30** indicates **identity** Score **> 17** indicates **homology** | U | K.DFSATDLTEFAAR.A |
| 59986 | 26 | – | 38 | 722.3406 | 1442.6667 | 1442.6678 | -0.74 | 0 | 76 | 7e-08 | 1Score **> 30** indicates **identity** Score **> 17** indicates **homology** | U | K.DFSATDLTEFAAR.A |
| 59987 | 26 | – | 38 | 722.3408 | 1442.6670 | 1442.6678 | -0.50 | 0 | 32 | 0.001 | 1Score **> 30** indicates **identity** Score **> 14** indicates **homology** | U | K.DFSATDLTEFAAR.A |
| 59988 | 26 | – | 38 | 722.3409 | 1442.6672 | 1442.6678 | -0.42 | 0 | 86 | 7.8e-09 | 1Score **> 30** indicates **identity** Score **> 18** indicates **homology** | U | K.DFSATDLTEFAAR.A |
| 59989 | 26 | – | 38 | 722.3409 | 1442.6672 | 1442.6678 | -0.41 | 0 | 77 | 6.6e-08 | 1Score **> 30** indicates **identity** Score **> 17** indicates **homology** | U | K.DFSATDLTEFAAR.A |
| 59990 | 26 | – | 38 | 722.3410 | 1442.6674 | 1442.6678 | -0.27 | 0 | 43 | 9.3e-05 | 1Score **> 30** indicates **identity** Score **> 15** indicates **homology** | U | K.DFSATDLTEFAAR.A |
| 59991 | 26 | – | 38 | 722.3411 | 1442.6677 | 1442.6678 | -0.057 | 0 | 78 | 5.2e-08 | 1Score **> 30** indicates **identity** Score **> 17** indicates **homology** | U | K.DFSATDLTEFAAR.A |
| 59993 | 26 | – | 38 | 722.3412 | 1442.6678 | 1442.6678 | 0.021 | 0 | 76 | 7.1e-08 | 1Score **> 30** indicates **identity** Score **> 17** indicates **homology** | U | K.DFSATDLTEFAAR.A |
| 59997 | 26 | – | 38 | 722.3426 | 1442.6707 | 1442.6678 | 2.01 | 0 | 41 | 0.00015 | 1Score **> 31** indicates **identity** Score **> 15** indicates **homology** | U | K.DFSATDLTEFAAR.A |
| 185438 | 39 | – | 71 | 1106.5744 | 3316.7015 | 3316.6973 | 1.27 | 1 | 53 | 2e-05 | 1Score **> 37** indicates **identity** Score **> 19** indicates **homology** | U | R.AALSAGKVPPETIDSVIVGNVMQSSSDAAYLAR.H |
| 185439 | 39 | – | 71 | 830.1837 | 3316.7055 | 3316.6973 | 2.49 | 1 | 80 | 3.1e-08 | 1Score **> 37** indicates **identity** Score **> 18** indicates **homology** | U | R.AALSAGKVPPETIDSVIVGNVMQSSSDAAYLAR.H |
| 185440 | 39 | – | 71 | 1106.5761 | 3316.7066 | 3316.6973 | 2.81 | 1 | 116 | 1.2e-11 | 1Score **> 37** indicates **identity** Score **> 20** indicates **homology** | U | R.AALSAGKVPPETIDSVIVGNVMQSSSDAAYLAR.H |
| 185441 | 39 | – | 71 | 1106.5772 | 3316.7098 | 3316.6973 | 3.78 | 1 | 81 | 2.5e-08 | 1Score **> 37** indicates **identity** Score **> 18** indicates **homology** | U | R.AALSAGKVPPETIDSVIVGNVMQSSSDAAYLAR.H |
| 169862 | 46 | – | 71 | 907.1251 | 2718.3535 | 2718.3534 | 0.036 | 0 | 75 | 9.2e-08 | 1Score **> 37** indicates **identity** Score **> 17** indicates **homology** | U | K.VPPETIDSVIVGNVMQSSSDAAYLAR.H |
| 58115 | 77 | – | 90 | 714.3963 | 1426.7780 | 1426.7780 | -0.0042 | 0 | 49 | 2.6e-05 | 1Score **> 34** indicates **identity** Score **> 16** indicates **homology** | U | R.VGVPTETGALTLNR.L |
| 58116 | 77 | – | 90 | 714.3963 | 1426.7780 | 1426.7780 | 0.020 | 0 | 81 | 3.5e-08 | 1Score **> 34** indicates **identity** Score **> 19** indicates **homology** | U | R.VGVPTETGALTLNR.L |
| 58118 | 77 | – | 90 | 714.3965 | 1426.7785 | 1426.7780 | 0.36 | 0 | 90 | 4.7e-09 | 1Score **> 34** indicates **identity** Score **> 19** indicates **homology** | U | R.VGVPTETGALTLNR.L |
| 58119 | 77 | – | 90 | 714.3966 | 1426.7786 | 1426.7780 | 0.41 | 0 | 80 | 4.3e-08 | 1Score **> 33** indicates **identity** Score **> 19** indicates **homology** | U | R.VGVPTETGALTLNR.L |
| 58120 | 77 | – | 90 | 714.3966 | 1426.7786 | 1426.7780 | 0.42 | 0 | 44 | 7.7e-05 | 1Score **> 33** indicates **identity** Score **> 15** indicates **homology** | U | R.VGVPTETGALTLNR.L |
| 58125 | 77 | – | 90 | 714.3973 | 1426.7801 | 1426.7780 | 1.46 | 0 | 87 | 7.7e-09 | 1Score **> 34** indicates **identity** Score **> 19** indicates **homology** | U | R.VGVPTETGALTLNR.L |
| 58127 | 77 | – | 90 | 714.3974 | 1426.7803 | 1426.7780 | 1.63 | 0 | 63 | 1.1e-06 | 1Score **> 34** indicates **identity** Score **> 16** indicates **homology** | U | R.VGVPTETGALTLNR.L |
| 58132 | 77 | – | 90 | 714.3984 | 1426.7822 | 1426.7780 | 2.94 | 0 | 68 | 5.2e-07 | 1Score **> 33** indicates **identity** Score **> 17** indicates **homology** | U | R.VGVPTETGALTLNR.L |
| 133835 | 91 | – | 109 | 1058.9759 | 2115.9372 | 2115.9388 | -0.74 | 0 | 115 | 1.7e-11 | 1Score **> 30** indicates **identity** Score **> 20** indicates **homology** | U | R.LCGSGFQSIVSGCQEICSK.D |
| 133836 | 91 | – | 109 | 706.3197 | 2115.9374 | 2115.9388 | -0.65 | 0 | 82 | 2e-08 | 1Score **> 30** indicates **identity** Score **> 18** indicates **homology** | U | R.LCGSGFQSIVSGCQEICSK.D |
| 133837 | 91 | – | 109 | 1058.9761 | 2115.9376 | 2115.9388 | -0.55 | 0 | 107 | 9.1e-11 | 1Score **> 30** indicates **identity** Score **> 19** indicates **homology** | U | R.LCGSGFQSIVSGCQEICSK.D |
| 133838 | 91 | – | 109 | 1058.9765 | 2115.9384 | 2115.9388 | -0.17 | 0 | 89 | 4.8e-09 | 1Score **> 30** indicates **identity** Score **> 18** indicates **homology** | U | R.LCGSGFQSIVSGCQEICSK.D |
| 133840 | 91 | – | 109 | 706.3202 | 2115.9387 | 2115.9388 | -0.034 | 0 | 64 | 9.3e-07 | 1Score **> 30** indicates **identity** Score **> 17** indicates **homology** | U | R.LCGSGFQSIVSGCQEICSK.D |
| 133841 | 91 | – | 109 | 1058.9774 | 2115.9402 | 2115.9388 | 0.68 | 0 | 112 | 3.1e-11 | 1Score **> 30** indicates **identity** Score **> 20** indicates **homology** | U | R.LCGSGFQSIVSGCQEICSK.D |
| 133842 | 91 | – | 109 | 1058.9774 | 2115.9402 | 2115.9388 | 0.69 | 0 | 92 | 2.3e-09 | 1Score **> 30** indicates **identity** Score **> 18** indicates **homology** | U | R.LCGSGFQSIVSGCQEICSK.D |
| 133843 | 91 | – | 109 | 1058.9777 | 2115.9409 | 2115.9388 | 1.00 | 0 | 97 | 7.6e-10 | 1Score **> 30** indicates **identity** Score **> 19** indicates **homology** | U | R.LCGSGFQSIVSGCQEICSK.D |
| 133844 | 91 | – | 109 | 1058.9778 | 2115.9410 | 2115.9388 | 1.04 | 0 | 87 | 7.3e-09 | 1Score **> 30** indicates **identity** Score **> 18** indicates **homology** | U | R.LCGSGFQSIVSGCQEICSK.D |
| 133845 | 91 | – | 109 | 706.3212 | 2115.9418 | 2115.9388 | 1.43 | 0 | 49 | 2.8e-05 | 1Score **> 30** indicates **identity** Score **> 16** indicates **homology** | U | R.LCGSGFQSIVSGCQEICSK.D |
| 133846 | 91 | – | 109 | 1058.9782 | 2115.9419 | 2115.9388 | 1.47 | 0 | 69 | 3.1e-07 | 1Score **> 30** indicates **identity** Score **> 17** indicates **homology** | U | R.LCGSGFQSIVSGCQEICSK.D |
| 133847 | 91 | – | 109 | 706.3213 | 2115.9420 | 2115.9388 | 1.54 | 0 | 82 | 2e-08 | 1Score **> 30** indicates **identity** Score **> 18** indicates **homology** | U | R.LCGSGFQSIVSGCQEICSK.D |
| 133848 | 91 | – | 109 | 1058.9783 | 2115.9421 | 2115.9388 | 1.57 | 0 | 89 | 5e-09 | 1Score **> 30** indicates **identity** Score **> 18** indicates **homology** | U | R.LCGSGFQSIVSGCQEICSK.D |
| 133849 | 91 | – | 109 | 1058.9784 | 2115.9421 | 2115.9388 | 1.60 | 0 | 100 | 4.5e-10 | 1Score **> 30** indicates **identity** Score **> 19** indicates **homology** | U | R.LCGSGFQSIVSGCQEICSK.D |
| 133850 | 91 | – | 109 | 706.3218 | 2115.9437 | 2115.9388 | 2.33 | 0 | 39 | 0.00024 | 1Score **> 30** indicates **identity** Score **> 15** indicates **homology** | U | R.LCGSGFQSIVSGCQEICSK.D |
| 133851 | 91 | – | 109 | 1058.9795 | 2115.9444 | 2115.9388 | 2.64 | 0 | 36 | 0.0004 | 1Score **> 30** indicates **identity** Score **> 15** indicates **homology** | U | R.LCGSGFQSIVSGCQEICSK.D |
| 133852 | 91 | – | 109 | 706.3222 | 2115.9447 | 2115.9388 | 2.81 | 0 | 28 | 0.0026 | 1Score **> 30** indicates **identity** Score **> 14** indicates **homology** | U | R.LCGSGFQSIVSGCQEICSK.D |
| 133853 | 91 | – | 109 | 1058.9804 | 2115.9462 | 2115.9388 | 3.52 | 0 | 88 | 5.7e-09 | 1Score **> 30** indicates **identity** Score **> 18** indicates **homology** | U | R.LCGSGFQSIVSGCQEICSK.D |
| 133854 | 91 | – | 109 | 1058.9810 | 2115.9474 | 2115.9388 | 4.10 | 0 | 84 | 1.2e-08 | 1Score **> 31** indicates **identity** Score **> 18** indicates **homology** | U | R.LCGSGFQSIVSGCQEICSK.D |
| 194053 | 91 | – | 130 | 1140.2644 | 4557.0284 | 4557.0301 | -0.37 | 1 | 33 | 0.00086 | 1Score **> 31** indicates **identity** Score **> 15** indicates **homology** | U | R.LCGSGFQSIVSGCQEICSKDAEVVLCGGTESMSQSPYCVR.N  + HNE (C); Oxidation (M) |
| 194066 | 91 | – | 130 | 1140.5049 | 4557.9904 | 4558.0141 | -5.19 | 1 | 19 | 0.018 | 1Score **> 29** indicates **identity** Score **> 14** indicates **homology** | U | R.LCGSGFQSIVSGCQEICSKDAEVVLCGGTESMSQSPYCVR.N  + Deamidated (NQ); HNE (C); Oxidation (M) |
| 151456 | 110 | – | 130 | 782.3460 | 2344.0161 | 2344.0134 | 1.18 | 0 | 20 | 0.014 | 1Score **> 29** indicates **identity** Score **> 14** indicates **homology** | U | K.DAEVVLCGGTESMSQSPYCVR.N |
| 151457 | 110 | – | 130 | 1173.0187 | 2344.0229 | 2344.0134 | 4.07 | 0 | 93 | 1.9e-09 | 1Score **> 30** indicates **identity** Score **> 18** indicates **homology** | U | K.DAEVVLCGGTESMSQSPYCVR.N |
| 25451 | 134 | – | 143 | 375.8818 | 1124.6235 | 1124.6230 | 0.46 | 1 | 25 | 0.021 | 1Score **> 31** indicates **identity** Score **> 20** indicates **homology** | U | R.FGTKFGLDLK.L |
| 25452 | 134 | – | 143 | 375.8818 | 1124.6236 | 1124.6230 | 0.50 | 1 | 36 | 0.0024 | 1Score **> 31** indicates **identity** Score **> 22** indicates **homology** | U | R.FGTKFGLDLK.L |
| 25453 | 134 | – | 143 | 375.8820 | 1124.6241 | 1124.6230 | 1.01 | 1 | 31 | 0.0065 | 1Score **> 31** indicates **identity** Score **> 22** indicates **homology** | U | R.FGTKFGLDLK.L |
| 174297 | 134 | – | 158 | 567.3044 | 2831.4855 | 2831.4858 | -0.12 | 2 | 16 | 0.03 | 1Score **> 37** indicates **identity** Score **> 14** indicates **homology** | U | R.FGTKFGLDLKLEDTLWAGLTDQHVK.L |
| 174299 | 134 | – | 158 | 708.8801 | 2831.4912 | 2831.4858 | 1.90 | 2 | 37 | 0.00035 | 1Score **> 37** indicates **identity** Score **> 15** indicates **homology** | U | R.FGTKFGLDLKLEDTLWAGLTDQHVK.L |
| 155091 | 138 | – | 158 | 600.5700 | 2398.2507 | 2398.2533 | -1.06 | 1 | 30 | 0.0014 | 1Score **> 37** indicates **identity** Score **> 14** indicates **homology** | U | K.FGLDLKLEDTLWAGLTDQHVK.L |
| 155093 | 138 | – | 158 | 600.5702 | 2398.2516 | 2398.2533 | -0.68 | 1 | 30 | 0.0017 | 1Score **> 37** indicates **identity** Score **> 14** indicates **homology** | U | K.FGLDLKLEDTLWAGLTDQHVK.L |
| 155096 | 138 | – | 158 | 600.5712 | 2398.2558 | 2398.2533 | 1.06 | 1 | 51 | 1.6e-05 | 1Score **> 37** indicates **identity** Score **> 16** indicates **homology** | U | K.FGLDLKLEDTLWAGLTDQHVK.L |
| 155097 | 138 | – | 158 | 800.4259 | 2398.2558 | 2398.2533 | 1.07 | 1 | 22 | 0.0083 | 1Score **> 37** indicates **identity** Score **> 14** indicates **homology** | U | K.FGLDLKLEDTLWAGLTDQHVK.L |
| 155099 | 138 | – | 158 | 600.5713 | 2398.2562 | 2398.2533 | 1.23 | 1 | 43 | 8.8e-05 | 1Score **> 37** indicates **identity** Score **> 15** indicates **homology** | U | K.FGLDLKLEDTLWAGLTDQHVK.L |
| 155100 | 138 | – | 158 | 800.4263 | 2398.2571 | 2398.2533 | 1.59 | 1 | 42 | 0.00012 | 1Score **> 37** indicates **identity** Score **> 15** indicates **homology** | U | K.FGLDLKLEDTLWAGLTDQHVK.L |
| 96110 | 144 | – | 158 | 575.9649 | 1724.8730 | 1724.8734 | -0.21 | 0 | 20 | 0.015 | 1Score **> 35** indicates **identity** Score **> 14** indicates **homology** | U | K.LEDTLWAGLTDQHVK.L |
| 96112 | 144 | – | 158 | 575.9653 | 1724.8740 | 1724.8734 | 0.36 | 0 | 44 | 7.7e-05 | 1Score **> 35** indicates **identity** Score **> 15** indicates **homology** | U | K.LEDTLWAGLTDQHVK.L |
| 96113 | 144 | – | 158 | 863.4443 | 1724.8741 | 1724.8734 | 0.42 | 0 | 29 | 0.0021 | 1Score **> 35** indicates **identity** Score **> 14** indicates **homology** | U | K.LEDTLWAGLTDQHVK.L |
| 96114 | 144 | – | 158 | 575.9654 | 1724.8745 | 1724.8734 | 0.66 | 0 | 55 | 6.4e-06 | 1Score **> 35** indicates **identity** Score **> 16** indicates **homology** | U | K.LEDTLWAGLTDQHVK.L |
| 96115 | 144 | – | 158 | 863.4456 | 1724.8766 | 1724.8734 | 1.91 | 0 | 61 | 1.8e-06 | 1Score **> 35** indicates **identity** Score **> 16** indicates **homology** | U | K.LEDTLWAGLTDQHVK.L |
| 47567 | 172 | – | 181 | 664.2884 | 1326.5622 | 1326.5622 | -0.026 | 1 | 19 | 0.015 | 1Score **> 25** indicates **identity** Score **> 14** indicates **homology** | U | K.YNISREDCDR.Y |
| 47568 | 172 | – | 181 | 664.2886 | 1326.5627 | 1326.5622 | 0.34 | 1 | 15 | 0.042 | 1Score **> 25** indicates **identity** Score **> 13** indicates **homology** | U | K.YNISREDCDR.Y |
| 148596 | 172 | – | 189 | 768.0261 | 2301.0565 | 2301.0556 | 0.38 | 2 | 26 | 0.0034 | 1Score **> 33** indicates **identity** Score **> 14** indicates **homology** | U | K.YNISREDCDRYALQSQQR.W |
| 148597 | 172 | – | 189 | 768.0262 | 2301.0568 | 2301.0556 | 0.52 | 2 | 23 | 0.0074 | 1Score **> 33** indicates **identity** Score **> 14** indicates **homology** | U | K.YNISREDCDRYALQSQQR.W |
| 148599 | 172 | – | 189 | 768.0264 | 2301.0574 | 2301.0556 | 0.77 | 2 | 23 | 0.0066 | 1Score **> 33** indicates **identity** Score **> 14** indicates **homology** | U | K.YNISREDCDRYALQSQQR.W |
| 88835 | 177 | – | 189 | 556.9179 | 1667.7319 | 1667.7322 | -0.18 | 1 | 17 | 0.027 | 1Score **> 28** indicates **identity** Score **> 14** indicates **homology** | U | R.EDCDRYALQSQQR.W |
| 88836 | 177 | – | 189 | 834.8734 | 1667.7322 | 1667.7322 | 0.052 | 1 | 48 | 3.2e-05 | 1Score **> 28** indicates **identity** Score **> 15** indicates **homology** | U | R.EDCDRYALQSQQR.W |
| 88838 | 177 | – | 189 | 556.9184 | 1667.7333 | 1667.7322 | 0.70 | 1 | 36 | 0.00044 | 1Score **> 29** indicates **identity** Score **> 15** indicates **homology** | U | R.EDCDRYALQSQQR.W |
| 88840 | 177 | – | 189 | 556.9185 | 1667.7337 | 1667.7322 | 0.93 | 1 | 36 | 0.00042 | 1Score **> 29** indicates **identity** Score **> 15** indicates **homology** | U | R.EDCDRYALQSQQR.W |
| 88841 | 177 | – | 189 | 834.8744 | 1667.7343 | 1667.7322 | 1.29 | 1 | 37 | 0.00054 | 1Score **> 29** indicates **identity** Score **> 16** indicates **homology** | U | R.EDCDRYALQSQQR.W |
| 14401 | 182 | – | 189 | 497.2578 | 992.5011 | 992.5039 | -2.88 | 0 | 22 | 0.0087 | 1Score **> 31** indicates **identity** Score **> 14** indicates **homology** | U | R.YALQSQQR.W |
| 14403 | 182 | – | 189 | 497.2584 | 992.5022 | 992.5039 | -1.74 | 0 | 42 | 0.00026 | 1Score **> 32** indicates **identity** Score **> 19** indicates **homology** | U | R.YALQSQQR.W |
| 14406 | 182 | – | 189 | 497.2596 | 992.5047 | 992.5039 | 0.80 | 0 | 21 | 0.011 | 1Score **> 31** indicates **identity** Score **> 14** indicates **homology** | U | R.YALQSQQR.W |
| 148265 | 190 | – | 209 | 766.3648 | 2296.0727 | 2296.0834 | -4.65 | 1 | 20 | 0.012 | 1Score **> 34** indicates **identity** Score **> 14** indicates **homology** | U | R.WKAANEAGYFNEEMAPIEVK.T |
| 148267 | 190 | – | 209 | 766.3683 | 2296.0832 | 2296.0834 | -0.091 | 1 | 32 | 0.00098 | 1Score **> 35** indicates **identity** Score **> 15** indicates **homology** | U | R.WKAANEAGYFNEEMAPIEVK.T |
| 148269 | 190 | – | 209 | 766.3703 | 2296.0892 | 2296.0834 | 2.54 | 1 | 24 | 0.0061 | 1Score **> 35** indicates **identity** Score **> 14** indicates **homology** | U | R.WKAANEAGYFNEEMAPIEVK.T |
| 121578 | 192 | – | 209 | 991.9623 | 1981.9100 | 1981.9091 | 0.44 | 0 | 69 | 3.3e-07 | 1Score **> 33** indicates **identity** Score **> 17** indicates **homology** | U | K.AANEAGYFNEEMAPIEVK.T |
| 121579 | 192 | – | 209 | 991.9629 | 1981.9113 | 1981.9091 | 1.13 | 0 | 78 | 4.6e-08 | 1Score **> 33** indicates **identity** Score **> 17** indicates **homology** | U | K.AANEAGYFNEEMAPIEVK.T |
| 121580 | 192 | – | 209 | 991.9642 | 1981.9139 | 1981.9091 | 2.43 | 0 | 58 | 3.8e-06 | 1Score **> 33** indicates **identity** Score **> 16** indicates **homology** | U | K.AANEAGYFNEEMAPIEVK.T |
| 153804 | 215 | – | 234 | 596.0523 | 2380.1800 | 2380.1805 | -0.21 | 1 | 15 | 0.042 | 1Score **> 36** indicates **identity** Score **> 13** indicates **homology** | U | K.QTMQVDEHARPQTTLEQLQK.L |
| 153807 | 215 | – | 234 | 794.4026 | 2380.1861 | 2380.1805 | 2.35 | 1 | 17 | 0.023 | 1Score **> 37** indicates **identity** Score **> 14** indicates **homology** | U | K.QTMQVDEHARPQTTLEQLQK.L |
| 153814 | 215 | – | 234 | 596.0549 | 2380.1905 | 2380.1805 | 4.20 | 1 | 16 | 0.03 | 1Score **> 37** indicates **identity** Score **> 14** indicates **homology** | U | K.QTMQVDEHARPQTTLEQLQK.L |
| 153816 | 215 | – | 234 | 596.0552 | 2380.1919 | 2380.1805 | 4.78 | 1 | 16 | 0.029 | 1Score **> 37** indicates **identity** Score **> 14** indicates **homology** | U | K.QTMQVDEHARPQTTLEQLQK.L |
| 153824 | 215 | – | 234 | 794.4054 | 2380.1944 | 2380.1805 | 5.82 | 1 | 28 | 0.0024 | 1Score **> 37** indicates **identity** Score **> 14** indicates **homology** | U | K.QTMQVDEHARPQTTLEQLQK.L |
| 153825 | 215 | – | 234 | 794.4064 | 2380.1974 | 2380.1805 | 7.09 | 1 | 37 | 0.00033 | 1Score **> 37** indicates **identity** Score **> 15** indicates **homology** | U | K.QTMQVDEHARPQTTLEQLQK.L |
| 4617 | 235 | – | 241 | 409.7602 | 817.5059 | 817.5062 | -0.29 | 1 | 31 | 0.0063 | 1Score **> 22** indicates **identity** | U | K.LPSVFKK.D |
| 4618 | 235 | – | 241 | 409.7603 | 817.5061 | 817.5062 | -0.12 | 1 | 28 | 0.012 | 1Score **> 22** indicates **identity** | U | K.LPSVFKK.D |
| 162094 | 242 | – | 269 | 844.7500 | 2531.2283 | 2531.2351 | -2.71 | 0 | 14 | 0.044 | 1Score **> 36** indicates **identity** Score **> 13** indicates **homology** | U | K.DGTVTAGNASGVSDGAGAVIIASEDAVK.K |
| 162095 | 242 | – | 269 | 1266.6217 | 2531.2289 | 2531.2351 | -2.48 | 0 | 139 | 8.9e-14 | 1Score **> 36** indicates **identity** Score **> 21** indicates **homology** | U | K.DGTVTAGNASGVSDGAGAVIIASEDAVK.K |
| 162096 | 242 | – | 269 | 1266.6240 | 2531.2334 | 2531.2351 | -0.68 | 0 | 19 | 0.018 | 1Score **> 36** indicates **identity** Score **> 14** indicates **homology** | U | K.DGTVTAGNASGVSDGAGAVIIASEDAVK.K |
| 162097 | 242 | – | 269 | 1266.6240 | 2531.2335 | 2531.2351 | -0.66 | 0 | 150 | 8e-15 | 1Score **> 36** indicates **identity** Score **> 22** indicates **homology** | U | K.DGTVTAGNASGVSDGAGAVIIASEDAVK.K |
| 162098 | 242 | – | 269 | 844.7524 | 2531.2354 | 2531.2351 | 0.092 | 0 | 37 | 0.00033 | 1Score **> 36** indicates **identity** Score **> 15** indicates **homology** | U | K.DGTVTAGNASGVSDGAGAVIIASEDAVK.K |
| 162099 | 242 | – | 269 | 1266.6258 | 2531.2370 | 2531.2351 | 0.76 | 0 | 91 | 3.1e-09 | 1Score **> 36** indicates **identity** Score **> 18** indicates **homology** | U | K.DGTVTAGNASGVSDGAGAVIIASEDAVK.K |
| 162101 | 242 | – | 269 | 844.7532 | 2531.2376 | 2531.2351 | 0.99 | 0 | 79 | 4.1e-08 | 1Score **> 37** indicates **identity** Score **> 17** indicates **homology** | U | K.DGTVTAGNASGVSDGAGAVIIASEDAVK.K |
| 162102 | 242 | – | 269 | 1266.6265 | 2531.2385 | 2531.2351 | 1.34 | 0 | 143 | 4.3e-14 | 1Score **> 37** indicates **identity** Score **> 21** indicates **homology** | U | K.DGTVTAGNASGVSDGAGAVIIASEDAVK.K |
| 162103 | 242 | – | 269 | 1266.6271 | 2531.2396 | 2531.2351 | 1.75 | 0 | 138 | 1.1e-13 | 1Score **> 37** indicates **identity** Score **> 21** indicates **homology** | U | K.DGTVTAGNASGVSDGAGAVIIASEDAVK.K |
| 162109 | 242 | – | 269 | 844.7550 | 2531.2432 | 2531.2351 | 3.18 | 0 | 27 | 0.003 | 1Score **> 37** indicates **identity** Score **> 14** indicates **homology** | U | K.DGTVTAGNASGVSDGAGAVIIASEDAVK.K |
| 167557 | 242 | – | 270 | 887.4477 | 2659.3211 | 2659.3301 | -3.37 | 1 | 47 | 3.7e-05 | 1Score **> 37** indicates **identity** Score **> 15** indicates **homology** | U | K.DGTVTAGNASGVSDGAGAVIIASEDAVKK.H |
| 167558 | 242 | – | 270 | 887.4484 | 2659.3234 | 2659.3301 | -2.50 | 1 | 26 | 0.0034 | 1Score **> 37** indicates **identity** Score **> 14** indicates **homology** | U | K.DGTVTAGNASGVSDGAGAVIIASEDAVKK.H |
| 167561 | 242 | – | 270 | 887.4505 | 2659.3296 | 2659.3301 | -0.19 | 1 | 53 | 1e-05 | 1Score **> 37** indicates **identity** Score **> 16** indicates **homology** | U | K.DGTVTAGNASGVSDGAGAVIIASEDAVKK.H |
| 167562 | 242 | – | 270 | 887.4505 | 2659.3296 | 2659.3301 | -0.18 | 1 | 37 | 0.00033 | 1Score **> 37** indicates **identity** Score **> 15** indicates **homology** | U | K.DGTVTAGNASGVSDGAGAVIIASEDAVKK.H |
| 167564 | 242 | – | 270 | 887.4516 | 2659.3331 | 2659.3301 | 1.12 | 1 | 119 | 7e-12 | 1Score **> 37** indicates **identity** Score **> 20** indicates **homology** | U | K.DGTVTAGNASGVSDGAGAVIIASEDAVKK.H |
| 167565 | 242 | – | 270 | 887.4516 | 2659.3331 | 2659.3301 | 1.14 | 1 | 122 | 3.6e-12 | 1Score **> 37** indicates **identity** Score **> 20** indicates **homology** | U | K.DGTVTAGNASGVSDGAGAVIIASEDAVKK.H |
| 167566 | 242 | – | 270 | 1330.6740 | 2659.3335 | 2659.3301 | 1.30 | 1 | 126 | 1.5e-12 | 1Score **> 37** indicates **identity** Score **> 20** indicates **homology** | U | K.DGTVTAGNASGVSDGAGAVIIASEDAVKK.H |
| 167567 | 242 | – | 270 | 887.4522 | 2659.3347 | 2659.3301 | 1.73 | 1 | 78 | 4.6e-08 | 1Score **> 37** indicates **identity** Score **> 17** indicates **homology** | U | K.DGTVTAGNASGVSDGAGAVIIASEDAVKK.H |
| 167568 | 242 | – | 270 | 887.4525 | 2659.3355 | 2659.3301 | 2.05 | 1 | 75 | 9.8e-08 | 1Score **> 37** indicates **identity** Score **> 17** indicates **homology** | U | K.DGTVTAGNASGVSDGAGAVIIASEDAVKK.H |
| 167570 | 242 | – | 270 | 887.4535 | 2659.3386 | 2659.3301 | 3.20 | 1 | 65 | 8.7e-07 | 1Score **> 37** indicates **identity** Score **> 17** indicates **homology** | U | K.DGTVTAGNASGVSDGAGAVIIASEDAVKK.H |
| 167571 | 242 | – | 270 | 887.4547 | 2659.3422 | 2659.3301 | 4.56 | 1 | 54 | 8.7e-06 | 1Score **> 37** indicates **identity** Score **> 16** indicates **homology** | U | K.DGTVTAGNASGVSDGAGAVIIASEDAVKK.H |
| 167573 | 242 | – | 270 | 887.4560 | 2659.3461 | 2659.3301 | 6.03 | 1 | 96 | 9.3e-10 | 1Score **> 37** indicates **identity** Score **> 19** indicates **homology** | U | K.DGTVTAGNASGVSDGAGAVIIASEDAVKK.H |
| 167576 | 242 | – | 270 | 887.4577 | 2659.3511 | 2659.3301 | 7.92 | 1 | 39 | 0.00023 | 1Score **> 37** indicates **identity** Score **> 15** indicates **homology** | U | K.DGTVTAGNASGVSDGAGAVIIASEDAVKK.H |
| 167599 | 242 | – | 270 | 887.7831 | 2660.3274 | 2660.3141 | 4.99 | 1 | 21 | 0.011 | 1Score **> 37** indicates **identity** Score **> 14** indicates **homology** | U | K.DGTVTAGNASGVSDGAGAVIIASEDAVKK.H  + Deamidated (NQ) |
| 167600 | 242 | – | 270 | 887.7831 | 2660.3274 | 2660.3141 | 5.00 | 1 | 64 | 9e-07 | 1Score **> 37** indicates **identity** Score **> 17** indicates **homology** | U | K.DGTVTAGNASGVSDGAGAVIIASEDAVKK.H  + Deamidated (NQ) |
| 167603 | 242 | – | 270 | 1331.1734 | 2660.3323 | 2660.3141 | 6.85 | 1 | 103 | 2e-10 | 1Score **> 37** indicates **identity** Score **> 19** indicates **homology** | U | K.DGTVTAGNASGVSDGAGAVIIASEDAVKK.H  + Deamidated (NQ) |
| 167604 | 242 | – | 270 | 887.7852 | 2660.3338 | 2660.3141 | 7.41 | 1 | 47 | 4e-05 | 1Score **> 37** indicates **identity** Score **> 15** indicates **homology** | U | K.DGTVTAGNASGVSDGAGAVIIASEDAVKK.H  + Deamidated (NQ) |
| 167607 | 242 | – | 270 | 1331.1767 | 2660.3389 | 2660.3141 | 9.31 | 1 | 61 | 2e-06 | 1Score **> 37** indicates **identity** Score **> 16** indicates **homology** | U | K.DGTVTAGNASGVSDGAGAVIIASEDAVKK.H  + Deamidated (NQ) |
| 167608 | 242 | – | 270 | 887.7871 | 2660.3394 | 2660.3141 | 9.53 | 1 | 28 | 0.0022 | 1Score **> 37** indicates **identity** Score **> 14** indicates **homology** | U | K.DGTVTAGNASGVSDGAGAVIIASEDAVKK.H  + Deamidated (NQ) |
| 21296 | 270 | – | 278 | 361.8734 | 1082.5985 | 1082.5985 | 0.0028 | 1 | 38 | 0.00082 | 1Score **> 30** indicates **identity** Score **> 20** indicates **homology** | U | K.KHNFTPLAR.V |
| 21299 | 270 | – | 278 | 542.3068 | 1082.5991 | 1082.5985 | 0.56 | 1 | 31 | 0.0039 | 1Score **> 30** indicates **identity** Score **> 19** indicates **homology** | U | K.KHNFTPLAR.V |
| 21300 | 270 | – | 278 | 361.8738 | 1082.5995 | 1082.5985 | 0.97 | 1 | 25 | 0.0049 | 1Score **> 30** indicates **identity** Score **> 14** indicates **homology** | U | K.KHNFTPLAR.V |
| 12064 | 271 | – | 278 | 478.2595 | 954.5044 | 954.5035 | 0.91 | 0 | 34 | 0.0027 | 1Score **> 30** indicates **identity** Score **> 21** indicates **homology** | U | K.HNFTPLAR.V |
| 12065 | 271 | – | 278 | 478.2596 | 954.5045 | 954.5035 | 1.06 | 0 | 27 | 0.0074 | 1Score **> 30** indicates **identity** Score **> 18** indicates **homology** | U | K.HNFTPLAR.V |
| 12066 | 271 | – | 278 | 478.2598 | 954.5050 | 954.5035 | 1.51 | 0 | 31 | 0.0042 | 1Score **> 30** indicates **identity** Score **> 19** indicates **homology** | U | K.HNFTPLAR.V |
| 12067 | 271 | – | 278 | 478.2601 | 954.5057 | 954.5035 | 2.27 | 0 | 18 | 0.045 | 1Score **> 29** indicates **identity** Score **> 18** indicates **homology** | U | K.HNFTPLAR.V |
| 12069 | 271 | – | 278 | 478.2604 | 954.5062 | 954.5035 | 2.77 | 0 | 18 | 0.022 | 1Score **> 29** indicates **identity** Score **> 14** indicates **homology** | U | K.HNFTPLAR.V |
| 12070 | 271 | – | 278 | 478.2604 | 954.5062 | 954.5035 | 2.84 | 0 | 28 | 0.0067 | 1Score **> 29** indicates **identity** Score **> 19** indicates **homology** | U | K.HNFTPLAR.V |
| 12072 | 271 | – | 278 | 478.2623 | 954.5100 | 954.5035 | 6.74 | 0 | 24 | 0.0053 | 1Score **> 29** indicates **identity** Score **> 14** indicates **homology** | U | K.HNFTPLAR.V |
| 175154 | 279 | – | 306 | 954.1742 | 2859.5009 | 2859.5027 | -0.64 | 1 | 54 | 8.2e-06 | 1Score **> 37** indicates **identity** Score **> 16** indicates **homology** | U | R.VVGYFVSGCDPTIMGIGPVPAINGALKK.A |
| 175180 | 279 | – | 306 | 954.5047 | 2860.4924 | 2860.4867 | 1.98 | 1 | 65 | 8.8e-07 | 1Score **> 37** indicates **identity** Score **> 17** indicates **homology** | U | R.VVGYFVSGCDPTIMGIGPVPAINGALKK.A  + Deamidated (NQ) |
| 175181 | 279 | – | 306 | 954.5052 | 2860.4939 | 2860.4867 | 2.50 | 1 | 26 | 0.0037 | 1Score **> 37** indicates **identity** Score **> 14** indicates **homology** | U | R.VVGYFVSGCDPTIMGIGPVPAINGALKK.A  + Deamidated (NQ) |
| 174877 | 307 | – | 332 | 950.4994 | 2848.4765 | 2848.4681 | 2.95 | 1 | 70 | 2.9e-07 | 1Score **> 37** indicates **identity** Score **> 17** indicates **homology** | U | K.AGLSLKDMDLIDVNEAFAPQFLSVQK.A |
| 6661 | 333 | – | 340 | 429.7312 | 857.4479 | 857.4494 | -1.81 | 0 | 39 | 0.00058 | 1Score **> 28** indicates **identity** Score **> 19** indicates **homology** | U | K.ALDLDPSK.T |
| 6662 | 333 | – | 340 | 429.7315 | 857.4484 | 857.4494 | -1.15 | 0 | 43 | 0.00046 | 1Score **> 27** indicates **identity** Score **> 22** indicates **homology** | U | K.ALDLDPSK.T |
| 6663 | 333 | – | 340 | 429.7316 | 857.4487 | 857.4494 | -0.81 | 0 | 32 | 0.002 | 1Score **> 27** indicates **identity** Score **> 18** indicates **homology** | U | K.ALDLDPSK.T |
| 167108 | 333 | – | 360 | 883.1320 | 2646.3743 | 2646.3725 | 0.66 | 1 | 36 | 0.00043 | 1Score **> 37** indicates **identity** Score **> 15** indicates **homology** | U | K.ALDLDPSKTNVSGGAIALGHPLGGSGSR.I |
| 167109 | 333 | – | 360 | 883.1322 | 2646.3746 | 2646.3725 | 0.79 | 1 | 50 | 2e-05 | 1Score **> 37** indicates **identity** Score **> 16** indicates **homology** | U | K.ALDLDPSKTNVSGGAIALGHPLGGSGSR.I |
| 167110 | 333 | – | 360 | 662.6009 | 2646.3746 | 2646.3725 | 0.79 | 1 | 40 | 0.00017 | 1Score **> 37** indicates **identity** Score **> 15** indicates **homology** | U | K.ALDLDPSKTNVSGGAIALGHPLGGSGSR.I |
| 167111 | 333 | – | 360 | 883.1326 | 2646.3758 | 2646.3725 | 1.24 | 1 | 71 | 2.1e-07 | 1Score **> 37** indicates **identity** Score **> 17** indicates **homology** | U | K.ALDLDPSKTNVSGGAIALGHPLGGSGSR.I |
| 167112 | 333 | – | 360 | 1324.1953 | 2646.3760 | 2646.3725 | 1.31 | 1 | 45 | 5.6e-05 | 1Score **> 37** indicates **identity** Score **> 15** indicates **homology** | U | K.ALDLDPSKTNVSGGAIALGHPLGGSGSR.I |
| 167113 | 333 | – | 360 | 883.1327 | 2646.3761 | 2646.3725 | 1.36 | 1 | 54 | 8.3e-06 | 1Score **> 37** indicates **identity** Score **> 16** indicates **homology** | U | K.ALDLDPSKTNVSGGAIALGHPLGGSGSR.I |
| 167114 | 333 | – | 360 | 662.6014 | 2646.3767 | 2646.3725 | 1.55 | 1 | 45 | 5.7e-05 | 1Score **> 37** indicates **identity** Score **> 15** indicates **homology** | U | K.ALDLDPSKTNVSGGAIALGHPLGGSGSR.I |
| 167115 | 333 | – | 360 | 883.1328 | 2646.3767 | 2646.3725 | 1.58 | 1 | 21 | 0.011 | 1Score **> 37** indicates **identity** Score **> 14** indicates **homology** | U | K.ALDLDPSKTNVSGGAIALGHPLGGSGSR.I |
| 167116 | 333 | – | 360 | 662.6017 | 2646.3776 | 2646.3725 | 1.93 | 1 | 46 | 5.2e-05 | 1Score **> 37** indicates **identity** Score **> 15** indicates **homology** | U | K.ALDLDPSKTNVSGGAIALGHPLGGSGSR.I |
| 167117 | 333 | – | 360 | 662.6022 | 2646.3795 | 2646.3725 | 2.65 | 1 | 41 | 0.00015 | 1Score **> 37** indicates **identity** Score **> 15** indicates **homology** | U | K.ALDLDPSKTNVSGGAIALGHPLGGSGSR.I |
| 167118 | 333 | – | 360 | 883.1338 | 2646.3795 | 2646.3725 | 2.65 | 1 | 43 | 9e-05 | 1Score **> 37** indicates **identity** Score **> 15** indicates **homology** | U | K.ALDLDPSKTNVSGGAIALGHPLGGSGSR.I |
| 167119 | 333 | – | 360 | 662.6044 | 2646.3884 | 2646.3725 | 6.00 | 1 | 23 | 0.0064 | 1Score **> 37** indicates **identity** Score **> 14** indicates **homology** | U | K.ALDLDPSKTNVSGGAIALGHPLGGSGSR.I |
| 105034 | 341 | – | 360 | 904.4726 | 1806.9306 | 1806.9337 | -1.72 | 0 | 65 | 8.4e-07 | 1Score **> 35** indicates **identity** Score **> 17** indicates **homology** | U | K.TNVSGGAIALGHPLGGSGSR.I |
| 105037 | 341 | – | 360 | 904.4742 | 1806.9339 | 1806.9337 | 0.14 | 0 | 122 | 3.7e-12 | 1Score **> 36** indicates **identity** Score **> 20** indicates **homology** | U | K.TNVSGGAIALGHPLGGSGSR.I |
| 105038 | 341 | – | 360 | 603.3186 | 1806.9340 | 1806.9337 | 0.19 | 0 | 44 | 0.0026 | 1Score **> 36** indicates **identity** Score **> 31** indicates **homology** | U | K.TNVSGGAIALGHPLGGSGSR.I |
| 105039 | 341 | – | 360 | 603.3187 | 1806.9342 | 1806.9337 | 0.28 | 0 | 43 | 0.0014 | 1Score **> 36** indicates **identity** Score **> 27** indicates **homology** | U | K.TNVSGGAIALGHPLGGSGSR.I |
| 105040 | 341 | – | 360 | 603.3192 | 1806.9358 | 1806.9337 | 1.18 | 0 | 17 | 0.025 | 1Score **> 36** indicates **identity** Score **> 14** indicates **homology** | U | K.TNVSGGAIALGHPLGGSGSR.I |
| 105041 | 341 | – | 360 | 603.3194 | 1806.9365 | 1806.9337 | 1.55 | 0 | 26 | 0.0048 | 1Score **> 36** indicates **identity** Score **> 15** indicates **homology** | U | K.TNVSGGAIALGHPLGGSGSR.I |
| 105042 | 341 | – | 360 | 603.3196 | 1806.9371 | 1806.9337 | 1.88 | 0 | 28 | 0.0031 | 1Score **> 36** indicates **identity** Score **> 16** indicates **homology** | U | K.TNVSGGAIALGHPLGGSGSR.I |
| 105043 | 341 | – | 360 | 603.3198 | 1806.9376 | 1806.9337 | 2.17 | 0 | 57 | 1e-05 | 1Score **> 36** indicates **identity** Score **> 20** indicates **homology** | U | K.TNVSGGAIALGHPLGGSGSR.I |
| 105046 | 341 | – | 360 | 603.3202 | 1806.9389 | 1806.9337 | 2.89 | 0 | 18 | 0.022 | 1Score **> 36** indicates **identity** Score **> 14** indicates **homology** | U | K.TNVSGGAIALGHPLGGSGSR.I |
| 105048 | 341 | – | 360 | 603.3205 | 1806.9398 | 1806.9337 | 3.38 | 0 | 34 | 0.00076 | 1Score **> 35** indicates **identity** Score **> 16** indicates **homology** | U | K.TNVSGGAIALGHPLGGSGSR.I |
| 105051 | 341 | – | 360 | 603.3230 | 1806.9472 | 1806.9337 | 7.51 | 0 | 27 | 0.0027 | 1Score **> 35** indicates **identity** Score **> 14** indicates **homology** | U | K.TNVSGGAIALGHPLGGSGSR.I |
| 32196 | 361 | – | 370 | 396.8995 | 1187.6766 | 1187.6775 | -0.75 | 0 | 33 | 0.0054 | 1Score **> 34** indicates **identity** Score **> 22** indicates **homology** | U | R.ITAHLVHELR.R |
| 32197 | 361 | – | 370 | 594.8458 | 1187.6771 | 1187.6775 | -0.33 | 0 | 41 | 0.01 | 1Score **> 33** indicates **identity** | U | R.ITAHLVHELR.R |
| 32198 | 361 | – | 370 | 396.9001 | 1187.6785 | 1187.6775 | 0.87 | 0 | 34 | 0.0077 | 1Score **> 33** indicates **identity** Score **> 25** indicates **homology** | U | R.ITAHLVHELR.R |
| 32199 | 361 | – | 370 | 594.8469 | 1187.6793 | 1187.6775 | 1.55 | 0 | 58 | 0.00016 | 1Score **> 33** indicates **identity** | U | R.ITAHLVHELR.R |
| 49464 | 361 | – | 371 | 448.9334 | 1343.7783 | 1343.7786 | -0.22 | 1 | 35 | 0.0011 | 1Score **> 34** indicates **identity** Score **> 18** indicates **homology** | U | R.ITAHLVHELRR.R |
| 49465 | 361 | – | 371 | 448.9336 | 1343.7788 | 1343.7786 | 0.19 | 1 | 32 | 0.0018 | 1Score **> 34** indicates **identity** Score **> 17** indicates **homology** | U | R.ITAHLVHELRR.R |
| 49466 | 361 | – | 371 | 448.9336 | 1343.7788 | 1343.7786 | 0.20 | 1 | 29 | 0.0017 | 1Score **> 34** indicates **identity** Score **> 14** indicates **homology** | U | R.ITAHLVHELRR.R |
| 49468 | 361 | – | 371 | 672.8971 | 1343.7796 | 1343.7786 | 0.77 | 1 | 26 | 0.045 | 1Score **> 33** indicates **identity** Score **> 25** indicates **homology** | U | R.ITAHLVHELRR.R |

---

```
ID   THIM_MOUSE              Reviewed;         397 AA.
AC   Q8BWT1; Q3TIT9; Q8JZR8;
DT   30-AUG-2005, integrated into UniProtKB/Swiss-Prot.
DT   27-JUL-2011, sequence version 3.
DT   28-JUN-2023, entry version 149.
DE   RecName: Full=3-ketoacyl-CoA thiolase, mitochondrial {ECO:0000305};
DE            EC=2.3.1.16 {ECO:0000250|UniProtKB:P42765};
DE   AltName: Full=Acetyl-CoA acetyltransferase {ECO:0000305};
DE            EC=2.3.1.9 {ECO:0000255|PROSITE-ProRule:PRU10020};
DE   AltName: Full=Acetyl-CoA acyltransferase;
DE   AltName: Full=Acyl-CoA hydrolase, mitochondrial {ECO:0000305};
DE            EC=3.1.2.- {ECO:0000250|UniProtKB:P42765};
DE            EC=3.1.2.1 {ECO:0000250|UniProtKB:P42765};
DE            EC=3.1.2.2 {ECO:0000250|UniProtKB:P13437};
DE   AltName: Full=Beta-ketothiolase;
DE   AltName: Full=Mitochondrial 3-oxoacyl-CoA thiolase;
GN   Name=Acaa2;
OS   Mus musculus (Mouse).
OC   Eukaryota; Metazoa; Chordata; Craniata; Vertebrata; Euteleostomi; Mammalia;
OC   Eutheria; Euarchontoglires; Glires; Rodentia; Myomorpha; Muroidea; Muridae;
OC   Murinae; Mus; Mus.
OX   NCBI_TaxID=10090;
RN   [1]
RP   NUCLEOTIDE SEQUENCE [LARGE SCALE MRNA].
RC   STRAIN=C57BL/6J; TISSUE=Amnion, Heart, and Liver;
RX   PubMed=16141072; DOI=10.1126/science.1112014;
RA   Carninci P., Kasukawa T., Katayama S., Gough J., Frith M.C., Maeda N.,
RA   Oyama R., Ravasi T., Lenhard B., Wells C., Kodzius R., Shimokawa K.,
RA   Bajic V.B., Brenner S.E., Batalov S., Forrest A.R., Zavolan M., Davis M.J.,
RA   Wilming L.G., Aidinis V., Allen J.E., Ambesi-Impiombato A., Apweiler R.,
RA   Aturaliya R.N., Bailey T.L., Bansal M., Baxter L., Beisel K.W., Bersano T.,
RA   Bono H., Chalk A.M., Chiu K.P., Choudhary V., Christoffels A.,
RA   Clutterbuck D.R., Crowe M.L., Dalla E., Dalrymple B.P., de Bono B.,
RA   Della Gatta G., di Bernardo D., Down T., Engstrom P., Fagiolini M.,
RA   Faulkner G., Fletcher C.F., Fukushima T., Furuno M., Futaki S.,
RA   Gariboldi M., Georgii-Hemming P., Gingeras T.R., Gojobori T., Green R.E.,
RA   Gustincich S., Harbers M., Hayashi Y., Hensch T.K., Hirokawa N., Hill D.,
RA   Huminiecki L., Iacono M., Ikeo K., Iwama A., Ishikawa T., Jakt M.,
RA   Kanapin A., Katoh M., Kawasawa Y., Kelso J., Kitamura H., Kitano H.,
RA   Kollias G., Krishnan S.P., Kruger A., Kummerfeld S.K., Kurochkin I.V.,
RA   Lareau L.F., Lazarevic D., Lipovich L., Liu J., Liuni S., McWilliam S.,
RA   Madan Babu M., Madera M., Marchionni L., Matsuda H., Matsuzawa S., Miki H.,
RA   Mignone F., Miyake S., Morris K., Mottagui-Tabar S., Mulder N., Nakano N.,
RA   Nakauchi H., Ng P., Nilsson R., Nishiguchi S., Nishikawa S., Nori F.,
RA   Ohara O., Okazaki Y., Orlando V., Pang K.C., Pavan W.J., Pavesi G.,
RA   Pesole G., Petrovsky N., Piazza S., Reed J., Reid J.F., Ring B.Z.,
RA   Ringwald M., Rost B., Ruan Y., Salzberg S.L., Sandelin A., Schneider C.,
RA   Schoenbach C., Sekiguchi K., Semple C.A., Seno S., Sessa L., Sheng Y.,
RA   Shibata Y., Shimada H., Shimada K., Silva D., Sinclair B., Sperling S.,
RA   Stupka E., Sugiura K., Sultana R., Takenaka Y., Taki K., Tammoja K.,
RA   Tan S.L., Tang S., Taylor M.S., Tegner J., Teichmann S.A., Ueda H.R.,
RA   van Nimwegen E., Verardo R., Wei C.L., Yagi K., Yamanishi H.,
RA   Zabarovsky E., Zhu S., Zimmer A., Hide W., Bult C., Grimmond S.M.,
RA   Teasdale R.D., Liu E.T., Brusic V., Quackenbush J., Wahlestedt C.,
RA   Mattick J.S., Hume D.A., Kai C., Sasaki D., Tomaru Y., Fukuda S.,
RA   Kanamori-Katayama M., Suzuki M., Aoki J., Arakawa T., Iida J., Imamura K.,
RA   Itoh M., Kato T., Kawaji H., Kawagashira N., Kawashima T., Kojima M.,
RA   Kondo S., Konno H., Nakano K., Ninomiya N., Nishio T., Okada M., Plessy C.,
RA   Shibata K., Shiraki T., Suzuki S., Tagami M., Waki K., Watahiki A.,
RA   Okamura-Oho Y., Suzuki H., Kawai J., Hayashizaki Y.;
RT   "The transcriptional landscape of the mammalian genome.";
RL   Science 309:1559-1563(2005).
RN   [2]
RP   NUCLEOTIDE SEQUENCE [LARGE SCALE GENOMIC DNA].
RA   Mural R.J., Adams M.D., Myers E.W., Smith H.O., Venter J.C.;
RL   Submitted (SEP-2005) to the EMBL/GenBank/DDBJ databases.
RN   [3]
RP   NUCLEOTIDE SEQUENCE [LARGE SCALE MRNA].
RC   STRAIN=FVB/N; TISSUE=Liver;
RX   PubMed=15489334; DOI=10.1101/gr.2596504;
RG   The MGC Project Team;
RT   "The status, quality, and expansion of the NIH full-length cDNA project:
RT   the Mammalian Gene Collection (MGC).";
RL   Genome Res. 14:2121-2127(2004).
RN   [4]
RP   PHOSPHORYLATION [LARGE SCALE ANALYSIS] AT SER-344, AND IDENTIFICATION BY
RP   MASS SPECTROMETRY [LARGE SCALE ANALYSIS].
RC   TISSUE=Liver;
RX   PubMed=17208939; DOI=10.1074/mcp.m600218-mcp200;
RA   Lee J., Xu Y., Chen Y., Sprung R., Kim S.C., Xie S., Zhao Y.;
RT   "Mitochondrial phosphoproteome revealed by an improved IMAC method and
RT   MS/MS/MS.";
RL   Mol. Cell. Proteomics 6:669-676(2007).
RN   [5]
RP   PHOSPHORYLATION [LARGE SCALE ANALYSIS] AT SER-28, AND IDENTIFICATION BY
RP   MASS SPECTROMETRY [LARGE SCALE ANALYSIS].
RC   TISSUE=Liver;
RX   PubMed=17242355; DOI=10.1073/pnas.0609836104;
RA   Villen J., Beausoleil S.A., Gerber S.A., Gygi S.P.;
RT   "Large-scale phosphorylation analysis of mouse liver.";
RL   Proc. Natl. Acad. Sci. U.S.A. 104:1488-1493(2007).
RN   [6]
RP   PHOSPHORYLATION [LARGE SCALE ANALYSIS] AT SER-28; THR-136 AND SER-310, AND
RP   IDENTIFICATION BY MASS SPECTROMETRY [LARGE SCALE ANALYSIS].
RC   TISSUE=Brain, Brown adipose tissue, Heart, Kidney, Liver, Lung,
RC   Pancreas, Spleen, and Testis;
RX   PubMed=21183079; DOI=10.1016/j.cell.2010.12.001;
RA   Huttlin E.L., Jedrychowski M.P., Elias J.E., Goswami T., Rad R.,
RA   Beausoleil S.A., Villen J., Haas W., Sowa M.E., Gygi S.P.;
RT   "A tissue-specific atlas of mouse protein phosphorylation and expression.";
RL   Cell 143:1174-1189(2010).
RN   [7]
RP   ACETYLATION [LARGE SCALE ANALYSIS] AT LYS-191, SUCCINYLATION [LARGE SCALE
RP   ANALYSIS] AT LYS-25; LYS-45; LYS-137; LYS-143; LYS-158; LYS-171; LYS-191;
RP   LYS-209; LYS-211; LYS-212; LYS-214; LYS-234; LYS-240; LYS-305 AND LYS-312,
RP   AND IDENTIFICATION BY MASS SPECTROMETRY [LARGE SCALE ANALYSIS].
RC   TISSUE=Liver;
RX   PubMed=23806337; DOI=10.1016/j.molcel.2013.06.001;
RA   Park J., Chen Y., Tishkoff D.X., Peng C., Tan M., Dai L., Xie Z., Zhang Y.,
RA   Zwaans B.M., Skinner M.E., Lombard D.B., Zhao Y.;
RT   "SIRT5-mediated lysine desuccinylation impacts diverse metabolic
RT   pathways.";
RL   Mol. Cell 50:919-930(2013).
RN   [8]
RP   ACETYLATION [LARGE SCALE ANALYSIS] AT LYS-25; LYS-137; LYS-143; LYS-158;
RP   LYS-171; LYS-191; LYS-209; LYS-234; LYS-241; LYS-269; LYS-270; LYS-305;
RP   LYS-312; LYS-340 AND LYS-375, AND IDENTIFICATION BY MASS SPECTROMETRY
RP   [LARGE SCALE ANALYSIS].
RC   TISSUE=Liver;
RX   PubMed=23576753; DOI=10.1073/pnas.1302961110;
RA   Rardin M.J., Newman J.C., Held J.M., Cusack M.P., Sorensen D.J., Li B.,
RA   Schilling B., Mooney S.D., Kahn C.R., Verdin E., Gibson B.W.;
RT   "Label-free quantitative proteomics of the lysine acetylome in mitochondria
RT   identifies substrates of SIRT3 in metabolic pathways.";
RL   Proc. Natl. Acad. Sci. U.S.A. 110:6601-6606(2013).
CC   -!- FUNCTION: In the production of energy from fats, this is one of the
CC       enzymes that catalyzes the last step of the mitochondrial beta-
CC       oxidation pathway, an aerobic process breaking down fatty acids into
CC       acetyl-CoA. Using free coenzyme A/CoA, catalyzes the thiolytic cleavage
CC       of medium- to long-chain unbranched 3-oxoacyl-CoAs into acetyl-CoA and
CC       a fatty acyl-CoA shortened by two carbon atoms. Also catalyzes the
CC       condensation of two acetyl-CoA molecules into acetoacetyl-CoA and could
CC       be involved in the production of ketone bodies. Also displays hydrolase
CC       activity on various fatty acyl-CoAs (By similarity). Thereby, could be
CC       responsible for the production of acetate in a side reaction to beta-
CC       oxidation (By similarity). Abolishes BNIP3-mediated apoptosis and
CC       mitochondrial damage (By similarity). {ECO:0000250|UniProtKB:P13437,
CC       ECO:0000250|UniProtKB:P42765}.
CC   -!- CATALYTIC ACTIVITY:
CC       Reaction=acetyl-CoA + an acyl-CoA = a 3-oxoacyl-CoA + CoA;
CC         Xref=Rhea:RHEA:21564, ChEBI:CHEBI:57287, ChEBI:CHEBI:57288,
CC         ChEBI:CHEBI:58342, ChEBI:CHEBI:90726; EC=2.3.1.16;
CC         Evidence={ECO:0000250|UniProtKB:P42765};
CC       PhysiologicalDirection=left-to-right; Xref=Rhea:RHEA:21565;
CC         Evidence={ECO:0000250|UniProtKB:P42765};
CC       PhysiologicalDirection=right-to-left; Xref=Rhea:RHEA:21566;
CC         Evidence={ECO:0000250|UniProtKB:P42765};
CC   -!- CATALYTIC ACTIVITY:
CC       Reaction=2 acetyl-CoA = acetoacetyl-CoA + CoA; Xref=Rhea:RHEA:21036,
CC         ChEBI:CHEBI:57286, ChEBI:CHEBI:57287, ChEBI:CHEBI:57288; EC=2.3.1.9;
CC         Evidence={ECO:0000255|PROSITE-ProRule:PRU10020};
CC       PhysiologicalDirection=left-to-right; Xref=Rhea:RHEA:21037;
CC         Evidence={ECO:0000250|UniProtKB:P42765};
CC       PhysiologicalDirection=right-to-left; Xref=Rhea:RHEA:21038;
CC         Evidence={ECO:0000250|UniProtKB:P42765};
CC   -!- CATALYTIC ACTIVITY:
CC       Reaction=acetyl-CoA + H2O = acetate + CoA + H(+); Xref=Rhea:RHEA:20289,
CC         ChEBI:CHEBI:15377, ChEBI:CHEBI:15378, ChEBI:CHEBI:30089,
CC         ChEBI:CHEBI:57287, ChEBI:CHEBI:57288; EC=3.1.2.1;
CC         Evidence={ECO:0000250|UniProtKB:P42765};
CC       PhysiologicalDirection=left-to-right; Xref=Rhea:RHEA:20290;
CC         Evidence={ECO:0000250|UniProtKB:P42765};
CC   -!- CATALYTIC ACTIVITY:
CC       Reaction=H2O + propanoyl-CoA = CoA + H(+) + propanoate;
CC         Xref=Rhea:RHEA:40103, ChEBI:CHEBI:15377, ChEBI:CHEBI:15378,
CC         ChEBI:CHEBI:17272, ChEBI:CHEBI:57287, ChEBI:CHEBI:57392;
CC         Evidence={ECO:0000250|UniProtKB:P42765};
CC       PhysiologicalDirection=left-to-right; Xref=Rhea:RHEA:40104;
CC         Evidence={ECO:0000250|UniProtKB:P42765};
CC   -!- CATALYTIC ACTIVITY:
CC       Reaction=butanoyl-CoA + H2O = butanoate + CoA + H(+);
CC         Xref=Rhea:RHEA:40111, ChEBI:CHEBI:15377, ChEBI:CHEBI:15378,
CC         ChEBI:CHEBI:17968, ChEBI:CHEBI:57287, ChEBI:CHEBI:57371;
CC         Evidence={ECO:0000250|UniProtKB:P42765};
CC       PhysiologicalDirection=left-to-right; Xref=Rhea:RHEA:40112;
CC         Evidence={ECO:0000250|UniProtKB:P42765};
CC   -!- CATALYTIC ACTIVITY:
CC       Reaction=H2O + hexanoyl-CoA = CoA + H(+) + hexanoate;
CC         Xref=Rhea:RHEA:40115, ChEBI:CHEBI:15377, ChEBI:CHEBI:15378,
CC         ChEBI:CHEBI:17120, ChEBI:CHEBI:57287, ChEBI:CHEBI:62620;
CC         Evidence={ECO:0000250|UniProtKB:P42765};
CC       PhysiologicalDirection=left-to-right; Xref=Rhea:RHEA:40116;
CC         Evidence={ECO:0000250|UniProtKB:P42765};
CC   -!- CATALYTIC ACTIVITY:
CC       Reaction=H2O + octanoyl-CoA = CoA + H(+) + octanoate;
CC         Xref=Rhea:RHEA:30143, ChEBI:CHEBI:15377, ChEBI:CHEBI:15378,
CC         ChEBI:CHEBI:25646, ChEBI:CHEBI:57287, ChEBI:CHEBI:57386;
CC         Evidence={ECO:0000250|UniProtKB:P42765};
CC       PhysiologicalDirection=left-to-right; Xref=Rhea:RHEA:30144;
CC         Evidence={ECO:0000250|UniProtKB:P42765};
CC   -!- CATALYTIC ACTIVITY:
CC       Reaction=decanoyl-CoA + H2O = CoA + decanoate + H(+);
CC         Xref=Rhea:RHEA:40059, ChEBI:CHEBI:15377, ChEBI:CHEBI:15378,
CC         ChEBI:CHEBI:27689, ChEBI:CHEBI:57287, ChEBI:CHEBI:61430;
CC         Evidence={ECO:0000250|UniProtKB:P42765};
CC       PhysiologicalDirection=left-to-right; Xref=Rhea:RHEA:40060;
CC         Evidence={ECO:0000250|UniProtKB:P42765};
CC   -!- CATALYTIC ACTIVITY:
CC       Reaction=dodecanoyl-CoA + H2O = CoA + dodecanoate + H(+);
CC         Xref=Rhea:RHEA:30135, ChEBI:CHEBI:15377, ChEBI:CHEBI:15378,
CC         ChEBI:CHEBI:18262, ChEBI:CHEBI:57287, ChEBI:CHEBI:57375;
CC         Evidence={ECO:0000250|UniProtKB:P42765};
CC       PhysiologicalDirection=left-to-right; Xref=Rhea:RHEA:30136;
CC         Evidence={ECO:0000250|UniProtKB:P42765};
CC   -!- CATALYTIC ACTIVITY:
CC       Reaction=H2O + tetradecanoyl-CoA = CoA + H(+) + tetradecanoate;
CC         Xref=Rhea:RHEA:40119, ChEBI:CHEBI:15377, ChEBI:CHEBI:15378,
CC         ChEBI:CHEBI:30807, ChEBI:CHEBI:57287, ChEBI:CHEBI:57385;
CC         Evidence={ECO:0000250|UniProtKB:P42765};
CC       PhysiologicalDirection=left-to-right; Xref=Rhea:RHEA:40120;
CC         Evidence={ECO:0000250|UniProtKB:P42765};
CC   -!- CATALYTIC ACTIVITY:
CC       Reaction=H2O + hexadecanoyl-CoA = CoA + H(+) + hexadecanoate;
CC         Xref=Rhea:RHEA:16645, ChEBI:CHEBI:7896, ChEBI:CHEBI:15377,
CC         ChEBI:CHEBI:15378, ChEBI:CHEBI:57287, ChEBI:CHEBI:57379; EC=3.1.2.2;
CC         Evidence={ECO:0000250|UniProtKB:P13437};
CC       PhysiologicalDirection=left-to-right; Xref=Rhea:RHEA:16646;
CC         Evidence={ECO:0000250|UniProtKB:P42765};
CC   -!- PATHWAY: Lipid metabolism; fatty acid beta-oxidation.
CC       {ECO:0000250|UniProtKB:P42765}.
CC   -!- SUBUNIT: Homotetramer. Interacts with BNIP3.
CC       {ECO:0000250|UniProtKB:P42765}.
CC   -!- SUBCELLULAR LOCATION: Mitochondrion {ECO:0000250|UniProtKB:P42765}.
CC   -!- SIMILARITY: Belongs to the thiolase-like superfamily. Thiolase family.
CC       {ECO:0000305}.
CC   ---------------------------------------------------------------------------
CC   Copyrighted by the UniProt Consortium, see https://www.uniprot.org/terms
CC   Distributed under the Creative Commons Attribution (CC BY 4.0) License
CC   ---------------------------------------------------------------------------
DR   EMBL; AK050101; BAC34067.1; -; mRNA.
DR   EMBL; AK167567; BAE39630.1; -; mRNA.
DR   EMBL; AK167715; BAE39757.1; -; mRNA.
DR   EMBL; AK169359; BAE41108.1; -; mRNA.
DR   EMBL; CH466528; EDL09521.1; -; Genomic_DNA.
DR   EMBL; BC028901; AAH28901.1; -; mRNA.
DR   CCDS; CCDS29342.1; -.
DR   RefSeq; NP_803421.1; NM_177470.3.
DR   AlphaFoldDB; Q8BWT1; -.
DR   SMR; Q8BWT1; -.
DR   BioGRID; 206649; 30.
DR   IntAct; Q8BWT1; 9.
DR   MINT; Q8BWT1; -.
DR   STRING; 10090.ENSMUSP00000037348; -.
DR   GlyGen; Q8BWT1; 1 site, 1 O-linked glycan (1 site).
DR   iPTMnet; Q8BWT1; -.
DR   PhosphoSitePlus; Q8BWT1; -.
DR   SwissPalm; Q8BWT1; -.
DR   REPRODUCTION-2DPAGE; Q8BWT1; -.
DR   CPTAC; non-CPTAC-3618; -.
DR   CPTAC; non-CPTAC-3883; -.
DR   EPD; Q8BWT1; -.
DR   jPOST; Q8BWT1; -.
DR   MaxQB; Q8BWT1; -.
DR   PaxDb; Q8BWT1; -.
DR   PeptideAtlas; Q8BWT1; -.
DR   ProteomicsDB; 258872; -.
DR   Antibodypedia; 22617; 409 antibodies from 30 providers.
DR   Ensembl; ENSMUST00000041053; ENSMUSP00000037348; ENSMUSG00000036880.
DR   GeneID; 52538; -.
DR   KEGG; mmu:52538; -.
DR   UCSC; uc008fpt.1; mouse.
DR   AGR; MGI:1098623; -.
DR   CTD; 10449; -.
DR   MGI; MGI:1098623; Acaa2.
DR   VEuPathDB; HostDB:ENSMUSG00000036880; -.
DR   eggNOG; KOG1391; Eukaryota.
DR   GeneTree; ENSGT01030000234626; -.
DR   HOGENOM; CLU_031026_0_0_1; -.
DR   InParanoid; Q8BWT1; -.
DR   OMA; DYYWGMG; -.
DR   OrthoDB; 5481312at2759; -.
DR   PhylomeDB; Q8BWT1; -.
DR   TreeFam; TF105696; -.
DR   BRENDA; 2.3.1.16; 3474.
DR   Reactome; R-MMU-77289; Mitochondrial Fatty Acid Beta-Oxidation.
DR   UniPathway; UPA00659; -.
DR   BioGRID-ORCS; 52538; 1 hit in 80 CRISPR screens.
DR   ChiTaRS; Acaa2; mouse.
DR   PRO; PR:Q8BWT1; -.
DR   Proteomes; UP000000589; Chromosome 18.
DR   RNAct; Q8BWT1; protein.
DR   Bgee; ENSMUSG00000036880; Expressed in gall bladder and 285 other tissues.
DR   ExpressionAtlas; Q8BWT1; baseline and differential.
DR   Genevisible; Q8BWT1; MM.
DR   GO; GO:0005743; C:mitochondrial inner membrane; HDA:MGI.
DR   GO; GO:0005759; C:mitochondrial matrix; ISO:MGI.
DR   GO; GO:0005739; C:mitochondrion; HDA:MGI.
DR   GO; GO:0003985; F:acetyl-CoA C-acetyltransferase activity; ISS:UniProtKB.
DR   GO; GO:0003988; F:acetyl-CoA C-acyltransferase activity; ISS:UniProtKB.
DR   GO; GO:0003986; F:acetyl-CoA hydrolase activity; IEA:UniProtKB-EC.
DR   GO; GO:0047617; F:acyl-CoA hydrolase activity; ISS:UniProtKB.
DR   GO; GO:0102991; F:myristoyl-CoA hydrolase activity; IEA:UniProtKB-EC.
DR   GO; GO:0016290; F:palmitoyl-CoA hydrolase activity; IEA:UniProtKB-EC.
DR   GO; GO:0006084; P:acetyl-CoA metabolic process; ISO:MGI.
DR   GO; GO:0071456; P:cellular response to hypoxia; ISO:MGI.
DR   GO; GO:0006635; P:fatty acid beta-oxidation; ISO:MGI.
DR   GO; GO:1902109; P:negative regulation of mitochondrial membrane permeability involved in apoptotic process; ISO:MGI.
DR   GO; GO:1901029; P:negative regulation of mitochondrial outer membrane permeabilization involved in apoptotic signaling pathway; ISS:UniProtKB.
DR   CDD; cd00751; thiolase; 1.
DR   Gene3D; 3.40.47.10; -; 2.
DR   InterPro; IPR002155; Thiolase.
DR   InterPro; IPR016039; Thiolase-like.
DR   InterPro; IPR020615; Thiolase_acyl_enz_int_AS.
DR   InterPro; IPR020610; Thiolase_AS.
DR   InterPro; IPR020617; Thiolase_C.
DR   InterPro; IPR020613; Thiolase_CS.
DR   InterPro; IPR020616; Thiolase_N.
DR   PANTHER; PTHR18919:SF107; 3-KETOACYL-COA THIOLASE, MITOCHONDRIAL; 1.
DR   PANTHER; PTHR18919; ACETYL-COA C-ACYLTRANSFERASE; 1.
DR   Pfam; PF02803; Thiolase_C; 1.
DR   Pfam; PF00108; Thiolase_N; 1.
DR   PIRSF; PIRSF000429; Ac-CoA_Ac_transf; 1.
DR   SUPFAM; SSF53901; Thiolase-like; 2.
DR   PROSITE; PS00098; THIOLASE_1; 1.
DR   PROSITE; PS00737; THIOLASE_2; 1.
DR   PROSITE; PS00099; THIOLASE_3; 1.
DR   TIGRFAMs; TIGR01930; AcCoA-C-Actrans; 1.
PE   1: Evidence at protein level;
KW   Acetylation; Acyltransferase; Fatty acid metabolism; Hydrolase;
KW   Lipid metabolism; Mitochondrion; Phosphoprotein; Reference proteome;
KW   Transferase; Transit peptide.
FT   CHAIN           1..397
FT                   /note="3-ketoacyl-CoA thiolase, mitochondrial"
FT                   /id="PRO_0000223300"
FT   TRANSIT         1..16
FT                   /note="Mitochondrion; not cleaved"
FT                   /evidence="ECO:0000250"
FT   ACT_SITE        92
FT                   /note="Acyl-thioester intermediate"
FT                   /evidence="ECO:0000250|UniProtKB:P42765"
FT   ACT_SITE        382
FT                   /note="Proton donor/acceptor"
FT                   /evidence="ECO:0000250|UniProtKB:P42765"
FT   BINDING         224
FT                   /ligand="CoA"
FT                   /ligand_id="ChEBI:CHEBI:57287"
FT                   /evidence="ECO:0000250|UniProtKB:P42765"
FT   BINDING         227
FT                   /ligand="CoA"
FT                   /ligand_id="ChEBI:CHEBI:57287"
FT                   /evidence="ECO:0000250|UniProtKB:P42765"
FT   BINDING         251
FT                   /ligand="CoA"
FT                   /ligand_id="ChEBI:CHEBI:57287"
FT                   /evidence="ECO:0000250|UniProtKB:P42765"
FT   SITE            352
FT                   /note="Increases nucleophilicity of active site Cys"
FT                   /evidence="ECO:0000250|UniProtKB:P42765"
FT   MOD_RES         25
FT                   /note="N6-acetyllysine; alternate"
FT                   /evidence="ECO:0007744|PubMed:23576753"
FT   MOD_RES         25
FT                   /note="N6-succinyllysine; alternate"
FT                   /evidence="ECO:0007744|PubMed:23806337"
FT   MOD_RES         28
FT                   /note="Phosphoserine"
FT                   /evidence="ECO:0007744|PubMed:17242355,
FT                   ECO:0007744|PubMed:21183079"
FT   MOD_RES         45
FT                   /note="N6-succinyllysine"
FT                   /evidence="ECO:0007744|PubMed:23806337"
FT   MOD_RES         119
FT                   /note="Phosphothreonine"
FT                   /evidence="ECO:0000250|UniProtKB:P42765"
FT   MOD_RES         121
FT                   /note="Phosphoserine"
FT                   /evidence="ECO:0000250|UniProtKB:P42765"
FT   MOD_RES         127
FT                   /note="Phosphotyrosine"
FT                   /evidence="ECO:0000250|UniProtKB:P42765"
FT   MOD_RES         136
FT                   /note="Phosphothreonine"
FT                   /evidence="ECO:0007744|PubMed:21183079"
FT   MOD_RES         137
FT                   /note="N6-acetyllysine; alternate"
FT                   /evidence="ECO:0007744|PubMed:23576753"
FT   MOD_RES         137
FT                   /note="N6-succinyllysine; alternate"
FT                   /evidence="ECO:0007744|PubMed:23806337"
FT   MOD_RES         143
FT                   /note="N6-acetyllysine; alternate"
FT                   /evidence="ECO:0007744|PubMed:23576753"
FT   MOD_RES         143
FT                   /note="N6-succinyllysine; alternate"
FT                   /evidence="ECO:0007744|PubMed:23806337"
FT   MOD_RES         158
FT                   /note="N6-acetyllysine; alternate"
FT                   /evidence="ECO:0007744|PubMed:23576753"
FT   MOD_RES         158
FT                   /note="N6-succinyllysine; alternate"
FT                   /evidence="ECO:0007744|PubMed:23806337"
FT   MOD_RES         171
FT                   /note="N6-acetyllysine; alternate"
FT                   /evidence="ECO:0007744|PubMed:23576753"
FT   MOD_RES         171
FT                   /note="N6-succinyllysine; alternate"
FT                   /evidence="ECO:0007744|PubMed:23806337"
FT   MOD_RES         191
FT                   /note="N6-acetyllysine; alternate"
FT                   /evidence="ECO:0007744|PubMed:23576753,
FT                   ECO:0007744|PubMed:23806337"
FT   MOD_RES         191
FT                   /note="N6-succinyllysine; alternate"
FT                   /evidence="ECO:0007744|PubMed:23806337"
FT   MOD_RES         209
FT                   /note="N6-acetyllysine; alternate"
FT                   /evidence="ECO:0007744|PubMed:23576753"
FT   MOD_RES         209
FT                   /note="N6-succinyllysine; alternate"
FT                   /evidence="ECO:0007744|PubMed:23806337"
FT   MOD_RES         211
FT                   /note="N6-succinyllysine"
FT                   /evidence="ECO:0007744|PubMed:23806337"
FT   MOD_RES         212
FT                   /note="N6-succinyllysine"
FT                   /evidence="ECO:0007744|PubMed:23806337"
FT   MOD_RES         214
FT                   /note="N6-succinyllysine"
FT                   /evidence="ECO:0007744|PubMed:23806337"
FT   MOD_RES         234
FT                   /note="N6-acetyllysine; alternate"
FT                   /evidence="ECO:0007744|PubMed:23576753"
FT   MOD_RES         234
FT                   /note="N6-succinyllysine; alternate"
FT                   /evidence="ECO:0007744|PubMed:23806337"
FT   MOD_RES         240
FT                   /note="N6-succinyllysine"
FT                   /evidence="ECO:0007744|PubMed:23806337"
FT   MOD_RES         241
FT                   /note="N6-acetyllysine"
FT                   /evidence="ECO:0007744|PubMed:23576753"
FT   MOD_RES         269
FT                   /note="N6-acetyllysine"
FT                   /evidence="ECO:0007744|PubMed:23576753"
FT   MOD_RES         270
FT                   /note="N6-acetyllysine"
FT                   /evidence="ECO:0007744|PubMed:23576753"
FT   MOD_RES         305
FT                   /note="N6-acetyllysine; alternate"
FT                   /evidence="ECO:0007744|PubMed:23576753"
FT   MOD_RES         305
FT                   /note="N6-succinyllysine; alternate"
FT                   /evidence="ECO:0007744|PubMed:23806337"
FT   MOD_RES         310
FT                   /note="Phosphoserine"
FT                   /evidence="ECO:0007744|PubMed:21183079"
FT   MOD_RES         312
FT                   /note="N6-acetyllysine; alternate"
FT                   /evidence="ECO:0007744|PubMed:23576753"
FT   MOD_RES         312
FT                   /note="N6-succinyllysine; alternate"
FT                   /evidence="ECO:0007744|PubMed:23806337"
FT   MOD_RES         340
FT                   /note="N6-acetyllysine"
FT                   /evidence="ECO:0007744|PubMed:23576753"
FT   MOD_RES         344
FT                   /note="Phosphoserine"
FT                   /evidence="ECO:0007744|PubMed:17208939"
FT   MOD_RES         375
FT                   /note="N6-acetyllysine"
FT                   /evidence="ECO:0007744|PubMed:23576753"
FT   CONFLICT        397
FT                   /note="A -> V (in Ref. 3; AAH28901)"
FT                   /evidence="ECO:0000305"
SQ   SEQUENCE   397 AA;  41830 MW;  856840546F5CB8DA CRC64;
     MALLRGVFIV AAKRTPFGAY GGLLKDFSAT DLTEFAARAA LSAGKVPPET IDSVIVGNVM
     QSSSDAAYLA RHVGLRVGVP TETGALTLNR LCGSGFQSIV SGCQEICSKD AEVVLCGGTE
     SMSQSPYCVR NVRFGTKFGL DLKLEDTLWA GLTDQHVKLP MGMTAENLAA KYNISREDCD
     RYALQSQQRW KAANEAGYFN EEMAPIEVKT KKGKQTMQVD EHARPQTTLE QLQKLPSVFK
     KDGTVTAGNA SGVSDGAGAV IIASEDAVKK HNFTPLARVV GYFVSGCDPT IMGIGPVPAI
     NGALKKAGLS LKDMDLIDVN EAFAPQFLSV QKALDLDPSK TNVSGGAIAL GHPLGGSGSR
     ITAHLVHELR RRGGKYAVGS ACIGGGQGIA LIIQNTA
//
```

|  |
| --- |
| **Mascot:** http://www.matrixscience.com/ |
