# Supplementary material for: A common East-Asian ALDH2 mutation causes metabolic disorders and the therapeutic effect of ALDH2 activators
Source: Nat Commun. 2023 Sep 25;14:5971. doi: 10.1038/s41467-023-41570-6 (PMC10520061; doi:10.1038/s41467-023-41570-6)
Supplement: Supplementary file 4 — Supplementary Data 1 [file 41467_2023_41570_MOESM4_ESM.zip › Table S5b/Q8K2B3/Q8K2B3_WTO-1_C438_C574.html]

Mascot Search Results: Q8K2B3
 

# MASCOT Search Results

## Protein View: Q8K2B3

### Succinate dehydrogenase [ubiquinone] flavoprotein subunit, mitochondrial OS=Mus musculus OX=10090 GN=Sdha PE=1 SV=1

|  |  |
| --- | --- |
| Database: | Mouse\_UniProt\_proteomes |
| Score: | 11698 |
| Monoisotopic mass (Mr): | 73623 |
| Calculated pI: | 7.06 |

Sequence similarity is available as an NCBI BLAST search of Q8K2B3 against nr.

### Search parameters

|  |  |
| --- | --- |
| MS data file: | `D:\LCMSMS\2023 Users' data\230529-1\230529-1-WTO-1.raw` |
| Enzyme: | Trypsin/P: cuts C-term side of KR. |
| Fixed modifications: | Carbamidomethyl (C) |
| Variable modifications: | Deamidated (NQ), HNE (C), HNE (H), HNE (K), Oxidation (M) |

### Protein sequence coverage: 59%

Matched peptides shown in ***bold red***.

|  |  |  |  |  |  |
| --- | --- | --- | --- | --- | --- |
| `1` | `MAGVGAVSRL` | `LRGRRLALTG` | `AWPGTLQKQT` | `CGFHFSVGEN` | `KKASAKVSDA` |
| `51` | `ISTQYPVVDH` | `EFDAVVVGAG` | `GAGLRAAFGL` | `SEAGFNTACL` | `TKLFPTRSHT` |
| `101` | `VAAQGGINAA` | `LGNMEEDNWR` | `WHFYDTVKGS` | `DWLGDQDAIH` | `YMTEQAPASV` |
| `151` | `VELENYGMPF` | `SRTEDGKIYQ` | `RAFGGQSLKF` | `GKGGQAHRCC` | `CVADRTGHSL` |
| `201` | `LHTLYGRSLR` | `YDTSYFVEYF` | `ALDLLMENGE` | `CRGVIALCIE` | `DGSIHRIRAK` |
| `251` | `NTVIATGGYG` | `RTYFSCTSAH` | `TSTGDGTAMV` | `TRAGLPCQDL` | `EFVQFHPTGI` |
| `301` | `YGAGCLITEG` | `CRGEGGILIN` | `SQGERFMERY` | `APVAKDLASR` | `DVVSRSMTLE` |
| `351` | `IREGRGCGPE` | `KDHVYLQLHH` | `LPPEQLATRL` | `PGISETAMIF` | `AGVDVTKEPI` |
| `401` | `PVLPTVHYNM` | `GGIPTNYKGQ` | `VLKHVNGQDQ` | `IVPGLYACGE` | `AACASVHGAN` |
| `451` | `RLGANSLLDL` | `VVFGRACALS` | `IAESCRPGDK` | `VPSIKANAGE` | `ESVMNLDKLR` |
| `501` | `FADGSIRTSE` | `LRLNMQKSMQ` | `NHAAVFRVGS` | `VLQEGCEKIS` | `QLYGDLKHLK` |
| `551` | `TFDRGMVWNT` | `DLVETLELQN` | `LMLCALQTIY` | `GAEARKESRG` | `AHAREDYKVR` |
| `601` | `VDEYDYSKPI` | `QGQQKKPFGE` | `HWRKHTLSYV` | `DIKTGKVTLE` | `YRPVIDKTLN` |
| `651` | `EADCATVPPA` | `IRSY` |  |  |  |

Unformatted sequence string: 664 residues (for pasting into other applications).

|  |  |  |  |
| --- | --- | --- | --- |
| Sort by | residue number | increasing mass | decreasing mass |
| Show | matched peptides only | predicted peptides also |  |

| Query | Start | – | End | Observed | Mr(expt) | Mr(calc) | ppm | M | Score | Expect | Rank | U | Peptide |
| --- | --- | --- | --- | --- | --- | --- | --- | --- | --- | --- | --- | --- | --- |
| 171074 | 47 | – | 75 | 977.1554 | 2928.4445 | 2928.4618 | -5.90 | 0 | 25 | 0.0049 | 1Score **> 37** indicates **identity** Score **> 14** indicates **homology** | U | K.VSDAISTQYPVVDHEFDAVVVGAGGAGLR.A |
| 171076 | 47 | – | 75 | 977.1567 | 2928.4484 | 2928.4618 | -4.57 | 0 | 32 | 0.001 | 1Score **> 37** indicates **identity** Score **> 14** indicates **homology** | U | K.VSDAISTQYPVVDHEFDAVVVGAGGAGLR.A |
| 171077 | 47 | – | 75 | 977.1572 | 2928.4496 | 2928.4618 | -4.15 | 0 | 23 | 0.0063 | 1Score **> 37** indicates **identity** Score **> 14** indicates **homology** | U | K.VSDAISTQYPVVDHEFDAVVVGAGGAGLR.A |
| 171078 | 47 | – | 75 | 977.1584 | 2928.4533 | 2928.4618 | -2.88 | 0 | 50 | 2.2e-05 | 1Score **> 37** indicates **identity** Score **> 16** indicates **homology** | U | K.VSDAISTQYPVVDHEFDAVVVGAGGAGLR.A |
| 171079 | 47 | – | 75 | 977.1586 | 2928.4539 | 2928.4618 | -2.68 | 0 | 47 | 3.7e-05 | 1Score **> 37** indicates **identity** Score **> 15** indicates **homology** | U | K.VSDAISTQYPVVDHEFDAVVVGAGGAGLR.A |
| 171080 | 47 | – | 75 | 977.1597 | 2928.4573 | 2928.4618 | -1.55 | 0 | 33 | 0.00088 | 1Score **> 37** indicates **identity** Score **> 15** indicates **homology** | U | K.VSDAISTQYPVVDHEFDAVVVGAGGAGLR.A |
| 171083 | 47 | – | 75 | 977.1605 | 2928.4597 | 2928.4618 | -0.71 | 0 | 65 | 8.7e-07 | 1Score **> 37** indicates **identity** Score **> 17** indicates **homology** | U | K.VSDAISTQYPVVDHEFDAVVVGAGGAGLR.A |
| 171084 | 47 | – | 75 | 977.1605 | 2928.4598 | 2928.4618 | -0.68 | 0 | 62 | 1.5e-06 | 1Score **> 37** indicates **identity** Score **> 16** indicates **homology** | U | K.VSDAISTQYPVVDHEFDAVVVGAGGAGLR.A |
| 171086 | 47 | – | 75 | 733.1224 | 2928.4607 | 2928.4618 | -0.39 | 0 | 24 | 0.0051 | 1Score **> 37** indicates **identity** Score **> 14** indicates **homology** | U | K.VSDAISTQYPVVDHEFDAVVVGAGGAGLR.A |
| 171088 | 47 | – | 75 | 733.1226 | 2928.4615 | 2928.4618 | -0.11 | 0 | 17 | 0.032 | 1Score **> 37** indicates **identity** Score **> 15** indicates **homology** | U | K.VSDAISTQYPVVDHEFDAVVVGAGGAGLR.A |
| 171090 | 47 | – | 75 | 977.1612 | 2928.4617 | 2928.4618 | -0.043 | 0 | 52 | 1.3e-05 | 1Score **> 37** indicates **identity** Score **> 16** indicates **homology** | U | K.VSDAISTQYPVVDHEFDAVVVGAGGAGLR.A |
| 171091 | 47 | – | 75 | 977.1612 | 2928.4618 | 2928.4618 | -0.0058 | 0 | 60 | 2.4e-06 | 1Score **> 37** indicates **identity** Score **> 16** indicates **homology** | U | K.VSDAISTQYPVVDHEFDAVVVGAGGAGLR.A |
| 171092 | 47 | – | 75 | 977.1612 | 2928.4618 | 2928.4618 | 0.014 | 0 | 58 | 3.9e-06 | 1Score **> 37** indicates **identity** Score **> 16** indicates **homology** | U | K.VSDAISTQYPVVDHEFDAVVVGAGGAGLR.A |
| 171094 | 47 | – | 75 | 977.1614 | 2928.4623 | 2928.4618 | 0.16 | 0 | 68 | 4e-07 | 1Score **> 37** indicates **identity** Score **> 17** indicates **homology** | U | K.VSDAISTQYPVVDHEFDAVVVGAGGAGLR.A |
| 171095 | 47 | – | 75 | 733.1228 | 2928.4623 | 2928.4618 | 0.16 | 0 | 22 | 0.011 | 1Score **> 37** indicates **identity** Score **> 15** indicates **homology** | U | K.VSDAISTQYPVVDHEFDAVVVGAGGAGLR.A |
| 171097 | 47 | – | 75 | 1465.2386 | 2928.4626 | 2928.4618 | 0.27 | 0 | 53 | 9.9e-06 | 1Score **> 37** indicates **identity** Score **> 16** indicates **homology** | U | K.VSDAISTQYPVVDHEFDAVVVGAGGAGLR.A |
| 171098 | 47 | – | 75 | 977.1616 | 2928.4630 | 2928.4618 | 0.43 | 0 | 59 | 2.9e-06 | 1Score **> 37** indicates **identity** Score **> 16** indicates **homology** | U | K.VSDAISTQYPVVDHEFDAVVVGAGGAGLR.A |
| 171099 | 47 | – | 75 | 977.1617 | 2928.4632 | 2928.4618 | 0.49 | 0 | 63 | 1.2e-06 | 1Score **> 37** indicates **identity** Score **> 16** indicates **homology** | U | K.VSDAISTQYPVVDHEFDAVVVGAGGAGLR.A |
| 171100 | 47 | – | 75 | 733.1231 | 2928.4634 | 2928.4618 | 0.54 | 0 | 15 | 0.037 | 1Score **> 37** indicates **identity** Score **> 13** indicates **homology** | U | K.VSDAISTQYPVVDHEFDAVVVGAGGAGLR.A |
| 171101 | 47 | – | 75 | 977.1617 | 2928.4634 | 2928.4618 | 0.55 | 0 | 69 | 3.1e-07 | 1Score **> 37** indicates **identity** Score **> 17** indicates **homology** | U | K.VSDAISTQYPVVDHEFDAVVVGAGGAGLR.A |
| 171102 | 47 | – | 75 | 733.1231 | 2928.4634 | 2928.4618 | 0.55 | 0 | 18 | 0.022 | 1Score **> 37** indicates **identity** Score **> 14** indicates **homology** | U | K.VSDAISTQYPVVDHEFDAVVVGAGGAGLR.A |
| 171103 | 47 | – | 75 | 977.1618 | 2928.4636 | 2928.4618 | 0.61 | 0 | 53 | 9.9e-06 | 1Score **> 37** indicates **identity** Score **> 16** indicates **homology** | U | K.VSDAISTQYPVVDHEFDAVVVGAGGAGLR.A |
| 171104 | 47 | – | 75 | 977.1618 | 2928.4637 | 2928.4618 | 0.65 | 0 | 57 | 4.1e-06 | 1Score **> 37** indicates **identity** Score **> 16** indicates **homology** | U | K.VSDAISTQYPVVDHEFDAVVVGAGGAGLR.A |
| 171105 | 47 | – | 75 | 977.1619 | 2928.4638 | 2928.4618 | 0.67 | 0 | 58 | 3.3e-06 | 1Score **> 37** indicates **identity** Score **> 16** indicates **homology** | U | K.VSDAISTQYPVVDHEFDAVVVGAGGAGLR.A |
| 171106 | 47 | – | 75 | 733.1232 | 2928.4638 | 2928.4618 | 0.67 | 0 | 21 | 0.013 | 1Score **> 37** indicates **identity** Score **> 15** indicates **homology** | U | K.VSDAISTQYPVVDHEFDAVVVGAGGAGLR.A |
| 171109 | 47 | – | 75 | 977.1622 | 2928.4649 | 2928.4618 | 1.05 | 0 | 36 | 0.00039 | 1Score **> 37** indicates **identity** Score **> 15** indicates **homology** | U | K.VSDAISTQYPVVDHEFDAVVVGAGGAGLR.A |
| 171110 | 47 | – | 75 | 977.1623 | 2928.4650 | 2928.4618 | 1.09 | 0 | 54 | 9e-06 | 1Score **> 37** indicates **identity** Score **> 16** indicates **homology** | U | K.VSDAISTQYPVVDHEFDAVVVGAGGAGLR.A |
| 171111 | 47 | – | 75 | 977.1625 | 2928.4658 | 2928.4618 | 1.36 | 0 | 50 | 1.9e-05 | 1Score **> 37** indicates **identity** Score **> 16** indicates **homology** | U | K.VSDAISTQYPVVDHEFDAVVVGAGGAGLR.A |
| 171117 | 47 | – | 75 | 977.1629 | 2928.4669 | 2928.4618 | 1.75 | 0 | 48 | 3e-05 | 1Score **> 37** indicates **identity** Score **> 16** indicates **homology** | U | K.VSDAISTQYPVVDHEFDAVVVGAGGAGLR.A |
| 171121 | 47 | – | 75 | 977.1635 | 2928.4686 | 2928.4618 | 2.34 | 0 | 49 | 2.7e-05 | 1Score **> 37** indicates **identity** Score **> 16** indicates **homology** | U | K.VSDAISTQYPVVDHEFDAVVVGAGGAGLR.A |
| 171122 | 47 | – | 75 | 977.1638 | 2928.4694 | 2928.4618 | 2.61 | 0 | 61 | 2e-06 | 1Score **> 37** indicates **identity** Score **> 16** indicates **homology** | U | K.VSDAISTQYPVVDHEFDAVVVGAGGAGLR.A |
| 171123 | 47 | – | 75 | 977.1638 | 2928.4695 | 2928.4618 | 2.62 | 0 | 51 | 1.6e-05 | 1Score **> 37** indicates **identity** Score **> 16** indicates **homology** | U | K.VSDAISTQYPVVDHEFDAVVVGAGGAGLR.A |
| 171125 | 47 | – | 75 | 977.1642 | 2928.4708 | 2928.4618 | 3.09 | 0 | 21 | 0.012 | 1Score **> 37** indicates **identity** Score **> 14** indicates **homology** | U | K.VSDAISTQYPVVDHEFDAVVVGAGGAGLR.A |
| 171127 | 47 | – | 75 | 977.1651 | 2928.4734 | 2928.4618 | 3.96 | 0 | 39 | 0.00023 | 1Score **> 37** indicates **identity** Score **> 15** indicates **homology** | U | K.VSDAISTQYPVVDHEFDAVVVGAGGAGLR.A |
| 171181 | 47 | – | 75 | 977.4863 | 2929.4372 | 2929.4458 | -2.93 | 0 | 31 | 0.0013 | 1Score **> 37** indicates **identity** Score **> 14** indicates **homology** | U | K.VSDAISTQYPVVDHEFDAVVVGAGGAGLR.A  + Deamidated (NQ) |
| 171186 | 47 | – | 75 | 977.4942 | 2929.4609 | 2929.4458 | 5.16 | 0 | 33 | 0.00079 | 1Score **> 37** indicates **identity** Score **> 15** indicates **homology** | U | K.VSDAISTQYPVVDHEFDAVVVGAGGAGLR.A  + Deamidated (NQ) |
| 171188 | 47 | – | 75 | 977.4944 | 2929.4613 | 2929.4458 | 5.28 | 0 | 24 | 0.0055 | 1Score **> 37** indicates **identity** Score **> 14** indicates **homology** | U | K.VSDAISTQYPVVDHEFDAVVVGAGGAGLR.A  + Deamidated (NQ) |
| 171190 | 47 | – | 75 | 977.4945 | 2929.4616 | 2929.4458 | 5.40 | 0 | 37 | 0.00036 | 1Score **> 37** indicates **identity** Score **> 15** indicates **homology** | U | K.VSDAISTQYPVVDHEFDAVVVGAGGAGLR.A  + Deamidated (NQ) |
| 171191 | 47 | – | 75 | 977.4947 | 2929.4624 | 2929.4458 | 5.66 | 0 | 55 | 6.8e-06 | 1Score **> 37** indicates **identity** Score **> 16** indicates **homology** | U | K.VSDAISTQYPVVDHEFDAVVVGAGGAGLR.A  + Deamidated (NQ) |
| 171192 | 47 | – | 75 | 977.4948 | 2929.4625 | 2929.4458 | 5.72 | 0 | 20 | 0.012 | 1Score **> 37** indicates **identity** Score **> 14** indicates **homology** | U | K.VSDAISTQYPVVDHEFDAVVVGAGGAGLR.A  + Deamidated (NQ) |
| 171194 | 47 | – | 75 | 977.4951 | 2929.4635 | 2929.4458 | 6.05 | 0 | 15 | 0.037 | 1Score **> 37** indicates **identity** Score **> 13** indicates **homology** | U | K.VSDAISTQYPVVDHEFDAVVVGAGGAGLR.A  + Deamidated (NQ) |
| 171197 | 47 | – | 75 | 977.4956 | 2929.4650 | 2929.4458 | 6.54 | 0 | 41 | 0.00014 | 1Score **> 37** indicates **identity** Score **> 15** indicates **homology** | U | K.VSDAISTQYPVVDHEFDAVVVGAGGAGLR.A  + Deamidated (NQ) |
| 171199 | 47 | – | 75 | 1465.7403 | 2929.4661 | 2929.4458 | 6.94 | 0 | 67 | 4.9e-07 | 1Score **> 37** indicates **identity** Score **> 17** indicates **homology** | U | K.VSDAISTQYPVVDHEFDAVVVGAGGAGLR.A  + Deamidated (NQ) |
| 171203 | 47 | – | 75 | 977.4963 | 2929.4670 | 2929.4458 | 7.24 | 0 | 42 | 0.00011 | 1Score **> 38** indicates **identity** Score **> 15** indicates **homology** | U | K.VSDAISTQYPVVDHEFDAVVVGAGGAGLR.A  + Deamidated (NQ) |
| 171207 | 47 | – | 75 | 977.4972 | 2929.4697 | 2929.4458 | 8.17 | 0 | 23 | 0.01 | 1Score **> 37** indicates **identity** Score **> 16** indicates **homology** | U | K.VSDAISTQYPVVDHEFDAVVVGAGGAGLR.A  + Deamidated (NQ) |
| 171209 | 47 | – | 75 | 977.4974 | 2929.4705 | 2929.4458 | 8.42 | 0 | 47 | 3.6e-05 | 1Score **> 38** indicates **identity** Score **> 15** indicates **homology** | U | K.VSDAISTQYPVVDHEFDAVVVGAGGAGLR.A  + Deamidated (NQ) |
| 171212 | 47 | – | 75 | 977.4981 | 2929.4726 | 2929.4458 | 9.15 | 0 | 60 | 2.2e-06 | 1Score **> 38** indicates **identity** Score **> 16** indicates **homology** | U | K.VSDAISTQYPVVDHEFDAVVVGAGGAGLR.A  + Deamidated (NQ) |
| 171213 | 47 | – | 75 | 977.4982 | 2929.4729 | 2929.4458 | 9.25 | 0 | 23 | 0.0067 | 1Score **> 38** indicates **identity** Score **> 14** indicates **homology** | U | K.VSDAISTQYPVVDHEFDAVVVGAGGAGLR.A  + Deamidated (NQ) |
| 96985 | 76 | – | 92 | 879.4245 | 1756.8345 | 1756.8454 | -6.20 | 0 | 51 | 1.7e-05 | 1Score **> 33** indicates **identity** Score **> 16** indicates **homology** | U | R.AAFGLSEAGFNTACLTK.L |
| 96989 | 76 | – | 92 | 879.4270 | 1756.8395 | 1756.8454 | -3.34 | 0 | 78 | 4.6e-08 | 1Score **> 34** indicates **identity** Score **> 17** indicates **homology** | U | R.AAFGLSEAGFNTACLTK.L |
| 96991 | 76 | – | 92 | 879.4282 | 1756.8418 | 1756.8454 | -2.08 | 0 | 25 | 0.0046 | 1Score **> 34** indicates **identity** Score **> 14** indicates **homology** | U | R.AAFGLSEAGFNTACLTK.L |
| 96992 | 76 | – | 92 | 879.4286 | 1756.8427 | 1756.8454 | -1.52 | 0 | 104 | 1.8e-10 | 1Score **> 34** indicates **identity** Score **> 19** indicates **homology** | U | R.AAFGLSEAGFNTACLTK.L |
| 96994 | 76 | – | 92 | 879.4296 | 1756.8446 | 1756.8454 | -0.48 | 0 | 54 | 8.8e-06 | 1Score **> 34** indicates **identity** Score **> 16** indicates **homology** | U | R.AAFGLSEAGFNTACLTK.L |
| 96995 | 76 | – | 92 | 879.4296 | 1756.8446 | 1756.8454 | -0.44 | 0 | 80 | 3.1e-08 | 1Score **> 34** indicates **identity** Score **> 18** indicates **homology** | U | R.AAFGLSEAGFNTACLTK.L |
| 96996 | 76 | – | 92 | 879.4297 | 1756.8448 | 1756.8454 | -0.34 | 0 | 76 | 7.9e-08 | 1Score **> 34** indicates **identity** Score **> 17** indicates **homology** | U | R.AAFGLSEAGFNTACLTK.L |
| 96997 | 76 | – | 92 | 879.4298 | 1756.8451 | 1756.8454 | -0.15 | 0 | 47 | 4e-05 | 1Score **> 34** indicates **identity** Score **> 15** indicates **homology** | U | R.AAFGLSEAGFNTACLTK.L |
| 96999 | 76 | – | 92 | 879.4300 | 1756.8454 | 1756.8454 | -0.0097 | 0 | 108 | 8e-11 | 1Score **> 34** indicates **identity** Score **> 19** indicates **homology** | U | R.AAFGLSEAGFNTACLTK.L |
| 97000 | 76 | – | 92 | 879.4301 | 1756.8457 | 1756.8454 | 0.14 | 0 | 72 | 1.9e-07 | 1Score **> 34** indicates **identity** Score **> 17** indicates **homology** | U | R.AAFGLSEAGFNTACLTK.L |
| 97001 | 76 | – | 92 | 879.4301 | 1756.8457 | 1756.8454 | 0.16 | 0 | 79 | 3.7e-08 | 1Score **> 34** indicates **identity** Score **> 17** indicates **homology** | U | R.AAFGLSEAGFNTACLTK.L |
| 97002 | 76 | – | 92 | 879.4302 | 1756.8459 | 1756.8454 | 0.29 | 0 | 83 | 1.5e-08 | 1Score **> 34** indicates **identity** Score **> 18** indicates **homology** | U | R.AAFGLSEAGFNTACLTK.L |
| 97003 | 76 | – | 92 | 879.4303 | 1756.8460 | 1756.8454 | 0.35 | 0 | 92 | 2.5e-09 | 1Score **> 34** indicates **identity** Score **> 18** indicates **homology** | U | R.AAFGLSEAGFNTACLTK.L |
| 97004 | 76 | – | 92 | 879.4306 | 1756.8466 | 1756.8454 | 0.66 | 0 | 88 | 5.3e-09 | 1Score **> 34** indicates **identity** Score **> 18** indicates **homology** | U | R.AAFGLSEAGFNTACLTK.L |
| 97005 | 76 | – | 92 | 879.4315 | 1756.8485 | 1756.8454 | 1.78 | 0 | 85 | 1e-08 | 1Score **> 34** indicates **identity** Score **> 18** indicates **homology** | U | R.AAFGLSEAGFNTACLTK.L |
| 97006 | 76 | – | 92 | 879.4319 | 1756.8492 | 1756.8454 | 2.15 | 0 | 58 | 4e-06 | 1Score **> 34** indicates **identity** Score **> 16** indicates **homology** | U | R.AAFGLSEAGFNTACLTK.L |
| 97007 | 76 | – | 92 | 879.4329 | 1756.8513 | 1756.8454 | 3.34 | 0 | 93 | 2.1e-09 | 1Score **> 34** indicates **identity** Score **> 18** indicates **homology** | U | R.AAFGLSEAGFNTACLTK.L |
| 97008 | 76 | – | 92 | 879.4330 | 1756.8515 | 1756.8454 | 3.49 | 0 | 20 | 0.012 | 1Score **> 34** indicates **identity** Score **> 14** indicates **homology** | U | R.AAFGLSEAGFNTACLTK.L |
| 97009 | 76 | – | 92 | 879.4332 | 1756.8519 | 1756.8454 | 3.69 | 0 | 58 | 3.6e-06 | 1Score **> 34** indicates **identity** Score **> 16** indicates **homology** | U | R.AAFGLSEAGFNTACLTK.L |
| 97015 | 76 | – | 92 | 879.4373 | 1756.8601 | 1756.8454 | 8.34 | 0 | 15 | 0.035 | 1Score **> 34** indicates **identity** Score **> 13** indicates **homology** | U | R.AAFGLSEAGFNTACLTK.L |
| 21562 | 121 | – | 128 | 548.2659 | 1094.5172 | 1094.5185 | -1.24 | 0 | 25 | 0.0049 | 1Score **> 30** indicates **identity** Score **> 14** indicates **homology** | U | R.WHFYDTVK.G |
| 21564 | 121 | – | 128 | 365.8465 | 1094.5176 | 1094.5185 | -0.89 | 0 | 19 | 0.015 | 1Score **> 30** indicates **identity** Score **> 14** indicates **homology** | U | R.WHFYDTVK.G |
| 21565 | 121 | – | 128 | 365.8465 | 1094.5177 | 1094.5185 | -0.80 | 0 | 31 | 0.0012 | 1Score **> 30** indicates **identity** Score **> 14** indicates **homology** | U | R.WHFYDTVK.G |
| 21566 | 121 | – | 128 | 548.2661 | 1094.5177 | 1094.5185 | -0.75 | 0 | 28 | 0.0074 | 1Score **> 30** indicates **identity** Score **> 19** indicates **homology** | U | R.WHFYDTVK.G |
| 21569 | 121 | – | 128 | 365.8465 | 1094.5178 | 1094.5185 | -0.71 | 0 | 27 | 0.014 | 1Score **> 30** indicates **identity** Score **> 21** indicates **homology** | U | R.WHFYDTVK.G |
| 21570 | 121 | – | 128 | 365.8466 | 1094.5178 | 1094.5185 | -0.65 | 0 | 14 | 0.049 | 1Score **> 30** indicates **identity** Score **> 13** indicates **homology** | U | R.WHFYDTVK.G |
| 21571 | 121 | – | 128 | 365.8466 | 1094.5179 | 1094.5185 | -0.62 | 0 | 29 | 0.0019 | 1Score **> 30** indicates **identity** Score **> 14** indicates **homology** | U | R.WHFYDTVK.G |
| 21572 | 121 | – | 128 | 548.2662 | 1094.5179 | 1094.5185 | -0.59 | 0 | 34 | 0.00063 | 1Score **> 30** indicates **identity** Score **> 15** indicates **homology** | U | R.WHFYDTVK.G |
| 21575 | 121 | – | 128 | 365.8466 | 1094.5180 | 1094.5185 | -0.50 | 0 | 14 | 0.048 | 1Score **> 30** indicates **identity** Score **> 13** indicates **homology** | U | R.WHFYDTVK.G |
| 21576 | 121 | – | 128 | 548.2663 | 1094.5180 | 1094.5185 | -0.49 | 0 | 28 | 0.0026 | 1Score **> 30** indicates **identity** Score **> 14** indicates **homology** | U | R.WHFYDTVK.G |
| 21578 | 121 | – | 128 | 365.8466 | 1094.5181 | 1094.5185 | -0.38 | 0 | 35 | 0.00058 | 1Score **> 30** indicates **identity** Score **> 15** indicates **homology** | U | R.WHFYDTVK.G |
| 21579 | 121 | – | 128 | 365.8467 | 1094.5183 | 1094.5185 | -0.25 | 0 | 30 | 0.0016 | 1Score **> 30** indicates **identity** Score **> 14** indicates **homology** | U | R.WHFYDTVK.G |
| 21580 | 121 | – | 128 | 365.8467 | 1094.5183 | 1094.5185 | -0.25 | 0 | 24 | 0.0062 | 1Score **> 30** indicates **identity** Score **> 14** indicates **homology** | U | R.WHFYDTVK.G |
| 21582 | 121 | – | 128 | 365.8467 | 1094.5184 | 1094.5185 | -0.15 | 0 | 19 | 0.015 | 1Score **> 30** indicates **identity** Score **> 14** indicates **homology** | U | R.WHFYDTVK.G |
| 21584 | 121 | – | 128 | 365.8468 | 1094.5185 | 1094.5185 | -0.060 | 0 | 28 | 0.0024 | 1Score **> 30** indicates **identity** Score **> 14** indicates **homology** | U | R.WHFYDTVK.G |
| 21585 | 121 | – | 128 | 548.2665 | 1094.5185 | 1094.5185 | -0.039 | 0 | 30 | 0.0058 | 1Score **> 30** indicates **identity** Score **> 20** indicates **homology** | U | R.WHFYDTVK.G |
| 21589 | 121 | – | 128 | 365.8469 | 1094.5188 | 1094.5185 | 0.26 | 0 | 27 | 0.003 | 1Score **> 30** indicates **identity** Score **> 14** indicates **homology** | U | R.WHFYDTVK.G |
| 21590 | 121 | – | 128 | 365.8469 | 1094.5189 | 1094.5185 | 0.31 | 0 | 29 | 0.0019 | 1Score **> 30** indicates **identity** Score **> 14** indicates **homology** | U | R.WHFYDTVK.G |
| 21591 | 121 | – | 128 | 365.8469 | 1094.5190 | 1094.5185 | 0.42 | 0 | 29 | 0.0017 | 1Score **> 30** indicates **identity** Score **> 14** indicates **homology** | U | R.WHFYDTVK.G |
| 21592 | 121 | – | 128 | 548.2668 | 1094.5191 | 1094.5185 | 0.48 | 0 | 29 | 0.0019 | 1Score **> 30** indicates **identity** Score **> 14** indicates **homology** | U | R.WHFYDTVK.G |
| 21593 | 121 | – | 128 | 365.8470 | 1094.5191 | 1094.5185 | 0.52 | 0 | 40 | 0.00019 | 1Score **> 30** indicates **identity** Score **> 15** indicates **homology** | U | R.WHFYDTVK.G |
| 21594 | 121 | – | 128 | 365.8470 | 1094.5191 | 1094.5185 | 0.54 | 0 | 29 | 0.0019 | 1Score **> 30** indicates **identity** Score **> 14** indicates **homology** | U | R.WHFYDTVK.G |
| 21595 | 121 | – | 128 | 365.8470 | 1094.5192 | 1094.5185 | 0.57 | 0 | 28 | 0.0024 | 1Score **> 30** indicates **identity** Score **> 14** indicates **homology** | U | R.WHFYDTVK.G |
| 21597 | 121 | – | 128 | 548.2670 | 1094.5194 | 1094.5185 | 0.74 | 0 | 29 | 0.0074 | 1Score **> 30** indicates **identity** Score **> 20** indicates **homology** | U | R.WHFYDTVK.G |
| 21598 | 121 | – | 128 | 548.2670 | 1094.5195 | 1094.5185 | 0.87 | 0 | 33 | 0.0026 | 1Score **> 30** indicates **identity** Score **> 20** indicates **homology** | U | R.WHFYDTVK.G |
| 21601 | 121 | – | 128 | 365.8473 | 1094.5201 | 1094.5185 | 1.45 | 0 | 14 | 0.046 | 1Score **> 30** indicates **identity** Score **> 13** indicates **homology** | U | R.WHFYDTVK.G |
| 22947 | 163 | – | 171 | 555.2822 | 1108.5498 | 1108.5513 | -1.36 | 1 | 47 | 0.00012 | 1Score **> 31** indicates **identity** Score **> 20** indicates **homology** | U | R.TEDGKIYQR.A |
| 22948 | 163 | – | 171 | 555.2822 | 1108.5498 | 1108.5513 | -1.33 | 1 | 64 | 2.5e-06 | 1Score **> 31** indicates **identity** Score **> 20** indicates **homology** | U | R.TEDGKIYQR.A |
| 22949 | 163 | – | 171 | 370.5241 | 1108.5506 | 1108.5513 | -0.60 | 1 | 41 | 0.00013 | 1Score **> 31** indicates **identity** Score **> 15** indicates **homology** | U | R.TEDGKIYQR.A |
| 22950 | 163 | – | 171 | 370.5242 | 1108.5507 | 1108.5513 | -0.55 | 1 | 41 | 0.00016 | 1Score **> 31** indicates **identity** Score **> 15** indicates **homology** | U | R.TEDGKIYQR.A |
| 22951 | 163 | – | 171 | 555.2827 | 1108.5508 | 1108.5513 | -0.42 | 1 | 62 | 3.4e-06 | 1Score **> 31** indicates **identity** Score **> 20** indicates **homology** | U | R.TEDGKIYQR.A |
| 22953 | 163 | – | 171 | 370.5245 | 1108.5517 | 1108.5513 | 0.37 | 1 | 33 | 0.00087 | 1Score **> 32** indicates **identity** Score **> 15** indicates **homology** | U | R.TEDGKIYQR.A |
| 3820 | 172 | – | 179 | 404.2213 | 806.4281 | 806.4286 | -0.69 | 0 | 36 | 0.0025 | 1Score **> 26** indicates **identity** Score **> 22** indicates **homology** | U | R.AFGGQSLK.F |
| 3821 | 172 | – | 179 | 404.2214 | 806.4282 | 806.4286 | -0.57 | 0 | 46 | 0.00025 | 1Score **> 26** indicates **identity** Score **> 23** indicates **homology** | U | R.AFGGQSLK.F |
| 26003 | 172 | – | 182 | 380.5452 | 1138.6137 | 1138.6135 | 0.22 | 1 | 18 | 0.026 | 1Score **> 32** indicates **identity** Score **> 15** indicates **homology** | U | R.AFGGQSLKFGK.G |
| 142200 | 189 | – | 207 | 569.7678 | 2275.0421 | 2275.0409 | 0.55 | 1 | 14 | 0.047 | 1Score **> 33** indicates **identity** Score **> 13** indicates **homology** | U | R.CCCVADRTGHSLLHTLYGR.S |
| 142201 | 189 | – | 207 | 569.7679 | 2275.0424 | 2275.0409 | 0.68 | 1 | 19 | 0.017 | 1Score **> 33** indicates **identity** Score **> 14** indicates **homology** | U | R.CCCVADRTGHSLLHTLYGR.S |
| 49198 | 196 | – | 207 | 677.8647 | 1353.7149 | 1353.7153 | -0.33 | 0 | 67 | 9.4e-06 | 1Score **> 33** indicates **identity** Score **> 29** indicates **homology** | U | R.TGHSLLHTLYGR.S |
| 49200 | 196 | – | 207 | 677.8649 | 1353.7152 | 1353.7153 | -0.068 | 0 | 44 | 7.4e-05 | 1Score **> 33** indicates **identity** Score **> 15** indicates **homology** | U | R.TGHSLLHTLYGR.S |
| 70091 | 233 | – | 246 | 513.9343 | 1538.7811 | 1538.7875 | -4.15 | 0 | 27 | 0.0032 | 1Score **> 34** indicates **identity** Score **> 14** indicates **homology** | U | R.GVIALCIEDGSIHR.I |
| 70092 | 233 | – | 246 | 513.9344 | 1538.7813 | 1538.7875 | -4.01 | 0 | 42 | 0.00011 | 1Score **> 34** indicates **identity** Score **> 15** indicates **homology** | U | R.GVIALCIEDGSIHR.I |
| 70097 | 233 | – | 246 | 513.9352 | 1538.7836 | 1538.7875 | -2.51 | 0 | 45 | 5.6e-05 | 1Score **> 34** indicates **identity** Score **> 15** indicates **homology** | U | R.GVIALCIEDGSIHR.I |
| 70106 | 233 | – | 246 | 513.9364 | 1538.7875 | 1538.7875 | -0.011 | 0 | 39 | 0.00024 | 1Score **> 34** indicates **identity** Score **> 15** indicates **homology** | U | R.GVIALCIEDGSIHR.I |
| 70107 | 233 | – | 246 | 770.4013 | 1538.7880 | 1538.7875 | 0.30 | 0 | 86 | 3.3e-08 | 1Score **> 34** indicates **identity** Score **> 24** indicates **homology** | U | R.GVIALCIEDGSIHR.I |
| 70108 | 233 | – | 246 | 770.4013 | 1538.7881 | 1538.7875 | 0.36 | 0 | 99 | 2.7e-09 | 1Score **> 34** indicates **identity** Score **> 25** indicates **homology** | U | R.GVIALCIEDGSIHR.I |
| 70109 | 233 | – | 246 | 770.4014 | 1538.7882 | 1538.7875 | 0.45 | 0 | 99 | 2.7e-09 | 1Score **> 34** indicates **identity** Score **> 25** indicates **homology** | U | R.GVIALCIEDGSIHR.I |
| 70110 | 233 | – | 246 | 513.9367 | 1538.7882 | 1538.7875 | 0.47 | 0 | 58 | 4e-06 | 1Score **> 34** indicates **identity** Score **> 16** indicates **homology** | U | R.GVIALCIEDGSIHR.I |
| 70111 | 233 | – | 246 | 513.9367 | 1538.7884 | 1538.7875 | 0.57 | 0 | 57 | 4.2e-06 | 1Score **> 34** indicates **identity** Score **> 16** indicates **homology** | U | R.GVIALCIEDGSIHR.I |
| 70112 | 233 | – | 246 | 770.4015 | 1538.7884 | 1538.7875 | 0.57 | 0 | 98 | 7.8e-10 | 1Score **> 34** indicates **identity** Score **> 20** indicates **homology** | U | R.GVIALCIEDGSIHR.I |
| 70113 | 233 | – | 246 | 513.9368 | 1538.7885 | 1538.7875 | 0.66 | 0 | 66 | 6.3e-07 | 1Score **> 34** indicates **identity** Score **> 17** indicates **homology** | U | R.GVIALCIEDGSIHR.I |
| 70114 | 233 | – | 246 | 513.9368 | 1538.7886 | 1538.7875 | 0.73 | 0 | 39 | 0.00023 | 1Score **> 34** indicates **identity** Score **> 15** indicates **homology** | U | R.GVIALCIEDGSIHR.I |
| 70115 | 233 | – | 246 | 513.9368 | 1538.7887 | 1538.7875 | 0.76 | 0 | 50 | 2.3e-05 | 1Score **> 34** indicates **identity** Score **> 16** indicates **homology** | U | R.GVIALCIEDGSIHR.I |
| 70117 | 233 | – | 246 | 770.4018 | 1538.7890 | 1538.7875 | 0.96 | 0 | 59 | 1e-05 | 1Score **> 34** indicates **identity** Score **> 22** indicates **homology** | U | R.GVIALCIEDGSIHR.I |
| 44324 | 249 | – | 261 | 654.3567 | 1306.6988 | 1306.6993 | -0.43 | 1 | 53 | 1.1e-05 | 1Score **> 33** indicates **identity** Score **> 16** indicates **homology** | U | R.AKNTVIATGGYGR.T |
| 44325 | 249 | – | 261 | 436.5736 | 1306.6991 | 1306.6993 | -0.21 | 1 | 56 | 5.9e-06 | 1Score **> 33** indicates **identity** Score **> 16** indicates **homology** | U | R.AKNTVIATGGYGR.T |
| 44326 | 249 | – | 261 | 654.3570 | 1306.6994 | 1306.6993 | 0.037 | 1 | 38 | 0.00028 | 1Score **> 33** indicates **identity** Score **> 15** indicates **homology** | U | R.AKNTVIATGGYGR.T |
| 44327 | 249 | – | 261 | 436.5739 | 1306.6999 | 1306.6993 | 0.41 | 1 | 37 | 0.00032 | 1Score **> 33** indicates **identity** Score **> 15** indicates **homology** | U | R.AKNTVIATGGYGR.T |
| 44330 | 249 | – | 261 | 436.5743 | 1306.7010 | 1306.6993 | 1.25 | 1 | 34 | 0.00071 | 1Score **> 34** indicates **identity** Score **> 15** indicates **homology** | U | R.AKNTVIATGGYGR.T |
| 22869 | 251 | – | 261 | 554.7886 | 1107.5626 | 1107.5673 | -4.25 | 0 | 23 | 0.022 | 1Score **> 32** indicates **identity** Score **> 19** indicates **homology** | U | K.NTVIATGGYGR.T |
| 22874 | 251 | – | 261 | 554.7903 | 1107.5660 | 1107.5673 | -1.19 | 0 | 23 | 0.0076 | 1Score **> 32** indicates **identity** Score **> 14** indicates **homology** | U | K.NTVIATGGYGR.T |
| 22875 | 251 | – | 261 | 554.7904 | 1107.5662 | 1107.5673 | -0.97 | 0 | 56 | 1.3e-05 | 1Score **> 32** indicates **identity** Score **> 19** indicates **homology** | U | K.NTVIATGGYGR.T |
| 22877 | 251 | – | 261 | 554.7909 | 1107.5673 | 1107.5673 | 0.018 | 0 | 55 | 2.3e-05 | 1Score **> 31** indicates **identity** Score **> 22** indicates **homology** | U | K.NTVIATGGYGR.T |
| 22878 | 251 | – | 261 | 554.7910 | 1107.5675 | 1107.5673 | 0.24 | 0 | 39 | 0.00021 | 1Score **> 32** indicates **identity** Score **> 15** indicates **homology** | U | K.NTVIATGGYGR.T |
| 22879 | 251 | – | 261 | 554.7911 | 1107.5677 | 1107.5673 | 0.38 | 0 | 45 | 6.4e-05 | 1Score **> 32** indicates **identity** Score **> 16** indicates **homology** | U | K.NTVIATGGYGR.T |
| 22880 | 251 | – | 261 | 554.7916 | 1107.5686 | 1107.5673 | 1.18 | 0 | 22 | 0.0084 | 1Score **> 32** indicates **identity** Score **> 14** indicates **homology** | U | K.NTVIATGGYGR.T |
| 22881 | 251 | – | 261 | 554.7922 | 1107.5699 | 1107.5673 | 2.38 | 0 | 26 | 0.0037 | 1Score **> 32** indicates **identity** Score **> 14** indicates **homology** | U | K.NTVIATGGYGR.T |
| 140506 | 262 | – | 282 | 1125.9902 | 2249.9658 | 2249.9682 | -1.07 | 0 | 82 | 2.1e-08 | 1Score **> 28** indicates **identity** Score **> 18** indicates **homology** | U | R.TYFSCTSAHTSTGDGTAMVTR.A |
| 140507 | 262 | – | 282 | 750.9960 | 2249.9661 | 2249.9682 | -0.94 | 0 | 52 | 1.3e-05 | 1Score **> 28** indicates **identity** Score **> 16** indicates **homology** | U | R.TYFSCTSAHTSTGDGTAMVTR.A |
| 140508 | 262 | – | 282 | 1125.9904 | 2249.9663 | 2249.9682 | -0.83 | 0 | 38 | 0.00029 | 1Score **> 28** indicates **identity** Score **> 15** indicates **homology** | U | R.TYFSCTSAHTSTGDGTAMVTR.A |
| 140509 | 262 | – | 282 | 750.9962 | 2249.9669 | 2249.9682 | -0.58 | 0 | 65 | 8e-07 | 1Score **> 28** indicates **identity** Score **> 17** indicates **homology** | U | R.TYFSCTSAHTSTGDGTAMVTR.A |
| 140510 | 262 | – | 282 | 750.9966 | 2249.9679 | 2249.9682 | -0.14 | 0 | 61 | 2e-06 | 1Score **> 28** indicates **identity** Score **> 16** indicates **homology** | U | R.TYFSCTSAHTSTGDGTAMVTR.A |
| 140511 | 262 | – | 282 | 750.9969 | 2249.9688 | 2249.9682 | 0.27 | 0 | 43 | 8.8e-05 | 1Score **> 28** indicates **identity** Score **> 15** indicates **homology** | U | R.TYFSCTSAHTSTGDGTAMVTR.A |
| 140512 | 262 | – | 282 | 751.0019 | 2249.9838 | 2249.9682 | 6.94 | 0 | 47 | 3.7e-05 | 1Score **> 29** indicates **identity** Score **> 15** indicates **homology** | U | R.TYFSCTSAHTSTGDGTAMVTR.A |
| 141489 | 262 | – | 282 | 756.3284 | 2265.9634 | 2265.9631 | 0.16 | 0 | 18 | 0.023 | 1Score **> 26** indicates **identity** Score **> 14** indicates **homology** | U | R.TYFSCTSAHTSTGDGTAMVTR.A  + Oxidation (M) |
| 46685 | 313 | – | 325 | 665.3380 | 1328.6614 | 1328.6684 | -5.32 | 0 | 53 | 1.1e-05 | 1Score **> 32** indicates **identity** Score **> 16** indicates **homology** | U | R.GEGGILINSQGER.F |
| 46686 | 313 | – | 325 | 665.3382 | 1328.6619 | 1328.6684 | -4.91 | 0 | 27 | 0.0032 | 1Score **> 32** indicates **identity** Score **> 14** indicates **homology** | U | R.GEGGILINSQGER.F |
| 46688 | 313 | – | 325 | 665.3404 | 1328.6663 | 1328.6684 | -1.61 | 0 | 39 | 0.00024 | 1Score **> 32** indicates **identity** Score **> 15** indicates **homology** | U | R.GEGGILINSQGER.F |
| 46690 | 313 | – | 325 | 665.3409 | 1328.6673 | 1328.6684 | -0.84 | 0 | 35 | 0.00058 | 1Score **> 33** indicates **identity** Score **> 15** indicates **homology** | U | R.GEGGILINSQGER.F |
| 46692 | 313 | – | 325 | 665.3412 | 1328.6679 | 1328.6684 | -0.41 | 0 | 61 | 2.2e-06 | 1Score **> 33** indicates **identity** Score **> 17** indicates **homology** | U | R.GEGGILINSQGER.F |
| 46693 | 313 | – | 325 | 665.3413 | 1328.6680 | 1328.6684 | -0.30 | 0 | 55 | 8e-06 | 1Score **> 32** indicates **identity** Score **> 17** indicates **homology** | U | R.GEGGILINSQGER.F |
| 46694 | 313 | – | 325 | 665.3416 | 1328.6686 | 1328.6684 | 0.15 | 0 | 57 | 5.9e-06 | 1Score **> 32** indicates **identity** Score **> 17** indicates **homology** | U | R.GEGGILINSQGER.F |
| 46789 | 313 | – | 325 | 665.8328 | 1329.6511 | 1329.6524 | -1.02 | 0 | 29 | 0.0018 | 1Score **> 32** indicates **identity** Score **> 14** indicates **homology** | U | R.GEGGILINSQGER.F  + Deamidated (NQ) |
| 6570 | 346 | – | 352 | 433.2235 | 864.4325 | 864.4375 | -5.76 | 0 | 14 | 0.045 | 1Score **> 28** indicates **identity** Score **> 13** indicates **homology** | U | R.SMTLEIR.E  + Oxidation (M) |
| 6577 | 346 | – | 352 | 433.2265 | 864.4384 | 864.4375 | 1.10 | 0 | 23 | 0.0072 | 1Score **> 29** indicates **identity** Score **> 14** indicates **homology** | U | R.SMTLEIR.E  + Oxidation (M) |
| 167158 | 356 | – | 379 | 699.6054 | 2794.3924 | 2794.3973 | -1.77 | 1 | 27 | 0.0031 | 1Score **> 37** indicates **identity** Score **> 15** indicates **homology** | U | R.GCGPEKDHVYLQLHHLPPEQLATR.L |
| 167162 | 356 | – | 379 | 699.6060 | 2794.3947 | 2794.3973 | -0.92 | 1 | 25 | 0.0049 | 1Score **> 37** indicates **identity** Score **> 14** indicates **homology** | U | R.GCGPEKDHVYLQLHHLPPEQLATR.L |
| 167163 | 356 | – | 379 | 699.6061 | 2794.3951 | 2794.3973 | -0.78 | 1 | 18 | 0.02 | 1Score **> 37** indicates **identity** Score **> 14** indicates **homology** | U | R.GCGPEKDHVYLQLHHLPPEQLATR.L |
| 167164 | 356 | – | 379 | 699.6063 | 2794.3961 | 2794.3973 | -0.42 | 1 | 27 | 0.0028 | 1Score **> 37** indicates **identity** Score **> 14** indicates **homology** | U | R.GCGPEKDHVYLQLHHLPPEQLATR.L |
| 167169 | 356 | – | 379 | 699.6065 | 2794.3968 | 2794.3973 | -0.18 | 1 | 18 | 0.021 | 1Score **> 37** indicates **identity** Score **> 14** indicates **homology** | U | R.GCGPEKDHVYLQLHHLPPEQLATR.L |
| 167170 | 356 | – | 379 | 699.6065 | 2794.3969 | 2794.3973 | -0.14 | 1 | 23 | 0.0069 | 1Score **> 37** indicates **identity** Score **> 14** indicates **homology** | U | R.GCGPEKDHVYLQLHHLPPEQLATR.L |
| 167172 | 356 | – | 379 | 699.6066 | 2794.3975 | 2794.3973 | 0.057 | 1 | 29 | 0.0019 | 1Score **> 37** indicates **identity** Score **> 14** indicates **homology** | U | R.GCGPEKDHVYLQLHHLPPEQLATR.L |
| 167173 | 356 | – | 379 | 699.6066 | 2794.3975 | 2794.3973 | 0.063 | 1 | 22 | 0.0094 | 1Score **> 37** indicates **identity** Score **> 14** indicates **homology** | U | R.GCGPEKDHVYLQLHHLPPEQLATR.L |
| 167175 | 356 | – | 379 | 559.8869 | 2794.3981 | 2794.3973 | 0.30 | 1 | 18 | 0.021 | 1Score **> 37** indicates **identity** Score **> 14** indicates **homology** | U | R.GCGPEKDHVYLQLHHLPPEQLATR.L |
| 167184 | 356 | – | 379 | 559.8873 | 2794.4001 | 2794.3973 | 0.99 | 1 | 21 | 0.011 | 1Score **> 37** indicates **identity** Score **> 14** indicates **homology** | U | R.GCGPEKDHVYLQLHHLPPEQLATR.L |
| 167186 | 356 | – | 379 | 699.6084 | 2794.4045 | 2794.3973 | 2.57 | 1 | 16 | 0.031 | 1Score **> 37** indicates **identity** Score **> 14** indicates **homology** | U | R.GCGPEKDHVYLQLHHLPPEQLATR.L |
| 171768 | 424 | – | 451 | 984.4557 | 2950.3452 | 2950.3563 | -3.75 | 0 | 46 | 5.4e-05 | 1Score **> 34** indicates **identity** Score **> 15** indicates **homology** | U | K.HVNGQDQIVPGLYACGEAACASVHGANR.L |
| 171770 | 424 | – | 451 | 984.4580 | 2950.3522 | 2950.3563 | -1.38 | 0 | 32 | 0.001 | 1Score **> 34** indicates **identity** Score **> 14** indicates **homology** | U | K.HVNGQDQIVPGLYACGEAACASVHGANR.L |
| 171771 | 424 | – | 451 | 738.5955 | 2950.3530 | 2950.3563 | -1.12 | 0 | 27 | 0.0031 | 1Score **> 34** indicates **identity** Score **> 14** indicates **homology** | U | K.HVNGQDQIVPGLYACGEAACASVHGANR.L |
| 171772 | 424 | – | 451 | 738.5960 | 2950.3549 | 2950.3563 | -0.46 | 0 | 74 | 1.1e-07 | 1Score **> 34** indicates **identity** Score **> 17** indicates **homology** | U | K.HVNGQDQIVPGLYACGEAACASVHGANR.L |
| 171773 | 424 | – | 451 | 738.5961 | 2950.3552 | 2950.3563 | -0.37 | 0 | 68 | 4.4e-07 | 1Score **> 34** indicates **identity** Score **> 17** indicates **homology** | U | K.HVNGQDQIVPGLYACGEAACASVHGANR.L |
| 171774 | 424 | – | 451 | 984.4590 | 2950.3552 | 2950.3563 | -0.36 | 0 | 106 | 1.2e-10 | 1Score **> 34** indicates **identity** Score **> 19** indicates **homology** | U | K.HVNGQDQIVPGLYACGEAACASVHGANR.L |
| 171775 | 424 | – | 451 | 984.4591 | 2950.3554 | 2950.3563 | -0.29 | 0 | 101 | 3.6e-10 | 1Score **> 34** indicates **identity** Score **> 19** indicates **homology** | U | K.HVNGQDQIVPGLYACGEAACASVHGANR.L |
| 171776 | 424 | – | 451 | 984.4591 | 2950.3554 | 2950.3563 | -0.29 | 0 | 73 | 1.5e-07 | 1Score **> 34** indicates **identity** Score **> 17** indicates **homology** | U | K.HVNGQDQIVPGLYACGEAACASVHGANR.L |
| 171777 | 424 | – | 451 | 984.4591 | 2950.3554 | 2950.3563 | -0.29 | 0 | 66 | 6.2e-07 | 1Score **> 34** indicates **identity** Score **> 17** indicates **homology** | U | K.HVNGQDQIVPGLYACGEAACASVHGANR.L |
| 171778 | 424 | – | 451 | 984.4593 | 2950.3561 | 2950.3563 | -0.064 | 0 | 114 | 1.9e-11 | 1Score **> 34** indicates **identity** Score **> 20** indicates **homology** | U | K.HVNGQDQIVPGLYACGEAACASVHGANR.L |
| 171779 | 424 | – | 451 | 738.5971 | 2950.3592 | 2950.3563 | 0.98 | 0 | 44 | 7.2e-05 | 1Score **> 34** indicates **identity** Score **> 15** indicates **homology** | U | K.HVNGQDQIVPGLYACGEAACASVHGANR.L |
| 171781 | 424 | – | 451 | 738.5973 | 2950.3601 | 2950.3563 | 1.32 | 0 | 47 | 3.9e-05 | 1Score **> 34** indicates **identity** Score **> 15** indicates **homology** | U | K.HVNGQDQIVPGLYACGEAACASVHGANR.L |
| 171782 | 424 | – | 451 | 738.5974 | 2950.3604 | 2950.3563 | 1.39 | 0 | 15 | 0.036 | 1Score **> 34** indicates **identity** Score **> 13** indicates **homology** | U | K.HVNGQDQIVPGLYACGEAACASVHGANR.L |
| 171783 | 424 | – | 451 | 738.5975 | 2950.3608 | 2950.3563 | 1.55 | 0 | 58 | 3.8e-06 | 1Score **> 34** indicates **identity** Score **> 16** indicates **homology** | U | K.HVNGQDQIVPGLYACGEAACASVHGANR.L |
| 171784 | 424 | – | 451 | 738.5975 | 2950.3609 | 2950.3563 | 1.56 | 0 | 26 | 0.0039 | 1Score **> 34** indicates **identity** Score **> 14** indicates **homology** | U | K.HVNGQDQIVPGLYACGEAACASVHGANR.L |
| 171786 | 424 | – | 451 | 738.5988 | 2950.3662 | 2950.3563 | 3.35 | 0 | 31 | 0.0013 | 1Score **> 35** indicates **identity** Score **> 14** indicates **homology** | U | K.HVNGQDQIVPGLYACGEAACASVHGANR.L |
| 171788 | 424 | – | 451 | 738.5989 | 2950.3665 | 2950.3563 | 3.49 | 0 | 26 | 0.0035 | 1Score **> 35** indicates **identity** Score **> 14** indicates **homology** | U | K.HVNGQDQIVPGLYACGEAACASVHGANR.L |
| 171812 | 424 | – | 451 | 738.8393 | 2951.3283 | 2951.3403 | -4.06 | 0 | 44 | 8e-05 | 1Score **> 32** indicates **identity** Score **> 15** indicates **homology** | U | K.HVNGQDQIVPGLYACGEAACASVHGANR.L  + Deamidated (NQ) |
| 171813 | 424 | – | 451 | 738.8404 | 2951.3327 | 2951.3403 | -2.58 | 0 | 24 | 0.0052 | 1Score **> 33** indicates **identity** Score **> 14** indicates **homology** | U | K.HVNGQDQIVPGLYACGEAACASVHGANR.L  + Deamidated (NQ) |
| 171814 | 424 | – | 451 | 738.8412 | 2951.3359 | 2951.3403 | -1.49 | 0 | 18 | 0.019 | 1Score **> 33** indicates **identity** Score **> 14** indicates **homology** | U | K.HVNGQDQIVPGLYACGEAACASVHGANR.L  + Deamidated (NQ) |
| 171815 | 424 | – | 451 | 738.8424 | 2951.3407 | 2951.3403 | 0.13 | 0 | 52 | 1.3e-05 | 1Score **> 33** indicates **identity** Score **> 16** indicates **homology** | U | K.HVNGQDQIVPGLYACGEAACASVHGANR.L  + Deamidated (NQ) |
| 171816 | 424 | – | 451 | 738.8426 | 2951.3412 | 2951.3403 | 0.33 | 0 | 76 | 8e-08 | 1Score **> 33** indicates **identity** Score **> 17** indicates **homology** | U | K.HVNGQDQIVPGLYACGEAACASVHGANR.L  + Deamidated (NQ) |
| 171817 | 424 | – | 451 | 984.7877 | 2951.3413 | 2951.3403 | 0.34 | 0 | 103 | 2e-10 | 1Score **> 33** indicates **identity** Score **> 19** indicates **homology** | U | K.HVNGQDQIVPGLYACGEAACASVHGANR.L  + Deamidated (NQ) |
| 171819 | 424 | – | 451 | 984.7884 | 2951.3434 | 2951.3403 | 1.06 | 0 | 104 | 1.6e-10 | 1Score **> 33** indicates **identity** Score **> 19** indicates **homology** | U | K.HVNGQDQIVPGLYACGEAACASVHGANR.L  + Deamidated (NQ) |
| 171820 | 424 | – | 451 | 984.7884 | 2951.3435 | 2951.3403 | 1.09 | 0 | 33 | 0.00079 | 1Score **> 33** indicates **identity** Score **> 15** indicates **homology** | U | K.HVNGQDQIVPGLYACGEAACASVHGANR.L  + Deamidated (NQ) |
| 171821 | 424 | – | 451 | 984.7886 | 2951.3439 | 2951.3403 | 1.24 | 0 | 89 | 4.1e-09 | 1Score **> 33** indicates **identity** Score **> 18** indicates **homology** | U | K.HVNGQDQIVPGLYACGEAACASVHGANR.L  + Deamidated (NQ) |
| 171822 | 424 | – | 451 | 738.8433 | 2951.3440 | 2951.3403 | 1.27 | 0 | 77 | 5.9e-08 | 1Score **> 33** indicates **identity** Score **> 17** indicates **homology** | U | K.HVNGQDQIVPGLYACGEAACASVHGANR.L  + Deamidated (NQ) |
| 171823 | 424 | – | 451 | 738.8433 | 2951.3443 | 2951.3403 | 1.35 | 0 | 72 | 1.9e-07 | 1Score **> 33** indicates **identity** Score **> 17** indicates **homology** | U | K.HVNGQDQIVPGLYACGEAACASVHGANR.L  + Deamidated (NQ) |
| 171824 | 424 | – | 451 | 738.8434 | 2951.3444 | 2951.3403 | 1.40 | 0 | 59 | 3.1e-06 | 1Score **> 33** indicates **identity** Score **> 16** indicates **homology** | U | K.HVNGQDQIVPGLYACGEAACASVHGANR.L  + Deamidated (NQ) |
| 171825 | 424 | – | 451 | 984.7889 | 2951.3448 | 2951.3403 | 1.53 | 0 | 85 | 1.1e-08 | 1Score **> 33** indicates **identity** Score **> 18** indicates **homology** | U | K.HVNGQDQIVPGLYACGEAACASVHGANR.L  + Deamidated (NQ) |
| 171826 | 424 | – | 451 | 984.7889 | 2951.3449 | 2951.3403 | 1.58 | 0 | 30 | 0.0016 | 1Score **> 33** indicates **identity** Score **> 14** indicates **homology** | U | K.HVNGQDQIVPGLYACGEAACASVHGANR.L  + Deamidated (NQ) |
| 171827 | 424 | – | 451 | 738.8436 | 2951.3453 | 2951.3403 | 1.72 | 0 | 47 | 4.2e-05 | 1Score **> 33** indicates **identity** Score **> 15** indicates **homology** | U | K.HVNGQDQIVPGLYACGEAACASVHGANR.L  + Deamidated (NQ) |
| 171828 | 424 | – | 451 | 738.8436 | 2951.3454 | 2951.3403 | 1.75 | 0 | 21 | 0.011 | 1Score **> 33** indicates **identity** Score **> 14** indicates **homology** | U | K.HVNGQDQIVPGLYACGEAACASVHGANR.L  + Deamidated (NQ) |
| 171830 | 424 | – | 451 | 738.8437 | 2951.3457 | 2951.3403 | 1.84 | 0 | 63 | 1.2e-06 | 1Score **> 33** indicates **identity** Score **> 16** indicates **homology** | U | K.HVNGQDQIVPGLYACGEAACASVHGANR.L  + Deamidated (NQ) |
| 171831 | 424 | – | 451 | 738.8438 | 2951.3461 | 2951.3403 | 1.99 | 0 | 21 | 0.01 | 1Score **> 33** indicates **identity** Score **> 14** indicates **homology** | U | K.HVNGQDQIVPGLYACGEAACASVHGANR.L  + Deamidated (NQ) |
| 171832 | 424 | – | 451 | 738.8440 | 2951.3469 | 2951.3403 | 2.23 | 0 | 41 | 0.00016 | 1Score **> 34** indicates **identity** Score **> 15** indicates **homology** | U | K.HVNGQDQIVPGLYACGEAACASVHGANR.L  + Deamidated (NQ) |
| 171833 | 424 | – | 451 | 984.7896 | 2951.3471 | 2951.3403 | 2.31 | 0 | 35 | 0.00056 | 1Score **> 34** indicates **identity** Score **> 15** indicates **homology** | U | K.HVNGQDQIVPGLYACGEAACASVHGANR.L  + Deamidated (NQ) |
| 171835 | 424 | – | 451 | 984.7919 | 2951.3539 | 2951.3403 | 4.63 | 0 | 57 | 4.1e-06 | 1Score **> 34** indicates **identity** Score **> 16** indicates **homology** | U | K.HVNGQDQIVPGLYACGEAACASVHGANR.L  + Deamidated (NQ) |
| 171836 | 424 | – | 451 | 738.8460 | 2951.3548 | 2951.3403 | 4.94 | 0 | 42 | 0.00011 | 1Score **> 34** indicates **identity** Score **> 15** indicates **homology** | U | K.HVNGQDQIVPGLYACGEAACASVHGANR.L  + Deamidated (NQ) |
| 174585 | 424 | – | 451 | 1018.1392 | 3051.3958 | 3051.4178 | -7.21 | 0 | 15 | 0.039 | 1Score **> 33** indicates **identity** Score **> 13** indicates **homology** | U | K.HVNGQDQIVPGLYACGEAACASVHGANR.L  + 2 Deamidated (NQ); HNE (C) |
| 61987 | 452 | – | 465 | 737.4206 | 1472.8267 | 1472.8351 | -5.69 | 0 | 51 | 1.6e-05 | 1Score **> 34** indicates **identity** Score **> 16** indicates **homology** | U | R.LGANSLLDLVVFGR.A |
| 61994 | 452 | – | 465 | 737.4244 | 1472.8342 | 1472.8351 | -0.59 | 0 | 128 | 9.8e-13 | 1Score **> 34** indicates **identity** Score **> 21** indicates **homology** | U | R.LGANSLLDLVVFGR.A |
| 62000 | 452 | – | 465 | 491.9524 | 1472.8355 | 1472.8351 | 0.27 | 0 | 77 | 6.6e-08 | 1Score **> 34** indicates **identity** Score **> 17** indicates **homology** | U | R.LGANSLLDLVVFGR.A |
| 62001 | 452 | – | 465 | 737.4251 | 1472.8357 | 1472.8351 | 0.43 | 0 | 120 | 5.3e-12 | 1Score **> 34** indicates **identity** Score **> 20** indicates **homology** | U | R.LGANSLLDLVVFGR.A |
| 62004 | 452 | – | 465 | 491.9527 | 1472.8363 | 1472.8351 | 0.83 | 0 | 50 | 1.9e-05 | 1Score **> 34** indicates **identity** Score **> 16** indicates **homology** | U | R.LGANSLLDLVVFGR.A |
| 62006 | 452 | – | 465 | 737.4259 | 1472.8373 | 1472.8351 | 1.48 | 0 | 106 | 1.3e-10 | 1Score **> 33** indicates **identity** Score **> 19** indicates **homology** | U | R.LGANSLLDLVVFGR.A |
| 62009 | 452 | – | 465 | 491.9533 | 1472.8381 | 1472.8351 | 2.03 | 0 | 41 | 0.00015 | 1Score **> 34** indicates **identity** Score **> 15** indicates **homology** | U | R.LGANSLLDLVVFGR.A |
| 62010 | 452 | – | 465 | 491.9533 | 1472.8381 | 1472.8351 | 2.04 | 0 | 38 | 0.00029 | 1Score **> 34** indicates **identity** Score **> 15** indicates **homology** | U | R.LGANSLLDLVVFGR.A |
| 62012 | 452 | – | 465 | 491.9534 | 1472.8383 | 1472.8351 | 2.19 | 0 | 59 | 2.7e-06 | 1Score **> 34** indicates **identity** Score **> 16** indicates **homology** | U | R.LGANSLLDLVVFGR.A |
| 62014 | 452 | – | 465 | 737.4265 | 1472.8384 | 1472.8351 | 2.21 | 0 | 81 | 2.3e-08 | 1Score **> 34** indicates **identity** Score **> 18** indicates **homology** | U | R.LGANSLLDLVVFGR.A |
| 62015 | 452 | – | 465 | 737.4265 | 1472.8384 | 1472.8351 | 2.25 | 0 | 98 | 6.8e-10 | 1Score **> 34** indicates **identity** Score **> 19** indicates **homology** | U | R.LGANSLLDLVVFGR.A |
| 62016 | 452 | – | 465 | 737.4265 | 1472.8385 | 1472.8351 | 2.31 | 0 | 128 | 2.5e-12 | 1Score **> 34** indicates **identity** Score **> 24** indicates **homology** | U | R.LGANSLLDLVVFGR.A |
| 62017 | 452 | – | 465 | 737.4267 | 1472.8388 | 1472.8351 | 2.51 | 0 | 76 | 8.2e-08 | 1Score **> 33** indicates **identity** Score **> 17** indicates **homology** | U | R.LGANSLLDLVVFGR.A |
| 62018 | 452 | – | 465 | 737.4268 | 1472.8391 | 1472.8351 | 2.68 | 0 | 120 | 5.6e-12 | 1Score **> 33** indicates **identity** Score **> 20** indicates **homology** | U | R.LGANSLLDLVVFGR.A |
| 62019 | 452 | – | 465 | 491.9537 | 1472.8394 | 1472.8351 | 2.89 | 0 | 31 | 0.0014 | 1Score **> 33** indicates **identity** Score **> 14** indicates **homology** | U | R.LGANSLLDLVVFGR.A |
| 62020 | 452 | – | 465 | 737.4271 | 1472.8397 | 1472.8351 | 3.09 | 0 | 114 | 2.3e-11 | 1Score **> 33** indicates **identity** Score **> 20** indicates **homology** | U | R.LGANSLLDLVVFGR.A |
| 62021 | 452 | – | 465 | 737.4271 | 1472.8397 | 1472.8351 | 3.10 | 0 | 105 | 1.8e-10 | 1Score **> 33** indicates **identity** Score **> 21** indicates **homology** | U | R.LGANSLLDLVVFGR.A |
| 62022 | 452 | – | 465 | 737.4272 | 1472.8398 | 1472.8351 | 3.19 | 0 | 120 | 5.7e-12 | 1Score **> 33** indicates **identity** Score **> 20** indicates **homology** | U | R.LGANSLLDLVVFGR.A |
| 62024 | 452 | – | 465 | 737.4274 | 1472.8402 | 1472.8351 | 3.43 | 0 | 99 | 5.3e-10 | 1Score **> 33** indicates **identity** Score **> 19** indicates **homology** | U | R.LGANSLLDLVVFGR.A |
| 62027 | 452 | – | 465 | 491.9542 | 1472.8407 | 1472.8351 | 3.79 | 0 | 35 | 0.00054 | 1Score **> 33** indicates **identity** Score **> 15** indicates **homology** | U | R.LGANSLLDLVVFGR.A |
| 62030 | 452 | – | 465 | 737.4314 | 1472.8483 | 1472.8351 | 8.98 | 0 | 58 | 4e-06 | 1Score **> 33** indicates **identity** Score **> 16** indicates **homology** | U | R.LGANSLLDLVVFGR.A |
| 62031 | 452 | – | 465 | 737.4315 | 1472.8484 | 1472.8351 | 9.04 | 0 | 47 | 3.7e-05 | 1Score **> 33** indicates **identity** Score **> 15** indicates **homology** | U | R.LGANSLLDLVVFGR.A |
| 62103 | 452 | – | 465 | 737.9227 | 1473.8309 | 1473.8191 | 7.98 | 0 | 27 | 0.0028 | 1Score **> 34** indicates **identity** Score **> 14** indicates **homology** | U | R.LGANSLLDLVVFGR.A  + Deamidated (NQ) |
| 82312 | 466 | – | 480 | 545.5918 | 1633.7536 | 1633.7552 | -1.00 | 1 | 24 | 0.005 | 1Score **> 33** indicates **identity** Score **> 14** indicates **homology** | U | R.ACALSIAESCRPGDK.V |
| 82314 | 466 | – | 480 | 545.5928 | 1633.7564 | 1633.7552 | 0.75 | 1 | 23 | 0.0077 | 1Score **> 33** indicates **identity** Score **> 14** indicates **homology** | U | R.ACALSIAESCRPGDK.V |
| 82315 | 466 | – | 480 | 545.5937 | 1633.7593 | 1633.7552 | 2.52 | 1 | 18 | 0.019 | 1Score **> 33** indicates **identity** Score **> 14** indicates **homology** | U | R.ACALSIAESCRPGDK.V |
| 133497 | 466 | – | 485 | 540.5293 | 2158.0881 | 2158.0874 | 0.29 | 2 | 24 | 0.0056 | 1Score **> 36** indicates **identity** Score **> 14** indicates **homology** | U | R.ACALSIAESCRPGDKVPSIK.A |
| 133498 | 466 | – | 485 | 720.3700 | 2158.0882 | 2158.0874 | 0.36 | 2 | 15 | 0.037 | 1Score **> 36** indicates **identity** Score **> 13** indicates **homology** | U | R.ACALSIAESCRPGDKVPSIK.A |
| 133499 | 466 | – | 485 | 540.5294 | 2158.0884 | 2158.0874 | 0.46 | 2 | 30 | 0.0016 | 1Score **> 36** indicates **identity** Score **> 14** indicates **homology** | U | R.ACALSIAESCRPGDKVPSIK.A |
| 133500 | 466 | – | 485 | 540.5295 | 2158.0888 | 2158.0874 | 0.62 | 2 | 35 | 0.00048 | 1Score **> 36** indicates **identity** Score **> 15** indicates **homology** | U | R.ACALSIAESCRPGDKVPSIK.A |
| 133502 | 466 | – | 485 | 720.3702 | 2158.0888 | 2158.0874 | 0.66 | 2 | 27 | 0.0027 | 1Score **> 36** indicates **identity** Score **> 14** indicates **homology** | U | R.ACALSIAESCRPGDKVPSIK.A |
| 133504 | 466 | – | 485 | 720.3702 | 2158.0889 | 2158.0874 | 0.68 | 2 | 41 | 0.00015 | 1Score **> 36** indicates **identity** Score **> 15** indicates **homology** | U | R.ACALSIAESCRPGDKVPSIK.A |
| 133505 | 466 | – | 485 | 540.5295 | 2158.0890 | 2158.0874 | 0.73 | 2 | 30 | 0.0016 | 1Score **> 36** indicates **identity** Score **> 14** indicates **homology** | U | R.ACALSIAESCRPGDKVPSIK.A |
| 133508 | 466 | – | 485 | 540.5298 | 2158.0903 | 2158.0874 | 1.32 | 2 | 24 | 0.0055 | 1Score **> 37** indicates **identity** Score **> 14** indicates **homology** | U | R.ACALSIAESCRPGDKVPSIK.A |
| 51343 | 486 | – | 498 | 689.3192 | 1376.6238 | 1376.6242 | -0.24 | 0 | 82 | 2e-08 | 1Score **> 30** indicates **identity** Score **> 18** indicates **homology** | U | K.ANAGEESVMNLDK.L |
| 51344 | 486 | – | 498 | 689.3192 | 1376.6239 | 1376.6242 | -0.22 | 0 | 86 | 2.6e-08 | 1Score **> 30** indicates **identity** Score **> 23** indicates **homology** | U | K.ANAGEESVMNLDK.L |
| 51345 | 486 | – | 498 | 689.3193 | 1376.6240 | 1376.6242 | -0.15 | 0 | 77 | 2.6e-07 | 1Score **> 30** indicates **identity** Score **> 24** indicates **homology** | U | K.ANAGEESVMNLDK.L |
| 52866 | 486 | – | 498 | 697.3187 | 1392.6229 | 1392.6191 | 2.73 | 0 | 44 | 7.1e-05 | 1Score **> 29** indicates **identity** Score **> 15** indicates **homology** | U | K.ANAGEESVMNLDK.L  + Oxidation (M) |
| 83733 | 486 | – | 500 | 549.6074 | 1645.8004 | 1645.8093 | -5.45 | 1 | 20 | 0.015 | 1Score **> 34** indicates **identity** Score **> 14** indicates **homology** | U | K.ANAGEESVMNLDKLR.F |
| 83777 | 486 | – | 500 | 549.6106 | 1645.8099 | 1645.8093 | 0.36 | 1 | 51 | 1.8e-05 | 1Score **> 34** indicates **identity** Score **> 16** indicates **homology** | U | K.ANAGEESVMNLDKLR.F |
| 83778 | 486 | – | 500 | 549.6107 | 1645.8101 | 1645.8093 | 0.49 | 1 | 30 | 0.0015 | 1Score **> 34** indicates **identity** Score **> 14** indicates **homology** | U | K.ANAGEESVMNLDKLR.F |
| 83779 | 486 | – | 500 | 549.6108 | 1645.8105 | 1645.8093 | 0.72 | 1 | 54 | 8.1e-06 | 1Score **> 34** indicates **identity** Score **> 16** indicates **homology** | U | K.ANAGEESVMNLDKLR.F |
| 83780 | 486 | – | 500 | 823.9126 | 1645.8105 | 1645.8093 | 0.74 | 1 | 63 | 1.3e-06 | 1Score **> 34** indicates **identity** Score **> 16** indicates **homology** | U | K.ANAGEESVMNLDKLR.F |
| 83782 | 486 | – | 500 | 823.9128 | 1645.8111 | 1645.8093 | 1.08 | 1 | 107 | 1.1e-10 | 1Score **> 34** indicates **identity** Score **> 20** indicates **homology** | U | K.ANAGEESVMNLDKLR.F |
| 83783 | 486 | – | 500 | 823.9128 | 1645.8111 | 1645.8093 | 1.09 | 1 | 107 | 9.5e-11 | 1Score **> 34** indicates **identity** Score **> 19** indicates **homology** | U | K.ANAGEESVMNLDKLR.F |
| 83786 | 486 | – | 500 | 549.6112 | 1645.8117 | 1645.8093 | 1.45 | 1 | 61 | 2e-06 | 1Score **> 34** indicates **identity** Score **> 16** indicates **homology** | U | K.ANAGEESVMNLDKLR.F |
| 83787 | 486 | – | 500 | 549.6113 | 1645.8120 | 1645.8093 | 1.60 | 1 | 47 | 4.1e-05 | 1Score **> 34** indicates **identity** Score **> 15** indicates **homology** | U | K.ANAGEESVMNLDKLR.F |
| 85767 | 486 | – | 500 | 554.9406 | 1661.8001 | 1661.8042 | -2.49 | 1 | 43 | 9.2e-05 | 1Score **> 34** indicates **identity** Score **> 15** indicates **homology** | U | K.ANAGEESVMNLDKLR.F  + Oxidation (M) |
| 85769 | 486 | – | 500 | 554.9421 | 1661.8045 | 1661.8042 | 0.16 | 1 | 26 | 0.0033 | 1Score **> 34** indicates **identity** Score **> 14** indicates **homology** | U | K.ANAGEESVMNLDKLR.F  + Oxidation (M) |
| 85770 | 486 | – | 500 | 554.9425 | 1661.8056 | 1661.8042 | 0.84 | 1 | 18 | 0.019 | 1Score **> 34** indicates **identity** Score **> 14** indicates **homology** | U | K.ANAGEESVMNLDKLR.F  + Oxidation (M) |
| 85772 | 486 | – | 500 | 554.9433 | 1661.8079 | 1661.8042 | 2.22 | 1 | 27 | 0.0029 | 1Score **> 34** indicates **identity** Score **> 14** indicates **homology** | U | K.ANAGEESVMNLDKLR.F  + Oxidation (M) |
| 2231 | 501 | – | 507 | 383.1974 | 764.3803 | 764.3817 | -1.85 | 0 | 21 | 0.029 | 1Score **> 24** indicates **identity** Score **> 18** indicates **homology** | U | R.FADGSIR.T |
| 2232 | 501 | – | 507 | 383.1981 | 764.3817 | 764.3817 | 0.041 | 0 | 38 | 0.0011 | 1Score **> 21** indicates **identity** | U | R.FADGSIR.T |
| 2233 | 501 | – | 507 | 383.1983 | 764.3820 | 764.3817 | 0.34 | 0 | 40 | 0.00071 | 1Score **> 21** indicates **identity** | U | R.FADGSIR.T |
| 28272 | 518 | – | 527 | 580.7856 | 1159.5567 | 1159.5556 | 0.93 | 0 | 29 | 0.0082 | 1Score **> 30** indicates **identity** Score **> 20** indicates **homology** | U | K.SMQNHAAVFR.V |
| 30009 | 518 | – | 527 | 392.8569 | 1175.5488 | 1175.5506 | -1.51 | 0 | 20 | 0.014 | 1Score **> 31** indicates **identity** Score **> 14** indicates **homology** | U | K.SMQNHAAVFR.V  + Oxidation (M) |
| 30010 | 518 | – | 527 | 392.8569 | 1175.5490 | 1175.5506 | -1.32 | 0 | 37 | 0.00034 | 1Score **> 31** indicates **identity** Score **> 15** indicates **homology** | U | K.SMQNHAAVFR.V  + Oxidation (M) |
| 30011 | 518 | – | 527 | 392.8570 | 1175.5491 | 1175.5506 | -1.23 | 0 | 35 | 0.00056 | 1Score **> 31** indicates **identity** Score **> 15** indicates **homology** | U | K.SMQNHAAVFR.V  + Oxidation (M) |
| 30012 | 518 | – | 527 | 392.8570 | 1175.5491 | 1175.5506 | -1.22 | 0 | 22 | 0.0096 | 1Score **> 31** indicates **identity** Score **> 14** indicates **homology** | U | K.SMQNHAAVFR.V  + Oxidation (M) |
| 30013 | 518 | – | 527 | 392.8572 | 1175.5498 | 1175.5506 | -0.68 | 0 | 30 | 0.0016 | 1Score **> 31** indicates **identity** Score **> 14** indicates **homology** | U | K.SMQNHAAVFR.V  + Oxidation (M) |
| 30014 | 518 | – | 527 | 392.8574 | 1175.5505 | 1175.5506 | -0.054 | 0 | 32 | 0.00095 | 1Score **> 31** indicates **identity** Score **> 15** indicates **homology** | U | K.SMQNHAAVFR.V  + Oxidation (M) |
| 33244 | 528 | – | 538 | 603.2947 | 1204.5749 | 1204.5758 | -0.72 | 0 | 45 | 0.00014 | 1Score **> 32** indicates **identity** Score **> 19** indicates **homology** | U | R.VGSVLQEGCEK.I |
| 33245 | 528 | – | 538 | 603.2948 | 1204.5751 | 1204.5758 | -0.56 | 0 | 68 | 2.1e-06 | 1Score **> 32** indicates **identity** Score **> 24** indicates **homology** | U | R.VGSVLQEGCEK.I |
| 33246 | 528 | – | 538 | 603.2948 | 1204.5751 | 1204.5758 | -0.56 | 0 | 65 | 1.2e-06 | 1Score **> 32** indicates **identity** Score **> 19** indicates **homology** | U | R.VGSVLQEGCEK.I |
| 33247 | 528 | – | 538 | 603.2953 | 1204.5760 | 1204.5758 | 0.16 | 0 | 74 | 5.5e-07 | 1Score **> 32** indicates **identity** Score **> 24** indicates **homology** | U | R.VGSVLQEGCEK.I |
| 33251 | 528 | – | 538 | 603.2957 | 1204.5768 | 1204.5758 | 0.86 | 0 | 68 | 2e-06 | 1Score **> 32** indicates **identity** Score **> 23** indicates **homology** | U | R.VGSVLQEGCEK.I |
| 33255 | 528 | – | 538 | 603.2959 | 1204.5773 | 1204.5758 | 1.29 | 0 | 60 | 6.8e-06 | 1Score **> 32** indicates **identity** Score **> 21** indicates **homology** | U | R.VGSVLQEGCEK.I |
| 33256 | 528 | – | 538 | 603.2961 | 1204.5776 | 1204.5758 | 1.49 | 0 | 55 | 9e-06 | 1Score **> 32** indicates **identity** Score **> 17** indicates **homology** | U | R.VGSVLQEGCEK.I |
| 33257 | 528 | – | 538 | 603.2964 | 1204.5782 | 1204.5758 | 2.01 | 0 | 48 | 8.6e-05 | 1Score **> 32** indicates **identity** Score **> 20** indicates **homology** | U | R.VGSVLQEGCEK.I |
| 33258 | 528 | – | 538 | 603.2967 | 1204.5789 | 1204.5758 | 2.59 | 0 | 31 | 0.0011 | 1Score **> 32** indicates **identity** Score **> 14** indicates **homology** | U | R.VGSVLQEGCEK.I |
| 16482 | 539 | – | 547 | 518.7871 | 1035.5596 | 1035.5600 | -0.44 | 0 | 54 | 1.7e-05 | 1Score **> 30** indicates **identity** Score **> 19** indicates **homology** | U | K.ISQLYGDLK.H |
| 16483 | 539 | – | 547 | 518.7871 | 1035.5597 | 1035.5600 | -0.36 | 0 | 56 | 1.2e-05 | 1Score **> 30** indicates **identity** Score **> 19** indicates **homology** | U | K.ISQLYGDLK.H |
| 16484 | 539 | – | 547 | 518.7873 | 1035.5600 | 1035.5600 | 0.00097 | 0 | 47 | 8.5e-05 | 1Score **> 30** indicates **identity** Score **> 19** indicates **homology** | U | K.ISQLYGDLK.H |
| 16485 | 539 | – | 547 | 518.7874 | 1035.5603 | 1035.5600 | 0.22 | 0 | 28 | 0.0024 | 1Score **> 30** indicates **identity** Score **> 14** indicates **homology** | U | K.ISQLYGDLK.H |
| 16486 | 539 | – | 547 | 518.7876 | 1035.5607 | 1035.5600 | 0.65 | 0 | 32 | 0.00094 | 1Score **> 30** indicates **identity** Score **> 15** indicates **homology** | U | K.ISQLYGDLK.H |
| 16487 | 539 | – | 547 | 518.7878 | 1035.5611 | 1035.5600 | 0.99 | 0 | 39 | 0.00021 | 1Score **> 32** indicates **identity** Score **> 15** indicates **homology** | U | K.ISQLYGDLK.H |
| 16488 | 539 | – | 547 | 518.7880 | 1035.5614 | 1035.5600 | 1.27 | 0 | 21 | 0.011 | 1Score **> 32** indicates **identity** Score **> 14** indicates **homology** | U | K.ISQLYGDLK.H |
| 16489 | 539 | – | 547 | 518.7881 | 1035.5617 | 1035.5600 | 1.59 | 0 | 37 | 0.00035 | 1Score **> 32** indicates **identity** Score **> 15** indicates **homology** | U | K.ISQLYGDLK.H |
| 16490 | 539 | – | 547 | 518.7894 | 1035.5642 | 1035.5600 | 3.98 | 0 | 33 | 0.00076 | 1Score **> 31** indicates **identity** Score **> 15** indicates **homology** | U | K.ISQLYGDLK.H |
| 185837 | 551 | – | 585 | 838.4130 | 4187.0284 | 4187.0629 | -8.22 | 1 | 44 | 7.1e-05 | 1Score **> 37** indicates **identity** Score **> 15** indicates **homology** | U | K.TFDRGMVWNTDLVETLELQNLMLCALQTIYGAEAR.K  + Deamidated (NQ); HNE (C); Oxidation (M) |
| 183572 | 555 | – | 585 | 1223.2760 | 3666.8062 | 3666.8347 | -7.76 | 0 | 19 | 0.017 | 1Score **> 38** indicates **identity** Score **> 14** indicates **homology** | U | R.GMVWNTDLVETLELQNLMLCALQTIYGAEAR.K  + HNE (C); Oxidation (M) |
| 183600 | 555 | – | 585 | 918.4578 | 3669.8022 | 3669.7867 | 4.21 | 0 | 25 | 0.0044 | 1Score **> 37** indicates **identity** Score **> 14** indicates **homology** | U | R.GMVWNTDLVETLELQNLMLCALQTIYGAEAR.K  + 3 Deamidated (NQ); HNE (C); Oxidation (M) |
| 124488 | 599 | – | 615 | 685.0156 | 2052.0251 | 2052.0276 | -1.24 | 2 | 15 | 0.041 | 1Score **> 36** indicates **identity** Score **> 14** indicates **homology** | U | K.VRVDEYDYSKPIQGQQK.K |
| 124490 | 599 | – | 615 | 1027.0206 | 2052.0267 | 2052.0276 | -0.44 | 2 | 26 | 0.0036 | 1Score **> 36** indicates **identity** Score **> 14** indicates **homology** | U | K.VRVDEYDYSKPIQGQQK.K |
| 124492 | 599 | – | 615 | 685.0166 | 2052.0279 | 2052.0276 | 0.16 | 2 | 26 | 0.0034 | 1Score **> 36** indicates **identity** Score **> 14** indicates **homology** | U | K.VRVDEYDYSKPIQGQQK.K |
| 124494 | 599 | – | 615 | 685.0170 | 2052.0291 | 2052.0276 | 0.75 | 2 | 21 | 0.02 | 1Score **> 36** indicates **identity** Score **> 17** indicates **homology** | U | K.VRVDEYDYSKPIQGQQK.K |
| 124496 | 599 | – | 615 | 685.0173 | 2052.0300 | 2052.0276 | 1.18 | 2 | 38 | 0.0003 | 1Score **> 36** indicates **identity** Score **> 15** indicates **homology** | U | K.VRVDEYDYSKPIQGQQK.K |
| 124497 | 599 | – | 615 | 685.0173 | 2052.0302 | 2052.0276 | 1.25 | 2 | 40 | 0.00016 | 1Score **> 36** indicates **identity** Score **> 15** indicates **homology** | U | K.VRVDEYDYSKPIQGQQK.K |
| 124498 | 599 | – | 615 | 514.0149 | 2052.0307 | 2052.0276 | 1.49 | 2 | 33 | 0.00072 | 1Score **> 36** indicates **identity** Score **> 15** indicates **homology** | U | K.VRVDEYDYSKPIQGQQK.K |
| 124499 | 599 | – | 615 | 514.0153 | 2052.0319 | 2052.0276 | 2.10 | 2 | 34 | 0.00061 | 1Score **> 36** indicates **identity** Score **> 15** indicates **homology** | U | K.VRVDEYDYSKPIQGQQK.K |
| 124500 | 599 | – | 615 | 514.0157 | 2052.0336 | 2052.0276 | 2.93 | 2 | 22 | 0.0081 | 1Score **> 36** indicates **identity** Score **> 14** indicates **homology** | U | K.VRVDEYDYSKPIQGQQK.K |
| 101054 | 601 | – | 615 | 899.4353 | 1796.8561 | 1796.8581 | -1.09 | 1 | 41 | 0.00015 | 1Score **> 34** indicates **identity** Score **> 15** indicates **homology** | U | R.VDEYDYSKPIQGQQK.K |
| 101055 | 601 | – | 615 | 899.4354 | 1796.8562 | 1796.8581 | -1.03 | 1 | 26 | 0.0033 | 1Score **> 34** indicates **identity** Score **> 14** indicates **homology** | U | R.VDEYDYSKPIQGQQK.K |
| 101056 | 601 | – | 615 | 599.9595 | 1796.8568 | 1796.8581 | -0.74 | 1 | 24 | 0.0051 | 1Score **> 34** indicates **identity** Score **> 14** indicates **homology** | U | R.VDEYDYSKPIQGQQK.K |
| 101057 | 601 | – | 615 | 599.9595 | 1796.8568 | 1796.8581 | -0.73 | 1 | 17 | 0.027 | 1Score **> 34** indicates **identity** Score **> 14** indicates **homology** | U | R.VDEYDYSKPIQGQQK.K |
| 101058 | 601 | – | 615 | 599.9596 | 1796.8569 | 1796.8581 | -0.64 | 1 | 49 | 2.4e-05 | 1Score **> 34** indicates **identity** Score **> 16** indicates **homology** | U | R.VDEYDYSKPIQGQQK.K |
| 101059 | 601 | – | 615 | 899.4358 | 1796.8571 | 1796.8581 | -0.52 | 1 | 44 | 8.3e-05 | 1Score **> 33** indicates **identity** Score **> 15** indicates **homology** | U | R.VDEYDYSKPIQGQQK.K |
| 101060 | 601 | – | 615 | 899.4365 | 1796.8584 | 1796.8581 | 0.16 | 1 | 84 | 1.2e-08 | 1Score **> 33** indicates **identity** Score **> 18** indicates **homology** | U | R.VDEYDYSKPIQGQQK.K |
| 101061 | 601 | – | 615 | 899.4365 | 1796.8585 | 1796.8581 | 0.23 | 1 | 81 | 2.7e-08 | 1Score **> 33** indicates **identity** Score **> 18** indicates **homology** | U | R.VDEYDYSKPIQGQQK.K |
| 101062 | 601 | – | 615 | 599.9601 | 1796.8585 | 1796.8581 | 0.26 | 1 | 46 | 5e-05 | 1Score **> 33** indicates **identity** Score **> 15** indicates **homology** | U | R.VDEYDYSKPIQGQQK.K |
| 101063 | 601 | – | 615 | 599.9601 | 1796.8585 | 1796.8581 | 0.26 | 1 | 39 | 0.00021 | 1Score **> 33** indicates **identity** Score **> 15** indicates **homology** | U | R.VDEYDYSKPIQGQQK.K |
| 101064 | 601 | – | 615 | 599.9602 | 1796.8589 | 1796.8581 | 0.45 | 1 | 33 | 0.00083 | 1Score **> 34** indicates **identity** Score **> 15** indicates **homology** | U | R.VDEYDYSKPIQGQQK.K |
| 101065 | 601 | – | 615 | 599.9603 | 1796.8590 | 1796.8581 | 0.49 | 1 | 42 | 0.00011 | 1Score **> 34** indicates **identity** Score **> 15** indicates **homology** | U | R.VDEYDYSKPIQGQQK.K |
| 101066 | 601 | – | 615 | 599.9607 | 1796.8604 | 1796.8581 | 1.28 | 1 | 16 | 0.03 | 1Score **> 34** indicates **identity** Score **> 14** indicates **homology** | U | R.VDEYDYSKPIQGQQK.K |
| 101069 | 601 | – | 615 | 599.9620 | 1796.8643 | 1796.8581 | 3.45 | 1 | 15 | 0.039 | 1Score **> 34** indicates **identity** Score **> 13** indicates **homology** | U | R.VDEYDYSKPIQGQQK.K |
| 18002 | 616 | – | 623 | 352.8506 | 1055.5299 | 1055.5301 | -0.17 | 1 | 30 | 0.0014 | 1Score **> 30** indicates **identity** Score **> 14** indicates **homology** | U | K.KPFGEHWR.K |
| 18003 | 616 | – | 623 | 352.8506 | 1055.5301 | 1055.5301 | -0.012 | 1 | 15 | 0.04 | 1Score **> 30** indicates **identity** Score **> 13** indicates **homology** | U | K.KPFGEHWR.K |
| 18004 | 616 | – | 623 | 352.8506 | 1055.5301 | 1055.5301 | 0.0019 | 1 | 17 | 0.028 | 1Score **> 30** indicates **identity** Score **> 14** indicates **homology** | U | K.KPFGEHWR.K |
| 18005 | 616 | – | 623 | 352.8507 | 1055.5303 | 1055.5301 | 0.15 | 1 | 42 | 0.00013 | 1Score **> 30** indicates **identity** Score **> 15** indicates **homology** | U | K.KPFGEHWR.K |
| 18006 | 616 | – | 623 | 352.8507 | 1055.5303 | 1055.5301 | 0.17 | 1 | 29 | 0.0018 | 1Score **> 30** indicates **identity** Score **> 14** indicates **homology** | U | K.KPFGEHWR.K |
| 18008 | 616 | – | 623 | 352.8508 | 1055.5306 | 1055.5301 | 0.44 | 1 | 44 | 8.4e-05 | 1Score **> 30** indicates **identity** Score **> 16** indicates **homology** | U | K.KPFGEHWR.K |
| 18010 | 616 | – | 623 | 352.8509 | 1055.5309 | 1055.5301 | 0.78 | 1 | 42 | 0.00012 | 1Score **> 30** indicates **identity** Score **> 15** indicates **homology** | U | K.KPFGEHWR.K |
| 30939 | 616 | – | 624 | 395.5492 | 1183.6258 | 1183.6250 | 0.64 | 2 | 26 | 0.0038 | 1Score **> 32** indicates **identity** Score **> 14** indicates **homology** | U | K.KPFGEHWRK.H |
| 30940 | 616 | – | 624 | 395.5495 | 1183.6267 | 1183.6250 | 1.37 | 2 | 21 | 0.011 | 1Score **> 32** indicates **identity** Score **> 14** indicates **homology** | U | K.KPFGEHWRK.H |
| 9773 | 617 | – | 623 | 464.7240 | 927.4334 | 927.4351 | -1.91 | 0 | 25 | 0.0044 | 1Score **> 26** indicates **identity** Score **> 14** indicates **homology** | U | K.PFGEHWR.K |
| 9775 | 617 | – | 623 | 464.7248 | 927.4351 | 927.4351 | -0.066 | 0 | 25 | 0.0041 | 1Score **> 26** indicates **identity** Score **> 14** indicates **homology** | U | K.PFGEHWR.K |
| 33083 | 624 | – | 633 | 401.8949 | 1202.6628 | 1202.6659 | -2.62 | 1 | 19 | 0.017 | 1Score **> 33** indicates **identity** Score **> 14** indicates **homology** | U | R.KHTLSYVDIK.T |
| 33084 | 624 | – | 633 | 401.8952 | 1202.6639 | 1202.6659 | -1.71 | 1 | 39 | 0.0032 | 1Score **> 33** indicates **identity** Score **> 27** indicates **homology** | U | R.KHTLSYVDIK.T |
| 33085 | 624 | – | 633 | 401.8953 | 1202.6639 | 1202.6659 | -1.65 | 1 | 37 | 0.00062 | 1Score **> 33** indicates **identity** Score **> 18** indicates **homology** | U | R.KHTLSYVDIK.T |
| 33086 | 624 | – | 633 | 602.3395 | 1202.6644 | 1202.6659 | -1.24 | 1 | 24 | 0.028 | 1Score **> 33** indicates **identity** Score **> 21** indicates **homology** | U | R.KHTLSYVDIK.T |
| 33087 | 624 | – | 633 | 602.3395 | 1202.6645 | 1202.6659 | -1.15 | 1 | 66 | 7.7e-06 | 1Score **> 33** indicates **identity** Score **> 27** indicates **homology** | U | R.KHTLSYVDIK.T |
| 33088 | 624 | – | 633 | 602.3396 | 1202.6647 | 1202.6659 | -0.97 | 1 | 49 | 0.00012 | 1Score **> 33** indicates **identity** Score **> 22** indicates **homology** | U | R.KHTLSYVDIK.T |
| 33089 | 624 | – | 633 | 401.8955 | 1202.6648 | 1202.6659 | -0.91 | 1 | 43 | 0.0029 | 1Score **> 33** indicates **identity** Score **> 30** indicates **homology** | U | R.KHTLSYVDIK.T |
| 33090 | 624 | – | 633 | 401.8956 | 1202.6649 | 1202.6659 | -0.84 | 1 | 41 | 0.0033 | 1Score **> 33** indicates **identity** Score **> 29** indicates **homology** | U | R.KHTLSYVDIK.T |
| 33091 | 624 | – | 633 | 401.8956 | 1202.6649 | 1202.6659 | -0.82 | 1 | 46 | 0.0015 | 1Score **> 33** indicates **identity** Score **> 30** indicates **homology** | U | R.KHTLSYVDIK.T |
| 33092 | 624 | – | 633 | 401.8956 | 1202.6650 | 1202.6659 | -0.73 | 1 | 30 | 0.0034 | 1Score **> 33** indicates **identity** Score **> 17** indicates **homology** | U | R.KHTLSYVDIK.T |
| 33093 | 624 | – | 633 | 401.8958 | 1202.6655 | 1202.6659 | -0.36 | 1 | 52 | 0.00022 | 1Score **> 34** indicates **identity** Score **> 28** indicates **homology** | U | R.KHTLSYVDIK.T |
| 64052 | 624 | – | 636 | 373.2146 | 1488.8293 | 1488.8300 | -0.49 | 2 | 17 | 0.025 | 1Score **> 34** indicates **identity** Score **> 14** indicates **homology** | U | R.KHTLSYVDIKTGK.V |
| 64056 | 624 | – | 636 | 373.2149 | 1488.8304 | 1488.8300 | 0.26 | 2 | 27 | 0.0029 | 1Score **> 34** indicates **identity** Score **> 14** indicates **homology** | U | R.KHTLSYVDIKTGK.V |
| 19682 | 625 | – | 633 | 538.2926 | 1074.5707 | 1074.5710 | -0.20 | 0 | 41 | 0.00073 | 1Score **> 33** indicates **identity** Score **> 22** indicates **homology** | U | K.HTLSYVDIK.T |
| 19685 | 625 | – | 633 | 538.2928 | 1074.5710 | 1074.5710 | 0.0065 | 0 | 25 | 0.013 | 1Score **> 33** indicates **identity** Score **> 18** indicates **homology** | U | K.HTLSYVDIK.T |
| 19686 | 625 | – | 633 | 538.2930 | 1074.5714 | 1074.5710 | 0.38 | 0 | 67 | 3.9e-06 | 1Score **> 33** indicates **identity** Score **> 25** indicates **homology** | U | K.HTLSYVDIK.T |
| 19692 | 625 | – | 633 | 538.2942 | 1074.5738 | 1074.5710 | 2.63 | 0 | 35 | 0.007 | 1Score **> 33** indicates **identity** Score **> 26** indicates **homology** | U | K.HTLSYVDIK.T |
| 19693 | 625 | – | 633 | 538.2942 | 1074.5738 | 1074.5710 | 2.66 | 0 | 23 | 0.016 | 1Score **> 33** indicates **identity** Score **> 17** indicates **homology** | U | K.HTLSYVDIK.T |
| 80316 | 634 | – | 647 | 540.3100 | 1617.9081 | 1617.9090 | -0.52 | 2 | 16 | 0.03 | 1Score **> 33** indicates **identity** Score **> 14** indicates **homology** | U | K.TGKVTLEYRPVIDK.T |
| 47029 | 637 | – | 647 | 444.9223 | 1331.7449 | 1331.7449 | 0.041 | 1 | 18 | 0.022 | 1Score **> 34** indicates **identity** Score **> 14** indicates **homology** | U | K.VTLEYRPVIDK.T |
| 47030 | 637 | – | 647 | 444.9223 | 1331.7451 | 1331.7449 | 0.18 | 1 | 31 | 0.0089 | 1Score **> 34** indicates **identity** Score **> 23** indicates **homology** | U | K.VTLEYRPVIDK.T |
| 47031 | 637 | – | 647 | 666.8802 | 1331.7458 | 1331.7449 | 0.71 | 1 | 63 | 5.3e-05 | 1Score **> 34** indicates **identity** Score **> 33** indicates **homology** | U | K.VTLEYRPVIDK.T |
| 47032 | 637 | – | 647 | 666.8803 | 1331.7460 | 1331.7449 | 0.82 | 1 | 65 | 3.7e-05 | 1Score **> 34** indicates **identity** Score **> 33** indicates **homology** | U | K.VTLEYRPVIDK.T |
| 47033 | 637 | – | 647 | 444.9227 | 1331.7461 | 1331.7449 | 0.94 | 1 | 30 | 0.011 | 1Score **> 34** indicates **identity** Score **> 22** indicates **homology** | U | K.VTLEYRPVIDK.T |
| 171491 | 637 | – | 662 | 736.1411 | 2940.5353 | 2940.5379 | -0.89 | 2 | 24 | 0.0054 | 1Score **> 37** indicates **identity** Score **> 14** indicates **homology** | U | K.VTLEYRPVIDKTLNEADCATVPPAIR.S |
| 171492 | 637 | – | 662 | 981.1862 | 2940.5369 | 2940.5379 | -0.34 | 2 | 111 | 3.6e-11 | 1Score **> 37** indicates **identity** Score **> 19** indicates **homology** | U | K.VTLEYRPVIDKTLNEADCATVPPAIR.S |
| 171495 | 637 | – | 662 | 736.1421 | 2940.5391 | 2940.5379 | 0.42 | 2 | 37 | 0.00032 | 1Score **> 37** indicates **identity** Score **> 15** indicates **homology** | U | K.VTLEYRPVIDKTLNEADCATVPPAIR.S |
| 171496 | 637 | – | 662 | 736.1422 | 2940.5395 | 2940.5379 | 0.55 | 2 | 44 | 8.1e-05 | 1Score **> 37** indicates **identity** Score **> 15** indicates **homology** | U | K.VTLEYRPVIDKTLNEADCATVPPAIR.S |
| 171497 | 637 | – | 662 | 736.1422 | 2940.5397 | 2940.5379 | 0.61 | 2 | 47 | 4.1e-05 | 1Score **> 37** indicates **identity** Score **> 15** indicates **homology** | U | K.VTLEYRPVIDKTLNEADCATVPPAIR.S |
| 171498 | 637 | – | 662 | 736.1422 | 2940.5399 | 2940.5379 | 0.69 | 2 | 44 | 7.8e-05 | 1Score **> 37** indicates **identity** Score **> 15** indicates **homology** | U | K.VTLEYRPVIDKTLNEADCATVPPAIR.S |
| 171499 | 637 | – | 662 | 981.1873 | 2940.5400 | 2940.5379 | 0.73 | 2 | 117 | 1.1e-11 | 1Score **> 37** indicates **identity** Score **> 20** indicates **homology** | U | K.VTLEYRPVIDKTLNEADCATVPPAIR.S |
| 171500 | 637 | – | 662 | 981.1874 | 2940.5405 | 2940.5379 | 0.88 | 2 | 111 | 3.6e-11 | 1Score **> 37** indicates **identity** Score **> 19** indicates **homology** | U | K.VTLEYRPVIDKTLNEADCATVPPAIR.S |
| 171501 | 637 | – | 662 | 981.1874 | 2940.5405 | 2940.5379 | 0.90 | 2 | 82 | 1.9e-08 | 1Score **> 37** indicates **identity** Score **> 18** indicates **homology** | U | K.VTLEYRPVIDKTLNEADCATVPPAIR.S |
| 171504 | 637 | – | 662 | 981.1879 | 2940.5419 | 2940.5379 | 1.36 | 2 | 100 | 4.5e-10 | 1Score **> 37** indicates **identity** Score **> 19** indicates **homology** | U | K.VTLEYRPVIDKTLNEADCATVPPAIR.S |
| 171505 | 637 | – | 662 | 981.1895 | 2940.5468 | 2940.5379 | 3.04 | 2 | 60 | 2.3e-06 | 1Score **> 36** indicates **identity** Score **> 16** indicates **homology** | U | K.VTLEYRPVIDKTLNEADCATVPPAIR.S |
| 171506 | 637 | – | 662 | 981.1931 | 2940.5576 | 2940.5379 | 6.71 | 2 | 48 | 3e-05 | 1Score **> 36** indicates **identity** Score **> 16** indicates **homology** | U | K.VTLEYRPVIDKTLNEADCATVPPAIR.S |
| 171530 | 637 | – | 662 | 981.5224 | 2941.5454 | 2941.5219 | 8.01 | 2 | 61 | 1.8e-06 | 1Score **> 37** indicates **identity** Score **> 16** indicates **homology** | U | K.VTLEYRPVIDKTLNEADCATVPPAIR.S  + Deamidated (NQ) |
| 171531 | 637 | – | 662 | 981.5233 | 2941.5482 | 2941.5219 | 8.95 | 2 | 43 | 8.5e-05 | 1Score **> 37** indicates **identity** Score **> 15** indicates **homology** | U | K.VTLEYRPVIDKTLNEADCATVPPAIR.S  + Deamidated (NQ) |
| 81465 | 648 | – | 662 | 814.4072 | 1626.7998 | 1626.8035 | -2.28 | 0 | 45 | 6.1e-05 | 1Score **> 33** indicates **identity** Score **> 15** indicates **homology** | U | K.TLNEADCATVPPAIR.S |
| 81466 | 648 | – | 662 | 814.4078 | 1626.8010 | 1626.8035 | -1.59 | 0 | 60 | 2.4e-06 | 1Score **> 33** indicates **identity** Score **> 16** indicates **homology** | U | K.TLNEADCATVPPAIR.S |
| 81470 | 648 | – | 662 | 814.4085 | 1626.8025 | 1626.8035 | -0.67 | 0 | 75 | 9.1e-08 | 1Score **> 34** indicates **identity** Score **> 17** indicates **homology** | U | K.TLNEADCATVPPAIR.S |
| 81471 | 648 | – | 662 | 814.4086 | 1626.8026 | 1626.8035 | -0.61 | 0 | 80 | 3.2e-08 | 1Score **> 34** indicates **identity** Score **> 17** indicates **homology** | U | K.TLNEADCATVPPAIR.S |
| 81472 | 648 | – | 662 | 814.4089 | 1626.8032 | 1626.8035 | -0.24 | 0 | 71 | 2.3e-07 | 1Score **> 34** indicates **identity** Score **> 17** indicates **homology** | U | K.TLNEADCATVPPAIR.S |
| 81474 | 648 | – | 662 | 814.4089 | 1626.8033 | 1626.8035 | -0.17 | 0 | 73 | 1.5e-07 | 1Score **> 34** indicates **identity** Score **> 17** indicates **homology** | U | K.TLNEADCATVPPAIR.S |
| 81475 | 648 | – | 662 | 814.4089 | 1626.8033 | 1626.8035 | -0.17 | 0 | 80 | 3.1e-08 | 1Score **> 34** indicates **identity** Score **> 18** indicates **homology** | U | K.TLNEADCATVPPAIR.S |
| 81476 | 648 | – | 662 | 814.4089 | 1626.8033 | 1626.8035 | -0.15 | 0 | 73 | 1.5e-07 | 1Score **> 34** indicates **identity** Score **> 17** indicates **homology** | U | K.TLNEADCATVPPAIR.S |
| 81477 | 648 | – | 662 | 814.4090 | 1626.8034 | 1626.8035 | -0.083 | 0 | 93 | 1.7e-09 | 1Score **> 34** indicates **identity** Score **> 18** indicates **homology** | U | K.TLNEADCATVPPAIR.S |
| 81478 | 648 | – | 662 | 814.4090 | 1626.8035 | 1626.8035 | -0.012 | 0 | 63 | 1.2e-06 | 1Score **> 34** indicates **identity** Score **> 16** indicates **homology** | U | K.TLNEADCATVPPAIR.S |
| 81480 | 648 | – | 662 | 814.4090 | 1626.8035 | 1626.8035 | 0.0018 | 0 | 83 | 1.8e-08 | 1Score **> 34** indicates **identity** Score **> 18** indicates **homology** | U | K.TLNEADCATVPPAIR.S |
| 81481 | 648 | – | 662 | 814.4092 | 1626.8039 | 1626.8035 | 0.23 | 0 | 76 | 7.1e-08 | 1Score **> 34** indicates **identity** Score **> 17** indicates **homology** | U | K.TLNEADCATVPPAIR.S |
| 81482 | 648 | – | 662 | 814.4093 | 1626.8040 | 1626.8035 | 0.30 | 0 | 89 | 4.2e-09 | 1Score **> 34** indicates **identity** Score **> 18** indicates **homology** | U | K.TLNEADCATVPPAIR.S |
| 81484 | 648 | – | 662 | 814.4094 | 1626.8042 | 1626.8035 | 0.43 | 0 | 79 | 3.8e-08 | 1Score **> 34** indicates **identity** Score **> 17** indicates **homology** | U | K.TLNEADCATVPPAIR.S |
| 81485 | 648 | – | 662 | 814.4097 | 1626.8048 | 1626.8035 | 0.75 | 0 | 89 | 4.3e-09 | 1Score **> 34** indicates **identity** Score **> 18** indicates **homology** | U | K.TLNEADCATVPPAIR.S |
| 81486 | 648 | – | 662 | 814.4098 | 1626.8050 | 1626.8035 | 0.89 | 0 | 76 | 7.2e-08 | 1Score **> 34** indicates **identity** Score **> 17** indicates **homology** | U | K.TLNEADCATVPPAIR.S |
| 81491 | 648 | – | 662 | 814.4108 | 1626.8070 | 1626.8035 | 2.11 | 0 | 65 | 8.1e-07 | 1Score **> 34** indicates **identity** Score **> 17** indicates **homology** | U | K.TLNEADCATVPPAIR.S |
| 81493 | 648 | – | 662 | 814.4108 | 1626.8071 | 1626.8035 | 2.19 | 0 | 56 | 6e-06 | 1Score **> 34** indicates **identity** Score **> 16** indicates **homology** | U | K.TLNEADCATVPPAIR.S |
| 81494 | 648 | – | 662 | 814.4109 | 1626.8072 | 1626.8035 | 2.25 | 0 | 69 | 3.3e-07 | 1Score **> 34** indicates **identity** Score **> 17** indicates **homology** | U | K.TLNEADCATVPPAIR.S |
| 81499 | 648 | – | 662 | 814.4144 | 1626.8142 | 1626.8035 | 6.53 | 0 | 34 | 0.0006 | 1Score **> 34** indicates **identity** Score **> 15** indicates **homology** | U | K.TLNEADCATVPPAIR.S |
| 81614 | 648 | – | 662 | 814.9071 | 1627.7997 | 1627.7876 | 7.49 | 0 | 14 | 0.045 | 1Score **> 34** indicates **identity** Score **> 13** indicates **homology** | U | K.TLNEADCATVPPAIR.S  + Deamidated (NQ) |

---

```
ID   SDHA_MOUSE              Reviewed;         664 AA.
AC   Q8K2B3; Q0QF19; Q3UH25; Q3UKP7; Q3V4B1; Q921P5; Q9Z1Z4;
DT   27-SEP-2004, integrated into UniProtKB/Swiss-Prot.
DT   01-OCT-2002, sequence version 1.
DT   28-JUN-2023, entry version 166.
DE   RecName: Full=Succinate dehydrogenase [ubiquinone] flavoprotein subunit, mitochondrial;
DE            EC=1.3.5.1 {ECO:0000250|UniProtKB:P31040};
DE   AltName: Full=Flavoprotein subunit of complex II;
DE            Short=Fp;
DE   Flags: Precursor;
GN   Name=Sdha;
OS   Mus musculus (Mouse).
OC   Eukaryota; Metazoa; Chordata; Craniata; Vertebrata; Euteleostomi; Mammalia;
OC   Eutheria; Euarchontoglires; Glires; Rodentia; Myomorpha; Muroidea; Muridae;
OC   Murinae; Mus; Mus.
OX   NCBI_TaxID=10090;
RN   [1]
RP   NUCLEOTIDE SEQUENCE [LARGE SCALE MRNA].
RC   STRAIN=C57BL/6J; TISSUE=Bone marrow, Egg, Heart, Pancreas, and Testis;
RX   PubMed=16141072; DOI=10.1126/science.1112014;
RA   Carninci P., Kasukawa T., Katayama S., Gough J., Frith M.C., Maeda N.,
RA   Oyama R., Ravasi T., Lenhard B., Wells C., Kodzius R., Shimokawa K.,
RA   Bajic V.B., Brenner S.E., Batalov S., Forrest A.R., Zavolan M., Davis M.J.,
RA   Wilming L.G., Aidinis V., Allen J.E., Ambesi-Impiombato A., Apweiler R.,
RA   Aturaliya R.N., Bailey T.L., Bansal M., Baxter L., Beisel K.W., Bersano T.,
RA   Bono H., Chalk A.M., Chiu K.P., Choudhary V., Christoffels A.,
RA   Clutterbuck D.R., Crowe M.L., Dalla E., Dalrymple B.P., de Bono B.,
RA   Della Gatta G., di Bernardo D., Down T., Engstrom P., Fagiolini M.,
RA   Faulkner G., Fletcher C.F., Fukushima T., Furuno M., Futaki S.,
RA   Gariboldi M., Georgii-Hemming P., Gingeras T.R., Gojobori T., Green R.E.,
RA   Gustincich S., Harbers M., Hayashi Y., Hensch T.K., Hirokawa N., Hill D.,
RA   Huminiecki L., Iacono M., Ikeo K., Iwama A., Ishikawa T., Jakt M.,
RA   Kanapin A., Katoh M., Kawasawa Y., Kelso J., Kitamura H., Kitano H.,
RA   Kollias G., Krishnan S.P., Kruger A., Kummerfeld S.K., Kurochkin I.V.,
RA   Lareau L.F., Lazarevic D., Lipovich L., Liu J., Liuni S., McWilliam S.,
RA   Madan Babu M., Madera M., Marchionni L., Matsuda H., Matsuzawa S., Miki H.,
RA   Mignone F., Miyake S., Morris K., Mottagui-Tabar S., Mulder N., Nakano N.,
RA   Nakauchi H., Ng P., Nilsson R., Nishiguchi S., Nishikawa S., Nori F.,
RA   Ohara O., Okazaki Y., Orlando V., Pang K.C., Pavan W.J., Pavesi G.,
RA   Pesole G., Petrovsky N., Piazza S., Reed J., Reid J.F., Ring B.Z.,
RA   Ringwald M., Rost B., Ruan Y., Salzberg S.L., Sandelin A., Schneider C.,
RA   Schoenbach C., Sekiguchi K., Semple C.A., Seno S., Sessa L., Sheng Y.,
RA   Shibata Y., Shimada H., Shimada K., Silva D., Sinclair B., Sperling S.,
RA   Stupka E., Sugiura K., Sultana R., Takenaka Y., Taki K., Tammoja K.,
RA   Tan S.L., Tang S., Taylor M.S., Tegner J., Teichmann S.A., Ueda H.R.,
RA   van Nimwegen E., Verardo R., Wei C.L., Yagi K., Yamanishi H.,
RA   Zabarovsky E., Zhu S., Zimmer A., Hide W., Bult C., Grimmond S.M.,
RA   Teasdale R.D., Liu E.T., Brusic V., Quackenbush J., Wahlestedt C.,
RA   Mattick J.S., Hume D.A., Kai C., Sasaki D., Tomaru Y., Fukuda S.,
RA   Kanamori-Katayama M., Suzuki M., Aoki J., Arakawa T., Iida J., Imamura K.,
RA   Itoh M., Kato T., Kawaji H., Kawagashira N., Kawashima T., Kojima M.,
RA   Kondo S., Konno H., Nakano K., Ninomiya N., Nishio T., Okada M., Plessy C.,
RA   Shibata K., Shiraki T., Suzuki S., Tagami M., Waki K., Watahiki A.,
RA   Okamura-Oho Y., Suzuki H., Kawai J., Hayashizaki Y.;
RT   "The transcriptional landscape of the mammalian genome.";
RL   Science 309:1559-1563(2005).
RN   [2]
RP   NUCLEOTIDE SEQUENCE [LARGE SCALE MRNA].
RC   STRAIN=Czech II, and FVB/N; TISSUE=Mammary gland, and Mammary tumor;
RX   PubMed=15489334; DOI=10.1101/gr.2596504;
RG   The MGC Project Team;
RT   "The status, quality, and expansion of the NIH full-length cDNA project:
RT   the Mammalian Gene Collection (MGC).";
RL   Genome Res. 14:2121-2127(2004).
RN   [3]
RP   PROTEIN SEQUENCE OF 1-14; 47-92; 121-128; 196-207; 233-246; 251-282;
RP   313-325; 362-418; 452-480; 486-498; 528-547; 601-615; 624-633 AND 637-647.
RC   STRAIN=C57BL/6J; TISSUE=Brain, and Hippocampus;
RA   Lubec G., Klug S., Kang S.U.;
RL   Submitted (APR-2007) to UniProtKB.
RN   [4]
RP   NUCLEOTIDE SEQUENCE [MRNA] OF 59-609.
RC   TISSUE=Liver;
RX   PubMed=16751257; DOI=10.1093/molbev/msl027;
RA   Kullberg M., Nilsson M.A., Arnason U., Harley E.H., Janke A.;
RT   "Housekeeping genes for phylogenetic analysis of eutherian relationships.";
RL   Mol. Biol. Evol. 23:1493-1503(2006).
RN   [5]
RP   NUCLEOTIDE SEQUENCE [MRNA] OF 74-605.
RC   TISSUE=Heart;
RA   Weinreich D.M.;
RT   "OXPHOS genes in mammals and the molecular clock.";
RL   Submitted (OCT-1998) to the EMBL/GenBank/DDBJ databases.
RN   [6]
RP   IDENTIFICATION BY MASS SPECTROMETRY [LARGE SCALE ANALYSIS].
RC   TISSUE=Brain, Brown adipose tissue, Heart, Kidney, Liver, Lung,
RC   Pancreas, Spleen, and Testis;
RX   PubMed=21183079; DOI=10.1016/j.cell.2010.12.001;
RA   Huttlin E.L., Jedrychowski M.P., Elias J.E., Goswami T., Rad R.,
RA   Beausoleil S.A., Villen J., Haas W., Sowa M.E., Gygi S.P.;
RT   "A tissue-specific atlas of mouse protein phosphorylation and expression.";
RL   Cell 143:1174-1189(2010).
RN   [7]
RP   ACETYLATION AT LYS-179; LYS-182; LYS-250; LYS-335; LYS-480; LYS-485;
RP   LYS-498; LYS-547; LYS-550; LYS-598; LYS-608; LYS-624 AND LYS-633, AND
RP   DEACETYLATION BY SIRT3.
RX   PubMed=21858060; DOI=10.1371/journal.pone.0023295;
RA   Finley L.W., Haas W., Desquiret-Dumas V., Wallace D.C., Procaccio V.,
RA   Gygi S.P., Haigis M.C.;
RT   "Succinate dehydrogenase is a direct target of sirtuin 3 deacetylase
RT   activity.";
RL   PLoS ONE 6:E23295-E23295(2011).
RN   [8]
RP   ACETYLATION [LARGE SCALE ANALYSIS] AT LYS-179 AND LYS-547, SUCCINYLATION
RP   [LARGE SCALE ANALYSIS] AT LYS-179; LYS-250; LYS-335; LYS-485; LYS-498;
RP   LYS-538; LYS-547 AND LYS-615, AND IDENTIFICATION BY MASS SPECTROMETRY
RP   [LARGE SCALE ANALYSIS].
RC   TISSUE=Embryonic fibroblast, and Liver;
RX   PubMed=23806337; DOI=10.1016/j.molcel.2013.06.001;
RA   Park J., Chen Y., Tishkoff D.X., Peng C., Tan M., Dai L., Xie Z., Zhang Y.,
RA   Zwaans B.M., Skinner M.E., Lombard D.B., Zhao Y.;
RT   "SIRT5-mediated lysine desuccinylation impacts diverse metabolic
RT   pathways.";
RL   Mol. Cell 50:919-930(2013).
RN   [9]
RP   ACETYLATION [LARGE SCALE ANALYSIS] AT LYS-167; LYS-179; LYS-182; LYS-335;
RP   LYS-423; LYS-498; LYS-517; LYS-538; LYS-547; LYS-608; LYS-636 AND LYS-647,
RP   AND IDENTIFICATION BY MASS SPECTROMETRY [LARGE SCALE ANALYSIS].
RC   TISSUE=Liver;
RX   PubMed=23576753; DOI=10.1073/pnas.1302961110;
RA   Rardin M.J., Newman J.C., Held J.M., Cusack M.P., Sorensen D.J., Li B.,
RA   Schilling B., Mooney S.D., Kahn C.R., Verdin E., Gibson B.W.;
RT   "Label-free quantitative proteomics of the lysine acetylome in mitochondria
RT   identifies substrates of SIRT3 in metabolic pathways.";
RL   Proc. Natl. Acad. Sci. U.S.A. 110:6601-6606(2013).
CC   -!- FUNCTION: Flavoprotein (FP) subunit of succinate dehydrogenase (SDH)
CC       that is involved in complex II of the mitochondrial electron transport
CC       chain and is responsible for transferring electrons from succinate to
CC       ubiquinone (coenzyme Q). Can act as a tumor suppressor.
CC       {ECO:0000250|UniProtKB:P31040}.
CC   -!- CATALYTIC ACTIVITY:
CC       Reaction=a quinone + succinate = a quinol + fumarate;
CC         Xref=Rhea:RHEA:40523, ChEBI:CHEBI:24646, ChEBI:CHEBI:29806,
CC         ChEBI:CHEBI:30031, ChEBI:CHEBI:132124; EC=1.3.5.1;
CC         Evidence={ECO:0000250|UniProtKB:P31040};
CC   -!- COFACTOR:
CC       Name=FAD; Xref=ChEBI:CHEBI:57692;
CC         Evidence={ECO:0000250|UniProtKB:Q0QF01};
CC   -!- PATHWAY: Carbohydrate metabolism; tricarboxylic acid cycle; fumarate
CC       from succinate (eukaryal route): step 1/1.
CC       {ECO:0000250|UniProtKB:P31040}.
CC   -!- SUBUNIT: Component of complex II composed of four subunits: the
CC       flavoprotein (FP) SDHA, iron-sulfur protein (IP) SDHB, and a cytochrome
CC       b560 composed of SDHC and SDHD (By similarity). Interacts with
CC       SDHAF2/SDH5; interaction is required for FAD attachment (By
CC       similarity). Interacts with TRAP1 (By similarity). Interacts with LACC1
CC       (By similarity). {ECO:0000250|UniProtKB:P31040,
CC       ECO:0000250|UniProtKB:Q0QF01}.
CC   -!- SUBCELLULAR LOCATION: Mitochondrion inner membrane
CC       {ECO:0000250|UniProtKB:Q0QF01}; Peripheral membrane protein
CC       {ECO:0000250|UniProtKB:Q0QF01}; Matrix side
CC       {ECO:0000250|UniProtKB:Q0QF01}.
CC   -!- PTM: Acetylation of Lys-498 and Lys-538 is observed in liver
CC       mitochondria from fasted mice but not from fed mice. Deacetylated by
CC       SIRT3. {ECO:0000269|PubMed:21858060}.
CC   -!- PTM: Phosphorylation at Tyr-215 is important for efficient electron
CC       transfer in complex II and the prevention of ROS generation.
CC       {ECO:0000250|UniProtKB:P31040}.
CC   -!- SIMILARITY: Belongs to the FAD-dependent oxidoreductase 2 family.
CC       FRD/SDH subfamily. {ECO:0000305}.
CC   ---------------------------------------------------------------------------
CC   Copyrighted by the UniProt Consortium, see https://www.uniprot.org/terms
CC   Distributed under the Creative Commons Attribution (CC BY 4.0) License
CC   ---------------------------------------------------------------------------
DR   EMBL; AK029520; BAC26491.1; -; mRNA.
DR   EMBL; AK034928; BAC28884.1; -; mRNA.
DR   EMBL; AK049590; BAC33831.1; -; mRNA.
DR   EMBL; AK050475; BAC34276.1; -; mRNA.
DR   EMBL; AK075990; BAC36101.1; -; mRNA.
DR   EMBL; AK145923; BAE26754.1; -; mRNA.
DR   EMBL; AK147286; BAE27822.1; -; mRNA.
DR   EMBL; AK147624; BAE28032.1; -; mRNA.
DR   EMBL; AK153085; BAE31710.1; -; mRNA.
DR   EMBL; AK162148; BAE36754.1; -; mRNA.
DR   EMBL; AK169254; BAE41018.1; -; mRNA.
DR   EMBL; AK004362; BAE43173.1; -; mRNA.
DR   EMBL; BC011301; AAH11301.1; -; mRNA.
DR   EMBL; BC031849; AAH31849.1; -; mRNA.
DR   EMBL; DQ402975; ABD77308.1; -; mRNA.
DR   EMBL; AF095938; AAC72373.1; -; mRNA.
DR   CCDS; CCDS26643.1; -.
DR   RefSeq; NP_075770.1; NM_023281.1.
DR   AlphaFoldDB; Q8K2B3; -.
DR   SMR; Q8K2B3; -.
DR   BioGRID; 211828; 75.
DR   ComplexPortal; CPX-562; Mitochondrial respiratory chain complex II.
DR   CORUM; Q8K2B3; -.
DR   IntAct; Q8K2B3; 22.
DR   MINT; Q8K2B3; -.
DR   STRING; 10090.ENSMUSP00000022062; -.
DR   CarbonylDB; Q8K2B3; -.
DR   GlyGen; Q8K2B3; 1 site, 1 O-linked glycan (1 site).
DR   iPTMnet; Q8K2B3; -.
DR   PhosphoSitePlus; Q8K2B3; -.
DR   SwissPalm; Q8K2B3; -.
DR   REPRODUCTION-2DPAGE; Q8K2B3; -.
DR   EPD; Q8K2B3; -.
DR   jPOST; Q8K2B3; -.
DR   MaxQB; Q8K2B3; -.
DR   PaxDb; Q8K2B3; -.
DR   PeptideAtlas; Q8K2B3; -.
DR   ProteomicsDB; 255377; -.
DR   DNASU; 66945; -.
DR   Ensembl; ENSMUST00000022062; ENSMUSP00000022062; ENSMUSG00000021577.
DR   GeneID; 66945; -.
DR   KEGG; mmu:66945; -.
DR   UCSC; uc007rfa.1; mouse.
DR   AGR; MGI:1914195; -.
DR   CTD; 6389; -.
DR   MGI; MGI:1914195; Sdha.
DR   VEuPathDB; HostDB:ENSMUSG00000021577; -.
DR   eggNOG; KOG2403; Eukaryota.
DR   GeneTree; ENSGT00910000144277; -.
DR   HOGENOM; CLU_014312_6_1_1; -.
DR   InParanoid; Q8K2B3; -.
DR   OMA; DPIPIQP; -.
DR   OrthoDB; 551958at2759; -.
DR   PhylomeDB; Q8K2B3; -.
DR   TreeFam; TF300763; -.
DR   Reactome; R-MMU-71403; Citric acid cycle (TCA cycle).
DR   UniPathway; UPA00223; UER01006.
DR   BioGRID-ORCS; 66945; 18 hits in 80 CRISPR screens.
DR   ChiTaRS; Sdha; mouse.
DR   PRO; PR:Q8K2B3; -.
DR   Proteomes; UP000000589; Chromosome 13.
DR   RNAct; Q8K2B3; protein.
DR   Bgee; ENSMUSG00000021577; Expressed in heart right ventricle and 270 other tissues.
DR   ExpressionAtlas; Q8K2B3; baseline and differential.
DR   Genevisible; Q8K2B3; MM.
DR   GO; GO:0005743; C:mitochondrial inner membrane; HDA:MGI.
DR   GO; GO:0005749; C:mitochondrial respiratory chain complex II, succinate dehydrogenase complex (ubiquinone); ISS:UniProtKB.
DR   GO; GO:0005739; C:mitochondrion; IDA:MGI.
DR   GO; GO:0043209; C:myelin sheath; HDA:UniProtKB.
DR   GO; GO:0005730; C:nucleolus; ISO:MGI.
DR   GO; GO:0045257; C:succinate dehydrogenase complex (ubiquinone); IDA:MGI.
DR   GO; GO:0009055; F:electron transfer activity; IBA:GO_Central.
DR   GO; GO:0050660; F:flavin adenine dinucleotide binding; IBA:GO_Central.
DR   GO; GO:0102040; F:fumarate reductase (menaquinone); IEA:UniProtKB-EC.
DR   GO; GO:0008177; F:succinate dehydrogenase (ubiquinone) activity; ISS:UniProtKB.
DR   GO; GO:0000104; F:succinate dehydrogenase activity; ISO:MGI.
DR   GO; GO:0006121; P:mitochondrial electron transport, succinate to ubiquinone; IBA:GO_Central.
DR   GO; GO:0007399; P:nervous system development; ISO:MGI.
DR   GO; GO:0042776; P:proton motive force-driven mitochondrial ATP synthesis; NAS:ComplexPortal.
DR   GO; GO:0022904; P:respiratory electron transport chain; ISS:UniProtKB.
DR   GO; GO:0006105; P:succinate metabolic process; ISS:UniProtKB.
DR   GO; GO:0006099; P:tricarboxylic acid cycle; NAS:ComplexPortal.
DR   Gene3D; 3.50.50.60; FAD/NAD(P)-binding domain; 1.
DR   Gene3D; 1.20.58.100; Fumarate reductase/succinate dehydrogenase flavoprotein-like, C-terminal domain; 1.
DR   Gene3D; 4.10.80.40; succinate dehydrogenase protein domain; 1.
DR   Gene3D; 3.90.700.10; Succinate dehydrogenase/fumarate reductase flavoprotein, catalytic domain; 1.
DR   InterPro; IPR003953; FAD-binding_2.
DR   InterPro; IPR036188; FAD/NAD-bd_sf.
DR   InterPro; IPR003952; FRD_SDH_FAD_BS.
DR   InterPro; IPR037099; Fum_R/Succ_DH_flav-like_C_sf.
DR   InterPro; IPR015939; Fum_Rdtase/Succ_DH_flav-like_C.
DR   InterPro; IPR030664; SdhA/FrdA/AprA.
DR   InterPro; IPR027477; Succ_DH/fumarate_Rdtase_cat_sf.
DR   InterPro; IPR011281; Succ_DH_flav_su_fwd.
DR   InterPro; IPR014006; Succ_Dhase_FrdA_Gneg.
DR   PANTHER; PTHR11632; SUCCINATE DEHYDROGENASE 2 FLAVOPROTEIN SUBUNIT; 1.
DR   PANTHER; PTHR11632:SF51; SUCCINATE DEHYDROGENASE [UBIQUINONE] FLAVOPROTEIN SUBUNIT, MITOCHONDRIAL; 1.
DR   Pfam; PF00890; FAD_binding_2; 1.
DR   Pfam; PF02910; Succ_DH_flav_C; 1.
DR   PIRSF; PIRSF000171; SDHA_APRA_LASPO; 1.
DR   SUPFAM; SSF51905; FAD/NAD(P)-binding domain; 1.
DR   SUPFAM; SSF46977; Succinate dehydrogenase/fumarate reductase flavoprotein C-terminal domain; 1.
DR   SUPFAM; SSF56425; Succinate dehydrogenase/fumarate reductase flavoprotein, catalytic domain; 1.
DR   PROSITE; PS00504; FRD_SDH_FAD_BINDING; 1.
DR   TIGRFAMs; TIGR01816; sdhA_forward; 1.
DR   TIGRFAMs; TIGR01812; sdhA_frdA_Gneg; 1.
PE   1: Evidence at protein level;
KW   Acetylation; Direct protein sequencing; Electron transport; FAD;
KW   Flavoprotein; Membrane; Mitochondrion; Mitochondrion inner membrane;
KW   Oxidoreductase; Phosphoprotein; Reference proteome; Transit peptide;
KW   Transport; Tricarboxylic acid cycle; Tumor suppressor.
FT   TRANSIT         1..42
FT                   /note="Mitochondrion"
FT                   /evidence="ECO:0000250|UniProtKB:Q0QF01"
FT   CHAIN           43..664
FT                   /note="Succinate dehydrogenase [ubiquinone] flavoprotein
FT                   subunit, mitochondrial"
FT                   /id="PRO_0000010337"
FT   ACT_SITE        340
FT                   /note="Proton acceptor"
FT                   /evidence="ECO:0000250|UniProtKB:Q9YHT1"
FT   BINDING         68..73
FT                   /ligand="FAD"
FT                   /ligand_id="ChEBI:CHEBI:57692"
FT                   /evidence="ECO:0000250|UniProtKB:Q0QF01"
FT   BINDING         91..106
FT                   /ligand="FAD"
FT                   /ligand_id="ChEBI:CHEBI:57692"
FT                   /evidence="ECO:0000250|UniProtKB:Q0QF01"
FT   BINDING         275
FT                   /ligand="FAD"
FT                   /ligand_id="ChEBI:CHEBI:57692"
FT                   /evidence="ECO:0000250|UniProtKB:Q0QF01"
FT   BINDING         296
FT                   /ligand="substrate"
FT                   /evidence="ECO:0000250|UniProtKB:Q0QF01"
FT   BINDING         308
FT                   /ligand="substrate"
FT                   /evidence="ECO:0000250|UniProtKB:Q0QF01"
FT   BINDING         407
FT                   /ligand="substrate"
FT                   /evidence="ECO:0000250|UniProtKB:Q0QF01"
FT   BINDING         440
FT                   /ligand="FAD"
FT                   /ligand_id="ChEBI:CHEBI:57692"
FT                   /evidence="ECO:0000250|UniProtKB:Q0QF01"
FT   BINDING         451
FT                   /ligand="substrate"
FT                   /evidence="ECO:0000250|UniProtKB:Q0QF01"
FT   BINDING         456..457
FT                   /ligand="FAD"
FT                   /ligand_id="ChEBI:CHEBI:57692"
FT                   /evidence="ECO:0000250|UniProtKB:Q0QF01"
FT   MOD_RES         99
FT                   /note="Tele-8alpha-FAD histidine"
FT                   /evidence="ECO:0000250|UniProtKB:Q0QF01"
FT   MOD_RES         167
FT                   /note="N6-acetyllysine"
FT                   /evidence="ECO:0007744|PubMed:23576753"
FT   MOD_RES         179
FT                   /note="N6-acetyllysine; alternate"
FT                   /evidence="ECO:0000269|PubMed:21858060,
FT                   ECO:0007744|PubMed:23576753, ECO:0007744|PubMed:23806337"
FT   MOD_RES         179
FT                   /note="N6-succinyllysine; alternate"
FT                   /evidence="ECO:0007744|PubMed:23806337"
FT   MOD_RES         182
FT                   /note="N6-acetyllysine"
FT                   /evidence="ECO:0000269|PubMed:21858060,
FT                   ECO:0007744|PubMed:23576753"
FT   MOD_RES         215
FT                   /note="Phosphotyrosine; by SRC"
FT                   /evidence="ECO:0000250|UniProtKB:P31040"
FT   MOD_RES         250
FT                   /note="N6-acetyllysine; alternate"
FT                   /evidence="ECO:0000269|PubMed:21858060"
FT   MOD_RES         250
FT                   /note="N6-succinyllysine; alternate"
FT                   /evidence="ECO:0007744|PubMed:23806337"
FT   MOD_RES         335
FT                   /note="N6-acetyllysine; alternate"
FT                   /evidence="ECO:0000269|PubMed:21858060,
FT                   ECO:0007744|PubMed:23576753"
FT   MOD_RES         335
FT                   /note="N6-succinyllysine; alternate"
FT                   /evidence="ECO:0007744|PubMed:23806337"
FT   MOD_RES         423
FT                   /note="N6-acetyllysine"
FT                   /evidence="ECO:0007744|PubMed:23576753"
FT   MOD_RES         480
FT                   /note="N6-acetyllysine"
FT                   /evidence="ECO:0000269|PubMed:21858060"
FT   MOD_RES         485
FT                   /note="N6-acetyllysine; alternate"
FT                   /evidence="ECO:0000269|PubMed:21858060"
FT   MOD_RES         485
FT                   /note="N6-succinyllysine; alternate"
FT                   /evidence="ECO:0007744|PubMed:23806337"
FT   MOD_RES         498
FT                   /note="N6-acetyllysine; alternate"
FT                   /evidence="ECO:0000269|PubMed:21858060,
FT                   ECO:0007744|PubMed:23576753"
FT   MOD_RES         498
FT                   /note="N6-succinyllysine; alternate"
FT                   /evidence="ECO:0007744|PubMed:23806337"
FT   MOD_RES         517
FT                   /note="N6-acetyllysine"
FT                   /evidence="ECO:0007744|PubMed:23576753"
FT   MOD_RES         538
FT                   /note="N6-acetyllysine; alternate"
FT                   /evidence="ECO:0007744|PubMed:23576753"
FT   MOD_RES         538
FT                   /note="N6-succinyllysine; alternate"
FT                   /evidence="ECO:0007744|PubMed:23806337"
FT   MOD_RES         547
FT                   /note="N6-acetyllysine; alternate"
FT                   /evidence="ECO:0000269|PubMed:21858060,
FT                   ECO:0007744|PubMed:23576753, ECO:0007744|PubMed:23806337"
FT   MOD_RES         547
FT                   /note="N6-succinyllysine; alternate"
FT                   /evidence="ECO:0007744|PubMed:23806337"
FT   MOD_RES         550
FT                   /note="N6-acetyllysine"
FT                   /evidence="ECO:0000269|PubMed:21858060"
FT   MOD_RES         598
FT                   /note="N6-acetyllysine"
FT                   /evidence="ECO:0000269|PubMed:21858060"
FT   MOD_RES         608
FT                   /note="N6-acetyllysine"
FT                   /evidence="ECO:0000269|PubMed:21858060,
FT                   ECO:0007744|PubMed:23576753"
FT   MOD_RES         615
FT                   /note="N6-succinyllysine"
FT                   /evidence="ECO:0007744|PubMed:23806337"
FT   MOD_RES         624
FT                   /note="N6-acetyllysine"
FT                   /evidence="ECO:0000269|PubMed:21858060"
FT   MOD_RES         633
FT                   /note="N6-acetyllysine"
FT                   /evidence="ECO:0000269|PubMed:21858060"
FT   MOD_RES         636
FT                   /note="N6-acetyllysine"
FT                   /evidence="ECO:0007744|PubMed:23576753"
FT   MOD_RES         647
FT                   /note="N6-acetyllysine"
FT                   /evidence="ECO:0007744|PubMed:23576753"
FT   CONFLICT        69
FT                   /note="A -> V (in Ref. 1; BAE26754)"
FT                   /evidence="ECO:0000305"
FT   CONFLICT        246
FT                   /note="R -> Q (in Ref. 1; BAE26754)"
FT                   /evidence="ECO:0000305"
FT   CONFLICT        428
FT                   /note="Q -> R (in Ref. 1; BAE26754)"
FT                   /evidence="ECO:0000305"
FT   CONFLICT        501
FT                   /note="F -> L (in Ref. 4; ABD77308)"
FT                   /evidence="ECO:0000305"
FT   CONFLICT        517
FT                   /note="K -> M (in Ref. 4; ABD77308)"
FT                   /evidence="ECO:0000305"
FT   CONFLICT        571
FT                   /note="L -> M (in Ref. 4; ABD77308)"
FT                   /evidence="ECO:0000305"
SQ   SEQUENCE   664 AA;  72585 MW;  DDCE1535163C9449 CRC64;
     MAGVGAVSRL LRGRRLALTG AWPGTLQKQT CGFHFSVGEN KKASAKVSDA ISTQYPVVDH
     EFDAVVVGAG GAGLRAAFGL SEAGFNTACL TKLFPTRSHT VAAQGGINAA LGNMEEDNWR
     WHFYDTVKGS DWLGDQDAIH YMTEQAPASV VELENYGMPF SRTEDGKIYQ RAFGGQSLKF
     GKGGQAHRCC CVADRTGHSL LHTLYGRSLR YDTSYFVEYF ALDLLMENGE CRGVIALCIE
     DGSIHRIRAK NTVIATGGYG RTYFSCTSAH TSTGDGTAMV TRAGLPCQDL EFVQFHPTGI
     YGAGCLITEG CRGEGGILIN SQGERFMERY APVAKDLASR DVVSRSMTLE IREGRGCGPE
     KDHVYLQLHH LPPEQLATRL PGISETAMIF AGVDVTKEPI PVLPTVHYNM GGIPTNYKGQ
     VLKHVNGQDQ IVPGLYACGE AACASVHGAN RLGANSLLDL VVFGRACALS IAESCRPGDK
     VPSIKANAGE ESVMNLDKLR FADGSIRTSE LRLNMQKSMQ NHAAVFRVGS VLQEGCEKIS
     QLYGDLKHLK TFDRGMVWNT DLVETLELQN LMLCALQTIY GAEARKESRG AHAREDYKVR
     VDEYDYSKPI QGQQKKPFGE HWRKHTLSYV DIKTGKVTLE YRPVIDKTLN EADCATVPPA
     IRSY
//
```

|  |
| --- |
| **Mascot:** http://www.matrixscience.com/ |

Oxidation (M) (+15.9949)
