# Supplementary material for: A common East-Asian ALDH2 mutation causes metabolic disorders and the therapeutic effect of ALDH2 activators
Source: Nat Commun. 2023 Sep 25;14:5971. doi: 10.1038/s41467-023-41570-6 (PMC10520061; doi:10.1038/s41467-023-41570-6)
Supplement: Supplementary file 4 — Supplementary Data 1 [file 41467_2023_41570_MOESM4_ESM.zip › Table S5b/Q924L1/Q924L1_WTO-1-H184.html]

Mascot Search Results: Q924L1
 

# MASCOT Search Results

## Protein View: Q924L1

### LETM1 domain-containing protein 1 OS=Mus musculus OX=10090 GN=Letmd1 PE=1 SV=1

|  |  |
| --- | --- |
| Database: | Mouse\_UniProt\_proteomes |
| Score: | 2304 |
| Monoisotopic mass (Mr): | 42073 |
| Calculated pI: | 10.54 |

Sequence similarity is available as an NCBI BLAST search of Q924L1 against nr.

### Search parameters

|  |  |
| --- | --- |
| MS data file: | `D:\LCMSMS\2023 Users' data\230529-1\230529-1-WTO-1.raw` |
| Enzyme: | Trypsin/P: cuts C-term side of KR. |
| Fixed modifications: | Carbamidomethyl (C) |
| Variable modifications: | Deamidated (NQ), HNE (C), HNE (H), HNE (K), Oxidation (M) |

### Protein sequence coverage: 38%

Matched peptides shown in ***bold red***.

|  |  |  |  |  |  |
| --- | --- | --- | --- | --- | --- |
| `1` | `MALSRVCWAR` | `AALWGSTVAP` | `GPFVTRRLQL` | `GRSGPAWRAP` | `RSSKLHLSPK` |
| `51` | `ADVKNLISYV` | `VTKTRAINGS` | `YHRFLGRHFP` | `RFYALYTTFM` | `KGIQMLWADG` |
| `101` | `KKARRIKADM` | `WKQNLKFHQL` | `SYREMEHLRQ` | `FRRDITKCLF` | `VGLISIPPFA` |
| `151` | `NYLVFLLMYL` | `FPRQLLVKHF` | `WTPKQQIDFL` | `DVYHGLRRRS` | `HSEVITHLRR` |
| `201` | `ASTFVSHEKL` | `RRQLTDLCTK` | `VQSGTHPAAQ` | `DVLALRDCFS` | `TYPLGFSQLQ` |
| `251` | `ASQMRALSQA` | `MLLTPYLPPP` | `LLRQRLKSHT` | `TVIHQLDRAL` | `AKLGIGQLTA` |
| `301` | `QEVKSACYLR` | `GLNSTHIADD` | `RCRAWLGEWL` | `HISCSLKEPE` | `LSLLLHNVVL` |
| `351` | `LSTNYLETRR` |  |  |  |  |

Unformatted sequence string: 360 residues (for pasting into other applications).

|  |  |  |  |
| --- | --- | --- | --- |
| Sort by | residue number | increasing mass | decreasing mass |
| Show | matched peptides only | predicted peptides also |  |

| Query | Start | – | End | Observed | Mr(expt) | Mr(calc) | ppm | M | Score | Expect | Rank | U | Peptide |
| --- | --- | --- | --- | --- | --- | --- | --- | --- | --- | --- | --- | --- | --- |
| 16491 | 55 | – | 63 | 518.8056 | 1035.5966 | 1035.5964 | 0.16 | 0 | 29 | 0.0039 | 1Score **> 28** indicates **identity** Score **> 17** indicates **homology** | U | K.NLISYVVTK.T |
| 16492 | 55 | – | 63 | 518.8058 | 1035.5971 | 1035.5964 | 0.65 | 0 | 27 | 0.0029 | 1Score **> 27** indicates **identity** Score **> 14** indicates **homology** | U | K.NLISYVVTK.T |
| 16493 | 55 | – | 63 | 518.8059 | 1035.5972 | 1035.5964 | 0.77 | 0 | 15 | 0.043 | 1Score **> 27** indicates **identity** Score **> 13** indicates **homology** | U | K.NLISYVVTK.T |
| 11120 | 117 | – | 123 | 475.7444 | 949.4742 | 949.4770 | -2.92 | 0 | 17 | 0.024 | 1Score **> 31** indicates **identity** Score **> 14** indicates **homology** | U | K.FHQLSYR.E |
| 11123 | 117 | – | 123 | 475.7456 | 949.4766 | 949.4770 | -0.44 | 0 | 15 | 0.036 | 1Score **> 30** indicates **identity** Score **> 13** indicates **homology** | U | K.FHQLSYR.E |
| 78430 | 175 | – | 187 | 535.2775 | 1602.8108 | 1602.8154 | -2.93 | 0 | 17 | 0.025 | 1Score **> 35** indicates **identity** Score **> 14** indicates **homology** | U | K.QQIDFLDVYHGLR.R |
| 78432 | 175 | – | 187 | 535.2783 | 1602.8129 | 1602.8154 | -1.57 | 0 | 37 | 0.00036 | 1Score **> 35** indicates **identity** Score **> 15** indicates **homology** | U | K.QQIDFLDVYHGLR.R |
| 78435 | 175 | – | 187 | 535.2792 | 1602.8156 | 1602.8154 | 0.11 | 0 | 43 | 8.7e-05 | 1Score **> 35** indicates **identity** Score **> 15** indicates **homology** | U | K.QQIDFLDVYHGLR.R |
| 78436 | 175 | – | 187 | 535.2793 | 1602.8162 | 1602.8154 | 0.46 | 0 | 61 | 4.2e-06 | 1Score **> 35** indicates **identity** Score **> 20** indicates **homology** | U | K.QQIDFLDVYHGLR.R |
| 78437 | 175 | – | 187 | 535.2793 | 1602.8162 | 1602.8154 | 0.46 | 0 | 23 | 0.0088 | 1Score **> 35** indicates **identity** Score **> 15** indicates **homology** | U | K.QQIDFLDVYHGLR.R |
| 78439 | 175 | – | 187 | 535.2794 | 1602.8163 | 1602.8154 | 0.56 | 0 | 45 | 6.4e-05 | 1Score **> 35** indicates **identity** Score **> 15** indicates **homology** | U | K.QQIDFLDVYHGLR.R |
| 78441 | 175 | – | 187 | 535.2795 | 1602.8166 | 1602.8154 | 0.69 | 0 | 62 | 1.6e-06 | 1Score **> 35** indicates **identity** Score **> 16** indicates **homology** | U | K.QQIDFLDVYHGLR.R |
| 78443 | 175 | – | 187 | 535.2796 | 1602.8169 | 1602.8154 | 0.89 | 0 | 57 | 4.2e-06 | 1Score **> 35** indicates **identity** Score **> 16** indicates **homology** | U | K.QQIDFLDVYHGLR.R |
| 78444 | 175 | – | 187 | 535.2796 | 1602.8169 | 1602.8154 | 0.90 | 0 | 52 | 2.1e-05 | 1Score **> 35** indicates **identity** Score **> 18** indicates **homology** | U | K.QQIDFLDVYHGLR.R |
| 78448 | 175 | – | 187 | 802.4164 | 1602.8182 | 1602.8154 | 1.72 | 0 | 64 | 9.6e-07 | 1Score **> 35** indicates **identity** Score **> 17** indicates **homology** | U | K.QQIDFLDVYHGLR.R |
| 78450 | 175 | – | 187 | 535.2801 | 1602.8185 | 1602.8154 | 1.91 | 0 | 19 | 0.017 | 1Score **> 35** indicates **identity** Score **> 14** indicates **homology** | U | K.QQIDFLDVYHGLR.R |
| 78452 | 175 | – | 187 | 535.2803 | 1602.8190 | 1602.8154 | 2.20 | 0 | 29 | 0.0021 | 1Score **> 35** indicates **identity** Score **> 15** indicates **homology** | U | K.QQIDFLDVYHGLR.R |
| 78455 | 175 | – | 187 | 535.2804 | 1602.8193 | 1602.8154 | 2.44 | 0 | 48 | 3.2e-05 | 1Score **> 35** indicates **identity** Score **> 15** indicates **homology** | U | K.QQIDFLDVYHGLR.R |
| 78458 | 175 | – | 187 | 802.4174 | 1602.8203 | 1602.8154 | 3.01 | 0 | 65 | 3.7e-06 | 1Score **> 35** indicates **identity** Score **> 24** indicates **homology** | U | K.QQIDFLDVYHGLR.R |
| 78461 | 175 | – | 187 | 535.2811 | 1602.8215 | 1602.8154 | 3.80 | 0 | 21 | 0.011 | 1Score **> 35** indicates **identity** Score **> 14** indicates **homology** | U | K.QQIDFLDVYHGLR.R |
| 97245 | 175 | – | 187 | 440.7359 | 1758.9146 | 1758.9305 | -9.03 | 0 | 22 | 0.0081 | 1Score **> 35** indicates **identity** Score **> 14** indicates **homology** | U | K.QQIDFLDVYHGLR.R  + HNE (H) |
| 97248 | 175 | – | 187 | 440.7362 | 1758.9157 | 1758.9305 | -8.43 | 0 | 34 | 0.00066 | 1Score **> 35** indicates **identity** Score **> 15** indicates **homology** | U | K.QQIDFLDVYHGLR.R  + HNE (H) |
| 97249 | 175 | – | 187 | 440.7364 | 1758.9163 | 1758.9305 | -8.05 | 0 | 30 | 0.0015 | 1Score **> 36** indicates **identity** Score **> 14** indicates **homology** | U | K.QQIDFLDVYHGLR.R  + HNE (H) |
| 97250 | 175 | – | 187 | 587.3127 | 1758.9163 | 1758.9305 | -8.04 | 0 | 28 | 0.0023 | 1Score **> 36** indicates **identity** Score **> 14** indicates **homology** | U | K.QQIDFLDVYHGLR.R  + HNE (H) |
| 97251 | 175 | – | 187 | 440.7365 | 1758.9170 | 1758.9305 | -7.65 | 0 | 35 | 0.00051 | 1Score **> 36** indicates **identity** Score **> 15** indicates **homology** | U | K.QQIDFLDVYHGLR.R  + HNE (H) |
| 97252 | 175 | – | 187 | 440.7366 | 1758.9173 | 1758.9305 | -7.47 | 0 | 34 | 0.0011 | 1Score **> 36** indicates **identity** Score **> 17** indicates **homology** | U | K.QQIDFLDVYHGLR.R  + HNE (H) |
| 30293 | 190 | – | 199 | 393.5469 | 1177.6189 | 1177.6204 | -1.25 | 0 | 14 | 0.049 | 1Score **> 33** indicates **identity** Score **> 13** indicates **homology** | U | R.SHSEVITHLR.R |
| 30294 | 190 | – | 199 | 393.5474 | 1177.6204 | 1177.6204 | 0.040 | 0 | 31 | 0.0013 | 1Score **> 34** indicates **identity** Score **> 14** indicates **homology** | U | R.SHSEVITHLR.R |
| 30295 | 190 | – | 199 | 589.8175 | 1177.6204 | 1177.6204 | 0.065 | 0 | 26 | 0.0033 | 1Score **> 33** indicates **identity** Score **> 14** indicates **homology** | U | R.SHSEVITHLR.R |
| 30299 | 190 | – | 199 | 393.5478 | 1177.6217 | 1177.6204 | 1.14 | 0 | 20 | 0.013 | 1Score **> 33** indicates **identity** Score **> 14** indicates **homology** | U | R.SHSEVITHLR.R |
| 47204 | 190 | – | 200 | 445.5812 | 1333.7219 | 1333.7215 | 0.31 | 1 | 18 | 0.021 | 1Score **> 34** indicates **identity** Score **> 14** indicates **homology** | U | R.SHSEVITHLRR.A |
| 47205 | 190 | – | 200 | 445.5813 | 1333.7220 | 1333.7215 | 0.38 | 1 | 25 | 0.0044 | 1Score **> 34** indicates **identity** Score **> 14** indicates **homology** | U | R.SHSEVITHLRR.A |
| 25490 | 212 | – | 220 | 567.8005 | 1133.5864 | 1133.5863 | 0.10 | 1 | 44 | 0.0036 | 1Score **> 33** indicates **identity** | U | R.RQLTDLCTK.V |
| 12801 | 213 | – | 220 | 489.7493 | 977.4840 | 977.4852 | -1.26 | 0 | 36 | 0.0072 | 1Score **> 31** indicates **identity** Score **> 27** indicates **homology** | U | R.QLTDLCTK.V |
| 12802 | 213 | – | 220 | 489.7496 | 977.4846 | 977.4852 | -0.57 | 0 | 46 | 0.00021 | 1Score **> 31** indicates **identity** Score **> 22** indicates **homology** | U | R.QLTDLCTK.V |
| 12804 | 213 | – | 220 | 489.7503 | 977.4861 | 977.4852 | 0.96 | 0 | 42 | 0.00069 | 1Score **> 31** indicates **identity** Score **> 23** indicates **homology** | U | R.QLTDLCTK.V |
| 12805 | 213 | – | 220 | 489.7507 | 977.4869 | 977.4852 | 1.71 | 0 | 40 | 0.0014 | 1Score **> 32** indicates **identity** Score **> 24** indicates **homology** | U | R.QLTDLCTK.V |
| 85833 | 221 | – | 236 | 831.9499 | 1661.8852 | 1661.8849 | 0.16 | 0 | 44 | 0.00013 | 1Score **> 35** indicates **identity** Score **> 17** indicates **homology** | U | K.VQSGTHPAAQDVLALR.D |
| 85834 | 221 | – | 236 | 831.9499 | 1661.8852 | 1661.8849 | 0.19 | 0 | 77 | 1.4e-07 | 1Score **> 35** indicates **identity** Score **> 21** indicates **homology** | U | K.VQSGTHPAAQDVLALR.D |
| 85835 | 221 | – | 236 | 831.9499 | 1661.8852 | 1661.8849 | 0.20 | 0 | 100 | 4.5e-10 | 1Score **> 35** indicates **identity** Score **> 19** indicates **homology** | U | K.VQSGTHPAAQDVLALR.D |
| 85836 | 221 | – | 236 | 831.9501 | 1661.8857 | 1661.8849 | 0.47 | 0 | 103 | 2e-10 | 1Score **> 35** indicates **identity** Score **> 19** indicates **homology** | U | K.VQSGTHPAAQDVLALR.D |
| 85838 | 221 | – | 236 | 554.9692 | 1661.8858 | 1661.8849 | 0.55 | 0 | 43 | 8.7e-05 | 1Score **> 35** indicates **identity** Score **> 15** indicates **homology** | U | K.VQSGTHPAAQDVLALR.D |
| 85839 | 221 | – | 236 | 554.9693 | 1661.8860 | 1661.8849 | 0.63 | 0 | 33 | 0.00078 | 1Score **> 35** indicates **identity** Score **> 15** indicates **homology** | U | K.VQSGTHPAAQDVLALR.D |
| 85840 | 221 | – | 236 | 831.9503 | 1661.8861 | 1661.8849 | 0.73 | 0 | 27 | 0.0031 | 1Score **> 35** indicates **identity** Score **> 14** indicates **homology** | U | K.VQSGTHPAAQDVLALR.D |
| 85841 | 221 | – | 236 | 554.9693 | 1661.8862 | 1661.8849 | 0.74 | 0 | 50 | 2.2e-05 | 1Score **> 35** indicates **identity** Score **> 16** indicates **homology** | U | K.VQSGTHPAAQDVLALR.D |
| 85842 | 221 | – | 236 | 554.9693 | 1661.8862 | 1661.8849 | 0.76 | 0 | 68 | 4e-07 | 1Score **> 35** indicates **identity** Score **> 17** indicates **homology** | U | K.VQSGTHPAAQDVLALR.D |
| 85843 | 221 | – | 236 | 554.9694 | 1661.8864 | 1661.8849 | 0.87 | 0 | 57 | 4.8e-06 | 1Score **> 35** indicates **identity** Score **> 16** indicates **homology** | U | K.VQSGTHPAAQDVLALR.D |
| 85844 | 221 | – | 236 | 554.9696 | 1661.8870 | 1661.8849 | 1.23 | 0 | 66 | 7e-07 | 1Score **> 35** indicates **identity** Score **> 17** indicates **homology** | U | K.VQSGTHPAAQDVLALR.D |
| 85845 | 221 | – | 236 | 554.9697 | 1661.8873 | 1661.8849 | 1.42 | 0 | 67 | 4.9e-07 | 1Score **> 35** indicates **identity** Score **> 17** indicates **homology** | U | K.VQSGTHPAAQDVLALR.D |
| 85849 | 221 | – | 236 | 554.9708 | 1661.8906 | 1661.8849 | 3.41 | 0 | 43 | 8.7e-05 | 1Score **> 35** indicates **identity** Score **> 15** indicates **homology** | U | K.VQSGTHPAAQDVLALR.D |
| 85851 | 221 | – | 236 | 554.9711 | 1661.8914 | 1661.8849 | 3.88 | 0 | 19 | 0.016 | 1Score **> 35** indicates **identity** Score **> 14** indicates **homology** | U | K.VQSGTHPAAQDVLALR.D |
| 85852 | 221 | – | 236 | 554.9712 | 1661.8918 | 1661.8849 | 4.12 | 0 | 25 | 0.0042 | 1Score **> 35** indicates **identity** Score **> 14** indicates **homology** | U | K.VQSGTHPAAQDVLALR.D |
| 184767 | 221 | – | 255 | 970.7306 | 3878.8935 | 3878.8832 | 2.64 | 1 | 18 | 0.02 | 1Score **> 37** indicates **identity** Score **> 14** indicates **homology** | U | K.VQSGTHPAAQDVLALRDCFSTYPLGFSQLQASQMR.A |
| 139259 | 237 | – | 255 | 746.0087 | 2235.0042 | 2235.0089 | -2.09 | 0 | 48 | 3.5e-05 | 1Score **> 32** indicates **identity** Score **> 15** indicates **homology** | U | R.DCFSTYPLGFSQLQASQMR.A |
| 139260 | 237 | – | 255 | 1118.5128 | 2235.0110 | 2235.0089 | 0.95 | 0 | 87 | 6.8e-09 | 1Score **> 32** indicates **identity** Score **> 18** indicates **homology** | U | R.DCFSTYPLGFSQLQASQMR.A |
| 139261 | 237 | – | 255 | 1118.5173 | 2235.0201 | 2235.0089 | 5.00 | 0 | 75 | 9e-08 | 1Score **> 33** indicates **identity** Score **> 17** indicates **homology** | U | R.DCFSTYPLGFSQLQASQMR.A |
| 139373 | 237 | – | 255 | 1119.0098 | 2236.0051 | 2235.9929 | 5.46 | 0 | 20 | 0.013 | 1Score **> 31** indicates **identity** Score **> 14** indicates **homology** | U | R.DCFSTYPLGFSQLQASQMR.A  + Deamidated (NQ) |
| 119212 | 256 | – | 273 | 997.5788 | 1993.1430 | 1993.1434 | -0.18 | 0 | 38 | 0.00028 | 1Score **> 33** indicates **identity** Score **> 15** indicates **homology** | U | R.ALSQAMLLTPYLPPPLLR.Q |
| 119214 | 256 | – | 273 | 665.3895 | 1993.1465 | 1993.1434 | 1.57 | 0 | 38 | 0.00027 | 1Score **> 32** indicates **identity** Score **> 15** indicates **homology** | U | R.ALSQAMLLTPYLPPPLLR.Q |
| 119215 | 256 | – | 273 | 997.5808 | 1993.1470 | 1993.1434 | 1.82 | 0 | 36 | 0.00041 | 1Score **> 32** indicates **identity** Score **> 15** indicates **homology** | U | R.ALSQAMLLTPYLPPPLLR.Q |
| 119216 | 256 | – | 273 | 997.5815 | 1993.1484 | 1993.1434 | 2.53 | 0 | 39 | 0.00023 | 1Score **> 32** indicates **identity** Score **> 15** indicates **homology** | U | R.ALSQAMLLTPYLPPPLLR.Q |
| 119217 | 256 | – | 273 | 997.5817 | 1993.1488 | 1993.1434 | 2.72 | 0 | 53 | 1e-05 | 1Score **> 32** indicates **identity** Score **> 16** indicates **homology** | U | R.ALSQAMLLTPYLPPPLLR.Q |
| 119218 | 256 | – | 273 | 665.3908 | 1993.1505 | 1993.1434 | 3.56 | 0 | 44 | 7.8e-05 | 1Score **> 32** indicates **identity** Score **> 15** indicates **homology** | U | R.ALSQAMLLTPYLPPPLLR.Q |
| 119305 | 256 | – | 273 | 665.7212 | 1994.1418 | 1994.1274 | 7.24 | 0 | 14 | 0.048 | 1Score **> 33** indicates **identity** Score **> 13** indicates **homology** | U | R.ALSQAMLLTPYLPPPLLR.Q  + Deamidated (NQ) |
| 71243 | 276 | – | 288 | 387.7216 | 1546.8571 | 1546.8580 | -0.55 | 1 | 35 | 0.00056 | 1Score **> 34** indicates **identity** Score **> 15** indicates **homology** | U | R.LKSHTTVIHQLDR.A |
| 71244 | 276 | – | 288 | 516.6263 | 1546.8571 | 1546.8580 | -0.54 | 1 | 44 | 7.8e-05 | 1Score **> 34** indicates **identity** Score **> 15** indicates **homology** | U | R.LKSHTTVIHQLDR.A |
| 71245 | 276 | – | 288 | 387.7216 | 1546.8574 | 1546.8580 | -0.38 | 1 | 59 | 2.9e-06 | 1Score **> 34** indicates **identity** Score **> 16** indicates **homology** | U | R.LKSHTTVIHQLDR.A |
| 71246 | 276 | – | 288 | 516.6265 | 1546.8578 | 1546.8580 | -0.11 | 1 | 23 | 0.0072 | 1Score **> 34** indicates **identity** Score **> 14** indicates **homology** | U | R.LKSHTTVIHQLDR.A |
| 71248 | 276 | – | 288 | 387.7218 | 1546.8582 | 1546.8580 | 0.13 | 1 | 51 | 1.8e-05 | 1Score **> 34** indicates **identity** Score **> 16** indicates **homology** | U | R.LKSHTTVIHQLDR.A |
| 44175 | 278 | – | 288 | 436.2333 | 1305.6780 | 1305.6790 | -0.73 | 0 | 45 | 6.9e-05 | 1Score **> 34** indicates **identity** Score **> 16** indicates **homology** | U | K.SHTTVIHQLDR.A |
| 44176 | 278 | – | 288 | 436.2334 | 1305.6783 | 1305.6790 | -0.53 | 0 | 50 | 2e-05 | 1Score **> 33** indicates **identity** Score **> 16** indicates **homology** | U | K.SHTTVIHQLDR.A |
| 44177 | 278 | – | 288 | 653.8466 | 1305.6786 | 1305.6790 | -0.30 | 0 | 23 | 0.0072 | 1Score **> 34** indicates **identity** Score **> 14** indicates **homology** | U | K.SHTTVIHQLDR.A |
| 44178 | 278 | – | 288 | 653.8466 | 1305.6787 | 1305.6790 | -0.18 | 0 | 55 | 7.7e-06 | 1Score **> 34** indicates **identity** Score **> 16** indicates **homology** | U | K.SHTTVIHQLDR.A |
| 44179 | 278 | – | 288 | 436.2335 | 1305.6787 | 1305.6790 | -0.17 | 0 | 49 | 2.6e-05 | 1Score **> 34** indicates **identity** Score **> 16** indicates **homology** | U | K.SHTTVIHQLDR.A |
| 38808 | 293 | – | 304 | 628.8617 | 1255.7088 | 1255.7136 | -3.81 | 0 | 47 | 4.8e-05 | 1Score **> 32** indicates **identity** Score **> 17** indicates **homology** | U | K.LGIGQLTAQEVK.S |
| 38809 | 293 | – | 304 | 628.8617 | 1255.7089 | 1255.7136 | -3.70 | 0 | 47 | 3.8e-05 | 1Score **> 32** indicates **identity** Score **> 16** indicates **homology** | U | K.LGIGQLTAQEVK.S |
| 38811 | 293 | – | 304 | 628.8640 | 1255.7135 | 1255.7136 | -0.100 | 0 | 102 | 1.3e-09 | 1Score **> 33** indicates **identity** Score **> 26** indicates **homology** | U | K.LGIGQLTAQEVK.S |
| 38812 | 293 | – | 304 | 628.8642 | 1255.7138 | 1255.7136 | 0.19 | 0 | 84 | 7.2e-08 | 1Score **> 33** indicates **identity** Score **> 25** indicates **homology** | U | K.LGIGQLTAQEVK.S |
| 38813 | 293 | – | 304 | 628.8643 | 1255.7141 | 1255.7136 | 0.37 | 0 | 81 | 9.5e-08 | 1Score **> 33** indicates **identity** Score **> 24** indicates **homology** | U | K.LGIGQLTAQEVK.S |
| 32492 | 311 | – | 321 | 400.1963 | 1197.5670 | 1197.5738 | -5.69 | 0 | 40 | 0.00017 | 1Score **> 30** indicates **identity** Score **> 15** indicates **homology** | U | R.GLNSTHIADDR.C |
| 32499 | 311 | – | 321 | 400.1979 | 1197.5719 | 1197.5738 | -1.60 | 0 | 42 | 0.00011 | 1Score **> 30** indicates **identity** Score **> 15** indicates **homology** | U | R.GLNSTHIADDR.C |
| 32501 | 311 | – | 321 | 599.7941 | 1197.5737 | 1197.5738 | -0.051 | 0 | 71 | 6.7e-07 | 1Score **> 30** indicates **identity** Score **> 22** indicates **homology** | U | R.GLNSTHIADDR.C |
| 66956 | 311 | – | 323 | 505.5754 | 1513.7042 | 1513.7056 | -0.88 | 1 | 27 | 0.0028 | 1Score **> 31** indicates **identity** Score **> 14** indicates **homology** | U | R.GLNSTHIADDRCR.A |
| 66957 | 311 | – | 323 | 379.4335 | 1513.7050 | 1513.7056 | -0.36 | 1 | 15 | 0.038 | 1Score **> 31** indicates **identity** Score **> 13** indicates **homology** | U | R.GLNSTHIADDRCR.A |
| 66958 | 311 | – | 323 | 757.8599 | 1513.7052 | 1513.7056 | -0.23 | 1 | 16 | 0.045 | 1Score **> 31** indicates **identity** Score **> 15** indicates **homology** | U | R.GLNSTHIADDRCR.A |
| 66959 | 311 | – | 323 | 505.5757 | 1513.7054 | 1513.7056 | -0.12 | 1 | 51 | 1.6e-05 | 1Score **> 31** indicates **identity** Score **> 16** indicates **homology** | U | R.GLNSTHIADDRCR.A |
| 66960 | 311 | – | 323 | 505.5758 | 1513.7057 | 1513.7056 | 0.10 | 1 | 38 | 0.00026 | 1Score **> 31** indicates **identity** Score **> 15** indicates **homology** | U | R.GLNSTHIADDRCR.A |
| 66961 | 311 | – | 323 | 379.4338 | 1513.7060 | 1513.7056 | 0.29 | 1 | 31 | 0.0013 | 1Score **> 31** indicates **identity** Score **> 14** indicates **homology** | U | R.GLNSTHIADDRCR.A |

---

```
ID   LTMD1_MOUSE             Reviewed;         360 AA.
AC   Q924L1; Q3TF05; Q3U3M7; Q8BLG9; Q8K1F7; Q8VDS9;
DT   13-NOV-2007, integrated into UniProtKB/Swiss-Prot.
DT   01-DEC-2001, sequence version 1.
DT   28-JUN-2023, entry version 132.
DE   RecName: Full=LETM1 domain-containing protein 1 {ECO:0000305};
DE   AltName: Full=Cervical cancer receptor;
DE   AltName: Full=MCC-32;
GN   Name=Letmd1 {ECO:0000312|MGI:MGI:1915864}; Synonyms=Mccr;
OS   Mus musculus (Mouse).
OC   Eukaryota; Metazoa; Chordata; Craniata; Vertebrata; Euteleostomi; Mammalia;
OC   Eutheria; Euarchontoglires; Glires; Rodentia; Myomorpha; Muroidea; Muridae;
OC   Murinae; Mus; Mus.
OX   NCBI_TaxID=10090;
RN   [1]
RP   NUCLEOTIDE SEQUENCE [MRNA] (ISOFORM 1).
RC   STRAIN=C3H/He;
RA   Hwang J.H., Kim J.W., Hwang S.Y.;
RT   "Mouse cervical cancer receptor.";
RL   Submitted (JUL-2000) to the EMBL/GenBank/DDBJ databases.
RN   [2]
RP   NUCLEOTIDE SEQUENCE [MRNA] (ISOFORM 2).
RC   STRAIN=FVB/NJ; TISSUE=Kidney;
RA   Kim J.W.;
RT   "Identification of a mouse cDNA (MCC) homologous to human HCCR-1 and HCCR-2
RT   gene.";
RL   Submitted (JUL-2001) to the EMBL/GenBank/DDBJ databases.
RN   [3]
RP   NUCLEOTIDE SEQUENCE [LARGE SCALE MRNA] (ISOFORMS 1 AND 2).
RC   STRAIN=C57BL/6J, and NOD; TISSUE=Aorta, Embryo, and Vein;
RX   PubMed=16141072; DOI=10.1126/science.1112014;
RA   Carninci P., Kasukawa T., Katayama S., Gough J., Frith M.C., Maeda N.,
RA   Oyama R., Ravasi T., Lenhard B., Wells C., Kodzius R., Shimokawa K.,
RA   Bajic V.B., Brenner S.E., Batalov S., Forrest A.R., Zavolan M., Davis M.J.,
RA   Wilming L.G., Aidinis V., Allen J.E., Ambesi-Impiombato A., Apweiler R.,
RA   Aturaliya R.N., Bailey T.L., Bansal M., Baxter L., Beisel K.W., Bersano T.,
RA   Bono H., Chalk A.M., Chiu K.P., Choudhary V., Christoffels A.,
RA   Clutterbuck D.R., Crowe M.L., Dalla E., Dalrymple B.P., de Bono B.,
RA   Della Gatta G., di Bernardo D., Down T., Engstrom P., Fagiolini M.,
RA   Faulkner G., Fletcher C.F., Fukushima T., Furuno M., Futaki S.,
RA   Gariboldi M., Georgii-Hemming P., Gingeras T.R., Gojobori T., Green R.E.,
RA   Gustincich S., Harbers M., Hayashi Y., Hensch T.K., Hirokawa N., Hill D.,
RA   Huminiecki L., Iacono M., Ikeo K., Iwama A., Ishikawa T., Jakt M.,
RA   Kanapin A., Katoh M., Kawasawa Y., Kelso J., Kitamura H., Kitano H.,
RA   Kollias G., Krishnan S.P., Kruger A., Kummerfeld S.K., Kurochkin I.V.,
RA   Lareau L.F., Lazarevic D., Lipovich L., Liu J., Liuni S., McWilliam S.,
RA   Madan Babu M., Madera M., Marchionni L., Matsuda H., Matsuzawa S., Miki H.,
RA   Mignone F., Miyake S., Morris K., Mottagui-Tabar S., Mulder N., Nakano N.,
RA   Nakauchi H., Ng P., Nilsson R., Nishiguchi S., Nishikawa S., Nori F.,
RA   Ohara O., Okazaki Y., Orlando V., Pang K.C., Pavan W.J., Pavesi G.,
RA   Pesole G., Petrovsky N., Piazza S., Reed J., Reid J.F., Ring B.Z.,
RA   Ringwald M., Rost B., Ruan Y., Salzberg S.L., Sandelin A., Schneider C.,
RA   Schoenbach C., Sekiguchi K., Semple C.A., Seno S., Sessa L., Sheng Y.,
RA   Shibata Y., Shimada H., Shimada K., Silva D., Sinclair B., Sperling S.,
RA   Stupka E., Sugiura K., Sultana R., Takenaka Y., Taki K., Tammoja K.,
RA   Tan S.L., Tang S., Taylor M.S., Tegner J., Teichmann S.A., Ueda H.R.,
RA   van Nimwegen E., Verardo R., Wei C.L., Yagi K., Yamanishi H.,
RA   Zabarovsky E., Zhu S., Zimmer A., Hide W., Bult C., Grimmond S.M.,
RA   Teasdale R.D., Liu E.T., Brusic V., Quackenbush J., Wahlestedt C.,
RA   Mattick J.S., Hume D.A., Kai C., Sasaki D., Tomaru Y., Fukuda S.,
RA   Kanamori-Katayama M., Suzuki M., Aoki J., Arakawa T., Iida J., Imamura K.,
RA   Itoh M., Kato T., Kawaji H., Kawagashira N., Kawashima T., Kojima M.,
RA   Kondo S., Konno H., Nakano K., Ninomiya N., Nishio T., Okada M., Plessy C.,
RA   Shibata K., Shiraki T., Suzuki S., Tagami M., Waki K., Watahiki A.,
RA   Okamura-Oho Y., Suzuki H., Kawai J., Hayashizaki Y.;
RT   "The transcriptional landscape of the mammalian genome.";
RL   Science 309:1559-1563(2005).
RN   [4]
RP   NUCLEOTIDE SEQUENCE [LARGE SCALE MRNA] (ISOFORM 3).
RC   STRAIN=FVB/N-3; TISSUE=Mammary tumor;
RX   PubMed=15489334; DOI=10.1101/gr.2596504;
RG   The MGC Project Team;
RT   "The status, quality, and expansion of the NIH full-length cDNA project:
RT   the Mammalian Gene Collection (MGC).";
RL   Genome Res. 14:2121-2127(2004).
RN   [5]
RP   FUNCTION.
RX   PubMed=12879013; DOI=10.1038/sj.onc.1206624;
RA   Ko J., Lee Y.H., Hwang S.Y., Lee Y.S., Shin S.M., Hwang J.H., Kim J.,
RA   Kim Y.W., Jang S.-W., Ryoo Z.Y., Kim I.-K., Namkoong S.E., Kim J.W.;
RT   "Identification and differential expression of novel human cervical cancer
RT   oncogene HCCR-2 in human cancers and its involvement in p53
RT   stabilization.";
RL   Oncogene 22:4679-4689(2003).
RN   [6]
RP   PHOSPHORYLATION [LARGE SCALE ANALYSIS] AT SER-192, AND IDENTIFICATION BY
RP   MASS SPECTROMETRY [LARGE SCALE ANALYSIS].
RC   TISSUE=Brown adipose tissue, Heart, and Kidney;
RX   PubMed=21183079; DOI=10.1016/j.cell.2010.12.001;
RA   Huttlin E.L., Jedrychowski M.P., Elias J.E., Goswami T., Rad R.,
RA   Beausoleil S.A., Villen J., Haas W., Sowa M.E., Gygi S.P.;
RT   "A tissue-specific atlas of mouse protein phosphorylation and expression.";
RL   Cell 143:1174-1189(2010).
RN   [7]
RP   DISRUPTION PHENOTYPE, TISSUE SPECIFICITY, INDUCTION, AND FUNCTION.
RX   PubMed=34669999; DOI=10.1096/fj.202100597r;
RA   Snyder M.M., Yue F., Zhang L., Shang R., Qiu J., Chen J., Kim K.H.,
RA   Peng Y., Oprescu S.N., Donkin S.S., Bi P., Kuang S.;
RT   "LETMD1 is required for mitochondrial structure and thermogenic function of
RT   brown adipocytes.";
RL   FASEB J. 35:e21965-e21965(2021).
RN   [8]
RP   FUNCTION, DISRUPTION PHENOTYPE, SUBCELLULAR LOCATION, TISSUE SPECIFICITY,
RP   AND INTERACTION WITH SMARCA4.
RX   PubMed=34910916; DOI=10.1016/j.celrep.2021.110104;
RA   Choi K.M., Kim J.H., Kong X., Isik M., Zhang J., Lim H.W., Yoon J.C.;
RT   "Defective brown adipose tissue thermogenesis and impaired glucose
RT   metabolism in mice lacking Letmd1.";
RL   Cell Rep. 37:110104-110104(2021).
RN   [9]
RP   FUNCTION, DISRUPTION PHENOTYPE, SUBCELLULAR LOCATION, AND TISSUE
RP   SPECIFICITY.
RX   PubMed=36334589; DOI=10.1016/j.cell.2022.10.003;
RA   Xiao H., Bozi L.H.M., Sun Y., Riley C.L., Philip V.M., Chen M., Li J.,
RA   Zhang T., Mills E.L., Emont M.P., Sun W., Reddy A., Garrity R., Long J.,
RA   Becher T., Vitas L.P., Laznik-Bogoslavski D., Ordonez M., Liu X., Chen X.,
RA   Wang Y., Liu W., Tran N., Liu Y., Zhang Y., Cypess A.M., White A.P., He Y.,
RA   Deng R., Schoeder H., Paulo J.A., Jedrychowski M.P., Banks A.S.,
RA   Tseng Y.H., Cohen P., Tsai L.T., Rosen E.D., Klein S., Chondronikola M.,
RA   McAllister F.E., Van Bruggen N., Huttlin E.L., Spiegelman B.M.,
RA   Churchill G.A., Gygi S.P., Chouchani E.T.;
RT   "Architecture of the outbred brown fat proteome defines regulators of
RT   metabolic physiology.";
RL   Cell 0:0-0(2022).
CC   -!- FUNCTION: Plays an essential role for mitochondrial structure and
CC       function, as well as thermogenesis of brown adipocytes
CC       (PubMed:34669999, PubMed:34910916, PubMed:36334589). In brown adipose
CC       tissue also localizes in the nucleus where it interacts with the
CC       chromatin remodeler SMARCA4 to regulate thermogenic genes expression,
CC       such as UCP1 (PubMed:34910916). May regulate phagocytosis and
CC       inflammatory responses to lipopolysaccharide in macrophages (By
CC       similarity). Involved in tumorigenesis and may function as a negative
CC       regulator of the p53/TP53 (PubMed:12879013).
CC       {ECO:0000250|UniProtKB:Q6P1Q0, ECO:0000269|PubMed:12879013,
CC       ECO:0000269|PubMed:34669999, ECO:0000269|PubMed:34910916,
CC       ECO:0000269|PubMed:36334589}.
CC   -!- SUBUNIT: Interacts with BRI3BP. Interacts (via C-terminal) with
CC       SMARCA4; the interaction regulates transcriptional expression of
CC       thermogenic genes in brown adipose tissue (PubMed:34910916).
CC       {ECO:0000250|UniProtKB:Q6P1Q0, ECO:0000269|PubMed:34910916}.
CC   -!- SUBCELLULAR LOCATION: Mitochondrion outer membrane
CC       {ECO:0000250|UniProtKB:Q6P1Q0}; Single-pass membrane protein
CC       {ECO:0000250|UniProtKB:Q6P1Q0}. Nucleus {ECO:0000269|PubMed:34910916}.
CC       Mitochondrion inner membrane {ECO:0000269|PubMed:36334589}; Single-pass
CC       membrane protein {ECO:0000255}.
CC   -!- ALTERNATIVE PRODUCTS:
CC       Event=Alternative splicing; Named isoforms=3;
CC       Name=1;
CC         IsoId=Q924L1-1; Sequence=Displayed;
CC       Name=2;
CC         IsoId=Q924L1-2; Sequence=VSP_029281;
CC       Name=3;
CC         IsoId=Q924L1-3; Sequence=VSP_029280;
CC   -!- TISSUE SPECIFICITY: Highly expressed in brown adipose tissue (BAT)
CC       (PubMed:34669999, PubMed:34910916, PubMed:36334589). Also detected at
CC       low levels in white adipose tissue (PubMed:34910916).
CC       {ECO:0000269|PubMed:34669999, ECO:0000269|PubMed:34910916,
CC       ECO:0000269|PubMed:36334589}.
CC   -!- INDUCTION: Up-regulated in white and brown adipose tissues upon cold
CC       exposure and beta-adrenergic signaling. {ECO:0000269|PubMed:34669999,
CC       ECO:0000269|PubMed:34910916, ECO:0000269|PubMed:36334589}.
CC   -!- DISRUPTION PHENOTYPE: Homozygous mice for Letmd1 gene are born at
CC       normal Mendelian ratios, with normal morphology, bodyweight and body
CC       composition at 2-months old (PubMed:34669999). Mice could not tolerate
CC       cold exposure without food (PubMed:34669999). Mutants exhibit impaired
CC       thermogenesis and are prone to diet-induced obesity and defective
CC       glucose disposal (PubMed:34910916). {ECO:0000269|PubMed:34669999,
CC       ECO:0000269|PubMed:34910916}.
CC   ---------------------------------------------------------------------------
CC   Copyrighted by the UniProt Consortium, see https://www.uniprot.org/terms
CC   Distributed under the Creative Commons Attribution (CC BY 4.0) License
CC   ---------------------------------------------------------------------------
DR   EMBL; AF287293; AAK83032.1; -; mRNA.
DR   EMBL; AF401483; AAM90666.1; -; mRNA.
DR   EMBL; AK045256; BAC32283.1; -; mRNA.
DR   EMBL; AK080221; BAC37851.1; -; mRNA.
DR   EMBL; AK154676; BAE32758.1; -; mRNA.
DR   EMBL; AK169341; BAE41093.1; -; mRNA.
DR   EMBL; BC021361; AAH21361.1; -; mRNA.
DR   CCDS; CCDS27838.1; -. [Q924L1-1]
DR   CCDS; CCDS88844.1; -. [Q924L1-2]
DR   CCDS; CCDS88845.1; -. [Q924L1-3]
DR   RefSeq; NP_598854.1; NM_134093.2. [Q924L1-1]
DR   RefSeq; XP_006521384.1; XM_006521321.3.
DR   RefSeq; XP_006521385.1; XM_006521322.1. [Q924L1-3]
DR   RefSeq; XP_006521386.1; XM_006521323.3.
DR   RefSeq; XP_017172228.1; XM_017316739.1. [Q924L1-3]
DR   AlphaFoldDB; Q924L1; -.
DR   SMR; Q924L1; -.
DR   BioGRID; 212953; 4.
DR   STRING; 10090.ENSMUSP00000037546; -.
DR   iPTMnet; Q924L1; -.
DR   PhosphoSitePlus; Q924L1; -.
DR   SwissPalm; Q924L1; -.
DR   EPD; Q924L1; -.
DR   jPOST; Q924L1; -.
DR   MaxQB; Q924L1; -.
DR   PaxDb; Q924L1; -.
DR   PeptideAtlas; Q924L1; -.
DR   ProteomicsDB; 252683; -. [Q924L1-1]
DR   ProteomicsDB; 252684; -. [Q924L1-2]
DR   ProteomicsDB; 252685; -. [Q924L1-3]
DR   Antibodypedia; 26304; 170 antibodies from 21 providers.
DR   DNASU; 68614; -.
DR   Ensembl; ENSMUST00000037001; ENSMUSP00000037546; ENSMUSG00000037353. [Q924L1-1]
DR   Ensembl; ENSMUST00000229648; ENSMUSP00000155084; ENSMUSG00000037353. [Q924L1-2]
DR   Ensembl; ENSMUST00000230294; ENSMUSP00000155807; ENSMUSG00000037353. [Q924L1-3]
DR   GeneID; 68614; -.
DR   KEGG; mmu:68614; -.
DR   UCSC; uc007xrg.2; mouse. [Q924L1-1]
DR   AGR; MGI:1915864; -.
DR   CTD; 25875; -.
DR   MGI; MGI:1915864; Letmd1.
DR   VEuPathDB; HostDB:ENSMUSG00000037353; -.
DR   eggNOG; KOG4263; Eukaryota.
DR   GeneTree; ENSGT00950000183167; -.
DR   HOGENOM; CLU_049801_1_0_1; -.
DR   InParanoid; Q924L1; -.
DR   OMA; LVFWYPR; -.
DR   OrthoDB; 5399546at2759; -.
DR   PhylomeDB; Q924L1; -.
DR   TreeFam; TF314047; -.
DR   BioGRID-ORCS; 68614; 1 hit in 77 CRISPR screens.
DR   ChiTaRS; Letmd1; mouse.
DR   PRO; PR:Q924L1; -.
DR   Proteomes; UP000000589; Chromosome 15.
DR   RNAct; Q924L1; protein.
DR   Bgee; ENSMUSG00000037353; Expressed in brown adipose tissue and 250 other tissues.
DR   ExpressionAtlas; Q924L1; baseline and differential.
DR   Genevisible; Q924L1; MM.
DR   GO; GO:0005743; C:mitochondrial inner membrane; IDA:UniProtKB.
DR   GO; GO:0005741; C:mitochondrial outer membrane; IEA:UniProtKB-SubCell.
DR   GO; GO:0005739; C:mitochondrion; HDA:MGI.
DR   GO; GO:0005730; C:nucleolus; ISO:MGI.
DR   GO; GO:0005654; C:nucleoplasm; ISO:MGI.
DR   GO; GO:0005634; C:nucleus; IDA:UniProt.
DR   GO; GO:0001671; F:ATPase activator activity; IDA:UniProt.
DR   GO; GO:0008047; F:enzyme activator activity; IDA:UniProt.
DR   GO; GO:0060090; F:molecular adaptor activity; IDA:UniProtKB.
DR   GO; GO:0043022; F:ribosome binding; IEA:InterPro.
DR   GO; GO:0030003; P:intracellular monoatomic cation homeostasis; IBA:GO_Central.
DR   GO; GO:0007005; P:mitochondrion organization; IMP:UniProtKB.
DR   GO; GO:0120162; P:positive regulation of cold-induced thermogenesis; IDA:UniProtKB.
DR   GO; GO:0050727; P:regulation of inflammatory response; ISO:MGI.
DR   GO; GO:0050764; P:regulation of phagocytosis; ISO:MGI.
DR   InterPro; IPR033122; LETM1-like_RBD.
DR   InterPro; IPR044202; LETM1/MDM38-like.
DR   PANTHER; PTHR14009:SF13; LETM1 DOMAIN-CONTAINING PROTEIN 1; 1.
DR   PANTHER; PTHR14009; LEUCINE ZIPPER-EF-HAND CONTAINING TRANSMEMBRANE PROTEIN; 1.
DR   Pfam; PF07766; LETM1_RBD; 1.
DR   PROSITE; PS51758; LETM1_RBD; 1.
PE   1: Evidence at protein level;
KW   Alternative splicing; Membrane; Mitochondrion;
KW   Mitochondrion inner membrane; Mitochondrion outer membrane; Nucleus;
KW   Phosphoprotein; Proto-oncogene; Reference proteome; Transmembrane;
KW   Transmembrane helix.
FT   CHAIN           1..360
FT                   /note="LETM1 domain-containing protein 1"
FT                   /id="PRO_0000310420"
FT   TOPO_DOM        1..137
FT                   /note="Cytoplasmic"
FT                   /evidence="ECO:0000255"
FT   TRANSMEM        138..158
FT                   /note="Helical"
FT                   /evidence="ECO:0000255"
FT   TOPO_DOM        159..360
FT                   /note="Mitochondrial intermembrane"
FT                   /evidence="ECO:0000255"
FT   DOMAIN          178..360
FT                   /note="Letm1 RBD"
FT                   /evidence="ECO:0000255|PROSITE-ProRule:PRU01094"
FT   REGION          1..110
FT                   /note="Required and sufficient for mitochondrial import"
FT                   /evidence="ECO:0000250"
FT   MOD_RES         192
FT                   /note="Phosphoserine"
FT                   /evidence="ECO:0007744|PubMed:21183079"
FT   VAR_SEQ         1..157
FT                   /note="Missing (in isoform 3)"
FT                   /evidence="ECO:0000303|PubMed:15489334"
FT                   /id="VSP_029280"
FT   VAR_SEQ         1..89
FT                   /note="Missing (in isoform 2)"
FT                   /evidence="ECO:0000303|PubMed:16141072, ECO:0000303|Ref.2"
FT                   /id="VSP_029281"
FT   CONFLICT        20
FT                   /note="P -> T (in Ref. 3; BAE41093)"
FT                   /evidence="ECO:0000305"
FT   CONFLICT        207
FT                   /note="H -> Q (in Ref. 2; AAM90666)"
FT                   /evidence="ECO:0000305"
FT   CONFLICT        316
FT                   /note="H -> N (in Ref. 3; BAE32758)"
FT                   /evidence="ECO:0000305"
SQ   SEQUENCE   360 AA;  41701 MW;  4689BC4F3D2A0BBD CRC64;
     MALSRVCWAR AALWGSTVAP GPFVTRRLQL GRSGPAWRAP RSSKLHLSPK ADVKNLISYV
     VTKTRAINGS YHRFLGRHFP RFYALYTTFM KGIQMLWADG KKARRIKADM WKQNLKFHQL
     SYREMEHLRQ FRRDITKCLF VGLISIPPFA NYLVFLLMYL FPRQLLVKHF WTPKQQIDFL
     DVYHGLRRRS HSEVITHLRR ASTFVSHEKL RRQLTDLCTK VQSGTHPAAQ DVLALRDCFS
     TYPLGFSQLQ ASQMRALSQA MLLTPYLPPP LLRQRLKSHT TVIHQLDRAL AKLGIGQLTA
     QEVKSACYLR GLNSTHIADD RCRAWLGEWL HISCSLKEPE LSLLLHNVVL LSTNYLETRR
//
```

|  |
| --- |
| **Mascot:** http://www.matrixscience.com/ |

HNE (H) (+156.1150)
