# Supplementary material for: A common East-Asian ALDH2 mutation causes metabolic disorders and the therapeutic effect of ALDH2 activators
Source: Nat Commun. 2023 Sep 25;14:5971. doi: 10.1038/s41467-023-41570-6 (PMC10520061; doi:10.1038/s41467-023-41570-6)
Supplement: Supplementary file 4 — Supplementary Data 1 [file 41467_2023_41570_MOESM4_ESM.zip › Table S5b/Q99JY0/Q99JY0_WTO-1_H133.html]

Mascot Search Results: Q99JY0
 

# MASCOT Search Results

## Protein View: Q99JY0

### Trifunctional enzyme subunit beta, mitochondrial OS=Mus musculus OX=10090 GN=Hadhb PE=1 SV=1

|  |  |
| --- | --- |
| Database: | Mouse\_UniProt\_proteomes |
| Score: | 5485 |
| Monoisotopic mass (Mr): | 51639 |
| Calculated pI: | 9.43 |

Sequence similarity is available as an NCBI BLAST search of Q99JY0 against nr.

### Search parameters

|  |  |
| --- | --- |
| MS data file: | `D:\LCMSMS\2023 Users' data\230529-1\230529-1-WTO-1.raw` |
| Enzyme: | Trypsin/P: cuts C-term side of KR. |
| Fixed modifications: | Carbamidomethyl (C) |
| Variable modifications: | Deamidated (NQ), HNE (C), HNE (H), HNE (K), Oxidation (M) |

### Protein sequence coverage: 62%

Matched peptides shown in ***bold red***.

|  |  |  |  |  |  |
| --- | --- | --- | --- | --- | --- |
| `1` | `MTTILTSTFR` | `NLSTTSKWAL` | `RSSIRPLSCS` | `SQLHSAPAVQ` | `TKSKKTLAKP` |
| `51` | `NMKNIVVVEG` | `VRIPFLLSGT` | `SYKDLMPHDL` | `ARAALSGLLH` | `RTNIPKDVVD` |
| `101` | `YIIFGTVIQE` | `VKTSNVAREA` | `ALGAGFSDKT` | `PAHTVTMACI` | `SSNQAMTTAV` |
| `151` | `GLIASGQCDV` | `VVAGGVELMS` | `DVPIRHSRNM` | `RKMMLDLNKA` | `KTLGQRLSLL` |
| `201` | `SKFRLNFLSP` | `ELPAVAEFST` | `NETMGHSADR` | `LAAAFAVSRM` | `EQDEYALRSH` |
| `251` | `SLAKKAQDEG` | `HLSDIVPFKV` | `PGKDTVTKDN` | `GIRPSSLEQM` | `AKLKPAFIKP` |
| `301` | `YGTVTAANSS` | `FLTDGASAML` | `IMSEDRALAM` | `GYKPKAYLRD` | `FIYVSQDPKD` |
| `351` | `QLLLGPTYAT` | `PKVLEKAGLT` | `MNDIDAFEFH` | `EAFSGQILAN` | `FKAMDSDWFA` |
| `401` | `QNYMGRKTKV` | `GSPPLEKFNI` | `WGGSLSLGHP` | `FGATGCRLVM` | `AAANRLRKDG` |
| `451` | `GQYALVAACA` | `AGGQGHAMIV` | `EAYPK` |  |  |

Unformatted sequence string: 475 residues (for pasting into other applications).

|  |  |  |  |
| --- | --- | --- | --- |
| Sort by | residue number | increasing mass | decreasing mass |
| Show | matched peptides only | predicted peptides also |  |

| Query | Start | – | End | Observed | Mr(expt) | Mr(calc) | ppm | M | Score | Expect | Rank | U | Peptide |
| --- | --- | --- | --- | --- | --- | --- | --- | --- | --- | --- | --- | --- | --- |
| 13122 | 54 | – | 62 | 492.7939 | 983.5732 | 983.5764 | -3.22 | 0 | 26 | 0.0036 | 1Score **> 29** indicates **identity** Score **> 14** indicates **homology** | U | K.NIVVVEGVR.I |
| 13123 | 54 | – | 62 | 492.7947 | 983.5749 | 983.5764 | -1.56 | 0 | 29 | 0.0018 | 1Score **> 29** indicates **identity** Score **> 14** indicates **homology** | U | K.NIVVVEGVR.I |
| 13124 | 54 | – | 62 | 492.7950 | 983.5754 | 983.5764 | -0.99 | 0 | 35 | 0.0011 | 1Score **> 27** indicates **identity** Score **> 18** indicates **homology** | U | K.NIVVVEGVR.I |
| 13125 | 54 | – | 62 | 492.7951 | 983.5757 | 983.5764 | -0.69 | 0 | 26 | 0.0067 | 1Score **> 27** indicates **identity** Score **> 17** indicates **homology** | U | K.NIVVVEGVR.I |
| 13126 | 54 | – | 62 | 492.7952 | 983.5759 | 983.5764 | -0.48 | 0 | 31 | 0.0013 | 1Score **> 27** indicates **identity** Score **> 14** indicates **homology** | U | K.NIVVVEGVR.I |
| 13127 | 54 | – | 62 | 492.7953 | 983.5761 | 983.5764 | -0.27 | 0 | 42 | 0.00011 | 1Score **> 27** indicates **identity** Score **> 15** indicates **homology** | U | K.NIVVVEGVR.I |
| 13128 | 54 | – | 62 | 492.7953 | 983.5761 | 983.5764 | -0.26 | 0 | 45 | 0.00012 | 1Score **> 27** indicates **identity** Score **> 19** indicates **homology** | U | K.NIVVVEGVR.I |
| 13129 | 54 | – | 62 | 492.7954 | 983.5762 | 983.5764 | -0.21 | 0 | 32 | 0.0009 | 1Score **> 27** indicates **identity** Score **> 15** indicates **homology** | U | K.NIVVVEGVR.I |
| 13130 | 54 | – | 62 | 492.7954 | 983.5762 | 983.5764 | -0.15 | 0 | 29 | 0.0041 | 1Score **> 27** indicates **identity** Score **> 18** indicates **homology** | U | K.NIVVVEGVR.I |
| 13131 | 54 | – | 62 | 492.7955 | 983.5765 | 983.5764 | 0.16 | 0 | 32 | 0.00092 | 1Score **> 27** indicates **identity** Score **> 15** indicates **homology** | U | K.NIVVVEGVR.I |
| 13132 | 54 | – | 62 | 492.7956 | 983.5767 | 983.5764 | 0.30 | 0 | 41 | 0.00028 | 1Score **> 27** indicates **identity** Score **> 18** indicates **homology** | U | K.NIVVVEGVR.I |
| 13133 | 54 | – | 62 | 492.7957 | 983.5768 | 983.5764 | 0.44 | 0 | 27 | 0.0029 | 1Score **> 27** indicates **identity** Score **> 14** indicates **homology** | U | K.NIVVVEGVR.I |
| 13134 | 54 | – | 62 | 492.7957 | 983.5768 | 983.5764 | 0.47 | 0 | 38 | 0.00027 | 1Score **> 27** indicates **identity** Score **> 15** indicates **homology** | U | K.NIVVVEGVR.I |
| 13136 | 54 | – | 62 | 492.7957 | 983.5769 | 983.5764 | 0.56 | 0 | 27 | 0.0029 | 1Score **> 27** indicates **identity** Score **> 14** indicates **homology** | U | K.NIVVVEGVR.I |
| 13137 | 54 | – | 62 | 492.7958 | 983.5771 | 983.5764 | 0.75 | 0 | 30 | 0.0017 | 1Score **> 27** indicates **identity** Score **> 14** indicates **homology** | U | K.NIVVVEGVR.I |
| 13138 | 54 | – | 62 | 492.7959 | 983.5772 | 983.5764 | 0.80 | 0 | 21 | 0.0098 | 1Score **> 27** indicates **identity** Score **> 14** indicates **homology** | U | K.NIVVVEGVR.I |
| 13139 | 54 | – | 62 | 492.7959 | 983.5772 | 983.5764 | 0.87 | 0 | 38 | 0.00029 | 1Score **> 27** indicates **identity** Score **> 16** indicates **homology** | U | K.NIVVVEGVR.I |
| 13140 | 54 | – | 62 | 492.7970 | 983.5795 | 983.5764 | 3.21 | 0 | 28 | 0.0067 | 1Score **> 28** indicates **identity** Score **> 19** indicates **homology** | U | K.NIVVVEGVR.I |
| 136312 | 54 | – | 73 | 1096.1317 | 2190.2489 | 2190.2412 | 3.51 | 1 | 86 | 9.2e-09 | 1Score **> 32** indicates **identity** Score **> 18** indicates **homology** | U | K.NIVVVEGVRIPFLLSGTSYK.D |
| 136313 | 54 | – | 73 | 731.0906 | 2190.2498 | 2190.2412 | 3.92 | 1 | 24 | 0.0057 | 1Score **> 32** indicates **identity** Score **> 14** indicates **homology** | U | K.NIVVVEGVRIPFLLSGTSYK.D |
| 136314 | 54 | – | 73 | 731.0920 | 2190.2540 | 2190.2412 | 5.84 | 1 | 53 | 9.8e-06 | 1Score **> 32** indicates **identity** Score **> 16** indicates **homology** | U | K.NIVVVEGVRIPFLLSGTSYK.D |
| 35417 | 63 | – | 73 | 613.3439 | 1224.6733 | 1224.6754 | -1.72 | 0 | 48 | 3.3e-05 | 1Score **> 32** indicates **identity** Score **> 15** indicates **homology** | U | R.IPFLLSGTSYK.D |
| 35418 | 63 | – | 73 | 613.3443 | 1224.6740 | 1224.6754 | -1.16 | 0 | 42 | 0.00012 | 1Score **> 32** indicates **identity** Score **> 15** indicates **homology** | U | R.IPFLLSGTSYK.D |
| 35419 | 63 | – | 73 | 613.3445 | 1224.6745 | 1224.6754 | -0.74 | 0 | 59 | 3.3e-06 | 1Score **> 32** indicates **identity** Score **> 16** indicates **homology** | U | R.IPFLLSGTSYK.D |
| 35420 | 63 | – | 73 | 613.3446 | 1224.6747 | 1224.6754 | -0.56 | 0 | 53 | 1e-05 | 1Score **> 32** indicates **identity** Score **> 16** indicates **homology** | U | R.IPFLLSGTSYK.D |
| 35421 | 63 | – | 73 | 613.3450 | 1224.6755 | 1224.6754 | 0.037 | 0 | 47 | 3.9e-05 | 1Score **> 32** indicates **identity** Score **> 15** indicates **homology** | U | R.IPFLLSGTSYK.D |
| 35422 | 63 | – | 73 | 613.3454 | 1224.6762 | 1224.6754 | 0.65 | 0 | 63 | 1.2e-06 | 1Score **> 32** indicates **identity** Score **> 16** indicates **homology** | U | R.IPFLLSGTSYK.D |
| 35423 | 63 | – | 73 | 613.3455 | 1224.6765 | 1224.6754 | 0.87 | 0 | 69 | 3.3e-07 | 1Score **> 32** indicates **identity** Score **> 17** indicates **homology** | U | R.IPFLLSGTSYK.D |
| 35424 | 63 | – | 73 | 613.3455 | 1224.6765 | 1224.6754 | 0.87 | 0 | 77 | 6.1e-08 | 1Score **> 32** indicates **identity** Score **> 17** indicates **homology** | U | R.IPFLLSGTSYK.D |
| 35425 | 63 | – | 73 | 613.3456 | 1224.6767 | 1224.6754 | 1.04 | 0 | 60 | 2.2e-06 | 1Score **> 32** indicates **identity** Score **> 16** indicates **homology** | U | R.IPFLLSGTSYK.D |
| 35426 | 63 | – | 73 | 613.3465 | 1224.6784 | 1224.6754 | 2.46 | 0 | 53 | 9.8e-06 | 1Score **> 32** indicates **identity** Score **> 16** indicates **homology** | U | R.IPFLLSGTSYK.D |
| 142111 | 63 | – | 82 | 569.3033 | 2273.1840 | 2273.1878 | -1.69 | 1 | 32 | 0.001 | 1Score **> 37** indicates **identity** Score **> 14** indicates **homology** | U | R.IPFLLSGTSYKDLMPHDLAR.A |
| 142116 | 63 | – | 82 | 569.3041 | 2273.1875 | 2273.1878 | -0.14 | 1 | 32 | 0.0011 | 1Score **> 37** indicates **identity** Score **> 14** indicates **homology** | U | R.IPFLLSGTSYKDLMPHDLAR.A |
| 142118 | 63 | – | 82 | 569.3043 | 2273.1883 | 2273.1878 | 0.21 | 1 | 48 | 3.2e-05 | 1Score **> 37** indicates **identity** Score **> 15** indicates **homology** | U | R.IPFLLSGTSYKDLMPHDLAR.A |
| 142119 | 63 | – | 82 | 569.3046 | 2273.1892 | 2273.1878 | 0.61 | 1 | 47 | 3.9e-05 | 1Score **> 37** indicates **identity** Score **> 15** indicates **homology** | U | R.IPFLLSGTSYKDLMPHDLAR.A |
| 142120 | 63 | – | 82 | 569.3046 | 2273.1893 | 2273.1878 | 0.64 | 1 | 29 | 0.002 | 1Score **> 37** indicates **identity** Score **> 14** indicates **homology** | U | R.IPFLLSGTSYKDLMPHDLAR.A |
| 142121 | 63 | – | 82 | 758.7371 | 2273.1895 | 2273.1878 | 0.75 | 1 | 51 | 1.8e-05 | 1Score **> 37** indicates **identity** Score **> 16** indicates **homology** | U | R.IPFLLSGTSYKDLMPHDLAR.A |
| 142122 | 63 | – | 82 | 569.3047 | 2273.1897 | 2273.1878 | 0.82 | 1 | 37 | 0.00036 | 1Score **> 37** indicates **identity** Score **> 15** indicates **homology** | U | R.IPFLLSGTSYKDLMPHDLAR.A |
| 142123 | 63 | – | 82 | 569.3048 | 2273.1902 | 2273.1878 | 1.07 | 1 | 39 | 0.00024 | 1Score **> 37** indicates **identity** Score **> 15** indicates **homology** | U | R.IPFLLSGTSYKDLMPHDLAR.A |
| 142124 | 63 | – | 82 | 758.7379 | 2273.1919 | 2273.1878 | 1.82 | 1 | 39 | 0.00021 | 1Score **> 37** indicates **identity** Score **> 15** indicates **homology** | U | R.IPFLLSGTSYKDLMPHDLAR.A |
| 142125 | 63 | – | 82 | 758.7381 | 2273.1924 | 2273.1878 | 2.01 | 1 | 15 | 0.036 | 1Score **> 37** indicates **identity** Score **> 13** indicates **homology** | U | R.IPFLLSGTSYKDLMPHDLAR.A |
| 142126 | 63 | – | 82 | 758.7386 | 2273.1941 | 2273.1878 | 2.77 | 1 | 47 | 4.4e-05 | 1Score **> 37** indicates **identity** Score **> 15** indicates **homology** | U | R.IPFLLSGTSYKDLMPHDLAR.A |
| 143053 | 63 | – | 82 | 573.3064 | 2289.1966 | 2289.1827 | 6.06 | 1 | 29 | 0.0018 | 1Score **> 37** indicates **identity** Score **> 14** indicates **homology** | U | R.IPFLLSGTSYKDLMPHDLAR.A  + Oxidation (M) |
| 143054 | 63 | – | 82 | 764.0730 | 2289.1970 | 2289.1827 | 6.25 | 1 | 29 | 0.0021 | 1Score **> 37** indicates **identity** Score **> 14** indicates **homology** | U | R.IPFLLSGTSYKDLMPHDLAR.A  + Oxidation (M) |
| 143055 | 63 | – | 82 | 764.0731 | 2289.1976 | 2289.1827 | 6.51 | 1 | 14 | 0.044 | 1Score **> 37** indicates **identity** Score **> 13** indicates **homology** | U | R.IPFLLSGTSYKDLMPHDLAR.A  + Oxidation (M) |
| 18971 | 74 | – | 82 | 356.5147 | 1066.5224 | 1066.5229 | -0.51 | 0 | 22 | 0.0096 | 1Score **> 30** indicates **identity** Score **> 14** indicates **homology** | U | K.DLMPHDLAR.A |
| 18972 | 74 | – | 82 | 356.5148 | 1066.5225 | 1066.5229 | -0.38 | 0 | 16 | 0.034 | 1Score **> 30** indicates **identity** Score **> 13** indicates **homology** | U | K.DLMPHDLAR.A |
| 10340 | 83 | – | 91 | 469.2824 | 936.5502 | 936.5505 | -0.32 | 0 | 49 | 0.00017 | 1Score **> 23** indicates **identity** | U | R.AALSGLLHR.T |
| 10341 | 83 | – | 91 | 469.2825 | 936.5504 | 936.5505 | -0.12 | 0 | 73 | 1.4e-07 | 1Score **> 23** indicates **identity** Score **> 17** indicates **homology** | U | R.AALSGLLHR.T |
| 10342 | 83 | – | 91 | 469.2826 | 936.5506 | 936.5505 | 0.088 | 0 | 63 | 1.2e-06 | 1Score **> 23** indicates **identity** Score **> 16** indicates **homology** | U | R.AALSGLLHR.T |
| 10343 | 83 | – | 91 | 469.2826 | 936.5507 | 936.5505 | 0.29 | 0 | 76 | 8e-08 | 1Score **> 23** indicates **identity** Score **> 17** indicates **homology** | U | R.AALSGLLHR.T |
| 10344 | 83 | – | 91 | 469.2827 | 936.5509 | 936.5505 | 0.42 | 0 | 73 | 1.4e-07 | 1Score **> 23** indicates **identity** Score **> 17** indicates **homology** | U | R.AALSGLLHR.T |
| 149438 | 92 | – | 112 | 797.7758 | 2390.3056 | 2390.3097 | -1.72 | 1 | 23 | 0.007 | 1Score **> 35** indicates **identity** Score **> 14** indicates **homology** | U | R.TNIPKDVVDYIIFGTVIQEVK.T |
| 149440 | 92 | – | 112 | 797.7760 | 2390.3062 | 2390.3097 | -1.48 | 1 | 47 | 4.1e-05 | 1Score **> 35** indicates **identity** Score **> 15** indicates **homology** | U | R.TNIPKDVVDYIIFGTVIQEVK.T |
| 149441 | 92 | – | 112 | 797.7765 | 2390.3078 | 2390.3097 | -0.81 | 1 | 52 | 1.4e-05 | 1Score **> 35** indicates **identity** Score **> 16** indicates **homology** | U | R.TNIPKDVVDYIIFGTVIQEVK.T |
| 149443 | 92 | – | 112 | 598.5844 | 2390.3085 | 2390.3097 | -0.50 | 1 | 39 | 0.00023 | 1Score **> 35** indicates **identity** Score **> 15** indicates **homology** | U | R.TNIPKDVVDYIIFGTVIQEVK.T |
| 149444 | 92 | – | 112 | 1196.1620 | 2390.3094 | 2390.3097 | -0.13 | 1 | 46 | 5.1e-05 | 1Score **> 35** indicates **identity** Score **> 15** indicates **homology** | U | R.TNIPKDVVDYIIFGTVIQEVK.T |
| 149446 | 92 | – | 112 | 1196.1621 | 2390.3097 | 2390.3097 | 0.0071 | 1 | 92 | 2.3e-09 | 1Score **> 35** indicates **identity** Score **> 18** indicates **homology** | U | R.TNIPKDVVDYIIFGTVIQEVK.T |
| 149449 | 92 | – | 112 | 797.7773 | 2390.3101 | 2390.3097 | 0.15 | 1 | 61 | 1.8e-06 | 1Score **> 35** indicates **identity** Score **> 16** indicates **homology** | U | R.TNIPKDVVDYIIFGTVIQEVK.T |
| 149452 | 92 | – | 112 | 598.5853 | 2390.3121 | 2390.3097 | 0.98 | 1 | 47 | 3.9e-05 | 1Score **> 35** indicates **identity** Score **> 15** indicates **homology** | U | R.TNIPKDVVDYIIFGTVIQEVK.T |
| 149455 | 92 | – | 112 | 797.7782 | 2390.3127 | 2390.3097 | 1.24 | 1 | 45 | 6.2e-05 | 1Score **> 35** indicates **identity** Score **> 15** indicates **homology** | U | R.TNIPKDVVDYIIFGTVIQEVK.T |
| 149457 | 92 | – | 112 | 797.7783 | 2390.3130 | 2390.3097 | 1.36 | 1 | 48 | 3.1e-05 | 1Score **> 35** indicates **identity** Score **> 16** indicates **homology** | U | R.TNIPKDVVDYIIFGTVIQEVK.T |
| 149458 | 92 | – | 112 | 797.7786 | 2390.3141 | 2390.3097 | 1.83 | 1 | 57 | 4.9e-06 | 1Score **> 35** indicates **identity** Score **> 16** indicates **homology** | U | R.TNIPKDVVDYIIFGTVIQEVK.T |
| 149459 | 92 | – | 112 | 797.7788 | 2390.3144 | 2390.3097 | 1.97 | 1 | 55 | 6.7e-06 | 1Score **> 35** indicates **identity** Score **> 16** indicates **homology** | U | R.TNIPKDVVDYIIFGTVIQEVK.T |
| 149460 | 92 | – | 112 | 797.7788 | 2390.3145 | 2390.3097 | 2.01 | 1 | 44 | 8.2e-05 | 1Score **> 35** indicates **identity** Score **> 15** indicates **homology** | U | R.TNIPKDVVDYIIFGTVIQEVK.T |
| 149462 | 92 | – | 112 | 797.7792 | 2390.3158 | 2390.3097 | 2.56 | 1 | 61 | 2.1e-06 | 1Score **> 35** indicates **identity** Score **> 16** indicates **homology** | U | R.TNIPKDVVDYIIFGTVIQEVK.T |
| 149463 | 92 | – | 112 | 797.7793 | 2390.3160 | 2390.3097 | 2.61 | 1 | 30 | 0.0017 | 1Score **> 35** indicates **identity** Score **> 14** indicates **homology** | U | R.TNIPKDVVDYIIFGTVIQEVK.T |
| 149464 | 92 | – | 112 | 797.7802 | 2390.3187 | 2390.3097 | 3.77 | 1 | 20 | 0.013 | 1Score **> 35** indicates **identity** Score **> 14** indicates **homology** | U | R.TNIPKDVVDYIIFGTVIQEVK.T |
| 149537 | 92 | – | 112 | 1196.6636 | 2391.3127 | 2391.2937 | 7.94 | 1 | 84 | 1.5e-08 | 1Score **> 35** indicates **identity** Score **> 18** indicates **homology** | U | R.TNIPKDVVDYIIFGTVIQEVK.T  + Deamidated (NQ) |
| 173845 | 92 | – | 118 | 755.6717 | 3018.6577 | 3018.6390 | 6.20 | 2 | 18 | 0.021 | 1Score **> 34** indicates **identity** Score **> 14** indicates **homology** | U | R.TNIPKDVVDYIIFGTVIQEVKTSNVAR.E |
| 173873 | 92 | – | 118 | 1007.5543 | 3019.6409 | 3019.6230 | 5.94 | 2 | 46 | 5.1e-05 | 1Score **> 34** indicates **identity** Score **> 15** indicates **homology** | U | R.TNIPKDVVDYIIFGTVIQEVKTSNVAR.E  + Deamidated (NQ) |
| 105202 | 97 | – | 112 | 613.3364 | 1836.9874 | 1836.9873 | 0.041 | 0 | 55 | 7.7e-06 | 1Score **> 35** indicates **identity** Score **> 16** indicates **homology** | U | K.DVVDYIIFGTVIQEVK.T |
| 105204 | 97 | – | 112 | 613.3366 | 1836.9881 | 1836.9873 | 0.42 | 0 | 43 | 9.3e-05 | 1Score **> 35** indicates **identity** Score **> 15** indicates **homology** | U | K.DVVDYIIFGTVIQEVK.T |
| 105205 | 97 | – | 112 | 919.5013 | 1836.9881 | 1836.9873 | 0.43 | 0 | 67 | 7.3e-07 | 1Score **> 35** indicates **identity** Score **> 19** indicates **homology** | U | K.DVVDYIIFGTVIQEVK.T |
| 105206 | 97 | – | 112 | 613.3367 | 1836.9882 | 1836.9873 | 0.46 | 0 | 18 | 0.021 | 1Score **> 35** indicates **identity** Score **> 14** indicates **homology** | U | K.DVVDYIIFGTVIQEVK.T |
| 105207 | 97 | – | 112 | 613.3367 | 1836.9883 | 1836.9873 | 0.50 | 0 | 24 | 0.0056 | 1Score **> 35** indicates **identity** Score **> 14** indicates **homology** | U | K.DVVDYIIFGTVIQEVK.T |
| 105209 | 97 | – | 112 | 613.3369 | 1836.9887 | 1836.9873 | 0.76 | 0 | 26 | 0.0038 | 1Score **> 35** indicates **identity** Score **> 14** indicates **homology** | U | K.DVVDYIIFGTVIQEVK.T |
| 105210 | 97 | – | 112 | 613.3369 | 1836.9888 | 1836.9873 | 0.82 | 0 | 19 | 0.018 | 1Score **> 35** indicates **identity** Score **> 14** indicates **homology** | U | K.DVVDYIIFGTVIQEVK.T |
| 105211 | 97 | – | 112 | 919.5022 | 1836.9899 | 1836.9873 | 1.41 | 0 | 31 | 0.0011 | 1Score **> 35** indicates **identity** Score **> 14** indicates **homology** | U | K.DVVDYIIFGTVIQEVK.T |
| 105212 | 97 | – | 112 | 613.3380 | 1836.9923 | 1836.9873 | 2.71 | 0 | 51 | 1.7e-05 | 1Score **> 35** indicates **identity** Score **> 16** indicates **homology** | U | K.DVVDYIIFGTVIQEVK.T |
| 105214 | 97 | – | 112 | 919.5038 | 1836.9931 | 1836.9873 | 3.16 | 0 | 24 | 0.0053 | 1Score **> 35** indicates **identity** Score **> 14** indicates **homology** | U | K.DVVDYIIFGTVIQEVK.T |
| 105220 | 97 | – | 112 | 613.3401 | 1836.9986 | 1836.9873 | 6.13 | 0 | 32 | 0.0011 | 1Score **> 35** indicates **identity** Score **> 14** indicates **homology** | U | K.DVVDYIIFGTVIQEVK.T |
| 18830 | 119 | – | 129 | 533.2627 | 1064.5109 | 1064.5138 | -2.71 | 0 | 31 | 0.0012 | 1Score **> 31** indicates **identity** Score **> 14** indicates **homology** | U | R.EAALGAGFSDK.T |
| 18832 | 119 | – | 129 | 533.2633 | 1064.5121 | 1064.5138 | -1.59 | 0 | 61 | 1.6e-05 | 1Score **> 31** indicates **identity** Score **> 26** indicates **homology** | U | R.EAALGAGFSDK.T |
| 188947 | 119 | – | 175 | 989.8230 | 5932.8942 | 5932.9030 | -1.48 | 1 | 30 | 0.0017 | 1Score **> 35** indicates **identity** Score **> 14** indicates **homology** | U | R.EAALGAGFSDKTPAHTVTMACISSNQAMTTAVGLIASGQCDVVVAGGVELMSDVPIR.H  + HNE (H); Oxidation (M) |
| 188950 | 119 | – | 175 | 1187.7866 | 5933.8967 | 5933.8870 | 1.64 | 1 | 24 | 0.0052 | 1Score **> 35** indicates **identity** Score **> 14** indicates **homology** | U | R.EAALGAGFSDKTPAHTVTMACISSNQAMTTAVGLIASGQCDVVVAGGVELMSDVPIR.H  + Deamidated (NQ); HNE (H); Oxidation (M) |
| 176050 | 203 | – | 230 | 1046.1756 | 3135.5050 | 3135.5084 | -1.09 | 1 | 60 | 2.5e-06 | 1Score **> 37** indicates **identity** Score **> 16** indicates **homology** | U | K.FRLNFLSPELPAVAEFSTNETMGHSADR.L |
| 176051 | 203 | – | 230 | 784.8856 | 3135.5133 | 3135.5084 | 1.59 | 1 | 47 | 3.6e-05 | 1Score **> 37** indicates **identity** Score **> 15** indicates **homology** | U | K.FRLNFLSPELPAVAEFSTNETMGHSADR.L |
| 168402 | 205 | – | 230 | 945.1206 | 2832.3399 | 2832.3388 | 0.37 | 0 | 52 | 1.5e-05 | 1Score **> 36** indicates **identity** Score **> 16** indicates **homology** | U | R.LNFLSPELPAVAEFSTNETMGHSADR.L |
| 168404 | 205 | – | 230 | 945.1212 | 2832.3417 | 2832.3388 | 1.00 | 0 | 59 | 3e-06 | 1Score **> 36** indicates **identity** Score **> 16** indicates **homology** | U | R.LNFLSPELPAVAEFSTNETMGHSADR.L |
| 168917 | 205 | – | 230 | 950.4509 | 2848.3309 | 2848.3338 | -1.00 | 0 | 30 | 0.0015 | 1Score **> 35** indicates **identity** Score **> 14** indicates **homology** | U | R.LNFLSPELPAVAEFSTNETMGHSADR.L  + Oxidation (M) |
| 8546 | 231 | – | 239 | 453.2633 | 904.5120 | 904.5130 | -1.15 | 0 | 30 | 0.035 | 1Score **> 29** indicates **identity** Score **> 28** indicates **homology** | U | R.LAAAFAVSR.M |
| 8547 | 231 | – | 239 | 453.2634 | 904.5122 | 904.5130 | -0.89 | 0 | 66 | 1e-05 | 1Score **> 29** indicates **identity** | U | R.LAAAFAVSR.M |
| 8548 | 231 | – | 239 | 453.2634 | 904.5122 | 904.5130 | -0.88 | 0 | 58 | 5.6e-05 | 1Score **> 29** indicates **identity** Score **> 28** indicates **homology** | U | R.LAAAFAVSR.M |
| 8549 | 231 | – | 239 | 453.2635 | 904.5124 | 904.5130 | -0.66 | 0 | 66 | 1e-05 | 1Score **> 29** indicates **identity** Score **> 28** indicates **homology** | U | R.LAAAFAVSR.M |
| 8551 | 231 | – | 239 | 453.2635 | 904.5125 | 904.5130 | -0.60 | 0 | 29 | 0.013 | 1Score **> 29** indicates **identity** Score **> 22** indicates **homology** | U | R.LAAAFAVSR.M |
| 8552 | 231 | – | 239 | 453.2636 | 904.5126 | 904.5130 | -0.47 | 0 | 53 | 0.0002 | 1Score **> 29** indicates **identity** | U | R.LAAAFAVSR.M |
| 8553 | 231 | – | 239 | 453.2636 | 904.5127 | 904.5130 | -0.41 | 0 | 29 | 0.042 | 1Score **> 29** indicates **identity** Score **> 28** indicates **homology** | U | R.LAAAFAVSR.M |
| 8554 | 231 | – | 239 | 453.2638 | 904.5130 | 904.5130 | -0.0077 | 0 | 38 | 0.0029 | 1Score **> 29** indicates **identity** Score **> 25** indicates **homology** | U | R.LAAAFAVSR.M |
| 8555 | 231 | – | 239 | 453.2638 | 904.5130 | 904.5130 | -0.0011 | 0 | 68 | 6.5e-06 | 1Score **> 29** indicates **identity** | U | R.LAAAFAVSR.M |
| 8557 | 231 | – | 239 | 453.2640 | 904.5135 | 904.5130 | 0.54 | 0 | 49 | 0.00049 | 1Score **> 29** indicates **identity** | U | R.LAAAFAVSR.M |
| 8558 | 231 | – | 239 | 453.2641 | 904.5136 | 904.5130 | 0.60 | 0 | 55 | 0.00012 | 1Score **> 29** indicates **identity** | U | R.LAAAFAVSR.M |
| 8559 | 231 | – | 239 | 453.2642 | 904.5138 | 904.5130 | 0.87 | 0 | 47 | 0.00086 | 1Score **> 29** indicates **identity** | U | R.LAAAFAVSR.M |
| 8560 | 231 | – | 239 | 453.2642 | 904.5139 | 904.5130 | 0.95 | 0 | 30 | 0.014 | 1Score **> 29** indicates **identity** Score **> 24** indicates **homology** | U | R.LAAAFAVSR.M |
| 8561 | 231 | – | 239 | 453.2644 | 904.5142 | 904.5130 | 1.33 | 0 | 27 | 0.038 | 1Score **> 28** indicates **identity** Score **> 26** indicates **homology** | U | R.LAAAFAVSR.M |
| 8563 | 231 | – | 239 | 453.2646 | 904.5147 | 904.5130 | 1.83 | 0 | 40 | 0.0025 | 1Score **> 27** indicates **identity** Score **> 27** indicates **homology** | U | R.LAAAFAVSR.M |
| 8564 | 231 | – | 239 | 453.2648 | 904.5151 | 904.5130 | 2.29 | 0 | 29 | 0.035 | 1Score **> 27** indicates **identity** Score **> 27** indicates **homology** | U | R.LAAAFAVSR.M |
| 29328 | 240 | – | 248 | 585.7575 | 1169.5004 | 1169.5023 | -1.58 | 0 | 19 | 0.015 | 1Score **> 26** indicates **identity** Score **> 14** indicates **homology** | U | R.MEQDEYALR.S  + Oxidation (M) |
| 29329 | 240 | – | 248 | 585.7583 | 1169.5020 | 1169.5023 | -0.22 | 0 | 34 | 0.00082 | 1Score **> 26** indicates **identity** Score **> 15** indicates **homology** | U | R.MEQDEYALR.S  + Oxidation (M) |
| 29330 | 240 | – | 248 | 585.7584 | 1169.5022 | 1169.5023 | -0.082 | 0 | 42 | 0.00012 | 1Score **> 26** indicates **identity** Score **> 15** indicates **homology** | U | R.MEQDEYALR.S  + Oxidation (M) |
| 29331 | 240 | – | 248 | 585.7584 | 1169.5023 | 1169.5023 | -0.0051 | 0 | 42 | 0.00021 | 1Score **> 26** indicates **identity** Score **> 18** indicates **homology** | U | R.MEQDEYALR.S  + Oxidation (M) |
| 29332 | 240 | – | 248 | 585.7585 | 1169.5024 | 1169.5023 | 0.10 | 0 | 52 | 1.5e-05 | 1Score **> 26** indicates **identity** Score **> 17** indicates **homology** | U | R.MEQDEYALR.S  + Oxidation (M) |
| 29333 | 240 | – | 248 | 585.7587 | 1169.5029 | 1169.5023 | 0.51 | 0 | 58 | 5.9e-06 | 1Score **> 26** indicates **identity** Score **> 19** indicates **homology** | U | R.MEQDEYALR.S  + Oxidation (M) |
| 29334 | 240 | – | 248 | 585.7587 | 1169.5029 | 1169.5023 | 0.52 | 0 | 35 | 0.00057 | 1Score **> 26** indicates **identity** Score **> 15** indicates **homology** | U | R.MEQDEYALR.S  + Oxidation (M) |
| 29335 | 240 | – | 248 | 585.7589 | 1169.5033 | 1169.5023 | 0.86 | 0 | 43 | 8.7e-05 | 1Score **> 26** indicates **identity** Score **> 15** indicates **homology** | U | R.MEQDEYALR.S  + Oxidation (M) |
| 29336 | 240 | – | 248 | 585.7591 | 1169.5036 | 1169.5023 | 1.16 | 0 | 25 | 0.005 | 1Score **> 26** indicates **identity** Score **> 14** indicates **homology** | U | R.MEQDEYALR.S  + Oxidation (M) |
| 29338 | 240 | – | 248 | 585.7591 | 1169.5037 | 1169.5023 | 1.23 | 0 | 44 | 8.5e-05 | 1Score **> 26** indicates **identity** Score **> 15** indicates **homology** | U | R.MEQDEYALR.S  + Oxidation (M) |
| 29339 | 240 | – | 248 | 585.7592 | 1169.5037 | 1169.5023 | 1.27 | 0 | 38 | 0.00053 | 1Score **> 26** indicates **identity** Score **> 18** indicates **homology** | U | R.MEQDEYALR.S  + Oxidation (M) |
| 29340 | 240 | – | 248 | 585.7592 | 1169.5039 | 1169.5023 | 1.39 | 0 | 22 | 0.0083 | 1Score **> 26** indicates **identity** Score **> 14** indicates **homology** | U | R.MEQDEYALR.S  + Oxidation (M) |
| 29341 | 240 | – | 248 | 585.7593 | 1169.5040 | 1169.5023 | 1.47 | 0 | 44 | 7.2e-05 | 1Score **> 26** indicates **identity** Score **> 15** indicates **homology** | U | R.MEQDEYALR.S  + Oxidation (M) |
| 29342 | 240 | – | 248 | 585.7593 | 1169.5041 | 1169.5023 | 1.60 | 0 | 25 | 0.0049 | 1Score **> 26** indicates **identity** Score **> 14** indicates **homology** | U | R.MEQDEYALR.S  + Oxidation (M) |
| 29343 | 240 | – | 248 | 585.7599 | 1169.5052 | 1169.5023 | 2.49 | 0 | 46 | 7e-05 | 1Score **> 26** indicates **identity** Score **> 17** indicates **homology** | U | R.MEQDEYALR.S  + Oxidation (M) |
| 29344 | 240 | – | 248 | 585.7599 | 1169.5052 | 1169.5023 | 2.51 | 0 | 40 | 0.00016 | 1Score **> 26** indicates **identity** Score **> 15** indicates **homology** | U | R.MEQDEYALR.S  + Oxidation (M) |
| 29345 | 240 | – | 248 | 585.7608 | 1169.5071 | 1169.5023 | 4.17 | 0 | 31 | 0.0022 | 1Score **> 26** indicates **identity** Score **> 16** indicates **homology** | U | R.MEQDEYALR.S  + Oxidation (M) |
| 125344 | 255 | – | 273 | 689.0400 | 2064.0983 | 2064.1004 | -1.01 | 2 | 24 | 0.0055 | 1Score **> 36** indicates **identity** Score **> 14** indicates **homology** | U | K.KAQDEGHLSDIVPFKVPGK.D |
| 125345 | 255 | – | 273 | 517.0328 | 2064.1020 | 2064.1004 | 0.78 | 2 | 34 | 0.00058 | 1Score **> 36** indicates **identity** Score **> 15** indicates **homology** | U | K.KAQDEGHLSDIVPFKVPGK.D |
| 125350 | 255 | – | 273 | 517.0330 | 2064.1028 | 2064.1004 | 1.17 | 2 | 25 | 0.0041 | 1Score **> 36** indicates **identity** Score **> 14** indicates **homology** | U | K.KAQDEGHLSDIVPFKVPGK.D |
| 114051 | 256 | – | 273 | 485.0088 | 1936.0062 | 1936.0054 | 0.40 | 1 | 15 | 0.038 | 1Score **> 36** indicates **identity** Score **> 13** indicates **homology** | U | K.AQDEGHLSDIVPFKVPGK.D |
| 154318 | 256 | – | 278 | 621.0804 | 2480.2925 | 2480.2911 | 0.54 | 2 | 28 | 0.0024 | 1Score **> 37** indicates **identity** Score **> 14** indicates **homology** | U | K.AQDEGHLSDIVPFKVPGKDTVTK.D |
| 154320 | 256 | – | 278 | 497.0660 | 2480.2938 | 2480.2911 | 1.08 | 2 | 15 | 0.04 | 1Score **> 37** indicates **identity** Score **> 13** indicates **homology** | U | K.AQDEGHLSDIVPFKVPGKDTVTK.D |
| 154323 | 256 | – | 278 | 621.0809 | 2480.2946 | 2480.2911 | 1.39 | 2 | 20 | 0.012 | 1Score **> 37** indicates **identity** Score **> 14** indicates **homology** | U | K.AQDEGHLSDIVPFKVPGKDTVTK.D |
| 70918 | 279 | – | 292 | 515.9257 | 1544.7551 | 1544.7617 | -4.22 | 1 | 26 | 0.0035 | 1Score **> 33** indicates **identity** Score **> 14** indicates **homology** | U | K.DNGIRPSSLEQMAK.L |
| 70919 | 279 | – | 292 | 773.3851 | 1544.7556 | 1544.7617 | -3.95 | 1 | 39 | 0.0039 | 1Score **> 33** indicates **identity** Score **> 28** indicates **homology** | U | K.DNGIRPSSLEQMAK.L |
| 72785 | 279 | – | 292 | 781.3853 | 1560.7560 | 1560.7566 | -0.34 | 1 | 26 | 0.027 | 1Score **> 33** indicates **identity** Score **> 23** indicates **homology** | U | K.DNGIRPSSLEQMAK.L  + Oxidation (M) |
| 92168 | 336 | – | 349 | 572.2984 | 1713.8733 | 1713.8726 | 0.39 | 1 | 20 | 0.014 | 1Score **> 35** indicates **identity** Score **> 14** indicates **homology** | U | K.AYLRDFIYVSQDPK.D |
| 92170 | 336 | – | 349 | 857.9440 | 1713.8735 | 1713.8726 | 0.53 | 1 | 49 | 0.00012 | 1Score **> 35** indicates **identity** Score **> 22** indicates **homology** | U | K.AYLRDFIYVSQDPK.D |
| 92174 | 336 | – | 349 | 857.9447 | 1713.8748 | 1713.8726 | 1.29 | 1 | 23 | 0.018 | 1Score **> 35** indicates **identity** Score **> 18** indicates **homology** | U | K.AYLRDFIYVSQDPK.D |
| 92182 | 336 | – | 349 | 572.3003 | 1713.8792 | 1713.8726 | 3.86 | 1 | 19 | 0.015 | 1Score **> 35** indicates **identity** Score **> 14** indicates **homology** | U | K.AYLRDFIYVSQDPK.D |
| 175698 | 336 | – | 362 | 778.9127 | 3111.6217 | 3111.6281 | -2.05 | 2 | 41 | 0.00014 | 1Score **> 37** indicates **identity** Score **> 15** indicates **homology** | U | K.AYLRDFIYVSQDPKDQLLLGPTYATPK.V |
| 175699 | 336 | – | 362 | 1038.2151 | 3111.6234 | 3111.6281 | -1.52 | 2 | 17 | 0.025 | 1Score **> 37** indicates **identity** Score **> 14** indicates **homology** | U | K.AYLRDFIYVSQDPKDQLLLGPTYATPK.V |
| 175700 | 336 | – | 362 | 1038.2167 | 3111.6283 | 3111.6281 | 0.090 | 2 | 28 | 0.0024 | 1Score **> 37** indicates **identity** Score **> 14** indicates **homology** | U | K.AYLRDFIYVSQDPKDQLLLGPTYATPK.V |
| 175701 | 336 | – | 362 | 778.9145 | 3111.6288 | 3111.6281 | 0.25 | 2 | 39 | 0.00021 | 1Score **> 37** indicates **identity** Score **> 15** indicates **homology** | U | K.AYLRDFIYVSQDPKDQLLLGPTYATPK.V |
| 175702 | 336 | – | 362 | 1038.2171 | 3111.6293 | 3111.6281 | 0.41 | 2 | 75 | 9.9e-08 | 1Score **> 37** indicates **identity** Score **> 17** indicates **homology** | U | K.AYLRDFIYVSQDPKDQLLLGPTYATPK.V |
| 175703 | 336 | – | 362 | 1038.2171 | 3111.6294 | 3111.6281 | 0.43 | 2 | 61 | 1.8e-06 | 1Score **> 37** indicates **identity** Score **> 16** indicates **homology** | U | K.AYLRDFIYVSQDPKDQLLLGPTYATPK.V |
| 175704 | 336 | – | 362 | 1038.2173 | 3111.6299 | 3111.6281 | 0.60 | 2 | 75 | 9e-08 | 1Score **> 37** indicates **identity** Score **> 17** indicates **homology** | U | K.AYLRDFIYVSQDPKDQLLLGPTYATPK.V |
| 175705 | 336 | – | 362 | 778.9150 | 3111.6308 | 3111.6281 | 0.88 | 2 | 50 | 2.1e-05 | 1Score **> 37** indicates **identity** Score **> 16** indicates **homology** | U | K.AYLRDFIYVSQDPKDQLLLGPTYATPK.V |
| 175706 | 336 | – | 362 | 778.9150 | 3111.6309 | 3111.6281 | 0.90 | 2 | 54 | 8.8e-06 | 1Score **> 37** indicates **identity** Score **> 16** indicates **homology** | U | K.AYLRDFIYVSQDPKDQLLLGPTYATPK.V |
| 175707 | 336 | – | 362 | 778.9150 | 3111.6310 | 3111.6281 | 0.93 | 2 | 40 | 0.00019 | 1Score **> 37** indicates **identity** Score **> 15** indicates **homology** | U | K.AYLRDFIYVSQDPKDQLLLGPTYATPK.V |
| 175708 | 336 | – | 362 | 778.9161 | 3111.6351 | 3111.6281 | 2.27 | 2 | 35 | 0.00052 | 1Score **> 37** indicates **identity** Score **> 15** indicates **homology** | U | K.AYLRDFIYVSQDPKDQLLLGPTYATPK.V |
| 175725 | 336 | – | 362 | 779.1649 | 3112.6306 | 3112.6121 | 5.94 | 2 | 14 | 0.045 | 1Score **> 37** indicates **identity** Score **> 13** indicates **homology** | U | K.AYLRDFIYVSQDPKDQLLLGPTYATPK.V  + Deamidated (NQ) |
| 175726 | 336 | – | 362 | 1038.5510 | 3112.6312 | 3112.6121 | 6.13 | 2 | 22 | 0.008 | 1Score **> 37** indicates **identity** Score **> 14** indicates **homology** | U | K.AYLRDFIYVSQDPKDQLLLGPTYATPK.V  + Deamidated (NQ) |
| 175727 | 336 | – | 362 | 779.1659 | 3112.6345 | 3112.6121 | 7.21 | 2 | 16 | 0.031 | 1Score **> 37** indicates **identity** Score **> 14** indicates **homology** | U | K.AYLRDFIYVSQDPKDQLLLGPTYATPK.V  + Deamidated (NQ) |
| 175728 | 336 | – | 362 | 1038.5523 | 3112.6352 | 3112.6121 | 7.41 | 2 | 32 | 0.0011 | 1Score **> 37** indicates **identity** Score **> 15** indicates **homology** | U | K.AYLRDFIYVSQDPKDQLLLGPTYATPK.V  + Deamidated (NQ) |
| 175730 | 336 | – | 362 | 1038.5528 | 3112.6367 | 3112.6121 | 7.91 | 2 | 23 | 0.0076 | 1Score **> 37** indicates **identity** Score **> 14** indicates **homology** | U | K.AYLRDFIYVSQDPKDQLLLGPTYATPK.V  + Deamidated (NQ) |
| 33833 | 340 | – | 349 | 606.3002 | 1210.5859 | 1210.5870 | -0.93 | 0 | 31 | 0.0013 | 1Score **> 31** indicates **identity** Score **> 14** indicates **homology** | U | R.DFIYVSQDPK.D |
| 33834 | 340 | – | 349 | 606.3007 | 1210.5869 | 1210.5870 | -0.10 | 0 | 22 | 0.0085 | 1Score **> 32** indicates **identity** Score **> 14** indicates **homology** | U | R.DFIYVSQDPK.D |
| 159991 | 340 | – | 362 | 870.4502 | 2608.3287 | 2608.3425 | -5.26 | 1 | 26 | 0.0035 | 1Score **> 37** indicates **identity** Score **> 14** indicates **homology** | U | R.DFIYVSQDPKDQLLLGPTYATPK.V |
| 159992 | 340 | – | 362 | 870.4513 | 2608.3322 | 2608.3425 | -3.94 | 1 | 25 | 0.0046 | 1Score **> 37** indicates **identity** Score **> 14** indicates **homology** | U | R.DFIYVSQDPKDQLLLGPTYATPK.V |
| 159994 | 340 | – | 362 | 870.4520 | 2608.3342 | 2608.3425 | -3.17 | 1 | 25 | 0.0053 | 1Score **> 37** indicates **identity** Score **> 15** indicates **homology** | U | R.DFIYVSQDPKDQLLLGPTYATPK.V |
| 159996 | 340 | – | 362 | 870.4531 | 2608.3374 | 2608.3425 | -1.95 | 1 | 27 | 0.003 | 1Score **> 37** indicates **identity** Score **> 14** indicates **homology** | U | R.DFIYVSQDPKDQLLLGPTYATPK.V |
| 159998 | 340 | – | 362 | 870.4535 | 2608.3388 | 2608.3425 | -1.43 | 1 | 57 | 4.1e-06 | 1Score **> 37** indicates **identity** Score **> 16** indicates **homology** | U | R.DFIYVSQDPKDQLLLGPTYATPK.V |
| 160000 | 340 | – | 362 | 870.4540 | 2608.3403 | 2608.3425 | -0.84 | 1 | 39 | 0.00024 | 1Score **> 37** indicates **identity** Score **> 15** indicates **homology** | U | R.DFIYVSQDPKDQLLLGPTYATPK.V |
| 160001 | 340 | – | 362 | 870.4542 | 2608.3408 | 2608.3425 | -0.65 | 1 | 27 | 0.0031 | 1Score **> 37** indicates **identity** Score **> 14** indicates **homology** | U | R.DFIYVSQDPKDQLLLGPTYATPK.V |
| 160002 | 340 | – | 362 | 870.4543 | 2608.3412 | 2608.3425 | -0.49 | 1 | 21 | 0.01 | 1Score **> 37** indicates **identity** Score **> 14** indicates **homology** | U | R.DFIYVSQDPKDQLLLGPTYATPK.V |
| 160003 | 340 | – | 362 | 1305.1779 | 2608.3412 | 2608.3425 | -0.49 | 1 | 75 | 8.8e-08 | 1Score **> 37** indicates **identity** Score **> 17** indicates **homology** | U | R.DFIYVSQDPKDQLLLGPTYATPK.V |
| 160004 | 340 | – | 362 | 870.4544 | 2608.3415 | 2608.3425 | -0.39 | 1 | 54 | 8.6e-06 | 1Score **> 37** indicates **identity** Score **> 16** indicates **homology** | U | R.DFIYVSQDPKDQLLLGPTYATPK.V |
| 160006 | 340 | – | 362 | 870.4546 | 2608.3421 | 2608.3425 | -0.15 | 1 | 54 | 8.2e-06 | 1Score **> 37** indicates **identity** Score **> 16** indicates **homology** | U | R.DFIYVSQDPKDQLLLGPTYATPK.V |
| 160009 | 340 | – | 362 | 870.4549 | 2608.3430 | 2608.3425 | 0.18 | 1 | 27 | 0.0035 | 1Score **> 37** indicates **identity** Score **> 15** indicates **homology** | U | R.DFIYVSQDPKDQLLLGPTYATPK.V |
| 160010 | 340 | – | 362 | 870.4550 | 2608.3433 | 2608.3425 | 0.31 | 1 | 65 | 8.9e-07 | 1Score **> 37** indicates **identity** Score **> 17** indicates **homology** | U | R.DFIYVSQDPKDQLLLGPTYATPK.V |
| 160011 | 340 | – | 362 | 1305.1790 | 2608.3434 | 2608.3425 | 0.37 | 1 | 54 | 8.7e-06 | 1Score **> 37** indicates **identity** Score **> 16** indicates **homology** | U | R.DFIYVSQDPKDQLLLGPTYATPK.V |
| 160012 | 340 | – | 362 | 870.4552 | 2608.3437 | 2608.3425 | 0.46 | 1 | 68 | 4e-07 | 1Score **> 37** indicates **identity** Score **> 17** indicates **homology** | U | R.DFIYVSQDPKDQLLLGPTYATPK.V |
| 160013 | 340 | – | 362 | 1305.1792 | 2608.3439 | 2608.3425 | 0.55 | 1 | 61 | 2.1e-06 | 1Score **> 37** indicates **identity** Score **> 16** indicates **homology** | U | R.DFIYVSQDPKDQLLLGPTYATPK.V |
| 160015 | 340 | – | 362 | 870.4555 | 2608.3448 | 2608.3425 | 0.89 | 1 | 44 | 6.8e-05 | 1Score **> 37** indicates **identity** Score **> 15** indicates **homology** | U | R.DFIYVSQDPKDQLLLGPTYATPK.V |
| 160017 | 340 | – | 362 | 870.4560 | 2608.3461 | 2608.3425 | 1.38 | 1 | 51 | 1.8e-05 | 1Score **> 37** indicates **identity** Score **> 16** indicates **homology** | U | R.DFIYVSQDPKDQLLLGPTYATPK.V |
| 160019 | 340 | – | 362 | 870.4560 | 2608.3462 | 2608.3425 | 1.44 | 1 | 56 | 6.2e-06 | 1Score **> 37** indicates **identity** Score **> 16** indicates **homology** | U | R.DFIYVSQDPKDQLLLGPTYATPK.V |
| 160020 | 340 | – | 362 | 870.4561 | 2608.3464 | 2608.3425 | 1.51 | 1 | 34 | 0.00062 | 1Score **> 37** indicates **identity** Score **> 15** indicates **homology** | U | R.DFIYVSQDPKDQLLLGPTYATPK.V |
| 160021 | 340 | – | 362 | 870.4562 | 2608.3467 | 2608.3425 | 1.63 | 1 | 23 | 0.0089 | 1Score **> 37** indicates **identity** Score **> 15** indicates **homology** | U | R.DFIYVSQDPKDQLLLGPTYATPK.V |
| 160022 | 340 | – | 362 | 870.4562 | 2608.3468 | 2608.3425 | 1.67 | 1 | 29 | 0.0019 | 1Score **> 37** indicates **identity** Score **> 14** indicates **homology** | U | R.DFIYVSQDPKDQLLLGPTYATPK.V |
| 160027 | 340 | – | 362 | 870.4568 | 2608.3485 | 2608.3425 | 2.32 | 1 | 35 | 0.00048 | 1Score **> 37** indicates **identity** Score **> 15** indicates **homology** | U | R.DFIYVSQDPKDQLLLGPTYATPK.V |
| 160029 | 340 | – | 362 | 870.4572 | 2608.3499 | 2608.3425 | 2.86 | 1 | 24 | 0.0061 | 1Score **> 37** indicates **identity** Score **> 14** indicates **homology** | U | R.DFIYVSQDPKDQLLLGPTYATPK.V |
| 160030 | 340 | – | 362 | 1305.1824 | 2608.3503 | 2608.3425 | 2.99 | 1 | 42 | 0.00014 | 1Score **> 37** indicates **identity** Score **> 16** indicates **homology** | U | R.DFIYVSQDPKDQLLLGPTYATPK.V |
| 160032 | 340 | – | 362 | 870.4576 | 2608.3511 | 2608.3425 | 3.30 | 1 | 47 | 3.5e-05 | 1Score **> 37** indicates **identity** Score **> 15** indicates **homology** | U | R.DFIYVSQDPKDQLLLGPTYATPK.V |
| 160034 | 340 | – | 362 | 870.4586 | 2608.3539 | 2608.3425 | 4.37 | 1 | 18 | 0.021 | 1Score **> 37** indicates **identity** Score **> 14** indicates **homology** | U | R.DFIYVSQDPKDQLLLGPTYATPK.V |
| 160036 | 340 | – | 362 | 870.4592 | 2608.3558 | 2608.3425 | 5.11 | 1 | 30 | 0.0015 | 1Score **> 37** indicates **identity** Score **> 14** indicates **homology** | U | R.DFIYVSQDPKDQLLLGPTYATPK.V |
| 160038 | 340 | – | 362 | 870.4609 | 2608.3607 | 2608.3425 | 7.00 | 1 | 41 | 0.00013 | 1Score **> 37** indicates **identity** Score **> 15** indicates **homology** | U | R.DFIYVSQDPKDQLLLGPTYATPK.V |
| 160063 | 340 | – | 362 | 870.7901 | 2609.3484 | 2609.3265 | 8.41 | 1 | 16 | 0.031 | 1Score **> 37** indicates **identity** Score **> 14** indicates **homology** | U | R.DFIYVSQDPKDQLLLGPTYATPK.V  + Deamidated (NQ) |
| 55508 | 350 | – | 362 | 708.8896 | 1415.7647 | 1415.7660 | -0.90 | 0 | 35 | 0.00055 | 1Score **> 35** indicates **identity** Score **> 15** indicates **homology** | U | K.DQLLLGPTYATPK.V |
| 55510 | 350 | – | 362 | 708.8898 | 1415.7651 | 1415.7660 | -0.66 | 0 | 52 | 2.3e-05 | 1Score **> 35** indicates **identity** Score **> 18** indicates **homology** | U | K.DQLLLGPTYATPK.V |
| 55511 | 350 | – | 362 | 708.8900 | 1415.7655 | 1415.7660 | -0.39 | 0 | 63 | 2.4e-06 | 1Score **> 35** indicates **identity** Score **> 20** indicates **homology** | U | K.DQLLLGPTYATPK.V |
| 55512 | 350 | – | 362 | 708.8901 | 1415.7656 | 1415.7660 | -0.31 | 0 | 56 | 1.3e-05 | 1Score **> 35** indicates **identity** Score **> 19** indicates **homology** | U | K.DQLLLGPTYATPK.V |
| 55513 | 350 | – | 362 | 708.8902 | 1415.7658 | 1415.7660 | -0.14 | 0 | 64 | 1.9e-06 | 1Score **> 34** indicates **identity** Score **> 20** indicates **homology** | U | K.DQLLLGPTYATPK.V |
| 55514 | 350 | – | 362 | 708.8905 | 1415.7664 | 1415.7660 | 0.28 | 0 | 60 | 4.2e-06 | 1Score **> 34** indicates **identity** Score **> 19** indicates **homology** | U | K.DQLLLGPTYATPK.V |
| 55515 | 350 | – | 362 | 708.8914 | 1415.7682 | 1415.7660 | 1.57 | 0 | 28 | 0.0087 | 1Score **> 35** indicates **identity** Score **> 20** indicates **homology** | U | K.DQLLLGPTYATPK.V |
| 55518 | 350 | – | 362 | 708.8943 | 1415.7740 | 1415.7660 | 5.65 | 0 | 24 | 0.027 | 1Score **> 35** indicates **identity** Score **> 21** indicates **homology** | U | K.DQLLLGPTYATPK.V |
| 55522 | 350 | – | 362 | 708.8963 | 1415.7780 | 1415.7660 | 8.45 | 0 | 21 | 0.016 | 1Score **> 35** indicates **identity** Score **> 16** indicates **homology** | U | K.DQLLLGPTYATPK.V |
| 17953 | 408 | – | 417 | 528.3083 | 1054.6021 | 1054.6022 | -0.15 | 1 | 22 | 0.0088 | 1Score **> 28** indicates **identity** Score **> 14** indicates **homology** | U | K.TKVGSPPLEK.F |
| 17954 | 408 | – | 417 | 352.5416 | 1054.6030 | 1054.6022 | 0.74 | 1 | 48 | 0.00012 | 1Score **> 29** indicates **identity** Score **> 21** indicates **homology** | U | K.TKVGSPPLEK.F |
| 17955 | 408 | – | 417 | 528.3089 | 1054.6033 | 1054.6022 | 0.99 | 1 | 24 | 0.0076 | 1Score **> 29** indicates **identity** Score **> 15** indicates **homology** | U | K.TKVGSPPLEK.F |
| 17956 | 408 | – | 417 | 528.3089 | 1054.6033 | 1054.6022 | 1.03 | 1 | 31 | 0.002 | 1Score **> 29** indicates **identity** Score **> 16** indicates **homology** | U | K.TKVGSPPLEK.F |
| 17958 | 408 | – | 417 | 352.5419 | 1054.6038 | 1054.6022 | 1.46 | 1 | 43 | 0.00016 | 1Score **> 29** indicates **identity** Score **> 18** indicates **homology** | U | K.TKVGSPPLEK.F |
| 17960 | 408 | – | 417 | 352.5421 | 1054.6044 | 1054.6022 | 2.05 | 1 | 17 | 0.026 | 1Score **> 29** indicates **identity** Score **> 14** indicates **homology** | U | K.TKVGSPPLEK.F |
| 17961 | 408 | – | 417 | 352.5422 | 1054.6047 | 1054.6022 | 2.32 | 1 | 32 | 0.0035 | 1Score **> 29** indicates **identity** Score **> 20** indicates **homology** | U | K.TKVGSPPLEK.F |
| 17963 | 408 | – | 417 | 528.3102 | 1054.6059 | 1054.6022 | 3.42 | 1 | 36 | 0.00051 | 1Score **> 29** indicates **identity** Score **> 16** indicates **homology** | U | K.TKVGSPPLEK.F |
| 176673 | 408 | – | 437 | 634.9308 | 3169.6174 | 3169.6131 | 1.34 | 2 | 18 | 0.02 | 1Score **> 38** indicates **identity** Score **> 14** indicates **homology** | U | K.TKVGSPPLEKFNIWGGSLSLGHPFGATGCR.L |
| 4716 | 410 | – | 417 | 413.7369 | 825.4593 | 825.4596 | -0.37 | 0 | 52 | 7.5e-05 | 1Score **> 23** indicates **identity** | U | K.VGSPPLEK.F |
| 4717 | 410 | – | 417 | 413.7372 | 825.4598 | 825.4596 | 0.19 | 0 | 44 | 0.00047 | 1Score **> 23** indicates **identity** | U | K.VGSPPLEK.F |
| 4718 | 410 | – | 417 | 413.7376 | 825.4607 | 825.4596 | 1.27 | 0 | 33 | 0.004 | 1Score **> 24** indicates **identity** Score **> 21** indicates **homology** | U | K.VGSPPLEK.F |
| 4719 | 410 | – | 417 | 413.7393 | 825.4640 | 825.4596 | 5.27 | 0 | 25 | 0.0059 | 1Score **> 26** indicates **identity** Score **> 16** indicates **homology** | U | K.VGSPPLEK.F |
| 171489 | 410 | – | 437 | 736.1261 | 2940.4752 | 2940.4705 | 1.60 | 1 | 30 | 0.0017 | 1Score **> 38** indicates **identity** Score **> 14** indicates **homology** | U | K.VGSPPLEKFNIWGGSLSLGHPFGATGCR.L |
| 171490 | 410 | – | 437 | 981.1689 | 2940.4849 | 2940.4705 | 4.90 | 1 | 34 | 0.00066 | 1Score **> 37** indicates **identity** Score **> 15** indicates **homology** | U | K.VGSPPLEKFNIWGGSLSLGHPFGATGCR.L |
| 171518 | 410 | – | 437 | 981.4993 | 2941.4760 | 2941.4545 | 7.29 | 1 | 22 | 0.0088 | 1Score **> 37** indicates **identity** Score **> 14** indicates **homology** | U | K.VGSPPLEKFNIWGGSLSLGHPFGATGCR.L  + Deamidated (NQ) |
| 131163 | 418 | – | 437 | 1067.5183 | 2133.0220 | 2133.0215 | 0.24 | 0 | 110 | 4.6e-11 | 1Score **> 35** indicates **identity** Score **> 19** indicates **homology** | U | K.FNIWGGSLSLGHPFGATGCR.L |
| 131164 | 418 | – | 437 | 1067.5183 | 2133.0220 | 2133.0215 | 0.26 | 0 | 92 | 2.4e-09 | 1Score **> 35** indicates **identity** Score **> 18** indicates **homology** | U | K.FNIWGGSLSLGHPFGATGCR.L |
| 131166 | 418 | – | 437 | 712.0148 | 2133.0225 | 2133.0215 | 0.50 | 0 | 72 | 2.1e-07 | 1Score **> 35** indicates **identity** Score **> 18** indicates **homology** | U | K.FNIWGGSLSLGHPFGATGCR.L |
| 131168 | 418 | – | 437 | 712.0150 | 2133.0231 | 2133.0215 | 0.79 | 0 | 16 | 0.035 | 1Score **> 35** indicates **identity** Score **> 13** indicates **homology** | U | K.FNIWGGSLSLGHPFGATGCR.L |
| 131169 | 418 | – | 437 | 712.0150 | 2133.0232 | 2133.0215 | 0.80 | 0 | 43 | 8.8e-05 | 1Score **> 35** indicates **identity** Score **> 15** indicates **homology** | U | K.FNIWGGSLSLGHPFGATGCR.L |
| 131170 | 418 | – | 437 | 1067.5190 | 2133.0234 | 2133.0215 | 0.93 | 0 | 84 | 1.2e-08 | 1Score **> 35** indicates **identity** Score **> 18** indicates **homology** | U | K.FNIWGGSLSLGHPFGATGCR.L |
| 131173 | 418 | – | 437 | 712.0152 | 2133.0237 | 2133.0215 | 1.03 | 0 | 25 | 0.0043 | 1Score **> 35** indicates **identity** Score **> 14** indicates **homology** | U | K.FNIWGGSLSLGHPFGATGCR.L |
| 131174 | 418 | – | 437 | 712.0152 | 2133.0237 | 2133.0215 | 1.05 | 0 | 41 | 0.00013 | 1Score **> 35** indicates **identity** Score **> 15** indicates **homology** | U | K.FNIWGGSLSLGHPFGATGCR.L |
| 131176 | 418 | – | 437 | 712.0156 | 2133.0250 | 2133.0215 | 1.64 | 0 | 72 | 1.7e-07 | 1Score **> 35** indicates **identity** Score **> 17** indicates **homology** | U | K.FNIWGGSLSLGHPFGATGCR.L |
| 131177 | 418 | – | 437 | 712.0159 | 2133.0259 | 2133.0215 | 2.07 | 0 | 20 | 0.013 | 1Score **> 35** indicates **identity** Score **> 14** indicates **homology** | U | K.FNIWGGSLSLGHPFGATGCR.L |
| 5615 | 438 | – | 445 | 423.2365 | 844.4585 | 844.4589 | -0.49 | 0 | 19 | 0.018 | 1Score **> 32** indicates **identity** Score **> 14** indicates **homology** | U | R.LVMAAANR.L |
| 6379 | 438 | – | 445 | 431.2331 | 860.4517 | 860.4538 | -2.47 | 0 | 21 | 0.01 | 1Score **> 29** indicates **identity** Score **> 14** indicates **homology** | U | R.LVMAAANR.L  + Oxidation (M) |
| 6381 | 438 | – | 445 | 431.2335 | 860.4525 | 860.4538 | -1.46 | 0 | 33 | 0.0015 | 1Score **> 31** indicates **identity** Score **> 17** indicates **homology** | U | R.LVMAAANR.L  + Oxidation (M) |
| 6382 | 438 | – | 445 | 431.2336 | 860.4527 | 860.4538 | -1.27 | 0 | 28 | 0.0039 | 1Score **> 31** indicates **identity** Score **> 16** indicates **homology** | U | R.LVMAAANR.L  + Oxidation (M) |
| 6384 | 438 | – | 445 | 431.2341 | 860.4537 | 860.4538 | -0.067 | 0 | 28 | 0.0087 | 1Score **> 31** indicates **identity** Score **> 20** indicates **homology** | U | R.LVMAAANR.L  + Oxidation (M) |
| 6385 | 438 | – | 445 | 431.2343 | 860.4540 | 860.4538 | 0.25 | 0 | 18 | 0.022 | 1Score **> 31** indicates **identity** Score **> 14** indicates **homology** | U | R.LVMAAANR.L  + Oxidation (M) |
| 6386 | 438 | – | 445 | 431.2343 | 860.4541 | 860.4538 | 0.30 | 0 | 23 | 0.012 | 1Score **> 31** indicates **identity** Score **> 17** indicates **homology** | U | R.LVMAAANR.L  + Oxidation (M) |
| 6387 | 438 | – | 445 | 431.2343 | 860.4541 | 860.4538 | 0.38 | 0 | 29 | 0.0058 | 1Score **> 31** indicates **identity** Score **> 19** indicates **homology** | U | R.LVMAAANR.L  + Oxidation (M) |
| 6388 | 438 | – | 445 | 431.2344 | 860.4542 | 860.4538 | 0.47 | 0 | 23 | 0.0079 | 1Score **> 31** indicates **identity** Score **> 14** indicates **homology** | U | R.LVMAAANR.L  + Oxidation (M) |
| 6389 | 438 | – | 445 | 431.2346 | 860.4546 | 860.4538 | 0.93 | 0 | 22 | 0.023 | 1Score **> 31** indicates **identity** Score **> 19** indicates **homology** | U | R.LVMAAANR.L  + Oxidation (M) |
| 6390 | 438 | – | 445 | 431.2350 | 860.4554 | 860.4538 | 1.86 | 0 | 26 | 0.006 | 1Score **> 31** indicates **identity** Score **> 16** indicates **homology** | U | R.LVMAAANR.L  + Oxidation (M) |
| 168405 | 448 | – | 475 | 709.0987 | 2832.3656 | 2832.3687 | -1.10 | 1 | 55 | 7.3e-06 | 1Score **> 37** indicates **identity** Score **> 16** indicates **homology** | U | R.KDGGQYALVAACAAGGQGHAMIVEAYPK.- |
| 163762 | 449 | – | 475 | 902.4350 | 2704.2831 | 2704.2737 | 3.44 | 0 | 46 | 4.8e-05 | 1Score **> 36** indicates **identity** Score **> 15** indicates **homology** | U | K.DGGQYALVAACAAGGQGHAMIVEAYPK.- |
| 163763 | 449 | – | 475 | 902.4358 | 2704.2857 | 2704.2737 | 4.43 | 0 | 44 | 7.3e-05 | 1Score **> 36** indicates **identity** Score **> 15** indicates **homology** | U | K.DGGQYALVAACAAGGQGHAMIVEAYPK.- |
| 164281 | 449 | – | 475 | 908.0992 | 2721.2756 | 2721.2527 | 8.44 | 0 | 17 | 0.027 | 1Score **> 35** indicates **identity** Score **> 14** indicates **homology** | U | K.DGGQYALVAACAAGGQGHAMIVEAYPK.-  + Deamidated (NQ); Oxidation (M) |

---

```
ID   ECHB_MOUSE              Reviewed;         475 AA.
AC   Q99JY0; Q3TEH9; Q8BJI5; Q8BJM0; Q8BK52;
DT   16-AUG-2004, integrated into UniProtKB/Swiss-Prot.
DT   01-JUN-2001, sequence version 1.
DT   28-JUN-2023, entry version 165.
DE   RecName: Full=Trifunctional enzyme subunit beta, mitochondrial;
DE   AltName: Full=TP-beta;
DE   Includes:
DE     RecName: Full=3-ketoacyl-CoA thiolase;
DE              EC=2.3.1.155 {ECO:0000250|UniProtKB:P55084};
DE              EC=2.3.1.16 {ECO:0000250|UniProtKB:P55084};
DE     AltName: Full=Acetyl-CoA acyltransferase;
DE     AltName: Full=Beta-ketothiolase;
DE   Flags: Precursor;
GN   Name=Hadhb;
OS   Mus musculus (Mouse).
OC   Eukaryota; Metazoa; Chordata; Craniata; Vertebrata; Euteleostomi; Mammalia;
OC   Eutheria; Euarchontoglires; Glires; Rodentia; Myomorpha; Muroidea; Muridae;
OC   Murinae; Mus; Mus.
OX   NCBI_TaxID=10090;
RN   [1]
RP   NUCLEOTIDE SEQUENCE [LARGE SCALE MRNA].
RC   STRAIN=C57BL/6J;
RC   TISSUE=Bone marrow, Colon, Hippocampus, Spinal ganglion, Testis, and
RC   Thymus;
RX   PubMed=16141072; DOI=10.1126/science.1112014;
RA   Carninci P., Kasukawa T., Katayama S., Gough J., Frith M.C., Maeda N.,
RA   Oyama R., Ravasi T., Lenhard B., Wells C., Kodzius R., Shimokawa K.,
RA   Bajic V.B., Brenner S.E., Batalov S., Forrest A.R., Zavolan M., Davis M.J.,
RA   Wilming L.G., Aidinis V., Allen J.E., Ambesi-Impiombato A., Apweiler R.,
RA   Aturaliya R.N., Bailey T.L., Bansal M., Baxter L., Beisel K.W., Bersano T.,
RA   Bono H., Chalk A.M., Chiu K.P., Choudhary V., Christoffels A.,
RA   Clutterbuck D.R., Crowe M.L., Dalla E., Dalrymple B.P., de Bono B.,
RA   Della Gatta G., di Bernardo D., Down T., Engstrom P., Fagiolini M.,
RA   Faulkner G., Fletcher C.F., Fukushima T., Furuno M., Futaki S.,
RA   Gariboldi M., Georgii-Hemming P., Gingeras T.R., Gojobori T., Green R.E.,
RA   Gustincich S., Harbers M., Hayashi Y., Hensch T.K., Hirokawa N., Hill D.,
RA   Huminiecki L., Iacono M., Ikeo K., Iwama A., Ishikawa T., Jakt M.,
RA   Kanapin A., Katoh M., Kawasawa Y., Kelso J., Kitamura H., Kitano H.,
RA   Kollias G., Krishnan S.P., Kruger A., Kummerfeld S.K., Kurochkin I.V.,
RA   Lareau L.F., Lazarevic D., Lipovich L., Liu J., Liuni S., McWilliam S.,
RA   Madan Babu M., Madera M., Marchionni L., Matsuda H., Matsuzawa S., Miki H.,
RA   Mignone F., Miyake S., Morris K., Mottagui-Tabar S., Mulder N., Nakano N.,
RA   Nakauchi H., Ng P., Nilsson R., Nishiguchi S., Nishikawa S., Nori F.,
RA   Ohara O., Okazaki Y., Orlando V., Pang K.C., Pavan W.J., Pavesi G.,
RA   Pesole G., Petrovsky N., Piazza S., Reed J., Reid J.F., Ring B.Z.,
RA   Ringwald M., Rost B., Ruan Y., Salzberg S.L., Sandelin A., Schneider C.,
RA   Schoenbach C., Sekiguchi K., Semple C.A., Seno S., Sessa L., Sheng Y.,
RA   Shibata Y., Shimada H., Shimada K., Silva D., Sinclair B., Sperling S.,
RA   Stupka E., Sugiura K., Sultana R., Takenaka Y., Taki K., Tammoja K.,
RA   Tan S.L., Tang S., Taylor M.S., Tegner J., Teichmann S.A., Ueda H.R.,
RA   van Nimwegen E., Verardo R., Wei C.L., Yagi K., Yamanishi H.,
RA   Zabarovsky E., Zhu S., Zimmer A., Hide W., Bult C., Grimmond S.M.,
RA   Teasdale R.D., Liu E.T., Brusic V., Quackenbush J., Wahlestedt C.,
RA   Mattick J.S., Hume D.A., Kai C., Sasaki D., Tomaru Y., Fukuda S.,
RA   Kanamori-Katayama M., Suzuki M., Aoki J., Arakawa T., Iida J., Imamura K.,
RA   Itoh M., Kato T., Kawaji H., Kawagashira N., Kawashima T., Kojima M.,
RA   Kondo S., Konno H., Nakano K., Ninomiya N., Nishio T., Okada M., Plessy C.,
RA   Shibata K., Shiraki T., Suzuki S., Tagami M., Waki K., Watahiki A.,
RA   Okamura-Oho Y., Suzuki H., Kawai J., Hayashizaki Y.;
RT   "The transcriptional landscape of the mammalian genome.";
RL   Science 309:1559-1563(2005).
RN   [2]
RP   NUCLEOTIDE SEQUENCE [LARGE SCALE MRNA].
RC   STRAIN=FVB/N; TISSUE=Mammary tumor;
RX   PubMed=15489334; DOI=10.1101/gr.2596504;
RG   The MGC Project Team;
RT   "The status, quality, and expansion of the NIH full-length cDNA project:
RT   the Mammalian Gene Collection (MGC).";
RL   Genome Res. 14:2121-2127(2004).
RN   [3]
RP   IDENTIFICATION BY MASS SPECTROMETRY [LARGE SCALE ANALYSIS].
RC   TISSUE=Brain, Brown adipose tissue, Heart, Kidney, Liver, Lung,
RC   Pancreas, Spleen, and Testis;
RX   PubMed=21183079; DOI=10.1016/j.cell.2010.12.001;
RA   Huttlin E.L., Jedrychowski M.P., Elias J.E., Goswami T., Rad R.,
RA   Beausoleil S.A., Villen J., Haas W., Sowa M.E., Gygi S.P.;
RT   "A tissue-specific atlas of mouse protein phosphorylation and expression.";
RL   Cell 143:1174-1189(2010).
RN   [4]
RP   SUCCINYLATION [LARGE SCALE ANALYSIS] AT LYS-53; LYS-73; LYS-189; LYS-191;
RP   LYS-273; LYS-292; LYS-294 AND LYS-333, AND IDENTIFICATION BY MASS
RP   SPECTROMETRY [LARGE SCALE ANALYSIS].
RC   TISSUE=Embryonic fibroblast, and Liver;
RX   PubMed=23806337; DOI=10.1016/j.molcel.2013.06.001;
RA   Park J., Chen Y., Tishkoff D.X., Peng C., Tan M., Dai L., Xie Z., Zhang Y.,
RA   Zwaans B.M., Skinner M.E., Lombard D.B., Zhao Y.;
RT   "SIRT5-mediated lysine desuccinylation impacts diverse metabolic
RT   pathways.";
RL   Mol. Cell 50:919-930(2013).
RN   [5]
RP   ACETYLATION [LARGE SCALE ANALYSIS] AT LYS-73; LYS-189; LYS-294; LYS-299;
RP   LYS-333; LYS-349 AND LYS-362, AND IDENTIFICATION BY MASS SPECTROMETRY
RP   [LARGE SCALE ANALYSIS].
RC   TISSUE=Liver;
RX   PubMed=23576753; DOI=10.1073/pnas.1302961110;
RA   Rardin M.J., Newman J.C., Held J.M., Cusack M.P., Sorensen D.J., Li B.,
RA   Schilling B., Mooney S.D., Kahn C.R., Verdin E., Gibson B.W.;
RT   "Label-free quantitative proteomics of the lysine acetylome in mitochondria
RT   identifies substrates of SIRT3 in metabolic pathways.";
RL   Proc. Natl. Acad. Sci. U.S.A. 110:6601-6606(2013).
RN   [6]
RP   INTERACTION WITH MTLN.
RX   PubMed=29949755; DOI=10.1016/j.celrep.2018.05.058;
RA   Makarewich C.A., Baskin K.K., Munir A.Z., Bezprozvannaya S., Sharma G.,
RA   Khemtong C., Shah A.M., McAnally J.R., Malloy C.R., Szweda L.I.,
RA   Bassel-Duby R., Olson E.N.;
RT   "MOXI Is a Mitochondrial Micropeptide That Enhances Fatty Acid beta-
RT   Oxidation.";
RL   Cell Rep. 23:3701-3709(2018).
CC   -!- FUNCTION: Mitochondrial trifunctional enzyme catalyzes the last three
CC       of the four reactions of the mitochondrial beta-oxidation pathway. The
CC       mitochondrial beta-oxidation pathway is the major energy-producing
CC       process in tissues and is performed through four consecutive reactions
CC       breaking down fatty acids into acetyl-CoA. Among the enzymes involved
CC       in this pathway, the trifunctional enzyme exhibits specificity for
CC       long-chain fatty acids. Mitochondrial trifunctional enzyme is a
CC       heterotetrameric complex composed of two proteins, the trifunctional
CC       enzyme subunit alpha/HADHA carries the 2,3-enoyl-CoA hydratase and the
CC       3-hydroxyacyl-CoA dehydrogenase activities, while the trifunctional
CC       enzyme subunit beta/HADHB described here bears the 3-ketoacyl-CoA
CC       thiolase activity. {ECO:0000250|UniProtKB:P55084}.
CC   -!- CATALYTIC ACTIVITY:
CC       Reaction=acetyl-CoA + an acyl-CoA = a 3-oxoacyl-CoA + CoA;
CC         Xref=Rhea:RHEA:21564, ChEBI:CHEBI:57287, ChEBI:CHEBI:57288,
CC         ChEBI:CHEBI:58342, ChEBI:CHEBI:90726; EC=2.3.1.16;
CC         Evidence={ECO:0000250|UniProtKB:P55084};
CC       PhysiologicalDirection=right-to-left; Xref=Rhea:RHEA:21566;
CC         Evidence={ECO:0000250|UniProtKB:P55084};
CC   -!- CATALYTIC ACTIVITY:
CC       Reaction=acetyl-CoA + butanoyl-CoA = 3-oxohexanoyl-CoA + CoA;
CC         Xref=Rhea:RHEA:31111, ChEBI:CHEBI:57287, ChEBI:CHEBI:57288,
CC         ChEBI:CHEBI:57371, ChEBI:CHEBI:62418;
CC         Evidence={ECO:0000250|UniProtKB:P55084};
CC       PhysiologicalDirection=right-to-left; Xref=Rhea:RHEA:31113;
CC         Evidence={ECO:0000250|UniProtKB:P55084};
CC   -!- CATALYTIC ACTIVITY:
CC       Reaction=acetyl-CoA + hexanoyl-CoA = 3-oxooctanoyl-CoA + CoA;
CC         Xref=Rhea:RHEA:31203, ChEBI:CHEBI:57287, ChEBI:CHEBI:57288,
CC         ChEBI:CHEBI:62619, ChEBI:CHEBI:62620;
CC         Evidence={ECO:0000250|UniProtKB:P55084};
CC       PhysiologicalDirection=right-to-left; Xref=Rhea:RHEA:31205;
CC         Evidence={ECO:0000250|UniProtKB:P55084};
CC   -!- CATALYTIC ACTIVITY:
CC       Reaction=acetyl-CoA + octanoyl-CoA = 3-oxodecanoyl-CoA + CoA;
CC         Xref=Rhea:RHEA:31087, ChEBI:CHEBI:57287, ChEBI:CHEBI:57288,
CC         ChEBI:CHEBI:57386, ChEBI:CHEBI:62548;
CC         Evidence={ECO:0000250|UniProtKB:P55084};
CC       PhysiologicalDirection=right-to-left; Xref=Rhea:RHEA:31089;
CC         Evidence={ECO:0000250|UniProtKB:P55084};
CC   -!- CATALYTIC ACTIVITY:
CC       Reaction=acetyl-CoA + decanoyl-CoA = 3-oxododecanoyl-CoA + CoA;
CC         Xref=Rhea:RHEA:31183, ChEBI:CHEBI:57287, ChEBI:CHEBI:57288,
CC         ChEBI:CHEBI:61430, ChEBI:CHEBI:62615;
CC         Evidence={ECO:0000250|UniProtKB:P55084};
CC       PhysiologicalDirection=right-to-left; Xref=Rhea:RHEA:31185;
CC         Evidence={ECO:0000250|UniProtKB:P55084};
CC   -!- CATALYTIC ACTIVITY:
CC       Reaction=acetyl-CoA + dodecanoyl-CoA = 3-oxotetradecanoyl-CoA + CoA;
CC         Xref=Rhea:RHEA:31091, ChEBI:CHEBI:57287, ChEBI:CHEBI:57288,
CC         ChEBI:CHEBI:57375, ChEBI:CHEBI:62543;
CC         Evidence={ECO:0000250|UniProtKB:P55084};
CC       PhysiologicalDirection=right-to-left; Xref=Rhea:RHEA:31093;
CC         Evidence={ECO:0000250|UniProtKB:P55084};
CC   -!- CATALYTIC ACTIVITY:
CC       Reaction=acetyl-CoA + tetradecanoyl-CoA = 3-oxohexadecanoyl-CoA + CoA;
CC         Xref=Rhea:RHEA:18161, ChEBI:CHEBI:57287, ChEBI:CHEBI:57288,
CC         ChEBI:CHEBI:57349, ChEBI:CHEBI:57385; EC=2.3.1.155;
CC         Evidence={ECO:0000250|UniProtKB:P55084};
CC       PhysiologicalDirection=right-to-left; Xref=Rhea:RHEA:18163;
CC         Evidence={ECO:0000250|UniProtKB:P55084};
CC   -!- PATHWAY: Lipid metabolism; fatty acid beta-oxidation.
CC       {ECO:0000250|UniProtKB:P55084}.
CC   -!- SUBUNIT: Heterotetramer of 2 alpha/HADHA and 2 beta/HADHB subunits;
CC       forms the mitochondrial trifunctional enzyme (By similarity). Also
CC       purified as higher order heterooligomers including a 4 alpha/HADHA and
CC       4 beta/HADHB heterooligomer which physiological significance remains
CC       unclear (By similarity). The mitochondrial trifunctional enzyme
CC       interacts with MTLN (PubMed:29949755). Interacts with RSAD2/viperin (By
CC       similarity). {ECO:0000250|UniProtKB:P55084,
CC       ECO:0000269|PubMed:29949755}.
CC   -!- SUBCELLULAR LOCATION: Mitochondrion {ECO:0000250|UniProtKB:P55084}.
CC       Mitochondrion inner membrane {ECO:0000250|UniProtKB:P55084}.
CC       Mitochondrion outer membrane {ECO:0000250|UniProtKB:P55084}.
CC       Endoplasmic reticulum {ECO:0000250|UniProtKB:P55084}. Note=Protein
CC       stability and association with membranes require HADHA.
CC       {ECO:0000250|UniProtKB:P55084}.
CC   -!- PTM: Acetylation of Lys-202 is observed in liver mitochondria from
CC       fasted mice but not from fed mice.
CC   -!- SIMILARITY: Belongs to the thiolase-like superfamily. Thiolase family.
CC       {ECO:0000305}.
CC   ---------------------------------------------------------------------------
CC   Copyrighted by the UniProt Consortium, see https://www.uniprot.org/terms
CC   Distributed under the Creative Commons Attribution (CC BY 4.0) License
CC   ---------------------------------------------------------------------------
DR   EMBL; AK033462; BAC28300.1; -; mRNA.
DR   EMBL; AK076814; BAC36493.1; -; mRNA.
DR   EMBL; AK083164; BAC38790.1; -; mRNA.
DR   EMBL; AK083767; BAC39015.1; -; mRNA.
DR   EMBL; AK150889; BAE29936.1; -; mRNA.
DR   EMBL; AK169637; BAE41269.1; -; mRNA.
DR   EMBL; BC005585; AAH05585.1; -; mRNA.
DR   CCDS; CCDS39045.1; -.
DR   RefSeq; NP_001276727.1; NM_001289798.1.
DR   RefSeq; NP_001276728.1; NM_001289799.1.
DR   RefSeq; NP_663533.1; NM_145558.2.
DR   RefSeq; XP_017176317.1; XM_017320828.1.
DR   RefSeq; XP_017176318.1; XM_017320829.1.
DR   AlphaFoldDB; Q99JY0; -.
DR   SMR; Q99JY0; -.
DR   BioGRID; 231080; 33.
DR   IntAct; Q99JY0; 7.
DR   MINT; Q99JY0; -.
DR   STRING; 10090.ENSMUSP00000110434; -.
DR   iPTMnet; Q99JY0; -.
DR   PhosphoSitePlus; Q99JY0; -.
DR   SwissPalm; Q99JY0; -.
DR   EPD; Q99JY0; -.
DR   jPOST; Q99JY0; -.
DR   MaxQB; Q99JY0; -.
DR   PaxDb; Q99JY0; -.
DR   PeptideAtlas; Q99JY0; -.
DR   ProteomicsDB; 277669; -.
DR   Antibodypedia; 27848; 265 antibodies from 30 providers.
DR   DNASU; 231086; -.
DR   Ensembl; ENSMUST00000026841; ENSMUSP00000026841; ENSMUSG00000059447.
DR   Ensembl; ENSMUST00000114783; ENSMUSP00000110431; ENSMUSG00000059447.
DR   Ensembl; ENSMUST00000114786; ENSMUSP00000110434; ENSMUSG00000059447.
DR   GeneID; 231086; -.
DR   KEGG; mmu:231086; -.
DR   UCSC; uc008wve.2; mouse.
DR   AGR; MGI:2136381; -.
DR   CTD; 3032; -.
DR   MGI; MGI:2136381; Hadhb.
DR   VEuPathDB; HostDB:ENSMUSG00000059447; -.
DR   eggNOG; KOG1392; Eukaryota.
DR   GeneTree; ENSGT01030000234626; -.
DR   HOGENOM; CLU_031026_2_0_1; -.
DR   InParanoid; Q99JY0; -.
DR   OMA; MTAFPEP; -.
DR   OrthoDB; 1826604at2759; -.
DR   PhylomeDB; Q99JY0; -.
DR   TreeFam; TF315243; -.
DR   Reactome; R-MMU-1482798; Acyl chain remodeling of CL.
DR   Reactome; R-MMU-77285; Beta oxidation of myristoyl-CoA to lauroyl-CoA.
DR   Reactome; R-MMU-77305; Beta oxidation of palmitoyl-CoA to myristoyl-CoA.
DR   Reactome; R-MMU-77310; Beta oxidation of lauroyl-CoA to decanoyl-CoA-CoA.
DR   Reactome; R-MMU-77346; Beta oxidation of decanoyl-CoA to octanoyl-CoA-CoA.
DR   Reactome; R-MMU-77348; Beta oxidation of octanoyl-CoA to hexanoyl-CoA.
DR   Reactome; R-MMU-77350; Beta oxidation of hexanoyl-CoA to butanoyl-CoA.
DR   UniPathway; UPA00659; -.
DR   BioGRID-ORCS; 231086; 1 hit in 77 CRISPR screens.
DR   ChiTaRS; Hadhb; mouse.
DR   PRO; PR:Q99JY0; -.
DR   Proteomes; UP000000589; Chromosome 5.
DR   RNAct; Q99JY0; protein.
DR   Bgee; ENSMUSG00000059447; Expressed in spermatocyte and 170 other tissues.
DR   ExpressionAtlas; Q99JY0; baseline and differential.
DR   Genevisible; Q99JY0; MM.
DR   GO; GO:0005783; C:endoplasmic reticulum; ISS:UniProtKB.
DR   GO; GO:0016507; C:mitochondrial fatty acid beta-oxidation multienzyme complex; ISO:MGI.
DR   GO; GO:0005743; C:mitochondrial inner membrane; HDA:MGI.
DR   GO; GO:0042645; C:mitochondrial nucleoid; ISO:MGI.
DR   GO; GO:0005741; C:mitochondrial outer membrane; ISS:UniProtKB.
DR   GO; GO:0005739; C:mitochondrion; IDA:MGI.
DR   GO; GO:0003985; F:acetyl-CoA C-acetyltransferase activity; IDA:MGI.
DR   GO; GO:0003988; F:acetyl-CoA C-acyltransferase activity; IDA:MGI.
DR   GO; GO:0050633; F:acetyl-CoA C-myristoyltransferase activity; IEA:UniProtKB-EC.
DR   GO; GO:0106222; F:lncRNA binding; IDA:MGI.
DR   GO; GO:0044877; F:protein-containing complex binding; ISO:MGI.
DR   GO; GO:0071222; P:cellular response to lipopolysaccharide; IDA:MGI.
DR   GO; GO:0006635; P:fatty acid beta-oxidation; ISO:MGI.
DR   GO; GO:0010467; P:gene expression; IMP:MGI.
DR   CDD; cd00751; thiolase; 1.
DR   Gene3D; 3.40.47.10; -; 1.
DR   InterPro; IPR002155; Thiolase.
DR   InterPro; IPR016039; Thiolase-like.
DR   InterPro; IPR020615; Thiolase_acyl_enz_int_AS.
DR   InterPro; IPR020610; Thiolase_AS.
DR   InterPro; IPR020617; Thiolase_C.
DR   InterPro; IPR020613; Thiolase_CS.
DR   InterPro; IPR020616; Thiolase_N.
DR   PANTHER; PTHR18919; ACETYL-COA C-ACYLTRANSFERASE; 1.
DR   PANTHER; PTHR18919:SF153; TRIFUNCTIONAL ENZYME SUBUNIT BETA, MITOCHONDRIAL; 1.
DR   Pfam; PF02803; Thiolase_C; 1.
DR   Pfam; PF00108; Thiolase_N; 1.
DR   SUPFAM; SSF53901; Thiolase-like; 2.
DR   PROSITE; PS00098; THIOLASE_1; 1.
DR   PROSITE; PS00737; THIOLASE_2; 1.
DR   PROSITE; PS00099; THIOLASE_3; 1.
DR   TIGRFAMs; TIGR01930; AcCoA-C-Actrans; 1.
PE   1: Evidence at protein level;
KW   Acetylation; Acyltransferase; Endoplasmic reticulum; Fatty acid metabolism;
KW   Lipid metabolism; Membrane; Mitochondrion; Mitochondrion inner membrane;
KW   Mitochondrion outer membrane; Reference proteome; Transferase;
KW   Transit peptide.
FT   TRANSIT         1..34
FT                   /note="Mitochondrion"
FT                   /evidence="ECO:0000250"
FT   CHAIN           35..475
FT                   /note="Trifunctional enzyme subunit beta, mitochondrial"
FT                   /id="PRO_0000034082"
FT   INTRAMEM        174..221
FT                   /evidence="ECO:0000250|UniProtKB:P55084"
FT   ACT_SITE        139
FT                   /note="Acyl-thioester intermediate"
FT                   /evidence="ECO:0000250|UniProtKB:P55084"
FT   ACT_SITE        459
FT                   /note="Proton donor/acceptor"
FT                   /evidence="ECO:0000250|UniProtKB:P55084"
FT   SITE            429
FT                   /note="Increases nucleophilicity of active site Cys"
FT                   /evidence="ECO:0000250|UniProtKB:P55084"
FT   MOD_RES         53
FT                   /note="N6-succinyllysine"
FT                   /evidence="ECO:0007744|PubMed:23806337"
FT   MOD_RES         73
FT                   /note="N6-acetyllysine; alternate"
FT                   /evidence="ECO:0007744|PubMed:23576753"
FT   MOD_RES         73
FT                   /note="N6-succinyllysine; alternate"
FT                   /evidence="ECO:0007744|PubMed:23806337"
FT   MOD_RES         189
FT                   /note="N6-acetyllysine; alternate"
FT                   /evidence="ECO:0007744|PubMed:23576753"
FT   MOD_RES         189
FT                   /note="N6-succinyllysine; alternate"
FT                   /evidence="ECO:0007744|PubMed:23806337"
FT   MOD_RES         191
FT                   /note="N6-succinyllysine"
FT                   /evidence="ECO:0007744|PubMed:23806337"
FT   MOD_RES         273
FT                   /note="N6-succinyllysine"
FT                   /evidence="ECO:0007744|PubMed:23806337"
FT   MOD_RES         292
FT                   /note="N6-succinyllysine"
FT                   /evidence="ECO:0007744|PubMed:23806337"
FT   MOD_RES         294
FT                   /note="N6-acetyllysine; alternate"
FT                   /evidence="ECO:0007744|PubMed:23576753"
FT   MOD_RES         294
FT                   /note="N6-succinyllysine; alternate"
FT                   /evidence="ECO:0007744|PubMed:23806337"
FT   MOD_RES         299
FT                   /note="N6-acetyllysine"
FT                   /evidence="ECO:0007744|PubMed:23576753"
FT   MOD_RES         333
FT                   /note="N6-acetyllysine; alternate"
FT                   /evidence="ECO:0007744|PubMed:23576753"
FT   MOD_RES         333
FT                   /note="N6-succinyllysine; alternate"
FT                   /evidence="ECO:0007744|PubMed:23806337"
FT   MOD_RES         349
FT                   /note="N6-acetyllysine"
FT                   /evidence="ECO:0007744|PubMed:23576753"
FT   MOD_RES         362
FT                   /note="N6-acetyllysine"
FT                   /evidence="ECO:0007744|PubMed:23576753"
FT   CONFLICT        24..25
FT                   /note="IR -> HK (in Ref. 1; BAC36493)"
FT                   /evidence="ECO:0000305"
FT   CONFLICT        425
FT                   /note="L -> M (in Ref. 1; BAC38790)"
FT                   /evidence="ECO:0000305"
FT   CONFLICT        450
FT                   /note="G -> R (in Ref. 1; BAC38790)"
FT                   /evidence="ECO:0000305"
FT   CONFLICT        450
FT                   /note="G -> V (in Ref. 1; BAC39015)"
FT                   /evidence="ECO:0000305"
SQ   SEQUENCE   475 AA;  51386 MW;  F131B497C4F5FAF4 CRC64;
     MTTILTSTFR NLSTTSKWAL RSSIRPLSCS SQLHSAPAVQ TKSKKTLAKP NMKNIVVVEG
     VRIPFLLSGT SYKDLMPHDL ARAALSGLLH RTNIPKDVVD YIIFGTVIQE VKTSNVAREA
     ALGAGFSDKT PAHTVTMACI SSNQAMTTAV GLIASGQCDV VVAGGVELMS DVPIRHSRNM
     RKMMLDLNKA KTLGQRLSLL SKFRLNFLSP ELPAVAEFST NETMGHSADR LAAAFAVSRM
     EQDEYALRSH SLAKKAQDEG HLSDIVPFKV PGKDTVTKDN GIRPSSLEQM AKLKPAFIKP
     YGTVTAANSS FLTDGASAML IMSEDRALAM GYKPKAYLRD FIYVSQDPKD QLLLGPTYAT
     PKVLEKAGLT MNDIDAFEFH EAFSGQILAN FKAMDSDWFA QNYMGRKTKV GSPPLEKFNI
     WGGSLSLGHP FGATGCRLVM AAANRLRKDG GQYALVAACA AGGQGHAMIV EAYPK
//
```

|  |
| --- |
| **Mascot:** http://www.matrixscience.com/ |

HNE (H) (+156.1150)
