# Supplementary material for: A common East-Asian ALDH2 mutation causes metabolic disorders and the therapeutic effect of ALDH2 activators
Source: Nat Commun. 2023 Sep 25;14:5971. doi: 10.1038/s41467-023-41570-6 (PMC10520061; doi:10.1038/s41467-023-41570-6)
Supplement: Supplementary file 4 — Supplementary Data 1 [file 41467_2023_41570_MOESM4_ESM.zip › Table S5b/Q99JY0/Q99JY0_WTO-2_C549.html]

Mascot Search Results: Q99JY0
 

# MASCOT Search Results

## Protein View: Q99JY0

### Trifunctional enzyme subunit beta, mitochondrial OS=Mus musculus OX=10090 GN=Hadhb PE=1 SV=1

|  |  |
| --- | --- |
| Database: | Mouse\_UniProt\_proteomes |
| Score: | 8324 |
| Monoisotopic mass (Mr): | 51639 |
| Calculated pI: | 9.43 |

Sequence similarity is available as an NCBI BLAST search of Q99JY0 against nr.

### Search parameters

|  |  |
| --- | --- |
| MS data file: | `D:\LCMSMS\2023 Users' data\230529-1\230529-1-WTO-2.raw` |
| Enzyme: | Trypsin/P: cuts C-term side of KR. |
| Fixed modifications: | Carbamidomethyl (C) |
| Variable modifications: | Deamidated (NQ), HNE (C), HNE (H), HNE (K), Oxidation (M) |

### Protein sequence coverage: 69%

Matched peptides shown in ***bold red***.

|  |  |  |  |  |  |
| --- | --- | --- | --- | --- | --- |
| `1` | `MTTILTSTFR` | `NLSTTSKWAL` | `RSSIRPLSCS` | `SQLHSAPAVQ` | `TKSKKTLAKP` |
| `51` | `NMKNIVVVEG` | `VRIPFLLSGT` | `SYKDLMPHDL` | `ARAALSGLLH` | `RTNIPKDVVD` |
| `101` | `YIIFGTVIQE` | `VKTSNVAREA` | `ALGAGFSDKT` | `PAHTVTMACI` | `SSNQAMTTAV` |
| `151` | `GLIASGQCDV` | `VVAGGVELMS` | `DVPIRHSRNM` | `RKMMLDLNKA` | `KTLGQRLSLL` |
| `201` | `SKFRLNFLSP` | `ELPAVAEFST` | `NETMGHSADR` | `LAAAFAVSRM` | `EQDEYALRSH` |
| `251` | `SLAKKAQDEG` | `HLSDIVPFKV` | `PGKDTVTKDN` | `GIRPSSLEQM` | `AKLKPAFIKP` |
| `301` | `YGTVTAANSS` | `FLTDGASAML` | `IMSEDRALAM` | `GYKPKAYLRD` | `FIYVSQDPKD` |
| `351` | `QLLLGPTYAT` | `PKVLEKAGLT` | `MNDIDAFEFH` | `EAFSGQILAN` | `FKAMDSDWFA` |
| `401` | `QNYMGRKTKV` | `GSPPLEKFNI` | `WGGSLSLGHP` | `FGATGCRLVM` | `AAANRLRKDG` |
| `451` | `GQYALVAACA` | `AGGQGHAMIV` | `EAYPK` |  |  |

Unformatted sequence string: 475 residues (for pasting into other applications).

|  |  |  |  |
| --- | --- | --- | --- |
| Sort by | residue number | increasing mass | decreasing mass |
| Show | matched peptides only | predicted peptides also |  |

| Query | Start | – | End | Observed | Mr(expt) | Mr(calc) | ppm | M | Score | Expect | Rank | U | Peptide |
| --- | --- | --- | --- | --- | --- | --- | --- | --- | --- | --- | --- | --- | --- |
| 13803 | 54 | – | 62 | 492.7941 | 983.5737 | 983.5764 | -2.74 | 0 | 30 | 0.0017 | 1Score **> 29** indicates **identity** Score **> 14** indicates **homology** | U | K.NIVVVEGVR.I |
| 13804 | 54 | – | 62 | 492.7949 | 983.5753 | 983.5764 | -1.13 | 0 | 26 | 0.0033 | 1Score **> 27** indicates **identity** Score **> 14** indicates **homology** | U | K.NIVVVEGVR.I |
| 13805 | 54 | – | 62 | 492.7949 | 983.5753 | 983.5764 | -1.06 | 0 | 35 | 0.0016 | 1Score **> 27** indicates **identity** Score **> 19** indicates **homology** | U | K.NIVVVEGVR.I |
| 13806 | 54 | – | 62 | 492.7951 | 983.5756 | 983.5764 | -0.81 | 0 | 32 | 0.0011 | 1Score **> 27** indicates **identity** Score **> 14** indicates **homology** | U | K.NIVVVEGVR.I |
| 13807 | 54 | – | 62 | 492.7952 | 983.5758 | 983.5764 | -0.58 | 0 | 26 | 0.0037 | 1Score **> 27** indicates **identity** Score **> 14** indicates **homology** | U | K.NIVVVEGVR.I |
| 13808 | 54 | – | 62 | 492.7952 | 983.5758 | 983.5764 | -0.55 | 0 | 28 | 0.0023 | 1Score **> 27** indicates **identity** Score **> 14** indicates **homology** | U | K.NIVVVEGVR.I |
| 13809 | 54 | – | 62 | 492.7953 | 983.5760 | 983.5764 | -0.39 | 0 | 27 | 0.0029 | 1Score **> 27** indicates **identity** Score **> 14** indicates **homology** | U | K.NIVVVEGVR.I |
| 13810 | 54 | – | 62 | 492.7953 | 983.5761 | 983.5764 | -0.30 | 0 | 35 | 0.0011 | 1Score **> 27** indicates **identity** Score **> 18** indicates **homology** | U | K.NIVVVEGVR.I |
| 13811 | 54 | – | 62 | 492.7954 | 983.5762 | 983.5764 | -0.18 | 0 | 36 | 0.00041 | 1Score **> 27** indicates **identity** Score **> 15** indicates **homology** | U | K.NIVVVEGVR.I |
| 13812 | 54 | – | 62 | 492.7954 | 983.5763 | 983.5764 | -0.11 | 0 | 44 | 0.00015 | 1Score **> 27** indicates **identity** Score **> 19** indicates **homology** | U | K.NIVVVEGVR.I |
| 13814 | 54 | – | 62 | 492.7957 | 983.5769 | 983.5764 | 0.52 | 0 | 34 | 0.00066 | 1Score **> 27** indicates **identity** Score **> 15** indicates **homology** | U | K.NIVVVEGVR.I |
| 13815 | 54 | – | 62 | 492.7957 | 983.5769 | 983.5764 | 0.54 | 0 | 38 | 0.00025 | 1Score **> 27** indicates **identity** Score **> 15** indicates **homology** | U | K.NIVVVEGVR.I |
| 13816 | 54 | – | 62 | 492.7958 | 983.5770 | 983.5764 | 0.68 | 0 | 32 | 0.002 | 1Score **> 27** indicates **identity** Score **> 18** indicates **homology** | U | K.NIVVVEGVR.I |
| 13817 | 54 | – | 62 | 492.7958 | 983.5771 | 983.5764 | 0.68 | 0 | 36 | 0.00043 | 1Score **> 27** indicates **identity** Score **> 15** indicates **homology** | U | K.NIVVVEGVR.I |
| 13818 | 54 | – | 62 | 492.7958 | 983.5771 | 983.5764 | 0.69 | 0 | 42 | 0.00011 | 1Score **> 27** indicates **identity** Score **> 15** indicates **homology** | U | K.NIVVVEGVR.I |
| 13819 | 54 | – | 62 | 492.7959 | 983.5772 | 983.5764 | 0.79 | 0 | 45 | 0.00017 | 1Score **> 27** indicates **identity** Score **> 20** indicates **homology** | U | K.NIVVVEGVR.I |
| 13820 | 54 | – | 62 | 492.7959 | 983.5772 | 983.5764 | 0.87 | 0 | 31 | 0.0013 | 1Score **> 27** indicates **identity** Score **> 14** indicates **homology** | U | K.NIVVVEGVR.I |
| 13822 | 54 | – | 62 | 492.7969 | 983.5792 | 983.5764 | 2.85 | 0 | 28 | 0.0064 | 1Score **> 28** indicates **identity** Score **> 18** indicates **homology** | U | K.NIVVVEGVR.I |
| 13823 | 54 | – | 62 | 492.7972 | 983.5798 | 983.5764 | 3.44 | 0 | 29 | 0.0041 | 1Score **> 28** indicates **identity** Score **> 17** indicates **homology** | U | K.NIVVVEGVR.I |
| 13824 | 54 | – | 62 | 492.7978 | 983.5811 | 983.5764 | 4.78 | 0 | 37 | 0.0027 | 1Score **> 29** indicates **identity** Score **> 24** indicates **homology** | U | K.NIVVVEGVR.I |
| 13826 | 54 | – | 62 | 492.7982 | 983.5819 | 983.5764 | 5.62 | 0 | 35 | 0.0043 | 1Score **> 29** indicates **identity** Score **> 24** indicates **homology** | U | K.NIVVVEGVR.I |
| 140812 | 54 | – | 73 | 731.0872 | 2190.2398 | 2190.2412 | -0.68 | 1 | 34 | 0.00058 | 1Score **> 33** indicates **identity** Score **> 15** indicates **homology** | U | K.NIVVVEGVRIPFLLSGTSYK.D |
| 140814 | 54 | – | 73 | 1096.1317 | 2190.2488 | 2190.2412 | 3.45 | 1 | 106 | 1.2e-10 | 1Score **> 32** indicates **identity** Score **> 19** indicates **homology** | U | K.NIVVVEGVRIPFLLSGTSYK.D |
| 140815 | 54 | – | 73 | 1096.1320 | 2190.2494 | 2190.2412 | 3.73 | 1 | 36 | 0.00046 | 1Score **> 32** indicates **identity** Score **> 15** indicates **homology** | U | K.NIVVVEGVRIPFLLSGTSYK.D |
| 140817 | 54 | – | 73 | 731.0908 | 2190.2506 | 2190.2412 | 4.27 | 1 | 24 | 0.005 | 1Score **> 32** indicates **identity** Score **> 14** indicates **homology** | U | K.NIVVVEGVRIPFLLSGTSYK.D |
| 36156 | 63 | – | 73 | 613.3446 | 1224.6747 | 1224.6754 | -0.59 | 0 | 50 | 1.9e-05 | 1Score **> 32** indicates **identity** Score **> 16** indicates **homology** | U | R.IPFLLSGTSYK.D |
| 36157 | 63 | – | 73 | 613.3452 | 1224.6759 | 1224.6754 | 0.36 | 0 | 77 | 6e-08 | 1Score **> 32** indicates **identity** Score **> 17** indicates **homology** | U | R.IPFLLSGTSYK.D |
| 36158 | 63 | – | 73 | 613.3452 | 1224.6759 | 1224.6754 | 0.38 | 0 | 53 | 1.2e-05 | 1Score **> 32** indicates **identity** Score **> 16** indicates **homology** | U | R.IPFLLSGTSYK.D |
| 36159 | 63 | – | 73 | 613.3454 | 1224.6762 | 1224.6754 | 0.66 | 0 | 77 | 6e-08 | 1Score **> 32** indicates **identity** Score **> 17** indicates **homology** | U | R.IPFLLSGTSYK.D |
| 36160 | 63 | – | 73 | 613.3457 | 1224.6768 | 1224.6754 | 1.15 | 0 | 64 | 9.4e-07 | 1Score **> 32** indicates **identity** Score **> 17** indicates **homology** | U | R.IPFLLSGTSYK.D |
| 36161 | 63 | – | 73 | 613.3458 | 1224.6771 | 1224.6754 | 1.35 | 0 | 62 | 1.6e-06 | 1Score **> 32** indicates **identity** Score **> 16** indicates **homology** | U | R.IPFLLSGTSYK.D |
| 36162 | 63 | – | 73 | 613.3458 | 1224.6771 | 1224.6754 | 1.37 | 0 | 77 | 6e-08 | 1Score **> 32** indicates **identity** Score **> 17** indicates **homology** | U | R.IPFLLSGTSYK.D |
| 36163 | 63 | – | 73 | 613.3459 | 1224.6773 | 1224.6754 | 1.53 | 0 | 60 | 2.5e-06 | 1Score **> 32** indicates **identity** Score **> 16** indicates **homology** | U | R.IPFLLSGTSYK.D |
| 36164 | 63 | – | 73 | 613.3460 | 1224.6774 | 1224.6754 | 1.65 | 0 | 58 | 3.8e-06 | 1Score **> 32** indicates **identity** Score **> 16** indicates **homology** | U | R.IPFLLSGTSYK.D |
| 36166 | 63 | – | 73 | 613.3463 | 1224.6780 | 1224.6754 | 2.14 | 0 | 59 | 2.9e-06 | 1Score **> 32** indicates **identity** Score **> 16** indicates **homology** | U | R.IPFLLSGTSYK.D |
| 36167 | 63 | – | 73 | 613.3464 | 1224.6782 | 1224.6754 | 2.24 | 0 | 59 | 2.9e-06 | 1Score **> 32** indicates **identity** Score **> 16** indicates **homology** | U | R.IPFLLSGTSYK.D |
| 36168 | 63 | – | 73 | 613.3481 | 1224.6817 | 1224.6754 | 5.13 | 0 | 52 | 2.4e-05 | 1Score **> 32** indicates **identity** Score **> 18** indicates **homology** | U | R.IPFLLSGTSYK.D |
| 146826 | 63 | – | 82 | 758.7349 | 2273.1828 | 2273.1878 | -2.20 | 1 | 38 | 0.00028 | 1Score **> 37** indicates **identity** Score **> 15** indicates **homology** | U | R.IPFLLSGTSYKDLMPHDLAR.A |
| 146827 | 63 | – | 82 | 569.3037 | 2273.1857 | 2273.1878 | -0.91 | 1 | 21 | 0.011 | 1Score **> 37** indicates **identity** Score **> 14** indicates **homology** | U | R.IPFLLSGTSYKDLMPHDLAR.A |
| 146828 | 63 | – | 82 | 569.3038 | 2273.1859 | 2273.1878 | -0.82 | 1 | 39 | 0.00022 | 1Score **> 37** indicates **identity** Score **> 15** indicates **homology** | U | R.IPFLLSGTSYKDLMPHDLAR.A |
| 146829 | 63 | – | 82 | 569.3040 | 2273.1868 | 2273.1878 | -0.44 | 1 | 47 | 3.6e-05 | 1Score **> 37** indicates **identity** Score **> 15** indicates **homology** | U | R.IPFLLSGTSYKDLMPHDLAR.A |
| 146831 | 63 | – | 82 | 569.3041 | 2273.1872 | 2273.1878 | -0.28 | 1 | 49 | 2.6e-05 | 1Score **> 37** indicates **identity** Score **> 16** indicates **homology** | U | R.IPFLLSGTSYKDLMPHDLAR.A |
| 146832 | 63 | – | 82 | 569.3041 | 2273.1875 | 2273.1878 | -0.15 | 1 | 20 | 0.013 | 1Score **> 37** indicates **identity** Score **> 14** indicates **homology** | U | R.IPFLLSGTSYKDLMPHDLAR.A |
| 146833 | 63 | – | 82 | 758.7367 | 2273.1882 | 2273.1878 | 0.18 | 1 | 64 | 1.4e-06 | 1Score **> 37** indicates **identity** Score **> 19** indicates **homology** | U | R.IPFLLSGTSYKDLMPHDLAR.A |
| 146835 | 63 | – | 82 | 569.3045 | 2273.1887 | 2273.1878 | 0.40 | 1 | 48 | 3.4e-05 | 1Score **> 37** indicates **identity** Score **> 15** indicates **homology** | U | R.IPFLLSGTSYKDLMPHDLAR.A |
| 146836 | 63 | – | 82 | 569.3045 | 2273.1888 | 2273.1878 | 0.43 | 1 | 53 | 1.1e-05 | 1Score **> 37** indicates **identity** Score **> 16** indicates **homology** | U | R.IPFLLSGTSYKDLMPHDLAR.A |
| 146840 | 63 | – | 82 | 758.7372 | 2273.1897 | 2273.1878 | 0.82 | 1 | 20 | 0.013 | 1Score **> 37** indicates **identity** Score **> 14** indicates **homology** | U | R.IPFLLSGTSYKDLMPHDLAR.A |
| 146841 | 63 | – | 82 | 758.7373 | 2273.1901 | 2273.1878 | 1.00 | 1 | 52 | 1.4e-05 | 1Score **> 37** indicates **identity** Score **> 16** indicates **homology** | U | R.IPFLLSGTSYKDLMPHDLAR.A |
| 146843 | 63 | – | 82 | 758.7377 | 2273.1913 | 2273.1878 | 1.56 | 1 | 45 | 6.4e-05 | 1Score **> 37** indicates **identity** Score **> 15** indicates **homology** | U | R.IPFLLSGTSYKDLMPHDLAR.A |
| 146844 | 63 | – | 82 | 569.3051 | 2273.1914 | 2273.1878 | 1.57 | 1 | 54 | 8.6e-06 | 1Score **> 37** indicates **identity** Score **> 16** indicates **homology** | U | R.IPFLLSGTSYKDLMPHDLAR.A |
| 146846 | 63 | – | 82 | 569.3052 | 2273.1918 | 2273.1878 | 1.78 | 1 | 24 | 0.0057 | 1Score **> 37** indicates **identity** Score **> 14** indicates **homology** | U | R.IPFLLSGTSYKDLMPHDLAR.A |
| 147788 | 63 | – | 82 | 573.3031 | 2289.1833 | 2289.1827 | 0.23 | 1 | 18 | 0.02 | 1Score **> 37** indicates **identity** Score **> 14** indicates **homology** | U | R.IPFLLSGTSYKDLMPHDLAR.A  + Oxidation (M) |
| 147791 | 63 | – | 82 | 573.3035 | 2289.1849 | 2289.1827 | 0.96 | 1 | 30 | 0.0014 | 1Score **> 37** indicates **identity** Score **> 14** indicates **homology** | U | R.IPFLLSGTSYKDLMPHDLAR.A  + Oxidation (M) |
| 147792 | 63 | – | 82 | 764.0701 | 2289.1883 | 2289.1827 | 2.45 | 1 | 17 | 0.026 | 1Score **> 37** indicates **identity** Score **> 14** indicates **homology** | U | R.IPFLLSGTSYKDLMPHDLAR.A  + Oxidation (M) |
| 147793 | 63 | – | 82 | 764.0705 | 2289.1897 | 2289.1827 | 3.06 | 1 | 39 | 0.00021 | 1Score **> 37** indicates **identity** Score **> 15** indicates **homology** | U | R.IPFLLSGTSYKDLMPHDLAR.A  + Oxidation (M) |
| 147794 | 63 | – | 82 | 573.3049 | 2289.1905 | 2289.1827 | 3.38 | 1 | 36 | 0.00044 | 1Score **> 37** indicates **identity** Score **> 15** indicates **homology** | U | R.IPFLLSGTSYKDLMPHDLAR.A  + Oxidation (M) |
| 147798 | 63 | – | 82 | 573.3055 | 2289.1929 | 2289.1827 | 4.44 | 1 | 42 | 0.00013 | 1Score **> 37** indicates **identity** Score **> 15** indicates **homology** | U | R.IPFLLSGTSYKDLMPHDLAR.A  + Oxidation (M) |
| 147801 | 63 | – | 82 | 764.0757 | 2289.2052 | 2289.1827 | 9.81 | 1 | 15 | 0.043 | 1Score **> 37** indicates **identity** Score **> 13** indicates **homology** | U | R.IPFLLSGTSYKDLMPHDLAR.A  + Oxidation (M) |
| 19779 | 74 | – | 82 | 356.5145 | 1066.5216 | 1066.5229 | -1.31 | 0 | 15 | 0.035 | 1Score **> 31** indicates **identity** Score **> 13** indicates **homology** | U | K.DLMPHDLAR.A |
| 19780 | 74 | – | 82 | 356.5148 | 1066.5225 | 1066.5229 | -0.38 | 0 | 25 | 0.005 | 1Score **> 30** indicates **identity** Score **> 14** indicates **homology** | U | K.DLMPHDLAR.A |
| 19782 | 74 | – | 82 | 356.5151 | 1066.5234 | 1066.5229 | 0.41 | 0 | 21 | 0.011 | 1Score **> 30** indicates **identity** Score **> 14** indicates **homology** | U | K.DLMPHDLAR.A |
| 19783 | 74 | – | 82 | 356.5151 | 1066.5235 | 1066.5229 | 0.49 | 0 | 30 | 0.0014 | 1Score **> 30** indicates **identity** Score **> 14** indicates **homology** | U | K.DLMPHDLAR.A |
| 19784 | 74 | – | 82 | 356.5151 | 1066.5236 | 1066.5229 | 0.59 | 0 | 21 | 0.01 | 1Score **> 30** indicates **identity** Score **> 14** indicates **homology** | U | K.DLMPHDLAR.A |
| 19785 | 74 | – | 82 | 534.2692 | 1066.5239 | 1066.5229 | 0.89 | 0 | 18 | 0.022 | 1Score **> 30** indicates **identity** Score **> 14** indicates **homology** | U | K.DLMPHDLAR.A |
| 21238 | 74 | – | 82 | 542.2664 | 1082.5182 | 1082.5179 | 0.35 | 0 | 16 | 0.03 | 1Score **> 29** indicates **identity** Score **> 14** indicates **homology** | U | K.DLMPHDLAR.A  + Oxidation (M) |
| 10946 | 83 | – | 91 | 469.2825 | 936.5504 | 936.5505 | -0.051 | 0 | 49 | 0.00016 | 1Score **> 23** indicates **identity** | U | R.AALSGLLHR.T |
| 10947 | 83 | – | 91 | 469.2825 | 936.5504 | 936.5505 | -0.043 | 0 | 71 | 2.3e-07 | 1Score **> 23** indicates **identity** Score **> 17** indicates **homology** | U | R.AALSGLLHR.T |
| 10948 | 83 | – | 91 | 469.2826 | 936.5506 | 936.5505 | 0.15 | 0 | 76 | 8.2e-08 | 1Score **> 23** indicates **identity** Score **> 17** indicates **homology** | U | R.AALSGLLHR.T |
| 10949 | 83 | – | 91 | 469.2826 | 936.5507 | 936.5505 | 0.27 | 0 | 76 | 7.8e-08 | 1Score **> 23** indicates **identity** Score **> 17** indicates **homology** | U | R.AALSGLLHR.T |
| 10950 | 83 | – | 91 | 469.2826 | 936.5507 | 936.5505 | 0.28 | 0 | 46 | 4.9e-05 | 1Score **> 23** indicates **identity** Score **> 15** indicates **homology** | U | R.AALSGLLHR.T |
| 10951 | 83 | – | 91 | 469.2830 | 936.5515 | 936.5505 | 1.13 | 0 | 64 | 1.1e-06 | 1Score **> 23** indicates **identity** Score **> 16** indicates **homology** | U | R.AALSGLLHR.T |
| 154498 | 92 | – | 112 | 797.7763 | 2390.3071 | 2390.3097 | -1.09 | 1 | 52 | 1.3e-05 | 1Score **> 35** indicates **identity** Score **> 16** indicates **homology** | U | R.TNIPKDVVDYIIFGTVIQEVK.T |
| 154500 | 92 | – | 112 | 797.7772 | 2390.3097 | 2390.3097 | -0.030 | 1 | 57 | 4.8e-06 | 1Score **> 35** indicates **identity** Score **> 16** indicates **homology** | U | R.TNIPKDVVDYIIFGTVIQEVK.T |
| 154504 | 92 | – | 112 | 797.7775 | 2390.3106 | 2390.3097 | 0.35 | 1 | 30 | 0.0015 | 1Score **> 35** indicates **identity** Score **> 14** indicates **homology** | U | R.TNIPKDVVDYIIFGTVIQEVK.T |
| 154505 | 92 | – | 112 | 598.5850 | 2390.3109 | 2390.3097 | 0.50 | 1 | 52 | 1.4e-05 | 1Score **> 35** indicates **identity** Score **> 16** indicates **homology** | U | R.TNIPKDVVDYIIFGTVIQEVK.T |
| 154506 | 92 | – | 112 | 598.5850 | 2390.3110 | 2390.3097 | 0.53 | 1 | 50 | 1.9e-05 | 1Score **> 35** indicates **identity** Score **> 16** indicates **homology** | U | R.TNIPKDVVDYIIFGTVIQEVK.T |
| 154507 | 92 | – | 112 | 1196.1631 | 2390.3116 | 2390.3097 | 0.79 | 1 | 92 | 2.3e-09 | 1Score **> 35** indicates **identity** Score **> 18** indicates **homology** | U | R.TNIPKDVVDYIIFGTVIQEVK.T |
| 154508 | 92 | – | 112 | 797.7780 | 2390.3121 | 2390.3097 | 0.98 | 1 | 48 | 3.4e-05 | 1Score **> 35** indicates **identity** Score **> 15** indicates **homology** | U | R.TNIPKDVVDYIIFGTVIQEVK.T |
| 154510 | 92 | – | 112 | 797.7781 | 2390.3124 | 2390.3097 | 1.12 | 1 | 61 | 1.8e-06 | 1Score **> 35** indicates **identity** Score **> 16** indicates **homology** | U | R.TNIPKDVVDYIIFGTVIQEVK.T |
| 154511 | 92 | – | 112 | 797.7782 | 2390.3127 | 2390.3097 | 1.24 | 1 | 48 | 2.9e-05 | 1Score **> 35** indicates **identity** Score **> 16** indicates **homology** | U | R.TNIPKDVVDYIIFGTVIQEVK.T |
| 154514 | 92 | – | 112 | 797.7784 | 2390.3134 | 2390.3097 | 1.54 | 1 | 20 | 0.012 | 1Score **> 35** indicates **identity** Score **> 14** indicates **homology** | U | R.TNIPKDVVDYIIFGTVIQEVK.T |
| 154515 | 92 | – | 112 | 1196.1641 | 2390.3136 | 2390.3097 | 1.64 | 1 | 84 | 1.3e-08 | 1Score **> 35** indicates **identity** Score **> 18** indicates **homology** | U | R.TNIPKDVVDYIIFGTVIQEVK.T |
| 154516 | 92 | – | 112 | 797.7786 | 2390.3139 | 2390.3097 | 1.73 | 1 | 61 | 2e-06 | 1Score **> 35** indicates **identity** Score **> 16** indicates **homology** | U | R.TNIPKDVVDYIIFGTVIQEVK.T |
| 154517 | 92 | – | 112 | 797.7789 | 2390.3149 | 2390.3097 | 2.15 | 1 | 43 | 8.9e-05 | 1Score **> 35** indicates **identity** Score **> 15** indicates **homology** | U | R.TNIPKDVVDYIIFGTVIQEVK.T |
| 154519 | 92 | – | 112 | 797.7789 | 2390.3150 | 2390.3097 | 2.21 | 1 | 58 | 3.9e-06 | 1Score **> 35** indicates **identity** Score **> 16** indicates **homology** | U | R.TNIPKDVVDYIIFGTVIQEVK.T |
| 154520 | 92 | – | 112 | 797.7790 | 2390.3152 | 2390.3097 | 2.30 | 1 | 43 | 8.7e-05 | 1Score **> 35** indicates **identity** Score **> 15** indicates **homology** | U | R.TNIPKDVVDYIIFGTVIQEVK.T |
| 154521 | 92 | – | 112 | 797.7791 | 2390.3155 | 2390.3097 | 2.41 | 1 | 61 | 1.9e-06 | 1Score **> 35** indicates **identity** Score **> 16** indicates **homology** | U | R.TNIPKDVVDYIIFGTVIQEVK.T |
| 154523 | 92 | – | 112 | 1196.1651 | 2390.3157 | 2390.3097 | 2.49 | 1 | 98 | 6.2e-10 | 1Score **> 35** indicates **identity** Score **> 19** indicates **homology** | U | R.TNIPKDVVDYIIFGTVIQEVK.T |
| 154525 | 92 | – | 112 | 797.7800 | 2390.3181 | 2390.3097 | 3.50 | 1 | 44 | 8.2e-05 | 1Score **> 35** indicates **identity** Score **> 15** indicates **homology** | U | R.TNIPKDVVDYIIFGTVIQEVK.T |
| 154528 | 92 | – | 112 | 797.7821 | 2390.3246 | 2390.3097 | 6.22 | 1 | 51 | 1.7e-05 | 1Score **> 34** indicates **identity** Score **> 16** indicates **homology** | U | R.TNIPKDVVDYIIFGTVIQEVK.T |
| 154529 | 92 | – | 112 | 797.7825 | 2390.3257 | 2390.3097 | 6.70 | 1 | 32 | 0.00093 | 1Score **> 34** indicates **identity** Score **> 15** indicates **homology** | U | R.TNIPKDVVDYIIFGTVIQEVK.T |
| 154530 | 92 | – | 112 | 797.7836 | 2390.3289 | 2390.3097 | 8.01 | 1 | 23 | 0.0063 | 1Score **> 34** indicates **identity** Score **> 14** indicates **homology** | U | R.TNIPKDVVDYIIFGTVIQEVK.T |
| 154532 | 92 | – | 112 | 797.7836 | 2390.3290 | 2390.3097 | 8.07 | 1 | 24 | 0.0061 | 1Score **> 34** indicates **identity** Score **> 14** indicates **homology** | U | R.TNIPKDVVDYIIFGTVIQEVK.T |
| 154533 | 92 | – | 112 | 797.7838 | 2390.3296 | 2390.3097 | 8.32 | 1 | 43 | 9.3e-05 | 1Score **> 34** indicates **identity** Score **> 15** indicates **homology** | U | R.TNIPKDVVDYIIFGTVIQEVK.T |
| 180038 | 92 | – | 118 | 755.6679 | 3018.6427 | 3018.6390 | 1.22 | 2 | 25 | 0.0045 | 1Score **> 35** indicates **identity** Score **> 14** indicates **homology** | U | R.TNIPKDVVDYIIFGTVIQEVKTSNVAR.E |
| 180068 | 92 | – | 118 | 1007.5552 | 3019.6439 | 3019.6230 | 6.92 | 2 | 39 | 0.00024 | 1Score **> 34** indicates **identity** Score **> 15** indicates **homology** | U | R.TNIPKDVVDYIIFGTVIQEVKTSNVAR.E  + Deamidated (NQ) |
| 108150 | 97 | – | 112 | 919.4941 | 1836.9736 | 1836.9873 | -7.49 | 0 | 27 | 0.0032 | 1Score **> 35** indicates **identity** Score **> 14** indicates **homology** | U | K.DVVDYIIFGTVIQEVK.T |
| 108170 | 97 | – | 112 | 919.5004 | 1836.9862 | 1836.9873 | -0.59 | 0 | 31 | 0.0013 | 1Score **> 35** indicates **identity** Score **> 14** indicates **homology** | U | K.DVVDYIIFGTVIQEVK.T |
| 108171 | 97 | – | 112 | 613.3363 | 1836.9870 | 1836.9873 | -0.16 | 0 | 42 | 0.00012 | 1Score **> 35** indicates **identity** Score **> 15** indicates **homology** | U | K.DVVDYIIFGTVIQEVK.T |
| 108174 | 97 | – | 112 | 613.3367 | 1836.9882 | 1836.9873 | 0.50 | 0 | 45 | 5.7e-05 | 1Score **> 35** indicates **identity** Score **> 15** indicates **homology** | U | K.DVVDYIIFGTVIQEVK.T |
| 108176 | 97 | – | 112 | 613.3367 | 1836.9884 | 1836.9873 | 0.56 | 0 | 55 | 7.1e-06 | 1Score **> 35** indicates **identity** Score **> 16** indicates **homology** | U | K.DVVDYIIFGTVIQEVK.T |
| 108178 | 97 | – | 112 | 919.5016 | 1836.9887 | 1836.9873 | 0.75 | 0 | 75 | 1.1e-07 | 1Score **> 35** indicates **identity** Score **> 18** indicates **homology** | U | K.DVVDYIIFGTVIQEVK.T |
| 108179 | 97 | – | 112 | 613.3369 | 1836.9889 | 1836.9873 | 0.83 | 0 | 37 | 0.00033 | 1Score **> 35** indicates **identity** Score **> 15** indicates **homology** | U | K.DVVDYIIFGTVIQEVK.T |
| 108180 | 97 | – | 112 | 613.3370 | 1836.9891 | 1836.9873 | 0.94 | 0 | 48 | 3.4e-05 | 1Score **> 35** indicates **identity** Score **> 15** indicates **homology** | U | K.DVVDYIIFGTVIQEVK.T |
| 108185 | 97 | – | 112 | 613.3372 | 1836.9898 | 1836.9873 | 1.32 | 0 | 42 | 0.0001 | 1Score **> 35** indicates **identity** Score **> 15** indicates **homology** | U | K.DVVDYIIFGTVIQEVK.T |
| 108189 | 97 | – | 112 | 613.3374 | 1836.9905 | 1836.9873 | 1.70 | 0 | 31 | 0.0013 | 1Score **> 35** indicates **identity** Score **> 14** indicates **homology** | U | K.DVVDYIIFGTVIQEVK.T |
| 108193 | 97 | – | 112 | 613.3379 | 1836.9918 | 1836.9873 | 2.41 | 0 | 31 | 0.0014 | 1Score **> 35** indicates **identity** Score **> 14** indicates **homology** | U | K.DVVDYIIFGTVIQEVK.T |
| 108201 | 97 | – | 112 | 919.5041 | 1836.9936 | 1836.9873 | 3.39 | 0 | 16 | 0.03 | 1Score **> 35** indicates **identity** Score **> 14** indicates **homology** | U | K.DVVDYIIFGTVIQEVK.T |
| 108202 | 97 | – | 112 | 919.5041 | 1836.9936 | 1836.9873 | 3.41 | 0 | 30 | 0.0014 | 1Score **> 35** indicates **identity** Score **> 14** indicates **homology** | U | K.DVVDYIIFGTVIQEVK.T |
| 108203 | 97 | – | 112 | 613.3386 | 1836.9938 | 1836.9873 | 3.53 | 0 | 45 | 6.3e-05 | 1Score **> 35** indicates **identity** Score **> 15** indicates **homology** | U | K.DVVDYIIFGTVIQEVK.T |
| 108207 | 97 | – | 112 | 613.3393 | 1836.9960 | 1836.9873 | 4.71 | 0 | 48 | 3.5e-05 | 1Score **> 35** indicates **identity** Score **> 15** indicates **homology** | U | K.DVVDYIIFGTVIQEVK.T |
| 108208 | 97 | – | 112 | 919.5056 | 1836.9967 | 1836.9873 | 5.08 | 0 | 33 | 0.00082 | 1Score **> 35** indicates **identity** Score **> 15** indicates **homology** | U | K.DVVDYIIFGTVIQEVK.T |
| 108214 | 97 | – | 112 | 613.3402 | 1836.9989 | 1836.9873 | 6.30 | 0 | 43 | 9.5e-05 | 1Score **> 35** indicates **identity** Score **> 15** indicates **homology** | U | K.DVVDYIIFGTVIQEVK.T |
| 108218 | 97 | – | 112 | 613.3421 | 1837.0044 | 1836.9873 | 9.28 | 0 | 18 | 0.022 | 1Score **> 35** indicates **identity** Score **> 14** indicates **homology** | U | K.DVVDYIIFGTVIQEVK.T |
| 19616 | 119 | – | 129 | 533.2622 | 1064.5098 | 1064.5138 | -3.77 | 0 | 37 | 0.00037 | 1Score **> 31** indicates **identity** Score **> 15** indicates **homology** | U | R.EAALGAGFSDK.T |
| 19617 | 119 | – | 129 | 533.2632 | 1064.5118 | 1064.5138 | -1.91 | 0 | 61 | 1.6e-05 | 1Score **> 31** indicates **identity** Score **> 26** indicates **homology** | U | R.EAALGAGFSDK.T |
| 19619 | 119 | – | 129 | 533.2639 | 1064.5133 | 1064.5138 | -0.49 | 0 | 57 | 7e-06 | 1Score **> 31** indicates **identity** Score **> 18** indicates **homology** | U | R.EAALGAGFSDK.T |
| 19621 | 119 | – | 129 | 533.2641 | 1064.5136 | 1064.5138 | -0.17 | 0 | 51 | 1.7e-05 | 1Score **> 31** indicates **identity** Score **> 16** indicates **homology** | U | R.EAALGAGFSDK.T |
| 19622 | 119 | – | 129 | 533.2648 | 1064.5151 | 1064.5138 | 1.18 | 0 | 41 | 0.00015 | 1Score **> 31** indicates **identity** Score **> 15** indicates **homology** | U | R.EAALGAGFSDK.T |
| 195802 | 119 | – | 175 | 980.3249 | 5875.9056 | 5875.8815 | 4.11 | 1 | 22 | 0.0088 | 1Score **> 35** indicates **identity** Score **> 14** indicates **homology** | U | R.EAALGAGFSDKTPAHTVTMACISSNQAMTTAVGLIASGQCDVVVAGGVELMSDVPIR.H  + HNE (C); Oxidation (M) |
| 195846 | 119 | – | 175 | 1187.5870 | 5932.8989 | 5932.9030 | -0.69 | 1 | 26 | 0.0034 | 1Score **> 35** indicates **identity** Score **> 14** indicates **homology** | U | R.EAALGAGFSDKTPAHTVTMACISSNQAMTTAVGLIASGQCDVVVAGGVELMSDVPIR.H  + HNE (H); Oxidation (M) |
| 195847 | 119 | – | 175 | 989.8268 | 5932.9174 | 5932.9030 | 2.44 | 1 | 19 | 0.018 | 1Score **> 35** indicates **identity** Score **> 14** indicates **homology** | U | R.EAALGAGFSDKTPAHTVTMACISSNQAMTTAVGLIASGQCDVVVAGGVELMSDVPIR.H  + HNE (H); Oxidation (M) |
| 195855 | 119 | – | 175 | 990.3248 | 5935.9053 | 5935.8550 | 8.48 | 1 | 14 | 0.049 | 1Score **> 35** indicates **identity** Score **> 13** indicates **homology** | U | R.EAALGAGFSDKTPAHTVTMACISSNQAMTTAVGLIASGQCDVVVAGGVELMSDVPIR.H  + 3 Deamidated (NQ); HNE (H); Oxidation (M) |
| 15459 | 182 | – | 189 | 504.7634 | 1007.5123 | 1007.5143 | -1.99 | 1 | 18 | 0.04 | 1Score **> 32** indicates **identity** Score **> 17** indicates **homology** | U | R.KMMLDLNK.A  + Oxidation (M) |
| 182120 | 203 | – | 230 | 784.8842 | 3135.5079 | 3135.5084 | -0.16 | 1 | 46 | 4.5e-05 | 1Score **> 37** indicates **identity** Score **> 15** indicates **homology** | U | K.FRLNFLSPELPAVAEFSTNETMGHSADR.L |
| 182121 | 203 | – | 230 | 784.8850 | 3135.5108 | 3135.5084 | 0.77 | 1 | 51 | 1.6e-05 | 1Score **> 37** indicates **identity** Score **> 16** indicates **homology** | U | K.FRLNFLSPELPAVAEFSTNETMGHSADR.L |
| 182123 | 203 | – | 230 | 1046.1799 | 3135.5180 | 3135.5084 | 3.06 | 1 | 63 | 1.2e-06 | 1Score **> 37** indicates **identity** Score **> 16** indicates **homology** | U | K.FRLNFLSPELPAVAEFSTNETMGHSADR.L |
| 191981 | 203 | – | 239 | 805.4110 | 4022.0185 | 4022.0108 | 1.89 | 2 | 17 | 0.025 | 1Score **> 37** indicates **identity** Score **> 14** indicates **homology** | U | K.FRLNFLSPELPAVAEFSTNETMGHSADRLAAAFAVSR.M |
| 174318 | 205 | – | 230 | 945.1208 | 2832.3406 | 2832.3388 | 0.61 | 0 | 77 | 5.6e-08 | 1Score **> 36** indicates **identity** Score **> 17** indicates **homology** | U | R.LNFLSPELPAVAEFSTNETMGHSADR.L |
| 174394 | 205 | – | 230 | 945.4544 | 2833.3414 | 2833.3228 | 6.53 | 0 | 19 | 0.016 | 1Score **> 36** indicates **identity** Score **> 14** indicates **homology** | U | R.LNFLSPELPAVAEFSTNETMGHSADR.L  + Deamidated (NQ) |
| 174864 | 205 | – | 230 | 950.4545 | 2848.3416 | 2848.3338 | 2.74 | 0 | 43 | 8.6e-05 | 1Score **> 36** indicates **identity** Score **> 15** indicates **homology** | U | R.LNFLSPELPAVAEFSTNETMGHSADR.L  + Oxidation (M) |
| 9085 | 231 | – | 239 | 453.2631 | 904.5116 | 904.5130 | -1.63 | 0 | 40 | 0.0042 | 1Score **> 29** indicates **identity** | U | R.LAAAFAVSR.M |
| 9086 | 231 | – | 239 | 453.2635 | 904.5125 | 904.5130 | -0.56 | 0 | 46 | 0.0011 | 1Score **> 29** indicates **identity** Score **> 28** indicates **homology** | U | R.LAAAFAVSR.M |
| 9087 | 231 | – | 239 | 453.2637 | 904.5128 | 904.5130 | -0.22 | 0 | 58 | 5.7e-05 | 1Score **> 29** indicates **identity** Score **> 28** indicates **homology** | U | R.LAAAFAVSR.M |
| 9088 | 231 | – | 239 | 453.2637 | 904.5129 | 904.5130 | -0.14 | 0 | 62 | 2.7e-05 | 1Score **> 29** indicates **identity** | U | R.LAAAFAVSR.M |
| 9089 | 231 | – | 239 | 453.2637 | 904.5129 | 904.5130 | -0.094 | 0 | 38 | 0.0059 | 1Score **> 29** indicates **identity** | U | R.LAAAFAVSR.M |
| 9090 | 231 | – | 239 | 453.2638 | 904.5130 | 904.5130 | -0.076 | 0 | 47 | 0.00088 | 1Score **> 29** indicates **identity** | U | R.LAAAFAVSR.M |
| 9092 | 231 | – | 239 | 453.2638 | 904.5131 | 904.5130 | 0.054 | 0 | 78 | 6.4e-07 | 1Score **> 29** indicates **identity** | U | R.LAAAFAVSR.M |
| 9093 | 231 | – | 239 | 453.2638 | 904.5131 | 904.5130 | 0.065 | 0 | 40 | 0.0029 | 1Score **> 29** indicates **identity** Score **> 27** indicates **homology** | U | R.LAAAFAVSR.M |
| 9094 | 231 | – | 239 | 453.2639 | 904.5132 | 904.5130 | 0.19 | 0 | 46 | 0.00083 | 1Score **> 29** indicates **identity** Score **> 27** indicates **homology** | U | R.LAAAFAVSR.M |
| 9095 | 231 | – | 239 | 453.2639 | 904.5133 | 904.5130 | 0.31 | 0 | 45 | 0.00084 | 1Score **> 29** indicates **identity** Score **> 27** indicates **homology** | U | R.LAAAFAVSR.M |
| 9096 | 231 | – | 239 | 453.2639 | 904.5133 | 904.5130 | 0.32 | 0 | 39 | 0.0043 | 1Score **> 29** indicates **identity** Score **> 28** indicates **homology** | U | R.LAAAFAVSR.M |
| 9097 | 231 | – | 239 | 453.2639 | 904.5133 | 904.5130 | 0.34 | 0 | 51 | 0.00033 | 1Score **> 29** indicates **identity** | U | R.LAAAFAVSR.M |
| 9098 | 231 | – | 239 | 453.2640 | 904.5135 | 904.5130 | 0.48 | 0 | 50 | 0.00036 | 1Score **> 29** indicates **identity** | U | R.LAAAFAVSR.M |
| 9100 | 231 | – | 239 | 453.2641 | 904.5136 | 904.5130 | 0.59 | 0 | 45 | 0.0012 | 1Score **> 29** indicates **identity** | U | R.LAAAFAVSR.M |
| 9101 | 231 | – | 239 | 453.2641 | 904.5136 | 904.5130 | 0.64 | 0 | 60 | 4.4e-05 | 1Score **> 29** indicates **identity** | U | R.LAAAFAVSR.M |
| 9102 | 231 | – | 239 | 453.2641 | 904.5136 | 904.5130 | 0.67 | 0 | 41 | 0.0032 | 1Score **> 29** indicates **identity** | U | R.LAAAFAVSR.M |
| 9103 | 231 | – | 239 | 453.2642 | 904.5138 | 904.5130 | 0.86 | 0 | 46 | 0.00099 | 1Score **> 29** indicates **identity** | U | R.LAAAFAVSR.M |
| 9104 | 231 | – | 239 | 453.2642 | 904.5138 | 904.5130 | 0.88 | 0 | 47 | 0.00087 | 1Score **> 29** indicates **identity** | U | R.LAAAFAVSR.M |
| 9105 | 231 | – | 239 | 453.2646 | 904.5147 | 904.5130 | 1.88 | 0 | 35 | 0.0014 | 1Score **> 27** indicates **identity** Score **> 19** indicates **homology** | U | R.LAAAFAVSR.M |
| 9106 | 231 | – | 239 | 453.2648 | 904.5151 | 904.5130 | 2.34 | 0 | 39 | 0.00098 | 1Score **> 27** indicates **identity** Score **> 21** indicates **homology** | U | R.LAAAFAVSR.M |
| 30087 | 240 | – | 248 | 585.7577 | 1169.5008 | 1169.5023 | -1.26 | 0 | 20 | 0.013 | 1Score **> 26** indicates **identity** Score **> 14** indicates **homology** | U | R.MEQDEYALR.S  + Oxidation (M) |
| 30088 | 240 | – | 248 | 585.7580 | 1169.5014 | 1169.5023 | -0.73 | 0 | 42 | 0.00012 | 1Score **> 26** indicates **identity** Score **> 15** indicates **homology** | U | R.MEQDEYALR.S  + Oxidation (M) |
| 30090 | 240 | – | 248 | 585.7581 | 1169.5017 | 1169.5023 | -0.50 | 0 | 47 | 5.9e-05 | 1Score **> 26** indicates **identity** Score **> 17** indicates **homology** | U | R.MEQDEYALR.S  + Oxidation (M) |
| 30092 | 240 | – | 248 | 585.7582 | 1169.5019 | 1169.5023 | -0.29 | 0 | 30 | 0.0018 | 1Score **> 26** indicates **identity** Score **> 15** indicates **homology** | U | R.MEQDEYALR.S  + Oxidation (M) |
| 30093 | 240 | – | 248 | 585.7584 | 1169.5022 | 1169.5023 | -0.087 | 0 | 19 | 0.015 | 1Score **> 26** indicates **identity** Score **> 14** indicates **homology** | U | R.MEQDEYALR.S  + Oxidation (M) |
| 30094 | 240 | – | 248 | 585.7585 | 1169.5024 | 1169.5023 | 0.13 | 0 | 26 | 0.0081 | 1Score **> 26** indicates **identity** Score **> 17** indicates **homology** | U | R.MEQDEYALR.S  + Oxidation (M) |
| 30095 | 240 | – | 248 | 585.7586 | 1169.5026 | 1169.5023 | 0.31 | 0 | 42 | 0.00017 | 1Score **> 26** indicates **identity** Score **> 17** indicates **homology** | U | R.MEQDEYALR.S  + Oxidation (M) |
| 30096 | 240 | – | 248 | 585.7586 | 1169.5027 | 1169.5023 | 0.41 | 0 | 41 | 0.00015 | 1Score **> 26** indicates **identity** Score **> 15** indicates **homology** | U | R.MEQDEYALR.S  + Oxidation (M) |
| 30097 | 240 | – | 248 | 585.7592 | 1169.5039 | 1169.5023 | 1.39 | 0 | 28 | 0.0025 | 1Score **> 26** indicates **identity** Score **> 14** indicates **homology** | U | R.MEQDEYALR.S  + Oxidation (M) |
| 129129 | 255 | – | 273 | 689.0417 | 2064.1032 | 2064.1004 | 1.37 | 2 | 30 | 0.0016 | 1Score **> 36** indicates **identity** Score **> 14** indicates **homology** | U | K.KAQDEGHLSDIVPFKVPGK.D |
| 129130 | 255 | – | 273 | 413.8279 | 2064.1033 | 2064.1004 | 1.42 | 2 | 37 | 0.00032 | 1Score **> 36** indicates **identity** Score **> 15** indicates **homology** | U | K.KAQDEGHLSDIVPFKVPGK.D |
| 129131 | 255 | – | 273 | 413.8281 | 2064.1040 | 2064.1004 | 1.75 | 2 | 25 | 0.0045 | 1Score **> 36** indicates **identity** Score **> 14** indicates **homology** | U | K.KAQDEGHLSDIVPFKVPGK.D |
| 129132 | 255 | – | 273 | 517.0333 | 2064.1043 | 2064.1004 | 1.89 | 2 | 21 | 0.012 | 1Score **> 36** indicates **identity** Score **> 14** indicates **homology** | U | K.KAQDEGHLSDIVPFKVPGK.D |
| 129133 | 255 | – | 273 | 517.0333 | 2064.1043 | 2064.1004 | 1.89 | 2 | 46 | 5.3e-05 | 1Score **> 36** indicates **identity** Score **> 15** indicates **homology** | U | K.KAQDEGHLSDIVPFKVPGK.D |
| 129134 | 255 | – | 273 | 689.0421 | 2064.1045 | 2064.1004 | 1.99 | 2 | 18 | 0.019 | 1Score **> 36** indicates **identity** Score **> 14** indicates **homology** | U | K.KAQDEGHLSDIVPFKVPGK.D |
| 129135 | 255 | – | 273 | 517.0335 | 2064.1047 | 2064.1004 | 2.09 | 2 | 32 | 0.00094 | 1Score **> 36** indicates **identity** Score **> 15** indicates **homology** | U | K.KAQDEGHLSDIVPFKVPGK.D |
| 117335 | 256 | – | 273 | 646.3431 | 1936.0076 | 1936.0054 | 1.12 | 1 | 25 | 0.0043 | 1Score **> 36** indicates **identity** Score **> 14** indicates **homology** | U | K.AQDEGHLSDIVPFKVPGK.D |
| 117336 | 256 | – | 273 | 485.0094 | 1936.0087 | 1936.0054 | 1.68 | 1 | 21 | 0.012 | 1Score **> 36** indicates **identity** Score **> 14** indicates **homology** | U | K.AQDEGHLSDIVPFKVPGK.D |
| 117338 | 256 | – | 273 | 485.0099 | 1936.0105 | 1936.0054 | 2.63 | 1 | 17 | 0.028 | 1Score **> 36** indicates **identity** Score **> 14** indicates **homology** | U | K.AQDEGHLSDIVPFKVPGK.D |
| 159628 | 256 | – | 278 | 621.0805 | 2480.2929 | 2480.2911 | 0.71 | 2 | 25 | 0.0047 | 1Score **> 37** indicates **identity** Score **> 14** indicates **homology** | U | K.AQDEGHLSDIVPFKVPGKDTVTK.D |
| 159629 | 256 | – | 278 | 497.0659 | 2480.2930 | 2480.2911 | 0.77 | 2 | 22 | 0.0089 | 1Score **> 37** indicates **identity** Score **> 14** indicates **homology** | U | K.AQDEGHLSDIVPFKVPGKDTVTK.D |
| 159630 | 256 | – | 278 | 827.7717 | 2480.2932 | 2480.2911 | 0.82 | 2 | 24 | 0.0058 | 1Score **> 37** indicates **identity** Score **> 14** indicates **homology** | U | K.AQDEGHLSDIVPFKVPGKDTVTK.D |
| 159631 | 256 | – | 278 | 827.7717 | 2480.2933 | 2480.2911 | 0.87 | 2 | 25 | 0.0043 | 1Score **> 37** indicates **identity** Score **> 14** indicates **homology** | U | K.AQDEGHLSDIVPFKVPGKDTVTK.D |
| 159632 | 256 | – | 278 | 621.0807 | 2480.2936 | 2480.2911 | 0.99 | 2 | 31 | 0.0013 | 1Score **> 37** indicates **identity** Score **> 14** indicates **homology** | U | K.AQDEGHLSDIVPFKVPGKDTVTK.D |
| 159633 | 256 | – | 278 | 621.0809 | 2480.2943 | 2480.2911 | 1.28 | 2 | 25 | 0.0048 | 1Score **> 37** indicates **identity** Score **> 14** indicates **homology** | U | K.AQDEGHLSDIVPFKVPGKDTVTK.D |
| 72719 | 279 | – | 292 | 515.9268 | 1544.7584 | 1544.7617 | -2.09 | 1 | 14 | 0.044 | 1Score **> 34** indicates **identity** Score **> 13** indicates **homology** | U | K.DNGIRPSSLEQMAK.L |
| 13495 | 327 | – | 335 | 489.7750 | 977.5354 | 977.5368 | -1.38 | 1 | 27 | 0.0027 | 1Score **> 30** indicates **identity** Score **> 14** indicates **homology** | U | R.ALAMGYKPK.A |
| 94771 | 336 | – | 349 | 572.2983 | 1713.8731 | 1713.8726 | 0.32 | 1 | 25 | 0.0048 | 1Score **> 35** indicates **identity** Score **> 14** indicates **homology** | U | K.AYLRDFIYVSQDPK.D |
| 94772 | 336 | – | 349 | 857.9439 | 1713.8732 | 1713.8726 | 0.36 | 1 | 56 | 1.3e-05 | 1Score **> 35** indicates **identity** Score **> 20** indicates **homology** | U | K.AYLRDFIYVSQDPK.D |
| 94773 | 336 | – | 349 | 572.2984 | 1713.8733 | 1713.8726 | 0.41 | 1 | 27 | 0.0032 | 1Score **> 35** indicates **identity** Score **> 14** indicates **homology** | U | K.AYLRDFIYVSQDPK.D |
| 94777 | 336 | – | 349 | 572.2988 | 1713.8747 | 1713.8726 | 1.22 | 1 | 18 | 0.019 | 1Score **> 35** indicates **identity** Score **> 14** indicates **homology** | U | K.AYLRDFIYVSQDPK.D |
| 94780 | 336 | – | 349 | 857.9457 | 1713.8769 | 1713.8726 | 2.51 | 1 | 34 | 0.0014 | 1Score **> 35** indicates **identity** Score **> 18** indicates **homology** | U | K.AYLRDFIYVSQDPK.D |
| 94781 | 336 | – | 349 | 572.2996 | 1713.8770 | 1713.8726 | 2.54 | 1 | 17 | 0.027 | 1Score **> 35** indicates **identity** Score **> 14** indicates **homology** | U | K.AYLRDFIYVSQDPK.D |
| 181734 | 336 | – | 362 | 1038.2157 | 3111.6251 | 3111.6281 | -0.94 | 2 | 19 | 0.017 | 1Score **> 37** indicates **identity** Score **> 14** indicates **homology** | U | K.AYLRDFIYVSQDPKDQLLLGPTYATPK.V |
| 181735 | 336 | – | 362 | 1038.2162 | 3111.6266 | 3111.6281 | -0.46 | 2 | 64 | 9.2e-07 | 1Score **> 37** indicates **identity** Score **> 17** indicates **homology** | U | K.AYLRDFIYVSQDPKDQLLLGPTYATPK.V |
| 181737 | 336 | – | 362 | 778.9142 | 3111.6278 | 3111.6281 | -0.096 | 2 | 42 | 0.00012 | 1Score **> 37** indicates **identity** Score **> 15** indicates **homology** | U | K.AYLRDFIYVSQDPKDQLLLGPTYATPK.V |
| 181738 | 336 | – | 362 | 778.9143 | 3111.6281 | 3111.6281 | 0.023 | 2 | 47 | 4.2e-05 | 1Score **> 37** indicates **identity** Score **> 15** indicates **homology** | U | K.AYLRDFIYVSQDPKDQLLLGPTYATPK.V |
| 181739 | 336 | – | 362 | 778.9144 | 3111.6285 | 3111.6281 | 0.15 | 2 | 39 | 0.00022 | 1Score **> 37** indicates **identity** Score **> 15** indicates **homology** | U | K.AYLRDFIYVSQDPKDQLLLGPTYATPK.V |
| 181740 | 336 | – | 362 | 778.9144 | 3111.6286 | 3111.6281 | 0.17 | 2 | 42 | 0.00011 | 1Score **> 37** indicates **identity** Score **> 15** indicates **homology** | U | K.AYLRDFIYVSQDPKDQLLLGPTYATPK.V |
| 181741 | 336 | – | 362 | 778.9145 | 3111.6291 | 3111.6281 | 0.33 | 2 | 40 | 0.00018 | 1Score **> 37** indicates **identity** Score **> 15** indicates **homology** | U | K.AYLRDFIYVSQDPKDQLLLGPTYATPK.V |
| 181742 | 336 | – | 362 | 1038.2170 | 3111.6293 | 3111.6281 | 0.39 | 2 | 86 | 8e-09 | 1Score **> 37** indicates **identity** Score **> 18** indicates **homology** | U | K.AYLRDFIYVSQDPKDQLLLGPTYATPK.V |
| 181743 | 336 | – | 362 | 1038.2171 | 3111.6295 | 3111.6281 | 0.46 | 2 | 78 | 4.5e-08 | 1Score **> 37** indicates **identity** Score **> 17** indicates **homology** | U | K.AYLRDFIYVSQDPKDQLLLGPTYATPK.V |
| 181744 | 336 | – | 362 | 1038.2174 | 3111.6304 | 3111.6281 | 0.75 | 2 | 69 | 3.5e-07 | 1Score **> 37** indicates **identity** Score **> 17** indicates **homology** | U | K.AYLRDFIYVSQDPKDQLLLGPTYATPK.V |
| 181745 | 336 | – | 362 | 1038.2184 | 3111.6333 | 3111.6281 | 1.67 | 2 | 78 | 4.9e-08 | 1Score **> 37** indicates **identity** Score **> 17** indicates **homology** | U | K.AYLRDFIYVSQDPKDQLLLGPTYATPK.V |
| 181746 | 336 | – | 362 | 778.9158 | 3111.6341 | 3111.6281 | 1.93 | 2 | 49 | 2.7e-05 | 1Score **> 37** indicates **identity** Score **> 16** indicates **homology** | U | K.AYLRDFIYVSQDPKDQLLLGPTYATPK.V |
| 181747 | 336 | – | 362 | 778.9159 | 3111.6344 | 3111.6281 | 2.03 | 2 | 30 | 0.0015 | 1Score **> 37** indicates **identity** Score **> 14** indicates **homology** | U | K.AYLRDFIYVSQDPKDQLLLGPTYATPK.V |
| 181748 | 336 | – | 362 | 1038.2192 | 3111.6356 | 3111.6281 | 2.43 | 2 | 59 | 3.3e-06 | 1Score **> 37** indicates **identity** Score **> 17** indicates **homology** | U | K.AYLRDFIYVSQDPKDQLLLGPTYATPK.V |
| 181750 | 336 | – | 362 | 778.9168 | 3111.6381 | 3111.6281 | 3.21 | 2 | 19 | 0.015 | 1Score **> 37** indicates **identity** Score **> 14** indicates **homology** | U | K.AYLRDFIYVSQDPKDQLLLGPTYATPK.V |
| 181769 | 336 | – | 362 | 779.1633 | 3112.6240 | 3112.6121 | 3.82 | 2 | 33 | 0.00087 | 1Score **> 37** indicates **identity** Score **> 15** indicates **homology** | U | K.AYLRDFIYVSQDPKDQLLLGPTYATPK.V  + Deamidated (NQ) |
| 181770 | 336 | – | 362 | 1038.5486 | 3112.6241 | 3112.6121 | 3.87 | 2 | 41 | 0.00015 | 1Score **> 37** indicates **identity** Score **> 15** indicates **homology** | U | K.AYLRDFIYVSQDPKDQLLLGPTYATPK.V  + Deamidated (NQ) |
| 181771 | 336 | – | 362 | 1038.5488 | 3112.6247 | 3112.6121 | 4.06 | 2 | 43 | 9.9e-05 | 1Score **> 37** indicates **identity** Score **> 15** indicates **homology** | U | K.AYLRDFIYVSQDPKDQLLLGPTYATPK.V  + Deamidated (NQ) |
| 181773 | 336 | – | 362 | 1038.5527 | 3112.6362 | 3112.6121 | 7.75 | 2 | 30 | 0.0016 | 1Score **> 37** indicates **identity** Score **> 14** indicates **homology** | U | K.AYLRDFIYVSQDPKDQLLLGPTYATPK.V  + Deamidated (NQ) |
| 34542 | 340 | – | 349 | 606.3007 | 1210.5868 | 1210.5870 | -0.19 | 0 | 23 | 0.0076 | 1Score **> 31** indicates **identity** Score **> 14** indicates **homology** | U | R.DFIYVSQDPK.D |
| 34545 | 340 | – | 349 | 606.3012 | 1210.5878 | 1210.5870 | 0.69 | 0 | 35 | 0.00052 | 1Score **> 32** indicates **identity** Score **> 15** indicates **homology** | U | R.DFIYVSQDPK.D |
| 34546 | 340 | – | 349 | 606.3023 | 1210.5901 | 1210.5870 | 2.52 | 0 | 36 | 0.00046 | 1Score **> 32** indicates **identity** Score **> 15** indicates **homology** | U | R.DFIYVSQDPK.D |
| 165405 | 340 | – | 362 | 870.4496 | 2608.3269 | 2608.3425 | -5.95 | 1 | 24 | 0.007 | 1Score **> 37** indicates **identity** Score **> 15** indicates **homology** | U | R.DFIYVSQDPKDQLLLGPTYATPK.V |
| 165407 | 340 | – | 362 | 870.4514 | 2608.3324 | 2608.3425 | -3.87 | 1 | 47 | 4e-05 | 1Score **> 37** indicates **identity** Score **> 15** indicates **homology** | U | R.DFIYVSQDPKDQLLLGPTYATPK.V |
| 165408 | 340 | – | 362 | 870.4519 | 2608.3339 | 2608.3425 | -3.29 | 1 | 46 | 4.7e-05 | 1Score **> 37** indicates **identity** Score **> 15** indicates **homology** | U | R.DFIYVSQDPKDQLLLGPTYATPK.V |
| 165409 | 340 | – | 362 | 870.4540 | 2608.3402 | 2608.3425 | -0.86 | 1 | 21 | 0.01 | 1Score **> 37** indicates **identity** Score **> 14** indicates **homology** | U | R.DFIYVSQDPKDQLLLGPTYATPK.V |
| 165411 | 340 | – | 362 | 870.4542 | 2608.3406 | 2608.3425 | -0.70 | 1 | 44 | 7e-05 | 1Score **> 37** indicates **identity** Score **> 15** indicates **homology** | U | R.DFIYVSQDPKDQLLLGPTYATPK.V |
| 165412 | 340 | – | 362 | 870.4542 | 2608.3409 | 2608.3425 | -0.60 | 1 | 51 | 1.7e-05 | 1Score **> 37** indicates **identity** Score **> 16** indicates **homology** | U | R.DFIYVSQDPKDQLLLGPTYATPK.V |
| 165414 | 340 | – | 362 | 1305.1785 | 2608.3425 | 2608.3425 | -0.0061 | 1 | 85 | 1.1e-08 | 1Score **> 37** indicates **identity** Score **> 18** indicates **homology** | U | R.DFIYVSQDPKDQLLLGPTYATPK.V |
| 165416 | 340 | – | 362 | 870.4548 | 2608.3427 | 2608.3425 | 0.081 | 1 | 48 | 3.5e-05 | 1Score **> 37** indicates **identity** Score **> 15** indicates **homology** | U | R.DFIYVSQDPKDQLLLGPTYATPK.V |
| 165418 | 340 | – | 362 | 870.4549 | 2608.3428 | 2608.3425 | 0.12 | 1 | 47 | 4.2e-05 | 1Score **> 37** indicates **identity** Score **> 15** indicates **homology** | U | R.DFIYVSQDPKDQLLLGPTYATPK.V |
| 165419 | 340 | – | 362 | 870.4550 | 2608.3430 | 2608.3425 | 0.22 | 1 | 46 | 5.2e-05 | 1Score **> 37** indicates **identity** Score **> 15** indicates **homology** | U | R.DFIYVSQDPKDQLLLGPTYATPK.V |
| 165420 | 340 | – | 362 | 1305.1789 | 2608.3432 | 2608.3425 | 0.28 | 1 | 59 | 2.8e-06 | 1Score **> 37** indicates **identity** Score **> 16** indicates **homology** | U | R.DFIYVSQDPKDQLLLGPTYATPK.V |
| 165422 | 340 | – | 362 | 870.4552 | 2608.3436 | 2608.3425 | 0.45 | 1 | 45 | 6.1e-05 | 1Score **> 37** indicates **identity** Score **> 15** indicates **homology** | U | R.DFIYVSQDPKDQLLLGPTYATPK.V |
| 165423 | 340 | – | 362 | 653.0932 | 2608.3437 | 2608.3425 | 0.48 | 1 | 15 | 0.042 | 1Score **> 37** indicates **identity** Score **> 13** indicates **homology** | U | R.DFIYVSQDPKDQLLLGPTYATPK.V |
| 165424 | 340 | – | 362 | 1305.1792 | 2608.3439 | 2608.3425 | 0.53 | 1 | 42 | 0.00012 | 1Score **> 37** indicates **identity** Score **> 15** indicates **homology** | U | R.DFIYVSQDPKDQLLLGPTYATPK.V |
| 165426 | 340 | – | 362 | 1305.1793 | 2608.3440 | 2608.3425 | 0.60 | 1 | 80 | 3.1e-08 | 1Score **> 37** indicates **identity** Score **> 18** indicates **homology** | U | R.DFIYVSQDPKDQLLLGPTYATPK.V |
| 165427 | 340 | – | 362 | 870.4553 | 2608.3441 | 2608.3425 | 0.62 | 1 | 59 | 3e-06 | 1Score **> 37** indicates **identity** Score **> 16** indicates **homology** | U | R.DFIYVSQDPKDQLLLGPTYATPK.V |
| 165428 | 340 | – | 362 | 870.4553 | 2608.3441 | 2608.3425 | 0.64 | 1 | 17 | 0.028 | 1Score **> 37** indicates **identity** Score **> 14** indicates **homology** | U | R.DFIYVSQDPKDQLLLGPTYATPK.V |
| 165429 | 340 | – | 362 | 870.4554 | 2608.3442 | 2608.3425 | 0.68 | 1 | 60 | 2.6e-06 | 1Score **> 37** indicates **identity** Score **> 16** indicates **homology** | U | R.DFIYVSQDPKDQLLLGPTYATPK.V |
| 165430 | 340 | – | 362 | 870.4554 | 2608.3443 | 2608.3425 | 0.72 | 1 | 45 | 6.9e-05 | 1Score **> 37** indicates **identity** Score **> 16** indicates **homology** | U | R.DFIYVSQDPKDQLLLGPTYATPK.V |
| 165432 | 340 | – | 362 | 1305.1796 | 2608.3446 | 2608.3425 | 0.82 | 1 | 37 | 0.00036 | 1Score **> 37** indicates **identity** Score **> 15** indicates **homology** | U | R.DFIYVSQDPKDQLLLGPTYATPK.V |
| 165433 | 340 | – | 362 | 1305.1796 | 2608.3446 | 2608.3425 | 0.83 | 1 | 81 | 2.6e-08 | 1Score **> 37** indicates **identity** Score **> 18** indicates **homology** | U | R.DFIYVSQDPKDQLLLGPTYATPK.V |
| 165434 | 340 | – | 362 | 870.4555 | 2608.3447 | 2608.3425 | 0.85 | 1 | 47 | 4.1e-05 | 1Score **> 37** indicates **identity** Score **> 15** indicates **homology** | U | R.DFIYVSQDPKDQLLLGPTYATPK.V |
| 165435 | 340 | – | 362 | 870.4556 | 2608.3448 | 2608.3425 | 0.91 | 1 | 15 | 0.04 | 1Score **> 37** indicates **identity** Score **> 13** indicates **homology** | U | R.DFIYVSQDPKDQLLLGPTYATPK.V |
| 165436 | 340 | – | 362 | 870.4556 | 2608.3448 | 2608.3425 | 0.91 | 1 | 53 | 1e-05 | 1Score **> 37** indicates **identity** Score **> 16** indicates **homology** | U | R.DFIYVSQDPKDQLLLGPTYATPK.V |
| 165437 | 340 | – | 362 | 870.4557 | 2608.3453 | 2608.3425 | 1.07 | 1 | 56 | 6e-06 | 1Score **> 37** indicates **identity** Score **> 16** indicates **homology** | U | R.DFIYVSQDPKDQLLLGPTYATPK.V |
| 165438 | 340 | – | 362 | 870.4557 | 2608.3453 | 2608.3425 | 1.09 | 1 | 52 | 1.2e-05 | 1Score **> 37** indicates **identity** Score **> 16** indicates **homology** | U | R.DFIYVSQDPKDQLLLGPTYATPK.V |
| 165439 | 340 | – | 362 | 870.4559 | 2608.3458 | 2608.3425 | 1.26 | 1 | 58 | 3.7e-06 | 1Score **> 37** indicates **identity** Score **> 16** indicates **homology** | U | R.DFIYVSQDPKDQLLLGPTYATPK.V |
| 165440 | 340 | – | 362 | 870.4561 | 2608.3465 | 2608.3425 | 1.54 | 1 | 42 | 0.00011 | 1Score **> 37** indicates **identity** Score **> 15** indicates **homology** | U | R.DFIYVSQDPKDQLLLGPTYATPK.V |
| 165441 | 340 | – | 362 | 870.4562 | 2608.3467 | 2608.3425 | 1.63 | 1 | 50 | 2e-05 | 1Score **> 37** indicates **identity** Score **> 16** indicates **homology** | U | R.DFIYVSQDPKDQLLLGPTYATPK.V |
| 165442 | 340 | – | 362 | 870.4564 | 2608.3474 | 2608.3425 | 1.89 | 1 | 17 | 0.028 | 1Score **> 37** indicates **identity** Score **> 14** indicates **homology** | U | R.DFIYVSQDPKDQLLLGPTYATPK.V |
| 165444 | 340 | – | 362 | 870.4568 | 2608.3485 | 2608.3425 | 2.30 | 1 | 47 | 4.3e-05 | 1Score **> 37** indicates **identity** Score **> 15** indicates **homology** | U | R.DFIYVSQDPKDQLLLGPTYATPK.V |
| 165445 | 340 | – | 362 | 870.4572 | 2608.3498 | 2608.3425 | 2.82 | 1 | 55 | 7.7e-06 | 1Score **> 37** indicates **identity** Score **> 16** indicates **homology** | U | R.DFIYVSQDPKDQLLLGPTYATPK.V |
| 165446 | 340 | – | 362 | 870.4574 | 2608.3505 | 2608.3425 | 3.08 | 1 | 23 | 0.0076 | 1Score **> 37** indicates **identity** Score **> 14** indicates **homology** | U | R.DFIYVSQDPKDQLLLGPTYATPK.V |
| 165448 | 340 | – | 362 | 870.4577 | 2608.3511 | 2608.3425 | 3.33 | 1 | 23 | 0.0064 | 1Score **> 37** indicates **identity** Score **> 14** indicates **homology** | U | R.DFIYVSQDPKDQLLLGPTYATPK.V |
| 165449 | 340 | – | 362 | 870.4581 | 2608.3525 | 2608.3425 | 3.85 | 1 | 59 | 3e-06 | 1Score **> 37** indicates **identity** Score **> 16** indicates **homology** | U | R.DFIYVSQDPKDQLLLGPTYATPK.V |
| 165450 | 340 | – | 362 | 870.4583 | 2608.3530 | 2608.3425 | 4.03 | 1 | 31 | 0.0012 | 1Score **> 37** indicates **identity** Score **> 14** indicates **homology** | U | R.DFIYVSQDPKDQLLLGPTYATPK.V |
| 165451 | 340 | – | 362 | 870.4584 | 2608.3534 | 2608.3425 | 4.20 | 1 | 25 | 0.0044 | 1Score **> 37** indicates **identity** Score **> 14** indicates **homology** | U | R.DFIYVSQDPKDQLLLGPTYATPK.V |
| 165452 | 340 | – | 362 | 870.4584 | 2608.3535 | 2608.3425 | 4.23 | 1 | 26 | 0.0033 | 1Score **> 37** indicates **identity** Score **> 14** indicates **homology** | U | R.DFIYVSQDPKDQLLLGPTYATPK.V |
| 165485 | 340 | – | 362 | 870.7858 | 2609.3354 | 2609.3265 | 3.44 | 1 | 30 | 0.0014 | 1Score **> 37** indicates **identity** Score **> 14** indicates **homology** | U | R.DFIYVSQDPKDQLLLGPTYATPK.V  + Deamidated (NQ) |
| 165488 | 340 | – | 362 | 1305.6769 | 2609.3393 | 2609.3265 | 4.93 | 1 | 41 | 0.00013 | 1Score **> 37** indicates **identity** Score **> 15** indicates **homology** | U | R.DFIYVSQDPKDQLLLGPTYATPK.V  + Deamidated (NQ) |
| 165489 | 340 | – | 362 | 1305.6809 | 2609.3472 | 2609.3265 | 7.94 | 1 | 50 | 2e-05 | 1Score **> 37** indicates **identity** Score **> 16** indicates **homology** | U | R.DFIYVSQDPKDQLLLGPTYATPK.V  + Deamidated (NQ) |
| 165490 | 340 | – | 362 | 870.7904 | 2609.3493 | 2609.3265 | 8.75 | 1 | 23 | 0.0075 | 1Score **> 37** indicates **identity** Score **> 14** indicates **homology** | U | R.DFIYVSQDPKDQLLLGPTYATPK.V  + Deamidated (NQ) |
| 56918 | 350 | – | 362 | 708.8902 | 1415.7658 | 1415.7660 | -0.13 | 0 | 33 | 0.0008 | 1Score **> 34** indicates **identity** Score **> 15** indicates **homology** | U | K.DQLLLGPTYATPK.V |
| 56920 | 350 | – | 362 | 708.8904 | 1415.7662 | 1415.7660 | 0.10 | 0 | 61 | 4e-06 | 1Score **> 34** indicates **identity** Score **> 19** indicates **homology** | U | K.DQLLLGPTYATPK.V |
| 56921 | 350 | – | 362 | 708.8905 | 1415.7665 | 1415.7660 | 0.36 | 0 | 53 | 2.5e-05 | 1Score **> 35** indicates **identity** Score **> 19** indicates **homology** | U | K.DQLLLGPTYATPK.V |
| 56922 | 350 | – | 362 | 708.8907 | 1415.7669 | 1415.7660 | 0.62 | 0 | 61 | 4.1e-06 | 1Score **> 35** indicates **identity** Score **> 20** indicates **homology** | U | K.DQLLLGPTYATPK.V |
| 56924 | 350 | – | 362 | 708.8907 | 1415.7669 | 1415.7660 | 0.65 | 0 | 65 | 1.7e-06 | 1Score **> 35** indicates **identity** Score **> 20** indicates **homology** | U | K.DQLLLGPTYATPK.V |
| 56926 | 350 | – | 362 | 708.8931 | 1415.7716 | 1415.7660 | 3.92 | 0 | 34 | 0.00084 | 1Score **> 35** indicates **identity** Score **> 16** indicates **homology** | U | K.DQLLLGPTYATPK.V |
| 56928 | 350 | – | 362 | 708.8936 | 1415.7726 | 1415.7660 | 4.66 | 0 | 53 | 5.4e-05 | 1Score **> 34** indicates **identity** Score **> 23** indicates **homology** | U | K.DQLLLGPTYATPK.V |
| 56930 | 350 | – | 362 | 708.8948 | 1415.7750 | 1415.7660 | 6.36 | 0 | 34 | 0.0034 | 1Score **> 35** indicates **identity** Score **> 22** indicates **homology** | U | K.DQLLLGPTYATPK.V |
| 56933 | 350 | – | 362 | 708.8958 | 1415.7771 | 1415.7660 | 7.82 | 0 | 34 | 0.0017 | 1Score **> 35** indicates **identity** Score **> 19** indicates **homology** | U | K.DQLLLGPTYATPK.V |
| 91737 | 393 | – | 406 | 846.3537 | 1690.6928 | 1690.6868 | 3.54 | 0 | 40 | 0.00019 | 1Score **> 23** indicates **identity** Score **> 15** indicates **homology** | U | K.AMDSDWFAQNYMGR.K |
| 91740 | 393 | – | 406 | 846.3551 | 1690.6956 | 1690.6868 | 5.22 | 0 | 52 | 1.4e-05 | 1Score **> 23** indicates **identity** Score **> 16** indicates **homology** | U | K.AMDSDWFAQNYMGR.K |
| 106287 | 393 | – | 407 | 607.2674 | 1818.7805 | 1818.7818 | -0.70 | 1 | 60 | 2.4e-06 | 1Score **> 27** indicates **identity** Score **> 16** indicates **homology** | U | K.AMDSDWFAQNYMGRK.T |
| 106288 | 393 | – | 407 | 607.2678 | 1818.7816 | 1818.7818 | -0.084 | 1 | 27 | 0.0029 | 1Score **> 27** indicates **identity** Score **> 14** indicates **homology** | U | K.AMDSDWFAQNYMGRK.T |
| 18727 | 408 | – | 417 | 528.3082 | 1054.6018 | 1054.6022 | -0.46 | 1 | 26 | 0.0034 | 1Score **> 28** indicates **identity** Score **> 14** indicates **homology** | U | K.TKVGSPPLEK.F |
| 18729 | 408 | – | 417 | 528.3085 | 1054.6025 | 1054.6022 | 0.21 | 1 | 39 | 0.00068 | 1Score **> 28** indicates **identity** Score **> 20** indicates **homology** | U | K.TKVGSPPLEK.F |
| 18730 | 408 | – | 417 | 352.5415 | 1054.6028 | 1054.6022 | 0.54 | 1 | 46 | 0.00033 | 1Score **> 28** indicates **identity** Score **> 24** indicates **homology** | U | K.TKVGSPPLEK.F |
| 18731 | 408 | – | 417 | 352.5416 | 1054.6031 | 1054.6022 | 0.78 | 1 | 46 | 0.00016 | 1Score **> 29** indicates **identity** Score **> 20** indicates **homology** | U | K.TKVGSPPLEK.F |
| 18732 | 408 | – | 417 | 528.3089 | 1054.6032 | 1054.6022 | 0.87 | 1 | 37 | 0.0012 | 1Score **> 29** indicates **identity** Score **> 21** indicates **homology** | U | K.TKVGSPPLEK.F |
| 18733 | 408 | – | 417 | 528.3089 | 1054.6033 | 1054.6022 | 0.99 | 1 | 28 | 0.0031 | 1Score **> 29** indicates **identity** Score **> 15** indicates **homology** | U | K.TKVGSPPLEK.F |
| 18734 | 408 | – | 417 | 352.5419 | 1054.6038 | 1054.6022 | 1.44 | 1 | 21 | 0.033 | 1Score **> 29** indicates **identity** Score **> 19** indicates **homology** | U | K.TKVGSPPLEK.F |
| 18736 | 408 | – | 417 | 352.5423 | 1054.6049 | 1054.6022 | 2.54 | 1 | 42 | 0.00052 | 1Score **> 29** indicates **identity** Score **> 21** indicates **homology** | U | K.TKVGSPPLEK.F |
| 18737 | 408 | – | 417 | 528.3102 | 1054.6058 | 1054.6022 | 3.36 | 1 | 30 | 0.0019 | 1Score **> 29** indicates **identity** Score **> 15** indicates **homology** | U | K.TKVGSPPLEK.F |
| 18738 | 408 | – | 417 | 352.5426 | 1054.6059 | 1054.6022 | 3.43 | 1 | 26 | 0.0069 | 1Score **> 29** indicates **identity** Score **> 17** indicates **homology** | U | K.TKVGSPPLEK.F |
| 182679 | 408 | – | 437 | 634.9292 | 3169.6094 | 3169.6131 | -1.18 | 2 | 21 | 0.0099 | 1Score **> 38** indicates **identity** Score **> 14** indicates **homology** | U | K.TKVGSPPLEKFNIWGGSLSLGHPFGATGCR.L |
| 4996 | 410 | – | 417 | 413.7363 | 825.4580 | 825.4596 | -1.91 | 0 | 35 | 0.0026 | 1Score **> 23** indicates **identity** Score **> 22** indicates **homology** | U | K.VGSPPLEK.F |
| 4997 | 410 | – | 417 | 413.7369 | 825.4592 | 825.4596 | -0.50 | 0 | 49 | 0.00015 | 1Score **> 23** indicates **identity** | U | K.VGSPPLEK.F |
| 4998 | 410 | – | 417 | 413.7369 | 825.4593 | 825.4596 | -0.37 | 0 | 47 | 0.00022 | 1Score **> 23** indicates **identity** | U | K.VGSPPLEK.F |
| 4999 | 410 | – | 417 | 413.7372 | 825.4599 | 825.4596 | 0.30 | 0 | 42 | 0.00084 | 1Score **> 23** indicates **identity** | U | K.VGSPPLEK.F |
| 5000 | 410 | – | 417 | 413.7373 | 825.4600 | 825.4596 | 0.44 | 0 | 28 | 0.0023 | 1Score **> 24** indicates **identity** Score **> 14** indicates **homology** | U | K.VGSPPLEK.F |
| 177599 | 410 | – | 437 | 736.1223 | 2940.4601 | 2940.4705 | -3.53 | 1 | 17 | 0.025 | 1Score **> 37** indicates **identity** Score **> 14** indicates **homology** | U | K.VGSPPLEKFNIWGGSLSLGHPFGATGCR.L |
| 135337 | 418 | – | 437 | 712.0143 | 2133.0210 | 2133.0215 | -0.23 | 0 | 77 | 9.6e-08 | 1Score **> 35** indicates **identity** Score **> 19** indicates **homology** | U | K.FNIWGGSLSLGHPFGATGCR.L |
| 135340 | 418 | – | 437 | 1067.5181 | 2133.0216 | 2133.0215 | 0.048 | 0 | 124 | 2.3e-12 | 1Score **> 35** indicates **identity** Score **> 20** indicates **homology** | U | K.FNIWGGSLSLGHPFGATGCR.L |
| 135342 | 418 | – | 437 | 712.0146 | 2133.0220 | 2133.0215 | 0.27 | 0 | 27 | 0.0028 | 1Score **> 35** indicates **identity** Score **> 14** indicates **homology** | U | K.FNIWGGSLSLGHPFGATGCR.L |
| 135344 | 418 | – | 437 | 712.0148 | 2133.0224 | 2133.0215 | 0.45 | 0 | 15 | 0.043 | 1Score **> 35** indicates **identity** Score **> 13** indicates **homology** | U | K.FNIWGGSLSLGHPFGATGCR.L |
| 135345 | 418 | – | 437 | 712.0148 | 2133.0225 | 2133.0215 | 0.50 | 0 | 72 | 2.2e-07 | 1Score **> 35** indicates **identity** Score **> 18** indicates **homology** | U | K.FNIWGGSLSLGHPFGATGCR.L |
| 135346 | 418 | – | 437 | 1067.5186 | 2133.0227 | 2133.0215 | 0.57 | 0 | 108 | 7.8e-11 | 1Score **> 35** indicates **identity** Score **> 19** indicates **homology** | U | K.FNIWGGSLSLGHPFGATGCR.L |
| 135347 | 418 | – | 437 | 712.0150 | 2133.0231 | 2133.0215 | 0.79 | 0 | 22 | 0.0087 | 1Score **> 35** indicates **identity** Score **> 14** indicates **homology** | U | K.FNIWGGSLSLGHPFGATGCR.L |
| 135349 | 418 | – | 437 | 1067.5190 | 2133.0234 | 2133.0215 | 0.90 | 0 | 84 | 1.4e-08 | 1Score **> 35** indicates **identity** Score **> 18** indicates **homology** | U | K.FNIWGGSLSLGHPFGATGCR.L |
| 135350 | 418 | – | 437 | 1067.5190 | 2133.0234 | 2133.0215 | 0.92 | 0 | 84 | 1.2e-08 | 1Score **> 35** indicates **identity** Score **> 18** indicates **homology** | U | K.FNIWGGSLSLGHPFGATGCR.L |
| 135351 | 418 | – | 437 | 712.0152 | 2133.0237 | 2133.0215 | 1.07 | 0 | 24 | 0.006 | 1Score **> 35** indicates **identity** Score **> 14** indicates **homology** | U | K.FNIWGGSLSLGHPFGATGCR.L |
| 135352 | 418 | – | 437 | 712.0152 | 2133.0239 | 2133.0215 | 1.15 | 0 | 54 | 9.3e-06 | 1Score **> 35** indicates **identity** Score **> 16** indicates **homology** | U | K.FNIWGGSLSLGHPFGATGCR.L |
| 135353 | 418 | – | 437 | 1067.5193 | 2133.0240 | 2133.0215 | 1.19 | 0 | 121 | 4.5e-12 | 1Score **> 35** indicates **identity** Score **> 20** indicates **homology** | U | K.FNIWGGSLSLGHPFGATGCR.L |
| 135355 | 418 | – | 437 | 712.0154 | 2133.0242 | 2133.0215 | 1.31 | 0 | 23 | 0.0064 | 1Score **> 35** indicates **identity** Score **> 14** indicates **homology** | U | K.FNIWGGSLSLGHPFGATGCR.L |
| 135356 | 418 | – | 437 | 1067.5194 | 2133.0242 | 2133.0215 | 1.31 | 0 | 95 | 1.2e-09 | 1Score **> 35** indicates **identity** Score **> 18** indicates **homology** | U | K.FNIWGGSLSLGHPFGATGCR.L |
| 135364 | 418 | – | 437 | 1067.5238 | 2133.0330 | 2133.0215 | 5.44 | 0 | 107 | 8.8e-11 | 1Score **> 36** indicates **identity** Score **> 19** indicates **homology** | U | K.FNIWGGSLSLGHPFGATGCR.L |
| 135366 | 418 | – | 437 | 1067.5263 | 2133.0381 | 2133.0215 | 7.80 | 0 | 112 | 3e-11 | 1Score **> 36** indicates **identity** Score **> 20** indicates **homology** | U | K.FNIWGGSLSLGHPFGATGCR.L |
| 5910 | 438 | – | 445 | 423.2362 | 844.4579 | 844.4589 | -1.21 | 0 | 41 | 0.0007 | 1Score **> 32** indicates **identity** Score **> 22** indicates **homology** | U | R.LVMAAANR.L |
| 5911 | 438 | – | 445 | 423.2368 | 844.4590 | 844.4589 | 0.13 | 0 | 53 | 0.00016 | 1Score **> 32** indicates **identity** Score **> 27** indicates **homology** | U | R.LVMAAANR.L |
| 6849 | 438 | – | 445 | 431.2330 | 860.4515 | 860.4538 | -2.68 | 0 | 24 | 0.0053 | 1Score **> 29** indicates **identity** Score **> 14** indicates **homology** | U | R.LVMAAANR.L  + Oxidation (M) |
| 6852 | 438 | – | 445 | 431.2337 | 860.4528 | 860.4538 | -1.11 | 0 | 26 | 0.016 | 1Score **> 31** indicates **identity** Score **> 20** indicates **homology** | U | R.LVMAAANR.L  + Oxidation (M) |
| 6853 | 438 | – | 445 | 431.2338 | 860.4530 | 860.4538 | -0.88 | 0 | 19 | 0.033 | 1Score **> 31** indicates **identity** Score **> 16** indicates **homology** | U | R.LVMAAANR.L  + Oxidation (M) |
| 6854 | 438 | – | 445 | 431.2338 | 860.4531 | 860.4538 | -0.75 | 0 | 30 | 0.0048 | 1Score **> 31** indicates **identity** Score **> 19** indicates **homology** | U | R.LVMAAANR.L  + Oxidation (M) |
| 6855 | 438 | – | 445 | 431.2340 | 860.4534 | 860.4538 | -0.40 | 0 | 26 | 0.01 | 1Score **> 31** indicates **identity** Score **> 19** indicates **homology** | U | R.LVMAAANR.L  + Oxidation (M) |
| 6856 | 438 | – | 445 | 431.2341 | 860.4537 | 860.4538 | -0.13 | 0 | 15 | 0.039 | 1Score **> 31** indicates **identity** Score **> 13** indicates **homology** | U | R.LVMAAANR.L  + Oxidation (M) |
| 6857 | 438 | – | 445 | 431.2342 | 860.4538 | 860.4538 | -0.042 | 0 | 23 | 0.015 | 1Score **> 31** indicates **identity** Score **> 17** indicates **homology** | U | R.LVMAAANR.L  + Oxidation (M) |
| 6858 | 438 | – | 445 | 431.2343 | 860.4540 | 860.4538 | 0.25 | 0 | 17 | 0.025 | 1Score **> 31** indicates **identity** Score **> 14** indicates **homology** | U | R.LVMAAANR.L  + Oxidation (M) |
| 6859 | 438 | – | 445 | 431.2343 | 860.4540 | 860.4538 | 0.25 | 0 | 20 | 0.029 | 1Score **> 31** indicates **identity** Score **> 17** indicates **homology** | U | R.LVMAAANR.L  + Oxidation (M) |
| 6861 | 438 | – | 445 | 431.2344 | 860.4543 | 860.4538 | 0.59 | 0 | 23 | 0.013 | 1Score **> 31** indicates **identity** Score **> 17** indicates **homology** | U | R.LVMAAANR.L  + Oxidation (M) |
| 6862 | 438 | – | 445 | 431.2352 | 860.4559 | 860.4538 | 2.45 | 0 | 20 | 0.012 | 1Score **> 31** indicates **identity** Score **> 14** indicates **homology** | U | R.LVMAAANR.L  + Oxidation (M) |
| 174320 | 448 | – | 475 | 709.0996 | 2832.3693 | 2832.3687 | 0.21 | 1 | 39 | 0.00024 | 1Score **> 37** indicates **identity** Score **> 15** indicates **homology** | U | R.KDGGQYALVAACAAGGQGHAMIVEAYPK.- |
| 174321 | 448 | – | 475 | 945.1306 | 2832.3700 | 2832.3687 | 0.47 | 1 | 40 | 0.00017 | 1Score **> 37** indicates **identity** Score **> 15** indicates **homology** | U | R.KDGGQYALVAACAAGGQGHAMIVEAYPK.- |
| 174323 | 448 | – | 475 | 709.1026 | 2832.3811 | 2832.3687 | 4.38 | 1 | 45 | 5.5e-05 | 1Score **> 37** indicates **identity** Score **> 15** indicates **homology** | U | R.KDGGQYALVAACAAGGQGHAMIVEAYPK.- |
| 174400 | 448 | – | 475 | 709.3492 | 2833.3675 | 2833.3527 | 5.23 | 1 | 41 | 0.00014 | 1Score **> 37** indicates **identity** Score **> 15** indicates **homology** | U | R.KDGGQYALVAACAAGGQGHAMIVEAYPK.-  + Deamidated (NQ) |
| 169391 | 449 | – | 475 | 902.4291 | 2704.2653 | 2704.2737 | -3.11 | 0 | 16 | 0.034 | 1Score **> 35** indicates **identity** Score **> 13** indicates **homology** | U | K.DGGQYALVAACAAGGQGHAMIVEAYPK.- |
| 169395 | 449 | – | 475 | 902.4322 | 2704.2748 | 2704.2737 | 0.38 | 0 | 70 | 2.8e-07 | 1Score **> 35** indicates **identity** Score **> 17** indicates **homology** | U | K.DGGQYALVAACAAGGQGHAMIVEAYPK.- |
| 169396 | 449 | – | 475 | 902.4322 | 2704.2749 | 2704.2737 | 0.42 | 0 | 53 | 1e-05 | 1Score **> 35** indicates **identity** Score **> 16** indicates **homology** | U | K.DGGQYALVAACAAGGQGHAMIVEAYPK.- |
| 169397 | 449 | – | 475 | 1353.1466 | 2704.2786 | 2704.2737 | 1.81 | 0 | 105 | 1.6e-10 | 1Score **> 35** indicates **identity** Score **> 19** indicates **homology** | U | K.DGGQYALVAACAAGGQGHAMIVEAYPK.- |
| 169399 | 449 | – | 475 | 902.4338 | 2704.2797 | 2704.2737 | 2.19 | 0 | 80 | 3.1e-08 | 1Score **> 36** indicates **identity** Score **> 18** indicates **homology** | U | K.DGGQYALVAACAAGGQGHAMIVEAYPK.- |
| 169400 | 449 | – | 475 | 902.4339 | 2704.2798 | 2704.2737 | 2.24 | 0 | 66 | 6.7e-07 | 1Score **> 36** indicates **identity** Score **> 17** indicates **homology** | U | K.DGGQYALVAACAAGGQGHAMIVEAYPK.- |
| 169402 | 449 | – | 475 | 902.4341 | 2704.2806 | 2704.2737 | 2.52 | 0 | 34 | 0.00064 | 1Score **> 36** indicates **identity** Score **> 15** indicates **homology** | U | K.DGGQYALVAACAAGGQGHAMIVEAYPK.- |
| 169404 | 449 | – | 475 | 902.4373 | 2704.2901 | 2704.2737 | 6.06 | 0 | 18 | 0.021 | 1Score **> 36** indicates **identity** Score **> 14** indicates **homology** | U | K.DGGQYALVAACAAGGQGHAMIVEAYPK.- |
| 169407 | 449 | – | 475 | 1353.1537 | 2704.2929 | 2704.2737 | 7.08 | 0 | 55 | 6.4e-06 | 1Score **> 36** indicates **identity** Score **> 16** indicates **homology** | U | K.DGGQYALVAACAAGGQGHAMIVEAYPK.- |
| 169428 | 449 | – | 475 | 902.7642 | 2705.2708 | 2705.2578 | 4.84 | 0 | 53 | 1.1e-05 | 1Score **> 35** indicates **identity** Score **> 16** indicates **homology** | U | K.DGGQYALVAACAAGGQGHAMIVEAYPK.-  + Deamidated (NQ) |
| 173974 | 449 | – | 475 | 1411.1715 | 2820.3284 | 2820.3462 | -6.31 | 0 | 19 | 0.015 | 1Score **> 36** indicates **identity** Score **> 14** indicates **homology** | U | K.DGGQYALVAACAAGGQGHAMIVEAYPK.-  + Deamidated (NQ); HNE (C); Oxidation (M) |

---

```
ID   ECHB_MOUSE              Reviewed;         475 AA.
AC   Q99JY0; Q3TEH9; Q8BJI5; Q8BJM0; Q8BK52;
DT   16-AUG-2004, integrated into UniProtKB/Swiss-Prot.
DT   01-JUN-2001, sequence version 1.
DT   28-JUN-2023, entry version 165.
DE   RecName: Full=Trifunctional enzyme subunit beta, mitochondrial;
DE   AltName: Full=TP-beta;
DE   Includes:
DE     RecName: Full=3-ketoacyl-CoA thiolase;
DE              EC=2.3.1.155 {ECO:0000250|UniProtKB:P55084};
DE              EC=2.3.1.16 {ECO:0000250|UniProtKB:P55084};
DE     AltName: Full=Acetyl-CoA acyltransferase;
DE     AltName: Full=Beta-ketothiolase;
DE   Flags: Precursor;
GN   Name=Hadhb;
OS   Mus musculus (Mouse).
OC   Eukaryota; Metazoa; Chordata; Craniata; Vertebrata; Euteleostomi; Mammalia;
OC   Eutheria; Euarchontoglires; Glires; Rodentia; Myomorpha; Muroidea; Muridae;
OC   Murinae; Mus; Mus.
OX   NCBI_TaxID=10090;
RN   [1]
RP   NUCLEOTIDE SEQUENCE [LARGE SCALE MRNA].
RC   STRAIN=C57BL/6J;
RC   TISSUE=Bone marrow, Colon, Hippocampus, Spinal ganglion, Testis, and
RC   Thymus;
RX   PubMed=16141072; DOI=10.1126/science.1112014;
RA   Carninci P., Kasukawa T., Katayama S., Gough J., Frith M.C., Maeda N.,
RA   Oyama R., Ravasi T., Lenhard B., Wells C., Kodzius R., Shimokawa K.,
RA   Bajic V.B., Brenner S.E., Batalov S., Forrest A.R., Zavolan M., Davis M.J.,
RA   Wilming L.G., Aidinis V., Allen J.E., Ambesi-Impiombato A., Apweiler R.,
RA   Aturaliya R.N., Bailey T.L., Bansal M., Baxter L., Beisel K.W., Bersano T.,
RA   Bono H., Chalk A.M., Chiu K.P., Choudhary V., Christoffels A.,
RA   Clutterbuck D.R., Crowe M.L., Dalla E., Dalrymple B.P., de Bono B.,
RA   Della Gatta G., di Bernardo D., Down T., Engstrom P., Fagiolini M.,
RA   Faulkner G., Fletcher C.F., Fukushima T., Furuno M., Futaki S.,
RA   Gariboldi M., Georgii-Hemming P., Gingeras T.R., Gojobori T., Green R.E.,
RA   Gustincich S., Harbers M., Hayashi Y., Hensch T.K., Hirokawa N., Hill D.,
RA   Huminiecki L., Iacono M., Ikeo K., Iwama A., Ishikawa T., Jakt M.,
RA   Kanapin A., Katoh M., Kawasawa Y., Kelso J., Kitamura H., Kitano H.,
RA   Kollias G., Krishnan S.P., Kruger A., Kummerfeld S.K., Kurochkin I.V.,
RA   Lareau L.F., Lazarevic D., Lipovich L., Liu J., Liuni S., McWilliam S.,
RA   Madan Babu M., Madera M., Marchionni L., Matsuda H., Matsuzawa S., Miki H.,
RA   Mignone F., Miyake S., Morris K., Mottagui-Tabar S., Mulder N., Nakano N.,
RA   Nakauchi H., Ng P., Nilsson R., Nishiguchi S., Nishikawa S., Nori F.,
RA   Ohara O., Okazaki Y., Orlando V., Pang K.C., Pavan W.J., Pavesi G.,
RA   Pesole G., Petrovsky N., Piazza S., Reed J., Reid J.F., Ring B.Z.,
RA   Ringwald M., Rost B., Ruan Y., Salzberg S.L., Sandelin A., Schneider C.,
RA   Schoenbach C., Sekiguchi K., Semple C.A., Seno S., Sessa L., Sheng Y.,
RA   Shibata Y., Shimada H., Shimada K., Silva D., Sinclair B., Sperling S.,
RA   Stupka E., Sugiura K., Sultana R., Takenaka Y., Taki K., Tammoja K.,
RA   Tan S.L., Tang S., Taylor M.S., Tegner J., Teichmann S.A., Ueda H.R.,
RA   van Nimwegen E., Verardo R., Wei C.L., Yagi K., Yamanishi H.,
RA   Zabarovsky E., Zhu S., Zimmer A., Hide W., Bult C., Grimmond S.M.,
RA   Teasdale R.D., Liu E.T., Brusic V., Quackenbush J., Wahlestedt C.,
RA   Mattick J.S., Hume D.A., Kai C., Sasaki D., Tomaru Y., Fukuda S.,
RA   Kanamori-Katayama M., Suzuki M., Aoki J., Arakawa T., Iida J., Imamura K.,
RA   Itoh M., Kato T., Kawaji H., Kawagashira N., Kawashima T., Kojima M.,
RA   Kondo S., Konno H., Nakano K., Ninomiya N., Nishio T., Okada M., Plessy C.,
RA   Shibata K., Shiraki T., Suzuki S., Tagami M., Waki K., Watahiki A.,
RA   Okamura-Oho Y., Suzuki H., Kawai J., Hayashizaki Y.;
RT   "The transcriptional landscape of the mammalian genome.";
RL   Science 309:1559-1563(2005).
RN   [2]
RP   NUCLEOTIDE SEQUENCE [LARGE SCALE MRNA].
RC   STRAIN=FVB/N; TISSUE=Mammary tumor;
RX   PubMed=15489334; DOI=10.1101/gr.2596504;
RG   The MGC Project Team;
RT   "The status, quality, and expansion of the NIH full-length cDNA project:
RT   the Mammalian Gene Collection (MGC).";
RL   Genome Res. 14:2121-2127(2004).
RN   [3]
RP   IDENTIFICATION BY MASS SPECTROMETRY [LARGE SCALE ANALYSIS].
RC   TISSUE=Brain, Brown adipose tissue, Heart, Kidney, Liver, Lung,
RC   Pancreas, Spleen, and Testis;
RX   PubMed=21183079; DOI=10.1016/j.cell.2010.12.001;
RA   Huttlin E.L., Jedrychowski M.P., Elias J.E., Goswami T., Rad R.,
RA   Beausoleil S.A., Villen J., Haas W., Sowa M.E., Gygi S.P.;
RT   "A tissue-specific atlas of mouse protein phosphorylation and expression.";
RL   Cell 143:1174-1189(2010).
RN   [4]
RP   SUCCINYLATION [LARGE SCALE ANALYSIS] AT LYS-53; LYS-73; LYS-189; LYS-191;
RP   LYS-273; LYS-292; LYS-294 AND LYS-333, AND IDENTIFICATION BY MASS
RP   SPECTROMETRY [LARGE SCALE ANALYSIS].
RC   TISSUE=Embryonic fibroblast, and Liver;
RX   PubMed=23806337; DOI=10.1016/j.molcel.2013.06.001;
RA   Park J., Chen Y., Tishkoff D.X., Peng C., Tan M., Dai L., Xie Z., Zhang Y.,
RA   Zwaans B.M., Skinner M.E., Lombard D.B., Zhao Y.;
RT   "SIRT5-mediated lysine desuccinylation impacts diverse metabolic
RT   pathways.";
RL   Mol. Cell 50:919-930(2013).
RN   [5]
RP   ACETYLATION [LARGE SCALE ANALYSIS] AT LYS-73; LYS-189; LYS-294; LYS-299;
RP   LYS-333; LYS-349 AND LYS-362, AND IDENTIFICATION BY MASS SPECTROMETRY
RP   [LARGE SCALE ANALYSIS].
RC   TISSUE=Liver;
RX   PubMed=23576753; DOI=10.1073/pnas.1302961110;
RA   Rardin M.J., Newman J.C., Held J.M., Cusack M.P., Sorensen D.J., Li B.,
RA   Schilling B., Mooney S.D., Kahn C.R., Verdin E., Gibson B.W.;
RT   "Label-free quantitative proteomics of the lysine acetylome in mitochondria
RT   identifies substrates of SIRT3 in metabolic pathways.";
RL   Proc. Natl. Acad. Sci. U.S.A. 110:6601-6606(2013).
RN   [6]
RP   INTERACTION WITH MTLN.
RX   PubMed=29949755; DOI=10.1016/j.celrep.2018.05.058;
RA   Makarewich C.A., Baskin K.K., Munir A.Z., Bezprozvannaya S., Sharma G.,
RA   Khemtong C., Shah A.M., McAnally J.R., Malloy C.R., Szweda L.I.,
RA   Bassel-Duby R., Olson E.N.;
RT   "MOXI Is a Mitochondrial Micropeptide That Enhances Fatty Acid beta-
RT   Oxidation.";
RL   Cell Rep. 23:3701-3709(2018).
CC   -!- FUNCTION: Mitochondrial trifunctional enzyme catalyzes the last three
CC       of the four reactions of the mitochondrial beta-oxidation pathway. The
CC       mitochondrial beta-oxidation pathway is the major energy-producing
CC       process in tissues and is performed through four consecutive reactions
CC       breaking down fatty acids into acetyl-CoA. Among the enzymes involved
CC       in this pathway, the trifunctional enzyme exhibits specificity for
CC       long-chain fatty acids. Mitochondrial trifunctional enzyme is a
CC       heterotetrameric complex composed of two proteins, the trifunctional
CC       enzyme subunit alpha/HADHA carries the 2,3-enoyl-CoA hydratase and the
CC       3-hydroxyacyl-CoA dehydrogenase activities, while the trifunctional
CC       enzyme subunit beta/HADHB described here bears the 3-ketoacyl-CoA
CC       thiolase activity. {ECO:0000250|UniProtKB:P55084}.
CC   -!- CATALYTIC ACTIVITY:
CC       Reaction=acetyl-CoA + an acyl-CoA = a 3-oxoacyl-CoA + CoA;
CC         Xref=Rhea:RHEA:21564, ChEBI:CHEBI:57287, ChEBI:CHEBI:57288,
CC         ChEBI:CHEBI:58342, ChEBI:CHEBI:90726; EC=2.3.1.16;
CC         Evidence={ECO:0000250|UniProtKB:P55084};
CC       PhysiologicalDirection=right-to-left; Xref=Rhea:RHEA:21566;
CC         Evidence={ECO:0000250|UniProtKB:P55084};
CC   -!- CATALYTIC ACTIVITY:
CC       Reaction=acetyl-CoA + butanoyl-CoA = 3-oxohexanoyl-CoA + CoA;
CC         Xref=Rhea:RHEA:31111, ChEBI:CHEBI:57287, ChEBI:CHEBI:57288,
CC         ChEBI:CHEBI:57371, ChEBI:CHEBI:62418;
CC         Evidence={ECO:0000250|UniProtKB:P55084};
CC       PhysiologicalDirection=right-to-left; Xref=Rhea:RHEA:31113;
CC         Evidence={ECO:0000250|UniProtKB:P55084};
CC   -!- CATALYTIC ACTIVITY:
CC       Reaction=acetyl-CoA + hexanoyl-CoA = 3-oxooctanoyl-CoA + CoA;
CC         Xref=Rhea:RHEA:31203, ChEBI:CHEBI:57287, ChEBI:CHEBI:57288,
CC         ChEBI:CHEBI:62619, ChEBI:CHEBI:62620;
CC         Evidence={ECO:0000250|UniProtKB:P55084};
CC       PhysiologicalDirection=right-to-left; Xref=Rhea:RHEA:31205;
CC         Evidence={ECO:0000250|UniProtKB:P55084};
CC   -!- CATALYTIC ACTIVITY:
CC       Reaction=acetyl-CoA + octanoyl-CoA = 3-oxodecanoyl-CoA + CoA;
CC         Xref=Rhea:RHEA:31087, ChEBI:CHEBI:57287, ChEBI:CHEBI:57288,
CC         ChEBI:CHEBI:57386, ChEBI:CHEBI:62548;
CC         Evidence={ECO:0000250|UniProtKB:P55084};
CC       PhysiologicalDirection=right-to-left; Xref=Rhea:RHEA:31089;
CC         Evidence={ECO:0000250|UniProtKB:P55084};
CC   -!- CATALYTIC ACTIVITY:
CC       Reaction=acetyl-CoA + decanoyl-CoA = 3-oxododecanoyl-CoA + CoA;
CC         Xref=Rhea:RHEA:31183, ChEBI:CHEBI:57287, ChEBI:CHEBI:57288,
CC         ChEBI:CHEBI:61430, ChEBI:CHEBI:62615;
CC         Evidence={ECO:0000250|UniProtKB:P55084};
CC       PhysiologicalDirection=right-to-left; Xref=Rhea:RHEA:31185;
CC         Evidence={ECO:0000250|UniProtKB:P55084};
CC   -!- CATALYTIC ACTIVITY:
CC       Reaction=acetyl-CoA + dodecanoyl-CoA = 3-oxotetradecanoyl-CoA + CoA;
CC         Xref=Rhea:RHEA:31091, ChEBI:CHEBI:57287, ChEBI:CHEBI:57288,
CC         ChEBI:CHEBI:57375, ChEBI:CHEBI:62543;
CC         Evidence={ECO:0000250|UniProtKB:P55084};
CC       PhysiologicalDirection=right-to-left; Xref=Rhea:RHEA:31093;
CC         Evidence={ECO:0000250|UniProtKB:P55084};
CC   -!- CATALYTIC ACTIVITY:
CC       Reaction=acetyl-CoA + tetradecanoyl-CoA = 3-oxohexadecanoyl-CoA + CoA;
CC         Xref=Rhea:RHEA:18161, ChEBI:CHEBI:57287, ChEBI:CHEBI:57288,
CC         ChEBI:CHEBI:57349, ChEBI:CHEBI:57385; EC=2.3.1.155;
CC         Evidence={ECO:0000250|UniProtKB:P55084};
CC       PhysiologicalDirection=right-to-left; Xref=Rhea:RHEA:18163;
CC         Evidence={ECO:0000250|UniProtKB:P55084};
CC   -!- PATHWAY: Lipid metabolism; fatty acid beta-oxidation.
CC       {ECO:0000250|UniProtKB:P55084}.
CC   -!- SUBUNIT: Heterotetramer of 2 alpha/HADHA and 2 beta/HADHB subunits;
CC       forms the mitochondrial trifunctional enzyme (By similarity). Also
CC       purified as higher order heterooligomers including a 4 alpha/HADHA and
CC       4 beta/HADHB heterooligomer which physiological significance remains
CC       unclear (By similarity). The mitochondrial trifunctional enzyme
CC       interacts with MTLN (PubMed:29949755). Interacts with RSAD2/viperin (By
CC       similarity). {ECO:0000250|UniProtKB:P55084,
CC       ECO:0000269|PubMed:29949755}.
CC   -!- SUBCELLULAR LOCATION: Mitochondrion {ECO:0000250|UniProtKB:P55084}.
CC       Mitochondrion inner membrane {ECO:0000250|UniProtKB:P55084}.
CC       Mitochondrion outer membrane {ECO:0000250|UniProtKB:P55084}.
CC       Endoplasmic reticulum {ECO:0000250|UniProtKB:P55084}. Note=Protein
CC       stability and association with membranes require HADHA.
CC       {ECO:0000250|UniProtKB:P55084}.
CC   -!- PTM: Acetylation of Lys-202 is observed in liver mitochondria from
CC       fasted mice but not from fed mice.
CC   -!- SIMILARITY: Belongs to the thiolase-like superfamily. Thiolase family.
CC       {ECO:0000305}.
CC   ---------------------------------------------------------------------------
CC   Copyrighted by the UniProt Consortium, see https://www.uniprot.org/terms
CC   Distributed under the Creative Commons Attribution (CC BY 4.0) License
CC   ---------------------------------------------------------------------------
DR   EMBL; AK033462; BAC28300.1; -; mRNA.
DR   EMBL; AK076814; BAC36493.1; -; mRNA.
DR   EMBL; AK083164; BAC38790.1; -; mRNA.
DR   EMBL; AK083767; BAC39015.1; -; mRNA.
DR   EMBL; AK150889; BAE29936.1; -; mRNA.
DR   EMBL; AK169637; BAE41269.1; -; mRNA.
DR   EMBL; BC005585; AAH05585.1; -; mRNA.
DR   CCDS; CCDS39045.1; -.
DR   RefSeq; NP_001276727.1; NM_001289798.1.
DR   RefSeq; NP_001276728.1; NM_001289799.1.
DR   RefSeq; NP_663533.1; NM_145558.2.
DR   RefSeq; XP_017176317.1; XM_017320828.1.
DR   RefSeq; XP_017176318.1; XM_017320829.1.
DR   AlphaFoldDB; Q99JY0; -.
DR   SMR; Q99JY0; -.
DR   BioGRID; 231080; 33.
DR   IntAct; Q99JY0; 7.
DR   MINT; Q99JY0; -.
DR   STRING; 10090.ENSMUSP00000110434; -.
DR   iPTMnet; Q99JY0; -.
DR   PhosphoSitePlus; Q99JY0; -.
DR   SwissPalm; Q99JY0; -.
DR   EPD; Q99JY0; -.
DR   jPOST; Q99JY0; -.
DR   MaxQB; Q99JY0; -.
DR   PaxDb; Q99JY0; -.
DR   PeptideAtlas; Q99JY0; -.
DR   ProteomicsDB; 277669; -.
DR   Antibodypedia; 27848; 265 antibodies from 30 providers.
DR   DNASU; 231086; -.
DR   Ensembl; ENSMUST00000026841; ENSMUSP00000026841; ENSMUSG00000059447.
DR   Ensembl; ENSMUST00000114783; ENSMUSP00000110431; ENSMUSG00000059447.
DR   Ensembl; ENSMUST00000114786; ENSMUSP00000110434; ENSMUSG00000059447.
DR   GeneID; 231086; -.
DR   KEGG; mmu:231086; -.
DR   UCSC; uc008wve.2; mouse.
DR   AGR; MGI:2136381; -.
DR   CTD; 3032; -.
DR   MGI; MGI:2136381; Hadhb.
DR   VEuPathDB; HostDB:ENSMUSG00000059447; -.
DR   eggNOG; KOG1392; Eukaryota.
DR   GeneTree; ENSGT01030000234626; -.
DR   HOGENOM; CLU_031026_2_0_1; -.
DR   InParanoid; Q99JY0; -.
DR   OMA; MTAFPEP; -.
DR   OrthoDB; 1826604at2759; -.
DR   PhylomeDB; Q99JY0; -.
DR   TreeFam; TF315243; -.
DR   Reactome; R-MMU-1482798; Acyl chain remodeling of CL.
DR   Reactome; R-MMU-77285; Beta oxidation of myristoyl-CoA to lauroyl-CoA.
DR   Reactome; R-MMU-77305; Beta oxidation of palmitoyl-CoA to myristoyl-CoA.
DR   Reactome; R-MMU-77310; Beta oxidation of lauroyl-CoA to decanoyl-CoA-CoA.
DR   Reactome; R-MMU-77346; Beta oxidation of decanoyl-CoA to octanoyl-CoA-CoA.
DR   Reactome; R-MMU-77348; Beta oxidation of octanoyl-CoA to hexanoyl-CoA.
DR   Reactome; R-MMU-77350; Beta oxidation of hexanoyl-CoA to butanoyl-CoA.
DR   UniPathway; UPA00659; -.
DR   BioGRID-ORCS; 231086; 1 hit in 77 CRISPR screens.
DR   ChiTaRS; Hadhb; mouse.
DR   PRO; PR:Q99JY0; -.
DR   Proteomes; UP000000589; Chromosome 5.
DR   RNAct; Q99JY0; protein.
DR   Bgee; ENSMUSG00000059447; Expressed in spermatocyte and 170 other tissues.
DR   ExpressionAtlas; Q99JY0; baseline and differential.
DR   Genevisible; Q99JY0; MM.
DR   GO; GO:0005783; C:endoplasmic reticulum; ISS:UniProtKB.
DR   GO; GO:0016507; C:mitochondrial fatty acid beta-oxidation multienzyme complex; ISO:MGI.
DR   GO; GO:0005743; C:mitochondrial inner membrane; HDA:MGI.
DR   GO; GO:0042645; C:mitochondrial nucleoid; ISO:MGI.
DR   GO; GO:0005741; C:mitochondrial outer membrane; ISS:UniProtKB.
DR   GO; GO:0005739; C:mitochondrion; IDA:MGI.
DR   GO; GO:0003985; F:acetyl-CoA C-acetyltransferase activity; IDA:MGI.
DR   GO; GO:0003988; F:acetyl-CoA C-acyltransferase activity; IDA:MGI.
DR   GO; GO:0050633; F:acetyl-CoA C-myristoyltransferase activity; IEA:UniProtKB-EC.
DR   GO; GO:0106222; F:lncRNA binding; IDA:MGI.
DR   GO; GO:0044877; F:protein-containing complex binding; ISO:MGI.
DR   GO; GO:0071222; P:cellular response to lipopolysaccharide; IDA:MGI.
DR   GO; GO:0006635; P:fatty acid beta-oxidation; ISO:MGI.
DR   GO; GO:0010467; P:gene expression; IMP:MGI.
DR   CDD; cd00751; thiolase; 1.
DR   Gene3D; 3.40.47.10; -; 1.
DR   InterPro; IPR002155; Thiolase.
DR   InterPro; IPR016039; Thiolase-like.
DR   InterPro; IPR020615; Thiolase_acyl_enz_int_AS.
DR   InterPro; IPR020610; Thiolase_AS.
DR   InterPro; IPR020617; Thiolase_C.
DR   InterPro; IPR020613; Thiolase_CS.
DR   InterPro; IPR020616; Thiolase_N.
DR   PANTHER; PTHR18919; ACETYL-COA C-ACYLTRANSFERASE; 1.
DR   PANTHER; PTHR18919:SF153; TRIFUNCTIONAL ENZYME SUBUNIT BETA, MITOCHONDRIAL; 1.
DR   Pfam; PF02803; Thiolase_C; 1.
DR   Pfam; PF00108; Thiolase_N; 1.
DR   SUPFAM; SSF53901; Thiolase-like; 2.
DR   PROSITE; PS00098; THIOLASE_1; 1.
DR   PROSITE; PS00737; THIOLASE_2; 1.
DR   PROSITE; PS00099; THIOLASE_3; 1.
DR   TIGRFAMs; TIGR01930; AcCoA-C-Actrans; 1.
PE   1: Evidence at protein level;
KW   Acetylation; Acyltransferase; Endoplasmic reticulum; Fatty acid metabolism;
KW   Lipid metabolism; Membrane; Mitochondrion; Mitochondrion inner membrane;
KW   Mitochondrion outer membrane; Reference proteome; Transferase;
KW   Transit peptide.
FT   TRANSIT         1..34
FT                   /note="Mitochondrion"
FT                   /evidence="ECO:0000250"
FT   CHAIN           35..475
FT                   /note="Trifunctional enzyme subunit beta, mitochondrial"
FT                   /id="PRO_0000034082"
FT   INTRAMEM        174..221
FT                   /evidence="ECO:0000250|UniProtKB:P55084"
FT   ACT_SITE        139
FT                   /note="Acyl-thioester intermediate"
FT                   /evidence="ECO:0000250|UniProtKB:P55084"
FT   ACT_SITE        459
FT                   /note="Proton donor/acceptor"
FT                   /evidence="ECO:0000250|UniProtKB:P55084"
FT   SITE            429
FT                   /note="Increases nucleophilicity of active site Cys"
FT                   /evidence="ECO:0000250|UniProtKB:P55084"
FT   MOD_RES         53
FT                   /note="N6-succinyllysine"
FT                   /evidence="ECO:0007744|PubMed:23806337"
FT   MOD_RES         73
FT                   /note="N6-acetyllysine; alternate"
FT                   /evidence="ECO:0007744|PubMed:23576753"
FT   MOD_RES         73
FT                   /note="N6-succinyllysine; alternate"
FT                   /evidence="ECO:0007744|PubMed:23806337"
FT   MOD_RES         189
FT                   /note="N6-acetyllysine; alternate"
FT                   /evidence="ECO:0007744|PubMed:23576753"
FT   MOD_RES         189
FT                   /note="N6-succinyllysine; alternate"
FT                   /evidence="ECO:0007744|PubMed:23806337"
FT   MOD_RES         191
FT                   /note="N6-succinyllysine"
FT                   /evidence="ECO:0007744|PubMed:23806337"
FT   MOD_RES         273
FT                   /note="N6-succinyllysine"
FT                   /evidence="ECO:0007744|PubMed:23806337"
FT   MOD_RES         292
FT                   /note="N6-succinyllysine"
FT                   /evidence="ECO:0007744|PubMed:23806337"
FT   MOD_RES         294
FT                   /note="N6-acetyllysine; alternate"
FT                   /evidence="ECO:0007744|PubMed:23576753"
FT   MOD_RES         294
FT                   /note="N6-succinyllysine; alternate"
FT                   /evidence="ECO:0007744|PubMed:23806337"
FT   MOD_RES         299
FT                   /note="N6-acetyllysine"
FT                   /evidence="ECO:0007744|PubMed:23576753"
FT   MOD_RES         333
FT                   /note="N6-acetyllysine; alternate"
FT                   /evidence="ECO:0007744|PubMed:23576753"
FT   MOD_RES         333
FT                   /note="N6-succinyllysine; alternate"
FT                   /evidence="ECO:0007744|PubMed:23806337"
FT   MOD_RES         349
FT                   /note="N6-acetyllysine"
FT                   /evidence="ECO:0007744|PubMed:23576753"
FT   MOD_RES         362
FT                   /note="N6-acetyllysine"
FT                   /evidence="ECO:0007744|PubMed:23576753"
FT   CONFLICT        24..25
FT                   /note="IR -> HK (in Ref. 1; BAC36493)"
FT                   /evidence="ECO:0000305"
FT   CONFLICT        425
FT                   /note="L -> M (in Ref. 1; BAC38790)"
FT                   /evidence="ECO:0000305"
FT   CONFLICT        450
FT                   /note="G -> R (in Ref. 1; BAC38790)"
FT                   /evidence="ECO:0000305"
FT   CONFLICT        450
FT                   /note="G -> V (in Ref. 1; BAC39015)"
FT                   /evidence="ECO:0000305"
SQ   SEQUENCE   475 AA;  51386 MW;  F131B497C4F5FAF4 CRC64;
     MTTILTSTFR NLSTTSKWAL RSSIRPLSCS SQLHSAPAVQ TKSKKTLAKP NMKNIVVVEG
     VRIPFLLSGT SYKDLMPHDL ARAALSGLLH RTNIPKDVVD YIIFGTVIQE VKTSNVAREA
     ALGAGFSDKT PAHTVTMACI SSNQAMTTAV GLIASGQCDV VVAGGVELMS DVPIRHSRNM
     RKMMLDLNKA KTLGQRLSLL SKFRLNFLSP ELPAVAEFST NETMGHSADR LAAAFAVSRM
     EQDEYALRSH SLAKKAQDEG HLSDIVPFKV PGKDTVTKDN GIRPSSLEQM AKLKPAFIKP
     YGTVTAANSS FLTDGASAML IMSEDRALAM GYKPKAYLRD FIYVSQDPKD QLLLGPTYAT
     PKVLEKAGLT MNDIDAFEFH EAFSGQILAN FKAMDSDWFA QNYMGRKTKV GSPPLEKFNI
     WGGSLSLGHP FGATGCRLVM AAANRLRKDG GQYALVAACA AGGQGHAMIV EAYPK
//
```

|  |
| --- |
| **Mascot:** http://www.matrixscience.com/ |

Oxidation (M) (+15.9949)
