# Supplementary material for: A common East-Asian ALDH2 mutation causes metabolic disorders and the therapeutic effect of ALDH2 activators
Source: Nat Commun. 2023 Sep 25;14:5971. doi: 10.1038/s41467-023-41570-6 (PMC10520061; doi:10.1038/s41467-023-41570-6)
Supplement: Supplementary file 4 — Supplementary Data 1 [file 41467_2023_41570_MOESM4_ESM.zip › Table S5b/Q99JY0/Q99JY0_WTO60-1_C139_C158.html]

Mascot Search Results: Q99JY0
 

# MASCOT Search Results

## Protein View: Q99JY0

### Trifunctional enzyme subunit beta, mitochondrial OS=Mus musculus OX=10090 GN=Hadhb PE=1 SV=1

|  |  |
| --- | --- |
| Database: | Mouse\_UniProt\_proteomes |
| Score: | 8796 |
| Monoisotopic mass (Mr): | 51639 |
| Calculated pI: | 9.43 |

Sequence similarity is available as an NCBI BLAST search of Q99JY0 against nr.

### Search parameters

|  |  |
| --- | --- |
| MS data file: | `D:\LCMSMS\2023 Users' data\230529-1\230529-1-WT60-2.raw` |
| Enzyme: | Trypsin/P: cuts C-term side of KR. |
| Fixed modifications: | Carbamidomethyl (C) |
| Variable modifications: | Deamidated (NQ), HNE (C), HNE (H), HNE (K), Oxidation (M) |

### Protein sequence coverage: 75%

Matched peptides shown in ***bold red***.

|  |  |  |  |  |  |
| --- | --- | --- | --- | --- | --- |
| `1` | `MTTILTSTFR` | `NLSTTSKWAL` | `RSSIRPLSCS` | `SQLHSAPAVQ` | `TKSKKTLAKP` |
| `51` | `NMKNIVVVEG` | `VRIPFLLSGT` | `SYKDLMPHDL` | `ARAALSGLLH` | `RTNIPKDVVD` |
| `101` | `YIIFGTVIQE` | `VKTSNVAREA` | `ALGAGFSDKT` | `PAHTVTMACI` | `SSNQAMTTAV` |
| `151` | `GLIASGQCDV` | `VVAGGVELMS` | `DVPIRHSRNM` | `RKMMLDLNKA` | `KTLGQRLSLL` |
| `201` | `SKFRLNFLSP` | `ELPAVAEFST` | `NETMGHSADR` | `LAAAFAVSRM` | `EQDEYALRSH` |
| `251` | `SLAKKAQDEG` | `HLSDIVPFKV` | `PGKDTVTKDN` | `GIRPSSLEQM` | `AKLKPAFIKP` |
| `301` | `YGTVTAANSS` | `FLTDGASAML` | `IMSEDRALAM` | `GYKPKAYLRD` | `FIYVSQDPKD` |
| `351` | `QLLLGPTYAT` | `PKVLEKAGLT` | `MNDIDAFEFH` | `EAFSGQILAN` | `FKAMDSDWFA` |
| `401` | `QNYMGRKTKV` | `GSPPLEKFNI` | `WGGSLSLGHP` | `FGATGCRLVM` | `AAANRLRKDG` |
| `451` | `GQYALVAACA` | `AGGQGHAMIV` | `EAYPK` |  |  |

Unformatted sequence string: 475 residues (for pasting into other applications).

|  |  |  |  |
| --- | --- | --- | --- |
| Sort by | residue number | increasing mass | decreasing mass |
| Show | matched peptides only | predicted peptides also |  |

| Query | Start | – | End | Observed | Mr(expt) | Mr(calc) | ppm | M | Score | Expect | Rank | U | Peptide |
| --- | --- | --- | --- | --- | --- | --- | --- | --- | --- | --- | --- | --- | --- |
| 13965 | 54 | – | 62 | 492.7929 | 983.5712 | 983.5764 | -5.23 | 0 | 37 | 0.0006 | 1Score **> 29** indicates **identity** Score **> 17** indicates **homology** | U | K.NIVVVEGVR.I |
| 13966 | 54 | – | 62 | 492.7942 | 983.5739 | 983.5764 | -2.50 | 0 | 35 | 0.0005 | 1Score **> 29** indicates **identity** Score **> 15** indicates **homology** | U | K.NIVVVEGVR.I |
| 13967 | 54 | – | 62 | 492.7944 | 983.5742 | 983.5764 | -2.17 | 0 | 35 | 0.00051 | 1Score **> 29** indicates **identity** Score **> 15** indicates **homology** | U | K.NIVVVEGVR.I |
| 13969 | 54 | – | 62 | 492.7947 | 983.5748 | 983.5764 | -1.64 | 0 | 37 | 0.00035 | 1Score **> 29** indicates **identity** Score **> 15** indicates **homology** | U | K.NIVVVEGVR.I |
| 13970 | 54 | – | 62 | 492.7949 | 983.5752 | 983.5764 | -1.20 | 0 | 35 | 0.0013 | 1Score **> 27** indicates **identity** Score **> 19** indicates **homology** | U | K.NIVVVEGVR.I |
| 13971 | 54 | – | 62 | 492.7951 | 983.5756 | 983.5764 | -0.82 | 0 | 42 | 0.00054 | 1Score **> 27** indicates **identity** Score **> 22** indicates **homology** | U | K.NIVVVEGVR.I |
| 13972 | 54 | – | 62 | 492.7951 | 983.5756 | 983.5764 | -0.77 | 0 | 28 | 0.0023 | 1Score **> 27** indicates **identity** Score **> 14** indicates **homology** | U | K.NIVVVEGVR.I |
| 13973 | 54 | – | 62 | 492.7951 | 983.5757 | 983.5764 | -0.69 | 0 | 29 | 0.0045 | 1Score **> 27** indicates **identity** Score **> 18** indicates **homology** | U | K.NIVVVEGVR.I |
| 13974 | 54 | – | 62 | 492.7952 | 983.5758 | 983.5764 | -0.57 | 0 | 31 | 0.0024 | 1Score **> 27** indicates **identity** Score **> 17** indicates **homology** | U | K.NIVVVEGVR.I |
| 13975 | 54 | – | 62 | 492.7952 | 983.5759 | 983.5764 | -0.51 | 0 | 35 | 0.00051 | 1Score **> 27** indicates **identity** Score **> 15** indicates **homology** | U | K.NIVVVEGVR.I |
| 13976 | 54 | – | 62 | 492.7953 | 983.5761 | 983.5764 | -0.30 | 0 | 48 | 8.5e-05 | 1Score **> 27** indicates **identity** Score **> 20** indicates **homology** | U | K.NIVVVEGVR.I |
| 13977 | 54 | – | 62 | 492.7953 | 983.5761 | 983.5764 | -0.27 | 0 | 28 | 0.0022 | 1Score **> 27** indicates **identity** Score **> 14** indicates **homology** | U | K.NIVVVEGVR.I |
| 13978 | 54 | – | 62 | 492.7954 | 983.5761 | 983.5764 | -0.24 | 0 | 44 | 0.00017 | 1Score **> 27** indicates **identity** Score **> 19** indicates **homology** | U | K.NIVVVEGVR.I |
| 13979 | 54 | – | 62 | 492.7955 | 983.5764 | 983.5764 | -0.0092 | 0 | 39 | 0.00038 | 1Score **> 27** indicates **identity** Score **> 18** indicates **homology** | U | K.NIVVVEGVR.I |
| 13980 | 54 | – | 62 | 492.7955 | 983.5765 | 983.5764 | 0.076 | 0 | 43 | 0.00042 | 1Score **> 27** indicates **identity** Score **> 21** indicates **homology** | U | K.NIVVVEGVR.I |
| 13981 | 54 | – | 62 | 492.7955 | 983.5765 | 983.5764 | 0.13 | 0 | 28 | 0.0026 | 1Score **> 27** indicates **identity** Score **> 14** indicates **homology** | U | K.NIVVVEGVR.I |
| 13982 | 54 | – | 62 | 492.7956 | 983.5766 | 983.5764 | 0.21 | 0 | 30 | 0.0032 | 1Score **> 27** indicates **identity** Score **> 18** indicates **homology** | U | K.NIVVVEGVR.I |
| 13983 | 54 | – | 62 | 492.7956 | 983.5767 | 983.5764 | 0.30 | 0 | 35 | 0.00053 | 1Score **> 27** indicates **identity** Score **> 15** indicates **homology** | U | K.NIVVVEGVR.I |
| 13984 | 54 | – | 62 | 492.7957 | 983.5768 | 983.5764 | 0.43 | 0 | 27 | 0.0029 | 1Score **> 27** indicates **identity** Score **> 14** indicates **homology** | U | K.NIVVVEGVR.I |
| 13985 | 54 | – | 62 | 492.7959 | 983.5773 | 983.5764 | 0.94 | 0 | 30 | 0.0017 | 1Score **> 27** indicates **identity** Score **> 14** indicates **homology** | U | K.NIVVVEGVR.I |
| 13986 | 54 | – | 62 | 492.7960 | 983.5774 | 983.5764 | 0.99 | 0 | 28 | 0.0041 | 1Score **> 27** indicates **identity** Score **> 17** indicates **homology** | U | K.NIVVVEGVR.I |
| 13987 | 54 | – | 62 | 492.7960 | 983.5774 | 983.5764 | 1.00 | 0 | 23 | 0.0067 | 1Score **> 27** indicates **identity** Score **> 14** indicates **homology** | U | K.NIVVVEGVR.I |
| 13988 | 54 | – | 62 | 492.7962 | 983.5778 | 983.5764 | 1.48 | 0 | 25 | 0.0068 | 1Score **> 29** indicates **identity** Score **> 16** indicates **homology** | U | K.NIVVVEGVR.I |
| 13989 | 54 | – | 62 | 492.7964 | 983.5782 | 983.5764 | 1.85 | 0 | 35 | 0.0041 | 1Score **> 29** indicates **identity** Score **> 24** indicates **homology** | U | K.NIVVVEGVR.I |
| 13992 | 54 | – | 62 | 492.7981 | 983.5817 | 983.5764 | 5.40 | 0 | 37 | 0.003 | 1Score **> 29** indicates **identity** Score **> 24** indicates **homology** | U | K.NIVVVEGVR.I |
| 142265 | 54 | – | 73 | 731.0864 | 2190.2374 | 2190.2412 | -1.75 | 1 | 29 | 0.0019 | 1Score **> 33** indicates **identity** Score **> 14** indicates **homology** | U | K.NIVVVEGVRIPFLLSGTSYK.D |
| 142266 | 54 | – | 73 | 731.0879 | 2190.2420 | 2190.2412 | 0.34 | 1 | 26 | 0.0034 | 1Score **> 33** indicates **identity** Score **> 14** indicates **homology** | U | K.NIVVVEGVRIPFLLSGTSYK.D |
| 142268 | 54 | – | 73 | 1096.1309 | 2190.2473 | 2190.2412 | 2.76 | 1 | 94 | 1.5e-09 | 1Score **> 32** indicates **identity** Score **> 18** indicates **homology** | U | K.NIVVVEGVRIPFLLSGTSYK.D |
| 36313 | 63 | – | 73 | 613.3438 | 1224.6731 | 1224.6754 | -1.92 | 0 | 30 | 0.0016 | 1Score **> 32** indicates **identity** Score **> 14** indicates **homology** | U | R.IPFLLSGTSYK.D |
| 36315 | 63 | – | 73 | 613.3450 | 1224.6755 | 1224.6754 | 0.074 | 0 | 77 | 6.1e-08 | 1Score **> 32** indicates **identity** Score **> 17** indicates **homology** | U | R.IPFLLSGTSYK.D |
| 36316 | 63 | – | 73 | 613.3450 | 1224.6755 | 1224.6754 | 0.096 | 0 | 57 | 4.3e-06 | 1Score **> 32** indicates **identity** Score **> 16** indicates **homology** | U | R.IPFLLSGTSYK.D |
| 36317 | 63 | – | 73 | 613.3452 | 1224.6758 | 1224.6754 | 0.35 | 0 | 77 | 6.1e-08 | 1Score **> 32** indicates **identity** Score **> 17** indicates **homology** | U | R.IPFLLSGTSYK.D |
| 36318 | 63 | – | 73 | 613.3453 | 1224.6760 | 1224.6754 | 0.44 | 0 | 66 | 6.5e-07 | 1Score **> 32** indicates **identity** Score **> 17** indicates **homology** | U | R.IPFLLSGTSYK.D |
| 36319 | 63 | – | 73 | 613.3454 | 1224.6763 | 1224.6754 | 0.73 | 0 | 58 | 3.5e-06 | 1Score **> 32** indicates **identity** Score **> 16** indicates **homology** | U | R.IPFLLSGTSYK.D |
| 36320 | 63 | – | 73 | 613.3456 | 1224.6766 | 1224.6754 | 0.97 | 0 | 62 | 1.6e-06 | 1Score **> 32** indicates **identity** Score **> 16** indicates **homology** | U | R.IPFLLSGTSYK.D |
| 36321 | 63 | – | 73 | 613.3456 | 1224.6767 | 1224.6754 | 1.07 | 0 | 60 | 2.6e-06 | 1Score **> 32** indicates **identity** Score **> 16** indicates **homology** | U | R.IPFLLSGTSYK.D |
| 36322 | 63 | – | 73 | 613.3458 | 1224.6771 | 1224.6754 | 1.37 | 0 | 60 | 2.4e-06 | 1Score **> 32** indicates **identity** Score **> 16** indicates **homology** | U | R.IPFLLSGTSYK.D |
| 36323 | 63 | – | 73 | 613.3460 | 1224.6775 | 1224.6754 | 1.67 | 0 | 58 | 3.9e-06 | 1Score **> 32** indicates **identity** Score **> 16** indicates **homology** | U | R.IPFLLSGTSYK.D |
| 36324 | 63 | – | 73 | 613.3462 | 1224.6778 | 1224.6754 | 1.93 | 0 | 57 | 4.6e-06 | 1Score **> 32** indicates **identity** Score **> 16** indicates **homology** | U | R.IPFLLSGTSYK.D |
| 36330 | 63 | – | 73 | 613.3500 | 1224.6854 | 1224.6754 | 8.13 | 0 | 56 | 5.8e-06 | 1Score **> 32** indicates **identity** Score **> 16** indicates **homology** | U | R.IPFLLSGTSYK.D |
| 148190 | 63 | – | 82 | 569.3034 | 2273.1844 | 2273.1878 | -1.50 | 1 | 38 | 0.00028 | 1Score **> 37** indicates **identity** Score **> 15** indicates **homology** | U | R.IPFLLSGTSYKDLMPHDLAR.A |
| 148196 | 63 | – | 82 | 758.7366 | 2273.1878 | 2273.1878 | 0.011 | 1 | 60 | 4.8e-06 | 1Score **> 37** indicates **identity** Score **> 19** indicates **homology** | U | R.IPFLLSGTSYKDLMPHDLAR.A |
| 148197 | 63 | – | 82 | 569.3044 | 2273.1885 | 2273.1878 | 0.29 | 1 | 15 | 0.038 | 1Score **> 37** indicates **identity** Score **> 13** indicates **homology** | U | R.IPFLLSGTSYKDLMPHDLAR.A |
| 148198 | 63 | – | 82 | 569.3045 | 2273.1889 | 2273.1878 | 0.47 | 1 | 49 | 2.4e-05 | 1Score **> 37** indicates **identity** Score **> 16** indicates **homology** | U | R.IPFLLSGTSYKDLMPHDLAR.A |
| 148199 | 63 | – | 82 | 569.3046 | 2273.1892 | 2273.1878 | 0.63 | 1 | 37 | 0.00035 | 1Score **> 37** indicates **identity** Score **> 15** indicates **homology** | U | R.IPFLLSGTSYKDLMPHDLAR.A |
| 148201 | 63 | – | 82 | 1137.6020 | 2273.1894 | 2273.1878 | 0.72 | 1 | 118 | 9.6e-11 | 1Score **> 37** indicates **identity** Score **> 30** indicates **homology** | U | R.IPFLLSGTSYKDLMPHDLAR.A |
| 148203 | 63 | – | 82 | 758.7372 | 2273.1897 | 2273.1878 | 0.83 | 1 | 66 | 1.1e-06 | 1Score **> 37** indicates **identity** Score **> 19** indicates **homology** | U | R.IPFLLSGTSYKDLMPHDLAR.A |
| 148204 | 63 | – | 82 | 569.3048 | 2273.1899 | 2273.1878 | 0.93 | 1 | 44 | 7.4e-05 | 1Score **> 37** indicates **identity** Score **> 15** indicates **homology** | U | R.IPFLLSGTSYKDLMPHDLAR.A |
| 148206 | 63 | – | 82 | 758.7373 | 2273.1900 | 2273.1878 | 0.98 | 1 | 75 | 9.3e-08 | 1Score **> 37** indicates **identity** Score **> 17** indicates **homology** | U | R.IPFLLSGTSYKDLMPHDLAR.A |
| 148207 | 63 | – | 82 | 569.3048 | 2273.1901 | 2273.1878 | 1.00 | 1 | 45 | 6.6e-05 | 1Score **> 37** indicates **identity** Score **> 15** indicates **homology** | U | R.IPFLLSGTSYKDLMPHDLAR.A |
| 148209 | 63 | – | 82 | 569.3049 | 2273.1906 | 2273.1878 | 1.23 | 1 | 46 | 4.9e-05 | 1Score **> 37** indicates **identity** Score **> 15** indicates **homology** | U | R.IPFLLSGTSYKDLMPHDLAR.A |
| 148210 | 63 | – | 82 | 569.3050 | 2273.1908 | 2273.1878 | 1.32 | 1 | 28 | 0.0022 | 1Score **> 37** indicates **identity** Score **> 14** indicates **homology** | U | R.IPFLLSGTSYKDLMPHDLAR.A |
| 148212 | 63 | – | 82 | 569.3050 | 2273.1910 | 2273.1878 | 1.41 | 1 | 42 | 0.00012 | 1Score **> 37** indicates **identity** Score **> 15** indicates **homology** | U | R.IPFLLSGTSYKDLMPHDLAR.A |
| 149168 | 63 | – | 82 | 573.3019 | 2289.1784 | 2289.1827 | -1.89 | 1 | 15 | 0.04 | 1Score **> 37** indicates **identity** Score **> 13** indicates **homology** | U | R.IPFLLSGTSYKDLMPHDLAR.A  + Oxidation (M) |
| 149178 | 63 | – | 82 | 573.3056 | 2289.1935 | 2289.1827 | 4.70 | 1 | 36 | 0.00041 | 1Score **> 37** indicates **identity** Score **> 15** indicates **homology** | U | R.IPFLLSGTSYKDLMPHDLAR.A  + Oxidation (M) |
| 149179 | 63 | – | 82 | 764.0719 | 2289.1939 | 2289.1827 | 4.89 | 1 | 49 | 2.4e-05 | 1Score **> 37** indicates **identity** Score **> 16** indicates **homology** | U | R.IPFLLSGTSYKDLMPHDLAR.A  + Oxidation (M) |
| 149181 | 63 | – | 82 | 573.3065 | 2289.1970 | 2289.1827 | 6.22 | 1 | 27 | 0.003 | 1Score **> 37** indicates **identity** Score **> 14** indicates **homology** | U | R.IPFLLSGTSYKDLMPHDLAR.A  + Oxidation (M) |
| 149182 | 63 | – | 82 | 573.3068 | 2289.1982 | 2289.1827 | 6.77 | 1 | 37 | 0.00031 | 1Score **> 37** indicates **identity** Score **> 15** indicates **homology** | U | R.IPFLLSGTSYKDLMPHDLAR.A  + Oxidation (M) |
| 149188 | 63 | – | 82 | 764.0756 | 2289.2050 | 2289.1827 | 9.73 | 1 | 33 | 0.00072 | 1Score **> 37** indicates **identity** Score **> 15** indicates **homology** | U | R.IPFLLSGTSYKDLMPHDLAR.A  + Oxidation (M) |
| 19940 | 74 | – | 82 | 356.5147 | 1066.5224 | 1066.5229 | -0.52 | 0 | 25 | 0.014 | 1Score **> 30** indicates **identity** Score **> 19** indicates **homology** | U | K.DLMPHDLAR.A |
| 19942 | 74 | – | 82 | 534.2686 | 1066.5226 | 1066.5229 | -0.32 | 0 | 16 | 0.034 | 1Score **> 30** indicates **identity** Score **> 13** indicates **homology** | U | K.DLMPHDLAR.A |
| 19944 | 74 | – | 82 | 356.5149 | 1066.5230 | 1066.5229 | 0.052 | 0 | 17 | 0.027 | 1Score **> 30** indicates **identity** Score **> 14** indicates **homology** | U | K.DLMPHDLAR.A |
| 19945 | 74 | – | 82 | 356.5149 | 1066.5230 | 1066.5229 | 0.066 | 0 | 27 | 0.003 | 1Score **> 30** indicates **identity** Score **> 14** indicates **homology** | U | K.DLMPHDLAR.A |
| 19946 | 74 | – | 82 | 356.5150 | 1066.5232 | 1066.5229 | 0.21 | 0 | 23 | 0.0066 | 1Score **> 30** indicates **identity** Score **> 14** indicates **homology** | U | K.DLMPHDLAR.A |
| 11051 | 83 | – | 91 | 469.2822 | 936.5497 | 936.5505 | -0.78 | 0 | 78 | 9e-08 | 1Score **> 23** indicates **identity** Score **> 20** indicates **homology** | U | R.AALSGLLHR.T |
| 11052 | 83 | – | 91 | 469.2822 | 936.5498 | 936.5505 | -0.75 | 0 | 67 | 1.2e-06 | 1Score **> 23** indicates **identity** Score **> 20** indicates **homology** | U | R.AALSGLLHR.T |
| 11053 | 83 | – | 91 | 469.2822 | 936.5499 | 936.5505 | -0.61 | 0 | 58 | 1.8e-05 | 1Score **> 23** indicates **identity** | U | R.AALSGLLHR.T |
| 11054 | 83 | – | 91 | 469.2826 | 936.5506 | 936.5505 | 0.094 | 0 | 47 | 0.00027 | 1Score **> 23** indicates **identity** | U | R.AALSGLLHR.T |
| 11055 | 83 | – | 91 | 469.2828 | 936.5510 | 936.5505 | 0.55 | 0 | 43 | 8.5e-05 | 1Score **> 23** indicates **identity** Score **> 15** indicates **homology** | U | R.AALSGLLHR.T |
| 11056 | 83 | – | 91 | 469.2828 | 936.5511 | 936.5505 | 0.66 | 0 | 58 | 2.1e-05 | 1Score **> 23** indicates **identity** | U | R.AALSGLLHR.T |
| 155704 | 92 | – | 112 | 797.7742 | 2390.3007 | 2390.3097 | -3.79 | 1 | 44 | 8.2e-05 | 1Score **> 36** indicates **identity** Score **> 15** indicates **homology** | U | R.TNIPKDVVDYIIFGTVIQEVK.T |
| 155708 | 92 | – | 112 | 797.7761 | 2390.3064 | 2390.3097 | -1.39 | 1 | 43 | 8.9e-05 | 1Score **> 35** indicates **identity** Score **> 15** indicates **homology** | U | R.TNIPKDVVDYIIFGTVIQEVK.T |
| 155709 | 92 | – | 112 | 797.7762 | 2390.3067 | 2390.3097 | -1.25 | 1 | 57 | 4.5e-06 | 1Score **> 35** indicates **identity** Score **> 16** indicates **homology** | U | R.TNIPKDVVDYIIFGTVIQEVK.T |
| 155712 | 92 | – | 112 | 797.7766 | 2390.3079 | 2390.3097 | -0.77 | 1 | 48 | 3.3e-05 | 1Score **> 35** indicates **identity** Score **> 15** indicates **homology** | U | R.TNIPKDVVDYIIFGTVIQEVK.T |
| 155715 | 92 | – | 112 | 1196.1617 | 2390.3088 | 2390.3097 | -0.38 | 1 | 93 | 1.8e-09 | 1Score **> 35** indicates **identity** Score **> 18** indicates **homology** | U | R.TNIPKDVVDYIIFGTVIQEVK.T |
| 155717 | 92 | – | 112 | 598.5847 | 2390.3095 | 2390.3097 | -0.078 | 1 | 52 | 1.4e-05 | 1Score **> 35** indicates **identity** Score **> 16** indicates **homology** | U | R.TNIPKDVVDYIIFGTVIQEVK.T |
| 155718 | 92 | – | 112 | 797.7772 | 2390.3099 | 2390.3097 | 0.057 | 1 | 49 | 2.7e-05 | 1Score **> 35** indicates **identity** Score **> 16** indicates **homology** | U | R.TNIPKDVVDYIIFGTVIQEVK.T |
| 155722 | 92 | – | 112 | 598.5851 | 2390.3114 | 2390.3097 | 0.71 | 1 | 51 | 1.7e-05 | 1Score **> 35** indicates **identity** Score **> 16** indicates **homology** | U | R.TNIPKDVVDYIIFGTVIQEVK.T |
| 155724 | 92 | – | 112 | 797.7781 | 2390.3123 | 2390.3097 | 1.09 | 1 | 61 | 2e-06 | 1Score **> 35** indicates **identity** Score **> 16** indicates **homology** | U | R.TNIPKDVVDYIIFGTVIQEVK.T |
| 155726 | 92 | – | 112 | 598.5854 | 2390.3125 | 2390.3097 | 1.14 | 1 | 19 | 0.032 | 1Score **> 35** indicates **identity** Score **> 17** indicates **homology** | U | R.TNIPKDVVDYIIFGTVIQEVK.T |
| 155728 | 92 | – | 112 | 797.7782 | 2390.3128 | 2390.3097 | 1.30 | 1 | 61 | 1.8e-06 | 1Score **> 35** indicates **identity** Score **> 16** indicates **homology** | U | R.TNIPKDVVDYIIFGTVIQEVK.T |
| 155729 | 92 | – | 112 | 1196.1640 | 2390.3134 | 2390.3097 | 1.52 | 1 | 69 | 3.4e-07 | 1Score **> 35** indicates **identity** Score **> 17** indicates **homology** | U | R.TNIPKDVVDYIIFGTVIQEVK.T |
| 155731 | 92 | – | 112 | 797.7786 | 2390.3140 | 2390.3097 | 1.77 | 1 | 44 | 7.6e-05 | 1Score **> 35** indicates **identity** Score **> 15** indicates **homology** | U | R.TNIPKDVVDYIIFGTVIQEVK.T |
| 155732 | 92 | – | 112 | 797.7786 | 2390.3140 | 2390.3097 | 1.80 | 1 | 35 | 0.00051 | 1Score **> 35** indicates **identity** Score **> 15** indicates **homology** | U | R.TNIPKDVVDYIIFGTVIQEVK.T |
| 155734 | 92 | – | 112 | 797.7789 | 2390.3149 | 2390.3097 | 2.18 | 1 | 23 | 0.0075 | 1Score **> 35** indicates **identity** Score **> 14** indicates **homology** | U | R.TNIPKDVVDYIIFGTVIQEVK.T |
| 155736 | 92 | – | 112 | 797.7789 | 2390.3150 | 2390.3097 | 2.21 | 1 | 61 | 1.9e-06 | 1Score **> 35** indicates **identity** Score **> 16** indicates **homology** | U | R.TNIPKDVVDYIIFGTVIQEVK.T |
| 155737 | 92 | – | 112 | 797.7792 | 2390.3158 | 2390.3097 | 2.53 | 1 | 48 | 3.2e-05 | 1Score **> 35** indicates **identity** Score **> 15** indicates **homology** | U | R.TNIPKDVVDYIIFGTVIQEVK.T |
| 155740 | 92 | – | 112 | 797.7795 | 2390.3167 | 2390.3097 | 2.91 | 1 | 52 | 1.5e-05 | 1Score **> 35** indicates **identity** Score **> 16** indicates **homology** | U | R.TNIPKDVVDYIIFGTVIQEVK.T |
| 155751 | 92 | – | 112 | 797.7848 | 2390.3327 | 2390.3097 | 9.60 | 1 | 32 | 0.001 | 1Score **> 34** indicates **identity** Score **> 15** indicates **homology** | U | R.TNIPKDVVDYIIFGTVIQEVK.T |
| 155844 | 92 | – | 112 | 798.1111 | 2391.3114 | 2391.2937 | 7.40 | 1 | 17 | 0.024 | 1Score **> 35** indicates **identity** Score **> 14** indicates **homology** | U | R.TNIPKDVVDYIIFGTVIQEVK.T  + Deamidated (NQ) |
| 155845 | 92 | – | 112 | 798.1114 | 2391.3123 | 2391.2937 | 7.78 | 1 | 20 | 0.014 | 1Score **> 35** indicates **identity** Score **> 14** indicates **homology** | U | R.TNIPKDVVDYIIFGTVIQEVK.T  + Deamidated (NQ) |
| 182045 | 92 | – | 118 | 1007.2206 | 3018.6398 | 3018.6390 | 0.28 | 2 | 54 | 8.8e-06 | 1Score **> 35** indicates **identity** Score **> 16** indicates **homology** | U | R.TNIPKDVVDYIIFGTVIQEVKTSNVAR.E |
| 182046 | 92 | – | 118 | 1007.2214 | 3018.6422 | 3018.6390 | 1.07 | 2 | 48 | 3.5e-05 | 1Score **> 35** indicates **identity** Score **> 15** indicates **homology** | U | R.TNIPKDVVDYIIFGTVIQEVKTSNVAR.E |
| 182047 | 92 | – | 118 | 755.6706 | 3018.6533 | 3018.6390 | 4.75 | 2 | 33 | 0.00074 | 1Score **> 34** indicates **identity** Score **> 15** indicates **homology** | U | R.TNIPKDVVDYIIFGTVIQEVKTSNVAR.E |
| 109401 | 97 | – | 112 | 919.4991 | 1836.9836 | 1836.9873 | -2.03 | 0 | 76 | 1.1e-07 | 1Score **> 35** indicates **identity** Score **> 19** indicates **homology** | U | K.DVVDYIIFGTVIQEVK.T |
| 109402 | 97 | – | 112 | 919.4997 | 1836.9848 | 1836.9873 | -1.41 | 0 | 38 | 0.00032 | 1Score **> 35** indicates **identity** Score **> 15** indicates **homology** | U | K.DVVDYIIFGTVIQEVK.T |
| 109405 | 97 | – | 112 | 613.3357 | 1836.9854 | 1836.9873 | -1.08 | 0 | 59 | 2.8e-06 | 1Score **> 35** indicates **identity** Score **> 16** indicates **homology** | U | K.DVVDYIIFGTVIQEVK.T |
| 109413 | 97 | – | 112 | 613.3367 | 1836.9882 | 1836.9873 | 0.46 | 0 | 45 | 5.7e-05 | 1Score **> 35** indicates **identity** Score **> 15** indicates **homology** | U | K.DVVDYIIFGTVIQEVK.T |
| 109416 | 97 | – | 112 | 919.5015 | 1836.9884 | 1836.9873 | 0.58 | 0 | 69 | 5.2e-07 | 1Score **> 35** indicates **identity** Score **> 18** indicates **homology** | U | K.DVVDYIIFGTVIQEVK.T |
| 109418 | 97 | – | 112 | 613.3368 | 1836.9887 | 1836.9873 | 0.74 | 0 | 55 | 6.3e-06 | 1Score **> 35** indicates **identity** Score **> 16** indicates **homology** | U | K.DVVDYIIFGTVIQEVK.T |
| 109420 | 97 | – | 112 | 919.5019 | 1836.9892 | 1836.9873 | 1.03 | 0 | 38 | 0.0018 | 1Score **> 35** indicates **identity** Score **> 23** indicates **homology** | U | K.DVVDYIIFGTVIQEVK.T |
| 109421 | 97 | – | 112 | 613.3371 | 1836.9894 | 1836.9873 | 1.14 | 0 | 45 | 6.3e-05 | 1Score **> 35** indicates **identity** Score **> 15** indicates **homology** | U | K.DVVDYIIFGTVIQEVK.T |
| 109424 | 97 | – | 112 | 613.3376 | 1836.9911 | 1836.9873 | 2.03 | 0 | 44 | 8.2e-05 | 1Score **> 35** indicates **identity** Score **> 15** indicates **homology** | U | K.DVVDYIIFGTVIQEVK.T |
| 109429 | 97 | – | 112 | 919.5032 | 1836.9917 | 1836.9873 | 2.40 | 0 | 52 | 6.6e-05 | 1Score **> 35** indicates **identity** Score **> 23** indicates **homology** | U | K.DVVDYIIFGTVIQEVK.T |
| 109430 | 97 | – | 112 | 613.3379 | 1836.9918 | 1836.9873 | 2.42 | 0 | 39 | 0.00022 | 1Score **> 35** indicates **identity** Score **> 15** indicates **homology** | U | K.DVVDYIIFGTVIQEVK.T |
| 109432 | 97 | – | 112 | 613.3379 | 1836.9918 | 1836.9873 | 2.45 | 0 | 45 | 5.6e-05 | 1Score **> 35** indicates **identity** Score **> 15** indicates **homology** | U | K.DVVDYIIFGTVIQEVK.T |
| 109438 | 97 | – | 112 | 613.3384 | 1836.9934 | 1836.9873 | 3.31 | 0 | 49 | 2.4e-05 | 1Score **> 35** indicates **identity** Score **> 16** indicates **homology** | U | K.DVVDYIIFGTVIQEVK.T |
| 109439 | 97 | – | 112 | 613.3385 | 1836.9936 | 1836.9873 | 3.42 | 0 | 48 | 3.3e-05 | 1Score **> 35** indicates **identity** Score **> 15** indicates **homology** | U | K.DVVDYIIFGTVIQEVK.T |
| 109440 | 97 | – | 112 | 613.3385 | 1836.9937 | 1836.9873 | 3.45 | 0 | 19 | 0.017 | 1Score **> 35** indicates **identity** Score **> 14** indicates **homology** | U | K.DVVDYIIFGTVIQEVK.T |
| 109442 | 97 | – | 112 | 613.3385 | 1836.9938 | 1836.9873 | 3.53 | 0 | 44 | 7.3e-05 | 1Score **> 35** indicates **identity** Score **> 15** indicates **homology** | U | K.DVVDYIIFGTVIQEVK.T |
| 109443 | 97 | – | 112 | 919.5044 | 1836.9942 | 1836.9873 | 3.76 | 0 | 35 | 0.0011 | 1Score **> 35** indicates **identity** Score **> 18** indicates **homology** | U | K.DVVDYIIFGTVIQEVK.T |
| 109446 | 97 | – | 112 | 613.3392 | 1836.9956 | 1836.9873 | 4.52 | 0 | 36 | 0.00041 | 1Score **> 35** indicates **identity** Score **> 15** indicates **homology** | U | K.DVVDYIIFGTVIQEVK.T |
| 109452 | 97 | – | 112 | 919.5056 | 1836.9967 | 1836.9873 | 5.09 | 0 | 20 | 0.015 | 1Score **> 35** indicates **identity** Score **> 14** indicates **homology** | U | K.DVVDYIIFGTVIQEVK.T |
| 109457 | 97 | – | 112 | 613.3408 | 1837.0006 | 1836.9873 | 7.21 | 0 | 33 | 0.00076 | 1Score **> 35** indicates **identity** Score **> 15** indicates **homology** | U | K.DVVDYIIFGTVIQEVK.T |
| 19794 | 119 | – | 129 | 533.2627 | 1064.5109 | 1064.5138 | -2.71 | 0 | 62 | 2.6e-06 | 1Score **> 31** indicates **identity** Score **> 19** indicates **homology** | U | R.EAALGAGFSDK.T |
| 19798 | 119 | – | 129 | 533.2641 | 1064.5136 | 1064.5138 | -0.19 | 0 | 68 | 8.2e-07 | 1Score **> 31** indicates **identity** Score **> 19** indicates **homology** | U | R.EAALGAGFSDK.T |
| 19799 | 119 | – | 129 | 533.2642 | 1064.5138 | 1064.5138 | 0.013 | 0 | 61 | 5.6e-06 | 1Score **> 31** indicates **identity** Score **> 21** indicates **homology** | U | R.EAALGAGFSDK.T |
| 19800 | 119 | – | 129 | 533.2644 | 1064.5142 | 1064.5138 | 0.38 | 0 | 43 | 0.00011 | 1Score **> 31** indicates **identity** Score **> 16** indicates **homology** | U | R.EAALGAGFSDK.T |
| 197624 | 119 | – | 175 | 989.8339 | 5932.9595 | 5932.9030 | 9.54 | 1 | 20 | 0.012 | 1Score **> 35** indicates **identity** Score **> 14** indicates **homology** | U | R.EAALGAGFSDKTPAHTVTMACISSNQAMTTAVGLIASGQCDVVVAGGVELMSDVPIR.H  + HNE (H); Oxidation (M) |
| 197626 | 119 | – | 175 | 989.9851 | 5933.8669 | 5933.8870 | -3.38 | 1 | 25 | 0.0043 | 1Score **> 35** indicates **identity** Score **> 14** indicates **homology** | U | R.EAALGAGFSDKTPAHTVTMACISSNQAMTTAVGLIASGQCDVVVAGGVELMSDVPIR.H  + Deamidated (NQ); HNE (H); Oxidation (M) |
| 197632 | 119 | – | 175 | 990.1546 | 5934.8837 | 5934.8710 | 2.14 | 1 | 16 | 0.033 | 1Score **> 35** indicates **identity** Score **> 13** indicates **homology** | U | R.EAALGAGFSDKTPAHTVTMACISSNQAMTTAVGLIASGQCDVVVAGGVELMSDVPIR.H  + 2 Deamidated (NQ); HNE (H); Oxidation (M) |
| 197634 | 119 | – | 175 | 990.3214 | 5935.8844 | 5935.8550 | 4.96 | 1 | 17 | 0.023 | 1Score **> 35** indicates **identity** Score **> 14** indicates **homology** | U | R.EAALGAGFSDKTPAHTVTMACISSNQAMTTAVGLIASGQCDVVVAGGVELMSDVPIR.H  + 3 Deamidated (NQ); HNE (H); Oxidation (M) |
| 197711 | 119 | – | 175 | 1196.3870 | 5976.8987 | 5976.9431 | -7.43 | 1 | 21 | 0.01 | 1Score **> 35** indicates **identity** Score **> 14** indicates **homology** | U | R.EAALGAGFSDKTPAHTVTMACISSNQAMTTAVGLIASGQCDVVVAGGVELMSDVPIR.H  + 2 Deamidated (NQ); 2 HNE (C); Oxidation (M) |
| 197730 | 119 | – | 175 | 999.5004 | 5990.9587 | 5990.9700 | -1.88 | 1 | 21 | 0.01 | 1Score **> 35** indicates **identity** Score **> 14** indicates **homology** | U | R.EAALGAGFSDKTPAHTVTMACISSNQAMTTAVGLIASGQCDVVVAGGVELMSDVPIR.H  + 2 HNE (C); 2 Oxidation (M) |
| 184320 | 203 | – | 230 | 784.8830 | 3135.5031 | 3135.5084 | -1.69 | 1 | 53 | 1.1e-05 | 1Score **> 37** indicates **identity** Score **> 16** indicates **homology** | U | K.FRLNFLSPELPAVAEFSTNETMGHSADR.L |
| 184321 | 203 | – | 230 | 1046.1772 | 3135.5099 | 3135.5084 | 0.49 | 1 | 87 | 7.4e-09 | 1Score **> 37** indicates **identity** Score **> 18** indicates **homology** | U | K.FRLNFLSPELPAVAEFSTNETMGHSADR.L |
| 184322 | 203 | – | 230 | 784.8860 | 3135.5148 | 3135.5084 | 2.06 | 1 | 51 | 1.7e-05 | 1Score **> 37** indicates **identity** Score **> 16** indicates **homology** | U | K.FRLNFLSPELPAVAEFSTNETMGHSADR.L |
| 194059 | 203 | – | 239 | 805.4071 | 4021.9991 | 4022.0108 | -2.92 | 2 | 15 | 0.042 | 1Score **> 38** indicates **identity** Score **> 13** indicates **homology** | U | K.FRLNFLSPELPAVAEFSTNETMGHSADRLAAAFAVSR.M |
| 176264 | 205 | – | 230 | 945.1204 | 2832.3393 | 2832.3388 | 0.17 | 0 | 63 | 1.2e-06 | 1Score **> 35** indicates **identity** Score **> 16** indicates **homology** | U | R.LNFLSPELPAVAEFSTNETMGHSADR.L |
| 176335 | 205 | – | 230 | 945.4527 | 2833.3363 | 2833.3228 | 4.74 | 0 | 19 | 0.016 | 1Score **> 36** indicates **identity** Score **> 14** indicates **homology** | U | R.LNFLSPELPAVAEFSTNETMGHSADR.L  + Deamidated (NQ) |
| 176770 | 205 | – | 230 | 950.4522 | 2848.3348 | 2848.3338 | 0.36 | 0 | 41 | 0.00015 | 1Score **> 35** indicates **identity** Score **> 15** indicates **homology** | U | R.LNFLSPELPAVAEFSTNETMGHSADR.L  + Oxidation (M) |
| 176805 | 205 | – | 230 | 950.7825 | 2849.3257 | 2849.3178 | 2.79 | 0 | 15 | 0.039 | 1Score **> 35** indicates **identity** Score **> 13** indicates **homology** | U | R.LNFLSPELPAVAEFSTNETMGHSADR.L  + Deamidated (NQ); Oxidation (M) |
| 9121 | 231 | – | 239 | 453.2632 | 904.5119 | 904.5130 | -1.25 | 0 | 58 | 5.7e-05 | 1Score **> 29** indicates **identity** Score **> 28** indicates **homology** | U | R.LAAAFAVSR.M |
| 9122 | 231 | – | 239 | 453.2632 | 904.5119 | 904.5130 | -1.23 | 0 | 61 | 3.2e-05 | 1Score **> 29** indicates **identity** | U | R.LAAAFAVSR.M |
| 9124 | 231 | – | 239 | 453.2633 | 904.5121 | 904.5130 | -1.08 | 0 | 56 | 6.4e-05 | 1Score **> 29** indicates **identity** Score **> 26** indicates **homology** | U | R.LAAAFAVSR.M |
| 9125 | 231 | – | 239 | 453.2633 | 904.5121 | 904.5130 | -1.01 | 0 | 23 | 0.017 | 1Score **> 29** indicates **identity** Score **> 18** indicates **homology** | U | R.LAAAFAVSR.M |
| 9126 | 231 | – | 239 | 453.2634 | 904.5122 | 904.5130 | -0.90 | 0 | 26 | 0.015 | 1Score **> 29** indicates **identity** Score **> 21** indicates **homology** | U | R.LAAAFAVSR.M |
| 9127 | 231 | – | 239 | 453.2634 | 904.5122 | 904.5130 | -0.89 | 0 | 62 | 2.5e-05 | 1Score **> 29** indicates **identity** | U | R.LAAAFAVSR.M |
| 9128 | 231 | – | 239 | 453.2634 | 904.5123 | 904.5130 | -0.76 | 0 | 46 | 0.00094 | 1Score **> 29** indicates **identity** | U | R.LAAAFAVSR.M |
| 9130 | 231 | – | 239 | 453.2635 | 904.5124 | 904.5130 | -0.74 | 0 | 45 | 0.0011 | 1Score **> 29** indicates **identity** | U | R.LAAAFAVSR.M |
| 9131 | 231 | – | 239 | 453.2635 | 904.5125 | 904.5130 | -0.61 | 0 | 23 | 0.012 | 1Score **> 29** indicates **identity** Score **> 16** indicates **homology** | U | R.LAAAFAVSR.M |
| 9133 | 231 | – | 239 | 453.2636 | 904.5127 | 904.5130 | -0.40 | 0 | 48 | 0.00062 | 1Score **> 29** indicates **identity** | U | R.LAAAFAVSR.M |
| 9134 | 231 | – | 239 | 453.2636 | 904.5127 | 904.5130 | -0.34 | 0 | 53 | 0.00018 | 1Score **> 29** indicates **identity** | U | R.LAAAFAVSR.M |
| 9135 | 231 | – | 239 | 453.2637 | 904.5129 | 904.5130 | -0.14 | 0 | 46 | 0.00084 | 1Score **> 29** indicates **identity** Score **> 28** indicates **homology** | U | R.LAAAFAVSR.M |
| 9137 | 231 | – | 239 | 453.2637 | 904.5129 | 904.5130 | -0.096 | 0 | 41 | 0.0012 | 1Score **> 29** indicates **identity** Score **> 25** indicates **homology** | U | R.LAAAFAVSR.M |
| 9138 | 231 | – | 239 | 453.2638 | 904.5130 | 904.5130 | -0.090 | 0 | 46 | 0.0006 | 1Score **> 29** indicates **identity** Score **> 26** indicates **homology** | U | R.LAAAFAVSR.M |
| 9139 | 231 | – | 239 | 453.2638 | 904.5130 | 904.5130 | -0.085 | 0 | 63 | 2.2e-05 | 1Score **> 29** indicates **identity** | U | R.LAAAFAVSR.M |
| 9140 | 231 | – | 239 | 453.2638 | 904.5131 | 904.5130 | 0.045 | 0 | 46 | 0.00094 | 1Score **> 29** indicates **identity** | U | R.LAAAFAVSR.M |
| 9141 | 231 | – | 239 | 453.2638 | 904.5131 | 904.5130 | 0.072 | 0 | 18 | 0.035 | 1Score **> 29** indicates **identity** Score **> 16** indicates **homology** | U | R.LAAAFAVSR.M |
| 9142 | 231 | – | 239 | 453.2639 | 904.5132 | 904.5130 | 0.16 | 0 | 61 | 2.9e-05 | 1Score **> 29** indicates **identity** | U | R.LAAAFAVSR.M |
| 9143 | 231 | – | 239 | 453.2639 | 904.5132 | 904.5130 | 0.18 | 0 | 46 | 0.00096 | 1Score **> 29** indicates **identity** | U | R.LAAAFAVSR.M |
| 9144 | 231 | – | 239 | 453.2639 | 904.5132 | 904.5130 | 0.18 | 0 | 46 | 0.00097 | 1Score **> 29** indicates **identity** | U | R.LAAAFAVSR.M |
| 9149 | 231 | – | 239 | 453.2640 | 904.5135 | 904.5130 | 0.54 | 0 | 46 | 0.00055 | 1Score **> 29** indicates **identity** Score **> 26** indicates **homology** | U | R.LAAAFAVSR.M |
| 9150 | 231 | – | 239 | 453.2642 | 904.5139 | 904.5130 | 1.00 | 0 | 39 | 0.0015 | 1Score **> 29** indicates **identity** Score **> 24** indicates **homology** | U | R.LAAAFAVSR.M |
| 9152 | 231 | – | 239 | 453.2650 | 904.5154 | 904.5130 | 2.61 | 0 | 32 | 0.006 | 1Score **> 28** indicates **identity** Score **> 22** indicates **homology** | U | R.LAAAFAVSR.M |
| 9153 | 231 | – | 239 | 453.2653 | 904.5160 | 904.5130 | 3.29 | 0 | 46 | 0.00042 | 1Score **> 28** indicates **identity** Score **> 25** indicates **homology** | U | R.LAAAFAVSR.M |
| 28556 | 240 | – | 248 | 577.7606 | 1153.5067 | 1153.5073 | -0.57 | 0 | 46 | 0.0001 | 1Score **> 28** indicates **identity** Score **> 19** indicates **homology** | U | R.MEQDEYALR.S |
| 30277 | 240 | – | 248 | 585.7583 | 1169.5020 | 1169.5023 | -0.21 | 0 | 22 | 0.0078 | 1Score **> 26** indicates **identity** Score **> 14** indicates **homology** | U | R.MEQDEYALR.S  + Oxidation (M) |
| 30278 | 240 | – | 248 | 585.7585 | 1169.5024 | 1169.5023 | 0.11 | 0 | 18 | 0.021 | 1Score **> 26** indicates **identity** Score **> 14** indicates **homology** | U | R.MEQDEYALR.S  + Oxidation (M) |
| 30279 | 240 | – | 248 | 585.7585 | 1169.5025 | 1169.5023 | 0.21 | 0 | 33 | 0.00079 | 1Score **> 26** indicates **identity** Score **> 15** indicates **homology** | U | R.MEQDEYALR.S  + Oxidation (M) |
| 30280 | 240 | – | 248 | 585.7586 | 1169.5026 | 1169.5023 | 0.34 | 0 | 19 | 0.017 | 1Score **> 26** indicates **identity** Score **> 14** indicates **homology** | U | R.MEQDEYALR.S  + Oxidation (M) |
| 30281 | 240 | – | 248 | 585.7587 | 1169.5028 | 1169.5023 | 0.42 | 0 | 42 | 0.00011 | 1Score **> 26** indicates **identity** Score **> 15** indicates **homology** | U | R.MEQDEYALR.S  + Oxidation (M) |
| 30282 | 240 | – | 248 | 585.7587 | 1169.5029 | 1169.5023 | 0.53 | 0 | 31 | 0.0011 | 1Score **> 26** indicates **identity** Score **> 14** indicates **homology** | U | R.MEQDEYALR.S  + Oxidation (M) |
| 30283 | 240 | – | 248 | 585.7587 | 1169.5029 | 1169.5023 | 0.54 | 0 | 25 | 0.0049 | 1Score **> 26** indicates **identity** Score **> 14** indicates **homology** | U | R.MEQDEYALR.S  + Oxidation (M) |
| 30284 | 240 | – | 248 | 585.7590 | 1169.5034 | 1169.5023 | 0.97 | 0 | 42 | 0.0001 | 1Score **> 26** indicates **identity** Score **> 15** indicates **homology** | U | R.MEQDEYALR.S  + Oxidation (M) |
| 30285 | 240 | – | 248 | 585.7594 | 1169.5042 | 1169.5023 | 1.68 | 0 | 39 | 0.00038 | 1Score **> 26** indicates **identity** Score **> 17** indicates **homology** | U | R.MEQDEYALR.S  + Oxidation (M) |
| 30287 | 240 | – | 248 | 585.7602 | 1169.5059 | 1169.5023 | 3.12 | 0 | 30 | 0.0015 | 1Score **> 26** indicates **identity** Score **> 15** indicates **homology** | U | R.MEQDEYALR.S  + Oxidation (M) |
| 130702 | 255 | – | 273 | 517.0329 | 2064.1024 | 2064.1004 | 0.96 | 2 | 34 | 0.00071 | 1Score **> 36** indicates **identity** Score **> 15** indicates **homology** | U | K.KAQDEGHLSDIVPFKVPGK.D |
| 130703 | 255 | – | 273 | 517.0329 | 2064.1027 | 2064.1004 | 1.10 | 2 | 36 | 0.00046 | 1Score **> 36** indicates **identity** Score **> 15** indicates **homology** | U | K.KAQDEGHLSDIVPFKVPGK.D |
| 130704 | 255 | – | 273 | 413.8279 | 2064.1030 | 2064.1004 | 1.24 | 2 | 36 | 0.00045 | 1Score **> 36** indicates **identity** Score **> 15** indicates **homology** | U | K.KAQDEGHLSDIVPFKVPGK.D |
| 130707 | 255 | – | 273 | 413.8280 | 2064.1037 | 2064.1004 | 1.60 | 2 | 20 | 0.013 | 1Score **> 36** indicates **identity** Score **> 14** indicates **homology** | U | K.KAQDEGHLSDIVPFKVPGK.D |
| 130708 | 255 | – | 273 | 689.0419 | 2064.1038 | 2064.1004 | 1.67 | 2 | 20 | 0.013 | 1Score **> 36** indicates **identity** Score **> 14** indicates **homology** | U | K.KAQDEGHLSDIVPFKVPGK.D |
| 130709 | 255 | – | 273 | 689.0421 | 2064.1045 | 2064.1004 | 1.99 | 2 | 18 | 0.019 | 1Score **> 36** indicates **identity** Score **> 14** indicates **homology** | U | K.KAQDEGHLSDIVPFKVPGK.D |
| 130710 | 255 | – | 273 | 517.0335 | 2064.1049 | 2064.1004 | 2.20 | 2 | 37 | 0.00036 | 1Score **> 36** indicates **identity** Score **> 15** indicates **homology** | U | K.KAQDEGHLSDIVPFKVPGK.D |
| 118782 | 256 | – | 273 | 485.0088 | 1936.0061 | 1936.0054 | 0.35 | 1 | 18 | 0.019 | 1Score **> 36** indicates **identity** Score **> 14** indicates **homology** | U | K.AQDEGHLSDIVPFKVPGK.D |
| 118784 | 256 | – | 273 | 485.0093 | 1936.0081 | 1936.0054 | 1.38 | 1 | 16 | 0.028 | 1Score **> 36** indicates **identity** Score **> 14** indicates **homology** | U | K.AQDEGHLSDIVPFKVPGK.D |
| 160937 | 256 | – | 278 | 497.0653 | 2480.2900 | 2480.2911 | -0.46 | 2 | 16 | 0.03 | 1Score **> 37** indicates **identity** Score **> 14** indicates **homology** | U | K.AQDEGHLSDIVPFKVPGKDTVTK.D |
| 160938 | 256 | – | 278 | 621.0799 | 2480.2905 | 2480.2911 | -0.27 | 2 | 21 | 0.011 | 1Score **> 37** indicates **identity** Score **> 14** indicates **homology** | U | K.AQDEGHLSDIVPFKVPGKDTVTK.D |
| 160939 | 256 | – | 278 | 621.0799 | 2480.2907 | 2480.2911 | -0.18 | 2 | 22 | 0.0087 | 1Score **> 37** indicates **identity** Score **> 14** indicates **homology** | U | K.AQDEGHLSDIVPFKVPGKDTVTK.D |
| 160940 | 256 | – | 278 | 827.7709 | 2480.2910 | 2480.2911 | -0.042 | 2 | 50 | 2.2e-05 | 1Score **> 37** indicates **identity** Score **> 16** indicates **homology** | U | K.AQDEGHLSDIVPFKVPGKDTVTK.D |
| 160942 | 256 | – | 278 | 621.0802 | 2480.2918 | 2480.2911 | 0.29 | 2 | 28 | 0.0023 | 1Score **> 37** indicates **identity** Score **> 14** indicates **homology** | U | K.AQDEGHLSDIVPFKVPGKDTVTK.D |
| 160944 | 256 | – | 278 | 827.7716 | 2480.2929 | 2480.2911 | 0.72 | 2 | 20 | 0.013 | 1Score **> 37** indicates **identity** Score **> 14** indicates **homology** | U | K.AQDEGHLSDIVPFKVPGKDTVTK.D |
| 160992 | 256 | – | 278 | 828.1061 | 2481.2964 | 2481.2751 | 8.57 | 2 | 16 | 0.03 | 1Score **> 37** indicates **identity** Score **> 14** indicates **homology** | U | K.AQDEGHLSDIVPFKVPGKDTVTK.D  + Deamidated (NQ) |
| 73038 | 279 | – | 292 | 773.3894 | 1544.7643 | 1544.7617 | 1.71 | 1 | 31 | 0.011 | 1Score **> 34** indicates **identity** Score **> 24** indicates **homology** | U | K.DNGIRPSSLEQMAK.L |
| 75075 | 279 | – | 292 | 781.3839 | 1560.7533 | 1560.7566 | -2.07 | 1 | 31 | 0.024 | 1Score **> 33** indicates **identity** Score **> 27** indicates **homology** | U | K.DNGIRPSSLEQMAK.L  + Oxidation (M) |
| 191524 | 293 | – | 326 | 1201.6152 | 3601.8239 | 3601.8160 | 2.19 | 2 | 76 | 7.3e-08 | 1Score **> 38** indicates **identity** Score **> 17** indicates **homology** | U | K.LKPAFIKPYGTVTAANSSFLTDGASAMLIMSEDR.A |
| 191525 | 293 | – | 326 | 901.4639 | 3601.8265 | 3601.8160 | 2.92 | 2 | 58 | 3.4e-06 | 1Score **> 38** indicates **identity** Score **> 16** indicates **homology** | U | K.LKPAFIKPYGTVTAANSSFLTDGASAMLIMSEDR.A |
| 192809 | 293 | – | 326 | 944.4819 | 3773.8986 | 3773.9260 | -7.25 | 2 | 31 | 0.0013 | 1Score **> 38** indicates **identity** Score **> 14** indicates **homology** | U | K.LKPAFIKPYGTVTAANSSFLTDGASAMLIMSEDR.A  + HNE (K); Oxidation (M) |
| 13680 | 327 | – | 335 | 489.7766 | 977.5386 | 977.5368 | 1.86 | 1 | 25 | 0.0053 | 1Score **> 31** indicates **identity** Score **> 15** indicates **homology** | U | R.ALAMGYKPK.A |
| 14639 | 327 | – | 335 | 497.7742 | 993.5339 | 993.5317 | 2.25 | 1 | 16 | 0.033 | 1Score **> 30** indicates **identity** Score **> 13** indicates **homology** | U | R.ALAMGYKPK.A  + Oxidation (M) |
| 95809 | 336 | – | 349 | 572.2983 | 1713.8730 | 1713.8726 | 0.23 | 1 | 26 | 0.0033 | 1Score **> 35** indicates **identity** Score **> 14** indicates **homology** | U | K.AYLRDFIYVSQDPK.D |
| 95810 | 336 | – | 349 | 572.2983 | 1713.8731 | 1713.8726 | 0.31 | 1 | 28 | 0.0024 | 1Score **> 35** indicates **identity** Score **> 14** indicates **homology** | U | K.AYLRDFIYVSQDPK.D |
| 95813 | 336 | – | 349 | 857.9441 | 1713.8736 | 1713.8726 | 0.56 | 1 | 50 | 4.8e-05 | 1Score **> 35** indicates **identity** Score **> 19** indicates **homology** | U | K.AYLRDFIYVSQDPK.D |
| 95814 | 336 | – | 349 | 857.9445 | 1713.8745 | 1713.8726 | 1.09 | 1 | 56 | 1.4e-05 | 1Score **> 35** indicates **identity** Score **> 20** indicates **homology** | U | K.AYLRDFIYVSQDPK.D |
| 95818 | 336 | – | 349 | 572.2991 | 1713.8755 | 1713.8726 | 1.69 | 1 | 20 | 0.014 | 1Score **> 35** indicates **identity** Score **> 14** indicates **homology** | U | K.AYLRDFIYVSQDPK.D |
| 183942 | 336 | – | 362 | 778.9124 | 3111.6204 | 3111.6281 | -2.46 | 2 | 33 | 0.00074 | 1Score **> 37** indicates **identity** Score **> 15** indicates **homology** | U | K.AYLRDFIYVSQDPKDQLLLGPTYATPK.V |
| 183943 | 336 | – | 362 | 778.9125 | 3111.6208 | 3111.6281 | -2.34 | 2 | 20 | 0.013 | 1Score **> 37** indicates **identity** Score **> 14** indicates **homology** | U | K.AYLRDFIYVSQDPKDQLLLGPTYATPK.V |
| 183944 | 336 | – | 362 | 778.9129 | 3111.6226 | 3111.6281 | -1.75 | 2 | 18 | 0.02 | 1Score **> 37** indicates **identity** Score **> 14** indicates **homology** | U | K.AYLRDFIYVSQDPKDQLLLGPTYATPK.V |
| 183945 | 336 | – | 362 | 1038.2158 | 3111.6256 | 3111.6281 | -0.79 | 2 | 44 | 7.5e-05 | 1Score **> 37** indicates **identity** Score **> 15** indicates **homology** | U | K.AYLRDFIYVSQDPKDQLLLGPTYATPK.V |
| 183946 | 336 | – | 362 | 1038.2163 | 3111.6271 | 3111.6281 | -0.31 | 2 | 46 | 4.7e-05 | 1Score **> 37** indicates **identity** Score **> 15** indicates **homology** | U | K.AYLRDFIYVSQDPKDQLLLGPTYATPK.V |
| 183947 | 336 | – | 362 | 778.9142 | 3111.6278 | 3111.6281 | -0.095 | 2 | 39 | 0.0002 | 1Score **> 37** indicates **identity** Score **> 15** indicates **homology** | U | K.AYLRDFIYVSQDPKDQLLLGPTYATPK.V |
| 183948 | 336 | – | 362 | 778.9142 | 3111.6279 | 3111.6281 | -0.059 | 2 | 33 | 0.00077 | 1Score **> 37** indicates **identity** Score **> 15** indicates **homology** | U | K.AYLRDFIYVSQDPKDQLLLGPTYATPK.V |
| 183949 | 336 | – | 362 | 778.9143 | 3111.6279 | 3111.6281 | -0.041 | 2 | 34 | 0.0007 | 1Score **> 37** indicates **identity** Score **> 15** indicates **homology** | U | K.AYLRDFIYVSQDPKDQLLLGPTYATPK.V |
| 183950 | 336 | – | 362 | 778.9145 | 3111.6289 | 3111.6281 | 0.26 | 2 | 44 | 7.5e-05 | 1Score **> 37** indicates **identity** Score **> 15** indicates **homology** | U | K.AYLRDFIYVSQDPKDQLLLGPTYATPK.V |
| 183951 | 336 | – | 362 | 778.9146 | 3111.6293 | 3111.6281 | 0.39 | 2 | 43 | 9.6e-05 | 1Score **> 37** indicates **identity** Score **> 15** indicates **homology** | U | K.AYLRDFIYVSQDPKDQLLLGPTYATPK.V |
| 183952 | 336 | – | 362 | 1038.2171 | 3111.6295 | 3111.6281 | 0.46 | 2 | 77 | 6e-08 | 1Score **> 37** indicates **identity** Score **> 17** indicates **homology** | U | K.AYLRDFIYVSQDPKDQLLLGPTYATPK.V |
| 183953 | 336 | – | 362 | 1038.2172 | 3111.6296 | 3111.6281 | 0.50 | 2 | 80 | 3e-08 | 1Score **> 37** indicates **identity** Score **> 18** indicates **homology** | U | K.AYLRDFIYVSQDPKDQLLLGPTYATPK.V |
| 183954 | 336 | – | 362 | 778.9148 | 3111.6300 | 3111.6281 | 0.62 | 2 | 34 | 0.00065 | 1Score **> 37** indicates **identity** Score **> 15** indicates **homology** | U | K.AYLRDFIYVSQDPKDQLLLGPTYATPK.V |
| 183955 | 336 | – | 362 | 1038.2174 | 3111.6302 | 3111.6281 | 0.70 | 2 | 63 | 1.2e-06 | 1Score **> 37** indicates **identity** Score **> 16** indicates **homology** | U | K.AYLRDFIYVSQDPKDQLLLGPTYATPK.V |
| 183956 | 336 | – | 362 | 778.9156 | 3111.6334 | 3111.6281 | 1.70 | 2 | 43 | 8.9e-05 | 1Score **> 37** indicates **identity** Score **> 15** indicates **homology** | U | K.AYLRDFIYVSQDPKDQLLLGPTYATPK.V |
| 183957 | 336 | – | 362 | 1038.2186 | 3111.6339 | 3111.6281 | 1.88 | 2 | 71 | 2.4e-07 | 1Score **> 37** indicates **identity** Score **> 17** indicates **homology** | U | K.AYLRDFIYVSQDPKDQLLLGPTYATPK.V |
| 183958 | 336 | – | 362 | 1038.2198 | 3111.6376 | 3111.6281 | 3.06 | 2 | 68 | 4.4e-07 | 1Score **> 37** indicates **identity** Score **> 17** indicates **homology** | U | K.AYLRDFIYVSQDPKDQLLLGPTYATPK.V |
| 183979 | 336 | – | 362 | 1038.5504 | 3112.6293 | 3112.6121 | 5.53 | 2 | 62 | 1.5e-06 | 1Score **> 37** indicates **identity** Score **> 16** indicates **homology** | U | K.AYLRDFIYVSQDPKDQLLLGPTYATPK.V  + Deamidated (NQ) |
| 183981 | 336 | – | 362 | 779.1649 | 3112.6306 | 3112.6121 | 5.96 | 2 | 26 | 0.0036 | 1Score **> 37** indicates **identity** Score **> 14** indicates **homology** | U | K.AYLRDFIYVSQDPKDQLLLGPTYATPK.V  + Deamidated (NQ) |
| 186750 | 336 | – | 362 | 1090.9079 | 3269.7020 | 3269.7111 | -2.79 | 2 | 16 | 0.03 | 1Score **> 37** indicates **identity** Score **> 14** indicates **homology** | U | K.AYLRDFIYVSQDPKDQLLLGPTYATPK.V  + 2 Deamidated (NQ); HNE (K) |
| 34743 | 340 | – | 349 | 606.3007 | 1210.5868 | 1210.5870 | -0.13 | 0 | 40 | 0.00019 | 1Score **> 32** indicates **identity** Score **> 15** indicates **homology** | U | R.DFIYVSQDPK.D |
| 34744 | 340 | – | 349 | 606.3013 | 1210.5880 | 1210.5870 | 0.81 | 0 | 20 | 0.014 | 1Score **> 31** indicates **identity** Score **> 14** indicates **homology** | U | R.DFIYVSQDPK.D |
| 34745 | 340 | – | 349 | 606.3020 | 1210.5895 | 1210.5870 | 2.09 | 0 | 38 | 0.00025 | 1Score **> 32** indicates **identity** Score **> 15** indicates **homology** | U | R.DFIYVSQDPK.D |
| 167061 | 340 | – | 362 | 870.4508 | 2608.3306 | 2608.3425 | -4.57 | 1 | 36 | 0.00044 | 1Score **> 37** indicates **identity** Score **> 15** indicates **homology** | U | R.DFIYVSQDPKDQLLLGPTYATPK.V |
| 167064 | 340 | – | 362 | 870.4521 | 2608.3345 | 2608.3425 | -3.06 | 1 | 27 | 0.0027 | 1Score **> 37** indicates **identity** Score **> 14** indicates **homology** | U | R.DFIYVSQDPKDQLLLGPTYATPK.V |
| 167065 | 340 | – | 362 | 870.4523 | 2608.3352 | 2608.3425 | -2.80 | 1 | 23 | 0.0071 | 1Score **> 37** indicates **identity** Score **> 14** indicates **homology** | U | R.DFIYVSQDPKDQLLLGPTYATPK.V |
| 167066 | 340 | – | 362 | 870.4527 | 2608.3364 | 2608.3425 | -2.34 | 1 | 17 | 0.025 | 1Score **> 37** indicates **identity** Score **> 14** indicates **homology** | U | R.DFIYVSQDPKDQLLLGPTYATPK.V |
| 167067 | 340 | – | 362 | 870.4529 | 2608.3368 | 2608.3425 | -2.17 | 1 | 36 | 0.00042 | 1Score **> 37** indicates **identity** Score **> 15** indicates **homology** | U | R.DFIYVSQDPKDQLLLGPTYATPK.V |
| 167069 | 340 | – | 362 | 870.4538 | 2608.3395 | 2608.3425 | -1.15 | 1 | 54 | 9.1e-06 | 1Score **> 37** indicates **identity** Score **> 16** indicates **homology** | U | R.DFIYVSQDPKDQLLLGPTYATPK.V |
| 167072 | 340 | – | 362 | 870.4545 | 2608.3417 | 2608.3425 | -0.30 | 1 | 44 | 8.5e-05 | 1Score **> 37** indicates **identity** Score **> 15** indicates **homology** | U | R.DFIYVSQDPKDQLLLGPTYATPK.V |
| 167074 | 340 | – | 362 | 870.4552 | 2608.3437 | 2608.3425 | 0.47 | 1 | 36 | 0.00041 | 1Score **> 37** indicates **identity** Score **> 15** indicates **homology** | U | R.DFIYVSQDPKDQLLLGPTYATPK.V |
| 167075 | 340 | – | 362 | 870.4552 | 2608.3437 | 2608.3425 | 0.47 | 1 | 55 | 7.5e-06 | 1Score **> 37** indicates **identity** Score **> 16** indicates **homology** | U | R.DFIYVSQDPKDQLLLGPTYATPK.V |
| 167076 | 340 | – | 362 | 1305.1791 | 2608.3437 | 2608.3425 | 0.47 | 1 | 76 | 8.1e-08 | 1Score **> 37** indicates **identity** Score **> 17** indicates **homology** | U | R.DFIYVSQDPKDQLLLGPTYATPK.V |
| 167077 | 340 | – | 362 | 870.4553 | 2608.3440 | 2608.3425 | 0.58 | 1 | 64 | 1.1e-06 | 1Score **> 37** indicates **identity** Score **> 16** indicates **homology** | U | R.DFIYVSQDPKDQLLLGPTYATPK.V |
| 167078 | 340 | – | 362 | 870.4553 | 2608.3441 | 2608.3425 | 0.63 | 1 | 18 | 0.02 | 1Score **> 37** indicates **identity** Score **> 14** indicates **homology** | U | R.DFIYVSQDPKDQLLLGPTYATPK.V |
| 167080 | 340 | – | 362 | 653.0935 | 2608.3449 | 2608.3425 | 0.92 | 1 | 21 | 0.011 | 1Score **> 37** indicates **identity** Score **> 14** indicates **homology** | U | R.DFIYVSQDPKDQLLLGPTYATPK.V |
| 167081 | 340 | – | 362 | 870.4558 | 2608.3455 | 2608.3425 | 1.16 | 1 | 48 | 3.5e-05 | 1Score **> 37** indicates **identity** Score **> 15** indicates **homology** | U | R.DFIYVSQDPKDQLLLGPTYATPK.V |
| 167082 | 340 | – | 362 | 870.4558 | 2608.3455 | 2608.3425 | 1.16 | 1 | 59 | 3e-06 | 1Score **> 37** indicates **identity** Score **> 16** indicates **homology** | U | R.DFIYVSQDPKDQLLLGPTYATPK.V |
| 167083 | 340 | – | 362 | 1305.1800 | 2608.3455 | 2608.3425 | 1.16 | 1 | 77 | 6.7e-08 | 1Score **> 37** indicates **identity** Score **> 17** indicates **homology** | U | R.DFIYVSQDPKDQLLLGPTYATPK.V |
| 167084 | 340 | – | 362 | 1305.1801 | 2608.3456 | 2608.3425 | 1.18 | 1 | 47 | 3.6e-05 | 1Score **> 37** indicates **identity** Score **> 15** indicates **homology** | U | R.DFIYVSQDPKDQLLLGPTYATPK.V |
| 167085 | 340 | – | 362 | 870.4558 | 2608.3456 | 2608.3425 | 1.20 | 1 | 28 | 0.0026 | 1Score **> 37** indicates **identity** Score **> 14** indicates **homology** | U | R.DFIYVSQDPKDQLLLGPTYATPK.V |
| 167086 | 340 | – | 362 | 870.4559 | 2608.3458 | 2608.3425 | 1.27 | 1 | 36 | 0.00038 | 1Score **> 37** indicates **identity** Score **> 15** indicates **homology** | U | R.DFIYVSQDPKDQLLLGPTYATPK.V |
| 167087 | 340 | – | 362 | 870.4560 | 2608.3460 | 2608.3425 | 1.37 | 1 | 54 | 8e-06 | 1Score **> 37** indicates **identity** Score **> 16** indicates **homology** | U | R.DFIYVSQDPKDQLLLGPTYATPK.V |
| 167088 | 340 | – | 362 | 870.4560 | 2608.3463 | 2608.3425 | 1.45 | 1 | 54 | 7.9e-06 | 1Score **> 37** indicates **identity** Score **> 16** indicates **homology** | U | R.DFIYVSQDPKDQLLLGPTYATPK.V |
| 167090 | 340 | – | 362 | 1305.1808 | 2608.3470 | 2608.3425 | 1.74 | 1 | 71 | 2.1e-07 | 1Score **> 37** indicates **identity** Score **> 17** indicates **homology** | U | R.DFIYVSQDPKDQLLLGPTYATPK.V |
| 167091 | 340 | – | 362 | 1305.1808 | 2608.3471 | 2608.3425 | 1.76 | 1 | 56 | 5.7e-06 | 1Score **> 37** indicates **identity** Score **> 16** indicates **homology** | U | R.DFIYVSQDPKDQLLLGPTYATPK.V |
| 167092 | 340 | – | 362 | 870.4563 | 2608.3472 | 2608.3425 | 1.80 | 1 | 63 | 1.1e-06 | 1Score **> 37** indicates **identity** Score **> 16** indicates **homology** | U | R.DFIYVSQDPKDQLLLGPTYATPK.V |
| 167093 | 340 | – | 362 | 1305.1809 | 2608.3472 | 2608.3425 | 1.80 | 1 | 74 | 1.1e-07 | 1Score **> 37** indicates **identity** Score **> 17** indicates **homology** | U | R.DFIYVSQDPKDQLLLGPTYATPK.V |
| 167094 | 340 | – | 362 | 870.4564 | 2608.3473 | 2608.3425 | 1.85 | 1 | 38 | 0.00028 | 1Score **> 37** indicates **identity** Score **> 15** indicates **homology** | U | R.DFIYVSQDPKDQLLLGPTYATPK.V |
| 167095 | 340 | – | 362 | 1305.1812 | 2608.3478 | 2608.3425 | 2.06 | 1 | 61 | 2.1e-06 | 1Score **> 37** indicates **identity** Score **> 16** indicates **homology** | U | R.DFIYVSQDPKDQLLLGPTYATPK.V |
| 167096 | 340 | – | 362 | 870.4566 | 2608.3478 | 2608.3425 | 2.06 | 1 | 57 | 4.2e-06 | 1Score **> 37** indicates **identity** Score **> 16** indicates **homology** | U | R.DFIYVSQDPKDQLLLGPTYATPK.V |
| 167097 | 340 | – | 362 | 870.4566 | 2608.3479 | 2608.3425 | 2.08 | 1 | 57 | 4.9e-06 | 1Score **> 37** indicates **identity** Score **> 16** indicates **homology** | U | R.DFIYVSQDPKDQLLLGPTYATPK.V |
| 167098 | 340 | – | 362 | 870.4566 | 2608.3480 | 2608.3425 | 2.11 | 1 | 47 | 4e-05 | 1Score **> 37** indicates **identity** Score **> 15** indicates **homology** | U | R.DFIYVSQDPKDQLLLGPTYATPK.V |
| 167099 | 340 | – | 362 | 870.4567 | 2608.3482 | 2608.3425 | 2.18 | 1 | 51 | 1.8e-05 | 1Score **> 37** indicates **identity** Score **> 16** indicates **homology** | U | R.DFIYVSQDPKDQLLLGPTYATPK.V |
| 167100 | 340 | – | 362 | 870.4568 | 2608.3484 | 2608.3425 | 2.29 | 1 | 50 | 2.2e-05 | 1Score **> 37** indicates **identity** Score **> 16** indicates **homology** | U | R.DFIYVSQDPKDQLLLGPTYATPK.V |
| 167101 | 340 | – | 362 | 870.4568 | 2608.3486 | 2608.3425 | 2.35 | 1 | 59 | 3e-06 | 1Score **> 37** indicates **identity** Score **> 16** indicates **homology** | U | R.DFIYVSQDPKDQLLLGPTYATPK.V |
| 167102 | 340 | – | 362 | 870.4572 | 2608.3497 | 2608.3425 | 2.79 | 1 | 31 | 0.0013 | 1Score **> 37** indicates **identity** Score **> 14** indicates **homology** | U | R.DFIYVSQDPKDQLLLGPTYATPK.V |
| 167108 | 340 | – | 362 | 870.4592 | 2608.3559 | 2608.3425 | 5.16 | 1 | 23 | 0.0066 | 1Score **> 37** indicates **identity** Score **> 14** indicates **homology** | U | R.DFIYVSQDPKDQLLLGPTYATPK.V |
| 167111 | 340 | – | 362 | 870.4631 | 2608.3674 | 2608.3425 | 9.56 | 1 | 37 | 0.00031 | 1Score **> 37** indicates **identity** Score **> 15** indicates **homology** | U | R.DFIYVSQDPKDQLLLGPTYATPK.V |
| 167149 | 340 | – | 362 | 870.7879 | 2609.3418 | 2609.3265 | 5.87 | 1 | 36 | 0.00041 | 1Score **> 37** indicates **identity** Score **> 15** indicates **homology** | U | R.DFIYVSQDPKDQLLLGPTYATPK.V  + Deamidated (NQ) |
| 167150 | 340 | – | 362 | 870.7880 | 2609.3420 | 2609.3265 | 5.96 | 1 | 27 | 0.0031 | 1Score **> 37** indicates **identity** Score **> 14** indicates **homology** | U | R.DFIYVSQDPKDQLLLGPTYATPK.V  + Deamidated (NQ) |
| 167152 | 340 | – | 362 | 870.7893 | 2609.3462 | 2609.3265 | 7.56 | 1 | 22 | 0.0091 | 1Score **> 37** indicates **identity** Score **> 14** indicates **homology** | U | R.DFIYVSQDPKDQLLLGPTYATPK.V  + Deamidated (NQ) |
| 167153 | 340 | – | 362 | 870.7896 | 2609.3470 | 2609.3265 | 7.86 | 1 | 15 | 0.035 | 1Score **> 37** indicates **identity** Score **> 13** indicates **homology** | U | R.DFIYVSQDPKDQLLLGPTYATPK.V  + Deamidated (NQ) |
| 167154 | 340 | – | 362 | 870.7898 | 2609.3475 | 2609.3265 | 8.05 | 1 | 22 | 0.0096 | 1Score **> 37** indicates **identity** Score **> 14** indicates **homology** | U | R.DFIYVSQDPKDQLLLGPTYATPK.V  + Deamidated (NQ) |
| 167155 | 340 | – | 362 | 870.7902 | 2609.3488 | 2609.3265 | 8.54 | 1 | 27 | 0.0029 | 1Score **> 37** indicates **identity** Score **> 14** indicates **homology** | U | R.DFIYVSQDPKDQLLLGPTYATPK.V  + Deamidated (NQ) |
| 167158 | 340 | – | 362 | 870.7913 | 2609.3520 | 2609.3265 | 9.80 | 1 | 22 | 0.0081 | 1Score **> 37** indicates **identity** Score **> 14** indicates **homology** | U | R.DFIYVSQDPKDQLLLGPTYATPK.V  + Deamidated (NQ) |
| 57090 | 350 | – | 362 | 708.8898 | 1415.7650 | 1415.7660 | -0.74 | 0 | 26 | 0.0038 | 1Score **> 35** indicates **identity** Score **> 14** indicates **homology** | U | K.DQLLLGPTYATPK.V |
| 57091 | 350 | – | 362 | 708.8902 | 1415.7659 | 1415.7660 | -0.088 | 0 | 61 | 3.7e-06 | 1Score **> 34** indicates **identity** Score **> 20** indicates **homology** | U | K.DQLLLGPTYATPK.V |
| 57092 | 350 | – | 362 | 708.8902 | 1415.7659 | 1415.7660 | -0.082 | 0 | 65 | 1.7e-06 | 1Score **> 34** indicates **identity** Score **> 20** indicates **homology** | U | K.DQLLLGPTYATPK.V |
| 57094 | 350 | – | 362 | 708.8907 | 1415.7669 | 1415.7660 | 0.61 | 0 | 51 | 3.7e-05 | 1Score **> 35** indicates **identity** Score **> 19** indicates **homology** | U | K.DQLLLGPTYATPK.V |
| 57095 | 350 | – | 362 | 708.8911 | 1415.7676 | 1415.7660 | 1.13 | 0 | 37 | 0.00036 | 1Score **> 35** indicates **identity** Score **> 15** indicates **homology** | U | K.DQLLLGPTYATPK.V |
| 57097 | 350 | – | 362 | 708.8918 | 1415.7691 | 1415.7660 | 2.20 | 0 | 23 | 0.0069 | 1Score **> 35** indicates **identity** Score **> 14** indicates **homology** | U | K.DQLLLGPTYATPK.V |
| 57100 | 350 | – | 362 | 708.8924 | 1415.7703 | 1415.7660 | 3.03 | 0 | 52 | 2.7e-05 | 1Score **> 34** indicates **identity** Score **> 19** indicates **homology** | U | K.DQLLLGPTYATPK.V |
| 57101 | 350 | – | 362 | 708.8951 | 1415.7757 | 1415.7660 | 6.84 | 0 | 25 | 0.021 | 1Score **> 35** indicates **identity** Score **> 21** indicates **homology** | U | K.DQLLLGPTYATPK.V |
| 92698 | 393 | – | 406 | 846.3494 | 1690.6843 | 1690.6868 | -1.49 | 0 | 71 | 2.2e-07 | 1Score **> 23** indicates **identity** Score **> 17** indicates **homology** | U | K.AMDSDWFAQNYMGR.K |
| 107533 | 393 | – | 407 | 607.2679 | 1818.7820 | 1818.7818 | 0.13 | 1 | 53 | 1e-05 | 1Score **> 27** indicates **identity** Score **> 16** indicates **homology** | U | K.AMDSDWFAQNYMGRK.T |
| 18873 | 408 | – | 417 | 352.5413 | 1054.6021 | 1054.6022 | -0.17 | 1 | 49 | 0.00013 | 1Score **> 28** indicates **identity** Score **> 23** indicates **homology** | U | K.TKVGSPPLEK.F |
| 18874 | 408 | – | 417 | 352.5414 | 1054.6024 | 1054.6022 | 0.19 | 1 | 43 | 0.00024 | 1Score **> 28** indicates **identity** Score **> 19** indicates **homology** | U | K.TKVGSPPLEK.F |
| 18875 | 408 | – | 417 | 528.3087 | 1054.6028 | 1054.6022 | 0.51 | 1 | 29 | 0.0025 | 1Score **> 28** indicates **identity** Score **> 15** indicates **homology** | U | K.TKVGSPPLEK.F |
| 18876 | 408 | – | 417 | 528.3088 | 1054.6030 | 1054.6022 | 0.66 | 1 | 32 | 0.0014 | 1Score **> 29** indicates **identity** Score **> 16** indicates **homology** | U | K.TKVGSPPLEK.F |
| 18877 | 408 | – | 417 | 528.3088 | 1054.6030 | 1054.6022 | 0.67 | 1 | 49 | 9e-05 | 1Score **> 29** indicates **identity** Score **> 21** indicates **homology** | U | K.TKVGSPPLEK.F |
| 18878 | 408 | – | 417 | 352.5416 | 1054.6030 | 1054.6022 | 0.70 | 1 | 47 | 0.00033 | 1Score **> 29** indicates **identity** Score **> 24** indicates **homology** | U | K.TKVGSPPLEK.F |
| 18879 | 408 | – | 417 | 352.5417 | 1054.6032 | 1054.6022 | 0.93 | 1 | 16 | 0.029 | 1Score **> 29** indicates **identity** Score **> 14** indicates **homology** | U | K.TKVGSPPLEK.F |
| 18880 | 408 | – | 417 | 352.5422 | 1054.6046 | 1054.6022 | 2.28 | 1 | 40 | 0.00074 | 1Score **> 29** indicates **identity** Score **> 21** indicates **homology** | U | K.TKVGSPPLEK.F |
| 18881 | 408 | – | 417 | 528.3097 | 1054.6048 | 1054.6022 | 2.45 | 1 | 32 | 0.0014 | 1Score **> 29** indicates **identity** Score **> 16** indicates **homology** | U | K.TKVGSPPLEK.F |
| 184849 | 408 | – | 437 | 634.9313 | 3169.6202 | 3169.6131 | 2.24 | 2 | 16 | 0.033 | 1Score **> 38** indicates **identity** Score **> 13** indicates **homology** | U | K.TKVGSPPLEKFNIWGGSLSLGHPFGATGCR.L |
| 5024 | 410 | – | 417 | 413.7361 | 825.4576 | 825.4596 | -2.42 | 0 | 18 | 0.029 | 1Score **> 24** indicates **identity** Score **> 15** indicates **homology** | U | K.VGSPPLEK.F |
| 5025 | 410 | – | 417 | 413.7367 | 825.4589 | 825.4596 | -0.83 | 0 | 49 | 0.00016 | 1Score **> 23** indicates **identity** | U | K.VGSPPLEK.F |
| 5026 | 410 | – | 417 | 413.7368 | 825.4591 | 825.4596 | -0.67 | 0 | 49 | 0.00015 | 1Score **> 23** indicates **identity** | U | K.VGSPPLEK.F |
| 5027 | 410 | – | 417 | 413.7369 | 825.4592 | 825.4596 | -0.44 | 0 | 43 | 0.0006 | 1Score **> 23** indicates **identity** Score **> 23** indicates **homology** | U | K.VGSPPLEK.F |
| 5028 | 410 | – | 417 | 413.7383 | 825.4620 | 825.4596 | 2.95 | 0 | 20 | 0.015 | 1Score **> 23** indicates **identity** Score **> 14** indicates **homology** | U | K.VGSPPLEK.F |
| 179561 | 410 | – | 437 | 736.1262 | 2940.4755 | 2940.4705 | 1.71 | 1 | 21 | 0.011 | 1Score **> 37** indicates **identity** Score **> 14** indicates **homology** | U | K.VGSPPLEKFNIWGGSLSLGHPFGATGCR.L |
| 179563 | 410 | – | 437 | 981.1686 | 2940.4840 | 2940.4705 | 4.60 | 1 | 51 | 1.5e-05 | 1Score **> 37** indicates **identity** Score **> 16** indicates **homology** | U | K.VGSPPLEKFNIWGGSLSLGHPFGATGCR.L |
| 136866 | 418 | – | 437 | 712.0125 | 2133.0156 | 2133.0215 | -2.72 | 0 | 23 | 0.0071 | 1Score **> 35** indicates **identity** Score **> 14** indicates **homology** | U | K.FNIWGGSLSLGHPFGATGCR.L |
| 136876 | 418 | – | 437 | 712.0140 | 2133.0203 | 2133.0215 | -0.56 | 0 | 21 | 0.011 | 1Score **> 35** indicates **identity** Score **> 14** indicates **homology** | U | K.FNIWGGSLSLGHPFGATGCR.L |
| 136877 | 418 | – | 437 | 712.0141 | 2133.0206 | 2133.0215 | -0.41 | 0 | 40 | 0.00019 | 1Score **> 35** indicates **identity** Score **> 15** indicates **homology** | U | K.FNIWGGSLSLGHPFGATGCR.L |
| 136880 | 418 | – | 437 | 712.0144 | 2133.0215 | 2133.0215 | 0.014 | 0 | 29 | 0.0018 | 1Score **> 35** indicates **identity** Score **> 14** indicates **homology** | U | K.FNIWGGSLSLGHPFGATGCR.L |
| 136886 | 418 | – | 437 | 1067.5186 | 2133.0227 | 2133.0215 | 0.60 | 0 | 122 | 4e-12 | 1Score **> 35** indicates **identity** Score **> 20** indicates **homology** | U | K.FNIWGGSLSLGHPFGATGCR.L |
| 136887 | 418 | – | 437 | 712.0149 | 2133.0228 | 2133.0215 | 0.62 | 0 | 54 | 8.9e-06 | 1Score **> 35** indicates **identity** Score **> 16** indicates **homology** | U | K.FNIWGGSLSLGHPFGATGCR.L |
| 136890 | 418 | – | 437 | 712.0149 | 2133.0230 | 2133.0215 | 0.72 | 0 | 78 | 4.4e-08 | 1Score **> 35** indicates **identity** Score **> 17** indicates **homology** | U | K.FNIWGGSLSLGHPFGATGCR.L |
| 136891 | 418 | – | 437 | 1067.5188 | 2133.0230 | 2133.0215 | 0.74 | 0 | 71 | 2.2e-07 | 1Score **> 35** indicates **identity** Score **> 17** indicates **homology** | U | K.FNIWGGSLSLGHPFGATGCR.L |
| 136892 | 418 | – | 437 | 712.0150 | 2133.0230 | 2133.0215 | 0.74 | 0 | 41 | 0.00013 | 1Score **> 35** indicates **identity** Score **> 15** indicates **homology** | U | K.FNIWGGSLSLGHPFGATGCR.L |
| 136893 | 418 | – | 437 | 1067.5189 | 2133.0232 | 2133.0215 | 0.84 | 0 | 107 | 9.1e-11 | 1Score **> 35** indicates **identity** Score **> 19** indicates **homology** | U | K.FNIWGGSLSLGHPFGATGCR.L |
| 136895 | 418 | – | 437 | 712.0151 | 2133.0235 | 2133.0215 | 0.94 | 0 | 59 | 2.9e-06 | 1Score **> 35** indicates **identity** Score **> 16** indicates **homology** | U | K.FNIWGGSLSLGHPFGATGCR.L |
| 136898 | 418 | – | 437 | 712.0154 | 2133.0243 | 2133.0215 | 1.33 | 0 | 63 | 1.3e-06 | 1Score **> 35** indicates **identity** Score **> 16** indicates **homology** | U | K.FNIWGGSLSLGHPFGATGCR.L |
| 136899 | 418 | – | 437 | 1067.5194 | 2133.0243 | 2133.0215 | 1.35 | 0 | 108 | 7.7e-11 | 1Score **> 35** indicates **identity** Score **> 19** indicates **homology** | U | K.FNIWGGSLSLGHPFGATGCR.L |
| 136900 | 418 | – | 437 | 712.0154 | 2133.0244 | 2133.0215 | 1.39 | 0 | 54 | 9.3e-06 | 1Score **> 35** indicates **identity** Score **> 16** indicates **homology** | U | K.FNIWGGSLSLGHPFGATGCR.L |
| 136902 | 418 | – | 437 | 1067.5195 | 2133.0245 | 2133.0215 | 1.44 | 0 | 128 | 1.1e-12 | 1Score **> 35** indicates **identity** Score **> 20** indicates **homology** | U | K.FNIWGGSLSLGHPFGATGCR.L |
| 136904 | 418 | – | 437 | 712.0155 | 2133.0248 | 2133.0215 | 1.55 | 0 | 41 | 0.00015 | 1Score **> 35** indicates **identity** Score **> 15** indicates **homology** | U | K.FNIWGGSLSLGHPFGATGCR.L |
| 136905 | 418 | – | 437 | 1067.5197 | 2133.0248 | 2133.0215 | 1.57 | 0 | 67 | 4.7e-07 | 1Score **> 35** indicates **identity** Score **> 17** indicates **homology** | U | K.FNIWGGSLSLGHPFGATGCR.L |
| 136907 | 418 | – | 437 | 1067.5200 | 2133.0255 | 2133.0215 | 1.91 | 0 | 72 | 1.8e-07 | 1Score **> 35** indicates **identity** Score **> 17** indicates **homology** | U | K.FNIWGGSLSLGHPFGATGCR.L |
| 136908 | 418 | – | 437 | 1067.5201 | 2133.0256 | 2133.0215 | 1.96 | 0 | 99 | 5e-10 | 1Score **> 35** indicates **identity** Score **> 19** indicates **homology** | U | K.FNIWGGSLSLGHPFGATGCR.L |
| 136911 | 418 | – | 437 | 712.0160 | 2133.0262 | 2133.0215 | 2.25 | 0 | 31 | 0.0011 | 1Score **> 35** indicates **identity** Score **> 14** indicates **homology** | U | K.FNIWGGSLSLGHPFGATGCR.L |
| 136914 | 418 | – | 437 | 1067.5206 | 2133.0266 | 2133.0215 | 2.43 | 0 | 91 | 2.7e-09 | 1Score **> 35** indicates **identity** Score **> 18** indicates **homology** | U | K.FNIWGGSLSLGHPFGATGCR.L |
| 136919 | 418 | – | 437 | 1067.5218 | 2133.0291 | 2133.0215 | 3.57 | 0 | 75 | 9e-08 | 1Score **> 36** indicates **identity** Score **> 17** indicates **homology** | U | K.FNIWGGSLSLGHPFGATGCR.L |
| 136923 | 418 | – | 437 | 1067.5272 | 2133.0399 | 2133.0215 | 8.64 | 0 | 74 | 1.1e-07 | 1Score **> 36** indicates **identity** Score **> 17** indicates **homology** | U | K.FNIWGGSLSLGHPFGATGCR.L |
| 5904 | 438 | – | 445 | 423.2361 | 844.4577 | 844.4589 | -1.36 | 0 | 27 | 0.013 | 1Score **> 32** indicates **identity** Score **> 21** indicates **homology** | U | R.LVMAAANR.L |
| 5905 | 438 | – | 445 | 423.2365 | 844.4584 | 844.4589 | -0.59 | 0 | 52 | 0.00014 | 1Score **> 32** indicates **identity** Score **> 26** indicates **homology** | U | R.LVMAAANR.L |
| 5906 | 438 | – | 445 | 423.2366 | 844.4586 | 844.4589 | -0.30 | 0 | 34 | 0.0036 | 1Score **> 32** indicates **identity** Score **> 22** indicates **homology** | U | R.LVMAAANR.L |
| 6849 | 438 | – | 445 | 431.2330 | 860.4514 | 860.4538 | -2.81 | 0 | 27 | 0.0042 | 1Score **> 29** indicates **identity** Score **> 16** indicates **homology** | U | R.LVMAAANR.L  + Oxidation (M) |
| 6851 | 438 | – | 445 | 431.2332 | 860.4519 | 860.4538 | -2.19 | 0 | 18 | 0.028 | 1Score **> 31** indicates **identity** Score **> 15** indicates **homology** | U | R.LVMAAANR.L  + Oxidation (M) |
| 6853 | 438 | – | 445 | 431.2334 | 860.4523 | 860.4538 | -1.69 | 0 | 25 | 0.0079 | 1Score **> 31** indicates **identity** Score **> 17** indicates **homology** | U | R.LVMAAANR.L  + Oxidation (M) |
| 6855 | 438 | – | 445 | 431.2339 | 860.4533 | 860.4538 | -0.53 | 0 | 19 | 0.017 | 1Score **> 31** indicates **identity** Score **> 14** indicates **homology** | U | R.LVMAAANR.L  + Oxidation (M) |
| 6856 | 438 | – | 445 | 431.2341 | 860.4537 | 860.4538 | -0.12 | 0 | 23 | 0.015 | 1Score **> 31** indicates **identity** Score **> 17** indicates **homology** | U | R.LVMAAANR.L  + Oxidation (M) |
| 6857 | 438 | – | 445 | 431.2343 | 860.4540 | 860.4538 | 0.25 | 0 | 19 | 0.021 | 1Score **> 31** indicates **identity** Score **> 15** indicates **homology** | U | R.LVMAAANR.L  + Oxidation (M) |
| 6858 | 438 | – | 445 | 431.2345 | 860.4544 | 860.4538 | 0.73 | 0 | 28 | 0.0049 | 1Score **> 31** indicates **identity** Score **> 17** indicates **homology** | U | R.LVMAAANR.L  + Oxidation (M) |
| 6861 | 438 | – | 445 | 431.2359 | 860.4572 | 860.4538 | 3.93 | 0 | 17 | 0.025 | 1Score **> 31** indicates **identity** Score **> 14** indicates **homology** | U | R.LVMAAANR.L  + Oxidation (M) |
| 176266 | 448 | – | 475 | 709.0990 | 2832.3669 | 2832.3687 | -0.64 | 1 | 32 | 0.001 | 1Score **> 36** indicates **identity** Score **> 14** indicates **homology** | U | R.KDGGQYALVAACAAGGQGHAMIVEAYPK.- |
| 176267 | 448 | – | 475 | 709.1006 | 2832.3733 | 2832.3687 | 1.61 | 1 | 44 | 6.7e-05 | 1Score **> 37** indicates **identity** Score **> 15** indicates **homology** | U | R.KDGGQYALVAACAAGGQGHAMIVEAYPK.- |
| 176336 | 448 | – | 475 | 945.4618 | 2833.3635 | 2833.3527 | 3.80 | 1 | 43 | 8.9e-05 | 1Score **> 37** indicates **identity** Score **> 15** indicates **homology** | U | R.KDGGQYALVAACAAGGQGHAMIVEAYPK.-  + Deamidated (NQ) |
| 176338 | 448 | – | 475 | 945.4655 | 2833.3746 | 2833.3527 | 7.74 | 1 | 44 | 7.8e-05 | 1Score **> 37** indicates **identity** Score **> 15** indicates **homology** | U | R.KDGGQYALVAACAAGGQGHAMIVEAYPK.-  + Deamidated (NQ) |
| 171091 | 449 | – | 475 | 902.4300 | 2704.2681 | 2704.2737 | -2.10 | 0 | 60 | 2.3e-06 | 1Score **> 35** indicates **identity** Score **> 16** indicates **homology** | U | K.DGGQYALVAACAAGGQGHAMIVEAYPK.- |
| 171092 | 449 | – | 475 | 902.4325 | 2704.2757 | 2704.2737 | 0.74 | 0 | 34 | 0.00068 | 1Score **> 35** indicates **identity** Score **> 15** indicates **homology** | U | K.DGGQYALVAACAAGGQGHAMIVEAYPK.- |
| 171093 | 449 | – | 475 | 902.4325 | 2704.2758 | 2704.2737 | 0.75 | 0 | 54 | 9.6e-06 | 1Score **> 35** indicates **identity** Score **> 16** indicates **homology** | U | K.DGGQYALVAACAAGGQGHAMIVEAYPK.- |
| 171097 | 449 | – | 475 | 902.4333 | 2704.2781 | 2704.2737 | 1.61 | 0 | 66 | 7.1e-07 | 1Score **> 35** indicates **identity** Score **> 17** indicates **homology** | U | K.DGGQYALVAACAAGGQGHAMIVEAYPK.- |
| 171098 | 449 | – | 475 | 902.4336 | 2704.2790 | 2704.2737 | 1.95 | 0 | 79 | 4.3e-08 | 1Score **> 35** indicates **identity** Score **> 17** indicates **homology** | U | K.DGGQYALVAACAAGGQGHAMIVEAYPK.- |
| 171102 | 449 | – | 475 | 902.4342 | 2704.2808 | 2704.2737 | 2.62 | 0 | 70 | 2.8e-07 | 1Score **> 35** indicates **identity** Score **> 17** indicates **homology** | U | K.DGGQYALVAACAAGGQGHAMIVEAYPK.- |
| 171125 | 449 | – | 475 | 1353.6489 | 2705.2832 | 2705.2578 | 9.40 | 0 | 62 | 1.6e-06 | 1Score **> 36** indicates **identity** Score **> 16** indicates **homology** | U | K.DGGQYALVAACAAGGQGHAMIVEAYPK.-  + Deamidated (NQ) |
| 171629 | 449 | – | 475 | 907.7622 | 2720.2649 | 2720.2687 | -1.38 | 0 | 28 | 0.0025 | 1Score **> 34** indicates **identity** Score **> 14** indicates **homology** | U | K.DGGQYALVAACAAGGQGHAMIVEAYPK.-  + Oxidation (M) |
| 171663 | 449 | – | 475 | 908.1001 | 2721.2784 | 2721.2527 | 9.46 | 0 | 33 | 0.00076 | 1Score **> 35** indicates **identity** Score **> 15** indicates **homology** | U | K.DGGQYALVAACAAGGQGHAMIVEAYPK.-  + Deamidated (NQ); Oxidation (M) |

---

```
ID   ECHB_MOUSE              Reviewed;         475 AA.
AC   Q99JY0; Q3TEH9; Q8BJI5; Q8BJM0; Q8BK52;
DT   16-AUG-2004, integrated into UniProtKB/Swiss-Prot.
DT   01-JUN-2001, sequence version 1.
DT   28-JUN-2023, entry version 165.
DE   RecName: Full=Trifunctional enzyme subunit beta, mitochondrial;
DE   AltName: Full=TP-beta;
DE   Includes:
DE     RecName: Full=3-ketoacyl-CoA thiolase;
DE              EC=2.3.1.155 {ECO:0000250|UniProtKB:P55084};
DE              EC=2.3.1.16 {ECO:0000250|UniProtKB:P55084};
DE     AltName: Full=Acetyl-CoA acyltransferase;
DE     AltName: Full=Beta-ketothiolase;
DE   Flags: Precursor;
GN   Name=Hadhb;
OS   Mus musculus (Mouse).
OC   Eukaryota; Metazoa; Chordata; Craniata; Vertebrata; Euteleostomi; Mammalia;
OC   Eutheria; Euarchontoglires; Glires; Rodentia; Myomorpha; Muroidea; Muridae;
OC   Murinae; Mus; Mus.
OX   NCBI_TaxID=10090;
RN   [1]
RP   NUCLEOTIDE SEQUENCE [LARGE SCALE MRNA].
RC   STRAIN=C57BL/6J;
RC   TISSUE=Bone marrow, Colon, Hippocampus, Spinal ganglion, Testis, and
RC   Thymus;
RX   PubMed=16141072; DOI=10.1126/science.1112014;
RA   Carninci P., Kasukawa T., Katayama S., Gough J., Frith M.C., Maeda N.,
RA   Oyama R., Ravasi T., Lenhard B., Wells C., Kodzius R., Shimokawa K.,
RA   Bajic V.B., Brenner S.E., Batalov S., Forrest A.R., Zavolan M., Davis M.J.,
RA   Wilming L.G., Aidinis V., Allen J.E., Ambesi-Impiombato A., Apweiler R.,
RA   Aturaliya R.N., Bailey T.L., Bansal M., Baxter L., Beisel K.W., Bersano T.,
RA   Bono H., Chalk A.M., Chiu K.P., Choudhary V., Christoffels A.,
RA   Clutterbuck D.R., Crowe M.L., Dalla E., Dalrymple B.P., de Bono B.,
RA   Della Gatta G., di Bernardo D., Down T., Engstrom P., Fagiolini M.,
RA   Faulkner G., Fletcher C.F., Fukushima T., Furuno M., Futaki S.,
RA   Gariboldi M., Georgii-Hemming P., Gingeras T.R., Gojobori T., Green R.E.,
RA   Gustincich S., Harbers M., Hayashi Y., Hensch T.K., Hirokawa N., Hill D.,
RA   Huminiecki L., Iacono M., Ikeo K., Iwama A., Ishikawa T., Jakt M.,
RA   Kanapin A., Katoh M., Kawasawa Y., Kelso J., Kitamura H., Kitano H.,
RA   Kollias G., Krishnan S.P., Kruger A., Kummerfeld S.K., Kurochkin I.V.,
RA   Lareau L.F., Lazarevic D., Lipovich L., Liu J., Liuni S., McWilliam S.,
RA   Madan Babu M., Madera M., Marchionni L., Matsuda H., Matsuzawa S., Miki H.,
RA   Mignone F., Miyake S., Morris K., Mottagui-Tabar S., Mulder N., Nakano N.,
RA   Nakauchi H., Ng P., Nilsson R., Nishiguchi S., Nishikawa S., Nori F.,
RA   Ohara O., Okazaki Y., Orlando V., Pang K.C., Pavan W.J., Pavesi G.,
RA   Pesole G., Petrovsky N., Piazza S., Reed J., Reid J.F., Ring B.Z.,
RA   Ringwald M., Rost B., Ruan Y., Salzberg S.L., Sandelin A., Schneider C.,
RA   Schoenbach C., Sekiguchi K., Semple C.A., Seno S., Sessa L., Sheng Y.,
RA   Shibata Y., Shimada H., Shimada K., Silva D., Sinclair B., Sperling S.,
RA   Stupka E., Sugiura K., Sultana R., Takenaka Y., Taki K., Tammoja K.,
RA   Tan S.L., Tang S., Taylor M.S., Tegner J., Teichmann S.A., Ueda H.R.,
RA   van Nimwegen E., Verardo R., Wei C.L., Yagi K., Yamanishi H.,
RA   Zabarovsky E., Zhu S., Zimmer A., Hide W., Bult C., Grimmond S.M.,
RA   Teasdale R.D., Liu E.T., Brusic V., Quackenbush J., Wahlestedt C.,
RA   Mattick J.S., Hume D.A., Kai C., Sasaki D., Tomaru Y., Fukuda S.,
RA   Kanamori-Katayama M., Suzuki M., Aoki J., Arakawa T., Iida J., Imamura K.,
RA   Itoh M., Kato T., Kawaji H., Kawagashira N., Kawashima T., Kojima M.,
RA   Kondo S., Konno H., Nakano K., Ninomiya N., Nishio T., Okada M., Plessy C.,
RA   Shibata K., Shiraki T., Suzuki S., Tagami M., Waki K., Watahiki A.,
RA   Okamura-Oho Y., Suzuki H., Kawai J., Hayashizaki Y.;
RT   "The transcriptional landscape of the mammalian genome.";
RL   Science 309:1559-1563(2005).
RN   [2]
RP   NUCLEOTIDE SEQUENCE [LARGE SCALE MRNA].
RC   STRAIN=FVB/N; TISSUE=Mammary tumor;
RX   PubMed=15489334; DOI=10.1101/gr.2596504;
RG   The MGC Project Team;
RT   "The status, quality, and expansion of the NIH full-length cDNA project:
RT   the Mammalian Gene Collection (MGC).";
RL   Genome Res. 14:2121-2127(2004).
RN   [3]
RP   IDENTIFICATION BY MASS SPECTROMETRY [LARGE SCALE ANALYSIS].
RC   TISSUE=Brain, Brown adipose tissue, Heart, Kidney, Liver, Lung,
RC   Pancreas, Spleen, and Testis;
RX   PubMed=21183079; DOI=10.1016/j.cell.2010.12.001;
RA   Huttlin E.L., Jedrychowski M.P., Elias J.E., Goswami T., Rad R.,
RA   Beausoleil S.A., Villen J., Haas W., Sowa M.E., Gygi S.P.;
RT   "A tissue-specific atlas of mouse protein phosphorylation and expression.";
RL   Cell 143:1174-1189(2010).
RN   [4]
RP   SUCCINYLATION [LARGE SCALE ANALYSIS] AT LYS-53; LYS-73; LYS-189; LYS-191;
RP   LYS-273; LYS-292; LYS-294 AND LYS-333, AND IDENTIFICATION BY MASS
RP   SPECTROMETRY [LARGE SCALE ANALYSIS].
RC   TISSUE=Embryonic fibroblast, and Liver;
RX   PubMed=23806337; DOI=10.1016/j.molcel.2013.06.001;
RA   Park J., Chen Y., Tishkoff D.X., Peng C., Tan M., Dai L., Xie Z., Zhang Y.,
RA   Zwaans B.M., Skinner M.E., Lombard D.B., Zhao Y.;
RT   "SIRT5-mediated lysine desuccinylation impacts diverse metabolic
RT   pathways.";
RL   Mol. Cell 50:919-930(2013).
RN   [5]
RP   ACETYLATION [LARGE SCALE ANALYSIS] AT LYS-73; LYS-189; LYS-294; LYS-299;
RP   LYS-333; LYS-349 AND LYS-362, AND IDENTIFICATION BY MASS SPECTROMETRY
RP   [LARGE SCALE ANALYSIS].
RC   TISSUE=Liver;
RX   PubMed=23576753; DOI=10.1073/pnas.1302961110;
RA   Rardin M.J., Newman J.C., Held J.M., Cusack M.P., Sorensen D.J., Li B.,
RA   Schilling B., Mooney S.D., Kahn C.R., Verdin E., Gibson B.W.;
RT   "Label-free quantitative proteomics of the lysine acetylome in mitochondria
RT   identifies substrates of SIRT3 in metabolic pathways.";
RL   Proc. Natl. Acad. Sci. U.S.A. 110:6601-6606(2013).
RN   [6]
RP   INTERACTION WITH MTLN.
RX   PubMed=29949755; DOI=10.1016/j.celrep.2018.05.058;
RA   Makarewich C.A., Baskin K.K., Munir A.Z., Bezprozvannaya S., Sharma G.,
RA   Khemtong C., Shah A.M., McAnally J.R., Malloy C.R., Szweda L.I.,
RA   Bassel-Duby R., Olson E.N.;
RT   "MOXI Is a Mitochondrial Micropeptide That Enhances Fatty Acid beta-
RT   Oxidation.";
RL   Cell Rep. 23:3701-3709(2018).
CC   -!- FUNCTION: Mitochondrial trifunctional enzyme catalyzes the last three
CC       of the four reactions of the mitochondrial beta-oxidation pathway. The
CC       mitochondrial beta-oxidation pathway is the major energy-producing
CC       process in tissues and is performed through four consecutive reactions
CC       breaking down fatty acids into acetyl-CoA. Among the enzymes involved
CC       in this pathway, the trifunctional enzyme exhibits specificity for
CC       long-chain fatty acids. Mitochondrial trifunctional enzyme is a
CC       heterotetrameric complex composed of two proteins, the trifunctional
CC       enzyme subunit alpha/HADHA carries the 2,3-enoyl-CoA hydratase and the
CC       3-hydroxyacyl-CoA dehydrogenase activities, while the trifunctional
CC       enzyme subunit beta/HADHB described here bears the 3-ketoacyl-CoA
CC       thiolase activity. {ECO:0000250|UniProtKB:P55084}.
CC   -!- CATALYTIC ACTIVITY:
CC       Reaction=acetyl-CoA + an acyl-CoA = a 3-oxoacyl-CoA + CoA;
CC         Xref=Rhea:RHEA:21564, ChEBI:CHEBI:57287, ChEBI:CHEBI:57288,
CC         ChEBI:CHEBI:58342, ChEBI:CHEBI:90726; EC=2.3.1.16;
CC         Evidence={ECO:0000250|UniProtKB:P55084};
CC       PhysiologicalDirection=right-to-left; Xref=Rhea:RHEA:21566;
CC         Evidence={ECO:0000250|UniProtKB:P55084};
CC   -!- CATALYTIC ACTIVITY:
CC       Reaction=acetyl-CoA + butanoyl-CoA = 3-oxohexanoyl-CoA + CoA;
CC         Xref=Rhea:RHEA:31111, ChEBI:CHEBI:57287, ChEBI:CHEBI:57288,
CC         ChEBI:CHEBI:57371, ChEBI:CHEBI:62418;
CC         Evidence={ECO:0000250|UniProtKB:P55084};
CC       PhysiologicalDirection=right-to-left; Xref=Rhea:RHEA:31113;
CC         Evidence={ECO:0000250|UniProtKB:P55084};
CC   -!- CATALYTIC ACTIVITY:
CC       Reaction=acetyl-CoA + hexanoyl-CoA = 3-oxooctanoyl-CoA + CoA;
CC         Xref=Rhea:RHEA:31203, ChEBI:CHEBI:57287, ChEBI:CHEBI:57288,
CC         ChEBI:CHEBI:62619, ChEBI:CHEBI:62620;
CC         Evidence={ECO:0000250|UniProtKB:P55084};
CC       PhysiologicalDirection=right-to-left; Xref=Rhea:RHEA:31205;
CC         Evidence={ECO:0000250|UniProtKB:P55084};
CC   -!- CATALYTIC ACTIVITY:
CC       Reaction=acetyl-CoA + octanoyl-CoA = 3-oxodecanoyl-CoA + CoA;
CC         Xref=Rhea:RHEA:31087, ChEBI:CHEBI:57287, ChEBI:CHEBI:57288,
CC         ChEBI:CHEBI:57386, ChEBI:CHEBI:62548;
CC         Evidence={ECO:0000250|UniProtKB:P55084};
CC       PhysiologicalDirection=right-to-left; Xref=Rhea:RHEA:31089;
CC         Evidence={ECO:0000250|UniProtKB:P55084};
CC   -!- CATALYTIC ACTIVITY:
CC       Reaction=acetyl-CoA + decanoyl-CoA = 3-oxododecanoyl-CoA + CoA;
CC         Xref=Rhea:RHEA:31183, ChEBI:CHEBI:57287, ChEBI:CHEBI:57288,
CC         ChEBI:CHEBI:61430, ChEBI:CHEBI:62615;
CC         Evidence={ECO:0000250|UniProtKB:P55084};
CC       PhysiologicalDirection=right-to-left; Xref=Rhea:RHEA:31185;
CC         Evidence={ECO:0000250|UniProtKB:P55084};
CC   -!- CATALYTIC ACTIVITY:
CC       Reaction=acetyl-CoA + dodecanoyl-CoA = 3-oxotetradecanoyl-CoA + CoA;
CC         Xref=Rhea:RHEA:31091, ChEBI:CHEBI:57287, ChEBI:CHEBI:57288,
CC         ChEBI:CHEBI:57375, ChEBI:CHEBI:62543;
CC         Evidence={ECO:0000250|UniProtKB:P55084};
CC       PhysiologicalDirection=right-to-left; Xref=Rhea:RHEA:31093;
CC         Evidence={ECO:0000250|UniProtKB:P55084};
CC   -!- CATALYTIC ACTIVITY:
CC       Reaction=acetyl-CoA + tetradecanoyl-CoA = 3-oxohexadecanoyl-CoA + CoA;
CC         Xref=Rhea:RHEA:18161, ChEBI:CHEBI:57287, ChEBI:CHEBI:57288,
CC         ChEBI:CHEBI:57349, ChEBI:CHEBI:57385; EC=2.3.1.155;
CC         Evidence={ECO:0000250|UniProtKB:P55084};
CC       PhysiologicalDirection=right-to-left; Xref=Rhea:RHEA:18163;
CC         Evidence={ECO:0000250|UniProtKB:P55084};
CC   -!- PATHWAY: Lipid metabolism; fatty acid beta-oxidation.
CC       {ECO:0000250|UniProtKB:P55084}.
CC   -!- SUBUNIT: Heterotetramer of 2 alpha/HADHA and 2 beta/HADHB subunits;
CC       forms the mitochondrial trifunctional enzyme (By similarity). Also
CC       purified as higher order heterooligomers including a 4 alpha/HADHA and
CC       4 beta/HADHB heterooligomer which physiological significance remains
CC       unclear (By similarity). The mitochondrial trifunctional enzyme
CC       interacts with MTLN (PubMed:29949755). Interacts with RSAD2/viperin (By
CC       similarity). {ECO:0000250|UniProtKB:P55084,
CC       ECO:0000269|PubMed:29949755}.
CC   -!- SUBCELLULAR LOCATION: Mitochondrion {ECO:0000250|UniProtKB:P55084}.
CC       Mitochondrion inner membrane {ECO:0000250|UniProtKB:P55084}.
CC       Mitochondrion outer membrane {ECO:0000250|UniProtKB:P55084}.
CC       Endoplasmic reticulum {ECO:0000250|UniProtKB:P55084}. Note=Protein
CC       stability and association with membranes require HADHA.
CC       {ECO:0000250|UniProtKB:P55084}.
CC   -!- PTM: Acetylation of Lys-202 is observed in liver mitochondria from
CC       fasted mice but not from fed mice.
CC   -!- SIMILARITY: Belongs to the thiolase-like superfamily. Thiolase family.
CC       {ECO:0000305}.
CC   ---------------------------------------------------------------------------
CC   Copyrighted by the UniProt Consortium, see https://www.uniprot.org/terms
CC   Distributed under the Creative Commons Attribution (CC BY 4.0) License
CC   ---------------------------------------------------------------------------
DR   EMBL; AK033462; BAC28300.1; -; mRNA.
DR   EMBL; AK076814; BAC36493.1; -; mRNA.
DR   EMBL; AK083164; BAC38790.1; -; mRNA.
DR   EMBL; AK083767; BAC39015.1; -; mRNA.
DR   EMBL; AK150889; BAE29936.1; -; mRNA.
DR   EMBL; AK169637; BAE41269.1; -; mRNA.
DR   EMBL; BC005585; AAH05585.1; -; mRNA.
DR   CCDS; CCDS39045.1; -.
DR   RefSeq; NP_001276727.1; NM_001289798.1.
DR   RefSeq; NP_001276728.1; NM_001289799.1.
DR   RefSeq; NP_663533.1; NM_145558.2.
DR   RefSeq; XP_017176317.1; XM_017320828.1.
DR   RefSeq; XP_017176318.1; XM_017320829.1.
DR   AlphaFoldDB; Q99JY0; -.
DR   SMR; Q99JY0; -.
DR   BioGRID; 231080; 33.
DR   IntAct; Q99JY0; 7.
DR   MINT; Q99JY0; -.
DR   STRING; 10090.ENSMUSP00000110434; -.
DR   iPTMnet; Q99JY0; -.
DR   PhosphoSitePlus; Q99JY0; -.
DR   SwissPalm; Q99JY0; -.
DR   EPD; Q99JY0; -.
DR   jPOST; Q99JY0; -.
DR   MaxQB; Q99JY0; -.
DR   PaxDb; Q99JY0; -.
DR   PeptideAtlas; Q99JY0; -.
DR   ProteomicsDB; 277669; -.
DR   Antibodypedia; 27848; 265 antibodies from 30 providers.
DR   DNASU; 231086; -.
DR   Ensembl; ENSMUST00000026841; ENSMUSP00000026841; ENSMUSG00000059447.
DR   Ensembl; ENSMUST00000114783; ENSMUSP00000110431; ENSMUSG00000059447.
DR   Ensembl; ENSMUST00000114786; ENSMUSP00000110434; ENSMUSG00000059447.
DR   GeneID; 231086; -.
DR   KEGG; mmu:231086; -.
DR   UCSC; uc008wve.2; mouse.
DR   AGR; MGI:2136381; -.
DR   CTD; 3032; -.
DR   MGI; MGI:2136381; Hadhb.
DR   VEuPathDB; HostDB:ENSMUSG00000059447; -.
DR   eggNOG; KOG1392; Eukaryota.
DR   GeneTree; ENSGT01030000234626; -.
DR   HOGENOM; CLU_031026_2_0_1; -.
DR   InParanoid; Q99JY0; -.
DR   OMA; MTAFPEP; -.
DR   OrthoDB; 1826604at2759; -.
DR   PhylomeDB; Q99JY0; -.
DR   TreeFam; TF315243; -.
DR   Reactome; R-MMU-1482798; Acyl chain remodeling of CL.
DR   Reactome; R-MMU-77285; Beta oxidation of myristoyl-CoA to lauroyl-CoA.
DR   Reactome; R-MMU-77305; Beta oxidation of palmitoyl-CoA to myristoyl-CoA.
DR   Reactome; R-MMU-77310; Beta oxidation of lauroyl-CoA to decanoyl-CoA-CoA.
DR   Reactome; R-MMU-77346; Beta oxidation of decanoyl-CoA to octanoyl-CoA-CoA.
DR   Reactome; R-MMU-77348; Beta oxidation of octanoyl-CoA to hexanoyl-CoA.
DR   Reactome; R-MMU-77350; Beta oxidation of hexanoyl-CoA to butanoyl-CoA.
DR   UniPathway; UPA00659; -.
DR   BioGRID-ORCS; 231086; 1 hit in 77 CRISPR screens.
DR   ChiTaRS; Hadhb; mouse.
DR   PRO; PR:Q99JY0; -.
DR   Proteomes; UP000000589; Chromosome 5.
DR   RNAct; Q99JY0; protein.
DR   Bgee; ENSMUSG00000059447; Expressed in spermatocyte and 170 other tissues.
DR   ExpressionAtlas; Q99JY0; baseline and differential.
DR   Genevisible; Q99JY0; MM.
DR   GO; GO:0005783; C:endoplasmic reticulum; ISS:UniProtKB.
DR   GO; GO:0016507; C:mitochondrial fatty acid beta-oxidation multienzyme complex; ISO:MGI.
DR   GO; GO:0005743; C:mitochondrial inner membrane; HDA:MGI.
DR   GO; GO:0042645; C:mitochondrial nucleoid; ISO:MGI.
DR   GO; GO:0005741; C:mitochondrial outer membrane; ISS:UniProtKB.
DR   GO; GO:0005739; C:mitochondrion; IDA:MGI.
DR   GO; GO:0003985; F:acetyl-CoA C-acetyltransferase activity; IDA:MGI.
DR   GO; GO:0003988; F:acetyl-CoA C-acyltransferase activity; IDA:MGI.
DR   GO; GO:0050633; F:acetyl-CoA C-myristoyltransferase activity; IEA:UniProtKB-EC.
DR   GO; GO:0106222; F:lncRNA binding; IDA:MGI.
DR   GO; GO:0044877; F:protein-containing complex binding; ISO:MGI.
DR   GO; GO:0071222; P:cellular response to lipopolysaccharide; IDA:MGI.
DR   GO; GO:0006635; P:fatty acid beta-oxidation; ISO:MGI.
DR   GO; GO:0010467; P:gene expression; IMP:MGI.
DR   CDD; cd00751; thiolase; 1.
DR   Gene3D; 3.40.47.10; -; 1.
DR   InterPro; IPR002155; Thiolase.
DR   InterPro; IPR016039; Thiolase-like.
DR   InterPro; IPR020615; Thiolase_acyl_enz_int_AS.
DR   InterPro; IPR020610; Thiolase_AS.
DR   InterPro; IPR020617; Thiolase_C.
DR   InterPro; IPR020613; Thiolase_CS.
DR   InterPro; IPR020616; Thiolase_N.
DR   PANTHER; PTHR18919; ACETYL-COA C-ACYLTRANSFERASE; 1.
DR   PANTHER; PTHR18919:SF153; TRIFUNCTIONAL ENZYME SUBUNIT BETA, MITOCHONDRIAL; 1.
DR   Pfam; PF02803; Thiolase_C; 1.
DR   Pfam; PF00108; Thiolase_N; 1.
DR   SUPFAM; SSF53901; Thiolase-like; 2.
DR   PROSITE; PS00098; THIOLASE_1; 1.
DR   PROSITE; PS00737; THIOLASE_2; 1.
DR   PROSITE; PS00099; THIOLASE_3; 1.
DR   TIGRFAMs; TIGR01930; AcCoA-C-Actrans; 1.
PE   1: Evidence at protein level;
KW   Acetylation; Acyltransferase; Endoplasmic reticulum; Fatty acid metabolism;
KW   Lipid metabolism; Membrane; Mitochondrion; Mitochondrion inner membrane;
KW   Mitochondrion outer membrane; Reference proteome; Transferase;
KW   Transit peptide.
FT   TRANSIT         1..34
FT                   /note="Mitochondrion"
FT                   /evidence="ECO:0000250"
FT   CHAIN           35..475
FT                   /note="Trifunctional enzyme subunit beta, mitochondrial"
FT                   /id="PRO_0000034082"
FT   INTRAMEM        174..221
FT                   /evidence="ECO:0000250|UniProtKB:P55084"
FT   ACT_SITE        139
FT                   /note="Acyl-thioester intermediate"
FT                   /evidence="ECO:0000250|UniProtKB:P55084"
FT   ACT_SITE        459
FT                   /note="Proton donor/acceptor"
FT                   /evidence="ECO:0000250|UniProtKB:P55084"
FT   SITE            429
FT                   /note="Increases nucleophilicity of active site Cys"
FT                   /evidence="ECO:0000250|UniProtKB:P55084"
FT   MOD_RES         53
FT                   /note="N6-succinyllysine"
FT                   /evidence="ECO:0007744|PubMed:23806337"
FT   MOD_RES         73
FT                   /note="N6-acetyllysine; alternate"
FT                   /evidence="ECO:0007744|PubMed:23576753"
FT   MOD_RES         73
FT                   /note="N6-succinyllysine; alternate"
FT                   /evidence="ECO:0007744|PubMed:23806337"
FT   MOD_RES         189
FT                   /note="N6-acetyllysine; alternate"
FT                   /evidence="ECO:0007744|PubMed:23576753"
FT   MOD_RES         189
FT                   /note="N6-succinyllysine; alternate"
FT                   /evidence="ECO:0007744|PubMed:23806337"
FT   MOD_RES         191
FT                   /note="N6-succinyllysine"
FT                   /evidence="ECO:0007744|PubMed:23806337"
FT   MOD_RES         273
FT                   /note="N6-succinyllysine"
FT                   /evidence="ECO:0007744|PubMed:23806337"
FT   MOD_RES         292
FT                   /note="N6-succinyllysine"
FT                   /evidence="ECO:0007744|PubMed:23806337"
FT   MOD_RES         294
FT                   /note="N6-acetyllysine; alternate"
FT                   /evidence="ECO:0007744|PubMed:23576753"
FT   MOD_RES         294
FT                   /note="N6-succinyllysine; alternate"
FT                   /evidence="ECO:0007744|PubMed:23806337"
FT   MOD_RES         299
FT                   /note="N6-acetyllysine"
FT                   /evidence="ECO:0007744|PubMed:23576753"
FT   MOD_RES         333
FT                   /note="N6-acetyllysine; alternate"
FT                   /evidence="ECO:0007744|PubMed:23576753"
FT   MOD_RES         333
FT                   /note="N6-succinyllysine; alternate"
FT                   /evidence="ECO:0007744|PubMed:23806337"
FT   MOD_RES         349
FT                   /note="N6-acetyllysine"
FT                   /evidence="ECO:0007744|PubMed:23576753"
FT   MOD_RES         362
FT                   /note="N6-acetyllysine"
FT                   /evidence="ECO:0007744|PubMed:23576753"
FT   CONFLICT        24..25
FT                   /note="IR -> HK (in Ref. 1; BAC36493)"
FT                   /evidence="ECO:0000305"
FT   CONFLICT        425
FT                   /note="L -> M (in Ref. 1; BAC38790)"
FT                   /evidence="ECO:0000305"
FT   CONFLICT        450
FT                   /note="G -> R (in Ref. 1; BAC38790)"
FT                   /evidence="ECO:0000305"
FT   CONFLICT        450
FT                   /note="G -> V (in Ref. 1; BAC39015)"
FT                   /evidence="ECO:0000305"
SQ   SEQUENCE   475 AA;  51386 MW;  F131B497C4F5FAF4 CRC64;
     MTTILTSTFR NLSTTSKWAL RSSIRPLSCS SQLHSAPAVQ TKSKKTLAKP NMKNIVVVEG
     VRIPFLLSGT SYKDLMPHDL ARAALSGLLH RTNIPKDVVD YIIFGTVIQE VKTSNVAREA
     ALGAGFSDKT PAHTVTMACI SSNQAMTTAV GLIASGQCDV VVAGGVELMS DVPIRHSRNM
     RKMMLDLNKA KTLGQRLSLL SKFRLNFLSP ELPAVAEFST NETMGHSADR LAAAFAVSRM
     EQDEYALRSH SLAKKAQDEG HLSDIVPFKV PGKDTVTKDN GIRPSSLEQM AKLKPAFIKP
     YGTVTAANSS FLTDGASAML IMSEDRALAM GYKPKAYLRD FIYVSQDPKD QLLLGPTYAT
     PKVLEKAGLT MNDIDAFEFH EAFSGQILAN FKAMDSDWFA QNYMGRKTKV GSPPLEKFNI
     WGGSLSLGHP FGATGCRLVM AAANRLRKDG GQYALVAACA AGGQGHAMIV EAYPK
//
```

|  |
| --- |
| **Mascot:** http://www.matrixscience.com/ |

Oxidation (M) (+15.9949)
