# Supplementary material for: A common East-Asian ALDH2 mutation causes metabolic disorders and the therapeutic effect of ALDH2 activators
Source: Nat Commun. 2023 Sep 25;14:5971. doi: 10.1038/s41467-023-41570-6 (PMC10520061; doi:10.1038/s41467-023-41570-6)
Supplement: Supplementary file 4 — Supplementary Data 1 [file 41467_2023_41570_MOESM4_ESM.zip › Table S5b/Q99KIO/Q99KI0_WTO-2_H268_C277_C385.html]

Mascot Search Results: Q99KI0
 

# MASCOT Search Results

## Protein View: Q99KI0

### Aconitate hydratase, mitochondrial OS=Mus musculus OX=10090 GN=Aco2 PE=1 SV=1

|  |  |
| --- | --- |
| Database: | Mouse\_UniProt\_proteomes |
| Score: | 4878 |
| Monoisotopic mass (Mr): | 86151 |
| Calculated pI: | 8.08 |

Sequence similarity is available as an NCBI BLAST search of Q99KI0 against nr.

### Search parameters

|  |  |
| --- | --- |
| MS data file: | `D:\LCMSMS\2023 Users' data\230529-1\230529-1-WTO-2.raw` |
| Enzyme: | Trypsin/P: cuts C-term side of KR. |
| Fixed modifications: | Carbamidomethyl (C) |
| Variable modifications: | Deamidated (NQ), HNE (C), HNE (H), HNE (K), Oxidation (M) |

### Protein sequence coverage: 61%

Matched peptides shown in ***bold red***.

|  |  |  |  |  |  |
| --- | --- | --- | --- | --- | --- |
| `1` | `MAPYSLLVTR` | `LQKALGVRQY` | `HVASVLCQRA` | `KVAMSHFEPS` | `EYIRYDLLEK` |
| `51` | `NINIVRKRLN` | `RPLTLSEKIV` | `YGHLDDPANQ` | `EIERGKTYLR` | `LRPDRVAMQD` |
| `101` | `ATAQMAMLQF` | `ISSGLPKVAV` | `PSTIHCDHLI` | `EAQVGGEKDL` | `RRAKDINQEV` |
| `151` | `YNFLATAGAK` | `YGVGFWRPGS` | `GIIHQIILEN` | `YAYPGVLLIG` | `TDSHTPNGGG` |
| `201` | `LGGICIGVGG` | `ADAVDVMAGI` | `PWELKCPKVI` | `GVKLTGSLSG` | `WTSPKDVILK` |
| `251` | `VAGILTVKGG` | `TGAIVEYHGP` | `GVDSISCTGM` | `ATICNMGAEI` | `GATTSVFPYN` |
| `301` | `HRMKKYLSKT` | `GRTDIANLAE` | `EFKDHLVPDP` | `GCQYDQVIEI` | `NLNELKPHIN` |
| `351` | `GPFTPDLAHP` | `VADVGTVAEK` | `EGWPLDIRVG` | `LIGSCTNSSY` | `EDMGRSAAVA` |
| `401` | `KQALAHGLKC` | `KSQFTITPGS` | `EQIRATIERD` | `GYAQILRDVG` | `GIVLANACGP` |
| `451` | `CIGQWDRKDI` | `KKGEKNTIVT` | `SYNRNFTGRN` | `DANPETHAFV` | `TSPEIVTALA` |
| `501` | `IAGTLKFNPE` | `TDFLTGKDGK` | `KFKLEAPDAD` | `ELPRSDFDPG` | `QDTYQHPPKD` |
| `551` | `SSGQRVDVSP` | `TSQRLQLLEP` | `FDKWDGKDLE` | `DLQILIKVKG` | `KCTTDHISAA` |
| `601` | `GPWLKFRGHL` | `DNISNNLLIG` | `AINIENGKAN` | `SVRNAVTQEF` | `GPVPDTARYY` |
| `651` | `KKHGIRWVVI` | `GDENYGEGSS` | `REHAALEPRH` | `LGGRAIITKS` | `FARIHETNLK` |
| `701` | `KQGLLPLTFA` | `DPSDYNKIHP` | `VDKLTIQGLK` | `DFAPGKPLKC` | `VIKHPNGTQE` |
| `751` | `TILLNHTFNE` | `TQIEWFRAGS` | `ALNRMKELQQ` |  |  |

Unformatted sequence string: 780 residues (for pasting into other applications).

|  |  |  |  |
| --- | --- | --- | --- |
| Sort by | residue number | increasing mass | decreasing mass |
| Show | matched peptides only | predicted peptides also |  |

| Query | Start | – | End | Observed | Mr(expt) | Mr(calc) | ppm | M | Score | Expect | Rank | U | Peptide |
| --- | --- | --- | --- | --- | --- | --- | --- | --- | --- | --- | --- | --- | --- |
| 75301 | 32 | – | 44 | 522.5854 | 1564.7344 | 1564.7344 | -0.0077 | 0 | 32 | 0.00095 | 1Score **> 33** indicates **identity** Score **> 15** indicates **homology** | U | K.VAMSHFEPSEYIR.Y |
| 75304 | 32 | – | 44 | 522.5858 | 1564.7356 | 1564.7344 | 0.75 | 0 | 46 | 5.2e-05 | 1Score **> 32** indicates **identity** Score **> 15** indicates **homology** | U | K.VAMSHFEPSEYIR.Y |
| 75309 | 32 | – | 44 | 522.5869 | 1564.7390 | 1564.7344 | 2.95 | 0 | 28 | 0.0027 | 1Score **> 33** indicates **identity** Score **> 15** indicates **homology** | U | K.VAMSHFEPSEYIR.Y |
| 47554 | 58 | – | 68 | 442.9337 | 1325.7793 | 1325.7779 | 1.04 | 2 | 24 | 0.017 | 1Score **> 32** indicates **identity** Score **> 19** indicates **homology** | U | K.RLNRPLTLSEK.I |
| 30146 | 59 | – | 68 | 585.8454 | 1169.6762 | 1169.6768 | -0.51 | 1 | 38 | 0.006 | 1Score **> 32** indicates **identity** Score **> 28** indicates **homology** | U | R.LNRPLTLSEK.I |
| 30147 | 59 | – | 68 | 390.8994 | 1169.6764 | 1169.6768 | -0.33 | 1 | 17 | 0.039 | 1Score **> 32** indicates **identity** Score **> 16** indicates **homology** | U | R.LNRPLTLSEK.I |
| 30148 | 59 | – | 68 | 390.8994 | 1169.6764 | 1169.6768 | -0.33 | 1 | 17 | 0.031 | 1Score **> 32** indicates **identity** Score **> 14** indicates **homology** | U | R.LNRPLTLSEK.I |
| 30149 | 59 | – | 68 | 390.8995 | 1169.6766 | 1169.6768 | -0.20 | 1 | 22 | 0.036 | 1Score **> 32** indicates **identity** Score **> 20** indicates **homology** | U | R.LNRPLTLSEK.I |
| 30152 | 59 | – | 68 | 390.8995 | 1169.6768 | 1169.6768 | -0.015 | 1 | 17 | 0.04 | 1Score **> 32** indicates **identity** Score **> 16** indicates **homology** | U | R.LNRPLTLSEK.I |
| 30155 | 59 | – | 68 | 585.8463 | 1169.6780 | 1169.6768 | 1.06 | 1 | 33 | 0.0096 | 1Score **> 32** indicates **identity** Score **> 25** indicates **homology** | U | R.LNRPLTLSEK.I |
| 111106 | 69 | – | 84 | 623.6428 | 1867.9065 | 1867.9064 | 0.020 | 0 | 27 | 0.0028 | 1Score **> 35** indicates **identity** Score **> 14** indicates **homology** | U | K.IVYGHLDDPANQEIER.G |
| 111107 | 69 | – | 84 | 623.6432 | 1867.9077 | 1867.9064 | 0.70 | 0 | 74 | 2e-07 | 1Score **> 34** indicates **identity** Score **> 19** indicates **homology** | U | K.IVYGHLDDPANQEIER.G |
| 111108 | 69 | – | 84 | 934.9616 | 1867.9086 | 1867.9064 | 1.14 | 0 | 19 | 0.017 | 1Score **> 34** indicates **identity** Score **> 14** indicates **homology** | U | K.IVYGHLDDPANQEIER.G |
| 111109 | 69 | – | 84 | 934.9617 | 1867.9088 | 1867.9064 | 1.29 | 0 | 61 | 2e-06 | 1Score **> 35** indicates **identity** Score **> 16** indicates **homology** | U | K.IVYGHLDDPANQEIER.G |
| 111110 | 69 | – | 84 | 623.6438 | 1867.9096 | 1867.9064 | 1.71 | 0 | 74 | 1.1e-07 | 1Score **> 35** indicates **identity** Score **> 17** indicates **homology** | U | K.IVYGHLDDPANQEIER.G |
| 111111 | 69 | – | 84 | 623.6440 | 1867.9102 | 1867.9064 | 2.04 | 0 | 17 | 0.028 | 1Score **> 35** indicates **identity** Score **> 14** indicates **homology** | U | K.IVYGHLDDPANQEIER.G |
| 111112 | 69 | – | 84 | 623.6440 | 1867.9103 | 1867.9064 | 2.06 | 0 | 23 | 0.0074 | 1Score **> 35** indicates **identity** Score **> 14** indicates **homology** | U | K.IVYGHLDDPANQEIER.G |
| 111113 | 69 | – | 84 | 934.9625 | 1867.9104 | 1867.9064 | 2.11 | 0 | 52 | 1.3e-05 | 1Score **> 35** indicates **identity** Score **> 16** indicates **homology** | U | K.IVYGHLDDPANQEIER.G |
| 111114 | 69 | – | 84 | 623.6441 | 1867.9105 | 1867.9064 | 2.18 | 0 | 83 | 1.7e-08 | 1Score **> 35** indicates **identity** Score **> 18** indicates **homology** | U | K.IVYGHLDDPANQEIER.G |
| 111116 | 69 | – | 84 | 934.9628 | 1867.9110 | 1867.9064 | 2.47 | 0 | 65 | 7.3e-07 | 1Score **> 35** indicates **identity** Score **> 17** indicates **homology** | U | K.IVYGHLDDPANQEIER.G |
| 111117 | 69 | – | 84 | 623.6443 | 1867.9112 | 1867.9064 | 2.54 | 0 | 69 | 3.4e-07 | 1Score **> 35** indicates **identity** Score **> 17** indicates **homology** | U | K.IVYGHLDDPANQEIER.G |
| 111118 | 69 | – | 84 | 623.6447 | 1867.9122 | 1867.9064 | 3.11 | 0 | 21 | 0.011 | 1Score **> 35** indicates **identity** Score **> 14** indicates **homology** | U | K.IVYGHLDDPANQEIER.G |
| 145861 | 118 | – | 138 | 754.0520 | 2259.1342 | 2259.1318 | 1.09 | 0 | 23 | 0.0075 | 1Score **> 37** indicates **identity** Score **> 14** indicates **homology** | U | K.VAVPSTIHCDHLIEAQVGGEK.D |
| 145863 | 118 | – | 138 | 565.7909 | 2259.1345 | 2259.1318 | 1.22 | 0 | 43 | 9.6e-05 | 1Score **> 37** indicates **identity** Score **> 15** indicates **homology** | U | K.VAVPSTIHCDHLIEAQVGGEK.D |
| 145867 | 118 | – | 138 | 565.7910 | 2259.1347 | 2259.1318 | 1.30 | 0 | 46 | 5.3e-05 | 1Score **> 37** indicates **identity** Score **> 15** indicates **homology** | U | K.VAVPSTIHCDHLIEAQVGGEK.D |
| 145869 | 118 | – | 138 | 754.0526 | 2259.1359 | 2259.1318 | 1.81 | 0 | 49 | 2.3e-05 | 1Score **> 37** indicates **identity** Score **> 16** indicates **homology** | U | K.VAVPSTIHCDHLIEAQVGGEK.D |
| 145874 | 118 | – | 138 | 565.7915 | 2259.1369 | 2259.1318 | 2.28 | 0 | 36 | 0.00042 | 1Score **> 37** indicates **identity** Score **> 15** indicates **homology** | U | K.VAVPSTIHCDHLIEAQVGGEK.D |
| 166990 | 118 | – | 141 | 661.8424 | 2643.3405 | 2643.3439 | -1.28 | 1 | 26 | 0.0033 | 1Score **> 37** indicates **identity** Score **> 14** indicates **homology** | U | K.VAVPSTIHCDHLIEAQVGGEKDLR.R |
| 166991 | 118 | – | 141 | 529.6757 | 2643.3421 | 2643.3439 | -0.67 | 1 | 17 | 0.023 | 1Score **> 37** indicates **identity** Score **> 14** indicates **homology** | U | K.VAVPSTIHCDHLIEAQVGGEKDLR.R |
| 166994 | 118 | – | 141 | 661.8434 | 2643.3447 | 2643.3439 | 0.30 | 1 | 19 | 0.016 | 1Score **> 37** indicates **identity** Score **> 14** indicates **homology** | U | K.VAVPSTIHCDHLIEAQVGGEKDLR.R |
| 166995 | 118 | – | 141 | 661.8436 | 2643.3454 | 2643.3439 | 0.56 | 1 | 17 | 0.026 | 1Score **> 37** indicates **identity** Score **> 14** indicates **homology** | U | K.VAVPSTIHCDHLIEAQVGGEKDLR.R |
| 118667 | 143 | – | 160 | 651.6701 | 1951.9886 | 1952.0003 | -6.02 | 1 | 21 | 0.011 | 1Score **> 36** indicates **identity** Score **> 14** indicates **homology** | U | R.AKDINQEVYNFLATAGAK.Y |
| 118668 | 143 | – | 160 | 651.6701 | 1951.9886 | 1952.0003 | -6.02 | 1 | 45 | 6.1e-05 | 1Score **> 36** indicates **identity** Score **> 15** indicates **homology** | U | R.AKDINQEVYNFLATAGAK.Y |
| 118669 | 143 | – | 160 | 651.6705 | 1951.9898 | 1952.0003 | -5.41 | 1 | 24 | 0.0062 | 1Score **> 36** indicates **identity** Score **> 14** indicates **homology** | U | R.AKDINQEVYNFLATAGAK.Y |
| 118673 | 143 | – | 160 | 977.0066 | 1951.9986 | 1952.0003 | -0.88 | 1 | 78 | 5.4e-08 | 1Score **> 36** indicates **identity** Score **> 17** indicates **homology** | U | R.AKDINQEVYNFLATAGAK.Y |
| 118674 | 143 | – | 160 | 651.6744 | 1952.0015 | 1952.0003 | 0.62 | 1 | 68 | 4.6e-07 | 1Score **> 36** indicates **identity** Score **> 17** indicates **homology** | U | R.AKDINQEVYNFLATAGAK.Y |
| 118675 | 143 | – | 160 | 651.6748 | 1952.0026 | 1952.0003 | 1.20 | 1 | 71 | 2e-07 | 1Score **> 36** indicates **identity** Score **> 17** indicates **homology** | U | R.AKDINQEVYNFLATAGAK.Y |
| 118677 | 143 | – | 160 | 651.6751 | 1952.0035 | 1952.0003 | 1.61 | 1 | 73 | 1.5e-07 | 1Score **> 36** indicates **identity** Score **> 17** indicates **homology** | U | R.AKDINQEVYNFLATAGAK.Y |
| 99286 | 145 | – | 160 | 585.2974 | 1752.8702 | 1752.8682 | 1.14 | 0 | 64 | 1.1e-06 | 1Score **> 35** indicates **identity** Score **> 16** indicates **homology** | U | K.DINQEVYNFLATAGAK.Y |
| 99287 | 145 | – | 160 | 877.4426 | 1752.8706 | 1752.8682 | 1.36 | 0 | 82 | 2.3e-08 | 1Score **> 35** indicates **identity** Score **> 18** indicates **homology** | U | K.DINQEVYNFLATAGAK.Y |
| 99289 | 145 | – | 160 | 585.2983 | 1752.8731 | 1752.8682 | 2.78 | 0 | 56 | 5.4e-06 | 1Score **> 35** indicates **identity** Score **> 16** indicates **homology** | U | K.DINQEVYNFLATAGAK.Y |
| 37025 | 234 | – | 245 | 617.3274 | 1232.6402 | 1232.6401 | 0.068 | 0 | 80 | 2e-07 | 1Score **> 34** indicates **identity** Score **> 26** indicates **homology** |  | K.LTGSLSGWTSPK.D |
| 37027 | 234 | – | 245 | 617.3277 | 1232.6408 | 1232.6401 | 0.58 | 0 | 75 | 4.6e-07 | 1Score **> 34** indicates **identity** Score **> 24** indicates **homology** |  | K.LTGSLSGWTSPK.D |
| 104458 | 234 | – | 250 | 601.3391 | 1800.9956 | 1800.9986 | -1.63 | 1 | 19 | 0.015 | 1Score **> 34** indicates **identity** Score **> 14** indicates **homology** |  | K.LTGSLSGWTSPKDVILK.V |
| 104459 | 234 | – | 250 | 901.5055 | 1800.9965 | 1800.9986 | -1.16 | 1 | 53 | 9.6e-06 | 1Score **> 34** indicates **identity** Score **> 16** indicates **homology** |  | K.LTGSLSGWTSPKDVILK.V |
| 104460 | 234 | – | 250 | 901.5075 | 1801.0005 | 1800.9986 | 1.10 | 1 | 68 | 4.6e-07 | 1Score **> 34** indicates **identity** Score **> 17** indicates **homology** |  | K.LTGSLSGWTSPKDVILK.V |
| 104461 | 234 | – | 250 | 601.3409 | 1801.0010 | 1800.9986 | 1.36 | 1 | 43 | 9.5e-05 | 1Score **> 34** indicates **identity** Score **> 15** indicates **homology** |  | K.LTGSLSGWTSPKDVILK.V |
| 104463 | 234 | – | 250 | 601.3410 | 1801.0013 | 1800.9986 | 1.52 | 1 | 28 | 0.0022 | 1Score **> 34** indicates **identity** Score **> 14** indicates **homology** |  | K.LTGSLSGWTSPKDVILK.V |
| 104465 | 234 | – | 250 | 601.3413 | 1801.0021 | 1800.9986 | 1.97 | 1 | 66 | 6.6e-07 | 1Score **> 34** indicates **identity** Score **> 17** indicates **homology** |  | K.LTGSLSGWTSPKDVILK.V |
| 3828 | 251 | – | 258 | 400.7657 | 799.5168 | 799.5167 | 0.14 | 0 | 56 | 2.8e-05 | 1Score **> 23** indicates **identity** |  | K.VAGILTVK.G |
| 3829 | 251 | – | 258 | 400.7657 | 799.5169 | 799.5167 | 0.25 | 0 | 29 | 0.013 | 1Score **> 23** indicates **identity** Score **> 23** indicates **homology** |  | K.VAGILTVK.G |
| 3830 | 251 | – | 258 | 400.7658 | 799.5170 | 799.5167 | 0.30 | 0 | 38 | 0.0019 | 1Score **> 23** indicates **identity** |  | K.VAGILTVK.G |
| 3831 | 251 | – | 258 | 400.7659 | 799.5173 | 799.5167 | 0.70 | 0 | 54 | 5.1e-05 | 1Score **> 23** indicates **identity** |  | K.VAGILTVK.G |
| 194476 | 259 | – | 302 | 779.3587 | 4670.1086 | 4670.1550 | -9.95 | 0 | 21 | 0.012 | 1Score **> 32** indicates **identity** Score **> 14** indicates **homology** |  | K.GGTGAIVEYHGPGVDSISCTGMATICNMGAEIGATTSVFPYNHR.M  + HNE (C); Oxidation (M) |
| 194551 | 259 | – | 302 | 1179.0382 | 4712.1237 | 4712.1656 | -8.89 | 0 | 21 | 0.013 | 1Score **> 32** indicates **identity** Score **> 15** indicates **homology** |  | K.GGTGAIVEYHGPGVDSISCTGMATICNMGAEIGATTSVFPYNHR.M  + Deamidated (NQ); HNE (H) |
| 194580 | 259 | – | 302 | 946.4354 | 4727.1404 | 4727.1765 | -7.64 | 0 | 56 | 5.9e-06 | 1Score **> 32** indicates **identity** Score **> 16** indicates **homology** |  | K.GGTGAIVEYHGPGVDSISCTGMATICNMGAEIGATTSVFPYNHR.M  + HNE (H); Oxidation (M) |
| 108860 | 379 | – | 395 | 923.4100 | 1844.8054 | 1844.8033 | 1.16 | 0 | 98 | 6.6e-10 | 1Score **> 29** indicates **identity** Score **> 19** indicates **homology** | U | R.VGLIGSCTNSSYEDMGR.S |
| 108861 | 379 | – | 395 | 923.4100 | 1844.8054 | 1844.8033 | 1.17 | 0 | 72 | 2e-07 | 1Score **> 29** indicates **identity** Score **> 17** indicates **homology** | U | R.VGLIGSCTNSSYEDMGR.S |
| 108862 | 379 | – | 395 | 923.4104 | 1844.8062 | 1844.8033 | 1.58 | 0 | 105 | 1.5e-10 | 1Score **> 29** indicates **identity** Score **> 19** indicates **homology** | U | R.VGLIGSCTNSSYEDMGR.S |
| 119510 | 379 | – | 395 | 654.3085 | 1959.9037 | 1959.8918 | 6.08 | 0 | 16 | 0.031 | 1Score **> 32** indicates **identity** Score **> 14** indicates **homology** | U | R.VGLIGSCTNSSYEDMGR.S  + HNE (C); Oxidation (M) |
| 99044 | 410 | – | 424 | 584.6295 | 1750.8666 | 1750.8672 | -0.38 | 1 | 28 | 0.0022 | 1Score **> 34** indicates **identity** Score **> 14** indicates **homology** | U | K.CKSQFTITPGSEQIR.A |
| 99046 | 410 | – | 424 | 584.6303 | 1750.8691 | 1750.8672 | 1.07 | 1 | 50 | 0.00017 | 1Score **> 35** indicates **identity** Score **> 25** indicates **homology** | U | K.CKSQFTITPGSEQIR.A |
| 99193 | 410 | – | 424 | 876.9411 | 1751.8676 | 1751.8512 | 9.37 | 1 | 29 | 0.0019 | 1Score **> 35** indicates **identity** Score **> 14** indicates **homology** | U | K.CKSQFTITPGSEQIR.A  + Deamidated (NQ) |
| 62405 | 412 | – | 424 | 732.3782 | 1462.7419 | 1462.7416 | 0.21 | 0 | 57 | 5.8e-06 | 1Score **> 35** indicates **identity** Score **> 17** indicates **homology** | U | K.SQFTITPGSEQIR.A |
| 62406 | 412 | – | 424 | 732.3784 | 1462.7423 | 1462.7416 | 0.45 | 0 | 58 | 4.3e-06 | 1Score **> 35** indicates **identity** Score **> 17** indicates **homology** | U | K.SQFTITPGSEQIR.A |
| 62407 | 412 | – | 424 | 732.3787 | 1462.7428 | 1462.7416 | 0.84 | 0 | 53 | 1.3e-05 | 1Score **> 35** indicates **identity** Score **> 16** indicates **homology** | U | K.SQFTITPGSEQIR.A |
| 62412 | 412 | – | 424 | 732.3804 | 1462.7461 | 1462.7416 | 3.10 | 0 | 48 | 3.3e-05 | 1Score **> 35** indicates **identity** Score **> 15** indicates **homology** | U | K.SQFTITPGSEQIR.A |
| 67596 | 425 | – | 437 | 502.6078 | 1504.8015 | 1504.7998 | 1.15 | 1 | 44 | 0.00011 | 1Score **> 35** indicates **identity** Score **> 17** indicates **homology** | U | R.ATIERDGYAQILR.D |
| 67597 | 425 | – | 437 | 502.6090 | 1504.8051 | 1504.7998 | 3.53 | 1 | 27 | 0.0032 | 1Score **> 34** indicates **identity** Score **> 14** indicates **homology** | U | R.ATIERDGYAQILR.D |
| 10828 | 430 | – | 437 | 468.2512 | 934.4879 | 934.4872 | 0.78 | 0 | 41 | 0.00083 | 1Score **> 30** indicates **identity** Score **> 23** indicates **homology** | U | R.DGYAQILR.D |
| 10829 | 430 | – | 437 | 468.2512 | 934.4879 | 934.4872 | 0.78 | 0 | 35 | 0.0019 | 1Score **> 30** indicates **identity** Score **> 20** indicates **homology** | U | R.DGYAQILR.D |
| 10831 | 430 | – | 437 | 468.2514 | 934.4882 | 934.4872 | 1.08 | 0 | 56 | 3.5e-05 | 1Score **> 30** indicates **identity** Score **> 24** indicates **homology** | U | R.DGYAQILR.D |
| 10832 | 430 | – | 437 | 468.2514 | 934.4883 | 934.4872 | 1.16 | 0 | 59 | 2.4e-05 | 1Score **> 30** indicates **identity** Score **> 26** indicates **homology** | U | R.DGYAQILR.D |
| 137607 | 438 | – | 457 | 1079.5121 | 2157.0096 | 2157.0096 | 0.014 | 0 | 73 | 1.4e-07 | 1Score **> 34** indicates **identity** Score **> 17** indicates **homology** | U | R.DVGGIVLANACGPCIGQWDR.K |
| 137608 | 438 | – | 457 | 1079.5125 | 2157.0104 | 2157.0096 | 0.39 | 0 | 75 | 9.2e-08 | 1Score **> 34** indicates **identity** Score **> 17** indicates **homology** | U | R.DVGGIVLANACGPCIGQWDR.K |
| 137609 | 438 | – | 457 | 720.0114 | 2157.0123 | 2157.0096 | 1.28 | 0 | 34 | 0.00062 | 1Score **> 34** indicates **identity** Score **> 15** indicates **homology** | U | R.DVGGIVLANACGPCIGQWDR.K |
| 137611 | 438 | – | 457 | 1079.5136 | 2157.0126 | 2157.0096 | 1.39 | 0 | 52 | 1.2e-05 | 1Score **> 34** indicates **identity** Score **> 16** indicates **homology** | U | R.DVGGIVLANACGPCIGQWDR.K |
| 137612 | 438 | – | 457 | 720.0119 | 2157.0138 | 2157.0096 | 1.95 | 0 | 62 | 1.4e-06 | 1Score **> 34** indicates **identity** Score **> 16** indicates **homology** | U | R.DVGGIVLANACGPCIGQWDR.K |
| 137613 | 438 | – | 457 | 720.0120 | 2157.0143 | 2157.0096 | 2.20 | 0 | 63 | 1.2e-06 | 1Score **> 34** indicates **identity** Score **> 16** indicates **homology** | U | R.DVGGIVLANACGPCIGQWDR.K |
| 137614 | 438 | – | 457 | 1079.5146 | 2157.0146 | 2157.0096 | 2.34 | 0 | 41 | 0.00015 | 1Score **> 34** indicates **identity** Score **> 15** indicates **homology** | U | R.DVGGIVLANACGPCIGQWDR.K |
| 147544 | 438 | – | 458 | 762.7092 | 2285.1059 | 2285.1045 | 0.61 | 1 | 69 | 3.3e-07 | 1Score **> 36** indicates **identity** Score **> 17** indicates **homology** | U | R.DVGGIVLANACGPCIGQWDRK.D |
| 147545 | 438 | – | 458 | 1143.5605 | 2285.1065 | 2285.1045 | 0.86 | 1 | 76 | 6e-07 | 1Score **> 36** indicates **identity** Score **> 27** indicates **homology** | U | R.DVGGIVLANACGPCIGQWDRK.D |
| 147546 | 438 | – | 458 | 1143.5609 | 2285.1072 | 2285.1045 | 1.17 | 1 | 81 | 4e-07 | 1Score **> 36** indicates **identity** Score **> 29** indicates **homology** | U | R.DVGGIVLANACGPCIGQWDRK.D |
| 147547 | 438 | – | 458 | 762.7097 | 2285.1073 | 2285.1045 | 1.23 | 1 | 37 | 0.00032 | 1Score **> 36** indicates **identity** Score **> 15** indicates **homology** | U | R.DVGGIVLANACGPCIGQWDRK.D |
| 147578 | 438 | – | 458 | 1144.0610 | 2286.1075 | 2286.0885 | 8.28 | 1 | 46 | 5e-05 | 1Score **> 36** indicates **identity** Score **> 15** indicates **homology** | U | R.DVGGIVLANACGPCIGQWDRK.D  + Deamidated (NQ) |
| 19795 | 466 | – | 474 | 534.2775 | 1066.5405 | 1066.5407 | -0.22 | 0 | 42 | 0.00062 | 1Score **> 30** indicates **identity** Score **> 22** indicates **homology** | U | K.NTIVTSYNR.N |
| 19796 | 466 | – | 474 | 534.2776 | 1066.5407 | 1066.5407 | -0.0094 | 0 | 28 | 0.0023 | 1Score **> 30** indicates **identity** Score **> 14** indicates **homology** | U | K.NTIVTSYNR.N |
| 19797 | 466 | – | 474 | 534.2779 | 1066.5413 | 1066.5407 | 0.53 | 0 | 42 | 0.00062 | 1Score **> 31** indicates **identity** Score **> 22** indicates **homology** | U | K.NTIVTSYNR.N |
| 185921 | 475 | – | 506 | 839.6877 | 3354.7216 | 3354.7208 | 0.22 | 1 | 37 | 0.00031 | 1Score **> 37** indicates **identity** Score **> 15** indicates **homology** | U | R.NFTGRNDANPETHAFVTSPEIVTALAIAGTLK.F |
| 185923 | 475 | – | 506 | 1119.2481 | 3354.7224 | 3354.7208 | 0.49 | 1 | 47 | 4.1e-05 | 1Score **> 37** indicates **identity** Score **> 15** indicates **homology** | U | R.NFTGRNDANPETHAFVTSPEIVTALAIAGTLK.F |
| 185925 | 475 | – | 506 | 839.6883 | 3354.7240 | 3354.7208 | 0.94 | 1 | 42 | 0.00012 | 1Score **> 37** indicates **identity** Score **> 15** indicates **homology** | U | R.NFTGRNDANPETHAFVTSPEIVTALAIAGTLK.F |
| 185943 | 475 | – | 506 | 1119.5832 | 3355.7277 | 3355.7048 | 6.81 | 1 | 29 | 0.0018 | 1Score **> 37** indicates **identity** Score **> 14** indicates **homology** | U | R.NFTGRNDANPETHAFVTSPEIVTALAIAGTLK.F  + Deamidated (NQ) |
| 185944 | 475 | – | 506 | 839.9398 | 3355.7302 | 3355.7048 | 7.56 | 1 | 24 | 0.0058 | 1Score **> 37** indicates **identity** Score **> 14** indicates **homology** | U | R.NFTGRNDANPETHAFVTSPEIVTALAIAGTLK.F  + Deamidated (NQ) |
| 172352 | 480 | – | 506 | 1390.7275 | 2779.4405 | 2779.4392 | 0.46 | 0 | 93 | 2e-09 | 1Score **> 37** indicates **identity** Score **> 18** indicates **homology** | U | R.NDANPETHAFVTSPEIVTALAIAGTLK.F |
| 172353 | 480 | – | 506 | 1390.7287 | 2779.4429 | 2779.4392 | 1.33 | 0 | 49 | 2.8e-05 | 1Score **> 37** indicates **identity** Score **> 16** indicates **homology** | U | R.NDANPETHAFVTSPEIVTALAIAGTLK.F |
| 172354 | 480 | – | 506 | 927.4883 | 2779.4430 | 2779.4392 | 1.36 | 0 | 92 | 2.2e-09 | 1Score **> 37** indicates **identity** Score **> 18** indicates **homology** | U | R.NDANPETHAFVTSPEIVTALAIAGTLK.F |
| 172355 | 480 | – | 506 | 927.4888 | 2779.4446 | 2779.4392 | 1.94 | 0 | 49 | 2.7e-05 | 1Score **> 37** indicates **identity** Score **> 16** indicates **homology** | U | R.NDANPETHAFVTSPEIVTALAIAGTLK.F |
| 172358 | 480 | – | 506 | 927.4894 | 2779.4462 | 2779.4392 | 2.53 | 0 | 50 | 2.3e-05 | 1Score **> 37** indicates **identity** Score **> 16** indicates **homology** | U | R.NDANPETHAFVTSPEIVTALAIAGTLK.F |
| 172362 | 480 | – | 506 | 695.8695 | 2779.4489 | 2779.4392 | 3.50 | 0 | 48 | 3e-05 | 1Score **> 37** indicates **identity** Score **> 16** indicates **homology** | U | R.NDANPETHAFVTSPEIVTALAIAGTLK.F |
| 172392 | 480 | – | 506 | 927.8173 | 2780.4301 | 2780.4232 | 2.46 | 0 | 31 | 0.0012 | 1Score **> 37** indicates **identity** Score **> 14** indicates **homology** | U | R.NDANPETHAFVTSPEIVTALAIAGTLK.F  + Deamidated (NQ) |
| 172394 | 480 | – | 506 | 927.8211 | 2780.4415 | 2780.4232 | 6.57 | 0 | 35 | 0.00053 | 1Score **> 37** indicates **identity** Score **> 15** indicates **homology** | U | R.NDANPETHAFVTSPEIVTALAIAGTLK.F  + Deamidated (NQ) |
| 172395 | 480 | – | 506 | 927.8225 | 2780.4457 | 2780.4232 | 8.09 | 0 | 38 | 0.00025 | 1Score **> 37** indicates **identity** Score **> 15** indicates **homology** | U | R.NDANPETHAFVTSPEIVTALAIAGTLK.F  + Deamidated (NQ) |
| 40998 | 507 | – | 517 | 634.8090 | 1267.6035 | 1267.6085 | -3.95 | 0 | 49 | 2.7e-05 | 1Score **> 32** indicates **identity** Score **> 16** indicates **homology** | U | K.FNPETDFLTGK.D |
| 41000 | 507 | – | 517 | 634.8109 | 1267.6072 | 1267.6085 | -0.97 | 0 | 47 | 4.3e-05 | 1Score **> 31** indicates **identity** Score **> 16** indicates **homology** | U | K.FNPETDFLTGK.D |
| 41001 | 507 | – | 517 | 634.8111 | 1267.6077 | 1267.6085 | -0.61 | 0 | 42 | 0.00012 | 1Score **> 31** indicates **identity** Score **> 15** indicates **homology** | U | K.FNPETDFLTGK.D |
| 41002 | 507 | – | 517 | 634.8113 | 1267.6081 | 1267.6085 | -0.28 | 0 | 23 | 0.015 | 1Score **> 31** indicates **identity** Score **> 17** indicates **homology** | U | K.FNPETDFLTGK.D |
| 41003 | 507 | – | 517 | 634.8119 | 1267.6092 | 1267.6085 | 0.54 | 0 | 23 | 0.0063 | 1Score **> 31** indicates **identity** Score **> 14** indicates **homology** | U | K.FNPETDFLTGK.D |
| 41004 | 507 | – | 517 | 634.8124 | 1267.6102 | 1267.6085 | 1.35 | 0 | 14 | 0.048 | 1Score **> 31** indicates **identity** Score **> 13** indicates **homology** | U | K.FNPETDFLTGK.D |
| 75717 | 507 | – | 520 | 523.5911 | 1567.7516 | 1567.7518 | -0.15 | 1 | 28 | 0.0022 | 1Score **> 33** indicates **identity** Score **> 14** indicates **homology** | U | K.FNPETDFLTGKDGK.K |
| 75723 | 507 | – | 520 | 784.8842 | 1567.7538 | 1567.7518 | 1.25 | 1 | 40 | 0.00017 | 1Score **> 33** indicates **identity** Score **> 15** indicates **homology** | U | K.FNPETDFLTGKDGK.K |
| 92402 | 507 | – | 521 | 566.2886 | 1695.8440 | 1695.8468 | -1.65 | 2 | 31 | 0.0011 | 1Score **> 35** indicates **identity** Score **> 14** indicates **homology** | U | K.FNPETDFLTGKDGKK.F |
| 92407 | 507 | – | 521 | 424.9694 | 1695.8487 | 1695.8468 | 1.12 | 2 | 29 | 0.0018 | 1Score **> 35** indicates **identity** Score **> 14** indicates **homology** | U | K.FNPETDFLTGKDGKK.F |
| 92408 | 507 | – | 521 | 566.2902 | 1695.8489 | 1695.8468 | 1.23 | 2 | 29 | 0.002 | 1Score **> 35** indicates **identity** Score **> 14** indicates **homology** | U | K.FNPETDFLTGKDGKK.F |
| 92409 | 507 | – | 521 | 424.9695 | 1695.8489 | 1695.8468 | 1.24 | 2 | 33 | 0.00089 | 1Score **> 35** indicates **identity** Score **> 15** indicates **homology** | U | K.FNPETDFLTGKDGKK.F |
| 92410 | 507 | – | 521 | 566.2908 | 1695.8506 | 1695.8468 | 2.26 | 2 | 47 | 4.3e-05 | 1Score **> 34** indicates **identity** Score **> 15** indicates **homology** | U | K.FNPETDFLTGKDGKK.F |
| 67009 | 522 | – | 534 | 500.9278 | 1499.7616 | 1499.7620 | -0.25 | 1 | 30 | 0.0033 | 1Score **> 34** indicates **identity** Score **> 17** indicates **homology** | U | K.FKLEAPDADELPR.S |
| 67011 | 522 | – | 534 | 500.9282 | 1499.7627 | 1499.7620 | 0.49 | 1 | 25 | 0.009 | 1Score **> 34** indicates **identity** Score **> 17** indicates **homology** | U | K.FKLEAPDADELPR.S |
| 67013 | 522 | – | 534 | 500.9283 | 1499.7631 | 1499.7620 | 0.74 | 1 | 34 | 0.00084 | 1Score **> 34** indicates **identity** Score **> 16** indicates **homology** | U | K.FKLEAPDADELPR.S |
| 152399 | 535 | – | 555 | 788.0144 | 2361.0213 | 2361.0258 | -1.91 | 1 | 25 | 0.0046 | 1Score **> 28** indicates **identity** Score **> 14** indicates **homology** | U | R.SDFDPGQDTYQHPPKDSSGQR.V |
| 82323 | 550 | – | 564 | 540.2633 | 1617.7682 | 1617.7707 | -1.57 | 1 | 21 | 0.01 | 1Score **> 33** indicates **identity** Score **> 14** indicates **homology** | U | K.DSSGQRVDVSPTSQR.L |
| 82326 | 550 | – | 564 | 809.8932 | 1617.7718 | 1617.7707 | 0.67 | 1 | 16 | 0.039 | 1Score **> 33** indicates **identity** Score **> 14** indicates **homology** | U | K.DSSGQRVDVSPTSQR.L |
| 14066 | 556 | – | 564 | 494.7565 | 987.4984 | 987.4985 | -0.15 | 0 | 54 | 0.00023 | 1Score **> 30** indicates **identity** | U | R.VDVSPTSQR.L |
| 171799 | 565 | – | 587 | 693.1294 | 2768.4885 | 2768.5000 | -4.17 | 2 | 18 | 0.022 | 1Score **> 36** indicates **identity** Score **> 14** indicates **homology** | U | R.LQLLEPFDKWDGKDLEDLQILIK.V |
| 97862 | 590 | – | 605 | 581.2934 | 1740.8584 | 1740.8617 | -1.95 | 1 | 30 | 0.0014 | 1Score **> 34** indicates **identity** Score **> 14** indicates **homology** | U | K.GKCTTDHISAAGPWLK.F |
| 74013 | 592 | – | 605 | 778.8781 | 1555.7416 | 1555.7453 | -2.37 | 0 | 36 | 0.00042 | 1Score **> 33** indicates **identity** Score **> 15** indicates **homology** | U | K.CTTDHISAAGPWLK.F |
| 74017 | 592 | – | 605 | 519.5887 | 1555.7441 | 1555.7453 | -0.75 | 0 | 18 | 0.02 | 1Score **> 33** indicates **identity** Score **> 14** indicates **homology** | U | K.CTTDHISAAGPWLK.F |
| 161683 | 606 | – | 628 | 631.3400 | 2521.3310 | 2521.3401 | -3.61 | 1 | 24 | 0.0051 | 1Score **> 36** indicates **identity** Score **> 14** indicates **homology** | U | K.FRGHLDNISNNLLIGAINIENGK.A |
| 161684 | 606 | – | 628 | 631.3426 | 2521.3413 | 2521.3401 | 0.49 | 1 | 43 | 8.6e-05 | 1Score **> 36** indicates **identity** Score **> 15** indicates **homology** | U | K.FRGHLDNISNNLLIGAINIENGK.A |
| 161685 | 606 | – | 628 | 631.3435 | 2521.3448 | 2521.3401 | 1.86 | 1 | 42 | 0.00011 | 1Score **> 36** indicates **identity** Score **> 15** indicates **homology** | U | K.FRGHLDNISNNLLIGAINIENGK.A |
| 180642 | 606 | – | 633 | 763.4103 | 3049.6122 | 3049.6057 | 2.14 | 2 | 34 | 0.00065 | 1Score **> 37** indicates **identity** Score **> 15** indicates **homology** | U | K.FRGHLDNISNNLLIGAINIENGKANSVR.N  + Deamidated (NQ) |
| 180643 | 606 | – | 633 | 610.9301 | 3049.6140 | 3049.6057 | 2.75 | 2 | 20 | 0.014 | 1Score **> 37** indicates **identity** Score **> 14** indicates **homology** | U | K.FRGHLDNISNNLLIGAINIENGKANSVR.N  + Deamidated (NQ) |
| 180644 | 606 | – | 633 | 763.4125 | 3049.6210 | 3049.6057 | 5.04 | 2 | 46 | 4.5e-05 | 1Score **> 36** indicates **identity** Score **> 15** indicates **homology** | U | K.FRGHLDNISNNLLIGAINIENGKANSVR.N  + Deamidated (NQ) |
| 180673 | 606 | – | 633 | 611.1306 | 3050.6167 | 3050.5897 | 8.86 | 2 | 18 | 0.021 | 1Score **> 36** indicates **identity** Score **> 14** indicates **homology** | U | K.FRGHLDNISNNLLIGAINIENGKANSVR.N  + 2 Deamidated (NQ) |
| 142746 | 608 | – | 628 | 740.3969 | 2218.1687 | 2218.1705 | -0.82 | 0 | 57 | 4.4e-06 | 1Score **> 36** indicates **identity** Score **> 16** indicates **homology** | U | R.GHLDNISNNLLIGAINIENGK.A |
| 142749 | 608 | – | 628 | 740.3982 | 2218.1729 | 2218.1705 | 1.06 | 0 | 65 | 8.9e-07 | 1Score **> 36** indicates **identity** Score **> 17** indicates **homology** | U | R.GHLDNISNNLLIGAINIENGK.A |
| 142756 | 608 | – | 628 | 740.4008 | 2218.1804 | 2218.1705 | 4.46 | 0 | 25 | 0.0042 | 1Score **> 36** indicates **identity** Score **> 14** indicates **homology** | U | R.GHLDNISNNLLIGAINIENGK.A |
| 142789 | 608 | – | 628 | 740.7238 | 2219.1495 | 2219.1546 | -2.26 | 0 | 21 | 0.011 | 1Score **> 37** indicates **identity** Score **> 14** indicates **homology** | U | R.GHLDNISNNLLIGAINIENGK.A  + Deamidated (NQ) |
| 142802 | 608 | – | 628 | 1110.5897 | 2219.1648 | 2219.1546 | 4.63 | 0 | 66 | 6.3e-07 | 1Score **> 36** indicates **identity** Score **> 17** indicates **homology** | U | R.GHLDNISNNLLIGAINIENGK.A  + Deamidated (NQ) |
| 142804 | 608 | – | 628 | 1110.5925 | 2219.1705 | 2219.1546 | 7.18 | 0 | 30 | 0.0017 | 1Score **> 36** indicates **identity** Score **> 14** indicates **homology** | U | R.GHLDNISNNLLIGAINIENGK.A  + Deamidated (NQ) |
| 170948 | 608 | – | 633 | 687.3668 | 2745.4379 | 2745.4521 | -5.18 | 1 | 17 | 0.027 | 1Score **> 37** indicates **identity** Score **> 14** indicates **homology** | U | R.GHLDNISNNLLIGAINIENGKANSVR.N |
| 170951 | 608 | – | 633 | 687.3749 | 2745.4703 | 2745.4521 | 6.63 | 1 | 29 | 0.002 | 1Score **> 36** indicates **identity** Score **> 14** indicates **homology** | U | R.GHLDNISNNLLIGAINIENGKANSVR.N |
| 170996 | 608 | – | 633 | 916.4842 | 2746.4307 | 2746.4361 | -1.99 | 1 | 41 | 0.00014 | 1Score **> 37** indicates **identity** Score **> 15** indicates **homology** | U | R.GHLDNISNNLLIGAINIENGKANSVR.N  + Deamidated (NQ) |
| 170997 | 608 | – | 633 | 687.6173 | 2746.4401 | 2746.4361 | 1.46 | 1 | 33 | 0.00088 | 1Score **> 37** indicates **identity** Score **> 15** indicates **homology** | U | R.GHLDNISNNLLIGAINIENGKANSVR.N  + Deamidated (NQ) |
| 170998 | 608 | – | 633 | 916.4879 | 2746.4418 | 2746.4361 | 2.05 | 1 | 64 | 1e-06 | 1Score **> 37** indicates **identity** Score **> 16** indicates **homology** | U | R.GHLDNISNNLLIGAINIENGKANSVR.N  + Deamidated (NQ) |
| 170999 | 608 | – | 633 | 687.6179 | 2746.4423 | 2746.4361 | 2.25 | 1 | 41 | 0.00016 | 1Score **> 37** indicates **identity** Score **> 15** indicates **homology** | U | R.GHLDNISNNLLIGAINIENGKANSVR.N  + Deamidated (NQ) |
| 171063 | 608 | – | 633 | 916.8210 | 2747.4413 | 2747.4202 | 7.68 | 1 | 57 | 5e-06 | 1Score **> 37** indicates **identity** Score **> 16** indicates **homology** | U | R.GHLDNISNNLLIGAINIENGKANSVR.N  + 2 Deamidated (NQ) |
| 80098 | 634 | – | 648 | 801.3994 | 1600.7842 | 1600.7845 | -0.19 | 0 | 77 | 1.3e-07 | 1Score **> 34** indicates **identity** Score **> 20** indicates **homology** | U | R.NAVTQEFGPVPDTAR.Y |
| 80099 | 634 | – | 648 | 534.6021 | 1600.7845 | 1600.7845 | -0.059 | 0 | 45 | 6e-05 | 1Score **> 34** indicates **identity** Score **> 15** indicates **homology** | U | R.NAVTQEFGPVPDTAR.Y |
| 80100 | 634 | – | 648 | 801.3997 | 1600.7848 | 1600.7845 | 0.19 | 0 | 83 | 1.5e-08 | 1Score **> 34** indicates **identity** Score **> 18** indicates **homology** | U | R.NAVTQEFGPVPDTAR.Y |
| 80101 | 634 | – | 648 | 801.3997 | 1600.7849 | 1600.7845 | 0.21 | 0 | 58 | 4.5e-06 | 1Score **> 34** indicates **identity** Score **> 17** indicates **homology** | U | R.NAVTQEFGPVPDTAR.Y |
| 80103 | 634 | – | 648 | 801.4001 | 1600.7856 | 1600.7845 | 0.67 | 0 | 84 | 1.3e-08 | 1Score **> 34** indicates **identity** Score **> 18** indicates **homology** | U | R.NAVTQEFGPVPDTAR.Y |
| 80104 | 634 | – | 648 | 801.4001 | 1600.7857 | 1600.7845 | 0.73 | 0 | 83 | 3.4e-08 | 1Score **> 34** indicates **identity** Score **> 21** indicates **homology** | U | R.NAVTQEFGPVPDTAR.Y |
| 80105 | 634 | – | 648 | 801.4002 | 1600.7858 | 1600.7845 | 0.79 | 0 | 76 | 1.5e-07 | 1Score **> 34** indicates **identity** Score **> 21** indicates **homology** | U | R.NAVTQEFGPVPDTAR.Y |
| 80106 | 634 | – | 648 | 534.6027 | 1600.7862 | 1600.7845 | 1.01 | 0 | 17 | 0.026 | 1Score **> 34** indicates **identity** Score **> 14** indicates **homology** | U | R.NAVTQEFGPVPDTAR.Y |
| 80107 | 634 | – | 648 | 534.6029 | 1600.7870 | 1600.7845 | 1.52 | 0 | 48 | 2.8e-05 | 1Score **> 34** indicates **identity** Score **> 16** indicates **homology** | U | R.NAVTQEFGPVPDTAR.Y |
| 80118 | 634 | – | 648 | 801.4064 | 1600.7982 | 1600.7845 | 8.51 | 0 | 46 | 4.5e-05 | 1Score **> 34** indicates **identity** Score **> 15** indicates **homology** | U | R.NAVTQEFGPVPDTAR.Y |
| 80218 | 634 | – | 648 | 801.8896 | 1601.7647 | 1601.7686 | -2.39 | 0 | 62 | 3.1e-06 | 1Score **> 33** indicates **identity** Score **> 19** indicates **homology** | U | R.NAVTQEFGPVPDTAR.Y  + Deamidated (NQ) |
| 80220 | 634 | – | 648 | 801.8908 | 1601.7671 | 1601.7686 | -0.88 | 0 | 42 | 0.0002 | 1Score **> 33** indicates **identity** Score **> 17** indicates **homology** | U | R.NAVTQEFGPVPDTAR.Y  + Deamidated (NQ) |
| 88735 | 657 | – | 671 | 834.3874 | 1666.7602 | 1666.7587 | 0.90 | 0 | 46 | 4.4e-05 | 1Score **> 30** indicates **identity** Score **> 15** indicates **homology** | U | R.WVVIGDENYGEGSSR.E |
| 88736 | 657 | – | 671 | 834.3876 | 1666.7607 | 1666.7587 | 1.20 | 0 | 62 | 1.4e-06 | 1Score **> 31** indicates **identity** Score **> 16** indicates **homology** | U | R.WVVIGDENYGEGSSR.E |
| 88737 | 657 | – | 671 | 834.3877 | 1666.7609 | 1666.7587 | 1.34 | 0 | 46 | 5.1e-05 | 1Score **> 31** indicates **identity** Score **> 15** indicates **homology** | U | R.WVVIGDENYGEGSSR.E |
| 88740 | 657 | – | 671 | 834.3893 | 1666.7641 | 1666.7587 | 3.25 | 0 | 18 | 0.019 | 1Score **> 31** indicates **identity** Score **> 14** indicates **homology** | U | R.WVVIGDENYGEGSSR.E |
| 88741 | 657 | – | 671 | 834.3898 | 1666.7650 | 1666.7587 | 3.77 | 0 | 48 | 3.1e-05 | 1Score **> 31** indicates **identity** Score **> 16** indicates **homology** | U | R.WVVIGDENYGEGSSR.E |
| 163789 | 657 | – | 679 | 857.7451 | 2570.2136 | 2570.2150 | -0.53 | 1 | 23 | 0.0074 | 1Score **> 35** indicates **identity** Score **> 14** indicates **homology** | U | R.WVVIGDENYGEGSSREHAALEPR.H |
| 163824 | 657 | – | 679 | 858.0790 | 2571.2151 | 2571.1990 | 6.28 | 1 | 21 | 0.011 | 1Score **> 35** indicates **identity** Score **> 14** indicates **homology** | U | R.WVVIGDENYGEGSSREHAALEPR.H  + Deamidated (NQ) |
| 163826 | 657 | – | 679 | 858.0795 | 2571.2167 | 2571.1990 | 6.92 | 1 | 26 | 0.0039 | 1Score **> 35** indicates **identity** Score **> 14** indicates **homology** | U | R.WVVIGDENYGEGSSREHAALEPR.H  + Deamidated (NQ) |
| 114518 | 701 | – | 717 | 636.3374 | 1905.9905 | 1905.9836 | 3.62 | 1 | 38 | 0.00026 | 1Score **> 36** indicates **identity** Score **> 15** indicates **homology** | U | K.KQGLLPLTFADPSDYNK.I |
| 114522 | 701 | – | 717 | 636.3388 | 1905.9947 | 1905.9836 | 5.80 | 1 | 45 | 6.5e-05 | 1Score **> 36** indicates **identity** Score **> 15** indicates **homology** | U | K.KQGLLPLTFADPSDYNK.I |
| 114523 | 701 | – | 717 | 636.3401 | 1905.9986 | 1905.9836 | 7.85 | 1 | 23 | 0.0065 | 1Score **> 36** indicates **identity** Score **> 14** indicates **homology** | U | K.KQGLLPLTFADPSDYNK.I |
| 102002 | 702 | – | 717 | 889.9569 | 1777.8993 | 1777.8887 | 6.00 | 0 | 43 | 8.6e-05 | 1Score **> 35** indicates **identity** Score **> 15** indicates **homology** | U | K.QGLLPLTFADPSDYNK.I |
| 102003 | 702 | – | 717 | 889.9573 | 1777.9001 | 1777.8887 | 6.42 | 0 | 28 | 0.0026 | 1Score **> 35** indicates **identity** Score **> 14** indicates **homology** | U | K.QGLLPLTFADPSDYNK.I |
| 2633 | 724 | – | 730 | 386.7504 | 771.4862 | 771.4854 | 1.05 | 0 | 29 | 0.018 | 1Score **> 24** indicates **identity** | U | K.LTIQGLK.D |
| 96149 | 724 | – | 739 | 432.2622 | 1725.0198 | 1725.0189 | 0.56 | 2 | 39 | 0.00021 | 1Score **> 31** indicates **identity** Score **> 15** indicates **homology** | U | K.LTIQGLKDFAPGKPLK.C |
| 96150 | 724 | – | 739 | 432.2624 | 1725.0204 | 1725.0189 | 0.89 | 2 | 30 | 0.0015 | 1Score **> 31** indicates **identity** Score **> 14** indicates **homology** | U | K.LTIQGLKDFAPGKPLK.C |
| 13074 | 731 | – | 739 | 486.7800 | 971.5454 | 971.5440 | 1.48 | 1 | 25 | 0.0043 | 1Score **> 33** indicates **identity** Score **> 14** indicates **homology** | U | K.DFAPGKPLK.C |
| 187125 | 740 | – | 767 | 685.9446 | 3424.6865 | 3424.6986 | -3.55 | 1 | 20 | 0.014 | 1Score **> 38** indicates **identity** Score **> 14** indicates **homology** | U | K.CVIKHPNGTQETILLNHTFNETQIEWFR.A |
| 187126 | 740 | – | 767 | 857.1816 | 3424.6973 | 3424.6986 | -0.40 | 1 | 23 | 0.0084 | 1Score **> 38** indicates **identity** Score **> 15** indicates **homology** | U | K.CVIKHPNGTQETILLNHTFNETQIEWFR.A |
| 187127 | 740 | – | 767 | 685.9468 | 3424.6976 | 3424.6986 | -0.30 | 1 | 36 | 0.00043 | 1Score **> 38** indicates **identity** Score **> 15** indicates **homology** | U | K.CVIKHPNGTQETILLNHTFNETQIEWFR.A |
| 187128 | 740 | – | 767 | 857.1820 | 3424.6991 | 3424.6986 | 0.13 | 1 | 44 | 6.7e-05 | 1Score **> 38** indicates **identity** Score **> 15** indicates **homology** | U | K.CVIKHPNGTQETILLNHTFNETQIEWFR.A |
| 187130 | 740 | – | 767 | 685.9481 | 3424.7041 | 3424.6986 | 1.60 | 1 | 41 | 0.00017 | 1Score **> 38** indicates **identity** Score **> 16** indicates **homology** | U | K.CVIKHPNGTQETILLNHTFNETQIEWFR.A |
| 187150 | 740 | – | 767 | 857.4312 | 3425.6955 | 3425.6826 | 3.76 | 1 | 51 | 1.5e-05 | 1Score **> 37** indicates **identity** Score **> 16** indicates **homology** | U | K.CVIKHPNGTQETILLNHTFNETQIEWFR.A  + Deamidated (NQ) |
| 176987 | 744 | – | 767 | 732.1120 | 2924.4191 | 2924.4205 | -0.51 | 0 | 26 | 0.0039 | 1Score **> 37** indicates **identity** Score **> 14** indicates **homology** | U | K.HPNGTQETILLNHTFNETQIEWFR.A |

---

```
ID   ACON_MOUSE              Reviewed;         780 AA.
AC   Q99KI0; Q3UDK9; Q3ULG9; Q3UNH7; Q505P4;
DT   01-FEB-2005, integrated into UniProtKB/Swiss-Prot.
DT   01-JUN-2001, sequence version 1.
DT   28-JUN-2023, entry version 164.
DE   RecName: Full=Aconitate hydratase, mitochondrial;
DE            Short=Aconitase;
DE            EC=4.2.1.3 {ECO:0000250|UniProtKB:P16276};
DE   AltName: Full=Citrate hydro-lyase;
DE   Flags: Precursor;
GN   Name=Aco2;
OS   Mus musculus (Mouse).
OC   Eukaryota; Metazoa; Chordata; Craniata; Vertebrata; Euteleostomi; Mammalia;
OC   Eutheria; Euarchontoglires; Glires; Rodentia; Myomorpha; Muroidea; Muridae;
OC   Murinae; Mus; Mus.
OX   NCBI_TaxID=10090;
RN   [1]
RP   NUCLEOTIDE SEQUENCE [LARGE SCALE MRNA].
RC   STRAIN=C57BL/6J; TISSUE=Bone marrow, and Kidney;
RX   PubMed=16141072; DOI=10.1126/science.1112014;
RA   Carninci P., Kasukawa T., Katayama S., Gough J., Frith M.C., Maeda N.,
RA   Oyama R., Ravasi T., Lenhard B., Wells C., Kodzius R., Shimokawa K.,
RA   Bajic V.B., Brenner S.E., Batalov S., Forrest A.R., Zavolan M., Davis M.J.,
RA   Wilming L.G., Aidinis V., Allen J.E., Ambesi-Impiombato A., Apweiler R.,
RA   Aturaliya R.N., Bailey T.L., Bansal M., Baxter L., Beisel K.W., Bersano T.,
RA   Bono H., Chalk A.M., Chiu K.P., Choudhary V., Christoffels A.,
RA   Clutterbuck D.R., Crowe M.L., Dalla E., Dalrymple B.P., de Bono B.,
RA   Della Gatta G., di Bernardo D., Down T., Engstrom P., Fagiolini M.,
RA   Faulkner G., Fletcher C.F., Fukushima T., Furuno M., Futaki S.,
RA   Gariboldi M., Georgii-Hemming P., Gingeras T.R., Gojobori T., Green R.E.,
RA   Gustincich S., Harbers M., Hayashi Y., Hensch T.K., Hirokawa N., Hill D.,
RA   Huminiecki L., Iacono M., Ikeo K., Iwama A., Ishikawa T., Jakt M.,
RA   Kanapin A., Katoh M., Kawasawa Y., Kelso J., Kitamura H., Kitano H.,
RA   Kollias G., Krishnan S.P., Kruger A., Kummerfeld S.K., Kurochkin I.V.,
RA   Lareau L.F., Lazarevic D., Lipovich L., Liu J., Liuni S., McWilliam S.,
RA   Madan Babu M., Madera M., Marchionni L., Matsuda H., Matsuzawa S., Miki H.,
RA   Mignone F., Miyake S., Morris K., Mottagui-Tabar S., Mulder N., Nakano N.,
RA   Nakauchi H., Ng P., Nilsson R., Nishiguchi S., Nishikawa S., Nori F.,
RA   Ohara O., Okazaki Y., Orlando V., Pang K.C., Pavan W.J., Pavesi G.,
RA   Pesole G., Petrovsky N., Piazza S., Reed J., Reid J.F., Ring B.Z.,
RA   Ringwald M., Rost B., Ruan Y., Salzberg S.L., Sandelin A., Schneider C.,
RA   Schoenbach C., Sekiguchi K., Semple C.A., Seno S., Sessa L., Sheng Y.,
RA   Shibata Y., Shimada H., Shimada K., Silva D., Sinclair B., Sperling S.,
RA   Stupka E., Sugiura K., Sultana R., Takenaka Y., Taki K., Tammoja K.,
RA   Tan S.L., Tang S., Taylor M.S., Tegner J., Teichmann S.A., Ueda H.R.,
RA   van Nimwegen E., Verardo R., Wei C.L., Yagi K., Yamanishi H.,
RA   Zabarovsky E., Zhu S., Zimmer A., Hide W., Bult C., Grimmond S.M.,
RA   Teasdale R.D., Liu E.T., Brusic V., Quackenbush J., Wahlestedt C.,
RA   Mattick J.S., Hume D.A., Kai C., Sasaki D., Tomaru Y., Fukuda S.,
RA   Kanamori-Katayama M., Suzuki M., Aoki J., Arakawa T., Iida J., Imamura K.,
RA   Itoh M., Kato T., Kawaji H., Kawagashira N., Kawashima T., Kojima M.,
RA   Kondo S., Konno H., Nakano K., Ninomiya N., Nishio T., Okada M., Plessy C.,
RA   Shibata K., Shiraki T., Suzuki S., Tagami M., Waki K., Watahiki A.,
RA   Okamura-Oho Y., Suzuki H., Kawai J., Hayashizaki Y.;
RT   "The transcriptional landscape of the mammalian genome.";
RL   Science 309:1559-1563(2005).
RN   [2]
RP   NUCLEOTIDE SEQUENCE [LARGE SCALE MRNA].
RC   STRAIN=FVB/N; TISSUE=Kidney, and Mammary tumor;
RX   PubMed=15489334; DOI=10.1101/gr.2596504;
RG   The MGC Project Team;
RT   "The status, quality, and expansion of the NIH full-length cDNA project:
RT   the Mammalian Gene Collection (MGC).";
RL   Genome Res. 14:2121-2127(2004).
RN   [3]
RP   PROTEIN SEQUENCE OF 32-56; 59-84; 96-138; 143-160; 234-245; 251-258;
RP   313-323; 371-395; 402-409; 412-424; 430-457; 466-474; 480-517; 522-587;
RP   592-605; 608-628; 634-648; 657-671; 694-739 AND 744-767, AND IDENTIFICATION
RP   BY MASS SPECTROMETRY.
RC   STRAIN=C57BL/6J, and OF1; TISSUE=Brain, and Hippocampus;
RA   Lubec G., Klug S., Kang S.U., Sunyer B., Chen W.-Q.;
RL   Submitted (JAN-2009) to UniProtKB.
RN   [4]
RP   PHOSPHORYLATION [LARGE SCALE ANALYSIS] AT SER-670, AND IDENTIFICATION BY
RP   MASS SPECTROMETRY [LARGE SCALE ANALYSIS].
RC   TISSUE=Brain, Brown adipose tissue, Heart, Kidney, Liver, Lung,
RC   Pancreas, Spleen, and Testis;
RX   PubMed=21183079; DOI=10.1016/j.cell.2010.12.001;
RA   Huttlin E.L., Jedrychowski M.P., Elias J.E., Goswami T., Rad R.,
RA   Beausoleil S.A., Villen J., Haas W., Sowa M.E., Gygi S.P.;
RT   "A tissue-specific atlas of mouse protein phosphorylation and expression.";
RL   Cell 143:1174-1189(2010).
RN   [5]
RP   ACETYLATION [LARGE SCALE ANALYSIS] AT LYS-50 AND LYS-144, SUCCINYLATION
RP   [LARGE SCALE ANALYSIS] AT LYS-31; LYS-50; LYS-138; LYS-144; LYS-233;
RP   LYS-411; LYS-517; LYS-523; LYS-549; LYS-573; LYS-577; LYS-591; LYS-605;
RP   LYS-628; LYS-689; LYS-723 AND LYS-730, AND IDENTIFICATION BY MASS
RP   SPECTROMETRY [LARGE SCALE ANALYSIS].
RC   TISSUE=Embryonic fibroblast, and Liver;
RX   PubMed=23806337; DOI=10.1016/j.molcel.2013.06.001;
RA   Park J., Chen Y., Tishkoff D.X., Peng C., Tan M., Dai L., Xie Z., Zhang Y.,
RA   Zwaans B.M., Skinner M.E., Lombard D.B., Zhao Y.;
RT   "SIRT5-mediated lysine desuccinylation impacts diverse metabolic
RT   pathways.";
RL   Mol. Cell 50:919-930(2013).
RN   [6]
RP   ACETYLATION [LARGE SCALE ANALYSIS] AT LYS-50; LYS-138; LYS-144; LYS-233;
RP   LYS-517; LYS-523; LYS-605; LYS-723; LYS-730; LYS-736; LYS-739 AND LYS-743,
RP   AND IDENTIFICATION BY MASS SPECTROMETRY [LARGE SCALE ANALYSIS].
RC   TISSUE=Liver;
RX   PubMed=23576753; DOI=10.1073/pnas.1302961110;
RA   Rardin M.J., Newman J.C., Held J.M., Cusack M.P., Sorensen D.J., Li B.,
RA   Schilling B., Mooney S.D., Kahn C.R., Verdin E., Gibson B.W.;
RT   "Label-free quantitative proteomics of the lysine acetylome in mitochondria
RT   identifies substrates of SIRT3 in metabolic pathways.";
RL   Proc. Natl. Acad. Sci. U.S.A. 110:6601-6606(2013).
CC   -!- FUNCTION: Catalyzes the isomerization of citrate to isocitrate via cis-
CC       aconitate. {ECO:0000250|UniProtKB:P16276}.
CC   -!- CATALYTIC ACTIVITY:
CC       Reaction=citrate = D-threo-isocitrate; Xref=Rhea:RHEA:10336,
CC         ChEBI:CHEBI:15562, ChEBI:CHEBI:16947; EC=4.2.1.3;
CC         Evidence={ECO:0000250|UniProtKB:P16276};
CC   -!- COFACTOR:
CC       Name=[4Fe-4S] cluster; Xref=ChEBI:CHEBI:49883;
CC         Evidence={ECO:0000250|UniProtKB:P16276};
CC       Note=Binds 1 [4Fe-4S] cluster per subunit. Binding of a [3Fe-4S]
CC       cluster leads to an inactive enzyme. {ECO:0000250|UniProtKB:P16276};
CC   -!- PATHWAY: Carbohydrate metabolism; tricarboxylic acid cycle; isocitrate
CC       from oxaloacetate: step 2/2.
CC   -!- SUBUNIT: Monomer. {ECO:0000250|UniProtKB:P16276}.
CC   -!- SUBCELLULAR LOCATION: Mitochondrion {ECO:0000250|UniProtKB:P16276}.
CC   -!- PTM: Forms covalent cross-links mediated by transglutaminase TGM2,
CC       between a glutamine and the epsilon-amino group of a lysine residue,
CC       forming homopolymers and heteropolymers.
CC       {ECO:0000250|UniProtKB:Q9ER34}.
CC   -!- SIMILARITY: Belongs to the aconitase/IPM isomerase family.
CC       {ECO:0000305}.
CC   ---------------------------------------------------------------------------
CC   Copyrighted by the UniProt Consortium, see https://www.uniprot.org/terms
CC   Distributed under the Creative Commons Attribution (CC BY 4.0) License
CC   ---------------------------------------------------------------------------
DR   EMBL; AK143917; BAE25602.1; -; mRNA.
DR   EMBL; AK144207; BAE25770.1; -; mRNA.
DR   EMBL; AK145511; BAE26479.1; -; mRNA.
DR   EMBL; AK150027; BAE29252.1; -; mRNA.
DR   EMBL; AK165411; BAE38169.1; -; mRNA.
DR   EMBL; BC004645; AAH04645.1; -; mRNA.
DR   EMBL; BC094462; AAH94462.1; -; mRNA.
DR   CCDS; CCDS27675.1; -.
DR   RefSeq; NP_542364.1; NM_080633.2.
DR   AlphaFoldDB; Q99KI0; -.
DR   SMR; Q99KI0; -.
DR   BioGRID; 197925; 72.
DR   IntAct; Q99KI0; 8.
DR   MINT; Q99KI0; -.
DR   STRING; 10090.ENSMUSP00000023116; -.
DR   CarbonylDB; Q99KI0; -.
DR   GlyGen; Q99KI0; 1 site, 1 O-linked glycan (1 site).
DR   iPTMnet; Q99KI0; -.
DR   PhosphoSitePlus; Q99KI0; -.
DR   SwissPalm; Q99KI0; -.
DR   REPRODUCTION-2DPAGE; Q99KI0; -.
DR   EPD; Q99KI0; -.
DR   jPOST; Q99KI0; -.
DR   MaxQB; Q99KI0; -.
DR   PaxDb; Q99KI0; -.
DR   PeptideAtlas; Q99KI0; -.
DR   ProteomicsDB; 285596; -.
DR   Antibodypedia; 240; 582 antibodies from 39 providers.
DR   DNASU; 11429; -.
DR   Ensembl; ENSMUST00000023116; ENSMUSP00000023116; ENSMUSG00000022477.
DR   GeneID; 11429; -.
DR   KEGG; mmu:11429; -.
DR   UCSC; uc007wxp.1; mouse.
DR   AGR; MGI:87880; -.
DR   CTD; 50; -.
DR   MGI; MGI:87880; Aco2.
DR   VEuPathDB; HostDB:ENSMUSG00000022477; -.
DR   eggNOG; KOG0453; Eukaryota.
DR   GeneTree; ENSGT00940000154892; -.
DR   HOGENOM; CLU_006714_2_2_1; -.
DR   InParanoid; Q99KI0; -.
DR   OMA; GCIGMGQ; -.
DR   OrthoDB; 3266779at2759; -.
DR   PhylomeDB; Q99KI0; -.
DR   TreeFam; TF300627; -.
DR   BRENDA; 4.2.1.3; 3474.
DR   Reactome; R-MMU-71403; Citric acid cycle (TCA cycle).
DR   UniPathway; UPA00223; UER00718.
DR   BioGRID-ORCS; 11429; 16 hits in 78 CRISPR screens.
DR   ChiTaRS; Aco2; mouse.
DR   PRO; PR:Q99KI0; -.
DR   Proteomes; UP000000589; Chromosome 15.
DR   RNAct; Q99KI0; protein.
DR   Bgee; ENSMUSG00000022477; Expressed in cardiac muscle of left ventricle and 271 other tissues.
DR   ExpressionAtlas; Q99KI0; baseline and differential.
DR   Genevisible; Q99KI0; MM.
DR   GO; GO:0005829; C:cytosol; IBA:GO_Central.
DR   GO; GO:0005759; C:mitochondrial matrix; IDA:MGI.
DR   GO; GO:0005739; C:mitochondrion; IDA:MGI.
DR   GO; GO:0043209; C:myelin sheath; HDA:UniProtKB.
DR   GO; GO:0051538; F:3 iron, 4 sulfur cluster binding; ISO:MGI.
DR   GO; GO:0051539; F:4 iron, 4 sulfur cluster binding; ISO:MGI.
DR   GO; GO:0003994; F:aconitate hydratase activity; IDA:MGI.
DR   GO; GO:0047780; F:citrate dehydratase activity; IEA:UniProtKB-EC.
DR   GO; GO:0005506; F:iron ion binding; ISO:MGI.
DR   GO; GO:0006101; P:citrate metabolic process; ISO:MGI.
DR   GO; GO:0006102; P:isocitrate metabolic process; ISO:MGI.
DR   GO; GO:0001889; P:liver development; IEA:Ensembl.
DR   GO; GO:0035900; P:response to isolation stress; IEA:Ensembl.
DR   GO; GO:0006099; P:tricarboxylic acid cycle; IGI:MGI.
DR   CDD; cd01578; AcnA_Mitochon_Swivel; 1.
DR   CDD; cd01584; AcnA_Mitochondrial; 1.
DR   Gene3D; 3.40.1060.10; Aconitase, Domain 2; 1.
DR   Gene3D; 3.30.499.10; Aconitase, domain 3; 2.
DR   Gene3D; 3.20.19.10; Aconitase, domain 4; 1.
DR   InterPro; IPR015931; Acnase/IPM_dHydase_lsu_aba_1/3.
DR   InterPro; IPR001030; Acoase/IPM_deHydtase_lsu_aba.
DR   InterPro; IPR015928; Aconitase/3IPM_dehydase_swvl.
DR   InterPro; IPR018136; Aconitase_4Fe-4S_BS.
DR   InterPro; IPR036008; Aconitase_4Fe-4S_dom.
DR   InterPro; IPR015932; Aconitase_dom2.
DR   InterPro; IPR006248; Aconitase_mito-like.
DR   InterPro; IPR000573; AconitaseA/IPMdHydase_ssu_swvl.
DR   PANTHER; PTHR43160; ACONITATE HYDRATASE B; 1.
DR   PANTHER; PTHR43160:SF3; ACONITATE HYDRATASE, MITOCHONDRIAL; 1.
DR   Pfam; PF00330; Aconitase; 1.
DR   Pfam; PF00694; Aconitase_C; 1.
DR   PRINTS; PR00415; ACONITASE.
DR   SUPFAM; SSF53732; Aconitase iron-sulfur domain; 1.
DR   SUPFAM; SSF52016; LeuD/IlvD-like; 1.
DR   PROSITE; PS00450; ACONITASE_1; 1.
DR   PROSITE; PS01244; ACONITASE_2; 1.
DR   TIGRFAMs; TIGR01340; aconitase_mito; 1.
PE   1: Evidence at protein level;
KW   4Fe-4S; Acetylation; Direct protein sequencing; Iron; Iron-sulfur; Lyase;
KW   Metal-binding; Mitochondrion; Phosphoprotein; Reference proteome;
KW   Transit peptide; Tricarboxylic acid cycle.
FT   TRANSIT         1..27
FT                   /note="Mitochondrion"
FT                   /evidence="ECO:0000250"
FT   CHAIN           28..780
FT                   /note="Aconitate hydratase, mitochondrial"
FT                   /id="PRO_0000000542"
FT   REGION          524..560
FT                   /note="Disordered"
FT                   /evidence="ECO:0000256|SAM:MobiDB-lite"
FT   COMPBIAS        545..560
FT                   /note="Polar residues"
FT                   /evidence="ECO:0000256|SAM:MobiDB-lite"
FT   BINDING         99
FT                   /ligand="substrate"
FT                   /evidence="ECO:0000250"
FT   BINDING         192..194
FT                   /ligand="substrate"
FT                   /evidence="ECO:0000250"
FT   BINDING         385
FT                   /ligand="[4Fe-4S] cluster"
FT                   /ligand_id="ChEBI:CHEBI:49883"
FT                   /evidence="ECO:0000250"
FT   BINDING         448
FT                   /ligand="[4Fe-4S] cluster"
FT                   /ligand_id="ChEBI:CHEBI:49883"
FT                   /evidence="ECO:0000250"
FT   BINDING         451
FT                   /ligand="[4Fe-4S] cluster"
FT                   /ligand_id="ChEBI:CHEBI:49883"
FT                   /evidence="ECO:0000250"
FT   BINDING         474
FT                   /ligand="substrate"
FT                   /evidence="ECO:0000250"
FT   BINDING         479
FT                   /ligand="substrate"
FT                   /evidence="ECO:0000250"
FT   BINDING         607
FT                   /ligand="substrate"
FT                   /evidence="ECO:0000250"
FT   BINDING         670..671
FT                   /ligand="substrate"
FT                   /evidence="ECO:0000250"
FT   MOD_RES         31
FT                   /note="N6-succinyllysine"
FT                   /evidence="ECO:0007744|PubMed:23806337"
FT   MOD_RES         50
FT                   /note="N6-acetyllysine; alternate"
FT                   /evidence="ECO:0007744|PubMed:23576753,
FT                   ECO:0007744|PubMed:23806337"
FT   MOD_RES         50
FT                   /note="N6-succinyllysine; alternate"
FT                   /evidence="ECO:0007744|PubMed:23806337"
FT   MOD_RES         138
FT                   /note="N6-acetyllysine; alternate"
FT                   /evidence="ECO:0007744|PubMed:23576753"
FT   MOD_RES         138
FT                   /note="N6-succinyllysine; alternate"
FT                   /evidence="ECO:0007744|PubMed:23806337"
FT   MOD_RES         144
FT                   /note="N6-acetyllysine; alternate"
FT                   /evidence="ECO:0007744|PubMed:23576753,
FT                   ECO:0007744|PubMed:23806337"
FT   MOD_RES         144
FT                   /note="N6-succinyllysine; alternate"
FT                   /evidence="ECO:0007744|PubMed:23806337"
FT   MOD_RES         233
FT                   /note="N6-acetyllysine; alternate"
FT                   /evidence="ECO:0007744|PubMed:23576753"
FT   MOD_RES         233
FT                   /note="N6-succinyllysine; alternate"
FT                   /evidence="ECO:0007744|PubMed:23806337"
FT   MOD_RES         411
FT                   /note="N6-succinyllysine"
FT                   /evidence="ECO:0007744|PubMed:23806337"
FT   MOD_RES         517
FT                   /note="N6-acetyllysine; alternate"
FT                   /evidence="ECO:0007744|PubMed:23576753"
FT   MOD_RES         517
FT                   /note="N6-succinyllysine; alternate"
FT                   /evidence="ECO:0007744|PubMed:23806337"
FT   MOD_RES         523
FT                   /note="N6-acetyllysine; alternate"
FT                   /evidence="ECO:0007744|PubMed:23576753"
FT   MOD_RES         523
FT                   /note="N6-succinyllysine; alternate"
FT                   /evidence="ECO:0007744|PubMed:23806337"
FT   MOD_RES         549
FT                   /note="N6-succinyllysine"
FT                   /evidence="ECO:0007744|PubMed:23806337"
FT   MOD_RES         559
FT                   /note="Phosphoserine"
FT                   /evidence="ECO:0000250|UniProtKB:Q99798"
FT   MOD_RES         573
FT                   /note="N6-acetyllysine; alternate"
FT                   /evidence="ECO:0000250|UniProtKB:Q99798"
FT   MOD_RES         573
FT                   /note="N6-succinyllysine; alternate"
FT                   /evidence="ECO:0007744|PubMed:23806337"
FT   MOD_RES         577
FT                   /note="N6-succinyllysine"
FT                   /evidence="ECO:0007744|PubMed:23806337"
FT   MOD_RES         591
FT                   /note="N6-succinyllysine"
FT                   /evidence="ECO:0007744|PubMed:23806337"
FT   MOD_RES         605
FT                   /note="N6-acetyllysine; alternate"
FT                   /evidence="ECO:0007744|PubMed:23576753"
FT   MOD_RES         605
FT                   /note="N6-succinyllysine; alternate"
FT                   /evidence="ECO:0007744|PubMed:23806337"
FT   MOD_RES         628
FT                   /note="N6-succinyllysine"
FT                   /evidence="ECO:0007744|PubMed:23806337"
FT   MOD_RES         670
FT                   /note="Phosphoserine"
FT                   /evidence="ECO:0007744|PubMed:21183079"
FT   MOD_RES         689
FT                   /note="N6-succinyllysine"
FT                   /evidence="ECO:0007744|PubMed:23806337"
FT   MOD_RES         723
FT                   /note="N6-acetyllysine; alternate"
FT                   /evidence="ECO:0007744|PubMed:23576753"
FT   MOD_RES         723
FT                   /note="N6-succinyllysine; alternate"
FT                   /evidence="ECO:0007744|PubMed:23806337"
FT   MOD_RES         730
FT                   /note="N6-acetyllysine; alternate"
FT                   /evidence="ECO:0007744|PubMed:23576753"
FT   MOD_RES         730
FT                   /note="N6-succinyllysine; alternate"
FT                   /evidence="ECO:0007744|PubMed:23806337"
FT   MOD_RES         736
FT                   /note="N6-acetyllysine"
FT                   /evidence="ECO:0007744|PubMed:23576753"
FT   MOD_RES         739
FT                   /note="N6-acetyllysine"
FT                   /evidence="ECO:0007744|PubMed:23576753"
FT   MOD_RES         743
FT                   /note="N6-acetyllysine"
FT                   /evidence="ECO:0007744|PubMed:23576753"
FT   CONFLICT        7..8
FT                   /note="LV -> P (in Ref. 1; BAE25770)"
FT                   /evidence="ECO:0000305"
FT   CONFLICT        618
FT                   /note="L -> F (in Ref. 2; AAH94462)"
FT                   /evidence="ECO:0000305"
FT   CONFLICT        758
FT                   /note="F -> L (in Ref. 1; BAE29252)"
FT                   /evidence="ECO:0000305"
SQ   SEQUENCE   780 AA;  85464 MW;  9B515846E875D581 CRC64;
     MAPYSLLVTR LQKALGVRQY HVASVLCQRA KVAMSHFEPS EYIRYDLLEK NINIVRKRLN
     RPLTLSEKIV YGHLDDPANQ EIERGKTYLR LRPDRVAMQD ATAQMAMLQF ISSGLPKVAV
     PSTIHCDHLI EAQVGGEKDL RRAKDINQEV YNFLATAGAK YGVGFWRPGS GIIHQIILEN
     YAYPGVLLIG TDSHTPNGGG LGGICIGVGG ADAVDVMAGI PWELKCPKVI GVKLTGSLSG
     WTSPKDVILK VAGILTVKGG TGAIVEYHGP GVDSISCTGM ATICNMGAEI GATTSVFPYN
     HRMKKYLSKT GRTDIANLAE EFKDHLVPDP GCQYDQVIEI NLNELKPHIN GPFTPDLAHP
     VADVGTVAEK EGWPLDIRVG LIGSCTNSSY EDMGRSAAVA KQALAHGLKC KSQFTITPGS
     EQIRATIERD GYAQILRDVG GIVLANACGP CIGQWDRKDI KKGEKNTIVT SYNRNFTGRN
     DANPETHAFV TSPEIVTALA IAGTLKFNPE TDFLTGKDGK KFKLEAPDAD ELPRSDFDPG
     QDTYQHPPKD SSGQRVDVSP TSQRLQLLEP FDKWDGKDLE DLQILIKVKG KCTTDHISAA
     GPWLKFRGHL DNISNNLLIG AINIENGKAN SVRNAVTQEF GPVPDTARYY KKHGIRWVVI
     GDENYGEGSS REHAALEPRH LGGRAIITKS FARIHETNLK KQGLLPLTFA DPSDYNKIHP
     VDKLTIQGLK DFAPGKPLKC VIKHPNGTQE TILLNHTFNE TQIEWFRAGS ALNRMKELQQ
//
```

|  |
| --- |
| **Mascot:** http://www.matrixscience.com/ |

Oxidation (M) (+15.9949)
