# Supplementary material for: A common East-Asian ALDH2 mutation causes metabolic disorders and the therapeutic effect of ALDH2 activators
Source: Nat Commun. 2023 Sep 25;14:5971. doi: 10.1038/s41467-023-41570-6 (PMC10520061; doi:10.1038/s41467-023-41570-6)
Supplement: Supplementary file 4 — Supplementary Data 1 [file 41467_2023_41570_MOESM4_ESM.zip › Table S5b/Q99KIO/Q99KI0_WTO-4_C284_H268_C410.html]

Mascot Search Results: Q99KI0
 

# MASCOT Search Results

## Protein View: Q99KI0

### Aconitate hydratase, mitochondrial OS=Mus musculus OX=10090 GN=Aco2 PE=1 SV=1

|  |  |
| --- | --- |
| Database: | Mouse\_UniProt\_proteomes |
| Score: | 5971 |
| Monoisotopic mass (Mr): | 86151 |
| Calculated pI: | 8.08 |

Sequence similarity is available as an NCBI BLAST search of Q99KI0 against nr.

### Search parameters

|  |  |
| --- | --- |
| MS data file: | `D:\LCMSMS\2023 Users' data\230529-1\230529-1-WTO-4.raw` |
| Enzyme: | Trypsin/P: cuts C-term side of KR. |
| Fixed modifications: | Carbamidomethyl (C) |
| Variable modifications: | Deamidated (NQ), HNE (C), HNE (H), HNE (K), Oxidation (M) |

### Protein sequence coverage: 64%

Matched peptides shown in ***bold red***.

|  |  |  |  |  |  |
| --- | --- | --- | --- | --- | --- |
| `1` | `MAPYSLLVTR` | `LQKALGVRQY` | `HVASVLCQRA` | `KVAMSHFEPS` | `EYIRYDLLEK` |
| `51` | `NINIVRKRLN` | `RPLTLSEKIV` | `YGHLDDPANQ` | `EIERGKTYLR` | `LRPDRVAMQD` |
| `101` | `ATAQMAMLQF` | `ISSGLPKVAV` | `PSTIHCDHLI` | `EAQVGGEKDL` | `RRAKDINQEV` |
| `151` | `YNFLATAGAK` | `YGVGFWRPGS` | `GIIHQIILEN` | `YAYPGVLLIG` | `TDSHTPNGGG` |
| `201` | `LGGICIGVGG` | `ADAVDVMAGI` | `PWELKCPKVI` | `GVKLTGSLSG` | `WTSPKDVILK` |
| `251` | `VAGILTVKGG` | `TGAIVEYHGP` | `GVDSISCTGM` | `ATICNMGAEI` | `GATTSVFPYN` |
| `301` | `HRMKKYLSKT` | `GRTDIANLAE` | `EFKDHLVPDP` | `GCQYDQVIEI` | `NLNELKPHIN` |
| `351` | `GPFTPDLAHP` | `VADVGTVAEK` | `EGWPLDIRVG` | `LIGSCTNSSY` | `EDMGRSAAVA` |
| `401` | `KQALAHGLKC` | `KSQFTITPGS` | `EQIRATIERD` | `GYAQILRDVG` | `GIVLANACGP` |
| `451` | `CIGQWDRKDI` | `KKGEKNTIVT` | `SYNRNFTGRN` | `DANPETHAFV` | `TSPEIVTALA` |
| `501` | `IAGTLKFNPE` | `TDFLTGKDGK` | `KFKLEAPDAD` | `ELPRSDFDPG` | `QDTYQHPPKD` |
| `551` | `SSGQRVDVSP` | `TSQRLQLLEP` | `FDKWDGKDLE` | `DLQILIKVKG` | `KCTTDHISAA` |
| `601` | `GPWLKFRGHL` | `DNISNNLLIG` | `AINIENGKAN` | `SVRNAVTQEF` | `GPVPDTARYY` |
| `651` | `KKHGIRWVVI` | `GDENYGEGSS` | `REHAALEPRH` | `LGGRAIITKS` | `FARIHETNLK` |
| `701` | `KQGLLPLTFA` | `DPSDYNKIHP` | `VDKLTIQGLK` | `DFAPGKPLKC` | `VIKHPNGTQE` |
| `751` | `TILLNHTFNE` | `TQIEWFRAGS` | `ALNRMKELQQ` |  |  |

Unformatted sequence string: 780 residues (for pasting into other applications).

|  |  |  |  |
| --- | --- | --- | --- |
| Sort by | residue number | increasing mass | decreasing mass |
| Show | matched peptides only | predicted peptides also |  |

| Query | Start | – | End | Observed | Mr(expt) | Mr(calc) | ppm | M | Score | Expect | Rank | U | Peptide |
| --- | --- | --- | --- | --- | --- | --- | --- | --- | --- | --- | --- | --- | --- |
| 74775 | 32 | – | 44 | 522.5861 | 1564.7365 | 1564.7344 | 1.34 | 0 | 48 | 3.4e-05 | 1Score **> 32** indicates **identity** Score **> 15** indicates **homology** | U | K.VAMSHFEPSEYIR.Y |
| 74778 | 32 | – | 44 | 522.5868 | 1564.7385 | 1564.7344 | 2.61 | 0 | 31 | 0.0013 | 1Score **> 33** indicates **identity** Score **> 14** indicates **homology** | U | K.VAMSHFEPSEYIR.Y |
| 6507 | 51 | – | 57 | 428.7720 | 855.5294 | 855.5290 | 0.45 | 1 | 16 | 0.046 | 1Score **> 25** indicates **identity** Score **> 15** indicates **homology** | U | K.NINIVRK.R |
| 47047 | 58 | – | 68 | 442.9334 | 1325.7783 | 1325.7779 | 0.32 | 2 | 27 | 0.0091 | 1Score **> 31** indicates **identity** Score **> 19** indicates **homology** | U | K.RLNRPLTLSEK.I |
| 47048 | 58 | – | 68 | 442.9336 | 1325.7789 | 1325.7779 | 0.78 | 2 | 23 | 0.0064 | 1Score **> 32** indicates **identity** Score **> 14** indicates **homology** | U | K.RLNRPLTLSEK.I |
| 29914 | 59 | – | 68 | 390.8993 | 1169.6761 | 1169.6768 | -0.62 | 1 | 26 | 0.011 | 1Score **> 32** indicates **identity** Score **> 19** indicates **homology** | U | R.LNRPLTLSEK.I |
| 29915 | 59 | – | 68 | 390.8994 | 1169.6765 | 1169.6768 | -0.24 | 1 | 30 | 0.0046 | 1Score **> 32** indicates **identity** Score **> 19** indicates **homology** | U | R.LNRPLTLSEK.I |
| 29916 | 59 | – | 68 | 585.8456 | 1169.6767 | 1169.6768 | -0.10 | 1 | 44 | 0.0017 | 1Score **> 32** indicates **identity** Score **> 29** indicates **homology** | U | R.LNRPLTLSEK.I |
| 29917 | 59 | – | 68 | 390.8995 | 1169.6767 | 1169.6768 | -0.050 | 1 | 22 | 0.027 | 1Score **> 32** indicates **identity** Score **> 18** indicates **homology** | U | R.LNRPLTLSEK.I |
| 29918 | 59 | – | 68 | 585.8457 | 1169.6769 | 1169.6768 | 0.056 | 1 | 40 | 0.0015 | 1Score **> 32** indicates **identity** Score **> 24** indicates **homology** | U | R.LNRPLTLSEK.I |
| 110896 | 69 | – | 84 | 623.6422 | 1867.9048 | 1867.9064 | -0.88 | 0 | 56 | 6.2e-06 | 1Score **> 34** indicates **identity** Score **> 16** indicates **homology** | U | K.IVYGHLDDPANQEIER.G |
| 110897 | 69 | – | 84 | 623.6425 | 1867.9058 | 1867.9064 | -0.32 | 0 | 41 | 0.00016 | 1Score **> 35** indicates **identity** Score **> 15** indicates **homology** | U | K.IVYGHLDDPANQEIER.G |
| 110898 | 69 | – | 84 | 623.6426 | 1867.9059 | 1867.9064 | -0.27 | 0 | 89 | 4.2e-09 | 1Score **> 35** indicates **identity** Score **> 18** indicates **homology** | U | K.IVYGHLDDPANQEIER.G |
| 110899 | 69 | – | 84 | 623.6427 | 1867.9063 | 1867.9064 | -0.089 | 0 | 74 | 1.2e-07 | 1Score **> 34** indicates **identity** Score **> 17** indicates **homology** | U | K.IVYGHLDDPANQEIER.G |
| 110900 | 69 | – | 84 | 623.6427 | 1867.9063 | 1867.9064 | -0.086 | 0 | 55 | 6.6e-06 | 1Score **> 34** indicates **identity** Score **> 16** indicates **homology** | U | K.IVYGHLDDPANQEIER.G |
| 110901 | 69 | – | 84 | 623.6428 | 1867.9065 | 1867.9064 | 0.063 | 0 | 26 | 0.0035 | 1Score **> 35** indicates **identity** Score **> 14** indicates **homology** | U | K.IVYGHLDDPANQEIER.G |
| 110902 | 69 | – | 84 | 623.6428 | 1867.9066 | 1867.9064 | 0.082 | 0 | 52 | 1.3e-05 | 1Score **> 35** indicates **identity** Score **> 16** indicates **homology** | U | K.IVYGHLDDPANQEIER.G |
| 110903 | 69 | – | 84 | 934.9610 | 1867.9075 | 1867.9064 | 0.60 | 0 | 27 | 0.0026 | 1Score **> 34** indicates **identity** Score **> 14** indicates **homology** | U | K.IVYGHLDDPANQEIER.G |
| 110904 | 69 | – | 84 | 934.9612 | 1867.9078 | 1867.9064 | 0.73 | 0 | 77 | 5.8e-08 | 1Score **> 34** indicates **identity** Score **> 17** indicates **homology** | U | K.IVYGHLDDPANQEIER.G |
| 110905 | 69 | – | 84 | 623.6432 | 1867.9079 | 1867.9064 | 0.81 | 0 | 74 | 1.1e-07 | 1Score **> 34** indicates **identity** Score **> 17** indicates **homology** | U | K.IVYGHLDDPANQEIER.G |
| 110906 | 69 | – | 84 | 934.9613 | 1867.9080 | 1867.9064 | 0.84 | 0 | 60 | 2.4e-06 | 1Score **> 34** indicates **identity** Score **> 16** indicates **homology** | U | K.IVYGHLDDPANQEIER.G |
| 110907 | 69 | – | 84 | 623.6433 | 1867.9080 | 1867.9064 | 0.86 | 0 | 31 | 0.0012 | 1Score **> 34** indicates **identity** Score **> 14** indicates **homology** | U | K.IVYGHLDDPANQEIER.G |
| 110908 | 69 | – | 84 | 934.9613 | 1867.9081 | 1867.9064 | 0.92 | 0 | 76 | 8.1e-08 | 1Score **> 34** indicates **identity** Score **> 17** indicates **homology** | U | K.IVYGHLDDPANQEIER.G |
| 110910 | 69 | – | 84 | 623.6436 | 1867.9090 | 1867.9064 | 1.41 | 0 | 28 | 0.0024 | 1Score **> 35** indicates **identity** Score **> 14** indicates **homology** | U | K.IVYGHLDDPANQEIER.G |
| 110911 | 69 | – | 84 | 623.6437 | 1867.9092 | 1867.9064 | 1.50 | 0 | 39 | 0.00022 | 1Score **> 35** indicates **identity** Score **> 15** indicates **homology** | U | K.IVYGHLDDPANQEIER.G |
| 146209 | 118 | – | 138 | 565.7901 | 2259.1312 | 2259.1318 | -0.25 | 0 | 37 | 0.00038 | 1Score **> 37** indicates **identity** Score **> 15** indicates **homology** | U | K.VAVPSTIHCDHLIEAQVGGEK.D |
| 146219 | 118 | – | 138 | 754.0541 | 2259.1404 | 2259.1318 | 3.81 | 0 | 18 | 0.02 | 1Score **> 37** indicates **identity** Score **> 14** indicates **homology** | U | K.VAVPSTIHCDHLIEAQVGGEK.D |
| 146222 | 118 | – | 138 | 565.7933 | 2259.1440 | 2259.1318 | 5.40 | 0 | 28 | 0.0023 | 1Score **> 37** indicates **identity** Score **> 14** indicates **homology** | U | K.VAVPSTIHCDHLIEAQVGGEK.D |
| 146223 | 118 | – | 138 | 754.0554 | 2259.1443 | 2259.1318 | 5.56 | 0 | 26 | 0.0035 | 1Score **> 37** indicates **identity** Score **> 14** indicates **homology** | U | K.VAVPSTIHCDHLIEAQVGGEK.D |
| 167747 | 118 | – | 141 | 529.6764 | 2643.3457 | 2643.3439 | 0.67 | 1 | 16 | 0.03 | 1Score **> 37** indicates **identity** Score **> 14** indicates **homology** | U | K.VAVPSTIHCDHLIEAQVGGEKDLR.R |
| 167749 | 118 | – | 141 | 661.8441 | 2643.3471 | 2643.3439 | 1.22 | 1 | 14 | 0.05 | 1Score **> 37** indicates **identity** Score **> 13** indicates **homology** | U | K.VAVPSTIHCDHLIEAQVGGEKDLR.R |
| 173923 | 118 | – | 142 | 560.8969 | 2799.4481 | 2799.4450 | 1.12 | 2 | 39 | 0.035 | 1Score **> 37** indicates **identity** | U | K.VAVPSTIHCDHLIEAQVGGEKDLRR.A |
| 173925 | 118 | – | 142 | 467.5820 | 2799.4485 | 2799.4450 | 1.24 | 2 | 41 | 0.021 | 1Score **> 37** indicates **identity** | U | K.VAVPSTIHCDHLIEAQVGGEKDLRR.A |
| 133475 | 142 | – | 160 | 1055.5427 | 2109.0708 | 2109.0854 | -6.91 | 2 | 35 | 0.00056 | 1Score **> 36** indicates **identity** Score **> 15** indicates **homology** | U | R.RAKDINQEVYNFLATAGAK.Y  + Deamidated (NQ) |
| 118715 | 143 | – | 160 | 977.0051 | 1951.9956 | 1952.0003 | -2.43 | 1 | 139 | 9.8e-14 | 1Score **> 36** indicates **identity** Score **> 21** indicates **homology** | U | R.AKDINQEVYNFLATAGAK.Y |
| 118719 | 143 | – | 160 | 651.6734 | 1951.9985 | 1952.0003 | -0.94 | 1 | 26 | 0.0034 | 1Score **> 36** indicates **identity** Score **> 14** indicates **homology** | U | R.AKDINQEVYNFLATAGAK.Y |
| 118720 | 143 | – | 160 | 651.6741 | 1952.0006 | 1952.0003 | 0.13 | 1 | 72 | 1.8e-07 | 1Score **> 36** indicates **identity** Score **> 17** indicates **homology** | U | R.AKDINQEVYNFLATAGAK.Y |
| 118721 | 143 | – | 160 | 651.6741 | 1952.0006 | 1952.0003 | 0.14 | 1 | 57 | 4.9e-06 | 1Score **> 36** indicates **identity** Score **> 16** indicates **homology** | U | R.AKDINQEVYNFLATAGAK.Y |
| 118722 | 143 | – | 160 | 651.6746 | 1952.0018 | 1952.0003 | 0.78 | 1 | 67 | 5e-07 | 1Score **> 36** indicates **identity** Score **> 17** indicates **homology** | U | R.AKDINQEVYNFLATAGAK.Y |
| 118724 | 143 | – | 160 | 977.0089 | 1952.0033 | 1952.0003 | 1.55 | 1 | 90 | 3.7e-09 | 1Score **> 36** indicates **identity** Score **> 18** indicates **homology** | U | R.AKDINQEVYNFLATAGAK.Y |
| 118726 | 143 | – | 160 | 651.6753 | 1952.0041 | 1952.0003 | 1.92 | 1 | 83 | 1.7e-08 | 1Score **> 36** indicates **identity** Score **> 18** indicates **homology** | U | R.AKDINQEVYNFLATAGAK.Y |
| 118728 | 143 | – | 160 | 977.0115 | 1952.0085 | 1952.0003 | 4.19 | 1 | 97 | 8.8e-10 | 1Score **> 36** indicates **identity** Score **> 19** indicates **homology** | U | R.AKDINQEVYNFLATAGAK.Y |
| 99017 | 145 | – | 160 | 877.4409 | 1752.8672 | 1752.8682 | -0.57 | 0 | 75 | 8.6e-08 | 1Score **> 35** indicates **identity** Score **> 17** indicates **homology** | U | K.DINQEVYNFLATAGAK.Y |
| 99025 | 145 | – | 160 | 585.2973 | 1752.8700 | 1752.8682 | 1.03 | 0 | 84 | 1.4e-08 | 1Score **> 35** indicates **identity** Score **> 18** indicates **homology** | U | K.DINQEVYNFLATAGAK.Y |
| 36700 | 234 | – | 245 | 617.3267 | 1232.6388 | 1232.6401 | -1.09 | 0 | 62 | 3.4e-06 | 1Score **> 34** indicates **identity** Score **> 20** indicates **homology** |  | K.LTGSLSGWTSPK.D |
| 36705 | 234 | – | 245 | 617.3276 | 1232.6406 | 1232.6401 | 0.38 | 0 | 65 | 2.6e-06 | 1Score **> 34** indicates **identity** Score **> 22** indicates **homology** |  | K.LTGSLSGWTSPK.D |
| 36707 | 234 | – | 245 | 617.3279 | 1232.6413 | 1232.6401 | 0.95 | 0 | 80 | 2e-07 | 1Score **> 34** indicates **identity** Score **> 26** indicates **homology** |  | K.LTGSLSGWTSPK.D |
| 36712 | 234 | – | 245 | 617.3283 | 1232.6421 | 1232.6401 | 1.58 | 0 | 72 | 1.2e-06 | 1Score **> 33** indicates **identity** Score **> 25** indicates **homology** |  | K.LTGSLSGWTSPK.D |
| 104222 | 234 | – | 250 | 601.3399 | 1800.9980 | 1800.9986 | -0.31 | 1 | 33 | 0.00087 | 1Score **> 34** indicates **identity** Score **> 15** indicates **homology** |  | K.LTGSLSGWTSPKDVILK.V |
| 104223 | 234 | – | 250 | 901.5067 | 1800.9988 | 1800.9986 | 0.16 | 1 | 41 | 0.00013 | 1Score **> 34** indicates **identity** Score **> 15** indicates **homology** |  | K.LTGSLSGWTSPKDVILK.V |
| 104224 | 234 | – | 250 | 601.3407 | 1801.0003 | 1800.9986 | 0.95 | 1 | 56 | 6.2e-06 | 1Score **> 34** indicates **identity** Score **> 16** indicates **homology** |  | K.LTGSLSGWTSPKDVILK.V |
| 104225 | 234 | – | 250 | 601.3408 | 1801.0004 | 1800.9986 | 1.04 | 1 | 63 | 1.4e-06 | 1Score **> 34** indicates **identity** Score **> 16** indicates **homology** |  | K.LTGSLSGWTSPKDVILK.V |
| 104226 | 234 | – | 250 | 601.3411 | 1801.0015 | 1800.9986 | 1.64 | 1 | 37 | 0.00035 | 1Score **> 34** indicates **identity** Score **> 15** indicates **homology** |  | K.LTGSLSGWTSPKDVILK.V |
| 3824 | 251 | – | 258 | 400.7655 | 799.5165 | 799.5167 | -0.30 | 0 | 49 | 0.00013 | 1Score **> 23** indicates **identity** |  | K.VAGILTVK.G |
| 3825 | 251 | – | 258 | 400.7656 | 799.5166 | 799.5167 | -0.23 | 0 | 56 | 2.8e-05 | 1Score **> 23** indicates **identity** |  | K.VAGILTVK.G |
| 3826 | 251 | – | 258 | 400.7656 | 799.5166 | 799.5167 | -0.22 | 0 | 53 | 6.4e-05 | 1Score **> 23** indicates **identity** |  | K.VAGILTVK.G |
| 196289 | 259 | – | 302 | 935.2305 | 4671.1163 | 4671.1390 | -4.87 | 0 | 41 | 0.00015 | 1Score **> 33** indicates **identity** Score **> 15** indicates **homology** |  | K.GGTGAIVEYHGPGVDSISCTGMATICNMGAEIGATTSVFPYNHR.M  + Deamidated (NQ); HNE (C); Oxidation (M) |
| 196399 | 259 | – | 302 | 943.4320 | 4712.1236 | 4712.1656 | -8.91 | 0 | 27 | 0.0031 | 1Score **> 32** indicates **identity** Score **> 14** indicates **homology** |  | K.GGTGAIVEYHGPGVDSISCTGMATICNMGAEIGATTSVFPYNHR.M  + Deamidated (NQ); HNE (H) |
| 196452 | 259 | – | 302 | 946.4353 | 4727.1403 | 4727.1765 | -7.65 | 0 | 22 | 0.0082 | 1Score **> 32** indicates **identity** Score **> 14** indicates **homology** |  | K.GGTGAIVEYHGPGVDSISCTGMATICNMGAEIGATTSVFPYNHR.M  + HNE (H); Oxidation (M) |
| 196454 | 259 | – | 302 | 946.4360 | 4727.1436 | 4727.1765 | -6.95 | 0 | 25 | 0.0045 | 1Score **> 33** indicates **identity** Score **> 14** indicates **homology** |  | K.GGTGAIVEYHGPGVDSISCTGMATICNMGAEIGATTSVFPYNHR.M  + HNE (H); Oxidation (M) |
| 13849 | 371 | – | 378 | 493.2596 | 984.5046 | 984.5029 | 1.75 | 0 | 30 | 0.011 | 1Score **> 30** indicates **identity** Score **> 23** indicates **homology** | U | K.EGWPLDIR.V |
| 108652 | 379 | – | 395 | 923.4079 | 1844.8012 | 1844.8033 | -1.13 | 0 | 115 | 1.6e-11 | 1Score **> 29** indicates **identity** Score **> 20** indicates **homology** | U | R.VGLIGSCTNSSYEDMGR.S |
| 108653 | 379 | – | 395 | 923.4090 | 1844.8035 | 1844.8033 | 0.12 | 0 | 82 | 1.9e-08 | 1Score **> 29** indicates **identity** Score **> 18** indicates **homology** | U | R.VGLIGSCTNSSYEDMGR.S |
| 108654 | 379 | – | 395 | 923.4093 | 1844.8040 | 1844.8033 | 0.39 | 0 | 105 | 1.4e-10 | 1Score **> 29** indicates **identity** Score **> 19** indicates **homology** | U | R.VGLIGSCTNSSYEDMGR.S |
| 98762 | 410 | – | 424 | 584.6295 | 1750.8666 | 1750.8672 | -0.33 | 1 | 46 | 0.0004 | 1Score **> 34** indicates **identity** Score **> 25** indicates **homology** | U | K.CKSQFTITPGSEQIR.A |
| 98765 | 410 | – | 424 | 584.6297 | 1750.8674 | 1750.8672 | 0.087 | 1 | 32 | 0.0015 | 1Score **> 34** indicates **identity** Score **> 16** indicates **homology** | U | K.CKSQFTITPGSEQIR.A |
| 98766 | 410 | – | 424 | 876.4417 | 1750.8689 | 1750.8672 | 0.94 | 1 | 73 | 1.3e-07 | 1Score **> 35** indicates **identity** Score **> 17** indicates **homology** | U | K.CKSQFTITPGSEQIR.A |
| 109159 | 410 | – | 424 | 925.9786 | 1849.9426 | 1849.9608 | -9.84 | 1 | 17 | 0.025 | 1Score **> 36** indicates **identity** Score **> 14** indicates **homology** | U | K.CKSQFTITPGSEQIR.A  + HNE (C) |
| 61835 | 412 | – | 424 | 732.3770 | 1462.7394 | 1462.7416 | -1.50 | 0 | 46 | 5.2e-05 | 1Score **> 35** indicates **identity** Score **> 15** indicates **homology** | U | K.SQFTITPGSEQIR.A |
| 61836 | 412 | – | 424 | 732.3776 | 1462.7407 | 1462.7416 | -0.60 | 0 | 15 | 0.041 | 1Score **> 35** indicates **identity** Score **> 13** indicates **homology** | U | K.SQFTITPGSEQIR.A |
| 61837 | 412 | – | 424 | 732.3780 | 1462.7414 | 1462.7416 | -0.15 | 0 | 53 | 1.2e-05 | 1Score **> 35** indicates **identity** Score **> 17** indicates **homology** | U | K.SQFTITPGSEQIR.A |
| 61838 | 412 | – | 424 | 732.3783 | 1462.7421 | 1462.7416 | 0.37 | 0 | 59 | 4e-06 | 1Score **> 35** indicates **identity** Score **> 17** indicates **homology** | U | K.SQFTITPGSEQIR.A |
| 61839 | 412 | – | 424 | 732.3788 | 1462.7431 | 1462.7416 | 1.01 | 0 | 65 | 1e-06 | 1Score **> 35** indicates **identity** Score **> 18** indicates **homology** | U | K.SQFTITPGSEQIR.A |
| 61840 | 412 | – | 424 | 732.3791 | 1462.7436 | 1462.7416 | 1.36 | 0 | 44 | 7.7e-05 | 1Score **> 35** indicates **identity** Score **> 15** indicates **homology** | U | K.SQFTITPGSEQIR.A |
| 67082 | 425 | – | 437 | 502.6079 | 1504.8019 | 1504.7998 | 1.41 | 1 | 45 | 0.00011 | 1Score **> 35** indicates **identity** Score **> 18** indicates **homology** | U | R.ATIERDGYAQILR.D |
| 67083 | 425 | – | 437 | 502.6079 | 1504.8019 | 1504.7998 | 1.44 | 1 | 36 | 0.00043 | 1Score **> 35** indicates **identity** Score **> 15** indicates **homology** | U | R.ATIERDGYAQILR.D |
| 10861 | 430 | – | 437 | 468.2499 | 934.4853 | 934.4872 | -2.04 | 0 | 21 | 0.01 | 1Score **> 31** indicates **identity** Score **> 14** indicates **homology** | U | R.DGYAQILR.D |
| 10865 | 430 | – | 437 | 468.2504 | 934.4862 | 934.4872 | -1.07 | 0 | 41 | 0.00016 | 1Score **> 30** indicates **identity** Score **> 15** indicates **homology** | U | R.DGYAQILR.D |
| 10867 | 430 | – | 437 | 468.2507 | 934.4868 | 934.4872 | -0.39 | 0 | 56 | 3.5e-05 | 1Score **> 30** indicates **identity** Score **> 24** indicates **homology** | U | R.DGYAQILR.D |
| 10868 | 430 | – | 437 | 468.2508 | 934.4870 | 934.4872 | -0.19 | 0 | 42 | 0.00053 | 1Score **> 30** indicates **identity** Score **> 21** indicates **homology** | U | R.DGYAQILR.D |
| 10871 | 430 | – | 437 | 468.2511 | 934.4876 | 934.4872 | 0.37 | 0 | 59 | 2.5e-05 | 1Score **> 30** indicates **identity** Score **> 26** indicates **homology** | U | R.DGYAQILR.D |
| 10872 | 430 | – | 437 | 468.2511 | 934.4876 | 934.4872 | 0.38 | 0 | 59 | 2.5e-05 | 1Score **> 30** indicates **identity** Score **> 25** indicates **homology** | U | R.DGYAQILR.D |
| 10878 | 430 | – | 437 | 468.2529 | 934.4912 | 934.4872 | 4.29 | 0 | 30 | 0.0085 | 1Score **> 30** indicates **identity** Score **> 22** indicates **homology** | U | R.DGYAQILR.D |
| 137843 | 438 | – | 457 | 720.0107 | 2157.0101 | 2157.0096 | 0.26 | 0 | 43 | 9e-05 | 1Score **> 34** indicates **identity** Score **> 15** indicates **homology** | U | R.DVGGIVLANACGPCIGQWDR.K |
| 137844 | 438 | – | 457 | 1079.5128 | 2157.0109 | 2157.0096 | 0.64 | 0 | 80 | 3.2e-08 | 1Score **> 34** indicates **identity** Score **> 17** indicates **homology** | U | R.DVGGIVLANACGPCIGQWDR.K |
| 137845 | 438 | – | 457 | 720.0112 | 2157.0116 | 2157.0096 | 0.96 | 0 | 64 | 1.1e-06 | 1Score **> 34** indicates **identity** Score **> 16** indicates **homology** | U | R.DVGGIVLANACGPCIGQWDR.K |
| 137846 | 438 | – | 457 | 1079.5134 | 2157.0122 | 2157.0096 | 1.23 | 0 | 76 | 6.7e-08 | 1Score **> 34** indicates **identity** Score **> 17** indicates **homology** | U | R.DVGGIVLANACGPCIGQWDR.K |
| 137847 | 438 | – | 457 | 720.0114 | 2157.0125 | 2157.0096 | 1.35 | 0 | 89 | 4.5e-09 | 1Score **> 34** indicates **identity** Score **> 18** indicates **homology** | U | R.DVGGIVLANACGPCIGQWDR.K |
| 137848 | 438 | – | 457 | 1079.5140 | 2157.0135 | 2157.0096 | 1.83 | 0 | 65 | 8.2e-07 | 1Score **> 34** indicates **identity** Score **> 17** indicates **homology** | U | R.DVGGIVLANACGPCIGQWDR.K |
| 147942 | 438 | – | 458 | 762.7082 | 2285.1028 | 2285.1045 | -0.74 | 1 | 32 | 0.0011 | 1Score **> 35** indicates **identity** Score **> 14** indicates **homology** | U | R.DVGGIVLANACGPCIGQWDRK.D |
| 147944 | 438 | – | 458 | 1143.5590 | 2285.1035 | 2285.1045 | -0.45 | 1 | 64 | 1.6e-05 | 1Score **> 35** indicates **identity** Score **> 28** indicates **homology** | U | R.DVGGIVLANACGPCIGQWDRK.D |
| 147946 | 438 | – | 458 | 762.7091 | 2285.1054 | 2285.1045 | 0.38 | 1 | 31 | 0.0011 | 1Score **> 35** indicates **identity** Score **> 14** indicates **homology** | U | R.DVGGIVLANACGPCIGQWDRK.D |
| 147947 | 438 | – | 458 | 762.7092 | 2285.1058 | 2285.1045 | 0.55 | 1 | 65 | 7.3e-07 | 1Score **> 35** indicates **identity** Score **> 17** indicates **homology** | U | R.DVGGIVLANACGPCIGQWDRK.D |
| 147948 | 438 | – | 458 | 762.7094 | 2285.1065 | 2285.1045 | 0.85 | 1 | 28 | 0.0023 | 1Score **> 36** indicates **identity** Score **> 14** indicates **homology** | U | R.DVGGIVLANACGPCIGQWDRK.D |
| 147949 | 438 | – | 458 | 1143.5605 | 2285.1065 | 2285.1045 | 0.88 | 1 | 59 | 2e-05 | 1Score **> 36** indicates **identity** Score **> 24** indicates **homology** | U | R.DVGGIVLANACGPCIGQWDRK.D |
| 147950 | 438 | – | 458 | 762.7096 | 2285.1070 | 2285.1045 | 1.07 | 1 | 55 | 6.5e-06 | 1Score **> 36** indicates **identity** Score **> 16** indicates **homology** | U | R.DVGGIVLANACGPCIGQWDRK.D |
| 147951 | 438 | – | 458 | 1143.5608 | 2285.1070 | 2285.1045 | 1.08 | 1 | 77 | 7e-07 | 1Score **> 36** indicates **identity** Score **> 28** indicates **homology** | U | R.DVGGIVLANACGPCIGQWDRK.D |
| 67517 | 462 | – | 474 | 755.4049 | 1508.7953 | 1508.7947 | 0.41 | 2 | 56 | 1.9e-05 | 1Score **> 35** indicates **identity** Score **> 21** indicates **homology** | U | K.KGEKNTIVTSYNR.N |
| 19669 | 466 | – | 474 | 534.2753 | 1066.5360 | 1066.5407 | -4.37 | 0 | 16 | 0.029 | 1Score **> 30** indicates **identity** Score **> 14** indicates **homology** | U | K.NTIVTSYNR.N |
| 19670 | 466 | – | 474 | 534.2767 | 1066.5388 | 1066.5407 | -1.84 | 0 | 31 | 0.0013 | 1Score **> 31** indicates **identity** Score **> 14** indicates **homology** | U | K.NTIVTSYNR.N |
| 19672 | 466 | – | 474 | 534.2776 | 1066.5406 | 1066.5407 | -0.14 | 0 | 42 | 0.00062 | 1Score **> 30** indicates **identity** Score **> 22** indicates **homology** | U | K.NTIVTSYNR.N |
| 19673 | 466 | – | 474 | 534.2776 | 1066.5406 | 1066.5407 | -0.081 | 0 | 23 | 0.0088 | 1Score **> 30** indicates **identity** Score **> 15** indicates **homology** | U | K.NTIVTSYNR.N |
| 19674 | 466 | – | 474 | 534.2780 | 1066.5415 | 1066.5407 | 0.78 | 0 | 44 | 0.00013 | 1Score **> 31** indicates **identity** Score **> 17** indicates **homology** | U | K.NTIVTSYNR.N |
| 187164 | 475 | – | 506 | 1119.2465 | 3354.7176 | 3354.7208 | -0.95 | 1 | 69 | 3.3e-07 | 1Score **> 37** indicates **identity** Score **> 17** indicates **homology** | U | R.NFTGRNDANPETHAFVTSPEIVTALAIAGTLK.F |
| 187167 | 475 | – | 506 | 839.6878 | 3354.7222 | 3354.7208 | 0.42 | 1 | 39 | 0.00024 | 1Score **> 37** indicates **identity** Score **> 15** indicates **homology** | U | R.NFTGRNDANPETHAFVTSPEIVTALAIAGTLK.F |
| 187168 | 475 | – | 506 | 839.6881 | 3354.7232 | 3354.7208 | 0.71 | 1 | 50 | 2.2e-05 | 1Score **> 37** indicates **identity** Score **> 16** indicates **homology** | U | R.NFTGRNDANPETHAFVTSPEIVTALAIAGTLK.F |
| 187169 | 475 | – | 506 | 839.6886 | 3354.7254 | 3354.7208 | 1.38 | 1 | 42 | 0.0001 | 1Score **> 37** indicates **identity** Score **> 15** indicates **homology** | U | R.NFTGRNDANPETHAFVTSPEIVTALAIAGTLK.F |
| 187185 | 475 | – | 506 | 1119.5860 | 3355.7362 | 3355.7048 | 9.35 | 1 | 45 | 5.7e-05 | 1Score **> 37** indicates **identity** Score **> 15** indicates **homology** | U | R.NFTGRNDANPETHAFVTSPEIVTALAIAGTLK.F  + Deamidated (NQ) |
| 173107 | 480 | – | 506 | 927.4866 | 2779.4379 | 2779.4392 | -0.49 | 0 | 67 | 5e-07 | 1Score **> 37** indicates **identity** Score **> 17** indicates **homology** | U | R.NDANPETHAFVTSPEIVTALAIAGTLK.F |
| 173108 | 480 | – | 506 | 927.4874 | 2779.4404 | 2779.4392 | 0.42 | 0 | 97 | 8.6e-10 | 1Score **> 37** indicates **identity** Score **> 19** indicates **homology** | U | R.NDANPETHAFVTSPEIVTALAIAGTLK.F |
| 173109 | 480 | – | 506 | 695.8676 | 2779.4414 | 2779.4392 | 0.81 | 0 | 54 | 8.5e-06 | 1Score **> 37** indicates **identity** Score **> 16** indicates **homology** | U | R.NDANPETHAFVTSPEIVTALAIAGTLK.F |
| 173110 | 480 | – | 506 | 695.8677 | 2779.4419 | 2779.4392 | 0.96 | 0 | 15 | 0.036 | 1Score **> 37** indicates **identity** Score **> 13** indicates **homology** | U | R.NDANPETHAFVTSPEIVTALAIAGTLK.F |
| 173111 | 480 | – | 506 | 1390.7289 | 2779.4432 | 2779.4392 | 1.44 | 0 | 110 | 5.3e-11 | 1Score **> 37** indicates **identity** Score **> 19** indicates **homology** | U | R.NDANPETHAFVTSPEIVTALAIAGTLK.F |
| 173115 | 480 | – | 506 | 695.8695 | 2779.4488 | 2779.4392 | 3.46 | 0 | 51 | 1.8e-05 | 1Score **> 37** indicates **identity** Score **> 16** indicates **homology** | U | R.NDANPETHAFVTSPEIVTALAIAGTLK.F |
| 173116 | 480 | – | 506 | 927.4905 | 2779.4496 | 2779.4392 | 3.74 | 0 | 65 | 8.4e-07 | 1Score **> 37** indicates **identity** Score **> 17** indicates **homology** | U | R.NDANPETHAFVTSPEIVTALAIAGTLK.F |
| 173117 | 480 | – | 506 | 927.4905 | 2779.4498 | 2779.4392 | 3.80 | 0 | 73 | 1.6e-07 | 1Score **> 37** indicates **identity** Score **> 17** indicates **homology** | U | R.NDANPETHAFVTSPEIVTALAIAGTLK.F |
| 173132 | 480 | – | 506 | 927.4921 | 2779.4546 | 2779.4392 | 5.52 | 0 | 62 | 1.6e-06 | 1Score **> 37** indicates **identity** Score **> 16** indicates **homology** | U | R.NDANPETHAFVTSPEIVTALAIAGTLK.F |
| 40595 | 507 | – | 517 | 634.8108 | 1267.6071 | 1267.6085 | -1.08 | 0 | 40 | 0.00016 | 1Score **> 32** indicates **identity** Score **> 15** indicates **homology** | U | K.FNPETDFLTGK.D |
| 40596 | 507 | – | 517 | 634.8111 | 1267.6077 | 1267.6085 | -0.59 | 0 | 24 | 0.0056 | 1Score **> 31** indicates **identity** Score **> 14** indicates **homology** | U | K.FNPETDFLTGK.D |
| 40597 | 507 | – | 517 | 634.8113 | 1267.6080 | 1267.6085 | -0.39 | 0 | 38 | 0.00027 | 1Score **> 31** indicates **identity** Score **> 15** indicates **homology** | U | K.FNPETDFLTGK.D |
| 40598 | 507 | – | 517 | 634.8113 | 1267.6081 | 1267.6085 | -0.30 | 0 | 41 | 0.00026 | 1Score **> 31** indicates **identity** Score **> 18** indicates **homology** | U | K.FNPETDFLTGK.D |
| 40603 | 507 | – | 517 | 634.8133 | 1267.6120 | 1267.6085 | 2.78 | 0 | 19 | 0.018 | 1Score **> 32** indicates **identity** Score **> 14** indicates **homology** | U | K.FNPETDFLTGK.D |
| 75197 | 507 | – | 520 | 784.8824 | 1567.7502 | 1567.7518 | -1.06 | 1 | 42 | 0.00012 | 1Score **> 33** indicates **identity** Score **> 15** indicates **homology** | U | K.FNPETDFLTGKDGK.K |
| 75200 | 507 | – | 520 | 523.5918 | 1567.7536 | 1567.7518 | 1.10 | 1 | 47 | 3.9e-05 | 1Score **> 33** indicates **identity** Score **> 15** indicates **homology** | U | K.FNPETDFLTGKDGK.K |
| 75202 | 507 | – | 520 | 784.8846 | 1567.7546 | 1567.7518 | 1.74 | 1 | 56 | 5.3e-06 | 1Score **> 33** indicates **identity** Score **> 16** indicates **homology** | U | K.FNPETDFLTGKDGK.K |
| 91995 | 507 | – | 521 | 424.9691 | 1695.8474 | 1695.8468 | 0.37 | 2 | 30 | 0.0016 | 1Score **> 35** indicates **identity** Score **> 14** indicates **homology** | U | K.FNPETDFLTGKDGKK.F |
| 91996 | 507 | – | 521 | 424.9692 | 1695.8476 | 1695.8468 | 0.47 | 2 | 21 | 0.012 | 1Score **> 35** indicates **identity** Score **> 14** indicates **homology** | U | K.FNPETDFLTGKDGKK.F |
| 91997 | 507 | – | 521 | 566.2900 | 1695.8481 | 1695.8468 | 0.77 | 2 | 41 | 0.00013 | 1Score **> 35** indicates **identity** Score **> 15** indicates **homology** | U | K.FNPETDFLTGKDGKK.F |
| 91998 | 507 | – | 521 | 566.2900 | 1695.8483 | 1695.8468 | 0.88 | 2 | 47 | 4.2e-05 | 1Score **> 35** indicates **identity** Score **> 15** indicates **homology** | U | K.FNPETDFLTGKDGKK.F |
| 92002 | 507 | – | 521 | 566.2907 | 1695.8502 | 1695.8468 | 1.98 | 2 | 46 | 5e-05 | 1Score **> 34** indicates **identity** Score **> 15** indicates **homology** | U | K.FNPETDFLTGKDGKK.F |
| 66483 | 522 | – | 534 | 500.9284 | 1499.7633 | 1499.7620 | 0.85 | 1 | 15 | 0.037 | 1Score **> 34** indicates **identity** Score **> 13** indicates **homology** | U | K.FKLEAPDADELPR.S |
| 152872 | 535 | – | 555 | 591.2631 | 2361.0233 | 2361.0258 | -1.04 | 1 | 19 | 0.016 | 1Score **> 28** indicates **identity** Score **> 14** indicates **homology** | U | R.SDFDPGQDTYQHPPKDSSGQR.V |
| 186841 | 535 | – | 564 | 1111.1823 | 3330.5251 | 3330.5138 | 3.41 | 2 | 14 | 0.046 | 1Score **> 34** indicates **identity** Score **> 13** indicates **homology** | U | R.SDFDPGQDTYQHPPKDSSGQRVDVSPTSQR.L |
| 81885 | 550 | – | 564 | 540.2631 | 1617.7675 | 1617.7707 | -1.99 | 1 | 30 | 0.0014 | 1Score **> 33** indicates **identity** Score **> 14** indicates **homology** | U | K.DSSGQRVDVSPTSQR.L |
| 81888 | 550 | – | 564 | 540.2638 | 1617.7695 | 1617.7707 | -0.75 | 1 | 17 | 0.028 | 1Score **> 33** indicates **identity** Score **> 14** indicates **homology** | U | K.DSSGQRVDVSPTSQR.L |
| 81889 | 550 | – | 564 | 809.8931 | 1617.7717 | 1617.7707 | 0.60 | 1 | 19 | 0.02 | 1Score **> 33** indicates **identity** Score **> 15** indicates **homology** | U | K.DSSGQRVDVSPTSQR.L |
| 14065 | 556 | – | 564 | 494.7563 | 987.4981 | 987.4985 | -0.42 | 0 | 41 | 0.0026 | 1Score **> 30** indicates **identity** Score **> 28** indicates **homology** | U | R.VDVSPTSQR.L |
| 14070 | 556 | – | 564 | 494.7566 | 987.4986 | 987.4985 | 0.082 | 0 | 51 | 0.0005 | 1Score **> 30** indicates **identity** | U | R.VDVSPTSQR.L |
| 14074 | 556 | – | 564 | 494.7572 | 987.4998 | 987.4985 | 1.26 | 0 | 30 | 0.042 | 1Score **> 30** indicates **identity** Score **> 28** indicates **homology** | U | R.VDVSPTSQR.L |
| 172570 | 565 | – | 587 | 923.8384 | 2768.4934 | 2768.5000 | -2.37 | 2 | 47 | 4.1e-05 | 1Score **> 36** indicates **identity** Score **> 15** indicates **homology** | U | R.LQLLEPFDKWDGKDLEDLQILIK.V |
| 172571 | 565 | – | 587 | 923.8438 | 2768.5094 | 2768.5000 | 3.40 | 2 | 22 | 0.0078 | 1Score **> 35** indicates **identity** Score **> 14** indicates **homology** | U | R.LQLLEPFDKWDGKDLEDLQILIK.V |
| 172591 | 565 | – | 587 | 693.3793 | 2769.4882 | 2769.4840 | 1.52 | 2 | 23 | 0.0072 | 1Score **> 36** indicates **identity** Score **> 14** indicates **homology** | U | R.LQLLEPFDKWDGKDLEDLQILIK.V  + Deamidated (NQ) |
| 73483 | 592 | – | 605 | 778.8767 | 1555.7389 | 1555.7453 | -4.10 | 0 | 37 | 0.00035 | 1Score **> 32** indicates **identity** Score **> 15** indicates **homology** | U | K.CTTDHISAAGPWLK.F |
| 73484 | 592 | – | 605 | 778.8769 | 1555.7392 | 1555.7453 | -3.95 | 0 | 34 | 0.00089 | 1Score **> 32** indicates **identity** Score **> 16** indicates **homology** | U | K.CTTDHISAAGPWLK.F |
| 162376 | 606 | – | 628 | 631.3419 | 2521.3386 | 2521.3401 | -0.59 | 1 | 14 | 0.047 | 1Score **> 36** indicates **identity** Score **> 13** indicates **homology** | U | K.FRGHLDNISNNLLIGAINIENGK.A |
| 162377 | 606 | – | 628 | 631.3420 | 2521.3391 | 2521.3401 | -0.40 | 1 | 42 | 0.00011 | 1Score **> 36** indicates **identity** Score **> 15** indicates **homology** | U | K.FRGHLDNISNNLLIGAINIENGK.A |
| 162378 | 606 | – | 628 | 631.3428 | 2521.3420 | 2521.3401 | 0.76 | 1 | 40 | 0.00016 | 1Score **> 36** indicates **identity** Score **> 15** indicates **homology** | U | K.FRGHLDNISNNLLIGAINIENGK.A |
| 162408 | 606 | – | 628 | 631.5935 | 2522.3449 | 2522.3241 | 8.26 | 1 | 45 | 6.6e-05 | 1Score **> 37** indicates **identity** Score **> 15** indicates **homology** | U | K.FRGHLDNISNNLLIGAINIENGK.A  + Deamidated (NQ) |
| 181609 | 606 | – | 633 | 763.4101 | 3049.6115 | 3049.6057 | 1.91 | 2 | 50 | 2e-05 | 1Score **> 37** indicates **identity** Score **> 16** indicates **homology** | U | K.FRGHLDNISNNLLIGAINIENGKANSVR.N  + Deamidated (NQ) |
| 181610 | 606 | – | 633 | 610.9301 | 3049.6141 | 3049.6057 | 2.78 | 2 | 20 | 0.012 | 1Score **> 37** indicates **identity** Score **> 14** indicates **homology** | U | K.FRGHLDNISNNLLIGAINIENGKANSVR.N  + Deamidated (NQ) |
| 181611 | 606 | – | 633 | 610.9309 | 3049.6182 | 3049.6057 | 4.10 | 2 | 15 | 0.037 | 1Score **> 36** indicates **identity** Score **> 13** indicates **homology** | U | K.FRGHLDNISNNLLIGAINIENGKANSVR.N  + Deamidated (NQ) |
| 181612 | 606 | – | 633 | 610.9313 | 3049.6203 | 3049.6057 | 4.81 | 2 | 22 | 0.0091 | 1Score **> 36** indicates **identity** Score **> 14** indicates **homology** | U | K.FRGHLDNISNNLLIGAINIENGKANSVR.N  + Deamidated (NQ) |
| 181613 | 606 | – | 633 | 763.4127 | 3049.6218 | 3049.6057 | 5.28 | 2 | 37 | 0.00036 | 1Score **> 36** indicates **identity** Score **> 15** indicates **homology** | U | K.FRGHLDNISNNLLIGAINIENGKANSVR.N  + Deamidated (NQ) |
| 181614 | 606 | – | 633 | 763.4155 | 3049.6329 | 3049.6057 | 8.92 | 2 | 34 | 0.00071 | 1Score **> 36** indicates **identity** Score **> 15** indicates **homology** | U | K.FRGHLDNISNNLLIGAINIENGKANSVR.N  + Deamidated (NQ) |
| 143107 | 608 | – | 628 | 740.3953 | 2218.1640 | 2218.1705 | -2.93 | 0 | 43 | 9.2e-05 | 1Score **> 36** indicates **identity** Score **> 15** indicates **homology** | U | R.GHLDNISNNLLIGAINIENGK.A |
| 143130 | 608 | – | 628 | 740.3987 | 2218.1743 | 2218.1705 | 1.67 | 0 | 50 | 2.4e-05 | 1Score **> 36** indicates **identity** Score **> 16** indicates **homology** | U | R.GHLDNISNNLLIGAINIENGK.A |
| 143141 | 608 | – | 628 | 740.4004 | 2218.1795 | 2218.1705 | 4.03 | 0 | 31 | 0.0013 | 1Score **> 36** indicates **identity** Score **> 14** indicates **homology** | U | R.GHLDNISNNLLIGAINIENGK.A |
| 143145 | 608 | – | 628 | 740.4015 | 2218.1828 | 2218.1705 | 5.51 | 0 | 19 | 0.015 | 1Score **> 36** indicates **identity** Score **> 14** indicates **homology** | U | R.GHLDNISNNLLIGAINIENGK.A |
| 143200 | 608 | – | 628 | 1110.5931 | 2219.1717 | 2219.1546 | 7.73 | 0 | 41 | 0.00014 | 1Score **> 36** indicates **identity** Score **> 15** indicates **homology** | U | R.GHLDNISNNLLIGAINIENGK.A  + Deamidated (NQ) |
| 143205 | 608 | – | 628 | 1110.5945 | 2219.1744 | 2219.1546 | 8.92 | 0 | 45 | 6.5e-05 | 1Score **> 36** indicates **identity** Score **> 15** indicates **homology** | U | R.GHLDNISNNLLIGAINIENGK.A  + Deamidated (NQ) |
| 171702 | 608 | – | 633 | 687.3681 | 2745.4432 | 2745.4521 | -3.26 | 1 | 16 | 0.034 | 1Score **> 37** indicates **identity** Score **> 13** indicates **homology** | U | R.GHLDNISNNLLIGAINIENGKANSVR.N |
| 171704 | 608 | – | 633 | 687.3694 | 2745.4484 | 2745.4521 | -1.37 | 1 | 18 | 0.02 | 1Score **> 37** indicates **identity** Score **> 14** indicates **homology** | U | R.GHLDNISNNLLIGAINIENGKANSVR.N |
| 171753 | 608 | – | 633 | 687.6146 | 2746.4295 | 2746.4361 | -2.43 | 1 | 35 | 0.00057 | 1Score **> 37** indicates **identity** Score **> 15** indicates **homology** | U | R.GHLDNISNNLLIGAINIENGKANSVR.N  + Deamidated (NQ) |
| 171755 | 608 | – | 633 | 916.4890 | 2746.4451 | 2746.4361 | 3.27 | 1 | 83 | 1.7e-08 | 1Score **> 37** indicates **identity** Score **> 18** indicates **homology** | U | R.GHLDNISNNLLIGAINIENGKANSVR.N  + Deamidated (NQ) |
| 171756 | 608 | – | 633 | 687.6189 | 2746.4466 | 2746.4361 | 3.79 | 1 | 43 | 9.2e-05 | 1Score **> 37** indicates **identity** Score **> 15** indicates **homology** | U | R.GHLDNISNNLLIGAINIENGKANSVR.N  + Deamidated (NQ) |
| 171757 | 608 | – | 633 | 916.4896 | 2746.4470 | 2746.4361 | 3.97 | 1 | 71 | 2.2e-07 | 1Score **> 37** indicates **identity** Score **> 17** indicates **homology** | U | R.GHLDNISNNLLIGAINIENGKANSVR.N  + Deamidated (NQ) |
| 171758 | 608 | – | 633 | 687.6190 | 2746.4470 | 2746.4361 | 3.97 | 1 | 39 | 0.00023 | 1Score **> 37** indicates **identity** Score **> 15** indicates **homology** | U | R.GHLDNISNNLLIGAINIENGKANSVR.N  + Deamidated (NQ) |
| 171760 | 608 | – | 633 | 916.4902 | 2746.4487 | 2746.4361 | 4.59 | 1 | 75 | 8.4e-08 | 1Score **> 37** indicates **identity** Score **> 17** indicates **homology** | U | R.GHLDNISNNLLIGAINIENGKANSVR.N  + Deamidated (NQ) |
| 171761 | 608 | – | 633 | 687.6200 | 2746.4509 | 2746.4361 | 5.36 | 1 | 42 | 0.00012 | 1Score **> 37** indicates **identity** Score **> 15** indicates **homology** | U | R.GHLDNISNNLLIGAINIENGKANSVR.N  + Deamidated (NQ) |
| 171833 | 608 | – | 633 | 687.8670 | 2747.4389 | 2747.4202 | 6.83 | 1 | 15 | 0.04 | 1Score **> 37** indicates **identity** Score **> 13** indicates **homology** | U | R.GHLDNISNNLLIGAINIENGKANSVR.N  + 2 Deamidated (NQ) |
| 79609 | 634 | – | 648 | 801.3973 | 1600.7800 | 1600.7845 | -2.86 | 0 | 38 | 0.00044 | 1Score **> 34** indicates **identity** Score **> 17** indicates **homology** | U | R.NAVTQEFGPVPDTAR.Y |
| 79612 | 634 | – | 648 | 801.3996 | 1600.7847 | 1600.7845 | 0.11 | 0 | 67 | 4.9e-07 | 1Score **> 34** indicates **identity** Score **> 17** indicates **homology** | U | R.NAVTQEFGPVPDTAR.Y |
| 79614 | 634 | – | 648 | 801.4000 | 1600.7855 | 1600.7845 | 0.59 | 0 | 91 | 3.6e-09 | 1Score **> 34** indicates **identity** Score **> 19** indicates **homology** | U | R.NAVTQEFGPVPDTAR.Y |
| 79617 | 634 | – | 648 | 801.4002 | 1600.7858 | 1600.7845 | 0.81 | 0 | 80 | 7e-08 | 1Score **> 34** indicates **identity** Score **> 21** indicates **homology** | U | R.NAVTQEFGPVPDTAR.Y |
| 79619 | 634 | – | 648 | 801.4004 | 1600.7863 | 1600.7845 | 1.11 | 0 | 84 | 1.5e-08 | 1Score **> 34** indicates **identity** Score **> 19** indicates **homology** | U | R.NAVTQEFGPVPDTAR.Y |
| 79620 | 634 | – | 648 | 534.6029 | 1600.7869 | 1600.7845 | 1.46 | 0 | 47 | 3.6e-05 | 1Score **> 34** indicates **identity** Score **> 15** indicates **homology** | U | R.NAVTQEFGPVPDTAR.Y |
| 79621 | 634 | – | 648 | 534.6031 | 1600.7876 | 1600.7845 | 1.92 | 0 | 58 | 3.5e-06 | 1Score **> 34** indicates **identity** Score **> 16** indicates **homology** | U | R.NAVTQEFGPVPDTAR.Y |
| 79622 | 634 | – | 648 | 801.4012 | 1600.7879 | 1600.7845 | 2.11 | 0 | 37 | 0.00033 | 1Score **> 34** indicates **identity** Score **> 15** indicates **homology** | U | R.NAVTQEFGPVPDTAR.Y |
| 79624 | 634 | – | 648 | 801.4058 | 1600.7971 | 1600.7845 | 7.82 | 0 | 36 | 0.04 | 1Score **> 34** indicates **identity** | U | R.NAVTQEFGPVPDTAR.Y |
| 79625 | 634 | – | 648 | 801.4059 | 1600.7972 | 1600.7845 | 7.90 | 0 | 39 | 0.017 | 1Score **> 34** indicates **identity** | U | R.NAVTQEFGPVPDTAR.Y |
| 79712 | 634 | – | 648 | 801.8903 | 1601.7660 | 1601.7686 | -1.62 | 0 | 78 | 5.4e-08 | 1Score **> 33** indicates **identity** Score **> 18** indicates **homology** | U | R.NAVTQEFGPVPDTAR.Y  + Deamidated (NQ) |
| 79715 | 634 | – | 648 | 801.8926 | 1601.7707 | 1601.7686 | 1.35 | 0 | 60 | 6.1e-06 | 1Score **> 33** indicates **identity** Score **> 21** indicates **homology** | U | R.NAVTQEFGPVPDTAR.Y  + Deamidated (NQ) |
| 79716 | 634 | – | 648 | 801.8962 | 1601.7778 | 1601.7686 | 5.75 | 0 | 41 | 0.00013 | 1Score **> 33** indicates **identity** Score **> 15** indicates **homology** | U | R.NAVTQEFGPVPDTAR.Y  + Deamidated (NQ) |
| 88282 | 657 | – | 671 | 834.3864 | 1666.7582 | 1666.7587 | -0.34 | 0 | 51 | 1.7e-05 | 1Score **> 30** indicates **identity** Score **> 16** indicates **homology** | U | R.WVVIGDENYGEGSSR.E |
| 88283 | 657 | – | 671 | 834.3870 | 1666.7594 | 1666.7587 | 0.39 | 0 | 62 | 1.4e-06 | 1Score **> 30** indicates **identity** Score **> 16** indicates **homology** | U | R.WVVIGDENYGEGSSR.E |
| 88284 | 657 | – | 671 | 834.3871 | 1666.7596 | 1666.7587 | 0.52 | 0 | 15 | 0.04 | 1Score **> 30** indicates **identity** Score **> 13** indicates **homology** | U | R.WVVIGDENYGEGSSR.E |
| 88285 | 657 | – | 671 | 834.3872 | 1666.7599 | 1666.7587 | 0.69 | 0 | 63 | 1.3e-06 | 1Score **> 30** indicates **identity** Score **> 16** indicates **homology** | U | R.WVVIGDENYGEGSSR.E |
| 88286 | 657 | – | 671 | 834.3879 | 1666.7613 | 1666.7587 | 1.55 | 0 | 66 | 6.9e-07 | 1Score **> 30** indicates **identity** Score **> 17** indicates **homology** | U | R.WVVIGDENYGEGSSR.E |
| 88287 | 657 | – | 671 | 834.3884 | 1666.7623 | 1666.7587 | 2.14 | 0 | 56 | 6.2e-06 | 1Score **> 30** indicates **identity** Score **> 16** indicates **homology** | U | R.WVVIGDENYGEGSSR.E |
| 88288 | 657 | – | 671 | 834.3900 | 1666.7654 | 1666.7587 | 3.99 | 0 | 62 | 1.6e-06 | 1Score **> 31** indicates **identity** Score **> 16** indicates **homology** | U | R.WVVIGDENYGEGSSR.E |
| 88289 | 657 | – | 671 | 834.3910 | 1666.7675 | 1666.7587 | 5.28 | 0 | 36 | 0.00045 | 1Score **> 31** indicates **identity** Score **> 15** indicates **homology** | U | R.WVVIGDENYGEGSSR.E |
| 164640 | 657 | – | 679 | 857.7456 | 2570.2150 | 2570.2150 | 0.036 | 1 | 21 | 0.01 | 1Score **> 35** indicates **identity** Score **> 14** indicates **homology** | U | R.WVVIGDENYGEGSSREHAALEPR.H |
| 164645 | 657 | – | 679 | 857.7468 | 2570.2185 | 2570.2150 | 1.39 | 1 | 25 | 0.0045 | 1Score **> 35** indicates **identity** Score **> 14** indicates **homology** | U | R.WVVIGDENYGEGSSREHAALEPR.H |
| 114368 | 701 | – | 717 | 636.3359 | 1905.9859 | 1905.9836 | 1.23 | 1 | 34 | 0.00059 | 1Score **> 36** indicates **identity** Score **> 15** indicates **homology** | U | K.KQGLLPLTFADPSDYNK.I |
| 114372 | 701 | – | 717 | 636.3370 | 1905.9892 | 1905.9836 | 2.93 | 1 | 27 | 0.0032 | 1Score **> 36** indicates **identity** Score **> 14** indicates **homology** | U | K.KQGLLPLTFADPSDYNK.I |
| 95785 | 724 | – | 739 | 432.2624 | 1725.0203 | 1725.0189 | 0.83 | 2 | 35 | 0.00049 | 1Score **> 31** indicates **identity** Score **> 15** indicates **homology** | U | K.LTIQGLKDFAPGKPLK.C |
| 95786 | 724 | – | 739 | 432.2624 | 1725.0203 | 1725.0189 | 0.85 | 2 | 26 | 0.0038 | 1Score **> 31** indicates **identity** Score **> 14** indicates **homology** | U | K.LTIQGLKDFAPGKPLK.C |
| 13078 | 731 | – | 739 | 486.7797 | 971.5448 | 971.5440 | 0.84 | 1 | 15 | 0.038 | 1Score **> 33** indicates **identity** Score **> 13** indicates **homology** | U | K.DFAPGKPLK.C |
| 188421 | 740 | – | 767 | 857.1816 | 3424.6974 | 3424.6986 | -0.37 | 1 | 52 | 1.4e-05 | 1Score **> 38** indicates **identity** Score **> 16** indicates **homology** | U | K.CVIKHPNGTQETILLNHTFNETQIEWFR.A |
| 188422 | 740 | – | 767 | 685.9469 | 3424.6981 | 3424.6986 | -0.16 | 1 | 31 | 0.0013 | 1Score **> 38** indicates **identity** Score **> 14** indicates **homology** | U | K.CVIKHPNGTQETILLNHTFNETQIEWFR.A |
| 188424 | 740 | – | 767 | 685.9476 | 3424.7015 | 3424.6986 | 0.84 | 1 | 18 | 0.023 | 1Score **> 38** indicates **identity** Score **> 14** indicates **homology** | U | K.CVIKHPNGTQETILLNHTFNETQIEWFR.A |
| 188457 | 740 | – | 767 | 857.4281 | 3425.6831 | 3425.6826 | 0.13 | 1 | 47 | 3.5e-05 | 1Score **> 37** indicates **identity** Score **> 15** indicates **homology** | U | K.CVIKHPNGTQETILLNHTFNETQIEWFR.A  + Deamidated (NQ) |
| 188460 | 740 | – | 767 | 857.4288 | 3425.6860 | 3425.6826 | 0.97 | 1 | 55 | 6.7e-06 | 1Score **> 37** indicates **identity** Score **> 16** indicates **homology** | U | K.CVIKHPNGTQETILLNHTFNETQIEWFR.A  + Deamidated (NQ) |
| 177813 | 744 | – | 767 | 732.1090 | 2924.4070 | 2924.4205 | -4.62 | 0 | 16 | 0.032 | 1Score **> 36** indicates **identity** Score **> 13** indicates **homology** | U | K.HPNGTQETILLNHTFNETQIEWFR.A |

---

```
ID   ACON_MOUSE              Reviewed;         780 AA.
AC   Q99KI0; Q3UDK9; Q3ULG9; Q3UNH7; Q505P4;
DT   01-FEB-2005, integrated into UniProtKB/Swiss-Prot.
DT   01-JUN-2001, sequence version 1.
DT   28-JUN-2023, entry version 164.
DE   RecName: Full=Aconitate hydratase, mitochondrial;
DE            Short=Aconitase;
DE            EC=4.2.1.3 {ECO:0000250|UniProtKB:P16276};
DE   AltName: Full=Citrate hydro-lyase;
DE   Flags: Precursor;
GN   Name=Aco2;
OS   Mus musculus (Mouse).
OC   Eukaryota; Metazoa; Chordata; Craniata; Vertebrata; Euteleostomi; Mammalia;
OC   Eutheria; Euarchontoglires; Glires; Rodentia; Myomorpha; Muroidea; Muridae;
OC   Murinae; Mus; Mus.
OX   NCBI_TaxID=10090;
RN   [1]
RP   NUCLEOTIDE SEQUENCE [LARGE SCALE MRNA].
RC   STRAIN=C57BL/6J; TISSUE=Bone marrow, and Kidney;
RX   PubMed=16141072; DOI=10.1126/science.1112014;
RA   Carninci P., Kasukawa T., Katayama S., Gough J., Frith M.C., Maeda N.,
RA   Oyama R., Ravasi T., Lenhard B., Wells C., Kodzius R., Shimokawa K.,
RA   Bajic V.B., Brenner S.E., Batalov S., Forrest A.R., Zavolan M., Davis M.J.,
RA   Wilming L.G., Aidinis V., Allen J.E., Ambesi-Impiombato A., Apweiler R.,
RA   Aturaliya R.N., Bailey T.L., Bansal M., Baxter L., Beisel K.W., Bersano T.,
RA   Bono H., Chalk A.M., Chiu K.P., Choudhary V., Christoffels A.,
RA   Clutterbuck D.R., Crowe M.L., Dalla E., Dalrymple B.P., de Bono B.,
RA   Della Gatta G., di Bernardo D., Down T., Engstrom P., Fagiolini M.,
RA   Faulkner G., Fletcher C.F., Fukushima T., Furuno M., Futaki S.,
RA   Gariboldi M., Georgii-Hemming P., Gingeras T.R., Gojobori T., Green R.E.,
RA   Gustincich S., Harbers M., Hayashi Y., Hensch T.K., Hirokawa N., Hill D.,
RA   Huminiecki L., Iacono M., Ikeo K., Iwama A., Ishikawa T., Jakt M.,
RA   Kanapin A., Katoh M., Kawasawa Y., Kelso J., Kitamura H., Kitano H.,
RA   Kollias G., Krishnan S.P., Kruger A., Kummerfeld S.K., Kurochkin I.V.,
RA   Lareau L.F., Lazarevic D., Lipovich L., Liu J., Liuni S., McWilliam S.,
RA   Madan Babu M., Madera M., Marchionni L., Matsuda H., Matsuzawa S., Miki H.,
RA   Mignone F., Miyake S., Morris K., Mottagui-Tabar S., Mulder N., Nakano N.,
RA   Nakauchi H., Ng P., Nilsson R., Nishiguchi S., Nishikawa S., Nori F.,
RA   Ohara O., Okazaki Y., Orlando V., Pang K.C., Pavan W.J., Pavesi G.,
RA   Pesole G., Petrovsky N., Piazza S., Reed J., Reid J.F., Ring B.Z.,
RA   Ringwald M., Rost B., Ruan Y., Salzberg S.L., Sandelin A., Schneider C.,
RA   Schoenbach C., Sekiguchi K., Semple C.A., Seno S., Sessa L., Sheng Y.,
RA   Shibata Y., Shimada H., Shimada K., Silva D., Sinclair B., Sperling S.,
RA   Stupka E., Sugiura K., Sultana R., Takenaka Y., Taki K., Tammoja K.,
RA   Tan S.L., Tang S., Taylor M.S., Tegner J., Teichmann S.A., Ueda H.R.,
RA   van Nimwegen E., Verardo R., Wei C.L., Yagi K., Yamanishi H.,
RA   Zabarovsky E., Zhu S., Zimmer A., Hide W., Bult C., Grimmond S.M.,
RA   Teasdale R.D., Liu E.T., Brusic V., Quackenbush J., Wahlestedt C.,
RA   Mattick J.S., Hume D.A., Kai C., Sasaki D., Tomaru Y., Fukuda S.,
RA   Kanamori-Katayama M., Suzuki M., Aoki J., Arakawa T., Iida J., Imamura K.,
RA   Itoh M., Kato T., Kawaji H., Kawagashira N., Kawashima T., Kojima M.,
RA   Kondo S., Konno H., Nakano K., Ninomiya N., Nishio T., Okada M., Plessy C.,
RA   Shibata K., Shiraki T., Suzuki S., Tagami M., Waki K., Watahiki A.,
RA   Okamura-Oho Y., Suzuki H., Kawai J., Hayashizaki Y.;
RT   "The transcriptional landscape of the mammalian genome.";
RL   Science 309:1559-1563(2005).
RN   [2]
RP   NUCLEOTIDE SEQUENCE [LARGE SCALE MRNA].
RC   STRAIN=FVB/N; TISSUE=Kidney, and Mammary tumor;
RX   PubMed=15489334; DOI=10.1101/gr.2596504;
RG   The MGC Project Team;
RT   "The status, quality, and expansion of the NIH full-length cDNA project:
RT   the Mammalian Gene Collection (MGC).";
RL   Genome Res. 14:2121-2127(2004).
RN   [3]
RP   PROTEIN SEQUENCE OF 32-56; 59-84; 96-138; 143-160; 234-245; 251-258;
RP   313-323; 371-395; 402-409; 412-424; 430-457; 466-474; 480-517; 522-587;
RP   592-605; 608-628; 634-648; 657-671; 694-739 AND 744-767, AND IDENTIFICATION
RP   BY MASS SPECTROMETRY.
RC   STRAIN=C57BL/6J, and OF1; TISSUE=Brain, and Hippocampus;
RA   Lubec G., Klug S., Kang S.U., Sunyer B., Chen W.-Q.;
RL   Submitted (JAN-2009) to UniProtKB.
RN   [4]
RP   PHOSPHORYLATION [LARGE SCALE ANALYSIS] AT SER-670, AND IDENTIFICATION BY
RP   MASS SPECTROMETRY [LARGE SCALE ANALYSIS].
RC   TISSUE=Brain, Brown adipose tissue, Heart, Kidney, Liver, Lung,
RC   Pancreas, Spleen, and Testis;
RX   PubMed=21183079; DOI=10.1016/j.cell.2010.12.001;
RA   Huttlin E.L., Jedrychowski M.P., Elias J.E., Goswami T., Rad R.,
RA   Beausoleil S.A., Villen J., Haas W., Sowa M.E., Gygi S.P.;
RT   "A tissue-specific atlas of mouse protein phosphorylation and expression.";
RL   Cell 143:1174-1189(2010).
RN   [5]
RP   ACETYLATION [LARGE SCALE ANALYSIS] AT LYS-50 AND LYS-144, SUCCINYLATION
RP   [LARGE SCALE ANALYSIS] AT LYS-31; LYS-50; LYS-138; LYS-144; LYS-233;
RP   LYS-411; LYS-517; LYS-523; LYS-549; LYS-573; LYS-577; LYS-591; LYS-605;
RP   LYS-628; LYS-689; LYS-723 AND LYS-730, AND IDENTIFICATION BY MASS
RP   SPECTROMETRY [LARGE SCALE ANALYSIS].
RC   TISSUE=Embryonic fibroblast, and Liver;
RX   PubMed=23806337; DOI=10.1016/j.molcel.2013.06.001;
RA   Park J., Chen Y., Tishkoff D.X., Peng C., Tan M., Dai L., Xie Z., Zhang Y.,
RA   Zwaans B.M., Skinner M.E., Lombard D.B., Zhao Y.;
RT   "SIRT5-mediated lysine desuccinylation impacts diverse metabolic
RT   pathways.";
RL   Mol. Cell 50:919-930(2013).
RN   [6]
RP   ACETYLATION [LARGE SCALE ANALYSIS] AT LYS-50; LYS-138; LYS-144; LYS-233;
RP   LYS-517; LYS-523; LYS-605; LYS-723; LYS-730; LYS-736; LYS-739 AND LYS-743,
RP   AND IDENTIFICATION BY MASS SPECTROMETRY [LARGE SCALE ANALYSIS].
RC   TISSUE=Liver;
RX   PubMed=23576753; DOI=10.1073/pnas.1302961110;
RA   Rardin M.J., Newman J.C., Held J.M., Cusack M.P., Sorensen D.J., Li B.,
RA   Schilling B., Mooney S.D., Kahn C.R., Verdin E., Gibson B.W.;
RT   "Label-free quantitative proteomics of the lysine acetylome in mitochondria
RT   identifies substrates of SIRT3 in metabolic pathways.";
RL   Proc. Natl. Acad. Sci. U.S.A. 110:6601-6606(2013).
CC   -!- FUNCTION: Catalyzes the isomerization of citrate to isocitrate via cis-
CC       aconitate. {ECO:0000250|UniProtKB:P16276}.
CC   -!- CATALYTIC ACTIVITY:
CC       Reaction=citrate = D-threo-isocitrate; Xref=Rhea:RHEA:10336,
CC         ChEBI:CHEBI:15562, ChEBI:CHEBI:16947; EC=4.2.1.3;
CC         Evidence={ECO:0000250|UniProtKB:P16276};
CC   -!- COFACTOR:
CC       Name=[4Fe-4S] cluster; Xref=ChEBI:CHEBI:49883;
CC         Evidence={ECO:0000250|UniProtKB:P16276};
CC       Note=Binds 1 [4Fe-4S] cluster per subunit. Binding of a [3Fe-4S]
CC       cluster leads to an inactive enzyme. {ECO:0000250|UniProtKB:P16276};
CC   -!- PATHWAY: Carbohydrate metabolism; tricarboxylic acid cycle; isocitrate
CC       from oxaloacetate: step 2/2.
CC   -!- SUBUNIT: Monomer. {ECO:0000250|UniProtKB:P16276}.
CC   -!- SUBCELLULAR LOCATION: Mitochondrion {ECO:0000250|UniProtKB:P16276}.
CC   -!- PTM: Forms covalent cross-links mediated by transglutaminase TGM2,
CC       between a glutamine and the epsilon-amino group of a lysine residue,
CC       forming homopolymers and heteropolymers.
CC       {ECO:0000250|UniProtKB:Q9ER34}.
CC   -!- SIMILARITY: Belongs to the aconitase/IPM isomerase family.
CC       {ECO:0000305}.
CC   ---------------------------------------------------------------------------
CC   Copyrighted by the UniProt Consortium, see https://www.uniprot.org/terms
CC   Distributed under the Creative Commons Attribution (CC BY 4.0) License
CC   ---------------------------------------------------------------------------
DR   EMBL; AK143917; BAE25602.1; -; mRNA.
DR   EMBL; AK144207; BAE25770.1; -; mRNA.
DR   EMBL; AK145511; BAE26479.1; -; mRNA.
DR   EMBL; AK150027; BAE29252.1; -; mRNA.
DR   EMBL; AK165411; BAE38169.1; -; mRNA.
DR   EMBL; BC004645; AAH04645.1; -; mRNA.
DR   EMBL; BC094462; AAH94462.1; -; mRNA.
DR   CCDS; CCDS27675.1; -.
DR   RefSeq; NP_542364.1; NM_080633.2.
DR   AlphaFoldDB; Q99KI0; -.
DR   SMR; Q99KI0; -.
DR   BioGRID; 197925; 72.
DR   IntAct; Q99KI0; 8.
DR   MINT; Q99KI0; -.
DR   STRING; 10090.ENSMUSP00000023116; -.
DR   CarbonylDB; Q99KI0; -.
DR   GlyGen; Q99KI0; 1 site, 1 O-linked glycan (1 site).
DR   iPTMnet; Q99KI0; -.
DR   PhosphoSitePlus; Q99KI0; -.
DR   SwissPalm; Q99KI0; -.
DR   REPRODUCTION-2DPAGE; Q99KI0; -.
DR   EPD; Q99KI0; -.
DR   jPOST; Q99KI0; -.
DR   MaxQB; Q99KI0; -.
DR   PaxDb; Q99KI0; -.
DR   PeptideAtlas; Q99KI0; -.
DR   ProteomicsDB; 285596; -.
DR   Antibodypedia; 240; 582 antibodies from 39 providers.
DR   DNASU; 11429; -.
DR   Ensembl; ENSMUST00000023116; ENSMUSP00000023116; ENSMUSG00000022477.
DR   GeneID; 11429; -.
DR   KEGG; mmu:11429; -.
DR   UCSC; uc007wxp.1; mouse.
DR   AGR; MGI:87880; -.
DR   CTD; 50; -.
DR   MGI; MGI:87880; Aco2.
DR   VEuPathDB; HostDB:ENSMUSG00000022477; -.
DR   eggNOG; KOG0453; Eukaryota.
DR   GeneTree; ENSGT00940000154892; -.
DR   HOGENOM; CLU_006714_2_2_1; -.
DR   InParanoid; Q99KI0; -.
DR   OMA; GCIGMGQ; -.
DR   OrthoDB; 3266779at2759; -.
DR   PhylomeDB; Q99KI0; -.
DR   TreeFam; TF300627; -.
DR   BRENDA; 4.2.1.3; 3474.
DR   Reactome; R-MMU-71403; Citric acid cycle (TCA cycle).
DR   UniPathway; UPA00223; UER00718.
DR   BioGRID-ORCS; 11429; 16 hits in 78 CRISPR screens.
DR   ChiTaRS; Aco2; mouse.
DR   PRO; PR:Q99KI0; -.
DR   Proteomes; UP000000589; Chromosome 15.
DR   RNAct; Q99KI0; protein.
DR   Bgee; ENSMUSG00000022477; Expressed in cardiac muscle of left ventricle and 271 other tissues.
DR   ExpressionAtlas; Q99KI0; baseline and differential.
DR   Genevisible; Q99KI0; MM.
DR   GO; GO:0005829; C:cytosol; IBA:GO_Central.
DR   GO; GO:0005759; C:mitochondrial matrix; IDA:MGI.
DR   GO; GO:0005739; C:mitochondrion; IDA:MGI.
DR   GO; GO:0043209; C:myelin sheath; HDA:UniProtKB.
DR   GO; GO:0051538; F:3 iron, 4 sulfur cluster binding; ISO:MGI.
DR   GO; GO:0051539; F:4 iron, 4 sulfur cluster binding; ISO:MGI.
DR   GO; GO:0003994; F:aconitate hydratase activity; IDA:MGI.
DR   GO; GO:0047780; F:citrate dehydratase activity; IEA:UniProtKB-EC.
DR   GO; GO:0005506; F:iron ion binding; ISO:MGI.
DR   GO; GO:0006101; P:citrate metabolic process; ISO:MGI.
DR   GO; GO:0006102; P:isocitrate metabolic process; ISO:MGI.
DR   GO; GO:0001889; P:liver development; IEA:Ensembl.
DR   GO; GO:0035900; P:response to isolation stress; IEA:Ensembl.
DR   GO; GO:0006099; P:tricarboxylic acid cycle; IGI:MGI.
DR   CDD; cd01578; AcnA_Mitochon_Swivel; 1.
DR   CDD; cd01584; AcnA_Mitochondrial; 1.
DR   Gene3D; 3.40.1060.10; Aconitase, Domain 2; 1.
DR   Gene3D; 3.30.499.10; Aconitase, domain 3; 2.
DR   Gene3D; 3.20.19.10; Aconitase, domain 4; 1.
DR   InterPro; IPR015931; Acnase/IPM_dHydase_lsu_aba_1/3.
DR   InterPro; IPR001030; Acoase/IPM_deHydtase_lsu_aba.
DR   InterPro; IPR015928; Aconitase/3IPM_dehydase_swvl.
DR   InterPro; IPR018136; Aconitase_4Fe-4S_BS.
DR   InterPro; IPR036008; Aconitase_4Fe-4S_dom.
DR   InterPro; IPR015932; Aconitase_dom2.
DR   InterPro; IPR006248; Aconitase_mito-like.
DR   InterPro; IPR000573; AconitaseA/IPMdHydase_ssu_swvl.
DR   PANTHER; PTHR43160; ACONITATE HYDRATASE B; 1.
DR   PANTHER; PTHR43160:SF3; ACONITATE HYDRATASE, MITOCHONDRIAL; 1.
DR   Pfam; PF00330; Aconitase; 1.
DR   Pfam; PF00694; Aconitase_C; 1.
DR   PRINTS; PR00415; ACONITASE.
DR   SUPFAM; SSF53732; Aconitase iron-sulfur domain; 1.
DR   SUPFAM; SSF52016; LeuD/IlvD-like; 1.
DR   PROSITE; PS00450; ACONITASE_1; 1.
DR   PROSITE; PS01244; ACONITASE_2; 1.
DR   TIGRFAMs; TIGR01340; aconitase_mito; 1.
PE   1: Evidence at protein level;
KW   4Fe-4S; Acetylation; Direct protein sequencing; Iron; Iron-sulfur; Lyase;
KW   Metal-binding; Mitochondrion; Phosphoprotein; Reference proteome;
KW   Transit peptide; Tricarboxylic acid cycle.
FT   TRANSIT         1..27
FT                   /note="Mitochondrion"
FT                   /evidence="ECO:0000250"
FT   CHAIN           28..780
FT                   /note="Aconitate hydratase, mitochondrial"
FT                   /id="PRO_0000000542"
FT   REGION          524..560
FT                   /note="Disordered"
FT                   /evidence="ECO:0000256|SAM:MobiDB-lite"
FT   COMPBIAS        545..560
FT                   /note="Polar residues"
FT                   /evidence="ECO:0000256|SAM:MobiDB-lite"
FT   BINDING         99
FT                   /ligand="substrate"
FT                   /evidence="ECO:0000250"
FT   BINDING         192..194
FT                   /ligand="substrate"
FT                   /evidence="ECO:0000250"
FT   BINDING         385
FT                   /ligand="[4Fe-4S] cluster"
FT                   /ligand_id="ChEBI:CHEBI:49883"
FT                   /evidence="ECO:0000250"
FT   BINDING         448
FT                   /ligand="[4Fe-4S] cluster"
FT                   /ligand_id="ChEBI:CHEBI:49883"
FT                   /evidence="ECO:0000250"
FT   BINDING         451
FT                   /ligand="[4Fe-4S] cluster"
FT                   /ligand_id="ChEBI:CHEBI:49883"
FT                   /evidence="ECO:0000250"
FT   BINDING         474
FT                   /ligand="substrate"
FT                   /evidence="ECO:0000250"
FT   BINDING         479
FT                   /ligand="substrate"
FT                   /evidence="ECO:0000250"
FT   BINDING         607
FT                   /ligand="substrate"
FT                   /evidence="ECO:0000250"
FT   BINDING         670..671
FT                   /ligand="substrate"
FT                   /evidence="ECO:0000250"
FT   MOD_RES         31
FT                   /note="N6-succinyllysine"
FT                   /evidence="ECO:0007744|PubMed:23806337"
FT   MOD_RES         50
FT                   /note="N6-acetyllysine; alternate"
FT                   /evidence="ECO:0007744|PubMed:23576753,
FT                   ECO:0007744|PubMed:23806337"
FT   MOD_RES         50
FT                   /note="N6-succinyllysine; alternate"
FT                   /evidence="ECO:0007744|PubMed:23806337"
FT   MOD_RES         138
FT                   /note="N6-acetyllysine; alternate"
FT                   /evidence="ECO:0007744|PubMed:23576753"
FT   MOD_RES         138
FT                   /note="N6-succinyllysine; alternate"
FT                   /evidence="ECO:0007744|PubMed:23806337"
FT   MOD_RES         144
FT                   /note="N6-acetyllysine; alternate"
FT                   /evidence="ECO:0007744|PubMed:23576753,
FT                   ECO:0007744|PubMed:23806337"
FT   MOD_RES         144
FT                   /note="N6-succinyllysine; alternate"
FT                   /evidence="ECO:0007744|PubMed:23806337"
FT   MOD_RES         233
FT                   /note="N6-acetyllysine; alternate"
FT                   /evidence="ECO:0007744|PubMed:23576753"
FT   MOD_RES         233
FT                   /note="N6-succinyllysine; alternate"
FT                   /evidence="ECO:0007744|PubMed:23806337"
FT   MOD_RES         411
FT                   /note="N6-succinyllysine"
FT                   /evidence="ECO:0007744|PubMed:23806337"
FT   MOD_RES         517
FT                   /note="N6-acetyllysine; alternate"
FT                   /evidence="ECO:0007744|PubMed:23576753"
FT   MOD_RES         517
FT                   /note="N6-succinyllysine; alternate"
FT                   /evidence="ECO:0007744|PubMed:23806337"
FT   MOD_RES         523
FT                   /note="N6-acetyllysine; alternate"
FT                   /evidence="ECO:0007744|PubMed:23576753"
FT   MOD_RES         523
FT                   /note="N6-succinyllysine; alternate"
FT                   /evidence="ECO:0007744|PubMed:23806337"
FT   MOD_RES         549
FT                   /note="N6-succinyllysine"
FT                   /evidence="ECO:0007744|PubMed:23806337"
FT   MOD_RES         559
FT                   /note="Phosphoserine"
FT                   /evidence="ECO:0000250|UniProtKB:Q99798"
FT   MOD_RES         573
FT                   /note="N6-acetyllysine; alternate"
FT                   /evidence="ECO:0000250|UniProtKB:Q99798"
FT   MOD_RES         573
FT                   /note="N6-succinyllysine; alternate"
FT                   /evidence="ECO:0007744|PubMed:23806337"
FT   MOD_RES         577
FT                   /note="N6-succinyllysine"
FT                   /evidence="ECO:0007744|PubMed:23806337"
FT   MOD_RES         591
FT                   /note="N6-succinyllysine"
FT                   /evidence="ECO:0007744|PubMed:23806337"
FT   MOD_RES         605
FT                   /note="N6-acetyllysine; alternate"
FT                   /evidence="ECO:0007744|PubMed:23576753"
FT   MOD_RES         605
FT                   /note="N6-succinyllysine; alternate"
FT                   /evidence="ECO:0007744|PubMed:23806337"
FT   MOD_RES         628
FT                   /note="N6-succinyllysine"
FT                   /evidence="ECO:0007744|PubMed:23806337"
FT   MOD_RES         670
FT                   /note="Phosphoserine"
FT                   /evidence="ECO:0007744|PubMed:21183079"
FT   MOD_RES         689
FT                   /note="N6-succinyllysine"
FT                   /evidence="ECO:0007744|PubMed:23806337"
FT   MOD_RES         723
FT                   /note="N6-acetyllysine; alternate"
FT                   /evidence="ECO:0007744|PubMed:23576753"
FT   MOD_RES         723
FT                   /note="N6-succinyllysine; alternate"
FT                   /evidence="ECO:0007744|PubMed:23806337"
FT   MOD_RES         730
FT                   /note="N6-acetyllysine; alternate"
FT                   /evidence="ECO:0007744|PubMed:23576753"
FT   MOD_RES         730
FT                   /note="N6-succinyllysine; alternate"
FT                   /evidence="ECO:0007744|PubMed:23806337"
FT   MOD_RES         736
FT                   /note="N6-acetyllysine"
FT                   /evidence="ECO:0007744|PubMed:23576753"
FT   MOD_RES         739
FT                   /note="N6-acetyllysine"
FT                   /evidence="ECO:0007744|PubMed:23576753"
FT   MOD_RES         743
FT                   /note="N6-acetyllysine"
FT                   /evidence="ECO:0007744|PubMed:23576753"
FT   CONFLICT        7..8
FT                   /note="LV -> P (in Ref. 1; BAE25770)"
FT                   /evidence="ECO:0000305"
FT   CONFLICT        618
FT                   /note="L -> F (in Ref. 2; AAH94462)"
FT                   /evidence="ECO:0000305"
FT   CONFLICT        758
FT                   /note="F -> L (in Ref. 1; BAE29252)"
FT                   /evidence="ECO:0000305"
SQ   SEQUENCE   780 AA;  85464 MW;  9B515846E875D581 CRC64;
     MAPYSLLVTR LQKALGVRQY HVASVLCQRA KVAMSHFEPS EYIRYDLLEK NINIVRKRLN
     RPLTLSEKIV YGHLDDPANQ EIERGKTYLR LRPDRVAMQD ATAQMAMLQF ISSGLPKVAV
     PSTIHCDHLI EAQVGGEKDL RRAKDINQEV YNFLATAGAK YGVGFWRPGS GIIHQIILEN
     YAYPGVLLIG TDSHTPNGGG LGGICIGVGG ADAVDVMAGI PWELKCPKVI GVKLTGSLSG
     WTSPKDVILK VAGILTVKGG TGAIVEYHGP GVDSISCTGM ATICNMGAEI GATTSVFPYN
     HRMKKYLSKT GRTDIANLAE EFKDHLVPDP GCQYDQVIEI NLNELKPHIN GPFTPDLAHP
     VADVGTVAEK EGWPLDIRVG LIGSCTNSSY EDMGRSAAVA KQALAHGLKC KSQFTITPGS
     EQIRATIERD GYAQILRDVG GIVLANACGP CIGQWDRKDI KKGEKNTIVT SYNRNFTGRN
     DANPETHAFV TSPEIVTALA IAGTLKFNPE TDFLTGKDGK KFKLEAPDAD ELPRSDFDPG
     QDTYQHPPKD SSGQRVDVSP TSQRLQLLEP FDKWDGKDLE DLQILIKVKG KCTTDHISAA
     GPWLKFRGHL DNISNNLLIG AINIENGKAN SVRNAVTQEF GPVPDTARYY KKHGIRWVVI
     GDENYGEGSS REHAALEPRH LGGRAIITKS FARIHETNLK KQGLLPLTFA DPSDYNKIHP
     VDKLTIQGLK DFAPGKPLKC VIKHPNGTQE TILLNHTFNE TQIEWFRAGS ALNRMKELQQ
//
```

|  |
| --- |
| **Mascot:** http://www.matrixscience.com/ |

HNE (H) (+156.1150)
