# Supplementary material for: A common East-Asian ALDH2 mutation causes metabolic disorders and the therapeutic effect of ALDH2 activators
Source: Nat Commun. 2023 Sep 25;14:5971. doi: 10.1038/s41467-023-41570-6 (PMC10520061; doi:10.1038/s41467-023-41570-6)
Supplement: Supplementary file 4 — Supplementary Data 1 [file 41467_2023_41570_MOESM4_ESM.zip › Table S5b/Q99KIO/Q99KI0_WTO60-3_K144.html]

Mascot Search Results: Q99KI0
 

# MASCOT Search Results

## Protein View: Q99KI0

### Aconitate hydratase, mitochondrial OS=Mus musculus OX=10090 GN=Aco2 PE=1 SV=1

|  |  |
| --- | --- |
| Database: | Mouse\_UniProt\_proteomes |
| Score: | 7751 |
| Monoisotopic mass (Mr): | 86151 |
| Calculated pI: | 8.08 |

Sequence similarity is available as an NCBI BLAST search of Q99KI0 against nr.

### Search parameters

|  |  |
| --- | --- |
| MS data file: | `D:\LCMSMS\2023 Users' data\230529-1\230529-1-WT60-3.raw` |
| Enzyme: | Trypsin/P: cuts C-term side of KR. |
| Fixed modifications: | Carbamidomethyl (C) |
| Variable modifications: | Deamidated (NQ), HNE (C), HNE (H), HNE (K), Oxidation (M) |

### Protein sequence coverage: 66%

Matched peptides shown in ***bold red***.

|  |  |  |  |  |  |
| --- | --- | --- | --- | --- | --- |
| `1` | `MAPYSLLVTR` | `LQKALGVRQY` | `HVASVLCQRA` | `KVAMSHFEPS` | `EYIRYDLLEK` |
| `51` | `NINIVRKRLN` | `RPLTLSEKIV` | `YGHLDDPANQ` | `EIERGKTYLR` | `LRPDRVAMQD` |
| `101` | `ATAQMAMLQF` | `ISSGLPKVAV` | `PSTIHCDHLI` | `EAQVGGEKDL` | `RRAKDINQEV` |
| `151` | `YNFLATAGAK` | `YGVGFWRPGS` | `GIIHQIILEN` | `YAYPGVLLIG` | `TDSHTPNGGG` |
| `201` | `LGGICIGVGG` | `ADAVDVMAGI` | `PWELKCPKVI` | `GVKLTGSLSG` | `WTSPKDVILK` |
| `251` | `VAGILTVKGG` | `TGAIVEYHGP` | `GVDSISCTGM` | `ATICNMGAEI` | `GATTSVFPYN` |
| `301` | `HRMKKYLSKT` | `GRTDIANLAE` | `EFKDHLVPDP` | `GCQYDQVIEI` | `NLNELKPHIN` |
| `351` | `GPFTPDLAHP` | `VADVGTVAEK` | `EGWPLDIRVG` | `LIGSCTNSSY` | `EDMGRSAAVA` |
| `401` | `KQALAHGLKC` | `KSQFTITPGS` | `EQIRATIERD` | `GYAQILRDVG` | `GIVLANACGP` |
| `451` | `CIGQWDRKDI` | `KKGEKNTIVT` | `SYNRNFTGRN` | `DANPETHAFV` | `TSPEIVTALA` |
| `501` | `IAGTLKFNPE` | `TDFLTGKDGK` | `KFKLEAPDAD` | `ELPRSDFDPG` | `QDTYQHPPKD` |
| `551` | `SSGQRVDVSP` | `TSQRLQLLEP` | `FDKWDGKDLE` | `DLQILIKVKG` | `KCTTDHISAA` |
| `601` | `GPWLKFRGHL` | `DNISNNLLIG` | `AINIENGKAN` | `SVRNAVTQEF` | `GPVPDTARYY` |
| `651` | `KKHGIRWVVI` | `GDENYGEGSS` | `REHAALEPRH` | `LGGRAIITKS` | `FARIHETNLK` |
| `701` | `KQGLLPLTFA` | `DPSDYNKIHP` | `VDKLTIQGLK` | `DFAPGKPLKC` | `VIKHPNGTQE` |
| `751` | `TILLNHTFNE` | `TQIEWFRAGS` | `ALNRMKELQQ` |  |  |

Unformatted sequence string: 780 residues (for pasting into other applications).

|  |  |  |  |
| --- | --- | --- | --- |
| Sort by | residue number | increasing mass | decreasing mass |
| Show | matched peptides only | predicted peptides also |  |

| Query | Start | – | End | Observed | Mr(expt) | Mr(calc) | ppm | M | Score | Expect | Rank | U | Peptide |
| --- | --- | --- | --- | --- | --- | --- | --- | --- | --- | --- | --- | --- | --- |
| 77567 | 32 | – | 44 | 522.5856 | 1564.7350 | 1564.7344 | 0.39 | 0 | 22 | 0.0093 | 1Score **> 32** indicates **identity** Score **> 14** indicates **homology** | U | K.VAMSHFEPSEYIR.Y |
| 77568 | 32 | – | 44 | 522.5857 | 1564.7352 | 1564.7344 | 0.53 | 0 | 53 | 9.7e-06 | 1Score **> 32** indicates **identity** Score **> 16** indicates **homology** | U | K.VAMSHFEPSEYIR.Y |
| 77570 | 32 | – | 44 | 522.5858 | 1564.7357 | 1564.7344 | 0.85 | 0 | 42 | 0.00011 | 1Score **> 32** indicates **identity** Score **> 15** indicates **homology** | U | K.VAMSHFEPSEYIR.Y |
| 77572 | 32 | – | 44 | 522.5860 | 1564.7361 | 1564.7344 | 1.09 | 0 | 41 | 0.00014 | 1Score **> 32** indicates **identity** Score **> 15** indicates **homology** | U | K.VAMSHFEPSEYIR.Y |
| 77577 | 32 | – | 44 | 522.5862 | 1564.7369 | 1564.7344 | 1.58 | 0 | 32 | 0.00092 | 1Score **> 33** indicates **identity** Score **> 15** indicates **homology** | U | K.VAMSHFEPSEYIR.Y |
| 77579 | 32 | – | 44 | 522.5866 | 1564.7381 | 1564.7344 | 2.35 | 0 | 30 | 0.0015 | 1Score **> 33** indicates **identity** Score **> 14** indicates **homology** | U | K.VAMSHFEPSEYIR.Y |
| 79739 | 32 | – | 44 | 527.9164 | 1580.7272 | 1580.7293 | -1.31 | 0 | 15 | 0.043 | 1Score **> 31** indicates **identity** Score **> 13** indicates **homology** | U | K.VAMSHFEPSEYIR.Y  + Oxidation (M) |
| 68540 | 45 | – | 56 | 497.2843 | 1488.8311 | 1488.8300 | 0.77 | 1 | 47 | 4.1e-05 | 1Score **> 34** indicates **identity** Score **> 15** indicates **homology** | U | R.YDLLEKNINIVR.K |
| 49331 | 58 | – | 68 | 442.9335 | 1325.7787 | 1325.7779 | 0.59 | 2 | 31 | 0.0012 | 1Score **> 32** indicates **identity** Score **> 14** indicates **homology** | U | K.RLNRPLTLSEK.I |
| 31361 | 59 | – | 68 | 585.8461 | 1169.6776 | 1169.6768 | 0.65 | 1 | 43 | 0.0029 | 1Score **> 32** indicates **identity** Score **> 30** indicates **homology** | U | R.LNRPLTLSEK.I |
| 31362 | 59 | – | 68 | 390.8998 | 1169.6776 | 1169.6768 | 0.71 | 1 | 29 | 0.0044 | 1Score **> 32** indicates **identity** Score **> 18** indicates **homology** | U | R.LNRPLTLSEK.I |
| 31363 | 59 | – | 68 | 390.8999 | 1169.6778 | 1169.6768 | 0.87 | 1 | 26 | 0.01 | 1Score **> 32** indicates **identity** Score **> 19** indicates **homology** | U | R.LNRPLTLSEK.I |
| 31364 | 59 | – | 68 | 390.9000 | 1169.6780 | 1169.6768 | 1.08 | 1 | 25 | 0.013 | 1Score **> 32** indicates **identity** Score **> 19** indicates **homology** | U | R.LNRPLTLSEK.I |
| 114223 | 69 | – | 84 | 623.6401 | 1867.8986 | 1867.9064 | -4.20 | 0 | 26 | 0.0034 | 1Score **> 34** indicates **identity** Score **> 14** indicates **homology** | U | K.IVYGHLDDPANQEIER.G |
| 114224 | 69 | – | 84 | 623.6419 | 1867.9040 | 1867.9064 | -1.30 | 0 | 30 | 0.0017 | 1Score **> 34** indicates **identity** Score **> 14** indicates **homology** | U | K.IVYGHLDDPANQEIER.G |
| 114225 | 69 | – | 84 | 623.6420 | 1867.9042 | 1867.9064 | -1.18 | 0 | 15 | 0.035 | 1Score **> 34** indicates **identity** Score **> 13** indicates **homology** | U | K.IVYGHLDDPANQEIER.G |
| 114231 | 69 | – | 84 | 623.6430 | 1867.9072 | 1867.9064 | 0.44 | 0 | 18 | 0.019 | 1Score **> 35** indicates **identity** Score **> 14** indicates **homology** | U | K.IVYGHLDDPANQEIER.G |
| 114232 | 69 | – | 84 | 934.9610 | 1867.9074 | 1867.9064 | 0.54 | 0 | 77 | 6e-08 | 1Score **> 34** indicates **identity** Score **> 17** indicates **homology** | U | K.IVYGHLDDPANQEIER.G |
| 114233 | 69 | – | 84 | 623.6432 | 1867.9078 | 1867.9064 | 0.76 | 0 | 93 | 1.8e-09 | 1Score **> 34** indicates **identity** Score **> 18** indicates **homology** | U | K.IVYGHLDDPANQEIER.G |
| 114235 | 69 | – | 84 | 623.6434 | 1867.9084 | 1867.9064 | 1.07 | 0 | 72 | 1.9e-07 | 1Score **> 34** indicates **identity** Score **> 17** indicates **homology** | U | K.IVYGHLDDPANQEIER.G |
| 114236 | 69 | – | 84 | 623.6435 | 1867.9086 | 1867.9064 | 1.15 | 0 | 72 | 1.8e-07 | 1Score **> 34** indicates **identity** Score **> 17** indicates **homology** | U | K.IVYGHLDDPANQEIER.G |
| 114237 | 69 | – | 84 | 934.9623 | 1867.9101 | 1867.9064 | 1.99 | 0 | 64 | 1e-06 | 1Score **> 35** indicates **identity** Score **> 16** indicates **homology** | U | K.IVYGHLDDPANQEIER.G |
| 114238 | 69 | – | 84 | 934.9628 | 1867.9111 | 1867.9064 | 2.51 | 0 | 49 | 2.5e-05 | 1Score **> 35** indicates **identity** Score **> 16** indicates **homology** | U | K.IVYGHLDDPANQEIER.G |
| 114240 | 69 | – | 84 | 934.9636 | 1867.9126 | 1867.9064 | 3.33 | 0 | 40 | 0.00019 | 1Score **> 35** indicates **identity** Score **> 15** indicates **homology** | U | K.IVYGHLDDPANQEIER.G |
| 131703 | 69 | – | 86 | 685.3471 | 2053.0195 | 2053.0228 | -1.65 | 1 | 31 | 0.0013 | 1Score **> 36** indicates **identity** Score **> 14** indicates **homology** | U | K.IVYGHLDDPANQEIERGK.T |
| 153132 | 96 | – | 117 | 780.0595 | 2337.1565 | 2337.1531 | 1.48 | 0 | 84 | 1.3e-08 | 1Score **> 36** indicates **identity** Score **> 18** indicates **homology** | U | R.VAMQDATAQMAMLQFISSGLPK.V |
| 153133 | 96 | – | 117 | 1169.5856 | 2337.1566 | 2337.1531 | 1.53 | 0 | 94 | 1.6e-09 | 1Score **> 36** indicates **identity** Score **> 18** indicates **homology** | U | R.VAMQDATAQMAMLQFISSGLPK.V |
| 153135 | 96 | – | 117 | 780.0596 | 2337.1571 | 2337.1531 | 1.72 | 0 | 50 | 0.0024 | 1Score **> 36** indicates **identity** | U | R.VAMQDATAQMAMLQFISSGLPK.V |
| 154096 | 96 | – | 117 | 785.3907 | 2353.1501 | 2353.1480 | 0.92 | 0 | 30 | 0.0016 | 1Score **> 36** indicates **identity** Score **> 14** indicates **homology** | U | R.VAMQDATAQMAMLQFISSGLPK.V  + Oxidation (M) |
| 148252 | 118 | – | 138 | 754.0508 | 2259.1307 | 2259.1318 | -0.48 | 0 | 31 | 0.0011 | 1Score **> 37** indicates **identity** Score **> 14** indicates **homology** | U | K.VAVPSTIHCDHLIEAQVGGEK.D |
| 148254 | 118 | – | 138 | 754.0515 | 2259.1328 | 2259.1318 | 0.43 | 0 | 46 | 5.3e-05 | 1Score **> 37** indicates **identity** Score **> 15** indicates **homology** | U | K.VAVPSTIHCDHLIEAQVGGEK.D |
| 148256 | 118 | – | 138 | 754.0522 | 2259.1346 | 2259.1318 | 1.27 | 0 | 26 | 0.0033 | 1Score **> 37** indicates **identity** Score **> 14** indicates **homology** | U | K.VAVPSTIHCDHLIEAQVGGEK.D |
| 148257 | 118 | – | 138 | 565.7910 | 2259.1348 | 2259.1318 | 1.33 | 0 | 25 | 0.0046 | 1Score **> 37** indicates **identity** Score **> 14** indicates **homology** | U | K.VAVPSTIHCDHLIEAQVGGEK.D |
| 148258 | 118 | – | 138 | 565.7911 | 2259.1352 | 2259.1318 | 1.52 | 0 | 41 | 0.00015 | 1Score **> 37** indicates **identity** Score **> 15** indicates **homology** | U | K.VAVPSTIHCDHLIEAQVGGEK.D |
| 148260 | 118 | – | 138 | 565.7913 | 2259.1359 | 2259.1318 | 1.83 | 0 | 40 | 0.00017 | 1Score **> 37** indicates **identity** Score **> 15** indicates **homology** | U | K.VAVPSTIHCDHLIEAQVGGEK.D |
| 166998 | 118 | – | 141 | 661.8434 | 2643.3445 | 2643.3439 | 0.24 | 1 | 14 | 0.046 | 1Score **> 37** indicates **identity** Score **> 13** indicates **homology** | U | K.VAVPSTIHCDHLIEAQVGGEKDLR.R |
| 166999 | 118 | – | 141 | 882.1223 | 2643.3450 | 2643.3439 | 0.40 | 1 | 25 | 0.0043 | 1Score **> 37** indicates **identity** Score **> 14** indicates **homology** | U | K.VAVPSTIHCDHLIEAQVGGEKDLR.R |
| 167002 | 118 | – | 141 | 529.6764 | 2643.3457 | 2643.3439 | 0.67 | 1 | 33 | 0.00079 | 1Score **> 37** indicates **identity** Score **> 15** indicates **homology** | U | K.VAVPSTIHCDHLIEAQVGGEKDLR.R |
| 167003 | 118 | – | 141 | 529.6764 | 2643.3457 | 2643.3439 | 0.69 | 1 | 25 | 0.0044 | 1Score **> 37** indicates **identity** Score **> 14** indicates **homology** | U | K.VAVPSTIHCDHLIEAQVGGEKDLR.R |
| 167004 | 118 | – | 141 | 529.6764 | 2643.3458 | 2643.3439 | 0.73 | 1 | 32 | 0.00096 | 1Score **> 37** indicates **identity** Score **> 15** indicates **homology** | U | K.VAVPSTIHCDHLIEAQVGGEKDLR.R |
| 167007 | 118 | – | 141 | 529.6766 | 2643.3465 | 2643.3439 | 1.00 | 1 | 15 | 0.037 | 1Score **> 37** indicates **identity** Score **> 13** indicates **homology** | U | K.VAVPSTIHCDHLIEAQVGGEKDLR.R |
| 136452 | 142 | – | 160 | 703.7076 | 2108.1009 | 2108.1014 | -0.24 | 2 | 20 | 0.012 | 1Score **> 36** indicates **identity** Score **> 14** indicates **homology** | U | R.RAKDINQEVYNFLATAGAK.Y |
| 121614 | 143 | – | 160 | 651.6734 | 1951.9983 | 1952.0003 | -1.02 | 1 | 52 | 1.3e-05 | 1Score **> 36** indicates **identity** Score **> 16** indicates **homology** | U | R.AKDINQEVYNFLATAGAK.Y |
| 121615 | 143 | – | 160 | 651.6745 | 1952.0016 | 1952.0003 | 0.67 | 1 | 78 | 4.8e-08 | 1Score **> 36** indicates **identity** Score **> 17** indicates **homology** | U | R.AKDINQEVYNFLATAGAK.Y |
| 121616 | 143 | – | 160 | 651.6747 | 1952.0021 | 1952.0003 | 0.94 | 1 | 47 | 4.1e-05 | 1Score **> 36** indicates **identity** Score **> 15** indicates **homology** | U | R.AKDINQEVYNFLATAGAK.Y |
| 121617 | 143 | – | 160 | 651.6750 | 1952.0031 | 1952.0003 | 1.45 | 1 | 79 | 4.1e-08 | 1Score **> 36** indicates **identity** Score **> 17** indicates **homology** | U | R.AKDINQEVYNFLATAGAK.Y |
| 121618 | 143 | – | 160 | 651.6750 | 1952.0032 | 1952.0003 | 1.46 | 1 | 72 | 1.6e-07 | 1Score **> 36** indicates **identity** Score **> 17** indicates **homology** | U | R.AKDINQEVYNFLATAGAK.Y |
| 121620 | 143 | – | 160 | 977.0094 | 1952.0042 | 1952.0003 | 2.00 | 1 | 119 | 7.7e-12 | 1Score **> 36** indicates **identity** Score **> 20** indicates **homology** | U | R.AKDINQEVYNFLATAGAK.Y |
| 121621 | 143 | – | 160 | 977.0096 | 1952.0046 | 1952.0003 | 2.20 | 1 | 99 | 5.3e-10 | 1Score **> 36** indicates **identity** Score **> 19** indicates **homology** | U | R.AKDINQEVYNFLATAGAK.Y |
| 121623 | 143 | – | 160 | 977.0110 | 1952.0074 | 1952.0003 | 3.65 | 1 | 80 | 2.9e-08 | 1Score **> 36** indicates **identity** Score **> 18** indicates **homology** | U | R.AKDINQEVYNFLATAGAK.Y |
| 121625 | 143 | – | 160 | 977.0131 | 1952.0117 | 1952.0003 | 5.82 | 1 | 131 | 4.8e-13 | 1Score **> 36** indicates **identity** Score **> 21** indicates **homology** | U | R.AKDINQEVYNFLATAGAK.Y |
| 136535 | 143 | – | 160 | 704.0431 | 2109.1076 | 2109.0994 | 3.91 | 1 | 33 | 0.00084 | 1Score **> 36** indicates **identity** Score **> 15** indicates **homology** | U | R.AKDINQEVYNFLATAGAK.Y  + Deamidated (NQ); HNE (K) |
| 101776 | 145 | – | 160 | 877.4414 | 1752.8683 | 1752.8682 | 0.0080 | 0 | 76 | 8e-08 | 1Score **> 35** indicates **identity** Score **> 17** indicates **homology** | U | K.DINQEVYNFLATAGAK.Y |
| 101778 | 145 | – | 160 | 585.2980 | 1752.8722 | 1752.8682 | 2.27 | 0 | 67 | 4.9e-07 | 1Score **> 35** indicates **identity** Score **> 17** indicates **homology** | U | K.DINQEVYNFLATAGAK.Y |
| 101779 | 145 | – | 160 | 585.2982 | 1752.8729 | 1752.8682 | 2.66 | 0 | 39 | 0.00023 | 1Score **> 35** indicates **identity** Score **> 15** indicates **homology** | U | K.DINQEVYNFLATAGAK.Y |
| 101780 | 145 | – | 160 | 877.4438 | 1752.8730 | 1752.8682 | 2.73 | 0 | 89 | 4.5e-09 | 1Score **> 35** indicates **identity** Score **> 18** indicates **homology** | U | K.DINQEVYNFLATAGAK.Y |
| 38478 | 234 | – | 245 | 617.3273 | 1232.6401 | 1232.6401 | -0.037 | 0 | 69 | 2.6e-06 | 1Score **> 34** indicates **identity** Score **> 26** indicates **homology** |  | K.LTGSLSGWTSPK.D |
| 38479 | 234 | – | 245 | 617.3275 | 1232.6404 | 1232.6401 | 0.25 | 0 | 33 | 0.00085 | 1Score **> 34** indicates **identity** Score **> 15** indicates **homology** |  | K.LTGSLSGWTSPK.D |
| 38481 | 234 | – | 245 | 617.3276 | 1232.6406 | 1232.6401 | 0.38 | 0 | 72 | 1.1e-06 | 1Score **> 34** indicates **identity** Score **> 25** indicates **homology** |  | K.LTGSLSGWTSPK.D |
| 38485 | 234 | – | 245 | 617.3278 | 1232.6409 | 1232.6401 | 0.68 | 0 | 75 | 3.2e-07 | 1Score **> 34** indicates **identity** Score **> 22** indicates **homology** |  | K.LTGSLSGWTSPK.D |
| 38490 | 234 | – | 245 | 617.3282 | 1232.6419 | 1232.6401 | 1.49 | 0 | 38 | 0.00047 | 1Score **> 33** indicates **identity** Score **> 17** indicates **homology** |  | K.LTGSLSGWTSPK.D |
| 38493 | 234 | – | 245 | 617.3292 | 1232.6438 | 1232.6401 | 2.97 | 0 | 56 | 2.5e-05 | 1Score **> 34** indicates **identity** Score **> 23** indicates **homology** |  | K.LTGSLSGWTSPK.D |
| 38502 | 234 | – | 245 | 617.3326 | 1232.6506 | 1232.6401 | 8.48 | 0 | 58 | 4.8e-06 | 1Score **> 34** indicates **identity** Score **> 18** indicates **homology** |  | K.LTGSLSGWTSPK.D |
| 106964 | 234 | – | 250 | 901.5067 | 1800.9989 | 1800.9986 | 0.21 | 1 | 45 | 0.00011 | 1Score **> 34** indicates **identity** Score **> 18** indicates **homology** |  | K.LTGSLSGWTSPKDVILK.V |
| 106966 | 234 | – | 250 | 601.3409 | 1801.0010 | 1800.9986 | 1.35 | 1 | 56 | 5.3e-06 | 1Score **> 34** indicates **identity** Score **> 16** indicates **homology** |  | K.LTGSLSGWTSPKDVILK.V |
| 106968 | 234 | – | 250 | 601.3413 | 1801.0020 | 1800.9986 | 1.89 | 1 | 55 | 7.3e-06 | 1Score **> 34** indicates **identity** Score **> 16** indicates **homology** |  | K.LTGSLSGWTSPKDVILK.V |
| 106969 | 234 | – | 250 | 601.3413 | 1801.0021 | 1800.9986 | 1.97 | 1 | 55 | 7.2e-06 | 1Score **> 34** indicates **identity** Score **> 16** indicates **homology** |  | K.LTGSLSGWTSPKDVILK.V |
| 106970 | 234 | – | 250 | 901.5089 | 1801.0032 | 1800.9986 | 2.59 | 1 | 73 | 5.4e-07 | 1Score **> 34** indicates **identity** Score **> 23** indicates **homology** |  | K.LTGSLSGWTSPKDVILK.V |
| 106971 | 234 | – | 250 | 901.5091 | 1801.0037 | 1800.9986 | 2.86 | 1 | 47 | 0.00012 | 1Score **> 34** indicates **identity** Score **> 21** indicates **homology** |  | K.LTGSLSGWTSPKDVILK.V |
| 106974 | 234 | – | 250 | 601.3424 | 1801.0054 | 1800.9986 | 3.80 | 1 | 33 | 0.00083 | 1Score **> 34** indicates **identity** Score **> 15** indicates **homology** |  | K.LTGSLSGWTSPKDVILK.V |
| 3965 | 251 | – | 258 | 400.7652 | 799.5159 | 799.5167 | -1.07 | 0 | 27 | 0.021 | 1Score **> 23** indicates **identity** |  | K.VAGILTVK.G |
| 3966 | 251 | – | 258 | 400.7656 | 799.5166 | 799.5167 | -0.22 | 0 | 18 | 0.028 | 1Score **> 23** indicates **identity** Score **> 15** indicates **homology** |  | K.VAGILTVK.G |
| 3968 | 251 | – | 258 | 400.7659 | 799.5172 | 799.5167 | 0.52 | 0 | 54 | 4.4e-05 | 1Score **> 23** indicates **identity** |  | K.VAGILTVK.G |
| 3970 | 251 | – | 258 | 400.7659 | 799.5173 | 799.5167 | 0.76 | 0 | 52 | 7.9e-05 | 1Score **> 23** indicates **identity** |  | K.VAGILTVK.G |
| 3971 | 251 | – | 258 | 400.7660 | 799.5175 | 799.5167 | 0.99 | 0 | 54 | 4.3e-05 | 1Score **> 23** indicates **identity** |  | K.VAGILTVK.G |
| 193561 | 259 | – | 302 | 1139.7754 | 4555.0727 | 4555.0665 | 1.35 | 0 | 58 | 3.7e-06 | 1Score **> 33** indicates **identity** Score **> 16** indicates **homology** |  | K.GGTGAIVEYHGPGVDSISCTGMATICNMGAEIGATTSVFPYNHR.M |
| 14231 | 371 | – | 378 | 493.2593 | 984.5041 | 984.5029 | 1.25 | 0 | 24 | 0.043 | 1Score **> 30** indicates **identity** Score **> 23** indicates **homology** | U | K.EGWPLDIR.V |
| 111845 | 379 | – | 395 | 923.4054 | 1844.7963 | 1844.8033 | -3.78 | 0 | 71 | 2.3e-07 | 1Score **> 28** indicates **identity** Score **> 17** indicates **homology** | U | R.VGLIGSCTNSSYEDMGR.S |
| 111846 | 379 | – | 395 | 923.4070 | 1844.7995 | 1844.8033 | -2.05 | 0 | 48 | 3.3e-05 | 1Score **> 29** indicates **identity** Score **> 15** indicates **homology** | U | R.VGLIGSCTNSSYEDMGR.S |
| 111847 | 379 | – | 395 | 923.4073 | 1844.8001 | 1844.8033 | -1.75 | 0 | 83 | 1.6e-08 | 1Score **> 29** indicates **identity** Score **> 18** indicates **homology** | U | R.VGLIGSCTNSSYEDMGR.S |
| 111848 | 379 | – | 395 | 923.4087 | 1844.8029 | 1844.8033 | -0.23 | 0 | 75 | 9.5e-08 | 1Score **> 29** indicates **identity** Score **> 17** indicates **homology** | U | R.VGLIGSCTNSSYEDMGR.S |
| 111849 | 379 | – | 395 | 923.4089 | 1844.8033 | 1844.8033 | -0.021 | 0 | 60 | 2.4e-06 | 1Score **> 29** indicates **identity** Score **> 16** indicates **homology** | U | R.VGLIGSCTNSSYEDMGR.S |
| 111850 | 379 | – | 395 | 923.4093 | 1844.8040 | 1844.8033 | 0.38 | 0 | 84 | 1.2e-08 | 1Score **> 29** indicates **identity** Score **> 18** indicates **homology** | U | R.VGLIGSCTNSSYEDMGR.S |
| 111851 | 379 | – | 395 | 923.4094 | 1844.8042 | 1844.8033 | 0.46 | 0 | 88 | 5.5e-09 | 1Score **> 29** indicates **identity** Score **> 18** indicates **homology** | U | R.VGLIGSCTNSSYEDMGR.S |
| 111852 | 379 | – | 395 | 923.4097 | 1844.8049 | 1844.8033 | 0.87 | 0 | 135 | 2e-13 | 1Score **> 29** indicates **identity** Score **> 21** indicates **homology** | U | R.VGLIGSCTNSSYEDMGR.S |
| 111853 | 379 | – | 395 | 923.4098 | 1844.8050 | 1844.8033 | 0.91 | 0 | 113 | 2.4e-11 | 1Score **> 29** indicates **identity** Score **> 20** indicates **homology** | U | R.VGLIGSCTNSSYEDMGR.S |
| 111854 | 379 | – | 395 | 923.4099 | 1844.8053 | 1844.8033 | 1.06 | 0 | 124 | 2.4e-12 | 1Score **> 29** indicates **identity** Score **> 20** indicates **homology** | U | R.VGLIGSCTNSSYEDMGR.S |
| 111855 | 379 | – | 395 | 923.4100 | 1844.8053 | 1844.8033 | 1.11 | 0 | 88 | 5.3e-09 | 1Score **> 29** indicates **identity** Score **> 18** indicates **homology** | U | R.VGLIGSCTNSSYEDMGR.S |
| 111856 | 379 | – | 395 | 923.4101 | 1844.8056 | 1844.8033 | 1.25 | 0 | 127 | 1.1e-12 | 1Score **> 29** indicates **identity** Score **> 20** indicates **homology** | U | R.VGLIGSCTNSSYEDMGR.S |
| 111857 | 379 | – | 395 | 923.4103 | 1844.8061 | 1844.8033 | 1.52 | 0 | 83 | 1.5e-08 | 1Score **> 29** indicates **identity** Score **> 18** indicates **homology** | U | R.VGLIGSCTNSSYEDMGR.S |
| 111858 | 379 | – | 395 | 923.4107 | 1844.8068 | 1844.8033 | 1.92 | 0 | 93 | 1.8e-09 | 1Score **> 30** indicates **identity** Score **> 18** indicates **homology** | U | R.VGLIGSCTNSSYEDMGR.S |
| 111919 | 379 | – | 395 | 923.9022 | 1845.7899 | 1845.7873 | 1.43 | 0 | 26 | 0.0035 | 1Score **> 27** indicates **identity** Score **> 14** indicates **homology** | U | R.VGLIGSCTNSSYEDMGR.S  + Deamidated (NQ) |
| 111920 | 379 | – | 395 | 923.9034 | 1845.7922 | 1845.7873 | 2.63 | 0 | 83 | 1.7e-08 | 1Score **> 27** indicates **identity** Score **> 18** indicates **homology** | U | R.VGLIGSCTNSSYEDMGR.S  + Deamidated (NQ) |
| 113395 | 379 | – | 395 | 931.4075 | 1860.8005 | 1860.7982 | 1.22 | 0 | 78 | 4.4e-08 | 1Score **> 27** indicates **identity** Score **> 17** indicates **homology** | U | R.VGLIGSCTNSSYEDMGR.S  + Oxidation (M) |
| 113396 | 379 | – | 395 | 931.4076 | 1860.8006 | 1860.7982 | 1.28 | 0 | 41 | 0.00015 | 1Score **> 27** indicates **identity** Score **> 15** indicates **homology** | U | R.VGLIGSCTNSSYEDMGR.S  + Oxidation (M) |
| 113399 | 379 | – | 395 | 931.4107 | 1860.8068 | 1860.7982 | 4.63 | 0 | 95 | 1.2e-09 | 1Score **> 28** indicates **identity** Score **> 18** indicates **homology** | U | R.VGLIGSCTNSSYEDMGR.S  + Oxidation (M) |
| 101554 | 410 | – | 424 | 584.6279 | 1750.8619 | 1750.8672 | -3.05 | 1 | 22 | 0.008 | 1Score **> 35** indicates **identity** Score **> 14** indicates **homology** | U | K.CKSQFTITPGSEQIR.A |
| 101555 | 410 | – | 424 | 584.6296 | 1750.8669 | 1750.8672 | -0.19 | 1 | 47 | 6.9e-05 | 1Score **> 34** indicates **identity** Score **> 18** indicates **homology** | U | K.CKSQFTITPGSEQIR.A |
| 101558 | 410 | – | 424 | 876.4413 | 1750.8681 | 1750.8672 | 0.51 | 1 | 55 | 6.3e-06 | 1Score **> 35** indicates **identity** Score **> 16** indicates **homology** | U | K.CKSQFTITPGSEQIR.A |
| 65301 | 412 | – | 424 | 732.3761 | 1462.7376 | 1462.7416 | -2.71 | 0 | 48 | 0.00012 | 1Score **> 34** indicates **identity** Score **> 21** indicates **homology** | U | K.SQFTITPGSEQIR.A |
| 65304 | 412 | – | 424 | 732.3778 | 1462.7411 | 1462.7416 | -0.33 | 0 | 46 | 5e-05 | 1Score **> 35** indicates **identity** Score **> 16** indicates **homology** | U | K.SQFTITPGSEQIR.A |
| 65306 | 412 | – | 424 | 732.3782 | 1462.7418 | 1462.7416 | 0.13 | 0 | 29 | 0.002 | 1Score **> 35** indicates **identity** Score **> 14** indicates **homology** | U | K.SQFTITPGSEQIR.A |
| 65307 | 412 | – | 424 | 732.3783 | 1462.7420 | 1462.7416 | 0.27 | 0 | 64 | 1.3e-06 | 1Score **> 35** indicates **identity** Score **> 18** indicates **homology** | U | K.SQFTITPGSEQIR.A |
| 65308 | 412 | – | 424 | 732.3784 | 1462.7422 | 1462.7416 | 0.37 | 0 | 26 | 0.0034 | 1Score **> 35** indicates **identity** Score **> 14** indicates **homology** | U | K.SQFTITPGSEQIR.A |
| 65309 | 412 | – | 424 | 732.3785 | 1462.7425 | 1462.7416 | 0.63 | 0 | 50 | 2.7e-05 | 1Score **> 35** indicates **identity** Score **> 17** indicates **homology** | U | K.SQFTITPGSEQIR.A |
| 65310 | 412 | – | 424 | 732.3786 | 1462.7427 | 1462.7416 | 0.77 | 0 | 52 | 1.6e-05 | 1Score **> 35** indicates **identity** Score **> 17** indicates **homology** | U | K.SQFTITPGSEQIR.A |
| 70324 | 425 | – | 437 | 502.6074 | 1504.8005 | 1504.7998 | 0.47 | 1 | 32 | 0.0015 | 1Score **> 35** indicates **identity** Score **> 16** indicates **homology** | U | R.ATIERDGYAQILR.D |
| 70326 | 425 | – | 437 | 753.4078 | 1504.8011 | 1504.7998 | 0.90 | 1 | 25 | 0.012 | 1Score **> 35** indicates **identity** Score **> 18** indicates **homology** | U | R.ATIERDGYAQILR.D |
| 70328 | 425 | – | 437 | 502.6081 | 1504.8025 | 1504.7998 | 1.84 | 1 | 35 | 0.00057 | 1Score **> 35** indicates **identity** Score **> 15** indicates **homology** | U | R.ATIERDGYAQILR.D |
| 11254 | 430 | – | 437 | 468.2511 | 934.4877 | 934.4872 | 0.53 | 0 | 52 | 0.0001 | 1Score **> 30** indicates **identity** Score **> 25** indicates **homology** | U | R.DGYAQILR.D |
| 11255 | 430 | – | 437 | 468.2512 | 934.4878 | 934.4872 | 0.59 | 0 | 47 | 0.0003 | 1Score **> 30** indicates **identity** Score **> 24** indicates **homology** | U | R.DGYAQILR.D |
| 11257 | 430 | – | 437 | 468.2517 | 934.4888 | 934.4872 | 1.73 | 0 | 44 | 0.00021 | 1Score **> 29** indicates **identity** Score **> 20** indicates **homology** | U | R.DGYAQILR.D |
| 11258 | 430 | – | 437 | 468.2520 | 934.4895 | 934.4872 | 2.46 | 0 | 45 | 0.002 | 1Score **> 30** indicates **identity** | U | R.DGYAQILR.D |
| 140438 | 438 | – | 457 | 720.0100 | 2157.0080 | 2157.0096 | -0.70 | 0 | 39 | 0.00021 | 1Score **> 34** indicates **identity** Score **> 15** indicates **homology** | U | R.DVGGIVLANACGPCIGQWDR.K |
| 140440 | 438 | – | 457 | 720.0109 | 2157.0109 | 2157.0096 | 0.62 | 0 | 44 | 7.8e-05 | 1Score **> 34** indicates **identity** Score **> 15** indicates **homology** | U | R.DVGGIVLANACGPCIGQWDR.K |
| 140441 | 438 | – | 457 | 1079.5135 | 2157.0125 | 2157.0096 | 1.37 | 0 | 93 | 1.7e-09 | 1Score **> 34** indicates **identity** Score **> 18** indicates **homology** | U | R.DVGGIVLANACGPCIGQWDR.K |
| 140443 | 438 | – | 457 | 1079.5138 | 2157.0130 | 2157.0096 | 1.57 | 0 | 93 | 1.8e-09 | 1Score **> 34** indicates **identity** Score **> 18** indicates **homology** | U | R.DVGGIVLANACGPCIGQWDR.K |
| 140444 | 438 | – | 457 | 720.0116 | 2157.0130 | 2157.0096 | 1.60 | 0 | 74 | 1.3e-07 | 1Score **> 34** indicates **identity** Score **> 17** indicates **homology** | U | R.DVGGIVLANACGPCIGQWDR.K |
| 140538 | 438 | – | 457 | 1080.0142 | 2158.0139 | 2157.9936 | 9.43 | 0 | 62 | 1.7e-06 | 1Score **> 34** indicates **identity** Score **> 16** indicates **homology** | U | R.DVGGIVLANACGPCIGQWDR.K  + Deamidated (NQ) |
| 150012 | 438 | – | 458 | 762.7078 | 2285.1016 | 2285.1045 | -1.28 | 1 | 20 | 0.013 | 1Score **> 35** indicates **identity** Score **> 14** indicates **homology** | U | R.DVGGIVLANACGPCIGQWDRK.D |
| 150014 | 438 | – | 458 | 762.7084 | 2285.1034 | 2285.1045 | -0.49 | 1 | 36 | 0.0004 | 1Score **> 35** indicates **identity** Score **> 15** indicates **homology** | U | R.DVGGIVLANACGPCIGQWDRK.D |
| 150015 | 438 | – | 458 | 762.7091 | 2285.1055 | 2285.1045 | 0.41 | 1 | 70 | 2.8e-07 | 1Score **> 35** indicates **identity** Score **> 17** indicates **homology** | U | R.DVGGIVLANACGPCIGQWDRK.D |
| 150016 | 438 | – | 458 | 762.7092 | 2285.1056 | 2285.1045 | 0.48 | 1 | 44 | 7.6e-05 | 1Score **> 35** indicates **identity** Score **> 15** indicates **homology** | U | R.DVGGIVLANACGPCIGQWDRK.D |
| 150017 | 438 | – | 458 | 1143.5601 | 2285.1057 | 2285.1045 | 0.50 | 1 | 55 | 4.1e-05 | 1Score **> 35** indicates **identity** Score **> 23** indicates **homology** | U | R.DVGGIVLANACGPCIGQWDRK.D |
| 150018 | 438 | – | 458 | 762.7092 | 2285.1058 | 2285.1045 | 0.56 | 1 | 40 | 0.00018 | 1Score **> 35** indicates **identity** Score **> 15** indicates **homology** | U | R.DVGGIVLANACGPCIGQWDRK.D |
| 150019 | 438 | – | 458 | 1143.5603 | 2285.1060 | 2285.1045 | 0.64 | 1 | 42 | 0.00016 | 1Score **> 36** indicates **identity** Score **> 17** indicates **homology** | U | R.DVGGIVLANACGPCIGQWDRK.D |
| 150020 | 438 | – | 458 | 762.7093 | 2285.1061 | 2285.1045 | 0.67 | 1 | 70 | 2.5e-07 | 1Score **> 36** indicates **identity** Score **> 17** indicates **homology** | U | R.DVGGIVLANACGPCIGQWDRK.D |
| 150021 | 438 | – | 458 | 762.7094 | 2285.1065 | 2285.1045 | 0.87 | 1 | 70 | 2.8e-07 | 1Score **> 36** indicates **identity** Score **> 17** indicates **homology** | U | R.DVGGIVLANACGPCIGQWDRK.D |
| 150024 | 438 | – | 458 | 1143.5621 | 2285.1097 | 2285.1045 | 2.25 | 1 | 62 | 1.3e-05 | 1Score **> 36** indicates **identity** Score **> 26** indicates **homology** | U | R.DVGGIVLANACGPCIGQWDRK.D |
| 150092 | 438 | – | 458 | 763.0428 | 2286.1065 | 2286.0885 | 7.86 | 1 | 26 | 0.0037 | 1Score **> 36** indicates **identity** Score **> 14** indicates **homology** | U | R.DVGGIVLANACGPCIGQWDRK.D  + Deamidated (NQ) |
| 70752 | 462 | – | 474 | 503.9388 | 1508.7945 | 1508.7947 | -0.13 | 2 | 66 | 2.1e-06 | 1Score **> 35** indicates **identity** Score **> 22** indicates **homology** | U | K.KGEKNTIVTSYNR.N |
| 70755 | 462 | – | 474 | 755.4048 | 1508.7951 | 1508.7947 | 0.27 | 2 | 60 | 3.4e-06 | 1Score **> 35** indicates **identity** Score **> 18** indicates **homology** | U | K.KGEKNTIVTSYNR.N |
| 20361 | 466 | – | 474 | 534.2767 | 1066.5389 | 1066.5407 | -1.69 | 0 | 25 | 0.013 | 1Score **> 31** indicates **identity** Score **> 19** indicates **homology** | U | K.NTIVTSYNR.N |
| 20362 | 466 | – | 474 | 534.2779 | 1066.5412 | 1066.5407 | 0.44 | 0 | 41 | 0.00062 | 1Score **> 30** indicates **identity** Score **> 22** indicates **homology** | U | K.NTIVTSYNR.N |
| 20363 | 466 | – | 474 | 534.2779 | 1066.5413 | 1066.5407 | 0.52 | 0 | 39 | 0.00038 | 1Score **> 30** indicates **identity** Score **> 18** indicates **homology** | U | K.NTIVTSYNR.N |
| 185457 | 475 | – | 506 | 1119.2491 | 3354.7254 | 3354.7208 | 1.37 | 1 | 70 | 2.6e-07 | 1Score **> 37** indicates **identity** Score **> 17** indicates **homology** | U | R.NFTGRNDANPETHAFVTSPEIVTALAIAGTLK.F |
| 185458 | 475 | – | 506 | 1119.2492 | 3354.7256 | 3354.7208 | 1.44 | 1 | 42 | 0.00011 | 1Score **> 37** indicates **identity** Score **> 15** indicates **homology** | U | R.NFTGRNDANPETHAFVTSPEIVTALAIAGTLK.F |
| 185459 | 475 | – | 506 | 839.6891 | 3354.7271 | 3354.7208 | 1.88 | 1 | 44 | 8.1e-05 | 1Score **> 37** indicates **identity** Score **> 15** indicates **homology** | U | R.NFTGRNDANPETHAFVTSPEIVTALAIAGTLK.F |
| 171696 | 480 | – | 506 | 927.4881 | 2779.4424 | 2779.4392 | 1.15 | 0 | 58 | 4e-06 | 1Score **> 37** indicates **identity** Score **> 16** indicates **homology** | U | R.NDANPETHAFVTSPEIVTALAIAGTLK.F |
| 171697 | 480 | – | 506 | 927.4892 | 2779.4457 | 2779.4392 | 2.35 | 0 | 82 | 2.1e-08 | 1Score **> 37** indicates **identity** Score **> 18** indicates **homology** | U | R.NDANPETHAFVTSPEIVTALAIAGTLK.F |
| 171698 | 480 | – | 506 | 695.8690 | 2779.4470 | 2779.4392 | 2.82 | 0 | 38 | 0.00029 | 1Score **> 37** indicates **identity** Score **> 15** indicates **homology** | U | R.NDANPETHAFVTSPEIVTALAIAGTLK.F |
| 171700 | 480 | – | 506 | 927.4921 | 2779.4545 | 2779.4392 | 5.50 | 0 | 16 | 0.031 | 1Score **> 37** indicates **identity** Score **> 14** indicates **homology** | U | R.NDANPETHAFVTSPEIVTALAIAGTLK.F |
| 171732 | 480 | – | 506 | 927.8241 | 2780.4504 | 2780.4232 | 9.78 | 0 | 27 | 0.0028 | 1Score **> 37** indicates **identity** Score **> 14** indicates **homology** | U | R.NDANPETHAFVTSPEIVTALAIAGTLK.F  + Deamidated (NQ) |
| 42436 | 507 | – | 517 | 634.8119 | 1267.6093 | 1267.6085 | 0.65 | 0 | 45 | 5.6e-05 | 1Score **> 31** indicates **identity** Score **> 15** indicates **homology** | U | K.FNPETDFLTGK.D |
| 42437 | 507 | – | 517 | 634.8121 | 1267.6097 | 1267.6085 | 0.95 | 0 | 36 | 0.00045 | 1Score **> 31** indicates **identity** Score **> 15** indicates **homology** | U | K.FNPETDFLTGK.D |
| 42438 | 507 | – | 517 | 634.8121 | 1267.6097 | 1267.6085 | 0.96 | 0 | 48 | 2.9e-05 | 1Score **> 31** indicates **identity** Score **> 16** indicates **homology** | U | K.FNPETDFLTGK.D |
| 42439 | 507 | – | 517 | 634.8122 | 1267.6098 | 1267.6085 | 1.05 | 0 | 34 | 0.0011 | 1Score **> 31** indicates **identity** Score **> 17** indicates **homology** | U | K.FNPETDFLTGK.D |
| 77928 | 507 | – | 520 | 784.8841 | 1567.7537 | 1567.7518 | 1.20 | 1 | 67 | 5.8e-07 | 1Score **> 33** indicates **identity** Score **> 17** indicates **homology** | U | K.FNPETDFLTGKDGK.K |
| 77929 | 507 | – | 520 | 784.8849 | 1567.7553 | 1567.7518 | 2.20 | 1 | 42 | 0.00011 | 1Score **> 33** indicates **identity** Score **> 15** indicates **homology** | U | K.FNPETDFLTGKDGK.K |
| 77930 | 507 | – | 520 | 523.5925 | 1567.7557 | 1567.7518 | 2.44 | 1 | 50 | 2.3e-05 | 1Score **> 33** indicates **identity** Score **> 16** indicates **homology** | U | K.FNPETDFLTGKDGK.K |
| 94855 | 507 | – | 521 | 566.2873 | 1695.8402 | 1695.8468 | -3.88 | 2 | 29 | 0.0021 | 1Score **> 35** indicates **identity** Score **> 14** indicates **homology** | U | K.FNPETDFLTGKDGKK.F |
| 94860 | 507 | – | 521 | 848.9300 | 1695.8455 | 1695.8468 | -0.77 | 2 | 37 | 0.00036 | 1Score **> 35** indicates **identity** Score **> 15** indicates **homology** | U | K.FNPETDFLTGKDGKK.F |
| 94862 | 507 | – | 521 | 424.9690 | 1695.8471 | 1695.8468 | 0.17 | 2 | 19 | 0.016 | 1Score **> 34** indicates **identity** Score **> 14** indicates **homology** | U | K.FNPETDFLTGKDGKK.F |
| 94863 | 507 | – | 521 | 848.9310 | 1695.8473 | 1695.8468 | 0.32 | 2 | 33 | 0.001 | 1Score **> 35** indicates **identity** Score **> 15** indicates **homology** | U | K.FNPETDFLTGKDGKK.F |
| 94866 | 507 | – | 521 | 566.2901 | 1695.8484 | 1695.8468 | 0.94 | 2 | 36 | 0.00046 | 1Score **> 35** indicates **identity** Score **> 15** indicates **homology** | U | K.FNPETDFLTGKDGKK.F |
| 94867 | 507 | – | 521 | 566.2902 | 1695.8488 | 1695.8468 | 1.16 | 2 | 36 | 0.00038 | 1Score **> 35** indicates **identity** Score **> 15** indicates **homology** | U | K.FNPETDFLTGKDGKK.F |
| 94868 | 507 | – | 521 | 566.2904 | 1695.8494 | 1695.8468 | 1.52 | 2 | 29 | 0.0017 | 1Score **> 35** indicates **identity** Score **> 14** indicates **homology** | U | K.FNPETDFLTGKDGKK.F |
| 69743 | 522 | – | 534 | 750.8879 | 1499.7613 | 1499.7620 | -0.45 | 1 | 24 | 0.032 | 1Score **> 34** indicates **identity** Score **> 21** indicates **homology** | U | K.FKLEAPDADELPR.S |
| 69744 | 522 | – | 534 | 500.9279 | 1499.7618 | 1499.7620 | -0.12 | 1 | 55 | 2.5e-05 | 1Score **> 34** indicates **identity** Score **> 22** indicates **homology** | U | K.FKLEAPDADELPR.S |
| 69745 | 522 | – | 534 | 500.9281 | 1499.7625 | 1499.7620 | 0.38 | 1 | 40 | 0.0004 | 1Score **> 34** indicates **identity** Score **> 19** indicates **homology** | U | K.FKLEAPDADELPR.S |
| 84488 | 550 | – | 564 | 540.2652 | 1617.7739 | 1617.7707 | 1.97 | 1 | 28 | 0.0025 | 1Score **> 33** indicates **identity** Score **> 14** indicates **homology** | U | K.DSSGQRVDVSPTSQR.L |
| 14442 | 556 | – | 564 | 494.7568 | 987.4990 | 987.4985 | 0.52 | 0 | 54 | 0.00021 | 1Score **> 30** indicates **identity** | U | R.VDVSPTSQR.L |
| 171344 | 565 | – | 587 | 693.1326 | 2768.5011 | 2768.5000 | 0.40 | 2 | 26 | 0.0038 | 1Score **> 35** indicates **identity** Score **> 14** indicates **homology** | U | R.LQLLEPFDKWDGKDLEDLQILIK.V |
| 171345 | 565 | – | 587 | 923.8427 | 2768.5062 | 2768.5000 | 2.25 | 2 | 46 | 4.9e-05 | 1Score **> 35** indicates **identity** Score **> 15** indicates **homology** | U | R.LQLLEPFDKWDGKDLEDLQILIK.V |
| 171346 | 565 | – | 587 | 693.1359 | 2768.5144 | 2768.5000 | 5.20 | 2 | 18 | 0.02 | 1Score **> 35** indicates **identity** Score **> 14** indicates **homology** | U | R.LQLLEPFDKWDGKDLEDLQILIK.V |
| 100334 | 590 | – | 605 | 581.2948 | 1740.8626 | 1740.8617 | 0.47 | 1 | 19 | 0.016 | 1Score **> 34** indicates **identity** Score **> 14** indicates **homology** | U | K.GKCTTDHISAAGPWLK.F |
| 100335 | 590 | – | 605 | 581.2949 | 1740.8627 | 1740.8617 | 0.57 | 1 | 17 | 0.024 | 1Score **> 34** indicates **identity** Score **> 14** indicates **homology** | U | K.GKCTTDHISAAGPWLK.F |
| 100336 | 590 | – | 605 | 581.2950 | 1740.8631 | 1740.8617 | 0.80 | 1 | 56 | 5.6e-06 | 1Score **> 35** indicates **identity** Score **> 16** indicates **homology** | U | K.GKCTTDHISAAGPWLK.F |
| 100337 | 590 | – | 605 | 436.2232 | 1740.8636 | 1740.8617 | 1.05 | 1 | 23 | 0.0077 | 1Score **> 34** indicates **identity** Score **> 14** indicates **homology** | U | K.GKCTTDHISAAGPWLK.F |
| 100338 | 590 | – | 605 | 871.4395 | 1740.8645 | 1740.8617 | 1.56 | 1 | 91 | 3.2e-09 | 1Score **> 34** indicates **identity** Score **> 18** indicates **homology** | U | K.GKCTTDHISAAGPWLK.F |
| 76363 | 592 | – | 605 | 519.5851 | 1555.7335 | 1555.7453 | -7.59 | 0 | 25 | 0.0049 | 1Score **> 32** indicates **identity** Score **> 14** indicates **homology** | U | K.CTTDHISAAGPWLK.F |
| 76365 | 592 | – | 605 | 519.5874 | 1555.7403 | 1555.7453 | -3.23 | 0 | 23 | 0.0076 | 1Score **> 33** indicates **identity** Score **> 14** indicates **homology** | U | K.CTTDHISAAGPWLK.F |
| 76368 | 592 | – | 605 | 778.8781 | 1555.7416 | 1555.7453 | -2.37 | 0 | 49 | 0.00012 | 1Score **> 33** indicates **identity** Score **> 22** indicates **homology** | U | K.CTTDHISAAGPWLK.F |
| 76370 | 592 | – | 605 | 778.8791 | 1555.7437 | 1555.7453 | -1.05 | 0 | 48 | 0.00015 | 1Score **> 33** indicates **identity** Score **> 22** indicates **homology** | U | K.CTTDHISAAGPWLK.F |
| 76376 | 592 | – | 605 | 778.8816 | 1555.7486 | 1555.7453 | 2.10 | 0 | 50 | 1.9e-05 | 1Score **> 33** indicates **identity** Score **> 16** indicates **homology** | U | K.CTTDHISAAGPWLK.F |
| 162079 | 606 | – | 628 | 841.4553 | 2521.3439 | 2521.3401 | 1.54 | 1 | 48 | 3.1e-05 | 1Score **> 36** indicates **identity** Score **> 16** indicates **homology** | U | K.FRGHLDNISNNLLIGAINIENGK.A |
| 162080 | 606 | – | 628 | 631.3434 | 2521.3446 | 2521.3401 | 1.79 | 1 | 47 | 7.6e-05 | 1Score **> 36** indicates **identity** Score **> 19** indicates **homology** | U | K.FRGHLDNISNNLLIGAINIENGK.A |
| 162082 | 606 | – | 628 | 631.3436 | 2521.3452 | 2521.3401 | 2.03 | 1 | 39 | 0.00036 | 1Score **> 36** indicates **identity** Score **> 17** indicates **homology** | U | K.FRGHLDNISNNLLIGAINIENGK.A |
| 162083 | 606 | – | 628 | 631.3436 | 2521.3453 | 2521.3401 | 2.06 | 1 | 33 | 0.00077 | 1Score **> 36** indicates **identity** Score **> 15** indicates **homology** | U | K.FRGHLDNISNNLLIGAINIENGK.A |
| 162084 | 606 | – | 628 | 631.3437 | 2521.3458 | 2521.3401 | 2.29 | 1 | 36 | 0.00042 | 1Score **> 36** indicates **identity** Score **> 15** indicates **homology** | U | K.FRGHLDNISNNLLIGAINIENGK.A |
| 162085 | 606 | – | 628 | 631.3438 | 2521.3462 | 2521.3401 | 2.43 | 1 | 33 | 0.00079 | 1Score **> 36** indicates **identity** Score **> 15** indicates **homology** | U | K.FRGHLDNISNNLLIGAINIENGK.A |
| 162086 | 606 | – | 628 | 631.3440 | 2521.3469 | 2521.3401 | 2.70 | 1 | 17 | 0.026 | 1Score **> 36** indicates **identity** Score **> 14** indicates **homology** | U | K.FRGHLDNISNNLLIGAINIENGK.A |
| 162087 | 606 | – | 628 | 631.3443 | 2521.3480 | 2521.3401 | 3.13 | 1 | 21 | 0.012 | 1Score **> 36** indicates **identity** Score **> 14** indicates **homology** | U | K.FRGHLDNISNNLLIGAINIENGK.A |
| 162088 | 606 | – | 628 | 631.3444 | 2521.3484 | 2521.3401 | 3.32 | 1 | 19 | 0.016 | 1Score **> 36** indicates **identity** Score **> 14** indicates **homology** | U | K.FRGHLDNISNNLLIGAINIENGK.A |
| 179853 | 606 | – | 633 | 763.4029 | 3049.5824 | 3049.6057 | -7.62 | 2 | 24 | 0.0061 | 1Score **> 37** indicates **identity** Score **> 14** indicates **homology** | U | K.FRGHLDNISNNLLIGAINIENGKANSVR.N  + Deamidated (NQ) |
| 179953 | 606 | – | 633 | 763.4083 | 3049.6041 | 3049.6057 | -0.50 | 2 | 37 | 0.00037 | 1Score **> 37** indicates **identity** Score **> 15** indicates **homology** | U | K.FRGHLDNISNNLLIGAINIENGKANSVR.N  + Deamidated (NQ) |
| 179957 | 606 | – | 633 | 610.9291 | 3049.6090 | 3049.6057 | 1.10 | 2 | 33 | 0.00084 | 1Score **> 37** indicates **identity** Score **> 15** indicates **homology** | U | K.FRGHLDNISNNLLIGAINIENGKANSVR.N  + Deamidated (NQ) |
| 179959 | 606 | – | 633 | 610.9294 | 3049.6106 | 3049.6057 | 1.61 | 2 | 20 | 0.013 | 1Score **> 37** indicates **identity** Score **> 14** indicates **homology** | U | K.FRGHLDNISNNLLIGAINIENGKANSVR.N  + Deamidated (NQ) |
| 179961 | 606 | – | 633 | 763.4101 | 3049.6112 | 3049.6057 | 1.81 | 2 | 40 | 0.00017 | 1Score **> 37** indicates **identity** Score **> 15** indicates **homology** | U | K.FRGHLDNISNNLLIGAINIENGKANSVR.N  + Deamidated (NQ) |
| 179962 | 606 | – | 633 | 763.4102 | 3049.6118 | 3049.6057 | 2.03 | 2 | 62 | 1.5e-06 | 1Score **> 37** indicates **identity** Score **> 16** indicates **homology** | U | K.FRGHLDNISNNLLIGAINIENGKANSVR.N  + Deamidated (NQ) |
| 180015 | 606 | – | 633 | 1017.8788 | 3050.6147 | 3050.5897 | 8.21 | 2 | 48 | 3.2e-05 | 1Score **> 36** indicates **identity** Score **> 16** indicates **homology** | U | K.FRGHLDNISNNLLIGAINIENGKANSVR.N  + 2 Deamidated (NQ) |
| 145346 | 608 | – | 628 | 740.3988 | 2218.1745 | 2218.1705 | 1.78 | 0 | 61 | 2.3e-06 | 1Score **> 36** indicates **identity** Score **> 17** indicates **homology** | U | R.GHLDNISNNLLIGAINIENGK.A |
| 145347 | 608 | – | 628 | 740.3989 | 2218.1749 | 2218.1705 | 1.94 | 0 | 22 | 0.0094 | 1Score **> 36** indicates **identity** Score **> 14** indicates **homology** | U | R.GHLDNISNNLLIGAINIENGK.A |
| 145348 | 608 | – | 628 | 1110.0947 | 2218.1749 | 2218.1705 | 1.98 | 0 | 66 | 6.7e-07 | 1Score **> 36** indicates **identity** Score **> 17** indicates **homology** | U | R.GHLDNISNNLLIGAINIENGK.A |
| 145349 | 608 | – | 628 | 740.3992 | 2218.1759 | 2218.1705 | 2.39 | 0 | 50 | 2.6e-05 | 1Score **> 36** indicates **identity** Score **> 16** indicates **homology** | U | R.GHLDNISNNLLIGAINIENGK.A |
| 145352 | 608 | – | 628 | 1110.0968 | 2218.1790 | 2218.1705 | 3.82 | 0 | 66 | 6.5e-07 | 1Score **> 36** indicates **identity** Score **> 17** indicates **homology** | U | R.GHLDNISNNLLIGAINIENGK.A |
| 145353 | 608 | – | 628 | 740.4009 | 2218.1809 | 2218.1705 | 4.66 | 0 | 34 | 0.00061 | 1Score **> 36** indicates **identity** Score **> 15** indicates **homology** | U | R.GHLDNISNNLLIGAINIENGK.A |
| 170421 | 608 | – | 633 | 687.3714 | 2745.4563 | 2745.4521 | 1.52 | 1 | 24 | 0.0053 | 1Score **> 37** indicates **identity** Score **> 14** indicates **homology** | U | R.GHLDNISNNLLIGAINIENGKANSVR.N |
| 170422 | 608 | – | 633 | 916.1594 | 2745.4563 | 2745.4521 | 1.53 | 1 | 79 | 4.4e-08 | 1Score **> 37** indicates **identity** Score **> 18** indicates **homology** | U | R.GHLDNISNNLLIGAINIENGKANSVR.N |
| 170423 | 608 | – | 633 | 687.3714 | 2745.4566 | 2745.4521 | 1.64 | 1 | 40 | 0.00019 | 1Score **> 37** indicates **identity** Score **> 15** indicates **homology** | U | R.GHLDNISNNLLIGAINIENGKANSVR.N |
| 170425 | 608 | – | 633 | 916.1602 | 2745.4588 | 2745.4521 | 2.43 | 1 | 59 | 3e-06 | 1Score **> 37** indicates **identity** Score **> 16** indicates **homology** | U | R.GHLDNISNNLLIGAINIENGKANSVR.N |
| 170472 | 608 | – | 633 | 687.6161 | 2746.4352 | 2746.4361 | -0.33 | 1 | 47 | 0.00011 | 1Score **> 37** indicates **identity** Score **> 19** indicates **homology** | U | R.GHLDNISNNLLIGAINIENGKANSVR.N  + Deamidated (NQ) |
| 170473 | 608 | – | 633 | 916.4857 | 2746.4354 | 2746.4361 | -0.28 | 1 | 82 | 2.2e-08 | 1Score **> 37** indicates **identity** Score **> 18** indicates **homology** | U | R.GHLDNISNNLLIGAINIENGKANSVR.N  + Deamidated (NQ) |
| 170474 | 608 | – | 633 | 916.4866 | 2746.4381 | 2746.4361 | 0.70 | 1 | 72 | 1.6e-07 | 1Score **> 37** indicates **identity** Score **> 17** indicates **homology** | U | R.GHLDNISNNLLIGAINIENGKANSVR.N  + Deamidated (NQ) |
| 170475 | 608 | – | 633 | 687.6169 | 2746.4385 | 2746.4361 | 0.86 | 1 | 47 | 8.7e-05 | 1Score **> 37** indicates **identity** Score **> 19** indicates **homology** | U | R.GHLDNISNNLLIGAINIENGKANSVR.N  + Deamidated (NQ) |
| 170476 | 608 | – | 633 | 687.6170 | 2746.4389 | 2746.4361 | 1.00 | 1 | 52 | 2.1e-05 | 1Score **> 37** indicates **identity** Score **> 18** indicates **homology** | U | R.GHLDNISNNLLIGAINIENGKANSVR.N  + Deamidated (NQ) |
| 170477 | 608 | – | 633 | 916.4924 | 2746.4555 | 2746.4361 | 7.04 | 1 | 53 | 1.2e-05 | 1Score **> 37** indicates **identity** Score **> 16** indicates **homology** | U | R.GHLDNISNNLLIGAINIENGKANSVR.N  + Deamidated (NQ) |
| 170503 | 608 | – | 633 | 916.8198 | 2747.4374 | 2747.4202 | 6.29 | 1 | 37 | 0.00034 | 1Score **> 37** indicates **identity** Score **> 15** indicates **homology** | U | R.GHLDNISNNLLIGAINIENGKANSVR.N  + 2 Deamidated (NQ) |
| 82420 | 634 | – | 648 | 534.6015 | 1600.7826 | 1600.7845 | -1.19 | 0 | 20 | 0.014 | 1Score **> 34** indicates **identity** Score **> 14** indicates **homology** | U | R.NAVTQEFGPVPDTAR.Y |
| 82423 | 634 | – | 648 | 801.4000 | 1600.7855 | 1600.7845 | 0.59 | 0 | 79 | 8.8e-08 | 1Score **> 34** indicates **identity** Score **> 21** indicates **homology** | U | R.NAVTQEFGPVPDTAR.Y |
| 82424 | 634 | – | 648 | 534.6025 | 1600.7855 | 1600.7845 | 0.62 | 0 | 31 | 0.0013 | 1Score **> 34** indicates **identity** Score **> 14** indicates **homology** | U | R.NAVTQEFGPVPDTAR.Y |
| 82426 | 634 | – | 648 | 801.4002 | 1600.7858 | 1600.7845 | 0.79 | 0 | 84 | 2.8e-08 | 1Score **> 34** indicates **identity** Score **> 21** indicates **homology** | U | R.NAVTQEFGPVPDTAR.Y |
| 82427 | 634 | – | 648 | 801.4004 | 1600.7862 | 1600.7845 | 1.03 | 0 | 84 | 2.8e-08 | 1Score **> 34** indicates **identity** Score **> 21** indicates **homology** | U | R.NAVTQEFGPVPDTAR.Y |
| 82428 | 634 | – | 648 | 801.4012 | 1600.7878 | 1600.7845 | 2.01 | 0 | 35 | 0.00054 | 1Score **> 34** indicates **identity** Score **> 15** indicates **homology** | U | R.NAVTQEFGPVPDTAR.Y |
| 82429 | 634 | – | 648 | 534.6032 | 1600.7879 | 1600.7845 | 2.10 | 0 | 22 | 0.0081 | 1Score **> 34** indicates **identity** Score **> 14** indicates **homology** | U | R.NAVTQEFGPVPDTAR.Y |
| 82447 | 634 | – | 648 | 801.4065 | 1600.7985 | 1600.7845 | 8.75 | 0 | 44 | 0.0065 | 2Score **> 34** indicates **identity** | U | R.NAVTQEFGPVPDTAR.Y |
| 90966 | 657 | – | 671 | 834.3870 | 1666.7595 | 1666.7587 | 0.44 | 0 | 54 | 9.2e-06 | 1Score **> 30** indicates **identity** Score **> 16** indicates **homology** | U | R.WVVIGDENYGEGSSR.E |
| 90967 | 657 | – | 671 | 834.3871 | 1666.7597 | 1666.7587 | 0.58 | 0 | 67 | 5.8e-07 | 1Score **> 30** indicates **identity** Score **> 17** indicates **homology** | U | R.WVVIGDENYGEGSSR.E |
| 90968 | 657 | – | 671 | 834.3873 | 1666.7600 | 1666.7587 | 0.76 | 0 | 74 | 1.1e-07 | 1Score **> 30** indicates **identity** Score **> 17** indicates **homology** | U | R.WVVIGDENYGEGSSR.E |
| 90969 | 657 | – | 671 | 834.3874 | 1666.7602 | 1666.7587 | 0.88 | 0 | 41 | 0.00015 | 1Score **> 30** indicates **identity** Score **> 15** indicates **homology** | U | R.WVVIGDENYGEGSSR.E |
| 90970 | 657 | – | 671 | 834.3876 | 1666.7606 | 1666.7587 | 1.15 | 0 | 70 | 2.8e-07 | 1Score **> 30** indicates **identity** Score **> 17** indicates **homology** | U | R.WVVIGDENYGEGSSR.E |
| 164262 | 657 | – | 679 | 643.5610 | 2570.2148 | 2570.2150 | -0.065 | 1 | 17 | 0.024 | 1Score **> 35** indicates **identity** Score **> 14** indicates **homology** | U | R.WVVIGDENYGEGSSREHAALEPR.H |
| 164264 | 657 | – | 679 | 643.5611 | 2570.2152 | 2570.2150 | 0.080 | 1 | 16 | 0.031 | 1Score **> 35** indicates **identity** Score **> 14** indicates **homology** | U | R.WVVIGDENYGEGSSREHAALEPR.H |
| 164266 | 657 | – | 679 | 857.7457 | 2570.2154 | 2570.2150 | 0.18 | 1 | 18 | 0.02 | 1Score **> 35** indicates **identity** Score **> 14** indicates **homology** | U | R.WVVIGDENYGEGSSREHAALEPR.H |
| 164267 | 657 | – | 679 | 857.7458 | 2570.2156 | 2570.2150 | 0.24 | 1 | 32 | 0.00095 | 1Score **> 35** indicates **identity** Score **> 15** indicates **homology** | U | R.WVVIGDENYGEGSSREHAALEPR.H |
| 164268 | 657 | – | 679 | 643.5615 | 2570.2169 | 2570.2150 | 0.77 | 1 | 25 | 0.0043 | 1Score **> 35** indicates **identity** Score **> 14** indicates **homology** | U | R.WVVIGDENYGEGSSREHAALEPR.H |
| 164270 | 657 | – | 679 | 857.7463 | 2570.2172 | 2570.2150 | 0.87 | 1 | 17 | 0.026 | 1Score **> 35** indicates **identity** Score **> 14** indicates **homology** | U | R.WVVIGDENYGEGSSREHAALEPR.H |
| 164271 | 657 | – | 679 | 643.5617 | 2570.2175 | 2570.2150 | 1.00 | 1 | 15 | 0.042 | 1Score **> 35** indicates **identity** Score **> 13** indicates **homology** | U | R.WVVIGDENYGEGSSREHAALEPR.H |
| 117626 | 701 | – | 717 | 636.3348 | 1905.9825 | 1905.9836 | -0.60 | 1 | 56 | 5.6e-06 | 1Score **> 36** indicates **identity** Score **> 16** indicates **homology** | U | K.KQGLLPLTFADPSDYNK.I |
| 117629 | 701 | – | 717 | 636.3362 | 1905.9866 | 1905.9836 | 1.59 | 1 | 39 | 0.00023 | 1Score **> 36** indicates **identity** Score **> 15** indicates **homology** | U | K.KQGLLPLTFADPSDYNK.I |
| 117630 | 701 | – | 717 | 636.3364 | 1905.9874 | 1905.9836 | 1.97 | 1 | 29 | 0.0019 | 1Score **> 36** indicates **identity** Score **> 14** indicates **homology** | U | K.KQGLLPLTFADPSDYNK.I |
| 117635 | 701 | – | 717 | 636.3376 | 1905.9909 | 1905.9836 | 3.81 | 1 | 36 | 0.00045 | 1Score **> 36** indicates **identity** Score **> 15** indicates **homology** | U | K.KQGLLPLTFADPSDYNK.I |
| 104646 | 702 | – | 717 | 889.9479 | 1777.8812 | 1777.8887 | -4.20 | 0 | 42 | 0.011 | 1Score **> 35** indicates **identity** | U | K.QGLLPLTFADPSDYNK.I |
| 104649 | 702 | – | 717 | 889.9509 | 1777.8872 | 1777.8887 | -0.85 | 0 | 67 | 5.1e-07 | 1Score **> 35** indicates **identity** Score **> 17** indicates **homology** | U | K.QGLLPLTFADPSDYNK.I |
| 104650 | 702 | – | 717 | 889.9512 | 1777.8879 | 1777.8887 | -0.44 | 0 | 56 | 5.9e-06 | 1Score **> 35** indicates **identity** Score **> 16** indicates **homology** | U | K.QGLLPLTFADPSDYNK.I |
| 104651 | 702 | – | 717 | 889.9513 | 1777.8881 | 1777.8887 | -0.33 | 0 | 29 | 0.0021 | 1Score **> 35** indicates **identity** Score **> 14** indicates **homology** | U | K.QGLLPLTFADPSDYNK.I |
| 104653 | 702 | – | 717 | 889.9523 | 1777.8900 | 1777.8887 | 0.77 | 0 | 45 | 6.4e-05 | 1Score **> 35** indicates **identity** Score **> 15** indicates **homology** | U | K.QGLLPLTFADPSDYNK.I |
| 98430 | 724 | – | 739 | 432.2621 | 1725.0192 | 1725.0189 | 0.20 | 2 | 23 | 0.0068 | 1Score **> 31** indicates **identity** Score **> 14** indicates **homology** | U | K.LTIQGLKDFAPGKPLK.C |
| 98431 | 724 | – | 739 | 432.2623 | 1725.0202 | 1725.0189 | 0.78 | 2 | 33 | 0.00074 | 1Score **> 31** indicates **identity** Score **> 15** indicates **homology** | U | K.LTIQGLKDFAPGKPLK.C |
| 13446 | 731 | – | 739 | 486.7791 | 971.5437 | 971.5440 | -0.29 | 1 | 21 | 0.011 | 1Score **> 32** indicates **identity** Score **> 14** indicates **homology** | U | K.DFAPGKPLK.C |
| 13447 | 731 | – | 739 | 486.7793 | 971.5440 | 971.5440 | -0.054 | 1 | 19 | 0.017 | 1Score **> 32** indicates **identity** Score **> 14** indicates **homology** | U | K.DFAPGKPLK.C |
| 186420 | 740 | – | 767 | 857.1787 | 3424.6855 | 3424.6986 | -3.82 | 1 | 23 | 0.0067 | 1Score **> 38** indicates **identity** Score **> 14** indicates **homology** | U | K.CVIKHPNGTQETILLNHTFNETQIEWFR.A |
| 186421 | 740 | – | 767 | 857.1817 | 3424.6976 | 3424.6986 | -0.31 | 1 | 64 | 1.1e-06 | 1Score **> 38** indicates **identity** Score **> 16** indicates **homology** | U | K.CVIKHPNGTQETILLNHTFNETQIEWFR.A |
| 186422 | 740 | – | 767 | 685.9474 | 3424.7009 | 3424.6986 | 0.65 | 1 | 28 | 0.0026 | 1Score **> 38** indicates **identity** Score **> 14** indicates **homology** | U | K.CVIKHPNGTQETILLNHTFNETQIEWFR.A |
| 186423 | 740 | – | 767 | 685.9475 | 3424.7011 | 3424.6986 | 0.73 | 1 | 26 | 0.0039 | 1Score **> 38** indicates **identity** Score **> 14** indicates **homology** | U | K.CVIKHPNGTQETILLNHTFNETQIEWFR.A |
| 186424 | 740 | – | 767 | 857.1826 | 3424.7015 | 3424.6986 | 0.82 | 1 | 46 | 5e-05 | 1Score **> 38** indicates **identity** Score **> 15** indicates **homology** | U | K.CVIKHPNGTQETILLNHTFNETQIEWFR.A |
| 186425 | 740 | – | 767 | 1142.5748 | 3424.7026 | 3424.6986 | 1.17 | 1 | 26 | 0.0036 | 1Score **> 38** indicates **identity** Score **> 14** indicates **homology** | U | K.CVIKHPNGTQETILLNHTFNETQIEWFR.A |
| 186426 | 740 | – | 767 | 857.1830 | 3424.7027 | 3424.6986 | 1.19 | 1 | 47 | 3.8e-05 | 1Score **> 38** indicates **identity** Score **> 15** indicates **homology** | U | K.CVIKHPNGTQETILLNHTFNETQIEWFR.A |
| 186428 | 740 | – | 767 | 857.1853 | 3424.7120 | 3424.6986 | 3.92 | 1 | 22 | 0.0084 | 1Score **> 38** indicates **identity** Score **> 14** indicates **homology** | U | K.CVIKHPNGTQETILLNHTFNETQIEWFR.A |
| 186439 | 740 | – | 767 | 857.4251 | 3425.6712 | 3425.6826 | -3.33 | 1 | 17 | 0.024 | 1Score **> 37** indicates **identity** Score **> 14** indicates **homology** | U | K.CVIKHPNGTQETILLNHTFNETQIEWFR.A  + Deamidated (NQ) |
| 186440 | 740 | – | 767 | 1142.9092 | 3425.7057 | 3425.6826 | 6.73 | 1 | 22 | 0.0095 | 1Score **> 37** indicates **identity** Score **> 14** indicates **homology** | U | K.CVIKHPNGTQETILLNHTFNETQIEWFR.A  + Deamidated (NQ) |

---

```
ID   ACON_MOUSE              Reviewed;         780 AA.
AC   Q99KI0; Q3UDK9; Q3ULG9; Q3UNH7; Q505P4;
DT   01-FEB-2005, integrated into UniProtKB/Swiss-Prot.
DT   01-JUN-2001, sequence version 1.
DT   28-JUN-2023, entry version 164.
DE   RecName: Full=Aconitate hydratase, mitochondrial;
DE            Short=Aconitase;
DE            EC=4.2.1.3 {ECO:0000250|UniProtKB:P16276};
DE   AltName: Full=Citrate hydro-lyase;
DE   Flags: Precursor;
GN   Name=Aco2;
OS   Mus musculus (Mouse).
OC   Eukaryota; Metazoa; Chordata; Craniata; Vertebrata; Euteleostomi; Mammalia;
OC   Eutheria; Euarchontoglires; Glires; Rodentia; Myomorpha; Muroidea; Muridae;
OC   Murinae; Mus; Mus.
OX   NCBI_TaxID=10090;
RN   [1]
RP   NUCLEOTIDE SEQUENCE [LARGE SCALE MRNA].
RC   STRAIN=C57BL/6J; TISSUE=Bone marrow, and Kidney;
RX   PubMed=16141072; DOI=10.1126/science.1112014;
RA   Carninci P., Kasukawa T., Katayama S., Gough J., Frith M.C., Maeda N.,
RA   Oyama R., Ravasi T., Lenhard B., Wells C., Kodzius R., Shimokawa K.,
RA   Bajic V.B., Brenner S.E., Batalov S., Forrest A.R., Zavolan M., Davis M.J.,
RA   Wilming L.G., Aidinis V., Allen J.E., Ambesi-Impiombato A., Apweiler R.,
RA   Aturaliya R.N., Bailey T.L., Bansal M., Baxter L., Beisel K.W., Bersano T.,
RA   Bono H., Chalk A.M., Chiu K.P., Choudhary V., Christoffels A.,
RA   Clutterbuck D.R., Crowe M.L., Dalla E., Dalrymple B.P., de Bono B.,
RA   Della Gatta G., di Bernardo D., Down T., Engstrom P., Fagiolini M.,
RA   Faulkner G., Fletcher C.F., Fukushima T., Furuno M., Futaki S.,
RA   Gariboldi M., Georgii-Hemming P., Gingeras T.R., Gojobori T., Green R.E.,
RA   Gustincich S., Harbers M., Hayashi Y., Hensch T.K., Hirokawa N., Hill D.,
RA   Huminiecki L., Iacono M., Ikeo K., Iwama A., Ishikawa T., Jakt M.,
RA   Kanapin A., Katoh M., Kawasawa Y., Kelso J., Kitamura H., Kitano H.,
RA   Kollias G., Krishnan S.P., Kruger A., Kummerfeld S.K., Kurochkin I.V.,
RA   Lareau L.F., Lazarevic D., Lipovich L., Liu J., Liuni S., McWilliam S.,
RA   Madan Babu M., Madera M., Marchionni L., Matsuda H., Matsuzawa S., Miki H.,
RA   Mignone F., Miyake S., Morris K., Mottagui-Tabar S., Mulder N., Nakano N.,
RA   Nakauchi H., Ng P., Nilsson R., Nishiguchi S., Nishikawa S., Nori F.,
RA   Ohara O., Okazaki Y., Orlando V., Pang K.C., Pavan W.J., Pavesi G.,
RA   Pesole G., Petrovsky N., Piazza S., Reed J., Reid J.F., Ring B.Z.,
RA   Ringwald M., Rost B., Ruan Y., Salzberg S.L., Sandelin A., Schneider C.,
RA   Schoenbach C., Sekiguchi K., Semple C.A., Seno S., Sessa L., Sheng Y.,
RA   Shibata Y., Shimada H., Shimada K., Silva D., Sinclair B., Sperling S.,
RA   Stupka E., Sugiura K., Sultana R., Takenaka Y., Taki K., Tammoja K.,
RA   Tan S.L., Tang S., Taylor M.S., Tegner J., Teichmann S.A., Ueda H.R.,
RA   van Nimwegen E., Verardo R., Wei C.L., Yagi K., Yamanishi H.,
RA   Zabarovsky E., Zhu S., Zimmer A., Hide W., Bult C., Grimmond S.M.,
RA   Teasdale R.D., Liu E.T., Brusic V., Quackenbush J., Wahlestedt C.,
RA   Mattick J.S., Hume D.A., Kai C., Sasaki D., Tomaru Y., Fukuda S.,
RA   Kanamori-Katayama M., Suzuki M., Aoki J., Arakawa T., Iida J., Imamura K.,
RA   Itoh M., Kato T., Kawaji H., Kawagashira N., Kawashima T., Kojima M.,
RA   Kondo S., Konno H., Nakano K., Ninomiya N., Nishio T., Okada M., Plessy C.,
RA   Shibata K., Shiraki T., Suzuki S., Tagami M., Waki K., Watahiki A.,
RA   Okamura-Oho Y., Suzuki H., Kawai J., Hayashizaki Y.;
RT   "The transcriptional landscape of the mammalian genome.";
RL   Science 309:1559-1563(2005).
RN   [2]
RP   NUCLEOTIDE SEQUENCE [LARGE SCALE MRNA].
RC   STRAIN=FVB/N; TISSUE=Kidney, and Mammary tumor;
RX   PubMed=15489334; DOI=10.1101/gr.2596504;
RG   The MGC Project Team;
RT   "The status, quality, and expansion of the NIH full-length cDNA project:
RT   the Mammalian Gene Collection (MGC).";
RL   Genome Res. 14:2121-2127(2004).
RN   [3]
RP   PROTEIN SEQUENCE OF 32-56; 59-84; 96-138; 143-160; 234-245; 251-258;
RP   313-323; 371-395; 402-409; 412-424; 430-457; 466-474; 480-517; 522-587;
RP   592-605; 608-628; 634-648; 657-671; 694-739 AND 744-767, AND IDENTIFICATION
RP   BY MASS SPECTROMETRY.
RC   STRAIN=C57BL/6J, and OF1; TISSUE=Brain, and Hippocampus;
RA   Lubec G., Klug S., Kang S.U., Sunyer B., Chen W.-Q.;
RL   Submitted (JAN-2009) to UniProtKB.
RN   [4]
RP   PHOSPHORYLATION [LARGE SCALE ANALYSIS] AT SER-670, AND IDENTIFICATION BY
RP   MASS SPECTROMETRY [LARGE SCALE ANALYSIS].
RC   TISSUE=Brain, Brown adipose tissue, Heart, Kidney, Liver, Lung,
RC   Pancreas, Spleen, and Testis;
RX   PubMed=21183079; DOI=10.1016/j.cell.2010.12.001;
RA   Huttlin E.L., Jedrychowski M.P., Elias J.E., Goswami T., Rad R.,
RA   Beausoleil S.A., Villen J., Haas W., Sowa M.E., Gygi S.P.;
RT   "A tissue-specific atlas of mouse protein phosphorylation and expression.";
RL   Cell 143:1174-1189(2010).
RN   [5]
RP   ACETYLATION [LARGE SCALE ANALYSIS] AT LYS-50 AND LYS-144, SUCCINYLATION
RP   [LARGE SCALE ANALYSIS] AT LYS-31; LYS-50; LYS-138; LYS-144; LYS-233;
RP   LYS-411; LYS-517; LYS-523; LYS-549; LYS-573; LYS-577; LYS-591; LYS-605;
RP   LYS-628; LYS-689; LYS-723 AND LYS-730, AND IDENTIFICATION BY MASS
RP   SPECTROMETRY [LARGE SCALE ANALYSIS].
RC   TISSUE=Embryonic fibroblast, and Liver;
RX   PubMed=23806337; DOI=10.1016/j.molcel.2013.06.001;
RA   Park J., Chen Y., Tishkoff D.X., Peng C., Tan M., Dai L., Xie Z., Zhang Y.,
RA   Zwaans B.M., Skinner M.E., Lombard D.B., Zhao Y.;
RT   "SIRT5-mediated lysine desuccinylation impacts diverse metabolic
RT   pathways.";
RL   Mol. Cell 50:919-930(2013).
RN   [6]
RP   ACETYLATION [LARGE SCALE ANALYSIS] AT LYS-50; LYS-138; LYS-144; LYS-233;
RP   LYS-517; LYS-523; LYS-605; LYS-723; LYS-730; LYS-736; LYS-739 AND LYS-743,
RP   AND IDENTIFICATION BY MASS SPECTROMETRY [LARGE SCALE ANALYSIS].
RC   TISSUE=Liver;
RX   PubMed=23576753; DOI=10.1073/pnas.1302961110;
RA   Rardin M.J., Newman J.C., Held J.M., Cusack M.P., Sorensen D.J., Li B.,
RA   Schilling B., Mooney S.D., Kahn C.R., Verdin E., Gibson B.W.;
RT   "Label-free quantitative proteomics of the lysine acetylome in mitochondria
RT   identifies substrates of SIRT3 in metabolic pathways.";
RL   Proc. Natl. Acad. Sci. U.S.A. 110:6601-6606(2013).
CC   -!- FUNCTION: Catalyzes the isomerization of citrate to isocitrate via cis-
CC       aconitate. {ECO:0000250|UniProtKB:P16276}.
CC   -!- CATALYTIC ACTIVITY:
CC       Reaction=citrate = D-threo-isocitrate; Xref=Rhea:RHEA:10336,
CC         ChEBI:CHEBI:15562, ChEBI:CHEBI:16947; EC=4.2.1.3;
CC         Evidence={ECO:0000250|UniProtKB:P16276};
CC   -!- COFACTOR:
CC       Name=[4Fe-4S] cluster; Xref=ChEBI:CHEBI:49883;
CC         Evidence={ECO:0000250|UniProtKB:P16276};
CC       Note=Binds 1 [4Fe-4S] cluster per subunit. Binding of a [3Fe-4S]
CC       cluster leads to an inactive enzyme. {ECO:0000250|UniProtKB:P16276};
CC   -!- PATHWAY: Carbohydrate metabolism; tricarboxylic acid cycle; isocitrate
CC       from oxaloacetate: step 2/2.
CC   -!- SUBUNIT: Monomer. {ECO:0000250|UniProtKB:P16276}.
CC   -!- SUBCELLULAR LOCATION: Mitochondrion {ECO:0000250|UniProtKB:P16276}.
CC   -!- PTM: Forms covalent cross-links mediated by transglutaminase TGM2,
CC       between a glutamine and the epsilon-amino group of a lysine residue,
CC       forming homopolymers and heteropolymers.
CC       {ECO:0000250|UniProtKB:Q9ER34}.
CC   -!- SIMILARITY: Belongs to the aconitase/IPM isomerase family.
CC       {ECO:0000305}.
CC   ---------------------------------------------------------------------------
CC   Copyrighted by the UniProt Consortium, see https://www.uniprot.org/terms
CC   Distributed under the Creative Commons Attribution (CC BY 4.0) License
CC   ---------------------------------------------------------------------------
DR   EMBL; AK143917; BAE25602.1; -; mRNA.
DR   EMBL; AK144207; BAE25770.1; -; mRNA.
DR   EMBL; AK145511; BAE26479.1; -; mRNA.
DR   EMBL; AK150027; BAE29252.1; -; mRNA.
DR   EMBL; AK165411; BAE38169.1; -; mRNA.
DR   EMBL; BC004645; AAH04645.1; -; mRNA.
DR   EMBL; BC094462; AAH94462.1; -; mRNA.
DR   CCDS; CCDS27675.1; -.
DR   RefSeq; NP_542364.1; NM_080633.2.
DR   AlphaFoldDB; Q99KI0; -.
DR   SMR; Q99KI0; -.
DR   BioGRID; 197925; 72.
DR   IntAct; Q99KI0; 8.
DR   MINT; Q99KI0; -.
DR   STRING; 10090.ENSMUSP00000023116; -.
DR   CarbonylDB; Q99KI0; -.
DR   GlyGen; Q99KI0; 1 site, 1 O-linked glycan (1 site).
DR   iPTMnet; Q99KI0; -.
DR   PhosphoSitePlus; Q99KI0; -.
DR   SwissPalm; Q99KI0; -.
DR   REPRODUCTION-2DPAGE; Q99KI0; -.
DR   EPD; Q99KI0; -.
DR   jPOST; Q99KI0; -.
DR   MaxQB; Q99KI0; -.
DR   PaxDb; Q99KI0; -.
DR   PeptideAtlas; Q99KI0; -.
DR   ProteomicsDB; 285596; -.
DR   Antibodypedia; 240; 582 antibodies from 39 providers.
DR   DNASU; 11429; -.
DR   Ensembl; ENSMUST00000023116; ENSMUSP00000023116; ENSMUSG00000022477.
DR   GeneID; 11429; -.
DR   KEGG; mmu:11429; -.
DR   UCSC; uc007wxp.1; mouse.
DR   AGR; MGI:87880; -.
DR   CTD; 50; -.
DR   MGI; MGI:87880; Aco2.
DR   VEuPathDB; HostDB:ENSMUSG00000022477; -.
DR   eggNOG; KOG0453; Eukaryota.
DR   GeneTree; ENSGT00940000154892; -.
DR   HOGENOM; CLU_006714_2_2_1; -.
DR   InParanoid; Q99KI0; -.
DR   OMA; GCIGMGQ; -.
DR   OrthoDB; 3266779at2759; -.
DR   PhylomeDB; Q99KI0; -.
DR   TreeFam; TF300627; -.
DR   BRENDA; 4.2.1.3; 3474.
DR   Reactome; R-MMU-71403; Citric acid cycle (TCA cycle).
DR   UniPathway; UPA00223; UER00718.
DR   BioGRID-ORCS; 11429; 16 hits in 78 CRISPR screens.
DR   ChiTaRS; Aco2; mouse.
DR   PRO; PR:Q99KI0; -.
DR   Proteomes; UP000000589; Chromosome 15.
DR   RNAct; Q99KI0; protein.
DR   Bgee; ENSMUSG00000022477; Expressed in cardiac muscle of left ventricle and 271 other tissues.
DR   ExpressionAtlas; Q99KI0; baseline and differential.
DR   Genevisible; Q99KI0; MM.
DR   GO; GO:0005829; C:cytosol; IBA:GO_Central.
DR   GO; GO:0005759; C:mitochondrial matrix; IDA:MGI.
DR   GO; GO:0005739; C:mitochondrion; IDA:MGI.
DR   GO; GO:0043209; C:myelin sheath; HDA:UniProtKB.
DR   GO; GO:0051538; F:3 iron, 4 sulfur cluster binding; ISO:MGI.
DR   GO; GO:0051539; F:4 iron, 4 sulfur cluster binding; ISO:MGI.
DR   GO; GO:0003994; F:aconitate hydratase activity; IDA:MGI.
DR   GO; GO:0047780; F:citrate dehydratase activity; IEA:UniProtKB-EC.
DR   GO; GO:0005506; F:iron ion binding; ISO:MGI.
DR   GO; GO:0006101; P:citrate metabolic process; ISO:MGI.
DR   GO; GO:0006102; P:isocitrate metabolic process; ISO:MGI.
DR   GO; GO:0001889; P:liver development; IEA:Ensembl.
DR   GO; GO:0035900; P:response to isolation stress; IEA:Ensembl.
DR   GO; GO:0006099; P:tricarboxylic acid cycle; IGI:MGI.
DR   CDD; cd01578; AcnA_Mitochon_Swivel; 1.
DR   CDD; cd01584; AcnA_Mitochondrial; 1.
DR   Gene3D; 3.40.1060.10; Aconitase, Domain 2; 1.
DR   Gene3D; 3.30.499.10; Aconitase, domain 3; 2.
DR   Gene3D; 3.20.19.10; Aconitase, domain 4; 1.
DR   InterPro; IPR015931; Acnase/IPM_dHydase_lsu_aba_1/3.
DR   InterPro; IPR001030; Acoase/IPM_deHydtase_lsu_aba.
DR   InterPro; IPR015928; Aconitase/3IPM_dehydase_swvl.
DR   InterPro; IPR018136; Aconitase_4Fe-4S_BS.
DR   InterPro; IPR036008; Aconitase_4Fe-4S_dom.
DR   InterPro; IPR015932; Aconitase_dom2.
DR   InterPro; IPR006248; Aconitase_mito-like.
DR   InterPro; IPR000573; AconitaseA/IPMdHydase_ssu_swvl.
DR   PANTHER; PTHR43160; ACONITATE HYDRATASE B; 1.
DR   PANTHER; PTHR43160:SF3; ACONITATE HYDRATASE, MITOCHONDRIAL; 1.
DR   Pfam; PF00330; Aconitase; 1.
DR   Pfam; PF00694; Aconitase_C; 1.
DR   PRINTS; PR00415; ACONITASE.
DR   SUPFAM; SSF53732; Aconitase iron-sulfur domain; 1.
DR   SUPFAM; SSF52016; LeuD/IlvD-like; 1.
DR   PROSITE; PS00450; ACONITASE_1; 1.
DR   PROSITE; PS01244; ACONITASE_2; 1.
DR   TIGRFAMs; TIGR01340; aconitase_mito; 1.
PE   1: Evidence at protein level;
KW   4Fe-4S; Acetylation; Direct protein sequencing; Iron; Iron-sulfur; Lyase;
KW   Metal-binding; Mitochondrion; Phosphoprotein; Reference proteome;
KW   Transit peptide; Tricarboxylic acid cycle.
FT   TRANSIT         1..27
FT                   /note="Mitochondrion"
FT                   /evidence="ECO:0000250"
FT   CHAIN           28..780
FT                   /note="Aconitate hydratase, mitochondrial"
FT                   /id="PRO_0000000542"
FT   REGION          524..560
FT                   /note="Disordered"
FT                   /evidence="ECO:0000256|SAM:MobiDB-lite"
FT   COMPBIAS        545..560
FT                   /note="Polar residues"
FT                   /evidence="ECO:0000256|SAM:MobiDB-lite"
FT   BINDING         99
FT                   /ligand="substrate"
FT                   /evidence="ECO:0000250"
FT   BINDING         192..194
FT                   /ligand="substrate"
FT                   /evidence="ECO:0000250"
FT   BINDING         385
FT                   /ligand="[4Fe-4S] cluster"
FT                   /ligand_id="ChEBI:CHEBI:49883"
FT                   /evidence="ECO:0000250"
FT   BINDING         448
FT                   /ligand="[4Fe-4S] cluster"
FT                   /ligand_id="ChEBI:CHEBI:49883"
FT                   /evidence="ECO:0000250"
FT   BINDING         451
FT                   /ligand="[4Fe-4S] cluster"
FT                   /ligand_id="ChEBI:CHEBI:49883"
FT                   /evidence="ECO:0000250"
FT   BINDING         474
FT                   /ligand="substrate"
FT                   /evidence="ECO:0000250"
FT   BINDING         479
FT                   /ligand="substrate"
FT                   /evidence="ECO:0000250"
FT   BINDING         607
FT                   /ligand="substrate"
FT                   /evidence="ECO:0000250"
FT   BINDING         670..671
FT                   /ligand="substrate"
FT                   /evidence="ECO:0000250"
FT   MOD_RES         31
FT                   /note="N6-succinyllysine"
FT                   /evidence="ECO:0007744|PubMed:23806337"
FT   MOD_RES         50
FT                   /note="N6-acetyllysine; alternate"
FT                   /evidence="ECO:0007744|PubMed:23576753,
FT                   ECO:0007744|PubMed:23806337"
FT   MOD_RES         50
FT                   /note="N6-succinyllysine; alternate"
FT                   /evidence="ECO:0007744|PubMed:23806337"
FT   MOD_RES         138
FT                   /note="N6-acetyllysine; alternate"
FT                   /evidence="ECO:0007744|PubMed:23576753"
FT   MOD_RES         138
FT                   /note="N6-succinyllysine; alternate"
FT                   /evidence="ECO:0007744|PubMed:23806337"
FT   MOD_RES         144
FT                   /note="N6-acetyllysine; alternate"
FT                   /evidence="ECO:0007744|PubMed:23576753,
FT                   ECO:0007744|PubMed:23806337"
FT   MOD_RES         144
FT                   /note="N6-succinyllysine; alternate"
FT                   /evidence="ECO:0007744|PubMed:23806337"
FT   MOD_RES         233
FT                   /note="N6-acetyllysine; alternate"
FT                   /evidence="ECO:0007744|PubMed:23576753"
FT   MOD_RES         233
FT                   /note="N6-succinyllysine; alternate"
FT                   /evidence="ECO:0007744|PubMed:23806337"
FT   MOD_RES         411
FT                   /note="N6-succinyllysine"
FT                   /evidence="ECO:0007744|PubMed:23806337"
FT   MOD_RES         517
FT                   /note="N6-acetyllysine; alternate"
FT                   /evidence="ECO:0007744|PubMed:23576753"
FT   MOD_RES         517
FT                   /note="N6-succinyllysine; alternate"
FT                   /evidence="ECO:0007744|PubMed:23806337"
FT   MOD_RES         523
FT                   /note="N6-acetyllysine; alternate"
FT                   /evidence="ECO:0007744|PubMed:23576753"
FT   MOD_RES         523
FT                   /note="N6-succinyllysine; alternate"
FT                   /evidence="ECO:0007744|PubMed:23806337"
FT   MOD_RES         549
FT                   /note="N6-succinyllysine"
FT                   /evidence="ECO:0007744|PubMed:23806337"
FT   MOD_RES         559
FT                   /note="Phosphoserine"
FT                   /evidence="ECO:0000250|UniProtKB:Q99798"
FT   MOD_RES         573
FT                   /note="N6-acetyllysine; alternate"
FT                   /evidence="ECO:0000250|UniProtKB:Q99798"
FT   MOD_RES         573
FT                   /note="N6-succinyllysine; alternate"
FT                   /evidence="ECO:0007744|PubMed:23806337"
FT   MOD_RES         577
FT                   /note="N6-succinyllysine"
FT                   /evidence="ECO:0007744|PubMed:23806337"
FT   MOD_RES         591
FT                   /note="N6-succinyllysine"
FT                   /evidence="ECO:0007744|PubMed:23806337"
FT   MOD_RES         605
FT                   /note="N6-acetyllysine; alternate"
FT                   /evidence="ECO:0007744|PubMed:23576753"
FT   MOD_RES         605
FT                   /note="N6-succinyllysine; alternate"
FT                   /evidence="ECO:0007744|PubMed:23806337"
FT   MOD_RES         628
FT                   /note="N6-succinyllysine"
FT                   /evidence="ECO:0007744|PubMed:23806337"
FT   MOD_RES         670
FT                   /note="Phosphoserine"
FT                   /evidence="ECO:0007744|PubMed:21183079"
FT   MOD_RES         689
FT                   /note="N6-succinyllysine"
FT                   /evidence="ECO:0007744|PubMed:23806337"
FT   MOD_RES         723
FT                   /note="N6-acetyllysine; alternate"
FT                   /evidence="ECO:0007744|PubMed:23576753"
FT   MOD_RES         723
FT                   /note="N6-succinyllysine; alternate"
FT                   /evidence="ECO:0007744|PubMed:23806337"
FT   MOD_RES         730
FT                   /note="N6-acetyllysine; alternate"
FT                   /evidence="ECO:0007744|PubMed:23576753"
FT   MOD_RES         730
FT                   /note="N6-succinyllysine; alternate"
FT                   /evidence="ECO:0007744|PubMed:23806337"
FT   MOD_RES         736
FT                   /note="N6-acetyllysine"
FT                   /evidence="ECO:0007744|PubMed:23576753"
FT   MOD_RES         739
FT                   /note="N6-acetyllysine"
FT                   /evidence="ECO:0007744|PubMed:23576753"
FT   MOD_RES         743
FT                   /note="N6-acetyllysine"
FT                   /evidence="ECO:0007744|PubMed:23576753"
FT   CONFLICT        7..8
FT                   /note="LV -> P (in Ref. 1; BAE25770)"
FT                   /evidence="ECO:0000305"
FT   CONFLICT        618
FT                   /note="L -> F (in Ref. 2; AAH94462)"
FT                   /evidence="ECO:0000305"
FT   CONFLICT        758
FT                   /note="F -> L (in Ref. 1; BAE29252)"
FT                   /evidence="ECO:0000305"
SQ   SEQUENCE   780 AA;  85464 MW;  9B515846E875D581 CRC64;
     MAPYSLLVTR LQKALGVRQY HVASVLCQRA KVAMSHFEPS EYIRYDLLEK NINIVRKRLN
     RPLTLSEKIV YGHLDDPANQ EIERGKTYLR LRPDRVAMQD ATAQMAMLQF ISSGLPKVAV
     PSTIHCDHLI EAQVGGEKDL RRAKDINQEV YNFLATAGAK YGVGFWRPGS GIIHQIILEN
     YAYPGVLLIG TDSHTPNGGG LGGICIGVGG ADAVDVMAGI PWELKCPKVI GVKLTGSLSG
     WTSPKDVILK VAGILTVKGG TGAIVEYHGP GVDSISCTGM ATICNMGAEI GATTSVFPYN
     HRMKKYLSKT GRTDIANLAE EFKDHLVPDP GCQYDQVIEI NLNELKPHIN GPFTPDLAHP
     VADVGTVAEK EGWPLDIRVG LIGSCTNSSY EDMGRSAAVA KQALAHGLKC KSQFTITPGS
     EQIRATIERD GYAQILRDVG GIVLANACGP CIGQWDRKDI KKGEKNTIVT SYNRNFTGRN
     DANPETHAFV TSPEIVTALA IAGTLKFNPE TDFLTGKDGK KFKLEAPDAD ELPRSDFDPG
     QDTYQHPPKD SSGQRVDVSP TSQRLQLLEP FDKWDGKDLE DLQILIKVKG KCTTDHISAA
     GPWLKFRGHL DNISNNLLIG AINIENGKAN SVRNAVTQEF GPVPDTARYY KKHGIRWVVI
     GDENYGEGSS REHAALEPRH LGGRAIITKS FARIHETNLK KQGLLPLTFA DPSDYNKIHP
     VDKLTIQGLK DFAPGKPLKC VIKHPNGTQE TILLNHTFNE TQIEWFRAGS ALNRMKELQQ
//
```

|  |
| --- |
| **Mascot:** http://www.matrixscience.com/ |

Deamidated (NQ) (+0.9840)
