# Supplementary material for: A common East-Asian ALDH2 mutation causes metabolic disorders and the therapeutic effect of ALDH2 activators
Source: Nat Commun. 2023 Sep 25;14:5971. doi: 10.1038/s41467-023-41570-6 (PMC10520061; doi:10.1038/s41467-023-41570-6)
Supplement: Supplementary file 4 — Supplementary Data 1 [file 41467_2023_41570_MOESM4_ESM.zip › Table S5b/Q9CPQ1/Q9CPQ1_WTO1-1_H23.html]

Mascot Search Results: Q9CPQ1
 

# MASCOT Search Results

## Protein View: Q9CPQ1

### Cytochrome c oxidase subunit 6C OS=Mus musculus OX=10090 GN=Cox6c PE=1 SV=3

|  |  |
| --- | --- |
| Database: | Mouse\_UniProt\_proteomes |
| Score: | 1084 |
| Monoisotopic mass (Mr): | 8464 |
| Calculated pI: | 10.13 |

Sequence similarity is available as an NCBI BLAST search of Q9CPQ1 against nr.

### Search parameters

|  |  |
| --- | --- |
| MS data file: | `D:\LCMSMS\2023 Users' data\230529-1\230529-1-WTO-1.raw` |
| Enzyme: | Trypsin/P: cuts C-term side of KR. |
| Fixed modifications: | Carbamidomethyl (C) |
| Variable modifications: | Deamidated (NQ), HNE (C), HNE (H), HNE (K), Oxidation (M) |

### Protein sequence coverage: 75%

Matched peptides shown in ***bold red***.

|  |  |  |  |  |  |
| --- | --- | --- | --- | --- | --- |
| `1` | `MSSGALLPKP` | `QMRGLLAKRL` | `RVHIAGAFIV` | `ALGVAAAYKF` | `GVAEPRKKAY` |
| `51` | `AEFYRNYDSM` | `KDFEEMRKAG` | `IFQSAK` |  |  |

Unformatted sequence string: 76 residues (for pasting into other applications).

|  |  |  |  |
| --- | --- | --- | --- |
| Sort by | residue number | increasing mass | decreasing mass |
| Show | matched peptides only | predicted peptides also |  |

| Query | Start | – | End | Observed | Mr(expt) | Mr(calc) | ppm | M | Score | Expect | Rank | U | Peptide |
| --- | --- | --- | --- | --- | --- | --- | --- | --- | --- | --- | --- | --- | --- |
| 123138 | 20 | – | 39 | 510.8088 | 2039.2060 | 2039.2044 | 0.78 | 1 | 44 | 7.2e-05 | 1Score **> 29** indicates **identity** Score **> 15** indicates **homology** | U | R.LRVHIAGAFIVALGVAAAYK.F |
| 167269 | 20 | – | 46 | 699.9114 | 2795.6164 | 2795.5963 | 7.19 | 2 | 45 | 6.3e-05 | 1Score **> 29** indicates **identity** Score **> 15** indicates **homology** | U | R.LRVHIAGAFIVALGVAAAYKFGVAEPR.K |
| 167270 | 20 | – | 46 | 699.9117 | 2795.6178 | 2795.5963 | 7.71 | 2 | 52 | 1.4e-05 | 1Score **> 29** indicates **identity** Score **> 16** indicates **homology** | U | R.LRVHIAGAFIVALGVAAAYKFGVAEPR.K |
| 167271 | 20 | – | 46 | 560.1314 | 2795.6205 | 2795.5963 | 8.67 | 2 | 30 | 0.0015 | 1Score **> 28** indicates **identity** Score **> 14** indicates **homology** | U | R.LRVHIAGAFIVALGVAAAYKFGVAEPR.K |
| 171851 | 20 | – | 46 | 591.3466 | 2951.6967 | 2951.7113 | -4.94 | 2 | 21 | 0.01 | 1Score **> 29** indicates **identity** Score **> 14** indicates **homology** | U | R.LRVHIAGAFIVALGVAAAYKFGVAEPR.K  + HNE (H) |
| 171852 | 20 | – | 46 | 591.3470 | 2951.6987 | 2951.7113 | -4.28 | 2 | 15 | 0.04 | 1Score **> 29** indicates **identity** Score **> 13** indicates **homology** | U | R.LRVHIAGAFIVALGVAAAYKFGVAEPR.K  + HNE (H) |
| 98444 | 22 | – | 39 | 591.0125 | 1770.0157 | 1770.0192 | -2.00 | 0 | 45 | 6e-05 | 1Score **> 33** indicates **identity** Score **> 15** indicates **homology** | U | R.VHIAGAFIVALGVAAAYK.F |
| 98447 | 22 | – | 39 | 591.0126 | 1770.0160 | 1770.0192 | -1.84 | 0 | 37 | 0.00033 | 1Score **> 33** indicates **identity** Score **> 15** indicates **homology** | U | R.VHIAGAFIVALGVAAAYK.F |
| 98459 | 22 | – | 39 | 591.0139 | 1770.0197 | 1770.0192 | 0.30 | 0 | 16 | 0.031 | 1Score **> 33** indicates **identity** Score **> 14** indicates **homology** | U | R.VHIAGAFIVALGVAAAYK.F |
| 98460 | 22 | – | 39 | 591.0139 | 1770.0200 | 1770.0192 | 0.44 | 0 | 36 | 0.00042 | 1Score **> 33** indicates **identity** Score **> 15** indicates **homology** | U | R.VHIAGAFIVALGVAAAYK.F |
| 98462 | 22 | – | 39 | 591.0142 | 1770.0206 | 1770.0192 | 0.80 | 0 | 20 | 0.012 | 1Score **> 32** indicates **identity** Score **> 14** indicates **homology** | U | R.VHIAGAFIVALGVAAAYK.F |
| 98467 | 22 | – | 39 | 591.0146 | 1770.0219 | 1770.0192 | 1.51 | 0 | 22 | 0.009 | 1Score **> 32** indicates **identity** Score **> 14** indicates **homology** | U | R.VHIAGAFIVALGVAAAYK.F |
| 98468 | 22 | – | 39 | 591.0147 | 1770.0221 | 1770.0192 | 1.65 | 0 | 33 | 0.0008 | 1Score **> 32** indicates **identity** Score **> 15** indicates **homology** | U | R.VHIAGAFIVALGVAAAYK.F |
| 98487 | 22 | – | 39 | 591.0183 | 1770.0331 | 1770.0192 | 7.87 | 0 | 23 | 0.0071 | 1Score **> 31** indicates **identity** Score **> 14** indicates **homology** | U | R.VHIAGAFIVALGVAAAYK.F |
| 98490 | 22 | – | 39 | 591.0189 | 1770.0350 | 1770.0192 | 8.93 | 0 | 32 | 0.001 | 1Score **> 32** indicates **identity** Score **> 14** indicates **homology** | U | R.VHIAGAFIVALGVAAAYK.F |
| 2579 | 40 | – | 46 | 388.2083 | 774.4021 | 774.4024 | -0.41 | 0 | 31 | 0.0097 | 1Score **> 26** indicates **identity** Score **> 24** indicates **homology** | U | K.FGVAEPR.K |
| 2580 | 40 | – | 46 | 388.2084 | 774.4022 | 774.4024 | -0.33 | 0 | 29 | 0.0082 | 1Score **> 26** indicates **identity** Score **> 20** indicates **homology** | U | K.FGVAEPR.K |
| 2581 | 40 | – | 46 | 388.2088 | 774.4030 | 774.4024 | 0.69 | 0 | 49 | 0.00022 | 1Score **> 25** indicates **identity** | U | K.FGVAEPR.K |
| 2582 | 40 | – | 46 | 388.2088 | 774.4031 | 774.4024 | 0.87 | 0 | 25 | 0.0085 | 1Score **> 25** indicates **identity** Score **> 17** indicates **homology** | U | K.FGVAEPR.K |
| 29903 | 47 | – | 55 | 392.5449 | 1174.6130 | 1174.6134 | -0.37 | 2 | 48 | 3.4e-05 | 1Score **> 34** indicates **identity** Score **> 15** indicates **homology** | U | R.KKAYAEFYR.N |
| 29904 | 47 | – | 55 | 588.3138 | 1174.6131 | 1174.6134 | -0.28 | 2 | 41 | 0.0004 | 1Score **> 34** indicates **identity** Score **> 20** indicates **homology** | U | R.KKAYAEFYR.N |
| 29905 | 47 | – | 55 | 392.5451 | 1174.6135 | 1174.6134 | 0.017 | 2 | 48 | 3.4e-05 | 1Score **> 33** indicates **identity** Score **> 15** indicates **homology** | U | R.KKAYAEFYR.N |
| 29907 | 47 | – | 55 | 588.3140 | 1174.6135 | 1174.6134 | 0.062 | 2 | 17 | 0.028 | 1Score **> 33** indicates **identity** Score **> 14** indicates **homology** | U | R.KKAYAEFYR.N |
| 29909 | 47 | – | 55 | 588.3140 | 1174.6135 | 1174.6134 | 0.077 | 2 | 51 | 6.7e-05 | 1Score **> 33** indicates **identity** Score **> 22** indicates **homology** | U | R.KKAYAEFYR.N |
| 29910 | 47 | – | 55 | 588.3140 | 1174.6135 | 1174.6134 | 0.084 | 2 | 24 | 0.019 | 1Score **> 33** indicates **identity** Score **> 19** indicates **homology** | U | R.KKAYAEFYR.N |
| 29911 | 47 | – | 55 | 392.5451 | 1174.6136 | 1174.6134 | 0.11 | 2 | 20 | 0.013 | 1Score **> 33** indicates **identity** Score **> 14** indicates **homology** | U | R.KKAYAEFYR.N |
| 29912 | 47 | – | 55 | 392.5452 | 1174.6138 | 1174.6134 | 0.32 | 2 | 46 | 5e-05 | 1Score **> 33** indicates **identity** Score **> 15** indicates **homology** | U | R.KKAYAEFYR.N |
| 29913 | 47 | – | 55 | 392.5453 | 1174.6140 | 1174.6134 | 0.46 | 2 | 40 | 0.00019 | 1Score **> 33** indicates **identity** Score **> 15** indicates **homology** | U | R.KKAYAEFYR.N |
| 29914 | 47 | – | 55 | 588.3144 | 1174.6142 | 1174.6134 | 0.67 | 2 | 35 | 0.0021 | 1Score **> 33** indicates **identity** Score **> 20** indicates **homology** | U | R.KKAYAEFYR.N |
| 29916 | 47 | – | 55 | 392.5455 | 1174.6148 | 1174.6134 | 1.12 | 2 | 20 | 0.012 | 1Score **> 33** indicates **identity** Score **> 14** indicates **homology** | U | R.KKAYAEFYR.N |
| 29927 | 47 | – | 55 | 392.5475 | 1174.6206 | 1174.6134 | 6.11 | 2 | 17 | 0.023 | 1Score **> 34** indicates **identity** Score **> 14** indicates **homology** | U | R.KKAYAEFYR.N |
| 17099 | 48 | – | 55 | 349.8466 | 1046.5180 | 1046.5185 | -0.51 | 1 | 23 | 0.02 | 1Score **> 32** indicates **identity** Score **> 18** indicates **homology** | U | K.KAYAEFYR.N |
| 17100 | 48 | – | 55 | 524.2667 | 1046.5189 | 1046.5185 | 0.38 | 1 | 21 | 0.01 | 1Score **> 32** indicates **identity** Score **> 14** indicates **homology** | U | K.KAYAEFYR.N |
| 17101 | 48 | – | 55 | 524.2668 | 1046.5191 | 1046.5185 | 0.57 | 1 | 43 | 0.00038 | 1Score **> 32** indicates **identity** Score **> 21** indicates **homology** | U | K.KAYAEFYR.N |
| 17102 | 48 | – | 55 | 524.2677 | 1046.5208 | 1046.5185 | 2.24 | 1 | 52 | 7.9e-05 | 1Score **> 32** indicates **identity** Score **> 23** indicates **homology** | U | K.KAYAEFYR.N |
| 9369 | 49 | – | 55 | 460.2176 | 918.4206 | 918.4235 | -3.17 | 0 | 21 | 0.011 | 1Score **> 28** indicates **identity** Score **> 14** indicates **homology** | U | K.AYAEFYR.N |
| 9371 | 49 | – | 55 | 460.2185 | 918.4225 | 918.4235 | -1.18 | 0 | 16 | 0.032 | 1Score **> 28** indicates **identity** Score **> 13** indicates **homology** | U | K.AYAEFYR.N |
| 9373 | 49 | – | 55 | 460.2186 | 918.4227 | 918.4235 | -0.87 | 0 | 41 | 0.00015 | 1Score **> 28** indicates **identity** Score **> 15** indicates **homology** | U | K.AYAEFYR.N |
| 9374 | 49 | – | 55 | 460.2187 | 918.4229 | 918.4235 | -0.65 | 0 | 26 | 0.0034 | 1Score **> 28** indicates **identity** Score **> 14** indicates **homology** | U | K.AYAEFYR.N |
| 9375 | 49 | – | 55 | 460.2188 | 918.4231 | 918.4235 | -0.52 | 0 | 37 | 0.00036 | 1Score **> 28** indicates **identity** Score **> 15** indicates **homology** | U | K.AYAEFYR.N |
| 9376 | 49 | – | 55 | 460.2188 | 918.4231 | 918.4235 | -0.49 | 0 | 42 | 0.00012 | 1Score **> 28** indicates **identity** Score **> 15** indicates **homology** | U | K.AYAEFYR.N |
| 9377 | 49 | – | 55 | 460.2188 | 918.4231 | 918.4235 | -0.48 | 0 | 21 | 0.011 | 1Score **> 28** indicates **identity** Score **> 14** indicates **homology** | U | K.AYAEFYR.N |
| 9378 | 49 | – | 55 | 460.2189 | 918.4232 | 918.4235 | -0.41 | 0 | 42 | 0.00012 | 1Score **> 28** indicates **identity** Score **> 15** indicates **homology** | U | K.AYAEFYR.N |
| 9379 | 49 | – | 55 | 460.2189 | 918.4232 | 918.4235 | -0.33 | 0 | 33 | 0.00079 | 1Score **> 28** indicates **identity** Score **> 15** indicates **homology** | U | K.AYAEFYR.N |
| 9380 | 49 | – | 55 | 460.2189 | 918.4233 | 918.4235 | -0.24 | 0 | 39 | 0.00024 | 1Score **> 28** indicates **identity** Score **> 15** indicates **homology** | U | K.AYAEFYR.N |
| 9381 | 49 | – | 55 | 460.2190 | 918.4234 | 918.4235 | -0.20 | 0 | 39 | 0.00021 | 1Score **> 28** indicates **identity** Score **> 15** indicates **homology** | U | K.AYAEFYR.N |
| 9383 | 49 | – | 55 | 460.2191 | 918.4237 | 918.4235 | 0.21 | 0 | 35 | 0.00047 | 1Score **> 28** indicates **identity** Score **> 15** indicates **homology** | U | K.AYAEFYR.N |
| 9386 | 49 | – | 55 | 460.2195 | 918.4244 | 918.4235 | 0.90 | 0 | 38 | 0.00025 | 1Score **> 28** indicates **identity** Score **> 15** indicates **homology** | U | K.AYAEFYR.N |
| 89461 | 56 | – | 68 | 423.9391 | 1691.7274 | 1691.7283 | -0.52 | 2 | 23 | 0.0077 | 1Score **> 27** indicates **identity** Score **> 14** indicates **homology** | U | R.NYDSMKDFEEMRK.A |
| 89463 | 56 | – | 68 | 564.9167 | 1691.7283 | 1691.7283 | 0.025 | 2 | 27 | 0.0031 | 1Score **> 27** indicates **identity** Score **> 14** indicates **homology** | U | R.NYDSMKDFEEMRK.A |
| 11105 | 68 | – | 76 | 475.2764 | 948.5382 | 948.5392 | -1.15 | 1 | 42 | 0.00046 | 1Score **> 28** indicates **identity** Score **> 21** indicates **homology** | U | R.KAGIFQSAK.- |
| 4435 | 69 | – | 76 | 411.2286 | 820.4426 | 820.4443 | -2.07 | 0 | 44 | 0.00019 | 1Score **> 27** indicates **identity** Score **> 19** indicates **homology** |  | K.AGIFQSAK.- |
| 4436 | 69 | – | 76 | 411.2289 | 820.4432 | 820.4443 | -1.27 | 0 | 27 | 0.0036 | 1Score **> 27** indicates **identity** Score **> 15** indicates **homology** |  | K.AGIFQSAK.- |
| 4439 | 69 | – | 76 | 411.2293 | 820.4440 | 820.4443 | -0.39 | 0 | 48 | 8e-05 | 1Score **> 27** indicates **identity** Score **> 20** indicates **homology** |  | K.AGIFQSAK.- |
| 4442 | 69 | – | 76 | 411.2294 | 820.4442 | 820.4443 | -0.13 | 0 | 43 | 9.5e-05 | 1Score **> 27** indicates **identity** Score **> 15** indicates **homology** |  | K.AGIFQSAK.- |
| 4444 | 69 | – | 76 | 411.2294 | 820.4443 | 820.4443 | 0.049 | 0 | 51 | 5e-05 | 1Score **> 27** indicates **identity** Score **> 21** indicates **homology** |  | K.AGIFQSAK.- |
| 4446 | 69 | – | 76 | 411.2295 | 820.4444 | 820.4443 | 0.19 | 0 | 31 | 0.0038 | 1Score **> 27** indicates **identity** Score **> 19** indicates **homology** |  | K.AGIFQSAK.- |
| 4452 | 69 | – | 76 | 411.2296 | 820.4447 | 820.4443 | 0.52 | 0 | 47 | 0.0003 | 1Score **> 26** indicates **identity** Score **> 25** indicates **homology** |  | K.AGIFQSAK.- |
| 4453 | 69 | – | 76 | 411.2296 | 820.4447 | 820.4443 | 0.53 | 0 | 42 | 0.00011 | 1Score **> 26** indicates **identity** Score **> 15** indicates **homology** |  | K.AGIFQSAK.- |
| 4465 | 69 | – | 76 | 411.2300 | 820.4454 | 820.4443 | 1.41 | 0 | 42 | 0.00011 | 1Score **> 26** indicates **identity** Score **> 15** indicates **homology** |  | K.AGIFQSAK.- |

---

```
ID   COX6C_MOUSE             Reviewed;          76 AA.
AC   Q9CPQ1; Q52KC6;
DT   07-JUN-2004, integrated into UniProtKB/Swiss-Prot.
DT   23-JAN-2007, sequence version 3.
DT   28-JUN-2023, entry version 148.
DE   RecName: Full=Cytochrome c oxidase subunit 6C;
DE   AltName: Full=Cytochrome c oxidase polypeptide VIc;
GN   Name=Cox6c;
OS   Mus musculus (Mouse).
OC   Eukaryota; Metazoa; Chordata; Craniata; Vertebrata; Euteleostomi; Mammalia;
OC   Eutheria; Euarchontoglires; Glires; Rodentia; Myomorpha; Muroidea; Muridae;
OC   Murinae; Mus; Mus.
OX   NCBI_TaxID=10090;
RN   [1]
RP   PRELIMINARY NUCLEOTIDE SEQUENCE.
RX   PubMed=1645653; DOI=10.1111/j.1432-1033.1991.tb15989.x;
RA   Schneyder B., Mell O., Anthony G., Kadenbach B.;
RT   "Cross reactivity of monoclonal antibodies and cDNA hybridization suggest
RT   evolutionary relationships between cytochrome c oxidase subunits VIa and
RT   VIc and between VIIa and VIIb.";
RL   Eur. J. Biochem. 198:85-92(1991).
RN   [2]
RP   NUCLEOTIDE SEQUENCE [LARGE SCALE MRNA].
RC   STRAIN=C57BL/6J; TISSUE=Embryo, and Hippocampus;
RX   PubMed=16141072; DOI=10.1126/science.1112014;
RA   Carninci P., Kasukawa T., Katayama S., Gough J., Frith M.C., Maeda N.,
RA   Oyama R., Ravasi T., Lenhard B., Wells C., Kodzius R., Shimokawa K.,
RA   Bajic V.B., Brenner S.E., Batalov S., Forrest A.R., Zavolan M., Davis M.J.,
RA   Wilming L.G., Aidinis V., Allen J.E., Ambesi-Impiombato A., Apweiler R.,
RA   Aturaliya R.N., Bailey T.L., Bansal M., Baxter L., Beisel K.W., Bersano T.,
RA   Bono H., Chalk A.M., Chiu K.P., Choudhary V., Christoffels A.,
RA   Clutterbuck D.R., Crowe M.L., Dalla E., Dalrymple B.P., de Bono B.,
RA   Della Gatta G., di Bernardo D., Down T., Engstrom P., Fagiolini M.,
RA   Faulkner G., Fletcher C.F., Fukushima T., Furuno M., Futaki S.,
RA   Gariboldi M., Georgii-Hemming P., Gingeras T.R., Gojobori T., Green R.E.,
RA   Gustincich S., Harbers M., Hayashi Y., Hensch T.K., Hirokawa N., Hill D.,
RA   Huminiecki L., Iacono M., Ikeo K., Iwama A., Ishikawa T., Jakt M.,
RA   Kanapin A., Katoh M., Kawasawa Y., Kelso J., Kitamura H., Kitano H.,
RA   Kollias G., Krishnan S.P., Kruger A., Kummerfeld S.K., Kurochkin I.V.,
RA   Lareau L.F., Lazarevic D., Lipovich L., Liu J., Liuni S., McWilliam S.,
RA   Madan Babu M., Madera M., Marchionni L., Matsuda H., Matsuzawa S., Miki H.,
RA   Mignone F., Miyake S., Morris K., Mottagui-Tabar S., Mulder N., Nakano N.,
RA   Nakauchi H., Ng P., Nilsson R., Nishiguchi S., Nishikawa S., Nori F.,
RA   Ohara O., Okazaki Y., Orlando V., Pang K.C., Pavan W.J., Pavesi G.,
RA   Pesole G., Petrovsky N., Piazza S., Reed J., Reid J.F., Ring B.Z.,
RA   Ringwald M., Rost B., Ruan Y., Salzberg S.L., Sandelin A., Schneider C.,
RA   Schoenbach C., Sekiguchi K., Semple C.A., Seno S., Sessa L., Sheng Y.,
RA   Shibata Y., Shimada H., Shimada K., Silva D., Sinclair B., Sperling S.,
RA   Stupka E., Sugiura K., Sultana R., Takenaka Y., Taki K., Tammoja K.,
RA   Tan S.L., Tang S., Taylor M.S., Tegner J., Teichmann S.A., Ueda H.R.,
RA   van Nimwegen E., Verardo R., Wei C.L., Yagi K., Yamanishi H.,
RA   Zabarovsky E., Zhu S., Zimmer A., Hide W., Bult C., Grimmond S.M.,
RA   Teasdale R.D., Liu E.T., Brusic V., Quackenbush J., Wahlestedt C.,
RA   Mattick J.S., Hume D.A., Kai C., Sasaki D., Tomaru Y., Fukuda S.,
RA   Kanamori-Katayama M., Suzuki M., Aoki J., Arakawa T., Iida J., Imamura K.,
RA   Itoh M., Kato T., Kawaji H., Kawagashira N., Kawashima T., Kojima M.,
RA   Kondo S., Konno H., Nakano K., Ninomiya N., Nishio T., Okada M., Plessy C.,
RA   Shibata K., Shiraki T., Suzuki S., Tagami M., Waki K., Watahiki A.,
RA   Okamura-Oho Y., Suzuki H., Kawai J., Hayashizaki Y.;
RT   "The transcriptional landscape of the mammalian genome.";
RL   Science 309:1559-1563(2005).
RN   [3]
RP   NUCLEOTIDE SEQUENCE [LARGE SCALE MRNA].
RC   STRAIN=C57BL/6J; TISSUE=Liver, and Mammary gland;
RX   PubMed=15489334; DOI=10.1101/gr.2596504;
RG   The MGC Project Team;
RT   "The status, quality, and expansion of the NIH full-length cDNA project:
RT   the Mammalian Gene Collection (MGC).";
RL   Genome Res. 14:2121-2127(2004).
RN   [4]
RP   PROTEIN SEQUENCE OF 21-38 AND 48-66, AND IDENTIFICATION BY MASS
RP   SPECTROMETRY.
RC   STRAIN=C57BL/6J; TISSUE=Brain;
RA   Lubec G., Kang S.U.;
RL   Submitted (APR-2007) to UniProtKB.
RN   [5]
RP   IDENTIFICATION BY MASS SPECTROMETRY [LARGE SCALE ANALYSIS].
RC   TISSUE=Brain, Brown adipose tissue, Heart, Kidney, Liver, Lung,
RC   Pancreas, Spleen, and Testis;
RX   PubMed=21183079; DOI=10.1016/j.cell.2010.12.001;
RA   Huttlin E.L., Jedrychowski M.P., Elias J.E., Goswami T., Rad R.,
RA   Beausoleil S.A., Villen J., Haas W., Sowa M.E., Gygi S.P.;
RT   "A tissue-specific atlas of mouse protein phosphorylation and expression.";
RL   Cell 143:1174-1189(2010).
CC   -!- FUNCTION: Component of the cytochrome c oxidase, the last enzyme in the
CC       mitochondrial electron transport chain which drives oxidative
CC       phosphorylation. The respiratory chain contains 3 multisubunit
CC       complexes succinate dehydrogenase (complex II, CII), ubiquinol-
CC       cytochrome c oxidoreductase (cytochrome b-c1 complex, complex III,
CC       CIII) and cytochrome c oxidase (complex IV, CIV), that cooperate to
CC       transfer electrons derived from NADH and succinate to molecular oxygen,
CC       creating an electrochemical gradient over the inner membrane that
CC       drives transmembrane transport and the ATP synthase. Cytochrome c
CC       oxidase is the component of the respiratory chain that catalyzes the
CC       reduction of oxygen to water. Electrons originating from reduced
CC       cytochrome c in the intermembrane space (IMS) are transferred via the
CC       dinuclear copper A center (CU(A)) of subunit 2 and heme A of subunit 1
CC       to the active site in subunit 1, a binuclear center (BNC) formed by
CC       heme A3 and copper B (CU(B)). The BNC reduces molecular oxygen to 2
CC       water molecules using 4 electrons from cytochrome c in the IMS and 4
CC       protons from the mitochondrial matrix. {ECO:0000250|UniProtKB:P04038}.
CC   -!- PATHWAY: Energy metabolism; oxidative phosphorylation.
CC       {ECO:0000250|UniProtKB:P04038}.
CC   -!- SUBUNIT: Component of the cytochrome c oxidase (complex IV, CIV), a
CC       multisubunit enzyme composed of 14 subunits. The complex is composed of
CC       a catalytic core of 3 subunits MT-CO1, MT-CO2 and MT-CO3, encoded in
CC       the mitochondrial DNA, and 11 supernumerary subunits COX4I, COX5A,
CC       COX5B, COX6A, COX6B, COX6C, COX7A, COX7B, COX7C, COX8 and NDUFA4, which
CC       are encoded in the nuclear genome. The complex exists as a monomer or a
CC       dimer and forms supercomplexes (SCs) in the inner mitochondrial
CC       membrane with NADH-ubiquinone oxidoreductase (complex I, CI) and
CC       ubiquinol-cytochrome c oxidoreductase (cytochrome b-c1 complex, complex
CC       III, CIII), resulting in different assemblies (supercomplex
CC       SCI(1)III(2)IV(1) and megacomplex MCI(2)III(2)IV(2)).
CC       {ECO:0000250|UniProtKB:P04038}.
CC   -!- SUBCELLULAR LOCATION: Mitochondrion inner membrane
CC       {ECO:0000250|UniProtKB:P04038}; Single-pass membrane protein
CC       {ECO:0000250|UniProtKB:P04038}.
CC   -!- PTM: Acetylation of Lys-61 is observed in liver mitochondria from
CC       fasted mice but not from fed mice.
CC   -!- SIMILARITY: Belongs to the cytochrome c oxidase subunit 6c family.
CC       {ECO:0000305}.
CC   ---------------------------------------------------------------------------
CC   Copyrighted by the UniProt Consortium, see https://www.uniprot.org/terms
CC   Distributed under the Creative Commons Attribution (CC BY 4.0) License
CC   ---------------------------------------------------------------------------
DR   EMBL; AK012602; BAB28348.1; -; mRNA.
DR   EMBL; AK013459; BAB28866.1; -; mRNA.
DR   EMBL; BC024666; AAH24666.1; -; mRNA.
DR   EMBL; BC094413; AAH94413.1; -; mRNA.
DR   CCDS; CCDS27424.1; -.
DR   PIR; S16083; S16083.
DR   RefSeq; NP_444301.1; NM_053071.2.
DR   PDB; 7O37; EM; 3.20 A; i=2-76.
DR   PDB; 7O3C; EM; 3.30 A; i=2-76.
DR   PDB; 7O3E; EM; 3.60 A; i=2-76.
DR   PDBsum; 7O37; -.
DR   PDBsum; 7O3C; -.
DR   PDBsum; 7O3E; -.
DR   AlphaFoldDB; Q9CPQ1; -.
DR   SMR; Q9CPQ1; -.
DR   BioGRID; 198845; 41.
DR   CORUM; Q9CPQ1; -.
DR   IntAct; Q9CPQ1; 4.
DR   STRING; 10090.ENSMUSP00000014457; -.
DR   GlyGen; Q9CPQ1; 1 site, 1 O-linked glycan (1 site).
DR   iPTMnet; Q9CPQ1; -.
DR   PhosphoSitePlus; Q9CPQ1; -.
DR   SwissPalm; Q9CPQ1; -.
DR   EPD; Q9CPQ1; -.
DR   jPOST; Q9CPQ1; -.
DR   MaxQB; Q9CPQ1; -.
DR   PaxDb; Q9CPQ1; -.
DR   PeptideAtlas; Q9CPQ1; -.
DR   ProteomicsDB; 283429; -.
DR   TopDownProteomics; Q9CPQ1; -.
DR   Antibodypedia; 4263; 279 antibodies from 30 providers.
DR   DNASU; 12864; -.
DR   Ensembl; ENSMUST00000014457; ENSMUSP00000014457; ENSMUSG00000014313.
DR   Ensembl; ENSMUST00000153512; ENSMUSP00000123609; ENSMUSG00000014313.
DR   Ensembl; ENSMUST00000156915; ENSMUSP00000122391; ENSMUSG00000014313.
DR   GeneID; 12864; -.
DR   KEGG; mmu:12864; -.
DR   UCSC; uc007vmi.1; mouse.
DR   AGR; MGI:104614; -.
DR   CTD; 1345; -.
DR   MGI; MGI:104614; Cox6c.
DR   VEuPathDB; HostDB:ENSMUSG00000014313; -.
DR   eggNOG; ENOG502SEI2; Eukaryota.
DR   GeneTree; ENSGT00940000162275; -.
DR   HOGENOM; CLU_196254_0_0_1; -.
DR   InParanoid; Q9CPQ1; -.
DR   OMA; KNYDAMK; -.
DR   OrthoDB; 4905011at2759; -.
DR   PhylomeDB; Q9CPQ1; -.
DR   TreeFam; TF353619; -.
DR   UniPathway; UPA00705; -.
DR   BioGRID-ORCS; 12864; 33 hits in 80 CRISPR screens.
DR   ChiTaRS; Cox6c; mouse.
DR   PRO; PR:Q9CPQ1; -.
DR   Proteomes; UP000000589; Chromosome 15.
DR   RNAct; Q9CPQ1; protein.
DR   Bgee; ENSMUSG00000014313; Expressed in gonadal fat pad and 266 other tissues.
DR   ExpressionAtlas; Q9CPQ1; baseline and differential.
DR   Genevisible; Q9CPQ1; MM.
DR   GO; GO:0005743; C:mitochondrial inner membrane; HDA:MGI.
DR   GO; GO:0005751; C:mitochondrial respiratory chain complex IV; ISO:MGI.
DR   GO; GO:0005739; C:mitochondrion; HDA:MGI.
DR   GO; GO:0006119; P:oxidative phosphorylation; IEA:UniProtKB-UniPathway.
DR   CDD; cd22901; CcO_VIc; 1.
DR   Gene3D; 4.10.93.10; Mitochondrial cytochrome c oxidase subunit VIc/VIIs; 1.
DR   InterPro; IPR034884; Cytochrome_c_oxidase_VIc/VIIs.
DR   InterPro; IPR037169; Cytochrome_c_oxidase_VIc_sf.
DR   PANTHER; PTHR28264:SF1; CYTOCHROME C OXIDASE SUBUNIT 6C; 1.
DR   PANTHER; PTHR28264; CYTOCHROME C OXIDASE SUBUNIT 7A; 1.
DR   Pfam; PF02937; COX6C; 1.
DR   SUPFAM; SSF81415; Mitochondrial cytochrome c oxidase subunit VIc; 1.
PE   1: Evidence at protein level;
KW   3D-structure; Acetylation; Direct protein sequencing; Membrane;
KW   Mitochondrion; Mitochondrion inner membrane; Reference proteome;
KW   Transmembrane; Transmembrane helix.
FT   CHAIN           1..76
FT                   /note="Cytochrome c oxidase subunit 6C"
FT                   /id="PRO_0000191302"
FT   TOPO_DOM        4..14
FT                   /note="Mitochondrial matrix"
FT                   /evidence="ECO:0000250|UniProtKB:P04038"
FT   TRANSMEM        15..55
FT                   /note="Helical"
FT                   /evidence="ECO:0000250|UniProtKB:P04038"
FT   TOPO_DOM        56..76
FT                   /note="Mitochondrial intermembrane"
FT                   /evidence="ECO:0000250|UniProtKB:P04038"
FT   HELIX           15..55
FT                   /evidence="ECO:0007829|PDB:7O37"
FT   HELIX           59..67
FT                   /evidence="ECO:0007829|PDB:7O37"
SQ   SEQUENCE   76 AA;  8469 MW;  BE02A028CF77D5F4 CRC64;
     MSSGALLPKP QMRGLLAKRL RVHIAGAFIV ALGVAAAYKF GVAEPRKKAY AEFYRNYDSM
     KDFEEMRKAG IFQSAK
//
```

|  |
| --- |
| **Mascot:** http://www.matrixscience.com/ |

HNE (H) (+156.1150)
