# Supplementary material for: A common East-Asian ALDH2 mutation causes metabolic disorders and the therapeutic effect of ALDH2 activators
Source: Nat Commun. 2023 Sep 25;14:5971. doi: 10.1038/s41467-023-41570-6 (PMC10520061; doi:10.1038/s41467-023-41570-6)
Supplement: Supplementary file 4 — Supplementary Data 1 [file 41467_2023_41570_MOESM4_ESM.zip › Table S5b/Q9CZ13/Q9CZ13_WTO-1_H188.html]

Mascot Search Results: Q9CZ13
 

# MASCOT Search Results

## Protein View: Q9CZ13

### Cytochrome b-c1 complex subunit 1, mitochondrial OS=Mus musculus OX=10090 GN=Uqcrc1 PE=1 SV=2

|  |  |
| --- | --- |
| Database: | Mouse\_UniProt\_proteomes |
| Score: | 11361 |
| Monoisotopic mass (Mr): | 53446 |
| Calculated pI: | 5.81 |

Sequence similarity is available as an NCBI BLAST search of Q9CZ13 against nr.

### Search parameters

|  |  |
| --- | --- |
| MS data file: | `D:\LCMSMS\2023 Users' data\230529-1\230529-1-WTO-1.raw` |
| Enzyme: | Trypsin/P: cuts C-term side of KR. |
| Fixed modifications: | Carbamidomethyl (C) |
| Variable modifications: | Deamidated (NQ), HNE (C), HNE (H), HNE (K), Oxidation (M) |

### Protein sequence coverage: 82%

Matched peptides shown in ***bold red***.

|  |  |  |  |  |  |
| --- | --- | --- | --- | --- | --- |
| `1` | `MAASAVCRAA` | `CSGTQVLLRT` | `RRSPALLRLP` | `ALRGTATFAQ` | `ALQSVPETQV` |
| `51` | `SILDNGLRVA` | `SEQSSHATCT` | `VGVWIDAGSR` | `YETEKNNGAG` | `YFLEHLAFKG` |
| `101` | `TKNRPGNALE` | `KEVESIGAHL` | `NAYSTREHTA` | `YLIKALSKDL` | `PKVVELLADI` |
| `151` | `VQNSSLEDSQ` | `IEKERDVILR` | `EMQENDASMQ` | `NVVFDYLHAT` | `AFQGTPLAQA` |
| `201` | `VEGPSENVRR` | `LSRTDLTDYL` | `NRHYKAPRMV` | `LAAAGGVEHQ` | `QLLDLAQKHL` |
| `251` | `SSVSRVYEED` | `AVPGLTPCRF` | `TGSEIRHRDD` | `ALPLAHVAIA` | `VEGPGWANPD` |
| `301` | `NVTLQVANAI` | `IGHYDCTYGG` | `GVHLSSPLAS` | `VAVANKLCQS` | `FQTFNISYSD` |
| `351` | `TGLLGAHFVC` | `DAMSIDDMVF` | `FLQGQWMRLC` | `TSATESEVTR` | `GKNILRNALV` |
| `401` | `SHLDGTTPVC` | `EDIGRSLLTY` | `GRRIPLAEWE` | `SRIQEVDAQM` | `LRDICSKYFY` |
| `451` | `DQCPAVAGYG` | `PIEQLPDYNR` | `IRSGMFWLRF` |  |  |

Unformatted sequence string: 480 residues (for pasting into other applications).

|  |  |  |  |
| --- | --- | --- | --- |
| Sort by | residue number | increasing mass | decreasing mass |
| Show | matched peptides only | predicted peptides also |  |

| Query | Start | – | End | Observed | Mr(expt) | Mr(calc) | ppm | M | Score | Expect | Rank | U | Peptide |
| --- | --- | --- | --- | --- | --- | --- | --- | --- | --- | --- | --- | --- | --- |
| 160398 | 34 | – | 58 | 872.7923 | 2615.3550 | 2615.3555 | -0.18 | 0 | 61 | 1.8e-06 | 1Score **> 37** indicates **identity** Score **> 16** indicates **homology** | U | R.GTATFAQALQSVPETQVSILDNGLR.V |
| 160400 | 34 | – | 58 | 872.7928 | 2615.3565 | 2615.3555 | 0.39 | 0 | 59 | 3.2e-06 | 1Score **> 37** indicates **identity** Score **> 16** indicates **homology** | U | R.GTATFAQALQSVPETQVSILDNGLR.V |
| 160403 | 34 | – | 58 | 872.7930 | 2615.3572 | 2615.3555 | 0.65 | 0 | 90 | 3.8e-09 | 1Score **> 37** indicates **identity** Score **> 18** indicates **homology** | U | R.GTATFAQALQSVPETQVSILDNGLR.V |
| 160404 | 34 | – | 58 | 1308.6859 | 2615.3573 | 2615.3555 | 0.68 | 0 | 136 | 1.7e-13 | 1Score **> 37** indicates **identity** Score **> 21** indicates **homology** | U | R.GTATFAQALQSVPETQVSILDNGLR.V |
| 160405 | 34 | – | 58 | 872.7937 | 2615.3594 | 2615.3555 | 1.50 | 0 | 93 | 2e-09 | 1Score **> 37** indicates **identity** Score **> 18** indicates **homology** | U | R.GTATFAQALQSVPETQVSILDNGLR.V |
| 160406 | 34 | – | 58 | 1308.6870 | 2615.3595 | 2615.3555 | 1.55 | 0 | 87 | 7.4e-09 | 1Score **> 37** indicates **identity** Score **> 18** indicates **homology** | U | R.GTATFAQALQSVPETQVSILDNGLR.V |
| 160458 | 34 | – | 58 | 873.1203 | 2616.3392 | 2616.3395 | -0.12 | 0 | 56 | 5.4e-06 | 1Score **> 37** indicates **identity** Score **> 16** indicates **homology** | U | R.GTATFAQALQSVPETQVSILDNGLR.V  + Deamidated (NQ) |
| 160460 | 34 | – | 58 | 873.1212 | 2616.3419 | 2616.3395 | 0.92 | 0 | 45 | 5.6e-05 | 1Score **> 37** indicates **identity** Score **> 15** indicates **homology** | U | R.GTATFAQALQSVPETQVSILDNGLR.V  + Deamidated (NQ) |
| 160461 | 34 | – | 58 | 1309.1814 | 2616.3482 | 2616.3395 | 3.31 | 0 | 111 | 3.7e-11 | 1Score **> 37** indicates **identity** Score **> 19** indicates **homology** | U | R.GTATFAQALQSVPETQVSILDNGLR.V  + Deamidated (NQ) |
| 160462 | 34 | – | 58 | 873.1234 | 2616.3484 | 2616.3395 | 3.40 | 0 | 74 | 1.1e-07 | 1Score **> 37** indicates **identity** Score **> 17** indicates **homology** | U | R.GTATFAQALQSVPETQVSILDNGLR.V  + Deamidated (NQ) |
| 160468 | 34 | – | 58 | 873.1260 | 2616.3561 | 2616.3395 | 6.34 | 0 | 49 | 2.7e-05 | 1Score **> 37** indicates **identity** Score **> 16** indicates **homology** | U | R.GTATFAQALQSVPETQVSILDNGLR.V  + Deamidated (NQ) |
| 160470 | 34 | – | 58 | 873.1260 | 2616.3562 | 2616.3395 | 6.36 | 0 | 22 | 0.013 | 1Score **> 37** indicates **identity** Score **> 16** indicates **homology** | U | R.GTATFAQALQSVPETQVSILDNGLR.V  + Deamidated (NQ) |
| 160477 | 34 | – | 58 | 873.1279 | 2616.3620 | 2616.3395 | 8.58 | 0 | 47 | 3.7e-05 | 1Score **> 37** indicates **identity** Score **> 15** indicates **homology** | U | R.GTATFAQALQSVPETQVSILDNGLR.V  + Deamidated (NQ) |
| 160524 | 34 | – | 58 | 873.4519 | 2617.3338 | 2617.3235 | 3.94 | 0 | 38 | 0.0003 | 1Score **> 37** indicates **identity** Score **> 15** indicates **homology** | U | R.GTATFAQALQSVPETQVSILDNGLR.V  + 2 Deamidated (NQ) |
| 160525 | 34 | – | 58 | 1309.6748 | 2617.3350 | 2617.3235 | 4.38 | 0 | 51 | 1.7e-05 | 1Score **> 37** indicates **identity** Score **> 16** indicates **homology** | U | R.GTATFAQALQSVPETQVSILDNGLR.V  + 2 Deamidated (NQ) |
| 160528 | 34 | – | 58 | 873.4556 | 2617.3451 | 2617.3235 | 8.23 | 0 | 44 | 8.1e-05 | 1Score **> 37** indicates **identity** Score **> 15** indicates **homology** | U | R.GTATFAQALQSVPETQVSILDNGLR.V  + 2 Deamidated (NQ) |
| 144900 | 59 | – | 80 | 1159.5414 | 2317.0682 | 2317.0757 | -3.24 | 0 | 106 | 1.2e-10 | 1Score **> 33** indicates **identity** Score **> 19** indicates **homology** | U | R.VASEQSSHATCTVGVWIDAGSR.Y |
| 144902 | 59 | – | 80 | 773.3640 | 2317.0702 | 2317.0757 | -2.39 | 0 | 23 | 0.0071 | 1Score **> 33** indicates **identity** Score **> 14** indicates **homology** | U | R.VASEQSSHATCTVGVWIDAGSR.Y |
| 144907 | 59 | – | 80 | 773.3650 | 2317.0731 | 2317.0757 | -1.15 | 0 | 62 | 1.6e-06 | 1Score **> 33** indicates **identity** Score **> 16** indicates **homology** | U | R.VASEQSSHATCTVGVWIDAGSR.Y |
| 144908 | 59 | – | 80 | 773.3650 | 2317.0731 | 2317.0757 | -1.14 | 0 | 72 | 1.6e-07 | 1Score **> 33** indicates **identity** Score **> 17** indicates **homology** | U | R.VASEQSSHATCTVGVWIDAGSR.Y |
| 144909 | 59 | – | 80 | 773.3651 | 2317.0734 | 2317.0757 | -1.00 | 0 | 38 | 0.00029 | 1Score **> 33** indicates **identity** Score **> 15** indicates **homology** | U | R.VASEQSSHATCTVGVWIDAGSR.Y |
| 144911 | 59 | – | 80 | 773.3652 | 2317.0737 | 2317.0757 | -0.86 | 0 | 53 | 1e-05 | 1Score **> 33** indicates **identity** Score **> 16** indicates **homology** | U | R.VASEQSSHATCTVGVWIDAGSR.Y |
| 144912 | 59 | – | 80 | 773.3654 | 2317.0744 | 2317.0757 | -0.57 | 0 | 42 | 0.00011 | 1Score **> 34** indicates **identity** Score **> 15** indicates **homology** | U | R.VASEQSSHATCTVGVWIDAGSR.Y |
| 144913 | 59 | – | 80 | 773.3654 | 2317.0745 | 2317.0757 | -0.54 | 0 | 24 | 0.0051 | 1Score **> 34** indicates **identity** Score **> 14** indicates **homology** | U | R.VASEQSSHATCTVGVWIDAGSR.Y |
| 144914 | 59 | – | 80 | 773.3656 | 2317.0749 | 2317.0757 | -0.34 | 0 | 39 | 0.00021 | 1Score **> 34** indicates **identity** Score **> 15** indicates **homology** | U | R.VASEQSSHATCTVGVWIDAGSR.Y |
| 144915 | 59 | – | 80 | 773.3660 | 2317.0762 | 2317.0757 | 0.20 | 0 | 32 | 0.00097 | 1Score **> 34** indicates **identity** Score **> 15** indicates **homology** | U | R.VASEQSSHATCTVGVWIDAGSR.Y |
| 144916 | 59 | – | 80 | 773.3661 | 2317.0766 | 2317.0757 | 0.36 | 0 | 49 | 2.7e-05 | 1Score **> 34** indicates **identity** Score **> 16** indicates **homology** | U | R.VASEQSSHATCTVGVWIDAGSR.Y |
| 144917 | 59 | – | 80 | 773.3669 | 2317.0789 | 2317.0757 | 1.36 | 0 | 47 | 4.2e-05 | 1Score **> 34** indicates **identity** Score **> 15** indicates **homology** | U | R.VASEQSSHATCTVGVWIDAGSR.Y |
| 144919 | 59 | – | 80 | 773.3679 | 2317.0819 | 2317.0757 | 2.67 | 0 | 28 | 0.0024 | 1Score **> 34** indicates **identity** Score **> 14** indicates **homology** | U | R.VASEQSSHATCTVGVWIDAGSR.Y |
| 144960 | 59 | – | 80 | 773.6977 | 2318.0712 | 2318.0597 | 4.95 | 0 | 22 | 0.0093 | 1Score **> 33** indicates **identity** Score **> 14** indicates **homology** | U | R.VASEQSSHATCTVGVWIDAGSR.Y  + Deamidated (NQ) |
| 144961 | 59 | – | 80 | 773.6980 | 2318.0723 | 2318.0597 | 5.41 | 0 | 22 | 0.0084 | 1Score **> 33** indicates **identity** Score **> 14** indicates **homology** | U | R.VASEQSSHATCTVGVWIDAGSR.Y  + Deamidated (NQ) |
| 144966 | 59 | – | 80 | 773.7004 | 2318.0794 | 2318.0597 | 8.50 | 0 | 26 | 0.0033 | 1Score **> 34** indicates **identity** Score **> 14** indicates **homology** | U | R.VASEQSSHATCTVGVWIDAGSR.Y  + Deamidated (NQ) |
| 172194 | 59 | – | 85 | 990.1282 | 2967.3628 | 2967.3669 | -1.38 | 1 | 47 | 3.9e-05 | 1Score **> 34** indicates **identity** Score **> 15** indicates **homology** | U | R.VASEQSSHATCTVGVWIDAGSRYETEK.N |
| 172197 | 59 | – | 85 | 742.8486 | 2967.3653 | 2967.3669 | -0.54 | 1 | 33 | 0.0008 | 1Score **> 34** indicates **identity** Score **> 15** indicates **homology** | U | R.VASEQSSHATCTVGVWIDAGSRYETEK.N |
| 172198 | 59 | – | 85 | 990.1293 | 2967.3661 | 2967.3669 | -0.25 | 1 | 51 | 1.8e-05 | 1Score **> 34** indicates **identity** Score **> 16** indicates **homology** | U | R.VASEQSSHATCTVGVWIDAGSRYETEK.N |
| 172200 | 59 | – | 85 | 742.8492 | 2967.3676 | 2967.3669 | 0.23 | 1 | 24 | 0.0061 | 1Score **> 34** indicates **identity** Score **> 14** indicates **homology** | U | R.VASEQSSHATCTVGVWIDAGSRYETEK.N |
| 172201 | 59 | – | 85 | 990.1302 | 2967.3688 | 2967.3669 | 0.64 | 1 | 60 | 2.6e-06 | 1Score **> 34** indicates **identity** Score **> 16** indicates **homology** | U | R.VASEQSSHATCTVGVWIDAGSRYETEK.N |
| 172202 | 59 | – | 85 | 990.1306 | 2967.3699 | 2967.3669 | 1.02 | 1 | 46 | 5.2e-05 | 1Score **> 35** indicates **identity** Score **> 15** indicates **homology** | U | R.VASEQSSHATCTVGVWIDAGSRYETEK.N |
| 187300 | 59 | – | 99 | 906.8377 | 4529.1520 | 4529.1346 | 3.84 | 2 | 17 | 0.023 | 1Score **> 35** indicates **identity** Score **> 14** indicates **homology** | U | R.VASEQSSHATCTVGVWIDAGSRYETEKNNGAGYFLEHLAFK.G |
| 187303 | 59 | – | 99 | 907.0332 | 4530.1297 | 4530.1186 | 2.44 | 2 | 14 | 0.049 | 1Score **> 35** indicates **identity** Score **> 13** indicates **homology** | U | R.VASEQSSHATCTVGVWIDAGSRYETEKNNGAGYFLEHLAFK.G  + Deamidated (NQ) |
| 138927 | 81 | – | 99 | 558.5258 | 2230.0741 | 2230.0694 | 2.09 | 1 | 41 | 0.00013 | 1Score **> 35** indicates **identity** Score **> 15** indicates **homology** | U | R.YETEKNNGAGYFLEHLAFK.G |
| 138929 | 81 | – | 99 | 744.3661 | 2230.0765 | 2230.0694 | 3.15 | 1 | 47 | 3.6e-05 | 1Score **> 35** indicates **identity** Score **> 15** indicates **homology** | U | R.YETEKNNGAGYFLEHLAFK.G |
| 138930 | 81 | – | 99 | 744.3665 | 2230.0778 | 2230.0694 | 3.74 | 1 | 49 | 2.5e-05 | 1Score **> 35** indicates **identity** Score **> 16** indicates **homology** | U | R.YETEKNNGAGYFLEHLAFK.G |
| 138931 | 81 | – | 99 | 558.5268 | 2230.0780 | 2230.0694 | 3.86 | 1 | 26 | 0.0036 | 1Score **> 35** indicates **identity** Score **> 14** indicates **homology** | U | R.YETEKNNGAGYFLEHLAFK.G |
| 138932 | 81 | – | 99 | 558.5269 | 2230.0785 | 2230.0694 | 4.06 | 1 | 52 | 1.3e-05 | 1Score **> 35** indicates **identity** Score **> 16** indicates **homology** | U | R.YETEKNNGAGYFLEHLAFK.G |
| 138933 | 81 | – | 99 | 558.5271 | 2230.0791 | 2230.0694 | 4.33 | 1 | 44 | 6.8e-05 | 1Score **> 35** indicates **identity** Score **> 15** indicates **homology** | U | R.YETEKNNGAGYFLEHLAFK.G |
| 138936 | 81 | – | 99 | 744.3676 | 2230.0810 | 2230.0694 | 5.18 | 1 | 27 | 0.0029 | 1Score **> 35** indicates **identity** Score **> 14** indicates **homology** | U | R.YETEKNNGAGYFLEHLAFK.G |
| 138938 | 81 | – | 99 | 558.5286 | 2230.0851 | 2230.0694 | 7.04 | 1 | 30 | 0.0015 | 1Score **> 35** indicates **identity** Score **> 14** indicates **homology** | U | R.YETEKNNGAGYFLEHLAFK.G |
| 139009 | 81 | – | 99 | 558.7706 | 2231.0533 | 2231.0535 | -0.054 | 1 | 20 | 0.014 | 1Score **> 34** indicates **identity** Score **> 14** indicates **homology** | U | R.YETEKNNGAGYFLEHLAFK.G  + Deamidated (NQ) |
| 139010 | 81 | – | 99 | 558.7706 | 2231.0535 | 2231.0535 | 0.020 | 1 | 16 | 0.031 | 1Score **> 34** indicates **identity** Score **> 14** indicates **homology** | U | R.YETEKNNGAGYFLEHLAFK.G  + Deamidated (NQ) |
| 139011 | 81 | – | 99 | 558.7708 | 2231.0541 | 2231.0535 | 0.28 | 1 | 50 | 2.2e-05 | 1Score **> 34** indicates **identity** Score **> 16** indicates **homology** | U | R.YETEKNNGAGYFLEHLAFK.G  + Deamidated (NQ) |
| 139012 | 81 | – | 99 | 558.7710 | 2231.0551 | 2231.0535 | 0.72 | 1 | 35 | 0.00058 | 1Score **> 34** indicates **identity** Score **> 15** indicates **homology** | U | R.YETEKNNGAGYFLEHLAFK.G  + Deamidated (NQ) |
| 139013 | 81 | – | 99 | 744.6924 | 2231.0555 | 2231.0535 | 0.90 | 1 | 31 | 0.0012 | 1Score **> 34** indicates **identity** Score **> 14** indicates **homology** | U | R.YETEKNNGAGYFLEHLAFK.G  + Deamidated (NQ) |
| 156149 | 81 | – | 102 | 630.0657 | 2516.2336 | 2516.2336 | 0.029 | 2 | 30 | 0.0015 | 1Score **> 36** indicates **identity** Score **> 14** indicates **homology** | U | R.YETEKNNGAGYFLEHLAFKGTK.N |
| 156150 | 81 | – | 102 | 630.0661 | 2516.2353 | 2516.2336 | 0.69 | 2 | 29 | 0.0019 | 1Score **> 36** indicates **identity** Score **> 14** indicates **homology** | U | R.YETEKNNGAGYFLEHLAFKGTK.N |
| 156202 | 81 | – | 102 | 630.3130 | 2517.2229 | 2517.2176 | 2.13 | 2 | 33 | 0.00086 | 1Score **> 36** indicates **identity** Score **> 15** indicates **homology** | U | R.YETEKNNGAGYFLEHLAFKGTK.N  + Deamidated (NQ) |
| 75248 | 86 | – | 99 | 790.8957 | 1579.7769 | 1579.7783 | -0.88 | 0 | 73 | 1.4e-07 | 1Score **> 34** indicates **identity** Score **> 17** indicates **homology** | U | K.NNGAGYFLEHLAFK.G |
| 75249 | 86 | – | 99 | 790.8962 | 1579.7779 | 1579.7783 | -0.26 | 0 | 65 | 1.1e-06 | 1Score **> 34** indicates **identity** Score **> 18** indicates **homology** | U | K.NNGAGYFLEHLAFK.G |
| 75250 | 86 | – | 99 | 527.6002 | 1579.7786 | 1579.7783 | 0.22 | 0 | 39 | 0.00021 | 1Score **> 34** indicates **identity** Score **> 15** indicates **homology** | U | K.NNGAGYFLEHLAFK.G |
| 75253 | 86 | – | 99 | 527.6004 | 1579.7793 | 1579.7783 | 0.62 | 0 | 37 | 0.00033 | 1Score **> 34** indicates **identity** Score **> 15** indicates **homology** | U | K.NNGAGYFLEHLAFK.G |
| 75256 | 86 | – | 99 | 527.6005 | 1579.7797 | 1579.7783 | 0.88 | 0 | 26 | 0.0034 | 1Score **> 34** indicates **identity** Score **> 14** indicates **homology** | U | K.NNGAGYFLEHLAFK.G |
| 75258 | 86 | – | 99 | 527.6009 | 1579.7807 | 1579.7783 | 1.53 | 0 | 40 | 0.00016 | 1Score **> 34** indicates **identity** Score **> 15** indicates **homology** | U | K.NNGAGYFLEHLAFK.G |
| 75261 | 86 | – | 99 | 527.6009 | 1579.7809 | 1579.7783 | 1.64 | 0 | 38 | 0.00029 | 1Score **> 34** indicates **identity** Score **> 15** indicates **homology** | U | K.NNGAGYFLEHLAFK.G |
| 75371 | 86 | – | 99 | 791.3833 | 1580.7520 | 1580.7623 | -6.52 | 0 | 71 | 2.1e-07 | 1Score **> 33** indicates **identity** Score **> 17** indicates **homology** | U | K.NNGAGYFLEHLAFK.G  + Deamidated (NQ) |
| 75381 | 86 | – | 99 | 527.9275 | 1580.7607 | 1580.7623 | -0.99 | 0 | 47 | 3.7e-05 | 1Score **> 33** indicates **identity** Score **> 15** indicates **homology** | U | K.NNGAGYFLEHLAFK.G  + Deamidated (NQ) |
| 75384 | 86 | – | 99 | 527.9279 | 1580.7620 | 1580.7623 | -0.22 | 0 | 36 | 0.00044 | 1Score **> 33** indicates **identity** Score **> 15** indicates **homology** | U | K.NNGAGYFLEHLAFK.G  + Deamidated (NQ) |
| 75388 | 86 | – | 99 | 527.9281 | 1580.7624 | 1580.7623 | 0.081 | 0 | 24 | 0.0052 | 1Score **> 33** indicates **identity** Score **> 14** indicates **homology** | U | K.NNGAGYFLEHLAFK.G  + Deamidated (NQ) |
| 75389 | 86 | – | 99 | 527.9281 | 1580.7625 | 1580.7623 | 0.14 | 0 | 49 | 2.4e-05 | 1Score **> 33** indicates **identity** Score **> 16** indicates **homology** | U | K.NNGAGYFLEHLAFK.G  + Deamidated (NQ) |
| 75390 | 86 | – | 99 | 527.9284 | 1580.7633 | 1580.7623 | 0.64 | 0 | 32 | 0.0011 | 1Score **> 33** indicates **identity** Score **> 14** indicates **homology** | U | K.NNGAGYFLEHLAFK.G  + Deamidated (NQ) |
| 75394 | 86 | – | 99 | 791.3896 | 1580.7646 | 1580.7623 | 1.46 | 0 | 39 | 0.00024 | 1Score **> 34** indicates **identity** Score **> 15** indicates **homology** | U | K.NNGAGYFLEHLAFK.G  + Deamidated (NQ) |
| 107854 | 86 | – | 102 | 622.9881 | 1865.9424 | 1865.9424 | -0.012 | 1 | 46 | 4.5e-05 | 1Score **> 36** indicates **identity** Score **> 15** indicates **homology** | U | K.NNGAGYFLEHLAFKGTK.N |
| 107855 | 86 | – | 102 | 622.9886 | 1865.9440 | 1865.9424 | 0.87 | 1 | 47 | 4.2e-05 | 1Score **> 36** indicates **identity** Score **> 15** indicates **homology** | U | K.NNGAGYFLEHLAFKGTK.N |
| 107869 | 86 | – | 102 | 622.9925 | 1865.9556 | 1865.9424 | 7.05 | 1 | 33 | 0.00087 | 1Score **> 36** indicates **identity** Score **> 15** indicates **homology** | U | K.NNGAGYFLEHLAFKGTK.N |
| 107943 | 86 | – | 102 | 623.3162 | 1866.9268 | 1866.9264 | 0.20 | 1 | 47 | 3.8e-05 | 1Score **> 35** indicates **identity** Score **> 15** indicates **homology** | U | K.NNGAGYFLEHLAFKGTK.N  + Deamidated (NQ) |
| 107947 | 86 | – | 102 | 623.3179 | 1866.9319 | 1866.9264 | 2.95 | 1 | 53 | 1e-05 | 1Score **> 35** indicates **identity** Score **> 16** indicates **homology** | U | K.NNGAGYFLEHLAFKGTK.N  + Deamidated (NQ) |
| 107949 | 86 | – | 102 | 623.3187 | 1866.9342 | 1866.9264 | 4.19 | 1 | 17 | 0.023 | 1Score **> 35** indicates **identity** Score **> 14** indicates **homology** | U | K.NNGAGYFLEHLAFKGTK.N  + Deamidated (NQ) |
| 160851 | 103 | – | 126 | 526.0699 | 2625.3130 | 2625.3259 | -4.88 | 2 | 17 | 0.025 | 1Score **> 37** indicates **identity** Score **> 14** indicates **homology** | U | K.NRPGNALEKEVESIGAHLNAYSTR.E |
| 160852 | 103 | – | 126 | 526.0702 | 2625.3145 | 2625.3259 | -4.31 | 2 | 26 | 0.0035 | 1Score **> 37** indicates **identity** Score **> 14** indicates **homology** | U | K.NRPGNALEKEVESIGAHLNAYSTR.E |
| 160853 | 103 | – | 126 | 526.0710 | 2625.3186 | 2625.3259 | -2.77 | 2 | 14 | 0.049 | 1Score **> 37** indicates **identity** Score **> 13** indicates **homology** | U | K.NRPGNALEKEVESIGAHLNAYSTR.E |
| 160855 | 103 | – | 126 | 657.3372 | 2625.3196 | 2625.3259 | -2.37 | 2 | 49 | 2.5e-05 | 1Score **> 37** indicates **identity** Score **> 16** indicates **homology** | U | K.NRPGNALEKEVESIGAHLNAYSTR.E |
| 160856 | 103 | – | 126 | 526.0714 | 2625.3207 | 2625.3259 | -1.97 | 2 | 36 | 0.00046 | 1Score **> 37** indicates **identity** Score **> 15** indicates **homology** | U | K.NRPGNALEKEVESIGAHLNAYSTR.E |
| 160858 | 103 | – | 126 | 657.3377 | 2625.3218 | 2625.3259 | -1.54 | 2 | 42 | 0.00011 | 1Score **> 37** indicates **identity** Score **> 15** indicates **homology** | U | K.NRPGNALEKEVESIGAHLNAYSTR.E |
| 160859 | 103 | – | 126 | 526.0718 | 2625.3226 | 2625.3259 | -1.24 | 2 | 31 | 0.0017 | 1Score **> 37** indicates **identity** Score **> 16** indicates **homology** | U | K.NRPGNALEKEVESIGAHLNAYSTR.E |
| 160860 | 103 | – | 126 | 657.3380 | 2625.3229 | 2625.3259 | -1.13 | 2 | 40 | 0.00019 | 1Score **> 37** indicates **identity** Score **> 15** indicates **homology** | U | K.NRPGNALEKEVESIGAHLNAYSTR.E |
| 160861 | 103 | – | 126 | 526.0720 | 2625.3235 | 2625.3259 | -0.92 | 2 | 30 | 0.0015 | 1Score **> 37** indicates **identity** Score **> 14** indicates **homology** | U | K.NRPGNALEKEVESIGAHLNAYSTR.E |
| 160862 | 103 | – | 126 | 657.3382 | 2625.3238 | 2625.3259 | -0.81 | 2 | 36 | 0.00043 | 1Score **> 37** indicates **identity** Score **> 15** indicates **homology** | U | K.NRPGNALEKEVESIGAHLNAYSTR.E |
| 160864 | 103 | – | 126 | 657.3384 | 2625.3243 | 2625.3259 | -0.59 | 2 | 42 | 0.00012 | 1Score **> 37** indicates **identity** Score **> 15** indicates **homology** | U | K.NRPGNALEKEVESIGAHLNAYSTR.E |
| 160865 | 103 | – | 126 | 876.1155 | 2625.3246 | 2625.3259 | -0.47 | 2 | 77 | 6.5e-08 | 1Score **> 37** indicates **identity** Score **> 17** indicates **homology** | U | K.NRPGNALEKEVESIGAHLNAYSTR.E |
| 160866 | 103 | – | 126 | 526.0722 | 2625.3247 | 2625.3259 | -0.45 | 2 | 53 | 1.1e-05 | 1Score **> 37** indicates **identity** Score **> 16** indicates **homology** | U | K.NRPGNALEKEVESIGAHLNAYSTR.E |
| 160867 | 103 | – | 126 | 876.1156 | 2625.3250 | 2625.3259 | -0.35 | 2 | 56 | 5.4e-06 | 1Score **> 37** indicates **identity** Score **> 16** indicates **homology** | U | K.NRPGNALEKEVESIGAHLNAYSTR.E |
| 160868 | 103 | – | 126 | 657.3385 | 2625.3250 | 2625.3259 | -0.35 | 2 | 63 | 1.2e-06 | 1Score **> 37** indicates **identity** Score **> 16** indicates **homology** | U | K.NRPGNALEKEVESIGAHLNAYSTR.E |
| 160869 | 103 | – | 126 | 526.0723 | 2625.3250 | 2625.3259 | -0.34 | 2 | 50 | 2.2e-05 | 1Score **> 37** indicates **identity** Score **> 16** indicates **homology** | U | K.NRPGNALEKEVESIGAHLNAYSTR.E |
| 160870 | 103 | – | 126 | 526.0723 | 2625.3250 | 2625.3259 | -0.33 | 2 | 56 | 5.3e-06 | 1Score **> 37** indicates **identity** Score **> 16** indicates **homology** | U | K.NRPGNALEKEVESIGAHLNAYSTR.E |
| 160871 | 103 | – | 126 | 526.0723 | 2625.3250 | 2625.3259 | -0.32 | 2 | 25 | 0.0046 | 1Score **> 37** indicates **identity** Score **> 14** indicates **homology** | U | K.NRPGNALEKEVESIGAHLNAYSTR.E |
| 160872 | 103 | – | 126 | 526.0723 | 2625.3250 | 2625.3259 | -0.32 | 2 | 25 | 0.0042 | 1Score **> 37** indicates **identity** Score **> 14** indicates **homology** | U | K.NRPGNALEKEVESIGAHLNAYSTR.E |
| 160873 | 103 | – | 126 | 526.0723 | 2625.3251 | 2625.3259 | -0.31 | 2 | 56 | 5.2e-06 | 1Score **> 37** indicates **identity** Score **> 16** indicates **homology** | U | K.NRPGNALEKEVESIGAHLNAYSTR.E |
| 160874 | 103 | – | 126 | 526.0723 | 2625.3252 | 2625.3259 | -0.26 | 2 | 52 | 1.3e-05 | 1Score **> 37** indicates **identity** Score **> 16** indicates **homology** | U | K.NRPGNALEKEVESIGAHLNAYSTR.E |
| 160876 | 103 | – | 126 | 657.3386 | 2625.3253 | 2625.3259 | -0.21 | 2 | 47 | 4.1e-05 | 1Score **> 37** indicates **identity** Score **> 15** indicates **homology** | U | K.NRPGNALEKEVESIGAHLNAYSTR.E |
| 160877 | 103 | – | 126 | 526.0724 | 2625.3256 | 2625.3259 | -0.098 | 2 | 62 | 1.6e-06 | 1Score **> 37** indicates **identity** Score **> 16** indicates **homology** | U | K.NRPGNALEKEVESIGAHLNAYSTR.E |
| 160878 | 103 | – | 126 | 657.3387 | 2625.3257 | 2625.3259 | -0.067 | 2 | 73 | 1.5e-07 | 1Score **> 37** indicates **identity** Score **> 17** indicates **homology** | U | K.NRPGNALEKEVESIGAHLNAYSTR.E |
| 160879 | 103 | – | 126 | 657.3387 | 2625.3258 | 2625.3259 | -0.022 | 2 | 59 | 2.9e-06 | 1Score **> 37** indicates **identity** Score **> 16** indicates **homology** | U | K.NRPGNALEKEVESIGAHLNAYSTR.E |
| 160880 | 103 | – | 126 | 876.1159 | 2625.3260 | 2625.3259 | 0.051 | 2 | 68 | 3.8e-07 | 1Score **> 37** indicates **identity** Score **> 17** indicates **homology** | U | K.NRPGNALEKEVESIGAHLNAYSTR.E |
| 160881 | 103 | – | 126 | 657.3388 | 2625.3261 | 2625.3259 | 0.081 | 2 | 62 | 1.6e-06 | 1Score **> 37** indicates **identity** Score **> 16** indicates **homology** | U | K.NRPGNALEKEVESIGAHLNAYSTR.E |
| 160882 | 103 | – | 126 | 657.3388 | 2625.3261 | 2625.3259 | 0.096 | 2 | 48 | 3e-05 | 1Score **> 37** indicates **identity** Score **> 16** indicates **homology** | U | K.NRPGNALEKEVESIGAHLNAYSTR.E |
| 160884 | 103 | – | 126 | 657.3388 | 2625.3263 | 2625.3259 | 0.15 | 2 | 51 | 1.6e-05 | 1Score **> 37** indicates **identity** Score **> 16** indicates **homology** | U | K.NRPGNALEKEVESIGAHLNAYSTR.E |
| 160885 | 103 | – | 126 | 876.1160 | 2625.3263 | 2625.3259 | 0.17 | 2 | 77 | 6.6e-08 | 1Score **> 37** indicates **identity** Score **> 17** indicates **homology** | U | K.NRPGNALEKEVESIGAHLNAYSTR.E |
| 160886 | 103 | – | 126 | 657.3389 | 2625.3267 | 2625.3259 | 0.31 | 2 | 41 | 0.00016 | 1Score **> 37** indicates **identity** Score **> 15** indicates **homology** | U | K.NRPGNALEKEVESIGAHLNAYSTR.E |
| 160887 | 103 | – | 126 | 876.1162 | 2625.3268 | 2625.3259 | 0.36 | 2 | 77 | 2.3e-07 | 1Score **> 37** indicates **identity** Score **> 23** indicates **homology** | U | K.NRPGNALEKEVESIGAHLNAYSTR.E |
| 160888 | 103 | – | 126 | 657.3390 | 2625.3268 | 2625.3259 | 0.37 | 2 | 60 | 2.6e-06 | 1Score **> 37** indicates **identity** Score **> 16** indicates **homology** | U | K.NRPGNALEKEVESIGAHLNAYSTR.E |
| 160889 | 103 | – | 126 | 526.0727 | 2625.3270 | 2625.3259 | 0.44 | 2 | 48 | 3.4e-05 | 1Score **> 37** indicates **identity** Score **> 15** indicates **homology** | U | K.NRPGNALEKEVESIGAHLNAYSTR.E |
| 160890 | 103 | – | 126 | 526.0727 | 2625.3271 | 2625.3259 | 0.48 | 2 | 44 | 6.9e-05 | 1Score **> 37** indicates **identity** Score **> 15** indicates **homology** | U | K.NRPGNALEKEVESIGAHLNAYSTR.E |
| 160891 | 103 | – | 126 | 657.3391 | 2625.3274 | 2625.3259 | 0.59 | 2 | 29 | 0.002 | 1Score **> 37** indicates **identity** Score **> 14** indicates **homology** | U | K.NRPGNALEKEVESIGAHLNAYSTR.E |
| 160892 | 103 | – | 126 | 657.3392 | 2625.3277 | 2625.3259 | 0.70 | 2 | 59 | 2.8e-06 | 1Score **> 37** indicates **identity** Score **> 16** indicates **homology** | U | K.NRPGNALEKEVESIGAHLNAYSTR.E |
| 160893 | 103 | – | 126 | 526.0730 | 2625.3284 | 2625.3259 | 0.96 | 2 | 28 | 0.0023 | 1Score **> 37** indicates **identity** Score **> 14** indicates **homology** | U | K.NRPGNALEKEVESIGAHLNAYSTR.E |
| 160894 | 103 | – | 126 | 876.1168 | 2625.3285 | 2625.3259 | 1.01 | 2 | 68 | 4.6e-07 | 1Score **> 37** indicates **identity** Score **> 17** indicates **homology** | U | K.NRPGNALEKEVESIGAHLNAYSTR.E |
| 160895 | 103 | – | 126 | 657.3394 | 2625.3285 | 2625.3259 | 1.01 | 2 | 73 | 1.5e-07 | 1Score **> 37** indicates **identity** Score **> 17** indicates **homology** | U | K.NRPGNALEKEVESIGAHLNAYSTR.E |
| 160896 | 103 | – | 126 | 526.0730 | 2625.3286 | 2625.3259 | 1.05 | 2 | 75 | 9.8e-08 | 1Score **> 37** indicates **identity** Score **> 17** indicates **homology** | U | K.NRPGNALEKEVESIGAHLNAYSTR.E |
| 160897 | 103 | – | 126 | 657.3394 | 2625.3286 | 2625.3259 | 1.06 | 2 | 36 | 0.00039 | 1Score **> 37** indicates **identity** Score **> 15** indicates **homology** | U | K.NRPGNALEKEVESIGAHLNAYSTR.E |
| 160898 | 103 | – | 126 | 657.3396 | 2625.3292 | 2625.3259 | 1.28 | 2 | 58 | 3.4e-06 | 1Score **> 37** indicates **identity** Score **> 16** indicates **homology** | U | K.NRPGNALEKEVESIGAHLNAYSTR.E |
| 160899 | 103 | – | 126 | 876.1170 | 2625.3292 | 2625.3259 | 1.28 | 2 | 38 | 0.0004 | 1Score **> 37** indicates **identity** Score **> 17** indicates **homology** | U | K.NRPGNALEKEVESIGAHLNAYSTR.E |
| 160900 | 103 | – | 126 | 526.0732 | 2625.3295 | 2625.3259 | 1.39 | 2 | 27 | 0.0031 | 1Score **> 37** indicates **identity** Score **> 14** indicates **homology** | U | K.NRPGNALEKEVESIGAHLNAYSTR.E |
| 160901 | 103 | – | 126 | 657.3398 | 2625.3299 | 2625.3259 | 1.54 | 2 | 48 | 3e-05 | 1Score **> 37** indicates **identity** Score **> 16** indicates **homology** | U | K.NRPGNALEKEVESIGAHLNAYSTR.E |
| 160902 | 103 | – | 126 | 657.3399 | 2625.3304 | 2625.3259 | 1.73 | 2 | 37 | 0.00033 | 1Score **> 37** indicates **identity** Score **> 15** indicates **homology** | U | K.NRPGNALEKEVESIGAHLNAYSTR.E |
| 160906 | 103 | – | 126 | 876.1199 | 2625.3378 | 2625.3259 | 4.55 | 2 | 29 | 0.0019 | 1Score **> 37** indicates **identity** Score **> 14** indicates **homology** | U | K.NRPGNALEKEVESIGAHLNAYSTR.E |
| 160907 | 103 | – | 126 | 657.3422 | 2625.3398 | 2625.3259 | 5.31 | 2 | 34 | 0.00058 | 1Score **> 37** indicates **identity** Score **> 15** indicates **homology** | U | K.NRPGNALEKEVESIGAHLNAYSTR.E |
| 160923 | 103 | – | 126 | 526.2677 | 2626.3021 | 2626.3099 | -2.98 | 2 | 17 | 0.027 | 1Score **> 37** indicates **identity** Score **> 14** indicates **homology** | U | K.NRPGNALEKEVESIGAHLNAYSTR.E  + Deamidated (NQ) |
| 160927 | 103 | – | 126 | 657.5832 | 2626.3038 | 2626.3099 | -2.32 | 2 | 18 | 0.021 | 1Score **> 37** indicates **identity** Score **> 14** indicates **homology** | U | K.NRPGNALEKEVESIGAHLNAYSTR.E  + Deamidated (NQ) |
| 160929 | 103 | – | 126 | 657.5840 | 2626.3071 | 2626.3099 | -1.07 | 2 | 20 | 0.014 | 1Score **> 37** indicates **identity** Score **> 14** indicates **homology** | U | K.NRPGNALEKEVESIGAHLNAYSTR.E  + Deamidated (NQ) |
| 160931 | 103 | – | 126 | 657.5842 | 2626.3076 | 2626.3099 | -0.88 | 2 | 38 | 0.00029 | 1Score **> 37** indicates **identity** Score **> 15** indicates **homology** | U | K.NRPGNALEKEVESIGAHLNAYSTR.E  + Deamidated (NQ) |
| 160932 | 103 | – | 126 | 526.2689 | 2626.3082 | 2626.3099 | -0.63 | 2 | 39 | 0.00023 | 1Score **> 37** indicates **identity** Score **> 15** indicates **homology** | U | K.NRPGNALEKEVESIGAHLNAYSTR.E  + Deamidated (NQ) |
| 160933 | 103 | – | 126 | 526.2690 | 2626.3086 | 2626.3099 | -0.49 | 2 | 30 | 0.0015 | 1Score **> 37** indicates **identity** Score **> 14** indicates **homology** | U | K.NRPGNALEKEVESIGAHLNAYSTR.E  + Deamidated (NQ) |
| 160934 | 103 | – | 126 | 657.5850 | 2626.3108 | 2626.3099 | 0.36 | 2 | 56 | 6e-06 | 1Score **> 37** indicates **identity** Score **> 16** indicates **homology** | U | K.NRPGNALEKEVESIGAHLNAYSTR.E  + Deamidated (NQ) |
| 160935 | 103 | – | 126 | 657.5851 | 2626.3113 | 2626.3099 | 0.55 | 2 | 62 | 1.7e-06 | 1Score **> 37** indicates **identity** Score **> 16** indicates **homology** | U | K.NRPGNALEKEVESIGAHLNAYSTR.E  + Deamidated (NQ) |
| 160936 | 103 | – | 126 | 876.4447 | 2626.3123 | 2626.3099 | 0.92 | 2 | 15 | 0.037 | 1Score **> 37** indicates **identity** Score **> 13** indicates **homology** | U | K.NRPGNALEKEVESIGAHLNAYSTR.E  + Deamidated (NQ) |
| 160938 | 103 | – | 126 | 657.5858 | 2626.3142 | 2626.3099 | 1.63 | 2 | 48 | 7e-05 | 1Score **> 37** indicates **identity** Score **> 19** indicates **homology** | U | K.NRPGNALEKEVESIGAHLNAYSTR.E  + Deamidated (NQ) |
| 160939 | 103 | – | 126 | 526.2703 | 2626.3152 | 2626.3099 | 2.04 | 2 | 21 | 0.011 | 1Score **> 37** indicates **identity** Score **> 14** indicates **homology** | U | K.NRPGNALEKEVESIGAHLNAYSTR.E  + Deamidated (NQ) |
| 160940 | 103 | – | 126 | 876.4459 | 2626.3159 | 2626.3099 | 2.29 | 2 | 34 | 0.00069 | 1Score **> 37** indicates **identity** Score **> 15** indicates **homology** | U | K.NRPGNALEKEVESIGAHLNAYSTR.E  + Deamidated (NQ) |
| 160941 | 103 | – | 126 | 657.5865 | 2626.3168 | 2626.3099 | 2.62 | 2 | 19 | 0.016 | 1Score **> 37** indicates **identity** Score **> 14** indicates **homology** | U | K.NRPGNALEKEVESIGAHLNAYSTR.E  + Deamidated (NQ) |
| 160942 | 103 | – | 126 | 526.2716 | 2626.3215 | 2626.3099 | 4.44 | 2 | 17 | 0.024 | 1Score **> 37** indicates **identity** Score **> 14** indicates **homology** | U | K.NRPGNALEKEVESIGAHLNAYSTR.E  + Deamidated (NQ) |
| 160943 | 103 | – | 126 | 876.4513 | 2626.3320 | 2626.3099 | 8.42 | 2 | 41 | 0.00014 | 1Score **> 37** indicates **identity** Score **> 15** indicates **homology** | U | K.NRPGNALEKEVESIGAHLNAYSTR.E  + Deamidated (NQ) |
| 160969 | 103 | – | 126 | 526.4700 | 2627.3138 | 2627.2939 | 7.57 | 2 | 23 | 0.0076 | 1Score **> 37** indicates **identity** Score **> 14** indicates **homology** | U | K.NRPGNALEKEVESIGAHLNAYSTR.E  + 2 Deamidated (NQ) |
| 160970 | 103 | – | 126 | 526.4703 | 2627.3150 | 2627.2939 | 8.04 | 2 | 20 | 0.024 | 1Score **> 37** indicates **identity** Score **> 16** indicates **homology** | U | K.NRPGNALEKEVESIGAHLNAYSTR.E  + 2 Deamidated (NQ) |
| 160971 | 103 | – | 126 | 657.8365 | 2627.3171 | 2627.2939 | 8.83 | 2 | 41 | 0.00019 | 1Score **> 37** indicates **identity** Score **> 16** indicates **homology** | U | K.NRPGNALEKEVESIGAHLNAYSTR.E  + 2 Deamidated (NQ) |
| 83743 | 112 | – | 126 | 823.9081 | 1645.8016 | 1645.8060 | -2.67 | 0 | 41 | 0.00029 | 1Score **> 34** indicates **identity** Score **> 18** indicates **homology** | U | K.EVESIGAHLNAYSTR.E |
| 83755 | 112 | – | 126 | 823.9093 | 1645.8041 | 1645.8060 | -1.12 | 0 | 68 | 1.6e-06 | 1Score **> 34** indicates **identity** Score **> 23** indicates **homology** | U | K.EVESIGAHLNAYSTR.E |
| 83758 | 112 | – | 126 | 549.6092 | 1645.8058 | 1645.8060 | -0.076 | 0 | 45 | 6.7e-05 | 1Score **> 34** indicates **identity** Score **> 15** indicates **homology** | U | K.EVESIGAHLNAYSTR.E |
| 83763 | 112 | – | 126 | 549.6094 | 1645.8063 | 1645.8060 | 0.20 | 0 | 49 | 2.8e-05 | 1Score **> 34** indicates **identity** Score **> 16** indicates **homology** | U | K.EVESIGAHLNAYSTR.E |
| 83764 | 112 | – | 126 | 823.9106 | 1645.8066 | 1645.8060 | 0.36 | 0 | 72 | 1.6e-06 | 1Score **> 34** indicates **identity** Score **> 27** indicates **homology** | U | K.EVESIGAHLNAYSTR.E |
| 83766 | 112 | – | 126 | 823.9108 | 1645.8071 | 1645.8060 | 0.72 | 0 | 72 | 5.7e-07 | 1Score **> 34** indicates **identity** Score **> 22** indicates **homology** | U | K.EVESIGAHLNAYSTR.E |
| 83768 | 112 | – | 126 | 549.6097 | 1645.8073 | 1645.8060 | 0.83 | 0 | 22 | 0.0094 | 1Score **> 34** indicates **identity** Score **> 14** indicates **homology** | U | K.EVESIGAHLNAYSTR.E |
| 83775 | 112 | – | 126 | 823.9119 | 1645.8092 | 1645.8060 | 1.95 | 0 | 63 | 3.8e-06 | 1Score **> 34** indicates **identity** Score **> 21** indicates **homology** | U | K.EVESIGAHLNAYSTR.E |
| 12538 | 127 | – | 134 | 487.7688 | 973.5231 | 973.5233 | -0.12 | 0 | 42 | 0.0016 | 1Score **> 31** indicates **identity** Score **> 27** indicates **homology** | U | R.EHTAYLIK.A |
| 12539 | 127 | – | 134 | 487.7689 | 973.5232 | 973.5233 | -0.051 | 0 | 28 | 0.0081 | 1Score **> 31** indicates **identity** Score **> 20** indicates **homology** | U | R.EHTAYLIK.A |
| 12540 | 127 | – | 134 | 487.7689 | 973.5233 | 973.5233 | 0.082 | 0 | 32 | 0.012 | 1Score **> 31** indicates **identity** Score **> 25** indicates **homology** | U | R.EHTAYLIK.A |
| 12542 | 127 | – | 134 | 487.7691 | 973.5237 | 973.5233 | 0.47 | 0 | 25 | 0.016 | 1Score **> 33** indicates **identity** Score **> 19** indicates **homology** | U | R.EHTAYLIK.A |
| 12544 | 127 | – | 134 | 487.7697 | 973.5249 | 973.5233 | 1.71 | 0 | 24 | 0.01 | 1Score **> 33** indicates **identity** Score **> 16** indicates **homology** | U | R.EHTAYLIK.A |
| 6900 | 135 | – | 142 | 436.2662 | 870.5179 | 870.5174 | 0.54 | 1 | 45 | 0.00025 | 1Score **> 27** indicates **identity** Score **> 22** indicates **homology** | U | K.ALSKDLPK.V |
| 174897 | 139 | – | 165 | 767.6668 | 3066.6379 | 3066.6084 | 9.62 | 2 | 57 | 4.8e-06 | 1Score **> 36** indicates **identity** Score **> 16** indicates **homology** | U | K.DLPKVVELLADIVQNSSLEDSQIEKER.D |
| 145623 | 143 | – | 163 | 1165.1123 | 2328.2101 | 2328.2060 | 1.76 | 0 | 94 | 1.5e-09 | 1Score **> 37** indicates **identity** Score **> 18** indicates **homology** | U | K.VVELLADIVQNSSLEDSQIEK.E |
| 145626 | 143 | – | 163 | 777.0789 | 2328.2147 | 2328.2060 | 3.75 | 0 | 64 | 9.2e-07 | 1Score **> 37** indicates **identity** Score **> 17** indicates **homology** | U | K.VVELLADIVQNSSLEDSQIEK.E |
| 145628 | 143 | – | 163 | 777.0800 | 2328.2181 | 2328.2060 | 5.18 | 0 | 51 | 1.5e-05 | 1Score **> 37** indicates **identity** Score **> 16** indicates **homology** | U | K.VVELLADIVQNSSLEDSQIEK.E |
| 160238 | 143 | – | 165 | 872.1188 | 2613.3344 | 2613.3497 | -5.84 | 1 | 36 | 0.00043 | 1Score **> 37** indicates **identity** Score **> 15** indicates **homology** | U | K.VVELLADIVQNSSLEDSQIEKER.D |
| 160241 | 143 | – | 165 | 872.1233 | 2613.3480 | 2613.3497 | -0.65 | 1 | 30 | 0.0014 | 1Score **> 37** indicates **identity** Score **> 14** indicates **homology** | U | K.VVELLADIVQNSSLEDSQIEKER.D |
| 160245 | 143 | – | 165 | 872.1236 | 2613.3488 | 2613.3497 | -0.33 | 1 | 39 | 0.00022 | 1Score **> 37** indicates **identity** Score **> 15** indicates **homology** | U | K.VVELLADIVQNSSLEDSQIEKER.D |
| 160246 | 143 | – | 165 | 872.1238 | 2613.3497 | 2613.3497 | -0.0011 | 1 | 33 | 0.00089 | 1Score **> 37** indicates **identity** Score **> 15** indicates **homology** | U | K.VVELLADIVQNSSLEDSQIEKER.D |
| 160247 | 143 | – | 165 | 872.1239 | 2613.3499 | 2613.3497 | 0.077 | 1 | 49 | 2.8e-05 | 1Score **> 37** indicates **identity** Score **> 16** indicates **homology** | U | K.VVELLADIVQNSSLEDSQIEKER.D |
| 160251 | 143 | – | 165 | 872.1244 | 2613.3515 | 2613.3497 | 0.68 | 1 | 75 | 9.5e-08 | 1Score **> 37** indicates **identity** Score **> 17** indicates **homology** | U | K.VVELLADIVQNSSLEDSQIEKER.D |
| 160253 | 143 | – | 165 | 872.1246 | 2613.3519 | 2613.3497 | 0.86 | 1 | 75 | 9e-08 | 1Score **> 37** indicates **identity** Score **> 17** indicates **homology** | U | K.VVELLADIVQNSSLEDSQIEKER.D |
| 160256 | 143 | – | 165 | 654.3456 | 2613.3533 | 2613.3497 | 1.38 | 1 | 58 | 3.7e-06 | 1Score **> 37** indicates **identity** Score **> 16** indicates **homology** | U | K.VVELLADIVQNSSLEDSQIEKER.D |
| 160257 | 143 | – | 165 | 872.1252 | 2613.3537 | 2613.3497 | 1.55 | 1 | 70 | 2.8e-07 | 1Score **> 37** indicates **identity** Score **> 17** indicates **homology** | U | K.VVELLADIVQNSSLEDSQIEKER.D |
| 160258 | 143 | – | 165 | 654.3458 | 2613.3541 | 2613.3497 | 1.67 | 1 | 33 | 0.00078 | 1Score **> 37** indicates **identity** Score **> 15** indicates **homology** | U | K.VVELLADIVQNSSLEDSQIEKER.D |
| 160259 | 143 | – | 165 | 872.1253 | 2613.3541 | 2613.3497 | 1.67 | 1 | 79 | 4.2e-08 | 1Score **> 37** indicates **identity** Score **> 17** indicates **homology** | U | K.VVELLADIVQNSSLEDSQIEKER.D |
| 160261 | 143 | – | 165 | 654.3462 | 2613.3558 | 2613.3497 | 2.32 | 1 | 29 | 0.0019 | 1Score **> 37** indicates **identity** Score **> 14** indicates **homology** | U | K.VVELLADIVQNSSLEDSQIEKER.D |
| 160268 | 143 | – | 165 | 872.1264 | 2613.3575 | 2613.3497 | 2.98 | 1 | 75 | 1e-07 | 1Score **> 37** indicates **identity** Score **> 17** indicates **homology** | U | K.VVELLADIVQNSSLEDSQIEKER.D |
| 160276 | 143 | – | 165 | 872.1280 | 2613.3623 | 2613.3497 | 4.82 | 1 | 77 | 6e-08 | 1Score **> 37** indicates **identity** Score **> 17** indicates **homology** | U | K.VVELLADIVQNSSLEDSQIEKER.D |
| 160320 | 143 | – | 165 | 872.4526 | 2614.3360 | 2614.3337 | 0.90 | 1 | 39 | 0.00022 | 1Score **> 37** indicates **identity** Score **> 15** indicates **homology** | U | K.VVELLADIVQNSSLEDSQIEKER.D  + Deamidated (NQ) |
| 160321 | 143 | – | 165 | 872.4529 | 2614.3370 | 2614.3337 | 1.25 | 1 | 38 | 0.00027 | 1Score **> 37** indicates **identity** Score **> 15** indicates **homology** | U | K.VVELLADIVQNSSLEDSQIEKER.D  + Deamidated (NQ) |
| 160323 | 143 | – | 165 | 872.4555 | 2614.3445 | 2614.3337 | 4.14 | 1 | 46 | 4.6e-05 | 1Score **> 37** indicates **identity** Score **> 15** indicates **homology** | U | K.VVELLADIVQNSSLEDSQIEKER.D  + Deamidated (NQ) |
| 160327 | 143 | – | 165 | 872.4567 | 2614.3482 | 2614.3337 | 5.55 | 1 | 24 | 0.006 | 1Score **> 37** indicates **identity** Score **> 14** indicates **homology** | U | K.VVELLADIVQNSSLEDSQIEKER.D  + Deamidated (NQ) |
| 160332 | 143 | – | 165 | 1308.1844 | 2614.3543 | 2614.3337 | 7.88 | 1 | 74 | 1.1e-07 | 1Score **> 37** indicates **identity** Score **> 17** indicates **homology** | U | K.VVELLADIVQNSSLEDSQIEKER.D  + Deamidated (NQ) |
| 160392 | 143 | – | 165 | 872.7876 | 2615.3411 | 2615.3177 | 8.93 | 1 | 34 | 0.00072 | 1Score **> 37** indicates **identity** Score **> 15** indicates **homology** | U | K.VVELLADIVQNSSLEDSQIEKER.D  + 2 Deamidated (NQ) |
| 177434 | 143 | – | 170 | 1070.9100 | 3209.7082 | 3209.7143 | -1.89 | 2 | 33 | 0.00078 | 1Score **> 36** indicates **identity** Score **> 15** indicates **homology** | U | K.VVELLADIVQNSSLEDSQIEKERDVILR.E |
| 177436 | 143 | – | 170 | 803.4364 | 3209.7165 | 3209.7143 | 0.70 | 2 | 48 | 3.4e-05 | 1Score **> 36** indicates **identity** Score **> 15** indicates **homology** | U | K.VVELLADIVQNSSLEDSQIEKERDVILR.E |
| 177437 | 143 | – | 170 | 1070.9131 | 3209.7175 | 3209.7143 | 0.99 | 2 | 40 | 0.00016 | 1Score **> 36** indicates **identity** Score **> 15** indicates **homology** | U | K.VVELLADIVQNSSLEDSQIEKERDVILR.E |
| 177438 | 143 | – | 170 | 803.4367 | 3209.7175 | 3209.7143 | 1.01 | 2 | 17 | 0.023 | 1Score **> 36** indicates **identity** Score **> 14** indicates **homology** | U | K.VVELLADIVQNSSLEDSQIEKERDVILR.E |
| 177444 | 143 | – | 170 | 803.4396 | 3209.7293 | 3209.7143 | 4.66 | 2 | 22 | 0.008 | 1Score **> 35** indicates **identity** Score **> 14** indicates **homology** | U | K.VVELLADIVQNSSLEDSQIEKERDVILR.E |
| 177466 | 143 | – | 170 | 1071.2486 | 3210.7239 | 3210.6983 | 7.98 | 2 | 43 | 8.6e-05 | 1Score **> 36** indicates **identity** Score **> 15** indicates **homology** | U | K.VVELLADIVQNSSLEDSQIEKERDVILR.E  + Deamidated (NQ) |
| 177469 | 143 | – | 170 | 803.6890 | 3210.7269 | 3210.6983 | 8.92 | 2 | 16 | 0.03 | 1Score **> 36** indicates **identity** Score **> 14** indicates **homology** | U | K.VVELLADIVQNSSLEDSQIEKERDVILR.E  + Deamidated (NQ) |
| 177494 | 143 | – | 170 | 1071.5711 | 3211.6914 | 3211.6823 | 2.84 | 2 | 34 | 0.00067 | 1Score **> 36** indicates **identity** Score **> 15** indicates **homology** | U | K.VVELLADIVQNSSLEDSQIEKERDVILR.E  + 2 Deamidated (NQ) |
| 186986 | 171 | – | 209 | 1489.6856 | 4466.0351 | 4466.0682 | -7.43 | 0 | 27 | 0.0029 | 1Score **> 33** indicates **identity** Score **> 14** indicates **homology** | U | R.EMQENDASMQNVVFDYLHATAFQGTPLAQAVEGPSENVR.R  + Deamidated (NQ); HNE (H); Oxidation (M) |
| 61151 | 211 | – | 222 | 489.5908 | 1465.7507 | 1465.7525 | -1.20 | 1 | 34 | 0.001 | 1Score **> 34** indicates **identity** Score **> 16** indicates **homology** | U | R.LSRTDLTDYLNR.H |
| 61152 | 211 | – | 222 | 489.5912 | 1465.7517 | 1465.7525 | -0.55 | 1 | 33 | 0.00083 | 1Score **> 34** indicates **identity** Score **> 15** indicates **homology** | U | R.LSRTDLTDYLNR.H |
| 61153 | 211 | – | 222 | 733.8832 | 1465.7519 | 1465.7525 | -0.41 | 1 | 20 | 0.014 | 1Score **> 34** indicates **identity** Score **> 14** indicates **homology** | U | R.LSRTDLTDYLNR.H |
| 61154 | 211 | – | 222 | 733.8833 | 1465.7521 | 1465.7525 | -0.26 | 1 | 14 | 0.05 | 1Score **> 34** indicates **identity** Score **> 13** indicates **homology** | U | R.LSRTDLTDYLNR.H |
| 61155 | 211 | – | 222 | 489.5913 | 1465.7522 | 1465.7525 | -0.22 | 1 | 25 | 0.0048 | 1Score **> 34** indicates **identity** Score **> 14** indicates **homology** | U | R.LSRTDLTDYLNR.H |
| 23016 | 214 | – | 222 | 555.7738 | 1109.5331 | 1109.5353 | -1.94 | 0 | 29 | 0.0093 | 1Score **> 30** indicates **identity** Score **> 21** indicates **homology** | U | R.TDLTDYLNR.H |
| 23026 | 214 | – | 222 | 555.7749 | 1109.5352 | 1109.5353 | -0.045 | 0 | 68 | 7.8e-06 | 1Score **> 30** indicates **identity** Score **> 30** indicates **homology** | U | R.TDLTDYLNR.H |
| 23027 | 214 | – | 222 | 555.7749 | 1109.5352 | 1109.5353 | -0.040 | 0 | 66 | 1.4e-05 | 1Score **> 30** indicates **identity** | U | R.TDLTDYLNR.H |
| 23028 | 214 | – | 222 | 555.7749 | 1109.5353 | 1109.5353 | -0.034 | 0 | 68 | 7.8e-06 | 1Score **> 30** indicates **identity** Score **> 30** indicates **homology** | U | R.TDLTDYLNR.H |
| 23029 | 214 | – | 222 | 555.7749 | 1109.5353 | 1109.5353 | 0.025 | 0 | 68 | 7.7e-06 | 1Score **> 30** indicates **identity** Score **> 30** indicates **homology** | U | R.TDLTDYLNR.H |
| 23030 | 214 | – | 222 | 555.7752 | 1109.5359 | 1109.5353 | 0.51 | 0 | 43 | 0.0011 | 1Score **> 31** indicates **identity** Score **> 26** indicates **homology** | U | R.TDLTDYLNR.H |
| 23032 | 214 | – | 222 | 555.7757 | 1109.5368 | 1109.5353 | 1.39 | 0 | 54 | 0.00013 | 1Score **> 31** indicates **identity** Score **> 28** indicates **homology** | U | R.TDLTDYLNR.H |
| 69972 | 214 | – | 225 | 513.5915 | 1537.7528 | 1537.7525 | 0.19 | 1 | 20 | 0.013 | 1Score **> 33** indicates **identity** Score **> 14** indicates **homology** | U | R.TDLTDYLNRHYK.A |
| 127561 | 229 | – | 248 | 698.0439 | 2091.1098 | 2091.1146 | -2.31 | 0 | 18 | 0.022 | 1Score **> 36** indicates **identity** Score **> 14** indicates **homology** | U | R.MVLAAAGGVEHQQLLDLAQK.H |
| 127570 | 229 | – | 248 | 698.0452 | 2091.1138 | 2091.1146 | -0.39 | 0 | 59 | 2.8e-06 | 1Score **> 36** indicates **identity** Score **> 16** indicates **homology** | U | R.MVLAAAGGVEHQQLLDLAQK.H |
| 127581 | 229 | – | 248 | 698.0461 | 2091.1163 | 2091.1146 | 0.81 | 0 | 19 | 0.017 | 1Score **> 36** indicates **identity** Score **> 14** indicates **homology** | U | R.MVLAAAGGVEHQQLLDLAQK.H |
| 127583 | 229 | – | 248 | 698.0461 | 2091.1164 | 2091.1146 | 0.86 | 0 | 33 | 0.00087 | 1Score **> 36** indicates **identity** Score **> 15** indicates **homology** | U | R.MVLAAAGGVEHQQLLDLAQK.H |
| 127590 | 229 | – | 248 | 698.0463 | 2091.1172 | 2091.1146 | 1.21 | 0 | 42 | 0.00012 | 1Score **> 36** indicates **identity** Score **> 15** indicates **homology** | U | R.MVLAAAGGVEHQQLLDLAQK.H |
| 127595 | 229 | – | 248 | 698.0469 | 2091.1190 | 2091.1146 | 2.08 | 0 | 30 | 0.0016 | 1Score **> 36** indicates **identity** Score **> 14** indicates **homology** | U | R.MVLAAAGGVEHQQLLDLAQK.H |
| 127598 | 229 | – | 248 | 698.0478 | 2091.1214 | 2091.1146 | 3.25 | 0 | 45 | 6.1e-05 | 1Score **> 36** indicates **identity** Score **> 15** indicates **homology** | U | R.MVLAAAGGVEHQQLLDLAQK.H |
| 127602 | 229 | – | 248 | 698.0491 | 2091.1255 | 2091.1146 | 5.20 | 0 | 36 | 0.0004 | 1Score **> 36** indicates **identity** Score **> 15** indicates **homology** | U | R.MVLAAAGGVEHQQLLDLAQK.H |
| 129088 | 229 | – | 248 | 703.3755 | 2107.1048 | 2107.1096 | -2.26 | 0 | 41 | 0.00013 | 1Score **> 36** indicates **identity** Score **> 15** indicates **homology** | U | R.MVLAAAGGVEHQQLLDLAQK.H  + Oxidation (M) |
| 129090 | 229 | – | 248 | 703.3764 | 2107.1073 | 2107.1096 | -1.07 | 0 | 23 | 0.012 | 1Score **> 36** indicates **identity** Score **> 17** indicates **homology** | U | R.MVLAAAGGVEHQQLLDLAQK.H  + Oxidation (M) |
| 2878 | 249 | – | 255 | 393.2168 | 784.4190 | 784.4191 | -0.14 | 0 | 23 | 0.034 | 1Score **> 23** indicates **identity** Score **> 21** indicates **homology** | U | K.HLSSVSR.V |
| 78691 | 256 | – | 269 | 803.3772 | 1604.7399 | 1604.7505 | -6.55 | 0 | 22 | 0.0081 | 1Score **> 31** indicates **identity** Score **> 14** indicates **homology** | U | R.VYEEDAVPGLTPCR.F |
| 78692 | 256 | – | 269 | 803.3782 | 1604.7418 | 1604.7505 | -5.41 | 0 | 40 | 0.00017 | 1Score **> 31** indicates **identity** Score **> 15** indicates **homology** | U | R.VYEEDAVPGLTPCR.F |
| 78695 | 256 | – | 269 | 803.3800 | 1604.7455 | 1604.7505 | -3.12 | 0 | 21 | 0.01 | 1Score **> 32** indicates **identity** Score **> 14** indicates **homology** | U | R.VYEEDAVPGLTPCR.F |
| 78697 | 256 | – | 269 | 803.3801 | 1604.7456 | 1604.7505 | -3.06 | 0 | 36 | 0.00039 | 1Score **> 32** indicates **identity** Score **> 15** indicates **homology** | U | R.VYEEDAVPGLTPCR.F |
| 78699 | 256 | – | 269 | 803.3804 | 1604.7463 | 1604.7505 | -2.57 | 0 | 29 | 0.002 | 1Score **> 32** indicates **identity** Score **> 14** indicates **homology** | U | R.VYEEDAVPGLTPCR.F |
| 78700 | 256 | – | 269 | 803.3809 | 1604.7473 | 1604.7505 | -1.99 | 0 | 61 | 1.8e-06 | 1Score **> 32** indicates **identity** Score **> 16** indicates **homology** | U | R.VYEEDAVPGLTPCR.F |
| 78701 | 256 | – | 269 | 535.9233 | 1604.7480 | 1604.7505 | -1.53 | 0 | 24 | 0.0061 | 1Score **> 32** indicates **identity** Score **> 14** indicates **homology** | U | R.VYEEDAVPGLTPCR.F |
| 78702 | 256 | – | 269 | 803.3820 | 1604.7495 | 1604.7505 | -0.62 | 0 | 52 | 1.4e-05 | 1Score **> 32** indicates **identity** Score **> 16** indicates **homology** | U | R.VYEEDAVPGLTPCR.F |
| 78703 | 256 | – | 269 | 803.3821 | 1604.7496 | 1604.7505 | -0.56 | 0 | 54 | 9.1e-06 | 1Score **> 32** indicates **identity** Score **> 16** indicates **homology** | U | R.VYEEDAVPGLTPCR.F |
| 78704 | 256 | – | 269 | 535.9240 | 1604.7501 | 1604.7505 | -0.21 | 0 | 25 | 0.0052 | 1Score **> 32** indicates **identity** Score **> 14** indicates **homology** | U | R.VYEEDAVPGLTPCR.F |
| 78705 | 256 | – | 269 | 803.3824 | 1604.7502 | 1604.7505 | -0.16 | 0 | 67 | 4.7e-07 | 1Score **> 32** indicates **identity** Score **> 17** indicates **homology** | U | R.VYEEDAVPGLTPCR.F |
| 78706 | 256 | – | 269 | 803.3827 | 1604.7509 | 1604.7505 | 0.28 | 0 | 63 | 1.1e-06 | 1Score **> 32** indicates **identity** Score **> 16** indicates **homology** | U | R.VYEEDAVPGLTPCR.F |
| 78707 | 256 | – | 269 | 803.3827 | 1604.7509 | 1604.7505 | 0.30 | 0 | 38 | 0.00027 | 1Score **> 32** indicates **identity** Score **> 15** indicates **homology** | U | R.VYEEDAVPGLTPCR.F |
| 78709 | 256 | – | 269 | 803.3828 | 1604.7511 | 1604.7505 | 0.38 | 0 | 75 | 1e-07 | 1Score **> 32** indicates **identity** Score **> 17** indicates **homology** | U | R.VYEEDAVPGLTPCR.F |
| 78710 | 256 | – | 269 | 535.9244 | 1604.7512 | 1604.7505 | 0.48 | 0 | 27 | 0.0034 | 1Score **> 32** indicates **identity** Score **> 15** indicates **homology** | U | R.VYEEDAVPGLTPCR.F |
| 78711 | 256 | – | 269 | 803.3829 | 1604.7513 | 1604.7505 | 0.51 | 0 | 54 | 9.1e-06 | 1Score **> 32** indicates **identity** Score **> 16** indicates **homology** | U | R.VYEEDAVPGLTPCR.F |
| 78712 | 256 | – | 269 | 803.3830 | 1604.7514 | 1604.7505 | 0.61 | 0 | 68 | 4.2e-07 | 1Score **> 32** indicates **identity** Score **> 17** indicates **homology** | U | R.VYEEDAVPGLTPCR.F |
| 78713 | 256 | – | 269 | 803.3830 | 1604.7514 | 1604.7505 | 0.61 | 0 | 55 | 6.3e-06 | 1Score **> 32** indicates **identity** Score **> 16** indicates **homology** | U | R.VYEEDAVPGLTPCR.F |
| 78716 | 256 | – | 269 | 803.3831 | 1604.7516 | 1604.7505 | 0.74 | 0 | 44 | 7.3e-05 | 1Score **> 32** indicates **identity** Score **> 15** indicates **homology** | U | R.VYEEDAVPGLTPCR.F |
| 78717 | 256 | – | 269 | 803.3832 | 1604.7519 | 1604.7505 | 0.87 | 0 | 53 | 1e-05 | 1Score **> 32** indicates **identity** Score **> 16** indicates **homology** | U | R.VYEEDAVPGLTPCR.F |
| 78718 | 256 | – | 269 | 803.3832 | 1604.7519 | 1604.7505 | 0.89 | 0 | 40 | 0.00019 | 1Score **> 32** indicates **identity** Score **> 15** indicates **homology** | U | R.VYEEDAVPGLTPCR.F |
| 78719 | 256 | – | 269 | 803.3833 | 1604.7521 | 1604.7505 | 1.05 | 0 | 68 | 4.5e-07 | 1Score **> 32** indicates **identity** Score **> 17** indicates **homology** | U | R.VYEEDAVPGLTPCR.F |
| 78721 | 256 | – | 269 | 803.3843 | 1604.7540 | 1604.7505 | 2.18 | 0 | 30 | 0.0016 | 1Score **> 32** indicates **identity** Score **> 14** indicates **homology** | U | R.VYEEDAVPGLTPCR.F |
| 78722 | 256 | – | 269 | 803.3861 | 1604.7577 | 1604.7505 | 4.49 | 0 | 37 | 0.00034 | 1Score **> 33** indicates **identity** Score **> 15** indicates **homology** | U | R.VYEEDAVPGLTPCR.F |
| 78724 | 256 | – | 269 | 803.3866 | 1604.7587 | 1604.7505 | 5.15 | 0 | 35 | 0.00056 | 1Score **> 32** indicates **identity** Score **> 15** indicates **homology** | U | R.VYEEDAVPGLTPCR.F |
| 78729 | 256 | – | 269 | 803.3884 | 1604.7622 | 1604.7505 | 7.34 | 0 | 61 | 2.1e-06 | 1Score **> 33** indicates **identity** Score **> 16** indicates **homology** | U | R.VYEEDAVPGLTPCR.F |
| 149712 | 256 | – | 276 | 799.3910 | 2395.1511 | 2395.1478 | 1.36 | 1 | 32 | 0.00096 | 1Score **> 35** indicates **identity** Score **> 15** indicates **homology** | U | R.VYEEDAVPGLTPCRFTGSEIR.H |
| 149713 | 256 | – | 276 | 799.3912 | 2395.1517 | 2395.1478 | 1.61 | 1 | 20 | 0.013 | 1Score **> 36** indicates **identity** Score **> 14** indicates **homology** | U | R.VYEEDAVPGLTPCRFTGSEIR.H |
| 3883 | 270 | – | 276 | 405.2100 | 808.4054 | 808.4079 | -3.15 | 0 | 38 | 0.00069 | 1Score **> 24** indicates **identity** Score **> 19** indicates **homology** | U | R.FTGSEIR.H |
| 3884 | 270 | – | 276 | 405.2100 | 808.4054 | 808.4079 | -3.11 | 0 | 27 | 0.003 | 1Score **> 24** indicates **identity** Score **> 14** indicates **homology** | U | R.FTGSEIR.H |
| 3886 | 270 | – | 276 | 405.2108 | 808.4070 | 808.4079 | -1.13 | 0 | 31 | 0.0011 | 1Score **> 24** indicates **identity** Score **> 14** indicates **homology** | U | R.FTGSEIR.H |
| 3888 | 270 | – | 276 | 405.2109 | 808.4072 | 808.4079 | -0.84 | 0 | 31 | 0.0012 | 1Score **> 25** indicates **identity** Score **> 14** indicates **homology** | U | R.FTGSEIR.H |
| 3889 | 270 | – | 276 | 405.2109 | 808.4073 | 808.4079 | -0.76 | 0 | 45 | 0.00012 | 1Score **> 25** indicates **identity** Score **> 18** indicates **homology** | U | R.FTGSEIR.H |
| 3890 | 270 | – | 276 | 405.2111 | 808.4076 | 808.4079 | -0.45 | 0 | 32 | 0.0045 | 1Score **> 25** indicates **identity** Score **> 21** indicates **homology** | U | R.FTGSEIR.H |
| 3892 | 270 | – | 276 | 405.2113 | 808.4081 | 808.4079 | 0.24 | 0 | 43 | 0.00015 | 1Score **> 25** indicates **identity** Score **> 17** indicates **homology** | U | R.FTGSEIR.H |
| 3893 | 270 | – | 276 | 405.2118 | 808.4090 | 808.4079 | 1.35 | 0 | 21 | 0.01 | 1Score **> 24** indicates **identity** Score **> 14** indicates **homology** | U | R.FTGSEIR.H |
| 189013 | 277 | – | 336 | 1240.6273 | 6198.1000 | 6198.1134 | -2.17 | 1 | 28 | 0.0021 | 1Score **> 34** indicates **identity** Score **> 14** indicates **homology** | U | R.HRDDALPLAHVAIAVEGPGWANPDNVTLQVANAIIGHYDCTYGGGVHLSSPLASVAVANK.L |
| 189016 | 277 | – | 336 | 1240.6321 | 6198.1240 | 6198.1134 | 1.71 | 1 | 28 | 0.0026 | 1Score **> 34** indicates **identity** Score **> 14** indicates **homology** | U | R.HRDDALPLAHVAIAVEGPGWANPDNVTLQVANAIIGHYDCTYGGGVHLSSPLASVAVANK.L |
| 49018 | 379 | – | 390 | 677.3157 | 1352.6168 | 1352.6242 | -5.47 | 0 | 55 | 7.4e-06 | 1Score **> 30** indicates **identity** Score **> 16** indicates **homology** | U | R.LCTSATESEVTR.G |
| 49019 | 379 | – | 390 | 677.3167 | 1352.6189 | 1352.6242 | -3.91 | 0 | 74 | 1.3e-07 | 1Score **> 31** indicates **identity** Score **> 17** indicates **homology** | U | R.LCTSATESEVTR.G |
| 49020 | 379 | – | 390 | 677.3173 | 1352.6201 | 1352.6242 | -3.06 | 0 | 48 | 3.1e-05 | 1Score **> 30** indicates **identity** Score **> 16** indicates **homology** | U | R.LCTSATESEVTR.G |
| 49021 | 379 | – | 390 | 677.3173 | 1352.6201 | 1352.6242 | -3.05 | 0 | 19 | 0.016 | 1Score **> 30** indicates **identity** Score **> 14** indicates **homology** | U | R.LCTSATESEVTR.G |
| 49024 | 379 | – | 390 | 677.3183 | 1352.6221 | 1352.6242 | -1.53 | 0 | 73 | 1.4e-07 | 1Score **> 32** indicates **identity** Score **> 17** indicates **homology** | U | R.LCTSATESEVTR.G |
| 49025 | 379 | – | 390 | 677.3184 | 1352.6222 | 1352.6242 | -1.43 | 0 | 23 | 0.0075 | 1Score **> 32** indicates **identity** Score **> 14** indicates **homology** | U | R.LCTSATESEVTR.G |
| 49026 | 379 | – | 390 | 677.3185 | 1352.6225 | 1352.6242 | -1.25 | 0 | 70 | 2.9e-07 | 1Score **> 32** indicates **identity** Score **> 17** indicates **homology** | U | R.LCTSATESEVTR.G |
| 49027 | 379 | – | 390 | 677.3186 | 1352.6227 | 1352.6242 | -1.08 | 0 | 25 | 0.035 | 1Score **> 32** indicates **identity** Score **> 23** indicates **homology** | U | R.LCTSATESEVTR.G |
| 49029 | 379 | – | 390 | 677.3187 | 1352.6229 | 1352.6242 | -0.96 | 0 | 50 | 8.2e-05 | 1Score **> 32** indicates **identity** Score **> 22** indicates **homology** | U | R.LCTSATESEVTR.G |
| 49030 | 379 | – | 390 | 677.3188 | 1352.6230 | 1352.6242 | -0.89 | 0 | 75 | 1e-07 | 1Score **> 32** indicates **identity** Score **> 17** indicates **homology** | U | R.LCTSATESEVTR.G |
| 49031 | 379 | – | 390 | 677.3188 | 1352.6230 | 1352.6242 | -0.88 | 0 | 73 | 1.4e-07 | 1Score **> 32** indicates **identity** Score **> 17** indicates **homology** | U | R.LCTSATESEVTR.G |
| 49032 | 379 | – | 390 | 677.3189 | 1352.6232 | 1352.6242 | -0.73 | 0 | 37 | 0.0025 | 1Score **> 32** indicates **identity** Score **> 24** indicates **homology** | U | R.LCTSATESEVTR.G |
| 49033 | 379 | – | 390 | 677.3189 | 1352.6232 | 1352.6242 | -0.72 | 0 | 70 | 3e-07 | 1Score **> 32** indicates **identity** Score **> 17** indicates **homology** | U | R.LCTSATESEVTR.G |
| 49034 | 379 | – | 390 | 677.3191 | 1352.6236 | 1352.6242 | -0.45 | 0 | 75 | 1e-07 | 1Score **> 32** indicates **identity** Score **> 17** indicates **homology** | U | R.LCTSATESEVTR.G |
| 49035 | 379 | – | 390 | 677.3192 | 1352.6239 | 1352.6242 | -0.18 | 0 | 80 | 1.5e-07 | 1Score **> 32** indicates **identity** Score **> 24** indicates **homology** | U | R.LCTSATESEVTR.G |
| 49036 | 379 | – | 390 | 677.3194 | 1352.6243 | 1352.6242 | 0.098 | 0 | 52 | 2.2e-05 | 1Score **> 31** indicates **identity** Score **> 18** indicates **homology** | U | R.LCTSATESEVTR.G |
| 49037 | 379 | – | 390 | 677.3199 | 1352.6252 | 1352.6242 | 0.76 | 0 | 31 | 0.0011 | 1Score **> 31** indicates **identity** Score **> 14** indicates **homology** | U | R.LCTSATESEVTR.G |
| 49038 | 379 | – | 390 | 677.3201 | 1352.6256 | 1352.6242 | 1.01 | 0 | 46 | 5e-05 | 1Score **> 32** indicates **identity** Score **> 15** indicates **homology** | U | R.LCTSATESEVTR.G |
| 124519 | 397 | – | 415 | 685.3362 | 2052.9866 | 2052.9899 | -1.58 | 0 | 18 | 0.022 | 1Score **> 35** indicates **identity** Score **> 14** indicates **homology** | U | R.NALVSHLDGTTPVCEDIGR.S |
| 124521 | 397 | – | 415 | 685.3363 | 2052.9870 | 2052.9899 | -1.37 | 0 | 56 | 6e-06 | 1Score **> 35** indicates **identity** Score **> 16** indicates **homology** | U | R.NALVSHLDGTTPVCEDIGR.S |
| 124522 | 397 | – | 415 | 1027.5013 | 2052.9880 | 2052.9899 | -0.90 | 0 | 63 | 1.2e-06 | 1Score **> 35** indicates **identity** Score **> 16** indicates **homology** | U | R.NALVSHLDGTTPVCEDIGR.S |
| 124523 | 397 | – | 415 | 685.3366 | 2052.9881 | 2052.9899 | -0.88 | 0 | 29 | 0.002 | 1Score **> 35** indicates **identity** Score **> 14** indicates **homology** | U | R.NALVSHLDGTTPVCEDIGR.S |
| 124524 | 397 | – | 415 | 1027.5013 | 2052.9881 | 2052.9899 | -0.86 | 0 | 35 | 0.00051 | 1Score **> 35** indicates **identity** Score **> 15** indicates **homology** | U | R.NALVSHLDGTTPVCEDIGR.S |
| 124525 | 397 | – | 415 | 685.3369 | 2052.9889 | 2052.9899 | -0.46 | 0 | 70 | 4.2e-07 | 1Score **> 35** indicates **identity** Score **> 19** indicates **homology** | U | R.NALVSHLDGTTPVCEDIGR.S |
| 124526 | 397 | – | 415 | 1027.5018 | 2052.9890 | 2052.9899 | -0.42 | 0 | 70 | 2.6e-07 | 1Score **> 35** indicates **identity** Score **> 17** indicates **homology** | U | R.NALVSHLDGTTPVCEDIGR.S |
| 124527 | 397 | – | 415 | 685.3370 | 2052.9892 | 2052.9899 | -0.30 | 0 | 52 | 1.3e-05 | 1Score **> 35** indicates **identity** Score **> 16** indicates **homology** | U | R.NALVSHLDGTTPVCEDIGR.S |
| 124528 | 397 | – | 415 | 685.3371 | 2052.9896 | 2052.9899 | -0.15 | 0 | 39 | 0.00025 | 1Score **> 35** indicates **identity** Score **> 15** indicates **homology** | U | R.NALVSHLDGTTPVCEDIGR.S |
| 124529 | 397 | – | 415 | 685.3371 | 2052.9896 | 2052.9899 | -0.13 | 0 | 69 | 4.9e-07 | 1Score **> 35** indicates **identity** Score **> 19** indicates **homology** | U | R.NALVSHLDGTTPVCEDIGR.S |
| 124530 | 397 | – | 415 | 1027.5021 | 2052.9896 | 2052.9899 | -0.13 | 0 | 85 | 1.1e-08 | 1Score **> 35** indicates **identity** Score **> 18** indicates **homology** | U | R.NALVSHLDGTTPVCEDIGR.S |
| 124531 | 397 | – | 415 | 685.3372 | 2052.9898 | 2052.9899 | -0.031 | 0 | 71 | 3.6e-07 | 1Score **> 35** indicates **identity** Score **> 19** indicates **homology** | U | R.NALVSHLDGTTPVCEDIGR.S |
| 124532 | 397 | – | 415 | 685.3373 | 2052.9901 | 2052.9899 | 0.11 | 0 | 77 | 1e-07 | 1Score **> 35** indicates **identity** Score **> 20** indicates **homology** | U | R.NALVSHLDGTTPVCEDIGR.S |
| 124533 | 397 | – | 415 | 685.3373 | 2052.9901 | 2052.9899 | 0.11 | 0 | 58 | 5.4e-06 | 1Score **> 35** indicates **identity** Score **> 18** indicates **homology** | U | R.NALVSHLDGTTPVCEDIGR.S |
| 124534 | 397 | – | 415 | 1027.5024 | 2052.9903 | 2052.9899 | 0.20 | 0 | 80 | 3.1e-08 | 1Score **> 35** indicates **identity** Score **> 18** indicates **homology** | U | R.NALVSHLDGTTPVCEDIGR.S |
| 124535 | 397 | – | 415 | 685.3374 | 2052.9903 | 2052.9899 | 0.22 | 0 | 34 | 0.00061 | 1Score **> 35** indicates **identity** Score **> 15** indicates **homology** | U | R.NALVSHLDGTTPVCEDIGR.S |
| 124536 | 397 | – | 415 | 1027.5024 | 2052.9903 | 2052.9899 | 0.22 | 0 | 94 | 1.7e-09 | 1Score **> 35** indicates **identity** Score **> 18** indicates **homology** | U | R.NALVSHLDGTTPVCEDIGR.S |
| 124537 | 397 | – | 415 | 1027.5025 | 2052.9904 | 2052.9899 | 0.24 | 0 | 94 | 1.5e-09 | 1Score **> 35** indicates **identity** Score **> 18** indicates **homology** | U | R.NALVSHLDGTTPVCEDIGR.S |
| 124538 | 397 | – | 415 | 685.3374 | 2052.9905 | 2052.9899 | 0.29 | 0 | 71 | 3.7e-07 | 1Score **> 35** indicates **identity** Score **> 19** indicates **homology** | U | R.NALVSHLDGTTPVCEDIGR.S |
| 124539 | 397 | – | 415 | 1027.5025 | 2052.9905 | 2052.9899 | 0.31 | 0 | 86 | 7.8e-09 | 1Score **> 35** indicates **identity** Score **> 18** indicates **homology** | U | R.NALVSHLDGTTPVCEDIGR.S |
| 124540 | 397 | – | 415 | 685.3374 | 2052.9905 | 2052.9899 | 0.31 | 0 | 66 | 8.2e-07 | 1Score **> 35** indicates **identity** Score **> 17** indicates **homology** | U | R.NALVSHLDGTTPVCEDIGR.S |
| 124541 | 397 | – | 415 | 685.3375 | 2052.9905 | 2052.9899 | 0.32 | 0 | 66 | 8.2e-07 | 1Score **> 35** indicates **identity** Score **> 18** indicates **homology** | U | R.NALVSHLDGTTPVCEDIGR.S |
| 124542 | 397 | – | 415 | 685.3375 | 2052.9907 | 2052.9899 | 0.38 | 0 | 67 | 7.4e-07 | 1Score **> 35** indicates **identity** Score **> 18** indicates **homology** | U | R.NALVSHLDGTTPVCEDIGR.S |
| 124543 | 397 | – | 415 | 1027.5027 | 2052.9909 | 2052.9899 | 0.51 | 0 | 96 | 1e-09 | 1Score **> 35** indicates **identity** Score **> 18** indicates **homology** | U | R.NALVSHLDGTTPVCEDIGR.S |
| 124544 | 397 | – | 415 | 1027.5028 | 2052.9910 | 2052.9899 | 0.57 | 0 | 84 | 1.4e-08 | 1Score **> 35** indicates **identity** Score **> 18** indicates **homology** | U | R.NALVSHLDGTTPVCEDIGR.S |
| 124545 | 397 | – | 415 | 685.3378 | 2052.9916 | 2052.9899 | 0.84 | 0 | 71 | 3.4e-07 | 1Score **> 35** indicates **identity** Score **> 19** indicates **homology** | U | R.NALVSHLDGTTPVCEDIGR.S |
| 124546 | 397 | – | 415 | 1027.5031 | 2052.9916 | 2052.9899 | 0.85 | 0 | 73 | 1.3e-07 | 1Score **> 35** indicates **identity** Score **> 17** indicates **homology** | U | R.NALVSHLDGTTPVCEDIGR.S |
| 124547 | 397 | – | 415 | 685.3378 | 2052.9917 | 2052.9899 | 0.88 | 0 | 71 | 3.6e-07 | 1Score **> 35** indicates **identity** Score **> 19** indicates **homology** | U | R.NALVSHLDGTTPVCEDIGR.S |
| 124548 | 397 | – | 415 | 685.3379 | 2052.9918 | 2052.9899 | 0.96 | 0 | 71 | 3.9e-07 | 1Score **> 35** indicates **identity** Score **> 19** indicates **homology** | U | R.NALVSHLDGTTPVCEDIGR.S |
| 124549 | 397 | – | 415 | 1027.5034 | 2052.9922 | 2052.9899 | 1.12 | 0 | 22 | 0.0096 | 1Score **> 35** indicates **identity** Score **> 14** indicates **homology** | U | R.NALVSHLDGTTPVCEDIGR.S |
| 124550 | 397 | – | 415 | 685.3381 | 2052.9925 | 2052.9899 | 1.30 | 0 | 70 | 3.4e-07 | 1Score **> 35** indicates **identity** Score **> 18** indicates **homology** | U | R.NALVSHLDGTTPVCEDIGR.S |
| 124551 | 397 | – | 415 | 685.3385 | 2052.9936 | 2052.9899 | 1.81 | 0 | 31 | 0.0012 | 1Score **> 35** indicates **identity** Score **> 14** indicates **homology** | U | R.NALVSHLDGTTPVCEDIGR.S |
| 124552 | 397 | – | 415 | 1027.5041 | 2052.9936 | 2052.9899 | 1.84 | 0 | 41 | 0.00014 | 1Score **> 35** indicates **identity** Score **> 15** indicates **homology** | U | R.NALVSHLDGTTPVCEDIGR.S |
| 124553 | 397 | – | 415 | 685.3386 | 2052.9939 | 2052.9899 | 1.97 | 0 | 30 | 0.0014 | 1Score **> 35** indicates **identity** Score **> 14** indicates **homology** | U | R.NALVSHLDGTTPVCEDIGR.S |
| 124554 | 397 | – | 415 | 685.3387 | 2052.9944 | 2052.9899 | 2.19 | 0 | 29 | 0.0019 | 1Score **> 35** indicates **identity** Score **> 14** indicates **homology** | U | R.NALVSHLDGTTPVCEDIGR.S |
| 124555 | 397 | – | 415 | 1027.5045 | 2052.9944 | 2052.9899 | 2.19 | 0 | 29 | 0.002 | 1Score **> 35** indicates **identity** Score **> 14** indicates **homology** | U | R.NALVSHLDGTTPVCEDIGR.S |
| 124557 | 397 | – | 415 | 685.3393 | 2052.9959 | 2052.9899 | 2.96 | 0 | 40 | 0.00017 | 1Score **> 35** indicates **identity** Score **> 15** indicates **homology** | U | R.NALVSHLDGTTPVCEDIGR.S |
| 124605 | 397 | – | 415 | 1027.9951 | 2053.9757 | 2053.9739 | 0.88 | 0 | 78 | 4.7e-08 | 1Score **> 34** indicates **identity** Score **> 17** indicates **homology** | U | R.NALVSHLDGTTPVCEDIGR.S  + Deamidated (NQ) |
| 124606 | 397 | – | 415 | 685.6658 | 2053.9757 | 2053.9739 | 0.88 | 0 | 49 | 6.5e-05 | 1Score **> 34** indicates **identity** Score **> 19** indicates **homology** | U | R.NALVSHLDGTTPVCEDIGR.S  + Deamidated (NQ) |
| 124607 | 397 | – | 415 | 685.6664 | 2053.9775 | 2053.9739 | 1.74 | 0 | 31 | 0.0011 | 1Score **> 34** indicates **identity** Score **> 14** indicates **homology** | U | R.NALVSHLDGTTPVCEDIGR.S  + Deamidated (NQ) |
| 124608 | 397 | – | 415 | 1027.9966 | 2053.9786 | 2053.9739 | 2.31 | 0 | 71 | 2.1e-07 | 1Score **> 35** indicates **identity** Score **> 17** indicates **homology** | U | R.NALVSHLDGTTPVCEDIGR.S  + Deamidated (NQ) |
| 124609 | 397 | – | 415 | 685.6689 | 2053.9850 | 2053.9739 | 5.39 | 0 | 37 | 0.00032 | 1Score **> 35** indicates **identity** Score **> 15** indicates **homology** | U | R.NALVSHLDGTTPVCEDIGR.S  + Deamidated (NQ) |
| 124610 | 397 | – | 415 | 685.6706 | 2053.9900 | 2053.9739 | 7.83 | 0 | 41 | 0.00014 | 1Score **> 35** indicates **identity** Score **> 15** indicates **homology** | U | R.NALVSHLDGTTPVCEDIGR.S  + Deamidated (NQ) |
| 124611 | 397 | – | 415 | 1028.0040 | 2053.9935 | 2053.9739 | 9.54 | 0 | 57 | 4.8e-06 | 1Score **> 35** indicates **identity** Score **> 16** indicates **homology** | U | R.NALVSHLDGTTPVCEDIGR.S  + Deamidated (NQ) |
| 3903 | 416 | – | 422 | 405.2282 | 808.4419 | 808.4443 | -2.94 | 0 | 20 | 0.015 | 1Score **> 22** indicates **identity** Score **> 14** indicates **homology** | U | R.SLLTYGR.R |
| 3904 | 416 | – | 422 | 405.2284 | 808.4423 | 808.4443 | -2.42 | 0 | 20 | 0.012 | 1Score **> 22** indicates **identity** Score **> 14** indicates **homology** | U | R.SLLTYGR.R |
| 3907 | 416 | – | 422 | 405.2290 | 808.4434 | 808.4443 | -1.04 | 0 | 51 | 2e-05 | 1Score **> 22** indicates **identity** Score **> 16** indicates **homology** | U | R.SLLTYGR.R |
| 3908 | 416 | – | 422 | 405.2291 | 808.4436 | 808.4443 | -0.88 | 0 | 39 | 0.00022 | 1Score **> 22** indicates **identity** Score **> 15** indicates **homology** | U | R.SLLTYGR.R |
| 3909 | 416 | – | 422 | 405.2291 | 808.4437 | 808.4443 | -0.68 | 0 | 36 | 0.00038 | 1Score **> 22** indicates **identity** Score **> 15** indicates **homology** | U | R.SLLTYGR.R |
| 3910 | 416 | – | 422 | 405.2292 | 808.4438 | 808.4443 | -0.58 | 0 | 20 | 0.015 | 1Score **> 22** indicates **identity** Score **> 14** indicates **homology** | U | R.SLLTYGR.R |
| 3911 | 416 | – | 422 | 405.2292 | 808.4439 | 808.4443 | -0.45 | 0 | 30 | 0.0017 | 1Score **> 22** indicates **identity** Score **> 14** indicates **homology** | U | R.SLLTYGR.R |
| 3912 | 416 | – | 422 | 405.2294 | 808.4442 | 808.4443 | -0.16 | 0 | 39 | 0.00024 | 1Score **> 22** indicates **identity** Score **> 15** indicates **homology** | U | R.SLLTYGR.R |
| 3913 | 416 | – | 422 | 405.2294 | 808.4443 | 808.4443 | 0.015 | 0 | 51 | 4.4e-05 | 1Score **> 22** indicates **identity** Score **> 20** indicates **homology** | U | R.SLLTYGR.R |
| 3914 | 416 | – | 422 | 405.2296 | 808.4447 | 808.4443 | 0.47 | 0 | 49 | 2.6e-05 | 1Score **> 22** indicates **identity** Score **> 16** indicates **homology** | U | R.SLLTYGR.R |
| 3915 | 416 | – | 422 | 405.2301 | 808.4457 | 808.4443 | 1.75 | 0 | 31 | 0.0012 | 1Score **> 22** indicates **identity** Score **> 14** indicates **homology** | U | R.SLLTYGR.R |
| 38740 | 423 | – | 432 | 419.5615 | 1255.6625 | 1255.6673 | -3.78 | 1 | 24 | 0.0052 | 1Score **> 33** indicates **identity** Score **> 14** indicates **homology** | U | R.RIPLAEWESR.I |
| 38741 | 423 | – | 432 | 419.5615 | 1255.6628 | 1255.6673 | -3.56 | 1 | 31 | 0.0013 | 1Score **> 32** indicates **identity** Score **> 14** indicates **homology** | U | R.RIPLAEWESR.I |
| 38743 | 423 | – | 432 | 419.5618 | 1255.6636 | 1255.6673 | -2.96 | 1 | 24 | 0.0058 | 1Score **> 32** indicates **identity** Score **> 14** indicates **homology** | U | R.RIPLAEWESR.I |
| 38744 | 423 | – | 432 | 419.5618 | 1255.6637 | 1255.6673 | -2.85 | 1 | 31 | 0.0013 | 1Score **> 32** indicates **identity** Score **> 14** indicates **homology** | U | R.RIPLAEWESR.I |
| 38745 | 423 | – | 432 | 419.5619 | 1255.6639 | 1255.6673 | -2.68 | 1 | 29 | 0.0018 | 1Score **> 32** indicates **identity** Score **> 14** indicates **homology** | U | R.RIPLAEWESR.I |
| 38746 | 423 | – | 432 | 419.5622 | 1255.6647 | 1255.6673 | -2.08 | 1 | 15 | 0.036 | 1Score **> 33** indicates **identity** Score **> 13** indicates **homology** | U | R.RIPLAEWESR.I |
| 38748 | 423 | – | 432 | 419.5625 | 1255.6658 | 1255.6673 | -1.17 | 1 | 35 | 0.00053 | 1Score **> 33** indicates **identity** Score **> 15** indicates **homology** | U | R.RIPLAEWESR.I |
| 38749 | 423 | – | 432 | 419.5626 | 1255.6659 | 1255.6673 | -1.09 | 1 | 27 | 0.0029 | 1Score **> 33** indicates **identity** Score **> 14** indicates **homology** | U | R.RIPLAEWESR.I |
| 38750 | 423 | – | 432 | 419.5626 | 1255.6661 | 1255.6673 | -0.92 | 1 | 29 | 0.0019 | 1Score **> 33** indicates **identity** Score **> 14** indicates **homology** | U | R.RIPLAEWESR.I |
| 38751 | 423 | – | 432 | 419.5627 | 1255.6664 | 1255.6673 | -0.70 | 1 | 30 | 0.0015 | 1Score **> 33** indicates **identity** Score **> 14** indicates **homology** | U | R.RIPLAEWESR.I |
| 38753 | 423 | – | 432 | 419.5628 | 1255.6666 | 1255.6673 | -0.57 | 1 | 28 | 0.0025 | 1Score **> 33** indicates **identity** Score **> 14** indicates **homology** | U | R.RIPLAEWESR.I |
| 38754 | 423 | – | 432 | 628.8406 | 1255.6666 | 1255.6673 | -0.55 | 1 | 24 | 0.034 | 1Score **> 33** indicates **identity** Score **> 22** indicates **homology** | U | R.RIPLAEWESR.I |
| 38755 | 423 | – | 432 | 419.5628 | 1255.6666 | 1255.6673 | -0.55 | 1 | 25 | 0.0044 | 1Score **> 33** indicates **identity** Score **> 14** indicates **homology** | U | R.RIPLAEWESR.I |
| 38756 | 423 | – | 432 | 628.8406 | 1255.6666 | 1255.6673 | -0.52 | 1 | 46 | 0.0028 | 1Score **> 33** indicates **identity** | U | R.RIPLAEWESR.I |
| 38757 | 423 | – | 432 | 628.8406 | 1255.6667 | 1255.6673 | -0.43 | 1 | 34 | 0.0074 | 1Score **> 33** indicates **identity** Score **> 26** indicates **homology** | U | R.RIPLAEWESR.I |
| 38759 | 423 | – | 432 | 419.5629 | 1255.6669 | 1255.6673 | -0.30 | 1 | 26 | 0.0033 | 1Score **> 33** indicates **identity** Score **> 14** indicates **homology** | U | R.RIPLAEWESR.I |
| 38760 | 423 | – | 432 | 419.5629 | 1255.6670 | 1255.6673 | -0.22 | 1 | 33 | 0.0008 | 1Score **> 33** indicates **identity** Score **> 15** indicates **homology** | U | R.RIPLAEWESR.I |
| 38761 | 423 | – | 432 | 419.5630 | 1255.6670 | 1255.6673 | -0.20 | 1 | 35 | 0.00053 | 1Score **> 33** indicates **identity** Score **> 15** indicates **homology** | U | R.RIPLAEWESR.I |
| 38762 | 423 | – | 432 | 628.8408 | 1255.6671 | 1255.6673 | -0.15 | 1 | 33 | 0.0043 | 1Score **> 33** indicates **identity** Score **> 22** indicates **homology** | U | R.RIPLAEWESR.I |
| 38764 | 423 | – | 432 | 419.5630 | 1255.6672 | 1255.6673 | -0.100 | 1 | 27 | 0.0029 | 1Score **> 33** indicates **identity** Score **> 14** indicates **homology** | U | R.RIPLAEWESR.I |
| 38765 | 423 | – | 432 | 628.8409 | 1255.6672 | 1255.6673 | -0.083 | 1 | 61 | 4.5e-05 | 1Score **> 33** indicates **identity** Score **> 30** indicates **homology** | U | R.RIPLAEWESR.I |
| 38767 | 423 | – | 432 | 419.5630 | 1255.6673 | 1255.6673 | 0.0032 | 1 | 31 | 0.0013 | 1Score **> 33** indicates **identity** Score **> 14** indicates **homology** | U | R.RIPLAEWESR.I |
| 38769 | 423 | – | 432 | 419.5631 | 1255.6675 | 1255.6673 | 0.17 | 1 | 38 | 0.0003 | 1Score **> 33** indicates **identity** Score **> 15** indicates **homology** | U | R.RIPLAEWESR.I |
| 38770 | 423 | – | 432 | 419.5631 | 1255.6676 | 1255.6673 | 0.25 | 1 | 20 | 0.013 | 1Score **> 33** indicates **identity** Score **> 14** indicates **homology** | U | R.RIPLAEWESR.I |
| 38771 | 423 | – | 432 | 419.5632 | 1255.6677 | 1255.6673 | 0.31 | 1 | 32 | 0.001 | 1Score **> 33** indicates **identity** Score **> 14** indicates **homology** | U | R.RIPLAEWESR.I |
| 38772 | 423 | – | 432 | 419.5632 | 1255.6677 | 1255.6673 | 0.32 | 1 | 16 | 0.039 | 1Score **> 33** indicates **identity** Score **> 14** indicates **homology** | U | R.RIPLAEWESR.I |
| 38774 | 423 | – | 432 | 419.5632 | 1255.6677 | 1255.6673 | 0.32 | 1 | 46 | 4.6e-05 | 1Score **> 33** indicates **identity** Score **> 15** indicates **homology** | U | R.RIPLAEWESR.I |
| 38776 | 423 | – | 432 | 419.5634 | 1255.6683 | 1255.6673 | 0.82 | 1 | 29 | 0.0018 | 1Score **> 33** indicates **identity** Score **> 14** indicates **homology** | U | R.RIPLAEWESR.I |
| 38777 | 423 | – | 432 | 628.8414 | 1255.6683 | 1255.6673 | 0.82 | 1 | 50 | 0.001 | 1Score **> 33** indicates **identity** | U | R.RIPLAEWESR.I |
| 38780 | 423 | – | 432 | 628.8415 | 1255.6684 | 1255.6673 | 0.90 | 1 | 31 | 0.014 | 1Score **> 33** indicates **identity** Score **> 25** indicates **homology** | U | R.RIPLAEWESR.I |
| 38781 | 423 | – | 432 | 419.5634 | 1255.6685 | 1255.6673 | 0.96 | 1 | 18 | 0.021 | 1Score **> 33** indicates **identity** Score **> 14** indicates **homology** | U | R.RIPLAEWESR.I |
| 38784 | 423 | – | 432 | 419.5635 | 1255.6687 | 1255.6673 | 1.11 | 1 | 24 | 0.0062 | 1Score **> 33** indicates **identity** Score **> 14** indicates **homology** | U | R.RIPLAEWESR.I |
| 38785 | 423 | – | 432 | 419.5635 | 1255.6688 | 1255.6673 | 1.20 | 1 | 36 | 0.00042 | 1Score **> 33** indicates **identity** Score **> 15** indicates **homology** | U | R.RIPLAEWESR.I |
| 38786 | 423 | – | 432 | 419.5635 | 1255.6688 | 1255.6673 | 1.21 | 1 | 29 | 0.0018 | 1Score **> 33** indicates **identity** Score **> 14** indicates **homology** | U | R.RIPLAEWESR.I |
| 38787 | 423 | – | 432 | 628.8418 | 1255.6690 | 1255.6673 | 1.36 | 1 | 20 | 0.027 | 1Score **> 33** indicates **identity** Score **> 17** indicates **homology** | U | R.RIPLAEWESR.I |
| 38789 | 423 | – | 432 | 628.8418 | 1255.6691 | 1255.6673 | 1.47 | 1 | 34 | 0.044 | 1Score **> 33** indicates **identity** | U | R.RIPLAEWESR.I |
| 38790 | 423 | – | 432 | 628.8418 | 1255.6691 | 1255.6673 | 1.47 | 1 | 24 | 0.031 | 1Score **> 33** indicates **identity** Score **> 22** indicates **homology** | U | R.RIPLAEWESR.I |
| 38792 | 423 | – | 432 | 419.5639 | 1255.6700 | 1255.6673 | 2.14 | 1 | 35 | 0.00049 | 1Score **> 33** indicates **identity** Score **> 15** indicates **homology** | U | R.RIPLAEWESR.I |
| 22107 | 424 | – | 432 | 550.7903 | 1099.5661 | 1099.5662 | -0.070 | 0 | 47 | 0.00064 | 1Score **> 31** indicates **identity** Score **> 28** indicates **homology** | U | R.IPLAEWESR.I |
| 22108 | 424 | – | 432 | 550.7906 | 1099.5667 | 1099.5662 | 0.49 | 0 | 46 | 0.00068 | 1Score **> 32** indicates **identity** Score **> 27** indicates **homology** | U | R.IPLAEWESR.I |
| 32974 | 433 | – | 442 | 601.8128 | 1201.6111 | 1201.6125 | -1.14 | 0 | 39 | 0.00077 | 1Score **> 33** indicates **identity** Score **> 20** indicates **homology** | U | R.IQEVDAQMLR.D |
| 32975 | 433 | – | 442 | 601.8133 | 1201.6121 | 1201.6125 | -0.28 | 0 | 53 | 0.00039 | 1Score **> 33** indicates **identity** Score **> 31** indicates **homology** | U | R.IQEVDAQMLR.D |
| 32977 | 433 | – | 442 | 601.8136 | 1201.6127 | 1201.6125 | 0.21 | 0 | 58 | 5.3e-05 | 1Score **> 33** indicates **identity** Score **> 28** indicates **homology** | U | R.IQEVDAQMLR.D |
| 32978 | 433 | – | 442 | 601.8138 | 1201.6131 | 1201.6125 | 0.52 | 0 | 58 | 0.00018 | 1Score **> 33** indicates **identity** | U | R.IQEVDAQMLR.D |
| 34534 | 433 | – | 442 | 609.8092 | 1217.6038 | 1217.6074 | -2.96 | 0 | 21 | 0.012 | 1Score **> 33** indicates **identity** Score **> 14** indicates **homology** | U | R.IQEVDAQMLR.D  + Oxidation (M) |
| 34540 | 433 | – | 442 | 609.8105 | 1217.6064 | 1217.6074 | -0.81 | 0 | 17 | 0.027 | 1Score **> 32** indicates **identity** Score **> 14** indicates **homology** | U | R.IQEVDAQMLR.D  + Oxidation (M) |
| 101809 | 433 | – | 447 | 602.6332 | 1804.8779 | 1804.8811 | -1.81 | 1 | 16 | 0.029 | 1Score **> 35** indicates **identity** Score **> 14** indicates **homology** | U | R.IQEVDAQMLRDICSK.Y |
| 164873 | 448 | – | 470 | 1368.6231 | 2735.2316 | 2735.2326 | -0.36 | 0 | 84 | 1.2e-08 | 1Score **> 32** indicates **identity** Score **> 18** indicates **homology** | U | K.YFYDQCPAVAGYGPIEQLPDYNR.I |
| 164874 | 448 | – | 470 | 912.7512 | 2735.2318 | 2735.2326 | -0.28 | 0 | 86 | 8e-09 | 1Score **> 32** indicates **identity** Score **> 18** indicates **homology** | U | K.YFYDQCPAVAGYGPIEQLPDYNR.I |
| 164875 | 448 | – | 470 | 1368.6232 | 2735.2319 | 2735.2326 | -0.25 | 0 | 59 | 2.9e-06 | 1Score **> 32** indicates **identity** Score **> 16** indicates **homology** | U | K.YFYDQCPAVAGYGPIEQLPDYNR.I |
| 164876 | 448 | – | 470 | 912.7513 | 2735.2320 | 2735.2326 | -0.21 | 0 | 15 | 0.042 | 1Score **> 32** indicates **identity** Score **> 13** indicates **homology** | U | K.YFYDQCPAVAGYGPIEQLPDYNR.I |
| 164877 | 448 | – | 470 | 912.7514 | 2735.2323 | 2735.2326 | -0.088 | 0 | 69 | 3.5e-07 | 1Score **> 32** indicates **identity** Score **> 17** indicates **homology** | U | K.YFYDQCPAVAGYGPIEQLPDYNR.I |
| 164878 | 448 | – | 470 | 1368.6241 | 2735.2336 | 2735.2326 | 0.36 | 0 | 79 | 3.8e-08 | 1Score **> 33** indicates **identity** Score **> 17** indicates **homology** | U | K.YFYDQCPAVAGYGPIEQLPDYNR.I |
| 164879 | 448 | – | 470 | 1368.6243 | 2735.2340 | 2735.2326 | 0.52 | 0 | 92 | 2.3e-09 | 1Score **> 33** indicates **identity** Score **> 18** indicates **homology** | U | K.YFYDQCPAVAGYGPIEQLPDYNR.I |
| 164880 | 448 | – | 470 | 912.7522 | 2735.2346 | 2735.2326 | 0.75 | 0 | 72 | 1.8e-07 | 1Score **> 33** indicates **identity** Score **> 17** indicates **homology** | U | K.YFYDQCPAVAGYGPIEQLPDYNR.I |
| 164881 | 448 | – | 470 | 912.7522 | 2735.2347 | 2735.2326 | 0.76 | 0 | 34 | 0.00065 | 1Score **> 33** indicates **identity** Score **> 15** indicates **homology** | U | K.YFYDQCPAVAGYGPIEQLPDYNR.I |
| 164882 | 448 | – | 470 | 912.7523 | 2735.2350 | 2735.2326 | 0.90 | 0 | 21 | 0.0098 | 1Score **> 33** indicates **identity** Score **> 14** indicates **homology** | U | K.YFYDQCPAVAGYGPIEQLPDYNR.I |
| 164883 | 448 | – | 470 | 912.7528 | 2735.2366 | 2735.2326 | 1.46 | 0 | 59 | 3e-06 | 1Score **> 33** indicates **identity** Score **> 16** indicates **homology** | U | K.YFYDQCPAVAGYGPIEQLPDYNR.I |
| 164884 | 448 | – | 470 | 1368.6262 | 2735.2379 | 2735.2326 | 1.94 | 0 | 70 | 2.5e-07 | 1Score **> 33** indicates **identity** Score **> 17** indicates **homology** | U | K.YFYDQCPAVAGYGPIEQLPDYNR.I |
| 164886 | 448 | – | 470 | 912.7534 | 2735.2383 | 2735.2326 | 2.08 | 0 | 66 | 2.5e-06 | 1Score **> 33** indicates **identity** Score **> 22** indicates **homology** | U | K.YFYDQCPAVAGYGPIEQLPDYNR.I |
| 164887 | 448 | – | 470 | 912.7540 | 2735.2402 | 2735.2326 | 2.78 | 0 | 34 | 0.0011 | 1Score **> 33** indicates **identity** Score **> 17** indicates **homology** | U | K.YFYDQCPAVAGYGPIEQLPDYNR.I |
| 164888 | 448 | – | 470 | 912.7572 | 2735.2498 | 2735.2326 | 6.29 | 0 | 57 | 1.1e-05 | 1Score **> 34** indicates **identity** Score **> 19** indicates **homology** | U | K.YFYDQCPAVAGYGPIEQLPDYNR.I |
| 164963 | 448 | – | 470 | 913.0828 | 2736.2266 | 2736.2166 | 3.66 | 0 | 24 | 0.0057 | 1Score **> 32** indicates **identity** Score **> 14** indicates **homology** | U | K.YFYDQCPAVAGYGPIEQLPDYNR.I  + Deamidated (NQ) |
| 164964 | 448 | – | 470 | 913.0835 | 2736.2288 | 2736.2166 | 4.45 | 0 | 15 | 0.041 | 1Score **> 32** indicates **identity** Score **> 13** indicates **homology** | U | K.YFYDQCPAVAGYGPIEQLPDYNR.I  + Deamidated (NQ) |
| 164965 | 448 | – | 470 | 913.0852 | 2736.2338 | 2736.2166 | 6.28 | 0 | 35 | 0.0005 | 1Score **> 33** indicates **identity** Score **> 15** indicates **homology** | U | K.YFYDQCPAVAGYGPIEQLPDYNR.I  + Deamidated (NQ) |
| 164966 | 448 | – | 470 | 913.0852 | 2736.2339 | 2736.2166 | 6.31 | 0 | 37 | 0.00031 | 1Score **> 33** indicates **identity** Score **> 15** indicates **homology** | U | K.YFYDQCPAVAGYGPIEQLPDYNR.I  + Deamidated (NQ) |
| 164967 | 448 | – | 470 | 913.0853 | 2736.2341 | 2736.2166 | 6.41 | 0 | 23 | 0.0076 | 1Score **> 33** indicates **identity** Score **> 14** indicates **homology** | U | K.YFYDQCPAVAGYGPIEQLPDYNR.I  + Deamidated (NQ) |
| 164968 | 448 | – | 470 | 913.0866 | 2736.2379 | 2736.2166 | 7.79 | 0 | 22 | 0.0094 | 1Score **> 33** indicates **identity** Score **> 14** indicates **homology** | U | K.YFYDQCPAVAGYGPIEQLPDYNR.I  + Deamidated (NQ) |
| 164969 | 448 | – | 470 | 913.0869 | 2736.2389 | 2736.2166 | 8.16 | 0 | 45 | 6.2e-05 | 1Score **> 33** indicates **identity** Score **> 15** indicates **homology** | U | K.YFYDQCPAVAGYGPIEQLPDYNR.I  + Deamidated (NQ) |
| 164970 | 448 | – | 470 | 913.0876 | 2736.2411 | 2736.2166 | 8.95 | 0 | 26 | 0.0033 | 1Score **> 33** indicates **identity** Score **> 14** indicates **homology** | U | K.YFYDQCPAVAGYGPIEQLPDYNR.I  + Deamidated (NQ) |
| 173515 | 448 | – | 472 | 1002.4813 | 3004.4220 | 3004.4178 | 1.43 | 1 | 39 | 0.00023 | 1Score **> 36** indicates **identity** Score **> 15** indicates **homology** | U | K.YFYDQCPAVAGYGPIEQLPDYNRIR.S |
| 173516 | 448 | – | 472 | 1002.4817 | 3004.4232 | 3004.4178 | 1.80 | 1 | 61 | 2e-06 | 1Score **> 36** indicates **identity** Score **> 16** indicates **homology** | U | K.YFYDQCPAVAGYGPIEQLPDYNRIR.S |
| 173518 | 448 | – | 472 | 1002.4832 | 3004.4279 | 3004.4178 | 3.38 | 1 | 39 | 0.0002 | 1Score **> 36** indicates **identity** Score **> 15** indicates **homology** | U | K.YFYDQCPAVAGYGPIEQLPDYNRIR.S |
| 173523 | 448 | – | 472 | 1002.4877 | 3004.4412 | 3004.4178 | 7.80 | 1 | 17 | 0.025 | 1Score **> 37** indicates **identity** Score **> 14** indicates **homology** | U | K.YFYDQCPAVAGYGPIEQLPDYNRIR.S |
| 173534 | 448 | – | 472 | 1002.8142 | 3005.4208 | 3005.4018 | 6.33 | 1 | 17 | 0.023 | 1Score **> 36** indicates **identity** Score **> 14** indicates **homology** | U | K.YFYDQCPAVAGYGPIEQLPDYNRIR.S  + Deamidated (NQ) |
| 7956 | 473 | – | 479 | 448.7261 | 895.4376 | 895.4374 | 0.16 | 0 | 42 | 0.0018 | 1Score **> 27** indicates **identity** | U | R.SGMFWLR.F |
| 7957 | 473 | – | 479 | 448.7261 | 895.4377 | 895.4374 | 0.24 | 0 | 42 | 0.0014 | 1Score **> 26** indicates **identity** | U | R.SGMFWLR.F |
| 7958 | 473 | – | 479 | 448.7262 | 895.4379 | 895.4374 | 0.49 | 0 | 34 | 0.00081 | 1Score **> 25** indicates **identity** Score **> 16** indicates **homology** | U | R.SGMFWLR.F |
| 8976 | 473 | – | 479 | 456.7237 | 911.4328 | 911.4324 | 0.43 | 0 | 21 | 0.013 | 1Score **> 26** indicates **identity** Score **> 15** indicates **homology** | U | R.SGMFWLR.F  + Oxidation (M) |
| 16802 | 473 | – | 480 | 522.2594 | 1042.5043 | 1042.5059 | -1.45 | 1 | 53 | 1e-05 | 1Score **> 28** indicates **identity** Score **> 16** indicates **homology** | U | R.SGMFWLRF.- |
| 16803 | 473 | – | 480 | 522.2604 | 1042.5063 | 1042.5059 | 0.43 | 1 | 54 | 9.2e-06 | 1Score **> 28** indicates **identity** Score **> 16** indicates **homology** | U | R.SGMFWLRF.- |
| 16804 | 473 | – | 480 | 522.2606 | 1042.5066 | 1042.5059 | 0.67 | 1 | 52 | 1.3e-05 | 1Score **> 28** indicates **identity** Score **> 16** indicates **homology** | U | R.SGMFWLRF.- |

---

```
ID   QCR1_MOUSE              Reviewed;         480 AA.
AC   Q9CZ13; Q3TV75; Q9CWL6;
DT   20-JUN-2001, integrated into UniProtKB/Swiss-Prot.
DT   27-JUL-2011, sequence version 2.
DT   28-JUN-2023, entry version 182.
DE   RecName: Full=Cytochrome b-c1 complex subunit 1, mitochondrial;
DE   AltName: Full=Complex III subunit 1;
DE   AltName: Full=Core protein I;
DE   AltName: Full=Ubiquinol-cytochrome-c reductase complex core protein 1;
DE   Flags: Precursor;
GN   Name=Uqcrc1;
OS   Mus musculus (Mouse).
OC   Eukaryota; Metazoa; Chordata; Craniata; Vertebrata; Euteleostomi; Mammalia;
OC   Eutheria; Euarchontoglires; Glires; Rodentia; Myomorpha; Muroidea; Muridae;
OC   Murinae; Mus; Mus.
OX   NCBI_TaxID=10090;
RN   [1]
RP   NUCLEOTIDE SEQUENCE [LARGE SCALE MRNA].
RC   STRAIN=C57BL/6J; TISSUE=Bone marrow, and Embryo;
RX   PubMed=16141072; DOI=10.1126/science.1112014;
RA   Carninci P., Kasukawa T., Katayama S., Gough J., Frith M.C., Maeda N.,
RA   Oyama R., Ravasi T., Lenhard B., Wells C., Kodzius R., Shimokawa K.,
RA   Bajic V.B., Brenner S.E., Batalov S., Forrest A.R., Zavolan M., Davis M.J.,
RA   Wilming L.G., Aidinis V., Allen J.E., Ambesi-Impiombato A., Apweiler R.,
RA   Aturaliya R.N., Bailey T.L., Bansal M., Baxter L., Beisel K.W., Bersano T.,
RA   Bono H., Chalk A.M., Chiu K.P., Choudhary V., Christoffels A.,
RA   Clutterbuck D.R., Crowe M.L., Dalla E., Dalrymple B.P., de Bono B.,
RA   Della Gatta G., di Bernardo D., Down T., Engstrom P., Fagiolini M.,
RA   Faulkner G., Fletcher C.F., Fukushima T., Furuno M., Futaki S.,
RA   Gariboldi M., Georgii-Hemming P., Gingeras T.R., Gojobori T., Green R.E.,
RA   Gustincich S., Harbers M., Hayashi Y., Hensch T.K., Hirokawa N., Hill D.,
RA   Huminiecki L., Iacono M., Ikeo K., Iwama A., Ishikawa T., Jakt M.,
RA   Kanapin A., Katoh M., Kawasawa Y., Kelso J., Kitamura H., Kitano H.,
RA   Kollias G., Krishnan S.P., Kruger A., Kummerfeld S.K., Kurochkin I.V.,
RA   Lareau L.F., Lazarevic D., Lipovich L., Liu J., Liuni S., McWilliam S.,
RA   Madan Babu M., Madera M., Marchionni L., Matsuda H., Matsuzawa S., Miki H.,
RA   Mignone F., Miyake S., Morris K., Mottagui-Tabar S., Mulder N., Nakano N.,
RA   Nakauchi H., Ng P., Nilsson R., Nishiguchi S., Nishikawa S., Nori F.,
RA   Ohara O., Okazaki Y., Orlando V., Pang K.C., Pavan W.J., Pavesi G.,
RA   Pesole G., Petrovsky N., Piazza S., Reed J., Reid J.F., Ring B.Z.,
RA   Ringwald M., Rost B., Ruan Y., Salzberg S.L., Sandelin A., Schneider C.,
RA   Schoenbach C., Sekiguchi K., Semple C.A., Seno S., Sessa L., Sheng Y.,
RA   Shibata Y., Shimada H., Shimada K., Silva D., Sinclair B., Sperling S.,
RA   Stupka E., Sugiura K., Sultana R., Takenaka Y., Taki K., Tammoja K.,
RA   Tan S.L., Tang S., Taylor M.S., Tegner J., Teichmann S.A., Ueda H.R.,
RA   van Nimwegen E., Verardo R., Wei C.L., Yagi K., Yamanishi H.,
RA   Zabarovsky E., Zhu S., Zimmer A., Hide W., Bult C., Grimmond S.M.,
RA   Teasdale R.D., Liu E.T., Brusic V., Quackenbush J., Wahlestedt C.,
RA   Mattick J.S., Hume D.A., Kai C., Sasaki D., Tomaru Y., Fukuda S.,
RA   Kanamori-Katayama M., Suzuki M., Aoki J., Arakawa T., Iida J., Imamura K.,
RA   Itoh M., Kato T., Kawaji H., Kawagashira N., Kawashima T., Kojima M.,
RA   Kondo S., Konno H., Nakano K., Ninomiya N., Nishio T., Okada M., Plessy C.,
RA   Shibata K., Shiraki T., Suzuki S., Tagami M., Waki K., Watahiki A.,
RA   Okamura-Oho Y., Suzuki H., Kawai J., Hayashizaki Y.;
RT   "The transcriptional landscape of the mammalian genome.";
RL   Science 309:1559-1563(2005).
RN   [2]
RP   NUCLEOTIDE SEQUENCE [LARGE SCALE GENOMIC DNA].
RA   Mural R.J., Adams M.D., Myers E.W., Smith H.O., Venter J.C.;
RL   Submitted (JUL-2005) to the EMBL/GenBank/DDBJ databases.
RN   [3]
RP   PROTEIN SEQUENCE OF 59-80; 86-99; 112-134; 143-163; 214-222; 229-248;
RP   256-276; 379-390; 397-442; 448-470 AND 473-479, AND IDENTIFICATION BY MASS
RP   SPECTROMETRY.
RC   STRAIN=C57BL/6J; TISSUE=Brain, and Hippocampus;
RA   Lubec G., Kang S.U., Klug S., Yang J.W., Zigmond M.;
RL   Submitted (JUL-2007) to UniProtKB.
RN   [4]
RP   SUBUNIT.
RX   PubMed=19026783; DOI=10.1016/j.molcel.2008.10.021;
RA   Acin-Perez R., Fernandez-Silva P., Peleato M.L., Perez-Martos A.,
RA   Enriquez J.A.;
RT   "Respiratory active mitochondrial supercomplexes.";
RL   Mol. Cell 32:529-539(2008).
RN   [5]
RP   IDENTIFICATION BY MASS SPECTROMETRY [LARGE SCALE ANALYSIS].
RC   TISSUE=Brain, Brown adipose tissue, Heart, Kidney, Liver, Lung,
RC   Pancreas, Spleen, and Testis;
RX   PubMed=21183079; DOI=10.1016/j.cell.2010.12.001;
RA   Huttlin E.L., Jedrychowski M.P., Elias J.E., Goswami T., Rad R.,
RA   Beausoleil S.A., Villen J., Haas W., Sowa M.E., Gygi S.P.;
RT   "A tissue-specific atlas of mouse protein phosphorylation and expression.";
RL   Cell 143:1174-1189(2010).
RN   [6]
RP   SUCCINYLATION [LARGE SCALE ANALYSIS] AT LYS-163, AND IDENTIFICATION BY MASS
RP   SPECTROMETRY [LARGE SCALE ANALYSIS].
RC   TISSUE=Liver;
RX   PubMed=23806337; DOI=10.1016/j.molcel.2013.06.001;
RA   Park J., Chen Y., Tishkoff D.X., Peng C., Tan M., Dai L., Xie Z., Zhang Y.,
RA   Zwaans B.M., Skinner M.E., Lombard D.B., Zhao Y.;
RT   "SIRT5-mediated lysine desuccinylation impacts diverse metabolic
RT   pathways.";
RL   Mol. Cell 50:919-930(2013).
RN   [7]
RP   ACETYLATION [LARGE SCALE ANALYSIS] AT LYS-111; LYS-138; LYS-163 AND
RP   LYS-248, AND IDENTIFICATION BY MASS SPECTROMETRY [LARGE SCALE ANALYSIS].
RC   TISSUE=Liver;
RX   PubMed=23576753; DOI=10.1073/pnas.1302961110;
RA   Rardin M.J., Newman J.C., Held J.M., Cusack M.P., Sorensen D.J., Li B.,
RA   Schilling B., Mooney S.D., Kahn C.R., Verdin E., Gibson B.W.;
RT   "Label-free quantitative proteomics of the lysine acetylome in mitochondria
RT   identifies substrates of SIRT3 in metabolic pathways.";
RL   Proc. Natl. Acad. Sci. U.S.A. 110:6601-6606(2013).
RN   [8]
RP   TISSUE SPECIFICITY, AND DISRUPTION PHENOTYPE.
RX   PubMed=30666338; DOI=10.1007/s00018-019-03007-6;
RA   Shan W., Li J., Xu W., Li H., Zuo Z.;
RT   "Critical role of UQCRC1 in embryo survival, brain ischemic tolerance and
RT   normal cognition in mice.";
RL   Cell. Mol. Life Sci. 76:1381-1396(2019).
RN   [9]
RP   INTERACTION WITH UQCC6.
RX   PubMed=32161263; DOI=10.1038/s41467-020-14999-2;
RA   Zhang S., Reljic B., Liang C., Kerouanton B., Francisco J.C., Peh J.H.,
RA   Mary C., Jagannathan N.S., Olexiouk V., Tang C., Fidelito G., Nama S.,
RA   Cheng R.K., Wee C.L., Wang L.C., Duek Roggli P., Sampath P., Lane L.,
RA   Petretto E., Sobota R.M., Jesuthasan S., Tucker-Kellogg L., Reversade B.,
RA   Menschaert G., Sun L., Stroud D.A., Ho L.;
RT   "Mitochondrial peptide BRAWNIN is essential for vertebrate respiratory
RT   complex III assembly.";
RL   Nat. Commun. 11:1312-1312(2020).
RN   [10]
RP   INTERACTION WITH STMP1.
RX   PubMed=35101990; DOI=10.1073/pnas.2120476119;
RA   Makarewich C.A., Munir A.Z., Bezprozvannaya S., Gibson A.M., Young Kim S.,
RA   Martin-Sandoval M.S., Mathews T.P., Szweda L.I., Bassel-Duby R.,
RA   Olson E.N.;
RT   "The cardiac-enriched microprotein mitolamban regulates mitochondrial
RT   respiratory complex assembly and function in mice.";
RL   Proc. Natl. Acad. Sci. U.S.A. 119:0-0(2022).
CC   -!- FUNCTION: Component of the ubiquinol-cytochrome c oxidoreductase, a
CC       multisubunit transmembrane complex that is part of the mitochondrial
CC       electron transport chain which drives oxidative phosphorylation. The
CC       respiratory chain contains 3 multisubunit complexes succinate
CC       dehydrogenase (complex II, CII), ubiquinol-cytochrome c oxidoreductase
CC       (cytochrome b-c1 complex, complex III, CIII) and cytochrome c oxidase
CC       (complex IV, CIV), that cooperate to transfer electrons derived from
CC       NADH and succinate to molecular oxygen, creating an electrochemical
CC       gradient over the inner membrane that drives transmembrane transport
CC       and the ATP synthase. The cytochrome b-c1 complex catalyzes electron
CC       transfer from ubiquinol to cytochrome c, linking this redox reaction to
CC       translocation of protons across the mitochondrial inner membrane, with
CC       protons being carried across the membrane as hydrogens on the quinol.
CC       In the process called Q cycle, 2 protons are consumed from the matrix,
CC       4 protons are released into the intermembrane space and 2 electrons are
CC       passed to cytochrome c (By similarity). The 2 core subunits UQCRC1/QCR1
CC       and UQCRC2/QCR2 are homologous to the 2 mitochondrial-processing
CC       peptidase (MPP) subunits beta-MPP and alpha-MPP respectively, and they
CC       seem to have preserved their MPP processing properties. May be involved
CC       in the in situ processing of UQCRFS1 into the mature Rieske protein and
CC       its mitochondrial targeting sequence (MTS)/subunit 9 when incorporated
CC       into complex III (By similarity). Seems to play an important role in
CC       the maintenance of proper mitochondrial function in nigral dopaminergic
CC       neurons (By similarity). {ECO:0000250|UniProtKB:P07256,
CC       ECO:0000250|UniProtKB:P31800, ECO:0000250|UniProtKB:P31930}.
CC   -!- SUBUNIT: Component of the ubiquinol-cytochrome c oxidoreductase
CC       (cytochrome b-c1 complex, complex III, CIII), a multisubunit enzyme
CC       composed of 11 subunits. The complex is composed of 3 respiratory
CC       subunits cytochrome b, cytochrome c1 and Rieske protein UQCRFS1, 2 core
CC       protein subunits UQCRC1/QCR1 and UQCRC2/QCR2, and 6 low-molecular
CC       weight protein subunits UQCRH/QCR6, UQCRB/QCR7, UQCRQ/QCR8,
CC       UQCR10/QCR9, UQCR11/QCR10 and subunit 9, the cleavage product of Rieske
CC       protein UQCRFS1 (By similarity). The complex exists as an obligatory
CC       dimer and forms supercomplexes (SCs) in the inner mitochondrial
CC       membrane with NADH-ubiquinone oxidoreductase (complex I, CI) and
CC       cytochrome c oxidase (complex IV, CIV), resulting in different
CC       assemblies (supercomplex SCI(1)III(2)IV(1) and megacomplex
CC       MCI(2)III(2)IV(2)) (PubMed:19026783). Interacts with UQCC6
CC       (PubMed:32161263). Interacts with STMP1 (PubMed:35101990).
CC       {ECO:0000250|UniProtKB:P31800, ECO:0000269|PubMed:19026783,
CC       ECO:0000269|PubMed:32161263, ECO:0000269|PubMed:35101990}.
CC   -!- SUBCELLULAR LOCATION: Mitochondrion inner membrane
CC       {ECO:0000250|UniProtKB:P07256}; Peripheral membrane protein
CC       {ECO:0000250|UniProtKB:P07256}; Matrix side
CC       {ECO:0000250|UniProtKB:P07256}.
CC   -!- TISSUE SPECIFICITY: Expressed in neurons and astrocytes of the cerebral
CC       cortex and hippocampus (at protein level).
CC       {ECO:0000269|PubMed:30666338}.
CC   -!- PTM: Acetylation of Lys-138 is observed in liver mitochondria from
CC       fasted mice but not from fed mice.
CC   -!- DISRUPTION PHENOTYPE: Results in early embryonic lethality.
CC       {ECO:0000269|PubMed:30666338}.
CC   -!- SIMILARITY: Belongs to the peptidase M16 family. UQCRC1/QCR1 subfamily.
CC       {ECO:0000305}.
CC   ---------------------------------------------------------------------------
CC   Copyrighted by the UniProt Consortium, see https://www.uniprot.org/terms
CC   Distributed under the Creative Commons Attribution (CC BY 4.0) License
CC   ---------------------------------------------------------------------------
DR   EMBL; AK013128; BAB28666.1; -; mRNA.
DR   EMBL; AK010553; BAB27022.1; -; mRNA.
DR   EMBL; AK151764; BAE30670.1; -; mRNA.
DR   EMBL; AK160337; BAE35744.1; -; mRNA.
DR   EMBL; CH466560; EDL21324.1; -; Genomic_DNA.
DR   CCDS; CCDS23540.1; -.
DR   RefSeq; NP_079683.2; NM_025407.2.
DR   PDB; 7O37; EM; 3.20 A; A/L=35-480.
DR   PDB; 7O3C; EM; 3.30 A; A/L=35-480.
DR   PDB; 7O3E; EM; 3.60 A; A/L=35-480.
DR   PDB; 7O3H; EM; 2.60 A; A/L=35-480.
DR   PDBsum; 7O37; -.
DR   PDBsum; 7O3C; -.
DR   PDBsum; 7O3E; -.
DR   PDBsum; 7O3H; -.
DR   AlphaFoldDB; Q9CZ13; -.
DR   SMR; Q9CZ13; -.
DR   BioGRID; 204459; 58.
DR   ComplexPortal; CPX-563; Mitochondrial respiratory chain complex III.
DR   CORUM; Q9CZ13; -.
DR   IntAct; Q9CZ13; 11.
DR   MINT; Q9CZ13; -.
DR   STRING; 10090.ENSMUSP00000026743; -.
DR   MEROPS; M16.975; -.
DR   MEROPS; M16.981; -.
DR   GlyGen; Q9CZ13; 2 sites, 1 O-linked glycan (2 sites).
DR   iPTMnet; Q9CZ13; -.
DR   PhosphoSitePlus; Q9CZ13; -.
DR   SwissPalm; Q9CZ13; -.
DR   REPRODUCTION-2DPAGE; IPI00111885; -.
DR   REPRODUCTION-2DPAGE; Q9CZ13; -.
DR   UCD-2DPAGE; Q9CZ13; -.
DR   EPD; Q9CZ13; -.
DR   jPOST; Q9CZ13; -.
DR   MaxQB; Q9CZ13; -.
DR   PaxDb; Q9CZ13; -.
DR   PeptideAtlas; Q9CZ13; -.
DR   ProteomicsDB; 301902; -.
DR   TopDownProteomics; Q9CZ13; -.
DR   Antibodypedia; 1257; 399 antibodies from 29 providers.
DR   DNASU; 22273; -.
DR   Ensembl; ENSMUST00000026743; ENSMUSP00000026743; ENSMUSG00000025651.
DR   GeneID; 22273; -.
DR   KEGG; mmu:22273; -.
DR   UCSC; uc009rrg.1; mouse.
DR   AGR; MGI:107876; -.
DR   CTD; 7384; -.
DR   MGI; MGI:107876; Uqcrc1.
DR   VEuPathDB; HostDB:ENSMUSG00000025651; -.
DR   eggNOG; KOG0960; Eukaryota.
DR   GeneTree; ENSGT00940000158931; -.
DR   HOGENOM; CLU_009902_4_2_1; -.
DR   InParanoid; Q9CZ13; -.
DR   OMA; WSNPDNV; -.
DR   OrthoDB; 167798at2759; -.
DR   PhylomeDB; Q9CZ13; -.
DR   TreeFam; TF105032; -.
DR   Reactome; R-MMU-611105; Respiratory electron transport.
DR   BioGRID-ORCS; 22273; 24 hits in 79 CRISPR screens.
DR   ChiTaRS; Uqcrc1; mouse.
DR   PRO; PR:Q9CZ13; -.
DR   Proteomes; UP000000589; Chromosome 9.
DR   RNAct; Q9CZ13; protein.
DR   Bgee; ENSMUSG00000025651; Expressed in proximal tubule and 143 other tissues.
DR   ExpressionAtlas; Q9CZ13; baseline and differential.
DR   Genevisible; Q9CZ13; MM.
DR   GO; GO:0005743; C:mitochondrial inner membrane; HDA:MGI.
DR   GO; GO:0005750; C:mitochondrial respiratory chain complex III; ISO:MGI.
DR   GO; GO:0005739; C:mitochondrion; IDA:MGI.
DR   GO; GO:0043209; C:myelin sheath; HDA:UniProtKB.
DR   GO; GO:0046872; F:metal ion binding; IEA:InterPro.
DR   GO; GO:0044877; F:protein-containing complex binding; ISO:MGI.
DR   GO; GO:0031625; F:ubiquitin protein ligase binding; ISO:MGI.
DR   GO; GO:0045333; P:cellular respiration; NAS:ComplexPortal.
DR   GO; GO:0006122; P:mitochondrial electron transport, ubiquinol to cytochrome c; IDA:MGI.
DR   GO; GO:0034551; P:mitochondrial respiratory chain complex III assembly; ISS:UniProtKB.
DR   GO; GO:0014823; P:response to activity; IEA:Ensembl.
DR   GO; GO:0043279; P:response to alkaloid; IEA:Ensembl.
DR   Gene3D; 3.30.830.10; Metalloenzyme, LuxS/M16 peptidase-like; 2.
DR   InterPro; IPR011249; Metalloenz_LuxS/M16.
DR   InterPro; IPR011765; Pept_M16_N.
DR   InterPro; IPR007863; Peptidase_M16_C.
DR   PANTHER; PTHR11851:SF116; CYTOCHROME B-C1 COMPLEX SUBUNIT 1, MITOCHONDRIAL; 1.
DR   PANTHER; PTHR11851; METALLOPROTEASE; 1.
DR   Pfam; PF00675; Peptidase_M16; 1.
DR   Pfam; PF05193; Peptidase_M16_C; 1.
DR   SUPFAM; SSF63411; LuxS/MPP-like metallohydrolase; 2.
PE   1: Evidence at protein level;
KW   3D-structure; Acetylation; Direct protein sequencing; Electron transport;
KW   Membrane; Mitochondrion; Mitochondrion inner membrane; Phosphoprotein;
KW   Reference proteome; Respiratory chain; Transit peptide; Transport.
FT   TRANSIT         1..34
FT                   /note="Mitochondrion"
FT                   /evidence="ECO:0000250"
FT   CHAIN           35..480
FT                   /note="Cytochrome b-c1 complex subunit 1, mitochondrial"
FT                   /id="PRO_0000026787"
FT   MOD_RES         111
FT                   /note="N6-acetyllysine"
FT                   /evidence="ECO:0007744|PubMed:23576753"
FT   MOD_RES         138
FT                   /note="N6-acetyllysine"
FT                   /evidence="ECO:0007744|PubMed:23576753"
FT   MOD_RES         163
FT                   /note="N6-acetyllysine; alternate"
FT                   /evidence="ECO:0007744|PubMed:23576753"
FT   MOD_RES         163
FT                   /note="N6-succinyllysine; alternate"
FT                   /evidence="ECO:0007744|PubMed:23806337"
FT   MOD_RES         212
FT                   /note="Phosphoserine"
FT                   /evidence="ECO:0000250|UniProtKB:Q68FY0"
FT   MOD_RES         214
FT                   /note="Phosphothreonine"
FT                   /evidence="ECO:0000250|UniProtKB:Q68FY0"
FT   MOD_RES         248
FT                   /note="N6-acetyllysine"
FT                   /evidence="ECO:0007744|PubMed:23576753"
FT   CONFLICT        223
FT                   /note="H -> N (in Ref. 1; BAB28666)"
FT                   /evidence="ECO:0000305"
FT   CONFLICT        318
FT                   /note="Y -> C (in Ref. 1; BAB28666)"
FT                   /evidence="ECO:0000305"
FT   HELIX           38..43
FT                   /evidence="ECO:0007829|PDB:7O3H"
FT   STRAND          49..52
FT                   /evidence="ECO:0007829|PDB:7O3H"
FT   STRAND          58..63
FT                   /evidence="ECO:0007829|PDB:7O3H"
FT   STRAND          67..76
FT                   /evidence="ECO:0007829|PDB:7O3H"
FT   HELIX           79..81
FT                   /evidence="ECO:0007829|PDB:7O3H"
FT   STRAND          84..86
FT                   /evidence="ECO:0007829|PDB:7O3H"
FT   HELIX           89..96
FT                   /evidence="ECO:0007829|PDB:7O3H"
FT   STRAND          101..104
FT                   /evidence="ECO:0007829|PDB:7O3H"
FT   HELIX           107..115
FT                   /evidence="ECO:0007829|PDB:7O3H"
FT   STRAND          119..124
FT                   /evidence="ECO:0007829|PDB:7O3H"
FT   STRAND          129..137
FT                   /evidence="ECO:0007829|PDB:7O3H"
FT   HELIX           140..152
FT                   /evidence="ECO:0007829|PDB:7O3H"
FT   HELIX           158..175
FT                   /evidence="ECO:0007829|PDB:7O3H"
FT   HELIX           179..191
FT                   /evidence="ECO:0007829|PDB:7O3H"
FT   TURN            196..198
FT                   /evidence="ECO:0007829|PDB:7O3H"
FT   HELIX           205..210
FT                   /evidence="ECO:0007829|PDB:7O3H"
FT   HELIX           213..223
FT                   /evidence="ECO:0007829|PDB:7O3H"
FT   HELIX           226..228
FT                   /evidence="ECO:0007829|PDB:7O3H"
FT   STRAND          229..236
FT                   /evidence="ECO:0007829|PDB:7O3H"
FT   HELIX           239..249
FT                   /evidence="ECO:0007829|PDB:7O3H"
FT   STRAND          250..253
FT                   /evidence="ECO:0007829|PDB:7O37"
FT   STRAND          258..260
FT                   /evidence="ECO:0007829|PDB:7O3H"
FT   STRAND          273..278
FT                   /evidence="ECO:0007829|PDB:7O3H"
FT   STRAND          282..292
FT                   /evidence="ECO:0007829|PDB:7O3H"
FT   HELIX           300..311
FT                   /evidence="ECO:0007829|PDB:7O3H"
FT   STRAND          313..315
FT                   /evidence="ECO:0007829|PDB:7O3C"
FT   HELIX           321..323
FT                   /evidence="ECO:0007829|PDB:7O3H"
FT   HELIX           327..335
FT                   /evidence="ECO:0007829|PDB:7O3H"
FT   STRAND          339..348
FT                   /evidence="ECO:0007829|PDB:7O3H"
FT   STRAND          351..360
FT                   /evidence="ECO:0007829|PDB:7O3H"
FT   HELIX           362..364
FT                   /evidence="ECO:0007829|PDB:7O3H"
FT   HELIX           365..381
FT                   /evidence="ECO:0007829|PDB:7O3H"
FT   HELIX           385..402
FT                   /evidence="ECO:0007829|PDB:7O3H"
FT   HELIX           406..418
FT                   /evidence="ECO:0007829|PDB:7O3H"
FT   HELIX           426..434
FT                   /evidence="ECO:0007829|PDB:7O3H"
FT   HELIX           438..448
FT                   /evidence="ECO:0007829|PDB:7O3H"
FT   TURN            449..451
FT                   /evidence="ECO:0007829|PDB:7O3H"
FT   STRAND          455..461
FT                   /evidence="ECO:0007829|PDB:7O3H"
FT   STRAND          463..465
FT                   /evidence="ECO:0007829|PDB:7O3H"
FT   HELIX           468..474
FT                   /evidence="ECO:0007829|PDB:7O3H"
SQ   SEQUENCE   480 AA;  52852 MW;  8150C97C655A91C9 CRC64;
     MAASAVCRAA CSGTQVLLRT RRSPALLRLP ALRGTATFAQ ALQSVPETQV SILDNGLRVA
     SEQSSHATCT VGVWIDAGSR YETEKNNGAG YFLEHLAFKG TKNRPGNALE KEVESIGAHL
     NAYSTREHTA YLIKALSKDL PKVVELLADI VQNSSLEDSQ IEKERDVILR EMQENDASMQ
     NVVFDYLHAT AFQGTPLAQA VEGPSENVRR LSRTDLTDYL NRHYKAPRMV LAAAGGVEHQ
     QLLDLAQKHL SSVSRVYEED AVPGLTPCRF TGSEIRHRDD ALPLAHVAIA VEGPGWANPD
     NVTLQVANAI IGHYDCTYGG GVHLSSPLAS VAVANKLCQS FQTFNISYSD TGLLGAHFVC
     DAMSIDDMVF FLQGQWMRLC TSATESEVTR GKNILRNALV SHLDGTTPVC EDIGRSLLTY
     GRRIPLAEWE SRIQEVDAQM LRDICSKYFY DQCPAVAGYG PIEQLPDYNR IRSGMFWLRF
//
```

|  |
| --- |
| **Mascot:** http://www.matrixscience.com/ |

Oxidation (M) (+15.9949)
