# Supplementary material for: A common East-Asian ALDH2 mutation causes metabolic disorders and the therapeutic effect of ALDH2 activators
Source: Nat Commun. 2023 Sep 25;14:5971. doi: 10.1038/s41467-023-41570-6 (PMC10520061; doi:10.1038/s41467-023-41570-6)
Supplement: Supplementary file 4 — Supplementary Data 1 [file 41467_2023_41570_MOESM4_ESM.zip › Table S5b/Q9CZ13/Q9CZ13_WTO-3_K111.html]

Mascot Search Results: Q9CZ13
 

# MASCOT Search Results

## Protein View: Q9CZ13

### Cytochrome b-c1 complex subunit 1, mitochondrial OS=Mus musculus OX=10090 GN=Uqcrc1 PE=1 SV=2

|  |  |
| --- | --- |
| Database: | Mouse\_UniProt\_proteomes |
| Score: | 13397 |
| Monoisotopic mass (Mr): | 53446 |
| Calculated pI: | 5.81 |

Sequence similarity is available as an NCBI BLAST search of Q9CZ13 against nr.

### Search parameters

|  |  |
| --- | --- |
| MS data file: | `D:\LCMSMS\2023 Users' data\230529-1\230529-1-WTO-3_20230601175054.raw` |
| Enzyme: | Trypsin/P: cuts C-term side of KR. |
| Fixed modifications: | Carbamidomethyl (C) |
| Variable modifications: | Deamidated (NQ), HNE (C), HNE (H), HNE (K), Oxidation (M) |

### Protein sequence coverage: 82%

Matched peptides shown in ***bold red***.

|  |  |  |  |  |  |
| --- | --- | --- | --- | --- | --- |
| `1` | `MAASAVCRAA` | `CSGTQVLLRT` | `RRSPALLRLP` | `ALRGTATFAQ` | `ALQSVPETQV` |
| `51` | `SILDNGLRVA` | `SEQSSHATCT` | `VGVWIDAGSR` | `YETEKNNGAG` | `YFLEHLAFKG` |
| `101` | `TKNRPGNALE` | `KEVESIGAHL` | `NAYSTREHTA` | `YLIKALSKDL` | `PKVVELLADI` |
| `151` | `VQNSSLEDSQ` | `IEKERDVILR` | `EMQENDASMQ` | `NVVFDYLHAT` | `AFQGTPLAQA` |
| `201` | `VEGPSENVRR` | `LSRTDLTDYL` | `NRHYKAPRMV` | `LAAAGGVEHQ` | `QLLDLAQKHL` |
| `251` | `SSVSRVYEED` | `AVPGLTPCRF` | `TGSEIRHRDD` | `ALPLAHVAIA` | `VEGPGWANPD` |
| `301` | `NVTLQVANAI` | `IGHYDCTYGG` | `GVHLSSPLAS` | `VAVANKLCQS` | `FQTFNISYSD` |
| `351` | `TGLLGAHFVC` | `DAMSIDDMVF` | `FLQGQWMRLC` | `TSATESEVTR` | `GKNILRNALV` |
| `401` | `SHLDGTTPVC` | `EDIGRSLLTY` | `GRRIPLAEWE` | `SRIQEVDAQM` | `LRDICSKYFY` |
| `451` | `DQCPAVAGYG` | `PIEQLPDYNR` | `IRSGMFWLRF` |  |  |

Unformatted sequence string: 480 residues (for pasting into other applications).

|  |  |  |  |
| --- | --- | --- | --- |
| Sort by | residue number | increasing mass | decreasing mass |
| Show | matched peptides only | predicted peptides also |  |

| Query | Start | – | End | Observed | Mr(expt) | Mr(calc) | ppm | M | Score | Expect | Rank | U | Peptide |
| --- | --- | --- | --- | --- | --- | --- | --- | --- | --- | --- | --- | --- | --- |
| 166179 | 34 | – | 58 | 872.7885 | 2615.3438 | 2615.3555 | -4.49 | 0 | 68 | 4.4e-07 | 1Score **> 37** indicates **identity** Score **> 17** indicates **homology** | U | R.GTATFAQALQSVPETQVSILDNGLR.V |
| 166186 | 34 | – | 58 | 872.7917 | 2615.3532 | 2615.3555 | -0.90 | 0 | 77 | 6.6e-08 | 1Score **> 37** indicates **identity** Score **> 17** indicates **homology** | U | R.GTATFAQALQSVPETQVSILDNGLR.V |
| 166189 | 34 | – | 58 | 872.7920 | 2615.3541 | 2615.3555 | -0.55 | 0 | 30 | 0.0016 | 1Score **> 37** indicates **identity** Score **> 14** indicates **homology** | U | R.GTATFAQALQSVPETQVSILDNGLR.V |
| 166192 | 34 | – | 58 | 872.7924 | 2615.3553 | 2615.3555 | -0.080 | 0 | 100 | 3.9e-10 | 1Score **> 37** indicates **identity** Score **> 19** indicates **homology** | U | R.GTATFAQALQSVPETQVSILDNGLR.V |
| 166194 | 34 | – | 58 | 1308.6859 | 2615.3573 | 2615.3555 | 0.70 | 0 | 92 | 2.4e-09 | 1Score **> 37** indicates **identity** Score **> 18** indicates **homology** | U | R.GTATFAQALQSVPETQVSILDNGLR.V |
| 166196 | 34 | – | 58 | 1308.6866 | 2615.3587 | 2615.3555 | 1.21 | 0 | 123 | 3e-12 | 1Score **> 37** indicates **identity** Score **> 20** indicates **homology** | U | R.GTATFAQALQSVPETQVSILDNGLR.V |
| 166232 | 34 | – | 58 | 873.1179 | 2616.3320 | 2616.3395 | -2.87 | 0 | 66 | 7.1e-07 | 1Score **> 37** indicates **identity** Score **> 17** indicates **homology** | U | R.GTATFAQALQSVPETQVSILDNGLR.V  + Deamidated (NQ) |
| 166246 | 34 | – | 58 | 873.1205 | 2616.3398 | 2616.3395 | 0.11 | 0 | 47 | 3.6e-05 | 1Score **> 37** indicates **identity** Score **> 15** indicates **homology** | U | R.GTATFAQALQSVPETQVSILDNGLR.V  + Deamidated (NQ) |
| 166249 | 34 | – | 58 | 1309.1779 | 2616.3413 | 2616.3395 | 0.70 | 0 | 20 | 0.014 | 1Score **> 37** indicates **identity** Score **> 14** indicates **homology** | U | R.GTATFAQALQSVPETQVSILDNGLR.V  + Deamidated (NQ) |
| 166252 | 34 | – | 58 | 1309.1791 | 2616.3437 | 2616.3395 | 1.61 | 0 | 117 | 1.2e-11 | 1Score **> 37** indicates **identity** Score **> 20** indicates **homology** | U | R.GTATFAQALQSVPETQVSILDNGLR.V  + Deamidated (NQ) |
| 166253 | 34 | – | 58 | 873.1226 | 2616.3459 | 2616.3395 | 2.44 | 0 | 80 | 3.3e-08 | 1Score **> 37** indicates **identity** Score **> 17** indicates **homology** | U | R.GTATFAQALQSVPETQVSILDNGLR.V  + Deamidated (NQ) |
| 166255 | 34 | – | 58 | 873.1235 | 2616.3485 | 2616.3395 | 3.45 | 0 | 16 | 0.03 | 1Score **> 37** indicates **identity** Score **> 14** indicates **homology** | U | R.GTATFAQALQSVPETQVSILDNGLR.V  + Deamidated (NQ) |
| 166256 | 34 | – | 58 | 873.1238 | 2616.3496 | 2616.3395 | 3.86 | 0 | 62 | 1.6e-06 | 1Score **> 37** indicates **identity** Score **> 16** indicates **homology** | U | R.GTATFAQALQSVPETQVSILDNGLR.V  + Deamidated (NQ) |
| 166258 | 34 | – | 58 | 873.1249 | 2616.3530 | 2616.3395 | 5.15 | 0 | 51 | 1.8e-05 | 1Score **> 37** indicates **identity** Score **> 16** indicates **homology** | U | R.GTATFAQALQSVPETQVSILDNGLR.V  + Deamidated (NQ) |
| 166259 | 34 | – | 58 | 1309.1853 | 2616.3560 | 2616.3395 | 6.29 | 0 | 70 | 2.7e-07 | 1Score **> 37** indicates **identity** Score **> 17** indicates **homology** | U | R.GTATFAQALQSVPETQVSILDNGLR.V  + Deamidated (NQ) |
| 166302 | 34 | – | 58 | 873.4535 | 2617.3386 | 2617.3235 | 5.77 | 0 | 33 | 0.00074 | 1Score **> 37** indicates **identity** Score **> 15** indicates **homology** | U | R.GTATFAQALQSVPETQVSILDNGLR.V  + 2 Deamidated (NQ) |
| 166304 | 34 | – | 58 | 1309.6778 | 2617.3410 | 2617.3235 | 6.70 | 0 | 71 | 2.4e-07 | 1Score **> 37** indicates **identity** Score **> 17** indicates **homology** | U | R.GTATFAQALQSVPETQVSILDNGLR.V  + 2 Deamidated (NQ) |
| 149754 | 59 | – | 80 | 773.3623 | 2317.0652 | 2317.0757 | -4.54 | 0 | 44 | 7e-05 | 1Score **> 33** indicates **identity** Score **> 15** indicates **homology** | U | R.VASEQSSHATCTVGVWIDAGSR.Y |
| 149756 | 59 | – | 80 | 773.3630 | 2317.0671 | 2317.0757 | -3.74 | 0 | 27 | 0.0031 | 1Score **> 33** indicates **identity** Score **> 14** indicates **homology** | U | R.VASEQSSHATCTVGVWIDAGSR.Y |
| 149757 | 59 | – | 80 | 1159.5412 | 2317.0679 | 2317.0757 | -3.37 | 0 | 91 | 3e-09 | 1Score **> 33** indicates **identity** Score **> 18** indicates **homology** | U | R.VASEQSSHATCTVGVWIDAGSR.Y |
| 149758 | 59 | – | 80 | 773.3638 | 2317.0697 | 2317.0757 | -2.60 | 0 | 27 | 0.0032 | 1Score **> 33** indicates **identity** Score **> 14** indicates **homology** | U | R.VASEQSSHATCTVGVWIDAGSR.Y |
| 149761 | 59 | – | 80 | 773.3650 | 2317.0731 | 2317.0757 | -1.15 | 0 | 25 | 0.0043 | 1Score **> 33** indicates **identity** Score **> 14** indicates **homology** | U | R.VASEQSSHATCTVGVWIDAGSR.Y |
| 149762 | 59 | – | 80 | 773.3650 | 2317.0733 | 2317.0757 | -1.06 | 0 | 24 | 0.0059 | 1Score **> 33** indicates **identity** Score **> 14** indicates **homology** | U | R.VASEQSSHATCTVGVWIDAGSR.Y |
| 149763 | 59 | – | 80 | 773.3651 | 2317.0734 | 2317.0757 | -1.01 | 0 | 51 | 1.7e-05 | 1Score **> 33** indicates **identity** Score **> 16** indicates **homology** | U | R.VASEQSSHATCTVGVWIDAGSR.Y |
| 149765 | 59 | – | 80 | 773.3652 | 2317.0737 | 2317.0757 | -0.86 | 0 | 55 | 6.8e-06 | 1Score **> 33** indicates **identity** Score **> 16** indicates **homology** | U | R.VASEQSSHATCTVGVWIDAGSR.Y |
| 149766 | 59 | – | 80 | 773.3653 | 2317.0741 | 2317.0757 | -0.69 | 0 | 63 | 1.3e-06 | 1Score **> 34** indicates **identity** Score **> 16** indicates **homology** | U | R.VASEQSSHATCTVGVWIDAGSR.Y |
| 149767 | 59 | – | 80 | 1159.5444 | 2317.0743 | 2317.0757 | -0.61 | 0 | 87 | 6.9e-09 | 1Score **> 34** indicates **identity** Score **> 18** indicates **homology** | U | R.VASEQSSHATCTVGVWIDAGSR.Y |
| 149768 | 59 | – | 80 | 773.3654 | 2317.0745 | 2317.0757 | -0.53 | 0 | 63 | 1.2e-06 | 1Score **> 34** indicates **identity** Score **> 16** indicates **homology** | U | R.VASEQSSHATCTVGVWIDAGSR.Y |
| 149769 | 59 | – | 80 | 773.3656 | 2317.0749 | 2317.0757 | -0.37 | 0 | 24 | 0.0056 | 1Score **> 34** indicates **identity** Score **> 14** indicates **homology** | U | R.VASEQSSHATCTVGVWIDAGSR.Y |
| 149770 | 59 | – | 80 | 773.3656 | 2317.0749 | 2317.0757 | -0.36 | 0 | 37 | 0.00038 | 1Score **> 34** indicates **identity** Score **> 15** indicates **homology** | U | R.VASEQSSHATCTVGVWIDAGSR.Y |
| 149773 | 59 | – | 80 | 1159.5453 | 2317.0760 | 2317.0757 | 0.11 | 0 | 83 | 2e-08 | 1Score **> 34** indicates **identity** Score **> 18** indicates **homology** | U | R.VASEQSSHATCTVGVWIDAGSR.Y |
| 149774 | 59 | – | 80 | 1159.5453 | 2317.0760 | 2317.0757 | 0.12 | 0 | 51 | 4.2e-05 | 1Score **> 34** indicates **identity** Score **> 19** indicates **homology** | U | R.VASEQSSHATCTVGVWIDAGSR.Y |
| 149776 | 59 | – | 80 | 773.3661 | 2317.0766 | 2317.0757 | 0.36 | 0 | 59 | 3e-06 | 1Score **> 34** indicates **identity** Score **> 16** indicates **homology** | U | R.VASEQSSHATCTVGVWIDAGSR.Y |
| 149777 | 59 | – | 80 | 1159.5463 | 2317.0781 | 2317.0757 | 1.04 | 0 | 41 | 0.00017 | 1Score **> 34** indicates **identity** Score **> 15** indicates **homology** | U | R.VASEQSSHATCTVGVWIDAGSR.Y |
| 149781 | 59 | – | 80 | 773.3711 | 2317.0915 | 2317.0757 | 6.79 | 0 | 32 | 0.0009 | 1Score **> 34** indicates **identity** Score **> 15** indicates **homology** | U | R.VASEQSSHATCTVGVWIDAGSR.Y |
| 149817 | 59 | – | 80 | 773.6995 | 2318.0765 | 2318.0597 | 7.25 | 0 | 26 | 0.0036 | 1Score **> 34** indicates **identity** Score **> 14** indicates **homology** | U | R.VASEQSSHATCTVGVWIDAGSR.Y  + Deamidated (NQ) |
| 149820 | 59 | – | 80 | 773.7007 | 2318.0804 | 2318.0597 | 8.92 | 0 | 16 | 0.034 | 1Score **> 34** indicates **identity** Score **> 13** indicates **homology** | U | R.VASEQSSHATCTVGVWIDAGSR.Y  + Deamidated (NQ) |
| 178674 | 59 | – | 85 | 742.8490 | 2967.3671 | 2967.3669 | 0.077 | 1 | 25 | 0.0047 | 1Score **> 34** indicates **identity** Score **> 14** indicates **homology** | U | R.VASEQSSHATCTVGVWIDAGSRYETEK.N |
| 178675 | 59 | – | 85 | 742.8491 | 2967.3672 | 2967.3669 | 0.11 | 1 | 31 | 0.0012 | 1Score **> 34** indicates **identity** Score **> 14** indicates **homology** | U | R.VASEQSSHATCTVGVWIDAGSRYETEK.N |
| 178677 | 59 | – | 85 | 990.1301 | 2967.3686 | 2967.3669 | 0.57 | 1 | 51 | 1.6e-05 | 1Score **> 34** indicates **identity** Score **> 16** indicates **homology** | U | R.VASEQSSHATCTVGVWIDAGSRYETEK.N |
| 178678 | 59 | – | 85 | 990.1303 | 2967.3690 | 2967.3669 | 0.73 | 1 | 49 | 2.6e-05 | 1Score **> 34** indicates **identity** Score **> 16** indicates **homology** | U | R.VASEQSSHATCTVGVWIDAGSRYETEK.N |
| 178679 | 59 | – | 85 | 990.1315 | 2967.3726 | 2967.3669 | 1.92 | 1 | 38 | 0.00026 | 1Score **> 34** indicates **identity** Score **> 15** indicates **homology** | U | R.VASEQSSHATCTVGVWIDAGSRYETEK.N |
| 194137 | 59 | – | 99 | 906.8352 | 4529.1398 | 4529.1346 | 1.14 | 2 | 16 | 0.033 | 1Score **> 35** indicates **identity** Score **> 13** indicates **homology** | U | R.VASEQSSHATCTVGVWIDAGSRYETEKNNGAGYFLEHLAFK.G |
| 194144 | 59 | – | 99 | 1133.5388 | 4530.1262 | 4530.1186 | 1.67 | 2 | 18 | 0.02 | 1Score **> 35** indicates **identity** Score **> 14** indicates **homology** | U | R.VASEQSSHATCTVGVWIDAGSRYETEKNNGAGYFLEHLAFK.G  + Deamidated (NQ) |
| 143474 | 81 | – | 99 | 558.5257 | 2230.0737 | 2230.0694 | 1.93 | 1 | 51 | 1.8e-05 | 1Score **> 35** indicates **identity** Score **> 16** indicates **homology** | U | R.YETEKNNGAGYFLEHLAFK.G |
| 143475 | 81 | – | 99 | 744.3658 | 2230.0756 | 2230.0694 | 2.74 | 1 | 57 | 4.7e-06 | 1Score **> 35** indicates **identity** Score **> 16** indicates **homology** | U | R.YETEKNNGAGYFLEHLAFK.G |
| 143476 | 81 | – | 99 | 744.3661 | 2230.0764 | 2230.0694 | 3.11 | 1 | 23 | 0.0063 | 1Score **> 35** indicates **identity** Score **> 14** indicates **homology** | U | R.YETEKNNGAGYFLEHLAFK.G |
| 143477 | 81 | – | 99 | 558.5264 | 2230.0767 | 2230.0694 | 3.24 | 1 | 33 | 0.00084 | 1Score **> 35** indicates **identity** Score **> 15** indicates **homology** | U | R.YETEKNNGAGYFLEHLAFK.G |
| 143478 | 81 | – | 99 | 558.5265 | 2230.0767 | 2230.0694 | 3.26 | 1 | 55 | 6.4e-06 | 1Score **> 35** indicates **identity** Score **> 16** indicates **homology** | U | R.YETEKNNGAGYFLEHLAFK.G |
| 143479 | 81 | – | 99 | 558.5265 | 2230.0769 | 2230.0694 | 3.33 | 1 | 19 | 0.018 | 1Score **> 35** indicates **identity** Score **> 14** indicates **homology** | U | R.YETEKNNGAGYFLEHLAFK.G |
| 143480 | 81 | – | 99 | 744.3663 | 2230.0770 | 2230.0694 | 3.38 | 1 | 48 | 3.2e-05 | 1Score **> 35** indicates **identity** Score **> 15** indicates **homology** | U | R.YETEKNNGAGYFLEHLAFK.G |
| 143481 | 81 | – | 99 | 558.5274 | 2230.0807 | 2230.0694 | 5.04 | 1 | 42 | 0.00013 | 1Score **> 35** indicates **identity** Score **> 15** indicates **homology** | U | R.YETEKNNGAGYFLEHLAFK.G |
| 143575 | 81 | – | 99 | 558.7703 | 2231.0523 | 2231.0535 | -0.53 | 1 | 18 | 0.019 | 1Score **> 34** indicates **identity** Score **> 14** indicates **homology** | U | R.YETEKNNGAGYFLEHLAFK.G  + Deamidated (NQ) |
| 143576 | 81 | – | 99 | 558.7708 | 2231.0539 | 2231.0535 | 0.22 | 1 | 17 | 0.025 | 1Score **> 34** indicates **identity** Score **> 14** indicates **homology** | U | R.YETEKNNGAGYFLEHLAFK.G  + Deamidated (NQ) |
| 143579 | 81 | – | 99 | 558.7709 | 2231.0547 | 2231.0535 | 0.54 | 1 | 35 | 0.00048 | 1Score **> 34** indicates **identity** Score **> 15** indicates **homology** | U | R.YETEKNNGAGYFLEHLAFK.G  + Deamidated (NQ) |
| 143580 | 81 | – | 99 | 558.7710 | 2231.0549 | 2231.0535 | 0.63 | 1 | 41 | 0.00014 | 1Score **> 34** indicates **identity** Score **> 15** indicates **homology** | U | R.YETEKNNGAGYFLEHLAFK.G  + Deamidated (NQ) |
| 143581 | 81 | – | 99 | 744.6924 | 2231.0555 | 2231.0535 | 0.90 | 1 | 35 | 0.00056 | 1Score **> 34** indicates **identity** Score **> 15** indicates **homology** | U | R.YETEKNNGAGYFLEHLAFK.G  + Deamidated (NQ) |
| 143582 | 81 | – | 99 | 744.6924 | 2231.0555 | 2231.0535 | 0.90 | 1 | 56 | 5.1e-06 | 1Score **> 34** indicates **identity** Score **> 16** indicates **homology** | U | R.YETEKNNGAGYFLEHLAFK.G  + Deamidated (NQ) |
| 143587 | 81 | – | 99 | 558.7733 | 2231.0642 | 2231.0535 | 4.82 | 1 | 33 | 0.00074 | 1Score **> 35** indicates **identity** Score **> 15** indicates **homology** | U | R.YETEKNNGAGYFLEHLAFK.G  + Deamidated (NQ) |
| 143588 | 81 | – | 99 | 744.6955 | 2231.0646 | 2231.0535 | 5.01 | 1 | 22 | 0.0089 | 1Score **> 35** indicates **identity** Score **> 14** indicates **homology** | U | R.YETEKNNGAGYFLEHLAFK.G  + Deamidated (NQ) |
| 161667 | 81 | – | 102 | 630.0652 | 2516.2319 | 2516.2336 | -0.68 | 2 | 19 | 0.018 | 1Score **> 36** indicates **identity** Score **> 14** indicates **homology** | U | R.YETEKNNGAGYFLEHLAFKGTK.N |
| 161670 | 81 | – | 102 | 630.0657 | 2516.2336 | 2516.2336 | 0.0068 | 2 | 31 | 0.0013 | 1Score **> 36** indicates **identity** Score **> 14** indicates **homology** | U | R.YETEKNNGAGYFLEHLAFKGTK.N |
| 161671 | 81 | – | 102 | 630.0660 | 2516.2348 | 2516.2336 | 0.51 | 2 | 19 | 0.015 | 1Score **> 36** indicates **identity** Score **> 14** indicates **homology** | U | R.YETEKNNGAGYFLEHLAFKGTK.N |
| 161672 | 81 | – | 102 | 630.0661 | 2516.2353 | 2516.2336 | 0.68 | 2 | 32 | 0.00092 | 1Score **> 36** indicates **identity** Score **> 15** indicates **homology** | U | R.YETEKNNGAGYFLEHLAFKGTK.N |
| 161740 | 81 | – | 102 | 630.3100 | 2517.2109 | 2517.2176 | -2.65 | 2 | 14 | 0.044 | 1Score **> 36** indicates **identity** Score **> 13** indicates **homology** | U | R.YETEKNNGAGYFLEHLAFKGTK.N  + Deamidated (NQ) |
| 161745 | 81 | – | 102 | 630.3128 | 2517.2222 | 2517.2176 | 1.83 | 2 | 33 | 0.00089 | 1Score **> 36** indicates **identity** Score **> 15** indicates **homology** | U | R.YETEKNNGAGYFLEHLAFKGTK.N  + Deamidated (NQ) |
| 76714 | 86 | – | 99 | 790.8943 | 1579.7740 | 1579.7783 | -2.71 | 0 | 60 | 2.1e-06 | 1Score **> 34** indicates **identity** Score **> 16** indicates **homology** | U | K.NNGAGYFLEHLAFK.G |
| 76715 | 86 | – | 99 | 790.8960 | 1579.7774 | 1579.7783 | -0.58 | 0 | 73 | 1.5e-07 | 1Score **> 34** indicates **identity** Score **> 17** indicates **homology** | U | K.NNGAGYFLEHLAFK.G |
| 76718 | 86 | – | 99 | 527.6002 | 1579.7788 | 1579.7783 | 0.33 | 0 | 46 | 5.3e-05 | 1Score **> 34** indicates **identity** Score **> 15** indicates **homology** | U | K.NNGAGYFLEHLAFK.G |
| 76719 | 86 | – | 99 | 527.6003 | 1579.7790 | 1579.7783 | 0.42 | 0 | 43 | 8.5e-05 | 1Score **> 34** indicates **identity** Score **> 15** indicates **homology** | U | K.NNGAGYFLEHLAFK.G |
| 76721 | 86 | – | 99 | 527.6005 | 1579.7795 | 1579.7783 | 0.78 | 0 | 20 | 0.012 | 1Score **> 34** indicates **identity** Score **> 14** indicates **homology** | U | K.NNGAGYFLEHLAFK.G |
| 76722 | 86 | – | 99 | 527.6005 | 1579.7797 | 1579.7783 | 0.91 | 0 | 56 | 5.7e-06 | 1Score **> 34** indicates **identity** Score **> 16** indicates **homology** | U | K.NNGAGYFLEHLAFK.G |
| 76723 | 86 | – | 99 | 527.6005 | 1579.7798 | 1579.7783 | 0.93 | 0 | 49 | 2.5e-05 | 1Score **> 34** indicates **identity** Score **> 16** indicates **homology** | U | K.NNGAGYFLEHLAFK.G |
| 76728 | 86 | – | 99 | 527.6010 | 1579.7813 | 1579.7783 | 1.90 | 0 | 20 | 0.013 | 1Score **> 34** indicates **identity** Score **> 14** indicates **homology** | U | K.NNGAGYFLEHLAFK.G |
| 76856 | 86 | – | 99 | 791.3833 | 1580.7520 | 1580.7623 | -6.53 | 0 | 72 | 1.9e-07 | 1Score **> 33** indicates **identity** Score **> 17** indicates **homology** | U | K.NNGAGYFLEHLAFK.G  + Deamidated (NQ) |
| 76864 | 86 | – | 99 | 527.9273 | 1580.7601 | 1580.7623 | -1.41 | 0 | 19 | 0.016 | 1Score **> 33** indicates **identity** Score **> 14** indicates **homology** | U | K.NNGAGYFLEHLAFK.G  + Deamidated (NQ) |
| 76869 | 86 | – | 99 | 527.9276 | 1580.7610 | 1580.7623 | -0.86 | 0 | 56 | 5.1e-06 | 1Score **> 33** indicates **identity** Score **> 16** indicates **homology** | U | K.NNGAGYFLEHLAFK.G  + Deamidated (NQ) |
| 76870 | 86 | – | 99 | 527.9277 | 1580.7614 | 1580.7623 | -0.59 | 0 | 28 | 0.0024 | 1Score **> 33** indicates **identity** Score **> 14** indicates **homology** | U | K.NNGAGYFLEHLAFK.G  + Deamidated (NQ) |
| 76871 | 86 | – | 99 | 527.9278 | 1580.7616 | 1580.7623 | -0.48 | 0 | 58 | 3.8e-06 | 1Score **> 33** indicates **identity** Score **> 16** indicates **homology** | U | K.NNGAGYFLEHLAFK.G  + Deamidated (NQ) |
| 76872 | 86 | – | 99 | 791.3882 | 1580.7618 | 1580.7623 | -0.32 | 0 | 52 | 1.5e-05 | 1Score **> 33** indicates **identity** Score **> 16** indicates **homology** | U | K.NNGAGYFLEHLAFK.G  + Deamidated (NQ) |
| 76874 | 86 | – | 99 | 527.9282 | 1580.7628 | 1580.7623 | 0.31 | 0 | 44 | 7.4e-05 | 1Score **> 33** indicates **identity** Score **> 15** indicates **homology** | U | K.NNGAGYFLEHLAFK.G  + Deamidated (NQ) |
| 76877 | 86 | – | 99 | 527.9285 | 1580.7637 | 1580.7623 | 0.87 | 0 | 20 | 0.014 | 1Score **> 34** indicates **identity** Score **> 14** indicates **homology** | U | K.NNGAGYFLEHLAFK.G  + Deamidated (NQ) |
| 76907 | 86 | – | 99 | 791.3952 | 1580.7758 | 1580.7623 | 8.54 | 0 | 71 | 2.3e-07 | 1Score **> 34** indicates **identity** Score **> 17** indicates **homology** | U | K.NNGAGYFLEHLAFK.G  + Deamidated (NQ) |
| 110776 | 86 | – | 102 | 622.9881 | 1865.9426 | 1865.9424 | 0.10 | 1 | 47 | 3.7e-05 | 1Score **> 36** indicates **identity** Score **> 15** indicates **homology** | U | K.NNGAGYFLEHLAFKGTK.N |
| 110777 | 86 | – | 102 | 622.9886 | 1865.9441 | 1865.9424 | 0.91 | 1 | 47 | 3.8e-05 | 1Score **> 36** indicates **identity** Score **> 15** indicates **homology** | U | K.NNGAGYFLEHLAFKGTK.N |
| 110778 | 86 | – | 102 | 622.9896 | 1865.9470 | 1865.9424 | 2.44 | 1 | 42 | 0.00011 | 1Score **> 36** indicates **identity** Score **> 15** indicates **homology** | U | K.NNGAGYFLEHLAFKGTK.N |
| 110855 | 86 | – | 102 | 623.3144 | 1866.9215 | 1866.9264 | -2.65 | 1 | 38 | 0.00027 | 1Score **> 35** indicates **identity** Score **> 15** indicates **homology** | U | K.NNGAGYFLEHLAFKGTK.N  + Deamidated (NQ) |
| 110858 | 86 | – | 102 | 623.3152 | 1866.9238 | 1866.9264 | -1.39 | 1 | 33 | 0.00082 | 1Score **> 35** indicates **identity** Score **> 15** indicates **homology** | U | K.NNGAGYFLEHLAFKGTK.N  + Deamidated (NQ) |
| 110864 | 86 | – | 102 | 623.3195 | 1866.9367 | 1866.9264 | 5.51 | 1 | 38 | 0.00029 | 1Score **> 36** indicates **identity** Score **> 15** indicates **homology** | U | K.NNGAGYFLEHLAFKGTK.N  + Deamidated (NQ) |
| 166646 | 103 | – | 126 | 526.0699 | 2625.3130 | 2625.3259 | -4.88 | 2 | 18 | 0.019 | 1Score **> 37** indicates **identity** Score **> 14** indicates **homology** | U | K.NRPGNALEKEVESIGAHLNAYSTR.E |
| 166649 | 103 | – | 126 | 526.0703 | 2625.3151 | 2625.3259 | -4.09 | 2 | 23 | 0.0071 | 1Score **> 37** indicates **identity** Score **> 14** indicates **homology** | U | K.NRPGNALEKEVESIGAHLNAYSTR.E |
| 166650 | 103 | – | 126 | 657.3365 | 2625.3170 | 2625.3259 | -3.39 | 2 | 55 | 7.6e-06 | 1Score **> 37** indicates **identity** Score **> 16** indicates **homology** | U | K.NRPGNALEKEVESIGAHLNAYSTR.E |
| 166651 | 103 | – | 126 | 526.0708 | 2625.3177 | 2625.3259 | -3.12 | 2 | 27 | 0.0032 | 1Score **> 37** indicates **identity** Score **> 14** indicates **homology** | U | K.NRPGNALEKEVESIGAHLNAYSTR.E |
| 166652 | 103 | – | 126 | 657.3369 | 2625.3185 | 2625.3259 | -2.79 | 2 | 43 | 8.4e-05 | 1Score **> 37** indicates **identity** Score **> 15** indicates **homology** | U | K.NRPGNALEKEVESIGAHLNAYSTR.E |
| 166653 | 103 | – | 126 | 526.0713 | 2625.3201 | 2625.3259 | -2.18 | 2 | 16 | 0.032 | 1Score **> 37** indicates **identity** Score **> 13** indicates **homology** | U | K.NRPGNALEKEVESIGAHLNAYSTR.E |
| 166654 | 103 | – | 126 | 657.3374 | 2625.3203 | 2625.3259 | -2.11 | 2 | 32 | 0.00092 | 1Score **> 37** indicates **identity** Score **> 15** indicates **homology** | U | K.NRPGNALEKEVESIGAHLNAYSTR.E |
| 166655 | 103 | – | 126 | 657.3376 | 2625.3212 | 2625.3259 | -1.79 | 2 | 47 | 3.6e-05 | 1Score **> 37** indicates **identity** Score **> 15** indicates **homology** | U | K.NRPGNALEKEVESIGAHLNAYSTR.E |
| 166656 | 103 | – | 126 | 657.3376 | 2625.3213 | 2625.3259 | -1.72 | 2 | 70 | 2.6e-07 | 1Score **> 37** indicates **identity** Score **> 17** indicates **homology** | U | K.NRPGNALEKEVESIGAHLNAYSTR.E |
| 166657 | 103 | – | 126 | 526.0716 | 2625.3214 | 2625.3259 | -1.71 | 2 | 20 | 0.015 | 1Score **> 37** indicates **identity** Score **> 14** indicates **homology** | U | K.NRPGNALEKEVESIGAHLNAYSTR.E |
| 166658 | 103 | – | 126 | 657.3376 | 2625.3215 | 2625.3259 | -1.67 | 2 | 46 | 5.1e-05 | 1Score **> 37** indicates **identity** Score **> 15** indicates **homology** | U | K.NRPGNALEKEVESIGAHLNAYSTR.E |
| 166659 | 103 | – | 126 | 657.3379 | 2625.3223 | 2625.3259 | -1.34 | 2 | 67 | 5.6e-07 | 1Score **> 37** indicates **identity** Score **> 17** indicates **homology** | U | K.NRPGNALEKEVESIGAHLNAYSTR.E |
| 166661 | 103 | – | 126 | 657.3379 | 2625.3224 | 2625.3259 | -1.32 | 2 | 49 | 2.7e-05 | 1Score **> 37** indicates **identity** Score **> 16** indicates **homology** | U | K.NRPGNALEKEVESIGAHLNAYSTR.E |
| 166664 | 103 | – | 126 | 526.0719 | 2625.3230 | 2625.3259 | -1.09 | 2 | 41 | 0.00015 | 1Score **> 37** indicates **identity** Score **> 15** indicates **homology** | U | K.NRPGNALEKEVESIGAHLNAYSTR.E |
| 166665 | 103 | – | 126 | 526.0719 | 2625.3230 | 2625.3259 | -1.08 | 2 | 25 | 0.0049 | 1Score **> 37** indicates **identity** Score **> 14** indicates **homology** | U | K.NRPGNALEKEVESIGAHLNAYSTR.E |
| 166666 | 103 | – | 126 | 876.1152 | 2625.3236 | 2625.3259 | -0.85 | 2 | 27 | 0.0032 | 1Score **> 37** indicates **identity** Score **> 14** indicates **homology** | U | K.NRPGNALEKEVESIGAHLNAYSTR.E |
| 166667 | 103 | – | 126 | 526.0721 | 2625.3240 | 2625.3259 | -0.72 | 2 | 49 | 2.3e-05 | 1Score **> 37** indicates **identity** Score **> 16** indicates **homology** | U | K.NRPGNALEKEVESIGAHLNAYSTR.E |
| 166668 | 103 | – | 126 | 526.0721 | 2625.3241 | 2625.3259 | -0.69 | 2 | 44 | 7.5e-05 | 1Score **> 37** indicates **identity** Score **> 15** indicates **homology** | U | K.NRPGNALEKEVESIGAHLNAYSTR.E |
| 166669 | 103 | – | 126 | 657.3383 | 2625.3243 | 2625.3259 | -0.61 | 2 | 58 | 4e-06 | 1Score **> 37** indicates **identity** Score **> 16** indicates **homology** | U | K.NRPGNALEKEVESIGAHLNAYSTR.E |
| 166670 | 103 | – | 126 | 526.0723 | 2625.3250 | 2625.3259 | -0.33 | 2 | 38 | 0.00028 | 1Score **> 37** indicates **identity** Score **> 15** indicates **homology** | U | K.NRPGNALEKEVESIGAHLNAYSTR.E |
| 166671 | 103 | – | 126 | 526.0723 | 2625.3252 | 2625.3259 | -0.24 | 2 | 54 | 9e-06 | 1Score **> 37** indicates **identity** Score **> 16** indicates **homology** | U | K.NRPGNALEKEVESIGAHLNAYSTR.E |
| 166672 | 103 | – | 126 | 657.3386 | 2625.3254 | 2625.3259 | -0.19 | 2 | 60 | 2.5e-06 | 1Score **> 37** indicates **identity** Score **> 16** indicates **homology** | U | K.NRPGNALEKEVESIGAHLNAYSTR.E |
| 166673 | 103 | – | 126 | 876.1157 | 2625.3254 | 2625.3259 | -0.18 | 2 | 77 | 6.7e-08 | 1Score **> 37** indicates **identity** Score **> 17** indicates **homology** | U | K.NRPGNALEKEVESIGAHLNAYSTR.E |
| 166674 | 103 | – | 126 | 876.1158 | 2625.3256 | 2625.3259 | -0.10 | 2 | 65 | 7.5e-07 | 1Score **> 37** indicates **identity** Score **> 17** indicates **homology** | U | K.NRPGNALEKEVESIGAHLNAYSTR.E |
| 166675 | 103 | – | 126 | 657.3387 | 2625.3257 | 2625.3259 | -0.077 | 2 | 63 | 1.2e-06 | 1Score **> 37** indicates **identity** Score **> 16** indicates **homology** | U | K.NRPGNALEKEVESIGAHLNAYSTR.E |
| 166676 | 103 | – | 126 | 657.3387 | 2625.3258 | 2625.3259 | -0.042 | 2 | 65 | 7.4e-07 | 1Score **> 37** indicates **identity** Score **> 17** indicates **homology** | U | K.NRPGNALEKEVESIGAHLNAYSTR.E |
| 166677 | 103 | – | 126 | 526.0725 | 2625.3259 | 2625.3259 | 0.018 | 2 | 54 | 8.9e-06 | 1Score **> 37** indicates **identity** Score **> 16** indicates **homology** | U | K.NRPGNALEKEVESIGAHLNAYSTR.E |
| 166678 | 103 | – | 126 | 657.3388 | 2625.3262 | 2625.3259 | 0.13 | 2 | 45 | 6e-05 | 1Score **> 37** indicates **identity** Score **> 15** indicates **homology** | U | K.NRPGNALEKEVESIGAHLNAYSTR.E |
| 166679 | 103 | – | 126 | 876.1161 | 2625.3264 | 2625.3259 | 0.21 | 2 | 77 | 6.4e-08 | 1Score **> 37** indicates **identity** Score **> 17** indicates **homology** | U | K.NRPGNALEKEVESIGAHLNAYSTR.E |
| 166680 | 103 | – | 126 | 876.1161 | 2625.3265 | 2625.3259 | 0.22 | 2 | 79 | 4.1e-08 | 1Score **> 37** indicates **identity** Score **> 17** indicates **homology** | U | K.NRPGNALEKEVESIGAHLNAYSTR.E |
| 166681 | 103 | – | 126 | 526.0726 | 2625.3267 | 2625.3259 | 0.32 | 2 | 57 | 4.8e-06 | 1Score **> 37** indicates **identity** Score **> 16** indicates **homology** | U | K.NRPGNALEKEVESIGAHLNAYSTR.E |
| 166682 | 103 | – | 126 | 526.0726 | 2625.3268 | 2625.3259 | 0.35 | 2 | 40 | 0.00017 | 1Score **> 37** indicates **identity** Score **> 15** indicates **homology** | U | K.NRPGNALEKEVESIGAHLNAYSTR.E |
| 166683 | 103 | – | 126 | 657.3390 | 2625.3269 | 2625.3259 | 0.38 | 2 | 65 | 8.9e-07 | 1Score **> 37** indicates **identity** Score **> 17** indicates **homology** | U | K.NRPGNALEKEVESIGAHLNAYSTR.E |
| 166684 | 103 | – | 126 | 657.3390 | 2625.3270 | 2625.3259 | 0.42 | 2 | 44 | 8.3e-05 | 1Score **> 37** indicates **identity** Score **> 15** indicates **homology** | U | K.NRPGNALEKEVESIGAHLNAYSTR.E |
| 166685 | 103 | – | 126 | 526.0727 | 2625.3270 | 2625.3259 | 0.42 | 2 | 61 | 2.1e-06 | 1Score **> 37** indicates **identity** Score **> 16** indicates **homology** | U | K.NRPGNALEKEVESIGAHLNAYSTR.E |
| 166686 | 103 | – | 126 | 657.3390 | 2625.3270 | 2625.3259 | 0.43 | 2 | 71 | 2.3e-07 | 1Score **> 37** indicates **identity** Score **> 17** indicates **homology** | U | K.NRPGNALEKEVESIGAHLNAYSTR.E |
| 166687 | 103 | – | 126 | 657.3390 | 2625.3271 | 2625.3259 | 0.46 | 2 | 64 | 1e-06 | 1Score **> 37** indicates **identity** Score **> 16** indicates **homology** | U | K.NRPGNALEKEVESIGAHLNAYSTR.E |
| 166689 | 103 | – | 126 | 526.0727 | 2625.3271 | 2625.3259 | 0.48 | 2 | 50 | 1.9e-05 | 1Score **> 37** indicates **identity** Score **> 16** indicates **homology** | U | K.NRPGNALEKEVESIGAHLNAYSTR.E |
| 166690 | 103 | – | 126 | 657.3391 | 2625.3272 | 2625.3259 | 0.50 | 2 | 39 | 0.00024 | 1Score **> 37** indicates **identity** Score **> 15** indicates **homology** | U | K.NRPGNALEKEVESIGAHLNAYSTR.E |
| 166691 | 103 | – | 126 | 526.0727 | 2625.3273 | 2625.3259 | 0.56 | 2 | 24 | 0.0053 | 1Score **> 37** indicates **identity** Score **> 14** indicates **homology** | U | K.NRPGNALEKEVESIGAHLNAYSTR.E |
| 166692 | 103 | – | 126 | 657.3391 | 2625.3274 | 2625.3259 | 0.59 | 2 | 63 | 1.2e-06 | 1Score **> 37** indicates **identity** Score **> 16** indicates **homology** | U | K.NRPGNALEKEVESIGAHLNAYSTR.E |
| 166694 | 103 | – | 126 | 876.1165 | 2625.3278 | 2625.3259 | 0.72 | 2 | 68 | 4.7e-07 | 1Score **> 37** indicates **identity** Score **> 17** indicates **homology** | U | K.NRPGNALEKEVESIGAHLNAYSTR.E |
| 166695 | 103 | – | 126 | 526.0729 | 2625.3279 | 2625.3259 | 0.78 | 2 | 17 | 0.028 | 1Score **> 37** indicates **identity** Score **> 14** indicates **homology** | U | K.NRPGNALEKEVESIGAHLNAYSTR.E |
| 166696 | 103 | – | 126 | 526.0729 | 2625.3281 | 2625.3259 | 0.86 | 2 | 25 | 0.0045 | 1Score **> 37** indicates **identity** Score **> 14** indicates **homology** | U | K.NRPGNALEKEVESIGAHLNAYSTR.E |
| 166697 | 103 | – | 126 | 876.1167 | 2625.3281 | 2625.3259 | 0.86 | 2 | 47 | 4.2e-05 | 1Score **> 37** indicates **identity** Score **> 15** indicates **homology** | U | K.NRPGNALEKEVESIGAHLNAYSTR.E |
| 166698 | 103 | – | 126 | 876.1167 | 2625.3283 | 2625.3259 | 0.92 | 2 | 62 | 1.4e-06 | 1Score **> 37** indicates **identity** Score **> 16** indicates **homology** | U | K.NRPGNALEKEVESIGAHLNAYSTR.E |
| 166699 | 103 | – | 126 | 876.1171 | 2625.3296 | 2625.3259 | 1.42 | 2 | 42 | 0.00013 | 1Score **> 37** indicates **identity** Score **> 15** indicates **homology** | U | K.NRPGNALEKEVESIGAHLNAYSTR.E |
| 166701 | 103 | – | 126 | 526.0732 | 2625.3297 | 2625.3259 | 1.44 | 2 | 61 | 1.9e-06 | 1Score **> 37** indicates **identity** Score **> 16** indicates **homology** | U | K.NRPGNALEKEVESIGAHLNAYSTR.E |
| 166702 | 103 | – | 126 | 657.3398 | 2625.3301 | 2625.3259 | 1.61 | 2 | 66 | 6e-07 | 1Score **> 37** indicates **identity** Score **> 17** indicates **homology** | U | K.NRPGNALEKEVESIGAHLNAYSTR.E |
| 166703 | 103 | – | 126 | 657.3399 | 2625.3307 | 2625.3259 | 1.83 | 2 | 37 | 0.00031 | 1Score **> 37** indicates **identity** Score **> 15** indicates **homology** | U | K.NRPGNALEKEVESIGAHLNAYSTR.E |
| 166705 | 103 | – | 126 | 526.0745 | 2625.3359 | 2625.3259 | 3.82 | 2 | 24 | 0.0056 | 1Score **> 37** indicates **identity** Score **> 14** indicates **homology** | U | K.NRPGNALEKEVESIGAHLNAYSTR.E |
| 166706 | 103 | – | 126 | 526.0746 | 2625.3364 | 2625.3259 | 4.01 | 2 | 15 | 0.042 | 1Score **> 37** indicates **identity** Score **> 13** indicates **homology** | U | K.NRPGNALEKEVESIGAHLNAYSTR.E |
| 166733 | 103 | – | 126 | 876.4400 | 2626.2981 | 2626.3099 | -4.48 | 2 | 15 | 0.037 | 1Score **> 37** indicates **identity** Score **> 13** indicates **homology** | U | K.NRPGNALEKEVESIGAHLNAYSTR.E  + Deamidated (NQ) |
| 166738 | 103 | – | 126 | 526.2688 | 2626.3078 | 2626.3099 | -0.78 | 2 | 30 | 0.0014 | 1Score **> 37** indicates **identity** Score **> 14** indicates **homology** | U | K.NRPGNALEKEVESIGAHLNAYSTR.E  + Deamidated (NQ) |
| 166739 | 103 | – | 126 | 657.5844 | 2626.3086 | 2626.3099 | -0.49 | 2 | 45 | 6e-05 | 1Score **> 37** indicates **identity** Score **> 15** indicates **homology** | U | K.NRPGNALEKEVESIGAHLNAYSTR.E  + Deamidated (NQ) |
| 166741 | 103 | – | 126 | 657.5848 | 2626.3100 | 2626.3099 | 0.052 | 2 | 52 | 1.3e-05 | 1Score **> 37** indicates **identity** Score **> 16** indicates **homology** | U | K.NRPGNALEKEVESIGAHLNAYSTR.E  + Deamidated (NQ) |
| 166742 | 103 | – | 126 | 526.2693 | 2626.3102 | 2626.3099 | 0.12 | 2 | 18 | 0.022 | 1Score **> 37** indicates **identity** Score **> 14** indicates **homology** | U | K.NRPGNALEKEVESIGAHLNAYSTR.E  + Deamidated (NQ) |
| 166743 | 103 | – | 126 | 657.5849 | 2626.3103 | 2626.3099 | 0.18 | 2 | 30 | 0.0015 | 1Score **> 37** indicates **identity** Score **> 14** indicates **homology** | U | K.NRPGNALEKEVESIGAHLNAYSTR.E  + Deamidated (NQ) |
| 166744 | 103 | – | 126 | 657.5849 | 2626.3106 | 2626.3099 | 0.28 | 2 | 63 | 1.2e-06 | 1Score **> 37** indicates **identity** Score **> 16** indicates **homology** | U | K.NRPGNALEKEVESIGAHLNAYSTR.E  + Deamidated (NQ) |
| 166745 | 103 | – | 126 | 657.5853 | 2626.3120 | 2626.3099 | 0.81 | 2 | 29 | 0.0018 | 1Score **> 37** indicates **identity** Score **> 14** indicates **homology** | U | K.NRPGNALEKEVESIGAHLNAYSTR.E  + Deamidated (NQ) |
| 166746 | 103 | – | 126 | 657.5854 | 2626.3125 | 2626.3099 | 1.01 | 2 | 70 | 2.5e-07 | 1Score **> 37** indicates **identity** Score **> 17** indicates **homology** | U | K.NRPGNALEKEVESIGAHLNAYSTR.E  + Deamidated (NQ) |
| 166749 | 103 | – | 126 | 526.2703 | 2626.3151 | 2626.3099 | 1.97 | 2 | 37 | 0.00031 | 1Score **> 37** indicates **identity** Score **> 15** indicates **homology** | U | K.NRPGNALEKEVESIGAHLNAYSTR.E  + Deamidated (NQ) |
| 166750 | 103 | – | 126 | 657.5862 | 2626.3159 | 2626.3099 | 2.28 | 2 | 30 | 0.0014 | 1Score **> 37** indicates **identity** Score **> 14** indicates **homology** | U | K.NRPGNALEKEVESIGAHLNAYSTR.E  + Deamidated (NQ) |
| 166774 | 103 | – | 126 | 876.7773 | 2627.3102 | 2627.2939 | 6.19 | 2 | 16 | 0.032 | 1Score **> 37** indicates **identity** Score **> 13** indicates **homology** | U | K.NRPGNALEKEVESIGAHLNAYSTR.E  + 2 Deamidated (NQ) |
| 166776 | 103 | – | 126 | 657.8354 | 2627.3124 | 2627.2939 | 7.06 | 2 | 39 | 0.00024 | 1Score **> 37** indicates **identity** Score **> 15** indicates **homology** | U | K.NRPGNALEKEVESIGAHLNAYSTR.E  + 2 Deamidated (NQ) |
| 166778 | 103 | – | 126 | 526.4700 | 2627.3134 | 2627.2939 | 7.43 | 2 | 17 | 0.024 | 1Score **> 37** indicates **identity** Score **> 14** indicates **homology** | U | K.NRPGNALEKEVESIGAHLNAYSTR.E  + 2 Deamidated (NQ) |
| 166779 | 103 | – | 126 | 657.8357 | 2627.3137 | 2627.2939 | 7.53 | 2 | 47 | 8.1e-05 | 1Score **> 37** indicates **identity** Score **> 18** indicates **homology** | U | K.NRPGNALEKEVESIGAHLNAYSTR.E  + 2 Deamidated (NQ) |
| 161451 | 105 | – | 126 | 628.8297 | 2511.2898 | 2511.2969 | -2.82 | 1 | 20 | 0.012 | 1Score **> 37** indicates **identity** Score **> 14** indicates **homology** | U | R.PGNALEKEVESIGAHLNAYSTR.E  + HNE (K) |
| 85715 | 112 | – | 126 | 549.6082 | 1645.8027 | 1645.8060 | -1.97 | 0 | 19 | 0.017 | 1Score **> 34** indicates **identity** Score **> 14** indicates **homology** | U | K.EVESIGAHLNAYSTR.E |
| 85722 | 112 | – | 126 | 823.9094 | 1645.8041 | 1645.8060 | -1.11 | 0 | 85 | 5.8e-08 | 1Score **> 34** indicates **identity** Score **> 26** indicates **homology** | U | K.EVESIGAHLNAYSTR.E |
| 85725 | 112 | – | 126 | 549.6094 | 1645.8064 | 1645.8060 | 0.28 | 0 | 43 | 8.5e-05 | 1Score **> 34** indicates **identity** Score **> 15** indicates **homology** | U | K.EVESIGAHLNAYSTR.E |
| 85726 | 112 | – | 126 | 823.9106 | 1645.8066 | 1645.8060 | 0.37 | 0 | 71 | 1.4e-06 | 1Score **> 34** indicates **identity** Score **> 25** indicates **homology** | U | K.EVESIGAHLNAYSTR.E |
| 85729 | 112 | – | 126 | 823.9109 | 1645.8072 | 1645.8060 | 0.76 | 0 | 91 | 3.7e-08 | 1Score **> 34** indicates **identity** Score **> 29** indicates **homology** | U | K.EVESIGAHLNAYSTR.E |
| 85731 | 112 | – | 126 | 549.6098 | 1645.8075 | 1645.8060 | 0.92 | 0 | 53 | 1.1e-05 | 1Score **> 34** indicates **identity** Score **> 16** indicates **homology** | U | K.EVESIGAHLNAYSTR.E |
| 85769 | 112 | – | 126 | 823.9183 | 1645.8221 | 1645.8060 | 9.81 | 0 | 74 | 4.2e-07 | 1Score **> 35** indicates **identity** Score **> 22** indicates **homology** | U | K.EVESIGAHLNAYSTR.E |
| 13007 | 127 | – | 134 | 487.7688 | 973.5230 | 973.5233 | -0.22 | 0 | 34 | 0.0096 | 1Score **> 31** indicates **identity** Score **> 26** indicates **homology** | U | R.EHTAYLIK.A |
| 13009 | 127 | – | 134 | 487.7689 | 973.5232 | 973.5233 | -0.0041 | 0 | 32 | 0.012 | 1Score **> 31** indicates **identity** Score **> 25** indicates **homology** | U | R.EHTAYLIK.A |
| 13010 | 127 | – | 134 | 487.7689 | 973.5233 | 973.5233 | 0.033 | 0 | 30 | 0.011 | 1Score **> 31** indicates **identity** Score **> 23** indicates **homology** | U | R.EHTAYLIK.A |
| 13011 | 127 | – | 134 | 487.7692 | 973.5238 | 973.5233 | 0.54 | 0 | 25 | 0.0049 | 1Score **> 33** indicates **identity** Score **> 15** indicates **homology** | U | R.EHTAYLIK.A |
| 13012 | 127 | – | 134 | 487.7693 | 973.5240 | 973.5233 | 0.73 | 0 | 37 | 0.0017 | 1Score **> 33** indicates **identity** Score **> 22** indicates **homology** | U | R.EHTAYLIK.A |
| 7149 | 135 | – | 142 | 436.2660 | 870.5175 | 870.5174 | 0.11 | 1 | 37 | 0.00078 | 1Score **> 27** indicates **identity** Score **> 19** indicates **homology** | U | K.ALSKDLPK.V |
| 7152 | 135 | – | 142 | 436.2662 | 870.5179 | 870.5174 | 0.51 | 1 | 46 | 0.0002 | 1Score **> 27** indicates **identity** Score **> 21** indicates **homology** | U | K.ALSKDLPK.V |
| 150625 | 143 | – | 163 | 1165.6124 | 2329.2103 | 2329.1900 | 8.69 | 0 | 79 | 3.6e-08 | 1Score **> 37** indicates **identity** Score **> 17** indicates **homology** | U | K.VVELLADIVQNSSLEDSQIEK.E  + Deamidated (NQ) |
| 165996 | 143 | – | 165 | 872.1220 | 2613.3441 | 2613.3497 | -2.14 | 1 | 26 | 0.0033 | 1Score **> 37** indicates **identity** Score **> 14** indicates **homology** | U | K.VVELLADIVQNSSLEDSQIEKER.D |
| 166000 | 143 | – | 165 | 872.1227 | 2613.3463 | 2613.3497 | -1.31 | 1 | 37 | 0.00036 | 1Score **> 37** indicates **identity** Score **> 15** indicates **homology** | U | K.VVELLADIVQNSSLEDSQIEKER.D |
| 166003 | 143 | – | 165 | 872.1231 | 2613.3475 | 2613.3497 | -0.84 | 1 | 62 | 1.6e-06 | 1Score **> 37** indicates **identity** Score **> 16** indicates **homology** | U | K.VVELLADIVQNSSLEDSQIEKER.D |
| 166006 | 143 | – | 165 | 872.1237 | 2613.3491 | 2613.3497 | -0.22 | 1 | 42 | 0.00012 | 1Score **> 37** indicates **identity** Score **> 15** indicates **homology** | U | K.VVELLADIVQNSSLEDSQIEKER.D |
| 166009 | 143 | – | 165 | 872.1240 | 2613.3501 | 2613.3497 | 0.13 | 1 | 57 | 4.8e-06 | 1Score **> 37** indicates **identity** Score **> 16** indicates **homology** | U | K.VVELLADIVQNSSLEDSQIEKER.D |
| 166013 | 143 | – | 165 | 654.3450 | 2613.3507 | 2613.3497 | 0.39 | 1 | 34 | 0.00059 | 1Score **> 37** indicates **identity** Score **> 15** indicates **homology** | U | K.VVELLADIVQNSSLEDSQIEKER.D |
| 166016 | 143 | – | 165 | 654.3451 | 2613.3512 | 2613.3497 | 0.56 | 1 | 68 | 4.3e-07 | 1Score **> 37** indicates **identity** Score **> 17** indicates **homology** | U | K.VVELLADIVQNSSLEDSQIEKER.D |
| 166017 | 143 | – | 165 | 1307.6829 | 2613.3512 | 2613.3497 | 0.57 | 1 | 97 | 1.6e-09 | 1Score **> 37** indicates **identity** Score **> 22** indicates **homology** | U | K.VVELLADIVQNSSLEDSQIEKER.D |
| 166018 | 143 | – | 165 | 872.1244 | 2613.3514 | 2613.3497 | 0.65 | 1 | 85 | 1e-08 | 1Score **> 37** indicates **identity** Score **> 18** indicates **homology** | U | K.VVELLADIVQNSSLEDSQIEKER.D |
| 166019 | 143 | – | 165 | 872.1245 | 2613.3516 | 2613.3497 | 0.74 | 1 | 54 | 9e-06 | 1Score **> 37** indicates **identity** Score **> 16** indicates **homology** | U | K.VVELLADIVQNSSLEDSQIEKER.D |
| 166024 | 143 | – | 165 | 654.3455 | 2613.3529 | 2613.3497 | 1.24 | 1 | 34 | 0.00066 | 1Score **> 37** indicates **identity** Score **> 15** indicates **homology** | U | K.VVELLADIVQNSSLEDSQIEKER.D |
| 166025 | 143 | – | 165 | 872.1250 | 2613.3533 | 2613.3497 | 1.38 | 1 | 53 | 1.1e-05 | 1Score **> 37** indicates **identity** Score **> 16** indicates **homology** | U | K.VVELLADIVQNSSLEDSQIEKER.D |
| 166028 | 143 | – | 165 | 872.1252 | 2613.3536 | 2613.3497 | 1.50 | 1 | 83 | 1.6e-08 | 1Score **> 37** indicates **identity** Score **> 18** indicates **homology** | U | K.VVELLADIVQNSSLEDSQIEKER.D |
| 166029 | 143 | – | 165 | 872.1252 | 2613.3537 | 2613.3497 | 1.52 | 1 | 71 | 2.3e-07 | 1Score **> 37** indicates **identity** Score **> 17** indicates **homology** | U | K.VVELLADIVQNSSLEDSQIEKER.D |
| 166030 | 143 | – | 165 | 654.3458 | 2613.3540 | 2613.3497 | 1.65 | 1 | 24 | 0.0052 | 1Score **> 37** indicates **identity** Score **> 14** indicates **homology** | U | K.VVELLADIVQNSSLEDSQIEKER.D |
| 166031 | 143 | – | 165 | 872.1253 | 2613.3540 | 2613.3497 | 1.65 | 1 | 64 | 1e-06 | 1Score **> 37** indicates **identity** Score **> 16** indicates **homology** | U | K.VVELLADIVQNSSLEDSQIEKER.D |
| 166033 | 143 | – | 165 | 872.1254 | 2613.3543 | 2613.3497 | 1.77 | 1 | 38 | 0.00025 | 1Score **> 37** indicates **identity** Score **> 15** indicates **homology** | U | K.VVELLADIVQNSSLEDSQIEKER.D |
| 166044 | 143 | – | 165 | 872.1264 | 2613.3575 | 2613.3497 | 2.98 | 1 | 29 | 0.0021 | 1Score **> 37** indicates **identity** Score **> 14** indicates **homology** | U | K.VVELLADIVQNSSLEDSQIEKER.D |
| 166046 | 143 | – | 165 | 872.1266 | 2613.3579 | 2613.3497 | 3.15 | 1 | 15 | 0.04 | 1Score **> 37** indicates **identity** Score **> 13** indicates **homology** | U | K.VVELLADIVQNSSLEDSQIEKER.D |
| 166050 | 143 | – | 165 | 872.1269 | 2613.3588 | 2613.3497 | 3.49 | 1 | 15 | 0.042 | 1Score **> 37** indicates **identity** Score **> 13** indicates **homology** | U | K.VVELLADIVQNSSLEDSQIEKER.D |
| 166051 | 143 | – | 165 | 872.1272 | 2613.3597 | 2613.3497 | 3.81 | 1 | 22 | 0.0082 | 1Score **> 37** indicates **identity** Score **> 14** indicates **homology** | U | K.VVELLADIVQNSSLEDSQIEKER.D |
| 166058 | 143 | – | 165 | 872.1288 | 2613.3644 | 2613.3497 | 5.64 | 1 | 28 | 0.0026 | 1Score **> 37** indicates **identity** Score **> 14** indicates **homology** | U | K.VVELLADIVQNSSLEDSQIEKER.D |
| 166068 | 143 | – | 165 | 872.1320 | 2613.3741 | 2613.3497 | 9.35 | 1 | 16 | 0.029 | 1Score **> 37** indicates **identity** Score **> 14** indicates **homology** | U | K.VVELLADIVQNSSLEDSQIEKER.D |
| 166111 | 143 | – | 165 | 872.4484 | 2614.3233 | 2614.3337 | -4.00 | 1 | 27 | 0.003 | 1Score **> 37** indicates **identity** Score **> 14** indicates **homology** | U | K.VVELLADIVQNSSLEDSQIEKER.D  + Deamidated (NQ) |
| 166112 | 143 | – | 165 | 872.4484 | 2614.3233 | 2614.3337 | -4.00 | 1 | 65 | 7.6e-07 | 1Score **> 37** indicates **identity** Score **> 17** indicates **homology** | U | K.VVELLADIVQNSSLEDSQIEKER.D  + Deamidated (NQ) |
| 166116 | 143 | – | 165 | 872.4542 | 2614.3408 | 2614.3337 | 2.72 | 1 | 31 | 0.0014 | 1Score **> 37** indicates **identity** Score **> 14** indicates **homology** | U | K.VVELLADIVQNSSLEDSQIEKER.D  + Deamidated (NQ) |
| 166121 | 143 | – | 165 | 872.4576 | 2614.3510 | 2614.3337 | 6.62 | 1 | 34 | 0.00059 | 1Score **> 37** indicates **identity** Score **> 15** indicates **homology** | U | K.VVELLADIVQNSSLEDSQIEKER.D  + Deamidated (NQ) |
| 166122 | 143 | – | 165 | 1308.1828 | 2614.3511 | 2614.3337 | 6.64 | 1 | 88 | 5.7e-09 | 1Score **> 37** indicates **identity** Score **> 18** indicates **homology** | U | K.VVELLADIVQNSSLEDSQIEKER.D  + Deamidated (NQ) |
| 184023 | 143 | – | 170 | 803.4316 | 3209.6973 | 3209.7143 | -5.30 | 2 | 27 | 0.0032 | 1Score **> 36** indicates **identity** Score **> 14** indicates **homology** | U | K.VVELLADIVQNSSLEDSQIEKERDVILR.E |
| 184028 | 143 | – | 170 | 1070.9104 | 3209.7095 | 3209.7143 | -1.50 | 2 | 47 | 3.5e-05 | 1Score **> 36** indicates **identity** Score **> 15** indicates **homology** | U | K.VVELLADIVQNSSLEDSQIEKERDVILR.E |
| 184031 | 143 | – | 170 | 1070.9113 | 3209.7120 | 3209.7143 | -0.72 | 2 | 49 | 2.8e-05 | 1Score **> 36** indicates **identity** Score **> 16** indicates **homology** | U | K.VVELLADIVQNSSLEDSQIEKERDVILR.E |
| 184033 | 143 | – | 170 | 1070.9118 | 3209.7134 | 3209.7143 | -0.27 | 2 | 57 | 4.7e-06 | 1Score **> 36** indicates **identity** Score **> 16** indicates **homology** | U | K.VVELLADIVQNSSLEDSQIEKERDVILR.E |
| 184034 | 143 | – | 170 | 803.4357 | 3209.7136 | 3209.7143 | -0.23 | 2 | 42 | 0.00012 | 1Score **> 36** indicates **identity** Score **> 15** indicates **homology** | U | K.VVELLADIVQNSSLEDSQIEKERDVILR.E |
| 184044 | 143 | – | 170 | 803.4380 | 3209.7231 | 3209.7143 | 2.74 | 2 | 23 | 0.0071 | 1Score **> 36** indicates **identity** Score **> 14** indicates **homology** | U | K.VVELLADIVQNSSLEDSQIEKERDVILR.E |
| 184049 | 143 | – | 170 | 803.4393 | 3209.7282 | 3209.7143 | 4.33 | 2 | 21 | 0.012 | 1Score **> 35** indicates **identity** Score **> 14** indicates **homology** | U | K.VVELLADIVQNSSLEDSQIEKERDVILR.E |
| 184070 | 143 | – | 170 | 803.6804 | 3210.6926 | 3210.6983 | -1.77 | 2 | 19 | 0.017 | 1Score **> 36** indicates **identity** Score **> 14** indicates **homology** | U | K.VVELLADIVQNSSLEDSQIEKERDVILR.E  + Deamidated (NQ) |
| 193741 | 171 | – | 209 | 1113.5146 | 4450.0292 | 4450.0733 | -9.92 | 0 | 32 | 0.0011 | 1Score **> 33** indicates **identity** Score **> 14** indicates **homology** | U | R.EMQENDASMQNVVFDYLHATAFQGTPLAQAVEGPSENVR.R  + Deamidated (NQ); HNE (H) |
| 193754 | 171 | – | 209 | 1484.6823 | 4451.0252 | 4451.0573 | -7.22 | 0 | 56 | 5.9e-06 | 1Score **> 33** indicates **identity** Score **> 16** indicates **homology** | U | R.EMQENDASMQNVVFDYLHATAFQGTPLAQAVEGPSENVR.R  + 2 Deamidated (NQ); HNE (H) |
| 193764 | 171 | – | 209 | 1485.0164 | 4452.0275 | 4452.0413 | -3.10 | 0 | 59 | 2.9e-06 | 1Score **> 33** indicates **identity** Score **> 16** indicates **homology** | U | R.EMQENDASMQNVVFDYLHATAFQGTPLAQAVEGPSENVR.R  + 3 Deamidated (NQ); HNE (H) |
| 62291 | 211 | – | 222 | 489.5913 | 1465.7520 | 1465.7525 | -0.35 | 1 | 31 | 0.0013 | 1Score **> 34** indicates **identity** Score **> 14** indicates **homology** | U | R.LSRTDLTDYLNR.H |
| 62293 | 211 | – | 222 | 489.5914 | 1465.7524 | 1465.7525 | -0.040 | 1 | 37 | 0.00058 | 1Score **> 34** indicates **identity** Score **> 17** indicates **homology** | U | R.LSRTDLTDYLNR.H |
| 62294 | 211 | – | 222 | 489.5918 | 1465.7535 | 1465.7525 | 0.66 | 1 | 19 | 0.018 | 1Score **> 34** indicates **identity** Score **> 14** indicates **homology** | U | R.LSRTDLTDYLNR.H |
| 23626 | 214 | – | 222 | 555.7732 | 1109.5318 | 1109.5353 | -3.17 | 0 | 30 | 0.0027 | 1Score **> 30** indicates **identity** Score **> 16** indicates **homology** | U | R.TDLTDYLNR.H |
| 23630 | 214 | – | 222 | 555.7744 | 1109.5343 | 1109.5353 | -0.92 | 0 | 32 | 0.0017 | 1Score **> 30** indicates **identity** Score **> 17** indicates **homology** | U | R.TDLTDYLNR.H |
| 23634 | 214 | – | 222 | 555.7747 | 1109.5348 | 1109.5353 | -0.48 | 0 | 49 | 0.00027 | 1Score **> 30** indicates **identity** Score **> 26** indicates **homology** | U | R.TDLTDYLNR.H |
| 23636 | 214 | – | 222 | 555.7750 | 1109.5355 | 1109.5353 | 0.19 | 0 | 64 | 2e-05 | 1Score **> 31** indicates **identity** Score **> 29** indicates **homology** | U | R.TDLTDYLNR.H |
| 23638 | 214 | – | 222 | 555.7751 | 1109.5356 | 1109.5353 | 0.28 | 0 | 41 | 0.0014 | 1Score **> 31** indicates **identity** Score **> 26** indicates **homology** | U | R.TDLTDYLNR.H |
| 23639 | 214 | – | 222 | 555.7751 | 1109.5356 | 1109.5353 | 0.29 | 0 | 64 | 2e-05 | 1Score **> 31** indicates **identity** Score **> 30** indicates **homology** | U | R.TDLTDYLNR.H |
| 23641 | 214 | – | 222 | 555.7752 | 1109.5358 | 1109.5353 | 0.50 | 0 | 68 | 7.6e-06 | 1Score **> 31** indicates **identity** Score **> 30** indicates **homology** | U | R.TDLTDYLNR.H |
| 23642 | 214 | – | 222 | 555.7753 | 1109.5360 | 1109.5353 | 0.66 | 0 | 57 | 5.9e-05 | 1Score **> 31** indicates **identity** Score **> 28** indicates **homology** | U | R.TDLTDYLNR.H |
| 23647 | 214 | – | 222 | 555.7760 | 1109.5374 | 1109.5353 | 1.91 | 0 | 35 | 0.0048 | 1Score **> 31** indicates **identity** Score **> 24** indicates **homology** | U | R.TDLTDYLNR.H |
| 23648 | 214 | – | 222 | 555.7762 | 1109.5379 | 1109.5353 | 2.33 | 0 | 17 | 0.034 | 1Score **> 31** indicates **identity** Score **> 15** indicates **homology** | U | R.TDLTDYLNR.H |
| 71149 | 214 | – | 225 | 513.5925 | 1537.7557 | 1537.7525 | 2.06 | 1 | 33 | 0.00081 | 1Score **> 34** indicates **identity** Score **> 15** indicates **homology** | U | R.TDLTDYLNRHYK.A |
| 131360 | 229 | – | 248 | 698.0433 | 2091.1079 | 2091.1146 | -3.21 | 0 | 33 | 0.00073 | 1Score **> 36** indicates **identity** Score **> 15** indicates **homology** | U | R.MVLAAAGGVEHQQLLDLAQK.H |
| 131361 | 229 | – | 248 | 698.0433 | 2091.1081 | 2091.1146 | -3.15 | 0 | 17 | 0.027 | 1Score **> 36** indicates **identity** Score **> 14** indicates **homology** | U | R.MVLAAAGGVEHQQLLDLAQK.H |
| 131378 | 229 | – | 248 | 698.0451 | 2091.1136 | 2091.1146 | -0.50 | 0 | 35 | 0.00055 | 1Score **> 36** indicates **identity** Score **> 15** indicates **homology** | U | R.MVLAAAGGVEHQQLLDLAQK.H |
| 131380 | 229 | – | 248 | 698.0452 | 2091.1137 | 2091.1146 | -0.46 | 0 | 27 | 0.0027 | 1Score **> 36** indicates **identity** Score **> 14** indicates **homology** | U | R.MVLAAAGGVEHQQLLDLAQK.H |
| 131389 | 229 | – | 248 | 1046.5655 | 2091.1164 | 2091.1146 | 0.86 | 0 | 95 | 1.3e-09 | 1Score **> 36** indicates **identity** Score **> 18** indicates **homology** | U | R.MVLAAAGGVEHQQLLDLAQK.H |
| 131396 | 229 | – | 248 | 698.0464 | 2091.1174 | 2091.1146 | 1.34 | 0 | 40 | 0.00018 | 1Score **> 36** indicates **identity** Score **> 15** indicates **homology** | U | R.MVLAAAGGVEHQQLLDLAQK.H |
| 131398 | 229 | – | 248 | 698.0465 | 2091.1177 | 2091.1146 | 1.44 | 0 | 25 | 0.0044 | 1Score **> 36** indicates **identity** Score **> 14** indicates **homology** | U | R.MVLAAAGGVEHQQLLDLAQK.H |
| 131405 | 229 | – | 248 | 698.0469 | 2091.1188 | 2091.1146 | 1.97 | 0 | 22 | 0.0091 | 1Score **> 36** indicates **identity** Score **> 14** indicates **homology** | U | R.MVLAAAGGVEHQQLLDLAQK.H |
| 131406 | 229 | – | 248 | 698.0469 | 2091.1189 | 2091.1146 | 2.03 | 0 | 59 | 3.2e-06 | 1Score **> 36** indicates **identity** Score **> 16** indicates **homology** | U | R.MVLAAAGGVEHQQLLDLAQK.H |
| 131407 | 229 | – | 248 | 698.0472 | 2091.1198 | 2091.1146 | 2.47 | 0 | 44 | 7.9e-05 | 1Score **> 36** indicates **identity** Score **> 15** indicates **homology** | U | R.MVLAAAGGVEHQQLLDLAQK.H |
| 131411 | 229 | – | 248 | 698.0477 | 2091.1214 | 2091.1146 | 3.22 | 0 | 42 | 0.00012 | 1Score **> 36** indicates **identity** Score **> 15** indicates **homology** | U | R.MVLAAAGGVEHQQLLDLAQK.H |
| 131413 | 229 | – | 248 | 698.0481 | 2091.1225 | 2091.1146 | 3.76 | 0 | 41 | 0.00014 | 1Score **> 36** indicates **identity** Score **> 15** indicates **homology** | U | R.MVLAAAGGVEHQQLLDLAQK.H |
| 132934 | 229 | – | 248 | 703.3761 | 2107.1064 | 2107.1096 | -1.49 | 0 | 21 | 0.011 | 1Score **> 36** indicates **identity** Score **> 14** indicates **homology** | U | R.MVLAAAGGVEHQQLLDLAQK.H  + Oxidation (M) |
| 3033 | 249 | – | 255 | 393.2162 | 784.4178 | 784.4191 | -1.77 | 0 | 27 | 0.02 | 1Score **> 23** indicates **identity** | U | K.HLSSVSR.V |
| 80374 | 256 | – | 269 | 803.3770 | 1604.7395 | 1604.7505 | -6.84 | 0 | 31 | 0.0013 | 1Score **> 31** indicates **identity** Score **> 14** indicates **homology** | U | R.VYEEDAVPGLTPCR.F |
| 80377 | 256 | – | 269 | 803.3785 | 1604.7424 | 1604.7505 | -5.02 | 0 | 37 | 0.00033 | 1Score **> 31** indicates **identity** Score **> 15** indicates **homology** | U | R.VYEEDAVPGLTPCR.F |
| 80379 | 256 | – | 269 | 803.3791 | 1604.7437 | 1604.7505 | -4.19 | 0 | 55 | 6.8e-06 | 1Score **> 32** indicates **identity** Score **> 16** indicates **homology** | U | R.VYEEDAVPGLTPCR.F |
| 80380 | 256 | – | 269 | 803.3793 | 1604.7441 | 1604.7505 | -3.98 | 0 | 33 | 0.00086 | 1Score **> 31** indicates **identity** Score **> 15** indicates **homology** | U | R.VYEEDAVPGLTPCR.F |
| 80381 | 256 | – | 269 | 803.3798 | 1604.7449 | 1604.7505 | -3.43 | 0 | 25 | 0.015 | 1Score **> 32** indicates **identity** Score **> 19** indicates **homology** | U | R.VYEEDAVPGLTPCR.F |
| 80383 | 256 | – | 269 | 803.3799 | 1604.7453 | 1604.7505 | -3.22 | 0 | 34 | 0.00065 | 1Score **> 32** indicates **identity** Score **> 15** indicates **homology** | U | R.VYEEDAVPGLTPCR.F |
| 80384 | 256 | – | 269 | 803.3802 | 1604.7459 | 1604.7505 | -2.85 | 0 | 41 | 0.00013 | 1Score **> 32** indicates **identity** Score **> 15** indicates **homology** | U | R.VYEEDAVPGLTPCR.F |
| 80385 | 256 | – | 269 | 803.3808 | 1604.7471 | 1604.7505 | -2.07 | 0 | 62 | 1.4e-06 | 1Score **> 32** indicates **identity** Score **> 16** indicates **homology** | U | R.VYEEDAVPGLTPCR.F |
| 80386 | 256 | – | 269 | 803.3812 | 1604.7479 | 1604.7505 | -1.62 | 0 | 59 | 3.2e-06 | 1Score **> 32** indicates **identity** Score **> 16** indicates **homology** | U | R.VYEEDAVPGLTPCR.F |
| 80387 | 256 | – | 269 | 803.3813 | 1604.7480 | 1604.7505 | -1.56 | 0 | 35 | 0.0005 | 1Score **> 32** indicates **identity** Score **> 15** indicates **homology** | U | R.VYEEDAVPGLTPCR.F |
| 80388 | 256 | – | 269 | 535.9235 | 1604.7488 | 1604.7505 | -1.03 | 0 | 18 | 0.019 | 1Score **> 32** indicates **identity** Score **> 14** indicates **homology** | U | R.VYEEDAVPGLTPCR.F |
| 80389 | 256 | – | 269 | 803.3820 | 1604.7495 | 1604.7505 | -0.62 | 0 | 25 | 0.0045 | 1Score **> 32** indicates **identity** Score **> 14** indicates **homology** | U | R.VYEEDAVPGLTPCR.F |
| 80390 | 256 | – | 269 | 803.3822 | 1604.7498 | 1604.7505 | -0.39 | 0 | 72 | 1.9e-07 | 1Score **> 32** indicates **identity** Score **> 17** indicates **homology** | U | R.VYEEDAVPGLTPCR.F |
| 80392 | 256 | – | 269 | 535.9239 | 1604.7499 | 1604.7505 | -0.32 | 0 | 26 | 0.0038 | 1Score **> 32** indicates **identity** Score **> 15** indicates **homology** | U | R.VYEEDAVPGLTPCR.F |
| 80393 | 256 | – | 269 | 535.9240 | 1604.7500 | 1604.7505 | -0.27 | 0 | 25 | 0.0049 | 1Score **> 32** indicates **identity** Score **> 15** indicates **homology** | U | R.VYEEDAVPGLTPCR.F |
| 80394 | 256 | – | 269 | 803.3823 | 1604.7501 | 1604.7505 | -0.24 | 0 | 72 | 2.2e-07 | 1Score **> 32** indicates **identity** Score **> 18** indicates **homology** | U | R.VYEEDAVPGLTPCR.F |
| 80395 | 256 | – | 269 | 535.9240 | 1604.7502 | 1604.7505 | -0.16 | 0 | 37 | 0.00039 | 1Score **> 32** indicates **identity** Score **> 15** indicates **homology** | U | R.VYEEDAVPGLTPCR.F |
| 80397 | 256 | – | 269 | 803.3829 | 1604.7512 | 1604.7505 | 0.44 | 0 | 68 | 5e-07 | 1Score **> 32** indicates **identity** Score **> 17** indicates **homology** | U | R.VYEEDAVPGLTPCR.F |
| 80398 | 256 | – | 269 | 803.3834 | 1604.7523 | 1604.7505 | 1.17 | 0 | 63 | 1.2e-06 | 1Score **> 32** indicates **identity** Score **> 16** indicates **homology** | U | R.VYEEDAVPGLTPCR.F |
| 80399 | 256 | – | 269 | 803.3835 | 1604.7525 | 1604.7505 | 1.28 | 0 | 60 | 2.2e-06 | 1Score **> 32** indicates **identity** Score **> 16** indicates **homology** | U | R.VYEEDAVPGLTPCR.F |
| 80401 | 256 | – | 269 | 803.3838 | 1604.7531 | 1604.7505 | 1.67 | 0 | 81 | 2.6e-08 | 1Score **> 32** indicates **identity** Score **> 18** indicates **homology** | U | R.VYEEDAVPGLTPCR.F |
| 80402 | 256 | – | 269 | 803.3839 | 1604.7532 | 1604.7505 | 1.72 | 0 | 66 | 6.3e-07 | 1Score **> 32** indicates **identity** Score **> 17** indicates **homology** | U | R.VYEEDAVPGLTPCR.F |
| 80403 | 256 | – | 269 | 803.3842 | 1604.7538 | 1604.7505 | 2.08 | 0 | 66 | 7.1e-07 | 1Score **> 32** indicates **identity** Score **> 17** indicates **homology** | U | R.VYEEDAVPGLTPCR.F |
| 80404 | 256 | – | 269 | 803.3843 | 1604.7540 | 1604.7505 | 2.19 | 0 | 74 | 1.1e-07 | 1Score **> 32** indicates **identity** Score **> 17** indicates **homology** | U | R.VYEEDAVPGLTPCR.F |
| 80405 | 256 | – | 269 | 803.3848 | 1604.7551 | 1604.7505 | 2.88 | 0 | 35 | 0.00052 | 1Score **> 32** indicates **identity** Score **> 15** indicates **homology** | U | R.VYEEDAVPGLTPCR.F |
| 154813 | 256 | – | 276 | 799.3861 | 2395.1364 | 2395.1478 | -4.76 | 1 | 20 | 0.012 | 1Score **> 35** indicates **identity** Score **> 14** indicates **homology** | U | R.VYEEDAVPGLTPCRFTGSEIR.H |
| 154819 | 256 | – | 276 | 799.3905 | 2395.1498 | 2395.1478 | 0.82 | 1 | 22 | 0.0082 | 1Score **> 36** indicates **identity** Score **> 14** indicates **homology** | U | R.VYEEDAVPGLTPCRFTGSEIR.H |
| 154822 | 256 | – | 276 | 799.3913 | 2395.1521 | 2395.1478 | 1.78 | 1 | 31 | 0.0012 | 1Score **> 36** indicates **identity** Score **> 14** indicates **homology** | U | R.VYEEDAVPGLTPCRFTGSEIR.H |
| 4079 | 270 | – | 276 | 405.2099 | 808.4053 | 808.4079 | -3.22 | 0 | 25 | 0.0096 | 1Score **> 24** indicates **identity** Score **> 17** indicates **homology** | U | R.FTGSEIR.H |
| 4080 | 270 | – | 276 | 405.2106 | 808.4066 | 808.4079 | -1.65 | 0 | 32 | 0.001 | 1Score **> 24** indicates **identity** Score **> 14** indicates **homology** | U | R.FTGSEIR.H |
| 4082 | 270 | – | 276 | 405.2108 | 808.4071 | 808.4079 | -0.99 | 0 | 31 | 0.0036 | 1Score **> 24** indicates **identity** Score **> 19** indicates **homology** | U | R.FTGSEIR.H |
| 4083 | 270 | – | 276 | 405.2109 | 808.4073 | 808.4079 | -0.75 | 0 | 41 | 0.00025 | 1Score **> 25** indicates **identity** Score **> 17** indicates **homology** | U | R.FTGSEIR.H |
| 4084 | 270 | – | 276 | 405.2109 | 808.4073 | 808.4079 | -0.73 | 0 | 28 | 0.0024 | 1Score **> 25** indicates **identity** Score **> 14** indicates **homology** | U | R.FTGSEIR.H |
| 4086 | 270 | – | 276 | 405.2111 | 808.4077 | 808.4079 | -0.22 | 0 | 43 | 0.00015 | 1Score **> 25** indicates **identity** Score **> 17** indicates **homology** | U | R.FTGSEIR.H |
| 4087 | 270 | – | 276 | 405.2112 | 808.4078 | 808.4079 | -0.15 | 0 | 36 | 0.00056 | 1Score **> 25** indicates **identity** Score **> 16** indicates **homology** | U | R.FTGSEIR.H |
| 195877 | 277 | – | 336 | 1240.6317 | 6198.1221 | 6198.1134 | 1.41 | 1 | 28 | 0.0026 | 1Score **> 34** indicates **identity** Score **> 14** indicates **homology** | U | R.HRDDALPLAHVAIAVEGPGWANPDNVTLQVANAIIGHYDCTYGGGVHLSSPLASVAVANK.L |
| 195878 | 277 | – | 336 | 1034.0277 | 6198.1228 | 6198.1134 | 1.51 | 1 | 14 | 0.047 | 1Score **> 34** indicates **identity** Score **> 13** indicates **homology** | U | R.HRDDALPLAHVAIAVEGPGWANPDNVTLQVANAIIGHYDCTYGGGVHLSSPLASVAVANK.L |
| 195879 | 277 | – | 336 | 1240.8346 | 6199.1367 | 6199.0974 | 6.34 | 1 | 25 | 0.0041 | 1Score **> 34** indicates **identity** Score **> 14** indicates **homology** | U | R.HRDDALPLAHVAIAVEGPGWANPDNVTLQVANAIIGHYDCTYGGGVHLSSPLASVAVANK.L  + Deamidated (NQ) |
| 49888 | 379 | – | 390 | 677.3163 | 1352.6181 | 1352.6242 | -4.48 | 0 | 32 | 0.00098 | 1Score **> 30** indicates **identity** Score **> 15** indicates **homology** | U | R.LCTSATESEVTR.G |
| 49889 | 379 | – | 390 | 677.3167 | 1352.6188 | 1352.6242 | -3.97 | 0 | 31 | 0.0013 | 1Score **> 31** indicates **identity** Score **> 14** indicates **homology** | U | R.LCTSATESEVTR.G |
| 49890 | 379 | – | 390 | 677.3172 | 1352.6198 | 1352.6242 | -3.24 | 0 | 27 | 0.0031 | 1Score **> 31** indicates **identity** Score **> 14** indicates **homology** | U | R.LCTSATESEVTR.G |
| 49891 | 379 | – | 390 | 677.3172 | 1352.6199 | 1352.6242 | -3.16 | 0 | 57 | 4.5e-06 | 1Score **> 31** indicates **identity** Score **> 16** indicates **homology** | U | R.LCTSATESEVTR.G |
| 49892 | 379 | – | 390 | 677.3179 | 1352.6212 | 1352.6242 | -2.23 | 0 | 56 | 1.1e-05 | 1Score **> 30** indicates **identity** Score **> 19** indicates **homology** | U | R.LCTSATESEVTR.G |
| 49894 | 379 | – | 390 | 677.3180 | 1352.6214 | 1352.6242 | -2.06 | 0 | 36 | 0.014 | 1Score **> 30** indicates **identity** | U | R.LCTSATESEVTR.G |
| 49896 | 379 | – | 390 | 677.3182 | 1352.6219 | 1352.6242 | -1.71 | 0 | 74 | 1.1e-07 | 1Score **> 30** indicates **identity** Score **> 17** indicates **homology** | U | R.LCTSATESEVTR.G |
| 49897 | 379 | – | 390 | 677.3183 | 1352.6220 | 1352.6242 | -1.65 | 0 | 25 | 0.0049 | 1Score **> 30** indicates **identity** Score **> 14** indicates **homology** | U | R.LCTSATESEVTR.G |
| 49898 | 379 | – | 390 | 677.3183 | 1352.6220 | 1352.6242 | -1.63 | 0 | 74 | 1e-07 | 1Score **> 30** indicates **identity** Score **> 17** indicates **homology** | U | R.LCTSATESEVTR.G |
| 49899 | 379 | – | 390 | 677.3183 | 1352.6220 | 1352.6242 | -1.60 | 0 | 60 | 1.7e-05 | 1Score **> 30** indicates **identity** Score **> 24** indicates **homology** | U | R.LCTSATESEVTR.G |
| 49902 | 379 | – | 390 | 677.3187 | 1352.6229 | 1352.6242 | -0.99 | 0 | 52 | 1.4e-05 | 1Score **> 32** indicates **identity** Score **> 16** indicates **homology** | U | R.LCTSATESEVTR.G |
| 49903 | 379 | – | 390 | 677.3190 | 1352.6235 | 1352.6242 | -0.53 | 0 | 86 | 2.8e-08 | 1Score **> 32** indicates **identity** Score **> 23** indicates **homology** | U | R.LCTSATESEVTR.G |
| 49904 | 379 | – | 390 | 677.3191 | 1352.6236 | 1352.6242 | -0.43 | 0 | 70 | 2.8e-07 | 1Score **> 32** indicates **identity** Score **> 17** indicates **homology** | U | R.LCTSATESEVTR.G |
| 49905 | 379 | – | 390 | 677.3191 | 1352.6237 | 1352.6242 | -0.34 | 0 | 62 | 1.4e-06 | 1Score **> 32** indicates **identity** Score **> 16** indicates **homology** | U | R.LCTSATESEVTR.G |
| 49906 | 379 | – | 390 | 677.3192 | 1352.6238 | 1352.6242 | -0.27 | 0 | 26 | 0.0033 | 1Score **> 32** indicates **identity** Score **> 14** indicates **homology** | U | R.LCTSATESEVTR.G |
| 49907 | 379 | – | 390 | 677.3192 | 1352.6239 | 1352.6242 | -0.24 | 0 | 81 | 3.3e-08 | 1Score **> 32** indicates **identity** Score **> 19** indicates **homology** | U | R.LCTSATESEVTR.G |
| 49908 | 379 | – | 390 | 677.3192 | 1352.6239 | 1352.6242 | -0.24 | 0 | 81 | 2.5e-08 | 1Score **> 32** indicates **identity** Score **> 18** indicates **homology** | U | R.LCTSATESEVTR.G |
| 49909 | 379 | – | 390 | 677.3192 | 1352.6239 | 1352.6242 | -0.23 | 0 | 75 | 1e-07 | 1Score **> 32** indicates **identity** Score **> 17** indicates **homology** | U | R.LCTSATESEVTR.G |
| 49911 | 379 | – | 390 | 677.3193 | 1352.6240 | 1352.6242 | -0.15 | 0 | 74 | 1e-07 | 1Score **> 32** indicates **identity** Score **> 17** indicates **homology** | U | R.LCTSATESEVTR.G |
| 49912 | 379 | – | 390 | 677.3196 | 1352.6246 | 1352.6242 | 0.31 | 0 | 47 | 3.9e-05 | 1Score **> 31** indicates **identity** Score **> 15** indicates **homology** | U | R.LCTSATESEVTR.G |
| 49913 | 379 | – | 390 | 677.3199 | 1352.6252 | 1352.6242 | 0.73 | 0 | 45 | 6.2e-05 | 1Score **> 31** indicates **identity** Score **> 15** indicates **homology** | U | R.LCTSATESEVTR.G |
| 49915 | 379 | – | 390 | 677.3205 | 1352.6265 | 1352.6242 | 1.71 | 0 | 26 | 0.0035 | 1Score **> 32** indicates **identity** Score **> 14** indicates **homology** | U | R.LCTSATESEVTR.G |
| 128188 | 397 | – | 415 | 1027.4989 | 2052.9832 | 2052.9899 | -3.23 | 0 | 46 | 4.4e-05 | 1Score **> 35** indicates **identity** Score **> 15** indicates **homology** | U | R.NALVSHLDGTTPVCEDIGR.S |
| 128190 | 397 | – | 415 | 685.3355 | 2052.9846 | 2052.9899 | -2.55 | 0 | 31 | 0.0013 | 1Score **> 35** indicates **identity** Score **> 14** indicates **homology** | U | R.NALVSHLDGTTPVCEDIGR.S |
| 128191 | 397 | – | 415 | 1027.5000 | 2052.9854 | 2052.9899 | -2.15 | 0 | 107 | 8.8e-11 | 1Score **> 35** indicates **identity** Score **> 19** indicates **homology** | U | R.NALVSHLDGTTPVCEDIGR.S |
| 128192 | 397 | – | 415 | 685.3364 | 2052.9872 | 2052.9899 | -1.29 | 0 | 52 | 1.4e-05 | 1Score **> 35** indicates **identity** Score **> 16** indicates **homology** | U | R.NALVSHLDGTTPVCEDIGR.S |
| 128193 | 397 | – | 415 | 685.3365 | 2052.9876 | 2052.9899 | -1.12 | 0 | 75 | 1.6e-07 | 1Score **> 35** indicates **identity** Score **> 19** indicates **homology** | U | R.NALVSHLDGTTPVCEDIGR.S |
| 128194 | 397 | – | 415 | 1027.5011 | 2052.9877 | 2052.9899 | -1.04 | 0 | 68 | 3.9e-07 | 1Score **> 35** indicates **identity** Score **> 17** indicates **homology** | U | R.NALVSHLDGTTPVCEDIGR.S |
| 128195 | 397 | – | 415 | 685.3365 | 2052.9878 | 2052.9899 | -1.01 | 0 | 77 | 1e-07 | 1Score **> 35** indicates **identity** Score **> 19** indicates **homology** | U | R.NALVSHLDGTTPVCEDIGR.S |
| 128196 | 397 | – | 415 | 685.3366 | 2052.9880 | 2052.9899 | -0.91 | 0 | 75 | 1.3e-07 | 1Score **> 35** indicates **identity** Score **> 18** indicates **homology** | U | R.NALVSHLDGTTPVCEDIGR.S |
| 128197 | 397 | – | 415 | 1027.5014 | 2052.9882 | 2052.9899 | -0.79 | 0 | 95 | 1.2e-09 | 1Score **> 35** indicates **identity** Score **> 18** indicates **homology** | U | R.NALVSHLDGTTPVCEDIGR.S |
| 128198 | 397 | – | 415 | 685.3367 | 2052.9883 | 2052.9899 | -0.76 | 0 | 71 | 3.2e-07 | 1Score **> 35** indicates **identity** Score **> 19** indicates **homology** | U | R.NALVSHLDGTTPVCEDIGR.S |
| 128199 | 397 | – | 415 | 685.3367 | 2052.9884 | 2052.9899 | -0.74 | 0 | 49 | 2.3e-05 | 1Score **> 35** indicates **identity** Score **> 16** indicates **homology** | U | R.NALVSHLDGTTPVCEDIGR.S |
| 128200 | 397 | – | 415 | 1027.5016 | 2052.9886 | 2052.9899 | -0.61 | 0 | 95 | 1.3e-09 | 1Score **> 35** indicates **identity** Score **> 18** indicates **homology** | U | R.NALVSHLDGTTPVCEDIGR.S |
| 128201 | 397 | – | 415 | 685.3369 | 2052.9888 | 2052.9899 | -0.52 | 0 | 45 | 5.6e-05 | 1Score **> 35** indicates **identity** Score **> 15** indicates **homology** | U | R.NALVSHLDGTTPVCEDIGR.S |
| 128202 | 397 | – | 415 | 1027.5017 | 2052.9889 | 2052.9899 | -0.49 | 0 | 82 | 2e-08 | 1Score **> 35** indicates **identity** Score **> 18** indicates **homology** | U | R.NALVSHLDGTTPVCEDIGR.S |
| 128204 | 397 | – | 415 | 685.3370 | 2052.9891 | 2052.9899 | -0.37 | 0 | 71 | 4.3e-07 | 1Score **> 35** indicates **identity** Score **> 20** indicates **homology** | U | R.NALVSHLDGTTPVCEDIGR.S |
| 128205 | 397 | – | 415 | 685.3372 | 2052.9899 | 2052.9899 | -0.0015 | 0 | 71 | 3.6e-07 | 1Score **> 35** indicates **identity** Score **> 19** indicates **homology** | U | R.NALVSHLDGTTPVCEDIGR.S |
| 128206 | 397 | – | 415 | 685.3373 | 2052.9900 | 2052.9899 | 0.077 | 0 | 71 | 3.5e-07 | 1Score **> 35** indicates **identity** Score **> 19** indicates **homology** | U | R.NALVSHLDGTTPVCEDIGR.S |
| 128207 | 397 | – | 415 | 685.3373 | 2052.9902 | 2052.9899 | 0.14 | 0 | 49 | 2.5e-05 | 1Score **> 35** indicates **identity** Score **> 16** indicates **homology** | U | R.NALVSHLDGTTPVCEDIGR.S |
| 128208 | 397 | – | 415 | 685.3374 | 2052.9903 | 2052.9899 | 0.23 | 0 | 71 | 2.9e-07 | 1Score **> 35** indicates **identity** Score **> 18** indicates **homology** | U | R.NALVSHLDGTTPVCEDIGR.S |
| 128209 | 397 | – | 415 | 685.3375 | 2052.9906 | 2052.9899 | 0.35 | 0 | 77 | 1e-07 | 1Score **> 35** indicates **identity** Score **> 19** indicates **homology** | U | R.NALVSHLDGTTPVCEDIGR.S |
| 128210 | 397 | – | 415 | 1027.5026 | 2052.9907 | 2052.9899 | 0.40 | 0 | 102 | 2.8e-10 | 1Score **> 35** indicates **identity** Score **> 19** indicates **homology** | U | R.NALVSHLDGTTPVCEDIGR.S |
| 128211 | 397 | – | 415 | 685.3376 | 2052.9910 | 2052.9899 | 0.55 | 0 | 71 | 3.4e-07 | 1Score **> 35** indicates **identity** Score **> 19** indicates **homology** | U | R.NALVSHLDGTTPVCEDIGR.S |
| 128212 | 397 | – | 415 | 1027.5028 | 2052.9910 | 2052.9899 | 0.56 | 0 | 81 | 2.8e-08 | 1Score **> 35** indicates **identity** Score **> 18** indicates **homology** | U | R.NALVSHLDGTTPVCEDIGR.S |
| 128213 | 397 | – | 415 | 1027.5028 | 2052.9911 | 2052.9899 | 0.58 | 0 | 71 | 2e-07 | 1Score **> 35** indicates **identity** Score **> 17** indicates **homology** | U | R.NALVSHLDGTTPVCEDIGR.S |
| 128214 | 397 | – | 415 | 685.3377 | 2052.9912 | 2052.9899 | 0.66 | 0 | 75 | 1.2e-07 | 1Score **> 35** indicates **identity** Score **> 19** indicates **homology** | U | R.NALVSHLDGTTPVCEDIGR.S |
| 128216 | 397 | – | 415 | 685.3378 | 2052.9916 | 2052.9899 | 0.86 | 0 | 54 | 1.3e-05 | 1Score **> 35** indicates **identity** Score **> 17** indicates **homology** | U | R.NALVSHLDGTTPVCEDIGR.S |
| 128217 | 397 | – | 415 | 685.3380 | 2052.9921 | 2052.9899 | 1.09 | 0 | 71 | 3.6e-07 | 1Score **> 35** indicates **identity** Score **> 19** indicates **homology** | U | R.NALVSHLDGTTPVCEDIGR.S |
| 128218 | 397 | – | 415 | 685.3381 | 2052.9925 | 2052.9899 | 1.27 | 0 | 40 | 0.00019 | 1Score **> 35** indicates **identity** Score **> 15** indicates **homology** | U | R.NALVSHLDGTTPVCEDIGR.S |
| 128219 | 397 | – | 415 | 1027.5035 | 2052.9925 | 2052.9899 | 1.28 | 0 | 85 | 1.1e-08 | 1Score **> 35** indicates **identity** Score **> 18** indicates **homology** | U | R.NALVSHLDGTTPVCEDIGR.S |
| 128220 | 397 | – | 415 | 1027.5039 | 2052.9933 | 2052.9899 | 1.69 | 0 | 76 | 7e-08 | 1Score **> 35** indicates **identity** Score **> 17** indicates **homology** | U | R.NALVSHLDGTTPVCEDIGR.S |
| 128221 | 397 | – | 415 | 1027.5046 | 2052.9946 | 2052.9899 | 2.31 | 0 | 71 | 6e-07 | 1Score **> 35** indicates **identity** Score **> 21** indicates **homology** | U | R.NALVSHLDGTTPVCEDIGR.S |
| 128222 | 397 | – | 415 | 685.3390 | 2052.9953 | 2052.9899 | 2.65 | 0 | 49 | 2.5e-05 | 1Score **> 35** indicates **identity** Score **> 16** indicates **homology** | U | R.NALVSHLDGTTPVCEDIGR.S |
| 128223 | 397 | – | 415 | 685.3392 | 2052.9956 | 2052.9899 | 2.80 | 0 | 21 | 0.012 | 1Score **> 35** indicates **identity** Score **> 14** indicates **homology** | U | R.NALVSHLDGTTPVCEDIGR.S |
| 128227 | 397 | – | 415 | 1027.5064 | 2052.9982 | 2052.9899 | 4.06 | 0 | 23 | 0.007 | 1Score **> 35** indicates **identity** Score **> 14** indicates **homology** | U | R.NALVSHLDGTTPVCEDIGR.S |
| 128228 | 397 | – | 415 | 685.3416 | 2053.0030 | 2052.9899 | 6.40 | 0 | 24 | 0.0069 | 1Score **> 35** indicates **identity** Score **> 15** indicates **homology** | U | R.NALVSHLDGTTPVCEDIGR.S |
| 128276 | 397 | – | 415 | 1027.9935 | 2053.9724 | 2053.9739 | -0.71 | 0 | 54 | 8.9e-06 | 1Score **> 34** indicates **identity** Score **> 16** indicates **homology** | U | R.NALVSHLDGTTPVCEDIGR.S  + Deamidated (NQ) |
| 128277 | 397 | – | 415 | 685.6651 | 2053.9736 | 2053.9739 | -0.14 | 0 | 43 | 8.4e-05 | 1Score **> 34** indicates **identity** Score **> 15** indicates **homology** | U | R.NALVSHLDGTTPVCEDIGR.S  + Deamidated (NQ) |
| 128278 | 397 | – | 415 | 685.6652 | 2053.9736 | 2053.9739 | -0.11 | 0 | 56 | 5.2e-06 | 1Score **> 34** indicates **identity** Score **> 16** indicates **homology** | U | R.NALVSHLDGTTPVCEDIGR.S  + Deamidated (NQ) |
| 128279 | 397 | – | 415 | 1027.9948 | 2053.9751 | 2053.9739 | 0.61 | 0 | 93 | 2.1e-09 | 1Score **> 34** indicates **identity** Score **> 18** indicates **homology** | U | R.NALVSHLDGTTPVCEDIGR.S  + Deamidated (NQ) |
| 128280 | 397 | – | 415 | 1027.9949 | 2053.9752 | 2053.9739 | 0.63 | 0 | 94 | 1.6e-09 | 1Score **> 34** indicates **identity** Score **> 18** indicates **homology** | U | R.NALVSHLDGTTPVCEDIGR.S  + Deamidated (NQ) |
| 128281 | 397 | – | 415 | 1027.9957 | 2053.9768 | 2053.9739 | 1.44 | 0 | 94 | 1.7e-09 | 1Score **> 34** indicates **identity** Score **> 18** indicates **homology** | U | R.NALVSHLDGTTPVCEDIGR.S  + Deamidated (NQ) |
| 128282 | 397 | – | 415 | 1028.0024 | 2053.9903 | 2053.9739 | 7.98 | 0 | 61 | 1.9e-06 | 1Score **> 35** indicates **identity** Score **> 16** indicates **homology** | U | R.NALVSHLDGTTPVCEDIGR.S  + Deamidated (NQ) |
| 4101 | 416 | – | 422 | 405.2287 | 808.4429 | 808.4443 | -1.73 | 0 | 22 | 0.0088 | 1Score **> 22** indicates **identity** Score **> 14** indicates **homology** | U | R.SLLTYGR.R |
| 4102 | 416 | – | 422 | 405.2288 | 808.4430 | 808.4443 | -1.58 | 0 | 35 | 0.00054 | 1Score **> 22** indicates **identity** Score **> 15** indicates **homology** | U | R.SLLTYGR.R |
| 4103 | 416 | – | 422 | 405.2290 | 808.4434 | 808.4443 | -1.05 | 0 | 33 | 0.00086 | 1Score **> 22** indicates **identity** Score **> 15** indicates **homology** | U | R.SLLTYGR.R |
| 4105 | 416 | – | 422 | 405.2290 | 808.4435 | 808.4443 | -0.92 | 0 | 25 | 0.0041 | 1Score **> 22** indicates **identity** Score **> 14** indicates **homology** | U | R.SLLTYGR.R |
| 4106 | 416 | – | 422 | 405.2292 | 808.4438 | 808.4443 | -0.58 | 0 | 51 | 2e-05 | 1Score **> 22** indicates **identity** Score **> 16** indicates **homology** | U | R.SLLTYGR.R |
| 4107 | 416 | – | 422 | 405.2292 | 808.4438 | 808.4443 | -0.56 | 0 | 51 | 1.7e-05 | 1Score **> 22** indicates **identity** Score **> 16** indicates **homology** | U | R.SLLTYGR.R |
| 4108 | 416 | – | 422 | 405.2292 | 808.4439 | 808.4443 | -0.44 | 0 | 45 | 6.3e-05 | 1Score **> 22** indicates **identity** Score **> 15** indicates **homology** | U | R.SLLTYGR.R |
| 4109 | 416 | – | 422 | 405.2294 | 808.4443 | 808.4443 | 0.0025 | 0 | 44 | 7.1e-05 | 1Score **> 22** indicates **identity** Score **> 15** indicates **homology** | U | R.SLLTYGR.R |
| 4110 | 416 | – | 422 | 405.2296 | 808.4446 | 808.4443 | 0.38 | 0 | 29 | 0.0021 | 1Score **> 22** indicates **identity** Score **> 14** indicates **homology** | U | R.SLLTYGR.R |
| 4111 | 416 | – | 422 | 405.2296 | 808.4447 | 808.4443 | 0.47 | 0 | 44 | 7.4e-05 | 1Score **> 22** indicates **identity** Score **> 15** indicates **homology** | U | R.SLLTYGR.R |
| 4112 | 416 | – | 422 | 405.2296 | 808.4447 | 808.4443 | 0.47 | 0 | 27 | 0.0031 | 1Score **> 22** indicates **identity** Score **> 14** indicates **homology** | U | R.SLLTYGR.R |
| 4113 | 416 | – | 422 | 405.2299 | 808.4452 | 808.4443 | 1.13 | 0 | 26 | 0.0039 | 1Score **> 22** indicates **identity** Score **> 14** indicates **homology** | U | R.SLLTYGR.R |
| 39292 | 423 | – | 432 | 419.5615 | 1255.6628 | 1255.6673 | -3.59 | 1 | 37 | 0.00034 | 1Score **> 32** indicates **identity** Score **> 15** indicates **homology** | U | R.RIPLAEWESR.I |
| 39293 | 423 | – | 432 | 419.5617 | 1255.6633 | 1255.6673 | -3.21 | 1 | 22 | 0.0079 | 1Score **> 32** indicates **identity** Score **> 14** indicates **homology** | U | R.RIPLAEWESR.I |
| 39295 | 423 | – | 432 | 419.5622 | 1255.6649 | 1255.6673 | -1.89 | 1 | 28 | 0.0024 | 1Score **> 33** indicates **identity** Score **> 14** indicates **homology** | U | R.RIPLAEWESR.I |
| 39296 | 423 | – | 432 | 419.5623 | 1255.6649 | 1255.6673 | -1.87 | 1 | 21 | 0.01 | 1Score **> 33** indicates **identity** Score **> 14** indicates **homology** | U | R.RIPLAEWESR.I |
| 39297 | 423 | – | 432 | 419.5623 | 1255.6652 | 1255.6673 | -1.68 | 1 | 21 | 0.011 | 1Score **> 33** indicates **identity** Score **> 14** indicates **homology** | U | R.RIPLAEWESR.I |
| 39299 | 423 | – | 432 | 628.8402 | 1255.6658 | 1255.6673 | -1.14 | 1 | 32 | 0.0077 | 1Score **> 33** indicates **identity** Score **> 24** indicates **homology** | U | R.RIPLAEWESR.I |
| 39300 | 423 | – | 432 | 419.5626 | 1255.6658 | 1255.6673 | -1.14 | 1 | 32 | 0.00096 | 1Score **> 33** indicates **identity** Score **> 15** indicates **homology** | U | R.RIPLAEWESR.I |
| 39301 | 423 | – | 432 | 628.8403 | 1255.6660 | 1255.6673 | -1.04 | 1 | 29 | 0.022 | 1Score **> 33** indicates **identity** Score **> 25** indicates **homology** | U | R.RIPLAEWESR.I |
| 39302 | 423 | – | 432 | 419.5626 | 1255.6660 | 1255.6673 | -1.01 | 1 | 35 | 0.00053 | 1Score **> 33** indicates **identity** Score **> 15** indicates **homology** | U | R.RIPLAEWESR.I |
| 39303 | 423 | – | 432 | 419.5626 | 1255.6660 | 1255.6673 | -0.99 | 1 | 28 | 0.0026 | 1Score **> 33** indicates **identity** Score **> 14** indicates **homology** | U | R.RIPLAEWESR.I |
| 39304 | 423 | – | 432 | 628.8404 | 1255.6662 | 1255.6673 | -0.86 | 1 | 21 | 0.032 | 1Score **> 33** indicates **identity** Score **> 19** indicates **homology** | U | R.RIPLAEWESR.I |
| 39306 | 423 | – | 432 | 419.5627 | 1255.6664 | 1255.6673 | -0.70 | 1 | 27 | 0.003 | 1Score **> 33** indicates **identity** Score **> 14** indicates **homology** | U | R.RIPLAEWESR.I |
| 39307 | 423 | – | 432 | 628.8405 | 1255.6665 | 1255.6673 | -0.66 | 1 | 26 | 0.019 | 1Score **> 33** indicates **identity** Score **> 21** indicates **homology** | U | R.RIPLAEWESR.I |
| 39308 | 423 | – | 432 | 419.5628 | 1255.6665 | 1255.6673 | -0.65 | 1 | 24 | 0.0051 | 1Score **> 33** indicates **identity** Score **> 14** indicates **homology** | U | R.RIPLAEWESR.I |
| 39309 | 423 | – | 432 | 419.5628 | 1255.6665 | 1255.6673 | -0.64 | 1 | 33 | 0.0008 | 1Score **> 33** indicates **identity** Score **> 15** indicates **homology** | U | R.RIPLAEWESR.I |
| 39310 | 423 | – | 432 | 419.5628 | 1255.6665 | 1255.6673 | -0.64 | 1 | 19 | 0.016 | 1Score **> 33** indicates **identity** Score **> 14** indicates **homology** | U | R.RIPLAEWESR.I |
| 39311 | 423 | – | 432 | 419.5628 | 1255.6666 | 1255.6673 | -0.56 | 1 | 28 | 0.0023 | 1Score **> 33** indicates **identity** Score **> 14** indicates **homology** | U | R.RIPLAEWESR.I |
| 39312 | 423 | – | 432 | 419.5628 | 1255.6667 | 1255.6673 | -0.48 | 1 | 49 | 2.5e-05 | 1Score **> 33** indicates **identity** Score **> 16** indicates **homology** | U | R.RIPLAEWESR.I |
| 39313 | 423 | – | 432 | 419.5628 | 1255.6667 | 1255.6673 | -0.47 | 1 | 28 | 0.0022 | 1Score **> 33** indicates **identity** Score **> 14** indicates **homology** | U | R.RIPLAEWESR.I |
| 39315 | 423 | – | 432 | 419.5629 | 1255.6668 | 1255.6673 | -0.40 | 1 | 31 | 0.0013 | 1Score **> 33** indicates **identity** Score **> 14** indicates **homology** | U | R.RIPLAEWESR.I |
| 39316 | 423 | – | 432 | 419.5629 | 1255.6668 | 1255.6673 | -0.36 | 1 | 49 | 5.2e-05 | 1Score **> 33** indicates **identity** Score **> 19** indicates **homology** | U | R.RIPLAEWESR.I |
| 39317 | 423 | – | 432 | 419.5629 | 1255.6668 | 1255.6673 | -0.36 | 1 | 34 | 0.00065 | 1Score **> 33** indicates **identity** Score **> 15** indicates **homology** | U | R.RIPLAEWESR.I |
| 39318 | 423 | – | 432 | 419.5629 | 1255.6668 | 1255.6673 | -0.35 | 1 | 15 | 0.042 | 1Score **> 33** indicates **identity** Score **> 13** indicates **homology** | U | R.RIPLAEWESR.I |
| 39319 | 423 | – | 432 | 419.5629 | 1255.6669 | 1255.6673 | -0.34 | 1 | 28 | 0.018 | 1Score **> 33** indicates **identity** Score **> 23** indicates **homology** | U | R.RIPLAEWESR.I |
| 39321 | 423 | – | 432 | 419.5629 | 1255.6669 | 1255.6673 | -0.27 | 1 | 19 | 0.02 | 1Score **> 33** indicates **identity** Score **> 14** indicates **homology** | U | R.RIPLAEWESR.I |
| 39322 | 423 | – | 432 | 628.8408 | 1255.6670 | 1255.6673 | -0.21 | 1 | 28 | 0.016 | 1Score **> 33** indicates **identity** Score **> 23** indicates **homology** | U | R.RIPLAEWESR.I |
| 39323 | 423 | – | 432 | 419.5630 | 1255.6671 | 1255.6673 | -0.17 | 1 | 31 | 0.0012 | 1Score **> 33** indicates **identity** Score **> 14** indicates **homology** | U | R.RIPLAEWESR.I |
| 39324 | 423 | – | 432 | 628.8408 | 1255.6671 | 1255.6673 | -0.16 | 1 | 50 | 0.001 | 1Score **> 33** indicates **identity** | U | R.RIPLAEWESR.I |
| 39326 | 423 | – | 432 | 628.8409 | 1255.6672 | 1255.6673 | -0.056 | 1 | 47 | 0.00089 | 1Score **> 33** indicates **identity** Score **> 29** indicates **homology** | U | R.RIPLAEWESR.I |
| 39327 | 423 | – | 432 | 628.8409 | 1255.6673 | 1255.6673 | -0.022 | 1 | 49 | 0.0014 | 1Score **> 33** indicates **identity** | U | R.RIPLAEWESR.I |
| 39329 | 423 | – | 432 | 628.8409 | 1255.6673 | 1255.6673 | 0.027 | 1 | 41 | 0.0059 | 1Score **> 33** indicates **identity** Score **> 31** indicates **homology** | U | R.RIPLAEWESR.I |
| 39330 | 423 | – | 432 | 628.8410 | 1255.6675 | 1255.6673 | 0.14 | 1 | 54 | 0.00043 | 1Score **> 33** indicates **identity** | U | R.RIPLAEWESR.I |
| 39331 | 423 | – | 432 | 628.8410 | 1255.6675 | 1255.6673 | 0.17 | 1 | 49 | 0.0013 | 1Score **> 33** indicates **identity** | U | R.RIPLAEWESR.I |
| 39332 | 423 | – | 432 | 419.5631 | 1255.6676 | 1255.6673 | 0.23 | 1 | 34 | 0.00059 | 1Score **> 33** indicates **identity** Score **> 15** indicates **homology** | U | R.RIPLAEWESR.I |
| 39333 | 423 | – | 432 | 419.5632 | 1255.6676 | 1255.6673 | 0.28 | 1 | 35 | 0.00048 | 1Score **> 33** indicates **identity** Score **> 15** indicates **homology** | U | R.RIPLAEWESR.I |
| 39334 | 423 | – | 432 | 419.5632 | 1255.6677 | 1255.6673 | 0.30 | 1 | 37 | 0.00034 | 1Score **> 33** indicates **identity** Score **> 15** indicates **homology** | U | R.RIPLAEWESR.I |
| 39335 | 423 | – | 432 | 419.5632 | 1255.6677 | 1255.6673 | 0.34 | 1 | 24 | 0.0056 | 1Score **> 33** indicates **identity** Score **> 14** indicates **homology** | U | R.RIPLAEWESR.I |
| 39336 | 423 | – | 432 | 419.5632 | 1255.6678 | 1255.6673 | 0.39 | 1 | 27 | 0.0032 | 1Score **> 33** indicates **identity** Score **> 14** indicates **homology** | U | R.RIPLAEWESR.I |
| 39337 | 423 | – | 432 | 628.8412 | 1255.6678 | 1255.6673 | 0.41 | 1 | 55 | 0.00016 | 1Score **> 33** indicates **identity** Score **> 30** indicates **homology** | U | R.RIPLAEWESR.I |
| 39338 | 423 | – | 432 | 419.5632 | 1255.6678 | 1255.6673 | 0.41 | 1 | 23 | 0.0077 | 1Score **> 33** indicates **identity** Score **> 14** indicates **homology** | U | R.RIPLAEWESR.I |
| 39339 | 423 | – | 432 | 419.5632 | 1255.6679 | 1255.6673 | 0.46 | 1 | 36 | 0.00046 | 1Score **> 33** indicates **identity** Score **> 15** indicates **homology** | U | R.RIPLAEWESR.I |
| 39341 | 423 | – | 432 | 419.5633 | 1255.6679 | 1255.6673 | 0.52 | 1 | 21 | 0.011 | 1Score **> 33** indicates **identity** Score **> 14** indicates **homology** | U | R.RIPLAEWESR.I |
| 39343 | 423 | – | 432 | 419.5633 | 1255.6681 | 1255.6673 | 0.67 | 1 | 38 | 0.00029 | 1Score **> 33** indicates **identity** Score **> 15** indicates **homology** | U | R.RIPLAEWESR.I |
| 39344 | 423 | – | 432 | 419.5633 | 1255.6682 | 1255.6673 | 0.74 | 1 | 38 | 0.011 | 1Score **> 33** indicates **identity** Score **> 31** indicates **homology** | U | R.RIPLAEWESR.I |
| 39345 | 423 | – | 432 | 419.5634 | 1255.6683 | 1255.6673 | 0.77 | 1 | 33 | 0.00084 | 1Score **> 33** indicates **identity** Score **> 15** indicates **homology** | U | R.RIPLAEWESR.I |
| 39347 | 423 | – | 432 | 419.5634 | 1255.6683 | 1255.6673 | 0.82 | 1 | 35 | 0.00055 | 1Score **> 33** indicates **identity** Score **> 15** indicates **homology** | U | R.RIPLAEWESR.I |
| 39348 | 423 | – | 432 | 419.5634 | 1255.6683 | 1255.6673 | 0.83 | 1 | 33 | 0.00088 | 1Score **> 33** indicates **identity** Score **> 15** indicates **homology** | U | R.RIPLAEWESR.I |
| 39352 | 423 | – | 432 | 628.8416 | 1255.6686 | 1255.6673 | 1.01 | 1 | 29 | 0.022 | 1Score **> 33** indicates **identity** Score **> 25** indicates **homology** | U | R.RIPLAEWESR.I |
| 39353 | 423 | – | 432 | 419.5635 | 1255.6686 | 1255.6673 | 1.03 | 1 | 23 | 0.0068 | 1Score **> 33** indicates **identity** Score **> 14** indicates **homology** | U | R.RIPLAEWESR.I |
| 39355 | 423 | – | 432 | 419.5636 | 1255.6690 | 1255.6673 | 1.40 | 1 | 31 | 0.0012 | 1Score **> 33** indicates **identity** Score **> 14** indicates **homology** | U | R.RIPLAEWESR.I |
| 39356 | 423 | – | 432 | 419.5637 | 1255.6693 | 1255.6673 | 1.59 | 1 | 20 | 0.013 | 1Score **> 33** indicates **identity** Score **> 14** indicates **homology** | U | R.RIPLAEWESR.I |
| 39358 | 423 | – | 432 | 419.5638 | 1255.6695 | 1255.6673 | 1.76 | 1 | 33 | 0.00088 | 1Score **> 33** indicates **identity** Score **> 15** indicates **homology** | U | R.RIPLAEWESR.I |
| 39360 | 423 | – | 432 | 419.5639 | 1255.6698 | 1255.6673 | 2.03 | 1 | 36 | 0.00047 | 1Score **> 33** indicates **identity** Score **> 15** indicates **homology** | U | R.RIPLAEWESR.I |
| 39361 | 423 | – | 432 | 419.5640 | 1255.6702 | 1255.6673 | 2.36 | 1 | 28 | 0.0038 | 1Score **> 33** indicates **identity** Score **> 16** indicates **homology** | U | R.RIPLAEWESR.I |
| 22696 | 424 | – | 432 | 550.7897 | 1099.5649 | 1099.5662 | -1.19 | 0 | 37 | 0.00037 | 1Score **> 31** indicates **identity** Score **> 15** indicates **homology** | U | R.IPLAEWESR.I |
| 22697 | 424 | – | 432 | 550.7903 | 1099.5661 | 1099.5662 | -0.075 | 0 | 58 | 7.7e-05 | 1Score **> 31** indicates **identity** Score **> 29** indicates **homology** | U | R.IPLAEWESR.I |
| 22698 | 424 | – | 432 | 550.7906 | 1099.5666 | 1099.5662 | 0.36 | 0 | 54 | 0.00014 | 1Score **> 32** indicates **identity** Score **> 28** indicates **homology** | U | R.IPLAEWESR.I |
| 33449 | 433 | – | 442 | 601.8136 | 1201.6126 | 1201.6125 | 0.11 | 0 | 51 | 0.00072 | 1Score **> 33** indicates **identity** Score **> 32** indicates **homology** | U | R.IQEVDAQMLR.D |
| 33450 | 433 | – | 442 | 601.8137 | 1201.6129 | 1201.6125 | 0.32 | 0 | 44 | 0.0031 | 1Score **> 33** indicates **identity** Score **> 31** indicates **homology** | U | R.IQEVDAQMLR.D |
| 33451 | 433 | – | 442 | 601.8141 | 1201.6137 | 1201.6125 | 1.03 | 0 | 60 | 0.00011 | 1Score **> 33** indicates **identity** | U | R.IQEVDAQMLR.D |
| 104554 | 433 | – | 447 | 602.6335 | 1804.8787 | 1804.8811 | -1.33 | 1 | 28 | 0.0025 | 1Score **> 35** indicates **identity** Score **> 14** indicates **homology** | U | R.IQEVDAQMLRDICSK.Y |
| 170883 | 448 | – | 470 | 1368.6183 | 2735.2220 | 2735.2326 | -3.87 | 0 | 77 | 5.7e-08 | 1Score **> 32** indicates **identity** Score **> 17** indicates **homology** | U | K.YFYDQCPAVAGYGPIEQLPDYNR.I |
| 170884 | 448 | – | 470 | 912.7492 | 2735.2256 | 2735.2326 | -2.54 | 0 | 30 | 0.0017 | 1Score **> 32** indicates **identity** Score **> 14** indicates **homology** | U | K.YFYDQCPAVAGYGPIEQLPDYNR.I |
| 170885 | 448 | – | 470 | 912.7494 | 2735.2262 | 2735.2326 | -2.32 | 0 | 33 | 0.00075 | 1Score **> 32** indicates **identity** Score **> 15** indicates **homology** | U | K.YFYDQCPAVAGYGPIEQLPDYNR.I |
| 170886 | 448 | – | 470 | 912.7500 | 2735.2281 | 2735.2326 | -1.64 | 0 | 22 | 0.0096 | 1Score **> 32** indicates **identity** Score **> 14** indicates **homology** | U | K.YFYDQCPAVAGYGPIEQLPDYNR.I |
| 170887 | 448 | – | 470 | 1368.6220 | 2735.2295 | 2735.2326 | -1.12 | 0 | 68 | 3.9e-07 | 1Score **> 32** indicates **identity** Score **> 17** indicates **homology** | U | K.YFYDQCPAVAGYGPIEQLPDYNR.I |
| 170888 | 448 | – | 470 | 1368.6223 | 2735.2300 | 2735.2326 | -0.93 | 0 | 69 | 3.5e-07 | 1Score **> 32** indicates **identity** Score **> 17** indicates **homology** | U | K.YFYDQCPAVAGYGPIEQLPDYNR.I |
| 170889 | 448 | – | 470 | 912.7509 | 2735.2308 | 2735.2326 | -0.64 | 0 | 45 | 6.2e-05 | 1Score **> 32** indicates **identity** Score **> 15** indicates **homology** | U | K.YFYDQCPAVAGYGPIEQLPDYNR.I |
| 170890 | 448 | – | 470 | 912.7513 | 2735.2319 | 2735.2326 | -0.24 | 0 | 53 | 1.1e-05 | 1Score **> 32** indicates **identity** Score **> 16** indicates **homology** | U | K.YFYDQCPAVAGYGPIEQLPDYNR.I |
| 170891 | 448 | – | 470 | 912.7516 | 2735.2329 | 2735.2326 | 0.13 | 0 | 77 | 6.1e-08 | 1Score **> 32** indicates **identity** Score **> 17** indicates **homology** | U | K.YFYDQCPAVAGYGPIEQLPDYNR.I |
| 170892 | 448 | – | 470 | 912.7517 | 2735.2332 | 2735.2326 | 0.22 | 0 | 78 | 4.7e-08 | 1Score **> 33** indicates **identity** Score **> 17** indicates **homology** | U | K.YFYDQCPAVAGYGPIEQLPDYNR.I |
| 170893 | 448 | – | 470 | 1368.6241 | 2735.2337 | 2735.2326 | 0.40 | 0 | 85 | 9.7e-09 | 1Score **> 32** indicates **identity** Score **> 18** indicates **homology** | U | K.YFYDQCPAVAGYGPIEQLPDYNR.I |
| 170894 | 448 | – | 470 | 912.7519 | 2735.2340 | 2735.2326 | 0.51 | 0 | 70 | 2.5e-07 | 1Score **> 33** indicates **identity** Score **> 17** indicates **homology** | U | K.YFYDQCPAVAGYGPIEQLPDYNR.I |
| 170895 | 448 | – | 470 | 912.7520 | 2735.2340 | 2735.2326 | 0.52 | 0 | 82 | 2e-08 | 1Score **> 33** indicates **identity** Score **> 18** indicates **homology** | U | K.YFYDQCPAVAGYGPIEQLPDYNR.I |
| 170896 | 448 | – | 470 | 1368.6243 | 2735.2340 | 2735.2326 | 0.53 | 0 | 93 | 2e-09 | 1Score **> 33** indicates **identity** Score **> 18** indicates **homology** | U | K.YFYDQCPAVAGYGPIEQLPDYNR.I |
| 170897 | 448 | – | 470 | 1368.6243 | 2735.2341 | 2735.2326 | 0.55 | 0 | 78 | 5.3e-08 | 1Score **> 33** indicates **identity** Score **> 17** indicates **homology** | U | K.YFYDQCPAVAGYGPIEQLPDYNR.I |
| 170898 | 448 | – | 470 | 1368.6244 | 2735.2342 | 2735.2326 | 0.58 | 0 | 87 | 7.2e-09 | 1Score **> 33** indicates **identity** Score **> 18** indicates **homology** | U | K.YFYDQCPAVAGYGPIEQLPDYNR.I |
| 170899 | 448 | – | 470 | 912.7520 | 2735.2343 | 2735.2326 | 0.62 | 0 | 84 | 1.3e-08 | 1Score **> 33** indicates **identity** Score **> 18** indicates **homology** | U | K.YFYDQCPAVAGYGPIEQLPDYNR.I |
| 170900 | 448 | – | 470 | 912.7521 | 2735.2345 | 2735.2326 | 0.71 | 0 | 42 | 0.00012 | 1Score **> 33** indicates **identity** Score **> 15** indicates **homology** | U | K.YFYDQCPAVAGYGPIEQLPDYNR.I |
| 170901 | 448 | – | 470 | 912.7522 | 2735.2349 | 2735.2326 | 0.85 | 0 | 77 | 6.4e-08 | 1Score **> 33** indicates **identity** Score **> 17** indicates **homology** | U | K.YFYDQCPAVAGYGPIEQLPDYNR.I |
| 170902 | 448 | – | 470 | 1368.6248 | 2735.2351 | 2735.2326 | 0.91 | 0 | 69 | 3.4e-07 | 1Score **> 33** indicates **identity** Score **> 17** indicates **homology** | U | K.YFYDQCPAVAGYGPIEQLPDYNR.I |
| 170903 | 448 | – | 470 | 912.7527 | 2735.2364 | 2735.2326 | 1.40 | 0 | 52 | 1.4e-05 | 1Score **> 33** indicates **identity** Score **> 16** indicates **homology** | U | K.YFYDQCPAVAGYGPIEQLPDYNR.I |
| 170904 | 448 | – | 470 | 912.7528 | 2735.2366 | 2735.2326 | 1.46 | 0 | 21 | 0.0099 | 1Score **> 33** indicates **identity** Score **> 14** indicates **homology** | U | K.YFYDQCPAVAGYGPIEQLPDYNR.I |
| 170905 | 448 | – | 470 | 912.7539 | 2735.2399 | 2735.2326 | 2.66 | 0 | 41 | 0.00027 | 1Score **> 33** indicates **identity** Score **> 18** indicates **homology** | U | K.YFYDQCPAVAGYGPIEQLPDYNR.I |
| 170955 | 448 | – | 470 | 913.0842 | 2736.2307 | 2736.2166 | 5.14 | 0 | 32 | 0.0011 | 1Score **> 32** indicates **identity** Score **> 14** indicates **homology** | U | K.YFYDQCPAVAGYGPIEQLPDYNR.I  + Deamidated (NQ) |
| 170956 | 448 | – | 470 | 1369.1235 | 2736.2325 | 2736.2166 | 5.80 | 0 | 64 | 1e-06 | 1Score **> 33** indicates **identity** Score **> 16** indicates **homology** | U | K.YFYDQCPAVAGYGPIEQLPDYNR.I  + Deamidated (NQ) |
| 170957 | 448 | – | 470 | 913.0851 | 2736.2335 | 2736.2166 | 6.19 | 0 | 21 | 0.011 | 1Score **> 33** indicates **identity** Score **> 14** indicates **homology** | U | K.YFYDQCPAVAGYGPIEQLPDYNR.I  + Deamidated (NQ) |
| 170960 | 448 | – | 470 | 913.0865 | 2736.2375 | 2736.2166 | 7.65 | 0 | 20 | 0.014 | 1Score **> 33** indicates **identity** Score **> 14** indicates **homology** | U | K.YFYDQCPAVAGYGPIEQLPDYNR.I  + Deamidated (NQ) |
| 170961 | 448 | – | 470 | 913.0872 | 2736.2399 | 2736.2166 | 8.52 | 0 | 17 | 0.027 | 1Score **> 33** indicates **identity** Score **> 14** indicates **homology** | U | K.YFYDQCPAVAGYGPIEQLPDYNR.I  + Deamidated (NQ) |
| 170962 | 448 | – | 470 | 913.0877 | 2736.2412 | 2736.2166 | 9.01 | 0 | 24 | 0.0054 | 1Score **> 33** indicates **identity** Score **> 14** indicates **homology** | U | K.YFYDQCPAVAGYGPIEQLPDYNR.I  + Deamidated (NQ) |
| 171014 | 448 | – | 470 | 1369.6209 | 2737.2273 | 2737.2006 | 9.75 | 0 | 18 | 0.019 | 1Score **> 32** indicates **identity** Score **> 14** indicates **homology** | U | K.YFYDQCPAVAGYGPIEQLPDYNR.I  + 2 Deamidated (NQ) |
| 180031 | 448 | – | 472 | 1002.4835 | 3004.4286 | 3004.4178 | 3.62 | 1 | 52 | 1.3e-05 | 1Score **> 36** indicates **identity** Score **> 16** indicates **homology** | U | K.YFYDQCPAVAGYGPIEQLPDYNRIR.S |
| 180032 | 448 | – | 472 | 1002.4836 | 3004.4289 | 3004.4178 | 3.71 | 1 | 50 | 1.9e-05 | 1Score **> 36** indicates **identity** Score **> 16** indicates **homology** | U | K.YFYDQCPAVAGYGPIEQLPDYNRIR.S |
| 180033 | 448 | – | 472 | 1002.4839 | 3004.4300 | 3004.4178 | 4.07 | 1 | 33 | 0.00089 | 1Score **> 36** indicates **identity** Score **> 15** indicates **homology** | U | K.YFYDQCPAVAGYGPIEQLPDYNRIR.S |
| 180050 | 448 | – | 472 | 1002.8098 | 3005.4074 | 3005.4018 | 1.89 | 1 | 28 | 0.0023 | 1Score **> 35** indicates **identity** Score **> 14** indicates **homology** | U | K.YFYDQCPAVAGYGPIEQLPDYNRIR.S  + Deamidated (NQ) |
| 180051 | 448 | – | 472 | 1002.8108 | 3005.4107 | 3005.4018 | 2.97 | 1 | 24 | 0.0051 | 1Score **> 35** indicates **identity** Score **> 14** indicates **homology** | U | K.YFYDQCPAVAGYGPIEQLPDYNRIR.S  + Deamidated (NQ) |
| 180087 | 448 | – | 472 | 1003.1403 | 3006.3989 | 3006.3858 | 4.38 | 1 | 24 | 0.0053 | 1Score **> 35** indicates **identity** Score **> 14** indicates **homology** | U | K.YFYDQCPAVAGYGPIEQLPDYNRIR.S  + 2 Deamidated (NQ) |
| 8264 | 473 | – | 479 | 448.7251 | 895.4357 | 895.4374 | -1.97 | 0 | 33 | 0.00077 | 1Score **> 27** indicates **identity** Score **> 15** indicates **homology** | U | R.SGMFWLR.F |
| 8265 | 473 | – | 479 | 448.7257 | 895.4369 | 895.4374 | -0.65 | 0 | 22 | 0.0096 | 1Score **> 27** indicates **identity** Score **> 14** indicates **homology** | U | R.SGMFWLR.F |
| 8266 | 473 | – | 479 | 448.7258 | 895.4370 | 895.4374 | -0.50 | 0 | 43 | 0.0015 | 1Score **> 27** indicates **identity** | U | R.SGMFWLR.F |
| 8267 | 473 | – | 479 | 448.7258 | 895.4370 | 895.4374 | -0.46 | 0 | 47 | 0.0006 | 1Score **> 27** indicates **identity** | U | R.SGMFWLR.F |
| 8268 | 473 | – | 479 | 448.7263 | 895.4381 | 895.4374 | 0.72 | 0 | 47 | 0.00042 | 1Score **> 25** indicates **identity** | U | R.SGMFWLR.F |
| 8269 | 473 | – | 479 | 448.7264 | 895.4382 | 895.4374 | 0.83 | 0 | 34 | 0.0012 | 1Score **> 25** indicates **identity** Score **> 17** indicates **homology** | U | R.SGMFWLR.F |
| 8270 | 473 | – | 479 | 448.7264 | 895.4383 | 895.4374 | 0.91 | 0 | 33 | 0.00084 | 1Score **> 25** indicates **identity** Score **> 15** indicates **homology** | U | R.SGMFWLR.F |
| 8271 | 473 | – | 479 | 448.7273 | 895.4401 | 895.4374 | 3.01 | 0 | 30 | 0.0015 | 1Score **> 27** indicates **identity** Score **> 14** indicates **homology** | U | R.SGMFWLR.F |
| 8280 | 473 | – | 479 | 448.7293 | 895.4441 | 895.4374 | 7.40 | 0 | 32 | 0.0022 | 1Score **> 27** indicates **identity** Score **> 18** indicates **homology** | U | R.SGMFWLR.F |
| 9334 | 473 | – | 479 | 456.7244 | 911.4342 | 911.4324 | 1.99 | 0 | 26 | 0.0036 | 1Score **> 26** indicates **identity** Score **> 14** indicates **homology** | U | R.SGMFWLR.F  + Oxidation (M) |
| 17349 | 473 | – | 480 | 522.2601 | 1042.5057 | 1042.5059 | -0.17 | 1 | 54 | 9.2e-06 | 1Score **> 28** indicates **identity** Score **> 16** indicates **homology** | U | R.SGMFWLRF.- |
| 17350 | 473 | – | 480 | 522.2605 | 1042.5065 | 1042.5059 | 0.63 | 1 | 37 | 0.00032 | 1Score **> 28** indicates **identity** Score **> 15** indicates **homology** | U | R.SGMFWLRF.- |
| 17351 | 473 | – | 480 | 522.2608 | 1042.5070 | 1042.5059 | 1.09 | 1 | 35 | 0.00049 | 1Score **> 28** indicates **identity** Score **> 15** indicates **homology** | U | R.SGMFWLRF.- |
| 17353 | 473 | – | 480 | 522.2615 | 1042.5084 | 1042.5059 | 2.39 | 1 | 52 | 1.3e-05 | 1Score **> 28** indicates **identity** Score **> 16** indicates **homology** | U | R.SGMFWLRF.- |

---

```
ID   QCR1_MOUSE              Reviewed;         480 AA.
AC   Q9CZ13; Q3TV75; Q9CWL6;
DT   20-JUN-2001, integrated into UniProtKB/Swiss-Prot.
DT   27-JUL-2011, sequence version 2.
DT   28-JUN-2023, entry version 182.
DE   RecName: Full=Cytochrome b-c1 complex subunit 1, mitochondrial;
DE   AltName: Full=Complex III subunit 1;
DE   AltName: Full=Core protein I;
DE   AltName: Full=Ubiquinol-cytochrome-c reductase complex core protein 1;
DE   Flags: Precursor;
GN   Name=Uqcrc1;
OS   Mus musculus (Mouse).
OC   Eukaryota; Metazoa; Chordata; Craniata; Vertebrata; Euteleostomi; Mammalia;
OC   Eutheria; Euarchontoglires; Glires; Rodentia; Myomorpha; Muroidea; Muridae;
OC   Murinae; Mus; Mus.
OX   NCBI_TaxID=10090;
RN   [1]
RP   NUCLEOTIDE SEQUENCE [LARGE SCALE MRNA].
RC   STRAIN=C57BL/6J; TISSUE=Bone marrow, and Embryo;
RX   PubMed=16141072; DOI=10.1126/science.1112014;
RA   Carninci P., Kasukawa T., Katayama S., Gough J., Frith M.C., Maeda N.,
RA   Oyama R., Ravasi T., Lenhard B., Wells C., Kodzius R., Shimokawa K.,
RA   Bajic V.B., Brenner S.E., Batalov S., Forrest A.R., Zavolan M., Davis M.J.,
RA   Wilming L.G., Aidinis V., Allen J.E., Ambesi-Impiombato A., Apweiler R.,
RA   Aturaliya R.N., Bailey T.L., Bansal M., Baxter L., Beisel K.W., Bersano T.,
RA   Bono H., Chalk A.M., Chiu K.P., Choudhary V., Christoffels A.,
RA   Clutterbuck D.R., Crowe M.L., Dalla E., Dalrymple B.P., de Bono B.,
RA   Della Gatta G., di Bernardo D., Down T., Engstrom P., Fagiolini M.,
RA   Faulkner G., Fletcher C.F., Fukushima T., Furuno M., Futaki S.,
RA   Gariboldi M., Georgii-Hemming P., Gingeras T.R., Gojobori T., Green R.E.,
RA   Gustincich S., Harbers M., Hayashi Y., Hensch T.K., Hirokawa N., Hill D.,
RA   Huminiecki L., Iacono M., Ikeo K., Iwama A., Ishikawa T., Jakt M.,
RA   Kanapin A., Katoh M., Kawasawa Y., Kelso J., Kitamura H., Kitano H.,
RA   Kollias G., Krishnan S.P., Kruger A., Kummerfeld S.K., Kurochkin I.V.,
RA   Lareau L.F., Lazarevic D., Lipovich L., Liu J., Liuni S., McWilliam S.,
RA   Madan Babu M., Madera M., Marchionni L., Matsuda H., Matsuzawa S., Miki H.,
RA   Mignone F., Miyake S., Morris K., Mottagui-Tabar S., Mulder N., Nakano N.,
RA   Nakauchi H., Ng P., Nilsson R., Nishiguchi S., Nishikawa S., Nori F.,
RA   Ohara O., Okazaki Y., Orlando V., Pang K.C., Pavan W.J., Pavesi G.,
RA   Pesole G., Petrovsky N., Piazza S., Reed J., Reid J.F., Ring B.Z.,
RA   Ringwald M., Rost B., Ruan Y., Salzberg S.L., Sandelin A., Schneider C.,
RA   Schoenbach C., Sekiguchi K., Semple C.A., Seno S., Sessa L., Sheng Y.,
RA   Shibata Y., Shimada H., Shimada K., Silva D., Sinclair B., Sperling S.,
RA   Stupka E., Sugiura K., Sultana R., Takenaka Y., Taki K., Tammoja K.,
RA   Tan S.L., Tang S., Taylor M.S., Tegner J., Teichmann S.A., Ueda H.R.,
RA   van Nimwegen E., Verardo R., Wei C.L., Yagi K., Yamanishi H.,
RA   Zabarovsky E., Zhu S., Zimmer A., Hide W., Bult C., Grimmond S.M.,
RA   Teasdale R.D., Liu E.T., Brusic V., Quackenbush J., Wahlestedt C.,
RA   Mattick J.S., Hume D.A., Kai C., Sasaki D., Tomaru Y., Fukuda S.,
RA   Kanamori-Katayama M., Suzuki M., Aoki J., Arakawa T., Iida J., Imamura K.,
RA   Itoh M., Kato T., Kawaji H., Kawagashira N., Kawashima T., Kojima M.,
RA   Kondo S., Konno H., Nakano K., Ninomiya N., Nishio T., Okada M., Plessy C.,
RA   Shibata K., Shiraki T., Suzuki S., Tagami M., Waki K., Watahiki A.,
RA   Okamura-Oho Y., Suzuki H., Kawai J., Hayashizaki Y.;
RT   "The transcriptional landscape of the mammalian genome.";
RL   Science 309:1559-1563(2005).
RN   [2]
RP   NUCLEOTIDE SEQUENCE [LARGE SCALE GENOMIC DNA].
RA   Mural R.J., Adams M.D., Myers E.W., Smith H.O., Venter J.C.;
RL   Submitted (JUL-2005) to the EMBL/GenBank/DDBJ databases.
RN   [3]
RP   PROTEIN SEQUENCE OF 59-80; 86-99; 112-134; 143-163; 214-222; 229-248;
RP   256-276; 379-390; 397-442; 448-470 AND 473-479, AND IDENTIFICATION BY MASS
RP   SPECTROMETRY.
RC   STRAIN=C57BL/6J; TISSUE=Brain, and Hippocampus;
RA   Lubec G., Kang S.U., Klug S., Yang J.W., Zigmond M.;
RL   Submitted (JUL-2007) to UniProtKB.
RN   [4]
RP   SUBUNIT.
RX   PubMed=19026783; DOI=10.1016/j.molcel.2008.10.021;
RA   Acin-Perez R., Fernandez-Silva P., Peleato M.L., Perez-Martos A.,
RA   Enriquez J.A.;
RT   "Respiratory active mitochondrial supercomplexes.";
RL   Mol. Cell 32:529-539(2008).
RN   [5]
RP   IDENTIFICATION BY MASS SPECTROMETRY [LARGE SCALE ANALYSIS].
RC   TISSUE=Brain, Brown adipose tissue, Heart, Kidney, Liver, Lung,
RC   Pancreas, Spleen, and Testis;
RX   PubMed=21183079; DOI=10.1016/j.cell.2010.12.001;
RA   Huttlin E.L., Jedrychowski M.P., Elias J.E., Goswami T., Rad R.,
RA   Beausoleil S.A., Villen J., Haas W., Sowa M.E., Gygi S.P.;
RT   "A tissue-specific atlas of mouse protein phosphorylation and expression.";
RL   Cell 143:1174-1189(2010).
RN   [6]
RP   SUCCINYLATION [LARGE SCALE ANALYSIS] AT LYS-163, AND IDENTIFICATION BY MASS
RP   SPECTROMETRY [LARGE SCALE ANALYSIS].
RC   TISSUE=Liver;
RX   PubMed=23806337; DOI=10.1016/j.molcel.2013.06.001;
RA   Park J., Chen Y., Tishkoff D.X., Peng C., Tan M., Dai L., Xie Z., Zhang Y.,
RA   Zwaans B.M., Skinner M.E., Lombard D.B., Zhao Y.;
RT   "SIRT5-mediated lysine desuccinylation impacts diverse metabolic
RT   pathways.";
RL   Mol. Cell 50:919-930(2013).
RN   [7]
RP   ACETYLATION [LARGE SCALE ANALYSIS] AT LYS-111; LYS-138; LYS-163 AND
RP   LYS-248, AND IDENTIFICATION BY MASS SPECTROMETRY [LARGE SCALE ANALYSIS].
RC   TISSUE=Liver;
RX   PubMed=23576753; DOI=10.1073/pnas.1302961110;
RA   Rardin M.J., Newman J.C., Held J.M., Cusack M.P., Sorensen D.J., Li B.,
RA   Schilling B., Mooney S.D., Kahn C.R., Verdin E., Gibson B.W.;
RT   "Label-free quantitative proteomics of the lysine acetylome in mitochondria
RT   identifies substrates of SIRT3 in metabolic pathways.";
RL   Proc. Natl. Acad. Sci. U.S.A. 110:6601-6606(2013).
RN   [8]
RP   TISSUE SPECIFICITY, AND DISRUPTION PHENOTYPE.
RX   PubMed=30666338; DOI=10.1007/s00018-019-03007-6;
RA   Shan W., Li J., Xu W., Li H., Zuo Z.;
RT   "Critical role of UQCRC1 in embryo survival, brain ischemic tolerance and
RT   normal cognition in mice.";
RL   Cell. Mol. Life Sci. 76:1381-1396(2019).
RN   [9]
RP   INTERACTION WITH UQCC6.
RX   PubMed=32161263; DOI=10.1038/s41467-020-14999-2;
RA   Zhang S., Reljic B., Liang C., Kerouanton B., Francisco J.C., Peh J.H.,
RA   Mary C., Jagannathan N.S., Olexiouk V., Tang C., Fidelito G., Nama S.,
RA   Cheng R.K., Wee C.L., Wang L.C., Duek Roggli P., Sampath P., Lane L.,
RA   Petretto E., Sobota R.M., Jesuthasan S., Tucker-Kellogg L., Reversade B.,
RA   Menschaert G., Sun L., Stroud D.A., Ho L.;
RT   "Mitochondrial peptide BRAWNIN is essential for vertebrate respiratory
RT   complex III assembly.";
RL   Nat. Commun. 11:1312-1312(2020).
RN   [10]
RP   INTERACTION WITH STMP1.
RX   PubMed=35101990; DOI=10.1073/pnas.2120476119;
RA   Makarewich C.A., Munir A.Z., Bezprozvannaya S., Gibson A.M., Young Kim S.,
RA   Martin-Sandoval M.S., Mathews T.P., Szweda L.I., Bassel-Duby R.,
RA   Olson E.N.;
RT   "The cardiac-enriched microprotein mitolamban regulates mitochondrial
RT   respiratory complex assembly and function in mice.";
RL   Proc. Natl. Acad. Sci. U.S.A. 119:0-0(2022).
CC   -!- FUNCTION: Component of the ubiquinol-cytochrome c oxidoreductase, a
CC       multisubunit transmembrane complex that is part of the mitochondrial
CC       electron transport chain which drives oxidative phosphorylation. The
CC       respiratory chain contains 3 multisubunit complexes succinate
CC       dehydrogenase (complex II, CII), ubiquinol-cytochrome c oxidoreductase
CC       (cytochrome b-c1 complex, complex III, CIII) and cytochrome c oxidase
CC       (complex IV, CIV), that cooperate to transfer electrons derived from
CC       NADH and succinate to molecular oxygen, creating an electrochemical
CC       gradient over the inner membrane that drives transmembrane transport
CC       and the ATP synthase. The cytochrome b-c1 complex catalyzes electron
CC       transfer from ubiquinol to cytochrome c, linking this redox reaction to
CC       translocation of protons across the mitochondrial inner membrane, with
CC       protons being carried across the membrane as hydrogens on the quinol.
CC       In the process called Q cycle, 2 protons are consumed from the matrix,
CC       4 protons are released into the intermembrane space and 2 electrons are
CC       passed to cytochrome c (By similarity). The 2 core subunits UQCRC1/QCR1
CC       and UQCRC2/QCR2 are homologous to the 2 mitochondrial-processing
CC       peptidase (MPP) subunits beta-MPP and alpha-MPP respectively, and they
CC       seem to have preserved their MPP processing properties. May be involved
CC       in the in situ processing of UQCRFS1 into the mature Rieske protein and
CC       its mitochondrial targeting sequence (MTS)/subunit 9 when incorporated
CC       into complex III (By similarity). Seems to play an important role in
CC       the maintenance of proper mitochondrial function in nigral dopaminergic
CC       neurons (By similarity). {ECO:0000250|UniProtKB:P07256,
CC       ECO:0000250|UniProtKB:P31800, ECO:0000250|UniProtKB:P31930}.
CC   -!- SUBUNIT: Component of the ubiquinol-cytochrome c oxidoreductase
CC       (cytochrome b-c1 complex, complex III, CIII), a multisubunit enzyme
CC       composed of 11 subunits. The complex is composed of 3 respiratory
CC       subunits cytochrome b, cytochrome c1 and Rieske protein UQCRFS1, 2 core
CC       protein subunits UQCRC1/QCR1 and UQCRC2/QCR2, and 6 low-molecular
CC       weight protein subunits UQCRH/QCR6, UQCRB/QCR7, UQCRQ/QCR8,
CC       UQCR10/QCR9, UQCR11/QCR10 and subunit 9, the cleavage product of Rieske
CC       protein UQCRFS1 (By similarity). The complex exists as an obligatory
CC       dimer and forms supercomplexes (SCs) in the inner mitochondrial
CC       membrane with NADH-ubiquinone oxidoreductase (complex I, CI) and
CC       cytochrome c oxidase (complex IV, CIV), resulting in different
CC       assemblies (supercomplex SCI(1)III(2)IV(1) and megacomplex
CC       MCI(2)III(2)IV(2)) (PubMed:19026783). Interacts with UQCC6
CC       (PubMed:32161263). Interacts with STMP1 (PubMed:35101990).
CC       {ECO:0000250|UniProtKB:P31800, ECO:0000269|PubMed:19026783,
CC       ECO:0000269|PubMed:32161263, ECO:0000269|PubMed:35101990}.
CC   -!- SUBCELLULAR LOCATION: Mitochondrion inner membrane
CC       {ECO:0000250|UniProtKB:P07256}; Peripheral membrane protein
CC       {ECO:0000250|UniProtKB:P07256}; Matrix side
CC       {ECO:0000250|UniProtKB:P07256}.
CC   -!- TISSUE SPECIFICITY: Expressed in neurons and astrocytes of the cerebral
CC       cortex and hippocampus (at protein level).
CC       {ECO:0000269|PubMed:30666338}.
CC   -!- PTM: Acetylation of Lys-138 is observed in liver mitochondria from
CC       fasted mice but not from fed mice.
CC   -!- DISRUPTION PHENOTYPE: Results in early embryonic lethality.
CC       {ECO:0000269|PubMed:30666338}.
CC   -!- SIMILARITY: Belongs to the peptidase M16 family. UQCRC1/QCR1 subfamily.
CC       {ECO:0000305}.
CC   ---------------------------------------------------------------------------
CC   Copyrighted by the UniProt Consortium, see https://www.uniprot.org/terms
CC   Distributed under the Creative Commons Attribution (CC BY 4.0) License
CC   ---------------------------------------------------------------------------
DR   EMBL; AK013128; BAB28666.1; -; mRNA.
DR   EMBL; AK010553; BAB27022.1; -; mRNA.
DR   EMBL; AK151764; BAE30670.1; -; mRNA.
DR   EMBL; AK160337; BAE35744.1; -; mRNA.
DR   EMBL; CH466560; EDL21324.1; -; Genomic_DNA.
DR   CCDS; CCDS23540.1; -.
DR   RefSeq; NP_079683.2; NM_025407.2.
DR   PDB; 7O37; EM; 3.20 A; A/L=35-480.
DR   PDB; 7O3C; EM; 3.30 A; A/L=35-480.
DR   PDB; 7O3E; EM; 3.60 A; A/L=35-480.
DR   PDB; 7O3H; EM; 2.60 A; A/L=35-480.
DR   PDBsum; 7O37; -.
DR   PDBsum; 7O3C; -.
DR   PDBsum; 7O3E; -.
DR   PDBsum; 7O3H; -.
DR   AlphaFoldDB; Q9CZ13; -.
DR   SMR; Q9CZ13; -.
DR   BioGRID; 204459; 58.
DR   ComplexPortal; CPX-563; Mitochondrial respiratory chain complex III.
DR   CORUM; Q9CZ13; -.
DR   IntAct; Q9CZ13; 11.
DR   MINT; Q9CZ13; -.
DR   STRING; 10090.ENSMUSP00000026743; -.
DR   MEROPS; M16.975; -.
DR   MEROPS; M16.981; -.
DR   GlyGen; Q9CZ13; 2 sites, 1 O-linked glycan (2 sites).
DR   iPTMnet; Q9CZ13; -.
DR   PhosphoSitePlus; Q9CZ13; -.
DR   SwissPalm; Q9CZ13; -.
DR   REPRODUCTION-2DPAGE; IPI00111885; -.
DR   REPRODUCTION-2DPAGE; Q9CZ13; -.
DR   UCD-2DPAGE; Q9CZ13; -.
DR   EPD; Q9CZ13; -.
DR   jPOST; Q9CZ13; -.
DR   MaxQB; Q9CZ13; -.
DR   PaxDb; Q9CZ13; -.
DR   PeptideAtlas; Q9CZ13; -.
DR   ProteomicsDB; 301902; -.
DR   TopDownProteomics; Q9CZ13; -.
DR   Antibodypedia; 1257; 399 antibodies from 29 providers.
DR   DNASU; 22273; -.
DR   Ensembl; ENSMUST00000026743; ENSMUSP00000026743; ENSMUSG00000025651.
DR   GeneID; 22273; -.
DR   KEGG; mmu:22273; -.
DR   UCSC; uc009rrg.1; mouse.
DR   AGR; MGI:107876; -.
DR   CTD; 7384; -.
DR   MGI; MGI:107876; Uqcrc1.
DR   VEuPathDB; HostDB:ENSMUSG00000025651; -.
DR   eggNOG; KOG0960; Eukaryota.
DR   GeneTree; ENSGT00940000158931; -.
DR   HOGENOM; CLU_009902_4_2_1; -.
DR   InParanoid; Q9CZ13; -.
DR   OMA; WSNPDNV; -.
DR   OrthoDB; 167798at2759; -.
DR   PhylomeDB; Q9CZ13; -.
DR   TreeFam; TF105032; -.
DR   Reactome; R-MMU-611105; Respiratory electron transport.
DR   BioGRID-ORCS; 22273; 24 hits in 79 CRISPR screens.
DR   ChiTaRS; Uqcrc1; mouse.
DR   PRO; PR:Q9CZ13; -.
DR   Proteomes; UP000000589; Chromosome 9.
DR   RNAct; Q9CZ13; protein.
DR   Bgee; ENSMUSG00000025651; Expressed in proximal tubule and 143 other tissues.
DR   ExpressionAtlas; Q9CZ13; baseline and differential.
DR   Genevisible; Q9CZ13; MM.
DR   GO; GO:0005743; C:mitochondrial inner membrane; HDA:MGI.
DR   GO; GO:0005750; C:mitochondrial respiratory chain complex III; ISO:MGI.
DR   GO; GO:0005739; C:mitochondrion; IDA:MGI.
DR   GO; GO:0043209; C:myelin sheath; HDA:UniProtKB.
DR   GO; GO:0046872; F:metal ion binding; IEA:InterPro.
DR   GO; GO:0044877; F:protein-containing complex binding; ISO:MGI.
DR   GO; GO:0031625; F:ubiquitin protein ligase binding; ISO:MGI.
DR   GO; GO:0045333; P:cellular respiration; NAS:ComplexPortal.
DR   GO; GO:0006122; P:mitochondrial electron transport, ubiquinol to cytochrome c; IDA:MGI.
DR   GO; GO:0034551; P:mitochondrial respiratory chain complex III assembly; ISS:UniProtKB.
DR   GO; GO:0014823; P:response to activity; IEA:Ensembl.
DR   GO; GO:0043279; P:response to alkaloid; IEA:Ensembl.
DR   Gene3D; 3.30.830.10; Metalloenzyme, LuxS/M16 peptidase-like; 2.
DR   InterPro; IPR011249; Metalloenz_LuxS/M16.
DR   InterPro; IPR011765; Pept_M16_N.
DR   InterPro; IPR007863; Peptidase_M16_C.
DR   PANTHER; PTHR11851:SF116; CYTOCHROME B-C1 COMPLEX SUBUNIT 1, MITOCHONDRIAL; 1.
DR   PANTHER; PTHR11851; METALLOPROTEASE; 1.
DR   Pfam; PF00675; Peptidase_M16; 1.
DR   Pfam; PF05193; Peptidase_M16_C; 1.
DR   SUPFAM; SSF63411; LuxS/MPP-like metallohydrolase; 2.
PE   1: Evidence at protein level;
KW   3D-structure; Acetylation; Direct protein sequencing; Electron transport;
KW   Membrane; Mitochondrion; Mitochondrion inner membrane; Phosphoprotein;
KW   Reference proteome; Respiratory chain; Transit peptide; Transport.
FT   TRANSIT         1..34
FT                   /note="Mitochondrion"
FT                   /evidence="ECO:0000250"
FT   CHAIN           35..480
FT                   /note="Cytochrome b-c1 complex subunit 1, mitochondrial"
FT                   /id="PRO_0000026787"
FT   MOD_RES         111
FT                   /note="N6-acetyllysine"
FT                   /evidence="ECO:0007744|PubMed:23576753"
FT   MOD_RES         138
FT                   /note="N6-acetyllysine"
FT                   /evidence="ECO:0007744|PubMed:23576753"
FT   MOD_RES         163
FT                   /note="N6-acetyllysine; alternate"
FT                   /evidence="ECO:0007744|PubMed:23576753"
FT   MOD_RES         163
FT                   /note="N6-succinyllysine; alternate"
FT                   /evidence="ECO:0007744|PubMed:23806337"
FT   MOD_RES         212
FT                   /note="Phosphoserine"
FT                   /evidence="ECO:0000250|UniProtKB:Q68FY0"
FT   MOD_RES         214
FT                   /note="Phosphothreonine"
FT                   /evidence="ECO:0000250|UniProtKB:Q68FY0"
FT   MOD_RES         248
FT                   /note="N6-acetyllysine"
FT                   /evidence="ECO:0007744|PubMed:23576753"
FT   CONFLICT        223
FT                   /note="H -> N (in Ref. 1; BAB28666)"
FT                   /evidence="ECO:0000305"
FT   CONFLICT        318
FT                   /note="Y -> C (in Ref. 1; BAB28666)"
FT                   /evidence="ECO:0000305"
FT   HELIX           38..43
FT                   /evidence="ECO:0007829|PDB:7O3H"
FT   STRAND          49..52
FT                   /evidence="ECO:0007829|PDB:7O3H"
FT   STRAND          58..63
FT                   /evidence="ECO:0007829|PDB:7O3H"
FT   STRAND          67..76
FT                   /evidence="ECO:0007829|PDB:7O3H"
FT   HELIX           79..81
FT                   /evidence="ECO:0007829|PDB:7O3H"
FT   STRAND          84..86
FT                   /evidence="ECO:0007829|PDB:7O3H"
FT   HELIX           89..96
FT                   /evidence="ECO:0007829|PDB:7O3H"
FT   STRAND          101..104
FT                   /evidence="ECO:0007829|PDB:7O3H"
FT   HELIX           107..115
FT                   /evidence="ECO:0007829|PDB:7O3H"
FT   STRAND          119..124
FT                   /evidence="ECO:0007829|PDB:7O3H"
FT   STRAND          129..137
FT                   /evidence="ECO:0007829|PDB:7O3H"
FT   HELIX           140..152
FT                   /evidence="ECO:0007829|PDB:7O3H"
FT   HELIX           158..175
FT                   /evidence="ECO:0007829|PDB:7O3H"
FT   HELIX           179..191
FT                   /evidence="ECO:0007829|PDB:7O3H"
FT   TURN            196..198
FT                   /evidence="ECO:0007829|PDB:7O3H"
FT   HELIX           205..210
FT                   /evidence="ECO:0007829|PDB:7O3H"
FT   HELIX           213..223
FT                   /evidence="ECO:0007829|PDB:7O3H"
FT   HELIX           226..228
FT                   /evidence="ECO:0007829|PDB:7O3H"
FT   STRAND          229..236
FT                   /evidence="ECO:0007829|PDB:7O3H"
FT   HELIX           239..249
FT                   /evidence="ECO:0007829|PDB:7O3H"
FT   STRAND          250..253
FT                   /evidence="ECO:0007829|PDB:7O37"
FT   STRAND          258..260
FT                   /evidence="ECO:0007829|PDB:7O3H"
FT   STRAND          273..278
FT                   /evidence="ECO:0007829|PDB:7O3H"
FT   STRAND          282..292
FT                   /evidence="ECO:0007829|PDB:7O3H"
FT   HELIX           300..311
FT                   /evidence="ECO:0007829|PDB:7O3H"
FT   STRAND          313..315
FT                   /evidence="ECO:0007829|PDB:7O3C"
FT   HELIX           321..323
FT                   /evidence="ECO:0007829|PDB:7O3H"
FT   HELIX           327..335
FT                   /evidence="ECO:0007829|PDB:7O3H"
FT   STRAND          339..348
FT                   /evidence="ECO:0007829|PDB:7O3H"
FT   STRAND          351..360
FT                   /evidence="ECO:0007829|PDB:7O3H"
FT   HELIX           362..364
FT                   /evidence="ECO:0007829|PDB:7O3H"
FT   HELIX           365..381
FT                   /evidence="ECO:0007829|PDB:7O3H"
FT   HELIX           385..402
FT                   /evidence="ECO:0007829|PDB:7O3H"
FT   HELIX           406..418
FT                   /evidence="ECO:0007829|PDB:7O3H"
FT   HELIX           426..434
FT                   /evidence="ECO:0007829|PDB:7O3H"
FT   HELIX           438..448
FT                   /evidence="ECO:0007829|PDB:7O3H"
FT   TURN            449..451
FT                   /evidence="ECO:0007829|PDB:7O3H"
FT   STRAND          455..461
FT                   /evidence="ECO:0007829|PDB:7O3H"
FT   STRAND          463..465
FT                   /evidence="ECO:0007829|PDB:7O3H"
FT   HELIX           468..474
FT                   /evidence="ECO:0007829|PDB:7O3H"
SQ   SEQUENCE   480 AA;  52852 MW;  8150C97C655A91C9 CRC64;
     MAASAVCRAA CSGTQVLLRT RRSPALLRLP ALRGTATFAQ ALQSVPETQV SILDNGLRVA
     SEQSSHATCT VGVWIDAGSR YETEKNNGAG YFLEHLAFKG TKNRPGNALE KEVESIGAHL
     NAYSTREHTA YLIKALSKDL PKVVELLADI VQNSSLEDSQ IEKERDVILR EMQENDASMQ
     NVVFDYLHAT AFQGTPLAQA VEGPSENVRR LSRTDLTDYL NRHYKAPRMV LAAAGGVEHQ
     QLLDLAQKHL SSVSRVYEED AVPGLTPCRF TGSEIRHRDD ALPLAHVAIA VEGPGWANPD
     NVTLQVANAI IGHYDCTYGG GVHLSSPLAS VAVANKLCQS FQTFNISYSD TGLLGAHFVC
     DAMSIDDMVF FLQGQWMRLC TSATESEVTR GKNILRNALV SHLDGTTPVC EDIGRSLLTY
     GRRIPLAEWE SRIQEVDAQM LRDICSKYFY DQCPAVAGYG PIEQLPDYNR IRSGMFWLRF
//
```

|  |
| --- |
| **Mascot:** http://www.matrixscience.com/ |

Deamidated (NQ) (+0.9840)
