# Supplementary figures and images for: A common East-Asian ALDH2 mutation causes metabolic disorders and the therapeutic effect of ALDH2 activators
Source: Nat Commun. 2023 Sep 25;14:5971. doi: 10.1038/s41467-023-41570-6 (PMC10520061; doi:10.1038/s41467-023-41570-6)

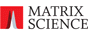

Supplement: Supplementary file 4 — Supplementary Data 1 [file 41467_2023_41570_MOESM4_ESM.zip › Table S5b/D3Z041/D3Z041_WTO-1_K264_files/88x31_logo_white.gif]

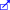

Supplement: Supplementary file 4 — Supplementary Data 1 [file 41467_2023_41570_MOESM4_ESM.zip › Table S5b/D3Z041/D3Z041_WTO-1_K264_files/external_arrow.png]

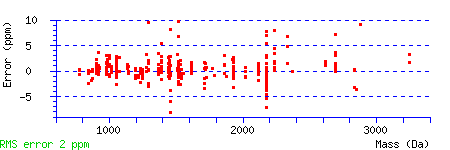

Supplement: Supplementary file 4 — Supplementary Data 1 [file 41467_2023_41570_MOESM4_ESM.zip › Table S5b/D3Z041/D3Z041_WTO-1_K264_files/mass_error.pl.ñU╕n]

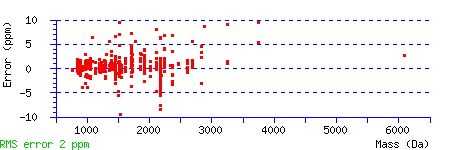

Supplement: Supplementary file 4 — Supplementary Data 1 [file 41467_2023_41570_MOESM4_ESM.zip › Table S5b/D3Z041/D3Z041_WTO-2_C168_files/mass_error.pl.ñU╕n]

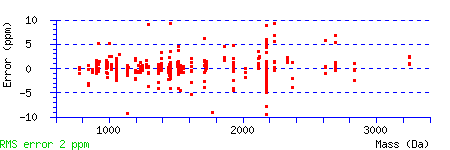

Supplement: Supplementary file 4 — Supplementary Data 1 [file 41467_2023_41570_MOESM4_ESM.zip › Table S5b/D3Z041/D3Z041_WTO-3_K633_files/mass_error.pl.ñU╕n]

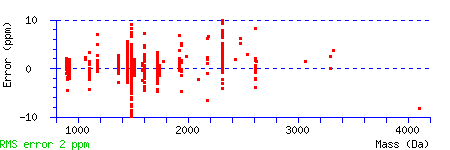

Supplement: Supplementary file 4 — Supplementary Data 1 [file 41467_2023_41570_MOESM4_ESM.zip › Table S5b/P12242/P12242_WTO-1_C25_files/mass_error.pl.ñU╕n]

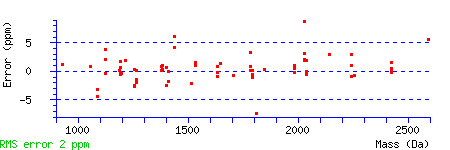

Supplement: Supplementary file 4 — Supplementary Data 1 [file 41467_2023_41570_MOESM4_ESM.zip › Table S5b/P58281-2/P58281-2_WTO-1_H495_files/mass_error.pl.ñU╕n]

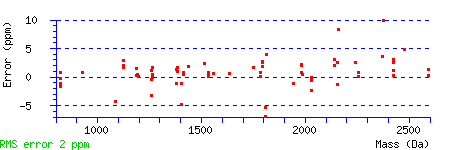

Supplement: Supplementary file 4 — Supplementary Data 1 [file 41467_2023_41570_MOESM4_ESM.zip › Table S5b/P58281-2/P58281-2_WTO60-1_files/mass_error.pl.ñU╕n]

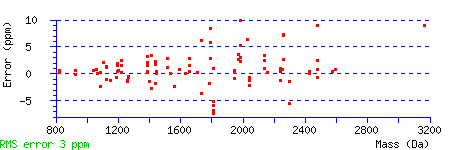

Supplement: Supplementary file 4 — Supplementary Data 1 [file 41467_2023_41570_MOESM4_ESM.zip › Table S5b/P58281-2/P58281-2_WTO60-3_H495_H879_files/mass_error.pl.ñU╕n]

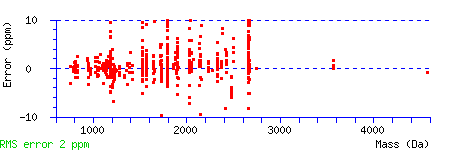

Supplement: Supplementary file 4 — Supplementary Data 1 [file 41467_2023_41570_MOESM4_ESM.zip › Table S5b/Q64521/Q64521_WTO-1_C309_H633_K634_files/mass_error.pl.ñU╕n]

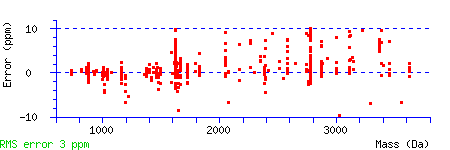

Supplement: Supplementary file 4 — Supplementary Data 1 [file 41467_2023_41570_MOESM4_ESM.zip › Table S5b/Q8BMS1/Q8BMS1_WTO-1_K66_K664_files/mass_error.pl.ñU╕n]

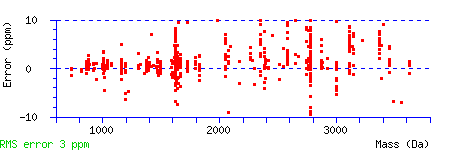

Supplement: Supplementary file 4 — Supplementary Data 1 [file 41467_2023_41570_MOESM4_ESM.zip › Table S5b/Q8BMS1/Q8BMS1_WTO-2_C97_files/mass_error.pl.ñU╕n]

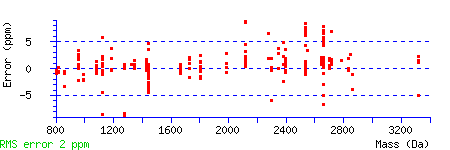

Supplement: Supplementary file 4 — Supplementary Data 1 [file 41467_2023_41570_MOESM4_ESM.zip › Table S5b/Q8BWT1/Q8BWT1_WTO-1-K25_files/mass_error.pl.ñU╕n]

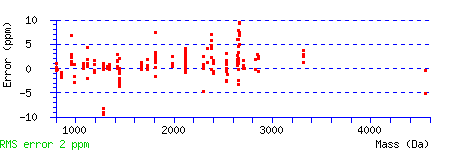

Supplement: Supplementary file 4 — Supplementary Data 1 [file 41467_2023_41570_MOESM4_ESM.zip › Table S5b/Q8BWT1/Q8BWT1_WTO-2_C103_C107_files/mass_error.pl.ñU╕n]

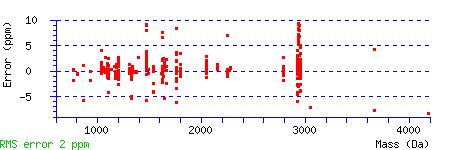

Supplement: Supplementary file 4 — Supplementary Data 1 [file 41467_2023_41570_MOESM4_ESM.zip › Table S5b/Q8K2B3/Q8K2B3_WTO-1_C438_C574_files/mass_error.pl.ñU╕n]

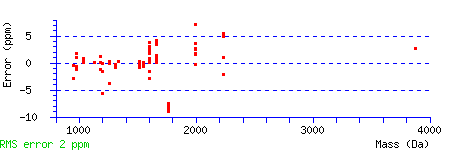

Supplement: Supplementary file 4 — Supplementary Data 1 [file 41467_2023_41570_MOESM4_ESM.zip › Table S5b/Q924L1/Q924L1_WTO-1-H184_files/mass_error.pl.ñU╕n]

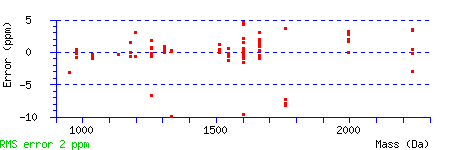

Supplement: Supplementary file 4 — Supplementary Data 1 [file 41467_2023_41570_MOESM4_ESM.zip › Table S5b/Q924L1/Q924L1_WTO-3-H197_files/mass_error.pl.ñU╕n]

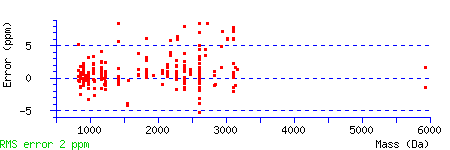

Supplement: Supplementary file 4 — Supplementary Data 1 [file 41467_2023_41570_MOESM4_ESM.zip › Table S5b/Q99JY0/Q99JY0_WTO-1_H133_files/mass_error.pl.ñU╕n]

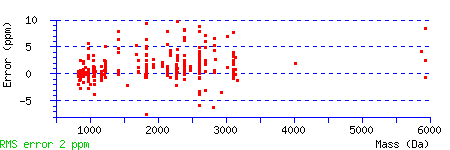

Supplement: Supplementary file 4 — Supplementary Data 1 [file 41467_2023_41570_MOESM4_ESM.zip › Table S5b/Q99JY0/Q99JY0_WTO-2_C549_files/mass_error.pl.ñU╕n]

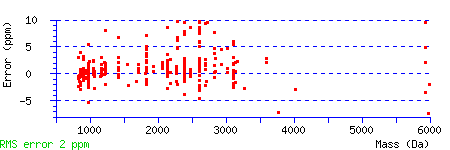

Supplement: Supplementary file 4 — Supplementary Data 1 [file 41467_2023_41570_MOESM4_ESM.zip › Table S5b/Q99JY0/Q99JY0_WTO60-1_C139_C158_files/mass_error.pl.ñU╕n]

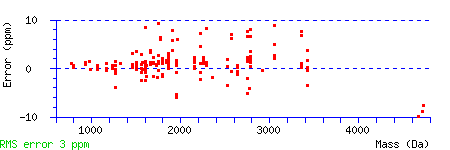

Supplement: Supplementary file 4 — Supplementary Data 1 [file 41467_2023_41570_MOESM4_ESM.zip › Table S5b/Q99KIO/Q99KI0_WTO-2_H268_C277_C385_files/mass_error.pl.ñU╕n]

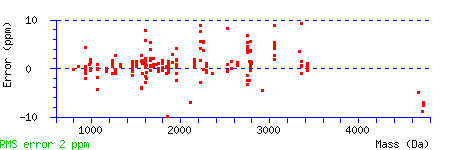

Supplement: Supplementary file 4 — Supplementary Data 1 [file 41467_2023_41570_MOESM4_ESM.zip › Table S5b/Q99KIO/Q99KI0_WTO-4_C284_H268_C410_files/mass_error.pl.ñU╕n]

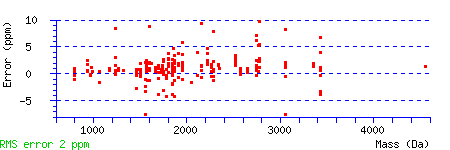

Supplement: Supplementary file 4 — Supplementary Data 1 [file 41467_2023_41570_MOESM4_ESM.zip › Table S5b/Q99KIO/Q99KI0_WTO60-3_K144_files/mass_error.pl.ñU╕n]

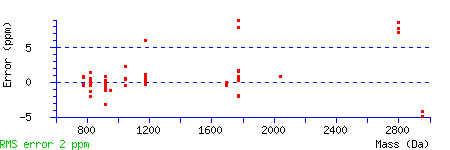

Supplement: Supplementary file 4 — Supplementary Data 1 [file 41467_2023_41570_MOESM4_ESM.zip › Table S5b/Q9CPQ1/Q9CPQ1_WTO1-1_H23_files/mass_error.pl.ñU╕n]

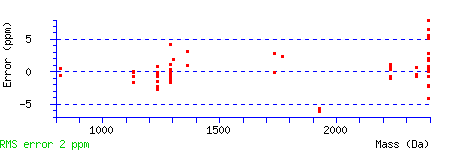

Supplement: Supplementary file 4 — Supplementary Data 1 [file 41467_2023_41570_MOESM4_ESM.zip › Table S5b/Q9CQB4/Q9CQB4_WTO-1-H39_files/mass_error.pl.ñU╕n]

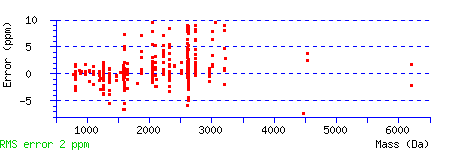

Supplement: Supplementary file 4 — Supplementary Data 1 [file 41467_2023_41570_MOESM4_ESM.zip › Table S5b/Q9CZ13/Q9CZ13_WTO-1_H188_files/mass_error.pl.ñU╕n]

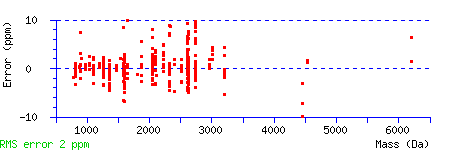

Supplement: Supplementary file 4 — Supplementary Data 1 [file 41467_2023_41570_MOESM4_ESM.zip › Table S5b/Q9CZ13/Q9CZ13_WTO-3_K111_files/mass_error.pl.ñU╕n]
