# Supplementary material for: A common East-Asian ALDH2 mutation causes metabolic disorders and the therapeutic effect of ALDH2 activators
Source: Nat Commun. 2023 Sep 25;14:5971. doi: 10.1038/s41467-023-41570-6 (PMC10520061; doi:10.1038/s41467-023-41570-6)
Supplement: Supplementary file 5 — Reporting Summary [file 41467_2023_41570_MOESM5_ESM.pdf]

nature portfolio

Corresponding author(s): Daria Monchly Rosen
Lee-Ming Chuang

Last updated by author(s): Aug 18, 2023

Reporting Summary

Nature Portfolio wishes to improve the reproducibility of the work that we publish. This form provides structure for consistency and transparency in reporting. For further information on Nature Portfolio policies, see our Editorial Policies and the Editorial Policy Checklist.

Statistics

For all statistical analyses, confirm that the following items are present in the figure legend, table legend, main text, or Methods section.

n/a	Confirmed	
☐	☒	The exact sample size (n) for each experimental group/condition, given as a discrete number and unit of measurement
☐	☒	A statement on whether measurements were taken from distinct samples or whether the same sample was measured repeatedly
☐	☒	The statistical test(s) used AND whether they are one- or two-sided<br>*Only common tests should be described solely by name; describe more complex techniques in the Methods section.*
☐	☒	A description of all covariates tested
☐	☒	A description of any assumptions or corrections, such as tests of normality and adjustment for multiple comparisons
☐	☒	A full description of the statistical parameters including central tendency (e.g. means) or other basic estimates (e.g. regression coefficient) AND variation (e.g. standard deviation) or associated estimates of uncertainty (e.g. confidence intervals)
☐	☒	For null hypothesis testing, the test statistic (e.g. F, t, r) with confidence intervals, effect sizes, degrees of freedom and P value noted<br>*Give P values as exact values whenever suitable.*
☒	☐	For Bayesian analysis, information on the choice of priors and Markov chain Monte Carlo settings
☒	☐	For hierarchical and complex designs, identification of the appropriate level for tests and full reporting of outcomes
☒	☐	Estimates of effect sizes (e.g. Cohen's d, Pearson's r), indicating how they were calculated

Our web collection on statistics for biologists contains articles on many of the points above.

Software and code

Policy information about availability of computer code

Data collection	In the Method section, we have stated that "Ligand energy was minimized using the PyRx 0.8 program before docking. Three-dimensional models are generated by using the PyMOL v7.4 program and the 2D protein-ligand interaction diagrams were generated by the LigPlot+ 2.2 program.".<br>The software used is listed as below:<br>PyRx 0.8<br>PyMOL v7.4<br>LigPlot+ v2.2
Data analysis	Ligand energy was minimized using the PyRx 0.8 program before docking. Three-dimensional models are generated by using the PyMOL v7.4 program and the 2D protein-ligand interaction diagrams were generated by the LigPlot+ 2.2 program.

For manuscripts utilizing custom algorithms or software that are central to the research but not yet described in published literature, software must be made available to editors and reviewers. We strongly encourage code deposition in a community repository (e.g. GitHub). See the Nature Portfolio guidelines for submitting code & software for further information.

Data

Policy information about availability of data

All manuscripts must include a data availability statement. This statement should provide the following information, where applicable:

- Accession codes, unique identifiers, or web links for publicly available datasets
- A description of any restrictions on data availability
- For clinical datasets or third party data, please ensure that the statement adheres to our policy

All raw mass spectrometry data was uploaded to the MassIVE (Mass Spectrometry Interactive Virtual Environment) database (https://massive.ucsd.edu/ProteoSAFe/static/massive.jsp). The accession code is MassIVE00091724. The uncropped immunoblot images and the Source data are deposited in the Zenodo database https://doi.org/10.5281/zenodo.8241510. To visualize the interaction between the ALDH2 with activators Alda-1 and AD-5591, the X-ray structure of human ALDH2 (PDB ID: 1NZX) and NAD+ were used (https://www.rcsb.org/structure/1NZX). All other raw data associated with this study will be provided upon request.

Research involving human participants, their data, or biological material

Policy information about studies with human participants or human data. See also policy information about sex, gender (identity/presentation), and sexual orientation and race, ethnicity and racism.

Reporting on sex and gender	Not applicable
Reporting on race, ethnicity, or other socially relevant groupings	Not applicable
Population characteristics	Not applicable
Recruitment	Not applicable
Ethics oversight	Not applicable

Note that full information on the approval of the study protocol must also be provided in the manuscript.

Field-specific reporting

Please select the one below that is the best fit for your research. If you are not sure, read the appropriate sections before making your selection.

☒ Life sciences ☐ Behavioural & social sciences ☐ Ecological, evolutionary & environmental sciences

For a reference copy of the document with all sections, see nature.com/documents/nr-reporting-summary-flat.pdf

Life sciences study design

All studies must disclose on these points even when the disclosure is negative.

Sample size	We did not pre-specified sample size because sample size and power calculation is almost impossible because of lack or prior data of effect size and standard deviation..<br>(2) Samples were collected until statistical significant was reached<br>(3) In our study, almost all animal sample size is larger than 30. If we did not observe any trend of difference, we stop increasing sample size. If we observe trend of difference, we kept increasing sample size.
Data exclusions	We did not exclude any outlier data
Replication	We reported biological repeats as "n" values and technical repeats as " in duplicates or triplicates" in the Figure legends.
Randomization	Animals are grouping by randomization. We randomized animal groups (such as control or experimental group) by their id number. The id number is randomly assigned when they were born.
Blinding	1. For animal experiments, the technicians or the core facilities were unaware of the genotype information and the whole study design<br>2. For molecular biological experiments, it is difficult for the operators to be blind to the experimental design.

Reporting for specific materials, systems and methods

We require information from authors about some types of materials, experimental systems and methods used in many studies. Here, indicate whether each material, system or method listed is relevant to your study. If you are not sure if a list item applies to your research, read the appropriate section before selecting a response.

Materials & experimental systems

n/a	Involved in the study
☐	☒ Antibodies
☒	☐ Eukaryotic cell lines
☒	☐ Palaeontology and archaeology
☐	☒ Animals and other organisms
☒	☐ Clinical data
☒	☐ Dual use research of concern
☒	☐ Plants

Methods

n/a	Involved in the study
☒	☐ ChIP-seq
☒	☐ Flow cytometry
☒	☐ MRI-based neuroimaging

Antibodies

Antibodies used

(1) We listed the detailed information of the primary and secondary antibodies in the method section
(2) The information of antibody used is also listed below
Primary antibodies
(a) rabbit polyclonal anti-Ucp1 antibody (1:1000, cat. no. GTX10983, GeneTex)
(b) rabbit polyclonal anti-Prdm16 (1:1000, cat. no. ab106410, Abcam)
(c) rabbit polyclonal anti-Pgc1α antibody (1:1000, cat. no. NBP1-04676PCP, Novus)
(d) rabbit polyclonal anti-Dio2 antibody (1:1000, cat. no. GTX81072, GeneTex)
(e) rabbit polycloncal anti-Cidea (1:1000, cat. no. ab8402, Abcam)
(f) rabbit monoclonocal anti-Hsp70antibody (1:4000. cat. no.ab45133, Abcam)
(g) rabbit polyclononal anti-Gapdh antibody (1: 5000,GTX100118, GeneTex)

secondary antibody
(a) goat polyclonal anti-rabbit IgG antibody with HRP(1:10000; cat. no. GTX26721, GeneTex)

Validation	We validated antibodies according to their molecular weight by ourself as well as the validation statements on the manufacturer's website as follows<br>(The image validation data was uploaded to the Zenodo database https://zenodo.org/record/8263030 because the image validation data cannot be presented in this Summary Report.)<br><br>(a) rabbit polyclonal anti-Ucp1 antibody (1:1000, cat. no. GTX10983, GeneTex)<br>(Theoretical MW =33 kDa)<br>Validation statements on the manufacturer's website: https://www.genetex.com/Product/Detail/UCP1-antibody/GTX10983<br><br>(b) rabbit polyclonal anti-Prdm16 (1:1000, cat. no. ab106410, Abcam)<br>(Theoretical MW =140kDa)<br>Validation statements on the manufacturer's website: https://www.citeab.com/antibodies/748339-ab106410-anti-prdm16-antibody<br><br>(c) rabbit polyclonal anti-Pgc1α antibody (1:1000, cat. no. NBP1-04676PCP, Novus)<br>PGC1α (Theoretical MW =91 kDa)<br>Validation statements on the manufacturer's website:<br>https://www.novusbio.com/products/pgc1-alpha-antibody_nbp1-04676pcp<br><br>(d) rabbit polyclonal anti-Dio2 antibody (1:1000, cat. no. GTX81072, GeneTex)<br>DIO2 (Theoretical MW =31 kDa)<br>Validation statements on the manufacturer's website:<br>https://www.genetex.com/Product/Detail/DIO2-antibody-Internal/GTX81072<br><br>(e) rabbit polycloncal anti-Cidea (1:1000, cat. no. ab8402, Abcam)<br>CIDEA (Theoretical MW =25 kDa)<br>Validation statements on the manufacturer's website:<br>https://www.abcam.com/products/primary-antibodies/cide-a-antibody-ab8402.html<br><br>(f) rabbit monoclonocal anti-Hsp70antibody (1:4000. cat. no.ab45133, Abcam)<br>HSP70 (Theoretical MW =70 kDa)<br>Validation statements on the manufacturer's website:<br>https://www.abcam.com/products/primary-antibodies/hsp70-antibody-ep1007y-ab45133.html<br><br>(g) rabbit polyclononal anti-Gapdh antibody (1: 5000,GTX100118, GeneTex)<br>GAPDH (Theoretical MW =36 kDa)<br>Validation statements on the manufacturer's website:<br>Https://www.genetex.com/Product/Detail/GAPDH-antibody/GTX100118

Animals and other research organisms

Policy information about studies involving animals; ARRIVE guidelines recommended for reporting animal research, and Sex and Gender in Research

Laboratory animals	Aldh2 knockin mice with C57BL6/J background are kind gifts from Dr. Daria Mochly-Rosen of the Stanford University. All animals used are male. The information of genetic manipulation is reported in Supplementary Figure S17. The mice were housed at 22-24°C with light: dark cycles of 12:12 hours. The mice were fed either a HFHSD (cat. no. D12331, Research Diets) which provided 58% kilocalorie from fat and 12.5% kilocalorie from sucrose or a chow diet (cat no. 5001, Lab Diet). Mice were bred by mating heterozygotes. The age for each experiment is list as blows:<br>(1) Fig. 1a: 4-24 weeks<br>(2) Fig.1b, 1c, 1d, 1e, 1f, 1g, 1h, 1i, 1j, 2g, 2i, 2j, 2k, 2l, 2m, 2n, 2o, 2p, 3a, 3b, 3d, 3e, 3f, 3g, 3h, 3i, 3j, 3k. 3n, Supplementary Fig. 4, 5, 6 ,7, 8, 9,10,11, &13: 24 weeks.<br>(3) Fig. 2a, 2b, 2c, 2d,2e, Supplementary Fig. 1,2, &3: 8-10 weeks<br>(4) Fig 3l, m, &o: 4 weeks<br>(5) Fig. 5b, 5c, & 6a-6f: 10-30 weeks<br>(6) Fig. 5d- 5m, 6g, Supplementary Table S2, S4 Supplementary Fig. 14,15,16 at the age of 30 weeks
Wild animals	We did not use wild animals
Reporting on sex	All animals used are male.
Field-collected samples	We did not used file-collected samples.
Ethics oversight	All animal experiments were performed according to institutional ethical guidelines and were approved by the Institutional Animal Care and Use Committee (IACUC)(IACUC Approval No: 20110418) of the National Taiwan University College of Medicine and College of Public Health, which is accredited by the Association for Assessment and Accreditation of Laboratory Animal Care International (AAALAC).

The full names of the boards/committees that approved the protocol are Shu-Wha Lin PhD, Huei-Wen Chen PhD, Chang Chia-Yi DVM, and Liu Yu-Ju DVM

Note that full information on the approval of the study protocol must also be provided in the manuscript.
