# Supplementary material for: A common East-Asian ALDH2 mutation causes metabolic disorders and the therapeutic effect of ALDH2 activators
Source: Nat Commun. 2023 Sep 25;14:5971. doi: 10.1038/s41467-023-41570-6 (PMC10520061; doi:10.1038/s41467-023-41570-6)
Supplement: Supplementary file 4 — Supplementary Data 1 [file 41467_2023_41570_MOESM4_ESM.zip › Table S5b/Q9DCS9/Q9DCS9_WTO60-2.html]

Mascot Search Results: Q9DCS9
 

# MASCOT Search Results

## Protein View: Q9DCS9

### NADH dehydrogenase [ubiquinone] 1 beta subcomplex subunit 10 OS=Mus musculus OX=10090 GN=Ndufb10 PE=1 SV=3

|  |  |
| --- | --- |
| Database: | Mouse\_UniProt\_proteomes |
| Score: | 1750 |
| Monoisotopic mass (M_r_): | 21296 |
| Calculated pI: | 8.19 |

Sequence similarity is available as an NCBI BLAST search of Q9DCS9 against nr.

### Search parameters

|  |  |
| --- | --- |
| MS data file: | `D:\LCMSMS\2023 Users' data\230529-1\230529-1-WT60-2.raw` |
| Enzyme: | Trypsin/P: cuts C-term side of KR. |
| Fixed modifications: | Carbamidomethyl (C) |
| Variable modifications: | Deamidated (NQ), HNE (C), HNE (H), HNE (K), Oxidation (M) |

### Protein sequence coverage: 54%

Matched peptides shown in ***bold red***.

|  |  |  |  |  |  |
| --- | --- | --- | --- | --- | --- |
| `1` | `MPDSWDKDVY` | `PEPPSRTPAP` | `SPQTSLPNPI` | `TYLTKAYDLV` | `VDWPVTLVRE` |
| `51` | `FIERQHAKNR` | `TYYYHRQYRR` | `VPDITECKEG` | `DVLCIYEAEM` | `QWRRDFKVDQ` |
| `101` | `EIMNIIQERL` | `KACQQREGEN` | `YQQNCAKELE` | `QFTKVTKAYQ` | `DRYLDLGAYY` |
| `151` | `SARKCLAKQK` | `QRMLEERKAA` | `RQEAAA` |  |  |

Unformatted sequence string: 176 residues (for pasting into other applications).

|  |  |  |  |
| --- | --- | --- | --- |
| Sort by | residue number | increasing mass | decreasing mass |
| Show | matched peptides only | predicted peptides also |  |

| Query | Start | – | End | Observed | Mr(expt) | Mr(calc) | ppm | M | Score | Expect | Rank | U | Peptide |
| --- | --- | --- | --- | --- | --- | --- | --- | --- | --- | --- | --- | --- | --- |
| 126983 | 17 | – | 35 | 1013.5455 | 2025.0765 | 2025.0782 | -0.86 | 0 | 90 | 3.6e-09 | 1Score **> 36** indicates **identity** Score **> 18** indicates **homology** | U | R.TPAPSPQTSLPNPITYLTK.A |
| 126984 | 17 | – | 35 | 1013.5475 | 2025.0804 | 2025.0782 | 1.04 | 0 | 69 | 3.1e-07 | 1Score **> 36** indicates **identity** Score **> 17** indicates **homology** | U | R.TPAPSPQTSLPNPITYLTK.A |
| 126985 | 17 | – | 35 | 1013.5475 | 2025.0805 | 2025.0782 | 1.13 | 0 | 83 | 2.5e-08 | 1Score **> 36** indicates **identity** Score **> 20** indicates **homology** | U | R.TPAPSPQTSLPNPITYLTK.A |
| 126986 | 17 | – | 35 | 676.0346 | 2025.0820 | 2025.0782 | 1.86 | 0 | 50 | 2.2e-05 | 1Score **> 36** indicates **identity** Score **> 16** indicates **homology** | U | R.TPAPSPQTSLPNPITYLTK.A |
| 126987 | 17 | – | 35 | 676.0347 | 2025.0824 | 2025.0782 | 2.06 | 0 | 56 | 5.2e-06 | 1Score **> 36** indicates **identity** Score **> 16** indicates **homology** | U | R.TPAPSPQTSLPNPITYLTK.A |
| 126988 | 17 | – | 35 | 1013.5486 | 2025.0827 | 2025.0782 | 2.19 | 0 | 72 | 1.8e-07 | 1Score **> 36** indicates **identity** Score **> 17** indicates **homology** | U | R.TPAPSPQTSLPNPITYLTK.A |
| 126989 | 17 | – | 35 | 1013.5488 | 2025.0830 | 2025.0782 | 2.36 | 0 | 82 | 2e-08 | 1Score **> 36** indicates **identity** Score **> 18** indicates **homology** | U | R.TPAPSPQTSLPNPITYLTK.A |
| 126990 | 17 | – | 35 | 1013.5489 | 2025.0832 | 2025.0782 | 2.44 | 0 | 90 | 4e-09 | 1Score **> 36** indicates **identity** Score **> 18** indicates **homology** | U | R.TPAPSPQTSLPNPITYLTK.A |
| 126991 | 17 | – | 35 | 676.0350 | 2025.0832 | 2025.0782 | 2.47 | 0 | 50 | 2.1e-05 | 1Score **> 36** indicates **identity** Score **> 16** indicates **homology** | U | R.TPAPSPQTSLPNPITYLTK.A |
| 126992 | 17 | – | 35 | 1013.5490 | 2025.0835 | 2025.0782 | 2.58 | 0 | 29 | 0.0018 | 1Score **> 36** indicates **identity** Score **> 14** indicates **homology** | U | R.TPAPSPQTSLPNPITYLTK.A |
| 126993 | 17 | – | 35 | 1013.5491 | 2025.0836 | 2025.0782 | 2.64 | 0 | 92 | 2.3e-09 | 1Score **> 36** indicates **identity** Score **> 18** indicates **homology** | U | R.TPAPSPQTSLPNPITYLTK.A |
| 126995 | 17 | – | 35 | 676.0352 | 2025.0837 | 2025.0782 | 2.71 | 0 | 29 | 0.0018 | 1Score **> 36** indicates **identity** Score **> 14** indicates **homology** | U | R.TPAPSPQTSLPNPITYLTK.A |
| 126996 | 17 | – | 35 | 1013.5493 | 2025.0841 | 2025.0782 | 2.89 | 0 | 66 | 7e-07 | 1Score **> 36** indicates **identity** Score **> 17** indicates **homology** | U | R.TPAPSPQTSLPNPITYLTK.A |
| 126998 | 17 | – | 35 | 1013.5508 | 2025.0870 | 2025.0782 | 4.30 | 0 | 64 | 1.1e-06 | 1Score **> 36** indicates **identity** Score **> 16** indicates **homology** | U | R.TPAPSPQTSLPNPITYLTK.A |
| 126999 | 17 | – | 35 | 676.0364 | 2025.0873 | 2025.0782 | 4.46 | 0 | 24 | 0.0054 | 1Score **> 36** indicates **identity** Score **> 14** indicates **homology** | U | R.TPAPSPQTSLPNPITYLTK.A |
| 127000 | 17 | – | 35 | 1013.5513 | 2025.0880 | 2025.0782 | 4.80 | 0 | 73 | 1.3e-07 | 1Score **> 36** indicates **identity** Score **> 17** indicates **homology** | U | R.TPAPSPQTSLPNPITYLTK.A |
| 86671 | 36 | – | 49 | 823.4483 | 1644.8820 | 1644.8876 | -3.36 | 0 | 48 | 3e-05 | 1Score **> 35** indicates **identity** Score **> 16** indicates **homology** | U | K.AYDLVVDWPVTLVR.E |
| 86683 | 36 | – | 49 | 823.4507 | 1644.8869 | 1644.8876 | -0.39 | 0 | 61 | 2.1e-06 | 1Score **> 35** indicates **identity** Score **> 16** indicates **homology** | U | K.AYDLVVDWPVTLVR.E |
| 86685 | 36 | – | 49 | 549.3032 | 1644.8877 | 1644.8876 | 0.070 | 0 | 53 | 1.1e-05 | 1Score **> 35** indicates **identity** Score **> 16** indicates **homology** | U | K.AYDLVVDWPVTLVR.E |
| 30490 | 59 | – | 66 | 391.5225 | 1171.5456 | 1171.5523 | -5.68 | 1 | 25 | 0.0047 | 1Score **> 29** indicates **identity** Score **> 14** indicates **homology** | U | K.NRTYYYHR.Q |
| 24683 | 70 | – | 78 | 559.2875 | 1116.5604 | 1116.5597 | 0.57 | 1 | 17 | 0.032 | 1Score **> 33** indicates **identity** Score **> 15** indicates **homology** | U | R.RVPDITECK.E |
| 127842 | 94 | – | 109 | 509.2651 | 2033.0314 | 2033.0364 | -2.44 | 2 | 43 | 0.0015 | 1Score **> 37** indicates **identity** Score **> 27** indicates **homology** | U | R.RDFKVDQEIMNIIQER.L |
| 127855 | 94 | – | 109 | 678.6879 | 2033.0417 | 2033.0364 | 2.63 | 2 | 69 | 3.1e-07 | 1Score **> 37** indicates **identity** Score **> 17** indicates **homology** | U | R.RDFKVDQEIMNIIQER.L |
| 113400 | 95 | – | 109 | 626.6519 | 1876.9338 | 1876.9353 | -0.76 | 1 | 20 | 0.014 | 1Score **> 36** indicates **identity** Score **> 14** indicates **homology** | U | R.DFKVDQEIMNIIQER.L |
| 113401 | 95 | – | 109 | 626.6527 | 1876.9364 | 1876.9353 | 0.60 | 1 | 34 | 0.00064 | 1Score **> 36** indicates **identity** Score **> 15** indicates **homology** | U | R.DFKVDQEIMNIIQER.L |
| 113402 | 95 | – | 109 | 939.4759 | 1876.9372 | 1876.9353 | 1.03 | 1 | 39 | 0.0002 | 1Score **> 36** indicates **identity** Score **> 15** indicates **homology** | U | R.DFKVDQEIMNIIQER.L |
| 113403 | 95 | – | 109 | 626.6532 | 1876.9376 | 1876.9353 | 1.26 | 1 | 27 | 0.0027 | 1Score **> 36** indicates **identity** Score **> 14** indicates **homology** | U | R.DFKVDQEIMNIIQER.L |
| 127847 | 95 | – | 109 | 678.6861 | 2033.0366 | 2033.0503 | -6.76 | 1 | 41 | 0.00015 | 1Score **> 37** indicates **identity** Score **> 15** indicates **homology** | U | R.DFKVDQEIMNIIQER.L  + HNE (K) |
| 127849 | 95 | – | 109 | 678.6866 | 2033.0381 | 2033.0503 | -6.03 | 1 | 61 | 2e-06 | 1Score **> 37** indicates **identity** Score **> 16** indicates **homology** | U | R.DFKVDQEIMNIIQER.L  + HNE (K) |
| 127850 | 95 | – | 109 | 509.2669 | 2033.0383 | 2033.0503 | -5.89 | 1 | 25 | 0.0049 | 1Score **> 37** indicates **identity** Score **> 14** indicates **homology** | U | R.DFKVDQEIMNIIQER.L  + HNE (K) |
| 127853 | 95 | – | 109 | 509.2674 | 2033.0404 | 2033.0503 | -4.85 | 1 | 48 | 6.6e-05 | 1Score **> 37** indicates **identity** Score **> 18** indicates **homology** | U | R.DFKVDQEIMNIIQER.L  + HNE (K) |
| 127854 | 95 | – | 109 | 678.6875 | 2033.0406 | 2033.0503 | -4.80 | 1 | 26 | 0.0057 | 1Score **> 37** indicates **identity** Score **> 16** indicates **homology** | U | R.DFKVDQEIMNIIQER.L  + HNE (K) |
| 49045 | 117 | – | 127 | 670.7796 | 1339.5446 | 1339.5462 | -1.22 | 0 | 37 | 0.00036 | 1Score **> 24** indicates **identity** Score **> 15** indicates **homology** | U | R.EGENYQQNCAK.E |
| 49046 | 117 | – | 127 | 670.7801 | 1339.5457 | 1339.5462 | -0.41 | 0 | 46 | 5.1e-05 | 1Score **> 24** indicates **identity** Score **> 15** indicates **homology** | U | R.EGENYQQNCAK.E |
| 143963 | 117 | – | 134 | 739.3351 | 2214.9834 | 2214.9851 | -0.76 | 1 | 34 | 0.0006 | 1Score **> 31** indicates **identity** Score **> 15** indicates **homology** | U | R.EGENYQQNCAKELEQFTK.V |
| 143964 | 117 | – | 134 | 739.3352 | 2214.9837 | 2214.9851 | -0.62 | 1 | 51 | 1.7e-05 | 1Score **> 31** indicates **identity** Score **> 16** indicates **homology** | U | R.EGENYQQNCAKELEQFTK.V |
| 143965 | 117 | – | 134 | 739.3355 | 2214.9846 | 2214.9851 | -0.22 | 1 | 47 | 3.8e-05 | 1Score **> 31** indicates **identity** Score **> 15** indicates **homology** | U | R.EGENYQQNCAKELEQFTK.V |
| 143966 | 117 | – | 134 | 1108.4998 | 2214.9850 | 2214.9851 | -0.033 | 1 | 69 | 3.4e-07 | 1Score **> 31** indicates **identity** Score **> 17** indicates **homology** | U | R.EGENYQQNCAKELEQFTK.V |
| 143967 | 117 | – | 134 | 739.3358 | 2214.9855 | 2214.9851 | 0.18 | 1 | 44 | 7.8e-05 | 1Score **> 31** indicates **identity** Score **> 15** indicates **homology** | U | R.EGENYQQNCAKELEQFTK.V |
| 143968 | 117 | – | 134 | 1108.5021 | 2214.9897 | 2214.9851 | 2.08 | 1 | 45 | 6.1e-05 | 1Score **> 31** indicates **identity** Score **> 15** indicates **homology** | U | R.EGENYQQNCAKELEQFTK.V |
| 144055 | 117 | – | 134 | 739.6623 | 2215.9652 | 2215.9691 | -1.79 | 1 | 27 | 0.0032 | 1Score **> 30** indicates **identity** Score **> 14** indicates **homology** | U | R.EGENYQQNCAKELEQFTK.V  + Deamidated (NQ) |
| 144095 | 117 | – | 134 | 739.9970 | 2216.9691 | 2216.9531 | 7.22 | 1 | 14 | 0.046 | 1Score **> 29** indicates **identity** Score **> 13** indicates **homology** | U | R.EGENYQQNCAKELEQFTK.V  + 2 Deamidated (NQ) |
| 8412 | 128 | – | 134 | 447.7315 | 893.4484 | 893.4494 | -1.11 | 0 | 32 | 0.02 | 1Score **> 28** indicates **identity** | U | K.ELEQFTK.V |
| 8413 | 128 | – | 134 | 447.7316 | 893.4486 | 893.4494 | -0.99 | 0 | 35 | 0.0096 | 1Score **> 28** indicates **identity** | U | K.ELEQFTK.V |
| 8415 | 128 | – | 134 | 447.7317 | 893.4489 | 893.4494 | -0.65 | 0 | 46 | 0.00083 | 1Score **> 28** indicates **identity** | U | K.ELEQFTK.V |
| 8417 | 128 | – | 134 | 447.7325 | 893.4505 | 893.4494 | 1.22 | 0 | 23 | 0.032 | 1Score **> 28** indicates **identity** Score **> 20** indicates **homology** | U | K.ELEQFTK.V |
| 43649 | 143 | – | 153 | 646.3152 | 1290.6159 | 1290.6244 | -6.62 | 0 | 30 | 0.0015 | 1Score **> 32** indicates **identity** Score **> 14** indicates **homology** | U | R.YLDLGAYYSAR.K |
| 43650 | 143 | – | 153 | 646.3155 | 1290.6164 | 1290.6244 | -6.21 | 0 | 21 | 0.027 | 1Score **> 32** indicates **identity** Score **> 18** indicates **homology** | U | R.YLDLGAYYSAR.K |
| 43671 | 143 | – | 153 | 646.3187 | 1290.6228 | 1290.6244 | -1.28 | 0 | 41 | 0.0011 | 1Score **> 33** indicates **identity** Score **> 24** indicates **homology** | U | R.YLDLGAYYSAR.K |
| 43677 | 143 | – | 153 | 646.3194 | 1290.6242 | 1290.6244 | -0.14 | 0 | 28 | 0.01 | 1Score **> 33** indicates **identity** Score **> 20** indicates **homology** | U | R.YLDLGAYYSAR.K |
| 43678 | 143 | – | 153 | 646.3194 | 1290.6243 | 1290.6244 | -0.082 | 0 | 26 | 0.019 | 1Score **> 33** indicates **identity** Score **> 21** indicates **homology** | U | R.YLDLGAYYSAR.K |
| 43679 | 143 | – | 153 | 646.3196 | 1290.6247 | 1290.6244 | 0.23 | 0 | 58 | 0.00015 | 1Score **> 33** indicates **identity** Score **> 32** indicates **homology** | U | R.YLDLGAYYSAR.K |
| 43680 | 143 | – | 153 | 646.3197 | 1290.6248 | 1290.6244 | 0.31 | 0 | 58 | 0.00015 | 1Score **> 33** indicates **identity** Score **> 32** indicates **homology** | U | R.YLDLGAYYSAR.K |
| 43681 | 143 | – | 153 | 646.3200 | 1290.6254 | 1290.6244 | 0.77 | 0 | 61 | 8.8e-05 | 1Score **> 33** indicates **identity** Score **> 33** indicates **homology** | U | R.YLDLGAYYSAR.K |
| 43682 | 143 | – | 153 | 646.3200 | 1290.6255 | 1290.6244 | 0.86 | 0 | 63 | 4.7e-05 | 1Score **> 33** indicates **identity** | U | R.YLDLGAYYSAR.K |
| 43683 | 143 | – | 153 | 646.3201 | 1290.6257 | 1290.6244 | 1.00 | 0 | 31 | 0.0069 | 1Score **> 33** indicates **identity** Score **> 22** indicates **homology** | U | R.YLDLGAYYSAR.K |
| 43684 | 143 | – | 153 | 646.3202 | 1290.6259 | 1290.6244 | 1.18 | 0 | 29 | 0.01 | 1Score **> 33** indicates **identity** Score **> 21** indicates **homology** | U | R.YLDLGAYYSAR.K |
| 43685 | 143 | – | 153 | 646.3204 | 1290.6262 | 1290.6244 | 1.35 | 0 | 42 | 0.00047 | 1Score **> 33** indicates **identity** Score **> 22** indicates **homology** | U | R.YLDLGAYYSAR.K |
| 43686 | 143 | – | 153 | 646.3205 | 1290.6264 | 1290.6244 | 1.56 | 0 | 35 | 0.0028 | 1Score **> 33** indicates **identity** Score **> 22** indicates **homology** | U | R.YLDLGAYYSAR.K |
| 43687 | 143 | – | 153 | 646.3205 | 1290.6265 | 1290.6244 | 1.61 | 0 | 51 | 0.00016 | 1Score **> 33** indicates **identity** Score **> 26** indicates **homology** | U | R.YLDLGAYYSAR.K |
| 43688 | 143 | – | 153 | 646.3215 | 1290.6285 | 1290.6244 | 3.14 | 0 | 22 | 0.021 | 1Score **> 33** indicates **identity** Score **> 18** indicates **homology** | U | R.YLDLGAYYSAR.K |
| 57408 | 143 | – | 154 | 473.9133 | 1418.7180 | 1418.7194 | -0.95 | 1 | 54 | 8.8e-06 | 1Score **> 34** indicates **identity** Score **> 16** indicates **homology** | U | R.YLDLGAYYSARK.C |
| 57409 | 143 | – | 154 | 473.9138 | 1418.7197 | 1418.7194 | 0.20 | 1 | 31 | 0.0012 | 1Score **> 34** indicates **identity** Score **> 14** indicates **homology** | U | R.YLDLGAYYSARK.C |
| 57410 | 143 | – | 154 | 473.9139 | 1418.7198 | 1418.7194 | 0.29 | 1 | 25 | 0.005 | 1Score **> 34** indicates **identity** Score **> 14** indicates **homology** | U | R.YLDLGAYYSARK.C |

---

```
ID   NDUBA_MOUSE             Reviewed;         176 AA.
AC   Q9DCS9; Q3UAE3;
DT   27-JUN-2003, integrated into UniProtKB/Swiss-Prot.
DT   23-JAN-2007, sequence version 3.
DT   28-JUN-2023, entry version 146.
DE   RecName: Full=NADH dehydrogenase [ubiquinone] 1 beta subcomplex subunit 10;
DE   AltName: Full=Complex I-PDSW;
DE            Short=CI-PDSW;
DE   AltName: Full=NADH-ubiquinone oxidoreductase PDSW subunit;
GN   Name=Ndufb10;
OS   Mus musculus (Mouse).
OC   Eukaryota; Metazoa; Chordata; Craniata; Vertebrata; Euteleostomi; Mammalia;
OC   Eutheria; Euarchontoglires; Glires; Rodentia; Myomorpha; Muroidea; Muridae;
OC   Murinae; Mus; Mus.
OX   NCBI_TaxID=10090;
RN   [1]
RP   NUCLEOTIDE SEQUENCE [LARGE SCALE MRNA].
RC   STRAIN=C57BL/6J; TISSUE=Bone marrow, and Kidney;
RX   PubMed=16141072; DOI=10.1126/science.1112014;
RA   Carninci P., Kasukawa T., Katayama S., Gough J., Frith M.C., Maeda N.,
RA   Oyama R., Ravasi T., Lenhard B., Wells C., Kodzius R., Shimokawa K.,
RA   Bajic V.B., Brenner S.E., Batalov S., Forrest A.R., Zavolan M., Davis M.J.,
RA   Wilming L.G., Aidinis V., Allen J.E., Ambesi-Impiombato A., Apweiler R.,
RA   Aturaliya R.N., Bailey T.L., Bansal M., Baxter L., Beisel K.W., Bersano T.,
RA   Bono H., Chalk A.M., Chiu K.P., Choudhary V., Christoffels A.,
RA   Clutterbuck D.R., Crowe M.L., Dalla E., Dalrymple B.P., de Bono B.,
RA   Della Gatta G., di Bernardo D., Down T., Engstrom P., Fagiolini M.,
RA   Faulkner G., Fletcher C.F., Fukushima T., Furuno M., Futaki S.,
RA   Gariboldi M., Georgii-Hemming P., Gingeras T.R., Gojobori T., Green R.E.,
RA   Gustincich S., Harbers M., Hayashi Y., Hensch T.K., Hirokawa N., Hill D.,
RA   Huminiecki L., Iacono M., Ikeo K., Iwama A., Ishikawa T., Jakt M.,
RA   Kanapin A., Katoh M., Kawasawa Y., Kelso J., Kitamura H., Kitano H.,
RA   Kollias G., Krishnan S.P., Kruger A., Kummerfeld S.K., Kurochkin I.V.,
RA   Lareau L.F., Lazarevic D., Lipovich L., Liu J., Liuni S., McWilliam S.,
RA   Madan Babu M., Madera M., Marchionni L., Matsuda H., Matsuzawa S., Miki H.,
RA   Mignone F., Miyake S., Morris K., Mottagui-Tabar S., Mulder N., Nakano N.,
RA   Nakauchi H., Ng P., Nilsson R., Nishiguchi S., Nishikawa S., Nori F.,
RA   Ohara O., Okazaki Y., Orlando V., Pang K.C., Pavan W.J., Pavesi G.,
RA   Pesole G., Petrovsky N., Piazza S., Reed J., Reid J.F., Ring B.Z.,
RA   Ringwald M., Rost B., Ruan Y., Salzberg S.L., Sandelin A., Schneider C.,
RA   Schoenbach C., Sekiguchi K., Semple C.A., Seno S., Sessa L., Sheng Y.,
RA   Shibata Y., Shimada H., Shimada K., Silva D., Sinclair B., Sperling S.,
RA   Stupka E., Sugiura K., Sultana R., Takenaka Y., Taki K., Tammoja K.,
RA   Tan S.L., Tang S., Taylor M.S., Tegner J., Teichmann S.A., Ueda H.R.,
RA   van Nimwegen E., Verardo R., Wei C.L., Yagi K., Yamanishi H.,
RA   Zabarovsky E., Zhu S., Zimmer A., Hide W., Bult C., Grimmond S.M.,
RA   Teasdale R.D., Liu E.T., Brusic V., Quackenbush J., Wahlestedt C.,
RA   Mattick J.S., Hume D.A., Kai C., Sasaki D., Tomaru Y., Fukuda S.,
RA   Kanamori-Katayama M., Suzuki M., Aoki J., Arakawa T., Iida J., Imamura K.,
RA   Itoh M., Kato T., Kawaji H., Kawagashira N., Kawashima T., Kojima M.,
RA   Kondo S., Konno H., Nakano K., Ninomiya N., Nishio T., Okada M., Plessy C.,
RA   Shibata K., Shiraki T., Suzuki S., Tagami M., Waki K., Watahiki A.,
RA   Okamura-Oho Y., Suzuki H., Kawai J., Hayashizaki Y.;
RT   "The transcriptional landscape of the mammalian genome.";
RL   Science 309:1559-1563(2005).
RN   [2]
RP   NUCLEOTIDE SEQUENCE [LARGE SCALE MRNA].
RC   STRAIN=C57BL/6J; TISSUE=Brain, and Colon;
RX   PubMed=15489334; DOI=10.1101/gr.2596504;
RG   The MGC Project Team;
RT   "The status, quality, and expansion of the NIH full-length cDNA project:
RT   the Mammalian Gene Collection (MGC).";
RL   Genome Res. 14:2121-2127(2004).
RN   [3]
RP   PROTEIN SEQUENCE OF 2-49; 71-93; 95-109; 128-134 AND 143-153, AND
RP   IDENTIFICATION BY MASS SPECTROMETRY.
RC   STRAIN=C57BL/6J; TISSUE=Brain;
RA   Lubec G., Kang S.U.;
RL   Submitted (APR-2007) to UniProtKB.
RN   [4]
RP   PHOSPHORYLATION [LARGE SCALE ANALYSIS] AT SER-21, AND IDENTIFICATION BY
RP   MASS SPECTROMETRY [LARGE SCALE ANALYSIS].
RC   TISSUE=Brain, Brown adipose tissue, Heart, Kidney, Liver, Lung,
RC   Pancreas, Spleen, and Testis;
RX   PubMed=21183079; DOI=10.1016/j.cell.2010.12.001;
RA   Huttlin E.L., Jedrychowski M.P., Elias J.E., Goswami T., Rad R.,
RA   Beausoleil S.A., Villen J., Haas W., Sowa M.E., Gygi S.P.;
RT   "A tissue-specific atlas of mouse protein phosphorylation and expression.";
RL   Cell 143:1174-1189(2010).
CC   -!- FUNCTION: Accessory subunit that is involved in the functional assembly
CC       of the mitochondrial respiratory chain complex I. Complex I has an NADH
CC       dehydrogenase activity with ubiquinone as an immediate electron
CC       acceptor and mediates the transfer of electrons from NADH to the
CC       respiratory chain. {ECO:0000250|UniProtKB:O96000}.
CC   -!- FUNCTION: Accessory subunit of the mitochondrial membrane respiratory
CC       chain NADH dehydrogenase (Complex I), that is believed not to be
CC       involved in catalysis. Complex I functions in the transfer of electrons
CC       from NADH to the respiratory chain. The immediate electron acceptor for
CC       the enzyme is believed to be ubiquinone.
CC       {ECO:0000250|UniProtKB:O96000}.
CC   -!- SUBUNIT: Complex I is composed of 45 different subunits. Interacts with
CC       CHCHD4. {ECO:0000250|UniProtKB:O96000}.
CC   -!- SUBCELLULAR LOCATION: Mitochondrion inner membrane
CC       {ECO:0000250|UniProtKB:O96000}; Peripheral membrane protein
CC       {ECO:0000250|UniProtKB:O96000}; Matrix side
CC       {ECO:0000250|UniProtKB:O96000}.
CC   -!- SIMILARITY: Belongs to the complex I NDUFB10 subunit family.
CC       {ECO:0000305}.
CC   ---------------------------------------------------------------------------
CC   Copyrighted by the UniProt Consortium, see https://www.uniprot.org/terms
CC   Distributed under the Creative Commons Attribution (CC BY 4.0) License
CC   ---------------------------------------------------------------------------
DR   EMBL; AK002517; BAB22156.1; -; mRNA.
DR   EMBL; AK151404; BAE30371.1; -; mRNA.
DR   EMBL; BC031664; AAH31664.1; -; mRNA.
DR   EMBL; BC043013; AAH43013.1; -; mRNA.
DR   CCDS; CCDS28494.1; -.
DR   RefSeq; NP_080960.1; NM_026684.2.
DR   PDB; 6G2J; EM; 3.30 A; p=1-176.
DR   PDB; 6G72; EM; 3.90 A; p=1-176.
DR   PDB; 6ZR2; EM; 3.10 A; p=1-176.
DR   PDB; 6ZTQ; EM; 3.00 A; p=1-176.
DR   PDB; 7AK5; EM; 3.17 A; p=1-176.
DR   PDB; 7AK6; EM; 3.82 A; p=1-176.
DR   PDB; 7B93; EM; 3.04 A; p=1-176.
DR   PDB; 7PSA; EM; 3.40 A; p=1-176.
DR   PDBsum; 6G2J; -.
DR   PDBsum; 6G72; -.
DR   PDBsum; 6ZR2; -.
DR   PDBsum; 6ZTQ; -.
DR   PDBsum; 7AK5; -.
DR   PDBsum; 7AK6; -.
DR   PDBsum; 7B93; -.
DR   PDBsum; 7PSA; -.
DR   AlphaFoldDB; Q9DCS9; -.
DR   SMR; Q9DCS9; -.
DR   BioGRID; 212811; 86.
DR   ComplexPortal; CPX-266; Mitochondrial respiratory chain complex I.
DR   CORUM; Q9DCS9; -.
DR   IntAct; Q9DCS9; 3.
DR   STRING; 10090.ENSMUSP00000043543; -.
DR   TCDB; 3.D.1.6.1; the h+ or na+-translocating nadh dehydrogenase (ndh) family.
DR   GlyGen; Q9DCS9; 2 sites, 1 O-linked glycan (2 sites).
DR   iPTMnet; Q9DCS9; -.
DR   PhosphoSitePlus; Q9DCS9; -.
DR   SwissPalm; Q9DCS9; -.
DR   EPD; Q9DCS9; -.
DR   jPOST; Q9DCS9; -.
DR   MaxQB; Q9DCS9; -.
DR   PaxDb; Q9DCS9; -.
DR   PeptideAtlas; Q9DCS9; -.
DR   ProteomicsDB; 252868; -.
DR   Antibodypedia; 23340; 378 antibodies from 31 providers.
DR   DNASU; 68342; -.
DR   Ensembl; ENSMUST00000045602; ENSMUSP00000043543; ENSMUSG00000040048.
DR   GeneID; 68342; -.
DR   KEGG; mmu:68342; -.
DR   UCSC; uc008ayd.1; mouse.
DR   AGR; MGI:1915592; -.
DR   CTD; 4716; -.
DR   MGI; MGI:1915592; Ndufb10.
DR   VEuPathDB; HostDB:ENSMUSG00000040048; -.
DR   eggNOG; KOG4009; Eukaryota.
DR   GeneTree; ENSGT00390000006348; -.
DR   InParanoid; Q9DCS9; -.
DR   OMA; TFVREWI; -.
DR   OrthoDB; 2871654at2759; -.
DR   PhylomeDB; Q9DCS9; -.
DR   TreeFam; TF105792; -.
DR   Reactome; R-MMU-611105; Respiratory electron transport.
DR   Reactome; R-MMU-6799198; Complex I biogenesis.
DR   BioGRID-ORCS; 68342; 25 hits in 80 CRISPR screens.
DR   ChiTaRS; Ndufb10; mouse.
DR   PRO; PR:Q9DCS9; -.
DR   Proteomes; UP000000589; Chromosome 17.
DR   RNAct; Q9DCS9; protein.
DR   Bgee; ENSMUSG00000040048; Expressed in interventricular septum and 261 other tissues.
DR   ExpressionAtlas; Q9DCS9; baseline and differential.
DR   Genevisible; Q9DCS9; MM.
DR   GO; GO:0005743; C:mitochondrial inner membrane; HDA:MGI.
DR   GO; GO:0005747; C:mitochondrial respiratory chain complex I; ISS:UniProtKB.
DR   GO; GO:0005739; C:mitochondrion; HDA:MGI.
DR   GO; GO:0009060; P:aerobic respiration; NAS:ComplexPortal.
DR   GO; GO:0032981; P:mitochondrial respiratory chain complex I assembly; ISS:UniProtKB.
DR   GO; GO:0042776; P:proton motive force-driven mitochondrial ATP synthesis; NAS:ComplexPortal.
DR   InterPro; IPR019377; NADH_UbQ_OxRdtase_su10.
DR   InterPro; IPR039993; NDUFB10.
DR   PANTHER; PTHR13094:SF1; NADH DEHYDROGENASE [UBIQUINONE] 1 BETA SUBCOMPLEX SUBUNIT 10; 1.
DR   PANTHER; PTHR13094; NADH-UBIQUINONE OXIDOREDUCTASE PDSW SUBUNIT; 1.
DR   Pfam; PF10249; NDUFB10; 1.
PE   1: Evidence at protein level;
KW   3D-structure; Direct protein sequencing; Electron transport; Membrane;
KW   Mitochondrion; Mitochondrion inner membrane; Phosphoprotein;
KW   Reference proteome; Respiratory chain; Transport.
FT   CHAIN           1..176
FT                   /note="NADH dehydrogenase [ubiquinone] 1 beta subcomplex
FT                   subunit 10"
FT                   /id="PRO_0000118831"
FT   REGION          1..27
FT                   /note="Disordered"
FT                   /evidence="ECO:0000256|SAM:MobiDB-lite"
FT   MOD_RES         21
FT                   /note="Phosphoserine"
FT                   /evidence="ECO:0007744|PubMed:21183079"
FT   MOD_RES         151
FT                   /note="Phosphoserine"
FT                   /evidence="ECO:0000250|UniProtKB:O96000"
FT   TURN            7..9
FT                   /evidence="ECO:0007829|PDB:6ZTQ"
FT   STRAND          24..26
FT                   /evidence="ECO:0007829|PDB:7B93"
FT   HELIX           29..58
FT                   /evidence="ECO:0007829|PDB:6ZTQ"
FT   HELIX           74..76
FT                   /evidence="ECO:0007829|PDB:6ZTQ"
FT   STRAND          77..80
FT                   /evidence="ECO:0007829|PDB:7AK5"
FT   HELIX           82..117
FT                   /evidence="ECO:0007829|PDB:6ZTQ"
FT   HELIX           118..120
FT                   /evidence="ECO:0007829|PDB:6ZR2"
FT   HELIX           121..124
FT                   /evidence="ECO:0007829|PDB:6ZTQ"
FT   HELIX           126..143
FT                   /evidence="ECO:0007829|PDB:6ZTQ"
FT   HELIX           152..170
FT                   /evidence="ECO:0007829|PDB:6ZTQ"
SQ   SEQUENCE   176 AA;  21024 MW;  1A3BA32E0F8551C4 CRC64;
     MPDSWDKDVY PEPPSRTPAP SPQTSLPNPI TYLTKAYDLV VDWPVTLVRE FIERQHAKNR
     TYYYHRQYRR VPDITECKEG DVLCIYEAEM QWRRDFKVDQ EIMNIIQERL KACQQREGEN
     YQQNCAKELE QFTKVTKAYQ DRYLDLGAYY SARKCLAKQK QRMLEERKAA RQEAAA
//
```

**Mascot:** http://www.matrixscience.com/


HNE (K) (+156.1150)
