# Supplementary material for: A common East-Asian ALDH2 mutation causes metabolic disorders and the therapeutic effect of ALDH2 activators
Source: Nat Commun. 2023 Sep 25;14:5971. doi: 10.1038/s41467-023-41570-6 (PMC10520061; doi:10.1038/s41467-023-41570-6)
Supplement: Supplementary file 4 — Supplementary Data 1 [file 41467_2023_41570_MOESM4_ESM.zip › Table S5c/P12242/P12242_KIO-1-_K16_C25_C189_H215.html]

Mascot Search Results: P12242
 

# MASCOT Search Results

## Protein View: P12242

### Mitochondrial brown fat uncoupling protein 1 OS=Mus musculus OX=10090 GN=Ucp1 PE=1 SV=2

|  |  |
| --- | --- |
| Database: | Mouse\_UniProt\_proteomes |
| Score: | 18845 |
| Monoisotopic mass (M_r_): | 33625 |
| Calculated pI: | 9.29 |

Sequence similarity is available as an NCBI BLAST search of P12242 against nr.

### Search parameters

|  |  |
| --- | --- |
| MS data file: | `D:\LCMSMS\2023 Users' data\230529-1\230529-1-KIO-1.raw` |
| Enzyme: | Trypsin/P: cuts C-term side of KR. |
| Fixed modifications: | Carbamidomethyl (C) |
| Variable modifications: | Deamidated (NQ), HNE (C), HNE (H), HNE (K), Oxidation (M) |

### Protein sequence coverage: 82%

Matched peptides shown in ***bold red***.

|  |  |  |  |  |  |
| --- | --- | --- | --- | --- | --- |
| `1` | `MVNPTTSEVQ` | `PTMGVKIFSA` | `GVSACLADII` | `TFPLDTAKVR` | `LQIQGEGQAS` |
| `51` | `STIRYKGVLG` | `TITTLAKTEG` | `LPKLYSGLPA` | `GIQRQISFAS` | `LRIGLYDSVQ` |
| `101` | `EYFSSGRETP` | `ASLGNKISAG` | `LMTGGVAVFI` | `GQPTEVVKVR` | `MQAQSHLHGI` |
| `151` | `KPRYTGTYNA` | `YRVIATTESL` | `STLWKGTTPN` | `LMRNVIINCT` | `ELVTYDLMKG` |
| `201` | `ALVNNKILAD` | `DVPCHLLSAL` | `VAGFCTTLLA` | `SPVDVVKTRF` | `INSLPGQYPS` |
| `251` | `VPSCAMSMYT` | `KEGPTAFFKG` | `FVASFLRLGS` | `WNVIMFVCFE` | `QLKKELMKSR` |
| `301` | `QTVDCTT` |  |  |  |  |

Unformatted sequence string: 307 residues (for pasting into other applications).

|  |  |  |  |
| --- | --- | --- | --- |
| Sort by | residue number | increasing mass | decreasing mass |
| Show | matched peptides only | predicted peptides also |  |

| Query | Start | – | End | Observed | Mr(expt) | Mr(calc) | ppm | M | Score | Expect | Rank | U | Peptide |
| --- | --- | --- | --- | --- | --- | --- | --- | --- | --- | --- | --- | --- | --- |
| 194198 | 1 | – | 38 | 1370.3710 | 4108.0913 | 4108.1186 | -6.66 | 1 | 29 | 0.0021 | 1Score **> 36** indicates **identity** Score **> 14** indicates **homology** | U | -.MVNPTTSEVQPTMGVKIFSAGVSACLADIITFPLDTAK.V  + HNE (C) |
| 73661 | 2 | – | 16 | 794.4067 | 1586.7988 | 1586.7974 | 0.86 | 0 | 80 | 5.8e-08 | 1Score **> 34** indicates **identity** Score **> 20** indicates **homology** | U | M.VNPTTSEVQPTMGVK.I |
| 73663 | 2 | – | 16 | 794.4070 | 1586.7994 | 1586.7974 | 1.22 | 0 | 76 | 8.1e-08 | 1Score **> 34** indicates **identity** Score **> 17** indicates **homology** | U | M.VNPTTSEVQPTMGVK.I |
| 73664 | 2 | – | 16 | 794.4073 | 1586.8000 | 1586.7974 | 1.63 | 0 | 50 | 2.1e-05 | 1Score **> 34** indicates **identity** Score **> 16** indicates **homology** | U | M.VNPTTSEVQPTMGVK.I |
| 73666 | 2 | – | 16 | 794.4083 | 1586.8020 | 1586.7974 | 2.91 | 0 | 33 | 0.00079 | 1Score **> 34** indicates **identity** Score **> 15** indicates **homology** | U | M.VNPTTSEVQPTMGVK.I |
| 75864 | 2 | – | 16 | 802.4020 | 1602.7894 | 1602.7923 | -1.85 | 0 | 35 | 0.00054 | 1Score **> 34** indicates **identity** Score **> 15** indicates **homology** | U | M.VNPTTSEVQPTMGVK.I  + Oxidation (M) |
| 75867 | 2 | – | 16 | 802.4031 | 1602.7916 | 1602.7923 | -0.49 | 0 | 26 | 0.0077 | 1Score **> 34** indicates **identity** Score **> 18** indicates **homology** | U | M.VNPTTSEVQPTMGVK.I  + Oxidation (M) |
| 75868 | 2 | – | 16 | 802.4035 | 1602.7924 | 1602.7923 | 0.063 | 0 | 74 | 1.1e-07 | 1Score **> 34** indicates **identity** Score **> 17** indicates **homology** | U | M.VNPTTSEVQPTMGVK.I  + Oxidation (M) |
| 75869 | 2 | – | 16 | 802.4043 | 1602.7940 | 1602.7923 | 1.03 | 0 | 35 | 0.00048 | 1Score **> 34** indicates **identity** Score **> 15** indicates **homology** | U | M.VNPTTSEVQPTMGVK.I  + Oxidation (M) |
| 75871 | 2 | – | 16 | 802.4043 | 1602.7941 | 1602.7923 | 1.11 | 0 | 32 | 0.0013 | 1Score **> 34** indicates **identity** Score **> 15** indicates **homology** | U | M.VNPTTSEVQPTMGVK.I  + Oxidation (M) |
| 75872 | 2 | – | 16 | 802.4044 | 1602.7942 | 1602.7923 | 1.18 | 0 | 47 | 5.4e-05 | 1Score **> 34** indicates **identity** Score **> 17** indicates **homology** | U | M.VNPTTSEVQPTMGVK.I  + Oxidation (M) |
| 75874 | 2 | – | 16 | 802.4045 | 1602.7945 | 1602.7923 | 1.33 | 0 | 33 | 0.00096 | 1Score **> 34** indicates **identity** Score **> 15** indicates **homology** | U | M.VNPTTSEVQPTMGVK.I  + Oxidation (M) |
| 75875 | 2 | – | 16 | 802.4047 | 1602.7948 | 1602.7923 | 1.52 | 0 | 29 | 0.0018 | 1Score **> 34** indicates **identity** Score **> 14** indicates **homology** | U | M.VNPTTSEVQPTMGVK.I  + Oxidation (M) |
| 75876 | 2 | – | 16 | 802.4050 | 1602.7955 | 1602.7923 | 1.97 | 0 | 72 | 1.8e-07 | 1Score **> 34** indicates **identity** Score **> 17** indicates **homology** | U | M.VNPTTSEVQPTMGVK.I  + Oxidation (M) |
| 75877 | 2 | – | 16 | 802.4052 | 1602.7959 | 1602.7923 | 2.24 | 0 | 26 | 0.0034 | 1Score **> 34** indicates **identity** Score **> 14** indicates **homology** | U | M.VNPTTSEVQPTMGVK.I  + Oxidation (M) |
| 75879 | 2 | – | 16 | 802.4057 | 1602.7969 | 1602.7923 | 2.86 | 0 | 26 | 0.0035 | 1Score **> 34** indicates **identity** Score **> 14** indicates **homology** | U | M.VNPTTSEVQPTMGVK.I  + Oxidation (M) |
| 75881 | 2 | – | 16 | 802.4064 | 1602.7983 | 1602.7923 | 3.71 | 0 | 22 | 0.0095 | 1Score **> 34** indicates **identity** Score **> 14** indicates **homology** | U | M.VNPTTSEVQPTMGVK.I  + Oxidation (M) |
| 193746 | 2 | – | 38 | 1332.0312 | 3993.0719 | 3993.0731 | -0.30 | 1 | 23 | 0.0073 | 1Score **> 36** indicates **identity** Score **> 14** indicates **homology** | U | M.VNPTTSEVQPTMGVKIFSAGVSACLADIITFPLDTAK.V  + HNE (C); Oxidation (M) |
| 193977 | 2 | – | 38 | 1351.0256 | 4050.0549 | 4050.0945 | -9.78 | 1 | 72 | 1.8e-07 | 1Score **> 37** indicates **identity** Score **> 17** indicates **homology** | U | M.VNPTTSEVQPTMGVKIFSAGVSACLADIITFPLDTAK.V  + HNE (K); Oxidation (M) |
| 193986 | 2 | – | 38 | 1351.3696 | 4051.0870 | 4051.0786 | 2.08 | 1 | 32 | 0.0011 | 1Score **> 36** indicates **identity** Score **> 14** indicates **homology** | U | M.VNPTTSEVQPTMGVKIFSAGVSACLADIITFPLDTAK.V  + Deamidated (NQ); HNE (K); Oxidation (M) |
| 194927 | 2 | – | 40 | 862.0538 | 4305.2328 | 4305.2641 | -7.27 | 2 | 20 | 0.013 | 1Score **> 36** indicates **identity** Score **> 14** indicates **homology** | U | M.VNPTTSEVQPTMGVKIFSAGVSACLADIITFPLDTAKVR.L  + HNE (K); Oxidation (M) |
| 194946 | 2 | – | 40 | 1077.5663 | 4306.2361 | 4306.2481 | -2.78 | 2 | 19 | 0.017 | 1Score **> 36** indicates **identity** Score **> 14** indicates **homology** | U | M.VNPTTSEVQPTMGVKIFSAGVSACLADIITFPLDTAKVR.L  + Deamidated (NQ); HNE (K); Oxidation (M) |
| 147190 | 17 | – | 38 | 770.7375 | 2309.1906 | 2309.1977 | -3.07 | 0 | 28 | 0.0026 | 1Score **> 37** indicates **identity** Score **> 14** indicates **homology** | U | K.IFSAGVSACLADIITFPLDTAK.V |
| 147193 | 17 | – | 38 | 1155.6036 | 2309.1926 | 2309.1977 | -2.24 | 0 | 57 | 4.2e-06 | 1Score **> 37** indicates **identity** Score **> 16** indicates **homology** | U | K.IFSAGVSACLADIITFPLDTAK.V |
| 147197 | 17 | – | 38 | 1155.6054 | 2309.1963 | 2309.1977 | -0.63 | 0 | 132 | 4.5e-13 | 1Score **> 37** indicates **identity** Score **> 21** indicates **homology** | U | K.IFSAGVSACLADIITFPLDTAK.V |
| 147200 | 17 | – | 38 | 1155.6056 | 2309.1967 | 2309.1977 | -0.43 | 0 | 16 | 0.035 | 1Score **> 37** indicates **identity** Score **> 13** indicates **homology** | U | K.IFSAGVSACLADIITFPLDTAK.V |
| 147201 | 17 | – | 38 | 770.7395 | 2309.1968 | 2309.1977 | -0.40 | 0 | 37 | 0.00032 | 1Score **> 37** indicates **identity** Score **> 15** indicates **homology** | U | K.IFSAGVSACLADIITFPLDTAK.V |
| 147202 | 17 | – | 38 | 770.7396 | 2309.1971 | 2309.1977 | -0.28 | 0 | 31 | 0.0013 | 1Score **> 37** indicates **identity** Score **> 14** indicates **homology** | U | K.IFSAGVSACLADIITFPLDTAK.V |
| 147203 | 17 | – | 38 | 770.7397 | 2309.1972 | 2309.1977 | -0.24 | 0 | 18 | 0.019 | 1Score **> 37** indicates **identity** Score **> 14** indicates **homology** | U | K.IFSAGVSACLADIITFPLDTAK.V |
| 147204 | 17 | – | 38 | 770.7397 | 2309.1973 | 2309.1977 | -0.18 | 0 | 15 | 0.041 | 1Score **> 37** indicates **identity** Score **> 13** indicates **homology** | U | K.IFSAGVSACLADIITFPLDTAK.V |
| 147205 | 17 | – | 38 | 770.7398 | 2309.1977 | 2309.1977 | -0.012 | 0 | 17 | 0.027 | 1Score **> 37** indicates **identity** Score **> 14** indicates **homology** | U | K.IFSAGVSACLADIITFPLDTAK.V |
| 147206 | 17 | – | 38 | 770.7399 | 2309.1980 | 2309.1977 | 0.12 | 0 | 27 | 0.0032 | 1Score **> 37** indicates **identity** Score **> 14** indicates **homology** | U | K.IFSAGVSACLADIITFPLDTAK.V |
| 147210 | 17 | – | 38 | 770.7400 | 2309.1983 | 2309.1977 | 0.24 | 0 | 28 | 0.0025 | 1Score **> 37** indicates **identity** Score **> 14** indicates **homology** | U | K.IFSAGVSACLADIITFPLDTAK.V |
| 147211 | 17 | – | 38 | 770.7401 | 2309.1984 | 2309.1977 | 0.29 | 0 | 18 | 0.025 | 1Score **> 37** indicates **identity** Score **> 15** indicates **homology** | U | K.IFSAGVSACLADIITFPLDTAK.V |
| 147212 | 17 | – | 38 | 770.7401 | 2309.1986 | 2309.1977 | 0.37 | 0 | 30 | 0.0014 | 1Score **> 37** indicates **identity** Score **> 14** indicates **homology** | U | K.IFSAGVSACLADIITFPLDTAK.V |
| 147214 | 17 | – | 38 | 1155.6066 | 2309.1987 | 2309.1977 | 0.41 | 0 | 59 | 3.2e-06 | 1Score **> 37** indicates **identity** Score **> 16** indicates **homology** | U | K.IFSAGVSACLADIITFPLDTAK.V |
| 147217 | 17 | – | 38 | 1155.6069 | 2309.1993 | 2309.1977 | 0.70 | 0 | 79 | 3.6e-08 | 1Score **> 37** indicates **identity** Score **> 17** indicates **homology** | U | K.IFSAGVSACLADIITFPLDTAK.V |
| 147218 | 17 | – | 38 | 770.7404 | 2309.1994 | 2309.1977 | 0.71 | 0 | 29 | 0.0019 | 1Score **> 37** indicates **identity** Score **> 14** indicates **homology** | U | K.IFSAGVSACLADIITFPLDTAK.V |
| 147224 | 17 | – | 38 | 1155.6074 | 2309.2003 | 2309.1977 | 1.10 | 0 | 46 | 5e-05 | 1Score **> 37** indicates **identity** Score **> 15** indicates **homology** | U | K.IFSAGVSACLADIITFPLDTAK.V |
| 147228 | 17 | – | 38 | 770.7410 | 2309.2011 | 2309.1977 | 1.47 | 0 | 19 | 0.024 | 1Score **> 37** indicates **identity** Score **> 15** indicates **homology** | U | K.IFSAGVSACLADIITFPLDTAK.V |
| 147229 | 17 | – | 38 | 1155.6079 | 2309.2012 | 2309.1977 | 1.52 | 0 | 50 | 2.2e-05 | 1Score **> 37** indicates **identity** Score **> 16** indicates **homology** | U | K.IFSAGVSACLADIITFPLDTAK.V |
| 147231 | 17 | – | 38 | 1155.6080 | 2309.2014 | 2309.1977 | 1.61 | 0 | 31 | 0.0011 | 1Score **> 37** indicates **identity** Score **> 14** indicates **homology** | U | K.IFSAGVSACLADIITFPLDTAK.V |
| 147232 | 17 | – | 38 | 1155.6082 | 2309.2019 | 2309.1977 | 1.81 | 0 | 17 | 0.024 | 1Score **> 37** indicates **identity** Score **> 14** indicates **homology** | U | K.IFSAGVSACLADIITFPLDTAK.V |
| 147233 | 17 | – | 38 | 770.7414 | 2309.2025 | 2309.1977 | 2.06 | 0 | 59 | 3e-06 | 1Score **> 37** indicates **identity** Score **> 16** indicates **homology** | U | K.IFSAGVSACLADIITFPLDTAK.V |
| 147234 | 17 | – | 38 | 1155.6085 | 2309.2025 | 2309.1977 | 2.06 | 0 | 114 | 2.1e-11 | 1Score **> 37** indicates **identity** Score **> 20** indicates **homology** | U | K.IFSAGVSACLADIITFPLDTAK.V |
| 147235 | 17 | – | 38 | 770.7415 | 2309.2026 | 2309.1977 | 2.11 | 0 | 18 | 0.019 | 1Score **> 37** indicates **identity** Score **> 14** indicates **homology** | U | K.IFSAGVSACLADIITFPLDTAK.V |
| 147236 | 17 | – | 38 | 1155.6086 | 2309.2026 | 2309.1977 | 2.13 | 0 | 113 | 2.8e-11 | 1Score **> 37** indicates **identity** Score **> 20** indicates **homology** | U | K.IFSAGVSACLADIITFPLDTAK.V |
| 147237 | 17 | – | 38 | 770.7415 | 2309.2026 | 2309.1977 | 2.13 | 0 | 48 | 3.2e-05 | 1Score **> 37** indicates **identity** Score **> 15** indicates **homology** | U | K.IFSAGVSACLADIITFPLDTAK.V |
| 147238 | 17 | – | 38 | 770.7415 | 2309.2027 | 2309.1977 | 2.16 | 0 | 37 | 0.00037 | 1Score **> 37** indicates **identity** Score **> 15** indicates **homology** | U | K.IFSAGVSACLADIITFPLDTAK.V |
| 147239 | 17 | – | 38 | 1155.6088 | 2309.2031 | 2309.1977 | 2.32 | 0 | 86 | 9.2e-09 | 1Score **> 36** indicates **identity** Score **> 18** indicates **homology** | U | K.IFSAGVSACLADIITFPLDTAK.V |
| 147243 | 17 | – | 38 | 770.7418 | 2309.2035 | 2309.1977 | 2.51 | 0 | 33 | 0.00074 | 1Score **> 36** indicates **identity** Score **> 15** indicates **homology** | U | K.IFSAGVSACLADIITFPLDTAK.V |
| 147244 | 17 | – | 38 | 770.7418 | 2309.2036 | 2309.1977 | 2.55 | 0 | 19 | 0.02 | 1Score **> 37** indicates **identity** Score **> 15** indicates **homology** | U | K.IFSAGVSACLADIITFPLDTAK.V |
| 147247 | 17 | – | 38 | 1155.6094 | 2309.2042 | 2309.1977 | 2.79 | 0 | 109 | 5.7e-11 | 1Score **> 37** indicates **identity** Score **> 19** indicates **homology** | U | K.IFSAGVSACLADIITFPLDTAK.V |
| 147248 | 17 | – | 38 | 770.7423 | 2309.2051 | 2309.1977 | 3.20 | 0 | 41 | 0.00015 | 1Score **> 37** indicates **identity** Score **> 15** indicates **homology** | U | K.IFSAGVSACLADIITFPLDTAK.V |
| 147250 | 17 | – | 38 | 770.7425 | 2309.2056 | 2309.1977 | 3.40 | 0 | 46 | 4.9e-05 | 1Score **> 37** indicates **identity** Score **> 15** indicates **homology** | U | K.IFSAGVSACLADIITFPLDTAK.V |
| 147251 | 17 | – | 38 | 770.7425 | 2309.2056 | 2309.1977 | 3.42 | 0 | 30 | 0.0015 | 1Score **> 37** indicates **identity** Score **> 14** indicates **homology** | U | K.IFSAGVSACLADIITFPLDTAK.V |
| 147252 | 17 | – | 38 | 1155.6101 | 2309.2056 | 2309.1977 | 3.43 | 0 | 73 | 1.6e-07 | 1Score **> 37** indicates **identity** Score **> 17** indicates **homology** | U | K.IFSAGVSACLADIITFPLDTAK.V |
| 147253 | 17 | – | 38 | 770.7431 | 2309.2075 | 2309.1977 | 4.23 | 0 | 20 | 0.015 | 1Score **> 37** indicates **identity** Score **> 14** indicates **homology** | U | K.IFSAGVSACLADIITFPLDTAK.V |
| 147255 | 17 | – | 38 | 1155.6112 | 2309.2079 | 2309.1977 | 4.40 | 0 | 102 | 2.8e-10 | 1Score **> 37** indicates **identity** Score **> 19** indicates **homology** | U | K.IFSAGVSACLADIITFPLDTAK.V |
| 147256 | 17 | – | 38 | 1155.6112 | 2309.2079 | 2309.1977 | 4.41 | 0 | 86 | 8.5e-09 | 1Score **> 37** indicates **identity** Score **> 18** indicates **homology** | U | K.IFSAGVSACLADIITFPLDTAK.V |
| 147258 | 17 | – | 38 | 770.7433 | 2309.2080 | 2309.1977 | 4.44 | 0 | 46 | 4.7e-05 | 1Score **> 37** indicates **identity** Score **> 15** indicates **homology** | U | K.IFSAGVSACLADIITFPLDTAK.V |
| 147259 | 17 | – | 38 | 1155.6114 | 2309.2083 | 2309.1977 | 4.59 | 0 | 45 | 5.7e-05 | 1Score **> 37** indicates **identity** Score **> 15** indicates **homology** | U | K.IFSAGVSACLADIITFPLDTAK.V |
| 147261 | 17 | – | 38 | 1155.6116 | 2309.2086 | 2309.1977 | 4.69 | 0 | 23 | 0.0065 | 1Score **> 37** indicates **identity** Score **> 14** indicates **homology** | U | K.IFSAGVSACLADIITFPLDTAK.V |
| 147262 | 17 | – | 38 | 1155.6118 | 2309.2090 | 2309.1977 | 4.90 | 0 | 24 | 0.0059 | 1Score **> 37** indicates **identity** Score **> 14** indicates **homology** | U | K.IFSAGVSACLADIITFPLDTAK.V |
| 147263 | 17 | – | 38 | 1155.6121 | 2309.2096 | 2309.1977 | 5.12 | 0 | 100 | 4.3e-10 | 1Score **> 37** indicates **identity** Score **> 19** indicates **homology** | U | K.IFSAGVSACLADIITFPLDTAK.V |
| 147264 | 17 | – | 38 | 770.7438 | 2309.2096 | 2309.1977 | 5.12 | 0 | 42 | 0.00011 | 1Score **> 37** indicates **identity** Score **> 15** indicates **homology** | U | K.IFSAGVSACLADIITFPLDTAK.V |
| 147265 | 17 | – | 38 | 1155.6125 | 2309.2104 | 2309.1977 | 5.50 | 0 | 74 | 1.1e-07 | 1Score **> 36** indicates **identity** Score **> 17** indicates **homology** | U | K.IFSAGVSACLADIITFPLDTAK.V |
| 147268 | 17 | – | 38 | 770.7446 | 2309.2118 | 2309.1977 | 6.11 | 0 | 25 | 0.0048 | 1Score **> 37** indicates **identity** Score **> 14** indicates **homology** | U | K.IFSAGVSACLADIITFPLDTAK.V |
| 147269 | 17 | – | 38 | 770.7448 | 2309.2125 | 2309.1977 | 6.41 | 0 | 33 | 0.00082 | 1Score **> 37** indicates **identity** Score **> 15** indicates **homology** | U | K.IFSAGVSACLADIITFPLDTAK.V |
| 147270 | 17 | – | 38 | 1155.6135 | 2309.2125 | 2309.1977 | 6.41 | 0 | 96 | 9.2e-10 | 1Score **> 37** indicates **identity** Score **> 19** indicates **homology** | U | K.IFSAGVSACLADIITFPLDTAK.V |
| 147271 | 17 | – | 38 | 770.7451 | 2309.2135 | 2309.1977 | 6.81 | 0 | 31 | 0.0013 | 1Score **> 36** indicates **identity** Score **> 14** indicates **homology** | U | K.IFSAGVSACLADIITFPLDTAK.V |
| 147272 | 17 | – | 38 | 1155.6140 | 2309.2135 | 2309.1977 | 6.84 | 0 | 99 | 5.6e-10 | 1Score **> 36** indicates **identity** Score **> 19** indicates **homology** | U | K.IFSAGVSACLADIITFPLDTAK.V |
| 147273 | 17 | – | 38 | 1155.6141 | 2309.2137 | 2309.1977 | 6.93 | 0 | 78 | 5.5e-08 | 1Score **> 36** indicates **identity** Score **> 18** indicates **homology** | U | K.IFSAGVSACLADIITFPLDTAK.V |
| 147274 | 17 | – | 38 | 1155.6141 | 2309.2137 | 2309.1977 | 6.93 | 0 | 60 | 2.3e-06 | 1Score **> 36** indicates **identity** Score **> 16** indicates **homology** | U | K.IFSAGVSACLADIITFPLDTAK.V |
| 147275 | 17 | – | 38 | 770.7452 | 2309.2139 | 2309.1977 | 7.00 | 0 | 14 | 0.048 | 1Score **> 36** indicates **identity** Score **> 13** indicates **homology** | U | K.IFSAGVSACLADIITFPLDTAK.V |
| 147276 | 17 | – | 38 | 1155.6143 | 2309.2140 | 2309.1977 | 7.05 | 0 | 69 | 3.1e-07 | 1Score **> 36** indicates **identity** Score **> 17** indicates **homology** | U | K.IFSAGVSACLADIITFPLDTAK.V |
| 147277 | 17 | – | 38 | 1155.6145 | 2309.2145 | 2309.1977 | 7.28 | 0 | 80 | 2.8e-08 | 1Score **> 36** indicates **identity** Score **> 18** indicates **homology** | U | K.IFSAGVSACLADIITFPLDTAK.V |
| 147279 | 17 | – | 38 | 770.7457 | 2309.2153 | 2309.1977 | 7.61 | 0 | 31 | 0.0013 | 1Score **> 37** indicates **identity** Score **> 14** indicates **homology** | U | K.IFSAGVSACLADIITFPLDTAK.V |
| 147280 | 17 | – | 38 | 770.7457 | 2309.2154 | 2309.1977 | 7.65 | 0 | 33 | 0.00087 | 1Score **> 37** indicates **identity** Score **> 15** indicates **homology** | U | K.IFSAGVSACLADIITFPLDTAK.V |
| 147281 | 17 | – | 38 | 1155.6151 | 2309.2157 | 2309.1977 | 7.77 | 0 | 36 | 0.00038 | 1Score **> 37** indicates **identity** Score **> 15** indicates **homology** | U | K.IFSAGVSACLADIITFPLDTAK.V |
| 147284 | 17 | – | 38 | 1155.6164 | 2309.2183 | 2309.1977 | 8.91 | 0 | 52 | 1.4e-05 | 1Score **> 37** indicates **identity** Score **> 16** indicates **homology** | U | K.IFSAGVSACLADIITFPLDTAK.V |
| 94131 | 39 | – | 54 | 581.6509 | 1741.9308 | 1741.9435 | -7.31 | 1 | 14 | 0.044 | 1Score **> 35** indicates **identity** Score **> 13** indicates **homology** | U | K.VRLQIQGEGQASSTIR.Y |
| 94136 | 39 | – | 54 | 581.6538 | 1741.9396 | 1741.9435 | -2.25 | 1 | 19 | 0.016 | 1Score **> 35** indicates **identity** Score **> 14** indicates **homology** | U | K.VRLQIQGEGQASSTIR.Y |
| 94137 | 39 | – | 54 | 581.6540 | 1741.9401 | 1741.9435 | -1.94 | 1 | 21 | 0.012 | 1Score **> 35** indicates **identity** Score **> 14** indicates **homology** | U | K.VRLQIQGEGQASSTIR.Y |
| 94139 | 39 | – | 54 | 581.6543 | 1741.9410 | 1741.9435 | -1.41 | 1 | 44 | 8.1e-05 | 1Score **> 35** indicates **identity** Score **> 15** indicates **homology** | U | K.VRLQIQGEGQASSTIR.Y |
| 94140 | 39 | – | 54 | 581.6544 | 1741.9415 | 1741.9435 | -1.15 | 1 | 20 | 0.013 | 1Score **> 35** indicates **identity** Score **> 14** indicates **homology** | U | K.VRLQIQGEGQASSTIR.Y |
| 94142 | 39 | – | 54 | 581.6545 | 1741.9417 | 1741.9435 | -1.02 | 1 | 17 | 0.025 | 1Score **> 35** indicates **identity** Score **> 14** indicates **homology** | U | K.VRLQIQGEGQASSTIR.Y |
| 94147 | 39 | – | 54 | 581.6552 | 1741.9436 | 1741.9435 | 0.083 | 1 | 20 | 0.015 | 1Score **> 35** indicates **identity** Score **> 14** indicates **homology** | U | K.VRLQIQGEGQASSTIR.Y |
| 94149 | 39 | – | 54 | 581.6552 | 1741.9437 | 1741.9435 | 0.14 | 1 | 15 | 0.037 | 1Score **> 35** indicates **identity** Score **> 13** indicates **homology** | U | K.VRLQIQGEGQASSTIR.Y |
| 94153 | 39 | – | 54 | 581.6553 | 1741.9442 | 1741.9435 | 0.41 | 1 | 18 | 0.019 | 1Score **> 35** indicates **identity** Score **> 14** indicates **homology** | U | K.VRLQIQGEGQASSTIR.Y |
| 94160 | 39 | – | 54 | 581.6557 | 1741.9452 | 1741.9435 | 1.00 | 1 | 14 | 0.05 | 1Score **> 35** indicates **identity** Score **> 13** indicates **homology** | U | K.VRLQIQGEGQASSTIR.Y |
| 94162 | 39 | – | 54 | 581.6557 | 1741.9454 | 1741.9435 | 1.11 | 1 | 58 | 4e-06 | 1Score **> 35** indicates **identity** Score **> 16** indicates **homology** | U | K.VRLQIQGEGQASSTIR.Y |
| 94163 | 39 | – | 54 | 581.6558 | 1741.9456 | 1741.9435 | 1.24 | 1 | 19 | 0.015 | 1Score **> 35** indicates **identity** Score **> 14** indicates **homology** | U | K.VRLQIQGEGQASSTIR.Y |
| 94165 | 39 | – | 54 | 581.6560 | 1741.9461 | 1741.9435 | 1.52 | 1 | 52 | 1.2e-05 | 1Score **> 35** indicates **identity** Score **> 16** indicates **homology** | U | K.VRLQIQGEGQASSTIR.Y |
| 94166 | 39 | – | 54 | 581.6560 | 1741.9463 | 1741.9435 | 1.62 | 1 | 26 | 0.0039 | 1Score **> 35** indicates **identity** Score **> 14** indicates **homology** | U | K.VRLQIQGEGQASSTIR.Y |
| 94169 | 39 | – | 54 | 581.6563 | 1741.9472 | 1741.9435 | 2.14 | 1 | 25 | 0.0043 | 1Score **> 35** indicates **identity** Score **> 14** indicates **homology** | U | K.VRLQIQGEGQASSTIR.Y |
| 94171 | 39 | – | 54 | 581.6568 | 1741.9487 | 1741.9435 | 2.98 | 1 | 19 | 0.015 | 1Score **> 35** indicates **identity** Score **> 14** indicates **homology** | U | K.VRLQIQGEGQASSTIR.Y |
| 94173 | 39 | – | 54 | 581.6572 | 1741.9496 | 1741.9435 | 3.53 | 1 | 25 | 0.0043 | 1Score **> 35** indicates **identity** Score **> 14** indicates **homology** | U | K.VRLQIQGEGQASSTIR.Y |
| 94175 | 39 | – | 54 | 581.6572 | 1741.9498 | 1741.9435 | 3.62 | 1 | 20 | 0.012 | 1Score **> 35** indicates **identity** Score **> 14** indicates **homology** | U | K.VRLQIQGEGQASSTIR.Y |
| 94328 | 39 | – | 54 | 582.3131 | 1743.9175 | 1743.9115 | 3.44 | 1 | 25 | 0.0044 | 1Score **> 36** indicates **identity** Score **> 14** indicates **homology** | U | K.VRLQIQGEGQASSTIR.Y  + 2 Deamidated (NQ) |
| 60342 | 41 | – | 54 | 744.3869 | 1486.7593 | 1486.7740 | -9.84 | 0 | 24 | 0.0061 | 1Score **> 34** indicates **identity** Score **> 14** indicates **homology** | U | R.LQIQGEGQASSTIR.Y |
| 60343 | 41 | – | 54 | 744.3882 | 1486.7619 | 1486.7740 | -8.12 | 0 | 47 | 3.9e-05 | 1Score **> 34** indicates **identity** Score **> 16** indicates **homology** | U | R.LQIQGEGQASSTIR.Y |
| 60344 | 41 | – | 54 | 744.3894 | 1486.7642 | 1486.7740 | -6.53 | 0 | 34 | 0.0022 | 1Score **> 34** indicates **identity** Score **> 20** indicates **homology** | U | R.LQIQGEGQASSTIR.Y |
| 60346 | 41 | – | 54 | 744.3900 | 1486.7655 | 1486.7740 | -5.66 | 0 | 21 | 0.011 | 1Score **> 34** indicates **identity** Score **> 14** indicates **homology** | U | R.LQIQGEGQASSTIR.Y |
| 60347 | 41 | – | 54 | 744.3906 | 1486.7666 | 1486.7740 | -4.97 | 0 | 60 | 1.7e-05 | 1Score **> 34** indicates **identity** Score **> 25** indicates **homology** | U | R.LQIQGEGQASSTIR.Y |
| 60349 | 41 | – | 54 | 744.3907 | 1486.7668 | 1486.7740 | -4.83 | 0 | 37 | 0.00037 | 1Score **> 34** indicates **identity** Score **> 15** indicates **homology** | U | R.LQIQGEGQASSTIR.Y |
| 60350 | 41 | – | 54 | 744.3909 | 1486.7673 | 1486.7740 | -4.51 | 0 | 40 | 0.00029 | 1Score **> 34** indicates **identity** Score **> 17** indicates **homology** | U | R.LQIQGEGQASSTIR.Y |
| 60351 | 41 | – | 54 | 744.3910 | 1486.7674 | 1486.7740 | -4.41 | 0 | 74 | 1.2e-06 | 1Score **> 34** indicates **identity** Score **> 27** indicates **homology** | U | R.LQIQGEGQASSTIR.Y |
| 60353 | 41 | – | 54 | 744.3911 | 1486.7676 | 1486.7740 | -4.25 | 0 | 16 | 0.034 | 1Score **> 34** indicates **identity** Score **> 13** indicates **homology** | U | R.LQIQGEGQASSTIR.Y |
| 60354 | 41 | – | 54 | 744.3912 | 1486.7678 | 1486.7740 | -4.16 | 0 | 23 | 0.022 | 1Score **> 34** indicates **identity** Score **> 19** indicates **homology** | U | R.LQIQGEGQASSTIR.Y |
| 60356 | 41 | – | 54 | 744.3912 | 1486.7679 | 1486.7740 | -4.06 | 0 | 25 | 0.0049 | 1Score **> 34** indicates **identity** Score **> 14** indicates **homology** | U | R.LQIQGEGQASSTIR.Y |
| 60357 | 41 | – | 54 | 744.3915 | 1486.7685 | 1486.7740 | -3.67 | 0 | 61 | 8.3e-06 | 1Score **> 34** indicates **identity** Score **> 22** indicates **homology** | U | R.LQIQGEGQASSTIR.Y |
| 60360 | 41 | – | 54 | 744.3917 | 1486.7689 | 1486.7740 | -3.42 | 0 | 20 | 0.044 | 1Score **> 34** indicates **identity** Score **> 19** indicates **homology** | U | R.LQIQGEGQASSTIR.Y |
| 60361 | 41 | – | 54 | 744.3919 | 1486.7692 | 1486.7740 | -3.20 | 0 | 45 | 0.00016 | 1Score **> 34** indicates **identity** Score **> 20** indicates **homology** | U | R.LQIQGEGQASSTIR.Y |
| 60365 | 41 | – | 54 | 744.3921 | 1486.7697 | 1486.7740 | -2.84 | 0 | 44 | 0.00051 | 1Score **> 34** indicates **identity** Score **> 24** indicates **homology** | U | R.LQIQGEGQASSTIR.Y |
| 60367 | 41 | – | 54 | 744.3925 | 1486.7705 | 1486.7740 | -2.34 | 0 | 45 | 0.00028 | 1Score **> 34** indicates **identity** Score **> 22** indicates **homology** | U | R.LQIQGEGQASSTIR.Y |
| 60368 | 41 | – | 54 | 744.3925 | 1486.7705 | 1486.7740 | -2.34 | 0 | 22 | 0.012 | 1Score **> 34** indicates **identity** Score **> 15** indicates **homology** | U | R.LQIQGEGQASSTIR.Y |
| 60370 | 41 | – | 54 | 744.3925 | 1486.7705 | 1486.7740 | -2.31 | 0 | 72 | 1.1e-06 | 1Score **> 34** indicates **identity** Score **> 25** indicates **homology** | U | R.LQIQGEGQASSTIR.Y |
| 60373 | 41 | – | 54 | 744.3928 | 1486.7710 | 1486.7740 | -2.01 | 0 | 55 | 4.5e-05 | 1Score **> 34** indicates **identity** Score **> 24** indicates **homology** | U | R.LQIQGEGQASSTIR.Y |
| 60374 | 41 | – | 54 | 744.3928 | 1486.7710 | 1486.7740 | -1.96 | 0 | 45 | 0.0005 | 1Score **> 34** indicates **identity** Score **> 25** indicates **homology** | U | R.LQIQGEGQASSTIR.Y |
| 60375 | 41 | – | 54 | 744.3928 | 1486.7710 | 1486.7740 | -1.96 | 0 | 37 | 0.00054 | 1Score **> 34** indicates **identity** Score **> 17** indicates **homology** | U | R.LQIQGEGQASSTIR.Y |
| 60376 | 41 | – | 54 | 744.3929 | 1486.7712 | 1486.7740 | -1.87 | 0 | 65 | 9.9e-06 | 1Score **> 34** indicates **identity** Score **> 27** indicates **homology** | U | R.LQIQGEGQASSTIR.Y |
| 60377 | 41 | – | 54 | 744.3930 | 1486.7714 | 1486.7740 | -1.70 | 0 | 45 | 0.00016 | 1Score **> 34** indicates **identity** Score **> 19** indicates **homology** | U | R.LQIQGEGQASSTIR.Y |
| 60379 | 41 | – | 54 | 744.3931 | 1486.7716 | 1486.7740 | -1.56 | 0 | 50 | 0.00015 | 1Score **> 34** indicates **identity** Score **> 24** indicates **homology** | U | R.LQIQGEGQASSTIR.Y |
| 60380 | 41 | – | 54 | 744.3932 | 1486.7718 | 1486.7740 | -1.47 | 0 | 34 | 0.00071 | 1Score **> 34** indicates **identity** Score **> 15** indicates **homology** | U | R.LQIQGEGQASSTIR.Y |
| 60381 | 41 | – | 54 | 744.3932 | 1486.7718 | 1486.7740 | -1.45 | 0 | 55 | 3.5e-05 | 1Score **> 34** indicates **identity** Score **> 23** indicates **homology** | U | R.LQIQGEGQASSTIR.Y |
| 60382 | 41 | – | 54 | 744.3932 | 1486.7718 | 1486.7740 | -1.45 | 0 | 20 | 0.013 | 1Score **> 34** indicates **identity** Score **> 14** indicates **homology** | U | R.LQIQGEGQASSTIR.Y |
| 60383 | 41 | – | 54 | 744.3932 | 1486.7719 | 1486.7740 | -1.35 | 0 | 56 | 5e-05 | 1Score **> 34** indicates **identity** Score **> 25** indicates **homology** | U | R.LQIQGEGQASSTIR.Y |
| 60384 | 41 | – | 54 | 744.3933 | 1486.7720 | 1486.7740 | -1.31 | 0 | 25 | 0.0041 | 1Score **> 34** indicates **identity** Score **> 14** indicates **homology** | U | R.LQIQGEGQASSTIR.Y |
| 60385 | 41 | – | 54 | 744.3933 | 1486.7720 | 1486.7740 | -1.30 | 0 | 72 | 8.1e-06 | 1Score **> 34** indicates **identity** Score **> 34** indicates **homology** | U | R.LQIQGEGQASSTIR.Y |
| 60386 | 41 | – | 54 | 744.3934 | 1486.7723 | 1486.7740 | -1.12 | 0 | 23 | 0.012 | 1Score **> 34** indicates **identity** Score **> 16** indicates **homology** | U | R.LQIQGEGQASSTIR.Y |
| 60387 | 41 | – | 54 | 744.3934 | 1486.7723 | 1486.7740 | -1.11 | 0 | 56 | 2.4e-05 | 1Score **> 34** indicates **identity** Score **> 22** indicates **homology** | U | R.LQIQGEGQASSTIR.Y |
| 60388 | 41 | – | 54 | 744.3935 | 1486.7724 | 1486.7740 | -1.05 | 0 | 63 | 7e-06 | 1Score **> 34** indicates **identity** Score **> 24** indicates **homology** | U | R.LQIQGEGQASSTIR.Y |
| 60389 | 41 | – | 54 | 744.3935 | 1486.7724 | 1486.7740 | -1.04 | 0 | 56 | 3.9e-05 | 1Score **> 34** indicates **identity** Score **> 25** indicates **homology** | U | R.LQIQGEGQASSTIR.Y |
| 60390 | 41 | – | 54 | 744.3935 | 1486.7724 | 1486.7740 | -1.03 | 0 | 70 | 2.8e-06 | 1Score **> 34** indicates **identity** Score **> 27** indicates **homology** | U | R.LQIQGEGQASSTIR.Y |
| 60391 | 41 | – | 54 | 744.3936 | 1486.7726 | 1486.7740 | -0.89 | 0 | 26 | 0.0037 | 1Score **> 34** indicates **identity** Score **> 14** indicates **homology** | U | R.LQIQGEGQASSTIR.Y |
| 60392 | 41 | – | 54 | 744.3936 | 1486.7727 | 1486.7740 | -0.86 | 0 | 31 | 0.0098 | 1Score **> 34** indicates **identity** Score **> 24** indicates **homology** | U | R.LQIQGEGQASSTIR.Y |
| 60393 | 41 | – | 54 | 744.3936 | 1486.7727 | 1486.7740 | -0.81 | 0 | 36 | 0.0021 | 1Score **> 34** indicates **identity** Score **> 21** indicates **homology** | U | R.LQIQGEGQASSTIR.Y |
| 60394 | 41 | – | 54 | 744.3937 | 1486.7728 | 1486.7740 | -0.81 | 0 | 77 | 1.8e-06 | 1Score **> 34** indicates **identity** Score **> 32** indicates **homology** | U | R.LQIQGEGQASSTIR.Y |
| 60395 | 41 | – | 54 | 744.3937 | 1486.7728 | 1486.7740 | -0.81 | 0 | 61 | 1.4e-05 | 1Score **> 34** indicates **identity** Score **> 25** indicates **homology** | U | R.LQIQGEGQASSTIR.Y |
| 60396 | 41 | – | 54 | 744.3937 | 1486.7728 | 1486.7740 | -0.78 | 0 | 68 | 6.2e-06 | 1Score **> 34** indicates **identity** Score **> 28** indicates **homology** | U | R.LQIQGEGQASSTIR.Y |
| 60397 | 41 | – | 54 | 744.3937 | 1486.7728 | 1486.7740 | -0.78 | 0 | 38 | 0.004 | 1Score **> 34** indicates **identity** Score **> 27** indicates **homology** | U | R.LQIQGEGQASSTIR.Y |
| 60398 | 41 | – | 54 | 744.3937 | 1486.7729 | 1486.7740 | -0.73 | 0 | 46 | 0.00028 | 1Score **> 34** indicates **identity** Score **> 23** indicates **homology** | U | R.LQIQGEGQASSTIR.Y |
| 60399 | 41 | – | 54 | 744.3937 | 1486.7729 | 1486.7740 | -0.73 | 0 | 15 | 0.037 | 1Score **> 34** indicates **identity** Score **> 13** indicates **homology** | U | R.LQIQGEGQASSTIR.Y |
| 60400 | 41 | – | 54 | 744.3937 | 1486.7729 | 1486.7740 | -0.73 | 0 | 31 | 0.0012 | 1Score **> 34** indicates **identity** Score **> 14** indicates **homology** | U | R.LQIQGEGQASSTIR.Y |
| 60401 | 41 | – | 54 | 744.3937 | 1486.7729 | 1486.7740 | -0.70 | 0 | 45 | 0.00059 | 1Score **> 34** indicates **identity** Score **> 26** indicates **homology** | U | R.LQIQGEGQASSTIR.Y |
| 60402 | 41 | – | 54 | 744.3937 | 1486.7729 | 1486.7740 | -0.70 | 0 | 64 | 9.9e-06 | 1Score **> 34** indicates **identity** Score **> 26** indicates **homology** | U | R.LQIQGEGQASSTIR.Y |
| 60403 | 41 | – | 54 | 744.3937 | 1486.7729 | 1486.7740 | -0.69 | 0 | 64 | 5.9e-06 | 1Score **> 34** indicates **identity** Score **> 24** indicates **homology** | U | R.LQIQGEGQASSTIR.Y |
| 60404 | 41 | – | 54 | 744.3938 | 1486.7730 | 1486.7740 | -0.65 | 0 | 66 | 2.1e-05 | 1Score **> 34** indicates **identity** Score **> 31** indicates **homology** | U | R.LQIQGEGQASSTIR.Y |
| 60405 | 41 | – | 54 | 744.3938 | 1486.7730 | 1486.7740 | -0.62 | 0 | 47 | 0.00048 | 1Score **> 34** indicates **identity** Score **> 26** indicates **homology** | U | R.LQIQGEGQASSTIR.Y |
| 60406 | 41 | – | 54 | 744.3938 | 1486.7730 | 1486.7740 | -0.62 | 0 | 68 | 5.5e-06 | 1Score **> 34** indicates **identity** Score **> 28** indicates **homology** | U | R.LQIQGEGQASSTIR.Y |
| 60407 | 41 | – | 54 | 744.3938 | 1486.7730 | 1486.7740 | -0.62 | 0 | 40 | 0.00097 | 1Score **> 34** indicates **identity** Score **> 22** indicates **homology** | U | R.LQIQGEGQASSTIR.Y |
| 60408 | 41 | – | 54 | 744.3938 | 1486.7731 | 1486.7740 | -0.58 | 0 | 17 | 0.026 | 1Score **> 34** indicates **identity** Score **> 14** indicates **homology** | U | R.LQIQGEGQASSTIR.Y |
| 60409 | 41 | – | 54 | 744.3938 | 1486.7731 | 1486.7740 | -0.57 | 0 | 25 | 0.0087 | 1Score **> 34** indicates **identity** Score **> 17** indicates **homology** | U | R.LQIQGEGQASSTIR.Y |
| 60410 | 41 | – | 54 | 744.3940 | 1486.7734 | 1486.7740 | -0.40 | 0 | 68 | 5.7e-06 | 1Score **> 34** indicates **identity** Score **> 28** indicates **homology** | U | R.LQIQGEGQASSTIR.Y |
| 60411 | 41 | – | 54 | 744.3940 | 1486.7734 | 1486.7740 | -0.40 | 0 | 65 | 2e-05 | 1Score **> 34** indicates **identity** Score **> 31** indicates **homology** | U | R.LQIQGEGQASSTIR.Y |
| 60412 | 41 | – | 54 | 744.3940 | 1486.7734 | 1486.7740 | -0.38 | 0 | 37 | 0.00087 | 1Score **> 34** indicates **identity** Score **> 19** indicates **homology** | U | R.LQIQGEGQASSTIR.Y |
| 60414 | 41 | – | 54 | 744.3940 | 1486.7735 | 1486.7740 | -0.31 | 0 | 65 | 5.1e-06 | 1Score **> 34** indicates **identity** Score **> 25** indicates **homology** | U | R.LQIQGEGQASSTIR.Y |
| 60415 | 41 | – | 54 | 744.3940 | 1486.7735 | 1486.7740 | -0.29 | 0 | 40 | 0.0074 | 1Score **> 34** indicates **identity** Score **> 31** indicates **homology** | U | R.LQIQGEGQASSTIR.Y |
| 60416 | 41 | – | 54 | 744.3940 | 1486.7735 | 1486.7740 | -0.28 | 0 | 14 | 0.045 | 1Score **> 34** indicates **identity** Score **> 13** indicates **homology** | U | R.LQIQGEGQASSTIR.Y |
| 60417 | 41 | – | 54 | 744.3941 | 1486.7736 | 1486.7740 | -0.24 | 0 | 60 | 1.4e-05 | 1Score **> 34** indicates **identity** Score **> 24** indicates **homology** | U | R.LQIQGEGQASSTIR.Y |
| 60418 | 41 | – | 54 | 744.3941 | 1486.7736 | 1486.7740 | -0.21 | 0 | 70 | 2.5e-06 | 1Score **> 34** indicates **identity** Score **> 27** indicates **homology** | U | R.LQIQGEGQASSTIR.Y |
| 60419 | 41 | – | 54 | 744.3941 | 1486.7737 | 1486.7740 | -0.16 | 0 | 67 | 3.7e-06 | 1Score **> 34** indicates **identity** Score **> 25** indicates **homology** | U | R.LQIQGEGQASSTIR.Y |
| 60420 | 41 | – | 54 | 744.3941 | 1486.7737 | 1486.7740 | -0.14 | 0 | 44 | 0.00036 | 1Score **> 34** indicates **identity** Score **> 23** indicates **homology** | U | R.LQIQGEGQASSTIR.Y |
| 60421 | 41 | – | 54 | 744.3942 | 1486.7738 | 1486.7740 | -0.14 | 0 | 35 | 0.00055 | 1Score **> 34** indicates **identity** Score **> 15** indicates **homology** | U | R.LQIQGEGQASSTIR.Y |
| 60423 | 41 | – | 54 | 744.3942 | 1486.7738 | 1486.7740 | -0.12 | 0 | 18 | 0.033 | 1Score **> 34** indicates **identity** Score **> 15** indicates **homology** | U | R.LQIQGEGQASSTIR.Y |
| 60424 | 41 | – | 54 | 744.3942 | 1486.7738 | 1486.7740 | -0.12 | 0 | 73 | 4.2e-06 | 1Score **> 34** indicates **identity** Score **> 32** indicates **homology** | U | R.LQIQGEGQASSTIR.Y |
| 60425 | 41 | – | 54 | 744.3942 | 1486.7738 | 1486.7740 | -0.073 | 0 | 60 | 1.6e-05 | 1Score **> 34** indicates **identity** Score **> 24** indicates **homology** | U | R.LQIQGEGQASSTIR.Y |
| 60426 | 41 | – | 54 | 744.3942 | 1486.7739 | 1486.7740 | -0.070 | 0 | 74 | 1.4e-06 | 1Score **> 34** indicates **identity** Score **> 28** indicates **homology** | U | R.LQIQGEGQASSTIR.Y |
| 60427 | 41 | – | 54 | 744.3942 | 1486.7739 | 1486.7740 | -0.063 | 0 | 73 | 1.8e-06 | 1Score **> 34** indicates **identity** Score **> 28** indicates **homology** | U | R.LQIQGEGQASSTIR.Y |
| 60428 | 41 | – | 54 | 744.3942 | 1486.7739 | 1486.7740 | -0.051 | 0 | 78 | 9.5e-07 | 1Score **> 34** indicates **identity** Score **> 30** indicates **homology** | U | R.LQIQGEGQASSTIR.Y |
| 60429 | 41 | – | 54 | 744.3942 | 1486.7739 | 1486.7740 | -0.048 | 0 | 38 | 0.00028 | 1Score **> 34** indicates **identity** Score **> 15** indicates **homology** | U | R.LQIQGEGQASSTIR.Y |
| 60431 | 41 | – | 54 | 744.3943 | 1486.7740 | 1486.7740 | 0.0067 | 0 | 69 | 4.2e-06 | 1Score **> 34** indicates **identity** Score **> 27** indicates **homology** | U | R.LQIQGEGQASSTIR.Y |
| 60432 | 41 | – | 54 | 744.3943 | 1486.7740 | 1486.7740 | 0.0067 | 0 | 40 | 0.00073 | 1Score **> 34** indicates **identity** Score **> 21** indicates **homology** | U | R.LQIQGEGQASSTIR.Y |
| 60433 | 41 | – | 54 | 744.3943 | 1486.7740 | 1486.7740 | 0.016 | 0 | 16 | 0.028 | 1Score **> 34** indicates **identity** Score **> 14** indicates **homology** | U | R.LQIQGEGQASSTIR.Y |
| 60434 | 41 | – | 54 | 744.3943 | 1486.7740 | 1486.7740 | 0.042 | 0 | 56 | 3.6e-05 | 1Score **> 34** indicates **identity** Score **> 24** indicates **homology** | U | R.LQIQGEGQASSTIR.Y |
| 60435 | 41 | – | 54 | 744.3943 | 1486.7740 | 1486.7740 | 0.044 | 0 | 37 | 0.00042 | 1Score **> 34** indicates **identity** Score **> 16** indicates **homology** | U | R.LQIQGEGQASSTIR.Y |
| 60436 | 41 | – | 54 | 744.3943 | 1486.7741 | 1486.7740 | 0.090 | 0 | 64 | 7.8e-06 | 1Score **> 34** indicates **identity** Score **> 25** indicates **homology** | U | R.LQIQGEGQASSTIR.Y |
| 60437 | 41 | – | 54 | 744.3943 | 1486.7741 | 1486.7740 | 0.12 | 0 | 63 | 8.3e-06 | 1Score **> 34** indicates **identity** Score **> 24** indicates **homology** | U | R.LQIQGEGQASSTIR.Y |
| 60438 | 41 | – | 54 | 744.3943 | 1486.7741 | 1486.7740 | 0.12 | 0 | 69 | 3.1e-06 | 1Score **> 34** indicates **identity** Score **> 26** indicates **homology** | U | R.LQIQGEGQASSTIR.Y |
| 60439 | 41 | – | 54 | 744.3944 | 1486.7742 | 1486.7740 | 0.17 | 0 | 72 | 2.4e-06 | 1Score **> 34** indicates **identity** Score **> 29** indicates **homology** | U | R.LQIQGEGQASSTIR.Y |
| 60440 | 41 | – | 54 | 744.3944 | 1486.7742 | 1486.7740 | 0.18 | 0 | 68 | 4.7e-06 | 1Score **> 34** indicates **identity** Score **> 27** indicates **homology** | U | R.LQIQGEGQASSTIR.Y |
| 60441 | 41 | – | 54 | 744.3944 | 1486.7742 | 1486.7740 | 0.18 | 0 | 68 | 5.9e-06 | 1Score **> 34** indicates **identity** Score **> 28** indicates **homology** | U | R.LQIQGEGQASSTIR.Y |
| 60442 | 41 | – | 54 | 744.3944 | 1486.7742 | 1486.7740 | 0.19 | 0 | 72 | 1.1e-06 | 1Score **> 34** indicates **identity** Score **> 25** indicates **homology** | U | R.LQIQGEGQASSTIR.Y |
| 60443 | 41 | – | 54 | 744.3944 | 1486.7743 | 1486.7740 | 0.20 | 0 | 49 | 0.00027 | 1Score **> 34** indicates **identity** Score **> 26** indicates **homology** | U | R.LQIQGEGQASSTIR.Y |
| 60444 | 41 | – | 54 | 744.3944 | 1486.7743 | 1486.7740 | 0.25 | 0 | 63 | 1.2e-05 | 1Score **> 35** indicates **identity** Score **> 26** indicates **homology** | U | R.LQIQGEGQASSTIR.Y |
| 60445 | 41 | – | 54 | 744.3944 | 1486.7743 | 1486.7740 | 0.26 | 0 | 86 | 1.4e-07 | 1Score **> 35** indicates **identity** Score **> 30** indicates **homology** | U | R.LQIQGEGQASSTIR.Y |
| 60446 | 41 | – | 54 | 744.3945 | 1486.7745 | 1486.7740 | 0.34 | 0 | 79 | 3.1e-07 | 1Score **> 35** indicates **identity** Score **> 26** indicates **homology** | U | R.LQIQGEGQASSTIR.Y |
| 60447 | 41 | – | 54 | 744.3945 | 1486.7745 | 1486.7740 | 0.34 | 0 | 74 | 2.2e-06 | 1Score **> 35** indicates **identity** Score **> 30** indicates **homology** | U | R.LQIQGEGQASSTIR.Y |
| 60448 | 41 | – | 54 | 744.3945 | 1486.7745 | 1486.7740 | 0.35 | 0 | 67 | 5.9e-06 | 1Score **> 35** indicates **identity** Score **> 27** indicates **homology** | U | R.LQIQGEGQASSTIR.Y |
| 60449 | 41 | – | 54 | 744.3945 | 1486.7745 | 1486.7740 | 0.36 | 0 | 75 | 6.3e-07 | 1Score **> 35** indicates **identity** Score **> 25** indicates **homology** | U | R.LQIQGEGQASSTIR.Y |
| 60450 | 41 | – | 54 | 744.3945 | 1486.7745 | 1486.7740 | 0.36 | 0 | 88 | 1.8e-07 | 1Score **> 35** indicates **identity** Score **> 34** indicates **homology** | U | R.LQIQGEGQASSTIR.Y |
| 60451 | 41 | – | 54 | 744.3946 | 1486.7746 | 1486.7740 | 0.45 | 0 | 42 | 0.00012 | 1Score **> 35** indicates **identity** Score **> 15** indicates **homology** | U | R.LQIQGEGQASSTIR.Y |
| 60453 | 41 | – | 54 | 744.3946 | 1486.7747 | 1486.7740 | 0.48 | 0 | 18 | 0.021 | 1Score **> 35** indicates **identity** Score **> 14** indicates **homology** | U | R.LQIQGEGQASSTIR.Y |
| 60454 | 41 | – | 54 | 744.3946 | 1486.7747 | 1486.7740 | 0.51 | 0 | 30 | 0.0017 | 1Score **> 35** indicates **identity** Score **> 14** indicates **homology** | U | R.LQIQGEGQASSTIR.Y |
| 60455 | 41 | – | 54 | 744.3946 | 1486.7747 | 1486.7740 | 0.51 | 0 | 65 | 8.6e-06 | 1Score **> 35** indicates **identity** Score **> 27** indicates **homology** | U | R.LQIQGEGQASSTIR.Y |
| 60456 | 41 | – | 54 | 744.3946 | 1486.7747 | 1486.7740 | 0.52 | 0 | 47 | 0.00023 | 1Score **> 35** indicates **identity** Score **> 23** indicates **homology** | U | R.LQIQGEGQASSTIR.Y |
| 60457 | 41 | – | 54 | 744.3946 | 1486.7747 | 1486.7740 | 0.53 | 0 | 46 | 0.00035 | 1Score **> 35** indicates **identity** Score **> 24** indicates **homology** | U | R.LQIQGEGQASSTIR.Y |
| 60458 | 41 | – | 54 | 744.3947 | 1486.7748 | 1486.7740 | 0.53 | 0 | 38 | 0.0011 | 1Score **> 35** indicates **identity** Score **> 21** indicates **homology** | U | R.LQIQGEGQASSTIR.Y |
| 60459 | 41 | – | 54 | 744.3947 | 1486.7748 | 1486.7740 | 0.58 | 0 | 54 | 7.7e-05 | 1Score **> 35** indicates **identity** Score **> 25** indicates **homology** | U | R.LQIQGEGQASSTIR.Y |
| 60460 | 41 | – | 54 | 744.3947 | 1486.7748 | 1486.7740 | 0.59 | 0 | 50 | 7.4e-05 | 1Score **> 35** indicates **identity** Score **> 21** indicates **homology** | U | R.LQIQGEGQASSTIR.Y |
| 60461 | 41 | – | 54 | 744.3947 | 1486.7748 | 1486.7740 | 0.59 | 0 | 76 | 2.3e-06 | 1Score **> 35** indicates **identity** Score **> 32** indicates **homology** | U | R.LQIQGEGQASSTIR.Y |
| 60462 | 41 | – | 54 | 744.3947 | 1486.7748 | 1486.7740 | 0.60 | 0 | 57 | 3.5e-05 | 1Score **> 35** indicates **identity** Score **> 25** indicates **homology** | U | R.LQIQGEGQASSTIR.Y |
| 60463 | 41 | – | 54 | 744.3947 | 1486.7748 | 1486.7740 | 0.60 | 0 | 74 | 1.6e-06 | 1Score **> 35** indicates **identity** Score **> 28** indicates **homology** | U | R.LQIQGEGQASSTIR.Y |
| 60464 | 41 | – | 54 | 744.3947 | 1486.7749 | 1486.7740 | 0.61 | 0 | 72 | 2.1e-06 | 1Score **> 35** indicates **identity** Score **> 28** indicates **homology** | U | R.LQIQGEGQASSTIR.Y |
| 60465 | 41 | – | 54 | 744.3947 | 1486.7749 | 1486.7740 | 0.62 | 0 | 56 | 3.3e-05 | 1Score **> 35** indicates **identity** Score **> 24** indicates **homology** | U | R.LQIQGEGQASSTIR.Y |
| 60466 | 41 | – | 54 | 744.3948 | 1486.7750 | 1486.7740 | 0.68 | 0 | 86 | 1.4e-07 | 1Score **> 35** indicates **identity** Score **> 30** indicates **homology** | U | R.LQIQGEGQASSTIR.Y |
| 60467 | 41 | – | 54 | 744.3948 | 1486.7750 | 1486.7740 | 0.69 | 0 | 80 | 3.4e-07 | 1Score **> 35** indicates **identity** Score **> 28** indicates **homology** | U | R.LQIQGEGQASSTIR.Y |
| 60468 | 41 | – | 54 | 744.3948 | 1486.7750 | 1486.7740 | 0.70 | 0 | 25 | 0.0098 | 1Score **> 35** indicates **identity** Score **> 18** indicates **homology** | U | R.LQIQGEGQASSTIR.Y |
| 60469 | 41 | – | 54 | 744.3948 | 1486.7750 | 1486.7740 | 0.70 | 0 | 30 | 0.0017 | 1Score **> 35** indicates **identity** Score **> 14** indicates **homology** | U | R.LQIQGEGQASSTIR.Y |
| 60470 | 41 | – | 54 | 744.3948 | 1486.7751 | 1486.7740 | 0.75 | 0 | 53 | 7.8e-05 | 1Score **> 35** indicates **identity** Score **> 24** indicates **homology** | U | R.LQIQGEGQASSTIR.Y |
| 60471 | 41 | – | 54 | 744.3948 | 1486.7751 | 1486.7740 | 0.75 | 0 | 70 | 4.4e-06 | 1Score **> 35** indicates **identity** Score **> 29** indicates **homology** | U | R.LQIQGEGQASSTIR.Y |
| 60472 | 41 | – | 54 | 744.3948 | 1486.7751 | 1486.7740 | 0.76 | 0 | 77 | 1.2e-06 | 1Score **> 35** indicates **identity** Score **> 30** indicates **homology** | U | R.LQIQGEGQASSTIR.Y |
| 60474 | 41 | – | 54 | 744.3949 | 1486.7752 | 1486.7740 | 0.83 | 0 | 19 | 0.026 | 1Score **> 35** indicates **identity** Score **> 16** indicates **homology** | U | R.LQIQGEGQASSTIR.Y |
| 60475 | 41 | – | 54 | 744.3949 | 1486.7752 | 1486.7740 | 0.84 | 0 | 67 | 6.7e-06 | 1Score **> 35** indicates **identity** Score **> 27** indicates **homology** | U | R.LQIQGEGQASSTIR.Y |
| 60476 | 41 | – | 54 | 744.3949 | 1486.7752 | 1486.7740 | 0.84 | 0 | 46 | 0.00017 | 1Score **> 35** indicates **identity** Score **> 21** indicates **homology** | U | R.LQIQGEGQASSTIR.Y |
| 60477 | 41 | – | 54 | 744.3949 | 1486.7752 | 1486.7740 | 0.85 | 0 | 74 | 1.5e-06 | 1Score **> 35** indicates **identity** Score **> 28** indicates **homology** | U | R.LQIQGEGQASSTIR.Y |
| 60478 | 41 | – | 54 | 744.3949 | 1486.7752 | 1486.7740 | 0.86 | 0 | 71 | 1.3e-05 | 1Score **> 35** indicates **identity** | U | R.LQIQGEGQASSTIR.Y |
| 60479 | 41 | – | 54 | 744.3949 | 1486.7752 | 1486.7740 | 0.86 | 0 | 56 | 6.3e-05 | 1Score **> 35** indicates **identity** Score **> 26** indicates **homology** | U | R.LQIQGEGQASSTIR.Y |
| 60480 | 41 | – | 54 | 744.3949 | 1486.7752 | 1486.7740 | 0.86 | 0 | 85 | 3.9e-07 | 1Score **> 35** indicates **identity** Score **> 33** indicates **homology** | U | R.LQIQGEGQASSTIR.Y |
| 60481 | 41 | – | 54 | 744.3949 | 1486.7752 | 1486.7740 | 0.86 | 0 | 70 | 2.8e-06 | 1Score **> 35** indicates **identity** Score **> 27** indicates **homology** | U | R.LQIQGEGQASSTIR.Y |
| 60482 | 41 | – | 54 | 744.3949 | 1486.7753 | 1486.7740 | 0.93 | 0 | 78 | 5.4e-07 | 1Score **> 35** indicates **identity** Score **> 28** indicates **homology** | U | R.LQIQGEGQASSTIR.Y |
| 60483 | 41 | – | 54 | 744.3949 | 1486.7753 | 1486.7740 | 0.93 | 0 | 78 | 1.2e-06 | 1Score **> 35** indicates **identity** Score **> 31** indicates **homology** | U | R.LQIQGEGQASSTIR.Y |
| 60484 | 41 | – | 54 | 744.3950 | 1486.7754 | 1486.7740 | 0.94 | 0 | 72 | 2e-06 | 1Score **> 35** indicates **identity** Score **> 28** indicates **homology** | U | R.LQIQGEGQASSTIR.Y |
| 60485 | 41 | – | 54 | 744.3950 | 1486.7754 | 1486.7740 | 0.95 | 0 | 74 | 9.6e-07 | 1Score **> 35** indicates **identity** Score **> 26** indicates **homology** | U | R.LQIQGEGQASSTIR.Y |
| 60487 | 41 | – | 54 | 744.3950 | 1486.7754 | 1486.7740 | 1.00 | 0 | 88 | 2.4e-07 | 1Score **> 35** indicates **identity** | U | R.LQIQGEGQASSTIR.Y |
| 60488 | 41 | – | 54 | 744.3950 | 1486.7754 | 1486.7740 | 1.00 | 0 | 70 | 3e-06 | 1Score **> 35** indicates **identity** Score **> 28** indicates **homology** | U | R.LQIQGEGQASSTIR.Y |
| 60489 | 41 | – | 54 | 744.3950 | 1486.7755 | 1486.7740 | 1.03 | 0 | 75 | 1.3e-06 | 1Score **> 35** indicates **identity** Score **> 28** indicates **homology** | U | R.LQIQGEGQASSTIR.Y |
| 60490 | 41 | – | 54 | 744.3950 | 1486.7755 | 1486.7740 | 1.03 | 0 | 51 | 2.4e-05 | 1Score **> 35** indicates **identity** Score **> 18** indicates **homology** | U | R.LQIQGEGQASSTIR.Y |
| 60491 | 41 | – | 54 | 744.3951 | 1486.7757 | 1486.7740 | 1.15 | 0 | 46 | 0.00033 | 1Score **> 35** indicates **identity** Score **> 24** indicates **homology** | U | R.LQIQGEGQASSTIR.Y |
| 60492 | 41 | – | 54 | 744.3951 | 1486.7757 | 1486.7740 | 1.15 | 0 | 55 | 2.6e-05 | 1Score **> 35** indicates **identity** Score **> 22** indicates **homology** | U | R.LQIQGEGQASSTIR.Y |
| 60493 | 41 | – | 54 | 744.3951 | 1486.7757 | 1486.7740 | 1.16 | 0 | 43 | 0.00059 | 1Score **> 35** indicates **identity** Score **> 23** indicates **homology** | U | R.LQIQGEGQASSTIR.Y |
| 60494 | 41 | – | 54 | 744.3951 | 1486.7757 | 1486.7740 | 1.17 | 0 | 79 | 2.5e-07 | 1Score **> 35** indicates **identity** Score **> 26** indicates **homology** | U | R.LQIQGEGQASSTIR.Y |
| 60495 | 41 | – | 54 | 744.3951 | 1486.7757 | 1486.7740 | 1.18 | 0 | 48 | 8.7e-05 | 1Score **> 35** indicates **identity** Score **> 20** indicates **homology** | U | R.LQIQGEGQASSTIR.Y |
| 60496 | 41 | – | 54 | 744.3951 | 1486.7757 | 1486.7740 | 1.18 | 0 | 61 | 2.2e-05 | 1Score **> 35** indicates **identity** Score **> 27** indicates **homology** | U | R.LQIQGEGQASSTIR.Y |
| 60497 | 41 | – | 54 | 744.3951 | 1486.7757 | 1486.7740 | 1.19 | 0 | 57 | 2.2e-05 | 1Score **> 35** indicates **identity** Score **> 23** indicates **homology** | U | R.LQIQGEGQASSTIR.Y |
| 60498 | 41 | – | 54 | 744.3951 | 1486.7757 | 1486.7740 | 1.19 | 0 | 52 | 5.9e-05 | 1Score **> 35** indicates **identity** Score **> 23** indicates **homology** | U | R.LQIQGEGQASSTIR.Y |
| 60499 | 41 | – | 54 | 744.3952 | 1486.7758 | 1486.7740 | 1.24 | 0 | 62 | 1.2e-05 | 1Score **> 35** indicates **identity** Score **> 25** indicates **homology** | U | R.LQIQGEGQASSTIR.Y |
| 60500 | 41 | – | 54 | 744.3952 | 1486.7758 | 1486.7740 | 1.25 | 0 | 52 | 5.6e-05 | 1Score **> 35** indicates **identity** Score **> 22** indicates **homology** | U | R.LQIQGEGQASSTIR.Y |
| 60502 | 41 | – | 54 | 744.3952 | 1486.7758 | 1486.7740 | 1.26 | 0 | 77 | 5.3e-07 | 1Score **> 35** indicates **identity** Score **> 27** indicates **homology** | U | R.LQIQGEGQASSTIR.Y |
| 60503 | 41 | – | 54 | 744.3952 | 1486.7759 | 1486.7740 | 1.34 | 0 | 66 | 8.2e-06 | 1Score **> 35** indicates **identity** Score **> 27** indicates **homology** | U | R.LQIQGEGQASSTIR.Y |
| 60504 | 41 | – | 54 | 744.3953 | 1486.7760 | 1486.7740 | 1.40 | 0 | 67 | 3.7e-06 | 1Score **> 35** indicates **identity** Score **> 25** indicates **homology** | U | R.LQIQGEGQASSTIR.Y |
| 60505 | 41 | – | 54 | 744.3953 | 1486.7761 | 1486.7740 | 1.43 | 0 | 23 | 0.0077 | 1Score **> 35** indicates **identity** Score **> 14** indicates **homology** | U | R.LQIQGEGQASSTIR.Y |
| 60506 | 41 | – | 54 | 744.3953 | 1486.7761 | 1486.7740 | 1.43 | 0 | 69 | 4.8e-06 | 1Score **> 35** indicates **identity** Score **> 28** indicates **homology** | U | R.LQIQGEGQASSTIR.Y |
| 60507 | 41 | – | 54 | 744.3953 | 1486.7761 | 1486.7740 | 1.44 | 0 | 34 | 0.00069 | 1Score **> 35** indicates **identity** Score **> 15** indicates **homology** | U | R.LQIQGEGQASSTIR.Y |
| 60509 | 41 | – | 54 | 744.3954 | 1486.7762 | 1486.7740 | 1.48 | 0 | 56 | 5.1e-05 | 1Score **> 35** indicates **identity** Score **> 26** indicates **homology** | U | R.LQIQGEGQASSTIR.Y |
| 60510 | 41 | – | 54 | 744.3954 | 1486.7762 | 1486.7740 | 1.50 | 0 | 76 | 6.8e-07 | 1Score **> 35** indicates **identity** Score **> 27** indicates **homology** | U | R.LQIQGEGQASSTIR.Y |
| 60512 | 41 | – | 54 | 744.3954 | 1486.7762 | 1486.7740 | 1.51 | 0 | 74 | 1.3e-06 | 1Score **> 35** indicates **identity** Score **> 28** indicates **homology** | U | R.LQIQGEGQASSTIR.Y |
| 60513 | 41 | – | 54 | 744.3954 | 1486.7762 | 1486.7740 | 1.51 | 0 | 39 | 0.00047 | 1Score **> 35** indicates **identity** Score **> 19** indicates **homology** | U | R.LQIQGEGQASSTIR.Y |
| 60514 | 41 | – | 54 | 744.3954 | 1486.7762 | 1486.7740 | 1.51 | 0 | 84 | 5.9e-07 | 1Score **> 35** indicates **identity** | U | R.LQIQGEGQASSTIR.Y |
| 60516 | 41 | – | 54 | 744.3954 | 1486.7762 | 1486.7740 | 1.52 | 0 | 65 | 7e-06 | 1Score **> 35** indicates **identity** Score **> 26** indicates **homology** | U | R.LQIQGEGQASSTIR.Y |
| 60517 | 41 | – | 54 | 744.3954 | 1486.7763 | 1486.7740 | 1.60 | 0 | 82 | 2.4e-07 | 1Score **> 35** indicates **identity** Score **> 28** indicates **homology** | U | R.LQIQGEGQASSTIR.Y |
| 60518 | 41 | – | 54 | 744.3955 | 1486.7764 | 1486.7740 | 1.65 | 0 | 56 | 6.1e-05 | 1Score **> 35** indicates **identity** Score **> 27** indicates **homology** | U | R.LQIQGEGQASSTIR.Y |
| 60519 | 41 | – | 54 | 744.3955 | 1486.7764 | 1486.7740 | 1.66 | 0 | 42 | 0.00071 | 1Score **> 35** indicates **identity** Score **> 23** indicates **homology** | U | R.LQIQGEGQASSTIR.Y |
| 60520 | 41 | – | 54 | 744.3955 | 1486.7764 | 1486.7740 | 1.67 | 0 | 42 | 0.00055 | 1Score **> 35** indicates **identity** Score **> 22** indicates **homology** | U | R.LQIQGEGQASSTIR.Y |
| 60521 | 41 | – | 54 | 744.3955 | 1486.7764 | 1486.7740 | 1.67 | 0 | 75 | 1.2e-06 | 1Score **> 35** indicates **identity** Score **> 28** indicates **homology** | U | R.LQIQGEGQASSTIR.Y |
| 60522 | 41 | – | 54 | 744.3955 | 1486.7764 | 1486.7740 | 1.68 | 0 | 56 | 4.4e-05 | 1Score **> 35** indicates **identity** Score **> 25** indicates **homology** | U | R.LQIQGEGQASSTIR.Y |
| 60523 | 41 | – | 54 | 744.3955 | 1486.7764 | 1486.7740 | 1.68 | 0 | 81 | 5.5e-07 | 1Score **> 35** indicates **identity** Score **> 31** indicates **homology** | U | R.LQIQGEGQASSTIR.Y |
| 60524 | 41 | – | 54 | 744.3955 | 1486.7765 | 1486.7740 | 1.69 | 0 | 52 | 5.2e-05 | 1Score **> 35** indicates **identity** Score **> 22** indicates **homology** | U | R.LQIQGEGQASSTIR.Y |
| 60525 | 41 | – | 54 | 744.3955 | 1486.7765 | 1486.7740 | 1.73 | 0 | 86 | 3.6e-07 | 1Score **> 35** indicates **identity** Score **> 34** indicates **homology** | U | R.LQIQGEGQASSTIR.Y |
| 60526 | 41 | – | 54 | 744.3956 | 1486.7766 | 1486.7740 | 1.75 | 0 | 69 | 5.8e-06 | 1Score **> 35** indicates **identity** Score **> 29** indicates **homology** | U | R.LQIQGEGQASSTIR.Y |
| 60527 | 41 | – | 54 | 744.3956 | 1486.7766 | 1486.7740 | 1.77 | 0 | 57 | 2.6e-05 | 1Score **> 35** indicates **identity** Score **> 24** indicates **homology** | U | R.LQIQGEGQASSTIR.Y |
| 60528 | 41 | – | 54 | 744.3956 | 1486.7766 | 1486.7740 | 1.77 | 0 | 65 | 9.1e-06 | 1Score **> 35** indicates **identity** Score **> 27** indicates **homology** | U | R.LQIQGEGQASSTIR.Y |
| 60529 | 41 | – | 54 | 744.3956 | 1486.7767 | 1486.7740 | 1.82 | 0 | 59 | 1.9e-05 | 1Score **> 35** indicates **identity** Score **> 24** indicates **homology** | U | R.LQIQGEGQASSTIR.Y |
| 60530 | 41 | – | 54 | 744.3956 | 1486.7767 | 1486.7740 | 1.82 | 0 | 43 | 0.00095 | 1Score **> 35** indicates **identity** Score **> 25** indicates **homology** | U | R.LQIQGEGQASSTIR.Y |
| 60531 | 41 | – | 54 | 744.3956 | 1486.7767 | 1486.7740 | 1.84 | 0 | 28 | 0.006 | 1Score **> 35** indicates **identity** Score **> 18** indicates **homology** | U | R.LQIQGEGQASSTIR.Y |
| 60532 | 41 | – | 54 | 744.3956 | 1486.7767 | 1486.7740 | 1.84 | 0 | 64 | 7e-06 | 1Score **> 35** indicates **identity** Score **> 25** indicates **homology** | U | R.LQIQGEGQASSTIR.Y |
| 60533 | 41 | – | 54 | 744.3957 | 1486.7768 | 1486.7740 | 1.89 | 0 | 61 | 1.5e-05 | 1Score **> 35** indicates **identity** Score **> 25** indicates **homology** | U | R.LQIQGEGQASSTIR.Y |
| 60534 | 41 | – | 54 | 744.3957 | 1486.7768 | 1486.7740 | 1.90 | 0 | 67 | 6.7e-06 | 1Score **> 35** indicates **identity** Score **> 28** indicates **homology** | U | R.LQIQGEGQASSTIR.Y |
| 60535 | 41 | – | 54 | 744.3957 | 1486.7768 | 1486.7740 | 1.90 | 0 | 81 | 2.9e-07 | 1Score **> 35** indicates **identity** Score **> 28** indicates **homology** | U | R.LQIQGEGQASSTIR.Y |
| 60536 | 41 | – | 54 | 744.3957 | 1486.7768 | 1486.7740 | 1.92 | 0 | 57 | 3.5e-05 | 1Score **> 35** indicates **identity** Score **> 25** indicates **homology** | U | R.LQIQGEGQASSTIR.Y |
| 60537 | 41 | – | 54 | 744.3957 | 1486.7768 | 1486.7740 | 1.92 | 0 | 51 | 8.4e-05 | 1Score **> 35** indicates **identity** Score **> 23** indicates **homology** | U | R.LQIQGEGQASSTIR.Y |
| 60538 | 41 | – | 54 | 744.3957 | 1486.7768 | 1486.7740 | 1.92 | 0 | 64 | 1.1e-05 | 1Score **> 35** indicates **identity** Score **> 27** indicates **homology** | U | R.LQIQGEGQASSTIR.Y |
| 60539 | 41 | – | 54 | 744.3957 | 1486.7768 | 1486.7740 | 1.92 | 0 | 48 | 0.00011 | 1Score **> 35** indicates **identity** Score **> 21** indicates **homology** | U | R.LQIQGEGQASSTIR.Y |
| 60540 | 41 | – | 54 | 744.3957 | 1486.7768 | 1486.7740 | 1.92 | 0 | 60 | 1.8e-05 | 1Score **> 35** indicates **identity** Score **> 25** indicates **homology** | U | R.LQIQGEGQASSTIR.Y |
| 60541 | 41 | – | 54 | 744.3957 | 1486.7768 | 1486.7740 | 1.93 | 0 | 39 | 0.00079 | 1Score **> 35** indicates **identity** Score **> 21** indicates **homology** | U | R.LQIQGEGQASSTIR.Y |
| 60542 | 41 | – | 54 | 744.3957 | 1486.7769 | 1486.7740 | 1.98 | 0 | 70 | 3.6e-06 | 1Score **> 35** indicates **identity** Score **> 29** indicates **homology** | U | R.LQIQGEGQASSTIR.Y |
| 60543 | 41 | – | 54 | 744.3957 | 1486.7769 | 1486.7740 | 2.01 | 0 | 55 | 3.6e-05 | 1Score **> 35** indicates **identity** Score **> 23** indicates **homology** | U | R.LQIQGEGQASSTIR.Y |
| 60544 | 41 | – | 54 | 744.3958 | 1486.7770 | 1486.7740 | 2.02 | 0 | 31 | 0.002 | 1Score **> 35** indicates **identity** Score **> 16** indicates **homology** | U | R.LQIQGEGQASSTIR.Y |
| 60545 | 41 | – | 54 | 744.3958 | 1486.7770 | 1486.7740 | 2.02 | 0 | 67 | 6.4e-06 | 1Score **> 35** indicates **identity** Score **> 27** indicates **homology** | U | R.LQIQGEGQASSTIR.Y |
| 60546 | 41 | – | 54 | 744.3958 | 1486.7770 | 1486.7740 | 2.07 | 0 | 45 | 6.3e-05 | 1Score **> 35** indicates **identity** Score **> 15** indicates **homology** | U | R.LQIQGEGQASSTIR.Y |
| 60547 | 41 | – | 54 | 744.3958 | 1486.7771 | 1486.7740 | 2.08 | 0 | 63 | 1.1e-05 | 1Score **> 35** indicates **identity** Score **> 26** indicates **homology** | U | R.LQIQGEGQASSTIR.Y |
| 60548 | 41 | – | 54 | 744.3958 | 1486.7771 | 1486.7740 | 2.09 | 0 | 69 | 3.3e-06 | 1Score **> 35** indicates **identity** Score **> 27** indicates **homology** | U | R.LQIQGEGQASSTIR.Y |
| 60549 | 41 | – | 54 | 744.3958 | 1486.7771 | 1486.7740 | 2.14 | 0 | 73 | 1.6e-06 | 1Score **> 35** indicates **identity** Score **> 28** indicates **homology** | U | R.LQIQGEGQASSTIR.Y |
| 60550 | 41 | – | 54 | 744.3958 | 1486.7771 | 1486.7740 | 2.14 | 0 | 43 | 0.00014 | 1Score **> 35** indicates **identity** Score **> 17** indicates **homology** | U | R.LQIQGEGQASSTIR.Y |
| 60551 | 41 | – | 54 | 744.3959 | 1486.7772 | 1486.7740 | 2.15 | 0 | 35 | 0.0014 | 1Score **> 35** indicates **identity** Score **> 19** indicates **homology** | U | R.LQIQGEGQASSTIR.Y |
| 60552 | 41 | – | 54 | 744.3959 | 1486.7772 | 1486.7740 | 2.15 | 0 | 56 | 3.6e-05 | 1Score **> 35** indicates **identity** Score **> 24** indicates **homology** | U | R.LQIQGEGQASSTIR.Y |
| 60553 | 41 | – | 54 | 744.3959 | 1486.7772 | 1486.7740 | 2.16 | 0 | 56 | 3.6e-05 | 1Score **> 35** indicates **identity** Score **> 24** indicates **homology** | U | R.LQIQGEGQASSTIR.Y |
| 60554 | 41 | – | 54 | 744.3959 | 1486.7772 | 1486.7740 | 2.17 | 0 | 43 | 0.00019 | 1Score **> 35** indicates **identity** Score **> 18** indicates **homology** | U | R.LQIQGEGQASSTIR.Y |
| 60555 | 41 | – | 54 | 744.3959 | 1486.7772 | 1486.7740 | 2.17 | 0 | 59 | 2.2e-05 | 1Score **> 35** indicates **identity** Score **> 25** indicates **homology** | U | R.LQIQGEGQASSTIR.Y |
| 60556 | 41 | – | 54 | 744.3959 | 1486.7772 | 1486.7740 | 2.18 | 0 | 57 | 1.9e-05 | 1Score **> 35** indicates **identity** Score **> 23** indicates **homology** | U | R.LQIQGEGQASSTIR.Y |
| 60557 | 41 | – | 54 | 744.3959 | 1486.7773 | 1486.7740 | 2.23 | 0 | 48 | 0.00018 | 1Score **> 35** indicates **identity** Score **> 23** indicates **homology** | U | R.LQIQGEGQASSTIR.Y |
| 60558 | 41 | – | 54 | 744.3959 | 1486.7773 | 1486.7740 | 2.24 | 0 | 56 | 4.8e-05 | 1Score **> 35** indicates **identity** Score **> 25** indicates **homology** | U | R.LQIQGEGQASSTIR.Y |
| 60559 | 41 | – | 54 | 744.3959 | 1486.7773 | 1486.7740 | 2.24 | 0 | 34 | 0.00075 | 1Score **> 35** indicates **identity** Score **> 15** indicates **homology** | U | R.LQIQGEGQASSTIR.Y |
| 60560 | 41 | – | 54 | 744.3959 | 1486.7773 | 1486.7740 | 2.25 | 0 | 76 | 1e-06 | 1Score **> 35** indicates **identity** Score **> 28** indicates **homology** | U | R.LQIQGEGQASSTIR.Y |
| 60561 | 41 | – | 54 | 744.3960 | 1486.7774 | 1486.7740 | 2.33 | 0 | 63 | 1.2e-05 | 1Score **> 35** indicates **identity** Score **> 26** indicates **homology** | U | R.LQIQGEGQASSTIR.Y |
| 60562 | 41 | – | 54 | 744.3960 | 1486.7774 | 1486.7740 | 2.33 | 0 | 46 | 0.00031 | 1Score **> 35** indicates **identity** Score **> 24** indicates **homology** | U | R.LQIQGEGQASSTIR.Y |
| 60564 | 41 | – | 54 | 744.3960 | 1486.7774 | 1486.7740 | 2.34 | 0 | 56 | 3.7e-05 | 1Score **> 35** indicates **identity** Score **> 24** indicates **homology** | U | R.LQIQGEGQASSTIR.Y |
| 60565 | 41 | – | 54 | 744.3961 | 1486.7776 | 1486.7740 | 2.42 | 0 | 34 | 0.0025 | 1Score **> 35** indicates **identity** Score **> 21** indicates **homology** | U | R.LQIQGEGQASSTIR.Y |
| 60566 | 41 | – | 54 | 744.3961 | 1486.7776 | 1486.7740 | 2.45 | 0 | 37 | 0.00036 | 1Score **> 34** indicates **identity** Score **> 15** indicates **homology** | U | R.LQIQGEGQASSTIR.Y |
| 60571 | 41 | – | 54 | 744.3962 | 1486.7779 | 1486.7740 | 2.64 | 0 | 52 | 8.5e-05 | 1Score **> 35** indicates **identity** Score **> 24** indicates **homology** | U | R.LQIQGEGQASSTIR.Y |
| 60572 | 41 | – | 54 | 744.3962 | 1486.7779 | 1486.7740 | 2.66 | 0 | 31 | 0.0022 | 1Score **> 35** indicates **identity** Score **> 17** indicates **homology** | U | R.LQIQGEGQASSTIR.Y |
| 60573 | 41 | – | 54 | 744.3963 | 1486.7780 | 1486.7740 | 2.73 | 0 | 78 | 2.4e-06 | 1Score **> 35** indicates **identity** Score **> 34** indicates **homology** | U | R.LQIQGEGQASSTIR.Y |
| 60574 | 41 | – | 54 | 744.3963 | 1486.7780 | 1486.7740 | 2.74 | 0 | 35 | 0.0017 | 1Score **> 35** indicates **identity** Score **> 20** indicates **homology** | U | R.LQIQGEGQASSTIR.Y |
| 60576 | 41 | – | 54 | 744.3963 | 1486.7781 | 1486.7740 | 2.80 | 0 | 41 | 0.00016 | 1Score **> 35** indicates **identity** Score **> 15** indicates **homology** | U | R.LQIQGEGQASSTIR.Y |
| 60577 | 41 | – | 54 | 744.3963 | 1486.7781 | 1486.7740 | 2.81 | 0 | 48 | 0.00011 | 1Score **> 35** indicates **identity** Score **> 21** indicates **homology** | U | R.LQIQGEGQASSTIR.Y |
| 60578 | 41 | – | 54 | 744.3963 | 1486.7781 | 1486.7740 | 2.82 | 0 | 60 | 1.4e-05 | 1Score **> 35** indicates **identity** Score **> 24** indicates **homology** | U | R.LQIQGEGQASSTIR.Y |
| 60579 | 41 | – | 54 | 744.3964 | 1486.7782 | 1486.7740 | 2.82 | 0 | 61 | 3.7e-05 | 1Score **> 35** indicates **identity** Score **> 30** indicates **homology** | U | R.LQIQGEGQASSTIR.Y |
| 60580 | 41 | – | 54 | 744.3965 | 1486.7784 | 1486.7740 | 2.98 | 0 | 58 | 1.7e-05 | 1Score **> 35** indicates **identity** Score **> 22** indicates **homology** | U | R.LQIQGEGQASSTIR.Y |
| 60581 | 41 | – | 54 | 744.3965 | 1486.7785 | 1486.7740 | 3.06 | 0 | 52 | 5.9e-05 | 1Score **> 35** indicates **identity** Score **> 22** indicates **homology** | U | R.LQIQGEGQASSTIR.Y |
| 60582 | 41 | – | 54 | 744.3965 | 1486.7785 | 1486.7740 | 3.07 | 0 | 55 | 3.5e-05 | 1Score **> 35** indicates **identity** Score **> 23** indicates **homology** | U | R.LQIQGEGQASSTIR.Y |
| 60583 | 41 | – | 54 | 744.3966 | 1486.7786 | 1486.7740 | 3.13 | 0 | 67 | 6.1e-06 | 1Score **> 35** indicates **identity** Score **> 27** indicates **homology** | U | R.LQIQGEGQASSTIR.Y |
| 60584 | 41 | – | 54 | 744.3966 | 1486.7786 | 1486.7740 | 3.13 | 0 | 31 | 0.013 | 1Score **> 35** indicates **identity** Score **> 25** indicates **homology** | U | R.LQIQGEGQASSTIR.Y |
| 60585 | 41 | – | 54 | 744.3966 | 1486.7786 | 1486.7740 | 3.14 | 0 | 38 | 0.0052 | 1Score **> 35** indicates **identity** Score **> 28** indicates **homology** | U | R.LQIQGEGQASSTIR.Y |
| 60586 | 41 | – | 54 | 744.3966 | 1486.7786 | 1486.7740 | 3.14 | 0 | 41 | 0.00053 | 1Score **> 35** indicates **identity** Score **> 21** indicates **homology** | U | R.LQIQGEGQASSTIR.Y |
| 60587 | 41 | – | 54 | 744.3966 | 1486.7787 | 1486.7740 | 3.16 | 0 | 40 | 0.00049 | 1Score **> 35** indicates **identity** Score **> 19** indicates **homology** | U | R.LQIQGEGQASSTIR.Y |
| 60588 | 41 | – | 54 | 744.3966 | 1486.7787 | 1486.7740 | 3.16 | 0 | 67 | 2.8e-06 | 1Score **> 35** indicates **identity** Score **> 24** indicates **homology** | U | R.LQIQGEGQASSTIR.Y |
| 60589 | 41 | – | 54 | 744.3968 | 1486.7790 | 1486.7740 | 3.38 | 0 | 31 | 0.0027 | 1Score **> 35** indicates **identity** Score **> 18** indicates **homology** | U | R.LQIQGEGQASSTIR.Y |
| 60590 | 41 | – | 54 | 744.3968 | 1486.7790 | 1486.7740 | 3.40 | 0 | 67 | 4.9e-06 | 1Score **> 35** indicates **identity** Score **> 27** indicates **homology** | U | R.LQIQGEGQASSTIR.Y |
| 60591 | 41 | – | 54 | 744.3968 | 1486.7791 | 1486.7740 | 3.47 | 0 | 39 | 0.00071 | 1Score **> 35** indicates **identity** Score **> 21** indicates **homology** | U | R.LQIQGEGQASSTIR.Y |
| 60592 | 41 | – | 54 | 744.3970 | 1486.7794 | 1486.7740 | 3.65 | 0 | 52 | 0.00013 | 1Score **> 35** indicates **identity** Score **> 25** indicates **homology** | U | R.LQIQGEGQASSTIR.Y |
| 60593 | 41 | – | 54 | 744.3970 | 1486.7794 | 1486.7740 | 3.66 | 0 | 61 | 0.00013 | 1Score **> 35** indicates **identity** | U | R.LQIQGEGQASSTIR.Y |
| 60594 | 41 | – | 54 | 744.3970 | 1486.7795 | 1486.7740 | 3.71 | 0 | 73 | 8e-07 | 1Score **> 35** indicates **identity** Score **> 25** indicates **homology** | U | R.LQIQGEGQASSTIR.Y |
| 60595 | 41 | – | 54 | 744.3971 | 1486.7796 | 1486.7740 | 3.79 | 0 | 21 | 0.011 | 1Score **> 35** indicates **identity** Score **> 14** indicates **homology** | U | R.LQIQGEGQASSTIR.Y |
| 60596 | 41 | – | 54 | 744.3971 | 1486.7796 | 1486.7740 | 3.81 | 0 | 20 | 0.046 | 1Score **> 35** indicates **identity** Score **> 19** indicates **homology** | U | R.LQIQGEGQASSTIR.Y |
| 60597 | 41 | – | 54 | 744.3972 | 1486.7799 | 1486.7740 | 3.98 | 0 | 56 | 4.7e-05 | 1Score **> 35** indicates **identity** Score **> 26** indicates **homology** | U | R.LQIQGEGQASSTIR.Y |
| 60598 | 41 | – | 54 | 744.3972 | 1486.7799 | 1486.7740 | 4.03 | 0 | 53 | 0.00033 | 1Score **> 35** indicates **identity** Score **> 31** indicates **homology** | U | R.LQIQGEGQASSTIR.Y |
| 60599 | 41 | – | 54 | 744.3973 | 1486.7800 | 1486.7740 | 4.07 | 0 | 39 | 0.00085 | 1Score **> 35** indicates **identity** Score **> 21** indicates **homology** | U | R.LQIQGEGQASSTIR.Y |
| 60600 | 41 | – | 54 | 744.3973 | 1486.7801 | 1486.7740 | 4.12 | 0 | 58 | 3.2e-05 | 1Score **> 35** indicates **identity** Score **> 25** indicates **homology** | U | R.LQIQGEGQASSTIR.Y |
| 60601 | 41 | – | 54 | 744.3973 | 1486.7801 | 1486.7740 | 4.13 | 0 | 56 | 3.5e-05 | 1Score **> 35** indicates **identity** Score **> 24** indicates **homology** | U | R.LQIQGEGQASSTIR.Y |
| 60602 | 41 | – | 54 | 744.3973 | 1486.7801 | 1486.7740 | 4.15 | 0 | 63 | 5e-06 | 1Score **> 35** indicates **identity** Score **> 23** indicates **homology** | U | R.LQIQGEGQASSTIR.Y |
| 60603 | 41 | – | 54 | 744.3974 | 1486.7802 | 1486.7740 | 4.19 | 0 | 52 | 5.8e-05 | 1Score **> 35** indicates **identity** Score **> 22** indicates **homology** | U | R.LQIQGEGQASSTIR.Y |
| 60604 | 41 | – | 54 | 744.3974 | 1486.7802 | 1486.7740 | 4.20 | 0 | 59 | 2.8e-05 | 1Score **> 35** indicates **identity** Score **> 26** indicates **homology** | U | R.LQIQGEGQASSTIR.Y |
| 60605 | 41 | – | 54 | 744.3975 | 1486.7804 | 1486.7740 | 4.30 | 0 | 54 | 2.3e-05 | 1Score **> 35** indicates **identity** Score **> 20** indicates **homology** | U | R.LQIQGEGQASSTIR.Y |
| 60606 | 41 | – | 54 | 744.3975 | 1486.7805 | 1486.7740 | 4.37 | 0 | 17 | 0.023 | 1Score **> 35** indicates **identity** Score **> 14** indicates **homology** | U | R.LQIQGEGQASSTIR.Y |
| 60607 | 41 | – | 54 | 744.3978 | 1486.7810 | 1486.7740 | 4.71 | 0 | 31 | 0.0012 | 1Score **> 35** indicates **identity** Score **> 14** indicates **homology** | U | R.LQIQGEGQASSTIR.Y |
| 60608 | 41 | – | 54 | 744.3978 | 1486.7811 | 1486.7740 | 4.78 | 0 | 40 | 0.00017 | 1Score **> 35** indicates **identity** Score **> 15** indicates **homology** | U | R.LQIQGEGQASSTIR.Y |
| 60609 | 41 | – | 54 | 744.3979 | 1486.7813 | 1486.7740 | 4.96 | 0 | 50 | 7e-05 | 1Score **> 35** indicates **identity** Score **> 21** indicates **homology** | U | R.LQIQGEGQASSTIR.Y |
| 60610 | 41 | – | 54 | 744.3980 | 1486.7814 | 1486.7740 | 5.03 | 0 | 31 | 0.0021 | 1Score **> 35** indicates **identity** Score **> 16** indicates **homology** | U | R.LQIQGEGQASSTIR.Y |
| 60611 | 41 | – | 54 | 744.3980 | 1486.7815 | 1486.7740 | 5.05 | 0 | 38 | 0.00096 | 1Score **> 35** indicates **identity** Score **> 20** indicates **homology** | U | R.LQIQGEGQASSTIR.Y |
| 60612 | 41 | – | 54 | 744.3981 | 1486.7816 | 1486.7740 | 5.13 | 0 | 51 | 0.00017 | 1Score **> 35** indicates **identity** Score **> 26** indicates **homology** | U | R.LQIQGEGQASSTIR.Y |
| 60613 | 41 | – | 54 | 744.3981 | 1486.7816 | 1486.7740 | 5.13 | 0 | 41 | 0.00018 | 1Score **> 35** indicates **identity** Score **> 16** indicates **homology** | U | R.LQIQGEGQASSTIR.Y |
| 60614 | 41 | – | 54 | 744.3982 | 1486.7819 | 1486.7740 | 5.34 | 0 | 60 | 1.9e-05 | 1Score **> 35** indicates **identity** Score **> 25** indicates **homology** | U | R.LQIQGEGQASSTIR.Y |
| 60615 | 41 | – | 54 | 744.3982 | 1486.7819 | 1486.7740 | 5.35 | 0 | 48 | 0.00013 | 1Score **> 35** indicates **identity** Score **> 22** indicates **homology** | U | R.LQIQGEGQASSTIR.Y |
| 60616 | 41 | – | 54 | 744.3982 | 1486.7819 | 1486.7740 | 5.36 | 0 | 63 | 4.6e-06 | 1Score **> 35** indicates **identity** Score **> 22** indicates **homology** | U | R.LQIQGEGQASSTIR.Y |
| 60617 | 41 | – | 54 | 744.3982 | 1486.7819 | 1486.7740 | 5.36 | 0 | 47 | 0.00026 | 1Score **> 35** indicates **identity** Score **> 24** indicates **homology** | U | R.LQIQGEGQASSTIR.Y |
| 60618 | 41 | – | 54 | 744.3983 | 1486.7820 | 1486.7740 | 5.41 | 0 | 40 | 0.00017 | 1Score **> 35** indicates **identity** Score **> 15** indicates **homology** | U | R.LQIQGEGQASSTIR.Y |
| 60619 | 41 | – | 54 | 744.3984 | 1486.7822 | 1486.7740 | 5.53 | 0 | 53 | 5.4e-05 | 1Score **> 35** indicates **identity** Score **> 22** indicates **homology** | U | R.LQIQGEGQASSTIR.Y |
| 60620 | 41 | – | 54 | 744.3985 | 1486.7824 | 1486.7740 | 5.70 | 0 | 40 | 0.00082 | 1Score **> 35** indicates **identity** Score **> 22** indicates **homology** | U | R.LQIQGEGQASSTIR.Y |
| 60624 | 41 | – | 54 | 744.3987 | 1486.7828 | 1486.7740 | 5.92 | 0 | 54 | 2.4e-05 | 1Score **> 34** indicates **identity** Score **> 20** indicates **homology** | U | R.LQIQGEGQASSTIR.Y |
| 60625 | 41 | – | 54 | 744.3987 | 1486.7829 | 1486.7740 | 6.01 | 0 | 43 | 0.0005 | 1Score **> 34** indicates **identity** Score **> 23** indicates **homology** | U | R.LQIQGEGQASSTIR.Y |
| 60626 | 41 | – | 54 | 744.3988 | 1486.7830 | 1486.7740 | 6.08 | 0 | 74 | 7.8e-07 | 1Score **> 34** indicates **identity** Score **> 25** indicates **homology** | U | R.LQIQGEGQASSTIR.Y |
| 60627 | 41 | – | 54 | 744.3989 | 1486.7832 | 1486.7740 | 6.20 | 0 | 42 | 0.00065 | 1Score **> 34** indicates **identity** Score **> 23** indicates **homology** | U | R.LQIQGEGQASSTIR.Y |
| 60628 | 41 | – | 54 | 744.3989 | 1486.7832 | 1486.7740 | 6.20 | 0 | 40 | 0.00076 | 1Score **> 34** indicates **identity** Score **> 21** indicates **homology** | U | R.LQIQGEGQASSTIR.Y |
| 60629 | 41 | – | 54 | 744.3989 | 1486.7833 | 1486.7740 | 6.26 | 0 | 31 | 0.003 | 1Score **> 34** indicates **identity** Score **> 18** indicates **homology** | U | R.LQIQGEGQASSTIR.Y |
| 60630 | 41 | – | 54 | 744.3991 | 1486.7837 | 1486.7740 | 6.57 | 0 | 55 | 5.7e-05 | 1Score **> 34** indicates **identity** Score **> 25** indicates **homology** | U | R.LQIQGEGQASSTIR.Y |
| 60631 | 41 | – | 54 | 744.3992 | 1486.7838 | 1486.7740 | 6.60 | 0 | 22 | 0.0085 | 1Score **> 34** indicates **identity** Score **> 14** indicates **homology** | U | R.LQIQGEGQASSTIR.Y |
| 60632 | 41 | – | 54 | 744.3992 | 1486.7838 | 1486.7740 | 6.63 | 0 | 43 | 0.00034 | 1Score **> 34** indicates **identity** Score **> 21** indicates **homology** | U | R.LQIQGEGQASSTIR.Y |
| 60633 | 41 | – | 54 | 744.3997 | 1486.7849 | 1486.7740 | 7.33 | 0 | 52 | 5.6e-05 | 1Score **> 35** indicates **identity** Score **> 22** indicates **homology** | U | R.LQIQGEGQASSTIR.Y |
| 60634 | 41 | – | 54 | 744.4002 | 1486.7859 | 1486.7740 | 8.05 | 0 | 31 | 0.0098 | 1Score **> 35** indicates **identity** Score **> 23** indicates **homology** | U | R.LQIQGEGQASSTIR.Y |
| 60635 | 41 | – | 54 | 744.4003 | 1486.7860 | 1486.7740 | 8.09 | 0 | 36 | 0.00084 | 1Score **> 35** indicates **identity** Score **> 17** indicates **homology** | U | R.LQIQGEGQASSTIR.Y |
| 60636 | 41 | – | 54 | 744.4004 | 1486.7862 | 1486.7740 | 8.22 | 0 | 30 | 0.0062 | 1Score **> 35** indicates **identity** Score **> 20** indicates **homology** | U | R.LQIQGEGQASSTIR.Y |
| 60637 | 41 | – | 54 | 744.4007 | 1486.7868 | 1486.7740 | 8.63 | 0 | 65 | 3.7e-06 | 1Score **> 35** indicates **identity** Score **> 23** indicates **homology** | U | R.LQIQGEGQASSTIR.Y |
| 60638 | 41 | – | 54 | 744.4007 | 1486.7868 | 1486.7740 | 8.64 | 0 | 51 | 1.6e-05 | 1Score **> 35** indicates **identity** Score **> 16** indicates **homology** | U | R.LQIQGEGQASSTIR.Y |
| 60639 | 41 | – | 54 | 744.4008 | 1486.7871 | 1486.7740 | 8.83 | 0 | 42 | 0.00042 | 1Score **> 35** indicates **identity** Score **> 20** indicates **homology** | U | R.LQIQGEGQASSTIR.Y |
| 60640 | 41 | – | 54 | 744.4009 | 1486.7872 | 1486.7740 | 8.91 | 0 | 19 | 0.02 | 1Score **> 35** indicates **identity** Score **> 15** indicates **homology** | U | R.LQIQGEGQASSTIR.Y |
| 60641 | 41 | – | 54 | 744.4011 | 1486.7876 | 1486.7740 | 9.15 | 0 | 32 | 0.007 | 1Score **> 35** indicates **identity** Score **> 23** indicates **homology** | U | R.LQIQGEGQASSTIR.Y |
| 60642 | 41 | – | 54 | 744.4012 | 1486.7878 | 1486.7740 | 9.31 | 0 | 16 | 0.034 | 1Score **> 35** indicates **identity** Score **> 13** indicates **homology** | U | R.LQIQGEGQASSTIR.Y |
| 60725 | 41 | – | 54 | 744.8922 | 1487.7698 | 1487.7580 | 7.92 | 0 | 31 | 0.0011 | 1Score **> 35** indicates **identity** Score **> 14** indicates **homology** | U | R.LQIQGEGQASSTIR.Y  + Deamidated (NQ) |
| 46891 | 55 | – | 67 | 682.9109 | 1363.8073 | 1363.8075 | -0.13 | 1 | 56 | 3.5e-05 | 1Score **> 30** indicates **identity** Score **> 24** indicates **homology** | U | R.YKGVLGTITTLAK.T |
| 46892 | 55 | – | 67 | 455.6098 | 1363.8074 | 1363.8075 | -0.038 | 1 | 34 | 0.0044 | 1Score **> 30** indicates **identity** Score **> 23** indicates **homology** | U | R.YKGVLGTITTLAK.T |
| 46894 | 55 | – | 67 | 682.9111 | 1363.8077 | 1363.8075 | 0.14 | 1 | 89 | 3.8e-08 | 1Score **> 30** indicates **identity** Score **> 28** indicates **homology** | U | R.YKGVLGTITTLAK.T |
| 46896 | 55 | – | 67 | 455.6099 | 1363.8077 | 1363.8075 | 0.18 | 1 | 51 | 0.00038 | 1Score **> 30** indicates **identity** Score **> 29** indicates **homology** | U | R.YKGVLGTITTLAK.T |
| 46897 | 55 | – | 67 | 455.6099 | 1363.8078 | 1363.8075 | 0.19 | 1 | 27 | 0.028 | 1Score **> 30** indicates **identity** Score **> 24** indicates **homology** | U | R.YKGVLGTITTLAK.T |
| 46899 | 55 | – | 67 | 455.6099 | 1363.8078 | 1363.8075 | 0.20 | 1 | 37 | 0.0033 | 1Score **> 30** indicates **identity** Score **> 24** indicates **homology** | U | R.YKGVLGTITTLAK.T |
| 46900 | 55 | – | 67 | 682.9112 | 1363.8078 | 1363.8075 | 0.23 | 1 | 39 | 0.00034 | 1Score **> 30** indicates **identity** Score **> 17** indicates **homology** | U | R.YKGVLGTITTLAK.T |
| 46909 | 55 | – | 67 | 455.6101 | 1363.8084 | 1363.8075 | 0.64 | 1 | 15 | 0.037 | 1Score **> 30** indicates **identity** Score **> 13** indicates **homology** | U | R.YKGVLGTITTLAK.T |
| 46910 | 55 | – | 67 | 455.6101 | 1363.8084 | 1363.8075 | 0.64 | 1 | 40 | 0.0023 | 1Score **> 30** indicates **identity** Score **> 26** indicates **homology** | U | R.YKGVLGTITTLAK.T |
| 46912 | 55 | – | 67 | 682.9115 | 1363.8085 | 1363.8075 | 0.71 | 1 | 32 | 0.0051 | 1Score **> 30** indicates **identity** Score **> 22** indicates **homology** | U | R.YKGVLGTITTLAK.T |
| 46913 | 55 | – | 67 | 455.6101 | 1363.8086 | 1363.8075 | 0.80 | 1 | 25 | 0.005 | 1Score **> 30** indicates **identity** Score **> 14** indicates **homology** | U | R.YKGVLGTITTLAK.T |
| 46915 | 55 | – | 67 | 455.6102 | 1363.8087 | 1363.8075 | 0.87 | 1 | 56 | 0.00014 | 1Score **> 30** indicates **identity** | U | R.YKGVLGTITTLAK.T |
| 46916 | 55 | – | 67 | 682.9117 | 1363.8089 | 1363.8075 | 1.05 | 1 | 92 | 2.9e-08 | 1Score **> 30** indicates **identity** Score **> 29** indicates **homology** | U | R.YKGVLGTITTLAK.T |
| 46917 | 55 | – | 67 | 682.9118 | 1363.8090 | 1363.8075 | 1.07 | 1 | 88 | 7.2e-08 | 1Score **> 30** indicates **identity** Score **> 29** indicates **homology** | U | R.YKGVLGTITTLAK.T |
| 46918 | 55 | – | 67 | 455.6103 | 1363.8090 | 1363.8075 | 1.09 | 1 | 29 | 0.0067 | 1Score **> 30** indicates **identity** Score **> 20** indicates **homology** | U | R.YKGVLGTITTLAK.T |
| 46919 | 55 | – | 67 | 682.9118 | 1363.8090 | 1363.8075 | 1.10 | 1 | 92 | 2.8e-08 | 1Score **> 30** indicates **identity** Score **> 29** indicates **homology** | U | R.YKGVLGTITTLAK.T |
| 46922 | 55 | – | 67 | 682.9119 | 1363.8092 | 1363.8075 | 1.28 | 1 | 64 | 6e-06 | 1Score **> 30** indicates **identity** Score **> 24** indicates **homology** | U | R.YKGVLGTITTLAK.T |
| 46923 | 55 | – | 67 | 455.6104 | 1363.8093 | 1363.8075 | 1.29 | 1 | 46 | 0.0016 | 1Score **> 30** indicates **identity** | U | R.YKGVLGTITTLAK.T |
| 46924 | 55 | – | 67 | 455.6104 | 1363.8093 | 1363.8075 | 1.35 | 1 | 43 | 0.0034 | 1Score **> 30** indicates **identity** | U | R.YKGVLGTITTLAK.T |
| 46926 | 55 | – | 67 | 455.6107 | 1363.8102 | 1363.8075 | 2.01 | 1 | 39 | 0.00021 | 1Score **> 30** indicates **identity** Score **> 15** indicates **homology** | U | R.YKGVLGTITTLAK.T |
| 46928 | 55 | – | 67 | 455.6108 | 1363.8106 | 1363.8075 | 2.29 | 1 | 28 | 0.0026 | 1Score **> 30** indicates **identity** Score **> 15** indicates **homology** | U | R.YKGVLGTITTLAK.T |
| 46929 | 55 | – | 67 | 455.6109 | 1363.8108 | 1363.8075 | 2.40 | 1 | 32 | 0.0029 | 1Score **> 30** indicates **identity** Score **> 19** indicates **homology** | U | R.YKGVLGTITTLAK.T |
| 46933 | 55 | – | 67 | 682.9139 | 1363.8133 | 1363.8075 | 4.22 | 1 | 59 | 9e-06 | 1Score **> 30** indicates **identity** Score **> 22** indicates **homology** | U | R.YKGVLGTITTLAK.T |
| 46935 | 55 | – | 67 | 455.6121 | 1363.8144 | 1363.8075 | 5.03 | 1 | 18 | 0.022 | 1Score **> 30** indicates **identity** Score **> 14** indicates **homology** | U | R.YKGVLGTITTLAK.T |
| 119564 | 55 | – | 73 | 498.2983 | 1989.1641 | 1989.1510 | 6.57 | 2 | 16 | 0.03 | 1Score **> 30** indicates **identity** Score **> 14** indicates **homology** | U | R.YKGVLGTITTLAKTEGLPK.L |
| 119565 | 55 | – | 73 | 664.0622 | 1989.1649 | 1989.1510 | 6.96 | 2 | 100 | 4.4e-10 | 1Score **> 30** indicates **identity** Score **> 19** indicates **homology** | U | R.YKGVLGTITTLAKTEGLPK.L |
| 119566 | 55 | – | 73 | 498.2986 | 1989.1654 | 1989.1510 | 7.23 | 2 | 16 | 0.03 | 1Score **> 30** indicates **identity** Score **> 14** indicates **homology** | U | R.YKGVLGTITTLAKTEGLPK.L |
| 119567 | 55 | – | 73 | 664.0627 | 1989.1663 | 1989.1510 | 7.69 | 2 | 94 | 1.6e-09 | 1Score **> 30** indicates **identity** Score **> 18** indicates **homology** | U | R.YKGVLGTITTLAKTEGLPK.L |
| 119568 | 55 | – | 73 | 664.0628 | 1989.1667 | 1989.1510 | 7.86 | 2 | 41 | 0.00014 | 1Score **> 30** indicates **identity** Score **> 15** indicates **homology** | U | R.YKGVLGTITTLAKTEGLPK.L |
| 119569 | 55 | – | 73 | 664.0636 | 1989.1691 | 1989.1510 | 9.10 | 2 | 20 | 0.013 | 1Score **> 30** indicates **identity** Score **> 14** indicates **homology** | U | R.YKGVLGTITTLAKTEGLPK.L |
| 119570 | 55 | – | 73 | 664.0639 | 1989.1698 | 1989.1510 | 9.44 | 2 | 69 | 3.1e-07 | 1Score **> 30** indicates **identity** Score **> 17** indicates **homology** | U | R.YKGVLGTITTLAKTEGLPK.L |
| 18322 | 57 | – | 67 | 537.3330 | 1072.6514 | 1072.6492 | 2.05 | 0 | 37 | 0.00065 | 1Score **> 29** indicates **identity** Score **> 18** indicates **homology** | U | K.GVLGTITTLAK.T |
| 18323 | 57 | – | 67 | 537.3334 | 1072.6522 | 1072.6492 | 2.74 | 0 | 41 | 0.0016 | 1Score **> 29** indicates **identity** Score **> 26** indicates **homology** | U | K.GVLGTITTLAK.T |
| 88653 | 57 | – | 73 | 567.0048 | 1697.9926 | 1697.9927 | -0.092 | 1 | 22 | 0.0091 | 1Score **> 32** indicates **identity** Score **> 14** indicates **homology** | U | K.GVLGTITTLAKTEGLPK.L |
| 88654 | 57 | – | 73 | 567.0060 | 1697.9962 | 1697.9927 | 2.05 | 1 | 45 | 5.8e-05 | 1Score **> 32** indicates **identity** Score **> 15** indicates **homology** | U | K.GVLGTITTLAKTEGLPK.L |
| 27712 | 74 | – | 84 | 587.8327 | 1173.6509 | 1173.6506 | 0.29 | 0 | 64 | 5.3e-06 | 1Score **> 35** indicates **identity** Score **> 23** indicates **homology** | U | K.LYSGLPAGIQR.Q |
| 27716 | 74 | – | 84 | 587.8331 | 1173.6516 | 1173.6506 | 0.85 | 0 | 57 | 1e-05 | 1Score **> 34** indicates **identity** Score **> 19** indicates **homology** | U | K.LYSGLPAGIQR.Q |
| 27718 | 74 | – | 84 | 587.8332 | 1173.6518 | 1173.6506 | 1.05 | 0 | 53 | 1.3e-05 | 1Score **> 34** indicates **identity** Score **> 16** indicates **homology** | U | K.LYSGLPAGIQR.Q |
| 27719 | 74 | – | 84 | 587.8332 | 1173.6519 | 1173.6506 | 1.14 | 0 | 23 | 0.0077 | 1Score **> 34** indicates **identity** Score **> 14** indicates **homology** | U | K.LYSGLPAGIQR.Q |
| 27720 | 74 | – | 84 | 587.8333 | 1173.6520 | 1173.6506 | 1.23 | 0 | 27 | 0.003 | 1Score **> 34** indicates **identity** Score **> 14** indicates **homology** | U | K.LYSGLPAGIQR.Q |
| 27722 | 74 | – | 84 | 587.8334 | 1173.6522 | 1173.6506 | 1.34 | 0 | 32 | 0.00097 | 1Score **> 34** indicates **identity** Score **> 15** indicates **homology** | U | K.LYSGLPAGIQR.Q |
| 27723 | 74 | – | 84 | 587.8334 | 1173.6522 | 1173.6506 | 1.35 | 0 | 61 | 4e-06 | 1Score **> 34** indicates **identity** Score **> 20** indicates **homology** | U | K.LYSGLPAGIQR.Q |
| 27724 | 74 | – | 84 | 587.8336 | 1173.6527 | 1173.6506 | 1.77 | 0 | 57 | 5.2e-06 | 1Score **> 34** indicates **identity** Score **> 17** indicates **homology** | U | K.LYSGLPAGIQR.Q |
| 27725 | 74 | – | 84 | 587.8337 | 1173.6529 | 1173.6506 | 1.95 | 0 | 28 | 0.0026 | 1Score **> 34** indicates **identity** Score **> 14** indicates **homology** | U | K.LYSGLPAGIQR.Q |
| 27727 | 74 | – | 84 | 587.8341 | 1173.6536 | 1173.6506 | 2.57 | 0 | 40 | 0.00017 | 1Score **> 34** indicates **identity** Score **> 15** indicates **homology** | U | K.LYSGLPAGIQR.Q |
| 27728 | 74 | – | 84 | 587.8343 | 1173.6541 | 1173.6506 | 3.02 | 0 | 36 | 0.00042 | 1Score **> 34** indicates **identity** Score **> 15** indicates **homology** | U | K.LYSGLPAGIQR.Q |
| 27730 | 74 | – | 84 | 587.8345 | 1173.6544 | 1173.6506 | 3.22 | 0 | 18 | 0.023 | 1Score **> 34** indicates **identity** Score **> 14** indicates **homology** | U | K.LYSGLPAGIQR.Q |
| 27731 | 74 | – | 84 | 587.8346 | 1173.6546 | 1173.6506 | 3.40 | 0 | 17 | 0.026 | 1Score **> 34** indicates **identity** Score **> 14** indicates **homology** | U | K.LYSGLPAGIQR.Q |
| 27732 | 74 | – | 84 | 587.8348 | 1173.6549 | 1173.6506 | 3.71 | 0 | 30 | 0.0015 | 1Score **> 34** indicates **identity** Score **> 14** indicates **homology** | U | K.LYSGLPAGIQR.Q |
| 27733 | 74 | – | 84 | 587.8348 | 1173.6551 | 1173.6506 | 3.82 | 0 | 29 | 0.002 | 1Score **> 34** indicates **identity** Score **> 14** indicates **homology** | U | K.LYSGLPAGIQR.Q |
| 27734 | 74 | – | 84 | 587.8351 | 1173.6556 | 1173.6506 | 4.26 | 0 | 27 | 0.0046 | 1Score **> 34** indicates **identity** Score **> 17** indicates **homology** | U | K.LYSGLPAGIQR.Q |
| 27736 | 74 | – | 84 | 587.8352 | 1173.6558 | 1173.6506 | 4.46 | 0 | 27 | 0.0026 | 1Score **> 34** indicates **identity** Score **> 14** indicates **homology** | U | K.LYSGLPAGIQR.Q |
| 27737 | 74 | – | 84 | 587.8354 | 1173.6562 | 1173.6506 | 4.77 | 0 | 23 | 0.0074 | 1Score **> 34** indicates **identity** Score **> 14** indicates **homology** | U | K.LYSGLPAGIQR.Q |
| 27738 | 74 | – | 84 | 587.8356 | 1173.6566 | 1173.6506 | 5.11 | 0 | 29 | 0.0021 | 1Score **> 34** indicates **identity** Score **> 14** indicates **homology** | U | K.LYSGLPAGIQR.Q |
| 27742 | 74 | – | 84 | 587.8369 | 1173.6592 | 1173.6506 | 7.38 | 0 | 25 | 0.0048 | 1Score **> 34** indicates **identity** Score **> 14** indicates **homology** | U | K.LYSGLPAGIQR.Q |
| 8773 | 85 | – | 92 | 461.2607 | 920.5069 | 920.5080 | -1.14 | 0 | 33 | 0.0053 | 1Score **> 29** indicates **identity** Score **> 23** indicates **homology** | U | R.QISFASLR.I |
| 8774 | 85 | – | 92 | 461.2609 | 920.5072 | 920.5080 | -0.78 | 0 | 40 | 0.0028 | 1Score **> 29** indicates **identity** Score **> 27** indicates **homology** | U | R.QISFASLR.I |
| 8775 | 85 | – | 92 | 461.2611 | 920.5076 | 920.5080 | -0.38 | 0 | 40 | 0.0029 | 1Score **> 29** indicates **identity** Score **> 27** indicates **homology** | U | R.QISFASLR.I |
| 8776 | 85 | – | 92 | 461.2612 | 920.5078 | 920.5080 | -0.22 | 0 | 38 | 0.0034 | 1Score **> 29** indicates **identity** Score **> 25** indicates **homology** | U | R.QISFASLR.I |
| 8777 | 85 | – | 92 | 461.2612 | 920.5078 | 920.5080 | -0.11 | 0 | 40 | 0.0023 | 1Score **> 29** indicates **identity** Score **> 26** indicates **homology** | U | R.QISFASLR.I |
| 8778 | 85 | – | 92 | 461.2613 | 920.5081 | 920.5080 | 0.19 | 0 | 20 | 0.03 | 1Score **> 29** indicates **identity** Score **> 17** indicates **homology** | U | R.QISFASLR.I |
| 8779 | 85 | – | 92 | 461.2614 | 920.5082 | 920.5080 | 0.28 | 0 | 41 | 0.0017 | 1Score **> 29** indicates **identity** Score **> 26** indicates **homology** | U | R.QISFASLR.I |
| 8780 | 85 | – | 92 | 461.2615 | 920.5084 | 920.5080 | 0.46 | 0 | 40 | 0.0021 | 1Score **> 29** indicates **identity** Score **> 26** indicates **homology** | U | R.QISFASLR.I |
| 8781 | 85 | – | 92 | 461.2615 | 920.5085 | 920.5080 | 0.58 | 0 | 38 | 0.0061 | 1Score **> 29** indicates **identity** | U | R.QISFASLR.I |
| 8782 | 85 | – | 92 | 461.2616 | 920.5087 | 920.5080 | 0.78 | 0 | 43 | 0.002 | 1Score **> 29** indicates **identity** Score **> 28** indicates **homology** | U | R.QISFASLR.I |
| 8784 | 85 | – | 92 | 461.2617 | 920.5089 | 920.5080 | 1.00 | 0 | 44 | 0.00096 | 1Score **> 29** indicates **identity** Score **> 26** indicates **homology** | U | R.QISFASLR.I |
| 8785 | 85 | – | 92 | 461.2617 | 920.5089 | 920.5080 | 1.01 | 0 | 46 | 0.001 | 1Score **> 29** indicates **identity** Score **> 28** indicates **homology** | U | R.QISFASLR.I |
| 8786 | 85 | – | 92 | 461.2617 | 920.5089 | 920.5080 | 1.07 | 0 | 25 | 0.024 | 1Score **> 29** indicates **identity** Score **> 22** indicates **homology** | U | R.QISFASLR.I |
| 8787 | 85 | – | 92 | 461.2618 | 920.5091 | 920.5080 | 1.21 | 0 | 45 | 0.0011 | 1Score **> 29** indicates **identity** Score **> 28** indicates **homology** | U | R.QISFASLR.I |
| 8788 | 85 | – | 92 | 461.2619 | 920.5092 | 920.5080 | 1.34 | 0 | 40 | 0.0029 | 1Score **> 29** indicates **identity** Score **> 27** indicates **homology** | U | R.QISFASLR.I |
| 8789 | 85 | – | 92 | 461.2619 | 920.5092 | 920.5080 | 1.41 | 0 | 43 | 0.0012 | 1Score **> 29** indicates **identity** Score **> 27** indicates **homology** | U | R.QISFASLR.I |
| 8790 | 85 | – | 92 | 461.2619 | 920.5093 | 920.5080 | 1.46 | 0 | 33 | 0.0056 | 1Score **> 28** indicates **identity** Score **> 23** indicates **homology** | U | R.QISFASLR.I |
| 8792 | 85 | – | 92 | 461.2620 | 920.5095 | 920.5080 | 1.73 | 0 | 15 | 0.037 | 1Score **> 27** indicates **identity** Score **> 13** indicates **homology** | U | R.QISFASLR.I |
| 8793 | 85 | – | 92 | 461.2621 | 920.5097 | 920.5080 | 1.94 | 0 | 36 | 0.0042 | 1Score **> 27** indicates **identity** Score **> 25** indicates **homology** | U | R.QISFASLR.I |
| 8794 | 85 | – | 92 | 461.2623 | 920.5101 | 920.5080 | 2.31 | 0 | 40 | 0.0029 | 1Score **> 29** indicates **identity** Score **> 27** indicates **homology** | U | R.QISFASLR.I |
| 8795 | 85 | – | 92 | 461.2624 | 920.5102 | 920.5080 | 2.45 | 0 | 21 | 0.023 | 1Score **> 29** indicates **identity** Score **> 17** indicates **homology** | U | R.QISFASLR.I |
| 8797 | 85 | – | 92 | 461.2625 | 920.5104 | 920.5080 | 2.65 | 0 | 37 | 0.0034 | 1Score **> 29** indicates **identity** Score **> 25** indicates **homology** | U | R.QISFASLR.I |
| 8798 | 85 | – | 92 | 461.2625 | 920.5104 | 920.5080 | 2.66 | 0 | 19 | 0.037 | 1Score **> 29** indicates **identity** Score **> 17** indicates **homology** | U | R.QISFASLR.I |
| 8799 | 85 | – | 92 | 461.2628 | 920.5110 | 920.5080 | 3.27 | 0 | 17 | 0.028 | 1Score **> 29** indicates **identity** Score **> 14** indicates **homology** | U | R.QISFASLR.I |
| 91395 | 93 | – | 107 | 860.9089 | 1719.8032 | 1719.8104 | -4.20 | 0 | 69 | 3.5e-07 | 1Score **> 33** indicates **identity** Score **> 17** indicates **homology** | U | R.IGLYDSVQEYFSSGR.E |
| 91398 | 93 | – | 107 | 860.9097 | 1719.8048 | 1719.8104 | -3.24 | 0 | 46 | 5.2e-05 | 1Score **> 33** indicates **identity** Score **> 15** indicates **homology** | U | R.IGLYDSVQEYFSSGR.E |
| 91399 | 93 | – | 107 | 860.9099 | 1719.8052 | 1719.8104 | -3.02 | 0 | 69 | 3.1e-07 | 1Score **> 33** indicates **identity** Score **> 17** indicates **homology** | U | R.IGLYDSVQEYFSSGR.E |
| 91400 | 93 | – | 107 | 860.9106 | 1719.8066 | 1719.8104 | -2.24 | 0 | 53 | 9.8e-06 | 1Score **> 33** indicates **identity** Score **> 16** indicates **homology** | U | R.IGLYDSVQEYFSSGR.E |
| 91401 | 93 | – | 107 | 574.2762 | 1719.8066 | 1719.8104 | -2.19 | 0 | 39 | 0.0002 | 1Score **> 33** indicates **identity** Score **> 15** indicates **homology** | U | R.IGLYDSVQEYFSSGR.E |
| 91403 | 93 | – | 107 | 574.2766 | 1719.8080 | 1719.8104 | -1.43 | 0 | 37 | 0.00032 | 1Score **> 33** indicates **identity** Score **> 15** indicates **homology** | U | R.IGLYDSVQEYFSSGR.E |
| 91405 | 93 | – | 107 | 860.9114 | 1719.8083 | 1719.8104 | -1.22 | 0 | 68 | 3.9e-07 | 1Score **> 33** indicates **identity** Score **> 17** indicates **homology** | U | R.IGLYDSVQEYFSSGR.E |
| 91406 | 93 | – | 107 | 860.9118 | 1719.8090 | 1719.8104 | -0.80 | 0 | 21 | 0.011 | 1Score **> 33** indicates **identity** Score **> 14** indicates **homology** | U | R.IGLYDSVQEYFSSGR.E |
| 91407 | 93 | – | 107 | 860.9119 | 1719.8093 | 1719.8104 | -0.65 | 0 | 68 | 4.5e-07 | 1Score **> 33** indicates **identity** Score **> 17** indicates **homology** | U | R.IGLYDSVQEYFSSGR.E |
| 91408 | 93 | – | 107 | 860.9120 | 1719.8094 | 1719.8104 | -0.58 | 0 | 57 | 4.5e-06 | 1Score **> 33** indicates **identity** Score **> 16** indicates **homology** | U | R.IGLYDSVQEYFSSGR.E |
| 91409 | 93 | – | 107 | 860.9124 | 1719.8102 | 1719.8104 | -0.12 | 0 | 48 | 3.4e-05 | 1Score **> 33** indicates **identity** Score **> 15** indicates **homology** | U | R.IGLYDSVQEYFSSGR.E |
| 91410 | 93 | – | 107 | 860.9124 | 1719.8103 | 1719.8104 | -0.070 | 0 | 79 | 4e-08 | 1Score **> 33** indicates **identity** Score **> 17** indicates **homology** | U | R.IGLYDSVQEYFSSGR.E |
| 91411 | 93 | – | 107 | 860.9125 | 1719.8104 | 1719.8104 | -0.023 | 0 | 91 | 2.8e-09 | 1Score **> 33** indicates **identity** Score **> 18** indicates **homology** | U | R.IGLYDSVQEYFSSGR.E |
| 91412 | 93 | – | 107 | 860.9125 | 1719.8104 | 1719.8104 | 0.014 | 0 | 50 | 2.3e-05 | 1Score **> 33** indicates **identity** Score **> 16** indicates **homology** | U | R.IGLYDSVQEYFSSGR.E |
| 91413 | 93 | – | 107 | 860.9126 | 1719.8106 | 1719.8104 | 0.11 | 0 | 77 | 5.5e-08 | 1Score **> 33** indicates **identity** Score **> 17** indicates **homology** | U | R.IGLYDSVQEYFSSGR.E |
| 91414 | 93 | – | 107 | 860.9126 | 1719.8106 | 1719.8104 | 0.13 | 0 | 73 | 1.3e-07 | 1Score **> 33** indicates **identity** Score **> 17** indicates **homology** | U | R.IGLYDSVQEYFSSGR.E |
| 91415 | 93 | – | 107 | 860.9127 | 1719.8108 | 1719.8104 | 0.20 | 0 | 42 | 0.00012 | 1Score **> 33** indicates **identity** Score **> 15** indicates **homology** | U | R.IGLYDSVQEYFSSGR.E |
| 91416 | 93 | – | 107 | 860.9127 | 1719.8109 | 1719.8104 | 0.27 | 0 | 94 | 1.4e-09 | 1Score **> 33** indicates **identity** Score **> 18** indicates **homology** | U | R.IGLYDSVQEYFSSGR.E |
| 91417 | 93 | – | 107 | 860.9128 | 1719.8111 | 1719.8104 | 0.42 | 0 | 75 | 1e-07 | 1Score **> 33** indicates **identity** Score **> 17** indicates **homology** | U | R.IGLYDSVQEYFSSGR.E |
| 91418 | 93 | – | 107 | 860.9131 | 1719.8116 | 1719.8104 | 0.68 | 0 | 16 | 0.029 | 1Score **> 33** indicates **identity** Score **> 14** indicates **homology** | U | R.IGLYDSVQEYFSSGR.E |
| 91419 | 93 | – | 107 | 860.9132 | 1719.8119 | 1719.8104 | 0.85 | 0 | 91 | 2.9e-09 | 1Score **> 33** indicates **identity** Score **> 18** indicates **homology** | U | R.IGLYDSVQEYFSSGR.E |
| 91421 | 93 | – | 107 | 860.9132 | 1719.8119 | 1719.8104 | 0.88 | 0 | 69 | 3.8e-07 | 1Score **> 33** indicates **identity** Score **> 17** indicates **homology** | U | R.IGLYDSVQEYFSSGR.E |
| 91422 | 93 | – | 107 | 574.2780 | 1719.8121 | 1719.8104 | 0.99 | 0 | 20 | 0.012 | 1Score **> 33** indicates **identity** Score **> 14** indicates **homology** | U | R.IGLYDSVQEYFSSGR.E |
| 91423 | 93 | – | 107 | 860.9134 | 1719.8123 | 1719.8104 | 1.10 | 0 | 67 | 5.3e-07 | 1Score **> 33** indicates **identity** Score **> 17** indicates **homology** | U | R.IGLYDSVQEYFSSGR.E |
| 91424 | 93 | – | 107 | 860.9135 | 1719.8124 | 1719.8104 | 1.17 | 0 | 85 | 9.9e-09 | 1Score **> 33** indicates **identity** Score **> 18** indicates **homology** | U | R.IGLYDSVQEYFSSGR.E |
| 91425 | 93 | – | 107 | 860.9136 | 1719.8127 | 1719.8104 | 1.31 | 0 | 38 | 0.00028 | 1Score **> 33** indicates **identity** Score **> 15** indicates **homology** | U | R.IGLYDSVQEYFSSGR.E |
| 91429 | 93 | – | 107 | 860.9138 | 1719.8131 | 1719.8104 | 1.54 | 0 | 90 | 3.7e-09 | 1Score **> 33** indicates **identity** Score **> 18** indicates **homology** | U | R.IGLYDSVQEYFSSGR.E |
| 91431 | 93 | – | 107 | 860.9139 | 1719.8132 | 1719.8104 | 1.60 | 0 | 53 | 1e-05 | 1Score **> 33** indicates **identity** Score **> 16** indicates **homology** | U | R.IGLYDSVQEYFSSGR.E |
| 91433 | 93 | – | 107 | 860.9142 | 1719.8139 | 1719.8104 | 2.04 | 0 | 36 | 0.00046 | 1Score **> 33** indicates **identity** Score **> 15** indicates **homology** | U | R.IGLYDSVQEYFSSGR.E |
| 91435 | 93 | – | 107 | 860.9144 | 1719.8142 | 1719.8104 | 2.19 | 0 | 96 | 1e-09 | 1Score **> 33** indicates **identity** Score **> 18** indicates **homology** | U | R.IGLYDSVQEYFSSGR.E |
| 91436 | 93 | – | 107 | 860.9146 | 1719.8146 | 1719.8104 | 2.46 | 0 | 69 | 3.6e-07 | 1Score **> 33** indicates **identity** Score **> 17** indicates **homology** | U | R.IGLYDSVQEYFSSGR.E |
| 91438 | 93 | – | 107 | 860.9146 | 1719.8147 | 1719.8104 | 2.51 | 0 | 67 | 5.3e-07 | 1Score **> 33** indicates **identity** Score **> 17** indicates **homology** | U | R.IGLYDSVQEYFSSGR.E |
| 91446 | 93 | – | 107 | 860.9194 | 1719.8243 | 1719.8104 | 8.09 | 0 | 47 | 3.5e-05 | 1Score **> 34** indicates **identity** Score **> 15** indicates **homology** | U | R.IGLYDSVQEYFSSGR.E |
| 164454 | 93 | – | 116 | 873.4245 | 2617.2518 | 2617.2660 | -5.42 | 1 | 52 | 1.5e-05 | 1Score **> 36** indicates **identity** Score **> 16** indicates **homology** | U | R.IGLYDSVQEYFSSGRETPASLGNK.I |
| 164455 | 93 | – | 116 | 873.4252 | 2617.2538 | 2617.2660 | -4.66 | 1 | 59 | 2.9e-06 | 1Score **> 36** indicates **identity** Score **> 16** indicates **homology** | U | R.IGLYDSVQEYFSSGRETPASLGNK.I |
| 164457 | 93 | – | 116 | 1309.6362 | 2617.2578 | 2617.2660 | -3.12 | 1 | 83 | 1.7e-08 | 1Score **> 36** indicates **identity** Score **> 18** indicates **homology** | U | R.IGLYDSVQEYFSSGRETPASLGNK.I |
| 164458 | 93 | – | 116 | 873.4266 | 2617.2579 | 2617.2660 | -3.11 | 1 | 56 | 5.4e-06 | 1Score **> 36** indicates **identity** Score **> 16** indicates **homology** | U | R.IGLYDSVQEYFSSGRETPASLGNK.I |
| 164460 | 93 | – | 116 | 873.4269 | 2617.2588 | 2617.2660 | -2.76 | 1 | 15 | 0.037 | 1Score **> 36** indicates **identity** Score **> 13** indicates **homology** | U | R.IGLYDSVQEYFSSGRETPASLGNK.I |
| 164461 | 93 | – | 116 | 873.4270 | 2617.2592 | 2617.2660 | -2.61 | 1 | 48 | 2.8e-05 | 1Score **> 36** indicates **identity** Score **> 16** indicates **homology** | U | R.IGLYDSVQEYFSSGRETPASLGNK.I |
| 164463 | 93 | – | 116 | 873.4274 | 2617.2604 | 2617.2660 | -2.14 | 1 | 46 | 4.7e-05 | 1Score **> 36** indicates **identity** Score **> 15** indicates **homology** | U | R.IGLYDSVQEYFSSGRETPASLGNK.I |
| 164465 | 93 | – | 116 | 873.4280 | 2617.2622 | 2617.2660 | -1.45 | 1 | 48 | 3.2e-05 | 1Score **> 36** indicates **identity** Score **> 15** indicates **homology** | U | R.IGLYDSVQEYFSSGRETPASLGNK.I |
| 164467 | 93 | – | 116 | 873.4284 | 2617.2635 | 2617.2660 | -0.96 | 1 | 34 | 0.00068 | 1Score **> 36** indicates **identity** Score **> 15** indicates **homology** | U | R.IGLYDSVQEYFSSGRETPASLGNK.I |
| 164468 | 93 | – | 116 | 873.4287 | 2617.2642 | 2617.2660 | -0.69 | 1 | 36 | 0.00042 | 1Score **> 36** indicates **identity** Score **> 15** indicates **homology** | U | R.IGLYDSVQEYFSSGRETPASLGNK.I |
| 164469 | 93 | – | 116 | 873.4289 | 2617.2648 | 2617.2660 | -0.47 | 1 | 52 | 1.3e-05 | 1Score **> 36** indicates **identity** Score **> 16** indicates **homology** | U | R.IGLYDSVQEYFSSGRETPASLGNK.I |
| 164470 | 93 | – | 116 | 873.4293 | 2617.2661 | 2617.2660 | 0.023 | 1 | 53 | 1e-05 | 1Score **> 36** indicates **identity** Score **> 16** indicates **homology** | U | R.IGLYDSVQEYFSSGRETPASLGNK.I |
| 164471 | 93 | – | 116 | 873.4294 | 2617.2663 | 2617.2660 | 0.12 | 1 | 31 | 0.0011 | 1Score **> 36** indicates **identity** Score **> 14** indicates **homology** | U | R.IGLYDSVQEYFSSGRETPASLGNK.I |
| 164472 | 93 | – | 116 | 873.4294 | 2617.2664 | 2617.2660 | 0.15 | 1 | 60 | 2.3e-06 | 1Score **> 36** indicates **identity** Score **> 16** indicates **homology** | U | R.IGLYDSVQEYFSSGRETPASLGNK.I |
| 164473 | 93 | – | 116 | 1309.6408 | 2617.2671 | 2617.2660 | 0.43 | 1 | 92 | 2.3e-09 | 1Score **> 36** indicates **identity** Score **> 18** indicates **homology** | U | R.IGLYDSVQEYFSSGRETPASLGNK.I |
| 164474 | 93 | – | 116 | 873.4297 | 2617.2671 | 2617.2660 | 0.44 | 1 | 52 | 1.5e-05 | 1Score **> 36** indicates **identity** Score **> 16** indicates **homology** | U | R.IGLYDSVQEYFSSGRETPASLGNK.I |
| 164475 | 93 | – | 116 | 1309.6412 | 2617.2678 | 2617.2660 | 0.69 | 1 | 102 | 2.8e-10 | 1Score **> 36** indicates **identity** Score **> 19** indicates **homology** | U | R.IGLYDSVQEYFSSGRETPASLGNK.I |
| 164476 | 93 | – | 116 | 873.4300 | 2617.2682 | 2617.2660 | 0.84 | 1 | 51 | 1.8e-05 | 1Score **> 36** indicates **identity** Score **> 16** indicates **homology** | U | R.IGLYDSVQEYFSSGRETPASLGNK.I |
| 164477 | 93 | – | 116 | 873.4301 | 2617.2684 | 2617.2660 | 0.94 | 1 | 15 | 0.043 | 1Score **> 36** indicates **identity** Score **> 13** indicates **homology** | U | R.IGLYDSVQEYFSSGRETPASLGNK.I |
| 164478 | 93 | – | 116 | 873.4301 | 2617.2686 | 2617.2660 | 0.98 | 1 | 53 | 1.1e-05 | 1Score **> 36** indicates **identity** Score **> 16** indicates **homology** | U | R.IGLYDSVQEYFSSGRETPASLGNK.I |
| 164479 | 93 | – | 116 | 873.4305 | 2617.2696 | 2617.2660 | 1.38 | 1 | 30 | 0.0017 | 1Score **> 36** indicates **identity** Score **> 14** indicates **homology** | U | R.IGLYDSVQEYFSSGRETPASLGNK.I |
| 164482 | 93 | – | 116 | 873.4321 | 2617.2744 | 2617.2660 | 3.20 | 1 | 14 | 0.045 | 1Score **> 37** indicates **identity** Score **> 13** indicates **homology** | U | R.IGLYDSVQEYFSSGRETPASLGNK.I |
| 164483 | 93 | – | 116 | 873.4322 | 2617.2749 | 2617.2660 | 3.41 | 1 | 40 | 0.00019 | 1Score **> 36** indicates **identity** Score **> 15** indicates **homology** | U | R.IGLYDSVQEYFSSGRETPASLGNK.I |
| 164522 | 93 | – | 116 | 873.7653 | 2618.2741 | 2618.2500 | 9.20 | 1 | 39 | 0.0002 | 1Score **> 36** indicates **identity** Score **> 15** indicates **homology** | U | R.IGLYDSVQEYFSSGRETPASLGNK.I  + Deamidated (NQ) |
| 8492 | 108 | – | 116 | 458.7397 | 915.4649 | 915.4661 | -1.38 | 0 | 23 | 0.0064 | 1Score **> 29** indicates **identity** Score **> 14** indicates **homology** | U | R.ETPASLGNK.I |
| 8493 | 108 | – | 116 | 458.7401 | 915.4657 | 915.4661 | -0.47 | 0 | 40 | 0.00019 | 1Score **> 29** indicates **identity** Score **> 15** indicates **homology** | U | R.ETPASLGNK.I |
| 186121 | 108 | – | 140 | 832.4616 | 3325.8174 | 3325.8068 | 3.18 | 2 | 32 | 0.00098 | 1Score **> 34** indicates **identity** Score **> 15** indicates **homology** | U | R.ETPASLGNKISAGLMTGGVAVFIGQPTEVVKVR.M |
| 136874 | 117 | – | 138 | 1087.5968 | 2173.1790 | 2173.1817 | -1.26 | 0 | 55 | 7e-06 | 1Score **> 35** indicates **identity** Score **> 16** indicates **homology** | U | K.ISAGLMTGGVAVFIGQPTEVVK.V |
| 136878 | 117 | – | 138 | 1087.5989 | 2173.1832 | 2173.1817 | 0.68 | 0 | 69 | 3.1e-07 | 1Score **> 35** indicates **identity** Score **> 17** indicates **homology** | U | K.ISAGLMTGGVAVFIGQPTEVVK.V |
| 136879 | 117 | – | 138 | 725.4022 | 2173.1848 | 2173.1817 | 1.44 | 0 | 17 | 0.028 | 1Score **> 35** indicates **identity** Score **> 14** indicates **homology** | U | K.ISAGLMTGGVAVFIGQPTEVVK.V |
| 136880 | 117 | – | 138 | 1087.6006 | 2173.1866 | 2173.1817 | 2.24 | 0 | 75 | 1e-07 | 1Score **> 35** indicates **identity** Score **> 17** indicates **homology** | U | K.ISAGLMTGGVAVFIGQPTEVVK.V |
| 154793 | 117 | – | 140 | 810.4582 | 2428.3527 | 2428.3512 | 0.59 | 1 | 35 | 0.00056 | 1Score **> 34** indicates **identity** Score **> 15** indicates **homology** | U | K.ISAGLMTGGVAVFIGQPTEVVKVR.M |
| 62440 | 141 | – | 153 | 376.4550 | 1501.7909 | 1501.7936 | -1.78 | 1 | 27 | 0.0028 | 1Score **> 35** indicates **identity** Score **> 14** indicates **homology** | U | R.MQAQSHLHGIKPR.Y |
| 62442 | 141 | – | 153 | 376.4553 | 1501.7921 | 1501.7936 | -1.02 | 1 | 21 | 0.01 | 1Score **> 35** indicates **identity** Score **> 14** indicates **homology** | U | R.MQAQSHLHGIKPR.Y |
| 62445 | 141 | – | 153 | 501.6052 | 1501.7937 | 1501.7936 | 0.10 | 1 | 30 | 0.0015 | 1Score **> 35** indicates **identity** Score **> 14** indicates **homology** | U | R.MQAQSHLHGIKPR.Y |
| 62446 | 141 | – | 153 | 751.9044 | 1501.7942 | 1501.7936 | 0.44 | 1 | 76 | 7e-08 | 1Score **> 35** indicates **identity** Score **> 17** indicates **homology** | U | R.MQAQSHLHGIKPR.Y |
| 62448 | 141 | – | 153 | 376.4559 | 1501.7947 | 1501.7936 | 0.73 | 1 | 37 | 0.00034 | 1Score **> 35** indicates **identity** Score **> 15** indicates **homology** | U | R.MQAQSHLHGIKPR.Y |
| 64377 | 141 | – | 153 | 380.4540 | 1517.7869 | 1517.7885 | -1.05 | 1 | 25 | 0.0043 | 1Score **> 35** indicates **identity** Score **> 14** indicates **homology** | U | R.MQAQSHLHGIKPR.Y  + Oxidation (M) |
| 64379 | 141 | – | 153 | 506.9364 | 1517.7875 | 1517.7885 | -0.66 | 1 | 23 | 0.0075 | 1Score **> 35** indicates **identity** Score **> 14** indicates **homology** | U | R.MQAQSHLHGIKPR.Y  + Oxidation (M) |
| 64382 | 141 | – | 153 | 380.4543 | 1517.7880 | 1517.7885 | -0.33 | 1 | 26 | 0.0036 | 1Score **> 35** indicates **identity** Score **> 14** indicates **homology** | U | R.MQAQSHLHGIKPR.Y  + Oxidation (M) |
| 64383 | 141 | – | 153 | 380.4544 | 1517.7885 | 1517.7885 | 0.012 | 1 | 21 | 0.01 | 1Score **> 35** indicates **identity** Score **> 14** indicates **homology** | U | R.MQAQSHLHGIKPR.Y  + Oxidation (M) |
| 64385 | 141 | – | 153 | 380.4545 | 1517.7888 | 1517.7885 | 0.20 | 1 | 27 | 0.0032 | 1Score **> 35** indicates **identity** Score **> 14** indicates **homology** | U | R.MQAQSHLHGIKPR.Y  + Oxidation (M) |
| 64386 | 141 | – | 153 | 506.9370 | 1517.7893 | 1517.7885 | 0.54 | 1 | 27 | 0.0027 | 1Score **> 35** indicates **identity** Score **> 14** indicates **homology** | U | R.MQAQSHLHGIKPR.Y  + Oxidation (M) |
| 64389 | 141 | – | 153 | 380.4547 | 1517.7897 | 1517.7885 | 0.79 | 1 | 14 | 0.047 | 1Score **> 35** indicates **identity** Score **> 13** indicates **homology** | U | R.MQAQSHLHGIKPR.Y  + Oxidation (M) |
| 64390 | 141 | – | 153 | 506.9372 | 1517.7898 | 1517.7885 | 0.87 | 1 | 32 | 0.001 | 1Score **> 35** indicates **identity** Score **> 14** indicates **homology** | U | R.MQAQSHLHGIKPR.Y  + Oxidation (M) |
| 64391 | 141 | – | 153 | 380.4549 | 1517.7905 | 1517.7885 | 1.30 | 1 | 22 | 0.008 | 1Score **> 35** indicates **identity** Score **> 14** indicates **homology** | U | R.MQAQSHLHGIKPR.Y  + Oxidation (M) |
| 64392 | 141 | – | 153 | 506.9375 | 1517.7907 | 1517.7885 | 1.43 | 1 | 43 | 9.4e-05 | 1Score **> 35** indicates **identity** Score **> 15** indicates **homology** | U | R.MQAQSHLHGIKPR.Y  + Oxidation (M) |
| 64393 | 141 | – | 153 | 380.4549 | 1517.7907 | 1517.7885 | 1.45 | 1 | 15 | 0.036 | 1Score **> 35** indicates **identity** Score **> 13** indicates **homology** | U | R.MQAQSHLHGIKPR.Y  + Oxidation (M) |
| 64397 | 141 | – | 153 | 506.9388 | 1517.7946 | 1517.7885 | 4.00 | 1 | 30 | 0.0016 | 1Score **> 35** indicates **identity** Score **> 14** indicates **homology** | U | R.MQAQSHLHGIKPR.Y  + Oxidation (M) |
| 21400 | 154 | – | 162 | 554.7555 | 1107.4964 | 1107.4985 | -1.86 | 0 | 31 | 0.0012 | 1Score **> 29** indicates **identity** Score **> 14** indicates **homology** | U | R.YTGTYNAYR.V |
| 21401 | 154 | – | 162 | 554.7560 | 1107.4974 | 1107.4985 | -1.02 | 0 | 47 | 3.9e-05 | 1Score **> 29** indicates **identity** Score **> 15** indicates **homology** | U | R.YTGTYNAYR.V |
| 21402 | 154 | – | 162 | 554.7560 | 1107.4975 | 1107.4985 | -0.89 | 0 | 40 | 0.00018 | 1Score **> 29** indicates **identity** Score **> 15** indicates **homology** | U | R.YTGTYNAYR.V |
| 21403 | 154 | – | 162 | 554.7563 | 1107.4981 | 1107.4985 | -0.35 | 0 | 46 | 4.9e-05 | 1Score **> 28** indicates **identity** Score **> 15** indicates **homology** | U | R.YTGTYNAYR.V |
| 21404 | 154 | – | 162 | 554.7564 | 1107.4983 | 1107.4985 | -0.15 | 0 | 47 | 4e-05 | 1Score **> 28** indicates **identity** Score **> 15** indicates **homology** | U | R.YTGTYNAYR.V |
| 21405 | 154 | – | 162 | 554.7566 | 1107.4986 | 1107.4985 | 0.11 | 0 | 32 | 0.001 | 1Score **> 28** indicates **identity** Score **> 14** indicates **homology** | U | R.YTGTYNAYR.V |
| 21406 | 154 | – | 162 | 554.7566 | 1107.4986 | 1107.4985 | 0.11 | 0 | 62 | 1.4e-06 | 1Score **> 28** indicates **identity** Score **> 16** indicates **homology** | U | R.YTGTYNAYR.V |
| 21407 | 154 | – | 162 | 554.7569 | 1107.4992 | 1107.4985 | 0.63 | 0 | 47 | 3.9e-05 | 1Score **> 28** indicates **identity** Score **> 15** indicates **homology** | U | R.YTGTYNAYR.V |
| 21408 | 154 | – | 162 | 554.7569 | 1107.4992 | 1107.4985 | 0.66 | 0 | 62 | 4.8e-06 | 1Score **> 28** indicates **identity** Score **> 21** indicates **homology** | U | R.YTGTYNAYR.V |
| 21409 | 154 | – | 162 | 554.7574 | 1107.5002 | 1107.4985 | 1.52 | 0 | 45 | 6.3e-05 | 1Score **> 28** indicates **identity** Score **> 15** indicates **homology** | U | R.YTGTYNAYR.V |
| 21410 | 154 | – | 162 | 554.7576 | 1107.5007 | 1107.4985 | 2.02 | 0 | 30 | 0.0014 | 1Score **> 29** indicates **identity** Score **> 14** indicates **homology** | U | R.YTGTYNAYR.V |
| 21411 | 154 | – | 162 | 554.7577 | 1107.5009 | 1107.4985 | 2.12 | 0 | 42 | 0.00012 | 1Score **> 29** indicates **identity** Score **> 15** indicates **homology** | U | R.YTGTYNAYR.V |
| 21412 | 154 | – | 162 | 554.7579 | 1107.5013 | 1107.4985 | 2.53 | 0 | 26 | 0.004 | 1Score **> 29** indicates **identity** Score **> 14** indicates **homology** | U | R.YTGTYNAYR.V |
| 21413 | 154 | – | 162 | 554.7587 | 1107.5028 | 1107.4985 | 3.91 | 0 | 31 | 0.0028 | 1Score **> 29** indicates **identity** Score **> 18** indicates **homology** | U | R.YTGTYNAYR.V |
| 21482 | 154 | – | 162 | 555.2500 | 1108.4855 | 1108.4825 | 2.65 | 0 | 17 | 0.025 | 1Score **> 28** indicates **identity** Score **> 14** indicates **homology** | U | R.YTGTYNAYR.V  + Deamidated (NQ) |
| 21483 | 154 | – | 162 | 555.2505 | 1108.4864 | 1108.4825 | 3.52 | 0 | 41 | 0.00013 | 1Score **> 28** indicates **identity** Score **> 15** indicates **homology** | U | R.YTGTYNAYR.V  + Deamidated (NQ) |
| 160752 | 154 | – | 175 | 846.7681 | 2537.2824 | 2537.2802 | 0.87 | 1 | 42 | 0.00013 | 1Score **> 37** indicates **identity** Score **> 15** indicates **homology** | U | R.YTGTYNAYRVIATTESLSTLWK.G |
| 55977 | 163 | – | 175 | 724.8999 | 1447.7852 | 1447.7922 | -4.87 | 0 | 14 | 0.047 | 1Score **> 34** indicates **identity** Score **> 13** indicates **homology** | U | R.VIATTESLSTLWK.G |
| 55982 | 163 | – | 175 | 724.9016 | 1447.7887 | 1447.7922 | -2.47 | 0 | 61 | 1.8e-06 | 1Score **> 34** indicates **identity** Score **> 16** indicates **homology** | U | R.VIATTESLSTLWK.G |
| 55985 | 163 | – | 175 | 724.9018 | 1447.7891 | 1447.7922 | -2.16 | 0 | 33 | 0.00076 | 1Score **> 34** indicates **identity** Score **> 15** indicates **homology** | U | R.VIATTESLSTLWK.G |
| 55987 | 163 | – | 175 | 724.9023 | 1447.7900 | 1447.7922 | -1.55 | 0 | 62 | 1.6e-06 | 1Score **> 34** indicates **identity** Score **> 16** indicates **homology** | U | R.VIATTESLSTLWK.G |
| 55988 | 163 | – | 175 | 724.9024 | 1447.7903 | 1447.7922 | -1.31 | 0 | 18 | 0.022 | 1Score **> 34** indicates **identity** Score **> 14** indicates **homology** | U | R.VIATTESLSTLWK.G |
| 55989 | 163 | – | 175 | 724.9026 | 1447.7906 | 1447.7922 | -1.11 | 0 | 65 | 1.9e-06 | 1Score **> 34** indicates **identity** Score **> 20** indicates **homology** | U | R.VIATTESLSTLWK.G |
| 55992 | 163 | – | 175 | 724.9029 | 1447.7913 | 1447.7922 | -0.63 | 0 | 26 | 0.004 | 1Score **> 34** indicates **identity** Score **> 14** indicates **homology** | U | R.VIATTESLSTLWK.G |
| 55995 | 163 | – | 175 | 483.6044 | 1447.7914 | 1447.7922 | -0.58 | 0 | 16 | 0.033 | 1Score **> 34** indicates **identity** Score **> 13** indicates **homology** | U | R.VIATTESLSTLWK.G |
| 55996 | 163 | – | 175 | 724.9034 | 1447.7923 | 1447.7922 | 0.029 | 0 | 50 | 2.2e-05 | 1Score **> 34** indicates **identity** Score **> 16** indicates **homology** | U | R.VIATTESLSTLWK.G |
| 55997 | 163 | – | 175 | 724.9035 | 1447.7924 | 1447.7922 | 0.15 | 0 | 97 | 8.3e-10 | 1Score **> 34** indicates **identity** Score **> 19** indicates **homology** | U | R.VIATTESLSTLWK.G |
| 56000 | 163 | – | 175 | 724.9037 | 1447.7929 | 1447.7922 | 0.49 | 0 | 48 | 3.1e-05 | 1Score **> 33** indicates **identity** Score **> 16** indicates **homology** | U | R.VIATTESLSTLWK.G |
| 56001 | 163 | – | 175 | 724.9038 | 1447.7930 | 1447.7922 | 0.54 | 0 | 65 | 7.3e-07 | 1Score **> 34** indicates **identity** Score **> 17** indicates **homology** | U | R.VIATTESLSTLWK.G |
| 56002 | 163 | – | 175 | 724.9039 | 1447.7933 | 1447.7922 | 0.74 | 0 | 98 | 8.6e-10 | 1Score **> 34** indicates **identity** Score **> 20** indicates **homology** | U | R.VIATTESLSTLWK.G |
| 56003 | 163 | – | 175 | 724.9040 | 1447.7934 | 1447.7922 | 0.82 | 0 | 102 | 4.6e-10 | 1Score **> 34** indicates **identity** Score **> 21** indicates **homology** | U | R.VIATTESLSTLWK.G |
| 56004 | 163 | – | 175 | 724.9040 | 1447.7935 | 1447.7922 | 0.87 | 0 | 84 | 1.5e-08 | 1Score **> 34** indicates **identity** Score **> 18** indicates **homology** | U | R.VIATTESLSTLWK.G |
| 56005 | 163 | – | 175 | 724.9041 | 1447.7937 | 1447.7922 | 0.98 | 0 | 98 | 8.5e-10 | 1Score **> 34** indicates **identity** Score **> 20** indicates **homology** | U | R.VIATTESLSTLWK.G |
| 56006 | 163 | – | 175 | 724.9041 | 1447.7937 | 1447.7922 | 0.99 | 0 | 84 | 1.4e-08 | 1Score **> 34** indicates **identity** Score **> 18** indicates **homology** | U | R.VIATTESLSTLWK.G |
| 56007 | 163 | – | 175 | 724.9042 | 1447.7937 | 1447.7922 | 1.04 | 0 | 48 | 3e-05 | 1Score **> 34** indicates **identity** Score **> 16** indicates **homology** | U | R.VIATTESLSTLWK.G |
| 56008 | 163 | – | 175 | 724.9042 | 1447.7938 | 1447.7922 | 1.07 | 0 | 99 | 8.6e-10 | 1Score **> 34** indicates **identity** Score **> 21** indicates **homology** | U | R.VIATTESLSTLWK.G |
| 56009 | 163 | – | 175 | 724.9042 | 1447.7938 | 1447.7922 | 1.07 | 0 | 97 | 8.9e-10 | 1Score **> 34** indicates **identity** Score **> 19** indicates **homology** | U | R.VIATTESLSTLWK.G |
| 56010 | 163 | – | 175 | 724.9042 | 1447.7938 | 1447.7922 | 1.07 | 0 | 78 | 4.4e-08 | 1Score **> 34** indicates **identity** Score **> 17** indicates **homology** | U | R.VIATTESLSTLWK.G |
| 56011 | 163 | – | 175 | 724.9042 | 1447.7939 | 1447.7922 | 1.15 | 0 | 62 | 1.6e-06 | 1Score **> 34** indicates **identity** Score **> 16** indicates **homology** | U | R.VIATTESLSTLWK.G |
| 56012 | 163 | – | 175 | 724.9043 | 1447.7940 | 1447.7922 | 1.21 | 0 | 109 | 9.8e-11 | 1Score **> 34** indicates **identity** Score **> 21** indicates **homology** | U | R.VIATTESLSTLWK.G |
| 56013 | 163 | – | 175 | 724.9043 | 1447.7941 | 1447.7922 | 1.25 | 0 | 119 | 7.6e-12 | 1Score **> 34** indicates **identity** Score **> 20** indicates **homology** | U | R.VIATTESLSTLWK.G |
| 56014 | 163 | – | 175 | 483.6053 | 1447.7941 | 1447.7922 | 1.26 | 0 | 45 | 6e-05 | 1Score **> 34** indicates **identity** Score **> 15** indicates **homology** | U | R.VIATTESLSTLWK.G |
| 56015 | 163 | – | 175 | 724.9043 | 1447.7941 | 1447.7922 | 1.30 | 0 | 99 | 8.2e-10 | 1Score **> 34** indicates **identity** Score **> 20** indicates **homology** | U | R.VIATTESLSTLWK.G |
| 56016 | 163 | – | 175 | 724.9044 | 1447.7942 | 1447.7922 | 1.39 | 0 | 99 | 8.7e-10 | 1Score **> 34** indicates **identity** Score **> 20** indicates **homology** | U | R.VIATTESLSTLWK.G |
| 56017 | 163 | – | 175 | 724.9044 | 1447.7943 | 1447.7922 | 1.40 | 0 | 61 | 1.8e-06 | 1Score **> 34** indicates **identity** Score **> 16** indicates **homology** | U | R.VIATTESLSTLWK.G |
| 56019 | 163 | – | 175 | 724.9045 | 1447.7944 | 1447.7922 | 1.47 | 0 | 103 | 2.4e-10 | 1Score **> 34** indicates **identity** Score **> 19** indicates **homology** | U | R.VIATTESLSTLWK.G |
| 56020 | 163 | – | 175 | 724.9045 | 1447.7944 | 1447.7922 | 1.49 | 0 | 78 | 1.2e-07 | 1Score **> 34** indicates **identity** Score **> 22** indicates **homology** | U | R.VIATTESLSTLWK.G |
| 56021 | 163 | – | 175 | 724.9046 | 1447.7946 | 1447.7922 | 1.63 | 0 | 119 | 7.3e-12 | 1Score **> 34** indicates **identity** Score **> 20** indicates **homology** | U | R.VIATTESLSTLWK.G |
| 56022 | 163 | – | 175 | 724.9046 | 1447.7946 | 1447.7922 | 1.63 | 0 | 118 | 8.2e-12 | 1Score **> 34** indicates **identity** Score **> 20** indicates **homology** | U | R.VIATTESLSTLWK.G |
| 56023 | 163 | – | 175 | 724.9046 | 1447.7946 | 1447.7922 | 1.63 | 0 | 99 | 8.5e-10 | 1Score **> 34** indicates **identity** Score **> 20** indicates **homology** | U | R.VIATTESLSTLWK.G |
| 56024 | 163 | – | 175 | 483.6055 | 1447.7947 | 1447.7922 | 1.70 | 0 | 45 | 5.7e-05 | 1Score **> 34** indicates **identity** Score **> 15** indicates **homology** | U | R.VIATTESLSTLWK.G |
| 56026 | 163 | – | 175 | 724.9046 | 1447.7947 | 1447.7922 | 1.74 | 0 | 79 | 4.1e-08 | 1Score **> 34** indicates **identity** Score **> 17** indicates **homology** | U | R.VIATTESLSTLWK.G |
| 56027 | 163 | – | 175 | 724.9047 | 1447.7949 | 1447.7922 | 1.83 | 0 | 79 | 4e-08 | 1Score **> 34** indicates **identity** Score **> 17** indicates **homology** | U | R.VIATTESLSTLWK.G |
| 56028 | 163 | – | 175 | 724.9048 | 1447.7950 | 1447.7922 | 1.90 | 0 | 64 | 1.1e-06 | 1Score **> 34** indicates **identity** Score **> 16** indicates **homology** | U | R.VIATTESLSTLWK.G |
| 56029 | 163 | – | 175 | 724.9048 | 1447.7950 | 1447.7922 | 1.91 | 0 | 91 | 3.1e-09 | 1Score **> 34** indicates **identity** Score **> 18** indicates **homology** | U | R.VIATTESLSTLWK.G |
| 56031 | 163 | – | 175 | 724.9048 | 1447.7951 | 1447.7922 | 2.00 | 0 | 119 | 7.5e-12 | 1Score **> 34** indicates **identity** Score **> 20** indicates **homology** | U | R.VIATTESLSTLWK.G |
| 56032 | 163 | – | 175 | 724.9048 | 1447.7951 | 1447.7922 | 2.00 | 0 | 65 | 7.7e-07 | 1Score **> 34** indicates **identity** Score **> 17** indicates **homology** | U | R.VIATTESLSTLWK.G |
| 56033 | 163 | – | 175 | 724.9049 | 1447.7953 | 1447.7922 | 2.14 | 0 | 98 | 8.3e-10 | 1Score **> 34** indicates **identity** Score **> 20** indicates **homology** | U | R.VIATTESLSTLWK.G |
| 56034 | 163 | – | 175 | 724.9051 | 1447.7956 | 1447.7922 | 2.31 | 0 | 97 | 8.4e-10 | 1Score **> 33** indicates **identity** Score **> 19** indicates **homology** | U | R.VIATTESLSTLWK.G |
| 56035 | 163 | – | 175 | 724.9055 | 1447.7965 | 1447.7922 | 2.93 | 0 | 83 | 1.8e-08 | 1Score **> 33** indicates **identity** Score **> 18** indicates **homology** | U | R.VIATTESLSTLWK.G |
| 56036 | 163 | – | 175 | 724.9062 | 1447.7979 | 1447.7922 | 3.94 | 0 | 78 | 4.7e-08 | 1Score **> 33** indicates **identity** Score **> 17** indicates **homology** | U | R.VIATTESLSTLWK.G |
| 56038 | 163 | – | 175 | 724.9068 | 1447.7990 | 1447.7922 | 4.70 | 0 | 78 | 4.5e-08 | 1Score **> 33** indicates **identity** Score **> 17** indicates **homology** | U | R.VIATTESLSTLWK.G |
| 56039 | 163 | – | 175 | 724.9073 | 1447.8001 | 1447.7922 | 5.46 | 0 | 82 | 1.9e-08 | 1Score **> 33** indicates **identity** Score **> 18** indicates **homology** | U | R.VIATTESLSTLWK.G |
| 56040 | 163 | – | 175 | 724.9076 | 1447.8006 | 1447.7922 | 5.80 | 0 | 22 | 0.0085 | 1Score **> 33** indicates **identity** Score **> 14** indicates **homology** | U | R.VIATTESLSTLWK.G |
| 56041 | 163 | – | 175 | 724.9076 | 1447.8007 | 1447.7922 | 5.85 | 0 | 83 | 1.8e-08 | 1Score **> 33** indicates **identity** Score **> 18** indicates **homology** | U | R.VIATTESLSTLWK.G |
| 56042 | 163 | – | 175 | 724.9081 | 1447.8016 | 1447.7922 | 6.45 | 0 | 36 | 0.00038 | 1Score **> 33** indicates **identity** Score **> 15** indicates **homology** | U | R.VIATTESLSTLWK.G |
| 56043 | 163 | – | 175 | 724.9085 | 1447.8025 | 1447.7922 | 7.11 | 0 | 53 | 1.1e-05 | 1Score **> 33** indicates **identity** Score **> 16** indicates **homology** | U | R.VIATTESLSTLWK.G |
| 56044 | 163 | – | 175 | 724.9096 | 1447.8047 | 1447.7922 | 8.58 | 0 | 83 | 1.6e-08 | 1Score **> 33** indicates **identity** Score **> 18** indicates **homology** | U | R.VIATTESLSTLWK.G |
| 147957 | 163 | – | 183 | 773.7518 | 2318.2336 | 2318.2304 | 1.38 | 1 | 36 | 0.00044 | 1Score **> 36** indicates **identity** Score **> 15** indicates **homology** | U | R.VIATTESLSTLWKGTTPNLMR.N |
| 7143 | 176 | – | 183 | 445.2307 | 888.4469 | 888.4487 | -2.09 | 0 | 28 | 0.0022 | 1Score **> 32** indicates **identity** Score **> 14** indicates **homology** | U | K.GTTPNLMR.N |
| 7144 | 176 | – | 183 | 445.2310 | 888.4475 | 888.4487 | -1.35 | 0 | 34 | 0.00062 | 1Score **> 31** indicates **identity** Score **> 15** indicates **homology** | U | K.GTTPNLMR.N |
| 7820 | 176 | – | 183 | 453.2284 | 904.4423 | 904.4436 | -1.45 | 0 | 22 | 0.0088 | 1Score **> 30** indicates **identity** Score **> 14** indicates **homology** | U | K.GTTPNLMR.N  + Oxidation (M) |
| 7825 | 176 | – | 183 | 453.2300 | 904.4455 | 904.4436 | 2.00 | 0 | 20 | 0.036 | 1Score **> 30** indicates **identity** Score **> 18** indicates **homology** | U | K.GTTPNLMR.N  + Oxidation (M) |
| 113071 | 184 | – | 199 | 963.4819 | 1924.9492 | 1924.9638 | -7.59 | 0 | 15 | 0.041 | 1Score **> 35** indicates **identity** Score **> 13** indicates **homology** | U | R.NVIINCTELVTYDLMK.G |
| 113081 | 184 | – | 199 | 642.6618 | 1924.9635 | 1924.9638 | -0.16 | 0 | 51 | 1.7e-05 | 1Score **> 35** indicates **identity** Score **> 16** indicates **homology** | U | R.NVIINCTELVTYDLMK.G |
| 113084 | 184 | – | 199 | 642.6625 | 1924.9656 | 1924.9638 | 0.91 | 0 | 64 | 1.1e-06 | 1Score **> 35** indicates **identity** Score **> 16** indicates **homology** | U | R.NVIINCTELVTYDLMK.G |
| 113085 | 184 | – | 199 | 963.4902 | 1924.9659 | 1924.9638 | 1.09 | 0 | 83 | 1.5e-08 | 1Score **> 35** indicates **identity** Score **> 18** indicates **homology** | U | R.NVIINCTELVTYDLMK.G |
| 113086 | 184 | – | 199 | 642.6626 | 1924.9659 | 1924.9638 | 1.10 | 0 | 52 | 1.4e-05 | 1Score **> 35** indicates **identity** Score **> 16** indicates **homology** | U | R.NVIINCTELVTYDLMK.G |
| 113087 | 184 | – | 199 | 963.4907 | 1924.9668 | 1924.9638 | 1.57 | 0 | 64 | 9.5e-07 | 1Score **> 35** indicates **identity** Score **> 17** indicates **homology** | U | R.NVIINCTELVTYDLMK.G |
| 113088 | 184 | – | 199 | 963.4907 | 1924.9669 | 1924.9638 | 1.58 | 0 | 68 | 4.4e-07 | 1Score **> 35** indicates **identity** Score **> 17** indicates **homology** | U | R.NVIINCTELVTYDLMK.G |
| 113095 | 184 | – | 199 | 963.4955 | 1924.9764 | 1924.9638 | 6.54 | 0 | 66 | 6e-07 | 1Score **> 35** indicates **identity** Score **> 17** indicates **homology** | U | R.NVIINCTELVTYDLMK.G |
| 198392 | 184 | – | 237 | 1003.5362 | 6015.1735 | 6015.1260 | 7.90 | 2 | 21 | 0.01 | 1Score **> 31** indicates **identity** Score **> 14** indicates **homology** | U | R.NVIINCTELVTYDLMKGALVNNKILADDVPCHLLSALVAGFCTTLLASPVDVVK.T  + 3 Deamidated (NQ); HNE (C); Oxidation (M) |
| 198408 | 184 | – | 237 | 1012.5384 | 6069.1867 | 6069.1954 | -1.43 | 2 | 18 | 0.023 | 1Score **> 32** indicates **identity** Score **> 14** indicates **homology** | U | R.NVIINCTELVTYDLMKGALVNNKILADDVPCHLLSALVAGFCTTLLASPVDVVK.T  + HNE (H); Oxidation (M) |
| 198409 | 184 | – | 237 | 1012.5442 | 6069.2215 | 6069.1954 | 4.29 | 2 | 18 | 0.02 | 1Score **> 31** indicates **identity** Score **> 14** indicates **homology** | U | R.NVIINCTELVTYDLMKGALVNNKILADDVPCHLLSALVAGFCTTLLASPVDVVK.T  + HNE (H); Oxidation (M) |
| 185344 | 207 | – | 237 | 1098.9243 | 3293.7510 | 3293.7404 | 3.23 | 0 | 86 | 8.4e-09 | 1Score **> 36** indicates **identity** Score **> 18** indicates **homology** | U | K.ILADDVPCHLLSALVAGFCTTLLASPVDVVK.T |
| 185345 | 207 | – | 237 | 824.4472 | 3293.7596 | 3293.7404 | 5.85 | 0 | 57 | 4.6e-06 | 1Score **> 36** indicates **identity** Score **> 16** indicates **homology** | U | K.ILADDVPCHLLSALVAGFCTTLLASPVDVVK.T |
| 7360 | 262 | – | 269 | 448.7287 | 895.4428 | 895.4440 | -1.26 | 0 | 40 | 0.0021 | 1Score **> 27** indicates **identity** Score **> 26** indicates **homology** | U | K.EGPTAFFK.G |
| 7361 | 262 | – | 269 | 448.7292 | 895.4438 | 895.4440 | -0.17 | 0 | 42 | 0.0016 | 1Score **> 27** indicates **identity** Score **> 27** indicates **homology** | U | K.EGPTAFFK.G |
| 97850 | 262 | – | 277 | 591.9824 | 1772.9253 | 1772.9250 | 0.18 | 1 | 28 | 0.0025 | 1Score **> 36** indicates **identity** Score **> 14** indicates **homology** | U | K.EGPTAFFKGFVASFLR.L |
| 7372 | 270 | – | 277 | 448.7528 | 895.4910 | 895.4916 | -0.66 | 0 | 15 | 0.036 | 1Score **> 27** indicates **identity** Score **> 13** indicates **homology** | U | K.GFVASFLR.L |
| 7374 | 270 | – | 277 | 448.7533 | 895.4920 | 895.4916 | 0.47 | 0 | 16 | 0.035 | 1Score **> 27** indicates **identity** Score **> 13** indicates **homology** | U | K.GFVASFLR.L |
| 7375 | 270 | – | 277 | 448.7534 | 895.4922 | 895.4916 | 0.67 | 0 | 36 | 0.00052 | 1Score **> 27** indicates **identity** Score **> 16** indicates **homology** | U | K.GFVASFLR.L |
| 7377 | 270 | – | 277 | 448.7540 | 895.4934 | 895.4916 | 1.98 | 0 | 37 | 0.0013 | 1Score **> 27** indicates **identity** Score **> 21** indicates **homology** | U | K.GFVASFLR.L |
| 7378 | 270 | – | 277 | 448.7542 | 895.4939 | 895.4916 | 2.59 | 0 | 35 | 0.0018 | 1Score **> 27** indicates **identity** Score **> 20** indicates **homology** | U | K.GFVASFLR.L |
| 7379 | 270 | – | 277 | 448.7545 | 895.4945 | 895.4916 | 3.20 | 0 | 20 | 0.013 | 1Score **> 27** indicates **identity** Score **> 14** indicates **homology** | U | K.GFVASFLR.L |

---

```
ID   UCP1_MOUSE              Reviewed;         307 AA.
AC   P12242;
DT   01-OCT-1989, integrated into UniProtKB/Swiss-Prot.
DT   23-JAN-2007, sequence version 2.
DT   28-JUN-2023, entry version 182.
DE   RecName: Full=Mitochondrial brown fat uncoupling protein 1 {ECO:0000305};
DE            Short=UCP 1 {ECO:0000305};
DE   AltName: Full=Solute carrier family 25 member 7 {ECO:0000250|UniProtKB:P25874};
DE   AltName: Full=Thermogenin {ECO:0000250|UniProtKB:P04575};
GN   Name=Ucp1 {ECO:0000312|MGI:MGI:98894};
GN   Synonyms=Slc25a7 {ECO:0000250|UniProtKB:P25874}, Ucp
GN   {ECO:0000303|PubMed:3410843};
OS   Mus musculus (Mouse).
OC   Eukaryota; Metazoa; Chordata; Craniata; Vertebrata; Euteleostomi; Mammalia;
OC   Eutheria; Euarchontoglires; Glires; Rodentia; Myomorpha; Muroidea; Muridae;
OC   Murinae; Mus; Mus.
OX   NCBI_TaxID=10090;
RN   [1]
RP   NUCLEOTIDE SEQUENCE [GENOMIC DNA].
RX   PubMed=3410843; DOI=10.1016/s0021-9258(18)37751-2;
RA   Kozak L.P., Britton J.H., Kozak U.C., Wells J.M.;
RT   "The mitochondrial uncoupling protein gene. Correlation of exon structure
RT   to transmembrane domains.";
RL   J. Biol. Chem. 263:12274-12277(1988).
RN   [2]
RP   NUCLEOTIDE SEQUENCE, AND TISSUE SPECIFICITY.
RC   STRAIN=129/SvJ;
RX   PubMed=8264627; DOI=10.1128/mcb.14.1.59-67.1994;
RA   Kozak U.C., Kopecky J., Teisinger J., Enerbaeck S., Boyer B., Kozak L.P.;
RT   "An upstream enhancer regulating brown-fat-specific expression of the
RT   mitochondrial uncoupling protein gene.";
RL   Mol. Cell. Biol. 14:59-67(1994).
RN   [3]
RP   NUCLEOTIDE SEQUENCE [LARGE SCALE MRNA].
RC   STRAIN=FVB/N; TISSUE=Salivary gland;
RX   PubMed=15489334; DOI=10.1101/gr.2596504;
RG   The MGC Project Team;
RT   "The status, quality, and expansion of the NIH full-length cDNA project:
RT   the Mammalian Gene Collection (MGC).";
RL   Genome Res. 14:2121-2127(2004).
RN   [4]
RP   FUNCTION, AND DISRUPTION PHENOTYPE.
RX   PubMed=9139827; DOI=10.1038/387090a0;
RA   Enerbaeck S., Jacobsson A., Simpson E.M., Guerra C., Yamashita H.,
RA   Harper M.E., Kozak L.P.;
RT   "Mice lacking mitochondrial uncoupling protein are cold-sensitive but not
RT   obese.";
RL   Nature 387:90-94(1997).
RN   [5]
RP   FUNCTION, INDUCTION BY HIGH-FAT DIET, AND DISRUPTION PHENOTYPE.
RX   PubMed=19187776; DOI=10.1016/j.cmet.2008.12.014;
RA   Feldmann H.M., Golozoubova V., Cannon B., Nedergaard J.;
RT   "UCP1 ablation induces obesity and abolishes diet-induced thermogenesis in
RT   mice exempt from thermal stress by living at thermoneutrality.";
RL   Cell Metab. 9:203-209(2009).
RN   [6]
RP   FUNCTION, AND DISRUPTION PHENOTYPE.
RX   PubMed=20416274; DOI=10.1016/j.bbabio.2010.04.008;
RA   Dlaskova A., Clarke K.J., Porter R.K.;
RT   "The role of UCP 1 in production of reactive oxygen species by mitochondria
RT   isolated from brown adipose tissue.";
RL   Biochim. Biophys. Acta 1797:1470-1476(2010).
RN   [7]
RP   IDENTIFICATION BY MASS SPECTROMETRY [LARGE SCALE ANALYSIS].
RC   TISSUE=Brown adipose tissue, Heart, Kidney, Liver, Lung, and Spleen;
RX   PubMed=21183079; DOI=10.1016/j.cell.2010.12.001;
RA   Huttlin E.L., Jedrychowski M.P., Elias J.E., Goswami T., Rad R.,
RA   Beausoleil S.A., Villen J., Haas W., Sowa M.E., Gygi S.P.;
RT   "A tissue-specific atlas of mouse protein phosphorylation and expression.";
RL   Cell 143:1174-1189(2010).
RN   [8]
RP   FUNCTION, DISRUPTION PHENOTYPE, AND INDUCTION BY COLD.
RX   PubMed=20466728; DOI=10.1074/jbc.m110.122861;
RA   Oelkrug R., Kutschke M., Meyer C.W., Heldmaier G., Jastroch M.;
RT   "Uncoupling protein 1 decreases superoxide production in brown adipose
RT   tissue mitochondria.";
RL   J. Biol. Chem. 285:21961-21968(2010).
RN   [9]
RP   FUNCTION, TRANSPORTER ACTIVITY, ACTIVITY REGULATION, AND SUBCELLULAR
RP   LOCATION.
RX   PubMed=23063128; DOI=10.1016/j.cell.2012.09.010;
RA   Fedorenko A., Lishko P.V., Kirichok Y.;
RT   "Mechanism of fatty-acid-dependent UCP1 uncoupling in brown fat
RT   mitochondria.";
RL   Cell 151:400-413(2012).
RN   [10]
RP   INDUCTION BY COLD.
RX   PubMed=25578880; DOI=10.1016/j.molcel.2014.12.005;
RA   Dempersmier J., Sambeat A., Gulyaeva O., Paul S.M., Hudak C.S.,
RA   Raposo H.F., Kwan H.Y., Kang C., Wong R.H., Sul H.S.;
RT   "Cold-inducible Zfp516 activates UCP1 transcription to promote browning of
RT   white fat and development of brown fat.";
RL   Mol. Cell 57:235-246(2015).
RN   [11]
RP   FUNCTION, ACTIVITY REGULATION, OXIDATION AT CYS-254, AND MUTAGENESIS OF
RP   CYS-25; CYS-189; CYS-214; CYS-225; CYS-254; CYS-288 AND CYS-305.
RX   PubMed=27027295; DOI=10.1038/nature17399;
RA   Chouchani E.T., Kazak L., Jedrychowski M.P., Lu G.Z., Erickson B.K.,
RA   Szpyt J., Pierce K.A., Laznik-Bogoslavski D., Vetrivelan R., Clish C.B.,
RA   Robinson A.J., Gygi S.P., Spiegelman B.M.;
RT   "Mitochondrial ROS regulate thermogenic energy expenditure and
RT   sulfenylation of UCP1.";
RL   Nature 532:112-116(2016).
RN   [12]
RP   ACTIVITY REGULATION.
RX   PubMed=32358195; DOI=10.1073/pnas.2001387117;
RA   Jedrychowski M.P., Lu G.Z., Szpyt J., Mariotti M., Garrity R., Paulo J.A.,
RA   Schweppe D.K., Laznik-Bogoslavski D., Kazak L., Murphy M.P.,
RA   Gladyshev V.N., Gygi S.P., Chouchani E.T., Spiegelman B.M.;
RT   "Facultative protein selenation regulates redox sensitivity, adipose tissue
RT   thermogenesis, and obesity.";
RL   Proc. Natl. Acad. Sci. U.S.A. 117:10789-10796(2020).
CC   -!- FUNCTION: Mitochondrial transporter that functions as a long-chain
CC       fatty acid/LCFA and proton symporter, simultaneously transporting one
CC       LCFA and one proton through the inner mitochondrial membrane. However,
CC       LCFAs remaining associated with the transporter via their hydrophobic
CC       tails, it results in an apparent transport of protons activated by
CC       LCFAs. Thereby, dissipates the mitochondrial proton gradient and
CC       converts the energy of substrate oxydation into heat instead of ATP
CC       (PubMed:23063128). Responsible for thermogenic respiration, a
CC       specialized capacity of brown adipose tissue and beige fat that
CC       participates in non-shivering adaptive thermogenesis to temperature and
CC       diet variations and more generally to the regulation of energy balance
CC       (PubMed:9139827, PubMed:19187776, PubMed:23063128, PubMed:27027295).
CC       Regulates the production of reactive oxygen species/ROS by mitochondria
CC       (PubMed:20416274, PubMed:20466728). {ECO:0000269|PubMed:19187776,
CC       ECO:0000269|PubMed:20416274, ECO:0000269|PubMed:20466728,
CC       ECO:0000269|PubMed:23063128, ECO:0000269|PubMed:27027295,
CC       ECO:0000269|PubMed:9139827}.
CC   -!- CATALYTIC ACTIVITY:
CC       Reaction=H(+)(in) = H(+)(out); Xref=Rhea:RHEA:34979, ChEBI:CHEBI:15378;
CC         Evidence={ECO:0000269|PubMed:23063128};
CC   -!- ACTIVITY REGULATION: Has no constitutive proton transporter activity
CC       and has to be activated by long-chain fatty acids/LCFAs. Inhibited by
CC       purine nucleotides. Both purine nucleotides and LCFAs bind the
CC       cytosolic side of the transporter and directly compete to activate or
CC       inhibit it (PubMed:23063128). Activated by noradrenaline and reactive
CC       oxygen species (PubMed:27027295). Despite lacking canonical
CC       translational encoding for selenocysteine, a small pool of the protein
CC       has been observed to selectively incorporate selenocysteine at 'Cys-
CC       254' (PubMed:32358195). Selenocysteine-modified protein is highly
CC       sensitive to redox modification and may constitute a pool of protein
CC       highly sensitive to activation by elevated levels of reactive oxygen
CC       species (ROS) (PubMed:32358195). {ECO:0000269|PubMed:23063128,
CC       ECO:0000269|PubMed:27027295, ECO:0000269|PubMed:32358195}.
CC   -!- SUBUNIT: Most probably functions as a monomer. Binds one purine
CC       nucleotide per monomer. However, has also been suggested to function as
CC       a homodimer or a homotetramer. Tightly associates with cardiolipin in
CC       the mitochondrion inner membrane; may stabilize and regulate its
CC       activity. {ECO:0000250|UniProtKB:P25874, ECO:0000250|UniProtKB:W5PSH7}.
CC   -!- SUBCELLULAR LOCATION: Mitochondrion inner membrane
CC       {ECO:0000269|PubMed:23063128}; Multi-pass membrane protein
CC       {ECO:0000250|UniProtKB:P04633}.
CC   -!- TISSUE SPECIFICITY: Expressed in brown adipose tissue.
CC       {ECO:0000269|PubMed:8264627}.
CC   -!- INDUCTION: Up-regulated in response to cold in brown adipose tissue
CC       where it may regulate non-shivering thermogenesis (at protein level)
CC       (PubMed:20466728, PubMed:25578880). Up-regulated by high-fat diet (at
CC       protein level) (PubMed:19187776). {ECO:0000269|PubMed:19187776,
CC       ECO:0000269|PubMed:20466728, ECO:0000269|PubMed:25578880}.
CC   -!- PTM: Sulfenylation at Cys-254 is increased upon cold exposure. It
CC       increases the sensitivity of UCP1 thermogenic function to the
CC       activation by noradrenaline probably through structural effects.
CC       {ECO:0000269|PubMed:27027295}.
CC   -!- PTM: May undergo ubiquitin-mediated proteasomal degradation.
CC       {ECO:0000250|UniProtKB:P04633}.
CC   -!- DISRUPTION PHENOTYPE: Mice lacking Ucp1 display an absence of adaptive
CC       thermogenesis in response to cold. Compared to wild-type mice, they are
CC       sensitive to cold and consume less oxygen upon treatment with beta-3-
CC       adrenergic-receptor agonists that normally activate thermogenesis
CC       (PubMed:9139827, PubMed:19187776). They also display impaired adaptive
CC       thermogenesis in response to diet variation (PubMed:19187776). If they
CC       display lipid accumulation in adipocytes of brown adipose tissues, no
CC       overt obesity is observed when mice are housed under classical
CC       conditions, i.e. 18 to 20 degrees Celsius (PubMed:9139827). However,
CC       when mice are housed at thermoneutrality, i.e. at 30 degrees Celsius,
CC       obesity is clearly observed and exacerbated by high fat diet
CC       (PubMed:19187776). The brown adipose tissue of mice lacking Ucp1
CC       produce higher levels of reactive oxygen species (PubMed:20416274,
CC       PubMed:20466728). {ECO:0000269|PubMed:19187776,
CC       ECO:0000269|PubMed:20416274, ECO:0000269|PubMed:20466728,
CC       ECO:0000269|PubMed:9139827}.
CC   -!- SIMILARITY: Belongs to the mitochondrial carrier (TC 2.A.29) family.
CC       {ECO:0000305}.
CC   ---------------------------------------------------------------------------
CC   Copyrighted by the UniProt Consortium, see https://www.uniprot.org/terms
CC   Distributed under the Creative Commons Attribution (CC BY 4.0) License
CC   ---------------------------------------------------------------------------
DR   EMBL; M21247; AAA40521.1; -; Genomic_DNA.
DR   EMBL; M21222; AAA40521.1; JOINED; Genomic_DNA.
DR   EMBL; M21244; AAA40521.1; JOINED; Genomic_DNA.
DR   EMBL; M21245; AAA40521.1; JOINED; Genomic_DNA.
DR   EMBL; M21246; AAA40521.1; JOINED; Genomic_DNA.
DR   EMBL; U63419; AAB05870.1; -; mRNA.
DR   EMBL; U63418; AAB07367.1; -; Genomic_DNA.
DR   EMBL; BC012701; AAH12701.1; -; mRNA.
DR   CCDS; CCDS22449.1; -.
DR   PIR; A31106; A31106.
DR   RefSeq; NP_033489.1; NM_009463.3.
DR   AlphaFoldDB; P12242; -.
DR   SMR; P12242; -.
DR   STRING; 10090.ENSMUSP00000034146; -.
DR   iPTMnet; P12242; -.
DR   PhosphoSitePlus; P12242; -.
DR   MaxQB; P12242; -.
DR   PaxDb; P12242; -.
DR   ProteomicsDB; 297718; -.
DR   Antibodypedia; 16250; 477 antibodies from 38 providers.
DR   DNASU; 22227; -.
DR   Ensembl; ENSMUST00000034146; ENSMUSP00000034146; ENSMUSG00000031710.
DR   GeneID; 22227; -.
DR   KEGG; mmu:22227; -.
DR   UCSC; uc009mjx.2; mouse.
DR   AGR; MGI:98894; -.
DR   CTD; 7350; -.
DR   MGI; MGI:98894; Ucp1.
DR   VEuPathDB; HostDB:ENSMUSG00000031710; -.
DR   eggNOG; KOG0753; Eukaryota.
DR   GeneTree; ENSGT00940000160382; -.
DR   HOGENOM; CLU_015166_14_2_1; -.
DR   InParanoid; P12242; -.
DR   OMA; NCAMKMF; -.
DR   OrthoDB; 1832865at2759; -.
DR   PhylomeDB; P12242; -.
DR   TreeFam; TF323211; -.
DR   Reactome; R-MMU-167826; The fatty acid cycling model.
DR   Reactome; R-MMU-167827; The proton buffering model.
DR   BioGRID-ORCS; 22227; 2 hits in 76 CRISPR screens.
DR   ChiTaRS; Ucp1; mouse.
DR   PRO; PR:P12242; -.
DR   Proteomes; UP000000589; Chromosome 8.
DR   RNAct; P12242; protein.
DR   Bgee; ENSMUSG00000031710; Expressed in intercostal muscle and 56 other tissues.
DR   Genevisible; P12242; MM.
DR   GO; GO:0005740; C:mitochondrial envelope; IDA:MGI.
DR   GO; GO:0005743; C:mitochondrial inner membrane; IDA:UniProtKB.
DR   GO; GO:0005739; C:mitochondrion; IDA:UniProtKB.
DR   GO; GO:1901612; F:cardiolipin binding; ISS:UniProtKB.
DR   GO; GO:0019003; F:GDP binding; ISO:MGI.
DR   GO; GO:0005525; F:GTP binding; ISO:MGI.
DR   GO; GO:0036041; F:long-chain fatty acid binding; IDA:UniProtKB.
DR   GO; GO:0017077; F:oxidative phosphorylation uncoupler activity; IDA:UniProtKB.
DR   GO; GO:0032555; F:purine ribonucleotide binding; IDA:UniProtKB.
DR   GO; GO:0022857; F:transmembrane transporter activity; ISO:MGI.
DR   GO; GO:1990845; P:adaptive thermogenesis; IMP:UniProtKB.
DR   GO; GO:0050873; P:brown fat cell differentiation; IDA:MGI.
DR   GO; GO:0070417; P:cellular response to cold; IDA:MGI.
DR   GO; GO:1903495; P:cellular response to dehydroepiandrosterone; IEA:Ensembl.
DR   GO; GO:0071398; P:cellular response to fatty acid; IDA:UniProtKB.
DR   GO; GO:0032870; P:cellular response to hormone stimulus; IMP:UniProtKB.
DR   GO; GO:0034614; P:cellular response to reactive oxygen species; IMP:UniProtKB.
DR   GO; GO:0002024; P:diet induced thermogenesis; IMP:UniProtKB.
DR   GO; GO:1990542; P:mitochondrial transmembrane transport; IDA:UniProtKB.
DR   GO; GO:0006839; P:mitochondrial transport; ISO:MGI.
DR   GO; GO:0120162; P:positive regulation of cold-induced thermogenesis; IMP:YuBioLab.
DR   GO; GO:1902600; P:proton transmembrane transport; IDA:UniProtKB.
DR   GO; GO:1903426; P:regulation of reactive oxygen species biosynthetic process; IMP:UniProtKB.
DR   GO; GO:0006357; P:regulation of transcription by RNA polymerase II; IDA:MGI.
DR   GO; GO:0009409; P:response to cold; IBA:GO_Central.
DR   GO; GO:0031667; P:response to nutrient levels; IMP:UniProtKB.
DR   GO; GO:0009266; P:response to temperature stimulus; IMP:UniProtKB.
DR   Gene3D; 1.50.40.10; Mitochondrial carrier domain; 1.
DR   InterPro; IPR002030; Mit_uncoupling_UCP-like.
DR   InterPro; IPR018108; Mitochondrial_sb/sol_carrier.
DR   InterPro; IPR023395; Mt_carrier_dom_sf.
DR   PANTHER; PTHR45618:SF19; MITOCHONDRIAL BROWN FAT UNCOUPLING PROTEIN 1; 1.
DR   PANTHER; PTHR45618; MITOCHONDRIAL DICARBOXYLATE CARRIER-RELATED; 1.
DR   Pfam; PF00153; Mito_carr; 3.
DR   PRINTS; PR00784; MTUNCOUPLING.
DR   SUPFAM; SSF103506; Mitochondrial carrier; 1.
DR   PROSITE; PS50920; SOLCAR; 3.
PE   1: Evidence at protein level;
KW   Ion channel; Ion transport; Membrane; Mitochondrion;
KW   Mitochondrion inner membrane; Oxidation; Reference proteome; Repeat;
KW   Selenium; Selenocysteine; Transmembrane; Transmembrane helix; Transport.
FT   CHAIN           1..307
FT                   /note="Mitochondrial brown fat uncoupling protein 1"
FT                   /id="PRO_0000090659"
FT   TOPO_DOM        1..10
FT                   /note="Mitochondrial intermembrane"
FT                   /evidence="ECO:0000250|UniProtKB:P04633"
FT   TRANSMEM        11..32
FT                   /note="Helical; Name=1"
FT                   /evidence="ECO:0000255"
FT   TOPO_DOM        33..73
FT                   /note="Mitochondrial matrix"
FT                   /evidence="ECO:0000250|UniProtKB:P04633"
FT   TRANSMEM        74..96
FT                   /note="Helical; Name=2"
FT                   /evidence="ECO:0000255"
FT   TOPO_DOM        97..116
FT                   /note="Mitochondrial intermembrane"
FT                   /evidence="ECO:0000250|UniProtKB:P04633"
FT   TRANSMEM        117..133
FT                   /note="Helical; Name=3"
FT                   /evidence="ECO:0000255"
FT   TOPO_DOM        134..178
FT                   /note="Mitochondrial matrix"
FT                   /evidence="ECO:0000250|UniProtKB:P04633"
FT   TRANSMEM        179..195
FT                   /note="Helical; Name=4"
FT                   /evidence="ECO:0000255"
FT   TOPO_DOM        196..212
FT                   /note="Mitochondrial intermembrane"
FT                   /evidence="ECO:0000250|UniProtKB:P04633"
FT   TRANSMEM        213..232
FT                   /note="Helical; Name=5"
FT                   /evidence="ECO:0000255"
FT   TOPO_DOM        233..266
FT                   /note="Mitochondrial matrix"
FT                   /evidence="ECO:0000250|UniProtKB:P04633"
FT   TRANSMEM        267..289
FT                   /note="Helical; Name=6"
FT                   /evidence="ECO:0000255"
FT   TOPO_DOM        290..307
FT                   /note="Mitochondrial intermembrane"
FT                   /evidence="ECO:0000250|UniProtKB:P04633"
FT   REPEAT          11..102
FT                   /note="Solcar 1"
FT   REPEAT          111..201
FT                   /note="Solcar 2"
FT   REPEAT          210..295
FT                   /note="Solcar 3"
FT   BINDING         56
FT                   /ligand="fatty acid 16:0"
FT                   /ligand_id="ChEBI:CHEBI:78123"
FT                   /evidence="ECO:0000250|UniProtKB:P25874"
FT   BINDING         269
FT                   /ligand="fatty acid 16:0"
FT                   /ligand_id="ChEBI:CHEBI:78123"
FT                   /evidence="ECO:0000250|UniProtKB:P25874"
FT   MOD_RES         254
FT                   /note="Cysteine sulfenic acid (-SOH)"
FT                   /evidence="ECO:0000269|PubMed:27027295"
FT   MUTAGEN         25
FT                   /note="C->A: No effect on UCP1 activity in thermogenic
FT                   respiration."
FT                   /evidence="ECO:0000269|PubMed:27027295"
FT   MUTAGEN         189
FT                   /note="C->A: No effect on UCP1 activity in thermogenic
FT                   respiration."
FT                   /evidence="ECO:0000269|PubMed:27027295"
FT   MUTAGEN         214
FT                   /note="C->A: No effect on UCP1 activity in thermogenic
FT                   respiration."
FT                   /evidence="ECO:0000269|PubMed:27027295"
FT   MUTAGEN         225
FT                   /note="C->A: Decreased UCP1 activity in thermogenic
FT                   respiration."
FT                   /evidence="ECO:0000269|PubMed:27027295"
FT   MUTAGEN         254
FT                   /note="C->A: Decreased sensitivity to activation by
FT                   noradrenaline in thermogenic respiration."
FT                   /evidence="ECO:0000269|PubMed:27027295"
FT   MUTAGEN         288
FT                   /note="C->A: No effect on UCP1 activity in thermogenic
FT                   respiration."
FT                   /evidence="ECO:0000269|PubMed:27027295"
FT   MUTAGEN         305
FT                   /note="C->A: No effect on UCP1 activity in thermogenic
FT                   respiration."
FT                   /evidence="ECO:0000269|PubMed:27027295"
SQ   SEQUENCE   307 AA;  33248 MW;  33D107EDF04BD1AF CRC64;
     MVNPTTSEVQ PTMGVKIFSA GVSACLADII TFPLDTAKVR LQIQGEGQAS STIRYKGVLG
     TITTLAKTEG LPKLYSGLPA GIQRQISFAS LRIGLYDSVQ EYFSSGRETP ASLGNKISAG
     LMTGGVAVFI GQPTEVVKVR MQAQSHLHGI KPRYTGTYNA YRVIATTESL STLWKGTTPN
     LMRNVIINCT ELVTYDLMKG ALVNNKILAD DVPCHLLSAL VAGFCTTLLA SPVDVVKTRF
     INSLPGQYPS VPSCAMSMYT KEGPTAFFKG FVASFLRLGS WNVIMFVCFE QLKKELMKSR
     QTVDCTT
//
```

**Mascot:** http://www.matrixscience.com/


Oxidation (M) (+15.9949)
