# Supplementary material for: A common East-Asian ALDH2 mutation causes metabolic disorders and the therapeutic effect of ALDH2 activators
Source: Nat Commun. 2023 Sep 25;14:5971. doi: 10.1038/s41467-023-41570-6 (PMC10520061; doi:10.1038/s41467-023-41570-6)
Supplement: Supplementary file 4 — Supplementary Data 1 [file 41467_2023_41570_MOESM4_ESM.zip › Table S5c/P15105/P15105_KIO-1-H304.html]

Mascot Search Results: P15105
 

# MASCOT Search Results

## Protein View: P15105

### Glutamine synthetase OS=Mus musculus OX=10090 GN=Glul PE=1 SV=6

|  |  |
| --- | --- |
| Database: | Mouse\_UniProt\_proteomes |
| Score: | 212 |
| Monoisotopic mass (M_r_): | 42834 |
| Calculated pI: | 6.64 |

Sequence similarity is available as an NCBI BLAST search of P15105 against nr.

### Search parameters

|  |  |
| --- | --- |
| MS data file: | `D:\LCMSMS\2023 Users' data\230529-1\230529-1-KIO-1.raw` |
| Enzyme: | Trypsin/P: cuts C-term side of KR. |
| Fixed modifications: | Carbamidomethyl (C) |
| Variable modifications: | Deamidated (NQ), HNE (C), HNE (H), HNE (K), Oxidation (M) |

### Protein sequence coverage: 19%

Matched peptides shown in ***bold red***.

|  |  |  |  |  |  |
| --- | --- | --- | --- | --- | --- |
| `1` | `MATSASSHLN` | `KGIKQMYMSL` | `PQGEKVQAMY` | `IWVDGTGEGL` | `RCKTRTLDCE` |
| `51` | `PKCVEELPEW` | `NFDGSSTFQS` | `EGSNSDMYLH` | `PVAMFRDPFR` | `KDPNKLVLCE` |
| `101` | `VFKYNRKPAE` | `TNLRHICKRI` | `MDMVSNQHPW` | `FGMEQEYTLM` | `GTDGHPFGWP` |
| `151` | `SNGFPGPQGP` | `YYCGVGADKA` | `YGRDIVEAHY` | `RACLYAGVKI` | `TGTNAEVMPA` |
| `201` | `QWEFQIGPCE` | `GIRMGDHLWI` | `ARFILHRVCE` | `DFGVIATFDP` | `KPIPGNWNGA` |
| `251` | `GCHTNFSTKA` | `MREENGLKCI` | `EEAIDKLSKR` | `HQYHIRAYDP` | `KGGLDNARRL` |
| `301` | `TGFHETSNIN` | `DFSAGVANRG` | `ASIRIPRTVG` | `QEKKGYFEDR` | `RPSANCDPYA` |
| `351` | `VTEAIVRTCL` | `LNETGDEPFQ` | `YKN` |  |  |

Unformatted sequence string: 373 residues (for pasting into other applications).

|  |  |  |  |
| --- | --- | --- | --- |
| Sort by | residue number | increasing mass | decreasing mass |
| Show | matched peptides only | predicted peptides also |  |

| Query | Start | – | End | Observed | Mr(expt) | Mr(calc) | ppm | M | Score | Expect | Rank | U | Peptide |
| --- | --- | --- | --- | --- | --- | --- | --- | --- | --- | --- | --- | --- | --- |
| 56119 | 170 | – | 181 | 363.1863 | 1448.7162 | 1448.7160 | 0.13 | 1 | 30 | 0.0018 | 1Score **> 34** indicates **identity** Score **> 15** indicates **homology** | U | K.AYGRDIVEAHYR.A |
| 13256 | 174 | – | 181 | 501.7541 | 1001.4936 | 1001.4930 | 0.59 | 0 | 28 | 0.0054 | 1Score **> 31** indicates **identity** Score **> 17** indicates **homology** | U | R.DIVEAHYR.A |
| 6831 | 182 | – | 189 | 441.2314 | 880.4482 | 880.4477 | 0.67 | 0 | 21 | 0.01 | 1Score **> 27** indicates **identity** Score **> 14** indicates **homology** | U | R.ACLYAGVK.I |
| 146915 | 299 | – | 319 | 577.2867 | 2305.1178 | 2305.1199 | -0.92 | 1 | 18 | 0.02 | 1Score **> 36** indicates **identity** Score **> 14** indicates **homology** | U | R.RLTGFHETSNINDFSAGVANR.G |
| 146916 | 299 | – | 319 | 769.3802 | 2305.1188 | 2305.1199 | -0.49 | 1 | 16 | 0.03 | 1Score **> 36** indicates **identity** Score **> 14** indicates **homology** | U | R.RLTGFHETSNINDFSAGVANR.G |
| 146921 | 299 | – | 319 | 769.3828 | 2305.1265 | 2305.1199 | 2.86 | 1 | 37 | 0.00032 | 1Score **> 36** indicates **identity** Score **> 15** indicates **homology** | U | R.RLTGFHETSNINDFSAGVANR.G |
| 146980 | 299 | – | 319 | 577.5389 | 2306.1266 | 2306.1040 | 9.81 | 1 | 20 | 0.014 | 1Score **> 36** indicates **identity** Score **> 14** indicates **homology** | U | R.RLTGFHETSNINDFSAGVANR.G  + Deamidated (NQ) |
| 146919 | 300 | – | 319 | 577.2882 | 2305.1239 | 2305.1339 | -4.34 | 0 | 48 | 0.0001 | 1Score **> 36** indicates **identity** Score **> 21** indicates **homology** | U | R.LTGFHETSNINDFSAGVANR.G  + HNE (H) |
| 112274 | 341 | – | 357 | 640.3200 | 1917.9381 | 1917.9367 | 0.75 | 1 | 14 | 0.047 | 1Score **> 35** indicates **identity** Score **> 13** indicates **homology** | U | R.RPSANCDPYAVTEAIVR.T |
| 112275 | 341 | – | 357 | 640.3207 | 1917.9402 | 1917.9367 | 1.82 | 1 | 22 | 0.0087 | 1Score **> 35** indicates **identity** Score **> 14** indicates **homology** | U | R.RPSANCDPYAVTEAIVR.T |
| 113340 | 358 | – | 373 | 964.9420 | 1927.8694 | 1927.8622 | 3.72 | 1 | 68 | 4.2e-07 | 1Score **> 31** indicates **identity** Score **> 17** indicates **homology** | U | R.TCLLNETGDEPFQYKN.- |
| 113341 | 358 | – | 373 | 964.9421 | 1927.8697 | 1927.8622 | 3.90 | 1 | 58 | 3.6e-06 | 1Score **> 31** indicates **identity** Score **> 16** indicates **homology** | U | R.TCLLNETGDEPFQYKN.- |

---

```
ID   GLNA_MOUSE              Reviewed;         373 AA.
AC   P15105; Q3TRK7; Q64432; Q91VC6;
DT   01-APR-1990, integrated into UniProtKB/Swiss-Prot.
DT   17-APR-2007, sequence version 6.
DT   28-JUN-2023, entry version 195.
DE   RecName: Full=Glutamine synthetase {ECO:0000303|PubMed:30158707};
DE            Short=GS {ECO:0000303|PubMed:30158707};
DE            EC=6.3.1.2 {ECO:0000250|UniProtKB:P15104};
DE   AltName: Full=Glutamate--ammonia ligase {ECO:0000305};
DE   AltName: Full=Palmitoyltransferase GLUL {ECO:0000305};
DE            EC=2.3.1.225 {ECO:0000250|UniProtKB:P15104};
GN   Name=Glul {ECO:0000303|PubMed:30158707, ECO:0000312|MGI:MGI:95739};
GN   Synonyms=Glns;
OS   Mus musculus (Mouse).
OC   Eukaryota; Metazoa; Chordata; Craniata; Vertebrata; Euteleostomi; Mammalia;
OC   Eutheria; Euarchontoglires; Glires; Rodentia; Myomorpha; Muroidea; Muridae;
OC   Murinae; Mus; Mus.
OX   NCBI_TaxID=10090;
RN   [1]
RP   NUCLEOTIDE SEQUENCE [MRNA].
RX   PubMed=2475638; DOI=10.1016/0022-2836(89)90086-7;
RA   Kuo C.F., Darnell J.E. Jr.;
RT   "Mouse glutamine synthetase is encoded by a single gene that can be
RT   expressed in a localized fashion.";
RL   J. Mol. Biol. 208:45-56(1989).
RN   [2]
RP   NUCLEOTIDE SEQUENCE [MRNA].
RC   TISSUE=Thymus;
RA   Lindemann A.E., Tempest P.R.;
RT   "Sequence of a mouse glutamine synthetase cDNA.";
RL   Submitted (APR-1994) to the EMBL/GenBank/DDBJ databases.
RN   [3]
RP   NUCLEOTIDE SEQUENCE [MRNA].
RC   STRAIN=FVB/NJ; TISSUE=Liver;
RA   Labruyere W.T., van Hemert F.J., Lamers W.H.;
RT   "Glutamine synthetase mRNA in rodents.";
RL   Submitted (JUL-2001) to the EMBL/GenBank/DDBJ databases.
RN   [4]
RP   NUCLEOTIDE SEQUENCE [LARGE SCALE MRNA].
RC   STRAIN=C57BL/6J; TISSUE=Amnion, Hippocampus, and Vagina;
RX   PubMed=16141072; DOI=10.1126/science.1112014;
RA   Carninci P., Kasukawa T., Katayama S., Gough J., Frith M.C., Maeda N.,
RA   Oyama R., Ravasi T., Lenhard B., Wells C., Kodzius R., Shimokawa K.,
RA   Bajic V.B., Brenner S.E., Batalov S., Forrest A.R., Zavolan M., Davis M.J.,
RA   Wilming L.G., Aidinis V., Allen J.E., Ambesi-Impiombato A., Apweiler R.,
RA   Aturaliya R.N., Bailey T.L., Bansal M., Baxter L., Beisel K.W., Bersano T.,
RA   Bono H., Chalk A.M., Chiu K.P., Choudhary V., Christoffels A.,
RA   Clutterbuck D.R., Crowe M.L., Dalla E., Dalrymple B.P., de Bono B.,
RA   Della Gatta G., di Bernardo D., Down T., Engstrom P., Fagiolini M.,
RA   Faulkner G., Fletcher C.F., Fukushima T., Furuno M., Futaki S.,
RA   Gariboldi M., Georgii-Hemming P., Gingeras T.R., Gojobori T., Green R.E.,
RA   Gustincich S., Harbers M., Hayashi Y., Hensch T.K., Hirokawa N., Hill D.,
RA   Huminiecki L., Iacono M., Ikeo K., Iwama A., Ishikawa T., Jakt M.,
RA   Kanapin A., Katoh M., Kawasawa Y., Kelso J., Kitamura H., Kitano H.,
RA   Kollias G., Krishnan S.P., Kruger A., Kummerfeld S.K., Kurochkin I.V.,
RA   Lareau L.F., Lazarevic D., Lipovich L., Liu J., Liuni S., McWilliam S.,
RA   Madan Babu M., Madera M., Marchionni L., Matsuda H., Matsuzawa S., Miki H.,
RA   Mignone F., Miyake S., Morris K., Mottagui-Tabar S., Mulder N., Nakano N.,
RA   Nakauchi H., Ng P., Nilsson R., Nishiguchi S., Nishikawa S., Nori F.,
RA   Ohara O., Okazaki Y., Orlando V., Pang K.C., Pavan W.J., Pavesi G.,
RA   Pesole G., Petrovsky N., Piazza S., Reed J., Reid J.F., Ring B.Z.,
RA   Ringwald M., Rost B., Ruan Y., Salzberg S.L., Sandelin A., Schneider C.,
RA   Schoenbach C., Sekiguchi K., Semple C.A., Seno S., Sessa L., Sheng Y.,
RA   Shibata Y., Shimada H., Shimada K., Silva D., Sinclair B., Sperling S.,
RA   Stupka E., Sugiura K., Sultana R., Takenaka Y., Taki K., Tammoja K.,
RA   Tan S.L., Tang S., Taylor M.S., Tegner J., Teichmann S.A., Ueda H.R.,
RA   van Nimwegen E., Verardo R., Wei C.L., Yagi K., Yamanishi H.,
RA   Zabarovsky E., Zhu S., Zimmer A., Hide W., Bult C., Grimmond S.M.,
RA   Teasdale R.D., Liu E.T., Brusic V., Quackenbush J., Wahlestedt C.,
RA   Mattick J.S., Hume D.A., Kai C., Sasaki D., Tomaru Y., Fukuda S.,
RA   Kanamori-Katayama M., Suzuki M., Aoki J., Arakawa T., Iida J., Imamura K.,
RA   Itoh M., Kato T., Kawaji H., Kawagashira N., Kawashima T., Kojima M.,
RA   Kondo S., Konno H., Nakano K., Ninomiya N., Nishio T., Okada M., Plessy C.,
RA   Shibata K., Shiraki T., Suzuki S., Tagami M., Waki K., Watahiki A.,
RA   Okamura-Oho Y., Suzuki H., Kawai J., Hayashizaki Y.;
RT   "The transcriptional landscape of the mammalian genome.";
RL   Science 309:1559-1563(2005).
RN   [5]
RP   NUCLEOTIDE SEQUENCE [LARGE SCALE MRNA].
RC   STRAIN=FVB/N; TISSUE=Colon;
RX   PubMed=15489334; DOI=10.1101/gr.2596504;
RG   The MGC Project Team;
RT   "The status, quality, and expansion of the NIH full-length cDNA project:
RT   the Mammalian Gene Collection (MGC).";
RL   Genome Res. 14:2121-2127(2004).
RN   [6]
RP   PROTEIN SEQUENCE OF 2-11; 15-25; 96-103; 174-181; 292-298 AND 341-357,
RP   CLEAVAGE OF INITIATOR METHIONINE, ACETYLATION AT ALA-2, AND IDENTIFICATION
RP   BY MASS SPECTROMETRY.
RC   STRAIN=C57BL/6J; TISSUE=Liver;
RA   Bienvenut W.V.;
RL   Submitted (JUL-2005) to UniProtKB.
RN   [7]
RP   INTERACTION WITH PALMD.
RX   PubMed=16323283; DOI=10.1016/j.ejcb.2005.07.002;
RA   Hu B., Petrasch-Parwez E., Laue M.M., Kilimann M.W.;
RT   "Molecular characterization and immunohistochemical localization of
RT   palmdelphin, a cytosolic isoform of the paralemmin protein family
RT   implicated in membrane dynamics.";
RL   Eur. J. Cell Biol. 84:853-866(2005).
RN   [8]
RP   PROTEIN SEQUENCE OF 26-41; 46-52; 174-222 AND 341-357, AND IDENTIFICATION
RP   BY MASS SPECTROMETRY.
RC   STRAIN=C57BL/6J, and OF1; TISSUE=Brain, and Hippocampus;
RA   Lubec G., Kang S.U., Klug S., Sunyer B., Chen W.-Q.;
RL   Submitted (JAN-2009) to UniProtKB.
RN   [9]
RP   DISRUPTION PHENOTYPE.
RX   PubMed=17557305; DOI=10.1002/dvdy.21185;
RA   He Y., Hakvoort T.B., Vermeulen J.L., Lamers W.H., Van Roon M.A.;
RT   "Glutamine synthetase is essential in early mouse embryogenesis.";
RL   Dev. Dyn. 236:1865-1875(2007).
RN   [10]
RP   PHOSPHORYLATION [LARGE SCALE ANALYSIS] AT TYR-104, AND IDENTIFICATION BY
RP   MASS SPECTROMETRY [LARGE SCALE ANALYSIS].
RC   TISSUE=Brain;
RX   PubMed=18034455; DOI=10.1021/pr0701254;
RA   Ballif B.A., Carey G.R., Sunyaev S.R., Gygi S.P.;
RT   "Large-scale identification and evolution indexing of tyrosine
RT   phosphorylation sites from murine brain.";
RL   J. Proteome Res. 7:311-318(2008).
RN   [11]
RP   PHOSPHORYLATION [LARGE SCALE ANALYSIS] AT SER-343, AND IDENTIFICATION BY
RP   MASS SPECTROMETRY [LARGE SCALE ANALYSIS].
RC   TISSUE=Brain, Brown adipose tissue, Heart, Kidney, Liver, Lung,
RC   Pancreas, Spleen, and Testis;
RX   PubMed=21183079; DOI=10.1016/j.cell.2010.12.001;
RA   Huttlin E.L., Jedrychowski M.P., Elias J.E., Goswami T., Rad R.,
RA   Beausoleil S.A., Villen J., Haas W., Sowa M.E., Gygi S.P.;
RT   "A tissue-specific atlas of mouse protein phosphorylation and expression.";
RL   Cell 143:1174-1189(2010).
RN   [12]
RP   UBIQUITINATION.
RX   PubMed=20107048; DOI=10.1523/jneurosci.3591-09.2010;
RA   Saitoh F., Araki T.;
RT   "Proteasomal degradation of glutamine synthetase regulates schwann cell
RT   differentiation.";
RL   J. Neurosci. 30:1204-1212(2010).
RN   [13]
RP   FUNCTION, AND DISRUPTION PHENOTYPE.
RX   PubMed=25870278; DOI=10.1073/pnas.1423968112;
RA   Qvartskhava N., Lang P.A., Goerg B., Pozdeev V.I., Ortiz M.P., Lang K.S.,
RA   Bidmon H.J., Lang E., Leibrock C.B., Herebian D., Bode J.G., Lang F.,
RA   Haeussinger D.;
RT   "Hyperammonemia in gene-targeted mice lacking functional hepatic glutamine
RT   synthetase.";
RL   Proc. Natl. Acad. Sci. U.S.A. 112:5521-5526(2015).
RN   [14]
RP   FUNCTION, TISSUE SPECIFICITY, AND DISRUPTION PHENOTYPE.
RX   PubMed=30158707; DOI=10.1038/s41586-018-0466-7;
RA   Eelen G., Dubois C., Cantelmo A.R., Goveia J., Bruening U., DeRan M.,
RA   Jarugumilli G., van Rijssel J., Saladino G., Comitani F., Zecchin A.,
RA   Rocha S., Chen R., Huang H., Vandekeere S., Kalucka J., Lange C.,
RA   Morales-Rodriguez F., Cruys B., Treps L., Ramer L., Vinckier S.,
RA   Brepoels K., Wyns S., Souffreau J., Schoonjans L., Lamers W.H., Wu Y.,
RA   Haustraete J., Hofkens J., Liekens S., Cubbon R., Ghesquiere B.,
RA   Dewerchin M., Gervasio F.L., Li X., van Buul J.D., Wu X., Carmeliet P.;
RT   "Role of glutamine synthetase in angiogenesis beyond glutamine synthesis.";
RL   Nature 561:63-69(2018).
CC   -!- FUNCTION: Glutamine synthetase that catalyzes the ATP-dependent
CC       conversion of glutamate and ammonia to glutamine (By similarity). Its
CC       role depends on tissue localization: in the brain, it regulates the
CC       levels of toxic ammonia and converts neurotoxic glutamate to harmless
CC       glutamine, whereas in the liver, it is one of the enzymes responsible
CC       for the removal of ammonia (PubMed:25870278). Essential for
CC       proliferation of fetal skin fibroblasts (By similarity). Independently
CC       of its glutamine synthetase activity, required for endothelial cell
CC       migration during vascular development (PubMed:30158707). Involved in
CC       angiogenesis by regulating membrane localization and activation of the
CC       GTPase RHOJ, possibly by promoting RHOJ palmitoylation (By similarity).
CC       May act as a palmitoyltransferase for RHOJ: able to autopalmitoylate
CC       and then transfer the palmitoyl group to RHOJ (By similarity). Plays a
CC       role in ribosomal 40S subunit biogenesis (By similarity).
CC       {ECO:0000250|UniProtKB:P15104, ECO:0000269|PubMed:25870278,
CC       ECO:0000269|PubMed:30158707}.
CC   -!- CATALYTIC ACTIVITY:
CC       Reaction=ATP + L-glutamate + NH4(+) = ADP + H(+) + L-glutamine +
CC         phosphate; Xref=Rhea:RHEA:16169, ChEBI:CHEBI:15378,
CC         ChEBI:CHEBI:28938, ChEBI:CHEBI:29985, ChEBI:CHEBI:30616,
CC         ChEBI:CHEBI:43474, ChEBI:CHEBI:58359, ChEBI:CHEBI:456216; EC=6.3.1.2;
CC         Evidence={ECO:0000250|UniProtKB:P15104};
CC   -!- CATALYTIC ACTIVITY:
CC       Reaction=hexadecanoyl-CoA + L-cysteinyl-[protein] = CoA + S-
CC         hexadecanoyl-L-cysteinyl-[protein]; Xref=Rhea:RHEA:36683, Rhea:RHEA-
CC         COMP:10131, Rhea:RHEA-COMP:11032, ChEBI:CHEBI:29950,
CC         ChEBI:CHEBI:57287, ChEBI:CHEBI:57379, ChEBI:CHEBI:74151;
CC         EC=2.3.1.225; Evidence={ECO:0000250|UniProtKB:P15104};
CC   -!- COFACTOR:
CC       Name=Mg(2+); Xref=ChEBI:CHEBI:18420;
CC         Evidence={ECO:0000250|UniProtKB:P09606};
CC       Name=Mn(2+); Xref=ChEBI:CHEBI:29035;
CC         Evidence={ECO:0000250|UniProtKB:P15104};
CC   -!- ACTIVITY REGULATION: Glutamine synthetase activity is inhibited by
CC       methionine sulfoximine (MSO). {ECO:0000250|UniProtKB:P15104}.
CC   -!- SUBUNIT: Decamer; composed of two pentamers (By similarity). Interacts
CC       with PALMD (PubMed:16323283). Interacts with RHOJ (By similarity).
CC       {ECO:0000250|UniProtKB:P15104, ECO:0000269|PubMed:16323283}.
CC   -!- SUBCELLULAR LOCATION: Cytoplasm, cytosol
CC       {ECO:0000250|UniProtKB:P15104}. Microsome
CC       {ECO:0000250|UniProtKB:P09606}. Mitochondrion
CC       {ECO:0000250|UniProtKB:P09606}. Cell membrane
CC       {ECO:0000250|UniProtKB:P15104}; Lipid-anchor
CC       {ECO:0000250|UniProtKB:P15104}. Note=Mainly localizes in the cytosol,
CC       with a fraction associated with the cell membrane.
CC       {ECO:0000250|UniProtKB:P15104}.
CC   -!- TISSUE SPECIFICITY: Expressed in microvascular endothelial cells.
CC       {ECO:0000269|PubMed:30158707}.
CC   -!- PTM: Palmitoylated; undergoes autopalmitoylation.
CC       {ECO:0000250|UniProtKB:P15104}.
CC   -!- PTM: Ubiquitinated by ZNRF1. {ECO:0000269|PubMed:20107048}.
CC   -!- DISRUPTION PHENOTYPE: Embryonic lethality when embryos move from the
CC       uterine tube to the uterine environment (PubMed:17557305). Conditional
CC       deletion in the liver leads to a marked increase of plasma ammonia
CC       levels, causing increased locomotion, impaired fear memory and a
CC       slightly reduced life span (PubMed:25870278). Conditional deletion in
CC       endothelial cells impairs vessel sprouting during vascular development
CC       due to defects in endothelial cell migration (PubMed:30158707).
CC       {ECO:0000269|PubMed:17557305, ECO:0000269|PubMed:25870278,
CC       ECO:0000269|PubMed:30158707}.
CC   -!- SIMILARITY: Belongs to the glutamine synthetase family. {ECO:0000305}.
CC   ---------------------------------------------------------------------------
CC   Copyrighted by the UniProt Consortium, see https://www.uniprot.org/terms
CC   Distributed under the Creative Commons Attribution (CC BY 4.0) License
CC   ---------------------------------------------------------------------------
DR   EMBL; X16314; CAA34381.1; -; mRNA.
DR   EMBL; U09114; AAA17989.1; -; mRNA.
DR   EMBL; AY044241; AAK95328.1; -; mRNA.
DR   EMBL; AK159106; BAE34822.1; -; mRNA.
DR   EMBL; AK160670; BAE35950.1; -; mRNA.
DR   EMBL; AK162685; BAE37022.1; -; mRNA.
DR   EMBL; AK168493; BAE40380.1; -; mRNA.
DR   EMBL; BC015086; AAH15086.1; -; mRNA.
DR   CCDS; CCDS15381.1; -.
DR   PIR; S04991; AJMSQ.
DR   RefSeq; NP_032157.2; NM_008131.4.
DR   AlphaFoldDB; P15105; -.
DR   SMR; P15105; -.
DR   BioGRID; 199947; 21.
DR   IntAct; P15105; 15.
DR   MINT; P15105; -.
DR   STRING; 10090.ENSMUSP00000083375; -.
DR   GlyGen; P15105; 2 sites, 1 O-linked glycan (2 sites).
DR   iPTMnet; P15105; -.
DR   PhosphoSitePlus; P15105; -.
DR   SwissPalm; P15105; -.
DR   REPRODUCTION-2DPAGE; IPI00626790; -.
DR   REPRODUCTION-2DPAGE; P15105; -.
DR   SWISS-2DPAGE; P15105; -.
DR   UCD-2DPAGE; P15105; -.
DR   CPTAC; non-CPTAC-3816; -.
DR   EPD; P15105; -.
DR   jPOST; P15105; -.
DR   MaxQB; P15105; -.
DR   PaxDb; P15105; -.
DR   PeptideAtlas; P15105; -.
DR   ProteomicsDB; 266809; -.
DR   Antibodypedia; 1536; 739 antibodies from 42 providers.
DR   DNASU; 14645; -.
DR   Ensembl; ENSMUST00000086199; ENSMUSP00000083375; ENSMUSG00000026473.
DR   Ensembl; ENSMUST00000140685; ENSMUSP00000123157; ENSMUSG00000026473.
DR   GeneID; 14645; -.
DR   KEGG; mmu:14645; -.
DR   UCSC; uc007daq.2; mouse.
DR   AGR; MGI:95739; -.
DR   CTD; 2752; -.
DR   MGI; MGI:95739; Glul.
DR   VEuPathDB; HostDB:ENSMUSG00000026473; -.
DR   eggNOG; KOG0683; Eukaryota.
DR   GeneTree; ENSGT00390000010047; -.
DR   HOGENOM; CLU_036762_1_1_1; -.
DR   InParanoid; P15105; -.
DR   OMA; DRRPNAN; -.
DR   OrthoDB; 1115057at2759; -.
DR   PhylomeDB; P15105; -.
DR   TreeFam; TF300491; -.
DR   BRENDA; 6.3.1.2; 3474.
DR   Reactome; R-MMU-210455; Astrocytic Glutamate-Glutamine Uptake And Metabolism.
DR   Reactome; R-MMU-8964539; Glutamate and glutamine metabolism.
DR   BioGRID-ORCS; 14645; 7 hits in 78 CRISPR screens.
DR   ChiTaRS; Glul; mouse.
DR   PRO; PR:P15105; -.
DR   Proteomes; UP000000589; Chromosome 1.
DR   RNAct; P15105; protein.
DR   Bgee; ENSMUSG00000026473; Expressed in efferent duct and 260 other tissues.
DR   ExpressionAtlas; P15105; baseline and differential.
DR   Genevisible; P15105; MM.
DR   GO; GO:0043679; C:axon terminus; ISO:MGI.
DR   GO; GO:0044297; C:cell body; IDA:MGI.
DR   GO; GO:0042995; C:cell projection; ISO:MGI.
DR   GO; GO:0005737; C:cytoplasm; IDA:MGI.
DR   GO; GO:0005829; C:cytosol; ISS:UniProtKB.
DR   GO; GO:0097386; C:glial cell projection; IDA:MGI.
DR   GO; GO:0043231; C:intracellular membrane-bounded organelle; ISO:MGI.
DR   GO; GO:0005739; C:mitochondrion; HDA:MGI.
DR   GO; GO:0043209; C:myelin sheath; HDA:UniProtKB.
DR   GO; GO:0043005; C:neuron projection; ISO:MGI.
DR   GO; GO:0043204; C:perikaryon; ISO:MGI.
DR   GO; GO:0005886; C:plasma membrane; ISS:UniProtKB.
DR   GO; GO:0032991; C:protein-containing complex; ISO:MGI.
DR   GO; GO:0005791; C:rough endoplasmic reticulum; ISO:MGI.
DR   GO; GO:0005524; F:ATP binding; ISO:MGI.
DR   GO; GO:0045503; F:dynein light chain binding; ISO:MGI.
DR   GO; GO:0016595; F:glutamate binding; ISO:MGI.
DR   GO; GO:0004356; F:glutamate-ammonia ligase activity; ISS:UniProtKB.
DR   GO; GO:0042802; F:identical protein binding; ISO:MGI.
DR   GO; GO:0000287; F:magnesium ion binding; ISO:MGI.
DR   GO; GO:0030145; F:manganese ion binding; ISO:MGI.
DR   GO; GO:0019706; F:protein-cysteine S-palmitoyltransferase activity; ISS:UniProtKB.
DR   GO; GO:0019676; P:ammonia assimilation cycle; ISO:MGI.
DR   GO; GO:0001525; P:angiogenesis; IEA:UniProtKB-KW.
DR   GO; GO:0008283; P:cell population proliferation; ISO:MGI.
DR   GO; GO:0009267; P:cellular response to starvation; IEP:MGI.
DR   GO; GO:0006536; P:glutamate metabolic process; ISO:MGI.
DR   GO; GO:0006542; P:glutamine biosynthetic process; ISO:MGI.
DR   GO; GO:0050679; P:positive regulation of epithelial cell proliferation; ISO:MGI.
DR   GO; GO:0032024; P:positive regulation of insulin secretion; ISO:MGI.
DR   GO; GO:0051968; P:positive regulation of synaptic transmission, glutamatergic; ISO:MGI.
DR   GO; GO:0018345; P:protein palmitoylation; ISS:UniProtKB.
DR   GO; GO:0010594; P:regulation of endothelial cell migration; IMP:UniProtKB.
DR   GO; GO:1904749; P:regulation of protein localization to nucleolus; ISO:MGI.
DR   GO; GO:1903670; P:regulation of sprouting angiogenesis; IMP:UniProtKB.
DR   GO; GO:0009749; P:response to glucose; IMP:MGI.
DR   GO; GO:0042254; P:ribosome biogenesis; ISO:MGI.
DR   Gene3D; 3.10.20.70; Glutamine synthetase, N-terminal domain; 1.
DR   Gene3D; 3.30.590.10; Glutamine synthetase/guanido kinase, catalytic domain; 2.
DR   InterPro; IPR008147; Gln_synt_N.
DR   InterPro; IPR036651; Gln_synt_N_sf.
DR   InterPro; IPR014746; Gln_synth/guanido_kin_cat_dom.
DR   InterPro; IPR008146; Gln_synth_cat_dom.
DR   InterPro; IPR027303; Gln_synth_gly_rich_site.
DR   InterPro; IPR027302; Gln_synth_N_conserv_site.
DR   PANTHER; PTHR20852; GLUTAMINE SYNTHETASE; 1.
DR   PANTHER; PTHR20852:SF45; GLUTAMINE SYNTHETASE; 1.
DR   Pfam; PF00120; Gln-synt_C; 1.
DR   Pfam; PF03951; Gln-synt_N; 1.
DR   SMART; SM01230; Gln-synt_C; 1.
DR   SUPFAM; SSF54368; Glutamine synthetase, N-terminal domain; 1.
DR   SUPFAM; SSF55931; Glutamine synthetase/guanido kinase; 1.
DR   PROSITE; PS00180; GLNA_1; 1.
DR   PROSITE; PS00181; GLNA_ATP; 1.
DR   PROSITE; PS51986; GS_BETA_GRASP; 1.
DR   PROSITE; PS51987; GS_CATALYTIC; 1.
PE   1: Evidence at protein level;
KW   Acetylation; Angiogenesis; ATP-binding; Cell membrane; Cytoplasm;
KW   Direct protein sequencing; Endoplasmic reticulum; Ligase; Lipoprotein;
KW   Magnesium; Manganese; Membrane; Metal-binding; Microsome; Mitochondrion;
KW   Nucleotide-binding; Palmitate; Phosphoprotein; Reference proteome;
KW   Transferase; Ubl conjugation.
FT   INIT_MET        1
FT                   /note="Removed"
FT                   /evidence="ECO:0000269|Ref.6"
FT   CHAIN           2..373
FT                   /note="Glutamine synthetase"
FT                   /id="PRO_0000153141"
FT   DOMAIN          24..106
FT                   /note="GS beta-grasp"
FT                   /evidence="ECO:0000255|PROSITE-ProRule:PRU01330"
FT   DOMAIN          113..373
FT                   /note="GS catalytic"
FT                   /evidence="ECO:0000255|PROSITE-ProRule:PRU01331"
FT   BINDING         134
FT                   /ligand="ATP"
FT                   /ligand_id="ChEBI:CHEBI:30616"
FT                   /evidence="ECO:0000250|UniProtKB:P15104"
FT   BINDING         134
FT                   /ligand="Mn(2+)"
FT                   /ligand_id="ChEBI:CHEBI:29035"
FT                   /ligand_label="1"
FT                   /evidence="ECO:0000250|UniProtKB:P15104"
FT   BINDING         136
FT                   /ligand="Mn(2+)"
FT                   /ligand_id="ChEBI:CHEBI:29035"
FT                   /ligand_label="2"
FT                   /evidence="ECO:0000250|UniProtKB:P15104"
FT   BINDING         196
FT                   /ligand="Mn(2+)"
FT                   /ligand_id="ChEBI:CHEBI:29035"
FT                   /ligand_label="2"
FT                   /evidence="ECO:0000250|UniProtKB:P15104"
FT   BINDING         203..208
FT                   /ligand="ATP"
FT                   /ligand_id="ChEBI:CHEBI:30616"
FT                   /evidence="ECO:0000250|UniProtKB:P15104"
FT   BINDING         203
FT                   /ligand="Mn(2+)"
FT                   /ligand_id="ChEBI:CHEBI:29035"
FT                   /ligand_label="2"
FT                   /evidence="ECO:0000250|UniProtKB:P15104"
FT   BINDING         246..247
FT                   /ligand="L-glutamate"
FT                   /ligand_id="ChEBI:CHEBI:29985"
FT                   /evidence="ECO:0000250|UniProtKB:P9WN39"
FT   BINDING         253
FT                   /ligand="Mn(2+)"
FT                   /ligand_id="ChEBI:CHEBI:29035"
FT                   /ligand_label="1"
FT                   /evidence="ECO:0000250|UniProtKB:P15104"
FT   BINDING         255..257
FT                   /ligand="ATP"
FT                   /ligand_id="ChEBI:CHEBI:30616"
FT                   /evidence="ECO:0000250|UniProtKB:P15104"
FT   BINDING         319
FT                   /ligand="ATP"
FT                   /ligand_id="ChEBI:CHEBI:30616"
FT                   /evidence="ECO:0000250|UniProtKB:P15104"
FT   BINDING         319
FT                   /ligand="L-glutamate"
FT                   /ligand_id="ChEBI:CHEBI:29985"
FT                   /evidence="ECO:0000250|UniProtKB:P9WN39"
FT   BINDING         324
FT                   /ligand="ATP"
FT                   /ligand_id="ChEBI:CHEBI:30616"
FT                   /evidence="ECO:0000250|UniProtKB:P15104"
FT   BINDING         336..338
FT                   /ligand="ADP"
FT                   /ligand_id="ChEBI:CHEBI:456216"
FT                   /evidence="ECO:0000250|UniProtKB:P15104"
FT   BINDING         338
FT                   /ligand="Mn(2+)"
FT                   /ligand_id="ChEBI:CHEBI:29035"
FT                   /ligand_label="1"
FT                   /evidence="ECO:0000250|UniProtKB:P15104"
FT   BINDING         340
FT                   /ligand="L-glutamate"
FT                   /ligand_id="ChEBI:CHEBI:29985"
FT                   /evidence="ECO:0000250|UniProtKB:P9WN39"
FT   MOD_RES         2
FT                   /note="N-acetylalanine"
FT                   /evidence="ECO:0000269|Ref.6"
FT   MOD_RES         104
FT                   /note="Phosphotyrosine"
FT                   /evidence="ECO:0007744|PubMed:18034455"
FT   MOD_RES         343
FT                   /note="Phosphoserine"
FT                   /evidence="ECO:0007744|PubMed:21183079"
FT   CONFLICT        91
FT                   /note="K -> R (in Ref. 1; CAA34381)"
FT                   /evidence="ECO:0000305"
FT   CONFLICT        111
FT                   /note="T -> S (in Ref. 2; AAA17989)"
FT                   /evidence="ECO:0000305"
FT   CONFLICT        133
FT                   /note="M -> L (in Ref. 4; BAE37022)"
FT                   /evidence="ECO:0000305"
FT   CONFLICT        249
FT                   /note="G -> V (in Ref. 1; CAA34381)"
FT                   /evidence="ECO:0000305"
FT   CONFLICT        269
FT                   /note="C -> W (in Ref. 2; AAA17989)"
FT                   /evidence="ECO:0000305"
FT   CONFLICT        299
FT                   /note="R -> A (in Ref. 1; CAA34381)"
FT                   /evidence="ECO:0000305"
FT   CONFLICT        342..343
FT                   /note="PS -> LR (in Ref. 1; CAA34381)"
FT                   /evidence="ECO:0000305"
SQ   SEQUENCE   373 AA;  42120 MW;  1EC9CDC5D81DE63F CRC64;
     MATSASSHLN KGIKQMYMSL PQGEKVQAMY IWVDGTGEGL RCKTRTLDCE PKCVEELPEW
     NFDGSSTFQS EGSNSDMYLH PVAMFRDPFR KDPNKLVLCE VFKYNRKPAE TNLRHICKRI
     MDMVSNQHPW FGMEQEYTLM GTDGHPFGWP SNGFPGPQGP YYCGVGADKA YGRDIVEAHY
     RACLYAGVKI TGTNAEVMPA QWEFQIGPCE GIRMGDHLWI ARFILHRVCE DFGVIATFDP
     KPIPGNWNGA GCHTNFSTKA MREENGLKCI EEAIDKLSKR HQYHIRAYDP KGGLDNARRL
     TGFHETSNIN DFSAGVANRG ASIRIPRTVG QEKKGYFEDR RPSANCDPYA VTEAIVRTCL
     LNETGDEPFQ YKN
//
```

**Mascot:** http://www.matrixscience.com/


HNE (H) (+156.1150)
