# Supplementary material for: A common East-Asian ALDH2 mutation causes metabolic disorders and the therapeutic effect of ALDH2 activators
Source: Nat Commun. 2023 Sep 25;14:5971. doi: 10.1038/s41467-023-41570-6 (PMC10520061; doi:10.1038/s41467-023-41570-6)
Supplement: Supplementary file 4 — Supplementary Data 1 [file 41467_2023_41570_MOESM4_ESM.zip › Table S5c/P19096/P19096_KIO60-1_C2187.html]

Mascot Search Results: P19096
 

# MASCOT Search Results

## Protein View: P19096

### Fatty acid synthase OS=Mus musculus OX=10090 GN=Fasn PE=1 SV=2

|  |  |
| --- | --- |
| Database: | Mouse\_UniProt\_proteomes |
| Score: | 19141 |
| Monoisotopic mass (M_r_): | 274994 |
| Calculated pI: | 6.13 |

Sequence similarity is available as an NCBI BLAST search of P19096 against nr.

### Search parameters

|  |  |
| --- | --- |
| MS data file: | `D:\LCMSMS\2023 Users' data\230529-1\230529-1-KI60-1.raw` |
| Enzyme: | Trypsin/P: cuts C-term side of KR. |
| Fixed modifications: | Carbamidomethyl (C) |
| Variable modifications: | Deamidated (NQ), HNE (C), HNE (H), HNE (K), Oxidation (M) |

### Protein sequence coverage: 67%

Matched peptides shown in ***bold red***.

|  |  |  |  |  |  |
| --- | --- | --- | --- | --- | --- |
| `1` | `MEEVVIAGMS` | `GKLPESENLQ` | `EFWANLIGGV` | `DMVTDDDRRW` | `KAGLYGLPKR` |
| `51` | `SGKLKDLSKF` | `DASFFGVHPK` | `QAHTMDPQLR` | `LLLEVSYEAI` | `VDGGINPASL` |
| `101` | `RGTNTGVWVG` | `VSGSEASEAL` | `SRDPETLLGY` | `SMVGCQRAMM` | `ANRLSFFFDF` |
| `151` | `KGPSIALDTA` | `CSSSLLALQN` | `AYQAIRSGEC` | `PAALVGGINL` | `LLKPNTSVQF` |
| `201` | `MKLGMLSPDG` | `TCRSFDDSGS` | `GYCRSEAVVA` | `VLLTKKSLAR` | `RVYATILNAG` |
| `251` | `TNTDGSKEQG` | `VTFPSGEVQE` | `QLICSLYQPA` | `GLAPESLEYI` | `EAHGTGTKVG` |
| `301` | `DPQELNGITR` | `SLCAFRQAPL` | `LIGSTKSNMG` | `HPEPASGLAA` | `LTKVLLSLEH` |
| `351` | `GVWAPNLHFH` | `NPNPEIPALL` | `DGRLQVVDRP` | `LPVRGGNVGI` | `NSFGFGGSNV` |
| `401` | `HVILQPNTRQ` | `APAPTAHAAL` | `PHLLHASGRT` | `LEAVQDLLEQ` | `GRQHSQDLAF` |
| `451` | `VSMLNDIAAT` | `PTAAMPFRGY` | `TVLGVEGRVQ` | `EVQQVSTNKR` | `PLWFICSGMG` |
| `501` | `TQWRGMGLSL` | `MRLDSFRESI` | `LRSDEAVKPL` | `GVKVSDLLLS` | `TDERTFDDIV` |
| `551` | `HAFVSLTAIQ` | `IALIDLLTSV` | `GLKPDGIIGH` | `SLGEVACGYA` | `DGCLSQREAV` |
| `601` | `LAAYWRGQCI` | `KDAHLPPGSM` | `AAVGLSWEEC` | `KQRCPAGVVP` | `ACHNSEDTVT` |
| `651` | `ISGPQAAVNE` | `FVEQLKQEGV` | `FAKEVRTGGL` | `AFHSYFMEGI` | `APTLLQALKK` |
| `701` | `VIREPRPRSA` | `RWLSTSIPEA` | `QWQSSLARTS` | `SAEYNVNNLV` | `SPVLFQEALW` |
| `751` | `HIPEHAVVLE` | `IAPHALLQAV` | `LKRGVKSSCT` | `IIPLMKRDHK` | `DNLEFFLTNL` |
| `801` | `GKVHLTGINV` | `NPNALFPPVE` | `FPAPRGTPLI` | `SPHIKWDHSQ` | `TWDVPVAEDF` |
| `851` | `PNGSSSSSAT` | `VYSIDASPES` | `PDHYLVDHCI` | `DGRVIFPGTG` | `YLCLVWKTLA` |
| `901` | `RSLGLSLEET` | `PVVFENVSFH` | `QATILPKTGT` | `VALEVRLLEA` | `SHAFEVSDTG` |
| `951` | `NLIVSGKVYL` | `WEDPNSKLFD` | `HPEVPTPPES` | `ASVSRLTQGE` | `VYKELRLRGY` |
| `1001` | `DYGPQFQGIC` | `EATLEGEQGK` | `LLWKDNWVTF` | `MDTMLQVSIL` | `GSSQQSLQLP` |
| `1051` | `TRVTAIYIDP` | `ATHRQKVYRL` | `KEDTQVADVT` | `TSRCLGITVS` | `GGIHISRLQT` |
| `1101` | `TATSRRQQEQ` | `LVPTLEKFVF` | `TPHMEAECLS` | `ESTALQKELQ` | `LCKGLARALQ` |
| `1151` | `TKATQQGLKA` | `AMLGQEDPPQ` | `HGLPRLLAAA` | `CQLQLNGNLQ` | `LELGEALAQE` |
| `1201` | `RLLLPEDPLI` | `SGLLNSQALK` | `ACVDTALENL` | `STLKMKVAEV` | `LAGEGHLYSR` |
| `1251` | `IPALLNTQPM` | `LQLEYTATDR` | `HPQALKDVQT` | `KLQQHDVAQG` | `QWNPSDPAPS` |
| `1301` | `SLGALDLLVC` | `NCALATLGDP` | `ALALDNMVAA` | `LKEGGFLLVH` | `TVLKGHALGE` |
| `1351` | `TLACLPSEVQ` | `PAPSLLSQEE` | `WESLFSRKAL` | `HLVGLKRSFY` | `GTALFLCRRA` |
| `1401` | `IPQEKPIFLS` | `VEDTSFQWVD` | `SLKSTLATSS` | `SQPVWLTAMD` | `CPTSGVVGLV` |
| `1451` | `NCLRKEPGGH` | `RIRCILLSNL` | `SNTSHAPKLD` | `PGSPELQQVL` | `KHDLVMNVYR` |
| `1501` | `DGAWGAFRHF` | `QLEQDKPKEQ` | `TAHAFVNVLT` | `RGDLASIRWV` | `SSPLKHTQPS` |
| `1551` | `SSGAQLCTVY` | `YASLNFRDIM` | `LATGKLSPDA` | `IPGKWASRDC` | `MLGMEFSGRD` |
| `1601` | `RCGRRVMGLV` | `PAEGLATSVL` | `LSSDFLWDVP` | `SSWTLEEAAS` | `VPVVYTTAYY` |
| `1651` | `SLVVRGRIQR` | `GETVLIHSGS` | `GGVGQAAISI` | `ALSLGCRVFT` | `TVGSAEKRAY` |
| `1701` | `LQARFPQLDD` | `TSFANSRDTS` | `FEQHVLLHTG` | `GKGVDLVLNS` | `LAEEKLQASV` |
| `1751` | `RCLAQHGRFL` | `EIGKFDLSNN` | `HPLGMAIFLK` | `NVTFHGILLD` | `ALFEEANDSW` |
| `1801` | `REVAALLKAG` | `IRDGVVKPLK` | `CTVFPKAQVE` | `DAFRYMAQGK` | `HIGKVLVQVR` |
| `1851` | `EEEPEAVLPG` | `AQPTLISAIS` | `KTFCPAHKSY` | `IITGGLGGFG` | `LELARWLVLR` |
| `1901` | `GAQRLVLTSR` | `SGIRTGYQAK` | `HIREWRRQGI` | `QVLVSTSNVS` | `SLEGARALIA` |
| `1951` | `EATKLGPVGG` | `VFNLAMVLRD` | `AMLENQTPEL` | `FQDVNKPKYN` | `GTLNLDRATR` |
| `2001` | `EACPELDYFV` | `AFSSVSCGRG` | `NAGQTNYGFA` | `NSTMERICEQ` | `RRHDGLPGLA` |
| `2051` | `VQWGAIGDVG` | `IVLEAMGTND` | `TVIGGTLPQR` | `ISSCMEVLDL` | `FLNQPHAVLS` |
| `2101` | `SFVLAEKKAV` | `AHGDGDTQRD` | `LVKAVAHILG` | `IRDLAGINLD` | `STLADLGLDS` |
| `2151` | `LMGVEVRQIL` | `EREHDLVLPM` | `REVRQLTLRK` | `LQEMSSKTDS` | `ATDTTAPKSR` |
| `2201` | `SDTSLKQNQL` | `NLSTLLVNPE` | `GPTLTQLNSV` | `QSSERPLFLV` | `HPIEGSTTVF` |
| `2251` | `HSLAAKLSVP` | `TYGLQCTQAA` | `PLDSIPNLAA` | `YYIDCIKQVQ` | `PEGPYRIAGY` |
| `2301` | `SFGACVAFEM` | `CSQLQAQQGP` | `APTHNNLFLF` | `DGSHTYVLAY` | `TQSYRAKMTP` |
| `2351` | `GCEAEAEAEA` | `LCFFIKQFLD` | `VEHSKVLEAL` | `LPLKSLEDRV` | `AASVDLITKS` |
| `2401` | `HHSLDRRELS` | `FAAVSFYHKL` | `RAADQYKPKA` | `KYHGNVTLLR` | `AKTGGTYGED` |
| `2451` | `LGADYNLSQV` | `CDGKVSVHII` | `EGDHRTLLEG` | `SGLESIINII` | `HSSLAEPRVS` |
| `2501` | `VREG` |  |  |  |  |

Unformatted sequence string: 2504 residues (for pasting into other applications).

|  |  |  |  |
| --- | --- | --- | --- |
| Sort by | residue number | increasing mass | decreasing mass |
| Show | matched peptides only | predicted peptides also |  |

| Query | Start | – | End | Observed | Mr(expt) | Mr(calc) | ppm | M | Score | Expect | Rank | U | Peptide |
| --- | --- | --- | --- | --- | --- | --- | --- | --- | --- | --- | --- | --- | --- |
| 24326 | 40 | – | 49 | 378.2219 | 1131.6438 | 1131.6440 | -0.17 | 1 | 33 | 0.00074 | 1Score **> 32** indicates **identity** Score **> 15** indicates **homology** | U | R.WKAGLYGLPK.R |
| 24327 | 40 | – | 49 | 378.2219 | 1131.6439 | 1131.6440 | -0.12 | 1 | 34 | 0.00069 | 1Score **> 32** indicates **identity** Score **> 15** indicates **homology** | U | R.WKAGLYGLPK.R |
| 24328 | 40 | – | 49 | 378.2219 | 1131.6440 | 1131.6440 | -0.021 | 1 | 33 | 0.00073 | 1Score **> 32** indicates **identity** Score **> 15** indicates **homology** | U | R.WKAGLYGLPK.R |
| 24330 | 40 | – | 49 | 378.2221 | 1131.6444 | 1131.6440 | 0.29 | 1 | 31 | 0.0013 | 1Score **> 32** indicates **identity** Score **> 14** indicates **homology** | U | R.WKAGLYGLPK.R |
| 24332 | 40 | – | 49 | 378.2224 | 1131.6454 | 1131.6440 | 1.18 | 1 | 23 | 0.0071 | 1Score **> 32** indicates **identity** Score **> 14** indicates **homology** | U | R.WKAGLYGLPK.R |
| 40756 | 40 | – | 50 | 430.2554 | 1287.7444 | 1287.7451 | -0.62 | 2 | 14 | 0.045 | 1Score **> 32** indicates **identity** Score **> 13** indicates **homology** | U | R.WKAGLYGLPKR.S |
| 40757 | 40 | – | 50 | 430.2555 | 1287.7446 | 1287.7451 | -0.46 | 2 | 14 | 0.05 | 1Score **> 32** indicates **identity** Score **> 13** indicates **homology** | U | R.WKAGLYGLPKR.S |
| 40758 | 40 | – | 50 | 430.2560 | 1287.7463 | 1287.7451 | 0.87 | 2 | 46 | 0.0023 | 1Score **> 32** indicates **identity** | U | R.WKAGLYGLPKR.S |
| 40759 | 40 | – | 50 | 430.2564 | 1287.7475 | 1287.7451 | 1.82 | 2 | 36 | 0.02 | 1Score **> 32** indicates **identity** Score **> 31** indicates **homology** | U | R.WKAGLYGLPKR.S |
| 4038 | 42 | – | 49 | 409.7419 | 817.4693 | 817.4698 | -0.60 | 0 | 44 | 0.0015 | 1Score **> 28** indicates **identity** | U | K.AGLYGLPK.R |
| 4039 | 42 | – | 49 | 409.7422 | 817.4699 | 817.4698 | 0.15 | 0 | 43 | 0.0018 | 1Score **> 28** indicates **identity** | U | K.AGLYGLPK.R |
| 116797 | 54 | – | 70 | 484.7635 | 1935.0250 | 1935.0254 | -0.24 | 2 | 20 | 0.013 | 1Score **> 36** indicates **identity** Score **> 14** indicates **homology** | U | K.LKDLSKFDASFFGVHPK.Q |
| 116801 | 54 | – | 70 | 484.7639 | 1935.0263 | 1935.0254 | 0.45 | 2 | 20 | 0.013 | 1Score **> 36** indicates **identity** Score **> 14** indicates **homology** | U | K.LKDLSKFDASFFGVHPK.Q |
| 90583 | 56 | – | 70 | 565.6227 | 1693.8463 | 1693.8464 | -0.064 | 1 | 51 | 0.00037 | 1Score **> 35** indicates **identity** Score **> 29** indicates **homology** | U | K.DLSKFDASFFGVHPK.Q |
| 90586 | 56 | – | 70 | 565.6229 | 1693.8469 | 1693.8464 | 0.28 | 1 | 44 | 0.00033 | 1Score **> 35** indicates **identity** Score **> 22** indicates **homology** | U | K.DLSKFDASFFGVHPK.Q |
| 90588 | 56 | – | 70 | 565.6230 | 1693.8473 | 1693.8464 | 0.53 | 1 | 35 | 0.001 | 1Score **> 35** indicates **identity** Score **> 17** indicates **homology** | U | K.DLSKFDASFFGVHPK.Q |
| 36740 | 60 | – | 70 | 417.8765 | 1250.6078 | 1250.6084 | -0.50 | 0 | 14 | 0.046 | 1Score **> 32** indicates **identity** Score **> 13** indicates **homology** | U | K.FDASFFGVHPK.Q |
| 36742 | 60 | – | 70 | 417.8768 | 1250.6086 | 1250.6084 | 0.11 | 0 | 29 | 0.0019 | 1Score **> 32** indicates **identity** Score **> 14** indicates **homology** | U | K.FDASFFGVHPK.Q |
| 36745 | 60 | – | 70 | 417.8769 | 1250.6087 | 1250.6084 | 0.25 | 0 | 27 | 0.0029 | 1Score **> 32** indicates **identity** Score **> 14** indicates **homology** | U | K.FDASFFGVHPK.Q |
| 36747 | 60 | – | 70 | 417.8769 | 1250.6088 | 1250.6084 | 0.32 | 0 | 42 | 0.00011 | 1Score **> 32** indicates **identity** Score **> 15** indicates **homology** | U | K.FDASFFGVHPK.Q |
| 36748 | 60 | – | 70 | 626.3117 | 1250.6088 | 1250.6084 | 0.34 | 0 | 40 | 0.00019 | 1Score **> 32** indicates **identity** Score **> 15** indicates **homology** | U | K.FDASFFGVHPK.Q |
| 36749 | 60 | – | 70 | 626.3117 | 1250.6089 | 1250.6084 | 0.35 | 0 | 38 | 0.00026 | 1Score **> 32** indicates **identity** Score **> 15** indicates **homology** | U | K.FDASFFGVHPK.Q |
| 36751 | 60 | – | 70 | 417.8770 | 1250.6093 | 1250.6084 | 0.70 | 0 | 30 | 0.0016 | 1Score **> 32** indicates **identity** Score **> 14** indicates **homology** | U | K.FDASFFGVHPK.Q |
| 36752 | 60 | – | 70 | 417.8772 | 1250.6096 | 1250.6084 | 0.96 | 0 | 23 | 0.0069 | 1Score **> 32** indicates **identity** Score **> 14** indicates **homology** | U | K.FDASFFGVHPK.Q |
| 36753 | 60 | – | 70 | 417.8772 | 1250.6096 | 1250.6084 | 0.96 | 0 | 39 | 0.00022 | 1Score **> 32** indicates **identity** Score **> 15** indicates **homology** | U | K.FDASFFGVHPK.Q |
| 36754 | 60 | – | 70 | 417.8772 | 1250.6097 | 1250.6084 | 1.03 | 0 | 22 | 0.0095 | 1Score **> 32** indicates **identity** Score **> 14** indicates **homology** | U | K.FDASFFGVHPK.Q |
| 31044 | 71 | – | 80 | 399.5324 | 1195.5754 | 1195.5768 | -1.19 | 0 | 40 | 0.0002 | 1Score **> 31** indicates **identity** Score **> 15** indicates **homology** | U | K.QAHTMDPQLR.L |
| 144033 | 81 | – | 101 | 1115.1051 | 2228.1956 | 2228.2052 | -4.33 | 0 | 19 | 0.018 | 1Score **> 36** indicates **identity** Score **> 14** indicates **homology** | U | R.LLLEVSYEAIVDGGINPASLR.G |
| 16660 | 144 | – | 151 | 525.7679 | 1049.5212 | 1049.5222 | -1.00 | 0 | 28 | 0.0023 | 1Score **> 32** indicates **identity** Score **> 14** indicates **homology** | U | R.LSFFFDFK.G |
| 16663 | 144 | – | 151 | 525.7689 | 1049.5232 | 1049.5222 | 0.95 | 0 | 47 | 3.9e-05 | 1Score **> 32** indicates **identity** Score **> 15** indicates **homology** | U | R.LSFFFDFK.G |
| 166696 | 152 | – | 176 | 874.1191 | 2619.3355 | 2619.3326 | 1.11 | 0 | 100 | 5.8e-10 | 1Score **> 37** indicates **identity** Score **> 20** indicates **homology** | U | K.GPSIALDTACSSSLLALQNAYQAIR.S |
| 166697 | 152 | – | 176 | 874.1198 | 2619.3377 | 2619.3326 | 1.95 | 0 | 66 | 6.2e-07 | 1Score **> 37** indicates **identity** Score **> 17** indicates **homology** | U | K.GPSIALDTACSSSLLALQNAYQAIR.S |
| 166698 | 152 | – | 176 | 1310.6772 | 2619.3398 | 2619.3326 | 2.74 | 0 | 144 | 2.9e-14 | 1Score **> 37** indicates **identity** Score **> 22** indicates **homology** | U | K.GPSIALDTACSSSLLALQNAYQAIR.S |
| 36601 | 214 | – | 224 | 625.7407 | 1249.4668 | 1249.4670 | -0.17 | 0 | 22 | 0.014 | 1Score **> 16** indicates **identity** | U | R.SFDDSGSGYCR.S |
| 36603 | 214 | – | 224 | 625.7408 | 1249.4671 | 1249.4670 | 0.12 | 0 | 30 | 0.0023 | 1Score **> 16** indicates **identity** | U | R.SFDDSGSGYCR.S |
| 36604 | 214 | – | 224 | 625.7410 | 1249.4675 | 1249.4670 | 0.40 | 0 | 74 | 9.9e-08 | 1Score **> 16** indicates **identity** | U | R.SFDDSGSGYCR.S |
| 36605 | 214 | – | 224 | 625.7415 | 1249.4684 | 1249.4670 | 1.10 | 0 | 34 | 0.00083 | 1Score **> 16** indicates **identity** | U | R.SFDDSGSGYCR.S |
| 36606 | 214 | – | 224 | 625.7415 | 1249.4684 | 1249.4670 | 1.17 | 0 | 59 | 3e-06 | 1Score **> 16** indicates **identity** | U | R.SFDDSGSGYCR.S |
| 160402 | 214 | – | 236 | 623.0644 | 2488.2283 | 2488.2268 | 0.62 | 2 | 16 | 0.031 | 1Score **> 37** indicates **identity** Score **> 14** indicates **homology** | U | R.SFDDSGSGYCRSEAVVAVLLTKK.S |
| 24046 | 225 | – | 235 | 565.3445 | 1128.6745 | 1128.6754 | -0.80 | 0 | 14 | 0.05 | 1Score **> 32** indicates **identity** Score **> 13** indicates **homology** | U | R.SEAVVAVLLTK.K |
| 24048 | 225 | – | 235 | 565.3447 | 1128.6748 | 1128.6754 | -0.50 | 0 | 63 | 1.3e-06 | 1Score **> 32** indicates **identity** Score **> 16** indicates **homology** | U | R.SEAVVAVLLTK.K |
| 24050 | 225 | – | 235 | 565.3453 | 1128.6760 | 1128.6754 | 0.50 | 0 | 69 | 3.8e-07 | 1Score **> 32** indicates **identity** Score **> 17** indicates **homology** | U | R.SEAVVAVLLTK.K |
| 24051 | 225 | – | 235 | 565.3453 | 1128.6761 | 1128.6754 | 0.62 | 0 | 64 | 1e-06 | 1Score **> 32** indicates **identity** Score **> 16** indicates **homology** | U | R.SEAVVAVLLTK.K |
| 24052 | 225 | – | 235 | 565.3454 | 1128.6763 | 1128.6754 | 0.82 | 0 | 47 | 3.9e-05 | 1Score **> 32** indicates **identity** Score **> 15** indicates **homology** | U | R.SEAVVAVLLTK.K |
| 24053 | 225 | – | 235 | 565.3459 | 1128.6773 | 1128.6754 | 1.67 | 0 | 44 | 8.2e-05 | 1Score **> 31** indicates **identity** Score **> 15** indicates **homology** | U | R.SEAVVAVLLTK.K |
| 37536 | 225 | – | 236 | 419.9303 | 1256.7691 | 1256.7704 | -1.04 | 1 | 21 | 0.0097 | 1Score **> 30** indicates **identity** Score **> 14** indicates **homology** | U | R.SEAVVAVLLTKK.S |
| 37537 | 225 | – | 236 | 629.3919 | 1256.7693 | 1256.7704 | -0.83 | 1 | 36 | 0.00046 | 1Score **> 30** indicates **identity** Score **> 15** indicates **homology** | U | R.SEAVVAVLLTKK.S |
| 37538 | 225 | – | 236 | 419.9306 | 1256.7700 | 1256.7704 | -0.27 | 1 | 47 | 4.1e-05 | 1Score **> 30** indicates **identity** Score **> 15** indicates **homology** | U | R.SEAVVAVLLTKK.S |
| 37539 | 225 | – | 236 | 419.9306 | 1256.7701 | 1256.7704 | -0.25 | 1 | 32 | 0.0011 | 1Score **> 30** indicates **identity** Score **> 14** indicates **homology** | U | R.SEAVVAVLLTKK.S |
| 37540 | 225 | – | 236 | 419.9306 | 1256.7701 | 1256.7704 | -0.24 | 1 | 34 | 0.00061 | 1Score **> 30** indicates **identity** Score **> 15** indicates **homology** | U | R.SEAVVAVLLTKK.S |
| 37541 | 225 | – | 236 | 419.9308 | 1256.7705 | 1256.7704 | 0.12 | 1 | 27 | 0.0028 | 1Score **> 30** indicates **identity** Score **> 14** indicates **homology** | U | R.SEAVVAVLLTKK.S |
| 37542 | 225 | – | 236 | 419.9308 | 1256.7706 | 1256.7704 | 0.17 | 1 | 23 | 0.0069 | 1Score **> 30** indicates **identity** Score **> 14** indicates **homology** | U | R.SEAVVAVLLTKK.S |
| 37543 | 225 | – | 236 | 419.9309 | 1256.7708 | 1256.7704 | 0.31 | 1 | 31 | 0.0012 | 1Score **> 30** indicates **identity** Score **> 14** indicates **homology** | U | R.SEAVVAVLLTKK.S |
| 37544 | 225 | – | 236 | 419.9310 | 1256.7713 | 1256.7704 | 0.71 | 1 | 31 | 0.0012 | 1Score **> 30** indicates **identity** Score **> 14** indicates **homology** | U | R.SEAVVAVLLTKK.S |
| 37545 | 225 | – | 236 | 629.3929 | 1256.7713 | 1256.7704 | 0.73 | 1 | 47 | 4.1e-05 | 1Score **> 30** indicates **identity** Score **> 15** indicates **homology** | U | R.SEAVVAVLLTKK.S |
| 37546 | 225 | – | 236 | 629.3930 | 1256.7715 | 1256.7704 | 0.93 | 1 | 44 | 7.5e-05 | 1Score **> 30** indicates **identity** Score **> 15** indicates **homology** | U | R.SEAVVAVLLTKK.S |
| 37547 | 225 | – | 236 | 629.3932 | 1256.7718 | 1256.7704 | 1.12 | 1 | 48 | 3.2e-05 | 1Score **> 30** indicates **identity** Score **> 15** indicates **homology** | U | R.SEAVVAVLLTKK.S |
| 37548 | 225 | – | 236 | 629.3933 | 1256.7720 | 1256.7704 | 1.26 | 1 | 48 | 3e-05 | 1Score **> 30** indicates **identity** Score **> 16** indicates **homology** | U | R.SEAVVAVLLTKK.S |
| 37549 | 225 | – | 236 | 419.9313 | 1256.7721 | 1256.7704 | 1.34 | 1 | 26 | 0.0034 | 1Score **> 30** indicates **identity** Score **> 14** indicates **homology** | U | R.SEAVVAVLLTKK.S |
| 37550 | 225 | – | 236 | 419.9313 | 1256.7722 | 1256.7704 | 1.44 | 1 | 29 | 0.0019 | 1Score **> 30** indicates **identity** Score **> 14** indicates **homology** | U | R.SEAVVAVLLTKK.S |
| 37551 | 225 | – | 236 | 629.3934 | 1256.7723 | 1256.7704 | 1.51 | 1 | 45 | 5.6e-05 | 1Score **> 30** indicates **identity** Score **> 15** indicates **homology** | U | R.SEAVVAVLLTKK.S |
| 37552 | 225 | – | 236 | 419.9315 | 1256.7728 | 1256.7704 | 1.91 | 1 | 48 | 3.3e-05 | 1Score **> 30** indicates **identity** Score **> 15** indicates **homology** | U | R.SEAVVAVLLTKK.S |
| 37553 | 225 | – | 236 | 419.9316 | 1256.7731 | 1256.7704 | 2.19 | 1 | 48 | 3.1e-05 | 1Score **> 30** indicates **identity** Score **> 16** indicates **homology** | U | R.SEAVVAVLLTKK.S |
| 37554 | 225 | – | 236 | 629.3939 | 1256.7732 | 1256.7704 | 2.26 | 1 | 47 | 3.9e-05 | 1Score **> 30** indicates **identity** Score **> 15** indicates **homology** | U | R.SEAVVAVLLTKK.S |
| 37555 | 225 | – | 236 | 419.9322 | 1256.7747 | 1256.7704 | 3.45 | 1 | 65 | 7.9e-07 | 1Score **> 30** indicates **identity** Score **> 17** indicates **homology** | U | R.SEAVVAVLLTKK.S |
| 37556 | 225 | – | 236 | 419.9329 | 1256.7767 | 1256.7704 | 5.07 | 1 | 25 | 0.0043 | 1Score **> 30** indicates **identity** Score **> 14** indicates **homology** | U | R.SEAVVAVLLTKK.S |
| 198952 | 241 | – | 298 | 1240.2177 | 6196.0523 | 6196.0223 | 4.85 | 2 | 25 | 0.0049 | 1Score **> 34** indicates **identity** Score **> 14** indicates **homology** | U | R.RVYATILNAGTNTDGSKEQGVTFPSGEVQEQLICSLYQPAGLAPESLEYIEAHGTGTK.V  + Deamidated (NQ) |
| 198953 | 241 | – | 298 | 1240.2195 | 6196.0613 | 6196.0223 | 6.31 | 2 | 19 | 0.016 | 1Score **> 34** indicates **identity** Score **> 14** indicates **homology** | U | R.RVYATILNAGTNTDGSKEQGVTFPSGEVQEQLICSLYQPAGLAPESLEYIEAHGTGTK.V  + Deamidated (NQ) |
| 41806 | 299 | – | 310 | 649.8384 | 1297.6622 | 1297.6626 | -0.36 | 0 | 61 | 2.2e-06 | 1Score **> 32** indicates **identity** Score **> 17** indicates **homology** | U | K.VGDPQELNGITR.S |
| 41809 | 299 | – | 310 | 649.8387 | 1297.6628 | 1297.6626 | 0.12 | 0 | 91 | 1.1e-08 | 1Score **> 32** indicates **identity** Score **> 24** indicates **homology** | U | K.VGDPQELNGITR.S |
| 41810 | 299 | – | 310 | 649.8389 | 1297.6633 | 1297.6626 | 0.49 | 0 | 88 | 6.8e-09 | 1Score **> 32** indicates **identity** Score **> 19** indicates **homology** | U | K.VGDPQELNGITR.S |
| 41942 | 299 | – | 310 | 650.3284 | 1298.6422 | 1298.6466 | -3.43 | 0 | 74 | 1.4e-06 | 1Score **> 31** indicates **identity** Score **> 28** indicates **homology** | U | K.VGDPQELNGITR.S  + Deamidated (NQ) |
| 41944 | 299 | – | 310 | 650.3293 | 1298.6441 | 1298.6466 | -1.96 | 0 | 50 | 4.6e-05 | 1Score **> 31** indicates **identity** Score **> 19** indicates **homology** | U | K.VGDPQELNGITR.S  + Deamidated (NQ) |
| 41945 | 299 | – | 310 | 650.3304 | 1298.6463 | 1298.6466 | -0.27 | 0 | 84 | 8.2e-08 | 1Score **> 32** indicates **identity** Score **> 25** indicates **homology** | U | K.VGDPQELNGITR.S  + Deamidated (NQ) |
| 41946 | 299 | – | 310 | 650.3307 | 1298.6469 | 1298.6466 | 0.22 | 0 | 70 | 1.5e-06 | 1Score **> 32** indicates **identity** Score **> 24** indicates **homology** | U | K.VGDPQELNGITR.S  + Deamidated (NQ) |
| 41948 | 299 | – | 310 | 650.3310 | 1298.6474 | 1298.6466 | 0.58 | 0 | 74 | 3.6e-07 | 1Score **> 32** indicates **identity** Score **> 22** indicates **homology** | U | K.VGDPQELNGITR.S  + Deamidated (NQ) |
| 41950 | 299 | – | 310 | 650.3315 | 1298.6485 | 1298.6466 | 1.46 | 0 | 71 | 1e-06 | 1Score **> 32** indicates **identity** Score **> 24** indicates **homology** | U | K.VGDPQELNGITR.S  + Deamidated (NQ) |
| 41951 | 299 | – | 310 | 650.3327 | 1298.6508 | 1298.6466 | 3.23 | 0 | 45 | 0.00013 | 1Score **> 32** indicates **identity** Score **> 19** indicates **homology** | U | K.VGDPQELNGITR.S  + Deamidated (NQ) |
| 15077 | 317 | – | 326 | 514.3110 | 1026.6074 | 1026.6073 | 0.076 | 0 | 41 | 0.00014 | 1Score **> 28** indicates **identity** Score **> 15** indicates **homology** | U | R.QAPLLIGSTK.S |
| 15078 | 317 | – | 326 | 514.3110 | 1026.6075 | 1026.6073 | 0.12 | 0 | 36 | 0.00039 | 1Score **> 28** indicates **identity** Score **> 15** indicates **homology** | U | R.QAPLLIGSTK.S |
| 15079 | 317 | – | 326 | 514.3111 | 1026.6076 | 1026.6073 | 0.23 | 0 | 41 | 0.00014 | 1Score **> 28** indicates **identity** Score **> 15** indicates **homology** | U | R.QAPLLIGSTK.S |
| 15080 | 317 | – | 326 | 514.3112 | 1026.6078 | 1026.6073 | 0.41 | 0 | 38 | 0.00029 | 1Score **> 28** indicates **identity** Score **> 15** indicates **homology** | U | R.QAPLLIGSTK.S |
| 88751 | 327 | – | 343 | 560.9506 | 1679.8300 | 1679.8301 | -0.022 | 0 | 69 | 3.6e-07 | 1Score **> 34** indicates **identity** Score **> 17** indicates **homology** | U | K.SNMGHPEPASGLAALTK.V |
| 88752 | 327 | – | 343 | 560.9507 | 1679.8304 | 1679.8301 | 0.20 | 0 | 53 | 1.1e-05 | 1Score **> 34** indicates **identity** Score **> 16** indicates **homology** | U | K.SNMGHPEPASGLAALTK.V |
| 88755 | 327 | – | 343 | 840.9245 | 1679.8344 | 1679.8301 | 2.56 | 0 | 38 | 0.00027 | 1Score **> 34** indicates **identity** Score **> 15** indicates **homology** | U | K.SNMGHPEPASGLAALTK.V |
| 188345 | 344 | – | 373 | 839.6988 | 3354.7663 | 3354.7625 | 1.11 | 0 | 42 | 0.00012 | 1Score **> 37** indicates **identity** Score **> 15** indicates **homology** | U | K.VLLSLEHGVWAPNLHFHNPNPEIPALLDGR.L |
| 188346 | 344 | – | 373 | 671.9605 | 3354.7663 | 3354.7625 | 1.13 | 0 | 15 | 0.04 | 1Score **> 37** indicates **identity** Score **> 13** indicates **homology** | U | K.VLLSLEHGVWAPNLHFHNPNPEIPALLDGR.L |
| 188349 | 344 | – | 373 | 671.9615 | 3354.7713 | 3354.7625 | 2.62 | 0 | 15 | 0.042 | 1Score **> 36** indicates **identity** Score **> 13** indicates **homology** | U | K.VLLSLEHGVWAPNLHFHNPNPEIPALLDGR.L |
| 188350 | 344 | – | 373 | 1119.2658 | 3354.7756 | 3354.7625 | 3.90 | 0 | 20 | 0.013 | 1Score **> 36** indicates **identity** Score **> 14** indicates **homology** | U | K.VLLSLEHGVWAPNLHFHNPNPEIPALLDGR.L |
| 41138 | 374 | – | 384 | 431.2664 | 1290.7773 | 1290.7772 | 0.075 | 1 | 23 | 0.0067 | 1Score **> 29** indicates **identity** Score **> 14** indicates **homology** | U | R.LQVVDRPLPVR.G |
| 41139 | 374 | – | 384 | 431.2664 | 1290.7775 | 1290.7772 | 0.23 | 1 | 17 | 0.023 | 1Score **> 29** indicates **identity** Score **> 14** indicates **homology** | U | R.LQVVDRPLPVR.G |
| 41142 | 374 | – | 384 | 646.3963 | 1290.7781 | 1290.7772 | 0.70 | 1 | 29 | 0.0018 | 1Score **> 29** indicates **identity** Score **> 14** indicates **homology** | U | R.LQVVDRPLPVR.G |
| 41143 | 374 | – | 384 | 646.3964 | 1290.7782 | 1290.7772 | 0.75 | 1 | 32 | 0.0011 | 1Score **> 29** indicates **identity** Score **> 14** indicates **homology** | U | R.LQVVDRPLPVR.G |
| 41144 | 374 | – | 384 | 431.2667 | 1290.7782 | 1290.7772 | 0.76 | 1 | 17 | 0.029 | 1Score **> 30** indicates **identity** Score **> 14** indicates **homology** | U | R.LQVVDRPLPVR.G |
| 41146 | 374 | – | 384 | 431.2667 | 1290.7783 | 1290.7772 | 0.85 | 1 | 17 | 0.024 | 1Score **> 30** indicates **identity** Score **> 14** indicates **homology** | U | R.LQVVDRPLPVR.G |
| 41147 | 374 | – | 384 | 431.2668 | 1290.7785 | 1290.7772 | 0.98 | 1 | 18 | 0.019 | 1Score **> 30** indicates **identity** Score **> 14** indicates **homology** | U | R.LQVVDRPLPVR.G |
| 193498 | 374 | – | 409 | 954.2711 | 3813.0553 | 3813.0551 | 0.064 | 2 | 18 | 0.023 | 1Score **> 34** indicates **identity** Score **> 14** indicates **homology** | U | R.LQVVDRPLPVRGGNVGINSFGFGGSNVHVILQPNTR.Q |
| 193499 | 374 | – | 409 | 763.6185 | 3813.0563 | 3813.0551 | 0.31 | 2 | 18 | 0.021 | 1Score **> 34** indicates **identity** Score **> 14** indicates **homology** | U | R.LQVVDRPLPVRGGNVGINSFGFGGSNVHVILQPNTR.Q |
| 163026 | 385 | – | 409 | 847.7647 | 2540.2724 | 2540.2885 | -6.32 | 0 | 15 | 0.037 | 1Score **> 37** indicates **identity** Score **> 13** indicates **homology** | U | R.GGNVGINSFGFGGSNVHVILQPNTR.Q |
| 163028 | 385 | – | 409 | 847.7691 | 2540.2855 | 2540.2885 | -1.15 | 0 | 22 | 0.0081 | 1Score **> 37** indicates **identity** Score **> 14** indicates **homology** | U | R.GGNVGINSFGFGGSNVHVILQPNTR.Q |
| 163033 | 385 | – | 409 | 847.7708 | 2540.2907 | 2540.2885 | 0.89 | 0 | 85 | 1.1e-08 | 1Score **> 37** indicates **identity** Score **> 18** indicates **homology** | U | R.GGNVGINSFGFGGSNVHVILQPNTR.Q |
| 163034 | 385 | – | 409 | 847.7712 | 2540.2916 | 2540.2885 | 1.25 | 0 | 60 | 2.2e-06 | 1Score **> 37** indicates **identity** Score **> 16** indicates **homology** | U | R.GGNVGINSFGFGGSNVHVILQPNTR.Q |
| 163036 | 385 | – | 409 | 1271.1542 | 2540.2938 | 2540.2885 | 2.10 | 0 | 111 | 4.3e-11 | 1Score **> 37** indicates **identity** Score **> 19** indicates **homology** | U | R.GGNVGINSFGFGGSNVHVILQPNTR.Q |
| 163038 | 385 | – | 409 | 847.7722 | 2540.2947 | 2540.2885 | 2.46 | 0 | 56 | 5.7e-06 | 1Score **> 37** indicates **identity** Score **> 16** indicates **homology** | U | R.GGNVGINSFGFGGSNVHVILQPNTR.Q |
| 163039 | 385 | – | 409 | 847.7723 | 2540.2952 | 2540.2885 | 2.66 | 0 | 90 | 3.8e-09 | 1Score **> 37** indicates **identity** Score **> 18** indicates **homology** | U | R.GGNVGINSFGFGGSNVHVILQPNTR.Q |
| 163040 | 385 | – | 409 | 847.7724 | 2540.2952 | 2540.2885 | 2.67 | 0 | 35 | 0.00051 | 1Score **> 37** indicates **identity** Score **> 15** indicates **homology** | U | R.GGNVGINSFGFGGSNVHVILQPNTR.Q |
| 163041 | 385 | – | 409 | 847.7725 | 2540.2955 | 2540.2885 | 2.79 | 0 | 20 | 0.015 | 1Score **> 37** indicates **identity** Score **> 14** indicates **homology** | U | R.GGNVGINSFGFGGSNVHVILQPNTR.Q |
| 163042 | 385 | – | 409 | 1271.1552 | 2540.2958 | 2540.2885 | 2.91 | 0 | 61 | 1.7e-06 | 1Score **> 37** indicates **identity** Score **> 16** indicates **homology** | U | R.GGNVGINSFGFGGSNVHVILQPNTR.Q |
| 163043 | 385 | – | 409 | 847.7726 | 2540.2961 | 2540.2885 | 2.99 | 0 | 17 | 0.025 | 1Score **> 37** indicates **identity** Score **> 14** indicates **homology** | U | R.GGNVGINSFGFGGSNVHVILQPNTR.Q |
| 163044 | 385 | – | 409 | 847.7728 | 2540.2964 | 2540.2885 | 3.14 | 0 | 86 | 9.2e-09 | 1Score **> 37** indicates **identity** Score **> 18** indicates **homology** | U | R.GGNVGINSFGFGGSNVHVILQPNTR.Q |
| 163045 | 385 | – | 409 | 847.7728 | 2540.2967 | 2540.2885 | 3.23 | 0 | 38 | 0.00029 | 1Score **> 37** indicates **identity** Score **> 15** indicates **homology** | U | R.GGNVGINSFGFGGSNVHVILQPNTR.Q |
| 163046 | 385 | – | 409 | 847.7735 | 2540.2986 | 2540.2885 | 4.00 | 0 | 71 | 2.3e-07 | 1Score **> 37** indicates **identity** Score **> 17** indicates **homology** | U | R.GGNVGINSFGFGGSNVHVILQPNTR.Q |
| 163047 | 385 | – | 409 | 847.7735 | 2540.2988 | 2540.2885 | 4.07 | 0 | 69 | 3.1e-07 | 1Score **> 37** indicates **identity** Score **> 17** indicates **homology** | U | R.GGNVGINSFGFGGSNVHVILQPNTR.Q |
| 163049 | 385 | – | 409 | 847.7738 | 2540.2996 | 2540.2885 | 4.39 | 0 | 69 | 3.2e-07 | 1Score **> 37** indicates **identity** Score **> 17** indicates **homology** | U | R.GGNVGINSFGFGGSNVHVILQPNTR.Q |
| 163050 | 385 | – | 409 | 1271.1571 | 2540.2997 | 2540.2885 | 4.42 | 0 | 93 | 1.9e-09 | 1Score **> 37** indicates **identity** Score **> 18** indicates **homology** | U | R.GGNVGINSFGFGGSNVHVILQPNTR.Q |
| 163051 | 385 | – | 409 | 847.7742 | 2540.3008 | 2540.2885 | 4.85 | 0 | 35 | 0.00055 | 1Score **> 37** indicates **identity** Score **> 15** indicates **homology** | U | R.GGNVGINSFGFGGSNVHVILQPNTR.Q |
| 163052 | 385 | – | 409 | 847.7742 | 2540.3008 | 2540.2885 | 4.87 | 0 | 33 | 0.00072 | 1Score **> 37** indicates **identity** Score **> 15** indicates **homology** | U | R.GGNVGINSFGFGGSNVHVILQPNTR.Q |
| 163053 | 385 | – | 409 | 847.7744 | 2540.3014 | 2540.2885 | 5.08 | 0 | 28 | 0.0025 | 1Score **> 37** indicates **identity** Score **> 14** indicates **homology** | U | R.GGNVGINSFGFGGSNVHVILQPNTR.Q |
| 163054 | 385 | – | 409 | 847.7748 | 2540.3025 | 2540.2885 | 5.55 | 0 | 15 | 0.037 | 1Score **> 37** indicates **identity** Score **> 13** indicates **homology** | U | R.GGNVGINSFGFGGSNVHVILQPNTR.Q |
| 163055 | 385 | – | 409 | 847.7760 | 2540.3061 | 2540.2885 | 6.94 | 0 | 27 | 0.0028 | 1Score **> 37** indicates **identity** Score **> 14** indicates **homology** | U | R.GGNVGINSFGFGGSNVHVILQPNTR.Q |
| 163093 | 385 | – | 409 | 848.1035 | 2541.2887 | 2541.2725 | 6.37 | 0 | 32 | 0.001 | 1Score **> 37** indicates **identity** Score **> 14** indicates **homology** | U | R.GGNVGINSFGFGGSNVHVILQPNTR.Q  + Deamidated (NQ) |
| 163098 | 385 | – | 409 | 848.1055 | 2541.2946 | 2541.2725 | 8.70 | 0 | 29 | 0.0021 | 1Score **> 37** indicates **identity** Score **> 14** indicates **homology** | U | R.GGNVGINSFGFGGSNVHVILQPNTR.Q  + Deamidated (NQ) |
| 163099 | 385 | – | 409 | 848.1063 | 2541.2971 | 2541.2725 | 9.69 | 0 | 15 | 0.044 | 1Score **> 37** indicates **identity** Score **> 13** indicates **homology** | U | R.GGNVGINSFGFGGSNVHVILQPNTR.Q  + Deamidated (NQ) |
| 124685 | 410 | – | 429 | 672.7013 | 2015.0821 | 2015.0813 | 0.40 | 0 | 24 | 0.0055 | 1Score **> 36** indicates **identity** Score **> 14** indicates **homology** | U | R.QAPAPTAHAALPHLLHASGR.T |
| 124686 | 410 | – | 429 | 404.0238 | 2015.0824 | 2015.0813 | 0.57 | 0 | 39 | 0.0002 | 1Score **> 36** indicates **identity** Score **> 15** indicates **homology** | U | R.QAPAPTAHAALPHLLHASGR.T |
| 124687 | 410 | – | 429 | 504.7780 | 2015.0829 | 2015.0813 | 0.78 | 0 | 50 | 1.9e-05 | 1Score **> 36** indicates **identity** Score **> 16** indicates **homology** | U | R.QAPAPTAHAALPHLLHASGR.T |
| 124688 | 410 | – | 429 | 404.0240 | 2015.0834 | 2015.0813 | 1.04 | 0 | 40 | 0.00018 | 1Score **> 36** indicates **identity** Score **> 15** indicates **homology** | U | R.QAPAPTAHAALPHLLHASGR.T |
| 124689 | 410 | – | 429 | 672.7018 | 2015.0834 | 2015.0813 | 1.05 | 0 | 35 | 0.00058 | 1Score **> 36** indicates **identity** Score **> 15** indicates **homology** | U | R.QAPAPTAHAALPHLLHASGR.T |
| 124793 | 410 | – | 429 | 1009.0485 | 2016.0825 | 2016.0653 | 8.51 | 0 | 18 | 0.02 | 1Score **> 36** indicates **identity** Score **> 14** indicates **homology** | U | R.QAPAPTAHAALPHLLHASGR.T  + Deamidated (NQ) |
| 60556 | 430 | – | 442 | 736.3884 | 1470.7622 | 1470.7678 | -3.84 | 0 | 56 | 5.3e-06 | 1Score **> 34** indicates **identity** Score **> 16** indicates **homology** | U | R.TLEAVQDLLEQGR.Q |
| 60558 | 430 | – | 442 | 736.3900 | 1470.7654 | 1470.7678 | -1.62 | 0 | 29 | 0.0019 | 1Score **> 34** indicates **identity** Score **> 14** indicates **homology** | U | R.TLEAVQDLLEQGR.Q |
| 60559 | 430 | – | 442 | 736.3905 | 1470.7665 | 1470.7678 | -0.92 | 0 | 40 | 0.00017 | 1Score **> 34** indicates **identity** Score **> 15** indicates **homology** | U | R.TLEAVQDLLEQGR.Q |
| 60560 | 430 | – | 442 | 736.3907 | 1470.7669 | 1470.7678 | -0.62 | 0 | 72 | 7.3e-07 | 1Score **> 34** indicates **identity** Score **> 23** indicates **homology** | U | R.TLEAVQDLLEQGR.Q |
| 60563 | 430 | – | 442 | 736.3913 | 1470.7680 | 1470.7678 | 0.13 | 0 | 71 | 5.8e-06 | 1Score **> 34** indicates **identity** Score **> 31** indicates **homology** | U | R.TLEAVQDLLEQGR.Q |
| 60564 | 430 | – | 442 | 736.3913 | 1470.7680 | 1470.7678 | 0.13 | 0 | 79 | 2.1e-07 | 1Score **> 34** indicates **identity** Score **> 25** indicates **homology** | U | R.TLEAVQDLLEQGR.Q |
| 60565 | 430 | – | 442 | 736.3915 | 1470.7685 | 1470.7678 | 0.47 | 0 | 46 | 5.3e-05 | 1Score **> 34** indicates **identity** Score **> 15** indicates **homology** | U | R.TLEAVQDLLEQGR.Q |
| 60566 | 430 | – | 442 | 736.3919 | 1470.7692 | 1470.7678 | 0.97 | 0 | 76 | 3.8e-07 | 1Score **> 34** indicates **identity** Score **> 24** indicates **homology** | U | R.TLEAVQDLLEQGR.Q |
| 60567 | 430 | – | 442 | 736.3920 | 1470.7695 | 1470.7678 | 1.13 | 0 | 40 | 0.00017 | 1Score **> 34** indicates **identity** Score **> 15** indicates **homology** | U | R.TLEAVQDLLEQGR.Q |
| 60569 | 430 | – | 442 | 736.3921 | 1470.7697 | 1470.7678 | 1.29 | 0 | 33 | 0.00081 | 1Score **> 34** indicates **identity** Score **> 15** indicates **homology** | U | R.TLEAVQDLLEQGR.Q |
| 60571 | 430 | – | 442 | 736.3923 | 1470.7700 | 1470.7678 | 1.51 | 0 | 48 | 2.9e-05 | 1Score **> 34** indicates **identity** Score **> 16** indicates **homology** | U | R.TLEAVQDLLEQGR.Q |
| 60572 | 430 | – | 442 | 736.3923 | 1470.7701 | 1470.7678 | 1.54 | 0 | 30 | 0.0016 | 1Score **> 34** indicates **identity** Score **> 14** indicates **homology** | U | R.TLEAVQDLLEQGR.Q |
| 60577 | 430 | – | 442 | 736.3941 | 1470.7736 | 1470.7678 | 3.94 | 0 | 44 | 8.3e-05 | 1Score **> 34** indicates **identity** Score **> 15** indicates **homology** | U | R.TLEAVQDLLEQGR.Q |
| 60592 | 430 | – | 442 | 736.3957 | 1470.7769 | 1470.7678 | 6.21 | 0 | 27 | 0.003 | 1Score **> 34** indicates **identity** Score **> 14** indicates **homology** | U | R.TLEAVQDLLEQGR.Q |
| 60595 | 430 | – | 442 | 736.3964 | 1470.7782 | 1470.7678 | 7.04 | 0 | 42 | 0.00011 | 1Score **> 34** indicates **identity** Score **> 15** indicates **homology** | U | R.TLEAVQDLLEQGR.Q |
| 174918 | 443 | – | 468 | 945.1348 | 2832.3826 | 2832.3575 | 8.87 | 0 | 35 | 0.00054 | 1Score **> 37** indicates **identity** Score **> 15** indicates **homology** | U | R.QHSQDLAFVSMLNDIAATPTAAMPFR.G  + Deamidated (NQ) |
| 16699 | 469 | – | 478 | 525.7827 | 1049.5508 | 1049.5506 | 0.18 | 0 | 56 | 4.1e-05 | 1Score **> 31** indicates **identity** Score **> 25** indicates **homology** | U | R.GYTVLGVEGR.V |
| 16701 | 469 | – | 478 | 525.7828 | 1049.5510 | 1049.5506 | 0.39 | 0 | 55 | 4e-05 | 1Score **> 31** indicates **identity** Score **> 24** indicates **homology** | U | R.GYTVLGVEGR.V |
| 16704 | 469 | – | 478 | 525.7828 | 1049.5511 | 1049.5506 | 0.49 | 0 | 19 | 0.016 | 1Score **> 31** indicates **identity** Score **> 14** indicates **homology** | U | R.GYTVLGVEGR.V |
| 16708 | 469 | – | 478 | 525.7830 | 1049.5514 | 1049.5506 | 0.84 | 0 | 47 | 0.00031 | 1Score **> 32** indicates **identity** Score **> 24** indicates **homology** | U | R.GYTVLGVEGR.V |
| 16715 | 469 | – | 478 | 525.7834 | 1049.5523 | 1049.5506 | 1.68 | 0 | 43 | 0.00036 | 1Score **> 32** indicates **identity** Score **> 21** indicates **homology** | U | R.GYTVLGVEGR.V |
| 16718 | 469 | – | 478 | 525.7850 | 1049.5554 | 1049.5506 | 4.60 | 0 | 40 | 0.00055 | 1Score **> 32** indicates **identity** Score **> 20** indicates **homology** | U | R.GYTVLGVEGR.V |
| 37706 | 479 | – | 489 | 630.3318 | 1258.6491 | 1258.6517 | -2.07 | 0 | 61 | 7e-06 | 1Score **> 34** indicates **identity** Score **> 22** indicates **homology** | U | R.VQEVQQVSTNK.R |
| 183497 | 479 | – | 504 | 784.6462 | 3134.5559 | 3134.5542 | 0.52 | 2 | 28 | 0.0021 | 1Score **> 37** indicates **identity** Score **> 14** indicates **homology** | U | R.VQEVQQVSTNKRPLWFICSGMGTQWR.G |
| 183499 | 479 | – | 504 | 784.6471 | 3134.5593 | 3134.5542 | 1.60 | 2 | 28 | 0.0025 | 1Score **> 37** indicates **identity** Score **> 14** indicates **homology** | U | R.VQEVQQVSTNKRPLWFICSGMGTQWR.G |
| 183548 | 479 | – | 504 | 784.8973 | 3135.5602 | 3135.5383 | 7.00 | 2 | 24 | 0.0056 | 1Score **> 37** indicates **identity** Score **> 14** indicates **homology** | U | R.VQEVQQVSTNKRPLWFICSGMGTQWR.G  + Deamidated (NQ) |
| 112813 | 490 | – | 504 | 632.3104 | 1893.9095 | 1893.9131 | -1.89 | 1 | 27 | 0.0031 | 1Score **> 34** indicates **identity** Score **> 14** indicates **homology** | U | K.RPLWFICSGMGTQWR.G |
| 112815 | 490 | – | 504 | 632.3121 | 1893.9145 | 1893.9131 | 0.78 | 1 | 36 | 0.00042 | 1Score **> 35** indicates **identity** Score **> 15** indicates **homology** | U | K.RPLWFICSGMGTQWR.G |
| 6079 | 505 | – | 512 | 432.7250 | 863.4355 | 863.4357 | -0.30 | 0 | 20 | 0.013 | 1Score **> 28** indicates **identity** Score **> 14** indicates **homology** | U | R.GMGLSLMR.L |
| 35189 | 513 | – | 522 | 412.5626 | 1234.6660 | 1234.6670 | -0.74 | 1 | 34 | 0.0049 | 1Score **> 33** indicates **identity** Score **> 23** indicates **homology** | U | R.LDSFRESILR.S |
| 35191 | 513 | – | 522 | 618.3406 | 1234.6667 | 1234.6670 | -0.23 | 1 | 34 | 0.027 | 1Score **> 33** indicates **identity** Score **> 31** indicates **homology** | U | R.LDSFRESILR.S |
| 25267 | 523 | – | 533 | 381.5520 | 1141.6343 | 1141.6343 | 0.021 | 1 | 35 | 0.00078 | 1Score **> 32** indicates **identity** Score **> 16** indicates **homology** | U | R.SDEAVKPLGVK.V |
| 25268 | 523 | – | 533 | 571.8246 | 1141.6347 | 1141.6343 | 0.35 | 1 | 39 | 0.00039 | 1Score **> 32** indicates **identity** Score **> 17** indicates **homology** | U | R.SDEAVKPLGVK.V |
| 25269 | 523 | – | 533 | 381.5524 | 1141.6355 | 1141.6343 | 1.05 | 1 | 41 | 0.00016 | 1Score **> 32** indicates **identity** Score **> 15** indicates **homology** | U | R.SDEAVKPLGVK.V |
| 25270 | 523 | – | 533 | 571.8251 | 1141.6356 | 1141.6343 | 1.18 | 1 | 69 | 3.4e-06 | 1Score **> 32** indicates **identity** Score **> 26** indicates **homology** | U | R.SDEAVKPLGVK.V |
| 36346 | 534 | – | 544 | 624.3268 | 1246.6391 | 1246.6405 | -1.07 | 0 | 70 | 7.9e-07 | 1Score **> 33** indicates **identity** Score **> 21** indicates **homology** | U | K.VSDLLLSTDER.T |
| 36353 | 534 | – | 544 | 624.3289 | 1246.6433 | 1246.6405 | 2.24 | 0 | 61 | 8.9e-06 | 1Score **> 34** indicates **identity** Score **> 23** indicates **homology** | U | K.VSDLLLSTDER.T |
| 36354 | 534 | – | 544 | 624.3291 | 1246.6436 | 1246.6405 | 2.51 | 0 | 70 | 7.1e-07 | 1Score **> 34** indicates **identity** Score **> 21** indicates **homology** | U | K.VSDLLLSTDER.T |
| 19117 | 598 | – | 606 | 539.7872 | 1077.5599 | 1077.5607 | -0.77 | 0 | 41 | 0.00015 | 1Score **> 33** indicates **identity** Score **> 15** indicates **homology** | U | R.EAVLAAYWR.G |
| 19118 | 598 | – | 606 | 539.7877 | 1077.5608 | 1077.5607 | 0.049 | 0 | 36 | 0.00043 | 1Score **> 33** indicates **identity** Score **> 15** indicates **homology** | U | R.EAVLAAYWR.G |
| 19119 | 598 | – | 606 | 539.7878 | 1077.5610 | 1077.5607 | 0.28 | 0 | 34 | 0.00064 | 1Score **> 32** indicates **identity** Score **> 15** indicates **homology** | U | R.EAVLAAYWR.G |
| 19120 | 598 | – | 606 | 539.7878 | 1077.5611 | 1077.5607 | 0.36 | 0 | 59 | 1.7e-05 | 1Score **> 32** indicates **identity** Score **> 23** indicates **homology** | U | R.EAVLAAYWR.G |
| 19121 | 598 | – | 606 | 539.7880 | 1077.5614 | 1077.5607 | 0.61 | 0 | 68 | 4.5e-06 | 1Score **> 32** indicates **identity** Score **> 27** indicates **homology** | U | R.EAVLAAYWR.G |
| 19122 | 598 | – | 606 | 539.7881 | 1077.5616 | 1077.5607 | 0.85 | 0 | 59 | 1.7e-05 | 1Score **> 32** indicates **identity** Score **> 24** indicates **homology** | U | R.EAVLAAYWR.G |
| 19123 | 598 | – | 606 | 539.7884 | 1077.5622 | 1077.5607 | 1.41 | 0 | 27 | 0.0028 | 1Score **> 32** indicates **identity** Score **> 14** indicates **homology** | U | R.EAVLAAYWR.G |
| 19124 | 598 | – | 606 | 539.7885 | 1077.5625 | 1077.5607 | 1.64 | 0 | 39 | 0.00023 | 1Score **> 32** indicates **identity** Score **> 15** indicates **homology** | U | R.EAVLAAYWR.G |
| 19127 | 598 | – | 606 | 539.7893 | 1077.5640 | 1077.5607 | 3.10 | 0 | 27 | 0.0027 | 1Score **> 33** indicates **identity** Score **> 14** indicates **homology** | U | R.EAVLAAYWR.G |
| 195616 | 634 | – | 673 | 1428.6983 | 4283.0731 | 4283.0627 | 2.41 | 1 | 58 | 3.6e-06 | 1Score **> 37** indicates **identity** Score **> 16** indicates **homology** | U | R.CPAGVVPACHNSEDTVTISGPQAAVNEFVEQLKQEGVFAK.E |
| 195617 | 634 | – | 673 | 1071.7770 | 4283.0790 | 4283.0627 | 3.80 | 1 | 50 | 2.2e-05 | 1Score **> 37** indicates **identity** Score **> 16** indicates **homology** | U | R.CPAGVVPACHNSEDTVTISGPQAAVNEFVEQLKQEGVFAK.E |
| 195618 | 634 | – | 673 | 1428.7035 | 4283.0887 | 4283.0627 | 6.06 | 1 | 23 | 0.0071 | 1Score **> 37** indicates **identity** Score **> 14** indicates **homology** | U | R.CPAGVVPACHNSEDTVTISGPQAAVNEFVEQLKQEGVFAK.E |
| 159136 | 677 | – | 699 | 822.4358 | 2464.2857 | 2464.2824 | 1.33 | 0 | 65 | 8.9e-07 | 1Score **> 37** indicates **identity** Score **> 17** indicates **homology** | U | R.TGGLAFHSYFMEGIAPTLLQALK.K |
| 165488 | 677 | – | 700 | 865.1349 | 2592.3829 | 2592.3774 | 2.14 | 1 | 22 | 0.0095 | 1Score **> 36** indicates **identity** Score **> 14** indicates **homology** | U | R.TGGLAFHSYFMEGIAPTLLQALKK.V |
| 165489 | 677 | – | 700 | 649.1040 | 2592.3867 | 2592.3774 | 3.60 | 1 | 23 | 0.0077 | 1Score **> 36** indicates **identity** Score **> 14** indicates **homology** | U | R.TGGLAFHSYFMEGIAPTLLQALKK.V |
| 165490 | 677 | – | 700 | 865.1362 | 2592.3867 | 2592.3774 | 3.60 | 1 | 17 | 0.028 | 1Score **> 36** indicates **identity** Score **> 14** indicates **homology** | U | R.TGGLAFHSYFMEGIAPTLLQALKK.V |
| 119154 | 712 | – | 728 | 980.4949 | 1958.9753 | 1958.9850 | -4.95 | 0 | 55 | 1.2e-05 | 1Score **> 35** indicates **identity** Score **> 19** indicates **homology** | U | R.WLSTSIPEAQWQSSLAR.T |
| 119155 | 712 | – | 728 | 980.4975 | 1958.9804 | 1958.9850 | -2.34 | 0 | 59 | 5.1e-05 | 1Score **> 35** indicates **identity** Score **> 29** indicates **homology** | U | R.WLSTSIPEAQWQSSLAR.T |
| 119157 | 712 | – | 728 | 980.4978 | 1958.9810 | 1958.9850 | -2.03 | 0 | 70 | 4.9e-07 | 1Score **> 35** indicates **identity** Score **> 20** indicates **homology** | U | R.WLSTSIPEAQWQSSLAR.T |
| 119159 | 712 | – | 728 | 980.4987 | 1958.9829 | 1958.9850 | -1.08 | 0 | 49 | 0.00029 | 1Score **> 35** indicates **identity** Score **> 26** indicates **homology** | U | R.WLSTSIPEAQWQSSLAR.T |
| 119160 | 712 | – | 728 | 980.4994 | 1958.9842 | 1958.9850 | -0.43 | 0 | 30 | 0.0021 | 1Score **> 35** indicates **identity** Score **> 15** indicates **homology** | U | R.WLSTSIPEAQWQSSLAR.T |
| 119162 | 712 | – | 728 | 980.5006 | 1958.9867 | 1958.9850 | 0.89 | 0 | 18 | 0.021 | 1Score **> 36** indicates **identity** Score **> 14** indicates **homology** | U | R.WLSTSIPEAQWQSSLAR.T |
| 119163 | 712 | – | 728 | 980.5008 | 1958.9870 | 1958.9850 | 1.03 | 0 | 71 | 2.4e-07 | 1Score **> 35** indicates **identity** Score **> 17** indicates **homology** | U | R.WLSTSIPEAQWQSSLAR.T |
| 119164 | 712 | – | 728 | 654.0030 | 1958.9872 | 1958.9850 | 1.13 | 0 | 42 | 0.00011 | 1Score **> 35** indicates **identity** Score **> 15** indicates **homology** | U | R.WLSTSIPEAQWQSSLAR.T |
| 119167 | 712 | – | 728 | 980.5018 | 1958.9891 | 1958.9850 | 2.07 | 0 | 40 | 0.00018 | 1Score **> 35** indicates **identity** Score **> 15** indicates **homology** | U | R.WLSTSIPEAQWQSSLAR.T |
| 119170 | 712 | – | 728 | 980.5020 | 1958.9894 | 1958.9850 | 2.24 | 0 | 33 | 0.00087 | 1Score **> 35** indicates **identity** Score **> 15** indicates **homology** | U | R.WLSTSIPEAQWQSSLAR.T |
| 119171 | 712 | – | 728 | 654.0039 | 1958.9898 | 1958.9850 | 2.47 | 0 | 41 | 0.00013 | 1Score **> 35** indicates **identity** Score **> 15** indicates **homology** | U | R.WLSTSIPEAQWQSSLAR.T |
| 119173 | 712 | – | 728 | 980.5025 | 1958.9905 | 1958.9850 | 2.82 | 0 | 79 | 5.8e-08 | 1Score **> 36** indicates **identity** Score **> 19** indicates **homology** | U | R.WLSTSIPEAQWQSSLAR.T |
| 119177 | 712 | – | 728 | 980.5037 | 1958.9928 | 1958.9850 | 4.00 | 0 | 54 | 8.1e-06 | 1Score **> 36** indicates **identity** Score **> 16** indicates **homology** | U | R.WLSTSIPEAQWQSSLAR.T |
| 119179 | 712 | – | 728 | 980.5046 | 1958.9946 | 1958.9850 | 4.90 | 0 | 71 | 2.1e-07 | 1Score **> 36** indicates **identity** Score **> 17** indicates **homology** | U | R.WLSTSIPEAQWQSSLAR.T |
| 119180 | 712 | – | 728 | 654.0058 | 1958.9957 | 1958.9850 | 5.45 | 0 | 22 | 0.0085 | 1Score **> 36** indicates **identity** Score **> 14** indicates **homology** | U | R.WLSTSIPEAQWQSSLAR.T |
| 119183 | 712 | – | 728 | 980.5078 | 1959.0011 | 1958.9850 | 8.20 | 0 | 52 | 1.4e-05 | 1Score **> 36** indicates **identity** Score **> 16** indicates **homology** | U | R.WLSTSIPEAQWQSSLAR.T |
| 119184 | 712 | – | 728 | 980.5080 | 1959.0015 | 1958.9850 | 8.43 | 0 | 72 | 1.7e-07 | 1Score **> 36** indicates **identity** Score **> 17** indicates **homology** | U | R.WLSTSIPEAQWQSSLAR.T |
| 119248 | 712 | – | 728 | 980.9982 | 1959.9818 | 1959.9690 | 6.53 | 0 | 25 | 0.005 | 1Score **> 36** indicates **identity** Score **> 14** indicates **homology** | U | R.WLSTSIPEAQWQSSLAR.T  + Deamidated (NQ) |
| 26006 | 777 | – | 786 | 575.3033 | 1148.5920 | 1148.5933 | -1.15 | 0 | 23 | 0.0072 | 1Score **> 33** indicates **identity** Score **> 14** indicates **homology** | U | K.SSCTIIPLMK.R |
| 26007 | 777 | – | 786 | 575.3042 | 1148.5938 | 1148.5933 | 0.40 | 0 | 42 | 0.00011 | 1Score **> 34** indicates **identity** Score **> 15** indicates **homology** | U | K.SSCTIIPLMK.R |
| 26012 | 777 | – | 786 | 575.3052 | 1148.5958 | 1148.5933 | 2.11 | 0 | 23 | 0.0069 | 1Score **> 33** indicates **identity** Score **> 14** indicates **homology** | U | K.SSCTIIPLMK.R |
| 117792 | 787 | – | 802 | 487.5066 | 1945.9973 | 1946.0010 | -1.88 | 2 | 31 | 0.0013 | 1Score **> 36** indicates **identity** Score **> 14** indicates **homology** | U | K.RDHKDNLEFFLTNLGK.V |
| 117795 | 787 | – | 802 | 487.5071 | 1945.9993 | 1946.0010 | -0.88 | 2 | 40 | 0.00018 | 1Score **> 36** indicates **identity** Score **> 15** indicates **homology** | U | K.RDHKDNLEFFLTNLGK.V |
| 117797 | 787 | – | 802 | 487.5073 | 1946.0001 | 1946.0010 | -0.47 | 2 | 63 | 1.4e-06 | 1Score **> 36** indicates **identity** Score **> 16** indicates **homology** | U | K.RDHKDNLEFFLTNLGK.V |
| 117802 | 787 | – | 802 | 487.5084 | 1946.0045 | 1946.0010 | 1.82 | 2 | 20 | 0.013 | 1Score **> 36** indicates **identity** Score **> 14** indicates **homology** | U | K.RDHKDNLEFFLTNLGK.V |
| 117804 | 787 | – | 802 | 487.5089 | 1946.0065 | 1946.0010 | 2.81 | 2 | 16 | 0.031 | 1Score **> 36** indicates **identity** Score **> 14** indicates **homology** | U | K.RDHKDNLEFFLTNLGK.V |
| 102063 | 788 | – | 802 | 895.9567 | 1789.8989 | 1789.8999 | -0.57 | 1 | 63 | 1.2e-06 | 1Score **> 35** indicates **identity** Score **> 16** indicates **homology** | U | R.DHKDNLEFFLTNLGK.V |
| 102064 | 788 | – | 802 | 448.4820 | 1789.8990 | 1789.8999 | -0.49 | 1 | 37 | 0.00052 | 1Score **> 35** indicates **identity** Score **> 17** indicates **homology** | U | R.DHKDNLEFFLTNLGK.V |
| 102065 | 788 | – | 802 | 448.4821 | 1789.8992 | 1789.8999 | -0.37 | 1 | 37 | 0.0017 | 1Score **> 35** indicates **identity** Score **> 22** indicates **homology** | U | R.DHKDNLEFFLTNLGK.V |
| 102066 | 788 | – | 802 | 597.6404 | 1789.8994 | 1789.8999 | -0.27 | 1 | 22 | 0.0078 | 1Score **> 35** indicates **identity** Score **> 14** indicates **homology** | U | R.DHKDNLEFFLTNLGK.V |
| 102069 | 788 | – | 802 | 448.4823 | 1789.8999 | 1789.8999 | 0.032 | 1 | 22 | 0.0094 | 1Score **> 35** indicates **identity** Score **> 14** indicates **homology** | U | R.DHKDNLEFFLTNLGK.V |
| 102071 | 788 | – | 802 | 597.6407 | 1789.9003 | 1789.8999 | 0.21 | 1 | 48 | 3.1e-05 | 1Score **> 35** indicates **identity** Score **> 16** indicates **homology** | U | R.DHKDNLEFFLTNLGK.V |
| 102072 | 788 | – | 802 | 597.6408 | 1789.9004 | 1789.8999 | 0.30 | 1 | 49 | 2.7e-05 | 1Score **> 35** indicates **identity** Score **> 16** indicates **homology** | U | R.DHKDNLEFFLTNLGK.V |
| 102073 | 788 | – | 802 | 895.9577 | 1789.9008 | 1789.8999 | 0.53 | 1 | 85 | 1.1e-08 | 1Score **> 35** indicates **identity** Score **> 18** indicates **homology** | U | R.DHKDNLEFFLTNLGK.V |
| 102074 | 788 | – | 802 | 597.6410 | 1789.9013 | 1789.8999 | 0.78 | 1 | 14 | 0.045 | 1Score **> 35** indicates **identity** Score **> 13** indicates **homology** | U | R.DHKDNLEFFLTNLGK.V |
| 102075 | 788 | – | 802 | 448.4827 | 1789.9018 | 1789.8999 | 1.04 | 1 | 19 | 0.016 | 1Score **> 35** indicates **identity** Score **> 14** indicates **homology** | U | R.DHKDNLEFFLTNLGK.V |
| 102078 | 788 | – | 802 | 597.6413 | 1789.9021 | 1789.8999 | 1.26 | 1 | 37 | 0.00032 | 1Score **> 35** indicates **identity** Score **> 15** indicates **homology** | U | R.DHKDNLEFFLTNLGK.V |
| 102080 | 788 | – | 802 | 597.6414 | 1789.9022 | 1789.8999 | 1.31 | 1 | 32 | 0.0011 | 1Score **> 35** indicates **identity** Score **> 14** indicates **homology** | U | R.DHKDNLEFFLTNLGK.V |
| 102081 | 788 | – | 802 | 597.6414 | 1789.9023 | 1789.8999 | 1.33 | 1 | 43 | 8.8e-05 | 1Score **> 35** indicates **identity** Score **> 15** indicates **homology** | U | R.DHKDNLEFFLTNLGK.V |
| 102087 | 788 | – | 802 | 895.9604 | 1789.9062 | 1789.8999 | 3.51 | 1 | 82 | 1.9e-08 | 1Score **> 35** indicates **identity** Score **> 18** indicates **homology** | U | R.DHKDNLEFFLTNLGK.V |
| 53381 | 791 | – | 802 | 705.8653 | 1409.7161 | 1409.7191 | -2.09 | 0 | 59 | 2.7e-06 | 1Score **> 34** indicates **identity** Score **> 16** indicates **homology** | U | K.DNLEFFLTNLGK.V |
| 161031 | 803 | – | 825 | 833.7895 | 2498.3467 | 2498.3434 | 1.30 | 0 | 61 | 1.8e-06 | 1Score **> 36** indicates **identity** Score **> 16** indicates **homology** | U | K.VHLTGINVNPNALFPPVEFPAPR.G |
| 161032 | 803 | – | 825 | 833.7900 | 2498.3481 | 2498.3434 | 1.86 | 0 | 40 | 0.00019 | 1Score **> 36** indicates **identity** Score **> 15** indicates **homology** | U | K.VHLTGINVNPNALFPPVEFPAPR.G |
| 161033 | 803 | – | 825 | 833.7900 | 2498.3482 | 2498.3434 | 1.93 | 0 | 39 | 0.00024 | 1Score **> 36** indicates **identity** Score **> 15** indicates **homology** | U | K.VHLTGINVNPNALFPPVEFPAPR.G |
| 161034 | 803 | – | 825 | 1250.1819 | 2498.3492 | 2498.3434 | 2.30 | 0 | 113 | 2.8e-11 | 1Score **> 36** indicates **identity** Score **> 20** indicates **homology** | U | K.VHLTGINVNPNALFPPVEFPAPR.G |
| 161035 | 803 | – | 825 | 1250.1819 | 2498.3493 | 2498.3434 | 2.35 | 0 | 47 | 3.9e-05 | 1Score **> 36** indicates **identity** Score **> 15** indicates **homology** | U | K.VHLTGINVNPNALFPPVEFPAPR.G |
| 161038 | 803 | – | 825 | 833.7927 | 2498.3563 | 2498.3434 | 5.15 | 0 | 41 | 0.00014 | 1Score **> 36** indicates **identity** Score **> 15** indicates **homology** | U | K.VHLTGINVNPNALFPPVEFPAPR.G |
| 161073 | 803 | – | 825 | 834.1208 | 2499.3404 | 2499.3274 | 5.21 | 0 | 26 | 0.0035 | 1Score **> 36** indicates **identity** Score **> 14** indicates **homology** | U | K.VHLTGINVNPNALFPPVEFPAPR.G  + Deamidated (NQ) |
| 161074 | 803 | – | 825 | 1250.6805 | 2499.3465 | 2499.3274 | 7.63 | 0 | 99 | 5.4e-10 | 1Score **> 36** indicates **identity** Score **> 19** indicates **homology** | U | K.VHLTGINVNPNALFPPVEFPAPR.G  + Deamidated (NQ) |
| 17803 | 826 | – | 835 | 354.8817 | 1061.6234 | 1061.6233 | 0.055 | 0 | 19 | 0.018 | 1Score **> 28** indicates **identity** Score **> 14** indicates **homology** | U | R.GTPLISPHIK.W |
| 17804 | 826 | – | 835 | 354.8818 | 1061.6234 | 1061.6233 | 0.12 | 0 | 25 | 0.0045 | 1Score **> 28** indicates **identity** Score **> 14** indicates **homology** | U | R.GTPLISPHIK.W |
| 17805 | 826 | – | 835 | 354.8818 | 1061.6236 | 1061.6233 | 0.23 | 0 | 22 | 0.0094 | 1Score **> 28** indicates **identity** Score **> 14** indicates **homology** | U | R.GTPLISPHIK.W |
| 17806 | 826 | – | 835 | 354.8819 | 1061.6239 | 1061.6233 | 0.50 | 0 | 25 | 0.0042 | 1Score **> 28** indicates **identity** Score **> 14** indicates **homology** | U | R.GTPLISPHIK.W |
| 17807 | 826 | – | 835 | 354.8819 | 1061.6239 | 1061.6233 | 0.56 | 0 | 19 | 0.015 | 1Score **> 29** indicates **identity** Score **> 14** indicates **homology** | U | R.GTPLISPHIK.W |
| 17808 | 826 | – | 835 | 354.8819 | 1061.6240 | 1061.6233 | 0.61 | 0 | 24 | 0.005 | 1Score **> 29** indicates **identity** Score **> 14** indicates **homology** | U | R.GTPLISPHIK.W |
| 17810 | 826 | – | 835 | 354.8821 | 1061.6246 | 1061.6233 | 1.18 | 0 | 18 | 0.022 | 1Score **> 29** indicates **identity** Score **> 14** indicates **homology** | U | R.GTPLISPHIK.W |
| 198593 | 836 | – | 883 | 1334.1012 | 5332.3758 | 5332.3229 | 9.92 | 0 | 30 | 0.0016 | 1Score **> 30** indicates **identity** Score **> 14** indicates **homology** | U | K.WDHSQTWDVPVAEDFPNGSSSSSATVYSIDASPESPDHYLVDHCIDGR.V |
| 85096 | 884 | – | 897 | 551.6341 | 1651.8806 | 1651.8796 | 0.58 | 0 | 15 | 0.036 | 1Score **> 35** indicates **identity** Score **> 13** indicates **homology** | U | R.VIFPGTGYLCLVWK.T |
| 85097 | 884 | – | 897 | 826.9487 | 1651.8828 | 1651.8796 | 1.89 | 0 | 66 | 5.9e-07 | 1Score **> 35** indicates **identity** Score **> 17** indicates **homology** | U | R.VIFPGTGYLCLVWK.T |
| 85098 | 884 | – | 897 | 826.9494 | 1651.8842 | 1651.8796 | 2.76 | 0 | 70 | 3.4e-07 | 1Score **> 35** indicates **identity** Score **> 18** indicates **homology** | U | R.VIFPGTGYLCLVWK.T |
| 85099 | 884 | – | 897 | 551.6355 | 1651.8846 | 1651.8796 | 2.98 | 0 | 51 | 1.5e-05 | 1Score **> 35** indicates **identity** Score **> 16** indicates **homology** | U | R.VIFPGTGYLCLVWK.T |
| 175627 | 902 | – | 927 | 1428.2632 | 2854.5119 | 2854.5117 | 0.10 | 0 | 85 | 1.2e-08 | 1Score **> 37** indicates **identity** Score **> 18** indicates **homology** | U | R.SLGLSLEETPVVFENVSFHQATILPK.T |
| 175628 | 902 | – | 927 | 952.5119 | 2854.5137 | 2854.5117 | 0.73 | 0 | 48 | 3.1e-05 | 1Score **> 37** indicates **identity** Score **> 16** indicates **homology** | U | R.SLGLSLEETPVVFENVSFHQATILPK.T |
| 10055 | 928 | – | 936 | 473.2675 | 944.5205 | 944.5291 | -9.06 | 0 | 49 | 0.0013 | 1Score **> 33** indicates **identity** | U | K.TGTVALEVR.L |
| 10056 | 928 | – | 936 | 473.2696 | 944.5247 | 944.5291 | -4.67 | 0 | 39 | 0.0018 | 1Score **> 32** indicates **identity** Score **> 24** indicates **homology** | U | K.TGTVALEVR.L |
| 10057 | 928 | – | 936 | 473.2697 | 944.5248 | 944.5291 | -4.55 | 0 | 43 | 0.00062 | 1Score **> 32** indicates **identity** Score **> 23** indicates **homology** | U | K.TGTVALEVR.L |
| 10059 | 928 | – | 936 | 473.2712 | 944.5278 | 944.5291 | -1.34 | 0 | 62 | 3.1e-05 | 1Score **> 31** indicates **identity** Score **> 30** indicates **homology** | U | K.TGTVALEVR.L |
| 10061 | 928 | – | 936 | 473.2719 | 944.5292 | 944.5291 | 0.090 | 0 | 61 | 1.9e-05 | 1Score **> 31** indicates **identity** Score **> 26** indicates **homology** | U | K.TGTVALEVR.L |
| 10062 | 928 | – | 936 | 473.2720 | 944.5295 | 944.5291 | 0.45 | 0 | 76 | 5.8e-07 | 1Score **> 31** indicates **identity** Score **> 26** indicates **homology** | U | K.TGTVALEVR.L |
| 10064 | 928 | – | 936 | 473.2721 | 944.5297 | 944.5291 | 0.59 | 0 | 69 | 4e-06 | 1Score **> 31** indicates **identity** Score **> 27** indicates **homology** | U | K.TGTVALEVR.L |
| 140859 | 937 | – | 957 | 729.7127 | 2186.1162 | 2186.1219 | -2.59 | 0 | 45 | 5.7e-05 | 1Score **> 37** indicates **identity** Score **> 15** indicates **homology** | U | R.LLEASHAFEVSDTGNLIVSGK.V |
| 140861 | 937 | – | 957 | 729.7132 | 2186.1179 | 2186.1219 | -1.84 | 0 | 27 | 0.0029 | 1Score **> 37** indicates **identity** Score **> 14** indicates **homology** | U | R.LLEASHAFEVSDTGNLIVSGK.V |
| 140863 | 937 | – | 957 | 729.7140 | 2186.1201 | 2186.1219 | -0.84 | 0 | 41 | 0.00016 | 1Score **> 37** indicates **identity** Score **> 15** indicates **homology** | U | R.LLEASHAFEVSDTGNLIVSGK.V |
| 140869 | 937 | – | 957 | 729.7149 | 2186.1228 | 2186.1219 | 0.43 | 0 | 15 | 0.043 | 1Score **> 37** indicates **identity** Score **> 13** indicates **homology** | U | R.LLEASHAFEVSDTGNLIVSGK.V |
| 140873 | 937 | – | 957 | 729.7151 | 2186.1236 | 2186.1219 | 0.77 | 0 | 49 | 2.7e-05 | 1Score **> 37** indicates **identity** Score **> 16** indicates **homology** | U | R.LLEASHAFEVSDTGNLIVSGK.V |
| 140876 | 937 | – | 957 | 729.7153 | 2186.1240 | 2186.1219 | 0.95 | 0 | 52 | 1.2e-05 | 1Score **> 37** indicates **identity** Score **> 16** indicates **homology** | U | R.LLEASHAFEVSDTGNLIVSGK.V |
| 140877 | 937 | – | 957 | 729.7154 | 2186.1243 | 2186.1219 | 1.08 | 0 | 45 | 5.4e-05 | 1Score **> 37** indicates **identity** Score **> 15** indicates **homology** | U | R.LLEASHAFEVSDTGNLIVSGK.V |
| 140878 | 937 | – | 957 | 729.7155 | 2186.1246 | 2186.1219 | 1.25 | 0 | 62 | 1.6e-06 | 1Score **> 37** indicates **identity** Score **> 16** indicates **homology** | U | R.LLEASHAFEVSDTGNLIVSGK.V |
| 140881 | 937 | – | 957 | 729.7157 | 2186.1254 | 2186.1219 | 1.59 | 0 | 63 | 1.1e-06 | 1Score **> 37** indicates **identity** Score **> 16** indicates **homology** | U | R.LLEASHAFEVSDTGNLIVSGK.V |
| 140884 | 937 | – | 957 | 729.7160 | 2186.1262 | 2186.1219 | 1.98 | 0 | 50 | 2.2e-05 | 1Score **> 37** indicates **identity** Score **> 16** indicates **homology** | U | R.LLEASHAFEVSDTGNLIVSGK.V |
| 140885 | 937 | – | 957 | 729.7160 | 2186.1263 | 2186.1219 | 1.99 | 0 | 42 | 0.00012 | 1Score **> 37** indicates **identity** Score **> 15** indicates **homology** | U | R.LLEASHAFEVSDTGNLIVSGK.V |
| 140893 | 937 | – | 957 | 729.7168 | 2186.1285 | 2186.1219 | 3.00 | 0 | 16 | 0.031 | 1Score **> 37** indicates **identity** Score **> 14** indicates **homology** | U | R.LLEASHAFEVSDTGNLIVSGK.V |
| 140900 | 937 | – | 957 | 729.7177 | 2186.1313 | 2186.1219 | 4.32 | 0 | 47 | 4.2e-05 | 1Score **> 37** indicates **identity** Score **> 15** indicates **homology** | U | R.LLEASHAFEVSDTGNLIVSGK.V |
| 140910 | 937 | – | 957 | 729.7213 | 2186.1419 | 2186.1219 | 9.16 | 0 | 25 | 0.0049 | 1Score **> 37** indicates **identity** Score **> 14** indicates **homology** | U | R.LLEASHAFEVSDTGNLIVSGK.V |
| 36623 | 958 | – | 967 | 625.8059 | 1249.5972 | 1249.5979 | -0.55 | 0 | 26 | 0.0033 | 1Score **> 32** indicates **identity** Score **> 14** indicates **homology** | U | K.VYLWEDPNSK.L |
| 36624 | 958 | – | 967 | 625.8060 | 1249.5974 | 1249.5979 | -0.42 | 0 | 42 | 0.00012 | 1Score **> 32** indicates **identity** Score **> 15** indicates **homology** | U | K.VYLWEDPNSK.L |
| 36625 | 958 | – | 967 | 625.8061 | 1249.5977 | 1249.5979 | -0.15 | 0 | 41 | 0.00014 | 1Score **> 32** indicates **identity** Score **> 15** indicates **homology** | U | K.VYLWEDPNSK.L |
| 185008 | 958 | – | 985 | 1066.1894 | 3195.5463 | 3195.5513 | -1.55 | 1 | 44 | 7.7e-05 | 1Score **> 37** indicates **identity** Score **> 15** indicates **homology** | U | K.VYLWEDPNSKLFDHPEVPTPPESASVSR.L |
| 185009 | 958 | – | 985 | 799.8943 | 3195.5481 | 3195.5513 | -0.98 | 1 | 20 | 0.012 | 1Score **> 37** indicates **identity** Score **> 14** indicates **homology** | U | K.VYLWEDPNSKLFDHPEVPTPPESASVSR.L |
| 185010 | 958 | – | 985 | 799.8948 | 3195.5502 | 3195.5513 | -0.35 | 1 | 28 | 0.0022 | 1Score **> 37** indicates **identity** Score **> 14** indicates **homology** | U | K.VYLWEDPNSKLFDHPEVPTPPESASVSR.L |
| 185011 | 958 | – | 985 | 1066.1907 | 3195.5504 | 3195.5513 | -0.27 | 1 | 35 | 0.00047 | 1Score **> 37** indicates **identity** Score **> 15** indicates **homology** | U | K.VYLWEDPNSKLFDHPEVPTPPESASVSR.L |
| 185013 | 958 | – | 985 | 799.8952 | 3195.5515 | 3195.5513 | 0.077 | 1 | 23 | 0.0066 | 1Score **> 37** indicates **identity** Score **> 14** indicates **homology** | U | K.VYLWEDPNSKLFDHPEVPTPPESASVSR.L |
| 185015 | 958 | – | 985 | 1066.1920 | 3195.5541 | 3195.5513 | 0.88 | 1 | 28 | 0.0023 | 1Score **> 37** indicates **identity** Score **> 14** indicates **homology** | U | K.VYLWEDPNSKLFDHPEVPTPPESASVSR.L |
| 185016 | 958 | – | 985 | 1066.1921 | 3195.5545 | 3195.5513 | 1.00 | 1 | 27 | 0.0029 | 1Score **> 37** indicates **identity** Score **> 14** indicates **homology** | U | K.VYLWEDPNSKLFDHPEVPTPPESASVSR.L |
| 185043 | 958 | – | 985 | 1066.5273 | 3196.5601 | 3196.5353 | 7.76 | 1 | 19 | 0.018 | 1Score **> 37** indicates **identity** Score **> 14** indicates **homology** | U | K.VYLWEDPNSKLFDHPEVPTPPESASVSR.L  + Deamidated (NQ) |
| 185044 | 958 | – | 985 | 1066.5288 | 3196.5645 | 3196.5353 | 9.13 | 1 | 28 | 0.0025 | 1Score **> 37** indicates **identity** Score **> 14** indicates **homology** | U | K.VYLWEDPNSKLFDHPEVPTPPESASVSR.L  + Deamidated (NQ) |
| 119564 | 968 | – | 985 | 982.9891 | 1963.9637 | 1963.9640 | -0.15 | 0 | 41 | 0.00013 | 1Score **> 35** indicates **identity** Score **> 15** indicates **homology** | U | K.LFDHPEVPTPPESASVSR.L |
| 119566 | 968 | – | 985 | 655.6620 | 1963.9643 | 1963.9640 | 0.15 | 0 | 18 | 0.022 | 1Score **> 35** indicates **identity** Score **> 14** indicates **homology** | U | K.LFDHPEVPTPPESASVSR.L |
| 119567 | 968 | – | 985 | 655.6626 | 1963.9661 | 1963.9640 | 1.07 | 0 | 25 | 0.0042 | 1Score **> 35** indicates **identity** Score **> 14** indicates **homology** | U | K.LFDHPEVPTPPESASVSR.L |
| 119569 | 968 | – | 985 | 982.9917 | 1963.9689 | 1963.9640 | 2.49 | 0 | 29 | 0.003 | 1Score **> 35** indicates **identity** Score **> 16** indicates **homology** | U | K.LFDHPEVPTPPESASVSR.L |
| 119570 | 968 | – | 985 | 655.6637 | 1963.9692 | 1963.9640 | 2.67 | 0 | 25 | 0.0045 | 1Score **> 35** indicates **identity** Score **> 14** indicates **homology** | U | K.LFDHPEVPTPPESASVSR.L |
| 9619 | 986 | – | 993 | 469.2531 | 936.4917 | 936.4916 | 0.096 | 0 | 38 | 0.0033 | 1Score **> 29** indicates **identity** Score **> 26** indicates **homology** | U | R.LTQGEVYK.E |
| 9623 | 986 | – | 993 | 469.2547 | 936.4948 | 936.4916 | 3.34 | 0 | 26 | 0.015 | 1Score **> 29** indicates **identity** Score **> 20** indicates **homology** | U | R.LTQGEVYK.E |
| 45825 | 986 | – | 996 | 445.9132 | 1334.7177 | 1334.7194 | -1.28 | 1 | 34 | 0.00095 | 1Score **> 34** indicates **identity** Score **> 17** indicates **homology** | U | R.LTQGEVYKELR.L |
| 45826 | 986 | – | 996 | 445.9133 | 1334.7181 | 1334.7194 | -0.96 | 1 | 44 | 6.9e-05 | 1Score **> 34** indicates **identity** Score **> 15** indicates **homology** | U | R.LTQGEVYKELR.L |
| 45827 | 986 | – | 996 | 445.9133 | 1334.7182 | 1334.7194 | -0.88 | 1 | 21 | 0.0099 | 1Score **> 34** indicates **identity** Score **> 14** indicates **homology** | U | R.LTQGEVYKELR.L |
| 45828 | 986 | – | 996 | 445.9134 | 1334.7183 | 1334.7194 | -0.79 | 1 | 36 | 0.00044 | 1Score **> 34** indicates **identity** Score **> 15** indicates **homology** | U | R.LTQGEVYKELR.L |
| 45829 | 986 | – | 996 | 668.3668 | 1334.7190 | 1334.7194 | -0.30 | 1 | 46 | 5.3e-05 | 1Score **> 33** indicates **identity** Score **> 15** indicates **homology** | U | R.LTQGEVYKELR.L |
| 45830 | 986 | – | 996 | 445.9136 | 1334.7190 | 1334.7194 | -0.26 | 1 | 30 | 0.0015 | 1Score **> 33** indicates **identity** Score **> 14** indicates **homology** | U | R.LTQGEVYKELR.L |
| 45832 | 986 | – | 996 | 445.9137 | 1334.7194 | 1334.7194 | -0.031 | 1 | 22 | 0.0084 | 1Score **> 33** indicates **identity** Score **> 14** indicates **homology** | U | R.LTQGEVYKELR.L |
| 45833 | 986 | – | 996 | 445.9138 | 1334.7196 | 1334.7194 | 0.14 | 1 | 43 | 9.3e-05 | 1Score **> 33** indicates **identity** Score **> 15** indicates **homology** | U | R.LTQGEVYKELR.L |
| 45834 | 986 | – | 996 | 445.9139 | 1334.7200 | 1334.7194 | 0.43 | 1 | 39 | 0.00023 | 1Score **> 34** indicates **identity** Score **> 15** indicates **homology** | U | R.LTQGEVYKELR.L |
| 170385 | 997 | – | 1020 | 906.0937 | 2715.2593 | 2715.2599 | -0.20 | 1 | 68 | 4.4e-07 | 1Score **> 34** indicates **identity** Score **> 17** indicates **homology** | U | R.LRGYDYGPQFQGICEATLEGEQGK.L |
| 170386 | 997 | – | 1020 | 906.0937 | 2715.2594 | 2715.2599 | -0.18 | 1 | 43 | 8.9e-05 | 1Score **> 34** indicates **identity** Score **> 15** indicates **homology** | U | R.LRGYDYGPQFQGICEATLEGEQGK.L |
| 170387 | 997 | – | 1020 | 1358.6384 | 2715.2623 | 2715.2599 | 0.89 | 1 | 69 | 2.1e-06 | 1Score **> 34** indicates **identity** Score **> 24** indicates **homology** | U | R.LRGYDYGPQFQGICEATLEGEQGK.L |
| 170389 | 997 | – | 1020 | 906.0950 | 2715.2632 | 2715.2599 | 1.21 | 1 | 65 | 8.9e-07 | 1Score **> 34** indicates **identity** Score **> 17** indicates **homology** | U | R.LRGYDYGPQFQGICEATLEGEQGK.L |
| 170390 | 997 | – | 1020 | 906.0951 | 2715.2636 | 2715.2599 | 1.37 | 1 | 80 | 3.4e-08 | 1Score **> 34** indicates **identity** Score **> 17** indicates **homology** | U | R.LRGYDYGPQFQGICEATLEGEQGK.L |
| 170392 | 997 | – | 1020 | 906.0959 | 2715.2660 | 2715.2599 | 2.26 | 1 | 68 | 4.7e-07 | 1Score **> 35** indicates **identity** Score **> 17** indicates **homology** | U | R.LRGYDYGPQFQGICEATLEGEQGK.L |
| 170422 | 997 | – | 1020 | 906.4308 | 2716.2705 | 2716.2439 | 9.81 | 1 | 52 | 1.2e-05 | 1Score **> 35** indicates **identity** Score **> 16** indicates **homology** | U | R.LRGYDYGPQFQGICEATLEGEQGK.L  + Deamidated (NQ) |
| 186411 | 997 | – | 1024 | 814.9058 | 3255.5939 | 3255.6023 | -2.56 | 2 | 32 | 0.001 | 1Score **> 37** indicates **identity** Score **> 14** indicates **homology** | U | R.LRGYDYGPQFQGICEATLEGEQGKLLWK.D |
| 186434 | 997 | – | 1024 | 815.1571 | 3256.5993 | 3256.5863 | 4.01 | 2 | 38 | 0.00028 | 1Score **> 37** indicates **identity** Score **> 15** indicates **homology** | U | R.LRGYDYGPQFQGICEATLEGEQGKLLWK.D  + Deamidated (NQ) |
| 158080 | 999 | – | 1020 | 1224.0433 | 2446.0721 | 2446.0747 | -1.06 | 0 | 89 | 4.9e-09 | 1Score **> 29** indicates **identity** Score **> 18** indicates **homology** | U | R.GYDYGPQFQGICEATLEGEQGK.L |
| 158081 | 999 | – | 1020 | 816.3648 | 2446.0725 | 2446.0747 | -0.92 | 0 | 42 | 0.00012 | 1Score **> 30** indicates **identity** Score **> 15** indicates **homology** | U | R.GYDYGPQFQGICEATLEGEQGK.L |
| 158082 | 999 | – | 1020 | 816.3657 | 2446.0752 | 2446.0747 | 0.22 | 0 | 51 | 1.8e-05 | 1Score **> 30** indicates **identity** Score **> 16** indicates **homology** | U | R.GYDYGPQFQGICEATLEGEQGK.L |
| 158083 | 999 | – | 1020 | 1224.0451 | 2446.0757 | 2446.0747 | 0.39 | 0 | 100 | 5.3e-10 | 1Score **> 30** indicates **identity** Score **> 19** indicates **homology** | U | R.GYDYGPQFQGICEATLEGEQGK.L |
| 158085 | 999 | – | 1020 | 816.3661 | 2446.0765 | 2446.0747 | 0.74 | 0 | 58 | 3.7e-06 | 1Score **> 30** indicates **identity** Score **> 16** indicates **homology** | U | R.GYDYGPQFQGICEATLEGEQGK.L |
| 158086 | 999 | – | 1020 | 816.3664 | 2446.0772 | 2446.0747 | 1.04 | 0 | 30 | 0.0014 | 1Score **> 30** indicates **identity** Score **> 14** indicates **homology** | U | R.GYDYGPQFQGICEATLEGEQGK.L |
| 158087 | 999 | – | 1020 | 1224.0460 | 2446.0775 | 2446.0747 | 1.13 | 0 | 111 | 4.3e-11 | 1Score **> 30** indicates **identity** Score **> 19** indicates **homology** | U | R.GYDYGPQFQGICEATLEGEQGK.L |
| 158088 | 999 | – | 1020 | 1224.0467 | 2446.0789 | 2446.0747 | 1.70 | 0 | 93 | 1.9e-09 | 1Score **> 30** indicates **identity** Score **> 18** indicates **homology** | U | R.GYDYGPQFQGICEATLEGEQGK.L |
| 158134 | 999 | – | 1020 | 816.7004 | 2447.0793 | 2447.0587 | 8.40 | 0 | 44 | 8.3e-05 | 1Score **> 31** indicates **identity** Score **> 15** indicates **homology** | U | R.GYDYGPQFQGICEATLEGEQGK.L  + Deamidated (NQ) |
| 47935 | 1053 | – | 1064 | 452.9129 | 1355.7168 | 1355.7197 | -2.20 | 0 | 26 | 0.0034 | 1Score **> 34** indicates **identity** Score **> 14** indicates **homology** | U | R.VTAIYIDPATHR.Q |
| 47936 | 1053 | – | 1064 | 678.8667 | 1355.7188 | 1355.7197 | -0.72 | 0 | 52 | 1.5e-05 | 1Score **> 34** indicates **identity** Score **> 16** indicates **homology** | U | R.VTAIYIDPATHR.Q |
| 47938 | 1053 | – | 1064 | 678.8667 | 1355.7189 | 1355.7197 | -0.64 | 0 | 51 | 2.1e-05 | 1Score **> 34** indicates **identity** Score **> 16** indicates **homology** | U | R.VTAIYIDPATHR.Q |
| 47939 | 1053 | – | 1064 | 452.9136 | 1355.7189 | 1355.7197 | -0.61 | 0 | 43 | 9.2e-05 | 1Score **> 34** indicates **identity** Score **> 15** indicates **homology** | U | R.VTAIYIDPATHR.Q |
| 47942 | 1053 | – | 1064 | 452.9136 | 1355.7190 | 1355.7197 | -0.55 | 0 | 40 | 0.00019 | 1Score **> 33** indicates **identity** Score **> 15** indicates **homology** | U | R.VTAIYIDPATHR.Q |
| 47943 | 1053 | – | 1064 | 678.8668 | 1355.7190 | 1355.7197 | -0.55 | 0 | 56 | 2.5e-05 | 1Score **> 33** indicates **identity** Score **> 22** indicates **homology** | U | R.VTAIYIDPATHR.Q |
| 47944 | 1053 | – | 1064 | 452.9137 | 1355.7192 | 1355.7197 | -0.37 | 0 | 42 | 0.00011 | 1Score **> 33** indicates **identity** Score **> 15** indicates **homology** | U | R.VTAIYIDPATHR.Q |
| 47945 | 1053 | – | 1064 | 678.8669 | 1355.7192 | 1355.7197 | -0.37 | 0 | 51 | 3.2e-05 | 1Score **> 33** indicates **identity** Score **> 19** indicates **homology** | U | R.VTAIYIDPATHR.Q |
| 47954 | 1053 | – | 1064 | 678.8679 | 1355.7213 | 1355.7197 | 1.18 | 0 | 38 | 0.0003 | 1Score **> 34** indicates **identity** Score **> 15** indicates **homology** | U | R.VTAIYIDPATHR.Q |
| 72588 | 1070 | – | 1083 | 781.9033 | 1561.7921 | 1561.7948 | -1.73 | 1 | 93 | 5.7e-08 | 1Score **> 35** indicates **identity** Score **> 33** indicates **homology** | U | R.LKEDTQVADVTTSR.C |
| 72589 | 1070 | – | 1083 | 781.9038 | 1561.7930 | 1561.7948 | -1.10 | 1 | 103 | 5.5e-09 | 1Score **> 35** indicates **identity** Score **> 33** indicates **homology** | U | R.LKEDTQVADVTTSR.C |
| 72592 | 1070 | – | 1083 | 521.6057 | 1561.7953 | 1561.7948 | 0.34 | 1 | 58 | 4.4e-06 | 1Score **> 35** indicates **identity** Score **> 17** indicates **homology** | U | R.LKEDTQVADVTTSR.C |
| 72594 | 1070 | – | 1083 | 521.6058 | 1561.7956 | 1561.7948 | 0.52 | 1 | 60 | 3.2e-06 | 1Score **> 35** indicates **identity** Score **> 17** indicates **homology** | U | R.LKEDTQVADVTTSR.C |
| 72595 | 1070 | – | 1083 | 521.6059 | 1561.7959 | 1561.7948 | 0.70 | 1 | 19 | 0.015 | 1Score **> 35** indicates **identity** Score **> 14** indicates **homology** | U | R.LKEDTQVADVTTSR.C |
| 72596 | 1070 | – | 1083 | 781.9053 | 1561.7960 | 1561.7948 | 0.76 | 1 | 44 | 0.005 | 1Score **> 35** indicates **identity** Score **> 33** indicates **homology** | U | R.LKEDTQVADVTTSR.C |
| 72598 | 1070 | – | 1083 | 521.6069 | 1561.7988 | 1561.7948 | 2.58 | 1 | 18 | 0.021 | 1Score **> 35** indicates **identity** Score **> 14** indicates **homology** | U | R.LKEDTQVADVTTSR.C |
| 60363 | 1084 | – | 1097 | 490.5997 | 1468.7774 | 1468.7820 | -3.18 | 0 | 44 | 7.1e-05 | 1Score **> 34** indicates **identity** Score **> 15** indicates **homology** | U | R.CLGITVSGGIHISR.L |
| 60365 | 1084 | – | 1097 | 490.6001 | 1468.7785 | 1468.7820 | -2.44 | 0 | 34 | 0.0007 | 1Score **> 34** indicates **identity** Score **> 15** indicates **homology** | U | R.CLGITVSGGIHISR.L |
| 60367 | 1084 | – | 1097 | 490.6003 | 1468.7790 | 1468.7820 | -2.09 | 0 | 17 | 0.027 | 1Score **> 34** indicates **identity** Score **> 14** indicates **homology** | U | R.CLGITVSGGIHISR.L |
| 60368 | 1084 | – | 1097 | 490.6005 | 1468.7795 | 1468.7820 | -1.71 | 0 | 20 | 0.014 | 1Score **> 34** indicates **identity** Score **> 14** indicates **homology** | U | R.CLGITVSGGIHISR.L |
| 60370 | 1084 | – | 1097 | 490.6010 | 1468.7812 | 1468.7820 | -0.58 | 0 | 28 | 0.006 | 1Score **> 34** indicates **identity** Score **> 18** indicates **homology** | U | R.CLGITVSGGIHISR.L |
| 60371 | 1084 | – | 1097 | 490.6010 | 1468.7812 | 1468.7820 | -0.57 | 0 | 17 | 0.023 | 1Score **> 34** indicates **identity** Score **> 14** indicates **homology** | U | R.CLGITVSGGIHISR.L |
| 60373 | 1084 | – | 1097 | 490.6011 | 1468.7815 | 1468.7820 | -0.39 | 0 | 38 | 0.00026 | 1Score **> 34** indicates **identity** Score **> 15** indicates **homology** | U | R.CLGITVSGGIHISR.L |
| 60374 | 1084 | – | 1097 | 490.6012 | 1468.7817 | 1468.7820 | -0.25 | 0 | 28 | 0.0024 | 1Score **> 34** indicates **identity** Score **> 14** indicates **homology** | U | R.CLGITVSGGIHISR.L |
| 60376 | 1084 | – | 1097 | 490.6013 | 1468.7821 | 1468.7820 | 0.057 | 0 | 26 | 0.0035 | 1Score **> 34** indicates **identity** Score **> 14** indicates **homology** | U | R.CLGITVSGGIHISR.L |
| 60377 | 1084 | – | 1097 | 490.6013 | 1468.7821 | 1468.7820 | 0.057 | 0 | 23 | 0.022 | 1Score **> 34** indicates **identity** Score **> 19** indicates **homology** | U | R.CLGITVSGGIHISR.L |
| 60379 | 1084 | – | 1097 | 490.6013 | 1468.7822 | 1468.7820 | 0.12 | 0 | 28 | 0.006 | 1Score **> 34** indicates **identity** Score **> 18** indicates **homology** | U | R.CLGITVSGGIHISR.L |
| 60380 | 1084 | – | 1097 | 490.6014 | 1468.7824 | 1468.7820 | 0.22 | 0 | 36 | 0.0004 | 1Score **> 34** indicates **identity** Score **> 15** indicates **homology** | U | R.CLGITVSGGIHISR.L |
| 60382 | 1084 | – | 1097 | 490.6015 | 1468.7826 | 1468.7820 | 0.35 | 0 | 42 | 0.00034 | 1Score **> 34** indicates **identity** Score **> 20** indicates **homology** | U | R.CLGITVSGGIHISR.L |
| 60384 | 1084 | – | 1097 | 490.6016 | 1468.7830 | 1468.7820 | 0.62 | 0 | 16 | 0.031 | 1Score **> 34** indicates **identity** Score **> 14** indicates **homology** | U | R.CLGITVSGGIHISR.L |
| 60385 | 1084 | – | 1097 | 490.6018 | 1468.7836 | 1468.7820 | 1.04 | 0 | 36 | 0.0015 | 1Score **> 34** indicates **identity** Score **> 20** indicates **homology** | U | R.CLGITVSGGIHISR.L |
| 60386 | 1084 | – | 1097 | 490.6018 | 1468.7836 | 1468.7820 | 1.05 | 0 | 38 | 0.00027 | 1Score **> 34** indicates **identity** Score **> 15** indicates **homology** | U | R.CLGITVSGGIHISR.L |
| 60387 | 1084 | – | 1097 | 490.6018 | 1468.7836 | 1468.7820 | 1.05 | 0 | 17 | 0.027 | 1Score **> 34** indicates **identity** Score **> 14** indicates **homology** | U | R.CLGITVSGGIHISR.L |
| 60391 | 1084 | – | 1097 | 490.6023 | 1468.7850 | 1468.7820 | 1.99 | 0 | 31 | 0.0044 | 1Score **> 34** indicates **identity** Score **> 20** indicates **homology** | U | R.CLGITVSGGIHISR.L |
| 60254 | 1106 | – | 1117 | 490.2749 | 1467.8030 | 1467.8045 | -1.06 | 1 | 16 | 0.031 | 1Score **> 33** indicates **identity** Score **> 14** indicates **homology** | U | R.RQQEQLVPTLEK.F |
| 60255 | 1106 | – | 1117 | 734.9089 | 1467.8032 | 1467.8045 | -0.89 | 1 | 26 | 0.0081 | 1Score **> 33** indicates **identity** Score **> 18** indicates **homology** | U | R.RQQEQLVPTLEK.F |
| 60256 | 1106 | – | 1117 | 734.9093 | 1467.8040 | 1467.8045 | -0.36 | 1 | 49 | 0.00027 | 1Score **> 34** indicates **identity** Score **> 26** indicates **homology** | U | R.RQQEQLVPTLEK.F |
| 60258 | 1106 | – | 1117 | 490.2755 | 1467.8046 | 1467.8045 | 0.059 | 1 | 34 | 0.00061 | 1Score **> 34** indicates **identity** Score **> 15** indicates **homology** | U | R.RQQEQLVPTLEK.F |
| 60259 | 1106 | – | 1117 | 734.9096 | 1467.8047 | 1467.8045 | 0.13 | 1 | 57 | 5.5e-05 | 1Score **> 34** indicates **identity** Score **> 27** indicates **homology** | U | R.RQQEQLVPTLEK.F |
| 60261 | 1106 | – | 1117 | 490.2757 | 1467.8052 | 1467.8045 | 0.49 | 1 | 34 | 0.00062 | 1Score **> 34** indicates **identity** Score **> 15** indicates **homology** | U | R.RQQEQLVPTLEK.F |
| 60262 | 1106 | – | 1117 | 734.9105 | 1467.8065 | 1467.8045 | 1.34 | 1 | 28 | 0.0088 | 1Score **> 34** indicates **identity** Score **> 20** indicates **homology** | U | R.RQQEQLVPTLEK.F |
| 43431 | 1107 | – | 1117 | 656.8590 | 1311.7035 | 1311.7034 | 0.031 | 0 | 59 | 3.1e-06 | 1Score **> 33** indicates **identity** Score **> 16** indicates **homology** | U | R.QQEQLVPTLEK.F |
| 43433 | 1107 | – | 1117 | 656.8595 | 1311.7045 | 1311.7034 | 0.86 | 0 | 37 | 0.00035 | 1Score **> 33** indicates **identity** Score **> 15** indicates **homology** | U | R.QQEQLVPTLEK.F |
| 150706 | 1118 | – | 1137 | 775.7002 | 2324.0788 | 2324.0817 | -1.23 | 0 | 37 | 0.00035 | 1Score **> 34** indicates **identity** Score **> 15** indicates **homology** | U | K.FVFTPHMEAECLSESTALQK.E |
| 150708 | 1118 | – | 1137 | 775.7027 | 2324.0863 | 2324.0817 | 1.98 | 0 | 66 | 6.1e-07 | 1Score **> 34** indicates **identity** Score **> 17** indicates **homology** | U | K.FVFTPHMEAECLSESTALQK.E |
| 150709 | 1118 | – | 1137 | 775.7028 | 2324.0867 | 2324.0817 | 2.14 | 0 | 23 | 0.0067 | 1Score **> 34** indicates **identity** Score **> 14** indicates **homology** | U | K.FVFTPHMEAECLSESTALQK.E |
| 150710 | 1118 | – | 1137 | 1163.0519 | 2324.0892 | 2324.0817 | 3.22 | 0 | 27 | 0.0029 | 1Score **> 35** indicates **identity** Score **> 14** indicates **homology** | U | K.FVFTPHMEAECLSESTALQK.E |
| 93489 | 1160 | – | 1175 | 572.9541 | 1715.8404 | 1715.8413 | -0.52 | 0 | 34 | 0.00059 | 1Score **> 34** indicates **identity** Score **> 15** indicates **homology** | U | K.AAMLGQEDPPQHGLPR.L |
| 93490 | 1160 | – | 1175 | 572.9546 | 1715.8421 | 1715.8413 | 0.46 | 0 | 42 | 0.00011 | 1Score **> 34** indicates **identity** Score **> 15** indicates **homology** | U | K.AAMLGQEDPPQHGLPR.L |
| 93491 | 1160 | – | 1175 | 858.9287 | 1715.8428 | 1715.8413 | 0.86 | 0 | 28 | 0.0025 | 1Score **> 34** indicates **identity** Score **> 14** indicates **homology** | U | K.AAMLGQEDPPQHGLPR.L |
| 93492 | 1160 | – | 1175 | 572.9550 | 1715.8432 | 1715.8413 | 1.07 | 0 | 27 | 0.0027 | 1Score **> 34** indicates **identity** Score **> 14** indicates **homology** | U | K.AAMLGQEDPPQHGLPR.L |
| 93494 | 1160 | – | 1175 | 858.9297 | 1715.8448 | 1715.8413 | 2.02 | 0 | 44 | 7.3e-05 | 1Score **> 34** indicates **identity** Score **> 15** indicates **homology** | U | K.AAMLGQEDPPQHGLPR.L |
| 175085 | 1176 | – | 1201 | 946.4919 | 2836.4537 | 2836.4752 | -7.58 | 0 | 22 | 0.008 | 1Score **> 37** indicates **identity** Score **> 14** indicates **homology** | U | R.LLAAACQLQLNGNLQLELGEALAQER.L  + Deamidated (NQ) |
| 175091 | 1176 | – | 1201 | 946.4960 | 2836.4661 | 2836.4752 | -3.21 | 0 | 32 | 0.001 | 1Score **> 37** indicates **identity** Score **> 14** indicates **homology** | U | R.LLAAACQLQLNGNLQLELGEALAQER.L  + Deamidated (NQ) |
| 175098 | 1176 | – | 1201 | 946.5009 | 2836.4809 | 2836.4752 | 2.00 | 0 | 29 | 0.002 | 1Score **> 37** indicates **identity** Score **> 14** indicates **homology** | U | R.LLAAACQLQLNGNLQLELGEALAQER.L  + Deamidated (NQ) |
| 175101 | 1176 | – | 1201 | 946.5015 | 2836.4827 | 2836.4752 | 2.63 | 0 | 28 | 0.0023 | 1Score **> 37** indicates **identity** Score **> 14** indicates **homology** | U | R.LLAAACQLQLNGNLQLELGEALAQER.L  + Deamidated (NQ) |
| 175112 | 1176 | – | 1201 | 946.5056 | 2836.4950 | 2836.4752 | 6.98 | 0 | 32 | 0.00095 | 1Score **> 37** indicates **identity** Score **> 15** indicates **homology** | U | R.LLAAACQLQLNGNLQLELGEALAQER.L  + Deamidated (NQ) |
| 175137 | 1176 | – | 1201 | 946.8322 | 2837.4748 | 2837.4592 | 5.47 | 0 | 26 | 0.0038 | 1Score **> 37** indicates **identity** Score **> 14** indicates **homology** | U | R.LLAAACQLQLNGNLQLELGEALAQER.L  + 2 Deamidated (NQ) |
| 126353 | 1202 | – | 1220 | 678.7332 | 2033.1778 | 2033.1772 | 0.29 | 0 | 20 | 0.015 | 1Score **> 31** indicates **identity** Score **> 14** indicates **homology** | U | R.LLLPEDPLISGLLNSQALK.A |
| 126354 | 1202 | – | 1220 | 678.7339 | 2033.1799 | 2033.1772 | 1.34 | 0 | 90 | 3.8e-09 | 1Score **> 31** indicates **identity** Score **> 18** indicates **homology** | U | R.LLLPEDPLISGLLNSQALK.A |
| 126355 | 1202 | – | 1220 | 678.7343 | 2033.1812 | 2033.1772 | 1.97 | 0 | 65 | 8.7e-07 | 1Score **> 31** indicates **identity** Score **> 17** indicates **homology** | U | R.LLLPEDPLISGLLNSQALK.A |
| 126356 | 1202 | – | 1220 | 1017.5980 | 2033.1815 | 2033.1772 | 2.12 | 0 | 89 | 4.3e-09 | 1Score **> 31** indicates **identity** Score **> 18** indicates **homology** | U | R.LLLPEDPLISGLLNSQALK.A |
| 126357 | 1202 | – | 1220 | 1017.5984 | 2033.1823 | 2033.1772 | 2.50 | 0 | 66 | 7.1e-07 | 1Score **> 31** indicates **identity** Score **> 17** indicates **homology** | U | R.LLLPEDPLISGLLNSQALK.A |
| 126358 | 1202 | – | 1220 | 1017.5985 | 2033.1825 | 2033.1772 | 2.58 | 0 | 59 | 2.6e-06 | 1Score **> 31** indicates **identity** Score **> 16** indicates **homology** | U | R.LLLPEDPLISGLLNSQALK.A |
| 126410 | 1202 | – | 1220 | 1018.0929 | 2034.1712 | 2034.1612 | 4.92 | 0 | 80 | 3e-08 | 1Score **> 32** indicates **identity** Score **> 18** indicates **homology** | U | R.LLLPEDPLISGLLNSQALK.A  + Deamidated (NQ) |
| 126414 | 1202 | – | 1220 | 1018.0970 | 2034.1795 | 2034.1612 | 9.00 | 0 | 50 | 2.1e-05 | 1Score **> 31** indicates **identity** Score **> 16** indicates **homology** | U | R.LLLPEDPLISGLLNSQALK.A  + Deamidated (NQ) |
| 68622 | 1221 | – | 1234 | 767.8904 | 1533.7663 | 1533.7708 | -2.95 | 0 | 56 | 6.6e-06 | 1Score **> 34** indicates **identity** Score **> 16** indicates **homology** | U | K.ACVDTALENLSTLK.M |
| 68624 | 1221 | – | 1234 | 767.8927 | 1533.7709 | 1533.7708 | 0.010 | 0 | 34 | 0.00071 | 1Score **> 35** indicates **identity** Score **> 15** indicates **homology** | U | K.ACVDTALENLSTLK.M |
| 68625 | 1221 | – | 1234 | 767.8929 | 1533.7712 | 1533.7708 | 0.23 | 0 | 70 | 4.6e-07 | 1Score **> 35** indicates **identity** Score **> 19** indicates **homology** | U | K.ACVDTALENLSTLK.M |
| 68628 | 1221 | – | 1234 | 767.8932 | 1533.7719 | 1533.7708 | 0.69 | 0 | 78 | 3.5e-07 | 1Score **> 35** indicates **identity** Score **> 26** indicates **homology** | U | K.ACVDTALENLSTLK.M |
| 68629 | 1221 | – | 1234 | 767.8933 | 1533.7721 | 1533.7708 | 0.80 | 0 | 44 | 0.00025 | 1Score **> 35** indicates **identity** Score **> 21** indicates **homology** | U | K.ACVDTALENLSTLK.M |
| 68631 | 1221 | – | 1234 | 767.8935 | 1533.7725 | 1533.7708 | 1.10 | 0 | 73 | 3.4e-07 | 1Score **> 35** indicates **identity** Score **> 21** indicates **homology** | U | K.ACVDTALENLSTLK.M |
| 68632 | 1221 | – | 1234 | 512.2648 | 1533.7726 | 1533.7708 | 1.15 | 0 | 23 | 0.0071 | 1Score **> 35** indicates **identity** Score **> 14** indicates **homology** | U | K.ACVDTALENLSTLK.M |
| 68633 | 1221 | – | 1234 | 767.8936 | 1533.7726 | 1533.7708 | 1.16 | 0 | 78 | 3.4e-07 | 1Score **> 35** indicates **identity** Score **> 26** indicates **homology** | U | K.ACVDTALENLSTLK.M |
| 68634 | 1221 | – | 1234 | 512.2650 | 1533.7731 | 1533.7708 | 1.49 | 0 | 53 | 1.1e-05 | 1Score **> 34** indicates **identity** Score **> 16** indicates **homology** | U | K.ACVDTALENLSTLK.M |
| 68635 | 1221 | – | 1234 | 767.8941 | 1533.7736 | 1533.7708 | 1.81 | 0 | 67 | 2.2e-06 | 1Score **> 35** indicates **identity** Score **> 23** indicates **homology** | U | K.ACVDTALENLSTLK.M |
| 68637 | 1221 | – | 1234 | 767.8949 | 1533.7752 | 1533.7708 | 2.83 | 0 | 71 | 2.4e-07 | 1Score **> 35** indicates **identity** Score **> 17** indicates **homology** | U | K.ACVDTALENLSTLK.M |
| 98794 | 1235 | – | 1250 | 587.3105 | 1758.9096 | 1758.9087 | 0.52 | 1 | 25 | 0.0047 | 1Score **> 35** indicates **identity** Score **> 14** indicates **homology** | U | K.MKVAEVLAGEGHLYSR.I |
| 98796 | 1235 | – | 1250 | 587.3107 | 1758.9104 | 1758.9087 | 0.99 | 1 | 33 | 0.00089 | 1Score **> 35** indicates **identity** Score **> 15** indicates **homology** | U | K.MKVAEVLAGEGHLYSR.I |
| 64352 | 1237 | – | 1250 | 500.9318 | 1499.7736 | 1499.7732 | 0.26 | 0 | 52 | 1.3e-05 | 1Score **> 34** indicates **identity** Score **> 16** indicates **homology** | U | K.VAEVLAGEGHLYSR.I |
| 64353 | 1237 | – | 1250 | 750.8942 | 1499.7738 | 1499.7732 | 0.41 | 0 | 56 | 7.4e-06 | 1Score **> 34** indicates **identity** Score **> 17** indicates **homology** | U | K.VAEVLAGEGHLYSR.I |
| 64354 | 1237 | – | 1250 | 750.8942 | 1499.7739 | 1499.7732 | 0.47 | 0 | 58 | 3.7e-06 | 1Score **> 34** indicates **identity** Score **> 17** indicates **homology** | U | K.VAEVLAGEGHLYSR.I |
| 64356 | 1237 | – | 1250 | 500.9321 | 1499.7743 | 1499.7732 | 0.75 | 0 | 50 | 2e-05 | 1Score **> 34** indicates **identity** Score **> 16** indicates **homology** | U | K.VAEVLAGEGHLYSR.I |
| 64357 | 1237 | – | 1250 | 750.8945 | 1499.7744 | 1499.7732 | 0.80 | 0 | 79 | 9.6e-08 | 1Score **> 34** indicates **identity** Score **> 22** indicates **homology** | U | K.VAEVLAGEGHLYSR.I |
| 64358 | 1237 | – | 1250 | 500.9322 | 1499.7746 | 1499.7732 | 0.93 | 0 | 62 | 1.4e-06 | 1Score **> 34** indicates **identity** Score **> 16** indicates **homology** | U | K.VAEVLAGEGHLYSR.I |
| 64359 | 1237 | – | 1250 | 750.8950 | 1499.7754 | 1499.7732 | 1.48 | 0 | 59 | 3.1e-06 | 1Score **> 34** indicates **identity** Score **> 16** indicates **homology** | U | K.VAEVLAGEGHLYSR.I |
| 191294 | 1251 | – | 1281 | 884.2256 | 3532.8733 | 3532.8712 | 0.60 | 2 | 17 | 0.026 | 1Score **> 36** indicates **identity** Score **> 14** indicates **homology** | U | R.IPALLNTQPMLQLEYTATDRHPQALKDVQTK.L |
| 191295 | 1251 | – | 1281 | 707.5822 | 3532.8744 | 3532.8712 | 0.92 | 2 | 15 | 0.036 | 1Score **> 36** indicates **identity** Score **> 13** indicates **homology** | U | R.IPALLNTQPMLQLEYTATDRHPQALKDVQTK.L |
| 191296 | 1251 | – | 1281 | 884.2262 | 3532.8759 | 3532.8712 | 1.34 | 2 | 15 | 0.037 | 1Score **> 36** indicates **identity** Score **> 13** indicates **homology** | U | R.IPALLNTQPMLQLEYTATDRHPQALKDVQTK.L |
| 191298 | 1251 | – | 1281 | 884.2267 | 3532.8777 | 3532.8712 | 1.85 | 2 | 32 | 0.0009 | 1Score **> 36** indicates **identity** Score **> 15** indicates **homology** | U | R.IPALLNTQPMLQLEYTATDRHPQALKDVQTK.L |
| 191299 | 1251 | – | 1281 | 884.2267 | 3532.8778 | 3532.8712 | 1.88 | 2 | 31 | 0.0014 | 1Score **> 36** indicates **identity** Score **> 14** indicates **homology** | U | R.IPALLNTQPMLQLEYTATDRHPQALKDVQTK.L |
| 43442 | 1333 | – | 1344 | 438.2580 | 1311.7522 | 1311.7551 | -2.19 | 0 | 18 | 0.022 | 1Score **> 33** indicates **identity** Score **> 14** indicates **homology** | U | K.EGGFLLVHTVLK.G |
| 43443 | 1333 | – | 1344 | 656.8843 | 1311.7540 | 1311.7551 | -0.84 | 0 | 18 | 0.019 | 1Score **> 32** indicates **identity** Score **> 14** indicates **homology** | U | K.EGGFLLVHTVLK.G |
| 43444 | 1333 | – | 1344 | 438.2590 | 1311.7551 | 1311.7551 | -0.0061 | 0 | 21 | 0.011 | 1Score **> 32** indicates **identity** Score **> 14** indicates **homology** | U | K.EGGFLLVHTVLK.G |
| 43445 | 1333 | – | 1344 | 438.2590 | 1311.7553 | 1311.7551 | 0.18 | 0 | 26 | 0.0039 | 1Score **> 32** indicates **identity** Score **> 14** indicates **homology** | U | K.EGGFLLVHTVLK.G |
| 43447 | 1333 | – | 1344 | 438.2591 | 1311.7555 | 1311.7551 | 0.29 | 0 | 29 | 0.0018 | 1Score **> 32** indicates **identity** Score **> 14** indicates **homology** | U | K.EGGFLLVHTVLK.G |
| 43448 | 1333 | – | 1344 | 438.2592 | 1311.7559 | 1311.7551 | 0.60 | 0 | 30 | 0.0015 | 1Score **> 32** indicates **identity** Score **> 14** indicates **homology** | U | K.EGGFLLVHTVLK.G |
| 43449 | 1333 | – | 1344 | 656.8852 | 1311.7559 | 1311.7551 | 0.62 | 0 | 65 | 7.6e-07 | 1Score **> 32** indicates **identity** Score **> 17** indicates **homology** | U | K.EGGFLLVHTVLK.G |
| 43451 | 1333 | – | 1344 | 438.2598 | 1311.7575 | 1311.7551 | 1.81 | 0 | 17 | 0.026 | 1Score **> 32** indicates **identity** Score **> 14** indicates **homology** | U | K.EGGFLLVHTVLK.G |
| 43452 | 1333 | – | 1344 | 656.8860 | 1311.7575 | 1311.7551 | 1.85 | 0 | 36 | 0.00043 | 1Score **> 32** indicates **identity** Score **> 15** indicates **homology** | U | K.EGGFLLVHTVLK.G |
| 192334 | 1345 | – | 1377 | 910.4534 | 3637.7845 | 3637.7722 | 3.37 | 0 | 47 | 3.9e-05 | 1Score **> 37** indicates **identity** Score **> 15** indicates **homology** | U | K.GHALGETLACLPSEVQPAPSLLSQEEWESLFSR.K |
| 192335 | 1345 | – | 1377 | 1213.6024 | 3637.7853 | 3637.7722 | 3.58 | 0 | 88 | 6.1e-09 | 1Score **> 37** indicates **identity** Score **> 18** indicates **homology** | U | K.GHALGETLACLPSEVQPAPSLLSQEEWESLFSR.K |
| 193264 | 1345 | – | 1378 | 1256.2955 | 3765.8646 | 3765.8672 | -0.70 | 1 | 47 | 4.3e-05 | 1Score **> 38** indicates **identity** Score **> 15** indicates **homology** | U | K.GHALGETLACLPSEVQPAPSLLSQEEWESLFSRK.A |
| 193265 | 1345 | – | 1378 | 942.4735 | 3765.8650 | 3765.8672 | -0.57 | 1 | 64 | 9.1e-07 | 1Score **> 38** indicates **identity** Score **> 17** indicates **homology** | U | K.GHALGETLACLPSEVQPAPSLLSQEEWESLFSRK.A |
| 193272 | 1345 | – | 1378 | 1256.6296 | 3766.8671 | 3766.8512 | 4.21 | 1 | 62 | 1.6e-06 | 1Score **> 37** indicates **identity** Score **> 16** indicates **homology** | U | K.GHALGETLACLPSEVQPAPSLLSQEEWESLFSRK.A  + Deamidated (NQ) |
| 24541 | 1378 | – | 1387 | 378.9207 | 1133.7404 | 1133.7397 | 0.63 | 2 | 17 | 0.025 | 1Score **> 20** indicates **identity** Score **> 14** indicates **homology** | U | R.KALHLVGLKR.S |
| 24543 | 1378 | – | 1387 | 567.8780 | 1133.7414 | 1133.7397 | 1.57 | 2 | 23 | 0.0073 | 1Score **> 20** indicates **identity** Score **> 14** indicates **homology** | U | R.KALHLVGLKR.S |
| 63082 | 1387 | – | 1398 | 497.5899 | 1489.7479 | 1489.7500 | -1.42 | 1 | 23 | 0.0066 | 1Score **> 34** indicates **identity** Score **> 14** indicates **homology** | U | K.RSFYGTALFLCR.R |
| 63086 | 1387 | – | 1398 | 497.5909 | 1489.7509 | 1489.7500 | 0.61 | 1 | 15 | 0.037 | 1Score **> 34** indicates **identity** Score **> 13** indicates **homology** | U | K.RSFYGTALFLCR.R |
| 45688 | 1388 | – | 1398 | 667.8308 | 1333.6471 | 1333.6489 | -1.32 | 0 | 38 | 0.0003 | 1Score **> 32** indicates **identity** Score **> 15** indicates **homology** | U | R.SFYGTALFLCR.R |
| 45689 | 1388 | – | 1398 | 667.8311 | 1333.6476 | 1333.6489 | -0.94 | 0 | 52 | 1.4e-05 | 1Score **> 32** indicates **identity** Score **> 16** indicates **homology** | U | R.SFYGTALFLCR.R |
| 45691 | 1388 | – | 1398 | 667.8324 | 1333.6503 | 1333.6489 | 1.06 | 0 | 68 | 3.9e-07 | 1Score **> 33** indicates **identity** Score **> 17** indicates **homology** | U | R.SFYGTALFLCR.R |
| 45693 | 1388 | – | 1398 | 667.8328 | 1333.6511 | 1333.6489 | 1.62 | 0 | 74 | 1.2e-07 | 1Score **> 33** indicates **identity** Score **> 17** indicates **homology** | U | R.SFYGTALFLCR.R |
| 45694 | 1388 | – | 1398 | 667.8333 | 1333.6520 | 1333.6489 | 2.36 | 0 | 68 | 4.7e-07 | 1Score **> 33** indicates **identity** Score **> 17** indicates **homology** | U | R.SFYGTALFLCR.R |
| 45695 | 1388 | – | 1398 | 667.8334 | 1333.6522 | 1333.6489 | 2.47 | 0 | 79 | 3.9e-08 | 1Score **> 33** indicates **identity** Score **> 17** indicates **homology** | U | R.SFYGTALFLCR.R |
| 45696 | 1388 | – | 1398 | 667.8335 | 1333.6525 | 1333.6489 | 2.71 | 0 | 68 | 4e-07 | 1Score **> 33** indicates **identity** Score **> 17** indicates **homology** | U | R.SFYGTALFLCR.R |
| 45697 | 1388 | – | 1398 | 667.8337 | 1333.6529 | 1333.6489 | 3.02 | 0 | 74 | 1.2e-07 | 1Score **> 33** indicates **identity** Score **> 17** indicates **homology** | U | R.SFYGTALFLCR.R |
| 45698 | 1388 | – | 1398 | 667.8340 | 1333.6535 | 1333.6489 | 3.44 | 0 | 74 | 1.2e-07 | 1Score **> 33** indicates **identity** Score **> 17** indicates **homology** | U | R.SFYGTALFLCR.R |
| 45699 | 1388 | – | 1398 | 667.8340 | 1333.6535 | 1333.6489 | 3.47 | 0 | 14 | 0.047 | 1Score **> 33** indicates **identity** Score **> 13** indicates **homology** | U | R.SFYGTALFLCR.R |
| 45701 | 1388 | – | 1398 | 667.8350 | 1333.6554 | 1333.6489 | 4.91 | 0 | 40 | 0.00018 | 1Score **> 33** indicates **identity** Score **> 15** indicates **homology** | U | R.SFYGTALFLCR.R |
| 45702 | 1388 | – | 1398 | 667.8351 | 1333.6557 | 1333.6489 | 5.13 | 0 | 37 | 0.00031 | 1Score **> 33** indicates **identity** Score **> 15** indicates **homology** | U | R.SFYGTALFLCR.R |
| 45703 | 1388 | – | 1398 | 667.8352 | 1333.6559 | 1333.6489 | 5.22 | 0 | 67 | 4.9e-07 | 1Score **> 33** indicates **identity** Score **> 17** indicates **homology** | U | R.SFYGTALFLCR.R |
| 45705 | 1388 | – | 1398 | 667.8355 | 1333.6565 | 1333.6489 | 5.68 | 0 | 49 | 2.4e-05 | 1Score **> 33** indicates **identity** Score **> 16** indicates **homology** | U | R.SFYGTALFLCR.R |
| 178046 | 1399 | – | 1423 | 734.1426 | 2932.5415 | 2932.5334 | 2.74 | 2 | 69 | 3.1e-07 | 1Score **> 37** indicates **identity** Score **> 17** indicates **homology** | U | R.RAIPQEKPIFLSVEDTSFQWVDSLK.S |
| 178047 | 1399 | – | 1423 | 734.1465 | 2932.5570 | 2932.5334 | 8.02 | 2 | 57 | 4.1e-06 | 1Score **> 36** indicates **identity** Score **> 16** indicates **homology** | U | R.RAIPQEKPIFLSVEDTSFQWVDSLK.S |
| 172917 | 1400 | – | 1423 | 926.4819 | 2776.4239 | 2776.4323 | -3.03 | 1 | 16 | 0.033 | 1Score **> 37** indicates **identity** Score **> 13** indicates **homology** | U | R.AIPQEKPIFLSVEDTSFQWVDSLK.S |
| 172921 | 1400 | – | 1423 | 926.4893 | 2776.4459 | 2776.4323 | 4.90 | 1 | 55 | 7.4e-06 | 1Score **> 37** indicates **identity** Score **> 16** indicates **homology** | U | R.AIPQEKPIFLSVEDTSFQWVDSLK.S |
| 85300 | 1464 | – | 1478 | 552.2893 | 1653.8461 | 1653.8508 | -2.86 | 0 | 16 | 0.034 | 1Score **> 34** indicates **identity** Score **> 13** indicates **homology** | U | R.CILLSNLSNTSHAPK.L |
| 85303 | 1464 | – | 1478 | 552.2907 | 1653.8503 | 1653.8508 | -0.33 | 0 | 66 | 7.2e-07 | 1Score **> 35** indicates **identity** Score **> 17** indicates **homology** | U | R.CILLSNLSNTSHAPK.L |
| 85306 | 1464 | – | 1478 | 552.2908 | 1653.8506 | 1653.8508 | -0.11 | 0 | 29 | 0.0019 | 1Score **> 35** indicates **identity** Score **> 14** indicates **homology** | U | R.CILLSNLSNTSHAPK.L |
| 85307 | 1464 | – | 1478 | 827.9327 | 1653.8508 | 1653.8508 | -0.0042 | 0 | 67 | 5.7e-07 | 1Score **> 35** indicates **identity** Score **> 17** indicates **homology** | U | R.CILLSNLSNTSHAPK.L |
| 85308 | 1464 | – | 1478 | 827.9330 | 1653.8514 | 1653.8508 | 0.33 | 0 | 86 | 3.4e-08 | 1Score **> 35** indicates **identity** Score **> 24** indicates **homology** | U | R.CILLSNLSNTSHAPK.L |
| 85309 | 1464 | – | 1478 | 552.2912 | 1653.8516 | 1653.8508 | 0.50 | 0 | 64 | 1e-06 | 1Score **> 34** indicates **identity** Score **> 16** indicates **homology** | U | R.CILLSNLSNTSHAPK.L |
| 85310 | 1464 | – | 1478 | 827.9337 | 1653.8528 | 1653.8508 | 1.22 | 0 | 97 | 3.8e-09 | 1Score **> 34** indicates **identity** Score **> 25** indicates **homology** | U | R.CILLSNLSNTSHAPK.L |
| 85311 | 1464 | – | 1478 | 552.2916 | 1653.8530 | 1653.8508 | 1.33 | 0 | 24 | 0.0059 | 1Score **> 35** indicates **identity** Score **> 14** indicates **homology** | U | R.CILLSNLSNTSHAPK.L |
| 85314 | 1464 | – | 1478 | 552.2922 | 1653.8548 | 1653.8508 | 2.42 | 0 | 25 | 0.0044 | 1Score **> 35** indicates **identity** Score **> 14** indicates **homology** | U | R.CILLSNLSNTSHAPK.L |
| 85315 | 1464 | – | 1478 | 552.2923 | 1653.8552 | 1653.8508 | 2.64 | 0 | 39 | 0.00021 | 1Score **> 35** indicates **identity** Score **> 15** indicates **homology** | U | R.CILLSNLSNTSHAPK.L |
| 85318 | 1464 | – | 1478 | 552.2934 | 1653.8583 | 1653.8508 | 4.51 | 0 | 16 | 0.029 | 1Score **> 35** indicates **identity** Score **> 14** indicates **homology** | U | R.CILLSNLSNTSHAPK.L |
| 181915 | 1464 | – | 1491 | 765.6603 | 3058.6121 | 3058.6121 | 0.0016 | 1 | 21 | 0.011 | 1Score **> 36** indicates **identity** Score **> 14** indicates **homology** | U | R.CILLSNLSNTSHAPKLDPGSPELQQVLK.H |
| 54975 | 1479 | – | 1491 | 712.3931 | 1422.7717 | 1422.7718 | -0.13 | 0 | 15 | 0.039 | 1Score **> 33** indicates **identity** Score **> 13** indicates **homology** | U | K.LDPGSPELQQVLK.H |
| 54976 | 1479 | – | 1491 | 712.3934 | 1422.7723 | 1422.7718 | 0.33 | 0 | 76 | 7.2e-08 | 1Score **> 33** indicates **identity** Score **> 17** indicates **homology** | U | K.LDPGSPELQQVLK.H |
| 54977 | 1479 | – | 1491 | 712.3935 | 1422.7724 | 1422.7718 | 0.40 | 0 | 80 | 3.5e-08 | 1Score **> 33** indicates **identity** Score **> 17** indicates **homology** | U | K.LDPGSPELQQVLK.H |
| 54978 | 1479 | – | 1491 | 712.3938 | 1422.7731 | 1422.7718 | 0.90 | 0 | 90 | 1.4e-08 | 1Score **> 33** indicates **identity** Score **> 24** indicates **homology** | U | K.LDPGSPELQQVLK.H |
| 54980 | 1479 | – | 1491 | 712.3946 | 1422.7746 | 1422.7718 | 1.94 | 0 | 78 | 4.7e-08 | 1Score **> 33** indicates **identity** Score **> 17** indicates **homology** | U | K.LDPGSPELQQVLK.H |
| 54981 | 1479 | – | 1491 | 712.3948 | 1422.7750 | 1422.7718 | 2.21 | 0 | 40 | 0.00017 | 1Score **> 33** indicates **identity** Score **> 15** indicates **homology** | U | K.LDPGSPELQQVLK.H |
| 163496 | 1479 | – | 1500 | 851.1148 | 2550.3227 | 2550.3264 | -1.45 | 1 | 21 | 0.01 | 1Score **> 37** indicates **identity** Score **> 14** indicates **homology** | U | K.LDPGSPELQQVLKHDLVMNVYR.D |
| 163498 | 1479 | – | 1500 | 638.5883 | 2550.3240 | 2550.3264 | -0.94 | 1 | 54 | 8e-06 | 1Score **> 37** indicates **identity** Score **> 16** indicates **homology** | U | K.LDPGSPELQQVLKHDLVMNVYR.D |
| 163557 | 1479 | – | 1500 | 851.4489 | 2551.3248 | 2551.3104 | 5.64 | 1 | 26 | 0.0038 | 1Score **> 37** indicates **identity** Score **> 14** indicates **homology** | U | K.LDPGSPELQQVLKHDLVMNVYR.D  + Deamidated (NQ) |
| 25643 | 1492 | – | 1500 | 573.7895 | 1145.5644 | 1145.5652 | -0.67 | 0 | 53 | 1e-05 | 1Score **> 32** indicates **identity** Score **> 16** indicates **homology** | U | K.HDLVMNVYR.D |
| 25645 | 1492 | – | 1500 | 573.7899 | 1145.5652 | 1145.5652 | 0.0044 | 0 | 30 | 0.0015 | 1Score **> 32** indicates **identity** Score **> 14** indicates **homology** | U | K.HDLVMNVYR.D |
| 25646 | 1492 | – | 1500 | 573.7905 | 1145.5665 | 1145.5652 | 1.15 | 0 | 29 | 0.002 | 1Score **> 32** indicates **identity** Score **> 14** indicates **homology** | U | K.HDLVMNVYR.D |
| 25647 | 1492 | – | 1500 | 573.7908 | 1145.5670 | 1145.5652 | 1.57 | 0 | 47 | 4.2e-05 | 1Score **> 32** indicates **identity** Score **> 15** indicates **homology** | U | K.HDLVMNVYR.D |
| 6727 | 1501 | – | 1508 | 440.2100 | 878.4055 | 878.4035 | 2.23 | 0 | 45 | 0.00068 | 1Score **> 28** indicates **identity** Score **> 26** indicates **homology** | U | R.DGAWGAFR.H |
| 6728 | 1501 | – | 1508 | 440.2101 | 878.4057 | 878.4035 | 2.45 | 0 | 47 | 0.00046 | 1Score **> 28** indicates **identity** Score **> 26** indicates **homology** | U | R.DGAWGAFR.H |
| 6729 | 1501 | – | 1508 | 440.2107 | 878.4069 | 878.4035 | 3.85 | 0 | 30 | 0.0026 | 1Score **> 28** indicates **identity** Score **> 17** indicates **homology** | U | R.DGAWGAFR.H |
| 38767 | 1509 | – | 1518 | 423.8906 | 1268.6498 | 1268.6513 | -1.18 | 1 | 34 | 0.00064 | 1Score **> 32** indicates **identity** Score **> 15** indicates **homology** | U | R.HFQLEQDKPK.E |
| 38769 | 1509 | – | 1518 | 635.3323 | 1268.6500 | 1268.6513 | -1.01 | 1 | 30 | 0.0015 | 1Score **> 33** indicates **identity** Score **> 14** indicates **homology** | U | R.HFQLEQDKPK.E |
| 38772 | 1509 | – | 1518 | 423.8912 | 1268.6517 | 1268.6513 | 0.29 | 1 | 24 | 0.005 | 1Score **> 32** indicates **identity** Score **> 14** indicates **homology** | U | R.HFQLEQDKPK.E |
| 171132 | 1509 | – | 1531 | 684.8598 | 2735.4103 | 2735.4143 | -1.48 | 2 | 19 | 0.015 | 1Score **> 37** indicates **identity** Score **> 14** indicates **homology** | U | R.HFQLEQDKPKEQTAHAFVNVLTR.G |
| 171136 | 1509 | – | 1531 | 684.8604 | 2735.4124 | 2735.4143 | -0.72 | 2 | 55 | 6.9e-06 | 1Score **> 37** indicates **identity** Score **> 16** indicates **homology** | U | R.HFQLEQDKPKEQTAHAFVNVLTR.G |
| 171137 | 1509 | – | 1531 | 548.0898 | 2735.4124 | 2735.4143 | -0.71 | 2 | 15 | 0.039 | 1Score **> 37** indicates **identity** Score **> 13** indicates **homology** | U | R.HFQLEQDKPKEQTAHAFVNVLTR.G |
| 171139 | 1509 | – | 1531 | 912.8116 | 2735.4129 | 2735.4143 | -0.54 | 2 | 30 | 0.0016 | 1Score **> 37** indicates **identity** Score **> 14** indicates **homology** | U | R.HFQLEQDKPKEQTAHAFVNVLTR.G |
| 171142 | 1509 | – | 1531 | 684.8610 | 2735.4148 | 2735.4143 | 0.18 | 2 | 50 | 2.1e-05 | 1Score **> 37** indicates **identity** Score **> 16** indicates **homology** | U | R.HFQLEQDKPKEQTAHAFVNVLTR.G |
| 171148 | 1509 | – | 1531 | 684.8618 | 2735.4182 | 2735.4143 | 1.40 | 2 | 15 | 0.036 | 1Score **> 37** indicates **identity** Score **> 13** indicates **homology** | U | R.HFQLEQDKPKEQTAHAFVNVLTR.G |
| 62300 | 1519 | – | 1531 | 495.9322 | 1484.7749 | 1484.7736 | 0.89 | 0 | 39 | 0.00022 | 1Score **> 34** indicates **identity** Score **> 15** indicates **homology** | U | K.EQTAHAFVNVLTR.G |
| 62301 | 1519 | – | 1531 | 495.9323 | 1484.7751 | 1484.7736 | 1.01 | 0 | 23 | 0.0066 | 1Score **> 34** indicates **identity** Score **> 14** indicates **homology** | U | K.EQTAHAFVNVLTR.G |
| 62302 | 1519 | – | 1531 | 495.9323 | 1484.7752 | 1484.7736 | 1.09 | 0 | 37 | 0.00042 | 1Score **> 34** indicates **identity** Score **> 16** indicates **homology** | U | K.EQTAHAFVNVLTR.G |
| 62303 | 1519 | – | 1531 | 743.3949 | 1484.7752 | 1484.7736 | 1.13 | 0 | 70 | 2.9e-07 | 1Score **> 34** indicates **identity** Score **> 17** indicates **homology** | U | K.EQTAHAFVNVLTR.G |
| 62304 | 1519 | – | 1531 | 743.3950 | 1484.7754 | 1484.7736 | 1.23 | 0 | 67 | 9.8e-07 | 1Score **> 34** indicates **identity** Score **> 19** indicates **homology** | U | K.EQTAHAFVNVLTR.G |
| 62305 | 1519 | – | 1531 | 495.9329 | 1484.7770 | 1484.7736 | 2.30 | 0 | 15 | 0.038 | 1Score **> 34** indicates **identity** Score **> 13** indicates **homology** | U | K.EQTAHAFVNVLTR.G |
| 62306 | 1519 | – | 1531 | 495.9331 | 1484.7775 | 1484.7736 | 2.67 | 0 | 38 | 0.0003 | 1Score **> 34** indicates **identity** Score **> 15** indicates **homology** | U | K.EQTAHAFVNVLTR.G |
| 62308 | 1519 | – | 1531 | 495.9341 | 1484.7804 | 1484.7736 | 4.61 | 0 | 42 | 0.00014 | 1Score **> 35** indicates **identity** Score **> 16** indicates **homology** | U | K.EQTAHAFVNVLTR.G |
| 1013 | 1532 | – | 1538 | 366.2060 | 730.3974 | 730.3973 | 0.15 | 0 | 39 | 0.0024 | 1Score **> 25** indicates **identity** | U | R.GDLASIR.W |
| 1015 | 1532 | – | 1538 | 366.2071 | 730.3997 | 730.3973 | 3.21 | 0 | 31 | 0.015 | 1Score **> 25** indicates **identity** | U | R.GDLASIR.W |
| 3964 | 1539 | – | 1545 | 408.7338 | 815.4531 | 815.4541 | -1.26 | 0 | 24 | 0.0058 | 1Score **> 30** indicates **identity** Score **> 15** indicates **homology** | U | R.WVSSPLK.H |
| 160247 | 1546 | – | 1567 | 829.7264 | 2486.1572 | 2486.1649 | -3.07 | 0 | 16 | 0.028 | 1Score **> 35** indicates **identity** Score **> 14** indicates **homology** | U | K.HTQPSSSGAQLCTVYYASLNFR.D |
| 160252 | 1546 | – | 1567 | 829.7277 | 2486.1614 | 2486.1649 | -1.40 | 0 | 50 | 2.1e-05 | 1Score **> 35** indicates **identity** Score **> 16** indicates **homology** | U | K.HTQPSSSGAQLCTVYYASLNFR.D |
| 160257 | 1546 | – | 1567 | 829.7282 | 2486.1627 | 2486.1649 | -0.85 | 0 | 20 | 0.013 | 1Score **> 35** indicates **identity** Score **> 14** indicates **homology** | U | K.HTQPSSSGAQLCTVYYASLNFR.D |
| 160264 | 1546 | – | 1567 | 829.7291 | 2486.1656 | 2486.1649 | 0.28 | 0 | 33 | 0.00088 | 1Score **> 35** indicates **identity** Score **> 15** indicates **homology** | U | K.HTQPSSSGAQLCTVYYASLNFR.D |
| 160269 | 1546 | – | 1567 | 829.7299 | 2486.1678 | 2486.1649 | 1.18 | 0 | 56 | 5.7e-06 | 1Score **> 35** indicates **identity** Score **> 16** indicates **homology** | U | K.HTQPSSSGAQLCTVYYASLNFR.D |
| 160273 | 1546 | – | 1567 | 829.7309 | 2486.1709 | 2486.1649 | 2.42 | 0 | 60 | 2.2e-06 | 1Score **> 35** indicates **identity** Score **> 16** indicates **homology** | U | K.HTQPSSSGAQLCTVYYASLNFR.D |
| 160307 | 1546 | – | 1567 | 830.0615 | 2487.1627 | 2487.1489 | 5.56 | 0 | 30 | 0.0017 | 1Score **> 35** indicates **identity** Score **> 14** indicates **homology** | U | K.HTQPSSSGAQLCTVYYASLNFR.D  + Deamidated (NQ) |
| 160308 | 1546 | – | 1567 | 830.0616 | 2487.1630 | 2487.1489 | 5.69 | 0 | 36 | 0.00043 | 1Score **> 35** indicates **identity** Score **> 15** indicates **homology** | U | K.HTQPSSSGAQLCTVYYASLNFR.D  + Deamidated (NQ) |
| 160314 | 1546 | – | 1567 | 830.0627 | 2487.1663 | 2487.1489 | 7.03 | 0 | 35 | 0.00055 | 1Score **> 35** indicates **identity** Score **> 15** indicates **homology** | U | K.HTQPSSSGAQLCTVYYASLNFR.D  + Deamidated (NQ) |
| 160318 | 1546 | – | 1567 | 830.0637 | 2487.1692 | 2487.1489 | 8.20 | 0 | 17 | 0.028 | 1Score **> 35** indicates **identity** Score **> 14** indicates **homology** | U | K.HTQPSSSGAQLCTVYYASLNFR.D  + Deamidated (NQ) |
| 160320 | 1546 | – | 1567 | 830.0639 | 2487.1698 | 2487.1489 | 8.44 | 0 | 32 | 0.001 | 1Score **> 35** indicates **identity** Score **> 14** indicates **homology** | U | K.HTQPSSSGAQLCTVYYASLNFR.D  + Deamidated (NQ) |
| 187753 | 1546 | – | 1575 | 830.4023 | 3317.5800 | 3317.5696 | 3.13 | 1 | 24 | 0.0057 | 1Score **> 36** indicates **identity** Score **> 14** indicates **homology** | U | K.HTQPSSSGAQLCTVYYASLNFRDIMLATGK.L  + 2 Deamidated (NQ) |
| 7405 | 1576 | – | 1584 | 449.2554 | 896.4963 | 896.4967 | -0.47 | 0 | 46 | 6.8e-05 | 1Score **> 25** indicates **identity** Score **> 17** indicates **homology** | U | K.LSPDAIPGK.W |
| 7406 | 1576 | – | 1584 | 449.2557 | 896.4968 | 896.4967 | 0.097 | 0 | 49 | 8.9e-05 | 1Score **> 25** indicates **identity** Score **> 21** indicates **homology** | U | K.LSPDAIPGK.W |
| 7407 | 1576 | – | 1584 | 449.2565 | 896.4985 | 896.4967 | 1.98 | 0 | 18 | 0.023 | 1Score **> 25** indicates **identity** Score **> 14** indicates **homology** | U | K.LSPDAIPGK.W |
| 166131 | 1661 | – | 1687 | 870.7926 | 2609.3561 | 2609.3595 | -1.32 | 0 | 36 | 0.00038 | 1Score **> 37** indicates **identity** Score **> 15** indicates **homology** | U | R.GETVLIHSGSGGVGQAAISIALSLGCR.V |
| 166132 | 1661 | – | 1687 | 870.7931 | 2609.3575 | 2609.3595 | -0.78 | 0 | 29 | 0.0018 | 1Score **> 37** indicates **identity** Score **> 14** indicates **homology** | U | R.GETVLIHSGSGGVGQAAISIALSLGCR.V |
| 166133 | 1661 | – | 1687 | 870.7942 | 2609.3609 | 2609.3595 | 0.53 | 0 | 65 | 8.1e-07 | 1Score **> 37** indicates **identity** Score **> 17** indicates **homology** | U | R.GETVLIHSGSGGVGQAAISIALSLGCR.V |
| 15753 | 1688 | – | 1697 | 519.7769 | 1037.5392 | 1037.5393 | -0.093 | 0 | 15 | 0.039 | 1Score **> 30** indicates **identity** Score **> 13** indicates **homology** | U | R.VFTTVGSAEK.R |
| 15755 | 1688 | – | 1697 | 519.7773 | 1037.5400 | 1037.5393 | 0.63 | 0 | 48 | 3e-05 | 1Score **> 30** indicates **identity** Score **> 16** indicates **homology** | U | R.VFTTVGSAEK.R |
| 30902 | 1688 | – | 1698 | 597.8279 | 1193.6412 | 1193.6404 | 0.62 | 1 | 52 | 5.6e-05 | 1Score **> 32** indicates **identity** Score **> 22** indicates **homology** | U | R.VFTTVGSAEKR.A |
| 30903 | 1688 | – | 1698 | 398.8877 | 1193.6412 | 1193.6404 | 0.66 | 1 | 51 | 7.2e-05 | 1Score **> 32** indicates **identity** Score **> 22** indicates **homology** | U | R.VFTTVGSAEKR.A |
| 30904 | 1688 | – | 1698 | 398.8877 | 1193.6412 | 1193.6404 | 0.67 | 1 | 24 | 0.0052 | 1Score **> 32** indicates **identity** Score **> 14** indicates **homology** | U | R.VFTTVGSAEKR.A |
| 30905 | 1688 | – | 1698 | 597.8281 | 1193.6417 | 1193.6404 | 1.03 | 1 | 34 | 0.0012 | 1Score **> 32** indicates **identity** Score **> 18** indicates **homology** | U | R.VFTTVGSAEKR.A |
| 30906 | 1688 | – | 1698 | 398.8878 | 1193.6417 | 1193.6404 | 1.03 | 1 | 43 | 9.7e-05 | 1Score **> 32** indicates **identity** Score **> 15** indicates **homology** | U | R.VFTTVGSAEKR.A |
| 63860 | 1705 | – | 1717 | 749.3495 | 1496.6844 | 1496.6896 | -3.46 | 0 | 50 | 2.2e-05 | 1Score **> 30** indicates **identity** Score **> 16** indicates **homology** | U | R.FPQLDDTSFANSR.D |
| 63861 | 1705 | – | 1717 | 749.3519 | 1496.6893 | 1496.6896 | -0.20 | 0 | 80 | 3.1e-08 | 1Score **> 31** indicates **identity** Score **> 18** indicates **homology** | U | R.FPQLDDTSFANSR.D |
| 63862 | 1705 | – | 1717 | 749.3520 | 1496.6894 | 1496.6896 | -0.14 | 0 | 83 | 1.8e-08 | 1Score **> 31** indicates **identity** Score **> 18** indicates **homology** | U | R.FPQLDDTSFANSR.D |
| 63863 | 1705 | – | 1717 | 749.3522 | 1496.6898 | 1496.6896 | 0.15 | 0 | 65 | 8.7e-07 | 1Score **> 31** indicates **identity** Score **> 17** indicates **homology** | U | R.FPQLDDTSFANSR.D |
| 63864 | 1705 | – | 1717 | 749.3522 | 1496.6899 | 1496.6896 | 0.23 | 0 | 66 | 7e-07 | 1Score **> 31** indicates **identity** Score **> 17** indicates **homology** | U | R.FPQLDDTSFANSR.D |
| 63865 | 1705 | – | 1717 | 749.3525 | 1496.6904 | 1496.6896 | 0.53 | 0 | 88 | 5.8e-09 | 1Score **> 31** indicates **identity** Score **> 18** indicates **homology** | U | R.FPQLDDTSFANSR.D |
| 63866 | 1705 | – | 1717 | 749.3527 | 1496.6909 | 1496.6896 | 0.88 | 0 | 38 | 0.00026 | 1Score **> 31** indicates **identity** Score **> 15** indicates **homology** | U | R.FPQLDDTSFANSR.D |
| 183733 | 1705 | – | 1732 | 630.3086 | 3146.5064 | 3146.5058 | 0.22 | 1 | 14 | 0.048 | 1Score **> 37** indicates **identity** Score **> 13** indicates **homology** | U | R.FPQLDDTSFANSRDTSFEQHVLLHTGGK.G |
| 183736 | 1705 | – | 1732 | 1049.8447 | 3146.5123 | 3146.5058 | 2.06 | 1 | 18 | 0.022 | 1Score **> 37** indicates **identity** Score **> 14** indicates **homology** | U | R.FPQLDDTSFANSRDTSFEQHVLLHTGGK.G |
| 183737 | 1705 | – | 1732 | 630.3098 | 3146.5125 | 3146.5058 | 2.14 | 1 | 22 | 0.0086 | 1Score **> 37** indicates **identity** Score **> 14** indicates **homology** | U | R.FPQLDDTSFANSRDTSFEQHVLLHTGGK.G |
| 183738 | 1705 | – | 1732 | 787.6357 | 3146.5136 | 3146.5058 | 2.48 | 1 | 17 | 0.028 | 1Score **> 37** indicates **identity** Score **> 14** indicates **homology** | U | R.FPQLDDTSFANSRDTSFEQHVLLHTGGK.G |
| 87169 | 1718 | – | 1732 | 556.9491 | 1667.8254 | 1667.8268 | -0.80 | 0 | 14 | 0.044 | 1Score **> 34** indicates **identity** Score **> 13** indicates **homology** | U | R.DTSFEQHVLLHTGGK.G |
| 87170 | 1718 | – | 1732 | 556.9491 | 1667.8256 | 1667.8268 | -0.70 | 0 | 30 | 0.0015 | 1Score **> 34** indicates **identity** Score **> 14** indicates **homology** | U | R.DTSFEQHVLLHTGGK.G |
| 87173 | 1718 | – | 1732 | 556.9498 | 1667.8276 | 1667.8268 | 0.50 | 0 | 54 | 8.2e-06 | 1Score **> 35** indicates **identity** Score **> 16** indicates **homology** | U | R.DTSFEQHVLLHTGGK.G |
| 87174 | 1718 | – | 1732 | 834.9212 | 1667.8278 | 1667.8268 | 0.61 | 0 | 66 | 7.1e-07 | 1Score **> 34** indicates **identity** Score **> 17** indicates **homology** | U | R.DTSFEQHVLLHTGGK.G |
| 87179 | 1718 | – | 1732 | 556.9509 | 1667.8310 | 1667.8268 | 2.55 | 0 | 48 | 3.4e-05 | 1Score **> 34** indicates **identity** Score **> 15** indicates **homology** | U | R.DTSFEQHVLLHTGGK.G |
| 87180 | 1718 | – | 1732 | 556.9510 | 1667.8312 | 1667.8268 | 2.70 | 0 | 50 | 2.1e-05 | 1Score **> 35** indicates **identity** Score **> 16** indicates **homology** | U | R.DTSFEQHVLLHTGGK.G |
| 87186 | 1718 | – | 1732 | 556.9525 | 1667.8357 | 1667.8268 | 5.34 | 0 | 42 | 0.00011 | 1Score **> 34** indicates **identity** Score **> 15** indicates **homology** | U | R.DTSFEQHVLLHTGGK.G |
| 87306 | 1718 | – | 1732 | 835.4195 | 1668.8245 | 1668.8108 | 8.26 | 0 | 48 | 3.8e-05 | 1Score **> 34** indicates **identity** Score **> 16** indicates **homology** | U | R.DTSFEQHVLLHTGGK.G  + Deamidated (NQ) |
| 50825 | 1733 | – | 1745 | 693.8777 | 1385.7408 | 1385.7402 | 0.45 | 0 | 55 | 7.5e-06 | 1Score **> 34** indicates **identity** Score **> 16** indicates **homology** | U | K.GVDLVLNSLAEEK.L |
| 50826 | 1733 | – | 1745 | 693.8778 | 1385.7410 | 1385.7402 | 0.61 | 0 | 74 | 1.5e-07 | 1Score **> 34** indicates **identity** Score **> 18** indicates **homology** | U | K.GVDLVLNSLAEEK.L |
| 50827 | 1733 | – | 1745 | 693.8779 | 1385.7412 | 1385.7402 | 0.71 | 0 | 59 | 3e-06 | 1Score **> 34** indicates **identity** Score **> 16** indicates **homology** | U | K.GVDLVLNSLAEEK.L |
| 50833 | 1733 | – | 1745 | 693.8808 | 1385.7470 | 1385.7402 | 4.94 | 0 | 38 | 0.0003 | 1Score **> 34** indicates **identity** Score **> 15** indicates **homology** | U | K.GVDLVLNSLAEEK.L |
| 127094 | 1733 | – | 1751 | 681.0465 | 2040.1176 | 2040.1215 | -1.89 | 1 | 30 | 0.0014 | 1Score **> 35** indicates **identity** Score **> 14** indicates **homology** | U | K.GVDLVLNSLAEEKLQASVR.C |
| 127096 | 1733 | – | 1751 | 681.0468 | 2040.1187 | 2040.1215 | -1.38 | 1 | 81 | 2.6e-08 | 1Score **> 35** indicates **identity** Score **> 18** indicates **homology** | U | K.GVDLVLNSLAEEKLQASVR.C |
| 127099 | 1733 | – | 1751 | 681.0473 | 2040.1202 | 2040.1215 | -0.63 | 1 | 29 | 0.0018 | 1Score **> 35** indicates **identity** Score **> 14** indicates **homology** | U | K.GVDLVLNSLAEEKLQASVR.C |
| 127102 | 1733 | – | 1751 | 681.0476 | 2040.1209 | 2040.1215 | -0.29 | 1 | 69 | 3.3e-07 | 1Score **> 35** indicates **identity** Score **> 17** indicates **homology** | U | K.GVDLVLNSLAEEKLQASVR.C |
| 127103 | 1733 | – | 1751 | 681.0478 | 2040.1214 | 2040.1215 | -0.029 | 1 | 18 | 0.02 | 1Score **> 35** indicates **identity** Score **> 14** indicates **homology** | U | K.GVDLVLNSLAEEKLQASVR.C |
| 127106 | 1733 | – | 1751 | 681.0480 | 2040.1220 | 2040.1215 | 0.27 | 1 | 31 | 0.0012 | 1Score **> 35** indicates **identity** Score **> 14** indicates **homology** | U | K.GVDLVLNSLAEEKLQASVR.C |
| 127110 | 1733 | – | 1751 | 681.0486 | 2040.1239 | 2040.1215 | 1.17 | 1 | 17 | 0.028 | 1Score **> 35** indicates **identity** Score **> 14** indicates **homology** | U | K.GVDLVLNSLAEEKLQASVR.C |
| 127111 | 1733 | – | 1751 | 681.0486 | 2040.1239 | 2040.1215 | 1.17 | 1 | 20 | 0.013 | 1Score **> 35** indicates **identity** Score **> 14** indicates **homology** | U | K.GVDLVLNSLAEEKLQASVR.C |
| 127116 | 1733 | – | 1751 | 681.0499 | 2040.1279 | 2040.1215 | 3.12 | 1 | 49 | 2.4e-05 | 1Score **> 35** indicates **identity** Score **> 16** indicates **homology** | U | K.GVDLVLNSLAEEKLQASVR.C |
| 127124 | 1733 | – | 1751 | 681.0540 | 2040.1402 | 2040.1215 | 9.15 | 1 | 40 | 0.00019 | 1Score **> 34** indicates **identity** Score **> 15** indicates **homology** | U | K.GVDLVLNSLAEEKLQASVR.C |
| 127247 | 1733 | – | 1751 | 681.3800 | 2041.1181 | 2041.1055 | 6.19 | 1 | 47 | 0.00016 | 1Score **> 35** indicates **identity** Score **> 21** indicates **homology** | U | K.GVDLVLNSLAEEKLQASVR.C  + Deamidated (NQ) |
| 127254 | 1733 | – | 1751 | 681.3824 | 2041.1252 | 2041.1055 | 9.67 | 1 | 64 | 4e-06 | 1Score **> 35** indicates **identity** Score **> 23** indicates **homology** | U | K.GVDLVLNSLAEEKLQASVR.C  + Deamidated (NQ) |
| 161269 | 1759 | – | 1780 | 835.4501 | 2503.3286 | 2503.3297 | -0.46 | 1 | 37 | 0.00035 | 1Score **> 37** indicates **identity** Score **> 15** indicates **homology** | U | R.FLEIGKFDLSNNHPLGMAIFLK.N |
| 161272 | 1759 | – | 1780 | 835.4517 | 2503.3334 | 2503.3297 | 1.46 | 1 | 25 | 0.0042 | 1Score **> 37** indicates **identity** Score **> 14** indicates **homology** | U | R.FLEIGKFDLSNNHPLGMAIFLK.N |
| 1417 | 1802 | – | 1808 | 372.2371 | 742.4596 | 742.4589 | 0.98 | 0 | 31 | 0.0053 | 1Score **> 21** indicates **identity** | U | R.EVAALLK.A |
| 36940 | 1809 | – | 1820 | 418.2628 | 1251.7664 | 1251.7663 | 0.10 | 2 | 17 | 0.026 | 1Score **> 27** indicates **identity** Score **> 14** indicates **homology** | U | K.AGIRDGVVKPLK.C |
| 9516 | 1827 | – | 1834 | 468.2326 | 934.4506 | 934.4508 | -0.28 | 0 | 37 | 0.00098 | 1Score **> 30** indicates **identity** Score **> 19** indicates **homology** | U | K.AQVEDAFR.Y |
| 9517 | 1827 | – | 1834 | 468.2327 | 934.4509 | 934.4508 | 0.032 | 0 | 34 | 0.0019 | 1Score **> 30** indicates **identity** Score **> 19** indicates **homology** | U | K.AQVEDAFR.Y |
| 176217 | 1845 | – | 1871 | 958.5384 | 2872.5935 | 2872.5909 | 0.89 | 1 | 33 | 0.00082 | 1Score **> 34** indicates **identity** Score **> 15** indicates **homology** | U | K.VLVQVREEEPEAVLPGAQPTLISAISK.T |
| 176221 | 1845 | – | 1871 | 719.1564 | 2872.5964 | 2872.5909 | 1.90 | 1 | 43 | 9.8e-05 | 1Score **> 34** indicates **identity** Score **> 15** indicates **homology** | U | K.VLVQVREEEPEAVLPGAQPTLISAISK.T |
| 176222 | 1845 | – | 1871 | 958.5394 | 2872.5965 | 2872.5909 | 1.94 | 1 | 57 | 4.1e-06 | 1Score **> 34** indicates **identity** Score **> 16** indicates **homology** | U | K.VLVQVREEEPEAVLPGAQPTLISAISK.T |
| 176223 | 1845 | – | 1871 | 1437.3057 | 2872.5969 | 2872.5909 | 2.08 | 1 | 40 | 0.00017 | 1Score **> 34** indicates **identity** Score **> 15** indicates **homology** | U | K.VLVQVREEEPEAVLPGAQPTLISAISK.T |
| 176224 | 1845 | – | 1871 | 958.5399 | 2872.5979 | 2872.5909 | 2.42 | 1 | 56 | 6.1e-06 | 1Score **> 34** indicates **identity** Score **> 16** indicates **homology** | U | K.VLVQVREEEPEAVLPGAQPTLISAISK.T |
| 176226 | 1845 | – | 1871 | 719.1568 | 2872.5983 | 2872.5909 | 2.56 | 1 | 33 | 0.00078 | 1Score **> 34** indicates **identity** Score **> 15** indicates **homology** | U | K.VLVQVREEEPEAVLPGAQPTLISAISK.T |
| 176227 | 1845 | – | 1871 | 958.5410 | 2872.6011 | 2872.5909 | 3.55 | 1 | 58 | 3.6e-06 | 1Score **> 33** indicates **identity** Score **> 16** indicates **homology** | U | K.VLVQVREEEPEAVLPGAQPTLISAISK.T |
| 176252 | 1845 | – | 1871 | 1437.8072 | 2873.5999 | 2873.5750 | 8.69 | 1 | 35 | 0.00058 | 1Score **> 33** indicates **identity** Score **> 15** indicates **homology** | U | K.VLVQVREEEPEAVLPGAQPTLISAISK.T  + Deamidated (NQ) |
| 140040 | 1851 | – | 1871 | 1090.0836 | 2178.1526 | 2178.1419 | 4.87 | 0 | 62 | 1.4e-06 | 1Score **> 36** indicates **identity** Score **> 16** indicates **homology** | U | R.EEEPEAVLPGAQPTLISAISK.T |
| 140041 | 1851 | – | 1871 | 727.0585 | 2178.1536 | 2178.1419 | 5.34 | 0 | 44 | 7.1e-05 | 1Score **> 36** indicates **identity** Score **> 15** indicates **homology** | U | R.EEEPEAVLPGAQPTLISAISK.T |
| 140042 | 1851 | – | 1871 | 1090.0842 | 2178.1538 | 2178.1419 | 5.42 | 0 | 38 | 0.00027 | 1Score **> 36** indicates **identity** Score **> 15** indicates **homology** | U | R.EEEPEAVLPGAQPTLISAISK.T |
| 140044 | 1851 | – | 1871 | 1090.0848 | 2178.1550 | 2178.1419 | 6.01 | 0 | 30 | 0.0016 | 1Score **> 36** indicates **identity** Score **> 14** indicates **homology** | U | R.EEEPEAVLPGAQPTLISAISK.T |
| 140045 | 1851 | – | 1871 | 727.0594 | 2178.1565 | 2178.1419 | 6.69 | 0 | 49 | 2.8e-05 | 1Score **> 36** indicates **identity** Score **> 16** indicates **homology** | U | R.EEEPEAVLPGAQPTLISAISK.T |
| 140051 | 1851 | – | 1871 | 1090.0885 | 2178.1625 | 2178.1419 | 9.43 | 0 | 26 | 0.0038 | 1Score **> 36** indicates **identity** Score **> 14** indicates **homology** | U | R.EEEPEAVLPGAQPTLISAISK.T |
| 94424 | 1879 | – | 1895 | 862.4713 | 1722.9281 | 1722.9305 | -1.35 | 0 | 60 | 2.3e-06 | 1Score **> 35** indicates **identity** Score **> 16** indicates **homology** | U | K.SYIITGGLGGFGLELAR.W |
| 94434 | 1879 | – | 1895 | 862.4735 | 1722.9325 | 1722.9305 | 1.15 | 0 | 88 | 2.8e-08 | 1Score **> 35** indicates **identity** Score **> 25** indicates **homology** | U | K.SYIITGGLGGFGLELAR.W |
| 132939 | 1927 | – | 1946 | 701.0495 | 2100.1268 | 2100.1287 | -0.91 | 1 | 102 | 3.8e-10 | 1Score **> 36** indicates **identity** Score **> 21** indicates **homology** | U | R.RQGIQVLVSTSNVSSLEGAR.A |
| 132940 | 1927 | – | 1946 | 701.0497 | 2100.1272 | 2100.1287 | -0.73 | 1 | 30 | 0.0017 | 1Score **> 36** indicates **identity** Score **> 14** indicates **homology** | U | R.RQGIQVLVSTSNVSSLEGAR.A |
| 132942 | 1927 | – | 1946 | 701.0499 | 2100.1280 | 2100.1287 | -0.37 | 1 | 108 | 1.1e-10 | 1Score **> 36** indicates **identity** Score **> 21** indicates **homology** | U | R.RQGIQVLVSTSNVSSLEGAR.A |
| 132951 | 1927 | – | 1946 | 701.0511 | 2100.1315 | 2100.1287 | 1.31 | 1 | 52 | 1.2e-05 | 1Score **> 36** indicates **identity** Score **> 16** indicates **homology** | U | R.RQGIQVLVSTSNVSSLEGAR.A |
| 132957 | 1927 | – | 1946 | 701.0515 | 2100.1328 | 2100.1287 | 1.94 | 1 | 53 | 9.7e-06 | 1Score **> 36** indicates **identity** Score **> 16** indicates **homology** | U | R.RQGIQVLVSTSNVSSLEGAR.A |
| 132972 | 1927 | – | 1946 | 701.0532 | 2100.1379 | 2100.1287 | 4.37 | 1 | 78 | 5e-08 | 1Score **> 36** indicates **identity** Score **> 17** indicates **homology** | U | R.RQGIQVLVSTSNVSSLEGAR.A |
| 132973 | 1927 | – | 1946 | 701.0533 | 2100.1380 | 2100.1287 | 4.40 | 1 | 35 | 0.00055 | 1Score **> 36** indicates **identity** Score **> 15** indicates **homology** | U | R.RQGIQVLVSTSNVSSLEGAR.A |
| 117532 | 1928 | – | 1946 | 973.0201 | 1944.0257 | 1944.0276 | -1.01 | 0 | 64 | 9.3e-07 | 1Score **> 36** indicates **identity** Score **> 17** indicates **homology** | U | R.QGIQVLVSTSNVSSLEGAR.A |
| 117533 | 1928 | – | 1946 | 973.0202 | 1944.0259 | 1944.0276 | -0.87 | 0 | 40 | 0.00017 | 1Score **> 36** indicates **identity** Score **> 15** indicates **homology** | U | R.QGIQVLVSTSNVSSLEGAR.A |
| 117535 | 1928 | – | 1946 | 973.0209 | 1944.0272 | 1944.0276 | -0.23 | 0 | 57 | 4.3e-06 | 1Score **> 36** indicates **identity** Score **> 16** indicates **homology** | U | R.QGIQVLVSTSNVSSLEGAR.A |
| 117536 | 1928 | – | 1946 | 973.0218 | 1944.0291 | 1944.0276 | 0.78 | 0 | 62 | 1.6e-06 | 1Score **> 36** indicates **identity** Score **> 16** indicates **homology** | U | R.QGIQVLVSTSNVSSLEGAR.A |
| 117537 | 1928 | – | 1946 | 973.0222 | 1944.0298 | 1944.0276 | 1.13 | 0 | 60 | 2.2e-06 | 1Score **> 36** indicates **identity** Score **> 16** indicates **homology** | U | R.QGIQVLVSTSNVSSLEGAR.A |
| 117538 | 1928 | – | 1946 | 973.0223 | 1944.0301 | 1944.0276 | 1.29 | 0 | 81 | 2.7e-08 | 1Score **> 36** indicates **identity** Score **> 18** indicates **homology** | U | R.QGIQVLVSTSNVSSLEGAR.A |
| 117539 | 1928 | – | 1946 | 973.0226 | 1944.0307 | 1944.0276 | 1.58 | 0 | 78 | 5.4e-08 | 1Score **> 36** indicates **identity** Score **> 17** indicates **homology** | U | R.QGIQVLVSTSNVSSLEGAR.A |
| 117540 | 1928 | – | 1946 | 973.0227 | 1944.0308 | 1944.0276 | 1.64 | 0 | 75 | 8.7e-08 | 1Score **> 36** indicates **identity** Score **> 17** indicates **homology** | U | R.QGIQVLVSTSNVSSLEGAR.A |
| 117542 | 1928 | – | 1946 | 973.0238 | 1944.0330 | 1944.0276 | 2.75 | 0 | 76 | 7.8e-08 | 1Score **> 36** indicates **identity** Score **> 17** indicates **homology** | U | R.QGIQVLVSTSNVSSLEGAR.A |
| 117543 | 1928 | – | 1946 | 973.0239 | 1944.0332 | 1944.0276 | 2.86 | 0 | 47 | 4.2e-05 | 1Score **> 36** indicates **identity** Score **> 15** indicates **homology** | U | R.QGIQVLVSTSNVSSLEGAR.A |
| 117545 | 1928 | – | 1946 | 973.0239 | 1944.0333 | 1944.0276 | 2.90 | 0 | 43 | 9.7e-05 | 1Score **> 36** indicates **identity** Score **> 15** indicates **homology** | U | R.QGIQVLVSTSNVSSLEGAR.A |
| 117706 | 1928 | – | 1946 | 973.5215 | 1945.0285 | 1945.0116 | 8.69 | 0 | 54 | 8.7e-06 | 1Score **> 36** indicates **identity** Score **> 16** indicates **homology** | U | R.QGIQVLVSTSNVSSLEGAR.A  + Deamidated (NQ) |
| 3972 | 1947 | – | 1954 | 408.7451 | 815.4757 | 815.4752 | 0.54 | 0 | 34 | 0.0015 | 1Score **> 28** indicates **identity** Score **> 18** indicates **homology** | U | R.ALIAEATK.L |
| 3973 | 1947 | – | 1954 | 408.7452 | 815.4758 | 815.4752 | 0.73 | 0 | 61 | 2.9e-05 | 1Score **> 28** indicates **identity** | U | R.ALIAEATK.L |
| 69797 | 1955 | – | 1969 | 771.9469 | 1541.8793 | 1541.8752 | 2.66 | 0 | 86 | 6.7e-08 | 1Score **> 34** indicates **identity** Score **> 26** indicates **homology** | U | K.LGPVGGVFNLAMVLR.D |
| 143166 | 1970 | – | 1988 | 739.7011 | 2216.0813 | 2216.0783 | 1.37 | 1 | 46 | 5.2e-05 | 1Score **> 36** indicates **identity** Score **> 15** indicates **homology** | U | R.DAMLENQTPELFQDVNKPK.Y |
| 143170 | 1970 | – | 1988 | 739.7030 | 2216.0870 | 2216.0783 | 3.94 | 1 | 42 | 0.00011 | 1Score **> 36** indicates **identity** Score **> 15** indicates **homology** | U | R.DAMLENQTPELFQDVNKPK.Y |
| 143171 | 1970 | – | 1988 | 1109.0518 | 2216.0891 | 2216.0783 | 4.87 | 1 | 69 | 3.1e-07 | 1Score **> 36** indicates **identity** Score **> 17** indicates **homology** | U | R.DAMLENQTPELFQDVNKPK.Y |
| 143172 | 1970 | – | 1988 | 739.7049 | 2216.0929 | 2216.0783 | 6.59 | 1 | 16 | 0.03 | 1Score **> 36** indicates **identity** Score **> 14** indicates **homology** | U | R.DAMLENQTPELFQDVNKPK.Y |
| 17978 | 1989 | – | 1997 | 533.2667 | 1064.5189 | 1064.5251 | -5.81 | 0 | 19 | 0.018 | 1Score **> 31** indicates **identity** Score **> 14** indicates **homology** | U | K.YNGTLNLDR.A |
| 17981 | 1989 | – | 1997 | 533.2699 | 1064.5252 | 1064.5251 | 0.15 | 0 | 52 | 7.9e-05 | 1Score **> 31** indicates **identity** Score **> 23** indicates **homology** | U | K.YNGTLNLDR.A |
| 17982 | 1989 | – | 1997 | 533.2699 | 1064.5253 | 1064.5251 | 0.22 | 0 | 48 | 0.00026 | 1Score **> 31** indicates **identity** Score **> 25** indicates **homology** | U | K.YNGTLNLDR.A |
| 18047 | 1989 | – | 1997 | 533.7615 | 1065.5085 | 1065.5091 | -0.51 | 0 | 45 | 7.4e-05 | 1Score **> 30** indicates **identity** Score **> 16** indicates **homology** | U | K.YNGTLNLDR.A  + Deamidated (NQ) |
| 18048 | 1989 | – | 1997 | 533.7617 | 1065.5088 | 1065.5091 | -0.29 | 0 | 44 | 0.00034 | 1Score **> 30** indicates **identity** Score **> 22** indicates **homology** | U | K.YNGTLNLDR.A  + Deamidated (NQ) |
| 18049 | 1989 | – | 1997 | 533.7621 | 1065.5096 | 1065.5091 | 0.52 | 0 | 65 | 1.6e-06 | 1Score **> 31** indicates **identity** Score **> 20** indicates **homology** | U | K.YNGTLNLDR.A  + Deamidated (NQ) |
| 18050 | 1989 | – | 1997 | 533.7621 | 1065.5097 | 1065.5091 | 0.60 | 0 | 65 | 1.3e-05 | 1Score **> 31** indicates **identity** Score **> 29** indicates **homology** | U | K.YNGTLNLDR.A  + Deamidated (NQ) |
| 18053 | 1989 | – | 1997 | 533.7627 | 1065.5109 | 1065.5091 | 1.72 | 0 | 32 | 0.0034 | 1Score **> 31** indicates **identity** Score **> 20** indicates **homology** | U | K.YNGTLNLDR.A  + Deamidated (NQ) |
| 18054 | 1989 | – | 1997 | 533.7629 | 1065.5113 | 1065.5091 | 2.12 | 0 | 29 | 0.0035 | 1Score **> 31** indicates **identity** Score **> 17** indicates **homology** | U | K.YNGTLNLDR.A  + Deamidated (NQ) |
| 18055 | 1989 | – | 1997 | 533.7632 | 1065.5118 | 1065.5091 | 2.58 | 0 | 41 | 0.00065 | 1Score **> 31** indicates **identity** Score **> 22** indicates **homology** | U | K.YNGTLNLDR.A  + Deamidated (NQ) |
| 141437 | 2001 | – | 2019 | 731.9887 | 2192.9443 | 2192.9507 | -2.92 | 0 | 17 | 0.023 | 1Score **> 28** indicates **identity** Score **> 14** indicates **homology** | U | R.EACPELDYFVAFSSVSCGR.G |
| 141438 | 2001 | – | 2019 | 1097.4807 | 2192.9468 | 2192.9507 | -1.80 | 0 | 106 | 1.2e-10 | 1Score **> 28** indicates **identity** Score **> 19** indicates **homology** | U | R.EACPELDYFVAFSSVSCGR.G |
| 141440 | 2001 | – | 2019 | 1097.4836 | 2192.9527 | 2192.9507 | 0.93 | 0 | 67 | 4.8e-07 | 1Score **> 29** indicates **identity** Score **> 17** indicates **homology** | U | R.EACPELDYFVAFSSVSCGR.G |
| 141441 | 2001 | – | 2019 | 1097.4837 | 2192.9529 | 2192.9507 | 1.02 | 0 | 95 | 1.2e-09 | 1Score **> 29** indicates **identity** Score **> 18** indicates **homology** | U | R.EACPELDYFVAFSSVSCGR.G |
| 141442 | 2001 | – | 2019 | 1097.4844 | 2192.9542 | 2192.9507 | 1.58 | 0 | 81 | 2.5e-08 | 1Score **> 29** indicates **identity** Score **> 18** indicates **homology** | U | R.EACPELDYFVAFSSVSCGR.G |
| 141443 | 2001 | – | 2019 | 1097.4847 | 2192.9549 | 2192.9507 | 1.90 | 0 | 76 | 7.4e-08 | 1Score **> 29** indicates **identity** Score **> 17** indicates **homology** | U | R.EACPELDYFVAFSSVSCGR.G |
| 105009 | 2020 | – | 2036 | 909.3973 | 1816.7801 | 1816.7798 | 0.15 | 0 | 88 | 5.2e-09 | 1Score **> 28** indicates **identity** Score **> 18** indicates **homology** | U | R.GNAGQTNYGFANSTMER.I |
| 105010 | 2020 | – | 2036 | 909.3978 | 1816.7810 | 1816.7798 | 0.62 | 0 | 104 | 1.8e-10 | 1Score **> 28** indicates **identity** Score **> 19** indicates **homology** | U | R.GNAGQTNYGFANSTMER.I |
| 105011 | 2020 | – | 2036 | 909.3990 | 1816.7834 | 1816.7798 | 1.95 | 0 | 72 | 1.6e-07 | 1Score **> 28** indicates **identity** Score **> 17** indicates **homology** | U | R.GNAGQTNYGFANSTMER.I |
| 105085 | 2020 | – | 2036 | 909.8910 | 1817.7675 | 1817.7639 | 2.03 | 0 | 53 | 1e-05 | 1Score **> 25** indicates **identity** Score **> 16** indicates **homology** | U | R.GNAGQTNYGFANSTMER.I  + Deamidated (NQ) |
| 106652 | 2020 | – | 2036 | 917.3924 | 1832.7702 | 1832.7748 | -2.50 | 0 | 46 | 5e-05 | 1Score **> 25** indicates **identity** Score **> 15** indicates **homology** | U | R.GNAGQTNYGFANSTMER.I  + Oxidation (M) |
| 184429 | 2081 | – | 2108 | 794.6730 | 3174.6629 | 3174.6457 | 5.43 | 1 | 39 | 0.00023 | 1Score **> 37** indicates **identity** Score **> 15** indicates **homology** | U | R.ISSCMEVLDLFLNQPHAVLSSFVLAEKK.A |
| 184430 | 2081 | – | 2108 | 1059.2293 | 3174.6661 | 3174.6457 | 6.41 | 1 | 20 | 0.014 | 1Score **> 37** indicates **identity** Score **> 14** indicates **homology** | U | R.ISSCMEVLDLFLNQPHAVLSSFVLAEKK.A |
| 92607 | 2108 | – | 2123 | 428.2287 | 1708.8858 | 1708.8857 | 0.080 | 2 | 17 | 0.028 | 1Score **> 35** indicates **identity** Score **> 14** indicates **homology** | U | K.KAVAHGDGDTQRDLVK.A |
| 92608 | 2108 | – | 2123 | 855.4504 | 1708.8862 | 1708.8857 | 0.35 | 2 | 24 | 0.0055 | 1Score **> 35** indicates **identity** Score **> 14** indicates **homology** | U | K.KAVAHGDGDTQRDLVK.A |
| 75136 | 2109 | – | 2123 | 527.9376 | 1580.7911 | 1580.7907 | 0.25 | 1 | 31 | 0.0014 | 1Score **> 34** indicates **identity** Score **> 14** indicates **homology** | U | K.AVAHGDGDTQRDLVK.A |
| 75137 | 2109 | – | 2123 | 791.4030 | 1580.7915 | 1580.7907 | 0.49 | 1 | 44 | 7e-05 | 1Score **> 34** indicates **identity** Score **> 15** indicates **homology** | U | K.AVAHGDGDTQRDLVK.A |
| 10373 | 2124 | – | 2132 | 475.3005 | 948.5865 | 948.5869 | -0.40 | 0 | 25 | 0.0043 | 1Score **> 20** indicates **identity** Score **> 14** indicates **homology** | U | K.AVAHILGIR.D |
| 97361 | 2158 | – | 2171 | 437.9924 | 1747.9405 | 1747.9403 | 0.11 | 1 | 21 | 0.042 | 1Score **> 35** indicates **identity** Score **> 20** indicates **homology** | U | R.QILEREHDLVLPMR.E |
| 97362 | 2158 | – | 2171 | 437.9933 | 1747.9442 | 1747.9403 | 2.21 | 1 | 18 | 0.021 | 1Score **> 35** indicates **identity** Score **> 14** indicates **homology** | U | R.QILEREHDLVLPMR.E |
| 126808 | 2180 | – | 2198 | 1019.9984 | 2037.9822 | 2037.9888 | -3.23 | 2 | 16 | 0.033 | 1Score **> 35** indicates **identity** Score **> 14** indicates **homology** | U | R.KLQEMSSKTDSATDTTAPK.S |
| 126811 | 2180 | – | 2198 | 680.3368 | 2037.9887 | 2037.9888 | -0.061 | 2 | 58 | 3.7e-06 | 1Score **> 35** indicates **identity** Score **> 16** indicates **homology** | U | R.KLQEMSSKTDSATDTTAPK.S |
| 126813 | 2180 | – | 2198 | 510.5051 | 2037.9911 | 2037.9888 | 1.12 | 2 | 25 | 0.0041 | 1Score **> 35** indicates **identity** Score **> 14** indicates **homology** | U | R.KLQEMSSKTDSATDTTAPK.S |
| 114233 | 2181 | – | 2198 | 637.6394 | 1909.8964 | 1909.8939 | 1.31 | 1 | 55 | 7e-06 | 1Score **> 33** indicates **identity** Score **> 16** indicates **homology** | U | K.LQEMSSKTDSATDTTAPK.S |
| 131048 | 2181 | – | 2198 | 695.3435 | 2083.0087 | 2082.9878 | 10.00 | 1 | 28 | 0.0023 | 1Score **> 35** indicates **identity** Score **> 14** indicates **homology** | U | K.LQEMSSKTDSATDTTAPK.S  + Deamidated (NQ); HNE (K); Oxidation (M) |
| 190142 | 2257 | – | 2287 | 1152.5810 | 3454.7211 | 3454.7152 | 1.70 | 0 | 44 | 7.2e-05 | 1Score **> 38** indicates **identity** Score **> 15** indicates **homology** | U | K.LSVPTYGLQCTQAAPLDSIPNLAAYYIDCIK.Q |
| 18633 | 2288 | – | 2296 | 537.2728 | 1072.5311 | 1072.5302 | 0.88 | 0 | 63 | 8.5e-06 | 1Score **> 31** indicates **identity** Score **> 25** indicates **homology** | U | K.QVQPEGPYR.I |
| 139369 | 2348 | – | 2366 | 725.3246 | 2172.9519 | 2172.9530 | -0.50 | 0 | 38 | 0.0003 | 1Score **> 29** indicates **identity** Score **> 15** indicates **homology** | U | K.MTPGCEAEAEAEALCFFIK.Q |
| 139370 | 2348 | – | 2366 | 1087.4867 | 2172.9588 | 2172.9530 | 2.70 | 0 | 52 | 1.4e-05 | 1Score **> 30** indicates **identity** Score **> 16** indicates **homology** | U | K.MTPGCEAEAEAEALCFFIK.Q |
| 21323 | 2367 | – | 2375 | 368.1890 | 1101.5453 | 1101.5455 | -0.18 | 0 | 36 | 0.0039 | 1Score **> 31** indicates **identity** Score **> 24** indicates **homology** | U | K.QFLDVEHSK.V |
| 21325 | 2367 | – | 2375 | 551.7805 | 1101.5465 | 1101.5455 | 0.91 | 0 | 30 | 0.0014 | 1Score **> 32** indicates **identity** Score **> 14** indicates **homology** | U | K.QFLDVEHSK.V |
| 21326 | 2367 | – | 2375 | 368.1894 | 1101.5465 | 1101.5455 | 0.93 | 0 | 38 | 0.0023 | 1Score **> 32** indicates **identity** Score **> 25** indicates **homology** | U | K.QFLDVEHSK.V |
| 77233 | 2376 | – | 2389 | 532.6497 | 1594.9273 | 1594.9294 | -1.33 | 1 | 60 | 2.4e-06 | 1Score **> 31** indicates **identity** Score **> 16** indicates **homology** | U | K.VLEALLPLKSLEDR.V |
| 77238 | 2376 | – | 2389 | 532.6510 | 1594.9311 | 1594.9294 | 1.09 | 1 | 26 | 0.0039 | 1Score **> 31** indicates **identity** Score **> 14** indicates **homology** | U | K.VLEALLPLKSLEDR.V |
| 77239 | 2376 | – | 2389 | 532.6510 | 1594.9311 | 1594.9294 | 1.09 | 1 | 62 | 1.7e-06 | 1Score **> 31** indicates **identity** Score **> 16** indicates **homology** | U | K.VLEALLPLKSLEDR.V |
| 77240 | 2376 | – | 2389 | 798.4729 | 1594.9312 | 1594.9294 | 1.15 | 1 | 49 | 2.8e-05 | 1Score **> 31** indicates **identity** Score **> 16** indicates **homology** | U | K.VLEALLPLKSLEDR.V |
| 77243 | 2376 | – | 2389 | 532.6512 | 1594.9316 | 1594.9294 | 1.42 | 1 | 51 | 1.5e-05 | 1Score **> 31** indicates **identity** Score **> 16** indicates **homology** | U | K.VLEALLPLKSLEDR.V |
| 77246 | 2376 | – | 2389 | 532.6515 | 1594.9326 | 1594.9294 | 2.00 | 1 | 63 | 1.4e-06 | 1Score **> 31** indicates **identity** Score **> 16** indicates **homology** | U | K.VLEALLPLKSLEDR.V |
| 165499 | 2376 | – | 2399 | 865.1857 | 2592.5351 | 2592.5102 | 9.64 | 2 | 18 | 0.023 | 1Score **> 26** indicates **identity** Score **> 14** indicates **homology** | U | K.VLEALLPLKSLEDRVAASVDLITK.S |
| 80161 | 2385 | – | 2399 | 539.6321 | 1615.8746 | 1615.8781 | -2.16 | 1 | 19 | 0.018 | 1Score **> 35** indicates **identity** Score **> 14** indicates **homology** | U | K.SLEDRVAASVDLITK.S |
| 80162 | 2385 | – | 2399 | 539.6322 | 1615.8749 | 1615.8781 | -1.96 | 1 | 31 | 0.0012 | 1Score **> 35** indicates **identity** Score **> 14** indicates **homology** | U | K.SLEDRVAASVDLITK.S |
| 80166 | 2385 | – | 2399 | 539.6336 | 1615.8789 | 1615.8781 | 0.51 | 1 | 45 | 6.2e-05 | 1Score **> 35** indicates **identity** Score **> 15** indicates **homology** | U | K.SLEDRVAASVDLITK.S |
| 80168 | 2385 | – | 2399 | 539.6337 | 1615.8793 | 1615.8781 | 0.76 | 1 | 34 | 0.00069 | 1Score **> 35** indicates **identity** Score **> 15** indicates **homology** | U | K.SLEDRVAASVDLITK.S |
| 80171 | 2385 | – | 2399 | 808.9473 | 1615.8801 | 1615.8781 | 1.24 | 1 | 34 | 0.0073 | 1Score **> 35** indicates **identity** Score **> 25** indicates **homology** | U | K.SLEDRVAASVDLITK.S |
| 80172 | 2385 | – | 2399 | 539.6340 | 1615.8802 | 1615.8781 | 1.32 | 1 | 24 | 0.0061 | 1Score **> 35** indicates **identity** Score **> 14** indicates **homology** | U | K.SLEDRVAASVDLITK.S |
| 80173 | 2385 | – | 2399 | 539.6342 | 1615.8808 | 1615.8781 | 1.69 | 1 | 36 | 0.00042 | 1Score **> 35** indicates **identity** Score **> 15** indicates **homology** | U | K.SLEDRVAASVDLITK.S |
| 80175 | 2385 | – | 2399 | 808.9484 | 1615.8822 | 1615.8781 | 2.57 | 1 | 37 | 0.0027 | 1Score **> 35** indicates **identity** Score **> 24** indicates **homology** | U | K.SLEDRVAASVDLITK.S |
| 14413 | 2390 | – | 2399 | 508.8021 | 1015.5897 | 1015.5914 | -1.65 | 0 | 29 | 0.007 | 1Score **> 34** indicates **identity** Score **> 20** indicates **homology** | U | R.VAASVDLITK.S |
| 14414 | 2390 | – | 2399 | 508.8029 | 1015.5913 | 1015.5914 | -0.081 | 0 | 47 | 0.00026 | 1Score **> 32** indicates **identity** Score **> 24** indicates **homology** | U | R.VAASVDLITK.S |
| 14416 | 2390 | – | 2399 | 508.8032 | 1015.5918 | 1015.5914 | 0.39 | 0 | 63 | 5.6e-06 | 1Score **> 32** indicates **identity** Score **> 23** indicates **homology** | U | R.VAASVDLITK.S |
| 14417 | 2390 | – | 2399 | 508.8032 | 1015.5919 | 1015.5914 | 0.50 | 0 | 71 | 3.5e-06 | 1Score **> 32** indicates **identity** Score **> 29** indicates **homology** | U | R.VAASVDLITK.S |
| 14418 | 2390 | – | 2399 | 508.8033 | 1015.5921 | 1015.5914 | 0.75 | 0 | 71 | 3.5e-06 | 1Score **> 32** indicates **identity** Score **> 29** indicates **homology** | U | R.VAASVDLITK.S |
| 14420 | 2390 | – | 2399 | 508.8034 | 1015.5922 | 1015.5914 | 0.82 | 0 | 71 | 3.5e-06 | 1Score **> 32** indicates **identity** Score **> 29** indicates **homology** | U | R.VAASVDLITK.S |
| 14423 | 2390 | – | 2399 | 508.8040 | 1015.5934 | 1015.5914 | 1.97 | 0 | 42 | 0.00035 | 1Score **> 33** indicates **identity** Score **> 20** indicates **homology** | U | R.VAASVDLITK.S |
| 52023 | 2408 | – | 2419 | 466.9063 | 1397.6970 | 1397.6979 | -0.66 | 0 | 39 | 0.00024 | 1Score **> 33** indicates **identity** Score **> 15** indicates **homology** | U | R.ELSFAAVSFYHK.L |
| 52025 | 2408 | – | 2419 | 466.9064 | 1397.6974 | 1397.6979 | -0.40 | 0 | 14 | 0.05 | 1Score **> 33** indicates **identity** Score **> 13** indicates **homology** | U | R.ELSFAAVSFYHK.L |
| 52026 | 2408 | – | 2419 | 466.9064 | 1397.6975 | 1397.6979 | -0.34 | 0 | 27 | 0.0029 | 1Score **> 33** indicates **identity** Score **> 14** indicates **homology** | U | R.ELSFAAVSFYHK.L |
| 52027 | 2408 | – | 2419 | 466.9065 | 1397.6978 | 1397.6979 | -0.087 | 0 | 42 | 0.00011 | 1Score **> 33** indicates **identity** Score **> 15** indicates **homology** | U | R.ELSFAAVSFYHK.L |
| 52028 | 2408 | – | 2419 | 466.9066 | 1397.6980 | 1397.6979 | 0.059 | 0 | 23 | 0.0063 | 1Score **> 33** indicates **identity** Score **> 14** indicates **homology** | U | R.ELSFAAVSFYHK.L |
| 52030 | 2408 | – | 2419 | 699.8566 | 1397.6987 | 1397.6979 | 0.57 | 0 | 67 | 5.1e-07 | 1Score **> 33** indicates **identity** Score **> 17** indicates **homology** | U | R.ELSFAAVSFYHK.L |
| 52031 | 2408 | – | 2419 | 466.9069 | 1397.6988 | 1397.6979 | 0.63 | 0 | 22 | 0.0082 | 1Score **> 33** indicates **identity** Score **> 14** indicates **homology** | U | R.ELSFAAVSFYHK.L |
| 52032 | 2408 | – | 2419 | 466.9071 | 1397.6994 | 1397.6979 | 1.02 | 0 | 38 | 0.00027 | 1Score **> 33** indicates **identity** Score **> 15** indicates **homology** | U | R.ELSFAAVSFYHK.L |
| 52033 | 2408 | – | 2419 | 466.9071 | 1397.6994 | 1397.6979 | 1.07 | 0 | 34 | 0.00064 | 1Score **> 33** indicates **identity** Score **> 15** indicates **homology** | U | R.ELSFAAVSFYHK.L |
| 52034 | 2408 | – | 2419 | 466.9071 | 1397.6996 | 1397.6979 | 1.18 | 0 | 14 | 0.048 | 1Score **> 33** indicates **identity** Score **> 13** indicates **homology** | U | R.ELSFAAVSFYHK.L |
| 52035 | 2408 | – | 2419 | 466.9073 | 1397.7000 | 1397.6979 | 1.47 | 0 | 20 | 0.015 | 1Score **> 33** indicates **identity** Score **> 14** indicates **homology** | U | R.ELSFAAVSFYHK.L |
| 52037 | 2408 | – | 2419 | 466.9078 | 1397.7016 | 1397.6979 | 2.61 | 0 | 16 | 0.035 | 1Score **> 33** indicates **identity** Score **> 13** indicates **homology** | U | R.ELSFAAVSFYHK.L |
| 52038 | 2408 | – | 2419 | 466.9078 | 1397.7017 | 1397.6979 | 2.67 | 0 | 22 | 0.0086 | 1Score **> 33** indicates **identity** Score **> 14** indicates **homology** | U | R.ELSFAAVSFYHK.L |
| 39057 | 2430 | – | 2440 | 424.5806 | 1270.7201 | 1270.7146 | 4.35 | 1 | 37 | 0.00079 | 1Score **> 33** indicates **identity** Score **> 18** indicates **homology** | U | K.AKYHGNVTLLR.A |
| 18570 | 2432 | – | 2440 | 536.7986 | 1071.5825 | 1071.5825 | 0.026 | 0 | 36 | 0.00046 | 1Score **> 33** indicates **identity** Score **> 15** indicates **homology** | U | K.YHGNVTLLR.A |
| 18571 | 2432 | – | 2440 | 358.2015 | 1071.5826 | 1071.5825 | 0.061 | 0 | 40 | 0.0066 | 1Score **> 33** indicates **identity** Score **> 30** indicates **homology** | U | K.YHGNVTLLR.A |
| 18572 | 2432 | – | 2440 | 358.2016 | 1071.5828 | 1071.5825 | 0.31 | 0 | 43 | 0.0049 | 1Score **> 33** indicates **identity** Score **> 33** indicates **homology** | U | K.YHGNVTLLR.A |
| 18575 | 2432 | – | 2440 | 536.7989 | 1071.5833 | 1071.5825 | 0.76 | 0 | 17 | 0.027 | 1Score **> 33** indicates **identity** Score **> 14** indicates **homology** | U | K.YHGNVTLLR.A |
| 18577 | 2432 | – | 2440 | 536.7991 | 1071.5836 | 1071.5825 | 1.05 | 0 | 20 | 0.014 | 1Score **> 33** indicates **identity** Score **> 14** indicates **homology** | U | K.YHGNVTLLR.A |
| 161954 | 2441 | – | 2464 | 840.3830 | 2518.1273 | 2518.1282 | -0.36 | 1 | 54 | 8.2e-06 | 1Score **> 31** indicates **identity** Score **> 16** indicates **homology** | U | R.AKTGGTYGEDLGADYNLSQVCDGK.V |
| 161955 | 2441 | – | 2464 | 840.3846 | 2518.1319 | 2518.1282 | 1.47 | 1 | 40 | 0.00019 | 1Score **> 32** indicates **identity** Score **> 15** indicates **homology** | U | R.AKTGGTYGEDLGADYNLSQVCDGK.V |
| 162016 | 2441 | – | 2464 | 840.7177 | 2519.1311 | 2519.1122 | 7.51 | 1 | 21 | 0.01 | 1Score **> 32** indicates **identity** Score **> 14** indicates **homology** | U | R.AKTGGTYGEDLGADYNLSQVCDGK.V  + Deamidated (NQ) |
| 162017 | 2441 | – | 2464 | 840.7182 | 2519.1329 | 2519.1122 | 8.21 | 1 | 26 | 0.004 | 1Score **> 32** indicates **identity** Score **> 14** indicates **homology** | U | R.AKTGGTYGEDLGADYNLSQVCDGK.V  + Deamidated (NQ) |
| 193241 | 2441 | – | 2475 | 941.2018 | 3760.7780 | 3760.7751 | 0.75 | 2 | 33 | 0.00084 | 1Score **> 36** indicates **identity** Score **> 15** indicates **homology** | U | R.AKTGGTYGEDLGADYNLSQVCDGKVSVHIIEGDHR.T |
| 193242 | 2441 | – | 2475 | 753.1639 | 3760.7831 | 3760.7751 | 2.12 | 2 | 28 | 0.0026 | 1Score **> 36** indicates **identity** Score **> 14** indicates **homology** | U | R.AKTGGTYGEDLGADYNLSQVCDGKVSVHIIEGDHR.T |
| 193251 | 2441 | – | 2475 | 753.3649 | 3761.7880 | 3761.7591 | 7.66 | 2 | 32 | 0.00091 | 1Score **> 36** indicates **identity** Score **> 15** indicates **homology** | U | R.AKTGGTYGEDLGADYNLSQVCDGKVSVHIIEGDHR.T  + Deamidated (NQ) |
| 150350 | 2443 | – | 2464 | 774.0058 | 2318.9956 | 2318.9961 | -0.25 | 0 | 19 | 0.015 | 1Score **> 28** indicates **identity** Score **> 14** indicates **homology** | U | K.TGGTYGEDLGADYNLSQVCDGK.V |
| 150351 | 2443 | – | 2464 | 1160.5068 | 2318.9991 | 2318.9961 | 1.28 | 0 | 111 | 4.1e-11 | 1Score **> 28** indicates **identity** Score **> 19** indicates **homology** | U | K.TGGTYGEDLGADYNLSQVCDGK.V |
| 150353 | 2443 | – | 2464 | 1160.5081 | 2319.0017 | 2318.9961 | 2.41 | 0 | 158 | 1.6e-15 | 1Score **> 28** indicates **identity** Score **> 22** indicates **homology** | U | K.TGGTYGEDLGADYNLSQVCDGK.V |
| 191599 | 2443 | – | 2475 | 891.4180 | 3561.6427 | 3561.6431 | -0.10 | 1 | 35 | 0.00057 | 1Score **> 34** indicates **identity** Score **> 15** indicates **homology** | U | K.TGGTYGEDLGADYNLSQVCDGKVSVHIIEGDHR.T |
| 191600 | 2443 | – | 2475 | 713.3364 | 3561.6455 | 3561.6431 | 0.67 | 1 | 28 | 0.0023 | 1Score **> 35** indicates **identity** Score **> 14** indicates **homology** | U | K.TGGTYGEDLGADYNLSQVCDGKVSVHIIEGDHR.T |
| 191601 | 2443 | – | 2475 | 713.3377 | 3561.6519 | 3561.6431 | 2.49 | 1 | 25 | 0.0046 | 1Score **> 35** indicates **identity** Score **> 14** indicates **homology** | U | K.TGGTYGEDLGADYNLSQVCDGKVSVHIIEGDHR.T |
| 191609 | 2443 | – | 2475 | 1188.5568 | 3562.6487 | 3562.6271 | 6.06 | 1 | 47 | 4.5e-05 | 1Score **> 35** indicates **identity** Score **> 16** indicates **homology** | U | K.TGGTYGEDLGADYNLSQVCDGKVSVHIIEGDHR.T  + Deamidated (NQ) |
| 37928 | 2465 | – | 2475 | 421.2263 | 1260.6571 | 1260.6575 | -0.35 | 0 | 35 | 0.0042 | 1Score **> 34** indicates **identity** Score **> 23** indicates **homology** | U | K.VSVHIIEGDHR.T |
| 37931 | 2465 | – | 2475 | 631.3361 | 1260.6577 | 1260.6575 | 0.15 | 0 | 29 | 0.0038 | 1Score **> 34** indicates **identity** Score **> 17** indicates **homology** | U | K.VSVHIIEGDHR.T |
| 37932 | 2465 | – | 2475 | 631.3365 | 1260.6585 | 1260.6575 | 0.82 | 0 | 45 | 9.4e-05 | 1Score **> 34** indicates **identity** Score **> 17** indicates **homology** | U | K.VSVHIIEGDHR.T |
| 37933 | 2465 | – | 2475 | 421.2269 | 1260.6589 | 1260.6575 | 1.12 | 0 | 30 | 0.0097 | 1Score **> 34** indicates **identity** Score **> 22** indicates **homology** | U | K.VSVHIIEGDHR.T |
| 158239 | 2476 | – | 2498 | 613.0885 | 2448.3251 | 2448.3224 | 1.10 | 0 | 29 | 0.0018 | 1Score **> 36** indicates **identity** Score **> 14** indicates **homology** | U | R.TLLEGSGLESIINIIHSSLAEPR.V |
| 158240 | 2476 | – | 2498 | 817.1158 | 2448.3255 | 2448.3224 | 1.28 | 0 | 24 | 0.012 | 1Score **> 36** indicates **identity** Score **> 17** indicates **homology** | U | R.TLLEGSGLESIINIIHSSLAEPR.V |
| 158246 | 2476 | – | 2498 | 817.1163 | 2448.3272 | 2448.3224 | 1.97 | 0 | 68 | 3.9e-07 | 1Score **> 36** indicates **identity** Score **> 17** indicates **homology** | U | R.TLLEGSGLESIINIIHSSLAEPR.V |
| 158248 | 2476 | – | 2498 | 817.1172 | 2448.3298 | 2448.3224 | 3.05 | 0 | 20 | 0.015 | 1Score **> 36** indicates **identity** Score **> 14** indicates **homology** | U | R.TLLEGSGLESIINIIHSSLAEPR.V |
| 158252 | 2476 | – | 2498 | 1225.1727 | 2448.3309 | 2448.3224 | 3.50 | 0 | 136 | 1.7e-13 | 1Score **> 36** indicates **identity** Score **> 21** indicates **homology** | U | R.TLLEGSGLESIINIIHSSLAEPR.V |
| 158262 | 2476 | – | 2498 | 817.1193 | 2448.3362 | 2448.3224 | 5.65 | 0 | 80 | 3.1e-08 | 1Score **> 36** indicates **identity** Score **> 18** indicates **homology** | U | R.TLLEGSGLESIINIIHSSLAEPR.V |
| 158263 | 2476 | – | 2498 | 1225.1754 | 2448.3362 | 2448.3224 | 5.65 | 0 | 123 | 3.2e-12 | 1Score **> 36** indicates **identity** Score **> 20** indicates **homology** | U | R.TLLEGSGLESIINIIHSSLAEPR.V |
| 158265 | 2476 | – | 2498 | 817.1194 | 2448.3364 | 2448.3224 | 5.74 | 0 | 16 | 0.035 | 1Score **> 36** indicates **identity** Score **> 13** indicates **homology** | U | R.TLLEGSGLESIINIIHSSLAEPR.V |

---

```
ID   FAS_MOUSE               Reviewed;        2504 AA.
AC   P19096; B1ATU8; Q6PB72; Q8C4Z0; Q9EQR0;
DT   01-NOV-1990, integrated into UniProtKB/Swiss-Prot.
DT   11-OCT-2004, sequence version 2.
DT   28-JUN-2023, entry version 215.
DE   RecName: Full=Fatty acid synthase;
DE            EC=2.3.1.85 {ECO:0000269|PubMed:29328619};
DE   AltName: Full=Type I Fatty Acid Synthase {ECO:0000303|PubMed:31811668};
DE   Includes:
DE     RecName: Full=[Acyl-carrier-protein] S-acetyltransferase;
DE              EC=2.3.1.38 {ECO:0000269|PubMed:29328619};
DE   Includes:
DE     RecName: Full=[Acyl-carrier-protein] S-malonyltransferase;
DE              EC=2.3.1.39 {ECO:0000269|PubMed:29328619};
DE   Includes:
DE     RecName: Full=3-oxoacyl-[acyl-carrier-protein] synthase;
DE              EC=2.3.1.41 {ECO:0000269|PubMed:31811668};
DE   Includes:
DE     RecName: Full=3-oxoacyl-[acyl-carrier-protein] reductase;
DE              EC=1.1.1.100 {ECO:0000250|UniProtKB:P49327};
DE   Includes:
DE     RecName: Full=3-hydroxyacyl-[acyl-carrier-protein] dehydratase;
DE              EC=4.2.1.59 {ECO:0000250|UniProtKB:P49327};
DE   Includes:
DE     RecName: Full=Enoyl-[acyl-carrier-protein] reductase;
DE              EC=1.3.1.39 {ECO:0000250|UniProtKB:P49327};
DE   Includes:
DE     RecName: Full=Acyl-[acyl-carrier-protein] hydrolase;
DE              EC=3.1.2.14 {ECO:0000250|UniProtKB:P49327};
GN   Name=Fasn;
OS   Mus musculus (Mouse).
OC   Eukaryota; Metazoa; Chordata; Craniata; Vertebrata; Euteleostomi; Mammalia;
OC   Eutheria; Euarchontoglires; Glires; Rodentia; Myomorpha; Muroidea; Muridae;
OC   Murinae; Mus; Mus.
OX   NCBI_TaxID=10090;
RN   [1]
RP   NUCLEOTIDE SEQUENCE [MRNA].
RC   STRAIN=C57BL/6J;
RX   PubMed=11029661; DOI=10.1046/j.1365-2443.2000.00369.x;
RA   Ueno K.;
RT   "Involvement of fatty acid synthase in axonal development in mouse
RT   embryos.";
RL   Genes Cells 5:859-869(2000).
RN   [2]
RP   NUCLEOTIDE SEQUENCE [LARGE SCALE GENOMIC DNA].
RC   STRAIN=C57BL/6J;
RX   PubMed=19468303; DOI=10.1371/journal.pbio.1000112;
RA   Church D.M., Goodstadt L., Hillier L.W., Zody M.C., Goldstein S., She X.,
RA   Bult C.J., Agarwala R., Cherry J.L., DiCuccio M., Hlavina W., Kapustin Y.,
RA   Meric P., Maglott D., Birtle Z., Marques A.C., Graves T., Zhou S.,
RA   Teague B., Potamousis K., Churas C., Place M., Herschleb J., Runnheim R.,
RA   Forrest D., Amos-Landgraf J., Schwartz D.C., Cheng Z., Lindblad-Toh K.,
RA   Eichler E.E., Ponting C.P.;
RT   "Lineage-specific biology revealed by a finished genome assembly of the
RT   mouse.";
RL   PLoS Biol. 7:E1000112-E1000112(2009).
RN   [3]
RP   NUCLEOTIDE SEQUENCE [LARGE SCALE MRNA].
RC   STRAIN=FVB/N; TISSUE=Brain, and Liver;
RX   PubMed=15489334; DOI=10.1101/gr.2596504;
RG   The MGC Project Team;
RT   "The status, quality, and expansion of the NIH full-length cDNA project:
RT   the Mammalian Gene Collection (MGC).";
RL   Genome Res. 14:2121-2127(2004).
RN   [4]
RP   PROTEIN SEQUENCE OF 1-12; 225-235; 299-310; 385-409; 469-478; 1252-1270;
RP   1333-1344; 1388-1398; 1501-1508; 1705-1717; 1733-1745; 2124-2132; 2376-2384
RP   AND 2476-2498, ACETYLATION AT MET-1, AND IDENTIFICATION BY MASS
RP   SPECTROMETRY.
RC   STRAIN=C57BL/6J; TISSUE=Liver;
RA   Bienvenut W.V.;
RL   Submitted (JUL-2005) to UniProtKB.
RN   [5]
RP   NUCLEOTIDE SEQUENCE [LARGE SCALE MRNA] OF 1527-2504.
RC   STRAIN=C57BL/6J; TISSUE=Thymus;
RX   PubMed=16141072; DOI=10.1126/science.1112014;
RA   Carninci P., Kasukawa T., Katayama S., Gough J., Frith M.C., Maeda N.,
RA   Oyama R., Ravasi T., Lenhard B., Wells C., Kodzius R., Shimokawa K.,
RA   Bajic V.B., Brenner S.E., Batalov S., Forrest A.R., Zavolan M., Davis M.J.,
RA   Wilming L.G., Aidinis V., Allen J.E., Ambesi-Impiombato A., Apweiler R.,
RA   Aturaliya R.N., Bailey T.L., Bansal M., Baxter L., Beisel K.W., Bersano T.,
RA   Bono H., Chalk A.M., Chiu K.P., Choudhary V., Christoffels A.,
RA   Clutterbuck D.R., Crowe M.L., Dalla E., Dalrymple B.P., de Bono B.,
RA   Della Gatta G., di Bernardo D., Down T., Engstrom P., Fagiolini M.,
RA   Faulkner G., Fletcher C.F., Fukushima T., Furuno M., Futaki S.,
RA   Gariboldi M., Georgii-Hemming P., Gingeras T.R., Gojobori T., Green R.E.,
RA   Gustincich S., Harbers M., Hayashi Y., Hensch T.K., Hirokawa N., Hill D.,
RA   Huminiecki L., Iacono M., Ikeo K., Iwama A., Ishikawa T., Jakt M.,
RA   Kanapin A., Katoh M., Kawasawa Y., Kelso J., Kitamura H., Kitano H.,
RA   Kollias G., Krishnan S.P., Kruger A., Kummerfeld S.K., Kurochkin I.V.,
RA   Lareau L.F., Lazarevic D., Lipovich L., Liu J., Liuni S., McWilliam S.,
RA   Madan Babu M., Madera M., Marchionni L., Matsuda H., Matsuzawa S., Miki H.,
RA   Mignone F., Miyake S., Morris K., Mottagui-Tabar S., Mulder N., Nakano N.,
RA   Nakauchi H., Ng P., Nilsson R., Nishiguchi S., Nishikawa S., Nori F.,
RA   Ohara O., Okazaki Y., Orlando V., Pang K.C., Pavan W.J., Pavesi G.,
RA   Pesole G., Petrovsky N., Piazza S., Reed J., Reid J.F., Ring B.Z.,
RA   Ringwald M., Rost B., Ruan Y., Salzberg S.L., Sandelin A., Schneider C.,
RA   Schoenbach C., Sekiguchi K., Semple C.A., Seno S., Sessa L., Sheng Y.,
RA   Shibata Y., Shimada H., Shimada K., Silva D., Sinclair B., Sperling S.,
RA   Stupka E., Sugiura K., Sultana R., Takenaka Y., Taki K., Tammoja K.,
RA   Tan S.L., Tang S., Taylor M.S., Tegner J., Teichmann S.A., Ueda H.R.,
RA   van Nimwegen E., Verardo R., Wei C.L., Yagi K., Yamanishi H.,
RA   Zabarovsky E., Zhu S., Zimmer A., Hide W., Bult C., Grimmond S.M.,
RA   Teasdale R.D., Liu E.T., Brusic V., Quackenbush J., Wahlestedt C.,
RA   Mattick J.S., Hume D.A., Kai C., Sasaki D., Tomaru Y., Fukuda S.,
RA   Kanamori-Katayama M., Suzuki M., Aoki J., Arakawa T., Iida J., Imamura K.,
RA   Itoh M., Kato T., Kawaji H., Kawagashira N., Kawashima T., Kojima M.,
RA   Kondo S., Konno H., Nakano K., Ninomiya N., Nishio T., Okada M., Plessy C.,
RA   Shibata K., Shiraki T., Suzuki S., Tagami M., Waki K., Watahiki A.,
RA   Okamura-Oho Y., Suzuki H., Kawai J., Hayashizaki Y.;
RT   "The transcriptional landscape of the mammalian genome.";
RL   Science 309:1559-1563(2005).
RN   [6]
RP   NUCLEOTIDE SEQUENCE [MRNA] OF 1666-2504.
RC   STRAIN=CD-1; TISSUE=Liver;
RX   PubMed=2920037; DOI=10.1016/0006-291x(89)92776-9;
RA   Paulauskis J.D., Sul H.S.;
RT   "Structure of mouse fatty acid synthase mRNA. Identification of the two
RT   NADPH binding sites.";
RL   Biochem. Biophys. Res. Commun. 158:690-695(1989).
RN   [7]
RP   PHOSPHORYLATION [LARGE SCALE ANALYSIS] AT SER-725; SER-1577; SER-1587 AND
RP   SER-2190, AND IDENTIFICATION BY MASS SPECTROMETRY [LARGE SCALE ANALYSIS].
RC   TISSUE=Brain, Brown adipose tissue, Heart, Kidney, Liver, Lung,
RC   Pancreas, Spleen, and Testis;
RX   PubMed=21183079; DOI=10.1016/j.cell.2010.12.001;
RA   Huttlin E.L., Jedrychowski M.P., Elias J.E., Goswami T., Rad R.,
RA   Beausoleil S.A., Villen J., Haas W., Sowa M.E., Gygi S.P.;
RT   "A tissue-specific atlas of mouse protein phosphorylation and expression.";
RL   Cell 143:1174-1189(2010).
RN   [8]
RP   INDUCTION BY ENDOCANNABINOID ANANDAMIDE.
RX   PubMed=21987372; DOI=10.1002/hep.24733;
RA   Jourdan T., Demizieux L., Gresti J., Djaouti L., Gaba L., Verges B.,
RA   Degrace P.;
RT   "Antagonism of peripheral hepatic cannabinoid receptor-1 improves liver
RT   lipid metabolism in mice: evidence from cultured explants.";
RL   Hepatology 55:790-799(2012).
RN   [9]
RP   FUNCTION, CATALYTIC ACTIVITY, BIOPHYSICOCHEMICAL PROPERTIES, AND PATHWAY.
RX   PubMed=29328619; DOI=10.1021/acschembio.7b00718;
RA   Rittner A., Paithankar K.S., Huu K.V., Grininger M.;
RT   "Characterization of the Polyspecific Transferase of Murine Type I Fatty
RT   Acid Synthase (FAS) and Implications for Polyketide Synthase (PKS)
RT   Engineering.";
RL   ACS Chem. Biol. 13:723-732(2018).
RN   [10]
RP   ACETYLATION [LARGE SCALE ANALYSIS] AT LYS-59; LYS-790; LYS-993; LYS-1071;
RP   LYS-1276; LYS-1840 AND LYS-2384, AND IDENTIFICATION BY MASS SPECTROMETRY
RP   [LARGE SCALE ANALYSIS].
RC   TISSUE=Embryonic fibroblast;
RX   PubMed=23806337; DOI=10.1016/j.molcel.2013.06.001;
RA   Park J., Chen Y., Tishkoff D.X., Peng C., Tan M., Dai L., Xie Z., Zhang Y.,
RA   Zwaans B.M., Skinner M.E., Lombard D.B., Zhao Y.;
RT   "SIRT5-mediated lysine desuccinylation impacts diverse metabolic
RT   pathways.";
RL   Mol. Cell 50:919-930(2013).
RN   [11] {ECO:0007744|PDB:6ROP}
RP   X-RAY CRYSTALLOGRAPHY (2.70 ANGSTROMS) OF 2-852 IN COMPLEX WITH OCTANOATE
RP   AND OCTANOYL-COA, FUNCTION, AND CATALYTIC ACTIVITY.
RX   PubMed=31811668; DOI=10.1002/pro.3797;
RA   Rittner A., Paithankar K.S., Himmler A., Grininger M.;
RT   "Type I fatty acid synthase trapped in the octanoyl-bound state.";
RL   Protein Sci. 29:589-605(2020).
CC   -!- FUNCTION: Fatty acid synthetase is a multifunctional enzyme that
CC       catalyzes the de novo biosynthesis of long-chain saturated fatty acids
CC       starting from acetyl-CoA and malonyl-CoA in the presence of NADPH. This
CC       multifunctional protein contains 7 catalytic activities and a site for
CC       the binding of the prosthetic group 4'-phosphopantetheine of the acyl
CC       carrier protein ([ACP]) domain. {ECO:0000269|PubMed:29328619,
CC       ECO:0000269|PubMed:31811668}.
CC   -!- CATALYTIC ACTIVITY:
CC       Reaction=acetyl-CoA + 2n H(+) + n malonyl-CoA + 2n NADPH = a long-chain
CC         fatty acid + n CO2 + (n+1) CoA + 2n NADP(+); Xref=Rhea:RHEA:14993,
CC         ChEBI:CHEBI:15378, ChEBI:CHEBI:16526, ChEBI:CHEBI:57287,
CC         ChEBI:CHEBI:57288, ChEBI:CHEBI:57384, ChEBI:CHEBI:57560,
CC         ChEBI:CHEBI:57783, ChEBI:CHEBI:58349; EC=2.3.1.85;
CC         Evidence={ECO:0000269|PubMed:29328619};
CC       PhysiologicalDirection=left-to-right; Xref=Rhea:RHEA:14994;
CC         Evidence={ECO:0000269|PubMed:29328619};
CC   -!- CATALYTIC ACTIVITY:
CC       Reaction=acetyl-CoA + holo-[ACP] = acetyl-[ACP] + CoA;
CC         Xref=Rhea:RHEA:41788, Rhea:RHEA-COMP:9621, Rhea:RHEA-COMP:9685,
CC         ChEBI:CHEBI:57287, ChEBI:CHEBI:57288, ChEBI:CHEBI:64479,
CC         ChEBI:CHEBI:78446; EC=2.3.1.38;
CC         Evidence={ECO:0000269|PubMed:29328619};
CC       PhysiologicalDirection=left-to-right; Xref=Rhea:RHEA:41789;
CC         Evidence={ECO:0000269|PubMed:29328619};
CC   -!- CATALYTIC ACTIVITY:
CC       Reaction=holo-[ACP] + malonyl-CoA = CoA + malonyl-[ACP];
CC         Xref=Rhea:RHEA:41792, Rhea:RHEA-COMP:9623, Rhea:RHEA-COMP:9685,
CC         ChEBI:CHEBI:57287, ChEBI:CHEBI:57384, ChEBI:CHEBI:64479,
CC         ChEBI:CHEBI:78449; EC=2.3.1.39;
CC         Evidence={ECO:0000269|PubMed:29328619};
CC       PhysiologicalDirection=left-to-right; Xref=Rhea:RHEA:41793;
CC         Evidence={ECO:0000269|PubMed:29328619};
CC   -!- CATALYTIC ACTIVITY:
CC       Reaction=a fatty acyl-[ACP] + H(+) + malonyl-[ACP] = a 3-oxoacyl-[ACP]
CC         + CO2 + holo-[ACP]; Xref=Rhea:RHEA:22836, Rhea:RHEA-COMP:9623,
CC         Rhea:RHEA-COMP:9685, Rhea:RHEA-COMP:9916, Rhea:RHEA-COMP:14125,
CC         ChEBI:CHEBI:15378, ChEBI:CHEBI:16526, ChEBI:CHEBI:64479,
CC         ChEBI:CHEBI:78449, ChEBI:CHEBI:78776, ChEBI:CHEBI:138651;
CC         EC=2.3.1.41; Evidence={ECO:0000269|PubMed:31811668};
CC       PhysiologicalDirection=left-to-right; Xref=Rhea:RHEA:22837;
CC         Evidence={ECO:0000269|PubMed:31811668};
CC   -!- CATALYTIC ACTIVITY:
CC       Reaction=a (3R)-hydroxyacyl-[ACP] + NADP(+) = a 3-oxoacyl-[ACP] + H(+)
CC         + NADPH; Xref=Rhea:RHEA:17397, Rhea:RHEA-COMP:9916, Rhea:RHEA-
CC         COMP:9945, ChEBI:CHEBI:15378, ChEBI:CHEBI:57783, ChEBI:CHEBI:58349,
CC         ChEBI:CHEBI:78776, ChEBI:CHEBI:78827; EC=1.1.1.100;
CC         Evidence={ECO:0000250|UniProtKB:P49327};
CC       PhysiologicalDirection=right-to-left; Xref=Rhea:RHEA:17399;
CC         Evidence={ECO:0000250|UniProtKB:P49327};
CC   -!- CATALYTIC ACTIVITY:
CC       Reaction=a (3R)-hydroxyacyl-[ACP] = a (2E)-enoyl-[ACP] + H2O;
CC         Xref=Rhea:RHEA:13097, Rhea:RHEA-COMP:9925, Rhea:RHEA-COMP:9945,
CC         ChEBI:CHEBI:15377, ChEBI:CHEBI:78784, ChEBI:CHEBI:78827; EC=4.2.1.59;
CC         Evidence={ECO:0000250|UniProtKB:P49327};
CC       PhysiologicalDirection=left-to-right; Xref=Rhea:RHEA:13098;
CC         Evidence={ECO:0000250|UniProtKB:P49327};
CC   -!- CATALYTIC ACTIVITY:
CC       Reaction=a 2,3-saturated acyl-[ACP] + NADP(+) = a (2E)-enoyl-[ACP] +
CC         H(+) + NADPH; Xref=Rhea:RHEA:22564, Rhea:RHEA-COMP:9925, Rhea:RHEA-
CC         COMP:9926, ChEBI:CHEBI:15378, ChEBI:CHEBI:57783, ChEBI:CHEBI:58349,
CC         ChEBI:CHEBI:78784, ChEBI:CHEBI:78785; EC=1.3.1.39;
CC         Evidence={ECO:0000250|UniProtKB:P49327};
CC       PhysiologicalDirection=right-to-left; Xref=Rhea:RHEA:22566;
CC         Evidence={ECO:0000250|UniProtKB:P49327};
CC   -!- CATALYTIC ACTIVITY:
CC       Reaction=H2O + hexadecanoyl-[ACP] = H(+) + hexadecanoate + holo-[ACP];
CC         Xref=Rhea:RHEA:41932, Rhea:RHEA-COMP:9652, Rhea:RHEA-COMP:9685,
CC         ChEBI:CHEBI:7896, ChEBI:CHEBI:15377, ChEBI:CHEBI:15378,
CC         ChEBI:CHEBI:64479, ChEBI:CHEBI:78483; EC=3.1.2.14;
CC         Evidence={ECO:0000250|UniProtKB:P49327};
CC       PhysiologicalDirection=left-to-right; Xref=Rhea:RHEA:41933;
CC         Evidence={ECO:0000250|UniProtKB:P49327};
CC   -!- CATALYTIC ACTIVITY:
CC       Reaction=acetyl-[ACP] + H(+) + malonyl-[ACP] = 3-oxobutanoyl-[ACP] +
CC         CO2 + holo-[ACP]; Xref=Rhea:RHEA:41800, Rhea:RHEA-COMP:9621,
CC         Rhea:RHEA-COMP:9623, Rhea:RHEA-COMP:9625, Rhea:RHEA-COMP:9685,
CC         ChEBI:CHEBI:15378, ChEBI:CHEBI:16526, ChEBI:CHEBI:64479,
CC         ChEBI:CHEBI:78446, ChEBI:CHEBI:78449, ChEBI:CHEBI:78450;
CC         Evidence={ECO:0000305|PubMed:31811668};
CC       PhysiologicalDirection=left-to-right; Xref=Rhea:RHEA:41801;
CC         Evidence={ECO:0000305|PubMed:31811668};
CC   -!- CATALYTIC ACTIVITY:
CC       Reaction=3-oxobutanoyl-[ACP] + H(+) + NADPH = (3R)-hydroxybutanoyl-
CC         [ACP] + NADP(+); Xref=Rhea:RHEA:41804, Rhea:RHEA-COMP:9625,
CC         Rhea:RHEA-COMP:9626, ChEBI:CHEBI:15378, ChEBI:CHEBI:57783,
CC         ChEBI:CHEBI:58349, ChEBI:CHEBI:78450, ChEBI:CHEBI:78451;
CC         Evidence={ECO:0000250|UniProtKB:P49327};
CC       PhysiologicalDirection=left-to-right; Xref=Rhea:RHEA:41805;
CC         Evidence={ECO:0000250|UniProtKB:P49327};
CC   -!- CATALYTIC ACTIVITY:
CC       Reaction=(3R)-hydroxybutanoyl-[ACP] = (2E)-butenoyl-[ACP] + H2O;
CC         Xref=Rhea:RHEA:41808, Rhea:RHEA-COMP:9626, Rhea:RHEA-COMP:9627,
CC         ChEBI:CHEBI:15377, ChEBI:CHEBI:78451, ChEBI:CHEBI:78453;
CC         Evidence={ECO:0000250|UniProtKB:P49327};
CC       PhysiologicalDirection=left-to-right; Xref=Rhea:RHEA:41809;
CC         Evidence={ECO:0000250|UniProtKB:P49327};
CC   -!- CATALYTIC ACTIVITY:
CC       Reaction=(2E)-butenoyl-[ACP] + H(+) + NADPH = butanoyl-[ACP] + NADP(+);
CC         Xref=Rhea:RHEA:41812, Rhea:RHEA-COMP:9627, Rhea:RHEA-COMP:9628,
CC         ChEBI:CHEBI:15378, ChEBI:CHEBI:57783, ChEBI:CHEBI:58349,
CC         ChEBI:CHEBI:78453, ChEBI:CHEBI:78454;
CC         Evidence={ECO:0000250|UniProtKB:P49327};
CC       PhysiologicalDirection=left-to-right; Xref=Rhea:RHEA:41813;
CC         Evidence={ECO:0000250|UniProtKB:P49327};
CC   -!- CATALYTIC ACTIVITY:
CC       Reaction=butanoyl-[ACP] + H(+) + malonyl-[ACP] = 3-oxohexanoyl-[ACP] +
CC         CO2 + holo-[ACP]; Xref=Rhea:RHEA:41820, Rhea:RHEA-COMP:9623,
CC         Rhea:RHEA-COMP:9628, Rhea:RHEA-COMP:9629, Rhea:RHEA-COMP:9685,
CC         ChEBI:CHEBI:15378, ChEBI:CHEBI:16526, ChEBI:CHEBI:64479,
CC         ChEBI:CHEBI:78449, ChEBI:CHEBI:78454, ChEBI:CHEBI:78456;
CC         Evidence={ECO:0000305|PubMed:31811668};
CC       PhysiologicalDirection=left-to-right; Xref=Rhea:RHEA:41821;
CC         Evidence={ECO:0000305|PubMed:31811668};
CC   -!- CATALYTIC ACTIVITY:
CC       Reaction=3-oxohexanoyl-[ACP] + H(+) + NADPH = (3R)-hydroxyhexanoyl-
CC         [ACP] + NADP(+); Xref=Rhea:RHEA:41824, Rhea:RHEA-COMP:9629,
CC         Rhea:RHEA-COMP:9630, ChEBI:CHEBI:15378, ChEBI:CHEBI:57783,
CC         ChEBI:CHEBI:58349, ChEBI:CHEBI:78456, ChEBI:CHEBI:78457;
CC         Evidence={ECO:0000250|UniProtKB:P49327};
CC       PhysiologicalDirection=left-to-right; Xref=Rhea:RHEA:41825;
CC         Evidence={ECO:0000250|UniProtKB:P49327};
CC   -!- CATALYTIC ACTIVITY:
CC       Reaction=(3R)-hydroxyhexanoyl-[ACP] = (2E)-hexenoyl-[ACP] + H2O;
CC         Xref=Rhea:RHEA:41828, Rhea:RHEA-COMP:9630, Rhea:RHEA-COMP:9631,
CC         ChEBI:CHEBI:15377, ChEBI:CHEBI:78457, ChEBI:CHEBI:78458;
CC         Evidence={ECO:0000250|UniProtKB:P49327};
CC       PhysiologicalDirection=left-to-right; Xref=Rhea:RHEA:41829;
CC         Evidence={ECO:0000250|UniProtKB:P49327};
CC   -!- CATALYTIC ACTIVITY:
CC       Reaction=(2E)-hexenoyl-[ACP] + H(+) + NADPH = hexanoyl-[ACP] + NADP(+);
CC         Xref=Rhea:RHEA:41832, Rhea:RHEA-COMP:9631, Rhea:RHEA-COMP:9632,
CC         ChEBI:CHEBI:15378, ChEBI:CHEBI:57783, ChEBI:CHEBI:58349,
CC         ChEBI:CHEBI:78458, ChEBI:CHEBI:78459;
CC         Evidence={ECO:0000250|UniProtKB:P49327};
CC       PhysiologicalDirection=left-to-right; Xref=Rhea:RHEA:41833;
CC         Evidence={ECO:0000250|UniProtKB:P49327};
CC   -!- CATALYTIC ACTIVITY:
CC       Reaction=H(+) + hexanoyl-[ACP] + malonyl-[ACP] = 3-oxooctanoyl-[ACP] +
CC         CO2 + holo-[ACP]; Xref=Rhea:RHEA:41836, Rhea:RHEA-COMP:9623,
CC         Rhea:RHEA-COMP:9632, Rhea:RHEA-COMP:9633, Rhea:RHEA-COMP:9685,
CC         ChEBI:CHEBI:15378, ChEBI:CHEBI:16526, ChEBI:CHEBI:64479,
CC         ChEBI:CHEBI:78449, ChEBI:CHEBI:78459, ChEBI:CHEBI:78460;
CC         Evidence={ECO:0000269|PubMed:31811668};
CC       PhysiologicalDirection=left-to-right; Xref=Rhea:RHEA:41837;
CC         Evidence={ECO:0000269|PubMed:31811668};
CC   -!- CATALYTIC ACTIVITY:
CC       Reaction=3-oxooctanoyl-[ACP] + H(+) + NADPH = (3R)-hydroxyoctanoyl-
CC         [ACP] + NADP(+); Xref=Rhea:RHEA:41840, Rhea:RHEA-COMP:9633,
CC         Rhea:RHEA-COMP:9634, ChEBI:CHEBI:15378, ChEBI:CHEBI:57783,
CC         ChEBI:CHEBI:58349, ChEBI:CHEBI:78460, ChEBI:CHEBI:78461;
CC         Evidence={ECO:0000250|UniProtKB:P49327};
CC       PhysiologicalDirection=left-to-right; Xref=Rhea:RHEA:41841;
CC         Evidence={ECO:0000250|UniProtKB:P49327};
CC   -!- CATALYTIC ACTIVITY:
CC       Reaction=(3R)-hydroxyoctanoyl-[ACP] = (2E)-octenoyl-[ACP] + H2O;
CC         Xref=Rhea:RHEA:41844, Rhea:RHEA-COMP:9634, Rhea:RHEA-COMP:9635,
CC         ChEBI:CHEBI:15377, ChEBI:CHEBI:78461, ChEBI:CHEBI:78462;
CC         Evidence={ECO:0000250|UniProtKB:P49327};
CC       PhysiologicalDirection=left-to-right; Xref=Rhea:RHEA:41845;
CC         Evidence={ECO:0000250|UniProtKB:P49327};
CC   -!- CATALYTIC ACTIVITY:
CC       Reaction=(2E)-octenoyl-[ACP] + H(+) + NADPH = NADP(+) + octanoyl-[ACP];
CC         Xref=Rhea:RHEA:41848, Rhea:RHEA-COMP:9635, Rhea:RHEA-COMP:9636,
CC         ChEBI:CHEBI:15378, ChEBI:CHEBI:57783, ChEBI:CHEBI:58349,
CC         ChEBI:CHEBI:78462, ChEBI:CHEBI:78463;
CC         Evidence={ECO:0000250|UniProtKB:P49327};
CC       PhysiologicalDirection=left-to-right; Xref=Rhea:RHEA:41849;
CC         Evidence={ECO:0000250|UniProtKB:P49327};
CC   -!- CATALYTIC ACTIVITY:
CC       Reaction=H(+) + malonyl-[ACP] + octanoyl-[ACP] = 3-oxodecanoyl-[ACP] +
CC         CO2 + holo-[ACP]; Xref=Rhea:RHEA:41852, Rhea:RHEA-COMP:9623,
CC         Rhea:RHEA-COMP:9636, Rhea:RHEA-COMP:9637, Rhea:RHEA-COMP:9685,
CC         ChEBI:CHEBI:15378, ChEBI:CHEBI:16526, ChEBI:CHEBI:64479,
CC         ChEBI:CHEBI:78449, ChEBI:CHEBI:78463, ChEBI:CHEBI:78464;
CC         Evidence={ECO:0000269|PubMed:31811668};
CC       PhysiologicalDirection=left-to-right; Xref=Rhea:RHEA:41853;
CC         Evidence={ECO:0000269|PubMed:31811668};
CC   -!- CATALYTIC ACTIVITY:
CC       Reaction=3-oxodecanoyl-[ACP] + H(+) + NADPH = (3R)-hydroxydecanoyl-
CC         [ACP] + NADP(+); Xref=Rhea:RHEA:41856, Rhea:RHEA-COMP:9637,
CC         Rhea:RHEA-COMP:9638, ChEBI:CHEBI:15378, ChEBI:CHEBI:57783,
CC         ChEBI:CHEBI:58349, ChEBI:CHEBI:78464, ChEBI:CHEBI:78466;
CC         Evidence={ECO:0000250|UniProtKB:P49327};
CC       PhysiologicalDirection=left-to-right; Xref=Rhea:RHEA:41857;
CC         Evidence={ECO:0000250|UniProtKB:P49327};
CC   -!- CATALYTIC ACTIVITY:
CC       Reaction=(3R)-hydroxydecanoyl-[ACP] = (2E)-decenoyl-[ACP] + H2O;
CC         Xref=Rhea:RHEA:41860, Rhea:RHEA-COMP:9638, Rhea:RHEA-COMP:9639,
CC         ChEBI:CHEBI:15377, ChEBI:CHEBI:78466, ChEBI:CHEBI:78467;
CC         Evidence={ECO:0000250|UniProtKB:P49327};
CC       PhysiologicalDirection=left-to-right; Xref=Rhea:RHEA:41861;
CC         Evidence={ECO:0000250|UniProtKB:P49327};
CC   -!- CATALYTIC ACTIVITY:
CC       Reaction=(2E)-decenoyl-[ACP] + H(+) + NADPH = decanoyl-[ACP] + NADP(+);
CC         Xref=Rhea:RHEA:41864, Rhea:RHEA-COMP:9639, Rhea:RHEA-COMP:9640,
CC         ChEBI:CHEBI:15378, ChEBI:CHEBI:57783, ChEBI:CHEBI:58349,
CC         ChEBI:CHEBI:78467, ChEBI:CHEBI:78468;
CC         Evidence={ECO:0000250|UniProtKB:P49327};
CC       PhysiologicalDirection=left-to-right; Xref=Rhea:RHEA:41865;
CC         Evidence={ECO:0000250|UniProtKB:P49327};
CC   -!- CATALYTIC ACTIVITY:
CC       Reaction=decanoyl-[ACP] + H(+) + malonyl-[ACP] = 3-oxododecanoyl-[ACP]
CC         + CO2 + holo-[ACP]; Xref=Rhea:RHEA:41868, Rhea:RHEA-COMP:9623,
CC         Rhea:RHEA-COMP:9640, Rhea:RHEA-COMP:9641, Rhea:RHEA-COMP:9685,
CC         ChEBI:CHEBI:15378, ChEBI:CHEBI:16526, ChEBI:CHEBI:64479,
CC         ChEBI:CHEBI:78449, ChEBI:CHEBI:78468, ChEBI:CHEBI:78469;
CC         Evidence={ECO:0000269|PubMed:31811668};
CC       PhysiologicalDirection=left-to-right; Xref=Rhea:RHEA:41869;
CC         Evidence={ECO:0000269|PubMed:31811668};
CC   -!- CATALYTIC ACTIVITY:
CC       Reaction=3-oxododecanoyl-[ACP] + H(+) + NADPH = (3R)-hydroxydodecanoyl-
CC         [ACP] + NADP(+); Xref=Rhea:RHEA:41872, Rhea:RHEA-COMP:9641,
CC         Rhea:RHEA-COMP:9642, ChEBI:CHEBI:15378, ChEBI:CHEBI:57783,
CC         ChEBI:CHEBI:58349, ChEBI:CHEBI:78469, ChEBI:CHEBI:78470;
CC         Evidence={ECO:0000250|UniProtKB:P49327};
CC       PhysiologicalDirection=left-to-right; Xref=Rhea:RHEA:41873;
CC         Evidence={ECO:0000250|UniProtKB:P49327};
CC   -!- CATALYTIC ACTIVITY:
CC       Reaction=(3R)-hydroxydodecanoyl-[ACP] = (2E)-dodecenoyl-[ACP] + H2O;
CC         Xref=Rhea:RHEA:41876, Rhea:RHEA-COMP:9642, Rhea:RHEA-COMP:9643,
CC         ChEBI:CHEBI:15377, ChEBI:CHEBI:78470, ChEBI:CHEBI:78472;
CC         Evidence={ECO:0000250|UniProtKB:P49327};
CC       PhysiologicalDirection=left-to-right; Xref=Rhea:RHEA:41877;
CC         Evidence={ECO:0000250|UniProtKB:P49327};
CC   -!- CATALYTIC ACTIVITY:
CC       Reaction=(2E)-dodecenoyl-[ACP] + H(+) + NADPH = dodecanoyl-[ACP] +
CC         NADP(+); Xref=Rhea:RHEA:41880, Rhea:RHEA-COMP:9643, Rhea:RHEA-
CC         COMP:9644, ChEBI:CHEBI:15378, ChEBI:CHEBI:57783, ChEBI:CHEBI:58349,
CC         ChEBI:CHEBI:65264, ChEBI:CHEBI:78472;
CC         Evidence={ECO:0000250|UniProtKB:P49327};
CC       PhysiologicalDirection=left-to-right; Xref=Rhea:RHEA:41881;
CC         Evidence={ECO:0000250|UniProtKB:P49327};
CC   -!- CATALYTIC ACTIVITY:
CC       Reaction=dodecanoyl-[ACP] + H(+) + malonyl-[ACP] = 3-oxotetradecanoyl-
CC         [ACP] + CO2 + holo-[ACP]; Xref=Rhea:RHEA:41884, Rhea:RHEA-COMP:9623,
CC         Rhea:RHEA-COMP:9644, Rhea:RHEA-COMP:9645, Rhea:RHEA-COMP:9685,
CC         ChEBI:CHEBI:15378, ChEBI:CHEBI:16526, ChEBI:CHEBI:64479,
CC         ChEBI:CHEBI:65264, ChEBI:CHEBI:78449, ChEBI:CHEBI:78473;
CC         Evidence={ECO:0000269|PubMed:31811668};
CC       PhysiologicalDirection=left-to-right; Xref=Rhea:RHEA:41885;
CC         Evidence={ECO:0000269|PubMed:31811668};
CC   -!- CATALYTIC ACTIVITY:
CC       Reaction=3-oxotetradecanoyl-[ACP] + H(+) + NADPH = (3R)-
CC         hydroxytetradecanoyl-[ACP] + NADP(+); Xref=Rhea:RHEA:41888,
CC         Rhea:RHEA-COMP:9645, Rhea:RHEA-COMP:9646, ChEBI:CHEBI:15378,
CC         ChEBI:CHEBI:57783, ChEBI:CHEBI:58349, ChEBI:CHEBI:78473,
CC         ChEBI:CHEBI:78474; Evidence={ECO:0000250|UniProtKB:P49327};
CC       PhysiologicalDirection=left-to-right; Xref=Rhea:RHEA:41889;
CC         Evidence={ECO:0000250|UniProtKB:P49327};
CC   -!- CATALYTIC ACTIVITY:
CC       Reaction=(3R)-hydroxytetradecanoyl-[ACP] = (2E)-tetradecenoyl-[ACP] +
CC         H2O; Xref=Rhea:RHEA:41892, Rhea:RHEA-COMP:9646, Rhea:RHEA-COMP:9647,
CC         ChEBI:CHEBI:15377, ChEBI:CHEBI:78474, ChEBI:CHEBI:78475;
CC         Evidence={ECO:0000250|UniProtKB:P49327};
CC       PhysiologicalDirection=left-to-right; Xref=Rhea:RHEA:41893;
CC         Evidence={ECO:0000250|UniProtKB:P49327};
CC   -!- CATALYTIC ACTIVITY:
CC       Reaction=(2E)-tetradecenoyl-[ACP] + H(+) + NADPH = NADP(+) +
CC         tetradecanoyl-[ACP]; Xref=Rhea:RHEA:41896, Rhea:RHEA-COMP:9647,
CC         Rhea:RHEA-COMP:9648, ChEBI:CHEBI:15378, ChEBI:CHEBI:57783,
CC         ChEBI:CHEBI:58349, ChEBI:CHEBI:78475, ChEBI:CHEBI:78477;
CC         Evidence={ECO:0000250|UniProtKB:P49327};
CC       PhysiologicalDirection=left-to-right; Xref=Rhea:RHEA:41897;
CC         Evidence={ECO:0000250|UniProtKB:P49327};
CC   -!- CATALYTIC ACTIVITY:
CC       Reaction=H(+) + malonyl-[ACP] + tetradecanoyl-[ACP] = 3-
CC         oxohexadecanoyl-[ACP] + CO2 + holo-[ACP]; Xref=Rhea:RHEA:41900,
CC         Rhea:RHEA-COMP:9623, Rhea:RHEA-COMP:9648, Rhea:RHEA-COMP:9649,
CC         Rhea:RHEA-COMP:9685, ChEBI:CHEBI:15378, ChEBI:CHEBI:16526,
CC         ChEBI:CHEBI:64479, ChEBI:CHEBI:78449, ChEBI:CHEBI:78477,
CC         ChEBI:CHEBI:78478; Evidence={ECO:0000269|PubMed:31811668};
CC       PhysiologicalDirection=left-to-right; Xref=Rhea:RHEA:41901;
CC         Evidence={ECO:0000269|PubMed:31811668};
CC   -!- CATALYTIC ACTIVITY:
CC       Reaction=3-oxohexadecanoyl-[ACP] + H(+) + NADPH = (3R)-
CC         hydroxyhexadecanoyl-[ACP] + NADP(+); Xref=Rhea:RHEA:41904, Rhea:RHEA-
CC         COMP:9649, Rhea:RHEA-COMP:9650, ChEBI:CHEBI:15378, ChEBI:CHEBI:57783,
CC         ChEBI:CHEBI:58349, ChEBI:CHEBI:78478, ChEBI:CHEBI:78480;
CC         Evidence={ECO:0000250|UniProtKB:P49327};
CC       PhysiologicalDirection=left-to-right; Xref=Rhea:RHEA:41905;
CC         Evidence={ECO:0000250|UniProtKB:P49327};
CC   -!- CATALYTIC ACTIVITY:
CC       Reaction=(3R)-hydroxyhexadecanoyl-[ACP] = (2E)-hexadecenoyl-[ACP] +
CC         H2O; Xref=Rhea:RHEA:41908, Rhea:RHEA-COMP:9650, Rhea:RHEA-COMP:9651,
CC         ChEBI:CHEBI:15377, ChEBI:CHEBI:78480, ChEBI:CHEBI:78481;
CC         Evidence={ECO:0000250|UniProtKB:P49327};
CC       PhysiologicalDirection=left-to-right; Xref=Rhea:RHEA:41909;
CC         Evidence={ECO:0000250|UniProtKB:P49327};
CC   -!- CATALYTIC ACTIVITY:
CC       Reaction=(2E)-hexadecenoyl-[ACP] + H(+) + NADPH = hexadecanoyl-[ACP] +
CC         NADP(+); Xref=Rhea:RHEA:41912, Rhea:RHEA-COMP:9651, Rhea:RHEA-
CC         COMP:9652, ChEBI:CHEBI:15378, ChEBI:CHEBI:57783, ChEBI:CHEBI:58349,
CC         ChEBI:CHEBI:78481, ChEBI:CHEBI:78483;
CC         Evidence={ECO:0000250|UniProtKB:P49327};
CC       PhysiologicalDirection=left-to-right; Xref=Rhea:RHEA:41913;
CC         Evidence={ECO:0000250|UniProtKB:P49327};
CC   -!- CATALYTIC ACTIVITY:
CC       Reaction=H(+) + hexadecanoyl-[ACP] + malonyl-[ACP] = 3-oxooctadecanoyl-
CC         [ACP] + CO2 + holo-[ACP]; Xref=Rhea:RHEA:41916, Rhea:RHEA-COMP:9623,
CC         Rhea:RHEA-COMP:9652, Rhea:RHEA-COMP:9653, Rhea:RHEA-COMP:9685,
CC         ChEBI:CHEBI:15378, ChEBI:CHEBI:16526, ChEBI:CHEBI:64479,
CC         ChEBI:CHEBI:78449, ChEBI:CHEBI:78483, ChEBI:CHEBI:78487;
CC         Evidence={ECO:0000250|UniProtKB:P49327};
CC       PhysiologicalDirection=left-to-right; Xref=Rhea:RHEA:41917;
CC         Evidence={ECO:0000250|UniProtKB:P49327};
CC   -!- CATALYTIC ACTIVITY:
CC       Reaction=3-oxooctadecanoyl-[ACP] + H(+) + NADPH = (3R)-
CC         hydroxyoctadecanoyl-[ACP] + NADP(+); Xref=Rhea:RHEA:41920, Rhea:RHEA-
CC         COMP:9653, Rhea:RHEA-COMP:9654, ChEBI:CHEBI:15378, ChEBI:CHEBI:57783,
CC         ChEBI:CHEBI:58349, ChEBI:CHEBI:78487, ChEBI:CHEBI:78488;
CC         Evidence={ECO:0000250|UniProtKB:P49327};
CC       PhysiologicalDirection=left-to-right; Xref=Rhea:RHEA:41921;
CC         Evidence={ECO:0000250|UniProtKB:P49327};
CC   -!- CATALYTIC ACTIVITY:
CC       Reaction=(3R)-hydroxyoctadecanoyl-[ACP] = (2E)-octadecenoyl-[ACP] +
CC         H2O; Xref=Rhea:RHEA:41924, Rhea:RHEA-COMP:9654, Rhea:RHEA-COMP:9655,
CC         ChEBI:CHEBI:15377, ChEBI:CHEBI:78488, ChEBI:CHEBI:78489;
CC         Evidence={ECO:0000250|UniProtKB:P49327};
CC       PhysiologicalDirection=left-to-right; Xref=Rhea:RHEA:41925;
CC         Evidence={ECO:0000250|UniProtKB:P49327};
CC   -!- CATALYTIC ACTIVITY:
CC       Reaction=(2E)-octadecenoyl-[ACP] + H(+) + NADPH = NADP(+) +
CC         octadecanoyl-[ACP]; Xref=Rhea:RHEA:41928, Rhea:RHEA-COMP:9655,
CC         Rhea:RHEA-COMP:9656, ChEBI:CHEBI:15378, ChEBI:CHEBI:57783,
CC         ChEBI:CHEBI:58349, ChEBI:CHEBI:78489, ChEBI:CHEBI:78495;
CC         Evidence={ECO:0000250|UniProtKB:P49327};
CC       PhysiologicalDirection=left-to-right; Xref=Rhea:RHEA:41929;
CC         Evidence={ECO:0000250|UniProtKB:P49327};
CC   -!- CATALYTIC ACTIVITY:
CC       Reaction=H2O + tetradecanoyl-[ACP] = H(+) + holo-[ACP] +
CC         tetradecanoate; Xref=Rhea:RHEA:30123, Rhea:RHEA-COMP:9648, Rhea:RHEA-
CC         COMP:9685, ChEBI:CHEBI:15377, ChEBI:CHEBI:15378, ChEBI:CHEBI:30807,
CC         ChEBI:CHEBI:64479, ChEBI:CHEBI:78477; EC=3.1.2.14;
CC         Evidence={ECO:0000250|UniProtKB:P49327};
CC       PhysiologicalDirection=left-to-right; Xref=Rhea:RHEA:30124;
CC         Evidence={ECO:0000250|UniProtKB:P49327};
CC   -!- BIOPHYSICOCHEMICAL PROPERTIES:
CC       Kinetic parameters:
CC         KM=1.28 uM for malonyl-CoA {ECO:0000269|PubMed:29328619};
CC         KM=1.63 uM for acetyl-CoA {ECO:0000269|PubMed:29328619};
CC   -!- PATHWAY: Lipid metabolism; fatty acid biosynthesis.
CC       {ECO:0000269|PubMed:29328619}.
CC   -!- SUBUNIT: Homodimer which is arranged in a head to tail fashion (By
CC       similarity). Interacts with CEACAM1; this interaction is insulin and
CC       phosphorylation-dependent; reduces fatty-acid synthase activity (By
CC       similarity). {ECO:0000250|UniProtKB:P12785,
CC       ECO:0000250|UniProtKB:P49327}.
CC   -!- SUBCELLULAR LOCATION: Cytoplasm {ECO:0000250}. Melanosome
CC       {ECO:0000250}.
CC   -!- INDUCTION: Up-regulated by endocannabinoid anandamide/AEA.
CC       {ECO:0000269|PubMed:21987372}.
CC   -!- PTM: S-nitrosylation of Fatty acid synthase at cysteine residues Cys-
CC       1464 or Cys-2084 is important for the enzyme dimerization. In
CC       adipocytes, S-nitrosylation of Fatty acid synthase occurs under
CC       physiological conditions and gradually increases during adipogenesis.
CC       {ECO:0000250|UniProtKB:P49327}.
CC   -!- SEQUENCE CAUTION:
CC       Sequence=CAA31525.1; Type=Frameshift; Evidence={ECO:0000305};
CC   ---------------------------------------------------------------------------
CC   Copyrighted by the UniProt Consortium, see https://www.uniprot.org/terms
CC   Distributed under the Creative Commons Attribution (CC BY 4.0) License
CC   ---------------------------------------------------------------------------
DR   EMBL; AF127033; AAG02285.1; -; mRNA.
DR   EMBL; AL663090; -; NOT_ANNOTATED_CDS; Genomic_DNA.
DR   EMBL; BC046513; AAH46513.1; -; mRNA.
DR   EMBL; BC059850; AAH59850.1; -; mRNA.
DR   EMBL; AK080374; BAC37895.1; -; mRNA.
DR   EMBL; X13135; CAA31525.1; ALT_FRAME; mRNA.
DR   CCDS; CCDS25759.1; -.
DR   PIR; A32262; A32262.
DR   RefSeq; NP_032014.3; NM_007988.3.
DR   PDB; 5MY0; X-ray; 2.94 A; A/B/C/D=2-852.
DR   PDB; 5MY2; X-ray; 2.70 A; A/B/C/D=2-852.
DR   PDB; 6ROP; X-ray; 2.70 A; A/B/C/D=2-852.
DR   PDBsum; 5MY0; -.
DR   PDBsum; 5MY2; -.
DR   PDBsum; 6ROP; -.
DR   AlphaFoldDB; P19096; -.
DR   SMR; P19096; -.
DR   BioGRID; 199596; 29.
DR   IntAct; P19096; 6.
DR   MINT; P19096; -.
DR   STRING; 10090.ENSMUSP00000052872; -.
DR   BindingDB; P19096; -.
DR   ChEMBL; CHEMBL1795189; -.
DR   ESTHER; mouse-FASN; Thioesterase.
DR   GlyGen; P19096; 1 site, 1 O-linked glycan (1 site).
DR   iPTMnet; P19096; -.
DR   PhosphoSitePlus; P19096; -.
DR   SwissPalm; P19096; -.
DR   CPTAC; non-CPTAC-3808; -.
DR   EPD; P19096; -.
DR   jPOST; P19096; -.
DR   MaxQB; P19096; -.
DR   PaxDb; P19096; -.
DR   PeptideAtlas; P19096; -.
DR   ProteomicsDB; 275589; -.
DR   Antibodypedia; 1650; 820 antibodies from 40 providers.
DR   DNASU; 14104; -.
DR   Ensembl; ENSMUST00000055655; ENSMUSP00000052872; ENSMUSG00000025153.
DR   GeneID; 14104; -.
DR   KEGG; mmu:14104; -.
DR   UCSC; uc007mut.1; mouse.
DR   AGR; MGI:95485; -.
DR   CTD; 2194; -.
DR   MGI; MGI:95485; Fasn.
DR   VEuPathDB; HostDB:ENSMUSG00000025153; -.
DR   eggNOG; KOG1202; Eukaryota.
DR   GeneTree; ENSGT00940000157276; -.
DR   HOGENOM; CLU_000022_31_7_1; -.
DR   InParanoid; P19096; -.
DR   OMA; KMRGGEF; -.
DR   OrthoDB; 3378513at2759; -.
DR   PhylomeDB; P19096; -.
DR   TreeFam; TF300549; -.
DR   BRENDA; 2.3.1.85; 3474.
DR   Reactome; R-MMU-163765; ChREBP activates metabolic gene expression.
DR   Reactome; R-MMU-199220; Vitamin B5 (pantothenate) metabolism.
DR   Reactome; R-MMU-75105; Fatty acyl-CoA biosynthesis.
DR   UniPathway; UPA00094; -.
DR   BioGRID-ORCS; 14104; 23 hits in 80 CRISPR screens.
DR   ChiTaRS; Fasn; mouse.
DR   PRO; PR:P19096; -.
DR   Proteomes; UP000000589; Chromosome 11.
DR   RNAct; P19096; protein.
DR   Bgee; ENSMUSG00000025153; Expressed in aorta tunica adventitia and 292 other tissues.
DR   ExpressionAtlas; P19096; baseline and differential.
DR   Genevisible; P19096; MM.
DR   GO; GO:0005737; C:cytoplasm; ISO:MGI.
DR   GO; GO:0005829; C:cytosol; ISO:MGI.
DR   GO; GO:0042587; C:glycogen granule; IDA:MGI.
DR   GO; GO:0005794; C:Golgi apparatus; ISO:MGI.
DR   GO; GO:0042470; C:melanosome; IEA:UniProtKB-SubCell.
DR   GO; GO:0005739; C:mitochondrion; HDA:MGI.
DR   GO; GO:0005886; C:plasma membrane; ISO:MGI.
DR   GO; GO:0008693; F:(3R)-3-hydroxydecanoyl-[acyl-carrier-protein] dehydratase activity; IEA:RHEA.
DR   GO; GO:0047451; F:(3R)-3-hydroxyoctanoyl-[acyl-carrier-protein] dehydratase activity; IEA:RHEA.
DR   GO; GO:0008659; F:(3R)-hydroxymyristoyl-[acyl-carrier-protein] dehydratase activity; IEA:UniProtKB-EC.
DR   GO; GO:0004317; F:(3R)-hydroxypalmitoyl-[acyl-carrier-protein] dehydratase activity; IEA:RHEA.
DR   GO; GO:0004316; F:3-oxoacyl-[acyl-carrier-protein] reductase (NADPH) activity; IEA:UniProtKB-EC.
DR   GO; GO:0004315; F:3-oxoacyl-[acyl-carrier-protein] synthase activity; IEA:UniProtKB-EC.
DR   GO; GO:0004313; F:[acyl-carrier-protein] S-acetyltransferase activity; IEA:UniProtKB-EC.
DR   GO; GO:0004314; F:[acyl-carrier-protein] S-malonyltransferase activity; ISO:MGI.
DR   GO; GO:0047117; F:enoyl-[acyl-carrier-protein] reductase (NADPH, A-specific) activity; IEA:UniProtKB-EC.
DR   GO; GO:0004312; F:fatty acid synthase activity; IDA:MGI.
DR   GO; GO:0042802; F:identical protein binding; ISO:MGI.
DR   GO; GO:0016295; F:myristoyl-[acyl-carrier-protein] hydrolase activity; IEA:UniProtKB-EC.
DR   GO; GO:0004320; F:oleoyl-[acyl-carrier-protein] hydrolase activity; IEA:UniProtKB-EC.
DR   GO; GO:0016296; F:palmitoyl-[acyl-carrier-protein] hydrolase activity; IEA:UniProtKB-EC.
DR   GO; GO:0031177; F:phosphopantetheine binding; IEA:InterPro.
DR   GO; GO:0006084; P:acetyl-CoA metabolic process; ISO:MGI.
DR   GO; GO:0071353; P:cellular response to interleukin-4; IDA:MGI.
DR   GO; GO:0090557; P:establishment of endothelial intestinal barrier; IMP:MGI.
DR   GO; GO:0008611; P:ether lipid biosynthetic process; IMP:MGI.
DR   GO; GO:0006633; P:fatty acid biosynthetic process; IDA:MGI.
DR   GO; GO:0002068; P:glandular epithelial cell development; IMP:MGI.
DR   GO; GO:0006954; P:inflammatory response; IMP:MGI.
DR   GO; GO:0008610; P:lipid biosynthetic process; IDA:MGI.
DR   GO; GO:0030879; P:mammary gland development; IMP:CACAO.
DR   GO; GO:0044788; P:modulation by host of viral process; ISO:MGI.
DR   GO; GO:0030224; P:monocyte differentiation; IMP:MGI.
DR   GO; GO:0030223; P:neutrophil differentiation; IMP:MGI.
DR   GO; GO:0009888; P:tissue development; IMP:MGI.
DR   CDD; cd05195; enoyl_red; 1.
DR   CDD; cd08954; KR_1_FAS_SDR_x; 2.
DR   CDD; cd00833; PKS; 1.
DR   Gene3D; 3.30.70.3290; -; 1.
DR   Gene3D; 3.40.47.10; -; 1.
DR   Gene3D; 1.10.1200.10; ACP-like; 1.
DR   Gene3D; 3.40.50.1820; alpha/beta hydrolase; 2.
DR   Gene3D; 3.40.366.10; Malonyl-Coenzyme A Acyl Carrier Protein, domain 2; 1.
DR   Gene3D; 3.90.180.10; Medium-chain alcohol dehydrogenases, catalytic domain; 1.
DR   Gene3D; 3.40.50.720; NAD(P)-binding Rossmann-like Domain; 1.
DR   Gene3D; 3.10.129.110; Polyketide synthase dehydratase; 1.
DR   Gene3D; 3.40.50.150; Vaccinia Virus protein VP39; 1.
DR   InterPro; IPR029058; AB_hydrolase.
DR   InterPro; IPR001227; Ac_transferase_dom_sf.
DR   InterPro; IPR036736; ACP-like_sf.
DR   InterPro; IPR014043; Acyl_transferase.
DR   InterPro; IPR016035; Acyl_Trfase/lysoPLipase.
DR   InterPro; IPR013149; ADH-like_C.
DR   InterPro; IPR011032; GroES-like_sf.
DR   InterPro; IPR018201; Ketoacyl_synth_AS.
DR   InterPro; IPR014031; Ketoacyl_synth_C.
DR   InterPro; IPR014030; Ketoacyl_synth_N.
DR   InterPro; IPR016036; Malonyl_transacylase_ACP-bd.
DR   InterPro; IPR013217; Methyltransf_12.
DR   InterPro; IPR036291; NAD(P)-bd_dom_sf.
DR   InterPro; IPR032821; PKS_assoc.
DR   InterPro; IPR020841; PKS_Beta-ketoAc_synthase_dom.
DR   InterPro; IPR042104; PKS_dehydratase_sf.
DR   InterPro; IPR020807; PKS_DH.
DR   InterPro; IPR020843; PKS_ER.
DR   InterPro; IPR013968; PKS_KR.
DR   InterPro; IPR020806; PKS_PP-bd.
DR   InterPro; IPR009081; PP-bd_ACP.
DR   InterPro; IPR006162; Ppantetheine_attach_site.
DR   InterPro; IPR029063; SAM-dependent_MTases_sf.
DR   InterPro; IPR001031; Thioesterase.
DR   InterPro; IPR016039; Thiolase-like.
DR   PANTHER; PTHR43775; FATTY ACID SYNTHASE; 1.
DR   PANTHER; PTHR43775:SF7; FATTY ACID SYNTHASE; 1.
DR   Pfam; PF00698; Acyl_transf_1; 1.
DR   Pfam; PF00107; ADH_zinc_N; 1.
DR   Pfam; PF16197; KAsynt_C_assoc; 1.
DR   Pfam; PF00109; ketoacyl-synt; 1.
DR   Pfam; PF02801; Ketoacyl-synt_C; 1.
DR   Pfam; PF08659; KR; 1.
DR   Pfam; PF08242; Methyltransf_12; 1.
DR   Pfam; PF00550; PP-binding; 1.
DR   Pfam; PF14765; PS-DH; 1.
DR   Pfam; PF00975; Thioesterase; 1.
DR   SMART; SM00827; PKS_AT; 1.
DR   SMART; SM00826; PKS_DH; 1.
DR   SMART; SM00829; PKS_ER; 1.
DR   SMART; SM00822; PKS_KR; 1.
DR   SMART; SM00825; PKS_KS; 1.
DR   SMART; SM00823; PKS_PP; 1.
DR   SUPFAM; SSF47336; ACP-like; 1.
DR   SUPFAM; SSF53474; alpha/beta-Hydrolases; 1.
DR   SUPFAM; SSF52151; FabD/lysophospholipase-like; 1.
DR   SUPFAM; SSF50129; GroES-like; 1.
DR   SUPFAM; SSF51735; NAD(P)-binding Rossmann-fold domains; 2.
DR   SUPFAM; SSF55048; Probable ACP-binding domain of malonyl-CoA ACP transacylase; 1.
DR   SUPFAM; SSF53335; S-adenosyl-L-methionine-dependent methyltransferases; 1.
DR   SUPFAM; SSF53901; Thiolase-like; 1.
DR   PROSITE; PS50075; CARRIER; 1.
DR   PROSITE; PS00606; KS3_1; 1.
DR   PROSITE; PS52004; KS3_2; 1.
DR   PROSITE; PS00012; PHOSPHOPANTETHEINE; 1.
DR   PROSITE; PS52019; PKS_MFAS_DH; 1.
PE   1: Evidence at protein level;
KW   3D-structure; Acetylation; Cytoplasm; Direct protein sequencing;
KW   Fatty acid biosynthesis; Fatty acid metabolism; Hydrolase; Isopeptide bond;
KW   Lipid biosynthesis; Lipid metabolism; Lyase; Multifunctional enzyme; NAD;
KW   NADP; Oxidoreductase; Phosphopantetheine; Phosphoprotein;
KW   Pyridoxal phosphate; Reference proteome; S-nitrosylation; Transferase;
KW   Ubl conjugation.
FT   CHAIN           1..2504
FT                   /note="Fatty acid synthase"
FT                   /id="PRO_0000180277"
FT   DOMAIN          1..406
FT                   /note="Ketosynthase family 3 (KS3)"
FT                   /evidence="ECO:0000255|PROSITE-ProRule:PRU01348"
FT   DOMAIN          844..1104
FT                   /note="PKS/mFAS DH"
FT                   /evidence="ECO:0000255|PROSITE-ProRule:PRU01363"
FT   DOMAIN          2112..2192
FT                   /note="Carrier"
FT                   /evidence="ECO:0000255|PROSITE-ProRule:PRU00258"
FT   REGION          429..817
FT                   /note="Acyl and malonyl transferases"
FT   REGION          844..967
FT                   /note="N-terminal hotdog fold"
FT                   /evidence="ECO:0000255|PROSITE-ProRule:PRU01363"
FT   REGION          982..1104
FT                   /note="C-terminal hotdog fold"
FT                   /evidence="ECO:0000255|PROSITE-ProRule:PRU01363"
FT   REGION          1628..1856
FT                   /note="Enoyl reductase"
FT   REGION          1857..2111
FT                   /note="Beta-ketoacyl reductase"
FT   REGION          2181..2205
FT                   /note="Disordered"
FT                   /evidence="ECO:0000256|SAM:MobiDB-lite"
FT   REGION          2201..2504
FT                   /note="Thioesterase"
FT   ACT_SITE        161
FT                   /note="For beta-ketoacyl synthase activity"
FT                   /evidence="ECO:0000255|PROSITE-ProRule:PRU01348"
FT   ACT_SITE        293
FT                   /note="For beta-ketoacyl synthase activity"
FT                   /evidence="ECO:0000255|PROSITE-ProRule:PRU01348"
FT   ACT_SITE        331
FT                   /note="For beta-ketoacyl synthase activity"
FT                   /evidence="ECO:0000255|PROSITE-ProRule:PRU01348"
FT   ACT_SITE        581
FT                   /note="For malonyltransferase activity"
FT                   /evidence="ECO:0000255|PROSITE-ProRule:PRU10022"
FT   ACT_SITE        878
FT                   /note="Proton acceptor; for dehydratase activity"
FT                   /evidence="ECO:0000255|PROSITE-ProRule:PRU01363"
FT   ACT_SITE        1032
FT                   /note="Proton donor; for dehydratase activity"
FT                   /evidence="ECO:0000255|PROSITE-ProRule:PRU01363"
FT   ACT_SITE        2301
FT                   /note="For thioesterase activity"
FT                   /evidence="ECO:0000255|PROSITE-ProRule:PRU10022"
FT   ACT_SITE        2474
FT                   /note="For thioesterase activity"
FT                   /evidence="ECO:0000255|PROSITE-ProRule:PRU10022"
FT   BINDING         647..648
FT                   /ligand="an acyl-CoA"
FT                   /ligand_id="ChEBI:CHEBI:58342"
FT                   /evidence="ECO:0000269|PubMed:31811668,
FT                   ECO:0007744|PDB:6ROP"
FT   BINDING         671
FT                   /ligand="an acyl-CoA"
FT                   /ligand_id="ChEBI:CHEBI:58342"
FT                   /evidence="ECO:0000269|PubMed:31811668,
FT                   ECO:0007744|PDB:6ROP"
FT   BINDING         773
FT                   /ligand="an acyl-CoA"
FT                   /ligand_id="ChEBI:CHEBI:58342"
FT                   /evidence="ECO:0000269|PubMed:31811668,
FT                   ECO:0007744|PDB:6ROP"
FT   BINDING         1664..1681
FT                   /ligand="NADP(+)"
FT                   /ligand_id="ChEBI:CHEBI:58349"
FT                   /ligand_label="1"
FT                   /ligand_note="for enoyl reductase activity"
FT                   /evidence="ECO:0000250"
FT   BINDING         1879..1894
FT                   /ligand="NADP(+)"
FT                   /ligand_id="ChEBI:CHEBI:58349"
FT                   /ligand_label="2"
FT                   /ligand_note="for ketoreductase activity"
FT                   /evidence="ECO:0000250"
FT   MOD_RES         1
FT                   /note="N-acetylmethionine"
FT                   /evidence="ECO:0000269|Ref.4"
FT   MOD_RES         59
FT                   /note="N6-acetyllysine"
FT                   /evidence="ECO:0007744|PubMed:23806337"
FT   MOD_RES         63
FT                   /note="Phosphoserine"
FT                   /evidence="ECO:0000250|UniProtKB:P49327"
FT   MOD_RES         70
FT                   /note="N6-acetyllysine"
FT                   /evidence="ECO:0000250|UniProtKB:P49327"
FT   MOD_RES         207
FT                   /note="Phosphoserine"
FT                   /evidence="ECO:0000250|UniProtKB:P49327"
FT   MOD_RES         298
FT                   /note="N6-acetyllysine"
FT                   /evidence="ECO:0000250|UniProtKB:P49327"
FT   MOD_RES         528
FT                   /note="N6-acetyllysine"
FT                   /evidence="ECO:0000250|UniProtKB:P49327"
FT   MOD_RES         673
FT                   /note="N6-acetyllysine"
FT                   /evidence="ECO:0000250|UniProtKB:P49327"
FT   MOD_RES         725
FT                   /note="Phosphoserine"
FT                   /evidence="ECO:0007744|PubMed:21183079"
FT   MOD_RES         790
FT                   /note="N6-acetyllysine"
FT                   /evidence="ECO:0007744|PubMed:23806337"
FT   MOD_RES         993
FT                   /note="N6-acetyllysine"
FT                   /evidence="ECO:0007744|PubMed:23806337"
FT   MOD_RES         1071
FT                   /note="N6-acetyllysine"
FT                   /evidence="ECO:0007744|PubMed:23806337"
FT   MOD_RES         1276
FT                   /note="N6-acetyllysine"
FT                   /evidence="ECO:0007744|PubMed:23806337"
FT   MOD_RES         1464
FT                   /note="S-nitrosocysteine"
FT                   /evidence="ECO:0000250|UniProtKB:P49327"
FT   MOD_RES         1577
FT                   /note="Phosphoserine"
FT                   /evidence="ECO:0007744|PubMed:21183079"
FT   MOD_RES         1587
FT                   /note="Phosphoserine"
FT                   /evidence="ECO:0007744|PubMed:21183079"
FT   MOD_RES         1697
FT                   /note="N6-(pyridoxal phosphate)lysine; alternate"
FT                   /evidence="ECO:0000250"
FT   MOD_RES         1697
FT                   /note="N6-acetyllysine; alternate"
FT                   /evidence="ECO:0000250|UniProtKB:P49327"
FT   MOD_RES         1764
FT                   /note="N6-acetyllysine"
FT                   /evidence="ECO:0000250|UniProtKB:P49327"
FT   MOD_RES         1840
FT                   /note="N6-acetyllysine"
FT                   /evidence="ECO:0007744|PubMed:23806337"
FT   MOD_RES         1988
FT                   /note="N6-acetyllysine"
FT                   /evidence="ECO:0000250|UniProtKB:P49327"
FT   MOD_RES         2084
FT                   /note="S-nitrosocysteine"
FT                   /evidence="ECO:0000250|UniProtKB:P49327"
FT   MOD_RES         2150
FT                   /note="O-(pantetheine 4'-phosphoryl)serine; alternate"
FT                   /evidence="ECO:0000255|PROSITE-ProRule:PRU00258"
FT   MOD_RES         2150
FT                   /note="Phosphoserine; alternate"
FT                   /evidence="ECO:0000250|UniProtKB:P12785"
FT   MOD_RES         2190
FT                   /note="Phosphoserine"
FT                   /evidence="ECO:0007744|PubMed:21183079"
FT   MOD_RES         2229
FT                   /note="Phosphoserine"
FT                   /evidence="ECO:0000250|UniProtKB:P49327"
FT   MOD_RES         2384
FT                   /note="N6-acetyllysine"
FT                   /evidence="ECO:0007744|PubMed:23806337"
FT   CROSSLNK        2442
FT                   /note="Glycyl lysine isopeptide (Lys-Gly) (interchain with
FT                   G-Cter in SUMO2)"
FT                   /evidence="ECO:0000250|UniProtKB:P49327"
FT   CONFLICT        1992
FT                   /note="T -> N (in Ref. 6; CAA31525)"
FT                   /evidence="ECO:0000305"
FT   CONFLICT        2028
FT                   /note="G -> C (in Ref. 6; CAA31525)"
FT                   /evidence="ECO:0000305"
FT   CONFLICT        2045
FT                   /note="G -> V (in Ref. 6; CAA31525)"
FT                   /evidence="ECO:0000305"
FT   CONFLICT        2117
FT                   /note="T -> N (in Ref. 6; CAA31525)"
FT                   /evidence="ECO:0000305"
FT   CONFLICT        2175
FT                   /note="Q -> R (in Ref. 6; CAA31525)"
FT                   /evidence="ECO:0000305"
FT   CONFLICT        2295
FT                   /note="Y -> H (in Ref. 6; CAA31525)"
FT                   /evidence="ECO:0000305"
FT   STRAND          4..13
FT                   /evidence="ECO:0007829|PDB:5MY2"
FT   STRAND          16..18
FT                   /evidence="ECO:0007829|PDB:5MY2"
FT   HELIX           19..27
FT                   /evidence="ECO:0007829|PDB:5MY2"
FT   STRAND          36..40
FT                   /evidence="ECO:0007829|PDB:5MY2"
FT   HELIX           44..46
FT                   /evidence="ECO:0007829|PDB:5MY2"
FT   HELIX           62..65
FT                   /evidence="ECO:0007829|PDB:5MY2"
FT   HELIX           69..73
FT                   /evidence="ECO:0007829|PDB:5MY2"
FT   HELIX           77..92
FT                   /evidence="ECO:0007829|PDB:5MY2"
FT   HELIX           97..100
FT                   /evidence="ECO:0007829|PDB:5MY2"
FT   STRAND          106..111
FT                   /evidence="ECO:0007829|PDB:5MY2"
FT   HELIX           115..120
FT                   /evidence="ECO:0007829|PDB:5MY2"
FT   TURN            124..126
FT                   /evidence="ECO:0007829|PDB:5MY2"
FT   HELIX           130..135
FT                   /evidence="ECO:0007829|PDB:5MY2"
FT   HELIX           139..148
FT                   /evidence="ECO:0007829|PDB:5MY2"
FT   STRAND          154..158
FT                   /evidence="ECO:0007829|PDB:5MY2"
FT   HELIX           160..162
FT                   /evidence="ECO:0007829|PDB:5MY2"
FT   HELIX           163..176
FT                   /evidence="ECO:0007829|PDB:5MY2"
FT   STRAND          181..189
FT                   /evidence="ECO:0007829|PDB:5MY2"
FT   HELIX           194..202
FT                   /evidence="ECO:0007829|PDB:5MY2"
FT   STRAND          227..235
FT                   /evidence="ECO:0007829|PDB:5MY2"
FT   HELIX           236..238
FT                   /evidence="ECO:0007829|PDB:5MY2"
FT   STRAND          243..253
FT                   /evidence="ECO:0007829|PDB:5MY2"
FT   HELIX           266..276
FT                   /evidence="ECO:0007829|PDB:5MY2"
FT   HELIX           278..280
FT                   /evidence="ECO:0007829|PDB:5MY2"
FT   HELIX           284..286
FT                   /evidence="ECO:0007829|PDB:5MY2"
FT   STRAND          287..291
FT                   /evidence="ECO:0007829|PDB:5MY2"
FT   HELIX           300..311
FT                   /evidence="ECO:0007829|PDB:5MY2"
FT   HELIX           313..315
FT                   /evidence="ECO:0007829|PDB:5MY2"
FT   STRAND          320..323
FT                   /evidence="ECO:0007829|PDB:5MY2"
FT   HELIX           326..329
FT                   /evidence="ECO:0007829|PDB:5MY2"
FT   HELIX           333..335
FT                   /evidence="ECO:0007829|PDB:5MY2"
FT   HELIX           336..349
FT                   /evidence="ECO:0007829|PDB:5MY2"
FT   HELIX           367..370
FT                   /evidence="ECO:0007829|PDB:5MY2"
FT   STRAND          373..376
FT                   /evidence="ECO:0007829|PDB:5MY2"
FT   STRAND          387..393
FT                   /evidence="ECO:0007829|PDB:5MY2"
FT   STRAND          397..406
FT                   /evidence="ECO:0007829|PDB:5MY2"
FT   HELIX           416..418
FT                   /evidence="ECO:0007829|PDB:5MY2"
FT   STRAND          422..430
FT                   /evidence="ECO:0007829|PDB:5MY2"
FT   HELIX           431..443
FT                   /evidence="ECO:0007829|PDB:5MY2"
FT   TURN            444..446
FT                   /evidence="ECO:0007829|PDB:5MY2"
FT   HELIX           448..456
FT                   /evidence="ECO:0007829|PDB:5MY2"
FT   TURN            462..464
FT                   /evidence="ECO:0007829|PDB:5MY2"
FT   STRAND          467..477
FT                   /evidence="ECO:0007829|PDB:5MY2"
FT   STRAND          480..484
FT                   /evidence="ECO:0007829|PDB:5MY2"
FT   STRAND          492..496
FT                   /evidence="ECO:0007829|PDB:5MY2"
FT   TURN            504..507
FT                   /evidence="ECO:0007829|PDB:5MY2"
FT   HELIX           509..512
FT                   /evidence="ECO:0007829|PDB:5MY2"
FT   HELIX           514..527
FT                   /evidence="ECO:0007829|PDB:5MY2"
FT   TURN            528..531
FT                   /evidence="ECO:0007829|PDB:5MY2"
FT   HELIX           534..539
FT                   /evidence="ECO:0007829|PDB:5MY2"
FT   HELIX           545..547
FT                   /evidence="ECO:0007829|PDB:5MY2"
FT   HELIX           549..569
FT                   /evidence="ECO:0007829|PDB:5MY2"
FT   STRAND          575..579
FT                   /evidence="ECO:0007829|PDB:5MY2"
FT   HELIX           583..590
FT                   /evidence="ECO:0007829|PDB:5MY2"
FT   HELIX           596..611
FT                   /evidence="ECO:0007829|PDB:5MY2"
FT   TURN            612..614
FT                   /evidence="ECO:0007829|PDB:5MY0"
FT   STRAND          618..625
FT                   /evidence="ECO:0007829|PDB:5MY2"
FT   HELIX           627..633
FT                   /evidence="ECO:0007829|PDB:5MY2"
FT   STRAND          639..643
FT                   /evidence="ECO:0007829|PDB:5MY2"
FT   STRAND          645..654
FT                   /evidence="ECO:0007829|PDB:5MY2"
FT   HELIX           655..658
FT                   /evidence="ECO:0007829|PDB:5MY2"
FT   HELIX           660..666
FT                   /evidence="ECO:0007829|PDB:5MY2"
FT   TURN            667..669
FT                   /evidence="ECO:0007829|PDB:5MY2"
FT   STRAND          672..675
FT                   /evidence="ECO:0007829|PDB:5MY2"
FT   HELIX           685..687
FT                   /evidence="ECO:0007829|PDB:5MY2"
FT   TURN            688..690
FT                   /evidence="ECO:0007829|PDB:5MY2"
FT   HELIX           691..701
FT                   /evidence="ECO:0007829|PDB:5MY2"
FT   STRAND          702..704
FT                   /evidence="ECO:0007829|PDB:5MY2"
FT   STRAND          715..717
FT                   /evidence="ECO:0007829|PDB:5MY2"
FT   HELIX           719..721
FT                   /evidence="ECO:0007829|PDB:5MY2"
FT   STRAND          722..724
FT                   /evidence="ECO:0007829|PDB:5MY2"
FT   HELIX           725..728
FT                   /evidence="ECO:0007829|PDB:5MY2"
FT   HELIX           732..740
FT                   /evidence="ECO:0007829|PDB:5MY2"
FT   HELIX           745..749
FT                   /evidence="ECO:0007829|PDB:5MY2"
FT   STRAND          757..764
FT                   /evidence="ECO:0007829|PDB:5MY2"
FT   HELIX           768..774
FT                   /evidence="ECO:0007829|PDB:5MY2"
FT   STRAND          779..782
FT                   /evidence="ECO:0007829|PDB:5MY2"
FT   HELIX           792..804
FT                   /evidence="ECO:0007829|PDB:5MY2"
FT   TURN            805..807
FT                   /evidence="ECO:0007829|PDB:5MY2"
FT   HELIX           812..815
FT                   /evidence="ECO:0007829|PDB:5MY2"
FT   HELIX           831..833
FT                   /evidence="ECO:0007829|PDB:5MY2"
FT   HELIX           847..849
FT                   /evidence="ECO:0007829|PDB:5MY2"
SQ   SEQUENCE   2504 AA;  272428 MW;  2B48068B9D370C6F CRC64;
     MEEVVIAGMS GKLPESENLQ EFWANLIGGV DMVTDDDRRW KAGLYGLPKR SGKLKDLSKF
     DASFFGVHPK QAHTMDPQLR LLLEVSYEAI VDGGINPASL RGTNTGVWVG VSGSEASEAL
     SRDPETLLGY SMVGCQRAMM ANRLSFFFDF KGPSIALDTA CSSSLLALQN AYQAIRSGEC
     PAALVGGINL LLKPNTSVQF MKLGMLSPDG TCRSFDDSGS GYCRSEAVVA VLLTKKSLAR
     RVYATILNAG TNTDGSKEQG VTFPSGEVQE QLICSLYQPA GLAPESLEYI EAHGTGTKVG
     DPQELNGITR SLCAFRQAPL LIGSTKSNMG HPEPASGLAA LTKVLLSLEH GVWAPNLHFH
     NPNPEIPALL DGRLQVVDRP LPVRGGNVGI NSFGFGGSNV HVILQPNTRQ APAPTAHAAL
     PHLLHASGRT LEAVQDLLEQ GRQHSQDLAF VSMLNDIAAT PTAAMPFRGY TVLGVEGRVQ
     EVQQVSTNKR PLWFICSGMG TQWRGMGLSL MRLDSFRESI LRSDEAVKPL GVKVSDLLLS
     TDERTFDDIV HAFVSLTAIQ IALIDLLTSV GLKPDGIIGH SLGEVACGYA DGCLSQREAV
     LAAYWRGQCI KDAHLPPGSM AAVGLSWEEC KQRCPAGVVP ACHNSEDTVT ISGPQAAVNE
     FVEQLKQEGV FAKEVRTGGL AFHSYFMEGI APTLLQALKK VIREPRPRSA RWLSTSIPEA
     QWQSSLARTS SAEYNVNNLV SPVLFQEALW HIPEHAVVLE IAPHALLQAV LKRGVKSSCT
     IIPLMKRDHK DNLEFFLTNL GKVHLTGINV NPNALFPPVE FPAPRGTPLI SPHIKWDHSQ
     TWDVPVAEDF PNGSSSSSAT VYSIDASPES PDHYLVDHCI DGRVIFPGTG YLCLVWKTLA
     RSLGLSLEET PVVFENVSFH QATILPKTGT VALEVRLLEA SHAFEVSDTG NLIVSGKVYL
     WEDPNSKLFD HPEVPTPPES ASVSRLTQGE VYKELRLRGY DYGPQFQGIC EATLEGEQGK
     LLWKDNWVTF MDTMLQVSIL GSSQQSLQLP TRVTAIYIDP ATHRQKVYRL KEDTQVADVT
     TSRCLGITVS GGIHISRLQT TATSRRQQEQ LVPTLEKFVF TPHMEAECLS ESTALQKELQ
     LCKGLARALQ TKATQQGLKA AMLGQEDPPQ HGLPRLLAAA CQLQLNGNLQ LELGEALAQE
     RLLLPEDPLI SGLLNSQALK ACVDTALENL STLKMKVAEV LAGEGHLYSR IPALLNTQPM
     LQLEYTATDR HPQALKDVQT KLQQHDVAQG QWNPSDPAPS SLGALDLLVC NCALATLGDP
     ALALDNMVAA LKEGGFLLVH TVLKGHALGE TLACLPSEVQ PAPSLLSQEE WESLFSRKAL
     HLVGLKRSFY GTALFLCRRA IPQEKPIFLS VEDTSFQWVD SLKSTLATSS SQPVWLTAMD
     CPTSGVVGLV NCLRKEPGGH RIRCILLSNL SNTSHAPKLD PGSPELQQVL KHDLVMNVYR
     DGAWGAFRHF QLEQDKPKEQ TAHAFVNVLT RGDLASIRWV SSPLKHTQPS SSGAQLCTVY
     YASLNFRDIM LATGKLSPDA IPGKWASRDC MLGMEFSGRD RCGRRVMGLV PAEGLATSVL
     LSSDFLWDVP SSWTLEEAAS VPVVYTTAYY SLVVRGRIQR GETVLIHSGS GGVGQAAISI
     ALSLGCRVFT TVGSAEKRAY LQARFPQLDD TSFANSRDTS FEQHVLLHTG GKGVDLVLNS
     LAEEKLQASV RCLAQHGRFL EIGKFDLSNN HPLGMAIFLK NVTFHGILLD ALFEEANDSW
     REVAALLKAG IRDGVVKPLK CTVFPKAQVE DAFRYMAQGK HIGKVLVQVR EEEPEAVLPG
     AQPTLISAIS KTFCPAHKSY IITGGLGGFG LELARWLVLR GAQRLVLTSR SGIRTGYQAK
     HIREWRRQGI QVLVSTSNVS SLEGARALIA EATKLGPVGG VFNLAMVLRD AMLENQTPEL
     FQDVNKPKYN GTLNLDRATR EACPELDYFV AFSSVSCGRG NAGQTNYGFA NSTMERICEQ
     RRHDGLPGLA VQWGAIGDVG IVLEAMGTND TVIGGTLPQR ISSCMEVLDL FLNQPHAVLS
     SFVLAEKKAV AHGDGDTQRD LVKAVAHILG IRDLAGINLD STLADLGLDS LMGVEVRQIL
     EREHDLVLPM REVRQLTLRK LQEMSSKTDS ATDTTAPKSR SDTSLKQNQL NLSTLLVNPE
     GPTLTQLNSV QSSERPLFLV HPIEGSTTVF HSLAAKLSVP TYGLQCTQAA PLDSIPNLAA
     YYIDCIKQVQ PEGPYRIAGY SFGACVAFEM CSQLQAQQGP APTHNNLFLF DGSHTYVLAY
     TQSYRAKMTP GCEAEAEAEA LCFFIKQFLD VEHSKVLEAL LPLKSLEDRV AASVDLITKS
     HHSLDRRELS FAAVSFYHKL RAADQYKPKA KYHGNVTLLR AKTGGTYGED LGADYNLSQV
     CDGKVSVHII EGDHRTLLEG SGLESIINII HSSLAEPRVS VREG
//
```

**Mascot:** http://www.matrixscience.com/


Oxidation (M) (+15.9949)
