# Supplementary material for: A common East-Asian ALDH2 mutation causes metabolic disorders and the therapeutic effect of ALDH2 activators
Source: Nat Commun. 2023 Sep 25;14:5971. doi: 10.1038/s41467-023-41570-6 (PMC10520061; doi:10.1038/s41467-023-41570-6)
Supplement: Supplementary file 4 — Supplementary Data 1 [file 41467_2023_41570_MOESM4_ESM.zip › Table S5c/P19096/P19096_KIO60-2_C2084.html]

Mascot Search Results: P19096
 

# MASCOT Search Results

## Protein View: P19096

### Fatty acid synthase OS=Mus musculus OX=10090 GN=Fasn PE=1 SV=2

|  |  |
| --- | --- |
| Database: | Mouse\_UniProt\_proteomes |
| Score: | 11711 |
| Monoisotopic mass (M_r_): | 274994 |
| Calculated pI: | 6.13 |

Sequence similarity is available as an NCBI BLAST search of P19096 against nr.

### Search parameters

|  |  |
| --- | --- |
| MS data file: | `D:\LCMSMS\2023 Users' data\230529-1\230529-1-KI60-2.raw` |
| Enzyme: | Trypsin/P: cuts C-term side of KR. |
| Fixed modifications: | Carbamidomethyl (C) |
| Variable modifications: | Deamidated (NQ), HNE (C), HNE (H), HNE (K), Oxidation (M) |

### Protein sequence coverage: 55%

Matched peptides shown in ***bold red***.

|  |  |  |  |  |  |
| --- | --- | --- | --- | --- | --- |
| `1` | `MEEVVIAGMS` | `GKLPESENLQ` | `EFWANLIGGV` | `DMVTDDDRRW` | `KAGLYGLPKR` |
| `51` | `SGKLKDLSKF` | `DASFFGVHPK` | `QAHTMDPQLR` | `LLLEVSYEAI` | `VDGGINPASL` |
| `101` | `RGTNTGVWVG` | `VSGSEASEAL` | `SRDPETLLGY` | `SMVGCQRAMM` | `ANRLSFFFDF` |
| `151` | `KGPSIALDTA` | `CSSSLLALQN` | `AYQAIRSGEC` | `PAALVGGINL` | `LLKPNTSVQF` |
| `201` | `MKLGMLSPDG` | `TCRSFDDSGS` | `GYCRSEAVVA` | `VLLTKKSLAR` | `RVYATILNAG` |
| `251` | `TNTDGSKEQG` | `VTFPSGEVQE` | `QLICSLYQPA` | `GLAPESLEYI` | `EAHGTGTKVG` |
| `301` | `DPQELNGITR` | `SLCAFRQAPL` | `LIGSTKSNMG` | `HPEPASGLAA` | `LTKVLLSLEH` |
| `351` | `GVWAPNLHFH` | `NPNPEIPALL` | `DGRLQVVDRP` | `LPVRGGNVGI` | `NSFGFGGSNV` |
| `401` | `HVILQPNTRQ` | `APAPTAHAAL` | `PHLLHASGRT` | `LEAVQDLLEQ` | `GRQHSQDLAF` |
| `451` | `VSMLNDIAAT` | `PTAAMPFRGY` | `TVLGVEGRVQ` | `EVQQVSTNKR` | `PLWFICSGMG` |
| `501` | `TQWRGMGLSL` | `MRLDSFRESI` | `LRSDEAVKPL` | `GVKVSDLLLS` | `TDERTFDDIV` |
| `551` | `HAFVSLTAIQ` | `IALIDLLTSV` | `GLKPDGIIGH` | `SLGEVACGYA` | `DGCLSQREAV` |
| `601` | `LAAYWRGQCI` | `KDAHLPPGSM` | `AAVGLSWEEC` | `KQRCPAGVVP` | `ACHNSEDTVT` |
| `651` | `ISGPQAAVNE` | `FVEQLKQEGV` | `FAKEVRTGGL` | `AFHSYFMEGI` | `APTLLQALKK` |
| `701` | `VIREPRPRSA` | `RWLSTSIPEA` | `QWQSSLARTS` | `SAEYNVNNLV` | `SPVLFQEALW` |
| `751` | `HIPEHAVVLE` | `IAPHALLQAV` | `LKRGVKSSCT` | `IIPLMKRDHK` | `DNLEFFLTNL` |
| `801` | `GKVHLTGINV` | `NPNALFPPVE` | `FPAPRGTPLI` | `SPHIKWDHSQ` | `TWDVPVAEDF` |
| `851` | `PNGSSSSSAT` | `VYSIDASPES` | `PDHYLVDHCI` | `DGRVIFPGTG` | `YLCLVWKTLA` |
| `901` | `RSLGLSLEET` | `PVVFENVSFH` | `QATILPKTGT` | `VALEVRLLEA` | `SHAFEVSDTG` |
| `951` | `NLIVSGKVYL` | `WEDPNSKLFD` | `HPEVPTPPES` | `ASVSRLTQGE` | `VYKELRLRGY` |
| `1001` | `DYGPQFQGIC` | `EATLEGEQGK` | `LLWKDNWVTF` | `MDTMLQVSIL` | `GSSQQSLQLP` |
| `1051` | `TRVTAIYIDP` | `ATHRQKVYRL` | `KEDTQVADVT` | `TSRCLGITVS` | `GGIHISRLQT` |
| `1101` | `TATSRRQQEQ` | `LVPTLEKFVF` | `TPHMEAECLS` | `ESTALQKELQ` | `LCKGLARALQ` |
| `1151` | `TKATQQGLKA` | `AMLGQEDPPQ` | `HGLPRLLAAA` | `CQLQLNGNLQ` | `LELGEALAQE` |
| `1201` | `RLLLPEDPLI` | `SGLLNSQALK` | `ACVDTALENL` | `STLKMKVAEV` | `LAGEGHLYSR` |
| `1251` | `IPALLNTQPM` | `LQLEYTATDR` | `HPQALKDVQT` | `KLQQHDVAQG` | `QWNPSDPAPS` |
| `1301` | `SLGALDLLVC` | `NCALATLGDP` | `ALALDNMVAA` | `LKEGGFLLVH` | `TVLKGHALGE` |
| `1351` | `TLACLPSEVQ` | `PAPSLLSQEE` | `WESLFSRKAL` | `HLVGLKRSFY` | `GTALFLCRRA` |
| `1401` | `IPQEKPIFLS` | `VEDTSFQWVD` | `SLKSTLATSS` | `SQPVWLTAMD` | `CPTSGVVGLV` |
| `1451` | `NCLRKEPGGH` | `RIRCILLSNL` | `SNTSHAPKLD` | `PGSPELQQVL` | `KHDLVMNVYR` |
| `1501` | `DGAWGAFRHF` | `QLEQDKPKEQ` | `TAHAFVNVLT` | `RGDLASIRWV` | `SSPLKHTQPS` |
| `1551` | `SSGAQLCTVY` | `YASLNFRDIM` | `LATGKLSPDA` | `IPGKWASRDC` | `MLGMEFSGRD` |
| `1601` | `RCGRRVMGLV` | `PAEGLATSVL` | `LSSDFLWDVP` | `SSWTLEEAAS` | `VPVVYTTAYY` |
| `1651` | `SLVVRGRIQR` | `GETVLIHSGS` | `GGVGQAAISI` | `ALSLGCRVFT` | `TVGSAEKRAY` |
| `1701` | `LQARFPQLDD` | `TSFANSRDTS` | `FEQHVLLHTG` | `GKGVDLVLNS` | `LAEEKLQASV` |
| `1751` | `RCLAQHGRFL` | `EIGKFDLSNN` | `HPLGMAIFLK` | `NVTFHGILLD` | `ALFEEANDSW` |
| `1801` | `REVAALLKAG` | `IRDGVVKPLK` | `CTVFPKAQVE` | `DAFRYMAQGK` | `HIGKVLVQVR` |
| `1851` | `EEEPEAVLPG` | `AQPTLISAIS` | `KTFCPAHKSY` | `IITGGLGGFG` | `LELARWLVLR` |
| `1901` | `GAQRLVLTSR` | `SGIRTGYQAK` | `HIREWRRQGI` | `QVLVSTSNVS` | `SLEGARALIA` |
| `1951` | `EATKLGPVGG` | `VFNLAMVLRD` | `AMLENQTPEL` | `FQDVNKPKYN` | `GTLNLDRATR` |
| `2001` | `EACPELDYFV` | `AFSSVSCGRG` | `NAGQTNYGFA` | `NSTMERICEQ` | `RRHDGLPGLA` |
| `2051` | `VQWGAIGDVG` | `IVLEAMGTND` | `TVIGGTLPQR` | `ISSCMEVLDL` | `FLNQPHAVLS` |
| `2101` | `SFVLAEKKAV` | `AHGDGDTQRD` | `LVKAVAHILG` | `IRDLAGINLD` | `STLADLGLDS` |
| `2151` | `LMGVEVRQIL` | `EREHDLVLPM` | `REVRQLTLRK` | `LQEMSSKTDS` | `ATDTTAPKSR` |
| `2201` | `SDTSLKQNQL` | `NLSTLLVNPE` | `GPTLTQLNSV` | `QSSERPLFLV` | `HPIEGSTTVF` |
| `2251` | `HSLAAKLSVP` | `TYGLQCTQAA` | `PLDSIPNLAA` | `YYIDCIKQVQ` | `PEGPYRIAGY` |
| `2301` | `SFGACVAFEM` | `CSQLQAQQGP` | `APTHNNLFLF` | `DGSHTYVLAY` | `TQSYRAKMTP` |
| `2351` | `GCEAEAEAEA` | `LCFFIKQFLD` | `VEHSKVLEAL` | `LPLKSLEDRV` | `AASVDLITKS` |
| `2401` | `HHSLDRRELS` | `FAAVSFYHKL` | `RAADQYKPKA` | `KYHGNVTLLR` | `AKTGGTYGED` |
| `2451` | `LGADYNLSQV` | `CDGKVSVHII` | `EGDHRTLLEG` | `SGLESIINII` | `HSSLAEPRVS` |
| `2501` | `VREG` |  |  |  |  |

Unformatted sequence string: 2504 residues (for pasting into other applications).

|  |  |  |  |
| --- | --- | --- | --- |
| Sort by | residue number | increasing mass | decreasing mass |
| Show | matched peptides only | predicted peptides also |  |

| Query | Start | – | End | Observed | Mr(expt) | Mr(calc) | ppm | M | Score | Expect | Rank | U | Peptide |
| --- | --- | --- | --- | --- | --- | --- | --- | --- | --- | --- | --- | --- | --- |
| 25763 | 40 | – | 49 | 378.2221 | 1131.6444 | 1131.6440 | 0.29 | 1 | 33 | 0.00074 | 1Score **> 32** indicates **identity** Score **> 15** indicates **homology** | U | R.WKAGLYGLPK.R |
| 25764 | 40 | – | 49 | 378.2221 | 1131.6445 | 1131.6440 | 0.37 | 1 | 33 | 0.0008 | 1Score **> 32** indicates **identity** Score **> 15** indicates **homology** | U | R.WKAGLYGLPK.R |
| 4329 | 42 | – | 49 | 409.7420 | 817.4694 | 817.4698 | -0.41 | 0 | 31 | 0.0045 | 1Score **> 28** indicates **identity** Score **> 20** indicates **homology** | U | K.AGLYGLPK.R |
| 93258 | 56 | – | 70 | 565.6230 | 1693.8471 | 1693.8464 | 0.42 | 1 | 36 | 0.0014 | 1Score **> 35** indicates **identity** Score **> 20** indicates **homology** | U | K.DLSKFDASFFGVHPK.Q |
| 93259 | 56 | – | 70 | 565.6235 | 1693.8488 | 1693.8464 | 1.39 | 1 | 23 | 0.007 | 1Score **> 35** indicates **identity** Score **> 14** indicates **homology** | U | K.DLSKFDASFFGVHPK.Q |
| 93265 | 56 | – | 70 | 565.6249 | 1693.8528 | 1693.8464 | 3.74 | 1 | 35 | 0.00088 | 1Score **> 35** indicates **identity** Score **> 17** indicates **homology** | U | K.DLSKFDASFFGVHPK.Q |
| 38439 | 60 | – | 70 | 417.8768 | 1250.6085 | 1250.6084 | 0.030 | 0 | 41 | 0.00014 | 1Score **> 32** indicates **identity** Score **> 15** indicates **homology** | U | K.FDASFFGVHPK.Q |
| 38442 | 60 | – | 70 | 417.8769 | 1250.6090 | 1250.6084 | 0.45 | 0 | 42 | 0.00011 | 1Score **> 32** indicates **identity** Score **> 15** indicates **homology** | U | K.FDASFFGVHPK.Q |
| 38443 | 60 | – | 70 | 417.8769 | 1250.6090 | 1250.6084 | 0.47 | 0 | 39 | 0.00021 | 1Score **> 32** indicates **identity** Score **> 15** indicates **homology** | U | K.FDASFFGVHPK.Q |
| 38444 | 60 | – | 70 | 417.8773 | 1250.6099 | 1250.6084 | 1.20 | 0 | 29 | 0.0019 | 1Score **> 32** indicates **identity** Score **> 14** indicates **homology** | U | K.FDASFFGVHPK.Q |
| 38446 | 60 | – | 70 | 417.8774 | 1250.6105 | 1250.6084 | 1.63 | 0 | 39 | 0.00023 | 1Score **> 32** indicates **identity** Score **> 15** indicates **homology** | U | K.FDASFFGVHPK.Q |
| 17827 | 144 | – | 151 | 525.7679 | 1049.5213 | 1049.5222 | -0.89 | 0 | 29 | 0.0019 | 1Score **> 32** indicates **identity** Score **> 14** indicates **homology** | U | R.LSFFFDFK.G |
| 17830 | 144 | – | 151 | 525.7690 | 1049.5235 | 1049.5222 | 1.19 | 0 | 46 | 5.1e-05 | 1Score **> 32** indicates **identity** Score **> 15** indicates **homology** | U | R.LSFFFDFK.G |
| 170801 | 152 | – | 176 | 874.1204 | 2619.3394 | 2619.3326 | 2.59 | 0 | 76 | 7.2e-08 | 1Score **> 37** indicates **identity** Score **> 17** indicates **homology** | U | K.GPSIALDTACSSSLLALQNAYQAIR.S |
| 170802 | 152 | – | 176 | 1310.6770 | 2619.3394 | 2619.3326 | 2.59 | 0 | 139 | 9.1e-14 | 1Score **> 37** indicates **identity** Score **> 21** indicates **homology** | U | K.GPSIALDTACSSSLLALQNAYQAIR.S |
| 175921 | 177 | – | 202 | 915.8221 | 2744.4446 | 2744.4241 | 7.47 | 1 | 41 | 0.00015 | 1Score **> 37** indicates **identity** Score **> 15** indicates **homology** | U | R.SGECPAALVGGINLLLKPNTSVQFMK.L  + Deamidated (NQ) |
| 38308 | 214 | – | 224 | 625.7406 | 1249.4667 | 1249.4670 | -0.19 | 0 | 67 | 4.7e-07 | 1Score **> 16** indicates **identity** | U | R.SFDDSGSGYCR.S |
| 38309 | 214 | – | 224 | 625.7411 | 1249.4676 | 1249.4670 | 0.50 | 0 | 73 | 1.2e-07 | 1Score **> 16** indicates **identity** | U | R.SFDDSGSGYCR.S |
| 38310 | 214 | – | 224 | 625.7415 | 1249.4685 | 1249.4670 | 1.18 | 0 | 28 | 0.0033 | 1Score **> 16** indicates **identity** | U | R.SFDDSGSGYCR.S |
| 25418 | 225 | – | 235 | 565.3450 | 1128.6755 | 1128.6754 | 0.052 | 0 | 68 | 4e-07 | 1Score **> 32** indicates **identity** Score **> 17** indicates **homology** | U | R.SEAVVAVLLTK.K |
| 25419 | 225 | – | 235 | 565.3452 | 1128.6759 | 1128.6754 | 0.40 | 0 | 34 | 0.00063 | 1Score **> 32** indicates **identity** Score **> 15** indicates **homology** | U | R.SEAVVAVLLTK.K |
| 25420 | 225 | – | 235 | 565.3454 | 1128.6762 | 1128.6754 | 0.71 | 0 | 68 | 3.8e-07 | 1Score **> 32** indicates **identity** Score **> 17** indicates **homology** | U | R.SEAVVAVLLTK.K |
| 25421 | 225 | – | 235 | 565.3454 | 1128.6762 | 1128.6754 | 0.72 | 0 | 30 | 0.0016 | 1Score **> 32** indicates **identity** Score **> 14** indicates **homology** | U | R.SEAVVAVLLTK.K |
| 25422 | 225 | – | 235 | 565.3457 | 1128.6768 | 1128.6754 | 1.24 | 0 | 45 | 5.4e-05 | 1Score **> 31** indicates **identity** Score **> 15** indicates **homology** | U | R.SEAVVAVLLTK.K |
| 25423 | 225 | – | 235 | 565.3460 | 1128.6775 | 1128.6754 | 1.87 | 0 | 68 | 4.2e-07 | 1Score **> 31** indicates **identity** Score **> 17** indicates **homology** | U | R.SEAVVAVLLTK.K |
| 39305 | 225 | – | 236 | 419.9310 | 1256.7711 | 1256.7704 | 0.56 | 1 | 30 | 0.0015 | 1Score **> 30** indicates **identity** Score **> 14** indicates **homology** | U | R.SEAVVAVLLTKK.S |
| 39306 | 225 | – | 236 | 419.9310 | 1256.7712 | 1256.7704 | 0.65 | 1 | 24 | 0.005 | 1Score **> 30** indicates **identity** Score **> 14** indicates **homology** | U | R.SEAVVAVLLTKK.S |
| 39307 | 225 | – | 236 | 629.3933 | 1256.7720 | 1256.7704 | 1.32 | 1 | 31 | 0.0012 | 1Score **> 30** indicates **identity** Score **> 14** indicates **homology** | U | R.SEAVVAVLLTKK.S |
| 39308 | 225 | – | 236 | 419.9313 | 1256.7720 | 1256.7704 | 1.33 | 1 | 18 | 0.022 | 1Score **> 30** indicates **identity** Score **> 14** indicates **homology** | U | R.SEAVVAVLLTKK.S |
| 39309 | 225 | – | 236 | 419.9315 | 1256.7728 | 1256.7704 | 1.93 | 1 | 30 | 0.0015 | 1Score **> 30** indicates **identity** Score **> 14** indicates **homology** | U | R.SEAVVAVLLTKK.S |
| 39310 | 225 | – | 236 | 419.9316 | 1256.7729 | 1256.7704 | 2.01 | 1 | 15 | 0.042 | 1Score **> 30** indicates **identity** Score **> 13** indicates **homology** | U | R.SEAVVAVLLTKK.S |
| 39312 | 225 | – | 236 | 629.3941 | 1256.7736 | 1256.7704 | 2.55 | 1 | 30 | 0.0014 | 1Score **> 30** indicates **identity** Score **> 14** indicates **homology** | U | R.SEAVVAVLLTKK.S |
| 39313 | 225 | – | 236 | 419.9318 | 1256.7737 | 1256.7704 | 2.67 | 1 | 30 | 0.0014 | 1Score **> 30** indicates **identity** Score **> 14** indicates **homology** | U | R.SEAVVAVLLTKK.S |
| 39314 | 225 | – | 236 | 419.9319 | 1256.7738 | 1256.7704 | 2.71 | 1 | 19 | 0.016 | 1Score **> 30** indicates **identity** Score **> 14** indicates **homology** | U | R.SEAVVAVLLTKK.S |
| 39316 | 225 | – | 236 | 629.3962 | 1256.7778 | 1256.7704 | 5.93 | 1 | 35 | 0.00055 | 1Score **> 30** indicates **identity** Score **> 15** indicates **homology** | U | R.SEAVVAVLLTKK.S |
| 39317 | 225 | – | 236 | 419.9333 | 1256.7780 | 1256.7704 | 6.09 | 1 | 30 | 0.0014 | 1Score **> 30** indicates **identity** Score **> 14** indicates **homology** | U | R.SEAVVAVLLTKK.S |
| 39318 | 225 | – | 236 | 629.3969 | 1256.7793 | 1256.7704 | 7.14 | 1 | 33 | 0.00088 | 1Score **> 30** indicates **identity** Score **> 15** indicates **homology** | U | R.SEAVVAVLLTKK.S |
| 43827 | 299 | – | 310 | 649.8380 | 1297.6615 | 1297.6626 | -0.89 | 0 | 58 | 3.5e-06 | 1Score **> 33** indicates **identity** Score **> 16** indicates **homology** | U | K.VGDPQELNGITR.S |
| 43829 | 299 | – | 310 | 649.8387 | 1297.6629 | 1297.6626 | 0.19 | 0 | 65 | 2.6e-06 | 1Score **> 32** indicates **identity** Score **> 22** indicates **homology** | U | K.VGDPQELNGITR.S |
| 43831 | 299 | – | 310 | 649.8397 | 1297.6649 | 1297.6626 | 1.74 | 0 | 53 | 6.7e-05 | 1Score **> 33** indicates **identity** Score **> 24** indicates **homology** | U | K.VGDPQELNGITR.S |
| 43957 | 299 | – | 310 | 650.3287 | 1298.6428 | 1298.6466 | -2.96 | 0 | 49 | 2.8e-05 | 1Score **> 31** indicates **identity** Score **> 16** indicates **homology** | U | K.VGDPQELNGITR.S  + Deamidated (NQ) |
| 43959 | 299 | – | 310 | 650.3301 | 1298.6457 | 1298.6466 | -0.72 | 0 | 68 | 1.2e-06 | 1Score **> 32** indicates **identity** Score **> 21** indicates **homology** | U | K.VGDPQELNGITR.S  + Deamidated (NQ) |
| 43960 | 299 | – | 310 | 650.3302 | 1298.6458 | 1298.6466 | -0.62 | 0 | 72 | 1.9e-06 | 1Score **> 32** indicates **identity** Score **> 27** indicates **homology** | U | K.VGDPQELNGITR.S  + Deamidated (NQ) |
| 43961 | 299 | – | 310 | 650.3304 | 1298.6463 | 1298.6466 | -0.27 | 0 | 54 | 1.7e-05 | 1Score **> 32** indicates **identity** Score **> 19** indicates **homology** | U | K.VGDPQELNGITR.S  + Deamidated (NQ) |
| 43962 | 299 | – | 310 | 650.3307 | 1298.6468 | 1298.6466 | 0.11 | 0 | 72 | 5.9e-07 | 1Score **> 32** indicates **identity** Score **> 22** indicates **homology** | U | K.VGDPQELNGITR.S  + Deamidated (NQ) |
| 43963 | 299 | – | 310 | 650.3311 | 1298.6476 | 1298.6466 | 0.70 | 0 | 47 | 0.00013 | 1Score **> 32** indicates **identity** Score **> 21** indicates **homology** | U | K.VGDPQELNGITR.S  + Deamidated (NQ) |
| 16219 | 317 | – | 326 | 514.3107 | 1026.6069 | 1026.6073 | -0.42 | 0 | 34 | 0.00059 | 1Score **> 28** indicates **identity** Score **> 15** indicates **homology** | U | R.QAPLLIGSTK.S |
| 16220 | 317 | – | 326 | 514.3109 | 1026.6072 | 1026.6073 | -0.15 | 0 | 43 | 8.8e-05 | 1Score **> 28** indicates **identity** Score **> 15** indicates **homology** | U | R.QAPLLIGSTK.S |
| 16221 | 317 | – | 326 | 514.3115 | 1026.6084 | 1026.6073 | 1.08 | 0 | 27 | 0.003 | 1Score **> 30** indicates **identity** Score **> 14** indicates **homology** | U | R.QAPLLIGSTK.S |
| 91411 | 327 | – | 343 | 560.9504 | 1679.8295 | 1679.8301 | -0.35 | 0 | 24 | 0.0062 | 1Score **> 34** indicates **identity** Score **> 14** indicates **homology** | U | K.SNMGHPEPASGLAALTK.V |
| 91413 | 327 | – | 343 | 560.9514 | 1679.8324 | 1679.8301 | 1.39 | 0 | 48 | 3.4e-05 | 1Score **> 35** indicates **identity** Score **> 15** indicates **homology** | U | K.SNMGHPEPASGLAALTK.V |
| 192758 | 344 | – | 373 | 839.6984 | 3354.7643 | 3354.7625 | 0.52 | 0 | 21 | 0.012 | 1Score **> 37** indicates **identity** Score **> 14** indicates **homology** | U | K.VLLSLEHGVWAPNLHFHNPNPEIPALLDGR.L |
| 192759 | 344 | – | 373 | 1119.2643 | 3354.7710 | 3354.7625 | 2.52 | 0 | 36 | 0.00046 | 1Score **> 36** indicates **identity** Score **> 15** indicates **homology** | U | K.VLLSLEHGVWAPNLHFHNPNPEIPALLDGR.L |
| 192760 | 344 | – | 373 | 671.9615 | 3354.7710 | 3354.7625 | 2.52 | 0 | 14 | 0.044 | 1Score **> 36** indicates **identity** Score **> 13** indicates **homology** | U | K.VLLSLEHGVWAPNLHFHNPNPEIPALLDGR.L |
| 43125 | 374 | – | 384 | 431.2659 | 1290.7757 | 1290.7772 | -1.13 | 1 | 25 | 0.0049 | 1Score **> 29** indicates **identity** Score **> 14** indicates **homology** | U | R.LQVVDRPLPVR.G |
| 43129 | 374 | – | 384 | 431.2665 | 1290.7778 | 1290.7772 | 0.44 | 1 | 26 | 0.0039 | 1Score **> 29** indicates **identity** Score **> 14** indicates **homology** | U | R.LQVVDRPLPVR.G |
| 43130 | 374 | – | 384 | 431.2665 | 1290.7778 | 1290.7772 | 0.46 | 1 | 24 | 0.0058 | 1Score **> 29** indicates **identity** Score **> 15** indicates **homology** | U | R.LQVVDRPLPVR.G |
| 43131 | 374 | – | 384 | 431.2666 | 1290.7779 | 1290.7772 | 0.51 | 1 | 19 | 0.018 | 1Score **> 29** indicates **identity** Score **> 14** indicates **homology** | U | R.LQVVDRPLPVR.G |
| 43132 | 374 | – | 384 | 646.3963 | 1290.7780 | 1290.7772 | 0.62 | 1 | 31 | 0.0011 | 1Score **> 29** indicates **identity** Score **> 14** indicates **homology** | U | R.LQVVDRPLPVR.G |
| 43133 | 374 | – | 384 | 431.2667 | 1290.7781 | 1290.7772 | 0.73 | 1 | 27 | 0.0029 | 1Score **> 29** indicates **identity** Score **> 14** indicates **homology** | U | R.LQVVDRPLPVR.G |
| 43134 | 374 | – | 384 | 646.3964 | 1290.7783 | 1290.7772 | 0.88 | 1 | 16 | 0.029 | 1Score **> 30** indicates **identity** Score **> 14** indicates **homology** | U | R.LQVVDRPLPVR.G |
| 43136 | 374 | – | 384 | 431.2669 | 1290.7790 | 1290.7772 | 1.36 | 1 | 17 | 0.025 | 1Score **> 29** indicates **identity** Score **> 14** indicates **homology** | U | R.LQVVDRPLPVR.G |
| 166999 | 385 | – | 409 | 1271.1554 | 2540.2962 | 2540.2885 | 3.05 | 0 | 76 | 6.9e-08 | 1Score **> 37** indicates **identity** Score **> 17** indicates **homology** | U | R.GGNVGINSFGFGGSNVHVILQPNTR.Q |
| 167002 | 385 | – | 409 | 847.7732 | 2540.2977 | 2540.2885 | 3.64 | 0 | 67 | 4.7e-07 | 1Score **> 37** indicates **identity** Score **> 17** indicates **homology** | U | R.GGNVGINSFGFGGSNVHVILQPNTR.Q |
| 167003 | 385 | – | 409 | 847.7733 | 2540.2980 | 2540.2885 | 3.74 | 0 | 86 | 9.1e-09 | 1Score **> 37** indicates **identity** Score **> 18** indicates **homology** | U | R.GGNVGINSFGFGGSNVHVILQPNTR.Q |
| 167004 | 385 | – | 409 | 1271.1563 | 2540.2980 | 2540.2885 | 3.75 | 0 | 49 | 2.5e-05 | 1Score **> 37** indicates **identity** Score **> 16** indicates **homology** | U | R.GGNVGINSFGFGGSNVHVILQPNTR.Q |
| 167005 | 385 | – | 409 | 1271.1569 | 2540.2993 | 2540.2885 | 4.27 | 0 | 76 | 8.3e-08 | 1Score **> 37** indicates **identity** Score **> 17** indicates **homology** | U | R.GGNVGINSFGFGGSNVHVILQPNTR.Q |
| 167007 | 385 | – | 409 | 847.7743 | 2540.3010 | 2540.2885 | 4.94 | 0 | 28 | 0.0025 | 1Score **> 37** indicates **identity** Score **> 14** indicates **homology** | U | R.GGNVGINSFGFGGSNVHVILQPNTR.Q |
| 167009 | 385 | – | 409 | 847.7746 | 2540.3019 | 2540.2885 | 5.28 | 0 | 90 | 3.8e-09 | 1Score **> 37** indicates **identity** Score **> 18** indicates **homology** | U | R.GGNVGINSFGFGGSNVHVILQPNTR.Q |
| 167011 | 385 | – | 409 | 847.7750 | 2540.3032 | 2540.2885 | 5.83 | 0 | 38 | 0.0003 | 1Score **> 37** indicates **identity** Score **> 15** indicates **homology** | U | R.GGNVGINSFGFGGSNVHVILQPNTR.Q |
| 167012 | 385 | – | 409 | 847.7758 | 2540.3055 | 2540.2885 | 6.73 | 0 | 47 | 3.6e-05 | 1Score **> 37** indicates **identity** Score **> 15** indicates **homology** | U | R.GGNVGINSFGFGGSNVHVILQPNTR.Q |
| 167014 | 385 | – | 409 | 847.7768 | 2540.3084 | 2540.2885 | 7.87 | 0 | 74 | 1.1e-07 | 1Score **> 37** indicates **identity** Score **> 17** indicates **homology** | U | R.GGNVGINSFGFGGSNVHVILQPNTR.Q |
| 167015 | 385 | – | 409 | 847.7768 | 2540.3085 | 2540.2885 | 7.87 | 0 | 29 | 0.0021 | 1Score **> 37** indicates **identity** Score **> 14** indicates **homology** | U | R.GGNVGINSFGFGGSNVHVILQPNTR.Q |
| 167016 | 385 | – | 409 | 847.7768 | 2540.3087 | 2540.2885 | 7.96 | 0 | 43 | 8.9e-05 | 1Score **> 37** indicates **identity** Score **> 15** indicates **homology** | U | R.GGNVGINSFGFGGSNVHVILQPNTR.Q |
| 167017 | 385 | – | 409 | 847.7769 | 2540.3088 | 2540.2885 | 8.03 | 0 | 46 | 5.4e-05 | 1Score **> 37** indicates **identity** Score **> 15** indicates **homology** | U | R.GGNVGINSFGFGGSNVHVILQPNTR.Q |
| 167042 | 385 | – | 409 | 848.1036 | 2541.2889 | 2541.2725 | 6.46 | 0 | 30 | 0.0017 | 1Score **> 37** indicates **identity** Score **> 14** indicates **homology** | U | R.GGNVGINSFGFGGSNVHVILQPNTR.Q  + Deamidated (NQ) |
| 167043 | 385 | – | 409 | 848.1041 | 2541.2905 | 2541.2725 | 7.11 | 0 | 21 | 0.011 | 1Score **> 37** indicates **identity** Score **> 14** indicates **homology** | U | R.GGNVGINSFGFGGSNVHVILQPNTR.Q  + Deamidated (NQ) |
| 167045 | 385 | – | 409 | 848.1043 | 2541.2910 | 2541.2725 | 7.30 | 0 | 25 | 0.0044 | 1Score **> 37** indicates **identity** Score **> 14** indicates **homology** | U | R.GGNVGINSFGFGGSNVHVILQPNTR.Q  + Deamidated (NQ) |
| 127804 | 410 | – | 429 | 504.7777 | 2015.0818 | 2015.0813 | 0.26 | 0 | 43 | 0.0001 | 1Score **> 36** indicates **identity** Score **> 15** indicates **homology** | U | R.QAPAPTAHAALPHLLHASGR.T |
| 127805 | 410 | – | 429 | 404.0237 | 2015.0821 | 2015.0813 | 0.41 | 0 | 39 | 0.0002 | 1Score **> 36** indicates **identity** Score **> 15** indicates **homology** | U | R.QAPAPTAHAALPHLLHASGR.T |
| 127806 | 410 | – | 429 | 504.7779 | 2015.0824 | 2015.0813 | 0.56 | 0 | 38 | 0.00028 | 1Score **> 36** indicates **identity** Score **> 15** indicates **homology** | U | R.QAPAPTAHAALPHLLHASGR.T |
| 127807 | 410 | – | 429 | 672.7016 | 2015.0831 | 2015.0813 | 0.88 | 0 | 28 | 0.0025 | 1Score **> 36** indicates **identity** Score **> 14** indicates **homology** | U | R.QAPAPTAHAALPHLLHASGR.T |
| 127808 | 410 | – | 429 | 672.7017 | 2015.0832 | 2015.0813 | 0.94 | 0 | 30 | 0.0014 | 1Score **> 36** indicates **identity** Score **> 14** indicates **homology** | U | R.QAPAPTAHAALPHLLHASGR.T |
| 127891 | 410 | – | 429 | 1009.0494 | 2016.0843 | 2016.0653 | 9.42 | 0 | 32 | 0.001 | 1Score **> 36** indicates **identity** Score **> 14** indicates **homology** | U | R.QAPAPTAHAALPHLLHASGR.T  + Deamidated (NQ) |
| 63300 | 430 | – | 442 | 736.3888 | 1470.7631 | 1470.7678 | -3.20 | 0 | 25 | 0.0047 | 1Score **> 34** indicates **identity** Score **> 14** indicates **homology** | U | R.TLEAVQDLLEQGR.Q |
| 63304 | 430 | – | 442 | 736.3904 | 1470.7662 | 1470.7678 | -1.10 | 0 | 76 | 3.8e-07 | 1Score **> 34** indicates **identity** Score **> 24** indicates **homology** | U | R.TLEAVQDLLEQGR.Q |
| 63305 | 430 | – | 442 | 736.3906 | 1470.7667 | 1470.7678 | -0.78 | 0 | 17 | 0.025 | 1Score **> 34** indicates **identity** Score **> 14** indicates **homology** | U | R.TLEAVQDLLEQGR.Q |
| 63306 | 430 | – | 442 | 736.3907 | 1470.7668 | 1470.7678 | -0.68 | 0 | 28 | 0.0026 | 1Score **> 34** indicates **identity** Score **> 14** indicates **homology** | U | R.TLEAVQDLLEQGR.Q |
| 63307 | 430 | – | 442 | 736.3907 | 1470.7669 | 1470.7678 | -0.60 | 0 | 74 | 3.1e-06 | 1Score **> 34** indicates **identity** Score **> 31** indicates **homology** | U | R.TLEAVQDLLEQGR.Q |
| 63308 | 430 | – | 442 | 736.3907 | 1470.7669 | 1470.7678 | -0.59 | 0 | 41 | 0.00014 | 1Score **> 34** indicates **identity** Score **> 15** indicates **homology** | U | R.TLEAVQDLLEQGR.Q |
| 63309 | 430 | – | 442 | 736.3911 | 1470.7677 | 1470.7678 | -0.11 | 0 | 73 | 7.1e-07 | 1Score **> 34** indicates **identity** Score **> 24** indicates **homology** | U | R.TLEAVQDLLEQGR.Q |
| 63314 | 430 | – | 442 | 736.3920 | 1470.7694 | 1470.7678 | 1.05 | 0 | 92 | 8.6e-09 | 1Score **> 34** indicates **identity** Score **> 24** indicates **homology** | U | R.TLEAVQDLLEQGR.Q |
| 63315 | 430 | – | 442 | 736.3922 | 1470.7698 | 1470.7678 | 1.32 | 0 | 19 | 0.015 | 1Score **> 34** indicates **identity** Score **> 14** indicates **homology** | U | R.TLEAVQDLLEQGR.Q |
| 63316 | 430 | – | 442 | 736.3923 | 1470.7700 | 1470.7678 | 1.46 | 0 | 59 | 1e-05 | 1Score **> 34** indicates **identity** Score **> 21** indicates **homology** | U | R.TLEAVQDLLEQGR.Q |
| 17863 | 469 | – | 478 | 525.7830 | 1049.5515 | 1049.5506 | 0.85 | 0 | 55 | 5.1e-05 | 1Score **> 32** indicates **identity** Score **> 25** indicates **homology** | U | R.GYTVLGVEGR.V |
| 17865 | 469 | – | 478 | 525.7831 | 1049.5516 | 1049.5506 | 1.00 | 0 | 52 | 8.1e-05 | 1Score **> 32** indicates **identity** Score **> 24** indicates **homology** | U | R.GYTVLGVEGR.V |
| 17867 | 469 | – | 478 | 525.7833 | 1049.5521 | 1049.5506 | 1.46 | 0 | 50 | 7.9e-05 | 1Score **> 32** indicates **identity** Score **> 22** indicates **homology** | U | R.GYTVLGVEGR.V |
| 17868 | 469 | – | 478 | 525.7834 | 1049.5522 | 1049.5506 | 1.55 | 0 | 45 | 0.00017 | 1Score **> 32** indicates **identity** Score **> 20** indicates **homology** | U | R.GYTVLGVEGR.V |
| 17871 | 469 | – | 478 | 525.7841 | 1049.5536 | 1049.5506 | 2.85 | 0 | 30 | 0.0016 | 1Score **> 31** indicates **identity** Score **> 14** indicates **homology** | U | R.GYTVLGVEGR.V |
| 39477 | 479 | – | 489 | 630.3324 | 1258.6503 | 1258.6517 | -1.16 | 0 | 24 | 0.0055 | 1Score **> 34** indicates **identity** Score **> 14** indicates **homology** | U | R.VQEVQQVSTNK.R |
| 188352 | 479 | – | 504 | 784.6451 | 3134.5512 | 3134.5542 | -0.96 | 2 | 15 | 0.039 | 1Score **> 37** indicates **identity** Score **> 13** indicates **homology** | U | R.VQEVQQVSTNKRPLWFICSGMGTQWR.G |
| 188378 | 479 | – | 504 | 784.8976 | 3135.5613 | 3135.5383 | 7.34 | 2 | 26 | 0.0035 | 1Score **> 37** indicates **identity** Score **> 14** indicates **homology** | U | R.VQEVQQVSTNKRPLWFICSGMGTQWR.G  + Deamidated (NQ) |
| 115870 | 490 | – | 504 | 632.3102 | 1893.9086 | 1893.9131 | -2.33 | 1 | 23 | 0.0074 | 1Score **> 34** indicates **identity** Score **> 14** indicates **homology** | U | K.RPLWFICSGMGTQWR.G |
| 36838 | 513 | – | 522 | 412.5629 | 1234.6668 | 1234.6670 | -0.11 | 1 | 26 | 0.024 | 1Score **> 33** indicates **identity** Score **> 22** indicates **homology** | U | R.LDSFRESILR.S |
| 26706 | 523 | – | 533 | 381.5518 | 1141.6336 | 1141.6343 | -0.55 | 1 | 30 | 0.0046 | 1Score **> 31** indicates **identity** Score **> 19** indicates **homology** | U | R.SDEAVKPLGVK.V |
| 26707 | 523 | – | 533 | 571.8243 | 1141.6340 | 1141.6343 | -0.20 | 1 | 57 | 2.4e-05 | 1Score **> 31** indicates **identity** Score **> 23** indicates **homology** | U | R.SDEAVKPLGVK.V |
| 26708 | 523 | – | 533 | 571.8246 | 1141.6346 | 1141.6343 | 0.26 | 1 | 75 | 1.5e-06 | 1Score **> 32** indicates **identity** Score **> 29** indicates **homology** | U | R.SDEAVKPLGVK.V |
| 26709 | 523 | – | 533 | 381.5523 | 1141.6351 | 1141.6343 | 0.73 | 1 | 40 | 0.00018 | 1Score **> 32** indicates **identity** Score **> 15** indicates **homology** | U | R.SDEAVKPLGVK.V |
| 37981 | 534 | – | 544 | 624.3281 | 1246.6417 | 1246.6405 | 0.94 | 0 | 63 | 5.9e-06 | 1Score **> 34** indicates **identity** Score **> 23** indicates **homology** | U | K.VSDLLLSTDER.T |
| 37983 | 534 | – | 544 | 624.3303 | 1246.6461 | 1246.6405 | 4.47 | 0 | 42 | 0.00012 | 1Score **> 34** indicates **identity** Score **> 15** indicates **homology** | U | K.VSDLLLSTDER.T |
| 37985 | 534 | – | 544 | 624.3315 | 1246.6485 | 1246.6405 | 6.41 | 0 | 64 | 3.6e-06 | 1Score **> 34** indicates **identity** Score **> 22** indicates **homology** | U | K.VSDLLLSTDER.T |
| 20438 | 598 | – | 606 | 539.7875 | 1077.5604 | 1077.5607 | -0.32 | 0 | 41 | 0.00015 | 1Score **> 32** indicates **identity** Score **> 15** indicates **homology** | U | R.EAVLAAYWR.G |
| 20439 | 598 | – | 606 | 539.7876 | 1077.5607 | 1077.5607 | 0.036 | 0 | 59 | 1.3e-05 | 1Score **> 33** indicates **identity** Score **> 23** indicates **homology** | U | R.EAVLAAYWR.G |
| 20441 | 598 | – | 606 | 539.7881 | 1077.5617 | 1077.5607 | 0.93 | 0 | 62 | 8.5e-06 | 1Score **> 32** indicates **identity** Score **> 24** indicates **homology** | U | R.EAVLAAYWR.G |
| 20442 | 598 | – | 606 | 539.7883 | 1077.5621 | 1077.5607 | 1.29 | 0 | 38 | 0.00026 | 1Score **> 32** indicates **identity** Score **> 15** indicates **homology** | U | R.EAVLAAYWR.G |
| 20444 | 598 | – | 606 | 539.7886 | 1077.5626 | 1077.5607 | 1.75 | 0 | 61 | 1.2e-05 | 1Score **> 32** indicates **identity** Score **> 24** indicates **homology** | U | R.EAVLAAYWR.G |
| 200029 | 634 | – | 673 | 1428.6971 | 4283.0696 | 4283.0627 | 1.60 | 1 | 17 | 0.023 | 1Score **> 36** indicates **identity** Score **> 14** indicates **homology** | U | R.CPAGVVPACHNSEDTVTISGPQAAVNEFVEQLKQEGVFAK.E |
| 200030 | 634 | – | 673 | 1071.7769 | 4283.0784 | 4283.0627 | 3.66 | 1 | 46 | 5.3e-05 | 1Score **> 37** indicates **identity** Score **> 15** indicates **homology** | U | R.CPAGVVPACHNSEDTVTISGPQAAVNEFVEQLKQEGVFAK.E |
| 200031 | 634 | – | 673 | 1428.7003 | 4283.0792 | 4283.0627 | 3.83 | 1 | 33 | 0.00081 | 1Score **> 37** indicates **identity** Score **> 15** indicates **homology** | U | R.CPAGVVPACHNSEDTVTISGPQAAVNEFVEQLKQEGVFAK.E |
| 162859 | 677 | – | 699 | 822.4301 | 2464.2685 | 2464.2824 | -5.65 | 0 | 31 | 0.0013 | 1Score **> 37** indicates **identity** Score **> 14** indicates **homology** | U | R.TGGLAFHSYFMEGIAPTLLQALK.K |
| 122253 | 712 | – | 728 | 654.0024 | 1958.9852 | 1958.9850 | 0.12 | 0 | 42 | 0.00011 | 1Score **> 35** indicates **identity** Score **> 15** indicates **homology** | U | R.WLSTSIPEAQWQSSLAR.T |
| 122255 | 712 | – | 728 | 980.5020 | 1958.9894 | 1958.9850 | 2.27 | 0 | 71 | 2.1e-07 | 1Score **> 35** indicates **identity** Score **> 17** indicates **homology** | U | R.WLSTSIPEAQWQSSLAR.T |
| 122256 | 712 | – | 728 | 980.5022 | 1958.9899 | 1958.9850 | 2.50 | 0 | 26 | 0.0033 | 1Score **> 35** indicates **identity** Score **> 14** indicates **homology** | U | R.WLSTSIPEAQWQSSLAR.T |
| 122257 | 712 | – | 728 | 980.5024 | 1958.9902 | 1958.9850 | 2.63 | 0 | 60 | 2.3e-06 | 1Score **> 36** indicates **identity** Score **> 16** indicates **homology** | U | R.WLSTSIPEAQWQSSLAR.T |
| 122258 | 712 | – | 728 | 980.5028 | 1958.9911 | 1958.9850 | 3.10 | 0 | 52 | 1.5e-05 | 1Score **> 36** indicates **identity** Score **> 16** indicates **homology** | U | R.WLSTSIPEAQWQSSLAR.T |
| 122260 | 712 | – | 728 | 654.0050 | 1958.9932 | 1958.9850 | 4.18 | 0 | 15 | 0.041 | 1Score **> 35** indicates **identity** Score **> 13** indicates **homology** | U | R.WLSTSIPEAQWQSSLAR.T |
| 122261 | 712 | – | 728 | 654.0051 | 1958.9936 | 1958.9850 | 4.36 | 0 | 40 | 0.00017 | 1Score **> 35** indicates **identity** Score **> 15** indicates **homology** | U | R.WLSTSIPEAQWQSSLAR.T |
| 122262 | 712 | – | 728 | 980.5045 | 1958.9944 | 1958.9850 | 4.80 | 0 | 57 | 6.9e-06 | 1Score **> 36** indicates **identity** Score **> 18** indicates **homology** | U | R.WLSTSIPEAQWQSSLAR.T |
| 122268 | 712 | – | 728 | 980.5084 | 1959.0022 | 1958.9850 | 8.79 | 0 | 57 | 4.1e-06 | 1Score **> 36** indicates **identity** Score **> 16** indicates **homology** | U | R.WLSTSIPEAQWQSSLAR.T |
| 27447 | 777 | – | 786 | 575.3038 | 1148.5930 | 1148.5933 | -0.31 | 0 | 23 | 0.0071 | 1Score **> 34** indicates **identity** Score **> 14** indicates **homology** | U | K.SSCTIIPLMK.R |
| 121003 | 787 | – | 802 | 487.5059 | 1945.9944 | 1946.0010 | -3.40 | 2 | 54 | 8.5e-06 | 1Score **> 36** indicates **identity** Score **> 16** indicates **homology** | U | K.RDHKDNLEFFLTNLGK.V |
| 121004 | 787 | – | 802 | 649.6722 | 1945.9948 | 1946.0010 | -3.19 | 2 | 25 | 0.0047 | 1Score **> 36** indicates **identity** Score **> 14** indicates **homology** | U | K.RDHKDNLEFFLTNLGK.V |
| 121012 | 787 | – | 802 | 487.5071 | 1945.9992 | 1946.0010 | -0.94 | 2 | 40 | 0.00016 | 1Score **> 36** indicates **identity** Score **> 15** indicates **homology** | U | K.RDHKDNLEFFLTNLGK.V |
| 121013 | 787 | – | 802 | 487.5072 | 1945.9996 | 1946.0010 | -0.71 | 2 | 16 | 0.035 | 1Score **> 36** indicates **identity** Score **> 13** indicates **homology** | U | K.RDHKDNLEFFLTNLGK.V |
| 121014 | 787 | – | 802 | 649.6738 | 1945.9997 | 1946.0010 | -0.68 | 2 | 28 | 0.0024 | 1Score **> 36** indicates **identity** Score **> 14** indicates **homology** | U | K.RDHKDNLEFFLTNLGK.V |
| 121017 | 787 | – | 802 | 487.5079 | 1946.0026 | 1946.0010 | 0.81 | 2 | 30 | 0.0016 | 1Score **> 36** indicates **identity** Score **> 14** indicates **homology** | U | K.RDHKDNLEFFLTNLGK.V |
| 104917 | 788 | – | 802 | 448.4825 | 1789.9007 | 1789.8999 | 0.46 | 1 | 25 | 0.0048 | 1Score **> 35** indicates **identity** Score **> 14** indicates **homology** | U | R.DHKDNLEFFLTNLGK.V |
| 104918 | 788 | – | 802 | 448.4825 | 1789.9008 | 1789.8999 | 0.49 | 1 | 23 | 0.0066 | 1Score **> 35** indicates **identity** Score **> 14** indicates **homology** | U | R.DHKDNLEFFLTNLGK.V |
| 104919 | 788 | – | 802 | 895.9579 | 1789.9012 | 1789.8999 | 0.72 | 1 | 58 | 3.8e-06 | 1Score **> 35** indicates **identity** Score **> 16** indicates **homology** | U | R.DHKDNLEFFLTNLGK.V |
| 104920 | 788 | – | 802 | 597.6413 | 1789.9020 | 1789.8999 | 1.18 | 1 | 40 | 0.00017 | 1Score **> 36** indicates **identity** Score **> 15** indicates **homology** | U | R.DHKDNLEFFLTNLGK.V |
| 104922 | 788 | – | 802 | 597.6415 | 1789.9027 | 1789.8999 | 1.56 | 1 | 47 | 3.9e-05 | 1Score **> 35** indicates **identity** Score **> 15** indicates **homology** | U | R.DHKDNLEFFLTNLGK.V |
| 104924 | 788 | – | 802 | 597.6417 | 1789.9034 | 1789.8999 | 1.96 | 1 | 36 | 0.00043 | 1Score **> 35** indicates **identity** Score **> 15** indicates **homology** | U | R.DHKDNLEFFLTNLGK.V |
| 104925 | 788 | – | 802 | 448.4832 | 1789.9037 | 1789.8999 | 2.11 | 1 | 29 | 0.0064 | 1Score **> 35** indicates **identity** Score **> 19** indicates **homology** | U | R.DHKDNLEFFLTNLGK.V |
| 104926 | 788 | – | 802 | 895.9596 | 1789.9046 | 1789.8999 | 2.62 | 1 | 82 | 1.9e-08 | 1Score **> 36** indicates **identity** Score **> 18** indicates **homology** | U | R.DHKDNLEFFLTNLGK.V |
| 104928 | 788 | – | 802 | 597.6425 | 1789.9057 | 1789.8999 | 3.23 | 1 | 16 | 0.03 | 1Score **> 36** indicates **identity** Score **> 14** indicates **homology** | U | R.DHKDNLEFFLTNLGK.V |
| 164930 | 803 | – | 825 | 833.7899 | 2498.3477 | 2498.3434 | 1.73 | 0 | 58 | 3.5e-06 | 1Score **> 36** indicates **identity** Score **> 16** indicates **homology** | U | K.VHLTGINVNPNALFPPVEFPAPR.G |
| 164931 | 803 | – | 825 | 1250.1820 | 2498.3494 | 2498.3434 | 2.39 | 0 | 89 | 4.2e-09 | 1Score **> 36** indicates **identity** Score **> 18** indicates **homology** | U | K.VHLTGINVNPNALFPPVEFPAPR.G |
| 164969 | 803 | – | 825 | 834.1235 | 2499.3486 | 2499.3274 | 8.47 | 0 | 42 | 0.00011 | 1Score **> 36** indicates **identity** Score **> 15** indicates **homology** | U | K.VHLTGINVNPNALFPPVEFPAPR.G  + Deamidated (NQ) |
| 19065 | 826 | – | 835 | 354.8821 | 1061.6244 | 1061.6233 | 1.02 | 0 | 22 | 0.0085 | 1Score **> 29** indicates **identity** Score **> 14** indicates **homology** | U | R.GTPLISPHIK.W |
| 87687 | 884 | – | 897 | 551.6354 | 1651.8845 | 1651.8796 | 2.95 | 0 | 39 | 0.00021 | 1Score **> 35** indicates **identity** Score **> 15** indicates **homology** | U | R.VIFPGTGYLCLVWK.T |
| 87688 | 884 | – | 897 | 826.9504 | 1651.8862 | 1651.8796 | 3.97 | 0 | 53 | 1.2e-05 | 1Score **> 35** indicates **identity** Score **> 16** indicates **homology** | U | R.VIFPGTGYLCLVWK.T |
| 87689 | 884 | – | 897 | 826.9512 | 1651.8879 | 1651.8796 | 5.01 | 0 | 59 | 3e-06 | 1Score **> 35** indicates **identity** Score **> 16** indicates **homology** | U | R.VIFPGTGYLCLVWK.T |
| 180273 | 902 | – | 927 | 1428.2634 | 2854.5123 | 2854.5117 | 0.21 | 0 | 94 | 1.5e-09 | 1Score **> 37** indicates **identity** Score **> 18** indicates **homology** | U | R.SLGLSLEETPVVFENVSFHQATILPK.T |
| 180274 | 902 | – | 927 | 952.5131 | 2854.5173 | 2854.5117 | 1.99 | 0 | 43 | 0.0001 | 1Score **> 37** indicates **identity** Score **> 15** indicates **homology** | U | R.SLGLSLEETPVVFENVSFHQATILPK.T |
| 180275 | 902 | – | 927 | 952.5147 | 2854.5224 | 2854.5117 | 3.76 | 0 | 23 | 0.0068 | 1Score **> 36** indicates **identity** Score **> 14** indicates **homology** | U | R.SLGLSLEETPVVFENVSFHQATILPK.T |
| 10886 | 928 | – | 936 | 473.2682 | 944.5218 | 944.5291 | -7.70 | 0 | 35 | 0.0025 | 1Score **> 33** indicates **identity** Score **> 21** indicates **homology** | U | K.TGTVALEVR.L |
| 10887 | 928 | – | 936 | 473.2691 | 944.5236 | 944.5291 | -5.86 | 0 | 48 | 0.0018 | 1Score **> 33** indicates **identity** | U | K.TGTVALEVR.L |
| 10892 | 928 | – | 936 | 473.2719 | 944.5292 | 944.5291 | 0.086 | 0 | 40 | 0.00016 | 1Score **> 31** indicates **identity** Score **> 15** indicates **homology** | U | K.TGTVALEVR.L |
| 10893 | 928 | – | 936 | 473.2719 | 944.5292 | 944.5291 | 0.096 | 0 | 68 | 4.5e-06 | 1Score **> 31** indicates **identity** Score **> 27** indicates **homology** | U | K.TGTVALEVR.L |
| 10894 | 928 | – | 936 | 473.2720 | 944.5294 | 944.5291 | 0.28 | 0 | 71 | 2.3e-06 | 1Score **> 31** indicates **identity** Score **> 27** indicates **homology** | U | K.TGTVALEVR.L |
| 10895 | 928 | – | 936 | 473.2722 | 944.5299 | 944.5291 | 0.82 | 0 | 71 | 2.3e-06 | 1Score **> 31** indicates **identity** Score **> 27** indicates **homology** | U | K.TGTVALEVR.L |
| 10896 | 928 | – | 936 | 473.2729 | 944.5313 | 944.5291 | 2.32 | 0 | 53 | 3.5e-05 | 1Score **> 32** indicates **identity** Score **> 21** indicates **homology** | U | K.TGTVALEVR.L |
| 10897 | 928 | – | 936 | 473.2730 | 944.5315 | 944.5291 | 2.54 | 0 | 37 | 0.00033 | 1Score **> 32** indicates **identity** Score **> 15** indicates **homology** | U | K.TGTVALEVR.L |
| 143887 | 937 | – | 957 | 729.7128 | 2186.1166 | 2186.1219 | -2.41 | 0 | 22 | 0.0095 | 1Score **> 37** indicates **identity** Score **> 14** indicates **homology** | U | R.LLEASHAFEVSDTGNLIVSGK.V |
| 143893 | 937 | – | 957 | 729.7139 | 2186.1198 | 2186.1219 | -0.95 | 0 | 21 | 0.011 | 1Score **> 37** indicates **identity** Score **> 14** indicates **homology** | U | R.LLEASHAFEVSDTGNLIVSGK.V |
| 143894 | 937 | – | 957 | 729.7145 | 2186.1216 | 2186.1219 | -0.15 | 0 | 16 | 0.034 | 1Score **> 37** indicates **identity** Score **> 13** indicates **homology** | U | R.LLEASHAFEVSDTGNLIVSGK.V |
| 143897 | 937 | – | 957 | 729.7160 | 2186.1261 | 2186.1219 | 1.90 | 0 | 27 | 0.0028 | 1Score **> 37** indicates **identity** Score **> 14** indicates **homology** | U | R.LLEASHAFEVSDTGNLIVSGK.V |
| 143900 | 937 | – | 957 | 729.7164 | 2186.1272 | 2186.1219 | 2.44 | 0 | 18 | 0.037 | 1Score **> 37** indicates **identity** Score **> 17** indicates **homology** | U | R.LLEASHAFEVSDTGNLIVSGK.V |
| 143904 | 937 | – | 957 | 729.7183 | 2186.1330 | 2186.1219 | 5.09 | 0 | 25 | 0.0043 | 1Score **> 37** indicates **identity** Score **> 14** indicates **homology** | U | R.LLEASHAFEVSDTGNLIVSGK.V |
| 143984 | 937 | – | 957 | 730.0488 | 2187.1245 | 2187.1059 | 8.49 | 0 | 22 | 0.008 | 1Score **> 37** indicates **identity** Score **> 14** indicates **homology** | U | R.LLEASHAFEVSDTGNLIVSGK.V  + Deamidated (NQ) |
| 38328 | 958 | – | 967 | 625.8057 | 1249.5969 | 1249.5979 | -0.81 | 0 | 36 | 0.00042 | 1Score **> 32** indicates **identity** Score **> 15** indicates **homology** | U | K.VYLWEDPNSK.L |
| 38329 | 958 | – | 967 | 625.8064 | 1249.5983 | 1249.5979 | 0.36 | 0 | 42 | 0.00012 | 1Score **> 32** indicates **identity** Score **> 15** indicates **homology** | U | K.VYLWEDPNSK.L |
| 189612 | 958 | – | 985 | 1066.1911 | 3195.5513 | 3195.5513 | 0.020 | 1 | 28 | 0.0023 | 1Score **> 37** indicates **identity** Score **> 14** indicates **homology** | U | K.VYLWEDPNSKLFDHPEVPTPPESASVSR.L |
| 189614 | 958 | – | 985 | 1066.1917 | 3195.5532 | 3195.5513 | 0.59 | 1 | 40 | 0.00019 | 1Score **> 37** indicates **identity** Score **> 15** indicates **homology** | U | K.VYLWEDPNSKLFDHPEVPTPPESASVSR.L |
| 189619 | 958 | – | 985 | 1066.1930 | 3195.5572 | 3195.5513 | 1.84 | 1 | 34 | 0.0007 | 1Score **> 37** indicates **identity** Score **> 15** indicates **homology** | U | K.VYLWEDPNSKLFDHPEVPTPPESASVSR.L |
| 189640 | 958 | – | 985 | 1066.5289 | 3196.5648 | 3196.5353 | 9.24 | 1 | 17 | 0.025 | 1Score **> 37** indicates **identity** Score **> 14** indicates **homology** | U | K.VYLWEDPNSKLFDHPEVPTPPESASVSR.L  + Deamidated (NQ) |
| 122677 | 968 | – | 985 | 655.6619 | 1963.9639 | 1963.9640 | -0.020 | 0 | 18 | 0.021 | 1Score **> 35** indicates **identity** Score **> 14** indicates **homology** | U | K.LFDHPEVPTPPESASVSR.L |
| 122678 | 968 | – | 985 | 655.6620 | 1963.9641 | 1963.9640 | 0.049 | 0 | 16 | 0.033 | 1Score **> 35** indicates **identity** Score **> 13** indicates **homology** | U | K.LFDHPEVPTPPESASVSR.L |
| 122679 | 968 | – | 985 | 982.9894 | 1963.9642 | 1963.9640 | 0.11 | 0 | 21 | 0.01 | 1Score **> 35** indicates **identity** Score **> 14** indicates **homology** | U | K.LFDHPEVPTPPESASVSR.L |
| 122680 | 968 | – | 985 | 655.6620 | 1963.9643 | 1963.9640 | 0.17 | 0 | 23 | 0.0069 | 1Score **> 35** indicates **identity** Score **> 14** indicates **homology** | U | K.LFDHPEVPTPPESASVSR.L |
| 122681 | 968 | – | 985 | 982.9901 | 1963.9656 | 1963.9640 | 0.84 | 0 | 16 | 0.03 | 1Score **> 35** indicates **identity** Score **> 14** indicates **homology** | U | K.LFDHPEVPTPPESASVSR.L |
| 10443 | 986 | – | 993 | 469.2527 | 936.4909 | 936.4916 | -0.77 | 0 | 24 | 0.014 | 1Score **> 29** indicates **identity** Score **> 18** indicates **homology** | U | R.LTQGEVYK.E |
| 48083 | 986 | – | 996 | 445.9134 | 1334.7185 | 1334.7194 | -0.68 | 1 | 38 | 0.00025 | 1Score **> 34** indicates **identity** Score **> 15** indicates **homology** | U | R.LTQGEVYKELR.L |
| 48085 | 986 | – | 996 | 668.3670 | 1334.7194 | 1334.7194 | -0.0097 | 1 | 49 | 2.8e-05 | 1Score **> 33** indicates **identity** Score **> 16** indicates **homology** | U | R.LTQGEVYKELR.L |
| 48087 | 986 | – | 996 | 445.9138 | 1334.7194 | 1334.7194 | 0.023 | 1 | 30 | 0.0024 | 1Score **> 33** indicates **identity** Score **> 17** indicates **homology** | U | R.LTQGEVYKELR.L |
| 48088 | 986 | – | 996 | 445.9142 | 1334.7206 | 1334.7194 | 0.92 | 1 | 46 | 5.2e-05 | 1Score **> 34** indicates **identity** Score **> 15** indicates **homology** | U | R.LTQGEVYKELR.L |
| 48091 | 986 | – | 996 | 445.9169 | 1334.7290 | 1334.7194 | 7.17 | 1 | 17 | 0.024 | 1Score **> 33** indicates **identity** Score **> 14** indicates **homology** | U | R.LTQGEVYKELR.L |
| 174700 | 997 | – | 1020 | 906.0875 | 2715.2407 | 2715.2599 | -7.07 | 1 | 56 | 5.8e-06 | 1Score **> 33** indicates **identity** Score **> 16** indicates **homology** | U | R.LRGYDYGPQFQGICEATLEGEQGK.L |
| 174702 | 997 | – | 1020 | 906.0932 | 2715.2579 | 2715.2599 | -0.72 | 1 | 80 | 3.5e-08 | 1Score **> 34** indicates **identity** Score **> 17** indicates **homology** | U | R.LRGYDYGPQFQGICEATLEGEQGK.L |
| 174703 | 997 | – | 1020 | 906.0936 | 2715.2588 | 2715.2599 | -0.39 | 1 | 67 | 5.2e-07 | 1Score **> 34** indicates **identity** Score **> 17** indicates **homology** | U | R.LRGYDYGPQFQGICEATLEGEQGK.L |
| 174705 | 997 | – | 1020 | 906.0949 | 2715.2628 | 2715.2599 | 1.09 | 1 | 52 | 1.3e-05 | 1Score **> 34** indicates **identity** Score **> 16** indicates **homology** | U | R.LRGYDYGPQFQGICEATLEGEQGK.L |
| 174731 | 997 | – | 1020 | 1359.1374 | 2716.2603 | 2716.2439 | 6.05 | 1 | 60 | 2.2e-06 | 1Score **> 34** indicates **identity** Score **> 16** indicates **homology** | U | R.LRGYDYGPQFQGICEATLEGEQGK.L  + Deamidated (NQ) |
| 190932 | 997 | – | 1024 | 814.9059 | 3255.5945 | 3255.6023 | -2.37 | 2 | 18 | 0.022 | 1Score **> 37** indicates **identity** Score **> 14** indicates **homology** | U | R.LRGYDYGPQFQGICEATLEGEQGKLLWK.D |
| 161672 | 999 | – | 1020 | 816.3655 | 2446.0747 | 2446.0747 | -0.017 | 0 | 21 | 0.011 | 1Score **> 30** indicates **identity** Score **> 14** indicates **homology** | U | R.GYDYGPQFQGICEATLEGEQGK.L |
| 161673 | 999 | – | 1020 | 1224.0455 | 2446.0764 | 2446.0747 | 0.71 | 0 | 79 | 4.2e-08 | 1Score **> 30** indicates **identity** Score **> 17** indicates **homology** | U | R.GYDYGPQFQGICEATLEGEQGK.L |
| 161674 | 999 | – | 1020 | 1224.0456 | 2446.0767 | 2446.0747 | 0.80 | 0 | 105 | 1.6e-10 | 1Score **> 30** indicates **identity** Score **> 19** indicates **homology** | U | R.GYDYGPQFQGICEATLEGEQGK.L |
| 50357 | 1053 | – | 1064 | 678.8668 | 1355.7190 | 1355.7197 | -0.53 | 0 | 55 | 6.8e-06 | 1Score **> 33** indicates **identity** Score **> 16** indicates **homology** | U | R.VTAIYIDPATHR.Q |
| 50358 | 1053 | – | 1064 | 678.8669 | 1355.7192 | 1355.7197 | -0.41 | 0 | 45 | 0.00011 | 1Score **> 33** indicates **identity** Score **> 18** indicates **homology** | U | R.VTAIYIDPATHR.Q |
| 50359 | 1053 | – | 1064 | 678.8669 | 1355.7192 | 1355.7197 | -0.40 | 0 | 52 | 2e-05 | 1Score **> 33** indicates **identity** Score **> 17** indicates **homology** | U | R.VTAIYIDPATHR.Q |
| 50368 | 1053 | – | 1064 | 678.8677 | 1355.7208 | 1355.7197 | 0.82 | 0 | 50 | 3.2e-05 | 1Score **> 34** indicates **identity** Score **> 18** indicates **homology** | U | R.VTAIYIDPATHR.Q |
| 75262 | 1070 | – | 1083 | 781.9045 | 1561.7945 | 1561.7948 | -0.18 | 1 | 106 | 2.2e-09 | 1Score **> 35** indicates **identity** Score **> 32** indicates **homology** | U | R.LKEDTQVADVTTSR.C |
| 75264 | 1070 | – | 1083 | 781.9047 | 1561.7948 | 1561.7948 | 0.051 | 1 | 88 | 1.4e-07 | 1Score **> 35** indicates **identity** Score **> 32** indicates **homology** | U | R.LKEDTQVADVTTSR.C |
| 75266 | 1070 | – | 1083 | 521.6058 | 1561.7956 | 1561.7948 | 0.55 | 1 | 58 | 4.1e-06 | 1Score **> 35** indicates **identity** Score **> 16** indicates **homology** | U | R.LKEDTQVADVTTSR.C |
| 75269 | 1070 | – | 1083 | 781.9056 | 1561.7967 | 1561.7948 | 1.21 | 1 | 54 | 6.8e-05 | 1Score **> 35** indicates **identity** Score **> 25** indicates **homology** | U | R.LKEDTQVADVTTSR.C |
| 75270 | 1070 | – | 1083 | 521.6065 | 1561.7978 | 1561.7948 | 1.95 | 1 | 43 | 8.6e-05 | 1Score **> 35** indicates **identity** Score **> 15** indicates **homology** | U | R.LKEDTQVADVTTSR.C |
| 63082 | 1084 | – | 1097 | 490.5996 | 1468.7769 | 1468.7820 | -3.53 | 0 | 17 | 0.025 | 1Score **> 34** indicates **identity** Score **> 14** indicates **homology** | U | R.CLGITVSGGIHISR.L |
| 63084 | 1084 | – | 1097 | 490.6000 | 1468.7781 | 1468.7820 | -2.72 | 0 | 26 | 0.0037 | 1Score **> 34** indicates **identity** Score **> 14** indicates **homology** | U | R.CLGITVSGGIHISR.L |
| 63087 | 1084 | – | 1097 | 490.6012 | 1468.7817 | 1468.7820 | -0.21 | 0 | 37 | 0.00035 | 1Score **> 34** indicates **identity** Score **> 15** indicates **homology** | U | R.CLGITVSGGIHISR.L |
| 63090 | 1084 | – | 1097 | 490.6016 | 1468.7829 | 1468.7820 | 0.59 | 0 | 23 | 0.0066 | 1Score **> 34** indicates **identity** Score **> 14** indicates **homology** | U | R.CLGITVSGGIHISR.L |
| 63094 | 1084 | – | 1097 | 490.6022 | 1468.7848 | 1468.7820 | 1.86 | 0 | 27 | 0.0031 | 1Score **> 34** indicates **identity** Score **> 14** indicates **homology** | U | R.CLGITVSGGIHISR.L |
| 62986 | 1106 | – | 1117 | 734.9094 | 1467.8043 | 1467.8045 | -0.12 | 1 | 36 | 0.0028 | 1Score **> 34** indicates **identity** Score **> 23** indicates **homology** | U | R.RQQEQLVPTLEK.F |
| 62989 | 1106 | – | 1117 | 734.9105 | 1467.8064 | 1467.8045 | 1.26 | 1 | 20 | 0.026 | 1Score **> 34** indicates **identity** Score **> 17** indicates **homology** | U | R.RQQEQLVPTLEK.F |
| 62991 | 1106 | – | 1117 | 734.9114 | 1467.8083 | 1467.8045 | 2.56 | 1 | 40 | 0.0012 | 1Score **> 33** indicates **identity** Score **> 23** indicates **homology** | U | R.RQQEQLVPTLEK.F |
| 62993 | 1106 | – | 1117 | 490.2776 | 1467.8110 | 1467.8045 | 4.41 | 1 | 26 | 0.0038 | 1Score **> 33** indicates **identity** Score **> 14** indicates **homology** | U | R.RQQEQLVPTLEK.F |
| 45523 | 1107 | – | 1117 | 656.8570 | 1311.6994 | 1311.7034 | -3.03 | 0 | 28 | 0.0025 | 1Score **> 33** indicates **identity** Score **> 14** indicates **homology** | U | R.QQEQLVPTLEK.F |
| 45525 | 1107 | – | 1117 | 656.8595 | 1311.7044 | 1311.7034 | 0.78 | 0 | 49 | 2.5e-05 | 1Score **> 33** indicates **identity** Score **> 16** indicates **homology** | U | R.QQEQLVPTLEK.F |
| 45526 | 1107 | – | 1117 | 656.8603 | 1311.7060 | 1311.7034 | 1.97 | 0 | 43 | 0.0001 | 1Score **> 33** indicates **identity** Score **> 15** indicates **homology** | U | R.QQEQLVPTLEK.F |
| 96179 | 1160 | – | 1175 | 572.9541 | 1715.8406 | 1715.8413 | -0.44 | 0 | 45 | 5.8e-05 | 1Score **> 34** indicates **identity** Score **> 15** indicates **homology** | U | K.AAMLGQEDPPQHGLPR.L |
| 96180 | 1160 | – | 1175 | 572.9547 | 1715.8423 | 1715.8413 | 0.58 | 0 | 21 | 0.014 | 1Score **> 34** indicates **identity** Score **> 15** indicates **homology** | U | K.AAMLGQEDPPQHGLPR.L |
| 96181 | 1160 | – | 1175 | 572.9554 | 1715.8443 | 1715.8413 | 1.73 | 0 | 17 | 0.026 | 1Score **> 34** indicates **identity** Score **> 14** indicates **homology** | U | K.AAMLGQEDPPQHGLPR.L |
| 129442 | 1202 | – | 1220 | 1017.5970 | 2033.1795 | 2033.1772 | 1.14 | 0 | 52 | 1.5e-05 | 1Score **> 31** indicates **identity** Score **> 16** indicates **homology** | U | R.LLLPEDPLISGLLNSQALK.A |
| 129443 | 1202 | – | 1220 | 678.7344 | 2033.1814 | 2033.1772 | 2.06 | 0 | 90 | 4e-09 | 1Score **> 31** indicates **identity** Score **> 18** indicates **homology** | U | R.LLLPEDPLISGLLNSQALK.A |
| 129444 | 1202 | – | 1220 | 1017.5981 | 2033.1816 | 2033.1772 | 2.16 | 0 | 86 | 8.2e-09 | 1Score **> 31** indicates **identity** Score **> 18** indicates **homology** | U | R.LLLPEDPLISGLLNSQALK.A |
| 129508 | 1202 | – | 1220 | 1018.0892 | 2034.1639 | 2034.1612 | 1.32 | 0 | 47 | 3.9e-05 | 1Score **> 32** indicates **identity** Score **> 15** indicates **homology** | U | R.LLLPEDPLISGLLNSQALK.A  + Deamidated (NQ) |
| 129509 | 1202 | – | 1220 | 679.0664 | 2034.1773 | 2034.1612 | 7.93 | 0 | 17 | 0.024 | 1Score **> 32** indicates **identity** Score **> 14** indicates **homology** | U | R.LLLPEDPLISGLLNSQALK.A  + Deamidated (NQ) |
| 71330 | 1221 | – | 1234 | 767.8916 | 1533.7687 | 1533.7708 | -1.41 | 0 | 24 | 0.0058 | 1Score **> 34** indicates **identity** Score **> 14** indicates **homology** | U | K.ACVDTALENLSTLK.M |
| 71332 | 1221 | – | 1234 | 767.8928 | 1533.7711 | 1533.7708 | 0.19 | 0 | 53 | 1.3e-05 | 1Score **> 35** indicates **identity** Score **> 17** indicates **homology** | U | K.ACVDTALENLSTLK.M |
| 71334 | 1221 | – | 1234 | 767.8933 | 1533.7720 | 1533.7708 | 0.78 | 0 | 74 | 6.3e-07 | 1Score **> 35** indicates **identity** Score **> 25** indicates **homology** | U | K.ACVDTALENLSTLK.M |
| 71335 | 1221 | – | 1234 | 767.8934 | 1533.7723 | 1533.7708 | 0.93 | 0 | 80 | 2.1e-07 | 1Score **> 35** indicates **identity** Score **> 26** indicates **homology** | U | K.ACVDTALENLSTLK.M |
| 71336 | 1221 | – | 1234 | 512.2648 | 1533.7724 | 1533.7708 | 1.03 | 0 | 37 | 0.00035 | 1Score **> 35** indicates **identity** Score **> 15** indicates **homology** | U | K.ACVDTALENLSTLK.M |
| 71337 | 1221 | – | 1234 | 767.8940 | 1533.7734 | 1533.7708 | 1.67 | 0 | 80 | 2.1e-07 | 1Score **> 35** indicates **identity** Score **> 26** indicates **homology** | U | K.ACVDTALENLSTLK.M |
| 71469 | 1221 | – | 1234 | 768.3913 | 1534.7680 | 1534.7549 | 8.59 | 0 | 27 | 0.0085 | 1Score **> 34** indicates **identity** Score **> 19** indicates **homology** | U | K.ACVDTALENLSTLK.M  + Deamidated (NQ) |
| 67044 | 1237 | – | 1250 | 500.9319 | 1499.7740 | 1499.7732 | 0.52 | 0 | 58 | 3.9e-06 | 1Score **> 34** indicates **identity** Score **> 16** indicates **homology** | U | K.VAEVLAGEGHLYSR.I |
| 67045 | 1237 | – | 1250 | 750.8943 | 1499.7741 | 1499.7732 | 0.61 | 0 | 84 | 3.5e-08 | 1Score **> 34** indicates **identity** Score **> 22** indicates **homology** | U | K.VAEVLAGEGHLYSR.I |
| 67047 | 1237 | – | 1250 | 500.9326 | 1499.7760 | 1499.7732 | 1.84 | 0 | 38 | 0.00026 | 1Score **> 34** indicates **identity** Score **> 15** indicates **homology** | U | K.VAEVLAGEGHLYSR.I |
| 45542 | 1333 | – | 1344 | 438.2582 | 1311.7529 | 1311.7551 | -1.68 | 0 | 32 | 0.0041 | 1Score **> 33** indicates **identity** Score **> 21** indicates **homology** | U | K.EGGFLLVHTVLK.G |
| 45545 | 1333 | – | 1344 | 438.2588 | 1311.7546 | 1311.7551 | -0.39 | 0 | 29 | 0.0019 | 1Score **> 32** indicates **identity** Score **> 14** indicates **homology** | U | K.EGGFLLVHTVLK.G |
| 45546 | 1333 | – | 1344 | 438.2588 | 1311.7546 | 1311.7551 | -0.37 | 0 | 24 | 0.0057 | 1Score **> 32** indicates **identity** Score **> 14** indicates **homology** | U | K.EGGFLLVHTVLK.G |
| 45547 | 1333 | – | 1344 | 438.2589 | 1311.7550 | 1311.7551 | -0.095 | 0 | 31 | 0.0012 | 1Score **> 32** indicates **identity** Score **> 14** indicates **homology** | U | K.EGGFLLVHTVLK.G |
| 45548 | 1333 | – | 1344 | 656.8851 | 1311.7556 | 1311.7551 | 0.42 | 0 | 24 | 0.0053 | 1Score **> 32** indicates **identity** Score **> 14** indicates **homology** | U | K.EGGFLLVHTVLK.G |
| 45550 | 1333 | – | 1344 | 438.2593 | 1311.7560 | 1311.7551 | 0.71 | 0 | 31 | 0.0012 | 1Score **> 32** indicates **identity** Score **> 14** indicates **homology** | U | K.EGGFLLVHTVLK.G |
| 45551 | 1333 | – | 1344 | 438.2596 | 1311.7570 | 1311.7551 | 1.47 | 0 | 25 | 0.0044 | 1Score **> 32** indicates **identity** Score **> 14** indicates **homology** | U | K.EGGFLLVHTVLK.G |
| 45552 | 1333 | – | 1344 | 438.2596 | 1311.7571 | 1311.7551 | 1.52 | 0 | 18 | 0.022 | 1Score **> 32** indicates **identity** Score **> 14** indicates **homology** | U | K.EGGFLLVHTVLK.G |
| 196964 | 1345 | – | 1377 | 1213.6053 | 3637.7941 | 3637.7722 | 6.01 | 0 | 66 | 6.4e-07 | 1Score **> 37** indicates **identity** Score **> 17** indicates **homology** | U | K.GHALGETLACLPSEVQPAPSLLSQEEWESLFSR.K |
| 196965 | 1345 | – | 1377 | 910.4559 | 3637.7945 | 3637.7722 | 6.12 | 0 | 21 | 0.011 | 1Score **> 37** indicates **identity** Score **> 14** indicates **homology** | U | K.GHALGETLACLPSEVQPAPSLLSQEEWESLFSR.K |
| 197852 | 1345 | – | 1378 | 942.4728 | 3765.8621 | 3765.8672 | -1.35 | 1 | 40 | 0.00019 | 1Score **> 38** indicates **identity** Score **> 15** indicates **homology** | U | K.GHALGETLACLPSEVQPAPSLLSQEEWESLFSRK.A |
| 65749 | 1387 | – | 1398 | 497.5862 | 1489.7367 | 1489.7500 | -8.92 | 1 | 18 | 0.018 | 1Score **> 34** indicates **identity** Score **> 14** indicates **homology** | U | K.RSFYGTALFLCR.R |
| 65761 | 1387 | – | 1398 | 497.5909 | 1489.7510 | 1489.7500 | 0.66 | 1 | 22 | 0.027 | 1Score **> 34** indicates **identity** Score **> 19** indicates **homology** | U | K.RSFYGTALFLCR.R |
| 65763 | 1387 | – | 1398 | 497.5913 | 1489.7522 | 1489.7500 | 1.47 | 1 | 20 | 0.015 | 1Score **> 34** indicates **identity** Score **> 14** indicates **homology** | U | K.RSFYGTALFLCR.R |
| 47941 | 1388 | – | 1398 | 667.8313 | 1333.6480 | 1333.6489 | -0.66 | 0 | 51 | 1.5e-05 | 1Score **> 33** indicates **identity** Score **> 16** indicates **homology** | U | R.SFYGTALFLCR.R |
| 47945 | 1388 | – | 1398 | 667.8335 | 1333.6524 | 1333.6489 | 2.61 | 0 | 74 | 1.3e-07 | 1Score **> 33** indicates **identity** Score **> 17** indicates **homology** | U | R.SFYGTALFLCR.R |
| 47946 | 1388 | – | 1398 | 667.8337 | 1333.6528 | 1333.6489 | 2.90 | 0 | 79 | 3.9e-08 | 1Score **> 33** indicates **identity** Score **> 17** indicates **homology** | U | R.SFYGTALFLCR.R |
| 47947 | 1388 | – | 1398 | 667.8338 | 1333.6530 | 1333.6489 | 3.10 | 0 | 79 | 4e-08 | 1Score **> 33** indicates **identity** Score **> 17** indicates **homology** | U | R.SFYGTALFLCR.R |
| 47948 | 1388 | – | 1398 | 667.8341 | 1333.6537 | 1333.6489 | 3.63 | 0 | 69 | 3.1e-07 | 1Score **> 33** indicates **identity** Score **> 17** indicates **homology** | U | R.SFYGTALFLCR.R |
| 47950 | 1388 | – | 1398 | 667.8343 | 1333.6541 | 1333.6489 | 3.93 | 0 | 69 | 3.5e-07 | 1Score **> 33** indicates **identity** Score **> 17** indicates **homology** | U | R.SFYGTALFLCR.R |
| 47952 | 1388 | – | 1398 | 667.8351 | 1333.6557 | 1333.6489 | 5.10 | 0 | 56 | 6.2e-06 | 1Score **> 33** indicates **identity** Score **> 16** indicates **homology** | U | R.SFYGTALFLCR.R |
| 47954 | 1388 | – | 1398 | 667.8355 | 1333.6564 | 1333.6489 | 5.63 | 0 | 66 | 5.9e-07 | 1Score **> 33** indicates **identity** Score **> 17** indicates **homology** | U | R.SFYGTALFLCR.R |
| 47955 | 1388 | – | 1398 | 667.8358 | 1333.6571 | 1333.6489 | 6.13 | 0 | 32 | 0.00094 | 1Score **> 33** indicates **identity** Score **> 15** indicates **homology** | U | R.SFYGTALFLCR.R |
| 47956 | 1388 | – | 1398 | 667.8363 | 1333.6580 | 1333.6489 | 6.83 | 0 | 51 | 1.8e-05 | 1Score **> 33** indicates **identity** Score **> 16** indicates **homology** | U | R.SFYGTALFLCR.R |
| 47958 | 1388 | – | 1398 | 667.8382 | 1333.6618 | 1333.6489 | 9.66 | 0 | 49 | 2.6e-05 | 1Score **> 33** indicates **identity** Score **> 16** indicates **homology** | U | R.SFYGTALFLCR.R |
| 47959 | 1388 | – | 1398 | 667.8384 | 1333.6622 | 1333.6489 | 9.96 | 0 | 30 | 0.0015 | 1Score **> 33** indicates **identity** Score **> 14** indicates **homology** | U | R.SFYGTALFLCR.R |
| 65762 | 1388 | – | 1399 | 497.5911 | 1489.7515 | 1489.7500 | 1.01 | 1 | 18 | 0.023 | 1Score **> 34** indicates **identity** Score **> 14** indicates **homology** | U | R.SFYGTALFLCRR.A |
| 182864 | 1399 | – | 1423 | 734.1406 | 2932.5335 | 2932.5334 | 0.015 | 2 | 58 | 3.5e-06 | 1Score **> 37** indicates **identity** Score **> 16** indicates **homology** | U | R.RAIPQEKPIFLSVEDTSFQWVDSLK.S |
| 182865 | 1399 | – | 1423 | 734.1432 | 2932.5438 | 2932.5334 | 3.54 | 2 | 70 | 2.6e-07 | 1Score **> 36** indicates **identity** Score **> 17** indicates **homology** | U | R.RAIPQEKPIFLSVEDTSFQWVDSLK.S |
| 191944 | 1424 | – | 1454 | 1103.5453 | 3307.6140 | 3307.5887 | 7.66 | 0 | 42 | 0.0001 | 1Score **> 37** indicates **identity** Score **> 15** indicates **homology** | U | K.STLATSSSQPVWLTAMDCPTSGVVGLVNCLR.K  + Deamidated (NQ) |
| 191945 | 1424 | – | 1454 | 1103.5455 | 3307.6147 | 3307.5887 | 7.86 | 0 | 36 | 0.00041 | 1Score **> 37** indicates **identity** Score **> 15** indicates **homology** | U | K.STLATSSSQPVWLTAMDCPTSGVVGLVNCLR.K  + Deamidated (NQ) |
| 87894 | 1464 | – | 1478 | 552.2870 | 1653.8392 | 1653.8508 | -6.99 | 0 | 23 | 0.0075 | 1Score **> 34** indicates **identity** Score **> 14** indicates **homology** | U | R.CILLSNLSNTSHAPK.L |
| 87904 | 1464 | – | 1478 | 552.2912 | 1653.8517 | 1653.8508 | 0.53 | 0 | 57 | 5e-06 | 1Score **> 34** indicates **identity** Score **> 16** indicates **homology** | U | R.CILLSNLSNTSHAPK.L |
| 87905 | 1464 | – | 1478 | 552.2915 | 1653.8527 | 1653.8508 | 1.14 | 0 | 65 | 7.5e-07 | 1Score **> 35** indicates **identity** Score **> 17** indicates **homology** | U | R.CILLSNLSNTSHAPK.L |
| 87908 | 1464 | – | 1478 | 552.2919 | 1653.8540 | 1653.8508 | 1.90 | 0 | 59 | 2.7e-06 | 1Score **> 35** indicates **identity** Score **> 16** indicates **homology** | U | R.CILLSNLSNTSHAPK.L |
| 87909 | 1464 | – | 1478 | 827.9343 | 1653.8541 | 1653.8508 | 1.97 | 0 | 83 | 1.8e-08 | 1Score **> 35** indicates **identity** Score **> 18** indicates **homology** | U | R.CILLSNLSNTSHAPK.L |
| 87910 | 1464 | – | 1478 | 552.2922 | 1653.8547 | 1653.8508 | 2.33 | 0 | 41 | 0.00014 | 1Score **> 35** indicates **identity** Score **> 15** indicates **homology** | U | R.CILLSNLSNTSHAPK.L |
| 87911 | 1464 | – | 1478 | 827.9346 | 1653.8547 | 1653.8508 | 2.34 | 0 | 74 | 5e-07 | 1Score **> 35** indicates **identity** Score **> 24** indicates **homology** | U | R.CILLSNLSNTSHAPK.L |
| 87914 | 1464 | – | 1478 | 552.2932 | 1653.8578 | 1653.8508 | 4.24 | 0 | 44 | 8.2e-05 | 1Score **> 35** indicates **identity** Score **> 15** indicates **homology** | U | R.CILLSNLSNTSHAPK.L |
| 87916 | 1464 | – | 1478 | 827.9376 | 1653.8607 | 1653.8508 | 5.95 | 0 | 51 | 1.7e-05 | 1Score **> 35** indicates **identity** Score **> 16** indicates **homology** | U | R.CILLSNLSNTSHAPK.L |
| 87917 | 1464 | – | 1478 | 552.2946 | 1653.8618 | 1653.8508 | 6.66 | 0 | 50 | 2e-05 | 1Score **> 35** indicates **identity** Score **> 16** indicates **homology** | U | R.CILLSNLSNTSHAPK.L |
| 57580 | 1479 | – | 1491 | 712.3932 | 1422.7719 | 1422.7718 | 0.050 | 0 | 49 | 2.6e-05 | 1Score **> 33** indicates **identity** Score **> 16** indicates **homology** | U | K.LDPGSPELQQVLK.H |
| 57581 | 1479 | – | 1491 | 712.3933 | 1422.7720 | 1422.7718 | 0.13 | 0 | 17 | 0.027 | 1Score **> 33** indicates **identity** Score **> 14** indicates **homology** | U | K.LDPGSPELQQVLK.H |
| 57583 | 1479 | – | 1491 | 712.3935 | 1422.7724 | 1422.7718 | 0.42 | 0 | 76 | 7.1e-08 | 1Score **> 33** indicates **identity** Score **> 17** indicates **homology** | U | K.LDPGSPELQQVLK.H |
| 57584 | 1479 | – | 1491 | 712.3939 | 1422.7733 | 1422.7718 | 1.01 | 0 | 77 | 6.1e-08 | 1Score **> 33** indicates **identity** Score **> 17** indicates **homology** | U | K.LDPGSPELQQVLK.H |
| 57585 | 1479 | – | 1491 | 712.3941 | 1422.7737 | 1422.7718 | 1.33 | 0 | 95 | 5.3e-09 | 1Score **> 33** indicates **identity** Score **> 24** indicates **homology** | U | K.LDPGSPELQQVLK.H |
| 57587 | 1479 | – | 1491 | 712.3952 | 1422.7759 | 1422.7718 | 2.82 | 0 | 66 | 6.1e-07 | 1Score **> 33** indicates **identity** Score **> 17** indicates **homology** | U | K.LDPGSPELQQVLK.H |
| 27066 | 1492 | – | 1500 | 573.7884 | 1145.5623 | 1145.5652 | -2.45 | 0 | 38 | 0.0003 | 1Score **> 32** indicates **identity** Score **> 15** indicates **homology** | U | K.HDLVMNVYR.D |
| 27067 | 1492 | – | 1500 | 573.7890 | 1145.5635 | 1145.5652 | -1.42 | 0 | 38 | 0.00029 | 1Score **> 32** indicates **identity** Score **> 15** indicates **homology** | U | K.HDLVMNVYR.D |
| 27069 | 1492 | – | 1500 | 573.7903 | 1145.5661 | 1145.5652 | 0.82 | 0 | 17 | 0.027 | 1Score **> 32** indicates **identity** Score **> 14** indicates **homology** | U | K.HDLVMNVYR.D |
| 7253 | 1501 | – | 1508 | 440.2092 | 878.4039 | 878.4035 | 0.43 | 0 | 42 | 0.0009 | 1Score **> 27** indicates **identity** Score **> 24** indicates **homology** | U | R.DGAWGAFR.H |
| 7254 | 1501 | – | 1508 | 440.2099 | 878.4052 | 878.4035 | 1.91 | 0 | 49 | 0.0004 | 1Score **> 28** indicates **identity** Score **> 27** indicates **homology** | U | R.DGAWGAFR.H |
| 7255 | 1501 | – | 1508 | 440.2103 | 878.4061 | 878.4035 | 2.97 | 0 | 43 | 0.00069 | 1Score **> 28** indicates **identity** Score **> 24** indicates **homology** | U | R.DGAWGAFR.H |
| 40640 | 1509 | – | 1518 | 635.3324 | 1268.6502 | 1268.6513 | -0.86 | 1 | 29 | 0.0019 | 1Score **> 32** indicates **identity** Score **> 14** indicates **homology** | U | R.HFQLEQDKPK.E |
| 40641 | 1509 | – | 1518 | 423.8911 | 1268.6514 | 1268.6513 | 0.069 | 1 | 23 | 0.0074 | 1Score **> 32** indicates **identity** Score **> 14** indicates **homology** | U | R.HFQLEQDKPK.E |
| 40642 | 1509 | – | 1518 | 423.8911 | 1268.6515 | 1268.6513 | 0.12 | 1 | 21 | 0.0098 | 1Score **> 32** indicates **identity** Score **> 14** indicates **homology** | U | R.HFQLEQDKPK.E |
| 175532 | 1509 | – | 1531 | 684.8609 | 2735.4144 | 2735.4143 | 0.022 | 2 | 26 | 0.0036 | 1Score **> 37** indicates **identity** Score **> 14** indicates **homology** | U | R.HFQLEQDKPKEQTAHAFVNVLTR.G |
| 175534 | 1509 | – | 1531 | 684.8614 | 2735.4166 | 2735.4143 | 0.84 | 2 | 38 | 0.00029 | 1Score **> 37** indicates **identity** Score **> 15** indicates **homology** | U | R.HFQLEQDKPKEQTAHAFVNVLTR.G |
| 175536 | 1509 | – | 1531 | 684.8616 | 2735.4174 | 2735.4143 | 1.13 | 2 | 43 | 9.8e-05 | 1Score **> 37** indicates **identity** Score **> 15** indicates **homology** | U | R.HFQLEQDKPKEQTAHAFVNVLTR.G |
| 65002 | 1519 | – | 1531 | 495.9316 | 1484.7730 | 1484.7736 | -0.37 | 0 | 34 | 0.00067 | 1Score **> 34** indicates **identity** Score **> 15** indicates **homology** | U | K.EQTAHAFVNVLTR.G |
| 65003 | 1519 | – | 1531 | 495.9316 | 1484.7731 | 1484.7736 | -0.34 | 0 | 41 | 0.0002 | 1Score **> 34** indicates **identity** Score **> 17** indicates **homology** | U | K.EQTAHAFVNVLTR.G |
| 65005 | 1519 | – | 1531 | 743.3941 | 1484.7736 | 1484.7736 | -0.013 | 0 | 56 | 5.7e-06 | 1Score **> 34** indicates **identity** Score **> 16** indicates **homology** | U | K.EQTAHAFVNVLTR.G |
| 65006 | 1519 | – | 1531 | 743.3942 | 1484.7739 | 1484.7736 | 0.25 | 0 | 54 | 7.8e-06 | 1Score **> 34** indicates **identity** Score **> 16** indicates **homology** | U | K.EQTAHAFVNVLTR.G |
| 65007 | 1519 | – | 1531 | 495.9322 | 1484.7747 | 1484.7736 | 0.78 | 0 | 36 | 0.00053 | 1Score **> 34** indicates **identity** Score **> 16** indicates **homology** | U | K.EQTAHAFVNVLTR.G |
| 65009 | 1519 | – | 1531 | 495.9335 | 1484.7788 | 1484.7736 | 3.49 | 0 | 35 | 0.00053 | 1Score **> 34** indicates **identity** Score **> 15** indicates **homology** | U | K.EQTAHAFVNVLTR.G |
| 65016 | 1519 | – | 1531 | 743.3985 | 1484.7825 | 1484.7736 | 6.02 | 0 | 44 | 8.2e-05 | 1Score **> 34** indicates **identity** Score **> 15** indicates **homology** | U | K.EQTAHAFVNVLTR.G |
| 1117 | 1532 | – | 1538 | 366.2061 | 730.3977 | 730.3973 | 0.44 | 0 | 36 | 0.0045 | 1Score **> 25** indicates **identity** | U | R.GDLASIR.W |
| 164083 | 1546 | – | 1567 | 829.7226 | 2486.1459 | 2486.1649 | -7.62 | 0 | 38 | 0.025 | 1Score **> 34** indicates **identity** | U | K.HTQPSSSGAQLCTVYYASLNFR.D |
| 164088 | 1546 | – | 1567 | 829.7270 | 2486.1591 | 2486.1649 | -2.32 | 0 | 31 | 0.0031 | 1Score **> 35** indicates **identity** Score **> 18** indicates **homology** | U | K.HTQPSSSGAQLCTVYYASLNFR.D |
| 164104 | 1546 | – | 1567 | 829.7282 | 2486.1628 | 2486.1649 | -0.81 | 0 | 30 | 0.0016 | 1Score **> 35** indicates **identity** Score **> 14** indicates **homology** | U | K.HTQPSSSGAQLCTVYYASLNFR.D |
| 164106 | 1546 | – | 1567 | 829.7286 | 2486.1638 | 2486.1649 | -0.41 | 0 | 42 | 0.00011 | 1Score **> 35** indicates **identity** Score **> 15** indicates **homology** | U | K.HTQPSSSGAQLCTVYYASLNFR.D |
| 164116 | 1546 | – | 1567 | 829.7293 | 2486.1661 | 2486.1649 | 0.49 | 0 | 49 | 2.6e-05 | 1Score **> 35** indicates **identity** Score **> 16** indicates **homology** | U | K.HTQPSSSGAQLCTVYYASLNFR.D |
| 164119 | 1546 | – | 1567 | 829.7297 | 2486.1672 | 2486.1649 | 0.95 | 0 | 42 | 0.00013 | 1Score **> 35** indicates **identity** Score **> 15** indicates **homology** | U | K.HTQPSSSGAQLCTVYYASLNFR.D |
| 164124 | 1546 | – | 1567 | 829.7304 | 2486.1695 | 2486.1649 | 1.87 | 0 | 44 | 7.1e-05 | 1Score **> 35** indicates **identity** Score **> 15** indicates **homology** | U | K.HTQPSSSGAQLCTVYYASLNFR.D |
| 164132 | 1546 | – | 1567 | 829.7318 | 2486.1736 | 2486.1649 | 3.51 | 0 | 44 | 7.6e-05 | 1Score **> 35** indicates **identity** Score **> 15** indicates **homology** | U | K.HTQPSSSGAQLCTVYYASLNFR.D |
| 164133 | 1546 | – | 1567 | 829.7337 | 2486.1792 | 2486.1649 | 5.78 | 0 | 14 | 0.044 | 1Score **> 35** indicates **identity** Score **> 13** indicates **homology** | U | K.HTQPSSSGAQLCTVYYASLNFR.D |
| 164155 | 1546 | – | 1567 | 830.0627 | 2487.1662 | 2487.1489 | 6.97 | 0 | 31 | 0.0012 | 1Score **> 35** indicates **identity** Score **> 14** indicates **homology** | U | K.HTQPSSSGAQLCTVYYASLNFR.D  + Deamidated (NQ) |
| 164156 | 1546 | – | 1567 | 830.0629 | 2487.1669 | 2487.1489 | 7.25 | 0 | 35 | 0.00049 | 1Score **> 35** indicates **identity** Score **> 15** indicates **homology** | U | K.HTQPSSSGAQLCTVYYASLNFR.D  + Deamidated (NQ) |
| 8043 | 1576 | – | 1584 | 449.2554 | 896.4962 | 896.4967 | -0.54 | 0 | 41 | 0.0012 | 1Score **> 25** indicates **identity** | U | K.LSPDAIPGK.W |
| 8045 | 1576 | – | 1584 | 449.2561 | 896.4976 | 896.4967 | 0.96 | 0 | 46 | 6.6e-05 | 1Score **> 25** indicates **identity** Score **> 17** indicates **homology** | U | K.LSPDAIPGK.W |
| 170223 | 1661 | – | 1687 | 870.7975 | 2609.3708 | 2609.3595 | 4.32 | 0 | 65 | 8.4e-07 | 1Score **> 37** indicates **identity** Score **> 17** indicates **homology** | U | R.GETVLIHSGSGGVGQAAISIALSLGCR.V |
| 170277 | 1661 | – | 1687 | 871.1285 | 2610.3637 | 2610.3436 | 7.70 | 0 | 52 | 1.4e-05 | 1Score **> 37** indicates **identity** Score **> 16** indicates **homology** | U | R.GETVLIHSGSGGVGQAAISIALSLGCR.V  + Deamidated (NQ) |
| 16923 | 1688 | – | 1697 | 519.7769 | 1037.5392 | 1037.5393 | -0.18 | 0 | 46 | 5e-05 | 1Score **> 30** indicates **identity** Score **> 15** indicates **homology** | U | R.VFTTVGSAEK.R |
| 16924 | 1688 | – | 1697 | 519.7772 | 1037.5399 | 1037.5393 | 0.53 | 0 | 31 | 0.0012 | 1Score **> 30** indicates **identity** Score **> 14** indicates **homology** | U | R.VFTTVGSAEK.R |
| 32435 | 1688 | – | 1698 | 597.8272 | 1193.6399 | 1193.6404 | -0.42 | 1 | 38 | 0.0018 | 1Score **> 33** indicates **identity** Score **> 23** indicates **homology** | U | R.VFTTVGSAEKR.A |
| 32437 | 1688 | – | 1698 | 398.8876 | 1193.6410 | 1193.6404 | 0.50 | 1 | 29 | 0.0019 | 1Score **> 33** indicates **identity** Score **> 15** indicates **homology** | U | R.VFTTVGSAEKR.A |
| 32440 | 1688 | – | 1698 | 398.8879 | 1193.6418 | 1193.6404 | 1.18 | 1 | 50 | 7.7e-05 | 1Score **> 32** indicates **identity** Score **> 21** indicates **homology** | U | R.VFTTVGSAEKR.A |
| 32441 | 1688 | – | 1698 | 597.8282 | 1193.6419 | 1193.6404 | 1.21 | 1 | 48 | 8.4e-05 | 1Score **> 32** indicates **identity** Score **> 20** indicates **homology** | U | R.VFTTVGSAEKR.A |
| 66514 | 1705 | – | 1717 | 749.3524 | 1496.6902 | 1496.6896 | 0.39 | 0 | 81 | 2.6e-08 | 1Score **> 31** indicates **identity** Score **> 18** indicates **homology** | U | R.FPQLDDTSFANSR.D |
| 66516 | 1705 | – | 1717 | 749.3526 | 1496.6906 | 1496.6896 | 0.70 | 0 | 75 | 1.9e-07 | 1Score **> 31** indicates **identity** Score **> 21** indicates **homology** | U | R.FPQLDDTSFANSR.D |
| 66517 | 1705 | – | 1717 | 749.3527 | 1496.6909 | 1496.6896 | 0.87 | 0 | 77 | 1.4e-07 | 1Score **> 31** indicates **identity** Score **> 21** indicates **homology** | U | R.FPQLDDTSFANSR.D |
| 66518 | 1705 | – | 1717 | 749.3527 | 1496.6909 | 1496.6896 | 0.88 | 0 | 56 | 6.1e-06 | 1Score **> 31** indicates **identity** Score **> 17** indicates **homology** | U | R.FPQLDDTSFANSR.D |
| 66520 | 1705 | – | 1717 | 749.3534 | 1496.6923 | 1496.6896 | 1.85 | 0 | 35 | 0.00076 | 1Score **> 31** indicates **identity** Score **> 16** indicates **homology** | U | R.FPQLDDTSFANSR.D |
| 66522 | 1705 | – | 1717 | 749.3539 | 1496.6933 | 1496.6896 | 2.48 | 0 | 18 | 0.02 | 1Score **> 32** indicates **identity** Score **> 14** indicates **homology** | U | R.FPQLDDTSFANSR.D |
| 188515 | 1705 | – | 1732 | 630.3085 | 3146.5063 | 3146.5058 | 0.17 | 1 | 15 | 0.04 | 1Score **> 37** indicates **identity** Score **> 13** indicates **homology** | U | R.FPQLDDTSFANSRDTSFEQHVLLHTGGK.G |
| 89817 | 1718 | – | 1732 | 834.9201 | 1667.8256 | 1667.8268 | -0.67 | 0 | 70 | 2.7e-07 | 1Score **> 34** indicates **identity** Score **> 17** indicates **homology** | U | R.DTSFEQHVLLHTGGK.G |
| 89820 | 1718 | – | 1732 | 556.9496 | 1667.8268 | 1667.8268 | 0.055 | 0 | 40 | 0.00018 | 1Score **> 34** indicates **identity** Score **> 15** indicates **homology** | U | R.DTSFEQHVLLHTGGK.G |
| 89823 | 1718 | – | 1732 | 556.9497 | 1667.8274 | 1667.8268 | 0.40 | 0 | 53 | 2.2e-05 | 1Score **> 35** indicates **identity** Score **> 19** indicates **homology** | U | R.DTSFEQHVLLHTGGK.G |
| 89824 | 1718 | – | 1732 | 834.9211 | 1667.8276 | 1667.8268 | 0.48 | 0 | 15 | 0.041 | 1Score **> 35** indicates **identity** Score **> 13** indicates **homology** | U | R.DTSFEQHVLLHTGGK.G |
| 89826 | 1718 | – | 1732 | 556.9504 | 1667.8295 | 1667.8268 | 1.62 | 0 | 49 | 2.6e-05 | 1Score **> 34** indicates **identity** Score **> 16** indicates **homology** | U | R.DTSFEQHVLLHTGGK.G |
| 89827 | 1718 | – | 1732 | 556.9504 | 1667.8295 | 1667.8268 | 1.64 | 0 | 49 | 2.4e-05 | 1Score **> 34** indicates **identity** Score **> 16** indicates **homology** | U | R.DTSFEQHVLLHTGGK.G |
| 89828 | 1718 | – | 1732 | 834.9222 | 1667.8298 | 1667.8268 | 1.81 | 0 | 63 | 1.3e-06 | 1Score **> 34** indicates **identity** Score **> 16** indicates **homology** | U | R.DTSFEQHVLLHTGGK.G |
| 53423 | 1733 | – | 1745 | 693.8780 | 1385.7415 | 1385.7402 | 0.91 | 0 | 36 | 0.00043 | 1Score **> 34** indicates **identity** Score **> 15** indicates **homology** | U | K.GVDLVLNSLAEEK.L |
| 53424 | 1733 | – | 1745 | 693.8782 | 1385.7418 | 1385.7402 | 1.15 | 0 | 40 | 0.00017 | 1Score **> 34** indicates **identity** Score **> 15** indicates **homology** | U | K.GVDLVLNSLAEEK.L |
| 53425 | 1733 | – | 1745 | 693.8784 | 1385.7422 | 1385.7402 | 1.48 | 0 | 53 | 1e-05 | 1Score **> 34** indicates **identity** Score **> 16** indicates **homology** | U | K.GVDLVLNSLAEEK.L |
| 53427 | 1733 | – | 1745 | 693.8788 | 1385.7430 | 1385.7402 | 2.02 | 0 | 52 | 1.3e-05 | 1Score **> 34** indicates **identity** Score **> 16** indicates **homology** | U | K.GVDLVLNSLAEEK.L |
| 53428 | 1733 | – | 1745 | 693.8792 | 1385.7438 | 1385.7402 | 2.61 | 0 | 55 | 7.6e-06 | 1Score **> 34** indicates **identity** Score **> 16** indicates **homology** | U | K.GVDLVLNSLAEEK.L |
| 1486 | 1802 | – | 1808 | 372.2371 | 742.4597 | 742.4589 | 1.09 | 0 | 34 | 0.003 | 1Score **> 21** indicates **identity** | U | R.EVAALLK.A |
| 1488 | 1802 | – | 1808 | 372.2372 | 742.4599 | 742.4589 | 1.34 | 0 | 42 | 0.00045 | 1Score **> 21** indicates **identity** | U | R.EVAALLK.A |
| 38614 | 1809 | – | 1820 | 418.2638 | 1251.7696 | 1251.7663 | 2.65 | 2 | 14 | 0.046 | 1Score **> 26** indicates **identity** Score **> 13** indicates **homology** | U | K.AGIRDGVVKPLK.C |
| 10312 | 1827 | – | 1834 | 468.2335 | 934.4524 | 934.4508 | 1.69 | 0 | 34 | 0.00063 | 1Score **> 29** indicates **identity** Score **> 15** indicates **homology** | U | K.AQVEDAFR.Y |
| 180887 | 1845 | – | 1871 | 958.5397 | 2872.5972 | 2872.5909 | 2.19 | 1 | 54 | 9.3e-06 | 1Score **> 34** indicates **identity** Score **> 16** indicates **homology** | U | K.VLVQVREEEPEAVLPGAQPTLISAISK.T |
| 180888 | 1845 | – | 1871 | 958.5399 | 2872.5978 | 2872.5909 | 2.40 | 1 | 60 | 2.6e-06 | 1Score **> 34** indicates **identity** Score **> 16** indicates **homology** | U | K.VLVQVREEEPEAVLPGAQPTLISAISK.T |
| 180889 | 1845 | – | 1871 | 958.5400 | 2872.5983 | 2872.5909 | 2.55 | 1 | 47 | 3.6e-05 | 1Score **> 34** indicates **identity** Score **> 15** indicates **homology** | U | K.VLVQVREEEPEAVLPGAQPTLISAISK.T |
| 180910 | 1845 | – | 1871 | 1437.8023 | 2873.5901 | 2873.5750 | 5.27 | 1 | 34 | 0.0007 | 1Score **> 34** indicates **identity** Score **> 15** indicates **homology** | U | K.VLVQVREEEPEAVLPGAQPTLISAISK.T  + Deamidated (NQ) |
| 143090 | 1851 | – | 1871 | 1090.0889 | 2178.1632 | 2178.1419 | 9.77 | 0 | 40 | 0.00018 | 1Score **> 36** indicates **identity** Score **> 15** indicates **homology** | U | R.EEEPEAVLPGAQPTLISAISK.T |
| 97150 | 1879 | – | 1895 | 862.4669 | 1722.9192 | 1722.9305 | -6.53 | 0 | 67 | 1e-06 | 1Score **> 35** indicates **identity** Score **> 19** indicates **homology** | U | K.SYIITGGLGGFGLELAR.W |
| 97167 | 1879 | – | 1895 | 862.4745 | 1722.9345 | 1722.9305 | 2.34 | 0 | 57 | 7.6e-06 | 1Score **> 35** indicates **identity** Score **> 18** indicates **homology** | U | K.SYIITGGLGGFGLELAR.W |
| 135752 | 1927 | – | 1946 | 701.0476 | 2100.1210 | 2100.1287 | -3.67 | 1 | 49 | 2.5e-05 | 1Score **> 36** indicates **identity** Score **> 16** indicates **homology** | U | R.RQGIQVLVSTSNVSSLEGAR.A |
| 135757 | 1927 | – | 1946 | 701.0497 | 2100.1273 | 2100.1287 | -0.67 | 1 | 35 | 0.00049 | 1Score **> 36** indicates **identity** Score **> 15** indicates **homology** | U | R.RQGIQVLVSTSNVSSLEGAR.A |
| 135761 | 1927 | – | 1946 | 701.0508 | 2100.1305 | 2100.1287 | 0.83 | 1 | 73 | 4.2e-07 | 1Score **> 36** indicates **identity** Score **> 22** indicates **homology** | U | R.RQGIQVLVSTSNVSSLEGAR.A |
| 135769 | 1927 | – | 1946 | 701.0513 | 2100.1320 | 2100.1287 | 1.56 | 1 | 89 | 1.5e-08 | 1Score **> 36** indicates **identity** Score **> 23** indicates **homology** | U | R.RQGIQVLVSTSNVSSLEGAR.A |
| 135770 | 1927 | – | 1946 | 701.0513 | 2100.1321 | 2100.1287 | 1.60 | 1 | 27 | 0.0031 | 1Score **> 36** indicates **identity** Score **> 14** indicates **homology** | U | R.RQGIQVLVSTSNVSSLEGAR.A |
| 135895 | 1927 | – | 1946 | 701.3850 | 2101.1331 | 2101.1127 | 9.71 | 1 | 26 | 0.0034 | 1Score **> 36** indicates **identity** Score **> 14** indicates **homology** | U | R.RQGIQVLVSTSNVSSLEGAR.A  + Deamidated (NQ) |
| 135896 | 1927 | – | 1946 | 701.3850 | 2101.1332 | 2101.1127 | 9.74 | 1 | 16 | 0.031 | 1Score **> 36** indicates **identity** Score **> 14** indicates **homology** | U | R.RQGIQVLVSTSNVSSLEGAR.A  + Deamidated (NQ) |
| 120742 | 1928 | – | 1946 | 973.0224 | 1944.0303 | 1944.0276 | 1.39 | 0 | 39 | 0.00023 | 1Score **> 36** indicates **identity** Score **> 15** indicates **homology** | U | R.QGIQVLVSTSNVSSLEGAR.A |
| 120743 | 1928 | – | 1946 | 973.0240 | 1944.0335 | 1944.0276 | 3.03 | 0 | 57 | 4.1e-06 | 1Score **> 36** indicates **identity** Score **> 16** indicates **homology** | U | R.QGIQVLVSTSNVSSLEGAR.A |
| 120746 | 1928 | – | 1946 | 973.0251 | 1944.0356 | 1944.0276 | 4.11 | 0 | 57 | 4.1e-06 | 1Score **> 36** indicates **identity** Score **> 16** indicates **homology** | U | R.QGIQVLVSTSNVSSLEGAR.A |
| 120747 | 1928 | – | 1946 | 973.0251 | 1944.0356 | 1944.0276 | 4.11 | 0 | 78 | 4.4e-08 | 1Score **> 36** indicates **identity** Score **> 17** indicates **homology** | U | R.QGIQVLVSTSNVSSLEGAR.A |
| 120933 | 1928 | – | 1946 | 973.5220 | 1945.0294 | 1945.0116 | 9.12 | 0 | 22 | 0.0078 | 1Score **> 36** indicates **identity** Score **> 14** indicates **homology** | U | R.QGIQVLVSTSNVSSLEGAR.A  + Deamidated (NQ) |
| 4236 | 1947 | – | 1954 | 408.7450 | 815.4754 | 815.4752 | 0.22 | 0 | 45 | 0.00056 | 1Score **> 28** indicates **identity** Score **> 25** indicates **homology** | U | R.ALIAEATK.L |
| 4237 | 1947 | – | 1954 | 408.7454 | 815.4763 | 815.4752 | 1.25 | 0 | 60 | 3.2e-05 | 1Score **> 28** indicates **identity** | U | R.ALIAEATK.L |
| 146250 | 1970 | – | 1988 | 739.7018 | 2216.0835 | 2216.0783 | 2.33 | 1 | 35 | 0.00055 | 1Score **> 36** indicates **identity** Score **> 15** indicates **homology** | U | R.DAMLENQTPELFQDVNKPK.Y |
| 146252 | 1970 | – | 1988 | 739.7025 | 2216.0855 | 2216.0783 | 3.26 | 1 | 28 | 0.0021 | 1Score **> 36** indicates **identity** Score **> 14** indicates **homology** | U | R.DAMLENQTPELFQDVNKPK.Y |
| 19269 | 1989 | – | 1997 | 533.2698 | 1064.5251 | 1064.5251 | 0.023 | 0 | 49 | 0.00021 | 1Score **> 31** indicates **identity** Score **> 25** indicates **homology** | U | K.YNGTLNLDR.A |
| 19270 | 1989 | – | 1997 | 533.2700 | 1064.5254 | 1064.5251 | 0.37 | 0 | 40 | 0.00034 | 1Score **> 31** indicates **identity** Score **> 18** indicates **homology** | U | K.YNGTLNLDR.A |
| 19331 | 1989 | – | 1997 | 533.7618 | 1065.5091 | 1065.5091 | 0.049 | 0 | 46 | 8.4e-05 | 1Score **> 30** indicates **identity** Score **> 18** indicates **homology** | U | K.YNGTLNLDR.A  + Deamidated (NQ) |
| 19332 | 1989 | – | 1997 | 533.7622 | 1065.5099 | 1065.5091 | 0.81 | 0 | 68 | 6.4e-06 | 1Score **> 31** indicates **identity** Score **> 28** indicates **homology** | U | K.YNGTLNLDR.A  + Deamidated (NQ) |
| 19333 | 1989 | – | 1997 | 533.7624 | 1065.5102 | 1065.5091 | 1.04 | 0 | 35 | 0.0087 | 1Score **> 31** indicates **identity** Score **> 27** indicates **homology** | U | K.YNGTLNLDR.A  + Deamidated (NQ) |
| 19335 | 1989 | – | 1997 | 533.7628 | 1065.5110 | 1065.5091 | 1.84 | 0 | 64 | 1.3e-05 | 1Score **> 31** indicates **identity** Score **> 28** indicates **homology** | U | K.YNGTLNLDR.A  + Deamidated (NQ) |
| 144492 | 2001 | – | 2019 | 1097.4801 | 2192.9457 | 2192.9507 | -2.30 | 0 | 65 | 8.8e-07 | 1Score **> 28** indicates **identity** Score **> 17** indicates **homology** | U | R.EACPELDYFVAFSSVSCGR.G |
| 144493 | 2001 | – | 2019 | 1097.4847 | 2192.9548 | 2192.9507 | 1.87 | 0 | 98 | 6.6e-10 | 1Score **> 29** indicates **identity** Score **> 19** indicates **homology** | U | R.EACPELDYFVAFSSVSCGR.G |
| 144496 | 2001 | – | 2019 | 1097.4872 | 2192.9598 | 2192.9507 | 4.14 | 0 | 88 | 5.5e-09 | 1Score **> 29** indicates **identity** Score **> 18** indicates **homology** | U | R.EACPELDYFVAFSSVSCGR.G |
| 107962 | 2020 | – | 2036 | 909.3953 | 1816.7761 | 1816.7798 | -2.07 | 0 | 93 | 1.9e-09 | 1Score **> 27** indicates **identity** Score **> 18** indicates **homology** | U | R.GNAGQTNYGFANSTMER.I |
| 107963 | 2020 | – | 2036 | 909.3967 | 1816.7789 | 1816.7798 | -0.54 | 0 | 115 | 1.7e-11 | 1Score **> 27** indicates **identity** Score **> 20** indicates **homology** | U | R.GNAGQTNYGFANSTMER.I |
| 107964 | 2020 | – | 2036 | 909.3981 | 1816.7817 | 1816.7798 | 1.00 | 0 | 57 | 5e-06 | 1Score **> 28** indicates **identity** Score **> 16** indicates **homology** | U | R.GNAGQTNYGFANSTMER.I |
| 108056 | 2020 | – | 2036 | 909.8969 | 1817.7792 | 1817.7639 | 8.42 | 0 | 42 | 0.00011 | 1Score **> 26** indicates **identity** Score **> 15** indicates **homology** | U | R.GNAGQTNYGFANSTMER.I  + Deamidated (NQ) |
| 188890 | 2081 | – | 2107 | 1055.2053 | 3162.5941 | 3162.6232 | -9.20 | 0 | 30 | 0.0015 | 1Score **> 38** indicates **identity** Score **> 14** indicates **homology** | U | R.ISSCMEVLDLFLNQPHAVLSSFVLAEK.K  + Deamidated (NQ); HNE (C); Oxidation (M) |
| 77811 | 2109 | – | 2123 | 791.4009 | 1580.7872 | 1580.7907 | -2.19 | 1 | 26 | 0.0036 | 1Score **> 34** indicates **identity** Score **> 14** indicates **homology** | U | K.AVAHGDGDTQRDLVK.A |
| 23089 | 2188 | – | 2198 | 554.2635 | 1106.5124 | 1106.5092 | 2.92 | 0 | 19 | 0.015 | 1Score **> 30** indicates **identity** Score **> 14** indicates **homology** | U | K.TDSATDTTAPK.S |
| 194712 | 2257 | – | 2287 | 1152.5828 | 3454.7267 | 3454.7152 | 3.31 | 0 | 16 | 0.034 | 1Score **> 38** indicates **identity** Score **> 13** indicates **homology** | U | K.LSVPTYGLQCTQAAPLDSIPNLAAYYIDCIK.Q |
| 19933 | 2288 | – | 2296 | 537.2723 | 1072.5301 | 1072.5302 | -0.025 | 0 | 54 | 2.2e-05 | 1Score **> 31** indicates **identity** Score **> 20** indicates **homology** | U | K.QVQPEGPYR.I |
| 142382 | 2348 | – | 2366 | 725.3268 | 2172.9585 | 2172.9530 | 2.53 | 0 | 24 | 0.0058 | 1Score **> 30** indicates **identity** Score **> 14** indicates **homology** | U | K.MTPGCEAEAEAEALCFFIK.Q |
| 22654 | 2367 | – | 2375 | 368.1891 | 1101.5453 | 1101.5455 | -0.13 | 0 | 24 | 0.0062 | 1Score **> 31** indicates **identity** Score **> 14** indicates **homology** | U | K.QFLDVEHSK.V |
| 22655 | 2367 | – | 2375 | 368.1891 | 1101.5454 | 1101.5455 | -0.037 | 0 | 36 | 0.004 | 1Score **> 31** indicates **identity** Score **> 24** indicates **homology** | U | K.QFLDVEHSK.V |
| 22657 | 2367 | – | 2375 | 551.7802 | 1101.5459 | 1101.5455 | 0.41 | 0 | 28 | 0.0039 | 1Score **> 32** indicates **identity** Score **> 16** indicates **homology** | U | K.QFLDVEHSK.V |
| 22659 | 2367 | – | 2375 | 368.1894 | 1101.5465 | 1101.5455 | 0.94 | 0 | 38 | 0.0033 | 1Score **> 32** indicates **identity** Score **> 25** indicates **homology** | U | K.QFLDVEHSK.V |
| 22660 | 2367 | – | 2375 | 551.7813 | 1101.5480 | 1101.5455 | 2.31 | 0 | 22 | 0.022 | 1Score **> 32** indicates **identity** Score **> 18** indicates **homology** | U | K.QFLDVEHSK.V |
| 79938 | 2376 | – | 2389 | 532.6511 | 1594.9313 | 1594.9294 | 1.24 | 1 | 55 | 7e-06 | 1Score **> 31** indicates **identity** Score **> 16** indicates **homology** | U | K.VLEALLPLKSLEDR.V |
| 79940 | 2376 | – | 2389 | 532.6513 | 1594.9321 | 1594.9294 | 1.69 | 1 | 66 | 7.2e-07 | 1Score **> 31** indicates **identity** Score **> 17** indicates **homology** | U | K.VLEALLPLKSLEDR.V |
| 79944 | 2376 | – | 2389 | 532.6517 | 1594.9333 | 1594.9294 | 2.47 | 1 | 24 | 0.0059 | 1Score **> 31** indicates **identity** Score **> 14** indicates **homology** | U | K.VLEALLPLKSLEDR.V |
| 82722 | 2385 | – | 2399 | 808.9425 | 1615.8704 | 1615.8781 | -4.75 | 1 | 17 | 0.028 | 1Score **> 35** indicates **identity** Score **> 14** indicates **homology** | U | K.SLEDRVAASVDLITK.S |
| 82725 | 2385 | – | 2399 | 539.6341 | 1615.8806 | 1615.8781 | 1.53 | 1 | 15 | 0.037 | 1Score **> 35** indicates **identity** Score **> 13** indicates **homology** | U | K.SLEDRVAASVDLITK.S |
| 82726 | 2385 | – | 2399 | 539.6341 | 1615.8806 | 1615.8781 | 1.56 | 1 | 37 | 0.00033 | 1Score **> 35** indicates **identity** Score **> 15** indicates **homology** | U | K.SLEDRVAASVDLITK.S |
| 82728 | 2385 | – | 2399 | 539.6343 | 1615.8812 | 1615.8781 | 1.92 | 1 | 34 | 0.00065 | 1Score **> 35** indicates **identity** Score **> 15** indicates **homology** | U | K.SLEDRVAASVDLITK.S |
| 82730 | 2385 | – | 2399 | 539.6352 | 1615.8839 | 1615.8781 | 3.58 | 1 | 36 | 0.00046 | 1Score **> 35** indicates **identity** Score **> 15** indicates **homology** | U | K.SLEDRVAASVDLITK.S |
| 15502 | 2390 | – | 2399 | 508.8031 | 1015.5917 | 1015.5914 | 0.34 | 0 | 64 | 5.8e-06 | 1Score **> 32** indicates **identity** Score **> 24** indicates **homology** | U | R.VAASVDLITK.S |
| 15504 | 2390 | – | 2399 | 508.8032 | 1015.5918 | 1015.5914 | 0.40 | 0 | 64 | 5.7e-06 | 1Score **> 32** indicates **identity** Score **> 24** indicates **homology** | U | R.VAASVDLITK.S |
| 15506 | 2390 | – | 2399 | 508.8037 | 1015.5929 | 1015.5914 | 1.52 | 0 | 42 | 0.00036 | 1Score **> 33** indicates **identity** Score **> 20** indicates **homology** | U | R.VAASVDLITK.S |
| 15507 | 2390 | – | 2399 | 508.8037 | 1015.5929 | 1015.5914 | 1.53 | 0 | 64 | 5.9e-06 | 1Score **> 33** indicates **identity** Score **> 25** indicates **homology** | U | R.VAASVDLITK.S |
| 15509 | 2390 | – | 2399 | 508.8039 | 1015.5932 | 1015.5914 | 1.83 | 0 | 62 | 1.8e-05 | 1Score **> 33** indicates **identity** Score **> 27** indicates **homology** | U | R.VAASVDLITK.S |
| 15510 | 2390 | – | 2399 | 508.8039 | 1015.5933 | 1015.5914 | 1.90 | 0 | 44 | 0.0018 | 1Score **> 33** indicates **identity** Score **> 29** indicates **homology** | U | R.VAASVDLITK.S |
| 54640 | 2408 | – | 2419 | 466.9068 | 1397.6984 | 1397.6979 | 0.36 | 0 | 23 | 0.0077 | 1Score **> 33** indicates **identity** Score **> 15** indicates **homology** | U | R.ELSFAAVSFYHK.L |
| 54641 | 2408 | – | 2419 | 466.9070 | 1397.6991 | 1397.6979 | 0.80 | 0 | 24 | 0.0055 | 1Score **> 33** indicates **identity** Score **> 14** indicates **homology** | U | R.ELSFAAVSFYHK.L |
| 54642 | 2408 | – | 2419 | 466.9072 | 1397.6998 | 1397.6979 | 1.36 | 0 | 25 | 0.0047 | 1Score **> 33** indicates **identity** Score **> 14** indicates **homology** | U | R.ELSFAAVSFYHK.L |
| 54643 | 2408 | – | 2419 | 699.8572 | 1397.6999 | 1397.6979 | 1.41 | 0 | 62 | 1.6e-06 | 1Score **> 33** indicates **identity** Score **> 16** indicates **homology** | U | R.ELSFAAVSFYHK.L |
| 40915 | 2430 | – | 2440 | 636.3642 | 1270.7138 | 1270.7146 | -0.62 | 1 | 14 | 0.044 | 1Score **> 34** indicates **identity** Score **> 13** indicates **homology** | U | K.AKYHGNVTLLR.A |
| 40916 | 2430 | – | 2440 | 424.5797 | 1270.7173 | 1270.7146 | 2.13 | 1 | 15 | 0.039 | 1Score **> 33** indicates **identity** Score **> 13** indicates **homology** | U | K.AKYHGNVTLLR.A |
| 19880 | 2432 | – | 2440 | 536.7994 | 1071.5843 | 1071.5825 | 1.63 | 0 | 25 | 0.0046 | 1Score **> 33** indicates **identity** Score **> 14** indicates **homology** | U | K.YHGNVTLLR.A |
| 165949 | 2441 | – | 2464 | 840.3834 | 2518.1282 | 2518.1282 | 0.011 | 1 | 54 | 7.8e-06 | 1Score **> 31** indicates **identity** Score **> 16** indicates **homology** | U | R.AKTGGTYGEDLGADYNLSQVCDGK.V |
| 166014 | 2441 | – | 2464 | 840.7183 | 2519.1332 | 2519.1122 | 8.32 | 1 | 38 | 0.00028 | 1Score **> 32** indicates **identity** Score **> 15** indicates **homology** | U | R.AKTGGTYGEDLGADYNLSQVCDGK.V  + Deamidated (NQ) |
| 153626 | 2443 | – | 2464 | 1160.5066 | 2318.9986 | 2318.9961 | 1.07 | 0 | 84 | 1.2e-08 | 1Score **> 28** indicates **identity** Score **> 18** indicates **homology** | U | K.TGGTYGEDLGADYNLSQVCDGK.V |
| 153627 | 2443 | – | 2464 | 1160.5079 | 2319.0013 | 2318.9961 | 2.21 | 0 | 92 | 2.4e-09 | 1Score **> 28** indicates **identity** Score **> 18** indicates **homology** | U | K.TGGTYGEDLGADYNLSQVCDGK.V |
| 153628 | 2443 | – | 2464 | 1160.5082 | 2319.0018 | 2318.9961 | 2.46 | 0 | 99 | 4.7e-10 | 1Score **> 28** indicates **identity** Score **> 19** indicates **homology** | U | K.TGGTYGEDLGADYNLSQVCDGK.V |
| 153671 | 2443 | – | 2464 | 1161.0066 | 2319.9986 | 2319.9801 | 7.94 | 0 | 37 | 0.00033 | 1Score **> 29** indicates **identity** Score **> 15** indicates **homology** | U | K.TGGTYGEDLGADYNLSQVCDGK.V  + Deamidated (NQ) |
| 39705 | 2465 | – | 2475 | 631.3356 | 1260.6567 | 1260.6575 | -0.65 | 0 | 19 | 0.018 | 1Score **> 34** indicates **identity** Score **> 14** indicates **homology** | U | K.VSVHIIEGDHR.T |
| 39708 | 2465 | – | 2475 | 631.3360 | 1260.6574 | 1260.6575 | -0.092 | 0 | 33 | 0.0041 | 1Score **> 34** indicates **identity** Score **> 21** indicates **homology** | U | K.VSVHIIEGDHR.T |
| 39709 | 2465 | – | 2475 | 421.2264 | 1260.6575 | 1260.6575 | 0.0024 | 0 | 19 | 0.017 | 1Score **> 34** indicates **identity** Score **> 14** indicates **homology** | U | K.VSVHIIEGDHR.T |
| 39710 | 2465 | – | 2475 | 421.2268 | 1260.6585 | 1260.6575 | 0.79 | 0 | 35 | 0.0036 | 1Score **> 34** indicates **identity** Score **> 23** indicates **homology** | U | K.VSVHIIEGDHR.T |
| 39712 | 2465 | – | 2475 | 421.2270 | 1260.6591 | 1260.6575 | 1.31 | 0 | 28 | 0.038 | 1Score **> 34** indicates **identity** Score **> 26** indicates **homology** | U | K.VSVHIIEGDHR.T |
| 161844 | 2476 | – | 2498 | 1225.1730 | 2448.3315 | 2448.3224 | 3.75 | 0 | 150 | 8.3e-15 | 1Score **> 36** indicates **identity** Score **> 22** indicates **homology** | U | R.TLLEGSGLESIINIIHSSLAEPR.V |
| 161846 | 2476 | – | 2498 | 817.1182 | 2448.3329 | 2448.3224 | 4.29 | 0 | 74 | 1.1e-07 | 1Score **> 36** indicates **identity** Score **> 17** indicates **homology** | U | R.TLLEGSGLESIINIIHSSLAEPR.V |
| 161847 | 2476 | – | 2498 | 817.1185 | 2448.3337 | 2448.3224 | 4.64 | 0 | 15 | 0.035 | 1Score **> 36** indicates **identity** Score **> 13** indicates **homology** | U | R.TLLEGSGLESIINIIHSSLAEPR.V |
| 161857 | 2476 | – | 2498 | 817.1210 | 2448.3413 | 2448.3224 | 7.73 | 0 | 71 | 2.4e-07 | 1Score **> 35** indicates **identity** Score **> 17** indicates **homology** | U | R.TLLEGSGLESIINIIHSSLAEPR.V |

---

```
ID   FAS_MOUSE               Reviewed;        2504 AA.
AC   P19096; B1ATU8; Q6PB72; Q8C4Z0; Q9EQR0;
DT   01-NOV-1990, integrated into UniProtKB/Swiss-Prot.
DT   11-OCT-2004, sequence version 2.
DT   28-JUN-2023, entry version 215.
DE   RecName: Full=Fatty acid synthase;
DE            EC=2.3.1.85 {ECO:0000269|PubMed:29328619};
DE   AltName: Full=Type I Fatty Acid Synthase {ECO:0000303|PubMed:31811668};
DE   Includes:
DE     RecName: Full=[Acyl-carrier-protein] S-acetyltransferase;
DE              EC=2.3.1.38 {ECO:0000269|PubMed:29328619};
DE   Includes:
DE     RecName: Full=[Acyl-carrier-protein] S-malonyltransferase;
DE              EC=2.3.1.39 {ECO:0000269|PubMed:29328619};
DE   Includes:
DE     RecName: Full=3-oxoacyl-[acyl-carrier-protein] synthase;
DE              EC=2.3.1.41 {ECO:0000269|PubMed:31811668};
DE   Includes:
DE     RecName: Full=3-oxoacyl-[acyl-carrier-protein] reductase;
DE              EC=1.1.1.100 {ECO:0000250|UniProtKB:P49327};
DE   Includes:
DE     RecName: Full=3-hydroxyacyl-[acyl-carrier-protein] dehydratase;
DE              EC=4.2.1.59 {ECO:0000250|UniProtKB:P49327};
DE   Includes:
DE     RecName: Full=Enoyl-[acyl-carrier-protein] reductase;
DE              EC=1.3.1.39 {ECO:0000250|UniProtKB:P49327};
DE   Includes:
DE     RecName: Full=Acyl-[acyl-carrier-protein] hydrolase;
DE              EC=3.1.2.14 {ECO:0000250|UniProtKB:P49327};
GN   Name=Fasn;
OS   Mus musculus (Mouse).
OC   Eukaryota; Metazoa; Chordata; Craniata; Vertebrata; Euteleostomi; Mammalia;
OC   Eutheria; Euarchontoglires; Glires; Rodentia; Myomorpha; Muroidea; Muridae;
OC   Murinae; Mus; Mus.
OX   NCBI_TaxID=10090;
RN   [1]
RP   NUCLEOTIDE SEQUENCE [MRNA].
RC   STRAIN=C57BL/6J;
RX   PubMed=11029661; DOI=10.1046/j.1365-2443.2000.00369.x;
RA   Ueno K.;
RT   "Involvement of fatty acid synthase in axonal development in mouse
RT   embryos.";
RL   Genes Cells 5:859-869(2000).
RN   [2]
RP   NUCLEOTIDE SEQUENCE [LARGE SCALE GENOMIC DNA].
RC   STRAIN=C57BL/6J;
RX   PubMed=19468303; DOI=10.1371/journal.pbio.1000112;
RA   Church D.M., Goodstadt L., Hillier L.W., Zody M.C., Goldstein S., She X.,
RA   Bult C.J., Agarwala R., Cherry J.L., DiCuccio M., Hlavina W., Kapustin Y.,
RA   Meric P., Maglott D., Birtle Z., Marques A.C., Graves T., Zhou S.,
RA   Teague B., Potamousis K., Churas C., Place M., Herschleb J., Runnheim R.,
RA   Forrest D., Amos-Landgraf J., Schwartz D.C., Cheng Z., Lindblad-Toh K.,
RA   Eichler E.E., Ponting C.P.;
RT   "Lineage-specific biology revealed by a finished genome assembly of the
RT   mouse.";
RL   PLoS Biol. 7:E1000112-E1000112(2009).
RN   [3]
RP   NUCLEOTIDE SEQUENCE [LARGE SCALE MRNA].
RC   STRAIN=FVB/N; TISSUE=Brain, and Liver;
RX   PubMed=15489334; DOI=10.1101/gr.2596504;
RG   The MGC Project Team;
RT   "The status, quality, and expansion of the NIH full-length cDNA project:
RT   the Mammalian Gene Collection (MGC).";
RL   Genome Res. 14:2121-2127(2004).
RN   [4]
RP   PROTEIN SEQUENCE OF 1-12; 225-235; 299-310; 385-409; 469-478; 1252-1270;
RP   1333-1344; 1388-1398; 1501-1508; 1705-1717; 1733-1745; 2124-2132; 2376-2384
RP   AND 2476-2498, ACETYLATION AT MET-1, AND IDENTIFICATION BY MASS
RP   SPECTROMETRY.
RC   STRAIN=C57BL/6J; TISSUE=Liver;
RA   Bienvenut W.V.;
RL   Submitted (JUL-2005) to UniProtKB.
RN   [5]
RP   NUCLEOTIDE SEQUENCE [LARGE SCALE MRNA] OF 1527-2504.
RC   STRAIN=C57BL/6J; TISSUE=Thymus;
RX   PubMed=16141072; DOI=10.1126/science.1112014;
RA   Carninci P., Kasukawa T., Katayama S., Gough J., Frith M.C., Maeda N.,
RA   Oyama R., Ravasi T., Lenhard B., Wells C., Kodzius R., Shimokawa K.,
RA   Bajic V.B., Brenner S.E., Batalov S., Forrest A.R., Zavolan M., Davis M.J.,
RA   Wilming L.G., Aidinis V., Allen J.E., Ambesi-Impiombato A., Apweiler R.,
RA   Aturaliya R.N., Bailey T.L., Bansal M., Baxter L., Beisel K.W., Bersano T.,
RA   Bono H., Chalk A.M., Chiu K.P., Choudhary V., Christoffels A.,
RA   Clutterbuck D.R., Crowe M.L., Dalla E., Dalrymple B.P., de Bono B.,
RA   Della Gatta G., di Bernardo D., Down T., Engstrom P., Fagiolini M.,
RA   Faulkner G., Fletcher C.F., Fukushima T., Furuno M., Futaki S.,
RA   Gariboldi M., Georgii-Hemming P., Gingeras T.R., Gojobori T., Green R.E.,
RA   Gustincich S., Harbers M., Hayashi Y., Hensch T.K., Hirokawa N., Hill D.,
RA   Huminiecki L., Iacono M., Ikeo K., Iwama A., Ishikawa T., Jakt M.,
RA   Kanapin A., Katoh M., Kawasawa Y., Kelso J., Kitamura H., Kitano H.,
RA   Kollias G., Krishnan S.P., Kruger A., Kummerfeld S.K., Kurochkin I.V.,
RA   Lareau L.F., Lazarevic D., Lipovich L., Liu J., Liuni S., McWilliam S.,
RA   Madan Babu M., Madera M., Marchionni L., Matsuda H., Matsuzawa S., Miki H.,
RA   Mignone F., Miyake S., Morris K., Mottagui-Tabar S., Mulder N., Nakano N.,
RA   Nakauchi H., Ng P., Nilsson R., Nishiguchi S., Nishikawa S., Nori F.,
RA   Ohara O., Okazaki Y., Orlando V., Pang K.C., Pavan W.J., Pavesi G.,
RA   Pesole G., Petrovsky N., Piazza S., Reed J., Reid J.F., Ring B.Z.,
RA   Ringwald M., Rost B., Ruan Y., Salzberg S.L., Sandelin A., Schneider C.,
RA   Schoenbach C., Sekiguchi K., Semple C.A., Seno S., Sessa L., Sheng Y.,
RA   Shibata Y., Shimada H., Shimada K., Silva D., Sinclair B., Sperling S.,
RA   Stupka E., Sugiura K., Sultana R., Takenaka Y., Taki K., Tammoja K.,
RA   Tan S.L., Tang S., Taylor M.S., Tegner J., Teichmann S.A., Ueda H.R.,
RA   van Nimwegen E., Verardo R., Wei C.L., Yagi K., Yamanishi H.,
RA   Zabarovsky E., Zhu S., Zimmer A., Hide W., Bult C., Grimmond S.M.,
RA   Teasdale R.D., Liu E.T., Brusic V., Quackenbush J., Wahlestedt C.,
RA   Mattick J.S., Hume D.A., Kai C., Sasaki D., Tomaru Y., Fukuda S.,
RA   Kanamori-Katayama M., Suzuki M., Aoki J., Arakawa T., Iida J., Imamura K.,
RA   Itoh M., Kato T., Kawaji H., Kawagashira N., Kawashima T., Kojima M.,
RA   Kondo S., Konno H., Nakano K., Ninomiya N., Nishio T., Okada M., Plessy C.,
RA   Shibata K., Shiraki T., Suzuki S., Tagami M., Waki K., Watahiki A.,
RA   Okamura-Oho Y., Suzuki H., Kawai J., Hayashizaki Y.;
RT   "The transcriptional landscape of the mammalian genome.";
RL   Science 309:1559-1563(2005).
RN   [6]
RP   NUCLEOTIDE SEQUENCE [MRNA] OF 1666-2504.
RC   STRAIN=CD-1; TISSUE=Liver;
RX   PubMed=2920037; DOI=10.1016/0006-291x(89)92776-9;
RA   Paulauskis J.D., Sul H.S.;
RT   "Structure of mouse fatty acid synthase mRNA. Identification of the two
RT   NADPH binding sites.";
RL   Biochem. Biophys. Res. Commun. 158:690-695(1989).
RN   [7]
RP   PHOSPHORYLATION [LARGE SCALE ANALYSIS] AT SER-725; SER-1577; SER-1587 AND
RP   SER-2190, AND IDENTIFICATION BY MASS SPECTROMETRY [LARGE SCALE ANALYSIS].
RC   TISSUE=Brain, Brown adipose tissue, Heart, Kidney, Liver, Lung,
RC   Pancreas, Spleen, and Testis;
RX   PubMed=21183079; DOI=10.1016/j.cell.2010.12.001;
RA   Huttlin E.L., Jedrychowski M.P., Elias J.E., Goswami T., Rad R.,
RA   Beausoleil S.A., Villen J., Haas W., Sowa M.E., Gygi S.P.;
RT   "A tissue-specific atlas of mouse protein phosphorylation and expression.";
RL   Cell 143:1174-1189(2010).
RN   [8]
RP   INDUCTION BY ENDOCANNABINOID ANANDAMIDE.
RX   PubMed=21987372; DOI=10.1002/hep.24733;
RA   Jourdan T., Demizieux L., Gresti J., Djaouti L., Gaba L., Verges B.,
RA   Degrace P.;
RT   "Antagonism of peripheral hepatic cannabinoid receptor-1 improves liver
RT   lipid metabolism in mice: evidence from cultured explants.";
RL   Hepatology 55:790-799(2012).
RN   [9]
RP   FUNCTION, CATALYTIC ACTIVITY, BIOPHYSICOCHEMICAL PROPERTIES, AND PATHWAY.
RX   PubMed=29328619; DOI=10.1021/acschembio.7b00718;
RA   Rittner A., Paithankar K.S., Huu K.V., Grininger M.;
RT   "Characterization of the Polyspecific Transferase of Murine Type I Fatty
RT   Acid Synthase (FAS) and Implications for Polyketide Synthase (PKS)
RT   Engineering.";
RL   ACS Chem. Biol. 13:723-732(2018).
RN   [10]
RP   ACETYLATION [LARGE SCALE ANALYSIS] AT LYS-59; LYS-790; LYS-993; LYS-1071;
RP   LYS-1276; LYS-1840 AND LYS-2384, AND IDENTIFICATION BY MASS SPECTROMETRY
RP   [LARGE SCALE ANALYSIS].
RC   TISSUE=Embryonic fibroblast;
RX   PubMed=23806337; DOI=10.1016/j.molcel.2013.06.001;
RA   Park J., Chen Y., Tishkoff D.X., Peng C., Tan M., Dai L., Xie Z., Zhang Y.,
RA   Zwaans B.M., Skinner M.E., Lombard D.B., Zhao Y.;
RT   "SIRT5-mediated lysine desuccinylation impacts diverse metabolic
RT   pathways.";
RL   Mol. Cell 50:919-930(2013).
RN   [11] {ECO:0007744|PDB:6ROP}
RP   X-RAY CRYSTALLOGRAPHY (2.70 ANGSTROMS) OF 2-852 IN COMPLEX WITH OCTANOATE
RP   AND OCTANOYL-COA, FUNCTION, AND CATALYTIC ACTIVITY.
RX   PubMed=31811668; DOI=10.1002/pro.3797;
RA   Rittner A., Paithankar K.S., Himmler A., Grininger M.;
RT   "Type I fatty acid synthase trapped in the octanoyl-bound state.";
RL   Protein Sci. 29:589-605(2020).
CC   -!- FUNCTION: Fatty acid synthetase is a multifunctional enzyme that
CC       catalyzes the de novo biosynthesis of long-chain saturated fatty acids
CC       starting from acetyl-CoA and malonyl-CoA in the presence of NADPH. This
CC       multifunctional protein contains 7 catalytic activities and a site for
CC       the binding of the prosthetic group 4'-phosphopantetheine of the acyl
CC       carrier protein ([ACP]) domain. {ECO:0000269|PubMed:29328619,
CC       ECO:0000269|PubMed:31811668}.
CC   -!- CATALYTIC ACTIVITY:
CC       Reaction=acetyl-CoA + 2n H(+) + n malonyl-CoA + 2n NADPH = a long-chain
CC         fatty acid + n CO2 + (n+1) CoA + 2n NADP(+); Xref=Rhea:RHEA:14993,
CC         ChEBI:CHEBI:15378, ChEBI:CHEBI:16526, ChEBI:CHEBI:57287,
CC         ChEBI:CHEBI:57288, ChEBI:CHEBI:57384, ChEBI:CHEBI:57560,
CC         ChEBI:CHEBI:57783, ChEBI:CHEBI:58349; EC=2.3.1.85;
CC         Evidence={ECO:0000269|PubMed:29328619};
CC       PhysiologicalDirection=left-to-right; Xref=Rhea:RHEA:14994;
CC         Evidence={ECO:0000269|PubMed:29328619};
CC   -!- CATALYTIC ACTIVITY:
CC       Reaction=acetyl-CoA + holo-[ACP] = acetyl-[ACP] + CoA;
CC         Xref=Rhea:RHEA:41788, Rhea:RHEA-COMP:9621, Rhea:RHEA-COMP:9685,
CC         ChEBI:CHEBI:57287, ChEBI:CHEBI:57288, ChEBI:CHEBI:64479,
CC         ChEBI:CHEBI:78446; EC=2.3.1.38;
CC         Evidence={ECO:0000269|PubMed:29328619};
CC       PhysiologicalDirection=left-to-right; Xref=Rhea:RHEA:41789;
CC         Evidence={ECO:0000269|PubMed:29328619};
CC   -!- CATALYTIC ACTIVITY:
CC       Reaction=holo-[ACP] + malonyl-CoA = CoA + malonyl-[ACP];
CC         Xref=Rhea:RHEA:41792, Rhea:RHEA-COMP:9623, Rhea:RHEA-COMP:9685,
CC         ChEBI:CHEBI:57287, ChEBI:CHEBI:57384, ChEBI:CHEBI:64479,
CC         ChEBI:CHEBI:78449; EC=2.3.1.39;
CC         Evidence={ECO:0000269|PubMed:29328619};
CC       PhysiologicalDirection=left-to-right; Xref=Rhea:RHEA:41793;
CC         Evidence={ECO:0000269|PubMed:29328619};
CC   -!- CATALYTIC ACTIVITY:
CC       Reaction=a fatty acyl-[ACP] + H(+) + malonyl-[ACP] = a 3-oxoacyl-[ACP]
CC         + CO2 + holo-[ACP]; Xref=Rhea:RHEA:22836, Rhea:RHEA-COMP:9623,
CC         Rhea:RHEA-COMP:9685, Rhea:RHEA-COMP:9916, Rhea:RHEA-COMP:14125,
CC         ChEBI:CHEBI:15378, ChEBI:CHEBI:16526, ChEBI:CHEBI:64479,
CC         ChEBI:CHEBI:78449, ChEBI:CHEBI:78776, ChEBI:CHEBI:138651;
CC         EC=2.3.1.41; Evidence={ECO:0000269|PubMed:31811668};
CC       PhysiologicalDirection=left-to-right; Xref=Rhea:RHEA:22837;
CC         Evidence={ECO:0000269|PubMed:31811668};
CC   -!- CATALYTIC ACTIVITY:
CC       Reaction=a (3R)-hydroxyacyl-[ACP] + NADP(+) = a 3-oxoacyl-[ACP] + H(+)
CC         + NADPH; Xref=Rhea:RHEA:17397, Rhea:RHEA-COMP:9916, Rhea:RHEA-
CC         COMP:9945, ChEBI:CHEBI:15378, ChEBI:CHEBI:57783, ChEBI:CHEBI:58349,
CC         ChEBI:CHEBI:78776, ChEBI:CHEBI:78827; EC=1.1.1.100;
CC         Evidence={ECO:0000250|UniProtKB:P49327};
CC       PhysiologicalDirection=right-to-left; Xref=Rhea:RHEA:17399;
CC         Evidence={ECO:0000250|UniProtKB:P49327};
CC   -!- CATALYTIC ACTIVITY:
CC       Reaction=a (3R)-hydroxyacyl-[ACP] = a (2E)-enoyl-[ACP] + H2O;
CC         Xref=Rhea:RHEA:13097, Rhea:RHEA-COMP:9925, Rhea:RHEA-COMP:9945,
CC         ChEBI:CHEBI:15377, ChEBI:CHEBI:78784, ChEBI:CHEBI:78827; EC=4.2.1.59;
CC         Evidence={ECO:0000250|UniProtKB:P49327};
CC       PhysiologicalDirection=left-to-right; Xref=Rhea:RHEA:13098;
CC         Evidence={ECO:0000250|UniProtKB:P49327};
CC   -!- CATALYTIC ACTIVITY:
CC       Reaction=a 2,3-saturated acyl-[ACP] + NADP(+) = a (2E)-enoyl-[ACP] +
CC         H(+) + NADPH; Xref=Rhea:RHEA:22564, Rhea:RHEA-COMP:9925, Rhea:RHEA-
CC         COMP:9926, ChEBI:CHEBI:15378, ChEBI:CHEBI:57783, ChEBI:CHEBI:58349,
CC         ChEBI:CHEBI:78784, ChEBI:CHEBI:78785; EC=1.3.1.39;
CC         Evidence={ECO:0000250|UniProtKB:P49327};
CC       PhysiologicalDirection=right-to-left; Xref=Rhea:RHEA:22566;
CC         Evidence={ECO:0000250|UniProtKB:P49327};
CC   -!- CATALYTIC ACTIVITY:
CC       Reaction=H2O + hexadecanoyl-[ACP] = H(+) + hexadecanoate + holo-[ACP];
CC         Xref=Rhea:RHEA:41932, Rhea:RHEA-COMP:9652, Rhea:RHEA-COMP:9685,
CC         ChEBI:CHEBI:7896, ChEBI:CHEBI:15377, ChEBI:CHEBI:15378,
CC         ChEBI:CHEBI:64479, ChEBI:CHEBI:78483; EC=3.1.2.14;
CC         Evidence={ECO:0000250|UniProtKB:P49327};
CC       PhysiologicalDirection=left-to-right; Xref=Rhea:RHEA:41933;
CC         Evidence={ECO:0000250|UniProtKB:P49327};
CC   -!- CATALYTIC ACTIVITY:
CC       Reaction=acetyl-[ACP] + H(+) + malonyl-[ACP] = 3-oxobutanoyl-[ACP] +
CC         CO2 + holo-[ACP]; Xref=Rhea:RHEA:41800, Rhea:RHEA-COMP:9621,
CC         Rhea:RHEA-COMP:9623, Rhea:RHEA-COMP:9625, Rhea:RHEA-COMP:9685,
CC         ChEBI:CHEBI:15378, ChEBI:CHEBI:16526, ChEBI:CHEBI:64479,
CC         ChEBI:CHEBI:78446, ChEBI:CHEBI:78449, ChEBI:CHEBI:78450;
CC         Evidence={ECO:0000305|PubMed:31811668};
CC       PhysiologicalDirection=left-to-right; Xref=Rhea:RHEA:41801;
CC         Evidence={ECO:0000305|PubMed:31811668};
CC   -!- CATALYTIC ACTIVITY:
CC       Reaction=3-oxobutanoyl-[ACP] + H(+) + NADPH = (3R)-hydroxybutanoyl-
CC         [ACP] + NADP(+); Xref=Rhea:RHEA:41804, Rhea:RHEA-COMP:9625,
CC         Rhea:RHEA-COMP:9626, ChEBI:CHEBI:15378, ChEBI:CHEBI:57783,
CC         ChEBI:CHEBI:58349, ChEBI:CHEBI:78450, ChEBI:CHEBI:78451;
CC         Evidence={ECO:0000250|UniProtKB:P49327};
CC       PhysiologicalDirection=left-to-right; Xref=Rhea:RHEA:41805;
CC         Evidence={ECO:0000250|UniProtKB:P49327};
CC   -!- CATALYTIC ACTIVITY:
CC       Reaction=(3R)-hydroxybutanoyl-[ACP] = (2E)-butenoyl-[ACP] + H2O;
CC         Xref=Rhea:RHEA:41808, Rhea:RHEA-COMP:9626, Rhea:RHEA-COMP:9627,
CC         ChEBI:CHEBI:15377, ChEBI:CHEBI:78451, ChEBI:CHEBI:78453;
CC         Evidence={ECO:0000250|UniProtKB:P49327};
CC       PhysiologicalDirection=left-to-right; Xref=Rhea:RHEA:41809;
CC         Evidence={ECO:0000250|UniProtKB:P49327};
CC   -!- CATALYTIC ACTIVITY:
CC       Reaction=(2E)-butenoyl-[ACP] + H(+) + NADPH = butanoyl-[ACP] + NADP(+);
CC         Xref=Rhea:RHEA:41812, Rhea:RHEA-COMP:9627, Rhea:RHEA-COMP:9628,
CC         ChEBI:CHEBI:15378, ChEBI:CHEBI:57783, ChEBI:CHEBI:58349,
CC         ChEBI:CHEBI:78453, ChEBI:CHEBI:78454;
CC         Evidence={ECO:0000250|UniProtKB:P49327};
CC       PhysiologicalDirection=left-to-right; Xref=Rhea:RHEA:41813;
CC         Evidence={ECO:0000250|UniProtKB:P49327};
CC   -!- CATALYTIC ACTIVITY:
CC       Reaction=butanoyl-[ACP] + H(+) + malonyl-[ACP] = 3-oxohexanoyl-[ACP] +
CC         CO2 + holo-[ACP]; Xref=Rhea:RHEA:41820, Rhea:RHEA-COMP:9623,
CC         Rhea:RHEA-COMP:9628, Rhea:RHEA-COMP:9629, Rhea:RHEA-COMP:9685,
CC         ChEBI:CHEBI:15378, ChEBI:CHEBI:16526, ChEBI:CHEBI:64479,
CC         ChEBI:CHEBI:78449, ChEBI:CHEBI:78454, ChEBI:CHEBI:78456;
CC         Evidence={ECO:0000305|PubMed:31811668};
CC       PhysiologicalDirection=left-to-right; Xref=Rhea:RHEA:41821;
CC         Evidence={ECO:0000305|PubMed:31811668};
CC   -!- CATALYTIC ACTIVITY:
CC       Reaction=3-oxohexanoyl-[ACP] + H(+) + NADPH = (3R)-hydroxyhexanoyl-
CC         [ACP] + NADP(+); Xref=Rhea:RHEA:41824, Rhea:RHEA-COMP:9629,
CC         Rhea:RHEA-COMP:9630, ChEBI:CHEBI:15378, ChEBI:CHEBI:57783,
CC         ChEBI:CHEBI:58349, ChEBI:CHEBI:78456, ChEBI:CHEBI:78457;
CC         Evidence={ECO:0000250|UniProtKB:P49327};
CC       PhysiologicalDirection=left-to-right; Xref=Rhea:RHEA:41825;
CC         Evidence={ECO:0000250|UniProtKB:P49327};
CC   -!- CATALYTIC ACTIVITY:
CC       Reaction=(3R)-hydroxyhexanoyl-[ACP] = (2E)-hexenoyl-[ACP] + H2O;
CC         Xref=Rhea:RHEA:41828, Rhea:RHEA-COMP:9630, Rhea:RHEA-COMP:9631,
CC         ChEBI:CHEBI:15377, ChEBI:CHEBI:78457, ChEBI:CHEBI:78458;
CC         Evidence={ECO:0000250|UniProtKB:P49327};
CC       PhysiologicalDirection=left-to-right; Xref=Rhea:RHEA:41829;
CC         Evidence={ECO:0000250|UniProtKB:P49327};
CC   -!- CATALYTIC ACTIVITY:
CC       Reaction=(2E)-hexenoyl-[ACP] + H(+) + NADPH = hexanoyl-[ACP] + NADP(+);
CC         Xref=Rhea:RHEA:41832, Rhea:RHEA-COMP:9631, Rhea:RHEA-COMP:9632,
CC         ChEBI:CHEBI:15378, ChEBI:CHEBI:57783, ChEBI:CHEBI:58349,
CC         ChEBI:CHEBI:78458, ChEBI:CHEBI:78459;
CC         Evidence={ECO:0000250|UniProtKB:P49327};
CC       PhysiologicalDirection=left-to-right; Xref=Rhea:RHEA:41833;
CC         Evidence={ECO:0000250|UniProtKB:P49327};
CC   -!- CATALYTIC ACTIVITY:
CC       Reaction=H(+) + hexanoyl-[ACP] + malonyl-[ACP] = 3-oxooctanoyl-[ACP] +
CC         CO2 + holo-[ACP]; Xref=Rhea:RHEA:41836, Rhea:RHEA-COMP:9623,
CC         Rhea:RHEA-COMP:9632, Rhea:RHEA-COMP:9633, Rhea:RHEA-COMP:9685,
CC         ChEBI:CHEBI:15378, ChEBI:CHEBI:16526, ChEBI:CHEBI:64479,
CC         ChEBI:CHEBI:78449, ChEBI:CHEBI:78459, ChEBI:CHEBI:78460;
CC         Evidence={ECO:0000269|PubMed:31811668};
CC       PhysiologicalDirection=left-to-right; Xref=Rhea:RHEA:41837;
CC         Evidence={ECO:0000269|PubMed:31811668};
CC   -!- CATALYTIC ACTIVITY:
CC       Reaction=3-oxooctanoyl-[ACP] + H(+) + NADPH = (3R)-hydroxyoctanoyl-
CC         [ACP] + NADP(+); Xref=Rhea:RHEA:41840, Rhea:RHEA-COMP:9633,
CC         Rhea:RHEA-COMP:9634, ChEBI:CHEBI:15378, ChEBI:CHEBI:57783,
CC         ChEBI:CHEBI:58349, ChEBI:CHEBI:78460, ChEBI:CHEBI:78461;
CC         Evidence={ECO:0000250|UniProtKB:P49327};
CC       PhysiologicalDirection=left-to-right; Xref=Rhea:RHEA:41841;
CC         Evidence={ECO:0000250|UniProtKB:P49327};
CC   -!- CATALYTIC ACTIVITY:
CC       Reaction=(3R)-hydroxyoctanoyl-[ACP] = (2E)-octenoyl-[ACP] + H2O;
CC         Xref=Rhea:RHEA:41844, Rhea:RHEA-COMP:9634, Rhea:RHEA-COMP:9635,
CC         ChEBI:CHEBI:15377, ChEBI:CHEBI:78461, ChEBI:CHEBI:78462;
CC         Evidence={ECO:0000250|UniProtKB:P49327};
CC       PhysiologicalDirection=left-to-right; Xref=Rhea:RHEA:41845;
CC         Evidence={ECO:0000250|UniProtKB:P49327};
CC   -!- CATALYTIC ACTIVITY:
CC       Reaction=(2E)-octenoyl-[ACP] + H(+) + NADPH = NADP(+) + octanoyl-[ACP];
CC         Xref=Rhea:RHEA:41848, Rhea:RHEA-COMP:9635, Rhea:RHEA-COMP:9636,
CC         ChEBI:CHEBI:15378, ChEBI:CHEBI:57783, ChEBI:CHEBI:58349,
CC         ChEBI:CHEBI:78462, ChEBI:CHEBI:78463;
CC         Evidence={ECO:0000250|UniProtKB:P49327};
CC       PhysiologicalDirection=left-to-right; Xref=Rhea:RHEA:41849;
CC         Evidence={ECO:0000250|UniProtKB:P49327};
CC   -!- CATALYTIC ACTIVITY:
CC       Reaction=H(+) + malonyl-[ACP] + octanoyl-[ACP] = 3-oxodecanoyl-[ACP] +
CC         CO2 + holo-[ACP]; Xref=Rhea:RHEA:41852, Rhea:RHEA-COMP:9623,
CC         Rhea:RHEA-COMP:9636, Rhea:RHEA-COMP:9637, Rhea:RHEA-COMP:9685,
CC         ChEBI:CHEBI:15378, ChEBI:CHEBI:16526, ChEBI:CHEBI:64479,
CC         ChEBI:CHEBI:78449, ChEBI:CHEBI:78463, ChEBI:CHEBI:78464;
CC         Evidence={ECO:0000269|PubMed:31811668};
CC       PhysiologicalDirection=left-to-right; Xref=Rhea:RHEA:41853;
CC         Evidence={ECO:0000269|PubMed:31811668};
CC   -!- CATALYTIC ACTIVITY:
CC       Reaction=3-oxodecanoyl-[ACP] + H(+) + NADPH = (3R)-hydroxydecanoyl-
CC         [ACP] + NADP(+); Xref=Rhea:RHEA:41856, Rhea:RHEA-COMP:9637,
CC         Rhea:RHEA-COMP:9638, ChEBI:CHEBI:15378, ChEBI:CHEBI:57783,
CC         ChEBI:CHEBI:58349, ChEBI:CHEBI:78464, ChEBI:CHEBI:78466;
CC         Evidence={ECO:0000250|UniProtKB:P49327};
CC       PhysiologicalDirection=left-to-right; Xref=Rhea:RHEA:41857;
CC         Evidence={ECO:0000250|UniProtKB:P49327};
CC   -!- CATALYTIC ACTIVITY:
CC       Reaction=(3R)-hydroxydecanoyl-[ACP] = (2E)-decenoyl-[ACP] + H2O;
CC         Xref=Rhea:RHEA:41860, Rhea:RHEA-COMP:9638, Rhea:RHEA-COMP:9639,
CC         ChEBI:CHEBI:15377, ChEBI:CHEBI:78466, ChEBI:CHEBI:78467;
CC         Evidence={ECO:0000250|UniProtKB:P49327};
CC       PhysiologicalDirection=left-to-right; Xref=Rhea:RHEA:41861;
CC         Evidence={ECO:0000250|UniProtKB:P49327};
CC   -!- CATALYTIC ACTIVITY:
CC       Reaction=(2E)-decenoyl-[ACP] + H(+) + NADPH = decanoyl-[ACP] + NADP(+);
CC         Xref=Rhea:RHEA:41864, Rhea:RHEA-COMP:9639, Rhea:RHEA-COMP:9640,
CC         ChEBI:CHEBI:15378, ChEBI:CHEBI:57783, ChEBI:CHEBI:58349,
CC         ChEBI:CHEBI:78467, ChEBI:CHEBI:78468;
CC         Evidence={ECO:0000250|UniProtKB:P49327};
CC       PhysiologicalDirection=left-to-right; Xref=Rhea:RHEA:41865;
CC         Evidence={ECO:0000250|UniProtKB:P49327};
CC   -!- CATALYTIC ACTIVITY:
CC       Reaction=decanoyl-[ACP] + H(+) + malonyl-[ACP] = 3-oxododecanoyl-[ACP]
CC         + CO2 + holo-[ACP]; Xref=Rhea:RHEA:41868, Rhea:RHEA-COMP:9623,
CC         Rhea:RHEA-COMP:9640, Rhea:RHEA-COMP:9641, Rhea:RHEA-COMP:9685,
CC         ChEBI:CHEBI:15378, ChEBI:CHEBI:16526, ChEBI:CHEBI:64479,
CC         ChEBI:CHEBI:78449, ChEBI:CHEBI:78468, ChEBI:CHEBI:78469;
CC         Evidence={ECO:0000269|PubMed:31811668};
CC       PhysiologicalDirection=left-to-right; Xref=Rhea:RHEA:41869;
CC         Evidence={ECO:0000269|PubMed:31811668};
CC   -!- CATALYTIC ACTIVITY:
CC       Reaction=3-oxododecanoyl-[ACP] + H(+) + NADPH = (3R)-hydroxydodecanoyl-
CC         [ACP] + NADP(+); Xref=Rhea:RHEA:41872, Rhea:RHEA-COMP:9641,
CC         Rhea:RHEA-COMP:9642, ChEBI:CHEBI:15378, ChEBI:CHEBI:57783,
CC         ChEBI:CHEBI:58349, ChEBI:CHEBI:78469, ChEBI:CHEBI:78470;
CC         Evidence={ECO:0000250|UniProtKB:P49327};
CC       PhysiologicalDirection=left-to-right; Xref=Rhea:RHEA:41873;
CC         Evidence={ECO:0000250|UniProtKB:P49327};
CC   -!- CATALYTIC ACTIVITY:
CC       Reaction=(3R)-hydroxydodecanoyl-[ACP] = (2E)-dodecenoyl-[ACP] + H2O;
CC         Xref=Rhea:RHEA:41876, Rhea:RHEA-COMP:9642, Rhea:RHEA-COMP:9643,
CC         ChEBI:CHEBI:15377, ChEBI:CHEBI:78470, ChEBI:CHEBI:78472;
CC         Evidence={ECO:0000250|UniProtKB:P49327};
CC       PhysiologicalDirection=left-to-right; Xref=Rhea:RHEA:41877;
CC         Evidence={ECO:0000250|UniProtKB:P49327};
CC   -!- CATALYTIC ACTIVITY:
CC       Reaction=(2E)-dodecenoyl-[ACP] + H(+) + NADPH = dodecanoyl-[ACP] +
CC         NADP(+); Xref=Rhea:RHEA:41880, Rhea:RHEA-COMP:9643, Rhea:RHEA-
CC         COMP:9644, ChEBI:CHEBI:15378, ChEBI:CHEBI:57783, ChEBI:CHEBI:58349,
CC         ChEBI:CHEBI:65264, ChEBI:CHEBI:78472;
CC         Evidence={ECO:0000250|UniProtKB:P49327};
CC       PhysiologicalDirection=left-to-right; Xref=Rhea:RHEA:41881;
CC         Evidence={ECO:0000250|UniProtKB:P49327};
CC   -!- CATALYTIC ACTIVITY:
CC       Reaction=dodecanoyl-[ACP] + H(+) + malonyl-[ACP] = 3-oxotetradecanoyl-
CC         [ACP] + CO2 + holo-[ACP]; Xref=Rhea:RHEA:41884, Rhea:RHEA-COMP:9623,
CC         Rhea:RHEA-COMP:9644, Rhea:RHEA-COMP:9645, Rhea:RHEA-COMP:9685,
CC         ChEBI:CHEBI:15378, ChEBI:CHEBI:16526, ChEBI:CHEBI:64479,
CC         ChEBI:CHEBI:65264, ChEBI:CHEBI:78449, ChEBI:CHEBI:78473;
CC         Evidence={ECO:0000269|PubMed:31811668};
CC       PhysiologicalDirection=left-to-right; Xref=Rhea:RHEA:41885;
CC         Evidence={ECO:0000269|PubMed:31811668};
CC   -!- CATALYTIC ACTIVITY:
CC       Reaction=3-oxotetradecanoyl-[ACP] + H(+) + NADPH = (3R)-
CC         hydroxytetradecanoyl-[ACP] + NADP(+); Xref=Rhea:RHEA:41888,
CC         Rhea:RHEA-COMP:9645, Rhea:RHEA-COMP:9646, ChEBI:CHEBI:15378,
CC         ChEBI:CHEBI:57783, ChEBI:CHEBI:58349, ChEBI:CHEBI:78473,
CC         ChEBI:CHEBI:78474; Evidence={ECO:0000250|UniProtKB:P49327};
CC       PhysiologicalDirection=left-to-right; Xref=Rhea:RHEA:41889;
CC         Evidence={ECO:0000250|UniProtKB:P49327};
CC   -!- CATALYTIC ACTIVITY:
CC       Reaction=(3R)-hydroxytetradecanoyl-[ACP] = (2E)-tetradecenoyl-[ACP] +
CC         H2O; Xref=Rhea:RHEA:41892, Rhea:RHEA-COMP:9646, Rhea:RHEA-COMP:9647,
CC         ChEBI:CHEBI:15377, ChEBI:CHEBI:78474, ChEBI:CHEBI:78475;
CC         Evidence={ECO:0000250|UniProtKB:P49327};
CC       PhysiologicalDirection=left-to-right; Xref=Rhea:RHEA:41893;
CC         Evidence={ECO:0000250|UniProtKB:P49327};
CC   -!- CATALYTIC ACTIVITY:
CC       Reaction=(2E)-tetradecenoyl-[ACP] + H(+) + NADPH = NADP(+) +
CC         tetradecanoyl-[ACP]; Xref=Rhea:RHEA:41896, Rhea:RHEA-COMP:9647,
CC         Rhea:RHEA-COMP:9648, ChEBI:CHEBI:15378, ChEBI:CHEBI:57783,
CC         ChEBI:CHEBI:58349, ChEBI:CHEBI:78475, ChEBI:CHEBI:78477;
CC         Evidence={ECO:0000250|UniProtKB:P49327};
CC       PhysiologicalDirection=left-to-right; Xref=Rhea:RHEA:41897;
CC         Evidence={ECO:0000250|UniProtKB:P49327};
CC   -!- CATALYTIC ACTIVITY:
CC       Reaction=H(+) + malonyl-[ACP] + tetradecanoyl-[ACP] = 3-
CC         oxohexadecanoyl-[ACP] + CO2 + holo-[ACP]; Xref=Rhea:RHEA:41900,
CC         Rhea:RHEA-COMP:9623, Rhea:RHEA-COMP:9648, Rhea:RHEA-COMP:9649,
CC         Rhea:RHEA-COMP:9685, ChEBI:CHEBI:15378, ChEBI:CHEBI:16526,
CC         ChEBI:CHEBI:64479, ChEBI:CHEBI:78449, ChEBI:CHEBI:78477,
CC         ChEBI:CHEBI:78478; Evidence={ECO:0000269|PubMed:31811668};
CC       PhysiologicalDirection=left-to-right; Xref=Rhea:RHEA:41901;
CC         Evidence={ECO:0000269|PubMed:31811668};
CC   -!- CATALYTIC ACTIVITY:
CC       Reaction=3-oxohexadecanoyl-[ACP] + H(+) + NADPH = (3R)-
CC         hydroxyhexadecanoyl-[ACP] + NADP(+); Xref=Rhea:RHEA:41904, Rhea:RHEA-
CC         COMP:9649, Rhea:RHEA-COMP:9650, ChEBI:CHEBI:15378, ChEBI:CHEBI:57783,
CC         ChEBI:CHEBI:58349, ChEBI:CHEBI:78478, ChEBI:CHEBI:78480;
CC         Evidence={ECO:0000250|UniProtKB:P49327};
CC       PhysiologicalDirection=left-to-right; Xref=Rhea:RHEA:41905;
CC         Evidence={ECO:0000250|UniProtKB:P49327};
CC   -!- CATALYTIC ACTIVITY:
CC       Reaction=(3R)-hydroxyhexadecanoyl-[ACP] = (2E)-hexadecenoyl-[ACP] +
CC         H2O; Xref=Rhea:RHEA:41908, Rhea:RHEA-COMP:9650, Rhea:RHEA-COMP:9651,
CC         ChEBI:CHEBI:15377, ChEBI:CHEBI:78480, ChEBI:CHEBI:78481;
CC         Evidence={ECO:0000250|UniProtKB:P49327};
CC       PhysiologicalDirection=left-to-right; Xref=Rhea:RHEA:41909;
CC         Evidence={ECO:0000250|UniProtKB:P49327};
CC   -!- CATALYTIC ACTIVITY:
CC       Reaction=(2E)-hexadecenoyl-[ACP] + H(+) + NADPH = hexadecanoyl-[ACP] +
CC         NADP(+); Xref=Rhea:RHEA:41912, Rhea:RHEA-COMP:9651, Rhea:RHEA-
CC         COMP:9652, ChEBI:CHEBI:15378, ChEBI:CHEBI:57783, ChEBI:CHEBI:58349,
CC         ChEBI:CHEBI:78481, ChEBI:CHEBI:78483;
CC         Evidence={ECO:0000250|UniProtKB:P49327};
CC       PhysiologicalDirection=left-to-right; Xref=Rhea:RHEA:41913;
CC         Evidence={ECO:0000250|UniProtKB:P49327};
CC   -!- CATALYTIC ACTIVITY:
CC       Reaction=H(+) + hexadecanoyl-[ACP] + malonyl-[ACP] = 3-oxooctadecanoyl-
CC         [ACP] + CO2 + holo-[ACP]; Xref=Rhea:RHEA:41916, Rhea:RHEA-COMP:9623,
CC         Rhea:RHEA-COMP:9652, Rhea:RHEA-COMP:9653, Rhea:RHEA-COMP:9685,
CC         ChEBI:CHEBI:15378, ChEBI:CHEBI:16526, ChEBI:CHEBI:64479,
CC         ChEBI:CHEBI:78449, ChEBI:CHEBI:78483, ChEBI:CHEBI:78487;
CC         Evidence={ECO:0000250|UniProtKB:P49327};
CC       PhysiologicalDirection=left-to-right; Xref=Rhea:RHEA:41917;
CC         Evidence={ECO:0000250|UniProtKB:P49327};
CC   -!- CATALYTIC ACTIVITY:
CC       Reaction=3-oxooctadecanoyl-[ACP] + H(+) + NADPH = (3R)-
CC         hydroxyoctadecanoyl-[ACP] + NADP(+); Xref=Rhea:RHEA:41920, Rhea:RHEA-
CC         COMP:9653, Rhea:RHEA-COMP:9654, ChEBI:CHEBI:15378, ChEBI:CHEBI:57783,
CC         ChEBI:CHEBI:58349, ChEBI:CHEBI:78487, ChEBI:CHEBI:78488;
CC         Evidence={ECO:0000250|UniProtKB:P49327};
CC       PhysiologicalDirection=left-to-right; Xref=Rhea:RHEA:41921;
CC         Evidence={ECO:0000250|UniProtKB:P49327};
CC   -!- CATALYTIC ACTIVITY:
CC       Reaction=(3R)-hydroxyoctadecanoyl-[ACP] = (2E)-octadecenoyl-[ACP] +
CC         H2O; Xref=Rhea:RHEA:41924, Rhea:RHEA-COMP:9654, Rhea:RHEA-COMP:9655,
CC         ChEBI:CHEBI:15377, ChEBI:CHEBI:78488, ChEBI:CHEBI:78489;
CC         Evidence={ECO:0000250|UniProtKB:P49327};
CC       PhysiologicalDirection=left-to-right; Xref=Rhea:RHEA:41925;
CC         Evidence={ECO:0000250|UniProtKB:P49327};
CC   -!- CATALYTIC ACTIVITY:
CC       Reaction=(2E)-octadecenoyl-[ACP] + H(+) + NADPH = NADP(+) +
CC         octadecanoyl-[ACP]; Xref=Rhea:RHEA:41928, Rhea:RHEA-COMP:9655,
CC         Rhea:RHEA-COMP:9656, ChEBI:CHEBI:15378, ChEBI:CHEBI:57783,
CC         ChEBI:CHEBI:58349, ChEBI:CHEBI:78489, ChEBI:CHEBI:78495;
CC         Evidence={ECO:0000250|UniProtKB:P49327};
CC       PhysiologicalDirection=left-to-right; Xref=Rhea:RHEA:41929;
CC         Evidence={ECO:0000250|UniProtKB:P49327};
CC   -!- CATALYTIC ACTIVITY:
CC       Reaction=H2O + tetradecanoyl-[ACP] = H(+) + holo-[ACP] +
CC         tetradecanoate; Xref=Rhea:RHEA:30123, Rhea:RHEA-COMP:9648, Rhea:RHEA-
CC         COMP:9685, ChEBI:CHEBI:15377, ChEBI:CHEBI:15378, ChEBI:CHEBI:30807,
CC         ChEBI:CHEBI:64479, ChEBI:CHEBI:78477; EC=3.1.2.14;
CC         Evidence={ECO:0000250|UniProtKB:P49327};
CC       PhysiologicalDirection=left-to-right; Xref=Rhea:RHEA:30124;
CC         Evidence={ECO:0000250|UniProtKB:P49327};
CC   -!- BIOPHYSICOCHEMICAL PROPERTIES:
CC       Kinetic parameters:
CC         KM=1.28 uM for malonyl-CoA {ECO:0000269|PubMed:29328619};
CC         KM=1.63 uM for acetyl-CoA {ECO:0000269|PubMed:29328619};
CC   -!- PATHWAY: Lipid metabolism; fatty acid biosynthesis.
CC       {ECO:0000269|PubMed:29328619}.
CC   -!- SUBUNIT: Homodimer which is arranged in a head to tail fashion (By
CC       similarity). Interacts with CEACAM1; this interaction is insulin and
CC       phosphorylation-dependent; reduces fatty-acid synthase activity (By
CC       similarity). {ECO:0000250|UniProtKB:P12785,
CC       ECO:0000250|UniProtKB:P49327}.
CC   -!- SUBCELLULAR LOCATION: Cytoplasm {ECO:0000250}. Melanosome
CC       {ECO:0000250}.
CC   -!- INDUCTION: Up-regulated by endocannabinoid anandamide/AEA.
CC       {ECO:0000269|PubMed:21987372}.
CC   -!- PTM: S-nitrosylation of Fatty acid synthase at cysteine residues Cys-
CC       1464 or Cys-2084 is important for the enzyme dimerization. In
CC       adipocytes, S-nitrosylation of Fatty acid synthase occurs under
CC       physiological conditions and gradually increases during adipogenesis.
CC       {ECO:0000250|UniProtKB:P49327}.
CC   -!- SEQUENCE CAUTION:
CC       Sequence=CAA31525.1; Type=Frameshift; Evidence={ECO:0000305};
CC   ---------------------------------------------------------------------------
CC   Copyrighted by the UniProt Consortium, see https://www.uniprot.org/terms
CC   Distributed under the Creative Commons Attribution (CC BY 4.0) License
CC   ---------------------------------------------------------------------------
DR   EMBL; AF127033; AAG02285.1; -; mRNA.
DR   EMBL; AL663090; -; NOT_ANNOTATED_CDS; Genomic_DNA.
DR   EMBL; BC046513; AAH46513.1; -; mRNA.
DR   EMBL; BC059850; AAH59850.1; -; mRNA.
DR   EMBL; AK080374; BAC37895.1; -; mRNA.
DR   EMBL; X13135; CAA31525.1; ALT_FRAME; mRNA.
DR   CCDS; CCDS25759.1; -.
DR   PIR; A32262; A32262.
DR   RefSeq; NP_032014.3; NM_007988.3.
DR   PDB; 5MY0; X-ray; 2.94 A; A/B/C/D=2-852.
DR   PDB; 5MY2; X-ray; 2.70 A; A/B/C/D=2-852.
DR   PDB; 6ROP; X-ray; 2.70 A; A/B/C/D=2-852.
DR   PDBsum; 5MY0; -.
DR   PDBsum; 5MY2; -.
DR   PDBsum; 6ROP; -.
DR   AlphaFoldDB; P19096; -.
DR   SMR; P19096; -.
DR   BioGRID; 199596; 29.
DR   IntAct; P19096; 6.
DR   MINT; P19096; -.
DR   STRING; 10090.ENSMUSP00000052872; -.
DR   BindingDB; P19096; -.
DR   ChEMBL; CHEMBL1795189; -.
DR   ESTHER; mouse-FASN; Thioesterase.
DR   GlyGen; P19096; 1 site, 1 O-linked glycan (1 site).
DR   iPTMnet; P19096; -.
DR   PhosphoSitePlus; P19096; -.
DR   SwissPalm; P19096; -.
DR   CPTAC; non-CPTAC-3808; -.
DR   EPD; P19096; -.
DR   jPOST; P19096; -.
DR   MaxQB; P19096; -.
DR   PaxDb; P19096; -.
DR   PeptideAtlas; P19096; -.
DR   ProteomicsDB; 275589; -.
DR   Antibodypedia; 1650; 820 antibodies from 40 providers.
DR   DNASU; 14104; -.
DR   Ensembl; ENSMUST00000055655; ENSMUSP00000052872; ENSMUSG00000025153.
DR   GeneID; 14104; -.
DR   KEGG; mmu:14104; -.
DR   UCSC; uc007mut.1; mouse.
DR   AGR; MGI:95485; -.
DR   CTD; 2194; -.
DR   MGI; MGI:95485; Fasn.
DR   VEuPathDB; HostDB:ENSMUSG00000025153; -.
DR   eggNOG; KOG1202; Eukaryota.
DR   GeneTree; ENSGT00940000157276; -.
DR   HOGENOM; CLU_000022_31_7_1; -.
DR   InParanoid; P19096; -.
DR   OMA; KMRGGEF; -.
DR   OrthoDB; 3378513at2759; -.
DR   PhylomeDB; P19096; -.
DR   TreeFam; TF300549; -.
DR   BRENDA; 2.3.1.85; 3474.
DR   Reactome; R-MMU-163765; ChREBP activates metabolic gene expression.
DR   Reactome; R-MMU-199220; Vitamin B5 (pantothenate) metabolism.
DR   Reactome; R-MMU-75105; Fatty acyl-CoA biosynthesis.
DR   UniPathway; UPA00094; -.
DR   BioGRID-ORCS; 14104; 23 hits in 80 CRISPR screens.
DR   ChiTaRS; Fasn; mouse.
DR   PRO; PR:P19096; -.
DR   Proteomes; UP000000589; Chromosome 11.
DR   RNAct; P19096; protein.
DR   Bgee; ENSMUSG00000025153; Expressed in aorta tunica adventitia and 292 other tissues.
DR   ExpressionAtlas; P19096; baseline and differential.
DR   Genevisible; P19096; MM.
DR   GO; GO:0005737; C:cytoplasm; ISO:MGI.
DR   GO; GO:0005829; C:cytosol; ISO:MGI.
DR   GO; GO:0042587; C:glycogen granule; IDA:MGI.
DR   GO; GO:0005794; C:Golgi apparatus; ISO:MGI.
DR   GO; GO:0042470; C:melanosome; IEA:UniProtKB-SubCell.
DR   GO; GO:0005739; C:mitochondrion; HDA:MGI.
DR   GO; GO:0005886; C:plasma membrane; ISO:MGI.
DR   GO; GO:0008693; F:(3R)-3-hydroxydecanoyl-[acyl-carrier-protein] dehydratase activity; IEA:RHEA.
DR   GO; GO:0047451; F:(3R)-3-hydroxyoctanoyl-[acyl-carrier-protein] dehydratase activity; IEA:RHEA.
DR   GO; GO:0008659; F:(3R)-hydroxymyristoyl-[acyl-carrier-protein] dehydratase activity; IEA:UniProtKB-EC.
DR   GO; GO:0004317; F:(3R)-hydroxypalmitoyl-[acyl-carrier-protein] dehydratase activity; IEA:RHEA.
DR   GO; GO:0004316; F:3-oxoacyl-[acyl-carrier-protein] reductase (NADPH) activity; IEA:UniProtKB-EC.
DR   GO; GO:0004315; F:3-oxoacyl-[acyl-carrier-protein] synthase activity; IEA:UniProtKB-EC.
DR   GO; GO:0004313; F:[acyl-carrier-protein] S-acetyltransferase activity; IEA:UniProtKB-EC.
DR   GO; GO:0004314; F:[acyl-carrier-protein] S-malonyltransferase activity; ISO:MGI.
DR   GO; GO:0047117; F:enoyl-[acyl-carrier-protein] reductase (NADPH, A-specific) activity; IEA:UniProtKB-EC.
DR   GO; GO:0004312; F:fatty acid synthase activity; IDA:MGI.
DR   GO; GO:0042802; F:identical protein binding; ISO:MGI.
DR   GO; GO:0016295; F:myristoyl-[acyl-carrier-protein] hydrolase activity; IEA:UniProtKB-EC.
DR   GO; GO:0004320; F:oleoyl-[acyl-carrier-protein] hydrolase activity; IEA:UniProtKB-EC.
DR   GO; GO:0016296; F:palmitoyl-[acyl-carrier-protein] hydrolase activity; IEA:UniProtKB-EC.
DR   GO; GO:0031177; F:phosphopantetheine binding; IEA:InterPro.
DR   GO; GO:0006084; P:acetyl-CoA metabolic process; ISO:MGI.
DR   GO; GO:0071353; P:cellular response to interleukin-4; IDA:MGI.
DR   GO; GO:0090557; P:establishment of endothelial intestinal barrier; IMP:MGI.
DR   GO; GO:0008611; P:ether lipid biosynthetic process; IMP:MGI.
DR   GO; GO:0006633; P:fatty acid biosynthetic process; IDA:MGI.
DR   GO; GO:0002068; P:glandular epithelial cell development; IMP:MGI.
DR   GO; GO:0006954; P:inflammatory response; IMP:MGI.
DR   GO; GO:0008610; P:lipid biosynthetic process; IDA:MGI.
DR   GO; GO:0030879; P:mammary gland development; IMP:CACAO.
DR   GO; GO:0044788; P:modulation by host of viral process; ISO:MGI.
DR   GO; GO:0030224; P:monocyte differentiation; IMP:MGI.
DR   GO; GO:0030223; P:neutrophil differentiation; IMP:MGI.
DR   GO; GO:0009888; P:tissue development; IMP:MGI.
DR   CDD; cd05195; enoyl_red; 1.
DR   CDD; cd08954; KR_1_FAS_SDR_x; 2.
DR   CDD; cd00833; PKS; 1.
DR   Gene3D; 3.30.70.3290; -; 1.
DR   Gene3D; 3.40.47.10; -; 1.
DR   Gene3D; 1.10.1200.10; ACP-like; 1.
DR   Gene3D; 3.40.50.1820; alpha/beta hydrolase; 2.
DR   Gene3D; 3.40.366.10; Malonyl-Coenzyme A Acyl Carrier Protein, domain 2; 1.
DR   Gene3D; 3.90.180.10; Medium-chain alcohol dehydrogenases, catalytic domain; 1.
DR   Gene3D; 3.40.50.720; NAD(P)-binding Rossmann-like Domain; 1.
DR   Gene3D; 3.10.129.110; Polyketide synthase dehydratase; 1.
DR   Gene3D; 3.40.50.150; Vaccinia Virus protein VP39; 1.
DR   InterPro; IPR029058; AB_hydrolase.
DR   InterPro; IPR001227; Ac_transferase_dom_sf.
DR   InterPro; IPR036736; ACP-like_sf.
DR   InterPro; IPR014043; Acyl_transferase.
DR   InterPro; IPR016035; Acyl_Trfase/lysoPLipase.
DR   InterPro; IPR013149; ADH-like_C.
DR   InterPro; IPR011032; GroES-like_sf.
DR   InterPro; IPR018201; Ketoacyl_synth_AS.
DR   InterPro; IPR014031; Ketoacyl_synth_C.
DR   InterPro; IPR014030; Ketoacyl_synth_N.
DR   InterPro; IPR016036; Malonyl_transacylase_ACP-bd.
DR   InterPro; IPR013217; Methyltransf_12.
DR   InterPro; IPR036291; NAD(P)-bd_dom_sf.
DR   InterPro; IPR032821; PKS_assoc.
DR   InterPro; IPR020841; PKS_Beta-ketoAc_synthase_dom.
DR   InterPro; IPR042104; PKS_dehydratase_sf.
DR   InterPro; IPR020807; PKS_DH.
DR   InterPro; IPR020843; PKS_ER.
DR   InterPro; IPR013968; PKS_KR.
DR   InterPro; IPR020806; PKS_PP-bd.
DR   InterPro; IPR009081; PP-bd_ACP.
DR   InterPro; IPR006162; Ppantetheine_attach_site.
DR   InterPro; IPR029063; SAM-dependent_MTases_sf.
DR   InterPro; IPR001031; Thioesterase.
DR   InterPro; IPR016039; Thiolase-like.
DR   PANTHER; PTHR43775; FATTY ACID SYNTHASE; 1.
DR   PANTHER; PTHR43775:SF7; FATTY ACID SYNTHASE; 1.
DR   Pfam; PF00698; Acyl_transf_1; 1.
DR   Pfam; PF00107; ADH_zinc_N; 1.
DR   Pfam; PF16197; KAsynt_C_assoc; 1.
DR   Pfam; PF00109; ketoacyl-synt; 1.
DR   Pfam; PF02801; Ketoacyl-synt_C; 1.
DR   Pfam; PF08659; KR; 1.
DR   Pfam; PF08242; Methyltransf_12; 1.
DR   Pfam; PF00550; PP-binding; 1.
DR   Pfam; PF14765; PS-DH; 1.
DR   Pfam; PF00975; Thioesterase; 1.
DR   SMART; SM00827; PKS_AT; 1.
DR   SMART; SM00826; PKS_DH; 1.
DR   SMART; SM00829; PKS_ER; 1.
DR   SMART; SM00822; PKS_KR; 1.
DR   SMART; SM00825; PKS_KS; 1.
DR   SMART; SM00823; PKS_PP; 1.
DR   SUPFAM; SSF47336; ACP-like; 1.
DR   SUPFAM; SSF53474; alpha/beta-Hydrolases; 1.
DR   SUPFAM; SSF52151; FabD/lysophospholipase-like; 1.
DR   SUPFAM; SSF50129; GroES-like; 1.
DR   SUPFAM; SSF51735; NAD(P)-binding Rossmann-fold domains; 2.
DR   SUPFAM; SSF55048; Probable ACP-binding domain of malonyl-CoA ACP transacylase; 1.
DR   SUPFAM; SSF53335; S-adenosyl-L-methionine-dependent methyltransferases; 1.
DR   SUPFAM; SSF53901; Thiolase-like; 1.
DR   PROSITE; PS50075; CARRIER; 1.
DR   PROSITE; PS00606; KS3_1; 1.
DR   PROSITE; PS52004; KS3_2; 1.
DR   PROSITE; PS00012; PHOSPHOPANTETHEINE; 1.
DR   PROSITE; PS52019; PKS_MFAS_DH; 1.
PE   1: Evidence at protein level;
KW   3D-structure; Acetylation; Cytoplasm; Direct protein sequencing;
KW   Fatty acid biosynthesis; Fatty acid metabolism; Hydrolase; Isopeptide bond;
KW   Lipid biosynthesis; Lipid metabolism; Lyase; Multifunctional enzyme; NAD;
KW   NADP; Oxidoreductase; Phosphopantetheine; Phosphoprotein;
KW   Pyridoxal phosphate; Reference proteome; S-nitrosylation; Transferase;
KW   Ubl conjugation.
FT   CHAIN           1..2504
FT                   /note="Fatty acid synthase"
FT                   /id="PRO_0000180277"
FT   DOMAIN          1..406
FT                   /note="Ketosynthase family 3 (KS3)"
FT                   /evidence="ECO:0000255|PROSITE-ProRule:PRU01348"
FT   DOMAIN          844..1104
FT                   /note="PKS/mFAS DH"
FT                   /evidence="ECO:0000255|PROSITE-ProRule:PRU01363"
FT   DOMAIN          2112..2192
FT                   /note="Carrier"
FT                   /evidence="ECO:0000255|PROSITE-ProRule:PRU00258"
FT   REGION          429..817
FT                   /note="Acyl and malonyl transferases"
FT   REGION          844..967
FT                   /note="N-terminal hotdog fold"
FT                   /evidence="ECO:0000255|PROSITE-ProRule:PRU01363"
FT   REGION          982..1104
FT                   /note="C-terminal hotdog fold"
FT                   /evidence="ECO:0000255|PROSITE-ProRule:PRU01363"
FT   REGION          1628..1856
FT                   /note="Enoyl reductase"
FT   REGION          1857..2111
FT                   /note="Beta-ketoacyl reductase"
FT   REGION          2181..2205
FT                   /note="Disordered"
FT                   /evidence="ECO:0000256|SAM:MobiDB-lite"
FT   REGION          2201..2504
FT                   /note="Thioesterase"
FT   ACT_SITE        161
FT                   /note="For beta-ketoacyl synthase activity"
FT                   /evidence="ECO:0000255|PROSITE-ProRule:PRU01348"
FT   ACT_SITE        293
FT                   /note="For beta-ketoacyl synthase activity"
FT                   /evidence="ECO:0000255|PROSITE-ProRule:PRU01348"
FT   ACT_SITE        331
FT                   /note="For beta-ketoacyl synthase activity"
FT                   /evidence="ECO:0000255|PROSITE-ProRule:PRU01348"
FT   ACT_SITE        581
FT                   /note="For malonyltransferase activity"
FT                   /evidence="ECO:0000255|PROSITE-ProRule:PRU10022"
FT   ACT_SITE        878
FT                   /note="Proton acceptor; for dehydratase activity"
FT                   /evidence="ECO:0000255|PROSITE-ProRule:PRU01363"
FT   ACT_SITE        1032
FT                   /note="Proton donor; for dehydratase activity"
FT                   /evidence="ECO:0000255|PROSITE-ProRule:PRU01363"
FT   ACT_SITE        2301
FT                   /note="For thioesterase activity"
FT                   /evidence="ECO:0000255|PROSITE-ProRule:PRU10022"
FT   ACT_SITE        2474
FT                   /note="For thioesterase activity"
FT                   /evidence="ECO:0000255|PROSITE-ProRule:PRU10022"
FT   BINDING         647..648
FT                   /ligand="an acyl-CoA"
FT                   /ligand_id="ChEBI:CHEBI:58342"
FT                   /evidence="ECO:0000269|PubMed:31811668,
FT                   ECO:0007744|PDB:6ROP"
FT   BINDING         671
FT                   /ligand="an acyl-CoA"
FT                   /ligand_id="ChEBI:CHEBI:58342"
FT                   /evidence="ECO:0000269|PubMed:31811668,
FT                   ECO:0007744|PDB:6ROP"
FT   BINDING         773
FT                   /ligand="an acyl-CoA"
FT                   /ligand_id="ChEBI:CHEBI:58342"
FT                   /evidence="ECO:0000269|PubMed:31811668,
FT                   ECO:0007744|PDB:6ROP"
FT   BINDING         1664..1681
FT                   /ligand="NADP(+)"
FT                   /ligand_id="ChEBI:CHEBI:58349"
FT                   /ligand_label="1"
FT                   /ligand_note="for enoyl reductase activity"
FT                   /evidence="ECO:0000250"
FT   BINDING         1879..1894
FT                   /ligand="NADP(+)"
FT                   /ligand_id="ChEBI:CHEBI:58349"
FT                   /ligand_label="2"
FT                   /ligand_note="for ketoreductase activity"
FT                   /evidence="ECO:0000250"
FT   MOD_RES         1
FT                   /note="N-acetylmethionine"
FT                   /evidence="ECO:0000269|Ref.4"
FT   MOD_RES         59
FT                   /note="N6-acetyllysine"
FT                   /evidence="ECO:0007744|PubMed:23806337"
FT   MOD_RES         63
FT                   /note="Phosphoserine"
FT                   /evidence="ECO:0000250|UniProtKB:P49327"
FT   MOD_RES         70
FT                   /note="N6-acetyllysine"
FT                   /evidence="ECO:0000250|UniProtKB:P49327"
FT   MOD_RES         207
FT                   /note="Phosphoserine"
FT                   /evidence="ECO:0000250|UniProtKB:P49327"
FT   MOD_RES         298
FT                   /note="N6-acetyllysine"
FT                   /evidence="ECO:0000250|UniProtKB:P49327"
FT   MOD_RES         528
FT                   /note="N6-acetyllysine"
FT                   /evidence="ECO:0000250|UniProtKB:P49327"
FT   MOD_RES         673
FT                   /note="N6-acetyllysine"
FT                   /evidence="ECO:0000250|UniProtKB:P49327"
FT   MOD_RES         725
FT                   /note="Phosphoserine"
FT                   /evidence="ECO:0007744|PubMed:21183079"
FT   MOD_RES         790
FT                   /note="N6-acetyllysine"
FT                   /evidence="ECO:0007744|PubMed:23806337"
FT   MOD_RES         993
FT                   /note="N6-acetyllysine"
FT                   /evidence="ECO:0007744|PubMed:23806337"
FT   MOD_RES         1071
FT                   /note="N6-acetyllysine"
FT                   /evidence="ECO:0007744|PubMed:23806337"
FT   MOD_RES         1276
FT                   /note="N6-acetyllysine"
FT                   /evidence="ECO:0007744|PubMed:23806337"
FT   MOD_RES         1464
FT                   /note="S-nitrosocysteine"
FT                   /evidence="ECO:0000250|UniProtKB:P49327"
FT   MOD_RES         1577
FT                   /note="Phosphoserine"
FT                   /evidence="ECO:0007744|PubMed:21183079"
FT   MOD_RES         1587
FT                   /note="Phosphoserine"
FT                   /evidence="ECO:0007744|PubMed:21183079"
FT   MOD_RES         1697
FT                   /note="N6-(pyridoxal phosphate)lysine; alternate"
FT                   /evidence="ECO:0000250"
FT   MOD_RES         1697
FT                   /note="N6-acetyllysine; alternate"
FT                   /evidence="ECO:0000250|UniProtKB:P49327"
FT   MOD_RES         1764
FT                   /note="N6-acetyllysine"
FT                   /evidence="ECO:0000250|UniProtKB:P49327"
FT   MOD_RES         1840
FT                   /note="N6-acetyllysine"
FT                   /evidence="ECO:0007744|PubMed:23806337"
FT   MOD_RES         1988
FT                   /note="N6-acetyllysine"
FT                   /evidence="ECO:0000250|UniProtKB:P49327"
FT   MOD_RES         2084
FT                   /note="S-nitrosocysteine"
FT                   /evidence="ECO:0000250|UniProtKB:P49327"
FT   MOD_RES         2150
FT                   /note="O-(pantetheine 4'-phosphoryl)serine; alternate"
FT                   /evidence="ECO:0000255|PROSITE-ProRule:PRU00258"
FT   MOD_RES         2150
FT                   /note="Phosphoserine; alternate"
FT                   /evidence="ECO:0000250|UniProtKB:P12785"
FT   MOD_RES         2190
FT                   /note="Phosphoserine"
FT                   /evidence="ECO:0007744|PubMed:21183079"
FT   MOD_RES         2229
FT                   /note="Phosphoserine"
FT                   /evidence="ECO:0000250|UniProtKB:P49327"
FT   MOD_RES         2384
FT                   /note="N6-acetyllysine"
FT                   /evidence="ECO:0007744|PubMed:23806337"
FT   CROSSLNK        2442
FT                   /note="Glycyl lysine isopeptide (Lys-Gly) (interchain with
FT                   G-Cter in SUMO2)"
FT                   /evidence="ECO:0000250|UniProtKB:P49327"
FT   CONFLICT        1992
FT                   /note="T -> N (in Ref. 6; CAA31525)"
FT                   /evidence="ECO:0000305"
FT   CONFLICT        2028
FT                   /note="G -> C (in Ref. 6; CAA31525)"
FT                   /evidence="ECO:0000305"
FT   CONFLICT        2045
FT                   /note="G -> V (in Ref. 6; CAA31525)"
FT                   /evidence="ECO:0000305"
FT   CONFLICT        2117
FT                   /note="T -> N (in Ref. 6; CAA31525)"
FT                   /evidence="ECO:0000305"
FT   CONFLICT        2175
FT                   /note="Q -> R (in Ref. 6; CAA31525)"
FT                   /evidence="ECO:0000305"
FT   CONFLICT        2295
FT                   /note="Y -> H (in Ref. 6; CAA31525)"
FT                   /evidence="ECO:0000305"
FT   STRAND          4..13
FT                   /evidence="ECO:0007829|PDB:5MY2"
FT   STRAND          16..18
FT                   /evidence="ECO:0007829|PDB:5MY2"
FT   HELIX           19..27
FT                   /evidence="ECO:0007829|PDB:5MY2"
FT   STRAND          36..40
FT                   /evidence="ECO:0007829|PDB:5MY2"
FT   HELIX           44..46
FT                   /evidence="ECO:0007829|PDB:5MY2"
FT   HELIX           62..65
FT                   /evidence="ECO:0007829|PDB:5MY2"
FT   HELIX           69..73
FT                   /evidence="ECO:0007829|PDB:5MY2"
FT   HELIX           77..92
FT                   /evidence="ECO:0007829|PDB:5MY2"
FT   HELIX           97..100
FT                   /evidence="ECO:0007829|PDB:5MY2"
FT   STRAND          106..111
FT                   /evidence="ECO:0007829|PDB:5MY2"
FT   HELIX           115..120
FT                   /evidence="ECO:0007829|PDB:5MY2"
FT   TURN            124..126
FT                   /evidence="ECO:0007829|PDB:5MY2"
FT   HELIX           130..135
FT                   /evidence="ECO:0007829|PDB:5MY2"
FT   HELIX           139..148
FT                   /evidence="ECO:0007829|PDB:5MY2"
FT   STRAND          154..158
FT                   /evidence="ECO:0007829|PDB:5MY2"
FT   HELIX           160..162
FT                   /evidence="ECO:0007829|PDB:5MY2"
FT   HELIX           163..176
FT                   /evidence="ECO:0007829|PDB:5MY2"
FT   STRAND          181..189
FT                   /evidence="ECO:0007829|PDB:5MY2"
FT   HELIX           194..202
FT                   /evidence="ECO:0007829|PDB:5MY2"
FT   STRAND          227..235
FT                   /evidence="ECO:0007829|PDB:5MY2"
FT   HELIX           236..238
FT                   /evidence="ECO:0007829|PDB:5MY2"
FT   STRAND          243..253
FT                   /evidence="ECO:0007829|PDB:5MY2"
FT   HELIX           266..276
FT                   /evidence="ECO:0007829|PDB:5MY2"
FT   HELIX           278..280
FT                   /evidence="ECO:0007829|PDB:5MY2"
FT   HELIX           284..286
FT                   /evidence="ECO:0007829|PDB:5MY2"
FT   STRAND          287..291
FT                   /evidence="ECO:0007829|PDB:5MY2"
FT   HELIX           300..311
FT                   /evidence="ECO:0007829|PDB:5MY2"
FT   HELIX           313..315
FT                   /evidence="ECO:0007829|PDB:5MY2"
FT   STRAND          320..323
FT                   /evidence="ECO:0007829|PDB:5MY2"
FT   HELIX           326..329
FT                   /evidence="ECO:0007829|PDB:5MY2"
FT   HELIX           333..335
FT                   /evidence="ECO:0007829|PDB:5MY2"
FT   HELIX           336..349
FT                   /evidence="ECO:0007829|PDB:5MY2"
FT   HELIX           367..370
FT                   /evidence="ECO:0007829|PDB:5MY2"
FT   STRAND          373..376
FT                   /evidence="ECO:0007829|PDB:5MY2"
FT   STRAND          387..393
FT                   /evidence="ECO:0007829|PDB:5MY2"
FT   STRAND          397..406
FT                   /evidence="ECO:0007829|PDB:5MY2"
FT   HELIX           416..418
FT                   /evidence="ECO:0007829|PDB:5MY2"
FT   STRAND          422..430
FT                   /evidence="ECO:0007829|PDB:5MY2"
FT   HELIX           431..443
FT                   /evidence="ECO:0007829|PDB:5MY2"
FT   TURN            444..446
FT                   /evidence="ECO:0007829|PDB:5MY2"
FT   HELIX           448..456
FT                   /evidence="ECO:0007829|PDB:5MY2"
FT   TURN            462..464
FT                   /evidence="ECO:0007829|PDB:5MY2"
FT   STRAND          467..477
FT                   /evidence="ECO:0007829|PDB:5MY2"
FT   STRAND          480..484
FT                   /evidence="ECO:0007829|PDB:5MY2"
FT   STRAND          492..496
FT                   /evidence="ECO:0007829|PDB:5MY2"
FT   TURN            504..507
FT                   /evidence="ECO:0007829|PDB:5MY2"
FT   HELIX           509..512
FT                   /evidence="ECO:0007829|PDB:5MY2"
FT   HELIX           514..527
FT                   /evidence="ECO:0007829|PDB:5MY2"
FT   TURN            528..531
FT                   /evidence="ECO:0007829|PDB:5MY2"
FT   HELIX           534..539
FT                   /evidence="ECO:0007829|PDB:5MY2"
FT   HELIX           545..547
FT                   /evidence="ECO:0007829|PDB:5MY2"
FT   HELIX           549..569
FT                   /evidence="ECO:0007829|PDB:5MY2"
FT   STRAND          575..579
FT                   /evidence="ECO:0007829|PDB:5MY2"
FT   HELIX           583..590
FT                   /evidence="ECO:0007829|PDB:5MY2"
FT   HELIX           596..611
FT                   /evidence="ECO:0007829|PDB:5MY2"
FT   TURN            612..614
FT                   /evidence="ECO:0007829|PDB:5MY0"
FT   STRAND          618..625
FT                   /evidence="ECO:0007829|PDB:5MY2"
FT   HELIX           627..633
FT                   /evidence="ECO:0007829|PDB:5MY2"
FT   STRAND          639..643
FT                   /evidence="ECO:0007829|PDB:5MY2"
FT   STRAND          645..654
FT                   /evidence="ECO:0007829|PDB:5MY2"
FT   HELIX           655..658
FT                   /evidence="ECO:0007829|PDB:5MY2"
FT   HELIX           660..666
FT                   /evidence="ECO:0007829|PDB:5MY2"
FT   TURN            667..669
FT                   /evidence="ECO:0007829|PDB:5MY2"
FT   STRAND          672..675
FT                   /evidence="ECO:0007829|PDB:5MY2"
FT   HELIX           685..687
FT                   /evidence="ECO:0007829|PDB:5MY2"
FT   TURN            688..690
FT                   /evidence="ECO:0007829|PDB:5MY2"
FT   HELIX           691..701
FT                   /evidence="ECO:0007829|PDB:5MY2"
FT   STRAND          702..704
FT                   /evidence="ECO:0007829|PDB:5MY2"
FT   STRAND          715..717
FT                   /evidence="ECO:0007829|PDB:5MY2"
FT   HELIX           719..721
FT                   /evidence="ECO:0007829|PDB:5MY2"
FT   STRAND          722..724
FT                   /evidence="ECO:0007829|PDB:5MY2"
FT   HELIX           725..728
FT                   /evidence="ECO:0007829|PDB:5MY2"
FT   HELIX           732..740
FT                   /evidence="ECO:0007829|PDB:5MY2"
FT   HELIX           745..749
FT                   /evidence="ECO:0007829|PDB:5MY2"
FT   STRAND          757..764
FT                   /evidence="ECO:0007829|PDB:5MY2"
FT   HELIX           768..774
FT                   /evidence="ECO:0007829|PDB:5MY2"
FT   STRAND          779..782
FT                   /evidence="ECO:0007829|PDB:5MY2"
FT   HELIX           792..804
FT                   /evidence="ECO:0007829|PDB:5MY2"
FT   TURN            805..807
FT                   /evidence="ECO:0007829|PDB:5MY2"
FT   HELIX           812..815
FT                   /evidence="ECO:0007829|PDB:5MY2"
FT   HELIX           831..833
FT                   /evidence="ECO:0007829|PDB:5MY2"
FT   HELIX           847..849
FT                   /evidence="ECO:0007829|PDB:5MY2"
SQ   SEQUENCE   2504 AA;  272428 MW;  2B48068B9D370C6F CRC64;
     MEEVVIAGMS GKLPESENLQ EFWANLIGGV DMVTDDDRRW KAGLYGLPKR SGKLKDLSKF
     DASFFGVHPK QAHTMDPQLR LLLEVSYEAI VDGGINPASL RGTNTGVWVG VSGSEASEAL
     SRDPETLLGY SMVGCQRAMM ANRLSFFFDF KGPSIALDTA CSSSLLALQN AYQAIRSGEC
     PAALVGGINL LLKPNTSVQF MKLGMLSPDG TCRSFDDSGS GYCRSEAVVA VLLTKKSLAR
     RVYATILNAG TNTDGSKEQG VTFPSGEVQE QLICSLYQPA GLAPESLEYI EAHGTGTKVG
     DPQELNGITR SLCAFRQAPL LIGSTKSNMG HPEPASGLAA LTKVLLSLEH GVWAPNLHFH
     NPNPEIPALL DGRLQVVDRP LPVRGGNVGI NSFGFGGSNV HVILQPNTRQ APAPTAHAAL
     PHLLHASGRT LEAVQDLLEQ GRQHSQDLAF VSMLNDIAAT PTAAMPFRGY TVLGVEGRVQ
     EVQQVSTNKR PLWFICSGMG TQWRGMGLSL MRLDSFRESI LRSDEAVKPL GVKVSDLLLS
     TDERTFDDIV HAFVSLTAIQ IALIDLLTSV GLKPDGIIGH SLGEVACGYA DGCLSQREAV
     LAAYWRGQCI KDAHLPPGSM AAVGLSWEEC KQRCPAGVVP ACHNSEDTVT ISGPQAAVNE
     FVEQLKQEGV FAKEVRTGGL AFHSYFMEGI APTLLQALKK VIREPRPRSA RWLSTSIPEA
     QWQSSLARTS SAEYNVNNLV SPVLFQEALW HIPEHAVVLE IAPHALLQAV LKRGVKSSCT
     IIPLMKRDHK DNLEFFLTNL GKVHLTGINV NPNALFPPVE FPAPRGTPLI SPHIKWDHSQ
     TWDVPVAEDF PNGSSSSSAT VYSIDASPES PDHYLVDHCI DGRVIFPGTG YLCLVWKTLA
     RSLGLSLEET PVVFENVSFH QATILPKTGT VALEVRLLEA SHAFEVSDTG NLIVSGKVYL
     WEDPNSKLFD HPEVPTPPES ASVSRLTQGE VYKELRLRGY DYGPQFQGIC EATLEGEQGK
     LLWKDNWVTF MDTMLQVSIL GSSQQSLQLP TRVTAIYIDP ATHRQKVYRL KEDTQVADVT
     TSRCLGITVS GGIHISRLQT TATSRRQQEQ LVPTLEKFVF TPHMEAECLS ESTALQKELQ
     LCKGLARALQ TKATQQGLKA AMLGQEDPPQ HGLPRLLAAA CQLQLNGNLQ LELGEALAQE
     RLLLPEDPLI SGLLNSQALK ACVDTALENL STLKMKVAEV LAGEGHLYSR IPALLNTQPM
     LQLEYTATDR HPQALKDVQT KLQQHDVAQG QWNPSDPAPS SLGALDLLVC NCALATLGDP
     ALALDNMVAA LKEGGFLLVH TVLKGHALGE TLACLPSEVQ PAPSLLSQEE WESLFSRKAL
     HLVGLKRSFY GTALFLCRRA IPQEKPIFLS VEDTSFQWVD SLKSTLATSS SQPVWLTAMD
     CPTSGVVGLV NCLRKEPGGH RIRCILLSNL SNTSHAPKLD PGSPELQQVL KHDLVMNVYR
     DGAWGAFRHF QLEQDKPKEQ TAHAFVNVLT RGDLASIRWV SSPLKHTQPS SSGAQLCTVY
     YASLNFRDIM LATGKLSPDA IPGKWASRDC MLGMEFSGRD RCGRRVMGLV PAEGLATSVL
     LSSDFLWDVP SSWTLEEAAS VPVVYTTAYY SLVVRGRIQR GETVLIHSGS GGVGQAAISI
     ALSLGCRVFT TVGSAEKRAY LQARFPQLDD TSFANSRDTS FEQHVLLHTG GKGVDLVLNS
     LAEEKLQASV RCLAQHGRFL EIGKFDLSNN HPLGMAIFLK NVTFHGILLD ALFEEANDSW
     REVAALLKAG IRDGVVKPLK CTVFPKAQVE DAFRYMAQGK HIGKVLVQVR EEEPEAVLPG
     AQPTLISAIS KTFCPAHKSY IITGGLGGFG LELARWLVLR GAQRLVLTSR SGIRTGYQAK
     HIREWRRQGI QVLVSTSNVS SLEGARALIA EATKLGPVGG VFNLAMVLRD AMLENQTPEL
     FQDVNKPKYN GTLNLDRATR EACPELDYFV AFSSVSCGRG NAGQTNYGFA NSTMERICEQ
     RRHDGLPGLA VQWGAIGDVG IVLEAMGTND TVIGGTLPQR ISSCMEVLDL FLNQPHAVLS
     SFVLAEKKAV AHGDGDTQRD LVKAVAHILG IRDLAGINLD STLADLGLDS LMGVEVRQIL
     EREHDLVLPM REVRQLTLRK LQEMSSKTDS ATDTTAPKSR SDTSLKQNQL NLSTLLVNPE
     GPTLTQLNSV QSSERPLFLV HPIEGSTTVF HSLAAKLSVP TYGLQCTQAA PLDSIPNLAA
     YYIDCIKQVQ PEGPYRIAGY SFGACVAFEM CSQLQAQQGP APTHNNLFLF DGSHTYVLAY
     TQSYRAKMTP GCEAEAEAEA LCFFIKQFLD VEHSKVLEAL LPLKSLEDRV AASVDLITKS
     HHSLDRRELS FAAVSFYHKL RAADQYKPKA KYHGNVTLLR AKTGGTYGED LGADYNLSQV
     CDGKVSVHII EGDHRTLLEG SGLESIINII HSSLAEPRVS VREG
//
```

**Mascot:** http://www.matrixscience.com/


HNE (C) (+156.1150)
