# Supplementary material for: A common East-Asian ALDH2 mutation causes metabolic disorders and the therapeutic effect of ALDH2 activators
Source: Nat Commun. 2023 Sep 25;14:5971. doi: 10.1038/s41467-023-41570-6 (PMC10520061; doi:10.1038/s41467-023-41570-6)
Supplement: Supplementary file 4 — Supplementary Data 1 [file 41467_2023_41570_MOESM4_ESM.zip › Table S5c/P19096/P19096_KIO60-3_H789.html]

Mascot Search Results: P19096
 

# MASCOT Search Results

## Protein View: P19096

### Fatty acid synthase OS=Mus musculus OX=10090 GN=Fasn PE=1 SV=2

|  |  |
| --- | --- |
| Database: | Mouse\_UniProt\_proteomes |
| Score: | 5520 |
| Monoisotopic mass (M_r_): | 274994 |
| Calculated pI: | 6.13 |

Sequence similarity is available as an NCBI BLAST search of P19096 against nr.

### Search parameters

|  |  |
| --- | --- |
| MS data file: | `D:\LCMSMS\2023 Users' data\230529-1\230529-1-KI60-4_20230609100525.raw` |
| Enzyme: | Trypsin/P: cuts C-term side of KR. |
| Fixed modifications: | Carbamidomethyl (C) |
| Variable modifications: | Deamidated (NQ), HNE (C), HNE (H), HNE (K), Oxidation (M) |

### Protein sequence coverage: 33%

Matched peptides shown in ***bold red***.

|  |  |  |  |  |  |
| --- | --- | --- | --- | --- | --- |
| `1` | `MEEVVIAGMS` | `GKLPESENLQ` | `EFWANLIGGV` | `DMVTDDDRRW` | `KAGLYGLPKR` |
| `51` | `SGKLKDLSKF` | `DASFFGVHPK` | `QAHTMDPQLR` | `LLLEVSYEAI` | `VDGGINPASL` |
| `101` | `RGTNTGVWVG` | `VSGSEASEAL` | `SRDPETLLGY` | `SMVGCQRAMM` | `ANRLSFFFDF` |
| `151` | `KGPSIALDTA` | `CSSSLLALQN` | `AYQAIRSGEC` | `PAALVGGINL` | `LLKPNTSVQF` |
| `201` | `MKLGMLSPDG` | `TCRSFDDSGS` | `GYCRSEAVVA` | `VLLTKKSLAR` | `RVYATILNAG` |
| `251` | `TNTDGSKEQG` | `VTFPSGEVQE` | `QLICSLYQPA` | `GLAPESLEYI` | `EAHGTGTKVG` |
| `301` | `DPQELNGITR` | `SLCAFRQAPL` | `LIGSTKSNMG` | `HPEPASGLAA` | `LTKVLLSLEH` |
| `351` | `GVWAPNLHFH` | `NPNPEIPALL` | `DGRLQVVDRP` | `LPVRGGNVGI` | `NSFGFGGSNV` |
| `401` | `HVILQPNTRQ` | `APAPTAHAAL` | `PHLLHASGRT` | `LEAVQDLLEQ` | `GRQHSQDLAF` |
| `451` | `VSMLNDIAAT` | `PTAAMPFRGY` | `TVLGVEGRVQ` | `EVQQVSTNKR` | `PLWFICSGMG` |
| `501` | `TQWRGMGLSL` | `MRLDSFRESI` | `LRSDEAVKPL` | `GVKVSDLLLS` | `TDERTFDDIV` |
| `551` | `HAFVSLTAIQ` | `IALIDLLTSV` | `GLKPDGIIGH` | `SLGEVACGYA` | `DGCLSQREAV` |
| `601` | `LAAYWRGQCI` | `KDAHLPPGSM` | `AAVGLSWEEC` | `KQRCPAGVVP` | `ACHNSEDTVT` |
| `651` | `ISGPQAAVNE` | `FVEQLKQEGV` | `FAKEVRTGGL` | `AFHSYFMEGI` | `APTLLQALKK` |
| `701` | `VIREPRPRSA` | `RWLSTSIPEA` | `QWQSSLARTS` | `SAEYNVNNLV` | `SPVLFQEALW` |
| `751` | `HIPEHAVVLE` | `IAPHALLQAV` | `LKRGVKSSCT` | `IIPLMKRDHK` | `DNLEFFLTNL` |
| `801` | `GKVHLTGINV` | `NPNALFPPVE` | `FPAPRGTPLI` | `SPHIKWDHSQ` | `TWDVPVAEDF` |
| `851` | `PNGSSSSSAT` | `VYSIDASPES` | `PDHYLVDHCI` | `DGRVIFPGTG` | `YLCLVWKTLA` |
| `901` | `RSLGLSLEET` | `PVVFENVSFH` | `QATILPKTGT` | `VALEVRLLEA` | `SHAFEVSDTG` |
| `951` | `NLIVSGKVYL` | `WEDPNSKLFD` | `HPEVPTPPES` | `ASVSRLTQGE` | `VYKELRLRGY` |
| `1001` | `DYGPQFQGIC` | `EATLEGEQGK` | `LLWKDNWVTF` | `MDTMLQVSIL` | `GSSQQSLQLP` |
| `1051` | `TRVTAIYIDP` | `ATHRQKVYRL` | `KEDTQVADVT` | `TSRCLGITVS` | `GGIHISRLQT` |
| `1101` | `TATSRRQQEQ` | `LVPTLEKFVF` | `TPHMEAECLS` | `ESTALQKELQ` | `LCKGLARALQ` |
| `1151` | `TKATQQGLKA` | `AMLGQEDPPQ` | `HGLPRLLAAA` | `CQLQLNGNLQ` | `LELGEALAQE` |
| `1201` | `RLLLPEDPLI` | `SGLLNSQALK` | `ACVDTALENL` | `STLKMKVAEV` | `LAGEGHLYSR` |
| `1251` | `IPALLNTQPM` | `LQLEYTATDR` | `HPQALKDVQT` | `KLQQHDVAQG` | `QWNPSDPAPS` |
| `1301` | `SLGALDLLVC` | `NCALATLGDP` | `ALALDNMVAA` | `LKEGGFLLVH` | `TVLKGHALGE` |
| `1351` | `TLACLPSEVQ` | `PAPSLLSQEE` | `WESLFSRKAL` | `HLVGLKRSFY` | `GTALFLCRRA` |
| `1401` | `IPQEKPIFLS` | `VEDTSFQWVD` | `SLKSTLATSS` | `SQPVWLTAMD` | `CPTSGVVGLV` |
| `1451` | `NCLRKEPGGH` | `RIRCILLSNL` | `SNTSHAPKLD` | `PGSPELQQVL` | `KHDLVMNVYR` |
| `1501` | `DGAWGAFRHF` | `QLEQDKPKEQ` | `TAHAFVNVLT` | `RGDLASIRWV` | `SSPLKHTQPS` |
| `1551` | `SSGAQLCTVY` | `YASLNFRDIM` | `LATGKLSPDA` | `IPGKWASRDC` | `MLGMEFSGRD` |
| `1601` | `RCGRRVMGLV` | `PAEGLATSVL` | `LSSDFLWDVP` | `SSWTLEEAAS` | `VPVVYTTAYY` |
| `1651` | `SLVVRGRIQR` | `GETVLIHSGS` | `GGVGQAAISI` | `ALSLGCRVFT` | `TVGSAEKRAY` |
| `1701` | `LQARFPQLDD` | `TSFANSRDTS` | `FEQHVLLHTG` | `GKGVDLVLNS` | `LAEEKLQASV` |
| `1751` | `RCLAQHGRFL` | `EIGKFDLSNN` | `HPLGMAIFLK` | `NVTFHGILLD` | `ALFEEANDSW` |
| `1801` | `REVAALLKAG` | `IRDGVVKPLK` | `CTVFPKAQVE` | `DAFRYMAQGK` | `HIGKVLVQVR` |
| `1851` | `EEEPEAVLPG` | `AQPTLISAIS` | `KTFCPAHKSY` | `IITGGLGGFG` | `LELARWLVLR` |
| `1901` | `GAQRLVLTSR` | `SGIRTGYQAK` | `HIREWRRQGI` | `QVLVSTSNVS` | `SLEGARALIA` |
| `1951` | `EATKLGPVGG` | `VFNLAMVLRD` | `AMLENQTPEL` | `FQDVNKPKYN` | `GTLNLDRATR` |
| `2001` | `EACPELDYFV` | `AFSSVSCGRG` | `NAGQTNYGFA` | `NSTMERICEQ` | `RRHDGLPGLA` |
| `2051` | `VQWGAIGDVG` | `IVLEAMGTND` | `TVIGGTLPQR` | `ISSCMEVLDL` | `FLNQPHAVLS` |
| `2101` | `SFVLAEKKAV` | `AHGDGDTQRD` | `LVKAVAHILG` | `IRDLAGINLD` | `STLADLGLDS` |
| `2151` | `LMGVEVRQIL` | `EREHDLVLPM` | `REVRQLTLRK` | `LQEMSSKTDS` | `ATDTTAPKSR` |
| `2201` | `SDTSLKQNQL` | `NLSTLLVNPE` | `GPTLTQLNSV` | `QSSERPLFLV` | `HPIEGSTTVF` |
| `2251` | `HSLAAKLSVP` | `TYGLQCTQAA` | `PLDSIPNLAA` | `YYIDCIKQVQ` | `PEGPYRIAGY` |
| `2301` | `SFGACVAFEM` | `CSQLQAQQGP` | `APTHNNLFLF` | `DGSHTYVLAY` | `TQSYRAKMTP` |
| `2351` | `GCEAEAEAEA` | `LCFFIKQFLD` | `VEHSKVLEAL` | `LPLKSLEDRV` | `AASVDLITKS` |
| `2401` | `HHSLDRRELS` | `FAAVSFYHKL` | `RAADQYKPKA` | `KYHGNVTLLR` | `AKTGGTYGED` |
| `2451` | `LGADYNLSQV` | `CDGKVSVHII` | `EGDHRTLLEG` | `SGLESIINII` | `HSSLAEPRVS` |
| `2501` | `VREG` |  |  |  |  |

Unformatted sequence string: 2504 residues (for pasting into other applications).

|  |  |  |  |
| --- | --- | --- | --- |
| Sort by | residue number | increasing mass | decreasing mass |
| Show | matched peptides only | predicted peptides also |  |

| Query | Start | – | End | Observed | Mr(expt) | Mr(calc) | ppm | M | Score | Expect | Rank | U | Peptide |
| --- | --- | --- | --- | --- | --- | --- | --- | --- | --- | --- | --- | --- | --- |
| 3950 | 42 | – | 49 | 409.7423 | 817.4700 | 817.4698 | 0.27 | 0 | 43 | 0.0015 | 1Score **> 28** indicates **identity** | U | K.AGLYGLPK.R |
| 3951 | 42 | – | 49 | 409.7424 | 817.4703 | 817.4698 | 0.64 | 0 | 33 | 0.0034 | 1Score **> 28** indicates **identity** Score **> 21** indicates **homology** | U | K.AGLYGLPK.R |
| 3952 | 42 | – | 49 | 409.7426 | 817.4706 | 817.4698 | 1.05 | 0 | 43 | 0.0015 | 1Score **> 28** indicates **identity** | U | K.AGLYGLPK.R |
| 3953 | 42 | – | 49 | 409.7429 | 817.4712 | 817.4698 | 1.78 | 0 | 27 | 0.0075 | 1Score **> 28** indicates **identity** Score **> 18** indicates **homology** | U | K.AGLYGLPK.R |
| 93462 | 54 | – | 70 | 484.7642 | 1935.0279 | 1935.0254 | 1.27 | 2 | 26 | 0.0039 | 1Score **> 36** indicates **identity** Score **> 14** indicates **homology** | U | K.LKDLSKFDASFFGVHPK.Q |
| 93466 | 54 | – | 70 | 484.7645 | 1935.0287 | 1935.0254 | 1.71 | 2 | 23 | 0.0077 | 1Score **> 36** indicates **identity** Score **> 14** indicates **homology** | U | K.LKDLSKFDASFFGVHPK.Q |
| 33638 | 60 | – | 70 | 417.8774 | 1250.6102 | 1250.6084 | 1.45 | 0 | 39 | 0.00021 | 1Score **> 32** indicates **identity** Score **> 15** indicates **homology** | U | K.FDASFFGVHPK.Q |
| 33639 | 60 | – | 70 | 626.3124 | 1250.6103 | 1250.6084 | 1.50 | 0 | 41 | 0.00015 | 1Score **> 32** indicates **identity** Score **> 15** indicates **homology** | U | K.FDASFFGVHPK.Q |
| 33640 | 60 | – | 70 | 417.8774 | 1250.6104 | 1250.6084 | 1.56 | 0 | 35 | 0.00057 | 1Score **> 32** indicates **identity** Score **> 15** indicates **homology** | U | K.FDASFFGVHPK.Q |
| 33641 | 60 | – | 70 | 626.3125 | 1250.6104 | 1250.6084 | 1.58 | 0 | 57 | 4.3e-06 | 1Score **> 32** indicates **identity** Score **> 16** indicates **homology** | U | K.FDASFFGVHPK.Q |
| 33646 | 60 | – | 70 | 626.3128 | 1250.6111 | 1250.6084 | 2.13 | 0 | 34 | 0.00069 | 1Score **> 32** indicates **identity** Score **> 15** indicates **homology** | U | K.FDASFFGVHPK.Q |
| 33647 | 60 | – | 70 | 417.8777 | 1250.6112 | 1250.6084 | 2.23 | 0 | 25 | 0.0045 | 1Score **> 32** indicates **identity** Score **> 14** indicates **homology** | U | K.FDASFFGVHPK.Q |
| 28596 | 71 | – | 80 | 399.5321 | 1195.5745 | 1195.5768 | -1.94 | 0 | 27 | 0.0032 | 1Score **> 30** indicates **identity** Score **> 14** indicates **homology** | U | K.QAHTMDPQLR.L |
| 33515 | 214 | – | 224 | 625.7413 | 1249.4681 | 1249.4670 | 0.88 | 0 | 30 | 0.002 | 1Score **> 16** indicates **identity** | U | R.SFDDSGSGYCR.S |
| 33516 | 214 | – | 224 | 625.7417 | 1249.4688 | 1249.4670 | 1.46 | 0 | 60 | 2.4e-06 | 1Score **> 16** indicates **identity** | U | R.SFDDSGSGYCR.S |
| 33517 | 214 | – | 224 | 625.7418 | 1249.4690 | 1249.4670 | 1.58 | 0 | 35 | 0.00072 | 1Score **> 16** indicates **identity** | U | R.SFDDSGSGYCR.S |
| 119181 | 214 | – | 236 | 623.0645 | 2488.2287 | 2488.2268 | 0.78 | 2 | 33 | 0.00079 | 1Score **> 37** indicates **identity** Score **> 15** indicates **homology** | U | R.SFDDSGSGYCRSEAVVAVLLTKK.S |
| 119184 | 214 | – | 236 | 623.0653 | 2488.2320 | 2488.2268 | 2.09 | 2 | 34 | 0.00066 | 1Score **> 37** indicates **identity** Score **> 15** indicates **homology** | U | R.SFDDSGSGYCRSEAVVAVLLTKK.S |
| 22090 | 225 | – | 235 | 565.3458 | 1128.6771 | 1128.6754 | 1.48 | 0 | 58 | 3.4e-06 | 1Score **> 31** indicates **identity** Score **> 16** indicates **homology** | U | R.SEAVVAVLLTK.K |
| 22092 | 225 | – | 235 | 565.3459 | 1128.6773 | 1128.6754 | 1.68 | 0 | 54 | 8.2e-06 | 1Score **> 31** indicates **identity** Score **> 16** indicates **homology** | U | R.SEAVVAVLLTK.K |
| 22093 | 225 | – | 235 | 565.3461 | 1128.6777 | 1128.6754 | 2.02 | 0 | 40 | 0.00016 | 1Score **> 31** indicates **identity** Score **> 15** indicates **homology** | U | R.SEAVVAVLLTK.K |
| 34302 | 225 | – | 236 | 629.3931 | 1256.7717 | 1256.7704 | 1.04 | 1 | 26 | 0.0039 | 1Score **> 30** indicates **identity** Score **> 14** indicates **homology** | U | R.SEAVVAVLLTKK.S |
| 34304 | 225 | – | 236 | 419.9316 | 1256.7730 | 1256.7704 | 2.05 | 1 | 48 | 3.3e-05 | 1Score **> 30** indicates **identity** Score **> 15** indicates **homology** | U | R.SEAVVAVLLTKK.S |
| 34305 | 225 | – | 236 | 419.9316 | 1256.7730 | 1256.7704 | 2.06 | 1 | 18 | 0.022 | 1Score **> 30** indicates **identity** Score **> 14** indicates **homology** | U | R.SEAVVAVLLTKK.S |
| 34306 | 225 | – | 236 | 629.3938 | 1256.7730 | 1256.7704 | 2.08 | 1 | 47 | 4e-05 | 1Score **> 30** indicates **identity** Score **> 15** indicates **homology** | U | R.SEAVVAVLLTKK.S |
| 34307 | 225 | – | 236 | 629.3939 | 1256.7732 | 1256.7704 | 2.22 | 1 | 68 | 3.8e-07 | 1Score **> 30** indicates **identity** Score **> 17** indicates **homology** | U | R.SEAVVAVLLTKK.S |
| 34309 | 225 | – | 236 | 419.9321 | 1256.7743 | 1256.7704 | 3.16 | 1 | 45 | 5.8e-05 | 1Score **> 30** indicates **identity** Score **> 15** indicates **homology** | U | R.SEAVVAVLLTKK.S |
| 68072 | 242 | – | 257 | 812.9114 | 1623.8082 | 1623.8104 | -1.36 | 0 | 15 | 0.037 | 1Score **> 34** indicates **identity** Score **> 13** indicates **homology** | U | R.VYATILNAGTNTDGSK.E |
| 68076 | 242 | – | 257 | 812.9135 | 1623.8124 | 1623.8104 | 1.19 | 0 | 48 | 3e-05 | 1Score **> 34** indicates **identity** Score **> 16** indicates **homology** | U | R.VYATILNAGTNTDGSK.E |
| 38091 | 299 | – | 310 | 649.8392 | 1297.6638 | 1297.6626 | 0.89 | 0 | 79 | 5e-08 | 1Score **> 33** indicates **identity** Score **> 18** indicates **homology** | U | K.VGDPQELNGITR.S |
| 38093 | 299 | – | 310 | 649.8394 | 1297.6642 | 1297.6626 | 1.23 | 0 | 75 | 9.1e-08 | 1Score **> 33** indicates **identity** Score **> 18** indicates **homology** | U | K.VGDPQELNGITR.S |
| 38094 | 299 | – | 310 | 649.8398 | 1297.6651 | 1297.6626 | 1.90 | 0 | 43 | 0.00019 | 1Score **> 33** indicates **identity** Score **> 18** indicates **homology** | U | K.VGDPQELNGITR.S |
| 38185 | 299 | – | 310 | 650.3309 | 1298.6472 | 1298.6466 | 0.41 | 0 | 40 | 0.00019 | 1Score **> 32** indicates **identity** Score **> 16** indicates **homology** | U | K.VGDPQELNGITR.S  + Deamidated (NQ) |
| 38186 | 299 | – | 310 | 650.3312 | 1298.6478 | 1298.6466 | 0.89 | 0 | 37 | 0.00032 | 1Score **> 32** indicates **identity** Score **> 15** indicates **homology** | U | K.VGDPQELNGITR.S  + Deamidated (NQ) |
| 38187 | 299 | – | 310 | 650.3319 | 1298.6492 | 1298.6466 | 2.01 | 0 | 65 | 3.6e-06 | 1Score **> 32** indicates **identity** Score **> 23** indicates **homology** | U | K.VGDPQELNGITR.S  + Deamidated (NQ) |
| 14438 | 317 | – | 326 | 514.3116 | 1026.6087 | 1026.6073 | 1.30 | 0 | 50 | 2.3e-05 | 1Score **> 30** indicates **identity** Score **> 16** indicates **homology** | U | R.QAPLLIGSTK.S |
| 14439 | 317 | – | 326 | 514.3116 | 1026.6087 | 1026.6073 | 1.32 | 0 | 51 | 1.8e-05 | 1Score **> 30** indicates **identity** Score **> 16** indicates **homology** | U | R.QAPLLIGSTK.S |
| 14440 | 317 | – | 326 | 514.3119 | 1026.6092 | 1026.6073 | 1.84 | 0 | 31 | 0.0012 | 1Score **> 30** indicates **identity** Score **> 14** indicates **homology** | U | R.QAPLLIGSTK.S |
| 73734 | 327 | – | 343 | 560.9519 | 1679.8339 | 1679.8301 | 2.26 | 0 | 83 | 1.8e-08 | 1Score **> 35** indicates **identity** Score **> 18** indicates **homology** | U | K.SNMGHPEPASGLAALTK.V |
| 75335 | 327 | – | 343 | 566.2846 | 1695.8320 | 1695.8250 | 4.11 | 0 | 23 | 0.0076 | 1Score **> 35** indicates **identity** Score **> 14** indicates **homology** | U | K.SNMGHPEPASGLAALTK.V  + Oxidation (M) |
| 37502 | 374 | – | 384 | 431.2662 | 1290.7769 | 1290.7772 | -0.27 | 1 | 15 | 0.04 | 1Score **> 29** indicates **identity** Score **> 13** indicates **homology** | U | R.LQVVDRPLPVR.G |
| 37503 | 374 | – | 384 | 431.2665 | 1290.7777 | 1290.7772 | 0.35 | 1 | 35 | 0.00049 | 1Score **> 29** indicates **identity** Score **> 15** indicates **homology** | U | R.LQVVDRPLPVR.G |
| 37504 | 374 | – | 384 | 431.2666 | 1290.7778 | 1290.7772 | 0.50 | 1 | 18 | 0.02 | 1Score **> 29** indicates **identity** Score **> 14** indicates **homology** | U | R.LQVVDRPLPVR.G |
| 37507 | 374 | – | 384 | 431.2667 | 1290.7783 | 1290.7772 | 0.84 | 1 | 16 | 0.032 | 1Score **> 30** indicates **identity** Score **> 14** indicates **homology** | U | R.LQVVDRPLPVR.G |
| 37508 | 374 | – | 384 | 431.2671 | 1290.7796 | 1290.7772 | 1.84 | 1 | 14 | 0.047 | 1Score **> 29** indicates **identity** Score **> 13** indicates **homology** | U | R.LQVVDRPLPVR.G |
| 120276 | 385 | – | 409 | 847.7705 | 2540.2896 | 2540.2885 | 0.47 | 0 | 41 | 0.00014 | 1Score **> 37** indicates **identity** Score **> 15** indicates **homology** | U | R.GGNVGINSFGFGGSNVHVILQPNTR.Q |
| 120279 | 385 | – | 409 | 847.7722 | 2540.2947 | 2540.2885 | 2.47 | 0 | 38 | 0.00029 | 1Score **> 37** indicates **identity** Score **> 15** indicates **homology** | U | R.GGNVGINSFGFGGSNVHVILQPNTR.Q |
| 98733 | 410 | – | 429 | 504.7781 | 2015.0831 | 2015.0813 | 0.91 | 0 | 35 | 0.00054 | 1Score **> 36** indicates **identity** Score **> 15** indicates **homology** | U | R.QAPAPTAHAALPHLLHASGR.T |
| 98735 | 410 | – | 429 | 404.0241 | 2015.0841 | 2015.0813 | 1.39 | 0 | 37 | 0.00036 | 1Score **> 36** indicates **identity** Score **> 15** indicates **homology** | U | R.QAPAPTAHAALPHLLHASGR.T |
| 98737 | 410 | – | 429 | 504.7784 | 2015.0844 | 2015.0813 | 1.52 | 0 | 50 | 2e-05 | 1Score **> 36** indicates **identity** Score **> 16** indicates **homology** | U | R.QAPAPTAHAALPHLLHASGR.T |
| 98738 | 410 | – | 429 | 404.0242 | 2015.0845 | 2015.0813 | 1.60 | 0 | 44 | 7.6e-05 | 1Score **> 36** indicates **identity** Score **> 15** indicates **homology** | U | R.QAPAPTAHAALPHLLHASGR.T |
| 98740 | 410 | – | 429 | 404.0242 | 2015.0847 | 2015.0813 | 1.67 | 0 | 27 | 0.0029 | 1Score **> 36** indicates **identity** Score **> 14** indicates **homology** | U | R.QAPAPTAHAALPHLLHASGR.T |
| 98741 | 410 | – | 429 | 504.7784 | 2015.0847 | 2015.0813 | 1.69 | 0 | 33 | 0.00082 | 1Score **> 36** indicates **identity** Score **> 15** indicates **homology** | U | R.QAPAPTAHAALPHLLHASGR.T |
| 98742 | 410 | – | 429 | 404.0243 | 2015.0850 | 2015.0813 | 1.82 | 0 | 38 | 0.00027 | 1Score **> 36** indicates **identity** Score **> 15** indicates **homology** | U | R.QAPAPTAHAALPHLLHASGR.T |
| 98745 | 410 | – | 429 | 404.0245 | 2015.0862 | 2015.0813 | 2.42 | 0 | 44 | 7.4e-05 | 1Score **> 36** indicates **identity** Score **> 15** indicates **homology** | U | R.QAPAPTAHAALPHLLHASGR.T |
| 53382 | 430 | – | 442 | 736.3893 | 1470.7640 | 1470.7678 | -2.58 | 0 | 56 | 2.3e-05 | 1Score **> 34** indicates **identity** Score **> 22** indicates **homology** | U | R.TLEAVQDLLEQGR.Q |
| 53383 | 430 | – | 442 | 736.3895 | 1470.7645 | 1470.7678 | -2.27 | 0 | 26 | 0.0035 | 1Score **> 34** indicates **identity** Score **> 14** indicates **homology** | U | R.TLEAVQDLLEQGR.Q |
| 53392 | 430 | – | 442 | 736.3917 | 1470.7688 | 1470.7678 | 0.70 | 0 | 68 | 5.3e-07 | 1Score **> 34** indicates **identity** Score **> 18** indicates **homology** | U | R.TLEAVQDLLEQGR.Q |
| 53395 | 430 | – | 442 | 736.3924 | 1470.7702 | 1470.7678 | 1.65 | 0 | 80 | 3.8e-08 | 1Score **> 34** indicates **identity** Score **> 18** indicates **homology** | U | R.TLEAVQDLLEQGR.Q |
| 53396 | 430 | – | 442 | 736.3926 | 1470.7707 | 1470.7678 | 1.96 | 0 | 79 | 1.9e-07 | 1Score **> 34** indicates **identity** Score **> 25** indicates **homology** | U | R.TLEAVQDLLEQGR.Q |
| 53397 | 430 | – | 442 | 736.3926 | 1470.7707 | 1470.7678 | 1.96 | 0 | 87 | 3.1e-08 | 1Score **> 34** indicates **identity** Score **> 25** indicates **homology** | U | R.TLEAVQDLLEQGR.Q |
| 53398 | 430 | – | 442 | 736.3928 | 1470.7711 | 1470.7678 | 2.22 | 0 | 88 | 9.3e-09 | 1Score **> 34** indicates **identity** Score **> 20** indicates **homology** | U | R.TLEAVQDLLEQGR.Q |
| 53399 | 430 | – | 442 | 736.3929 | 1470.7713 | 1470.7678 | 2.39 | 0 | 76 | 4.4e-07 | 1Score **> 34** indicates **identity** Score **> 25** indicates **homology** | U | R.TLEAVQDLLEQGR.Q |
| 15675 | 469 | – | 478 | 525.7828 | 1049.5511 | 1049.5506 | 0.52 | 0 | 41 | 0.00016 | 1Score **> 31** indicates **identity** Score **> 15** indicates **homology** | U | R.GYTVLGVEGR.V |
| 15677 | 469 | – | 478 | 525.7830 | 1049.5514 | 1049.5506 | 0.83 | 0 | 44 | 0.00029 | 1Score **> 32** indicates **identity** Score **> 21** indicates **homology** | U | R.GYTVLGVEGR.V |
| 15687 | 469 | – | 478 | 525.7834 | 1049.5522 | 1049.5506 | 1.54 | 0 | 52 | 7.3e-05 | 1Score **> 32** indicates **identity** Score **> 23** indicates **homology** | U | R.GYTVLGVEGR.V |
| 34452 | 479 | – | 489 | 630.3321 | 1258.6496 | 1258.6517 | -1.69 | 0 | 61 | 1.1e-05 | 1Score **> 34** indicates **identity** Score **> 24** indicates **homology** | U | R.VQEVQQVSTNK.R |
| 34453 | 479 | – | 489 | 630.3326 | 1258.6507 | 1258.6517 | -0.80 | 0 | 26 | 0.0067 | 1Score **> 34** indicates **identity** Score **> 17** indicates **homology** | U | R.VQEVQQVSTNK.R |
| 32355 | 513 | – | 522 | 412.5633 | 1234.6682 | 1234.6670 | 1.01 | 1 | 32 | 0.0093 | 1Score **> 32** indicates **identity** Score **> 24** indicates **homology** | U | R.LDSFRESILR.S |
| 23328 | 523 | – | 533 | 571.8238 | 1141.6331 | 1141.6343 | -1.01 | 1 | 19 | 0.015 | 1Score **> 31** indicates **identity** Score **> 14** indicates **homology** | U | R.SDEAVKPLGVK.V |
| 23329 | 523 | – | 533 | 381.5520 | 1141.6341 | 1141.6343 | -0.16 | 1 | 40 | 0.00016 | 1Score **> 32** indicates **identity** Score **> 15** indicates **homology** | U | R.SDEAVKPLGVK.V |
| 23330 | 523 | – | 533 | 571.8245 | 1141.6344 | 1141.6343 | 0.11 | 1 | 55 | 2.2e-05 | 1Score **> 32** indicates **identity** Score **> 21** indicates **homology** | U | R.SDEAVKPLGVK.V |
| 23331 | 523 | – | 533 | 571.8249 | 1141.6353 | 1141.6343 | 0.86 | 1 | 36 | 0.00069 | 1Score **> 32** indicates **identity** Score **> 17** indicates **homology** | U | R.SDEAVKPLGVK.V |
| 33334 | 534 | – | 544 | 624.3283 | 1246.6420 | 1246.6405 | 1.25 | 0 | 29 | 0.0027 | 1Score **> 33** indicates **identity** Score **> 16** indicates **homology** | U | K.VSDLLLSTDER.T |
| 33335 | 534 | – | 544 | 624.3287 | 1246.6428 | 1246.6405 | 1.84 | 0 | 47 | 0.00019 | 1Score **> 33** indicates **identity** Score **> 22** indicates **homology** | U | K.VSDLLLSTDER.T |
| 17733 | 598 | – | 606 | 539.7885 | 1077.5625 | 1077.5607 | 1.70 | 0 | 60 | 1.1e-05 | 1Score **> 32** indicates **identity** Score **> 23** indicates **homology** | U | R.EAVLAAYWR.G |
| 17734 | 598 | – | 606 | 539.7888 | 1077.5630 | 1077.5607 | 2.16 | 0 | 26 | 0.0073 | 1Score **> 32** indicates **identity** Score **> 17** indicates **homology** | U | R.EAVLAAYWR.G |
| 17736 | 598 | – | 606 | 539.7890 | 1077.5634 | 1077.5607 | 2.53 | 0 | 65 | 4e-06 | 1Score **> 33** indicates **identity** Score **> 24** indicates **homology** | U | R.EAVLAAYWR.G |
| 95066 | 712 | – | 728 | 980.5023 | 1958.9901 | 1958.9850 | 2.59 | 0 | 28 | 0.0023 | 1Score **> 36** indicates **identity** Score **> 14** indicates **homology** | U | R.WLSTSIPEAQWQSSLAR.T |
| 24111 | 777 | – | 786 | 575.3058 | 1148.5971 | 1148.5933 | 3.31 | 0 | 19 | 0.016 | 1Score **> 34** indicates **identity** Score **> 14** indicates **homology** | U | K.SSCTIIPLMK.R |
| 24114 | 777 | – | 786 | 575.3066 | 1148.5987 | 1148.5933 | 4.66 | 0 | 26 | 0.0083 | 1Score **> 33** indicates **identity** Score **> 17** indicates **homology** | U | K.SSCTIIPLMK.R |
| 24116 | 777 | – | 786 | 575.3072 | 1148.5998 | 1148.5933 | 5.63 | 0 | 33 | 0.0012 | 1Score **> 34** indicates **identity** Score **> 16** indicates **homology** | U | K.SSCTIIPLMK.R |
| 38865 | 777 | – | 786 | 435.9061 | 1304.6966 | 1304.7084 | -9.03 | 0 | 34 | 0.00072 | 1Score **> 34** indicates **identity** Score **> 15** indicates **homology** | U | K.SSCTIIPLMK.R  + HNE (K) |
| 83312 | 788 | – | 802 | 597.6412 | 1789.9017 | 1789.8999 | 1.00 | 1 | 24 | 0.0059 | 1Score **> 35** indicates **identity** Score **> 14** indicates **homology** | U | R.DHKDNLEFFLTNLGK.V |
| 83313 | 788 | – | 802 | 597.6415 | 1789.9027 | 1789.8999 | 1.56 | 1 | 32 | 0.001 | 1Score **> 35** indicates **identity** Score **> 14** indicates **homology** | U | R.DHKDNLEFFLTNLGK.V |
| 83314 | 788 | – | 802 | 597.6417 | 1789.9033 | 1789.8999 | 1.92 | 1 | 19 | 0.015 | 1Score **> 35** indicates **identity** Score **> 14** indicates **homology** | U | R.DHKDNLEFFLTNLGK.V |
| 94253 | 788 | – | 802 | 487.5072 | 1945.9999 | 1946.0149 | -7.72 | 1 | 24 | 0.0052 | 1Score **> 36** indicates **identity** Score **> 14** indicates **homology** | U | R.DHKDNLEFFLTNLGK.V  + HNE (H) |
| 119484 | 803 | – | 825 | 833.7898 | 2498.3475 | 2498.3434 | 1.65 | 0 | 40 | 0.00024 | 1Score **> 36** indicates **identity** Score **> 16** indicates **homology** | U | K.VHLTGINVNPNALFPPVEFPAPR.G |
| 17033 | 826 | – | 835 | 531.8194 | 1061.6243 | 1061.6233 | 0.92 | 0 | 42 | 0.00012 | 1Score **> 29** indicates **identity** Score **> 15** indicates **homology** | U | R.GTPLISPHIK.W |
| 17034 | 826 | – | 835 | 354.8821 | 1061.6245 | 1061.6233 | 1.10 | 0 | 18 | 0.019 | 1Score **> 29** indicates **identity** Score **> 14** indicates **homology** | U | R.GTPLISPHIK.W |
| 17035 | 826 | – | 835 | 531.8196 | 1061.6246 | 1061.6233 | 1.21 | 0 | 36 | 0.00038 | 1Score **> 29** indicates **identity** Score **> 15** indicates **homology** | U | R.GTPLISPHIK.W |
| 17036 | 826 | – | 835 | 531.8196 | 1061.6247 | 1061.6233 | 1.30 | 0 | 36 | 0.00038 | 1Score **> 29** indicates **identity** Score **> 15** indicates **homology** | U | R.GTPLISPHIK.W |
| 17037 | 826 | – | 835 | 354.8822 | 1061.6248 | 1061.6233 | 1.41 | 0 | 22 | 0.0087 | 1Score **> 29** indicates **identity** Score **> 14** indicates **homology** | U | R.GTPLISPHIK.W |
| 9498 | 928 | – | 936 | 473.2713 | 944.5280 | 944.5291 | -1.12 | 0 | 32 | 0.0013 | 1Score **> 31** indicates **identity** Score **> 15** indicates **homology** | U | K.TGTVALEVR.L |
| 9499 | 928 | – | 936 | 473.2723 | 944.5300 | 944.5291 | 0.99 | 0 | 63 | 6.1e-06 | 1Score **> 31** indicates **identity** Score **> 23** indicates **homology** | U | K.TGTVALEVR.L |
| 9500 | 928 | – | 936 | 473.2724 | 944.5302 | 944.5291 | 1.14 | 0 | 64 | 4.6e-06 | 1Score **> 31** indicates **identity** Score **> 23** indicates **homology** | U | K.TGTVALEVR.L |
| 9501 | 928 | – | 936 | 473.2724 | 944.5302 | 944.5291 | 1.21 | 0 | 50 | 4.5e-05 | 1Score **> 31** indicates **identity** Score **> 19** indicates **homology** | U | K.TGTVALEVR.L |
| 9502 | 928 | – | 936 | 473.2725 | 944.5305 | 944.5291 | 1.51 | 0 | 65 | 5e-06 | 1Score **> 31** indicates **identity** Score **> 24** indicates **homology** | U | K.TGTVALEVR.L |
| 108280 | 937 | – | 957 | 729.7148 | 2186.1227 | 2186.1219 | 0.35 | 0 | 19 | 0.016 | 1Score **> 37** indicates **identity** Score **> 14** indicates **homology** | U | R.LLEASHAFEVSDTGNLIVSGK.V |
| 33528 | 958 | – | 967 | 625.8040 | 1249.5934 | 1249.5979 | -3.63 | 0 | 29 | 0.0019 | 1Score **> 32** indicates **identity** Score **> 14** indicates **homology** | U | K.VYLWEDPNSK.L |
| 33530 | 958 | – | 967 | 625.8051 | 1249.5957 | 1249.5979 | -1.79 | 0 | 30 | 0.0016 | 1Score **> 32** indicates **identity** Score **> 15** indicates **homology** | U | K.VYLWEDPNSK.L |
| 95332 | 968 | – | 985 | 655.6621 | 1963.9646 | 1963.9640 | 0.30 | 0 | 23 | 0.0073 | 1Score **> 35** indicates **identity** Score **> 14** indicates **homology** | U | K.LFDHPEVPTPPESASVSR.L |
| 95334 | 968 | – | 985 | 655.6648 | 1963.9725 | 1963.9640 | 4.35 | 0 | 22 | 0.0091 | 1Score **> 35** indicates **identity** Score **> 14** indicates **homology** | U | K.LFDHPEVPTPPESASVSR.L |
| 95336 | 968 | – | 985 | 655.6651 | 1963.9736 | 1963.9640 | 4.91 | 0 | 16 | 0.032 | 1Score **> 35** indicates **identity** Score **> 13** indicates **homology** | U | K.LFDHPEVPTPPESASVSR.L |
| 95337 | 968 | – | 985 | 655.6657 | 1963.9753 | 1963.9640 | 5.79 | 0 | 20 | 0.015 | 1Score **> 36** indicates **identity** Score **> 14** indicates **homology** | U | K.LFDHPEVPTPPESASVSR.L |
| 9036 | 986 | – | 993 | 469.2533 | 936.4920 | 936.4916 | 0.34 | 0 | 48 | 0.00014 | 1Score **> 29** indicates **identity** Score **> 22** indicates **homology** | U | R.LTQGEVYK.E |
| 41596 | 986 | – | 996 | 445.9141 | 1334.7204 | 1334.7194 | 0.77 | 1 | 34 | 0.0007 | 1Score **> 34** indicates **identity** Score **> 15** indicates **homology** | U | R.LTQGEVYKELR.L |
| 41597 | 986 | – | 996 | 445.9143 | 1334.7212 | 1334.7194 | 1.33 | 1 | 26 | 0.0036 | 1Score **> 34** indicates **identity** Score **> 14** indicates **homology** | U | R.LTQGEVYKELR.L |
| 41598 | 986 | – | 996 | 445.9144 | 1334.7214 | 1334.7194 | 1.53 | 1 | 37 | 0.00031 | 1Score **> 34** indicates **identity** Score **> 15** indicates **homology** | U | R.LTQGEVYKELR.L |
| 41599 | 986 | – | 996 | 668.3681 | 1334.7216 | 1334.7194 | 1.66 | 1 | 53 | 2.5e-05 | 1Score **> 33** indicates **identity** Score **> 19** indicates **homology** | U | R.LTQGEVYKELR.L |
| 41600 | 986 | – | 996 | 445.9146 | 1334.7220 | 1334.7194 | 1.97 | 1 | 37 | 0.00035 | 1Score **> 33** indicates **identity** Score **> 15** indicates **homology** | U | R.LTQGEVYKELR.L |
| 43261 | 1053 | – | 1064 | 678.8668 | 1355.7190 | 1355.7197 | -0.51 | 0 | 23 | 0.0071 | 1Score **> 33** indicates **identity** Score **> 14** indicates **homology** | U | R.VTAIYIDPATHR.Q |
| 43267 | 1053 | – | 1064 | 678.8677 | 1355.7209 | 1355.7197 | 0.82 | 0 | 39 | 0.00021 | 1Score **> 34** indicates **identity** Score **> 15** indicates **homology** | U | R.VTAIYIDPATHR.Q |
| 43268 | 1053 | – | 1064 | 452.9142 | 1355.7209 | 1355.7197 | 0.86 | 0 | 22 | 0.0092 | 1Score **> 34** indicates **identity** Score **> 14** indicates **homology** | U | R.VTAIYIDPATHR.Q |
| 43271 | 1053 | – | 1064 | 452.9144 | 1355.7214 | 1355.7197 | 1.22 | 0 | 41 | 0.00014 | 1Score **> 34** indicates **identity** Score **> 15** indicates **homology** | U | R.VTAIYIDPATHR.Q |
| 43272 | 1053 | – | 1064 | 678.8680 | 1355.7215 | 1355.7197 | 1.31 | 0 | 44 | 8.1e-05 | 1Score **> 34** indicates **identity** Score **> 15** indicates **homology** | U | R.VTAIYIDPATHR.Q |
| 43276 | 1053 | – | 1064 | 452.9145 | 1355.7217 | 1355.7197 | 1.44 | 0 | 41 | 0.00014 | 1Score **> 34** indicates **identity** Score **> 15** indicates **homology** | U | R.VTAIYIDPATHR.Q |
| 62247 | 1070 | – | 1083 | 781.9053 | 1561.7960 | 1561.7948 | 0.76 | 1 | 80 | 9.6e-07 | 1Score **> 35** indicates **identity** Score **> 32** indicates **homology** | U | R.LKEDTQVADVTTSR.C |
| 62249 | 1070 | – | 1083 | 781.9057 | 1561.7968 | 1561.7948 | 1.31 | 1 | 86 | 3.9e-07 | 1Score **> 35** indicates **identity** | U | R.LKEDTQVADVTTSR.C |
| 62250 | 1070 | – | 1083 | 521.6062 | 1561.7969 | 1561.7948 | 1.35 | 1 | 45 | 6.4e-05 | 1Score **> 35** indicates **identity** Score **> 16** indicates **homology** | U | R.LKEDTQVADVTTSR.C |
| 62252 | 1070 | – | 1083 | 521.6064 | 1561.7973 | 1561.7948 | 1.63 | 1 | 27 | 0.003 | 1Score **> 35** indicates **identity** Score **> 14** indicates **homology** | U | R.LKEDTQVADVTTSR.C |
| 62253 | 1070 | – | 1083 | 521.6064 | 1561.7975 | 1561.7948 | 1.75 | 1 | 43 | 8.4e-05 | 1Score **> 35** indicates **identity** Score **> 15** indicates **homology** | U | R.LKEDTQVADVTTSR.C |
| 40483 | 1072 | – | 1083 | 661.3167 | 1320.6189 | 1320.6158 | 2.35 | 0 | 26 | 0.0037 | 1Score **> 32** indicates **identity** Score **> 14** indicates **homology** | U | K.EDTQVADVTTSR.C |
| 53149 | 1106 | – | 1117 | 490.2757 | 1467.8054 | 1467.8045 | 0.61 | 1 | 23 | 0.0064 | 1Score **> 34** indicates **identity** Score **> 14** indicates **homology** | U | R.RQQEQLVPTLEK.F |
| 53150 | 1106 | – | 1117 | 734.9101 | 1467.8056 | 1467.8045 | 0.77 | 1 | 49 | 0.00029 | 1Score **> 34** indicates **identity** Score **> 26** indicates **homology** | U | R.RQQEQLVPTLEK.F |
| 53152 | 1106 | – | 1117 | 734.9107 | 1467.8069 | 1467.8045 | 1.62 | 1 | 41 | 0.0014 | 1Score **> 34** indicates **identity** Score **> 25** indicates **homology** | U | R.RQQEQLVPTLEK.F |
| 53153 | 1106 | – | 1117 | 734.9111 | 1467.8076 | 1467.8045 | 2.08 | 1 | 49 | 0.00028 | 1Score **> 34** indicates **identity** Score **> 26** indicates **homology** | U | R.RQQEQLVPTLEK.F |
| 53154 | 1106 | – | 1117 | 490.2767 | 1467.8082 | 1467.8045 | 2.50 | 1 | 38 | 0.00025 | 1Score **> 33** indicates **identity** Score **> 15** indicates **homology** | U | R.RQQEQLVPTLEK.F |
| 39619 | 1107 | – | 1117 | 656.8613 | 1311.7080 | 1311.7034 | 3.46 | 0 | 60 | 2.3e-06 | 1Score **> 33** indicates **identity** Score **> 16** indicates **homology** | U | R.QQEQLVPTLEK.F |
| 77236 | 1160 | – | 1175 | 572.9550 | 1715.8432 | 1715.8413 | 1.07 | 0 | 37 | 0.00034 | 1Score **> 34** indicates **identity** Score **> 15** indicates **homology** | U | K.AAMLGQEDPPQHGLPR.L |
| 77238 | 1160 | – | 1175 | 572.9556 | 1715.8450 | 1715.8413 | 2.12 | 0 | 38 | 0.00055 | 1Score **> 34** indicates **identity** Score **> 18** indicates **homology** | U | K.AAMLGQEDPPQHGLPR.L |
| 77240 | 1160 | – | 1175 | 572.9558 | 1715.8456 | 1715.8413 | 2.49 | 0 | 38 | 0.0012 | 1Score **> 34** indicates **identity** Score **> 21** indicates **homology** | U | K.AAMLGQEDPPQHGLPR.L |
| 59717 | 1221 | – | 1234 | 767.8928 | 1533.7710 | 1533.7708 | 0.13 | 0 | 84 | 3.4e-08 | 1Score **> 35** indicates **identity** Score **> 22** indicates **homology** | U | K.ACVDTALENLSTLK.M |
| 59720 | 1221 | – | 1234 | 767.8931 | 1533.7717 | 1533.7708 | 0.54 | 0 | 78 | 1.3e-07 | 1Score **> 35** indicates **identity** Score **> 21** indicates **homology** | U | K.ACVDTALENLSTLK.M |
| 59721 | 1221 | – | 1234 | 767.8936 | 1533.7726 | 1533.7708 | 1.18 | 0 | 89 | 9.9e-09 | 1Score **> 35** indicates **identity** Score **> 22** indicates **homology** | U | K.ACVDTALENLSTLK.M |
| 59726 | 1221 | – | 1234 | 767.8948 | 1533.7750 | 1533.7708 | 2.68 | 0 | 25 | 0.006 | 1Score **> 35** indicates **identity** Score **> 16** indicates **homology** | U | K.ACVDTALENLSTLK.M |
| 56561 | 1237 | – | 1250 | 750.8942 | 1499.7738 | 1499.7732 | 0.38 | 0 | 89 | 3.3e-08 | 1Score **> 34** indicates **identity** Score **> 27** indicates **homology** | U | K.VAEVLAGEGHLYSR.I |
| 56564 | 1237 | – | 1250 | 750.8951 | 1499.7757 | 1499.7732 | 1.64 | 0 | 39 | 0.00044 | 1Score **> 34** indicates **identity** Score **> 18** indicates **homology** | U | K.VAEVLAGEGHLYSR.I |
| 22589 | 1378 | – | 1387 | 378.9209 | 1133.7407 | 1133.7397 | 0.94 | 2 | 17 | 0.024 | 1Score **> 20** indicates **identity** Score **> 14** indicates **homology** | U | R.KALHLVGLKR.S |
| 22591 | 1378 | – | 1387 | 378.9210 | 1133.7413 | 1133.7397 | 1.43 | 2 | 19 | 0.039 | 1Score **> 20** indicates **identity** Score **> 17** indicates **homology** | U | R.KALHLVGLKR.S |
| 41516 | 1388 | – | 1398 | 667.8313 | 1333.6480 | 1333.6489 | -0.68 | 0 | 34 | 0.00062 | 1Score **> 33** indicates **identity** Score **> 15** indicates **homology** | U | R.SFYGTALFLCR.R |
| 41517 | 1388 | – | 1398 | 667.8325 | 1333.6505 | 1333.6489 | 1.19 | 0 | 59 | 2.7e-06 | 1Score **> 33** indicates **identity** Score **> 16** indicates **homology** | U | R.SFYGTALFLCR.R |
| 41518 | 1388 | – | 1398 | 667.8331 | 1333.6516 | 1333.6489 | 2.01 | 0 | 59 | 2.8e-06 | 1Score **> 33** indicates **identity** Score **> 16** indicates **homology** | U | R.SFYGTALFLCR.R |
| 41519 | 1388 | – | 1398 | 667.8335 | 1333.6525 | 1333.6489 | 2.71 | 0 | 57 | 4.2e-06 | 1Score **> 33** indicates **identity** Score **> 16** indicates **homology** | U | R.SFYGTALFLCR.R |
| 55424 | 1388 | – | 1399 | 497.5912 | 1489.7517 | 1489.7500 | 1.15 | 1 | 19 | 0.025 | 1Score **> 35** indicates **identity** Score **> 16** indicates **homology** | U | R.SFYGTALFLCRR.A |
| 71182 | 1464 | – | 1478 | 827.9329 | 1653.8513 | 1653.8508 | 0.28 | 0 | 17 | 0.031 | 1Score **> 35** indicates **identity** Score **> 15** indicates **homology** | U | R.CILLSNLSNTSHAPK.L |
| 71184 | 1464 | – | 1478 | 552.2915 | 1653.8527 | 1653.8508 | 1.12 | 0 | 57 | 5.3e-06 | 1Score **> 35** indicates **identity** Score **> 16** indicates **homology** | U | R.CILLSNLSNTSHAPK.L |
| 71186 | 1464 | – | 1478 | 552.2918 | 1653.8537 | 1653.8508 | 1.72 | 0 | 54 | 9.8e-06 | 1Score **> 35** indicates **identity** Score **> 16** indicates **homology** | U | R.CILLSNLSNTSHAPK.L |
| 71187 | 1464 | – | 1478 | 552.2919 | 1653.8538 | 1653.8508 | 1.79 | 0 | 21 | 0.012 | 1Score **> 35** indicates **identity** Score **> 14** indicates **homology** | U | R.CILLSNLSNTSHAPK.L |
| 71191 | 1464 | – | 1478 | 552.2923 | 1653.8552 | 1653.8508 | 2.63 | 0 | 29 | 0.002 | 1Score **> 35** indicates **identity** Score **> 14** indicates **homology** | U | R.CILLSNLSNTSHAPK.L |
| 48771 | 1479 | – | 1491 | 712.3941 | 1422.7737 | 1422.7718 | 1.34 | 0 | 65 | 7.9e-07 | 1Score **> 33** indicates **identity** Score **> 17** indicates **homology** | U | K.LDPGSPELQQVLK.H |
| 48772 | 1479 | – | 1491 | 712.3947 | 1422.7748 | 1422.7718 | 2.10 | 0 | 65 | 1.4e-06 | 1Score **> 33** indicates **identity** Score **> 19** indicates **homology** | U | K.LDPGSPELQQVLK.H |
| 48774 | 1479 | – | 1491 | 712.3951 | 1422.7757 | 1422.7718 | 2.71 | 0 | 46 | 4.5e-05 | 1Score **> 33** indicates **identity** Score **> 15** indicates **homology** | U | K.LDPGSPELQQVLK.H |
| 6679 | 1501 | – | 1508 | 440.2098 | 878.4051 | 878.4035 | 1.82 | 0 | 43 | 0.00089 | 1Score **> 28** indicates **identity** Score **> 25** indicates **homology** | U | R.DGAWGAFR.H |
| 6680 | 1501 | – | 1508 | 440.2100 | 878.4055 | 878.4035 | 2.25 | 0 | 31 | 0.0031 | 1Score **> 28** indicates **identity** Score **> 18** indicates **homology** | U | R.DGAWGAFR.H |
| 54817 | 1519 | – | 1531 | 495.9319 | 1484.7738 | 1484.7736 | 0.16 | 0 | 42 | 0.00011 | 1Score **> 34** indicates **identity** Score **> 15** indicates **homology** | U | K.EQTAHAFVNVLTR.G |
| 54819 | 1519 | – | 1531 | 495.9321 | 1484.7745 | 1484.7736 | 0.60 | 0 | 47 | 4.2e-05 | 1Score **> 34** indicates **identity** Score **> 15** indicates **homology** | U | K.EQTAHAFVNVLTR.G |
| 54820 | 1519 | – | 1531 | 495.9331 | 1484.7776 | 1484.7736 | 2.69 | 0 | 23 | 0.0073 | 1Score **> 34** indicates **identity** Score **> 14** indicates **homology** | U | K.EQTAHAFVNVLTR.G |
| 998 | 1532 | – | 1538 | 366.2059 | 730.3973 | 730.3973 | -0.037 | 0 | 55 | 4.4e-05 | 1Score **> 24** indicates **identity** | U | R.GDLASIR.W |
| 999 | 1532 | – | 1538 | 366.2061 | 730.3977 | 730.3973 | 0.46 | 0 | 38 | 0.0026 | 1Score **> 25** indicates **identity** | U | R.GDLASIR.W |
| 7203 | 1576 | – | 1584 | 449.2564 | 896.4982 | 896.4967 | 1.70 | 0 | 54 | 3e-05 | 1Score **> 25** indicates **identity** Score **> 21** indicates **homology** | U | K.LSPDAIPGK.W |
| 7205 | 1576 | – | 1584 | 449.2578 | 896.5011 | 896.4967 | 4.90 | 0 | 26 | 0.0062 | 1Score **> 27** indicates **identity** Score **> 17** indicates **homology** | U | K.LSPDAIPGK.W |
| 28402 | 1688 | – | 1697 | 398.8884 | 1193.6434 | 1193.6544 | -9.20 | 0 | 31 | 0.0092 | 1Score **> 32** indicates **identity** Score **> 23** indicates **homology** | U | R.VFTTVGSAEK.R  + HNE (K) |
| 28396 | 1688 | – | 1698 | 597.8281 | 1193.6417 | 1193.6404 | 1.06 | 1 | 34 | 0.0022 | 1Score **> 32** indicates **identity** Score **> 20** indicates **homology** | U | R.VFTTVGSAEKR.A |
| 28398 | 1688 | – | 1698 | 398.8880 | 1193.6423 | 1193.6404 | 1.52 | 1 | 54 | 7e-05 | 1Score **> 32** indicates **identity** Score **> 25** indicates **homology** | U | R.VFTTVGSAEKR.A |
| 28400 | 1688 | – | 1698 | 597.8285 | 1193.6425 | 1193.6404 | 1.74 | 1 | 45 | 0.0033 | 1Score **> 32** indicates **identity** | U | R.VFTTVGSAEKR.A |
| 56142 | 1705 | – | 1717 | 749.3530 | 1496.6914 | 1496.6896 | 1.19 | 0 | 72 | 1.7e-07 | 1Score **> 31** indicates **identity** Score **> 17** indicates **homology** | U | R.FPQLDDTSFANSR.D |
| 56143 | 1705 | – | 1717 | 749.3531 | 1496.6917 | 1496.6896 | 1.42 | 0 | 70 | 3e-07 | 1Score **> 31** indicates **identity** Score **> 17** indicates **homology** | U | R.FPQLDDTSFANSR.D |
| 56144 | 1705 | – | 1717 | 749.3534 | 1496.6922 | 1496.6896 | 1.74 | 0 | 43 | 9.3e-05 | 1Score **> 31** indicates **identity** Score **> 15** indicates **homology** | U | R.FPQLDDTSFANSR.D |
| 56145 | 1705 | – | 1717 | 749.3560 | 1496.6975 | 1496.6896 | 5.33 | 0 | 17 | 0.024 | 1Score **> 32** indicates **identity** Score **> 14** indicates **homology** | U | R.FPQLDDTSFANSR.D |
| 72562 | 1718 | – | 1732 | 556.9499 | 1667.8280 | 1667.8268 | 0.73 | 0 | 43 | 0.00014 | 1Score **> 35** indicates **identity** Score **> 17** indicates **homology** | U | R.DTSFEQHVLLHTGGK.G |
| 72563 | 1718 | – | 1732 | 556.9501 | 1667.8283 | 1667.8268 | 0.96 | 0 | 51 | 1.5e-05 | 1Score **> 34** indicates **identity** Score **> 16** indicates **homology** | U | R.DTSFEQHVLLHTGGK.G |
| 72564 | 1718 | – | 1732 | 556.9506 | 1667.8300 | 1667.8268 | 1.96 | 0 | 37 | 0.00037 | 1Score **> 34** indicates **identity** Score **> 15** indicates **homology** | U | R.DTSFEQHVLLHTGGK.G |
| 72565 | 1718 | – | 1732 | 556.9513 | 1667.8319 | 1667.8268 | 3.11 | 0 | 15 | 0.038 | 1Score **> 34** indicates **identity** Score **> 13** indicates **homology** | U | R.DTSFEQHVLLHTGGK.G |
| 45458 | 1733 | – | 1745 | 693.8782 | 1385.7419 | 1385.7402 | 1.22 | 0 | 46 | 4.7e-05 | 1Score **> 34** indicates **identity** Score **> 15** indicates **homology** | U | K.GVDLVLNSLAEEK.L |
| 45459 | 1733 | – | 1745 | 693.8786 | 1385.7427 | 1385.7402 | 1.83 | 0 | 74 | 1.5e-07 | 1Score **> 34** indicates **identity** Score **> 19** indicates **homology** | U | K.GVDLVLNSLAEEK.L |
| 5751 | 1813 | – | 1820 | 428.2693 | 854.5241 | 854.5226 | 1.84 | 1 | 21 | 0.033 | 1Score **> 21** indicates **identity** Score **> 18** indicates **homology** | U | R.DGVVKPLK.C |
| 8941 | 1827 | – | 1834 | 468.2330 | 934.4515 | 934.4508 | 0.69 | 0 | 58 | 2.9e-05 | 1Score **> 31** indicates **identity** Score **> 25** indicates **homology** | U | K.AQVEDAFR.Y |
| 8942 | 1827 | – | 1834 | 468.2333 | 934.4520 | 934.4508 | 1.23 | 0 | 39 | 0.0002 | 1Score **> 31** indicates **identity** Score **> 15** indicates **homology** | U | K.AQVEDAFR.Y |
| 8943 | 1827 | – | 1834 | 468.2333 | 934.4520 | 934.4508 | 1.28 | 0 | 30 | 0.0043 | 1Score **> 31** indicates **identity** Score **> 19** indicates **homology** | U | K.AQVEDAFR.Y |
| 19834 | 1901 | – | 1910 | 367.5565 | 1099.6478 | 1099.6462 | 1.48 | 1 | 24 | 0.036 | 1Score **> 31** indicates **identity** Score **> 22** indicates **homology** | U | R.GAQRLVLTSR.S |
| 103939 | 1927 | – | 1946 | 701.0503 | 2100.1289 | 2100.1287 | 0.099 | 1 | 54 | 1.9e-05 | 1Score **> 36** indicates **identity** Score **> 19** indicates **homology** | U | R.RQGIQVLVSTSNVSSLEGAR.A |
| 103945 | 1927 | – | 1946 | 701.0522 | 2100.1346 | 2100.1287 | 2.82 | 1 | 65 | 1.2e-06 | 1Score **> 36** indicates **identity** Score **> 18** indicates **homology** | U | R.RQGIQVLVSTSNVSSLEGAR.A |
| 103957 | 1927 | – | 1946 | 701.0545 | 2100.1415 | 2100.1287 | 6.10 | 1 | 27 | 0.003 | 1Score **> 35** indicates **identity** Score **> 14** indicates **homology** | U | R.RQGIQVLVSTSNVSSLEGAR.A |
| 94079 | 1928 | – | 1946 | 973.0227 | 1944.0308 | 1944.0276 | 1.64 | 0 | 68 | 3.9e-07 | 1Score **> 36** indicates **identity** Score **> 17** indicates **homology** | U | R.QGIQVLVSTSNVSSLEGAR.A |
| 94084 | 1928 | – | 1946 | 973.0242 | 1944.0339 | 1944.0276 | 3.25 | 0 | 42 | 0.00013 | 1Score **> 36** indicates **identity** Score **> 15** indicates **homology** | U | R.QGIQVLVSTSNVSSLEGAR.A |
| 3847 | 1947 | – | 1954 | 408.7451 | 815.4757 | 815.4752 | 0.62 | 0 | 16 | 0.033 | 1Score **> 28** indicates **identity** Score **> 13** indicates **homology** | U | R.ALIAEATK.L |
| 3848 | 1947 | – | 1954 | 408.7455 | 815.4764 | 815.4752 | 1.42 | 0 | 60 | 3.2e-05 | 1Score **> 28** indicates **identity** | U | R.ALIAEATK.L |
| 60515 | 1955 | – | 1969 | 771.9463 | 1541.8780 | 1541.8752 | 1.80 | 0 | 19 | 0.015 | 1Score **> 34** indicates **identity** Score **> 14** indicates **homology** | U | K.LGPVGGVFNLAMVLR.D |
| 17174 | 1989 | – | 1997 | 533.2688 | 1064.5231 | 1064.5251 | -1.80 | 0 | 32 | 0.001 | 1Score **> 31** indicates **identity** Score **> 14** indicates **homology** | U | K.YNGTLNLDR.A |
| 17175 | 1989 | – | 1997 | 533.2699 | 1064.5252 | 1064.5251 | 0.16 | 0 | 46 | 0.00028 | 1Score **> 31** indicates **identity** Score **> 23** indicates **homology** | U | K.YNGTLNLDR.A |
| 17176 | 1989 | – | 1997 | 533.2703 | 1064.5260 | 1064.5251 | 0.91 | 0 | 47 | 0.00036 | 1Score **> 31** indicates **identity** Score **> 25** indicates **homology** | U | K.YNGTLNLDR.A |
| 17211 | 1989 | – | 1997 | 533.7607 | 1065.5068 | 1065.5091 | -2.16 | 0 | 21 | 0.012 | 1Score **> 31** indicates **identity** Score **> 14** indicates **homology** | U | K.YNGTLNLDR.A  + Deamidated (NQ) |
| 17212 | 1989 | – | 1997 | 533.7614 | 1065.5082 | 1065.5091 | -0.79 | 0 | 42 | 0.00011 | 1Score **> 31** indicates **identity** Score **> 15** indicates **homology** | U | K.YNGTLNLDR.A  + Deamidated (NQ) |
| 17214 | 1989 | – | 1997 | 533.7617 | 1065.5089 | 1065.5091 | -0.17 | 0 | 74 | 5.6e-07 | 1Score **> 30** indicates **identity** Score **> 24** indicates **homology** | U | K.YNGTLNLDR.A  + Deamidated (NQ) |
| 108620 | 2001 | – | 2019 | 1097.4825 | 2192.9504 | 2192.9507 | -0.11 | 0 | 14 | 0.048 | 1Score **> 29** indicates **identity** Score **> 13** indicates **homology** | U | R.EACPELDYFVAFSSVSCGR.G |
| 108622 | 2001 | – | 2019 | 1097.4881 | 2192.9616 | 2192.9507 | 4.95 | 0 | 25 | 0.0047 | 1Score **> 29** indicates **identity** Score **> 14** indicates **homology** | U | R.EACPELDYFVAFSSVSCGR.G |
| 85441 | 2020 | – | 2036 | 909.3984 | 1816.7823 | 1816.7798 | 1.34 | 0 | 76 | 8.7e-08 | 1Score **> 28** indicates **identity** Score **> 18** indicates **homology** | U | R.GNAGQTNYGFANSTMER.I |
| 85442 | 2020 | – | 2036 | 909.3993 | 1816.7841 | 1816.7798 | 2.36 | 0 | 100 | 3.8e-10 | 1Score **> 28** indicates **identity** Score **> 19** indicates **homology** | U | R.GNAGQTNYGFANSTMER.I |
| 64146 | 2109 | – | 2123 | 527.9380 | 1580.7923 | 1580.7907 | 1.03 | 1 | 29 | 0.002 | 1Score **> 34** indicates **identity** Score **> 14** indicates **homology** | U | K.AVAHGDGDTQRDLVK.A |
| 100242 | 2180 | – | 2198 | 680.3391 | 2037.9954 | 2037.9888 | 3.24 | 2 | 56 | 5.3e-06 | 1Score **> 36** indicates **identity** Score **> 16** indicates **homology** | U | R.KLQEMSSKTDSATDTTAPK.S |
| 100243 | 2180 | – | 2198 | 510.5063 | 2037.9961 | 2037.9888 | 3.56 | 2 | 44 | 7.7e-05 | 1Score **> 36** indicates **identity** Score **> 15** indicates **homology** | U | R.KLQEMSSKTDSATDTTAPK.S |
| 91656 | 2181 | – | 2198 | 637.6388 | 1909.8947 | 1909.8939 | 0.44 | 1 | 16 | 0.033 | 1Score **> 33** indicates **identity** Score **> 13** indicates **homology** | U | K.LQEMSSKTDSATDTTAPK.S |
| 19943 | 2367 | – | 2375 | 368.1900 | 1101.5481 | 1101.5455 | 2.36 | 0 | 27 | 0.02 | 1Score **> 32** indicates **identity** Score **> 23** indicates **homology** | U | K.QFLDVEHSK.V |
| 19944 | 2367 | – | 2375 | 551.7815 | 1101.5484 | 1101.5455 | 2.69 | 0 | 34 | 0.0042 | 1Score **> 32** indicates **identity** Score **> 23** indicates **homology** | U | K.QFLDVEHSK.V |
| 19945 | 2367 | – | 2375 | 368.1901 | 1101.5486 | 1101.5455 | 2.80 | 0 | 22 | 0.032 | 1Score **> 32** indicates **identity** Score **> 20** indicates **homology** | U | K.QFLDVEHSK.V |
| 12647 | 2376 | – | 2384 | 498.3288 | 994.6430 | 994.6426 | 0.33 | 0 | 55 | 8.3e-06 | 1Score **> 16** indicates **identity** | U | K.VLEALLPLK.S |
| 12649 | 2376 | – | 2384 | 498.3290 | 994.6435 | 994.6426 | 0.88 | 0 | 61 | 2e-06 | 1Score **> 16** indicates **identity** | U | K.VLEALLPLK.S |
| 12651 | 2376 | – | 2384 | 498.3293 | 994.6440 | 994.6426 | 1.37 | 0 | 60 | 6.6e-06 | 1Score **> 21** indicates **identity** | U | K.VLEALLPLK.S |
| 12652 | 2376 | – | 2384 | 498.3295 | 994.6444 | 994.6426 | 1.80 | 0 | 18 | 0.033 | 1Score **> 21** indicates **identity** Score **> 16** indicates **homology** | U | K.VLEALLPLK.S |
| 13772 | 2390 | – | 2399 | 508.8038 | 1015.5930 | 1015.5914 | 1.60 | 0 | 44 | 0.00035 | 1Score **> 33** indicates **identity** Score **> 22** indicates **homology** | U | R.VAASVDLITK.S |
| 13776 | 2390 | – | 2399 | 508.8042 | 1015.5938 | 1015.5914 | 2.37 | 0 | 48 | 0.00011 | 1Score **> 33** indicates **identity** Score **> 21** indicates **homology** | U | R.VAASVDLITK.S |
| 13780 | 2390 | – | 2399 | 508.8045 | 1015.5944 | 1015.5914 | 2.97 | 0 | 64 | 5.8e-06 | 1Score **> 33** indicates **identity** Score **> 24** indicates **homology** | U | R.VAASVDLITK.S |
| 46310 | 2408 | – | 2419 | 466.9071 | 1397.6995 | 1397.6979 | 1.10 | 0 | 39 | 0.0002 | 1Score **> 33** indicates **identity** Score **> 15** indicates **homology** | U | R.ELSFAAVSFYHK.L |
| 46311 | 2408 | – | 2419 | 466.9073 | 1397.7000 | 1397.6979 | 1.51 | 0 | 38 | 0.00025 | 1Score **> 33** indicates **identity** Score **> 15** indicates **homology** | U | R.ELSFAAVSFYHK.L |
| 46313 | 2408 | – | 2419 | 699.8574 | 1397.7002 | 1397.6979 | 1.62 | 0 | 35 | 0.00058 | 1Score **> 33** indicates **identity** Score **> 15** indicates **homology** | U | R.ELSFAAVSFYHK.L |
| 46314 | 2408 | – | 2419 | 699.8575 | 1397.7005 | 1397.6979 | 1.81 | 0 | 54 | 8.1e-06 | 1Score **> 33** indicates **identity** Score **> 16** indicates **homology** | U | R.ELSFAAVSFYHK.L |
| 46315 | 2408 | – | 2419 | 466.9075 | 1397.7007 | 1397.6979 | 2.00 | 0 | 36 | 0.0019 | 1Score **> 33** indicates **identity** Score **> 21** indicates **homology** | U | R.ELSFAAVSFYHK.L |
| 35668 | 2430 | – | 2440 | 424.5793 | 1270.7161 | 1270.7146 | 1.20 | 1 | 70 | 1.6e-06 | 1Score **> 33** indicates **identity** Score **> 25** indicates **homology** | U | K.AKYHGNVTLLR.A |
| 35669 | 2430 | – | 2440 | 424.5795 | 1270.7167 | 1270.7146 | 1.70 | 1 | 42 | 0.0011 | 1Score **> 33** indicates **identity** Score **> 25** indicates **homology** | U | K.AKYHGNVTLLR.A |
| 17469 | 2432 | – | 2440 | 358.2013 | 1071.5820 | 1071.5825 | -0.51 | 0 | 24 | 0.029 | 1Score **> 34** indicates **identity** Score **> 22** indicates **homology** | U | K.YHGNVTLLR.A |
| 17471 | 2432 | – | 2440 | 358.2014 | 1071.5823 | 1071.5825 | -0.19 | 0 | 29 | 0.012 | 1Score **> 33** indicates **identity** Score **> 22** indicates **homology** | U | K.YHGNVTLLR.A |
| 17473 | 2432 | – | 2440 | 358.2015 | 1071.5826 | 1071.5825 | 0.052 | 0 | 22 | 0.021 | 1Score **> 33** indicates **identity** Score **> 18** indicates **homology** | U | K.YHGNVTLLR.A |
| 17478 | 2432 | – | 2440 | 536.7990 | 1071.5834 | 1071.5825 | 0.79 | 0 | 42 | 0.00011 | 1Score **> 33** indicates **identity** Score **> 15** indicates **homology** | U | K.YHGNVTLLR.A |
| 17481 | 2432 | – | 2440 | 358.2019 | 1071.5838 | 1071.5825 | 1.20 | 0 | 36 | 0.0089 | 1Score **> 33** indicates **identity** Score **> 28** indicates **homology** | U | K.YHGNVTLLR.A |
| 17484 | 2432 | – | 2440 | 358.2021 | 1071.5843 | 1071.5825 | 1.70 | 0 | 31 | 0.047 | 1Score **> 33** indicates **identity** Score **> 30** indicates **homology** | U | K.YHGNVTLLR.A |
| 17485 | 2432 | – | 2440 | 358.2021 | 1071.5844 | 1071.5825 | 1.78 | 0 | 38 | 0.0077 | 1Score **> 33** indicates **identity** Score **> 30** indicates **homology** | U | K.YHGNVTLLR.A |
| 113952 | 2443 | – | 2464 | 1160.5067 | 2318.9989 | 2318.9961 | 1.17 | 0 | 16 | 0.029 | 1Score **> 28** indicates **identity** Score **> 14** indicates **homology** | U | K.TGGTYGEDLGADYNLSQVCDGK.V |
| 34649 | 2465 | – | 2475 | 421.2274 | 1260.6603 | 1260.6575 | 2.25 | 0 | 39 | 0.00023 | 1Score **> 34** indicates **identity** Score **> 15** indicates **homology** | U | K.VSVHIIEGDHR.T |
| 34651 | 2465 | – | 2475 | 421.2275 | 1260.6608 | 1260.6575 | 2.61 | 0 | 15 | 0.041 | 1Score **> 34** indicates **identity** Score **> 13** indicates **homology** | U | K.VSVHIIEGDHR.T |

---

```
ID   FAS_MOUSE               Reviewed;        2504 AA.
AC   P19096; B1ATU8; Q6PB72; Q8C4Z0; Q9EQR0;
DT   01-NOV-1990, integrated into UniProtKB/Swiss-Prot.
DT   11-OCT-2004, sequence version 2.
DT   28-JUN-2023, entry version 215.
DE   RecName: Full=Fatty acid synthase;
DE            EC=2.3.1.85 {ECO:0000269|PubMed:29328619};
DE   AltName: Full=Type I Fatty Acid Synthase {ECO:0000303|PubMed:31811668};
DE   Includes:
DE     RecName: Full=[Acyl-carrier-protein] S-acetyltransferase;
DE              EC=2.3.1.38 {ECO:0000269|PubMed:29328619};
DE   Includes:
DE     RecName: Full=[Acyl-carrier-protein] S-malonyltransferase;
DE              EC=2.3.1.39 {ECO:0000269|PubMed:29328619};
DE   Includes:
DE     RecName: Full=3-oxoacyl-[acyl-carrier-protein] synthase;
DE              EC=2.3.1.41 {ECO:0000269|PubMed:31811668};
DE   Includes:
DE     RecName: Full=3-oxoacyl-[acyl-carrier-protein] reductase;
DE              EC=1.1.1.100 {ECO:0000250|UniProtKB:P49327};
DE   Includes:
DE     RecName: Full=3-hydroxyacyl-[acyl-carrier-protein] dehydratase;
DE              EC=4.2.1.59 {ECO:0000250|UniProtKB:P49327};
DE   Includes:
DE     RecName: Full=Enoyl-[acyl-carrier-protein] reductase;
DE              EC=1.3.1.39 {ECO:0000250|UniProtKB:P49327};
DE   Includes:
DE     RecName: Full=Acyl-[acyl-carrier-protein] hydrolase;
DE              EC=3.1.2.14 {ECO:0000250|UniProtKB:P49327};
GN   Name=Fasn;
OS   Mus musculus (Mouse).
OC   Eukaryota; Metazoa; Chordata; Craniata; Vertebrata; Euteleostomi; Mammalia;
OC   Eutheria; Euarchontoglires; Glires; Rodentia; Myomorpha; Muroidea; Muridae;
OC   Murinae; Mus; Mus.
OX   NCBI_TaxID=10090;
RN   [1]
RP   NUCLEOTIDE SEQUENCE [MRNA].
RC   STRAIN=C57BL/6J;
RX   PubMed=11029661; DOI=10.1046/j.1365-2443.2000.00369.x;
RA   Ueno K.;
RT   "Involvement of fatty acid synthase in axonal development in mouse
RT   embryos.";
RL   Genes Cells 5:859-869(2000).
RN   [2]
RP   NUCLEOTIDE SEQUENCE [LARGE SCALE GENOMIC DNA].
RC   STRAIN=C57BL/6J;
RX   PubMed=19468303; DOI=10.1371/journal.pbio.1000112;
RA   Church D.M., Goodstadt L., Hillier L.W., Zody M.C., Goldstein S., She X.,
RA   Bult C.J., Agarwala R., Cherry J.L., DiCuccio M., Hlavina W., Kapustin Y.,
RA   Meric P., Maglott D., Birtle Z., Marques A.C., Graves T., Zhou S.,
RA   Teague B., Potamousis K., Churas C., Place M., Herschleb J., Runnheim R.,
RA   Forrest D., Amos-Landgraf J., Schwartz D.C., Cheng Z., Lindblad-Toh K.,
RA   Eichler E.E., Ponting C.P.;
RT   "Lineage-specific biology revealed by a finished genome assembly of the
RT   mouse.";
RL   PLoS Biol. 7:E1000112-E1000112(2009).
RN   [3]
RP   NUCLEOTIDE SEQUENCE [LARGE SCALE MRNA].
RC   STRAIN=FVB/N; TISSUE=Brain, and Liver;
RX   PubMed=15489334; DOI=10.1101/gr.2596504;
RG   The MGC Project Team;
RT   "The status, quality, and expansion of the NIH full-length cDNA project:
RT   the Mammalian Gene Collection (MGC).";
RL   Genome Res. 14:2121-2127(2004).
RN   [4]
RP   PROTEIN SEQUENCE OF 1-12; 225-235; 299-310; 385-409; 469-478; 1252-1270;
RP   1333-1344; 1388-1398; 1501-1508; 1705-1717; 1733-1745; 2124-2132; 2376-2384
RP   AND 2476-2498, ACETYLATION AT MET-1, AND IDENTIFICATION BY MASS
RP   SPECTROMETRY.
RC   STRAIN=C57BL/6J; TISSUE=Liver;
RA   Bienvenut W.V.;
RL   Submitted (JUL-2005) to UniProtKB.
RN   [5]
RP   NUCLEOTIDE SEQUENCE [LARGE SCALE MRNA] OF 1527-2504.
RC   STRAIN=C57BL/6J; TISSUE=Thymus;
RX   PubMed=16141072; DOI=10.1126/science.1112014;
RA   Carninci P., Kasukawa T., Katayama S., Gough J., Frith M.C., Maeda N.,
RA   Oyama R., Ravasi T., Lenhard B., Wells C., Kodzius R., Shimokawa K.,
RA   Bajic V.B., Brenner S.E., Batalov S., Forrest A.R., Zavolan M., Davis M.J.,
RA   Wilming L.G., Aidinis V., Allen J.E., Ambesi-Impiombato A., Apweiler R.,
RA   Aturaliya R.N., Bailey T.L., Bansal M., Baxter L., Beisel K.W., Bersano T.,
RA   Bono H., Chalk A.M., Chiu K.P., Choudhary V., Christoffels A.,
RA   Clutterbuck D.R., Crowe M.L., Dalla E., Dalrymple B.P., de Bono B.,
RA   Della Gatta G., di Bernardo D., Down T., Engstrom P., Fagiolini M.,
RA   Faulkner G., Fletcher C.F., Fukushima T., Furuno M., Futaki S.,
RA   Gariboldi M., Georgii-Hemming P., Gingeras T.R., Gojobori T., Green R.E.,
RA   Gustincich S., Harbers M., Hayashi Y., Hensch T.K., Hirokawa N., Hill D.,
RA   Huminiecki L., Iacono M., Ikeo K., Iwama A., Ishikawa T., Jakt M.,
RA   Kanapin A., Katoh M., Kawasawa Y., Kelso J., Kitamura H., Kitano H.,
RA   Kollias G., Krishnan S.P., Kruger A., Kummerfeld S.K., Kurochkin I.V.,
RA   Lareau L.F., Lazarevic D., Lipovich L., Liu J., Liuni S., McWilliam S.,
RA   Madan Babu M., Madera M., Marchionni L., Matsuda H., Matsuzawa S., Miki H.,
RA   Mignone F., Miyake S., Morris K., Mottagui-Tabar S., Mulder N., Nakano N.,
RA   Nakauchi H., Ng P., Nilsson R., Nishiguchi S., Nishikawa S., Nori F.,
RA   Ohara O., Okazaki Y., Orlando V., Pang K.C., Pavan W.J., Pavesi G.,
RA   Pesole G., Petrovsky N., Piazza S., Reed J., Reid J.F., Ring B.Z.,
RA   Ringwald M., Rost B., Ruan Y., Salzberg S.L., Sandelin A., Schneider C.,
RA   Schoenbach C., Sekiguchi K., Semple C.A., Seno S., Sessa L., Sheng Y.,
RA   Shibata Y., Shimada H., Shimada K., Silva D., Sinclair B., Sperling S.,
RA   Stupka E., Sugiura K., Sultana R., Takenaka Y., Taki K., Tammoja K.,
RA   Tan S.L., Tang S., Taylor M.S., Tegner J., Teichmann S.A., Ueda H.R.,
RA   van Nimwegen E., Verardo R., Wei C.L., Yagi K., Yamanishi H.,
RA   Zabarovsky E., Zhu S., Zimmer A., Hide W., Bult C., Grimmond S.M.,
RA   Teasdale R.D., Liu E.T., Brusic V., Quackenbush J., Wahlestedt C.,
RA   Mattick J.S., Hume D.A., Kai C., Sasaki D., Tomaru Y., Fukuda S.,
RA   Kanamori-Katayama M., Suzuki M., Aoki J., Arakawa T., Iida J., Imamura K.,
RA   Itoh M., Kato T., Kawaji H., Kawagashira N., Kawashima T., Kojima M.,
RA   Kondo S., Konno H., Nakano K., Ninomiya N., Nishio T., Okada M., Plessy C.,
RA   Shibata K., Shiraki T., Suzuki S., Tagami M., Waki K., Watahiki A.,
RA   Okamura-Oho Y., Suzuki H., Kawai J., Hayashizaki Y.;
RT   "The transcriptional landscape of the mammalian genome.";
RL   Science 309:1559-1563(2005).
RN   [6]
RP   NUCLEOTIDE SEQUENCE [MRNA] OF 1666-2504.
RC   STRAIN=CD-1; TISSUE=Liver;
RX   PubMed=2920037; DOI=10.1016/0006-291x(89)92776-9;
RA   Paulauskis J.D., Sul H.S.;
RT   "Structure of mouse fatty acid synthase mRNA. Identification of the two
RT   NADPH binding sites.";
RL   Biochem. Biophys. Res. Commun. 158:690-695(1989).
RN   [7]
RP   PHOSPHORYLATION [LARGE SCALE ANALYSIS] AT SER-725; SER-1577; SER-1587 AND
RP   SER-2190, AND IDENTIFICATION BY MASS SPECTROMETRY [LARGE SCALE ANALYSIS].
RC   TISSUE=Brain, Brown adipose tissue, Heart, Kidney, Liver, Lung,
RC   Pancreas, Spleen, and Testis;
RX   PubMed=21183079; DOI=10.1016/j.cell.2010.12.001;
RA   Huttlin E.L., Jedrychowski M.P., Elias J.E., Goswami T., Rad R.,
RA   Beausoleil S.A., Villen J., Haas W., Sowa M.E., Gygi S.P.;
RT   "A tissue-specific atlas of mouse protein phosphorylation and expression.";
RL   Cell 143:1174-1189(2010).
RN   [8]
RP   INDUCTION BY ENDOCANNABINOID ANANDAMIDE.
RX   PubMed=21987372; DOI=10.1002/hep.24733;
RA   Jourdan T., Demizieux L., Gresti J., Djaouti L., Gaba L., Verges B.,
RA   Degrace P.;
RT   "Antagonism of peripheral hepatic cannabinoid receptor-1 improves liver
RT   lipid metabolism in mice: evidence from cultured explants.";
RL   Hepatology 55:790-799(2012).
RN   [9]
RP   FUNCTION, CATALYTIC ACTIVITY, BIOPHYSICOCHEMICAL PROPERTIES, AND PATHWAY.
RX   PubMed=29328619; DOI=10.1021/acschembio.7b00718;
RA   Rittner A., Paithankar K.S., Huu K.V., Grininger M.;
RT   "Characterization of the Polyspecific Transferase of Murine Type I Fatty
RT   Acid Synthase (FAS) and Implications for Polyketide Synthase (PKS)
RT   Engineering.";
RL   ACS Chem. Biol. 13:723-732(2018).
RN   [10]
RP   ACETYLATION [LARGE SCALE ANALYSIS] AT LYS-59; LYS-790; LYS-993; LYS-1071;
RP   LYS-1276; LYS-1840 AND LYS-2384, AND IDENTIFICATION BY MASS SPECTROMETRY
RP   [LARGE SCALE ANALYSIS].
RC   TISSUE=Embryonic fibroblast;
RX   PubMed=23806337; DOI=10.1016/j.molcel.2013.06.001;
RA   Park J., Chen Y., Tishkoff D.X., Peng C., Tan M., Dai L., Xie Z., Zhang Y.,
RA   Zwaans B.M., Skinner M.E., Lombard D.B., Zhao Y.;
RT   "SIRT5-mediated lysine desuccinylation impacts diverse metabolic
RT   pathways.";
RL   Mol. Cell 50:919-930(2013).
RN   [11] {ECO:0007744|PDB:6ROP}
RP   X-RAY CRYSTALLOGRAPHY (2.70 ANGSTROMS) OF 2-852 IN COMPLEX WITH OCTANOATE
RP   AND OCTANOYL-COA, FUNCTION, AND CATALYTIC ACTIVITY.
RX   PubMed=31811668; DOI=10.1002/pro.3797;
RA   Rittner A., Paithankar K.S., Himmler A., Grininger M.;
RT   "Type I fatty acid synthase trapped in the octanoyl-bound state.";
RL   Protein Sci. 29:589-605(2020).
CC   -!- FUNCTION: Fatty acid synthetase is a multifunctional enzyme that
CC       catalyzes the de novo biosynthesis of long-chain saturated fatty acids
CC       starting from acetyl-CoA and malonyl-CoA in the presence of NADPH. This
CC       multifunctional protein contains 7 catalytic activities and a site for
CC       the binding of the prosthetic group 4'-phosphopantetheine of the acyl
CC       carrier protein ([ACP]) domain. {ECO:0000269|PubMed:29328619,
CC       ECO:0000269|PubMed:31811668}.
CC   -!- CATALYTIC ACTIVITY:
CC       Reaction=acetyl-CoA + 2n H(+) + n malonyl-CoA + 2n NADPH = a long-chain
CC         fatty acid + n CO2 + (n+1) CoA + 2n NADP(+); Xref=Rhea:RHEA:14993,
CC         ChEBI:CHEBI:15378, ChEBI:CHEBI:16526, ChEBI:CHEBI:57287,
CC         ChEBI:CHEBI:57288, ChEBI:CHEBI:57384, ChEBI:CHEBI:57560,
CC         ChEBI:CHEBI:57783, ChEBI:CHEBI:58349; EC=2.3.1.85;
CC         Evidence={ECO:0000269|PubMed:29328619};
CC       PhysiologicalDirection=left-to-right; Xref=Rhea:RHEA:14994;
CC         Evidence={ECO:0000269|PubMed:29328619};
CC   -!- CATALYTIC ACTIVITY:
CC       Reaction=acetyl-CoA + holo-[ACP] = acetyl-[ACP] + CoA;
CC         Xref=Rhea:RHEA:41788, Rhea:RHEA-COMP:9621, Rhea:RHEA-COMP:9685,
CC         ChEBI:CHEBI:57287, ChEBI:CHEBI:57288, ChEBI:CHEBI:64479,
CC         ChEBI:CHEBI:78446; EC=2.3.1.38;
CC         Evidence={ECO:0000269|PubMed:29328619};
CC       PhysiologicalDirection=left-to-right; Xref=Rhea:RHEA:41789;
CC         Evidence={ECO:0000269|PubMed:29328619};
CC   -!- CATALYTIC ACTIVITY:
CC       Reaction=holo-[ACP] + malonyl-CoA = CoA + malonyl-[ACP];
CC         Xref=Rhea:RHEA:41792, Rhea:RHEA-COMP:9623, Rhea:RHEA-COMP:9685,
CC         ChEBI:CHEBI:57287, ChEBI:CHEBI:57384, ChEBI:CHEBI:64479,
CC         ChEBI:CHEBI:78449; EC=2.3.1.39;
CC         Evidence={ECO:0000269|PubMed:29328619};
CC       PhysiologicalDirection=left-to-right; Xref=Rhea:RHEA:41793;
CC         Evidence={ECO:0000269|PubMed:29328619};
CC   -!- CATALYTIC ACTIVITY:
CC       Reaction=a fatty acyl-[ACP] + H(+) + malonyl-[ACP] = a 3-oxoacyl-[ACP]
CC         + CO2 + holo-[ACP]; Xref=Rhea:RHEA:22836, Rhea:RHEA-COMP:9623,
CC         Rhea:RHEA-COMP:9685, Rhea:RHEA-COMP:9916, Rhea:RHEA-COMP:14125,
CC         ChEBI:CHEBI:15378, ChEBI:CHEBI:16526, ChEBI:CHEBI:64479,
CC         ChEBI:CHEBI:78449, ChEBI:CHEBI:78776, ChEBI:CHEBI:138651;
CC         EC=2.3.1.41; Evidence={ECO:0000269|PubMed:31811668};
CC       PhysiologicalDirection=left-to-right; Xref=Rhea:RHEA:22837;
CC         Evidence={ECO:0000269|PubMed:31811668};
CC   -!- CATALYTIC ACTIVITY:
CC       Reaction=a (3R)-hydroxyacyl-[ACP] + NADP(+) = a 3-oxoacyl-[ACP] + H(+)
CC         + NADPH; Xref=Rhea:RHEA:17397, Rhea:RHEA-COMP:9916, Rhea:RHEA-
CC         COMP:9945, ChEBI:CHEBI:15378, ChEBI:CHEBI:57783, ChEBI:CHEBI:58349,
CC         ChEBI:CHEBI:78776, ChEBI:CHEBI:78827; EC=1.1.1.100;
CC         Evidence={ECO:0000250|UniProtKB:P49327};
CC       PhysiologicalDirection=right-to-left; Xref=Rhea:RHEA:17399;
CC         Evidence={ECO:0000250|UniProtKB:P49327};
CC   -!- CATALYTIC ACTIVITY:
CC       Reaction=a (3R)-hydroxyacyl-[ACP] = a (2E)-enoyl-[ACP] + H2O;
CC         Xref=Rhea:RHEA:13097, Rhea:RHEA-COMP:9925, Rhea:RHEA-COMP:9945,
CC         ChEBI:CHEBI:15377, ChEBI:CHEBI:78784, ChEBI:CHEBI:78827; EC=4.2.1.59;
CC         Evidence={ECO:0000250|UniProtKB:P49327};
CC       PhysiologicalDirection=left-to-right; Xref=Rhea:RHEA:13098;
CC         Evidence={ECO:0000250|UniProtKB:P49327};
CC   -!- CATALYTIC ACTIVITY:
CC       Reaction=a 2,3-saturated acyl-[ACP] + NADP(+) = a (2E)-enoyl-[ACP] +
CC         H(+) + NADPH; Xref=Rhea:RHEA:22564, Rhea:RHEA-COMP:9925, Rhea:RHEA-
CC         COMP:9926, ChEBI:CHEBI:15378, ChEBI:CHEBI:57783, ChEBI:CHEBI:58349,
CC         ChEBI:CHEBI:78784, ChEBI:CHEBI:78785; EC=1.3.1.39;
CC         Evidence={ECO:0000250|UniProtKB:P49327};
CC       PhysiologicalDirection=right-to-left; Xref=Rhea:RHEA:22566;
CC         Evidence={ECO:0000250|UniProtKB:P49327};
CC   -!- CATALYTIC ACTIVITY:
CC       Reaction=H2O + hexadecanoyl-[ACP] = H(+) + hexadecanoate + holo-[ACP];
CC         Xref=Rhea:RHEA:41932, Rhea:RHEA-COMP:9652, Rhea:RHEA-COMP:9685,
CC         ChEBI:CHEBI:7896, ChEBI:CHEBI:15377, ChEBI:CHEBI:15378,
CC         ChEBI:CHEBI:64479, ChEBI:CHEBI:78483; EC=3.1.2.14;
CC         Evidence={ECO:0000250|UniProtKB:P49327};
CC       PhysiologicalDirection=left-to-right; Xref=Rhea:RHEA:41933;
CC         Evidence={ECO:0000250|UniProtKB:P49327};
CC   -!- CATALYTIC ACTIVITY:
CC       Reaction=acetyl-[ACP] + H(+) + malonyl-[ACP] = 3-oxobutanoyl-[ACP] +
CC         CO2 + holo-[ACP]; Xref=Rhea:RHEA:41800, Rhea:RHEA-COMP:9621,
CC         Rhea:RHEA-COMP:9623, Rhea:RHEA-COMP:9625, Rhea:RHEA-COMP:9685,
CC         ChEBI:CHEBI:15378, ChEBI:CHEBI:16526, ChEBI:CHEBI:64479,
CC         ChEBI:CHEBI:78446, ChEBI:CHEBI:78449, ChEBI:CHEBI:78450;
CC         Evidence={ECO:0000305|PubMed:31811668};
CC       PhysiologicalDirection=left-to-right; Xref=Rhea:RHEA:41801;
CC         Evidence={ECO:0000305|PubMed:31811668};
CC   -!- CATALYTIC ACTIVITY:
CC       Reaction=3-oxobutanoyl-[ACP] + H(+) + NADPH = (3R)-hydroxybutanoyl-
CC         [ACP] + NADP(+); Xref=Rhea:RHEA:41804, Rhea:RHEA-COMP:9625,
CC         Rhea:RHEA-COMP:9626, ChEBI:CHEBI:15378, ChEBI:CHEBI:57783,
CC         ChEBI:CHEBI:58349, ChEBI:CHEBI:78450, ChEBI:CHEBI:78451;
CC         Evidence={ECO:0000250|UniProtKB:P49327};
CC       PhysiologicalDirection=left-to-right; Xref=Rhea:RHEA:41805;
CC         Evidence={ECO:0000250|UniProtKB:P49327};
CC   -!- CATALYTIC ACTIVITY:
CC       Reaction=(3R)-hydroxybutanoyl-[ACP] = (2E)-butenoyl-[ACP] + H2O;
CC         Xref=Rhea:RHEA:41808, Rhea:RHEA-COMP:9626, Rhea:RHEA-COMP:9627,
CC         ChEBI:CHEBI:15377, ChEBI:CHEBI:78451, ChEBI:CHEBI:78453;
CC         Evidence={ECO:0000250|UniProtKB:P49327};
CC       PhysiologicalDirection=left-to-right; Xref=Rhea:RHEA:41809;
CC         Evidence={ECO:0000250|UniProtKB:P49327};
CC   -!- CATALYTIC ACTIVITY:
CC       Reaction=(2E)-butenoyl-[ACP] + H(+) + NADPH = butanoyl-[ACP] + NADP(+);
CC         Xref=Rhea:RHEA:41812, Rhea:RHEA-COMP:9627, Rhea:RHEA-COMP:9628,
CC         ChEBI:CHEBI:15378, ChEBI:CHEBI:57783, ChEBI:CHEBI:58349,
CC         ChEBI:CHEBI:78453, ChEBI:CHEBI:78454;
CC         Evidence={ECO:0000250|UniProtKB:P49327};
CC       PhysiologicalDirection=left-to-right; Xref=Rhea:RHEA:41813;
CC         Evidence={ECO:0000250|UniProtKB:P49327};
CC   -!- CATALYTIC ACTIVITY:
CC       Reaction=butanoyl-[ACP] + H(+) + malonyl-[ACP] = 3-oxohexanoyl-[ACP] +
CC         CO2 + holo-[ACP]; Xref=Rhea:RHEA:41820, Rhea:RHEA-COMP:9623,
CC         Rhea:RHEA-COMP:9628, Rhea:RHEA-COMP:9629, Rhea:RHEA-COMP:9685,
CC         ChEBI:CHEBI:15378, ChEBI:CHEBI:16526, ChEBI:CHEBI:64479,
CC         ChEBI:CHEBI:78449, ChEBI:CHEBI:78454, ChEBI:CHEBI:78456;
CC         Evidence={ECO:0000305|PubMed:31811668};
CC       PhysiologicalDirection=left-to-right; Xref=Rhea:RHEA:41821;
CC         Evidence={ECO:0000305|PubMed:31811668};
CC   -!- CATALYTIC ACTIVITY:
CC       Reaction=3-oxohexanoyl-[ACP] + H(+) + NADPH = (3R)-hydroxyhexanoyl-
CC         [ACP] + NADP(+); Xref=Rhea:RHEA:41824, Rhea:RHEA-COMP:9629,
CC         Rhea:RHEA-COMP:9630, ChEBI:CHEBI:15378, ChEBI:CHEBI:57783,
CC         ChEBI:CHEBI:58349, ChEBI:CHEBI:78456, ChEBI:CHEBI:78457;
CC         Evidence={ECO:0000250|UniProtKB:P49327};
CC       PhysiologicalDirection=left-to-right; Xref=Rhea:RHEA:41825;
CC         Evidence={ECO:0000250|UniProtKB:P49327};
CC   -!- CATALYTIC ACTIVITY:
CC       Reaction=(3R)-hydroxyhexanoyl-[ACP] = (2E)-hexenoyl-[ACP] + H2O;
CC         Xref=Rhea:RHEA:41828, Rhea:RHEA-COMP:9630, Rhea:RHEA-COMP:9631,
CC         ChEBI:CHEBI:15377, ChEBI:CHEBI:78457, ChEBI:CHEBI:78458;
CC         Evidence={ECO:0000250|UniProtKB:P49327};
CC       PhysiologicalDirection=left-to-right; Xref=Rhea:RHEA:41829;
CC         Evidence={ECO:0000250|UniProtKB:P49327};
CC   -!- CATALYTIC ACTIVITY:
CC       Reaction=(2E)-hexenoyl-[ACP] + H(+) + NADPH = hexanoyl-[ACP] + NADP(+);
CC         Xref=Rhea:RHEA:41832, Rhea:RHEA-COMP:9631, Rhea:RHEA-COMP:9632,
CC         ChEBI:CHEBI:15378, ChEBI:CHEBI:57783, ChEBI:CHEBI:58349,
CC         ChEBI:CHEBI:78458, ChEBI:CHEBI:78459;
CC         Evidence={ECO:0000250|UniProtKB:P49327};
CC       PhysiologicalDirection=left-to-right; Xref=Rhea:RHEA:41833;
CC         Evidence={ECO:0000250|UniProtKB:P49327};
CC   -!- CATALYTIC ACTIVITY:
CC       Reaction=H(+) + hexanoyl-[ACP] + malonyl-[ACP] = 3-oxooctanoyl-[ACP] +
CC         CO2 + holo-[ACP]; Xref=Rhea:RHEA:41836, Rhea:RHEA-COMP:9623,
CC         Rhea:RHEA-COMP:9632, Rhea:RHEA-COMP:9633, Rhea:RHEA-COMP:9685,
CC         ChEBI:CHEBI:15378, ChEBI:CHEBI:16526, ChEBI:CHEBI:64479,
CC         ChEBI:CHEBI:78449, ChEBI:CHEBI:78459, ChEBI:CHEBI:78460;
CC         Evidence={ECO:0000269|PubMed:31811668};
CC       PhysiologicalDirection=left-to-right; Xref=Rhea:RHEA:41837;
CC         Evidence={ECO:0000269|PubMed:31811668};
CC   -!- CATALYTIC ACTIVITY:
CC       Reaction=3-oxooctanoyl-[ACP] + H(+) + NADPH = (3R)-hydroxyoctanoyl-
CC         [ACP] + NADP(+); Xref=Rhea:RHEA:41840, Rhea:RHEA-COMP:9633,
CC         Rhea:RHEA-COMP:9634, ChEBI:CHEBI:15378, ChEBI:CHEBI:57783,
CC         ChEBI:CHEBI:58349, ChEBI:CHEBI:78460, ChEBI:CHEBI:78461;
CC         Evidence={ECO:0000250|UniProtKB:P49327};
CC       PhysiologicalDirection=left-to-right; Xref=Rhea:RHEA:41841;
CC         Evidence={ECO:0000250|UniProtKB:P49327};
CC   -!- CATALYTIC ACTIVITY:
CC       Reaction=(3R)-hydroxyoctanoyl-[ACP] = (2E)-octenoyl-[ACP] + H2O;
CC         Xref=Rhea:RHEA:41844, Rhea:RHEA-COMP:9634, Rhea:RHEA-COMP:9635,
CC         ChEBI:CHEBI:15377, ChEBI:CHEBI:78461, ChEBI:CHEBI:78462;
CC         Evidence={ECO:0000250|UniProtKB:P49327};
CC       PhysiologicalDirection=left-to-right; Xref=Rhea:RHEA:41845;
CC         Evidence={ECO:0000250|UniProtKB:P49327};
CC   -!- CATALYTIC ACTIVITY:
CC       Reaction=(2E)-octenoyl-[ACP] + H(+) + NADPH = NADP(+) + octanoyl-[ACP];
CC         Xref=Rhea:RHEA:41848, Rhea:RHEA-COMP:9635, Rhea:RHEA-COMP:9636,
CC         ChEBI:CHEBI:15378, ChEBI:CHEBI:57783, ChEBI:CHEBI:58349,
CC         ChEBI:CHEBI:78462, ChEBI:CHEBI:78463;
CC         Evidence={ECO:0000250|UniProtKB:P49327};
CC       PhysiologicalDirection=left-to-right; Xref=Rhea:RHEA:41849;
CC         Evidence={ECO:0000250|UniProtKB:P49327};
CC   -!- CATALYTIC ACTIVITY:
CC       Reaction=H(+) + malonyl-[ACP] + octanoyl-[ACP] = 3-oxodecanoyl-[ACP] +
CC         CO2 + holo-[ACP]; Xref=Rhea:RHEA:41852, Rhea:RHEA-COMP:9623,
CC         Rhea:RHEA-COMP:9636, Rhea:RHEA-COMP:9637, Rhea:RHEA-COMP:9685,
CC         ChEBI:CHEBI:15378, ChEBI:CHEBI:16526, ChEBI:CHEBI:64479,
CC         ChEBI:CHEBI:78449, ChEBI:CHEBI:78463, ChEBI:CHEBI:78464;
CC         Evidence={ECO:0000269|PubMed:31811668};
CC       PhysiologicalDirection=left-to-right; Xref=Rhea:RHEA:41853;
CC         Evidence={ECO:0000269|PubMed:31811668};
CC   -!- CATALYTIC ACTIVITY:
CC       Reaction=3-oxodecanoyl-[ACP] + H(+) + NADPH = (3R)-hydroxydecanoyl-
CC         [ACP] + NADP(+); Xref=Rhea:RHEA:41856, Rhea:RHEA-COMP:9637,
CC         Rhea:RHEA-COMP:9638, ChEBI:CHEBI:15378, ChEBI:CHEBI:57783,
CC         ChEBI:CHEBI:58349, ChEBI:CHEBI:78464, ChEBI:CHEBI:78466;
CC         Evidence={ECO:0000250|UniProtKB:P49327};
CC       PhysiologicalDirection=left-to-right; Xref=Rhea:RHEA:41857;
CC         Evidence={ECO:0000250|UniProtKB:P49327};
CC   -!- CATALYTIC ACTIVITY:
CC       Reaction=(3R)-hydroxydecanoyl-[ACP] = (2E)-decenoyl-[ACP] + H2O;
CC         Xref=Rhea:RHEA:41860, Rhea:RHEA-COMP:9638, Rhea:RHEA-COMP:9639,
CC         ChEBI:CHEBI:15377, ChEBI:CHEBI:78466, ChEBI:CHEBI:78467;
CC         Evidence={ECO:0000250|UniProtKB:P49327};
CC       PhysiologicalDirection=left-to-right; Xref=Rhea:RHEA:41861;
CC         Evidence={ECO:0000250|UniProtKB:P49327};
CC   -!- CATALYTIC ACTIVITY:
CC       Reaction=(2E)-decenoyl-[ACP] + H(+) + NADPH = decanoyl-[ACP] + NADP(+);
CC         Xref=Rhea:RHEA:41864, Rhea:RHEA-COMP:9639, Rhea:RHEA-COMP:9640,
CC         ChEBI:CHEBI:15378, ChEBI:CHEBI:57783, ChEBI:CHEBI:58349,
CC         ChEBI:CHEBI:78467, ChEBI:CHEBI:78468;
CC         Evidence={ECO:0000250|UniProtKB:P49327};
CC       PhysiologicalDirection=left-to-right; Xref=Rhea:RHEA:41865;
CC         Evidence={ECO:0000250|UniProtKB:P49327};
CC   -!- CATALYTIC ACTIVITY:
CC       Reaction=decanoyl-[ACP] + H(+) + malonyl-[ACP] = 3-oxododecanoyl-[ACP]
CC         + CO2 + holo-[ACP]; Xref=Rhea:RHEA:41868, Rhea:RHEA-COMP:9623,
CC         Rhea:RHEA-COMP:9640, Rhea:RHEA-COMP:9641, Rhea:RHEA-COMP:9685,
CC         ChEBI:CHEBI:15378, ChEBI:CHEBI:16526, ChEBI:CHEBI:64479,
CC         ChEBI:CHEBI:78449, ChEBI:CHEBI:78468, ChEBI:CHEBI:78469;
CC         Evidence={ECO:0000269|PubMed:31811668};
CC       PhysiologicalDirection=left-to-right; Xref=Rhea:RHEA:41869;
CC         Evidence={ECO:0000269|PubMed:31811668};
CC   -!- CATALYTIC ACTIVITY:
CC       Reaction=3-oxododecanoyl-[ACP] + H(+) + NADPH = (3R)-hydroxydodecanoyl-
CC         [ACP] + NADP(+); Xref=Rhea:RHEA:41872, Rhea:RHEA-COMP:9641,
CC         Rhea:RHEA-COMP:9642, ChEBI:CHEBI:15378, ChEBI:CHEBI:57783,
CC         ChEBI:CHEBI:58349, ChEBI:CHEBI:78469, ChEBI:CHEBI:78470;
CC         Evidence={ECO:0000250|UniProtKB:P49327};
CC       PhysiologicalDirection=left-to-right; Xref=Rhea:RHEA:41873;
CC         Evidence={ECO:0000250|UniProtKB:P49327};
CC   -!- CATALYTIC ACTIVITY:
CC       Reaction=(3R)-hydroxydodecanoyl-[ACP] = (2E)-dodecenoyl-[ACP] + H2O;
CC         Xref=Rhea:RHEA:41876, Rhea:RHEA-COMP:9642, Rhea:RHEA-COMP:9643,
CC         ChEBI:CHEBI:15377, ChEBI:CHEBI:78470, ChEBI:CHEBI:78472;
CC         Evidence={ECO:0000250|UniProtKB:P49327};
CC       PhysiologicalDirection=left-to-right; Xref=Rhea:RHEA:41877;
CC         Evidence={ECO:0000250|UniProtKB:P49327};
CC   -!- CATALYTIC ACTIVITY:
CC       Reaction=(2E)-dodecenoyl-[ACP] + H(+) + NADPH = dodecanoyl-[ACP] +
CC         NADP(+); Xref=Rhea:RHEA:41880, Rhea:RHEA-COMP:9643, Rhea:RHEA-
CC         COMP:9644, ChEBI:CHEBI:15378, ChEBI:CHEBI:57783, ChEBI:CHEBI:58349,
CC         ChEBI:CHEBI:65264, ChEBI:CHEBI:78472;
CC         Evidence={ECO:0000250|UniProtKB:P49327};
CC       PhysiologicalDirection=left-to-right; Xref=Rhea:RHEA:41881;
CC         Evidence={ECO:0000250|UniProtKB:P49327};
CC   -!- CATALYTIC ACTIVITY:
CC       Reaction=dodecanoyl-[ACP] + H(+) + malonyl-[ACP] = 3-oxotetradecanoyl-
CC         [ACP] + CO2 + holo-[ACP]; Xref=Rhea:RHEA:41884, Rhea:RHEA-COMP:9623,
CC         Rhea:RHEA-COMP:9644, Rhea:RHEA-COMP:9645, Rhea:RHEA-COMP:9685,
CC         ChEBI:CHEBI:15378, ChEBI:CHEBI:16526, ChEBI:CHEBI:64479,
CC         ChEBI:CHEBI:65264, ChEBI:CHEBI:78449, ChEBI:CHEBI:78473;
CC         Evidence={ECO:0000269|PubMed:31811668};
CC       PhysiologicalDirection=left-to-right; Xref=Rhea:RHEA:41885;
CC         Evidence={ECO:0000269|PubMed:31811668};
CC   -!- CATALYTIC ACTIVITY:
CC       Reaction=3-oxotetradecanoyl-[ACP] + H(+) + NADPH = (3R)-
CC         hydroxytetradecanoyl-[ACP] + NADP(+); Xref=Rhea:RHEA:41888,
CC         Rhea:RHEA-COMP:9645, Rhea:RHEA-COMP:9646, ChEBI:CHEBI:15378,
CC         ChEBI:CHEBI:57783, ChEBI:CHEBI:58349, ChEBI:CHEBI:78473,
CC         ChEBI:CHEBI:78474; Evidence={ECO:0000250|UniProtKB:P49327};
CC       PhysiologicalDirection=left-to-right; Xref=Rhea:RHEA:41889;
CC         Evidence={ECO:0000250|UniProtKB:P49327};
CC   -!- CATALYTIC ACTIVITY:
CC       Reaction=(3R)-hydroxytetradecanoyl-[ACP] = (2E)-tetradecenoyl-[ACP] +
CC         H2O; Xref=Rhea:RHEA:41892, Rhea:RHEA-COMP:9646, Rhea:RHEA-COMP:9647,
CC         ChEBI:CHEBI:15377, ChEBI:CHEBI:78474, ChEBI:CHEBI:78475;
CC         Evidence={ECO:0000250|UniProtKB:P49327};
CC       PhysiologicalDirection=left-to-right; Xref=Rhea:RHEA:41893;
CC         Evidence={ECO:0000250|UniProtKB:P49327};
CC   -!- CATALYTIC ACTIVITY:
CC       Reaction=(2E)-tetradecenoyl-[ACP] + H(+) + NADPH = NADP(+) +
CC         tetradecanoyl-[ACP]; Xref=Rhea:RHEA:41896, Rhea:RHEA-COMP:9647,
CC         Rhea:RHEA-COMP:9648, ChEBI:CHEBI:15378, ChEBI:CHEBI:57783,
CC         ChEBI:CHEBI:58349, ChEBI:CHEBI:78475, ChEBI:CHEBI:78477;
CC         Evidence={ECO:0000250|UniProtKB:P49327};
CC       PhysiologicalDirection=left-to-right; Xref=Rhea:RHEA:41897;
CC         Evidence={ECO:0000250|UniProtKB:P49327};
CC   -!- CATALYTIC ACTIVITY:
CC       Reaction=H(+) + malonyl-[ACP] + tetradecanoyl-[ACP] = 3-
CC         oxohexadecanoyl-[ACP] + CO2 + holo-[ACP]; Xref=Rhea:RHEA:41900,
CC         Rhea:RHEA-COMP:9623, Rhea:RHEA-COMP:9648, Rhea:RHEA-COMP:9649,
CC         Rhea:RHEA-COMP:9685, ChEBI:CHEBI:15378, ChEBI:CHEBI:16526,
CC         ChEBI:CHEBI:64479, ChEBI:CHEBI:78449, ChEBI:CHEBI:78477,
CC         ChEBI:CHEBI:78478; Evidence={ECO:0000269|PubMed:31811668};
CC       PhysiologicalDirection=left-to-right; Xref=Rhea:RHEA:41901;
CC         Evidence={ECO:0000269|PubMed:31811668};
CC   -!- CATALYTIC ACTIVITY:
CC       Reaction=3-oxohexadecanoyl-[ACP] + H(+) + NADPH = (3R)-
CC         hydroxyhexadecanoyl-[ACP] + NADP(+); Xref=Rhea:RHEA:41904, Rhea:RHEA-
CC         COMP:9649, Rhea:RHEA-COMP:9650, ChEBI:CHEBI:15378, ChEBI:CHEBI:57783,
CC         ChEBI:CHEBI:58349, ChEBI:CHEBI:78478, ChEBI:CHEBI:78480;
CC         Evidence={ECO:0000250|UniProtKB:P49327};
CC       PhysiologicalDirection=left-to-right; Xref=Rhea:RHEA:41905;
CC         Evidence={ECO:0000250|UniProtKB:P49327};
CC   -!- CATALYTIC ACTIVITY:
CC       Reaction=(3R)-hydroxyhexadecanoyl-[ACP] = (2E)-hexadecenoyl-[ACP] +
CC         H2O; Xref=Rhea:RHEA:41908, Rhea:RHEA-COMP:9650, Rhea:RHEA-COMP:9651,
CC         ChEBI:CHEBI:15377, ChEBI:CHEBI:78480, ChEBI:CHEBI:78481;
CC         Evidence={ECO:0000250|UniProtKB:P49327};
CC       PhysiologicalDirection=left-to-right; Xref=Rhea:RHEA:41909;
CC         Evidence={ECO:0000250|UniProtKB:P49327};
CC   -!- CATALYTIC ACTIVITY:
CC       Reaction=(2E)-hexadecenoyl-[ACP] + H(+) + NADPH = hexadecanoyl-[ACP] +
CC         NADP(+); Xref=Rhea:RHEA:41912, Rhea:RHEA-COMP:9651, Rhea:RHEA-
CC         COMP:9652, ChEBI:CHEBI:15378, ChEBI:CHEBI:57783, ChEBI:CHEBI:58349,
CC         ChEBI:CHEBI:78481, ChEBI:CHEBI:78483;
CC         Evidence={ECO:0000250|UniProtKB:P49327};
CC       PhysiologicalDirection=left-to-right; Xref=Rhea:RHEA:41913;
CC         Evidence={ECO:0000250|UniProtKB:P49327};
CC   -!- CATALYTIC ACTIVITY:
CC       Reaction=H(+) + hexadecanoyl-[ACP] + malonyl-[ACP] = 3-oxooctadecanoyl-
CC         [ACP] + CO2 + holo-[ACP]; Xref=Rhea:RHEA:41916, Rhea:RHEA-COMP:9623,
CC         Rhea:RHEA-COMP:9652, Rhea:RHEA-COMP:9653, Rhea:RHEA-COMP:9685,
CC         ChEBI:CHEBI:15378, ChEBI:CHEBI:16526, ChEBI:CHEBI:64479,
CC         ChEBI:CHEBI:78449, ChEBI:CHEBI:78483, ChEBI:CHEBI:78487;
CC         Evidence={ECO:0000250|UniProtKB:P49327};
CC       PhysiologicalDirection=left-to-right; Xref=Rhea:RHEA:41917;
CC         Evidence={ECO:0000250|UniProtKB:P49327};
CC   -!- CATALYTIC ACTIVITY:
CC       Reaction=3-oxooctadecanoyl-[ACP] + H(+) + NADPH = (3R)-
CC         hydroxyoctadecanoyl-[ACP] + NADP(+); Xref=Rhea:RHEA:41920, Rhea:RHEA-
CC         COMP:9653, Rhea:RHEA-COMP:9654, ChEBI:CHEBI:15378, ChEBI:CHEBI:57783,
CC         ChEBI:CHEBI:58349, ChEBI:CHEBI:78487, ChEBI:CHEBI:78488;
CC         Evidence={ECO:0000250|UniProtKB:P49327};
CC       PhysiologicalDirection=left-to-right; Xref=Rhea:RHEA:41921;
CC         Evidence={ECO:0000250|UniProtKB:P49327};
CC   -!- CATALYTIC ACTIVITY:
CC       Reaction=(3R)-hydroxyoctadecanoyl-[ACP] = (2E)-octadecenoyl-[ACP] +
CC         H2O; Xref=Rhea:RHEA:41924, Rhea:RHEA-COMP:9654, Rhea:RHEA-COMP:9655,
CC         ChEBI:CHEBI:15377, ChEBI:CHEBI:78488, ChEBI:CHEBI:78489;
CC         Evidence={ECO:0000250|UniProtKB:P49327};
CC       PhysiologicalDirection=left-to-right; Xref=Rhea:RHEA:41925;
CC         Evidence={ECO:0000250|UniProtKB:P49327};
CC   -!- CATALYTIC ACTIVITY:
CC       Reaction=(2E)-octadecenoyl-[ACP] + H(+) + NADPH = NADP(+) +
CC         octadecanoyl-[ACP]; Xref=Rhea:RHEA:41928, Rhea:RHEA-COMP:9655,
CC         Rhea:RHEA-COMP:9656, ChEBI:CHEBI:15378, ChEBI:CHEBI:57783,
CC         ChEBI:CHEBI:58349, ChEBI:CHEBI:78489, ChEBI:CHEBI:78495;
CC         Evidence={ECO:0000250|UniProtKB:P49327};
CC       PhysiologicalDirection=left-to-right; Xref=Rhea:RHEA:41929;
CC         Evidence={ECO:0000250|UniProtKB:P49327};
CC   -!- CATALYTIC ACTIVITY:
CC       Reaction=H2O + tetradecanoyl-[ACP] = H(+) + holo-[ACP] +
CC         tetradecanoate; Xref=Rhea:RHEA:30123, Rhea:RHEA-COMP:9648, Rhea:RHEA-
CC         COMP:9685, ChEBI:CHEBI:15377, ChEBI:CHEBI:15378, ChEBI:CHEBI:30807,
CC         ChEBI:CHEBI:64479, ChEBI:CHEBI:78477; EC=3.1.2.14;
CC         Evidence={ECO:0000250|UniProtKB:P49327};
CC       PhysiologicalDirection=left-to-right; Xref=Rhea:RHEA:30124;
CC         Evidence={ECO:0000250|UniProtKB:P49327};
CC   -!- BIOPHYSICOCHEMICAL PROPERTIES:
CC       Kinetic parameters:
CC         KM=1.28 uM for malonyl-CoA {ECO:0000269|PubMed:29328619};
CC         KM=1.63 uM for acetyl-CoA {ECO:0000269|PubMed:29328619};
CC   -!- PATHWAY: Lipid metabolism; fatty acid biosynthesis.
CC       {ECO:0000269|PubMed:29328619}.
CC   -!- SUBUNIT: Homodimer which is arranged in a head to tail fashion (By
CC       similarity). Interacts with CEACAM1; this interaction is insulin and
CC       phosphorylation-dependent; reduces fatty-acid synthase activity (By
CC       similarity). {ECO:0000250|UniProtKB:P12785,
CC       ECO:0000250|UniProtKB:P49327}.
CC   -!- SUBCELLULAR LOCATION: Cytoplasm {ECO:0000250}. Melanosome
CC       {ECO:0000250}.
CC   -!- INDUCTION: Up-regulated by endocannabinoid anandamide/AEA.
CC       {ECO:0000269|PubMed:21987372}.
CC   -!- PTM: S-nitrosylation of Fatty acid synthase at cysteine residues Cys-
CC       1464 or Cys-2084 is important for the enzyme dimerization. In
CC       adipocytes, S-nitrosylation of Fatty acid synthase occurs under
CC       physiological conditions and gradually increases during adipogenesis.
CC       {ECO:0000250|UniProtKB:P49327}.
CC   -!- SEQUENCE CAUTION:
CC       Sequence=CAA31525.1; Type=Frameshift; Evidence={ECO:0000305};
CC   ---------------------------------------------------------------------------
CC   Copyrighted by the UniProt Consortium, see https://www.uniprot.org/terms
CC   Distributed under the Creative Commons Attribution (CC BY 4.0) License
CC   ---------------------------------------------------------------------------
DR   EMBL; AF127033; AAG02285.1; -; mRNA.
DR   EMBL; AL663090; -; NOT_ANNOTATED_CDS; Genomic_DNA.
DR   EMBL; BC046513; AAH46513.1; -; mRNA.
DR   EMBL; BC059850; AAH59850.1; -; mRNA.
DR   EMBL; AK080374; BAC37895.1; -; mRNA.
DR   EMBL; X13135; CAA31525.1; ALT_FRAME; mRNA.
DR   CCDS; CCDS25759.1; -.
DR   PIR; A32262; A32262.
DR   RefSeq; NP_032014.3; NM_007988.3.
DR   PDB; 5MY0; X-ray; 2.94 A; A/B/C/D=2-852.
DR   PDB; 5MY2; X-ray; 2.70 A; A/B/C/D=2-852.
DR   PDB; 6ROP; X-ray; 2.70 A; A/B/C/D=2-852.
DR   PDBsum; 5MY0; -.
DR   PDBsum; 5MY2; -.
DR   PDBsum; 6ROP; -.
DR   AlphaFoldDB; P19096; -.
DR   SMR; P19096; -.
DR   BioGRID; 199596; 29.
DR   IntAct; P19096; 6.
DR   MINT; P19096; -.
DR   STRING; 10090.ENSMUSP00000052872; -.
DR   BindingDB; P19096; -.
DR   ChEMBL; CHEMBL1795189; -.
DR   ESTHER; mouse-FASN; Thioesterase.
DR   GlyGen; P19096; 1 site, 1 O-linked glycan (1 site).
DR   iPTMnet; P19096; -.
DR   PhosphoSitePlus; P19096; -.
DR   SwissPalm; P19096; -.
DR   CPTAC; non-CPTAC-3808; -.
DR   EPD; P19096; -.
DR   jPOST; P19096; -.
DR   MaxQB; P19096; -.
DR   PaxDb; P19096; -.
DR   PeptideAtlas; P19096; -.
DR   ProteomicsDB; 275589; -.
DR   Antibodypedia; 1650; 820 antibodies from 40 providers.
DR   DNASU; 14104; -.
DR   Ensembl; ENSMUST00000055655; ENSMUSP00000052872; ENSMUSG00000025153.
DR   GeneID; 14104; -.
DR   KEGG; mmu:14104; -.
DR   UCSC; uc007mut.1; mouse.
DR   AGR; MGI:95485; -.
DR   CTD; 2194; -.
DR   MGI; MGI:95485; Fasn.
DR   VEuPathDB; HostDB:ENSMUSG00000025153; -.
DR   eggNOG; KOG1202; Eukaryota.
DR   GeneTree; ENSGT00940000157276; -.
DR   HOGENOM; CLU_000022_31_7_1; -.
DR   InParanoid; P19096; -.
DR   OMA; KMRGGEF; -.
DR   OrthoDB; 3378513at2759; -.
DR   PhylomeDB; P19096; -.
DR   TreeFam; TF300549; -.
DR   BRENDA; 2.3.1.85; 3474.
DR   Reactome; R-MMU-163765; ChREBP activates metabolic gene expression.
DR   Reactome; R-MMU-199220; Vitamin B5 (pantothenate) metabolism.
DR   Reactome; R-MMU-75105; Fatty acyl-CoA biosynthesis.
DR   UniPathway; UPA00094; -.
DR   BioGRID-ORCS; 14104; 23 hits in 80 CRISPR screens.
DR   ChiTaRS; Fasn; mouse.
DR   PRO; PR:P19096; -.
DR   Proteomes; UP000000589; Chromosome 11.
DR   RNAct; P19096; protein.
DR   Bgee; ENSMUSG00000025153; Expressed in aorta tunica adventitia and 292 other tissues.
DR   ExpressionAtlas; P19096; baseline and differential.
DR   Genevisible; P19096; MM.
DR   GO; GO:0005737; C:cytoplasm; ISO:MGI.
DR   GO; GO:0005829; C:cytosol; ISO:MGI.
DR   GO; GO:0042587; C:glycogen granule; IDA:MGI.
DR   GO; GO:0005794; C:Golgi apparatus; ISO:MGI.
DR   GO; GO:0042470; C:melanosome; IEA:UniProtKB-SubCell.
DR   GO; GO:0005739; C:mitochondrion; HDA:MGI.
DR   GO; GO:0005886; C:plasma membrane; ISO:MGI.
DR   GO; GO:0008693; F:(3R)-3-hydroxydecanoyl-[acyl-carrier-protein] dehydratase activity; IEA:RHEA.
DR   GO; GO:0047451; F:(3R)-3-hydroxyoctanoyl-[acyl-carrier-protein] dehydratase activity; IEA:RHEA.
DR   GO; GO:0008659; F:(3R)-hydroxymyristoyl-[acyl-carrier-protein] dehydratase activity; IEA:UniProtKB-EC.
DR   GO; GO:0004317; F:(3R)-hydroxypalmitoyl-[acyl-carrier-protein] dehydratase activity; IEA:RHEA.
DR   GO; GO:0004316; F:3-oxoacyl-[acyl-carrier-protein] reductase (NADPH) activity; IEA:UniProtKB-EC.
DR   GO; GO:0004315; F:3-oxoacyl-[acyl-carrier-protein] synthase activity; IEA:UniProtKB-EC.
DR   GO; GO:0004313; F:[acyl-carrier-protein] S-acetyltransferase activity; IEA:UniProtKB-EC.
DR   GO; GO:0004314; F:[acyl-carrier-protein] S-malonyltransferase activity; ISO:MGI.
DR   GO; GO:0047117; F:enoyl-[acyl-carrier-protein] reductase (NADPH, A-specific) activity; IEA:UniProtKB-EC.
DR   GO; GO:0004312; F:fatty acid synthase activity; IDA:MGI.
DR   GO; GO:0042802; F:identical protein binding; ISO:MGI.
DR   GO; GO:0016295; F:myristoyl-[acyl-carrier-protein] hydrolase activity; IEA:UniProtKB-EC.
DR   GO; GO:0004320; F:oleoyl-[acyl-carrier-protein] hydrolase activity; IEA:UniProtKB-EC.
DR   GO; GO:0016296; F:palmitoyl-[acyl-carrier-protein] hydrolase activity; IEA:UniProtKB-EC.
DR   GO; GO:0031177; F:phosphopantetheine binding; IEA:InterPro.
DR   GO; GO:0006084; P:acetyl-CoA metabolic process; ISO:MGI.
DR   GO; GO:0071353; P:cellular response to interleukin-4; IDA:MGI.
DR   GO; GO:0090557; P:establishment of endothelial intestinal barrier; IMP:MGI.
DR   GO; GO:0008611; P:ether lipid biosynthetic process; IMP:MGI.
DR   GO; GO:0006633; P:fatty acid biosynthetic process; IDA:MGI.
DR   GO; GO:0002068; P:glandular epithelial cell development; IMP:MGI.
DR   GO; GO:0006954; P:inflammatory response; IMP:MGI.
DR   GO; GO:0008610; P:lipid biosynthetic process; IDA:MGI.
DR   GO; GO:0030879; P:mammary gland development; IMP:CACAO.
DR   GO; GO:0044788; P:modulation by host of viral process; ISO:MGI.
DR   GO; GO:0030224; P:monocyte differentiation; IMP:MGI.
DR   GO; GO:0030223; P:neutrophil differentiation; IMP:MGI.
DR   GO; GO:0009888; P:tissue development; IMP:MGI.
DR   CDD; cd05195; enoyl_red; 1.
DR   CDD; cd08954; KR_1_FAS_SDR_x; 2.
DR   CDD; cd00833; PKS; 1.
DR   Gene3D; 3.30.70.3290; -; 1.
DR   Gene3D; 3.40.47.10; -; 1.
DR   Gene3D; 1.10.1200.10; ACP-like; 1.
DR   Gene3D; 3.40.50.1820; alpha/beta hydrolase; 2.
DR   Gene3D; 3.40.366.10; Malonyl-Coenzyme A Acyl Carrier Protein, domain 2; 1.
DR   Gene3D; 3.90.180.10; Medium-chain alcohol dehydrogenases, catalytic domain; 1.
DR   Gene3D; 3.40.50.720; NAD(P)-binding Rossmann-like Domain; 1.
DR   Gene3D; 3.10.129.110; Polyketide synthase dehydratase; 1.
DR   Gene3D; 3.40.50.150; Vaccinia Virus protein VP39; 1.
DR   InterPro; IPR029058; AB_hydrolase.
DR   InterPro; IPR001227; Ac_transferase_dom_sf.
DR   InterPro; IPR036736; ACP-like_sf.
DR   InterPro; IPR014043; Acyl_transferase.
DR   InterPro; IPR016035; Acyl_Trfase/lysoPLipase.
DR   InterPro; IPR013149; ADH-like_C.
DR   InterPro; IPR011032; GroES-like_sf.
DR   InterPro; IPR018201; Ketoacyl_synth_AS.
DR   InterPro; IPR014031; Ketoacyl_synth_C.
DR   InterPro; IPR014030; Ketoacyl_synth_N.
DR   InterPro; IPR016036; Malonyl_transacylase_ACP-bd.
DR   InterPro; IPR013217; Methyltransf_12.
DR   InterPro; IPR036291; NAD(P)-bd_dom_sf.
DR   InterPro; IPR032821; PKS_assoc.
DR   InterPro; IPR020841; PKS_Beta-ketoAc_synthase_dom.
DR   InterPro; IPR042104; PKS_dehydratase_sf.
DR   InterPro; IPR020807; PKS_DH.
DR   InterPro; IPR020843; PKS_ER.
DR   InterPro; IPR013968; PKS_KR.
DR   InterPro; IPR020806; PKS_PP-bd.
DR   InterPro; IPR009081; PP-bd_ACP.
DR   InterPro; IPR006162; Ppantetheine_attach_site.
DR   InterPro; IPR029063; SAM-dependent_MTases_sf.
DR   InterPro; IPR001031; Thioesterase.
DR   InterPro; IPR016039; Thiolase-like.
DR   PANTHER; PTHR43775; FATTY ACID SYNTHASE; 1.
DR   PANTHER; PTHR43775:SF7; FATTY ACID SYNTHASE; 1.
DR   Pfam; PF00698; Acyl_transf_1; 1.
DR   Pfam; PF00107; ADH_zinc_N; 1.
DR   Pfam; PF16197; KAsynt_C_assoc; 1.
DR   Pfam; PF00109; ketoacyl-synt; 1.
DR   Pfam; PF02801; Ketoacyl-synt_C; 1.
DR   Pfam; PF08659; KR; 1.
DR   Pfam; PF08242; Methyltransf_12; 1.
DR   Pfam; PF00550; PP-binding; 1.
DR   Pfam; PF14765; PS-DH; 1.
DR   Pfam; PF00975; Thioesterase; 1.
DR   SMART; SM00827; PKS_AT; 1.
DR   SMART; SM00826; PKS_DH; 1.
DR   SMART; SM00829; PKS_ER; 1.
DR   SMART; SM00822; PKS_KR; 1.
DR   SMART; SM00825; PKS_KS; 1.
DR   SMART; SM00823; PKS_PP; 1.
DR   SUPFAM; SSF47336; ACP-like; 1.
DR   SUPFAM; SSF53474; alpha/beta-Hydrolases; 1.
DR   SUPFAM; SSF52151; FabD/lysophospholipase-like; 1.
DR   SUPFAM; SSF50129; GroES-like; 1.
DR   SUPFAM; SSF51735; NAD(P)-binding Rossmann-fold domains; 2.
DR   SUPFAM; SSF55048; Probable ACP-binding domain of malonyl-CoA ACP transacylase; 1.
DR   SUPFAM; SSF53335; S-adenosyl-L-methionine-dependent methyltransferases; 1.
DR   SUPFAM; SSF53901; Thiolase-like; 1.
DR   PROSITE; PS50075; CARRIER; 1.
DR   PROSITE; PS00606; KS3_1; 1.
DR   PROSITE; PS52004; KS3_2; 1.
DR   PROSITE; PS00012; PHOSPHOPANTETHEINE; 1.
DR   PROSITE; PS52019; PKS_MFAS_DH; 1.
PE   1: Evidence at protein level;
KW   3D-structure; Acetylation; Cytoplasm; Direct protein sequencing;
KW   Fatty acid biosynthesis; Fatty acid metabolism; Hydrolase; Isopeptide bond;
KW   Lipid biosynthesis; Lipid metabolism; Lyase; Multifunctional enzyme; NAD;
KW   NADP; Oxidoreductase; Phosphopantetheine; Phosphoprotein;
KW   Pyridoxal phosphate; Reference proteome; S-nitrosylation; Transferase;
KW   Ubl conjugation.
FT   CHAIN           1..2504
FT                   /note="Fatty acid synthase"
FT                   /id="PRO_0000180277"
FT   DOMAIN          1..406
FT                   /note="Ketosynthase family 3 (KS3)"
FT                   /evidence="ECO:0000255|PROSITE-ProRule:PRU01348"
FT   DOMAIN          844..1104
FT                   /note="PKS/mFAS DH"
FT                   /evidence="ECO:0000255|PROSITE-ProRule:PRU01363"
FT   DOMAIN          2112..2192
FT                   /note="Carrier"
FT                   /evidence="ECO:0000255|PROSITE-ProRule:PRU00258"
FT   REGION          429..817
FT                   /note="Acyl and malonyl transferases"
FT   REGION          844..967
FT                   /note="N-terminal hotdog fold"
FT                   /evidence="ECO:0000255|PROSITE-ProRule:PRU01363"
FT   REGION          982..1104
FT                   /note="C-terminal hotdog fold"
FT                   /evidence="ECO:0000255|PROSITE-ProRule:PRU01363"
FT   REGION          1628..1856
FT                   /note="Enoyl reductase"
FT   REGION          1857..2111
FT                   /note="Beta-ketoacyl reductase"
FT   REGION          2181..2205
FT                   /note="Disordered"
FT                   /evidence="ECO:0000256|SAM:MobiDB-lite"
FT   REGION          2201..2504
FT                   /note="Thioesterase"
FT   ACT_SITE        161
FT                   /note="For beta-ketoacyl synthase activity"
FT                   /evidence="ECO:0000255|PROSITE-ProRule:PRU01348"
FT   ACT_SITE        293
FT                   /note="For beta-ketoacyl synthase activity"
FT                   /evidence="ECO:0000255|PROSITE-ProRule:PRU01348"
FT   ACT_SITE        331
FT                   /note="For beta-ketoacyl synthase activity"
FT                   /evidence="ECO:0000255|PROSITE-ProRule:PRU01348"
FT   ACT_SITE        581
FT                   /note="For malonyltransferase activity"
FT                   /evidence="ECO:0000255|PROSITE-ProRule:PRU10022"
FT   ACT_SITE        878
FT                   /note="Proton acceptor; for dehydratase activity"
FT                   /evidence="ECO:0000255|PROSITE-ProRule:PRU01363"
FT   ACT_SITE        1032
FT                   /note="Proton donor; for dehydratase activity"
FT                   /evidence="ECO:0000255|PROSITE-ProRule:PRU01363"
FT   ACT_SITE        2301
FT                   /note="For thioesterase activity"
FT                   /evidence="ECO:0000255|PROSITE-ProRule:PRU10022"
FT   ACT_SITE        2474
FT                   /note="For thioesterase activity"
FT                   /evidence="ECO:0000255|PROSITE-ProRule:PRU10022"
FT   BINDING         647..648
FT                   /ligand="an acyl-CoA"
FT                   /ligand_id="ChEBI:CHEBI:58342"
FT                   /evidence="ECO:0000269|PubMed:31811668,
FT                   ECO:0007744|PDB:6ROP"
FT   BINDING         671
FT                   /ligand="an acyl-CoA"
FT                   /ligand_id="ChEBI:CHEBI:58342"
FT                   /evidence="ECO:0000269|PubMed:31811668,
FT                   ECO:0007744|PDB:6ROP"
FT   BINDING         773
FT                   /ligand="an acyl-CoA"
FT                   /ligand_id="ChEBI:CHEBI:58342"
FT                   /evidence="ECO:0000269|PubMed:31811668,
FT                   ECO:0007744|PDB:6ROP"
FT   BINDING         1664..1681
FT                   /ligand="NADP(+)"
FT                   /ligand_id="ChEBI:CHEBI:58349"
FT                   /ligand_label="1"
FT                   /ligand_note="for enoyl reductase activity"
FT                   /evidence="ECO:0000250"
FT   BINDING         1879..1894
FT                   /ligand="NADP(+)"
FT                   /ligand_id="ChEBI:CHEBI:58349"
FT                   /ligand_label="2"
FT                   /ligand_note="for ketoreductase activity"
FT                   /evidence="ECO:0000250"
FT   MOD_RES         1
FT                   /note="N-acetylmethionine"
FT                   /evidence="ECO:0000269|Ref.4"
FT   MOD_RES         59
FT                   /note="N6-acetyllysine"
FT                   /evidence="ECO:0007744|PubMed:23806337"
FT   MOD_RES         63
FT                   /note="Phosphoserine"
FT                   /evidence="ECO:0000250|UniProtKB:P49327"
FT   MOD_RES         70
FT                   /note="N6-acetyllysine"
FT                   /evidence="ECO:0000250|UniProtKB:P49327"
FT   MOD_RES         207
FT                   /note="Phosphoserine"
FT                   /evidence="ECO:0000250|UniProtKB:P49327"
FT   MOD_RES         298
FT                   /note="N6-acetyllysine"
FT                   /evidence="ECO:0000250|UniProtKB:P49327"
FT   MOD_RES         528
FT                   /note="N6-acetyllysine"
FT                   /evidence="ECO:0000250|UniProtKB:P49327"
FT   MOD_RES         673
FT                   /note="N6-acetyllysine"
FT                   /evidence="ECO:0000250|UniProtKB:P49327"
FT   MOD_RES         725
FT                   /note="Phosphoserine"
FT                   /evidence="ECO:0007744|PubMed:21183079"
FT   MOD_RES         790
FT                   /note="N6-acetyllysine"
FT                   /evidence="ECO:0007744|PubMed:23806337"
FT   MOD_RES         993
FT                   /note="N6-acetyllysine"
FT                   /evidence="ECO:0007744|PubMed:23806337"
FT   MOD_RES         1071
FT                   /note="N6-acetyllysine"
FT                   /evidence="ECO:0007744|PubMed:23806337"
FT   MOD_RES         1276
FT                   /note="N6-acetyllysine"
FT                   /evidence="ECO:0007744|PubMed:23806337"
FT   MOD_RES         1464
FT                   /note="S-nitrosocysteine"
FT                   /evidence="ECO:0000250|UniProtKB:P49327"
FT   MOD_RES         1577
FT                   /note="Phosphoserine"
FT                   /evidence="ECO:0007744|PubMed:21183079"
FT   MOD_RES         1587
FT                   /note="Phosphoserine"
FT                   /evidence="ECO:0007744|PubMed:21183079"
FT   MOD_RES         1697
FT                   /note="N6-(pyridoxal phosphate)lysine; alternate"
FT                   /evidence="ECO:0000250"
FT   MOD_RES         1697
FT                   /note="N6-acetyllysine; alternate"
FT                   /evidence="ECO:0000250|UniProtKB:P49327"
FT   MOD_RES         1764
FT                   /note="N6-acetyllysine"
FT                   /evidence="ECO:0000250|UniProtKB:P49327"
FT   MOD_RES         1840
FT                   /note="N6-acetyllysine"
FT                   /evidence="ECO:0007744|PubMed:23806337"
FT   MOD_RES         1988
FT                   /note="N6-acetyllysine"
FT                   /evidence="ECO:0000250|UniProtKB:P49327"
FT   MOD_RES         2084
FT                   /note="S-nitrosocysteine"
FT                   /evidence="ECO:0000250|UniProtKB:P49327"
FT   MOD_RES         2150
FT                   /note="O-(pantetheine 4'-phosphoryl)serine; alternate"
FT                   /evidence="ECO:0000255|PROSITE-ProRule:PRU00258"
FT   MOD_RES         2150
FT                   /note="Phosphoserine; alternate"
FT                   /evidence="ECO:0000250|UniProtKB:P12785"
FT   MOD_RES         2190
FT                   /note="Phosphoserine"
FT                   /evidence="ECO:0007744|PubMed:21183079"
FT   MOD_RES         2229
FT                   /note="Phosphoserine"
FT                   /evidence="ECO:0000250|UniProtKB:P49327"
FT   MOD_RES         2384
FT                   /note="N6-acetyllysine"
FT                   /evidence="ECO:0007744|PubMed:23806337"
FT   CROSSLNK        2442
FT                   /note="Glycyl lysine isopeptide (Lys-Gly) (interchain with
FT                   G-Cter in SUMO2)"
FT                   /evidence="ECO:0000250|UniProtKB:P49327"
FT   CONFLICT        1992
FT                   /note="T -> N (in Ref. 6; CAA31525)"
FT                   /evidence="ECO:0000305"
FT   CONFLICT        2028
FT                   /note="G -> C (in Ref. 6; CAA31525)"
FT                   /evidence="ECO:0000305"
FT   CONFLICT        2045
FT                   /note="G -> V (in Ref. 6; CAA31525)"
FT                   /evidence="ECO:0000305"
FT   CONFLICT        2117
FT                   /note="T -> N (in Ref. 6; CAA31525)"
FT                   /evidence="ECO:0000305"
FT   CONFLICT        2175
FT                   /note="Q -> R (in Ref. 6; CAA31525)"
FT                   /evidence="ECO:0000305"
FT   CONFLICT        2295
FT                   /note="Y -> H (in Ref. 6; CAA31525)"
FT                   /evidence="ECO:0000305"
FT   STRAND          4..13
FT                   /evidence="ECO:0007829|PDB:5MY2"
FT   STRAND          16..18
FT                   /evidence="ECO:0007829|PDB:5MY2"
FT   HELIX           19..27
FT                   /evidence="ECO:0007829|PDB:5MY2"
FT   STRAND          36..40
FT                   /evidence="ECO:0007829|PDB:5MY2"
FT   HELIX           44..46
FT                   /evidence="ECO:0007829|PDB:5MY2"
FT   HELIX           62..65
FT                   /evidence="ECO:0007829|PDB:5MY2"
FT   HELIX           69..73
FT                   /evidence="ECO:0007829|PDB:5MY2"
FT   HELIX           77..92
FT                   /evidence="ECO:0007829|PDB:5MY2"
FT   HELIX           97..100
FT                   /evidence="ECO:0007829|PDB:5MY2"
FT   STRAND          106..111
FT                   /evidence="ECO:0007829|PDB:5MY2"
FT   HELIX           115..120
FT                   /evidence="ECO:0007829|PDB:5MY2"
FT   TURN            124..126
FT                   /evidence="ECO:0007829|PDB:5MY2"
FT   HELIX           130..135
FT                   /evidence="ECO:0007829|PDB:5MY2"
FT   HELIX           139..148
FT                   /evidence="ECO:0007829|PDB:5MY2"
FT   STRAND          154..158
FT                   /evidence="ECO:0007829|PDB:5MY2"
FT   HELIX           160..162
FT                   /evidence="ECO:0007829|PDB:5MY2"
FT   HELIX           163..176
FT                   /evidence="ECO:0007829|PDB:5MY2"
FT   STRAND          181..189
FT                   /evidence="ECO:0007829|PDB:5MY2"
FT   HELIX           194..202
FT                   /evidence="ECO:0007829|PDB:5MY2"
FT   STRAND          227..235
FT                   /evidence="ECO:0007829|PDB:5MY2"
FT   HELIX           236..238
FT                   /evidence="ECO:0007829|PDB:5MY2"
FT   STRAND          243..253
FT                   /evidence="ECO:0007829|PDB:5MY2"
FT   HELIX           266..276
FT                   /evidence="ECO:0007829|PDB:5MY2"
FT   HELIX           278..280
FT                   /evidence="ECO:0007829|PDB:5MY2"
FT   HELIX           284..286
FT                   /evidence="ECO:0007829|PDB:5MY2"
FT   STRAND          287..291
FT                   /evidence="ECO:0007829|PDB:5MY2"
FT   HELIX           300..311
FT                   /evidence="ECO:0007829|PDB:5MY2"
FT   HELIX           313..315
FT                   /evidence="ECO:0007829|PDB:5MY2"
FT   STRAND          320..323
FT                   /evidence="ECO:0007829|PDB:5MY2"
FT   HELIX           326..329
FT                   /evidence="ECO:0007829|PDB:5MY2"
FT   HELIX           333..335
FT                   /evidence="ECO:0007829|PDB:5MY2"
FT   HELIX           336..349
FT                   /evidence="ECO:0007829|PDB:5MY2"
FT   HELIX           367..370
FT                   /evidence="ECO:0007829|PDB:5MY2"
FT   STRAND          373..376
FT                   /evidence="ECO:0007829|PDB:5MY2"
FT   STRAND          387..393
FT                   /evidence="ECO:0007829|PDB:5MY2"
FT   STRAND          397..406
FT                   /evidence="ECO:0007829|PDB:5MY2"
FT   HELIX           416..418
FT                   /evidence="ECO:0007829|PDB:5MY2"
FT   STRAND          422..430
FT                   /evidence="ECO:0007829|PDB:5MY2"
FT   HELIX           431..443
FT                   /evidence="ECO:0007829|PDB:5MY2"
FT   TURN            444..446
FT                   /evidence="ECO:0007829|PDB:5MY2"
FT   HELIX           448..456
FT                   /evidence="ECO:0007829|PDB:5MY2"
FT   TURN            462..464
FT                   /evidence="ECO:0007829|PDB:5MY2"
FT   STRAND          467..477
FT                   /evidence="ECO:0007829|PDB:5MY2"
FT   STRAND          480..484
FT                   /evidence="ECO:0007829|PDB:5MY2"
FT   STRAND          492..496
FT                   /evidence="ECO:0007829|PDB:5MY2"
FT   TURN            504..507
FT                   /evidence="ECO:0007829|PDB:5MY2"
FT   HELIX           509..512
FT                   /evidence="ECO:0007829|PDB:5MY2"
FT   HELIX           514..527
FT                   /evidence="ECO:0007829|PDB:5MY2"
FT   TURN            528..531
FT                   /evidence="ECO:0007829|PDB:5MY2"
FT   HELIX           534..539
FT                   /evidence="ECO:0007829|PDB:5MY2"
FT   HELIX           545..547
FT                   /evidence="ECO:0007829|PDB:5MY2"
FT   HELIX           549..569
FT                   /evidence="ECO:0007829|PDB:5MY2"
FT   STRAND          575..579
FT                   /evidence="ECO:0007829|PDB:5MY2"
FT   HELIX           583..590
FT                   /evidence="ECO:0007829|PDB:5MY2"
FT   HELIX           596..611
FT                   /evidence="ECO:0007829|PDB:5MY2"
FT   TURN            612..614
FT                   /evidence="ECO:0007829|PDB:5MY0"
FT   STRAND          618..625
FT                   /evidence="ECO:0007829|PDB:5MY2"
FT   HELIX           627..633
FT                   /evidence="ECO:0007829|PDB:5MY2"
FT   STRAND          639..643
FT                   /evidence="ECO:0007829|PDB:5MY2"
FT   STRAND          645..654
FT                   /evidence="ECO:0007829|PDB:5MY2"
FT   HELIX           655..658
FT                   /evidence="ECO:0007829|PDB:5MY2"
FT   HELIX           660..666
FT                   /evidence="ECO:0007829|PDB:5MY2"
FT   TURN            667..669
FT                   /evidence="ECO:0007829|PDB:5MY2"
FT   STRAND          672..675
FT                   /evidence="ECO:0007829|PDB:5MY2"
FT   HELIX           685..687
FT                   /evidence="ECO:0007829|PDB:5MY2"
FT   TURN            688..690
FT                   /evidence="ECO:0007829|PDB:5MY2"
FT   HELIX           691..701
FT                   /evidence="ECO:0007829|PDB:5MY2"
FT   STRAND          702..704
FT                   /evidence="ECO:0007829|PDB:5MY2"
FT   STRAND          715..717
FT                   /evidence="ECO:0007829|PDB:5MY2"
FT   HELIX           719..721
FT                   /evidence="ECO:0007829|PDB:5MY2"
FT   STRAND          722..724
FT                   /evidence="ECO:0007829|PDB:5MY2"
FT   HELIX           725..728
FT                   /evidence="ECO:0007829|PDB:5MY2"
FT   HELIX           732..740
FT                   /evidence="ECO:0007829|PDB:5MY2"
FT   HELIX           745..749
FT                   /evidence="ECO:0007829|PDB:5MY2"
FT   STRAND          757..764
FT                   /evidence="ECO:0007829|PDB:5MY2"
FT   HELIX           768..774
FT                   /evidence="ECO:0007829|PDB:5MY2"
FT   STRAND          779..782
FT                   /evidence="ECO:0007829|PDB:5MY2"
FT   HELIX           792..804
FT                   /evidence="ECO:0007829|PDB:5MY2"
FT   TURN            805..807
FT                   /evidence="ECO:0007829|PDB:5MY2"
FT   HELIX           812..815
FT                   /evidence="ECO:0007829|PDB:5MY2"
FT   HELIX           831..833
FT                   /evidence="ECO:0007829|PDB:5MY2"
FT   HELIX           847..849
FT                   /evidence="ECO:0007829|PDB:5MY2"
SQ   SEQUENCE   2504 AA;  272428 MW;  2B48068B9D370C6F CRC64;
     MEEVVIAGMS GKLPESENLQ EFWANLIGGV DMVTDDDRRW KAGLYGLPKR SGKLKDLSKF
     DASFFGVHPK QAHTMDPQLR LLLEVSYEAI VDGGINPASL RGTNTGVWVG VSGSEASEAL
     SRDPETLLGY SMVGCQRAMM ANRLSFFFDF KGPSIALDTA CSSSLLALQN AYQAIRSGEC
     PAALVGGINL LLKPNTSVQF MKLGMLSPDG TCRSFDDSGS GYCRSEAVVA VLLTKKSLAR
     RVYATILNAG TNTDGSKEQG VTFPSGEVQE QLICSLYQPA GLAPESLEYI EAHGTGTKVG
     DPQELNGITR SLCAFRQAPL LIGSTKSNMG HPEPASGLAA LTKVLLSLEH GVWAPNLHFH
     NPNPEIPALL DGRLQVVDRP LPVRGGNVGI NSFGFGGSNV HVILQPNTRQ APAPTAHAAL
     PHLLHASGRT LEAVQDLLEQ GRQHSQDLAF VSMLNDIAAT PTAAMPFRGY TVLGVEGRVQ
     EVQQVSTNKR PLWFICSGMG TQWRGMGLSL MRLDSFRESI LRSDEAVKPL GVKVSDLLLS
     TDERTFDDIV HAFVSLTAIQ IALIDLLTSV GLKPDGIIGH SLGEVACGYA DGCLSQREAV
     LAAYWRGQCI KDAHLPPGSM AAVGLSWEEC KQRCPAGVVP ACHNSEDTVT ISGPQAAVNE
     FVEQLKQEGV FAKEVRTGGL AFHSYFMEGI APTLLQALKK VIREPRPRSA RWLSTSIPEA
     QWQSSLARTS SAEYNVNNLV SPVLFQEALW HIPEHAVVLE IAPHALLQAV LKRGVKSSCT
     IIPLMKRDHK DNLEFFLTNL GKVHLTGINV NPNALFPPVE FPAPRGTPLI SPHIKWDHSQ
     TWDVPVAEDF PNGSSSSSAT VYSIDASPES PDHYLVDHCI DGRVIFPGTG YLCLVWKTLA
     RSLGLSLEET PVVFENVSFH QATILPKTGT VALEVRLLEA SHAFEVSDTG NLIVSGKVYL
     WEDPNSKLFD HPEVPTPPES ASVSRLTQGE VYKELRLRGY DYGPQFQGIC EATLEGEQGK
     LLWKDNWVTF MDTMLQVSIL GSSQQSLQLP TRVTAIYIDP ATHRQKVYRL KEDTQVADVT
     TSRCLGITVS GGIHISRLQT TATSRRQQEQ LVPTLEKFVF TPHMEAECLS ESTALQKELQ
     LCKGLARALQ TKATQQGLKA AMLGQEDPPQ HGLPRLLAAA CQLQLNGNLQ LELGEALAQE
     RLLLPEDPLI SGLLNSQALK ACVDTALENL STLKMKVAEV LAGEGHLYSR IPALLNTQPM
     LQLEYTATDR HPQALKDVQT KLQQHDVAQG QWNPSDPAPS SLGALDLLVC NCALATLGDP
     ALALDNMVAA LKEGGFLLVH TVLKGHALGE TLACLPSEVQ PAPSLLSQEE WESLFSRKAL
     HLVGLKRSFY GTALFLCRRA IPQEKPIFLS VEDTSFQWVD SLKSTLATSS SQPVWLTAMD
     CPTSGVVGLV NCLRKEPGGH RIRCILLSNL SNTSHAPKLD PGSPELQQVL KHDLVMNVYR
     DGAWGAFRHF QLEQDKPKEQ TAHAFVNVLT RGDLASIRWV SSPLKHTQPS SSGAQLCTVY
     YASLNFRDIM LATGKLSPDA IPGKWASRDC MLGMEFSGRD RCGRRVMGLV PAEGLATSVL
     LSSDFLWDVP SSWTLEEAAS VPVVYTTAYY SLVVRGRIQR GETVLIHSGS GGVGQAAISI
     ALSLGCRVFT TVGSAEKRAY LQARFPQLDD TSFANSRDTS FEQHVLLHTG GKGVDLVLNS
     LAEEKLQASV RCLAQHGRFL EIGKFDLSNN HPLGMAIFLK NVTFHGILLD ALFEEANDSW
     REVAALLKAG IRDGVVKPLK CTVFPKAQVE DAFRYMAQGK HIGKVLVQVR EEEPEAVLPG
     AQPTLISAIS KTFCPAHKSY IITGGLGGFG LELARWLVLR GAQRLVLTSR SGIRTGYQAK
     HIREWRRQGI QVLVSTSNVS SLEGARALIA EATKLGPVGG VFNLAMVLRD AMLENQTPEL
     FQDVNKPKYN GTLNLDRATR EACPELDYFV AFSSVSCGRG NAGQTNYGFA NSTMERICEQ
     RRHDGLPGLA VQWGAIGDVG IVLEAMGTND TVIGGTLPQR ISSCMEVLDL FLNQPHAVLS
     SFVLAEKKAV AHGDGDTQRD LVKAVAHILG IRDLAGINLD STLADLGLDS LMGVEVRQIL
     EREHDLVLPM REVRQLTLRK LQEMSSKTDS ATDTTAPKSR SDTSLKQNQL NLSTLLVNPE
     GPTLTQLNSV QSSERPLFLV HPIEGSTTVF HSLAAKLSVP TYGLQCTQAA PLDSIPNLAA
     YYIDCIKQVQ PEGPYRIAGY SFGACVAFEM CSQLQAQQGP APTHNNLFLF DGSHTYVLAY
     TQSYRAKMTP GCEAEAEAEA LCFFIKQFLD VEHSKVLEAL LPLKSLEDRV AASVDLITKS
     HHSLDRRELS FAAVSFYHKL RAADQYKPKA KYHGNVTLLR AKTGGTYGED LGADYNLSQV
     CDGKVSVHII EGDHRTLLEG SGLESIINII HSSLAEPRVS VREG
//
```

**Mascot:** http://www.matrixscience.com/


HNE (H) (+156.1150)
