# Supplementary material for: A common East-Asian ALDH2 mutation causes metabolic disorders and the therapeutic effect of ALDH2 activators
Source: Nat Commun. 2023 Sep 25;14:5971. doi: 10.1038/s41467-023-41570-6 (PMC10520061; doi:10.1038/s41467-023-41570-6)
Supplement: Supplementary file 4 — Supplementary Data 1 [file 41467_2023_41570_MOESM4_ESM.zip › Table S5c/P52503/P52503_KIO-1_K53.html]

Mascot Search Results: P52503
 

# MASCOT Search Results

## Protein View: P52503

### NADH dehydrogenase [ubiquinone] iron-sulfur protein 6, mitochondrial OS=Mus musculus OX=10090 GN=Ndufs6 PE=1 SV=2

|  |  |
| --- | --- |
| Database: | Mouse\_UniProt\_proteomes |
| Score: | 1478 |
| Monoisotopic mass (M_r_): | 13183 |
| Calculated pI: | 8.87 |

Sequence similarity is available as an NCBI BLAST search of P52503 against nr.

### Search parameters

|  |  |
| --- | --- |
| MS data file: | `D:\LCMSMS\2023 Users' data\230529-1\230529-1-KIO-1.raw` |
| Enzyme: | Trypsin/P: cuts C-term side of KR. |
| Fixed modifications: | Carbamidomethyl (C) |
| Variable modifications: | Deamidated (NQ), HNE (C), HNE (H), HNE (K), Oxidation (M) |

### Protein sequence coverage: 68%

Matched peptides shown in ***bold red***.

|  |  |  |  |  |  |
| --- | --- | --- | --- | --- | --- |
| `1` | `MAAVLTFRRL` | `LTLPRAARGF` | `GVQVSPSGEK` | `ITHTGQVYDE` | `KDYRRVRFVD` |
| `51` | `RQKEVNENFA` | `IDLIAQQPVN` | `EVEHRIIACD` | `GGGGALGHPK` | `VYINLDKETK` |
| `101` | `TGTCGYCGLQ` | `FKQHHH` |  |  |  |

Unformatted sequence string: 116 residues (for pasting into other applications).

|  |  |  |  |
| --- | --- | --- | --- |
| Sort by | residue number | increasing mass | decreasing mass |
| Show | matched peptides only | predicted peptides also |  |

| Query | Start | – | End | Observed | Mr(expt) | Mr(calc) | ppm | M | Score | Expect | Rank | U | Peptide |
| --- | --- | --- | --- | --- | --- | --- | --- | --- | --- | --- | --- | --- | --- |
| 91922 | 31 | – | 44 | 431.9621 | 1723.8193 | 1723.8166 | 1.60 | 1 | 38 | 0.00029 | 1Score **> 33** indicates **identity** Score **> 15** indicates **homology** | U | K.ITHTGQVYDEKDYR.R |
| 91923 | 31 | – | 44 | 431.9621 | 1723.8194 | 1723.8166 | 1.63 | 1 | 22 | 0.0094 | 1Score **> 33** indicates **identity** Score **> 14** indicates **homology** | U | K.ITHTGQVYDEKDYR.R |
| 91924 | 31 | – | 44 | 575.6142 | 1723.8208 | 1723.8166 | 2.43 | 1 | 44 | 7e-05 | 1Score **> 33** indicates **identity** Score **> 15** indicates **homology** | U | K.ITHTGQVYDEKDYR.R |
| 91925 | 31 | – | 44 | 862.9183 | 1723.8220 | 1723.8166 | 3.15 | 1 | 29 | 0.0019 | 1Score **> 33** indicates **identity** Score **> 14** indicates **homology** | U | K.ITHTGQVYDEKDYR.R |
| 108943 | 31 | – | 45 | 940.9630 | 1879.9114 | 1879.9177 | -3.36 | 2 | 21 | 0.032 | 1Score **> 34** indicates **identity** Score **> 18** indicates **homology** | U | K.ITHTGQVYDEKDYRR.V |
| 108946 | 31 | – | 45 | 470.9861 | 1879.9153 | 1879.9177 | -1.26 | 2 | 39 | 0.02 | 1Score **> 35** indicates **identity** | U | K.ITHTGQVYDEKDYRR.V |
| 108954 | 31 | – | 45 | 470.9866 | 1879.9174 | 1879.9177 | -0.13 | 2 | 52 | 0.0011 | 1Score **> 35** indicates **identity** | U | K.ITHTGQVYDEKDYRR.V |
| 108956 | 31 | – | 45 | 470.9869 | 1879.9184 | 1879.9177 | 0.37 | 2 | 42 | 0.0096 | 1Score **> 35** indicates **identity** | U | K.ITHTGQVYDEKDYRR.V |
| 108964 | 31 | – | 45 | 376.9918 | 1879.9227 | 1879.9177 | 2.66 | 2 | 35 | 0.011 | 1Score **> 35** indicates **identity** Score **> 28** indicates **homology** | U | K.ITHTGQVYDEKDYRR.V |
| 186263 | 48 | – | 75 | 835.1739 | 3336.6665 | 3336.6851 | -5.57 | 2 | 43 | 0.0001 | 1Score **> 37** indicates **identity** Score **> 15** indicates **homology** | U | R.FVDRQKEVNENFAIDLIAQQPVNEVEHR.I |
| 186265 | 48 | – | 75 | 668.3434 | 3336.6806 | 3336.6851 | -1.33 | 2 | 18 | 0.02 | 1Score **> 38** indicates **identity** Score **> 14** indicates **homology** | U | R.FVDRQKEVNENFAIDLIAQQPVNEVEHR.I |
| 186266 | 48 | – | 75 | 668.3442 | 3336.6848 | 3336.6851 | -0.075 | 2 | 15 | 0.043 | 1Score **> 38** indicates **identity** Score **> 13** indicates **homology** | U | R.FVDRQKEVNENFAIDLIAQQPVNEVEHR.I |
| 186267 | 48 | – | 75 | 835.1792 | 3336.6878 | 3336.6851 | 0.81 | 2 | 47 | 4e-05 | 1Score **> 38** indicates **identity** Score **> 15** indicates **homology** | U | R.FVDRQKEVNENFAIDLIAQQPVNEVEHR.I |
| 186268 | 48 | – | 75 | 835.1800 | 3336.6908 | 3336.6851 | 1.71 | 2 | 34 | 0.00058 | 1Score **> 37** indicates **identity** Score **> 15** indicates **homology** | U | R.FVDRQKEVNENFAIDLIAQQPVNEVEHR.I |
| 186294 | 48 | – | 75 | 1113.5716 | 3337.6930 | 3337.6691 | 7.15 | 2 | 25 | 0.0047 | 1Score **> 37** indicates **identity** Score **> 14** indicates **homology** | U | R.FVDRQKEVNENFAIDLIAQQPVNEVEHR.I  + Deamidated (NQ) |
| 189549 | 48 | – | 75 | 874.9433 | 3495.7441 | 3495.7521 | -2.30 | 2 | 14 | 0.047 | 1Score **> 37** indicates **identity** Score **> 13** indicates **homology** | U | R.FVDRQKEVNENFAIDLIAQQPVNEVEHR.I  + 3 Deamidated (NQ); HNE (K) |
| 172532 | 52 | – | 75 | 705.8627 | 2819.4215 | 2819.4202 | 0.48 | 1 | 61 | 1.9e-06 | 1Score **> 37** indicates **identity** Score **> 16** indicates **homology** | U | R.QKEVNENFAIDLIAQQPVNEVEHR.I |
| 172534 | 52 | – | 75 | 940.8147 | 2819.4223 | 2819.4202 | 0.74 | 1 | 74 | 1.2e-07 | 1Score **> 37** indicates **identity** Score **> 17** indicates **homology** | U | R.QKEVNENFAIDLIAQQPVNEVEHR.I |
| 172536 | 52 | – | 75 | 940.8148 | 2819.4225 | 2819.4202 | 0.81 | 1 | 67 | 5.2e-07 | 1Score **> 37** indicates **identity** Score **> 17** indicates **homology** | U | R.QKEVNENFAIDLIAQQPVNEVEHR.I |
| 172537 | 52 | – | 75 | 940.8149 | 2819.4227 | 2819.4202 | 0.91 | 1 | 37 | 0.00033 | 1Score **> 37** indicates **identity** Score **> 15** indicates **homology** | U | R.QKEVNENFAIDLIAQQPVNEVEHR.I |
| 172538 | 52 | – | 75 | 705.8630 | 2819.4229 | 2819.4202 | 0.97 | 1 | 52 | 1.3e-05 | 1Score **> 37** indicates **identity** Score **> 16** indicates **homology** | U | R.QKEVNENFAIDLIAQQPVNEVEHR.I |
| 172539 | 52 | – | 75 | 940.8149 | 2819.4230 | 2819.4202 | 0.99 | 1 | 117 | 1e-11 | 1Score **> 37** indicates **identity** Score **> 20** indicates **homology** | U | R.QKEVNENFAIDLIAQQPVNEVEHR.I |
| 172540 | 52 | – | 75 | 705.8630 | 2819.4231 | 2819.4202 | 1.02 | 1 | 28 | 0.0022 | 1Score **> 37** indicates **identity** Score **> 14** indicates **homology** | U | R.QKEVNENFAIDLIAQQPVNEVEHR.I |
| 172541 | 52 | – | 75 | 705.8631 | 2819.4233 | 2819.4202 | 1.09 | 1 | 58 | 3.7e-06 | 1Score **> 37** indicates **identity** Score **> 16** indicates **homology** | U | R.QKEVNENFAIDLIAQQPVNEVEHR.I |
| 172543 | 52 | – | 75 | 705.8640 | 2819.4267 | 2819.4202 | 2.32 | 1 | 23 | 0.0066 | 1Score **> 37** indicates **identity** Score **> 14** indicates **homology** | U | R.QKEVNENFAIDLIAQQPVNEVEHR.I |
| 172544 | 52 | – | 75 | 705.8643 | 2819.4279 | 2819.4202 | 2.75 | 1 | 26 | 0.0036 | 1Score **> 37** indicates **identity** Score **> 14** indicates **homology** | U | R.QKEVNENFAIDLIAQQPVNEVEHR.I |
| 172580 | 52 | – | 75 | 941.1458 | 2820.4156 | 2820.4042 | 4.05 | 1 | 36 | 0.00041 | 1Score **> 37** indicates **identity** Score **> 15** indicates **homology** | U | R.QKEVNENFAIDLIAQQPVNEVEHR.I  + Deamidated (NQ) |
| 172581 | 52 | – | 75 | 706.1116 | 2820.4172 | 2820.4042 | 4.60 | 1 | 14 | 0.046 | 1Score **> 37** indicates **identity** Score **> 13** indicates **homology** | U | R.QKEVNENFAIDLIAQQPVNEVEHR.I  + Deamidated (NQ) |
| 177675 | 52 | – | 75 | 993.4989 | 2977.4748 | 2977.5032 | -9.54 | 1 | 32 | 0.001 | 1Score **> 37** indicates **identity** Score **> 14** indicates **homology** | U | R.QKEVNENFAIDLIAQQPVNEVEHR.I  + 2 Deamidated (NQ); HNE (K) |
| 162082 | 54 | – | 75 | 855.4331 | 2563.2774 | 2563.2666 | 4.18 | 0 | 51 | 1.7e-05 | 1Score **> 37** indicates **identity** Score **> 16** indicates **homology** | U | K.EVNENFAIDLIAQQPVNEVEHR.I |
| 52940 | 76 | – | 90 | 474.9092 | 1421.7057 | 1421.7085 | -2.00 | 0 | 55 | 7.7e-06 | 1Score **> 33** indicates **identity** Score **> 16** indicates **homology** | U | R.IIACDGGGGALGHPK.V |
| 52941 | 76 | – | 90 | 711.8614 | 1421.7082 | 1421.7085 | -0.22 | 0 | 54 | 9.2e-06 | 1Score **> 33** indicates **identity** Score **> 16** indicates **homology** | U | R.IIACDGGGGALGHPK.V |
| 52942 | 76 | – | 90 | 474.9102 | 1421.7089 | 1421.7085 | 0.22 | 0 | 80 | 2.9e-08 | 1Score **> 33** indicates **identity** Score **> 18** indicates **homology** | U | R.IIACDGGGGALGHPK.V |
| 52943 | 76 | – | 90 | 711.8618 | 1421.7089 | 1421.7085 | 0.29 | 0 | 98 | 6.5e-10 | 1Score **> 33** indicates **identity** Score **> 19** indicates **homology** | U | R.IIACDGGGGALGHPK.V |
| 52944 | 76 | – | 90 | 474.9106 | 1421.7099 | 1421.7085 | 0.96 | 0 | 23 | 0.0075 | 1Score **> 34** indicates **identity** Score **> 14** indicates **homology** | U | R.IIACDGGGGALGHPK.V |
| 52946 | 76 | – | 90 | 711.8626 | 1421.7106 | 1421.7085 | 1.41 | 0 | 40 | 0.00016 | 1Score **> 33** indicates **identity** Score **> 15** indicates **homology** | U | R.IIACDGGGGALGHPK.V |
| 52947 | 76 | – | 90 | 711.8626 | 1421.7106 | 1421.7085 | 1.43 | 0 | 26 | 0.0033 | 1Score **> 33** indicates **identity** Score **> 14** indicates **homology** | U | R.IIACDGGGGALGHPK.V |
| 52948 | 76 | – | 90 | 474.9108 | 1421.7106 | 1421.7085 | 1.48 | 0 | 91 | 2.9e-09 | 1Score **> 33** indicates **identity** Score **> 18** indicates **homology** | U | R.IIACDGGGGALGHPK.V |
| 52949 | 76 | – | 90 | 474.9112 | 1421.7119 | 1421.7085 | 2.36 | 0 | 32 | 0.00095 | 1Score **> 33** indicates **identity** Score **> 15** indicates **homology** | U | R.IIACDGGGGALGHPK.V |
| 32396 | 91 | – | 100 | 408.2276 | 1221.6610 | 1221.6605 | 0.41 | 1 | 19 | 0.017 | 1Score **> 33** indicates **identity** Score **> 14** indicates **homology** | U | K.VYINLDKETK.T |
| 32397 | 91 | – | 100 | 611.8379 | 1221.6613 | 1221.6605 | 0.68 | 1 | 56 | 7.1e-06 | 1Score **> 33** indicates **identity** Score **> 17** indicates **homology** | U | K.VYINLDKETK.T |
| 32398 | 91 | – | 100 | 611.8380 | 1221.6614 | 1221.6605 | 0.71 | 1 | 43 | 0.0001 | 1Score **> 33** indicates **identity** Score **> 16** indicates **homology** | U | K.VYINLDKETK.T |
| 32399 | 91 | – | 100 | 408.2279 | 1221.6620 | 1221.6605 | 1.21 | 1 | 37 | 0.0003 | 1Score **> 33** indicates **identity** Score **> 15** indicates **homology** | U | K.VYINLDKETK.T |
| 32400 | 91 | – | 100 | 408.2279 | 1221.6620 | 1221.6605 | 1.21 | 1 | 32 | 0.00092 | 1Score **> 33** indicates **identity** Score **> 15** indicates **homology** | U | K.VYINLDKETK.T |
| 49430 | 101 | – | 112 | 696.3071 | 1390.5997 | 1390.6010 | -0.95 | 0 | 28 | 0.0022 | 1Score **> 26** indicates **identity** Score **> 14** indicates **homology** | U | K.TGTCGYCGLQFK.Q |
| 49431 | 101 | – | 112 | 696.3073 | 1390.6000 | 1390.6010 | -0.74 | 0 | 14 | 0.044 | 1Score **> 26** indicates **identity** Score **> 13** indicates **homology** | U | K.TGTCGYCGLQFK.Q |
| 49433 | 101 | – | 112 | 696.3080 | 1390.6015 | 1390.6010 | 0.39 | 0 | 73 | 1.5e-07 | 1Score **> 27** indicates **identity** Score **> 17** indicates **homology** | U | K.TGTCGYCGLQFK.Q |
| 49434 | 101 | – | 112 | 696.3083 | 1390.6021 | 1390.6010 | 0.80 | 0 | 83 | 1.7e-08 | 1Score **> 27** indicates **identity** Score **> 18** indicates **homology** | U | K.TGTCGYCGLQFK.Q |
| 49435 | 101 | – | 112 | 696.3083 | 1390.6021 | 1390.6010 | 0.81 | 0 | 89 | 4.1e-09 | 1Score **> 27** indicates **identity** Score **> 18** indicates **homology** | U | K.TGTCGYCGLQFK.Q |
| 49436 | 101 | – | 112 | 696.3085 | 1390.6025 | 1390.6010 | 1.10 | 0 | 68 | 3.9e-07 | 1Score **> 27** indicates **identity** Score **> 17** indicates **homology** | U | K.TGTCGYCGLQFK.Q |
| 49437 | 101 | – | 112 | 696.3089 | 1390.6032 | 1390.6010 | 1.59 | 0 | 74 | 1.1e-07 | 1Score **> 27** indicates **identity** Score **> 17** indicates **homology** | U | K.TGTCGYCGLQFK.Q |
| 49438 | 101 | – | 112 | 696.3098 | 1390.6051 | 1390.6010 | 2.94 | 0 | 19 | 0.017 | 1Score **> 27** indicates **identity** Score **> 14** indicates **homology** | U | K.TGTCGYCGLQFK.Q |
| 49439 | 101 | – | 112 | 696.3119 | 1390.6092 | 1390.6010 | 5.90 | 0 | 20 | 0.015 | 1Score **> 28** indicates **identity** Score **> 14** indicates **homology** | U | K.TGTCGYCGLQFK.Q |

---

```
ID   NDUS6_MOUSE             Reviewed;         116 AA.
AC   P52503; Q5M9J7;
DT   01-OCT-1996, integrated into UniProtKB/Swiss-Prot.
DT   06-DEC-2005, sequence version 2.
DT   28-JUN-2023, entry version 154.
DE   RecName: Full=NADH dehydrogenase [ubiquinone] iron-sulfur protein 6, mitochondrial;
DE   AltName: Full=Complex I-13kD-A;
DE            Short=CI-13kD-A;
DE   AltName: Full=NADH-ubiquinone oxidoreductase 13 kDa-A subunit;
DE   Flags: Precursor;
GN   Name=Ndufs6; Synonyms=Ip13;
OS   Mus musculus (Mouse).
OC   Eukaryota; Metazoa; Chordata; Craniata; Vertebrata; Euteleostomi; Mammalia;
OC   Eutheria; Euarchontoglires; Glires; Rodentia; Myomorpha; Muroidea; Muridae;
OC   Murinae; Mus; Mus.
OX   NCBI_TaxID=10090;
RN   [1]
RP   NUCLEOTIDE SEQUENCE [LARGE SCALE MRNA].
RC   TISSUE=Liver;
RX   PubMed=15489334; DOI=10.1101/gr.2596504;
RG   The MGC Project Team;
RT   "The status, quality, and expansion of the NIH full-length cDNA project:
RT   the Mammalian Gene Collection (MGC).";
RL   Genome Res. 14:2121-2127(2004).
RN   [2]
RP   NUCLEOTIDE SEQUENCE [GENOMIC DNA] OF 34-113.
RC   STRAIN=CD-1;
RX   PubMed=7607554; DOI=10.1016/0378-1119(95)00029-6;
RA   Watson J.D., Beckett-Jones B., Roy R.N., Green N.C., Flynn T.G.;
RT   "Genomic sequence, structural organization and evolutionary conservation of
RT   the 13.2-kDa subunit of rat NADH:ubiquinone oxidoreductase.";
RL   Gene 158:275-280(1995).
RN   [3]
RP   IDENTIFICATION BY MASS SPECTROMETRY [LARGE SCALE ANALYSIS].
RC   TISSUE=Brain, Brown adipose tissue, Heart, Kidney, Liver, Lung,
RC   Pancreas, Spleen, and Testis;
RX   PubMed=21183079; DOI=10.1016/j.cell.2010.12.001;
RA   Huttlin E.L., Jedrychowski M.P., Elias J.E., Goswami T., Rad R.,
RA   Beausoleil S.A., Villen J., Haas W., Sowa M.E., Gygi S.P.;
RT   "A tissue-specific atlas of mouse protein phosphorylation and expression.";
RL   Cell 143:1174-1189(2010).
RN   [4]
RP   FUNCTION, AND DISRUPTION PHENOTYPE.
RX   PubMed=22474353; DOI=10.1073/pnas.1113987109;
RA   Ke B.X., Pepe S., Grubb D.R., Komen J.C., Laskowski A., Rodda F.A.,
RA   Hardman B.M., Pitt J.J., Ryan M.T., Lazarou M., Koleff J., Cheung M.M.,
RA   Smolich J.J., Thorburn D.R.;
RT   "Tissue-specific splicing of an Ndufs6 gene-trap insertion generates a
RT   mitochondrial complex I deficiency-specific cardiomyopathy.";
RL   Proc. Natl. Acad. Sci. U.S.A. 109:6165-6170(2012).
RN   [5]
RP   ACETYLATION [LARGE SCALE ANALYSIS] AT LYS-90 AND LYS-112, AND
RP   IDENTIFICATION BY MASS SPECTROMETRY [LARGE SCALE ANALYSIS].
RC   TISSUE=Liver;
RX   PubMed=23576753; DOI=10.1073/pnas.1302961110;
RA   Rardin M.J., Newman J.C., Held J.M., Cusack M.P., Sorensen D.J., Li B.,
RA   Schilling B., Mooney S.D., Kahn C.R., Verdin E., Gibson B.W.;
RT   "Label-free quantitative proteomics of the lysine acetylome in mitochondria
RT   identifies substrates of SIRT3 in metabolic pathways.";
RL   Proc. Natl. Acad. Sci. U.S.A. 110:6601-6606(2013).
CC   -!- FUNCTION: Accessory subunit of the mitochondrial membrane respiratory
CC       chain NADH dehydrogenase (Complex I), that is believed not to be
CC       involved in catalysis. Complex I functions in the transfer of electrons
CC       from NADH to the respiratory chain. The immediate electron acceptor for
CC       the enzyme is believed to be ubiquinone.
CC       {ECO:0000250|UniProtKB:O75380}.
CC   -!- SUBUNIT: Mammalian complex I is composed of 45 different subunits. This
CC       is a component of the iron-sulfur (IP) fragment of the enzyme.
CC       {ECO:0000269|PubMed:22474353}.
CC   -!- SUBCELLULAR LOCATION: Mitochondrion inner membrane
CC       {ECO:0000305|PubMed:22474353}; Peripheral membrane protein
CC       {ECO:0000305}; Matrix side {ECO:0000305}.
CC   -!- DISRUPTION PHENOTYPE: Male and female mice are fertile but produce
CC       smaller litters and pups have a lower neonatal survival rate
CC       (PubMed:22474353). While mice are normal during the first 4 months of
CC       life, they are prone to rapid onset weight loss and sudden death after
CC       this period (PubMed:22474353). They display cardiomyopathy associated
CC       with a doubling of heart weight, impaired systolic function and a
CC       reduction in functional capacity (PubMed:22474353). Males are most
CC       severely affected, with a propensity to develop cardiac failure and
CC       diminished survival after 4 months of age (PubMed:22474353). Defects
CC       are due to membrane respiratory chain NADH dehydrogenase (Complex I)
CC       deficiency (PubMed:22474353). In the knockout experiment described
CC       above, mice show a complete knockout of Ndufs6 subunit in heart
CC       resulting in marked complex I deficiency, but small amounts of wild-
CC       type Ndufs6 mRNA are still present in other tissues, probably due to
CC       tissue-specific mRNA splicing, resulting in milder complex I defects
CC       (PubMed:22474353). {ECO:0000269|PubMed:22474353}.
CC   -!- SIMILARITY: Belongs to the complex I NDUFS6 subunit family.
CC       {ECO:0000305}.
CC   ---------------------------------------------------------------------------
CC   Copyrighted by the UniProt Consortium, see https://www.uniprot.org/terms
CC   Distributed under the Creative Commons Attribution (CC BY 4.0) License
CC   ---------------------------------------------------------------------------
DR   EMBL; BC086933; AAH86933.1; -; mRNA.
DR   EMBL; L38438; AAB64010.1; -; Genomic_DNA.
DR   CCDS; CCDS26630.1; -.
DR   RefSeq; NP_035018.1; NM_010888.2.
DR   PDB; 6G2J; EM; 3.30 A; R=1-116.
DR   PDB; 6G72; EM; 3.90 A; R=1-116.
DR   PDB; 6ZR2; EM; 3.10 A; R=1-116.
DR   PDB; 6ZTQ; EM; 3.00 A; R=1-116.
DR   PDB; 7AK5; EM; 3.17 A; R=1-114.
DR   PDB; 7AK6; EM; 3.82 A; R=1-116.
DR   PDB; 7B93; EM; 3.04 A; R=1-116.
DR   PDB; 7PSA; EM; 3.40 A; R=1-116.
DR   PDBsum; 6G2J; -.
DR   PDBsum; 6G72; -.
DR   PDBsum; 6ZR2; -.
DR   PDBsum; 6ZTQ; -.
DR   PDBsum; 7AK5; -.
DR   PDBsum; 7AK6; -.
DR   PDBsum; 7B93; -.
DR   PDBsum; 7PSA; -.
DR   AlphaFoldDB; P52503; -.
DR   SMR; P52503; -.
DR   BioGRID; 240437; 35.
DR   ComplexPortal; CPX-266; Mitochondrial respiratory chain complex I.
DR   CORUM; P52503; -.
DR   IntAct; P52503; 3.
DR   STRING; 10090.ENSMUSP00000022097; -.
DR   iPTMnet; P52503; -.
DR   PhosphoSitePlus; P52503; -.
DR   SwissPalm; P52503; -.
DR   EPD; P52503; -.
DR   jPOST; P52503; -.
DR   MaxQB; P52503; -.
DR   PaxDb; P52503; -.
DR   PeptideAtlas; P52503; -.
DR   ProteomicsDB; 293647; -.
DR   Antibodypedia; 22354; 236 antibodies from 32 providers.
DR   DNASU; 407785; -.
DR   Ensembl; ENSMUST00000022097; ENSMUSP00000022097; ENSMUSG00000021606.
DR   GeneID; 407785; -.
DR   KEGG; mmu:407785; -.
DR   UCSC; uc007rdh.2; mouse.
DR   AGR; MGI:107932; -.
DR   CTD; 4726; -.
DR   MGI; MGI:107932; Ndufs6.
DR   VEuPathDB; HostDB:ENSMUSG00000021606; -.
DR   eggNOG; KOG3456; Eukaryota.
DR   GeneTree; ENSGT00390000015775; -.
DR   HOGENOM; CLU_083053_3_2_1; -.
DR   InParanoid; P52503; -.
DR   OrthoDB; 6826at2759; -.
DR   PhylomeDB; P52503; -.
DR   TreeFam; TF315128; -.
DR   Reactome; R-MMU-611105; Respiratory electron transport.
DR   Reactome; R-MMU-6799198; Complex I biogenesis.
DR   BioGRID-ORCS; 407785; 4 hits in 77 CRISPR screens.
DR   ChiTaRS; Ndufs6; mouse.
DR   PRO; PR:P52503; -.
DR   Proteomes; UP000000589; Chromosome 13.
DR   RNAct; P52503; protein.
DR   Bgee; ENSMUSG00000021606; Expressed in right kidney and 120 other tissues.
DR   ExpressionAtlas; P52503; baseline and differential.
DR   Genevisible; P52503; MM.
DR   GO; GO:0005747; C:mitochondrial respiratory chain complex I; IDA:MGI.
DR   GO; GO:0005739; C:mitochondrion; IDA:MGI.
DR   GO; GO:0009060; P:aerobic respiration; NAS:ComplexPortal.
DR   GO; GO:0072359; P:circulatory system development; IMP:MGI.
DR   GO; GO:0006631; P:fatty acid metabolic process; IMP:MGI.
DR   GO; GO:0001822; P:kidney development; IMP:MGI.
DR   GO; GO:0006120; P:mitochondrial electron transport, NADH to ubiquinone; IBA:GO_Central.
DR   GO; GO:0032981; P:mitochondrial respiratory chain complex I assembly; IMP:MGI.
DR   GO; GO:0070584; P:mitochondrion morphogenesis; IMP:MGI.
DR   GO; GO:0035264; P:multicellular organism growth; IMP:MGI.
DR   GO; GO:0006936; P:muscle contraction; IMP:MGI.
DR   GO; GO:0042776; P:proton motive force-driven mitochondrial ATP synthesis; NAS:ComplexPortal.
DR   GO; GO:0061458; P:reproductive system development; IMP:MGI.
DR   GO; GO:0022904; P:respiratory electron transport chain; IMP:MGI.
DR   Gene3D; 2.60.260.40; q5lls5 like domains; 1.
DR   InterPro; IPR016668; NDUFS6.
DR   InterPro; IPR019401; Znf_CHCC.
DR   PANTHER; PTHR13156:SF0; NADH DEHYDROGENASE [UBIQUINONE] IRON-SULFUR PROTEIN 6, MITOCHONDRIAL; 1.
DR   PANTHER; PTHR13156; NADH-UBIQUINONE OXIDOREDUCTASE 13 KD-A SUBUNIT; 1.
DR   Pfam; PF10276; zf-CHCC; 1.
DR   PIRSF; PIRSF016564; CI-13KD-A; 1.
PE   1: Evidence at protein level;
KW   3D-structure; Acetylation; Electron transport; Membrane; Mitochondrion;
KW   Mitochondrion inner membrane; Reference proteome; Respiratory chain;
KW   Transit peptide; Transport.
FT   TRANSIT         1..20
FT                   /note="Mitochondrion"
FT                   /evidence="ECO:0000250"
FT   CHAIN           21..116
FT                   /note="NADH dehydrogenase [ubiquinone] iron-sulfur protein
FT                   6, mitochondrial"
FT                   /id="PRO_0000043171"
FT   MOD_RES         90
FT                   /note="N6-acetyllysine"
FT                   /evidence="ECO:0007744|PubMed:23576753"
FT   MOD_RES         112
FT                   /note="N6-acetyllysine"
FT                   /evidence="ECO:0007744|PubMed:23576753"
FT   CONFLICT        37
FT                   /note="V -> VTM (in Ref. 2; AAB64010)"
FT                   /evidence="ECO:0000305"
FT   CONFLICT        75
FT                   /note="R -> H (in Ref. 2; AAB64010)"
FT                   /evidence="ECO:0000305"
FT   CONFLICT        85..87
FT                   /note="ALG -> SLV (in Ref. 2; AAB64010)"
FT                   /evidence="ECO:0000305"
FT   CONFLICT        105
FT                   /note="G -> A (in Ref. 2; AAB64010)"
FT                   /evidence="ECO:0000305"
FT   STRAND          26..28
FT                   /evidence="ECO:0007829|PDB:6G2J"
FT   HELIX           43..48
FT                   /evidence="ECO:0007829|PDB:6ZTQ"
FT   HELIX           60..65
FT                   /evidence="ECO:0007829|PDB:6ZTQ"
FT   STRAND          70..79
FT                   /evidence="ECO:0007829|PDB:6ZTQ"
FT   STRAND          82..84
FT                   /evidence="ECO:0007829|PDB:6ZTQ"
FT   STRAND          91..94
FT                   /evidence="ECO:0007829|PDB:6ZTQ"
FT   STRAND          98..100
FT                   /evidence="ECO:0007829|PDB:6ZTQ"
FT   STRAND          105..107
FT                   /evidence="ECO:0007829|PDB:6ZTQ"
FT   STRAND          111..113
FT                   /evidence="ECO:0007829|PDB:6ZTQ"
SQ   SEQUENCE   116 AA;  13020 MW;  29C5FDCB491C4B2C CRC64;
     MAAVLTFRRL LTLPRAARGF GVQVSPSGEK ITHTGQVYDE KDYRRVRFVD RQKEVNENFA
     IDLIAQQPVN EVEHRIIACD GGGGALGHPK VYINLDKETK TGTCGYCGLQ FKQHHH
//
```

**Mascot:** http://www.matrixscience.com/


Deamidated (NQ) (+0.9840)
