# Supplementary material for: A common East-Asian ALDH2 mutation causes metabolic disorders and the therapeutic effect of ALDH2 activators
Source: Nat Commun. 2023 Sep 25;14:5971. doi: 10.1038/s41467-023-41570-6 (PMC10520061; doi:10.1038/s41467-023-41570-6)
Supplement: Supplementary file 4 — Supplementary Data 1 [file 41467_2023_41570_MOESM4_ESM.zip › Table S5c/Q62425/Q62425_KIO-1_H12_K64_K56.html]

Mascot Search Results: Q62425
 

# MASCOT Search Results

## Protein View: Q62425

### Cytochrome c oxidase subunit NDUFA4 OS=Mus musculus OX=10090 GN=Ndufa4 PE=1 SV=2

|  |  |
| --- | --- |
| Database: | Mouse\_UniProt\_proteomes |
| Score: | 2894 |
| Monoisotopic mass (M_r_): | 9321 |
| Calculated pI: | 9.52 |

Sequence similarity is available as an NCBI BLAST search of Q62425 against nr.

### Search parameters

|  |  |
| --- | --- |
| MS data file: | `D:\LCMSMS\2023 Users' data\230529-1\230529-1-KIO-1.raw` |
| Enzyme: | Trypsin/P: cuts C-term side of KR. |
| Fixed modifications: | Carbamidomethyl (C) |
| Variable modifications: | Deamidated (NQ), HNE (C), HNE (H), HNE (K), Oxidation (M) |

### Protein sequence coverage: 96%

Matched peptides shown in ***bold red***.

|  |  |  |  |  |  |
| --- | --- | --- | --- | --- | --- |
| `1` | `MLRQILGQAK` | `KHPSLIPLFV` | `FIGAGGTGAA` | `LYVMRLALFN` | `PDVSWDRKNN` |
| `51` | `PEPWNKLGPN` | `EQYKFYSVNV` | `DYSKLKKEGP` | `DF` |  |

Unformatted sequence string: 82 residues (for pasting into other applications).

|  |  |  |  |
| --- | --- | --- | --- |
| Sort by | residue number | increasing mass | decreasing mass |
| Show | matched peptides only | predicted peptides also |  |

| Query | Start | – | End | Observed | Mr(expt) | Mr(calc) | ppm | M | Score | Expect | Rank | U | Peptide |
| --- | --- | --- | --- | --- | --- | --- | --- | --- | --- | --- | --- | --- | --- |
| 1783 | 4 | – | 10 | 379.2317 | 756.4489 | 756.4494 | -0.58 | 0 | 30 | 0.015 | 1Score **> 24** indicates **identity** | U | R.QILGQAK.K |
| 1784 | 4 | – | 10 | 379.2323 | 756.4500 | 756.4494 | 0.81 | 0 | 26 | 0.024 | 1Score **> 24** indicates **identity** Score **> 23** indicates **homology** | U | R.QILGQAK.K |
| 189928 | 4 | – | 35 | 705.9999 | 3524.9633 | 3524.9945 | -8.86 | 2 | 16 | 0.03 | 1Score **> 31** indicates **identity** Score **> 14** indicates **homology** | U | R.QILGQAKKHPSLIPLFVFIGAGGTGAALYVMR.L  + HNE (H); Oxidation (M) |
| 164320 | 11 | – | 35 | 654.6199 | 2614.4504 | 2614.4458 | 1.77 | 1 | 76 | 7.7e-08 | 1Score **> 34** indicates **identity** Score **> 17** indicates **homology** | U | K.KHPSLIPLFVFIGAGGTGAALYVMR.L |
| 164322 | 11 | – | 35 | 872.4915 | 2614.4526 | 2614.4458 | 2.60 | 1 | 37 | 0.00031 | 1Score **> 34** indicates **identity** Score **> 15** indicates **homology** | U | K.KHPSLIPLFVFIGAGGTGAALYVMR.L |
| 54035 | 36 | – | 47 | 716.8622 | 1431.7099 | 1431.7147 | -3.30 | 0 | 53 | 1.1e-05 | 1Score **> 34** indicates **identity** Score **> 16** indicates **homology** | U | R.LALFNPDVSWDR.K |
| 54040 | 36 | – | 47 | 716.8633 | 1431.7120 | 1431.7147 | -1.86 | 0 | 38 | 0.00029 | 1Score **> 34** indicates **identity** Score **> 15** indicates **homology** | U | R.LALFNPDVSWDR.K |
| 54046 | 36 | – | 47 | 716.8647 | 1431.7148 | 1431.7147 | 0.11 | 0 | 59 | 4.2e-06 | 1Score **> 34** indicates **identity** Score **> 18** indicates **homology** | U | R.LALFNPDVSWDR.K |
| 54047 | 36 | – | 47 | 716.8647 | 1431.7149 | 1431.7147 | 0.15 | 0 | 57 | 4.5e-06 | 1Score **> 34** indicates **identity** Score **> 16** indicates **homology** | U | R.LALFNPDVSWDR.K |
| 54048 | 36 | – | 47 | 716.8647 | 1431.7149 | 1431.7147 | 0.19 | 0 | 34 | 0.00068 | 1Score **> 34** indicates **identity** Score **> 15** indicates **homology** | U | R.LALFNPDVSWDR.K |
| 54050 | 36 | – | 47 | 716.8651 | 1431.7157 | 1431.7147 | 0.76 | 0 | 80 | 5.1e-08 | 1Score **> 34** indicates **identity** Score **> 20** indicates **homology** | U | R.LALFNPDVSWDR.K |
| 54052 | 36 | – | 47 | 716.8654 | 1431.7162 | 1431.7147 | 1.11 | 0 | 80 | 5.1e-08 | 1Score **> 34** indicates **identity** Score **> 20** indicates **homology** | U | R.LALFNPDVSWDR.K |
| 54053 | 36 | – | 47 | 716.8655 | 1431.7165 | 1431.7147 | 1.29 | 0 | 29 | 0.002 | 1Score **> 34** indicates **identity** Score **> 14** indicates **homology** | U | R.LALFNPDVSWDR.K |
| 54054 | 36 | – | 47 | 716.8657 | 1431.7168 | 1431.7147 | 1.47 | 0 | 75 | 1.2e-07 | 1Score **> 34** indicates **identity** Score **> 19** indicates **homology** | U | R.LALFNPDVSWDR.K |
| 54055 | 36 | – | 47 | 716.8661 | 1431.7176 | 1431.7147 | 2.05 | 0 | 23 | 0.0074 | 1Score **> 34** indicates **identity** Score **> 14** indicates **homology** | U | R.LALFNPDVSWDR.K |
| 54057 | 36 | – | 47 | 716.8662 | 1431.7178 | 1431.7147 | 2.20 | 0 | 20 | 0.013 | 1Score **> 34** indicates **identity** Score **> 14** indicates **homology** | U | R.LALFNPDVSWDR.K |
| 54059 | 36 | – | 47 | 716.8665 | 1431.7184 | 1431.7147 | 2.58 | 0 | 20 | 0.014 | 1Score **> 34** indicates **identity** Score **> 14** indicates **homology** | U | R.LALFNPDVSWDR.K |
| 69883 | 36 | – | 48 | 520.9432 | 1559.8076 | 1559.8096 | -1.26 | 1 | 23 | 0.0063 | 1Score **> 35** indicates **identity** Score **> 14** indicates **homology** | U | R.LALFNPDVSWDRK.N |
| 69885 | 36 | – | 48 | 780.9114 | 1559.8082 | 1559.8096 | -0.93 | 1 | 56 | 8.9e-05 | 1Score **> 35** indicates **identity** Score **> 28** indicates **homology** | U | R.LALFNPDVSWDRK.N |
| 69887 | 36 | – | 48 | 520.9436 | 1559.8091 | 1559.8096 | -0.34 | 1 | 31 | 0.0012 | 1Score **> 35** indicates **identity** Score **> 14** indicates **homology** | U | R.LALFNPDVSWDRK.N |
| 69888 | 36 | – | 48 | 780.9119 | 1559.8092 | 1559.8096 | -0.29 | 1 | 77 | 1.6e-06 | 1Score **> 35** indicates **identity** Score **> 31** indicates **homology** | U | R.LALFNPDVSWDRK.N |
| 69892 | 36 | – | 48 | 780.9122 | 1559.8098 | 1559.8096 | 0.15 | 1 | 76 | 1.2e-07 | 1Score **> 35** indicates **identity** Score **> 20** indicates **homology** | U | R.LALFNPDVSWDRK.N |
| 69893 | 36 | – | 48 | 520.9439 | 1559.8099 | 1559.8096 | 0.20 | 1 | 20 | 0.013 | 1Score **> 35** indicates **identity** Score **> 14** indicates **homology** | U | R.LALFNPDVSWDRK.N |
| 69894 | 36 | – | 48 | 520.9439 | 1559.8100 | 1559.8096 | 0.25 | 1 | 43 | 9.7e-05 | 1Score **> 35** indicates **identity** Score **> 15** indicates **homology** | U | R.LALFNPDVSWDRK.N |
| 69896 | 36 | – | 48 | 520.9442 | 1559.8107 | 1559.8096 | 0.71 | 1 | 42 | 0.00011 | 1Score **> 35** indicates **identity** Score **> 15** indicates **homology** | U | R.LALFNPDVSWDRK.N |
| 69899 | 36 | – | 48 | 520.9442 | 1559.8107 | 1559.8096 | 0.72 | 1 | 43 | 9.9e-05 | 1Score **> 35** indicates **identity** Score **> 15** indicates **homology** | U | R.LALFNPDVSWDRK.N |
| 69900 | 36 | – | 48 | 520.9442 | 1559.8107 | 1559.8096 | 0.72 | 1 | 43 | 9.7e-05 | 1Score **> 35** indicates **identity** Score **> 15** indicates **homology** | U | R.LALFNPDVSWDRK.N |
| 69901 | 36 | – | 48 | 780.9127 | 1559.8109 | 1559.8096 | 0.80 | 1 | 19 | 0.016 | 1Score **> 35** indicates **identity** Score **> 14** indicates **homology** | U | R.LALFNPDVSWDRK.N |
| 69902 | 36 | – | 48 | 520.9444 | 1559.8113 | 1559.8096 | 1.07 | 1 | 47 | 4.9e-05 | 1Score **> 35** indicates **identity** Score **> 16** indicates **homology** | U | R.LALFNPDVSWDRK.N |
| 69903 | 36 | – | 48 | 520.9444 | 1559.8113 | 1559.8096 | 1.08 | 1 | 23 | 0.0066 | 1Score **> 35** indicates **identity** Score **> 14** indicates **homology** | U | R.LALFNPDVSWDRK.N |
| 69904 | 36 | – | 48 | 520.9445 | 1559.8117 | 1559.8096 | 1.33 | 1 | 42 | 0.00011 | 1Score **> 35** indicates **identity** Score **> 15** indicates **homology** | U | R.LALFNPDVSWDRK.N |
| 69905 | 36 | – | 48 | 520.9445 | 1559.8117 | 1559.8096 | 1.34 | 1 | 19 | 0.017 | 1Score **> 35** indicates **identity** Score **> 14** indicates **homology** | U | R.LALFNPDVSWDRK.N |
| 69906 | 36 | – | 48 | 520.9447 | 1559.8122 | 1559.8096 | 1.65 | 1 | 24 | 0.0054 | 1Score **> 35** indicates **identity** Score **> 14** indicates **homology** | U | R.LALFNPDVSWDRK.N |
| 69908 | 36 | – | 48 | 520.9450 | 1559.8132 | 1559.8096 | 2.28 | 1 | 27 | 0.0032 | 1Score **> 35** indicates **identity** Score **> 14** indicates **homology** | U | R.LALFNPDVSWDRK.N |
| 69913 | 36 | – | 48 | 520.9454 | 1559.8145 | 1559.8096 | 3.10 | 1 | 25 | 0.0043 | 1Score **> 35** indicates **identity** Score **> 14** indicates **homology** | U | R.LALFNPDVSWDRK.N |
| 69915 | 36 | – | 48 | 520.9458 | 1559.8157 | 1559.8096 | 3.89 | 1 | 21 | 0.012 | 1Score **> 35** indicates **identity** Score **> 14** indicates **homology** | U | R.LALFNPDVSWDRK.N |
| 160856 | 36 | – | 56 | 635.8212 | 2539.2558 | 2539.2608 | -1.97 | 2 | 26 | 0.0038 | 1Score **> 37** indicates **identity** Score **> 14** indicates **homology** | U | R.LALFNPDVSWDRKNNPEPWNK.L |
| 160858 | 36 | – | 56 | 847.4276 | 2539.2608 | 2539.2608 | 0.028 | 2 | 21 | 0.011 | 1Score **> 37** indicates **identity** Score **> 14** indicates **homology** | U | R.LALFNPDVSWDRKNNPEPWNK.L |
| 160860 | 36 | – | 56 | 847.4280 | 2539.2621 | 2539.2608 | 0.52 | 2 | 22 | 0.0081 | 1Score **> 37** indicates **identity** Score **> 14** indicates **homology** | U | R.LALFNPDVSWDRKNNPEPWNK.L |
| 160861 | 36 | – | 56 | 635.8230 | 2539.2630 | 2539.2608 | 0.89 | 2 | 27 | 0.0029 | 1Score **> 37** indicates **identity** Score **> 14** indicates **homology** | U | R.LALFNPDVSWDRKNNPEPWNK.L |
| 160866 | 36 | – | 56 | 635.8240 | 2539.2670 | 2539.2608 | 2.44 | 2 | 20 | 0.014 | 1Score **> 37** indicates **identity** Score **> 14** indicates **homology** | U | R.LALFNPDVSWDRKNNPEPWNK.L |
| 23044 | 48 | – | 56 | 376.1927 | 1125.5564 | 1125.5567 | -0.27 | 1 | 14 | 0.045 | 1Score **> 31** indicates **identity** Score **> 13** indicates **homology** | U | R.KNNPEPWNK.L |
| 23045 | 48 | – | 56 | 376.1928 | 1125.5565 | 1125.5567 | -0.19 | 1 | 30 | 0.0014 | 1Score **> 31** indicates **identity** Score **> 14** indicates **homology** | U | R.KNNPEPWNK.L |
| 23046 | 48 | – | 56 | 376.1929 | 1125.5569 | 1125.5567 | 0.21 | 1 | 19 | 0.017 | 1Score **> 31** indicates **identity** Score **> 14** indicates **homology** | U | R.KNNPEPWNK.L |
| 23049 | 48 | – | 56 | 563.7858 | 1125.5570 | 1125.5567 | 0.29 | 1 | 66 | 3.9e-06 | 1Score **> 31** indicates **identity** Score **> 24** indicates **homology** | U | R.KNNPEPWNK.L |
| 23051 | 48 | – | 56 | 563.7860 | 1125.5574 | 1125.5567 | 0.64 | 1 | 42 | 0.00038 | 1Score **> 31** indicates **identity** Score **> 21** indicates **homology** | U | R.KNNPEPWNK.L |
| 23052 | 48 | – | 56 | 563.7860 | 1125.5575 | 1125.5567 | 0.73 | 1 | 32 | 0.0013 | 1Score **> 31** indicates **identity** Score **> 16** indicates **homology** | U | R.KNNPEPWNK.L |
| 125839 | 48 | – | 64 | 686.0120 | 2055.0140 | 2055.0173 | -1.60 | 2 | 66 | 6.9e-07 | 1Score **> 36** indicates **identity** Score **> 17** indicates **homology** | U | R.KNNPEPWNKLGPNEQYK.F |
| 125841 | 48 | – | 64 | 1028.5152 | 2055.0159 | 2055.0173 | -0.69 | 2 | 87 | 3.8e-08 | 1Score **> 36** indicates **identity** Score **> 25** indicates **homology** | U | R.KNNPEPWNKLGPNEQYK.F |
| 125844 | 48 | – | 64 | 1028.5162 | 2055.0179 | 2055.0173 | 0.28 | 2 | 60 | 2.7e-06 | 1Score **> 36** indicates **identity** Score **> 17** indicates **homology** | U | R.KNNPEPWNKLGPNEQYK.F |
| 125845 | 48 | – | 64 | 514.7618 | 2055.0181 | 2055.0173 | 0.36 | 2 | 22 | 0.0086 | 1Score **> 36** indicates **identity** Score **> 14** indicates **homology** | U | R.KNNPEPWNKLGPNEQYK.F |
| 125847 | 48 | – | 64 | 686.0133 | 2055.0182 | 2055.0173 | 0.41 | 2 | 75 | 8.4e-08 | 1Score **> 36** indicates **identity** Score **> 17** indicates **homology** | U | R.KNNPEPWNKLGPNEQYK.F |
| 125848 | 48 | – | 64 | 686.0135 | 2055.0185 | 2055.0173 | 0.59 | 2 | 71 | 2.1e-07 | 1Score **> 36** indicates **identity** Score **> 17** indicates **homology** | U | R.KNNPEPWNKLGPNEQYK.F |
| 125849 | 48 | – | 64 | 686.0138 | 2055.0194 | 2055.0173 | 1.03 | 2 | 65 | 8.2e-07 | 1Score **> 36** indicates **identity** Score **> 17** indicates **homology** | U | R.KNNPEPWNKLGPNEQYK.F |
| 125851 | 48 | – | 64 | 514.7625 | 2055.0207 | 2055.0173 | 1.64 | 2 | 26 | 0.0033 | 1Score **> 36** indicates **identity** Score **> 14** indicates **homology** | U | R.KNNPEPWNKLGPNEQYK.F |
| 125852 | 48 | – | 64 | 1028.5178 | 2055.0210 | 2055.0173 | 1.80 | 2 | 89 | 7.9e-08 | 1Score **> 36** indicates **identity** Score **> 30** indicates **homology** | U | R.KNNPEPWNKLGPNEQYK.F |
| 125853 | 48 | – | 64 | 686.0145 | 2055.0217 | 2055.0173 | 2.12 | 2 | 15 | 0.042 | 1Score **> 36** indicates **identity** Score **> 13** indicates **homology** | U | R.KNNPEPWNKLGPNEQYK.F |
| 125919 | 48 | – | 64 | 686.3474 | 2056.0203 | 2056.0013 | 9.24 | 2 | 40 | 0.00018 | 1Score **> 36** indicates **identity** Score **> 15** indicates **homology** | U | R.KNNPEPWNKLGPNEQYK.F  + Deamidated (NQ) |
| 12985 | 49 | – | 56 | 499.7383 | 997.4620 | 997.4617 | 0.27 | 0 | 26 | 0.0033 | 1Score **> 27** indicates **identity** Score **> 14** indicates **homology** | U | K.NNPEPWNK.L |
| 12987 | 49 | – | 56 | 499.7387 | 997.4628 | 997.4617 | 1.06 | 0 | 25 | 0.0046 | 1Score **> 28** indicates **identity** Score **> 14** indicates **homology** | U | K.NNPEPWNK.L |
| 12988 | 49 | – | 56 | 499.7387 | 997.4629 | 997.4617 | 1.20 | 0 | 18 | 0.021 | 1Score **> 28** indicates **identity** Score **> 14** indicates **homology** | U | K.NNPEPWNK.L |
| 113250 | 49 | – | 64 | 643.3141 | 1926.9204 | 1926.9224 | -1.01 | 1 | 30 | 0.0016 | 1Score **> 34** indicates **identity** Score **> 14** indicates **homology** | U | K.NNPEPWNKLGPNEQYK.F |
| 113252 | 49 | – | 64 | 643.3158 | 1926.9257 | 1926.9224 | 1.73 | 1 | 53 | 9.8e-06 | 1Score **> 34** indicates **identity** Score **> 16** indicates **homology** | U | K.NNPEPWNKLGPNEQYK.F |
| 113254 | 49 | – | 64 | 643.3160 | 1926.9262 | 1926.9224 | 2.00 | 1 | 46 | 5.2e-05 | 1Score **> 34** indicates **identity** Score **> 15** indicates **homology** | U | K.NNPEPWNKLGPNEQYK.F |
| 113255 | 49 | – | 64 | 964.4704 | 1926.9263 | 1926.9224 | 2.05 | 1 | 60 | 2.6e-06 | 1Score **> 34** indicates **identity** Score **> 16** indicates **homology** | U | K.NNPEPWNKLGPNEQYK.F |
| 113256 | 49 | – | 64 | 964.4706 | 1926.9266 | 1926.9224 | 2.17 | 1 | 80 | 3.1e-08 | 1Score **> 34** indicates **identity** Score **> 18** indicates **homology** | U | K.NNPEPWNKLGPNEQYK.F |
| 113257 | 49 | – | 64 | 643.3166 | 1926.9281 | 1926.9224 | 2.97 | 1 | 52 | 1.4e-05 | 1Score **> 34** indicates **identity** Score **> 16** indicates **homology** | U | K.NNPEPWNKLGPNEQYK.F |
| 113258 | 49 | – | 64 | 964.4715 | 1926.9284 | 1926.9224 | 3.14 | 1 | 59 | 2.7e-06 | 1Score **> 34** indicates **identity** Score **> 16** indicates **homology** | U | K.NNPEPWNKLGPNEQYK.F |
| 181768 | 49 | – | 74 | 783.3756 | 3129.4732 | 3129.4832 | -3.20 | 2 | 21 | 0.011 | 1Score **> 36** indicates **identity** Score **> 14** indicates **homology** | U | K.NNPEPWNKLGPNEQYKFYSVNVDYSK.L |
| 181769 | 49 | – | 74 | 783.3761 | 3129.4754 | 3129.4832 | -2.49 | 2 | 33 | 0.0008 | 1Score **> 36** indicates **identity** Score **> 15** indicates **homology** | U | K.NNPEPWNKLGPNEQYKFYSVNVDYSK.L |
| 181770 | 49 | – | 74 | 783.3782 | 3129.4836 | 3129.4832 | 0.14 | 2 | 30 | 0.0016 | 1Score **> 36** indicates **identity** Score **> 14** indicates **homology** | U | K.NNPEPWNKLGPNEQYKFYSVNVDYSK.L |
| 181771 | 49 | – | 74 | 1044.1685 | 3129.4837 | 3129.4832 | 0.16 | 2 | 57 | 4.5e-06 | 1Score **> 36** indicates **identity** Score **> 16** indicates **homology** | U | K.NNPEPWNKLGPNEQYKFYSVNVDYSK.L |
| 181772 | 49 | – | 74 | 783.3786 | 3129.4852 | 3129.4832 | 0.66 | 2 | 34 | 0.00071 | 1Score **> 36** indicates **identity** Score **> 15** indicates **homology** | U | K.NNPEPWNKLGPNEQYKFYSVNVDYSK.L |
| 181773 | 49 | – | 74 | 1044.1694 | 3129.4863 | 3129.4832 | 1.01 | 2 | 34 | 0.00059 | 1Score **> 36** indicates **identity** Score **> 15** indicates **homology** | U | K.NNPEPWNKLGPNEQYKFYSVNVDYSK.L |
| 181774 | 49 | – | 74 | 1044.1698 | 3129.4876 | 3129.4832 | 1.42 | 2 | 48 | 3.3e-05 | 1Score **> 36** indicates **identity** Score **> 15** indicates **homology** | U | K.NNPEPWNKLGPNEQYKFYSVNVDYSK.L |
| 181792 | 49 | – | 74 | 1044.5040 | 3130.4902 | 3130.4672 | 7.35 | 2 | 28 | 0.0022 | 1Score **> 36** indicates **identity** Score **> 14** indicates **homology** | U | K.NNPEPWNKLGPNEQYKFYSVNVDYSK.L  + Deamidated (NQ) |
| 185201 | 49 | – | 74 | 1096.2001 | 3285.5784 | 3285.5982 | -6.04 | 2 | 23 | 0.0066 | 1Score **> 37** indicates **identity** Score **> 14** indicates **homology** | U | K.NNPEPWNKLGPNEQYKFYSVNVDYSK.L  + HNE (K) |
| 185210 | 49 | – | 74 | 1096.5260 | 3286.5562 | 3286.5822 | -7.92 | 2 | 24 | 0.0059 | 1Score **> 36** indicates **identity** Score **> 14** indicates **homology** | U | K.NNPEPWNKLGPNEQYKFYSVNVDYSK.L  + Deamidated (NQ); HNE (K) |
| 185211 | 49 | – | 74 | 1096.5279 | 3286.5620 | 3286.5822 | -6.16 | 2 | 21 | 0.01 | 1Score **> 36** indicates **identity** Score **> 14** indicates **homology** | U | K.NNPEPWNKLGPNEQYKFYSVNVDYSK.L  + Deamidated (NQ); HNE (K) |
| 185219 | 49 | – | 74 | 1096.8533 | 3287.5380 | 3287.5662 | -8.59 | 2 | 20 | 0.012 | 1Score **> 35** indicates **identity** Score **> 14** indicates **homology** | U | K.NNPEPWNKLGPNEQYKFYSVNVDYSK.L  + 2 Deamidated (NQ); HNE (K) |
| 185220 | 49 | – | 74 | 1096.8535 | 3287.5387 | 3287.5662 | -8.38 | 2 | 19 | 0.015 | 1Score **> 35** indicates **identity** Score **> 14** indicates **homology** | U | K.NNPEPWNKLGPNEQYKFYSVNVDYSK.L  + 2 Deamidated (NQ); HNE (K) |
| 185229 | 49 | – | 74 | 1096.8608 | 3287.5606 | 3287.5662 | -1.71 | 2 | 18 | 0.019 | 1Score **> 36** indicates **identity** Score **> 14** indicates **homology** | U | K.NNPEPWNKLGPNEQYKFYSVNVDYSK.L  + 2 Deamidated (NQ); HNE (K) |
| 10205 | 57 | – | 64 | 474.7433 | 947.4720 | 947.4712 | 0.79 | 0 | 46 | 0.00065 | 1Score **> 31** indicates **identity** Score **> 26** indicates **homology** | U | K.LGPNEQYK.F |
| 10206 | 57 | – | 64 | 474.7433 | 947.4721 | 947.4712 | 0.89 | 0 | 52 | 0.00011 | 1Score **> 31** indicates **identity** Score **> 26** indicates **homology** | U | K.LGPNEQYK.F |
| 10207 | 57 | – | 64 | 474.7437 | 947.4728 | 947.4712 | 1.66 | 0 | 21 | 0.024 | 1Score **> 31** indicates **identity** Score **> 17** indicates **homology** | U | K.LGPNEQYK.F |
| 10209 | 57 | – | 64 | 474.7443 | 947.4741 | 947.4712 | 3.01 | 0 | 21 | 0.012 | 1Score **> 31** indicates **identity** Score **> 14** indicates **homology** | U | K.LGPNEQYK.F |
| 134915 | 57 | – | 74 | 717.6816 | 2150.0229 | 2150.0320 | -4.24 | 1 | 29 | 0.002 | 1Score **> 35** indicates **identity** Score **> 14** indicates **homology** | U | K.LGPNEQYKFYSVNVDYSK.L |
| 134916 | 57 | – | 74 | 717.6838 | 2150.0294 | 2150.0320 | -1.20 | 1 | 16 | 0.029 | 1Score **> 35** indicates **identity** Score **> 14** indicates **homology** | U | K.LGPNEQYKFYSVNVDYSK.L |
| 134917 | 57 | – | 74 | 717.6848 | 2150.0326 | 2150.0320 | 0.26 | 1 | 20 | 0.012 | 1Score **> 35** indicates **identity** Score **> 14** indicates **homology** | U | K.LGPNEQYKFYSVNVDYSK.L |
| 134918 | 57 | – | 74 | 717.6857 | 2150.0351 | 2150.0320 | 1.45 | 1 | 56 | 6.1e-06 | 1Score **> 35** indicates **identity** Score **> 16** indicates **homology** | U | K.LGPNEQYKFYSVNVDYSK.L |
| 134919 | 57 | – | 74 | 717.6858 | 2150.0354 | 2150.0320 | 1.59 | 1 | 90 | 4e-09 | 1Score **> 35** indicates **identity** Score **> 18** indicates **homology** | U | K.LGPNEQYKFYSVNVDYSK.L |
| 134920 | 57 | – | 74 | 1076.0252 | 2150.0358 | 2150.0320 | 1.78 | 1 | 88 | 5.7e-09 | 1Score **> 35** indicates **identity** Score **> 18** indicates **homology** | U | K.LGPNEQYKFYSVNVDYSK.L |
| 134921 | 57 | – | 74 | 1076.0252 | 2150.0359 | 2150.0320 | 1.79 | 1 | 81 | 2.8e-08 | 1Score **> 35** indicates **identity** Score **> 18** indicates **homology** | U | K.LGPNEQYKFYSVNVDYSK.L |
| 134922 | 57 | – | 74 | 717.6860 | 2150.0361 | 2150.0320 | 1.91 | 1 | 56 | 5.6e-06 | 1Score **> 35** indicates **identity** Score **> 16** indicates **homology** | U | K.LGPNEQYKFYSVNVDYSK.L |
| 134923 | 57 | – | 74 | 1076.0256 | 2150.0367 | 2150.0320 | 2.20 | 1 | 87 | 7.5e-09 | 1Score **> 35** indicates **identity** Score **> 18** indicates **homology** | U | K.LGPNEQYKFYSVNVDYSK.L |
| 134924 | 57 | – | 74 | 717.6863 | 2150.0371 | 2150.0320 | 2.36 | 1 | 70 | 2.6e-07 | 1Score **> 35** indicates **identity** Score **> 17** indicates **homology** | U | K.LGPNEQYKFYSVNVDYSK.L |
| 134925 | 57 | – | 74 | 1076.0259 | 2150.0373 | 2150.0320 | 2.44 | 1 | 89 | 4.2e-09 | 1Score **> 35** indicates **identity** Score **> 18** indicates **homology** | U | K.LGPNEQYKFYSVNVDYSK.L |
| 134926 | 57 | – | 74 | 717.6869 | 2150.0388 | 2150.0320 | 3.15 | 1 | 24 | 0.0058 | 1Score **> 35** indicates **identity** Score **> 14** indicates **homology** | U | K.LGPNEQYKFYSVNVDYSK.L |
| 134933 | 57 | – | 74 | 717.6884 | 2150.0433 | 2150.0320 | 5.27 | 1 | 33 | 0.0008 | 1Score **> 36** indicates **identity** Score **> 15** indicates **homology** | U | K.LGPNEQYKFYSVNVDYSK.L |
| 134934 | 57 | – | 74 | 717.6889 | 2150.0450 | 2150.0320 | 6.03 | 1 | 25 | 0.0047 | 1Score **> 36** indicates **identity** Score **> 14** indicates **homology** | U | K.LGPNEQYKFYSVNVDYSK.L |
| 32233 | 65 | – | 74 | 611.2899 | 1220.5652 | 1220.5714 | -5.00 | 0 | 38 | 0.00029 | 1Score **> 30** indicates **identity** Score **> 15** indicates **homology** | U | K.FYSVNVDYSK.L |
| 32236 | 65 | – | 74 | 611.2919 | 1220.5693 | 1220.5714 | -1.67 | 0 | 41 | 0.00014 | 1Score **> 31** indicates **identity** Score **> 15** indicates **homology** | U | K.FYSVNVDYSK.L |
| 32237 | 65 | – | 74 | 611.2925 | 1220.5705 | 1220.5714 | -0.69 | 0 | 54 | 8.6e-06 | 1Score **> 31** indicates **identity** Score **> 16** indicates **homology** | U | K.FYSVNVDYSK.L |
| 32238 | 65 | – | 74 | 611.2926 | 1220.5706 | 1220.5714 | -0.65 | 0 | 39 | 0.00023 | 1Score **> 31** indicates **identity** Score **> 15** indicates **homology** | U | K.FYSVNVDYSK.L |
| 32239 | 65 | – | 74 | 611.2926 | 1220.5706 | 1220.5714 | -0.59 | 0 | 41 | 0.00015 | 1Score **> 31** indicates **identity** Score **> 15** indicates **homology** | U | K.FYSVNVDYSK.L |
| 32242 | 65 | – | 74 | 611.2929 | 1220.5713 | 1220.5714 | -0.0082 | 0 | 34 | 0.0015 | 1Score **> 31** indicates **identity** Score **> 18** indicates **homology** | U | K.FYSVNVDYSK.L |
| 32244 | 65 | – | 74 | 611.2933 | 1220.5721 | 1220.5714 | 0.59 | 0 | 41 | 0.00015 | 1Score **> 31** indicates **identity** Score **> 15** indicates **homology** | U | K.FYSVNVDYSK.L |
| 32246 | 65 | – | 74 | 611.2937 | 1220.5728 | 1220.5714 | 1.22 | 0 | 41 | 0.00017 | 1Score **> 31** indicates **identity** Score **> 15** indicates **homology** | U | K.FYSVNVDYSK.L |
| 32247 | 65 | – | 74 | 611.2937 | 1220.5729 | 1220.5714 | 1.28 | 0 | 32 | 0.001 | 2Score **> 31** indicates **identity** Score **> 15** indicates **homology** | U | K.FYSVNVDYSK.L |
| 32248 | 65 | – | 74 | 611.2939 | 1220.5732 | 1220.5714 | 1.48 | 0 | 14 | 0.045 | 1Score **> 31** indicates **identity** Score **> 13** indicates **homology** | U | K.FYSVNVDYSK.L |
| 32249 | 65 | – | 74 | 611.2939 | 1220.5732 | 1220.5714 | 1.50 | 0 | 45 | 7.2e-05 | 1Score **> 31** indicates **identity** Score **> 16** indicates **homology** | U | K.FYSVNVDYSK.L |
| 32250 | 65 | – | 74 | 611.2939 | 1220.5732 | 1220.5714 | 1.56 | 0 | 39 | 0.00023 | 1Score **> 31** indicates **identity** Score **> 15** indicates **homology** | U | K.FYSVNVDYSK.L |
| 32251 | 65 | – | 74 | 611.2939 | 1220.5733 | 1220.5714 | 1.62 | 0 | 37 | 0.00033 | 1Score **> 31** indicates **identity** Score **> 15** indicates **homology** | U | K.FYSVNVDYSK.L |
| 32256 | 65 | – | 74 | 611.2954 | 1220.5763 | 1220.5714 | 4.03 | 0 | 49 | 2.8e-05 | 1Score **> 31** indicates **identity** Score **> 16** indicates **homology** | U | K.FYSVNVDYSK.L |
| 9324 | 75 | – | 82 | 467.2547 | 932.4948 | 932.4967 | -2.08 | 2 | 26 | 0.012 | 1Score **> 32** indicates **identity** Score **> 19** indicates **homology** | U | K.LKKEGPDF.- |
| 9328 | 75 | – | 82 | 467.2562 | 932.4978 | 932.4967 | 1.11 | 2 | 41 | 0.0013 | 1Score **> 32** indicates **identity** Score **> 25** indicates **homology** | U | K.LKKEGPDF.- |

---

```
ID   NDUA4_MOUSE             Reviewed;          82 AA.
AC   Q62425; Q9CQP6;
DT   01-NOV-1997, integrated into UniProtKB/Swiss-Prot.
DT   19-SEP-2002, sequence version 2.
DT   28-JUN-2023, entry version 176.
DE   RecName: Full=Cytochrome c oxidase subunit NDUFA4;
GN   Name=Ndufa4;
OS   Mus musculus (Mouse).
OC   Eukaryota; Metazoa; Chordata; Craniata; Vertebrata; Euteleostomi; Mammalia;
OC   Eutheria; Euarchontoglires; Glires; Rodentia; Myomorpha; Muroidea; Muridae;
OC   Murinae; Mus; Mus.
OX   NCBI_TaxID=10090;
RN   [1]
RP   NUCLEOTIDE SEQUENCE [LARGE SCALE MRNA].
RC   STRAIN=C57BL/6J; TISSUE=Cerebellum, and Small intestine;
RX   PubMed=16141072; DOI=10.1126/science.1112014;
RA   Carninci P., Kasukawa T., Katayama S., Gough J., Frith M.C., Maeda N.,
RA   Oyama R., Ravasi T., Lenhard B., Wells C., Kodzius R., Shimokawa K.,
RA   Bajic V.B., Brenner S.E., Batalov S., Forrest A.R., Zavolan M., Davis M.J.,
RA   Wilming L.G., Aidinis V., Allen J.E., Ambesi-Impiombato A., Apweiler R.,
RA   Aturaliya R.N., Bailey T.L., Bansal M., Baxter L., Beisel K.W., Bersano T.,
RA   Bono H., Chalk A.M., Chiu K.P., Choudhary V., Christoffels A.,
RA   Clutterbuck D.R., Crowe M.L., Dalla E., Dalrymple B.P., de Bono B.,
RA   Della Gatta G., di Bernardo D., Down T., Engstrom P., Fagiolini M.,
RA   Faulkner G., Fletcher C.F., Fukushima T., Furuno M., Futaki S.,
RA   Gariboldi M., Georgii-Hemming P., Gingeras T.R., Gojobori T., Green R.E.,
RA   Gustincich S., Harbers M., Hayashi Y., Hensch T.K., Hirokawa N., Hill D.,
RA   Huminiecki L., Iacono M., Ikeo K., Iwama A., Ishikawa T., Jakt M.,
RA   Kanapin A., Katoh M., Kawasawa Y., Kelso J., Kitamura H., Kitano H.,
RA   Kollias G., Krishnan S.P., Kruger A., Kummerfeld S.K., Kurochkin I.V.,
RA   Lareau L.F., Lazarevic D., Lipovich L., Liu J., Liuni S., McWilliam S.,
RA   Madan Babu M., Madera M., Marchionni L., Matsuda H., Matsuzawa S., Miki H.,
RA   Mignone F., Miyake S., Morris K., Mottagui-Tabar S., Mulder N., Nakano N.,
RA   Nakauchi H., Ng P., Nilsson R., Nishiguchi S., Nishikawa S., Nori F.,
RA   Ohara O., Okazaki Y., Orlando V., Pang K.C., Pavan W.J., Pavesi G.,
RA   Pesole G., Petrovsky N., Piazza S., Reed J., Reid J.F., Ring B.Z.,
RA   Ringwald M., Rost B., Ruan Y., Salzberg S.L., Sandelin A., Schneider C.,
RA   Schoenbach C., Sekiguchi K., Semple C.A., Seno S., Sessa L., Sheng Y.,
RA   Shibata Y., Shimada H., Shimada K., Silva D., Sinclair B., Sperling S.,
RA   Stupka E., Sugiura K., Sultana R., Takenaka Y., Taki K., Tammoja K.,
RA   Tan S.L., Tang S., Taylor M.S., Tegner J., Teichmann S.A., Ueda H.R.,
RA   van Nimwegen E., Verardo R., Wei C.L., Yagi K., Yamanishi H.,
RA   Zabarovsky E., Zhu S., Zimmer A., Hide W., Bult C., Grimmond S.M.,
RA   Teasdale R.D., Liu E.T., Brusic V., Quackenbush J., Wahlestedt C.,
RA   Mattick J.S., Hume D.A., Kai C., Sasaki D., Tomaru Y., Fukuda S.,
RA   Kanamori-Katayama M., Suzuki M., Aoki J., Arakawa T., Iida J., Imamura K.,
RA   Itoh M., Kato T., Kawaji H., Kawagashira N., Kawashima T., Kojima M.,
RA   Kondo S., Konno H., Nakano K., Ninomiya N., Nishio T., Okada M., Plessy C.,
RA   Shibata K., Shiraki T., Suzuki S., Tagami M., Waki K., Watahiki A.,
RA   Okamura-Oho Y., Suzuki H., Kawai J., Hayashizaki Y.;
RT   "The transcriptional landscape of the mammalian genome.";
RL   Science 309:1559-1563(2005).
RN   [2]
RP   NUCLEOTIDE SEQUENCE [LARGE SCALE MRNA].
RC   TISSUE=Mammary tumor;
RX   PubMed=15489334; DOI=10.1101/gr.2596504;
RG   The MGC Project Team;
RT   "The status, quality, and expansion of the NIH full-length cDNA project:
RT   the Mammalian Gene Collection (MGC).";
RL   Genome Res. 14:2121-2127(2004).
RN   [3]
RP   NUCLEOTIDE SEQUENCE [MRNA] OF 8-82, AND PROTEIN SEQUENCE OF 61-68.
RC   STRAIN=C57BL/6J;
RX   PubMed=9064319; DOI=10.1084/jem.184.3.1017;
RA   Tallquist M.D., Yun T.J., Pease L.R.;
RT   "A single T cell receptor recognizes structurally distinct MHC/peptide
RT   complexes with high specificity.";
RL   J. Exp. Med. 184:1017-1026(1996).
RN   [4]
RP   PROTEIN SEQUENCE OF 36-74, AND IDENTIFICATION BY MASS SPECTROMETRY.
RC   STRAIN=C57BL/6J; TISSUE=Brain;
RA   Lubec G., Kang S.U.;
RL   Submitted (APR-2007) to UniProtKB.
RN   [5]
RP   IDENTIFICATION BY MASS SPECTROMETRY [LARGE SCALE ANALYSIS].
RC   TISSUE=Brain;
RX   PubMed=16452087; DOI=10.1074/mcp.t500041-mcp200;
RA   Trinidad J.C., Specht C.G., Thalhammer A., Schoepfer R., Burlingame A.L.;
RT   "Comprehensive identification of phosphorylation sites in postsynaptic
RT   density preparations.";
RL   Mol. Cell. Proteomics 5:914-922(2006).
RN   [6]
RP   PHOSPHORYLATION [LARGE SCALE ANALYSIS] AT SER-67, AND IDENTIFICATION BY
RP   MASS SPECTROMETRY [LARGE SCALE ANALYSIS].
RC   TISSUE=Brain, Brown adipose tissue, Heart, Kidney, Liver, Lung,
RC   Pancreas, and Testis;
RX   PubMed=21183079; DOI=10.1016/j.cell.2010.12.001;
RA   Huttlin E.L., Jedrychowski M.P., Elias J.E., Goswami T., Rad R.,
RA   Beausoleil S.A., Villen J., Haas W., Sowa M.E., Gygi S.P.;
RT   "A tissue-specific atlas of mouse protein phosphorylation and expression.";
RL   Cell 143:1174-1189(2010).
RN   [7]
RP   IDENTIFICATION AS CYTOCHROME C OXIDASE SUBUNIT.
RX   PubMed=22902835; DOI=10.1016/j.cmet.2012.07.015;
RA   Balsa E., Marco R., Perales-Clemente E., Szklarczyk R., Calvo E.,
RA   Landazuri M.O., Enriquez J.A.;
RT   "NDUFA4 is a subunit of complex IV of the mammalian electron transport
RT   chain.";
RL   Cell Metab. 16:378-386(2012).
RN   [8]
RP   ACETYLATION [LARGE SCALE ANALYSIS] AT LYS-10, AND IDENTIFICATION BY MASS
RP   SPECTROMETRY [LARGE SCALE ANALYSIS].
RC   TISSUE=Liver;
RX   PubMed=23576753; DOI=10.1073/pnas.1302961110;
RA   Rardin M.J., Newman J.C., Held J.M., Cusack M.P., Sorensen D.J., Li B.,
RA   Schilling B., Mooney S.D., Kahn C.R., Verdin E., Gibson B.W.;
RT   "Label-free quantitative proteomics of the lysine acetylome in mitochondria
RT   identifies substrates of SIRT3 in metabolic pathways.";
RL   Proc. Natl. Acad. Sci. U.S.A. 110:6601-6606(2013).
RN   [9]
RP   INTERACTION WITH FLVCR2.
RX   PubMed=32973183; DOI=10.1038/s41467-020-18607-1;
RA   Li Y., Ivica N.A., Dong T., Papageorgiou D.P., He Y., Brown D.R.,
RA   Kleyman M., Hu G., Chen W.W., Sullivan L.B., Del Rosario A., Hammond P.T.,
RA   Vander Heiden M.G., Chen J.;
RT   "MFSD7C switches mitochondrial ATP synthesis to thermogenesis in response
RT   to heme.";
RL   Nat. Commun. 11:4837-4837(2020).
RN   [10]
RP   X-RAY CRYSTALLOGRAPHY (1.75 ANGSTROMS) OF 61-68 IN COMPLEX WITH THE MAJOR
RP   HISTOCOMPATIBILITY COMPLEX.
RX   PubMed=11994422; DOI=10.1084/jem.20011644;
RA   Luz J.G., Huang M., Garcia K.C., Rudolph M.G., Apostolopoulos V.,
RA   Teyton L., Wilson I.A.;
RT   "Structural comparison of allogeneic and syngeneic T cell receptor-peptide-
RT   major histocompatibility complex complexes: a buried alloreactive mutation
RT   subtly alters peptide presentation substantially increasing V(beta)
RT   interactions.";
RL   J. Exp. Med. 195:1175-1186(2002).
CC   -!- FUNCTION: Component of the cytochrome c oxidase, the last enzyme in the
CC       mitochondrial electron transport chain which drives oxidative
CC       phosphorylation. The respiratory chain contains 3 multisubunit
CC       complexes succinate dehydrogenase (complex II, CII), ubiquinol-
CC       cytochrome c oxidoreductase (cytochrome b-c1 complex, complex III,
CC       CIII) and cytochrome c oxidase (complex IV, CIV), that cooperate to
CC       transfer electrons derived from NADH and succinate to molecular oxygen,
CC       creating an electrochemical gradient over the inner membrane that
CC       drives transmembrane transport and the ATP synthase. Cytochrome c
CC       oxidase is the component of the respiratory chain that catalyzes the
CC       reduction of oxygen to water. Electrons originating from reduced
CC       cytochrome c in the intermembrane space (IMS) are transferred via the
CC       dinuclear copper A center (CU(A)) of subunit 2 and heme A of subunit 1
CC       to the active site in subunit 1, a binuclear center (BNC) formed by
CC       heme A3 and copper B (CU(B)). The BNC reduces molecular oxygen to 2
CC       water molecules unsing 4 electrons from cytochrome c in the IMS and 4
CC       protons from the mitochondrial matrix. NDUFA4 is required for complex
CC       IV maintenance. {ECO:0000250|UniProtKB:O00483}.
CC   -!- SUBUNIT: Component of the cytochrome c oxidase (complex IV, CIV), a
CC       multisubunit enzyme composed of 14 subunits. The complex is composed of
CC       a catalytic core of 3 subunits MT-CO1, MT-CO2 and MT-CO3, encoded in
CC       the mitochondrial DNA, and 11 supernumerary subunits COX4I, COX5A,
CC       COX5B, COX6A, COX6B, COX6C, COX7A, COX7B, COX7C, COX8 and NDUFA4, which
CC       are encoded in the nuclear genome. The complex exists as a monomer or a
CC       dimer and forms supercomplexes (SCs) in the inner mitochondrial
CC       membrane with NADH-ubiquinone oxidoreductase (complex I, CI) and
CC       ubiquinol-cytochrome c oxidoreductase (cytochrome b-c1 complex, complex
CC       III, CIII), resulting in different assemblies (supercomplex
CC       SCI(1)III(2)IV(1) and megacomplex MCI(2)III(2)IV(2)) (By similarity).
CC       Interacts with RAB5IF (By similarity). Interacts with FLVCR2; this
CC       interaction occurs in the absence of heme and is disrupted upon heme
CC       binding. {ECO:0000250|UniProtKB:O00483, ECO:0000269|PubMed:32973183}.
CC   -!- SUBCELLULAR LOCATION: Mitochondrion inner membrane
CC       {ECO:0000250|UniProtKB:O00483}; Single-pass membrane protein
CC       {ECO:0000250|UniProtKB:O00483}.
CC   -!- SIMILARITY: Belongs to the complex IV NDUFA4 subunit family.
CC       {ECO:0000305}.
CC   ---------------------------------------------------------------------------
CC   Copyrighted by the UniProt Consortium, see https://www.uniprot.org/terms
CC   Distributed under the Creative Commons Attribution (CC BY 4.0) License
CC   ---------------------------------------------------------------------------
DR   EMBL; AK005084; -; NOT_ANNOTATED_CDS; mRNA.
DR   EMBL; AK008357; BAB25627.1; -; mRNA.
DR   EMBL; AK008506; BAB25706.1; -; mRNA.
DR   EMBL; AK076036; BAC36137.1; -; mRNA.
DR   EMBL; BC011114; AAH11114.1; -; mRNA.
DR   EMBL; U59509; AAB03395.1; -; mRNA.
DR   CCDS; CCDS39427.1; -.
DR   RefSeq; NP_035016.1; NM_010886.2.
DR   PDB; 1LEG; X-ray; 1.75 A; P=61-68.
DR   PDB; 1LEK; X-ray; 2.15 A; P=61-68.
DR   PDB; 1MWA; X-ray; 2.40 A; P/Q=61-68.
DR   PDB; 2CKB; X-ray; 3.00 A; P/Q=61-68.
DR   PDBsum; 1LEG; -.
DR   PDBsum; 1LEK; -.
DR   PDBsum; 1MWA; -.
DR   PDBsum; 2CKB; -.
DR   AlphaFoldDB; Q62425; -.
DR   SMR; Q62425; -.
DR   BioGRID; 201717; 68.
DR   CORUM; Q62425; -.
DR   IntAct; Q62425; 9.
DR   MINT; Q62425; -.
DR   STRING; 10090.ENSMUSP00000031637; -.
DR   GlyGen; Q62425; 1 site, 1 O-linked glycan (1 site).
DR   iPTMnet; Q62425; -.
DR   PhosphoSitePlus; Q62425; -.
DR   SwissPalm; Q62425; -.
DR   EPD; Q62425; -.
DR   jPOST; Q62425; -.
DR   MaxQB; Q62425; -.
DR   PaxDb; Q62425; -.
DR   PeptideAtlas; Q62425; -.
DR   ProteomicsDB; 286165; -.
DR   TopDownProteomics; Q62425; -.
DR   Antibodypedia; 56007; 171 antibodies from 27 providers.
DR   DNASU; 17992; -.
DR   Ensembl; ENSMUST00000204714; ENSMUSP00000145413; ENSMUSG00000029632.
DR   Ensembl; ENSMUST00000204978; ENSMUSP00000144932; ENSMUSG00000029632.
DR   GeneID; 17992; -.
DR   KEGG; mmu:17992; -.
DR   UCSC; uc009ayd.1; mouse.
DR   AGR; MGI:107686; -.
DR   CTD; 4697; -.
DR   MGI; MGI:107686; Ndufa4.
DR   VEuPathDB; HostDB:ENSMUSG00000029632; -.
DR   eggNOG; ENOG502S65P; Eukaryota.
DR   GeneTree; ENSGT00940000154268; -.
DR   HOGENOM; CLU_181002_0_0_1; -.
DR   InParanoid; Q62425; -.
DR   OMA; QHKFYSP; -.
DR   OrthoDB; 1347256at2759; -.
DR   PhylomeDB; Q62425; -.
DR   TreeFam; TF106383; -.
DR   Reactome; R-MMU-5628897; TP53 Regulates Metabolic Genes.
DR   Reactome; R-MMU-611105; Respiratory electron transport.
DR   Reactome; R-MMU-9707564; Cytoprotection by HMOX1.
DR   BioGRID-ORCS; 17992; 6 hits in 75 CRISPR screens.
DR   ChiTaRS; Ndufa4; mouse.
DR   EvolutionaryTrace; Q62425; -.
DR   PRO; PR:Q62425; -.
DR   Proteomes; UP000000589; Chromosome 6.
DR   RNAct; Q62425; protein.
DR   Bgee; ENSMUSG00000029632; Expressed in right kidney and 279 other tissues.
DR   ExpressionAtlas; Q62425; baseline and differential.
DR   Genevisible; Q62425; MM.
DR   GO; GO:0005743; C:mitochondrial inner membrane; HDA:MGI.
DR   GO; GO:0005747; C:mitochondrial respiratory chain complex I; IDA:UniProtKB.
DR   GO; GO:0005751; C:mitochondrial respiratory chain complex IV; IDA:UniProtKB.
DR   GO; GO:0005739; C:mitochondrion; HDA:MGI.
DR   GO; GO:0044877; F:protein-containing complex binding; ISO:MGI.
DR   GO; GO:1904960; P:positive regulation of cytochrome-c oxidase activity; ISS:UniProtKB.
DR   InterPro; IPR010530; B12D.
DR   PANTHER; PTHR14256:SF4; CYTOCHROME C OXIDASE SUBUNIT NDUFA4; 1.
DR   PANTHER; PTHR14256; NADH-UBIQUINONE OXIDOREDUCTASE MLRQ SUBUNIT; 1.
DR   Pfam; PF06522; B12D; 1.
PE   1: Evidence at protein level;
KW   3D-structure; Acetylation; Direct protein sequencing; Electron transport;
KW   Membrane; Mitochondrion; Mitochondrion inner membrane; Phosphoprotein;
KW   Reference proteome; Respiratory chain; Transmembrane; Transmembrane helix;
KW   Transport.
FT   CHAIN           1..82
FT                   /note="Cytochrome c oxidase subunit NDUFA4"
FT                   /id="PRO_0000118822"
FT   TOPO_DOM        1..14
FT                   /note="Mitochondrial matrix"
FT                   /evidence="ECO:0000250|UniProtKB:O00483"
FT   TRANSMEM        15..37
FT                   /note="Helical"
FT                   /evidence="ECO:0000250|UniProtKB:O00483"
FT   TOPO_DOM        38..82
FT                   /note="Mitochondrial intermembrane"
FT                   /evidence="ECO:0000250|UniProtKB:O00483"
FT   MOD_RES         10
FT                   /note="N6-acetyllysine"
FT                   /evidence="ECO:0007744|PubMed:23576753"
FT   MOD_RES         67
FT                   /note="Phosphoserine"
FT                   /evidence="ECO:0007744|PubMed:21183079"
SQ   SEQUENCE   82 AA;  9327 MW;  6E29865845807C7B CRC64;
     MLRQILGQAK KHPSLIPLFV FIGAGGTGAA LYVMRLALFN PDVSWDRKNN PEPWNKLGPN
     EQYKFYSVNV DYSKLKKEGP DF
//
```

**Mascot:** http://www.matrixscience.com/
