# Supplementary material for: A common East-Asian ALDH2 mutation causes metabolic disorders and the therapeutic effect of ALDH2 activators
Source: Nat Commun. 2023 Sep 25;14:5971. doi: 10.1038/s41467-023-41570-6 (PMC10520061; doi:10.1038/s41467-023-41570-6)
Supplement: Supplementary file 4 — Supplementary Data 1 [file 41467_2023_41570_MOESM4_ESM.zip › Table S5c/Q64521/Q64521_KIO-1_K65_H633_K634_K124.html]

Mascot Search Results: Q64521
 

# MASCOT Search Results

## Protein View: Q64521

### Glycerol-3-phosphate dehydrogenase, mitochondrial OS=Mus musculus OX=10090 GN=Gpd2 PE=1 SV=2

|  |  |
| --- | --- |
| Database: | Mouse\_UniProt\_proteomes |
| Score: | 15457 |
| Monoisotopic mass (M_r_): | 81416 |
| Calculated pI: | 6.17 |

Sequence similarity is available as an NCBI BLAST search of Q64521 against nr.

### Search parameters

|  |  |
| --- | --- |
| MS data file: | `D:\LCMSMS\2023 Users' data\230529-1\230529-1-KIO-1.raw` |
| Enzyme: | Trypsin/P: cuts C-term side of KR. |
| Fixed modifications: | Carbamidomethyl (C) |
| Variable modifications: | Deamidated (NQ), HNE (C), HNE (H), HNE (K), Oxidation (M) |

### Protein sequence coverage: 65%

Matched peptides shown in ***bold red***.

|  |  |  |  |  |  |
| --- | --- | --- | --- | --- | --- |
| `1` | `MAFQKAVKGT` | `ILVGGGALAT` | `VLGLSPFAHY` | `RRKQVSLAYV` | `EAAGYLTEPV` |
| `51` | `NREPPSREAQ` | `LMTLKNTPEF` | `DILVIGGGAT` | `GCGCALDAVT` | `RGLKTALVER` |
| `101` | `DDFSSGTSSR` | `STKLIHGGVR` | `YLQKAIMNLD` | `VEQYRMVKEA` | `LHERANLLEI` |
| `151` | `APHLSAPLPI` | `MLPLYKWWQL` | `PYYWVGIKMY` | `DLVAGSQCLK` | `SSYVLSKSRA` |
| `201` | `LEHFPMLQKD` | `KLVGAIVYYD` | `GQHNDARMNL` | `AIALTAARYG` | `AATANYMEVV` |
| `251` | `SLLKKTDPET` | `GKERVSGARC` | `KDVLTGQEFD` | `VRAKCVINAS` | `GPFTDSVRKM` |
| `301` | `DDKNVVPICQ` | `PSAGVHIVMP` | `GYYSPENMGL` | `LDPATSDGRV` | `IFFLPWEKMT` |
| `351` | `IAGTTDTPTD` | `VTHHPIPSEE` | `DINFILNEVR` | `NYLSSDVEVR` | `RGDVLAAWSG` |
| `401` | `IRPLVTDPKS` | `ADTQSISRNH` | `VVDISDSGLI` | `TIAGGKWTTY` | `RSMAEDTVDA` |
| `451` | `AVKFHNLNAG` | `PSRTVGLFLQ` | `GGKDWSPTLY` | `IRLVQDYGLE` | `SEVAQHLAKT` |
| `501` | `YGDKAFEVAK` | `MASVTGKRWP` | `VVGVRLVSEF` | `PYIEAEVKYG` | `IKEYACTAVD` |
| `551` | `MISRRTRLAF` | `LNVQAAEEAL` | `PRIVELMGRE` | `LNWSELRKQE` | `ELETATRFLY` |
| `601` | `YEMGYKSRTE` | `QLTDSTEISL` | `LPSDIDRYKK` | `RFHKFDEDEK` | `GFITIVDVQR` |
| `651` | `VLESINVQMD` | `ENTLHEILCE` | `VDLNKNGQVE` | `LHEFLQLMSA` | `VQKGRVSGSR` |
| `701` | `LAILMKTAEE` | `NLDRRVPIPV` | `DRSCGGL` |  |  |

Unformatted sequence string: 727 residues (for pasting into other applications).

|  |  |  |  |
| --- | --- | --- | --- |
| Sort by | residue number | increasing mass | decreasing mass |
| Show | matched peptides only | predicted peptides also |  |

| Query | Start | – | End | Observed | Mr(expt) | Mr(calc) | ppm | M | Score | Expect | Rank | U | Peptide |
| --- | --- | --- | --- | --- | --- | --- | --- | --- | --- | --- | --- | --- | --- |
| 9330 | 58 | – | 65 | 467.2575 | 932.5004 | 932.5001 | 0.34 | 0 | 36 | 0.00085 | 1Score **> 32** indicates **identity** Score **> 18** indicates **homology** | U | R.EAQLMTLK.N |
| 9331 | 58 | – | 65 | 467.2576 | 932.5006 | 932.5001 | 0.58 | 0 | 34 | 0.00094 | 1Score **> 32** indicates **identity** Score **> 16** indicates **homology** | U | R.EAQLMTLK.N |
| 9333 | 58 | – | 65 | 467.2585 | 932.5025 | 932.5001 | 2.62 | 0 | 37 | 0.0008 | 1Score **> 32** indicates **identity** Score **> 18** indicates **homology** | U | R.EAQLMTLK.N |
| 192178 | 58 | – | 91 | 1250.9516 | 3749.8331 | 3749.8678 | -9.27 | 1 | 64 | 9.5e-07 | 1Score **> 38** indicates **identity** Score **> 17** indicates **homology** | U | R.EAQLMTLKNTPEFDILVIGGGATGCGCALDAVTR.G  + HNE (K); Oxidation (M) |
| 166242 | 66 | – | 91 | 888.7575 | 2663.2505 | 2663.2684 | -6.69 | 0 | 48 | 3.4e-05 | 1Score **> 35** indicates **identity** Score **> 15** indicates **homology** | U | K.NTPEFDILVIGGGATGCGCALDAVTR.G |
| 166243 | 66 | – | 91 | 888.7595 | 2663.2566 | 2663.2684 | -4.42 | 0 | 110 | 5.1e-11 | 1Score **> 35** indicates **identity** Score **> 19** indicates **homology** | U | K.NTPEFDILVIGGGATGCGCALDAVTR.G |
| 166245 | 66 | – | 91 | 888.7611 | 2663.2616 | 2663.2684 | -2.56 | 0 | 37 | 0.00032 | 1Score **> 36** indicates **identity** Score **> 15** indicates **homology** | U | K.NTPEFDILVIGGGATGCGCALDAVTR.G |
| 166249 | 66 | – | 91 | 1332.6384 | 2663.2622 | 2663.2684 | -2.30 | 0 | 103 | 2.2e-10 | 1Score **> 36** indicates **identity** Score **> 19** indicates **homology** | U | K.NTPEFDILVIGGGATGCGCALDAVTR.G |
| 166250 | 66 | – | 91 | 888.7615 | 2663.2627 | 2663.2684 | -2.13 | 0 | 29 | 0.0021 | 1Score **> 36** indicates **identity** Score **> 14** indicates **homology** | U | K.NTPEFDILVIGGGATGCGCALDAVTR.G |
| 166256 | 66 | – | 91 | 888.7626 | 2663.2660 | 2663.2684 | -0.90 | 0 | 42 | 0.00011 | 1Score **> 36** indicates **identity** Score **> 15** indicates **homology** | U | K.NTPEFDILVIGGGATGCGCALDAVTR.G |
| 166257 | 66 | – | 91 | 888.7627 | 2663.2664 | 2663.2684 | -0.73 | 0 | 62 | 1.7e-06 | 1Score **> 36** indicates **identity** Score **> 16** indicates **homology** | U | K.NTPEFDILVIGGGATGCGCALDAVTR.G |
| 166258 | 66 | – | 91 | 1332.6417 | 2663.2688 | 2663.2684 | 0.15 | 0 | 95 | 1.3e-09 | 1Score **> 36** indicates **identity** Score **> 18** indicates **homology** | U | K.NTPEFDILVIGGGATGCGCALDAVTR.G |
| 166260 | 66 | – | 91 | 888.7638 | 2663.2694 | 2663.2684 | 0.40 | 0 | 109 | 5.9e-11 | 1Score **> 36** indicates **identity** Score **> 19** indicates **homology** | U | K.NTPEFDILVIGGGATGCGCALDAVTR.G |
| 166264 | 66 | – | 91 | 1332.6423 | 2663.2700 | 2663.2684 | 0.63 | 0 | 99 | 4.9e-10 | 1Score **> 36** indicates **identity** Score **> 19** indicates **homology** | U | K.NTPEFDILVIGGGATGCGCALDAVTR.G |
| 166265 | 66 | – | 91 | 888.7641 | 2663.2703 | 2663.2684 | 0.73 | 0 | 39 | 0.0002 | 1Score **> 36** indicates **identity** Score **> 15** indicates **homology** | U | K.NTPEFDILVIGGGATGCGCALDAVTR.G |
| 166266 | 66 | – | 91 | 888.7641 | 2663.2706 | 2663.2684 | 0.84 | 0 | 23 | 0.0064 | 1Score **> 36** indicates **identity** Score **> 14** indicates **homology** | U | K.NTPEFDILVIGGGATGCGCALDAVTR.G |
| 166269 | 66 | – | 91 | 888.7643 | 2663.2712 | 2663.2684 | 1.06 | 0 | 41 | 0.00015 | 1Score **> 36** indicates **identity** Score **> 15** indicates **homology** | U | K.NTPEFDILVIGGGATGCGCALDAVTR.G |
| 166270 | 66 | – | 91 | 888.7644 | 2663.2714 | 2663.2684 | 1.14 | 0 | 38 | 0.00025 | 1Score **> 36** indicates **identity** Score **> 15** indicates **homology** | U | K.NTPEFDILVIGGGATGCGCALDAVTR.G |
| 166272 | 66 | – | 91 | 1332.6433 | 2663.2721 | 2663.2684 | 1.40 | 0 | 110 | 5.1e-11 | 1Score **> 36** indicates **identity** Score **> 19** indicates **homology** | U | K.NTPEFDILVIGGGATGCGCALDAVTR.G |
| 166273 | 66 | – | 91 | 888.7647 | 2663.2723 | 2663.2684 | 1.47 | 0 | 76 | 8.3e-08 | 1Score **> 36** indicates **identity** Score **> 17** indicates **homology** | U | K.NTPEFDILVIGGGATGCGCALDAVTR.G |
| 166275 | 66 | – | 91 | 888.7649 | 2663.2728 | 2663.2684 | 1.67 | 0 | 55 | 6.4e-06 | 1Score **> 36** indicates **identity** Score **> 16** indicates **homology** | U | K.NTPEFDILVIGGGATGCGCALDAVTR.G |
| 166276 | 66 | – | 91 | 888.7654 | 2663.2745 | 2663.2684 | 2.30 | 0 | 55 | 7.6e-06 | 1Score **> 36** indicates **identity** Score **> 16** indicates **homology** | U | K.NTPEFDILVIGGGATGCGCALDAVTR.G |
| 166279 | 66 | – | 91 | 888.7661 | 2663.2764 | 2663.2684 | 3.03 | 0 | 111 | 4.1e-11 | 1Score **> 36** indicates **identity** Score **> 19** indicates **homology** | U | K.NTPEFDILVIGGGATGCGCALDAVTR.G |
| 166282 | 66 | – | 91 | 888.7665 | 2663.2776 | 2663.2684 | 3.47 | 0 | 37 | 0.00037 | 1Score **> 36** indicates **identity** Score **> 15** indicates **homology** | U | K.NTPEFDILVIGGGATGCGCALDAVTR.G |
| 166284 | 66 | – | 91 | 888.7667 | 2663.2781 | 2663.2684 | 3.67 | 0 | 57 | 4.7e-06 | 1Score **> 36** indicates **identity** Score **> 16** indicates **homology** | U | K.NTPEFDILVIGGGATGCGCALDAVTR.G |
| 166286 | 66 | – | 91 | 888.7669 | 2663.2788 | 2663.2684 | 3.91 | 0 | 54 | 8e-06 | 1Score **> 36** indicates **identity** Score **> 16** indicates **homology** | U | K.NTPEFDILVIGGGATGCGCALDAVTR.G |
| 166290 | 66 | – | 91 | 888.7680 | 2663.2821 | 2663.2684 | 5.14 | 0 | 26 | 0.0039 | 1Score **> 36** indicates **identity** Score **> 15** indicates **homology** | U | K.NTPEFDILVIGGGATGCGCALDAVTR.G |
| 166291 | 66 | – | 91 | 888.7680 | 2663.2821 | 2663.2684 | 5.14 | 0 | 75 | 8.6e-08 | 1Score **> 36** indicates **identity** Score **> 17** indicates **homology** | U | K.NTPEFDILVIGGGATGCGCALDAVTR.G |
| 166292 | 66 | – | 91 | 888.7681 | 2663.2824 | 2663.2684 | 5.29 | 0 | 39 | 0.00023 | 1Score **> 36** indicates **identity** Score **> 15** indicates **homology** | U | K.NTPEFDILVIGGGATGCGCALDAVTR.G |
| 166295 | 66 | – | 91 | 888.7684 | 2663.2834 | 2663.2684 | 5.64 | 0 | 36 | 0.00042 | 1Score **> 36** indicates **identity** Score **> 15** indicates **homology** | U | K.NTPEFDILVIGGGATGCGCALDAVTR.G |
| 166298 | 66 | – | 91 | 888.7713 | 2663.2921 | 2663.2684 | 8.90 | 0 | 37 | 0.00038 | 1Score **> 37** indicates **identity** Score **> 15** indicates **homology** | U | K.NTPEFDILVIGGGATGCGCALDAVTR.G |
| 166299 | 66 | – | 91 | 888.7721 | 2663.2944 | 2663.2684 | 9.76 | 0 | 31 | 0.0012 | 1Score **> 37** indicates **identity** Score **> 14** indicates **homology** | U | K.NTPEFDILVIGGGATGCGCALDAVTR.G |
| 166332 | 66 | – | 91 | 889.0947 | 2664.2624 | 2664.2524 | 3.76 | 0 | 46 | 5.2e-05 | 1Score **> 35** indicates **identity** Score **> 15** indicates **homology** | U | K.NTPEFDILVIGGGATGCGCALDAVTR.G  + Deamidated (NQ) |
| 166335 | 66 | – | 91 | 889.0967 | 2664.2682 | 2664.2524 | 5.93 | 0 | 48 | 3.2e-05 | 1Score **> 36** indicates **identity** Score **> 15** indicates **homology** | U | K.NTPEFDILVIGGGATGCGCALDAVTR.G  + Deamidated (NQ) |
| 166337 | 66 | – | 91 | 889.0974 | 2664.2704 | 2664.2524 | 6.77 | 0 | 31 | 0.0012 | 1Score **> 36** indicates **identity** Score **> 14** indicates **homology** | U | K.NTPEFDILVIGGGATGCGCALDAVTR.G  + Deamidated (NQ) |
| 166338 | 66 | – | 91 | 889.0976 | 2664.2709 | 2664.2524 | 6.96 | 0 | 21 | 0.012 | 1Score **> 36** indicates **identity** Score **> 14** indicates **homology** | U | K.NTPEFDILVIGGGATGCGCALDAVTR.G  + Deamidated (NQ) |
| 166339 | 66 | – | 91 | 889.0980 | 2664.2722 | 2664.2524 | 7.42 | 0 | 30 | 0.0016 | 1Score **> 36** indicates **identity** Score **> 14** indicates **homology** | U | K.NTPEFDILVIGGGATGCGCALDAVTR.G  + Deamidated (NQ) |
| 166342 | 66 | – | 91 | 889.0991 | 2664.2755 | 2664.2524 | 8.69 | 0 | 50 | 2e-05 | 1Score **> 36** indicates **identity** Score **> 16** indicates **homology** | U | K.NTPEFDILVIGGGATGCGCALDAVTR.G  + Deamidated (NQ) |
| 166344 | 66 | – | 91 | 889.0992 | 2664.2757 | 2664.2524 | 8.76 | 0 | 35 | 0.00051 | 1Score **> 36** indicates **identity** Score **> 15** indicates **homology** | U | K.NTPEFDILVIGGGATGCGCALDAVTR.G  + Deamidated (NQ) |
| 166345 | 66 | – | 91 | 889.0994 | 2664.2765 | 2664.2524 | 9.06 | 0 | 42 | 0.00013 | 1Score **> 36** indicates **identity** Score **> 15** indicates **homology** | U | K.NTPEFDILVIGGGATGCGCALDAVTR.G  + Deamidated (NQ) |
| 122735 | 92 | – | 110 | 676.0093 | 2025.0061 | 2025.0127 | -3.27 | 2 | 29 | 0.002 | 1Score **> 35** indicates **identity** Score **> 14** indicates **homology** | U | R.GLKTALVERDDFSSGTSSR.S |
| 122742 | 92 | – | 110 | 676.0106 | 2025.0100 | 2025.0127 | -1.32 | 2 | 14 | 0.045 | 1Score **> 35** indicates **identity** Score **> 13** indicates **homology** | U | R.GLKTALVERDDFSSGTSSR.S |
| 92244 | 95 | – | 110 | 576.6080 | 1726.8022 | 1726.8122 | -5.80 | 1 | 32 | 0.0011 | 1Score **> 32** indicates **identity** Score **> 14** indicates **homology** | U | K.TALVERDDFSSGTSSR.S |
| 92248 | 95 | – | 110 | 864.4111 | 1726.8077 | 1726.8122 | -2.61 | 1 | 73 | 6.3e-07 | 1Score **> 33** indicates **identity** Score **> 23** indicates **homology** | U | K.TALVERDDFSSGTSSR.S |
| 92249 | 95 | – | 110 | 864.4112 | 1726.8079 | 1726.8122 | -2.49 | 1 | 59 | 3e-06 | 1Score **> 32** indicates **identity** Score **> 16** indicates **homology** | U | K.TALVERDDFSSGTSSR.S |
| 92250 | 95 | – | 110 | 576.6099 | 1726.8079 | 1726.8122 | -2.47 | 1 | 23 | 0.0064 | 1Score **> 32** indicates **identity** Score **> 14** indicates **homology** | U | K.TALVERDDFSSGTSSR.S |
| 92252 | 95 | – | 110 | 576.6107 | 1726.8102 | 1726.8122 | -1.14 | 1 | 23 | 0.0065 | 1Score **> 33** indicates **identity** Score **> 14** indicates **homology** | U | K.TALVERDDFSSGTSSR.S |
| 92253 | 95 | – | 110 | 864.4125 | 1726.8105 | 1726.8122 | -1.01 | 1 | 21 | 0.011 | 1Score **> 33** indicates **identity** Score **> 14** indicates **homology** | U | K.TALVERDDFSSGTSSR.S |
| 92255 | 95 | – | 110 | 576.6110 | 1726.8111 | 1726.8122 | -0.67 | 1 | 36 | 0.00043 | 1Score **> 33** indicates **identity** Score **> 15** indicates **homology** | U | K.TALVERDDFSSGTSSR.S |
| 92258 | 95 | – | 110 | 576.6111 | 1726.8116 | 1726.8122 | -0.36 | 1 | 41 | 0.00016 | 1Score **> 33** indicates **identity** Score **> 15** indicates **homology** | U | K.TALVERDDFSSGTSSR.S |
| 92259 | 95 | – | 110 | 864.4132 | 1726.8118 | 1726.8122 | -0.21 | 1 | 35 | 0.00049 | 1Score **> 33** indicates **identity** Score **> 15** indicates **homology** | U | K.TALVERDDFSSGTSSR.S |
| 92260 | 95 | – | 110 | 576.6114 | 1726.8122 | 1726.8122 | 0.0069 | 1 | 21 | 0.012 | 1Score **> 33** indicates **identity** Score **> 14** indicates **homology** | U | K.TALVERDDFSSGTSSR.S |
| 92261 | 95 | – | 110 | 864.4136 | 1726.8127 | 1726.8122 | 0.30 | 1 | 96 | 9.2e-10 | 1Score **> 32** indicates **identity** Score **> 19** indicates **homology** | U | K.TALVERDDFSSGTSSR.S |
| 92262 | 95 | – | 110 | 864.4137 | 1726.8129 | 1726.8122 | 0.42 | 1 | 54 | 8.8e-06 | 1Score **> 33** indicates **identity** Score **> 16** indicates **homology** | U | K.TALVERDDFSSGTSSR.S |
| 92263 | 95 | – | 110 | 576.6116 | 1726.8129 | 1726.8122 | 0.42 | 1 | 58 | 3.5e-06 | 1Score **> 33** indicates **identity** Score **> 16** indicates **homology** | U | K.TALVERDDFSSGTSSR.S |
| 92264 | 95 | – | 110 | 576.6116 | 1726.8130 | 1726.8122 | 0.47 | 1 | 60 | 2.5e-06 | 1Score **> 33** indicates **identity** Score **> 16** indicates **homology** | U | K.TALVERDDFSSGTSSR.S |
| 92265 | 95 | – | 110 | 576.6116 | 1726.8130 | 1726.8122 | 0.47 | 1 | 14 | 0.045 | 1Score **> 33** indicates **identity** Score **> 13** indicates **homology** | U | K.TALVERDDFSSGTSSR.S |
| 92266 | 95 | – | 110 | 576.6116 | 1726.8130 | 1726.8122 | 0.48 | 1 | 60 | 2.5e-06 | 1Score **> 33** indicates **identity** Score **> 16** indicates **homology** | U | K.TALVERDDFSSGTSSR.S |
| 92267 | 95 | – | 110 | 864.4139 | 1726.8132 | 1726.8122 | 0.56 | 1 | 89 | 5.7e-09 | 1Score **> 33** indicates **identity** Score **> 19** indicates **homology** | U | K.TALVERDDFSSGTSSR.S |
| 92268 | 95 | – | 110 | 576.6117 | 1726.8132 | 1726.8122 | 0.56 | 1 | 25 | 0.0047 | 1Score **> 33** indicates **identity** Score **> 14** indicates **homology** | U | K.TALVERDDFSSGTSSR.S |
| 92270 | 95 | – | 110 | 864.4142 | 1726.8138 | 1726.8122 | 0.95 | 1 | 70 | 2.6e-07 | 1Score **> 33** indicates **identity** Score **> 17** indicates **homology** | U | K.TALVERDDFSSGTSSR.S |
| 92272 | 95 | – | 110 | 864.4142 | 1726.8139 | 1726.8122 | 1.00 | 1 | 24 | 0.0078 | 1Score **> 33** indicates **identity** Score **> 15** indicates **homology** | U | K.TALVERDDFSSGTSSR.S |
| 92273 | 95 | – | 110 | 576.6120 | 1726.8141 | 1726.8122 | 1.12 | 1 | 24 | 0.0051 | 1Score **> 33** indicates **identity** Score **> 14** indicates **homology** | U | K.TALVERDDFSSGTSSR.S |
| 92274 | 95 | – | 110 | 864.4144 | 1726.8142 | 1726.8122 | 1.17 | 1 | 90 | 3.3e-09 | 1Score **> 33** indicates **identity** Score **> 18** indicates **homology** | U | K.TALVERDDFSSGTSSR.S |
| 92277 | 95 | – | 110 | 864.4144 | 1726.8143 | 1726.8122 | 1.19 | 1 | 94 | 2.2e-09 | 1Score **> 33** indicates **identity** Score **> 20** indicates **homology** | U | K.TALVERDDFSSGTSSR.S |
| 92278 | 95 | – | 110 | 576.6120 | 1726.8143 | 1726.8122 | 1.21 | 1 | 63 | 1.4e-06 | 1Score **> 33** indicates **identity** Score **> 16** indicates **homology** | U | K.TALVERDDFSSGTSSR.S |
| 92280 | 95 | – | 110 | 864.4145 | 1726.8144 | 1726.8122 | 1.28 | 1 | 85 | 1.4e-08 | 1Score **> 33** indicates **identity** Score **> 19** indicates **homology** | U | K.TALVERDDFSSGTSSR.S |
| 92281 | 95 | – | 110 | 576.6121 | 1726.8145 | 1726.8122 | 1.31 | 1 | 55 | 7.2e-06 | 1Score **> 33** indicates **identity** Score **> 16** indicates **homology** | U | K.TALVERDDFSSGTSSR.S |
| 92282 | 95 | – | 110 | 576.6121 | 1726.8145 | 1726.8122 | 1.32 | 1 | 60 | 2.4e-06 | 1Score **> 33** indicates **identity** Score **> 16** indicates **homology** | U | K.TALVERDDFSSGTSSR.S |
| 92283 | 95 | – | 110 | 576.6122 | 1726.8146 | 1726.8122 | 1.41 | 1 | 69 | 3.5e-07 | 1Score **> 33** indicates **identity** Score **> 17** indicates **homology** | U | K.TALVERDDFSSGTSSR.S |
| 92286 | 95 | – | 110 | 576.6122 | 1726.8149 | 1726.8122 | 1.53 | 1 | 61 | 2.1e-06 | 1Score **> 33** indicates **identity** Score **> 16** indicates **homology** | U | K.TALVERDDFSSGTSSR.S |
| 92290 | 95 | – | 110 | 576.6125 | 1726.8156 | 1726.8122 | 1.97 | 1 | 28 | 0.0022 | 1Score **> 33** indicates **identity** Score **> 14** indicates **homology** | U | K.TALVERDDFSSGTSSR.S |
| 92291 | 95 | – | 110 | 864.4157 | 1726.8168 | 1726.8122 | 2.65 | 1 | 21 | 0.011 | 1Score **> 33** indicates **identity** Score **> 14** indicates **homology** | U | K.TALVERDDFSSGTSSR.S |
| 92292 | 95 | – | 110 | 576.6131 | 1726.8174 | 1726.8122 | 3.01 | 1 | 17 | 0.025 | 1Score **> 33** indicates **identity** Score **> 14** indicates **homology** | U | K.TALVERDDFSSGTSSR.S |
| 92293 | 95 | – | 110 | 864.4164 | 1726.8182 | 1726.8122 | 3.47 | 1 | 31 | 0.0017 | 1Score **> 33** indicates **identity** Score **> 15** indicates **homology** | U | K.TALVERDDFSSGTSSR.S |
| 92295 | 95 | – | 110 | 576.6138 | 1726.8195 | 1726.8122 | 4.20 | 1 | 19 | 0.033 | 1Score **> 33** indicates **identity** Score **> 17** indicates **homology** | U | K.TALVERDDFSSGTSSR.S |
| 16908 | 101 | – | 110 | 529.7228 | 1057.4311 | 1057.4312 | -0.13 | 0 | 59 | 2.9e-06 | 1Score **> 22** indicates **identity** Score **> 16** indicates **homology** | U | R.DDFSSGTSSR.S |
| 16909 | 101 | – | 110 | 529.7234 | 1057.4323 | 1057.4312 | 1.00 | 0 | 22 | 0.0082 | 1Score **> 22** indicates **identity** Score **> 14** indicates **homology** | U | R.DDFSSGTSSR.S |
| 16910 | 101 | – | 110 | 529.7237 | 1057.4328 | 1057.4312 | 1.46 | 0 | 38 | 0.00026 | 1Score **> 22** indicates **identity** Score **> 15** indicates **homology** | U | R.DDFSSGTSSR.S |
| 17727 | 111 | – | 120 | 356.5489 | 1066.6248 | 1066.6247 | 0.096 | 1 | 33 | 0.00088 | 1Score **> 27** indicates **identity** Score **> 15** indicates **homology** | U | R.STKLIHGGVR.Y |
| 17728 | 111 | – | 120 | 356.5490 | 1066.6253 | 1066.6247 | 0.50 | 1 | 23 | 0.0074 | 1Score **> 27** indicates **identity** Score **> 14** indicates **homology** | U | R.STKLIHGGVR.Y |
| 17729 | 111 | – | 120 | 534.3203 | 1066.6261 | 1066.6247 | 1.31 | 1 | 38 | 0.0003 | 1Score **> 28** indicates **identity** Score **> 15** indicates **homology** | U | R.STKLIHGGVR.Y |
| 1609 | 114 | – | 120 | 376.2324 | 750.4502 | 750.4501 | 0.13 | 0 | 23 | 0.0045 | 1Score **> 13** indicates **identity** | U | K.LIHGGVR.Y |
| 125935 | 121 | – | 135 | 686.3546 | 2056.0420 | 2056.0550 | -6.33 | 1 | 25 | 0.0049 | 1Score **> 36** indicates **identity** Score **> 14** indicates **homology** | U | R.YLQKAIMNLDVEQYR.M  + Deamidated (NQ); HNE (K); Oxidation (M) |
| 47148 | 125 | – | 135 | 684.3346 | 1366.6547 | 1366.6551 | -0.25 | 0 | 48 | 3.2e-05 | 1Score **> 32** indicates **identity** Score **> 15** indicates **homology** | U | K.AIMNLDVEQYR.M  + Oxidation (M) |
| 153847 | 145 | – | 166 | 805.4669 | 2413.3788 | 2413.3806 | -0.75 | 0 | 56 | 5.3e-06 | 1Score **> 32** indicates **identity** Score **> 16** indicates **homology** | U | R.ANLLEIAPHLSAPLPIMLPLYK.W |
| 153873 | 145 | – | 166 | 805.8003 | 2414.3791 | 2414.3647 | 5.99 | 0 | 42 | 0.00011 | 1Score **> 31** indicates **identity** Score **> 15** indicates **homology** | U | R.ANLLEIAPHLSAPLPIMLPLYK.W  + Deamidated (NQ) |
| 80665 | 167 | – | 178 | 819.9293 | 1637.8440 | 1637.8395 | 2.78 | 0 | 35 | 0.00055 | 1Score **> 35** indicates **identity** Score **> 15** indicates **homology** | U | K.WWQLPYYWVGIK.M |
| 48773 | 179 | – | 190 | 692.8323 | 1383.6500 | 1383.6526 | -1.92 | 0 | 46 | 5.1e-05 | 1Score **> 31** indicates **identity** Score **> 15** indicates **homology** | U | K.MYDLVAGSQCLK.S |
| 48777 | 179 | – | 190 | 692.8348 | 1383.6551 | 1383.6526 | 1.76 | 0 | 47 | 3.9e-05 | 1Score **> 32** indicates **identity** Score **> 15** indicates **homology** | U | K.MYDLVAGSQCLK.S |
| 2614 | 191 | – | 197 | 392.2161 | 782.4176 | 782.4174 | 0.19 | 0 | 46 | 6.5e-05 | 1Score **> 17** indicates **identity** | U | K.SSYVLSK.S |
| 2615 | 191 | – | 197 | 392.2166 | 782.4186 | 782.4174 | 1.55 | 0 | 39 | 0.00031 | 1Score **> 17** indicates **identity** | U | K.SSYVLSK.S |
| 123559 | 210 | – | 227 | 678.6709 | 2032.9908 | 2032.9966 | -2.88 | 1 | 30 | 0.0016 | 1Score **> 36** indicates **identity** Score **> 14** indicates **homology** | U | K.DKLVGAIVYYDGQHNDAR.M |
| 123561 | 210 | – | 227 | 1017.5062 | 2032.9978 | 2032.9966 | 0.55 | 1 | 79 | 4.2e-08 | 1Score **> 36** indicates **identity** Score **> 18** indicates **homology** | U | K.DKLVGAIVYYDGQHNDAR.M |
| 123564 | 210 | – | 227 | 678.6736 | 2032.9989 | 2032.9966 | 1.12 | 1 | 59 | 2.9e-06 | 1Score **> 36** indicates **identity** Score **> 16** indicates **homology** | U | K.DKLVGAIVYYDGQHNDAR.M |
| 123565 | 210 | – | 227 | 678.6739 | 2032.9999 | 2032.9966 | 1.61 | 1 | 46 | 4.4e-05 | 1Score **> 36** indicates **identity** Score **> 15** indicates **homology** | U | K.DKLVGAIVYYDGQHNDAR.M |
| 123566 | 210 | – | 227 | 509.2573 | 2033.0000 | 2032.9966 | 1.65 | 1 | 29 | 0.0019 | 1Score **> 36** indicates **identity** Score **> 14** indicates **homology** | U | K.DKLVGAIVYYDGQHNDAR.M |
| 123567 | 210 | – | 227 | 678.6740 | 2033.0002 | 2032.9966 | 1.74 | 1 | 57 | 4.3e-06 | 1Score **> 36** indicates **identity** Score **> 16** indicates **homology** | U | K.DKLVGAIVYYDGQHNDAR.M |
| 123568 | 210 | – | 227 | 509.2574 | 2033.0004 | 2032.9966 | 1.87 | 1 | 26 | 0.004 | 1Score **> 36** indicates **identity** Score **> 14** indicates **homology** | U | K.DKLVGAIVYYDGQHNDAR.M |
| 123569 | 210 | – | 227 | 678.6741 | 2033.0006 | 2032.9966 | 1.94 | 1 | 19 | 0.016 | 1Score **> 36** indicates **identity** Score **> 14** indicates **homology** | U | K.DKLVGAIVYYDGQHNDAR.M |
| 123570 | 210 | – | 227 | 1017.5077 | 2033.0008 | 2032.9966 | 2.03 | 1 | 63 | 1.1e-06 | 1Score **> 36** indicates **identity** Score **> 16** indicates **homology** | U | K.DKLVGAIVYYDGQHNDAR.M |
| 123571 | 210 | – | 227 | 1017.5078 | 2033.0010 | 2032.9966 | 2.12 | 1 | 67 | 5.2e-07 | 1Score **> 36** indicates **identity** Score **> 17** indicates **homology** | U | K.DKLVGAIVYYDGQHNDAR.M |
| 123575 | 210 | – | 227 | 678.6748 | 2033.0027 | 2032.9966 | 2.99 | 1 | 18 | 0.02 | 1Score **> 36** indicates **identity** Score **> 14** indicates **homology** | U | K.DKLVGAIVYYDGQHNDAR.M |
| 123576 | 210 | – | 227 | 678.6749 | 2033.0028 | 2032.9966 | 3.03 | 1 | 22 | 0.0082 | 1Score **> 36** indicates **identity** Score **> 14** indicates **homology** | U | K.DKLVGAIVYYDGQHNDAR.M |
| 123577 | 210 | – | 227 | 509.2580 | 2033.0029 | 2032.9966 | 3.07 | 1 | 26 | 0.0038 | 1Score **> 36** indicates **identity** Score **> 14** indicates **homology** | U | K.DKLVGAIVYYDGQHNDAR.M |
| 99547 | 212 | – | 227 | 597.6318 | 1789.8735 | 1789.8747 | -0.70 | 0 | 28 | 0.0026 | 1Score **> 35** indicates **identity** Score **> 14** indicates **homology** | U | K.LVGAIVYYDGQHNDAR.M |
| 99548 | 212 | – | 227 | 597.6324 | 1789.8755 | 1789.8747 | 0.43 | 0 | 35 | 0.00055 | 1Score **> 35** indicates **identity** Score **> 15** indicates **homology** | U | K.LVGAIVYYDGQHNDAR.M |
| 99549 | 212 | – | 227 | 895.9455 | 1789.8765 | 1789.8747 | 0.99 | 0 | 77 | 6e-08 | 1Score **> 35** indicates **identity** Score **> 17** indicates **homology** | U | K.LVGAIVYYDGQHNDAR.M |
| 99550 | 212 | – | 227 | 597.6331 | 1789.8775 | 1789.8747 | 1.54 | 0 | 38 | 0.00025 | 1Score **> 35** indicates **identity** Score **> 15** indicates **homology** | U | K.LVGAIVYYDGQHNDAR.M |
| 99551 | 212 | – | 227 | 597.6335 | 1789.8788 | 1789.8747 | 2.25 | 0 | 57 | 4.9e-06 | 1Score **> 35** indicates **identity** Score **> 16** indicates **homology** | U | K.LVGAIVYYDGQHNDAR.M |
| 99552 | 212 | – | 227 | 597.6341 | 1789.8804 | 1789.8747 | 3.18 | 0 | 27 | 0.0026 | 1Score **> 35** indicates **identity** Score **> 14** indicates **homology** | U | K.LVGAIVYYDGQHNDAR.M |
| 99554 | 212 | – | 227 | 895.9478 | 1789.8810 | 1789.8747 | 3.51 | 0 | 63 | 1.2e-06 | 1Score **> 35** indicates **identity** Score **> 16** indicates **homology** | U | K.LVGAIVYYDGQHNDAR.M |
| 24796 | 228 | – | 238 | 572.8292 | 1143.6438 | 1143.6434 | 0.33 | 0 | 31 | 0.0026 | 1Score **> 34** indicates **identity** Score **> 17** indicates **homology** | U | R.MNLAIALTAAR.Y |
| 24797 | 228 | – | 238 | 572.8292 | 1143.6439 | 1143.6434 | 0.45 | 0 | 54 | 4.9e-05 | 1Score **> 34** indicates **identity** Score **> 23** indicates **homology** | U | R.MNLAIALTAAR.Y |
| 24798 | 228 | – | 238 | 572.8295 | 1143.6445 | 1143.6434 | 0.99 | 0 | 34 | 0.0014 | 1Score **> 34** indicates **identity** Score **> 18** indicates **homology** | U | R.MNLAIALTAAR.Y |
| 24799 | 228 | – | 238 | 572.8297 | 1143.6449 | 1143.6434 | 1.31 | 0 | 51 | 7e-05 | 1Score **> 34** indicates **identity** Score **> 22** indicates **homology** | U | R.MNLAIALTAAR.Y |
| 24800 | 228 | – | 238 | 572.8301 | 1143.6456 | 1143.6434 | 1.90 | 0 | 68 | 1.1e-05 | 1Score **> 34** indicates **identity** Score **> 31** indicates **homology** | U | R.MNLAIALTAAR.Y |
| 24803 | 228 | – | 238 | 572.8307 | 1143.6469 | 1143.6434 | 3.08 | 0 | 65 | 4.2e-05 | 1Score **> 34** indicates **identity** | U | R.MNLAIALTAAR.Y |
| 24804 | 228 | – | 238 | 572.8309 | 1143.6472 | 1143.6434 | 3.31 | 0 | 44 | 0.00067 | 1Score **> 34** indicates **identity** Score **> 25** indicates **homology** | U | R.MNLAIALTAAR.Y |
| 92573 | 239 | – | 254 | 865.4384 | 1728.8623 | 1728.8756 | -7.72 | 0 | 37 | 0.00037 | 1Score **> 35** indicates **identity** Score **> 15** indicates **homology** | U | R.YGAATANYMEVVSLLK.K |
| 92598 | 239 | – | 254 | 865.4480 | 1728.8814 | 1728.8756 | 3.35 | 0 | 106 | 1.1e-10 | 1Score **> 35** indicates **identity** Score **> 19** indicates **homology** | U | R.YGAATANYMEVVSLLK.K |
| 106502 | 239 | – | 255 | 619.9998 | 1856.9776 | 1856.9706 | 3.78 | 1 | 56 | 5.2e-06 | 1Score **> 36** indicates **identity** Score **> 16** indicates **homology** | U | R.YGAATANYMEVVSLLKK.T |
| 106503 | 239 | – | 255 | 620.0002 | 1856.9788 | 1856.9706 | 4.44 | 1 | 57 | 4.7e-06 | 1Score **> 36** indicates **identity** Score **> 16** indicates **homology** | U | R.YGAATANYMEVVSLLKK.T |
| 70675 | 270 | – | 282 | 783.8812 | 1565.7478 | 1565.7508 | -1.94 | 1 | 58 | 4.2e-06 | 1Score **> 33** indicates **identity** Score **> 17** indicates **homology** | U | R.CKDVLTGQEFDVR.A |
| 70676 | 270 | – | 282 | 783.8821 | 1565.7497 | 1565.7508 | -0.70 | 1 | 42 | 0.00011 | 1Score **> 33** indicates **identity** Score **> 15** indicates **homology** | U | R.CKDVLTGQEFDVR.A |
| 70677 | 270 | – | 282 | 783.8827 | 1565.7508 | 1565.7508 | -0.0045 | 1 | 43 | 0.0001 | 1Score **> 33** indicates **identity** Score **> 15** indicates **homology** | U | R.CKDVLTGQEFDVR.A |
| 70678 | 270 | – | 282 | 522.9244 | 1565.7515 | 1565.7508 | 0.42 | 1 | 76 | 1.5e-07 | 1Score **> 33** indicates **identity** Score **> 20** indicates **homology** | U | R.CKDVLTGQEFDVR.A |
| 70679 | 270 | – | 282 | 522.9245 | 1565.7517 | 1565.7508 | 0.55 | 1 | 30 | 0.0015 | 1Score **> 33** indicates **identity** Score **> 14** indicates **homology** | U | R.CKDVLTGQEFDVR.A |
| 70680 | 270 | – | 282 | 522.9248 | 1565.7524 | 1565.7508 | 1.03 | 1 | 25 | 0.0048 | 1Score **> 33** indicates **identity** Score **> 14** indicates **homology** | U | R.CKDVLTGQEFDVR.A |
| 70681 | 270 | – | 282 | 522.9248 | 1565.7525 | 1565.7508 | 1.10 | 1 | 20 | 0.013 | 1Score **> 33** indicates **identity** Score **> 14** indicates **homology** | U | R.CKDVLTGQEFDVR.A |
| 70682 | 270 | – | 282 | 522.9248 | 1565.7526 | 1565.7508 | 1.12 | 1 | 15 | 0.036 | 1Score **> 33** indicates **identity** Score **> 13** indicates **homology** | U | R.CKDVLTGQEFDVR.A |
| 70683 | 270 | – | 282 | 522.9249 | 1565.7527 | 1565.7508 | 1.23 | 1 | 18 | 0.019 | 1Score **> 33** indicates **identity** Score **> 14** indicates **homology** | U | R.CKDVLTGQEFDVR.A |
| 70684 | 270 | – | 282 | 783.8837 | 1565.7528 | 1565.7508 | 1.25 | 1 | 68 | 4.7e-07 | 1Score **> 33** indicates **identity** Score **> 18** indicates **homology** | U | R.CKDVLTGQEFDVR.A |
| 70685 | 270 | – | 282 | 522.9251 | 1565.7534 | 1565.7508 | 1.68 | 1 | 63 | 1.1e-06 | 1Score **> 33** indicates **identity** Score **> 16** indicates **homology** | U | R.CKDVLTGQEFDVR.A |
| 70686 | 270 | – | 282 | 783.8840 | 1565.7535 | 1565.7508 | 1.72 | 1 | 46 | 4.8e-05 | 1Score **> 33** indicates **identity** Score **> 15** indicates **homology** | U | R.CKDVLTGQEFDVR.A |
| 70687 | 270 | – | 282 | 522.9252 | 1565.7537 | 1565.7508 | 1.84 | 1 | 22 | 0.0092 | 1Score **> 33** indicates **identity** Score **> 14** indicates **homology** | U | R.CKDVLTGQEFDVR.A |
| 70688 | 270 | – | 282 | 522.9254 | 1565.7544 | 1565.7508 | 2.28 | 1 | 60 | 2.2e-06 | 1Score **> 33** indicates **identity** Score **> 16** indicates **homology** | U | R.CKDVLTGQEFDVR.A |
| 70689 | 270 | – | 282 | 522.9260 | 1565.7562 | 1565.7508 | 3.42 | 1 | 49 | 2.7e-05 | 1Score **> 33** indicates **identity** Score **> 16** indicates **homology** | U | R.CKDVLTGQEFDVR.A |
| 70690 | 270 | – | 282 | 783.8858 | 1565.7569 | 1565.7508 | 3.92 | 1 | 32 | 0.0011 | 1Score **> 33** indicates **identity** Score **> 14** indicates **homology** | U | R.CKDVLTGQEFDVR.A |
| 70691 | 270 | – | 282 | 783.8883 | 1565.7621 | 1565.7508 | 7.19 | 1 | 37 | 0.00036 | 1Score **> 34** indicates **identity** Score **> 15** indicates **homology** | U | R.CKDVLTGQEFDVR.A |
| 38255 | 272 | – | 282 | 639.8200 | 1277.6254 | 1277.6252 | 0.17 | 0 | 57 | 9.7e-06 | 1Score **> 33** indicates **identity** Score **> 19** indicates **homology** | U | K.DVLTGQEFDVR.A |
| 38256 | 272 | – | 282 | 639.8209 | 1277.6272 | 1277.6252 | 1.53 | 0 | 65 | 9.9e-07 | 1Score **> 33** indicates **identity** Score **> 18** indicates **homology** | U | K.DVLTGQEFDVR.A |
| 38265 | 272 | – | 282 | 639.8228 | 1277.6311 | 1277.6252 | 4.59 | 0 | 40 | 0.00016 | 1Score **> 33** indicates **identity** Score **> 15** indicates **homology** | U | K.DVLTGQEFDVR.A |
| 91553 | 283 | – | 298 | 861.4323 | 1720.8501 | 1720.8567 | -3.81 | 1 | 54 | 9e-06 | 1Score **> 35** indicates **identity** Score **> 16** indicates **homology** | U | R.AKCVINASGPFTDSVR.K |
| 91558 | 283 | – | 298 | 861.4355 | 1720.8565 | 1720.8567 | -0.11 | 1 | 65 | 8.8e-07 | 1Score **> 35** indicates **identity** Score **> 17** indicates **homology** | U | R.AKCVINASGPFTDSVR.K |
| 91560 | 283 | – | 298 | 574.6266 | 1720.8579 | 1720.8567 | 0.74 | 1 | 68 | 4.6e-07 | 1Score **> 35** indicates **identity** Score **> 17** indicates **homology** | U | R.AKCVINASGPFTDSVR.K |
| 91562 | 283 | – | 298 | 574.6271 | 1720.8595 | 1720.8567 | 1.65 | 1 | 61 | 2e-06 | 1Score **> 35** indicates **identity** Score **> 16** indicates **homology** | U | R.AKCVINASGPFTDSVR.K |
| 91565 | 283 | – | 298 | 861.4377 | 1720.8608 | 1720.8567 | 2.40 | 1 | 64 | 1.1e-06 | 1Score **> 35** indicates **identity** Score **> 16** indicates **homology** | U | R.AKCVINASGPFTDSVR.K |
| 91567 | 283 | – | 298 | 574.6280 | 1720.8623 | 1720.8567 | 3.28 | 1 | 22 | 0.0084 | 1Score **> 35** indicates **identity** Score **> 14** indicates **homology** | U | R.AKCVINASGPFTDSVR.K |
| 91569 | 283 | – | 298 | 861.4388 | 1720.8631 | 1720.8567 | 3.76 | 1 | 62 | 1.5e-06 | 1Score **> 35** indicates **identity** Score **> 16** indicates **homology** | U | R.AKCVINASGPFTDSVR.K |
| 64862 | 285 | – | 298 | 761.8656 | 1521.7166 | 1521.7246 | -5.26 | 0 | 56 | 5.9e-06 | 1Score **> 32** indicates **identity** Score **> 16** indicates **homology** | U | K.CVINASGPFTDSVR.K |
| 64863 | 285 | – | 298 | 761.8669 | 1521.7192 | 1521.7246 | -3.57 | 0 | 60 | 2.4e-06 | 1Score **> 32** indicates **identity** Score **> 16** indicates **homology** | U | K.CVINASGPFTDSVR.K |
| 64864 | 285 | – | 298 | 761.8672 | 1521.7198 | 1521.7246 | -3.16 | 0 | 69 | 3.1e-07 | 1Score **> 32** indicates **identity** Score **> 17** indicates **homology** | U | K.CVINASGPFTDSVR.K |
| 64866 | 285 | – | 298 | 761.8683 | 1521.7221 | 1521.7246 | -1.66 | 0 | 59 | 3.2e-06 | 1Score **> 32** indicates **identity** Score **> 16** indicates **homology** | U | K.CVINASGPFTDSVR.K |
| 64867 | 285 | – | 298 | 761.8685 | 1521.7224 | 1521.7246 | -1.47 | 0 | 76 | 1.1e-07 | 1Score **> 32** indicates **identity** Score **> 19** indicates **homology** | U | K.CVINASGPFTDSVR.K |
| 64868 | 285 | – | 298 | 761.8685 | 1521.7225 | 1521.7246 | -1.40 | 0 | 75 | 1.2e-07 | 1Score **> 32** indicates **identity** Score **> 19** indicates **homology** | U | K.CVINASGPFTDSVR.K |
| 64871 | 285 | – | 298 | 761.8691 | 1521.7237 | 1521.7246 | -0.60 | 0 | 46 | 4.7e-05 | 1Score **> 32** indicates **identity** Score **> 15** indicates **homology** | U | K.CVINASGPFTDSVR.K |
| 64872 | 285 | – | 298 | 761.8693 | 1521.7240 | 1521.7246 | -0.42 | 0 | 57 | 4.3e-06 | 1Score **> 32** indicates **identity** Score **> 16** indicates **homology** | U | K.CVINASGPFTDSVR.K |
| 64873 | 285 | – | 298 | 761.8693 | 1521.7240 | 1521.7246 | -0.42 | 0 | 87 | 4.6e-08 | 1Score **> 32** indicates **identity** Score **> 26** indicates **homology** | U | K.CVINASGPFTDSVR.K |
| 64874 | 285 | – | 298 | 761.8693 | 1521.7241 | 1521.7246 | -0.36 | 0 | 60 | 2.2e-06 | 1Score **> 32** indicates **identity** Score **> 16** indicates **homology** | U | K.CVINASGPFTDSVR.K |
| 64875 | 285 | – | 298 | 761.8694 | 1521.7243 | 1521.7246 | -0.17 | 0 | 78 | 9.3e-08 | 1Score **> 33** indicates **identity** Score **> 20** indicates **homology** | U | K.CVINASGPFTDSVR.K |
| 64876 | 285 | – | 298 | 761.8695 | 1521.7244 | 1521.7246 | -0.10 | 0 | 83 | 7.1e-08 | 1Score **> 33** indicates **identity** Score **> 24** indicates **homology** | U | K.CVINASGPFTDSVR.K |
| 64877 | 285 | – | 298 | 761.8700 | 1521.7255 | 1521.7246 | 0.61 | 0 | 85 | 7.3e-08 | 1Score **> 32** indicates **identity** Score **> 26** indicates **homology** | U | K.CVINASGPFTDSVR.K |
| 64878 | 285 | – | 298 | 761.8701 | 1521.7256 | 1521.7246 | 0.69 | 0 | 98 | 3.7e-09 | 1Score **> 32** indicates **identity** Score **> 26** indicates **homology** | U | K.CVINASGPFTDSVR.K |
| 64879 | 285 | – | 298 | 761.8701 | 1521.7257 | 1521.7246 | 0.75 | 0 | 64 | 1.3e-06 | 1Score **> 32** indicates **identity** Score **> 18** indicates **homology** | U | K.CVINASGPFTDSVR.K |
| 64880 | 285 | – | 298 | 761.8702 | 1521.7257 | 1521.7246 | 0.76 | 0 | 32 | 0.0011 | 1Score **> 32** indicates **identity** Score **> 14** indicates **homology** | U | K.CVINASGPFTDSVR.K |
| 64881 | 285 | – | 298 | 761.8702 | 1521.7259 | 1521.7246 | 0.84 | 0 | 41 | 0.0002 | 1Score **> 32** indicates **identity** Score **> 16** indicates **homology** | U | K.CVINASGPFTDSVR.K |
| 64885 | 285 | – | 298 | 761.8711 | 1521.7276 | 1521.7246 | 1.97 | 0 | 71 | 2.4e-07 | 1Score **> 33** indicates **identity** Score **> 17** indicates **homology** | U | K.CVINASGPFTDSVR.K |
| 64886 | 285 | – | 298 | 761.8715 | 1521.7284 | 1521.7246 | 2.47 | 0 | 87 | 3e-08 | 1Score **> 33** indicates **identity** Score **> 25** indicates **homology** | U | K.CVINASGPFTDSVR.K |
| 64887 | 285 | – | 298 | 761.8718 | 1521.7290 | 1521.7246 | 2.87 | 0 | 73 | 1.3e-07 | 1Score **> 33** indicates **identity** Score **> 17** indicates **homology** | U | K.CVINASGPFTDSVR.K |
| 64888 | 285 | – | 298 | 761.8734 | 1521.7322 | 1521.7246 | 5.03 | 0 | 44 | 8.5e-05 | 1Score **> 33** indicates **identity** Score **> 16** indicates **homology** | U | K.CVINASGPFTDSVR.K |
| 64995 | 285 | – | 298 | 762.3677 | 1522.7208 | 1522.7086 | 7.98 | 0 | 60 | 2.3e-06 | 1Score **> 32** indicates **identity** Score **> 16** indicates **homology** | U | K.CVINASGPFTDSVR.K  + Deamidated (NQ) |
| 82312 | 285 | – | 299 | 550.9462 | 1649.8169 | 1649.8196 | -1.63 | 1 | 27 | 0.0032 | 1Score **> 34** indicates **identity** Score **> 14** indicates **homology** | U | K.CVINASGPFTDSVRK.M |
| 82315 | 285 | – | 299 | 550.9470 | 1649.8192 | 1649.8196 | -0.19 | 1 | 52 | 1.2e-05 | 1Score **> 34** indicates **identity** Score **> 16** indicates **homology** | U | K.CVINASGPFTDSVRK.M |
| 82316 | 285 | – | 299 | 550.9471 | 1649.8196 | 1649.8196 | 0.013 | 1 | 51 | 1.7e-05 | 1Score **> 34** indicates **identity** Score **> 16** indicates **homology** | U | K.CVINASGPFTDSVRK.M |
| 82317 | 285 | – | 299 | 550.9478 | 1649.8214 | 1649.8196 | 1.14 | 1 | 60 | 2.4e-06 | 1Score **> 35** indicates **identity** Score **> 16** indicates **homology** | U | K.CVINASGPFTDSVRK.M |
| 82320 | 285 | – | 299 | 825.9191 | 1649.8237 | 1649.8196 | 2.50 | 1 | 26 | 0.0034 | 1Score **> 35** indicates **identity** Score **> 14** indicates **homology** | U | K.CVINASGPFTDSVRK.M |
| 28154 | 340 | – | 348 | 589.8338 | 1177.6530 | 1177.6536 | -0.45 | 0 | 29 | 0.0058 | 1Score **> 32** indicates **identity** Score **> 19** indicates **homology** | U | R.VIFFLPWEK.M |
| 28157 | 340 | – | 348 | 589.8344 | 1177.6543 | 1177.6536 | 0.65 | 0 | 40 | 0.00032 | 1Score **> 32** indicates **identity** Score **> 17** indicates **homology** | U | R.VIFFLPWEK.M |
| 28159 | 340 | – | 348 | 589.8345 | 1177.6545 | 1177.6536 | 0.76 | 0 | 32 | 0.0045 | 1Score **> 32** indicates **identity** Score **> 21** indicates **homology** | U | R.VIFFLPWEK.M |
| 28163 | 340 | – | 348 | 589.8352 | 1177.6558 | 1177.6536 | 1.92 | 0 | 36 | 0.0035 | 1Score **> 32** indicates **identity** Score **> 24** indicates **homology** | U | R.VIFFLPWEK.M |
| 28167 | 340 | – | 348 | 589.8360 | 1177.6575 | 1177.6536 | 3.38 | 0 | 31 | 0.0043 | 1Score **> 31** indicates **identity** Score **> 20** indicates **homology** | U | R.VIFFLPWEK.M |
| 28169 | 340 | – | 348 | 589.8365 | 1177.6584 | 1177.6536 | 4.10 | 0 | 15 | 0.035 | 1Score **> 31** indicates **identity** Score **> 13** indicates **homology** | U | R.VIFFLPWEK.M |
| 28170 | 340 | – | 348 | 589.8366 | 1177.6587 | 1177.6536 | 4.33 | 0 | 21 | 0.011 | 1Score **> 31** indicates **identity** Score **> 14** indicates **homology** | U | R.VIFFLPWEK.M |
| 28174 | 340 | – | 348 | 589.8389 | 1177.6633 | 1177.6536 | 8.27 | 0 | 16 | 0.04 | 1Score **> 32** indicates **identity** Score **> 14** indicates **homology** | U | R.VIFFLPWEK.M |
| 28360 | 381 | – | 390 | 591.2906 | 1180.5667 | 1180.5724 | -4.84 | 0 | 18 | 0.022 | 1Score **> 31** indicates **identity** Score **> 14** indicates **homology** | U | R.NYLSSDVEVR.R |
| 28361 | 381 | – | 390 | 591.2907 | 1180.5668 | 1180.5724 | -4.73 | 0 | 48 | 3.5e-05 | 1Score **> 31** indicates **identity** Score **> 15** indicates **homology** | U | R.NYLSSDVEVR.R |
| 28365 | 381 | – | 390 | 591.2925 | 1180.5704 | 1180.5724 | -1.72 | 0 | 37 | 0.00037 | 1Score **> 31** indicates **identity** Score **> 15** indicates **homology** | U | R.NYLSSDVEVR.R |
| 28366 | 381 | – | 390 | 591.2928 | 1180.5711 | 1180.5724 | -1.12 | 0 | 42 | 0.00011 | 1Score **> 31** indicates **identity** Score **> 15** indicates **homology** | U | R.NYLSSDVEVR.R |
| 28367 | 381 | – | 390 | 591.2928 | 1180.5711 | 1180.5724 | -1.08 | 0 | 46 | 5.4e-05 | 1Score **> 31** indicates **identity** Score **> 15** indicates **homology** | U | R.NYLSSDVEVR.R |
| 28369 | 381 | – | 390 | 591.2933 | 1180.5720 | 1180.5724 | -0.35 | 0 | 29 | 0.0019 | 1Score **> 32** indicates **identity** Score **> 14** indicates **homology** | U | R.NYLSSDVEVR.R |
| 28370 | 381 | – | 390 | 591.2933 | 1180.5720 | 1180.5724 | -0.35 | 0 | 16 | 0.035 | 1Score **> 32** indicates **identity** Score **> 13** indicates **homology** | U | R.NYLSSDVEVR.R |
| 28371 | 381 | – | 390 | 591.2939 | 1180.5733 | 1180.5724 | 0.79 | 0 | 50 | 2e-05 | 1Score **> 32** indicates **identity** Score **> 16** indicates **homology** | U | R.NYLSSDVEVR.R |
| 28372 | 381 | – | 390 | 591.2940 | 1180.5734 | 1180.5724 | 0.86 | 0 | 62 | 1.7e-06 | 1Score **> 32** indicates **identity** Score **> 16** indicates **homology** | U | R.NYLSSDVEVR.R |
| 28373 | 381 | – | 390 | 591.2942 | 1180.5738 | 1180.5724 | 1.17 | 0 | 56 | 1.7e-05 | 1Score **> 32** indicates **identity** Score **> 20** indicates **homology** | U | R.NYLSSDVEVR.R |
| 28374 | 381 | – | 390 | 591.2942 | 1180.5739 | 1180.5724 | 1.27 | 0 | 57 | 4.2e-06 | 1Score **> 31** indicates **identity** Score **> 16** indicates **homology** | U | R.NYLSSDVEVR.R |
| 28376 | 381 | – | 390 | 591.2943 | 1180.5740 | 1180.5724 | 1.34 | 0 | 55 | 6.3e-06 | 1Score **> 31** indicates **identity** Score **> 16** indicates **homology** | U | R.NYLSSDVEVR.R |
| 28377 | 381 | – | 390 | 591.2943 | 1180.5740 | 1180.5724 | 1.36 | 0 | 55 | 6.8e-06 | 1Score **> 31** indicates **identity** Score **> 16** indicates **homology** | U | R.NYLSSDVEVR.R |
| 28378 | 381 | – | 390 | 591.2943 | 1180.5740 | 1180.5724 | 1.37 | 0 | 30 | 0.0014 | 1Score **> 31** indicates **identity** Score **> 14** indicates **homology** | U | R.NYLSSDVEVR.R |
| 28379 | 381 | – | 390 | 591.2944 | 1180.5742 | 1180.5724 | 1.51 | 0 | 57 | 4.9e-06 | 1Score **> 31** indicates **identity** Score **> 16** indicates **homology** | U | R.NYLSSDVEVR.R |
| 28380 | 381 | – | 390 | 591.2944 | 1180.5743 | 1180.5724 | 1.61 | 0 | 52 | 1.3e-05 | 1Score **> 31** indicates **identity** Score **> 16** indicates **homology** | U | R.NYLSSDVEVR.R |
| 28381 | 381 | – | 390 | 591.2946 | 1180.5746 | 1180.5724 | 1.85 | 0 | 57 | 4.6e-06 | 1Score **> 32** indicates **identity** Score **> 16** indicates **homology** | U | R.NYLSSDVEVR.R |
| 28382 | 381 | – | 390 | 591.2949 | 1180.5752 | 1180.5724 | 2.33 | 0 | 65 | 7.8e-07 | 1Score **> 32** indicates **identity** Score **> 17** indicates **homology** | U | R.NYLSSDVEVR.R |
| 28383 | 381 | – | 390 | 591.2951 | 1180.5756 | 1180.5724 | 2.71 | 0 | 43 | 9.9e-05 | 1Score **> 32** indicates **identity** Score **> 15** indicates **homology** | U | R.NYLSSDVEVR.R |
| 28385 | 381 | – | 390 | 591.2952 | 1180.5758 | 1180.5724 | 2.91 | 0 | 48 | 3.3e-05 | 1Score **> 32** indicates **identity** Score **> 15** indicates **homology** | U | R.NYLSSDVEVR.R |
| 28389 | 381 | – | 390 | 591.2958 | 1180.5771 | 1180.5724 | 3.96 | 0 | 24 | 0.007 | 1Score **> 32** indicates **identity** Score **> 15** indicates **homology** | U | R.NYLSSDVEVR.R |
| 28393 | 381 | – | 390 | 591.2966 | 1180.5786 | 1180.5724 | 5.26 | 0 | 26 | 0.029 | 1Score **> 31** indicates **identity** Score **> 23** indicates **homology** | U | R.NYLSSDVEVR.R |
| 28408 | 381 | – | 390 | 591.2979 | 1180.5812 | 1180.5724 | 7.42 | 0 | 15 | 0.04 | 1Score **> 32** indicates **identity** Score **> 13** indicates **homology** | U | R.NYLSSDVEVR.R |
| 28492 | 381 | – | 390 | 591.7870 | 1181.5594 | 1181.5564 | 2.51 | 0 | 37 | 0.00035 | 1Score **> 30** indicates **identity** Score **> 15** indicates **homology** | U | R.NYLSSDVEVR.R  + Deamidated (NQ) |
| 44226 | 381 | – | 391 | 669.3417 | 1336.6689 | 1336.6735 | -3.46 | 1 | 21 | 0.022 | 1Score **> 33** indicates **identity** Score **> 17** indicates **homology** | U | R.NYLSSDVEVRR.G |
| 44230 | 381 | – | 391 | 669.3427 | 1336.6708 | 1336.6735 | -2.00 | 1 | 36 | 0.00054 | 1Score **> 33** indicates **identity** Score **> 16** indicates **homology** | U | R.NYLSSDVEVRR.G |
| 44231 | 381 | – | 391 | 446.5643 | 1336.6712 | 1336.6735 | -1.72 | 1 | 16 | 0.031 | 1Score **> 33** indicates **identity** Score **> 14** indicates **homology** | U | R.NYLSSDVEVRR.G |
| 44233 | 381 | – | 391 | 446.5646 | 1336.6719 | 1336.6735 | -1.23 | 1 | 21 | 0.011 | 1Score **> 33** indicates **identity** Score **> 14** indicates **homology** | U | R.NYLSSDVEVRR.G |
| 40456 | 391 | – | 402 | 434.2428 | 1299.7066 | 1299.7048 | 1.42 | 1 | 39 | 0.00024 | 1Score **> 34** indicates **identity** Score **> 15** indicates **homology** | U | R.RGDVLAAWSGIR.P |
| 125475 | 391 | – | 409 | 513.5400 | 2050.1309 | 2050.1324 | -0.71 | 2 | 21 | 0.025 | 1Score **> 35** indicates **identity** Score **> 17** indicates **homology** | U | R.RGDVLAAWSGIRPLVTDPK.S |
| 125476 | 391 | – | 409 | 1026.0729 | 2050.1313 | 2050.1324 | -0.54 | 2 | 18 | 0.024 | 1Score **> 35** indicates **identity** Score **> 15** indicates **homology** | U | R.RGDVLAAWSGIRPLVTDPK.S |
| 125477 | 391 | – | 409 | 513.5401 | 2050.1314 | 2050.1324 | -0.46 | 2 | 26 | 0.0062 | 1Score **> 35** indicates **identity** Score **> 17** indicates **homology** | U | R.RGDVLAAWSGIRPLVTDPK.S |
| 125478 | 391 | – | 409 | 513.5402 | 2050.1318 | 2050.1324 | -0.29 | 2 | 16 | 0.035 | 1Score **> 35** indicates **identity** Score **> 13** indicates **homology** | U | R.RGDVLAAWSGIRPLVTDPK.S |
| 125479 | 391 | – | 409 | 684.3846 | 2050.1318 | 2050.1324 | -0.26 | 2 | 39 | 0.00036 | 1Score **> 35** indicates **identity** Score **> 17** indicates **homology** | U | R.RGDVLAAWSGIRPLVTDPK.S |
| 125480 | 391 | – | 409 | 684.3846 | 2050.1319 | 2050.1324 | -0.22 | 2 | 36 | 0.00083 | 1Score **> 35** indicates **identity** Score **> 17** indicates **homology** | U | R.RGDVLAAWSGIRPLVTDPK.S |
| 125483 | 391 | – | 409 | 513.5404 | 2050.1324 | 2050.1324 | 0.045 | 2 | 23 | 0.0095 | 1Score **> 35** indicates **identity** Score **> 15** indicates **homology** | U | R.RGDVLAAWSGIRPLVTDPK.S |
| 125484 | 391 | – | 409 | 684.3848 | 2050.1326 | 2050.1324 | 0.11 | 2 | 32 | 0.0052 | 1Score **> 35** indicates **identity** Score **> 21** indicates **homology** | U | R.RGDVLAAWSGIRPLVTDPK.S |
| 125485 | 391 | – | 409 | 684.3848 | 2050.1327 | 2050.1324 | 0.15 | 2 | 41 | 0.00035 | 1Score **> 35** indicates **identity** Score **> 19** indicates **homology** | U | R.RGDVLAAWSGIRPLVTDPK.S |
| 125487 | 391 | – | 409 | 513.5405 | 2050.1330 | 2050.1324 | 0.31 | 2 | 26 | 0.022 | 1Score **> 35** indicates **identity** Score **> 22** indicates **homology** | U | R.RGDVLAAWSGIRPLVTDPK.S |
| 125489 | 391 | – | 409 | 513.5406 | 2050.1335 | 2050.1324 | 0.55 | 2 | 20 | 0.037 | 1Score **> 35** indicates **identity** Score **> 18** indicates **homology** | U | R.RGDVLAAWSGIRPLVTDPK.S |
| 125490 | 391 | – | 409 | 513.5407 | 2050.1338 | 2050.1324 | 0.72 | 2 | 20 | 0.018 | 1Score **> 34** indicates **identity** Score **> 15** indicates **homology** | U | R.RGDVLAAWSGIRPLVTDPK.S |
| 125491 | 391 | – | 409 | 684.3853 | 2050.1341 | 2050.1324 | 0.85 | 2 | 46 | 0.00026 | 1Score **> 34** indicates **identity** Score **> 23** indicates **homology** | U | R.RGDVLAAWSGIRPLVTDPK.S |
| 125493 | 391 | – | 409 | 513.5409 | 2050.1347 | 2050.1324 | 1.14 | 2 | 19 | 0.042 | 1Score **> 34** indicates **identity** Score **> 17** indicates **homology** | U | R.RGDVLAAWSGIRPLVTDPK.S |
| 125494 | 391 | – | 409 | 513.5410 | 2050.1347 | 2050.1324 | 1.15 | 2 | 18 | 0.037 | 1Score **> 34** indicates **identity** Score **> 17** indicates **homology** | U | R.RGDVLAAWSGIRPLVTDPK.S |
| 125497 | 391 | – | 409 | 513.5411 | 2050.1354 | 2050.1324 | 1.50 | 2 | 22 | 0.046 | 1Score **> 34** indicates **identity** Score **> 22** indicates **homology** | U | R.RGDVLAAWSGIRPLVTDPK.S |
| 125499 | 391 | – | 409 | 513.5412 | 2050.1359 | 2050.1324 | 1.72 | 2 | 20 | 0.012 | 1Score **> 34** indicates **identity** Score **> 14** indicates **homology** | U | R.RGDVLAAWSGIRPLVTDPK.S |
| 125503 | 391 | – | 409 | 684.3872 | 2050.1399 | 2050.1324 | 3.66 | 2 | 22 | 0.0085 | 1Score **> 34** indicates **identity** Score **> 14** indicates **homology** | U | R.RGDVLAAWSGIRPLVTDPK.S |
| 125507 | 391 | – | 409 | 513.5426 | 2050.1412 | 2050.1324 | 4.29 | 2 | 16 | 0.033 | 1Score **> 34** indicates **identity** Score **> 13** indicates **homology** | U | R.RGDVLAAWSGIRPLVTDPK.S |
| 125510 | 391 | – | 409 | 513.5431 | 2050.1434 | 2050.1324 | 5.41 | 2 | 18 | 0.029 | 1Score **> 34** indicates **identity** Score **> 15** indicates **homology** | U | R.RGDVLAAWSGIRPLVTDPK.S |
| 125511 | 391 | – | 409 | 513.5432 | 2050.1437 | 2050.1324 | 5.53 | 2 | 22 | 0.0086 | 1Score **> 34** indicates **identity** Score **> 14** indicates **homology** | U | R.RGDVLAAWSGIRPLVTDPK.S |
| 100083 | 419 | – | 436 | 599.3211 | 1794.9415 | 1794.9476 | -3.40 | 0 | 29 | 0.0021 | 1Score **> 36** indicates **identity** Score **> 14** indicates **homology** | U | R.NHVVDISDSGLITIAGGK.W |
| 100086 | 419 | – | 436 | 898.4792 | 1794.9438 | 1794.9476 | -2.10 | 0 | 64 | 1.1e-06 | 1Score **> 36** indicates **identity** Score **> 16** indicates **homology** | U | R.NHVVDISDSGLITIAGGK.W |
| 100092 | 419 | – | 436 | 599.3227 | 1794.9462 | 1794.9476 | -0.76 | 0 | 14 | 0.047 | 1Score **> 36** indicates **identity** Score **> 13** indicates **homology** | U | R.NHVVDISDSGLITIAGGK.W |
| 100101 | 419 | – | 436 | 599.3233 | 1794.9482 | 1794.9476 | 0.34 | 0 | 49 | 2.3e-05 | 1Score **> 35** indicates **identity** Score **> 16** indicates **homology** | U | R.NHVVDISDSGLITIAGGK.W |
| 100102 | 419 | – | 436 | 599.3234 | 1794.9483 | 1794.9476 | 0.38 | 0 | 65 | 7.8e-07 | 1Score **> 36** indicates **identity** Score **> 17** indicates **homology** | U | R.NHVVDISDSGLITIAGGK.W |
| 100104 | 419 | – | 436 | 599.3235 | 1794.9488 | 1794.9476 | 0.68 | 0 | 69 | 3.7e-07 | 1Score **> 36** indicates **identity** Score **> 17** indicates **homology** | U | R.NHVVDISDSGLITIAGGK.W |
| 100105 | 419 | – | 436 | 599.3236 | 1794.9490 | 1794.9476 | 0.77 | 0 | 44 | 7.4e-05 | 1Score **> 36** indicates **identity** Score **> 15** indicates **homology** | U | R.NHVVDISDSGLITIAGGK.W |
| 100106 | 419 | – | 436 | 898.4818 | 1794.9490 | 1794.9476 | 0.78 | 0 | 75 | 9.4e-08 | 1Score **> 36** indicates **identity** Score **> 17** indicates **homology** | U | R.NHVVDISDSGLITIAGGK.W |
| 100107 | 419 | – | 436 | 599.3236 | 1794.9491 | 1794.9476 | 0.82 | 0 | 28 | 0.0023 | 1Score **> 36** indicates **identity** Score **> 14** indicates **homology** | U | R.NHVVDISDSGLITIAGGK.W |
| 100110 | 419 | – | 436 | 599.3238 | 1794.9495 | 1794.9476 | 1.05 | 0 | 89 | 6.5e-09 | 1Score **> 36** indicates **identity** Score **> 20** indicates **homology** | U | R.NHVVDISDSGLITIAGGK.W |
| 100111 | 419 | – | 436 | 898.4820 | 1794.9495 | 1794.9476 | 1.07 | 0 | 101 | 3.7e-10 | 1Score **> 36** indicates **identity** Score **> 19** indicates **homology** | U | R.NHVVDISDSGLITIAGGK.W |
| 100112 | 419 | – | 436 | 898.4821 | 1794.9497 | 1794.9476 | 1.16 | 0 | 76 | 7.2e-08 | 1Score **> 36** indicates **identity** Score **> 17** indicates **homology** | U | R.NHVVDISDSGLITIAGGK.W |
| 100114 | 419 | – | 436 | 599.3239 | 1794.9499 | 1794.9476 | 1.30 | 0 | 84 | 1.8e-08 | 1Score **> 36** indicates **identity** Score **> 19** indicates **homology** | U | R.NHVVDISDSGLITIAGGK.W |
| 100116 | 419 | – | 436 | 599.3241 | 1794.9504 | 1794.9476 | 1.57 | 0 | 41 | 0.00013 | 1Score **> 36** indicates **identity** Score **> 15** indicates **homology** | U | R.NHVVDISDSGLITIAGGK.W |
| 100119 | 419 | – | 436 | 898.4825 | 1794.9505 | 1794.9476 | 1.61 | 0 | 89 | 4.5e-09 | 1Score **> 36** indicates **identity** Score **> 18** indicates **homology** | U | R.NHVVDISDSGLITIAGGK.W |
| 100122 | 419 | – | 436 | 898.4827 | 1794.9509 | 1794.9476 | 1.82 | 0 | 97 | 8.6e-10 | 1Score **> 36** indicates **identity** Score **> 19** indicates **homology** | U | R.NHVVDISDSGLITIAGGK.W |
| 100123 | 419 | – | 436 | 599.3243 | 1794.9509 | 1794.9476 | 1.87 | 0 | 54 | 9.2e-06 | 1Score **> 36** indicates **identity** Score **> 16** indicates **homology** | U | R.NHVVDISDSGLITIAGGK.W |
| 100124 | 419 | – | 436 | 898.4828 | 1794.9510 | 1794.9476 | 1.89 | 0 | 47 | 4e-05 | 1Score **> 36** indicates **identity** Score **> 15** indicates **homology** | U | R.NHVVDISDSGLITIAGGK.W |
| 100125 | 419 | – | 436 | 599.3244 | 1794.9513 | 1794.9476 | 2.07 | 0 | 40 | 0.00018 | 1Score **> 36** indicates **identity** Score **> 15** indicates **homology** | U | R.NHVVDISDSGLITIAGGK.W |
| 100127 | 419 | – | 436 | 599.3248 | 1794.9525 | 1794.9476 | 2.72 | 0 | 19 | 0.017 | 1Score **> 36** indicates **identity** Score **> 14** indicates **homology** | U | R.NHVVDISDSGLITIAGGK.W |
| 100128 | 419 | – | 436 | 599.3248 | 1794.9526 | 1794.9476 | 2.80 | 0 | 99 | 7.3e-10 | 1Score **> 36** indicates **identity** Score **> 20** indicates **homology** | U | R.NHVVDISDSGLITIAGGK.W |
| 100129 | 419 | – | 436 | 599.3249 | 1794.9530 | 1794.9476 | 3.00 | 0 | 57 | 4.3e-06 | 1Score **> 36** indicates **identity** Score **> 16** indicates **homology** | U | R.NHVVDISDSGLITIAGGK.W |
| 100131 | 419 | – | 436 | 599.3251 | 1794.9535 | 1794.9476 | 3.30 | 0 | 95 | 1.6e-09 | 1Score **> 36** indicates **identity** Score **> 19** indicates **homology** | U | R.NHVVDISDSGLITIAGGK.W |
| 100133 | 419 | – | 436 | 599.3252 | 1794.9537 | 1794.9476 | 3.41 | 0 | 54 | 8.5e-06 | 1Score **> 36** indicates **identity** Score **> 16** indicates **homology** | U | R.NHVVDISDSGLITIAGGK.W |
| 100134 | 419 | – | 436 | 599.3252 | 1794.9538 | 1794.9476 | 3.44 | 0 | 55 | 6.8e-06 | 1Score **> 36** indicates **identity** Score **> 16** indicates **homology** | U | R.NHVVDISDSGLITIAGGK.W |
| 100135 | 419 | – | 436 | 599.3252 | 1794.9539 | 1794.9476 | 3.51 | 0 | 36 | 0.00039 | 1Score **> 36** indicates **identity** Score **> 15** indicates **homology** | U | R.NHVVDISDSGLITIAGGK.W |
| 100136 | 419 | – | 436 | 599.3252 | 1794.9539 | 1794.9476 | 3.52 | 0 | 61 | 1.8e-06 | 1Score **> 36** indicates **identity** Score **> 16** indicates **homology** | U | R.NHVVDISDSGLITIAGGK.W |
| 100139 | 419 | – | 436 | 599.3253 | 1794.9541 | 1794.9476 | 3.65 | 0 | 49 | 2.6e-05 | 1Score **> 36** indicates **identity** Score **> 16** indicates **homology** | U | R.NHVVDISDSGLITIAGGK.W |
| 100141 | 419 | – | 436 | 599.3258 | 1794.9556 | 1794.9476 | 4.45 | 0 | 14 | 0.046 | 1Score **> 35** indicates **identity** Score **> 13** indicates **homology** | U | R.NHVVDISDSGLITIAGGK.W |
| 100142 | 419 | – | 436 | 599.3264 | 1794.9572 | 1794.9476 | 5.38 | 0 | 32 | 0.0011 | 1Score **> 35** indicates **identity** Score **> 14** indicates **homology** | U | R.NHVVDISDSGLITIAGGK.W |
| 100147 | 419 | – | 436 | 599.3274 | 1794.9605 | 1794.9476 | 7.18 | 0 | 77 | 6.5e-08 | 1Score **> 35** indicates **identity** Score **> 17** indicates **homology** | U | R.NHVVDISDSGLITIAGGK.W |
| 100149 | 419 | – | 436 | 599.3279 | 1794.9618 | 1794.9476 | 7.91 | 0 | 15 | 0.037 | 1Score **> 35** indicates **identity** Score **> 13** indicates **homology** | U | R.NHVVDISDSGLITIAGGK.W |
| 100150 | 419 | – | 436 | 599.3282 | 1794.9629 | 1794.9476 | 8.53 | 0 | 76 | 7.6e-08 | 1Score **> 35** indicates **identity** Score **> 17** indicates **homology** | U | R.NHVVDISDSGLITIAGGK.W |
| 100211 | 419 | – | 436 | 599.6569 | 1795.9489 | 1795.9316 | 9.66 | 0 | 16 | 0.031 | 1Score **> 36** indicates **identity** Score **> 14** indicates **homology** | U | R.NHVVDISDSGLITIAGGK.W  + Deamidated (NQ) |
| 159050 | 419 | – | 441 | 835.1023 | 2502.2850 | 2502.2867 | -0.67 | 1 | 29 | 0.0021 | 1Score **> 37** indicates **identity** Score **> 14** indicates **homology** | U | R.NHVVDISDSGLITIAGGKWTTYR.S |
| 159052 | 419 | – | 441 | 835.1029 | 2502.2868 | 2502.2867 | 0.045 | 1 | 78 | 4.9e-08 | 1Score **> 37** indicates **identity** Score **> 17** indicates **homology** | U | R.NHVVDISDSGLITIAGGKWTTYR.S |
| 159053 | 419 | – | 441 | 835.1034 | 2502.2885 | 2502.2867 | 0.72 | 1 | 77 | 5.6e-08 | 1Score **> 37** indicates **identity** Score **> 17** indicates **homology** | U | R.NHVVDISDSGLITIAGGKWTTYR.S |
| 159057 | 419 | – | 441 | 626.5804 | 2502.2925 | 2502.2867 | 2.30 | 1 | 61 | 1.8e-06 | 1Score **> 37** indicates **identity** Score **> 16** indicates **homology** | U | R.NHVVDISDSGLITIAGGKWTTYR.S |
| 159058 | 419 | – | 441 | 626.5806 | 2502.2932 | 2502.2867 | 2.59 | 1 | 56 | 6.2e-06 | 1Score **> 37** indicates **identity** Score **> 16** indicates **homology** | U | R.NHVVDISDSGLITIAGGKWTTYR.S |
| 159059 | 419 | – | 441 | 835.1055 | 2502.2947 | 2502.2867 | 3.22 | 1 | 86 | 8e-09 | 1Score **> 37** indicates **identity** Score **> 18** indicates **homology** | U | R.NHVVDISDSGLITIAGGKWTTYR.S |
| 159060 | 419 | – | 441 | 626.5825 | 2502.3008 | 2502.2867 | 5.64 | 1 | 32 | 0.00091 | 1Score **> 37** indicates **identity** Score **> 15** indicates **homology** | U | R.NHVVDISDSGLITIAGGKWTTYR.S |
| 159112 | 419 | – | 441 | 626.8286 | 2503.2852 | 2503.2707 | 5.77 | 1 | 31 | 0.0024 | 1Score **> 37** indicates **identity** Score **> 17** indicates **homology** | U | R.NHVVDISDSGLITIAGGKWTTYR.S  + Deamidated (NQ) |
| 33850 | 442 | – | 453 | 618.7937 | 1235.5728 | 1235.5704 | 1.98 | 0 | 44 | 7e-05 | 1Score **> 30** indicates **identity** Score **> 15** indicates **homology** | U | R.SMAEDTVDAAVK.F |
| 148646 | 442 | – | 463 | 777.3799 | 2329.1180 | 2329.1121 | 2.54 | 1 | 42 | 0.00011 | 1Score **> 35** indicates **identity** Score **> 15** indicates **homology** | U | R.SMAEDTVDAAVKFHNLNAGPSR.T |
| 148650 | 442 | – | 463 | 583.2875 | 2329.1208 | 2329.1121 | 3.74 | 1 | 34 | 0.00061 | 1Score **> 35** indicates **identity** Score **> 15** indicates **homology** | U | R.SMAEDTVDAAVKFHNLNAGPSR.T |
| 21744 | 454 | – | 463 | 556.7830 | 1111.5514 | 1111.5523 | -0.77 | 0 | 32 | 0.0015 | 1Score **> 31** indicates **identity** Score **> 16** indicates **homology** | U | K.FHNLNAGPSR.T |
| 21746 | 454 | – | 463 | 371.5247 | 1111.5523 | 1111.5523 | -0.0018 | 0 | 38 | 0.00029 | 1Score **> 31** indicates **identity** Score **> 15** indicates **homology** | U | K.FHNLNAGPSR.T |
| 21747 | 454 | – | 463 | 556.7834 | 1111.5523 | 1111.5523 | 0.0027 | 0 | 31 | 0.0019 | 1Score **> 31** indicates **identity** Score **> 16** indicates **homology** | U | K.FHNLNAGPSR.T |
| 21748 | 454 | – | 463 | 371.5249 | 1111.5528 | 1111.5523 | 0.49 | 0 | 31 | 0.0013 | 1Score **> 31** indicates **identity** Score **> 14** indicates **homology** | U | K.FHNLNAGPSR.T |
| 21749 | 454 | – | 463 | 556.7838 | 1111.5530 | 1111.5523 | 0.61 | 0 | 48 | 0.00014 | 1Score **> 31** indicates **identity** Score **> 22** indicates **homology** | U | K.FHNLNAGPSR.T |
| 21750 | 454 | – | 463 | 371.5250 | 1111.5532 | 1111.5523 | 0.79 | 0 | 51 | 1.7e-05 | 1Score **> 31** indicates **identity** Score **> 16** indicates **homology** | U | K.FHNLNAGPSR.T |
| 21751 | 454 | – | 463 | 556.7841 | 1111.5536 | 1111.5523 | 1.23 | 0 | 44 | 0.0004 | 1Score **> 31** indicates **identity** Score **> 23** indicates **homology** | U | K.FHNLNAGPSR.T |
| 21752 | 454 | – | 463 | 371.5253 | 1111.5541 | 1111.5523 | 1.66 | 0 | 26 | 0.0034 | 1Score **> 31** indicates **identity** Score **> 14** indicates **homology** | U | K.FHNLNAGPSR.T |
| 14306 | 464 | – | 473 | 510.2980 | 1018.5814 | 1018.5811 | 0.23 | 0 | 18 | 0.02 | 1Score **> 31** indicates **identity** Score **> 14** indicates **homology** | U | R.TVGLFLQGGK.D |
| 14307 | 464 | – | 473 | 510.2992 | 1018.5839 | 1018.5811 | 2.67 | 0 | 29 | 0.002 | 1Score **> 30** indicates **identity** Score **> 14** indicates **homology** | U | R.TVGLFLQGGK.D |
| 134981 | 464 | – | 482 | 717.7220 | 2150.1441 | 2150.1524 | -3.87 | 1 | 29 | 0.0018 | 1Score **> 36** indicates **identity** Score **> 14** indicates **homology** | U | R.TVGLFLQGGKDWSPTLYIR.L |
| 134985 | 464 | – | 482 | 1076.0824 | 2150.1502 | 2150.1524 | -1.04 | 1 | 107 | 8.8e-11 | 1Score **> 36** indicates **identity** Score **> 19** indicates **homology** | U | R.TVGLFLQGGKDWSPTLYIR.L |
| 134986 | 464 | – | 482 | 717.7243 | 2150.1512 | 2150.1524 | -0.58 | 1 | 66 | 5.9e-07 | 1Score **> 36** indicates **identity** Score **> 17** indicates **homology** | U | R.TVGLFLQGGKDWSPTLYIR.L |
| 134989 | 464 | – | 482 | 717.7245 | 2150.1518 | 2150.1524 | -0.31 | 1 | 29 | 0.0021 | 1Score **> 36** indicates **identity** Score **> 14** indicates **homology** | U | R.TVGLFLQGGKDWSPTLYIR.L |
| 134990 | 464 | – | 482 | 1076.0834 | 2150.1522 | 2150.1524 | -0.12 | 1 | 86 | 9.1e-09 | 1Score **> 36** indicates **identity** Score **> 18** indicates **homology** | U | R.TVGLFLQGGKDWSPTLYIR.L |
| 134991 | 464 | – | 482 | 717.7247 | 2150.1523 | 2150.1524 | -0.060 | 1 | 28 | 0.0026 | 1Score **> 36** indicates **identity** Score **> 14** indicates **homology** | U | R.TVGLFLQGGKDWSPTLYIR.L |
| 134993 | 464 | – | 482 | 717.7249 | 2150.1529 | 2150.1524 | 0.21 | 1 | 77 | 5.6e-08 | 1Score **> 36** indicates **identity** Score **> 17** indicates **homology** | U | R.TVGLFLQGGKDWSPTLYIR.L |
| 134994 | 464 | – | 482 | 717.7253 | 2150.1541 | 2150.1524 | 0.76 | 1 | 77 | 5.7e-08 | 1Score **> 36** indicates **identity** Score **> 17** indicates **homology** | U | R.TVGLFLQGGKDWSPTLYIR.L |
| 134995 | 464 | – | 482 | 1076.0843 | 2150.1541 | 2150.1524 | 0.79 | 1 | 94 | 1.4e-09 | 1Score **> 36** indicates **identity** Score **> 18** indicates **homology** | U | R.TVGLFLQGGKDWSPTLYIR.L |
| 134996 | 464 | – | 482 | 717.7256 | 2150.1551 | 2150.1524 | 1.22 | 1 | 62 | 1.6e-06 | 1Score **> 36** indicates **identity** Score **> 16** indicates **homology** | U | R.TVGLFLQGGKDWSPTLYIR.L |
| 135110 | 464 | – | 482 | 718.0592 | 2151.1559 | 2151.1364 | 9.05 | 1 | 29 | 0.0018 | 1Score **> 36** indicates **identity** Score **> 14** indicates **homology** | U | R.TVGLFLQGGKDWSPTLYIR.L  + Deamidated (NQ) |
| 25340 | 474 | – | 482 | 575.7983 | 1149.5821 | 1149.5818 | 0.22 | 0 | 34 | 0.00067 | 1Score **> 32** indicates **identity** Score **> 15** indicates **homology** | U | K.DWSPTLYIR.L |
| 25341 | 474 | – | 482 | 575.7992 | 1149.5839 | 1149.5818 | 1.80 | 0 | 27 | 0.0031 | 1Score **> 33** indicates **identity** Score **> 14** indicates **homology** | U | K.DWSPTLYIR.L |
| 25342 | 474 | – | 482 | 575.7994 | 1149.5842 | 1149.5818 | 2.04 | 0 | 33 | 0.00077 | 1Score **> 33** indicates **identity** Score **> 15** indicates **homology** | U | K.DWSPTLYIR.L |
| 25346 | 474 | – | 482 | 575.8008 | 1149.5870 | 1149.5818 | 4.45 | 0 | 19 | 0.017 | 1Score **> 33** indicates **identity** Score **> 14** indicates **homology** | U | K.DWSPTLYIR.L |
| 110675 | 483 | – | 499 | 633.9968 | 1898.9686 | 1898.9738 | -2.72 | 0 | 16 | 0.033 | 1Score **> 35** indicates **identity** Score **> 13** indicates **homology** | U | R.LVQDYGLESEVAQHLAK.T |
| 110677 | 483 | – | 499 | 950.4951 | 1898.9757 | 1898.9738 | 1.03 | 0 | 84 | 1.2e-08 | 1Score **> 35** indicates **identity** Score **> 18** indicates **homology** | U | R.LVQDYGLESEVAQHLAK.T |
| 110678 | 483 | – | 499 | 633.9992 | 1898.9758 | 1898.9738 | 1.09 | 0 | 75 | 8.9e-08 | 1Score **> 35** indicates **identity** Score **> 17** indicates **homology** | U | R.LVQDYGLESEVAQHLAK.T |
| 110679 | 483 | – | 499 | 950.4953 | 1898.9760 | 1898.9738 | 1.15 | 0 | 90 | 3.7e-09 | 1Score **> 36** indicates **identity** Score **> 18** indicates **homology** | U | R.LVQDYGLESEVAQHLAK.T |
| 110680 | 483 | – | 499 | 950.4953 | 1898.9760 | 1898.9738 | 1.17 | 0 | 73 | 1.3e-07 | 1Score **> 36** indicates **identity** Score **> 17** indicates **homology** | U | R.LVQDYGLESEVAQHLAK.T |
| 110681 | 483 | – | 499 | 633.9993 | 1898.9760 | 1898.9738 | 1.18 | 0 | 85 | 1.1e-08 | 1Score **> 36** indicates **identity** Score **> 18** indicates **homology** | U | R.LVQDYGLESEVAQHLAK.T |
| 110682 | 483 | – | 499 | 633.9993 | 1898.9761 | 1898.9738 | 1.21 | 0 | 70 | 2.7e-07 | 1Score **> 36** indicates **identity** Score **> 17** indicates **homology** | U | R.LVQDYGLESEVAQHLAK.T |
| 110683 | 483 | – | 499 | 633.9993 | 1898.9761 | 1898.9738 | 1.25 | 0 | 102 | 2.5e-10 | 1Score **> 36** indicates **identity** Score **> 19** indicates **homology** | U | R.LVQDYGLESEVAQHLAK.T |
| 110685 | 483 | – | 499 | 950.4960 | 1898.9775 | 1898.9738 | 1.94 | 0 | 53 | 1.2e-05 | 1Score **> 36** indicates **identity** Score **> 16** indicates **homology** | U | R.LVQDYGLESEVAQHLAK.T |
| 110686 | 483 | – | 499 | 633.9999 | 1898.9777 | 1898.9738 | 2.10 | 0 | 51 | 1.7e-05 | 1Score **> 36** indicates **identity** Score **> 16** indicates **homology** | U | R.LVQDYGLESEVAQHLAK.T |
| 110687 | 483 | – | 499 | 633.9999 | 1898.9779 | 1898.9738 | 2.16 | 0 | 87 | 6.7e-09 | 1Score **> 36** indicates **identity** Score **> 18** indicates **homology** | U | R.LVQDYGLESEVAQHLAK.T |
| 110689 | 483 | – | 499 | 634.0012 | 1898.9818 | 1898.9738 | 4.23 | 0 | 42 | 0.00011 | 1Score **> 36** indicates **identity** Score **> 15** indicates **homology** | U | R.LVQDYGLESEVAQHLAK.T |
| 110690 | 483 | – | 499 | 634.0021 | 1898.9845 | 1898.9738 | 5.66 | 0 | 65 | 7.8e-07 | 1Score **> 36** indicates **identity** Score **> 17** indicates **homology** | U | R.LVQDYGLESEVAQHLAK.T |
| 110691 | 483 | – | 499 | 634.0027 | 1898.9863 | 1898.9738 | 6.60 | 0 | 50 | 2e-05 | 1Score **> 36** indicates **identity** Score **> 16** indicates **homology** | U | R.LVQDYGLESEVAQHLAK.T |
| 110774 | 483 | – | 499 | 634.3313 | 1899.9722 | 1899.9578 | 7.59 | 0 | 16 | 0.031 | 1Score **> 36** indicates **identity** Score **> 14** indicates **homology** | U | R.LVQDYGLESEVAQHLAK.T  + Deamidated (NQ) |
| 33005 | 500 | – | 510 | 410.2113 | 1227.6121 | 1227.6135 | -1.18 | 1 | 24 | 0.0055 | 1Score **> 31** indicates **identity** Score **> 14** indicates **homology** | U | K.TYGDKAFEVAK.M |
| 33008 | 500 | – | 510 | 410.2119 | 1227.6138 | 1227.6135 | 0.24 | 1 | 50 | 2.3e-05 | 1Score **> 31** indicates **identity** Score **> 16** indicates **homology** | U | K.TYGDKAFEVAK.M |
| 33009 | 500 | – | 510 | 614.8145 | 1227.6145 | 1227.6135 | 0.81 | 1 | 73 | 1.4e-07 | 1Score **> 31** indicates **identity** Score **> 17** indicates **homology** | U | K.TYGDKAFEVAK.M |
| 33010 | 500 | – | 510 | 410.2121 | 1227.6145 | 1227.6135 | 0.81 | 1 | 57 | 4.1e-06 | 1Score **> 31** indicates **identity** Score **> 16** indicates **homology** | U | K.TYGDKAFEVAK.M |
| 33012 | 500 | – | 510 | 614.8146 | 1227.6147 | 1227.6135 | 0.98 | 1 | 74 | 1.5e-07 | 1Score **> 31** indicates **identity** Score **> 18** indicates **homology** | U | K.TYGDKAFEVAK.M |
| 33013 | 500 | – | 510 | 410.2123 | 1227.6151 | 1227.6135 | 1.25 | 1 | 37 | 0.00033 | 1Score **> 31** indicates **identity** Score **> 15** indicates **homology** | U | K.TYGDKAFEVAK.M |
| 33014 | 500 | – | 510 | 410.2124 | 1227.6154 | 1227.6135 | 1.50 | 1 | 53 | 1.2e-05 | 1Score **> 31** indicates **identity** Score **> 16** indicates **homology** | U | K.TYGDKAFEVAK.M |
| 33015 | 500 | – | 510 | 614.8151 | 1227.6156 | 1227.6135 | 1.68 | 1 | 69 | 1.8e-06 | 1Score **> 31** indicates **identity** Score **> 24** indicates **homology** | U | K.TYGDKAFEVAK.M |
| 11253 | 518 | – | 525 | 484.7930 | 967.5715 | 967.5716 | -0.080 | 1 | 30 | 0.019 | 1Score **> 26** indicates **identity** Score **> 25** indicates **homology** | U | K.RWPVVGVR.L |
| 11254 | 518 | – | 525 | 484.7931 | 967.5716 | 967.5716 | -0.018 | 1 | 35 | 0.0033 | 1Score **> 26** indicates **identity** Score **> 23** indicates **homology** | U | K.RWPVVGVR.L |
| 11255 | 518 | – | 525 | 484.7931 | 967.5716 | 967.5716 | 0.032 | 1 | 18 | 0.039 | 1Score **> 26** indicates **identity** Score **> 16** indicates **homology** | U | K.RWPVVGVR.L |
| 11256 | 518 | – | 525 | 484.7932 | 967.5719 | 967.5716 | 0.34 | 1 | 27 | 0.016 | 1Score **> 28** indicates **identity** Score **> 22** indicates **homology** | U | K.RWPVVGVR.L |
| 11257 | 518 | – | 525 | 484.7932 | 967.5719 | 967.5716 | 0.36 | 1 | 33 | 0.0049 | 1Score **> 28** indicates **identity** Score **> 22** indicates **homology** | U | K.RWPVVGVR.L |
| 11258 | 518 | – | 525 | 484.7933 | 967.5720 | 967.5716 | 0.42 | 1 | 40 | 0.0025 | 1Score **> 28** indicates **identity** Score **> 26** indicates **homology** | U | K.RWPVVGVR.L |
| 11259 | 518 | – | 525 | 484.7934 | 967.5723 | 967.5716 | 0.79 | 1 | 34 | 0.0058 | 1Score **> 28** indicates **identity** Score **> 24** indicates **homology** | U | K.RWPVVGVR.L |
| 11260 | 518 | – | 525 | 484.7936 | 967.5726 | 967.5716 | 1.10 | 1 | 32 | 0.006 | 1Score **> 28** indicates **identity** Score **> 23** indicates **homology** | U | K.RWPVVGVR.L |
| 11261 | 518 | – | 525 | 484.7938 | 967.5730 | 967.5716 | 1.43 | 1 | 40 | 0.0025 | 1Score **> 28** indicates **identity** Score **> 26** indicates **homology** | U | K.RWPVVGVR.L |
| 3723 | 519 | – | 525 | 406.7419 | 811.4692 | 811.4705 | -1.59 | 0 | 21 | 0.011 | 1Score **> 21** indicates **identity** Score **> 14** indicates **homology** | U | R.WPVVGVR.L |
| 3725 | 519 | – | 525 | 406.7425 | 811.4704 | 811.4705 | -0.081 | 0 | 17 | 0.024 | 1Score **> 21** indicates **identity** Score **> 14** indicates **homology** | U | R.WPVVGVR.L |
| 3726 | 519 | – | 525 | 406.7425 | 811.4704 | 811.4705 | -0.076 | 0 | 15 | 0.041 | 1Score **> 21** indicates **identity** Score **> 14** indicates **homology** | U | R.WPVVGVR.L |
| 3727 | 519 | – | 525 | 406.7425 | 811.4705 | 811.4705 | 0.0074 | 0 | 22 | 0.0083 | 1Score **> 21** indicates **identity** Score **> 14** indicates **homology** | U | R.WPVVGVR.L |
| 3728 | 519 | – | 525 | 406.7426 | 811.4706 | 811.4705 | 0.10 | 0 | 16 | 0.032 | 1Score **> 21** indicates **identity** Score **> 13** indicates **homology** | U | R.WPVVGVR.L |
| 3729 | 519 | – | 525 | 406.7427 | 811.4708 | 811.4705 | 0.37 | 0 | 21 | 0.011 | 1Score **> 21** indicates **identity** Score **> 14** indicates **homology** | U | R.WPVVGVR.L |
| 3730 | 519 | – | 525 | 406.7427 | 811.4708 | 811.4705 | 0.38 | 0 | 23 | 0.0072 | 1Score **> 21** indicates **identity** Score **> 14** indicates **homology** | U | R.WPVVGVR.L |
| 3731 | 519 | – | 525 | 406.7427 | 811.4709 | 811.4705 | 0.49 | 0 | 34 | 0.0006 | 1Score **> 21** indicates **identity** Score **> 15** indicates **homology** | U | R.WPVVGVR.L |
| 3732 | 519 | – | 525 | 406.7427 | 811.4709 | 811.4705 | 0.57 | 0 | 22 | 0.0078 | 1Score **> 21** indicates **identity** Score **> 14** indicates **homology** | U | R.WPVVGVR.L |
| 3733 | 519 | – | 525 | 406.7428 | 811.4710 | 811.4705 | 0.69 | 0 | 20 | 0.013 | 1Score **> 21** indicates **identity** Score **> 14** indicates **homology** | U | R.WPVVGVR.L |
| 3734 | 519 | – | 525 | 406.7428 | 811.4711 | 811.4705 | 0.83 | 0 | 19 | 0.015 | 1Score **> 21** indicates **identity** Score **> 14** indicates **homology** | U | R.WPVVGVR.L |
| 3735 | 519 | – | 525 | 406.7429 | 811.4712 | 811.4705 | 0.85 | 0 | 19 | 0.017 | 1Score **> 21** indicates **identity** Score **> 14** indicates **homology** | U | R.WPVVGVR.L |
| 3736 | 519 | – | 525 | 406.7431 | 811.4716 | 811.4705 | 1.35 | 0 | 34 | 0.00061 | 1Score **> 21** indicates **identity** Score **> 15** indicates **homology** | U | R.WPVVGVR.L |
| 3738 | 519 | – | 525 | 406.7432 | 811.4718 | 811.4705 | 1.66 | 0 | 18 | 0.021 | 1Score **> 21** indicates **identity** Score **> 14** indicates **homology** | U | R.WPVVGVR.L |
| 3739 | 519 | – | 525 | 406.7432 | 811.4718 | 811.4705 | 1.67 | 0 | 31 | 0.0013 | 1Score **> 21** indicates **identity** Score **> 14** indicates **homology** | U | R.WPVVGVR.L |
| 3740 | 519 | – | 525 | 406.7434 | 811.4722 | 811.4705 | 2.10 | 0 | 22 | 0.0083 | 1Score **> 21** indicates **identity** Score **> 14** indicates **homology** | U | R.WPVVGVR.L |
| 3741 | 519 | – | 525 | 406.7435 | 811.4724 | 811.4705 | 2.43 | 0 | 20 | 0.013 | 1Score **> 24** indicates **identity** Score **> 14** indicates **homology** | U | R.WPVVGVR.L |
| 3742 | 519 | – | 525 | 406.7435 | 811.4724 | 811.4705 | 2.43 | 0 | 18 | 0.021 | 1Score **> 24** indicates **identity** Score **> 14** indicates **homology** | U | R.WPVVGVR.L |
| 3743 | 519 | – | 525 | 406.7436 | 811.4727 | 811.4705 | 2.79 | 0 | 17 | 0.027 | 1Score **> 24** indicates **identity** Score **> 14** indicates **homology** | U | R.WPVVGVR.L |
| 65054 | 526 | – | 538 | 762.4023 | 1522.7900 | 1522.7919 | -1.23 | 0 | 20 | 0.014 | 1Score **> 35** indicates **identity** Score **> 14** indicates **homology** | U | R.LVSEFPYIEAEVK.Y |
| 65058 | 526 | – | 538 | 508.6052 | 1522.7938 | 1522.7919 | 1.26 | 0 | 16 | 0.031 | 1Score **> 35** indicates **identity** Score **> 14** indicates **homology** | U | R.LVSEFPYIEAEVK.Y |
| 65059 | 526 | – | 538 | 762.4042 | 1522.7939 | 1522.7919 | 1.33 | 0 | 60 | 7.3e-06 | 1Score **> 35** indicates **identity** Score **> 22** indicates **homology** | U | R.LVSEFPYIEAEVK.Y |
| 65060 | 526 | – | 538 | 762.4043 | 1522.7940 | 1522.7919 | 1.40 | 0 | 67 | 1.8e-06 | 1Score **> 35** indicates **identity** Score **> 22** indicates **homology** | U | R.LVSEFPYIEAEVK.Y |
| 65062 | 526 | – | 538 | 762.4051 | 1522.7956 | 1522.7919 | 2.42 | 0 | 40 | 0.00017 | 1Score **> 35** indicates **identity** Score **> 15** indicates **homology** | U | R.LVSEFPYIEAEVK.Y |
| 65068 | 526 | – | 538 | 762.4066 | 1522.7985 | 1522.7919 | 4.38 | 0 | 64 | 2.9e-06 | 1Score **> 35** indicates **identity** Score **> 21** indicates **homology** | U | R.LVSEFPYIEAEVK.Y |
| 65069 | 526 | – | 538 | 762.4087 | 1522.8029 | 1522.7919 | 7.26 | 0 | 42 | 0.00012 | 1Score **> 35** indicates **identity** Score **> 15** indicates **homology** | U | R.LVSEFPYIEAEVK.Y |
| 123444 | 539 | – | 555 | 678.3354 | 2031.9843 | 2031.9870 | -1.32 | 2 | 35 | 0.00054 | 1Score **> 35** indicates **identity** Score **> 15** indicates **homology** | U | K.YGIKEYACTAVDMISRR.T |
| 123445 | 539 | – | 555 | 509.0037 | 2031.9855 | 2031.9870 | -0.73 | 2 | 31 | 0.0012 | 1Score **> 35** indicates **identity** Score **> 14** indicates **homology** | U | K.YGIKEYACTAVDMISRR.T |
| 123446 | 539 | – | 555 | 678.3359 | 2031.9858 | 2031.9870 | -0.57 | 2 | 19 | 0.018 | 1Score **> 35** indicates **identity** Score **> 14** indicates **homology** | U | K.YGIKEYACTAVDMISRR.T |
| 123447 | 539 | – | 555 | 509.0040 | 2031.9870 | 2031.9870 | 0.014 | 2 | 41 | 0.00015 | 1Score **> 35** indicates **identity** Score **> 15** indicates **homology** | U | K.YGIKEYACTAVDMISRR.T |
| 123449 | 539 | – | 555 | 509.0046 | 2031.9894 | 2031.9870 | 1.18 | 2 | 41 | 0.00013 | 1Score **> 35** indicates **identity** Score **> 15** indicates **homology** | U | K.YGIKEYACTAVDMISRR.T |
| 52115 | 543 | – | 554 | 708.3183 | 1414.6221 | 1414.6221 | 0.047 | 0 | 26 | 0.0039 | 1Score **> 27** indicates **identity** Score **> 14** indicates **homology** | U | K.EYACTAVDMISR.R |
| 71265 | 543 | – | 555 | 524.5822 | 1570.7248 | 1570.7232 | 1.02 | 1 | 39 | 0.0002 | 1Score **> 31** indicates **identity** Score **> 15** indicates **homology** | U | K.EYACTAVDMISRR.T |
| 71274 | 543 | – | 555 | 524.5834 | 1570.7284 | 1570.7232 | 3.30 | 1 | 21 | 0.0097 | 1Score **> 31** indicates **identity** Score **> 14** indicates **homology** | U | K.EYACTAVDMISRR.T |
| 110558 | 556 | – | 572 | 950.0245 | 1898.0345 | 1898.0374 | -1.51 | 1 | 68 | 4.4e-07 | 1Score **> 35** indicates **identity** Score **> 17** indicates **homology** | U | R.TRLAFLNVQAAEEALPR.I |
| 110562 | 556 | – | 572 | 633.6869 | 1898.0388 | 1898.0374 | 0.78 | 1 | 71 | 2.4e-07 | 1Score **> 35** indicates **identity** Score **> 18** indicates **homology** | U | R.TRLAFLNVQAAEEALPR.I |
| 110566 | 556 | – | 572 | 633.6875 | 1898.0405 | 1898.0374 | 1.67 | 1 | 82 | 2.5e-08 | 1Score **> 35** indicates **identity** Score **> 18** indicates **homology** | U | R.TRLAFLNVQAAEEALPR.I |
| 110567 | 556 | – | 572 | 633.6875 | 1898.0406 | 1898.0374 | 1.72 | 1 | 44 | 8.2e-05 | 1Score **> 35** indicates **identity** Score **> 15** indicates **homology** | U | R.TRLAFLNVQAAEEALPR.I |
| 110568 | 556 | – | 572 | 633.6876 | 1898.0410 | 1898.0374 | 1.90 | 1 | 84 | 1.2e-08 | 1Score **> 35** indicates **identity** Score **> 18** indicates **homology** | U | R.TRLAFLNVQAAEEALPR.I |
| 110569 | 556 | – | 572 | 950.0278 | 1898.0410 | 1898.0374 | 1.94 | 1 | 74 | 1.3e-07 | 1Score **> 35** indicates **identity** Score **> 17** indicates **homology** | U | R.TRLAFLNVQAAEEALPR.I |
| 110571 | 556 | – | 572 | 950.0281 | 1898.0416 | 1898.0374 | 2.22 | 1 | 93 | 2e-09 | 1Score **> 35** indicates **identity** Score **> 18** indicates **homology** | U | R.TRLAFLNVQAAEEALPR.I |
| 110572 | 556 | – | 572 | 950.0281 | 1898.0417 | 1898.0374 | 2.29 | 1 | 22 | 0.0089 | 1Score **> 35** indicates **identity** Score **> 14** indicates **homology** | U | R.TRLAFLNVQAAEEALPR.I |
| 110574 | 556 | – | 572 | 633.6879 | 1898.0418 | 1898.0374 | 2.33 | 1 | 62 | 1.5e-06 | 1Score **> 35** indicates **identity** Score **> 16** indicates **homology** | U | R.TRLAFLNVQAAEEALPR.I |
| 110575 | 556 | – | 572 | 633.6879 | 1898.0419 | 1898.0374 | 2.39 | 1 | 65 | 7.9e-07 | 1Score **> 35** indicates **identity** Score **> 17** indicates **homology** | U | R.TRLAFLNVQAAEEALPR.I |
| 110576 | 556 | – | 572 | 633.6879 | 1898.0419 | 1898.0374 | 2.40 | 1 | 59 | 2.9e-06 | 1Score **> 35** indicates **identity** Score **> 17** indicates **homology** | U | R.TRLAFLNVQAAEEALPR.I |
| 110577 | 556 | – | 572 | 633.6880 | 1898.0421 | 1898.0374 | 2.50 | 1 | 88 | 5.3e-09 | 1Score **> 35** indicates **identity** Score **> 18** indicates **homology** | U | R.TRLAFLNVQAAEEALPR.I |
| 110578 | 556 | – | 572 | 633.6880 | 1898.0421 | 1898.0374 | 2.50 | 1 | 57 | 4.9e-06 | 1Score **> 35** indicates **identity** Score **> 16** indicates **homology** | U | R.TRLAFLNVQAAEEALPR.I |
| 110581 | 556 | – | 572 | 633.6883 | 1898.0429 | 1898.0374 | 2.94 | 1 | 56 | 6.2e-06 | 1Score **> 35** indicates **identity** Score **> 16** indicates **homology** | U | R.TRLAFLNVQAAEEALPR.I |
| 110582 | 556 | – | 572 | 950.0288 | 1898.0431 | 1898.0374 | 3.00 | 1 | 73 | 1.5e-07 | 1Score **> 35** indicates **identity** Score **> 17** indicates **homology** | U | R.TRLAFLNVQAAEEALPR.I |
| 110584 | 556 | – | 572 | 950.0290 | 1898.0434 | 1898.0374 | 3.19 | 1 | 32 | 0.00099 | 1Score **> 35** indicates **identity** Score **> 15** indicates **homology** | U | R.TRLAFLNVQAAEEALPR.I |
| 110585 | 556 | – | 572 | 633.6889 | 1898.0448 | 1898.0374 | 3.95 | 1 | 61 | 1.9e-06 | 1Score **> 35** indicates **identity** Score **> 16** indicates **homology** | U | R.TRLAFLNVQAAEEALPR.I |
| 110591 | 556 | – | 572 | 633.6898 | 1898.0474 | 1898.0374 | 5.32 | 1 | 61 | 2.1e-06 | 1Score **> 35** indicates **identity** Score **> 16** indicates **homology** | U | R.TRLAFLNVQAAEEALPR.I |
| 81081 | 558 | – | 572 | 821.4498 | 1640.8850 | 1640.8886 | -2.16 | 0 | 71 | 2.4e-07 | 1Score **> 35** indicates **identity** Score **> 17** indicates **homology** | U | R.LAFLNVQAAEEALPR.I |
| 81082 | 558 | – | 572 | 821.4501 | 1640.8856 | 1640.8886 | -1.82 | 0 | 82 | 1.9e-08 | 1Score **> 35** indicates **identity** Score **> 18** indicates **homology** | U | R.LAFLNVQAAEEALPR.I |
| 81086 | 558 | – | 572 | 547.9703 | 1640.8889 | 1640.8886 | 0.22 | 0 | 83 | 1.8e-08 | 1Score **> 35** indicates **identity** Score **> 18** indicates **homology** | U | R.LAFLNVQAAEEALPR.I |
| 81087 | 558 | – | 572 | 547.9703 | 1640.8892 | 1640.8886 | 0.38 | 0 | 56 | 5.6e-06 | 1Score **> 35** indicates **identity** Score **> 16** indicates **homology** | U | R.LAFLNVQAAEEALPR.I |
| 81088 | 558 | – | 572 | 547.9704 | 1640.8895 | 1640.8886 | 0.57 | 0 | 52 | 1.4e-05 | 1Score **> 35** indicates **identity** Score **> 16** indicates **homology** | U | R.LAFLNVQAAEEALPR.I |
| 81089 | 558 | – | 572 | 821.4521 | 1640.8896 | 1640.8886 | 0.61 | 0 | 82 | 2e-08 | 1Score **> 35** indicates **identity** Score **> 18** indicates **homology** | U | R.LAFLNVQAAEEALPR.I |
| 81093 | 558 | – | 572 | 547.9709 | 1640.8908 | 1640.8886 | 1.37 | 0 | 77 | 5.6e-08 | 1Score **> 35** indicates **identity** Score **> 17** indicates **homology** | U | R.LAFLNVQAAEEALPR.I |
| 81095 | 558 | – | 572 | 821.4530 | 1640.8914 | 1640.8886 | 1.74 | 0 | 59 | 3e-06 | 1Score **> 35** indicates **identity** Score **> 16** indicates **homology** | U | R.LAFLNVQAAEEALPR.I |
| 81096 | 558 | – | 572 | 547.9711 | 1640.8915 | 1640.8886 | 1.79 | 0 | 88 | 5.7e-09 | 1Score **> 35** indicates **identity** Score **> 18** indicates **homology** | U | R.LAFLNVQAAEEALPR.I |
| 81097 | 558 | – | 572 | 821.4531 | 1640.8916 | 1640.8886 | 1.87 | 0 | 88 | 5.9e-09 | 1Score **> 35** indicates **identity** Score **> 18** indicates **homology** | U | R.LAFLNVQAAEEALPR.I |
| 81098 | 558 | – | 572 | 821.4531 | 1640.8917 | 1640.8886 | 1.93 | 0 | 117 | 1.2e-11 | 1Score **> 35** indicates **identity** Score **> 20** indicates **homology** | U | R.LAFLNVQAAEEALPR.I |
| 81099 | 558 | – | 572 | 821.4545 | 1640.8945 | 1640.8886 | 3.59 | 0 | 66 | 6.3e-07 | 1Score **> 35** indicates **identity** Score **> 17** indicates **homology** | U | R.LAFLNVQAAEEALPR.I |
| 81104 | 558 | – | 572 | 821.4548 | 1640.8951 | 1640.8886 | 3.96 | 0 | 61 | 2e-06 | 1Score **> 35** indicates **identity** Score **> 16** indicates **homology** | U | R.LAFLNVQAAEEALPR.I |
| 81114 | 558 | – | 572 | 821.4572 | 1640.8999 | 1640.8886 | 6.89 | 0 | 18 | 0.022 | 1Score **> 35** indicates **identity** Score **> 14** indicates **homology** | U | R.LAFLNVQAAEEALPR.I |
| 15823 | 580 | – | 587 | 523.7669 | 1045.5193 | 1045.5192 | 0.11 | 0 | 26 | 0.009 | 1Score **> 33** indicates **identity** Score **> 18** indicates **homology** | U | R.ELNWSELR.K |
| 15824 | 580 | – | 587 | 523.7672 | 1045.5198 | 1045.5192 | 0.58 | 0 | 41 | 0.00093 | 1Score **> 33** indicates **identity** Score **> 24** indicates **homology** | U | R.ELNWSELR.K |
| 15825 | 580 | – | 587 | 523.7673 | 1045.5200 | 1045.5192 | 0.75 | 0 | 41 | 0.00094 | 1Score **> 33** indicates **identity** Score **> 23** indicates **homology** | U | R.ELNWSELR.K |
| 15826 | 580 | – | 587 | 523.7673 | 1045.5201 | 1045.5192 | 0.81 | 0 | 43 | 0.00067 | 1Score **> 33** indicates **identity** Score **> 24** indicates **homology** | U | R.ELNWSELR.K |
| 15827 | 580 | – | 587 | 523.7675 | 1045.5204 | 1045.5192 | 1.08 | 0 | 26 | 0.0075 | 1Score **> 33** indicates **identity** Score **> 17** indicates **homology** | U | R.ELNWSELR.K |
| 15828 | 580 | – | 587 | 523.7678 | 1045.5210 | 1045.5192 | 1.66 | 0 | 30 | 0.0056 | 1Score **> 33** indicates **identity** Score **> 20** indicates **homology** | U | R.ELNWSELR.K |
| 15829 | 580 | – | 587 | 523.7679 | 1045.5212 | 1045.5192 | 1.90 | 0 | 36 | 0.0019 | 1Score **> 33** indicates **identity** Score **> 21** indicates **homology** | U | R.ELNWSELR.K |
| 15830 | 580 | – | 587 | 523.7680 | 1045.5214 | 1045.5192 | 2.13 | 0 | 41 | 0.0049 | 1Score **> 33** indicates **identity** Score **> 30** indicates **homology** | U | R.ELNWSELR.K |
| 27680 | 580 | – | 588 | 392.2124 | 1173.6153 | 1173.6142 | 0.98 | 1 | 18 | 0.022 | 1Score **> 33** indicates **identity** Score **> 14** indicates **homology** | U | R.ELNWSELRK.Q |
| 27682 | 580 | – | 588 | 392.2126 | 1173.6160 | 1173.6142 | 1.53 | 1 | 16 | 0.029 | 1Score **> 33** indicates **identity** Score **> 14** indicates **homology** | U | R.ELNWSELRK.Q |
| 141659 | 580 | – | 597 | 558.7868 | 2231.1181 | 2231.1182 | -0.023 | 2 | 29 | 0.0018 | 1Score **> 37** indicates **identity** Score **> 14** indicates **homology** | U | R.ELNWSELRKQEELETATR.F |
| 141661 | 580 | – | 597 | 1116.5674 | 2231.1202 | 2231.1182 | 0.92 | 2 | 20 | 0.023 | 1Score **> 37** indicates **identity** Score **> 16** indicates **homology** | U | R.ELNWSELRKQEELETATR.F |
| 141662 | 580 | – | 597 | 558.7874 | 2231.1204 | 2231.1182 | 1.02 | 2 | 36 | 0.00038 | 1Score **> 37** indicates **identity** Score **> 15** indicates **homology** | U | R.ELNWSELRKQEELETATR.F |
| 141663 | 580 | – | 597 | 744.7142 | 2231.1208 | 2231.1182 | 1.17 | 2 | 49 | 2.4e-05 | 1Score **> 37** indicates **identity** Score **> 16** indicates **homology** | U | R.ELNWSELRKQEELETATR.F |
| 141664 | 580 | – | 597 | 744.7142 | 2231.1208 | 2231.1182 | 1.18 | 2 | 41 | 0.00015 | 1Score **> 37** indicates **identity** Score **> 15** indicates **homology** | U | R.ELNWSELRKQEELETATR.F |
| 141665 | 580 | – | 597 | 558.7875 | 2231.1209 | 2231.1182 | 1.21 | 2 | 40 | 0.00024 | 1Score **> 37** indicates **identity** Score **> 17** indicates **homology** | U | R.ELNWSELRKQEELETATR.F |
| 141666 | 580 | – | 597 | 558.7875 | 2231.1209 | 2231.1182 | 1.24 | 2 | 34 | 0.00066 | 1Score **> 37** indicates **identity** Score **> 15** indicates **homology** | U | R.ELNWSELRKQEELETATR.F |
| 141667 | 580 | – | 597 | 744.7143 | 2231.1211 | 2231.1182 | 1.30 | 2 | 36 | 0.0004 | 1Score **> 37** indicates **identity** Score **> 15** indicates **homology** | U | R.ELNWSELRKQEELETATR.F |
| 141668 | 580 | – | 597 | 558.7877 | 2231.1215 | 2231.1182 | 1.50 | 2 | 21 | 0.011 | 1Score **> 37** indicates **identity** Score **> 14** indicates **homology** | U | R.ELNWSELRKQEELETATR.F |
| 141669 | 580 | – | 597 | 558.7877 | 2231.1215 | 2231.1182 | 1.51 | 2 | 20 | 0.013 | 1Score **> 37** indicates **identity** Score **> 14** indicates **homology** | U | R.ELNWSELRKQEELETATR.F |
| 141672 | 580 | – | 597 | 744.7147 | 2231.1223 | 2231.1182 | 1.85 | 2 | 34 | 0.0006 | 1Score **> 37** indicates **identity** Score **> 15** indicates **homology** | U | R.ELNWSELRKQEELETATR.F |
| 141673 | 580 | – | 597 | 1116.5685 | 2231.1224 | 2231.1182 | 1.88 | 2 | 24 | 0.011 | 1Score **> 37** indicates **identity** Score **> 17** indicates **homology** | U | R.ELNWSELRKQEELETATR.F |
| 141675 | 580 | – | 597 | 744.7151 | 2231.1234 | 2231.1182 | 2.33 | 2 | 18 | 0.023 | 1Score **> 37** indicates **identity** Score **> 14** indicates **homology** | U | R.ELNWSELRKQEELETATR.F |
| 30734 | 588 | – | 597 | 602.8094 | 1203.6043 | 1203.6095 | -4.31 | 1 | 52 | 0.00038 | 1Score **> 33** indicates **identity** Score **> 30** indicates **homology** | U | R.KQEELETATR.F |
| 30738 | 588 | – | 597 | 602.8119 | 1203.6092 | 1203.6095 | -0.21 | 1 | 68 | 2e-05 | 1Score **> 34** indicates **identity** | U | R.KQEELETATR.F |
| 30739 | 588 | – | 597 | 402.2105 | 1203.6097 | 1203.6095 | 0.15 | 1 | 43 | 0.0046 | 1Score **> 34** indicates **identity** Score **> 32** indicates **homology** | U | R.KQEELETATR.F |
| 30744 | 588 | – | 597 | 402.2114 | 1203.6123 | 1203.6095 | 2.35 | 1 | 19 | 0.016 | 1Score **> 34** indicates **identity** Score **> 14** indicates **homology** | U | R.KQEELETATR.F |
| 153882 | 588 | – | 606 | 604.7852 | 2415.1117 | 2415.1304 | -7.74 | 2 | 17 | 0.027 | 1Score **> 34** indicates **identity** Score **> 14** indicates **homology** | U | R.KQEELETATRFLYYEMGYK.S  + Deamidated (NQ); Oxidation (M) |
| 153884 | 588 | – | 606 | 604.7882 | 2415.1239 | 2415.1304 | -2.69 | 2 | 15 | 0.04 | 1Score **> 34** indicates **identity** Score **> 13** indicates **homology** | U | R.KQEELETATRFLYYEMGYK.S  + Deamidated (NQ); Oxidation (M) |
| 18526 | 589 | – | 597 | 538.7675 | 1075.5204 | 1075.5145 | 5.48 | 0 | 47 | 0.00033 | 1Score **> 31** indicates **identity** Score **> 25** indicates **homology** | U | K.QEELETATR.F |
| 31513 | 598 | – | 606 | 607.2834 | 1212.5523 | 1212.5525 | -0.17 | 0 | 38 | 0.0003 | 1Score **> 28** indicates **identity** Score **> 15** indicates **homology** | U | R.FLYYEMGYK.S |
| 31514 | 598 | – | 606 | 607.2837 | 1212.5529 | 1212.5525 | 0.33 | 0 | 38 | 0.00025 | 1Score **> 28** indicates **identity** Score **> 15** indicates **homology** | U | R.FLYYEMGYK.S |
| 31515 | 598 | – | 606 | 607.2846 | 1212.5546 | 1212.5525 | 1.75 | 0 | 37 | 0.00037 | 1Score **> 28** indicates **identity** Score **> 15** indicates **homology** | U | R.FLYYEMGYK.S |
| 151492 | 607 | – | 627 | 792.7350 | 2375.1831 | 2375.1816 | 0.64 | 1 | 59 | 6.9e-06 | 1Score **> 37** indicates **identity** Score **> 20** indicates **homology** | U | K.SRTEQLTDSTEISLLPSDIDR.Y |
| 151496 | 607 | – | 627 | 1188.5995 | 2375.1844 | 2375.1816 | 1.18 | 1 | 77 | 8.6e-08 | 1Score **> 37** indicates **identity** Score **> 19** indicates **homology** | U | K.SRTEQLTDSTEISLLPSDIDR.Y |
| 151497 | 607 | – | 627 | 1188.6001 | 2375.1856 | 2375.1816 | 1.67 | 1 | 67 | 1.3e-06 | 1Score **> 37** indicates **identity** Score **> 20** indicates **homology** | U | K.SRTEQLTDSTEISLLPSDIDR.Y |
| 151498 | 607 | – | 627 | 792.7359 | 2375.1859 | 2375.1816 | 1.82 | 1 | 68 | 5.5e-07 | 1Score **> 37** indicates **identity** Score **> 18** indicates **homology** | U | K.SRTEQLTDSTEISLLPSDIDR.Y |
| 151499 | 607 | – | 627 | 1188.6005 | 2375.1864 | 2375.1816 | 2.00 | 1 | 64 | 6.2e-06 | 1Score **> 37** indicates **identity** Score **> 24** indicates **homology** | U | K.SRTEQLTDSTEISLLPSDIDR.Y |
| 151501 | 607 | – | 627 | 792.7367 | 2375.1881 | 2375.1816 | 2.76 | 1 | 74 | 1.2e-07 | 1Score **> 37** indicates **identity** Score **> 17** indicates **homology** | U | K.SRTEQLTDSTEISLLPSDIDR.Y |
| 166518 | 607 | – | 629 | 889.7796 | 2666.3169 | 2666.3399 | -8.61 | 2 | 52 | 2.2e-05 | 1Score **> 37** indicates **identity** Score **> 17** indicates **homology** | U | K.SRTEQLTDSTEISLLPSDIDRYK.K |
| 166527 | 607 | – | 629 | 667.5918 | 2666.3383 | 2666.3399 | -0.60 | 2 | 17 | 0.027 | 1Score **> 37** indicates **identity** Score **> 14** indicates **homology** | U | K.SRTEQLTDSTEISLLPSDIDRYK.K |
| 166528 | 607 | – | 629 | 889.7879 | 2666.3418 | 2666.3399 | 0.73 | 2 | 55 | 1.4e-05 | 1Score **> 37** indicates **identity** Score **> 19** indicates **homology** | U | K.SRTEQLTDSTEISLLPSDIDRYK.K |
| 166529 | 607 | – | 629 | 667.5927 | 2666.3418 | 2666.3399 | 0.73 | 2 | 46 | 0.00024 | 1Score **> 37** indicates **identity** Score **> 23** indicates **homology** | U | K.SRTEQLTDSTEISLLPSDIDRYK.K |
| 166530 | 607 | – | 629 | 667.5928 | 2666.3422 | 2666.3399 | 0.86 | 2 | 51 | 1.6e-05 | 1Score **> 37** indicates **identity** Score **> 16** indicates **homology** | U | K.SRTEQLTDSTEISLLPSDIDRYK.K |
| 166531 | 607 | – | 629 | 667.5930 | 2666.3429 | 2666.3399 | 1.15 | 2 | 40 | 0.00031 | 1Score **> 37** indicates **identity** Score **> 17** indicates **homology** | U | K.SRTEQLTDSTEISLLPSDIDRYK.K |
| 166532 | 607 | – | 629 | 889.7883 | 2666.3430 | 2666.3399 | 1.17 | 2 | 63 | 2.2e-06 | 1Score **> 37** indicates **identity** Score **> 19** indicates **homology** | U | K.SRTEQLTDSTEISLLPSDIDRYK.K |
| 166533 | 607 | – | 629 | 889.7890 | 2666.3451 | 2666.3399 | 1.95 | 2 | 50 | 2.8e-05 | 1Score **> 37** indicates **identity** Score **> 17** indicates **homology** | U | K.SRTEQLTDSTEISLLPSDIDRYK.K |
| 133050 | 609 | – | 627 | 1067.0292 | 2132.0438 | 2132.0485 | -2.19 | 0 | 108 | 1.8e-09 | 1Score **> 36** indicates **identity** Score **> 33** indicates **homology** | U | R.TEQLTDSTEISLLPSDIDR.Y |
| 133052 | 609 | – | 627 | 1067.0316 | 2132.0487 | 2132.0485 | 0.099 | 0 | 108 | 1.8e-09 | 1Score **> 36** indicates **identity** Score **> 33** indicates **homology** | U | R.TEQLTDSTEISLLPSDIDR.Y |
| 133053 | 609 | – | 627 | 1067.0320 | 2132.0494 | 2132.0485 | 0.45 | 0 | 102 | 5.4e-09 | 1Score **> 36** indicates **identity** Score **> 32** indicates **homology** | U | R.TEQLTDSTEISLLPSDIDR.Y |
| 133055 | 609 | – | 627 | 1067.0325 | 2132.0505 | 2132.0485 | 0.94 | 0 | 121 | 8.2e-11 | 1Score **> 36** indicates **identity** Score **> 33** indicates **homology** | U | R.TEQLTDSTEISLLPSDIDR.Y |
| 133056 | 609 | – | 627 | 1067.0328 | 2132.0511 | 2132.0485 | 1.22 | 0 | 116 | 3.5e-11 | 1Score **> 36** indicates **identity** Score **> 24** indicates **homology** | U | R.TEQLTDSTEISLLPSDIDR.Y |
| 133058 | 609 | – | 627 | 1067.0328 | 2132.0511 | 2132.0485 | 1.25 | 0 | 108 | 1.8e-09 | 1Score **> 36** indicates **identity** Score **> 33** indicates **homology** | U | R.TEQLTDSTEISLLPSDIDR.Y |
| 133059 | 609 | – | 627 | 711.6914 | 2132.0523 | 2132.0485 | 1.81 | 0 | 35 | 0.00053 | 1Score **> 36** indicates **identity** Score **> 15** indicates **homology** | U | R.TEQLTDSTEISLLPSDIDR.Y |
| 133060 | 609 | – | 627 | 1067.0340 | 2132.0534 | 2132.0485 | 2.30 | 0 | 102 | 6.3e-09 | 1Score **> 36** indicates **identity** Score **> 33** indicates **homology** | U | R.TEQLTDSTEISLLPSDIDR.Y |
| 133062 | 609 | – | 627 | 1067.0350 | 2132.0555 | 2132.0485 | 3.28 | 0 | 99 | 2.5e-09 | 1Score **> 36** indicates **identity** Score **> 26** indicates **homology** | U | R.TEQLTDSTEISLLPSDIDR.Y |
| 154404 | 609 | – | 629 | 808.7426 | 2423.2060 | 2423.2067 | -0.30 | 1 | 40 | 0.00018 | 1Score **> 37** indicates **identity** Score **> 15** indicates **homology** | U | R.TEQLTDSTEISLLPSDIDRYK.K |
| 154406 | 609 | – | 629 | 1212.6112 | 2423.2078 | 2423.2067 | 0.45 | 1 | 96 | 1.1e-09 | 1Score **> 37** indicates **identity** Score **> 18** indicates **homology** | U | R.TEQLTDSTEISLLPSDIDRYK.K |
| 154408 | 609 | – | 629 | 1212.6114 | 2423.2083 | 2423.2067 | 0.63 | 1 | 103 | 2.2e-10 | 1Score **> 37** indicates **identity** Score **> 19** indicates **homology** | U | R.TEQLTDSTEISLLPSDIDRYK.K |
| 154409 | 609 | – | 629 | 808.7436 | 2423.2089 | 2423.2067 | 0.89 | 1 | 33 | 0.00076 | 1Score **> 37** indicates **identity** Score **> 15** indicates **homology** | U | R.TEQLTDSTEISLLPSDIDRYK.K |
| 154410 | 609 | – | 629 | 808.7436 | 2423.2091 | 2423.2067 | 0.96 | 1 | 28 | 0.0025 | 1Score **> 37** indicates **identity** Score **> 14** indicates **homology** | U | R.TEQLTDSTEISLLPSDIDRYK.K |
| 154412 | 609 | – | 629 | 808.7437 | 2423.2094 | 2423.2067 | 1.09 | 1 | 18 | 0.02 | 1Score **> 37** indicates **identity** Score **> 14** indicates **homology** | U | R.TEQLTDSTEISLLPSDIDRYK.K |
| 154414 | 609 | – | 629 | 1212.6126 | 2423.2106 | 2423.2067 | 1.59 | 1 | 83 | 1.7e-08 | 1Score **> 37** indicates **identity** Score **> 18** indicates **homology** | U | R.TEQLTDSTEISLLPSDIDRYK.K |
| 154458 | 609 | – | 629 | 1213.1093 | 2424.2041 | 2424.1908 | 5.50 | 1 | 71 | 2.1e-07 | 1Score **> 37** indicates **identity** Score **> 17** indicates **homology** | U | R.TEQLTDSTEISLLPSDIDRYK.K  + Deamidated (NQ) |
| 161442 | 609 | – | 630 | 638.8327 | 2551.3017 | 2551.3017 | -0.0035 | 2 | 19 | 0.024 | 1Score **> 37** indicates **identity** Score **> 15** indicates **homology** | U | R.TEQLTDSTEISLLPSDIDRYKK.R |
| 148182 | 632 | – | 650 | 581.5471 | 2322.1593 | 2322.1645 | -2.24 | 2 | 39 | 0.00024 | 1Score **> 37** indicates **identity** Score **> 15** indicates **homology** | U | R.FHKFDEDEKGFITIVDVQR.V |
| 148183 | 632 | – | 650 | 581.5477 | 2322.1617 | 2322.1645 | -1.17 | 2 | 15 | 0.037 | 1Score **> 37** indicates **identity** Score **> 13** indicates **homology** | U | R.FHKFDEDEKGFITIVDVQR.V |
| 148184 | 632 | – | 650 | 581.5477 | 2322.1618 | 2322.1645 | -1.13 | 2 | 36 | 0.00043 | 1Score **> 37** indicates **identity** Score **> 15** indicates **homology** | U | R.FHKFDEDEKGFITIVDVQR.V |
| 148186 | 632 | – | 650 | 581.5478 | 2322.1620 | 2322.1645 | -1.04 | 2 | 39 | 0.00021 | 1Score **> 37** indicates **identity** Score **> 15** indicates **homology** | U | R.FHKFDEDEKGFITIVDVQR.V |
| 148189 | 632 | – | 650 | 775.0617 | 2322.1633 | 2322.1645 | -0.48 | 2 | 34 | 0.0007 | 1Score **> 37** indicates **identity** Score **> 15** indicates **homology** | U | R.FHKFDEDEKGFITIVDVQR.V |
| 148190 | 632 | – | 650 | 581.5483 | 2322.1639 | 2322.1645 | -0.24 | 2 | 33 | 0.0008 | 1Score **> 37** indicates **identity** Score **> 15** indicates **homology** | U | R.FHKFDEDEKGFITIVDVQR.V |
| 148191 | 632 | – | 650 | 581.5483 | 2322.1643 | 2322.1645 | -0.072 | 2 | 40 | 0.00016 | 1Score **> 37** indicates **identity** Score **> 15** indicates **homology** | U | R.FHKFDEDEKGFITIVDVQR.V |
| 148193 | 632 | – | 650 | 581.5487 | 2322.1659 | 2322.1645 | 0.62 | 2 | 27 | 0.0031 | 1Score **> 37** indicates **identity** Score **> 14** indicates **homology** | U | R.FHKFDEDEKGFITIVDVQR.V |
| 148194 | 632 | – | 650 | 775.0627 | 2322.1662 | 2322.1645 | 0.77 | 2 | 39 | 0.00022 | 1Score **> 37** indicates **identity** Score **> 15** indicates **homology** | U | R.FHKFDEDEKGFITIVDVQR.V |
| 148195 | 632 | – | 650 | 581.5489 | 2322.1663 | 2322.1645 | 0.81 | 2 | 49 | 2.4e-05 | 1Score **> 37** indicates **identity** Score **> 16** indicates **homology** | U | R.FHKFDEDEKGFITIVDVQR.V |
| 148196 | 632 | – | 650 | 581.5489 | 2322.1666 | 2322.1645 | 0.93 | 2 | 44 | 8.1e-05 | 1Score **> 37** indicates **identity** Score **> 16** indicates **homology** | U | R.FHKFDEDEKGFITIVDVQR.V |
| 148198 | 632 | – | 650 | 581.5491 | 2322.1673 | 2322.1645 | 1.23 | 2 | 43 | 9.9e-05 | 1Score **> 37** indicates **identity** Score **> 15** indicates **homology** | U | R.FHKFDEDEKGFITIVDVQR.V |
| 148199 | 632 | – | 650 | 775.0631 | 2322.1674 | 2322.1645 | 1.27 | 2 | 54 | 9.2e-06 | 1Score **> 37** indicates **identity** Score **> 16** indicates **homology** | U | R.FHKFDEDEKGFITIVDVQR.V |
| 148200 | 632 | – | 650 | 581.5492 | 2322.1677 | 2322.1645 | 1.40 | 2 | 19 | 0.017 | 1Score **> 37** indicates **identity** Score **> 14** indicates **homology** | U | R.FHKFDEDEKGFITIVDVQR.V |
| 148204 | 632 | – | 650 | 775.0632 | 2322.1679 | 2322.1645 | 1.46 | 2 | 33 | 0.00078 | 1Score **> 37** indicates **identity** Score **> 15** indicates **homology** | U | R.FHKFDEDEKGFITIVDVQR.V |
| 148205 | 632 | – | 650 | 581.5493 | 2322.1680 | 2322.1645 | 1.51 | 2 | 43 | 9.5e-05 | 1Score **> 37** indicates **identity** Score **> 15** indicates **homology** | U | R.FHKFDEDEKGFITIVDVQR.V |
| 148208 | 632 | – | 650 | 581.5494 | 2322.1686 | 2322.1645 | 1.80 | 2 | 33 | 0.00083 | 1Score **> 37** indicates **identity** Score **> 15** indicates **homology** | U | R.FHKFDEDEKGFITIVDVQR.V |
| 148209 | 632 | – | 650 | 775.0636 | 2322.1688 | 2322.1645 | 1.89 | 2 | 39 | 0.00021 | 1Score **> 37** indicates **identity** Score **> 15** indicates **homology** | U | R.FHKFDEDEKGFITIVDVQR.V |
| 148210 | 632 | – | 650 | 775.0636 | 2322.1689 | 2322.1645 | 1.92 | 2 | 27 | 0.0032 | 1Score **> 37** indicates **identity** Score **> 14** indicates **homology** | U | R.FHKFDEDEKGFITIVDVQR.V |
| 148211 | 632 | – | 650 | 581.5495 | 2322.1691 | 2322.1645 | 1.98 | 2 | 29 | 0.0017 | 1Score **> 37** indicates **identity** Score **> 14** indicates **homology** | U | R.FHKFDEDEKGFITIVDVQR.V |
| 148212 | 632 | – | 650 | 581.5496 | 2322.1692 | 2322.1645 | 2.05 | 2 | 33 | 0.00073 | 1Score **> 37** indicates **identity** Score **> 15** indicates **homology** | U | R.FHKFDEDEKGFITIVDVQR.V |
| 148213 | 632 | – | 650 | 581.5496 | 2322.1694 | 2322.1645 | 2.11 | 2 | 45 | 7.3e-05 | 1Score **> 37** indicates **identity** Score **> 16** indicates **homology** | U | R.FHKFDEDEKGFITIVDVQR.V |
| 148214 | 632 | – | 650 | 775.0638 | 2322.1695 | 2322.1645 | 2.18 | 2 | 30 | 0.0017 | 1Score **> 37** indicates **identity** Score **> 14** indicates **homology** | U | R.FHKFDEDEKGFITIVDVQR.V |
| 148216 | 632 | – | 650 | 581.5498 | 2322.1699 | 2322.1645 | 2.35 | 2 | 18 | 0.022 | 1Score **> 37** indicates **identity** Score **> 14** indicates **homology** | U | R.FHKFDEDEKGFITIVDVQR.V |
| 148218 | 632 | – | 650 | 775.0642 | 2322.1708 | 2322.1645 | 2.71 | 2 | 29 | 0.0018 | 1Score **> 37** indicates **identity** Score **> 14** indicates **homology** | U | R.FHKFDEDEKGFITIVDVQR.V |
| 148219 | 632 | – | 650 | 581.5505 | 2322.1728 | 2322.1645 | 3.60 | 2 | 32 | 0.00095 | 1Score **> 37** indicates **identity** Score **> 15** indicates **homology** | U | R.FHKFDEDEKGFITIVDVQR.V |
| 148222 | 632 | – | 650 | 581.5521 | 2322.1795 | 2322.1645 | 6.47 | 2 | 24 | 0.0057 | 1Score **> 37** indicates **identity** Score **> 14** indicates **homology** | U | R.FHKFDEDEKGFITIVDVQR.V |
| 157651 | 632 | – | 650 | 496.6606 | 2478.2665 | 2478.2795 | -5.23 | 2 | 23 | 0.0069 | 1Score **> 37** indicates **identity** Score **> 14** indicates **homology** | U | R.FHKFDEDEKGFITIVDVQR.V  + HNE (H) |
| 157652 | 632 | – | 650 | 620.5741 | 2478.2672 | 2478.2795 | -4.95 | 2 | 28 | 0.0021 | 1Score **> 37** indicates **identity** Score **> 14** indicates **homology** | U | R.FHKFDEDEKGFITIVDVQR.V  + HNE (H) |
| 157653 | 632 | – | 650 | 496.6607 | 2478.2673 | 2478.2795 | -4.91 | 2 | 30 | 0.0015 | 1Score **> 37** indicates **identity** Score **> 14** indicates **homology** | U | R.FHKFDEDEKGFITIVDVQR.V  + HNE (H) |
| 157654 | 632 | – | 650 | 620.5743 | 2478.2679 | 2478.2795 | -4.67 | 2 | 47 | 4.1e-05 | 1Score **> 37** indicates **identity** Score **> 15** indicates **homology** | U | R.FHKFDEDEKGFITIVDVQR.V  + HNE (K) |
| 157655 | 632 | – | 650 | 496.6609 | 2478.2680 | 2478.2795 | -4.64 | 2 | 33 | 0.00084 | 1Score **> 37** indicates **identity** Score **> 15** indicates **homology** | U | R.FHKFDEDEKGFITIVDVQR.V  + HNE (H) |
| 157656 | 632 | – | 650 | 620.5743 | 2478.2681 | 2478.2795 | -4.61 | 2 | 47 | 4e-05 | 1Score **> 37** indicates **identity** Score **> 15** indicates **homology** | U | R.FHKFDEDEKGFITIVDVQR.V  + HNE (K) |
| 157657 | 632 | – | 650 | 496.6609 | 2478.2682 | 2478.2795 | -4.56 | 2 | 15 | 0.039 | 1Score **> 37** indicates **identity** Score **> 13** indicates **homology** | U | R.FHKFDEDEKGFITIVDVQR.V  + HNE (H) |
| 157658 | 632 | – | 650 | 496.6610 | 2478.2685 | 2478.2795 | -4.43 | 2 | 28 | 0.0021 | 1Score **> 37** indicates **identity** Score **> 14** indicates **homology** | U | R.FHKFDEDEKGFITIVDVQR.V  + HNE (H) |
| 157659 | 632 | – | 650 | 827.0968 | 2478.2686 | 2478.2795 | -4.40 | 2 | 37 | 0.00031 | 1Score **> 37** indicates **identity** Score **> 15** indicates **homology** | U | R.FHKFDEDEKGFITIVDVQR.V  + HNE (H) |
| 157660 | 632 | – | 650 | 620.5745 | 2478.2688 | 2478.2795 | -4.32 | 2 | 22 | 0.008 | 1Score **> 37** indicates **identity** Score **> 14** indicates **homology** | U | R.FHKFDEDEKGFITIVDVQR.V  + HNE (K) |
| 157661 | 632 | – | 650 | 620.5745 | 2478.2689 | 2478.2795 | -4.27 | 2 | 43 | 9.9e-05 | 1Score **> 37** indicates **identity** Score **> 15** indicates **homology** | U | R.FHKFDEDEKGFITIVDVQR.V  + HNE (K) |
| 157662 | 632 | – | 650 | 827.0970 | 2478.2691 | 2478.2795 | -4.18 | 2 | 43 | 9.7e-05 | 1Score **> 37** indicates **identity** Score **> 15** indicates **homology** | U | R.FHKFDEDEKGFITIVDVQR.V  + HNE (H) |
| 157663 | 632 | – | 650 | 496.6615 | 2478.2710 | 2478.2795 | -3.42 | 2 | 24 | 0.0058 | 1Score **> 37** indicates **identity** Score **> 14** indicates **homology** | U | R.FHKFDEDEKGFITIVDVQR.V  + HNE (H) |
| 157664 | 632 | – | 650 | 620.5753 | 2478.2720 | 2478.2795 | -3.01 | 2 | 22 | 0.0083 | 1Score **> 37** indicates **identity** Score **> 14** indicates **homology** | U | R.FHKFDEDEKGFITIVDVQR.V  + HNE (K) |
| 157666 | 632 | – | 650 | 827.0988 | 2478.2747 | 2478.2795 | -1.93 | 2 | 32 | 0.001 | 1Score **> 37** indicates **identity** Score **> 14** indicates **homology** | U | R.FHKFDEDEKGFITIVDVQR.V  + HNE (H) |
| 157669 | 632 | – | 650 | 620.5776 | 2478.2814 | 2478.2795 | 0.78 | 2 | 36 | 0.0005 | 1Score **> 37** indicates **identity** Score **> 15** indicates **homology** | U | R.FHKFDEDEKGFITIVDVQR.V  + HNE (H) |
| 111584 | 635 | – | 650 | 637.6555 | 1909.9446 | 1909.9422 | 1.30 | 1 | 18 | 0.023 | 1Score **> 35** indicates **identity** Score **> 14** indicates **homology** | U | K.FDEDEKGFITIVDVQR.V |
| 10020 | 707 | – | 714 | 474.2239 | 946.4332 | 946.4356 | -2.45 | 0 | 34 | 0.00059 | 1Score **> 28** indicates **identity** Score **> 15** indicates **homology** | U | K.TAEENLDR.R |
| 20936 | 707 | – | 715 | 368.5200 | 1102.5383 | 1102.5367 | 1.49 | 1 | 24 | 0.017 | 1Score **> 31** indicates **identity** Score **> 19** indicates **homology** | U | K.TAEENLDRR.V |
| 108858 | 707 | – | 722 | 627.3384 | 1878.9932 | 1878.9912 | 1.10 | 2 | 15 | 0.042 | 1Score **> 35** indicates **identity** Score **> 14** indicates **homology** | U | K.TAEENLDRRVPIPVDR.S |
| 108860 | 707 | – | 722 | 627.3386 | 1878.9941 | 1878.9912 | 1.54 | 2 | 17 | 0.027 | 1Score **> 35** indicates **identity** Score **> 14** indicates **homology** | U | K.TAEENLDRRVPIPVDR.S |
| 10380 | 715 | – | 722 | 476.2911 | 950.5676 | 950.5661 | 1.58 | 1 | 23 | 0.036 | 1Score **> 23** indicates **identity** Score **> 21** indicates **homology** | U | R.RVPIPVDR.S |
| 10381 | 715 | – | 722 | 476.2915 | 950.5685 | 950.5661 | 2.48 | 1 | 42 | 0.00094 | 1Score **> 24** indicates **identity** | U | R.RVPIPVDR.S |
| 10382 | 715 | – | 722 | 476.2917 | 950.5689 | 950.5661 | 2.94 | 1 | 29 | 0.02 | 1Score **> 24** indicates **identity** | U | R.RVPIPVDR.S |
| 3136 | 716 | – | 722 | 398.2402 | 794.4658 | 794.4650 | 1.01 | 0 | 28 | 0.016 | 1Score **> 23** indicates **identity** | U | R.VPIPVDR.S |
| 3140 | 716 | – | 722 | 398.2404 | 794.4663 | 794.4650 | 1.55 | 0 | 32 | 0.006 | 1Score **> 23** indicates **identity** | U | R.VPIPVDR.S |

---

```
ID   GPDM_MOUSE              Reviewed;         727 AA.
AC   Q64521; Q3TK51; Q3UDY8; Q61507; Q8CBX6; Q8K4U5; Q8VDT0; Q9ERP0;
DT   01-NOV-1997, integrated into UniProtKB/Swiss-Prot.
DT   01-MAY-2007, sequence version 2.
DT   28-JUN-2023, entry version 186.
DE   RecName: Full=Glycerol-3-phosphate dehydrogenase, mitochondrial;
DE            Short=GPD-M;
DE            Short=GPDH-M;
DE            EC=1.1.5.3 {ECO:0000250|UniProtKB:P43304};
DE   AltName: Full=Protein TISP38;
DE   Flags: Precursor;
GN   Name=Gpd2 {ECO:0000312|MGI:MGI:99778}; Synonyms=Gdm1;
OS   Mus musculus (Mouse).
OC   Eukaryota; Metazoa; Chordata; Craniata; Vertebrata; Euteleostomi; Mammalia;
OC   Eutheria; Euarchontoglires; Glires; Rodentia; Myomorpha; Muroidea; Muridae;
OC   Murinae; Mus; Mus.
OX   NCBI_TaxID=10090;
RN   [1]
RP   NUCLEOTIDE SEQUENCE [MRNA].
RC   STRAIN=C57BL/6J; TISSUE=Adipocyte;
RX   PubMed=8951039; DOI=10.1006/abbi.1996.0536;
RA   Koza R.A., Kozak U.C., Brown L.J., Leiter E.H., Macdonald M.J., Kozak L.P.;
RT   "Sequence and tissue-dependent RNA expression of mouse FAD-linked glycerol-
RT   3-phosphate dehydrogenase.";
RL   Arch. Biochem. Biophys. 336:97-104(1996).
RN   [2]
RP   NUCLEOTIDE SEQUENCE [MRNA].
RC   STRAIN=C57BL/6J;
RX   PubMed=8772729; DOI=10.2337/diab.45.9.1238;
RA   Ishihara H., Nakazaki M., Kanegae Y., Inukai K., Asano T., Katagiri H.,
RA   Yazaki Y., Kikuchi M., Miyazaki J., Saito I., Oka Y.;
RT   "Effect of mitochondrial and/or cytosolic glycerol 3-phosphate
RT   dehydrogenase overexpression on glucose-stimulated insulin secretion from
RT   MIN6 and HIT cells.";
RL   Diabetes 45:1238-1244(1996).
RN   [3]
RP   NUCLEOTIDE SEQUENCE [LARGE SCALE MRNA].
RC   STRAIN=C57BL/6J; TISSUE=Bone marrow, Diencephalon, and Lung;
RX   PubMed=16141072; DOI=10.1126/science.1112014;
RA   Carninci P., Kasukawa T., Katayama S., Gough J., Frith M.C., Maeda N.,
RA   Oyama R., Ravasi T., Lenhard B., Wells C., Kodzius R., Shimokawa K.,
RA   Bajic V.B., Brenner S.E., Batalov S., Forrest A.R., Zavolan M., Davis M.J.,
RA   Wilming L.G., Aidinis V., Allen J.E., Ambesi-Impiombato A., Apweiler R.,
RA   Aturaliya R.N., Bailey T.L., Bansal M., Baxter L., Beisel K.W., Bersano T.,
RA   Bono H., Chalk A.M., Chiu K.P., Choudhary V., Christoffels A.,
RA   Clutterbuck D.R., Crowe M.L., Dalla E., Dalrymple B.P., de Bono B.,
RA   Della Gatta G., di Bernardo D., Down T., Engstrom P., Fagiolini M.,
RA   Faulkner G., Fletcher C.F., Fukushima T., Furuno M., Futaki S.,
RA   Gariboldi M., Georgii-Hemming P., Gingeras T.R., Gojobori T., Green R.E.,
RA   Gustincich S., Harbers M., Hayashi Y., Hensch T.K., Hirokawa N., Hill D.,
RA   Huminiecki L., Iacono M., Ikeo K., Iwama A., Ishikawa T., Jakt M.,
RA   Kanapin A., Katoh M., Kawasawa Y., Kelso J., Kitamura H., Kitano H.,
RA   Kollias G., Krishnan S.P., Kruger A., Kummerfeld S.K., Kurochkin I.V.,
RA   Lareau L.F., Lazarevic D., Lipovich L., Liu J., Liuni S., McWilliam S.,
RA   Madan Babu M., Madera M., Marchionni L., Matsuda H., Matsuzawa S., Miki H.,
RA   Mignone F., Miyake S., Morris K., Mottagui-Tabar S., Mulder N., Nakano N.,
RA   Nakauchi H., Ng P., Nilsson R., Nishiguchi S., Nishikawa S., Nori F.,
RA   Ohara O., Okazaki Y., Orlando V., Pang K.C., Pavan W.J., Pavesi G.,
RA   Pesole G., Petrovsky N., Piazza S., Reed J., Reid J.F., Ring B.Z.,
RA   Ringwald M., Rost B., Ruan Y., Salzberg S.L., Sandelin A., Schneider C.,
RA   Schoenbach C., Sekiguchi K., Semple C.A., Seno S., Sessa L., Sheng Y.,
RA   Shibata Y., Shimada H., Shimada K., Silva D., Sinclair B., Sperling S.,
RA   Stupka E., Sugiura K., Sultana R., Takenaka Y., Taki K., Tammoja K.,
RA   Tan S.L., Tang S., Taylor M.S., Tegner J., Teichmann S.A., Ueda H.R.,
RA   van Nimwegen E., Verardo R., Wei C.L., Yagi K., Yamanishi H.,
RA   Zabarovsky E., Zhu S., Zimmer A., Hide W., Bult C., Grimmond S.M.,
RA   Teasdale R.D., Liu E.T., Brusic V., Quackenbush J., Wahlestedt C.,
RA   Mattick J.S., Hume D.A., Kai C., Sasaki D., Tomaru Y., Fukuda S.,
RA   Kanamori-Katayama M., Suzuki M., Aoki J., Arakawa T., Iida J., Imamura K.,
RA   Itoh M., Kato T., Kawaji H., Kawagashira N., Kawashima T., Kojima M.,
RA   Kondo S., Konno H., Nakano K., Ninomiya N., Nishio T., Okada M., Plessy C.,
RA   Shibata K., Shiraki T., Suzuki S., Tagami M., Waki K., Watahiki A.,
RA   Okamura-Oho Y., Suzuki H., Kawai J., Hayashizaki Y.;
RT   "The transcriptional landscape of the mammalian genome.";
RL   Science 309:1559-1563(2005).
RN   [4]
RP   NUCLEOTIDE SEQUENCE [LARGE SCALE MRNA].
RC   STRAIN=Czech II; TISSUE=Mammary tumor;
RX   PubMed=15489334; DOI=10.1101/gr.2596504;
RG   The MGC Project Team;
RT   "The status, quality, and expansion of the NIH full-length cDNA project:
RT   the Mammalian Gene Collection (MGC).";
RL   Genome Res. 14:2121-2127(2004).
RN   [5]
RP   PROTEIN SEQUENCE OF 58-91; 95-110; 125-135; 167-178; 200-209; 212-254;
RP   285-298; 340-348; 381-390; 392-409; 442-453; 464-473; 483-499; 519-538;
RP   558-579; 598-606; 609-627 AND 635-693.
RC   STRAIN=C57BL/6J; TISSUE=Brain, and Hippocampus;
RA   Lubec G., Klug S., Kang S.U.;
RL   Submitted (APR-2007) to UniProtKB.
RN   [6]
RP   NUCLEOTIDE SEQUENCE [GENOMIC DNA] OF 390-686.
RC   STRAIN=129/Sv; TISSUE=Liver;
RA   Weitzel J.M.;
RT   "Genomic sequence of mouse mitochondrial glycerol-3-phosphate dehydrogenase
RT   (mGPDH).";
RL   Submitted (AUG-2000) to the EMBL/GenBank/DDBJ databases.
RN   [7]
RP   NUCLEOTIDE SEQUENCE [MRNA] OF 611-727.
RC   TISSUE=Testis;
RA   Tamura K., Nishimune Y., Nojima H.;
RT   "Mus musculus TISP38 mRNA.";
RL   Submitted (JUL-2000) to the EMBL/GenBank/DDBJ databases.
RN   [8]
RP   PHOSPHORYLATION [LARGE SCALE ANALYSIS] AT TYR-601, AND IDENTIFICATION BY
RP   MASS SPECTROMETRY [LARGE SCALE ANALYSIS].
RC   TISSUE=Brain;
RX   PubMed=18034455; DOI=10.1021/pr0701254;
RA   Ballif B.A., Carey G.R., Sunyaev S.R., Gygi S.P.;
RT   "Large-scale identification and evolution indexing of tyrosine
RT   phosphorylation sites from murine brain.";
RL   J. Proteome Res. 7:311-318(2008).
RN   [9]
RP   IDENTIFICATION BY MASS SPECTROMETRY [LARGE SCALE ANALYSIS].
RC   TISSUE=Brain, Brown adipose tissue, Heart, Kidney, Liver, Lung,
RC   Pancreas, Spleen, and Testis;
RX   PubMed=21183079; DOI=10.1016/j.cell.2010.12.001;
RA   Huttlin E.L., Jedrychowski M.P., Elias J.E., Goswami T., Rad R.,
RA   Beausoleil S.A., Villen J., Haas W., Sowa M.E., Gygi S.P.;
RT   "A tissue-specific atlas of mouse protein phosphorylation and expression.";
RL   Cell 143:1174-1189(2010).
CC   -!- FUNCTION: Calcium-responsive mitochondrial glycerol-3-phosphate
CC       dehydrogenase which seems to be a key component of the pancreatic beta-
CC       cell glucose-sensing device. {ECO:0000250|UniProtKB:P43304}.
CC   -!- CATALYTIC ACTIVITY:
CC       Reaction=a quinone + sn-glycerol 3-phosphate = a quinol +
CC         dihydroxyacetone phosphate; Xref=Rhea:RHEA:18977, ChEBI:CHEBI:24646,
CC         ChEBI:CHEBI:57597, ChEBI:CHEBI:57642, ChEBI:CHEBI:132124; EC=1.1.5.3;
CC         Evidence={ECO:0000250|UniProtKB:P43304};
CC       PhysiologicalDirection=left-to-right; Xref=Rhea:RHEA:18978;
CC         Evidence={ECO:0000250|UniProtKB:P43304};
CC   -!- COFACTOR:
CC       Name=FAD; Xref=ChEBI:CHEBI:57692;
CC   -!- ACTIVITY REGULATION: Calcium-binding enhance the activity of the
CC       enzyme. {ECO:0000250|UniProtKB:P43304}.
CC   -!- PATHWAY: Polyol metabolism; glycerol degradation via glycerol kinase
CC       pathway; glycerone phosphate from sn-glycerol 3-phosphate (anaerobic
CC       route): step 1/1.
CC   -!- SUBCELLULAR LOCATION: Mitochondrion inner membrane.
CC   -!- SIMILARITY: Belongs to the FAD-dependent glycerol-3-phosphate
CC       dehydrogenase family. {ECO:0000305}.
CC   ---------------------------------------------------------------------------
CC   Copyrighted by the UniProt Consortium, see https://www.uniprot.org/terms
CC   Distributed under the Creative Commons Attribution (CC BY 4.0) License
CC   ---------------------------------------------------------------------------
DR   EMBL; U60987; AAB50545.1; -; mRNA.
DR   EMBL; D50430; BAA08926.1; -; mRNA.
DR   EMBL; AK034353; BAC28685.1; -; mRNA.
DR   EMBL; AK144716; BAE26028.1; -; mRNA.
DR   EMBL; AK149851; BAE29123.1; -; mRNA.
DR   EMBL; AK167152; BAE39294.1; -; mRNA.
DR   EMBL; BC021359; AAH21359.1; -; mRNA.
DR   EMBL; AH009802; AAG12342.1; -; Genomic_DNA.
DR   EMBL; AB045714; BAB97201.1; -; mRNA.
DR   CCDS; CCDS16045.1; -.
DR   RefSeq; NP_001139292.1; NM_001145820.1.
DR   RefSeq; NP_034404.3; NM_010274.3.
DR   AlphaFoldDB; Q64521; -.
DR   SMR; Q64521; -.
DR   BioGRID; 199894; 21.
DR   IntAct; Q64521; 2.
DR   STRING; 10090.ENSMUSP00000108237; -.
DR   GlyGen; Q64521; 1 site, 1 O-linked glycan (1 site).
DR   iPTMnet; Q64521; -.
DR   PhosphoSitePlus; Q64521; -.
DR   SwissPalm; Q64521; -.
DR   REPRODUCTION-2DPAGE; Q64521; -.
DR   EPD; Q64521; -.
DR   jPOST; Q64521; -.
DR   MaxQB; Q64521; -.
DR   PaxDb; Q64521; -.
DR   PeptideAtlas; Q64521; -.
DR   ProteomicsDB; 271263; -.
DR   Antibodypedia; 2370; 316 antibodies from 30 providers.
DR   DNASU; 14571; -.
DR   Ensembl; ENSMUST00000028167; ENSMUSP00000028167; ENSMUSG00000026827.
DR   Ensembl; ENSMUST00000169687; ENSMUSP00000130992; ENSMUSG00000026827.
DR   GeneID; 14571; -.
DR   KEGG; mmu:14571; -.
DR   UCSC; uc008jsc.2; mouse.
DR   AGR; MGI:99778; -.
DR   CTD; 2820; -.
DR   MGI; MGI:99778; Gpd2.
DR   VEuPathDB; HostDB:ENSMUSG00000026827; -.
DR   eggNOG; KOG0042; Eukaryota.
DR   GeneTree; ENSGT00390000001718; -.
DR   InParanoid; Q64521; -.
DR   OMA; WAMAPHI; -.
DR   OrthoDB; 989271at2759; -.
DR   BRENDA; 1.1.5.3; 3474.
DR   Reactome; R-MMU-1483166; Synthesis of PA.
DR   Reactome; R-MMU-163560; Triglyceride catabolism.
DR   UniPathway; UPA00618; UER00673.
DR   BioGRID-ORCS; 14571; 2 hits in 78 CRISPR screens.
DR   ChiTaRS; Gpd2; mouse.
DR   PRO; PR:Q64521; -.
DR   Proteomes; UP000000589; Chromosome 2.
DR   RNAct; Q64521; protein.
DR   Bgee; ENSMUSG00000026827; Expressed in seminiferous tubule of testis and 269 other tissues.
DR   ExpressionAtlas; Q64521; baseline and differential.
DR   Genevisible; Q64521; MM.
DR   GO; GO:0009331; C:glycerol-3-phosphate dehydrogenase complex; TAS:MGI.
DR   GO; GO:0005743; C:mitochondrial inner membrane; HDA:MGI.
DR   GO; GO:0005739; C:mitochondrion; HDA:MGI.
DR   GO; GO:0005509; F:calcium ion binding; IEA:InterPro.
DR   GO; GO:0004368; F:glycerol-3-phosphate dehydrogenase (quinone) activity; ISO:MGI.
DR   GO; GO:0047952; F:glycerol-3-phosphate dehydrogenase [NAD(P)+] activity; ISO:MGI.
DR   GO; GO:0052591; F:sn-glycerol-3-phosphate:ubiquinone-8 oxidoreductase activity; IEA:UniProtKB-EC.
DR   GO; GO:0043010; P:camera-type eye development; IGI:MGI.
DR   GO; GO:0006094; P:gluconeogenesis; IMP:MGI.
DR   GO; GO:0019563; P:glycerol catabolic process; IEA:UniProtKB-UniPathway.
DR   GO; GO:0006072; P:glycerol-3-phosphate metabolic process; IMP:MGI.
DR   GO; GO:0035264; P:multicellular organism growth; IGI:MGI.
DR   GO; GO:0006734; P:NADH metabolic process; ISO:MGI.
DR   CDD; cd00051; EFh; 1.
DR   Gene3D; 1.10.8.870; Alpha-glycerophosphate oxidase, cap domain; 1.
DR   Gene3D; 3.30.9.10; D-Amino Acid Oxidase, subunit A, domain 2; 1.
DR   Gene3D; 1.10.238.10; EF-hand; 1.
DR   Gene3D; 3.50.50.60; FAD/NAD(P)-binding domain; 1.
DR   InterPro; IPR031656; DAO_C.
DR   InterPro; IPR038299; DAO_C_sf.
DR   InterPro; IPR011992; EF-hand-dom_pair.
DR   InterPro; IPR018247; EF_Hand_1_Ca_BS.
DR   InterPro; IPR002048; EF_hand_dom.
DR   InterPro; IPR006076; FAD-dep_OxRdtase.
DR   InterPro; IPR036188; FAD/NAD-bd_sf.
DR   InterPro; IPR000447; G3P_DH_FAD-dep.
DR   PANTHER; PTHR11985; GLYCEROL-3-PHOSPHATE DEHYDROGENASE; 1.
DR   PANTHER; PTHR11985:SF15; GLYCEROL-3-PHOSPHATE DEHYDROGENASE, MITOCHONDRIAL; 1.
DR   Pfam; PF01266; DAO; 1.
DR   Pfam; PF16901; DAO_C; 1.
DR   Pfam; PF13499; EF-hand_7; 1.
DR   PRINTS; PR01001; FADG3PDH.
DR   SMART; SM00054; EFh; 2.
DR   SUPFAM; SSF47473; EF-hand; 1.
DR   SUPFAM; SSF54373; FAD-linked reductases, C-terminal domain; 1.
DR   SUPFAM; SSF51905; FAD/NAD(P)-binding domain; 1.
DR   PROSITE; PS00018; EF_HAND_1; 1.
DR   PROSITE; PS50222; EF_HAND_2; 2.
DR   PROSITE; PS00977; FAD_G3PDH_1; 1.
DR   PROSITE; PS00978; FAD_G3PDH_2; 1.
PE   1: Evidence at protein level;
KW   Calcium; Direct protein sequencing; FAD; Flavoprotein; Membrane;
KW   Metal-binding; Mitochondrion; Mitochondrion inner membrane; Oxidoreductase;
KW   Phosphoprotein; Reference proteome; Repeat; Transit peptide.
FT   TRANSIT         1..42
FT                   /note="Mitochondrion"
FT                   /evidence="ECO:0000250"
FT   CHAIN           43..727
FT                   /note="Glycerol-3-phosphate dehydrogenase, mitochondrial"
FT                   /id="PRO_0000010430"
FT   DOMAIN          623..658
FT                   /note="EF-hand 1"
FT                   /evidence="ECO:0000255|PROSITE-ProRule:PRU00448"
FT   DOMAIN          659..694
FT                   /note="EF-hand 2"
FT                   /evidence="ECO:0000255|PROSITE-ProRule:PRU00448"
FT   BINDING         71..99
FT                   /ligand="FAD"
FT                   /ligand_id="ChEBI:CHEBI:57692"
FT                   /evidence="ECO:0000255"
FT   BINDING         672
FT                   /ligand="Ca(2+)"
FT                   /ligand_id="ChEBI:CHEBI:29108"
FT                   /evidence="ECO:0000255|PROSITE-ProRule:PRU00448"
FT   BINDING         674
FT                   /ligand="Ca(2+)"
FT                   /ligand_id="ChEBI:CHEBI:29108"
FT                   /evidence="ECO:0000255|PROSITE-ProRule:PRU00448"
FT   BINDING         676
FT                   /ligand="Ca(2+)"
FT                   /ligand_id="ChEBI:CHEBI:29108"
FT                   /evidence="ECO:0000255|PROSITE-ProRule:PRU00448"
FT   BINDING         678
FT                   /ligand="Ca(2+)"
FT                   /ligand_id="ChEBI:CHEBI:29108"
FT                   /evidence="ECO:0000255|PROSITE-ProRule:PRU00448"
FT   BINDING         683
FT                   /ligand="Ca(2+)"
FT                   /ligand_id="ChEBI:CHEBI:29108"
FT                   /evidence="ECO:0000255|PROSITE-ProRule:PRU00448"
FT   MOD_RES         601
FT                   /note="Phosphotyrosine"
FT                   /evidence="ECO:0007744|PubMed:18034455"
FT   CONFLICT        26
FT                   /note="P -> Q (in Ref. 1; AAB50545 and 4; AAH21359)"
FT                   /evidence="ECO:0000305"
FT   CONFLICT        42
FT                   /note="Missing (in Ref. 3; BAC28685)"
FT                   /evidence="ECO:0000305"
FT   CONFLICT        158
FT                   /note="L -> V (in Ref. 1; AAB50545)"
FT                   /evidence="ECO:0000305"
FT   CONFLICT        295
FT                   /note="D -> E (in Ref. 2; BAA08926)"
FT                   /evidence="ECO:0000305"
FT   CONFLICT        449
FT                   /note="D -> N (in Ref. 4; AAH21359)"
FT                   /evidence="ECO:0000305"
FT   CONFLICT        498
FT                   /note="A -> P (in Ref. 2; BAA08926)"
FT                   /evidence="ECO:0000305"
FT   CONFLICT        582
FT                   /note="N -> D (in Ref. 1; AAB50545)"
FT                   /evidence="ECO:0000305"
FT   CONFLICT        593
FT                   /note="E -> G (in Ref. 1; AAB50545)"
FT                   /evidence="ECO:0000305"
SQ   SEQUENCE   727 AA;  80954 MW;  319F52E31CFDFC44 CRC64;
     MAFQKAVKGT ILVGGGALAT VLGLSPFAHY RRKQVSLAYV EAAGYLTEPV NREPPSREAQ
     LMTLKNTPEF DILVIGGGAT GCGCALDAVT RGLKTALVER DDFSSGTSSR STKLIHGGVR
     YLQKAIMNLD VEQYRMVKEA LHERANLLEI APHLSAPLPI MLPLYKWWQL PYYWVGIKMY
     DLVAGSQCLK SSYVLSKSRA LEHFPMLQKD KLVGAIVYYD GQHNDARMNL AIALTAARYG
     AATANYMEVV SLLKKTDPET GKERVSGARC KDVLTGQEFD VRAKCVINAS GPFTDSVRKM
     DDKNVVPICQ PSAGVHIVMP GYYSPENMGL LDPATSDGRV IFFLPWEKMT IAGTTDTPTD
     VTHHPIPSEE DINFILNEVR NYLSSDVEVR RGDVLAAWSG IRPLVTDPKS ADTQSISRNH
     VVDISDSGLI TIAGGKWTTY RSMAEDTVDA AVKFHNLNAG PSRTVGLFLQ GGKDWSPTLY
     IRLVQDYGLE SEVAQHLAKT YGDKAFEVAK MASVTGKRWP VVGVRLVSEF PYIEAEVKYG
     IKEYACTAVD MISRRTRLAF LNVQAAEEAL PRIVELMGRE LNWSELRKQE ELETATRFLY
     YEMGYKSRTE QLTDSTEISL LPSDIDRYKK RFHKFDEDEK GFITIVDVQR VLESINVQMD
     ENTLHEILCE VDLNKNGQVE LHEFLQLMSA VQKGRVSGSR LAILMKTAEE NLDRRVPIPV
     DRSCGGL
//
```

**Mascot:** http://www.matrixscience.com/


HNE (K) (+156.1150)
