# Supplementary material for: A common East-Asian ALDH2 mutation causes metabolic disorders and the therapeutic effect of ALDH2 activators
Source: Nat Commun. 2023 Sep 25;14:5971. doi: 10.1038/s41467-023-41570-6 (PMC10520061; doi:10.1038/s41467-023-41570-6)
Supplement: Supplementary file 4 — Supplementary Data 1 [file 41467_2023_41570_MOESM4_ESM.zip › Table S5c/Q64521/Q64521_KIO60-1-_H633.html]

Mascot Search Results: Q64521
 

# MASCOT Search Results

## Protein View: Q64521

### Glycerol-3-phosphate dehydrogenase, mitochondrial OS=Mus musculus OX=10090 GN=Gpd2 PE=1 SV=2

|  |  |
| --- | --- |
| Database: | Mouse\_UniProt\_proteomes |
| Score: | 20331 |
| Monoisotopic mass (M_r_): | 81416 |
| Calculated pI: | 6.17 |

Sequence similarity is available as an NCBI BLAST search of Q64521 against nr.

### Search parameters

|  |  |
| --- | --- |
| MS data file: | `D:\LCMSMS\2023 Users' data\230529-1\230529-1-KI60-1.raw` |
| Enzyme: | Trypsin/P: cuts C-term side of KR. |
| Fixed modifications: | Carbamidomethyl (C) |
| Variable modifications: | Deamidated (NQ), HNE (C), HNE (H), HNE (K), Oxidation (M) |

### Protein sequence coverage: 80%

Matched peptides shown in ***bold red***.

|  |  |  |  |  |  |
| --- | --- | --- | --- | --- | --- |
| `1` | `MAFQKAVKGT` | `ILVGGGALAT` | `VLGLSPFAHY` | `RRKQVSLAYV` | `EAAGYLTEPV` |
| `51` | `NREPPSREAQ` | `LMTLKNTPEF` | `DILVIGGGAT` | `GCGCALDAVT` | `RGLKTALVER` |
| `101` | `DDFSSGTSSR` | `STKLIHGGVR` | `YLQKAIMNLD` | `VEQYRMVKEA` | `LHERANLLEI` |
| `151` | `APHLSAPLPI` | `MLPLYKWWQL` | `PYYWVGIKMY` | `DLVAGSQCLK` | `SSYVLSKSRA` |
| `201` | `LEHFPMLQKD` | `KLVGAIVYYD` | `GQHNDARMNL` | `AIALTAARYG` | `AATANYMEVV` |
| `251` | `SLLKKTDPET` | `GKERVSGARC` | `KDVLTGQEFD` | `VRAKCVINAS` | `GPFTDSVRKM` |
| `301` | `DDKNVVPICQ` | `PSAGVHIVMP` | `GYYSPENMGL` | `LDPATSDGRV` | `IFFLPWEKMT` |
| `351` | `IAGTTDTPTD` | `VTHHPIPSEE` | `DINFILNEVR` | `NYLSSDVEVR` | `RGDVLAAWSG` |
| `401` | `IRPLVTDPKS` | `ADTQSISRNH` | `VVDISDSGLI` | `TIAGGKWTTY` | `RSMAEDTVDA` |
| `451` | `AVKFHNLNAG` | `PSRTVGLFLQ` | `GGKDWSPTLY` | `IRLVQDYGLE` | `SEVAQHLAKT` |
| `501` | `YGDKAFEVAK` | `MASVTGKRWP` | `VVGVRLVSEF` | `PYIEAEVKYG` | `IKEYACTAVD` |
| `551` | `MISRRTRLAF` | `LNVQAAEEAL` | `PRIVELMGRE` | `LNWSELRKQE` | `ELETATRFLY` |
| `601` | `YEMGYKSRTE` | `QLTDSTEISL` | `LPSDIDRYKK` | `RFHKFDEDEK` | `GFITIVDVQR` |
| `651` | `VLESINVQMD` | `ENTLHEILCE` | `VDLNKNGQVE` | `LHEFLQLMSA` | `VQKGRVSGSR` |
| `701` | `LAILMKTAEE` | `NLDRRVPIPV` | `DRSCGGL` |  |  |

Unformatted sequence string: 727 residues (for pasting into other applications).

|  |  |  |  |
| --- | --- | --- | --- |
| Sort by | residue number | increasing mass | decreasing mass |
| Show | matched peptides only | predicted peptides also |  |

| Query | Start | – | End | Observed | Mr(expt) | Mr(calc) | ppm | M | Score | Expect | Rank | U | Peptide |
| --- | --- | --- | --- | --- | --- | --- | --- | --- | --- | --- | --- | --- | --- |
| 147092 | 9 | – | 31 | 757.4288 | 2269.2646 | 2269.2583 | 2.79 | 0 | 39 | 0.00024 | 1Score **> 35** indicates **identity** Score **> 15** indicates **homology** | U | K.GTILVGGGALATVLGLSPFAHYR.R |
| 9410 | 58 | – | 65 | 467.2562 | 932.4978 | 932.5001 | -2.43 | 0 | 35 | 0.0013 | 1Score **> 32** indicates **identity** Score **> 18** indicates **homology** | U | R.EAQLMTLK.N |
| 9414 | 58 | – | 65 | 467.2566 | 932.4986 | 932.5001 | -1.56 | 0 | 39 | 0.00043 | 1Score **> 32** indicates **identity** Score **> 18** indicates **homology** | U | R.EAQLMTLK.N |
| 9415 | 58 | – | 65 | 467.2572 | 932.4998 | 932.5001 | -0.34 | 0 | 43 | 0.00026 | 1Score **> 32** indicates **identity** Score **> 20** indicates **homology** | U | R.EAQLMTLK.N |
| 9416 | 58 | – | 65 | 467.2576 | 932.5006 | 932.5001 | 0.51 | 0 | 38 | 0.00063 | 1Score **> 32** indicates **identity** Score **> 19** indicates **homology** | U | R.EAQLMTLK.N |
| 168313 | 66 | – | 91 | 888.7592 | 2663.2558 | 2663.2684 | -4.74 | 0 | 44 | 7.5e-05 | 1Score **> 35** indicates **identity** Score **> 15** indicates **homology** | U | K.NTPEFDILVIGGGATGCGCALDAVTR.G |
| 168314 | 66 | – | 91 | 888.7597 | 2663.2572 | 2663.2684 | -4.19 | 0 | 32 | 0.001 | 1Score **> 36** indicates **identity** Score **> 14** indicates **homology** | U | K.NTPEFDILVIGGGATGCGCALDAVTR.G |
| 168315 | 66 | – | 91 | 888.7599 | 2663.2579 | 2663.2684 | -3.92 | 0 | 53 | 1.1e-05 | 1Score **> 36** indicates **identity** Score **> 16** indicates **homology** | U | K.NTPEFDILVIGGGATGCGCALDAVTR.G |
| 168321 | 66 | – | 91 | 888.7617 | 2663.2633 | 2663.2684 | -1.91 | 0 | 60 | 2.5e-06 | 1Score **> 36** indicates **identity** Score **> 16** indicates **homology** | U | K.NTPEFDILVIGGGATGCGCALDAVTR.G |
| 168322 | 66 | – | 91 | 888.7624 | 2663.2653 | 2663.2684 | -1.15 | 0 | 123 | 2.7e-12 | 1Score **> 36** indicates **identity** Score **> 20** indicates **homology** | U | K.NTPEFDILVIGGGATGCGCALDAVTR.G |
| 168325 | 66 | – | 91 | 1332.6411 | 2663.2676 | 2663.2684 | -0.29 | 0 | 66 | 5.9e-07 | 1Score **> 36** indicates **identity** Score **> 17** indicates **homology** | U | K.NTPEFDILVIGGGATGCGCALDAVTR.G |
| 168329 | 66 | – | 91 | 888.7636 | 2663.2689 | 2663.2684 | 0.18 | 0 | 26 | 0.0045 | 1Score **> 36** indicates **identity** Score **> 15** indicates **homology** | U | K.NTPEFDILVIGGGATGCGCALDAVTR.G |
| 168330 | 66 | – | 91 | 1332.6419 | 2663.2693 | 2663.2684 | 0.35 | 0 | 98 | 7.2e-10 | 1Score **> 36** indicates **identity** Score **> 19** indicates **homology** | U | K.NTPEFDILVIGGGATGCGCALDAVTR.G |
| 168331 | 66 | – | 91 | 888.7637 | 2663.2693 | 2663.2684 | 0.35 | 0 | 136 | 1.8e-13 | 1Score **> 36** indicates **identity** Score **> 21** indicates **homology** | U | K.NTPEFDILVIGGGATGCGCALDAVTR.G |
| 168333 | 66 | – | 91 | 1332.6420 | 2663.2695 | 2663.2684 | 0.42 | 0 | 135 | 2.4e-13 | 1Score **> 36** indicates **identity** Score **> 21** indicates **homology** | U | K.NTPEFDILVIGGGATGCGCALDAVTR.G |
| 168334 | 66 | – | 91 | 888.7640 | 2663.2701 | 2663.2684 | 0.63 | 0 | 20 | 0.032 | 1Score **> 36** indicates **identity** Score **> 18** indicates **homology** | U | K.NTPEFDILVIGGGATGCGCALDAVTR.G |
| 168335 | 66 | – | 91 | 888.7640 | 2663.2702 | 2663.2684 | 0.69 | 0 | 37 | 0.00032 | 1Score **> 36** indicates **identity** Score **> 15** indicates **homology** | U | K.NTPEFDILVIGGGATGCGCALDAVTR.G |
| 168336 | 66 | – | 91 | 888.7641 | 2663.2704 | 2663.2684 | 0.75 | 0 | 59 | 2.7e-06 | 1Score **> 36** indicates **identity** Score **> 16** indicates **homology** | U | K.NTPEFDILVIGGGATGCGCALDAVTR.G |
| 168337 | 66 | – | 91 | 1332.6426 | 2663.2707 | 2663.2684 | 0.88 | 0 | 112 | 3.5e-11 | 1Score **> 36** indicates **identity** Score **> 19** indicates **homology** | U | K.NTPEFDILVIGGGATGCGCALDAVTR.G |
| 168338 | 66 | – | 91 | 888.7646 | 2663.2720 | 2663.2684 | 1.37 | 0 | 57 | 4.2e-06 | 1Score **> 36** indicates **identity** Score **> 16** indicates **homology** | U | K.NTPEFDILVIGGGATGCGCALDAVTR.G |
| 168339 | 66 | – | 91 | 888.7647 | 2663.2723 | 2663.2684 | 1.49 | 0 | 125 | 1.8e-12 | 1Score **> 36** indicates **identity** Score **> 20** indicates **homology** | U | K.NTPEFDILVIGGGATGCGCALDAVTR.G |
| 168342 | 66 | – | 91 | 888.7655 | 2663.2745 | 2663.2684 | 2.31 | 0 | 37 | 0.00031 | 1Score **> 36** indicates **identity** Score **> 15** indicates **homology** | U | K.NTPEFDILVIGGGATGCGCALDAVTR.G |
| 168345 | 66 | – | 91 | 888.7657 | 2663.2753 | 2663.2684 | 2.59 | 0 | 45 | 5.4e-05 | 1Score **> 36** indicates **identity** Score **> 15** indicates **homology** | U | K.NTPEFDILVIGGGATGCGCALDAVTR.G |
| 168347 | 66 | – | 91 | 888.7662 | 2663.2766 | 2663.2684 | 3.11 | 0 | 71 | 2.3e-07 | 1Score **> 36** indicates **identity** Score **> 17** indicates **homology** | U | K.NTPEFDILVIGGGATGCGCALDAVTR.G |
| 168348 | 66 | – | 91 | 888.7663 | 2663.2771 | 2663.2684 | 3.28 | 0 | 48 | 3.5e-05 | 1Score **> 36** indicates **identity** Score **> 15** indicates **homology** | U | K.NTPEFDILVIGGGATGCGCALDAVTR.G |
| 168349 | 66 | – | 91 | 888.7668 | 2663.2784 | 2663.2684 | 3.77 | 0 | 52 | 1.3e-05 | 1Score **> 36** indicates **identity** Score **> 16** indicates **homology** | U | K.NTPEFDILVIGGGATGCGCALDAVTR.G |
| 168350 | 66 | – | 91 | 888.7668 | 2663.2785 | 2663.2684 | 3.79 | 0 | 45 | 6.3e-05 | 1Score **> 36** indicates **identity** Score **> 15** indicates **homology** | U | K.NTPEFDILVIGGGATGCGCALDAVTR.G |
| 168352 | 66 | – | 91 | 888.7670 | 2663.2791 | 2663.2684 | 4.01 | 0 | 39 | 0.00023 | 1Score **> 36** indicates **identity** Score **> 15** indicates **homology** | U | K.NTPEFDILVIGGGATGCGCALDAVTR.G |
| 168356 | 66 | – | 91 | 888.7672 | 2663.2799 | 2663.2684 | 4.32 | 0 | 18 | 0.021 | 1Score **> 36** indicates **identity** Score **> 14** indicates **homology** | U | K.NTPEFDILVIGGGATGCGCALDAVTR.G |
| 168357 | 66 | – | 91 | 888.7678 | 2663.2817 | 2663.2684 | 4.99 | 0 | 27 | 0.0029 | 1Score **> 36** indicates **identity** Score **> 14** indicates **homology** | U | K.NTPEFDILVIGGGATGCGCALDAVTR.G |
| 168358 | 66 | – | 91 | 888.7680 | 2663.2820 | 2663.2684 | 5.13 | 0 | 53 | 1.1e-05 | 1Score **> 36** indicates **identity** Score **> 16** indicates **homology** | U | K.NTPEFDILVIGGGATGCGCALDAVTR.G |
| 168359 | 66 | – | 91 | 888.7688 | 2663.2845 | 2663.2684 | 6.04 | 0 | 42 | 0.00012 | 1Score **> 36** indicates **identity** Score **> 15** indicates **homology** | U | K.NTPEFDILVIGGGATGCGCALDAVTR.G |
| 168360 | 66 | – | 91 | 888.7688 | 2663.2846 | 2663.2684 | 6.08 | 0 | 28 | 0.0024 | 1Score **> 36** indicates **identity** Score **> 14** indicates **homology** | U | K.NTPEFDILVIGGGATGCGCALDAVTR.G |
| 168361 | 66 | – | 91 | 888.7691 | 2663.2855 | 2663.2684 | 6.41 | 0 | 97 | 8.1e-10 | 1Score **> 36** indicates **identity** Score **> 19** indicates **homology** | U | K.NTPEFDILVIGGGATGCGCALDAVTR.G |
| 168363 | 66 | – | 91 | 888.7694 | 2663.2865 | 2663.2684 | 6.81 | 0 | 45 | 6.4e-05 | 1Score **> 36** indicates **identity** Score **> 15** indicates **homology** | U | K.NTPEFDILVIGGGATGCGCALDAVTR.G |
| 168364 | 66 | – | 91 | 888.7695 | 2663.2867 | 2663.2684 | 6.89 | 0 | 58 | 4e-06 | 1Score **> 36** indicates **identity** Score **> 16** indicates **homology** | U | K.NTPEFDILVIGGGATGCGCALDAVTR.G |
| 168365 | 66 | – | 91 | 888.7702 | 2663.2887 | 2663.2684 | 7.62 | 0 | 39 | 0.00021 | 1Score **> 37** indicates **identity** Score **> 15** indicates **homology** | U | K.NTPEFDILVIGGGATGCGCALDAVTR.G |
| 168366 | 66 | – | 91 | 888.7702 | 2663.2887 | 2663.2684 | 7.62 | 0 | 19 | 0.015 | 1Score **> 37** indicates **identity** Score **> 14** indicates **homology** | U | K.NTPEFDILVIGGGATGCGCALDAVTR.G |
| 168367 | 66 | – | 91 | 888.7703 | 2663.2890 | 2663.2684 | 7.73 | 0 | 46 | 5.1e-05 | 1Score **> 37** indicates **identity** Score **> 15** indicates **homology** | U | K.NTPEFDILVIGGGATGCGCALDAVTR.G |
| 168368 | 66 | – | 91 | 888.7706 | 2663.2900 | 2663.2684 | 8.12 | 0 | 30 | 0.0014 | 1Score **> 37** indicates **identity** Score **> 14** indicates **homology** | U | K.NTPEFDILVIGGGATGCGCALDAVTR.G |
| 168369 | 66 | – | 91 | 888.7716 | 2663.2931 | 2663.2684 | 9.29 | 0 | 35 | 0.00055 | 1Score **> 37** indicates **identity** Score **> 15** indicates **homology** | U | K.NTPEFDILVIGGGATGCGCALDAVTR.G |
| 168386 | 66 | – | 91 | 889.0944 | 2664.2612 | 2664.2524 | 3.32 | 0 | 45 | 6.2e-05 | 1Score **> 35** indicates **identity** Score **> 15** indicates **homology** | U | K.NTPEFDILVIGGGATGCGCALDAVTR.G  + Deamidated (NQ) |
| 168387 | 66 | – | 91 | 1333.1391 | 2664.2637 | 2664.2524 | 4.26 | 0 | 99 | 5.5e-10 | 1Score **> 35** indicates **identity** Score **> 19** indicates **homology** | U | K.NTPEFDILVIGGGATGCGCALDAVTR.G  + Deamidated (NQ) |
| 168389 | 66 | – | 91 | 889.0957 | 2664.2652 | 2664.2524 | 4.81 | 0 | 39 | 0.00024 | 1Score **> 35** indicates **identity** Score **> 15** indicates **homology** | U | K.NTPEFDILVIGGGATGCGCALDAVTR.G  + Deamidated (NQ) |
| 168390 | 66 | – | 91 | 889.0960 | 2664.2663 | 2664.2524 | 5.23 | 0 | 31 | 0.0014 | 1Score **> 36** indicates **identity** Score **> 14** indicates **homology** | U | K.NTPEFDILVIGGGATGCGCALDAVTR.G  + Deamidated (NQ) |
| 168392 | 66 | – | 91 | 889.0969 | 2664.2688 | 2664.2524 | 6.15 | 0 | 20 | 0.012 | 1Score **> 36** indicates **identity** Score **> 14** indicates **homology** | U | K.NTPEFDILVIGGGATGCGCALDAVTR.G  + Deamidated (NQ) |
| 168393 | 66 | – | 91 | 1333.1425 | 2664.2705 | 2664.2524 | 6.81 | 0 | 65 | 7.7e-07 | 1Score **> 36** indicates **identity** Score **> 17** indicates **homology** | U | K.NTPEFDILVIGGGATGCGCALDAVTR.G  + Deamidated (NQ) |
| 168396 | 66 | – | 91 | 889.0981 | 2664.2725 | 2664.2524 | 7.56 | 0 | 94 | 1.7e-09 | 1Score **> 36** indicates **identity** Score **> 18** indicates **homology** | U | K.NTPEFDILVIGGGATGCGCALDAVTR.G  + Deamidated (NQ) |
| 168397 | 66 | – | 91 | 889.0982 | 2664.2727 | 2664.2524 | 7.62 | 0 | 18 | 0.019 | 1Score **> 36** indicates **identity** Score **> 14** indicates **homology** | U | K.NTPEFDILVIGGGATGCGCALDAVTR.G  + Deamidated (NQ) |
| 168399 | 66 | – | 91 | 889.0990 | 2664.2751 | 2664.2524 | 8.55 | 0 | 66 | 7.1e-07 | 1Score **> 36** indicates **identity** Score **> 17** indicates **homology** | U | K.NTPEFDILVIGGGATGCGCALDAVTR.G  + Deamidated (NQ) |
| 168401 | 66 | – | 91 | 1333.1451 | 2664.2757 | 2664.2524 | 8.74 | 0 | 82 | 2.1e-08 | 1Score **> 36** indicates **identity** Score **> 18** indicates **homology** | U | K.NTPEFDILVIGGGATGCGCALDAVTR.G  + Deamidated (NQ) |
| 168403 | 66 | – | 91 | 889.0995 | 2664.2768 | 2664.2524 | 9.17 | 0 | 43 | 8.9e-05 | 1Score **> 36** indicates **identity** Score **> 15** indicates **homology** | U | K.NTPEFDILVIGGGATGCGCALDAVTR.G  + Deamidated (NQ) |
| 168404 | 66 | – | 91 | 889.0997 | 2664.2773 | 2664.2524 | 9.35 | 0 | 52 | 1.4e-05 | 1Score **> 36** indicates **identity** Score **> 16** indicates **homology** | U | K.NTPEFDILVIGGGATGCGCALDAVTR.G  + Deamidated (NQ) |
| 125460 | 92 | – | 110 | 676.0093 | 2025.0061 | 2025.0127 | -3.24 | 2 | 27 | 0.0049 | 1Score **> 35** indicates **identity** Score **> 17** indicates **homology** | U | R.GLKTALVERDDFSSGTSSR.S |
| 125463 | 92 | – | 110 | 676.0106 | 2025.0100 | 2025.0127 | -1.32 | 2 | 26 | 0.0036 | 1Score **> 35** indicates **identity** Score **> 14** indicates **homology** | U | R.GLKTALVERDDFSSGTSSR.S |
| 125466 | 92 | – | 110 | 676.0115 | 2025.0125 | 2025.0127 | -0.078 | 2 | 15 | 0.042 | 1Score **> 36** indicates **identity** Score **> 13** indicates **homology** | U | R.GLKTALVERDDFSSGTSSR.S |
| 94858 | 95 | – | 110 | 864.4097 | 1726.8049 | 1726.8122 | -4.25 | 1 | 24 | 0.0054 | 1Score **> 32** indicates **identity** Score **> 14** indicates **homology** | U | K.TALVERDDFSSGTSSR.S |
| 94859 | 95 | – | 110 | 576.6096 | 1726.8070 | 1726.8122 | -3.00 | 1 | 34 | 0.0007 | 1Score **> 32** indicates **identity** Score **> 15** indicates **homology** | U | K.TALVERDDFSSGTSSR.S |
| 94863 | 95 | – | 110 | 576.6104 | 1726.8095 | 1726.8122 | -1.57 | 1 | 30 | 0.0016 | 1Score **> 32** indicates **identity** Score **> 14** indicates **homology** | U | K.TALVERDDFSSGTSSR.S |
| 94864 | 95 | – | 110 | 576.6105 | 1726.8096 | 1726.8122 | -1.50 | 1 | 46 | 5.3e-05 | 1Score **> 32** indicates **identity** Score **> 15** indicates **homology** | U | K.TALVERDDFSSGTSSR.S |
| 94865 | 95 | – | 110 | 864.4122 | 1726.8099 | 1726.8122 | -1.33 | 1 | 52 | 1.9e-05 | 1Score **> 33** indicates **identity** Score **> 17** indicates **homology** | U | K.TALVERDDFSSGTSSR.S |
| 94868 | 95 | – | 110 | 576.6109 | 1726.8109 | 1726.8122 | -0.77 | 1 | 15 | 0.036 | 1Score **> 33** indicates **identity** Score **> 13** indicates **homology** | U | K.TALVERDDFSSGTSSR.S |
| 94869 | 95 | – | 110 | 864.4128 | 1726.8111 | 1726.8122 | -0.62 | 1 | 73 | 1.6e-07 | 1Score **> 33** indicates **identity** Score **> 18** indicates **homology** | U | K.TALVERDDFSSGTSSR.S |
| 94870 | 95 | – | 110 | 576.6110 | 1726.8112 | 1726.8122 | -0.59 | 1 | 43 | 0.0001 | 1Score **> 33** indicates **identity** Score **> 15** indicates **homology** | U | K.TALVERDDFSSGTSSR.S |
| 94871 | 95 | – | 110 | 864.4129 | 1726.8112 | 1726.8122 | -0.58 | 1 | 26 | 0.0038 | 1Score **> 33** indicates **identity** Score **> 14** indicates **homology** | U | K.TALVERDDFSSGTSSR.S |
| 94872 | 95 | – | 110 | 576.6111 | 1726.8116 | 1726.8122 | -0.38 | 1 | 35 | 0.00055 | 1Score **> 33** indicates **identity** Score **> 15** indicates **homology** | U | K.TALVERDDFSSGTSSR.S |
| 94873 | 95 | – | 110 | 864.4131 | 1726.8116 | 1726.8122 | -0.34 | 1 | 31 | 0.0011 | 1Score **> 33** indicates **identity** Score **> 14** indicates **homology** | U | K.TALVERDDFSSGTSSR.S |
| 94874 | 95 | – | 110 | 864.4131 | 1726.8117 | 1726.8122 | -0.30 | 1 | 94 | 1.5e-09 | 1Score **> 33** indicates **identity** Score **> 18** indicates **homology** | U | K.TALVERDDFSSGTSSR.S |
| 94875 | 95 | – | 110 | 576.6112 | 1726.8118 | 1726.8122 | -0.25 | 1 | 39 | 0.0002 | 1Score **> 33** indicates **identity** Score **> 15** indicates **homology** | U | K.TALVERDDFSSGTSSR.S |
| 94876 | 95 | – | 110 | 864.4132 | 1726.8118 | 1726.8122 | -0.23 | 1 | 98 | 1.9e-09 | 1Score **> 33** indicates **identity** Score **> 24** indicates **homology** | U | K.TALVERDDFSSGTSSR.S |
| 94877 | 95 | – | 110 | 576.6113 | 1726.8121 | 1726.8122 | -0.071 | 1 | 43 | 8.7e-05 | 1Score **> 33** indicates **identity** Score **> 15** indicates **homology** | U | K.TALVERDDFSSGTSSR.S |
| 94878 | 95 | – | 110 | 576.6113 | 1726.8121 | 1726.8122 | -0.036 | 1 | 55 | 7.2e-06 | 1Score **> 33** indicates **identity** Score **> 16** indicates **homology** | U | K.TALVERDDFSSGTSSR.S |
| 94879 | 95 | – | 110 | 576.6114 | 1726.8123 | 1726.8122 | 0.066 | 1 | 66 | 6.4e-07 | 1Score **> 33** indicates **identity** Score **> 17** indicates **homology** | U | K.TALVERDDFSSGTSSR.S |
| 94881 | 95 | – | 110 | 576.6114 | 1726.8125 | 1726.8122 | 0.17 | 1 | 63 | 1.3e-06 | 1Score **> 32** indicates **identity** Score **> 16** indicates **homology** | U | K.TALVERDDFSSGTSSR.S |
| 94882 | 95 | – | 110 | 576.6115 | 1726.8126 | 1726.8122 | 0.25 | 1 | 64 | 1.1e-06 | 1Score **> 32** indicates **identity** Score **> 16** indicates **homology** | U | K.TALVERDDFSSGTSSR.S |
| 94883 | 95 | – | 110 | 864.4137 | 1726.8128 | 1726.8122 | 0.33 | 1 | 77 | 6.1e-08 | 1Score **> 32** indicates **identity** Score **> 17** indicates **homology** | U | K.TALVERDDFSSGTSSR.S |
| 94884 | 95 | – | 110 | 864.4137 | 1726.8128 | 1726.8122 | 0.35 | 1 | 94 | 3.2e-09 | 1Score **> 32** indicates **identity** Score **> 21** indicates **homology** | U | K.TALVERDDFSSGTSSR.S |
| 94885 | 95 | – | 110 | 864.4137 | 1726.8129 | 1726.8122 | 0.40 | 1 | 99 | 5.8e-10 | 1Score **> 33** indicates **identity** Score **> 19** indicates **homology** | U | K.TALVERDDFSSGTSSR.S |
| 94886 | 95 | – | 110 | 576.6117 | 1726.8132 | 1726.8122 | 0.60 | 1 | 59 | 2.7e-06 | 1Score **> 33** indicates **identity** Score **> 16** indicates **homology** | U | K.TALVERDDFSSGTSSR.S |
| 94888 | 95 | – | 110 | 576.6118 | 1726.8137 | 1726.8122 | 0.84 | 1 | 55 | 6.4e-06 | 1Score **> 33** indicates **identity** Score **> 16** indicates **homology** | U | K.TALVERDDFSSGTSSR.S |
| 94889 | 95 | – | 110 | 576.6119 | 1726.8139 | 1726.8122 | 0.99 | 1 | 64 | 1.1e-06 | 1Score **> 33** indicates **identity** Score **> 16** indicates **homology** | U | K.TALVERDDFSSGTSSR.S |
| 94890 | 95 | – | 110 | 576.6119 | 1726.8139 | 1726.8122 | 0.99 | 1 | 66 | 6e-07 | 1Score **> 33** indicates **identity** Score **> 17** indicates **homology** | U | K.TALVERDDFSSGTSSR.S |
| 94892 | 95 | – | 110 | 576.6121 | 1726.8143 | 1726.8122 | 1.24 | 1 | 21 | 0.01 | 1Score **> 33** indicates **identity** Score **> 14** indicates **homology** | U | K.TALVERDDFSSGTSSR.S |
| 94893 | 95 | – | 110 | 864.4146 | 1726.8146 | 1726.8122 | 1.36 | 1 | 60 | 1.3e-05 | 1Score **> 33** indicates **identity** Score **> 23** indicates **homology** | U | K.TALVERDDFSSGTSSR.S |
| 94894 | 95 | – | 110 | 864.4146 | 1726.8146 | 1726.8122 | 1.39 | 1 | 47 | 3.6e-05 | 1Score **> 33** indicates **identity** Score **> 15** indicates **homology** | U | K.TALVERDDFSSGTSSR.S |
| 94895 | 95 | – | 110 | 576.6122 | 1726.8146 | 1726.8122 | 1.40 | 1 | 27 | 0.0028 | 1Score **> 33** indicates **identity** Score **> 14** indicates **homology** | U | K.TALVERDDFSSGTSSR.S |
| 94896 | 95 | – | 110 | 864.4152 | 1726.8159 | 1726.8122 | 2.16 | 1 | 18 | 0.021 | 1Score **> 33** indicates **identity** Score **> 14** indicates **homology** | U | K.TALVERDDFSSGTSSR.S |
| 17363 | 101 | – | 110 | 529.7222 | 1057.4299 | 1057.4312 | -1.29 | 0 | 40 | 0.00018 | 1Score **> 22** indicates **identity** Score **> 15** indicates **homology** | U | R.DDFSSGTSSR.S |
| 17364 | 101 | – | 110 | 529.7225 | 1057.4305 | 1057.4312 | -0.74 | 0 | 46 | 4.7e-05 | 1Score **> 22** indicates **identity** Score **> 15** indicates **homology** | U | R.DDFSSGTSSR.S |
| 17365 | 101 | – | 110 | 529.7227 | 1057.4308 | 1057.4312 | -0.40 | 0 | 24 | 0.0059 | 1Score **> 22** indicates **identity** Score **> 14** indicates **homology** | U | R.DDFSSGTSSR.S |
| 18174 | 111 | – | 120 | 356.5492 | 1066.6259 | 1066.6247 | 1.12 | 1 | 29 | 0.0021 | 1Score **> 28** indicates **identity** Score **> 14** indicates **homology** | U | R.STKLIHGGVR.Y |
| 18175 | 111 | – | 120 | 534.3203 | 1066.6261 | 1066.6247 | 1.29 | 1 | 28 | 0.0022 | 1Score **> 28** indicates **identity** Score **> 14** indicates **homology** | U | R.STKLIHGGVR.Y |
| 1701 | 114 | – | 120 | 376.2326 | 750.4507 | 750.4501 | 0.84 | 0 | 24 | 0.0038 | 1Score **> 13** indicates **identity** | U | K.LIHGGVR.Y |
| 47405 | 125 | – | 135 | 676.3373 | 1350.6600 | 1350.6602 | -0.13 | 0 | 23 | 0.0063 | 1Score **> 32** indicates **identity** Score **> 14** indicates **homology** | U | K.AIMNLDVEQYR.M |
| 47406 | 125 | – | 135 | 676.3378 | 1350.6610 | 1350.6602 | 0.66 | 0 | 24 | 0.0051 | 1Score **> 33** indicates **identity** Score **> 14** indicates **homology** | U | K.AIMNLDVEQYR.M |
| 48953 | 125 | – | 135 | 684.3345 | 1366.6545 | 1366.6551 | -0.44 | 0 | 36 | 0.0004 | 1Score **> 32** indicates **identity** Score **> 15** indicates **homology** | U | K.AIMNLDVEQYR.M  + Oxidation (M) |
| 156189 | 145 | – | 166 | 1207.7028 | 2413.3911 | 2413.3806 | 4.32 | 0 | 75 | 9.8e-08 | 1Score **> 31** indicates **identity** Score **> 17** indicates **homology** | U | R.ANLLEIAPHLSAPLPIMLPLYK.W |
| 83119 | 167 | – | 178 | 819.9277 | 1637.8409 | 1637.8395 | 0.85 | 0 | 23 | 0.0068 | 1Score **> 35** indicates **identity** Score **> 14** indicates **homology** | U | K.WWQLPYYWVGIK.M |
| 83122 | 167 | – | 178 | 819.9285 | 1637.8424 | 1637.8395 | 1.76 | 0 | 31 | 0.0013 | 1Score **> 35** indicates **identity** Score **> 14** indicates **homology** | U | K.WWQLPYYWVGIK.M |
| 50607 | 179 | – | 190 | 692.8316 | 1383.6487 | 1383.6526 | -2.83 | 0 | 58 | 3.7e-06 | 1Score **> 31** indicates **identity** Score **> 16** indicates **homology** | U | K.MYDLVAGSQCLK.S |
| 50609 | 179 | – | 190 | 692.8332 | 1383.6519 | 1383.6526 | -0.56 | 0 | 21 | 0.011 | 1Score **> 31** indicates **identity** Score **> 14** indicates **homology** | U | K.MYDLVAGSQCLK.S |
| 50610 | 179 | – | 190 | 692.8332 | 1383.6519 | 1383.6526 | -0.53 | 0 | 61 | 2.1e-06 | 1Score **> 31** indicates **identity** Score **> 17** indicates **homology** | U | K.MYDLVAGSQCLK.S |
| 2646 | 191 | – | 197 | 392.2157 | 782.4168 | 782.4174 | -0.79 | 0 | 46 | 6.5e-05 | 1Score **> 17** indicates **identity** | U | K.SSYVLSK.S |
| 2647 | 191 | – | 197 | 392.2162 | 782.4179 | 782.4174 | 0.67 | 0 | 22 | 0.016 | 1Score **> 17** indicates **identity** | U | K.SSYVLSK.S |
| 2652 | 191 | – | 197 | 392.2174 | 782.4203 | 782.4174 | 3.71 | 0 | 39 | 0.00024 | 1Score **> 16** indicates **identity** | U | K.SSYVLSK.S |
| 32782 | 200 | – | 209 | 405.2161 | 1212.6264 | 1212.6325 | -4.99 | 0 | 15 | 0.039 | 1Score **> 32** indicates **identity** Score **> 13** indicates **homology** | U | R.ALEHFPMLQK.D |
| 32786 | 200 | – | 209 | 405.2163 | 1212.6270 | 1212.6325 | -4.55 | 0 | 22 | 0.0088 | 1Score **> 32** indicates **identity** Score **> 14** indicates **homology** | U | R.ALEHFPMLQK.D |
| 32791 | 200 | – | 209 | 405.2168 | 1212.6284 | 1212.6325 | -3.34 | 0 | 21 | 0.011 | 1Score **> 32** indicates **identity** Score **> 14** indicates **homology** | U | R.ALEHFPMLQK.D |
| 32792 | 200 | – | 209 | 405.2170 | 1212.6293 | 1212.6325 | -2.66 | 0 | 21 | 0.011 | 1Score **> 32** indicates **identity** Score **> 14** indicates **homology** | U | R.ALEHFPMLQK.D |
| 32795 | 200 | – | 209 | 405.2174 | 1212.6304 | 1212.6325 | -1.69 | 0 | 27 | 0.0028 | 1Score **> 32** indicates **identity** Score **> 14** indicates **homology** | U | R.ALEHFPMLQK.D |
| 32796 | 200 | – | 209 | 405.2176 | 1212.6310 | 1212.6325 | -1.20 | 0 | 18 | 0.02 | 1Score **> 32** indicates **identity** Score **> 14** indicates **homology** | U | R.ALEHFPMLQK.D |
| 32797 | 200 | – | 209 | 405.2178 | 1212.6316 | 1212.6325 | -0.71 | 0 | 22 | 0.0085 | 1Score **> 32** indicates **identity** Score **> 14** indicates **homology** | U | R.ALEHFPMLQK.D |
| 126266 | 210 | – | 227 | 678.6714 | 2032.9923 | 2032.9966 | -2.12 | 1 | 47 | 3.7e-05 | 1Score **> 36** indicates **identity** Score **> 15** indicates **homology** | U | K.DKLVGAIVYYDGQHNDAR.M |
| 126268 | 210 | – | 227 | 1017.5051 | 2032.9957 | 2032.9966 | -0.47 | 1 | 78 | 4.8e-08 | 1Score **> 36** indicates **identity** Score **> 17** indicates **homology** | U | K.DKLVGAIVYYDGQHNDAR.M |
| 126270 | 210 | – | 227 | 1017.5056 | 2032.9967 | 2032.9966 | 0.016 | 1 | 75 | 8.7e-08 | 1Score **> 36** indicates **identity** Score **> 17** indicates **homology** | U | K.DKLVGAIVYYDGQHNDAR.M |
| 126273 | 210 | – | 227 | 509.2565 | 2032.9968 | 2032.9966 | 0.087 | 1 | 39 | 0.00022 | 1Score **> 36** indicates **identity** Score **> 15** indicates **homology** | U | K.DKLVGAIVYYDGQHNDAR.M |
| 126274 | 210 | – | 227 | 678.6729 | 2032.9970 | 2032.9966 | 0.17 | 1 | 57 | 4.3e-06 | 1Score **> 36** indicates **identity** Score **> 16** indicates **homology** | U | K.DKLVGAIVYYDGQHNDAR.M |
| 126275 | 210 | – | 227 | 678.6730 | 2032.9972 | 2032.9966 | 0.28 | 1 | 34 | 0.00069 | 1Score **> 36** indicates **identity** Score **> 15** indicates **homology** | U | K.DKLVGAIVYYDGQHNDAR.M |
| 126276 | 210 | – | 227 | 509.2566 | 2032.9972 | 2032.9966 | 0.29 | 1 | 44 | 8.3e-05 | 1Score **> 36** indicates **identity** Score **> 15** indicates **homology** | U | K.DKLVGAIVYYDGQHNDAR.M |
| 126277 | 210 | – | 227 | 678.6731 | 2032.9974 | 2032.9966 | 0.39 | 1 | 77 | 5.9e-08 | 1Score **> 36** indicates **identity** Score **> 17** indicates **homology** | U | K.DKLVGAIVYYDGQHNDAR.M |
| 126278 | 210 | – | 227 | 678.6732 | 2032.9978 | 2032.9966 | 0.56 | 1 | 57 | 4.7e-06 | 1Score **> 36** indicates **identity** Score **> 16** indicates **homology** | U | K.DKLVGAIVYYDGQHNDAR.M |
| 126280 | 210 | – | 227 | 509.2570 | 2032.9987 | 2032.9966 | 1.02 | 1 | 27 | 0.003 | 1Score **> 36** indicates **identity** Score **> 14** indicates **homology** | U | K.DKLVGAIVYYDGQHNDAR.M |
| 126283 | 210 | – | 227 | 678.6736 | 2032.9991 | 2032.9966 | 1.19 | 1 | 30 | 0.0014 | 1Score **> 36** indicates **identity** Score **> 14** indicates **homology** | U | K.DKLVGAIVYYDGQHNDAR.M |
| 126285 | 210 | – | 227 | 678.6741 | 2033.0003 | 2032.9966 | 1.82 | 1 | 27 | 0.0026 | 1Score **> 36** indicates **identity** Score **> 14** indicates **homology** | U | K.DKLVGAIVYYDGQHNDAR.M |
| 126287 | 210 | – | 227 | 678.6742 | 2033.0006 | 2032.9966 | 1.96 | 1 | 34 | 0.00063 | 1Score **> 36** indicates **identity** Score **> 15** indicates **homology** | U | K.DKLVGAIVYYDGQHNDAR.M |
| 126288 | 210 | – | 227 | 678.6742 | 2033.0008 | 2032.9966 | 2.06 | 1 | 28 | 0.0025 | 1Score **> 36** indicates **identity** Score **> 14** indicates **homology** | U | K.DKLVGAIVYYDGQHNDAR.M |
| 126296 | 210 | – | 227 | 1017.5097 | 2033.0049 | 2032.9966 | 4.04 | 1 | 55 | 6.7e-06 | 1Score **> 36** indicates **identity** Score **> 16** indicates **homology** | U | K.DKLVGAIVYYDGQHNDAR.M |
| 126304 | 210 | – | 227 | 509.2597 | 2033.0096 | 2032.9966 | 6.39 | 1 | 29 | 0.0018 | 1Score **> 36** indicates **identity** Score **> 14** indicates **homology** | U | K.DKLVGAIVYYDGQHNDAR.M |
| 126307 | 210 | – | 227 | 509.2602 | 2033.0116 | 2032.9966 | 7.36 | 1 | 27 | 0.0027 | 1Score **> 36** indicates **identity** Score **> 14** indicates **homology** | U | K.DKLVGAIVYYDGQHNDAR.M |
| 126370 | 210 | – | 227 | 679.0070 | 2033.9992 | 2033.9807 | 9.13 | 1 | 23 | 0.0068 | 1Score **> 36** indicates **identity** Score **> 14** indicates **homology** | U | K.DKLVGAIVYYDGQHNDAR.M  + Deamidated (NQ) |
| 102035 | 212 | – | 227 | 895.9441 | 1789.8736 | 1789.8747 | -0.64 | 0 | 42 | 0.00012 | 1Score **> 35** indicates **identity** Score **> 15** indicates **homology** | U | K.LVGAIVYYDGQHNDAR.M |
| 102036 | 212 | – | 227 | 895.9443 | 1789.8740 | 1789.8747 | -0.44 | 0 | 61 | 1.9e-06 | 1Score **> 35** indicates **identity** Score **> 16** indicates **homology** | U | K.LVGAIVYYDGQHNDAR.M |
| 102037 | 212 | – | 227 | 597.6320 | 1789.8742 | 1789.8747 | -0.32 | 0 | 41 | 0.00013 | 1Score **> 35** indicates **identity** Score **> 15** indicates **homology** | U | K.LVGAIVYYDGQHNDAR.M |
| 102038 | 212 | – | 227 | 895.9446 | 1789.8746 | 1789.8747 | -0.10 | 0 | 62 | 1.5e-06 | 1Score **> 35** indicates **identity** Score **> 16** indicates **homology** | U | K.LVGAIVYYDGQHNDAR.M |
| 102039 | 212 | – | 227 | 597.6325 | 1789.8756 | 1789.8747 | 0.48 | 0 | 46 | 4.9e-05 | 1Score **> 35** indicates **identity** Score **> 15** indicates **homology** | U | K.LVGAIVYYDGQHNDAR.M |
| 102040 | 212 | – | 227 | 895.9451 | 1789.8757 | 1789.8747 | 0.52 | 0 | 63 | 1.3e-06 | 1Score **> 35** indicates **identity** Score **> 16** indicates **homology** | U | K.LVGAIVYYDGQHNDAR.M |
| 102042 | 212 | – | 227 | 597.6331 | 1789.8774 | 1789.8747 | 1.49 | 0 | 35 | 0.00061 | 1Score **> 35** indicates **identity** Score **> 16** indicates **homology** | U | K.LVGAIVYYDGQHNDAR.M |
| 102043 | 212 | – | 227 | 895.9462 | 1789.8779 | 1789.8747 | 1.77 | 0 | 73 | 1.6e-07 | 1Score **> 35** indicates **identity** Score **> 17** indicates **homology** | U | K.LVGAIVYYDGQHNDAR.M |
| 102045 | 212 | – | 227 | 597.6337 | 1789.8792 | 1789.8747 | 2.47 | 0 | 27 | 0.0027 | 1Score **> 35** indicates **identity** Score **> 14** indicates **homology** | U | K.LVGAIVYYDGQHNDAR.M |
| 25502 | 228 | – | 238 | 572.8274 | 1143.6403 | 1143.6434 | -2.67 | 0 | 20 | 0.014 | 1Score **> 34** indicates **identity** Score **> 14** indicates **homology** | U | R.MNLAIALTAAR.Y |
| 25508 | 228 | – | 238 | 572.8279 | 1143.6412 | 1143.6434 | -1.94 | 0 | 36 | 0.00096 | 1Score **> 34** indicates **identity** Score **> 18** indicates **homology** | U | R.MNLAIALTAAR.Y |
| 25517 | 228 | – | 238 | 572.8290 | 1143.6434 | 1143.6434 | -0.012 | 0 | 71 | 6.4e-06 | 1Score **> 34** indicates **identity** Score **> 32** indicates **homology** | U | R.MNLAIALTAAR.Y |
| 25518 | 228 | – | 238 | 572.8290 | 1143.6434 | 1143.6434 | 0.028 | 0 | 74 | 3.2e-06 | 1Score **> 34** indicates **identity** Score **> 32** indicates **homology** | U | R.MNLAIALTAAR.Y |
| 25519 | 228 | – | 238 | 572.8291 | 1143.6436 | 1143.6434 | 0.19 | 0 | 31 | 0.0026 | 1Score **> 34** indicates **identity** Score **> 18** indicates **homology** | U | R.MNLAIALTAAR.Y |
| 25521 | 228 | – | 238 | 572.8291 | 1143.6437 | 1143.6434 | 0.32 | 0 | 68 | 9.2e-06 | 1Score **> 34** indicates **identity** Score **> 30** indicates **homology** | U | R.MNLAIALTAAR.Y |
| 25522 | 228 | – | 238 | 572.8293 | 1143.6440 | 1143.6434 | 0.54 | 0 | 18 | 0.048 | 1Score **> 34** indicates **identity** Score **> 17** indicates **homology** | U | R.MNLAIALTAAR.Y |
| 25523 | 228 | – | 238 | 572.8293 | 1143.6441 | 1143.6434 | 0.64 | 0 | 62 | 2.3e-05 | 1Score **> 34** indicates **identity** Score **> 28** indicates **homology** | U | R.MNLAIALTAAR.Y |
| 25526 | 228 | – | 238 | 572.8299 | 1143.6452 | 1143.6434 | 1.60 | 0 | 37 | 0.00087 | 1Score **> 34** indicates **identity** Score **> 19** indicates **homology** | U | R.MNLAIALTAAR.Y |
| 25527 | 228 | – | 238 | 572.8300 | 1143.6454 | 1143.6434 | 1.80 | 0 | 33 | 0.0019 | 1Score **> 34** indicates **identity** Score **> 18** indicates **homology** | U | R.MNLAIALTAAR.Y |
| 25529 | 228 | – | 238 | 572.8308 | 1143.6470 | 1143.6434 | 3.20 | 0 | 35 | 0.0014 | 1Score **> 34** indicates **identity** Score **> 19** indicates **homology** | U | R.MNLAIALTAAR.Y |
| 25530 | 228 | – | 238 | 572.8309 | 1143.6473 | 1143.6434 | 3.40 | 0 | 37 | 0.0017 | 1Score **> 34** indicates **identity** Score **> 22** indicates **homology** | U | R.MNLAIALTAAR.Y |
| 95176 | 239 | – | 254 | 865.4430 | 1728.8714 | 1728.8756 | -2.44 | 0 | 92 | 2.6e-09 | 1Score **> 35** indicates **identity** Score **> 18** indicates **homology** | U | R.YGAATANYMEVVSLLK.K |
| 95184 | 239 | – | 254 | 865.4458 | 1728.8770 | 1728.8756 | 0.80 | 0 | 108 | 7.1e-11 | 1Score **> 35** indicates **identity** Score **> 19** indicates **homology** | U | R.YGAATANYMEVVSLLK.K |
| 95190 | 239 | – | 254 | 865.4476 | 1728.8807 | 1728.8756 | 2.92 | 0 | 72 | 1.8e-07 | 1Score **> 35** indicates **identity** Score **> 17** indicates **homology** | U | R.YGAATANYMEVVSLLK.K |
| 95303 | 239 | – | 254 | 865.9448 | 1729.8750 | 1729.8596 | 8.86 | 0 | 44 | 7e-05 | 1Score **> 35** indicates **identity** Score **> 15** indicates **homology** | U | R.YGAATANYMEVVSLLK.K  + Deamidated (NQ) |
| 109059 | 239 | – | 255 | 619.9977 | 1856.9713 | 1856.9706 | 0.38 | 1 | 63 | 1.2e-06 | 1Score **> 36** indicates **identity** Score **> 16** indicates **homology** | U | R.YGAATANYMEVVSLLKK.T |
| 109060 | 239 | – | 255 | 619.9979 | 1856.9719 | 1856.9706 | 0.69 | 1 | 61 | 1.8e-06 | 1Score **> 36** indicates **identity** Score **> 16** indicates **homology** | U | R.YGAATANYMEVVSLLKK.T |
| 109063 | 239 | – | 255 | 619.9996 | 1856.9770 | 1856.9706 | 3.46 | 1 | 73 | 1.5e-07 | 1Score **> 36** indicates **identity** Score **> 17** indicates **homology** | U | R.YGAATANYMEVVSLLKK.T |
| 73022 | 270 | – | 282 | 522.9222 | 1565.7446 | 1565.7508 | -3.94 | 1 | 22 | 0.0092 | 1Score **> 33** indicates **identity** Score **> 14** indicates **homology** | U | R.CKDVLTGQEFDVR.A |
| 73024 | 270 | – | 282 | 783.8812 | 1565.7478 | 1565.7508 | -1.94 | 1 | 66 | 6.9e-07 | 1Score **> 33** indicates **identity** Score **> 17** indicates **homology** | U | R.CKDVLTGQEFDVR.A |
| 73025 | 270 | – | 282 | 783.8815 | 1565.7484 | 1565.7508 | -1.52 | 1 | 48 | 3.1e-05 | 1Score **> 33** indicates **identity** Score **> 16** indicates **homology** | U | R.CKDVLTGQEFDVR.A |
| 73026 | 270 | – | 282 | 783.8816 | 1565.7486 | 1565.7508 | -1.42 | 1 | 82 | 2.8e-08 | 1Score **> 33** indicates **identity** Score **> 19** indicates **homology** | U | R.CKDVLTGQEFDVR.A |
| 73027 | 270 | – | 282 | 783.8820 | 1565.7494 | 1565.7508 | -0.93 | 1 | 58 | 6.2e-06 | 1Score **> 33** indicates **identity** Score **> 19** indicates **homology** | U | R.CKDVLTGQEFDVR.A |
| 73028 | 270 | – | 282 | 783.8821 | 1565.7497 | 1565.7508 | -0.73 | 1 | 70 | 5.7e-07 | 1Score **> 33** indicates **identity** Score **> 20** indicates **homology** | U | R.CKDVLTGQEFDVR.A |
| 73029 | 270 | – | 282 | 783.8826 | 1565.7506 | 1565.7508 | -0.15 | 1 | 45 | 8.9e-05 | 1Score **> 33** indicates **identity** Score **> 17** indicates **homology** | U | R.CKDVLTGQEFDVR.A |
| 73031 | 270 | – | 282 | 783.8829 | 1565.7512 | 1565.7508 | 0.24 | 1 | 73 | 1.4e-07 | 1Score **> 33** indicates **identity** Score **> 17** indicates **homology** | U | R.CKDVLTGQEFDVR.A |
| 73032 | 270 | – | 282 | 783.8830 | 1565.7515 | 1565.7508 | 0.45 | 1 | 68 | 4.4e-07 | 1Score **> 33** indicates **identity** Score **> 17** indicates **homology** | U | R.CKDVLTGQEFDVR.A |
| 73033 | 270 | – | 282 | 522.9245 | 1565.7516 | 1565.7508 | 0.50 | 1 | 63 | 1.1e-06 | 1Score **> 33** indicates **identity** Score **> 16** indicates **homology** | U | R.CKDVLTGQEFDVR.A |
| 73034 | 270 | – | 282 | 522.9245 | 1565.7516 | 1565.7508 | 0.54 | 1 | 59 | 3.1e-06 | 1Score **> 33** indicates **identity** Score **> 16** indicates **homology** | U | R.CKDVLTGQEFDVR.A |
| 73036 | 270 | – | 282 | 522.9248 | 1565.7527 | 1565.7508 | 1.21 | 1 | 52 | 1.3e-05 | 1Score **> 33** indicates **identity** Score **> 16** indicates **homology** | U | R.CKDVLTGQEFDVR.A |
| 73037 | 270 | – | 282 | 522.9249 | 1565.7529 | 1565.7508 | 1.32 | 1 | 47 | 3.6e-05 | 1Score **> 33** indicates **identity** Score **> 15** indicates **homology** | U | R.CKDVLTGQEFDVR.A |
| 73038 | 270 | – | 282 | 522.9249 | 1565.7529 | 1565.7508 | 1.34 | 1 | 61 | 2.1e-06 | 1Score **> 33** indicates **identity** Score **> 16** indicates **homology** | U | R.CKDVLTGQEFDVR.A |
| 73039 | 270 | – | 282 | 522.9254 | 1565.7542 | 1565.7508 | 2.19 | 1 | 31 | 0.0012 | 1Score **> 33** indicates **identity** Score **> 14** indicates **homology** | U | R.CKDVLTGQEFDVR.A |
| 39798 | 272 | – | 282 | 639.8197 | 1277.6248 | 1277.6252 | -0.31 | 0 | 73 | 3.6e-07 | 1Score **> 33** indicates **identity** Score **> 21** indicates **homology** | U | K.DVLTGQEFDVR.A |
| 39799 | 272 | – | 282 | 639.8198 | 1277.6251 | 1277.6252 | -0.088 | 0 | 76 | 2.2e-07 | 1Score **> 33** indicates **identity** Score **> 22** indicates **homology** | U | K.DVLTGQEFDVR.A |
| 39801 | 272 | – | 282 | 639.8202 | 1277.6258 | 1277.6252 | 0.49 | 0 | 55 | 1.2e-05 | 1Score **> 33** indicates **identity** Score **> 18** indicates **homology** | U | K.DVLTGQEFDVR.A |
| 39807 | 272 | – | 282 | 639.8218 | 1277.6290 | 1277.6252 | 2.98 | 0 | 27 | 0.003 | 1Score **> 33** indicates **identity** Score **> 14** indicates **homology** | U | K.DVLTGQEFDVR.A |
| 39818 | 272 | – | 282 | 639.8235 | 1277.6324 | 1277.6252 | 5.67 | 0 | 27 | 0.0028 | 1Score **> 33** indicates **identity** Score **> 14** indicates **homology** | U | K.DVLTGQEFDVR.A |
| 94112 | 283 | – | 298 | 574.6260 | 1720.8563 | 1720.8567 | -0.22 | 1 | 62 | 1.6e-06 | 1Score **> 35** indicates **identity** Score **> 16** indicates **homology** | U | R.AKCVINASGPFTDSVR.K |
| 94113 | 283 | – | 298 | 861.4357 | 1720.8569 | 1720.8567 | 0.12 | 1 | 84 | 1.4e-08 | 1Score **> 35** indicates **identity** Score **> 18** indicates **homology** | U | R.AKCVINASGPFTDSVR.K |
| 94114 | 283 | – | 298 | 574.6263 | 1720.8572 | 1720.8567 | 0.30 | 1 | 65 | 8.9e-07 | 1Score **> 35** indicates **identity** Score **> 17** indicates **homology** | U | R.AKCVINASGPFTDSVR.K |
| 94116 | 283 | – | 298 | 574.6266 | 1720.8581 | 1720.8567 | 0.81 | 1 | 15 | 0.037 | 1Score **> 35** indicates **identity** Score **> 13** indicates **homology** | U | R.AKCVINASGPFTDSVR.K |
| 94118 | 283 | – | 298 | 861.4368 | 1720.8590 | 1720.8567 | 1.34 | 1 | 64 | 1e-06 | 1Score **> 35** indicates **identity** Score **> 16** indicates **homology** | U | R.AKCVINASGPFTDSVR.K |
| 94122 | 283 | – | 298 | 861.4392 | 1720.8638 | 1720.8567 | 4.18 | 1 | 40 | 0.00018 | 1Score **> 35** indicates **identity** Score **> 15** indicates **homology** | U | R.AKCVINASGPFTDSVR.K |
| 67080 | 285 | – | 298 | 761.8651 | 1521.7156 | 1521.7246 | -5.93 | 0 | 49 | 2.6e-05 | 1Score **> 32** indicates **identity** Score **> 16** indicates **homology** | U | K.CVINASGPFTDSVR.K |
| 67081 | 285 | – | 298 | 761.8653 | 1521.7160 | 1521.7246 | -5.68 | 0 | 74 | 1e-07 | 1Score **> 32** indicates **identity** Score **> 17** indicates **homology** | U | K.CVINASGPFTDSVR.K |
| 67084 | 285 | – | 298 | 761.8658 | 1521.7171 | 1521.7246 | -4.93 | 0 | 70 | 2.7e-07 | 1Score **> 32** indicates **identity** Score **> 17** indicates **homology** | U | K.CVINASGPFTDSVR.K |
| 67085 | 285 | – | 298 | 761.8670 | 1521.7195 | 1521.7246 | -3.32 | 0 | 74 | 1.9e-07 | 1Score **> 32** indicates **identity** Score **> 19** indicates **homology** | U | K.CVINASGPFTDSVR.K |
| 67086 | 285 | – | 298 | 761.8684 | 1521.7222 | 1521.7246 | -1.55 | 0 | 60 | 2.3e-06 | 1Score **> 32** indicates **identity** Score **> 16** indicates **homology** | U | K.CVINASGPFTDSVR.K |
| 67087 | 285 | – | 298 | 761.8684 | 1521.7222 | 1521.7246 | -1.55 | 0 | 63 | 1.4e-06 | 1Score **> 32** indicates **identity** Score **> 17** indicates **homology** | U | K.CVINASGPFTDSVR.K |
| 67088 | 285 | – | 298 | 761.8688 | 1521.7230 | 1521.7246 | -1.08 | 0 | 94 | 6.2e-09 | 1Score **> 32** indicates **identity** Score **> 25** indicates **homology** | U | K.CVINASGPFTDSVR.K |
| 67089 | 285 | – | 298 | 761.8688 | 1521.7230 | 1521.7246 | -1.07 | 0 | 50 | 2.2e-05 | 1Score **> 32** indicates **identity** Score **> 16** indicates **homology** | U | K.CVINASGPFTDSVR.K |
| 67090 | 285 | – | 298 | 761.8693 | 1521.7240 | 1521.7246 | -0.41 | 0 | 89 | 7.2e-09 | 1Score **> 32** indicates **identity** Score **> 21** indicates **homology** | U | K.CVINASGPFTDSVR.K |
| 67091 | 285 | – | 298 | 761.8693 | 1521.7240 | 1521.7246 | -0.36 | 0 | 70 | 2.7e-07 | 1Score **> 32** indicates **identity** Score **> 17** indicates **homology** | U | K.CVINASGPFTDSVR.K |
| 67092 | 285 | – | 298 | 761.8693 | 1521.7241 | 1521.7246 | -0.34 | 0 | 85 | 1.9e-08 | 1Score **> 32** indicates **identity** Score **> 21** indicates **homology** | U | K.CVINASGPFTDSVR.K |
| 67094 | 285 | – | 298 | 761.8695 | 1521.7245 | 1521.7246 | -0.043 | 0 | 99 | 2.3e-09 | 1Score **> 32** indicates **identity** Score **> 25** indicates **homology** | U | K.CVINASGPFTDSVR.K |
| 67097 | 285 | – | 298 | 761.8697 | 1521.7248 | 1521.7246 | 0.12 | 0 | 42 | 0.00012 | 1Score **> 32** indicates **identity** Score **> 15** indicates **homology** | U | K.CVINASGPFTDSVR.K |
| 67098 | 285 | – | 298 | 761.8698 | 1521.7250 | 1521.7246 | 0.29 | 0 | 96 | 3.3e-09 | 1Score **> 32** indicates **identity** Score **> 24** indicates **homology** | U | K.CVINASGPFTDSVR.K |
| 67099 | 285 | – | 298 | 761.8701 | 1521.7256 | 1521.7246 | 0.68 | 0 | 83 | 2.7e-08 | 1Score **> 32** indicates **identity** Score **> 20** indicates **homology** | U | K.CVINASGPFTDSVR.K |
| 67103 | 285 | – | 298 | 761.8707 | 1521.7268 | 1521.7246 | 1.43 | 0 | 28 | 0.0026 | 1Score **> 33** indicates **identity** Score **> 14** indicates **homology** | U | K.CVINASGPFTDSVR.K |
| 67106 | 285 | – | 298 | 761.8716 | 1521.7286 | 1521.7246 | 2.62 | 0 | 56 | 5.7e-06 | 1Score **> 33** indicates **identity** Score **> 16** indicates **homology** | U | K.CVINASGPFTDSVR.K |
| 67107 | 285 | – | 298 | 761.8716 | 1521.7287 | 1521.7246 | 2.68 | 0 | 49 | 2.5e-05 | 1Score **> 33** indicates **identity** Score **> 16** indicates **homology** | U | K.CVINASGPFTDSVR.K |
| 67108 | 285 | – | 298 | 761.8723 | 1521.7301 | 1521.7246 | 3.65 | 0 | 42 | 0.00013 | 1Score **> 33** indicates **identity** Score **> 16** indicates **homology** | U | K.CVINASGPFTDSVR.K |
| 67112 | 285 | – | 298 | 761.8738 | 1521.7331 | 1521.7246 | 5.59 | 0 | 44 | 0.00011 | 1Score **> 33** indicates **identity** Score **> 16** indicates **homology** | U | K.CVINASGPFTDSVR.K |
| 67219 | 285 | – | 298 | 762.3689 | 1522.7233 | 1522.7086 | 9.67 | 0 | 41 | 0.00014 | 1Score **> 33** indicates **identity** Score **> 15** indicates **homology** | U | K.CVINASGPFTDSVR.K  + Deamidated (NQ) |
| 84773 | 285 | – | 299 | 825.9122 | 1649.8099 | 1649.8196 | -5.82 | 1 | 19 | 0.016 | 1Score **> 34** indicates **identity** Score **> 14** indicates **homology** | U | K.CVINASGPFTDSVRK.M |
| 84774 | 285 | – | 299 | 550.9442 | 1649.8107 | 1649.8196 | -5.34 | 1 | 34 | 0.00067 | 1Score **> 34** indicates **identity** Score **> 15** indicates **homology** | U | K.CVINASGPFTDSVRK.M |
| 84775 | 285 | – | 299 | 550.9462 | 1649.8166 | 1649.8196 | -1.77 | 1 | 34 | 0.00062 | 1Score **> 34** indicates **identity** Score **> 15** indicates **homology** | U | K.CVINASGPFTDSVRK.M |
| 84776 | 285 | – | 299 | 550.9464 | 1649.8174 | 1649.8196 | -1.30 | 1 | 59 | 2.8e-06 | 1Score **> 34** indicates **identity** Score **> 16** indicates **homology** | U | K.CVINASGPFTDSVRK.M |
| 84777 | 285 | – | 299 | 550.9467 | 1649.8181 | 1649.8196 | -0.86 | 1 | 45 | 6.1e-05 | 1Score **> 34** indicates **identity** Score **> 15** indicates **homology** | U | K.CVINASGPFTDSVRK.M |
| 29182 | 340 | – | 348 | 589.8334 | 1177.6523 | 1177.6536 | -1.09 | 0 | 35 | 0.0034 | 1Score **> 32** indicates **identity** Score **> 23** indicates **homology** | U | R.VIFFLPWEK.M |
| 29185 | 340 | – | 348 | 589.8336 | 1177.6526 | 1177.6536 | -0.79 | 0 | 21 | 0.012 | 1Score **> 32** indicates **identity** Score **> 14** indicates **homology** | U | R.VIFFLPWEK.M |
| 29190 | 340 | – | 348 | 589.8339 | 1177.6533 | 1177.6536 | -0.26 | 0 | 31 | 0.0044 | 1Score **> 32** indicates **identity** Score **> 20** indicates **homology** | U | R.VIFFLPWEK.M |
| 29192 | 340 | – | 348 | 589.8340 | 1177.6534 | 1177.6536 | -0.13 | 0 | 26 | 0.0038 | 1Score **> 32** indicates **identity** Score **> 14** indicates **homology** | U | R.VIFFLPWEK.M |
| 29193 | 340 | – | 348 | 589.8340 | 1177.6535 | 1177.6536 | -0.058 | 0 | 24 | 0.0055 | 1Score **> 32** indicates **identity** Score **> 14** indicates **homology** | U | R.VIFFLPWEK.M |
| 29195 | 340 | – | 348 | 589.8342 | 1177.6539 | 1177.6536 | 0.27 | 0 | 25 | 0.0041 | 1Score **> 32** indicates **identity** Score **> 14** indicates **homology** | U | R.VIFFLPWEK.M |
| 29196 | 340 | – | 348 | 589.8343 | 1177.6540 | 1177.6536 | 0.35 | 0 | 26 | 0.0051 | 1Score **> 32** indicates **identity** Score **> 15** indicates **homology** | U | R.VIFFLPWEK.M |
| 29198 | 340 | – | 348 | 589.8344 | 1177.6542 | 1177.6536 | 0.55 | 0 | 23 | 0.0063 | 1Score **> 32** indicates **identity** Score **> 14** indicates **homology** | U | R.VIFFLPWEK.M |
| 29203 | 340 | – | 348 | 589.8348 | 1177.6550 | 1177.6536 | 1.20 | 0 | 21 | 0.01 | 1Score **> 32** indicates **identity** Score **> 14** indicates **homology** | U | R.VIFFLPWEK.M |
| 29204 | 340 | – | 348 | 589.8348 | 1177.6550 | 1177.6536 | 1.25 | 0 | 40 | 0.00032 | 1Score **> 32** indicates **identity** Score **> 18** indicates **homology** | U | R.VIFFLPWEK.M |
| 29209 | 340 | – | 348 | 589.8350 | 1177.6555 | 1177.6536 | 1.61 | 0 | 21 | 0.011 | 1Score **> 32** indicates **identity** Score **> 14** indicates **homology** | U | R.VIFFLPWEK.M |
| 29210 | 340 | – | 348 | 589.8350 | 1177.6555 | 1177.6536 | 1.63 | 0 | 26 | 0.0036 | 1Score **> 32** indicates **identity** Score **> 14** indicates **homology** | U | R.VIFFLPWEK.M |
| 29211 | 340 | – | 348 | 589.8352 | 1177.6558 | 1177.6536 | 1.92 | 0 | 16 | 0.029 | 1Score **> 32** indicates **identity** Score **> 14** indicates **homology** | U | R.VIFFLPWEK.M |
| 29212 | 340 | – | 348 | 589.8352 | 1177.6559 | 1177.6536 | 2.01 | 0 | 16 | 0.035 | 1Score **> 32** indicates **identity** Score **> 14** indicates **homology** | U | R.VIFFLPWEK.M |
| 29213 | 340 | – | 348 | 589.8353 | 1177.6560 | 1177.6536 | 2.04 | 0 | 32 | 0.0043 | 1Score **> 32** indicates **identity** Score **> 20** indicates **homology** | U | R.VIFFLPWEK.M |
| 29214 | 340 | – | 348 | 589.8353 | 1177.6560 | 1177.6536 | 2.11 | 0 | 20 | 0.012 | 1Score **> 32** indicates **identity** Score **> 14** indicates **homology** | U | R.VIFFLPWEK.M |
| 29215 | 340 | – | 348 | 589.8353 | 1177.6561 | 1177.6536 | 2.16 | 0 | 34 | 0.0028 | 1Score **> 32** indicates **identity** Score **> 21** indicates **homology** | U | R.VIFFLPWEK.M |
| 29216 | 340 | – | 348 | 589.8354 | 1177.6562 | 1177.6536 | 2.23 | 0 | 33 | 0.0047 | 1Score **> 32** indicates **identity** Score **> 22** indicates **homology** | U | R.VIFFLPWEK.M |
| 29217 | 340 | – | 348 | 589.8354 | 1177.6563 | 1177.6536 | 2.31 | 0 | 18 | 0.019 | 1Score **> 32** indicates **identity** Score **> 14** indicates **homology** | U | R.VIFFLPWEK.M |
| 29218 | 340 | – | 348 | 589.8354 | 1177.6563 | 1177.6536 | 2.32 | 0 | 36 | 0.0033 | 1Score **> 32** indicates **identity** Score **> 24** indicates **homology** | U | R.VIFFLPWEK.M |
| 29221 | 340 | – | 348 | 589.8358 | 1177.6570 | 1177.6536 | 2.94 | 0 | 25 | 0.0047 | 1Score **> 32** indicates **identity** Score **> 14** indicates **homology** | U | R.VIFFLPWEK.M |
| 29222 | 340 | – | 348 | 589.8359 | 1177.6572 | 1177.6536 | 3.07 | 0 | 14 | 0.046 | 1Score **> 32** indicates **identity** Score **> 13** indicates **homology** | U | R.VIFFLPWEK.M |
| 29223 | 340 | – | 348 | 589.8360 | 1177.6574 | 1177.6536 | 3.26 | 0 | 24 | 0.0076 | 1Score **> 31** indicates **identity** Score **> 15** indicates **homology** | U | R.VIFFLPWEK.M |
| 29228 | 340 | – | 348 | 589.8364 | 1177.6583 | 1177.6536 | 4.02 | 0 | 37 | 0.0019 | 1Score **> 31** indicates **identity** Score **> 22** indicates **homology** | U | R.VIFFLPWEK.M |
| 29230 | 340 | – | 348 | 589.8370 | 1177.6594 | 1177.6536 | 4.94 | 0 | 18 | 0.022 | 1Score **> 32** indicates **identity** Score **> 14** indicates **homology** | U | R.VIFFLPWEK.M |
| 29232 | 340 | – | 348 | 589.8372 | 1177.6598 | 1177.6536 | 5.25 | 0 | 21 | 0.012 | 1Score **> 32** indicates **identity** Score **> 14** indicates **homology** | U | R.VIFFLPWEK.M |
| 29234 | 340 | – | 348 | 589.8386 | 1177.6627 | 1177.6536 | 7.75 | 0 | 18 | 0.021 | 1Score **> 32** indicates **identity** Score **> 14** indicates **homology** | U | R.VIFFLPWEK.M |
| 191612 | 349 | – | 380 | 1188.5829 | 3562.7270 | 3562.7250 | 0.56 | 0 | 21 | 0.011 | 1Score **> 37** indicates **identity** Score **> 14** indicates **homology** | U | K.MTIAGTTDTPTDVTHHPIPSEEDINFILNEVR.N |
| 191613 | 349 | – | 380 | 891.6899 | 3562.7304 | 3562.7250 | 1.52 | 0 | 32 | 0.00091 | 1Score **> 37** indicates **identity** Score **> 15** indicates **homology** | U | K.MTIAGTTDTPTDVTHHPIPSEEDINFILNEVR.N |
| 191614 | 349 | – | 380 | 1188.5846 | 3562.7319 | 3562.7250 | 1.93 | 0 | 26 | 0.0038 | 1Score **> 37** indicates **identity** Score **> 14** indicates **homology** | U | K.MTIAGTTDTPTDVTHHPIPSEEDINFILNEVR.N |
| 191615 | 349 | – | 380 | 1188.5859 | 3562.7360 | 3562.7250 | 3.09 | 0 | 39 | 0.00022 | 1Score **> 37** indicates **identity** Score **> 15** indicates **homology** | U | K.MTIAGTTDTPTDVTHHPIPSEEDINFILNEVR.N |
| 191621 | 349 | – | 380 | 1188.9208 | 3563.7405 | 3563.7090 | 8.83 | 0 | 35 | 0.0005 | 1Score **> 37** indicates **identity** Score **> 15** indicates **homology** | U | K.MTIAGTTDTPTDVTHHPIPSEEDINFILNEVR.N  + Deamidated (NQ) |
| 29428 | 381 | – | 390 | 591.2925 | 1180.5705 | 1180.5724 | -1.65 | 0 | 14 | 0.048 | 1Score **> 31** indicates **identity** Score **> 13** indicates **homology** | U | R.NYLSSDVEVR.R |
| 29429 | 381 | – | 390 | 591.2928 | 1180.5711 | 1180.5724 | -1.10 | 0 | 57 | 4.9e-06 | 1Score **> 31** indicates **identity** Score **> 16** indicates **homology** | U | R.NYLSSDVEVR.R |
| 29431 | 381 | – | 390 | 591.2929 | 1180.5713 | 1180.5724 | -0.97 | 0 | 48 | 3e-05 | 1Score **> 31** indicates **identity** Score **> 16** indicates **homology** | U | R.NYLSSDVEVR.R |
| 29432 | 381 | – | 390 | 591.2929 | 1180.5713 | 1180.5724 | -0.90 | 0 | 58 | 3.3e-06 | 1Score **> 31** indicates **identity** Score **> 16** indicates **homology** | U | R.NYLSSDVEVR.R |
| 29433 | 381 | – | 390 | 591.2930 | 1180.5714 | 1180.5724 | -0.83 | 0 | 46 | 4.8e-05 | 1Score **> 32** indicates **identity** Score **> 15** indicates **homology** | U | R.NYLSSDVEVR.R |
| 29435 | 381 | – | 390 | 591.2933 | 1180.5720 | 1180.5724 | -0.34 | 0 | 56 | 5.2e-06 | 1Score **> 32** indicates **identity** Score **> 16** indicates **homology** | U | R.NYLSSDVEVR.R |
| 29436 | 381 | – | 390 | 591.2933 | 1180.5721 | 1180.5724 | -0.25 | 0 | 57 | 1.2e-05 | 1Score **> 32** indicates **identity** Score **> 20** indicates **homology** | U | R.NYLSSDVEVR.R |
| 29437 | 381 | – | 390 | 591.2936 | 1180.5726 | 1180.5724 | 0.15 | 0 | 57 | 4.2e-06 | 1Score **> 32** indicates **identity** Score **> 16** indicates **homology** | U | R.NYLSSDVEVR.R |
| 29439 | 381 | – | 390 | 591.2938 | 1180.5730 | 1180.5724 | 0.47 | 0 | 49 | 2.8e-05 | 1Score **> 32** indicates **identity** Score **> 16** indicates **homology** | U | R.NYLSSDVEVR.R |
| 29440 | 381 | – | 390 | 591.2938 | 1180.5730 | 1180.5724 | 0.53 | 0 | 20 | 0.015 | 1Score **> 32** indicates **identity** Score **> 14** indicates **homology** | U | R.NYLSSDVEVR.R |
| 29441 | 381 | – | 390 | 591.2938 | 1180.5731 | 1180.5724 | 0.58 | 0 | 54 | 9.5e-06 | 1Score **> 32** indicates **identity** Score **> 16** indicates **homology** | U | R.NYLSSDVEVR.R |
| 29442 | 381 | – | 390 | 591.2940 | 1180.5734 | 1180.5724 | 0.86 | 0 | 54 | 9.6e-06 | 1Score **> 32** indicates **identity** Score **> 16** indicates **homology** | U | R.NYLSSDVEVR.R |
| 29444 | 381 | – | 390 | 591.2943 | 1180.5741 | 1180.5724 | 1.47 | 0 | 57 | 4.8e-06 | 1Score **> 31** indicates **identity** Score **> 16** indicates **homology** | U | R.NYLSSDVEVR.R |
| 29445 | 381 | – | 390 | 591.2944 | 1180.5743 | 1180.5724 | 1.61 | 0 | 36 | 0.00041 | 1Score **> 31** indicates **identity** Score **> 15** indicates **homology** | U | R.NYLSSDVEVR.R |
| 29448 | 381 | – | 390 | 591.2947 | 1180.5748 | 1180.5724 | 2.02 | 0 | 26 | 0.0036 | 1Score **> 32** indicates **identity** Score **> 14** indicates **homology** | U | R.NYLSSDVEVR.R |
| 29452 | 381 | – | 390 | 591.2950 | 1180.5755 | 1180.5724 | 2.62 | 0 | 44 | 7.4e-05 | 1Score **> 32** indicates **identity** Score **> 15** indicates **homology** | U | R.NYLSSDVEVR.R |
| 29455 | 381 | – | 390 | 591.2954 | 1180.5762 | 1180.5724 | 3.25 | 0 | 31 | 0.0012 | 1Score **> 32** indicates **identity** Score **> 14** indicates **homology** | U | R.NYLSSDVEVR.R |
| 29458 | 381 | – | 390 | 591.2957 | 1180.5769 | 1180.5724 | 3.79 | 0 | 25 | 0.0068 | 1Score **> 32** indicates **identity** Score **> 16** indicates **homology** | U | R.NYLSSDVEVR.R |
| 29465 | 381 | – | 390 | 591.2961 | 1180.5777 | 1180.5724 | 4.49 | 0 | 26 | 0.022 | 1Score **> 31** indicates **identity** Score **> 22** indicates **homology** | U | R.NYLSSDVEVR.R |
| 29466 | 381 | – | 390 | 591.2964 | 1180.5782 | 1180.5724 | 4.87 | 0 | 24 | 0.0089 | 1Score **> 31** indicates **identity** Score **> 16** indicates **homology** | U | R.NYLSSDVEVR.R |
| 29467 | 381 | – | 390 | 591.2964 | 1180.5782 | 1180.5724 | 4.88 | 0 | 27 | 0.003 | 1Score **> 31** indicates **identity** Score **> 14** indicates **homology** | U | R.NYLSSDVEVR.R |
| 29469 | 381 | – | 390 | 591.2964 | 1180.5783 | 1180.5724 | 4.97 | 0 | 28 | 0.0022 | 1Score **> 31** indicates **identity** Score **> 14** indicates **homology** | U | R.NYLSSDVEVR.R |
| 29494 | 381 | – | 390 | 591.2984 | 1180.5822 | 1180.5724 | 8.30 | 0 | 16 | 0.03 | 1Score **> 32** indicates **identity** Score **> 14** indicates **homology** | U | R.NYLSSDVEVR.R |
| 29569 | 381 | – | 390 | 591.7901 | 1181.5656 | 1181.5564 | 7.78 | 0 | 17 | 0.027 | 1Score **> 31** indicates **identity** Score **> 14** indicates **homology** | U | R.NYLSSDVEVR.R  + Deamidated (NQ) |
| 45963 | 381 | – | 391 | 669.3422 | 1336.6698 | 1336.6735 | -2.79 | 1 | 22 | 0.021 | 1Score **> 33** indicates **identity** Score **> 17** indicates **homology** | U | R.NYLSSDVEVRR.G |
| 45965 | 381 | – | 391 | 446.5642 | 1336.6709 | 1336.6735 | -1.97 | 1 | 24 | 0.0059 | 1Score **> 33** indicates **identity** Score **> 14** indicates **homology** | U | R.NYLSSDVEVRR.G |
| 45967 | 381 | – | 391 | 446.5644 | 1336.6714 | 1336.6735 | -1.61 | 1 | 18 | 0.02 | 1Score **> 33** indicates **identity** Score **> 14** indicates **homology** | U | R.NYLSSDVEVRR.G |
| 45969 | 381 | – | 391 | 669.3442 | 1336.6738 | 1336.6735 | 0.24 | 1 | 28 | 0.0078 | 1Score **> 33** indicates **identity** Score **> 19** indicates **homology** | U | R.NYLSSDVEVRR.G |
| 42099 | 391 | – | 402 | 434.2414 | 1299.7023 | 1299.7048 | -1.92 | 1 | 34 | 0.00071 | 1Score **> 35** indicates **identity** Score **> 15** indicates **homology** | U | R.RGDVLAAWSGIR.P |
| 42101 | 391 | – | 402 | 434.2424 | 1299.7053 | 1299.7048 | 0.41 | 1 | 38 | 0.00028 | 1Score **> 34** indicates **identity** Score **> 15** indicates **homology** | U | R.RGDVLAAWSGIR.P |
| 128230 | 391 | – | 409 | 513.5403 | 2050.1323 | 2050.1324 | -0.040 | 2 | 24 | 0.012 | 1Score **> 35** indicates **identity** Score **> 17** indicates **homology** | U | R.RGDVLAAWSGIRPLVTDPK.S |
| 128232 | 391 | – | 409 | 513.5404 | 2050.1325 | 2050.1324 | 0.084 | 2 | 24 | 0.012 | 1Score **> 35** indicates **identity** Score **> 17** indicates **homology** | U | R.RGDVLAAWSGIRPLVTDPK.S |
| 128235 | 391 | – | 409 | 513.5405 | 2050.1330 | 2050.1324 | 0.31 | 2 | 26 | 0.0083 | 1Score **> 35** indicates **identity** Score **> 18** indicates **homology** | U | R.RGDVLAAWSGIRPLVTDPK.S |
| 128236 | 391 | – | 409 | 684.3849 | 2050.1330 | 2050.1324 | 0.31 | 2 | 41 | 0.00023 | 1Score **> 35** indicates **identity** Score **> 17** indicates **homology** | U | R.RGDVLAAWSGIRPLVTDPK.S |
| 128240 | 391 | – | 409 | 513.5406 | 2050.1334 | 2050.1324 | 0.49 | 2 | 20 | 0.033 | 1Score **> 35** indicates **identity** Score **> 18** indicates **homology** | U | R.RGDVLAAWSGIRPLVTDPK.S |
| 128241 | 391 | – | 409 | 684.3851 | 2050.1334 | 2050.1324 | 0.49 | 2 | 36 | 0.00083 | 1Score **> 35** indicates **identity** Score **> 17** indicates **homology** | U | R.RGDVLAAWSGIRPLVTDPK.S |
| 128242 | 391 | – | 409 | 684.3851 | 2050.1336 | 2050.1324 | 0.61 | 2 | 37 | 0.00061 | 1Score **> 35** indicates **identity** Score **> 18** indicates **homology** | U | R.RGDVLAAWSGIRPLVTDPK.S |
| 128243 | 391 | – | 409 | 513.5407 | 2050.1336 | 2050.1324 | 0.62 | 2 | 26 | 0.022 | 1Score **> 35** indicates **identity** Score **> 22** indicates **homology** | U | R.RGDVLAAWSGIRPLVTDPK.S |
| 128244 | 391 | – | 409 | 684.3852 | 2050.1336 | 2050.1324 | 0.62 | 2 | 39 | 0.00045 | 1Score **> 35** indicates **identity** Score **> 18** indicates **homology** | U | R.RGDVLAAWSGIRPLVTDPK.S |
| 128245 | 391 | – | 409 | 513.5407 | 2050.1336 | 2050.1324 | 0.62 | 2 | 17 | 0.03 | 1Score **> 35** indicates **identity** Score **> 15** indicates **homology** | U | R.RGDVLAAWSGIRPLVTDPK.S |
| 128246 | 391 | – | 409 | 684.3852 | 2050.1337 | 2050.1324 | 0.65 | 2 | 34 | 0.00094 | 1Score **> 35** indicates **identity** Score **> 16** indicates **homology** | U | R.RGDVLAAWSGIRPLVTDPK.S |
| 128252 | 391 | – | 409 | 513.5408 | 2050.1340 | 2050.1324 | 0.80 | 2 | 21 | 0.014 | 1Score **> 34** indicates **identity** Score **> 15** indicates **homology** | U | R.RGDVLAAWSGIRPLVTDPK.S |
| 128253 | 391 | – | 409 | 684.3853 | 2050.1340 | 2050.1324 | 0.80 | 2 | 48 | 6.9e-05 | 1Score **> 34** indicates **identity** Score **> 19** indicates **homology** | U | R.RGDVLAAWSGIRPLVTDPK.S |
| 128254 | 391 | – | 409 | 513.5408 | 2050.1341 | 2050.1324 | 0.83 | 2 | 22 | 0.019 | 1Score **> 34** indicates **identity** Score **> 17** indicates **homology** | U | R.RGDVLAAWSGIRPLVTDPK.S |
| 128255 | 391 | – | 409 | 1026.0744 | 2050.1342 | 2050.1324 | 0.89 | 2 | 21 | 0.015 | 1Score **> 34** indicates **identity** Score **> 16** indicates **homology** | U | R.RGDVLAAWSGIRPLVTDPK.S |
| 128256 | 391 | – | 409 | 513.5410 | 2050.1347 | 2050.1324 | 1.16 | 2 | 19 | 0.038 | 1Score **> 34** indicates **identity** Score **> 18** indicates **homology** | U | R.RGDVLAAWSGIRPLVTDPK.S |
| 128257 | 391 | – | 409 | 1026.0748 | 2050.1350 | 2050.1324 | 1.30 | 2 | 35 | 0.00057 | 1Score **> 34** indicates **identity** Score **> 15** indicates **homology** | U | R.RGDVLAAWSGIRPLVTDPK.S |
| 128258 | 391 | – | 409 | 684.3856 | 2050.1351 | 2050.1324 | 1.35 | 2 | 21 | 0.012 | 1Score **> 34** indicates **identity** Score **> 15** indicates **homology** | U | R.RGDVLAAWSGIRPLVTDPK.S |
| 128259 | 391 | – | 409 | 684.3858 | 2050.1356 | 2050.1324 | 1.59 | 2 | 15 | 0.041 | 1Score **> 34** indicates **identity** Score **> 13** indicates **homology** | U | R.RGDVLAAWSGIRPLVTDPK.S |
| 128263 | 391 | – | 409 | 513.5416 | 2050.1371 | 2050.1324 | 2.33 | 2 | 24 | 0.0055 | 1Score **> 35** indicates **identity** Score **> 14** indicates **homology** | U | R.RGDVLAAWSGIRPLVTDPK.S |
| 128266 | 391 | – | 409 | 513.5418 | 2050.1382 | 2050.1324 | 2.85 | 2 | 23 | 0.013 | 1Score **> 34** indicates **identity** Score **> 17** indicates **homology** | U | R.RGDVLAAWSGIRPLVTDPK.S |
| 128267 | 391 | – | 409 | 513.5419 | 2050.1384 | 2050.1324 | 2.96 | 2 | 22 | 0.008 | 1Score **> 34** indicates **identity** Score **> 14** indicates **homology** | U | R.RGDVLAAWSGIRPLVTDPK.S |
| 112880 | 392 | – | 409 | 632.3516 | 1894.0329 | 1894.0312 | 0.86 | 1 | 23 | 0.0068 | 1Score **> 35** indicates **identity** Score **> 14** indicates **homology** | U | R.GDVLAAWSGIRPLVTDPK.S |
| 112882 | 392 | – | 409 | 948.0241 | 1894.0336 | 1894.0312 | 1.23 | 1 | 23 | 0.011 | 1Score **> 35** indicates **identity** Score **> 16** indicates **homology** | U | R.GDVLAAWSGIRPLVTDPK.S |
| 112885 | 392 | – | 409 | 632.3521 | 1894.0346 | 1894.0312 | 1.75 | 1 | 15 | 0.037 | 1Score **> 35** indicates **identity** Score **> 13** indicates **homology** | U | R.GDVLAAWSGIRPLVTDPK.S |
| 112888 | 392 | – | 409 | 948.0254 | 1894.0363 | 1894.0312 | 2.64 | 1 | 26 | 0.0034 | 1Score **> 35** indicates **identity** Score **> 14** indicates **homology** | U | R.GDVLAAWSGIRPLVTDPK.S |
| 102580 | 419 | – | 436 | 599.3199 | 1794.9380 | 1794.9476 | -5.36 | 0 | 21 | 0.012 | 1Score **> 36** indicates **identity** Score **> 14** indicates **homology** | U | R.NHVVDISDSGLITIAGGK.W |
| 102582 | 419 | – | 436 | 599.3202 | 1794.9389 | 1794.9476 | -4.84 | 0 | 57 | 4.3e-06 | 1Score **> 36** indicates **identity** Score **> 16** indicates **homology** | U | R.NHVVDISDSGLITIAGGK.W |
| 102584 | 419 | – | 436 | 599.3205 | 1794.9398 | 1794.9476 | -4.33 | 0 | 20 | 0.012 | 1Score **> 36** indicates **identity** Score **> 14** indicates **homology** | U | R.NHVVDISDSGLITIAGGK.W |
| 102589 | 419 | – | 436 | 599.3215 | 1794.9427 | 1794.9476 | -2.72 | 0 | 47 | 3.9e-05 | 1Score **> 36** indicates **identity** Score **> 15** indicates **homology** | U | R.NHVVDISDSGLITIAGGK.W |
| 102592 | 419 | – | 436 | 599.3217 | 1794.9434 | 1794.9476 | -2.32 | 0 | 61 | 1.8e-06 | 1Score **> 36** indicates **identity** Score **> 16** indicates **homology** | U | R.NHVVDISDSGLITIAGGK.W |
| 102593 | 419 | – | 436 | 599.3219 | 1794.9440 | 1794.9476 | -2.01 | 0 | 22 | 0.009 | 1Score **> 36** indicates **identity** Score **> 14** indicates **homology** | U | R.NHVVDISDSGLITIAGGK.W |
| 102596 | 419 | – | 436 | 599.3221 | 1794.9445 | 1794.9476 | -1.69 | 0 | 58 | 3.4e-06 | 1Score **> 35** indicates **identity** Score **> 16** indicates **homology** | U | R.NHVVDISDSGLITIAGGK.W |
| 102599 | 419 | – | 436 | 898.4801 | 1794.9456 | 1794.9476 | -1.12 | 0 | 46 | 4.7e-05 | 1Score **> 36** indicates **identity** Score **> 15** indicates **homology** | U | R.NHVVDISDSGLITIAGGK.W |
| 102600 | 419 | – | 436 | 599.3225 | 1794.9456 | 1794.9476 | -1.11 | 0 | 38 | 0.00029 | 1Score **> 36** indicates **identity** Score **> 15** indicates **homology** | U | R.NHVVDISDSGLITIAGGK.W |
| 102603 | 419 | – | 436 | 599.3227 | 1794.9462 | 1794.9476 | -0.79 | 0 | 61 | 1.9e-06 | 1Score **> 36** indicates **identity** Score **> 16** indicates **homology** | U | R.NHVVDISDSGLITIAGGK.W |
| 102604 | 419 | – | 436 | 599.3227 | 1794.9462 | 1794.9476 | -0.78 | 0 | 17 | 0.023 | 1Score **> 36** indicates **identity** Score **> 14** indicates **homology** | U | R.NHVVDISDSGLITIAGGK.W |
| 102605 | 419 | – | 436 | 898.4804 | 1794.9462 | 1794.9476 | -0.75 | 0 | 87 | 7.5e-09 | 1Score **> 36** indicates **identity** Score **> 18** indicates **homology** | U | R.NHVVDISDSGLITIAGGK.W |
| 102606 | 419 | – | 436 | 599.3228 | 1794.9464 | 1794.9476 | -0.65 | 0 | 24 | 0.0057 | 1Score **> 36** indicates **identity** Score **> 14** indicates **homology** | U | R.NHVVDISDSGLITIAGGK.W |
| 102609 | 419 | – | 436 | 599.3229 | 1794.9467 | 1794.9476 | -0.47 | 0 | 17 | 0.025 | 1Score **> 36** indicates **identity** Score **> 14** indicates **homology** | U | R.NHVVDISDSGLITIAGGK.W |
| 102612 | 419 | – | 436 | 599.3229 | 1794.9469 | 1794.9476 | -0.37 | 0 | 51 | 1.6e-05 | 1Score **> 36** indicates **identity** Score **> 16** indicates **homology** | U | R.NHVVDISDSGLITIAGGK.W |
| 102614 | 419 | – | 436 | 599.3229 | 1794.9470 | 1794.9476 | -0.34 | 0 | 80 | 3.3e-08 | 1Score **> 36** indicates **identity** Score **> 17** indicates **homology** | U | R.NHVVDISDSGLITIAGGK.W |
| 102615 | 419 | – | 436 | 898.4808 | 1794.9470 | 1794.9476 | -0.30 | 0 | 31 | 0.0014 | 1Score **> 36** indicates **identity** Score **> 14** indicates **homology** | U | R.NHVVDISDSGLITIAGGK.W |
| 102617 | 419 | – | 436 | 599.3230 | 1794.9471 | 1794.9476 | -0.25 | 0 | 78 | 5.3e-08 | 1Score **> 36** indicates **identity** Score **> 17** indicates **homology** | U | R.NHVVDISDSGLITIAGGK.W |
| 102619 | 419 | – | 436 | 599.3232 | 1794.9477 | 1794.9476 | 0.038 | 0 | 67 | 5.2e-07 | 1Score **> 36** indicates **identity** Score **> 17** indicates **homology** | U | R.NHVVDISDSGLITIAGGK.W |
| 102623 | 419 | – | 436 | 599.3232 | 1794.9479 | 1794.9476 | 0.18 | 0 | 67 | 5.5e-07 | 1Score **> 35** indicates **identity** Score **> 17** indicates **homology** | U | R.NHVVDISDSGLITIAGGK.W |
| 102624 | 419 | – | 436 | 898.4814 | 1794.9482 | 1794.9476 | 0.32 | 0 | 81 | 2.4e-08 | 1Score **> 35** indicates **identity** Score **> 18** indicates **homology** | U | R.NHVVDISDSGLITIAGGK.W |
| 102625 | 419 | – | 436 | 599.3234 | 1794.9482 | 1794.9476 | 0.37 | 0 | 65 | 7.7e-07 | 1Score **> 36** indicates **identity** Score **> 17** indicates **homology** | U | R.NHVVDISDSGLITIAGGK.W |
| 102626 | 419 | – | 436 | 599.3234 | 1794.9483 | 1794.9476 | 0.37 | 0 | 16 | 0.03 | 1Score **> 36** indicates **identity** Score **> 14** indicates **homology** | U | R.NHVVDISDSGLITIAGGK.W |
| 102627 | 419 | – | 436 | 599.3235 | 1794.9488 | 1794.9476 | 0.68 | 0 | 22 | 0.0095 | 1Score **> 36** indicates **identity** Score **> 14** indicates **homology** | U | R.NHVVDISDSGLITIAGGK.W |
| 102628 | 419 | – | 436 | 599.3236 | 1794.9490 | 1794.9476 | 0.78 | 0 | 77 | 5.5e-08 | 1Score **> 36** indicates **identity** Score **> 17** indicates **homology** | U | R.NHVVDISDSGLITIAGGK.W |
| 102630 | 419 | – | 436 | 599.3237 | 1794.9493 | 1794.9476 | 0.94 | 0 | 77 | 6.9e-08 | 1Score **> 36** indicates **identity** Score **> 18** indicates **homology** | U | R.NHVVDISDSGLITIAGGK.W |
| 102632 | 419 | – | 436 | 599.3238 | 1794.9495 | 1794.9476 | 1.05 | 0 | 19 | 0.022 | 1Score **> 36** indicates **identity** Score **> 15** indicates **homology** | U | R.NHVVDISDSGLITIAGGK.W |
| 102633 | 419 | – | 436 | 599.3238 | 1794.9495 | 1794.9476 | 1.08 | 0 | 75 | 9.6e-08 | 1Score **> 36** indicates **identity** Score **> 17** indicates **homology** | U | R.NHVVDISDSGLITIAGGK.W |
| 102634 | 419 | – | 436 | 599.3238 | 1794.9496 | 1794.9476 | 1.14 | 0 | 109 | 8.2e-11 | 1Score **> 36** indicates **identity** Score **> 21** indicates **homology** | U | R.NHVVDISDSGLITIAGGK.W |
| 102635 | 419 | – | 436 | 599.3238 | 1794.9497 | 1794.9476 | 1.18 | 0 | 95 | 1.4e-09 | 1Score **> 36** indicates **identity** Score **> 19** indicates **homology** | U | R.NHVVDISDSGLITIAGGK.W |
| 102637 | 419 | – | 436 | 599.3239 | 1794.9500 | 1794.9476 | 1.35 | 0 | 109 | 7.7e-11 | 1Score **> 36** indicates **identity** Score **> 20** indicates **homology** | U | R.NHVVDISDSGLITIAGGK.W |
| 102638 | 419 | – | 436 | 599.3240 | 1794.9500 | 1794.9476 | 1.37 | 0 | 32 | 0.0011 | 1Score **> 36** indicates **identity** Score **> 14** indicates **homology** | U | R.NHVVDISDSGLITIAGGK.W |
| 102639 | 419 | – | 436 | 898.4823 | 1794.9501 | 1794.9476 | 1.41 | 0 | 99 | 5.4e-10 | 1Score **> 36** indicates **identity** Score **> 19** indicates **homology** | U | R.NHVVDISDSGLITIAGGK.W |
| 102641 | 419 | – | 436 | 898.4825 | 1794.9504 | 1794.9476 | 1.60 | 0 | 94 | 1.5e-09 | 1Score **> 36** indicates **identity** Score **> 18** indicates **homology** | U | R.NHVVDISDSGLITIAGGK.W |
| 102642 | 419 | – | 436 | 599.3241 | 1794.9505 | 1794.9476 | 1.60 | 0 | 113 | 3.5e-11 | 1Score **> 36** indicates **identity** Score **> 21** indicates **homology** | U | R.NHVVDISDSGLITIAGGK.W |
| 102643 | 419 | – | 436 | 898.4826 | 1794.9506 | 1794.9476 | 1.66 | 0 | 92 | 2.5e-09 | 1Score **> 36** indicates **identity** Score **> 18** indicates **homology** | U | R.NHVVDISDSGLITIAGGK.W |
| 102645 | 419 | – | 436 | 599.3241 | 1794.9506 | 1794.9476 | 1.69 | 0 | 32 | 0.0011 | 1Score **> 36** indicates **identity** Score **> 14** indicates **homology** | U | R.NHVVDISDSGLITIAGGK.W |
| 102647 | 419 | – | 436 | 898.4826 | 1794.9507 | 1794.9476 | 1.75 | 0 | 45 | 5.4e-05 | 1Score **> 36** indicates **identity** Score **> 15** indicates **homology** | U | R.NHVVDISDSGLITIAGGK.W |
| 102648 | 419 | – | 436 | 599.3242 | 1794.9508 | 1794.9476 | 1.77 | 0 | 87 | 6.7e-09 | 1Score **> 36** indicates **identity** Score **> 18** indicates **homology** | U | R.NHVVDISDSGLITIAGGK.W |
| 102649 | 419 | – | 436 | 599.3242 | 1794.9508 | 1794.9476 | 1.77 | 0 | 89 | 5.4e-09 | 1Score **> 36** indicates **identity** Score **> 19** indicates **homology** | U | R.NHVVDISDSGLITIAGGK.W |
| 102650 | 419 | – | 436 | 599.3242 | 1794.9508 | 1794.9476 | 1.81 | 0 | 35 | 0.0005 | 1Score **> 36** indicates **identity** Score **> 15** indicates **homology** | U | R.NHVVDISDSGLITIAGGK.W |
| 102651 | 419 | – | 436 | 898.4827 | 1794.9509 | 1794.9476 | 1.85 | 0 | 36 | 0.00038 | 1Score **> 36** indicates **identity** Score **> 15** indicates **homology** | U | R.NHVVDISDSGLITIAGGK.W |
| 102652 | 419 | – | 436 | 599.3243 | 1794.9509 | 1794.9476 | 1.86 | 0 | 88 | 5.7e-09 | 1Score **> 36** indicates **identity** Score **> 18** indicates **homology** | U | R.NHVVDISDSGLITIAGGK.W |
| 102653 | 419 | – | 436 | 599.3243 | 1794.9510 | 1794.9476 | 1.89 | 0 | 31 | 0.0013 | 1Score **> 36** indicates **identity** Score **> 14** indicates **homology** | U | R.NHVVDISDSGLITIAGGK.W |
| 102655 | 419 | – | 436 | 898.4828 | 1794.9511 | 1794.9476 | 1.97 | 0 | 82 | 2.2e-08 | 1Score **> 36** indicates **identity** Score **> 18** indicates **homology** | U | R.NHVVDISDSGLITIAGGK.W |
| 102656 | 419 | – | 436 | 898.4829 | 1794.9512 | 1794.9476 | 2.03 | 0 | 94 | 1.6e-09 | 1Score **> 36** indicates **identity** Score **> 18** indicates **homology** | U | R.NHVVDISDSGLITIAGGK.W |
| 102657 | 419 | – | 436 | 599.3244 | 1794.9513 | 1794.9476 | 2.05 | 0 | 30 | 0.0014 | 1Score **> 36** indicates **identity** Score **> 14** indicates **homology** | U | R.NHVVDISDSGLITIAGGK.W |
| 102658 | 419 | – | 436 | 599.3244 | 1794.9513 | 1794.9476 | 2.07 | 0 | 60 | 2.4e-06 | 1Score **> 36** indicates **identity** Score **> 16** indicates **homology** | U | R.NHVVDISDSGLITIAGGK.W |
| 102659 | 419 | – | 436 | 599.3244 | 1794.9513 | 1794.9476 | 2.09 | 0 | 25 | 0.0049 | 1Score **> 36** indicates **identity** Score **> 14** indicates **homology** | U | R.NHVVDISDSGLITIAGGK.W |
| 102663 | 419 | – | 436 | 898.4832 | 1794.9519 | 1794.9476 | 2.43 | 0 | 72 | 4.9e-07 | 1Score **> 35** indicates **identity** Score **> 22** indicates **homology** | U | R.NHVVDISDSGLITIAGGK.W |
| 102664 | 419 | – | 436 | 599.3247 | 1794.9524 | 1794.9476 | 2.69 | 0 | 57 | 4.1e-06 | 1Score **> 36** indicates **identity** Score **> 16** indicates **homology** | U | R.NHVVDISDSGLITIAGGK.W |
| 102666 | 419 | – | 436 | 599.3248 | 1794.9525 | 1794.9476 | 2.72 | 0 | 55 | 6.9e-06 | 1Score **> 36** indicates **identity** Score **> 16** indicates **homology** | U | R.NHVVDISDSGLITIAGGK.W |
| 102667 | 419 | – | 436 | 898.4838 | 1794.9531 | 1794.9476 | 3.07 | 0 | 77 | 6e-08 | 1Score **> 36** indicates **identity** Score **> 17** indicates **homology** | U | R.NHVVDISDSGLITIAGGK.W |
| 102668 | 419 | – | 436 | 599.3250 | 1794.9531 | 1794.9476 | 3.10 | 0 | 19 | 0.016 | 1Score **> 36** indicates **identity** Score **> 14** indicates **homology** | U | R.NHVVDISDSGLITIAGGK.W |
| 102669 | 419 | – | 436 | 599.3250 | 1794.9532 | 1794.9476 | 3.12 | 0 | 38 | 0.00025 | 1Score **> 36** indicates **identity** Score **> 15** indicates **homology** | U | R.NHVVDISDSGLITIAGGK.W |
| 102673 | 419 | – | 436 | 599.3253 | 1794.9540 | 1794.9476 | 3.60 | 0 | 71 | 2.4e-07 | 1Score **> 36** indicates **identity** Score **> 17** indicates **homology** | U | R.NHVVDISDSGLITIAGGK.W |
| 102674 | 419 | – | 436 | 599.3254 | 1794.9543 | 1794.9476 | 3.74 | 0 | 22 | 0.0086 | 1Score **> 35** indicates **identity** Score **> 14** indicates **homology** | U | R.NHVVDISDSGLITIAGGK.W |
| 102675 | 419 | – | 436 | 599.3257 | 1794.9554 | 1794.9476 | 4.36 | 0 | 35 | 0.00049 | 1Score **> 35** indicates **identity** Score **> 15** indicates **homology** | U | R.NHVVDISDSGLITIAGGK.W |
| 102676 | 419 | – | 436 | 599.3259 | 1794.9559 | 1794.9476 | 4.61 | 0 | 58 | 3.8e-06 | 1Score **> 35** indicates **identity** Score **> 16** indicates **homology** | U | R.NHVVDISDSGLITIAGGK.W |
| 102677 | 419 | – | 436 | 599.3259 | 1794.9559 | 1794.9476 | 4.62 | 0 | 57 | 4.7e-06 | 1Score **> 35** indicates **identity** Score **> 16** indicates **homology** | U | R.NHVVDISDSGLITIAGGK.W |
| 102679 | 419 | – | 436 | 599.3263 | 1794.9570 | 1794.9476 | 5.23 | 0 | 43 | 0.0001 | 1Score **> 35** indicates **identity** Score **> 15** indicates **homology** | U | R.NHVVDISDSGLITIAGGK.W |
| 102681 | 419 | – | 436 | 599.3266 | 1794.9581 | 1794.9476 | 5.84 | 0 | 56 | 5.2e-06 | 1Score **> 35** indicates **identity** Score **> 16** indicates **homology** | U | R.NHVVDISDSGLITIAGGK.W |
| 102682 | 419 | – | 436 | 599.3266 | 1794.9581 | 1794.9476 | 5.87 | 0 | 35 | 0.00049 | 1Score **> 35** indicates **identity** Score **> 15** indicates **homology** | U | R.NHVVDISDSGLITIAGGK.W |
| 102683 | 419 | – | 436 | 599.3270 | 1794.9591 | 1794.9476 | 6.39 | 0 | 37 | 0.00032 | 1Score **> 35** indicates **identity** Score **> 15** indicates **homology** | U | R.NHVVDISDSGLITIAGGK.W |
| 102684 | 419 | – | 436 | 599.3273 | 1794.9599 | 1794.9476 | 6.88 | 0 | 56 | 8.6e-06 | 1Score **> 35** indicates **identity** Score **> 18** indicates **homology** | U | R.NHVVDISDSGLITIAGGK.W |
| 102685 | 419 | – | 436 | 599.3275 | 1794.9607 | 1794.9476 | 7.31 | 0 | 21 | 0.01 | 1Score **> 35** indicates **identity** Score **> 14** indicates **homology** | U | R.NHVVDISDSGLITIAGGK.W |
| 102687 | 419 | – | 436 | 599.3276 | 1794.9609 | 1794.9476 | 7.44 | 0 | 16 | 0.029 | 1Score **> 35** indicates **identity** Score **> 14** indicates **homology** | U | R.NHVVDISDSGLITIAGGK.W |
| 102689 | 419 | – | 436 | 599.3276 | 1794.9610 | 1794.9476 | 7.50 | 0 | 23 | 0.0075 | 1Score **> 35** indicates **identity** Score **> 14** indicates **homology** | U | R.NHVVDISDSGLITIAGGK.W |
| 102690 | 419 | – | 436 | 599.3280 | 1794.9623 | 1794.9476 | 8.19 | 0 | 67 | 4.8e-07 | 1Score **> 35** indicates **identity** Score **> 17** indicates **homology** | U | R.NHVVDISDSGLITIAGGK.W |
| 102692 | 419 | – | 436 | 599.3287 | 1794.9642 | 1794.9476 | 9.25 | 0 | 26 | 0.0034 | 1Score **> 35** indicates **identity** Score **> 14** indicates **homology** | U | R.NHVVDISDSGLITIAGGK.W |
| 102745 | 419 | – | 436 | 599.6561 | 1795.9465 | 1795.9316 | 8.30 | 0 | 19 | 0.018 | 1Score **> 36** indicates **identity** Score **> 14** indicates **homology** | U | R.NHVVDISDSGLITIAGGK.W  + Deamidated (NQ) |
| 102750 | 419 | – | 436 | 599.6571 | 1795.9495 | 1795.9316 | 9.96 | 0 | 26 | 0.0038 | 1Score **> 35** indicates **identity** Score **> 14** indicates **homology** | U | R.NHVVDISDSGLITIAGGK.W  + Deamidated (NQ) |
| 161206 | 419 | – | 441 | 835.1001 | 2502.2784 | 2502.2867 | -3.32 | 1 | 20 | 0.013 | 1Score **> 37** indicates **identity** Score **> 14** indicates **homology** | U | R.NHVVDISDSGLITIAGGKWTTYR.S |
| 161210 | 419 | – | 441 | 835.1035 | 2502.2886 | 2502.2867 | 0.74 | 1 | 28 | 0.0025 | 1Score **> 37** indicates **identity** Score **> 14** indicates **homology** | U | R.NHVVDISDSGLITIAGGKWTTYR.S |
| 161211 | 419 | – | 441 | 835.1038 | 2502.2895 | 2502.2867 | 1.10 | 1 | 35 | 0.00051 | 1Score **> 37** indicates **identity** Score **> 15** indicates **homology** | U | R.NHVVDISDSGLITIAGGKWTTYR.S |
| 161212 | 419 | – | 441 | 626.5797 | 2502.2899 | 2502.2867 | 1.26 | 1 | 71 | 2.4e-07 | 1Score **> 37** indicates **identity** Score **> 17** indicates **homology** | U | R.NHVVDISDSGLITIAGGKWTTYR.S |
| 161213 | 419 | – | 441 | 835.1040 | 2502.2902 | 2502.2867 | 1.39 | 1 | 51 | 1.7e-05 | 1Score **> 37** indicates **identity** Score **> 16** indicates **homology** | U | R.NHVVDISDSGLITIAGGKWTTYR.S |
| 161214 | 419 | – | 441 | 835.1041 | 2502.2904 | 2502.2867 | 1.47 | 1 | 83 | 1.6e-08 | 1Score **> 37** indicates **identity** Score **> 18** indicates **homology** | U | R.NHVVDISDSGLITIAGGKWTTYR.S |
| 161215 | 419 | – | 441 | 626.5801 | 2502.2914 | 2502.2867 | 1.89 | 1 | 65 | 8.3e-07 | 1Score **> 37** indicates **identity** Score **> 17** indicates **homology** | U | R.NHVVDISDSGLITIAGGKWTTYR.S |
| 161216 | 419 | – | 441 | 835.1047 | 2502.2923 | 2502.2867 | 2.23 | 1 | 68 | 4e-07 | 1Score **> 37** indicates **identity** Score **> 17** indicates **homology** | U | R.NHVVDISDSGLITIAGGKWTTYR.S |
| 161217 | 419 | – | 441 | 835.1052 | 2502.2938 | 2502.2867 | 2.83 | 1 | 85 | 1.1e-08 | 1Score **> 37** indicates **identity** Score **> 18** indicates **homology** | U | R.NHVVDISDSGLITIAGGKWTTYR.S |
| 161218 | 419 | – | 441 | 626.5809 | 2502.2945 | 2502.2867 | 3.10 | 1 | 15 | 0.038 | 1Score **> 37** indicates **identity** Score **> 13** indicates **homology** | U | R.NHVVDISDSGLITIAGGKWTTYR.S |
| 161260 | 419 | – | 441 | 626.8301 | 2503.2912 | 2503.2707 | 8.17 | 1 | 15 | 0.035 | 1Score **> 37** indicates **identity** Score **> 13** indicates **homology** | U | R.NHVVDISDSGLITIAGGKWTTYR.S  + Deamidated (NQ) |
| 35215 | 442 | – | 453 | 618.7921 | 1235.5696 | 1235.5704 | -0.60 | 0 | 50 | 1.9e-05 | 1Score **> 30** indicates **identity** Score **> 16** indicates **homology** | U | R.SMAEDTVDAAVK.F |
| 35217 | 442 | – | 453 | 618.7925 | 1235.5704 | 1235.5704 | 0.0065 | 0 | 55 | 7.6e-06 | 1Score **> 30** indicates **identity** Score **> 16** indicates **homology** | U | R.SMAEDTVDAAVK.F |
| 151004 | 442 | – | 463 | 777.3762 | 2329.1068 | 2329.1121 | -2.27 | 1 | 42 | 0.00012 | 1Score **> 35** indicates **identity** Score **> 15** indicates **homology** | U | R.SMAEDTVDAAVKFHNLNAGPSR.T |
| 151005 | 442 | – | 463 | 583.2841 | 2329.1075 | 2329.1121 | -1.97 | 1 | 30 | 0.0015 | 1Score **> 35** indicates **identity** Score **> 14** indicates **homology** | U | R.SMAEDTVDAAVKFHNLNAGPSR.T |
| 151009 | 442 | – | 463 | 777.3770 | 2329.1092 | 2329.1121 | -1.25 | 1 | 37 | 0.00031 | 1Score **> 35** indicates **identity** Score **> 15** indicates **homology** | U | R.SMAEDTVDAAVKFHNLNAGPSR.T |
| 151013 | 442 | – | 463 | 777.3790 | 2329.1151 | 2329.1121 | 1.29 | 1 | 58 | 4e-06 | 1Score **> 35** indicates **identity** Score **> 16** indicates **homology** | U | R.SMAEDTVDAAVKFHNLNAGPSR.T |
| 151015 | 442 | – | 463 | 583.2862 | 2329.1157 | 2329.1121 | 1.56 | 1 | 48 | 3.5e-05 | 1Score **> 35** indicates **identity** Score **> 15** indicates **homology** | U | R.SMAEDTVDAAVKFHNLNAGPSR.T |
| 151016 | 442 | – | 463 | 777.3792 | 2329.1157 | 2329.1121 | 1.56 | 1 | 64 | 9.8e-07 | 1Score **> 35** indicates **identity** Score **> 17** indicates **homology** | U | R.SMAEDTVDAAVKFHNLNAGPSR.T |
| 22252 | 454 | – | 463 | 371.5245 | 1111.5517 | 1111.5523 | -0.53 | 0 | 21 | 0.012 | 1Score **> 31** indicates **identity** Score **> 14** indicates **homology** | U | K.FHNLNAGPSR.T |
| 22253 | 454 | – | 463 | 556.7832 | 1111.5519 | 1111.5523 | -0.34 | 0 | 37 | 0.00063 | 1Score **> 31** indicates **identity** Score **> 18** indicates **homology** | U | K.FHNLNAGPSR.T |
| 22254 | 454 | – | 463 | 371.5248 | 1111.5525 | 1111.5523 | 0.22 | 0 | 36 | 0.00043 | 1Score **> 31** indicates **identity** Score **> 15** indicates **homology** | U | K.FHNLNAGPSR.T |
| 22255 | 454 | – | 463 | 371.5249 | 1111.5527 | 1111.5523 | 0.41 | 0 | 51 | 1.7e-05 | 1Score **> 31** indicates **identity** Score **> 16** indicates **homology** | U | K.FHNLNAGPSR.T |
| 22256 | 454 | – | 463 | 371.5249 | 1111.5528 | 1111.5523 | 0.46 | 0 | 38 | 0.00026 | 1Score **> 31** indicates **identity** Score **> 15** indicates **homology** | U | K.FHNLNAGPSR.T |
| 22257 | 454 | – | 463 | 556.7839 | 1111.5532 | 1111.5523 | 0.86 | 0 | 34 | 0.0013 | 1Score **> 31** indicates **identity** Score **> 18** indicates **homology** | U | K.FHNLNAGPSR.T |
| 22258 | 454 | – | 463 | 556.7839 | 1111.5533 | 1111.5523 | 0.93 | 0 | 51 | 7.1e-05 | 1Score **> 31** indicates **identity** Score **> 22** indicates **homology** | U | K.FHNLNAGPSR.T |
| 22259 | 454 | – | 463 | 371.5251 | 1111.5535 | 1111.5523 | 1.07 | 0 | 32 | 0.00097 | 1Score **> 31** indicates **identity** Score **> 15** indicates **homology** | U | K.FHNLNAGPSR.T |
| 22260 | 454 | – | 463 | 556.7841 | 1111.5536 | 1111.5523 | 1.23 | 0 | 28 | 0.0031 | 1Score **> 31** indicates **identity** Score **> 16** indicates **homology** | U | K.FHNLNAGPSR.T |
| 22261 | 454 | – | 463 | 556.7841 | 1111.5537 | 1111.5523 | 1.31 | 0 | 33 | 0.0019 | 1Score **> 31** indicates **identity** Score **> 18** indicates **homology** | U | K.FHNLNAGPSR.T |
| 22309 | 454 | – | 463 | 557.2747 | 1112.5348 | 1112.5363 | -1.35 | 0 | 27 | 0.0029 | 1Score **> 29** indicates **identity** Score **> 14** indicates **homology** | U | K.FHNLNAGPSR.T  + Deamidated (NQ) |
| 22310 | 454 | – | 463 | 371.8524 | 1112.5354 | 1112.5363 | -0.79 | 0 | 17 | 0.026 | 1Score **> 30** indicates **identity** Score **> 14** indicates **homology** | U | K.FHNLNAGPSR.T  + Deamidated (NQ) |
| 14613 | 464 | – | 473 | 510.2989 | 1018.5832 | 1018.5811 | 1.98 | 0 | 29 | 0.002 | 1Score **> 32** indicates **identity** Score **> 14** indicates **homology** | U | R.TVGLFLQGGK.D |
| 14614 | 464 | – | 473 | 510.2993 | 1018.5841 | 1018.5811 | 2.94 | 0 | 49 | 2.7e-05 | 1Score **> 30** indicates **identity** Score **> 16** indicates **homology** | U | R.TVGLFLQGGK.D |
| 14615 | 464 | – | 473 | 510.2994 | 1018.5843 | 1018.5811 | 3.11 | 0 | 32 | 0.0011 | 1Score **> 30** indicates **identity** Score **> 14** indicates **homology** | U | R.TVGLFLQGGK.D |
| 137671 | 464 | – | 482 | 1076.0839 | 2150.1532 | 2150.1524 | 0.34 | 1 | 86 | 8.9e-09 | 1Score **> 36** indicates **identity** Score **> 18** indicates **homology** | U | R.TVGLFLQGGKDWSPTLYIR.L |
| 137672 | 464 | – | 482 | 1076.0841 | 2150.1537 | 2150.1524 | 0.58 | 1 | 80 | 2.8e-08 | 1Score **> 36** indicates **identity** Score **> 18** indicates **homology** | U | R.TVGLFLQGGKDWSPTLYIR.L |
| 137673 | 464 | – | 482 | 717.7253 | 2150.1541 | 2150.1524 | 0.79 | 1 | 77 | 5.7e-08 | 1Score **> 36** indicates **identity** Score **> 17** indicates **homology** | U | R.TVGLFLQGGKDWSPTLYIR.L |
| 137674 | 464 | – | 482 | 717.7254 | 2150.1544 | 2150.1524 | 0.92 | 1 | 74 | 1.2e-07 | 1Score **> 36** indicates **identity** Score **> 17** indicates **homology** | U | R.TVGLFLQGGKDWSPTLYIR.L |
| 137676 | 464 | – | 482 | 717.7256 | 2150.1549 | 2150.1524 | 1.16 | 1 | 77 | 5.5e-08 | 1Score **> 36** indicates **identity** Score **> 17** indicates **homology** | U | R.TVGLFLQGGKDWSPTLYIR.L |
| 137677 | 464 | – | 482 | 1076.0849 | 2150.1553 | 2150.1524 | 1.31 | 1 | 42 | 0.0001 | 1Score **> 36** indicates **identity** Score **> 15** indicates **homology** | U | R.TVGLFLQGGKDWSPTLYIR.L |
| 137678 | 464 | – | 482 | 1076.0850 | 2150.1554 | 2150.1524 | 1.36 | 1 | 65 | 8e-07 | 1Score **> 36** indicates **identity** Score **> 17** indicates **homology** | U | R.TVGLFLQGGKDWSPTLYIR.L |
| 137679 | 464 | – | 482 | 717.7258 | 2150.1555 | 2150.1524 | 1.42 | 1 | 20 | 0.012 | 1Score **> 36** indicates **identity** Score **> 14** indicates **homology** | U | R.TVGLFLQGGKDWSPTLYIR.L |
| 137680 | 464 | – | 482 | 717.7259 | 2150.1560 | 2150.1524 | 1.66 | 1 | 82 | 2e-08 | 1Score **> 36** indicates **identity** Score **> 18** indicates **homology** | U | R.TVGLFLQGGKDWSPTLYIR.L |
| 137681 | 464 | – | 482 | 1076.0855 | 2150.1565 | 2150.1524 | 1.89 | 1 | 86 | 9.3e-09 | 1Score **> 36** indicates **identity** Score **> 18** indicates **homology** | U | R.TVGLFLQGGKDWSPTLYIR.L |
| 137683 | 464 | – | 482 | 717.7263 | 2150.1571 | 2150.1524 | 2.16 | 1 | 65 | 8.8e-07 | 1Score **> 36** indicates **identity** Score **> 17** indicates **homology** | U | R.TVGLFLQGGKDWSPTLYIR.L |
| 137684 | 464 | – | 482 | 717.7271 | 2150.1596 | 2150.1524 | 3.33 | 1 | 61 | 1.7e-06 | 1Score **> 36** indicates **identity** Score **> 16** indicates **homology** | U | R.TVGLFLQGGKDWSPTLYIR.L |
| 26107 | 474 | – | 482 | 575.7988 | 1149.5831 | 1149.5818 | 1.09 | 0 | 35 | 0.00049 | 1Score **> 32** indicates **identity** Score **> 15** indicates **homology** | U | K.DWSPTLYIR.L |
| 26108 | 474 | – | 482 | 575.7989 | 1149.5832 | 1149.5818 | 1.16 | 0 | 33 | 0.00083 | 1Score **> 32** indicates **identity** Score **> 15** indicates **homology** | U | K.DWSPTLYIR.L |
| 26109 | 474 | – | 482 | 575.7990 | 1149.5835 | 1149.5818 | 1.40 | 0 | 32 | 0.00097 | 1Score **> 32** indicates **identity** Score **> 15** indicates **homology** | U | K.DWSPTLYIR.L |
| 113310 | 483 | – | 499 | 633.9937 | 1898.9592 | 1898.9738 | -7.65 | 0 | 53 | 1e-05 | 1Score **> 36** indicates **identity** Score **> 16** indicates **homology** | U | R.LVQDYGLESEVAQHLAK.T |
| 113314 | 483 | – | 499 | 633.9958 | 1898.9655 | 1898.9738 | -4.37 | 0 | 34 | 0.0007 | 1Score **> 35** indicates **identity** Score **> 15** indicates **homology** | U | R.LVQDYGLESEVAQHLAK.T |
| 113319 | 483 | – | 499 | 633.9980 | 1898.9721 | 1898.9738 | -0.88 | 0 | 91 | 2.9e-09 | 1Score **> 35** indicates **identity** Score **> 18** indicates **homology** | U | R.LVQDYGLESEVAQHLAK.T |
| 113320 | 483 | – | 499 | 950.4943 | 1898.9741 | 1898.9738 | 0.15 | 0 | 82 | 2.3e-08 | 1Score **> 36** indicates **identity** Score **> 18** indicates **homology** | U | R.LVQDYGLESEVAQHLAK.T |
| 113321 | 483 | – | 499 | 633.9988 | 1898.9744 | 1898.9738 | 0.36 | 0 | 83 | 1.6e-08 | 1Score **> 36** indicates **identity** Score **> 18** indicates **homology** | U | R.LVQDYGLESEVAQHLAK.T |
| 113322 | 483 | – | 499 | 950.4945 | 1898.9745 | 1898.9738 | 0.39 | 0 | 98 | 6.5e-10 | 1Score **> 36** indicates **identity** Score **> 19** indicates **homology** | U | R.LVQDYGLESEVAQHLAK.T |
| 113323 | 483 | – | 499 | 633.9988 | 1898.9747 | 1898.9738 | 0.49 | 0 | 74 | 1.2e-07 | 1Score **> 36** indicates **identity** Score **> 17** indicates **homology** | U | R.LVQDYGLESEVAQHLAK.T |
| 113325 | 483 | – | 499 | 950.4948 | 1898.9750 | 1898.9738 | 0.67 | 0 | 96 | 1e-09 | 1Score **> 35** indicates **identity** Score **> 18** indicates **homology** | U | R.LVQDYGLESEVAQHLAK.T |
| 113326 | 483 | – | 499 | 633.9990 | 1898.9753 | 1898.9738 | 0.78 | 0 | 70 | 2.6e-07 | 1Score **> 35** indicates **identity** Score **> 17** indicates **homology** | U | R.LVQDYGLESEVAQHLAK.T |
| 113327 | 483 | – | 499 | 950.4951 | 1898.9756 | 1898.9738 | 0.96 | 0 | 94 | 1.5e-09 | 1Score **> 35** indicates **identity** Score **> 18** indicates **homology** | U | R.LVQDYGLESEVAQHLAK.T |
| 113328 | 483 | – | 499 | 633.9993 | 1898.9761 | 1898.9738 | 1.21 | 0 | 50 | 2e-05 | 1Score **> 36** indicates **identity** Score **> 16** indicates **homology** | U | R.LVQDYGLESEVAQHLAK.T |
| 113329 | 483 | – | 499 | 633.9994 | 1898.9762 | 1898.9738 | 1.30 | 0 | 88 | 5.7e-09 | 1Score **> 36** indicates **identity** Score **> 18** indicates **homology** | U | R.LVQDYGLESEVAQHLAK.T |
| 113330 | 483 | – | 499 | 633.9994 | 1898.9764 | 1898.9738 | 1.37 | 0 | 61 | 1.9e-06 | 1Score **> 36** indicates **identity** Score **> 16** indicates **homology** | U | R.LVQDYGLESEVAQHLAK.T |
| 113331 | 483 | – | 499 | 633.9994 | 1898.9765 | 1898.9738 | 1.43 | 0 | 77 | 6.7e-08 | 1Score **> 36** indicates **identity** Score **> 17** indicates **homology** | U | R.LVQDYGLESEVAQHLAK.T |
| 113332 | 483 | – | 499 | 633.9995 | 1898.9768 | 1898.9738 | 1.58 | 0 | 88 | 6e-09 | 1Score **> 36** indicates **identity** Score **> 18** indicates **homology** | U | R.LVQDYGLESEVAQHLAK.T |
| 113333 | 483 | – | 499 | 633.9997 | 1898.9773 | 1898.9738 | 1.84 | 0 | 62 | 1.5e-06 | 1Score **> 36** indicates **identity** Score **> 16** indicates **homology** | U | R.LVQDYGLESEVAQHLAK.T |
| 113334 | 483 | – | 499 | 950.4960 | 1898.9775 | 1898.9738 | 1.97 | 0 | 57 | 4.8e-06 | 1Score **> 36** indicates **identity** Score **> 16** indicates **homology** | U | R.LVQDYGLESEVAQHLAK.T |
| 113335 | 483 | – | 499 | 633.9999 | 1898.9777 | 1898.9738 | 2.09 | 0 | 71 | 2.3e-07 | 1Score **> 36** indicates **identity** Score **> 17** indicates **homology** | U | R.LVQDYGLESEVAQHLAK.T |
| 113336 | 483 | – | 499 | 634.0004 | 1898.9794 | 1898.9738 | 2.94 | 0 | 18 | 0.022 | 1Score **> 35** indicates **identity** Score **> 14** indicates **homology** | U | R.LVQDYGLESEVAQHLAK.T |
| 113337 | 483 | – | 499 | 634.0005 | 1898.9795 | 1898.9738 | 3.04 | 0 | 55 | 6.7e-06 | 1Score **> 35** indicates **identity** Score **> 16** indicates **homology** | U | R.LVQDYGLESEVAQHLAK.T |
| 113339 | 483 | – | 499 | 634.0006 | 1898.9799 | 1898.9738 | 3.20 | 0 | 75 | 9.9e-08 | 1Score **> 35** indicates **identity** Score **> 17** indicates **homology** | U | R.LVQDYGLESEVAQHLAK.T |
| 113340 | 483 | – | 499 | 634.0008 | 1898.9807 | 1898.9738 | 3.66 | 0 | 73 | 1.3e-07 | 1Score **> 36** indicates **identity** Score **> 17** indicates **homology** | U | R.LVQDYGLESEVAQHLAK.T |
| 113342 | 483 | – | 499 | 950.4980 | 1898.9815 | 1898.9738 | 4.08 | 0 | 94 | 1.6e-09 | 1Score **> 36** indicates **identity** Score **> 18** indicates **homology** | U | R.LVQDYGLESEVAQHLAK.T |
| 34321 | 500 | – | 510 | 410.2113 | 1227.6121 | 1227.6135 | -1.21 | 1 | 26 | 0.0033 | 1Score **> 31** indicates **identity** Score **> 14** indicates **homology** | U | K.TYGDKAFEVAK.M |
| 34323 | 500 | – | 510 | 614.8135 | 1227.6124 | 1227.6135 | -0.97 | 1 | 55 | 7.4e-06 | 1Score **> 31** indicates **identity** Score **> 16** indicates **homology** | U | K.TYGDKAFEVAK.M |
| 34324 | 500 | – | 510 | 614.8135 | 1227.6124 | 1227.6135 | -0.97 | 1 | 14 | 0.046 | 1Score **> 31** indicates **identity** Score **> 13** indicates **homology** | U | K.TYGDKAFEVAK.M |
| 34325 | 500 | – | 510 | 614.8141 | 1227.6136 | 1227.6135 | 0.019 | 1 | 80 | 4.5e-08 | 1Score **> 31** indicates **identity** Score **> 19** indicates **homology** | U | K.TYGDKAFEVAK.M |
| 34326 | 500 | – | 510 | 410.2118 | 1227.6137 | 1227.6135 | 0.11 | 1 | 54 | 8.6e-06 | 1Score **> 31** indicates **identity** Score **> 16** indicates **homology** | U | K.TYGDKAFEVAK.M |
| 34327 | 500 | – | 510 | 614.8141 | 1227.6137 | 1227.6135 | 0.11 | 1 | 76 | 2.3e-07 | 1Score **> 31** indicates **identity** Score **> 22** indicates **homology** | U | K.TYGDKAFEVAK.M |
| 34328 | 500 | – | 510 | 410.2118 | 1227.6137 | 1227.6135 | 0.13 | 1 | 44 | 6.7e-05 | 1Score **> 31** indicates **identity** Score **> 15** indicates **homology** | U | K.TYGDKAFEVAK.M |
| 34329 | 500 | – | 510 | 410.2120 | 1227.6141 | 1227.6135 | 0.45 | 1 | 35 | 0.00055 | 1Score **> 31** indicates **identity** Score **> 15** indicates **homology** | U | K.TYGDKAFEVAK.M |
| 34330 | 500 | – | 510 | 410.2120 | 1227.6142 | 1227.6135 | 0.55 | 1 | 53 | 1.2e-05 | 1Score **> 31** indicates **identity** Score **> 16** indicates **homology** | U | K.TYGDKAFEVAK.M |
| 34331 | 500 | – | 510 | 614.8146 | 1227.6146 | 1227.6135 | 0.83 | 1 | 73 | 2.6e-07 | 1Score **> 31** indicates **identity** Score **> 20** indicates **homology** | U | K.TYGDKAFEVAK.M |
| 34334 | 500 | – | 510 | 614.8160 | 1227.6175 | 1227.6135 | 3.19 | 1 | 52 | 5.5e-05 | 1Score **> 32** indicates **identity** Score **> 22** indicates **homology** | U | K.TYGDKAFEVAK.M |
| 34342 | 500 | – | 510 | 410.2146 | 1227.6218 | 1227.6135 | 6.75 | 1 | 20 | 0.014 | 1Score **> 31** indicates **identity** Score **> 14** indicates **homology** | U | K.TYGDKAFEVAK.M |
| 11407 | 518 | – | 525 | 484.7925 | 967.5704 | 967.5716 | -1.23 | 1 | 24 | 0.0098 | 1Score **> 26** indicates **identity** Score **> 17** indicates **homology** | U | K.RWPVVGVR.L |
| 11409 | 518 | – | 525 | 484.7928 | 967.5710 | 967.5716 | -0.59 | 1 | 26 | 0.015 | 1Score **> 26** indicates **identity** Score **> 20** indicates **homology** | U | K.RWPVVGVR.L |
| 11410 | 518 | – | 525 | 484.7929 | 967.5712 | 967.5716 | -0.34 | 1 | 35 | 0.0034 | 1Score **> 26** indicates **identity** Score **> 23** indicates **homology** | U | K.RWPVVGVR.L |
| 11412 | 518 | – | 525 | 484.7929 | 967.5713 | 967.5716 | -0.27 | 1 | 36 | 0.0041 | 1Score **> 26** indicates **identity** Score **> 24** indicates **homology** | U | K.RWPVVGVR.L |
| 11413 | 518 | – | 525 | 484.7931 | 967.5717 | 967.5716 | 0.098 | 1 | 40 | 0.0025 | 1Score **> 26** indicates **identity** Score **> 26** indicates **homology** | U | K.RWPVVGVR.L |
| 11414 | 518 | – | 525 | 484.7931 | 967.5717 | 967.5716 | 0.16 | 1 | 30 | 0.0074 | 1Score **> 26** indicates **identity** Score **> 21** indicates **homology** | U | K.RWPVVGVR.L |
| 11415 | 518 | – | 525 | 484.7933 | 967.5720 | 967.5716 | 0.47 | 1 | 35 | 0.0039 | 1Score **> 28** indicates **identity** Score **> 24** indicates **homology** | U | K.RWPVVGVR.L |
| 11416 | 518 | – | 525 | 484.7934 | 967.5722 | 967.5716 | 0.61 | 1 | 38 | 0.0035 | 1Score **> 28** indicates **identity** Score **> 26** indicates **homology** | U | K.RWPVVGVR.L |
| 11417 | 518 | – | 525 | 484.7934 | 967.5723 | 967.5716 | 0.72 | 1 | 36 | 0.0028 | 1Score **> 28** indicates **identity** Score **> 23** indicates **homology** | U | K.RWPVVGVR.L |
| 11418 | 518 | – | 525 | 484.7937 | 967.5728 | 967.5716 | 1.23 | 1 | 40 | 0.0025 | 1Score **> 28** indicates **identity** Score **> 26** indicates **homology** | U | K.RWPVVGVR.L |
| 3746 | 519 | – | 525 | 406.7421 | 811.4696 | 811.4705 | -1.13 | 0 | 18 | 0.022 | 1Score **> 21** indicates **identity** Score **> 14** indicates **homology** | U | R.WPVVGVR.L |
| 3747 | 519 | – | 525 | 406.7422 | 811.4699 | 811.4705 | -0.67 | 0 | 19 | 0.015 | 1Score **> 21** indicates **identity** Score **> 14** indicates **homology** | U | R.WPVVGVR.L |
| 3748 | 519 | – | 525 | 406.7423 | 811.4700 | 811.4705 | -0.60 | 0 | 15 | 0.042 | 1Score **> 21** indicates **identity** Score **> 13** indicates **homology** | U | R.WPVVGVR.L |
| 3749 | 519 | – | 525 | 406.7423 | 811.4700 | 811.4705 | -0.52 | 0 | 19 | 0.016 | 1Score **> 21** indicates **identity** Score **> 14** indicates **homology** | U | R.WPVVGVR.L |
| 3750 | 519 | – | 525 | 406.7424 | 811.4702 | 811.4705 | -0.29 | 0 | 19 | 0.017 | 1Score **> 21** indicates **identity** Score **> 14** indicates **homology** | U | R.WPVVGVR.L |
| 3751 | 519 | – | 525 | 406.7424 | 811.4703 | 811.4705 | -0.22 | 0 | 23 | 0.0067 | 1Score **> 21** indicates **identity** Score **> 14** indicates **homology** | U | R.WPVVGVR.L |
| 3752 | 519 | – | 525 | 406.7424 | 811.4703 | 811.4705 | -0.22 | 0 | 22 | 0.0086 | 1Score **> 21** indicates **identity** Score **> 14** indicates **homology** | U | R.WPVVGVR.L |
| 3753 | 519 | – | 525 | 406.7424 | 811.4703 | 811.4705 | -0.21 | 0 | 34 | 0.0006 | 1Score **> 21** indicates **identity** Score **> 15** indicates **homology** | U | R.WPVVGVR.L |
| 3754 | 519 | – | 525 | 406.7424 | 811.4703 | 811.4705 | -0.21 | 0 | 25 | 0.0041 | 1Score **> 21** indicates **identity** Score **> 14** indicates **homology** | U | R.WPVVGVR.L |
| 3755 | 519 | – | 525 | 406.7425 | 811.4704 | 811.4705 | -0.13 | 0 | 26 | 0.0039 | 1Score **> 21** indicates **identity** Score **> 14** indicates **homology** | U | R.WPVVGVR.L |
| 3756 | 519 | – | 525 | 406.7426 | 811.4706 | 811.4705 | 0.12 | 0 | 17 | 0.023 | 1Score **> 21** indicates **identity** Score **> 14** indicates **homology** | U | R.WPVVGVR.L |
| 3757 | 519 | – | 525 | 406.7426 | 811.4706 | 811.4705 | 0.22 | 0 | 21 | 0.0097 | 1Score **> 21** indicates **identity** Score **> 14** indicates **homology** | U | R.WPVVGVR.L |
| 3758 | 519 | – | 525 | 406.7426 | 811.4707 | 811.4705 | 0.22 | 0 | 20 | 0.015 | 1Score **> 21** indicates **identity** Score **> 14** indicates **homology** | U | R.WPVVGVR.L |
| 3759 | 519 | – | 525 | 406.7426 | 811.4707 | 811.4705 | 0.33 | 0 | 20 | 0.012 | 1Score **> 21** indicates **identity** Score **> 14** indicates **homology** | U | R.WPVVGVR.L |
| 3760 | 519 | – | 525 | 406.7426 | 811.4707 | 811.4705 | 0.33 | 0 | 20 | 0.012 | 1Score **> 21** indicates **identity** Score **> 14** indicates **homology** | U | R.WPVVGVR.L |
| 3761 | 519 | – | 525 | 406.7427 | 811.4708 | 811.4705 | 0.40 | 0 | 17 | 0.026 | 1Score **> 21** indicates **identity** Score **> 14** indicates **homology** | U | R.WPVVGVR.L |
| 3762 | 519 | – | 525 | 406.7427 | 811.4709 | 811.4705 | 0.52 | 0 | 21 | 0.0096 | 1Score **> 21** indicates **identity** Score **> 14** indicates **homology** | U | R.WPVVGVR.L |
| 3763 | 519 | – | 525 | 406.7434 | 811.4723 | 811.4705 | 2.26 | 0 | 22 | 0.0081 | 1Score **> 24** indicates **identity** Score **> 14** indicates **homology** | U | R.WPVVGVR.L |
| 67277 | 526 | – | 538 | 508.6045 | 1522.7918 | 1522.7919 | -0.057 | 0 | 33 | 0.0013 | 1Score **> 35** indicates **identity** Score **> 17** indicates **homology** | U | R.LVSEFPYIEAEVK.Y |
| 67278 | 526 | – | 538 | 762.4035 | 1522.7925 | 1522.7919 | 0.37 | 0 | 64 | 2e-06 | 1Score **> 35** indicates **identity** Score **> 20** indicates **homology** | U | R.LVSEFPYIEAEVK.Y |
| 67279 | 526 | – | 538 | 762.4035 | 1522.7925 | 1522.7919 | 0.42 | 0 | 65 | 1.4e-06 | 1Score **> 35** indicates **identity** Score **> 19** indicates **homology** | U | R.LVSEFPYIEAEVK.Y |
| 67281 | 526 | – | 538 | 762.4047 | 1522.7948 | 1522.7919 | 1.89 | 0 | 66 | 1.4e-06 | 1Score **> 35** indicates **identity** Score **> 20** indicates **homology** | U | R.LVSEFPYIEAEVK.Y |
| 67284 | 526 | – | 538 | 762.4057 | 1522.7969 | 1522.7919 | 3.31 | 0 | 26 | 0.0036 | 1Score **> 35** indicates **identity** Score **> 14** indicates **homology** | U | R.LVSEFPYIEAEVK.Y |
| 67287 | 526 | – | 538 | 762.4092 | 1522.8039 | 1522.7919 | 7.90 | 0 | 48 | 2.9e-05 | 1Score **> 35** indicates **identity** Score **> 16** indicates **homology** | U | R.LVSEFPYIEAEVK.Y |
| 110966 | 539 | – | 554 | 938.9498 | 1875.8850 | 1875.8859 | -0.49 | 1 | 59 | 2.8e-06 | 1Score **> 34** indicates **identity** Score **> 16** indicates **homology** | U | K.YGIKEYACTAVDMISR.R |
| 110969 | 539 | – | 554 | 626.3025 | 1875.8855 | 1875.8859 | -0.19 | 1 | 35 | 0.00052 | 1Score **> 34** indicates **identity** Score **> 15** indicates **homology** | U | K.YGIKEYACTAVDMISR.R |
| 110974 | 539 | – | 554 | 626.3028 | 1875.8866 | 1875.8859 | 0.41 | 1 | 39 | 0.00023 | 1Score **> 34** indicates **identity** Score **> 15** indicates **homology** | U | K.YGIKEYACTAVDMISR.R |
| 126122 | 539 | – | 555 | 678.3349 | 2031.9828 | 2031.9870 | -2.08 | 2 | 15 | 0.039 | 1Score **> 35** indicates **identity** Score **> 13** indicates **homology** | U | K.YGIKEYACTAVDMISRR.T |
| 126124 | 539 | – | 555 | 678.3357 | 2031.9851 | 2031.9870 | -0.91 | 2 | 19 | 0.017 | 1Score **> 35** indicates **identity** Score **> 14** indicates **homology** | U | K.YGIKEYACTAVDMISRR.T |
| 126125 | 539 | – | 555 | 678.3359 | 2031.9859 | 2031.9870 | -0.53 | 2 | 32 | 0.00092 | 1Score **> 35** indicates **identity** Score **> 15** indicates **homology** | U | K.YGIKEYACTAVDMISRR.T |
| 126126 | 539 | – | 555 | 509.0038 | 2031.9860 | 2031.9870 | -0.50 | 2 | 23 | 0.007 | 1Score **> 35** indicates **identity** Score **> 14** indicates **homology** | U | K.YGIKEYACTAVDMISRR.T |
| 126127 | 539 | – | 555 | 509.0039 | 2031.9865 | 2031.9870 | -0.26 | 2 | 33 | 0.0008 | 1Score **> 35** indicates **identity** Score **> 15** indicates **homology** | U | K.YGIKEYACTAVDMISRR.T |
| 126128 | 539 | – | 555 | 509.0041 | 2031.9873 | 2031.9870 | 0.14 | 2 | 55 | 1.4e-05 | 1Score **> 35** indicates **identity** Score **> 19** indicates **homology** | U | K.YGIKEYACTAVDMISRR.T |
| 126129 | 539 | – | 555 | 509.0042 | 2031.9879 | 2031.9870 | 0.44 | 2 | 63 | 9e-06 | 1Score **> 35** indicates **identity** Score **> 25** indicates **homology** | U | K.YGIKEYACTAVDMISRR.T |
| 126130 | 539 | – | 555 | 678.3371 | 2031.9893 | 2031.9870 | 1.16 | 2 | 23 | 0.0067 | 1Score **> 35** indicates **identity** Score **> 14** indicates **homology** | U | K.YGIKEYACTAVDMISRR.T |
| 126131 | 539 | – | 555 | 509.0050 | 2031.9909 | 2031.9870 | 1.90 | 2 | 27 | 0.0026 | 1Score **> 35** indicates **identity** Score **> 14** indicates **homology** | U | K.YGIKEYACTAVDMISRR.T |
| 54007 | 543 | – | 554 | 708.3185 | 1414.6225 | 1414.6221 | 0.31 | 0 | 65 | 8.7e-07 | 1Score **> 28** indicates **identity** Score **> 17** indicates **homology** | U | K.EYACTAVDMISR.R |
| 54008 | 543 | – | 554 | 708.3190 | 1414.6234 | 1414.6221 | 0.91 | 0 | 57 | 4.6e-06 | 1Score **> 28** indicates **identity** Score **> 16** indicates **homology** | U | K.EYACTAVDMISR.R |
| 54009 | 543 | – | 554 | 708.3203 | 1414.6261 | 1414.6221 | 2.83 | 0 | 25 | 0.0048 | 1Score **> 28** indicates **identity** Score **> 14** indicates **homology** | U | K.EYACTAVDMISR.R |
| 73602 | 543 | – | 555 | 524.5817 | 1570.7232 | 1570.7232 | -0.013 | 1 | 55 | 7.4e-06 | 1Score **> 31** indicates **identity** Score **> 16** indicates **homology** | U | K.EYACTAVDMISRR.T |
| 73603 | 543 | – | 555 | 524.5819 | 1570.7239 | 1570.7232 | 0.46 | 1 | 26 | 0.0039 | 1Score **> 31** indicates **identity** Score **> 14** indicates **homology** | U | K.EYACTAVDMISRR.T |
| 73604 | 543 | – | 555 | 524.5823 | 1570.7249 | 1570.7232 | 1.12 | 1 | 36 | 0.00044 | 1Score **> 31** indicates **identity** Score **> 15** indicates **homology** | U | K.EYACTAVDMISRR.T |
| 113184 | 556 | – | 572 | 633.6857 | 1898.0352 | 1898.0374 | -1.11 | 1 | 14 | 0.048 | 1Score **> 35** indicates **identity** Score **> 13** indicates **homology** | U | R.TRLAFLNVQAAEEALPR.I |
| 113185 | 556 | – | 572 | 950.0254 | 1898.0363 | 1898.0374 | -0.54 | 1 | 79 | 4.1e-08 | 1Score **> 35** indicates **identity** Score **> 17** indicates **homology** | U | R.TRLAFLNVQAAEEALPR.I |
| 113187 | 556 | – | 572 | 633.6865 | 1898.0376 | 1898.0374 | 0.12 | 1 | 15 | 0.041 | 1Score **> 35** indicates **identity** Score **> 13** indicates **homology** | U | R.TRLAFLNVQAAEEALPR.I |
| 113188 | 556 | – | 572 | 950.0263 | 1898.0381 | 1898.0374 | 0.42 | 1 | 78 | 4.9e-08 | 1Score **> 35** indicates **identity** Score **> 17** indicates **homology** | U | R.TRLAFLNVQAAEEALPR.I |
| 113190 | 556 | – | 572 | 950.0265 | 1898.0384 | 1898.0374 | 0.56 | 1 | 43 | 9.6e-05 | 1Score **> 35** indicates **identity** Score **> 15** indicates **homology** | U | R.TRLAFLNVQAAEEALPR.I |
| 113191 | 556 | – | 572 | 950.0267 | 1898.0388 | 1898.0374 | 0.75 | 1 | 77 | 6.4e-08 | 1Score **> 35** indicates **identity** Score **> 17** indicates **homology** | U | R.TRLAFLNVQAAEEALPR.I |
| 113192 | 556 | – | 572 | 633.6869 | 1898.0388 | 1898.0374 | 0.77 | 1 | 55 | 7e-06 | 1Score **> 35** indicates **identity** Score **> 16** indicates **homology** | U | R.TRLAFLNVQAAEEALPR.I |
| 113193 | 556 | – | 572 | 633.6870 | 1898.0391 | 1898.0374 | 0.93 | 1 | 79 | 4e-08 | 1Score **> 35** indicates **identity** Score **> 18** indicates **homology** | U | R.TRLAFLNVQAAEEALPR.I |
| 113194 | 556 | – | 572 | 950.0270 | 1898.0394 | 1898.0374 | 1.06 | 1 | 106 | 1.3e-10 | 1Score **> 35** indicates **identity** Score **> 19** indicates **homology** | U | R.TRLAFLNVQAAEEALPR.I |
| 113196 | 556 | – | 572 | 633.6872 | 1898.0398 | 1898.0374 | 1.31 | 1 | 65 | 1.1e-06 | 1Score **> 35** indicates **identity** Score **> 19** indicates **homology** | U | R.TRLAFLNVQAAEEALPR.I |
| 113197 | 556 | – | 572 | 633.6873 | 1898.0401 | 1898.0374 | 1.44 | 1 | 70 | 3.2e-07 | 1Score **> 35** indicates **identity** Score **> 17** indicates **homology** | U | R.TRLAFLNVQAAEEALPR.I |
| 113200 | 556 | – | 572 | 633.6875 | 1898.0406 | 1898.0374 | 1.70 | 1 | 63 | 1.2e-06 | 1Score **> 35** indicates **identity** Score **> 16** indicates **homology** | U | R.TRLAFLNVQAAEEALPR.I |
| 113203 | 556 | – | 572 | 633.6875 | 1898.0407 | 1898.0374 | 1.75 | 1 | 37 | 0.00035 | 1Score **> 35** indicates **identity** Score **> 15** indicates **homology** | U | R.TRLAFLNVQAAEEALPR.I |
| 113204 | 556 | – | 572 | 633.6877 | 1898.0413 | 1898.0374 | 2.10 | 1 | 55 | 7.5e-06 | 1Score **> 35** indicates **identity** Score **> 16** indicates **homology** | U | R.TRLAFLNVQAAEEALPR.I |
| 113214 | 556 | – | 572 | 950.0309 | 1898.0472 | 1898.0374 | 5.18 | 1 | 37 | 0.00037 | 1Score **> 35** indicates **identity** Score **> 15** indicates **homology** | U | R.TRLAFLNVQAAEEALPR.I |
| 113251 | 556 | – | 572 | 633.6919 | 1898.0538 | 1898.0374 | 8.65 | 1 | 59 | 2.9e-06 | 1Score **> 35** indicates **identity** Score **> 16** indicates **homology** | U | R.TRLAFLNVQAAEEALPR.I |
| 83492 | 558 | – | 572 | 821.4509 | 1640.8872 | 1640.8886 | -0.83 | 0 | 63 | 1.3e-06 | 1Score **> 35** indicates **identity** Score **> 16** indicates **homology** | U | R.LAFLNVQAAEEALPR.I |
| 83493 | 558 | – | 572 | 547.9698 | 1640.8877 | 1640.8886 | -0.55 | 0 | 51 | 1.7e-05 | 1Score **> 35** indicates **identity** Score **> 16** indicates **homology** | U | R.LAFLNVQAAEEALPR.I |
| 83494 | 558 | – | 572 | 821.4511 | 1640.8877 | 1640.8886 | -0.50 | 0 | 79 | 4.2e-08 | 1Score **> 35** indicates **identity** Score **> 17** indicates **homology** | U | R.LAFLNVQAAEEALPR.I |
| 83496 | 558 | – | 572 | 821.4514 | 1640.8882 | 1640.8886 | -0.23 | 0 | 84 | 1.2e-08 | 1Score **> 35** indicates **identity** Score **> 18** indicates **homology** | U | R.LAFLNVQAAEEALPR.I |
| 83498 | 558 | – | 572 | 821.4514 | 1640.8883 | 1640.8886 | -0.18 | 0 | 36 | 0.00045 | 1Score **> 35** indicates **identity** Score **> 15** indicates **homology** | U | R.LAFLNVQAAEEALPR.I |
| 83501 | 558 | – | 572 | 547.9704 | 1640.8893 | 1640.8886 | 0.48 | 0 | 91 | 3.2e-09 | 1Score **> 35** indicates **identity** Score **> 19** indicates **homology** | U | R.LAFLNVQAAEEALPR.I |
| 83502 | 558 | – | 572 | 547.9707 | 1640.8904 | 1640.8886 | 1.11 | 0 | 102 | 3.1e-10 | 1Score **> 35** indicates **identity** Score **> 19** indicates **homology** | U | R.LAFLNVQAAEEALPR.I |
| 83503 | 558 | – | 572 | 821.4525 | 1640.8904 | 1640.8886 | 1.12 | 0 | 111 | 4.2e-11 | 1Score **> 35** indicates **identity** Score **> 19** indicates **homology** | U | R.LAFLNVQAAEEALPR.I |
| 83504 | 558 | – | 572 | 547.9708 | 1640.8905 | 1640.8886 | 1.17 | 0 | 78 | 4.7e-08 | 1Score **> 35** indicates **identity** Score **> 17** indicates **homology** | U | R.LAFLNVQAAEEALPR.I |
| 83506 | 558 | – | 572 | 547.9711 | 1640.8913 | 1640.8886 | 1.69 | 0 | 91 | 2.8e-09 | 1Score **> 35** indicates **identity** Score **> 18** indicates **homology** | U | R.LAFLNVQAAEEALPR.I |
| 83508 | 558 | – | 572 | 821.4532 | 1640.8918 | 1640.8886 | 1.95 | 0 | 98 | 6.7e-10 | 1Score **> 35** indicates **identity** Score **> 19** indicates **homology** | U | R.LAFLNVQAAEEALPR.I |
| 83511 | 558 | – | 572 | 821.4539 | 1640.8933 | 1640.8886 | 2.91 | 0 | 61 | 1.9e-06 | 1Score **> 35** indicates **identity** Score **> 16** indicates **homology** | U | R.LAFLNVQAAEEALPR.I |
| 83513 | 558 | – | 572 | 547.9719 | 1640.8939 | 1640.8886 | 3.22 | 0 | 56 | 6.2e-06 | 1Score **> 35** indicates **identity** Score **> 16** indicates **homology** | U | R.LAFLNVQAAEEALPR.I |
| 83516 | 558 | – | 572 | 547.9722 | 1640.8948 | 1640.8886 | 3.81 | 0 | 56 | 5.8e-06 | 1Score **> 35** indicates **identity** Score **> 16** indicates **homology** | U | R.LAFLNVQAAEEALPR.I |
| 83527 | 558 | – | 572 | 821.4566 | 1640.8986 | 1640.8886 | 6.13 | 0 | 82 | 2.2e-08 | 1Score **> 35** indicates **identity** Score **> 18** indicates **homology** | U | R.LAFLNVQAAEEALPR.I |
| 4007 | 573 | – | 579 | 409.2335 | 816.4524 | 816.4527 | -0.48 | 0 | 34 | 0.024 | 1Score **> 30** indicates **identity** | U | R.IVELMGR.E |
| 4008 | 573 | – | 579 | 409.2338 | 816.4530 | 816.4527 | 0.36 | 0 | 47 | 0.0013 | 1Score **> 30** indicates **identity** | U | R.IVELMGR.E |
| 16254 | 580 | – | 587 | 523.7653 | 1045.5160 | 1045.5192 | -3.06 | 0 | 19 | 0.016 | 1Score **> 31** indicates **identity** Score **> 14** indicates **homology** | U | R.ELNWSELR.K |
| 16257 | 580 | – | 587 | 523.7667 | 1045.5188 | 1045.5192 | -0.37 | 0 | 32 | 0.0099 | 1Score **> 33** indicates **identity** Score **> 24** indicates **homology** | U | R.ELNWSELR.K |
| 16258 | 580 | – | 587 | 523.7667 | 1045.5189 | 1045.5192 | -0.36 | 0 | 47 | 0.00026 | 1Score **> 33** indicates **identity** Score **> 24** indicates **homology** | U | R.ELNWSELR.K |
| 16259 | 580 | – | 587 | 523.7668 | 1045.5191 | 1045.5192 | -0.14 | 0 | 26 | 0.0085 | 1Score **> 33** indicates **identity** Score **> 18** indicates **homology** | U | R.ELNWSELR.K |
| 16260 | 580 | – | 587 | 523.7669 | 1045.5192 | 1045.5192 | 0.0096 | 0 | 29 | 0.013 | 1Score **> 33** indicates **identity** Score **> 23** indicates **homology** | U | R.ELNWSELR.K |
| 16261 | 580 | – | 587 | 523.7671 | 1045.5196 | 1045.5192 | 0.33 | 0 | 32 | 0.0024 | 1Score **> 33** indicates **identity** Score **> 18** indicates **homology** | U | R.ELNWSELR.K |
| 16262 | 580 | – | 587 | 523.7672 | 1045.5199 | 1045.5192 | 0.60 | 0 | 38 | 0.00099 | 1Score **> 33** indicates **identity** Score **> 20** indicates **homology** | U | R.ELNWSELR.K |
| 16263 | 580 | – | 587 | 523.7673 | 1045.5200 | 1045.5192 | 0.73 | 0 | 42 | 0.0014 | 1Score **> 33** indicates **identity** Score **> 26** indicates **homology** | U | R.ELNWSELR.K |
| 16264 | 580 | – | 587 | 523.7673 | 1045.5201 | 1045.5192 | 0.81 | 0 | 46 | 0.00058 | 1Score **> 33** indicates **identity** Score **> 26** indicates **homology** | U | R.ELNWSELR.K |
| 16265 | 580 | – | 587 | 523.7674 | 1045.5203 | 1045.5192 | 1.04 | 0 | 33 | 0.0099 | 1Score **> 33** indicates **identity** Score **> 26** indicates **homology** | U | R.ELNWSELR.K |
| 16266 | 580 | – | 587 | 523.7678 | 1045.5210 | 1045.5192 | 1.66 | 0 | 43 | 0.00059 | 1Score **> 33** indicates **identity** Score **> 23** indicates **homology** | U | R.ELNWSELR.K |
| 28630 | 580 | – | 588 | 392.2120 | 1173.6141 | 1173.6142 | -0.029 | 1 | 24 | 0.0092 | 1Score **> 34** indicates **identity** Score **> 16** indicates **homology** | U | R.ELNWSELRK.Q |
| 28631 | 580 | – | 588 | 392.2121 | 1173.6145 | 1173.6142 | 0.25 | 1 | 25 | 0.0091 | 1Score **> 34** indicates **identity** Score **> 17** indicates **homology** | U | R.ELNWSELRK.Q |
| 144207 | 580 | – | 597 | 558.7867 | 2231.1178 | 2231.1182 | -0.15 | 2 | 38 | 0.00033 | 1Score **> 37** indicates **identity** Score **> 16** indicates **homology** | U | R.ELNWSELRKQEELETATR.F |
| 144208 | 580 | – | 597 | 558.7868 | 2231.1181 | 2231.1182 | -0.022 | 2 | 29 | 0.0023 | 1Score **> 37** indicates **identity** Score **> 15** indicates **homology** | U | R.ELNWSELRKQEELETATR.F |
| 144209 | 580 | – | 597 | 558.7869 | 2231.1184 | 2231.1182 | 0.086 | 2 | 38 | 0.00043 | 1Score **> 37** indicates **identity** Score **> 17** indicates **homology** | U | R.ELNWSELRKQEELETATR.F |
| 144210 | 580 | – | 597 | 744.7134 | 2231.1185 | 2231.1182 | 0.13 | 2 | 32 | 0.00091 | 1Score **> 37** indicates **identity** Score **> 15** indicates **homology** | U | R.ELNWSELRKQEELETATR.F |
| 144212 | 580 | – | 597 | 744.7136 | 2231.1189 | 2231.1182 | 0.35 | 2 | 34 | 0.00066 | 1Score **> 37** indicates **identity** Score **> 15** indicates **homology** | U | R.ELNWSELRKQEELETATR.F |
| 144215 | 580 | – | 597 | 744.7139 | 2231.1197 | 2231.1182 | 0.71 | 2 | 35 | 0.00054 | 1Score **> 37** indicates **identity** Score **> 15** indicates **homology** | U | R.ELNWSELRKQEELETATR.F |
| 144216 | 580 | – | 597 | 744.7139 | 2231.1198 | 2231.1182 | 0.74 | 2 | 24 | 0.0061 | 1Score **> 37** indicates **identity** Score **> 14** indicates **homology** | U | R.ELNWSELRKQEELETATR.F |
| 144217 | 580 | – | 597 | 558.7876 | 2231.1213 | 2231.1182 | 1.40 | 2 | 22 | 0.0095 | 1Score **> 37** indicates **identity** Score **> 14** indicates **homology** | U | R.ELNWSELRKQEELETATR.F |
| 144218 | 580 | – | 597 | 558.7876 | 2231.1215 | 2231.1182 | 1.49 | 2 | 39 | 0.00027 | 1Score **> 37** indicates **identity** Score **> 16** indicates **homology** | U | R.ELNWSELRKQEELETATR.F |
| 144220 | 580 | – | 597 | 558.7883 | 2231.1240 | 2231.1182 | 2.60 | 2 | 19 | 0.016 | 1Score **> 37** indicates **identity** Score **> 14** indicates **homology** | U | R.ELNWSELRKQEELETATR.F |
| 31922 | 588 | – | 597 | 402.2099 | 1203.6078 | 1203.6095 | -1.40 | 1 | 27 | 0.029 | 1Score **> 34** indicates **identity** Score **> 24** indicates **homology** | U | R.KQEELETATR.F |
| 31923 | 588 | – | 597 | 602.8112 | 1203.6079 | 1203.6095 | -1.29 | 1 | 35 | 0.028 | 1Score **> 34** indicates **identity** Score **> 32** indicates **homology** | U | R.KQEELETATR.F |
| 31924 | 588 | – | 597 | 602.8113 | 1203.6081 | 1203.6095 | -1.17 | 1 | 68 | 2.1e-05 | 1Score **> 34** indicates **identity** | U | R.KQEELETATR.F |
| 31928 | 588 | – | 597 | 402.2103 | 1203.6090 | 1203.6095 | -0.45 | 1 | 37 | 0.0084 | 1Score **> 34** indicates **identity** Score **> 29** indicates **homology** | U | R.KQEELETATR.F |
| 31930 | 588 | – | 597 | 602.8126 | 1203.6106 | 1203.6095 | 0.88 | 1 | 35 | 0.0038 | 1Score **> 33** indicates **identity** Score **> 23** indicates **homology** | U | R.KQEELETATR.F |
| 31932 | 588 | – | 597 | 602.8128 | 1203.6110 | 1203.6095 | 1.27 | 1 | 36 | 0.0042 | 1Score **> 33** indicates **identity** Score **> 25** indicates **homology** | U | R.KQEELETATR.F |
| 156235 | 588 | – | 606 | 604.7855 | 2415.1129 | 2415.1304 | -7.22 | 2 | 17 | 0.027 | 1Score **> 34** indicates **identity** Score **> 14** indicates **homology** | U | R.KQEELETATRFLYYEMGYK.S  + Deamidated (NQ); Oxidation (M) |
| 18896 | 589 | – | 597 | 538.7650 | 1075.5154 | 1075.5145 | 0.84 | 0 | 46 | 0.00066 | 1Score **> 31** indicates **identity** Score **> 27** indicates **homology** | U | K.QEELETATR.F |
| 32744 | 598 | – | 606 | 607.2832 | 1212.5518 | 1212.5525 | -0.54 | 0 | 24 | 0.0055 | 1Score **> 28** indicates **identity** Score **> 14** indicates **homology** | U | R.FLYYEMGYK.S |
| 32745 | 598 | – | 606 | 607.2832 | 1212.5518 | 1212.5525 | -0.54 | 0 | 41 | 0.00015 | 1Score **> 28** indicates **identity** Score **> 15** indicates **homology** | U | R.FLYYEMGYK.S |
| 32746 | 598 | – | 606 | 607.2836 | 1212.5526 | 1212.5525 | 0.065 | 0 | 42 | 0.00012 | 1Score **> 28** indicates **identity** Score **> 15** indicates **homology** | U | R.FLYYEMGYK.S |
| 153764 | 607 | – | 627 | 792.7336 | 2375.1790 | 2375.1816 | -1.08 | 1 | 23 | 0.0069 | 1Score **> 37** indicates **identity** Score **> 14** indicates **homology** | U | K.SRTEQLTDSTEISLLPSDIDR.Y |
| 153766 | 607 | – | 627 | 792.7353 | 2375.1840 | 2375.1816 | 1.03 | 1 | 73 | 1.6e-07 | 1Score **> 37** indicates **identity** Score **> 17** indicates **homology** | U | K.SRTEQLTDSTEISLLPSDIDR.Y |
| 153767 | 607 | – | 627 | 792.7354 | 2375.1843 | 2375.1816 | 1.13 | 1 | 71 | 2.4e-07 | 1Score **> 37** indicates **identity** Score **> 17** indicates **homology** | U | K.SRTEQLTDSTEISLLPSDIDR.Y |
| 153768 | 607 | – | 627 | 792.7354 | 2375.1843 | 2375.1816 | 1.16 | 1 | 62 | 1.6e-06 | 1Score **> 37** indicates **identity** Score **> 16** indicates **homology** | U | K.SRTEQLTDSTEISLLPSDIDR.Y |
| 153769 | 607 | – | 627 | 1188.5997 | 2375.1848 | 2375.1816 | 1.34 | 1 | 96 | 3.3e-09 | 1Score **> 37** indicates **identity** Score **> 24** indicates **homology** | U | K.SRTEQLTDSTEISLLPSDIDR.Y |
| 153770 | 607 | – | 627 | 1188.5998 | 2375.1851 | 2375.1816 | 1.48 | 1 | 89 | 1.6e-08 | 1Score **> 37** indicates **identity** Score **> 23** indicates **homology** | U | K.SRTEQLTDSTEISLLPSDIDR.Y |
| 153771 | 607 | – | 627 | 792.7357 | 2375.1853 | 2375.1816 | 1.56 | 1 | 44 | 8e-05 | 1Score **> 37** indicates **identity** Score **> 15** indicates **homology** | U | K.SRTEQLTDSTEISLLPSDIDR.Y |
| 153772 | 607 | – | 627 | 792.7358 | 2375.1857 | 2375.1816 | 1.72 | 1 | 23 | 0.0064 | 1Score **> 37** indicates **identity** Score **> 14** indicates **homology** | U | K.SRTEQLTDSTEISLLPSDIDR.Y |
| 153775 | 607 | – | 627 | 1188.6004 | 2375.1863 | 2375.1816 | 1.97 | 1 | 31 | 0.0013 | 1Score **> 37** indicates **identity** Score **> 14** indicates **homology** | U | K.SRTEQLTDSTEISLLPSDIDR.Y |
| 153826 | 607 | – | 627 | 793.0677 | 2376.1812 | 2376.1656 | 6.58 | 1 | 18 | 0.021 | 1Score **> 37** indicates **identity** Score **> 14** indicates **homology** | U | K.SRTEQLTDSTEISLLPSDIDR.Y  + Deamidated (NQ) |
| 168546 | 607 | – | 629 | 1334.1701 | 2666.3257 | 2666.3399 | -5.32 | 2 | 21 | 0.012 | 1Score **> 37** indicates **identity** Score **> 14** indicates **homology** | U | K.SRTEQLTDSTEISLLPSDIDRYK.K |
| 168547 | 607 | – | 629 | 889.7840 | 2666.3301 | 2666.3399 | -3.68 | 2 | 21 | 0.0098 | 1Score **> 37** indicates **identity** Score **> 14** indicates **homology** | U | K.SRTEQLTDSTEISLLPSDIDRYK.K |
| 168549 | 607 | – | 629 | 889.7863 | 2666.3372 | 2666.3399 | -1.01 | 2 | 40 | 0.00018 | 1Score **> 37** indicates **identity** Score **> 15** indicates **homology** | U | K.SRTEQLTDSTEISLLPSDIDRYK.K |
| 168550 | 607 | – | 629 | 667.5921 | 2666.3392 | 2666.3399 | -0.26 | 2 | 35 | 0.0034 | 1Score **> 37** indicates **identity** Score **> 22** indicates **homology** | U | K.SRTEQLTDSTEISLLPSDIDRYK.K |
| 168551 | 607 | – | 629 | 667.5922 | 2666.3395 | 2666.3399 | -0.14 | 2 | 45 | 0.00013 | 1Score **> 37** indicates **identity** Score **> 19** indicates **homology** | U | K.SRTEQLTDSTEISLLPSDIDRYK.K |
| 168553 | 607 | – | 629 | 889.7875 | 2666.3406 | 2666.3399 | 0.26 | 2 | 49 | 5.5e-05 | 1Score **> 37** indicates **identity** Score **> 19** indicates **homology** | U | K.SRTEQLTDSTEISLLPSDIDRYK.K |
| 168554 | 607 | – | 629 | 667.5924 | 2666.3406 | 2666.3399 | 0.26 | 2 | 47 | 9.4e-05 | 1Score **> 37** indicates **identity** Score **> 19** indicates **homology** | U | K.SRTEQLTDSTEISLLPSDIDRYK.K |
| 168556 | 607 | – | 629 | 667.5927 | 2666.3416 | 2666.3399 | 0.66 | 2 | 44 | 0.00015 | 1Score **> 37** indicates **identity** Score **> 18** indicates **homology** | U | K.SRTEQLTDSTEISLLPSDIDRYK.K |
| 168557 | 607 | – | 629 | 889.7878 | 2666.3416 | 2666.3399 | 0.66 | 2 | 55 | 1.3e-05 | 1Score **> 37** indicates **identity** Score **> 19** indicates **homology** | U | K.SRTEQLTDSTEISLLPSDIDRYK.K |
| 168558 | 607 | – | 629 | 889.7880 | 2666.3422 | 2666.3399 | 0.87 | 2 | 49 | 4.5e-05 | 1Score **> 37** indicates **identity** Score **> 18** indicates **homology** | U | K.SRTEQLTDSTEISLLPSDIDRYK.K |
| 168559 | 607 | – | 629 | 667.5928 | 2666.3422 | 2666.3399 | 0.87 | 2 | 40 | 0.00027 | 1Score **> 37** indicates **identity** Score **> 17** indicates **homology** | U | K.SRTEQLTDSTEISLLPSDIDRYK.K |
| 168560 | 607 | – | 629 | 889.7880 | 2666.3422 | 2666.3399 | 0.89 | 2 | 50 | 3.1e-05 | 1Score **> 37** indicates **identity** Score **> 18** indicates **homology** | U | K.SRTEQLTDSTEISLLPSDIDRYK.K |
| 168561 | 607 | – | 629 | 889.7881 | 2666.3424 | 2666.3399 | 0.95 | 2 | 24 | 0.006 | 1Score **> 37** indicates **identity** Score **> 14** indicates **homology** | U | K.SRTEQLTDSTEISLLPSDIDRYK.K |
| 168562 | 607 | – | 629 | 667.5931 | 2666.3434 | 2666.3399 | 1.33 | 2 | 18 | 0.032 | 1Score **> 37** indicates **identity** Score **> 15** indicates **homology** | U | K.SRTEQLTDSTEISLLPSDIDRYK.K |
| 168563 | 607 | – | 629 | 667.5933 | 2666.3443 | 2666.3399 | 1.65 | 2 | 22 | 0.041 | 1Score **> 37** indicates **identity** Score **> 21** indicates **homology** | U | K.SRTEQLTDSTEISLLPSDIDRYK.K |
| 168564 | 607 | – | 629 | 667.5934 | 2666.3445 | 2666.3399 | 1.73 | 2 | 20 | 0.02 | 1Score **> 37** indicates **identity** Score **> 16** indicates **homology** | U | K.SRTEQLTDSTEISLLPSDIDRYK.K |
| 135799 | 609 | – | 627 | 1067.0321 | 2132.0497 | 2132.0485 | 0.56 | 0 | 109 | 1.6e-09 | 1Score **> 36** indicates **identity** Score **> 33** indicates **homology** | U | R.TEQLTDSTEISLLPSDIDR.Y |
| 135800 | 609 | – | 627 | 1067.0323 | 2132.0501 | 2132.0485 | 0.79 | 0 | 129 | 2.2e-11 | 1Score **> 36** indicates **identity** Score **> 35** indicates **homology** | U | R.TEQLTDSTEISLLPSDIDR.Y |
| 135801 | 609 | – | 627 | 1067.0326 | 2132.0506 | 2132.0485 | 0.99 | 0 | 94 | 3.1e-09 | 1Score **> 36** indicates **identity** Score **> 22** indicates **homology** | U | R.TEQLTDSTEISLLPSDIDR.Y |
| 135802 | 609 | – | 627 | 1067.0328 | 2132.0510 | 2132.0485 | 1.20 | 0 | 108 | 1.8e-09 | 1Score **> 36** indicates **identity** Score **> 33** indicates **homology** | U | R.TEQLTDSTEISLLPSDIDR.Y |
| 135803 | 609 | – | 627 | 1067.0328 | 2132.0511 | 2132.0485 | 1.22 | 0 | 17 | 0.031 | 1Score **> 36** indicates **identity** Score **> 14** indicates **homology** | U | R.TEQLTDSTEISLLPSDIDR.Y |
| 135804 | 609 | – | 627 | 1067.0330 | 2132.0515 | 2132.0485 | 1.43 | 0 | 62 | 3.4e-06 | 1Score **> 36** indicates **identity** Score **> 20** indicates **homology** | U | R.TEQLTDSTEISLLPSDIDR.Y |
| 135805 | 609 | – | 627 | 711.6912 | 2132.0516 | 2132.0485 | 1.48 | 0 | 33 | 0.00079 | 1Score **> 36** indicates **identity** Score **> 15** indicates **homology** | U | R.TEQLTDSTEISLLPSDIDR.Y |
| 135806 | 609 | – | 627 | 1067.0332 | 2132.0518 | 2132.0485 | 1.55 | 0 | 122 | 7.9e-11 | 1Score **> 36** indicates **identity** Score **> 33** indicates **homology** | U | R.TEQLTDSTEISLLPSDIDR.Y |
| 135807 | 609 | – | 627 | 1067.0336 | 2132.0526 | 2132.0485 | 1.94 | 0 | 75 | 1.3e-07 | 1Score **> 36** indicates **identity** Score **> 19** indicates **homology** | U | R.TEQLTDSTEISLLPSDIDR.Y |
| 135808 | 609 | – | 627 | 1067.0338 | 2132.0530 | 2132.0485 | 2.13 | 0 | 108 | 1.8e-09 | 1Score **> 36** indicates **identity** Score **> 33** indicates **homology** | U | R.TEQLTDSTEISLLPSDIDR.Y |
| 135810 | 609 | – | 627 | 1067.0340 | 2132.0535 | 2132.0485 | 2.37 | 0 | 106 | 2e-09 | 1Score **> 36** indicates **identity** Score **> 32** indicates **homology** | U | R.TEQLTDSTEISLLPSDIDR.Y |
| 135811 | 609 | – | 627 | 1067.0343 | 2132.0540 | 2132.0485 | 2.62 | 0 | 109 | 3.2e-09 | 1Score **> 36** indicates **identity** | U | R.TEQLTDSTEISLLPSDIDR.Y |
| 135813 | 609 | – | 627 | 1067.0353 | 2132.0560 | 2132.0485 | 3.55 | 0 | 120 | 1.3e-10 | 1Score **> 36** indicates **identity** Score **> 34** indicates **homology** | U | R.TEQLTDSTEISLLPSDIDR.Y |
| 135816 | 609 | – | 627 | 1067.0388 | 2132.0631 | 2132.0485 | 6.89 | 0 | 92 | 3.4e-09 | 1Score **> 36** indicates **identity** Score **> 20** indicates **homology** | U | R.TEQLTDSTEISLLPSDIDR.Y |
| 135917 | 609 | – | 627 | 1067.5292 | 2133.0438 | 2133.0325 | 5.30 | 0 | 52 | 1.2e-05 | 1Score **> 36** indicates **identity** Score **> 16** indicates **homology** | U | R.TEQLTDSTEISLLPSDIDR.Y  + Deamidated (NQ) |
| 156750 | 609 | – | 629 | 1212.6077 | 2423.2008 | 2423.2067 | -2.46 | 1 | 83 | 1.8e-08 | 1Score **> 36** indicates **identity** Score **> 18** indicates **homology** | U | R.TEQLTDSTEISLLPSDIDRYK.K |
| 156753 | 609 | – | 629 | 808.7433 | 2423.2082 | 2423.2067 | 0.59 | 1 | 43 | 0.0001 | 1Score **> 37** indicates **identity** Score **> 15** indicates **homology** | U | R.TEQLTDSTEISLLPSDIDRYK.K |
| 156754 | 609 | – | 629 | 808.7434 | 2423.2083 | 2423.2067 | 0.64 | 1 | 15 | 0.037 | 1Score **> 37** indicates **identity** Score **> 13** indicates **homology** | U | R.TEQLTDSTEISLLPSDIDRYK.K |
| 156755 | 609 | – | 629 | 1212.6115 | 2423.2085 | 2423.2067 | 0.71 | 1 | 93 | 1.8e-09 | 1Score **> 37** indicates **identity** Score **> 18** indicates **homology** | U | R.TEQLTDSTEISLLPSDIDRYK.K |
| 156756 | 609 | – | 629 | 1212.6115 | 2423.2085 | 2423.2067 | 0.72 | 1 | 103 | 2.2e-10 | 1Score **> 37** indicates **identity** Score **> 19** indicates **homology** | U | R.TEQLTDSTEISLLPSDIDRYK.K |
| 156757 | 609 | – | 629 | 808.7435 | 2423.2087 | 2423.2067 | 0.80 | 1 | 38 | 0.00026 | 1Score **> 37** indicates **identity** Score **> 15** indicates **homology** | U | R.TEQLTDSTEISLLPSDIDRYK.K |
| 156758 | 609 | – | 629 | 808.7435 | 2423.2087 | 2423.2067 | 0.80 | 1 | 56 | 5.8e-06 | 1Score **> 37** indicates **identity** Score **> 16** indicates **homology** | U | R.TEQLTDSTEISLLPSDIDRYK.K |
| 156759 | 609 | – | 629 | 1212.6119 | 2423.2092 | 2423.2067 | 1.03 | 1 | 17 | 0.025 | 1Score **> 37** indicates **identity** Score **> 14** indicates **homology** | U | R.TEQLTDSTEISLLPSDIDRYK.K |
| 156760 | 609 | – | 629 | 1212.6121 | 2423.2097 | 2423.2067 | 1.23 | 1 | 97 | 7.3e-10 | 1Score **> 37** indicates **identity** Score **> 19** indicates **homology** | U | R.TEQLTDSTEISLLPSDIDRYK.K |
| 156762 | 609 | – | 629 | 808.7442 | 2423.2109 | 2423.2067 | 1.71 | 1 | 74 | 1e-07 | 1Score **> 37** indicates **identity** Score **> 17** indicates **homology** | U | R.TEQLTDSTEISLLPSDIDRYK.K |
| 156766 | 609 | – | 629 | 808.7447 | 2423.2124 | 2423.2067 | 2.33 | 1 | 26 | 0.0037 | 1Score **> 37** indicates **identity** Score **> 14** indicates **homology** | U | R.TEQLTDSTEISLLPSDIDRYK.K |
| 156767 | 609 | – | 629 | 808.7452 | 2423.2138 | 2423.2067 | 2.91 | 1 | 22 | 0.0096 | 1Score **> 37** indicates **identity** Score **> 14** indicates **homology** | U | R.TEQLTDSTEISLLPSDIDRYK.K |
| 156817 | 609 | – | 629 | 809.0755 | 2424.2047 | 2424.1908 | 5.74 | 1 | 19 | 0.016 | 1Score **> 37** indicates **identity** Score **> 14** indicates **homology** | U | R.TEQLTDSTEISLLPSDIDRYK.K  + Deamidated (NQ) |
| 156820 | 609 | – | 629 | 1213.1145 | 2424.2144 | 2424.1908 | 9.76 | 1 | 40 | 0.00018 | 1Score **> 37** indicates **identity** Score **> 15** indicates **homology** | U | R.TEQLTDSTEISLLPSDIDRYK.K  + Deamidated (NQ) |
| 150565 | 632 | – | 650 | 581.5473 | 2322.1601 | 2322.1645 | -1.89 | 2 | 16 | 0.034 | 1Score **> 37** indicates **identity** Score **> 13** indicates **homology** | U | R.FHKFDEDEKGFITIVDVQR.V |
| 150570 | 632 | – | 650 | 581.5480 | 2322.1630 | 2322.1645 | -0.62 | 2 | 32 | 0.001 | 1Score **> 37** indicates **identity** Score **> 14** indicates **homology** | U | R.FHKFDEDEKGFITIVDVQR.V |
| 150573 | 632 | – | 650 | 581.5483 | 2322.1639 | 2322.1645 | -0.24 | 2 | 42 | 0.00011 | 1Score **> 37** indicates **identity** Score **> 15** indicates **homology** | U | R.FHKFDEDEKGFITIVDVQR.V |
| 150575 | 632 | – | 650 | 581.5483 | 2322.1643 | 2322.1645 | -0.079 | 2 | 36 | 0.0004 | 1Score **> 37** indicates **identity** Score **> 15** indicates **homology** | U | R.FHKFDEDEKGFITIVDVQR.V |
| 150577 | 632 | – | 650 | 581.5484 | 2322.1646 | 2322.1645 | 0.050 | 2 | 35 | 0.00054 | 1Score **> 37** indicates **identity** Score **> 15** indicates **homology** | U | R.FHKFDEDEKGFITIVDVQR.V |
| 150578 | 632 | – | 650 | 775.0622 | 2322.1648 | 2322.1645 | 0.14 | 2 | 45 | 5.5e-05 | 1Score **> 37** indicates **identity** Score **> 15** indicates **homology** | U | R.FHKFDEDEKGFITIVDVQR.V |
| 150579 | 632 | – | 650 | 775.0622 | 2322.1648 | 2322.1645 | 0.15 | 2 | 37 | 0.00033 | 1Score **> 37** indicates **identity** Score **> 15** indicates **homology** | U | R.FHKFDEDEKGFITIVDVQR.V |
| 150581 | 632 | – | 650 | 775.0623 | 2322.1652 | 2322.1645 | 0.32 | 2 | 50 | 2.1e-05 | 1Score **> 37** indicates **identity** Score **> 16** indicates **homology** | U | R.FHKFDEDEKGFITIVDVQR.V |
| 150582 | 632 | – | 650 | 581.5486 | 2322.1652 | 2322.1645 | 0.32 | 2 | 33 | 0.00077 | 1Score **> 37** indicates **identity** Score **> 15** indicates **homology** | U | R.FHKFDEDEKGFITIVDVQR.V |
| 150585 | 632 | – | 650 | 775.0624 | 2322.1654 | 2322.1645 | 0.40 | 2 | 34 | 0.00061 | 1Score **> 37** indicates **identity** Score **> 15** indicates **homology** | U | R.FHKFDEDEKGFITIVDVQR.V |
| 150586 | 632 | – | 650 | 581.5486 | 2322.1655 | 2322.1645 | 0.44 | 2 | 17 | 0.023 | 1Score **> 37** indicates **identity** Score **> 14** indicates **homology** | U | R.FHKFDEDEKGFITIVDVQR.V |
| 150587 | 632 | – | 650 | 581.5487 | 2322.1655 | 2322.1645 | 0.45 | 2 | 34 | 0.00066 | 1Score **> 37** indicates **identity** Score **> 15** indicates **homology** | U | R.FHKFDEDEKGFITIVDVQR.V |
| 150588 | 632 | – | 650 | 465.4404 | 2322.1657 | 2322.1645 | 0.53 | 2 | 18 | 0.028 | 1Score **> 37** indicates **identity** Score **> 15** indicates **homology** | U | R.FHKFDEDEKGFITIVDVQR.V |
| 150589 | 632 | – | 650 | 581.5488 | 2322.1659 | 2322.1645 | 0.63 | 2 | 40 | 0.0002 | 1Score **> 37** indicates **identity** Score **> 15** indicates **homology** | U | R.FHKFDEDEKGFITIVDVQR.V |
| 150590 | 632 | – | 650 | 465.4405 | 2322.1661 | 2322.1645 | 0.71 | 2 | 18 | 0.036 | 1Score **> 37** indicates **identity** Score **> 16** indicates **homology** | U | R.FHKFDEDEKGFITIVDVQR.V |
| 150591 | 632 | – | 650 | 581.5488 | 2322.1662 | 2322.1645 | 0.77 | 2 | 39 | 0.0002 | 1Score **> 37** indicates **identity** Score **> 15** indicates **homology** | U | R.FHKFDEDEKGFITIVDVQR.V |
| 150592 | 632 | – | 650 | 581.5488 | 2322.1663 | 2322.1645 | 0.78 | 2 | 17 | 0.027 | 1Score **> 37** indicates **identity** Score **> 14** indicates **homology** | U | R.FHKFDEDEKGFITIVDVQR.V |
| 150594 | 632 | – | 650 | 581.5490 | 2322.1668 | 2322.1645 | 1.01 | 2 | 51 | 2.1e-05 | 1Score **> 37** indicates **identity** Score **> 16** indicates **homology** | U | R.FHKFDEDEKGFITIVDVQR.V |
| 150596 | 632 | – | 650 | 581.5490 | 2322.1669 | 2322.1645 | 1.06 | 2 | 38 | 0.00026 | 1Score **> 37** indicates **identity** Score **> 15** indicates **homology** | U | R.FHKFDEDEKGFITIVDVQR.V |
| 150597 | 632 | – | 650 | 581.5490 | 2322.1669 | 2322.1645 | 1.06 | 2 | 18 | 0.03 | 1Score **> 37** indicates **identity** Score **> 15** indicates **homology** | U | R.FHKFDEDEKGFITIVDVQR.V |
| 150598 | 632 | – | 650 | 581.5491 | 2322.1672 | 2322.1645 | 1.17 | 2 | 18 | 0.023 | 1Score **> 37** indicates **identity** Score **> 15** indicates **homology** | U | R.FHKFDEDEKGFITIVDVQR.V |
| 150599 | 632 | – | 650 | 581.5491 | 2322.1672 | 2322.1645 | 1.19 | 2 | 20 | 0.014 | 1Score **> 37** indicates **identity** Score **> 14** indicates **homology** | U | R.FHKFDEDEKGFITIVDVQR.V |
| 150601 | 632 | – | 650 | 775.0631 | 2322.1674 | 2322.1645 | 1.28 | 2 | 53 | 1.2e-05 | 1Score **> 37** indicates **identity** Score **> 16** indicates **homology** | U | R.FHKFDEDEKGFITIVDVQR.V |
| 150603 | 632 | – | 650 | 581.5493 | 2322.1680 | 2322.1645 | 1.51 | 2 | 40 | 0.00018 | 1Score **> 37** indicates **identity** Score **> 15** indicates **homology** | U | R.FHKFDEDEKGFITIVDVQR.V |
| 150604 | 632 | – | 650 | 775.0634 | 2322.1684 | 2322.1645 | 1.70 | 2 | 45 | 6.6e-05 | 1Score **> 37** indicates **identity** Score **> 15** indicates **homology** | U | R.FHKFDEDEKGFITIVDVQR.V |
| 150605 | 632 | – | 650 | 581.5495 | 2322.1688 | 2322.1645 | 1.89 | 2 | 37 | 0.00038 | 1Score **> 37** indicates **identity** Score **> 15** indicates **homology** | U | R.FHKFDEDEKGFITIVDVQR.V |
| 150606 | 632 | – | 650 | 581.5495 | 2322.1689 | 2322.1645 | 1.93 | 2 | 43 | 9.3e-05 | 1Score **> 37** indicates **identity** Score **> 15** indicates **homology** | U | R.FHKFDEDEKGFITIVDVQR.V |
| 150609 | 632 | – | 650 | 581.5496 | 2322.1692 | 2322.1645 | 2.06 | 2 | 16 | 0.029 | 1Score **> 37** indicates **identity** Score **> 14** indicates **homology** | U | R.FHKFDEDEKGFITIVDVQR.V |
| 150610 | 632 | – | 650 | 581.5496 | 2322.1693 | 2322.1645 | 2.09 | 2 | 25 | 0.0041 | 1Score **> 37** indicates **identity** Score **> 14** indicates **homology** | U | R.FHKFDEDEKGFITIVDVQR.V |
| 150612 | 632 | – | 650 | 581.5499 | 2322.1705 | 2322.1645 | 2.62 | 2 | 39 | 0.00023 | 1Score **> 37** indicates **identity** Score **> 15** indicates **homology** | U | R.FHKFDEDEKGFITIVDVQR.V |
| 150617 | 632 | – | 650 | 581.5506 | 2322.1733 | 2322.1645 | 3.79 | 2 | 41 | 0.00013 | 1Score **> 37** indicates **identity** Score **> 15** indicates **homology** | U | R.FHKFDEDEKGFITIVDVQR.V |
| 150618 | 632 | – | 650 | 775.0661 | 2322.1764 | 2322.1645 | 5.16 | 2 | 36 | 0.00039 | 1Score **> 37** indicates **identity** Score **> 15** indicates **homology** | U | R.FHKFDEDEKGFITIVDVQR.V |
| 150620 | 632 | – | 650 | 581.5515 | 2322.1767 | 2322.1645 | 5.28 | 2 | 26 | 0.004 | 1Score **> 37** indicates **identity** Score **> 14** indicates **homology** | U | R.FHKFDEDEKGFITIVDVQR.V |
| 150624 | 632 | – | 650 | 775.0692 | 2322.1858 | 2322.1645 | 9.17 | 2 | 28 | 0.0026 | 1Score **> 37** indicates **identity** Score **> 14** indicates **homology** | U | R.FHKFDEDEKGFITIVDVQR.V |
| 159841 | 632 | – | 650 | 620.5726 | 2478.2612 | 2478.2795 | -7.39 | 2 | 29 | 0.0021 | 1Score **> 37** indicates **identity** Score **> 14** indicates **homology** | U | R.FHKFDEDEKGFITIVDVQR.V  + HNE (H) |
| 159843 | 632 | – | 650 | 620.5735 | 2478.2649 | 2478.2795 | -5.88 | 2 | 32 | 0.001 | 1Score **> 37** indicates **identity** Score **> 14** indicates **homology** | U | R.FHKFDEDEKGFITIVDVQR.V  + HNE (H) |
| 159846 | 632 | – | 650 | 496.6605 | 2478.2661 | 2478.2795 | -5.40 | 2 | 37 | 0.00033 | 1Score **> 37** indicates **identity** Score **> 15** indicates **homology** | U | R.FHKFDEDEKGFITIVDVQR.V  + HNE (H) |
| 159848 | 632 | – | 650 | 620.5740 | 2478.2668 | 2478.2795 | -5.11 | 2 | 48 | 3e-05 | 1Score **> 37** indicates **identity** Score **> 16** indicates **homology** | U | R.FHKFDEDEKGFITIVDVQR.V  + HNE (H) |
| 159849 | 632 | – | 650 | 496.6607 | 2478.2670 | 2478.2795 | -5.02 | 2 | 47 | 4.3e-05 | 1Score **> 37** indicates **identity** Score **> 15** indicates **homology** | U | R.FHKFDEDEKGFITIVDVQR.V  + HNE (H) |
| 159850 | 632 | – | 650 | 496.6608 | 2478.2674 | 2478.2795 | -4.88 | 2 | 22 | 0.0082 | 1Score **> 37** indicates **identity** Score **> 14** indicates **homology** | U | R.FHKFDEDEKGFITIVDVQR.V  + HNE (H) |
| 159852 | 632 | – | 650 | 620.5743 | 2478.2681 | 2478.2795 | -4.59 | 2 | 48 | 3.2e-05 | 1Score **> 37** indicates **identity** Score **> 15** indicates **homology** | U | R.FHKFDEDEKGFITIVDVQR.V  + HNE (H) |
| 159853 | 632 | – | 650 | 827.0970 | 2478.2691 | 2478.2795 | -4.20 | 2 | 25 | 0.0041 | 1Score **> 37** indicates **identity** Score **> 14** indicates **homology** | U | R.FHKFDEDEKGFITIVDVQR.V  + HNE (H) |
| 159854 | 632 | – | 650 | 496.6612 | 2478.2694 | 2478.2795 | -4.05 | 2 | 29 | 0.0018 | 1Score **> 37** indicates **identity** Score **> 14** indicates **homology** | U | R.FHKFDEDEKGFITIVDVQR.V  + HNE (H) |
| 159855 | 632 | – | 650 | 827.0972 | 2478.2696 | 2478.2795 | -3.97 | 2 | 30 | 0.0015 | 1Score **> 37** indicates **identity** Score **> 14** indicates **homology** | U | R.FHKFDEDEKGFITIVDVQR.V  + HNE (H) |
| 159856 | 632 | – | 650 | 620.5748 | 2478.2700 | 2478.2795 | -3.82 | 2 | 32 | 0.001 | 1Score **> 37** indicates **identity** Score **> 14** indicates **homology** | U | R.FHKFDEDEKGFITIVDVQR.V  + HNE (K) |
| 159857 | 632 | – | 650 | 620.5748 | 2478.2701 | 2478.2795 | -3.77 | 2 | 46 | 4.7e-05 | 1Score **> 37** indicates **identity** Score **> 15** indicates **homology** | U | R.FHKFDEDEKGFITIVDVQR.V  + HNE (H) |
| 159859 | 632 | – | 650 | 620.5748 | 2478.2703 | 2478.2795 | -3.72 | 2 | 47 | 4.4e-05 | 1Score **> 37** indicates **identity** Score **> 16** indicates **homology** | U | R.FHKFDEDEKGFITIVDVQR.V  + HNE (K) |
| 159860 | 632 | – | 650 | 496.6614 | 2478.2707 | 2478.2795 | -3.55 | 2 | 19 | 0.016 | 1Score **> 37** indicates **identity** Score **> 14** indicates **homology** | U | R.FHKFDEDEKGFITIVDVQR.V  + HNE (K) |
| 114242 | 635 | – | 650 | 637.6530 | 1909.9373 | 1909.9422 | -2.54 | 1 | 30 | 0.0016 | 1Score **> 35** indicates **identity** Score **> 14** indicates **homology** | U | K.FDEDEKGFITIVDVQR.V |
| 114244 | 635 | – | 650 | 637.6556 | 1909.9449 | 1909.9422 | 1.44 | 1 | 20 | 0.015 | 1Score **> 35** indicates **identity** Score **> 14** indicates **homology** | U | K.FDEDEKGFITIVDVQR.V |
| 178857 | 651 | – | 675 | 986.4844 | 2956.4314 | 2956.4045 | 9.10 | 0 | 41 | 0.00016 | 1Score **> 37** indicates **identity** Score **> 15** indicates **homology** | U | R.VLESINVQMDENTLHEILCEVDLNK.N  + 2 Deamidated (NQ) |
| 130009 | 676 | – | 693 | 691.0263 | 2070.0572 | 2070.0568 | 0.20 | 0 | 29 | 0.0018 | 1Score **> 36** indicates **identity** Score **> 14** indicates **homology** | U | K.NGQVELHEFLQLMSAVQK.G |
| 10133 | 707 | – | 714 | 474.2236 | 946.4327 | 946.4356 | -3.02 | 0 | 38 | 0.00029 | 1Score **> 28** indicates **identity** Score **> 15** indicates **homology** | U | K.TAEENLDR.R |
| 21400 | 707 | – | 715 | 368.5196 | 1102.5370 | 1102.5367 | 0.34 | 1 | 24 | 0.021 | 1Score **> 31** indicates **identity** Score **> 20** indicates **homology** | U | K.TAEENLDRR.V |
| 10499 | 715 | – | 722 | 476.2905 | 950.5665 | 950.5661 | 0.35 | 1 | 32 | 0.0082 | 1Score **> 23** indicates **identity** | U | R.RVPIPVDR.S |
| 10501 | 715 | – | 722 | 476.2906 | 950.5667 | 950.5661 | 0.62 | 1 | 33 | 0.004 | 1Score **> 23** indicates **identity** Score **> 22** indicates **homology** | U | R.RVPIPVDR.S |
| 3139 | 716 | – | 722 | 398.2395 | 794.4645 | 794.4650 | -0.70 | 0 | 36 | 0.0029 | 1Score **> 23** indicates **identity** | U | R.VPIPVDR.S |
| 3143 | 716 | – | 722 | 398.2399 | 794.4653 | 794.4650 | 0.32 | 0 | 33 | 0.0052 | 1Score **> 23** indicates **identity** | U | R.VPIPVDR.S |

---

```
ID   GPDM_MOUSE              Reviewed;         727 AA.
AC   Q64521; Q3TK51; Q3UDY8; Q61507; Q8CBX6; Q8K4U5; Q8VDT0; Q9ERP0;
DT   01-NOV-1997, integrated into UniProtKB/Swiss-Prot.
DT   01-MAY-2007, sequence version 2.
DT   28-JUN-2023, entry version 186.
DE   RecName: Full=Glycerol-3-phosphate dehydrogenase, mitochondrial;
DE            Short=GPD-M;
DE            Short=GPDH-M;
DE            EC=1.1.5.3 {ECO:0000250|UniProtKB:P43304};
DE   AltName: Full=Protein TISP38;
DE   Flags: Precursor;
GN   Name=Gpd2 {ECO:0000312|MGI:MGI:99778}; Synonyms=Gdm1;
OS   Mus musculus (Mouse).
OC   Eukaryota; Metazoa; Chordata; Craniata; Vertebrata; Euteleostomi; Mammalia;
OC   Eutheria; Euarchontoglires; Glires; Rodentia; Myomorpha; Muroidea; Muridae;
OC   Murinae; Mus; Mus.
OX   NCBI_TaxID=10090;
RN   [1]
RP   NUCLEOTIDE SEQUENCE [MRNA].
RC   STRAIN=C57BL/6J; TISSUE=Adipocyte;
RX   PubMed=8951039; DOI=10.1006/abbi.1996.0536;
RA   Koza R.A., Kozak U.C., Brown L.J., Leiter E.H., Macdonald M.J., Kozak L.P.;
RT   "Sequence and tissue-dependent RNA expression of mouse FAD-linked glycerol-
RT   3-phosphate dehydrogenase.";
RL   Arch. Biochem. Biophys. 336:97-104(1996).
RN   [2]
RP   NUCLEOTIDE SEQUENCE [MRNA].
RC   STRAIN=C57BL/6J;
RX   PubMed=8772729; DOI=10.2337/diab.45.9.1238;
RA   Ishihara H., Nakazaki M., Kanegae Y., Inukai K., Asano T., Katagiri H.,
RA   Yazaki Y., Kikuchi M., Miyazaki J., Saito I., Oka Y.;
RT   "Effect of mitochondrial and/or cytosolic glycerol 3-phosphate
RT   dehydrogenase overexpression on glucose-stimulated insulin secretion from
RT   MIN6 and HIT cells.";
RL   Diabetes 45:1238-1244(1996).
RN   [3]
RP   NUCLEOTIDE SEQUENCE [LARGE SCALE MRNA].
RC   STRAIN=C57BL/6J; TISSUE=Bone marrow, Diencephalon, and Lung;
RX   PubMed=16141072; DOI=10.1126/science.1112014;
RA   Carninci P., Kasukawa T., Katayama S., Gough J., Frith M.C., Maeda N.,
RA   Oyama R., Ravasi T., Lenhard B., Wells C., Kodzius R., Shimokawa K.,
RA   Bajic V.B., Brenner S.E., Batalov S., Forrest A.R., Zavolan M., Davis M.J.,
RA   Wilming L.G., Aidinis V., Allen J.E., Ambesi-Impiombato A., Apweiler R.,
RA   Aturaliya R.N., Bailey T.L., Bansal M., Baxter L., Beisel K.W., Bersano T.,
RA   Bono H., Chalk A.M., Chiu K.P., Choudhary V., Christoffels A.,
RA   Clutterbuck D.R., Crowe M.L., Dalla E., Dalrymple B.P., de Bono B.,
RA   Della Gatta G., di Bernardo D., Down T., Engstrom P., Fagiolini M.,
RA   Faulkner G., Fletcher C.F., Fukushima T., Furuno M., Futaki S.,
RA   Gariboldi M., Georgii-Hemming P., Gingeras T.R., Gojobori T., Green R.E.,
RA   Gustincich S., Harbers M., Hayashi Y., Hensch T.K., Hirokawa N., Hill D.,
RA   Huminiecki L., Iacono M., Ikeo K., Iwama A., Ishikawa T., Jakt M.,
RA   Kanapin A., Katoh M., Kawasawa Y., Kelso J., Kitamura H., Kitano H.,
RA   Kollias G., Krishnan S.P., Kruger A., Kummerfeld S.K., Kurochkin I.V.,
RA   Lareau L.F., Lazarevic D., Lipovich L., Liu J., Liuni S., McWilliam S.,
RA   Madan Babu M., Madera M., Marchionni L., Matsuda H., Matsuzawa S., Miki H.,
RA   Mignone F., Miyake S., Morris K., Mottagui-Tabar S., Mulder N., Nakano N.,
RA   Nakauchi H., Ng P., Nilsson R., Nishiguchi S., Nishikawa S., Nori F.,
RA   Ohara O., Okazaki Y., Orlando V., Pang K.C., Pavan W.J., Pavesi G.,
RA   Pesole G., Petrovsky N., Piazza S., Reed J., Reid J.F., Ring B.Z.,
RA   Ringwald M., Rost B., Ruan Y., Salzberg S.L., Sandelin A., Schneider C.,
RA   Schoenbach C., Sekiguchi K., Semple C.A., Seno S., Sessa L., Sheng Y.,
RA   Shibata Y., Shimada H., Shimada K., Silva D., Sinclair B., Sperling S.,
RA   Stupka E., Sugiura K., Sultana R., Takenaka Y., Taki K., Tammoja K.,
RA   Tan S.L., Tang S., Taylor M.S., Tegner J., Teichmann S.A., Ueda H.R.,
RA   van Nimwegen E., Verardo R., Wei C.L., Yagi K., Yamanishi H.,
RA   Zabarovsky E., Zhu S., Zimmer A., Hide W., Bult C., Grimmond S.M.,
RA   Teasdale R.D., Liu E.T., Brusic V., Quackenbush J., Wahlestedt C.,
RA   Mattick J.S., Hume D.A., Kai C., Sasaki D., Tomaru Y., Fukuda S.,
RA   Kanamori-Katayama M., Suzuki M., Aoki J., Arakawa T., Iida J., Imamura K.,
RA   Itoh M., Kato T., Kawaji H., Kawagashira N., Kawashima T., Kojima M.,
RA   Kondo S., Konno H., Nakano K., Ninomiya N., Nishio T., Okada M., Plessy C.,
RA   Shibata K., Shiraki T., Suzuki S., Tagami M., Waki K., Watahiki A.,
RA   Okamura-Oho Y., Suzuki H., Kawai J., Hayashizaki Y.;
RT   "The transcriptional landscape of the mammalian genome.";
RL   Science 309:1559-1563(2005).
RN   [4]
RP   NUCLEOTIDE SEQUENCE [LARGE SCALE MRNA].
RC   STRAIN=Czech II; TISSUE=Mammary tumor;
RX   PubMed=15489334; DOI=10.1101/gr.2596504;
RG   The MGC Project Team;
RT   "The status, quality, and expansion of the NIH full-length cDNA project:
RT   the Mammalian Gene Collection (MGC).";
RL   Genome Res. 14:2121-2127(2004).
RN   [5]
RP   PROTEIN SEQUENCE OF 58-91; 95-110; 125-135; 167-178; 200-209; 212-254;
RP   285-298; 340-348; 381-390; 392-409; 442-453; 464-473; 483-499; 519-538;
RP   558-579; 598-606; 609-627 AND 635-693.
RC   STRAIN=C57BL/6J; TISSUE=Brain, and Hippocampus;
RA   Lubec G., Klug S., Kang S.U.;
RL   Submitted (APR-2007) to UniProtKB.
RN   [6]
RP   NUCLEOTIDE SEQUENCE [GENOMIC DNA] OF 390-686.
RC   STRAIN=129/Sv; TISSUE=Liver;
RA   Weitzel J.M.;
RT   "Genomic sequence of mouse mitochondrial glycerol-3-phosphate dehydrogenase
RT   (mGPDH).";
RL   Submitted (AUG-2000) to the EMBL/GenBank/DDBJ databases.
RN   [7]
RP   NUCLEOTIDE SEQUENCE [MRNA] OF 611-727.
RC   TISSUE=Testis;
RA   Tamura K., Nishimune Y., Nojima H.;
RT   "Mus musculus TISP38 mRNA.";
RL   Submitted (JUL-2000) to the EMBL/GenBank/DDBJ databases.
RN   [8]
RP   PHOSPHORYLATION [LARGE SCALE ANALYSIS] AT TYR-601, AND IDENTIFICATION BY
RP   MASS SPECTROMETRY [LARGE SCALE ANALYSIS].
RC   TISSUE=Brain;
RX   PubMed=18034455; DOI=10.1021/pr0701254;
RA   Ballif B.A., Carey G.R., Sunyaev S.R., Gygi S.P.;
RT   "Large-scale identification and evolution indexing of tyrosine
RT   phosphorylation sites from murine brain.";
RL   J. Proteome Res. 7:311-318(2008).
RN   [9]
RP   IDENTIFICATION BY MASS SPECTROMETRY [LARGE SCALE ANALYSIS].
RC   TISSUE=Brain, Brown adipose tissue, Heart, Kidney, Liver, Lung,
RC   Pancreas, Spleen, and Testis;
RX   PubMed=21183079; DOI=10.1016/j.cell.2010.12.001;
RA   Huttlin E.L., Jedrychowski M.P., Elias J.E., Goswami T., Rad R.,
RA   Beausoleil S.A., Villen J., Haas W., Sowa M.E., Gygi S.P.;
RT   "A tissue-specific atlas of mouse protein phosphorylation and expression.";
RL   Cell 143:1174-1189(2010).
CC   -!- FUNCTION: Calcium-responsive mitochondrial glycerol-3-phosphate
CC       dehydrogenase which seems to be a key component of the pancreatic beta-
CC       cell glucose-sensing device. {ECO:0000250|UniProtKB:P43304}.
CC   -!- CATALYTIC ACTIVITY:
CC       Reaction=a quinone + sn-glycerol 3-phosphate = a quinol +
CC         dihydroxyacetone phosphate; Xref=Rhea:RHEA:18977, ChEBI:CHEBI:24646,
CC         ChEBI:CHEBI:57597, ChEBI:CHEBI:57642, ChEBI:CHEBI:132124; EC=1.1.5.3;
CC         Evidence={ECO:0000250|UniProtKB:P43304};
CC       PhysiologicalDirection=left-to-right; Xref=Rhea:RHEA:18978;
CC         Evidence={ECO:0000250|UniProtKB:P43304};
CC   -!- COFACTOR:
CC       Name=FAD; Xref=ChEBI:CHEBI:57692;
CC   -!- ACTIVITY REGULATION: Calcium-binding enhance the activity of the
CC       enzyme. {ECO:0000250|UniProtKB:P43304}.
CC   -!- PATHWAY: Polyol metabolism; glycerol degradation via glycerol kinase
CC       pathway; glycerone phosphate from sn-glycerol 3-phosphate (anaerobic
CC       route): step 1/1.
CC   -!- SUBCELLULAR LOCATION: Mitochondrion inner membrane.
CC   -!- SIMILARITY: Belongs to the FAD-dependent glycerol-3-phosphate
CC       dehydrogenase family. {ECO:0000305}.
CC   ---------------------------------------------------------------------------
CC   Copyrighted by the UniProt Consortium, see https://www.uniprot.org/terms
CC   Distributed under the Creative Commons Attribution (CC BY 4.0) License
CC   ---------------------------------------------------------------------------
DR   EMBL; U60987; AAB50545.1; -; mRNA.
DR   EMBL; D50430; BAA08926.1; -; mRNA.
DR   EMBL; AK034353; BAC28685.1; -; mRNA.
DR   EMBL; AK144716; BAE26028.1; -; mRNA.
DR   EMBL; AK149851; BAE29123.1; -; mRNA.
DR   EMBL; AK167152; BAE39294.1; -; mRNA.
DR   EMBL; BC021359; AAH21359.1; -; mRNA.
DR   EMBL; AH009802; AAG12342.1; -; Genomic_DNA.
DR   EMBL; AB045714; BAB97201.1; -; mRNA.
DR   CCDS; CCDS16045.1; -.
DR   RefSeq; NP_001139292.1; NM_001145820.1.
DR   RefSeq; NP_034404.3; NM_010274.3.
DR   AlphaFoldDB; Q64521; -.
DR   SMR; Q64521; -.
DR   BioGRID; 199894; 21.
DR   IntAct; Q64521; 2.
DR   STRING; 10090.ENSMUSP00000108237; -.
DR   GlyGen; Q64521; 1 site, 1 O-linked glycan (1 site).
DR   iPTMnet; Q64521; -.
DR   PhosphoSitePlus; Q64521; -.
DR   SwissPalm; Q64521; -.
DR   REPRODUCTION-2DPAGE; Q64521; -.
DR   EPD; Q64521; -.
DR   jPOST; Q64521; -.
DR   MaxQB; Q64521; -.
DR   PaxDb; Q64521; -.
DR   PeptideAtlas; Q64521; -.
DR   ProteomicsDB; 271263; -.
DR   Antibodypedia; 2370; 316 antibodies from 30 providers.
DR   DNASU; 14571; -.
DR   Ensembl; ENSMUST00000028167; ENSMUSP00000028167; ENSMUSG00000026827.
DR   Ensembl; ENSMUST00000169687; ENSMUSP00000130992; ENSMUSG00000026827.
DR   GeneID; 14571; -.
DR   KEGG; mmu:14571; -.
DR   UCSC; uc008jsc.2; mouse.
DR   AGR; MGI:99778; -.
DR   CTD; 2820; -.
DR   MGI; MGI:99778; Gpd2.
DR   VEuPathDB; HostDB:ENSMUSG00000026827; -.
DR   eggNOG; KOG0042; Eukaryota.
DR   GeneTree; ENSGT00390000001718; -.
DR   InParanoid; Q64521; -.
DR   OMA; WAMAPHI; -.
DR   OrthoDB; 989271at2759; -.
DR   BRENDA; 1.1.5.3; 3474.
DR   Reactome; R-MMU-1483166; Synthesis of PA.
DR   Reactome; R-MMU-163560; Triglyceride catabolism.
DR   UniPathway; UPA00618; UER00673.
DR   BioGRID-ORCS; 14571; 2 hits in 78 CRISPR screens.
DR   ChiTaRS; Gpd2; mouse.
DR   PRO; PR:Q64521; -.
DR   Proteomes; UP000000589; Chromosome 2.
DR   RNAct; Q64521; protein.
DR   Bgee; ENSMUSG00000026827; Expressed in seminiferous tubule of testis and 269 other tissues.
DR   ExpressionAtlas; Q64521; baseline and differential.
DR   Genevisible; Q64521; MM.
DR   GO; GO:0009331; C:glycerol-3-phosphate dehydrogenase complex; TAS:MGI.
DR   GO; GO:0005743; C:mitochondrial inner membrane; HDA:MGI.
DR   GO; GO:0005739; C:mitochondrion; HDA:MGI.
DR   GO; GO:0005509; F:calcium ion binding; IEA:InterPro.
DR   GO; GO:0004368; F:glycerol-3-phosphate dehydrogenase (quinone) activity; ISO:MGI.
DR   GO; GO:0047952; F:glycerol-3-phosphate dehydrogenase [NAD(P)+] activity; ISO:MGI.
DR   GO; GO:0052591; F:sn-glycerol-3-phosphate:ubiquinone-8 oxidoreductase activity; IEA:UniProtKB-EC.
DR   GO; GO:0043010; P:camera-type eye development; IGI:MGI.
DR   GO; GO:0006094; P:gluconeogenesis; IMP:MGI.
DR   GO; GO:0019563; P:glycerol catabolic process; IEA:UniProtKB-UniPathway.
DR   GO; GO:0006072; P:glycerol-3-phosphate metabolic process; IMP:MGI.
DR   GO; GO:0035264; P:multicellular organism growth; IGI:MGI.
DR   GO; GO:0006734; P:NADH metabolic process; ISO:MGI.
DR   CDD; cd00051; EFh; 1.
DR   Gene3D; 1.10.8.870; Alpha-glycerophosphate oxidase, cap domain; 1.
DR   Gene3D; 3.30.9.10; D-Amino Acid Oxidase, subunit A, domain 2; 1.
DR   Gene3D; 1.10.238.10; EF-hand; 1.
DR   Gene3D; 3.50.50.60; FAD/NAD(P)-binding domain; 1.
DR   InterPro; IPR031656; DAO_C.
DR   InterPro; IPR038299; DAO_C_sf.
DR   InterPro; IPR011992; EF-hand-dom_pair.
DR   InterPro; IPR018247; EF_Hand_1_Ca_BS.
DR   InterPro; IPR002048; EF_hand_dom.
DR   InterPro; IPR006076; FAD-dep_OxRdtase.
DR   InterPro; IPR036188; FAD/NAD-bd_sf.
DR   InterPro; IPR000447; G3P_DH_FAD-dep.
DR   PANTHER; PTHR11985; GLYCEROL-3-PHOSPHATE DEHYDROGENASE; 1.
DR   PANTHER; PTHR11985:SF15; GLYCEROL-3-PHOSPHATE DEHYDROGENASE, MITOCHONDRIAL; 1.
DR   Pfam; PF01266; DAO; 1.
DR   Pfam; PF16901; DAO_C; 1.
DR   Pfam; PF13499; EF-hand_7; 1.
DR   PRINTS; PR01001; FADG3PDH.
DR   SMART; SM00054; EFh; 2.
DR   SUPFAM; SSF47473; EF-hand; 1.
DR   SUPFAM; SSF54373; FAD-linked reductases, C-terminal domain; 1.
DR   SUPFAM; SSF51905; FAD/NAD(P)-binding domain; 1.
DR   PROSITE; PS00018; EF_HAND_1; 1.
DR   PROSITE; PS50222; EF_HAND_2; 2.
DR   PROSITE; PS00977; FAD_G3PDH_1; 1.
DR   PROSITE; PS00978; FAD_G3PDH_2; 1.
PE   1: Evidence at protein level;
KW   Calcium; Direct protein sequencing; FAD; Flavoprotein; Membrane;
KW   Metal-binding; Mitochondrion; Mitochondrion inner membrane; Oxidoreductase;
KW   Phosphoprotein; Reference proteome; Repeat; Transit peptide.
FT   TRANSIT         1..42
FT                   /note="Mitochondrion"
FT                   /evidence="ECO:0000250"
FT   CHAIN           43..727
FT                   /note="Glycerol-3-phosphate dehydrogenase, mitochondrial"
FT                   /id="PRO_0000010430"
FT   DOMAIN          623..658
FT                   /note="EF-hand 1"
FT                   /evidence="ECO:0000255|PROSITE-ProRule:PRU00448"
FT   DOMAIN          659..694
FT                   /note="EF-hand 2"
FT                   /evidence="ECO:0000255|PROSITE-ProRule:PRU00448"
FT   BINDING         71..99
FT                   /ligand="FAD"
FT                   /ligand_id="ChEBI:CHEBI:57692"
FT                   /evidence="ECO:0000255"
FT   BINDING         672
FT                   /ligand="Ca(2+)"
FT                   /ligand_id="ChEBI:CHEBI:29108"
FT                   /evidence="ECO:0000255|PROSITE-ProRule:PRU00448"
FT   BINDING         674
FT                   /ligand="Ca(2+)"
FT                   /ligand_id="ChEBI:CHEBI:29108"
FT                   /evidence="ECO:0000255|PROSITE-ProRule:PRU00448"
FT   BINDING         676
FT                   /ligand="Ca(2+)"
FT                   /ligand_id="ChEBI:CHEBI:29108"
FT                   /evidence="ECO:0000255|PROSITE-ProRule:PRU00448"
FT   BINDING         678
FT                   /ligand="Ca(2+)"
FT                   /ligand_id="ChEBI:CHEBI:29108"
FT                   /evidence="ECO:0000255|PROSITE-ProRule:PRU00448"
FT   BINDING         683
FT                   /ligand="Ca(2+)"
FT                   /ligand_id="ChEBI:CHEBI:29108"
FT                   /evidence="ECO:0000255|PROSITE-ProRule:PRU00448"
FT   MOD_RES         601
FT                   /note="Phosphotyrosine"
FT                   /evidence="ECO:0007744|PubMed:18034455"
FT   CONFLICT        26
FT                   /note="P -> Q (in Ref. 1; AAB50545 and 4; AAH21359)"
FT                   /evidence="ECO:0000305"
FT   CONFLICT        42
FT                   /note="Missing (in Ref. 3; BAC28685)"
FT                   /evidence="ECO:0000305"
FT   CONFLICT        158
FT                   /note="L -> V (in Ref. 1; AAB50545)"
FT                   /evidence="ECO:0000305"
FT   CONFLICT        295
FT                   /note="D -> E (in Ref. 2; BAA08926)"
FT                   /evidence="ECO:0000305"
FT   CONFLICT        449
FT                   /note="D -> N (in Ref. 4; AAH21359)"
FT                   /evidence="ECO:0000305"
FT   CONFLICT        498
FT                   /note="A -> P (in Ref. 2; BAA08926)"
FT                   /evidence="ECO:0000305"
FT   CONFLICT        582
FT                   /note="N -> D (in Ref. 1; AAB50545)"
FT                   /evidence="ECO:0000305"
FT   CONFLICT        593
FT                   /note="E -> G (in Ref. 1; AAB50545)"
FT                   /evidence="ECO:0000305"
SQ   SEQUENCE   727 AA;  80954 MW;  319F52E31CFDFC44 CRC64;
     MAFQKAVKGT ILVGGGALAT VLGLSPFAHY RRKQVSLAYV EAAGYLTEPV NREPPSREAQ
     LMTLKNTPEF DILVIGGGAT GCGCALDAVT RGLKTALVER DDFSSGTSSR STKLIHGGVR
     YLQKAIMNLD VEQYRMVKEA LHERANLLEI APHLSAPLPI MLPLYKWWQL PYYWVGIKMY
     DLVAGSQCLK SSYVLSKSRA LEHFPMLQKD KLVGAIVYYD GQHNDARMNL AIALTAARYG
     AATANYMEVV SLLKKTDPET GKERVSGARC KDVLTGQEFD VRAKCVINAS GPFTDSVRKM
     DDKNVVPICQ PSAGVHIVMP GYYSPENMGL LDPATSDGRV IFFLPWEKMT IAGTTDTPTD
     VTHHPIPSEE DINFILNEVR NYLSSDVEVR RGDVLAAWSG IRPLVTDPKS ADTQSISRNH
     VVDISDSGLI TIAGGKWTTY RSMAEDTVDA AVKFHNLNAG PSRTVGLFLQ GGKDWSPTLY
     IRLVQDYGLE SEVAQHLAKT YGDKAFEVAK MASVTGKRWP VVGVRLVSEF PYIEAEVKYG
     IKEYACTAVD MISRRTRLAF LNVQAAEEAL PRIVELMGRE LNWSELRKQE ELETATRFLY
     YEMGYKSRTE QLTDSTEISL LPSDIDRYKK RFHKFDEDEK GFITIVDVQR VLESINVQMD
     ENTLHEILCE VDLNKNGQVE LHEFLQLMSA VQKGRVSGSR LAILMKTAEE NLDRRVPIPV
     DRSCGGL
//
```

**Mascot:** http://www.matrixscience.com/


HNE (H) (+156.1150)
