# Supplementary material for: A common East-Asian ALDH2 mutation causes metabolic disorders and the therapeutic effect of ALDH2 activators
Source: Nat Commun. 2023 Sep 25;14:5971. doi: 10.1038/s41467-023-41570-6 (PMC10520061; doi:10.1038/s41467-023-41570-6)
Supplement: Supplementary file 4 — Supplementary Data 1 [file 41467_2023_41570_MOESM4_ESM.zip › Table S5c/Q64521/Q64521_KIO60-2-_H633_K65.html]

Mascot Search Results: Q64521
 

# MASCOT Search Results

## Protein View: Q64521

### Glycerol-3-phosphate dehydrogenase, mitochondrial OS=Mus musculus OX=10090 GN=Gpd2 PE=1 SV=2

|  |  |
| --- | --- |
| Database: | Mouse\_UniProt\_proteomes |
| Score: | 16515 |
| Monoisotopic mass (M_r_): | 81416 |
| Calculated pI: | 6.17 |

Sequence similarity is available as an NCBI BLAST search of Q64521 against nr.

### Search parameters

|  |  |
| --- | --- |
| MS data file: | `D:\LCMSMS\2023 Users' data\230529-1\230529-1-KI60-2.raw` |
| Enzyme: | Trypsin/P: cuts C-term side of KR. |
| Fixed modifications: | Carbamidomethyl (C) |
| Variable modifications: | Deamidated (NQ), HNE (C), HNE (H), HNE (K), Oxidation (M) |

### Protein sequence coverage: 74%

Matched peptides shown in ***bold red***.

|  |  |  |  |  |  |
| --- | --- | --- | --- | --- | --- |
| `1` | `MAFQKAVKGT` | `ILVGGGALAT` | `VLGLSPFAHY` | `RRKQVSLAYV` | `EAAGYLTEPV` |
| `51` | `NREPPSREAQ` | `LMTLKNTPEF` | `DILVIGGGAT` | `GCGCALDAVT` | `RGLKTALVER` |
| `101` | `DDFSSGTSSR` | `STKLIHGGVR` | `YLQKAIMNLD` | `VEQYRMVKEA` | `LHERANLLEI` |
| `151` | `APHLSAPLPI` | `MLPLYKWWQL` | `PYYWVGIKMY` | `DLVAGSQCLK` | `SSYVLSKSRA` |
| `201` | `LEHFPMLQKD` | `KLVGAIVYYD` | `GQHNDARMNL` | `AIALTAARYG` | `AATANYMEVV` |
| `251` | `SLLKKTDPET` | `GKERVSGARC` | `KDVLTGQEFD` | `VRAKCVINAS` | `GPFTDSVRKM` |
| `301` | `DDKNVVPICQ` | `PSAGVHIVMP` | `GYYSPENMGL` | `LDPATSDGRV` | `IFFLPWEKMT` |
| `351` | `IAGTTDTPTD` | `VTHHPIPSEE` | `DINFILNEVR` | `NYLSSDVEVR` | `RGDVLAAWSG` |
| `401` | `IRPLVTDPKS` | `ADTQSISRNH` | `VVDISDSGLI` | `TIAGGKWTTY` | `RSMAEDTVDA` |
| `451` | `AVKFHNLNAG` | `PSRTVGLFLQ` | `GGKDWSPTLY` | `IRLVQDYGLE` | `SEVAQHLAKT` |
| `501` | `YGDKAFEVAK` | `MASVTGKRWP` | `VVGVRLVSEF` | `PYIEAEVKYG` | `IKEYACTAVD` |
| `551` | `MISRRTRLAF` | `LNVQAAEEAL` | `PRIVELMGRE` | `LNWSELRKQE` | `ELETATRFLY` |
| `601` | `YEMGYKSRTE` | `QLTDSTEISL` | `LPSDIDRYKK` | `RFHKFDEDEK` | `GFITIVDVQR` |
| `651` | `VLESINVQMD` | `ENTLHEILCE` | `VDLNKNGQVE` | `LHEFLQLMSA` | `VQKGRVSGSR` |
| `701` | `LAILMKTAEE` | `NLDRRVPIPV` | `DRSCGGL` |  |  |

Unformatted sequence string: 727 residues (for pasting into other applications).

|  |  |  |  |
| --- | --- | --- | --- |
| Sort by | residue number | increasing mass | decreasing mass |
| Show | matched peptides only | predicted peptides also |  |

| Query | Start | – | End | Observed | Mr(expt) | Mr(calc) | ppm | M | Score | Expect | Rank | U | Peptide |
| --- | --- | --- | --- | --- | --- | --- | --- | --- | --- | --- | --- | --- | --- |
| 150336 | 9 | – | 31 | 757.4256 | 2269.2551 | 2269.2583 | -1.40 | 0 | 49 | 2.4e-05 | 1Score **> 35** indicates **identity** Score **> 16** indicates **homology** | U | K.GTILVGGGALATVLGLSPFAHYR.R |
| 150337 | 9 | – | 31 | 757.4305 | 2269.2695 | 2269.2583 | 4.95 | 0 | 34 | 0.00063 | 1Score **> 34** indicates **identity** Score **> 15** indicates **homology** | U | K.GTILVGGGALATVLGLSPFAHYR.R |
| 10217 | 58 | – | 65 | 467.2571 | 932.4996 | 932.5001 | -0.46 | 0 | 44 | 0.00019 | 1Score **> 32** indicates **identity** Score **> 20** indicates **homology** | U | R.EAQLMTLK.N |
| 10218 | 58 | – | 65 | 467.2571 | 932.4997 | 932.5001 | -0.39 | 0 | 41 | 0.00036 | 1Score **> 32** indicates **identity** Score **> 19** indicates **homology** | U | R.EAQLMTLK.N |
| 10219 | 58 | – | 65 | 467.2572 | 932.4998 | 932.5001 | -0.26 | 0 | 38 | 0.00071 | 1Score **> 32** indicates **identity** Score **> 19** indicates **homology** | U | R.EAQLMTLK.N |
| 10220 | 58 | – | 65 | 467.2584 | 932.5023 | 932.5001 | 2.35 | 0 | 32 | 0.0016 | 1Score **> 32** indicates **identity** Score **> 16** indicates **homology** | U | R.EAQLMTLK.N |
| 197778 | 58 | – | 91 | 1251.2822 | 3750.8247 | 3750.8519 | -7.24 | 1 | 61 | 1.9e-06 | 1Score **> 37** indicates **identity** Score **> 16** indicates **homology** | U | R.EAQLMTLKNTPEFDILVIGGGATGCGCALDAVTR.G  + Deamidated (NQ); HNE (K); Oxidation (M) |
| 197780 | 58 | – | 91 | 938.9645 | 3751.8291 | 3751.8359 | -1.81 | 1 | 34 | 0.0006 | 1Score **> 37** indicates **identity** Score **> 15** indicates **homology** | U | R.EAQLMTLKNTPEFDILVIGGGATGCGCALDAVTR.G  + 2 Deamidated (NQ); HNE (K); Oxidation (M) |
| 172513 | 66 | – | 91 | 888.7606 | 2663.2599 | 2663.2684 | -3.19 | 0 | 30 | 0.0014 | 1Score **> 36** indicates **identity** Score **> 14** indicates **homology** | U | K.NTPEFDILVIGGGATGCGCALDAVTR.G |
| 172514 | 66 | – | 91 | 888.7607 | 2663.2603 | 2663.2684 | -3.01 | 0 | 48 | 3.1e-05 | 1Score **> 36** indicates **identity** Score **> 16** indicates **homology** | U | K.NTPEFDILVIGGGATGCGCALDAVTR.G |
| 172518 | 66 | – | 91 | 888.7619 | 2663.2637 | 2663.2684 | -1.74 | 0 | 48 | 3.5e-05 | 1Score **> 36** indicates **identity** Score **> 15** indicates **homology** | U | K.NTPEFDILVIGGGATGCGCALDAVTR.G |
| 172519 | 66 | – | 91 | 888.7620 | 2663.2643 | 2663.2684 | -1.53 | 0 | 16 | 0.033 | 1Score **> 36** indicates **identity** Score **> 13** indicates **homology** | U | K.NTPEFDILVIGGGATGCGCALDAVTR.G |
| 172521 | 66 | – | 91 | 888.7624 | 2663.2653 | 2663.2684 | -1.14 | 0 | 25 | 0.0042 | 1Score **> 36** indicates **identity** Score **> 14** indicates **homology** | U | K.NTPEFDILVIGGGATGCGCALDAVTR.G |
| 172522 | 66 | – | 91 | 888.7626 | 2663.2658 | 2663.2684 | -0.96 | 0 | 15 | 0.043 | 1Score **> 36** indicates **identity** Score **> 13** indicates **homology** | U | K.NTPEFDILVIGGGATGCGCALDAVTR.G |
| 172524 | 66 | – | 91 | 888.7627 | 2663.2662 | 2663.2684 | -0.81 | 0 | 85 | 1.1e-08 | 1Score **> 36** indicates **identity** Score **> 18** indicates **homology** | U | K.NTPEFDILVIGGGATGCGCALDAVTR.G |
| 172525 | 66 | – | 91 | 1332.6408 | 2663.2670 | 2663.2684 | -0.50 | 0 | 14 | 0.05 | 1Score **> 36** indicates **identity** Score **> 13** indicates **homology** | U | K.NTPEFDILVIGGGATGCGCALDAVTR.G |
| 172529 | 66 | – | 91 | 1332.6410 | 2663.2675 | 2663.2684 | -0.33 | 0 | 62 | 1.4e-06 | 1Score **> 36** indicates **identity** Score **> 16** indicates **homology** | U | K.NTPEFDILVIGGGATGCGCALDAVTR.G |
| 172533 | 66 | – | 91 | 888.7640 | 2663.2703 | 2663.2684 | 0.73 | 0 | 26 | 0.0036 | 1Score **> 36** indicates **identity** Score **> 14** indicates **homology** | U | K.NTPEFDILVIGGGATGCGCALDAVTR.G |
| 172537 | 66 | – | 91 | 1332.6429 | 2663.2713 | 2663.2684 | 1.09 | 0 | 113 | 3.2e-11 | 1Score **> 36** indicates **identity** Score **> 20** indicates **homology** | U | K.NTPEFDILVIGGGATGCGCALDAVTR.G |
| 172539 | 66 | – | 91 | 888.7645 | 2663.2716 | 2663.2684 | 1.20 | 0 | 128 | 1.1e-12 | 1Score **> 36** indicates **identity** Score **> 20** indicates **homology** | U | K.NTPEFDILVIGGGATGCGCALDAVTR.G |
| 172542 | 66 | – | 91 | 888.7647 | 2663.2722 | 2663.2684 | 1.43 | 0 | 93 | 2.1e-09 | 1Score **> 36** indicates **identity** Score **> 18** indicates **homology** | U | K.NTPEFDILVIGGGATGCGCALDAVTR.G |
| 172543 | 66 | – | 91 | 888.7647 | 2663.2724 | 2663.2684 | 1.50 | 0 | 47 | 4.2e-05 | 1Score **> 36** indicates **identity** Score **> 15** indicates **homology** | U | K.NTPEFDILVIGGGATGCGCALDAVTR.G |
| 172544 | 66 | – | 91 | 888.7649 | 2663.2728 | 2663.2684 | 1.68 | 0 | 16 | 0.034 | 1Score **> 36** indicates **identity** Score **> 13** indicates **homology** | U | K.NTPEFDILVIGGGATGCGCALDAVTR.G |
| 172545 | 66 | – | 91 | 888.7650 | 2663.2731 | 2663.2684 | 1.79 | 0 | 117 | 1.2e-11 | 1Score **> 36** indicates **identity** Score **> 20** indicates **homology** | U | K.NTPEFDILVIGGGATGCGCALDAVTR.G |
| 172547 | 66 | – | 91 | 1332.6441 | 2663.2736 | 2663.2684 | 1.95 | 0 | 74 | 1.2e-07 | 1Score **> 36** indicates **identity** Score **> 17** indicates **homology** | U | K.NTPEFDILVIGGGATGCGCALDAVTR.G |
| 172549 | 66 | – | 91 | 1332.6442 | 2663.2739 | 2663.2684 | 2.09 | 0 | 106 | 1.3e-10 | 1Score **> 36** indicates **identity** Score **> 19** indicates **homology** | U | K.NTPEFDILVIGGGATGCGCALDAVTR.G |
| 172551 | 66 | – | 91 | 888.7654 | 2663.2743 | 2663.2684 | 2.24 | 0 | 42 | 0.00013 | 1Score **> 36** indicates **identity** Score **> 15** indicates **homology** | U | K.NTPEFDILVIGGGATGCGCALDAVTR.G |
| 172552 | 66 | – | 91 | 888.7655 | 2663.2748 | 2663.2684 | 2.40 | 0 | 109 | 5.9e-11 | 1Score **> 36** indicates **identity** Score **> 19** indicates **homology** | U | K.NTPEFDILVIGGGATGCGCALDAVTR.G |
| 172555 | 66 | – | 91 | 888.7661 | 2663.2763 | 2663.2684 | 2.99 | 0 | 42 | 0.00013 | 1Score **> 36** indicates **identity** Score **> 15** indicates **homology** | U | K.NTPEFDILVIGGGATGCGCALDAVTR.G |
| 172557 | 66 | – | 91 | 888.7661 | 2663.2766 | 2663.2684 | 3.09 | 0 | 97 | 7.3e-10 | 1Score **> 36** indicates **identity** Score **> 19** indicates **homology** | U | K.NTPEFDILVIGGGATGCGCALDAVTR.G |
| 172559 | 66 | – | 91 | 888.7668 | 2663.2786 | 2663.2684 | 3.86 | 0 | 41 | 0.00015 | 1Score **> 36** indicates **identity** Score **> 15** indicates **homology** | U | K.NTPEFDILVIGGGATGCGCALDAVTR.G |
| 172560 | 66 | – | 91 | 888.7670 | 2663.2793 | 2663.2684 | 4.11 | 0 | 35 | 0.00055 | 1Score **> 36** indicates **identity** Score **> 15** indicates **homology** | U | K.NTPEFDILVIGGGATGCGCALDAVTR.G |
| 172561 | 66 | – | 91 | 888.7672 | 2663.2797 | 2663.2684 | 4.26 | 0 | 47 | 3.7e-05 | 1Score **> 36** indicates **identity** Score **> 15** indicates **homology** | U | K.NTPEFDILVIGGGATGCGCALDAVTR.G |
| 172565 | 66 | – | 91 | 888.7675 | 2663.2807 | 2663.2684 | 4.62 | 0 | 41 | 0.00015 | 1Score **> 36** indicates **identity** Score **> 15** indicates **homology** | U | K.NTPEFDILVIGGGATGCGCALDAVTR.G |
| 172566 | 66 | – | 91 | 888.7676 | 2663.2808 | 2663.2684 | 4.68 | 0 | 52 | 1.5e-05 | 1Score **> 36** indicates **identity** Score **> 16** indicates **homology** | U | K.NTPEFDILVIGGGATGCGCALDAVTR.G |
| 172567 | 66 | – | 91 | 888.7676 | 2663.2808 | 2663.2684 | 4.68 | 0 | 25 | 0.0041 | 1Score **> 36** indicates **identity** Score **> 14** indicates **homology** | U | K.NTPEFDILVIGGGATGCGCALDAVTR.G |
| 172568 | 66 | – | 91 | 888.7679 | 2663.2818 | 2663.2684 | 5.05 | 0 | 37 | 0.00036 | 1Score **> 36** indicates **identity** Score **> 15** indicates **homology** | U | K.NTPEFDILVIGGGATGCGCALDAVTR.G |
| 172569 | 66 | – | 91 | 888.7679 | 2663.2819 | 2663.2684 | 5.07 | 0 | 31 | 0.0012 | 1Score **> 36** indicates **identity** Score **> 14** indicates **homology** | U | K.NTPEFDILVIGGGATGCGCALDAVTR.G |
| 172571 | 66 | – | 91 | 888.7682 | 2663.2829 | 2663.2684 | 5.45 | 0 | 50 | 1.8e-05 | 1Score **> 36** indicates **identity** Score **> 16** indicates **homology** | U | K.NTPEFDILVIGGGATGCGCALDAVTR.G |
| 172572 | 66 | – | 91 | 888.7686 | 2663.2839 | 2663.2684 | 5.82 | 0 | 35 | 0.0005 | 1Score **> 36** indicates **identity** Score **> 15** indicates **homology** | U | K.NTPEFDILVIGGGATGCGCALDAVTR.G |
| 172574 | 66 | – | 91 | 888.7688 | 2663.2847 | 2663.2684 | 6.14 | 0 | 28 | 0.0023 | 1Score **> 36** indicates **identity** Score **> 14** indicates **homology** | U | K.NTPEFDILVIGGGATGCGCALDAVTR.G |
| 172575 | 66 | – | 91 | 888.7695 | 2663.2868 | 2663.2684 | 6.90 | 0 | 30 | 0.0014 | 1Score **> 36** indicates **identity** Score **> 14** indicates **homology** | U | K.NTPEFDILVIGGGATGCGCALDAVTR.G |
| 172576 | 66 | – | 91 | 888.7697 | 2663.2872 | 2663.2684 | 7.08 | 0 | 21 | 0.01 | 1Score **> 36** indicates **identity** Score **> 14** indicates **homology** | U | K.NTPEFDILVIGGGATGCGCALDAVTR.G |
| 172577 | 66 | – | 91 | 1332.6511 | 2663.2876 | 2663.2684 | 7.23 | 0 | 71 | 4.1e-07 | 1Score **> 36** indicates **identity** Score **> 19** indicates **homology** | U | K.NTPEFDILVIGGGATGCGCALDAVTR.G |
| 172579 | 66 | – | 91 | 888.7700 | 2663.2882 | 2663.2684 | 7.43 | 0 | 33 | 0.00083 | 1Score **> 36** indicates **identity** Score **> 15** indicates **homology** | U | K.NTPEFDILVIGGGATGCGCALDAVTR.G |
| 172581 | 66 | – | 91 | 888.7704 | 2663.2893 | 2663.2684 | 7.87 | 0 | 64 | 1e-06 | 1Score **> 37** indicates **identity** Score **> 16** indicates **homology** | U | K.NTPEFDILVIGGGATGCGCALDAVTR.G |
| 172582 | 66 | – | 91 | 888.7704 | 2663.2894 | 2663.2684 | 7.88 | 0 | 14 | 0.047 | 1Score **> 37** indicates **identity** Score **> 13** indicates **homology** | U | K.NTPEFDILVIGGGATGCGCALDAVTR.G |
| 172583 | 66 | – | 91 | 888.7705 | 2663.2898 | 2663.2684 | 8.04 | 0 | 46 | 4.4e-05 | 1Score **> 37** indicates **identity** Score **> 15** indicates **homology** | U | K.NTPEFDILVIGGGATGCGCALDAVTR.G |
| 172584 | 66 | – | 91 | 888.7709 | 2663.2908 | 2663.2684 | 8.42 | 0 | 41 | 0.00014 | 1Score **> 37** indicates **identity** Score **> 15** indicates **homology** | U | K.NTPEFDILVIGGGATGCGCALDAVTR.G |
| 172586 | 66 | – | 91 | 888.7711 | 2663.2914 | 2663.2684 | 8.65 | 0 | 39 | 0.00021 | 1Score **> 37** indicates **identity** Score **> 15** indicates **homology** | U | K.NTPEFDILVIGGGATGCGCALDAVTR.G |
| 172587 | 66 | – | 91 | 888.7712 | 2663.2917 | 2663.2684 | 8.75 | 0 | 16 | 0.03 | 1Score **> 36** indicates **identity** Score **> 14** indicates **homology** | U | K.NTPEFDILVIGGGATGCGCALDAVTR.G |
| 172589 | 66 | – | 91 | 888.7717 | 2663.2934 | 2663.2684 | 9.38 | 0 | 31 | 0.0013 | 1Score **> 37** indicates **identity** Score **> 14** indicates **homology** | U | K.NTPEFDILVIGGGATGCGCALDAVTR.G |
| 172611 | 66 | – | 91 | 1333.1351 | 2664.2557 | 2664.2524 | 1.23 | 0 | 91 | 2.9e-09 | 1Score **> 35** indicates **identity** Score **> 18** indicates **homology** | U | K.NTPEFDILVIGGGATGCGCALDAVTR.G  + Deamidated (NQ) |
| 172615 | 66 | – | 91 | 889.0971 | 2664.2694 | 2664.2524 | 6.38 | 0 | 17 | 0.028 | 1Score **> 36** indicates **identity** Score **> 14** indicates **homology** | U | K.NTPEFDILVIGGGATGCGCALDAVTR.G  + Deamidated (NQ) |
| 172616 | 66 | – | 91 | 889.0981 | 2664.2724 | 2664.2524 | 7.50 | 0 | 39 | 0.00024 | 1Score **> 36** indicates **identity** Score **> 15** indicates **homology** | U | K.NTPEFDILVIGGGATGCGCALDAVTR.G  + Deamidated (NQ) |
| 172620 | 66 | – | 91 | 889.0995 | 2664.2766 | 2664.2524 | 9.08 | 0 | 40 | 0.00016 | 1Score **> 36** indicates **identity** Score **> 15** indicates **homology** | U | K.NTPEFDILVIGGGATGCGCALDAVTR.G  + Deamidated (NQ) |
| 172621 | 66 | – | 91 | 889.0996 | 2664.2770 | 2664.2524 | 9.25 | 0 | 56 | 6.2e-06 | 1Score **> 36** indicates **identity** Score **> 16** indicates **homology** | U | K.NTPEFDILVIGGGATGCGCALDAVTR.G  + Deamidated (NQ) |
| 172623 | 66 | – | 91 | 889.0999 | 2664.2779 | 2664.2524 | 9.59 | 0 | 43 | 8.5e-05 | 1Score **> 36** indicates **identity** Score **> 15** indicates **homology** | U | K.NTPEFDILVIGGGATGCGCALDAVTR.G  + Deamidated (NQ) |
| 172624 | 66 | – | 91 | 1333.1466 | 2664.2786 | 2664.2524 | 9.85 | 0 | 80 | 3e-08 | 1Score **> 36** indicates **identity** Score **> 18** indicates **homology** | U | K.NTPEFDILVIGGGATGCGCALDAVTR.G  + Deamidated (NQ) |
| 97591 | 95 | – | 110 | 864.4064 | 1726.7983 | 1726.8122 | -8.06 | 1 | 15 | 0.039 | 1Score **> 32** indicates **identity** Score **> 13** indicates **homology** | U | K.TALVERDDFSSGTSSR.S |
| 97596 | 95 | – | 110 | 576.6094 | 1726.8063 | 1726.8122 | -3.44 | 1 | 45 | 6.4e-05 | 1Score **> 32** indicates **identity** Score **> 15** indicates **homology** | U | K.TALVERDDFSSGTSSR.S |
| 97597 | 95 | – | 110 | 576.6106 | 1726.8101 | 1726.8122 | -1.22 | 1 | 43 | 8.8e-05 | 1Score **> 33** indicates **identity** Score **> 15** indicates **homology** | U | K.TALVERDDFSSGTSSR.S |
| 97598 | 95 | – | 110 | 576.6106 | 1726.8101 | 1726.8122 | -1.22 | 1 | 40 | 0.0002 | 1Score **> 33** indicates **identity** Score **> 15** indicates **homology** | U | K.TALVERDDFSSGTSSR.S |
| 97599 | 95 | – | 110 | 576.6107 | 1726.8101 | 1726.8122 | -1.20 | 1 | 16 | 0.032 | 1Score **> 33** indicates **identity** Score **> 14** indicates **homology** | U | K.TALVERDDFSSGTSSR.S |
| 97600 | 95 | – | 110 | 576.6107 | 1726.8102 | 1726.8122 | -1.16 | 1 | 33 | 0.00073 | 1Score **> 33** indicates **identity** Score **> 15** indicates **homology** | U | K.TALVERDDFSSGTSSR.S |
| 97601 | 95 | – | 110 | 576.6109 | 1726.8108 | 1726.8122 | -0.82 | 1 | 20 | 0.014 | 1Score **> 33** indicates **identity** Score **> 14** indicates **homology** | U | K.TALVERDDFSSGTSSR.S |
| 97602 | 95 | – | 110 | 576.6109 | 1726.8109 | 1726.8122 | -0.77 | 1 | 22 | 0.0084 | 1Score **> 33** indicates **identity** Score **> 14** indicates **homology** | U | K.TALVERDDFSSGTSSR.S |
| 97603 | 95 | – | 110 | 864.4127 | 1726.8109 | 1726.8122 | -0.75 | 1 | 30 | 0.0014 | 1Score **> 33** indicates **identity** Score **> 14** indicates **homology** | U | K.TALVERDDFSSGTSSR.S |
| 97604 | 95 | – | 110 | 576.6109 | 1726.8110 | 1726.8122 | -0.72 | 1 | 46 | 4.6e-05 | 1Score **> 33** indicates **identity** Score **> 15** indicates **homology** | U | K.TALVERDDFSSGTSSR.S |
| 97606 | 95 | – | 110 | 864.4130 | 1726.8114 | 1726.8122 | -0.49 | 1 | 77 | 2.6e-07 | 1Score **> 33** indicates **identity** Score **> 23** indicates **homology** | U | K.TALVERDDFSSGTSSR.S |
| 97607 | 95 | – | 110 | 576.6111 | 1726.8115 | 1726.8122 | -0.41 | 1 | 38 | 0.00025 | 1Score **> 33** indicates **identity** Score **> 15** indicates **homology** | U | K.TALVERDDFSSGTSSR.S |
| 97608 | 95 | – | 110 | 576.6111 | 1726.8116 | 1726.8122 | -0.36 | 1 | 37 | 0.00031 | 1Score **> 33** indicates **identity** Score **> 15** indicates **homology** | U | K.TALVERDDFSSGTSSR.S |
| 97610 | 95 | – | 110 | 576.6112 | 1726.8117 | 1726.8122 | -0.28 | 1 | 16 | 0.029 | 1Score **> 33** indicates **identity** Score **> 14** indicates **homology** | U | K.TALVERDDFSSGTSSR.S |
| 97611 | 95 | – | 110 | 576.6112 | 1726.8117 | 1726.8122 | -0.28 | 1 | 61 | 1.8e-06 | 1Score **> 33** indicates **identity** Score **> 16** indicates **homology** | U | K.TALVERDDFSSGTSSR.S |
| 97612 | 95 | – | 110 | 576.6112 | 1726.8119 | 1726.8122 | -0.17 | 1 | 66 | 7.1e-07 | 1Score **> 33** indicates **identity** Score **> 17** indicates **homology** | U | K.TALVERDDFSSGTSSR.S |
| 97613 | 95 | – | 110 | 576.6113 | 1726.8121 | 1726.8122 | -0.057 | 1 | 18 | 0.02 | 1Score **> 33** indicates **identity** Score **> 14** indicates **homology** | U | K.TALVERDDFSSGTSSR.S |
| 97614 | 95 | – | 110 | 576.6113 | 1726.8122 | 1726.8122 | -0.031 | 1 | 40 | 0.00019 | 1Score **> 33** indicates **identity** Score **> 15** indicates **homology** | U | K.TALVERDDFSSGTSSR.S |
| 97615 | 95 | – | 110 | 576.6114 | 1726.8123 | 1726.8122 | 0.045 | 1 | 62 | 1.6e-06 | 1Score **> 33** indicates **identity** Score **> 16** indicates **homology** | U | K.TALVERDDFSSGTSSR.S |
| 97617 | 95 | – | 110 | 576.6115 | 1726.8126 | 1726.8122 | 0.25 | 1 | 51 | 1.8e-05 | 1Score **> 32** indicates **identity** Score **> 16** indicates **homology** | U | K.TALVERDDFSSGTSSR.S |
| 97618 | 95 | – | 110 | 864.4136 | 1726.8127 | 1726.8122 | 0.28 | 1 | 109 | 5.9e-11 | 1Score **> 32** indicates **identity** Score **> 19** indicates **homology** | U | K.TALVERDDFSSGTSSR.S |
| 97619 | 95 | – | 110 | 576.6115 | 1726.8128 | 1726.8122 | 0.35 | 1 | 47 | 3.8e-05 | 1Score **> 32** indicates **identity** Score **> 15** indicates **homology** | U | K.TALVERDDFSSGTSSR.S |
| 97620 | 95 | – | 110 | 576.6116 | 1726.8130 | 1726.8122 | 0.47 | 1 | 57 | 4.3e-06 | 1Score **> 33** indicates **identity** Score **> 16** indicates **homology** | U | K.TALVERDDFSSGTSSR.S |
| 97621 | 95 | – | 110 | 864.4138 | 1726.8131 | 1726.8122 | 0.54 | 1 | 102 | 2.8e-10 | 1Score **> 33** indicates **identity** Score **> 19** indicates **homology** | U | K.TALVERDDFSSGTSSR.S |
| 97623 | 95 | – | 110 | 864.4139 | 1726.8133 | 1726.8122 | 0.62 | 1 | 61 | 2e-06 | 1Score **> 33** indicates **identity** Score **> 16** indicates **homology** | U | K.TALVERDDFSSGTSSR.S |
| 97624 | 95 | – | 110 | 576.6117 | 1726.8133 | 1726.8122 | 0.62 | 1 | 47 | 4.2e-05 | 1Score **> 33** indicates **identity** Score **> 15** indicates **homology** | U | K.TALVERDDFSSGTSSR.S |
| 97625 | 95 | – | 110 | 576.6117 | 1726.8134 | 1726.8122 | 0.67 | 1 | 67 | 5.3e-07 | 1Score **> 33** indicates **identity** Score **> 17** indicates **homology** | U | K.TALVERDDFSSGTSSR.S |
| 97626 | 95 | – | 110 | 864.4140 | 1726.8134 | 1726.8122 | 0.68 | 1 | 91 | 2.9e-09 | 1Score **> 33** indicates **identity** Score **> 18** indicates **homology** | U | K.TALVERDDFSSGTSSR.S |
| 97627 | 95 | – | 110 | 576.6118 | 1726.8136 | 1726.8122 | 0.80 | 1 | 18 | 0.021 | 1Score **> 33** indicates **identity** Score **> 14** indicates **homology** | U | K.TALVERDDFSSGTSSR.S |
| 97629 | 95 | – | 110 | 864.4143 | 1726.8141 | 1726.8122 | 1.11 | 1 | 85 | 1.1e-08 | 1Score **> 33** indicates **identity** Score **> 18** indicates **homology** | U | K.TALVERDDFSSGTSSR.S |
| 97630 | 95 | – | 110 | 576.6120 | 1726.8143 | 1726.8122 | 1.20 | 1 | 65 | 7.8e-07 | 1Score **> 33** indicates **identity** Score **> 17** indicates **homology** | U | K.TALVERDDFSSGTSSR.S |
| 97632 | 95 | – | 110 | 864.4146 | 1726.8146 | 1726.8122 | 1.37 | 1 | 78 | 5.2e-08 | 1Score **> 33** indicates **identity** Score **> 17** indicates **homology** | U | K.TALVERDDFSSGTSSR.S |
| 97633 | 95 | – | 110 | 576.6123 | 1726.8150 | 1726.8122 | 1.63 | 1 | 26 | 0.004 | 1Score **> 33** indicates **identity** Score **> 14** indicates **homology** | U | K.TALVERDDFSSGTSSR.S |
| 97634 | 95 | – | 110 | 576.6124 | 1726.8152 | 1726.8122 | 1.76 | 1 | 31 | 0.0013 | 1Score **> 33** indicates **identity** Score **> 14** indicates **homology** | U | K.TALVERDDFSSGTSSR.S |
| 97640 | 95 | – | 110 | 864.4155 | 1726.8165 | 1726.8122 | 2.48 | 1 | 38 | 0.00029 | 1Score **> 33** indicates **identity** Score **> 15** indicates **homology** | U | K.TALVERDDFSSGTSSR.S |
| 97641 | 95 | – | 110 | 864.4157 | 1726.8168 | 1726.8122 | 2.68 | 1 | 100 | 4.7e-10 | 1Score **> 33** indicates **identity** Score **> 19** indicates **homology** | U | K.TALVERDDFSSGTSSR.S |
| 97644 | 95 | – | 110 | 576.6135 | 1726.8187 | 1726.8122 | 3.79 | 1 | 15 | 0.039 | 1Score **> 33** indicates **identity** Score **> 13** indicates **homology** | U | K.TALVERDDFSSGTSSR.S |
| 18601 | 101 | – | 110 | 529.7228 | 1057.4311 | 1057.4312 | -0.14 | 0 | 29 | 0.0017 | 1Score **> 22** indicates **identity** Score **> 14** indicates **homology** | U | R.DDFSSGTSSR.S |
| 18602 | 101 | – | 110 | 529.7229 | 1057.4312 | 1057.4312 | -0.042 | 0 | 50 | 2.1e-05 | 1Score **> 22** indicates **identity** Score **> 16** indicates **homology** | U | R.DDFSSGTSSR.S |
| 19454 | 111 | – | 120 | 356.5490 | 1066.6253 | 1066.6247 | 0.50 | 1 | 36 | 0.00041 | 1Score **> 27** indicates **identity** Score **> 15** indicates **homology** | U | R.STKLIHGGVR.Y |
| 49801 | 125 | – | 135 | 676.3376 | 1350.6607 | 1350.6602 | 0.43 | 0 | 23 | 0.0065 | 1Score **> 33** indicates **identity** Score **> 14** indicates **homology** | U | K.AIMNLDVEQYR.M |
| 49803 | 125 | – | 135 | 676.3379 | 1350.6612 | 1350.6602 | 0.78 | 0 | 25 | 0.0049 | 1Score **> 33** indicates **identity** Score **> 14** indicates **homology** | U | K.AIMNLDVEQYR.M |
| 49806 | 125 | – | 135 | 676.3400 | 1350.6655 | 1350.6602 | 3.93 | 0 | 15 | 0.037 | 1Score **> 33** indicates **identity** Score **> 13** indicates **homology** | U | K.AIMNLDVEQYR.M |
| 51402 | 125 | – | 135 | 684.3365 | 1366.6585 | 1366.6551 | 2.52 | 0 | 48 | 3.4e-05 | 1Score **> 32** indicates **identity** Score **> 15** indicates **homology** | U | K.AIMNLDVEQYR.M  + Oxidation (M) |
| 51403 | 125 | – | 135 | 684.3366 | 1366.6587 | 1366.6551 | 2.63 | 0 | 47 | 3.5e-05 | 1Score **> 32** indicates **identity** Score **> 15** indicates **homology** | U | K.AIMNLDVEQYR.M  + Oxidation (M) |
| 159748 | 145 | – | 166 | 805.4667 | 2413.3782 | 2413.3806 | -1.01 | 0 | 66 | 6.6e-07 | 1Score **> 32** indicates **identity** Score **> 17** indicates **homology** | U | R.ANLLEIAPHLSAPLPIMLPLYK.W |
| 159749 | 145 | – | 166 | 1207.7072 | 2413.3999 | 2413.3806 | 8.00 | 0 | 76 | 6.8e-08 | 1Score **> 30** indicates **identity** Score **> 17** indicates **homology** | U | R.ANLLEIAPHLSAPLPIMLPLYK.W |
| 85711 | 167 | – | 178 | 819.9273 | 1637.8401 | 1637.8395 | 0.39 | 0 | 35 | 0.00051 | 1Score **> 35** indicates **identity** Score **> 15** indicates **homology** | U | K.WWQLPYYWVGIK.M |
| 53151 | 179 | – | 190 | 692.8325 | 1383.6504 | 1383.6526 | -1.60 | 0 | 58 | 4e-06 | 1Score **> 31** indicates **identity** Score **> 16** indicates **homology** | U | K.MYDLVAGSQCLK.S |
| 53152 | 179 | – | 190 | 692.8329 | 1383.6513 | 1383.6526 | -0.97 | 0 | 37 | 0.00035 | 1Score **> 31** indicates **identity** Score **> 15** indicates **homology** | U | K.MYDLVAGSQCLK.S |
| 53154 | 179 | – | 190 | 692.8341 | 1383.6537 | 1383.6526 | 0.73 | 0 | 43 | 8.6e-05 | 1Score **> 32** indicates **identity** Score **> 15** indicates **homology** | U | K.MYDLVAGSQCLK.S |
| 2848 | 191 | – | 197 | 392.2159 | 782.4173 | 782.4174 | -0.13 | 0 | 46 | 6.3e-05 | 1Score **> 17** indicates **identity** | U | K.SSYVLSK.S |
| 34366 | 200 | – | 209 | 405.2169 | 1212.6289 | 1212.6325 | -2.94 | 0 | 22 | 0.009 | 1Score **> 32** indicates **identity** Score **> 14** indicates **homology** | U | R.ALEHFPMLQK.D |
| 34369 | 200 | – | 209 | 405.2179 | 1212.6318 | 1212.6325 | -0.56 | 0 | 25 | 0.0044 | 1Score **> 32** indicates **identity** Score **> 14** indicates **homology** | U | R.ALEHFPMLQK.D |
| 129346 | 210 | – | 227 | 678.6722 | 2032.9949 | 2032.9966 | -0.87 | 1 | 23 | 0.0073 | 1Score **> 36** indicates **identity** Score **> 14** indicates **homology** | U | K.DKLVGAIVYYDGQHNDAR.M |
| 129350 | 210 | – | 227 | 509.2565 | 2032.9969 | 2032.9966 | 0.14 | 1 | 38 | 0.00027 | 1Score **> 36** indicates **identity** Score **> 15** indicates **homology** | U | K.DKLVGAIVYYDGQHNDAR.M |
| 129352 | 210 | – | 227 | 509.2567 | 2032.9976 | 2032.9966 | 0.48 | 1 | 36 | 0.00038 | 1Score **> 36** indicates **identity** Score **> 15** indicates **homology** | U | K.DKLVGAIVYYDGQHNDAR.M |
| 129353 | 210 | – | 227 | 678.6732 | 2032.9978 | 2032.9966 | 0.55 | 1 | 61 | 1.9e-06 | 1Score **> 36** indicates **identity** Score **> 16** indicates **homology** | U | K.DKLVGAIVYYDGQHNDAR.M |
| 129354 | 210 | – | 227 | 1017.5063 | 2032.9980 | 2032.9966 | 0.66 | 1 | 38 | 0.00027 | 1Score **> 36** indicates **identity** Score **> 15** indicates **homology** | U | K.DKLVGAIVYYDGQHNDAR.M |
| 129355 | 210 | – | 227 | 1017.5063 | 2032.9980 | 2032.9966 | 0.69 | 1 | 50 | 2e-05 | 1Score **> 36** indicates **identity** Score **> 16** indicates **homology** | U | K.DKLVGAIVYYDGQHNDAR.M |
| 129356 | 210 | – | 227 | 678.6733 | 2032.9982 | 2032.9966 | 0.77 | 1 | 52 | 1.5e-05 | 1Score **> 36** indicates **identity** Score **> 16** indicates **homology** | U | K.DKLVGAIVYYDGQHNDAR.M |
| 129357 | 210 | – | 227 | 678.6735 | 2032.9986 | 2032.9966 | 0.94 | 1 | 49 | 2.6e-05 | 1Score **> 36** indicates **identity** Score **> 16** indicates **homology** | U | K.DKLVGAIVYYDGQHNDAR.M |
| 129359 | 210 | – | 227 | 509.2571 | 2032.9993 | 2032.9966 | 1.31 | 1 | 17 | 0.025 | 1Score **> 36** indicates **identity** Score **> 14** indicates **homology** | U | K.DKLVGAIVYYDGQHNDAR.M |
| 129360 | 210 | – | 227 | 678.6740 | 2033.0001 | 2032.9966 | 1.70 | 1 | 16 | 0.031 | 1Score **> 36** indicates **identity** Score **> 14** indicates **homology** | U | K.DKLVGAIVYYDGQHNDAR.M |
| 129366 | 210 | – | 227 | 1017.5093 | 2033.0041 | 2032.9966 | 3.66 | 1 | 72 | 2.1e-07 | 1Score **> 36** indicates **identity** Score **> 18** indicates **homology** | U | K.DKLVGAIVYYDGQHNDAR.M |
| 129369 | 210 | – | 227 | 678.6756 | 2033.0050 | 2032.9966 | 4.10 | 1 | 27 | 0.0031 | 1Score **> 36** indicates **identity** Score **> 14** indicates **homology** | U | K.DKLVGAIVYYDGQHNDAR.M |
| 129371 | 210 | – | 227 | 678.6759 | 2033.0060 | 2032.9966 | 4.61 | 1 | 20 | 0.014 | 1Score **> 36** indicates **identity** Score **> 14** indicates **homology** | U | K.DKLVGAIVYYDGQHNDAR.M |
| 129372 | 210 | – | 227 | 678.6762 | 2033.0067 | 2032.9966 | 4.93 | 1 | 25 | 0.0044 | 1Score **> 36** indicates **identity** Score **> 14** indicates **homology** | U | K.DKLVGAIVYYDGQHNDAR.M |
| 129374 | 210 | – | 227 | 678.6772 | 2033.0097 | 2032.9966 | 6.43 | 1 | 14 | 0.049 | 1Score **> 36** indicates **identity** Score **> 13** indicates **homology** | U | K.DKLVGAIVYYDGQHNDAR.M |
| 129379 | 210 | – | 227 | 509.2603 | 2033.0123 | 2032.9966 | 7.68 | 1 | 31 | 0.0014 | 1Score **> 37** indicates **identity** Score **> 14** indicates **homology** | U | K.DKLVGAIVYYDGQHNDAR.M |
| 129465 | 210 | – | 227 | 1018.0065 | 2033.9985 | 2033.9807 | 8.76 | 1 | 27 | 0.003 | 1Score **> 36** indicates **identity** Score **> 14** indicates **homology** | U | K.DKLVGAIVYYDGQHNDAR.M  + Deamidated (NQ) |
| 104876 | 212 | – | 227 | 895.9393 | 1789.8640 | 1789.8747 | -5.98 | 0 | 52 | 1.4e-05 | 1Score **> 34** indicates **identity** Score **> 16** indicates **homology** | U | K.LVGAIVYYDGQHNDAR.M |
| 104877 | 212 | – | 227 | 597.6304 | 1789.8692 | 1789.8747 | -3.07 | 0 | 37 | 0.00036 | 1Score **> 35** indicates **identity** Score **> 15** indicates **homology** | U | K.LVGAIVYYDGQHNDAR.M |
| 104880 | 212 | – | 227 | 597.6319 | 1789.8738 | 1789.8747 | -0.54 | 0 | 31 | 0.0012 | 1Score **> 35** indicates **identity** Score **> 14** indicates **homology** | U | K.LVGAIVYYDGQHNDAR.M |
| 104881 | 212 | – | 227 | 895.9447 | 1789.8748 | 1789.8747 | 0.041 | 0 | 76 | 7.2e-08 | 1Score **> 35** indicates **identity** Score **> 17** indicates **homology** | U | K.LVGAIVYYDGQHNDAR.M |
| 104882 | 212 | – | 227 | 895.9449 | 1789.8753 | 1789.8747 | 0.31 | 0 | 59 | 2.8e-06 | 1Score **> 35** indicates **identity** Score **> 16** indicates **homology** | U | K.LVGAIVYYDGQHNDAR.M |
| 104883 | 212 | – | 227 | 597.6324 | 1789.8753 | 1789.8747 | 0.31 | 0 | 36 | 0.00043 | 1Score **> 35** indicates **identity** Score **> 15** indicates **homology** | U | K.LVGAIVYYDGQHNDAR.M |
| 104884 | 212 | – | 227 | 597.6325 | 1789.8757 | 1789.8747 | 0.52 | 0 | 16 | 0.03 | 1Score **> 35** indicates **identity** Score **> 14** indicates **homology** | U | K.LVGAIVYYDGQHNDAR.M |
| 104885 | 212 | – | 227 | 597.6327 | 1789.8764 | 1789.8747 | 0.94 | 0 | 46 | 4.7e-05 | 1Score **> 35** indicates **identity** Score **> 15** indicates **homology** | U | K.LVGAIVYYDGQHNDAR.M |
| 104886 | 212 | – | 227 | 597.6330 | 1789.8770 | 1789.8747 | 1.28 | 0 | 41 | 0.00014 | 1Score **> 35** indicates **identity** Score **> 15** indicates **homology** | U | K.LVGAIVYYDGQHNDAR.M |
| 104887 | 212 | – | 227 | 895.9464 | 1789.8782 | 1789.8747 | 1.94 | 0 | 76 | 7.3e-08 | 1Score **> 35** indicates **identity** Score **> 17** indicates **homology** | U | K.LVGAIVYYDGQHNDAR.M |
| 104888 | 212 | – | 227 | 597.6338 | 1789.8797 | 1789.8747 | 2.75 | 0 | 19 | 0.017 | 1Score **> 35** indicates **identity** Score **> 14** indicates **homology** | U | K.LVGAIVYYDGQHNDAR.M |
| 104892 | 212 | – | 227 | 895.9488 | 1789.8830 | 1789.8747 | 4.60 | 0 | 63 | 1.3e-06 | 1Score **> 35** indicates **identity** Score **> 16** indicates **homology** | U | K.LVGAIVYYDGQHNDAR.M |
| 26935 | 228 | – | 238 | 572.8278 | 1143.6411 | 1143.6434 | -2.00 | 0 | 32 | 0.0078 | 1Score **> 34** indicates **identity** Score **> 23** indicates **homology** | U | R.MNLAIALTAAR.Y |
| 26940 | 228 | – | 238 | 572.8291 | 1143.6437 | 1143.6434 | 0.31 | 0 | 25 | 0.0058 | 1Score **> 34** indicates **identity** Score **> 15** indicates **homology** | U | R.MNLAIALTAAR.Y |
| 26942 | 228 | – | 238 | 572.8297 | 1143.6449 | 1143.6434 | 1.37 | 0 | 71 | 5.4e-06 | 1Score **> 34** indicates **identity** Score **> 31** indicates **homology** | U | R.MNLAIALTAAR.Y |
| 26943 | 228 | – | 238 | 572.8298 | 1143.6450 | 1143.6434 | 1.39 | 0 | 51 | 6.9e-05 | 1Score **> 34** indicates **identity** Score **> 22** indicates **homology** | U | R.MNLAIALTAAR.Y |
| 26945 | 228 | – | 238 | 572.8307 | 1143.6468 | 1143.6434 | 3.01 | 0 | 71 | 6.7e-06 | 1Score **> 34** indicates **identity** Score **> 32** indicates **homology** | U | R.MNLAIALTAAR.Y |
| 26946 | 228 | – | 238 | 572.8317 | 1143.6489 | 1143.6434 | 4.83 | 0 | 55 | 2.5e-05 | 1Score **> 33** indicates **identity** Score **> 22** indicates **homology** | U | R.MNLAIALTAAR.Y |
| 97952 | 239 | – | 254 | 865.4401 | 1728.8657 | 1728.8756 | -5.77 | 0 | 41 | 0.00015 | 1Score **> 35** indicates **identity** Score **> 15** indicates **homology** | U | R.YGAATANYMEVVSLLK.K |
| 97969 | 239 | – | 254 | 865.4459 | 1728.8773 | 1728.8756 | 0.96 | 0 | 92 | 2.4e-09 | 1Score **> 35** indicates **identity** Score **> 18** indicates **homology** | U | R.YGAATANYMEVVSLLK.K |
| 97972 | 239 | – | 254 | 865.4471 | 1728.8797 | 1728.8756 | 2.34 | 0 | 59 | 3e-06 | 1Score **> 35** indicates **identity** Score **> 16** indicates **homology** | U | R.YGAATANYMEVVSLLK.K |
| 112032 | 239 | – | 255 | 619.9974 | 1856.9704 | 1856.9706 | -0.11 | 1 | 57 | 4.3e-06 | 1Score **> 36** indicates **identity** Score **> 16** indicates **homology** | U | R.YGAATANYMEVVSLLKK.T |
| 112033 | 239 | – | 255 | 619.9974 | 1856.9704 | 1856.9706 | -0.092 | 1 | 50 | 2.2e-05 | 1Score **> 36** indicates **identity** Score **> 16** indicates **homology** | U | R.YGAATANYMEVVSLLKK.T |
| 75716 | 270 | – | 282 | 783.8790 | 1565.7435 | 1565.7508 | -4.66 | 1 | 22 | 0.0086 | 1Score **> 33** indicates **identity** Score **> 14** indicates **homology** | U | R.CKDVLTGQEFDVR.A |
| 75718 | 270 | – | 282 | 783.8791 | 1565.7436 | 1565.7508 | -4.60 | 1 | 48 | 3.3e-05 | 1Score **> 33** indicates **identity** Score **> 15** indicates **homology** | U | R.CKDVLTGQEFDVR.A |
| 75721 | 270 | – | 282 | 783.8814 | 1565.7482 | 1565.7508 | -1.64 | 1 | 48 | 2.9e-05 | 1Score **> 33** indicates **identity** Score **> 16** indicates **homology** | U | R.CKDVLTGQEFDVR.A |
| 75722 | 270 | – | 282 | 783.8820 | 1565.7494 | 1565.7508 | -0.93 | 1 | 80 | 3.1e-08 | 1Score **> 33** indicates **identity** Score **> 18** indicates **homology** | U | R.CKDVLTGQEFDVR.A |
| 75723 | 270 | – | 282 | 522.9240 | 1565.7502 | 1565.7508 | -0.42 | 1 | 14 | 0.05 | 1Score **> 33** indicates **identity** Score **> 13** indicates **homology** | U | R.CKDVLTGQEFDVR.A |
| 75724 | 270 | – | 282 | 783.8824 | 1565.7502 | 1565.7508 | -0.39 | 1 | 40 | 0.00025 | 1Score **> 33** indicates **identity** Score **> 17** indicates **homology** | U | R.CKDVLTGQEFDVR.A |
| 75725 | 270 | – | 282 | 522.9243 | 1565.7509 | 1565.7508 | 0.082 | 1 | 32 | 0.00096 | 1Score **> 33** indicates **identity** Score **> 15** indicates **homology** | U | R.CKDVLTGQEFDVR.A |
| 75726 | 270 | – | 282 | 783.8833 | 1565.7521 | 1565.7508 | 0.84 | 1 | 65 | 1.5e-06 | 1Score **> 33** indicates **identity** Score **> 19** indicates **homology** | U | R.CKDVLTGQEFDVR.A |
| 75727 | 270 | – | 282 | 522.9247 | 1565.7522 | 1565.7508 | 0.91 | 1 | 75 | 9.7e-08 | 1Score **> 33** indicates **identity** Score **> 17** indicates **homology** | U | R.CKDVLTGQEFDVR.A |
| 75730 | 270 | – | 282 | 783.8836 | 1565.7527 | 1565.7508 | 1.24 | 1 | 64 | 9.4e-07 | 1Score **> 33** indicates **identity** Score **> 17** indicates **homology** | U | R.CKDVLTGQEFDVR.A |
| 75731 | 270 | – | 282 | 522.9250 | 1565.7531 | 1565.7508 | 1.44 | 1 | 76 | 7.6e-08 | 1Score **> 33** indicates **identity** Score **> 17** indicates **homology** | U | R.CKDVLTGQEFDVR.A |
| 75732 | 270 | – | 282 | 522.9250 | 1565.7532 | 1565.7508 | 1.55 | 1 | 61 | 2.1e-06 | 1Score **> 33** indicates **identity** Score **> 16** indicates **homology** | U | R.CKDVLTGQEFDVR.A |
| 75733 | 270 | – | 282 | 522.9251 | 1565.7534 | 1565.7508 | 1.67 | 1 | 64 | 1.1e-06 | 1Score **> 33** indicates **identity** Score **> 16** indicates **homology** | U | R.CKDVLTGQEFDVR.A |
| 41682 | 272 | – | 282 | 639.8192 | 1277.6239 | 1277.6252 | -0.98 | 0 | 60 | 4.3e-06 | 1Score **> 33** indicates **identity** Score **> 19** indicates **homology** | U | K.DVLTGQEFDVR.A |
| 41685 | 272 | – | 282 | 639.8199 | 1277.6253 | 1277.6252 | 0.089 | 0 | 73 | 3.5e-07 | 1Score **> 33** indicates **identity** Score **> 21** indicates **homology** | U | K.DVLTGQEFDVR.A |
| 41686 | 272 | – | 282 | 639.8200 | 1277.6255 | 1277.6252 | 0.27 | 0 | 75 | 2.1e-07 | 1Score **> 33** indicates **identity** Score **> 21** indicates **homology** | U | K.DVLTGQEFDVR.A |
| 41687 | 272 | – | 282 | 639.8201 | 1277.6256 | 1277.6252 | 0.29 | 0 | 30 | 0.0017 | 1Score **> 33** indicates **identity** Score **> 14** indicates **homology** | U | K.DVLTGQEFDVR.A |
| 96857 | 283 | – | 298 | 574.6273 | 1720.8599 | 1720.8567 | 1.91 | 1 | 29 | 0.0019 | 1Score **> 35** indicates **identity** Score **> 14** indicates **homology** | U | R.AKCVINASGPFTDSVR.K |
| 96858 | 283 | – | 298 | 574.6276 | 1720.8608 | 1720.8567 | 2.43 | 1 | 43 | 0.0001 | 1Score **> 35** indicates **identity** Score **> 15** indicates **homology** | U | R.AKCVINASGPFTDSVR.K |
| 69795 | 285 | – | 298 | 761.8648 | 1521.7150 | 1521.7246 | -6.32 | 0 | 69 | 3.3e-07 | 1Score **> 32** indicates **identity** Score **> 17** indicates **homology** | U | K.CVINASGPFTDSVR.K |
| 69798 | 285 | – | 298 | 761.8670 | 1521.7194 | 1521.7246 | -3.39 | 0 | 72 | 1.7e-07 | 1Score **> 32** indicates **identity** Score **> 17** indicates **homology** | U | K.CVINASGPFTDSVR.K |
| 69799 | 285 | – | 298 | 761.8674 | 1521.7203 | 1521.7246 | -2.82 | 0 | 71 | 3.3e-07 | 1Score **> 32** indicates **identity** Score **> 19** indicates **homology** | U | K.CVINASGPFTDSVR.K |
| 69801 | 285 | – | 298 | 761.8678 | 1521.7211 | 1521.7246 | -2.27 | 0 | 74 | 1.4e-07 | 1Score **> 32** indicates **identity** Score **> 18** indicates **homology** | U | K.CVINASGPFTDSVR.K |
| 69802 | 285 | – | 298 | 761.8685 | 1521.7224 | 1521.7246 | -1.41 | 0 | 59 | 3.2e-06 | 1Score **> 32** indicates **identity** Score **> 16** indicates **homology** | U | K.CVINASGPFTDSVR.K |
| 69803 | 285 | – | 298 | 761.8687 | 1521.7228 | 1521.7246 | -1.16 | 0 | 45 | 6.7e-05 | 1Score **> 32** indicates **identity** Score **> 15** indicates **homology** | U | K.CVINASGPFTDSVR.K |
| 69804 | 285 | – | 298 | 761.8688 | 1521.7230 | 1521.7246 | -1.07 | 0 | 79 | 6.2e-08 | 1Score **> 32** indicates **identity** Score **> 19** indicates **homology** | U | K.CVINASGPFTDSVR.K |
| 69805 | 285 | – | 298 | 761.8688 | 1521.7231 | 1521.7246 | -1.00 | 0 | 75 | 9.4e-08 | 1Score **> 32** indicates **identity** Score **> 17** indicates **homology** | U | K.CVINASGPFTDSVR.K |
| 69806 | 285 | – | 298 | 761.8692 | 1521.7238 | 1521.7246 | -0.55 | 0 | 42 | 0.00011 | 1Score **> 32** indicates **identity** Score **> 15** indicates **homology** | U | K.CVINASGPFTDSVR.K |
| 69807 | 285 | – | 298 | 761.8693 | 1521.7241 | 1521.7246 | -0.35 | 0 | 76 | 7.8e-08 | 1Score **> 32** indicates **identity** Score **> 17** indicates **homology** | U | K.CVINASGPFTDSVR.K |
| 69808 | 285 | – | 298 | 761.8694 | 1521.7242 | 1521.7246 | -0.27 | 0 | 95 | 6.3e-09 | 1Score **> 33** indicates **identity** Score **> 25** indicates **homology** | U | K.CVINASGPFTDSVR.K |
| 69809 | 285 | – | 298 | 761.8695 | 1521.7244 | 1521.7246 | -0.12 | 0 | 85 | 5.2e-08 | 1Score **> 33** indicates **identity** Score **> 24** indicates **homology** | U | K.CVINASGPFTDSVR.K |
| 69810 | 285 | – | 298 | 761.8696 | 1521.7246 | 1521.7246 | 0.037 | 0 | 99 | 1.6e-09 | 1Score **> 32** indicates **identity** Score **> 23** indicates **homology** | U | K.CVINASGPFTDSVR.K |
| 69811 | 285 | – | 298 | 761.8696 | 1521.7247 | 1521.7246 | 0.072 | 0 | 83 | 2.5e-08 | 1Score **> 32** indicates **identity** Score **> 20** indicates **homology** | U | K.CVINASGPFTDSVR.K |
| 69813 | 285 | – | 298 | 761.8699 | 1521.7252 | 1521.7246 | 0.37 | 0 | 94 | 5.2e-09 | 1Score **> 32** indicates **identity** Score **> 24** indicates **homology** | U | K.CVINASGPFTDSVR.K |
| 69815 | 285 | – | 298 | 761.8711 | 1521.7276 | 1521.7246 | 1.97 | 0 | 59 | 3.2e-06 | 1Score **> 33** indicates **identity** Score **> 16** indicates **homology** | U | K.CVINASGPFTDSVR.K |
| 69817 | 285 | – | 298 | 761.8717 | 1521.7289 | 1521.7246 | 2.86 | 0 | 49 | 3.7e-05 | 1Score **> 33** indicates **identity** Score **> 17** indicates **homology** | U | K.CVINASGPFTDSVR.K |
| 69924 | 285 | – | 298 | 762.3646 | 1522.7146 | 1522.7086 | 3.92 | 0 | 42 | 0.00012 | 1Score **> 32** indicates **identity** Score **> 15** indicates **homology** | U | K.CVINASGPFTDSVR.K  + Deamidated (NQ) |
| 69926 | 285 | – | 298 | 762.3652 | 1522.7159 | 1522.7086 | 4.79 | 0 | 72 | 1.8e-07 | 1Score **> 32** indicates **identity** Score **> 17** indicates **homology** | U | K.CVINASGPFTDSVR.K  + Deamidated (NQ) |
| 69928 | 285 | – | 298 | 762.3663 | 1522.7181 | 1522.7086 | 6.24 | 0 | 49 | 2.5e-05 | 1Score **> 32** indicates **identity** Score **> 16** indicates **homology** | U | K.CVINASGPFTDSVR.K  + Deamidated (NQ) |
| 69929 | 285 | – | 298 | 762.3665 | 1522.7185 | 1522.7086 | 6.47 | 0 | 57 | 5.9e-06 | 1Score **> 32** indicates **identity** Score **> 17** indicates **homology** | U | K.CVINASGPFTDSVR.K  + Deamidated (NQ) |
| 69932 | 285 | – | 298 | 762.3690 | 1522.7234 | 1522.7086 | 9.74 | 0 | 52 | 7.1e-05 | 1Score **> 33** indicates **identity** Score **> 23** indicates **homology** | U | K.CVINASGPFTDSVR.K  + Deamidated (NQ) |
| 87410 | 285 | – | 299 | 550.9469 | 1649.8189 | 1649.8196 | -0.41 | 1 | 61 | 1.8e-06 | 1Score **> 34** indicates **identity** Score **> 16** indicates **homology** | U | K.CVINASGPFTDSVRK.M |
| 87412 | 285 | – | 299 | 550.9472 | 1649.8198 | 1649.8196 | 0.14 | 1 | 46 | 4.7e-05 | 1Score **> 34** indicates **identity** Score **> 15** indicates **homology** | U | K.CVINASGPFTDSVRK.M |
| 30589 | 340 | – | 348 | 589.8330 | 1177.6515 | 1177.6536 | -1.76 | 0 | 35 | 0.0034 | 1Score **> 32** indicates **identity** Score **> 23** indicates **homology** | U | R.VIFFLPWEK.M |
| 30592 | 340 | – | 348 | 589.8336 | 1177.6526 | 1177.6536 | -0.78 | 0 | 29 | 0.0056 | 1Score **> 32** indicates **identity** Score **> 19** indicates **homology** | U | R.VIFFLPWEK.M |
| 30593 | 340 | – | 348 | 589.8337 | 1177.6528 | 1177.6536 | -0.67 | 0 | 32 | 0.0045 | 1Score **> 32** indicates **identity** Score **> 22** indicates **homology** | U | R.VIFFLPWEK.M |
| 30599 | 340 | – | 348 | 589.8344 | 1177.6543 | 1177.6536 | 0.60 | 0 | 25 | 0.0041 | 1Score **> 32** indicates **identity** Score **> 14** indicates **homology** | U | R.VIFFLPWEK.M |
| 30601 | 340 | – | 348 | 589.8345 | 1177.6545 | 1177.6536 | 0.79 | 0 | 35 | 0.0035 | 1Score **> 32** indicates **identity** Score **> 23** indicates **homology** | U | R.VIFFLPWEK.M |
| 30602 | 340 | – | 348 | 589.8345 | 1177.6545 | 1177.6536 | 0.81 | 0 | 19 | 0.016 | 1Score **> 32** indicates **identity** Score **> 14** indicates **homology** | U | R.VIFFLPWEK.M |
| 30604 | 340 | – | 348 | 589.8347 | 1177.6548 | 1177.6536 | 1.09 | 0 | 43 | 0.0005 | 1Score **> 32** indicates **identity** Score **> 22** indicates **homology** | U | R.VIFFLPWEK.M |
| 30608 | 340 | – | 348 | 589.8349 | 1177.6552 | 1177.6536 | 1.38 | 0 | 27 | 0.0063 | 1Score **> 32** indicates **identity** Score **> 18** indicates **homology** | U | R.VIFFLPWEK.M |
| 30609 | 340 | – | 348 | 589.8349 | 1177.6552 | 1177.6536 | 1.38 | 0 | 21 | 0.01 | 1Score **> 32** indicates **identity** Score **> 14** indicates **homology** | U | R.VIFFLPWEK.M |
| 30611 | 340 | – | 348 | 589.8350 | 1177.6555 | 1177.6536 | 1.63 | 0 | 16 | 0.028 | 1Score **> 32** indicates **identity** Score **> 14** indicates **homology** | U | R.VIFFLPWEK.M |
| 30615 | 340 | – | 348 | 589.8352 | 1177.6559 | 1177.6536 | 2.01 | 0 | 18 | 0.021 | 1Score **> 32** indicates **identity** Score **> 14** indicates **homology** | U | R.VIFFLPWEK.M |
| 30618 | 340 | – | 348 | 589.8354 | 1177.6562 | 1177.6536 | 2.23 | 0 | 20 | 0.012 | 1Score **> 32** indicates **identity** Score **> 14** indicates **homology** | U | R.VIFFLPWEK.M |
| 30620 | 340 | – | 348 | 589.8356 | 1177.6566 | 1177.6536 | 2.54 | 0 | 19 | 0.018 | 1Score **> 32** indicates **identity** Score **> 14** indicates **homology** | U | R.VIFFLPWEK.M |
| 30621 | 340 | – | 348 | 589.8356 | 1177.6567 | 1177.6536 | 2.65 | 0 | 29 | 0.002 | 1Score **> 32** indicates **identity** Score **> 14** indicates **homology** | U | R.VIFFLPWEK.M |
| 30622 | 340 | – | 348 | 589.8357 | 1177.6568 | 1177.6536 | 2.73 | 0 | 21 | 0.012 | 1Score **> 32** indicates **identity** Score **> 14** indicates **homology** | U | R.VIFFLPWEK.M |
| 30626 | 340 | – | 348 | 589.8367 | 1177.6589 | 1177.6536 | 4.50 | 0 | 20 | 0.014 | 1Score **> 32** indicates **identity** Score **> 14** indicates **homology** | U | R.VIFFLPWEK.M |
| 30627 | 340 | – | 348 | 589.8368 | 1177.6591 | 1177.6536 | 4.72 | 0 | 20 | 0.012 | 1Score **> 32** indicates **identity** Score **> 14** indicates **homology** | U | R.VIFFLPWEK.M |
| 30628 | 340 | – | 348 | 589.8369 | 1177.6592 | 1177.6536 | 4.75 | 0 | 16 | 0.031 | 1Score **> 32** indicates **identity** Score **> 14** indicates **homology** | U | R.VIFFLPWEK.M |
| 196274 | 349 | – | 380 | 891.6891 | 3562.7273 | 3562.7250 | 0.64 | 0 | 16 | 0.033 | 1Score **> 37** indicates **identity** Score **> 13** indicates **homology** | U | K.MTIAGTTDTPTDVTHHPIPSEEDINFILNEVR.N |
| 196275 | 349 | – | 380 | 891.6893 | 3562.7280 | 3562.7250 | 0.86 | 0 | 24 | 0.0062 | 1Score **> 37** indicates **identity** Score **> 14** indicates **homology** | U | K.MTIAGTTDTPTDVTHHPIPSEEDINFILNEVR.N |
| 196276 | 349 | – | 380 | 1188.5834 | 3562.7283 | 3562.7250 | 0.93 | 0 | 79 | 4.3e-08 | 1Score **> 37** indicates **identity** Score **> 17** indicates **homology** | U | K.MTIAGTTDTPTDVTHHPIPSEEDINFILNEVR.N |
| 196277 | 349 | – | 380 | 1188.5847 | 3562.7324 | 3562.7250 | 2.07 | 0 | 18 | 0.019 | 1Score **> 37** indicates **identity** Score **> 14** indicates **homology** | U | K.MTIAGTTDTPTDVTHHPIPSEEDINFILNEVR.N |
| 196280 | 349 | – | 380 | 1188.9173 | 3563.7301 | 3563.7090 | 5.92 | 0 | 55 | 7.3e-06 | 1Score **> 37** indicates **identity** Score **> 16** indicates **homology** | U | K.MTIAGTTDTPTDVTHHPIPSEEDINFILNEVR.N  + Deamidated (NQ) |
| 30848 | 381 | – | 390 | 591.2927 | 1180.5709 | 1180.5724 | -1.29 | 0 | 18 | 0.023 | 1Score **> 31** indicates **identity** Score **> 14** indicates **homology** | U | R.NYLSSDVEVR.R |
| 30849 | 381 | – | 390 | 591.2929 | 1180.5712 | 1180.5724 | -0.98 | 0 | 37 | 0.00036 | 1Score **> 31** indicates **identity** Score **> 15** indicates **homology** | U | R.NYLSSDVEVR.R |
| 30851 | 381 | – | 390 | 591.2929 | 1180.5713 | 1180.5724 | -0.96 | 0 | 27 | 0.0033 | 1Score **> 31** indicates **identity** Score **> 14** indicates **homology** | U | R.NYLSSDVEVR.R |
| 30852 | 381 | – | 390 | 591.2930 | 1180.5714 | 1180.5724 | -0.87 | 0 | 50 | 1.8e-05 | 1Score **> 32** indicates **identity** Score **> 16** indicates **homology** | U | R.NYLSSDVEVR.R |
| 30853 | 381 | – | 390 | 591.2931 | 1180.5717 | 1180.5724 | -0.60 | 0 | 27 | 0.0027 | 1Score **> 32** indicates **identity** Score **> 14** indicates **homology** | U | R.NYLSSDVEVR.R |
| 30854 | 381 | – | 390 | 591.2932 | 1180.5718 | 1180.5724 | -0.47 | 0 | 41 | 0.00015 | 1Score **> 32** indicates **identity** Score **> 15** indicates **homology** | U | R.NYLSSDVEVR.R |
| 30857 | 381 | – | 390 | 591.2935 | 1180.5724 | 1180.5724 | 0.019 | 0 | 33 | 0.00083 | 1Score **> 32** indicates **identity** Score **> 15** indicates **homology** | U | R.NYLSSDVEVR.R |
| 30859 | 381 | – | 390 | 591.2936 | 1180.5726 | 1180.5724 | 0.15 | 0 | 47 | 4.1e-05 | 1Score **> 32** indicates **identity** Score **> 15** indicates **homology** | U | R.NYLSSDVEVR.R |
| 30860 | 381 | – | 390 | 591.2938 | 1180.5730 | 1180.5724 | 0.47 | 0 | 58 | 3.6e-06 | 1Score **> 32** indicates **identity** Score **> 16** indicates **homology** | U | R.NYLSSDVEVR.R |
| 30861 | 381 | – | 390 | 591.2938 | 1180.5730 | 1180.5724 | 0.49 | 0 | 57 | 4.7e-06 | 1Score **> 32** indicates **identity** Score **> 16** indicates **homology** | U | R.NYLSSDVEVR.R |
| 30862 | 381 | – | 390 | 591.2938 | 1180.5730 | 1180.5724 | 0.51 | 0 | 46 | 4.9e-05 | 1Score **> 32** indicates **identity** Score **> 15** indicates **homology** | U | R.NYLSSDVEVR.R |
| 30863 | 381 | – | 390 | 591.2938 | 1180.5731 | 1180.5724 | 0.57 | 0 | 56 | 5.4e-06 | 1Score **> 32** indicates **identity** Score **> 16** indicates **homology** | U | R.NYLSSDVEVR.R |
| 30865 | 381 | – | 390 | 591.2940 | 1180.5735 | 1180.5724 | 0.96 | 0 | 53 | 9.9e-06 | 1Score **> 32** indicates **identity** Score **> 16** indicates **homology** | U | R.NYLSSDVEVR.R |
| 30867 | 381 | – | 390 | 591.2949 | 1180.5753 | 1180.5724 | 2.45 | 0 | 29 | 0.0019 | 1Score **> 32** indicates **identity** Score **> 14** indicates **homology** | U | R.NYLSSDVEVR.R |
| 30872 | 381 | – | 390 | 591.2952 | 1180.5759 | 1180.5724 | 2.92 | 0 | 49 | 2.5e-05 | 1Score **> 32** indicates **identity** Score **> 16** indicates **homology** | U | R.NYLSSDVEVR.R |
| 30873 | 381 | – | 390 | 591.2954 | 1180.5762 | 1180.5724 | 3.23 | 0 | 36 | 0.00044 | 1Score **> 32** indicates **identity** Score **> 15** indicates **homology** | U | R.NYLSSDVEVR.R |
| 30878 | 381 | – | 390 | 591.2963 | 1180.5781 | 1180.5724 | 4.79 | 0 | 21 | 0.035 | 1Score **> 31** indicates **identity** Score **> 19** indicates **homology** | U | R.NYLSSDVEVR.R |
| 30904 | 381 | – | 390 | 591.2971 | 1180.5796 | 1180.5724 | 6.12 | 0 | 18 | 0.022 | 1Score **> 32** indicates **identity** Score **> 14** indicates **homology** | U | R.NYLSSDVEVR.R |
| 30907 | 381 | – | 390 | 591.2972 | 1180.5798 | 1180.5724 | 6.30 | 0 | 27 | 0.0091 | 1Score **> 32** indicates **identity** Score **> 19** indicates **homology** | U | R.NYLSSDVEVR.R |
| 30923 | 381 | – | 390 | 591.2986 | 1180.5827 | 1180.5724 | 8.68 | 0 | 24 | 0.031 | 1Score **> 32** indicates **identity** Score **> 21** indicates **homology** | U | R.NYLSSDVEVR.R |
| 48276 | 381 | – | 391 | 669.3455 | 1336.6764 | 1336.6735 | 2.13 | 1 | 25 | 0.012 | 1Score **> 33** indicates **identity** Score **> 18** indicates **homology** | U | R.NYLSSDVEVRR.G |
| 44101 | 391 | – | 402 | 434.2426 | 1299.7061 | 1299.7048 | 1.02 | 1 | 45 | 5.4e-05 | 1Score **> 34** indicates **identity** Score **> 15** indicates **homology** | U | R.RGDVLAAWSGIR.P |
| 131248 | 391 | – | 409 | 513.5402 | 2050.1316 | 2050.1324 | -0.38 | 2 | 23 | 0.017 | 1Score **> 35** indicates **identity** Score **> 18** indicates **homology** | U | R.RGDVLAAWSGIRPLVTDPK.S |
| 131249 | 391 | – | 409 | 513.5403 | 2050.1321 | 2050.1324 | -0.14 | 2 | 21 | 0.022 | 1Score **> 35** indicates **identity** Score **> 17** indicates **homology** | U | R.RGDVLAAWSGIRPLVTDPK.S |
| 131250 | 391 | – | 409 | 1026.0733 | 2050.1321 | 2050.1324 | -0.12 | 2 | 45 | 8.5e-05 | 1Score **> 35** indicates **identity** Score **> 16** indicates **homology** | U | R.RGDVLAAWSGIRPLVTDPK.S |
| 131251 | 391 | – | 409 | 684.3847 | 2050.1322 | 2050.1324 | -0.099 | 2 | 42 | 0.00019 | 1Score **> 35** indicates **identity** Score **> 17** indicates **homology** | U | R.RGDVLAAWSGIRPLVTDPK.S |
| 131253 | 391 | – | 409 | 684.3847 | 2050.1323 | 2050.1324 | -0.0054 | 2 | 36 | 0.0025 | 1Score **> 35** indicates **identity** Score **> 22** indicates **homology** | U | R.RGDVLAAWSGIRPLVTDPK.S |
| 131254 | 391 | – | 409 | 684.3848 | 2050.1325 | 2050.1324 | 0.050 | 2 | 42 | 0.00023 | 1Score **> 35** indicates **identity** Score **> 18** indicates **homology** | U | R.RGDVLAAWSGIRPLVTDPK.S |
| 131256 | 391 | – | 409 | 1026.0737 | 2050.1328 | 2050.1324 | 0.20 | 2 | 17 | 0.024 | 1Score **> 35** indicates **identity** Score **> 14** indicates **homology** | U | R.RGDVLAAWSGIRPLVTDPK.S |
| 131257 | 391 | – | 409 | 513.5405 | 2050.1328 | 2050.1324 | 0.21 | 2 | 25 | 0.01 | 1Score **> 35** indicates **identity** Score **> 17** indicates **homology** | U | R.RGDVLAAWSGIRPLVTDPK.S |
| 131259 | 391 | – | 409 | 513.5405 | 2050.1330 | 2050.1324 | 0.33 | 2 | 25 | 0.0075 | 1Score **> 35** indicates **identity** Score **> 16** indicates **homology** | U | R.RGDVLAAWSGIRPLVTDPK.S |
| 131261 | 391 | – | 409 | 513.5406 | 2050.1333 | 2050.1324 | 0.46 | 2 | 15 | 0.037 | 1Score **> 35** indicates **identity** Score **> 13** indicates **homology** | U | R.RGDVLAAWSGIRPLVTDPK.S |
| 131262 | 391 | – | 409 | 684.3851 | 2050.1335 | 2050.1324 | 0.54 | 2 | 25 | 0.0064 | 1Score **> 35** indicates **identity** Score **> 16** indicates **homology** | U | R.RGDVLAAWSGIRPLVTDPK.S |
| 131263 | 391 | – | 409 | 1026.0741 | 2050.1336 | 2050.1324 | 0.62 | 2 | 34 | 0.0012 | 1Score **> 35** indicates **identity** Score **> 17** indicates **homology** | U | R.RGDVLAAWSGIRPLVTDPK.S |
| 131264 | 391 | – | 409 | 513.5407 | 2050.1338 | 2050.1324 | 0.71 | 2 | 26 | 0.0059 | 1Score **> 34** indicates **identity** Score **> 17** indicates **homology** | U | R.RGDVLAAWSGIRPLVTDPK.S |
| 131265 | 391 | – | 409 | 684.3852 | 2050.1338 | 2050.1324 | 0.72 | 2 | 42 | 0.00012 | 1Score **> 34** indicates **identity** Score **> 15** indicates **homology** | U | R.RGDVLAAWSGIRPLVTDPK.S |
| 131267 | 391 | – | 409 | 684.3853 | 2050.1341 | 2050.1324 | 0.83 | 2 | 21 | 0.027 | 1Score **> 34** indicates **identity** Score **> 18** indicates **homology** | U | R.RGDVLAAWSGIRPLVTDPK.S |
| 131269 | 391 | – | 409 | 513.5409 | 2050.1344 | 2050.1324 | 1.02 | 2 | 24 | 0.012 | 1Score **> 34** indicates **identity** Score **> 18** indicates **homology** | U | R.RGDVLAAWSGIRPLVTDPK.S |
| 131271 | 391 | – | 409 | 513.5411 | 2050.1352 | 2050.1324 | 1.38 | 2 | 26 | 0.022 | 1Score **> 34** indicates **identity** Score **> 22** indicates **homology** | U | R.RGDVLAAWSGIRPLVTDPK.S |
| 131272 | 391 | – | 409 | 513.5414 | 2050.1363 | 2050.1324 | 1.94 | 2 | 22 | 0.045 | 1Score **> 34** indicates **identity** Score **> 21** indicates **homology** | U | R.RGDVLAAWSGIRPLVTDPK.S |
| 131274 | 391 | – | 409 | 513.5416 | 2050.1372 | 2050.1324 | 2.35 | 2 | 14 | 0.047 | 1Score **> 35** indicates **identity** Score **> 13** indicates **homology** | U | R.RGDVLAAWSGIRPLVTDPK.S |
| 131275 | 391 | – | 409 | 513.5418 | 2050.1379 | 2050.1324 | 2.73 | 2 | 23 | 0.013 | 1Score **> 34** indicates **identity** Score **> 17** indicates **homology** | U | R.RGDVLAAWSGIRPLVTDPK.S |
| 131277 | 391 | – | 409 | 513.5420 | 2050.1389 | 2050.1324 | 3.17 | 2 | 15 | 0.042 | 1Score **> 34** indicates **identity** Score **> 13** indicates **homology** | U | R.RGDVLAAWSGIRPLVTDPK.S |
| 131278 | 391 | – | 409 | 513.5420 | 2050.1390 | 2050.1324 | 3.24 | 2 | 24 | 0.0055 | 1Score **> 34** indicates **identity** Score **> 14** indicates **homology** | U | R.RGDVLAAWSGIRPLVTDPK.S |
| 131280 | 391 | – | 409 | 513.5430 | 2050.1430 | 2050.1324 | 5.21 | 2 | 22 | 0.0095 | 1Score **> 34** indicates **identity** Score **> 14** indicates **homology** | U | R.RGDVLAAWSGIRPLVTDPK.S |
| 131281 | 391 | – | 409 | 513.5432 | 2050.1438 | 2050.1324 | 5.59 | 2 | 15 | 0.042 | 1Score **> 34** indicates **identity** Score **> 13** indicates **homology** | U | R.RGDVLAAWSGIRPLVTDPK.S |
| 105413 | 419 | – | 436 | 898.4742 | 1794.9338 | 1794.9476 | -7.71 | 0 | 48 | 3.5e-05 | 1Score **> 36** indicates **identity** Score **> 15** indicates **homology** | U | R.NHVVDISDSGLITIAGGK.W |
| 105414 | 419 | – | 436 | 599.3186 | 1794.9341 | 1794.9476 | -7.50 | 0 | 17 | 0.025 | 1Score **> 36** indicates **identity** Score **> 14** indicates **homology** | U | R.NHVVDISDSGLITIAGGK.W |
| 105417 | 419 | – | 436 | 599.3194 | 1794.9364 | 1794.9476 | -6.23 | 0 | 24 | 0.005 | 1Score **> 36** indicates **identity** Score **> 14** indicates **homology** | U | R.NHVVDISDSGLITIAGGK.W |
| 105418 | 419 | – | 436 | 599.3197 | 1794.9374 | 1794.9476 | -5.68 | 0 | 25 | 0.0043 | 1Score **> 36** indicates **identity** Score **> 14** indicates **homology** | U | R.NHVVDISDSGLITIAGGK.W |
| 105420 | 419 | – | 436 | 599.3205 | 1794.9396 | 1794.9476 | -4.45 | 0 | 19 | 0.018 | 1Score **> 36** indicates **identity** Score **> 14** indicates **homology** | U | R.NHVVDISDSGLITIAGGK.W |
| 105422 | 419 | – | 436 | 599.3210 | 1794.9413 | 1794.9476 | -3.50 | 0 | 37 | 0.00033 | 1Score **> 36** indicates **identity** Score **> 15** indicates **homology** | U | R.NHVVDISDSGLITIAGGK.W |
| 105424 | 419 | – | 436 | 599.3215 | 1794.9427 | 1794.9476 | -2.73 | 0 | 16 | 0.029 | 1Score **> 36** indicates **identity** Score **> 14** indicates **homology** | U | R.NHVVDISDSGLITIAGGK.W |
| 105428 | 419 | – | 436 | 599.3223 | 1794.9451 | 1794.9476 | -1.38 | 0 | 50 | 2e-05 | 1Score **> 36** indicates **identity** Score **> 16** indicates **homology** | U | R.NHVVDISDSGLITIAGGK.W |
| 105432 | 419 | – | 436 | 599.3227 | 1794.9462 | 1794.9476 | -0.77 | 0 | 23 | 0.0077 | 1Score **> 36** indicates **identity** Score **> 14** indicates **homology** | U | R.NHVVDISDSGLITIAGGK.W |
| 105433 | 419 | – | 436 | 599.3228 | 1794.9466 | 1794.9476 | -0.56 | 0 | 32 | 0.0009 | 1Score **> 36** indicates **identity** Score **> 15** indicates **homology** | U | R.NHVVDISDSGLITIAGGK.W |
| 105434 | 419 | – | 436 | 599.3230 | 1794.9472 | 1794.9476 | -0.24 | 0 | 54 | 8.5e-06 | 1Score **> 36** indicates **identity** Score **> 16** indicates **homology** | U | R.NHVVDISDSGLITIAGGK.W |
| 105440 | 419 | – | 436 | 599.3234 | 1794.9482 | 1794.9476 | 0.36 | 0 | 99 | 6.2e-10 | 1Score **> 36** indicates **identity** Score **> 20** indicates **homology** | U | R.NHVVDISDSGLITIAGGK.W |
| 105442 | 419 | – | 436 | 599.3235 | 1794.9488 | 1794.9476 | 0.65 | 0 | 32 | 0.0011 | 1Score **> 36** indicates **identity** Score **> 14** indicates **homology** | U | R.NHVVDISDSGLITIAGGK.W |
| 105444 | 419 | – | 436 | 599.3236 | 1794.9491 | 1794.9476 | 0.85 | 0 | 15 | 0.038 | 1Score **> 36** indicates **identity** Score **> 13** indicates **homology** | U | R.NHVVDISDSGLITIAGGK.W |
| 105446 | 419 | – | 436 | 599.3237 | 1794.9494 | 1794.9476 | 1.01 | 0 | 82 | 2e-08 | 1Score **> 36** indicates **identity** Score **> 18** indicates **homology** | U | R.NHVVDISDSGLITIAGGK.W |
| 105447 | 419 | – | 436 | 599.3238 | 1794.9495 | 1794.9476 | 1.06 | 0 | 67 | 5.4e-07 | 1Score **> 36** indicates **identity** Score **> 17** indicates **homology** | U | R.NHVVDISDSGLITIAGGK.W |
| 105448 | 419 | – | 436 | 599.3239 | 1794.9499 | 1794.9476 | 1.28 | 0 | 23 | 0.0066 | 1Score **> 36** indicates **identity** Score **> 14** indicates **homology** | U | R.NHVVDISDSGLITIAGGK.W |
| 105450 | 419 | – | 436 | 599.3240 | 1794.9502 | 1794.9476 | 1.46 | 0 | 87 | 8.5e-09 | 1Score **> 36** indicates **identity** Score **> 19** indicates **homology** | U | R.NHVVDISDSGLITIAGGK.W |
| 105453 | 419 | – | 436 | 898.4826 | 1794.9506 | 1794.9476 | 1.69 | 0 | 104 | 1.9e-10 | 1Score **> 36** indicates **identity** Score **> 19** indicates **homology** | U | R.NHVVDISDSGLITIAGGK.W |
| 105454 | 419 | – | 436 | 898.4827 | 1794.9508 | 1794.9476 | 1.77 | 0 | 98 | 6e-10 | 1Score **> 36** indicates **identity** Score **> 19** indicates **homology** | U | R.NHVVDISDSGLITIAGGK.W |
| 105455 | 419 | – | 436 | 599.3243 | 1794.9510 | 1794.9476 | 1.89 | 0 | 95 | 1.6e-09 | 1Score **> 36** indicates **identity** Score **> 20** indicates **homology** | U | R.NHVVDISDSGLITIAGGK.W |
| 105456 | 419 | – | 436 | 599.3243 | 1794.9510 | 1794.9476 | 1.92 | 0 | 25 | 0.0054 | 1Score **> 36** indicates **identity** Score **> 15** indicates **homology** | U | R.NHVVDISDSGLITIAGGK.W |
| 105457 | 419 | – | 436 | 599.3244 | 1794.9513 | 1794.9476 | 2.08 | 0 | 26 | 0.004 | 1Score **> 36** indicates **identity** Score **> 14** indicates **homology** | U | R.NHVVDISDSGLITIAGGK.W |
| 105459 | 419 | – | 436 | 898.4829 | 1794.9513 | 1794.9476 | 2.09 | 0 | 73 | 3.9e-07 | 1Score **> 36** indicates **identity** Score **> 22** indicates **homology** | U | R.NHVVDISDSGLITIAGGK.W |
| 105460 | 419 | – | 436 | 599.3244 | 1794.9513 | 1794.9476 | 2.09 | 0 | 31 | 0.0013 | 1Score **> 36** indicates **identity** Score **> 14** indicates **homology** | U | R.NHVVDISDSGLITIAGGK.W |
| 105462 | 419 | – | 436 | 599.3244 | 1794.9514 | 1794.9476 | 2.14 | 0 | 21 | 0.01 | 1Score **> 36** indicates **identity** Score **> 14** indicates **homology** | U | R.NHVVDISDSGLITIAGGK.W |
| 105463 | 419 | – | 436 | 599.3244 | 1794.9515 | 1794.9476 | 2.17 | 0 | 65 | 8.2e-07 | 1Score **> 36** indicates **identity** Score **> 17** indicates **homology** | U | R.NHVVDISDSGLITIAGGK.W |
| 105464 | 419 | – | 436 | 898.4832 | 1794.9518 | 1794.9476 | 2.35 | 0 | 85 | 1.1e-08 | 1Score **> 35** indicates **identity** Score **> 18** indicates **homology** | U | R.NHVVDISDSGLITIAGGK.W |
| 105466 | 419 | – | 436 | 599.3247 | 1794.9524 | 1794.9476 | 2.68 | 0 | 77 | 6.4e-08 | 1Score **> 36** indicates **identity** Score **> 17** indicates **homology** | U | R.NHVVDISDSGLITIAGGK.W |
| 105467 | 419 | – | 436 | 599.3248 | 1794.9525 | 1794.9476 | 2.72 | 0 | 59 | 3e-06 | 1Score **> 36** indicates **identity** Score **> 16** indicates **homology** | U | R.NHVVDISDSGLITIAGGK.W |
| 105468 | 419 | – | 436 | 599.3248 | 1794.9526 | 1794.9476 | 2.79 | 0 | 73 | 1.6e-07 | 1Score **> 36** indicates **identity** Score **> 17** indicates **homology** | U | R.NHVVDISDSGLITIAGGK.W |
| 105473 | 419 | – | 436 | 898.4839 | 1794.9532 | 1794.9476 | 3.12 | 0 | 79 | 3.5e-08 | 1Score **> 36** indicates **identity** Score **> 17** indicates **homology** | U | R.NHVVDISDSGLITIAGGK.W |
| 105474 | 419 | – | 436 | 898.4841 | 1794.9536 | 1794.9476 | 3.33 | 0 | 76 | 6.8e-08 | 1Score **> 36** indicates **identity** Score **> 17** indicates **homology** | U | R.NHVVDISDSGLITIAGGK.W |
| 105475 | 419 | – | 436 | 599.3252 | 1794.9537 | 1794.9476 | 3.42 | 0 | 49 | 2.7e-05 | 1Score **> 36** indicates **identity** Score **> 16** indicates **homology** | U | R.NHVVDISDSGLITIAGGK.W |
| 105478 | 419 | – | 436 | 599.3255 | 1794.9546 | 1794.9476 | 3.90 | 0 | 18 | 0.019 | 1Score **> 35** indicates **identity** Score **> 14** indicates **homology** | U | R.NHVVDISDSGLITIAGGK.W |
| 105480 | 419 | – | 436 | 599.3255 | 1794.9548 | 1794.9476 | 4.00 | 0 | 49 | 2.4e-05 | 1Score **> 36** indicates **identity** Score **> 16** indicates **homology** | U | R.NHVVDISDSGLITIAGGK.W |
| 105481 | 419 | – | 436 | 599.3255 | 1794.9548 | 1794.9476 | 4.00 | 0 | 20 | 0.014 | 1Score **> 36** indicates **identity** Score **> 14** indicates **homology** | U | R.NHVVDISDSGLITIAGGK.W |
| 105482 | 419 | – | 436 | 599.3255 | 1794.9548 | 1794.9476 | 4.03 | 0 | 38 | 0.00025 | 1Score **> 36** indicates **identity** Score **> 15** indicates **homology** | U | R.NHVVDISDSGLITIAGGK.W |
| 105483 | 419 | – | 436 | 599.3256 | 1794.9548 | 1794.9476 | 4.03 | 0 | 14 | 0.047 | 1Score **> 36** indicates **identity** Score **> 13** indicates **homology** | U | R.NHVVDISDSGLITIAGGK.W |
| 105486 | 419 | – | 436 | 599.3256 | 1794.9550 | 1794.9476 | 4.15 | 0 | 16 | 0.03 | 1Score **> 36** indicates **identity** Score **> 14** indicates **homology** | U | R.NHVVDISDSGLITIAGGK.W |
| 105487 | 419 | – | 436 | 599.3257 | 1794.9552 | 1794.9476 | 4.24 | 0 | 27 | 0.0037 | 1Score **> 35** indicates **identity** Score **> 15** indicates **homology** | U | R.NHVVDISDSGLITIAGGK.W |
| 105489 | 419 | – | 436 | 599.3258 | 1794.9555 | 1794.9476 | 4.40 | 0 | 39 | 0.00023 | 1Score **> 35** indicates **identity** Score **> 15** indicates **homology** | U | R.NHVVDISDSGLITIAGGK.W |
| 105491 | 419 | – | 436 | 599.3258 | 1794.9556 | 1794.9476 | 4.44 | 0 | 65 | 8e-07 | 1Score **> 35** indicates **identity** Score **> 17** indicates **homology** | U | R.NHVVDISDSGLITIAGGK.W |
| 105492 | 419 | – | 436 | 898.4851 | 1794.9556 | 1794.9476 | 4.48 | 0 | 99 | 5.7e-10 | 1Score **> 35** indicates **identity** Score **> 19** indicates **homology** | U | R.NHVVDISDSGLITIAGGK.W |
| 105493 | 419 | – | 436 | 599.3259 | 1794.9560 | 1794.9476 | 4.66 | 0 | 54 | 7.8e-06 | 1Score **> 35** indicates **identity** Score **> 16** indicates **homology** | U | R.NHVVDISDSGLITIAGGK.W |
| 105494 | 419 | – | 436 | 599.3260 | 1794.9562 | 1794.9476 | 4.82 | 0 | 68 | 4.6e-07 | 1Score **> 35** indicates **identity** Score **> 17** indicates **homology** | U | R.NHVVDISDSGLITIAGGK.W |
| 105497 | 419 | – | 436 | 599.3264 | 1794.9572 | 1794.9476 | 5.38 | 0 | 17 | 0.025 | 1Score **> 35** indicates **identity** Score **> 14** indicates **homology** | U | R.NHVVDISDSGLITIAGGK.W |
| 105498 | 419 | – | 436 | 599.3265 | 1794.9575 | 1794.9476 | 5.55 | 0 | 47 | 4.2e-05 | 1Score **> 35** indicates **identity** Score **> 15** indicates **homology** | U | R.NHVVDISDSGLITIAGGK.W |
| 105500 | 419 | – | 436 | 599.3266 | 1794.9579 | 1794.9476 | 5.74 | 0 | 42 | 0.00013 | 1Score **> 35** indicates **identity** Score **> 15** indicates **homology** | U | R.NHVVDISDSGLITIAGGK.W |
| 105501 | 419 | – | 436 | 599.3266 | 1794.9579 | 1794.9476 | 5.74 | 0 | 44 | 7.2e-05 | 1Score **> 35** indicates **identity** Score **> 15** indicates **homology** | U | R.NHVVDISDSGLITIAGGK.W |
| 105503 | 419 | – | 436 | 599.3271 | 1794.9596 | 1794.9476 | 6.68 | 0 | 76 | 7.3e-08 | 1Score **> 35** indicates **identity** Score **> 17** indicates **homology** | U | R.NHVVDISDSGLITIAGGK.W |
| 105504 | 419 | – | 436 | 599.3271 | 1794.9596 | 1794.9476 | 6.70 | 0 | 66 | 7.2e-07 | 1Score **> 35** indicates **identity** Score **> 17** indicates **homology** | U | R.NHVVDISDSGLITIAGGK.W |
| 105505 | 419 | – | 436 | 599.3272 | 1794.9599 | 1794.9476 | 6.86 | 0 | 24 | 0.0054 | 1Score **> 35** indicates **identity** Score **> 14** indicates **homology** | U | R.NHVVDISDSGLITIAGGK.W |
| 105506 | 419 | – | 436 | 898.4875 | 1794.9604 | 1794.9476 | 7.14 | 0 | 83 | 1.8e-08 | 1Score **> 35** indicates **identity** Score **> 18** indicates **homology** | U | R.NHVVDISDSGLITIAGGK.W |
| 105507 | 419 | – | 436 | 599.3275 | 1794.9607 | 1794.9476 | 7.29 | 0 | 31 | 0.0012 | 1Score **> 35** indicates **identity** Score **> 14** indicates **homology** | U | R.NHVVDISDSGLITIAGGK.W |
| 105508 | 419 | – | 436 | 599.3276 | 1794.9608 | 1794.9476 | 7.38 | 0 | 18 | 0.021 | 1Score **> 35** indicates **identity** Score **> 14** indicates **homology** | U | R.NHVVDISDSGLITIAGGK.W |
| 105509 | 419 | – | 436 | 898.4882 | 1794.9618 | 1794.9476 | 7.94 | 0 | 80 | 3.4e-08 | 1Score **> 35** indicates **identity** Score **> 17** indicates **homology** | U | R.NHVVDISDSGLITIAGGK.W |
| 105511 | 419 | – | 436 | 599.3281 | 1794.9624 | 1794.9476 | 8.23 | 0 | 81 | 2.5e-08 | 1Score **> 35** indicates **identity** Score **> 18** indicates **homology** | U | R.NHVVDISDSGLITIAGGK.W |
| 105513 | 419 | – | 436 | 599.3282 | 1794.9627 | 1794.9476 | 8.43 | 0 | 25 | 0.005 | 1Score **> 35** indicates **identity** Score **> 14** indicates **homology** | U | R.NHVVDISDSGLITIAGGK.W |
| 105516 | 419 | – | 436 | 599.3290 | 1794.9651 | 1794.9476 | 9.75 | 0 | 102 | 2.7e-10 | 1Score **> 35** indicates **identity** Score **> 19** indicates **homology** | U | R.NHVVDISDSGLITIAGGK.W |
| 105563 | 419 | – | 436 | 599.6554 | 1795.9444 | 1795.9316 | 7.11 | 0 | 37 | 0.00036 | 1Score **> 35** indicates **identity** Score **> 15** indicates **homology** | U | R.NHVVDISDSGLITIAGGK.W  + Deamidated (NQ) |
| 165132 | 419 | – | 441 | 835.0996 | 2502.2768 | 2502.2867 | -3.95 | 1 | 43 | 9.2e-05 | 1Score **> 37** indicates **identity** Score **> 15** indicates **homology** | U | R.NHVVDISDSGLITIAGGKWTTYR.S |
| 165144 | 419 | – | 441 | 835.1023 | 2502.2851 | 2502.2867 | -0.66 | 1 | 55 | 7.7e-06 | 1Score **> 37** indicates **identity** Score **> 16** indicates **homology** | U | R.NHVVDISDSGLITIAGGKWTTYR.S |
| 165150 | 419 | – | 441 | 626.5811 | 2502.2951 | 2502.2867 | 3.36 | 1 | 37 | 0.00036 | 1Score **> 37** indicates **identity** Score **> 15** indicates **homology** | U | R.NHVVDISDSGLITIAGGKWTTYR.S |
| 36866 | 442 | – | 453 | 618.7927 | 1235.5709 | 1235.5704 | 0.40 | 0 | 55 | 7.2e-06 | 1Score **> 30** indicates **identity** Score **> 16** indicates **homology** | U | R.SMAEDTVDAAVK.F |
| 36867 | 442 | – | 453 | 618.7928 | 1235.5710 | 1235.5704 | 0.53 | 0 | 24 | 0.0055 | 1Score **> 30** indicates **identity** Score **> 14** indicates **homology** | U | R.SMAEDTVDAAVK.F |
| 154271 | 442 | – | 463 | 777.3764 | 2329.1075 | 2329.1121 | -1.97 | 1 | 32 | 0.00097 | 1Score **> 35** indicates **identity** Score **> 15** indicates **homology** | U | R.SMAEDTVDAAVKFHNLNAGPSR.T |
| 23585 | 454 | – | 463 | 371.5246 | 1111.5520 | 1111.5523 | -0.29 | 0 | 38 | 0.00057 | 1Score **> 31** indicates **identity** Score **> 18** indicates **homology** | U | K.FHNLNAGPSR.T |
| 23586 | 454 | – | 463 | 371.5247 | 1111.5521 | 1111.5523 | -0.12 | 0 | 27 | 0.0027 | 1Score **> 31** indicates **identity** Score **> 14** indicates **homology** | U | K.FHNLNAGPSR.T |
| 23588 | 454 | – | 463 | 556.7837 | 1111.5529 | 1111.5523 | 0.56 | 0 | 44 | 0.00022 | 1Score **> 31** indicates **identity** Score **> 20** indicates **homology** | U | K.FHNLNAGPSR.T |
| 23589 | 454 | – | 463 | 371.5249 | 1111.5530 | 1111.5523 | 0.64 | 0 | 33 | 0.00076 | 1Score **> 31** indicates **identity** Score **> 15** indicates **homology** | U | K.FHNLNAGPSR.T |
| 23590 | 454 | – | 463 | 371.5250 | 1111.5533 | 1111.5523 | 0.89 | 0 | 54 | 8.6e-06 | 1Score **> 31** indicates **identity** Score **> 16** indicates **homology** | U | K.FHNLNAGPSR.T |
| 23591 | 454 | – | 463 | 556.7840 | 1111.5535 | 1111.5523 | 1.12 | 0 | 49 | 0.0009 | 1Score **> 31** indicates **identity** | U | K.FHNLNAGPSR.T |
| 23592 | 454 | – | 463 | 556.7841 | 1111.5536 | 1111.5523 | 1.22 | 0 | 29 | 0.0031 | 1Score **> 31** indicates **identity** Score **> 17** indicates **homology** | U | K.FHNLNAGPSR.T |
| 23593 | 454 | – | 463 | 371.5252 | 1111.5537 | 1111.5523 | 1.25 | 0 | 27 | 0.0029 | 1Score **> 31** indicates **identity** Score **> 14** indicates **homology** | U | K.FHNLNAGPSR.T |
| 23643 | 454 | – | 463 | 371.8522 | 1112.5348 | 1112.5363 | -1.30 | 0 | 37 | 0.0032 | 1Score **> 29** indicates **identity** Score **> 24** indicates **homology** | U | K.FHNLNAGPSR.T  + Deamidated (NQ) |
| 15728 | 464 | – | 473 | 510.2993 | 1018.5841 | 1018.5811 | 2.93 | 0 | 33 | 0.00087 | 1Score **> 30** indicates **identity** Score **> 15** indicates **homology** | U | R.TVGLFLQGGK.D |
| 15729 | 464 | – | 473 | 510.2995 | 1018.5844 | 1018.5811 | 3.22 | 0 | 23 | 0.0063 | 1Score **> 30** indicates **identity** Score **> 14** indicates **homology** | U | R.TVGLFLQGGK.D |
| 15730 | 464 | – | 473 | 510.2998 | 1018.5850 | 1018.5811 | 3.77 | 0 | 46 | 4.9e-05 | 1Score **> 31** indicates **identity** Score **> 15** indicates **homology** | U | R.TVGLFLQGGK.D |
| 15731 | 464 | – | 473 | 510.3004 | 1018.5863 | 1018.5811 | 5.09 | 0 | 32 | 0.0011 | 1Score **> 31** indicates **identity** Score **> 14** indicates **homology** | U | R.TVGLFLQGGK.D |
| 140613 | 464 | – | 482 | 1076.0838 | 2150.1530 | 2150.1524 | 0.26 | 1 | 75 | 9e-08 | 1Score **> 36** indicates **identity** Score **> 17** indicates **homology** | U | R.TVGLFLQGGKDWSPTLYIR.L |
| 140616 | 464 | – | 482 | 717.7252 | 2150.1539 | 2150.1524 | 0.68 | 1 | 90 | 3.9e-09 | 1Score **> 36** indicates **identity** Score **> 18** indicates **homology** | U | R.TVGLFLQGGKDWSPTLYIR.L |
| 140617 | 464 | – | 482 | 717.7254 | 2150.1543 | 2150.1524 | 0.87 | 1 | 94 | 1.6e-09 | 1Score **> 36** indicates **identity** Score **> 18** indicates **homology** | U | R.TVGLFLQGGKDWSPTLYIR.L |
| 140618 | 464 | – | 482 | 1076.0845 | 2150.1544 | 2150.1524 | 0.91 | 1 | 74 | 1.9e-07 | 1Score **> 36** indicates **identity** Score **> 19** indicates **homology** | U | R.TVGLFLQGGKDWSPTLYIR.L |
| 140619 | 464 | – | 482 | 717.7255 | 2150.1545 | 2150.1524 | 0.98 | 1 | 77 | 5.6e-08 | 1Score **> 36** indicates **identity** Score **> 17** indicates **homology** | U | R.TVGLFLQGGKDWSPTLYIR.L |
| 140620 | 464 | – | 482 | 1076.0849 | 2150.1553 | 2150.1524 | 1.35 | 1 | 60 | 2.2e-06 | 1Score **> 36** indicates **identity** Score **> 16** indicates **homology** | U | R.TVGLFLQGGKDWSPTLYIR.L |
| 140622 | 464 | – | 482 | 717.7260 | 2150.1562 | 2150.1524 | 1.74 | 1 | 94 | 1.7e-09 | 1Score **> 36** indicates **identity** Score **> 18** indicates **homology** | U | R.TVGLFLQGGKDWSPTLYIR.L |
| 140623 | 464 | – | 482 | 1076.0855 | 2150.1564 | 2150.1524 | 1.85 | 1 | 77 | 1.1e-07 | 1Score **> 36** indicates **identity** Score **> 19** indicates **homology** | U | R.TVGLFLQGGKDWSPTLYIR.L |
| 140625 | 464 | – | 482 | 717.7267 | 2150.1583 | 2150.1524 | 2.74 | 1 | 49 | 2.5e-05 | 1Score **> 36** indicates **identity** Score **> 16** indicates **homology** | U | R.TVGLFLQGGKDWSPTLYIR.L |
| 140734 | 464 | – | 482 | 718.0563 | 2151.1469 | 2151.1364 | 4.88 | 1 | 37 | 0.00038 | 1Score **> 36** indicates **identity** Score **> 15** indicates **homology** | U | R.TVGLFLQGGKDWSPTLYIR.L  + Deamidated (NQ) |
| 140736 | 464 | – | 482 | 718.0577 | 2151.1512 | 2151.1364 | 6.86 | 1 | 22 | 0.0082 | 1Score **> 36** indicates **identity** Score **> 14** indicates **homology** | U | R.TVGLFLQGGKDWSPTLYIR.L  + Deamidated (NQ) |
| 27541 | 474 | – | 482 | 575.7978 | 1149.5811 | 1149.5818 | -0.66 | 0 | 27 | 0.0029 | 1Score **> 33** indicates **identity** Score **> 14** indicates **homology** | U | K.DWSPTLYIR.L |
| 27542 | 474 | – | 482 | 575.7989 | 1149.5832 | 1149.5818 | 1.18 | 0 | 35 | 0.00047 | 1Score **> 32** indicates **identity** Score **> 15** indicates **homology** | U | K.DWSPTLYIR.L |
| 27543 | 474 | – | 482 | 575.7990 | 1149.5834 | 1149.5818 | 1.36 | 0 | 33 | 0.00083 | 1Score **> 32** indicates **identity** Score **> 15** indicates **homology** | U | K.DWSPTLYIR.L |
| 27548 | 474 | – | 482 | 575.8001 | 1149.5856 | 1149.5818 | 3.29 | 0 | 31 | 0.0014 | 1Score **> 33** indicates **identity** Score **> 14** indicates **homology** | U | K.DWSPTLYIR.L |
| 116395 | 483 | – | 499 | 633.9970 | 1898.9690 | 1898.9738 | -2.49 | 0 | 69 | 3.3e-07 | 1Score **> 35** indicates **identity** Score **> 17** indicates **homology** | U | R.LVQDYGLESEVAQHLAK.T |
| 116396 | 483 | – | 499 | 633.9975 | 1898.9706 | 1898.9738 | -1.68 | 0 | 25 | 0.0041 | 1Score **> 36** indicates **identity** Score **> 14** indicates **homology** | U | R.LVQDYGLESEVAQHLAK.T |
| 116398 | 483 | – | 499 | 950.4930 | 1898.9714 | 1898.9738 | -1.23 | 0 | 56 | 6e-06 | 1Score **> 35** indicates **identity** Score **> 16** indicates **homology** | U | R.LVQDYGLESEVAQHLAK.T |
| 116402 | 483 | – | 499 | 950.4944 | 1898.9743 | 1898.9738 | 0.30 | 0 | 68 | 4.6e-07 | 1Score **> 36** indicates **identity** Score **> 17** indicates **homology** | U | R.LVQDYGLESEVAQHLAK.T |
| 116403 | 483 | – | 499 | 633.9990 | 1898.9752 | 1898.9738 | 0.76 | 0 | 98 | 6.5e-10 | 1Score **> 35** indicates **identity** Score **> 19** indicates **homology** | U | R.LVQDYGLESEVAQHLAK.T |
| 116404 | 483 | – | 499 | 633.9991 | 1898.9754 | 1898.9738 | 0.87 | 0 | 40 | 0.00017 | 1Score **> 35** indicates **identity** Score **> 15** indicates **homology** | U | R.LVQDYGLESEVAQHLAK.T |
| 116405 | 483 | – | 499 | 950.4954 | 1898.9763 | 1898.9738 | 1.34 | 0 | 75 | 8.4e-08 | 1Score **> 36** indicates **identity** Score **> 17** indicates **homology** | U | R.LVQDYGLESEVAQHLAK.T |
| 116406 | 483 | – | 499 | 950.4955 | 1898.9764 | 1898.9738 | 1.38 | 0 | 89 | 4.8e-09 | 1Score **> 36** indicates **identity** Score **> 18** indicates **homology** | U | R.LVQDYGLESEVAQHLAK.T |
| 116407 | 483 | – | 499 | 633.9995 | 1898.9767 | 1898.9738 | 1.52 | 0 | 98 | 6.9e-10 | 1Score **> 36** indicates **identity** Score **> 19** indicates **homology** | U | R.LVQDYGLESEVAQHLAK.T |
| 116408 | 483 | – | 499 | 633.9996 | 1898.9768 | 1898.9738 | 1.61 | 0 | 99 | 5.6e-10 | 1Score **> 36** indicates **identity** Score **> 19** indicates **homology** | U | R.LVQDYGLESEVAQHLAK.T |
| 116409 | 483 | – | 499 | 950.4960 | 1898.9774 | 1898.9738 | 1.93 | 0 | 95 | 1.3e-09 | 1Score **> 36** indicates **identity** Score **> 18** indicates **homology** | U | R.LVQDYGLESEVAQHLAK.T |
| 116410 | 483 | – | 499 | 633.9999 | 1898.9779 | 1898.9738 | 2.16 | 0 | 46 | 4.9e-05 | 1Score **> 36** indicates **identity** Score **> 15** indicates **homology** | U | R.LVQDYGLESEVAQHLAK.T |
| 116411 | 483 | – | 499 | 634.0000 | 1898.9781 | 1898.9738 | 2.30 | 0 | 61 | 1.8e-06 | 1Score **> 36** indicates **identity** Score **> 16** indicates **homology** | U | R.LVQDYGLESEVAQHLAK.T |
| 116412 | 483 | – | 499 | 634.0001 | 1898.9785 | 1898.9738 | 2.49 | 0 | 72 | 1.8e-07 | 1Score **> 36** indicates **identity** Score **> 17** indicates **homology** | U | R.LVQDYGLESEVAQHLAK.T |
| 116414 | 483 | – | 499 | 634.0014 | 1898.9825 | 1898.9738 | 4.58 | 0 | 56 | 5.9e-06 | 1Score **> 36** indicates **identity** Score **> 16** indicates **homology** | U | R.LVQDYGLESEVAQHLAK.T |
| 116415 | 483 | – | 499 | 950.4988 | 1898.9830 | 1898.9738 | 4.85 | 0 | 19 | 0.018 | 1Score **> 36** indicates **identity** Score **> 14** indicates **homology** | U | R.LVQDYGLESEVAQHLAK.T |
| 116416 | 483 | – | 499 | 634.0016 | 1898.9831 | 1898.9738 | 4.89 | 0 | 61 | 2e-06 | 1Score **> 36** indicates **identity** Score **> 16** indicates **homology** | U | R.LVQDYGLESEVAQHLAK.T |
| 116485 | 483 | – | 499 | 950.9830 | 1899.9514 | 1899.9578 | -3.37 | 0 | 19 | 0.016 | 1Score **> 35** indicates **identity** Score **> 14** indicates **homology** | U | R.LVQDYGLESEVAQHLAK.T  + Deamidated (NQ) |
| 116493 | 483 | – | 499 | 634.3271 | 1899.9595 | 1899.9578 | 0.93 | 0 | 21 | 0.01 | 1Score **> 35** indicates **identity** Score **> 14** indicates **homology** | U | R.LVQDYGLESEVAQHLAK.T  + Deamidated (NQ) |
| 35957 | 500 | – | 510 | 410.2109 | 1227.6107 | 1227.6135 | -2.28 | 1 | 16 | 0.03 | 1Score **> 31** indicates **identity** Score **> 14** indicates **homology** | U | K.TYGDKAFEVAK.M |
| 35959 | 500 | – | 510 | 410.2112 | 1227.6118 | 1227.6135 | -1.41 | 1 | 33 | 0.00075 | 1Score **> 31** indicates **identity** Score **> 15** indicates **homology** | U | K.TYGDKAFEVAK.M |
| 35961 | 500 | – | 510 | 614.8135 | 1227.6125 | 1227.6135 | -0.83 | 1 | 50 | 2.2e-05 | 1Score **> 31** indicates **identity** Score **> 16** indicates **homology** | U | K.TYGDKAFEVAK.M |
| 35962 | 500 | – | 510 | 410.2117 | 1227.6134 | 1227.6135 | -0.15 | 1 | 37 | 0.00035 | 1Score **> 31** indicates **identity** Score **> 15** indicates **homology** | U | K.TYGDKAFEVAK.M |
| 35963 | 500 | – | 510 | 614.8140 | 1227.6134 | 1227.6135 | -0.15 | 1 | 36 | 0.001 | 1Score **> 31** indicates **identity** Score **> 19** indicates **homology** | U | K.TYGDKAFEVAK.M |
| 35965 | 500 | – | 510 | 614.8140 | 1227.6135 | 1227.6135 | -0.011 | 1 | 75 | 2.5e-07 | 1Score **> 31** indicates **identity** Score **> 21** indicates **homology** | U | K.TYGDKAFEVAK.M |
| 35966 | 500 | – | 510 | 410.2118 | 1227.6135 | 1227.6135 | -0.0090 | 1 | 41 | 0.00013 | 1Score **> 31** indicates **identity** Score **> 15** indicates **homology** | U | K.TYGDKAFEVAK.M |
| 35969 | 500 | – | 510 | 614.8144 | 1227.6143 | 1227.6135 | 0.61 | 1 | 87 | 3.7e-08 | 1Score **> 31** indicates **identity** Score **> 25** indicates **homology** | U | K.TYGDKAFEVAK.M |
| 35970 | 500 | – | 510 | 410.2120 | 1227.6143 | 1227.6135 | 0.61 | 1 | 44 | 7e-05 | 1Score **> 31** indicates **identity** Score **> 15** indicates **homology** | U | K.TYGDKAFEVAK.M |
| 35971 | 500 | – | 510 | 410.2122 | 1227.6147 | 1227.6135 | 0.92 | 1 | 42 | 0.00012 | 1Score **> 31** indicates **identity** Score **> 15** indicates **homology** | U | K.TYGDKAFEVAK.M |
| 35973 | 500 | – | 510 | 614.8150 | 1227.6154 | 1227.6135 | 1.51 | 1 | 25 | 0.0045 | 1Score **> 31** indicates **identity** Score **> 14** indicates **homology** | U | K.TYGDKAFEVAK.M |
| 35976 | 500 | – | 510 | 614.8160 | 1227.6174 | 1227.6135 | 3.12 | 1 | 33 | 0.00082 | 1Score **> 32** indicates **identity** Score **> 15** indicates **homology** | U | K.TYGDKAFEVAK.M |
| 12367 | 518 | – | 525 | 484.7928 | 967.5710 | 967.5716 | -0.60 | 1 | 21 | 0.022 | 1Score **> 26** indicates **identity** Score **> 17** indicates **homology** | U | K.RWPVVGVR.L |
| 12368 | 518 | – | 525 | 484.7928 | 967.5710 | 967.5716 | -0.58 | 1 | 36 | 0.0027 | 1Score **> 26** indicates **identity** Score **> 23** indicates **homology** | U | K.RWPVVGVR.L |
| 12369 | 518 | – | 525 | 484.7931 | 967.5717 | 967.5716 | 0.16 | 1 | 41 | 0.0016 | 1Score **> 26** indicates **identity** Score **> 26** indicates **homology** | U | K.RWPVVGVR.L |
| 12370 | 518 | – | 525 | 484.7932 | 967.5718 | 967.5716 | 0.23 | 1 | 37 | 0.0024 | 1Score **> 28** indicates **identity** Score **> 23** indicates **homology** | U | K.RWPVVGVR.L |
| 12371 | 518 | – | 525 | 484.7932 | 967.5719 | 967.5716 | 0.29 | 1 | 40 | 0.0025 | 1Score **> 28** indicates **identity** Score **> 26** indicates **homology** | U | K.RWPVVGVR.L |
| 12372 | 518 | – | 525 | 484.7933 | 967.5720 | 967.5716 | 0.41 | 1 | 40 | 0.0025 | 1Score **> 28** indicates **identity** Score **> 26** indicates **homology** | U | K.RWPVVGVR.L |
| 12373 | 518 | – | 525 | 484.7933 | 967.5720 | 967.5716 | 0.47 | 1 | 39 | 0.0017 | 1Score **> 28** indicates **identity** Score **> 24** indicates **homology** | U | K.RWPVVGVR.L |
| 12374 | 518 | – | 525 | 484.7933 | 967.5721 | 967.5716 | 0.53 | 1 | 37 | 0.0025 | 1Score **> 28** indicates **identity** Score **> 23** indicates **homology** | U | K.RWPVVGVR.L |
| 12375 | 518 | – | 525 | 484.7933 | 967.5721 | 967.5716 | 0.55 | 1 | 36 | 0.0029 | 1Score **> 28** indicates **identity** Score **> 23** indicates **homology** | U | K.RWPVVGVR.L |
| 12376 | 518 | – | 525 | 484.7934 | 967.5722 | 967.5716 | 0.59 | 1 | 32 | 0.0059 | 1Score **> 28** indicates **identity** Score **> 23** indicates **homology** | U | K.RWPVVGVR.L |
| 12377 | 518 | – | 525 | 484.7935 | 967.5724 | 967.5716 | 0.85 | 1 | 34 | 0.005 | 1Score **> 28** indicates **identity** Score **> 23** indicates **homology** | U | K.RWPVVGVR.L |
| 4016 | 519 | – | 525 | 406.7422 | 811.4698 | 811.4705 | -0.83 | 0 | 16 | 0.031 | 1Score **> 21** indicates **identity** Score **> 14** indicates **homology** | U | R.WPVVGVR.L |
| 4017 | 519 | – | 525 | 406.7423 | 811.4700 | 811.4705 | -0.60 | 0 | 15 | 0.036 | 1Score **> 21** indicates **identity** Score **> 13** indicates **homology** | U | R.WPVVGVR.L |
| 4018 | 519 | – | 525 | 406.7423 | 811.4701 | 811.4705 | -0.44 | 0 | 18 | 0.022 | 1Score **> 21** indicates **identity** Score **> 14** indicates **homology** | U | R.WPVVGVR.L |
| 4019 | 519 | – | 525 | 406.7424 | 811.4702 | 811.4705 | -0.29 | 0 | 22 | 0.0079 | 1Score **> 21** indicates **identity** Score **> 14** indicates **homology** | U | R.WPVVGVR.L |
| 4020 | 519 | – | 525 | 406.7427 | 811.4708 | 811.4705 | 0.39 | 0 | 20 | 0.012 | 1Score **> 21** indicates **identity** Score **> 14** indicates **homology** | U | R.WPVVGVR.L |
| 4021 | 519 | – | 525 | 406.7428 | 811.4710 | 811.4705 | 0.59 | 0 | 19 | 0.018 | 1Score **> 21** indicates **identity** Score **> 14** indicates **homology** | U | R.WPVVGVR.L |
| 4022 | 519 | – | 525 | 406.7428 | 811.4710 | 811.4705 | 0.68 | 0 | 19 | 0.017 | 1Score **> 21** indicates **identity** Score **> 14** indicates **homology** | U | R.WPVVGVR.L |
| 4023 | 519 | – | 525 | 406.7428 | 811.4711 | 811.4705 | 0.74 | 0 | 22 | 0.0081 | 1Score **> 21** indicates **identity** Score **> 14** indicates **homology** | U | R.WPVVGVR.L |
| 4024 | 519 | – | 525 | 406.7428 | 811.4711 | 811.4705 | 0.75 | 0 | 17 | 0.023 | 1Score **> 21** indicates **identity** Score **> 14** indicates **homology** | U | R.WPVVGVR.L |
| 4025 | 519 | – | 525 | 406.7428 | 811.4711 | 811.4705 | 0.78 | 0 | 34 | 0.0006 | 1Score **> 21** indicates **identity** Score **> 15** indicates **homology** | U | R.WPVVGVR.L |
| 4026 | 519 | – | 525 | 406.7429 | 811.4712 | 811.4705 | 0.91 | 0 | 20 | 0.012 | 1Score **> 21** indicates **identity** Score **> 14** indicates **homology** | U | R.WPVVGVR.L |
| 4027 | 519 | – | 525 | 406.7429 | 811.4713 | 811.4705 | 1.05 | 0 | 19 | 0.017 | 1Score **> 21** indicates **identity** Score **> 14** indicates **homology** | U | R.WPVVGVR.L |
| 4028 | 519 | – | 525 | 406.7430 | 811.4714 | 811.4705 | 1.12 | 0 | 20 | 0.012 | 1Score **> 21** indicates **identity** Score **> 14** indicates **homology** | U | R.WPVVGVR.L |
| 4029 | 519 | – | 525 | 406.7430 | 811.4714 | 811.4705 | 1.20 | 0 | 21 | 0.01 | 1Score **> 21** indicates **identity** Score **> 14** indicates **homology** | U | R.WPVVGVR.L |
| 4030 | 519 | – | 525 | 406.7433 | 811.4721 | 811.4705 | 1.97 | 0 | 15 | 0.036 | 1Score **> 21** indicates **identity** Score **> 13** indicates **homology** | U | R.WPVVGVR.L |
| 4032 | 519 | – | 525 | 406.7446 | 811.4746 | 811.4705 | 5.03 | 0 | 19 | 0.015 | 1Score **> 21** indicates **identity** Score **> 14** indicates **homology** | U | R.WPVVGVR.L |
| 4033 | 519 | – | 525 | 406.7446 | 811.4747 | 811.4705 | 5.19 | 0 | 19 | 0.018 | 1Score **> 21** indicates **identity** Score **> 14** indicates **homology** | U | R.WPVVGVR.L |
| 69986 | 526 | – | 538 | 762.4041 | 1522.7936 | 1522.7919 | 1.14 | 0 | 70 | 7.5e-07 | 1Score **> 35** indicates **identity** Score **> 21** indicates **homology** | U | R.LVSEFPYIEAEVK.Y |
| 69987 | 526 | – | 538 | 762.4043 | 1522.7940 | 1522.7919 | 1.36 | 0 | 70 | 7.6e-07 | 1Score **> 35** indicates **identity** Score **> 21** indicates **homology** | U | R.LVSEFPYIEAEVK.Y |
| 69988 | 526 | – | 538 | 762.4043 | 1522.7940 | 1522.7919 | 1.41 | 0 | 21 | 0.011 | 1Score **> 35** indicates **identity** Score **> 14** indicates **homology** | U | R.LVSEFPYIEAEVK.Y |
| 69990 | 526 | – | 538 | 762.4046 | 1522.7947 | 1522.7919 | 1.82 | 0 | 67 | 1.7e-06 | 1Score **> 35** indicates **identity** Score **> 22** indicates **homology** | U | R.LVSEFPYIEAEVK.Y |
| 69993 | 526 | – | 538 | 762.4079 | 1522.8012 | 1522.7919 | 6.11 | 0 | 40 | 0.00018 | 1Score **> 35** indicates **identity** Score **> 15** indicates **homology** | U | R.LVSEFPYIEAEVK.Y |
| 113967 | 539 | – | 554 | 626.3028 | 1875.8867 | 1875.8859 | 0.44 | 1 | 21 | 0.011 | 1Score **> 34** indicates **identity** Score **> 14** indicates **homology** | U | K.YGIKEYACTAVDMISR.R |
| 113971 | 539 | – | 554 | 626.3030 | 1875.8872 | 1875.8859 | 0.71 | 1 | 41 | 0.00014 | 1Score **> 34** indicates **identity** Score **> 15** indicates **homology** | U | K.YGIKEYACTAVDMISR.R |
| 129226 | 539 | – | 555 | 678.3364 | 2031.9875 | 2031.9870 | 0.26 | 2 | 14 | 0.044 | 1Score **> 35** indicates **identity** Score **> 13** indicates **homology** | U | K.YGIKEYACTAVDMISRR.T |
| 129228 | 539 | – | 555 | 509.0044 | 2031.9886 | 2031.9870 | 0.77 | 2 | 37 | 0.00034 | 1Score **> 35** indicates **identity** Score **> 15** indicates **homology** | U | K.YGIKEYACTAVDMISRR.T |
| 129229 | 539 | – | 555 | 509.0045 | 2031.9889 | 2031.9870 | 0.95 | 2 | 57 | 4.5e-06 | 1Score **> 35** indicates **identity** Score **> 16** indicates **homology** | U | K.YGIKEYACTAVDMISRR.T |
| 129230 | 539 | – | 555 | 678.3372 | 2031.9899 | 2031.9870 | 1.44 | 2 | 30 | 0.0015 | 1Score **> 35** indicates **identity** Score **> 14** indicates **homology** | U | K.YGIKEYACTAVDMISRR.T |
| 56643 | 543 | – | 554 | 708.3179 | 1414.6213 | 1414.6221 | -0.58 | 0 | 46 | 5.3e-05 | 1Score **> 27** indicates **identity** Score **> 15** indicates **homology** | U | K.EYACTAVDMISR.R |
| 56644 | 543 | – | 554 | 708.3180 | 1414.6214 | 1414.6221 | -0.49 | 0 | 23 | 0.0076 | 1Score **> 27** indicates **identity** Score **> 14** indicates **homology** | U | K.EYACTAVDMISR.R |
| 56645 | 543 | – | 554 | 708.3183 | 1414.6221 | 1414.6221 | 0.030 | 0 | 43 | 9.9e-05 | 1Score **> 27** indicates **identity** Score **> 15** indicates **homology** | U | K.EYACTAVDMISR.R |
| 76297 | 543 | – | 555 | 524.5824 | 1570.7253 | 1570.7232 | 1.32 | 1 | 38 | 0.00027 | 1Score **> 32** indicates **identity** Score **> 15** indicates **homology** | U | K.EYACTAVDMISRR.T |
| 116303 | 556 | – | 572 | 633.6865 | 1898.0377 | 1898.0374 | 0.17 | 1 | 62 | 1.6e-06 | 1Score **> 35** indicates **identity** Score **> 16** indicates **homology** | U | R.TRLAFLNVQAAEEALPR.I |
| 116305 | 556 | – | 572 | 633.6872 | 1898.0397 | 1898.0374 | 1.23 | 1 | 48 | 3.4e-05 | 1Score **> 35** indicates **identity** Score **> 15** indicates **homology** | U | R.TRLAFLNVQAAEEALPR.I |
| 116306 | 556 | – | 572 | 950.0272 | 1898.0398 | 1898.0374 | 1.30 | 1 | 96 | 9.5e-10 | 1Score **> 35** indicates **identity** Score **> 19** indicates **homology** | U | R.TRLAFLNVQAAEEALPR.I |
| 116307 | 556 | – | 572 | 633.6874 | 1898.0403 | 1898.0374 | 1.53 | 1 | 74 | 1.5e-07 | 1Score **> 35** indicates **identity** Score **> 18** indicates **homology** | U | R.TRLAFLNVQAAEEALPR.I |
| 116308 | 556 | – | 572 | 950.0275 | 1898.0405 | 1898.0374 | 1.68 | 1 | 30 | 0.0017 | 1Score **> 35** indicates **identity** Score **> 14** indicates **homology** | U | R.TRLAFLNVQAAEEALPR.I |
| 116309 | 556 | – | 572 | 633.6875 | 1898.0407 | 1898.0374 | 1.75 | 1 | 60 | 2.4e-06 | 1Score **> 35** indicates **identity** Score **> 17** indicates **homology** | U | R.TRLAFLNVQAAEEALPR.I |
| 116310 | 556 | – | 572 | 950.0277 | 1898.0408 | 1898.0374 | 1.83 | 1 | 59 | 2.8e-06 | 1Score **> 35** indicates **identity** Score **> 16** indicates **homology** | U | R.TRLAFLNVQAAEEALPR.I |
| 116312 | 556 | – | 572 | 633.6876 | 1898.0409 | 1898.0374 | 1.88 | 1 | 72 | 2.2e-07 | 1Score **> 35** indicates **identity** Score **> 18** indicates **homology** | U | R.TRLAFLNVQAAEEALPR.I |
| 116314 | 556 | – | 572 | 950.0279 | 1898.0412 | 1898.0374 | 2.00 | 1 | 34 | 0.00072 | 1Score **> 35** indicates **identity** Score **> 15** indicates **homology** | U | R.TRLAFLNVQAAEEALPR.I |
| 116315 | 556 | – | 572 | 633.6879 | 1898.0419 | 1898.0374 | 2.41 | 1 | 69 | 3.7e-07 | 1Score **> 35** indicates **identity** Score **> 17** indicates **homology** | U | R.TRLAFLNVQAAEEALPR.I |
| 116316 | 556 | – | 572 | 633.6882 | 1898.0428 | 1898.0374 | 2.85 | 1 | 52 | 1.2e-05 | 1Score **> 35** indicates **identity** Score **> 16** indicates **homology** | U | R.TRLAFLNVQAAEEALPR.I |
| 116317 | 556 | – | 572 | 950.0287 | 1898.0429 | 1898.0374 | 2.93 | 1 | 73 | 1.5e-07 | 1Score **> 35** indicates **identity** Score **> 17** indicates **homology** | U | R.TRLAFLNVQAAEEALPR.I |
| 116318 | 556 | – | 572 | 633.6884 | 1898.0434 | 1898.0374 | 3.17 | 1 | 55 | 6.8e-06 | 1Score **> 35** indicates **identity** Score **> 16** indicates **homology** | U | R.TRLAFLNVQAAEEALPR.I |
| 116319 | 556 | – | 572 | 950.0290 | 1898.0435 | 1898.0374 | 3.25 | 1 | 55 | 7.7e-06 | 1Score **> 35** indicates **identity** Score **> 16** indicates **homology** | U | R.TRLAFLNVQAAEEALPR.I |
| 116322 | 556 | – | 572 | 950.0300 | 1898.0454 | 1898.0374 | 4.26 | 1 | 79 | 3.6e-08 | 1Score **> 35** indicates **identity** Score **> 17** indicates **homology** | U | R.TRLAFLNVQAAEEALPR.I |
| 86128 | 558 | – | 572 | 821.4461 | 1640.8776 | 1640.8886 | -6.67 | 0 | 35 | 0.00051 | 1Score **> 35** indicates **identity** Score **> 15** indicates **homology** | U | R.LAFLNVQAAEEALPR.I |
| 86135 | 558 | – | 572 | 547.9700 | 1640.8883 | 1640.8886 | -0.18 | 0 | 57 | 5e-06 | 1Score **> 35** indicates **identity** Score **> 16** indicates **homology** | U | R.LAFLNVQAAEEALPR.I |
| 86137 | 558 | – | 572 | 547.9705 | 1640.8896 | 1640.8886 | 0.63 | 0 | 75 | 1.3e-07 | 1Score **> 35** indicates **identity** Score **> 18** indicates **homology** | U | R.LAFLNVQAAEEALPR.I |
| 86138 | 558 | – | 572 | 547.9706 | 1640.8898 | 1640.8886 | 0.78 | 0 | 102 | 3.1e-10 | 1Score **> 35** indicates **identity** Score **> 20** indicates **homology** | U | R.LAFLNVQAAEEALPR.I |
| 86139 | 558 | – | 572 | 547.9707 | 1640.8902 | 1640.8886 | 1.02 | 0 | 96 | 9.6e-10 | 1Score **> 35** indicates **identity** Score **> 19** indicates **homology** | U | R.LAFLNVQAAEEALPR.I |
| 86140 | 558 | – | 572 | 821.4525 | 1640.8905 | 1640.8886 | 1.21 | 0 | 97 | 8.1e-10 | 1Score **> 35** indicates **identity** Score **> 19** indicates **homology** | U | R.LAFLNVQAAEEALPR.I |
| 86141 | 558 | – | 572 | 547.9708 | 1640.8906 | 1640.8886 | 1.27 | 0 | 85 | 1e-08 | 1Score **> 35** indicates **identity** Score **> 18** indicates **homology** | U | R.LAFLNVQAAEEALPR.I |
| 86142 | 558 | – | 572 | 821.4527 | 1640.8908 | 1640.8886 | 1.35 | 0 | 77 | 5.4e-08 | 1Score **> 35** indicates **identity** Score **> 17** indicates **homology** | U | R.LAFLNVQAAEEALPR.I |
| 86144 | 558 | – | 572 | 821.4528 | 1640.8911 | 1640.8886 | 1.57 | 0 | 70 | 3e-07 | 1Score **> 35** indicates **identity** Score **> 17** indicates **homology** | U | R.LAFLNVQAAEEALPR.I |
| 86145 | 558 | – | 572 | 821.4532 | 1640.8918 | 1640.8886 | 1.95 | 0 | 73 | 1.5e-07 | 1Score **> 35** indicates **identity** Score **> 17** indicates **homology** | U | R.LAFLNVQAAEEALPR.I |
| 86147 | 558 | – | 572 | 821.4537 | 1640.8929 | 1640.8886 | 2.64 | 0 | 65 | 7.5e-07 | 1Score **> 35** indicates **identity** Score **> 17** indicates **homology** | U | R.LAFLNVQAAEEALPR.I |
| 86149 | 558 | – | 572 | 547.9720 | 1640.8941 | 1640.8886 | 3.36 | 0 | 57 | 4.8e-06 | 1Score **> 35** indicates **identity** Score **> 16** indicates **homology** | U | R.LAFLNVQAAEEALPR.I |
| 86152 | 558 | – | 572 | 821.4563 | 1640.8980 | 1640.8886 | 5.78 | 0 | 47 | 4.1e-05 | 1Score **> 35** indicates **identity** Score **> 15** indicates **homology** | U | R.LAFLNVQAAEEALPR.I |
| 4282 | 573 | – | 579 | 409.2334 | 816.4522 | 816.4527 | -0.69 | 0 | 39 | 0.007 | 1Score **> 30** indicates **identity** | U | R.IVELMGR.E |
| 4283 | 573 | – | 579 | 409.2336 | 816.4527 | 816.4527 | -0.024 | 0 | 47 | 0.0014 | 1Score **> 30** indicates **identity** | U | R.IVELMGR.E |
| 17433 | 580 | – | 587 | 523.7666 | 1045.5186 | 1045.5192 | -0.55 | 0 | 23 | 0.0097 | 1Score **> 31** indicates **identity** Score **> 16** indicates **homology** | U | R.ELNWSELR.K |
| 17434 | 580 | – | 587 | 523.7668 | 1045.5190 | 1045.5192 | -0.25 | 0 | 38 | 0.0014 | 1Score **> 33** indicates **identity** Score **> 22** indicates **homology** | U | R.ELNWSELR.K |
| 17435 | 580 | – | 587 | 523.7668 | 1045.5191 | 1045.5192 | -0.14 | 0 | 40 | 0.0092 | 1Score **> 33** indicates **identity** | U | R.ELNWSELR.K |
| 17436 | 580 | – | 587 | 523.7671 | 1045.5197 | 1045.5192 | 0.46 | 0 | 39 | 0.001 | 1Score **> 33** indicates **identity** Score **> 22** indicates **homology** | U | R.ELNWSELR.K |
| 17437 | 580 | – | 587 | 523.7672 | 1045.5198 | 1045.5192 | 0.52 | 0 | 46 | 0.00043 | 1Score **> 33** indicates **identity** Score **> 24** indicates **homology** | U | R.ELNWSELR.K |
| 17438 | 580 | – | 587 | 523.7672 | 1045.5198 | 1045.5192 | 0.59 | 0 | 44 | 0.00074 | 1Score **> 33** indicates **identity** Score **> 25** indicates **homology** | U | R.ELNWSELR.K |
| 17440 | 580 | – | 587 | 523.7684 | 1045.5222 | 1045.5192 | 2.82 | 0 | 41 | 0.00091 | 1Score **> 33** indicates **identity** Score **> 23** indicates **homology** | U | R.ELNWSELR.K |
| 17441 | 580 | – | 587 | 523.7685 | 1045.5224 | 1045.5192 | 3.03 | 0 | 44 | 0.0026 | 1Score **> 33** indicates **identity** Score **> 31** indicates **homology** | U | R.ELNWSELR.K |
| 30038 | 580 | – | 588 | 392.2121 | 1173.6146 | 1173.6142 | 0.34 | 1 | 18 | 0.021 | 1Score **> 34** indicates **identity** Score **> 14** indicates **homology** | U | R.ELNWSELRK.Q |
| 30043 | 580 | – | 588 | 392.2126 | 1173.6160 | 1173.6142 | 1.55 | 1 | 20 | 0.014 | 1Score **> 33** indicates **identity** Score **> 14** indicates **homology** | U | R.ELNWSELRK.Q |
| 30044 | 580 | – | 588 | 392.2126 | 1173.6161 | 1173.6142 | 1.64 | 1 | 19 | 0.019 | 1Score **> 33** indicates **identity** Score **> 15** indicates **homology** | U | R.ELNWSELRK.Q |
| 147347 | 580 | – | 597 | 558.7865 | 2231.1167 | 2231.1182 | -0.65 | 2 | 14 | 0.049 | 1Score **> 37** indicates **identity** Score **> 13** indicates **homology** | U | R.ELNWSELRKQEELETATR.F |
| 147349 | 580 | – | 597 | 558.7869 | 2231.1183 | 2231.1182 | 0.081 | 2 | 26 | 0.0041 | 1Score **> 37** indicates **identity** Score **> 15** indicates **homology** | U | R.ELNWSELRKQEELETATR.F |
| 147350 | 580 | – | 597 | 744.7136 | 2231.1189 | 2231.1182 | 0.32 | 2 | 28 | 0.0023 | 1Score **> 37** indicates **identity** Score **> 14** indicates **homology** | U | R.ELNWSELRKQEELETATR.F |
| 147352 | 580 | – | 597 | 558.7871 | 2231.1194 | 2231.1182 | 0.56 | 2 | 18 | 0.022 | 1Score **> 37** indicates **identity** Score **> 14** indicates **homology** | U | R.ELNWSELRKQEELETATR.F |
| 147353 | 580 | – | 597 | 744.7138 | 2231.1194 | 2231.1182 | 0.57 | 2 | 29 | 0.0018 | 1Score **> 37** indicates **identity** Score **> 14** indicates **homology** | U | R.ELNWSELRKQEELETATR.F |
| 147354 | 580 | – | 597 | 558.7873 | 2231.1201 | 2231.1182 | 0.89 | 2 | 21 | 0.011 | 1Score **> 37** indicates **identity** Score **> 14** indicates **homology** | U | R.ELNWSELRKQEELETATR.F |
| 147355 | 580 | – | 597 | 744.7140 | 2231.1202 | 2231.1182 | 0.92 | 2 | 21 | 0.011 | 1Score **> 37** indicates **identity** Score **> 14** indicates **homology** | U | R.ELNWSELRKQEELETATR.F |
| 147356 | 580 | – | 597 | 558.7874 | 2231.1203 | 2231.1182 | 0.96 | 2 | 32 | 0.001 | 1Score **> 37** indicates **identity** Score **> 14** indicates **homology** | U | R.ELNWSELRKQEELETATR.F |
| 33467 | 588 | – | 597 | 602.8103 | 1203.6060 | 1203.6095 | -2.95 | 1 | 36 | 0.036 | 1Score **> 34** indicates **identity** | U | R.KQEELETATR.F |
| 33469 | 588 | – | 597 | 602.8117 | 1203.6089 | 1203.6095 | -0.47 | 1 | 37 | 0.03 | 1Score **> 34** indicates **identity** | U | R.KQEELETATR.F |
| 33474 | 588 | – | 597 | 402.2108 | 1203.6105 | 1203.6095 | 0.83 | 1 | 45 | 0.00024 | 1Score **> 33** indicates **identity** Score **> 21** indicates **homology** | U | R.KQEELETATR.F |
| 20203 | 589 | – | 597 | 538.7626 | 1075.5106 | 1075.5145 | -3.68 | 0 | 34 | 0.0022 | 1Score **> 31** indicates **identity** Score **> 20** indicates **homology** | U | K.QEELETATR.F |
| 34318 | 598 | – | 606 | 607.2826 | 1212.5506 | 1212.5525 | -1.57 | 0 | 39 | 0.00024 | 1Score **> 28** indicates **identity** Score **> 15** indicates **homology** | U | R.FLYYEMGYK.S |
| 34319 | 598 | – | 606 | 607.2840 | 1212.5535 | 1212.5525 | 0.85 | 0 | 24 | 0.0055 | 1Score **> 28** indicates **identity** Score **> 14** indicates **homology** | U | R.FLYYEMGYK.S |
| 34320 | 598 | – | 606 | 607.2843 | 1212.5540 | 1212.5525 | 1.27 | 0 | 36 | 0.00039 | 1Score **> 28** indicates **identity** Score **> 15** indicates **homology** | U | R.FLYYEMGYK.S |
| 157272 | 607 | – | 627 | 1188.5967 | 2375.1788 | 2375.1816 | -1.19 | 1 | 37 | 0.00064 | 1Score **> 37** indicates **identity** Score **> 18** indicates **homology** | U | K.SRTEQLTDSTEISLLPSDIDR.Y |
| 157276 | 607 | – | 627 | 792.7349 | 2375.1829 | 2375.1816 | 0.56 | 1 | 38 | 0.00029 | 1Score **> 37** indicates **identity** Score **> 15** indicates **homology** | U | K.SRTEQLTDSTEISLLPSDIDR.Y |
| 157277 | 607 | – | 627 | 792.7351 | 2375.1836 | 2375.1816 | 0.83 | 1 | 62 | 2e-06 | 1Score **> 37** indicates **identity** Score **> 18** indicates **homology** | U | K.SRTEQLTDSTEISLLPSDIDR.Y |
| 157278 | 607 | – | 627 | 792.7352 | 2375.1837 | 2375.1816 | 0.91 | 1 | 68 | 5.6e-07 | 1Score **> 37** indicates **identity** Score **> 18** indicates **homology** | U | K.SRTEQLTDSTEISLLPSDIDR.Y |
| 157279 | 607 | – | 627 | 1188.5998 | 2375.1851 | 2375.1816 | 1.47 | 1 | 80 | 3.6e-08 | 1Score **> 37** indicates **identity** Score **> 19** indicates **homology** | U | K.SRTEQLTDSTEISLLPSDIDR.Y |
| 157281 | 607 | – | 627 | 1188.5999 | 2375.1853 | 2375.1816 | 1.58 | 1 | 87 | 2.3e-08 | 1Score **> 37** indicates **identity** Score **> 23** indicates **homology** | U | K.SRTEQLTDSTEISLLPSDIDR.Y |
| 157282 | 607 | – | 627 | 792.7358 | 2375.1856 | 2375.1816 | 1.67 | 1 | 58 | 3.9e-06 | 1Score **> 37** indicates **identity** Score **> 16** indicates **homology** | U | K.SRTEQLTDSTEISLLPSDIDR.Y |
| 157283 | 607 | – | 627 | 1188.6015 | 2375.1884 | 2375.1816 | 2.87 | 1 | 70 | 4.2e-07 | 1Score **> 37** indicates **identity** Score **> 19** indicates **homology** | U | K.SRTEQLTDSTEISLLPSDIDR.Y |
| 157285 | 607 | – | 627 | 792.7380 | 2375.1922 | 2375.1816 | 4.47 | 1 | 30 | 0.0015 | 1Score **> 37** indicates **identity** Score **> 14** indicates **homology** | U | K.SRTEQLTDSTEISLLPSDIDR.Y |
| 157286 | 607 | – | 627 | 792.7418 | 2375.2037 | 2375.1816 | 9.29 | 1 | 39 | 0.00022 | 1Score **> 37** indicates **identity** Score **> 15** indicates **homology** | U | K.SRTEQLTDSTEISLLPSDIDR.Y |
| 172760 | 607 | – | 629 | 889.7804 | 2666.3195 | 2666.3399 | -7.65 | 2 | 31 | 0.011 | 1Score **> 37** indicates **identity** Score **> 24** indicates **homology** | U | K.SRTEQLTDSTEISLLPSDIDRYK.K |
| 172763 | 607 | – | 629 | 889.7876 | 2666.3410 | 2666.3399 | 0.42 | 2 | 43 | 0.00014 | 1Score **> 37** indicates **identity** Score **> 17** indicates **homology** | U | K.SRTEQLTDSTEISLLPSDIDRYK.K |
| 172764 | 607 | – | 629 | 889.7876 | 2666.3410 | 2666.3399 | 0.44 | 2 | 58 | 5.8e-06 | 1Score **> 37** indicates **identity** Score **> 18** indicates **homology** | U | K.SRTEQLTDSTEISLLPSDIDRYK.K |
| 172765 | 607 | – | 629 | 667.5927 | 2666.3415 | 2666.3399 | 0.62 | 2 | 59 | 7.1e-06 | 1Score **> 37** indicates **identity** Score **> 20** indicates **homology** | U | K.SRTEQLTDSTEISLLPSDIDRYK.K |
| 172766 | 607 | – | 629 | 667.5927 | 2666.3417 | 2666.3399 | 0.69 | 2 | 50 | 4.2e-05 | 1Score **> 37** indicates **identity** Score **> 18** indicates **homology** | U | K.SRTEQLTDSTEISLLPSDIDRYK.K |
| 172767 | 607 | – | 629 | 667.5930 | 2666.3430 | 2666.3399 | 1.18 | 2 | 24 | 0.0057 | 1Score **> 37** indicates **identity** Score **> 14** indicates **homology** | U | K.SRTEQLTDSTEISLLPSDIDRYK.K |
| 172768 | 607 | – | 629 | 889.7883 | 2666.3430 | 2666.3399 | 1.18 | 2 | 54 | 8.4e-06 | 1Score **> 37** indicates **identity** Score **> 16** indicates **homology** | U | K.SRTEQLTDSTEISLLPSDIDRYK.K |
| 138746 | 609 | – | 627 | 1067.0281 | 2132.0416 | 2132.0485 | -3.22 | 0 | 69 | 6.9e-07 | 1Score **> 36** indicates **identity** Score **> 20** indicates **homology** | U | R.TEQLTDSTEISLLPSDIDR.Y |
| 138752 | 609 | – | 627 | 1067.0322 | 2132.0499 | 2132.0485 | 0.66 | 0 | 108 | 1.5e-09 | 1Score **> 36** indicates **identity** Score **> 32** indicates **homology** | U | R.TEQLTDSTEISLLPSDIDR.Y |
| 138753 | 609 | – | 627 | 1067.0322 | 2132.0499 | 2132.0485 | 0.66 | 0 | 93 | 4.5e-08 | 1Score **> 36** indicates **identity** Score **> 32** indicates **homology** | U | R.TEQLTDSTEISLLPSDIDR.Y |
| 138755 | 609 | – | 627 | 711.6909 | 2132.0508 | 2132.0485 | 1.12 | 0 | 26 | 0.0039 | 1Score **> 36** indicates **identity** Score **> 14** indicates **homology** | U | R.TEQLTDSTEISLLPSDIDR.Y |
| 138756 | 609 | – | 627 | 1067.0327 | 2132.0509 | 2132.0485 | 1.13 | 0 | 102 | 6.6e-09 | 1Score **> 36** indicates **identity** Score **> 33** indicates **homology** | U | R.TEQLTDSTEISLLPSDIDR.Y |
| 138757 | 609 | – | 627 | 1067.0329 | 2132.0512 | 2132.0485 | 1.31 | 0 | 108 | 1.8e-09 | 1Score **> 36** indicates **identity** Score **> 33** indicates **homology** | U | R.TEQLTDSTEISLLPSDIDR.Y |
| 138759 | 609 | – | 627 | 1067.0338 | 2132.0531 | 2132.0485 | 2.18 | 0 | 103 | 4.7e-09 | 1Score **> 36** indicates **identity** Score **> 32** indicates **homology** | U | R.TEQLTDSTEISLLPSDIDR.Y |
| 138760 | 609 | – | 627 | 1067.0341 | 2132.0537 | 2132.0485 | 2.45 | 0 | 108 | 1.8e-09 | 1Score **> 36** indicates **identity** Score **> 33** indicates **homology** | U | R.TEQLTDSTEISLLPSDIDR.Y |
| 138761 | 609 | – | 627 | 1067.0348 | 2132.0550 | 2132.0485 | 3.04 | 0 | 80 | 1.7e-07 | 1Score **> 36** indicates **identity** Score **> 25** indicates **homology** | U | R.TEQLTDSTEISLLPSDIDR.Y |
| 138762 | 609 | – | 627 | 1067.0352 | 2132.0558 | 2132.0485 | 3.42 | 0 | 108 | 1.8e-09 | 1Score **> 36** indicates **identity** Score **> 33** indicates **homology** | U | R.TEQLTDSTEISLLPSDIDR.Y |
| 138763 | 609 | – | 627 | 1067.0356 | 2132.0566 | 2132.0485 | 3.83 | 0 | 104 | 3.2e-10 | 1Score **> 36** indicates **identity** Score **> 22** indicates **homology** | U | R.TEQLTDSTEISLLPSDIDR.Y |
| 138769 | 609 | – | 627 | 1067.0396 | 2132.0646 | 2132.0485 | 7.56 | 0 | 98 | 2.4e-09 | 1Score **> 36** indicates **identity** Score **> 25** indicates **homology** | U | R.TEQLTDSTEISLLPSDIDR.Y |
| 160295 | 609 | – | 629 | 808.7436 | 2423.2089 | 2423.2067 | 0.90 | 1 | 25 | 0.0047 | 1Score **> 37** indicates **identity** Score **> 14** indicates **homology** | U | R.TEQLTDSTEISLLPSDIDRYK.K |
| 160297 | 609 | – | 629 | 808.7436 | 2423.2090 | 2423.2067 | 0.94 | 1 | 35 | 0.00054 | 1Score **> 37** indicates **identity** Score **> 15** indicates **homology** | U | R.TEQLTDSTEISLLPSDIDRYK.K |
| 160298 | 609 | – | 629 | 1212.6118 | 2423.2091 | 2423.2067 | 0.99 | 1 | 101 | 3.3e-10 | 1Score **> 37** indicates **identity** Score **> 19** indicates **homology** | U | R.TEQLTDSTEISLLPSDIDRYK.K |
| 160299 | 609 | – | 629 | 808.7441 | 2423.2104 | 2423.2067 | 1.51 | 1 | 38 | 0.0003 | 1Score **> 37** indicates **identity** Score **> 15** indicates **homology** | U | R.TEQLTDSTEISLLPSDIDRYK.K |
| 160300 | 609 | – | 629 | 808.7442 | 2423.2107 | 2423.2067 | 1.62 | 1 | 52 | 1.4e-05 | 1Score **> 37** indicates **identity** Score **> 16** indicates **homology** | U | R.TEQLTDSTEISLLPSDIDRYK.K |
| 160301 | 609 | – | 629 | 1212.6127 | 2423.2109 | 2423.2067 | 1.70 | 1 | 95 | 1.2e-09 | 1Score **> 37** indicates **identity** Score **> 18** indicates **homology** | U | R.TEQLTDSTEISLLPSDIDRYK.K |
| 160303 | 609 | – | 629 | 808.7449 | 2423.2129 | 2423.2067 | 2.56 | 1 | 20 | 0.013 | 1Score **> 37** indicates **identity** Score **> 14** indicates **homology** | U | R.TEQLTDSTEISLLPSDIDRYK.K |
| 160305 | 609 | – | 629 | 1212.6142 | 2423.2138 | 2423.2067 | 2.91 | 1 | 71 | 2.3e-07 | 1Score **> 37** indicates **identity** Score **> 17** indicates **homology** | U | R.TEQLTDSTEISLLPSDIDRYK.K |
| 160367 | 609 | – | 629 | 1213.1130 | 2424.2115 | 2424.1908 | 8.55 | 1 | 55 | 6.7e-06 | 1Score **> 37** indicates **identity** Score **> 16** indicates **homology** | U | R.TEQLTDSTEISLLPSDIDRYK.K  + Deamidated (NQ) |
| 153823 | 632 | – | 650 | 581.5461 | 2322.1552 | 2322.1645 | -3.97 | 2 | 22 | 0.0085 | 1Score **> 37** indicates **identity** Score **> 14** indicates **homology** | U | R.FHKFDEDEKGFITIVDVQR.V |
| 153826 | 632 | – | 650 | 775.0606 | 2322.1601 | 2322.1645 | -1.88 | 2 | 25 | 0.0049 | 1Score **> 37** indicates **identity** Score **> 14** indicates **homology** | U | R.FHKFDEDEKGFITIVDVQR.V |
| 153829 | 632 | – | 650 | 581.5476 | 2322.1614 | 2322.1645 | -1.34 | 2 | 23 | 0.0069 | 1Score **> 37** indicates **identity** Score **> 14** indicates **homology** | U | R.FHKFDEDEKGFITIVDVQR.V |
| 153831 | 632 | – | 650 | 581.5479 | 2322.1626 | 2322.1645 | -0.79 | 2 | 34 | 0.00066 | 1Score **> 37** indicates **identity** Score **> 15** indicates **homology** | U | R.FHKFDEDEKGFITIVDVQR.V |
| 153833 | 632 | – | 650 | 775.0617 | 2322.1633 | 2322.1645 | -0.51 | 2 | 23 | 0.0065 | 1Score **> 37** indicates **identity** Score **> 14** indicates **homology** | U | R.FHKFDEDEKGFITIVDVQR.V |
| 153835 | 632 | – | 650 | 775.0618 | 2322.1635 | 2322.1645 | -0.42 | 2 | 38 | 0.00029 | 1Score **> 37** indicates **identity** Score **> 15** indicates **homology** | U | R.FHKFDEDEKGFITIVDVQR.V |
| 153836 | 632 | – | 650 | 581.5483 | 2322.1642 | 2322.1645 | -0.096 | 2 | 41 | 0.00014 | 1Score **> 37** indicates **identity** Score **> 15** indicates **homology** | U | R.FHKFDEDEKGFITIVDVQR.V |
| 153837 | 632 | – | 650 | 581.5484 | 2322.1645 | 2322.1645 | -0.0013 | 2 | 38 | 0.00025 | 1Score **> 37** indicates **identity** Score **> 15** indicates **homology** | U | R.FHKFDEDEKGFITIVDVQR.V |
| 153840 | 632 | – | 650 | 775.0623 | 2322.1652 | 2322.1645 | 0.30 | 2 | 15 | 0.041 | 1Score **> 37** indicates **identity** Score **> 13** indicates **homology** | U | R.FHKFDEDEKGFITIVDVQR.V |
| 153841 | 632 | – | 650 | 581.5487 | 2322.1657 | 2322.1645 | 0.55 | 2 | 30 | 0.0016 | 1Score **> 37** indicates **identity** Score **> 14** indicates **homology** | U | R.FHKFDEDEKGFITIVDVQR.V |
| 153842 | 632 | – | 650 | 581.5487 | 2322.1657 | 2322.1645 | 0.56 | 2 | 40 | 0.00017 | 1Score **> 37** indicates **identity** Score **> 15** indicates **homology** | U | R.FHKFDEDEKGFITIVDVQR.V |
| 153844 | 632 | – | 650 | 775.0626 | 2322.1660 | 2322.1645 | 0.67 | 2 | 18 | 0.019 | 1Score **> 37** indicates **identity** Score **> 14** indicates **homology** | U | R.FHKFDEDEKGFITIVDVQR.V |
| 153846 | 632 | – | 650 | 581.5489 | 2322.1664 | 2322.1645 | 0.83 | 2 | 32 | 0.00097 | 1Score **> 37** indicates **identity** Score **> 15** indicates **homology** | U | R.FHKFDEDEKGFITIVDVQR.V |
| 153847 | 632 | – | 650 | 581.5489 | 2322.1664 | 2322.1645 | 0.86 | 2 | 30 | 0.0017 | 1Score **> 37** indicates **identity** Score **> 14** indicates **homology** | U | R.FHKFDEDEKGFITIVDVQR.V |
| 153848 | 632 | – | 650 | 775.0628 | 2322.1666 | 2322.1645 | 0.93 | 2 | 57 | 4.6e-06 | 1Score **> 37** indicates **identity** Score **> 16** indicates **homology** | U | R.FHKFDEDEKGFITIVDVQR.V |
| 153849 | 632 | – | 650 | 581.5490 | 2322.1667 | 2322.1645 | 0.96 | 2 | 28 | 0.0026 | 1Score **> 37** indicates **identity** Score **> 14** indicates **homology** | U | R.FHKFDEDEKGFITIVDVQR.V |
| 153850 | 632 | – | 650 | 775.0629 | 2322.1667 | 2322.1645 | 0.98 | 2 | 34 | 0.00063 | 1Score **> 37** indicates **identity** Score **> 15** indicates **homology** | U | R.FHKFDEDEKGFITIVDVQR.V |
| 153851 | 632 | – | 650 | 775.0629 | 2322.1668 | 2322.1645 | 1.00 | 2 | 33 | 0.00086 | 1Score **> 37** indicates **identity** Score **> 15** indicates **homology** | U | R.FHKFDEDEKGFITIVDVQR.V |
| 153857 | 632 | – | 650 | 581.5491 | 2322.1674 | 2322.1645 | 1.27 | 2 | 37 | 0.00036 | 1Score **> 37** indicates **identity** Score **> 15** indicates **homology** | U | R.FHKFDEDEKGFITIVDVQR.V |
| 153858 | 632 | – | 650 | 581.5492 | 2322.1675 | 2322.1645 | 1.31 | 2 | 53 | 1.1e-05 | 1Score **> 37** indicates **identity** Score **> 16** indicates **homology** | U | R.FHKFDEDEKGFITIVDVQR.V |
| 153860 | 632 | – | 650 | 581.5493 | 2322.1682 | 2322.1645 | 1.60 | 2 | 29 | 0.0017 | 1Score **> 37** indicates **identity** Score **> 14** indicates **homology** | U | R.FHKFDEDEKGFITIVDVQR.V |
| 153861 | 632 | – | 650 | 581.5493 | 2322.1683 | 2322.1645 | 1.64 | 2 | 21 | 0.0098 | 1Score **> 37** indicates **identity** Score **> 14** indicates **homology** | U | R.FHKFDEDEKGFITIVDVQR.V |
| 153863 | 632 | – | 650 | 581.5502 | 2322.1718 | 2322.1645 | 3.16 | 2 | 17 | 0.026 | 1Score **> 37** indicates **identity** Score **> 14** indicates **homology** | U | R.FHKFDEDEKGFITIVDVQR.V |
| 153865 | 632 | – | 650 | 581.5503 | 2322.1721 | 2322.1645 | 3.28 | 2 | 42 | 0.00012 | 1Score **> 37** indicates **identity** Score **> 15** indicates **homology** | U | R.FHKFDEDEKGFITIVDVQR.V |
| 163634 | 632 | – | 650 | 496.6605 | 2478.2662 | 2478.2795 | -5.37 | 2 | 36 | 0.00041 | 1Score **> 37** indicates **identity** Score **> 15** indicates **homology** | U | R.FHKFDEDEKGFITIVDVQR.V  + HNE (H) |
| 163635 | 632 | – | 650 | 620.5739 | 2478.2663 | 2478.2795 | -5.31 | 2 | 33 | 0.00083 | 1Score **> 37** indicates **identity** Score **> 15** indicates **homology** | U | R.FHKFDEDEKGFITIVDVQR.V  + HNE (H) |
| 163637 | 632 | – | 650 | 496.6606 | 2478.2666 | 2478.2795 | -5.20 | 2 | 21 | 0.0099 | 1Score **> 37** indicates **identity** Score **> 14** indicates **homology** | U | R.FHKFDEDEKGFITIVDVQR.V  + HNE (H) |
| 163638 | 632 | – | 650 | 496.6606 | 2478.2667 | 2478.2795 | -5.14 | 2 | 16 | 0.031 | 1Score **> 37** indicates **identity** Score **> 14** indicates **homology** | U | R.FHKFDEDEKGFITIVDVQR.V  + HNE (H) |
| 163639 | 632 | – | 650 | 620.5742 | 2478.2675 | 2478.2795 | -4.83 | 2 | 33 | 0.00081 | 1Score **> 37** indicates **identity** Score **> 15** indicates **homology** | U | R.FHKFDEDEKGFITIVDVQR.V  + HNE (H) |
| 163644 | 632 | – | 650 | 620.5743 | 2478.2682 | 2478.2795 | -4.54 | 2 | 42 | 0.00012 | 1Score **> 37** indicates **identity** Score **> 15** indicates **homology** | U | R.FHKFDEDEKGFITIVDVQR.V  + HNE (H) |
| 163645 | 632 | – | 650 | 620.5744 | 2478.2683 | 2478.2795 | -4.50 | 2 | 28 | 0.0023 | 1Score **> 37** indicates **identity** Score **> 14** indicates **homology** | U | R.FHKFDEDEKGFITIVDVQR.V  + HNE (H) |
| 163646 | 632 | – | 650 | 827.0970 | 2478.2693 | 2478.2795 | -4.12 | 2 | 42 | 0.00012 | 1Score **> 37** indicates **identity** Score **> 15** indicates **homology** | U | R.FHKFDEDEKGFITIVDVQR.V  + HNE (H) |
| 117298 | 635 | – | 650 | 637.6546 | 1909.9419 | 1909.9422 | -0.12 | 1 | 28 | 0.0024 | 1Score **> 35** indicates **identity** Score **> 14** indicates **homology** | U | K.FDEDEKGFITIVDVQR.V |
| 117301 | 635 | – | 650 | 637.6573 | 1909.9500 | 1909.9422 | 4.12 | 1 | 23 | 0.0068 | 1Score **> 36** indicates **identity** Score **> 14** indicates **homology** | U | K.FDEDEKGFITIVDVQR.V |
| 117305 | 635 | – | 650 | 637.6590 | 1909.9551 | 1909.9422 | 6.78 | 1 | 18 | 0.02 | 1Score **> 36** indicates **identity** Score **> 14** indicates **homology** | U | K.FDEDEKGFITIVDVQR.V |
| 117308 | 635 | – | 650 | 637.6606 | 1909.9601 | 1909.9422 | 9.40 | 1 | 29 | 0.0018 | 1Score **> 36** indicates **identity** Score **> 14** indicates **homology** | U | K.FDEDEKGFITIVDVQR.V |
| 22763 | 707 | – | 715 | 368.5199 | 1102.5379 | 1102.5367 | 1.12 | 1 | 18 | 0.027 | 1Score **> 31** indicates **identity** Score **> 15** indicates **homology** | U | K.TAEENLDRR.V |
| 11376 | 715 | – | 722 | 476.2906 | 950.5667 | 950.5661 | 0.56 | 1 | 28 | 0.01 | 1Score **> 23** indicates **identity** Score **> 21** indicates **homology** | U | R.RVPIPVDR.S |
| 11378 | 715 | – | 722 | 476.2907 | 950.5668 | 950.5661 | 0.69 | 1 | 33 | 0.0065 | 1Score **> 23** indicates **identity** | U | R.RVPIPVDR.S |
| 3413 | 716 | – | 722 | 398.2398 | 794.4651 | 794.4650 | 0.088 | 0 | 42 | 0.00069 | 1Score **> 23** indicates **identity** | U | R.VPIPVDR.S |
| 3416 | 716 | – | 722 | 398.2402 | 794.4658 | 794.4650 | 0.99 | 0 | 30 | 0.011 | 1Score **> 23** indicates **identity** | U | R.VPIPVDR.S |
| 3419 | 716 | – | 722 | 398.2406 | 794.4666 | 794.4650 | 1.93 | 0 | 35 | 0.0031 | 1Score **> 23** indicates **identity** | U | R.VPIPVDR.S |

---

```
ID   GPDM_MOUSE              Reviewed;         727 AA.
AC   Q64521; Q3TK51; Q3UDY8; Q61507; Q8CBX6; Q8K4U5; Q8VDT0; Q9ERP0;
DT   01-NOV-1997, integrated into UniProtKB/Swiss-Prot.
DT   01-MAY-2007, sequence version 2.
DT   28-JUN-2023, entry version 186.
DE   RecName: Full=Glycerol-3-phosphate dehydrogenase, mitochondrial;
DE            Short=GPD-M;
DE            Short=GPDH-M;
DE            EC=1.1.5.3 {ECO:0000250|UniProtKB:P43304};
DE   AltName: Full=Protein TISP38;
DE   Flags: Precursor;
GN   Name=Gpd2 {ECO:0000312|MGI:MGI:99778}; Synonyms=Gdm1;
OS   Mus musculus (Mouse).
OC   Eukaryota; Metazoa; Chordata; Craniata; Vertebrata; Euteleostomi; Mammalia;
OC   Eutheria; Euarchontoglires; Glires; Rodentia; Myomorpha; Muroidea; Muridae;
OC   Murinae; Mus; Mus.
OX   NCBI_TaxID=10090;
RN   [1]
RP   NUCLEOTIDE SEQUENCE [MRNA].
RC   STRAIN=C57BL/6J; TISSUE=Adipocyte;
RX   PubMed=8951039; DOI=10.1006/abbi.1996.0536;
RA   Koza R.A., Kozak U.C., Brown L.J., Leiter E.H., Macdonald M.J., Kozak L.P.;
RT   "Sequence and tissue-dependent RNA expression of mouse FAD-linked glycerol-
RT   3-phosphate dehydrogenase.";
RL   Arch. Biochem. Biophys. 336:97-104(1996).
RN   [2]
RP   NUCLEOTIDE SEQUENCE [MRNA].
RC   STRAIN=C57BL/6J;
RX   PubMed=8772729; DOI=10.2337/diab.45.9.1238;
RA   Ishihara H., Nakazaki M., Kanegae Y., Inukai K., Asano T., Katagiri H.,
RA   Yazaki Y., Kikuchi M., Miyazaki J., Saito I., Oka Y.;
RT   "Effect of mitochondrial and/or cytosolic glycerol 3-phosphate
RT   dehydrogenase overexpression on glucose-stimulated insulin secretion from
RT   MIN6 and HIT cells.";
RL   Diabetes 45:1238-1244(1996).
RN   [3]
RP   NUCLEOTIDE SEQUENCE [LARGE SCALE MRNA].
RC   STRAIN=C57BL/6J; TISSUE=Bone marrow, Diencephalon, and Lung;
RX   PubMed=16141072; DOI=10.1126/science.1112014;
RA   Carninci P., Kasukawa T., Katayama S., Gough J., Frith M.C., Maeda N.,
RA   Oyama R., Ravasi T., Lenhard B., Wells C., Kodzius R., Shimokawa K.,
RA   Bajic V.B., Brenner S.E., Batalov S., Forrest A.R., Zavolan M., Davis M.J.,
RA   Wilming L.G., Aidinis V., Allen J.E., Ambesi-Impiombato A., Apweiler R.,
RA   Aturaliya R.N., Bailey T.L., Bansal M., Baxter L., Beisel K.W., Bersano T.,
RA   Bono H., Chalk A.M., Chiu K.P., Choudhary V., Christoffels A.,
RA   Clutterbuck D.R., Crowe M.L., Dalla E., Dalrymple B.P., de Bono B.,
RA   Della Gatta G., di Bernardo D., Down T., Engstrom P., Fagiolini M.,
RA   Faulkner G., Fletcher C.F., Fukushima T., Furuno M., Futaki S.,
RA   Gariboldi M., Georgii-Hemming P., Gingeras T.R., Gojobori T., Green R.E.,
RA   Gustincich S., Harbers M., Hayashi Y., Hensch T.K., Hirokawa N., Hill D.,
RA   Huminiecki L., Iacono M., Ikeo K., Iwama A., Ishikawa T., Jakt M.,
RA   Kanapin A., Katoh M., Kawasawa Y., Kelso J., Kitamura H., Kitano H.,
RA   Kollias G., Krishnan S.P., Kruger A., Kummerfeld S.K., Kurochkin I.V.,
RA   Lareau L.F., Lazarevic D., Lipovich L., Liu J., Liuni S., McWilliam S.,
RA   Madan Babu M., Madera M., Marchionni L., Matsuda H., Matsuzawa S., Miki H.,
RA   Mignone F., Miyake S., Morris K., Mottagui-Tabar S., Mulder N., Nakano N.,
RA   Nakauchi H., Ng P., Nilsson R., Nishiguchi S., Nishikawa S., Nori F.,
RA   Ohara O., Okazaki Y., Orlando V., Pang K.C., Pavan W.J., Pavesi G.,
RA   Pesole G., Petrovsky N., Piazza S., Reed J., Reid J.F., Ring B.Z.,
RA   Ringwald M., Rost B., Ruan Y., Salzberg S.L., Sandelin A., Schneider C.,
RA   Schoenbach C., Sekiguchi K., Semple C.A., Seno S., Sessa L., Sheng Y.,
RA   Shibata Y., Shimada H., Shimada K., Silva D., Sinclair B., Sperling S.,
RA   Stupka E., Sugiura K., Sultana R., Takenaka Y., Taki K., Tammoja K.,
RA   Tan S.L., Tang S., Taylor M.S., Tegner J., Teichmann S.A., Ueda H.R.,
RA   van Nimwegen E., Verardo R., Wei C.L., Yagi K., Yamanishi H.,
RA   Zabarovsky E., Zhu S., Zimmer A., Hide W., Bult C., Grimmond S.M.,
RA   Teasdale R.D., Liu E.T., Brusic V., Quackenbush J., Wahlestedt C.,
RA   Mattick J.S., Hume D.A., Kai C., Sasaki D., Tomaru Y., Fukuda S.,
RA   Kanamori-Katayama M., Suzuki M., Aoki J., Arakawa T., Iida J., Imamura K.,
RA   Itoh M., Kato T., Kawaji H., Kawagashira N., Kawashima T., Kojima M.,
RA   Kondo S., Konno H., Nakano K., Ninomiya N., Nishio T., Okada M., Plessy C.,
RA   Shibata K., Shiraki T., Suzuki S., Tagami M., Waki K., Watahiki A.,
RA   Okamura-Oho Y., Suzuki H., Kawai J., Hayashizaki Y.;
RT   "The transcriptional landscape of the mammalian genome.";
RL   Science 309:1559-1563(2005).
RN   [4]
RP   NUCLEOTIDE SEQUENCE [LARGE SCALE MRNA].
RC   STRAIN=Czech II; TISSUE=Mammary tumor;
RX   PubMed=15489334; DOI=10.1101/gr.2596504;
RG   The MGC Project Team;
RT   "The status, quality, and expansion of the NIH full-length cDNA project:
RT   the Mammalian Gene Collection (MGC).";
RL   Genome Res. 14:2121-2127(2004).
RN   [5]
RP   PROTEIN SEQUENCE OF 58-91; 95-110; 125-135; 167-178; 200-209; 212-254;
RP   285-298; 340-348; 381-390; 392-409; 442-453; 464-473; 483-499; 519-538;
RP   558-579; 598-606; 609-627 AND 635-693.
RC   STRAIN=C57BL/6J; TISSUE=Brain, and Hippocampus;
RA   Lubec G., Klug S., Kang S.U.;
RL   Submitted (APR-2007) to UniProtKB.
RN   [6]
RP   NUCLEOTIDE SEQUENCE [GENOMIC DNA] OF 390-686.
RC   STRAIN=129/Sv; TISSUE=Liver;
RA   Weitzel J.M.;
RT   "Genomic sequence of mouse mitochondrial glycerol-3-phosphate dehydrogenase
RT   (mGPDH).";
RL   Submitted (AUG-2000) to the EMBL/GenBank/DDBJ databases.
RN   [7]
RP   NUCLEOTIDE SEQUENCE [MRNA] OF 611-727.
RC   TISSUE=Testis;
RA   Tamura K., Nishimune Y., Nojima H.;
RT   "Mus musculus TISP38 mRNA.";
RL   Submitted (JUL-2000) to the EMBL/GenBank/DDBJ databases.
RN   [8]
RP   PHOSPHORYLATION [LARGE SCALE ANALYSIS] AT TYR-601, AND IDENTIFICATION BY
RP   MASS SPECTROMETRY [LARGE SCALE ANALYSIS].
RC   TISSUE=Brain;
RX   PubMed=18034455; DOI=10.1021/pr0701254;
RA   Ballif B.A., Carey G.R., Sunyaev S.R., Gygi S.P.;
RT   "Large-scale identification and evolution indexing of tyrosine
RT   phosphorylation sites from murine brain.";
RL   J. Proteome Res. 7:311-318(2008).
RN   [9]
RP   IDENTIFICATION BY MASS SPECTROMETRY [LARGE SCALE ANALYSIS].
RC   TISSUE=Brain, Brown adipose tissue, Heart, Kidney, Liver, Lung,
RC   Pancreas, Spleen, and Testis;
RX   PubMed=21183079; DOI=10.1016/j.cell.2010.12.001;
RA   Huttlin E.L., Jedrychowski M.P., Elias J.E., Goswami T., Rad R.,
RA   Beausoleil S.A., Villen J., Haas W., Sowa M.E., Gygi S.P.;
RT   "A tissue-specific atlas of mouse protein phosphorylation and expression.";
RL   Cell 143:1174-1189(2010).
CC   -!- FUNCTION: Calcium-responsive mitochondrial glycerol-3-phosphate
CC       dehydrogenase which seems to be a key component of the pancreatic beta-
CC       cell glucose-sensing device. {ECO:0000250|UniProtKB:P43304}.
CC   -!- CATALYTIC ACTIVITY:
CC       Reaction=a quinone + sn-glycerol 3-phosphate = a quinol +
CC         dihydroxyacetone phosphate; Xref=Rhea:RHEA:18977, ChEBI:CHEBI:24646,
CC         ChEBI:CHEBI:57597, ChEBI:CHEBI:57642, ChEBI:CHEBI:132124; EC=1.1.5.3;
CC         Evidence={ECO:0000250|UniProtKB:P43304};
CC       PhysiologicalDirection=left-to-right; Xref=Rhea:RHEA:18978;
CC         Evidence={ECO:0000250|UniProtKB:P43304};
CC   -!- COFACTOR:
CC       Name=FAD; Xref=ChEBI:CHEBI:57692;
CC   -!- ACTIVITY REGULATION: Calcium-binding enhance the activity of the
CC       enzyme. {ECO:0000250|UniProtKB:P43304}.
CC   -!- PATHWAY: Polyol metabolism; glycerol degradation via glycerol kinase
CC       pathway; glycerone phosphate from sn-glycerol 3-phosphate (anaerobic
CC       route): step 1/1.
CC   -!- SUBCELLULAR LOCATION: Mitochondrion inner membrane.
CC   -!- SIMILARITY: Belongs to the FAD-dependent glycerol-3-phosphate
CC       dehydrogenase family. {ECO:0000305}.
CC   ---------------------------------------------------------------------------
CC   Copyrighted by the UniProt Consortium, see https://www.uniprot.org/terms
CC   Distributed under the Creative Commons Attribution (CC BY 4.0) License
CC   ---------------------------------------------------------------------------
DR   EMBL; U60987; AAB50545.1; -; mRNA.
DR   EMBL; D50430; BAA08926.1; -; mRNA.
DR   EMBL; AK034353; BAC28685.1; -; mRNA.
DR   EMBL; AK144716; BAE26028.1; -; mRNA.
DR   EMBL; AK149851; BAE29123.1; -; mRNA.
DR   EMBL; AK167152; BAE39294.1; -; mRNA.
DR   EMBL; BC021359; AAH21359.1; -; mRNA.
DR   EMBL; AH009802; AAG12342.1; -; Genomic_DNA.
DR   EMBL; AB045714; BAB97201.1; -; mRNA.
DR   CCDS; CCDS16045.1; -.
DR   RefSeq; NP_001139292.1; NM_001145820.1.
DR   RefSeq; NP_034404.3; NM_010274.3.
DR   AlphaFoldDB; Q64521; -.
DR   SMR; Q64521; -.
DR   BioGRID; 199894; 21.
DR   IntAct; Q64521; 2.
DR   STRING; 10090.ENSMUSP00000108237; -.
DR   GlyGen; Q64521; 1 site, 1 O-linked glycan (1 site).
DR   iPTMnet; Q64521; -.
DR   PhosphoSitePlus; Q64521; -.
DR   SwissPalm; Q64521; -.
DR   REPRODUCTION-2DPAGE; Q64521; -.
DR   EPD; Q64521; -.
DR   jPOST; Q64521; -.
DR   MaxQB; Q64521; -.
DR   PaxDb; Q64521; -.
DR   PeptideAtlas; Q64521; -.
DR   ProteomicsDB; 271263; -.
DR   Antibodypedia; 2370; 316 antibodies from 30 providers.
DR   DNASU; 14571; -.
DR   Ensembl; ENSMUST00000028167; ENSMUSP00000028167; ENSMUSG00000026827.
DR   Ensembl; ENSMUST00000169687; ENSMUSP00000130992; ENSMUSG00000026827.
DR   GeneID; 14571; -.
DR   KEGG; mmu:14571; -.
DR   UCSC; uc008jsc.2; mouse.
DR   AGR; MGI:99778; -.
DR   CTD; 2820; -.
DR   MGI; MGI:99778; Gpd2.
DR   VEuPathDB; HostDB:ENSMUSG00000026827; -.
DR   eggNOG; KOG0042; Eukaryota.
DR   GeneTree; ENSGT00390000001718; -.
DR   InParanoid; Q64521; -.
DR   OMA; WAMAPHI; -.
DR   OrthoDB; 989271at2759; -.
DR   BRENDA; 1.1.5.3; 3474.
DR   Reactome; R-MMU-1483166; Synthesis of PA.
DR   Reactome; R-MMU-163560; Triglyceride catabolism.
DR   UniPathway; UPA00618; UER00673.
DR   BioGRID-ORCS; 14571; 2 hits in 78 CRISPR screens.
DR   ChiTaRS; Gpd2; mouse.
DR   PRO; PR:Q64521; -.
DR   Proteomes; UP000000589; Chromosome 2.
DR   RNAct; Q64521; protein.
DR   Bgee; ENSMUSG00000026827; Expressed in seminiferous tubule of testis and 269 other tissues.
DR   ExpressionAtlas; Q64521; baseline and differential.
DR   Genevisible; Q64521; MM.
DR   GO; GO:0009331; C:glycerol-3-phosphate dehydrogenase complex; TAS:MGI.
DR   GO; GO:0005743; C:mitochondrial inner membrane; HDA:MGI.
DR   GO; GO:0005739; C:mitochondrion; HDA:MGI.
DR   GO; GO:0005509; F:calcium ion binding; IEA:InterPro.
DR   GO; GO:0004368; F:glycerol-3-phosphate dehydrogenase (quinone) activity; ISO:MGI.
DR   GO; GO:0047952; F:glycerol-3-phosphate dehydrogenase [NAD(P)+] activity; ISO:MGI.
DR   GO; GO:0052591; F:sn-glycerol-3-phosphate:ubiquinone-8 oxidoreductase activity; IEA:UniProtKB-EC.
DR   GO; GO:0043010; P:camera-type eye development; IGI:MGI.
DR   GO; GO:0006094; P:gluconeogenesis; IMP:MGI.
DR   GO; GO:0019563; P:glycerol catabolic process; IEA:UniProtKB-UniPathway.
DR   GO; GO:0006072; P:glycerol-3-phosphate metabolic process; IMP:MGI.
DR   GO; GO:0035264; P:multicellular organism growth; IGI:MGI.
DR   GO; GO:0006734; P:NADH metabolic process; ISO:MGI.
DR   CDD; cd00051; EFh; 1.
DR   Gene3D; 1.10.8.870; Alpha-glycerophosphate oxidase, cap domain; 1.
DR   Gene3D; 3.30.9.10; D-Amino Acid Oxidase, subunit A, domain 2; 1.
DR   Gene3D; 1.10.238.10; EF-hand; 1.
DR   Gene3D; 3.50.50.60; FAD/NAD(P)-binding domain; 1.
DR   InterPro; IPR031656; DAO_C.
DR   InterPro; IPR038299; DAO_C_sf.
DR   InterPro; IPR011992; EF-hand-dom_pair.
DR   InterPro; IPR018247; EF_Hand_1_Ca_BS.
DR   InterPro; IPR002048; EF_hand_dom.
DR   InterPro; IPR006076; FAD-dep_OxRdtase.
DR   InterPro; IPR036188; FAD/NAD-bd_sf.
DR   InterPro; IPR000447; G3P_DH_FAD-dep.
DR   PANTHER; PTHR11985; GLYCEROL-3-PHOSPHATE DEHYDROGENASE; 1.
DR   PANTHER; PTHR11985:SF15; GLYCEROL-3-PHOSPHATE DEHYDROGENASE, MITOCHONDRIAL; 1.
DR   Pfam; PF01266; DAO; 1.
DR   Pfam; PF16901; DAO_C; 1.
DR   Pfam; PF13499; EF-hand_7; 1.
DR   PRINTS; PR01001; FADG3PDH.
DR   SMART; SM00054; EFh; 2.
DR   SUPFAM; SSF47473; EF-hand; 1.
DR   SUPFAM; SSF54373; FAD-linked reductases, C-terminal domain; 1.
DR   SUPFAM; SSF51905; FAD/NAD(P)-binding domain; 1.
DR   PROSITE; PS00018; EF_HAND_1; 1.
DR   PROSITE; PS50222; EF_HAND_2; 2.
DR   PROSITE; PS00977; FAD_G3PDH_1; 1.
DR   PROSITE; PS00978; FAD_G3PDH_2; 1.
PE   1: Evidence at protein level;
KW   Calcium; Direct protein sequencing; FAD; Flavoprotein; Membrane;
KW   Metal-binding; Mitochondrion; Mitochondrion inner membrane; Oxidoreductase;
KW   Phosphoprotein; Reference proteome; Repeat; Transit peptide.
FT   TRANSIT         1..42
FT                   /note="Mitochondrion"
FT                   /evidence="ECO:0000250"
FT   CHAIN           43..727
FT                   /note="Glycerol-3-phosphate dehydrogenase, mitochondrial"
FT                   /id="PRO_0000010430"
FT   DOMAIN          623..658
FT                   /note="EF-hand 1"
FT                   /evidence="ECO:0000255|PROSITE-ProRule:PRU00448"
FT   DOMAIN          659..694
FT                   /note="EF-hand 2"
FT                   /evidence="ECO:0000255|PROSITE-ProRule:PRU00448"
FT   BINDING         71..99
FT                   /ligand="FAD"
FT                   /ligand_id="ChEBI:CHEBI:57692"
FT                   /evidence="ECO:0000255"
FT   BINDING         672
FT                   /ligand="Ca(2+)"
FT                   /ligand_id="ChEBI:CHEBI:29108"
FT                   /evidence="ECO:0000255|PROSITE-ProRule:PRU00448"
FT   BINDING         674
FT                   /ligand="Ca(2+)"
FT                   /ligand_id="ChEBI:CHEBI:29108"
FT                   /evidence="ECO:0000255|PROSITE-ProRule:PRU00448"
FT   BINDING         676
FT                   /ligand="Ca(2+)"
FT                   /ligand_id="ChEBI:CHEBI:29108"
FT                   /evidence="ECO:0000255|PROSITE-ProRule:PRU00448"
FT   BINDING         678
FT                   /ligand="Ca(2+)"
FT                   /ligand_id="ChEBI:CHEBI:29108"
FT                   /evidence="ECO:0000255|PROSITE-ProRule:PRU00448"
FT   BINDING         683
FT                   /ligand="Ca(2+)"
FT                   /ligand_id="ChEBI:CHEBI:29108"
FT                   /evidence="ECO:0000255|PROSITE-ProRule:PRU00448"
FT   MOD_RES         601
FT                   /note="Phosphotyrosine"
FT                   /evidence="ECO:0007744|PubMed:18034455"
FT   CONFLICT        26
FT                   /note="P -> Q (in Ref. 1; AAB50545 and 4; AAH21359)"
FT                   /evidence="ECO:0000305"
FT   CONFLICT        42
FT                   /note="Missing (in Ref. 3; BAC28685)"
FT                   /evidence="ECO:0000305"
FT   CONFLICT        158
FT                   /note="L -> V (in Ref. 1; AAB50545)"
FT                   /evidence="ECO:0000305"
FT   CONFLICT        295
FT                   /note="D -> E (in Ref. 2; BAA08926)"
FT                   /evidence="ECO:0000305"
FT   CONFLICT        449
FT                   /note="D -> N (in Ref. 4; AAH21359)"
FT                   /evidence="ECO:0000305"
FT   CONFLICT        498
FT                   /note="A -> P (in Ref. 2; BAA08926)"
FT                   /evidence="ECO:0000305"
FT   CONFLICT        582
FT                   /note="N -> D (in Ref. 1; AAB50545)"
FT                   /evidence="ECO:0000305"
FT   CONFLICT        593
FT                   /note="E -> G (in Ref. 1; AAB50545)"
FT                   /evidence="ECO:0000305"
SQ   SEQUENCE   727 AA;  80954 MW;  319F52E31CFDFC44 CRC64;
     MAFQKAVKGT ILVGGGALAT VLGLSPFAHY RRKQVSLAYV EAAGYLTEPV NREPPSREAQ
     LMTLKNTPEF DILVIGGGAT GCGCALDAVT RGLKTALVER DDFSSGTSSR STKLIHGGVR
     YLQKAIMNLD VEQYRMVKEA LHERANLLEI APHLSAPLPI MLPLYKWWQL PYYWVGIKMY
     DLVAGSQCLK SSYVLSKSRA LEHFPMLQKD KLVGAIVYYD GQHNDARMNL AIALTAARYG
     AATANYMEVV SLLKKTDPET GKERVSGARC KDVLTGQEFD VRAKCVINAS GPFTDSVRKM
     DDKNVVPICQ PSAGVHIVMP GYYSPENMGL LDPATSDGRV IFFLPWEKMT IAGTTDTPTD
     VTHHPIPSEE DINFILNEVR NYLSSDVEVR RGDVLAAWSG IRPLVTDPKS ADTQSISRNH
     VVDISDSGLI TIAGGKWTTY RSMAEDTVDA AVKFHNLNAG PSRTVGLFLQ GGKDWSPTLY
     IRLVQDYGLE SEVAQHLAKT YGDKAFEVAK MASVTGKRWP VVGVRLVSEF PYIEAEVKYG
     IKEYACTAVD MISRRTRLAF LNVQAAEEAL PRIVELMGRE LNWSELRKQE ELETATRFLY
     YEMGYKSRTE QLTDSTEISL LPSDIDRYKK RFHKFDEDEK GFITIVDVQR VLESINVQMD
     ENTLHEILCE VDLNKNGQVE LHEFLQLMSA VQKGRVSGSR LAILMKTAEE NLDRRVPIPV
     DRSCGGL
//
```

**Mascot:** http://www.matrixscience.com/


HNE (K) (+156.1150)
