# Supplementary material for: A common East-Asian ALDH2 mutation causes metabolic disorders and the therapeutic effect of ALDH2 activators
Source: Nat Commun. 2023 Sep 25;14:5971. doi: 10.1038/s41467-023-41570-6 (PMC10520061; doi:10.1038/s41467-023-41570-6)
Supplement: Supplementary file 4 — Supplementary Data 1 [file 41467_2023_41570_MOESM4_ESM.zip › Table S5c/Q8BMS1/Q8BMS1_KIO-1-K66-K664.html]

Mascot Search Results: Q8BMS1
 

# MASCOT Search Results

## Protein View: Q8BMS1

### Trifunctional enzyme subunit alpha, mitochondrial OS=Mus musculus OX=10090 GN=Hadha PE=1 SV=1

|  |  |
| --- | --- |
| Database: | Mouse\_UniProt\_proteomes |
| Score: | 12862 |
| Monoisotopic mass (M_r_): | 83302 |
| Calculated pI: | 9.24 |

Sequence similarity is available as an NCBI BLAST search of Q8BMS1 against nr.

### Search parameters

|  |  |
| --- | --- |
| MS data file: | `D:\LCMSMS\2023 Users' data\230529-1\230529-1-KIO-1.raw` |
| Enzyme: | Trypsin/P: cuts C-term side of KR. |
| Fixed modifications: | Carbamidomethyl (C) |
| Variable modifications: | Deamidated (NQ), HNE (C), HNE (H), HNE (K), Oxidation (M) |

### Protein sequence coverage: 68%

Matched peptides shown in ***bold red***.

|  |  |  |  |  |  |
| --- | --- | --- | --- | --- | --- |
| `1` | `MVASRAIGSL` | `SRFSAFRILR` | `SRGCICRSFT` | `TSSALLTRTH` | `INYGVKGDVA` |
| `51` | `VIRINSPNSK` | `VNTLNKEVQS` | `EFIEVMNEIW` | `ANDQIRSAVL` | `ISSKPGCFVA` |
| `101` | `GADINMLSSC` | `TTPQEATRIS` | `QEGQRMFEKL` | `EKSPKPVVAA` | `ISGSCLGGGL` |
| `151` | `ELAIACQYRI` | `ATKDRKTVLG` | `VPEVLLGILP` | `GAGGTQRLPK` | `MVGVPAAFDM` |
| `201` | `MLTGRNIRAD` | `RAKKMGLVDQ` | `LVEPLGPGIK` | `SPEERTIEYL` | `EEVAVNFAKG` |
| `251` | `LADRKVSAKQ` | `SKGLVEKLTT` | `YAMTVPFVRQ` | `QVYKTVEEKV` | `KKQTKGLYPA` |
| `301` | `PLKIIDAVKA` | `GLEQGSDAGY` | `LAESQKFGEL` | `ALTKESKALM` | `GLYNGQVLCK` |
| `351` | `KNKFGAPQKN` | `VQQLAILGAG` | `LMGAGIAQVS` | `VDKGLKTLLK` | `DTTVTGLGRG` |
| `401` | `QQQVFKGLND` | `KVKKKALTSF` | `ERDSIFSNLI` | `GQLDYKGFEK` | `ADMVIEAVFE` |
| `451` | `DLGVKHKVLK` | `EVESVTPEHC` | `IFASNTSALP` | `INQIAAVSKR` | `PEKVIGMHYF` |
| `501` | `SPVDKMQLLE` | `IITTDKTSKD` | `TTASAVAVGL` | `RQGKVIIVVK` | `DGPGFYTTRC` |
| `551` | `LAPMMSEVMR` | `ILQEGVDPKK` | `LDALTTGFGF` | `PVGAATLADE` | `VGVDVAQHVA` |
| `601` | `EDLGKAFGER` | `FGGGSVELLK` | `QMVSKGFLGR` | `KSGKGFYIYQ` | `EGSKNKSLNS` |
| `651` | `EMDNILANLR` | `LPAKPEVSSD` | `EDVQYRVITR` | `FVNEAVLCLQ` | `EGILATPAEG` |
| `701` | `DIGAVFGLGF` | `PPCLGGPFRF` | `VDLYGAQKVV` | `DRLRKYESAY` | `GTQFTPCQLL` |
| `751` | `LDHANNSSKK` | `FYQ` |  |  |  |

Unformatted sequence string: 763 residues (for pasting into other applications).

|  |  |  |  |
| --- | --- | --- | --- |
| Sort by | residue number | increasing mass | decreasing mass |
| Show | matched peptides only | predicted peptides also |  |

| Query | Start | – | End | Observed | Mr(expt) | Mr(calc) | ppm | M | Score | Expect | Rank | U | Peptide |
| --- | --- | --- | --- | --- | --- | --- | --- | --- | --- | --- | --- | --- | --- |
| 9206 | 39 | – | 46 | 466.2535 | 930.4924 | 930.4923 | 0.14 | 0 | 24 | 0.021 | 1Score **> 31** indicates **identity** Score **> 20** indicates **homology** | U | R.THINYGVK.G |
| 81084 | 39 | – | 53 | 411.2294 | 1640.8887 | 1640.8998 | -6.80 | 1 | 37 | 0.00034 | 1Score **> 35** indicates **identity** Score **> 15** indicates **homology** | U | R.THINYGVKGDVAVIR.I |
| 81108 | 39 | – | 53 | 411.2318 | 1640.8979 | 1640.8998 | -1.19 | 1 | 25 | 0.0043 | 1Score **> 35** indicates **identity** Score **> 14** indicates **homology** | U | R.THINYGVKGDVAVIR.I |
| 81117 | 39 | – | 53 | 547.9743 | 1640.9012 | 1640.8998 | 0.84 | 1 | 53 | 9.9e-06 | 1Score **> 34** indicates **identity** Score **> 16** indicates **homology** | U | R.THINYGVKGDVAVIR.I |
| 81118 | 39 | – | 53 | 547.9744 | 1640.9014 | 1640.8998 | 0.93 | 1 | 30 | 0.0015 | 1Score **> 34** indicates **identity** Score **> 14** indicates **homology** | U | R.THINYGVKGDVAVIR.I |
| 81119 | 39 | – | 53 | 547.9744 | 1640.9014 | 1640.8998 | 0.95 | 1 | 56 | 5.2e-06 | 1Score **> 34** indicates **identity** Score **> 16** indicates **homology** | U | R.THINYGVKGDVAVIR.I |
| 81120 | 39 | – | 53 | 547.9745 | 1640.9018 | 1640.8998 | 1.19 | 1 | 59 | 2.7e-06 | 1Score **> 34** indicates **identity** Score **> 16** indicates **homology** | U | R.THINYGVKGDVAVIR.I |
| 81121 | 39 | – | 53 | 547.9746 | 1640.9020 | 1640.8998 | 1.29 | 1 | 68 | 4.1e-07 | 1Score **> 34** indicates **identity** Score **> 17** indicates **homology** | U | R.THINYGVKGDVAVIR.I |
| 81123 | 39 | – | 53 | 411.2329 | 1640.9025 | 1640.8998 | 1.61 | 1 | 30 | 0.0015 | 1Score **> 34** indicates **identity** Score **> 14** indicates **homology** | U | R.THINYGVKGDVAVIR.I |
| 81126 | 39 | – | 53 | 411.2329 | 1640.9026 | 1640.8998 | 1.68 | 1 | 33 | 0.00081 | 1Score **> 34** indicates **identity** Score **> 15** indicates **homology** | U | R.THINYGVKGDVAVIR.I |
| 81128 | 39 | – | 53 | 547.9749 | 1640.9028 | 1640.8998 | 1.83 | 1 | 76 | 8.2e-08 | 1Score **> 34** indicates **identity** Score **> 17** indicates **homology** | U | R.THINYGVKGDVAVIR.I |
| 81129 | 39 | – | 53 | 547.9750 | 1640.9031 | 1640.8998 | 1.98 | 1 | 75 | 9.9e-08 | 1Score **> 34** indicates **identity** Score **> 17** indicates **homology** | U | R.THINYGVKGDVAVIR.I |
| 81135 | 39 | – | 53 | 411.2334 | 1640.9044 | 1640.8998 | 2.80 | 1 | 22 | 0.0081 | 1Score **> 34** indicates **identity** Score **> 14** indicates **homology** | U | R.THINYGVKGDVAVIR.I |
| 81136 | 39 | – | 53 | 411.2335 | 1640.9048 | 1640.8998 | 3.03 | 1 | 24 | 0.0068 | 1Score **> 34** indicates **identity** Score **> 15** indicates **homology** | U | R.THINYGVKGDVAVIR.I |
| 81147 | 39 | – | 53 | 821.4641 | 1640.9136 | 1640.8998 | 8.40 | 1 | 51 | 1.8e-05 | 1Score **> 34** indicates **identity** Score **> 16** indicates **homology** | U | R.THINYGVKGDVAVIR.I |
| 894 | 47 | – | 53 | 365.2163 | 728.4181 | 728.4181 | 0.059 | 0 | 46 | 0.00028 | 1Score **> 23** indicates **identity** | U | K.GDVAVIR.I |
| 895 | 47 | – | 53 | 365.2165 | 728.4185 | 728.4181 | 0.55 | 0 | 30 | 0.01 | 1Score **> 23** indicates **identity** | U | K.GDVAVIR.I |
| 185295 | 61 | – | 86 | 1098.2115 | 3291.6127 | 3291.6333 | -6.25 | 1 | 40 | 0.00016 | 1Score **> 37** indicates **identity** Score **> 15** indicates **homology** | U | K.VNTLNKEVQSEFIEVMNEIWANDQIR.S  + Deamidated (NQ); HNE (K); Oxidation (M) |
| 186895 | 87 | – | 118 | 1123.5539 | 3367.6398 | 3367.6210 | 5.56 | 1 | 96 | 9.9e-10 | 1Score **> 37** indicates **identity** Score **> 19** indicates **homology** | U | R.SAVLISSKPGCFVAGADINMLSSCTTPQEATR.I |
| 186896 | 87 | – | 118 | 1123.5553 | 3367.6440 | 3367.6210 | 6.83 | 1 | 98 | 7.1e-10 | 1Score **> 37** indicates **identity** Score **> 19** indicates **homology** | U | R.SAVLISSKPGCFVAGADINMLSSCTTPQEATR.I |
| 186898 | 87 | – | 118 | 1123.5577 | 3367.6512 | 3367.6210 | 8.95 | 1 | 77 | 6.3e-08 | 1Score **> 37** indicates **identity** Score **> 17** indicates **homology** | U | R.SAVLISSKPGCFVAGADINMLSSCTTPQEATR.I |
| 181980 | 130 | – | 159 | 786.9245 | 3143.6690 | 3143.6471 | 6.98 | 2 | 51 | 1.6e-05 | 1Score **> 36** indicates **identity** Score **> 16** indicates **homology** | U | K.LEKSPKPVVAAISGSCLGGGLELAIACQYR.I |
| 181981 | 130 | – | 159 | 786.9248 | 3143.6702 | 3143.6471 | 7.33 | 2 | 26 | 0.0033 | 1Score **> 36** indicates **identity** Score **> 14** indicates **homology** | U | K.LEKSPKPVVAAISGSCLGGGLELAIACQYR.I |
| 170685 | 133 | – | 159 | 925.4740 | 2773.4001 | 2773.4255 | -9.14 | 1 | 26 | 0.004 | 1Score **> 37** indicates **identity** Score **> 14** indicates **homology** | U | K.SPKPVVAAISGSCLGGGLELAIACQYR.I |
| 170686 | 133 | – | 159 | 925.4769 | 2773.4088 | 2773.4255 | -6.03 | 1 | 24 | 0.014 | 1Score **> 37** indicates **identity** Score **> 18** indicates **homology** | U | K.SPKPVVAAISGSCLGGGLELAIACQYR.I |
| 170687 | 133 | – | 159 | 925.4778 | 2773.4116 | 2773.4255 | -5.00 | 1 | 36 | 0.00082 | 1Score **> 37** indicates **identity** Score **> 18** indicates **homology** | U | K.SPKPVVAAISGSCLGGGLELAIACQYR.I |
| 170691 | 133 | – | 159 | 925.4788 | 2773.4147 | 2773.4255 | -3.89 | 1 | 27 | 0.0026 | 1Score **> 37** indicates **identity** Score **> 14** indicates **homology** | U | K.SPKPVVAAISGSCLGGGLELAIACQYR.I |
| 170692 | 133 | – | 159 | 925.4799 | 2773.4179 | 2773.4255 | -2.73 | 1 | 44 | 7.5e-05 | 1Score **> 37** indicates **identity** Score **> 15** indicates **homology** | U | K.SPKPVVAAISGSCLGGGLELAIACQYR.I |
| 170697 | 133 | – | 159 | 925.4814 | 2773.4223 | 2773.4255 | -1.17 | 1 | 102 | 3e-10 | 1Score **> 37** indicates **identity** Score **> 19** indicates **homology** | U | K.SPKPVVAAISGSCLGGGLELAIACQYR.I |
| 170698 | 133 | – | 159 | 925.4814 | 2773.4223 | 2773.4255 | -1.17 | 1 | 27 | 0.0031 | 1Score **> 37** indicates **identity** Score **> 14** indicates **homology** | U | K.SPKPVVAAISGSCLGGGLELAIACQYR.I |
| 170699 | 133 | – | 159 | 925.4816 | 2773.4228 | 2773.4255 | -0.95 | 1 | 43 | 9.2e-05 | 1Score **> 37** indicates **identity** Score **> 15** indicates **homology** | U | K.SPKPVVAAISGSCLGGGLELAIACQYR.I |
| 170701 | 133 | – | 159 | 925.4820 | 2773.4241 | 2773.4255 | -0.50 | 1 | 35 | 0.00052 | 1Score **> 37** indicates **identity** Score **> 15** indicates **homology** | U | K.SPKPVVAAISGSCLGGGLELAIACQYR.I |
| 170703 | 133 | – | 159 | 925.4823 | 2773.4250 | 2773.4255 | -0.18 | 1 | 89 | 4.7e-09 | 1Score **> 37** indicates **identity** Score **> 18** indicates **homology** | U | K.SPKPVVAAISGSCLGGGLELAIACQYR.I |
| 170704 | 133 | – | 159 | 925.4823 | 2773.4250 | 2773.4255 | -0.18 | 1 | 93 | 1.8e-09 | 1Score **> 37** indicates **identity** Score **> 18** indicates **homology** | U | K.SPKPVVAAISGSCLGGGLELAIACQYR.I |
| 170705 | 133 | – | 159 | 694.3637 | 2773.4257 | 2773.4255 | 0.084 | 1 | 25 | 0.0048 | 1Score **> 37** indicates **identity** Score **> 14** indicates **homology** | U | K.SPKPVVAAISGSCLGGGLELAIACQYR.I |
| 170707 | 133 | – | 159 | 925.4828 | 2773.4265 | 2773.4255 | 0.35 | 1 | 37 | 0.00033 | 1Score **> 37** indicates **identity** Score **> 15** indicates **homology** | U | K.SPKPVVAAISGSCLGGGLELAIACQYR.I |
| 170709 | 133 | – | 159 | 925.4828 | 2773.4266 | 2773.4255 | 0.41 | 1 | 90 | 3.9e-09 | 1Score **> 37** indicates **identity** Score **> 18** indicates **homology** | U | K.SPKPVVAAISGSCLGGGLELAIACQYR.I |
| 170710 | 133 | – | 159 | 925.4828 | 2773.4267 | 2773.4255 | 0.43 | 1 | 108 | 7.1e-11 | 1Score **> 37** indicates **identity** Score **> 19** indicates **homology** | U | K.SPKPVVAAISGSCLGGGLELAIACQYR.I |
| 170711 | 133 | – | 159 | 694.3640 | 2773.4267 | 2773.4255 | 0.44 | 1 | 54 | 9.1e-06 | 1Score **> 37** indicates **identity** Score **> 16** indicates **homology** | U | K.SPKPVVAAISGSCLGGGLELAIACQYR.I |
| 170714 | 133 | – | 159 | 925.4833 | 2773.4279 | 2773.4255 | 0.88 | 1 | 111 | 3.9e-11 | 1Score **> 37** indicates **identity** Score **> 19** indicates **homology** | U | K.SPKPVVAAISGSCLGGGLELAIACQYR.I |
| 170716 | 133 | – | 159 | 925.4834 | 2773.4283 | 2773.4255 | 1.01 | 1 | 38 | 0.00026 | 1Score **> 37** indicates **identity** Score **> 15** indicates **homology** | U | K.SPKPVVAAISGSCLGGGLELAIACQYR.I |
| 170718 | 133 | – | 159 | 694.3646 | 2773.4294 | 2773.4255 | 1.39 | 1 | 19 | 0.016 | 1Score **> 37** indicates **identity** Score **> 14** indicates **homology** | U | K.SPKPVVAAISGSCLGGGLELAIACQYR.I |
| 170719 | 133 | – | 159 | 925.4839 | 2773.4297 | 2773.4255 | 1.53 | 1 | 90 | 3.4e-09 | 1Score **> 37** indicates **identity** Score **> 18** indicates **homology** | U | K.SPKPVVAAISGSCLGGGLELAIACQYR.I |
| 170721 | 133 | – | 159 | 925.4839 | 2773.4298 | 2773.4255 | 1.55 | 1 | 104 | 2e-10 | 1Score **> 37** indicates **identity** Score **> 19** indicates **homology** | U | K.SPKPVVAAISGSCLGGGLELAIACQYR.I |
| 170722 | 133 | – | 159 | 694.3647 | 2773.4298 | 2773.4255 | 1.56 | 1 | 46 | 4.8e-05 | 1Score **> 37** indicates **identity** Score **> 15** indicates **homology** | U | K.SPKPVVAAISGSCLGGGLELAIACQYR.I |
| 170723 | 133 | – | 159 | 925.4841 | 2773.4304 | 2773.4255 | 1.76 | 1 | 44 | 7.2e-05 | 1Score **> 37** indicates **identity** Score **> 15** indicates **homology** | U | K.SPKPVVAAISGSCLGGGLELAIACQYR.I |
| 170725 | 133 | – | 159 | 925.4842 | 2773.4307 | 2773.4255 | 1.89 | 1 | 27 | 0.0032 | 1Score **> 37** indicates **identity** Score **> 14** indicates **homology** | U | K.SPKPVVAAISGSCLGGGLELAIACQYR.I |
| 170727 | 133 | – | 159 | 925.4845 | 2773.4318 | 2773.4255 | 2.26 | 1 | 81 | 2.5e-08 | 1Score **> 37** indicates **identity** Score **> 18** indicates **homology** | U | K.SPKPVVAAISGSCLGGGLELAIACQYR.I |
| 170728 | 133 | – | 159 | 925.4846 | 2773.4318 | 2773.4255 | 2.29 | 1 | 40 | 0.00016 | 1Score **> 37** indicates **identity** Score **> 15** indicates **homology** | U | K.SPKPVVAAISGSCLGGGLELAIACQYR.I |
| 170731 | 133 | – | 159 | 925.4848 | 2773.4325 | 2773.4255 | 2.53 | 1 | 82 | 2.1e-08 | 1Score **> 37** indicates **identity** Score **> 18** indicates **homology** | U | K.SPKPVVAAISGSCLGGGLELAIACQYR.I |
| 170733 | 133 | – | 159 | 925.4851 | 2773.4335 | 2773.4255 | 2.88 | 1 | 19 | 0.017 | 1Score **> 37** indicates **identity** Score **> 14** indicates **homology** | U | K.SPKPVVAAISGSCLGGGLELAIACQYR.I |
| 170734 | 133 | – | 159 | 925.4851 | 2773.4335 | 2773.4255 | 2.90 | 1 | 42 | 0.00011 | 1Score **> 37** indicates **identity** Score **> 15** indicates **homology** | U | K.SPKPVVAAISGSCLGGGLELAIACQYR.I |
| 170735 | 133 | – | 159 | 925.4854 | 2773.4342 | 2773.4255 | 3.15 | 1 | 47 | 3.8e-05 | 1Score **> 37** indicates **identity** Score **> 15** indicates **homology** | U | K.SPKPVVAAISGSCLGGGLELAIACQYR.I |
| 170738 | 133 | – | 159 | 925.4859 | 2773.4359 | 2773.4255 | 3.77 | 1 | 33 | 0.00081 | 1Score **> 37** indicates **identity** Score **> 15** indicates **homology** | U | K.SPKPVVAAISGSCLGGGLELAIACQYR.I |
| 170739 | 133 | – | 159 | 925.4860 | 2773.4362 | 2773.4255 | 3.84 | 1 | 40 | 0.00019 | 1Score **> 37** indicates **identity** Score **> 15** indicates **homology** | U | K.SPKPVVAAISGSCLGGGLELAIACQYR.I |
| 170740 | 133 | – | 159 | 925.4861 | 2773.4363 | 2773.4255 | 3.91 | 1 | 29 | 0.0021 | 1Score **> 37** indicates **identity** Score **> 14** indicates **homology** | U | K.SPKPVVAAISGSCLGGGLELAIACQYR.I |
| 170743 | 133 | – | 159 | 925.4864 | 2773.4375 | 2773.4255 | 4.32 | 1 | 65 | 7.3e-07 | 1Score **> 37** indicates **identity** Score **> 17** indicates **homology** | U | K.SPKPVVAAISGSCLGGGLELAIACQYR.I |
| 170744 | 133 | – | 159 | 925.4866 | 2773.4381 | 2773.4255 | 4.54 | 1 | 33 | 0.00088 | 1Score **> 37** indicates **identity** Score **> 15** indicates **homology** | U | K.SPKPVVAAISGSCLGGGLELAIACQYR.I |
| 170745 | 133 | – | 159 | 925.4869 | 2773.4389 | 2773.4255 | 4.82 | 1 | 38 | 0.0003 | 1Score **> 37** indicates **identity** Score **> 15** indicates **homology** | U | K.SPKPVVAAISGSCLGGGLELAIACQYR.I |
| 170747 | 133 | – | 159 | 925.4879 | 2773.4419 | 2773.4255 | 5.90 | 1 | 81 | 2.7e-08 | 1Score **> 37** indicates **identity** Score **> 18** indicates **homology** | U | K.SPKPVVAAISGSCLGGGLELAIACQYR.I |
| 170750 | 133 | – | 159 | 925.4883 | 2773.4431 | 2773.4255 | 6.34 | 1 | 24 | 0.0056 | 1Score **> 37** indicates **identity** Score **> 14** indicates **homology** | U | K.SPKPVVAAISGSCLGGGLELAIACQYR.I |
| 170752 | 133 | – | 159 | 925.4887 | 2773.4442 | 2773.4255 | 6.75 | 1 | 32 | 0.0018 | 1Score **> 37** indicates **identity** Score **> 17** indicates **homology** | U | K.SPKPVVAAISGSCLGGGLELAIACQYR.I |
| 170753 | 133 | – | 159 | 925.4890 | 2773.4453 | 2773.4255 | 7.14 | 1 | 38 | 0.00028 | 1Score **> 37** indicates **identity** Score **> 15** indicates **homology** | U | K.SPKPVVAAISGSCLGGGLELAIACQYR.I |
| 170754 | 133 | – | 159 | 925.4905 | 2773.4496 | 2773.4255 | 8.70 | 1 | 23 | 0.0071 | 1Score **> 37** indicates **identity** Score **> 14** indicates **homology** | U | K.SPKPVVAAISGSCLGGGLELAIACQYR.I |
| 170756 | 133 | – | 159 | 925.4908 | 2773.4505 | 2773.4255 | 9.01 | 1 | 26 | 0.0037 | 1Score **> 37** indicates **identity** Score **> 14** indicates **homology** | U | K.SPKPVVAAISGSCLGGGLELAIACQYR.I |
| 136929 | 166 | – | 187 | 725.7688 | 2174.2847 | 2174.2787 | 2.76 | 1 | 46 | 4.7e-05 | 1Score **> 29** indicates **identity** Score **> 15** indicates **homology** | U | R.KTVLGVPEVLLGILPGAGGTQR.L |
| 136930 | 166 | – | 187 | 725.7707 | 2174.2902 | 2174.2787 | 5.28 | 1 | 35 | 0.00055 | 1Score **> 28** indicates **identity** Score **> 15** indicates **homology** | U | R.KTVLGVPEVLLGILPGAGGTQR.L |
| 159532 | 166 | – | 190 | 629.1347 | 2512.5099 | 2512.5105 | -0.24 | 2 | 29 | 0.0019 | 1Score **> 24** indicates **identity** Score **> 14** indicates **homology** | U | R.KTVLGVPEVLLGILPGAGGTQRLPK.M |
| 159533 | 166 | – | 190 | 838.5116 | 2512.5128 | 2512.5105 | 0.93 | 2 | 62 | 1.4e-06 | 1Score **> 24** indicates **identity** Score **> 16** indicates **homology** | U | R.KTVLGVPEVLLGILPGAGGTQRLPK.M |
| 159534 | 166 | – | 190 | 629.1373 | 2512.5202 | 2512.5105 | 3.87 | 2 | 30 | 0.0016 | 1Score **> 24** indicates **identity** Score **> 14** indicates **homology** | U | R.KTVLGVPEVLLGILPGAGGTQRLPK.M |
| 159579 | 166 | – | 190 | 838.8464 | 2513.5174 | 2513.4945 | 9.12 | 2 | 37 | 0.00036 | 1Score **> 23** indicates **identity** Score **> 15** indicates **homology** | U | R.KTVLGVPEVLLGILPGAGGTQRLPK.M  + Deamidated (NQ) |
| 125015 | 167 | – | 187 | 683.0685 | 2046.1837 | 2046.1837 | -0.0098 | 0 | 48 | 3e-05 | 1Score **> 31** indicates **identity** Score **> 16** indicates **homology** | U | K.TVLGVPEVLLGILPGAGGTQR.L |
| 125016 | 167 | – | 187 | 683.0686 | 2046.1841 | 2046.1837 | 0.16 | 0 | 51 | 1.7e-05 | 1Score **> 31** indicates **identity** Score **> 16** indicates **homology** | U | K.TVLGVPEVLLGILPGAGGTQR.L |
| 125018 | 167 | – | 187 | 1024.1000 | 2046.1854 | 2046.1837 | 0.80 | 0 | 79 | 7.2e-08 | 1Score **> 31** indicates **identity** Score **> 20** indicates **homology** | U | K.TVLGVPEVLLGILPGAGGTQR.L |
| 125019 | 167 | – | 187 | 1024.1002 | 2046.1859 | 2046.1837 | 1.04 | 0 | 76 | 7e-08 | 1Score **> 31** indicates **identity** Score **> 17** indicates **homology** | U | K.TVLGVPEVLLGILPGAGGTQR.L |
| 125022 | 167 | – | 187 | 1024.1031 | 2046.1917 | 2046.1837 | 3.87 | 0 | 64 | 1e-06 | 1Score **> 31** indicates **identity** Score **> 16** indicates **homology** | U | K.TVLGVPEVLLGILPGAGGTQR.L |
| 125023 | 167 | – | 187 | 683.0720 | 2046.1941 | 2046.1837 | 5.08 | 0 | 55 | 6.7e-06 | 1Score **> 30** indicates **identity** Score **> 16** indicates **homology** | U | K.TVLGVPEVLLGILPGAGGTQR.L |
| 152039 | 167 | – | 190 | 795.8108 | 2384.4105 | 2384.4155 | -2.11 | 1 | 25 | 0.0046 | 1Score **> 28** indicates **identity** Score **> 14** indicates **homology** | U | K.TVLGVPEVLLGILPGAGGTQRLPK.M |
| 152040 | 167 | – | 190 | 1193.2147 | 2384.4148 | 2384.4155 | -0.33 | 1 | 42 | 0.00011 | 1Score **> 27** indicates **identity** Score **> 15** indicates **homology** | U | K.TVLGVPEVLLGILPGAGGTQRLPK.M |
| 152042 | 167 | – | 190 | 597.1114 | 2384.4165 | 2384.4155 | 0.39 | 1 | 15 | 0.038 | 1Score **> 27** indicates **identity** Score **> 13** indicates **homology** | U | K.TVLGVPEVLLGILPGAGGTQRLPK.M |
| 152043 | 167 | – | 190 | 1193.2156 | 2384.4166 | 2384.4155 | 0.44 | 1 | 29 | 0.0021 | 1Score **> 27** indicates **identity** Score **> 14** indicates **homology** | U | K.TVLGVPEVLLGILPGAGGTQRLPK.M |
| 152044 | 167 | – | 190 | 795.8133 | 2384.4181 | 2384.4155 | 1.06 | 1 | 28 | 0.0024 | 1Score **> 27** indicates **identity** Score **> 14** indicates **homology** | U | K.TVLGVPEVLLGILPGAGGTQRLPK.M |
| 152045 | 167 | – | 190 | 795.8133 | 2384.4182 | 2384.4155 | 1.12 | 1 | 37 | 0.00031 | 1Score **> 27** indicates **identity** Score **> 15** indicates **homology** | U | K.TVLGVPEVLLGILPGAGGTQRLPK.M |
| 152047 | 167 | – | 190 | 795.8137 | 2384.4194 | 2384.4155 | 1.62 | 1 | 26 | 0.0035 | 1Score **> 27** indicates **identity** Score **> 14** indicates **homology** | U | K.TVLGVPEVLLGILPGAGGTQRLPK.M |
| 99909 | 214 | – | 230 | 598.6787 | 1793.0143 | 1793.0121 | 1.25 | 1 | 23 | 0.0067 | 1Score **> 33** indicates **identity** Score **> 14** indicates **homology** | U | K.KMGLVDQLVEPLGPGIK.S |
| 152569 | 214 | – | 235 | 598.8296 | 2391.2893 | 2391.2832 | 2.55 | 2 | 38 | 0.0003 | 1Score **> 36** indicates **identity** Score **> 15** indicates **homology** | U | K.KMGLVDQLVEPLGPGIKSPEER.T |
| 152570 | 214 | – | 235 | 598.8300 | 2391.2907 | 2391.2832 | 3.16 | 2 | 55 | 7.3e-06 | 1Score **> 36** indicates **identity** Score **> 16** indicates **homology** | U | K.KMGLVDQLVEPLGPGIKSPEER.T |
| 152571 | 214 | – | 235 | 598.8301 | 2391.2912 | 2391.2832 | 3.36 | 2 | 17 | 0.024 | 1Score **> 36** indicates **identity** Score **> 14** indicates **homology** | U | K.KMGLVDQLVEPLGPGIKSPEER.T |
| 84425 | 215 | – | 230 | 555.9780 | 1664.9121 | 1664.9171 | -3.04 | 0 | 14 | 0.046 | 1Score **> 35** indicates **identity** Score **> 13** indicates **homology** | U | K.MGLVDQLVEPLGPGIK.S |
| 84428 | 215 | – | 230 | 555.9793 | 1664.9161 | 1664.9171 | -0.62 | 0 | 23 | 0.0067 | 1Score **> 34** indicates **identity** Score **> 14** indicates **homology** | U | K.MGLVDQLVEPLGPGIK.S |
| 84429 | 215 | – | 230 | 833.4659 | 1664.9173 | 1664.9171 | 0.11 | 0 | 39 | 0.00023 | 1Score **> 34** indicates **identity** Score **> 15** indicates **homology** | U | K.MGLVDQLVEPLGPGIK.S |
| 144261 | 215 | – | 235 | 755.3991 | 2263.1755 | 2263.1882 | -5.63 | 1 | 32 | 0.0009 | 1Score **> 37** indicates **identity** Score **> 15** indicates **homology** | U | K.MGLVDQLVEPLGPGIKSPEER.T |
| 144265 | 215 | – | 235 | 755.4035 | 2263.1886 | 2263.1882 | 0.16 | 1 | 65 | 8.1e-07 | 1Score **> 37** indicates **identity** Score **> 17** indicates **homology** | U | K.MGLVDQLVEPLGPGIKSPEER.T |
| 144268 | 215 | – | 235 | 755.4043 | 2263.1910 | 2263.1882 | 1.22 | 1 | 60 | 2.6e-06 | 1Score **> 37** indicates **identity** Score **> 16** indicates **homology** | U | K.MGLVDQLVEPLGPGIKSPEER.T |
| 144270 | 215 | – | 235 | 1132.6042 | 2263.1938 | 2263.1882 | 2.48 | 1 | 75 | 9.7e-08 | 1Score **> 37** indicates **identity** Score **> 18** indicates **homology** | U | K.MGLVDQLVEPLGPGIKSPEER.T |
| 78916 | 236 | – | 249 | 813.4174 | 1624.8203 | 1624.8348 | -8.91 | 0 | 43 | 9.8e-05 | 1Score **> 34** indicates **identity** Score **> 15** indicates **homology** | U | R.TIEYLEEVAVNFAK.G |
| 78917 | 236 | – | 249 | 813.4186 | 1624.8227 | 1624.8348 | -7.44 | 0 | 29 | 0.0018 | 1Score **> 34** indicates **identity** Score **> 14** indicates **homology** | U | R.TIEYLEEVAVNFAK.G |
| 78923 | 236 | – | 249 | 813.4238 | 1624.8330 | 1624.8348 | -1.13 | 0 | 36 | 0.00045 | 1Score **> 35** indicates **identity** Score **> 15** indicates **homology** | U | R.TIEYLEEVAVNFAK.G |
| 78926 | 236 | – | 249 | 813.4238 | 1624.8331 | 1624.8348 | -1.08 | 0 | 62 | 1.4e-06 | 1Score **> 35** indicates **identity** Score **> 16** indicates **homology** | U | R.TIEYLEEVAVNFAK.G |
| 78930 | 236 | – | 249 | 813.4242 | 1624.8338 | 1624.8348 | -0.61 | 0 | 62 | 1.4e-06 | 1Score **> 34** indicates **identity** Score **> 16** indicates **homology** | U | R.TIEYLEEVAVNFAK.G |
| 78931 | 236 | – | 249 | 542.6186 | 1624.8341 | 1624.8348 | -0.43 | 0 | 50 | 1.9e-05 | 1Score **> 34** indicates **identity** Score **> 16** indicates **homology** | U | R.TIEYLEEVAVNFAK.G |
| 78934 | 236 | – | 249 | 813.4244 | 1624.8343 | 1624.8348 | -0.31 | 0 | 42 | 0.00013 | 1Score **> 34** indicates **identity** Score **> 15** indicates **homology** | U | R.TIEYLEEVAVNFAK.G |
| 78939 | 236 | – | 249 | 542.6188 | 1624.8345 | 1624.8348 | -0.19 | 0 | 35 | 0.00055 | 1Score **> 34** indicates **identity** Score **> 15** indicates **homology** | U | R.TIEYLEEVAVNFAK.G |
| 78944 | 236 | – | 249 | 813.4247 | 1624.8349 | 1624.8348 | 0.057 | 0 | 59 | 2.9e-06 | 1Score **> 34** indicates **identity** Score **> 16** indicates **homology** | U | R.TIEYLEEVAVNFAK.G |
| 78946 | 236 | – | 249 | 813.4248 | 1624.8350 | 1624.8348 | 0.12 | 0 | 79 | 3.5e-08 | 1Score **> 34** indicates **identity** Score **> 17** indicates **homology** | U | R.TIEYLEEVAVNFAK.G |
| 78947 | 236 | – | 249 | 542.6191 | 1624.8353 | 1624.8348 | 0.32 | 0 | 33 | 0.00076 | 1Score **> 34** indicates **identity** Score **> 15** indicates **homology** | U | R.TIEYLEEVAVNFAK.G |
| 78948 | 236 | – | 249 | 813.4250 | 1624.8355 | 1624.8348 | 0.43 | 0 | 61 | 2e-06 | 1Score **> 34** indicates **identity** Score **> 16** indicates **homology** | U | R.TIEYLEEVAVNFAK.G |
| 78949 | 236 | – | 249 | 813.4250 | 1624.8355 | 1624.8348 | 0.43 | 0 | 62 | 1.5e-06 | 1Score **> 34** indicates **identity** Score **> 16** indicates **homology** | U | R.TIEYLEEVAVNFAK.G |
| 78950 | 236 | – | 249 | 542.6191 | 1624.8355 | 1624.8348 | 0.44 | 0 | 62 | 1.5e-06 | 1Score **> 34** indicates **identity** Score **> 16** indicates **homology** | U | R.TIEYLEEVAVNFAK.G |
| 78957 | 236 | – | 249 | 542.6192 | 1624.8358 | 1624.8348 | 0.60 | 0 | 67 | 4.9e-07 | 1Score **> 34** indicates **identity** Score **> 17** indicates **homology** | U | R.TIEYLEEVAVNFAK.G |
| 78958 | 236 | – | 249 | 813.4252 | 1624.8358 | 1624.8348 | 0.60 | 0 | 63 | 1.2e-06 | 1Score **> 34** indicates **identity** Score **> 16** indicates **homology** | U | R.TIEYLEEVAVNFAK.G |
| 78962 | 236 | – | 249 | 813.4254 | 1624.8362 | 1624.8348 | 0.87 | 0 | 32 | 0.001 | 1Score **> 34** indicates **identity** Score **> 15** indicates **homology** | U | R.TIEYLEEVAVNFAK.G |
| 78964 | 236 | – | 249 | 813.4254 | 1624.8363 | 1624.8348 | 0.90 | 0 | 73 | 1.5e-07 | 1Score **> 34** indicates **identity** Score **> 17** indicates **homology** | U | R.TIEYLEEVAVNFAK.G |
| 78965 | 236 | – | 249 | 542.6194 | 1624.8363 | 1624.8348 | 0.93 | 0 | 42 | 0.00011 | 1Score **> 34** indicates **identity** Score **> 15** indicates **homology** | U | R.TIEYLEEVAVNFAK.G |
| 78966 | 236 | – | 249 | 813.4255 | 1624.8365 | 1624.8348 | 1.05 | 0 | 84 | 1.4e-08 | 1Score **> 34** indicates **identity** Score **> 18** indicates **homology** | U | R.TIEYLEEVAVNFAK.G |
| 78968 | 236 | – | 249 | 542.6195 | 1624.8366 | 1624.8348 | 1.09 | 0 | 43 | 9.4e-05 | 1Score **> 34** indicates **identity** Score **> 15** indicates **homology** | U | R.TIEYLEEVAVNFAK.G |
| 78969 | 236 | – | 249 | 813.4256 | 1624.8366 | 1624.8348 | 1.09 | 0 | 41 | 0.00013 | 1Score **> 34** indicates **identity** Score **> 15** indicates **homology** | U | R.TIEYLEEVAVNFAK.G |
| 78970 | 236 | – | 249 | 813.4256 | 1624.8367 | 1624.8348 | 1.15 | 0 | 48 | 3.4e-05 | 1Score **> 34** indicates **identity** Score **> 15** indicates **homology** | U | R.TIEYLEEVAVNFAK.G |
| 78972 | 236 | – | 249 | 813.4257 | 1624.8369 | 1624.8348 | 1.27 | 0 | 26 | 0.0035 | 1Score **> 34** indicates **identity** Score **> 14** indicates **homology** | U | R.TIEYLEEVAVNFAK.G |
| 78974 | 236 | – | 249 | 542.6196 | 1624.8370 | 1624.8348 | 1.33 | 0 | 38 | 0.0003 | 1Score **> 34** indicates **identity** Score **> 15** indicates **homology** | U | R.TIEYLEEVAVNFAK.G |
| 78976 | 236 | – | 249 | 813.4258 | 1624.8371 | 1624.8348 | 1.41 | 0 | 64 | 1e-06 | 1Score **> 34** indicates **identity** Score **> 16** indicates **homology** | U | R.TIEYLEEVAVNFAK.G |
| 78977 | 236 | – | 249 | 813.4260 | 1624.8374 | 1624.8348 | 1.57 | 0 | 28 | 0.0023 | 1Score **> 34** indicates **identity** Score **> 14** indicates **homology** | U | R.TIEYLEEVAVNFAK.G |
| 78979 | 236 | – | 249 | 813.4261 | 1624.8376 | 1624.8348 | 1.72 | 0 | 46 | 5e-05 | 1Score **> 34** indicates **identity** Score **> 15** indicates **homology** | U | R.TIEYLEEVAVNFAK.G |
| 78980 | 236 | – | 249 | 813.4262 | 1624.8378 | 1624.8348 | 1.84 | 0 | 73 | 1.4e-07 | 1Score **> 34** indicates **identity** Score **> 17** indicates **homology** | U | R.TIEYLEEVAVNFAK.G |
| 78981 | 236 | – | 249 | 813.4262 | 1624.8378 | 1624.8348 | 1.85 | 0 | 63 | 1.3e-06 | 1Score **> 34** indicates **identity** Score **> 16** indicates **homology** | U | R.TIEYLEEVAVNFAK.G |
| 78982 | 236 | – | 249 | 813.4262 | 1624.8379 | 1624.8348 | 1.92 | 0 | 74 | 1.3e-07 | 1Score **> 34** indicates **identity** Score **> 17** indicates **homology** | U | R.TIEYLEEVAVNFAK.G |
| 78990 | 236 | – | 249 | 542.6201 | 1624.8384 | 1624.8348 | 2.22 | 0 | 50 | 2e-05 | 1Score **> 34** indicates **identity** Score **> 16** indicates **homology** | U | R.TIEYLEEVAVNFAK.G |
| 78991 | 236 | – | 249 | 813.4265 | 1624.8384 | 1624.8348 | 2.22 | 0 | 61 | 1.9e-06 | 1Score **> 34** indicates **identity** Score **> 16** indicates **homology** | U | R.TIEYLEEVAVNFAK.G |
| 78992 | 236 | – | 249 | 542.6202 | 1624.8386 | 1624.8348 | 2.36 | 0 | 52 | 1.4e-05 | 1Score **> 34** indicates **identity** Score **> 16** indicates **homology** | U | R.TIEYLEEVAVNFAK.G |
| 78994 | 236 | – | 249 | 813.4266 | 1624.8387 | 1624.8348 | 2.39 | 0 | 39 | 0.00021 | 1Score **> 34** indicates **identity** Score **> 15** indicates **homology** | U | R.TIEYLEEVAVNFAK.G |
| 78995 | 236 | – | 249 | 813.4266 | 1624.8387 | 1624.8348 | 2.40 | 0 | 35 | 0.00056 | 1Score **> 34** indicates **identity** Score **> 15** indicates **homology** | U | R.TIEYLEEVAVNFAK.G |
| 78996 | 236 | – | 249 | 813.4266 | 1624.8387 | 1624.8348 | 2.41 | 0 | 52 | 1.4e-05 | 1Score **> 34** indicates **identity** Score **> 16** indicates **homology** | U | R.TIEYLEEVAVNFAK.G |
| 78997 | 236 | – | 249 | 542.6202 | 1624.8387 | 1624.8348 | 2.42 | 0 | 41 | 0.00014 | 1Score **> 34** indicates **identity** Score **> 15** indicates **homology** | U | R.TIEYLEEVAVNFAK.G |
| 79000 | 236 | – | 249 | 813.4267 | 1624.8389 | 1624.8348 | 2.52 | 0 | 61 | 2.1e-06 | 1Score **> 34** indicates **identity** Score **> 16** indicates **homology** | U | R.TIEYLEEVAVNFAK.G |
| 79001 | 236 | – | 249 | 813.4268 | 1624.8390 | 1624.8348 | 2.60 | 0 | 84 | 1.5e-08 | 1Score **> 34** indicates **identity** Score **> 18** indicates **homology** | U | R.TIEYLEEVAVNFAK.G |
| 79002 | 236 | – | 249 | 813.4268 | 1624.8390 | 1624.8348 | 2.61 | 0 | 46 | 4.4e-05 | 1Score **> 34** indicates **identity** Score **> 15** indicates **homology** | U | R.TIEYLEEVAVNFAK.G |
| 79005 | 236 | – | 249 | 542.6205 | 1624.8395 | 1624.8348 | 2.91 | 0 | 53 | 1.1e-05 | 1Score **> 34** indicates **identity** Score **> 16** indicates **homology** | U | R.TIEYLEEVAVNFAK.G |
| 79006 | 236 | – | 249 | 542.6205 | 1624.8396 | 1624.8348 | 2.92 | 0 | 58 | 3.8e-06 | 1Score **> 34** indicates **identity** Score **> 16** indicates **homology** | U | R.TIEYLEEVAVNFAK.G |
| 79007 | 236 | – | 249 | 813.4271 | 1624.8397 | 1624.8348 | 2.99 | 0 | 37 | 0.00037 | 1Score **> 34** indicates **identity** Score **> 15** indicates **homology** | U | R.TIEYLEEVAVNFAK.G |
| 79008 | 236 | – | 249 | 813.4273 | 1624.8400 | 1624.8348 | 3.21 | 0 | 31 | 0.0012 | 1Score **> 35** indicates **identity** Score **> 14** indicates **homology** | U | R.TIEYLEEVAVNFAK.G |
| 79009 | 236 | – | 249 | 813.4274 | 1624.8402 | 1624.8348 | 3.30 | 0 | 82 | 1.9e-08 | 1Score **> 34** indicates **identity** Score **> 18** indicates **homology** | U | R.TIEYLEEVAVNFAK.G |
| 79011 | 236 | – | 249 | 813.4275 | 1624.8404 | 1624.8348 | 3.46 | 0 | 45 | 5.4e-05 | 1Score **> 34** indicates **identity** Score **> 15** indicates **homology** | U | R.TIEYLEEVAVNFAK.G |
| 79013 | 236 | – | 249 | 813.4275 | 1624.8405 | 1624.8348 | 3.52 | 0 | 53 | 1.2e-05 | 1Score **> 34** indicates **identity** Score **> 16** indicates **homology** | U | R.TIEYLEEVAVNFAK.G |
| 79014 | 236 | – | 249 | 813.4275 | 1624.8405 | 1624.8348 | 3.52 | 0 | 58 | 3.4e-06 | 1Score **> 34** indicates **identity** Score **> 16** indicates **homology** | U | R.TIEYLEEVAVNFAK.G |
| 79016 | 236 | – | 249 | 813.4276 | 1624.8407 | 1624.8348 | 3.61 | 0 | 80 | 3.4e-08 | 1Score **> 34** indicates **identity** Score **> 17** indicates **homology** | U | R.TIEYLEEVAVNFAK.G |
| 79018 | 236 | – | 249 | 813.4278 | 1624.8410 | 1624.8348 | 3.80 | 0 | 60 | 2.5e-06 | 1Score **> 34** indicates **identity** Score **> 16** indicates **homology** | U | R.TIEYLEEVAVNFAK.G |
| 79021 | 236 | – | 249 | 813.4279 | 1624.8413 | 1624.8348 | 4.02 | 0 | 63 | 1.4e-06 | 1Score **> 34** indicates **identity** Score **> 16** indicates **homology** | U | R.TIEYLEEVAVNFAK.G |
| 79022 | 236 | – | 249 | 813.4280 | 1624.8414 | 1624.8348 | 4.04 | 0 | 67 | 5.6e-07 | 1Score **> 34** indicates **identity** Score **> 17** indicates **homology** | U | R.TIEYLEEVAVNFAK.G |
| 79023 | 236 | – | 249 | 813.4285 | 1624.8425 | 1624.8348 | 4.73 | 0 | 40 | 0.00019 | 1Score **> 34** indicates **identity** Score **> 15** indicates **homology** | U | R.TIEYLEEVAVNFAK.G |
| 79024 | 236 | – | 249 | 813.4286 | 1624.8427 | 1624.8348 | 4.86 | 0 | 47 | 3.7e-05 | 1Score **> 34** indicates **identity** Score **> 15** indicates **homology** | U | R.TIEYLEEVAVNFAK.G |
| 79025 | 236 | – | 249 | 813.4287 | 1624.8428 | 1624.8348 | 4.95 | 0 | 75 | 9e-08 | 1Score **> 34** indicates **identity** Score **> 17** indicates **homology** | U | R.TIEYLEEVAVNFAK.G |
| 79027 | 236 | – | 249 | 813.4288 | 1624.8431 | 1624.8348 | 5.08 | 0 | 82 | 1.9e-08 | 1Score **> 34** indicates **identity** Score **> 18** indicates **homology** | U | R.TIEYLEEVAVNFAK.G |
| 79028 | 236 | – | 249 | 813.4290 | 1624.8434 | 1624.8348 | 5.30 | 0 | 23 | 0.0066 | 1Score **> 34** indicates **identity** Score **> 14** indicates **homology** | U | R.TIEYLEEVAVNFAK.G |
| 79029 | 236 | – | 249 | 813.4291 | 1624.8437 | 1624.8348 | 5.46 | 0 | 44 | 7.4e-05 | 1Score **> 34** indicates **identity** Score **> 15** indicates **homology** | U | R.TIEYLEEVAVNFAK.G |
| 79030 | 236 | – | 249 | 813.4293 | 1624.8440 | 1624.8348 | 5.63 | 0 | 37 | 0.00033 | 1Score **> 34** indicates **identity** Score **> 15** indicates **homology** | U | R.TIEYLEEVAVNFAK.G |
| 79031 | 236 | – | 249 | 813.4296 | 1624.8446 | 1624.8348 | 6.01 | 0 | 80 | 3.2e-08 | 1Score **> 34** indicates **identity** Score **> 17** indicates **homology** | U | R.TIEYLEEVAVNFAK.G |
| 79032 | 236 | – | 249 | 813.4300 | 1624.8454 | 1624.8348 | 6.52 | 0 | 40 | 0.00019 | 1Score **> 34** indicates **identity** Score **> 15** indicates **homology** | U | R.TIEYLEEVAVNFAK.G |
| 79035 | 236 | – | 249 | 813.4308 | 1624.8470 | 1624.8348 | 7.48 | 0 | 49 | 2.7e-05 | 1Score **> 34** indicates **identity** Score **> 16** indicates **homology** | U | R.TIEYLEEVAVNFAK.G |
| 79037 | 236 | – | 249 | 813.4316 | 1624.8487 | 1624.8348 | 8.57 | 0 | 77 | 5.9e-08 | 1Score **> 34** indicates **identity** Score **> 17** indicates **homology** | U | R.TIEYLEEVAVNFAK.G |
| 79039 | 236 | – | 249 | 813.4321 | 1624.8496 | 1624.8348 | 9.09 | 0 | 49 | 2.4e-05 | 1Score **> 34** indicates **identity** Score **> 16** indicates **homology** | U | R.TIEYLEEVAVNFAK.G |
| 79041 | 236 | – | 249 | 813.4322 | 1624.8498 | 1624.8348 | 9.24 | 0 | 47 | 4e-05 | 1Score **> 34** indicates **identity** Score **> 15** indicates **homology** | U | R.TIEYLEEVAVNFAK.G |
| 79043 | 236 | – | 249 | 813.4322 | 1624.8499 | 1624.8348 | 9.31 | 0 | 48 | 3e-05 | 1Score **> 34** indicates **identity** Score **> 16** indicates **homology** | U | R.TIEYLEEVAVNFAK.G |
| 50185 | 268 | – | 279 | 699.8768 | 1397.7391 | 1397.7377 | 0.97 | 0 | 66 | 9.2e-07 | 1Score **> 34** indicates **identity** Score **> 19** indicates **homology** | U | K.LTTYAMTVPFVR.Q |
| 50187 | 268 | – | 279 | 699.8772 | 1397.7398 | 1397.7377 | 1.48 | 0 | 70 | 1.1e-06 | 1Score **> 34** indicates **identity** Score **> 23** indicates **homology** | U | K.LTTYAMTVPFVR.Q |
| 50188 | 268 | – | 279 | 699.8772 | 1397.7399 | 1397.7377 | 1.56 | 0 | 70 | 1.1e-06 | 1Score **> 34** indicates **identity** Score **> 23** indicates **homology** | U | K.LTTYAMTVPFVR.Q |
| 50189 | 268 | – | 279 | 699.8778 | 1397.7411 | 1397.7377 | 2.39 | 0 | 34 | 0.00059 | 1Score **> 33** indicates **identity** Score **> 15** indicates **homology** | U | K.LTTYAMTVPFVR.Q |
| 52058 | 268 | – | 279 | 707.8753 | 1413.7361 | 1413.7326 | 2.43 | 0 | 30 | 0.0015 | 1Score **> 33** indicates **identity** Score **> 14** indicates **homology** | U | K.LTTYAMTVPFVR.Q  + Oxidation (M) |
| 52060 | 268 | – | 279 | 707.8758 | 1413.7371 | 1413.7326 | 3.17 | 0 | 40 | 0.00019 | 1Score **> 34** indicates **identity** Score **> 15** indicates **homology** | U | K.LTTYAMTVPFVR.Q  + Oxidation (M) |
| 35388 | 280 | – | 289 | 626.3324 | 1250.6502 | 1250.6506 | -0.34 | 1 | 46 | 0.0012 | 1Score **> 33** indicates **identity** Score **> 29** indicates **homology** | U | R.QQVYKTVEEK.V |
| 35390 | 280 | – | 289 | 417.8907 | 1250.6504 | 1250.6506 | -0.21 | 1 | 27 | 0.0045 | 1Score **> 33** indicates **identity** Score **> 16** indicates **homology** | U | R.QQVYKTVEEK.V |
| 35394 | 280 | – | 289 | 626.3334 | 1250.6523 | 1250.6506 | 1.33 | 1 | 22 | 0.0083 | 1Score **> 33** indicates **identity** Score **> 14** indicates **homology** | U | R.QQVYKTVEEK.V |
| 5809 | 296 | – | 303 | 429.7581 | 857.5017 | 857.5011 | 0.77 | 0 | 31 | 0.0024 | 1Score **> 32** indicates **identity** Score **> 18** indicates **homology** | U | K.GLYPAPLK.I |
| 5810 | 296 | – | 303 | 429.7587 | 857.5029 | 857.5011 | 2.14 | 0 | 32 | 0.0025 | 1Score **> 32** indicates **identity** Score **> 18** indicates **homology** | U | K.GLYPAPLK.I |
| 5811 | 296 | – | 303 | 429.7589 | 857.5032 | 857.5011 | 2.47 | 0 | 19 | 0.017 | 1Score **> 32** indicates **identity** Score **> 14** indicates **homology** | U | K.GLYPAPLK.I |
| 5812 | 296 | – | 303 | 429.7592 | 857.5039 | 857.5011 | 3.27 | 0 | 24 | 0.006 | 1Score **> 32** indicates **identity** Score **> 14** indicates **homology** | U | K.GLYPAPLK.I |
| 61929 | 296 | – | 309 | 499.9727 | 1496.8964 | 1496.8966 | -0.17 | 1 | 49 | 2.8e-05 | 1Score **> 31** indicates **identity** Score **> 16** indicates **homology** | U | K.GLYPAPLKIIDAVK.A |
| 61930 | 296 | – | 309 | 749.4556 | 1496.8967 | 1496.8966 | 0.047 | 1 | 37 | 0.00035 | 1Score **> 31** indicates **identity** Score **> 15** indicates **homology** | U | K.GLYPAPLKIIDAVK.A |
| 61931 | 296 | – | 309 | 499.9729 | 1496.8970 | 1496.8966 | 0.22 | 1 | 48 | 3.4e-05 | 1Score **> 31** indicates **identity** Score **> 16** indicates **homology** | U | K.GLYPAPLKIIDAVK.A |
| 61932 | 296 | – | 309 | 499.9731 | 1496.8976 | 1496.8966 | 0.63 | 1 | 40 | 0.00018 | 1Score **> 31** indicates **identity** Score **> 16** indicates **homology** | U | K.GLYPAPLKIIDAVK.A |
| 61933 | 296 | – | 309 | 499.9731 | 1496.8976 | 1496.8966 | 0.64 | 1 | 29 | 0.0019 | 1Score **> 31** indicates **identity** Score **> 14** indicates **homology** | U | K.GLYPAPLKIIDAVK.A |
| 61934 | 296 | – | 309 | 499.9734 | 1496.8983 | 1496.8966 | 1.14 | 1 | 36 | 0.00049 | 1Score **> 31** indicates **identity** Score **> 15** indicates **homology** | U | K.GLYPAPLKIIDAVK.A |
| 61936 | 296 | – | 309 | 499.9742 | 1496.9009 | 1496.8966 | 2.87 | 1 | 22 | 0.008 | 1Score **> 30** indicates **identity** Score **> 14** indicates **homology** | U | K.GLYPAPLKIIDAVK.A |
| 61937 | 296 | – | 309 | 499.9745 | 1496.9016 | 1496.8966 | 3.34 | 1 | 28 | 0.0023 | 1Score **> 30** indicates **identity** Score **> 14** indicates **homology** | U | K.GLYPAPLKIIDAVK.A |
| 61938 | 296 | – | 309 | 499.9748 | 1496.9026 | 1496.8966 | 4.00 | 1 | 18 | 0.02 | 1Score **> 30** indicates **identity** Score **> 14** indicates **homology** | U | K.GLYPAPLKIIDAVK.A |
| 61939 | 296 | – | 309 | 499.9750 | 1496.9032 | 1496.8966 | 4.42 | 1 | 25 | 0.0049 | 1Score **> 31** indicates **identity** Score **> 14** indicates **homology** | U | K.GLYPAPLKIIDAVK.A |
| 150519 | 304 | – | 326 | 788.4063 | 2362.1970 | 2362.2016 | -1.96 | 1 | 55 | 7.4e-06 | 1Score **> 37** indicates **identity** Score **> 16** indicates **homology** | U | K.IIDAVKAGLEQGSDAGYLAESQK.F |
| 150522 | 304 | – | 326 | 788.4081 | 2362.2025 | 2362.2016 | 0.40 | 1 | 63 | 1.2e-06 | 1Score **> 37** indicates **identity** Score **> 16** indicates **homology** | U | K.IIDAVKAGLEQGSDAGYLAESQK.F |
| 183813 | 304 | – | 334 | 1074.9029 | 3221.6868 | 3221.6819 | 1.52 | 2 | 46 | 5.3e-05 | 1Score **> 37** indicates **identity** Score **> 15** indicates **homology** | U | K.IIDAVKAGLEQGSDAGYLAESQKFGELALTK.E |
| 183814 | 304 | – | 334 | 806.4310 | 3221.6950 | 3221.6819 | 4.07 | 2 | 34 | 0.0006 | 1Score **> 37** indicates **identity** Score **> 15** indicates **homology** | U | K.IIDAVKAGLEQGSDAGYLAESQKFGELALTK.E |
| 91784 | 310 | – | 326 | 862.4091 | 1722.8036 | 1722.8060 | -1.39 | 0 | 75 | 2e-07 | 1Score **> 32** indicates **identity** Score **> 21** indicates **homology** | U | K.AGLEQGSDAGYLAESQK.F |
| 91785 | 310 | – | 326 | 862.4094 | 1722.8042 | 1722.8060 | -1.08 | 0 | 40 | 0.00017 | 1Score **> 32** indicates **identity** Score **> 15** indicates **homology** | U | K.AGLEQGSDAGYLAESQK.F |
| 91786 | 310 | – | 326 | 862.4098 | 1722.8051 | 1722.8060 | -0.52 | 0 | 77 | 5.7e-08 | 1Score **> 32** indicates **identity** Score **> 17** indicates **homology** | U | K.AGLEQGSDAGYLAESQK.F |
| 91788 | 310 | – | 326 | 862.4104 | 1722.8063 | 1722.8060 | 0.16 | 0 | 54 | 9e-06 | 1Score **> 32** indicates **identity** Score **> 16** indicates **homology** | U | K.AGLEQGSDAGYLAESQK.F |
| 91791 | 310 | – | 326 | 862.4112 | 1722.8078 | 1722.8060 | 1.02 | 0 | 109 | 1.4e-10 | 1Score **> 33** indicates **identity** Score **> 23** indicates **homology** | U | K.AGLEQGSDAGYLAESQK.F |
| 91792 | 310 | – | 326 | 575.2767 | 1722.8082 | 1722.8060 | 1.24 | 0 | 51 | 1.8e-05 | 1Score **> 33** indicates **identity** Score **> 16** indicates **homology** | U | K.AGLEQGSDAGYLAESQK.F |
| 91793 | 310 | – | 326 | 862.4117 | 1722.8088 | 1722.8060 | 1.60 | 0 | 56 | 5.3e-06 | 1Score **> 33** indicates **identity** Score **> 16** indicates **homology** | U | K.AGLEQGSDAGYLAESQK.F |
| 91794 | 310 | – | 326 | 862.4117 | 1722.8089 | 1722.8060 | 1.65 | 0 | 113 | 6.3e-11 | 1Score **> 33** indicates **identity** Score **> 23** indicates **homology** | U | K.AGLEQGSDAGYLAESQK.F |
| 91795 | 310 | – | 326 | 862.4117 | 1722.8089 | 1722.8060 | 1.66 | 0 | 113 | 6.2e-11 | 1Score **> 33** indicates **identity** Score **> 23** indicates **homology** | U | K.AGLEQGSDAGYLAESQK.F |
| 91796 | 310 | – | 326 | 575.2771 | 1722.8094 | 1722.8060 | 1.98 | 0 | 36 | 0.00042 | 1Score **> 33** indicates **identity** Score **> 15** indicates **homology** | U | K.AGLEQGSDAGYLAESQK.F |
| 91797 | 310 | – | 326 | 575.2772 | 1722.8098 | 1722.8060 | 2.18 | 0 | 41 | 0.00013 | 1Score **> 32** indicates **identity** Score **> 15** indicates **homology** | U | K.AGLEQGSDAGYLAESQK.F |
| 91798 | 310 | – | 326 | 862.4129 | 1722.8112 | 1722.8060 | 3.01 | 0 | 16 | 0.035 | 1Score **> 33** indicates **identity** Score **> 13** indicates **homology** | U | K.AGLEQGSDAGYLAESQK.F |
| 91799 | 310 | – | 326 | 862.4131 | 1722.8116 | 1722.8060 | 3.20 | 0 | 98 | 6.1e-10 | 1Score **> 33** indicates **identity** Score **> 19** indicates **homology** | U | K.AGLEQGSDAGYLAESQK.F |
| 91801 | 310 | – | 326 | 862.4142 | 1722.8139 | 1722.8060 | 4.54 | 0 | 57 | 4.8e-06 | 1Score **> 33** indicates **identity** Score **> 16** indicates **homology** | U | K.AGLEQGSDAGYLAESQK.F |
| 162836 | 310 | – | 334 | 861.7741 | 2582.3005 | 2582.2864 | 5.47 | 1 | 60 | 2.4e-06 | 1Score **> 37** indicates **identity** Score **> 16** indicates **homology** | U | K.AGLEQGSDAGYLAESQKFGELALTK.E |
| 162837 | 310 | – | 334 | 1292.1577 | 2582.3008 | 2582.2864 | 5.60 | 1 | 91 | 2.7e-09 | 1Score **> 37** indicates **identity** Score **> 18** indicates **homology** | U | K.AGLEQGSDAGYLAESQKFGELALTK.E |
| 162839 | 310 | – | 334 | 861.7747 | 2582.3023 | 2582.2864 | 6.16 | 1 | 61 | 1.9e-06 | 1Score **> 37** indicates **identity** Score **> 16** indicates **homology** | U | K.AGLEQGSDAGYLAESQKFGELALTK.E |
| 162840 | 310 | – | 334 | 861.7749 | 2582.3028 | 2582.2864 | 6.34 | 1 | 71 | 2e-07 | 1Score **> 37** indicates **identity** Score **> 17** indicates **homology** | U | K.AGLEQGSDAGYLAESQKFGELALTK.E |
| 162841 | 310 | – | 334 | 861.7749 | 2582.3029 | 2582.2864 | 6.39 | 1 | 95 | 1.3e-09 | 1Score **> 37** indicates **identity** Score **> 18** indicates **homology** | U | K.AGLEQGSDAGYLAESQKFGELALTK.E |
| 162842 | 310 | – | 334 | 1292.1611 | 2582.3077 | 2582.2864 | 8.26 | 1 | 99 | 4.9e-10 | 1Score **> 37** indicates **identity** Score **> 19** indicates **homology** | U | K.AGLEQGSDAGYLAESQKFGELALTK.E |
| 162843 | 310 | – | 334 | 861.7772 | 2582.3099 | 2582.2864 | 9.11 | 1 | 39 | 0.00021 | 1Score **> 37** indicates **identity** Score **> 15** indicates **homology** | U | K.AGLEQGSDAGYLAESQKFGELALTK.E |
| 162844 | 310 | – | 334 | 861.7773 | 2582.3099 | 2582.2864 | 9.12 | 1 | 47 | 4.2e-05 | 1Score **> 37** indicates **identity** Score **> 15** indicates **homology** | U | K.AGLEQGSDAGYLAESQKFGELALTK.E |
| 162845 | 310 | – | 334 | 861.7776 | 2582.3108 | 2582.2864 | 9.47 | 1 | 45 | 6.1e-05 | 1Score **> 37** indicates **identity** Score **> 15** indicates **homology** | U | K.AGLEQGSDAGYLAESQKFGELALTK.E |
| 162846 | 310 | – | 334 | 1292.1628 | 2582.3110 | 2582.2864 | 9.53 | 1 | 88 | 5.2e-09 | 1Score **> 37** indicates **identity** Score **> 18** indicates **homology** | U | K.AGLEQGSDAGYLAESQKFGELALTK.E |
| 162847 | 310 | – | 334 | 861.7776 | 2582.3111 | 2582.2864 | 9.57 | 1 | 55 | 7.2e-06 | 1Score **> 37** indicates **identity** Score **> 16** indicates **homology** | U | K.AGLEQGSDAGYLAESQKFGELALTK.E |
| 175926 | 310 | – | 337 | 732.6218 | 2926.4581 | 2926.4559 | 0.73 | 2 | 54 | 9.3e-06 | 1Score **> 38** indicates **identity** Score **> 16** indicates **homology** | U | K.AGLEQGSDAGYLAESQKFGELALTKESK.A |
| 175927 | 310 | – | 337 | 976.4934 | 2926.4584 | 2926.4559 | 0.84 | 2 | 42 | 0.00011 | 1Score **> 38** indicates **identity** Score **> 15** indicates **homology** | U | K.AGLEQGSDAGYLAESQKFGELALTKESK.A |
| 175928 | 310 | – | 337 | 732.6219 | 2926.4587 | 2926.4559 | 0.93 | 2 | 52 | 1.4e-05 | 1Score **> 38** indicates **identity** Score **> 16** indicates **homology** | U | K.AGLEQGSDAGYLAESQKFGELALTKESK.A |
| 175930 | 310 | – | 337 | 976.4939 | 2926.4598 | 2926.4559 | 1.32 | 2 | 28 | 0.0025 | 1Score **> 38** indicates **identity** Score **> 14** indicates **homology** | U | K.AGLEQGSDAGYLAESQKFGELALTKESK.A |
| 175931 | 310 | – | 337 | 732.6223 | 2926.4600 | 2926.4559 | 1.40 | 2 | 31 | 0.0012 | 1Score **> 38** indicates **identity** Score **> 14** indicates **homology** | U | K.AGLEQGSDAGYLAESQKFGELALTKESK.A |
| 6725 | 327 | – | 334 | 439.7528 | 877.4911 | 877.4909 | 0.21 | 0 | 44 | 0.00011 | 1Score **> 26** indicates **identity** Score **> 17** indicates **homology** | U | K.FGELALTK.E |
| 6727 | 327 | – | 334 | 439.7531 | 877.4916 | 877.4909 | 0.77 | 0 | 43 | 0.00015 | 1Score **> 26** indicates **identity** Score **> 17** indicates **homology** | U | K.FGELALTK.E |
| 6729 | 327 | – | 334 | 439.7533 | 877.4921 | 877.4909 | 1.34 | 0 | 40 | 0.00025 | 1Score **> 26** indicates **identity** Score **> 17** indicates **homology** | U | K.FGELALTK.E |
| 6730 | 327 | – | 334 | 439.7534 | 877.4922 | 877.4909 | 1.47 | 0 | 44 | 0.00023 | 1Score **> 26** indicates **identity** Score **> 20** indicates **homology** | U | K.FGELALTK.E |
| 6731 | 327 | – | 334 | 439.7535 | 877.4924 | 877.4909 | 1.68 | 0 | 59 | 5.1e-06 | 1Score **> 26** indicates **identity** Score **> 19** indicates **homology** | U | K.FGELALTK.E |
| 6732 | 327 | – | 334 | 439.7537 | 877.4928 | 877.4909 | 2.19 | 0 | 28 | 0.0053 | 1Score **> 26** indicates **identity** Score **> 18** indicates **homology** | U | K.FGELALTK.E |
| 6733 | 327 | – | 334 | 439.7539 | 877.4933 | 877.4909 | 2.74 | 0 | 40 | 0.00034 | 1Score **> 25** indicates **identity** Score **> 18** indicates **homology** | U | K.FGELALTK.E |
| 6735 | 327 | – | 334 | 439.7552 | 877.4958 | 877.4909 | 5.53 | 0 | 28 | 0.012 | 1Score **> 28** indicates **identity** Score **> 22** indicates **homology** | U | K.FGELALTK.E |
| 32384 | 327 | – | 337 | 408.2255 | 1221.6547 | 1221.6605 | -4.74 | 1 | 21 | 0.011 | 1Score **> 33** indicates **identity** Score **> 14** indicates **homology** | U | K.FGELALTKESK.A |
| 32386 | 327 | – | 337 | 611.8360 | 1221.6575 | 1221.6605 | -2.43 | 1 | 39 | 0.00023 | 1Score **> 33** indicates **identity** Score **> 15** indicates **homology** | U | K.FGELALTKESK.A |
| 32388 | 327 | – | 337 | 408.2269 | 1221.6588 | 1221.6605 | -1.34 | 1 | 37 | 0.00031 | 1Score **> 32** indicates **identity** Score **> 15** indicates **homology** | U | K.FGELALTKESK.A |
| 32389 | 327 | – | 337 | 611.8367 | 1221.6589 | 1221.6605 | -1.32 | 1 | 36 | 0.00041 | 1Score **> 32** indicates **identity** Score **> 15** indicates **homology** | U | K.FGELALTKESK.A |
| 32390 | 327 | – | 337 | 611.8370 | 1221.6594 | 1221.6605 | -0.90 | 1 | 66 | 6e-07 | 1Score **> 32** indicates **identity** Score **> 17** indicates **homology** | U | K.FGELALTKESK.A |
| 32393 | 327 | – | 337 | 408.2272 | 1221.6599 | 1221.6605 | -0.49 | 1 | 32 | 0.00092 | 1Score **> 33** indicates **identity** Score **> 15** indicates **homology** | U | K.FGELALTKESK.A |
| 32395 | 327 | – | 337 | 408.2276 | 1221.6610 | 1221.6605 | 0.40 | 1 | 48 | 2.9e-05 | 1Score **> 33** indicates **identity** Score **> 16** indicates **homology** | U | K.FGELALTKESK.A |
| 57969 | 338 | – | 350 | 733.8795 | 1465.7445 | 1465.7421 | 1.62 | 0 | 49 | 2.4e-05 | 1Score **> 34** indicates **identity** Score **> 16** indicates **homology** | U | K.ALMGLYNGQVLCK.K |
| 57971 | 338 | – | 350 | 733.8825 | 1465.7505 | 1465.7421 | 5.69 | 0 | 49 | 2.5e-05 | 1Score **> 34** indicates **identity** Score **> 16** indicates **homology** | U | K.ALMGLYNGQVLCK.K |
| 165805 | 360 | – | 386 | 884.5046 | 2650.4919 | 2650.4840 | 2.97 | 1 | 77 | 6.5e-08 | 1Score **> 33** indicates **identity** Score **> 17** indicates **homology** | U | K.NVQQLAILGAGLMGAGIAQVSVDKGLK.T |
| 47797 | 387 | – | 399 | 458.9365 | 1373.7878 | 1373.7878 | -0.021 | 1 | 34 | 0.00095 | 1Score **> 33** indicates **identity** Score **> 16** indicates **homology** | U | K.TLLKDTTVTGLGR.G |
| 47798 | 387 | – | 399 | 458.9366 | 1373.7880 | 1373.7878 | 0.084 | 1 | 39 | 0.0002 | 1Score **> 33** indicates **identity** Score **> 15** indicates **homology** | U | K.TLLKDTTVTGLGR.G |
| 47799 | 387 | – | 399 | 458.9366 | 1373.7880 | 1373.7878 | 0.11 | 1 | 25 | 0.0041 | 1Score **> 33** indicates **identity** Score **> 14** indicates **homology** | U | K.TLLKDTTVTGLGR.G |
| 47800 | 387 | – | 399 | 687.9015 | 1373.7884 | 1373.7878 | 0.40 | 1 | 49 | 2.7e-05 | 1Score **> 33** indicates **identity** Score **> 16** indicates **homology** | U | K.TLLKDTTVTGLGR.G |
| 47801 | 387 | – | 399 | 458.9368 | 1373.7885 | 1373.7878 | 0.46 | 1 | 40 | 0.00043 | 1Score **> 33** indicates **identity** Score **> 19** indicates **homology** | U | K.TLLKDTTVTGLGR.G |
| 47802 | 387 | – | 399 | 458.9368 | 1373.7886 | 1373.7878 | 0.57 | 1 | 43 | 0.00011 | 1Score **> 33** indicates **identity** Score **> 16** indicates **homology** | U | K.TLLKDTTVTGLGR.G |
| 47803 | 387 | – | 399 | 687.9016 | 1373.7886 | 1373.7878 | 0.59 | 1 | 17 | 0.025 | 1Score **> 33** indicates **identity** Score **> 14** indicates **homology** | U | K.TLLKDTTVTGLGR.G |
| 47804 | 387 | – | 399 | 458.9369 | 1373.7887 | 1373.7878 | 0.65 | 1 | 24 | 0.0061 | 1Score **> 33** indicates **identity** Score **> 14** indicates **homology** | U | K.TLLKDTTVTGLGR.G |
| 47805 | 387 | – | 399 | 687.9017 | 1373.7889 | 1373.7878 | 0.75 | 1 | 48 | 2.9e-05 | 1Score **> 33** indicates **identity** Score **> 16** indicates **homology** | U | K.TLLKDTTVTGLGR.G |
| 47806 | 387 | – | 399 | 458.9370 | 1373.7892 | 1373.7878 | 1.01 | 1 | 26 | 0.0038 | 1Score **> 33** indicates **identity** Score **> 14** indicates **homology** | U | K.TLLKDTTVTGLGR.G |
| 47807 | 387 | – | 399 | 458.9370 | 1373.7893 | 1373.7878 | 1.04 | 1 | 46 | 6.4e-05 | 1Score **> 33** indicates **identity** Score **> 17** indicates **homology** | U | K.TLLKDTTVTGLGR.G |
| 47808 | 387 | – | 399 | 458.9372 | 1373.7898 | 1373.7878 | 1.46 | 1 | 42 | 0.00014 | 1Score **> 34** indicates **identity** Score **> 16** indicates **homology** | U | K.TLLKDTTVTGLGR.G |
| 47809 | 387 | – | 399 | 458.9374 | 1373.7903 | 1373.7878 | 1.77 | 1 | 43 | 0.00016 | 1Score **> 34** indicates **identity** Score **> 18** indicates **homology** | U | K.TLLKDTTVTGLGR.G |
| 47810 | 387 | – | 399 | 458.9374 | 1373.7904 | 1373.7878 | 1.89 | 1 | 46 | 0.0002 | 1Score **> 33** indicates **identity** Score **> 22** indicates **homology** | U | K.TLLKDTTVTGLGR.G |
| 47811 | 387 | – | 399 | 458.9374 | 1373.7905 | 1373.7878 | 1.92 | 1 | 41 | 0.0005 | 1Score **> 33** indicates **identity** Score **> 21** indicates **homology** | U | K.TLLKDTTVTGLGR.G |
| 47812 | 387 | – | 399 | 458.9375 | 1373.7906 | 1373.7878 | 2.04 | 1 | 44 | 0.00016 | 1Score **> 33** indicates **identity** Score **> 18** indicates **homology** | U | K.TLLKDTTVTGLGR.G |
| 47813 | 387 | – | 399 | 458.9377 | 1373.7913 | 1373.7878 | 2.52 | 1 | 47 | 8.3e-05 | 1Score **> 33** indicates **identity** Score **> 19** indicates **homology** | U | K.TLLKDTTVTGLGR.G |
| 8671 | 391 | – | 399 | 460.2464 | 918.4783 | 918.4771 | 1.28 | 0 | 33 | 0.036 | 1Score **> 31** indicates **identity** | U | K.DTTVTGLGR.G |
| 8672 | 391 | – | 399 | 460.2468 | 918.4790 | 918.4771 | 2.12 | 0 | 24 | 0.039 | 1Score **> 31** indicates **identity** Score **> 22** indicates **homology** | U | K.DTTVTGLGR.G |
| 46594 | 400 | – | 411 | 454.5773 | 1360.7102 | 1360.7099 | 0.22 | 1 | 31 | 0.0011 | 1Score **> 35** indicates **identity** Score **> 14** indicates **homology** | U | R.GQQQVFKGLNDK.V |
| 46601 | 400 | – | 411 | 681.3631 | 1360.7117 | 1360.7099 | 1.28 | 1 | 20 | 0.014 | 1Score **> 35** indicates **identity** Score **> 14** indicates **homology** | U | R.GQQQVFKGLNDK.V |
| 73893 | 400 | – | 413 | 530.2988 | 1587.8745 | 1587.8733 | 0.74 | 2 | 27 | 0.0028 | 1Score **> 35** indicates **identity** Score **> 14** indicates **homology** | U | R.GQQQVFKGLNDKVK.K |
| 73894 | 400 | – | 413 | 397.9760 | 1587.8749 | 1587.8733 | 1.02 | 2 | 15 | 0.049 | 1Score **> 35** indicates **identity** Score **> 14** indicates **homology** | U | R.GQQQVFKGLNDKVK.K |
| 73895 | 400 | – | 413 | 530.2994 | 1587.8764 | 1587.8733 | 1.98 | 2 | 17 | 0.028 | 1Score **> 35** indicates **identity** Score **> 14** indicates **homology** | U | R.GQQQVFKGLNDKVK.K |
| 18887 | 414 | – | 422 | 360.5451 | 1078.6136 | 1078.6135 | 0.10 | 2 | 51 | 1.6e-05 | 1Score **> 29** indicates **identity** Score **> 16** indicates **homology** | U | K.KKALTSFER.D |
| 10365 | 415 | – | 422 | 476.2674 | 950.5202 | 950.5185 | 1.81 | 1 | 16 | 0.031 | 1Score **> 28** indicates **identity** Score **> 14** indicates **homology** | U | K.KALTSFER.D |
| 161105 | 415 | – | 436 | 637.0899 | 2544.3305 | 2544.3224 | 3.21 | 2 | 32 | 0.0011 | 1Score **> 37** indicates **identity** Score **> 15** indicates **homology** | U | K.KALTSFERDSIFSNLIGQLDYK.G |
| 4231 | 416 | – | 422 | 412.2199 | 822.4253 | 822.4236 | 2.18 | 0 | 36 | 0.0047 | 1Score **> 25** indicates **identity** | U | K.ALTSFER.D |
| 4232 | 416 | – | 422 | 412.2200 | 822.4254 | 822.4236 | 2.25 | 0 | 43 | 0.00086 | 1Score **> 25** indicates **identity** | U | K.ALTSFER.D |
| 153951 | 416 | – | 436 | 806.4172 | 2416.2297 | 2416.2274 | 0.93 | 1 | 38 | 0.00029 | 1Score **> 37** indicates **identity** Score **> 15** indicates **homology** | U | K.ALTSFERDSIFSNLIGQLDYK.G |
| 174495 | 416 | – | 440 | 960.1614 | 2877.4624 | 2877.4548 | 2.62 | 2 | 48 | 2.9e-05 | 1Score **> 37** indicates **identity** Score **> 16** indicates **homology** | U | K.ALTSFERDSIFSNLIGQLDYKGFEK.A |
| 77142 | 423 | – | 436 | 806.9182 | 1611.8218 | 1611.8144 | 4.57 | 0 | 73 | 1.5e-07 | 1Score **> 34** indicates **identity** Score **> 17** indicates **homology** | U | R.DSIFSNLIGQLDYK.G |
| 127494 | 423 | – | 440 | 692.0224 | 2073.0453 | 2073.0419 | 1.65 | 1 | 37 | 0.00043 | 1Score **> 36** indicates **identity** Score **> 15** indicates **homology** | U | R.DSIFSNLIGQLDYKGFEK.A |
| 127497 | 423 | – | 440 | 1037.5315 | 2073.0485 | 2073.0419 | 3.21 | 1 | 48 | 2.9e-05 | 1Score **> 36** indicates **identity** Score **> 16** indicates **homology** | U | R.DSIFSNLIGQLDYKGFEK.A |
| 80259 | 441 | – | 455 | 545.9477 | 1634.8212 | 1634.8226 | -0.86 | 0 | 51 | 1.6e-05 | 1Score **> 35** indicates **identity** Score **> 16** indicates **homology** | U | K.ADMVIEAVFEDLGVK.H |
| 80268 | 441 | – | 455 | 818.4207 | 1634.8268 | 1634.8226 | 2.61 | 0 | 79 | 3.8e-08 | 1Score **> 35** indicates **identity** Score **> 17** indicates **homology** | U | K.ADMVIEAVFEDLGVK.H |
| 188705 | 458 | – | 489 | 1151.6112 | 3451.8119 | 3451.8021 | 2.85 | 1 | 89 | 4.4e-09 | 1Score **> 37** indicates **identity** Score **> 18** indicates **homology** | U | K.VLKEVESVTPEHCIFASNTSALPINQIAAVSK.R |
| 188706 | 458 | – | 489 | 863.9605 | 3451.8130 | 3451.8021 | 3.17 | 1 | 38 | 0.00025 | 1Score **> 37** indicates **identity** Score **> 15** indicates **homology** | U | K.VLKEVESVTPEHCIFASNTSALPINQIAAVSK.R |
| 188708 | 458 | – | 489 | 1151.6125 | 3451.8156 | 3451.8021 | 3.92 | 1 | 24 | 0.0057 | 1Score **> 37** indicates **identity** Score **> 14** indicates **homology** | U | K.VLKEVESVTPEHCIFASNTSALPINQIAAVSK.R |
| 188709 | 458 | – | 489 | 863.9612 | 3451.8159 | 3451.8021 | 3.99 | 1 | 48 | 3.5e-05 | 1Score **> 37** indicates **identity** Score **> 15** indicates **homology** | U | K.VLKEVESVTPEHCIFASNTSALPINQIAAVSK.R |
| 188710 | 458 | – | 489 | 1151.6128 | 3451.8167 | 3451.8021 | 4.23 | 1 | 71 | 2.1e-07 | 1Score **> 37** indicates **identity** Score **> 17** indicates **homology** | U | K.VLKEVESVTPEHCIFASNTSALPINQIAAVSK.R |
| 188724 | 458 | – | 489 | 864.2119 | 3452.8185 | 3452.7861 | 9.38 | 1 | 17 | 0.025 | 1Score **> 36** indicates **identity** Score **> 14** indicates **homology** | U | K.VLKEVESVTPEHCIFASNTSALPINQIAAVSK.R  + Deamidated (NQ) |
| 181422 | 461 | – | 489 | 1038.1940 | 3111.5601 | 3111.5546 | 1.74 | 0 | 70 | 2.7e-07 | 1Score **> 38** indicates **identity** Score **> 17** indicates **homology** | U | K.EVESVTPEHCIFASNTSALPINQIAAVSK.R |
| 181423 | 461 | – | 489 | 1038.1948 | 3111.5625 | 3111.5546 | 2.52 | 0 | 72 | 1.9e-07 | 1Score **> 38** indicates **identity** Score **> 17** indicates **homology** | U | K.EVESVTPEHCIFASNTSALPINQIAAVSK.R |
| 181425 | 461 | – | 489 | 1038.1956 | 3111.5651 | 3111.5546 | 3.35 | 0 | 69 | 3.4e-07 | 1Score **> 38** indicates **identity** Score **> 17** indicates **homology** | U | K.EVESVTPEHCIFASNTSALPINQIAAVSK.R |
| 181426 | 461 | – | 489 | 1038.1969 | 3111.5689 | 3111.5546 | 4.57 | 0 | 23 | 0.0063 | 1Score **> 38** indicates **identity** Score **> 14** indicates **homology** | U | K.EVESVTPEHCIFASNTSALPINQIAAVSK.R |
| 181462 | 461 | – | 489 | 1557.2824 | 3112.5502 | 3112.5387 | 3.70 | 0 | 34 | 0.0006 | 1Score **> 38** indicates **identity** Score **> 15** indicates **homology** | U | K.EVESVTPEHCIFASNTSALPINQIAAVSK.R  + Deamidated (NQ) |
| 181464 | 461 | – | 489 | 1557.2844 | 3112.5542 | 3112.5387 | 5.00 | 0 | 23 | 0.007 | 1Score **> 38** indicates **identity** Score **> 14** indicates **homology** | U | K.EVESVTPEHCIFASNTSALPINQIAAVSK.R  + Deamidated (NQ) |
| 181471 | 461 | – | 489 | 1038.5290 | 3112.5652 | 3112.5387 | 8.52 | 0 | 27 | 0.0028 | 1Score **> 38** indicates **identity** Score **> 14** indicates **homology** | U | K.EVESVTPEHCIFASNTSALPINQIAAVSK.R  + Deamidated (NQ) |
| 191090 | 461 | – | 493 | 906.4696 | 3621.8491 | 3621.8461 | 0.84 | 2 | 40 | 0.00019 | 1Score **> 37** indicates **identity** Score **> 15** indicates **homology** | U | K.EVESVTPEHCIFASNTSALPINQIAAVSKRPEK.V |
| 191091 | 461 | – | 493 | 906.4699 | 3621.8507 | 3621.8461 | 1.27 | 2 | 27 | 0.003 | 1Score **> 37** indicates **identity** Score **> 14** indicates **homology** | U | K.EVESVTPEHCIFASNTSALPINQIAAVSKRPEK.V |
| 191092 | 461 | – | 493 | 725.3777 | 3621.8521 | 3621.8461 | 1.66 | 2 | 16 | 0.034 | 1Score **> 37** indicates **identity** Score **> 13** indicates **homology** | U | K.EVESVTPEHCIFASNTSALPINQIAAVSKRPEK.V |
| 191093 | 461 | – | 493 | 1208.2930 | 3621.8571 | 3621.8461 | 3.04 | 2 | 33 | 0.00076 | 1Score **> 37** indicates **identity** Score **> 15** indicates **homology** | U | K.EVESVTPEHCIFASNTSALPINQIAAVSKRPEK.V |
| 191116 | 461 | – | 493 | 906.7204 | 3622.8525 | 3622.8301 | 6.20 | 2 | 18 | 0.018 | 1Score **> 37** indicates **identity** Score **> 14** indicates **homology** | U | K.EVESVTPEHCIFASNTSALPINQIAAVSKRPEK.V  + Deamidated (NQ) |
| 40878 | 506 | – | 516 | 652.8636 | 1303.7126 | 1303.7057 | 5.28 | 0 | 31 | 0.0014 | 1Score **> 34** indicates **identity** Score **> 14** indicates **homology** | U | K.MQLLEIITTDK.T |
| 78351 | 506 | – | 519 | 540.9671 | 1619.8795 | 1619.8804 | -0.53 | 1 | 44 | 7.4e-05 | 1Score **> 34** indicates **identity** Score **> 15** indicates **homology** | U | K.MQLLEIITTDKTSK.D |
| 59093 | 517 | – | 531 | 738.9042 | 1475.7939 | 1475.7944 | -0.31 | 1 | 49 | 2.4e-05 | 1Score **> 35** indicates **identity** Score **> 16** indicates **homology** | U | K.TSKDTTASAVAVGLR.Q |
| 59094 | 517 | – | 531 | 738.9043 | 1475.7941 | 1475.7944 | -0.19 | 1 | 75 | 9.3e-08 | 1Score **> 34** indicates **identity** Score **> 17** indicates **homology** | U | K.TSKDTTASAVAVGLR.Q |
| 59095 | 517 | – | 531 | 738.9044 | 1475.7942 | 1475.7944 | -0.090 | 1 | 97 | 8.7e-10 | 1Score **> 34** indicates **identity** Score **> 19** indicates **homology** | U | K.TSKDTTASAVAVGLR.Q |
| 59096 | 517 | – | 531 | 492.9388 | 1475.7946 | 1475.7944 | 0.17 | 1 | 43 | 9.2e-05 | 1Score **> 35** indicates **identity** Score **> 15** indicates **homology** | U | K.TSKDTTASAVAVGLR.Q |
| 59099 | 517 | – | 531 | 492.9396 | 1475.7970 | 1475.7944 | 1.75 | 1 | 18 | 0.022 | 1Score **> 35** indicates **identity** Score **> 14** indicates **homology** | U | K.TSKDTTASAVAVGLR.Q |
| 59100 | 517 | – | 531 | 492.9398 | 1475.7974 | 1475.7944 | 2.07 | 1 | 29 | 0.0018 | 1Score **> 35** indicates **identity** Score **> 14** indicates **homology** | U | K.TSKDTTASAVAVGLR.Q |
| 26320 | 520 | – | 531 | 580.8165 | 1159.6185 | 1159.6197 | -1.05 | 0 | 61 | 2.1e-06 | 1Score **> 34** indicates **identity** Score **> 16** indicates **homology** | U | K.DTTASAVAVGLR.Q |
| 26324 | 520 | – | 531 | 580.8175 | 1159.6204 | 1159.6197 | 0.63 | 0 | 73 | 1.1e-06 | 1Score **> 34** indicates **identity** Score **> 26** indicates **homology** | U | K.DTTASAVAVGLR.Q |
| 26325 | 520 | – | 531 | 580.8178 | 1159.6211 | 1159.6197 | 1.17 | 0 | 54 | 1.5e-05 | 1Score **> 34** indicates **identity** Score **> 18** indicates **homology** | U | K.DTTASAVAVGLR.Q |
| 26326 | 520 | – | 531 | 580.8179 | 1159.6213 | 1159.6197 | 1.37 | 0 | 63 | 1.2e-05 | 1Score **> 34** indicates **identity** Score **> 26** indicates **homology** | U | K.DTTASAVAVGLR.Q |
| 26327 | 520 | – | 531 | 580.8180 | 1159.6215 | 1159.6197 | 1.57 | 0 | 73 | 1.1e-06 | 1Score **> 33** indicates **identity** Score **> 26** indicates **homology** | U | K.DTTASAVAVGLR.Q |
| 26328 | 520 | – | 531 | 580.8180 | 1159.6215 | 1159.6197 | 1.59 | 0 | 55 | 2e-05 | 1Score **> 33** indicates **identity** Score **> 21** indicates **homology** | U | K.DTTASAVAVGLR.Q |
| 84293 | 535 | – | 549 | 555.6506 | 1663.9299 | 1663.9298 | 0.064 | 1 | 15 | 0.039 | 1Score **> 34** indicates **identity** Score **> 13** indicates **homology** | U | K.VIIVVKDGPGFYTTR.C |
| 84294 | 535 | – | 549 | 832.9725 | 1663.9305 | 1663.9298 | 0.45 | 1 | 53 | 1.2e-05 | 1Score **> 34** indicates **identity** Score **> 16** indicates **homology** | U | K.VIIVVKDGPGFYTTR.C |
| 84295 | 535 | – | 549 | 832.9730 | 1663.9315 | 1663.9298 | 1.02 | 1 | 48 | 3e-05 | 1Score **> 33** indicates **identity** Score **> 16** indicates **homology** | U | K.VIIVVKDGPGFYTTR.C |
| 84296 | 535 | – | 549 | 832.9736 | 1663.9327 | 1663.9298 | 1.75 | 1 | 49 | 3.8e-05 | 1Score **> 33** indicates **identity** Score **> 17** indicates **homology** | U | K.VIIVVKDGPGFYTTR.C |
| 84297 | 535 | – | 549 | 555.6515 | 1663.9328 | 1663.9298 | 1.82 | 1 | 64 | 9.4e-07 | 1Score **> 33** indicates **identity** Score **> 17** indicates **homology** | U | K.VIIVVKDGPGFYTTR.C |
| 84298 | 535 | – | 549 | 555.6517 | 1663.9333 | 1663.9298 | 2.14 | 1 | 54 | 7.8e-06 | 1Score **> 33** indicates **identity** Score **> 16** indicates **homology** | U | K.VIIVVKDGPGFYTTR.C |
| 84299 | 535 | – | 549 | 555.6518 | 1663.9335 | 1663.9298 | 2.27 | 1 | 62 | 1.6e-06 | 1Score **> 33** indicates **identity** Score **> 16** indicates **homology** | U | K.VIIVVKDGPGFYTTR.C |
| 84300 | 535 | – | 549 | 832.9742 | 1663.9338 | 1663.9298 | 2.41 | 1 | 31 | 0.0012 | 1Score **> 33** indicates **identity** Score **> 14** indicates **homology** | U | K.VIIVVKDGPGFYTTR.C |
| 84301 | 535 | – | 549 | 555.6519 | 1663.9340 | 1663.9298 | 2.53 | 1 | 15 | 0.038 | 1Score **> 33** indicates **identity** Score **> 13** indicates **homology** | U | K.VIIVVKDGPGFYTTR.C |
| 84302 | 535 | – | 549 | 555.6524 | 1663.9353 | 1663.9298 | 3.32 | 1 | 47 | 4e-05 | 1Score **> 33** indicates **identity** Score **> 15** indicates **homology** | U | K.VIIVVKDGPGFYTTR.C |
| 84303 | 535 | – | 549 | 555.6526 | 1663.9359 | 1663.9298 | 3.69 | 1 | 36 | 0.00038 | 1Score **> 33** indicates **identity** Score **> 15** indicates **homology** | U | K.VIIVVKDGPGFYTTR.C |
| 84304 | 535 | – | 549 | 555.6526 | 1663.9361 | 1663.9298 | 3.79 | 1 | 36 | 0.00044 | 1Score **> 33** indicates **identity** Score **> 15** indicates **homology** | U | K.VIIVVKDGPGFYTTR.C |
| 84305 | 535 | – | 549 | 555.6535 | 1663.9388 | 1663.9298 | 5.42 | 1 | 27 | 0.003 | 1Score **> 33** indicates **identity** Score **> 14** indicates **homology** | U | K.VIIVVKDGPGFYTTR.C |
| 84306 | 535 | – | 549 | 555.6539 | 1663.9397 | 1663.9298 | 5.99 | 1 | 41 | 0.00014 | 1Score **> 33** indicates **identity** Score **> 15** indicates **homology** | U | K.VIIVVKDGPGFYTTR.C |
| 13882 | 541 | – | 549 | 507.2366 | 1012.4586 | 1012.4614 | -2.81 | 0 | 28 | 0.0026 | 1Score **> 27** indicates **identity** Score **> 14** indicates **homology** | U | K.DGPGFYTTR.C |
| 13884 | 541 | – | 549 | 507.2385 | 1012.4624 | 1012.4614 | 0.99 | 0 | 41 | 0.00013 | 1Score **> 27** indicates **identity** Score **> 15** indicates **homology** | U | K.DGPGFYTTR.C |
| 13885 | 541 | – | 549 | 507.2386 | 1012.4626 | 1012.4614 | 1.17 | 0 | 35 | 0.00056 | 1Score **> 27** indicates **identity** Score **> 15** indicates **homology** | U | K.DGPGFYTTR.C |
| 13886 | 541 | – | 549 | 507.2387 | 1012.4628 | 1012.4614 | 1.36 | 0 | 44 | 6.8e-05 | 1Score **> 27** indicates **identity** Score **> 15** indicates **homology** | U | K.DGPGFYTTR.C |
| 13887 | 541 | – | 549 | 507.2390 | 1012.4635 | 1012.4614 | 2.08 | 0 | 35 | 0.0005 | 1Score **> 27** indicates **identity** Score **> 15** indicates **homology** | U | K.DGPGFYTTR.C |
| 13014 | 561 | – | 569 | 499.7798 | 997.5450 | 997.5444 | 0.61 | 0 | 32 | 0.00092 | 1Score **> 28** indicates **identity** Score **> 15** indicates **homology** | U | R.ILQEGVDPK.K |
| 13015 | 561 | – | 569 | 499.7798 | 997.5451 | 997.5444 | 0.67 | 0 | 38 | 0.0003 | 1Score **> 28** indicates **identity** Score **> 15** indicates **homology** | U | R.ILQEGVDPK.K |
| 13016 | 561 | – | 569 | 499.7801 | 997.5456 | 997.5444 | 1.24 | 0 | 38 | 0.00026 | 1Score **> 29** indicates **identity** Score **> 15** indicates **homology** | U | R.ILQEGVDPK.K |
| 13017 | 561 | – | 569 | 499.7803 | 997.5460 | 997.5444 | 1.60 | 0 | 63 | 6.8e-06 | 1Score **> 30** indicates **identity** Score **> 23** indicates **homology** | U | R.ILQEGVDPK.K |
| 13527 | 611 | – | 620 | 503.7805 | 1005.5465 | 1005.5495 | -2.96 | 0 | 16 | 0.035 | 1Score **> 32** indicates **identity** Score **> 13** indicates **homology** | U | R.FGGGSVELLK.Q |
| 13533 | 611 | – | 620 | 503.7823 | 1005.5500 | 1005.5495 | 0.45 | 0 | 58 | 7.3e-06 | 1Score **> 31** indicates **identity** Score **> 19** indicates **homology** | U | R.FGGGSVELLK.Q |
| 13534 | 611 | – | 620 | 503.7824 | 1005.5503 | 1005.5495 | 0.81 | 0 | 60 | 4.6e-06 | 1Score **> 31** indicates **identity** Score **> 20** indicates **homology** | U | R.FGGGSVELLK.Q |
| 13535 | 611 | – | 620 | 503.7825 | 1005.5505 | 1005.5495 | 0.97 | 0 | 60 | 4.6e-06 | 1Score **> 31** indicates **identity** Score **> 19** indicates **homology** | U | R.FGGGSVELLK.Q |
| 13536 | 611 | – | 620 | 503.7826 | 1005.5506 | 1005.5495 | 1.05 | 0 | 63 | 2.1e-06 | 1Score **> 31** indicates **identity** Score **> 19** indicates **homology** | U | R.FGGGSVELLK.Q |
| 13537 | 611 | – | 620 | 503.7827 | 1005.5508 | 1005.5495 | 1.25 | 0 | 60 | 4.7e-06 | 1Score **> 32** indicates **identity** Score **> 19** indicates **homology** | U | R.FGGGSVELLK.Q |
| 13538 | 611 | – | 620 | 503.7827 | 1005.5508 | 1005.5495 | 1.28 | 0 | 43 | 9.2e-05 | 1Score **> 32** indicates **identity** Score **> 15** indicates **homology** | U | R.FGGGSVELLK.Q |
| 13539 | 611 | – | 620 | 503.7827 | 1005.5508 | 1005.5495 | 1.30 | 0 | 70 | 6e-07 | 1Score **> 32** indicates **identity** Score **> 20** indicates **homology** | U | R.FGGGSVELLK.Q |
| 13542 | 611 | – | 620 | 503.7831 | 1005.5516 | 1005.5495 | 2.07 | 0 | 45 | 6.6e-05 | 1Score **> 32** indicates **identity** Score **> 15** indicates **homology** | U | R.FGGGSVELLK.Q |
| 74270 | 631 | – | 644 | 531.2761 | 1590.8066 | 1590.8042 | 1.49 | 2 | 23 | 0.0077 | 1Score **> 34** indicates **identity** Score **> 14** indicates **homology** | U | R.KSGKGFYIYQEGSK.N |
| 57578 | 632 | – | 644 | 488.5774 | 1462.7103 | 1462.7092 | 0.76 | 1 | 21 | 0.01 | 1Score **> 33** indicates **identity** Score **> 14** indicates **homology** | U | K.SGKGFYIYQEGSK.N |
| 57579 | 632 | – | 644 | 732.3626 | 1462.7107 | 1462.7092 | 1.01 | 1 | 76 | 6.8e-08 | 1Score **> 33** indicates **identity** Score **> 17** indicates **homology** | U | K.SGKGFYIYQEGSK.N |
| 57580 | 632 | – | 644 | 732.3629 | 1462.7112 | 1462.7092 | 1.35 | 1 | 74 | 1.2e-07 | 1Score **> 33** indicates **identity** Score **> 17** indicates **homology** | U | K.SGKGFYIYQEGSK.N |
| 57581 | 632 | – | 644 | 488.5778 | 1462.7117 | 1462.7092 | 1.67 | 1 | 26 | 0.0034 | 1Score **> 33** indicates **identity** Score **> 14** indicates **homology** | U | K.SGKGFYIYQEGSK.N |
| 57582 | 632 | – | 644 | 732.3634 | 1462.7123 | 1462.7092 | 2.12 | 1 | 48 | 3.2e-05 | 1Score **> 33** indicates **identity** Score **> 15** indicates **homology** | U | K.SGKGFYIYQEGSK.N |
| 29399 | 635 | – | 644 | 596.2862 | 1190.5579 | 1190.5608 | -2.38 | 0 | 22 | 0.0078 | 1Score **> 31** indicates **identity** Score **> 14** indicates **homology** | U | K.GFYIYQEGSK.N |
| 29402 | 635 | – | 644 | 596.2871 | 1190.5597 | 1190.5608 | -0.94 | 0 | 28 | 0.0024 | 1Score **> 31** indicates **identity** Score **> 14** indicates **homology** | U | K.GFYIYQEGSK.N |
| 29406 | 635 | – | 644 | 596.2879 | 1190.5613 | 1190.5608 | 0.43 | 0 | 35 | 0.00053 | 1Score **> 31** indicates **identity** Score **> 15** indicates **homology** | U | K.GFYIYQEGSK.N |
| 29407 | 635 | – | 644 | 596.2880 | 1190.5614 | 1190.5608 | 0.55 | 0 | 39 | 0.00021 | 1Score **> 31** indicates **identity** Score **> 15** indicates **homology** | U | K.GFYIYQEGSK.N |
| 29408 | 635 | – | 644 | 596.2882 | 1190.5618 | 1190.5608 | 0.86 | 0 | 37 | 0.00031 | 1Score **> 31** indicates **identity** Score **> 15** indicates **homology** | U | K.GFYIYQEGSK.N |
| 29409 | 635 | – | 644 | 596.2884 | 1190.5622 | 1190.5608 | 1.19 | 0 | 29 | 0.0018 | 1Score **> 31** indicates **identity** Score **> 14** indicates **homology** | U | K.GFYIYQEGSK.N |
| 54159 | 635 | – | 646 | 717.3562 | 1432.6979 | 1432.6987 | -0.50 | 1 | 53 | 1e-05 | 1Score **> 33** indicates **identity** Score **> 16** indicates **homology** | U | K.GFYIYQEGSKNK.S |
| 54160 | 635 | – | 646 | 478.5734 | 1432.6985 | 1432.6987 | -0.094 | 1 | 35 | 0.0005 | 1Score **> 33** indicates **identity** Score **> 15** indicates **homology** | U | K.GFYIYQEGSKNK.S |
| 54163 | 635 | – | 646 | 717.3570 | 1432.6995 | 1432.6987 | 0.62 | 1 | 54 | 9.6e-06 | 1Score **> 33** indicates **identity** Score **> 16** indicates **homology** | U | K.GFYIYQEGSKNK.S |
| 54165 | 635 | – | 646 | 478.5742 | 1432.7007 | 1432.6987 | 1.45 | 1 | 27 | 0.0028 | 1Score **> 33** indicates **identity** Score **> 14** indicates **homology** | U | K.GFYIYQEGSKNK.S |
| 54166 | 635 | – | 646 | 717.3582 | 1432.7018 | 1432.6987 | 2.21 | 1 | 21 | 0.0099 | 1Score **> 33** indicates **identity** Score **> 14** indicates **homology** | U | K.GFYIYQEGSKNK.S |
| 54169 | 635 | – | 646 | 717.3596 | 1432.7046 | 1432.6987 | 4.17 | 1 | 24 | 0.0053 | 1Score **> 33** indicates **identity** Score **> 14** indicates **homology** | U | K.GFYIYQEGSKNK.S |
| 73961 | 647 | – | 660 | 795.3997 | 1588.7849 | 1588.7879 | -1.87 | 0 | 60 | 2.4e-06 | 1Score **> 34** indicates **identity** Score **> 16** indicates **homology** | U | K.SLNSEMDNILANLR.L |
| 187584 | 647 | – | 676 | 1135.2274 | 3402.6604 | 3402.6725 | -3.57 | 2 | 52 | 1.4e-05 | 1Score **> 37** indicates **identity** Score **> 16** indicates **homology** | U | K.SLNSEMDNILANLRLPAKPEVSSDEDVQYR.V |
| 187595 | 647 | – | 676 | 1135.5648 | 3403.6726 | 3403.6565 | 4.74 | 2 | 48 | 2.9e-05 | 1Score **> 37** indicates **identity** Score **> 16** indicates **homology** | U | K.SLNSEMDNILANLRLPAKPEVSSDEDVQYR.V  + Deamidated (NQ) |
| 190418 | 647 | – | 676 | 890.9418 | 3559.7381 | 3559.7715 | -9.39 | 2 | 19 | 0.017 | 1Score **> 37** indicates **identity** Score **> 14** indicates **homology** | U | K.SLNSEMDNILANLRLPAKPEVSSDEDVQYR.V  + Deamidated (NQ); HNE (K) |
| 190419 | 647 | – | 676 | 890.9418 | 3559.7381 | 3559.7715 | -9.38 | 2 | 18 | 0.022 | 1Score **> 37** indicates **identity** Score **> 14** indicates **homology** | U | K.SLNSEMDNILANLRLPAKPEVSSDEDVQYR.V  + Deamidated (NQ); HNE (K) |
| 190420 | 647 | – | 676 | 890.9424 | 3559.7405 | 3559.7715 | -8.71 | 2 | 21 | 0.012 | 1Score **> 37** indicates **identity** Score **> 14** indicates **homology** | U | K.SLNSEMDNILANLRLPAKPEVSSDEDVQYR.V  + Deamidated (NQ); HNE (K) |
| 104054 | 661 | – | 676 | 611.6378 | 1831.8915 | 1831.8952 | -2.00 | 1 | 64 | 9e-07 | 1Score **> 35** indicates **identity** Score **> 17** indicates **homology** | U | R.LPAKPEVSSDEDVQYR.V |
| 104057 | 661 | – | 676 | 611.6393 | 1831.8960 | 1831.8952 | 0.43 | 1 | 87 | 6.8e-09 | 1Score **> 35** indicates **identity** Score **> 18** indicates **homology** | U | R.LPAKPEVSSDEDVQYR.V |
| 104059 | 661 | – | 676 | 611.6397 | 1831.8972 | 1831.8952 | 1.07 | 1 | 84 | 1.3e-08 | 1Score **> 35** indicates **identity** Score **> 18** indicates **homology** | U | R.LPAKPEVSSDEDVQYR.V |
| 104060 | 661 | – | 676 | 611.6397 | 1831.8973 | 1831.8952 | 1.18 | 1 | 88 | 5.8e-09 | 1Score **> 35** indicates **identity** Score **> 18** indicates **homology** | U | R.LPAKPEVSSDEDVQYR.V |
| 104061 | 661 | – | 676 | 611.6399 | 1831.8978 | 1831.8952 | 1.42 | 1 | 55 | 7.3e-06 | 1Score **> 35** indicates **identity** Score **> 16** indicates **homology** | U | R.LPAKPEVSSDEDVQYR.V |
| 104062 | 661 | – | 676 | 916.9563 | 1831.8980 | 1831.8952 | 1.51 | 1 | 63 | 1.2e-06 | 1Score **> 35** indicates **identity** Score **> 16** indicates **homology** | U | R.LPAKPEVSSDEDVQYR.V |
| 104063 | 661 | – | 676 | 611.6401 | 1831.8984 | 1831.8952 | 1.75 | 1 | 94 | 1.5e-09 | 1Score **> 35** indicates **identity** Score **> 18** indicates **homology** | U | R.LPAKPEVSSDEDVQYR.V |
| 146751 | 661 | – | 680 | 576.3063 | 2301.1961 | 2301.1965 | -0.17 | 2 | 24 | 0.0057 | 1Score **> 37** indicates **identity** Score **> 14** indicates **homology** | U | R.LPAKPEVSSDEDVQYRVITR.F |
| 146753 | 661 | – | 680 | 768.0731 | 2301.1974 | 2301.1965 | 0.42 | 2 | 29 | 0.0018 | 1Score **> 37** indicates **identity** Score **> 14** indicates **homology** | U | R.LPAKPEVSSDEDVQYRVITR.F |
| 146755 | 661 | – | 680 | 576.3073 | 2301.1999 | 2301.1965 | 1.50 | 2 | 29 | 0.0018 | 1Score **> 37** indicates **identity** Score **> 14** indicates **homology** | U | R.LPAKPEVSSDEDVQYRVITR.F |
| 146756 | 661 | – | 680 | 768.0749 | 2301.2028 | 2301.1965 | 2.77 | 2 | 28 | 0.0022 | 1Score **> 37** indicates **identity** Score **> 14** indicates **homology** | U | R.LPAKPEVSSDEDVQYRVITR.F |
| 15470 | 720 | – | 728 | 520.7734 | 1039.5322 | 1039.5338 | -1.61 | 0 | 27 | 0.0032 | 1Score **> 31** indicates **identity** Score **> 14** indicates **homology** | U | R.FVDLYGAQK.V |
| 15471 | 720 | – | 728 | 520.7739 | 1039.5332 | 1039.5338 | -0.57 | 0 | 28 | 0.0026 | 1Score **> 31** indicates **identity** Score **> 14** indicates **homology** | U | R.FVDLYGAQK.V |
| 15472 | 720 | – | 728 | 520.7741 | 1039.5336 | 1039.5338 | -0.23 | 0 | 41 | 0.00013 | 1Score **> 31** indicates **identity** Score **> 15** indicates **homology** | U | R.FVDLYGAQK.V |
| 15473 | 720 | – | 728 | 520.7743 | 1039.5340 | 1039.5338 | 0.13 | 0 | 17 | 0.023 | 1Score **> 30** indicates **identity** Score **> 14** indicates **homology** | U | R.FVDLYGAQK.V |
| 15474 | 720 | – | 728 | 520.7745 | 1039.5344 | 1039.5338 | 0.53 | 0 | 25 | 0.0048 | 1Score **> 30** indicates **identity** Score **> 14** indicates **homology** | U | R.FVDLYGAQK.V |
| 15476 | 720 | – | 728 | 520.7745 | 1039.5345 | 1039.5338 | 0.63 | 0 | 33 | 0.00076 | 1Score **> 30** indicates **identity** Score **> 15** indicates **homology** | U | R.FVDLYGAQK.V |
| 15477 | 720 | – | 728 | 520.7746 | 1039.5346 | 1039.5338 | 0.74 | 0 | 43 | 8.6e-05 | 1Score **> 30** indicates **identity** Score **> 15** indicates **homology** | U | R.FVDLYGAQK.V |
| 15478 | 720 | – | 728 | 520.7747 | 1039.5349 | 1039.5338 | 1.00 | 0 | 43 | 8.8e-05 | 1Score **> 31** indicates **identity** Score **> 15** indicates **homology** | U | R.FVDLYGAQK.V |
| 15479 | 720 | – | 728 | 520.7748 | 1039.5349 | 1039.5338 | 1.06 | 0 | 45 | 6.6e-05 | 1Score **> 31** indicates **identity** Score **> 15** indicates **homology** | U | R.FVDLYGAQK.V |
| 15481 | 720 | – | 728 | 520.7750 | 1039.5355 | 1039.5338 | 1.57 | 0 | 42 | 0.00011 | 1Score **> 31** indicates **identity** Score **> 15** indicates **homology** | U | R.FVDLYGAQK.V |
| 15482 | 720 | – | 728 | 520.7751 | 1039.5356 | 1039.5338 | 1.70 | 0 | 20 | 0.012 | 1Score **> 31** indicates **identity** Score **> 14** indicates **homology** | U | R.FVDLYGAQK.V |
| 15483 | 720 | – | 728 | 520.7752 | 1039.5359 | 1039.5338 | 1.94 | 0 | 19 | 0.018 | 1Score **> 31** indicates **identity** Score **> 14** indicates **homology** | U | R.FVDLYGAQK.V |
| 15485 | 720 | – | 728 | 520.7754 | 1039.5362 | 1039.5338 | 2.27 | 0 | 36 | 0.00044 | 1Score **> 31** indicates **identity** Score **> 15** indicates **homology** | U | R.FVDLYGAQK.V |
| 63236 | 720 | – | 732 | 503.9409 | 1508.8010 | 1508.7987 | 1.51 | 1 | 15 | 0.043 | 1Score **> 34** indicates **identity** Score **> 13** indicates **homology** | U | R.FVDLYGAQKVVDR.L |
| 181943 | 733 | – | 759 | 786.1393 | 3140.5280 | 3140.5349 | -2.19 | 2 | 20 | 0.013 | 1Score **> 37** indicates **identity** Score **> 14** indicates **homology** | U | R.LRKYESAYGTQFTPCQLLLDHANNSSK.K |
| 181944 | 733 | – | 759 | 786.1427 | 3140.5418 | 3140.5349 | 2.19 | 2 | 25 | 0.0048 | 1Score **> 37** indicates **identity** Score **> 14** indicates **homology** | U | R.LRKYESAYGTQFTPCQLLLDHANNSSK.K |
| 174269 | 735 | – | 759 | 718.8424 | 2871.3403 | 2871.3497 | -3.28 | 1 | 46 | 5e-05 | 1Score **> 35** indicates **identity** Score **> 15** indicates **homology** | U | R.KYESAYGTQFTPCQLLLDHANNSSK.K |
| 174270 | 735 | – | 759 | 958.1216 | 2871.3429 | 2871.3497 | -2.38 | 1 | 61 | 2.1e-06 | 1Score **> 35** indicates **identity** Score **> 16** indicates **homology** | U | R.KYESAYGTQFTPCQLLLDHANNSSK.K |
| 174271 | 735 | – | 759 | 958.1234 | 2871.3484 | 2871.3497 | -0.47 | 1 | 77 | 5.6e-08 | 1Score **> 35** indicates **identity** Score **> 17** indicates **homology** | U | R.KYESAYGTQFTPCQLLLDHANNSSK.K |
| 174272 | 735 | – | 759 | 718.8445 | 2871.3490 | 2871.3497 | -0.26 | 1 | 21 | 0.01 | 1Score **> 35** indicates **identity** Score **> 14** indicates **homology** | U | R.KYESAYGTQFTPCQLLLDHANNSSK.K |
| 174273 | 735 | – | 759 | 958.1238 | 2871.3496 | 2871.3497 | -0.032 | 1 | 91 | 2.8e-09 | 1Score **> 35** indicates **identity** Score **> 18** indicates **homology** | U | R.KYESAYGTQFTPCQLLLDHANNSSK.K |
| 174274 | 735 | – | 759 | 1436.6823 | 2871.3501 | 2871.3497 | 0.14 | 1 | 70 | 2.7e-07 | 1Score **> 35** indicates **identity** Score **> 17** indicates **homology** | U | R.KYESAYGTQFTPCQLLLDHANNSSK.K |
| 174275 | 735 | – | 759 | 718.8450 | 2871.3509 | 2871.3497 | 0.42 | 1 | 55 | 6.5e-06 | 1Score **> 36** indicates **identity** Score **> 16** indicates **homology** | U | R.KYESAYGTQFTPCQLLLDHANNSSK.K |
| 174276 | 735 | – | 759 | 958.1243 | 2871.3511 | 2871.3497 | 0.48 | 1 | 91 | 2.7e-09 | 1Score **> 36** indicates **identity** Score **> 18** indicates **homology** | U | R.KYESAYGTQFTPCQLLLDHANNSSK.K |
| 174281 | 735 | – | 759 | 1436.6911 | 2871.3676 | 2871.3497 | 6.24 | 1 | 52 | 1.2e-05 | 1Score **> 36** indicates **identity** Score **> 16** indicates **homology** | U | R.KYESAYGTQFTPCQLLLDHANNSSK.K |
| 174328 | 735 | – | 759 | 958.4527 | 2872.3364 | 2872.3337 | 0.93 | 1 | 53 | 1.1e-05 | 1Score **> 35** indicates **identity** Score **> 16** indicates **homology** | U | R.KYESAYGTQFTPCQLLLDHANNSSK.K  + Deamidated (NQ) |
| 174329 | 735 | – | 759 | 719.0915 | 2872.3367 | 2872.3337 | 1.04 | 1 | 28 | 0.0022 | 1Score **> 35** indicates **identity** Score **> 14** indicates **homology** | U | R.KYESAYGTQFTPCQLLLDHANNSSK.K  + Deamidated (NQ) |
| 174331 | 735 | – | 759 | 719.0932 | 2872.3435 | 2872.3337 | 3.41 | 1 | 40 | 0.00019 | 1Score **> 35** indicates **identity** Score **> 15** indicates **homology** | U | R.KYESAYGTQFTPCQLLLDHANNSSK.K  + Deamidated (NQ) |
| 174332 | 735 | – | 759 | 958.4553 | 2872.3439 | 2872.3337 | 3.55 | 1 | 17 | 0.024 | 1Score **> 35** indicates **identity** Score **> 14** indicates **homology** | U | R.KYESAYGTQFTPCQLLLDHANNSSK.K  + Deamidated (NQ) |
| 174333 | 735 | – | 759 | 958.4557 | 2872.3454 | 2872.3337 | 4.05 | 1 | 61 | 1.9e-06 | 1Score **> 35** indicates **identity** Score **> 16** indicates **homology** | U | R.KYESAYGTQFTPCQLLLDHANNSSK.K  + Deamidated (NQ) |
| 174335 | 735 | – | 759 | 958.4566 | 2872.3480 | 2872.3337 | 4.97 | 1 | 83 | 1.7e-08 | 1Score **> 35** indicates **identity** Score **> 18** indicates **homology** | U | R.KYESAYGTQFTPCQLLLDHANNSSK.K  + Deamidated (NQ) |
| 174336 | 735 | – | 759 | 719.0943 | 2872.3483 | 2872.3337 | 5.05 | 1 | 28 | 0.0024 | 1Score **> 35** indicates **identity** Score **> 14** indicates **homology** | U | R.KYESAYGTQFTPCQLLLDHANNSSK.K  + Deamidated (NQ) |
| 174365 | 735 | – | 759 | 1437.6795 | 2873.3445 | 2873.3177 | 9.31 | 1 | 40 | 0.00018 | 1Score **> 35** indicates **identity** Score **> 15** indicates **homology** | U | R.KYESAYGTQFTPCQLLLDHANNSSK.K  + 2 Deamidated (NQ) |
| 178479 | 735 | – | 760 | 1000.8232 | 2999.4477 | 2999.4447 | 1.00 | 2 | 74 | 1.1e-07 | 1Score **> 37** indicates **identity** Score **> 17** indicates **homology** | U | R.KYESAYGTQFTPCQLLLDHANNSSKK.F |
| 178480 | 735 | – | 760 | 1000.8232 | 2999.4478 | 2999.4447 | 1.05 | 2 | 69 | 3.2e-07 | 1Score **> 37** indicates **identity** Score **> 17** indicates **homology** | U | R.KYESAYGTQFTPCQLLLDHANNSSKK.F |
| 178482 | 735 | – | 760 | 1000.8233 | 2999.4481 | 2999.4447 | 1.13 | 2 | 28 | 0.0023 | 1Score **> 37** indicates **identity** Score **> 14** indicates **homology** | U | R.KYESAYGTQFTPCQLLLDHANNSSKK.F |
| 178483 | 735 | – | 760 | 750.8694 | 2999.4487 | 2999.4447 | 1.34 | 2 | 22 | 0.0082 | 1Score **> 37** indicates **identity** Score **> 14** indicates **homology** | U | R.KYESAYGTQFTPCQLLLDHANNSSKK.F |
| 178484 | 735 | – | 760 | 750.8696 | 2999.4492 | 2999.4447 | 1.50 | 2 | 49 | 2.7e-05 | 1Score **> 37** indicates **identity** Score **> 16** indicates **homology** | U | R.KYESAYGTQFTPCQLLLDHANNSSKK.F |
| 178485 | 735 | – | 760 | 600.8972 | 2999.4496 | 2999.4447 | 1.62 | 2 | 51 | 1.7e-05 | 1Score **> 37** indicates **identity** Score **> 16** indicates **homology** | U | R.KYESAYGTQFTPCQLLLDHANNSSKK.F |
| 178486 | 735 | – | 760 | 750.8698 | 2999.4501 | 2999.4447 | 1.80 | 2 | 18 | 0.021 | 1Score **> 37** indicates **identity** Score **> 14** indicates **homology** | U | R.KYESAYGTQFTPCQLLLDHANNSSKK.F |
| 178487 | 735 | – | 760 | 600.8973 | 2999.4502 | 2999.4447 | 1.83 | 2 | 43 | 0.0001 | 1Score **> 37** indicates **identity** Score **> 15** indicates **homology** | U | R.KYESAYGTQFTPCQLLLDHANNSSKK.F |
| 178543 | 735 | – | 760 | 1001.1571 | 3000.4496 | 3000.4287 | 6.95 | 2 | 28 | 0.0021 | 1Score **> 37** indicates **identity** Score **> 14** indicates **homology** | U | R.KYESAYGTQFTPCQLLLDHANNSSKK.F  + Deamidated (NQ) |
| 169544 | 736 | – | 759 | 915.4261 | 2743.2564 | 2743.2548 | 0.59 | 0 | 58 | 3.5e-06 | 1Score **> 34** indicates **identity** Score **> 16** indicates **homology** | U | K.YESAYGTQFTPCQLLLDHANNSSK.K |
| 169545 | 736 | – | 759 | 915.4262 | 2743.2568 | 2743.2548 | 0.73 | 0 | 64 | 9.5e-07 | 1Score **> 34** indicates **identity** Score **> 17** indicates **homology** | U | K.YESAYGTQFTPCQLLLDHANNSSK.K |
| 169546 | 736 | – | 759 | 915.4281 | 2743.2624 | 2743.2548 | 2.77 | 0 | 33 | 0.00075 | 1Score **> 34** indicates **identity** Score **> 15** indicates **homology** | U | K.YESAYGTQFTPCQLLLDHANNSSK.K |

---

```
ID   ECHA_MOUSE              Reviewed;         763 AA.
AC   Q8BMS1; Q3TCY3; Q5U5Y5; Q8QZU4;
DT   18-MAR-2008, integrated into UniProtKB/Swiss-Prot.
DT   01-MAR-2003, sequence version 1.
DT   28-JUN-2023, entry version 183.
DE   RecName: Full=Trifunctional enzyme subunit alpha, mitochondrial;
DE   AltName: Full=Monolysocardiolipin acyltransferase {ECO:0000250|UniProtKB:P40939};
DE            EC=2.3.1.- {ECO:0000250|UniProtKB:P40939};
DE   AltName: Full=TP-alpha;
DE   Includes:
DE     RecName: Full=Long-chain enoyl-CoA hydratase;
DE              EC=4.2.1.17 {ECO:0000250|UniProtKB:P40939};
DE   Includes:
DE     RecName: Full=Long chain 3-hydroxyacyl-CoA dehydrogenase;
DE              EC=1.1.1.211 {ECO:0000250|UniProtKB:P40939};
DE   Flags: Precursor;
GN   Name=Hadha;
OS   Mus musculus (Mouse).
OC   Eukaryota; Metazoa; Chordata; Craniata; Vertebrata; Euteleostomi; Mammalia;
OC   Eutheria; Euarchontoglires; Glires; Rodentia; Myomorpha; Muroidea; Muridae;
OC   Murinae; Mus; Mus.
OX   NCBI_TaxID=10090;
RN   [1]
RP   NUCLEOTIDE SEQUENCE [LARGE SCALE MRNA].
RC   STRAIN=C57BL/6J, and NOD; TISSUE=Skin;
RX   PubMed=16141072; DOI=10.1126/science.1112014;
RA   Carninci P., Kasukawa T., Katayama S., Gough J., Frith M.C., Maeda N.,
RA   Oyama R., Ravasi T., Lenhard B., Wells C., Kodzius R., Shimokawa K.,
RA   Bajic V.B., Brenner S.E., Batalov S., Forrest A.R., Zavolan M., Davis M.J.,
RA   Wilming L.G., Aidinis V., Allen J.E., Ambesi-Impiombato A., Apweiler R.,
RA   Aturaliya R.N., Bailey T.L., Bansal M., Baxter L., Beisel K.W., Bersano T.,
RA   Bono H., Chalk A.M., Chiu K.P., Choudhary V., Christoffels A.,
RA   Clutterbuck D.R., Crowe M.L., Dalla E., Dalrymple B.P., de Bono B.,
RA   Della Gatta G., di Bernardo D., Down T., Engstrom P., Fagiolini M.,
RA   Faulkner G., Fletcher C.F., Fukushima T., Furuno M., Futaki S.,
RA   Gariboldi M., Georgii-Hemming P., Gingeras T.R., Gojobori T., Green R.E.,
RA   Gustincich S., Harbers M., Hayashi Y., Hensch T.K., Hirokawa N., Hill D.,
RA   Huminiecki L., Iacono M., Ikeo K., Iwama A., Ishikawa T., Jakt M.,
RA   Kanapin A., Katoh M., Kawasawa Y., Kelso J., Kitamura H., Kitano H.,
RA   Kollias G., Krishnan S.P., Kruger A., Kummerfeld S.K., Kurochkin I.V.,
RA   Lareau L.F., Lazarevic D., Lipovich L., Liu J., Liuni S., McWilliam S.,
RA   Madan Babu M., Madera M., Marchionni L., Matsuda H., Matsuzawa S., Miki H.,
RA   Mignone F., Miyake S., Morris K., Mottagui-Tabar S., Mulder N., Nakano N.,
RA   Nakauchi H., Ng P., Nilsson R., Nishiguchi S., Nishikawa S., Nori F.,
RA   Ohara O., Okazaki Y., Orlando V., Pang K.C., Pavan W.J., Pavesi G.,
RA   Pesole G., Petrovsky N., Piazza S., Reed J., Reid J.F., Ring B.Z.,
RA   Ringwald M., Rost B., Ruan Y., Salzberg S.L., Sandelin A., Schneider C.,
RA   Schoenbach C., Sekiguchi K., Semple C.A., Seno S., Sessa L., Sheng Y.,
RA   Shibata Y., Shimada H., Shimada K., Silva D., Sinclair B., Sperling S.,
RA   Stupka E., Sugiura K., Sultana R., Takenaka Y., Taki K., Tammoja K.,
RA   Tan S.L., Tang S., Taylor M.S., Tegner J., Teichmann S.A., Ueda H.R.,
RA   van Nimwegen E., Verardo R., Wei C.L., Yagi K., Yamanishi H.,
RA   Zabarovsky E., Zhu S., Zimmer A., Hide W., Bult C., Grimmond S.M.,
RA   Teasdale R.D., Liu E.T., Brusic V., Quackenbush J., Wahlestedt C.,
RA   Mattick J.S., Hume D.A., Kai C., Sasaki D., Tomaru Y., Fukuda S.,
RA   Kanamori-Katayama M., Suzuki M., Aoki J., Arakawa T., Iida J., Imamura K.,
RA   Itoh M., Kato T., Kawaji H., Kawagashira N., Kawashima T., Kojima M.,
RA   Kondo S., Konno H., Nakano K., Ninomiya N., Nishio T., Okada M., Plessy C.,
RA   Shibata K., Shiraki T., Suzuki S., Tagami M., Waki K., Watahiki A.,
RA   Okamura-Oho Y., Suzuki H., Kawai J., Hayashizaki Y.;
RT   "The transcriptional landscape of the mammalian genome.";
RL   Science 309:1559-1563(2005).
RN   [2]
RP   NUCLEOTIDE SEQUENCE [LARGE SCALE MRNA].
RC   STRAIN=FVB/N; TISSUE=Eye, Liver, and Olfactory epithelium;
RX   PubMed=15489334; DOI=10.1101/gr.2596504;
RG   The MGC Project Team;
RT   "The status, quality, and expansion of the NIH full-length cDNA project:
RT   the Mammalian Gene Collection (MGC).";
RL   Genome Res. 14:2121-2127(2004).
RN   [3]
RP   ACETYLATION [LARGE SCALE ANALYSIS] AT LYS-129, AND IDENTIFICATION BY MASS
RP   SPECTROMETRY [LARGE SCALE ANALYSIS].
RC   TISSUE=Liver;
RX   PubMed=16916647; DOI=10.1016/j.molcel.2006.06.026;
RA   Kim S.C., Sprung R., Chen Y., Xu Y., Ball H., Pei J., Cheng T., Kho Y.,
RA   Xiao H., Xiao L., Grishin N.V., White M., Yang X.-J., Zhao Y.;
RT   "Substrate and functional diversity of lysine acetylation revealed by a
RT   proteomics survey.";
RL   Mol. Cell 23:607-618(2006).
RN   [4]
RP   PHOSPHORYLATION [LARGE SCALE ANALYSIS] AT SER-231; SER-316; THR-395 AND
RP   SER-647, AND IDENTIFICATION BY MASS SPECTROMETRY [LARGE SCALE ANALYSIS].
RC   TISSUE=Brain, Brown adipose tissue, Heart, Kidney, Liver, Lung,
RC   Pancreas, Spleen, and Testis;
RX   PubMed=21183079; DOI=10.1016/j.cell.2010.12.001;
RA   Huttlin E.L., Jedrychowski M.P., Elias J.E., Goswami T., Rad R.,
RA   Beausoleil S.A., Villen J., Haas W., Sowa M.E., Gygi S.P.;
RT   "A tissue-specific atlas of mouse protein phosphorylation and expression.";
RL   Cell 143:1174-1189(2010).
RN   [5]
RP   ACETYLATION [LARGE SCALE ANALYSIS] AT LYS-60 AND LYS-406, SUCCINYLATION
RP   [LARGE SCALE ANALYSIS] AT LYS-46; LYS-60; LYS-166; LYS-213; LYS-214;
RP   LYS-230; LYS-249; LYS-303; LYS-326; LYS-334; LYS-350; LYS-406; LYS-411;
RP   LYS-415; LYS-436; LYS-440; LYS-460; LYS-505; LYS-519; LYS-569; LYS-620;
RP   LYS-634; LYS-644; LYS-646; LYS-664; LYS-728 AND LYS-759, AND IDENTIFICATION
RP   BY MASS SPECTROMETRY [LARGE SCALE ANALYSIS].
RC   TISSUE=Embryonic fibroblast, and Liver;
RX   PubMed=23806337; DOI=10.1016/j.molcel.2013.06.001;
RA   Park J., Chen Y., Tishkoff D.X., Peng C., Tan M., Dai L., Xie Z., Zhang Y.,
RA   Zwaans B.M., Skinner M.E., Lombard D.B., Zhao Y.;
RT   "SIRT5-mediated lysine desuccinylation impacts diverse metabolic
RT   pathways.";
RL   Mol. Cell 50:919-930(2013).
RN   [6]
RP   ACETYLATION [LARGE SCALE ANALYSIS] AT LYS-46; LYS-60; LYS-129; LYS-166;
RP   LYS-214; LYS-249; LYS-289; LYS-303; LYS-326; LYS-334; LYS-350; LYS-353;
RP   LYS-406; LYS-411; LYS-436; LYS-460; LYS-505; LYS-519; LYS-540; LYS-569;
RP   LYS-644; LYS-664; LYS-728; LYS-735 AND LYS-759, AND IDENTIFICATION BY MASS
RP   SPECTROMETRY [LARGE SCALE ANALYSIS].
RC   TISSUE=Liver;
RX   PubMed=23576753; DOI=10.1073/pnas.1302961110;
RA   Rardin M.J., Newman J.C., Held J.M., Cusack M.P., Sorensen D.J., Li B.,
RA   Schilling B., Mooney S.D., Kahn C.R., Verdin E., Gibson B.W.;
RT   "Label-free quantitative proteomics of the lysine acetylome in mitochondria
RT   identifies substrates of SIRT3 in metabolic pathways.";
RL   Proc. Natl. Acad. Sci. U.S.A. 110:6601-6606(2013).
RN   [7]
RP   METHYLATION [LARGE SCALE ANALYSIS] AT ARG-399, AND IDENTIFICATION BY MASS
RP   SPECTROMETRY [LARGE SCALE ANALYSIS].
RC   TISSUE=Brain;
RX   PubMed=24129315; DOI=10.1074/mcp.o113.027870;
RA   Guo A., Gu H., Zhou J., Mulhern D., Wang Y., Lee K.A., Yang V., Aguiar M.,
RA   Kornhauser J., Jia X., Ren J., Beausoleil S.A., Silva J.C., Vemulapalli V.,
RA   Bedford M.T., Comb M.J.;
RT   "Immunoaffinity enrichment and mass spectrometry analysis of protein
RT   methylation.";
RL   Mol. Cell. Proteomics 13:372-387(2014).
RN   [8]
RP   INTERACTION WITH MTLN, AND IDENTIFICATION BY MASS SPECTROMETRY.
RX   PubMed=29949755; DOI=10.1016/j.celrep.2018.05.058;
RA   Makarewich C.A., Baskin K.K., Munir A.Z., Bezprozvannaya S., Sharma G.,
RA   Khemtong C., Shah A.M., McAnally J.R., Malloy C.R., Szweda L.I.,
RA   Bassel-Duby R., Olson E.N.;
RT   "MOXI Is a Mitochondrial Micropeptide That Enhances Fatty Acid beta-
RT   Oxidation.";
RL   Cell Rep. 23:3701-3709(2018).
CC   -!- FUNCTION: Mitochondrial trifunctional enzyme catalyzes the last three
CC       of the four reactions of the mitochondrial beta-oxidation pathway. The
CC       mitochondrial beta-oxidation pathway is the major energy-producing
CC       process in tissues and is performed through four consecutive reactions
CC       breaking down fatty acids into acetyl-CoA. Among the enzymes involved
CC       in this pathway, the trifunctional enzyme exhibits specificity for
CC       long-chain fatty acids. Mitochondrial trifunctional enzyme is a
CC       heterotetrameric complex composed of two proteins, the trifunctional
CC       enzyme subunit alpha/HADHA described here carries the 2,3-enoyl-CoA
CC       hydratase and the 3-hydroxyacyl-CoA dehydrogenase activities while the
CC       trifunctional enzyme subunit beta/HADHB bears the 3-ketoacyl-CoA
CC       thiolase activity. Independently of the subunit beta, the trifunctional
CC       enzyme subunit alpha/HADHA also has a monolysocardiolipin
CC       acyltransferase activity. It acylates monolysocardiolipin into
CC       cardiolipin, a major mitochondrial membrane phospholipid which plays a
CC       key role in apoptosis and supports mitochondrial respiratory chain
CC       complexes in the generation of ATP. Allows the acylation of
CC       monolysocardiolipin with different acyl-CoA substrates including
CC       oleoyl-CoA for which it displays the highest activity.
CC       {ECO:0000250|UniProtKB:P40939}.
CC   -!- CATALYTIC ACTIVITY:
CC       Reaction=a (3S)-3-hydroxyacyl-CoA = a (2E)-enoyl-CoA + H2O;
CC         Xref=Rhea:RHEA:16105, ChEBI:CHEBI:15377, ChEBI:CHEBI:57318,
CC         ChEBI:CHEBI:58856; EC=4.2.1.17;
CC         Evidence={ECO:0000250|UniProtKB:P40939};
CC       PhysiologicalDirection=right-to-left; Xref=Rhea:RHEA:16107;
CC         Evidence={ECO:0000250|UniProtKB:P40939};
CC   -!- CATALYTIC ACTIVITY:
CC       Reaction=a 4-saturated-(3S)-3-hydroxyacyl-CoA = a (3E)-enoyl-CoA + H2O;
CC         Xref=Rhea:RHEA:20724, ChEBI:CHEBI:15377, ChEBI:CHEBI:58521,
CC         ChEBI:CHEBI:137480; EC=4.2.1.17;
CC         Evidence={ECO:0000250|UniProtKB:P40939};
CC       PhysiologicalDirection=right-to-left; Xref=Rhea:RHEA:20726;
CC         Evidence={ECO:0000250|UniProtKB:P40939};
CC   -!- CATALYTIC ACTIVITY:
CC       Reaction=(3S)-hydroxyoctanoyl-CoA = (2E)-octenoyl-CoA + H2O;
CC         Xref=Rhea:RHEA:31199, ChEBI:CHEBI:15377, ChEBI:CHEBI:62242,
CC         ChEBI:CHEBI:62617; Evidence={ECO:0000250|UniProtKB:P40939};
CC       PhysiologicalDirection=right-to-left; Xref=Rhea:RHEA:31201;
CC         Evidence={ECO:0000250|UniProtKB:P40939};
CC   -!- CATALYTIC ACTIVITY:
CC       Reaction=(3S)-3-hydroxydodecanoyl-CoA = (2E)-dodecenoyl-CoA + H2O;
CC         Xref=Rhea:RHEA:31075, ChEBI:CHEBI:15377, ChEBI:CHEBI:57330,
CC         ChEBI:CHEBI:62558; Evidence={ECO:0000250|UniProtKB:P40939};
CC       PhysiologicalDirection=right-to-left; Xref=Rhea:RHEA:31077;
CC         Evidence={ECO:0000250|UniProtKB:P40939};
CC   -!- CATALYTIC ACTIVITY:
CC       Reaction=(3S)-hydroxyhexadecanoyl-CoA = (2E)-hexadecenoyl-CoA + H2O;
CC         Xref=Rhea:RHEA:31163, ChEBI:CHEBI:15377, ChEBI:CHEBI:61526,
CC         ChEBI:CHEBI:62613; Evidence={ECO:0000250|UniProtKB:P40939};
CC       PhysiologicalDirection=right-to-left; Xref=Rhea:RHEA:31165;
CC         Evidence={ECO:0000250|UniProtKB:P40939};
CC   -!- CATALYTIC ACTIVITY:
CC       Reaction=a long-chain (3S)-3-hydroxy fatty acyl-CoA + NAD(+) = a long-
CC         chain 3-oxo-fatty acyl-CoA + H(+) + NADH; Xref=Rhea:RHEA:52656,
CC         ChEBI:CHEBI:15378, ChEBI:CHEBI:57540, ChEBI:CHEBI:57945,
CC         ChEBI:CHEBI:136757, ChEBI:CHEBI:136758; EC=1.1.1.211;
CC         Evidence={ECO:0000250|UniProtKB:P40939};
CC       PhysiologicalDirection=left-to-right; Xref=Rhea:RHEA:52657;
CC         Evidence={ECO:0000250|UniProtKB:P40939};
CC   -!- CATALYTIC ACTIVITY:
CC       Reaction=(3S)-hydroxyoctanoyl-CoA + NAD(+) = 3-oxooctanoyl-CoA + H(+) +
CC         NADH; Xref=Rhea:RHEA:31195, ChEBI:CHEBI:15378, ChEBI:CHEBI:57540,
CC         ChEBI:CHEBI:57945, ChEBI:CHEBI:62617, ChEBI:CHEBI:62619;
CC         Evidence={ECO:0000250|UniProtKB:P40939};
CC       PhysiologicalDirection=left-to-right; Xref=Rhea:RHEA:31196;
CC         Evidence={ECO:0000250|UniProtKB:P40939};
CC   -!- CATALYTIC ACTIVITY:
CC       Reaction=(3S)-hydroxydecanoyl-CoA + NAD(+) = 3-oxodecanoyl-CoA + H(+) +
CC         NADH; Xref=Rhea:RHEA:31187, ChEBI:CHEBI:15378, ChEBI:CHEBI:57540,
CC         ChEBI:CHEBI:57945, ChEBI:CHEBI:62548, ChEBI:CHEBI:62616;
CC         Evidence={ECO:0000250|UniProtKB:P40939};
CC       PhysiologicalDirection=left-to-right; Xref=Rhea:RHEA:31188;
CC         Evidence={ECO:0000250|UniProtKB:P40939};
CC   -!- CATALYTIC ACTIVITY:
CC       Reaction=(3S)-3-hydroxydodecanoyl-CoA + NAD(+) = 3-oxododecanoyl-CoA +
CC         H(+) + NADH; Xref=Rhea:RHEA:31179, ChEBI:CHEBI:15378,
CC         ChEBI:CHEBI:57540, ChEBI:CHEBI:57945, ChEBI:CHEBI:62558,
CC         ChEBI:CHEBI:62615; Evidence={ECO:0000250|UniProtKB:P40939};
CC       PhysiologicalDirection=left-to-right; Xref=Rhea:RHEA:31180;
CC         Evidence={ECO:0000250|UniProtKB:P40939};
CC   -!- CATALYTIC ACTIVITY:
CC       Reaction=(3S)-hydroxytetradecanoyl-CoA + NAD(+) = 3-oxotetradecanoyl-
CC         CoA + H(+) + NADH; Xref=Rhea:RHEA:31167, ChEBI:CHEBI:15378,
CC         ChEBI:CHEBI:57540, ChEBI:CHEBI:57945, ChEBI:CHEBI:62543,
CC         ChEBI:CHEBI:62614; Evidence={ECO:0000250|UniProtKB:P40939};
CC       PhysiologicalDirection=left-to-right; Xref=Rhea:RHEA:31168;
CC         Evidence={ECO:0000250|UniProtKB:P40939};
CC   -!- CATALYTIC ACTIVITY:
CC       Reaction=(3S)-hydroxyhexadecanoyl-CoA + NAD(+) = 3-oxohexadecanoyl-CoA
CC         + H(+) + NADH; Xref=Rhea:RHEA:31159, ChEBI:CHEBI:15378,
CC         ChEBI:CHEBI:57349, ChEBI:CHEBI:57540, ChEBI:CHEBI:57945,
CC         ChEBI:CHEBI:62613; Evidence={ECO:0000250|UniProtKB:P40939};
CC       PhysiologicalDirection=left-to-right; Xref=Rhea:RHEA:31160;
CC         Evidence={ECO:0000250|UniProtKB:P40939};
CC   -!- CATALYTIC ACTIVITY:
CC       Reaction=1'-[1,2-di-(9Z,12Z-octadecadienoyl)-sn-glycero-3-phospho]-3'-
CC         [1-(9Z,12Z-octadecadienoyl)-sn-glycero-3-phospho]-glycerol +
CC         hexadecanoyl-CoA = 1'-[1,2-di-(9Z,12Z-octadecadienoyl)-sn-glycero-3-
CC         phospho]-3'-[1-(9Z,12Z-octadecadienoyl)-2-hexadecanoyl-sn-glycero-3-
CC         phospho]-glycerol + CoA; Xref=Rhea:RHEA:43680, ChEBI:CHEBI:57287,
CC         ChEBI:CHEBI:57379, ChEBI:CHEBI:83580, ChEBI:CHEBI:83583;
CC         Evidence={ECO:0000250|UniProtKB:P40939};
CC       PhysiologicalDirection=left-to-right; Xref=Rhea:RHEA:43681;
CC         Evidence={ECO:0000250|UniProtKB:P40939};
CC   -!- CATALYTIC ACTIVITY:
CC       Reaction=(9Z)-octadecenoyl-CoA + 1'-[1,2-di-(9Z,12Z-octadecadienoyl)-
CC         sn-glycero-3-phospho]-3'-[1-(9Z,12Z-octadecadienoyl)-sn-glycero-3-
CC         phospho]-glycerol = 1'-[1,2-di-(9Z,12Z-octadecadienoyl)-sn-glycero-3-
CC         phospho]-3'-[1-(9Z,12Z-octadecadienoyl)-2-(9Z-octadecenoyl)-sn-
CC         glycero-3-phospho]-glycerol + CoA; Xref=Rhea:RHEA:43676,
CC         ChEBI:CHEBI:57287, ChEBI:CHEBI:57387, ChEBI:CHEBI:83580,
CC         ChEBI:CHEBI:83582; Evidence={ECO:0000250|UniProtKB:P40939};
CC       PhysiologicalDirection=left-to-right; Xref=Rhea:RHEA:43677;
CC         Evidence={ECO:0000250|UniProtKB:P40939};
CC   -!- CATALYTIC ACTIVITY:
CC       Reaction=(9Z,12Z)-octadecadienoyl-CoA + 1'-[1,2-di-(9Z,12Z-
CC         octadecadienoyl)-sn-glycero-3-phospho]-3'-[1-(9Z,12Z-
CC         octadecadienoyl)-sn-glycero-3-phospho]-glycerol = 1',3'-bis-[1,2-di-
CC         (9Z,12Z-octadecadienoyl)-sn-glycero-3-phospho]-glycerol + CoA;
CC         Xref=Rhea:RHEA:43672, ChEBI:CHEBI:57287, ChEBI:CHEBI:57383,
CC         ChEBI:CHEBI:83580, ChEBI:CHEBI:83581;
CC         Evidence={ECO:0000250|UniProtKB:P40939};
CC       PhysiologicalDirection=left-to-right; Xref=Rhea:RHEA:43673;
CC         Evidence={ECO:0000250|UniProtKB:P40939};
CC   -!- PATHWAY: Lipid metabolism; fatty acid beta-oxidation.
CC       {ECO:0000250|UniProtKB:P40939}.
CC   -!- SUBUNIT: Heterotetramer of 2 alpha/HADHA and 2 beta/HADHB subunits;
CC       forms the mitochondrial trifunctional enzyme (By similarity). Also
CC       purified as higher order heterooligomers including a 4 alpha/HADHA and
CC       4 beta/HADHB heterooligomer which physiological significance remains
CC       unclear (By similarity). The mitochondrial trifunctional enzyme
CC       interacts with MTLN (PubMed:29949755). {ECO:0000250|UniProtKB:P40939,
CC       ECO:0000269|PubMed:29949755}.
CC   -!- SUBCELLULAR LOCATION: Mitochondrion {ECO:0000250|UniProtKB:P40939}.
CC       Mitochondrion inner membrane {ECO:0000250|UniProtKB:P40939}.
CC       Note=Protein stability and association with mitochondrion inner
CC       membrane do not require HADHB. {ECO:0000250|UniProtKB:P40939}.
CC   -!- PTM: Acetylation of Lys-569 and Lys-728 is observed in liver
CC       mitochondria from fasted mice but not from fed mice.
CC   -!- SIMILARITY: In the N-terminal section; belongs to the enoyl-CoA
CC       hydratase/isomerase family. {ECO:0000305}.
CC   -!- SIMILARITY: In the central section; belongs to the 3-hydroxyacyl-CoA
CC       dehydrogenase family. {ECO:0000305}.
CC   ---------------------------------------------------------------------------
CC   Copyrighted by the UniProt Consortium, see https://www.uniprot.org/terms
CC   Distributed under the Creative Commons Attribution (CC BY 4.0) License
CC   ---------------------------------------------------------------------------
DR   EMBL; AK029017; BAC26245.1; -; mRNA.
DR   EMBL; AK170478; BAE41822.1; -; mRNA.
DR   EMBL; AK170683; BAE41956.1; -; mRNA.
DR   EMBL; BC027156; AAH27156.1; -; mRNA.
DR   EMBL; BC037009; AAH37009.1; -; mRNA.
DR   EMBL; BC046978; AAH46978.1; -; mRNA.
DR   EMBL; BC058569; AAH58569.1; -; mRNA.
DR   CCDS; CCDS19155.1; -.
DR   RefSeq; NP_849209.1; NM_178878.2.
DR   AlphaFoldDB; Q8BMS1; -.
DR   SMR; Q8BMS1; -.
DR   BioGRID; 220648; 43.
DR   IntAct; Q8BMS1; 8.
DR   MINT; Q8BMS1; -.
DR   STRING; 10090.ENSMUSP00000120976; -.
DR   GlyGen; Q8BMS1; 1 site, 1 O-linked glycan (1 site).
DR   iPTMnet; Q8BMS1; -.
DR   PhosphoSitePlus; Q8BMS1; -.
DR   SwissPalm; Q8BMS1; -.
DR   REPRODUCTION-2DPAGE; IPI00223092; -.
DR   EPD; Q8BMS1; -.
DR   jPOST; Q8BMS1; -.
DR   MaxQB; Q8BMS1; -.
DR   PaxDb; Q8BMS1; -.
DR   PeptideAtlas; Q8BMS1; -.
DR   ProteomicsDB; 277753; -.
DR   Antibodypedia; 3074; 238 antibodies from 32 providers.
DR   DNASU; 97212; -.
DR   Ensembl; ENSMUST00000156859; ENSMUSP00000120976; ENSMUSG00000025745.
DR   GeneID; 97212; -.
DR   KEGG; mmu:97212; -.
DR   UCSC; uc008wvc.1; mouse.
DR   AGR; MGI:2135593; -.
DR   CTD; 3030; -.
DR   MGI; MGI:2135593; Hadha.
DR   VEuPathDB; HostDB:ENSMUSG00000025745; -.
DR   eggNOG; KOG1683; Eukaryota.
DR   GeneTree; ENSGT00940000154677; -.
DR   HOGENOM; CLU_009834_16_1_1; -.
DR   InParanoid; Q8BMS1; -.
DR   OMA; PFRYMDT; -.
DR   OrthoDB; 622692at2759; -.
DR   PhylomeDB; Q8BMS1; -.
DR   TreeFam; TF352288; -.
DR   Reactome; R-MMU-1482798; Acyl chain remodeling of CL.
DR   Reactome; R-MMU-77285; Beta oxidation of myristoyl-CoA to lauroyl-CoA.
DR   Reactome; R-MMU-77305; Beta oxidation of palmitoyl-CoA to myristoyl-CoA.
DR   Reactome; R-MMU-77310; Beta oxidation of lauroyl-CoA to decanoyl-CoA-CoA.
DR   Reactome; R-MMU-77346; Beta oxidation of decanoyl-CoA to octanoyl-CoA-CoA.
DR   Reactome; R-MMU-77348; Beta oxidation of octanoyl-CoA to hexanoyl-CoA.
DR   Reactome; R-MMU-77350; Beta oxidation of hexanoyl-CoA to butanoyl-CoA.
DR   UniPathway; UPA00659; -.
DR   BioGRID-ORCS; 97212; 4 hits in 79 CRISPR screens.
DR   ChiTaRS; Hadha; mouse.
DR   PRO; PR:Q8BMS1; -.
DR   Proteomes; UP000000589; Chromosome 5.
DR   RNAct; Q8BMS1; protein.
DR   Bgee; ENSMUSG00000025745; Expressed in myocardium of ventricle and 247 other tissues.
DR   Genevisible; Q8BMS1; MM.
DR   GO; GO:0016507; C:mitochondrial fatty acid beta-oxidation multienzyme complex; ISO:MGI.
DR   GO; GO:0005743; C:mitochondrial inner membrane; HDA:MGI.
DR   GO; GO:0042645; C:mitochondrial nucleoid; ISO:MGI.
DR   GO; GO:0005739; C:mitochondrion; HDA:MGI.
DR   GO; GO:0003857; F:3-hydroxyacyl-CoA dehydrogenase activity; ISO:MGI.
DR   GO; GO:0003988; F:acetyl-CoA C-acyltransferase activity; ISO:MGI.
DR   GO; GO:0004300; F:enoyl-CoA hydratase activity; ISO:MGI.
DR   GO; GO:0000062; F:fatty-acyl-CoA binding; ISO:MGI.
DR   GO; GO:0016509; F:long-chain-3-hydroxyacyl-CoA dehydrogenase activity; IDA:MGI.
DR   GO; GO:0051287; F:NAD binding; ISO:MGI.
DR   GO; GO:0070403; F:NAD+ binding; IEA:InterPro.
DR   GO; GO:0044877; F:protein-containing complex binding; ISO:MGI.
DR   GO; GO:0035965; P:cardiolipin acyl-chain remodeling; ISS:UniProtKB.
DR   GO; GO:0006635; P:fatty acid beta-oxidation; IMP:MGI.
DR   GO; GO:0032868; P:response to insulin; IMP:MGI.
DR   GO; GO:0009410; P:response to xenobiotic stimulus; ISO:MGI.
DR   CDD; cd06558; crotonase-like; 1.
DR   Gene3D; 1.10.1040.50; -; 1.
DR   Gene3D; 3.40.50.720; NAD(P)-binding Rossmann-like Domain; 1.
DR   InterPro; IPR006180; 3-OHacyl-CoA_DH_CS.
DR   InterPro; IPR006176; 3-OHacyl-CoA_DH_NAD-bd.
DR   InterPro; IPR006108; 3HC_DH_C.
DR   InterPro; IPR008927; 6-PGluconate_DH-like_C_sf.
DR   InterPro; IPR029045; ClpP/crotonase-like_dom_sf.
DR   InterPro; IPR018376; Enoyl-CoA_hyd/isom_CS.
DR   InterPro; IPR001753; Enoyl-CoA_hydra/iso.
DR   InterPro; IPR012803; Fa_ox_alpha_mit.
DR   InterPro; IPR036291; NAD(P)-bd_dom_sf.
DR   PANTHER; PTHR43612; TRIFUNCTIONAL ENZYME SUBUNIT ALPHA; 1.
DR   PANTHER; PTHR43612:SF3; TRIFUNCTIONAL ENZYME SUBUNIT ALPHA, MITOCHONDRIAL; 1.
DR   Pfam; PF00725; 3HCDH; 2.
DR   Pfam; PF02737; 3HCDH_N; 1.
DR   Pfam; PF00378; ECH_1; 1.
DR   SUPFAM; SSF48179; 6-phosphogluconate dehydrogenase C-terminal domain-like; 2.
DR   SUPFAM; SSF52096; ClpP/crotonase; 1.
DR   SUPFAM; SSF51735; NAD(P)-binding Rossmann-fold domains; 1.
DR   PROSITE; PS00067; 3HCDH; 1.
DR   PROSITE; PS00166; ENOYL_COA_HYDRATASE; 1.
DR   TIGRFAMs; TIGR02441; fa_ox_alpha_mit; 1.
PE   1: Evidence at protein level;
KW   Acetylation; Fatty acid metabolism; Lipid metabolism; Lyase; Membrane;
KW   Methylation; Mitochondrion; Mitochondrion inner membrane;
KW   Multifunctional enzyme; NAD; Oxidoreductase; Phosphoprotein;
KW   Reference proteome; Transferase; Transit peptide.
FT   TRANSIT         1..36
FT                   /note="Mitochondrion"
FT                   /evidence="ECO:0000255"
FT   CHAIN           37..763
FT                   /note="Trifunctional enzyme subunit alpha, mitochondrial"
FT                   /id="PRO_0000322639"
FT   ACT_SITE        510
FT                   /note="For hydroxyacyl-coenzyme A dehydrogenase activity"
FT                   /evidence="ECO:0000250|UniProtKB:P40939"
FT   SITE            151
FT                   /note="Important for long-chain enoyl-CoA hydratase
FT                   activity"
FT                   /evidence="ECO:0000250|UniProtKB:P40939"
FT   SITE            173
FT                   /note="Important for long-chain enoyl-CoA hydratase
FT                   activity"
FT                   /evidence="ECO:0000250|UniProtKB:P40939"
FT   SITE            498
FT                   /note="Important for hydroxyacyl-coenzyme A dehydrogenase
FT                   activity"
FT                   /evidence="ECO:0000250|UniProtKB:P40939"
FT   MOD_RES         46
FT                   /note="N6-acetyllysine; alternate"
FT                   /evidence="ECO:0007744|PubMed:23576753"
FT   MOD_RES         46
FT                   /note="N6-succinyllysine; alternate"
FT                   /evidence="ECO:0007744|PubMed:23806337"
FT   MOD_RES         60
FT                   /note="N6-acetyllysine; alternate"
FT                   /evidence="ECO:0007744|PubMed:23576753,
FT                   ECO:0007744|PubMed:23806337"
FT   MOD_RES         60
FT                   /note="N6-succinyllysine; alternate"
FT                   /evidence="ECO:0007744|PubMed:23806337"
FT   MOD_RES         129
FT                   /note="N6-acetyllysine"
FT                   /evidence="ECO:0007744|PubMed:16916647,
FT                   ECO:0007744|PubMed:23576753"
FT   MOD_RES         166
FT                   /note="N6-acetyllysine; alternate"
FT                   /evidence="ECO:0007744|PubMed:23576753"
FT   MOD_RES         166
FT                   /note="N6-succinyllysine; alternate"
FT                   /evidence="ECO:0007744|PubMed:23806337"
FT   MOD_RES         213
FT                   /note="N6-succinyllysine"
FT                   /evidence="ECO:0007744|PubMed:23806337"
FT   MOD_RES         214
FT                   /note="N6-acetyllysine; alternate"
FT                   /evidence="ECO:0007744|PubMed:23576753"
FT   MOD_RES         214
FT                   /note="N6-succinyllysine; alternate"
FT                   /evidence="ECO:0007744|PubMed:23806337"
FT   MOD_RES         230
FT                   /note="N6-succinyllysine"
FT                   /evidence="ECO:0007744|PubMed:23806337"
FT   MOD_RES         231
FT                   /note="Phosphoserine"
FT                   /evidence="ECO:0007744|PubMed:21183079"
FT   MOD_RES         249
FT                   /note="N6-acetyllysine; alternate"
FT                   /evidence="ECO:0007744|PubMed:23576753"
FT   MOD_RES         249
FT                   /note="N6-succinyllysine; alternate"
FT                   /evidence="ECO:0007744|PubMed:23806337"
FT   MOD_RES         289
FT                   /note="N6-acetyllysine"
FT                   /evidence="ECO:0007744|PubMed:23576753"
FT   MOD_RES         295
FT                   /note="N6-acetyllysine"
FT                   /evidence="ECO:0000250|UniProtKB:P40939"
FT   MOD_RES         303
FT                   /note="N6-acetyllysine; alternate"
FT                   /evidence="ECO:0007744|PubMed:23576753"
FT   MOD_RES         303
FT                   /note="N6-succinyllysine; alternate"
FT                   /evidence="ECO:0007744|PubMed:23806337"
FT   MOD_RES         316
FT                   /note="Phosphoserine"
FT                   /evidence="ECO:0007744|PubMed:21183079"
FT   MOD_RES         326
FT                   /note="N6-acetyllysine; alternate"
FT                   /evidence="ECO:0007744|PubMed:23576753"
FT   MOD_RES         326
FT                   /note="N6-succinyllysine; alternate"
FT                   /evidence="ECO:0007744|PubMed:23806337"
FT   MOD_RES         334
FT                   /note="N6-acetyllysine; alternate"
FT                   /evidence="ECO:0007744|PubMed:23576753"
FT   MOD_RES         334
FT                   /note="N6-succinyllysine; alternate"
FT                   /evidence="ECO:0007744|PubMed:23806337"
FT   MOD_RES         350
FT                   /note="N6-acetyllysine; alternate"
FT                   /evidence="ECO:0007744|PubMed:23576753"
FT   MOD_RES         350
FT                   /note="N6-succinyllysine; alternate"
FT                   /evidence="ECO:0007744|PubMed:23806337"
FT   MOD_RES         353
FT                   /note="N6-acetyllysine"
FT                   /evidence="ECO:0007744|PubMed:23576753"
FT   MOD_RES         395
FT                   /note="Phosphothreonine"
FT                   /evidence="ECO:0007744|PubMed:21183079"
FT   MOD_RES         399
FT                   /note="Omega-N-methylarginine"
FT                   /evidence="ECO:0007744|PubMed:24129315"
FT   MOD_RES         406
FT                   /note="N6-acetyllysine; alternate"
FT                   /evidence="ECO:0007744|PubMed:23576753,
FT                   ECO:0007744|PubMed:23806337"
FT   MOD_RES         406
FT                   /note="N6-succinyllysine; alternate"
FT                   /evidence="ECO:0007744|PubMed:23806337"
FT   MOD_RES         411
FT                   /note="N6-acetyllysine; alternate"
FT                   /evidence="ECO:0007744|PubMed:23576753"
FT   MOD_RES         411
FT                   /note="N6-succinyllysine; alternate"
FT                   /evidence="ECO:0007744|PubMed:23806337"
FT   MOD_RES         415
FT                   /note="N6-succinyllysine"
FT                   /evidence="ECO:0007744|PubMed:23806337"
FT   MOD_RES         419
FT                   /note="Phosphoserine"
FT                   /evidence="ECO:0000250|UniProtKB:Q64428"
FT   MOD_RES         436
FT                   /note="N6-acetyllysine; alternate"
FT                   /evidence="ECO:0007744|PubMed:23576753"
FT   MOD_RES         436
FT                   /note="N6-succinyllysine; alternate"
FT                   /evidence="ECO:0007744|PubMed:23806337"
FT   MOD_RES         440
FT                   /note="N6-succinyllysine"
FT                   /evidence="ECO:0007744|PubMed:23806337"
FT   MOD_RES         460
FT                   /note="N6-acetyllysine; alternate"
FT                   /evidence="ECO:0007744|PubMed:23576753"
FT   MOD_RES         460
FT                   /note="N6-succinyllysine; alternate"
FT                   /evidence="ECO:0007744|PubMed:23806337"
FT   MOD_RES         505
FT                   /note="N6-acetyllysine; alternate"
FT                   /evidence="ECO:0007744|PubMed:23576753"
FT   MOD_RES         505
FT                   /note="N6-succinyllysine; alternate"
FT                   /evidence="ECO:0007744|PubMed:23806337"
FT   MOD_RES         519
FT                   /note="N6-acetyllysine; alternate"
FT                   /evidence="ECO:0007744|PubMed:23576753"
FT   MOD_RES         519
FT                   /note="N6-succinyllysine; alternate"
FT                   /evidence="ECO:0007744|PubMed:23806337"
FT   MOD_RES         540
FT                   /note="N6-acetyllysine"
FT                   /evidence="ECO:0007744|PubMed:23576753"
FT   MOD_RES         569
FT                   /note="N6-acetyllysine; alternate"
FT                   /evidence="ECO:0007744|PubMed:23576753"
FT   MOD_RES         569
FT                   /note="N6-succinyllysine; alternate"
FT                   /evidence="ECO:0007744|PubMed:23806337"
FT   MOD_RES         620
FT                   /note="N6-succinyllysine"
FT                   /evidence="ECO:0007744|PubMed:23806337"
FT   MOD_RES         634
FT                   /note="N6-succinyllysine"
FT                   /evidence="ECO:0007744|PubMed:23806337"
FT   MOD_RES         644
FT                   /note="N6-acetyllysine; alternate"
FT                   /evidence="ECO:0007744|PubMed:23576753"
FT   MOD_RES         644
FT                   /note="N6-succinyllysine; alternate"
FT                   /evidence="ECO:0007744|PubMed:23806337"
FT   MOD_RES         646
FT                   /note="N6-succinyllysine"
FT                   /evidence="ECO:0007744|PubMed:23806337"
FT   MOD_RES         647
FT                   /note="Phosphoserine"
FT                   /evidence="ECO:0007744|PubMed:21183079"
FT   MOD_RES         650
FT                   /note="Phosphoserine"
FT                   /evidence="ECO:0000250|UniProtKB:Q64428"
FT   MOD_RES         664
FT                   /note="N6-acetyllysine; alternate"
FT                   /evidence="ECO:0007744|PubMed:23576753"
FT   MOD_RES         664
FT                   /note="N6-succinyllysine; alternate"
FT                   /evidence="ECO:0007744|PubMed:23806337"
FT   MOD_RES         728
FT                   /note="N6-acetyllysine; alternate"
FT                   /evidence="ECO:0007744|PubMed:23576753"
FT   MOD_RES         728
FT                   /note="N6-succinyllysine; alternate"
FT                   /evidence="ECO:0007744|PubMed:23806337"
FT   MOD_RES         735
FT                   /note="N6-acetyllysine"
FT                   /evidence="ECO:0007744|PubMed:23576753"
FT   MOD_RES         759
FT                   /note="N6-acetyllysine; alternate"
FT                   /evidence="ECO:0007744|PubMed:23576753"
FT   MOD_RES         759
FT                   /note="N6-succinyllysine; alternate"
FT                   /evidence="ECO:0007744|PubMed:23806337"
FT   CONFLICT        196
FT                   /note="A -> D (in Ref. 1; BAE41822)"
FT                   /evidence="ECO:0000305"
FT   CONFLICT        459
FT                   /note="L -> S (in Ref. 2; AAH37009)"
FT                   /evidence="ECO:0000305"
SQ   SEQUENCE   763 AA;  82670 MW;  73D203795D5C1141 CRC64;
     MVASRAIGSL SRFSAFRILR SRGCICRSFT TSSALLTRTH INYGVKGDVA VIRINSPNSK
     VNTLNKEVQS EFIEVMNEIW ANDQIRSAVL ISSKPGCFVA GADINMLSSC TTPQEATRIS
     QEGQRMFEKL EKSPKPVVAA ISGSCLGGGL ELAIACQYRI ATKDRKTVLG VPEVLLGILP
     GAGGTQRLPK MVGVPAAFDM MLTGRNIRAD RAKKMGLVDQ LVEPLGPGIK SPEERTIEYL
     EEVAVNFAKG LADRKVSAKQ SKGLVEKLTT YAMTVPFVRQ QVYKTVEEKV KKQTKGLYPA
     PLKIIDAVKA GLEQGSDAGY LAESQKFGEL ALTKESKALM GLYNGQVLCK KNKFGAPQKN
     VQQLAILGAG LMGAGIAQVS VDKGLKTLLK DTTVTGLGRG QQQVFKGLND KVKKKALTSF
     ERDSIFSNLI GQLDYKGFEK ADMVIEAVFE DLGVKHKVLK EVESVTPEHC IFASNTSALP
     INQIAAVSKR PEKVIGMHYF SPVDKMQLLE IITTDKTSKD TTASAVAVGL RQGKVIIVVK
     DGPGFYTTRC LAPMMSEVMR ILQEGVDPKK LDALTTGFGF PVGAATLADE VGVDVAQHVA
     EDLGKAFGER FGGGSVELLK QMVSKGFLGR KSGKGFYIYQ EGSKNKSLNS EMDNILANLR
     LPAKPEVSSD EDVQYRVITR FVNEAVLCLQ EGILATPAEG DIGAVFGLGF PPCLGGPFRF
     VDLYGAQKVV DRLRKYESAY GTQFTPCQLL LDHANNSSKK FYQ
//
```

**Mascot:** http://www.matrixscience.com/


HNE (K) (+156.1150)
