# Supplementary material for: A common East-Asian ALDH2 mutation causes metabolic disorders and the therapeutic effect of ALDH2 activators
Source: Nat Commun. 2023 Sep 25;14:5971. doi: 10.1038/s41467-023-41570-6 (PMC10520061; doi:10.1038/s41467-023-41570-6)
Supplement: Supplementary file 4 — Supplementary Data 1 [file 41467_2023_41570_MOESM4_ESM.zip › Table S5c/Q8BMS1/Q8BMS1_KIO-2_C97_C747.html]

Mascot Search Results: Q8BMS1
 

# MASCOT Search Results

## Protein View: Q8BMS1

### Trifunctional enzyme subunit alpha, mitochondrial OS=Mus musculus OX=10090 GN=Hadha PE=1 SV=1

|  |  |
| --- | --- |
| Database: | Mouse\_UniProt\_proteomes |
| Score: | 17298 |
| Monoisotopic mass (M_r_): | 83302 |
| Calculated pI: | 9.24 |

Sequence similarity is available as an NCBI BLAST search of Q8BMS1 against nr.

### Search parameters

|  |  |
| --- | --- |
| MS data file: | `D:\LCMSMS\2023 Users' data\230529-1\230529-1-KIO-2.raw` |
| Enzyme: | Trypsin/P: cuts C-term side of KR. |
| Fixed modifications: | Carbamidomethyl (C) |
| Variable modifications: | Deamidated (NQ), HNE (C), HNE (H), HNE (K), Oxidation (M) |

### Protein sequence coverage: 69%

Matched peptides shown in ***bold red***.

|  |  |  |  |  |  |
| --- | --- | --- | --- | --- | --- |
| `1` | `MVASRAIGSL` | `SRFSAFRILR` | `SRGCICRSFT` | `TSSALLTRTH` | `INYGVKGDVA` |
| `51` | `VIRINSPNSK` | `VNTLNKEVQS` | `EFIEVMNEIW` | `ANDQIRSAVL` | `ISSKPGCFVA` |
| `101` | `GADINMLSSC` | `TTPQEATRIS` | `QEGQRMFEKL` | `EKSPKPVVAA` | `ISGSCLGGGL` |
| `151` | `ELAIACQYRI` | `ATKDRKTVLG` | `VPEVLLGILP` | `GAGGTQRLPK` | `MVGVPAAFDM` |
| `201` | `MLTGRNIRAD` | `RAKKMGLVDQ` | `LVEPLGPGIK` | `SPEERTIEYL` | `EEVAVNFAKG` |
| `251` | `LADRKVSAKQ` | `SKGLVEKLTT` | `YAMTVPFVRQ` | `QVYKTVEEKV` | `KKQTKGLYPA` |
| `301` | `PLKIIDAVKA` | `GLEQGSDAGY` | `LAESQKFGEL` | `ALTKESKALM` | `GLYNGQVLCK` |
| `351` | `KNKFGAPQKN` | `VQQLAILGAG` | `LMGAGIAQVS` | `VDKGLKTLLK` | `DTTVTGLGRG` |
| `401` | `QQQVFKGLND` | `KVKKKALTSF` | `ERDSIFSNLI` | `GQLDYKGFEK` | `ADMVIEAVFE` |
| `451` | `DLGVKHKVLK` | `EVESVTPEHC` | `IFASNTSALP` | `INQIAAVSKR` | `PEKVIGMHYF` |
| `501` | `SPVDKMQLLE` | `IITTDKTSKD` | `TTASAVAVGL` | `RQGKVIIVVK` | `DGPGFYTTRC` |
| `551` | `LAPMMSEVMR` | `ILQEGVDPKK` | `LDALTTGFGF` | `PVGAATLADE` | `VGVDVAQHVA` |
| `601` | `EDLGKAFGER` | `FGGGSVELLK` | `QMVSKGFLGR` | `KSGKGFYIYQ` | `EGSKNKSLNS` |
| `651` | `EMDNILANLR` | `LPAKPEVSSD` | `EDVQYRVITR` | `FVNEAVLCLQ` | `EGILATPAEG` |
| `701` | `DIGAVFGLGF` | `PPCLGGPFRF` | `VDLYGAQKVV` | `DRLRKYESAY` | `GTQFTPCQLL` |
| `751` | `LDHANNSSKK` | `FYQ` |  |  |  |

Unformatted sequence string: 763 residues (for pasting into other applications).

|  |  |  |  |
| --- | --- | --- | --- |
| Sort by | residue number | increasing mass | decreasing mass |
| Show | matched peptides only | predicted peptides also |  |

| Query | Start | – | End | Observed | Mr(expt) | Mr(calc) | ppm | M | Score | Expect | Rank | U | Peptide |
| --- | --- | --- | --- | --- | --- | --- | --- | --- | --- | --- | --- | --- | --- |
| 10408 | 39 | – | 46 | 466.2535 | 930.4925 | 930.4923 | 0.20 | 0 | 38 | 0.0015 | 1Score **> 31** indicates **identity** Score **> 23** indicates **homology** | U | R.THINYGVK.G |
| 10416 | 39 | – | 46 | 466.2542 | 930.4939 | 930.4923 | 1.73 | 0 | 38 | 0.0012 | 1Score **> 32** indicates **identity** Score **> 21** indicates **homology** | U | R.THINYGVK.G |
| 86877 | 39 | – | 53 | 411.2294 | 1640.8884 | 1640.8998 | -6.97 | 1 | 19 | 0.017 | 1Score **> 35** indicates **identity** Score **> 14** indicates **homology** | U | R.THINYGVKGDVAVIR.I |
| 86901 | 39 | – | 53 | 411.2317 | 1640.8975 | 1640.8998 | -1.41 | 1 | 20 | 0.015 | 1Score **> 35** indicates **identity** Score **> 14** indicates **homology** | U | R.THINYGVKGDVAVIR.I |
| 86902 | 39 | – | 53 | 411.2317 | 1640.8977 | 1640.8998 | -1.28 | 1 | 23 | 0.0074 | 1Score **> 35** indicates **identity** Score **> 14** indicates **homology** | U | R.THINYGVKGDVAVIR.I |
| 86906 | 39 | – | 53 | 547.9739 | 1640.8997 | 1640.8998 | -0.056 | 1 | 22 | 0.0095 | 1Score **> 35** indicates **identity** Score **> 14** indicates **homology** | U | R.THINYGVKGDVAVIR.I |
| 86907 | 39 | – | 53 | 547.9739 | 1640.8998 | 1640.8998 | -0.025 | 1 | 64 | 1e-06 | 1Score **> 35** indicates **identity** Score **> 17** indicates **homology** | U | R.THINYGVKGDVAVIR.I |
| 86908 | 39 | – | 53 | 547.9739 | 1640.8999 | 1640.8998 | 0.059 | 1 | 58 | 3.8e-06 | 1Score **> 35** indicates **identity** Score **> 16** indicates **homology** | U | R.THINYGVKGDVAVIR.I |
| 86909 | 39 | – | 53 | 547.9739 | 1640.9000 | 1640.8998 | 0.066 | 1 | 57 | 4.3e-06 | 1Score **> 35** indicates **identity** Score **> 16** indicates **homology** | U | R.THINYGVKGDVAVIR.I |
| 86912 | 39 | – | 53 | 547.9741 | 1640.9003 | 1640.8998 | 0.30 | 1 | 35 | 0.00048 | 1Score **> 35** indicates **identity** Score **> 15** indicates **homology** | U | R.THINYGVKGDVAVIR.I |
| 86913 | 39 | – | 53 | 547.9741 | 1640.9005 | 1640.8998 | 0.40 | 1 | 65 | 8.9e-07 | 1Score **> 35** indicates **identity** Score **> 17** indicates **homology** | U | R.THINYGVKGDVAVIR.I |
| 86914 | 39 | – | 53 | 547.9742 | 1640.9007 | 1640.8998 | 0.52 | 1 | 20 | 0.015 | 1Score **> 35** indicates **identity** Score **> 14** indicates **homology** | U | R.THINYGVKGDVAVIR.I |
| 86916 | 39 | – | 53 | 547.9744 | 1640.9013 | 1640.8998 | 0.92 | 1 | 27 | 0.0033 | 1Score **> 34** indicates **identity** Score **> 14** indicates **homology** | U | R.THINYGVKGDVAVIR.I |
| 86918 | 39 | – | 53 | 547.9746 | 1640.9019 | 1640.8998 | 1.26 | 1 | 64 | 9.5e-07 | 1Score **> 34** indicates **identity** Score **> 17** indicates **homology** | U | R.THINYGVKGDVAVIR.I |
| 86919 | 39 | – | 53 | 547.9747 | 1640.9022 | 1640.8998 | 1.42 | 1 | 66 | 7e-07 | 1Score **> 34** indicates **identity** Score **> 17** indicates **homology** | U | R.THINYGVKGDVAVIR.I |
| 86922 | 39 | – | 53 | 547.9750 | 1640.9031 | 1640.8998 | 1.98 | 1 | 24 | 0.0097 | 1Score **> 34** indicates **identity** Score **> 17** indicates **homology** | U | R.THINYGVKGDVAVIR.I |
| 86926 | 39 | – | 53 | 411.2333 | 1640.9043 | 1640.8998 | 2.71 | 1 | 19 | 0.015 | 1Score **> 34** indicates **identity** Score **> 14** indicates **homology** | U | R.THINYGVKGDVAVIR.I |
| 1105 | 47 | – | 53 | 365.2163 | 728.4180 | 728.4181 | -0.17 | 0 | 29 | 0.015 | 1Score **> 23** indicates **identity** | U | K.GDVAVIR.I |
| 1106 | 47 | – | 53 | 365.2164 | 728.4182 | 728.4181 | 0.11 | 0 | 50 | 0.0001 | 1Score **> 23** indicates **identity** | U | K.GDVAVIR.I |
| 197419 | 54 | – | 86 | 807.2058 | 4030.9927 | 4031.0309 | -9.49 | 2 | 34 | 0.0012 | 1Score **> 37** indicates **identity** Score **> 17** indicates **homology** | U | R.INSPNSKVNTLNKEVQSEFIEVMNEIWANDQIR.S  + HNE (K); Oxidation (M) |
| 189868 | 61 | – | 86 | 1097.8817 | 3290.6233 | 3290.6493 | -7.90 | 1 | 28 | 0.0025 | 1Score **> 38** indicates **identity** Score **> 14** indicates **homology** | U | K.VNTLNKEVQSEFIEVMNEIWANDQIR.S  + HNE (K); Oxidation (M) |
| 189885 | 61 | – | 86 | 823.9096 | 3291.6093 | 3291.6333 | -7.27 | 1 | 29 | 0.0018 | 1Score **> 37** indicates **identity** Score **> 14** indicates **homology** | U | K.VNTLNKEVQSEFIEVMNEIWANDQIR.S  + Deamidated (NQ); HNE (K); Oxidation (M) |
| 191170 | 87 | – | 118 | 1123.5500 | 3367.6282 | 3367.6210 | 2.12 | 1 | 54 | 8e-06 | 1Score **> 37** indicates **identity** Score **> 16** indicates **homology** | U | R.SAVLISSKPGCFVAGADINMLSSCTTPQEATR.I |
| 191171 | 87 | – | 118 | 1123.5516 | 3367.6329 | 3367.6210 | 3.52 | 1 | 29 | 0.0028 | 1Score **> 37** indicates **identity** Score **> 16** indicates **homology** | U | R.SAVLISSKPGCFVAGADINMLSSCTTPQEATR.I |
| 191172 | 87 | – | 118 | 1123.5522 | 3367.6349 | 3367.6210 | 4.12 | 1 | 59 | 2.7e-06 | 1Score **> 37** indicates **identity** Score **> 16** indicates **homology** | U | R.SAVLISSKPGCFVAGADINMLSSCTTPQEATR.I |
| 191173 | 87 | – | 118 | 1123.5536 | 3367.6389 | 3367.6210 | 5.29 | 1 | 83 | 1.5e-08 | 1Score **> 37** indicates **identity** Score **> 18** indicates **homology** | U | R.SAVLISSKPGCFVAGADINMLSSCTTPQEATR.I |
| 191174 | 87 | – | 118 | 1123.5557 | 3367.6452 | 3367.6210 | 7.17 | 1 | 107 | 9e-11 | 1Score **> 37** indicates **identity** Score **> 19** indicates **homology** | U | R.SAVLISSKPGCFVAGADINMLSSCTTPQEATR.I |
| 191175 | 87 | – | 118 | 1123.5564 | 3367.6472 | 3367.6210 | 7.78 | 1 | 75 | 9.2e-08 | 1Score **> 37** indicates **identity** Score **> 17** indicates **homology** | U | R.SAVLISSKPGCFVAGADINMLSSCTTPQEATR.I |
| 191176 | 87 | – | 118 | 842.9197 | 3367.6496 | 3367.6210 | 8.48 | 1 | 24 | 0.0057 | 1Score **> 37** indicates **identity** Score **> 14** indicates **homology** | U | R.SAVLISSKPGCFVAGADINMLSSCTTPQEATR.I |
| 191177 | 87 | – | 118 | 1123.5575 | 3367.6507 | 3367.6210 | 8.82 | 1 | 42 | 0.00012 | 1Score **> 37** indicates **identity** Score **> 15** indicates **homology** | U | R.SAVLISSKPGCFVAGADINMLSSCTTPQEATR.I |
| 191185 | 87 | – | 118 | 1123.8834 | 3368.6283 | 3368.6051 | 6.91 | 1 | 44 | 8.8e-05 | 1Score **> 37** indicates **identity** Score **> 16** indicates **homology** | U | R.SAVLISSKPGCFVAGADINMLSSCTTPQEATR.I  + Deamidated (NQ) |
| 191186 | 87 | – | 118 | 1123.8834 | 3368.6284 | 3368.6051 | 6.93 | 1 | 62 | 1.7e-06 | 1Score **> 37** indicates **identity** Score **> 16** indicates **homology** | U | R.SAVLISSKPGCFVAGADINMLSSCTTPQEATR.I  + Deamidated (NQ) |
| 191187 | 87 | – | 118 | 1123.8835 | 3368.6288 | 3368.6051 | 7.04 | 1 | 46 | 4.9e-05 | 1Score **> 37** indicates **identity** Score **> 15** indicates **homology** | U | R.SAVLISSKPGCFVAGADINMLSSCTTPQEATR.I  + Deamidated (NQ) |
| 191528 | 87 | – | 118 | 1128.8803 | 3383.6190 | 3383.6160 | 0.89 | 1 | 75 | 1e-07 | 1Score **> 37** indicates **identity** Score **> 17** indicates **homology** | U | R.SAVLISSKPGCFVAGADINMLSSCTTPQEATR.I  + Oxidation (M) |
| 191529 | 87 | – | 118 | 1128.8809 | 3383.6209 | 3383.6160 | 1.46 | 1 | 85 | 1e-08 | 1Score **> 37** indicates **identity** Score **> 18** indicates **homology** | U | R.SAVLISSKPGCFVAGADINMLSSCTTPQEATR.I  + Oxidation (M) |
| 191530 | 87 | – | 118 | 1128.8810 | 3383.6213 | 3383.6160 | 1.58 | 1 | 24 | 0.0058 | 1Score **> 37** indicates **identity** Score **> 14** indicates **homology** | U | R.SAVLISSKPGCFVAGADINMLSSCTTPQEATR.I  + Oxidation (M) |
| 191536 | 87 | – | 118 | 1129.2098 | 3384.6074 | 3384.6000 | 2.21 | 1 | 59 | 2.8e-06 | 1Score **> 36** indicates **identity** Score **> 16** indicates **homology** | U | R.SAVLISSKPGCFVAGADINMLSSCTTPQEATR.I  + Deamidated (NQ); Oxidation (M) |
| 193206 | 87 | – | 118 | 1161.9014 | 3482.6824 | 3482.7095 | -7.79 | 1 | 26 | 0.0035 | 1Score **> 37** indicates **identity** Score **> 14** indicates **homology** | U | R.SAVLISSKPGCFVAGADINMLSSCTTPQEATR.I  + HNE (C); Oxidation (M) |
| 10053 | 126 | – | 132 | 462.7463 | 923.4780 | 923.4786 | -0.68 | 1 | 22 | 0.022 | 1Score **> 29** indicates **identity** Score **> 18** indicates **homology** | U | R.MFEKLEK.S |
| 186866 | 130 | – | 159 | 786.9215 | 3143.6569 | 3143.6471 | 3.13 | 2 | 36 | 0.00047 | 1Score **> 36** indicates **identity** Score **> 15** indicates **homology** | U | K.LEKSPKPVVAAISGSCLGGGLELAIACQYR.I |
| 175621 | 133 | – | 159 | 925.4759 | 2773.4060 | 2773.4255 | -7.03 | 1 | 19 | 0.016 | 1Score **> 37** indicates **identity** Score **> 14** indicates **homology** | U | K.SPKPVVAAISGSCLGGGLELAIACQYR.I |
| 175622 | 133 | – | 159 | 925.4761 | 2773.4063 | 2773.4255 | -6.91 | 1 | 35 | 0.00049 | 1Score **> 37** indicates **identity** Score **> 15** indicates **homology** | U | K.SPKPVVAAISGSCLGGGLELAIACQYR.I |
| 175625 | 133 | – | 159 | 925.4792 | 2773.4159 | 2773.4255 | -3.45 | 1 | 28 | 0.0022 | 1Score **> 37** indicates **identity** Score **> 14** indicates **homology** | U | K.SPKPVVAAISGSCLGGGLELAIACQYR.I |
| 175626 | 133 | – | 159 | 925.4793 | 2773.4162 | 2773.4255 | -3.34 | 1 | 60 | 2.4e-06 | 1Score **> 37** indicates **identity** Score **> 16** indicates **homology** | U | K.SPKPVVAAISGSCLGGGLELAIACQYR.I |
| 175627 | 133 | – | 159 | 925.4797 | 2773.4174 | 2773.4255 | -2.93 | 1 | 67 | 4.8e-07 | 1Score **> 37** indicates **identity** Score **> 17** indicates **homology** | U | K.SPKPVVAAISGSCLGGGLELAIACQYR.I |
| 175631 | 133 | – | 159 | 925.4808 | 2773.4206 | 2773.4255 | -1.75 | 1 | 62 | 1.5e-06 | 1Score **> 37** indicates **identity** Score **> 16** indicates **homology** | U | K.SPKPVVAAISGSCLGGGLELAIACQYR.I |
| 175634 | 133 | – | 159 | 925.4817 | 2773.4234 | 2773.4255 | -0.77 | 1 | 94 | 1.5e-09 | 1Score **> 37** indicates **identity** Score **> 18** indicates **homology** | U | K.SPKPVVAAISGSCLGGGLELAIACQYR.I |
| 175635 | 133 | – | 159 | 694.3632 | 2773.4239 | 2773.4255 | -0.59 | 1 | 16 | 0.033 | 1Score **> 37** indicates **identity** Score **> 13** indicates **homology** | U | K.SPKPVVAAISGSCLGGGLELAIACQYR.I |
| 175636 | 133 | – | 159 | 925.4820 | 2773.4241 | 2773.4255 | -0.50 | 1 | 26 | 0.0038 | 1Score **> 37** indicates **identity** Score **> 14** indicates **homology** | U | K.SPKPVVAAISGSCLGGGLELAIACQYR.I |
| 175637 | 133 | – | 159 | 925.4821 | 2773.4246 | 2773.4255 | -0.33 | 1 | 40 | 0.00016 | 1Score **> 37** indicates **identity** Score **> 15** indicates **homology** | U | K.SPKPVVAAISGSCLGGGLELAIACQYR.I |
| 175638 | 133 | – | 159 | 925.4821 | 2773.4246 | 2773.4255 | -0.33 | 1 | 21 | 0.012 | 1Score **> 37** indicates **identity** Score **> 14** indicates **homology** | U | K.SPKPVVAAISGSCLGGGLELAIACQYR.I |
| 175640 | 133 | – | 159 | 925.4825 | 2773.4257 | 2773.4255 | 0.062 | 1 | 73 | 1.3e-07 | 1Score **> 37** indicates **identity** Score **> 17** indicates **homology** | U | K.SPKPVVAAISGSCLGGGLELAIACQYR.I |
| 175642 | 133 | – | 159 | 694.3638 | 2773.4259 | 2773.4255 | 0.15 | 1 | 33 | 0.00076 | 1Score **> 37** indicates **identity** Score **> 15** indicates **homology** | U | K.SPKPVVAAISGSCLGGGLELAIACQYR.I |
| 175643 | 133 | – | 159 | 694.3638 | 2773.4259 | 2773.4255 | 0.16 | 1 | 30 | 0.0016 | 1Score **> 37** indicates **identity** Score **> 14** indicates **homology** | U | K.SPKPVVAAISGSCLGGGLELAIACQYR.I |
| 175645 | 133 | – | 159 | 925.4826 | 2773.4261 | 2773.4255 | 0.20 | 1 | 117 | 9.7e-12 | 1Score **> 37** indicates **identity** Score **> 20** indicates **homology** | U | K.SPKPVVAAISGSCLGGGLELAIACQYR.I |
| 175646 | 133 | – | 159 | 925.4828 | 2773.4266 | 2773.4255 | 0.41 | 1 | 67 | 7.6e-07 | 1Score **> 37** indicates **identity** Score **> 18** indicates **homology** | U | K.SPKPVVAAISGSCLGGGLELAIACQYR.I |
| 175647 | 133 | – | 159 | 694.3640 | 2773.4267 | 2773.4255 | 0.44 | 1 | 30 | 0.0015 | 1Score **> 37** indicates **identity** Score **> 14** indicates **homology** | U | K.SPKPVVAAISGSCLGGGLELAIACQYR.I |
| 175648 | 133 | – | 159 | 925.4829 | 2773.4270 | 2773.4255 | 0.54 | 1 | 46 | 9.4e-05 | 1Score **> 37** indicates **identity** Score **> 18** indicates **homology** | U | K.SPKPVVAAISGSCLGGGLELAIACQYR.I |
| 175649 | 133 | – | 159 | 925.4829 | 2773.4270 | 2773.4255 | 0.55 | 1 | 82 | 1.8e-08 | 1Score **> 37** indicates **identity** Score **> 18** indicates **homology** | U | K.SPKPVVAAISGSCLGGGLELAIACQYR.I |
| 175651 | 133 | – | 159 | 694.3641 | 2773.4273 | 2773.4255 | 0.66 | 1 | 48 | 3.4e-05 | 1Score **> 37** indicates **identity** Score **> 15** indicates **homology** | U | K.SPKPVVAAISGSCLGGGLELAIACQYR.I |
| 175652 | 133 | – | 159 | 925.4831 | 2773.4274 | 2773.4255 | 0.68 | 1 | 101 | 3.5e-10 | 1Score **> 37** indicates **identity** Score **> 19** indicates **homology** | U | K.SPKPVVAAISGSCLGGGLELAIACQYR.I |
| 175655 | 133 | – | 159 | 925.4832 | 2773.4277 | 2773.4255 | 0.79 | 1 | 102 | 2.7e-10 | 1Score **> 37** indicates **identity** Score **> 19** indicates **homology** | U | K.SPKPVVAAISGSCLGGGLELAIACQYR.I |
| 175656 | 133 | – | 159 | 1387.7213 | 2773.4280 | 2773.4255 | 0.89 | 1 | 24 | 0.0061 | 1Score **> 37** indicates **identity** Score **> 14** indicates **homology** | U | K.SPKPVVAAISGSCLGGGLELAIACQYR.I |
| 175659 | 133 | – | 159 | 925.4834 | 2773.4284 | 2773.4255 | 1.05 | 1 | 93 | 2e-09 | 1Score **> 37** indicates **identity** Score **> 18** indicates **homology** | U | K.SPKPVVAAISGSCLGGGLELAIACQYR.I |
| 175660 | 133 | – | 159 | 925.4834 | 2773.4285 | 2773.4255 | 1.08 | 1 | 88 | 5.1e-09 | 1Score **> 37** indicates **identity** Score **> 18** indicates **homology** | U | K.SPKPVVAAISGSCLGGGLELAIACQYR.I |
| 175661 | 133 | – | 159 | 925.4835 | 2773.4286 | 2773.4255 | 1.13 | 1 | 41 | 0.00014 | 1Score **> 37** indicates **identity** Score **> 15** indicates **homology** | U | K.SPKPVVAAISGSCLGGGLELAIACQYR.I |
| 175663 | 133 | – | 159 | 694.3646 | 2773.4292 | 2773.4255 | 1.33 | 1 | 15 | 0.043 | 1Score **> 37** indicates **identity** Score **> 13** indicates **homology** | U | K.SPKPVVAAISGSCLGGGLELAIACQYR.I |
| 175664 | 133 | – | 159 | 925.4837 | 2773.4292 | 2773.4255 | 1.34 | 1 | 92 | 2.1e-09 | 1Score **> 37** indicates **identity** Score **> 18** indicates **homology** | U | K.SPKPVVAAISGSCLGGGLELAIACQYR.I |
| 175665 | 133 | – | 159 | 925.4837 | 2773.4292 | 2773.4255 | 1.34 | 1 | 53 | 1.1e-05 | 1Score **> 37** indicates **identity** Score **> 16** indicates **homology** | U | K.SPKPVVAAISGSCLGGGLELAIACQYR.I |
| 175666 | 133 | – | 159 | 925.4839 | 2773.4298 | 2773.4255 | 1.54 | 1 | 108 | 6.8e-11 | 1Score **> 37** indicates **identity** Score **> 19** indicates **homology** | U | K.SPKPVVAAISGSCLGGGLELAIACQYR.I |
| 175668 | 133 | – | 159 | 925.4839 | 2773.4298 | 2773.4255 | 1.56 | 1 | 80 | 3.5e-08 | 1Score **> 37** indicates **identity** Score **> 17** indicates **homology** | U | K.SPKPVVAAISGSCLGGGLELAIACQYR.I |
| 175670 | 133 | – | 159 | 925.4841 | 2773.4305 | 2773.4255 | 1.81 | 1 | 57 | 4.9e-06 | 1Score **> 37** indicates **identity** Score **> 16** indicates **homology** | U | K.SPKPVVAAISGSCLGGGLELAIACQYR.I |
| 175671 | 133 | – | 159 | 925.4841 | 2773.4306 | 2773.4255 | 1.83 | 1 | 46 | 5e-05 | 1Score **> 37** indicates **identity** Score **> 15** indicates **homology** | U | K.SPKPVVAAISGSCLGGGLELAIACQYR.I |
| 175672 | 133 | – | 159 | 925.4843 | 2773.4309 | 2773.4255 | 1.96 | 1 | 16 | 0.034 | 1Score **> 37** indicates **identity** Score **> 13** indicates **homology** | U | K.SPKPVVAAISGSCLGGGLELAIACQYR.I |
| 175675 | 133 | – | 159 | 925.4845 | 2773.4316 | 2773.4255 | 2.22 | 1 | 54 | 1.2e-05 | 1Score **> 37** indicates **identity** Score **> 17** indicates **homology** | U | K.SPKPVVAAISGSCLGGGLELAIACQYR.I |
| 175676 | 133 | – | 159 | 925.4845 | 2773.4318 | 2773.4255 | 2.28 | 1 | 63 | 3.7e-06 | 1Score **> 37** indicates **identity** Score **> 21** indicates **homology** | U | K.SPKPVVAAISGSCLGGGLELAIACQYR.I |
| 175677 | 133 | – | 159 | 925.4847 | 2773.4322 | 2773.4255 | 2.41 | 1 | 58 | 3.9e-06 | 1Score **> 37** indicates **identity** Score **> 16** indicates **homology** | U | K.SPKPVVAAISGSCLGGGLELAIACQYR.I |
| 175678 | 133 | – | 159 | 925.4850 | 2773.4330 | 2773.4255 | 2.73 | 1 | 56 | 5.9e-06 | 1Score **> 37** indicates **identity** Score **> 16** indicates **homology** | U | K.SPKPVVAAISGSCLGGGLELAIACQYR.I |
| 175679 | 133 | – | 159 | 925.4851 | 2773.4334 | 2773.4255 | 2.87 | 1 | 50 | 4.2e-05 | 1Score **> 37** indicates **identity** Score **> 19** indicates **homology** | U | K.SPKPVVAAISGSCLGGGLELAIACQYR.I |
| 175680 | 133 | – | 159 | 925.4851 | 2773.4335 | 2773.4255 | 2.88 | 1 | 40 | 0.00017 | 1Score **> 37** indicates **identity** Score **> 15** indicates **homology** | U | K.SPKPVVAAISGSCLGGGLELAIACQYR.I |
| 175681 | 133 | – | 159 | 925.4851 | 2773.4335 | 2773.4255 | 2.89 | 1 | 47 | 4.1e-05 | 1Score **> 37** indicates **identity** Score **> 15** indicates **homology** | U | K.SPKPVVAAISGSCLGGGLELAIACQYR.I |
| 175682 | 133 | – | 159 | 925.4852 | 2773.4338 | 2773.4255 | 3.00 | 1 | 64 | 9.8e-07 | 1Score **> 37** indicates **identity** Score **> 17** indicates **homology** | U | K.SPKPVVAAISGSCLGGGLELAIACQYR.I |
| 175683 | 133 | – | 159 | 925.4853 | 2773.4340 | 2773.4255 | 3.08 | 1 | 30 | 0.0023 | 1Score **> 37** indicates **identity** Score **> 16** indicates **homology** | U | K.SPKPVVAAISGSCLGGGLELAIACQYR.I |
| 175684 | 133 | – | 159 | 925.4853 | 2773.4342 | 2773.4255 | 3.15 | 1 | 18 | 0.019 | 1Score **> 37** indicates **identity** Score **> 14** indicates **homology** | U | K.SPKPVVAAISGSCLGGGLELAIACQYR.I |
| 175685 | 133 | – | 159 | 925.4854 | 2773.4342 | 2773.4255 | 3.15 | 1 | 79 | 4e-08 | 1Score **> 37** indicates **identity** Score **> 17** indicates **homology** | U | K.SPKPVVAAISGSCLGGGLELAIACQYR.I |
| 175686 | 133 | – | 159 | 925.4854 | 2773.4345 | 2773.4255 | 3.25 | 1 | 49 | 2.5e-05 | 1Score **> 37** indicates **identity** Score **> 16** indicates **homology** | U | K.SPKPVVAAISGSCLGGGLELAIACQYR.I |
| 175687 | 133 | – | 159 | 925.4857 | 2773.4354 | 2773.4255 | 3.56 | 1 | 61 | 1.8e-06 | 1Score **> 37** indicates **identity** Score **> 16** indicates **homology** | U | K.SPKPVVAAISGSCLGGGLELAIACQYR.I |
| 175689 | 133 | – | 159 | 925.4859 | 2773.4358 | 2773.4255 | 3.73 | 1 | 21 | 0.011 | 1Score **> 37** indicates **identity** Score **> 14** indicates **homology** | U | K.SPKPVVAAISGSCLGGGLELAIACQYR.I |
| 175690 | 133 | – | 159 | 925.4859 | 2773.4360 | 2773.4255 | 3.79 | 1 | 46 | 4.9e-05 | 1Score **> 37** indicates **identity** Score **> 15** indicates **homology** | U | K.SPKPVVAAISGSCLGGGLELAIACQYR.I |
| 175691 | 133 | – | 159 | 925.4860 | 2773.4362 | 2773.4255 | 3.87 | 1 | 17 | 0.023 | 1Score **> 37** indicates **identity** Score **> 14** indicates **homology** | U | K.SPKPVVAAISGSCLGGGLELAIACQYR.I |
| 175694 | 133 | – | 159 | 925.4862 | 2773.4369 | 2773.4255 | 4.12 | 1 | 44 | 7.3e-05 | 1Score **> 37** indicates **identity** Score **> 15** indicates **homology** | U | K.SPKPVVAAISGSCLGGGLELAIACQYR.I |
| 175695 | 133 | – | 159 | 1387.7262 | 2773.4378 | 2773.4255 | 4.42 | 1 | 41 | 0.00013 | 1Score **> 37** indicates **identity** Score **> 15** indicates **homology** | U | K.SPKPVVAAISGSCLGGGLELAIACQYR.I |
| 175696 | 133 | – | 159 | 925.4866 | 2773.4381 | 2773.4255 | 4.54 | 1 | 37 | 0.00036 | 1Score **> 37** indicates **identity** Score **> 15** indicates **homology** | U | K.SPKPVVAAISGSCLGGGLELAIACQYR.I |
| 175698 | 133 | – | 159 | 925.4871 | 2773.4395 | 2773.4255 | 5.06 | 1 | 49 | 2.7e-05 | 1Score **> 37** indicates **identity** Score **> 16** indicates **homology** | U | K.SPKPVVAAISGSCLGGGLELAIACQYR.I |
| 175700 | 133 | – | 159 | 925.4879 | 2773.4420 | 2773.4255 | 5.96 | 1 | 52 | 1.4e-05 | 1Score **> 37** indicates **identity** Score **> 16** indicates **homology** | U | K.SPKPVVAAISGSCLGGGLELAIACQYR.I |
| 175701 | 133 | – | 159 | 925.4884 | 2773.4434 | 2773.4255 | 6.44 | 1 | 58 | 3.8e-06 | 1Score **> 37** indicates **identity** Score **> 16** indicates **homology** | U | K.SPKPVVAAISGSCLGGGLELAIACQYR.I |
| 175704 | 133 | – | 159 | 925.4898 | 2773.4476 | 2773.4255 | 7.97 | 1 | 70 | 2.9e-07 | 1Score **> 37** indicates **identity** Score **> 17** indicates **homology** | U | K.SPKPVVAAISGSCLGGGLELAIACQYR.I |
| 175705 | 133 | – | 159 | 925.4899 | 2773.4477 | 2773.4255 | 8.02 | 1 | 38 | 0.00027 | 1Score **> 37** indicates **identity** Score **> 15** indicates **homology** | U | K.SPKPVVAAISGSCLGGGLELAIACQYR.I |
| 175706 | 133 | – | 159 | 925.4899 | 2773.4478 | 2773.4255 | 8.04 | 1 | 28 | 0.0028 | 1Score **> 37** indicates **identity** Score **> 15** indicates **homology** | U | K.SPKPVVAAISGSCLGGGLELAIACQYR.I |
| 175707 | 133 | – | 159 | 925.4905 | 2773.4495 | 2773.4255 | 8.67 | 1 | 44 | 7e-05 | 1Score **> 37** indicates **identity** Score **> 15** indicates **homology** | U | K.SPKPVVAAISGSCLGGGLELAIACQYR.I |
| 175708 | 133 | – | 159 | 925.4906 | 2773.4500 | 2773.4255 | 8.86 | 1 | 20 | 0.014 | 1Score **> 37** indicates **identity** Score **> 14** indicates **homology** | U | K.SPKPVVAAISGSCLGGGLELAIACQYR.I |
| 175709 | 133 | – | 159 | 925.4908 | 2773.4505 | 2773.4255 | 9.02 | 1 | 30 | 0.0034 | 1Score **> 37** indicates **identity** Score **> 18** indicates **homology** | U | K.SPKPVVAAISGSCLGGGLELAIACQYR.I |
| 175710 | 133 | – | 159 | 925.4910 | 2773.4513 | 2773.4255 | 9.31 | 1 | 59 | 3.2e-06 | 1Score **> 37** indicates **identity** Score **> 16** indicates **homology** | U | K.SPKPVVAAISGSCLGGGLELAIACQYR.I |
| 175711 | 133 | – | 159 | 925.4911 | 2773.4514 | 2773.4255 | 9.33 | 1 | 30 | 0.0067 | 1Score **> 37** indicates **identity** Score **> 21** indicates **homology** | U | K.SPKPVVAAISGSCLGGGLELAIACQYR.I |
| 175713 | 133 | – | 159 | 925.4916 | 2773.4530 | 2773.4255 | 9.93 | 1 | 71 | 2.3e-07 | 1Score **> 37** indicates **identity** Score **> 17** indicates **homology** | U | K.SPKPVVAAISGSCLGGGLELAIACQYR.I |
| 175766 | 133 | – | 159 | 925.8172 | 2774.4298 | 2774.4095 | 7.31 | 1 | 26 | 0.004 | 1Score **> 37** indicates **identity** Score **> 14** indicates **homology** | U | K.SPKPVVAAISGSCLGGGLELAIACQYR.I  + Deamidated (NQ) |
| 175769 | 133 | – | 159 | 925.8184 | 2774.4334 | 2774.4095 | 8.62 | 1 | 16 | 0.034 | 1Score **> 37** indicates **identity** Score **> 13** indicates **homology** | U | K.SPKPVVAAISGSCLGGGLELAIACQYR.I  + Deamidated (NQ) |
| 175772 | 133 | – | 159 | 925.8188 | 2774.4347 | 2774.4095 | 9.09 | 1 | 19 | 0.016 | 1Score **> 37** indicates **identity** Score **> 14** indicates **homology** | U | K.SPKPVVAAISGSCLGGGLELAIACQYR.I  + Deamidated (NQ) |
| 141989 | 166 | – | 187 | 725.7663 | 2174.2770 | 2174.2787 | -0.77 | 1 | 20 | 0.015 | 1Score **> 30** indicates **identity** Score **> 14** indicates **homology** | U | R.KTVLGVPEVLLGILPGAGGTQR.L |
| 141991 | 166 | – | 187 | 725.7673 | 2174.2801 | 2174.2787 | 0.66 | 1 | 47 | 3.8e-05 | 1Score **> 29** indicates **identity** Score **> 15** indicates **homology** | U | R.KTVLGVPEVLLGILPGAGGTQR.L |
| 141992 | 166 | – | 187 | 1088.1486 | 2174.2826 | 2174.2787 | 1.78 | 1 | 64 | 9e-07 | 1Score **> 29** indicates **identity** Score **> 17** indicates **homology** | U | R.KTVLGVPEVLLGILPGAGGTQR.L |
| 164596 | 166 | – | 190 | 629.1350 | 2512.5110 | 2512.5105 | 0.19 | 2 | 24 | 0.005 | 1Score **> 24** indicates **identity** Score **> 14** indicates **homology** | U | R.KTVLGVPEVLLGILPGAGGTQRLPK.M |
| 164597 | 166 | – | 190 | 629.1370 | 2512.5188 | 2512.5105 | 3.32 | 2 | 30 | 0.0015 | 1Score **> 24** indicates **identity** Score **> 14** indicates **homology** | U | R.KTVLGVPEVLLGILPGAGGTQRLPK.M |
| 164598 | 166 | – | 190 | 838.5137 | 2512.5192 | 2512.5105 | 3.47 | 2 | 46 | 5e-05 | 1Score **> 24** indicates **identity** Score **> 15** indicates **homology** | U | R.KTVLGVPEVLLGILPGAGGTQRLPK.M |
| 130602 | 167 | – | 187 | 683.0683 | 2046.1831 | 2046.1837 | -0.29 | 0 | 47 | 3.5e-05 | 1Score **> 31** indicates **identity** Score **> 15** indicates **homology** | U | K.TVLGVPEVLLGILPGAGGTQR.L |
| 130608 | 167 | – | 187 | 683.0687 | 2046.1842 | 2046.1837 | 0.20 | 0 | 42 | 0.0001 | 1Score **> 31** indicates **identity** Score **> 15** indicates **homology** | U | K.TVLGVPEVLLGILPGAGGTQR.L |
| 130610 | 167 | – | 187 | 1024.0995 | 2046.1845 | 2046.1837 | 0.36 | 0 | 65 | 7.3e-07 | 1Score **> 31** indicates **identity** Score **> 17** indicates **homology** | U | K.TVLGVPEVLLGILPGAGGTQR.L |
| 130611 | 167 | – | 187 | 1024.0996 | 2046.1846 | 2046.1837 | 0.42 | 0 | 34 | 0.00069 | 1Score **> 31** indicates **identity** Score **> 15** indicates **homology** | U | K.TVLGVPEVLLGILPGAGGTQR.L |
| 130614 | 167 | – | 187 | 683.0698 | 2046.1877 | 2046.1837 | 1.91 | 0 | 56 | 6.1e-06 | 1Score **> 31** indicates **identity** Score **> 16** indicates **homology** | U | K.TVLGVPEVLLGILPGAGGTQR.L |
| 130615 | 167 | – | 187 | 1024.1012 | 2046.1878 | 2046.1837 | 1.99 | 0 | 72 | 1.7e-07 | 1Score **> 31** indicates **identity** Score **> 17** indicates **homology** | U | K.TVLGVPEVLLGILPGAGGTQR.L |
| 130616 | 167 | – | 187 | 1024.1012 | 2046.1878 | 2046.1837 | 1.99 | 0 | 32 | 0.0027 | 1Score **> 31** indicates **identity** Score **> 18** indicates **homology** | U | K.TVLGVPEVLLGILPGAGGTQR.L |
| 130618 | 167 | – | 187 | 1024.1018 | 2046.1891 | 2046.1837 | 2.60 | 0 | 46 | 4.9e-05 | 1Score **> 31** indicates **identity** Score **> 15** indicates **homology** | U | K.TVLGVPEVLLGILPGAGGTQR.L |
| 130619 | 167 | – | 187 | 683.0705 | 2046.1898 | 2046.1837 | 2.97 | 0 | 20 | 0.013 | 1Score **> 31** indicates **identity** Score **> 14** indicates **homology** | U | K.TVLGVPEVLLGILPGAGGTQR.L |
| 130621 | 167 | – | 187 | 1024.1029 | 2046.1912 | 2046.1837 | 3.62 | 0 | 48 | 3e-05 | 1Score **> 31** indicates **identity** Score **> 16** indicates **homology** | U | K.TVLGVPEVLLGILPGAGGTQR.L |
| 130622 | 167 | – | 187 | 1024.1035 | 2046.1925 | 2046.1837 | 4.30 | 0 | 75 | 1.4e-07 | 1Score **> 31** indicates **identity** Score **> 19** indicates **homology** | U | K.TVLGVPEVLLGILPGAGGTQR.L |
| 130624 | 167 | – | 187 | 683.0720 | 2046.1941 | 2046.1837 | 5.07 | 0 | 34 | 0.0007 | 1Score **> 30** indicates **identity** Score **> 15** indicates **homology** | U | K.TVLGVPEVLLGILPGAGGTQR.L |
| 130625 | 167 | – | 187 | 683.0722 | 2046.1947 | 2046.1837 | 5.34 | 0 | 49 | 2.7e-05 | 1Score **> 30** indicates **identity** Score **> 16** indicates **homology** | U | K.TVLGVPEVLLGILPGAGGTQR.L |
| 130626 | 167 | – | 187 | 1024.1093 | 2046.2041 | 2046.1837 | 9.95 | 0 | 30 | 0.0019 | 1Score **> 30** indicates **identity** Score **> 16** indicates **homology** | U | K.TVLGVPEVLLGILPGAGGTQR.L |
| 157133 | 167 | – | 190 | 795.8087 | 2384.4043 | 2384.4155 | -4.73 | 1 | 32 | 0.0011 | 1Score **> 29** indicates **identity** Score **> 14** indicates **homology** | U | K.TVLGVPEVLLGILPGAGGTQRLPK.M |
| 157134 | 167 | – | 190 | 1193.2106 | 2384.4067 | 2384.4155 | -3.71 | 1 | 24 | 0.0062 | 1Score **> 29** indicates **identity** Score **> 14** indicates **homology** | U | K.TVLGVPEVLLGILPGAGGTQRLPK.M |
| 157135 | 167 | – | 190 | 795.8103 | 2384.4092 | 2384.4155 | -2.67 | 1 | 26 | 0.0037 | 1Score **> 28** indicates **identity** Score **> 14** indicates **homology** | U | K.TVLGVPEVLLGILPGAGGTQRLPK.M |
| 157136 | 167 | – | 190 | 795.8104 | 2384.4095 | 2384.4155 | -2.52 | 1 | 28 | 0.0025 | 1Score **> 28** indicates **identity** Score **> 14** indicates **homology** | U | K.TVLGVPEVLLGILPGAGGTQRLPK.M |
| 157137 | 167 | – | 190 | 597.1108 | 2384.4141 | 2384.4155 | -0.60 | 1 | 19 | 0.017 | 1Score **> 28** indicates **identity** Score **> 14** indicates **homology** | U | K.TVLGVPEVLLGILPGAGGTQRLPK.M |
| 157138 | 167 | – | 190 | 795.8124 | 2384.4153 | 2384.4155 | -0.10 | 1 | 32 | 0.00097 | 1Score **> 27** indicates **identity** Score **> 15** indicates **homology** | U | K.TVLGVPEVLLGILPGAGGTQRLPK.M |
| 157140 | 167 | – | 190 | 795.8150 | 2384.4233 | 2384.4155 | 3.27 | 1 | 20 | 0.015 | 1Score **> 27** indicates **identity** Score **> 14** indicates **homology** | U | K.TVLGVPEVLLGILPGAGGTQRLPK.M |
| 105761 | 214 | – | 230 | 598.6774 | 1793.0104 | 1793.0121 | -0.93 | 1 | 39 | 0.00022 | 1Score **> 34** indicates **identity** Score **> 15** indicates **homology** | U | K.KMGLVDQLVEPLGPGIK.S |
| 105763 | 214 | – | 230 | 598.6777 | 1793.0111 | 1793.0121 | -0.54 | 1 | 42 | 0.00012 | 1Score **> 33** indicates **identity** Score **> 15** indicates **homology** | U | K.KMGLVDQLVEPLGPGIK.S |
| 157606 | 214 | – | 235 | 598.8273 | 2391.2801 | 2391.2832 | -1.27 | 2 | 46 | 4.8e-05 | 1Score **> 36** indicates **identity** Score **> 15** indicates **homology** | U | K.KMGLVDQLVEPLGPGIKSPEER.T |
| 157607 | 214 | – | 235 | 798.1006 | 2391.2801 | 2391.2832 | -1.27 | 2 | 46 | 5.4e-05 | 1Score **> 36** indicates **identity** Score **> 15** indicates **homology** | U | K.KMGLVDQLVEPLGPGIKSPEER.T |
| 157609 | 214 | – | 235 | 598.8282 | 2391.2837 | 2391.2832 | 0.24 | 2 | 26 | 0.0038 | 1Score **> 36** indicates **identity** Score **> 14** indicates **homology** | U | K.KMGLVDQLVEPLGPGIKSPEER.T |
| 157612 | 214 | – | 235 | 598.8291 | 2391.2872 | 2391.2832 | 1.67 | 2 | 52 | 1.2e-05 | 1Score **> 36** indicates **identity** Score **> 16** indicates **homology** | U | K.KMGLVDQLVEPLGPGIKSPEER.T |
| 157620 | 214 | – | 235 | 798.1082 | 2391.3028 | 2391.2832 | 8.20 | 2 | 46 | 5.4e-05 | 1Score **> 35** indicates **identity** Score **> 15** indicates **homology** | U | K.KMGLVDQLVEPLGPGIKSPEER.T |
| 90196 | 215 | – | 230 | 833.4658 | 1664.9171 | 1664.9171 | -0.026 | 0 | 60 | 2.6e-06 | 1Score **> 34** indicates **identity** Score **> 16** indicates **homology** | U | K.MGLVDQLVEPLGPGIK.S |
| 90197 | 215 | – | 230 | 555.9798 | 1664.9176 | 1664.9171 | 0.26 | 0 | 45 | 5.6e-05 | 1Score **> 34** indicates **identity** Score **> 15** indicates **homology** | U | K.MGLVDQLVEPLGPGIK.S |
| 90198 | 215 | – | 230 | 833.4663 | 1664.9180 | 1664.9171 | 0.51 | 0 | 59 | 2.8e-06 | 1Score **> 34** indicates **identity** Score **> 16** indicates **homology** | U | K.MGLVDQLVEPLGPGIK.S |
| 90200 | 215 | – | 230 | 555.9801 | 1664.9184 | 1664.9171 | 0.77 | 0 | 49 | 2.3e-05 | 1Score **> 34** indicates **identity** Score **> 16** indicates **homology** | U | K.MGLVDQLVEPLGPGIK.S |
| 90201 | 215 | – | 230 | 833.4670 | 1664.9195 | 1664.9171 | 1.45 | 0 | 23 | 0.0063 | 1Score **> 34** indicates **identity** Score **> 14** indicates **homology** | U | K.MGLVDQLVEPLGPGIK.S |
| 92285 | 215 | – | 230 | 841.4656 | 1680.9166 | 1680.9120 | 2.69 | 0 | 19 | 0.015 | 1Score **> 34** indicates **identity** Score **> 14** indicates **homology** | U | K.MGLVDQLVEPLGPGIK.S  + Oxidation (M) |
| 92287 | 215 | – | 230 | 841.4687 | 1680.9228 | 1680.9120 | 6.39 | 0 | 17 | 0.023 | 1Score **> 34** indicates **identity** Score **> 14** indicates **homology** | U | K.MGLVDQLVEPLGPGIK.S  + Oxidation (M) |
| 149156 | 215 | – | 235 | 755.4045 | 2263.1917 | 2263.1882 | 1.53 | 1 | 32 | 0.0011 | 1Score **> 37** indicates **identity** Score **> 14** indicates **homology** | U | K.MGLVDQLVEPLGPGIKSPEER.T |
| 149157 | 215 | – | 235 | 755.4047 | 2263.1923 | 2263.1882 | 1.83 | 1 | 54 | 8.8e-06 | 1Score **> 37** indicates **identity** Score **> 16** indicates **homology** | U | K.MGLVDQLVEPLGPGIKSPEER.T |
| 149159 | 215 | – | 235 | 755.4067 | 2263.1983 | 2263.1882 | 4.47 | 1 | 54 | 9.2e-06 | 1Score **> 36** indicates **identity** Score **> 16** indicates **homology** | U | K.MGLVDQLVEPLGPGIKSPEER.T |
| 150263 | 215 | – | 235 | 760.7346 | 2279.1820 | 2279.1831 | -0.48 | 1 | 23 | 0.0091 | 1Score **> 37** indicates **identity** Score **> 15** indicates **homology** | U | K.MGLVDQLVEPLGPGIKSPEER.T  + Oxidation (M) |
| 150264 | 215 | – | 235 | 760.7349 | 2279.1829 | 2279.1831 | -0.091 | 1 | 44 | 8.1e-05 | 1Score **> 37** indicates **identity** Score **> 15** indicates **homology** | U | K.MGLVDQLVEPLGPGIKSPEER.T  + Oxidation (M) |
| 84794 | 236 | – | 249 | 813.4228 | 1624.8311 | 1624.8348 | -2.25 | 0 | 48 | 3.2e-05 | 1Score **> 35** indicates **identity** Score **> 15** indicates **homology** | U | R.TIEYLEEVAVNFAK.G |
| 84796 | 236 | – | 249 | 813.4231 | 1624.8316 | 1624.8348 | -1.97 | 0 | 32 | 0.001 | 1Score **> 35** indicates **identity** Score **> 14** indicates **homology** | U | R.TIEYLEEVAVNFAK.G |
| 84798 | 236 | – | 249 | 813.4237 | 1624.8328 | 1624.8348 | -1.23 | 0 | 60 | 2.3e-06 | 1Score **> 35** indicates **identity** Score **> 16** indicates **homology** | U | R.TIEYLEEVAVNFAK.G |
| 84799 | 236 | – | 249 | 542.6182 | 1624.8329 | 1624.8348 | -1.17 | 0 | 39 | 0.00024 | 1Score **> 35** indicates **identity** Score **> 15** indicates **homology** | U | R.TIEYLEEVAVNFAK.G |
| 84802 | 236 | – | 249 | 542.6186 | 1624.8339 | 1624.8348 | -0.58 | 0 | 48 | 3.4e-05 | 1Score **> 34** indicates **identity** Score **> 15** indicates **homology** | U | R.TIEYLEEVAVNFAK.G |
| 84806 | 236 | – | 249 | 542.6187 | 1624.8341 | 1624.8348 | -0.42 | 0 | 27 | 0.0027 | 1Score **> 34** indicates **identity** Score **> 14** indicates **homology** | U | R.TIEYLEEVAVNFAK.G |
| 84809 | 236 | – | 249 | 813.4245 | 1624.8344 | 1624.8348 | -0.24 | 0 | 63 | 1.3e-06 | 1Score **> 34** indicates **identity** Score **> 16** indicates **homology** | U | R.TIEYLEEVAVNFAK.G |
| 84810 | 236 | – | 249 | 542.6188 | 1624.8345 | 1624.8348 | -0.22 | 0 | 22 | 0.0081 | 1Score **> 34** indicates **identity** Score **> 14** indicates **homology** | U | R.TIEYLEEVAVNFAK.G |
| 84811 | 236 | – | 249 | 542.6188 | 1624.8345 | 1624.8348 | -0.22 | 0 | 62 | 1.6e-06 | 1Score **> 34** indicates **identity** Score **> 16** indicates **homology** | U | R.TIEYLEEVAVNFAK.G |
| 84812 | 236 | – | 249 | 813.4245 | 1624.8345 | 1624.8348 | -0.17 | 0 | 50 | 2.1e-05 | 1Score **> 34** indicates **identity** Score **> 16** indicates **homology** | U | R.TIEYLEEVAVNFAK.G |
| 84817 | 236 | – | 249 | 542.6188 | 1624.8347 | 1624.8348 | -0.095 | 0 | 45 | 6.1e-05 | 1Score **> 34** indicates **identity** Score **> 15** indicates **homology** | U | R.TIEYLEEVAVNFAK.G |
| 84819 | 236 | – | 249 | 813.4247 | 1624.8349 | 1624.8348 | 0.079 | 0 | 36 | 0.00042 | 1Score **> 34** indicates **identity** Score **> 15** indicates **homology** | U | R.TIEYLEEVAVNFAK.G |
| 84825 | 236 | – | 249 | 542.6191 | 1624.8354 | 1624.8348 | 0.37 | 0 | 71 | 2.4e-07 | 1Score **> 34** indicates **identity** Score **> 17** indicates **homology** | U | R.TIEYLEEVAVNFAK.G |
| 84826 | 236 | – | 249 | 813.4250 | 1624.8355 | 1624.8348 | 0.42 | 0 | 60 | 2.2e-06 | 1Score **> 34** indicates **identity** Score **> 16** indicates **homology** | U | R.TIEYLEEVAVNFAK.G |
| 84829 | 236 | – | 249 | 813.4251 | 1624.8357 | 1624.8348 | 0.57 | 0 | 64 | 1.1e-06 | 1Score **> 34** indicates **identity** Score **> 16** indicates **homology** | U | R.TIEYLEEVAVNFAK.G |
| 84830 | 236 | – | 249 | 542.6192 | 1624.8357 | 1624.8348 | 0.57 | 0 | 62 | 1.6e-06 | 1Score **> 34** indicates **identity** Score **> 16** indicates **homology** | U | R.TIEYLEEVAVNFAK.G |
| 84832 | 236 | – | 249 | 813.4253 | 1624.8361 | 1624.8348 | 0.80 | 0 | 70 | 2.7e-07 | 1Score **> 34** indicates **identity** Score **> 17** indicates **homology** | U | R.TIEYLEEVAVNFAK.G |
| 84834 | 236 | – | 249 | 813.4254 | 1624.8362 | 1624.8348 | 0.87 | 0 | 84 | 1.4e-08 | 1Score **> 34** indicates **identity** Score **> 18** indicates **homology** | U | R.TIEYLEEVAVNFAK.G |
| 84835 | 236 | – | 249 | 542.6194 | 1624.8362 | 1624.8348 | 0.88 | 0 | 57 | 4.4e-06 | 1Score **> 34** indicates **identity** Score **> 16** indicates **homology** | U | R.TIEYLEEVAVNFAK.G |
| 84838 | 236 | – | 249 | 542.6194 | 1624.8365 | 1624.8348 | 1.02 | 0 | 52 | 1.3e-05 | 1Score **> 34** indicates **identity** Score **> 16** indicates **homology** | U | R.TIEYLEEVAVNFAK.G |
| 84841 | 236 | – | 249 | 542.6196 | 1624.8370 | 1624.8348 | 1.33 | 0 | 36 | 0.00043 | 1Score **> 34** indicates **identity** Score **> 15** indicates **homology** | U | R.TIEYLEEVAVNFAK.G |
| 84842 | 236 | – | 249 | 813.4258 | 1624.8370 | 1624.8348 | 1.35 | 0 | 44 | 7.2e-05 | 1Score **> 34** indicates **identity** Score **> 15** indicates **homology** | U | R.TIEYLEEVAVNFAK.G |
| 84843 | 236 | – | 249 | 542.6196 | 1624.8370 | 1624.8348 | 1.37 | 0 | 57 | 4.5e-06 | 1Score **> 34** indicates **identity** Score **> 16** indicates **homology** | U | R.TIEYLEEVAVNFAK.G |
| 84844 | 236 | – | 249 | 813.4258 | 1624.8371 | 1624.8348 | 1.40 | 0 | 21 | 0.012 | 1Score **> 34** indicates **identity** Score **> 14** indicates **homology** | U | R.TIEYLEEVAVNFAK.G |
| 84845 | 236 | – | 249 | 813.4258 | 1624.8371 | 1624.8348 | 1.41 | 0 | 84 | 1.4e-08 | 1Score **> 34** indicates **identity** Score **> 18** indicates **homology** | U | R.TIEYLEEVAVNFAK.G |
| 84846 | 236 | – | 249 | 542.6197 | 1624.8372 | 1624.8348 | 1.46 | 0 | 59 | 2.8e-06 | 1Score **> 34** indicates **identity** Score **> 16** indicates **homology** | U | R.TIEYLEEVAVNFAK.G |
| 84849 | 236 | – | 249 | 542.6197 | 1624.8374 | 1624.8348 | 1.59 | 0 | 61 | 1.8e-06 | 1Score **> 34** indicates **identity** Score **> 16** indicates **homology** | U | R.TIEYLEEVAVNFAK.G |
| 84850 | 236 | – | 249 | 813.4260 | 1624.8375 | 1624.8348 | 1.66 | 0 | 61 | 1.8e-06 | 1Score **> 34** indicates **identity** Score **> 16** indicates **homology** | U | R.TIEYLEEVAVNFAK.G |
| 84854 | 236 | – | 249 | 813.4262 | 1624.8378 | 1624.8348 | 1.86 | 0 | 75 | 8.9e-08 | 1Score **> 34** indicates **identity** Score **> 17** indicates **homology** | U | R.TIEYLEEVAVNFAK.G |
| 84855 | 236 | – | 249 | 813.4262 | 1624.8379 | 1624.8348 | 1.88 | 0 | 44 | 6.8e-05 | 1Score **> 34** indicates **identity** Score **> 15** indicates **homology** | U | R.TIEYLEEVAVNFAK.G |
| 84857 | 236 | – | 249 | 813.4264 | 1624.8382 | 1624.8348 | 2.08 | 0 | 89 | 4.6e-09 | 1Score **> 34** indicates **identity** Score **> 18** indicates **homology** | U | R.TIEYLEEVAVNFAK.G |
| 84858 | 236 | – | 249 | 813.4264 | 1624.8382 | 1624.8348 | 2.10 | 0 | 21 | 0.011 | 1Score **> 34** indicates **identity** Score **> 14** indicates **homology** | U | R.TIEYLEEVAVNFAK.G |
| 84860 | 236 | – | 249 | 813.4264 | 1624.8383 | 1624.8348 | 2.17 | 0 | 81 | 2.5e-08 | 1Score **> 34** indicates **identity** Score **> 18** indicates **homology** | U | R.TIEYLEEVAVNFAK.G |
| 84861 | 236 | – | 249 | 813.4265 | 1624.8384 | 1624.8348 | 2.22 | 0 | 57 | 4.7e-06 | 1Score **> 34** indicates **identity** Score **> 16** indicates **homology** | U | R.TIEYLEEVAVNFAK.G |
| 84862 | 236 | – | 249 | 813.4265 | 1624.8384 | 1624.8348 | 2.24 | 0 | 81 | 2.4e-08 | 1Score **> 34** indicates **identity** Score **> 18** indicates **homology** | U | R.TIEYLEEVAVNFAK.G |
| 84863 | 236 | – | 249 | 813.4269 | 1624.8392 | 1624.8348 | 2.68 | 0 | 60 | 2.3e-06 | 1Score **> 34** indicates **identity** Score **> 16** indicates **homology** | U | R.TIEYLEEVAVNFAK.G |
| 84864 | 236 | – | 249 | 813.4269 | 1624.8392 | 1624.8348 | 2.70 | 0 | 61 | 1.9e-06 | 1Score **> 34** indicates **identity** Score **> 16** indicates **homology** | U | R.TIEYLEEVAVNFAK.G |
| 84866 | 236 | – | 249 | 813.4270 | 1624.8394 | 1624.8348 | 2.81 | 0 | 33 | 0.00077 | 1Score **> 34** indicates **identity** Score **> 15** indicates **homology** | U | R.TIEYLEEVAVNFAK.G |
| 84869 | 236 | – | 249 | 813.4272 | 1624.8399 | 1624.8348 | 3.16 | 0 | 75 | 9.5e-08 | 1Score **> 35** indicates **identity** Score **> 17** indicates **homology** | U | R.TIEYLEEVAVNFAK.G |
| 84870 | 236 | – | 249 | 813.4274 | 1624.8402 | 1624.8348 | 3.30 | 0 | 87 | 6.6e-09 | 1Score **> 34** indicates **identity** Score **> 18** indicates **homology** | U | R.TIEYLEEVAVNFAK.G |
| 84872 | 236 | – | 249 | 813.4274 | 1624.8403 | 1624.8348 | 3.35 | 0 | 64 | 9.1e-07 | 1Score **> 34** indicates **identity** Score **> 17** indicates **homology** | U | R.TIEYLEEVAVNFAK.G |
| 84873 | 236 | – | 249 | 813.4274 | 1624.8403 | 1624.8348 | 3.38 | 0 | 44 | 7.4e-05 | 1Score **> 34** indicates **identity** Score **> 15** indicates **homology** | U | R.TIEYLEEVAVNFAK.G |
| 84874 | 236 | – | 249 | 813.4275 | 1624.8404 | 1624.8348 | 3.44 | 0 | 61 | 2e-06 | 1Score **> 34** indicates **identity** Score **> 16** indicates **homology** | U | R.TIEYLEEVAVNFAK.G |
| 84876 | 236 | – | 249 | 813.4276 | 1624.8406 | 1624.8348 | 3.57 | 0 | 28 | 0.0023 | 1Score **> 34** indicates **identity** Score **> 14** indicates **homology** | U | R.TIEYLEEVAVNFAK.G |
| 84878 | 236 | – | 249 | 813.4277 | 1624.8408 | 1624.8348 | 3.67 | 0 | 75 | 1e-07 | 1Score **> 34** indicates **identity** Score **> 17** indicates **homology** | U | R.TIEYLEEVAVNFAK.G |
| 84881 | 236 | – | 249 | 813.4278 | 1624.8410 | 1624.8348 | 3.82 | 0 | 60 | 2.4e-06 | 1Score **> 34** indicates **identity** Score **> 16** indicates **homology** | U | R.TIEYLEEVAVNFAK.G |
| 84884 | 236 | – | 249 | 542.6213 | 1624.8419 | 1624.8348 | 4.38 | 0 | 37 | 0.00035 | 1Score **> 34** indicates **identity** Score **> 15** indicates **homology** | U | R.TIEYLEEVAVNFAK.G |
| 84886 | 236 | – | 249 | 813.4284 | 1624.8422 | 1624.8348 | 4.56 | 0 | 16 | 0.029 | 1Score **> 34** indicates **identity** Score **> 14** indicates **homology** | U | R.TIEYLEEVAVNFAK.G |
| 84888 | 236 | – | 249 | 813.4289 | 1624.8432 | 1624.8348 | 5.15 | 0 | 57 | 4.3e-06 | 1Score **> 34** indicates **identity** Score **> 16** indicates **homology** | U | R.TIEYLEEVAVNFAK.G |
| 84890 | 236 | – | 249 | 813.4290 | 1624.8435 | 1624.8348 | 5.34 | 0 | 81 | 2.5e-08 | 1Score **> 34** indicates **identity** Score **> 18** indicates **homology** | U | R.TIEYLEEVAVNFAK.G |
| 84892 | 236 | – | 249 | 813.4291 | 1624.8437 | 1624.8348 | 5.47 | 0 | 41 | 0.00014 | 1Score **> 34** indicates **identity** Score **> 15** indicates **homology** | U | R.TIEYLEEVAVNFAK.G |
| 84893 | 236 | – | 249 | 813.4291 | 1624.8437 | 1624.8348 | 5.49 | 0 | 48 | 3.5e-05 | 1Score **> 34** indicates **identity** Score **> 15** indicates **homology** | U | R.TIEYLEEVAVNFAK.G |
| 84895 | 236 | – | 249 | 813.4294 | 1624.8442 | 1624.8348 | 5.79 | 0 | 58 | 4e-06 | 1Score **> 34** indicates **identity** Score **> 16** indicates **homology** | U | R.TIEYLEEVAVNFAK.G |
| 84896 | 236 | – | 249 | 813.4294 | 1624.8443 | 1624.8348 | 5.84 | 0 | 88 | 5.7e-09 | 1Score **> 34** indicates **identity** Score **> 18** indicates **homology** | U | R.TIEYLEEVAVNFAK.G |
| 84899 | 236 | – | 249 | 813.4298 | 1624.8450 | 1624.8348 | 6.29 | 0 | 33 | 0.00081 | 1Score **> 34** indicates **identity** Score **> 15** indicates **homology** | U | R.TIEYLEEVAVNFAK.G |
| 84900 | 236 | – | 249 | 813.4299 | 1624.8452 | 1624.8348 | 6.41 | 0 | 66 | 7.2e-07 | 1Score **> 34** indicates **identity** Score **> 17** indicates **homology** | U | R.TIEYLEEVAVNFAK.G |
| 84901 | 236 | – | 249 | 813.4300 | 1624.8455 | 1624.8348 | 6.59 | 0 | 47 | 3.8e-05 | 1Score **> 34** indicates **identity** Score **> 15** indicates **homology** | U | R.TIEYLEEVAVNFAK.G |
| 84904 | 236 | – | 249 | 813.4314 | 1624.8482 | 1624.8348 | 8.27 | 0 | 89 | 4.9e-09 | 1Score **> 34** indicates **identity** Score **> 18** indicates **homology** | U | R.TIEYLEEVAVNFAK.G |
| 84905 | 236 | – | 249 | 813.4316 | 1624.8486 | 1624.8348 | 8.48 | 0 | 71 | 2.2e-07 | 1Score **> 34** indicates **identity** Score **> 17** indicates **homology** | U | R.TIEYLEEVAVNFAK.G |
| 84906 | 236 | – | 249 | 813.4318 | 1624.8490 | 1624.8348 | 8.71 | 0 | 64 | 1e-06 | 1Score **> 34** indicates **identity** Score **> 16** indicates **homology** | U | R.TIEYLEEVAVNFAK.G |
| 84907 | 236 | – | 249 | 813.4319 | 1624.8492 | 1624.8348 | 8.83 | 0 | 76 | 7.2e-08 | 1Score **> 34** indicates **identity** Score **> 17** indicates **homology** | U | R.TIEYLEEVAVNFAK.G |
| 84909 | 236 | – | 249 | 813.4322 | 1624.8499 | 1624.8348 | 9.28 | 0 | 34 | 0.00068 | 1Score **> 34** indicates **identity** Score **> 15** indicates **homology** | U | R.TIEYLEEVAVNFAK.G |
| 84910 | 236 | – | 249 | 813.4323 | 1624.8500 | 1624.8348 | 9.37 | 0 | 47 | 4e-05 | 1Score **> 34** indicates **identity** Score **> 15** indicates **homology** | U | R.TIEYLEEVAVNFAK.G |
| 84911 | 236 | – | 249 | 813.4328 | 1624.8510 | 1624.8348 | 9.96 | 0 | 52 | 1.3e-05 | 1Score **> 35** indicates **identity** Score **> 16** indicates **homology** | U | R.TIEYLEEVAVNFAK.G |
| 55438 | 268 | – | 279 | 699.8756 | 1397.7366 | 1397.7377 | -0.79 | 0 | 70 | 1.1e-06 | 1Score **> 33** indicates **identity** Score **> 23** indicates **homology** | U | K.LTTYAMTVPFVR.Q |
| 55440 | 268 | – | 279 | 699.8765 | 1397.7384 | 1397.7377 | 0.52 | 0 | 71 | 1e-06 | 1Score **> 34** indicates **identity** Score **> 23** indicates **homology** | U | K.LTTYAMTVPFVR.Q |
| 55441 | 268 | – | 279 | 699.8766 | 1397.7387 | 1397.7377 | 0.70 | 0 | 38 | 0.00029 | 1Score **> 34** indicates **identity** Score **> 15** indicates **homology** | U | K.LTTYAMTVPFVR.Q |
| 55443 | 268 | – | 279 | 699.8766 | 1397.7387 | 1397.7377 | 0.72 | 0 | 70 | 1.1e-06 | 1Score **> 34** indicates **identity** Score **> 23** indicates **homology** | U | K.LTTYAMTVPFVR.Q |
| 57389 | 268 | – | 279 | 707.8738 | 1413.7330 | 1413.7326 | 0.24 | 0 | 44 | 8.1e-05 | 1Score **> 34** indicates **identity** Score **> 15** indicates **homology** | U | K.LTTYAMTVPFVR.Q  + Oxidation (M) |
| 57395 | 268 | – | 279 | 707.8759 | 1413.7372 | 1413.7326 | 3.24 | 0 | 39 | 0.00026 | 1Score **> 34** indicates **identity** Score **> 16** indicates **homology** | U | K.LTTYAMTVPFVR.Q  + Oxidation (M) |
| 6519 | 296 | – | 303 | 429.7577 | 857.5008 | 857.5011 | -0.37 | 0 | 38 | 0.00079 | 1Score **> 32** indicates **identity** Score **> 20** indicates **homology** | U | K.GLYPAPLK.I |
| 6520 | 296 | – | 303 | 429.7577 | 857.5008 | 857.5011 | -0.30 | 0 | 29 | 0.0034 | 1Score **> 32** indicates **identity** Score **> 17** indicates **homology** | U | K.GLYPAPLK.I |
| 6521 | 296 | – | 303 | 429.7579 | 857.5013 | 857.5011 | 0.28 | 0 | 33 | 0.0021 | 1Score **> 32** indicates **identity** Score **> 19** indicates **homology** | U | K.GLYPAPLK.I |
| 6522 | 296 | – | 303 | 429.7580 | 857.5014 | 857.5011 | 0.40 | 0 | 35 | 0.0017 | 1Score **> 32** indicates **identity** Score **> 19** indicates **homology** | U | K.GLYPAPLK.I |
| 6523 | 296 | – | 303 | 429.7581 | 857.5017 | 857.5011 | 0.77 | 0 | 20 | 0.022 | 1Score **> 32** indicates **identity** Score **> 16** indicates **homology** | U | K.GLYPAPLK.I |
| 6524 | 296 | – | 303 | 429.7585 | 857.5025 | 857.5011 | 1.69 | 0 | 22 | 0.016 | 1Score **> 32** indicates **identity** Score **> 16** indicates **homology** | U | K.GLYPAPLK.I |
| 67624 | 296 | – | 309 | 499.9718 | 1496.8937 | 1496.8966 | -1.94 | 1 | 49 | 2.6e-05 | 1Score **> 32** indicates **identity** Score **> 16** indicates **homology** | U | K.GLYPAPLKIIDAVK.A |
| 67625 | 296 | – | 309 | 749.4546 | 1496.8947 | 1496.8966 | -1.26 | 1 | 20 | 0.012 | 1Score **> 31** indicates **identity** Score **> 14** indicates **homology** | U | K.GLYPAPLKIIDAVK.A |
| 67626 | 296 | – | 309 | 499.9723 | 1496.8950 | 1496.8966 | -1.06 | 1 | 39 | 0.0002 | 1Score **> 31** indicates **identity** Score **> 15** indicates **homology** | U | K.GLYPAPLKIIDAVK.A |
| 67627 | 296 | – | 309 | 499.9726 | 1496.8960 | 1496.8966 | -0.41 | 1 | 50 | 2.2e-05 | 1Score **> 32** indicates **identity** Score **> 16** indicates **homology** | U | K.GLYPAPLKIIDAVK.A |
| 67629 | 296 | – | 309 | 499.9730 | 1496.8971 | 1496.8966 | 0.35 | 1 | 41 | 0.00013 | 1Score **> 31** indicates **identity** Score **> 15** indicates **homology** | U | K.GLYPAPLKIIDAVK.A |
| 67630 | 296 | – | 309 | 499.9732 | 1496.8977 | 1496.8966 | 0.69 | 1 | 29 | 0.0021 | 1Score **> 31** indicates **identity** Score **> 14** indicates **homology** | U | K.GLYPAPLKIIDAVK.A |
| 67633 | 296 | – | 309 | 499.9736 | 1496.8990 | 1496.8966 | 1.61 | 1 | 22 | 0.009 | 1Score **> 31** indicates **identity** Score **> 14** indicates **homology** | U | K.GLYPAPLKIIDAVK.A |
| 97692 | 310 | – | 326 | 862.4078 | 1722.8010 | 1722.8060 | -2.92 | 0 | 66 | 6.3e-07 | 1Score **> 32** indicates **identity** Score **> 17** indicates **homology** | U | K.AGLEQGSDAGYLAESQK.F |
| 97693 | 310 | – | 326 | 862.4085 | 1722.8025 | 1722.8060 | -2.03 | 0 | 95 | 1.2e-09 | 1Score **> 32** indicates **identity** Score **> 18** indicates **homology** | U | K.AGLEQGSDAGYLAESQK.F |
| 97694 | 310 | – | 326 | 862.4086 | 1722.8027 | 1722.8060 | -1.95 | 0 | 65 | 7.7e-07 | 1Score **> 32** indicates **identity** Score **> 17** indicates **homology** | U | K.AGLEQGSDAGYLAESQK.F |
| 97695 | 310 | – | 326 | 862.4091 | 1722.8037 | 1722.8060 | -1.37 | 0 | 65 | 8.6e-07 | 1Score **> 32** indicates **identity** Score **> 17** indicates **homology** | U | K.AGLEQGSDAGYLAESQK.F |
| 97697 | 310 | – | 326 | 862.4096 | 1722.8047 | 1722.8060 | -0.80 | 0 | 83 | 1.7e-08 | 1Score **> 32** indicates **identity** Score **> 18** indicates **homology** | U | K.AGLEQGSDAGYLAESQK.F |
| 97698 | 310 | – | 326 | 862.4098 | 1722.8051 | 1722.8060 | -0.52 | 0 | 61 | 1.9e-06 | 1Score **> 32** indicates **identity** Score **> 16** indicates **homology** | U | K.AGLEQGSDAGYLAESQK.F |
| 97699 | 310 | – | 326 | 862.4099 | 1722.8052 | 1722.8060 | -0.46 | 0 | 101 | 1.2e-09 | 1Score **> 32** indicates **identity** Score **> 24** indicates **homology** | U | K.AGLEQGSDAGYLAESQK.F |
| 97700 | 310 | – | 326 | 862.4100 | 1722.8054 | 1722.8060 | -0.39 | 0 | 61 | 2e-06 | 1Score **> 32** indicates **identity** Score **> 16** indicates **homology** | U | K.AGLEQGSDAGYLAESQK.F |
| 97701 | 310 | – | 326 | 862.4100 | 1722.8055 | 1722.8060 | -0.29 | 0 | 44 | 6.7e-05 | 1Score **> 32** indicates **identity** Score **> 15** indicates **homology** | U | K.AGLEQGSDAGYLAESQK.F |
| 97702 | 310 | – | 326 | 862.4102 | 1722.8058 | 1722.8060 | -0.13 | 0 | 63 | 1.3e-06 | 1Score **> 32** indicates **identity** Score **> 16** indicates **homology** | U | K.AGLEQGSDAGYLAESQK.F |
| 97703 | 310 | – | 326 | 575.2760 | 1722.8061 | 1722.8060 | 0.060 | 0 | 58 | 3.6e-06 | 1Score **> 32** indicates **identity** Score **> 16** indicates **homology** | U | K.AGLEQGSDAGYLAESQK.F |
| 97704 | 310 | – | 326 | 575.2760 | 1722.8061 | 1722.8060 | 0.060 | 0 | 57 | 4.9e-06 | 1Score **> 32** indicates **identity** Score **> 16** indicates **homology** | U | K.AGLEQGSDAGYLAESQK.F |
| 97705 | 310 | – | 326 | 862.4105 | 1722.8064 | 1722.8060 | 0.24 | 0 | 113 | 5.3e-11 | 1Score **> 32** indicates **identity** Score **> 23** indicates **homology** | U | K.AGLEQGSDAGYLAESQK.F |
| 97706 | 310 | – | 326 | 862.4105 | 1722.8065 | 1722.8060 | 0.25 | 0 | 113 | 5.9e-11 | 1Score **> 32** indicates **identity** Score **> 23** indicates **homology** | U | K.AGLEQGSDAGYLAESQK.F |
| 97707 | 310 | – | 326 | 575.2761 | 1722.8065 | 1722.8060 | 0.29 | 0 | 48 | 3.2e-05 | 1Score **> 32** indicates **identity** Score **> 15** indicates **homology** | U | K.AGLEQGSDAGYLAESQK.F |
| 97708 | 310 | – | 326 | 575.2761 | 1722.8066 | 1722.8060 | 0.34 | 0 | 15 | 0.036 | 1Score **> 32** indicates **identity** Score **> 13** indicates **homology** | U | K.AGLEQGSDAGYLAESQK.F |
| 97709 | 310 | – | 326 | 862.4106 | 1722.8067 | 1722.8060 | 0.40 | 0 | 76 | 7.8e-08 | 1Score **> 32** indicates **identity** Score **> 17** indicates **homology** | U | K.AGLEQGSDAGYLAESQK.F |
| 97710 | 310 | – | 326 | 862.4108 | 1722.8071 | 1722.8060 | 0.61 | 0 | 99 | 9.6e-10 | 1Score **> 32** indicates **identity** Score **> 21** indicates **homology** | U | K.AGLEQGSDAGYLAESQK.F |
| 97711 | 310 | – | 326 | 862.4109 | 1722.8072 | 1722.8060 | 0.66 | 0 | 115 | 7.6e-11 | 1Score **> 32** indicates **identity** Score **> 26** indicates **homology** | U | K.AGLEQGSDAGYLAESQK.F |
| 97712 | 310 | – | 326 | 862.4111 | 1722.8077 | 1722.8060 | 0.95 | 0 | 53 | 1e-05 | 1Score **> 33** indicates **identity** Score **> 16** indicates **homology** | U | K.AGLEQGSDAGYLAESQK.F |
| 97837 | 310 | – | 326 | 862.9095 | 1723.8045 | 1723.7900 | 8.38 | 0 | 24 | 0.0052 | 1Score **> 32** indicates **identity** Score **> 14** indicates **homology** | U | K.AGLEQGSDAGYLAESQK.F  + Deamidated (NQ) |
| 97839 | 310 | – | 326 | 862.9101 | 1723.8057 | 1723.7900 | 9.10 | 0 | 30 | 0.0015 | 1Score **> 32** indicates **identity** Score **> 14** indicates **homology** | U | K.AGLEQGSDAGYLAESQK.F  + Deamidated (NQ) |
| 167821 | 310 | – | 334 | 861.7712 | 2582.2917 | 2582.2864 | 2.05 | 1 | 42 | 0.00013 | 1Score **> 37** indicates **identity** Score **> 15** indicates **homology** | U | K.AGLEQGSDAGYLAESQKFGELALTK.E |
| 167822 | 310 | – | 334 | 861.7714 | 2582.2925 | 2582.2864 | 2.37 | 1 | 67 | 5.3e-07 | 1Score **> 37** indicates **identity** Score **> 17** indicates **homology** | U | K.AGLEQGSDAGYLAESQKFGELALTK.E |
| 167823 | 310 | – | 334 | 1292.1535 | 2582.2925 | 2582.2864 | 2.37 | 1 | 96 | 9.8e-10 | 1Score **> 37** indicates **identity** Score **> 19** indicates **homology** | U | K.AGLEQGSDAGYLAESQKFGELALTK.E |
| 167824 | 310 | – | 334 | 861.7717 | 2582.2932 | 2582.2864 | 2.64 | 1 | 69 | 3.2e-07 | 1Score **> 37** indicates **identity** Score **> 17** indicates **homology** | U | K.AGLEQGSDAGYLAESQKFGELALTK.E |
| 167825 | 310 | – | 334 | 1292.1540 | 2582.2934 | 2582.2864 | 2.71 | 1 | 96 | 9.4e-10 | 1Score **> 37** indicates **identity** Score **> 19** indicates **homology** | U | K.AGLEQGSDAGYLAESQKFGELALTK.E |
| 167826 | 310 | – | 334 | 861.7719 | 2582.2940 | 2582.2864 | 2.94 | 1 | 83 | 1.8e-08 | 1Score **> 37** indicates **identity** Score **> 18** indicates **homology** | U | K.AGLEQGSDAGYLAESQKFGELALTK.E |
| 167827 | 310 | – | 334 | 1292.1549 | 2582.2952 | 2582.2864 | 3.41 | 1 | 106 | 1.2e-10 | 1Score **> 37** indicates **identity** Score **> 19** indicates **homology** | U | K.AGLEQGSDAGYLAESQKFGELALTK.E |
| 167828 | 310 | – | 334 | 861.7734 | 2582.2984 | 2582.2864 | 4.66 | 1 | 25 | 0.0041 | 1Score **> 37** indicates **identity** Score **> 14** indicates **homology** | U | K.AGLEQGSDAGYLAESQKFGELALTK.E |
| 167829 | 310 | – | 334 | 861.7737 | 2582.2992 | 2582.2864 | 4.95 | 1 | 73 | 1.5e-07 | 1Score **> 37** indicates **identity** Score **> 17** indicates **homology** | U | K.AGLEQGSDAGYLAESQKFGELALTK.E |
| 167830 | 310 | – | 334 | 861.7738 | 2582.2995 | 2582.2864 | 5.09 | 1 | 54 | 8.8e-06 | 1Score **> 37** indicates **identity** Score **> 16** indicates **homology** | U | K.AGLEQGSDAGYLAESQKFGELALTK.E |
| 167831 | 310 | – | 334 | 861.7740 | 2582.3001 | 2582.2864 | 5.32 | 1 | 45 | 6.1e-05 | 1Score **> 37** indicates **identity** Score **> 15** indicates **homology** | U | K.AGLEQGSDAGYLAESQKFGELALTK.E |
| 167833 | 310 | – | 334 | 861.7749 | 2582.3030 | 2582.2864 | 6.42 | 1 | 39 | 0.00022 | 1Score **> 37** indicates **identity** Score **> 15** indicates **homology** | U | K.AGLEQGSDAGYLAESQKFGELALTK.E |
| 167834 | 310 | – | 334 | 861.7767 | 2582.3083 | 2582.2864 | 8.51 | 1 | 25 | 0.0049 | 1Score **> 37** indicates **identity** Score **> 14** indicates **homology** | U | K.AGLEQGSDAGYLAESQKFGELALTK.E |
| 167904 | 310 | – | 334 | 1292.6527 | 2583.2908 | 2583.2704 | 7.90 | 1 | 80 | 2.9e-08 | 1Score **> 37** indicates **identity** Score **> 18** indicates **homology** | U | K.AGLEQGSDAGYLAESQKFGELALTK.E  + Deamidated (NQ) |
| 181149 | 310 | – | 337 | 732.6211 | 2926.4553 | 2926.4559 | -0.21 | 2 | 42 | 0.00013 | 1Score **> 38** indicates **identity** Score **> 15** indicates **homology** | U | K.AGLEQGSDAGYLAESQKFGELALTKESK.A |
| 7493 | 327 | – | 334 | 439.7510 | 877.4874 | 877.4909 | -3.95 | 0 | 27 | 0.0028 | 1Score **> 26** indicates **identity** Score **> 14** indicates **homology** | U | K.FGELALTK.E |
| 7494 | 327 | – | 334 | 439.7515 | 877.4884 | 877.4909 | -2.88 | 0 | 24 | 0.0054 | 1Score **> 26** indicates **identity** Score **> 14** indicates **homology** | U | K.FGELALTK.E |
| 7495 | 327 | – | 334 | 439.7526 | 877.4906 | 877.4909 | -0.30 | 0 | 35 | 0.00062 | 1Score **> 25** indicates **identity** Score **> 16** indicates **homology** | U | K.FGELALTK.E |
| 7496 | 327 | – | 334 | 439.7526 | 877.4907 | 877.4909 | -0.19 | 0 | 36 | 0.0011 | 1Score **> 26** indicates **identity** Score **> 19** indicates **homology** | U | K.FGELALTK.E |
| 7497 | 327 | – | 334 | 439.7526 | 877.4907 | 877.4909 | -0.18 | 0 | 35 | 0.00073 | 1Score **> 26** indicates **identity** Score **> 17** indicates **homology** | U | K.FGELALTK.E |
| 7498 | 327 | – | 334 | 439.7527 | 877.4908 | 877.4909 | -0.073 | 0 | 59 | 5.2e-06 | 1Score **> 26** indicates **identity** Score **> 19** indicates **homology** | U | K.FGELALTK.E |
| 7499 | 327 | – | 334 | 439.7527 | 877.4909 | 877.4909 | 0.0091 | 0 | 30 | 0.002 | 1Score **> 26** indicates **identity** Score **> 16** indicates **homology** | U | K.FGELALTK.E |
| 7500 | 327 | – | 334 | 439.7528 | 877.4911 | 877.4909 | 0.27 | 0 | 59 | 4.9e-06 | 1Score **> 26** indicates **identity** Score **> 18** indicates **homology** | U | K.FGELALTK.E |
| 7501 | 327 | – | 334 | 439.7529 | 877.4912 | 877.4909 | 0.37 | 0 | 59 | 5.2e-06 | 1Score **> 26** indicates **identity** Score **> 19** indicates **homology** | U | K.FGELALTK.E |
| 7502 | 327 | – | 334 | 439.7529 | 877.4912 | 877.4909 | 0.37 | 0 | 41 | 0.00014 | 1Score **> 26** indicates **identity** Score **> 15** indicates **homology** | U | K.FGELALTK.E |
| 7503 | 327 | – | 334 | 439.7529 | 877.4912 | 877.4909 | 0.38 | 0 | 41 | 0.00023 | 1Score **> 26** indicates **identity** Score **> 17** indicates **homology** | U | K.FGELALTK.E |
| 7505 | 327 | – | 334 | 439.7530 | 877.4914 | 877.4909 | 0.61 | 0 | 47 | 7.9e-05 | 1Score **> 26** indicates **identity** Score **> 18** indicates **homology** | U | K.FGELALTK.E |
| 7506 | 327 | – | 334 | 439.7530 | 877.4915 | 877.4909 | 0.72 | 0 | 42 | 0.00021 | 1Score **> 26** indicates **identity** Score **> 18** indicates **homology** | U | K.FGELALTK.E |
| 7507 | 327 | – | 334 | 439.7530 | 877.4915 | 877.4909 | 0.72 | 0 | 44 | 0.00024 | 1Score **> 26** indicates **identity** Score **> 20** indicates **homology** | U | K.FGELALTK.E |
| 7508 | 327 | – | 334 | 439.7531 | 877.4916 | 877.4909 | 0.80 | 0 | 35 | 0.00088 | 1Score **> 26** indicates **identity** Score **> 17** indicates **homology** | U | K.FGELALTK.E |
| 7509 | 327 | – | 334 | 439.7531 | 877.4916 | 877.4909 | 0.80 | 0 | 24 | 0.0092 | 1Score **> 26** indicates **identity** Score **> 16** indicates **homology** | U | K.FGELALTK.E |
| 7510 | 327 | – | 334 | 439.7531 | 877.4917 | 877.4909 | 0.94 | 0 | 37 | 0.00047 | 1Score **> 26** indicates **identity** Score **> 16** indicates **homology** | U | K.FGELALTK.E |
| 7511 | 327 | – | 334 | 439.7532 | 877.4918 | 877.4909 | 0.99 | 0 | 24 | 0.0085 | 1Score **> 26** indicates **identity** Score **> 16** indicates **homology** | U | K.FGELALTK.E |
| 7512 | 327 | – | 334 | 439.7532 | 877.4918 | 877.4909 | 1.00 | 0 | 31 | 0.0025 | 1Score **> 26** indicates **identity** Score **> 18** indicates **homology** | U | K.FGELALTK.E |
| 7513 | 327 | – | 334 | 439.7533 | 877.4921 | 877.4909 | 1.34 | 0 | 17 | 0.038 | 1Score **> 26** indicates **identity** Score **> 15** indicates **homology** | U | K.FGELALTK.E |
| 7514 | 327 | – | 334 | 439.7543 | 877.4940 | 877.4909 | 3.58 | 0 | 36 | 0.00094 | 1Score **> 28** indicates **identity** Score **> 18** indicates **homology** | U | K.FGELALTK.E |
| 36121 | 327 | – | 337 | 408.2258 | 1221.6555 | 1221.6605 | -4.11 | 1 | 32 | 0.001 | 1Score **> 33** indicates **identity** Score **> 14** indicates **homology** | U | K.FGELALTKESK.A |
| 36122 | 327 | – | 337 | 408.2260 | 1221.6562 | 1221.6605 | -3.49 | 1 | 29 | 0.0021 | 1Score **> 33** indicates **identity** Score **> 14** indicates **homology** | U | K.FGELALTKESK.A |
| 63581 | 338 | – | 350 | 733.8790 | 1465.7435 | 1465.7421 | 0.92 | 0 | 84 | 1.2e-08 | 1Score **> 34** indicates **identity** Score **> 18** indicates **homology** | U | K.ALMGLYNGQVLCK.K |
| 63582 | 338 | – | 350 | 733.8808 | 1465.7470 | 1465.7421 | 3.33 | 0 | 65 | 8.3e-07 | 1Score **> 34** indicates **identity** Score **> 17** indicates **homology** | U | K.ALMGLYNGQVLCK.K |
| 80463 | 338 | – | 351 | 797.9242 | 1593.8338 | 1593.8371 | -2.05 | 1 | 25 | 0.0042 | 1Score **> 35** indicates **identity** Score **> 14** indicates **homology** | U | K.ALMGLYNGQVLCKK.N |
| 82727 | 338 | – | 351 | 537.6189 | 1609.8350 | 1609.8320 | 1.85 | 1 | 14 | 0.044 | 1Score **> 34** indicates **identity** Score **> 13** indicates **homology** | U | K.ALMGLYNGQVLCKK.N  + Oxidation (M) |
| 155048 | 360 | – | 383 | 1177.1513 | 2352.2881 | 2352.2835 | 1.96 | 0 | 117 | 9.7e-12 | 1Score **> 35** indicates **identity** Score **> 20** indicates **homology** | U | K.NVQQLAILGAGLMGAGIAQVSVDK.G |
| 155051 | 360 | – | 383 | 785.1037 | 2352.2894 | 2352.2835 | 2.49 | 0 | 25 | 0.0042 | 1Score **> 35** indicates **identity** Score **> 14** indicates **homology** | U | K.NVQQLAILGAGLMGAGIAQVSVDK.G |
| 155055 | 360 | – | 383 | 785.1041 | 2352.2906 | 2352.2835 | 3.01 | 0 | 18 | 0.019 | 1Score **> 35** indicates **identity** Score **> 14** indicates **homology** | U | K.NVQQLAILGAGLMGAGIAQVSVDK.G |
| 170705 | 360 | – | 386 | 884.5023 | 2650.4851 | 2650.4840 | 0.43 | 1 | 61 | 1.9e-06 | 1Score **> 33** indicates **identity** Score **> 16** indicates **homology** | U | K.NVQQLAILGAGLMGAGIAQVSVDKGLK.T |
| 52993 | 387 | – | 399 | 458.9356 | 1373.7850 | 1373.7878 | -2.03 | 1 | 34 | 0.00082 | 1Score **> 33** indicates **identity** Score **> 16** indicates **homology** | U | K.TLLKDTTVTGLGR.G |
| 52994 | 387 | – | 399 | 458.9362 | 1373.7866 | 1373.7878 | -0.89 | 1 | 41 | 0.00014 | 1Score **> 33** indicates **identity** Score **> 15** indicates **homology** | U | K.TLLKDTTVTGLGR.G |
| 52995 | 387 | – | 399 | 458.9362 | 1373.7868 | 1373.7878 | -0.75 | 1 | 46 | 0.00017 | 1Score **> 33** indicates **identity** Score **> 21** indicates **homology** | U | K.TLLKDTTVTGLGR.G |
| 52996 | 387 | – | 399 | 458.9363 | 1373.7870 | 1373.7878 | -0.61 | 1 | 53 | 1.4e-05 | 1Score **> 33** indicates **identity** Score **> 17** indicates **homology** | U | K.TLLKDTTVTGLGR.G |
| 52997 | 387 | – | 399 | 687.9009 | 1373.7873 | 1373.7878 | -0.40 | 1 | 55 | 7.2e-06 | 1Score **> 33** indicates **identity** Score **> 16** indicates **homology** | U | K.TLLKDTTVTGLGR.G |
| 52998 | 387 | – | 399 | 687.9010 | 1373.7874 | 1373.7878 | -0.29 | 1 | 20 | 0.013 | 1Score **> 33** indicates **identity** Score **> 14** indicates **homology** | U | K.TLLKDTTVTGLGR.G |
| 52999 | 387 | – | 399 | 687.9010 | 1373.7875 | 1373.7878 | -0.25 | 1 | 17 | 0.024 | 1Score **> 33** indicates **identity** Score **> 14** indicates **homology** | U | K.TLLKDTTVTGLGR.G |
| 53000 | 387 | – | 399 | 458.9364 | 1373.7875 | 1373.7878 | -0.24 | 1 | 36 | 0.00046 | 1Score **> 33** indicates **identity** Score **> 15** indicates **homology** | U | K.TLLKDTTVTGLGR.G |
| 53001 | 387 | – | 399 | 458.9364 | 1373.7875 | 1373.7878 | -0.24 | 1 | 35 | 0.00064 | 1Score **> 33** indicates **identity** Score **> 15** indicates **homology** | U | K.TLLKDTTVTGLGR.G |
| 53002 | 387 | – | 399 | 458.9365 | 1373.7876 | 1373.7878 | -0.21 | 1 | 23 | 0.0071 | 1Score **> 33** indicates **identity** Score **> 14** indicates **homology** | U | K.TLLKDTTVTGLGR.G |
| 53003 | 387 | – | 399 | 458.9365 | 1373.7876 | 1373.7878 | -0.17 | 1 | 38 | 0.00026 | 1Score **> 33** indicates **identity** Score **> 15** indicates **homology** | U | K.TLLKDTTVTGLGR.G |
| 53004 | 387 | – | 399 | 458.9365 | 1373.7876 | 1373.7878 | -0.15 | 1 | 47 | 5.6e-05 | 1Score **> 33** indicates **identity** Score **> 17** indicates **homology** | U | K.TLLKDTTVTGLGR.G |
| 53005 | 387 | – | 399 | 458.9366 | 1373.7879 | 1373.7878 | 0.018 | 1 | 47 | 3.6e-05 | 1Score **> 33** indicates **identity** Score **> 15** indicates **homology** | U | K.TLLKDTTVTGLGR.G |
| 53006 | 387 | – | 399 | 458.9366 | 1373.7879 | 1373.7878 | 0.044 | 1 | 54 | 1.2e-05 | 1Score **> 33** indicates **identity** Score **> 18** indicates **homology** | U | K.TLLKDTTVTGLGR.G |
| 53008 | 387 | – | 399 | 458.9366 | 1373.7881 | 1373.7878 | 0.17 | 1 | 53 | 1.5e-05 | 1Score **> 33** indicates **identity** Score **> 17** indicates **homology** | U | K.TLLKDTTVTGLGR.G |
| 53009 | 387 | – | 399 | 687.9013 | 1373.7881 | 1373.7878 | 0.18 | 1 | 45 | 6.2e-05 | 1Score **> 33** indicates **identity** Score **> 15** indicates **homology** | U | K.TLLKDTTVTGLGR.G |
| 53010 | 387 | – | 399 | 458.9368 | 1373.7884 | 1373.7878 | 0.44 | 1 | 44 | 0.00027 | 1Score **> 33** indicates **identity** Score **> 21** indicates **homology** | U | K.TLLKDTTVTGLGR.G |
| 53011 | 387 | – | 399 | 458.9369 | 1373.7889 | 1373.7878 | 0.74 | 1 | 45 | 0.00016 | 1Score **> 33** indicates **identity** Score **> 20** indicates **homology** | U | K.TLLKDTTVTGLGR.G |
| 53013 | 387 | – | 399 | 458.9371 | 1373.7895 | 1373.7878 | 1.23 | 1 | 47 | 0.00018 | 1Score **> 34** indicates **identity** Score **> 22** indicates **homology** | U | K.TLLKDTTVTGLGR.G |
| 51685 | 400 | – | 411 | 454.5774 | 1360.7103 | 1360.7099 | 0.32 | 1 | 21 | 0.012 | 1Score **> 35** indicates **identity** Score **> 14** indicates **homology** | U | R.GQQQVFKGLNDK.V |
| 21042 | 414 | – | 422 | 360.5456 | 1078.6151 | 1078.6135 | 1.50 | 2 | 52 | 1.5e-05 | 1Score **> 30** indicates **identity** Score **> 16** indicates **homology** | U | K.KKALTSFER.D |
| 11688 | 415 | – | 422 | 476.2667 | 950.5188 | 950.5185 | 0.33 | 1 | 35 | 0.0015 | 1Score **> 28** indicates **identity** Score **> 20** indicates **homology** | U | K.KALTSFER.D |
| 166120 | 415 | – | 436 | 637.0898 | 2544.3300 | 2544.3224 | 2.99 | 2 | 19 | 0.017 | 1Score **> 37** indicates **identity** Score **> 14** indicates **homology** | U | K.KALTSFERDSIFSNLIGQLDYK.G |
| 4791 | 416 | – | 422 | 412.2190 | 822.4235 | 822.4236 | -0.12 | 0 | 39 | 0.003 | 1Score **> 26** indicates **identity** | U | K.ALTSFER.D |
| 4795 | 416 | – | 422 | 412.2199 | 822.4252 | 822.4236 | 1.95 | 0 | 44 | 0.00083 | 1Score **> 25** indicates **identity** | U | K.ALTSFER.D |
| 159078 | 416 | – | 436 | 806.4176 | 2416.2309 | 2416.2274 | 1.44 | 1 | 55 | 6.8e-06 | 1Score **> 37** indicates **identity** Score **> 16** indicates **homology** | U | K.ALTSFERDSIFSNLIGQLDYK.G |
| 179640 | 416 | – | 440 | 720.3723 | 2877.4600 | 2877.4548 | 1.80 | 2 | 26 | 0.0033 | 1Score **> 37** indicates **identity** Score **> 14** indicates **homology** | U | K.ALTSFERDSIFSNLIGQLDYKGFEK.A |
| 179672 | 416 | – | 440 | 960.4933 | 2878.4580 | 2878.4389 | 6.66 | 2 | 16 | 0.031 | 1Score **> 38** indicates **identity** Score **> 14** indicates **homology** | U | K.ALTSFERDSIFSNLIGQLDYKGFEK.A  + Deamidated (NQ) |
| 82963 | 423 | – | 436 | 538.2748 | 1611.8026 | 1611.8144 | -7.35 | 0 | 17 | 0.023 | 1Score **> 34** indicates **identity** Score **> 14** indicates **homology** | U | R.DSIFSNLIGQLDYK.G |
| 82967 | 423 | – | 436 | 806.9139 | 1611.8133 | 1611.8144 | -0.71 | 0 | 62 | 1.6e-06 | 1Score **> 34** indicates **identity** Score **> 16** indicates **homology** | U | R.DSIFSNLIGQLDYK.G |
| 82968 | 423 | – | 436 | 806.9156 | 1611.8167 | 1611.8144 | 1.41 | 0 | 91 | 2.7e-09 | 1Score **> 34** indicates **identity** Score **> 18** indicates **homology** | U | R.DSIFSNLIGQLDYK.G |
| 132818 | 423 | – | 440 | 692.0215 | 2073.0427 | 2073.0419 | 0.40 | 1 | 34 | 0.00065 | 1Score **> 36** indicates **identity** Score **> 15** indicates **homology** | U | R.DSIFSNLIGQLDYKGFEK.A |
| 132819 | 423 | – | 440 | 1037.5287 | 2073.0428 | 2073.0419 | 0.43 | 1 | 77 | 5.9e-08 | 1Score **> 36** indicates **identity** Score **> 17** indicates **homology** | U | R.DSIFSNLIGQLDYKGFEK.A |
| 132820 | 423 | – | 440 | 1037.5296 | 2073.0446 | 2073.0419 | 1.33 | 1 | 76 | 8.4e-08 | 1Score **> 36** indicates **identity** Score **> 18** indicates **homology** | U | R.DSIFSNLIGQLDYKGFEK.A |
| 132821 | 423 | – | 440 | 692.0230 | 2073.0472 | 2073.0419 | 2.58 | 1 | 28 | 0.0021 | 1Score **> 36** indicates **identity** Score **> 14** indicates **homology** | U | R.DSIFSNLIGQLDYKGFEK.A |
| 132823 | 423 | – | 440 | 692.0231 | 2073.0475 | 2073.0419 | 2.70 | 1 | 34 | 0.00072 | 1Score **> 36** indicates **identity** Score **> 15** indicates **homology** | U | R.DSIFSNLIGQLDYKGFEK.A |
| 86102 | 441 | – | 455 | 818.4194 | 1634.8243 | 1634.8226 | 1.05 | 0 | 60 | 2.4e-06 | 1Score **> 35** indicates **identity** Score **> 16** indicates **homology** | U | K.ADMVIEAVFEDLGVK.H |
| 86103 | 441 | – | 455 | 545.9488 | 1634.8247 | 1634.8226 | 1.29 | 0 | 52 | 1.3e-05 | 1Score **> 35** indicates **identity** Score **> 16** indicates **homology** | U | K.ADMVIEAVFEDLGVK.H |
| 86108 | 441 | – | 455 | 545.9493 | 1634.8259 | 1634.8226 | 2.06 | 0 | 65 | 8e-07 | 1Score **> 35** indicates **identity** Score **> 17** indicates **homology** | U | K.ADMVIEAVFEDLGVK.H |
| 86109 | 441 | – | 455 | 818.4206 | 1634.8266 | 1634.8226 | 2.49 | 0 | 93 | 2.1e-09 | 1Score **> 35** indicates **identity** Score **> 18** indicates **homology** | U | K.ADMVIEAVFEDLGVK.H |
| 88244 | 441 | – | 455 | 826.4187 | 1650.8229 | 1650.8175 | 3.31 | 0 | 59 | 3.2e-06 | 1Score **> 34** indicates **identity** Score **> 16** indicates **homology** | U | K.ADMVIEAVFEDLGVK.H  + Oxidation (M) |
| 192705 | 458 | – | 489 | 863.9581 | 3451.8031 | 3451.8021 | 0.30 | 1 | 14 | 0.045 | 1Score **> 37** indicates **identity** Score **> 13** indicates **homology** | U | K.VLKEVESVTPEHCIFASNTSALPINQIAAVSK.R |
| 192707 | 458 | – | 489 | 1151.6094 | 3451.8064 | 3451.8021 | 1.24 | 1 | 92 | 2.3e-09 | 1Score **> 37** indicates **identity** Score **> 18** indicates **homology** | U | K.VLKEVESVTPEHCIFASNTSALPINQIAAVSK.R |
| 192708 | 458 | – | 489 | 863.9589 | 3451.8065 | 3451.8021 | 1.28 | 1 | 44 | 6.8e-05 | 1Score **> 37** indicates **identity** Score **> 15** indicates **homology** | U | K.VLKEVESVTPEHCIFASNTSALPINQIAAVSK.R |
| 192709 | 458 | – | 489 | 1151.6098 | 3451.8076 | 3451.8021 | 1.59 | 1 | 76 | 7.7e-08 | 1Score **> 37** indicates **identity** Score **> 17** indicates **homology** | U | K.VLKEVESVTPEHCIFASNTSALPINQIAAVSK.R |
| 192710 | 458 | – | 489 | 863.9595 | 3451.8090 | 3451.8021 | 2.00 | 1 | 50 | 2e-05 | 1Score **> 37** indicates **identity** Score **> 16** indicates **homology** | U | K.VLKEVESVTPEHCIFASNTSALPINQIAAVSK.R |
| 192711 | 458 | – | 489 | 1151.6141 | 3451.8204 | 3451.8021 | 5.30 | 1 | 47 | 3.8e-05 | 1Score **> 37** indicates **identity** Score **> 15** indicates **homology** | U | K.VLKEVESVTPEHCIFASNTSALPINQIAAVSK.R |
| 192738 | 458 | – | 489 | 1152.2753 | 3453.8040 | 3453.7701 | 9.81 | 1 | 27 | 0.0029 | 1Score **> 37** indicates **identity** Score **> 14** indicates **homology** | U | K.VLKEVESVTPEHCIFASNTSALPINQIAAVSK.R  + 2 Deamidated (NQ) |
| 186363 | 461 | – | 489 | 1038.1928 | 3111.5565 | 3111.5546 | 0.59 | 0 | 70 | 2.5e-07 | 1Score **> 38** indicates **identity** Score **> 17** indicates **homology** | U | K.EVESVTPEHCIFASNTSALPINQIAAVSK.R |
| 186365 | 461 | – | 489 | 1038.1930 | 3111.5571 | 3111.5546 | 0.78 | 0 | 83 | 1.6e-08 | 1Score **> 38** indicates **identity** Score **> 18** indicates **homology** | U | K.EVESVTPEHCIFASNTSALPINQIAAVSK.R |
| 186366 | 461 | – | 489 | 1038.1931 | 3111.5576 | 3111.5546 | 0.95 | 0 | 77 | 6.3e-08 | 1Score **> 38** indicates **identity** Score **> 17** indicates **homology** | U | K.EVESVTPEHCIFASNTSALPINQIAAVSK.R |
| 186370 | 461 | – | 489 | 1556.7864 | 3111.5583 | 3111.5546 | 1.17 | 0 | 41 | 0.00015 | 1Score **> 38** indicates **identity** Score **> 15** indicates **homology** | U | K.EVESVTPEHCIFASNTSALPINQIAAVSK.R |
| 186371 | 461 | – | 489 | 1556.7864 | 3111.5583 | 3111.5546 | 1.18 | 0 | 54 | 9e-06 | 1Score **> 38** indicates **identity** Score **> 16** indicates **homology** | U | K.EVESVTPEHCIFASNTSALPINQIAAVSK.R |
| 186372 | 461 | – | 489 | 1038.1935 | 3111.5586 | 3111.5546 | 1.27 | 0 | 66 | 6.4e-07 | 1Score **> 38** indicates **identity** Score **> 17** indicates **homology** | U | K.EVESVTPEHCIFASNTSALPINQIAAVSK.R |
| 186373 | 461 | – | 489 | 1556.7868 | 3111.5590 | 3111.5546 | 1.38 | 0 | 82 | 2.2e-08 | 1Score **> 38** indicates **identity** Score **> 18** indicates **homology** | U | K.EVESVTPEHCIFASNTSALPINQIAAVSK.R |
| 186374 | 461 | – | 489 | 1038.1940 | 3111.5603 | 3111.5546 | 1.81 | 0 | 35 | 0.00051 | 1Score **> 38** indicates **identity** Score **> 15** indicates **homology** | U | K.EVESVTPEHCIFASNTSALPINQIAAVSK.R |
| 186375 | 461 | – | 489 | 1038.1947 | 3111.5623 | 3111.5546 | 2.45 | 0 | 49 | 2.7e-05 | 1Score **> 38** indicates **identity** Score **> 16** indicates **homology** | U | K.EVESVTPEHCIFASNTSALPINQIAAVSK.R |
| 186376 | 461 | – | 489 | 1038.1956 | 3111.5650 | 3111.5546 | 3.34 | 0 | 29 | 0.0021 | 1Score **> 38** indicates **identity** Score **> 14** indicates **homology** | U | K.EVESVTPEHCIFASNTSALPINQIAAVSK.R |
| 186377 | 461 | – | 489 | 1038.1957 | 3111.5651 | 3111.5546 | 3.37 | 0 | 45 | 6e-05 | 1Score **> 38** indicates **identity** Score **> 15** indicates **homology** | U | K.EVESVTPEHCIFASNTSALPINQIAAVSK.R |
| 186378 | 461 | – | 489 | 1038.1957 | 3111.5652 | 3111.5546 | 3.40 | 0 | 30 | 0.0015 | 1Score **> 38** indicates **identity** Score **> 14** indicates **homology** | U | K.EVESVTPEHCIFASNTSALPINQIAAVSK.R |
| 186407 | 461 | – | 489 | 1038.5241 | 3112.5505 | 3112.5387 | 3.82 | 0 | 22 | 0.0085 | 1Score **> 38** indicates **identity** Score **> 14** indicates **homology** | U | K.EVESVTPEHCIFASNTSALPINQIAAVSK.R  + Deamidated (NQ) |
| 186409 | 461 | – | 489 | 1038.5249 | 3112.5529 | 3112.5387 | 4.59 | 0 | 30 | 0.0014 | 1Score **> 38** indicates **identity** Score **> 14** indicates **homology** | U | K.EVESVTPEHCIFASNTSALPINQIAAVSK.R  + Deamidated (NQ) |
| 186410 | 461 | – | 489 | 1038.5260 | 3112.5560 | 3112.5387 | 5.59 | 0 | 24 | 0.0058 | 1Score **> 38** indicates **identity** Score **> 14** indicates **homology** | U | K.EVESVTPEHCIFASNTSALPINQIAAVSK.R  + Deamidated (NQ) |
| 186411 | 461 | – | 489 | 1557.2855 | 3112.5564 | 3112.5387 | 5.71 | 0 | 39 | 0.00021 | 1Score **> 38** indicates **identity** Score **> 15** indicates **homology** | U | K.EVESVTPEHCIFASNTSALPINQIAAVSK.R  + Deamidated (NQ) |
| 186413 | 461 | – | 489 | 1038.5269 | 3112.5590 | 3112.5387 | 6.53 | 0 | 29 | 0.0021 | 1Score **> 38** indicates **identity** Score **> 14** indicates **homology** | U | K.EVESVTPEHCIFASNTSALPINQIAAVSK.R  + Deamidated (NQ) |
| 186415 | 461 | – | 489 | 1038.5281 | 3112.5624 | 3112.5387 | 7.63 | 0 | 18 | 0.02 | 1Score **> 38** indicates **identity** Score **> 14** indicates **homology** | U | K.EVESVTPEHCIFASNTSALPINQIAAVSK.R  + Deamidated (NQ) |
| 186417 | 461 | – | 489 | 1038.5285 | 3112.5638 | 3112.5387 | 8.08 | 0 | 38 | 0.00027 | 1Score **> 38** indicates **identity** Score **> 15** indicates **homology** | U | K.EVESVTPEHCIFASNTSALPINQIAAVSK.R  + Deamidated (NQ) |
| 194825 | 461 | – | 493 | 906.4686 | 3621.8451 | 3621.8461 | -0.26 | 2 | 36 | 0.00043 | 1Score **> 37** indicates **identity** Score **> 15** indicates **homology** | U | K.EVESVTPEHCIFASNTSALPINQIAAVSKRPEK.V |
| 194827 | 461 | – | 493 | 1208.2896 | 3621.8471 | 3621.8461 | 0.28 | 2 | 22 | 0.0086 | 1Score **> 37** indicates **identity** Score **> 14** indicates **homology** | U | K.EVESVTPEHCIFASNTSALPINQIAAVSKRPEK.V |
| 194828 | 461 | – | 493 | 1208.2897 | 3621.8472 | 3621.8461 | 0.33 | 2 | 37 | 0.00034 | 1Score **> 37** indicates **identity** Score **> 15** indicates **homology** | U | K.EVESVTPEHCIFASNTSALPINQIAAVSKRPEK.V |
| 194829 | 461 | – | 493 | 906.4694 | 3621.8483 | 3621.8461 | 0.63 | 2 | 20 | 0.012 | 1Score **> 37** indicates **identity** Score **> 14** indicates **homology** | U | K.EVESVTPEHCIFASNTSALPINQIAAVSKRPEK.V |
| 194830 | 461 | – | 493 | 725.3770 | 3621.8487 | 3621.8461 | 0.72 | 2 | 14 | 0.048 | 1Score **> 37** indicates **identity** Score **> 13** indicates **homology** | U | K.EVESVTPEHCIFASNTSALPINQIAAVSKRPEK.V |
| 194831 | 461 | – | 493 | 725.3779 | 3621.8530 | 3621.8461 | 1.92 | 2 | 27 | 0.0032 | 1Score **> 37** indicates **identity** Score **> 14** indicates **homology** | U | K.EVESVTPEHCIFASNTSALPINQIAAVSKRPEK.V |
| 194832 | 461 | – | 493 | 906.4709 | 3621.8547 | 3621.8461 | 2.38 | 2 | 34 | 0.0007 | 1Score **> 37** indicates **identity** Score **> 15** indicates **homology** | U | K.EVESVTPEHCIFASNTSALPINQIAAVSKRPEK.V |
| 194833 | 461 | – | 493 | 906.4718 | 3621.8579 | 3621.8461 | 3.27 | 2 | 16 | 0.035 | 1Score **> 37** indicates **identity** Score **> 13** indicates **homology** | U | K.EVESVTPEHCIFASNTSALPINQIAAVSKRPEK.V |
| 54787 | 494 | – | 505 | 464.9044 | 1391.6913 | 1391.6908 | 0.39 | 0 | 25 | 0.0041 | 1Score **> 33** indicates **identity** Score **> 14** indicates **homology** | U | K.VIGMHYFSPVDK.M |
| 54789 | 494 | – | 505 | 696.8533 | 1391.6920 | 1391.6908 | 0.90 | 0 | 24 | 0.0051 | 1Score **> 33** indicates **identity** Score **> 14** indicates **homology** | U | K.VIGMHYFSPVDK.M |
| 54791 | 494 | – | 505 | 464.9051 | 1391.6935 | 1391.6908 | 1.96 | 0 | 28 | 0.0026 | 1Score **> 33** indicates **identity** Score **> 14** indicates **homology** | U | K.VIGMHYFSPVDK.M |
| 45477 | 506 | – | 516 | 652.8588 | 1303.7030 | 1303.7057 | -2.11 | 0 | 43 | 0.00017 | 1Score **> 34** indicates **identity** Score **> 18** indicates **homology** | U | K.MQLLEIITTDK.T |
| 45482 | 506 | – | 516 | 652.8620 | 1303.7094 | 1303.7057 | 2.84 | 0 | 55 | 7.5e-06 | 1Score **> 33** indicates **identity** Score **> 16** indicates **homology** | U | K.MQLLEIITTDK.T |
| 45484 | 506 | – | 516 | 652.8628 | 1303.7110 | 1303.7057 | 4.04 | 0 | 55 | 7.4e-06 | 1Score **> 34** indicates **identity** Score **> 16** indicates **homology** | U | K.MQLLEIITTDK.T |
| 45485 | 506 | – | 516 | 652.8628 | 1303.7111 | 1303.7057 | 4.15 | 0 | 36 | 0.0007 | 1Score **> 34** indicates **identity** Score **> 17** indicates **homology** | U | K.MQLLEIITTDK.T |
| 84144 | 506 | – | 519 | 540.9673 | 1619.8800 | 1619.8804 | -0.22 | 1 | 25 | 0.0041 | 1Score **> 34** indicates **identity** Score **> 14** indicates **homology** | U | K.MQLLEIITTDKTSK.D |
| 84146 | 506 | – | 519 | 540.9681 | 1619.8823 | 1619.8804 | 1.21 | 1 | 67 | 4.9e-07 | 1Score **> 34** indicates **identity** Score **> 17** indicates **homology** | U | K.MQLLEIITTDKTSK.D |
| 84147 | 506 | – | 519 | 540.9681 | 1619.8826 | 1619.8804 | 1.35 | 1 | 43 | 8.8e-05 | 1Score **> 34** indicates **identity** Score **> 15** indicates **homology** | U | K.MQLLEIITTDKTSK.D |
| 84148 | 506 | – | 519 | 540.9682 | 1619.8829 | 1619.8804 | 1.56 | 1 | 58 | 3.6e-06 | 1Score **> 34** indicates **identity** Score **> 16** indicates **homology** | U | K.MQLLEIITTDKTSK.D |
| 84150 | 506 | – | 519 | 810.9519 | 1619.8892 | 1619.8804 | 5.45 | 1 | 38 | 0.0003 | 1Score **> 34** indicates **identity** Score **> 15** indicates **homology** | U | K.MQLLEIITTDKTSK.D |
| 175214 | 506 | – | 531 | 691.3801 | 2761.4913 | 2761.4895 | 0.64 | 2 | 49 | 2.3e-05 | 1Score **> 36** indicates **identity** Score **> 16** indicates **homology** | U | K.MQLLEIITTDKTSKDTTASAVAVGLR.Q |
| 175215 | 506 | – | 531 | 691.3862 | 2761.5155 | 2761.4895 | 9.40 | 2 | 43 | 0.0001 | 1Score **> 35** indicates **identity** Score **> 15** indicates **homology** | U | K.MQLLEIITTDKTSKDTTASAVAVGLR.Q |
| 175249 | 506 | – | 531 | 921.8384 | 2762.4933 | 2762.4735 | 7.15 | 2 | 25 | 0.0045 | 1Score **> 35** indicates **identity** Score **> 14** indicates **homology** | U | K.MQLLEIITTDKTSKDTTASAVAVGLR.Q  + Deamidated (NQ) |
| 64832 | 517 | – | 531 | 492.9379 | 1475.7917 | 1475.7944 | -1.78 | 1 | 14 | 0.046 | 1Score **> 35** indicates **identity** Score **> 13** indicates **homology** | U | K.TSKDTTASAVAVGLR.Q |
| 64835 | 517 | – | 531 | 492.9382 | 1475.7928 | 1475.7944 | -1.09 | 1 | 20 | 0.012 | 1Score **> 35** indicates **identity** Score **> 14** indicates **homology** | U | K.TSKDTTASAVAVGLR.Q |
| 64836 | 517 | – | 531 | 492.9382 | 1475.7928 | 1475.7944 | -1.08 | 1 | 19 | 0.016 | 1Score **> 35** indicates **identity** Score **> 14** indicates **homology** | U | K.TSKDTTASAVAVGLR.Q |
| 64837 | 517 | – | 531 | 492.9383 | 1475.7931 | 1475.7944 | -0.88 | 1 | 60 | 1.3e-05 | 1Score **> 35** indicates **identity** Score **> 24** indicates **homology** | U | K.TSKDTTASAVAVGLR.Q |
| 64838 | 517 | – | 531 | 738.9039 | 1475.7933 | 1475.7944 | -0.71 | 1 | 89 | 4.4e-09 | 1Score **> 35** indicates **identity** Score **> 18** indicates **homology** | U | K.TSKDTTASAVAVGLR.Q |
| 64839 | 517 | – | 531 | 738.9040 | 1475.7935 | 1475.7944 | -0.59 | 1 | 85 | 1.2e-08 | 1Score **> 35** indicates **identity** Score **> 18** indicates **homology** | U | K.TSKDTTASAVAVGLR.Q |
| 64840 | 517 | – | 531 | 492.9385 | 1475.7938 | 1475.7944 | -0.38 | 1 | 46 | 4.4e-05 | 1Score **> 35** indicates **identity** Score **> 15** indicates **homology** | U | K.TSKDTTASAVAVGLR.Q |
| 64841 | 517 | – | 531 | 738.9054 | 1475.7962 | 1475.7944 | 1.26 | 1 | 61 | 1.9e-06 | 1Score **> 35** indicates **identity** Score **> 16** indicates **homology** | U | K.TSKDTTASAVAVGLR.Q |
| 64842 | 517 | – | 531 | 492.9394 | 1475.7965 | 1475.7944 | 1.44 | 1 | 32 | 0.00096 | 1Score **> 35** indicates **identity** Score **> 15** indicates **homology** | U | K.TSKDTTASAVAVGLR.Q |
| 29284 | 520 | – | 531 | 580.8162 | 1159.6178 | 1159.6197 | -1.67 | 0 | 46 | 4.7e-05 | 1Score **> 34** indicates **identity** Score **> 15** indicates **homology** | U | K.DTTASAVAVGLR.Q |
| 29285 | 520 | – | 531 | 580.8164 | 1159.6182 | 1159.6197 | -1.27 | 0 | 57 | 5e-06 | 1Score **> 34** indicates **identity** Score **> 16** indicates **homology** | U | K.DTTASAVAVGLR.Q |
| 29286 | 520 | – | 531 | 580.8167 | 1159.6189 | 1159.6197 | -0.72 | 0 | 19 | 0.022 | 1Score **> 34** indicates **identity** Score **> 15** indicates **homology** | U | K.DTTASAVAVGLR.Q |
| 29288 | 520 | – | 531 | 580.8169 | 1159.6192 | 1159.6197 | -0.40 | 0 | 46 | 4.9e-05 | 1Score **> 34** indicates **identity** Score **> 15** indicates **homology** | U | K.DTTASAVAVGLR.Q |
| 29289 | 520 | – | 531 | 580.8170 | 1159.6195 | 1159.6197 | -0.18 | 0 | 59 | 2.1e-05 | 1Score **> 34** indicates **identity** Score **> 25** indicates **homology** | U | K.DTTASAVAVGLR.Q |
| 29290 | 520 | – | 531 | 580.8170 | 1159.6195 | 1159.6197 | -0.17 | 0 | 74 | 1.1e-06 | 1Score **> 34** indicates **identity** Score **> 27** indicates **homology** | U | K.DTTASAVAVGLR.Q |
| 29291 | 520 | – | 531 | 580.8171 | 1159.6196 | 1159.6197 | -0.091 | 0 | 55 | 2.3e-05 | 1Score **> 34** indicates **identity** Score **> 21** indicates **homology** | U | K.DTTASAVAVGLR.Q |
| 29292 | 520 | – | 531 | 580.8172 | 1159.6198 | 1159.6197 | 0.058 | 0 | 66 | 5.3e-06 | 1Score **> 34** indicates **identity** Score **> 26** indicates **homology** | U | K.DTTASAVAVGLR.Q |
| 29293 | 520 | – | 531 | 580.8172 | 1159.6198 | 1159.6197 | 0.094 | 0 | 65 | 5.3e-06 | 1Score **> 34** indicates **identity** Score **> 24** indicates **homology** | U | K.DTTASAVAVGLR.Q |
| 29294 | 520 | – | 531 | 580.8172 | 1159.6199 | 1159.6197 | 0.20 | 0 | 17 | 0.024 | 1Score **> 34** indicates **identity** Score **> 14** indicates **homology** | U | K.DTTASAVAVGLR.Q |
| 29297 | 520 | – | 531 | 580.8176 | 1159.6207 | 1159.6197 | 0.82 | 0 | 62 | 1.2e-05 | 1Score **> 34** indicates **identity** Score **> 25** indicates **homology** | U | K.DTTASAVAVGLR.Q |
| 29298 | 520 | – | 531 | 580.8179 | 1159.6213 | 1159.6197 | 1.35 | 0 | 69 | 1.5e-06 | 1Score **> 34** indicates **identity** Score **> 23** indicates **homology** | U | K.DTTASAVAVGLR.Q |
| 29299 | 520 | – | 531 | 580.8188 | 1159.6230 | 1159.6197 | 2.84 | 0 | 56 | 2.1e-05 | 1Score **> 34** indicates **identity** Score **> 22** indicates **homology** | U | K.DTTASAVAVGLR.Q |
| 29300 | 520 | – | 531 | 580.8190 | 1159.6234 | 1159.6197 | 3.14 | 0 | 37 | 0.0013 | 1Score **> 34** indicates **identity** Score **> 21** indicates **homology** | U | K.DTTASAVAVGLR.Q |
| 90059 | 535 | – | 549 | 555.6506 | 1663.9298 | 1663.9298 | 0.046 | 1 | 26 | 0.0035 | 1Score **> 34** indicates **identity** Score **> 14** indicates **homology** | U | K.VIIVVKDGPGFYTTR.C |
| 90060 | 535 | – | 549 | 555.6507 | 1663.9302 | 1663.9298 | 0.26 | 1 | 45 | 5.6e-05 | 1Score **> 34** indicates **identity** Score **> 15** indicates **homology** | U | K.VIIVVKDGPGFYTTR.C |
| 90061 | 535 | – | 549 | 555.6508 | 1663.9306 | 1663.9298 | 0.50 | 1 | 23 | 0.0068 | 1Score **> 34** indicates **identity** Score **> 14** indicates **homology** | U | K.VIIVVKDGPGFYTTR.C |
| 90062 | 535 | – | 549 | 832.9728 | 1663.9310 | 1663.9298 | 0.76 | 1 | 44 | 7e-05 | 1Score **> 34** indicates **identity** Score **> 15** indicates **homology** | U | K.VIIVVKDGPGFYTTR.C |
| 90063 | 535 | – | 549 | 832.9732 | 1663.9319 | 1663.9298 | 1.27 | 1 | 41 | 0.00013 | 1Score **> 33** indicates **identity** Score **> 15** indicates **homology** | U | K.VIIVVKDGPGFYTTR.C |
| 90064 | 535 | – | 549 | 832.9733 | 1663.9320 | 1663.9298 | 1.33 | 1 | 30 | 0.0015 | 1Score **> 33** indicates **identity** Score **> 14** indicates **homology** | U | K.VIIVVKDGPGFYTTR.C |
| 90065 | 535 | – | 549 | 555.6513 | 1663.9321 | 1663.9298 | 1.41 | 1 | 50 | 1.9e-05 | 1Score **> 33** indicates **identity** Score **> 16** indicates **homology** | U | K.VIIVVKDGPGFYTTR.C |
| 90066 | 535 | – | 549 | 555.6515 | 1663.9327 | 1663.9298 | 1.77 | 1 | 21 | 0.0097 | 1Score **> 33** indicates **identity** Score **> 14** indicates **homology** | U | K.VIIVVKDGPGFYTTR.C |
| 90067 | 535 | – | 549 | 555.6515 | 1663.9327 | 1663.9298 | 1.79 | 1 | 48 | 3.3e-05 | 1Score **> 33** indicates **identity** Score **> 15** indicates **homology** | U | K.VIIVVKDGPGFYTTR.C |
| 90068 | 535 | – | 549 | 555.6516 | 1663.9329 | 1663.9298 | 1.88 | 1 | 46 | 4.5e-05 | 1Score **> 33** indicates **identity** Score **> 15** indicates **homology** | U | K.VIIVVKDGPGFYTTR.C |
| 90069 | 535 | – | 549 | 555.6517 | 1663.9333 | 1663.9298 | 2.11 | 1 | 20 | 0.012 | 1Score **> 33** indicates **identity** Score **> 14** indicates **homology** | U | K.VIIVVKDGPGFYTTR.C |
| 15651 | 541 | – | 549 | 507.2381 | 1012.4617 | 1012.4614 | 0.31 | 0 | 36 | 0.00044 | 1Score **> 27** indicates **identity** Score **> 15** indicates **homology** | U | K.DGPGFYTTR.C |
| 15652 | 541 | – | 549 | 507.2385 | 1012.4624 | 1012.4614 | 0.98 | 0 | 45 | 6.6e-05 | 1Score **> 27** indicates **identity** Score **> 15** indicates **homology** | U | K.DGPGFYTTR.C |
| 15653 | 541 | – | 549 | 507.2385 | 1012.4625 | 1012.4614 | 1.07 | 0 | 46 | 4.4e-05 | 1Score **> 27** indicates **identity** Score **> 15** indicates **homology** | U | K.DGPGFYTTR.C |
| 15654 | 541 | – | 549 | 507.2386 | 1012.4627 | 1012.4614 | 1.21 | 0 | 27 | 0.0029 | 1Score **> 27** indicates **identity** Score **> 14** indicates **homology** | U | K.DGPGFYTTR.C |
| 15655 | 541 | – | 549 | 507.2387 | 1012.4628 | 1012.4614 | 1.35 | 0 | 26 | 0.0034 | 1Score **> 27** indicates **identity** Score **> 14** indicates **homology** | U | K.DGPGFYTTR.C |
| 15656 | 541 | – | 549 | 507.2388 | 1012.4630 | 1012.4614 | 1.52 | 0 | 38 | 0.00027 | 1Score **> 27** indicates **identity** Score **> 15** indicates **homology** | U | K.DGPGFYTTR.C |
| 14702 | 561 | – | 569 | 499.7795 | 997.5444 | 997.5444 | -0.013 | 0 | 44 | 9.1e-05 | 1Score **> 29** indicates **identity** Score **> 17** indicates **homology** | U | R.ILQEGVDPK.K |
| 14703 | 561 | – | 569 | 499.7798 | 997.5450 | 997.5444 | 0.58 | 0 | 44 | 0.0001 | 1Score **> 28** indicates **identity** Score **> 17** indicates **homology** | U | R.ILQEGVDPK.K |
| 14704 | 561 | – | 569 | 499.7800 | 997.5454 | 997.5444 | 0.97 | 0 | 42 | 0.00012 | 1Score **> 28** indicates **identity** Score **> 15** indicates **homology** | U | R.ILQEGVDPK.K |
| 15275 | 611 | – | 620 | 503.7809 | 1005.5472 | 1005.5495 | -2.27 | 0 | 18 | 0.033 | 1Score **> 32** indicates **identity** Score **> 15** indicates **homology** | U | R.FGGGSVELLK.Q |
| 15278 | 611 | – | 620 | 503.7813 | 1005.5480 | 1005.5495 | -1.44 | 0 | 14 | 0.05 | 1Score **> 32** indicates **identity** Score **> 13** indicates **homology** | U | R.FGGGSVELLK.Q |
| 15279 | 611 | – | 620 | 503.7815 | 1005.5484 | 1005.5495 | -1.13 | 0 | 19 | 0.018 | 1Score **> 31** indicates **identity** Score **> 14** indicates **homology** | U | R.FGGGSVELLK.Q |
| 15283 | 611 | – | 620 | 503.7820 | 1005.5494 | 1005.5495 | -0.12 | 0 | 62 | 3.7e-06 | 1Score **> 31** indicates **identity** Score **> 20** indicates **homology** | U | R.FGGGSVELLK.Q |
| 15285 | 611 | – | 620 | 503.7821 | 1005.5496 | 1005.5495 | 0.12 | 0 | 60 | 4.7e-06 | 1Score **> 31** indicates **identity** Score **> 20** indicates **homology** | U | R.FGGGSVELLK.Q |
| 15287 | 611 | – | 620 | 503.7821 | 1005.5497 | 1005.5495 | 0.17 | 0 | 60 | 4.6e-06 | 1Score **> 31** indicates **identity** Score **> 20** indicates **homology** | U | R.FGGGSVELLK.Q |
| 15290 | 611 | – | 620 | 503.7822 | 1005.5498 | 1005.5495 | 0.32 | 0 | 42 | 0.00014 | 1Score **> 31** indicates **identity** Score **> 16** indicates **homology** | U | R.FGGGSVELLK.Q |
| 15293 | 611 | – | 620 | 503.7822 | 1005.5499 | 1005.5495 | 0.42 | 0 | 60 | 4.9e-06 | 1Score **> 31** indicates **identity** Score **> 20** indicates **homology** | U | R.FGGGSVELLK.Q |
| 15294 | 611 | – | 620 | 503.7823 | 1005.5501 | 1005.5495 | 0.61 | 0 | 59 | 5.8e-06 | 1Score **> 31** indicates **identity** Score **> 19** indicates **homology** | U | R.FGGGSVELLK.Q |
| 15295 | 611 | – | 620 | 503.7823 | 1005.5501 | 1005.5495 | 0.62 | 0 | 19 | 0.016 | 1Score **> 31** indicates **identity** Score **> 14** indicates **homology** | U | R.FGGGSVELLK.Q |
| 15296 | 611 | – | 620 | 503.7823 | 1005.5501 | 1005.5495 | 0.62 | 0 | 46 | 5.2e-05 | 1Score **> 31** indicates **identity** Score **> 15** indicates **homology** | U | R.FGGGSVELLK.Q |
| 15297 | 611 | – | 620 | 503.7823 | 1005.5501 | 1005.5495 | 0.62 | 0 | 64 | 1.6e-06 | 1Score **> 31** indicates **identity** Score **> 19** indicates **homology** | U | R.FGGGSVELLK.Q |
| 15299 | 611 | – | 620 | 503.7825 | 1005.5504 | 1005.5495 | 0.92 | 0 | 27 | 0.0029 | 1Score **> 31** indicates **identity** Score **> 14** indicates **homology** | U | R.FGGGSVELLK.Q |
| 15301 | 611 | – | 620 | 503.7826 | 1005.5506 | 1005.5495 | 1.05 | 0 | 58 | 7.5e-06 | 1Score **> 31** indicates **identity** Score **> 19** indicates **homology** | U | R.FGGGSVELLK.Q |
| 15304 | 611 | – | 620 | 503.7838 | 1005.5531 | 1005.5495 | 3.59 | 0 | 14 | 0.047 | 1Score **> 31** indicates **identity** Score **> 13** indicates **homology** | U | R.FGGGSVELLK.Q |
| 63198 | 632 | – | 644 | 488.5770 | 1462.7093 | 1462.7092 | 0.033 | 1 | 26 | 0.0039 | 1Score **> 34** indicates **identity** Score **> 14** indicates **homology** | U | K.SGKGFYIYQEGSK.N |
| 63199 | 632 | – | 644 | 732.3623 | 1462.7100 | 1462.7092 | 0.53 | 1 | 36 | 0.00042 | 1Score **> 33** indicates **identity** Score **> 15** indicates **homology** | U | K.SGKGFYIYQEGSK.N |
| 63200 | 632 | – | 644 | 732.3623 | 1462.7101 | 1462.7092 | 0.61 | 1 | 69 | 3.3e-07 | 1Score **> 33** indicates **identity** Score **> 17** indicates **homology** | U | K.SGKGFYIYQEGSK.N |
| 63201 | 632 | – | 644 | 488.5773 | 1462.7102 | 1462.7092 | 0.67 | 1 | 33 | 0.00089 | 1Score **> 33** indicates **identity** Score **> 15** indicates **homology** | U | K.SGKGFYIYQEGSK.N |
| 63202 | 632 | – | 644 | 732.3625 | 1462.7104 | 1462.7092 | 0.77 | 1 | 48 | 2.9e-05 | 1Score **> 33** indicates **identity** Score **> 16** indicates **homology** | U | K.SGKGFYIYQEGSK.N |
| 63204 | 632 | – | 644 | 488.5777 | 1462.7113 | 1462.7092 | 1.44 | 1 | 25 | 0.0042 | 1Score **> 33** indicates **identity** Score **> 14** indicates **homology** | U | K.SGKGFYIYQEGSK.N |
| 32730 | 635 | – | 644 | 596.2848 | 1190.5550 | 1190.5608 | -4.88 | 0 | 18 | 0.021 | 1Score **> 31** indicates **identity** Score **> 14** indicates **homology** | U | K.GFYIYQEGSK.N |
| 32738 | 635 | – | 644 | 596.2862 | 1190.5578 | 1190.5608 | -2.47 | 0 | 28 | 0.0023 | 1Score **> 31** indicates **identity** Score **> 14** indicates **homology** | U | K.GFYIYQEGSK.N |
| 32740 | 635 | – | 644 | 596.2862 | 1190.5579 | 1190.5608 | -2.38 | 0 | 22 | 0.0078 | 1Score **> 31** indicates **identity** Score **> 14** indicates **homology** | U | K.GFYIYQEGSK.N |
| 32745 | 635 | – | 644 | 596.2870 | 1190.5595 | 1190.5608 | -1.06 | 0 | 28 | 0.0024 | 1Score **> 31** indicates **identity** Score **> 14** indicates **homology** | U | K.GFYIYQEGSK.N |
| 32746 | 635 | – | 644 | 596.2872 | 1190.5598 | 1190.5608 | -0.84 | 0 | 29 | 0.002 | 1Score **> 31** indicates **identity** Score **> 14** indicates **homology** | U | K.GFYIYQEGSK.N |
| 32747 | 635 | – | 644 | 596.2873 | 1190.5600 | 1190.5608 | -0.62 | 0 | 34 | 0.00072 | 1Score **> 31** indicates **identity** Score **> 15** indicates **homology** | U | K.GFYIYQEGSK.N |
| 32749 | 635 | – | 644 | 596.2876 | 1190.5606 | 1190.5608 | -0.13 | 0 | 17 | 0.023 | 1Score **> 32** indicates **identity** Score **> 14** indicates **homology** | U | K.GFYIYQEGSK.N |
| 32750 | 635 | – | 644 | 596.2876 | 1190.5607 | 1190.5608 | -0.099 | 0 | 42 | 0.00011 | 1Score **> 32** indicates **identity** Score **> 15** indicates **homology** | U | K.GFYIYQEGSK.N |
| 32751 | 635 | – | 644 | 596.2878 | 1190.5610 | 1190.5608 | 0.15 | 0 | 22 | 0.0091 | 1Score **> 31** indicates **identity** Score **> 14** indicates **homology** | U | K.GFYIYQEGSK.N |
| 32752 | 635 | – | 644 | 596.2878 | 1190.5610 | 1190.5608 | 0.16 | 0 | 38 | 0.00028 | 1Score **> 31** indicates **identity** Score **> 15** indicates **homology** | U | K.GFYIYQEGSK.N |
| 32753 | 635 | – | 644 | 596.2881 | 1190.5617 | 1190.5608 | 0.79 | 0 | 22 | 0.0094 | 1Score **> 31** indicates **identity** Score **> 14** indicates **homology** | U | K.GFYIYQEGSK.N |
| 59603 | 635 | – | 646 | 717.3541 | 1432.6937 | 1432.6987 | -3.48 | 1 | 28 | 0.0021 | 1Score **> 33** indicates **identity** Score **> 14** indicates **homology** | U | K.GFYIYQEGSKNK.S |
| 59612 | 635 | – | 646 | 717.3573 | 1432.7001 | 1432.6987 | 1.04 | 1 | 30 | 0.0016 | 1Score **> 33** indicates **identity** Score **> 14** indicates **homology** | U | K.GFYIYQEGSKNK.S |
| 79743 | 647 | – | 660 | 795.3981 | 1588.7817 | 1588.7879 | -3.87 | 0 | 101 | 3.2e-10 | 1Score **> 34** indicates **identity** Score **> 19** indicates **homology** | U | K.SLNSEMDNILANLR.L |
| 79746 | 647 | – | 660 | 795.3998 | 1588.7850 | 1588.7879 | -1.81 | 0 | 74 | 1.2e-07 | 1Score **> 34** indicates **identity** Score **> 17** indicates **homology** | U | K.SLNSEMDNILANLR.L |
| 82100 | 647 | – | 660 | 803.3966 | 1604.7786 | 1604.7828 | -2.58 | 0 | 28 | 0.0026 | 1Score **> 33** indicates **identity** Score **> 14** indicates **homology** | U | K.SLNSEMDNILANLR.L  + Oxidation (M) |
| 191744 | 647 | – | 676 | 1135.2315 | 3402.6727 | 3402.6725 | 0.055 | 2 | 62 | 1.5e-06 | 1Score **> 37** indicates **identity** Score **> 16** indicates **homology** | U | K.SLNSEMDNILANLRLPAKPEVSSDEDVQYR.V |
| 194269 | 647 | – | 676 | 890.9451 | 3559.7513 | 3559.7715 | -5.69 | 2 | 14 | 0.048 | 1Score **> 37** indicates **identity** Score **> 13** indicates **homology** | U | K.SLNSEMDNILANLRLPAKPEVSSDEDVQYR.V  + Deamidated (NQ); HNE (K) |
| 109989 | 661 | – | 676 | 611.6360 | 1831.8862 | 1831.8952 | -4.89 | 1 | 56 | 5.4e-06 | 1Score **> 34** indicates **identity** Score **> 16** indicates **homology** | U | R.LPAKPEVSSDEDVQYR.V |
| 109990 | 661 | – | 676 | 611.6373 | 1831.8902 | 1831.8952 | -2.74 | 1 | 33 | 0.00089 | 1Score **> 34** indicates **identity** Score **> 15** indicates **homology** | U | R.LPAKPEVSSDEDVQYR.V |
| 109993 | 661 | – | 676 | 611.6384 | 1831.8935 | 1831.8952 | -0.94 | 1 | 88 | 6.1e-09 | 1Score **> 35** indicates **identity** Score **> 18** indicates **homology** | U | R.LPAKPEVSSDEDVQYR.V |
| 109994 | 661 | – | 676 | 611.6385 | 1831.8936 | 1831.8952 | -0.87 | 1 | 25 | 0.0057 | 1Score **> 35** indicates **identity** Score **> 15** indicates **homology** | U | R.LPAKPEVSSDEDVQYR.V |
| 109995 | 661 | – | 676 | 611.6387 | 1831.8943 | 1831.8952 | -0.47 | 1 | 41 | 0.00018 | 1Score **> 35** indicates **identity** Score **> 16** indicates **homology** | U | R.LPAKPEVSSDEDVQYR.V |
| 109996 | 661 | – | 676 | 611.6389 | 1831.8949 | 1831.8952 | -0.17 | 1 | 30 | 0.0017 | 1Score **> 35** indicates **identity** Score **> 14** indicates **homology** | U | R.LPAKPEVSSDEDVQYR.V |
| 109997 | 661 | – | 676 | 611.6390 | 1831.8951 | 1831.8952 | -0.051 | 1 | 90 | 3.3e-09 | 1Score **> 35** indicates **identity** Score **> 18** indicates **homology** | U | R.LPAKPEVSSDEDVQYR.V |
| 109998 | 661 | – | 676 | 611.6391 | 1831.8954 | 1831.8952 | 0.12 | 1 | 92 | 2.2e-09 | 1Score **> 35** indicates **identity** Score **> 18** indicates **homology** | U | R.LPAKPEVSSDEDVQYR.V |
| 109999 | 661 | – | 676 | 611.6391 | 1831.8956 | 1831.8952 | 0.20 | 1 | 87 | 6.8e-09 | 1Score **> 35** indicates **identity** Score **> 18** indicates **homology** | U | R.LPAKPEVSSDEDVQYR.V |
| 110000 | 661 | – | 676 | 611.6393 | 1831.8962 | 1831.8952 | 0.53 | 1 | 49 | 2.5e-05 | 1Score **> 35** indicates **identity** Score **> 16** indicates **homology** | U | R.LPAKPEVSSDEDVQYR.V |
| 110001 | 661 | – | 676 | 611.6395 | 1831.8967 | 1831.8952 | 0.81 | 1 | 31 | 0.0014 | 1Score **> 35** indicates **identity** Score **> 14** indicates **homology** | U | R.LPAKPEVSSDEDVQYR.V |
| 110003 | 661 | – | 676 | 916.9559 | 1831.8973 | 1831.8952 | 1.17 | 1 | 73 | 1.3e-07 | 1Score **> 35** indicates **identity** Score **> 17** indicates **homology** | U | R.LPAKPEVSSDEDVQYR.V |
| 110004 | 661 | – | 676 | 611.6401 | 1831.8985 | 1831.8952 | 1.83 | 1 | 34 | 0.00059 | 1Score **> 35** indicates **identity** Score **> 15** indicates **homology** | U | R.LPAKPEVSSDEDVQYR.V |
| 110005 | 661 | – | 676 | 916.9569 | 1831.8992 | 1831.8952 | 2.16 | 1 | 77 | 6.5e-08 | 1Score **> 35** indicates **identity** Score **> 17** indicates **homology** | U | R.LPAKPEVSSDEDVQYR.V |
| 110006 | 661 | – | 676 | 611.6406 | 1831.9000 | 1831.8952 | 2.64 | 1 | 43 | 9.7e-05 | 1Score **> 35** indicates **identity** Score **> 15** indicates **homology** | U | R.LPAKPEVSSDEDVQYR.V |
| 110007 | 661 | – | 676 | 916.9578 | 1831.9011 | 1831.8952 | 3.23 | 1 | 56 | 5.6e-06 | 1Score **> 35** indicates **identity** Score **> 16** indicates **homology** | U | R.LPAKPEVSSDEDVQYR.V |
| 110008 | 661 | – | 676 | 611.6412 | 1831.9019 | 1831.8952 | 3.64 | 1 | 92 | 2.6e-09 | 1Score **> 35** indicates **identity** Score **> 18** indicates **homology** | U | R.LPAKPEVSSDEDVQYR.V |
| 110009 | 661 | – | 676 | 611.6418 | 1831.9037 | 1831.8952 | 4.65 | 1 | 40 | 0.00022 | 1Score **> 35** indicates **identity** Score **> 16** indicates **homology** | U | R.LPAKPEVSSDEDVQYR.V |
| 110010 | 661 | – | 676 | 611.6423 | 1831.9051 | 1831.8952 | 5.40 | 1 | 16 | 0.03 | 1Score **> 35** indicates **identity** Score **> 14** indicates **homology** | U | R.LPAKPEVSSDEDVQYR.V |
| 110011 | 661 | – | 676 | 611.6424 | 1831.9053 | 1831.8952 | 5.52 | 1 | 75 | 9.2e-08 | 1Score **> 35** indicates **identity** Score **> 17** indicates **homology** | U | R.LPAKPEVSSDEDVQYR.V |
| 17471 | 720 | – | 728 | 520.7739 | 1039.5332 | 1039.5338 | -0.64 | 0 | 30 | 0.0016 | 1Score **> 31** indicates **identity** Score **> 14** indicates **homology** | U | R.FVDLYGAQK.V |
| 17472 | 720 | – | 728 | 520.7739 | 1039.5333 | 1039.5338 | -0.54 | 0 | 33 | 0.00083 | 1Score **> 31** indicates **identity** Score **> 15** indicates **homology** | U | R.FVDLYGAQK.V |
| 17473 | 720 | – | 728 | 520.7740 | 1039.5334 | 1039.5338 | -0.40 | 0 | 36 | 0.00038 | 1Score **> 31** indicates **identity** Score **> 15** indicates **homology** | U | R.FVDLYGAQK.V |
| 17474 | 720 | – | 728 | 520.7740 | 1039.5335 | 1039.5338 | -0.33 | 0 | 45 | 5.4e-05 | 1Score **> 31** indicates **identity** Score **> 15** indicates **homology** | U | R.FVDLYGAQK.V |
| 17475 | 720 | – | 728 | 520.7741 | 1039.5337 | 1039.5338 | -0.17 | 0 | 30 | 0.0014 | 1Score **> 31** indicates **identity** Score **> 14** indicates **homology** | U | R.FVDLYGAQK.V |
| 17476 | 720 | – | 728 | 520.7742 | 1039.5338 | 1039.5338 | -0.079 | 0 | 33 | 0.00083 | 1Score **> 30** indicates **identity** Score **> 15** indicates **homology** | U | R.FVDLYGAQK.V |
| 17477 | 720 | – | 728 | 520.7742 | 1039.5338 | 1039.5338 | -0.054 | 0 | 19 | 0.017 | 1Score **> 30** indicates **identity** Score **> 14** indicates **homology** | U | R.FVDLYGAQK.V |
| 17478 | 720 | – | 728 | 520.7742 | 1039.5338 | 1039.5338 | -0.017 | 0 | 46 | 5.3e-05 | 1Score **> 30** indicates **identity** Score **> 15** indicates **homology** | U | R.FVDLYGAQK.V |
| 17479 | 720 | – | 728 | 520.7742 | 1039.5338 | 1039.5338 | 0.0058 | 0 | 23 | 0.0065 | 1Score **> 30** indicates **identity** Score **> 14** indicates **homology** | U | R.FVDLYGAQK.V |
| 17481 | 720 | – | 728 | 520.7743 | 1039.5340 | 1039.5338 | 0.19 | 0 | 23 | 0.0066 | 1Score **> 30** indicates **identity** Score **> 14** indicates **homology** | U | R.FVDLYGAQK.V |
| 17483 | 720 | – | 728 | 520.7744 | 1039.5343 | 1039.5338 | 0.40 | 0 | 38 | 0.0003 | 1Score **> 30** indicates **identity** Score **> 15** indicates **homology** | U | R.FVDLYGAQK.V |
| 17484 | 720 | – | 728 | 520.7745 | 1039.5345 | 1039.5338 | 0.63 | 0 | 38 | 0.00028 | 1Score **> 30** indicates **identity** Score **> 15** indicates **homology** | U | R.FVDLYGAQK.V |
| 17486 | 720 | – | 728 | 520.7749 | 1039.5352 | 1039.5338 | 1.34 | 0 | 35 | 0.0005 | 1Score **> 31** indicates **identity** Score **> 15** indicates **homology** | U | R.FVDLYGAQK.V |
| 17487 | 720 | – | 728 | 520.7751 | 1039.5357 | 1039.5338 | 1.79 | 0 | 22 | 0.008 | 1Score **> 31** indicates **identity** Score **> 14** indicates **homology** | U | R.FVDLYGAQK.V |
| 17488 | 720 | – | 728 | 520.7756 | 1039.5367 | 1039.5338 | 2.73 | 0 | 17 | 0.026 | 1Score **> 30** indicates **identity** Score **> 14** indicates **homology** | U | R.FVDLYGAQK.V |
| 17490 | 720 | – | 728 | 520.7760 | 1039.5375 | 1039.5338 | 3.54 | 0 | 23 | 0.0071 | 1Score **> 30** indicates **identity** Score **> 14** indicates **homology** | U | R.FVDLYGAQK.V |
| 17491 | 720 | – | 728 | 520.7760 | 1039.5375 | 1039.5338 | 3.55 | 0 | 18 | 0.019 | 1Score **> 30** indicates **identity** Score **> 14** indicates **homology** | U | R.FVDLYGAQK.V |
| 17492 | 720 | – | 728 | 520.7768 | 1039.5390 | 1039.5338 | 4.97 | 0 | 15 | 0.042 | 1Score **> 31** indicates **identity** Score **> 13** indicates **homology** | U | R.FVDLYGAQK.V |
| 186835 | 733 | – | 759 | 786.1417 | 3140.5377 | 3140.5349 | 0.89 | 2 | 22 | 0.0091 | 1Score **> 37** indicates **identity** Score **> 14** indicates **homology** | U | R.LRKYESAYGTQFTPCQLLLDHANNSSK.K |
| 179443 | 735 | – | 759 | 958.1231 | 2871.3476 | 2871.3497 | -0.74 | 1 | 98 | 6.6e-10 | 1Score **> 35** indicates **identity** Score **> 19** indicates **homology** | U | R.KYESAYGTQFTPCQLLLDHANNSSK.K |
| 179444 | 735 | – | 759 | 718.8442 | 2871.3478 | 2871.3497 | -0.68 | 1 | 52 | 1.4e-05 | 1Score **> 35** indicates **identity** Score **> 16** indicates **homology** | U | R.KYESAYGTQFTPCQLLLDHANNSSK.K |
| 179446 | 735 | – | 759 | 718.8445 | 2871.3490 | 2871.3497 | -0.26 | 1 | 67 | 4.9e-07 | 1Score **> 35** indicates **identity** Score **> 17** indicates **homology** | U | R.KYESAYGTQFTPCQLLLDHANNSSK.K |
| 179447 | 735 | – | 759 | 718.8447 | 2871.3495 | 2871.3497 | -0.071 | 1 | 43 | 9.3e-05 | 1Score **> 35** indicates **identity** Score **> 15** indicates **homology** | U | R.KYESAYGTQFTPCQLLLDHANNSSK.K |
| 179448 | 735 | – | 759 | 958.1239 | 2871.3499 | 2871.3497 | 0.061 | 1 | 51 | 1.8e-05 | 1Score **> 35** indicates **identity** Score **> 16** indicates **homology** | U | R.KYESAYGTQFTPCQLLLDHANNSSK.K |
| 179450 | 735 | – | 759 | 958.1243 | 2871.3510 | 2871.3497 | 0.46 | 1 | 114 | 2e-11 | 1Score **> 36** indicates **identity** Score **> 20** indicates **homology** | U | R.KYESAYGTQFTPCQLLLDHANNSSK.K |
| 179452 | 735 | – | 759 | 958.1245 | 2871.3518 | 2871.3497 | 0.72 | 1 | 104 | 1.8e-10 | 1Score **> 36** indicates **identity** Score **> 19** indicates **homology** | U | R.KYESAYGTQFTPCQLLLDHANNSSK.K |
| 179453 | 735 | – | 759 | 1436.6834 | 2871.3522 | 2871.3497 | 0.85 | 1 | 73 | 1.3e-07 | 1Score **> 36** indicates **identity** Score **> 17** indicates **homology** | U | R.KYESAYGTQFTPCQLLLDHANNSSK.K |
| 179454 | 735 | – | 759 | 718.8453 | 2871.3523 | 2871.3497 | 0.89 | 1 | 26 | 0.0034 | 1Score **> 36** indicates **identity** Score **> 14** indicates **homology** | U | R.KYESAYGTQFTPCQLLLDHANNSSK.K |
| 179456 | 735 | – | 759 | 1436.6840 | 2871.3534 | 2871.3497 | 1.27 | 1 | 60 | 2.3e-06 | 1Score **> 36** indicates **identity** Score **> 16** indicates **homology** | U | R.KYESAYGTQFTPCQLLLDHANNSSK.K |
| 179458 | 735 | – | 759 | 718.8457 | 2871.3536 | 2871.3497 | 1.35 | 1 | 59 | 3e-06 | 1Score **> 36** indicates **identity** Score **> 16** indicates **homology** | U | R.KYESAYGTQFTPCQLLLDHANNSSK.K |
| 179459 | 735 | – | 759 | 718.8457 | 2871.3538 | 2871.3497 | 1.42 | 1 | 60 | 2.3e-06 | 1Score **> 36** indicates **identity** Score **> 16** indicates **homology** | U | R.KYESAYGTQFTPCQLLLDHANNSSK.K |
| 179461 | 735 | – | 759 | 958.1267 | 2871.3582 | 2871.3497 | 2.95 | 1 | 37 | 0.00036 | 1Score **> 36** indicates **identity** Score **> 15** indicates **homology** | U | R.KYESAYGTQFTPCQLLLDHANNSSK.K |
| 179491 | 735 | – | 759 | 719.0889 | 2872.3264 | 2872.3337 | -2.55 | 1 | 54 | 8.8e-06 | 1Score **> 34** indicates **identity** Score **> 16** indicates **homology** | U | R.KYESAYGTQFTPCQLLLDHANNSSK.K  + Deamidated (NQ) |
| 179492 | 735 | – | 759 | 958.4494 | 2872.3264 | 2872.3337 | -2.55 | 1 | 97 | 7.7e-10 | 1Score **> 34** indicates **identity** Score **> 19** indicates **homology** | U | R.KYESAYGTQFTPCQLLLDHANNSSK.K  + Deamidated (NQ) |
| 179494 | 735 | – | 759 | 958.4519 | 2872.3339 | 2872.3337 | 0.070 | 1 | 43 | 9.1e-05 | 1Score **> 35** indicates **identity** Score **> 15** indicates **homology** | U | R.KYESAYGTQFTPCQLLLDHANNSSK.K  + Deamidated (NQ) |
| 179495 | 735 | – | 759 | 719.0916 | 2872.3374 | 2872.3337 | 1.26 | 1 | 71 | 2.2e-07 | 1Score **> 35** indicates **identity** Score **> 17** indicates **homology** | U | R.KYESAYGTQFTPCQLLLDHANNSSK.K  + Deamidated (NQ) |
| 179496 | 735 | – | 759 | 958.4540 | 2872.3401 | 2872.3337 | 2.20 | 1 | 106 | 1.1e-10 | 1Score **> 35** indicates **identity** Score **> 19** indicates **homology** | U | R.KYESAYGTQFTPCQLLLDHANNSSK.K  + Deamidated (NQ) |
| 179497 | 735 | – | 759 | 719.0923 | 2872.3401 | 2872.3337 | 2.23 | 1 | 65 | 7.3e-07 | 1Score **> 35** indicates **identity** Score **> 17** indicates **homology** | U | R.KYESAYGTQFTPCQLLLDHANNSSK.K  + Deamidated (NQ) |
| 179498 | 735 | – | 759 | 958.4543 | 2872.3409 | 2872.3337 | 2.50 | 1 | 114 | 2.2e-11 | 1Score **> 35** indicates **identity** Score **> 20** indicates **homology** | U | R.KYESAYGTQFTPCQLLLDHANNSSK.K  + Deamidated (NQ) |
| 179499 | 735 | – | 759 | 719.0938 | 2872.3461 | 2872.3337 | 4.32 | 1 | 15 | 0.042 | 1Score **> 35** indicates **identity** Score **> 13** indicates **homology** | U | R.KYESAYGTQFTPCQLLLDHANNSSK.K  + Deamidated (NQ) |
| 179500 | 735 | – | 759 | 719.0951 | 2872.3511 | 2872.3337 | 6.05 | 1 | 30 | 0.0014 | 1Score **> 35** indicates **identity** Score **> 14** indicates **homology** | U | R.KYESAYGTQFTPCQLLLDHANNSSK.K  + Deamidated (NQ) |
| 179502 | 735 | – | 759 | 958.4583 | 2872.3530 | 2872.3337 | 6.70 | 1 | 61 | 2e-06 | 1Score **> 35** indicates **identity** Score **> 16** indicates **homology** | U | R.KYESAYGTQFTPCQLLLDHANNSSK.K  + Deamidated (NQ) |
| 182663 | 735 | – | 759 | 991.8017 | 2972.3831 | 2972.4113 | -9.48 | 1 | 15 | 0.041 | 1Score **> 35** indicates **identity** Score **> 13** indicates **homology** | U | R.KYESAYGTQFTPCQLLLDHANNSSK.K  + 2 Deamidated (NQ); HNE (C) |
| 182666 | 735 | – | 759 | 991.8036 | 2972.3889 | 2972.4113 | -7.55 | 1 | 16 | 0.032 | 1Score **> 35** indicates **identity** Score **> 13** indicates **homology** | U | R.KYESAYGTQFTPCQLLLDHANNSSK.K  + 2 Deamidated (NQ); HNE (C) |
| 184449 | 735 | – | 759 | 1010.8107 | 3029.4102 | 3029.4328 | -7.46 | 1 | 14 | 0.046 | 1Score **> 35** indicates **identity** Score **> 13** indicates **homology** | U | R.KYESAYGTQFTPCQLLLDHANNSSK.K  + 2 Deamidated (NQ); HNE (K) |
| 183726 | 735 | – | 760 | 750.8672 | 2999.4398 | 2999.4447 | -1.64 | 2 | 24 | 0.0053 | 1Score **> 37** indicates **identity** Score **> 14** indicates **homology** | U | R.KYESAYGTQFTPCQLLLDHANNSSKK.F |
| 183730 | 735 | – | 760 | 750.8684 | 2999.4445 | 2999.4447 | -0.073 | 2 | 26 | 0.004 | 1Score **> 37** indicates **identity** Score **> 14** indicates **homology** | U | R.KYESAYGTQFTPCQLLLDHANNSSKK.F |
| 183732 | 735 | – | 760 | 600.8965 | 2999.4460 | 2999.4447 | 0.44 | 2 | 30 | 0.0016 | 1Score **> 37** indicates **identity** Score **> 14** indicates **homology** | U | R.KYESAYGTQFTPCQLLLDHANNSSKK.F |
| 183733 | 735 | – | 760 | 750.8693 | 2999.4482 | 2999.4447 | 1.18 | 2 | 53 | 1.2e-05 | 1Score **> 37** indicates **identity** Score **> 16** indicates **homology** | U | R.KYESAYGTQFTPCQLLLDHANNSSKK.F |
| 183735 | 735 | – | 760 | 600.8970 | 2999.4486 | 2999.4447 | 1.30 | 2 | 42 | 0.00011 | 1Score **> 37** indicates **identity** Score **> 15** indicates **homology** | U | R.KYESAYGTQFTPCQLLLDHANNSSKK.F |
| 183738 | 735 | – | 760 | 600.8973 | 2999.4499 | 2999.4447 | 1.73 | 2 | 18 | 0.021 | 1Score **> 37** indicates **identity** Score **> 14** indicates **homology** | U | R.KYESAYGTQFTPCQLLLDHANNSSKK.F |
| 183739 | 735 | – | 760 | 1000.8241 | 2999.4504 | 2999.4447 | 1.90 | 2 | 73 | 1.5e-07 | 1Score **> 37** indicates **identity** Score **> 17** indicates **homology** | U | R.KYESAYGTQFTPCQLLLDHANNSSKK.F |
| 183741 | 735 | – | 760 | 600.8974 | 2999.4508 | 2999.4447 | 2.02 | 2 | 45 | 5.7e-05 | 1Score **> 37** indicates **identity** Score **> 15** indicates **homology** | U | R.KYESAYGTQFTPCQLLLDHANNSSKK.F |
| 183742 | 735 | – | 760 | 1000.8250 | 2999.4531 | 2999.4447 | 2.81 | 2 | 45 | 5.7e-05 | 1Score **> 37** indicates **identity** Score **> 15** indicates **homology** | U | R.KYESAYGTQFTPCQLLLDHANNSSKK.F |
| 183768 | 735 | – | 760 | 751.1119 | 3000.4184 | 3000.4287 | -3.43 | 2 | 17 | 0.025 | 1Score **> 36** indicates **identity** Score **> 14** indicates **homology** | U | R.KYESAYGTQFTPCQLLLDHANNSSKK.F  + Deamidated (NQ) |
| 183789 | 735 | – | 760 | 1001.1552 | 3000.4439 | 3000.4287 | 5.06 | 2 | 47 | 4.1e-05 | 1Score **> 37** indicates **identity** Score **> 15** indicates **homology** | U | R.KYESAYGTQFTPCQLLLDHANNSSKK.F  + Deamidated (NQ) |
| 183820 | 735 | – | 760 | 1001.4791 | 3001.4155 | 3001.4127 | 0.95 | 2 | 14 | 0.045 | 1Score **> 36** indicates **identity** Score **> 13** indicates **homology** | U | R.KYESAYGTQFTPCQLLLDHANNSSKK.F  + 2 Deamidated (NQ) |
| 174499 | 736 | – | 759 | 915.4253 | 2743.2540 | 2743.2548 | -0.28 | 0 | 58 | 3.8e-06 | 1Score **> 34** indicates **identity** Score **> 16** indicates **homology** | U | K.YESAYGTQFTPCQLLLDHANNSSK.K |
| 174500 | 736 | – | 759 | 915.4259 | 2743.2559 | 2743.2548 | 0.40 | 0 | 52 | 1.3e-05 | 1Score **> 34** indicates **identity** Score **> 16** indicates **homology** | U | K.YESAYGTQFTPCQLLLDHANNSSK.K |
| 174501 | 736 | – | 759 | 915.4275 | 2743.2606 | 2743.2548 | 2.14 | 0 | 61 | 1.8e-06 | 1Score **> 34** indicates **identity** Score **> 16** indicates **homology** | U | K.YESAYGTQFTPCQLLLDHANNSSK.K |
| 174533 | 736 | – | 759 | 1373.1349 | 2744.2552 | 2744.2388 | 5.98 | 0 | 56 | 5.9e-06 | 1Score **> 34** indicates **identity** Score **> 16** indicates **homology** | U | K.YESAYGTQFTPCQLLLDHANNSSK.K  + Deamidated (NQ) |
| 174538 | 736 | – | 759 | 915.7616 | 2744.2629 | 2744.2388 | 8.80 | 0 | 27 | 0.0032 | 1Score **> 34** indicates **identity** Score **> 14** indicates **homology** | U | K.YESAYGTQFTPCQLLLDHANNSSK.K  + Deamidated (NQ) |
| 174570 | 736 | – | 759 | 916.0881 | 2745.2424 | 2745.2228 | 7.14 | 0 | 35 | 0.00057 | 1Score **> 33** indicates **identity** Score **> 15** indicates **homology** | U | K.YESAYGTQFTPCQLLLDHANNSSK.K  + 2 Deamidated (NQ) |
| 174572 | 736 | – | 759 | 916.0901 | 2745.2483 | 2745.2228 | 9.30 | 0 | 29 | 0.0019 | 1Score **> 33** indicates **identity** Score **> 14** indicates **homology** | U | K.YESAYGTQFTPCQLLLDHANNSSK.K  + 2 Deamidated (NQ) |

---

```
ID   ECHA_MOUSE              Reviewed;         763 AA.
AC   Q8BMS1; Q3TCY3; Q5U5Y5; Q8QZU4;
DT   18-MAR-2008, integrated into UniProtKB/Swiss-Prot.
DT   01-MAR-2003, sequence version 1.
DT   28-JUN-2023, entry version 183.
DE   RecName: Full=Trifunctional enzyme subunit alpha, mitochondrial;
DE   AltName: Full=Monolysocardiolipin acyltransferase {ECO:0000250|UniProtKB:P40939};
DE            EC=2.3.1.- {ECO:0000250|UniProtKB:P40939};
DE   AltName: Full=TP-alpha;
DE   Includes:
DE     RecName: Full=Long-chain enoyl-CoA hydratase;
DE              EC=4.2.1.17 {ECO:0000250|UniProtKB:P40939};
DE   Includes:
DE     RecName: Full=Long chain 3-hydroxyacyl-CoA dehydrogenase;
DE              EC=1.1.1.211 {ECO:0000250|UniProtKB:P40939};
DE   Flags: Precursor;
GN   Name=Hadha;
OS   Mus musculus (Mouse).
OC   Eukaryota; Metazoa; Chordata; Craniata; Vertebrata; Euteleostomi; Mammalia;
OC   Eutheria; Euarchontoglires; Glires; Rodentia; Myomorpha; Muroidea; Muridae;
OC   Murinae; Mus; Mus.
OX   NCBI_TaxID=10090;
RN   [1]
RP   NUCLEOTIDE SEQUENCE [LARGE SCALE MRNA].
RC   STRAIN=C57BL/6J, and NOD; TISSUE=Skin;
RX   PubMed=16141072; DOI=10.1126/science.1112014;
RA   Carninci P., Kasukawa T., Katayama S., Gough J., Frith M.C., Maeda N.,
RA   Oyama R., Ravasi T., Lenhard B., Wells C., Kodzius R., Shimokawa K.,
RA   Bajic V.B., Brenner S.E., Batalov S., Forrest A.R., Zavolan M., Davis M.J.,
RA   Wilming L.G., Aidinis V., Allen J.E., Ambesi-Impiombato A., Apweiler R.,
RA   Aturaliya R.N., Bailey T.L., Bansal M., Baxter L., Beisel K.W., Bersano T.,
RA   Bono H., Chalk A.M., Chiu K.P., Choudhary V., Christoffels A.,
RA   Clutterbuck D.R., Crowe M.L., Dalla E., Dalrymple B.P., de Bono B.,
RA   Della Gatta G., di Bernardo D., Down T., Engstrom P., Fagiolini M.,
RA   Faulkner G., Fletcher C.F., Fukushima T., Furuno M., Futaki S.,
RA   Gariboldi M., Georgii-Hemming P., Gingeras T.R., Gojobori T., Green R.E.,
RA   Gustincich S., Harbers M., Hayashi Y., Hensch T.K., Hirokawa N., Hill D.,
RA   Huminiecki L., Iacono M., Ikeo K., Iwama A., Ishikawa T., Jakt M.,
RA   Kanapin A., Katoh M., Kawasawa Y., Kelso J., Kitamura H., Kitano H.,
RA   Kollias G., Krishnan S.P., Kruger A., Kummerfeld S.K., Kurochkin I.V.,
RA   Lareau L.F., Lazarevic D., Lipovich L., Liu J., Liuni S., McWilliam S.,
RA   Madan Babu M., Madera M., Marchionni L., Matsuda H., Matsuzawa S., Miki H.,
RA   Mignone F., Miyake S., Morris K., Mottagui-Tabar S., Mulder N., Nakano N.,
RA   Nakauchi H., Ng P., Nilsson R., Nishiguchi S., Nishikawa S., Nori F.,
RA   Ohara O., Okazaki Y., Orlando V., Pang K.C., Pavan W.J., Pavesi G.,
RA   Pesole G., Petrovsky N., Piazza S., Reed J., Reid J.F., Ring B.Z.,
RA   Ringwald M., Rost B., Ruan Y., Salzberg S.L., Sandelin A., Schneider C.,
RA   Schoenbach C., Sekiguchi K., Semple C.A., Seno S., Sessa L., Sheng Y.,
RA   Shibata Y., Shimada H., Shimada K., Silva D., Sinclair B., Sperling S.,
RA   Stupka E., Sugiura K., Sultana R., Takenaka Y., Taki K., Tammoja K.,
RA   Tan S.L., Tang S., Taylor M.S., Tegner J., Teichmann S.A., Ueda H.R.,
RA   van Nimwegen E., Verardo R., Wei C.L., Yagi K., Yamanishi H.,
RA   Zabarovsky E., Zhu S., Zimmer A., Hide W., Bult C., Grimmond S.M.,
RA   Teasdale R.D., Liu E.T., Brusic V., Quackenbush J., Wahlestedt C.,
RA   Mattick J.S., Hume D.A., Kai C., Sasaki D., Tomaru Y., Fukuda S.,
RA   Kanamori-Katayama M., Suzuki M., Aoki J., Arakawa T., Iida J., Imamura K.,
RA   Itoh M., Kato T., Kawaji H., Kawagashira N., Kawashima T., Kojima M.,
RA   Kondo S., Konno H., Nakano K., Ninomiya N., Nishio T., Okada M., Plessy C.,
RA   Shibata K., Shiraki T., Suzuki S., Tagami M., Waki K., Watahiki A.,
RA   Okamura-Oho Y., Suzuki H., Kawai J., Hayashizaki Y.;
RT   "The transcriptional landscape of the mammalian genome.";
RL   Science 309:1559-1563(2005).
RN   [2]
RP   NUCLEOTIDE SEQUENCE [LARGE SCALE MRNA].
RC   STRAIN=FVB/N; TISSUE=Eye, Liver, and Olfactory epithelium;
RX   PubMed=15489334; DOI=10.1101/gr.2596504;
RG   The MGC Project Team;
RT   "The status, quality, and expansion of the NIH full-length cDNA project:
RT   the Mammalian Gene Collection (MGC).";
RL   Genome Res. 14:2121-2127(2004).
RN   [3]
RP   ACETYLATION [LARGE SCALE ANALYSIS] AT LYS-129, AND IDENTIFICATION BY MASS
RP   SPECTROMETRY [LARGE SCALE ANALYSIS].
RC   TISSUE=Liver;
RX   PubMed=16916647; DOI=10.1016/j.molcel.2006.06.026;
RA   Kim S.C., Sprung R., Chen Y., Xu Y., Ball H., Pei J., Cheng T., Kho Y.,
RA   Xiao H., Xiao L., Grishin N.V., White M., Yang X.-J., Zhao Y.;
RT   "Substrate and functional diversity of lysine acetylation revealed by a
RT   proteomics survey.";
RL   Mol. Cell 23:607-618(2006).
RN   [4]
RP   PHOSPHORYLATION [LARGE SCALE ANALYSIS] AT SER-231; SER-316; THR-395 AND
RP   SER-647, AND IDENTIFICATION BY MASS SPECTROMETRY [LARGE SCALE ANALYSIS].
RC   TISSUE=Brain, Brown adipose tissue, Heart, Kidney, Liver, Lung,
RC   Pancreas, Spleen, and Testis;
RX   PubMed=21183079; DOI=10.1016/j.cell.2010.12.001;
RA   Huttlin E.L., Jedrychowski M.P., Elias J.E., Goswami T., Rad R.,
RA   Beausoleil S.A., Villen J., Haas W., Sowa M.E., Gygi S.P.;
RT   "A tissue-specific atlas of mouse protein phosphorylation and expression.";
RL   Cell 143:1174-1189(2010).
RN   [5]
RP   ACETYLATION [LARGE SCALE ANALYSIS] AT LYS-60 AND LYS-406, SUCCINYLATION
RP   [LARGE SCALE ANALYSIS] AT LYS-46; LYS-60; LYS-166; LYS-213; LYS-214;
RP   LYS-230; LYS-249; LYS-303; LYS-326; LYS-334; LYS-350; LYS-406; LYS-411;
RP   LYS-415; LYS-436; LYS-440; LYS-460; LYS-505; LYS-519; LYS-569; LYS-620;
RP   LYS-634; LYS-644; LYS-646; LYS-664; LYS-728 AND LYS-759, AND IDENTIFICATION
RP   BY MASS SPECTROMETRY [LARGE SCALE ANALYSIS].
RC   TISSUE=Embryonic fibroblast, and Liver;
RX   PubMed=23806337; DOI=10.1016/j.molcel.2013.06.001;
RA   Park J., Chen Y., Tishkoff D.X., Peng C., Tan M., Dai L., Xie Z., Zhang Y.,
RA   Zwaans B.M., Skinner M.E., Lombard D.B., Zhao Y.;
RT   "SIRT5-mediated lysine desuccinylation impacts diverse metabolic
RT   pathways.";
RL   Mol. Cell 50:919-930(2013).
RN   [6]
RP   ACETYLATION [LARGE SCALE ANALYSIS] AT LYS-46; LYS-60; LYS-129; LYS-166;
RP   LYS-214; LYS-249; LYS-289; LYS-303; LYS-326; LYS-334; LYS-350; LYS-353;
RP   LYS-406; LYS-411; LYS-436; LYS-460; LYS-505; LYS-519; LYS-540; LYS-569;
RP   LYS-644; LYS-664; LYS-728; LYS-735 AND LYS-759, AND IDENTIFICATION BY MASS
RP   SPECTROMETRY [LARGE SCALE ANALYSIS].
RC   TISSUE=Liver;
RX   PubMed=23576753; DOI=10.1073/pnas.1302961110;
RA   Rardin M.J., Newman J.C., Held J.M., Cusack M.P., Sorensen D.J., Li B.,
RA   Schilling B., Mooney S.D., Kahn C.R., Verdin E., Gibson B.W.;
RT   "Label-free quantitative proteomics of the lysine acetylome in mitochondria
RT   identifies substrates of SIRT3 in metabolic pathways.";
RL   Proc. Natl. Acad. Sci. U.S.A. 110:6601-6606(2013).
RN   [7]
RP   METHYLATION [LARGE SCALE ANALYSIS] AT ARG-399, AND IDENTIFICATION BY MASS
RP   SPECTROMETRY [LARGE SCALE ANALYSIS].
RC   TISSUE=Brain;
RX   PubMed=24129315; DOI=10.1074/mcp.o113.027870;
RA   Guo A., Gu H., Zhou J., Mulhern D., Wang Y., Lee K.A., Yang V., Aguiar M.,
RA   Kornhauser J., Jia X., Ren J., Beausoleil S.A., Silva J.C., Vemulapalli V.,
RA   Bedford M.T., Comb M.J.;
RT   "Immunoaffinity enrichment and mass spectrometry analysis of protein
RT   methylation.";
RL   Mol. Cell. Proteomics 13:372-387(2014).
RN   [8]
RP   INTERACTION WITH MTLN, AND IDENTIFICATION BY MASS SPECTROMETRY.
RX   PubMed=29949755; DOI=10.1016/j.celrep.2018.05.058;
RA   Makarewich C.A., Baskin K.K., Munir A.Z., Bezprozvannaya S., Sharma G.,
RA   Khemtong C., Shah A.M., McAnally J.R., Malloy C.R., Szweda L.I.,
RA   Bassel-Duby R., Olson E.N.;
RT   "MOXI Is a Mitochondrial Micropeptide That Enhances Fatty Acid beta-
RT   Oxidation.";
RL   Cell Rep. 23:3701-3709(2018).
CC   -!- FUNCTION: Mitochondrial trifunctional enzyme catalyzes the last three
CC       of the four reactions of the mitochondrial beta-oxidation pathway. The
CC       mitochondrial beta-oxidation pathway is the major energy-producing
CC       process in tissues and is performed through four consecutive reactions
CC       breaking down fatty acids into acetyl-CoA. Among the enzymes involved
CC       in this pathway, the trifunctional enzyme exhibits specificity for
CC       long-chain fatty acids. Mitochondrial trifunctional enzyme is a
CC       heterotetrameric complex composed of two proteins, the trifunctional
CC       enzyme subunit alpha/HADHA described here carries the 2,3-enoyl-CoA
CC       hydratase and the 3-hydroxyacyl-CoA dehydrogenase activities while the
CC       trifunctional enzyme subunit beta/HADHB bears the 3-ketoacyl-CoA
CC       thiolase activity. Independently of the subunit beta, the trifunctional
CC       enzyme subunit alpha/HADHA also has a monolysocardiolipin
CC       acyltransferase activity. It acylates monolysocardiolipin into
CC       cardiolipin, a major mitochondrial membrane phospholipid which plays a
CC       key role in apoptosis and supports mitochondrial respiratory chain
CC       complexes in the generation of ATP. Allows the acylation of
CC       monolysocardiolipin with different acyl-CoA substrates including
CC       oleoyl-CoA for which it displays the highest activity.
CC       {ECO:0000250|UniProtKB:P40939}.
CC   -!- CATALYTIC ACTIVITY:
CC       Reaction=a (3S)-3-hydroxyacyl-CoA = a (2E)-enoyl-CoA + H2O;
CC         Xref=Rhea:RHEA:16105, ChEBI:CHEBI:15377, ChEBI:CHEBI:57318,
CC         ChEBI:CHEBI:58856; EC=4.2.1.17;
CC         Evidence={ECO:0000250|UniProtKB:P40939};
CC       PhysiologicalDirection=right-to-left; Xref=Rhea:RHEA:16107;
CC         Evidence={ECO:0000250|UniProtKB:P40939};
CC   -!- CATALYTIC ACTIVITY:
CC       Reaction=a 4-saturated-(3S)-3-hydroxyacyl-CoA = a (3E)-enoyl-CoA + H2O;
CC         Xref=Rhea:RHEA:20724, ChEBI:CHEBI:15377, ChEBI:CHEBI:58521,
CC         ChEBI:CHEBI:137480; EC=4.2.1.17;
CC         Evidence={ECO:0000250|UniProtKB:P40939};
CC       PhysiologicalDirection=right-to-left; Xref=Rhea:RHEA:20726;
CC         Evidence={ECO:0000250|UniProtKB:P40939};
CC   -!- CATALYTIC ACTIVITY:
CC       Reaction=(3S)-hydroxyoctanoyl-CoA = (2E)-octenoyl-CoA + H2O;
CC         Xref=Rhea:RHEA:31199, ChEBI:CHEBI:15377, ChEBI:CHEBI:62242,
CC         ChEBI:CHEBI:62617; Evidence={ECO:0000250|UniProtKB:P40939};
CC       PhysiologicalDirection=right-to-left; Xref=Rhea:RHEA:31201;
CC         Evidence={ECO:0000250|UniProtKB:P40939};
CC   -!- CATALYTIC ACTIVITY:
CC       Reaction=(3S)-3-hydroxydodecanoyl-CoA = (2E)-dodecenoyl-CoA + H2O;
CC         Xref=Rhea:RHEA:31075, ChEBI:CHEBI:15377, ChEBI:CHEBI:57330,
CC         ChEBI:CHEBI:62558; Evidence={ECO:0000250|UniProtKB:P40939};
CC       PhysiologicalDirection=right-to-left; Xref=Rhea:RHEA:31077;
CC         Evidence={ECO:0000250|UniProtKB:P40939};
CC   -!- CATALYTIC ACTIVITY:
CC       Reaction=(3S)-hydroxyhexadecanoyl-CoA = (2E)-hexadecenoyl-CoA + H2O;
CC         Xref=Rhea:RHEA:31163, ChEBI:CHEBI:15377, ChEBI:CHEBI:61526,
CC         ChEBI:CHEBI:62613; Evidence={ECO:0000250|UniProtKB:P40939};
CC       PhysiologicalDirection=right-to-left; Xref=Rhea:RHEA:31165;
CC         Evidence={ECO:0000250|UniProtKB:P40939};
CC   -!- CATALYTIC ACTIVITY:
CC       Reaction=a long-chain (3S)-3-hydroxy fatty acyl-CoA + NAD(+) = a long-
CC         chain 3-oxo-fatty acyl-CoA + H(+) + NADH; Xref=Rhea:RHEA:52656,
CC         ChEBI:CHEBI:15378, ChEBI:CHEBI:57540, ChEBI:CHEBI:57945,
CC         ChEBI:CHEBI:136757, ChEBI:CHEBI:136758; EC=1.1.1.211;
CC         Evidence={ECO:0000250|UniProtKB:P40939};
CC       PhysiologicalDirection=left-to-right; Xref=Rhea:RHEA:52657;
CC         Evidence={ECO:0000250|UniProtKB:P40939};
CC   -!- CATALYTIC ACTIVITY:
CC       Reaction=(3S)-hydroxyoctanoyl-CoA + NAD(+) = 3-oxooctanoyl-CoA + H(+) +
CC         NADH; Xref=Rhea:RHEA:31195, ChEBI:CHEBI:15378, ChEBI:CHEBI:57540,
CC         ChEBI:CHEBI:57945, ChEBI:CHEBI:62617, ChEBI:CHEBI:62619;
CC         Evidence={ECO:0000250|UniProtKB:P40939};
CC       PhysiologicalDirection=left-to-right; Xref=Rhea:RHEA:31196;
CC         Evidence={ECO:0000250|UniProtKB:P40939};
CC   -!- CATALYTIC ACTIVITY:
CC       Reaction=(3S)-hydroxydecanoyl-CoA + NAD(+) = 3-oxodecanoyl-CoA + H(+) +
CC         NADH; Xref=Rhea:RHEA:31187, ChEBI:CHEBI:15378, ChEBI:CHEBI:57540,
CC         ChEBI:CHEBI:57945, ChEBI:CHEBI:62548, ChEBI:CHEBI:62616;
CC         Evidence={ECO:0000250|UniProtKB:P40939};
CC       PhysiologicalDirection=left-to-right; Xref=Rhea:RHEA:31188;
CC         Evidence={ECO:0000250|UniProtKB:P40939};
CC   -!- CATALYTIC ACTIVITY:
CC       Reaction=(3S)-3-hydroxydodecanoyl-CoA + NAD(+) = 3-oxododecanoyl-CoA +
CC         H(+) + NADH; Xref=Rhea:RHEA:31179, ChEBI:CHEBI:15378,
CC         ChEBI:CHEBI:57540, ChEBI:CHEBI:57945, ChEBI:CHEBI:62558,
CC         ChEBI:CHEBI:62615; Evidence={ECO:0000250|UniProtKB:P40939};
CC       PhysiologicalDirection=left-to-right; Xref=Rhea:RHEA:31180;
CC         Evidence={ECO:0000250|UniProtKB:P40939};
CC   -!- CATALYTIC ACTIVITY:
CC       Reaction=(3S)-hydroxytetradecanoyl-CoA + NAD(+) = 3-oxotetradecanoyl-
CC         CoA + H(+) + NADH; Xref=Rhea:RHEA:31167, ChEBI:CHEBI:15378,
CC         ChEBI:CHEBI:57540, ChEBI:CHEBI:57945, ChEBI:CHEBI:62543,
CC         ChEBI:CHEBI:62614; Evidence={ECO:0000250|UniProtKB:P40939};
CC       PhysiologicalDirection=left-to-right; Xref=Rhea:RHEA:31168;
CC         Evidence={ECO:0000250|UniProtKB:P40939};
CC   -!- CATALYTIC ACTIVITY:
CC       Reaction=(3S)-hydroxyhexadecanoyl-CoA + NAD(+) = 3-oxohexadecanoyl-CoA
CC         + H(+) + NADH; Xref=Rhea:RHEA:31159, ChEBI:CHEBI:15378,
CC         ChEBI:CHEBI:57349, ChEBI:CHEBI:57540, ChEBI:CHEBI:57945,
CC         ChEBI:CHEBI:62613; Evidence={ECO:0000250|UniProtKB:P40939};
CC       PhysiologicalDirection=left-to-right; Xref=Rhea:RHEA:31160;
CC         Evidence={ECO:0000250|UniProtKB:P40939};
CC   -!- CATALYTIC ACTIVITY:
CC       Reaction=1'-[1,2-di-(9Z,12Z-octadecadienoyl)-sn-glycero-3-phospho]-3'-
CC         [1-(9Z,12Z-octadecadienoyl)-sn-glycero-3-phospho]-glycerol +
CC         hexadecanoyl-CoA = 1'-[1,2-di-(9Z,12Z-octadecadienoyl)-sn-glycero-3-
CC         phospho]-3'-[1-(9Z,12Z-octadecadienoyl)-2-hexadecanoyl-sn-glycero-3-
CC         phospho]-glycerol + CoA; Xref=Rhea:RHEA:43680, ChEBI:CHEBI:57287,
CC         ChEBI:CHEBI:57379, ChEBI:CHEBI:83580, ChEBI:CHEBI:83583;
CC         Evidence={ECO:0000250|UniProtKB:P40939};
CC       PhysiologicalDirection=left-to-right; Xref=Rhea:RHEA:43681;
CC         Evidence={ECO:0000250|UniProtKB:P40939};
CC   -!- CATALYTIC ACTIVITY:
CC       Reaction=(9Z)-octadecenoyl-CoA + 1'-[1,2-di-(9Z,12Z-octadecadienoyl)-
CC         sn-glycero-3-phospho]-3'-[1-(9Z,12Z-octadecadienoyl)-sn-glycero-3-
CC         phospho]-glycerol = 1'-[1,2-di-(9Z,12Z-octadecadienoyl)-sn-glycero-3-
CC         phospho]-3'-[1-(9Z,12Z-octadecadienoyl)-2-(9Z-octadecenoyl)-sn-
CC         glycero-3-phospho]-glycerol + CoA; Xref=Rhea:RHEA:43676,
CC         ChEBI:CHEBI:57287, ChEBI:CHEBI:57387, ChEBI:CHEBI:83580,
CC         ChEBI:CHEBI:83582; Evidence={ECO:0000250|UniProtKB:P40939};
CC       PhysiologicalDirection=left-to-right; Xref=Rhea:RHEA:43677;
CC         Evidence={ECO:0000250|UniProtKB:P40939};
CC   -!- CATALYTIC ACTIVITY:
CC       Reaction=(9Z,12Z)-octadecadienoyl-CoA + 1'-[1,2-di-(9Z,12Z-
CC         octadecadienoyl)-sn-glycero-3-phospho]-3'-[1-(9Z,12Z-
CC         octadecadienoyl)-sn-glycero-3-phospho]-glycerol = 1',3'-bis-[1,2-di-
CC         (9Z,12Z-octadecadienoyl)-sn-glycero-3-phospho]-glycerol + CoA;
CC         Xref=Rhea:RHEA:43672, ChEBI:CHEBI:57287, ChEBI:CHEBI:57383,
CC         ChEBI:CHEBI:83580, ChEBI:CHEBI:83581;
CC         Evidence={ECO:0000250|UniProtKB:P40939};
CC       PhysiologicalDirection=left-to-right; Xref=Rhea:RHEA:43673;
CC         Evidence={ECO:0000250|UniProtKB:P40939};
CC   -!- PATHWAY: Lipid metabolism; fatty acid beta-oxidation.
CC       {ECO:0000250|UniProtKB:P40939}.
CC   -!- SUBUNIT: Heterotetramer of 2 alpha/HADHA and 2 beta/HADHB subunits;
CC       forms the mitochondrial trifunctional enzyme (By similarity). Also
CC       purified as higher order heterooligomers including a 4 alpha/HADHA and
CC       4 beta/HADHB heterooligomer which physiological significance remains
CC       unclear (By similarity). The mitochondrial trifunctional enzyme
CC       interacts with MTLN (PubMed:29949755). {ECO:0000250|UniProtKB:P40939,
CC       ECO:0000269|PubMed:29949755}.
CC   -!- SUBCELLULAR LOCATION: Mitochondrion {ECO:0000250|UniProtKB:P40939}.
CC       Mitochondrion inner membrane {ECO:0000250|UniProtKB:P40939}.
CC       Note=Protein stability and association with mitochondrion inner
CC       membrane do not require HADHB. {ECO:0000250|UniProtKB:P40939}.
CC   -!- PTM: Acetylation of Lys-569 and Lys-728 is observed in liver
CC       mitochondria from fasted mice but not from fed mice.
CC   -!- SIMILARITY: In the N-terminal section; belongs to the enoyl-CoA
CC       hydratase/isomerase family. {ECO:0000305}.
CC   -!- SIMILARITY: In the central section; belongs to the 3-hydroxyacyl-CoA
CC       dehydrogenase family. {ECO:0000305}.
CC   ---------------------------------------------------------------------------
CC   Copyrighted by the UniProt Consortium, see https://www.uniprot.org/terms
CC   Distributed under the Creative Commons Attribution (CC BY 4.0) License
CC   ---------------------------------------------------------------------------
DR   EMBL; AK029017; BAC26245.1; -; mRNA.
DR   EMBL; AK170478; BAE41822.1; -; mRNA.
DR   EMBL; AK170683; BAE41956.1; -; mRNA.
DR   EMBL; BC027156; AAH27156.1; -; mRNA.
DR   EMBL; BC037009; AAH37009.1; -; mRNA.
DR   EMBL; BC046978; AAH46978.1; -; mRNA.
DR   EMBL; BC058569; AAH58569.1; -; mRNA.
DR   CCDS; CCDS19155.1; -.
DR   RefSeq; NP_849209.1; NM_178878.2.
DR   AlphaFoldDB; Q8BMS1; -.
DR   SMR; Q8BMS1; -.
DR   BioGRID; 220648; 43.
DR   IntAct; Q8BMS1; 8.
DR   MINT; Q8BMS1; -.
DR   STRING; 10090.ENSMUSP00000120976; -.
DR   GlyGen; Q8BMS1; 1 site, 1 O-linked glycan (1 site).
DR   iPTMnet; Q8BMS1; -.
DR   PhosphoSitePlus; Q8BMS1; -.
DR   SwissPalm; Q8BMS1; -.
DR   REPRODUCTION-2DPAGE; IPI00223092; -.
DR   EPD; Q8BMS1; -.
DR   jPOST; Q8BMS1; -.
DR   MaxQB; Q8BMS1; -.
DR   PaxDb; Q8BMS1; -.
DR   PeptideAtlas; Q8BMS1; -.
DR   ProteomicsDB; 277753; -.
DR   Antibodypedia; 3074; 238 antibodies from 32 providers.
DR   DNASU; 97212; -.
DR   Ensembl; ENSMUST00000156859; ENSMUSP00000120976; ENSMUSG00000025745.
DR   GeneID; 97212; -.
DR   KEGG; mmu:97212; -.
DR   UCSC; uc008wvc.1; mouse.
DR   AGR; MGI:2135593; -.
DR   CTD; 3030; -.
DR   MGI; MGI:2135593; Hadha.
DR   VEuPathDB; HostDB:ENSMUSG00000025745; -.
DR   eggNOG; KOG1683; Eukaryota.
DR   GeneTree; ENSGT00940000154677; -.
DR   HOGENOM; CLU_009834_16_1_1; -.
DR   InParanoid; Q8BMS1; -.
DR   OMA; PFRYMDT; -.
DR   OrthoDB; 622692at2759; -.
DR   PhylomeDB; Q8BMS1; -.
DR   TreeFam; TF352288; -.
DR   Reactome; R-MMU-1482798; Acyl chain remodeling of CL.
DR   Reactome; R-MMU-77285; Beta oxidation of myristoyl-CoA to lauroyl-CoA.
DR   Reactome; R-MMU-77305; Beta oxidation of palmitoyl-CoA to myristoyl-CoA.
DR   Reactome; R-MMU-77310; Beta oxidation of lauroyl-CoA to decanoyl-CoA-CoA.
DR   Reactome; R-MMU-77346; Beta oxidation of decanoyl-CoA to octanoyl-CoA-CoA.
DR   Reactome; R-MMU-77348; Beta oxidation of octanoyl-CoA to hexanoyl-CoA.
DR   Reactome; R-MMU-77350; Beta oxidation of hexanoyl-CoA to butanoyl-CoA.
DR   UniPathway; UPA00659; -.
DR   BioGRID-ORCS; 97212; 4 hits in 79 CRISPR screens.
DR   ChiTaRS; Hadha; mouse.
DR   PRO; PR:Q8BMS1; -.
DR   Proteomes; UP000000589; Chromosome 5.
DR   RNAct; Q8BMS1; protein.
DR   Bgee; ENSMUSG00000025745; Expressed in myocardium of ventricle and 247 other tissues.
DR   Genevisible; Q8BMS1; MM.
DR   GO; GO:0016507; C:mitochondrial fatty acid beta-oxidation multienzyme complex; ISO:MGI.
DR   GO; GO:0005743; C:mitochondrial inner membrane; HDA:MGI.
DR   GO; GO:0042645; C:mitochondrial nucleoid; ISO:MGI.
DR   GO; GO:0005739; C:mitochondrion; HDA:MGI.
DR   GO; GO:0003857; F:3-hydroxyacyl-CoA dehydrogenase activity; ISO:MGI.
DR   GO; GO:0003988; F:acetyl-CoA C-acyltransferase activity; ISO:MGI.
DR   GO; GO:0004300; F:enoyl-CoA hydratase activity; ISO:MGI.
DR   GO; GO:0000062; F:fatty-acyl-CoA binding; ISO:MGI.
DR   GO; GO:0016509; F:long-chain-3-hydroxyacyl-CoA dehydrogenase activity; IDA:MGI.
DR   GO; GO:0051287; F:NAD binding; ISO:MGI.
DR   GO; GO:0070403; F:NAD+ binding; IEA:InterPro.
DR   GO; GO:0044877; F:protein-containing complex binding; ISO:MGI.
DR   GO; GO:0035965; P:cardiolipin acyl-chain remodeling; ISS:UniProtKB.
DR   GO; GO:0006635; P:fatty acid beta-oxidation; IMP:MGI.
DR   GO; GO:0032868; P:response to insulin; IMP:MGI.
DR   GO; GO:0009410; P:response to xenobiotic stimulus; ISO:MGI.
DR   CDD; cd06558; crotonase-like; 1.
DR   Gene3D; 1.10.1040.50; -; 1.
DR   Gene3D; 3.40.50.720; NAD(P)-binding Rossmann-like Domain; 1.
DR   InterPro; IPR006180; 3-OHacyl-CoA_DH_CS.
DR   InterPro; IPR006176; 3-OHacyl-CoA_DH_NAD-bd.
DR   InterPro; IPR006108; 3HC_DH_C.
DR   InterPro; IPR008927; 6-PGluconate_DH-like_C_sf.
DR   InterPro; IPR029045; ClpP/crotonase-like_dom_sf.
DR   InterPro; IPR018376; Enoyl-CoA_hyd/isom_CS.
DR   InterPro; IPR001753; Enoyl-CoA_hydra/iso.
DR   InterPro; IPR012803; Fa_ox_alpha_mit.
DR   InterPro; IPR036291; NAD(P)-bd_dom_sf.
DR   PANTHER; PTHR43612; TRIFUNCTIONAL ENZYME SUBUNIT ALPHA; 1.
DR   PANTHER; PTHR43612:SF3; TRIFUNCTIONAL ENZYME SUBUNIT ALPHA, MITOCHONDRIAL; 1.
DR   Pfam; PF00725; 3HCDH; 2.
DR   Pfam; PF02737; 3HCDH_N; 1.
DR   Pfam; PF00378; ECH_1; 1.
DR   SUPFAM; SSF48179; 6-phosphogluconate dehydrogenase C-terminal domain-like; 2.
DR   SUPFAM; SSF52096; ClpP/crotonase; 1.
DR   SUPFAM; SSF51735; NAD(P)-binding Rossmann-fold domains; 1.
DR   PROSITE; PS00067; 3HCDH; 1.
DR   PROSITE; PS00166; ENOYL_COA_HYDRATASE; 1.
DR   TIGRFAMs; TIGR02441; fa_ox_alpha_mit; 1.
PE   1: Evidence at protein level;
KW   Acetylation; Fatty acid metabolism; Lipid metabolism; Lyase; Membrane;
KW   Methylation; Mitochondrion; Mitochondrion inner membrane;
KW   Multifunctional enzyme; NAD; Oxidoreductase; Phosphoprotein;
KW   Reference proteome; Transferase; Transit peptide.
FT   TRANSIT         1..36
FT                   /note="Mitochondrion"
FT                   /evidence="ECO:0000255"
FT   CHAIN           37..763
FT                   /note="Trifunctional enzyme subunit alpha, mitochondrial"
FT                   /id="PRO_0000322639"
FT   ACT_SITE        510
FT                   /note="For hydroxyacyl-coenzyme A dehydrogenase activity"
FT                   /evidence="ECO:0000250|UniProtKB:P40939"
FT   SITE            151
FT                   /note="Important for long-chain enoyl-CoA hydratase
FT                   activity"
FT                   /evidence="ECO:0000250|UniProtKB:P40939"
FT   SITE            173
FT                   /note="Important for long-chain enoyl-CoA hydratase
FT                   activity"
FT                   /evidence="ECO:0000250|UniProtKB:P40939"
FT   SITE            498
FT                   /note="Important for hydroxyacyl-coenzyme A dehydrogenase
FT                   activity"
FT                   /evidence="ECO:0000250|UniProtKB:P40939"
FT   MOD_RES         46
FT                   /note="N6-acetyllysine; alternate"
FT                   /evidence="ECO:0007744|PubMed:23576753"
FT   MOD_RES         46
FT                   /note="N6-succinyllysine; alternate"
FT                   /evidence="ECO:0007744|PubMed:23806337"
FT   MOD_RES         60
FT                   /note="N6-acetyllysine; alternate"
FT                   /evidence="ECO:0007744|PubMed:23576753,
FT                   ECO:0007744|PubMed:23806337"
FT   MOD_RES         60
FT                   /note="N6-succinyllysine; alternate"
FT                   /evidence="ECO:0007744|PubMed:23806337"
FT   MOD_RES         129
FT                   /note="N6-acetyllysine"
FT                   /evidence="ECO:0007744|PubMed:16916647,
FT                   ECO:0007744|PubMed:23576753"
FT   MOD_RES         166
FT                   /note="N6-acetyllysine; alternate"
FT                   /evidence="ECO:0007744|PubMed:23576753"
FT   MOD_RES         166
FT                   /note="N6-succinyllysine; alternate"
FT                   /evidence="ECO:0007744|PubMed:23806337"
FT   MOD_RES         213
FT                   /note="N6-succinyllysine"
FT                   /evidence="ECO:0007744|PubMed:23806337"
FT   MOD_RES         214
FT                   /note="N6-acetyllysine; alternate"
FT                   /evidence="ECO:0007744|PubMed:23576753"
FT   MOD_RES         214
FT                   /note="N6-succinyllysine; alternate"
FT                   /evidence="ECO:0007744|PubMed:23806337"
FT   MOD_RES         230
FT                   /note="N6-succinyllysine"
FT                   /evidence="ECO:0007744|PubMed:23806337"
FT   MOD_RES         231
FT                   /note="Phosphoserine"
FT                   /evidence="ECO:0007744|PubMed:21183079"
FT   MOD_RES         249
FT                   /note="N6-acetyllysine; alternate"
FT                   /evidence="ECO:0007744|PubMed:23576753"
FT   MOD_RES         249
FT                   /note="N6-succinyllysine; alternate"
FT                   /evidence="ECO:0007744|PubMed:23806337"
FT   MOD_RES         289
FT                   /note="N6-acetyllysine"
FT                   /evidence="ECO:0007744|PubMed:23576753"
FT   MOD_RES         295
FT                   /note="N6-acetyllysine"
FT                   /evidence="ECO:0000250|UniProtKB:P40939"
FT   MOD_RES         303
FT                   /note="N6-acetyllysine; alternate"
FT                   /evidence="ECO:0007744|PubMed:23576753"
FT   MOD_RES         303
FT                   /note="N6-succinyllysine; alternate"
FT                   /evidence="ECO:0007744|PubMed:23806337"
FT   MOD_RES         316
FT                   /note="Phosphoserine"
FT                   /evidence="ECO:0007744|PubMed:21183079"
FT   MOD_RES         326
FT                   /note="N6-acetyllysine; alternate"
FT                   /evidence="ECO:0007744|PubMed:23576753"
FT   MOD_RES         326
FT                   /note="N6-succinyllysine; alternate"
FT                   /evidence="ECO:0007744|PubMed:23806337"
FT   MOD_RES         334
FT                   /note="N6-acetyllysine; alternate"
FT                   /evidence="ECO:0007744|PubMed:23576753"
FT   MOD_RES         334
FT                   /note="N6-succinyllysine; alternate"
FT                   /evidence="ECO:0007744|PubMed:23806337"
FT   MOD_RES         350
FT                   /note="N6-acetyllysine; alternate"
FT                   /evidence="ECO:0007744|PubMed:23576753"
FT   MOD_RES         350
FT                   /note="N6-succinyllysine; alternate"
FT                   /evidence="ECO:0007744|PubMed:23806337"
FT   MOD_RES         353
FT                   /note="N6-acetyllysine"
FT                   /evidence="ECO:0007744|PubMed:23576753"
FT   MOD_RES         395
FT                   /note="Phosphothreonine"
FT                   /evidence="ECO:0007744|PubMed:21183079"
FT   MOD_RES         399
FT                   /note="Omega-N-methylarginine"
FT                   /evidence="ECO:0007744|PubMed:24129315"
FT   MOD_RES         406
FT                   /note="N6-acetyllysine; alternate"
FT                   /evidence="ECO:0007744|PubMed:23576753,
FT                   ECO:0007744|PubMed:23806337"
FT   MOD_RES         406
FT                   /note="N6-succinyllysine; alternate"
FT                   /evidence="ECO:0007744|PubMed:23806337"
FT   MOD_RES         411
FT                   /note="N6-acetyllysine; alternate"
FT                   /evidence="ECO:0007744|PubMed:23576753"
FT   MOD_RES         411
FT                   /note="N6-succinyllysine; alternate"
FT                   /evidence="ECO:0007744|PubMed:23806337"
FT   MOD_RES         415
FT                   /note="N6-succinyllysine"
FT                   /evidence="ECO:0007744|PubMed:23806337"
FT   MOD_RES         419
FT                   /note="Phosphoserine"
FT                   /evidence="ECO:0000250|UniProtKB:Q64428"
FT   MOD_RES         436
FT                   /note="N6-acetyllysine; alternate"
FT                   /evidence="ECO:0007744|PubMed:23576753"
FT   MOD_RES         436
FT                   /note="N6-succinyllysine; alternate"
FT                   /evidence="ECO:0007744|PubMed:23806337"
FT   MOD_RES         440
FT                   /note="N6-succinyllysine"
FT                   /evidence="ECO:0007744|PubMed:23806337"
FT   MOD_RES         460
FT                   /note="N6-acetyllysine; alternate"
FT                   /evidence="ECO:0007744|PubMed:23576753"
FT   MOD_RES         460
FT                   /note="N6-succinyllysine; alternate"
FT                   /evidence="ECO:0007744|PubMed:23806337"
FT   MOD_RES         505
FT                   /note="N6-acetyllysine; alternate"
FT                   /evidence="ECO:0007744|PubMed:23576753"
FT   MOD_RES         505
FT                   /note="N6-succinyllysine; alternate"
FT                   /evidence="ECO:0007744|PubMed:23806337"
FT   MOD_RES         519
FT                   /note="N6-acetyllysine; alternate"
FT                   /evidence="ECO:0007744|PubMed:23576753"
FT   MOD_RES         519
FT                   /note="N6-succinyllysine; alternate"
FT                   /evidence="ECO:0007744|PubMed:23806337"
FT   MOD_RES         540
FT                   /note="N6-acetyllysine"
FT                   /evidence="ECO:0007744|PubMed:23576753"
FT   MOD_RES         569
FT                   /note="N6-acetyllysine; alternate"
FT                   /evidence="ECO:0007744|PubMed:23576753"
FT   MOD_RES         569
FT                   /note="N6-succinyllysine; alternate"
FT                   /evidence="ECO:0007744|PubMed:23806337"
FT   MOD_RES         620
FT                   /note="N6-succinyllysine"
FT                   /evidence="ECO:0007744|PubMed:23806337"
FT   MOD_RES         634
FT                   /note="N6-succinyllysine"
FT                   /evidence="ECO:0007744|PubMed:23806337"
FT   MOD_RES         644
FT                   /note="N6-acetyllysine; alternate"
FT                   /evidence="ECO:0007744|PubMed:23576753"
FT   MOD_RES         644
FT                   /note="N6-succinyllysine; alternate"
FT                   /evidence="ECO:0007744|PubMed:23806337"
FT   MOD_RES         646
FT                   /note="N6-succinyllysine"
FT                   /evidence="ECO:0007744|PubMed:23806337"
FT   MOD_RES         647
FT                   /note="Phosphoserine"
FT                   /evidence="ECO:0007744|PubMed:21183079"
FT   MOD_RES         650
FT                   /note="Phosphoserine"
FT                   /evidence="ECO:0000250|UniProtKB:Q64428"
FT   MOD_RES         664
FT                   /note="N6-acetyllysine; alternate"
FT                   /evidence="ECO:0007744|PubMed:23576753"
FT   MOD_RES         664
FT                   /note="N6-succinyllysine; alternate"
FT                   /evidence="ECO:0007744|PubMed:23806337"
FT   MOD_RES         728
FT                   /note="N6-acetyllysine; alternate"
FT                   /evidence="ECO:0007744|PubMed:23576753"
FT   MOD_RES         728
FT                   /note="N6-succinyllysine; alternate"
FT                   /evidence="ECO:0007744|PubMed:23806337"
FT   MOD_RES         735
FT                   /note="N6-acetyllysine"
FT                   /evidence="ECO:0007744|PubMed:23576753"
FT   MOD_RES         759
FT                   /note="N6-acetyllysine; alternate"
FT                   /evidence="ECO:0007744|PubMed:23576753"
FT   MOD_RES         759
FT                   /note="N6-succinyllysine; alternate"
FT                   /evidence="ECO:0007744|PubMed:23806337"
FT   CONFLICT        196
FT                   /note="A -> D (in Ref. 1; BAE41822)"
FT                   /evidence="ECO:0000305"
FT   CONFLICT        459
FT                   /note="L -> S (in Ref. 2; AAH37009)"
FT                   /evidence="ECO:0000305"
SQ   SEQUENCE   763 AA;  82670 MW;  73D203795D5C1141 CRC64;
     MVASRAIGSL SRFSAFRILR SRGCICRSFT TSSALLTRTH INYGVKGDVA VIRINSPNSK
     VNTLNKEVQS EFIEVMNEIW ANDQIRSAVL ISSKPGCFVA GADINMLSSC TTPQEATRIS
     QEGQRMFEKL EKSPKPVVAA ISGSCLGGGL ELAIACQYRI ATKDRKTVLG VPEVLLGILP
     GAGGTQRLPK MVGVPAAFDM MLTGRNIRAD RAKKMGLVDQ LVEPLGPGIK SPEERTIEYL
     EEVAVNFAKG LADRKVSAKQ SKGLVEKLTT YAMTVPFVRQ QVYKTVEEKV KKQTKGLYPA
     PLKIIDAVKA GLEQGSDAGY LAESQKFGEL ALTKESKALM GLYNGQVLCK KNKFGAPQKN
     VQQLAILGAG LMGAGIAQVS VDKGLKTLLK DTTVTGLGRG QQQVFKGLND KVKKKALTSF
     ERDSIFSNLI GQLDYKGFEK ADMVIEAVFE DLGVKHKVLK EVESVTPEHC IFASNTSALP
     INQIAAVSKR PEKVIGMHYF SPVDKMQLLE IITTDKTSKD TTASAVAVGL RQGKVIIVVK
     DGPGFYTTRC LAPMMSEVMR ILQEGVDPKK LDALTTGFGF PVGAATLADE VGVDVAQHVA
     EDLGKAFGER FGGGSVELLK QMVSKGFLGR KSGKGFYIYQ EGSKNKSLNS EMDNILANLR
     LPAKPEVSSD EDVQYRVITR FVNEAVLCLQ EGILATPAEG DIGAVFGLGF PPCLGGPFRF
     VDLYGAQKVV DRLRKYESAY GTQFTPCQLL LDHANNSSKK FYQ
//
```

**Mascot:** http://www.matrixscience.com/


HNE (C) (+156.1150)
