# Supplementary material for: A common East-Asian ALDH2 mutation causes metabolic disorders and the therapeutic effect of ALDH2 activators
Source: Nat Commun. 2023 Sep 25;14:5971. doi: 10.1038/s41467-023-41570-6 (PMC10520061; doi:10.1038/s41467-023-41570-6)
Supplement: Supplementary file 4 — Supplementary Data 1 [file 41467_2023_41570_MOESM4_ESM.zip › Table S5c/Q8BWT1/Q8BWT1_KIO-1-K25_109_K312.html]

Mascot Search Results: Q8BWT1
 

# MASCOT Search Results

## Protein View: Q8BWT1

### 3-ketoacyl-CoA thiolase, mitochondrial OS=Mus musculus OX=10090 GN=Acaa2 PE=1 SV=3

|  |  |
| --- | --- |
| Database: | Mouse\_UniProt\_proteomes |
| Score: | 6075 |
| Monoisotopic mass (M_r_): | 42260 |
| Calculated pI: | 8.33 |

Sequence similarity is available as an NCBI BLAST search of Q8BWT1 against nr.

### Search parameters

|  |  |
| --- | --- |
| MS data file: | `D:\LCMSMS\2023 Users' data\230529-1\230529-1-KIO-1.raw` |
| Enzyme: | Trypsin/P: cuts C-term side of KR. |
| Fixed modifications: | Carbamidomethyl (C) |
| Variable modifications: | Deamidated (NQ), HNE (C), HNE (H), HNE (K), Oxidation (M) |

### Protein sequence coverage: 73%

Matched peptides shown in ***bold red***.

|  |  |  |  |  |  |
| --- | --- | --- | --- | --- | --- |
| `1` | `MALLRGVFIV` | `AAKRTPFGAY` | `GGLLKDFSAT` | `DLTEFAARAA` | `LSAGKVPPET` |
| `51` | `IDSVIVGNVM` | `QSSSDAAYLA` | `RHVGLRVGVP` | `TETGALTLNR` | `LCGSGFQSIV` |
| `101` | `SGCQEICSKD` | `AEVVLCGGTE` | `SMSQSPYCVR` | `NVRFGTKFGL` | `DLKLEDTLWA` |
| `151` | `GLTDQHVKLP` | `MGMTAENLAA` | `KYNISREDCD` | `RYALQSQQRW` | `KAANEAGYFN` |
| `201` | `EEMAPIEVKT` | `KKGKQTMQVD` | `EHARPQTTLE` | `QLQKLPSVFK` | `KDGTVTAGNA` |
| `251` | `SGVSDGAGAV` | `IIASEDAVKK` | `HNFTPLARVV` | `GYFVSGCDPT` | `IMGIGPVPAI` |
| `301` | `NGALKKAGLS` | `LKDMDLIDVN` | `EAFAPQFLSV` | `QKALDLDPSK` | `TNVSGGAIAL` |
| `351` | `GHPLGGSGSR` | `ITAHLVHELR` | `RRGGKYAVGS` | `ACIGGGQGIA` | `LIIQNTA` |

Unformatted sequence string: 397 residues (for pasting into other applications).

|  |  |  |  |
| --- | --- | --- | --- |
| Sort by | residue number | increasing mass | decreasing mass |
| Show | matched peptides only | predicted peptides also |  |

| Query | Start | – | End | Observed | Mr(expt) | Mr(calc) | ppm | M | Score | Expect | Rank | U | Peptide |
| --- | --- | --- | --- | --- | --- | --- | --- | --- | --- | --- | --- | --- | --- |
| 3433 | 6 | – | 13 | 402.7525 | 803.4905 | 803.4905 | -0.042 | 0 | 27 | 0.0028 | 1Score **> 22** indicates **identity** Score **> 14** indicates **homology** | U | R.GVFIVAAK.R |
| 3434 | 6 | – | 13 | 402.7527 | 803.4908 | 803.4905 | 0.31 | 0 | 26 | 0.0037 | 1Score **> 22** indicates **identity** Score **> 14** indicates **homology** | U | R.GVFIVAAK.R |
| 3435 | 6 | – | 13 | 402.7527 | 803.4908 | 803.4905 | 0.38 | 0 | 30 | 0.0015 | 1Score **> 22** indicates **identity** Score **> 14** indicates **homology** | U | R.GVFIVAAK.R |
| 10894 | 6 | – | 14 | 480.8035 | 959.5924 | 959.5916 | 0.82 | 1 | 28 | 0.0021 | 1Score **> 26** indicates **identity** Score **> 14** indicates **homology** | U | R.GVFIVAAKR.T |
| 10895 | 6 | – | 14 | 480.8038 | 959.5930 | 959.5916 | 1.41 | 1 | 20 | 0.013 | 1Score **> 26** indicates **identity** Score **> 14** indicates **homology** | U | R.GVFIVAAKR.T |
| 38406 | 14 | – | 25 | 640.3624 | 1278.7102 | 1278.7084 | 1.34 | 1 | 50 | 0.00065 | 1Score **> 32** indicates **identity** Score **> 31** indicates **homology** | U | K.RTPFGAYGGLLK.D |
| 38407 | 14 | – | 25 | 640.3624 | 1278.7103 | 1278.7084 | 1.44 | 1 | 50 | 0.00064 | 1Score **> 32** indicates **identity** Score **> 30** indicates **homology** | U | K.RTPFGAYGGLLK.D |
| 38408 | 14 | – | 25 | 427.2441 | 1278.7104 | 1278.7084 | 1.53 | 1 | 40 | 0.0003 | 1Score **> 32** indicates **identity** Score **> 17** indicates **homology** | U | K.RTPFGAYGGLLK.D |
| 38409 | 14 | – | 25 | 427.2441 | 1278.7104 | 1278.7084 | 1.55 | 1 | 36 | 0.00085 | 1Score **> 32** indicates **identity** Score **> 17** indicates **homology** | U | K.RTPFGAYGGLLK.D |
| 38410 | 14 | – | 25 | 640.3627 | 1278.7108 | 1278.7084 | 1.86 | 1 | 41 | 0.0051 | 1Score **> 32** indicates **identity** Score **> 31** indicates **homology** | U | K.RTPFGAYGGLLK.D |
| 38413 | 14 | – | 25 | 427.2447 | 1278.7122 | 1278.7084 | 2.94 | 1 | 32 | 0.0019 | 1Score **> 32** indicates **identity** Score **> 18** indicates **homology** | U | K.RTPFGAYGGLLK.D |
| 167979 | 14 | – | 38 | 676.8478 | 2703.3623 | 2703.3657 | -1.26 | 2 | 56 | 5e-06 | 1Score **> 37** indicates **identity** Score **> 16** indicates **homology** | U | K.RTPFGAYGGLLKDFSATDLTEFAAR.A |
| 167981 | 14 | – | 38 | 902.1295 | 2703.3666 | 2703.3657 | 0.35 | 2 | 92 | 2.3e-09 | 1Score **> 37** indicates **identity** Score **> 18** indicates **homology** | U | K.RTPFGAYGGLLKDFSATDLTEFAAR.A |
| 167982 | 14 | – | 38 | 676.8492 | 2703.3677 | 2703.3657 | 0.75 | 2 | 89 | 4.9e-09 | 1Score **> 37** indicates **identity** Score **> 18** indicates **homology** | U | K.RTPFGAYGGLLKDFSATDLTEFAAR.A |
| 22824 | 15 | – | 25 | 562.3115 | 1122.6085 | 1122.6073 | 0.99 | 0 | 40 | 0.00017 | 1Score **> 32** indicates **identity** Score **> 15** indicates **homology** | U | R.TPFGAYGGLLK.D |
| 22825 | 15 | – | 25 | 562.3119 | 1122.6093 | 1122.6073 | 1.74 | 0 | 60 | 2.3e-06 | 1Score **> 32** indicates **identity** Score **> 16** indicates **homology** | U | R.TPFGAYGGLLK.D |
| 22826 | 15 | – | 25 | 562.3124 | 1122.6103 | 1122.6073 | 2.60 | 0 | 62 | 1.6e-06 | 1Score **> 32** indicates **identity** Score **> 17** indicates **homology** | U | R.TPFGAYGGLLK.D |
| 38411 | 15 | – | 25 | 427.2443 | 1278.7110 | 1278.7224 | -8.94 | 0 | 24 | 0.0061 | 1Score **> 32** indicates **identity** Score **> 14** indicates **homology** | U | R.TPFGAYGGLLK.D  + HNE (K) |
| 38412 | 15 | – | 25 | 427.2443 | 1278.7111 | 1278.7224 | -8.80 | 0 | 45 | 6e-05 | 1Score **> 32** indicates **identity** Score **> 15** indicates **homology** | U | R.TPFGAYGGLLK.D  + HNE (K) |
| 38417 | 15 | – | 25 | 427.2459 | 1278.7158 | 1278.7224 | -5.17 | 0 | 18 | 0.021 | 1Score **> 32** indicates **identity** Score **> 14** indicates **homology** | U | R.TPFGAYGGLLK.D  + HNE (K) |
| 161221 | 15 | – | 38 | 850.0947 | 2547.2623 | 2547.2646 | -0.89 | 1 | 84 | 1.4e-08 | 1Score **> 37** indicates **identity** Score **> 18** indicates **homology** | U | R.TPFGAYGGLLKDFSATDLTEFAAR.A |
| 161222 | 15 | – | 38 | 1274.6398 | 2547.2650 | 2547.2646 | 0.18 | 1 | 135 | 2e-13 | 1Score **> 37** indicates **identity** Score **> 21** indicates **homology** | U | R.TPFGAYGGLLKDFSATDLTEFAAR.A |
| 55300 | 26 | – | 38 | 722.3388 | 1442.6631 | 1442.6678 | -3.26 | 0 | 42 | 0.0001 | 1Score **> 30** indicates **identity** Score **> 15** indicates **homology** | U | K.DFSATDLTEFAAR.A |
| 55301 | 26 | – | 38 | 722.3395 | 1442.6644 | 1442.6678 | -2.37 | 0 | 46 | 4.9e-05 | 1Score **> 30** indicates **identity** Score **> 15** indicates **homology** | U | K.DFSATDLTEFAAR.A |
| 55303 | 26 | – | 38 | 722.3397 | 1442.6648 | 1442.6678 | -2.04 | 0 | 52 | 1.3e-05 | 1Score **> 30** indicates **identity** Score **> 16** indicates **homology** | U | K.DFSATDLTEFAAR.A |
| 55304 | 26 | – | 38 | 722.3398 | 1442.6650 | 1442.6678 | -1.95 | 0 | 35 | 0.00048 | 1Score **> 30** indicates **identity** Score **> 15** indicates **homology** | U | K.DFSATDLTEFAAR.A |
| 55305 | 26 | – | 38 | 722.3401 | 1442.6657 | 1442.6678 | -1.44 | 0 | 61 | 2.1e-06 | 1Score **> 31** indicates **identity** Score **> 16** indicates **homology** | U | K.DFSATDLTEFAAR.A |
| 55306 | 26 | – | 38 | 722.3405 | 1442.6665 | 1442.6678 | -0.91 | 0 | 78 | 5.4e-08 | 1Score **> 30** indicates **identity** Score **> 17** indicates **homology** | U | K.DFSATDLTEFAAR.A |
| 55307 | 26 | – | 38 | 722.3409 | 1442.6673 | 1442.6678 | -0.34 | 0 | 75 | 8.9e-08 | 1Score **> 30** indicates **identity** Score **> 17** indicates **homology** | U | K.DFSATDLTEFAAR.A |
| 55308 | 26 | – | 38 | 722.3409 | 1442.6673 | 1442.6678 | -0.32 | 0 | 86 | 7.9e-09 | 1Score **> 30** indicates **identity** Score **> 18** indicates **homology** | U | K.DFSATDLTEFAAR.A |
| 55309 | 26 | – | 38 | 722.3411 | 1442.6676 | 1442.6678 | -0.14 | 0 | 25 | 0.0044 | 1Score **> 30** indicates **identity** Score **> 14** indicates **homology** | U | K.DFSATDLTEFAAR.A |
| 55310 | 26 | – | 38 | 722.3411 | 1442.6676 | 1442.6678 | -0.13 | 0 | 84 | 1.5e-08 | 1Score **> 30** indicates **identity** Score **> 18** indicates **homology** | U | K.DFSATDLTEFAAR.A |
| 55311 | 26 | – | 38 | 722.3416 | 1442.6686 | 1442.6678 | 0.61 | 0 | 63 | 1.2e-06 | 1Score **> 31** indicates **identity** Score **> 16** indicates **homology** | U | K.DFSATDLTEFAAR.A |
| 55313 | 26 | – | 38 | 722.3417 | 1442.6688 | 1442.6678 | 0.71 | 0 | 85 | 1e-08 | 1Score **> 31** indicates **identity** Score **> 18** indicates **homology** | U | K.DFSATDLTEFAAR.A |
| 55315 | 26 | – | 38 | 722.3420 | 1442.6695 | 1442.6678 | 1.19 | 0 | 50 | 1.9e-05 | 1Score **> 31** indicates **identity** Score **> 16** indicates **homology** | U | K.DFSATDLTEFAAR.A |
| 185928 | 39 | – | 71 | 1106.5730 | 3316.6973 | 3316.6973 | 0.0069 | 1 | 77 | 6.1e-08 | 1Score **> 37** indicates **identity** Score **> 17** indicates **homology** | U | R.AALSAGKVPPETIDSVIVGNVMQSSSDAAYLAR.H |
| 168558 | 46 | – | 71 | 907.4610 | 2719.3613 | 2719.3374 | 8.77 | 0 | 55 | 6.4e-06 | 1Score **> 37** indicates **identity** Score **> 16** indicates **homology** | U | K.VPPETIDSVIVGNVMQSSSDAAYLAR.H  + Deamidated (NQ) |
| 53474 | 77 | – | 90 | 714.3964 | 1426.7782 | 1426.7780 | 0.11 | 0 | 64 | 1.4e-06 | 1Score **> 34** indicates **identity** Score **> 18** indicates **homology** | U | R.VGVPTETGALTLNR.L |
| 53476 | 77 | – | 90 | 714.3965 | 1426.7784 | 1426.7780 | 0.29 | 0 | 80 | 4.3e-08 | 1Score **> 34** indicates **identity** Score **> 19** indicates **homology** | U | R.VGVPTETGALTLNR.L |
| 53477 | 77 | – | 90 | 714.3965 | 1426.7785 | 1426.7780 | 0.36 | 0 | 21 | 0.022 | 1Score **> 34** indicates **identity** Score **> 17** indicates **homology** | U | R.VGVPTETGALTLNR.L |
| 53478 | 77 | – | 90 | 714.3965 | 1426.7785 | 1426.7780 | 0.37 | 0 | 79 | 1.3e-07 | 1Score **> 34** indicates **identity** Score **> 23** indicates **homology** | U | R.VGVPTETGALTLNR.L |
| 53479 | 77 | – | 90 | 476.6002 | 1426.7787 | 1426.7780 | 0.46 | 0 | 70 | 5.3e-07 | 1Score **> 33** indicates **identity** Score **> 20** indicates **homology** | U | R.VGVPTETGALTLNR.L |
| 53480 | 77 | – | 90 | 476.6003 | 1426.7790 | 1426.7780 | 0.69 | 0 | 71 | 3.2e-07 | 1Score **> 34** indicates **identity** Score **> 18** indicates **homology** | U | R.VGVPTETGALTLNR.L |
| 53481 | 77 | – | 90 | 714.3968 | 1426.7791 | 1426.7780 | 0.77 | 0 | 77 | 7.8e-08 | 1Score **> 34** indicates **identity** Score **> 19** indicates **homology** | U | R.VGVPTETGALTLNR.L |
| 53482 | 77 | – | 90 | 714.3969 | 1426.7792 | 1426.7780 | 0.81 | 0 | 63 | 1.1e-06 | 1Score **> 34** indicates **identity** Score **> 16** indicates **homology** | U | R.VGVPTETGALTLNR.L |
| 53484 | 77 | – | 90 | 714.3972 | 1426.7798 | 1426.7780 | 1.30 | 0 | 49 | 2.7e-05 | 1Score **> 34** indicates **identity** Score **> 16** indicates **homology** | U | R.VGVPTETGALTLNR.L |
| 53485 | 77 | – | 90 | 714.3975 | 1426.7805 | 1426.7780 | 1.74 | 0 | 64 | 1.1e-06 | 1Score **> 34** indicates **identity** Score **> 17** indicates **homology** | U | R.VGVPTETGALTLNR.L |
| 53487 | 77 | – | 90 | 714.3977 | 1426.7808 | 1426.7780 | 2.00 | 0 | 64 | 1.1e-06 | 1Score **> 34** indicates **identity** Score **> 17** indicates **homology** | U | R.VGVPTETGALTLNR.L |
| 131570 | 91 | – | 109 | 1058.9751 | 2115.9356 | 2115.9388 | -1.49 | 0 | 83 | 1.8e-08 | 1Score **> 30** indicates **identity** Score **> 18** indicates **homology** | U | R.LCGSGFQSIVSGCQEICSK.D |
| 131571 | 91 | – | 109 | 1058.9766 | 2115.9387 | 2115.9388 | -0.020 | 0 | 47 | 4e-05 | 1Score **> 30** indicates **identity** Score **> 15** indicates **homology** | U | R.LCGSGFQSIVSGCQEICSK.D |
| 131572 | 91 | – | 109 | 1058.9767 | 2115.9388 | 2115.9388 | 0.039 | 0 | 84 | 1.3e-08 | 1Score **> 30** indicates **identity** Score **> 18** indicates **homology** | U | R.LCGSGFQSIVSGCQEICSK.D |
| 131573 | 91 | – | 109 | 706.3208 | 2115.9406 | 2115.9388 | 0.87 | 0 | 49 | 2.4e-05 | 1Score **> 30** indicates **identity** Score **> 16** indicates **homology** | U | R.LCGSGFQSIVSGCQEICSK.D |
| 131575 | 91 | – | 109 | 1058.9785 | 2115.9424 | 2115.9388 | 1.73 | 0 | 104 | 1.8e-10 | 1Score **> 30** indicates **identity** Score **> 19** indicates **homology** | U | R.LCGSGFQSIVSGCQEICSK.D |
| 131576 | 91 | – | 109 | 1058.9785 | 2115.9425 | 2115.9388 | 1.75 | 0 | 95 | 1.2e-09 | 1Score **> 30** indicates **identity** Score **> 18** indicates **homology** | U | R.LCGSGFQSIVSGCQEICSK.D |
| 131577 | 91 | – | 109 | 1058.9788 | 2115.9431 | 2115.9388 | 2.04 | 0 | 78 | 5.1e-08 | 1Score **> 30** indicates **identity** Score **> 17** indicates **homology** | U | R.LCGSGFQSIVSGCQEICSK.D |
| 131578 | 91 | – | 109 | 706.3217 | 2115.9434 | 2115.9388 | 2.18 | 0 | 43 | 8.4e-05 | 1Score **> 30** indicates **identity** Score **> 15** indicates **homology** | U | R.LCGSGFQSIVSGCQEICSK.D |
| 131579 | 91 | – | 109 | 1058.9799 | 2115.9453 | 2115.9388 | 3.10 | 0 | 142 | 4.5e-14 | 1Score **> 30** indicates **identity** Score **> 21** indicates **homology** | U | R.LCGSGFQSIVSGCQEICSK.D |
| 131580 | 91 | – | 109 | 1058.9801 | 2115.9457 | 2115.9388 | 3.26 | 0 | 55 | 6.9e-06 | 1Score **> 30** indicates **identity** Score **> 16** indicates **homology** | U | R.LCGSGFQSIVSGCQEICSK.D |
| 131581 | 91 | – | 109 | 706.3228 | 2115.9465 | 2115.9388 | 3.64 | 0 | 22 | 0.0078 | 1Score **> 30** indicates **identity** Score **> 14** indicates **homology** | U | R.LCGSGFQSIVSGCQEICSK.D |
| 131583 | 91 | – | 109 | 1058.9817 | 2115.9488 | 2115.9388 | 4.72 | 0 | 97 | 8.5e-10 | 1Score **> 31** indicates **identity** Score **> 19** indicates **homology** | U | R.LCGSGFQSIVSGCQEICSK.D |
| 131584 | 91 | – | 109 | 1058.9819 | 2115.9493 | 2115.9388 | 4.99 | 0 | 107 | 1e-10 | 1Score **> 31** indicates **identity** Score **> 19** indicates **homology** | U | R.LCGSGFQSIVSGCQEICSK.D |
| 196531 | 91 | – | 130 | 1155.0133 | 4616.0241 | 4616.0196 | 0.99 | 1 | 22 | 0.0088 | 1Score **> 29** indicates **identity** Score **> 14** indicates **homology** | U | R.LCGSGFQSIVSGCQEICSKDAEVVLCGGTESMSQSPYCVR.N  + 2 Deamidated (NQ); HNE (K); Oxidation (M) |
| 196532 | 91 | – | 130 | 1155.0149 | 4616.0305 | 4616.0196 | 2.36 | 1 | 22 | 0.009 | 1Score **> 30** indicates **identity** Score **> 14** indicates **homology** | U | R.LCGSGFQSIVSGCQEICSKDAEVVLCGGTESMSQSPYCVR.N  + 2 Deamidated (NQ); HNE (K); Oxidation (M) |
| 23003 | 134 | – | 143 | 375.8812 | 1124.6219 | 1124.6230 | -0.96 | 1 | 26 | 0.014 | 1Score **> 31** indicates **identity** Score **> 20** indicates **homology** | U | R.FGTKFGLDLK.L |
| 23005 | 134 | – | 143 | 375.8820 | 1124.6241 | 1124.6230 | 0.99 | 1 | 36 | 0.0022 | 1Score **> 31** indicates **identity** Score **> 22** indicates **homology** | U | R.FGTKFGLDLK.L |
| 23007 | 134 | – | 143 | 375.8822 | 1124.6249 | 1124.6230 | 1.71 | 1 | 36 | 0.0023 | 1Score **> 31** indicates **identity** Score **> 22** indicates **homology** | U | R.FGTKFGLDLK.L |
| 23009 | 134 | – | 143 | 375.8824 | 1124.6253 | 1124.6230 | 2.04 | 1 | 33 | 0.0035 | 1Score **> 31** indicates **identity** Score **> 21** indicates **homology** | U | R.FGTKFGLDLK.L |
| 172995 | 134 | – | 158 | 567.5051 | 2832.4893 | 2832.4698 | 6.87 | 2 | 20 | 0.015 | 1Score **> 37** indicates **identity** Score **> 14** indicates **homology** | U | R.FGTKFGLDLKLEDTLWAGLTDQHVK.L  + Deamidated (NQ) |
| 153024 | 138 | – | 158 | 600.5708 | 2398.2541 | 2398.2533 | 0.34 | 1 | 25 | 0.005 | 1Score **> 37** indicates **identity** Score **> 14** indicates **homology** | U | K.FGLDLKLEDTLWAGLTDQHVK.L |
| 153026 | 138 | – | 158 | 600.5710 | 2398.2550 | 2398.2533 | 0.74 | 1 | 27 | 0.0032 | 1Score **> 37** indicates **identity** Score **> 14** indicates **homology** | U | K.FGLDLKLEDTLWAGLTDQHVK.L |
| 153028 | 138 | – | 158 | 800.4276 | 2398.2611 | 2398.2533 | 3.26 | 1 | 25 | 0.0049 | 1Score **> 37** indicates **identity** Score **> 14** indicates **homology** | U | K.FGLDLKLEDTLWAGLTDQHVK.L |
| 92054 | 144 | – | 158 | 575.9647 | 1724.8722 | 1724.8734 | -0.69 | 0 | 16 | 0.028 | 1Score **> 35** indicates **identity** Score **> 14** indicates **homology** | U | K.LEDTLWAGLTDQHVK.L |
| 92057 | 144 | – | 158 | 863.4449 | 1724.8752 | 1724.8734 | 1.07 | 0 | 53 | 1.1e-05 | 1Score **> 35** indicates **identity** Score **> 16** indicates **homology** | U | K.LEDTLWAGLTDQHVK.L |
| 92061 | 144 | – | 158 | 863.4472 | 1724.8799 | 1724.8734 | 3.82 | 0 | 35 | 0.00058 | 1Score **> 35** indicates **identity** Score **> 15** indicates **homology** | U | K.LEDTLWAGLTDQHVK.L |
| 146703 | 172 | – | 189 | 768.0224 | 2301.0455 | 2301.0556 | -4.38 | 2 | 16 | 0.034 | 1Score **> 32** indicates **identity** Score **> 13** indicates **homology** | U | K.YNISREDCDRYALQSQQR.W |
| 146707 | 172 | – | 189 | 768.0259 | 2301.0558 | 2301.0556 | 0.070 | 2 | 16 | 0.029 | 1Score **> 33** indicates **identity** Score **> 14** indicates **homology** | U | K.YNISREDCDRYALQSQQR.W |
| 146710 | 172 | – | 189 | 768.0264 | 2301.0572 | 2301.0556 | 0.71 | 2 | 15 | 0.038 | 1Score **> 33** indicates **identity** Score **> 13** indicates **homology** | U | K.YNISREDCDRYALQSQQR.W |
| 146712 | 172 | – | 189 | 768.0264 | 2301.0573 | 2301.0556 | 0.75 | 2 | 29 | 0.0021 | 1Score **> 33** indicates **identity** Score **> 14** indicates **homology** | U | K.YNISREDCDRYALQSQQR.W |
| 146713 | 172 | – | 189 | 768.0264 | 2301.0574 | 2301.0556 | 0.77 | 2 | 25 | 0.0048 | 1Score **> 33** indicates **identity** Score **> 14** indicates **homology** | U | K.YNISREDCDRYALQSQQR.W |
| 146715 | 172 | – | 189 | 768.0265 | 2301.0577 | 2301.0556 | 0.93 | 2 | 28 | 0.0025 | 1Score **> 33** indicates **identity** Score **> 14** indicates **homology** | U | K.YNISREDCDRYALQSQQR.W |
| 146723 | 172 | – | 189 | 768.0271 | 2301.0595 | 2301.0556 | 1.71 | 2 | 24 | 0.0057 | 1Score **> 33** indicates **identity** Score **> 14** indicates **homology** | U | K.YNISREDCDRYALQSQQR.W |
| 84654 | 177 | – | 189 | 834.8726 | 1667.7307 | 1667.7322 | -0.89 | 1 | 49 | 2.7e-05 | 1Score **> 28** indicates **identity** Score **> 16** indicates **homology** | U | R.EDCDRYALQSQQR.W |
| 84655 | 177 | – | 189 | 556.9178 | 1667.7314 | 1667.7322 | -0.44 | 1 | 15 | 0.042 | 1Score **> 28** indicates **identity** Score **> 13** indicates **homology** | U | R.EDCDRYALQSQQR.W |
| 84658 | 177 | – | 189 | 556.9183 | 1667.7332 | 1667.7322 | 0.60 | 1 | 34 | 0.00062 | 1Score **> 29** indicates **identity** Score **> 15** indicates **homology** | U | R.EDCDRYALQSQQR.W |
| 84659 | 177 | – | 189 | 556.9186 | 1667.7338 | 1667.7322 | 1.00 | 1 | 36 | 0.00038 | 1Score **> 29** indicates **identity** Score **> 15** indicates **homology** | U | R.EDCDRYALQSQQR.W |
| 84660 | 177 | – | 189 | 834.8746 | 1667.7346 | 1667.7322 | 1.45 | 1 | 35 | 0.00048 | 1Score **> 29** indicates **identity** Score **> 15** indicates **homology** | U | R.EDCDRYALQSQQR.W |
| 84661 | 177 | – | 189 | 556.9189 | 1667.7349 | 1667.7322 | 1.65 | 1 | 21 | 0.011 | 1Score **> 29** indicates **identity** Score **> 14** indicates **homology** | U | R.EDCDRYALQSQQR.W |
| 12706 | 182 | – | 189 | 497.2579 | 992.5012 | 992.5039 | -2.70 | 0 | 41 | 0.00024 | 1Score **> 32** indicates **identity** Score **> 17** indicates **homology** | U | R.YALQSQQR.W |
| 12707 | 182 | – | 189 | 497.2583 | 992.5020 | 992.5039 | -1.97 | 0 | 26 | 0.0051 | 1Score **> 32** indicates **identity** Score **> 15** indicates **homology** | U | R.YALQSQQR.W |
| 12708 | 182 | – | 189 | 497.2587 | 992.5029 | 992.5039 | -1.06 | 0 | 41 | 0.00014 | 1Score **> 31** indicates **identity** Score **> 15** indicates **homology** | U | R.YALQSQQR.W |
| 118756 | 192 | – | 209 | 991.9637 | 1981.9128 | 1981.9091 | 1.88 | 0 | 78 | 4.5e-08 | 1Score **> 33** indicates **identity** Score **> 17** indicates **homology** | U | K.AANEAGYFNEEMAPIEVK.T |
| 118757 | 192 | – | 209 | 991.9645 | 1981.9145 | 1981.9091 | 2.72 | 0 | 63 | 1.2e-06 | 1Score **> 33** indicates **identity** Score **> 16** indicates **homology** | U | K.AANEAGYFNEEMAPIEVK.T |
| 4029 | 235 | – | 241 | 409.7606 | 817.5067 | 817.5062 | 0.62 | 1 | 27 | 0.017 | 1Score **> 22** indicates **identity** | U | K.LPSVFKK.D |
| 4030 | 235 | – | 241 | 409.7606 | 817.5067 | 817.5062 | 0.70 | 1 | 22 | 0.047 | 1Score **> 22** indicates **identity** | U | K.LPSVFKK.D |
| 160395 | 242 | – | 269 | 844.7502 | 2531.2288 | 2531.2351 | -2.50 | 0 | 17 | 0.024 | 1Score **> 36** indicates **identity** Score **> 14** indicates **homology** | U | K.DGTVTAGNASGVSDGAGAVIIASEDAVK.K |
| 160397 | 242 | – | 269 | 1266.6234 | 2531.2322 | 2531.2351 | -1.15 | 0 | 99 | 5.3e-10 | 1Score **> 36** indicates **identity** Score **> 19** indicates **homology** | U | K.DGTVTAGNASGVSDGAGAVIIASEDAVK.K |
| 160400 | 242 | – | 269 | 844.7519 | 2531.2340 | 2531.2351 | -0.45 | 0 | 34 | 0.00064 | 1Score **> 36** indicates **identity** Score **> 15** indicates **homology** | U | K.DGTVTAGNASGVSDGAGAVIIASEDAVK.K |
| 160401 | 242 | – | 269 | 1266.6246 | 2531.2347 | 2531.2351 | -0.16 | 0 | 111 | 3.8e-11 | 1Score **> 36** indicates **identity** Score **> 19** indicates **homology** | U | K.DGTVTAGNASGVSDGAGAVIIASEDAVK.K |
| 160405 | 242 | – | 269 | 1266.6259 | 2531.2372 | 2531.2351 | 0.82 | 0 | 134 | 8.6e-13 | 1Score **> 37** indicates **identity** Score **> 26** indicates **homology** | U | K.DGTVTAGNASGVSDGAGAVIIASEDAVK.K |
| 160406 | 242 | – | 269 | 1266.6261 | 2531.2376 | 2531.2351 | 1.00 | 0 | 137 | 1.8e-13 | 1Score **> 37** indicates **identity** Score **> 22** indicates **homology** | U | K.DGTVTAGNASGVSDGAGAVIIASEDAVK.K |
| 160407 | 242 | – | 269 | 844.7534 | 2531.2384 | 2531.2351 | 1.31 | 0 | 77 | 7.4e-08 | 1Score **> 37** indicates **identity** Score **> 18** indicates **homology** | U | K.DGTVTAGNASGVSDGAGAVIIASEDAVK.K |
| 160409 | 242 | – | 269 | 844.7536 | 2531.2391 | 2531.2351 | 1.58 | 0 | 63 | 1.4e-06 | 1Score **> 37** indicates **identity** Score **> 17** indicates **homology** | U | K.DGTVTAGNASGVSDGAGAVIIASEDAVK.K |
| 160413 | 242 | – | 269 | 844.7542 | 2531.2408 | 2531.2351 | 2.26 | 0 | 36 | 0.00038 | 1Score **> 37** indicates **identity** Score **> 15** indicates **homology** | U | K.DGTVTAGNASGVSDGAGAVIIASEDAVK.K |
| 160414 | 242 | – | 269 | 1266.6277 | 2531.2409 | 2531.2351 | 2.27 | 0 | 36 | 0.00038 | 1Score **> 37** indicates **identity** Score **> 15** indicates **homology** | U | K.DGTVTAGNASGVSDGAGAVIIASEDAVK.K |
| 160417 | 242 | – | 269 | 1266.6284 | 2531.2422 | 2531.2351 | 2.80 | 0 | 134 | 2.5e-13 | 1Score **> 37** indicates **identity** Score **> 21** indicates **homology** | U | K.DGTVTAGNASGVSDGAGAVIIASEDAVK.K |
| 160418 | 242 | – | 269 | 1266.6285 | 2531.2425 | 2531.2351 | 2.91 | 0 | 137 | 1.3e-13 | 1Score **> 37** indicates **identity** Score **> 21** indicates **homology** | U | K.DGTVTAGNASGVSDGAGAVIIASEDAVK.K |
| 160419 | 242 | – | 269 | 1266.6286 | 2531.2427 | 2531.2351 | 2.98 | 0 | 75 | 1e-07 | 1Score **> 37** indicates **identity** Score **> 17** indicates **homology** | U | K.DGTVTAGNASGVSDGAGAVIIASEDAVK.K |
| 160422 | 242 | – | 269 | 844.7550 | 2531.2431 | 2531.2351 | 3.14 | 0 | 45 | 6.5e-05 | 1Score **> 37** indicates **identity** Score **> 15** indicates **homology** | U | K.DGTVTAGNASGVSDGAGAVIIASEDAVK.K |
| 160452 | 242 | – | 269 | 1267.1286 | 2532.2427 | 2532.2191 | 9.30 | 0 | 102 | 2.9e-10 | 1Score **> 37** indicates **identity** Score **> 19** indicates **homology** | U | K.DGTVTAGNASGVSDGAGAVIIASEDAVK.K  + Deamidated (NQ) |
| 166137 | 242 | – | 270 | 887.4493 | 2659.3260 | 2659.3301 | -1.54 | 1 | 52 | 1.3e-05 | 1Score **> 37** indicates **identity** Score **> 16** indicates **homology** | U | K.DGTVTAGNASGVSDGAGAVIIASEDAVKK.H |
| 166141 | 242 | – | 270 | 887.4518 | 2659.3335 | 2659.3301 | 1.30 | 1 | 82 | 2e-08 | 1Score **> 37** indicates **identity** Score **> 18** indicates **homology** | U | K.DGTVTAGNASGVSDGAGAVIIASEDAVKK.H |
| 166142 | 242 | – | 270 | 887.4520 | 2659.3341 | 2659.3301 | 1.52 | 1 | 106 | 1.1e-10 | 1Score **> 37** indicates **identity** Score **> 19** indicates **homology** | U | K.DGTVTAGNASGVSDGAGAVIIASEDAVKK.H |
| 166143 | 242 | – | 270 | 1330.6749 | 2659.3352 | 2659.3301 | 1.93 | 1 | 93 | 1.8e-09 | 1Score **> 37** indicates **identity** Score **> 18** indicates **homology** | U | K.DGTVTAGNASGVSDGAGAVIIASEDAVKK.H |
| 166144 | 242 | – | 270 | 887.4524 | 2659.3354 | 2659.3301 | 2.00 | 1 | 110 | 5.4e-11 | 1Score **> 37** indicates **identity** Score **> 19** indicates **homology** | U | K.DGTVTAGNASGVSDGAGAVIIASEDAVKK.H |
| 166147 | 242 | – | 270 | 887.4533 | 2659.3380 | 2659.3301 | 2.99 | 1 | 71 | 2.3e-07 | 1Score **> 37** indicates **identity** Score **> 17** indicates **homology** | U | K.DGTVTAGNASGVSDGAGAVIIASEDAVKK.H |
| 166148 | 242 | – | 270 | 887.4538 | 2659.3395 | 2659.3301 | 3.56 | 1 | 61 | 2.1e-06 | 1Score **> 37** indicates **identity** Score **> 16** indicates **homology** | U | K.DGTVTAGNASGVSDGAGAVIIASEDAVKK.H |
| 166150 | 242 | – | 270 | 887.4542 | 2659.3408 | 2659.3301 | 4.02 | 1 | 45 | 6.1e-05 | 1Score **> 37** indicates **identity** Score **> 15** indicates **homology** | U | K.DGTVTAGNASGVSDGAGAVIIASEDAVKK.H |
| 166153 | 242 | – | 270 | 887.4566 | 2659.3481 | 2659.3301 | 6.77 | 1 | 70 | 2.5e-07 | 1Score **> 37** indicates **identity** Score **> 17** indicates **homology** | U | K.DGTVTAGNASGVSDGAGAVIIASEDAVKK.H |
| 166154 | 242 | – | 270 | 887.4572 | 2659.3496 | 2659.3301 | 7.35 | 1 | 29 | 0.0018 | 1Score **> 37** indicates **identity** Score **> 14** indicates **homology** | U | K.DGTVTAGNASGVSDGAGAVIIASEDAVKK.H |
| 166169 | 242 | – | 270 | 887.7797 | 2660.3172 | 2660.3141 | 1.16 | 1 | 47 | 3.8e-05 | 1Score **> 37** indicates **identity** Score **> 15** indicates **homology** | U | K.DGTVTAGNASGVSDGAGAVIIASEDAVKK.H  + Deamidated (NQ) |
| 166170 | 242 | – | 270 | 887.7816 | 2660.3230 | 2660.3141 | 3.36 | 1 | 42 | 0.00011 | 1Score **> 37** indicates **identity** Score **> 15** indicates **homology** | U | K.DGTVTAGNASGVSDGAGAVIIASEDAVKK.H  + Deamidated (NQ) |
| 166171 | 242 | – | 270 | 887.7832 | 2660.3278 | 2660.3141 | 5.17 | 1 | 17 | 0.026 | 1Score **> 37** indicates **identity** Score **> 14** indicates **homology** | U | K.DGTVTAGNASGVSDGAGAVIIASEDAVKK.H  + Deamidated (NQ) |
| 166174 | 242 | – | 270 | 1331.1764 | 2660.3382 | 2660.3141 | 9.06 | 1 | 103 | 2.4e-10 | 1Score **> 37** indicates **identity** Score **> 19** indicates **homology** | U | K.DGTVTAGNASGVSDGAGAVIIASEDAVKK.H  + Deamidated (NQ) |
| 190772 | 242 | – | 278 | 720.1725 | 3595.8261 | 3595.8231 | 0.84 | 2 | 45 | 5.6e-05 | 1Score **> 37** indicates **identity** Score **> 15** indicates **homology** | U | K.DGTVTAGNASGVSDGAGAVIIASEDAVKKHNFTPLAR.V |
| 190773 | 242 | – | 278 | 899.9644 | 3595.8287 | 3595.8231 | 1.56 | 2 | 57 | 4.1e-06 | 1Score **> 37** indicates **identity** Score **> 16** indicates **homology** | U | K.DGTVTAGNASGVSDGAGAVIIASEDAVKKHNFTPLAR.V |
| 190774 | 242 | – | 278 | 899.9646 | 3595.8294 | 3595.8231 | 1.77 | 2 | 40 | 0.00019 | 1Score **> 37** indicates **identity** Score **> 15** indicates **homology** | U | K.DGTVTAGNASGVSDGAGAVIIASEDAVKKHNFTPLAR.V |
| 190775 | 242 | – | 278 | 720.1733 | 3595.8301 | 3595.8231 | 1.96 | 2 | 28 | 0.0024 | 1Score **> 37** indicates **identity** Score **> 14** indicates **homology** | U | K.DGTVTAGNASGVSDGAGAVIIASEDAVKKHNFTPLAR.V |
| 190776 | 242 | – | 278 | 899.9657 | 3595.8335 | 3595.8231 | 2.91 | 2 | 50 | 2.1e-05 | 1Score **> 37** indicates **identity** Score **> 16** indicates **homology** | U | K.DGTVTAGNASGVSDGAGAVIIASEDAVKKHNFTPLAR.V |
| 190790 | 242 | – | 278 | 1199.9497 | 3596.8272 | 3596.8071 | 5.59 | 2 | 66 | 6.5e-07 | 1Score **> 37** indicates **identity** Score **> 17** indicates **homology** | U | K.DGTVTAGNASGVSDGAGAVIIASEDAVKKHNFTPLAR.V  + Deamidated (NQ) |
| 19189 | 270 | – | 278 | 361.8735 | 1082.5988 | 1082.5985 | 0.28 | 1 | 36 | 0.0006 | 1Score **> 30** indicates **identity** Score **> 16** indicates **homology** | U | K.KHNFTPLAR.V |
| 19192 | 270 | – | 278 | 542.3069 | 1082.5993 | 1082.5985 | 0.73 | 1 | 31 | 0.015 | 1Score **> 30** indicates **identity** Score **> 26** indicates **homology** | U | K.KHNFTPLAR.V |
| 10584 | 271 | – | 278 | 478.2594 | 954.5042 | 954.5035 | 0.71 | 0 | 28 | 0.0071 | 1Score **> 30** indicates **identity** Score **> 19** indicates **homology** | U | K.HNFTPLAR.V |
| 10585 | 271 | – | 278 | 478.2594 | 954.5043 | 954.5035 | 0.80 | 0 | 38 | 0.00099 | 1Score **> 30** indicates **identity** Score **> 21** indicates **homology** | U | K.HNFTPLAR.V |
| 10586 | 271 | – | 278 | 478.2595 | 954.5045 | 954.5035 | 0.98 | 0 | 18 | 0.044 | 1Score **> 30** indicates **identity** Score **> 17** indicates **homology** | U | K.HNFTPLAR.V |
| 10588 | 271 | – | 278 | 478.2596 | 954.5047 | 954.5035 | 1.23 | 0 | 30 | 0.0068 | 1Score **> 30** indicates **identity** Score **> 20** indicates **homology** | U | K.HNFTPLAR.V |
| 10589 | 271 | – | 278 | 478.2598 | 954.5050 | 954.5035 | 1.49 | 0 | 31 | 0.0043 | 1Score **> 30** indicates **identity** Score **> 20** indicates **homology** | U | K.HNFTPLAR.V |
| 10593 | 271 | – | 278 | 478.2617 | 954.5089 | 954.5035 | 5.64 | 0 | 28 | 0.0065 | 1Score **> 30** indicates **identity** Score **> 18** indicates **homology** | U | K.HNFTPLAR.V |
| 173550 | 307 | – | 332 | 950.4943 | 2848.4611 | 2848.4681 | -2.45 | 1 | 61 | 2e-06 | 1Score **> 37** indicates **identity** Score **> 16** indicates **homology** | U | K.AGLSLKDMDLIDVNEAFAPQFLSVQK.A |
| 179201 | 307 | – | 332 | 1007.8589 | 3020.5550 | 3020.5780 | -7.63 | 1 | 15 | 0.038 | 1Score **> 38** indicates **identity** Score **> 13** indicates **homology** | U | K.AGLSLKDMDLIDVNEAFAPQFLSVQK.A  + HNE (K); Oxidation (M) |
| 5781 | 333 | – | 340 | 429.7316 | 857.4487 | 857.4494 | -0.87 | 0 | 36 | 0.0011 | 1Score **> 27** indicates **identity** Score **> 18** indicates **homology** | U | K.ALDLDPSK.T |
| 5782 | 333 | – | 340 | 429.7317 | 857.4488 | 857.4494 | -0.79 | 0 | 34 | 0.0041 | 1Score **> 27** indicates **identity** Score **> 23** indicates **homology** | U | K.ALDLDPSK.T |
| 165661 | 333 | – | 360 | 883.1325 | 2646.3758 | 2646.3725 | 1.23 | 1 | 23 | 0.0074 | 1Score **> 37** indicates **identity** Score **> 14** indicates **homology** | U | K.ALDLDPSKTNVSGGAIALGHPLGGSGSR.I |
| 165662 | 333 | – | 360 | 662.6013 | 2646.3759 | 2646.3725 | 1.27 | 1 | 18 | 0.019 | 1Score **> 37** indicates **identity** Score **> 14** indicates **homology** | U | K.ALDLDPSKTNVSGGAIALGHPLGGSGSR.I |
| 165663 | 333 | – | 360 | 662.6014 | 2646.3766 | 2646.3725 | 1.51 | 1 | 27 | 0.0027 | 1Score **> 37** indicates **identity** Score **> 14** indicates **homology** | U | K.ALDLDPSKTNVSGGAIALGHPLGGSGSR.I |
| 165664 | 333 | – | 360 | 662.6014 | 2646.3766 | 2646.3725 | 1.53 | 1 | 48 | 2.9e-05 | 1Score **> 37** indicates **identity** Score **> 16** indicates **homology** | U | K.ALDLDPSKTNVSGGAIALGHPLGGSGSR.I |
| 165665 | 333 | – | 360 | 662.6014 | 2646.3767 | 2646.3725 | 1.57 | 1 | 41 | 0.00013 | 1Score **> 37** indicates **identity** Score **> 15** indicates **homology** | U | K.ALDLDPSKTNVSGGAIALGHPLGGSGSR.I |
| 165666 | 333 | – | 360 | 1324.1957 | 2646.3768 | 2646.3725 | 1.61 | 1 | 49 | 2.3e-05 | 1Score **> 37** indicates **identity** Score **> 16** indicates **homology** | U | K.ALDLDPSKTNVSGGAIALGHPLGGSGSR.I |
| 165667 | 333 | – | 360 | 883.1329 | 2646.3768 | 2646.3725 | 1.61 | 1 | 55 | 6.8e-06 | 1Score **> 37** indicates **identity** Score **> 16** indicates **homology** | U | K.ALDLDPSKTNVSGGAIALGHPLGGSGSR.I |
| 165668 | 333 | – | 360 | 883.1330 | 2646.3771 | 2646.3725 | 1.72 | 1 | 67 | 5.4e-07 | 1Score **> 37** indicates **identity** Score **> 17** indicates **homology** | U | K.ALDLDPSKTNVSGGAIALGHPLGGSGSR.I |
| 165669 | 333 | – | 360 | 662.6016 | 2646.3773 | 2646.3725 | 1.81 | 1 | 40 | 0.00017 | 1Score **> 37** indicates **identity** Score **> 15** indicates **homology** | U | K.ALDLDPSKTNVSGGAIALGHPLGGSGSR.I |
| 165670 | 333 | – | 360 | 883.1331 | 2646.3775 | 2646.3725 | 1.86 | 1 | 75 | 8.9e-08 | 1Score **> 37** indicates **identity** Score **> 17** indicates **homology** | U | K.ALDLDPSKTNVSGGAIALGHPLGGSGSR.I |
| 165671 | 333 | – | 360 | 662.6018 | 2646.3781 | 2646.3725 | 2.09 | 1 | 48 | 3.5e-05 | 1Score **> 37** indicates **identity** Score **> 15** indicates **homology** | U | K.ALDLDPSKTNVSGGAIALGHPLGGSGSR.I |
| 165674 | 333 | – | 360 | 883.1341 | 2646.3804 | 2646.3725 | 2.98 | 1 | 36 | 0.00045 | 1Score **> 37** indicates **identity** Score **> 15** indicates **homology** | U | K.ALDLDPSKTNVSGGAIALGHPLGGSGSR.I |
| 165675 | 333 | – | 360 | 883.1344 | 2646.3814 | 2646.3725 | 3.36 | 1 | 20 | 0.013 | 1Score **> 37** indicates **identity** Score **> 14** indicates **homology** | U | K.ALDLDPSKTNVSGGAIALGHPLGGSGSR.I |
| 165676 | 333 | – | 360 | 662.6029 | 2646.3825 | 2646.3725 | 3.75 | 1 | 19 | 0.018 | 1Score **> 37** indicates **identity** Score **> 14** indicates **homology** | U | K.ALDLDPSKTNVSGGAIALGHPLGGSGSR.I |
| 165701 | 333 | – | 360 | 1324.6958 | 2647.3770 | 2647.3566 | 7.71 | 1 | 65 | 8.1e-07 | 1Score **> 37** indicates **identity** Score **> 17** indicates **homology** | U | K.ALDLDPSKTNVSGGAIALGHPLGGSGSR.I  + Deamidated (NQ) |
| 101345 | 341 | – | 360 | 904.4673 | 1806.9201 | 1806.9337 | -7.50 | 0 | 48 | 3.1e-05 | 1Score **> 36** indicates **identity** Score **> 16** indicates **homology** | U | K.TNVSGGAIALGHPLGGSGSR.I |
| 101380 | 341 | – | 360 | 904.4724 | 1806.9302 | 1806.9337 | -1.90 | 0 | 121 | 4.8e-12 | 1Score **> 35** indicates **identity** Score **> 20** indicates **homology** | U | K.TNVSGGAIALGHPLGGSGSR.I |
| 101387 | 341 | – | 360 | 603.3187 | 1806.9343 | 1806.9337 | 0.37 | 0 | 52 | 0.00018 | 1Score **> 36** indicates **identity** Score **> 27** indicates **homology** | U | K.TNVSGGAIALGHPLGGSGSR.I |
| 101389 | 341 | – | 360 | 904.4754 | 1806.9362 | 1806.9337 | 1.40 | 0 | 101 | 4.5e-10 | 1Score **> 36** indicates **identity** Score **> 20** indicates **homology** | U | K.TNVSGGAIALGHPLGGSGSR.I |
| 101390 | 341 | – | 360 | 904.4756 | 1806.9366 | 1806.9337 | 1.61 | 0 | 115 | 2.2e-11 | 1Score **> 36** indicates **identity** Score **> 21** indicates **homology** | U | K.TNVSGGAIALGHPLGGSGSR.I |
| 101392 | 341 | – | 360 | 603.3196 | 1806.9371 | 1806.9337 | 1.89 | 0 | 46 | 0.00053 | 1Score **> 36** indicates **identity** Score **> 26** indicates **homology** | U | K.TNVSGGAIALGHPLGGSGSR.I |
| 101393 | 341 | – | 360 | 603.3202 | 1806.9387 | 1806.9337 | 2.80 | 0 | 55 | 0.00011 | 1Score **> 36** indicates **identity** Score **> 28** indicates **homology** | U | K.TNVSGGAIALGHPLGGSGSR.I |
| 101394 | 341 | – | 360 | 603.3203 | 1806.9389 | 1806.9337 | 2.91 | 0 | 26 | 0.0038 | 1Score **> 36** indicates **identity** Score **> 14** indicates **homology** | U | K.TNVSGGAIALGHPLGGSGSR.I |
| 101397 | 341 | – | 360 | 603.3211 | 1806.9414 | 1806.9337 | 4.29 | 0 | 18 | 0.022 | 1Score **> 35** indicates **identity** Score **> 14** indicates **homology** | U | K.TNVSGGAIALGHPLGGSGSR.I |
| 101398 | 341 | – | 360 | 603.3215 | 1806.9426 | 1806.9337 | 4.93 | 0 | 25 | 0.0041 | 1Score **> 35** indicates **identity** Score **> 14** indicates **homology** | U | K.TNVSGGAIALGHPLGGSGSR.I |
| 29167 | 361 | – | 370 | 396.8999 | 1187.6779 | 1187.6775 | 0.40 | 0 | 33 | 0.011 | 1Score **> 33** indicates **identity** Score **> 26** indicates **homology** | U | R.ITAHLVHELR.R |
| 29168 | 361 | – | 370 | 594.8465 | 1187.6785 | 1187.6775 | 0.83 | 0 | 59 | 0.00012 | 1Score **> 33** indicates **identity** | U | R.ITAHLVHELR.R |
| 29171 | 361 | – | 370 | 594.8466 | 1187.6787 | 1187.6775 | 1.04 | 0 | 53 | 0.00058 | 1Score **> 33** indicates **identity** | U | R.ITAHLVHELR.R |
| 29172 | 361 | – | 370 | 396.9002 | 1187.6789 | 1187.6775 | 1.19 | 0 | 33 | 0.0019 | 1Score **> 33** indicates **identity** Score **> 18** indicates **homology** | U | R.ITAHLVHELR.R |
| 29173 | 361 | – | 370 | 396.9003 | 1187.6789 | 1187.6775 | 1.24 | 0 | 38 | 0.0045 | 1Score **> 33** indicates **identity** Score **> 27** indicates **homology** | U | R.ITAHLVHELR.R |
| 29175 | 361 | – | 370 | 396.9005 | 1187.6797 | 1187.6775 | 1.83 | 0 | 31 | 0.0076 | 1Score **> 33** indicates **identity** Score **> 23** indicates **homology** | U | R.ITAHLVHELR.R |
| 44995 | 361 | – | 371 | 448.9340 | 1343.7800 | 1343.7786 | 1.07 | 1 | 35 | 0.039 | 1Score **> 33** indicates **identity** | U | R.ITAHLVHELRR.R |
| 44999 | 361 | – | 371 | 448.9341 | 1343.7806 | 1343.7786 | 1.49 | 1 | 36 | 0.029 | 1Score **> 33** indicates **identity** | U | R.ITAHLVHELRR.R |
| 45000 | 361 | – | 371 | 672.8977 | 1343.7809 | 1343.7786 | 1.72 | 1 | 24 | 0.026 | 1Score **> 33** indicates **identity** Score **> 20** indicates **homology** | U | R.ITAHLVHELRR.R |

---

```
ID   THIM_MOUSE              Reviewed;         397 AA.
AC   Q8BWT1; Q3TIT9; Q8JZR8;
DT   30-AUG-2005, integrated into UniProtKB/Swiss-Prot.
DT   27-JUL-2011, sequence version 3.
DT   28-JUN-2023, entry version 149.
DE   RecName: Full=3-ketoacyl-CoA thiolase, mitochondrial {ECO:0000305};
DE            EC=2.3.1.16 {ECO:0000250|UniProtKB:P42765};
DE   AltName: Full=Acetyl-CoA acetyltransferase {ECO:0000305};
DE            EC=2.3.1.9 {ECO:0000255|PROSITE-ProRule:PRU10020};
DE   AltName: Full=Acetyl-CoA acyltransferase;
DE   AltName: Full=Acyl-CoA hydrolase, mitochondrial {ECO:0000305};
DE            EC=3.1.2.- {ECO:0000250|UniProtKB:P42765};
DE            EC=3.1.2.1 {ECO:0000250|UniProtKB:P42765};
DE            EC=3.1.2.2 {ECO:0000250|UniProtKB:P13437};
DE   AltName: Full=Beta-ketothiolase;
DE   AltName: Full=Mitochondrial 3-oxoacyl-CoA thiolase;
GN   Name=Acaa2;
OS   Mus musculus (Mouse).
OC   Eukaryota; Metazoa; Chordata; Craniata; Vertebrata; Euteleostomi; Mammalia;
OC   Eutheria; Euarchontoglires; Glires; Rodentia; Myomorpha; Muroidea; Muridae;
OC   Murinae; Mus; Mus.
OX   NCBI_TaxID=10090;
RN   [1]
RP   NUCLEOTIDE SEQUENCE [LARGE SCALE MRNA].
RC   STRAIN=C57BL/6J; TISSUE=Amnion, Heart, and Liver;
RX   PubMed=16141072; DOI=10.1126/science.1112014;
RA   Carninci P., Kasukawa T., Katayama S., Gough J., Frith M.C., Maeda N.,
RA   Oyama R., Ravasi T., Lenhard B., Wells C., Kodzius R., Shimokawa K.,
RA   Bajic V.B., Brenner S.E., Batalov S., Forrest A.R., Zavolan M., Davis M.J.,
RA   Wilming L.G., Aidinis V., Allen J.E., Ambesi-Impiombato A., Apweiler R.,
RA   Aturaliya R.N., Bailey T.L., Bansal M., Baxter L., Beisel K.W., Bersano T.,
RA   Bono H., Chalk A.M., Chiu K.P., Choudhary V., Christoffels A.,
RA   Clutterbuck D.R., Crowe M.L., Dalla E., Dalrymple B.P., de Bono B.,
RA   Della Gatta G., di Bernardo D., Down T., Engstrom P., Fagiolini M.,
RA   Faulkner G., Fletcher C.F., Fukushima T., Furuno M., Futaki S.,
RA   Gariboldi M., Georgii-Hemming P., Gingeras T.R., Gojobori T., Green R.E.,
RA   Gustincich S., Harbers M., Hayashi Y., Hensch T.K., Hirokawa N., Hill D.,
RA   Huminiecki L., Iacono M., Ikeo K., Iwama A., Ishikawa T., Jakt M.,
RA   Kanapin A., Katoh M., Kawasawa Y., Kelso J., Kitamura H., Kitano H.,
RA   Kollias G., Krishnan S.P., Kruger A., Kummerfeld S.K., Kurochkin I.V.,
RA   Lareau L.F., Lazarevic D., Lipovich L., Liu J., Liuni S., McWilliam S.,
RA   Madan Babu M., Madera M., Marchionni L., Matsuda H., Matsuzawa S., Miki H.,
RA   Mignone F., Miyake S., Morris K., Mottagui-Tabar S., Mulder N., Nakano N.,
RA   Nakauchi H., Ng P., Nilsson R., Nishiguchi S., Nishikawa S., Nori F.,
RA   Ohara O., Okazaki Y., Orlando V., Pang K.C., Pavan W.J., Pavesi G.,
RA   Pesole G., Petrovsky N., Piazza S., Reed J., Reid J.F., Ring B.Z.,
RA   Ringwald M., Rost B., Ruan Y., Salzberg S.L., Sandelin A., Schneider C.,
RA   Schoenbach C., Sekiguchi K., Semple C.A., Seno S., Sessa L., Sheng Y.,
RA   Shibata Y., Shimada H., Shimada K., Silva D., Sinclair B., Sperling S.,
RA   Stupka E., Sugiura K., Sultana R., Takenaka Y., Taki K., Tammoja K.,
RA   Tan S.L., Tang S., Taylor M.S., Tegner J., Teichmann S.A., Ueda H.R.,
RA   van Nimwegen E., Verardo R., Wei C.L., Yagi K., Yamanishi H.,
RA   Zabarovsky E., Zhu S., Zimmer A., Hide W., Bult C., Grimmond S.M.,
RA   Teasdale R.D., Liu E.T., Brusic V., Quackenbush J., Wahlestedt C.,
RA   Mattick J.S., Hume D.A., Kai C., Sasaki D., Tomaru Y., Fukuda S.,
RA   Kanamori-Katayama M., Suzuki M., Aoki J., Arakawa T., Iida J., Imamura K.,
RA   Itoh M., Kato T., Kawaji H., Kawagashira N., Kawashima T., Kojima M.,
RA   Kondo S., Konno H., Nakano K., Ninomiya N., Nishio T., Okada M., Plessy C.,
RA   Shibata K., Shiraki T., Suzuki S., Tagami M., Waki K., Watahiki A.,
RA   Okamura-Oho Y., Suzuki H., Kawai J., Hayashizaki Y.;
RT   "The transcriptional landscape of the mammalian genome.";
RL   Science 309:1559-1563(2005).
RN   [2]
RP   NUCLEOTIDE SEQUENCE [LARGE SCALE GENOMIC DNA].
RA   Mural R.J., Adams M.D., Myers E.W., Smith H.O., Venter J.C.;
RL   Submitted (SEP-2005) to the EMBL/GenBank/DDBJ databases.
RN   [3]
RP   NUCLEOTIDE SEQUENCE [LARGE SCALE MRNA].
RC   STRAIN=FVB/N; TISSUE=Liver;
RX   PubMed=15489334; DOI=10.1101/gr.2596504;
RG   The MGC Project Team;
RT   "The status, quality, and expansion of the NIH full-length cDNA project:
RT   the Mammalian Gene Collection (MGC).";
RL   Genome Res. 14:2121-2127(2004).
RN   [4]
RP   PHOSPHORYLATION [LARGE SCALE ANALYSIS] AT SER-344, AND IDENTIFICATION BY
RP   MASS SPECTROMETRY [LARGE SCALE ANALYSIS].
RC   TISSUE=Liver;
RX   PubMed=17208939; DOI=10.1074/mcp.m600218-mcp200;
RA   Lee J., Xu Y., Chen Y., Sprung R., Kim S.C., Xie S., Zhao Y.;
RT   "Mitochondrial phosphoproteome revealed by an improved IMAC method and
RT   MS/MS/MS.";
RL   Mol. Cell. Proteomics 6:669-676(2007).
RN   [5]
RP   PHOSPHORYLATION [LARGE SCALE ANALYSIS] AT SER-28, AND IDENTIFICATION BY
RP   MASS SPECTROMETRY [LARGE SCALE ANALYSIS].
RC   TISSUE=Liver;
RX   PubMed=17242355; DOI=10.1073/pnas.0609836104;
RA   Villen J., Beausoleil S.A., Gerber S.A., Gygi S.P.;
RT   "Large-scale phosphorylation analysis of mouse liver.";
RL   Proc. Natl. Acad. Sci. U.S.A. 104:1488-1493(2007).
RN   [6]
RP   PHOSPHORYLATION [LARGE SCALE ANALYSIS] AT SER-28; THR-136 AND SER-310, AND
RP   IDENTIFICATION BY MASS SPECTROMETRY [LARGE SCALE ANALYSIS].
RC   TISSUE=Brain, Brown adipose tissue, Heart, Kidney, Liver, Lung,
RC   Pancreas, Spleen, and Testis;
RX   PubMed=21183079; DOI=10.1016/j.cell.2010.12.001;
RA   Huttlin E.L., Jedrychowski M.P., Elias J.E., Goswami T., Rad R.,
RA   Beausoleil S.A., Villen J., Haas W., Sowa M.E., Gygi S.P.;
RT   "A tissue-specific atlas of mouse protein phosphorylation and expression.";
RL   Cell 143:1174-1189(2010).
RN   [7]
RP   ACETYLATION [LARGE SCALE ANALYSIS] AT LYS-191, SUCCINYLATION [LARGE SCALE
RP   ANALYSIS] AT LYS-25; LYS-45; LYS-137; LYS-143; LYS-158; LYS-171; LYS-191;
RP   LYS-209; LYS-211; LYS-212; LYS-214; LYS-234; LYS-240; LYS-305 AND LYS-312,
RP   AND IDENTIFICATION BY MASS SPECTROMETRY [LARGE SCALE ANALYSIS].
RC   TISSUE=Liver;
RX   PubMed=23806337; DOI=10.1016/j.molcel.2013.06.001;
RA   Park J., Chen Y., Tishkoff D.X., Peng C., Tan M., Dai L., Xie Z., Zhang Y.,
RA   Zwaans B.M., Skinner M.E., Lombard D.B., Zhao Y.;
RT   "SIRT5-mediated lysine desuccinylation impacts diverse metabolic
RT   pathways.";
RL   Mol. Cell 50:919-930(2013).
RN   [8]
RP   ACETYLATION [LARGE SCALE ANALYSIS] AT LYS-25; LYS-137; LYS-143; LYS-158;
RP   LYS-171; LYS-191; LYS-209; LYS-234; LYS-241; LYS-269; LYS-270; LYS-305;
RP   LYS-312; LYS-340 AND LYS-375, AND IDENTIFICATION BY MASS SPECTROMETRY
RP   [LARGE SCALE ANALYSIS].
RC   TISSUE=Liver;
RX   PubMed=23576753; DOI=10.1073/pnas.1302961110;
RA   Rardin M.J., Newman J.C., Held J.M., Cusack M.P., Sorensen D.J., Li B.,
RA   Schilling B., Mooney S.D., Kahn C.R., Verdin E., Gibson B.W.;
RT   "Label-free quantitative proteomics of the lysine acetylome in mitochondria
RT   identifies substrates of SIRT3 in metabolic pathways.";
RL   Proc. Natl. Acad. Sci. U.S.A. 110:6601-6606(2013).
CC   -!- FUNCTION: In the production of energy from fats, this is one of the
CC       enzymes that catalyzes the last step of the mitochondrial beta-
CC       oxidation pathway, an aerobic process breaking down fatty acids into
CC       acetyl-CoA. Using free coenzyme A/CoA, catalyzes the thiolytic cleavage
CC       of medium- to long-chain unbranched 3-oxoacyl-CoAs into acetyl-CoA and
CC       a fatty acyl-CoA shortened by two carbon atoms. Also catalyzes the
CC       condensation of two acetyl-CoA molecules into acetoacetyl-CoA and could
CC       be involved in the production of ketone bodies. Also displays hydrolase
CC       activity on various fatty acyl-CoAs (By similarity). Thereby, could be
CC       responsible for the production of acetate in a side reaction to beta-
CC       oxidation (By similarity). Abolishes BNIP3-mediated apoptosis and
CC       mitochondrial damage (By similarity). {ECO:0000250|UniProtKB:P13437,
CC       ECO:0000250|UniProtKB:P42765}.
CC   -!- CATALYTIC ACTIVITY:
CC       Reaction=acetyl-CoA + an acyl-CoA = a 3-oxoacyl-CoA + CoA;
CC         Xref=Rhea:RHEA:21564, ChEBI:CHEBI:57287, ChEBI:CHEBI:57288,
CC         ChEBI:CHEBI:58342, ChEBI:CHEBI:90726; EC=2.3.1.16;
CC         Evidence={ECO:0000250|UniProtKB:P42765};
CC       PhysiologicalDirection=left-to-right; Xref=Rhea:RHEA:21565;
CC         Evidence={ECO:0000250|UniProtKB:P42765};
CC       PhysiologicalDirection=right-to-left; Xref=Rhea:RHEA:21566;
CC         Evidence={ECO:0000250|UniProtKB:P42765};
CC   -!- CATALYTIC ACTIVITY:
CC       Reaction=2 acetyl-CoA = acetoacetyl-CoA + CoA; Xref=Rhea:RHEA:21036,
CC         ChEBI:CHEBI:57286, ChEBI:CHEBI:57287, ChEBI:CHEBI:57288; EC=2.3.1.9;
CC         Evidence={ECO:0000255|PROSITE-ProRule:PRU10020};
CC       PhysiologicalDirection=left-to-right; Xref=Rhea:RHEA:21037;
CC         Evidence={ECO:0000250|UniProtKB:P42765};
CC       PhysiologicalDirection=right-to-left; Xref=Rhea:RHEA:21038;
CC         Evidence={ECO:0000250|UniProtKB:P42765};
CC   -!- CATALYTIC ACTIVITY:
CC       Reaction=acetyl-CoA + H2O = acetate + CoA + H(+); Xref=Rhea:RHEA:20289,
CC         ChEBI:CHEBI:15377, ChEBI:CHEBI:15378, ChEBI:CHEBI:30089,
CC         ChEBI:CHEBI:57287, ChEBI:CHEBI:57288; EC=3.1.2.1;
CC         Evidence={ECO:0000250|UniProtKB:P42765};
CC       PhysiologicalDirection=left-to-right; Xref=Rhea:RHEA:20290;
CC         Evidence={ECO:0000250|UniProtKB:P42765};
CC   -!- CATALYTIC ACTIVITY:
CC       Reaction=H2O + propanoyl-CoA = CoA + H(+) + propanoate;
CC         Xref=Rhea:RHEA:40103, ChEBI:CHEBI:15377, ChEBI:CHEBI:15378,
CC         ChEBI:CHEBI:17272, ChEBI:CHEBI:57287, ChEBI:CHEBI:57392;
CC         Evidence={ECO:0000250|UniProtKB:P42765};
CC       PhysiologicalDirection=left-to-right; Xref=Rhea:RHEA:40104;
CC         Evidence={ECO:0000250|UniProtKB:P42765};
CC   -!- CATALYTIC ACTIVITY:
CC       Reaction=butanoyl-CoA + H2O = butanoate + CoA + H(+);
CC         Xref=Rhea:RHEA:40111, ChEBI:CHEBI:15377, ChEBI:CHEBI:15378,
CC         ChEBI:CHEBI:17968, ChEBI:CHEBI:57287, ChEBI:CHEBI:57371;
CC         Evidence={ECO:0000250|UniProtKB:P42765};
CC       PhysiologicalDirection=left-to-right; Xref=Rhea:RHEA:40112;
CC         Evidence={ECO:0000250|UniProtKB:P42765};
CC   -!- CATALYTIC ACTIVITY:
CC       Reaction=H2O + hexanoyl-CoA = CoA + H(+) + hexanoate;
CC         Xref=Rhea:RHEA:40115, ChEBI:CHEBI:15377, ChEBI:CHEBI:15378,
CC         ChEBI:CHEBI:17120, ChEBI:CHEBI:57287, ChEBI:CHEBI:62620;
CC         Evidence={ECO:0000250|UniProtKB:P42765};
CC       PhysiologicalDirection=left-to-right; Xref=Rhea:RHEA:40116;
CC         Evidence={ECO:0000250|UniProtKB:P42765};
CC   -!- CATALYTIC ACTIVITY:
CC       Reaction=H2O + octanoyl-CoA = CoA + H(+) + octanoate;
CC         Xref=Rhea:RHEA:30143, ChEBI:CHEBI:15377, ChEBI:CHEBI:15378,
CC         ChEBI:CHEBI:25646, ChEBI:CHEBI:57287, ChEBI:CHEBI:57386;
CC         Evidence={ECO:0000250|UniProtKB:P42765};
CC       PhysiologicalDirection=left-to-right; Xref=Rhea:RHEA:30144;
CC         Evidence={ECO:0000250|UniProtKB:P42765};
CC   -!- CATALYTIC ACTIVITY:
CC       Reaction=decanoyl-CoA + H2O = CoA + decanoate + H(+);
CC         Xref=Rhea:RHEA:40059, ChEBI:CHEBI:15377, ChEBI:CHEBI:15378,
CC         ChEBI:CHEBI:27689, ChEBI:CHEBI:57287, ChEBI:CHEBI:61430;
CC         Evidence={ECO:0000250|UniProtKB:P42765};
CC       PhysiologicalDirection=left-to-right; Xref=Rhea:RHEA:40060;
CC         Evidence={ECO:0000250|UniProtKB:P42765};
CC   -!- CATALYTIC ACTIVITY:
CC       Reaction=dodecanoyl-CoA + H2O = CoA + dodecanoate + H(+);
CC         Xref=Rhea:RHEA:30135, ChEBI:CHEBI:15377, ChEBI:CHEBI:15378,
CC         ChEBI:CHEBI:18262, ChEBI:CHEBI:57287, ChEBI:CHEBI:57375;
CC         Evidence={ECO:0000250|UniProtKB:P42765};
CC       PhysiologicalDirection=left-to-right; Xref=Rhea:RHEA:30136;
CC         Evidence={ECO:0000250|UniProtKB:P42765};
CC   -!- CATALYTIC ACTIVITY:
CC       Reaction=H2O + tetradecanoyl-CoA = CoA + H(+) + tetradecanoate;
CC         Xref=Rhea:RHEA:40119, ChEBI:CHEBI:15377, ChEBI:CHEBI:15378,
CC         ChEBI:CHEBI:30807, ChEBI:CHEBI:57287, ChEBI:CHEBI:57385;
CC         Evidence={ECO:0000250|UniProtKB:P42765};
CC       PhysiologicalDirection=left-to-right; Xref=Rhea:RHEA:40120;
CC         Evidence={ECO:0000250|UniProtKB:P42765};
CC   -!- CATALYTIC ACTIVITY:
CC       Reaction=H2O + hexadecanoyl-CoA = CoA + H(+) + hexadecanoate;
CC         Xref=Rhea:RHEA:16645, ChEBI:CHEBI:7896, ChEBI:CHEBI:15377,
CC         ChEBI:CHEBI:15378, ChEBI:CHEBI:57287, ChEBI:CHEBI:57379; EC=3.1.2.2;
CC         Evidence={ECO:0000250|UniProtKB:P13437};
CC       PhysiologicalDirection=left-to-right; Xref=Rhea:RHEA:16646;
CC         Evidence={ECO:0000250|UniProtKB:P42765};
CC   -!- PATHWAY: Lipid metabolism; fatty acid beta-oxidation.
CC       {ECO:0000250|UniProtKB:P42765}.
CC   -!- SUBUNIT: Homotetramer. Interacts with BNIP3.
CC       {ECO:0000250|UniProtKB:P42765}.
CC   -!- SUBCELLULAR LOCATION: Mitochondrion {ECO:0000250|UniProtKB:P42765}.
CC   -!- SIMILARITY: Belongs to the thiolase-like superfamily. Thiolase family.
CC       {ECO:0000305}.
CC   ---------------------------------------------------------------------------
CC   Copyrighted by the UniProt Consortium, see https://www.uniprot.org/terms
CC   Distributed under the Creative Commons Attribution (CC BY 4.0) License
CC   ---------------------------------------------------------------------------
DR   EMBL; AK050101; BAC34067.1; -; mRNA.
DR   EMBL; AK167567; BAE39630.1; -; mRNA.
DR   EMBL; AK167715; BAE39757.1; -; mRNA.
DR   EMBL; AK169359; BAE41108.1; -; mRNA.
DR   EMBL; CH466528; EDL09521.1; -; Genomic_DNA.
DR   EMBL; BC028901; AAH28901.1; -; mRNA.
DR   CCDS; CCDS29342.1; -.
DR   RefSeq; NP_803421.1; NM_177470.3.
DR   AlphaFoldDB; Q8BWT1; -.
DR   SMR; Q8BWT1; -.
DR   BioGRID; 206649; 30.
DR   IntAct; Q8BWT1; 9.
DR   MINT; Q8BWT1; -.
DR   STRING; 10090.ENSMUSP00000037348; -.
DR   GlyGen; Q8BWT1; 1 site, 1 O-linked glycan (1 site).
DR   iPTMnet; Q8BWT1; -.
DR   PhosphoSitePlus; Q8BWT1; -.
DR   SwissPalm; Q8BWT1; -.
DR   REPRODUCTION-2DPAGE; Q8BWT1; -.
DR   CPTAC; non-CPTAC-3618; -.
DR   CPTAC; non-CPTAC-3883; -.
DR   EPD; Q8BWT1; -.
DR   jPOST; Q8BWT1; -.
DR   MaxQB; Q8BWT1; -.
DR   PaxDb; Q8BWT1; -.
DR   PeptideAtlas; Q8BWT1; -.
DR   ProteomicsDB; 258872; -.
DR   Antibodypedia; 22617; 409 antibodies from 30 providers.
DR   Ensembl; ENSMUST00000041053; ENSMUSP00000037348; ENSMUSG00000036880.
DR   GeneID; 52538; -.
DR   KEGG; mmu:52538; -.
DR   UCSC; uc008fpt.1; mouse.
DR   AGR; MGI:1098623; -.
DR   CTD; 10449; -.
DR   MGI; MGI:1098623; Acaa2.
DR   VEuPathDB; HostDB:ENSMUSG00000036880; -.
DR   eggNOG; KOG1391; Eukaryota.
DR   GeneTree; ENSGT01030000234626; -.
DR   HOGENOM; CLU_031026_0_0_1; -.
DR   InParanoid; Q8BWT1; -.
DR   OMA; DYYWGMG; -.
DR   OrthoDB; 5481312at2759; -.
DR   PhylomeDB; Q8BWT1; -.
DR   TreeFam; TF105696; -.
DR   BRENDA; 2.3.1.16; 3474.
DR   Reactome; R-MMU-77289; Mitochondrial Fatty Acid Beta-Oxidation.
DR   UniPathway; UPA00659; -.
DR   BioGRID-ORCS; 52538; 1 hit in 80 CRISPR screens.
DR   ChiTaRS; Acaa2; mouse.
DR   PRO; PR:Q8BWT1; -.
DR   Proteomes; UP000000589; Chromosome 18.
DR   RNAct; Q8BWT1; protein.
DR   Bgee; ENSMUSG00000036880; Expressed in gall bladder and 285 other tissues.
DR   ExpressionAtlas; Q8BWT1; baseline and differential.
DR   Genevisible; Q8BWT1; MM.
DR   GO; GO:0005743; C:mitochondrial inner membrane; HDA:MGI.
DR   GO; GO:0005759; C:mitochondrial matrix; ISO:MGI.
DR   GO; GO:0005739; C:mitochondrion; HDA:MGI.
DR   GO; GO:0003985; F:acetyl-CoA C-acetyltransferase activity; ISS:UniProtKB.
DR   GO; GO:0003988; F:acetyl-CoA C-acyltransferase activity; ISS:UniProtKB.
DR   GO; GO:0003986; F:acetyl-CoA hydrolase activity; IEA:UniProtKB-EC.
DR   GO; GO:0047617; F:acyl-CoA hydrolase activity; ISS:UniProtKB.
DR   GO; GO:0102991; F:myristoyl-CoA hydrolase activity; IEA:UniProtKB-EC.
DR   GO; GO:0016290; F:palmitoyl-CoA hydrolase activity; IEA:UniProtKB-EC.
DR   GO; GO:0006084; P:acetyl-CoA metabolic process; ISO:MGI.
DR   GO; GO:0071456; P:cellular response to hypoxia; ISO:MGI.
DR   GO; GO:0006635; P:fatty acid beta-oxidation; ISO:MGI.
DR   GO; GO:1902109; P:negative regulation of mitochondrial membrane permeability involved in apoptotic process; ISO:MGI.
DR   GO; GO:1901029; P:negative regulation of mitochondrial outer membrane permeabilization involved in apoptotic signaling pathway; ISS:UniProtKB.
DR   CDD; cd00751; thiolase; 1.
DR   Gene3D; 3.40.47.10; -; 2.
DR   InterPro; IPR002155; Thiolase.
DR   InterPro; IPR016039; Thiolase-like.
DR   InterPro; IPR020615; Thiolase_acyl_enz_int_AS.
DR   InterPro; IPR020610; Thiolase_AS.
DR   InterPro; IPR020617; Thiolase_C.
DR   InterPro; IPR020613; Thiolase_CS.
DR   InterPro; IPR020616; Thiolase_N.
DR   PANTHER; PTHR18919:SF107; 3-KETOACYL-COA THIOLASE, MITOCHONDRIAL; 1.
DR   PANTHER; PTHR18919; ACETYL-COA C-ACYLTRANSFERASE; 1.
DR   Pfam; PF02803; Thiolase_C; 1.
DR   Pfam; PF00108; Thiolase_N; 1.
DR   PIRSF; PIRSF000429; Ac-CoA_Ac_transf; 1.
DR   SUPFAM; SSF53901; Thiolase-like; 2.
DR   PROSITE; PS00098; THIOLASE_1; 1.
DR   PROSITE; PS00737; THIOLASE_2; 1.
DR   PROSITE; PS00099; THIOLASE_3; 1.
DR   TIGRFAMs; TIGR01930; AcCoA-C-Actrans; 1.
PE   1: Evidence at protein level;
KW   Acetylation; Acyltransferase; Fatty acid metabolism; Hydrolase;
KW   Lipid metabolism; Mitochondrion; Phosphoprotein; Reference proteome;
KW   Transferase; Transit peptide.
FT   CHAIN           1..397
FT                   /note="3-ketoacyl-CoA thiolase, mitochondrial"
FT                   /id="PRO_0000223300"
FT   TRANSIT         1..16
FT                   /note="Mitochondrion; not cleaved"
FT                   /evidence="ECO:0000250"
FT   ACT_SITE        92
FT                   /note="Acyl-thioester intermediate"
FT                   /evidence="ECO:0000250|UniProtKB:P42765"
FT   ACT_SITE        382
FT                   /note="Proton donor/acceptor"
FT                   /evidence="ECO:0000250|UniProtKB:P42765"
FT   BINDING         224
FT                   /ligand="CoA"
FT                   /ligand_id="ChEBI:CHEBI:57287"
FT                   /evidence="ECO:0000250|UniProtKB:P42765"
FT   BINDING         227
FT                   /ligand="CoA"
FT                   /ligand_id="ChEBI:CHEBI:57287"
FT                   /evidence="ECO:0000250|UniProtKB:P42765"
FT   BINDING         251
FT                   /ligand="CoA"
FT                   /ligand_id="ChEBI:CHEBI:57287"
FT                   /evidence="ECO:0000250|UniProtKB:P42765"
FT   SITE            352
FT                   /note="Increases nucleophilicity of active site Cys"
FT                   /evidence="ECO:0000250|UniProtKB:P42765"
FT   MOD_RES         25
FT                   /note="N6-acetyllysine; alternate"
FT                   /evidence="ECO:0007744|PubMed:23576753"
FT   MOD_RES         25
FT                   /note="N6-succinyllysine; alternate"
FT                   /evidence="ECO:0007744|PubMed:23806337"
FT   MOD_RES         28
FT                   /note="Phosphoserine"
FT                   /evidence="ECO:0007744|PubMed:17242355,
FT                   ECO:0007744|PubMed:21183079"
FT   MOD_RES         45
FT                   /note="N6-succinyllysine"
FT                   /evidence="ECO:0007744|PubMed:23806337"
FT   MOD_RES         119
FT                   /note="Phosphothreonine"
FT                   /evidence="ECO:0000250|UniProtKB:P42765"
FT   MOD_RES         121
FT                   /note="Phosphoserine"
FT                   /evidence="ECO:0000250|UniProtKB:P42765"
FT   MOD_RES         127
FT                   /note="Phosphotyrosine"
FT                   /evidence="ECO:0000250|UniProtKB:P42765"
FT   MOD_RES         136
FT                   /note="Phosphothreonine"
FT                   /evidence="ECO:0007744|PubMed:21183079"
FT   MOD_RES         137
FT                   /note="N6-acetyllysine; alternate"
FT                   /evidence="ECO:0007744|PubMed:23576753"
FT   MOD_RES         137
FT                   /note="N6-succinyllysine; alternate"
FT                   /evidence="ECO:0007744|PubMed:23806337"
FT   MOD_RES         143
FT                   /note="N6-acetyllysine; alternate"
FT                   /evidence="ECO:0007744|PubMed:23576753"
FT   MOD_RES         143
FT                   /note="N6-succinyllysine; alternate"
FT                   /evidence="ECO:0007744|PubMed:23806337"
FT   MOD_RES         158
FT                   /note="N6-acetyllysine; alternate"
FT                   /evidence="ECO:0007744|PubMed:23576753"
FT   MOD_RES         158
FT                   /note="N6-succinyllysine; alternate"
FT                   /evidence="ECO:0007744|PubMed:23806337"
FT   MOD_RES         171
FT                   /note="N6-acetyllysine; alternate"
FT                   /evidence="ECO:0007744|PubMed:23576753"
FT   MOD_RES         171
FT                   /note="N6-succinyllysine; alternate"
FT                   /evidence="ECO:0007744|PubMed:23806337"
FT   MOD_RES         191
FT                   /note="N6-acetyllysine; alternate"
FT                   /evidence="ECO:0007744|PubMed:23576753,
FT                   ECO:0007744|PubMed:23806337"
FT   MOD_RES         191
FT                   /note="N6-succinyllysine; alternate"
FT                   /evidence="ECO:0007744|PubMed:23806337"
FT   MOD_RES         209
FT                   /note="N6-acetyllysine; alternate"
FT                   /evidence="ECO:0007744|PubMed:23576753"
FT   MOD_RES         209
FT                   /note="N6-succinyllysine; alternate"
FT                   /evidence="ECO:0007744|PubMed:23806337"
FT   MOD_RES         211
FT                   /note="N6-succinyllysine"
FT                   /evidence="ECO:0007744|PubMed:23806337"
FT   MOD_RES         212
FT                   /note="N6-succinyllysine"
FT                   /evidence="ECO:0007744|PubMed:23806337"
FT   MOD_RES         214
FT                   /note="N6-succinyllysine"
FT                   /evidence="ECO:0007744|PubMed:23806337"
FT   MOD_RES         234
FT                   /note="N6-acetyllysine; alternate"
FT                   /evidence="ECO:0007744|PubMed:23576753"
FT   MOD_RES         234
FT                   /note="N6-succinyllysine; alternate"
FT                   /evidence="ECO:0007744|PubMed:23806337"
FT   MOD_RES         240
FT                   /note="N6-succinyllysine"
FT                   /evidence="ECO:0007744|PubMed:23806337"
FT   MOD_RES         241
FT                   /note="N6-acetyllysine"
FT                   /evidence="ECO:0007744|PubMed:23576753"
FT   MOD_RES         269
FT                   /note="N6-acetyllysine"
FT                   /evidence="ECO:0007744|PubMed:23576753"
FT   MOD_RES         270
FT                   /note="N6-acetyllysine"
FT                   /evidence="ECO:0007744|PubMed:23576753"
FT   MOD_RES         305
FT                   /note="N6-acetyllysine; alternate"
FT                   /evidence="ECO:0007744|PubMed:23576753"
FT   MOD_RES         305
FT                   /note="N6-succinyllysine; alternate"
FT                   /evidence="ECO:0007744|PubMed:23806337"
FT   MOD_RES         310
FT                   /note="Phosphoserine"
FT                   /evidence="ECO:0007744|PubMed:21183079"
FT   MOD_RES         312
FT                   /note="N6-acetyllysine; alternate"
FT                   /evidence="ECO:0007744|PubMed:23576753"
FT   MOD_RES         312
FT                   /note="N6-succinyllysine; alternate"
FT                   /evidence="ECO:0007744|PubMed:23806337"
FT   MOD_RES         340
FT                   /note="N6-acetyllysine"
FT                   /evidence="ECO:0007744|PubMed:23576753"
FT   MOD_RES         344
FT                   /note="Phosphoserine"
FT                   /evidence="ECO:0007744|PubMed:17208939"
FT   MOD_RES         375
FT                   /note="N6-acetyllysine"
FT                   /evidence="ECO:0007744|PubMed:23576753"
FT   CONFLICT        397
FT                   /note="A -> V (in Ref. 3; AAH28901)"
FT                   /evidence="ECO:0000305"
SQ   SEQUENCE   397 AA;  41830 MW;  856840546F5CB8DA CRC64;
     MALLRGVFIV AAKRTPFGAY GGLLKDFSAT DLTEFAARAA LSAGKVPPET IDSVIVGNVM
     QSSSDAAYLA RHVGLRVGVP TETGALTLNR LCGSGFQSIV SGCQEICSKD AEVVLCGGTE
     SMSQSPYCVR NVRFGTKFGL DLKLEDTLWA GLTDQHVKLP MGMTAENLAA KYNISREDCD
     RYALQSQQRW KAANEAGYFN EEMAPIEVKT KKGKQTMQVD EHARPQTTLE QLQKLPSVFK
     KDGTVTAGNA SGVSDGAGAV IIASEDAVKK HNFTPLARVV GYFVSGCDPT IMGIGPVPAI
     NGALKKAGLS LKDMDLIDVN EAFAPQFLSV QKALDLDPSK TNVSGGAIAL GHPLGGSGSR
     ITAHLVHELR RRGGKYAVGS ACIGGGQGIA LIIQNTA
//
```

**Mascot:** http://www.matrixscience.com/


Score **> 27** indicates **identity**  
Score **> 18** indicates **homology**
