# Supplementary material for: A common East-Asian ALDH2 mutation causes metabolic disorders and the therapeutic effect of ALDH2 activators
Source: Nat Commun. 2023 Sep 25;14:5971. doi: 10.1038/s41467-023-41570-6 (PMC10520061; doi:10.1038/s41467-023-41570-6)
Supplement: Supplementary file 4 — Supplementary Data 1 [file 41467_2023_41570_MOESM4_ESM.zip › Table S5c/Q8BWT1/Q8BWT1_KIO-2_C103_H367_K109.html]

Mascot Search Results: Q8BWT1
 

# MASCOT Search Results

## Protein View: Q8BWT1

### 3-ketoacyl-CoA thiolase, mitochondrial OS=Mus musculus OX=10090 GN=Acaa2 PE=1 SV=3

|  |  |
| --- | --- |
| Database: | Mouse\_UniProt\_proteomes |
| Score: | 7944 |
| Monoisotopic mass (M_r_): | 42260 |
| Calculated pI: | 8.33 |

Sequence similarity is available as an NCBI BLAST search of Q8BWT1 against nr.

### Search parameters

|  |  |
| --- | --- |
| MS data file: | `D:\LCMSMS\2023 Users' data\230529-1\230529-1-KIO-2.raw` |
| Enzyme: | Trypsin/P: cuts C-term side of KR. |
| Fixed modifications: | Carbamidomethyl (C) |
| Variable modifications: | Deamidated (NQ), HNE (C), HNE (H), HNE (K), Oxidation (M) |

### Protein sequence coverage: 88%

Matched peptides shown in ***bold red***.

|  |  |  |  |  |  |
| --- | --- | --- | --- | --- | --- |
| `1` | `MALLRGVFIV` | `AAKRTPFGAY` | `GGLLKDFSAT` | `DLTEFAARAA` | `LSAGKVPPET` |
| `51` | `IDSVIVGNVM` | `QSSSDAAYLA` | `RHVGLRVGVP` | `TETGALTLNR` | `LCGSGFQSIV` |
| `101` | `SGCQEICSKD` | `AEVVLCGGTE` | `SMSQSPYCVR` | `NVRFGTKFGL` | `DLKLEDTLWA` |
| `151` | `GLTDQHVKLP` | `MGMTAENLAA` | `KYNISREDCD` | `RYALQSQQRW` | `KAANEAGYFN` |
| `201` | `EEMAPIEVKT` | `KKGKQTMQVD` | `EHARPQTTLE` | `QLQKLPSVFK` | `KDGTVTAGNA` |
| `251` | `SGVSDGAGAV` | `IIASEDAVKK` | `HNFTPLARVV` | `GYFVSGCDPT` | `IMGIGPVPAI` |
| `301` | `NGALKKAGLS` | `LKDMDLIDVN` | `EAFAPQFLSV` | `QKALDLDPSK` | `TNVSGGAIAL` |
| `351` | `GHPLGGSGSR` | `ITAHLVHELR` | `RRGGKYAVGS` | `ACIGGGQGIA` | `LIIQNTA` |

Unformatted sequence string: 397 residues (for pasting into other applications).

|  |  |  |  |
| --- | --- | --- | --- |
| Sort by | residue number | increasing mass | decreasing mass |
| Show | matched peptides only | predicted peptides also |  |

| Query | Start | – | End | Observed | Mr(expt) | Mr(calc) | ppm | M | Score | Expect | Rank | U | Peptide |
| --- | --- | --- | --- | --- | --- | --- | --- | --- | --- | --- | --- | --- | --- |
| 3890 | 6 | – | 13 | 402.7519 | 803.4893 | 803.4905 | -1.49 | 0 | 22 | 0.0088 | 1Score **> 22** indicates **identity** Score **> 14** indicates **homology** | U | R.GVFIVAAK.R |
| 3891 | 6 | – | 13 | 402.7523 | 803.4901 | 803.4905 | -0.56 | 0 | 30 | 0.0015 | 1Score **> 22** indicates **identity** Score **> 14** indicates **homology** | U | R.GVFIVAAK.R |
| 3893 | 6 | – | 13 | 402.7524 | 803.4903 | 803.4905 | -0.31 | 0 | 21 | 0.011 | 1Score **> 22** indicates **identity** Score **> 14** indicates **homology** | U | R.GVFIVAAK.R |
| 3894 | 6 | – | 13 | 402.7525 | 803.4905 | 803.4905 | -0.025 | 0 | 35 | 0.00083 | 1Score **> 22** indicates **identity** Score **> 17** indicates **homology** | U | R.GVFIVAAK.R |
| 12261 | 6 | – | 14 | 480.8035 | 959.5924 | 959.5916 | 0.84 | 1 | 24 | 0.005 | 1Score **> 26** indicates **identity** Score **> 14** indicates **homology** | U | R.GVFIVAAKR.T |
| 42759 | 14 | – | 25 | 427.2436 | 1278.7089 | 1278.7084 | 0.36 | 1 | 35 | 0.00064 | 1Score **> 32** indicates **identity** Score **> 16** indicates **homology** | U | K.RTPFGAYGGLLK.D |
| 42761 | 14 | – | 25 | 427.2437 | 1278.7093 | 1278.7084 | 0.69 | 1 | 47 | 5.3e-05 | 1Score **> 32** indicates **identity** Score **> 17** indicates **homology** | U | K.RTPFGAYGGLLK.D |
| 42762 | 14 | – | 25 | 640.3619 | 1278.7093 | 1278.7084 | 0.69 | 1 | 59 | 0.00011 | 1Score **> 32** indicates **identity** | U | K.RTPFGAYGGLLK.D |
| 42763 | 14 | – | 25 | 427.2437 | 1278.7094 | 1278.7084 | 0.70 | 1 | 40 | 0.00032 | 1Score **> 32** indicates **identity** Score **> 18** indicates **homology** | U | K.RTPFGAYGGLLK.D |
| 42764 | 14 | – | 25 | 640.3620 | 1278.7094 | 1278.7084 | 0.76 | 1 | 55 | 0.00032 | 1Score **> 32** indicates **identity** | U | K.RTPFGAYGGLLK.D |
| 42765 | 14 | – | 25 | 427.2438 | 1278.7097 | 1278.7084 | 0.95 | 1 | 37 | 0.00036 | 1Score **> 32** indicates **identity** Score **> 15** indicates **homology** | U | K.RTPFGAYGGLLK.D |
| 42766 | 14 | – | 25 | 640.3621 | 1278.7097 | 1278.7084 | 0.95 | 1 | 51 | 0.00044 | 1Score **> 32** indicates **identity** Score **> 30** indicates **homology** | U | K.RTPFGAYGGLLK.D |
| 42767 | 14 | – | 25 | 640.3621 | 1278.7097 | 1278.7084 | 1.00 | 1 | 55 | 0.0003 | 1Score **> 32** indicates **identity** | U | K.RTPFGAYGGLLK.D |
| 172925 | 14 | – | 38 | 902.1293 | 2703.3662 | 2703.3657 | 0.20 | 2 | 37 | 0.00034 | 1Score **> 37** indicates **identity** Score **> 15** indicates **homology** | U | K.RTPFGAYGGLLKDFSATDLTEFAAR.A |
| 172926 | 14 | – | 38 | 676.8488 | 2703.3662 | 2703.3657 | 0.20 | 2 | 57 | 5.1e-06 | 1Score **> 37** indicates **identity** Score **> 16** indicates **homology** | U | K.RTPFGAYGGLLKDFSATDLTEFAAR.A |
| 172927 | 14 | – | 38 | 676.8491 | 2703.3671 | 2703.3657 | 0.54 | 2 | 78 | 4.4e-08 | 1Score **> 37** indicates **identity** Score **> 17** indicates **homology** | U | K.RTPFGAYGGLLKDFSATDLTEFAAR.A |
| 172928 | 14 | – | 38 | 902.1296 | 2703.3671 | 2703.3657 | 0.54 | 2 | 66 | 6.8e-07 | 1Score **> 37** indicates **identity** Score **> 17** indicates **homology** | U | K.RTPFGAYGGLLKDFSATDLTEFAAR.A |
| 25365 | 15 | – | 25 | 562.3105 | 1122.6065 | 1122.6073 | -0.75 | 0 | 18 | 0.018 | 1Score **> 32** indicates **identity** Score **> 14** indicates **homology** | U | R.TPFGAYGGLLK.D |
| 25367 | 15 | – | 25 | 562.3117 | 1122.6088 | 1122.6073 | 1.33 | 0 | 65 | 1e-06 | 1Score **> 32** indicates **identity** Score **> 17** indicates **homology** | U | R.TPFGAYGGLLK.D |
| 25369 | 15 | – | 25 | 562.3120 | 1122.6095 | 1122.6073 | 1.96 | 0 | 60 | 7.5e-06 | 1Score **> 32** indicates **identity** Score **> 21** indicates **homology** | U | R.TPFGAYGGLLK.D |
| 25370 | 15 | – | 25 | 562.3132 | 1122.6119 | 1122.6073 | 4.02 | 0 | 66 | 1.1e-06 | 1Score **> 31** indicates **identity** Score **> 19** indicates **homology** | U | R.TPFGAYGGLLK.D |
| 25371 | 15 | – | 25 | 562.3143 | 1122.6140 | 1122.6073 | 5.91 | 0 | 35 | 0.00056 | 1Score **> 32** indicates **identity** Score **> 15** indicates **homology** | U | R.TPFGAYGGLLK.D |
| 42768 | 15 | – | 25 | 427.2441 | 1278.7104 | 1278.7224 | -9.40 | 0 | 51 | 1.8e-05 | 1Score **> 32** indicates **identity** Score **> 16** indicates **homology** | U | R.TPFGAYGGLLK.D  + HNE (K) |
| 42770 | 15 | – | 25 | 427.2442 | 1278.7109 | 1278.7224 | -9.01 | 0 | 47 | 4e-05 | 1Score **> 32** indicates **identity** Score **> 15** indicates **homology** | U | R.TPFGAYGGLLK.D  + HNE (K) |
| 166216 | 15 | – | 38 | 850.0934 | 2547.2583 | 2547.2646 | -2.47 | 1 | 37 | 0.00036 | 1Score **> 37** indicates **identity** Score **> 15** indicates **homology** | U | R.TPFGAYGGLLKDFSATDLTEFAAR.A |
| 60772 | 26 | – | 38 | 722.3364 | 1442.6583 | 1442.6678 | -6.57 | 0 | 46 | 5.1e-05 | 1Score **> 30** indicates **identity** Score **> 15** indicates **homology** | U | K.DFSATDLTEFAAR.A |
| 60773 | 26 | – | 38 | 722.3368 | 1442.6590 | 1442.6678 | -6.06 | 0 | 62 | 1.5e-06 | 1Score **> 30** indicates **identity** Score **> 16** indicates **homology** | U | K.DFSATDLTEFAAR.A |
| 60774 | 26 | – | 38 | 722.3390 | 1442.6634 | 1442.6678 | -3.02 | 0 | 61 | 1.8e-06 | 1Score **> 30** indicates **identity** Score **> 16** indicates **homology** | U | K.DFSATDLTEFAAR.A |
| 60775 | 26 | – | 38 | 722.3391 | 1442.6636 | 1442.6678 | -2.87 | 0 | 70 | 2.6e-07 | 1Score **> 30** indicates **identity** Score **> 17** indicates **homology** | U | K.DFSATDLTEFAAR.A |
| 60776 | 26 | – | 38 | 722.3392 | 1442.6639 | 1442.6678 | -2.69 | 0 | 54 | 8.8e-06 | 1Score **> 30** indicates **identity** Score **> 16** indicates **homology** | U | K.DFSATDLTEFAAR.A |
| 60777 | 26 | – | 38 | 722.3395 | 1442.6645 | 1442.6678 | -2.26 | 0 | 36 | 0.00043 | 1Score **> 30** indicates **identity** Score **> 15** indicates **homology** | U | K.DFSATDLTEFAAR.A |
| 60778 | 26 | – | 38 | 722.3396 | 1442.6646 | 1442.6678 | -2.20 | 0 | 44 | 7.5e-05 | 1Score **> 30** indicates **identity** Score **> 15** indicates **homology** | U | K.DFSATDLTEFAAR.A |
| 60779 | 26 | – | 38 | 722.3400 | 1442.6655 | 1442.6678 | -1.57 | 0 | 48 | 3.1e-05 | 1Score **> 31** indicates **identity** Score **> 16** indicates **homology** | U | K.DFSATDLTEFAAR.A |
| 60780 | 26 | – | 38 | 722.3401 | 1442.6656 | 1442.6678 | -1.50 | 0 | 49 | 2.8e-05 | 1Score **> 31** indicates **identity** Score **> 16** indicates **homology** | U | K.DFSATDLTEFAAR.A |
| 60781 | 26 | – | 38 | 722.3403 | 1442.6661 | 1442.6678 | -1.19 | 0 | 77 | 5.5e-08 | 1Score **> 30** indicates **identity** Score **> 17** indicates **homology** | U | K.DFSATDLTEFAAR.A |
| 60782 | 26 | – | 38 | 722.3407 | 1442.6668 | 1442.6678 | -0.66 | 0 | 86 | 7.9e-09 | 1Score **> 30** indicates **identity** Score **> 18** indicates **homology** | U | K.DFSATDLTEFAAR.A |
| 60783 | 26 | – | 38 | 722.3407 | 1442.6668 | 1442.6678 | -0.64 | 0 | 85 | 1.1e-08 | 1Score **> 30** indicates **identity** Score **> 18** indicates **homology** | U | K.DFSATDLTEFAAR.A |
| 60784 | 26 | – | 38 | 722.3410 | 1442.6674 | 1442.6678 | -0.25 | 0 | 87 | 7.7e-09 | 1Score **> 30** indicates **identity** Score **> 18** indicates **homology** | U | K.DFSATDLTEFAAR.A |
| 60785 | 26 | – | 38 | 722.3411 | 1442.6676 | 1442.6678 | -0.15 | 0 | 81 | 2.8e-08 | 1Score **> 30** indicates **identity** Score **> 18** indicates **homology** | U | K.DFSATDLTEFAAR.A |
| 60787 | 26 | – | 38 | 722.3412 | 1442.6679 | 1442.6678 | 0.080 | 0 | 90 | 3.3e-09 | 1Score **> 30** indicates **identity** Score **> 18** indicates **homology** | U | K.DFSATDLTEFAAR.A |
| 60789 | 26 | – | 38 | 722.3415 | 1442.6684 | 1442.6678 | 0.42 | 0 | 85 | 1.1e-08 | 1Score **> 31** indicates **identity** Score **> 18** indicates **homology** | U | K.DFSATDLTEFAAR.A |
| 60792 | 26 | – | 38 | 722.3423 | 1442.6701 | 1442.6678 | 1.62 | 0 | 51 | 1.6e-05 | 1Score **> 31** indicates **identity** Score **> 16** indicates **homology** | U | K.DFSATDLTEFAAR.A |
| 60797 | 26 | – | 38 | 722.3435 | 1442.6725 | 1442.6678 | 3.25 | 0 | 47 | 4e-05 | 1Score **> 31** indicates **identity** Score **> 15** indicates **homology** | U | K.DFSATDLTEFAAR.A |
| 190399 | 39 | – | 71 | 1106.5728 | 3316.6966 | 3316.6973 | -0.21 | 1 | 98 | 6.1e-10 | 1Score **> 37** indicates **identity** Score **> 19** indicates **homology** | U | R.AALSAGKVPPETIDSVIVGNVMQSSSDAAYLAR.H |
| 190400 | 39 | – | 71 | 830.1829 | 3316.7027 | 3316.6973 | 1.63 | 1 | 34 | 0.0006 | 1Score **> 37** indicates **identity** Score **> 15** indicates **homology** | U | R.AALSAGKVPPETIDSVIVGNVMQSSSDAAYLAR.H |
| 190417 | 39 | – | 71 | 1106.9090 | 3317.7052 | 3317.6813 | 7.21 | 1 | 28 | 0.0034 | 1Score **> 37** indicates **identity** Score **> 16** indicates **homology** | U | R.AALSAGKVPPETIDSVIVGNVMQSSSDAAYLAR.H  + Deamidated (NQ) |
| 190623 | 39 | – | 71 | 1112.2369 | 3333.6889 | 3333.6762 | 3.80 | 1 | 28 | 0.0022 | 1Score **> 38** indicates **identity** Score **> 14** indicates **homology** | U | R.AALSAGKVPPETIDSVIVGNVMQSSSDAAYLAR.H  + Deamidated (NQ); Oxidation (M) |
| 173443 | 46 | – | 71 | 907.1277 | 2718.3614 | 2718.3534 | 2.91 | 0 | 73 | 1.4e-07 | 1Score **> 37** indicates **identity** Score **> 17** indicates **homology** | U | K.VPPETIDSVIVGNVMQSSSDAAYLAR.H |
| 58838 | 77 | – | 90 | 476.5998 | 1426.7777 | 1426.7780 | -0.21 | 0 | 58 | 4e-06 | 1Score **> 34** indicates **identity** Score **> 16** indicates **homology** | U | R.VGVPTETGALTLNR.L |
| 58840 | 77 | – | 90 | 714.3963 | 1426.7780 | 1426.7780 | 0.017 | 0 | 80 | 4.8e-08 | 1Score **> 34** indicates **identity** Score **> 19** indicates **homology** | U | R.VGVPTETGALTLNR.L |
| 58841 | 77 | – | 90 | 714.3963 | 1426.7781 | 1426.7780 | 0.080 | 0 | 80 | 1.3e-07 | 1Score **> 34** indicates **identity** Score **> 23** indicates **homology** | U | R.VGVPTETGALTLNR.L |
| 58842 | 77 | – | 90 | 714.3965 | 1426.7784 | 1426.7780 | 0.26 | 0 | 78 | 6.9e-08 | 1Score **> 34** indicates **identity** Score **> 19** indicates **homology** | U | R.VGVPTETGALTLNR.L |
| 58843 | 77 | – | 90 | 476.6002 | 1426.7787 | 1426.7780 | 0.48 | 0 | 34 | 0.00062 | 1Score **> 33** indicates **identity** Score **> 15** indicates **homology** | U | R.VGVPTETGALTLNR.L |
| 58844 | 77 | – | 90 | 714.3968 | 1426.7790 | 1426.7780 | 0.68 | 0 | 64 | 1.1e-06 | 1Score **> 34** indicates **identity** Score **> 16** indicates **homology** | U | R.VGVPTETGALTLNR.L |
| 58849 | 77 | – | 90 | 714.3970 | 1426.7795 | 1426.7780 | 1.05 | 0 | 42 | 0.0001 | 1Score **> 34** indicates **identity** Score **> 15** indicates **homology** | U | R.VGVPTETGALTLNR.L |
| 58850 | 77 | – | 90 | 714.3973 | 1426.7800 | 1426.7780 | 1.37 | 0 | 57 | 4.5e-06 | 1Score **> 34** indicates **identity** Score **> 16** indicates **homology** | U | R.VGVPTETGALTLNR.L |
| 58852 | 77 | – | 90 | 714.3974 | 1426.7802 | 1426.7780 | 1.57 | 0 | 68 | 4.2e-07 | 1Score **> 34** indicates **identity** Score **> 17** indicates **homology** | U | R.VGVPTETGALTLNR.L |
| 136850 | 91 | – | 109 | 1058.9725 | 2115.9304 | 2115.9388 | -3.94 | 0 | 97 | 7.7e-10 | 1Score **> 29** indicates **identity** Score **> 19** indicates **homology** | U | R.LCGSGFQSIVSGCQEICSK.D |
| 136851 | 91 | – | 109 | 1058.9748 | 2115.9350 | 2115.9388 | -1.80 | 0 | 139 | 9.1e-14 | 1Score **> 30** indicates **identity** Score **> 21** indicates **homology** | U | R.LCGSGFQSIVSGCQEICSK.D |
| 136852 | 91 | – | 109 | 1058.9748 | 2115.9350 | 2115.9388 | -1.79 | 0 | 36 | 0.00041 | 1Score **> 30** indicates **identity** Score **> 15** indicates **homology** | U | R.LCGSGFQSIVSGCQEICSK.D |
| 136853 | 91 | – | 109 | 1058.9769 | 2115.9392 | 2115.9388 | 0.20 | 0 | 82 | 2e-08 | 1Score **> 30** indicates **identity** Score **> 18** indicates **homology** | U | R.LCGSGFQSIVSGCQEICSK.D |
| 136854 | 91 | – | 109 | 1058.9769 | 2115.9393 | 2115.9388 | 0.25 | 0 | 110 | 5.2e-11 | 1Score **> 30** indicates **identity** Score **> 19** indicates **homology** | U | R.LCGSGFQSIVSGCQEICSK.D |
| 136855 | 91 | – | 109 | 1058.9771 | 2115.9396 | 2115.9388 | 0.41 | 0 | 97 | 8.3e-10 | 1Score **> 30** indicates **identity** Score **> 19** indicates **homology** | U | R.LCGSGFQSIVSGCQEICSK.D |
| 136856 | 91 | – | 109 | 1058.9772 | 2115.9399 | 2115.9388 | 0.52 | 0 | 118 | 1.3e-11 | 1Score **> 30** indicates **identity** Score **> 22** indicates **homology** | U | R.LCGSGFQSIVSGCQEICSK.D |
| 136857 | 91 | – | 109 | 706.3207 | 2115.9403 | 2115.9388 | 0.74 | 0 | 75 | 9.1e-08 | 1Score **> 30** indicates **identity** Score **> 17** indicates **homology** | U | R.LCGSGFQSIVSGCQEICSK.D |
| 136858 | 91 | – | 109 | 1058.9775 | 2115.9405 | 2115.9388 | 0.82 | 0 | 126 | 1.5e-12 | 1Score **> 30** indicates **identity** Score **> 20** indicates **homology** | U | R.LCGSGFQSIVSGCQEICSK.D |
| 136859 | 91 | – | 109 | 1058.9778 | 2115.9410 | 2115.9388 | 1.03 | 0 | 94 | 1.5e-09 | 1Score **> 30** indicates **identity** Score **> 18** indicates **homology** | U | R.LCGSGFQSIVSGCQEICSK.D |
| 136860 | 91 | – | 109 | 1058.9778 | 2115.9410 | 2115.9388 | 1.04 | 0 | 97 | 8.4e-10 | 1Score **> 30** indicates **identity** Score **> 19** indicates **homology** | U | R.LCGSGFQSIVSGCQEICSK.D |
| 136861 | 91 | – | 109 | 706.3211 | 2115.9413 | 2115.9388 | 1.21 | 0 | 79 | 4.2e-08 | 1Score **> 30** indicates **identity** Score **> 17** indicates **homology** | U | R.LCGSGFQSIVSGCQEICSK.D |
| 136862 | 91 | – | 109 | 1058.9789 | 2115.9432 | 2115.9388 | 2.07 | 0 | 108 | 7e-11 | 1Score **> 30** indicates **identity** Score **> 19** indicates **homology** | U | R.LCGSGFQSIVSGCQEICSK.D |
| 136863 | 91 | – | 109 | 1058.9807 | 2115.9468 | 2115.9388 | 3.82 | 0 | 109 | 5.6e-11 | 1Score **> 31** indicates **identity** Score **> 19** indicates **homology** | U | R.LCGSGFQSIVSGCQEICSK.D |
| 136864 | 91 | – | 109 | 1058.9807 | 2115.9469 | 2115.9388 | 3.83 | 0 | 87 | 7.3e-09 | 1Score **> 31** indicates **identity** Score **> 18** indicates **homology** | U | R.LCGSGFQSIVSGCQEICSK.D |
| 136865 | 91 | – | 109 | 1058.9808 | 2115.9470 | 2115.9388 | 3.91 | 0 | 114 | 1.9e-11 | 1Score **> 31** indicates **identity** Score **> 20** indicates **homology** | U | R.LCGSGFQSIVSGCQEICSK.D |
| 199761 | 91 | – | 130 | 1140.7560 | 4558.9948 | 4558.9981 | -0.72 | 1 | 35 | 0.00053 | 1Score **> 29** indicates **identity** Score **> 15** indicates **homology** | U | R.LCGSGFQSIVSGCQEICSKDAEVVLCGGTESMSQSPYCVR.N  + 2 Deamidated (NQ); HNE (C); Oxidation (M) |
| 199762 | 91 | – | 130 | 1140.7568 | 4558.9982 | 4558.9981 | 0.032 | 1 | 23 | 0.007 | 1Score **> 29** indicates **identity** Score **> 14** indicates **homology** | U | R.LCGSGFQSIVSGCQEICSKDAEVVLCGGTESMSQSPYCVR.N  + 2 Deamidated (NQ); HNE (C); Oxidation (M) |
| 200065 | 91 | – | 130 | 1154.5153 | 4614.0319 | 4614.0515 | -4.25 | 1 | 27 | 0.0027 | 1Score **> 30** indicates **identity** Score **> 14** indicates **homology** | U | R.LCGSGFQSIVSGCQEICSKDAEVVLCGGTESMSQSPYCVR.N  + HNE (K); Oxidation (M) |
| 200067 | 91 | – | 130 | 1154.7600 | 4615.0109 | 4615.0356 | -5.33 | 1 | 48 | 3.2e-05 | 1Score **> 29** indicates **identity** Score **> 15** indicates **homology** | U | R.LCGSGFQSIVSGCQEICSKDAEVVLCGGTESMSQSPYCVR.N  + Deamidated (NQ); HNE (K); Oxidation (M) |
| 200068 | 91 | – | 130 | 1154.7660 | 4615.0349 | 4615.0356 | -0.15 | 1 | 33 | 0.00072 | 1Score **> 30** indicates **identity** Score **> 15** indicates **homology** | U | R.LCGSGFQSIVSGCQEICSKDAEVVLCGGTESMSQSPYCVR.N  + Deamidated (NQ); HNE (K); Oxidation (M) |
| 154486 | 110 | – | 130 | 1173.0191 | 2344.0237 | 2344.0134 | 4.40 | 0 | 100 | 4.6e-10 | 1Score **> 30** indicates **identity** Score **> 19** indicates **homology** | U | K.DAEVVLCGGTESMSQSPYCVR.N |
| 25576 | 134 | – | 143 | 375.8813 | 1124.6222 | 1124.6230 | -0.70 | 1 | 21 | 0.032 | 1Score **> 31** indicates **identity** Score **> 18** indicates **homology** | U | R.FGTKFGLDLK.L |
| 25577 | 134 | – | 143 | 375.8814 | 1124.6225 | 1124.6230 | -0.46 | 1 | 24 | 0.039 | 1Score **> 31** indicates **identity** Score **> 23** indicates **homology** | U | R.FGTKFGLDLK.L |
| 25578 | 134 | – | 143 | 375.8816 | 1124.6228 | 1124.6230 | -0.15 | 1 | 33 | 0.0037 | 1Score **> 31** indicates **identity** Score **> 21** indicates **homology** | U | R.FGTKFGLDLK.L |
| 25580 | 134 | – | 143 | 375.8818 | 1124.6236 | 1124.6230 | 0.57 | 1 | 33 | 0.0035 | 1Score **> 31** indicates **identity** Score **> 21** indicates **homology** | U | R.FGTKFGLDLK.L |
| 25581 | 134 | – | 143 | 375.8820 | 1124.6240 | 1124.6230 | 0.91 | 1 | 32 | 0.0034 | 1Score **> 31** indicates **identity** Score **> 20** indicates **homology** | U | R.FGTKFGLDLK.L |
| 25583 | 134 | – | 143 | 375.8820 | 1124.6243 | 1124.6230 | 1.17 | 1 | 36 | 0.0022 | 1Score **> 31** indicates **identity** Score **> 22** indicates **homology** | U | R.FGTKFGLDLK.L |
| 158081 | 138 | – | 158 | 600.5700 | 2398.2508 | 2398.2533 | -1.01 | 1 | 23 | 0.011 | 1Score **> 37** indicates **identity** Score **> 16** indicates **homology** | U | K.FGLDLKLEDTLWAGLTDQHVK.L |
| 158082 | 138 | – | 158 | 600.5703 | 2398.2520 | 2398.2533 | -0.51 | 1 | 22 | 0.0079 | 1Score **> 37** indicates **identity** Score **> 14** indicates **homology** | U | K.FGLDLKLEDTLWAGLTDQHVK.L |
| 158086 | 138 | – | 158 | 600.5710 | 2398.2549 | 2398.2533 | 0.66 | 1 | 21 | 0.012 | 1Score **> 37** indicates **identity** Score **> 14** indicates **homology** | U | K.FGLDLKLEDTLWAGLTDQHVK.L |
| 158089 | 138 | – | 158 | 600.5711 | 2398.2552 | 2398.2533 | 0.79 | 1 | 39 | 0.00022 | 1Score **> 37** indicates **identity** Score **> 15** indicates **homology** | U | K.FGLDLKLEDTLWAGLTDQHVK.L |
| 158092 | 138 | – | 158 | 800.4259 | 2398.2558 | 2398.2533 | 1.07 | 1 | 46 | 5.1e-05 | 1Score **> 37** indicates **identity** Score **> 15** indicates **homology** | U | K.FGLDLKLEDTLWAGLTDQHVK.L |
| 158094 | 138 | – | 158 | 600.5716 | 2398.2574 | 2398.2533 | 1.71 | 1 | 26 | 0.0036 | 1Score **> 37** indicates **identity** Score **> 14** indicates **homology** | U | K.FGLDLKLEDTLWAGLTDQHVK.L |
| 158097 | 138 | – | 158 | 800.4271 | 2398.2593 | 2398.2533 | 2.53 | 1 | 15 | 0.039 | 1Score **> 37** indicates **identity** Score **> 13** indicates **homology** | U | K.FGLDLKLEDTLWAGLTDQHVK.L |
| 158100 | 138 | – | 158 | 800.4288 | 2398.2647 | 2398.2533 | 4.78 | 1 | 39 | 0.00023 | 1Score **> 37** indicates **identity** Score **> 15** indicates **homology** | U | K.FGLDLKLEDTLWAGLTDQHVK.L |
| 98010 | 144 | – | 158 | 863.4431 | 1724.8716 | 1724.8734 | -0.99 | 0 | 65 | 8.1e-07 | 1Score **> 35** indicates **identity** Score **> 17** indicates **homology** | U | K.LEDTLWAGLTDQHVK.L |
| 98014 | 144 | – | 158 | 575.9652 | 1724.8739 | 1724.8734 | 0.32 | 0 | 50 | 2.2e-05 | 1Score **> 35** indicates **identity** Score **> 16** indicates **homology** | U | K.LEDTLWAGLTDQHVK.L |
| 98018 | 144 | – | 158 | 863.4450 | 1724.8754 | 1724.8734 | 1.19 | 0 | 61 | 1.8e-06 | 1Score **> 35** indicates **identity** Score **> 16** indicates **homology** | U | K.LEDTLWAGLTDQHVK.L |
| 98020 | 144 | – | 158 | 863.4457 | 1724.8768 | 1724.8734 | 1.99 | 0 | 49 | 2.6e-05 | 1Score **> 35** indicates **identity** Score **> 16** indicates **homology** | U | K.LEDTLWAGLTDQHVK.L |
| 98021 | 144 | – | 158 | 575.9662 | 1724.8769 | 1724.8734 | 2.04 | 0 | 29 | 0.0018 | 1Score **> 35** indicates **identity** Score **> 14** indicates **homology** | U | K.LEDTLWAGLTDQHVK.L |
| 98026 | 144 | – | 158 | 575.9668 | 1724.8786 | 1724.8734 | 3.06 | 0 | 41 | 0.00015 | 1Score **> 35** indicates **identity** Score **> 15** indicates **homology** | U | K.LEDTLWAGLTDQHVK.L |
| 53345 | 159 | – | 171 | 689.8389 | 1377.6633 | 1377.6632 | 0.079 | 0 | 30 | 0.0014 | 1Score **> 33** indicates **identity** Score **> 14** indicates **homology** | U | K.LPMGMTAENLAAK.Y  + 2 Oxidation (M) |
| 53346 | 159 | – | 171 | 689.8391 | 1377.6637 | 1377.6632 | 0.35 | 0 | 44 | 7.8e-05 | 1Score **> 33** indicates **identity** Score **> 15** indicates **homology** | U | K.LPMGMTAENLAAK.Y  + 2 Oxidation (M) |
| 53347 | 159 | – | 171 | 689.8405 | 1377.6664 | 1377.6632 | 2.36 | 0 | 26 | 0.004 | 1Score **> 33** indicates **identity** Score **> 14** indicates **homology** | U | K.LPMGMTAENLAAK.Y  + 2 Oxidation (M) |
| 48029 | 172 | – | 181 | 664.2885 | 1326.5625 | 1326.5622 | 0.23 | 1 | 20 | 0.022 | 1Score **> 25** indicates **identity** Score **> 15** indicates **homology** | U | K.YNISREDCDR.Y |
| 151739 | 172 | – | 189 | 768.0258 | 2301.0556 | 2301.0556 | -0.019 | 2 | 31 | 0.0013 | 1Score **> 33** indicates **identity** Score **> 14** indicates **homology** | U | K.YNISREDCDRYALQSQQR.W |
| 151741 | 172 | – | 189 | 768.0260 | 2301.0561 | 2301.0556 | 0.20 | 2 | 25 | 0.0041 | 1Score **> 33** indicates **identity** Score **> 14** indicates **homology** | U | K.YNISREDCDRYALQSQQR.W |
| 151744 | 172 | – | 189 | 768.0265 | 2301.0577 | 2301.0556 | 0.90 | 2 | 16 | 0.03 | 1Score **> 33** indicates **identity** Score **> 14** indicates **homology** | U | K.YNISREDCDRYALQSQQR.W |
| 90462 | 177 | – | 189 | 834.8727 | 1667.7309 | 1667.7322 | -0.74 | 1 | 39 | 0.00049 | 1Score **> 28** indicates **identity** Score **> 18** indicates **homology** | U | R.EDCDRYALQSQQR.W |
| 90463 | 177 | – | 189 | 834.8729 | 1667.7313 | 1667.7322 | -0.53 | 1 | 43 | 0.00033 | 1Score **> 28** indicates **identity** Score **> 21** indicates **homology** | U | R.EDCDRYALQSQQR.W |
| 90465 | 177 | – | 189 | 556.9178 | 1667.7316 | 1667.7322 | -0.32 | 1 | 41 | 0.00015 | 1Score **> 28** indicates **identity** Score **> 15** indicates **homology** | U | R.EDCDRYALQSQQR.W |
| 90466 | 177 | – | 189 | 556.9180 | 1667.7322 | 1667.7322 | 0.020 | 1 | 40 | 0.00019 | 1Score **> 28** indicates **identity** Score **> 15** indicates **homology** | U | R.EDCDRYALQSQQR.W |
| 90470 | 177 | – | 189 | 834.8743 | 1667.7340 | 1667.7322 | 1.08 | 1 | 28 | 0.0026 | 1Score **> 29** indicates **identity** Score **> 14** indicates **homology** | U | R.EDCDRYALQSQQR.W |
| 90471 | 177 | – | 189 | 834.8744 | 1667.7343 | 1667.7322 | 1.29 | 1 | 20 | 0.033 | 1Score **> 29** indicates **identity** Score **> 18** indicates **homology** | U | R.EDCDRYALQSQQR.W |
| 90472 | 177 | – | 189 | 556.9189 | 1667.7350 | 1667.7322 | 1.70 | 1 | 18 | 0.021 | 1Score **> 29** indicates **identity** Score **> 14** indicates **homology** | U | R.EDCDRYALQSQQR.W |
| 90473 | 177 | – | 189 | 834.8749 | 1667.7353 | 1667.7322 | 1.88 | 1 | 16 | 0.038 | 1Score **> 29** indicates **identity** Score **> 15** indicates **homology** | U | R.EDCDRYALQSQQR.W |
| 90474 | 177 | – | 189 | 556.9191 | 1667.7355 | 1667.7322 | 2.01 | 1 | 15 | 0.037 | 1Score **> 29** indicates **identity** Score **> 13** indicates **homology** | U | R.EDCDRYALQSQQR.W |
| 90475 | 177 | – | 189 | 834.8756 | 1667.7366 | 1667.7322 | 2.68 | 1 | 31 | 0.0021 | 1Score **> 29** indicates **identity** Score **> 17** indicates **homology** | U | R.EDCDRYALQSQQR.W |
| 90476 | 177 | – | 189 | 556.9206 | 1667.7401 | 1667.7322 | 4.74 | 1 | 18 | 0.022 | 1Score **> 29** indicates **identity** Score **> 14** indicates **homology** | U | R.EDCDRYALQSQQR.W |
| 14361 | 182 | – | 189 | 497.2579 | 992.5012 | 992.5039 | -2.69 | 0 | 30 | 0.003 | 1Score **> 32** indicates **identity** Score **> 17** indicates **homology** | U | R.YALQSQQR.W |
| 14362 | 182 | – | 189 | 497.2580 | 992.5014 | 992.5039 | -2.57 | 0 | 30 | 0.0017 | 1Score **> 31** indicates **identity** Score **> 15** indicates **homology** | U | R.YALQSQQR.W |
| 14363 | 182 | – | 189 | 497.2594 | 992.5043 | 992.5039 | 0.43 | 0 | 56 | 5.1e-06 | 1Score **> 31** indicates **identity** Score **> 16** indicates **homology** | U | R.YALQSQQR.W |
| 151476 | 190 | – | 209 | 766.7022 | 2297.0848 | 2297.0674 | 7.59 | 1 | 27 | 0.0029 | 1Score **> 35** indicates **identity** Score **> 14** indicates **homology** | U | R.WKAANEAGYFNEEMAPIEVK.T  + Deamidated (NQ) |
| 152395 | 190 | – | 209 | 771.7025 | 2312.0855 | 2312.0783 | 3.14 | 1 | 24 | 0.0054 | 1Score **> 35** indicates **identity** Score **> 14** indicates **homology** | U | R.WKAANEAGYFNEEMAPIEVK.T  + Oxidation (M) |
| 152396 | 190 | – | 209 | 771.7031 | 2312.0874 | 2312.0783 | 3.92 | 1 | 17 | 0.026 | 1Score **> 35** indicates **identity** Score **> 14** indicates **homology** | U | R.WKAANEAGYFNEEMAPIEVK.T  + Oxidation (M) |
| 124451 | 192 | – | 209 | 991.9627 | 1981.9108 | 1981.9091 | 0.88 | 0 | 58 | 3.5e-06 | 1Score **> 33** indicates **identity** Score **> 16** indicates **homology** | U | K.AANEAGYFNEEMAPIEVK.T |
| 124452 | 192 | – | 209 | 991.9633 | 1981.9121 | 1981.9091 | 1.50 | 0 | 60 | 2.6e-06 | 1Score **> 33** indicates **identity** Score **> 16** indicates **homology** | U | K.AANEAGYFNEEMAPIEVK.T |
| 124453 | 192 | – | 209 | 991.9637 | 1981.9128 | 1981.9091 | 1.88 | 0 | 81 | 2.8e-08 | 1Score **> 33** indicates **identity** Score **> 18** indicates **homology** | U | K.AANEAGYFNEEMAPIEVK.T |
| 124454 | 192 | – | 209 | 991.9640 | 1981.9135 | 1981.9091 | 2.22 | 0 | 61 | 2e-06 | 1Score **> 33** indicates **identity** Score **> 16** indicates **homology** | U | K.AANEAGYFNEEMAPIEVK.T |
| 124577 | 192 | – | 209 | 992.4542 | 1982.8939 | 1982.8931 | 0.39 | 0 | 42 | 0.00013 | 1Score **> 32** indicates **identity** Score **> 15** indicates **homology** | U | K.AANEAGYFNEEMAPIEVK.T  + Deamidated (NQ) |
| 126019 | 192 | – | 209 | 999.9585 | 1997.9024 | 1997.9040 | -0.80 | 0 | 58 | 3.4e-06 | 1Score **> 32** indicates **identity** Score **> 16** indicates **homology** | U | K.AANEAGYFNEEMAPIEVK.T  + Oxidation (M) |
| 126020 | 192 | – | 209 | 999.9594 | 1997.9042 | 1997.9040 | 0.097 | 0 | 43 | 8.4e-05 | 1Score **> 32** indicates **identity** Score **> 15** indicates **homology** | U | K.AANEAGYFNEEMAPIEVK.T  + Oxidation (M) |
| 126021 | 192 | – | 209 | 999.9610 | 1997.9075 | 1997.9040 | 1.77 | 0 | 76 | 7.4e-08 | 1Score **> 32** indicates **identity** Score **> 17** indicates **homology** | U | K.AANEAGYFNEEMAPIEVK.T  + Oxidation (M) |
| 156846 | 215 | – | 234 | 794.4009 | 2380.1809 | 2380.1805 | 0.19 | 1 | 15 | 0.042 | 1Score **> 36** indicates **identity** Score **> 13** indicates **homology** | U | K.QTMQVDEHARPQTTLEQLQK.L |
| 156847 | 215 | – | 234 | 794.4016 | 2380.1829 | 2380.1805 | 1.02 | 1 | 17 | 0.028 | 1Score **> 37** indicates **identity** Score **> 14** indicates **homology** | U | K.QTMQVDEHARPQTTLEQLQK.L |
| 156851 | 215 | – | 234 | 596.0544 | 2380.1886 | 2380.1805 | 3.41 | 1 | 15 | 0.038 | 1Score **> 37** indicates **identity** Score **> 13** indicates **homology** | U | K.QTMQVDEHARPQTTLEQLQK.L |
| 156852 | 215 | – | 234 | 794.4037 | 2380.1891 | 2380.1805 | 3.63 | 1 | 46 | 5.3e-05 | 1Score **> 37** indicates **identity** Score **> 15** indicates **homology** | U | K.QTMQVDEHARPQTTLEQLQK.L |
| 4552 | 235 | – | 241 | 409.7602 | 817.5059 | 817.5062 | -0.28 | 1 | 37 | 0.0015 | 1Score **> 22** indicates **identity** | U | K.LPSVFKK.D |
| 4553 | 235 | – | 241 | 409.7603 | 817.5060 | 817.5062 | -0.21 | 1 | 31 | 0.0065 | 1Score **> 22** indicates **identity** | U | K.LPSVFKK.D |
| 4554 | 235 | – | 241 | 409.7604 | 817.5062 | 817.5062 | 0.099 | 1 | 26 | 0.021 | 1Score **> 22** indicates **identity** | U | K.LPSVFKK.D |
| 4555 | 235 | – | 241 | 409.7607 | 817.5069 | 817.5062 | 0.91 | 1 | 28 | 0.013 | 1Score **> 22** indicates **identity** | U | K.LPSVFKK.D |
| 171015 | 241 | – | 269 | 887.4476 | 2659.3211 | 2659.3301 | -3.39 | 1 | 15 | 0.037 | 1Score **> 37** indicates **identity** Score **> 13** indicates **homology** | U | K.KDGTVTAGNASGVSDGAGAVIIASEDAVK.K |
| 165440 | 242 | – | 269 | 844.7508 | 2531.2305 | 2531.2351 | -1.82 | 0 | 16 | 0.029 | 1Score **> 36** indicates **identity** Score **> 14** indicates **homology** | U | K.DGTVTAGNASGVSDGAGAVIIASEDAVK.K |
| 165443 | 242 | – | 269 | 1266.6247 | 2531.2349 | 2531.2351 | -0.097 | 0 | 139 | 1.2e-13 | 1Score **> 36** indicates **identity** Score **> 23** indicates **homology** | U | K.DGTVTAGNASGVSDGAGAVIIASEDAVK.K |
| 165444 | 242 | – | 269 | 844.7523 | 2531.2352 | 2531.2351 | 0.037 | 0 | 70 | 3e-07 | 1Score **> 36** indicates **identity** Score **> 17** indicates **homology** | U | K.DGTVTAGNASGVSDGAGAVIIASEDAVK.K |
| 165445 | 242 | – | 269 | 1266.6251 | 2531.2356 | 2531.2351 | 0.17 | 0 | 112 | 3.5e-11 | 1Score **> 36** indicates **identity** Score **> 19** indicates **homology** | U | K.DGTVTAGNASGVSDGAGAVIIASEDAVK.K |
| 165446 | 242 | – | 269 | 1266.6251 | 2531.2356 | 2531.2351 | 0.19 | 0 | 126 | 1.6e-12 | 1Score **> 36** indicates **identity** Score **> 20** indicates **homology** | U | K.DGTVTAGNASGVSDGAGAVIIASEDAVK.K |
| 165447 | 242 | – | 269 | 1266.6251 | 2531.2356 | 2531.2351 | 0.20 | 0 | 112 | 3e-11 | 1Score **> 36** indicates **identity** Score **> 20** indicates **homology** | U | K.DGTVTAGNASGVSDGAGAVIIASEDAVK.K |
| 165448 | 242 | – | 269 | 844.7525 | 2531.2356 | 2531.2351 | 0.20 | 0 | 57 | 4.7e-06 | 1Score **> 36** indicates **identity** Score **> 16** indicates **homology** | U | K.DGTVTAGNASGVSDGAGAVIIASEDAVK.K |
| 165449 | 242 | – | 269 | 844.7525 | 2531.2357 | 2531.2351 | 0.24 | 0 | 44 | 6.7e-05 | 1Score **> 36** indicates **identity** Score **> 15** indicates **homology** | U | K.DGTVTAGNASGVSDGAGAVIIASEDAVK.K |
| 165450 | 242 | – | 269 | 844.7525 | 2531.2358 | 2531.2351 | 0.26 | 0 | 74 | 1.1e-07 | 1Score **> 36** indicates **identity** Score **> 17** indicates **homology** | U | K.DGTVTAGNASGVSDGAGAVIIASEDAVK.K |
| 165451 | 242 | – | 269 | 1266.6254 | 2531.2362 | 2531.2351 | 0.41 | 0 | 108 | 7.2e-11 | 1Score **> 36** indicates **identity** Score **> 19** indicates **homology** | U | K.DGTVTAGNASGVSDGAGAVIIASEDAVK.K |
| 165452 | 242 | – | 269 | 844.7527 | 2531.2363 | 2531.2351 | 0.48 | 0 | 17 | 0.023 | 1Score **> 36** indicates **identity** Score **> 14** indicates **homology** | U | K.DGTVTAGNASGVSDGAGAVIIASEDAVK.K |
| 165453 | 242 | – | 269 | 844.7527 | 2531.2364 | 2531.2351 | 0.49 | 0 | 36 | 0.00041 | 1Score **> 36** indicates **identity** Score **> 15** indicates **homology** | U | K.DGTVTAGNASGVSDGAGAVIIASEDAVK.K |
| 165456 | 242 | – | 269 | 1266.6271 | 2531.2397 | 2531.2351 | 1.80 | 0 | 176 | 4e-17 | 1Score **> 37** indicates **identity** Score **> 24** indicates **homology** | U | K.DGTVTAGNASGVSDGAGAVIIASEDAVK.K |
| 165457 | 242 | – | 269 | 844.7539 | 2531.2398 | 2531.2351 | 1.84 | 0 | 41 | 0.00016 | 1Score **> 37** indicates **identity** Score **> 15** indicates **homology** | U | K.DGTVTAGNASGVSDGAGAVIIASEDAVK.K |
| 165459 | 242 | – | 269 | 1266.6275 | 2531.2404 | 2531.2351 | 2.10 | 0 | 92 | 2.3e-09 | 1Score **> 37** indicates **identity** Score **> 18** indicates **homology** | U | K.DGTVTAGNASGVSDGAGAVIIASEDAVK.K |
| 165460 | 242 | – | 269 | 844.7547 | 2531.2423 | 2531.2351 | 2.84 | 0 | 58 | 3.7e-06 | 1Score **> 37** indicates **identity** Score **> 16** indicates **homology** | U | K.DGTVTAGNASGVSDGAGAVIIASEDAVK.K |
| 165464 | 242 | – | 269 | 1266.6290 | 2531.2434 | 2531.2351 | 3.28 | 0 | 154 | 3.8e-15 | 1Score **> 37** indicates **identity** Score **> 22** indicates **homology** | U | K.DGTVTAGNASGVSDGAGAVIIASEDAVK.K |
| 165494 | 242 | – | 269 | 1267.1242 | 2532.2339 | 2532.2191 | 5.83 | 0 | 69 | 3.5e-07 | 1Score **> 36** indicates **identity** Score **> 17** indicates **homology** | U | K.DGTVTAGNASGVSDGAGAVIIASEDAVK.K  + Deamidated (NQ) |
| 165498 | 242 | – | 269 | 1267.1265 | 2532.2385 | 2532.2191 | 7.65 | 0 | 76 | 1.2e-07 | 1Score **> 37** indicates **identity** Score **> 19** indicates **homology** | U | K.DGTVTAGNASGVSDGAGAVIIASEDAVK.K  + Deamidated (NQ) |
| 171019 | 242 | – | 270 | 887.4504 | 2659.3292 | 2659.3301 | -0.32 | 1 | 22 | 0.0091 | 1Score **> 37** indicates **identity** Score **> 14** indicates **homology** | U | K.DGTVTAGNASGVSDGAGAVIIASEDAVKK.H |
| 171021 | 242 | – | 270 | 1330.6736 | 2659.3326 | 2659.3301 | 0.97 | 1 | 131 | 5.4e-13 | 1Score **> 37** indicates **identity** Score **> 21** indicates **homology** | U | K.DGTVTAGNASGVSDGAGAVIIASEDAVKK.H |
| 171022 | 242 | – | 270 | 887.4517 | 2659.3334 | 2659.3301 | 1.24 | 1 | 106 | 1.2e-10 | 1Score **> 37** indicates **identity** Score **> 19** indicates **homology** | U | K.DGTVTAGNASGVSDGAGAVIIASEDAVKK.H |
| 171023 | 242 | – | 270 | 887.4525 | 2659.3357 | 2659.3301 | 2.13 | 1 | 101 | 3.2e-10 | 1Score **> 37** indicates **identity** Score **> 19** indicates **homology** | U | K.DGTVTAGNASGVSDGAGAVIIASEDAVKK.H |
| 171024 | 242 | – | 270 | 887.4526 | 2659.3360 | 2659.3301 | 2.22 | 1 | 32 | 0.0011 | 1Score **> 37** indicates **identity** Score **> 14** indicates **homology** | U | K.DGTVTAGNASGVSDGAGAVIIASEDAVKK.H |
| 171025 | 242 | – | 270 | 887.4530 | 2659.3372 | 2659.3301 | 2.68 | 1 | 51 | 1.7e-05 | 1Score **> 37** indicates **identity** Score **> 16** indicates **homology** | U | K.DGTVTAGNASGVSDGAGAVIIASEDAVKK.H |
| 171026 | 242 | – | 270 | 887.4530 | 2659.3372 | 2659.3301 | 2.68 | 1 | 97 | 7.9e-10 | 1Score **> 37** indicates **identity** Score **> 19** indicates **homology** | U | K.DGTVTAGNASGVSDGAGAVIIASEDAVKK.H |
| 171027 | 242 | – | 270 | 887.4532 | 2659.3379 | 2659.3301 | 2.94 | 1 | 36 | 0.00042 | 1Score **> 37** indicates **identity** Score **> 15** indicates **homology** | U | K.DGTVTAGNASGVSDGAGAVIIASEDAVKK.H |
| 171028 | 242 | – | 270 | 1330.6763 | 2659.3380 | 2659.3301 | 2.97 | 1 | 35 | 0.00051 | 1Score **> 37** indicates **identity** Score **> 15** indicates **homology** | U | K.DGTVTAGNASGVSDGAGAVIIASEDAVKK.H |
| 171030 | 242 | – | 270 | 887.4542 | 2659.3408 | 2659.3301 | 4.05 | 1 | 93 | 1.7e-09 | 1Score **> 37** indicates **identity** Score **> 18** indicates **homology** | U | K.DGTVTAGNASGVSDGAGAVIIASEDAVKK.H |
| 171032 | 242 | – | 270 | 887.4546 | 2659.3419 | 2659.3301 | 4.43 | 1 | 85 | 1.1e-08 | 1Score **> 37** indicates **identity** Score **> 18** indicates **homology** | U | K.DGTVTAGNASGVSDGAGAVIIASEDAVKK.H |
| 171053 | 242 | – | 270 | 887.7785 | 2660.3136 | 2660.3141 | -0.17 | 1 | 73 | 1.5e-07 | 1Score **> 37** indicates **identity** Score **> 17** indicates **homology** | U | K.DGTVTAGNASGVSDGAGAVIIASEDAVKK.H  + Deamidated (NQ) |
| 171056 | 242 | – | 270 | 887.7817 | 2660.3233 | 2660.3141 | 3.47 | 1 | 39 | 0.00023 | 1Score **> 37** indicates **identity** Score **> 15** indicates **homology** | U | K.DGTVTAGNASGVSDGAGAVIIASEDAVKK.H  + Deamidated (NQ) |
| 171060 | 242 | – | 270 | 1331.1769 | 2660.3392 | 2660.3141 | 9.43 | 1 | 53 | 1e-05 | 1Score **> 37** indicates **identity** Score **> 16** indicates **homology** | U | K.DGTVTAGNASGVSDGAGAVIIASEDAVKK.H  + Deamidated (NQ) |
| 21359 | 270 | – | 278 | 361.8733 | 1082.5982 | 1082.5985 | -0.31 | 1 | 30 | 0.0038 | 1Score **> 30** indicates **identity** Score **> 18** indicates **homology** | U | K.KHNFTPLAR.V |
| 21360 | 270 | – | 278 | 542.3065 | 1082.5985 | 1082.5985 | -0.0028 | 1 | 27 | 0.003 | 1Score **> 30** indicates **identity** Score **> 14** indicates **homology** | U | K.KHNFTPLAR.V |
| 21361 | 270 | – | 278 | 542.3066 | 1082.5986 | 1082.5985 | 0.066 | 1 | 29 | 0.002 | 1Score **> 30** indicates **identity** Score **> 15** indicates **homology** | U | K.KHNFTPLAR.V |
| 11926 | 271 | – | 278 | 478.2594 | 954.5042 | 954.5035 | 0.73 | 0 | 31 | 0.0043 | 1Score **> 30** indicates **identity** Score **> 20** indicates **homology** | U | K.HNFTPLAR.V |
| 11928 | 271 | – | 278 | 478.2596 | 954.5047 | 954.5035 | 1.25 | 0 | 34 | 0.0027 | 1Score **> 30** indicates **identity** Score **> 20** indicates **homology** | U | K.HNFTPLAR.V |
| 11930 | 271 | – | 278 | 478.2605 | 954.5065 | 954.5035 | 3.08 | 0 | 31 | 0.0042 | 1Score **> 29** indicates **identity** Score **> 19** indicates **homology** | U | K.HNFTPLAR.V |
| 11931 | 271 | – | 278 | 478.2605 | 954.5065 | 954.5035 | 3.08 | 0 | 25 | 0.011 | 1Score **> 29** indicates **identity** Score **> 18** indicates **homology** | U | K.HNFTPLAR.V |
| 11932 | 271 | – | 278 | 478.2608 | 954.5071 | 954.5035 | 3.73 | 0 | 18 | 0.044 | 1Score **> 29** indicates **identity** Score **> 16** indicates **homology** | U | K.HNFTPLAR.V |
| 179067 | 279 | – | 306 | 954.5034 | 2860.4884 | 2860.4867 | 0.59 | 1 | 65 | 8.2e-07 | 1Score **> 37** indicates **identity** Score **> 17** indicates **homology** | U | R.VVGYFVSGCDPTIMGIGPVPAINGALKK.A  + Deamidated (NQ) |
| 179616 | 279 | – | 306 | 959.8337 | 2876.4793 | 2876.4816 | -0.81 | 1 | 36 | 0.00042 | 1Score **> 37** indicates **identity** Score **> 15** indicates **homology** | U | R.VVGYFVSGCDPTIMGIGPVPAINGALKK.A  + Deamidated (NQ); Oxidation (M) |
| 178694 | 307 | – | 332 | 950.4954 | 2848.4645 | 2848.4681 | -1.25 | 1 | 67 | 4.8e-07 | 1Score **> 37** indicates **identity** Score **> 17** indicates **homology** | U | K.AGLSLKDMDLIDVNEAFAPQFLSVQK.A |
| 179224 | 307 | – | 332 | 955.8285 | 2864.4635 | 2864.4630 | 0.20 | 1 | 23 | 0.0068 | 1Score **> 38** indicates **identity** Score **> 14** indicates **homology** | U | K.AGLSLKDMDLIDVNEAFAPQFLSVQK.A  + Oxidation (M) |
| 150241 | 313 | – | 332 | 1140.5617 | 2279.1089 | 2279.1144 | -2.41 | 0 | 47 | 3.9e-05 | 1Score **> 36** indicates **identity** Score **> 15** indicates **homology** | U | K.DMDLIDVNEAFAPQFLSVQK.A |
| 6482 | 333 | – | 340 | 429.7316 | 857.4486 | 857.4494 | -0.94 | 0 | 39 | 0.0032 | 1Score **> 27** indicates **identity** Score **> 27** indicates **homology** | U | K.ALDLDPSK.T |
| 6484 | 333 | – | 340 | 429.7320 | 857.4495 | 857.4494 | 0.028 | 0 | 51 | 0.0002 | 1Score **> 27** indicates **identity** Score **> 27** indicates **homology** | U | K.ALDLDPSK.T |
| 6486 | 333 | – | 340 | 429.7321 | 857.4497 | 857.4494 | 0.30 | 0 | 33 | 0.0022 | 1Score **> 27** indicates **identity** Score **> 19** indicates **homology** | U | K.ALDLDPSK.T |
| 170551 | 333 | – | 360 | 883.1314 | 2646.3722 | 2646.3725 | -0.12 | 1 | 32 | 0.001 | 1Score **> 37** indicates **identity** Score **> 15** indicates **homology** | U | K.ALDLDPSKTNVSGGAIALGHPLGGSGSR.I |
| 170552 | 333 | – | 360 | 883.1319 | 2646.3737 | 2646.3725 | 0.45 | 1 | 40 | 0.00018 | 1Score **> 37** indicates **identity** Score **> 15** indicates **homology** | U | K.ALDLDPSKTNVSGGAIALGHPLGGSGSR.I |
| 170553 | 333 | – | 360 | 662.6008 | 2646.3742 | 2646.3725 | 0.61 | 1 | 20 | 0.015 | 1Score **> 37** indicates **identity** Score **> 14** indicates **homology** | U | K.ALDLDPSKTNVSGGAIALGHPLGGSGSR.I |
| 170554 | 333 | – | 360 | 662.6010 | 2646.3748 | 2646.3725 | 0.85 | 1 | 46 | 5.3e-05 | 1Score **> 37** indicates **identity** Score **> 15** indicates **homology** | U | K.ALDLDPSKTNVSGGAIALGHPLGGSGSR.I |
| 170555 | 333 | – | 360 | 883.1324 | 2646.3752 | 2646.3725 | 1.02 | 1 | 70 | 3e-07 | 1Score **> 37** indicates **identity** Score **> 17** indicates **homology** | U | K.ALDLDPSKTNVSGGAIALGHPLGGSGSR.I |
| 170556 | 333 | – | 360 | 662.6012 | 2646.3757 | 2646.3725 | 1.19 | 1 | 16 | 0.033 | 1Score **> 37** indicates **identity** Score **> 13** indicates **homology** | U | K.ALDLDPSKTNVSGGAIALGHPLGGSGSR.I |
| 170558 | 333 | – | 360 | 883.1327 | 2646.3763 | 2646.3725 | 1.41 | 1 | 78 | 4.6e-08 | 1Score **> 37** indicates **identity** Score **> 17** indicates **homology** | U | K.ALDLDPSKTNVSGGAIALGHPLGGSGSR.I |
| 170559 | 333 | – | 360 | 662.6014 | 2646.3765 | 2646.3725 | 1.49 | 1 | 42 | 0.00012 | 1Score **> 37** indicates **identity** Score **> 15** indicates **homology** | U | K.ALDLDPSKTNVSGGAIALGHPLGGSGSR.I |
| 170560 | 333 | – | 360 | 662.6014 | 2646.3766 | 2646.3725 | 1.52 | 1 | 40 | 0.00016 | 1Score **> 37** indicates **identity** Score **> 15** indicates **homology** | U | K.ALDLDPSKTNVSGGAIALGHPLGGSGSR.I |
| 170561 | 333 | – | 360 | 1324.1958 | 2646.3771 | 2646.3725 | 1.71 | 1 | 87 | 6.3e-09 | 1Score **> 37** indicates **identity** Score **> 18** indicates **homology** | U | K.ALDLDPSKTNVSGGAIALGHPLGGSGSR.I |
| 170562 | 333 | – | 360 | 883.1333 | 2646.3780 | 2646.3725 | 2.07 | 1 | 72 | 1.9e-07 | 1Score **> 37** indicates **identity** Score **> 17** indicates **homology** | U | K.ALDLDPSKTNVSGGAIALGHPLGGSGSR.I |
| 170563 | 333 | – | 360 | 662.6018 | 2646.3782 | 2646.3725 | 2.13 | 1 | 45 | 5.8e-05 | 1Score **> 37** indicates **identity** Score **> 15** indicates **homology** | U | K.ALDLDPSKTNVSGGAIALGHPLGGSGSR.I |
| 170564 | 333 | – | 360 | 1324.1975 | 2646.3804 | 2646.3725 | 2.96 | 1 | 52 | 1.4e-05 | 1Score **> 37** indicates **identity** Score **> 16** indicates **homology** | U | K.ALDLDPSKTNVSGGAIALGHPLGGSGSR.I |
| 170583 | 333 | – | 360 | 883.4646 | 2647.3721 | 2647.3566 | 5.86 | 1 | 23 | 0.0068 | 1Score **> 37** indicates **identity** Score **> 14** indicates **homology** | U | K.ALDLDPSKTNVSGGAIALGHPLGGSGSR.I  + Deamidated (NQ) |
| 170584 | 333 | – | 360 | 883.4647 | 2647.3723 | 2647.3566 | 5.96 | 1 | 19 | 0.017 | 1Score **> 37** indicates **identity** Score **> 14** indicates **homology** | U | K.ALDLDPSKTNVSGGAIALGHPLGGSGSR.I  + Deamidated (NQ) |
| 107255 | 341 | – | 360 | 904.4735 | 1806.9325 | 1806.9337 | -0.62 | 0 | 115 | 5.7e-11 | 1Score **> 36** indicates **identity** Score **> 25** indicates **homology** | U | K.TNVSGGAIALGHPLGGSGSR.I |
| 107256 | 341 | – | 360 | 904.4738 | 1806.9331 | 1806.9337 | -0.34 | 0 | 102 | 2.7e-10 | 1Score **> 35** indicates **identity** Score **> 19** indicates **homology** | U | K.TNVSGGAIALGHPLGGSGSR.I |
| 107257 | 341 | – | 360 | 904.4739 | 1806.9332 | 1806.9337 | -0.28 | 0 | 128 | 1.1e-11 | 1Score **> 35** indicates **identity** Score **> 31** indicates **homology** | U | K.TNVSGGAIALGHPLGGSGSR.I |
| 107261 | 341 | – | 360 | 603.3186 | 1806.9341 | 1806.9337 | 0.23 | 0 | 46 | 0.00087 | 1Score **> 36** indicates **identity** Score **> 27** indicates **homology** | U | K.TNVSGGAIALGHPLGGSGSR.I |
| 107262 | 341 | – | 360 | 904.4745 | 1806.9345 | 1806.9337 | 0.45 | 0 | 83 | 1.7e-08 | 1Score **> 36** indicates **identity** Score **> 18** indicates **homology** | U | K.TNVSGGAIALGHPLGGSGSR.I |
| 107263 | 341 | – | 360 | 603.3188 | 1806.9347 | 1806.9337 | 0.55 | 0 | 47 | 0.00047 | 1Score **> 36** indicates **identity** Score **> 26** indicates **homology** | U | K.TNVSGGAIALGHPLGGSGSR.I |
| 107264 | 341 | – | 360 | 603.3189 | 1806.9349 | 1806.9337 | 0.68 | 0 | 26 | 0.0037 | 1Score **> 36** indicates **identity** Score **> 14** indicates **homology** | U | K.TNVSGGAIALGHPLGGSGSR.I |
| 107266 | 341 | – | 360 | 603.3192 | 1806.9357 | 1806.9337 | 1.10 | 0 | 44 | 0.0027 | 1Score **> 36** indicates **identity** Score **> 31** indicates **homology** | U | K.TNVSGGAIALGHPLGGSGSR.I |
| 107267 | 341 | – | 360 | 603.3196 | 1806.9371 | 1806.9337 | 1.90 | 0 | 22 | 0.0078 | 1Score **> 36** indicates **identity** Score **> 14** indicates **homology** | U | K.TNVSGGAIALGHPLGGSGSR.I |
| 107268 | 341 | – | 360 | 603.3199 | 1806.9378 | 1806.9337 | 2.29 | 0 | 22 | 0.0092 | 1Score **> 36** indicates **identity** Score **> 14** indicates **homology** | U | K.TNVSGGAIALGHPLGGSGSR.I |
| 107270 | 341 | – | 360 | 603.3203 | 1806.9390 | 1806.9337 | 2.95 | 0 | 35 | 0.00082 | 1Score **> 36** indicates **identity** Score **> 17** indicates **homology** | U | K.TNVSGGAIALGHPLGGSGSR.I |
| 107277 | 341 | – | 360 | 603.3212 | 1806.9418 | 1806.9337 | 4.51 | 0 | 28 | 0.0026 | 1Score **> 35** indicates **identity** Score **> 14** indicates **homology** | U | K.TNVSGGAIALGHPLGGSGSR.I |
| 107278 | 341 | – | 360 | 603.3213 | 1806.9422 | 1806.9337 | 4.73 | 0 | 25 | 0.0043 | 1Score **> 35** indicates **identity** Score **> 14** indicates **homology** | U | K.TNVSGGAIALGHPLGGSGSR.I |
| 107280 | 341 | – | 360 | 603.3221 | 1806.9445 | 1806.9337 | 6.01 | 0 | 20 | 0.013 | 1Score **> 35** indicates **identity** Score **> 14** indicates **homology** | U | K.TNVSGGAIALGHPLGGSGSR.I |
| 32467 | 361 | – | 370 | 396.8993 | 1187.6760 | 1187.6775 | -1.21 | 0 | 21 | 0.04 | 1Score **> 34** indicates **identity** Score **> 20** indicates **homology** | U | R.ITAHLVHELR.R |
| 32470 | 361 | – | 370 | 396.8998 | 1187.6776 | 1187.6775 | 0.079 | 0 | 38 | 0.0052 | 1Score **> 33** indicates **identity** Score **> 28** indicates **homology** | U | R.ITAHLVHELR.R |
| 32471 | 361 | – | 370 | 396.8999 | 1187.6778 | 1187.6775 | 0.24 | 0 | 34 | 0.0056 | 1Score **> 33** indicates **identity** Score **> 24** indicates **homology** | U | R.ITAHLVHELR.R |
| 32472 | 361 | – | 370 | 594.8462 | 1187.6779 | 1187.6775 | 0.32 | 0 | 46 | 0.0027 | 1Score **> 33** indicates **identity** | U | R.ITAHLVHELR.R |
| 32473 | 361 | – | 370 | 594.8462 | 1187.6779 | 1187.6775 | 0.39 | 0 | 49 | 0.0016 | 1Score **> 33** indicates **identity** | U | R.ITAHLVHELR.R |
| 49952 | 361 | – | 370 | 448.9337 | 1343.7791 | 1343.7925 | -9.96 | 0 | 15 | 0.041 | 1Score **> 34** indicates **identity** Score **> 13** indicates **homology** | U | R.ITAHLVHELR.R  + HNE (H) |
| 49953 | 361 | – | 370 | 448.9338 | 1343.7797 | 1343.7925 | -9.57 | 0 | 17 | 0.027 | 1Score **> 33** indicates **identity** Score **> 14** indicates **homology** | U | R.ITAHLVHELR.R  + HNE (H) |
| 49948 | 361 | – | 371 | 448.9334 | 1343.7783 | 1343.7786 | -0.23 | 1 | 26 | 0.0036 | 1Score **> 34** indicates **identity** Score **> 14** indicates **homology** | U | R.ITAHLVHELRR.R |
| 49949 | 361 | – | 371 | 448.9335 | 1343.7787 | 1343.7786 | 0.11 | 1 | 33 | 0.00084 | 1Score **> 34** indicates **identity** Score **> 15** indicates **homology** | U | R.ITAHLVHELRR.R |
| 49951 | 361 | – | 371 | 672.8967 | 1343.7789 | 1343.7786 | 0.23 | 1 | 23 | 0.023 | 1Score **> 34** indicates **identity** Score **> 19** indicates **homology** | U | R.ITAHLVHELRR.R |
| 49954 | 361 | – | 371 | 448.9339 | 1343.7799 | 1343.7786 | 1.01 | 1 | 36 | 0.028 | 1Score **> 33** indicates **identity** | U | R.ITAHLVHELRR.R |

---

```
ID   THIM_MOUSE              Reviewed;         397 AA.
AC   Q8BWT1; Q3TIT9; Q8JZR8;
DT   30-AUG-2005, integrated into UniProtKB/Swiss-Prot.
DT   27-JUL-2011, sequence version 3.
DT   28-JUN-2023, entry version 149.
DE   RecName: Full=3-ketoacyl-CoA thiolase, mitochondrial {ECO:0000305};
DE            EC=2.3.1.16 {ECO:0000250|UniProtKB:P42765};
DE   AltName: Full=Acetyl-CoA acetyltransferase {ECO:0000305};
DE            EC=2.3.1.9 {ECO:0000255|PROSITE-ProRule:PRU10020};
DE   AltName: Full=Acetyl-CoA acyltransferase;
DE   AltName: Full=Acyl-CoA hydrolase, mitochondrial {ECO:0000305};
DE            EC=3.1.2.- {ECO:0000250|UniProtKB:P42765};
DE            EC=3.1.2.1 {ECO:0000250|UniProtKB:P42765};
DE            EC=3.1.2.2 {ECO:0000250|UniProtKB:P13437};
DE   AltName: Full=Beta-ketothiolase;
DE   AltName: Full=Mitochondrial 3-oxoacyl-CoA thiolase;
GN   Name=Acaa2;
OS   Mus musculus (Mouse).
OC   Eukaryota; Metazoa; Chordata; Craniata; Vertebrata; Euteleostomi; Mammalia;
OC   Eutheria; Euarchontoglires; Glires; Rodentia; Myomorpha; Muroidea; Muridae;
OC   Murinae; Mus; Mus.
OX   NCBI_TaxID=10090;
RN   [1]
RP   NUCLEOTIDE SEQUENCE [LARGE SCALE MRNA].
RC   STRAIN=C57BL/6J; TISSUE=Amnion, Heart, and Liver;
RX   PubMed=16141072; DOI=10.1126/science.1112014;
RA   Carninci P., Kasukawa T., Katayama S., Gough J., Frith M.C., Maeda N.,
RA   Oyama R., Ravasi T., Lenhard B., Wells C., Kodzius R., Shimokawa K.,
RA   Bajic V.B., Brenner S.E., Batalov S., Forrest A.R., Zavolan M., Davis M.J.,
RA   Wilming L.G., Aidinis V., Allen J.E., Ambesi-Impiombato A., Apweiler R.,
RA   Aturaliya R.N., Bailey T.L., Bansal M., Baxter L., Beisel K.W., Bersano T.,
RA   Bono H., Chalk A.M., Chiu K.P., Choudhary V., Christoffels A.,
RA   Clutterbuck D.R., Crowe M.L., Dalla E., Dalrymple B.P., de Bono B.,
RA   Della Gatta G., di Bernardo D., Down T., Engstrom P., Fagiolini M.,
RA   Faulkner G., Fletcher C.F., Fukushima T., Furuno M., Futaki S.,
RA   Gariboldi M., Georgii-Hemming P., Gingeras T.R., Gojobori T., Green R.E.,
RA   Gustincich S., Harbers M., Hayashi Y., Hensch T.K., Hirokawa N., Hill D.,
RA   Huminiecki L., Iacono M., Ikeo K., Iwama A., Ishikawa T., Jakt M.,
RA   Kanapin A., Katoh M., Kawasawa Y., Kelso J., Kitamura H., Kitano H.,
RA   Kollias G., Krishnan S.P., Kruger A., Kummerfeld S.K., Kurochkin I.V.,
RA   Lareau L.F., Lazarevic D., Lipovich L., Liu J., Liuni S., McWilliam S.,
RA   Madan Babu M., Madera M., Marchionni L., Matsuda H., Matsuzawa S., Miki H.,
RA   Mignone F., Miyake S., Morris K., Mottagui-Tabar S., Mulder N., Nakano N.,
RA   Nakauchi H., Ng P., Nilsson R., Nishiguchi S., Nishikawa S., Nori F.,
RA   Ohara O., Okazaki Y., Orlando V., Pang K.C., Pavan W.J., Pavesi G.,
RA   Pesole G., Petrovsky N., Piazza S., Reed J., Reid J.F., Ring B.Z.,
RA   Ringwald M., Rost B., Ruan Y., Salzberg S.L., Sandelin A., Schneider C.,
RA   Schoenbach C., Sekiguchi K., Semple C.A., Seno S., Sessa L., Sheng Y.,
RA   Shibata Y., Shimada H., Shimada K., Silva D., Sinclair B., Sperling S.,
RA   Stupka E., Sugiura K., Sultana R., Takenaka Y., Taki K., Tammoja K.,
RA   Tan S.L., Tang S., Taylor M.S., Tegner J., Teichmann S.A., Ueda H.R.,
RA   van Nimwegen E., Verardo R., Wei C.L., Yagi K., Yamanishi H.,
RA   Zabarovsky E., Zhu S., Zimmer A., Hide W., Bult C., Grimmond S.M.,
RA   Teasdale R.D., Liu E.T., Brusic V., Quackenbush J., Wahlestedt C.,
RA   Mattick J.S., Hume D.A., Kai C., Sasaki D., Tomaru Y., Fukuda S.,
RA   Kanamori-Katayama M., Suzuki M., Aoki J., Arakawa T., Iida J., Imamura K.,
RA   Itoh M., Kato T., Kawaji H., Kawagashira N., Kawashima T., Kojima M.,
RA   Kondo S., Konno H., Nakano K., Ninomiya N., Nishio T., Okada M., Plessy C.,
RA   Shibata K., Shiraki T., Suzuki S., Tagami M., Waki K., Watahiki A.,
RA   Okamura-Oho Y., Suzuki H., Kawai J., Hayashizaki Y.;
RT   "The transcriptional landscape of the mammalian genome.";
RL   Science 309:1559-1563(2005).
RN   [2]
RP   NUCLEOTIDE SEQUENCE [LARGE SCALE GENOMIC DNA].
RA   Mural R.J., Adams M.D., Myers E.W., Smith H.O., Venter J.C.;
RL   Submitted (SEP-2005) to the EMBL/GenBank/DDBJ databases.
RN   [3]
RP   NUCLEOTIDE SEQUENCE [LARGE SCALE MRNA].
RC   STRAIN=FVB/N; TISSUE=Liver;
RX   PubMed=15489334; DOI=10.1101/gr.2596504;
RG   The MGC Project Team;
RT   "The status, quality, and expansion of the NIH full-length cDNA project:
RT   the Mammalian Gene Collection (MGC).";
RL   Genome Res. 14:2121-2127(2004).
RN   [4]
RP   PHOSPHORYLATION [LARGE SCALE ANALYSIS] AT SER-344, AND IDENTIFICATION BY
RP   MASS SPECTROMETRY [LARGE SCALE ANALYSIS].
RC   TISSUE=Liver;
RX   PubMed=17208939; DOI=10.1074/mcp.m600218-mcp200;
RA   Lee J., Xu Y., Chen Y., Sprung R., Kim S.C., Xie S., Zhao Y.;
RT   "Mitochondrial phosphoproteome revealed by an improved IMAC method and
RT   MS/MS/MS.";
RL   Mol. Cell. Proteomics 6:669-676(2007).
RN   [5]
RP   PHOSPHORYLATION [LARGE SCALE ANALYSIS] AT SER-28, AND IDENTIFICATION BY
RP   MASS SPECTROMETRY [LARGE SCALE ANALYSIS].
RC   TISSUE=Liver;
RX   PubMed=17242355; DOI=10.1073/pnas.0609836104;
RA   Villen J., Beausoleil S.A., Gerber S.A., Gygi S.P.;
RT   "Large-scale phosphorylation analysis of mouse liver.";
RL   Proc. Natl. Acad. Sci. U.S.A. 104:1488-1493(2007).
RN   [6]
RP   PHOSPHORYLATION [LARGE SCALE ANALYSIS] AT SER-28; THR-136 AND SER-310, AND
RP   IDENTIFICATION BY MASS SPECTROMETRY [LARGE SCALE ANALYSIS].
RC   TISSUE=Brain, Brown adipose tissue, Heart, Kidney, Liver, Lung,
RC   Pancreas, Spleen, and Testis;
RX   PubMed=21183079; DOI=10.1016/j.cell.2010.12.001;
RA   Huttlin E.L., Jedrychowski M.P., Elias J.E., Goswami T., Rad R.,
RA   Beausoleil S.A., Villen J., Haas W., Sowa M.E., Gygi S.P.;
RT   "A tissue-specific atlas of mouse protein phosphorylation and expression.";
RL   Cell 143:1174-1189(2010).
RN   [7]
RP   ACETYLATION [LARGE SCALE ANALYSIS] AT LYS-191, SUCCINYLATION [LARGE SCALE
RP   ANALYSIS] AT LYS-25; LYS-45; LYS-137; LYS-143; LYS-158; LYS-171; LYS-191;
RP   LYS-209; LYS-211; LYS-212; LYS-214; LYS-234; LYS-240; LYS-305 AND LYS-312,
RP   AND IDENTIFICATION BY MASS SPECTROMETRY [LARGE SCALE ANALYSIS].
RC   TISSUE=Liver;
RX   PubMed=23806337; DOI=10.1016/j.molcel.2013.06.001;
RA   Park J., Chen Y., Tishkoff D.X., Peng C., Tan M., Dai L., Xie Z., Zhang Y.,
RA   Zwaans B.M., Skinner M.E., Lombard D.B., Zhao Y.;
RT   "SIRT5-mediated lysine desuccinylation impacts diverse metabolic
RT   pathways.";
RL   Mol. Cell 50:919-930(2013).
RN   [8]
RP   ACETYLATION [LARGE SCALE ANALYSIS] AT LYS-25; LYS-137; LYS-143; LYS-158;
RP   LYS-171; LYS-191; LYS-209; LYS-234; LYS-241; LYS-269; LYS-270; LYS-305;
RP   LYS-312; LYS-340 AND LYS-375, AND IDENTIFICATION BY MASS SPECTROMETRY
RP   [LARGE SCALE ANALYSIS].
RC   TISSUE=Liver;
RX   PubMed=23576753; DOI=10.1073/pnas.1302961110;
RA   Rardin M.J., Newman J.C., Held J.M., Cusack M.P., Sorensen D.J., Li B.,
RA   Schilling B., Mooney S.D., Kahn C.R., Verdin E., Gibson B.W.;
RT   "Label-free quantitative proteomics of the lysine acetylome in mitochondria
RT   identifies substrates of SIRT3 in metabolic pathways.";
RL   Proc. Natl. Acad. Sci. U.S.A. 110:6601-6606(2013).
CC   -!- FUNCTION: In the production of energy from fats, this is one of the
CC       enzymes that catalyzes the last step of the mitochondrial beta-
CC       oxidation pathway, an aerobic process breaking down fatty acids into
CC       acetyl-CoA. Using free coenzyme A/CoA, catalyzes the thiolytic cleavage
CC       of medium- to long-chain unbranched 3-oxoacyl-CoAs into acetyl-CoA and
CC       a fatty acyl-CoA shortened by two carbon atoms. Also catalyzes the
CC       condensation of two acetyl-CoA molecules into acetoacetyl-CoA and could
CC       be involved in the production of ketone bodies. Also displays hydrolase
CC       activity on various fatty acyl-CoAs (By similarity). Thereby, could be
CC       responsible for the production of acetate in a side reaction to beta-
CC       oxidation (By similarity). Abolishes BNIP3-mediated apoptosis and
CC       mitochondrial damage (By similarity). {ECO:0000250|UniProtKB:P13437,
CC       ECO:0000250|UniProtKB:P42765}.
CC   -!- CATALYTIC ACTIVITY:
CC       Reaction=acetyl-CoA + an acyl-CoA = a 3-oxoacyl-CoA + CoA;
CC         Xref=Rhea:RHEA:21564, ChEBI:CHEBI:57287, ChEBI:CHEBI:57288,
CC         ChEBI:CHEBI:58342, ChEBI:CHEBI:90726; EC=2.3.1.16;
CC         Evidence={ECO:0000250|UniProtKB:P42765};
CC       PhysiologicalDirection=left-to-right; Xref=Rhea:RHEA:21565;
CC         Evidence={ECO:0000250|UniProtKB:P42765};
CC       PhysiologicalDirection=right-to-left; Xref=Rhea:RHEA:21566;
CC         Evidence={ECO:0000250|UniProtKB:P42765};
CC   -!- CATALYTIC ACTIVITY:
CC       Reaction=2 acetyl-CoA = acetoacetyl-CoA + CoA; Xref=Rhea:RHEA:21036,
CC         ChEBI:CHEBI:57286, ChEBI:CHEBI:57287, ChEBI:CHEBI:57288; EC=2.3.1.9;
CC         Evidence={ECO:0000255|PROSITE-ProRule:PRU10020};
CC       PhysiologicalDirection=left-to-right; Xref=Rhea:RHEA:21037;
CC         Evidence={ECO:0000250|UniProtKB:P42765};
CC       PhysiologicalDirection=right-to-left; Xref=Rhea:RHEA:21038;
CC         Evidence={ECO:0000250|UniProtKB:P42765};
CC   -!- CATALYTIC ACTIVITY:
CC       Reaction=acetyl-CoA + H2O = acetate + CoA + H(+); Xref=Rhea:RHEA:20289,
CC         ChEBI:CHEBI:15377, ChEBI:CHEBI:15378, ChEBI:CHEBI:30089,
CC         ChEBI:CHEBI:57287, ChEBI:CHEBI:57288; EC=3.1.2.1;
CC         Evidence={ECO:0000250|UniProtKB:P42765};
CC       PhysiologicalDirection=left-to-right; Xref=Rhea:RHEA:20290;
CC         Evidence={ECO:0000250|UniProtKB:P42765};
CC   -!- CATALYTIC ACTIVITY:
CC       Reaction=H2O + propanoyl-CoA = CoA + H(+) + propanoate;
CC         Xref=Rhea:RHEA:40103, ChEBI:CHEBI:15377, ChEBI:CHEBI:15378,
CC         ChEBI:CHEBI:17272, ChEBI:CHEBI:57287, ChEBI:CHEBI:57392;
CC         Evidence={ECO:0000250|UniProtKB:P42765};
CC       PhysiologicalDirection=left-to-right; Xref=Rhea:RHEA:40104;
CC         Evidence={ECO:0000250|UniProtKB:P42765};
CC   -!- CATALYTIC ACTIVITY:
CC       Reaction=butanoyl-CoA + H2O = butanoate + CoA + H(+);
CC         Xref=Rhea:RHEA:40111, ChEBI:CHEBI:15377, ChEBI:CHEBI:15378,
CC         ChEBI:CHEBI:17968, ChEBI:CHEBI:57287, ChEBI:CHEBI:57371;
CC         Evidence={ECO:0000250|UniProtKB:P42765};
CC       PhysiologicalDirection=left-to-right; Xref=Rhea:RHEA:40112;
CC         Evidence={ECO:0000250|UniProtKB:P42765};
CC   -!- CATALYTIC ACTIVITY:
CC       Reaction=H2O + hexanoyl-CoA = CoA + H(+) + hexanoate;
CC         Xref=Rhea:RHEA:40115, ChEBI:CHEBI:15377, ChEBI:CHEBI:15378,
CC         ChEBI:CHEBI:17120, ChEBI:CHEBI:57287, ChEBI:CHEBI:62620;
CC         Evidence={ECO:0000250|UniProtKB:P42765};
CC       PhysiologicalDirection=left-to-right; Xref=Rhea:RHEA:40116;
CC         Evidence={ECO:0000250|UniProtKB:P42765};
CC   -!- CATALYTIC ACTIVITY:
CC       Reaction=H2O + octanoyl-CoA = CoA + H(+) + octanoate;
CC         Xref=Rhea:RHEA:30143, ChEBI:CHEBI:15377, ChEBI:CHEBI:15378,
CC         ChEBI:CHEBI:25646, ChEBI:CHEBI:57287, ChEBI:CHEBI:57386;
CC         Evidence={ECO:0000250|UniProtKB:P42765};
CC       PhysiologicalDirection=left-to-right; Xref=Rhea:RHEA:30144;
CC         Evidence={ECO:0000250|UniProtKB:P42765};
CC   -!- CATALYTIC ACTIVITY:
CC       Reaction=decanoyl-CoA + H2O = CoA + decanoate + H(+);
CC         Xref=Rhea:RHEA:40059, ChEBI:CHEBI:15377, ChEBI:CHEBI:15378,
CC         ChEBI:CHEBI:27689, ChEBI:CHEBI:57287, ChEBI:CHEBI:61430;
CC         Evidence={ECO:0000250|UniProtKB:P42765};
CC       PhysiologicalDirection=left-to-right; Xref=Rhea:RHEA:40060;
CC         Evidence={ECO:0000250|UniProtKB:P42765};
CC   -!- CATALYTIC ACTIVITY:
CC       Reaction=dodecanoyl-CoA + H2O = CoA + dodecanoate + H(+);
CC         Xref=Rhea:RHEA:30135, ChEBI:CHEBI:15377, ChEBI:CHEBI:15378,
CC         ChEBI:CHEBI:18262, ChEBI:CHEBI:57287, ChEBI:CHEBI:57375;
CC         Evidence={ECO:0000250|UniProtKB:P42765};
CC       PhysiologicalDirection=left-to-right; Xref=Rhea:RHEA:30136;
CC         Evidence={ECO:0000250|UniProtKB:P42765};
CC   -!- CATALYTIC ACTIVITY:
CC       Reaction=H2O + tetradecanoyl-CoA = CoA + H(+) + tetradecanoate;
CC         Xref=Rhea:RHEA:40119, ChEBI:CHEBI:15377, ChEBI:CHEBI:15378,
CC         ChEBI:CHEBI:30807, ChEBI:CHEBI:57287, ChEBI:CHEBI:57385;
CC         Evidence={ECO:0000250|UniProtKB:P42765};
CC       PhysiologicalDirection=left-to-right; Xref=Rhea:RHEA:40120;
CC         Evidence={ECO:0000250|UniProtKB:P42765};
CC   -!- CATALYTIC ACTIVITY:
CC       Reaction=H2O + hexadecanoyl-CoA = CoA + H(+) + hexadecanoate;
CC         Xref=Rhea:RHEA:16645, ChEBI:CHEBI:7896, ChEBI:CHEBI:15377,
CC         ChEBI:CHEBI:15378, ChEBI:CHEBI:57287, ChEBI:CHEBI:57379; EC=3.1.2.2;
CC         Evidence={ECO:0000250|UniProtKB:P13437};
CC       PhysiologicalDirection=left-to-right; Xref=Rhea:RHEA:16646;
CC         Evidence={ECO:0000250|UniProtKB:P42765};
CC   -!- PATHWAY: Lipid metabolism; fatty acid beta-oxidation.
CC       {ECO:0000250|UniProtKB:P42765}.
CC   -!- SUBUNIT: Homotetramer. Interacts with BNIP3.
CC       {ECO:0000250|UniProtKB:P42765}.
CC   -!- SUBCELLULAR LOCATION: Mitochondrion {ECO:0000250|UniProtKB:P42765}.
CC   -!- SIMILARITY: Belongs to the thiolase-like superfamily. Thiolase family.
CC       {ECO:0000305}.
CC   ---------------------------------------------------------------------------
CC   Copyrighted by the UniProt Consortium, see https://www.uniprot.org/terms
CC   Distributed under the Creative Commons Attribution (CC BY 4.0) License
CC   ---------------------------------------------------------------------------
DR   EMBL; AK050101; BAC34067.1; -; mRNA.
DR   EMBL; AK167567; BAE39630.1; -; mRNA.
DR   EMBL; AK167715; BAE39757.1; -; mRNA.
DR   EMBL; AK169359; BAE41108.1; -; mRNA.
DR   EMBL; CH466528; EDL09521.1; -; Genomic_DNA.
DR   EMBL; BC028901; AAH28901.1; -; mRNA.
DR   CCDS; CCDS29342.1; -.
DR   RefSeq; NP_803421.1; NM_177470.3.
DR   AlphaFoldDB; Q8BWT1; -.
DR   SMR; Q8BWT1; -.
DR   BioGRID; 206649; 30.
DR   IntAct; Q8BWT1; 9.
DR   MINT; Q8BWT1; -.
DR   STRING; 10090.ENSMUSP00000037348; -.
DR   GlyGen; Q8BWT1; 1 site, 1 O-linked glycan (1 site).
DR   iPTMnet; Q8BWT1; -.
DR   PhosphoSitePlus; Q8BWT1; -.
DR   SwissPalm; Q8BWT1; -.
DR   REPRODUCTION-2DPAGE; Q8BWT1; -.
DR   CPTAC; non-CPTAC-3618; -.
DR   CPTAC; non-CPTAC-3883; -.
DR   EPD; Q8BWT1; -.
DR   jPOST; Q8BWT1; -.
DR   MaxQB; Q8BWT1; -.
DR   PaxDb; Q8BWT1; -.
DR   PeptideAtlas; Q8BWT1; -.
DR   ProteomicsDB; 258872; -.
DR   Antibodypedia; 22617; 409 antibodies from 30 providers.
DR   Ensembl; ENSMUST00000041053; ENSMUSP00000037348; ENSMUSG00000036880.
DR   GeneID; 52538; -.
DR   KEGG; mmu:52538; -.
DR   UCSC; uc008fpt.1; mouse.
DR   AGR; MGI:1098623; -.
DR   CTD; 10449; -.
DR   MGI; MGI:1098623; Acaa2.
DR   VEuPathDB; HostDB:ENSMUSG00000036880; -.
DR   eggNOG; KOG1391; Eukaryota.
DR   GeneTree; ENSGT01030000234626; -.
DR   HOGENOM; CLU_031026_0_0_1; -.
DR   InParanoid; Q8BWT1; -.
DR   OMA; DYYWGMG; -.
DR   OrthoDB; 5481312at2759; -.
DR   PhylomeDB; Q8BWT1; -.
DR   TreeFam; TF105696; -.
DR   BRENDA; 2.3.1.16; 3474.
DR   Reactome; R-MMU-77289; Mitochondrial Fatty Acid Beta-Oxidation.
DR   UniPathway; UPA00659; -.
DR   BioGRID-ORCS; 52538; 1 hit in 80 CRISPR screens.
DR   ChiTaRS; Acaa2; mouse.
DR   PRO; PR:Q8BWT1; -.
DR   Proteomes; UP000000589; Chromosome 18.
DR   RNAct; Q8BWT1; protein.
DR   Bgee; ENSMUSG00000036880; Expressed in gall bladder and 285 other tissues.
DR   ExpressionAtlas; Q8BWT1; baseline and differential.
DR   Genevisible; Q8BWT1; MM.
DR   GO; GO:0005743; C:mitochondrial inner membrane; HDA:MGI.
DR   GO; GO:0005759; C:mitochondrial matrix; ISO:MGI.
DR   GO; GO:0005739; C:mitochondrion; HDA:MGI.
DR   GO; GO:0003985; F:acetyl-CoA C-acetyltransferase activity; ISS:UniProtKB.
DR   GO; GO:0003988; F:acetyl-CoA C-acyltransferase activity; ISS:UniProtKB.
DR   GO; GO:0003986; F:acetyl-CoA hydrolase activity; IEA:UniProtKB-EC.
DR   GO; GO:0047617; F:acyl-CoA hydrolase activity; ISS:UniProtKB.
DR   GO; GO:0102991; F:myristoyl-CoA hydrolase activity; IEA:UniProtKB-EC.
DR   GO; GO:0016290; F:palmitoyl-CoA hydrolase activity; IEA:UniProtKB-EC.
DR   GO; GO:0006084; P:acetyl-CoA metabolic process; ISO:MGI.
DR   GO; GO:0071456; P:cellular response to hypoxia; ISO:MGI.
DR   GO; GO:0006635; P:fatty acid beta-oxidation; ISO:MGI.
DR   GO; GO:1902109; P:negative regulation of mitochondrial membrane permeability involved in apoptotic process; ISO:MGI.
DR   GO; GO:1901029; P:negative regulation of mitochondrial outer membrane permeabilization involved in apoptotic signaling pathway; ISS:UniProtKB.
DR   CDD; cd00751; thiolase; 1.
DR   Gene3D; 3.40.47.10; -; 2.
DR   InterPro; IPR002155; Thiolase.
DR   InterPro; IPR016039; Thiolase-like.
DR   InterPro; IPR020615; Thiolase_acyl_enz_int_AS.
DR   InterPro; IPR020610; Thiolase_AS.
DR   InterPro; IPR020617; Thiolase_C.
DR   InterPro; IPR020613; Thiolase_CS.
DR   InterPro; IPR020616; Thiolase_N.
DR   PANTHER; PTHR18919:SF107; 3-KETOACYL-COA THIOLASE, MITOCHONDRIAL; 1.
DR   PANTHER; PTHR18919; ACETYL-COA C-ACYLTRANSFERASE; 1.
DR   Pfam; PF02803; Thiolase_C; 1.
DR   Pfam; PF00108; Thiolase_N; 1.
DR   PIRSF; PIRSF000429; Ac-CoA_Ac_transf; 1.
DR   SUPFAM; SSF53901; Thiolase-like; 2.
DR   PROSITE; PS00098; THIOLASE_1; 1.
DR   PROSITE; PS00737; THIOLASE_2; 1.
DR   PROSITE; PS00099; THIOLASE_3; 1.
DR   TIGRFAMs; TIGR01930; AcCoA-C-Actrans; 1.
PE   1: Evidence at protein level;
KW   Acetylation; Acyltransferase; Fatty acid metabolism; Hydrolase;
KW   Lipid metabolism; Mitochondrion; Phosphoprotein; Reference proteome;
KW   Transferase; Transit peptide.
FT   CHAIN           1..397
FT                   /note="3-ketoacyl-CoA thiolase, mitochondrial"
FT                   /id="PRO_0000223300"
FT   TRANSIT         1..16
FT                   /note="Mitochondrion; not cleaved"
FT                   /evidence="ECO:0000250"
FT   ACT_SITE        92
FT                   /note="Acyl-thioester intermediate"
FT                   /evidence="ECO:0000250|UniProtKB:P42765"
FT   ACT_SITE        382
FT                   /note="Proton donor/acceptor"
FT                   /evidence="ECO:0000250|UniProtKB:P42765"
FT   BINDING         224
FT                   /ligand="CoA"
FT                   /ligand_id="ChEBI:CHEBI:57287"
FT                   /evidence="ECO:0000250|UniProtKB:P42765"
FT   BINDING         227
FT                   /ligand="CoA"
FT                   /ligand_id="ChEBI:CHEBI:57287"
FT                   /evidence="ECO:0000250|UniProtKB:P42765"
FT   BINDING         251
FT                   /ligand="CoA"
FT                   /ligand_id="ChEBI:CHEBI:57287"
FT                   /evidence="ECO:0000250|UniProtKB:P42765"
FT   SITE            352
FT                   /note="Increases nucleophilicity of active site Cys"
FT                   /evidence="ECO:0000250|UniProtKB:P42765"
FT   MOD_RES         25
FT                   /note="N6-acetyllysine; alternate"
FT                   /evidence="ECO:0007744|PubMed:23576753"
FT   MOD_RES         25
FT                   /note="N6-succinyllysine; alternate"
FT                   /evidence="ECO:0007744|PubMed:23806337"
FT   MOD_RES         28
FT                   /note="Phosphoserine"
FT                   /evidence="ECO:0007744|PubMed:17242355,
FT                   ECO:0007744|PubMed:21183079"
FT   MOD_RES         45
FT                   /note="N6-succinyllysine"
FT                   /evidence="ECO:0007744|PubMed:23806337"
FT   MOD_RES         119
FT                   /note="Phosphothreonine"
FT                   /evidence="ECO:0000250|UniProtKB:P42765"
FT   MOD_RES         121
FT                   /note="Phosphoserine"
FT                   /evidence="ECO:0000250|UniProtKB:P42765"
FT   MOD_RES         127
FT                   /note="Phosphotyrosine"
FT                   /evidence="ECO:0000250|UniProtKB:P42765"
FT   MOD_RES         136
FT                   /note="Phosphothreonine"
FT                   /evidence="ECO:0007744|PubMed:21183079"
FT   MOD_RES         137
FT                   /note="N6-acetyllysine; alternate"
FT                   /evidence="ECO:0007744|PubMed:23576753"
FT   MOD_RES         137
FT                   /note="N6-succinyllysine; alternate"
FT                   /evidence="ECO:0007744|PubMed:23806337"
FT   MOD_RES         143
FT                   /note="N6-acetyllysine; alternate"
FT                   /evidence="ECO:0007744|PubMed:23576753"
FT   MOD_RES         143
FT                   /note="N6-succinyllysine; alternate"
FT                   /evidence="ECO:0007744|PubMed:23806337"
FT   MOD_RES         158
FT                   /note="N6-acetyllysine; alternate"
FT                   /evidence="ECO:0007744|PubMed:23576753"
FT   MOD_RES         158
FT                   /note="N6-succinyllysine; alternate"
FT                   /evidence="ECO:0007744|PubMed:23806337"
FT   MOD_RES         171
FT                   /note="N6-acetyllysine; alternate"
FT                   /evidence="ECO:0007744|PubMed:23576753"
FT   MOD_RES         171
FT                   /note="N6-succinyllysine; alternate"
FT                   /evidence="ECO:0007744|PubMed:23806337"
FT   MOD_RES         191
FT                   /note="N6-acetyllysine; alternate"
FT                   /evidence="ECO:0007744|PubMed:23576753,
FT                   ECO:0007744|PubMed:23806337"
FT   MOD_RES         191
FT                   /note="N6-succinyllysine; alternate"
FT                   /evidence="ECO:0007744|PubMed:23806337"
FT   MOD_RES         209
FT                   /note="N6-acetyllysine; alternate"
FT                   /evidence="ECO:0007744|PubMed:23576753"
FT   MOD_RES         209
FT                   /note="N6-succinyllysine; alternate"
FT                   /evidence="ECO:0007744|PubMed:23806337"
FT   MOD_RES         211
FT                   /note="N6-succinyllysine"
FT                   /evidence="ECO:0007744|PubMed:23806337"
FT   MOD_RES         212
FT                   /note="N6-succinyllysine"
FT                   /evidence="ECO:0007744|PubMed:23806337"
FT   MOD_RES         214
FT                   /note="N6-succinyllysine"
FT                   /evidence="ECO:0007744|PubMed:23806337"
FT   MOD_RES         234
FT                   /note="N6-acetyllysine; alternate"
FT                   /evidence="ECO:0007744|PubMed:23576753"
FT   MOD_RES         234
FT                   /note="N6-succinyllysine; alternate"
FT                   /evidence="ECO:0007744|PubMed:23806337"
FT   MOD_RES         240
FT                   /note="N6-succinyllysine"
FT                   /evidence="ECO:0007744|PubMed:23806337"
FT   MOD_RES         241
FT                   /note="N6-acetyllysine"
FT                   /evidence="ECO:0007744|PubMed:23576753"
FT   MOD_RES         269
FT                   /note="N6-acetyllysine"
FT                   /evidence="ECO:0007744|PubMed:23576753"
FT   MOD_RES         270
FT                   /note="N6-acetyllysine"
FT                   /evidence="ECO:0007744|PubMed:23576753"
FT   MOD_RES         305
FT                   /note="N6-acetyllysine; alternate"
FT                   /evidence="ECO:0007744|PubMed:23576753"
FT   MOD_RES         305
FT                   /note="N6-succinyllysine; alternate"
FT                   /evidence="ECO:0007744|PubMed:23806337"
FT   MOD_RES         310
FT                   /note="Phosphoserine"
FT                   /evidence="ECO:0007744|PubMed:21183079"
FT   MOD_RES         312
FT                   /note="N6-acetyllysine; alternate"
FT                   /evidence="ECO:0007744|PubMed:23576753"
FT   MOD_RES         312
FT                   /note="N6-succinyllysine; alternate"
FT                   /evidence="ECO:0007744|PubMed:23806337"
FT   MOD_RES         340
FT                   /note="N6-acetyllysine"
FT                   /evidence="ECO:0007744|PubMed:23576753"
FT   MOD_RES         344
FT                   /note="Phosphoserine"
FT                   /evidence="ECO:0007744|PubMed:17208939"
FT   MOD_RES         375
FT                   /note="N6-acetyllysine"
FT                   /evidence="ECO:0007744|PubMed:23576753"
FT   CONFLICT        397
FT                   /note="A -> V (in Ref. 3; AAH28901)"
FT                   /evidence="ECO:0000305"
SQ   SEQUENCE   397 AA;  41830 MW;  856840546F5CB8DA CRC64;
     MALLRGVFIV AAKRTPFGAY GGLLKDFSAT DLTEFAARAA LSAGKVPPET IDSVIVGNVM
     QSSSDAAYLA RHVGLRVGVP TETGALTLNR LCGSGFQSIV SGCQEICSKD AEVVLCGGTE
     SMSQSPYCVR NVRFGTKFGL DLKLEDTLWA GLTDQHVKLP MGMTAENLAA KYNISREDCD
     RYALQSQQRW KAANEAGYFN EEMAPIEVKT KKGKQTMQVD EHARPQTTLE QLQKLPSVFK
     KDGTVTAGNA SGVSDGAGAV IIASEDAVKK HNFTPLARVV GYFVSGCDPT IMGIGPVPAI
     NGALKKAGLS LKDMDLIDVN EAFAPQFLSV QKALDLDPSK TNVSGGAIAL GHPLGGSGSR
     ITAHLVHELR RRGGKYAVGS ACIGGGQGIA LIIQNTA
//
```

**Mascot:** http://www.matrixscience.com/


HNE (K) (+156.1150)
