# Supplementary material for: A common East-Asian ALDH2 mutation causes metabolic disorders and the therapeutic effect of ALDH2 activators
Source: Nat Commun. 2023 Sep 25;14:5971. doi: 10.1038/s41467-023-41570-6 (PMC10520061; doi:10.1038/s41467-023-41570-6)
Supplement: Supplementary file 4 — Supplementary Data 1 [file 41467_2023_41570_MOESM4_ESM.zip › Table S5c/Q8K2B3/Q8K2B3_KIO-1_C348_C574.html]

Mascot Search Results: Q8K2B3
 

# MASCOT Search Results

## Protein View: Q8K2B3

### Succinate dehydrogenase [ubiquinone] flavoprotein subunit, mitochondrial OS=Mus musculus OX=10090 GN=Sdha PE=1 SV=1

|  |  |
| --- | --- |
| Database: | Mouse\_UniProt\_proteomes |
| Score: | 11131 |
| Monoisotopic mass (M_r_): | 73623 |
| Calculated pI: | 7.06 |

Sequence similarity is available as an NCBI BLAST search of Q8K2B3 against nr.

### Search parameters

|  |  |
| --- | --- |
| MS data file: | `D:\LCMSMS\2023 Users' data\230529-1\230529-1-KIO-1.raw` |
| Enzyme: | Trypsin/P: cuts C-term side of KR. |
| Fixed modifications: | Carbamidomethyl (C) |
| Variable modifications: | Deamidated (NQ), HNE (C), HNE (H), HNE (K), Oxidation (M) |

### Protein sequence coverage: 66%

Matched peptides shown in ***bold red***.

|  |  |  |  |  |  |
| --- | --- | --- | --- | --- | --- |
| `1` | `MAGVGAVSRL` | `LRGRRLALTG` | `AWPGTLQKQT` | `CGFHFSVGEN` | `KKASAKVSDA` |
| `51` | `ISTQYPVVDH` | `EFDAVVVGAG` | `GAGLRAAFGL` | `SEAGFNTACL` | `TKLFPTRSHT` |
| `101` | `VAAQGGINAA` | `LGNMEEDNWR` | `WHFYDTVKGS` | `DWLGDQDAIH` | `YMTEQAPASV` |
| `151` | `VELENYGMPF` | `SRTEDGKIYQ` | `RAFGGQSLKF` | `GKGGQAHRCC` | `CVADRTGHSL` |
| `201` | `LHTLYGRSLR` | `YDTSYFVEYF` | `ALDLLMENGE` | `CRGVIALCIE` | `DGSIHRIRAK` |
| `251` | `NTVIATGGYG` | `RTYFSCTSAH` | `TSTGDGTAMV` | `TRAGLPCQDL` | `EFVQFHPTGI` |
| `301` | `YGAGCLITEG` | `CRGEGGILIN` | `SQGERFMERY` | `APVAKDLASR` | `DVVSRSMTLE` |
| `351` | `IREGRGCGPE` | `KDHVYLQLHH` | `LPPEQLATRL` | `PGISETAMIF` | `AGVDVTKEPI` |
| `401` | `PVLPTVHYNM` | `GGIPTNYKGQ` | `VLKHVNGQDQ` | `IVPGLYACGE` | `AACASVHGAN` |
| `451` | `RLGANSLLDL` | `VVFGRACALS` | `IAESCRPGDK` | `VPSIKANAGE` | `ESVMNLDKLR` |
| `501` | `FADGSIRTSE` | `LRLNMQKSMQ` | `NHAAVFRVGS` | `VLQEGCEKIS` | `QLYGDLKHLK` |
| `551` | `TFDRGMVWNT` | `DLVETLELQN` | `LMLCALQTIY` | `GAEARKESRG` | `AHAREDYKVR` |
| `601` | `VDEYDYSKPI` | `QGQQKKPFGE` | `HWRKHTLSYV` | `DIKTGKVTLE` | `YRPVIDKTLN` |
| `651` | `EADCATVPPA` | `IRSY` |  |  |  |

Unformatted sequence string: 664 residues (for pasting into other applications).

|  |  |  |  |
| --- | --- | --- | --- |
| Sort by | residue number | increasing mass | decreasing mass |
| Show | matched peptides only | predicted peptides also |  |

| Query | Start | – | End | Observed | Mr(expt) | Mr(calc) | ppm | M | Score | Expect | Rank | U | Peptide |
| --- | --- | --- | --- | --- | --- | --- | --- | --- | --- | --- | --- | --- | --- |
| 176014 | 47 | – | 75 | 977.1567 | 2928.4483 | 2928.4618 | -4.62 | 0 | 65 | 7.7e-07 | 1Score **> 37** indicates **identity** Score **> 17** indicates **homology** | U | K.VSDAISTQYPVVDHEFDAVVVGAGGAGLR.A |
| 176015 | 47 | – | 75 | 977.1568 | 2928.4487 | 2928.4618 | -4.46 | 0 | 54 | 8.7e-06 | 1Score **> 37** indicates **identity** Score **> 16** indicates **homology** | U | K.VSDAISTQYPVVDHEFDAVVVGAGGAGLR.A |
| 176016 | 47 | – | 75 | 977.1570 | 2928.4491 | 2928.4618 | -4.35 | 0 | 54 | 7.9e-06 | 1Score **> 37** indicates **identity** Score **> 16** indicates **homology** | U | K.VSDAISTQYPVVDHEFDAVVVGAGGAGLR.A |
| 176018 | 47 | – | 75 | 1465.2337 | 2928.4528 | 2928.4618 | -3.06 | 0 | 62 | 1.6e-06 | 1Score **> 37** indicates **identity** Score **> 16** indicates **homology** | U | K.VSDAISTQYPVVDHEFDAVVVGAGGAGLR.A |
| 176019 | 47 | – | 75 | 977.1597 | 2928.4571 | 2928.4618 | -1.59 | 0 | 51 | 1.8e-05 | 1Score **> 37** indicates **identity** Score **> 16** indicates **homology** | U | K.VSDAISTQYPVVDHEFDAVVVGAGGAGLR.A |
| 176021 | 47 | – | 75 | 977.1598 | 2928.4575 | 2928.4618 | -1.47 | 0 | 56 | 5.1e-06 | 1Score **> 37** indicates **identity** Score **> 16** indicates **homology** | U | K.VSDAISTQYPVVDHEFDAVVVGAGGAGLR.A |
| 176023 | 47 | – | 75 | 977.1603 | 2928.4591 | 2928.4618 | -0.91 | 0 | 60 | 2.5e-06 | 1Score **> 37** indicates **identity** Score **> 16** indicates **homology** | U | K.VSDAISTQYPVVDHEFDAVVVGAGGAGLR.A |
| 176024 | 47 | – | 75 | 977.1603 | 2928.4592 | 2928.4618 | -0.89 | 0 | 54 | 9e-06 | 1Score **> 37** indicates **identity** Score **> 16** indicates **homology** | U | K.VSDAISTQYPVVDHEFDAVVVGAGGAGLR.A |
| 176025 | 47 | – | 75 | 977.1606 | 2928.4600 | 2928.4618 | -0.60 | 0 | 45 | 5.7e-05 | 1Score **> 37** indicates **identity** Score **> 15** indicates **homology** | U | K.VSDAISTQYPVVDHEFDAVVVGAGGAGLR.A |
| 176026 | 47 | – | 75 | 977.1607 | 2928.4602 | 2928.4618 | -0.55 | 0 | 74 | 1.2e-07 | 1Score **> 37** indicates **identity** Score **> 17** indicates **homology** | U | K.VSDAISTQYPVVDHEFDAVVVGAGGAGLR.A |
| 176027 | 47 | – | 75 | 977.1610 | 2928.4612 | 2928.4618 | -0.19 | 0 | 27 | 0.0031 | 1Score **> 37** indicates **identity** Score **> 14** indicates **homology** | U | K.VSDAISTQYPVVDHEFDAVVVGAGGAGLR.A |
| 176028 | 47 | – | 75 | 977.1614 | 2928.4622 | 2928.4618 | 0.16 | 0 | 60 | 2.3e-06 | 1Score **> 37** indicates **identity** Score **> 16** indicates **homology** | U | K.VSDAISTQYPVVDHEFDAVVVGAGGAGLR.A |
| 176031 | 47 | – | 75 | 977.1616 | 2928.4630 | 2928.4618 | 0.41 | 0 | 66 | 6.4e-07 | 1Score **> 37** indicates **identity** Score **> 17** indicates **homology** | U | K.VSDAISTQYPVVDHEFDAVVVGAGGAGLR.A |
| 176032 | 47 | – | 75 | 977.1616 | 2928.4630 | 2928.4618 | 0.41 | 0 | 61 | 1.7e-06 | 1Score **> 37** indicates **identity** Score **> 16** indicates **homology** | U | K.VSDAISTQYPVVDHEFDAVVVGAGGAGLR.A |
| 176033 | 47 | – | 75 | 977.1616 | 2928.4630 | 2928.4618 | 0.42 | 0 | 48 | 3e-05 | 1Score **> 37** indicates **identity** Score **> 16** indicates **homology** | U | K.VSDAISTQYPVVDHEFDAVVVGAGGAGLR.A |
| 176034 | 47 | – | 75 | 977.1617 | 2928.4632 | 2928.4618 | 0.48 | 0 | 18 | 0.021 | 1Score **> 37** indicates **identity** Score **> 14** indicates **homology** | U | K.VSDAISTQYPVVDHEFDAVVVGAGGAGLR.A |
| 176035 | 47 | – | 75 | 977.1619 | 2928.4638 | 2928.4618 | 0.67 | 0 | 41 | 0.00014 | 1Score **> 37** indicates **identity** Score **> 15** indicates **homology** | U | K.VSDAISTQYPVVDHEFDAVVVGAGGAGLR.A |
| 176037 | 47 | – | 75 | 977.1619 | 2928.4639 | 2928.4618 | 0.71 | 0 | 59 | 2.8e-06 | 1Score **> 37** indicates **identity** Score **> 16** indicates **homology** | U | K.VSDAISTQYPVVDHEFDAVVVGAGGAGLR.A |
| 176039 | 47 | – | 75 | 733.1235 | 2928.4650 | 2928.4618 | 1.10 | 0 | 21 | 0.011 | 1Score **> 37** indicates **identity** Score **> 14** indicates **homology** | U | K.VSDAISTQYPVVDHEFDAVVVGAGGAGLR.A |
| 176040 | 47 | – | 75 | 977.1623 | 2928.4652 | 2928.4618 | 1.16 | 0 | 38 | 0.00027 | 1Score **> 37** indicates **identity** Score **> 15** indicates **homology** | U | K.VSDAISTQYPVVDHEFDAVVVGAGGAGLR.A |
| 176043 | 47 | – | 75 | 977.1626 | 2928.4659 | 2928.4618 | 1.42 | 0 | 64 | 1.1e-06 | 1Score **> 37** indicates **identity** Score **> 16** indicates **homology** | U | K.VSDAISTQYPVVDHEFDAVVVGAGGAGLR.A |
| 176044 | 47 | – | 75 | 977.1626 | 2928.4661 | 2928.4618 | 1.46 | 0 | 41 | 0.00015 | 1Score **> 37** indicates **identity** Score **> 15** indicates **homology** | U | K.VSDAISTQYPVVDHEFDAVVVGAGGAGLR.A |
| 176046 | 47 | – | 75 | 977.1628 | 2928.4666 | 2928.4618 | 1.64 | 0 | 43 | 9.2e-05 | 1Score **> 37** indicates **identity** Score **> 15** indicates **homology** | U | K.VSDAISTQYPVVDHEFDAVVVGAGGAGLR.A |
| 176048 | 47 | – | 75 | 977.1629 | 2928.4670 | 2928.4618 | 1.77 | 0 | 71 | 2e-07 | 1Score **> 37** indicates **identity** Score **> 17** indicates **homology** | U | K.VSDAISTQYPVVDHEFDAVVVGAGGAGLR.A |
| 176049 | 47 | – | 75 | 977.1630 | 2928.4671 | 2928.4618 | 1.80 | 0 | 19 | 0.024 | 1Score **> 37** indicates **identity** Score **> 15** indicates **homology** | U | K.VSDAISTQYPVVDHEFDAVVVGAGGAGLR.A |
| 176052 | 47 | – | 75 | 977.1632 | 2928.4679 | 2928.4618 | 2.08 | 0 | 19 | 0.016 | 1Score **> 37** indicates **identity** Score **> 14** indicates **homology** | U | K.VSDAISTQYPVVDHEFDAVVVGAGGAGLR.A |
| 176053 | 47 | – | 75 | 977.1633 | 2928.4680 | 2928.4618 | 2.12 | 0 | 46 | 4.9e-05 | 1Score **> 37** indicates **identity** Score **> 15** indicates **homology** | U | K.VSDAISTQYPVVDHEFDAVVVGAGGAGLR.A |
| 176054 | 47 | – | 75 | 1465.2413 | 2928.4680 | 2928.4618 | 2.13 | 0 | 74 | 1.1e-07 | 1Score **> 37** indicates **identity** Score **> 17** indicates **homology** | U | K.VSDAISTQYPVVDHEFDAVVVGAGGAGLR.A |
| 176055 | 47 | – | 75 | 977.1633 | 2928.4682 | 2928.4618 | 2.19 | 0 | 52 | 1.2e-05 | 1Score **> 37** indicates **identity** Score **> 16** indicates **homology** | U | K.VSDAISTQYPVVDHEFDAVVVGAGGAGLR.A |
| 176056 | 47 | – | 75 | 977.1635 | 2928.4686 | 2928.4618 | 2.33 | 0 | 56 | 6e-06 | 1Score **> 37** indicates **identity** Score **> 16** indicates **homology** | U | K.VSDAISTQYPVVDHEFDAVVVGAGGAGLR.A |
| 176058 | 47 | – | 75 | 977.1637 | 2928.4693 | 2928.4618 | 2.57 | 0 | 56 | 6.2e-06 | 1Score **> 37** indicates **identity** Score **> 16** indicates **homology** | U | K.VSDAISTQYPVVDHEFDAVVVGAGGAGLR.A |
| 176060 | 47 | – | 75 | 977.1639 | 2928.4698 | 2928.4618 | 2.74 | 0 | 33 | 0.00076 | 1Score **> 37** indicates **identity** Score **> 15** indicates **homology** | U | K.VSDAISTQYPVVDHEFDAVVVGAGGAGLR.A |
| 176062 | 47 | – | 75 | 977.1640 | 2928.4702 | 2928.4618 | 2.86 | 0 | 50 | 2.3e-05 | 1Score **> 37** indicates **identity** Score **> 16** indicates **homology** | U | K.VSDAISTQYPVVDHEFDAVVVGAGGAGLR.A |
| 176064 | 47 | – | 75 | 977.1643 | 2928.4710 | 2928.4618 | 3.13 | 0 | 57 | 4.8e-06 | 1Score **> 37** indicates **identity** Score **> 16** indicates **homology** | U | K.VSDAISTQYPVVDHEFDAVVVGAGGAGLR.A |
| 176066 | 47 | – | 75 | 977.1646 | 2928.4721 | 2928.4618 | 3.52 | 0 | 17 | 0.025 | 1Score **> 37** indicates **identity** Score **> 14** indicates **homology** | U | K.VSDAISTQYPVVDHEFDAVVVGAGGAGLR.A |
| 176067 | 47 | – | 75 | 1465.2434 | 2928.4723 | 2928.4618 | 3.59 | 0 | 23 | 0.0071 | 1Score **> 37** indicates **identity** Score **> 14** indicates **homology** | U | K.VSDAISTQYPVVDHEFDAVVVGAGGAGLR.A |
| 176069 | 47 | – | 75 | 977.1651 | 2928.4735 | 2928.4618 | 4.01 | 0 | 14 | 0.047 | 1Score **> 37** indicates **identity** Score **> 13** indicates **homology** | U | K.VSDAISTQYPVVDHEFDAVVVGAGGAGLR.A |
| 176071 | 47 | – | 75 | 977.1655 | 2928.4746 | 2928.4618 | 4.39 | 0 | 24 | 0.0057 | 1Score **> 37** indicates **identity** Score **> 14** indicates **homology** | U | K.VSDAISTQYPVVDHEFDAVVVGAGGAGLR.A |
| 176072 | 47 | – | 75 | 977.1656 | 2928.4749 | 2928.4618 | 4.48 | 0 | 21 | 0.01 | 1Score **> 37** indicates **identity** Score **> 14** indicates **homology** | U | K.VSDAISTQYPVVDHEFDAVVVGAGGAGLR.A |
| 176075 | 47 | – | 75 | 977.1659 | 2928.4758 | 2928.4618 | 4.80 | 0 | 35 | 0.00058 | 1Score **> 37** indicates **identity** Score **> 15** indicates **homology** | U | K.VSDAISTQYPVVDHEFDAVVVGAGGAGLR.A |
| 176076 | 47 | – | 75 | 977.1660 | 2928.4761 | 2928.4618 | 4.87 | 0 | 36 | 0.00046 | 1Score **> 37** indicates **identity** Score **> 15** indicates **homology** | U | K.VSDAISTQYPVVDHEFDAVVVGAGGAGLR.A |
| 176081 | 47 | – | 75 | 977.1670 | 2928.4792 | 2928.4618 | 5.93 | 0 | 17 | 0.025 | 1Score **> 37** indicates **identity** Score **> 14** indicates **homology** | U | K.VSDAISTQYPVVDHEFDAVVVGAGGAGLR.A |
| 176082 | 47 | – | 75 | 977.1674 | 2928.4803 | 2928.4618 | 6.32 | 0 | 20 | 0.012 | 1Score **> 37** indicates **identity** Score **> 14** indicates **homology** | U | K.VSDAISTQYPVVDHEFDAVVVGAGGAGLR.A |
| 176083 | 47 | – | 75 | 977.1676 | 2928.4809 | 2928.4618 | 6.54 | 0 | 57 | 4.6e-06 | 1Score **> 37** indicates **identity** Score **> 16** indicates **homology** | U | K.VSDAISTQYPVVDHEFDAVVVGAGGAGLR.A |
| 176135 | 47 | – | 75 | 977.4880 | 2929.4422 | 2929.4458 | -1.23 | 0 | 38 | 0.0003 | 1Score **> 37** indicates **identity** Score **> 15** indicates **homology** | U | K.VSDAISTQYPVVDHEFDAVVVGAGGAGLR.A  + Deamidated (NQ) |
| 176136 | 47 | – | 75 | 977.4904 | 2929.4494 | 2929.4458 | 1.23 | 0 | 15 | 0.035 | 1Score **> 37** indicates **identity** Score **> 13** indicates **homology** | U | K.VSDAISTQYPVVDHEFDAVVVGAGGAGLR.A  + Deamidated (NQ) |
| 176142 | 47 | – | 75 | 977.4950 | 2929.4633 | 2929.4458 | 5.96 | 0 | 58 | 3.4e-06 | 1Score **> 37** indicates **identity** Score **> 16** indicates **homology** | U | K.VSDAISTQYPVVDHEFDAVVVGAGGAGLR.A  + Deamidated (NQ) |
| 176143 | 47 | – | 75 | 977.4954 | 2929.4642 | 2929.4458 | 6.29 | 0 | 25 | 0.0044 | 1Score **> 37** indicates **identity** Score **> 14** indicates **homology** | U | K.VSDAISTQYPVVDHEFDAVVVGAGGAGLR.A  + Deamidated (NQ) |
| 176144 | 47 | – | 75 | 977.4957 | 2929.4651 | 2929.4458 | 6.60 | 0 | 63 | 1.3e-06 | 1Score **> 37** indicates **identity** Score **> 16** indicates **homology** | U | K.VSDAISTQYPVVDHEFDAVVVGAGGAGLR.A  + Deamidated (NQ) |
| 176145 | 47 | – | 75 | 1465.7401 | 2929.4656 | 2929.4458 | 6.75 | 0 | 66 | 7.1e-07 | 1Score **> 37** indicates **identity** Score **> 17** indicates **homology** | U | K.VSDAISTQYPVVDHEFDAVVVGAGGAGLR.A  + Deamidated (NQ) |
| 176149 | 47 | – | 75 | 977.4968 | 2929.4686 | 2929.4458 | 7.79 | 0 | 30 | 0.0014 | 1Score **> 38** indicates **identity** Score **> 14** indicates **homology** | U | K.VSDAISTQYPVVDHEFDAVVVGAGGAGLR.A  + Deamidated (NQ) |
| 176150 | 47 | – | 75 | 1465.7419 | 2929.4692 | 2929.4458 | 8.00 | 0 | 65 | 7.6e-07 | 1Score **> 38** indicates **identity** Score **> 17** indicates **homology** | U | K.VSDAISTQYPVVDHEFDAVVVGAGGAGLR.A  + Deamidated (NQ) |
| 176151 | 47 | – | 75 | 977.4972 | 2929.4697 | 2929.4458 | 8.16 | 0 | 37 | 0.00037 | 1Score **> 38** indicates **identity** Score **> 15** indicates **homology** | U | K.VSDAISTQYPVVDHEFDAVVVGAGGAGLR.A  + Deamidated (NQ) |
| 176154 | 47 | – | 75 | 977.4985 | 2929.4738 | 2929.4458 | 9.55 | 0 | 46 | 4.4e-05 | 1Score **> 38** indicates **identity** Score **> 15** indicates **homology** | U | K.VSDAISTQYPVVDHEFDAVVVGAGGAGLR.A  + Deamidated (NQ) |
| 176156 | 47 | – | 75 | 977.4990 | 2929.4750 | 2929.4458 | 9.98 | 0 | 28 | 0.0023 | 1Score **> 38** indicates **identity** Score **> 14** indicates **homology** | U | K.VSDAISTQYPVVDHEFDAVVVGAGGAGLR.A  + Deamidated (NQ) |
| 95870 | 76 | – | 92 | 879.4274 | 1756.8403 | 1756.8454 | -2.91 | 0 | 29 | 0.0019 | 1Score **> 34** indicates **identity** Score **> 14** indicates **homology** | U | R.AAFGLSEAGFNTACLTK.L |
| 95871 | 76 | – | 92 | 879.4284 | 1756.8422 | 1756.8454 | -1.82 | 0 | 19 | 0.017 | 1Score **> 34** indicates **identity** Score **> 14** indicates **homology** | U | R.AAFGLSEAGFNTACLTK.L |
| 95872 | 76 | – | 92 | 879.4289 | 1756.8433 | 1756.8454 | -1.19 | 0 | 60 | 2.3e-06 | 1Score **> 34** indicates **identity** Score **> 16** indicates **homology** | U | R.AAFGLSEAGFNTACLTK.L |
| 95875 | 76 | – | 92 | 879.4304 | 1756.8463 | 1756.8454 | 0.52 | 0 | 89 | 4.5e-09 | 1Score **> 34** indicates **identity** Score **> 18** indicates **homology** | U | R.AAFGLSEAGFNTACLTK.L |
| 95876 | 76 | – | 92 | 879.4306 | 1756.8467 | 1756.8454 | 0.71 | 0 | 89 | 4.5e-09 | 1Score **> 34** indicates **identity** Score **> 18** indicates **homology** | U | R.AAFGLSEAGFNTACLTK.L |
| 95880 | 76 | – | 92 | 879.4309 | 1756.8473 | 1756.8454 | 1.05 | 0 | 104 | 1.6e-10 | 1Score **> 34** indicates **identity** Score **> 19** indicates **homology** | U | R.AAFGLSEAGFNTACLTK.L |
| 95881 | 76 | – | 92 | 879.4312 | 1756.8479 | 1756.8454 | 1.40 | 0 | 102 | 2.5e-10 | 1Score **> 34** indicates **identity** Score **> 19** indicates **homology** | U | R.AAFGLSEAGFNTACLTK.L |
| 95882 | 76 | – | 92 | 879.4314 | 1756.8482 | 1756.8454 | 1.61 | 0 | 110 | 6.8e-11 | 1Score **> 34** indicates **identity** Score **> 21** indicates **homology** | U | R.AAFGLSEAGFNTACLTK.L |
| 95883 | 76 | – | 92 | 879.4316 | 1756.8487 | 1756.8454 | 1.88 | 0 | 105 | 1.5e-10 | 1Score **> 34** indicates **identity** Score **> 19** indicates **homology** | U | R.AAFGLSEAGFNTACLTK.L |
| 95884 | 76 | – | 92 | 879.4319 | 1756.8492 | 1756.8454 | 2.15 | 0 | 83 | 1.8e-08 | 1Score **> 34** indicates **identity** Score **> 18** indicates **homology** | U | R.AAFGLSEAGFNTACLTK.L |
| 95885 | 76 | – | 92 | 879.4319 | 1756.8493 | 1756.8454 | 2.24 | 0 | 74 | 2.4e-07 | 1Score **> 34** indicates **identity** Score **> 21** indicates **homology** | U | R.AAFGLSEAGFNTACLTK.L |
| 95887 | 76 | – | 92 | 879.4320 | 1756.8495 | 1756.8454 | 2.30 | 0 | 79 | 4.1e-08 | 1Score **> 34** indicates **identity** Score **> 17** indicates **homology** | U | R.AAFGLSEAGFNTACLTK.L |
| 95890 | 76 | – | 92 | 879.4326 | 1756.8507 | 1756.8454 | 3.00 | 0 | 71 | 2.1e-07 | 1Score **> 34** indicates **identity** Score **> 17** indicates **homology** | U | R.AAFGLSEAGFNTACLTK.L |
| 95891 | 76 | – | 92 | 879.4327 | 1756.8508 | 1756.8454 | 3.07 | 0 | 32 | 0.001 | 1Score **> 34** indicates **identity** Score **> 14** indicates **homology** | U | R.AAFGLSEAGFNTACLTK.L |
| 95893 | 76 | – | 92 | 879.4329 | 1756.8512 | 1756.8454 | 3.28 | 0 | 29 | 0.0021 | 1Score **> 34** indicates **identity** Score **> 14** indicates **homology** | U | R.AAFGLSEAGFNTACLTK.L |
| 95896 | 76 | – | 92 | 879.4344 | 1756.8543 | 1756.8454 | 5.05 | 0 | 62 | 1.4e-06 | 1Score **> 34** indicates **identity** Score **> 16** indicates **homology** | U | R.AAFGLSEAGFNTACLTK.L |
| 95897 | 76 | – | 92 | 879.4351 | 1756.8557 | 1756.8454 | 5.84 | 0 | 52 | 1.5e-05 | 1Score **> 34** indicates **identity** Score **> 17** indicates **homology** | U | R.AAFGLSEAGFNTACLTK.L |
| 95898 | 76 | – | 92 | 879.4356 | 1756.8566 | 1756.8454 | 6.38 | 0 | 20 | 0.014 | 1Score **> 34** indicates **identity** Score **> 14** indicates **homology** | U | R.AAFGLSEAGFNTACLTK.L |
| 20187 | 121 | – | 128 | 548.2660 | 1094.5174 | 1094.5185 | -1.07 | 0 | 23 | 0.0075 | 1Score **> 30** indicates **identity** Score **> 14** indicates **homology** | U | R.WHFYDTVK.G |
| 20188 | 121 | – | 128 | 365.8465 | 1094.5175 | 1094.5185 | -0.92 | 0 | 19 | 0.016 | 1Score **> 30** indicates **identity** Score **> 14** indicates **homology** | U | R.WHFYDTVK.G |
| 20189 | 121 | – | 128 | 365.8466 | 1094.5180 | 1094.5185 | -0.53 | 0 | 35 | 0.00047 | 1Score **> 30** indicates **identity** Score **> 15** indicates **homology** | U | R.WHFYDTVK.G |
| 20192 | 121 | – | 128 | 365.8469 | 1094.5188 | 1094.5185 | 0.25 | 0 | 15 | 0.043 | 1Score **> 30** indicates **identity** Score **> 13** indicates **homology** | U | R.WHFYDTVK.G |
| 20193 | 121 | – | 128 | 365.8469 | 1094.5189 | 1094.5185 | 0.30 | 0 | 27 | 0.0032 | 1Score **> 30** indicates **identity** Score **> 14** indicates **homology** | U | R.WHFYDTVK.G |
| 20194 | 121 | – | 128 | 548.2668 | 1094.5190 | 1094.5185 | 0.40 | 0 | 29 | 0.0065 | 1Score **> 30** indicates **identity** Score **> 20** indicates **homology** | U | R.WHFYDTVK.G |
| 20195 | 121 | – | 128 | 365.8469 | 1094.5190 | 1094.5185 | 0.43 | 0 | 23 | 0.0077 | 1Score **> 30** indicates **identity** Score **> 15** indicates **homology** | U | R.WHFYDTVK.G |
| 20197 | 121 | – | 128 | 365.8470 | 1094.5190 | 1094.5185 | 0.44 | 0 | 14 | 0.05 | 1Score **> 30** indicates **identity** Score **> 13** indicates **homology** | U | R.WHFYDTVK.G |
| 20199 | 121 | – | 128 | 365.8471 | 1094.5193 | 1094.5185 | 0.72 | 0 | 29 | 0.0019 | 1Score **> 30** indicates **identity** Score **> 14** indicates **homology** | U | R.WHFYDTVK.G |
| 20200 | 121 | – | 128 | 548.2670 | 1094.5194 | 1094.5185 | 0.78 | 0 | 31 | 0.0044 | 1Score **> 30** indicates **identity** Score **> 20** indicates **homology** | U | R.WHFYDTVK.G |
| 20202 | 121 | – | 128 | 365.8471 | 1094.5195 | 1094.5185 | 0.86 | 0 | 29 | 0.004 | 1Score **> 30** indicates **identity** Score **> 17** indicates **homology** | U | R.WHFYDTVK.G |
| 20203 | 121 | – | 128 | 548.2670 | 1094.5195 | 1094.5185 | 0.88 | 0 | 37 | 0.0013 | 1Score **> 30** indicates **identity** Score **> 20** indicates **homology** | U | R.WHFYDTVK.G |
| 20205 | 121 | – | 128 | 365.8472 | 1094.5199 | 1094.5185 | 1.26 | 0 | 37 | 0.00032 | 1Score **> 30** indicates **identity** Score **> 15** indicates **homology** | U | R.WHFYDTVK.G |
| 20208 | 121 | – | 128 | 548.2674 | 1094.5203 | 1094.5185 | 1.57 | 0 | 26 | 0.0045 | 1Score **> 30** indicates **identity** Score **> 15** indicates **homology** | U | R.WHFYDTVK.G |
| 20209 | 121 | – | 128 | 365.8475 | 1094.5206 | 1094.5185 | 1.85 | 0 | 16 | 0.034 | 1Score **> 30** indicates **identity** Score **> 13** indicates **homology** | U | R.WHFYDTVK.G |
| 20210 | 121 | – | 128 | 365.8475 | 1094.5208 | 1094.5185 | 2.06 | 0 | 30 | 0.0033 | 1Score **> 30** indicates **identity** Score **> 18** indicates **homology** | U | R.WHFYDTVK.G |
| 21497 | 163 | – | 171 | 555.2816 | 1108.5486 | 1108.5513 | -2.37 | 1 | 38 | 0.00029 | 1Score **> 31** indicates **identity** Score **> 15** indicates **homology** | U | R.TEDGKIYQR.A |
| 21498 | 163 | – | 171 | 370.5241 | 1108.5505 | 1108.5513 | -0.72 | 1 | 21 | 0.01 | 1Score **> 31** indicates **identity** Score **> 14** indicates **homology** | U | R.TEDGKIYQR.A |
| 21499 | 163 | – | 171 | 555.2827 | 1108.5509 | 1108.5513 | -0.34 | 1 | 65 | 2.5e-06 | 1Score **> 31** indicates **identity** Score **> 21** indicates **homology** | U | R.TEDGKIYQR.A |
| 21500 | 163 | – | 171 | 370.5244 | 1108.5514 | 1108.5513 | 0.078 | 1 | 56 | 5.5e-06 | 1Score **> 31** indicates **identity** Score **> 16** indicates **homology** | U | R.TEDGKIYQR.A |
| 21501 | 163 | – | 171 | 555.2831 | 1108.5517 | 1108.5513 | 0.40 | 1 | 17 | 0.027 | 1Score **> 32** indicates **identity** Score **> 14** indicates **homology** | U | R.TEDGKIYQR.A |
| 21502 | 163 | – | 171 | 555.2832 | 1108.5518 | 1108.5513 | 0.48 | 1 | 68 | 1.3e-06 | 1Score **> 32** indicates **identity** Score **> 21** indicates **homology** | U | R.TEDGKIYQR.A |
| 21503 | 163 | – | 171 | 370.5248 | 1108.5526 | 1108.5513 | 1.20 | 1 | 27 | 0.0029 | 1Score **> 31** indicates **identity** Score **> 14** indicates **homology** | U | R.TEDGKIYQR.A |
| 3571 | 172 | – | 179 | 404.2217 | 806.4289 | 806.4286 | 0.34 | 0 | 48 | 0.00021 | 1Score **> 25** indicates **identity** Score **> 24** indicates **homology** | U | R.AFGGQSLK.F |
| 3573 | 172 | – | 179 | 404.2221 | 806.4296 | 806.4286 | 1.23 | 0 | 24 | 0.016 | 1Score **> 25** indicates **identity** Score **> 19** indicates **homology** | U | R.AFGGQSLK.F |
| 24317 | 172 | – | 182 | 380.5456 | 1138.6151 | 1138.6135 | 1.40 | 1 | 24 | 0.0061 | 1Score **> 32** indicates **identity** Score **> 14** indicates **homology** | U | R.AFGGQSLKFGK.G |
| 24318 | 172 | – | 182 | 380.5459 | 1138.6159 | 1138.6135 | 2.07 | 1 | 21 | 0.011 | 1Score **> 32** indicates **identity** Score **> 14** indicates **homology** | U | R.AFGGQSLKFGK.G |
| 145070 | 189 | – | 207 | 759.3537 | 2275.0394 | 2275.0409 | -0.65 | 1 | 45 | 6.2e-05 | 1Score **> 33** indicates **identity** Score **> 15** indicates **homology** | U | R.CCCVADRTGHSLLHTLYGR.S |
| 145072 | 189 | – | 207 | 569.7681 | 2275.0433 | 2275.0409 | 1.08 | 1 | 24 | 0.0062 | 1Score **> 33** indicates **identity** Score **> 14** indicates **homology** | U | R.CCCVADRTGHSLLHTLYGR.S |
| 145073 | 189 | – | 207 | 759.3551 | 2275.0435 | 2275.0409 | 1.14 | 1 | 54 | 9.1e-06 | 1Score **> 33** indicates **identity** Score **> 16** indicates **homology** | U | R.CCCVADRTGHSLLHTLYGR.S |
| 145074 | 189 | – | 207 | 569.7682 | 2275.0439 | 2275.0409 | 1.31 | 1 | 23 | 0.0075 | 1Score **> 33** indicates **identity** Score **> 14** indicates **homology** | U | R.CCCVADRTGHSLLHTLYGR.S |
| 145075 | 189 | – | 207 | 456.0162 | 2275.0445 | 2275.0409 | 1.59 | 1 | 21 | 0.01 | 1Score **> 33** indicates **identity** Score **> 14** indicates **homology** | U | R.CCCVADRTGHSLLHTLYGR.S |
| 145078 | 189 | – | 207 | 456.0165 | 2275.0462 | 2275.0409 | 2.35 | 1 | 22 | 0.0088 | 1Score **> 33** indicates **identity** Score **> 14** indicates **homology** | U | R.CCCVADRTGHSLLHTLYGR.S |
| 45955 | 196 | – | 207 | 677.8649 | 1353.7153 | 1353.7153 | -0.031 | 0 | 31 | 0.0012 | 1Score **> 33** indicates **identity** Score **> 14** indicates **homology** | U | R.TGHSLLHTLYGR.S |
| 45957 | 196 | – | 207 | 677.8653 | 1353.7161 | 1353.7153 | 0.57 | 0 | 80 | 2.7e-07 | 1Score **> 33** indicates **identity** Score **> 27** indicates **homology** | U | R.TGHSLLHTLYGR.S |
| 45958 | 196 | – | 207 | 677.8654 | 1353.7163 | 1353.7153 | 0.69 | 0 | 68 | 3e-06 | 1Score **> 33** indicates **identity** Score **> 26** indicates **homology** | U | R.TGHSLLHTLYGR.S |
| 45962 | 196 | – | 207 | 677.8661 | 1353.7176 | 1353.7153 | 1.71 | 0 | 38 | 0.00026 | 1Score **> 33** indicates **identity** Score **> 15** indicates **homology** | U | R.TGHSLLHTLYGR.S |
| 66970 | 233 | – | 246 | 770.4003 | 1538.7861 | 1538.7875 | -0.90 | 0 | 91 | 1.1e-08 | 1Score **> 34** indicates **identity** Score **> 24** indicates **homology** | U | R.GVIALCIEDGSIHR.I |
| 66972 | 233 | – | 246 | 513.9362 | 1538.7866 | 1538.7875 | -0.56 | 0 | 33 | 0.00083 | 1Score **> 34** indicates **identity** Score **> 15** indicates **homology** | U | R.GVIALCIEDGSIHR.I |
| 66973 | 233 | – | 246 | 770.4006 | 1538.7867 | 1538.7875 | -0.55 | 0 | 24 | 0.0062 | 1Score **> 34** indicates **identity** Score **> 14** indicates **homology** | U | R.GVIALCIEDGSIHR.I |
| 66976 | 233 | – | 246 | 513.9363 | 1538.7870 | 1538.7875 | -0.35 | 0 | 16 | 0.029 | 1Score **> 34** indicates **identity** Score **> 14** indicates **homology** | U | R.GVIALCIEDGSIHR.I |
| 66978 | 233 | – | 246 | 513.9364 | 1538.7874 | 1538.7875 | -0.079 | 0 | 47 | 3.7e-05 | 1Score **> 34** indicates **identity** Score **> 15** indicates **homology** | U | R.GVIALCIEDGSIHR.I |
| 66981 | 233 | – | 246 | 513.9367 | 1538.7882 | 1538.7875 | 0.45 | 0 | 53 | 1e-05 | 1Score **> 34** indicates **identity** Score **> 16** indicates **homology** | U | R.GVIALCIEDGSIHR.I |
| 66982 | 233 | – | 246 | 513.9368 | 1538.7887 | 1538.7875 | 0.76 | 0 | 59 | 2.8e-06 | 1Score **> 34** indicates **identity** Score **> 16** indicates **homology** | U | R.GVIALCIEDGSIHR.I |
| 66983 | 233 | – | 246 | 513.9370 | 1538.7890 | 1538.7875 | 0.99 | 0 | 33 | 0.00073 | 1Score **> 34** indicates **identity** Score **> 15** indicates **homology** | U | R.GVIALCIEDGSIHR.I |
| 66984 | 233 | – | 246 | 513.9370 | 1538.7893 | 1538.7875 | 1.18 | 0 | 32 | 0.001 | 1Score **> 34** indicates **identity** Score **> 14** indicates **homology** | U | R.GVIALCIEDGSIHR.I |
| 66985 | 233 | – | 246 | 770.4020 | 1538.7895 | 1538.7875 | 1.29 | 0 | 49 | 6.3e-05 | 1Score **> 34** indicates **identity** Score **> 20** indicates **homology** | U | R.GVIALCIEDGSIHR.I |
| 66986 | 233 | – | 246 | 770.4021 | 1538.7897 | 1538.7875 | 1.40 | 0 | 92 | 1.1e-08 | 1Score **> 34** indicates **identity** Score **> 24** indicates **homology** | U | R.GVIALCIEDGSIHR.I |
| 66987 | 233 | – | 246 | 513.9372 | 1538.7897 | 1538.7875 | 1.45 | 0 | 54 | 8.1e-06 | 1Score **> 34** indicates **identity** Score **> 16** indicates **homology** | U | R.GVIALCIEDGSIHR.I |
| 66991 | 233 | – | 246 | 513.9374 | 1538.7904 | 1538.7875 | 1.90 | 0 | 52 | 1.3e-05 | 1Score **> 34** indicates **identity** Score **> 16** indicates **homology** | U | R.GVIALCIEDGSIHR.I |
| 66993 | 233 | – | 246 | 513.9375 | 1538.7907 | 1538.7875 | 2.11 | 0 | 31 | 0.0014 | 1Score **> 34** indicates **identity** Score **> 15** indicates **homology** | U | R.GVIALCIEDGSIHR.I |
| 66994 | 233 | – | 246 | 770.4028 | 1538.7911 | 1538.7875 | 2.32 | 0 | 48 | 3.1e-05 | 1Score **> 34** indicates **identity** Score **> 16** indicates **homology** | U | R.GVIALCIEDGSIHR.I |
| 66995 | 233 | – | 246 | 770.4028 | 1538.7911 | 1538.7875 | 2.34 | 0 | 73 | 3.8e-06 | 1Score **> 34** indicates **identity** Score **> 31** indicates **homology** | U | R.GVIALCIEDGSIHR.I |
| 66996 | 233 | – | 246 | 770.4029 | 1538.7912 | 1538.7875 | 2.38 | 0 | 74 | 4.9e-07 | 1Score **> 34** indicates **identity** Score **> 24** indicates **homology** | U | R.GVIALCIEDGSIHR.I |
| 66997 | 233 | – | 246 | 513.9377 | 1538.7914 | 1538.7875 | 2.50 | 0 | 59 | 3.2e-06 | 1Score **> 34** indicates **identity** Score **> 16** indicates **homology** | U | R.GVIALCIEDGSIHR.I |
| 67005 | 233 | – | 246 | 770.4047 | 1538.7949 | 1538.7875 | 4.80 | 0 | 20 | 0.014 | 1Score **> 34** indicates **identity** Score **> 14** indicates **homology** | U | R.GVIALCIEDGSIHR.I |
| 41202 | 249 | – | 261 | 436.5734 | 1306.6983 | 1306.6993 | -0.76 | 1 | 17 | 0.026 | 1Score **> 33** indicates **identity** Score **> 14** indicates **homology** | U | R.AKNTVIATGGYGR.T |
| 41204 | 249 | – | 261 | 654.3574 | 1306.7003 | 1306.6993 | 0.74 | 1 | 51 | 1.7e-05 | 1Score **> 34** indicates **identity** Score **> 16** indicates **homology** | U | R.AKNTVIATGGYGR.T |
| 41205 | 249 | – | 261 | 436.5740 | 1306.7003 | 1306.6993 | 0.75 | 1 | 53 | 1.2e-05 | 1Score **> 34** indicates **identity** Score **> 16** indicates **homology** | U | R.AKNTVIATGGYGR.T |
| 41206 | 249 | – | 261 | 654.3577 | 1306.7009 | 1306.6993 | 1.19 | 1 | 23 | 0.0071 | 1Score **> 34** indicates **identity** Score **> 14** indicates **homology** | U | R.AKNTVIATGGYGR.T |
| 41207 | 249 | – | 261 | 654.3580 | 1306.7014 | 1306.6993 | 1.55 | 1 | 38 | 0.0003 | 1Score **> 33** indicates **identity** Score **> 15** indicates **homology** | U | R.AKNTVIATGGYGR.T |
| 41209 | 249 | – | 261 | 436.5748 | 1306.7026 | 1306.6993 | 2.47 | 1 | 21 | 0.012 | 1Score **> 33** indicates **identity** Score **> 14** indicates **homology** | U | R.AKNTVIATGGYGR.T |
| 21428 | 251 | – | 261 | 554.7914 | 1107.5682 | 1107.5673 | 0.86 | 0 | 46 | 0.00019 | 1Score **> 32** indicates **identity** Score **> 21** indicates **homology** | U | K.NTVIATGGYGR.T |
| 21429 | 251 | – | 261 | 554.7914 | 1107.5683 | 1107.5673 | 0.96 | 0 | 29 | 0.002 | 1Score **> 32** indicates **identity** Score **> 14** indicates **homology** | U | K.NTVIATGGYGR.T |
| 21430 | 251 | – | 261 | 554.7918 | 1107.5690 | 1107.5673 | 1.57 | 0 | 41 | 0.00014 | 1Score **> 32** indicates **identity** Score **> 15** indicates **homology** | U | K.NTVIATGGYGR.T |
| 21432 | 251 | – | 261 | 554.7919 | 1107.5693 | 1107.5673 | 1.78 | 0 | 39 | 0.00021 | 1Score **> 32** indicates **identity** Score **> 15** indicates **homology** | U | K.NTVIATGGYGR.T |
| 21434 | 251 | – | 261 | 554.7925 | 1107.5705 | 1107.5673 | 2.89 | 0 | 22 | 0.0094 | 1Score **> 32** indicates **identity** Score **> 14** indicates **homology** | U | K.NTVIATGGYGR.T |
| 143321 | 262 | – | 282 | 750.9968 | 2249.9686 | 2249.9682 | 0.21 | 0 | 37 | 0.00034 | 1Score **> 28** indicates **identity** Score **> 15** indicates **homology** | U | R.TYFSCTSAHTSTGDGTAMVTR.A |
| 143322 | 262 | – | 282 | 750.9969 | 2249.9688 | 2249.9682 | 0.29 | 0 | 66 | 6.2e-07 | 1Score **> 28** indicates **identity** Score **> 17** indicates **homology** | U | R.TYFSCTSAHTSTGDGTAMVTR.A |
| 143323 | 262 | – | 282 | 1125.9922 | 2249.9699 | 2249.9682 | 0.76 | 0 | 93 | 1.8e-09 | 1Score **> 28** indicates **identity** Score **> 18** indicates **homology** | U | R.TYFSCTSAHTSTGDGTAMVTR.A |
| 143324 | 262 | – | 282 | 750.9978 | 2249.9715 | 2249.9682 | 1.49 | 0 | 65 | 7.6e-07 | 1Score **> 28** indicates **identity** Score **> 17** indicates **homology** | U | R.TYFSCTSAHTSTGDGTAMVTR.A |
| 143325 | 262 | – | 282 | 1125.9934 | 2249.9722 | 2249.9682 | 1.80 | 0 | 59 | 2.8e-06 | 1Score **> 28** indicates **identity** Score **> 16** indicates **homology** | U | R.TYFSCTSAHTSTGDGTAMVTR.A |
| 143326 | 262 | – | 282 | 750.9986 | 2249.9739 | 2249.9682 | 2.54 | 0 | 48 | 3.1e-05 | 1Score **> 28** indicates **identity** Score **> 16** indicates **homology** | U | R.TYFSCTSAHTSTGDGTAMVTR.A |
| 186818 | 283 | – | 312 | 1122.8650 | 3365.5732 | 3365.5632 | 2.98 | 0 | 24 | 0.005 | 1Score **> 35** indicates **identity** Score **> 14** indicates **homology** | U | R.AGLPCQDLEFVQFHPTGIYGAGCLITEGCR.G |
| 196778 | 283 | – | 325 | 1559.7513 | 4676.2322 | 4676.2210 | 2.38 | 1 | 30 | 0.0015 | 1Score **> 36** indicates **identity** Score **> 14** indicates **homology** | U | R.AGLPCQDLEFVQFHPTGIYGAGCLITEGCRGEGGILINSQGER.F |
| 43431 | 313 | – | 325 | 665.3414 | 1328.6682 | 1328.6684 | -0.13 | 0 | 63 | 1.4e-06 | 1Score **> 32** indicates **identity** Score **> 17** indicates **homology** | U | R.GEGGILINSQGER.F |
| 43433 | 313 | – | 325 | 665.3417 | 1328.6689 | 1328.6684 | 0.36 | 0 | 50 | 2.3e-05 | 1Score **> 32** indicates **identity** Score **> 16** indicates **homology** | U | R.GEGGILINSQGER.F |
| 43434 | 313 | – | 325 | 665.3418 | 1328.6691 | 1328.6684 | 0.54 | 0 | 40 | 0.00017 | 1Score **> 33** indicates **identity** Score **> 15** indicates **homology** | U | R.GEGGILINSQGER.F |
| 22148 | 336 | – | 345 | 373.2036 | 1116.5890 | 1116.5887 | 0.25 | 1 | 23 | 0.019 | 1Score **> 33** indicates **identity** Score **> 19** indicates **homology** | U | K.DLASRDVVSR.S |
| 22150 | 336 | – | 345 | 559.3022 | 1116.5899 | 1116.5887 | 1.05 | 1 | 41 | 0.0066 | 1Score **> 33** indicates **identity** Score **> 31** indicates **homology** | U | K.DLASRDVVSR.S |
| 22151 | 336 | – | 345 | 373.2041 | 1116.5906 | 1116.5887 | 1.66 | 1 | 26 | 0.0071 | 1Score **> 33** indicates **identity** Score **> 17** indicates **homology** | U | K.DLASRDVVSR.S |
| 5306 | 346 | – | 352 | 425.2286 | 848.4427 | 848.4426 | 0.18 | 0 | 41 | 0.0025 | 1Score **> 30** indicates **identity** Score **> 27** indicates **homology** | U | R.SMTLEIR.E |
| 5308 | 346 | – | 352 | 425.2290 | 848.4434 | 848.4426 | 0.97 | 0 | 34 | 0.013 | 1Score **> 30** indicates **identity** Score **> 28** indicates **homology** | U | R.SMTLEIR.E |
| 6121 | 346 | – | 352 | 433.2262 | 864.4378 | 864.4375 | 0.38 | 0 | 20 | 0.02 | 1Score **> 29** indicates **identity** Score **> 16** indicates **homology** | U | R.SMTLEIR.E  + Oxidation (M) |
| 6122 | 346 | – | 352 | 433.2265 | 864.4384 | 864.4375 | 1.12 | 0 | 18 | 0.022 | 1Score **> 29** indicates **identity** Score **> 14** indicates **homology** | U | R.SMTLEIR.E  + Oxidation (M) |
| 171577 | 356 | – | 379 | 559.8860 | 2794.3934 | 2794.3973 | -1.39 | 1 | 16 | 0.031 | 1Score **> 37** indicates **identity** Score **> 14** indicates **homology** | U | R.GCGPEKDHVYLQLHHLPPEQLATR.L |
| 171585 | 356 | – | 379 | 559.8881 | 2794.4044 | 2794.3973 | 2.53 | 1 | 23 | 0.0071 | 1Score **> 37** indicates **identity** Score **> 14** indicates **homology** | U | R.GCGPEKDHVYLQLHHLPPEQLATR.L |
| 176828 | 424 | – | 451 | 984.4592 | 2950.3559 | 2950.3563 | -0.14 | 0 | 75 | 9.5e-08 | 1Score **> 34** indicates **identity** Score **> 17** indicates **homology** | U | K.HVNGQDQIVPGLYACGEAACASVHGANR.L |
| 176829 | 424 | – | 451 | 984.4597 | 2950.3573 | 2950.3563 | 0.34 | 0 | 82 | 2.1e-08 | 1Score **> 34** indicates **identity** Score **> 18** indicates **homology** | U | K.HVNGQDQIVPGLYACGEAACASVHGANR.L |
| 176830 | 424 | – | 451 | 738.5971 | 2950.3592 | 2950.3563 | 0.99 | 0 | 60 | 2.4e-06 | 1Score **> 34** indicates **identity** Score **> 16** indicates **homology** | U | K.HVNGQDQIVPGLYACGEAACASVHGANR.L |
| 176831 | 424 | – | 451 | 984.4604 | 2950.3593 | 2950.3563 | 1.05 | 0 | 105 | 1.4e-10 | 1Score **> 34** indicates **identity** Score **> 19** indicates **homology** | U | K.HVNGQDQIVPGLYACGEAACASVHGANR.L |
| 176832 | 424 | – | 451 | 738.5973 | 2950.3600 | 2950.3563 | 1.26 | 0 | 51 | 1.7e-05 | 1Score **> 34** indicates **identity** Score **> 16** indicates **homology** | U | K.HVNGQDQIVPGLYACGEAACASVHGANR.L |
| 176833 | 424 | – | 451 | 984.4608 | 2950.3605 | 2950.3563 | 1.44 | 0 | 92 | 2.2e-09 | 1Score **> 34** indicates **identity** Score **> 18** indicates **homology** | U | K.HVNGQDQIVPGLYACGEAACASVHGANR.L |
| 176834 | 424 | – | 451 | 738.5974 | 2950.3606 | 2950.3563 | 1.46 | 0 | 43 | 9.1e-05 | 1Score **> 34** indicates **identity** Score **> 15** indicates **homology** | U | K.HVNGQDQIVPGLYACGEAACASVHGANR.L |
| 176835 | 424 | – | 451 | 738.5976 | 2950.3614 | 2950.3563 | 1.73 | 0 | 70 | 2.8e-07 | 1Score **> 35** indicates **identity** Score **> 17** indicates **homology** | U | K.HVNGQDQIVPGLYACGEAACASVHGANR.L |
| 176836 | 424 | – | 451 | 984.4613 | 2950.3620 | 2950.3563 | 1.96 | 0 | 44 | 8.2e-05 | 1Score **> 35** indicates **identity** Score **> 15** indicates **homology** | U | K.HVNGQDQIVPGLYACGEAACASVHGANR.L |
| 176837 | 424 | – | 451 | 738.5984 | 2950.3647 | 2950.3563 | 2.85 | 0 | 22 | 0.0082 | 1Score **> 35** indicates **identity** Score **> 14** indicates **homology** | U | K.HVNGQDQIVPGLYACGEAACASVHGANR.L |
| 176838 | 424 | – | 451 | 984.4638 | 2950.3695 | 2950.3563 | 4.50 | 0 | 52 | 1.2e-05 | 1Score **> 35** indicates **identity** Score **> 16** indicates **homology** | U | K.HVNGQDQIVPGLYACGEAACASVHGANR.L |
| 176882 | 424 | – | 451 | 738.8384 | 2951.3246 | 2951.3403 | -5.32 | 0 | 30 | 0.0016 | 1Score **> 32** indicates **identity** Score **> 14** indicates **homology** | U | K.HVNGQDQIVPGLYACGEAACASVHGANR.L  + Deamidated (NQ) |
| 176883 | 424 | – | 451 | 984.7846 | 2951.3320 | 2951.3403 | -2.81 | 0 | 75 | 9.9e-08 | 1Score **> 33** indicates **identity** Score **> 17** indicates **homology** | U | K.HVNGQDQIVPGLYACGEAACASVHGANR.L  + Deamidated (NQ) |
| 176884 | 424 | – | 451 | 738.8426 | 2951.3412 | 2951.3403 | 0.33 | 0 | 24 | 0.0052 | 1Score **> 33** indicates **identity** Score **> 14** indicates **homology** | U | K.HVNGQDQIVPGLYACGEAACASVHGANR.L  + Deamidated (NQ) |
| 176885 | 424 | – | 451 | 984.7877 | 2951.3412 | 2951.3403 | 0.33 | 0 | 57 | 4.6e-06 | 1Score **> 33** indicates **identity** Score **> 16** indicates **homology** | U | K.HVNGQDQIVPGLYACGEAACASVHGANR.L  + Deamidated (NQ) |
| 176886 | 424 | – | 451 | 738.8431 | 2951.3435 | 2951.3403 | 1.08 | 0 | 57 | 4.7e-06 | 1Score **> 33** indicates **identity** Score **> 16** indicates **homology** | U | K.HVNGQDQIVPGLYACGEAACASVHGANR.L  + Deamidated (NQ) |
| 176887 | 424 | – | 451 | 738.8432 | 2951.3435 | 2951.3403 | 1.10 | 0 | 63 | 1.2e-06 | 1Score **> 33** indicates **identity** Score **> 16** indicates **homology** | U | K.HVNGQDQIVPGLYACGEAACASVHGANR.L  + Deamidated (NQ) |
| 176888 | 424 | – | 451 | 984.7884 | 2951.3435 | 2951.3403 | 1.10 | 0 | 108 | 7.7e-11 | 1Score **> 33** indicates **identity** Score **> 19** indicates **homology** | U | K.HVNGQDQIVPGLYACGEAACASVHGANR.L  + Deamidated (NQ) |
| 176889 | 424 | – | 451 | 984.7890 | 2951.3451 | 2951.3403 | 1.64 | 0 | 84 | 1.3e-08 | 1Score **> 33** indicates **identity** Score **> 18** indicates **homology** | U | K.HVNGQDQIVPGLYACGEAACASVHGANR.L  + Deamidated (NQ) |
| 176890 | 424 | – | 451 | 984.7891 | 2951.3454 | 2951.3403 | 1.73 | 0 | 90 | 3.8e-09 | 1Score **> 33** indicates **identity** Score **> 18** indicates **homology** | U | K.HVNGQDQIVPGLYACGEAACASVHGANR.L  + Deamidated (NQ) |
| 176891 | 424 | – | 451 | 738.8437 | 2951.3457 | 2951.3403 | 1.83 | 0 | 67 | 4.9e-07 | 1Score **> 33** indicates **identity** Score **> 17** indicates **homology** | U | K.HVNGQDQIVPGLYACGEAACASVHGANR.L  + Deamidated (NQ) |
| 176892 | 424 | – | 451 | 984.7893 | 2951.3462 | 2951.3403 | 2.00 | 0 | 57 | 5e-06 | 1Score **> 33** indicates **identity** Score **> 16** indicates **homology** | U | K.HVNGQDQIVPGLYACGEAACASVHGANR.L  + Deamidated (NQ) |
| 176893 | 424 | – | 451 | 738.8439 | 2951.3466 | 2951.3403 | 2.14 | 0 | 78 | 5.3e-08 | 1Score **> 34** indicates **identity** Score **> 17** indicates **homology** | U | K.HVNGQDQIVPGLYACGEAACASVHGANR.L  + Deamidated (NQ) |
| 176894 | 424 | – | 451 | 738.8441 | 2951.3472 | 2951.3403 | 2.35 | 0 | 66 | 7e-07 | 1Score **> 34** indicates **identity** Score **> 17** indicates **homology** | U | K.HVNGQDQIVPGLYACGEAACASVHGANR.L  + Deamidated (NQ) |
| 176895 | 424 | – | 451 | 738.8441 | 2951.3473 | 2951.3403 | 2.39 | 0 | 46 | 4.9e-05 | 1Score **> 34** indicates **identity** Score **> 15** indicates **homology** | U | K.HVNGQDQIVPGLYACGEAACASVHGANR.L  + Deamidated (NQ) |
| 176896 | 424 | – | 451 | 984.7899 | 2951.3477 | 2951.3403 | 2.53 | 0 | 103 | 2.2e-10 | 1Score **> 34** indicates **identity** Score **> 19** indicates **homology** | U | K.HVNGQDQIVPGLYACGEAACASVHGANR.L  + Deamidated (NQ) |
| 176897 | 424 | – | 451 | 738.8443 | 2951.3481 | 2951.3403 | 2.66 | 0 | 23 | 0.007 | 1Score **> 34** indicates **identity** Score **> 14** indicates **homology** | U | K.HVNGQDQIVPGLYACGEAACASVHGANR.L  + Deamidated (NQ) |
| 176898 | 424 | – | 451 | 984.7901 | 2951.3485 | 2951.3403 | 2.79 | 0 | 57 | 4.2e-06 | 1Score **> 34** indicates **identity** Score **> 16** indicates **homology** | U | K.HVNGQDQIVPGLYACGEAACASVHGANR.L  + Deamidated (NQ) |
| 176899 | 424 | – | 451 | 984.7903 | 2951.3491 | 2951.3403 | 2.98 | 0 | 100 | 4.6e-10 | 1Score **> 34** indicates **identity** Score **> 19** indicates **homology** | U | K.HVNGQDQIVPGLYACGEAACASVHGANR.L  + Deamidated (NQ) |
| 176900 | 424 | – | 451 | 984.7908 | 2951.3506 | 2951.3403 | 3.49 | 0 | 33 | 0.0008 | 1Score **> 34** indicates **identity** Score **> 15** indicates **homology** | U | K.HVNGQDQIVPGLYACGEAACASVHGANR.L  + Deamidated (NQ) |
| 176901 | 424 | – | 451 | 738.8455 | 2951.3527 | 2951.3403 | 4.22 | 0 | 27 | 0.0028 | 1Score **> 34** indicates **identity** Score **> 14** indicates **homology** | U | K.HVNGQDQIVPGLYACGEAACASVHGANR.L  + Deamidated (NQ) |
| 176902 | 424 | – | 451 | 738.8456 | 2951.3535 | 2951.3403 | 4.47 | 0 | 23 | 0.007 | 1Score **> 34** indicates **identity** Score **> 14** indicates **homology** | U | K.HVNGQDQIVPGLYACGEAACASVHGANR.L  + Deamidated (NQ) |
| 176903 | 424 | – | 451 | 984.7935 | 2951.3587 | 2951.3403 | 6.25 | 0 | 51 | 1.6e-05 | 1Score **> 34** indicates **identity** Score **> 16** indicates **homology** | U | K.HVNGQDQIVPGLYACGEAACASVHGANR.L  + Deamidated (NQ) |
| 176904 | 424 | – | 451 | 738.8480 | 2951.3627 | 2951.3403 | 7.61 | 0 | 32 | 0.00095 | 1Score **> 34** indicates **identity** Score **> 15** indicates **homology** | U | K.HVNGQDQIVPGLYACGEAACASVHGANR.L  + Deamidated (NQ) |
| 176905 | 424 | – | 451 | 738.8481 | 2951.3631 | 2951.3403 | 7.74 | 0 | 26 | 0.0035 | 1Score **> 34** indicates **identity** Score **> 14** indicates **homology** | U | K.HVNGQDQIVPGLYACGEAACASVHGANR.L  + Deamidated (NQ) |
| 176926 | 424 | – | 451 | 985.1248 | 2952.3525 | 2952.3243 | 9.55 | 0 | 52 | 1.4e-05 | 1Score **> 34** indicates **identity** Score **> 16** indicates **homology** | U | K.HVNGQDQIVPGLYACGEAACASVHGANR.L  + 2 Deamidated (NQ) |
| 179991 | 424 | – | 451 | 1018.1365 | 3051.3878 | 3051.4178 | -9.86 | 0 | 21 | 0.011 | 1Score **> 33** indicates **identity** Score **> 14** indicates **homology** | U | K.HVNGQDQIVPGLYACGEAACASVHGANR.L  + 2 Deamidated (NQ); HNE (C) |
| 179993 | 424 | – | 451 | 1018.1391 | 3051.3956 | 3051.4178 | -7.30 | 0 | 14 | 0.05 | 1Score **> 33** indicates **identity** Score **> 13** indicates **homology** | U | K.HVNGQDQIVPGLYACGEAACASVHGANR.L  + 2 Deamidated (NQ); HNE (C) |
| 180004 | 424 | – | 451 | 1018.4686 | 3052.3840 | 3052.4019 | -5.84 | 0 | 26 | 0.0034 | 1Score **> 33** indicates **identity** Score **> 14** indicates **homology** | U | K.HVNGQDQIVPGLYACGEAACASVHGANR.L  + 3 Deamidated (NQ); HNE (C) |
| 195448 | 424 | – | 465 | 1102.5564 | 4406.1963 | 4406.1648 | 7.15 | 1 | 36 | 0.00045 | 1Score **> 37** indicates **identity** Score **> 15** indicates **homology** | U | K.HVNGQDQIVPGLYACGEAACASVHGANRLGANSLLDLVVFGR.A  + Deamidated (NQ) |
| 58755 | 452 | – | 465 | 737.4210 | 1472.8274 | 1472.8351 | -5.22 | 0 | 82 | 1.9e-08 | 1Score **> 34** indicates **identity** Score **> 18** indicates **homology** | U | R.LGANSLLDLVVFGR.A |
| 58756 | 452 | – | 465 | 737.4218 | 1472.8291 | 1472.8351 | -4.10 | 0 | 48 | 3.2e-05 | 1Score **> 34** indicates **identity** Score **> 15** indicates **homology** | U | R.LGANSLLDLVVFGR.A |
| 58761 | 452 | – | 465 | 491.9519 | 1472.8338 | 1472.8351 | -0.88 | 0 | 29 | 0.0018 | 1Score **> 34** indicates **identity** Score **> 14** indicates **homology** | U | R.LGANSLLDLVVFGR.A |
| 58763 | 452 | – | 465 | 737.4244 | 1472.8342 | 1472.8351 | -0.65 | 0 | 43 | 9.1e-05 | 1Score **> 34** indicates **identity** Score **> 15** indicates **homology** | U | R.LGANSLLDLVVFGR.A |
| 58766 | 452 | – | 465 | 737.4250 | 1472.8353 | 1472.8351 | 0.16 | 0 | 92 | 2.1e-09 | 1Score **> 34** indicates **identity** Score **> 18** indicates **homology** | U | R.LGANSLLDLVVFGR.A |
| 58768 | 452 | – | 465 | 491.9525 | 1472.8358 | 1472.8351 | 0.48 | 0 | 56 | 6e-06 | 1Score **> 34** indicates **identity** Score **> 16** indicates **homology** | U | R.LGANSLLDLVVFGR.A |
| 58770 | 452 | – | 465 | 737.4252 | 1472.8359 | 1472.8351 | 0.53 | 0 | 121 | 1e-11 | 1Score **> 34** indicates **identity** Score **> 24** indicates **homology** | U | R.LGANSLLDLVVFGR.A |
| 58773 | 452 | – | 465 | 491.9527 | 1472.8364 | 1472.8351 | 0.86 | 0 | 39 | 0.00021 | 1Score **> 34** indicates **identity** Score **> 15** indicates **homology** | U | R.LGANSLLDLVVFGR.A |
| 58775 | 452 | – | 465 | 737.4257 | 1472.8369 | 1472.8351 | 1.20 | 0 | 99 | 5.2e-10 | 1Score **> 34** indicates **identity** Score **> 19** indicates **homology** | U | R.LGANSLLDLVVFGR.A |
| 58776 | 452 | – | 465 | 491.9529 | 1472.8369 | 1472.8351 | 1.22 | 0 | 35 | 0.00049 | 1Score **> 34** indicates **identity** Score **> 15** indicates **homology** | U | R.LGANSLLDLVVFGR.A |
| 58777 | 452 | – | 465 | 737.4258 | 1472.8371 | 1472.8351 | 1.34 | 0 | 121 | 8.4e-12 | 1Score **> 34** indicates **identity** Score **> 22** indicates **homology** | U | R.LGANSLLDLVVFGR.A |
| 58779 | 452 | – | 465 | 737.4259 | 1472.8372 | 1472.8351 | 1.39 | 0 | 86 | 9.5e-09 | 1Score **> 34** indicates **identity** Score **> 18** indicates **homology** | U | R.LGANSLLDLVVFGR.A |
| 58781 | 452 | – | 465 | 737.4260 | 1472.8374 | 1472.8351 | 1.52 | 0 | 113 | 2.4e-11 | 1Score **> 33** indicates **identity** Score **> 20** indicates **homology** | U | R.LGANSLLDLVVFGR.A |
| 58783 | 452 | – | 465 | 737.4261 | 1472.8376 | 1472.8351 | 1.67 | 0 | 15 | 0.039 | 1Score **> 33** indicates **identity** Score **> 13** indicates **homology** | U | R.LGANSLLDLVVFGR.A |
| 58784 | 452 | – | 465 | 737.4263 | 1472.8380 | 1472.8351 | 1.94 | 0 | 19 | 0.018 | 1Score **> 34** indicates **identity** Score **> 14** indicates **homology** | U | R.LGANSLLDLVVFGR.A |
| 58785 | 452 | – | 465 | 737.4263 | 1472.8380 | 1472.8351 | 1.94 | 0 | 105 | 1.5e-10 | 1Score **> 34** indicates **identity** Score **> 19** indicates **homology** | U | R.LGANSLLDLVVFGR.A |
| 58786 | 452 | – | 465 | 491.9533 | 1472.8380 | 1472.8351 | 1.96 | 0 | 54 | 9.1e-06 | 1Score **> 34** indicates **identity** Score **> 16** indicates **homology** | U | R.LGANSLLDLVVFGR.A |
| 58788 | 452 | – | 465 | 491.9535 | 1472.8386 | 1472.8351 | 2.34 | 0 | 58 | 3.8e-06 | 1Score **> 34** indicates **identity** Score **> 16** indicates **homology** | U | R.LGANSLLDLVVFGR.A |
| 58789 | 452 | – | 465 | 491.9538 | 1472.8394 | 1472.8351 | 2.94 | 0 | 42 | 0.0001 | 1Score **> 33** indicates **identity** Score **> 15** indicates **homology** | U | R.LGANSLLDLVVFGR.A |
| 58791 | 452 | – | 465 | 737.4272 | 1472.8398 | 1472.8351 | 3.20 | 0 | 83 | 1.7e-08 | 1Score **> 33** indicates **identity** Score **> 18** indicates **homology** | U | R.LGANSLLDLVVFGR.A |
| 58792 | 452 | – | 465 | 737.4272 | 1472.8399 | 1472.8351 | 3.25 | 0 | 60 | 2.3e-06 | 1Score **> 33** indicates **identity** Score **> 16** indicates **homology** | U | R.LGANSLLDLVVFGR.A |
| 58794 | 452 | – | 465 | 737.4276 | 1472.8407 | 1472.8351 | 3.77 | 0 | 91 | 3.1e-09 | 1Score **> 33** indicates **identity** Score **> 18** indicates **homology** | U | R.LGANSLLDLVVFGR.A |
| 58796 | 452 | – | 465 | 737.4288 | 1472.8431 | 1472.8351 | 5.42 | 0 | 113 | 2.5e-11 | 1Score **> 33** indicates **identity** Score **> 20** indicates **homology** | U | R.LGANSLLDLVVFGR.A |
| 58797 | 452 | – | 465 | 737.4293 | 1472.8440 | 1472.8351 | 6.00 | 0 | 48 | 3.1e-05 | 1Score **> 33** indicates **identity** Score **> 16** indicates **homology** | U | R.LGANSLLDLVVFGR.A |
| 58798 | 452 | – | 465 | 737.4294 | 1472.8442 | 1472.8351 | 6.14 | 0 | 55 | 7.6e-06 | 1Score **> 33** indicates **identity** Score **> 16** indicates **homology** | U | R.LGANSLLDLVVFGR.A |
| 58800 | 452 | – | 465 | 737.4299 | 1472.8453 | 1472.8351 | 6.91 | 0 | 98 | 6.7e-10 | 1Score **> 33** indicates **identity** Score **> 19** indicates **homology** | U | R.LGANSLLDLVVFGR.A |
| 135695 | 466 | – | 485 | 540.5294 | 2158.0885 | 2158.0874 | 0.49 | 2 | 32 | 0.00092 | 1Score **> 36** indicates **identity** Score **> 15** indicates **homology** | U | R.ACALSIAESCRPGDKVPSIK.A |
| 135696 | 466 | – | 485 | 720.3701 | 2158.0886 | 2158.0874 | 0.52 | 2 | 25 | 0.0041 | 1Score **> 36** indicates **identity** Score **> 14** indicates **homology** | U | R.ACALSIAESCRPGDKVPSIK.A |
| 135698 | 466 | – | 485 | 1080.0517 | 2158.0889 | 2158.0874 | 0.66 | 2 | 16 | 0.04 | 1Score **> 36** indicates **identity** Score **> 14** indicates **homology** | U | R.ACALSIAESCRPGDKVPSIK.A |
| 135699 | 466 | – | 485 | 1080.0522 | 2158.0899 | 2158.0874 | 1.13 | 2 | 53 | 1.1e-05 | 1Score **> 36** indicates **identity** Score **> 16** indicates **homology** | U | R.ACALSIAESCRPGDKVPSIK.A |
| 135700 | 466 | – | 485 | 720.3706 | 2158.0899 | 2158.0874 | 1.14 | 2 | 28 | 0.0022 | 1Score **> 36** indicates **identity** Score **> 14** indicates **homology** | U | R.ACALSIAESCRPGDKVPSIK.A |
| 135701 | 466 | – | 485 | 540.5299 | 2158.0905 | 2158.0874 | 1.44 | 2 | 29 | 0.0018 | 1Score **> 36** indicates **identity** Score **> 14** indicates **homology** | U | R.ACALSIAESCRPGDKVPSIK.A |
| 135702 | 466 | – | 485 | 720.3709 | 2158.0909 | 2158.0874 | 1.60 | 2 | 29 | 0.0021 | 1Score **> 36** indicates **identity** Score **> 14** indicates **homology** | U | R.ACALSIAESCRPGDKVPSIK.A |
| 135703 | 466 | – | 485 | 540.5300 | 2158.0909 | 2158.0874 | 1.60 | 2 | 27 | 0.0027 | 1Score **> 36** indicates **identity** Score **> 14** indicates **homology** | U | R.ACALSIAESCRPGDKVPSIK.A |
| 135705 | 466 | – | 485 | 540.5302 | 2158.0919 | 2158.0874 | 2.06 | 2 | 25 | 0.0041 | 1Score **> 36** indicates **identity** Score **> 14** indicates **homology** | U | R.ACALSIAESCRPGDKVPSIK.A |
| 48030 | 486 | – | 498 | 689.3182 | 1376.6218 | 1376.6242 | -1.75 | 0 | 57 | 4.9e-06 | 1Score **> 30** indicates **identity** Score **> 16** indicates **homology** | U | K.ANAGEESVMNLDK.L |
| 48032 | 486 | – | 498 | 689.3199 | 1376.6253 | 1376.6242 | 0.83 | 0 | 51 | 1.6e-05 | 1Score **> 30** indicates **identity** Score **> 16** indicates **homology** | U | K.ANAGEESVMNLDK.L |
| 81795 | 486 | – | 500 | 549.6111 | 1645.8114 | 1645.8093 | 1.23 | 1 | 59 | 3e-06 | 1Score **> 34** indicates **identity** Score **> 16** indicates **homology** | U | K.ANAGEESVMNLDKLR.F |
| 81798 | 486 | – | 500 | 549.6111 | 1645.8116 | 1645.8093 | 1.39 | 1 | 35 | 0.00057 | 1Score **> 34** indicates **identity** Score **> 15** indicates **homology** | U | K.ANAGEESVMNLDKLR.F |
| 81799 | 486 | – | 500 | 823.9134 | 1645.8123 | 1645.8093 | 1.81 | 1 | 110 | 1.1e-10 | 1Score **> 34** indicates **identity** Score **> 23** indicates **homology** | U | K.ANAGEESVMNLDKLR.F |
| 81800 | 486 | – | 500 | 823.9135 | 1645.8124 | 1645.8093 | 1.88 | 1 | 77 | 6.2e-08 | 1Score **> 34** indicates **identity** Score **> 17** indicates **homology** | U | K.ANAGEESVMNLDKLR.F |
| 81802 | 486 | – | 500 | 549.6115 | 1645.8127 | 1645.8093 | 2.05 | 1 | 67 | 5.8e-07 | 1Score **> 34** indicates **identity** Score **> 17** indicates **homology** | U | K.ANAGEESVMNLDKLR.F |
| 81807 | 486 | – | 500 | 823.9142 | 1645.8139 | 1645.8093 | 2.79 | 1 | 79 | 6.2e-08 | 1Score **> 34** indicates **identity** Score **> 19** indicates **homology** | U | K.ANAGEESVMNLDKLR.F |
| 81810 | 486 | – | 500 | 549.6123 | 1645.8150 | 1645.8093 | 3.47 | 1 | 24 | 0.0057 | 1Score **> 34** indicates **identity** Score **> 14** indicates **homology** | U | K.ANAGEESVMNLDKLR.F |
| 81811 | 486 | – | 500 | 549.6125 | 1645.8156 | 1645.8093 | 3.80 | 1 | 48 | 3.1e-05 | 1Score **> 34** indicates **identity** Score **> 16** indicates **homology** | U | K.ANAGEESVMNLDKLR.F |
| 83954 | 486 | – | 500 | 554.9426 | 1661.8060 | 1661.8042 | 1.07 | 1 | 14 | 0.044 | 1Score **> 34** indicates **identity** Score **> 13** indicates **homology** | U | K.ANAGEESVMNLDKLR.F  + Oxidation (M) |
| 83957 | 486 | – | 500 | 554.9439 | 1661.8099 | 1661.8042 | 3.42 | 1 | 20 | 0.013 | 1Score **> 34** indicates **identity** Score **> 14** indicates **homology** | U | K.ANAGEESVMNLDKLR.F  + Oxidation (M) |
| 2031 | 501 | – | 507 | 383.1980 | 764.3814 | 764.3817 | -0.43 | 0 | 21 | 0.033 | 1Score **> 24** indicates **identity** Score **> 19** indicates **homology** | U | R.FADGSIR.T |
| 2032 | 501 | – | 507 | 383.1981 | 764.3817 | 764.3817 | -0.030 | 0 | 24 | 0.022 | 1Score **> 21** indicates **identity** Score **> 20** indicates **homology** | U | R.FADGSIR.T |
| 2034 | 501 | – | 507 | 383.1986 | 764.3826 | 764.3817 | 1.11 | 0 | 46 | 0.00018 | 1Score **> 21** indicates **identity** | U | R.FADGSIR.T |
| 2035 | 501 | – | 507 | 383.1988 | 764.3831 | 764.3817 | 1.81 | 0 | 29 | 0.011 | 1Score **> 21** indicates **identity** | U | R.FADGSIR.T |
| 2036 | 501 | – | 507 | 383.1988 | 764.3831 | 764.3817 | 1.82 | 0 | 33 | 0.0039 | 1Score **> 21** indicates **identity** | U | R.FADGSIR.T |
| 2037 | 501 | – | 507 | 383.1995 | 764.3845 | 764.3817 | 3.65 | 0 | 19 | 0.025 | 1Score **> 22** indicates **identity** Score **> 16** indicates **homology** | U | R.FADGSIR.T |
| 26286 | 518 | – | 527 | 580.7857 | 1159.5569 | 1159.5556 | 1.11 | 0 | 36 | 0.0004 | 1Score **> 30** indicates **identity** Score **> 15** indicates **homology** | U | K.SMQNHAAVFR.V |
| 27916 | 518 | – | 527 | 392.8578 | 1175.5515 | 1175.5506 | 0.82 | 0 | 33 | 0.00079 | 1Score **> 30** indicates **identity** Score **> 15** indicates **homology** | U | K.SMQNHAAVFR.V  + Oxidation (M) |
| 30795 | 528 | – | 538 | 603.2911 | 1204.5676 | 1204.5758 | -6.81 | 0 | 45 | 6.7e-05 | 1Score **> 31** indicates **identity** Score **> 15** indicates **homology** | U | R.VGSVLQEGCEK.I |
| 30797 | 528 | – | 538 | 603.2923 | 1204.5700 | 1204.5758 | -4.82 | 0 | 27 | 0.0074 | 1Score **> 31** indicates **identity** Score **> 18** indicates **homology** | U | R.VGSVLQEGCEK.I |
| 30799 | 528 | – | 538 | 603.2947 | 1204.5748 | 1204.5758 | -0.82 | 0 | 48 | 4.2e-05 | 1Score **> 32** indicates **identity** Score **> 17** indicates **homology** | U | R.VGSVLQEGCEK.I |
| 30802 | 528 | – | 538 | 603.2949 | 1204.5753 | 1204.5758 | -0.43 | 0 | 60 | 9.9e-06 | 1Score **> 32** indicates **identity** Score **> 23** indicates **homology** | U | R.VGSVLQEGCEK.I |
| 30804 | 528 | – | 538 | 603.2952 | 1204.5759 | 1204.5758 | 0.053 | 0 | 54 | 1.4e-05 | 1Score **> 32** indicates **identity** Score **> 18** indicates **homology** | U | R.VGSVLQEGCEK.I |
| 30807 | 528 | – | 538 | 603.2957 | 1204.5768 | 1204.5758 | 0.80 | 0 | 68 | 6.8e-07 | 1Score **> 32** indicates **identity** Score **> 19** indicates **homology** | U | R.VGSVLQEGCEK.I |
| 30808 | 528 | – | 538 | 603.2959 | 1204.5772 | 1204.5758 | 1.21 | 0 | 42 | 0.00011 | 1Score **> 32** indicates **identity** Score **> 15** indicates **homology** | U | R.VGSVLQEGCEK.I |
| 30809 | 528 | – | 538 | 603.2960 | 1204.5774 | 1204.5758 | 1.37 | 0 | 72 | 3.1e-07 | 1Score **> 32** indicates **identity** Score **> 20** indicates **homology** | U | R.VGSVLQEGCEK.I |
| 30812 | 528 | – | 538 | 603.2984 | 1204.5822 | 1204.5758 | 5.33 | 0 | 40 | 0.00018 | 1Score **> 32** indicates **identity** Score **> 15** indicates **homology** | U | R.VGSVLQEGCEK.I |
| 141119 | 528 | – | 547 | 1112.0732 | 2222.1319 | 2222.1253 | 2.99 | 1 | 57 | 4.3e-06 | 1Score **> 36** indicates **identity** Score **> 16** indicates **homology** | U | R.VGSVLQEGCEKISQLYGDLK.H |
| 141120 | 528 | – | 547 | 741.7182 | 2222.1329 | 2222.1253 | 3.42 | 1 | 54 | 7.8e-06 | 1Score **> 36** indicates **identity** Score **> 16** indicates **homology** | U | R.VGSVLQEGCEKISQLYGDLK.H |
| 15267 | 539 | – | 547 | 518.7874 | 1035.5602 | 1035.5600 | 0.20 | 0 | 31 | 0.0012 | 1Score **> 30** indicates **identity** Score **> 14** indicates **homology** | U | K.ISQLYGDLK.H |
| 15269 | 539 | – | 547 | 518.7878 | 1035.5610 | 1035.5600 | 0.89 | 0 | 40 | 0.00017 | 1Score **> 32** indicates **identity** Score **> 15** indicates **homology** | U | K.ISQLYGDLK.H |
| 15270 | 539 | – | 547 | 518.7879 | 1035.5612 | 1035.5600 | 1.17 | 0 | 47 | 6.1e-05 | 1Score **> 32** indicates **identity** Score **> 17** indicates **homology** | U | K.ISQLYGDLK.H |
| 15271 | 539 | – | 547 | 518.7880 | 1035.5614 | 1035.5600 | 1.35 | 0 | 43 | 0.00015 | 1Score **> 32** indicates **identity** Score **> 17** indicates **homology** | U | K.ISQLYGDLK.H |
| 15272 | 539 | – | 547 | 518.7880 | 1035.5615 | 1035.5600 | 1.41 | 0 | 32 | 0.0011 | 1Score **> 32** indicates **identity** Score **> 14** indicates **homology** | U | K.ISQLYGDLK.H |
| 52095 | 539 | – | 550 | 354.4568 | 1413.7979 | 1413.7980 | -0.050 | 1 | 24 | 0.0058 | 1Score **> 34** indicates **identity** Score **> 14** indicates **homology** | U | K.ISQLYGDLKHLK.T |
| 52097 | 539 | – | 550 | 354.4572 | 1413.7996 | 1413.7980 | 1.17 | 1 | 33 | 0.00089 | 1Score **> 34** indicates **identity** Score **> 15** indicates **homology** | U | K.ISQLYGDLKHLK.T |
| 52100 | 539 | – | 550 | 472.2742 | 1413.8007 | 1413.7980 | 1.90 | 1 | 40 | 0.00019 | 1Score **> 34** indicates **identity** Score **> 15** indicates **homology** | U | K.ISQLYGDLKHLK.T |
| 52101 | 539 | – | 550 | 472.2744 | 1413.8014 | 1413.7980 | 2.39 | 1 | 36 | 0.00051 | 1Score **> 34** indicates **identity** Score **> 16** indicates **homology** | U | K.ISQLYGDLKHLK.T |
| 52102 | 539 | – | 550 | 354.4577 | 1413.8015 | 1413.7980 | 2.53 | 1 | 34 | 0.00069 | 1Score **> 34** indicates **identity** Score **> 15** indicates **homology** | U | K.ISQLYGDLKHLK.T |
| 194429 | 551 | – | 585 | 1396.6856 | 4187.0350 | 4187.0629 | -6.65 | 1 | 34 | 0.00061 | 1Score **> 37** indicates **identity** Score **> 15** indicates **homology** | U | K.TFDRGMVWNTDLVETLELQNLMLCALQTIYGAEAR.K  + Deamidated (NQ); HNE (C); Oxidation (M) |
| 125642 | 599 | – | 615 | 685.0156 | 2052.0250 | 2052.0276 | -1.27 | 2 | 25 | 0.0045 | 1Score **> 36** indicates **identity** Score **> 14** indicates **homology** | U | K.VRVDEYDYSKPIQGQQK.K |
| 125644 | 599 | – | 615 | 685.0165 | 2052.0277 | 2052.0276 | 0.051 | 2 | 22 | 0.0084 | 1Score **> 36** indicates **identity** Score **> 14** indicates **homology** | U | K.VRVDEYDYSKPIQGQQK.K |
| 125646 | 599 | – | 615 | 685.0172 | 2052.0297 | 2052.0276 | 1.04 | 2 | 38 | 0.00025 | 1Score **> 36** indicates **identity** Score **> 15** indicates **homology** | U | K.VRVDEYDYSKPIQGQQK.K |
| 125647 | 599 | – | 615 | 1027.0222 | 2052.0299 | 2052.0276 | 1.13 | 2 | 28 | 0.0025 | 1Score **> 36** indicates **identity** Score **> 14** indicates **homology** | U | K.VRVDEYDYSKPIQGQQK.K |
| 125648 | 599 | – | 615 | 1027.0229 | 2052.0313 | 2052.0276 | 1.80 | 2 | 51 | 1.5e-05 | 1Score **> 36** indicates **identity** Score **> 16** indicates **homology** | U | K.VRVDEYDYSKPIQGQQK.K |
| 125649 | 599 | – | 615 | 514.0152 | 2052.0318 | 2052.0276 | 2.06 | 2 | 26 | 0.0038 | 1Score **> 36** indicates **identity** Score **> 14** indicates **homology** | U | K.VRVDEYDYSKPIQGQQK.K |
| 125650 | 599 | – | 615 | 514.0152 | 2052.0318 | 2052.0276 | 2.06 | 2 | 28 | 0.0025 | 1Score **> 36** indicates **identity** Score **> 14** indicates **homology** | U | K.VRVDEYDYSKPIQGQQK.K |
| 125651 | 599 | – | 615 | 685.0179 | 2052.0319 | 2052.0276 | 2.08 | 2 | 46 | 5e-05 | 1Score **> 36** indicates **identity** Score **> 15** indicates **homology** | U | K.VRVDEYDYSKPIQGQQK.K |
| 125652 | 599 | – | 615 | 685.0180 | 2052.0323 | 2052.0276 | 2.30 | 2 | 28 | 0.0026 | 1Score **> 36** indicates **identity** Score **> 14** indicates **homology** | U | K.VRVDEYDYSKPIQGQQK.K |
| 100280 | 601 | – | 615 | 599.9602 | 1796.8587 | 1796.8581 | 0.35 | 1 | 23 | 0.0068 | 1Score **> 33** indicates **identity** Score **> 14** indicates **homology** | U | R.VDEYDYSKPIQGQQK.K |
| 100281 | 601 | – | 615 | 599.9602 | 1796.8587 | 1796.8581 | 0.37 | 1 | 43 | 8.6e-05 | 1Score **> 34** indicates **identity** Score **> 15** indicates **homology** | U | R.VDEYDYSKPIQGQQK.K |
| 100282 | 601 | – | 615 | 899.4372 | 1796.8598 | 1796.8581 | 0.96 | 1 | 55 | 6.8e-06 | 1Score **> 34** indicates **identity** Score **> 16** indicates **homology** | U | R.VDEYDYSKPIQGQQK.K |
| 100283 | 601 | – | 615 | 899.4376 | 1796.8607 | 1796.8581 | 1.44 | 1 | 83 | 1.8e-08 | 1Score **> 34** indicates **identity** Score **> 18** indicates **homology** | U | R.VDEYDYSKPIQGQQK.K |
| 100284 | 601 | – | 615 | 899.4377 | 1796.8609 | 1796.8581 | 1.58 | 1 | 72 | 1.7e-07 | 1Score **> 34** indicates **identity** Score **> 17** indicates **homology** | U | R.VDEYDYSKPIQGQQK.K |
| 100285 | 601 | – | 615 | 599.9610 | 1796.8613 | 1796.8581 | 1.77 | 1 | 46 | 4.7e-05 | 1Score **> 34** indicates **identity** Score **> 15** indicates **homology** | U | R.VDEYDYSKPIQGQQK.K |
| 16738 | 616 | – | 623 | 352.8503 | 1055.5292 | 1055.5301 | -0.89 | 1 | 27 | 0.003 | 1Score **> 30** indicates **identity** Score **> 14** indicates **homology** | U | K.KPFGEHWR.K |
| 16739 | 616 | – | 623 | 352.8507 | 1055.5303 | 1055.5301 | 0.16 | 1 | 30 | 0.0014 | 1Score **> 30** indicates **identity** Score **> 14** indicates **homology** | U | K.KPFGEHWR.K |
| 16740 | 616 | – | 623 | 352.8507 | 1055.5304 | 1055.5301 | 0.30 | 1 | 42 | 0.00014 | 1Score **> 30** indicates **identity** Score **> 16** indicates **homology** | U | K.KPFGEHWR.K |
| 16742 | 616 | – | 623 | 352.8508 | 1055.5306 | 1055.5301 | 0.51 | 1 | 35 | 0.00052 | 1Score **> 30** indicates **identity** Score **> 15** indicates **homology** | U | K.KPFGEHWR.K |
| 16743 | 616 | – | 623 | 352.8508 | 1055.5306 | 1055.5301 | 0.52 | 1 | 40 | 0.00018 | 1Score **> 30** indicates **identity** Score **> 15** indicates **homology** | U | K.KPFGEHWR.K |
| 16745 | 616 | – | 623 | 352.8509 | 1055.5308 | 1055.5301 | 0.71 | 1 | 34 | 0.00063 | 1Score **> 30** indicates **identity** Score **> 15** indicates **homology** | U | K.KPFGEHWR.K |
| 16746 | 616 | – | 623 | 352.8509 | 1055.5309 | 1055.5301 | 0.80 | 1 | 32 | 0.0011 | 1Score **> 30** indicates **identity** Score **> 14** indicates **homology** | U | K.KPFGEHWR.K |
| 16747 | 616 | – | 623 | 528.7728 | 1055.5310 | 1055.5301 | 0.84 | 1 | 20 | 0.03 | 1Score **> 30** indicates **identity** Score **> 17** indicates **homology** | U | K.KPFGEHWR.K |
| 16748 | 616 | – | 623 | 352.8509 | 1055.5310 | 1055.5301 | 0.86 | 1 | 26 | 0.0033 | 1Score **> 30** indicates **identity** Score **> 14** indicates **homology** | U | K.KPFGEHWR.K |
| 28711 | 616 | – | 624 | 395.5497 | 1183.6272 | 1183.6250 | 1.83 | 2 | 23 | 0.0064 | 1Score **> 32** indicates **identity** Score **> 14** indicates **homology** | U | K.KPFGEHWRK.H |
| 28713 | 616 | – | 624 | 395.5502 | 1183.6286 | 1183.6250 | 3.04 | 2 | 18 | 0.02 | 1Score **> 32** indicates **identity** Score **> 14** indicates **homology** | U | K.KPFGEHWRK.H |
| 9049 | 617 | – | 623 | 464.7246 | 927.4346 | 927.4351 | -0.57 | 0 | 20 | 0.012 | 1Score **> 26** indicates **identity** Score **> 14** indicates **homology** | U | K.PFGEHWR.K |
| 30680 | 624 | – | 633 | 401.8961 | 1202.6664 | 1202.6659 | 0.39 | 1 | 43 | 0.0023 | 1Score **> 34** indicates **identity** Score **> 29** indicates **homology** | U | R.KHTLSYVDIK.T |
| 30681 | 624 | – | 633 | 401.8961 | 1202.6665 | 1202.6659 | 0.51 | 1 | 50 | 0.00056 | 1Score **> 34** indicates **identity** Score **> 30** indicates **homology** | U | R.KHTLSYVDIK.T |
| 30682 | 624 | – | 633 | 602.3407 | 1202.6668 | 1202.6659 | 0.72 | 1 | 58 | 7.1e-05 | 1Score **> 33** indicates **identity** Score **> 29** indicates **homology** | U | R.KHTLSYVDIK.T |
| 30683 | 624 | – | 633 | 602.3407 | 1202.6669 | 1202.6659 | 0.80 | 1 | 54 | 0.00037 | 1Score **> 33** indicates **identity** Score **> 32** indicates **homology** | U | R.KHTLSYVDIK.T |
| 30684 | 624 | – | 633 | 602.3409 | 1202.6673 | 1202.6659 | 1.14 | 1 | 33 | 0.0036 | 1Score **> 33** indicates **identity** Score **> 21** indicates **homology** | U | R.KHTLSYVDIK.T |
| 30685 | 624 | – | 633 | 401.8968 | 1202.6684 | 1202.6659 | 2.11 | 1 | 42 | 0.00065 | 1Score **> 33** indicates **identity** Score **> 22** indicates **homology** | U | R.KHTLSYVDIK.T |
| 30686 | 624 | – | 633 | 401.8968 | 1202.6687 | 1202.6659 | 2.32 | 1 | 29 | 0.0019 | 1Score **> 34** indicates **identity** Score **> 14** indicates **homology** | U | R.KHTLSYVDIK.T |
| 60876 | 624 | – | 636 | 373.2152 | 1488.8318 | 1488.8300 | 1.19 | 2 | 33 | 0.00083 | 1Score **> 33** indicates **identity** Score **> 15** indicates **homology** | U | R.KHTLSYVDIKTGK.V |
| 60877 | 624 | – | 636 | 373.2154 | 1488.8323 | 1488.8300 | 1.55 | 2 | 27 | 0.0031 | 1Score **> 33** indicates **identity** Score **> 14** indicates **homology** | U | R.KHTLSYVDIKTGK.V |
| 60879 | 624 | – | 636 | 373.2154 | 1488.8324 | 1488.8300 | 1.62 | 2 | 26 | 0.0035 | 1Score **> 34** indicates **identity** Score **> 14** indicates **homology** | U | R.KHTLSYVDIKTGK.V |
| 18482 | 625 | – | 633 | 538.2936 | 1074.5726 | 1074.5710 | 1.53 | 0 | 29 | 0.0072 | 1Score **> 33** indicates **identity** Score **> 20** indicates **homology** | U | K.HTLSYVDIK.T |
| 18489 | 625 | – | 633 | 538.2947 | 1074.5749 | 1074.5710 | 3.65 | 0 | 40 | 0.001 | 1Score **> 34** indicates **identity** Score **> 23** indicates **homology** | U | K.HTLSYVDIK.T |
| 46613 | 625 | – | 636 | 454.5856 | 1360.7348 | 1360.7351 | -0.17 | 1 | 20 | 0.015 | 1Score **> 34** indicates **identity** Score **> 14** indicates **homology** | U | K.HTLSYVDIKTGK.V |
| 78017 | 634 | – | 647 | 540.3108 | 1617.9106 | 1617.9090 | 1.02 | 2 | 16 | 0.033 | 1Score **> 34** indicates **identity** Score **> 13** indicates **homology** | U | K.TGKVTLEYRPVIDK.T |
| 43796 | 637 | – | 647 | 666.8803 | 1331.7460 | 1331.7449 | 0.81 | 1 | 55 | 0.00015 | 1Score **> 34** indicates **identity** Score **> 29** indicates **homology** | U | K.VTLEYRPVIDK.T |
| 43798 | 637 | – | 647 | 666.8807 | 1331.7469 | 1331.7449 | 1.49 | 1 | 55 | 0.00016 | 1Score **> 34** indicates **identity** Score **> 29** indicates **homology** | U | K.VTLEYRPVIDK.T |
| 43799 | 637 | – | 647 | 444.9229 | 1331.7469 | 1331.7449 | 1.50 | 1 | 22 | 0.027 | 1Score **> 34** indicates **identity** Score **> 18** indicates **homology** | U | K.VTLEYRPVIDK.T |
| 43800 | 637 | – | 647 | 666.8811 | 1331.7476 | 1331.7449 | 2.07 | 1 | 52 | 9.5e-05 | 1Score **> 34** indicates **identity** Score **> 24** indicates **homology** | U | K.VTLEYRPVIDK.T |
| 176481 | 637 | – | 662 | 981.1862 | 2940.5368 | 2940.5379 | -0.35 | 2 | 84 | 1.4e-08 | 1Score **> 37** indicates **identity** Score **> 18** indicates **homology** | U | K.VTLEYRPVIDKTLNEADCATVPPAIR.S |
| 176482 | 637 | – | 662 | 981.1864 | 2940.5373 | 2940.5379 | -0.19 | 2 | 23 | 0.0064 | 1Score **> 37** indicates **identity** Score **> 14** indicates **homology** | U | K.VTLEYRPVIDKTLNEADCATVPPAIR.S |
| 176484 | 637 | – | 662 | 736.1417 | 2940.5377 | 2940.5379 | -0.068 | 2 | 30 | 0.0014 | 1Score **> 37** indicates **identity** Score **> 14** indicates **homology** | U | K.VTLEYRPVIDKTLNEADCATVPPAIR.S |
| 176486 | 637 | – | 662 | 981.1868 | 2940.5385 | 2940.5379 | 0.20 | 2 | 111 | 3.6e-11 | 1Score **> 37** indicates **identity** Score **> 19** indicates **homology** | U | K.VTLEYRPVIDKTLNEADCATVPPAIR.S |
| 176488 | 637 | – | 662 | 981.1869 | 2940.5388 | 2940.5379 | 0.32 | 2 | 111 | 3.6e-11 | 1Score **> 37** indicates **identity** Score **> 19** indicates **homology** | U | K.VTLEYRPVIDKTLNEADCATVPPAIR.S |
| 176490 | 637 | – | 662 | 736.1421 | 2940.5393 | 2940.5379 | 0.47 | 2 | 40 | 0.00019 | 1Score **> 37** indicates **identity** Score **> 15** indicates **homology** | U | K.VTLEYRPVIDKTLNEADCATVPPAIR.S |
| 176492 | 637 | – | 662 | 736.1422 | 2940.5398 | 2940.5379 | 0.67 | 2 | 36 | 0.00042 | 1Score **> 37** indicates **identity** Score **> 15** indicates **homology** | U | K.VTLEYRPVIDKTLNEADCATVPPAIR.S |
| 176495 | 637 | – | 662 | 736.1427 | 2940.5418 | 2940.5379 | 1.32 | 2 | 44 | 7.5e-05 | 1Score **> 37** indicates **identity** Score **> 15** indicates **homology** | U | K.VTLEYRPVIDKTLNEADCATVPPAIR.S |
| 176496 | 637 | – | 662 | 981.1880 | 2940.5420 | 2940.5379 | 1.41 | 2 | 99 | 5.1e-10 | 1Score **> 37** indicates **identity** Score **> 19** indicates **homology** | U | K.VTLEYRPVIDKTLNEADCATVPPAIR.S |
| 176498 | 637 | – | 662 | 981.1880 | 2940.5423 | 2940.5379 | 1.51 | 2 | 102 | 2.8e-10 | 1Score **> 37** indicates **identity** Score **> 19** indicates **homology** | U | K.VTLEYRPVIDKTLNEADCATVPPAIR.S |
| 176501 | 637 | – | 662 | 981.1918 | 2940.5535 | 2940.5379 | 5.31 | 2 | 25 | 0.0041 | 1Score **> 36** indicates **identity** Score **> 14** indicates **homology** | U | K.VTLEYRPVIDKTLNEADCATVPPAIR.S |
| 176502 | 637 | – | 662 | 981.1918 | 2940.5535 | 2940.5379 | 5.31 | 2 | 42 | 0.00011 | 1Score **> 36** indicates **identity** Score **> 15** indicates **homology** | U | K.VTLEYRPVIDKTLNEADCATVPPAIR.S |
| 176535 | 637 | – | 662 | 981.5186 | 2941.5340 | 2941.5219 | 4.14 | 2 | 26 | 0.004 | 1Score **> 37** indicates **identity** Score **> 14** indicates **homology** | U | K.VTLEYRPVIDKTLNEADCATVPPAIR.S  + Deamidated (NQ) |
| 176538 | 637 | – | 662 | 981.5226 | 2941.5461 | 2941.5219 | 8.23 | 2 | 48 | 3.3e-05 | 1Score **> 37** indicates **identity** Score **> 15** indicates **homology** | U | K.VTLEYRPVIDKTLNEADCATVPPAIR.S  + Deamidated (NQ) |
| 176539 | 637 | – | 662 | 981.5232 | 2941.5477 | 2941.5219 | 8.77 | 2 | 63 | 1.3e-06 | 1Score **> 37** indicates **identity** Score **> 16** indicates **homology** | U | K.VTLEYRPVIDKTLNEADCATVPPAIR.S  + Deamidated (NQ) |
| 176540 | 637 | – | 662 | 981.5239 | 2941.5500 | 2941.5219 | 9.54 | 2 | 44 | 8.1e-05 | 1Score **> 36** indicates **identity** Score **> 15** indicates **homology** | U | K.VTLEYRPVIDKTLNEADCATVPPAIR.S  + Deamidated (NQ) |
| 79222 | 648 | – | 662 | 814.4074 | 1626.8003 | 1626.8035 | -2.02 | 0 | 68 | 4.3e-07 | 1Score **> 34** indicates **identity** Score **> 17** indicates **homology** | U | K.TLNEADCATVPPAIR.S |
| 79226 | 648 | – | 662 | 814.4089 | 1626.8032 | 1626.8035 | -0.22 | 0 | 76 | 6.8e-08 | 1Score **> 34** indicates **identity** Score **> 17** indicates **homology** | U | K.TLNEADCATVPPAIR.S |
| 79228 | 648 | – | 662 | 814.4091 | 1626.8036 | 1626.8035 | 0.063 | 0 | 78 | 4.6e-08 | 1Score **> 34** indicates **identity** Score **> 17** indicates **homology** | U | K.TLNEADCATVPPAIR.S |
| 79230 | 648 | – | 662 | 814.4094 | 1626.8042 | 1626.8035 | 0.43 | 0 | 76 | 6.9e-08 | 1Score **> 34** indicates **identity** Score **> 17** indicates **homology** | U | K.TLNEADCATVPPAIR.S |
| 79232 | 648 | – | 662 | 814.4097 | 1626.8048 | 1626.8035 | 0.76 | 0 | 89 | 4.4e-09 | 1Score **> 34** indicates **identity** Score **> 18** indicates **homology** | U | K.TLNEADCATVPPAIR.S |
| 79233 | 648 | – | 662 | 814.4097 | 1626.8049 | 1626.8035 | 0.83 | 0 | 94 | 1.7e-09 | 1Score **> 34** indicates **identity** Score **> 18** indicates **homology** | U | K.TLNEADCATVPPAIR.S |
| 79236 | 648 | – | 662 | 814.4101 | 1626.8056 | 1626.8035 | 1.25 | 0 | 81 | 2.6e-08 | 1Score **> 34** indicates **identity** Score **> 18** indicates **homology** | U | K.TLNEADCATVPPAIR.S |
| 79239 | 648 | – | 662 | 814.4102 | 1626.8059 | 1626.8035 | 1.44 | 0 | 85 | 1.2e-08 | 1Score **> 34** indicates **identity** Score **> 18** indicates **homology** | U | K.TLNEADCATVPPAIR.S |
| 79240 | 648 | – | 662 | 814.4103 | 1626.8060 | 1626.8035 | 1.48 | 0 | 68 | 3.8e-07 | 1Score **> 34** indicates **identity** Score **> 17** indicates **homology** | U | K.TLNEADCATVPPAIR.S |
| 79241 | 648 | – | 662 | 814.4104 | 1626.8063 | 1626.8035 | 1.72 | 0 | 81 | 2.8e-08 | 1Score **> 34** indicates **identity** Score **> 18** indicates **homology** | U | K.TLNEADCATVPPAIR.S |
| 79242 | 648 | – | 662 | 814.4105 | 1626.8064 | 1626.8035 | 1.73 | 0 | 78 | 4.7e-08 | 1Score **> 34** indicates **identity** Score **> 17** indicates **homology** | U | K.TLNEADCATVPPAIR.S |
| 79243 | 648 | – | 662 | 814.4105 | 1626.8064 | 1626.8035 | 1.74 | 0 | 80 | 3.2e-08 | 1Score **> 34** indicates **identity** Score **> 18** indicates **homology** | U | K.TLNEADCATVPPAIR.S |
| 79244 | 648 | – | 662 | 814.4106 | 1626.8066 | 1626.8035 | 1.85 | 0 | 81 | 2.6e-08 | 1Score **> 34** indicates **identity** Score **> 18** indicates **homology** | U | K.TLNEADCATVPPAIR.S |
| 79245 | 648 | – | 662 | 814.4107 | 1626.8068 | 1626.8035 | 2.03 | 0 | 89 | 4.3e-09 | 1Score **> 34** indicates **identity** Score **> 18** indicates **homology** | U | K.TLNEADCATVPPAIR.S |
| 79246 | 648 | – | 662 | 814.4108 | 1626.8070 | 1626.8035 | 2.10 | 0 | 83 | 1.8e-08 | 1Score **> 34** indicates **identity** Score **> 18** indicates **homology** | U | K.TLNEADCATVPPAIR.S |
| 79249 | 648 | – | 662 | 814.4126 | 1626.8106 | 1626.8035 | 4.34 | 0 | 46 | 5.3e-05 | 1Score **> 34** indicates **identity** Score **> 15** indicates **homology** | U | K.TLNEADCATVPPAIR.S |

---

```
ID   SDHA_MOUSE              Reviewed;         664 AA.
AC   Q8K2B3; Q0QF19; Q3UH25; Q3UKP7; Q3V4B1; Q921P5; Q9Z1Z4;
DT   27-SEP-2004, integrated into UniProtKB/Swiss-Prot.
DT   01-OCT-2002, sequence version 1.
DT   28-JUN-2023, entry version 166.
DE   RecName: Full=Succinate dehydrogenase [ubiquinone] flavoprotein subunit, mitochondrial;
DE            EC=1.3.5.1 {ECO:0000250|UniProtKB:P31040};
DE   AltName: Full=Flavoprotein subunit of complex II;
DE            Short=Fp;
DE   Flags: Precursor;
GN   Name=Sdha;
OS   Mus musculus (Mouse).
OC   Eukaryota; Metazoa; Chordata; Craniata; Vertebrata; Euteleostomi; Mammalia;
OC   Eutheria; Euarchontoglires; Glires; Rodentia; Myomorpha; Muroidea; Muridae;
OC   Murinae; Mus; Mus.
OX   NCBI_TaxID=10090;
RN   [1]
RP   NUCLEOTIDE SEQUENCE [LARGE SCALE MRNA].
RC   STRAIN=C57BL/6J; TISSUE=Bone marrow, Egg, Heart, Pancreas, and Testis;
RX   PubMed=16141072; DOI=10.1126/science.1112014;
RA   Carninci P., Kasukawa T., Katayama S., Gough J., Frith M.C., Maeda N.,
RA   Oyama R., Ravasi T., Lenhard B., Wells C., Kodzius R., Shimokawa K.,
RA   Bajic V.B., Brenner S.E., Batalov S., Forrest A.R., Zavolan M., Davis M.J.,
RA   Wilming L.G., Aidinis V., Allen J.E., Ambesi-Impiombato A., Apweiler R.,
RA   Aturaliya R.N., Bailey T.L., Bansal M., Baxter L., Beisel K.W., Bersano T.,
RA   Bono H., Chalk A.M., Chiu K.P., Choudhary V., Christoffels A.,
RA   Clutterbuck D.R., Crowe M.L., Dalla E., Dalrymple B.P., de Bono B.,
RA   Della Gatta G., di Bernardo D., Down T., Engstrom P., Fagiolini M.,
RA   Faulkner G., Fletcher C.F., Fukushima T., Furuno M., Futaki S.,
RA   Gariboldi M., Georgii-Hemming P., Gingeras T.R., Gojobori T., Green R.E.,
RA   Gustincich S., Harbers M., Hayashi Y., Hensch T.K., Hirokawa N., Hill D.,
RA   Huminiecki L., Iacono M., Ikeo K., Iwama A., Ishikawa T., Jakt M.,
RA   Kanapin A., Katoh M., Kawasawa Y., Kelso J., Kitamura H., Kitano H.,
RA   Kollias G., Krishnan S.P., Kruger A., Kummerfeld S.K., Kurochkin I.V.,
RA   Lareau L.F., Lazarevic D., Lipovich L., Liu J., Liuni S., McWilliam S.,
RA   Madan Babu M., Madera M., Marchionni L., Matsuda H., Matsuzawa S., Miki H.,
RA   Mignone F., Miyake S., Morris K., Mottagui-Tabar S., Mulder N., Nakano N.,
RA   Nakauchi H., Ng P., Nilsson R., Nishiguchi S., Nishikawa S., Nori F.,
RA   Ohara O., Okazaki Y., Orlando V., Pang K.C., Pavan W.J., Pavesi G.,
RA   Pesole G., Petrovsky N., Piazza S., Reed J., Reid J.F., Ring B.Z.,
RA   Ringwald M., Rost B., Ruan Y., Salzberg S.L., Sandelin A., Schneider C.,
RA   Schoenbach C., Sekiguchi K., Semple C.A., Seno S., Sessa L., Sheng Y.,
RA   Shibata Y., Shimada H., Shimada K., Silva D., Sinclair B., Sperling S.,
RA   Stupka E., Sugiura K., Sultana R., Takenaka Y., Taki K., Tammoja K.,
RA   Tan S.L., Tang S., Taylor M.S., Tegner J., Teichmann S.A., Ueda H.R.,
RA   van Nimwegen E., Verardo R., Wei C.L., Yagi K., Yamanishi H.,
RA   Zabarovsky E., Zhu S., Zimmer A., Hide W., Bult C., Grimmond S.M.,
RA   Teasdale R.D., Liu E.T., Brusic V., Quackenbush J., Wahlestedt C.,
RA   Mattick J.S., Hume D.A., Kai C., Sasaki D., Tomaru Y., Fukuda S.,
RA   Kanamori-Katayama M., Suzuki M., Aoki J., Arakawa T., Iida J., Imamura K.,
RA   Itoh M., Kato T., Kawaji H., Kawagashira N., Kawashima T., Kojima M.,
RA   Kondo S., Konno H., Nakano K., Ninomiya N., Nishio T., Okada M., Plessy C.,
RA   Shibata K., Shiraki T., Suzuki S., Tagami M., Waki K., Watahiki A.,
RA   Okamura-Oho Y., Suzuki H., Kawai J., Hayashizaki Y.;
RT   "The transcriptional landscape of the mammalian genome.";
RL   Science 309:1559-1563(2005).
RN   [2]
RP   NUCLEOTIDE SEQUENCE [LARGE SCALE MRNA].
RC   STRAIN=Czech II, and FVB/N; TISSUE=Mammary gland, and Mammary tumor;
RX   PubMed=15489334; DOI=10.1101/gr.2596504;
RG   The MGC Project Team;
RT   "The status, quality, and expansion of the NIH full-length cDNA project:
RT   the Mammalian Gene Collection (MGC).";
RL   Genome Res. 14:2121-2127(2004).
RN   [3]
RP   PROTEIN SEQUENCE OF 1-14; 47-92; 121-128; 196-207; 233-246; 251-282;
RP   313-325; 362-418; 452-480; 486-498; 528-547; 601-615; 624-633 AND 637-647.
RC   STRAIN=C57BL/6J; TISSUE=Brain, and Hippocampus;
RA   Lubec G., Klug S., Kang S.U.;
RL   Submitted (APR-2007) to UniProtKB.
RN   [4]
RP   NUCLEOTIDE SEQUENCE [MRNA] OF 59-609.
RC   TISSUE=Liver;
RX   PubMed=16751257; DOI=10.1093/molbev/msl027;
RA   Kullberg M., Nilsson M.A., Arnason U., Harley E.H., Janke A.;
RT   "Housekeeping genes for phylogenetic analysis of eutherian relationships.";
RL   Mol. Biol. Evol. 23:1493-1503(2006).
RN   [5]
RP   NUCLEOTIDE SEQUENCE [MRNA] OF 74-605.
RC   TISSUE=Heart;
RA   Weinreich D.M.;
RT   "OXPHOS genes in mammals and the molecular clock.";
RL   Submitted (OCT-1998) to the EMBL/GenBank/DDBJ databases.
RN   [6]
RP   IDENTIFICATION BY MASS SPECTROMETRY [LARGE SCALE ANALYSIS].
RC   TISSUE=Brain, Brown adipose tissue, Heart, Kidney, Liver, Lung,
RC   Pancreas, Spleen, and Testis;
RX   PubMed=21183079; DOI=10.1016/j.cell.2010.12.001;
RA   Huttlin E.L., Jedrychowski M.P., Elias J.E., Goswami T., Rad R.,
RA   Beausoleil S.A., Villen J., Haas W., Sowa M.E., Gygi S.P.;
RT   "A tissue-specific atlas of mouse protein phosphorylation and expression.";
RL   Cell 143:1174-1189(2010).
RN   [7]
RP   ACETYLATION AT LYS-179; LYS-182; LYS-250; LYS-335; LYS-480; LYS-485;
RP   LYS-498; LYS-547; LYS-550; LYS-598; LYS-608; LYS-624 AND LYS-633, AND
RP   DEACETYLATION BY SIRT3.
RX   PubMed=21858060; DOI=10.1371/journal.pone.0023295;
RA   Finley L.W., Haas W., Desquiret-Dumas V., Wallace D.C., Procaccio V.,
RA   Gygi S.P., Haigis M.C.;
RT   "Succinate dehydrogenase is a direct target of sirtuin 3 deacetylase
RT   activity.";
RL   PLoS ONE 6:E23295-E23295(2011).
RN   [8]
RP   ACETYLATION [LARGE SCALE ANALYSIS] AT LYS-179 AND LYS-547, SUCCINYLATION
RP   [LARGE SCALE ANALYSIS] AT LYS-179; LYS-250; LYS-335; LYS-485; LYS-498;
RP   LYS-538; LYS-547 AND LYS-615, AND IDENTIFICATION BY MASS SPECTROMETRY
RP   [LARGE SCALE ANALYSIS].
RC   TISSUE=Embryonic fibroblast, and Liver;
RX   PubMed=23806337; DOI=10.1016/j.molcel.2013.06.001;
RA   Park J., Chen Y., Tishkoff D.X., Peng C., Tan M., Dai L., Xie Z., Zhang Y.,
RA   Zwaans B.M., Skinner M.E., Lombard D.B., Zhao Y.;
RT   "SIRT5-mediated lysine desuccinylation impacts diverse metabolic
RT   pathways.";
RL   Mol. Cell 50:919-930(2013).
RN   [9]
RP   ACETYLATION [LARGE SCALE ANALYSIS] AT LYS-167; LYS-179; LYS-182; LYS-335;
RP   LYS-423; LYS-498; LYS-517; LYS-538; LYS-547; LYS-608; LYS-636 AND LYS-647,
RP   AND IDENTIFICATION BY MASS SPECTROMETRY [LARGE SCALE ANALYSIS].
RC   TISSUE=Liver;
RX   PubMed=23576753; DOI=10.1073/pnas.1302961110;
RA   Rardin M.J., Newman J.C., Held J.M., Cusack M.P., Sorensen D.J., Li B.,
RA   Schilling B., Mooney S.D., Kahn C.R., Verdin E., Gibson B.W.;
RT   "Label-free quantitative proteomics of the lysine acetylome in mitochondria
RT   identifies substrates of SIRT3 in metabolic pathways.";
RL   Proc. Natl. Acad. Sci. U.S.A. 110:6601-6606(2013).
CC   -!- FUNCTION: Flavoprotein (FP) subunit of succinate dehydrogenase (SDH)
CC       that is involved in complex II of the mitochondrial electron transport
CC       chain and is responsible for transferring electrons from succinate to
CC       ubiquinone (coenzyme Q). Can act as a tumor suppressor.
CC       {ECO:0000250|UniProtKB:P31040}.
CC   -!- CATALYTIC ACTIVITY:
CC       Reaction=a quinone + succinate = a quinol + fumarate;
CC         Xref=Rhea:RHEA:40523, ChEBI:CHEBI:24646, ChEBI:CHEBI:29806,
CC         ChEBI:CHEBI:30031, ChEBI:CHEBI:132124; EC=1.3.5.1;
CC         Evidence={ECO:0000250|UniProtKB:P31040};
CC   -!- COFACTOR:
CC       Name=FAD; Xref=ChEBI:CHEBI:57692;
CC         Evidence={ECO:0000250|UniProtKB:Q0QF01};
CC   -!- PATHWAY: Carbohydrate metabolism; tricarboxylic acid cycle; fumarate
CC       from succinate (eukaryal route): step 1/1.
CC       {ECO:0000250|UniProtKB:P31040}.
CC   -!- SUBUNIT: Component of complex II composed of four subunits: the
CC       flavoprotein (FP) SDHA, iron-sulfur protein (IP) SDHB, and a cytochrome
CC       b560 composed of SDHC and SDHD (By similarity). Interacts with
CC       SDHAF2/SDH5; interaction is required for FAD attachment (By
CC       similarity). Interacts with TRAP1 (By similarity). Interacts with LACC1
CC       (By similarity). {ECO:0000250|UniProtKB:P31040,
CC       ECO:0000250|UniProtKB:Q0QF01}.
CC   -!- SUBCELLULAR LOCATION: Mitochondrion inner membrane
CC       {ECO:0000250|UniProtKB:Q0QF01}; Peripheral membrane protein
CC       {ECO:0000250|UniProtKB:Q0QF01}; Matrix side
CC       {ECO:0000250|UniProtKB:Q0QF01}.
CC   -!- PTM: Acetylation of Lys-498 and Lys-538 is observed in liver
CC       mitochondria from fasted mice but not from fed mice. Deacetylated by
CC       SIRT3. {ECO:0000269|PubMed:21858060}.
CC   -!- PTM: Phosphorylation at Tyr-215 is important for efficient electron
CC       transfer in complex II and the prevention of ROS generation.
CC       {ECO:0000250|UniProtKB:P31040}.
CC   -!- SIMILARITY: Belongs to the FAD-dependent oxidoreductase 2 family.
CC       FRD/SDH subfamily. {ECO:0000305}.
CC   ---------------------------------------------------------------------------
CC   Copyrighted by the UniProt Consortium, see https://www.uniprot.org/terms
CC   Distributed under the Creative Commons Attribution (CC BY 4.0) License
CC   ---------------------------------------------------------------------------
DR   EMBL; AK029520; BAC26491.1; -; mRNA.
DR   EMBL; AK034928; BAC28884.1; -; mRNA.
DR   EMBL; AK049590; BAC33831.1; -; mRNA.
DR   EMBL; AK050475; BAC34276.1; -; mRNA.
DR   EMBL; AK075990; BAC36101.1; -; mRNA.
DR   EMBL; AK145923; BAE26754.1; -; mRNA.
DR   EMBL; AK147286; BAE27822.1; -; mRNA.
DR   EMBL; AK147624; BAE28032.1; -; mRNA.
DR   EMBL; AK153085; BAE31710.1; -; mRNA.
DR   EMBL; AK162148; BAE36754.1; -; mRNA.
DR   EMBL; AK169254; BAE41018.1; -; mRNA.
DR   EMBL; AK004362; BAE43173.1; -; mRNA.
DR   EMBL; BC011301; AAH11301.1; -; mRNA.
DR   EMBL; BC031849; AAH31849.1; -; mRNA.
DR   EMBL; DQ402975; ABD77308.1; -; mRNA.
DR   EMBL; AF095938; AAC72373.1; -; mRNA.
DR   CCDS; CCDS26643.1; -.
DR   RefSeq; NP_075770.1; NM_023281.1.
DR   AlphaFoldDB; Q8K2B3; -.
DR   SMR; Q8K2B3; -.
DR   BioGRID; 211828; 75.
DR   ComplexPortal; CPX-562; Mitochondrial respiratory chain complex II.
DR   CORUM; Q8K2B3; -.
DR   IntAct; Q8K2B3; 22.
DR   MINT; Q8K2B3; -.
DR   STRING; 10090.ENSMUSP00000022062; -.
DR   CarbonylDB; Q8K2B3; -.
DR   GlyGen; Q8K2B3; 1 site, 1 O-linked glycan (1 site).
DR   iPTMnet; Q8K2B3; -.
DR   PhosphoSitePlus; Q8K2B3; -.
DR   SwissPalm; Q8K2B3; -.
DR   REPRODUCTION-2DPAGE; Q8K2B3; -.
DR   EPD; Q8K2B3; -.
DR   jPOST; Q8K2B3; -.
DR   MaxQB; Q8K2B3; -.
DR   PaxDb; Q8K2B3; -.
DR   PeptideAtlas; Q8K2B3; -.
DR   ProteomicsDB; 255377; -.
DR   DNASU; 66945; -.
DR   Ensembl; ENSMUST00000022062; ENSMUSP00000022062; ENSMUSG00000021577.
DR   GeneID; 66945; -.
DR   KEGG; mmu:66945; -.
DR   UCSC; uc007rfa.1; mouse.
DR   AGR; MGI:1914195; -.
DR   CTD; 6389; -.
DR   MGI; MGI:1914195; Sdha.
DR   VEuPathDB; HostDB:ENSMUSG00000021577; -.
DR   eggNOG; KOG2403; Eukaryota.
DR   GeneTree; ENSGT00910000144277; -.
DR   HOGENOM; CLU_014312_6_1_1; -.
DR   InParanoid; Q8K2B3; -.
DR   OMA; DPIPIQP; -.
DR   OrthoDB; 551958at2759; -.
DR   PhylomeDB; Q8K2B3; -.
DR   TreeFam; TF300763; -.
DR   Reactome; R-MMU-71403; Citric acid cycle (TCA cycle).
DR   UniPathway; UPA00223; UER01006.
DR   BioGRID-ORCS; 66945; 18 hits in 80 CRISPR screens.
DR   ChiTaRS; Sdha; mouse.
DR   PRO; PR:Q8K2B3; -.
DR   Proteomes; UP000000589; Chromosome 13.
DR   RNAct; Q8K2B3; protein.
DR   Bgee; ENSMUSG00000021577; Expressed in heart right ventricle and 270 other tissues.
DR   ExpressionAtlas; Q8K2B3; baseline and differential.
DR   Genevisible; Q8K2B3; MM.
DR   GO; GO:0005743; C:mitochondrial inner membrane; HDA:MGI.
DR   GO; GO:0005749; C:mitochondrial respiratory chain complex II, succinate dehydrogenase complex (ubiquinone); ISS:UniProtKB.
DR   GO; GO:0005739; C:mitochondrion; IDA:MGI.
DR   GO; GO:0043209; C:myelin sheath; HDA:UniProtKB.
DR   GO; GO:0005730; C:nucleolus; ISO:MGI.
DR   GO; GO:0045257; C:succinate dehydrogenase complex (ubiquinone); IDA:MGI.
DR   GO; GO:0009055; F:electron transfer activity; IBA:GO_Central.
DR   GO; GO:0050660; F:flavin adenine dinucleotide binding; IBA:GO_Central.
DR   GO; GO:0102040; F:fumarate reductase (menaquinone); IEA:UniProtKB-EC.
DR   GO; GO:0008177; F:succinate dehydrogenase (ubiquinone) activity; ISS:UniProtKB.
DR   GO; GO:0000104; F:succinate dehydrogenase activity; ISO:MGI.
DR   GO; GO:0006121; P:mitochondrial electron transport, succinate to ubiquinone; IBA:GO_Central.
DR   GO; GO:0007399; P:nervous system development; ISO:MGI.
DR   GO; GO:0042776; P:proton motive force-driven mitochondrial ATP synthesis; NAS:ComplexPortal.
DR   GO; GO:0022904; P:respiratory electron transport chain; ISS:UniProtKB.
DR   GO; GO:0006105; P:succinate metabolic process; ISS:UniProtKB.
DR   GO; GO:0006099; P:tricarboxylic acid cycle; NAS:ComplexPortal.
DR   Gene3D; 3.50.50.60; FAD/NAD(P)-binding domain; 1.
DR   Gene3D; 1.20.58.100; Fumarate reductase/succinate dehydrogenase flavoprotein-like, C-terminal domain; 1.
DR   Gene3D; 4.10.80.40; succinate dehydrogenase protein domain; 1.
DR   Gene3D; 3.90.700.10; Succinate dehydrogenase/fumarate reductase flavoprotein, catalytic domain; 1.
DR   InterPro; IPR003953; FAD-binding_2.
DR   InterPro; IPR036188; FAD/NAD-bd_sf.
DR   InterPro; IPR003952; FRD_SDH_FAD_BS.
DR   InterPro; IPR037099; Fum_R/Succ_DH_flav-like_C_sf.
DR   InterPro; IPR015939; Fum_Rdtase/Succ_DH_flav-like_C.
DR   InterPro; IPR030664; SdhA/FrdA/AprA.
DR   InterPro; IPR027477; Succ_DH/fumarate_Rdtase_cat_sf.
DR   InterPro; IPR011281; Succ_DH_flav_su_fwd.
DR   InterPro; IPR014006; Succ_Dhase_FrdA_Gneg.
DR   PANTHER; PTHR11632; SUCCINATE DEHYDROGENASE 2 FLAVOPROTEIN SUBUNIT; 1.
DR   PANTHER; PTHR11632:SF51; SUCCINATE DEHYDROGENASE [UBIQUINONE] FLAVOPROTEIN SUBUNIT, MITOCHONDRIAL; 1.
DR   Pfam; PF00890; FAD_binding_2; 1.
DR   Pfam; PF02910; Succ_DH_flav_C; 1.
DR   PIRSF; PIRSF000171; SDHA_APRA_LASPO; 1.
DR   SUPFAM; SSF51905; FAD/NAD(P)-binding domain; 1.
DR   SUPFAM; SSF46977; Succinate dehydrogenase/fumarate reductase flavoprotein C-terminal domain; 1.
DR   SUPFAM; SSF56425; Succinate dehydrogenase/fumarate reductase flavoprotein, catalytic domain; 1.
DR   PROSITE; PS00504; FRD_SDH_FAD_BINDING; 1.
DR   TIGRFAMs; TIGR01816; sdhA_forward; 1.
DR   TIGRFAMs; TIGR01812; sdhA_frdA_Gneg; 1.
PE   1: Evidence at protein level;
KW   Acetylation; Direct protein sequencing; Electron transport; FAD;
KW   Flavoprotein; Membrane; Mitochondrion; Mitochondrion inner membrane;
KW   Oxidoreductase; Phosphoprotein; Reference proteome; Transit peptide;
KW   Transport; Tricarboxylic acid cycle; Tumor suppressor.
FT   TRANSIT         1..42
FT                   /note="Mitochondrion"
FT                   /evidence="ECO:0000250|UniProtKB:Q0QF01"
FT   CHAIN           43..664
FT                   /note="Succinate dehydrogenase [ubiquinone] flavoprotein
FT                   subunit, mitochondrial"
FT                   /id="PRO_0000010337"
FT   ACT_SITE        340
FT                   /note="Proton acceptor"
FT                   /evidence="ECO:0000250|UniProtKB:Q9YHT1"
FT   BINDING         68..73
FT                   /ligand="FAD"
FT                   /ligand_id="ChEBI:CHEBI:57692"
FT                   /evidence="ECO:0000250|UniProtKB:Q0QF01"
FT   BINDING         91..106
FT                   /ligand="FAD"
FT                   /ligand_id="ChEBI:CHEBI:57692"
FT                   /evidence="ECO:0000250|UniProtKB:Q0QF01"
FT   BINDING         275
FT                   /ligand="FAD"
FT                   /ligand_id="ChEBI:CHEBI:57692"
FT                   /evidence="ECO:0000250|UniProtKB:Q0QF01"
FT   BINDING         296
FT                   /ligand="substrate"
FT                   /evidence="ECO:0000250|UniProtKB:Q0QF01"
FT   BINDING         308
FT                   /ligand="substrate"
FT                   /evidence="ECO:0000250|UniProtKB:Q0QF01"
FT   BINDING         407
FT                   /ligand="substrate"
FT                   /evidence="ECO:0000250|UniProtKB:Q0QF01"
FT   BINDING         440
FT                   /ligand="FAD"
FT                   /ligand_id="ChEBI:CHEBI:57692"
FT                   /evidence="ECO:0000250|UniProtKB:Q0QF01"
FT   BINDING         451
FT                   /ligand="substrate"
FT                   /evidence="ECO:0000250|UniProtKB:Q0QF01"
FT   BINDING         456..457
FT                   /ligand="FAD"
FT                   /ligand_id="ChEBI:CHEBI:57692"
FT                   /evidence="ECO:0000250|UniProtKB:Q0QF01"
FT   MOD_RES         99
FT                   /note="Tele-8alpha-FAD histidine"
FT                   /evidence="ECO:0000250|UniProtKB:Q0QF01"
FT   MOD_RES         167
FT                   /note="N6-acetyllysine"
FT                   /evidence="ECO:0007744|PubMed:23576753"
FT   MOD_RES         179
FT                   /note="N6-acetyllysine; alternate"
FT                   /evidence="ECO:0000269|PubMed:21858060,
FT                   ECO:0007744|PubMed:23576753, ECO:0007744|PubMed:23806337"
FT   MOD_RES         179
FT                   /note="N6-succinyllysine; alternate"
FT                   /evidence="ECO:0007744|PubMed:23806337"
FT   MOD_RES         182
FT                   /note="N6-acetyllysine"
FT                   /evidence="ECO:0000269|PubMed:21858060,
FT                   ECO:0007744|PubMed:23576753"
FT   MOD_RES         215
FT                   /note="Phosphotyrosine; by SRC"
FT                   /evidence="ECO:0000250|UniProtKB:P31040"
FT   MOD_RES         250
FT                   /note="N6-acetyllysine; alternate"
FT                   /evidence="ECO:0000269|PubMed:21858060"
FT   MOD_RES         250
FT                   /note="N6-succinyllysine; alternate"
FT                   /evidence="ECO:0007744|PubMed:23806337"
FT   MOD_RES         335
FT                   /note="N6-acetyllysine; alternate"
FT                   /evidence="ECO:0000269|PubMed:21858060,
FT                   ECO:0007744|PubMed:23576753"
FT   MOD_RES         335
FT                   /note="N6-succinyllysine; alternate"
FT                   /evidence="ECO:0007744|PubMed:23806337"
FT   MOD_RES         423
FT                   /note="N6-acetyllysine"
FT                   /evidence="ECO:0007744|PubMed:23576753"
FT   MOD_RES         480
FT                   /note="N6-acetyllysine"
FT                   /evidence="ECO:0000269|PubMed:21858060"
FT   MOD_RES         485
FT                   /note="N6-acetyllysine; alternate"
FT                   /evidence="ECO:0000269|PubMed:21858060"
FT   MOD_RES         485
FT                   /note="N6-succinyllysine; alternate"
FT                   /evidence="ECO:0007744|PubMed:23806337"
FT   MOD_RES         498
FT                   /note="N6-acetyllysine; alternate"
FT                   /evidence="ECO:0000269|PubMed:21858060,
FT                   ECO:0007744|PubMed:23576753"
FT   MOD_RES         498
FT                   /note="N6-succinyllysine; alternate"
FT                   /evidence="ECO:0007744|PubMed:23806337"
FT   MOD_RES         517
FT                   /note="N6-acetyllysine"
FT                   /evidence="ECO:0007744|PubMed:23576753"
FT   MOD_RES         538
FT                   /note="N6-acetyllysine; alternate"
FT                   /evidence="ECO:0007744|PubMed:23576753"
FT   MOD_RES         538
FT                   /note="N6-succinyllysine; alternate"
FT                   /evidence="ECO:0007744|PubMed:23806337"
FT   MOD_RES         547
FT                   /note="N6-acetyllysine; alternate"
FT                   /evidence="ECO:0000269|PubMed:21858060,
FT                   ECO:0007744|PubMed:23576753, ECO:0007744|PubMed:23806337"
FT   MOD_RES         547
FT                   /note="N6-succinyllysine; alternate"
FT                   /evidence="ECO:0007744|PubMed:23806337"
FT   MOD_RES         550
FT                   /note="N6-acetyllysine"
FT                   /evidence="ECO:0000269|PubMed:21858060"
FT   MOD_RES         598
FT                   /note="N6-acetyllysine"
FT                   /evidence="ECO:0000269|PubMed:21858060"
FT   MOD_RES         608
FT                   /note="N6-acetyllysine"
FT                   /evidence="ECO:0000269|PubMed:21858060,
FT                   ECO:0007744|PubMed:23576753"
FT   MOD_RES         615
FT                   /note="N6-succinyllysine"
FT                   /evidence="ECO:0007744|PubMed:23806337"
FT   MOD_RES         624
FT                   /note="N6-acetyllysine"
FT                   /evidence="ECO:0000269|PubMed:21858060"
FT   MOD_RES         633
FT                   /note="N6-acetyllysine"
FT                   /evidence="ECO:0000269|PubMed:21858060"
FT   MOD_RES         636
FT                   /note="N6-acetyllysine"
FT                   /evidence="ECO:0007744|PubMed:23576753"
FT   MOD_RES         647
FT                   /note="N6-acetyllysine"
FT                   /evidence="ECO:0007744|PubMed:23576753"
FT   CONFLICT        69
FT                   /note="A -> V (in Ref. 1; BAE26754)"
FT                   /evidence="ECO:0000305"
FT   CONFLICT        246
FT                   /note="R -> Q (in Ref. 1; BAE26754)"
FT                   /evidence="ECO:0000305"
FT   CONFLICT        428
FT                   /note="Q -> R (in Ref. 1; BAE26754)"
FT                   /evidence="ECO:0000305"
FT   CONFLICT        501
FT                   /note="F -> L (in Ref. 4; ABD77308)"
FT                   /evidence="ECO:0000305"
FT   CONFLICT        517
FT                   /note="K -> M (in Ref. 4; ABD77308)"
FT                   /evidence="ECO:0000305"
FT   CONFLICT        571
FT                   /note="L -> M (in Ref. 4; ABD77308)"
FT                   /evidence="ECO:0000305"
SQ   SEQUENCE   664 AA;  72585 MW;  DDCE1535163C9449 CRC64;
     MAGVGAVSRL LRGRRLALTG AWPGTLQKQT CGFHFSVGEN KKASAKVSDA ISTQYPVVDH
     EFDAVVVGAG GAGLRAAFGL SEAGFNTACL TKLFPTRSHT VAAQGGINAA LGNMEEDNWR
     WHFYDTVKGS DWLGDQDAIH YMTEQAPASV VELENYGMPF SRTEDGKIYQ RAFGGQSLKF
     GKGGQAHRCC CVADRTGHSL LHTLYGRSLR YDTSYFVEYF ALDLLMENGE CRGVIALCIE
     DGSIHRIRAK NTVIATGGYG RTYFSCTSAH TSTGDGTAMV TRAGLPCQDL EFVQFHPTGI
     YGAGCLITEG CRGEGGILIN SQGERFMERY APVAKDLASR DVVSRSMTLE IREGRGCGPE
     KDHVYLQLHH LPPEQLATRL PGISETAMIF AGVDVTKEPI PVLPTVHYNM GGIPTNYKGQ
     VLKHVNGQDQ IVPGLYACGE AACASVHGAN RLGANSLLDL VVFGRACALS IAESCRPGDK
     VPSIKANAGE ESVMNLDKLR FADGSIRTSE LRLNMQKSMQ NHAAVFRVGS VLQEGCEKIS
     QLYGDLKHLK TFDRGMVWNT DLVETLELQN LMLCALQTIY GAEARKESRG AHAREDYKVR
     VDEYDYSKPI QGQQKKPFGE HWRKHTLSYV DIKTGKVTLE YRPVIDKTLN EADCATVPPA
     IRSY
//
```

**Mascot:** http://www.matrixscience.com/


HNE (C) (+156.1150)
