# Supplementary material for: A common East-Asian ALDH2 mutation causes metabolic disorders and the therapeutic effect of ALDH2 activators
Source: Nat Commun. 2023 Sep 25;14:5971. doi: 10.1038/s41467-023-41570-6 (PMC10520061; doi:10.1038/s41467-023-41570-6)
Supplement: Supplementary file 4 — Supplementary Data 1 [file 41467_2023_41570_MOESM4_ESM.zip › Table S5c/Q99JY0/Q99JY0_KIO-1_H133_C139_C158_C459.html]

Mascot Search Results: Q99JY0
 

# MASCOT Search Results

## Protein View: Q99JY0

### Trifunctional enzyme subunit beta, mitochondrial OS=Mus musculus OX=10090 GN=Hadhb PE=1 SV=1

|  |  |
| --- | --- |
| Database: | Mouse\_UniProt\_proteomes |
| Score: | 4458 |
| Monoisotopic mass (M_r_): | 51639 |
| Calculated pI: | 9.43 |

Sequence similarity is available as an NCBI BLAST search of Q99JY0 against nr.

### Search parameters

|  |  |
| --- | --- |
| MS data file: | `D:\LCMSMS\2023 Users' data\230529-1\230529-1-KIO-1.raw` |
| Enzyme: | Trypsin/P: cuts C-term side of KR. |
| Fixed modifications: | Carbamidomethyl (C) |
| Variable modifications: | Deamidated (NQ), HNE (C), HNE (H), HNE (K), Oxidation (M) |

### Protein sequence coverage: 59%

Matched peptides shown in ***bold red***.

|  |  |  |  |  |  |
| --- | --- | --- | --- | --- | --- |
| `1` | `MTTILTSTFR` | `NLSTTSKWAL` | `RSSIRPLSCS` | `SQLHSAPAVQ` | `TKSKKTLAKP` |
| `51` | `NMKNIVVVEG` | `VRIPFLLSGT` | `SYKDLMPHDL` | `ARAALSGLLH` | `RTNIPKDVVD` |
| `101` | `YIIFGTVIQE` | `VKTSNVAREA` | `ALGAGFSDKT` | `PAHTVTMACI` | `SSNQAMTTAV` |
| `151` | `GLIASGQCDV` | `VVAGGVELMS` | `DVPIRHSRNM` | `RKMMLDLNKA` | `KTLGQRLSLL` |
| `201` | `SKFRLNFLSP` | `ELPAVAEFST` | `NETMGHSADR` | `LAAAFAVSRM` | `EQDEYALRSH` |
| `251` | `SLAKKAQDEG` | `HLSDIVPFKV` | `PGKDTVTKDN` | `GIRPSSLEQM` | `AKLKPAFIKP` |
| `301` | `YGTVTAANSS` | `FLTDGASAML` | `IMSEDRALAM` | `GYKPKAYLRD` | `FIYVSQDPKD` |
| `351` | `QLLLGPTYAT` | `PKVLEKAGLT` | `MNDIDAFEFH` | `EAFSGQILAN` | `FKAMDSDWFA` |
| `401` | `QNYMGRKTKV` | `GSPPLEKFNI` | `WGGSLSLGHP` | `FGATGCRLVM` | `AAANRLRKDG` |
| `451` | `GQYALVAACA` | `AGGQGHAMIV` | `EAYPK` |  |  |

Unformatted sequence string: 475 residues (for pasting into other applications).

|  |  |  |  |
| --- | --- | --- | --- |
| Sort by | residue number | increasing mass | decreasing mass |
| Show | matched peptides only | predicted peptides also |  |

| Query | Start | – | End | Observed | Mr(expt) | Mr(calc) | ppm | M | Score | Expect | Rank | U | Peptide |
| --- | --- | --- | --- | --- | --- | --- | --- | --- | --- | --- | --- | --- | --- |
| 12186 | 54 | – | 62 | 492.7960 | 983.5774 | 983.5764 | 0.99 | 0 | 36 | 0.00038 | 1Score **> 27** indicates **identity** Score **> 15** indicates **homology** | U | K.NIVVVEGVR.I |
| 12187 | 54 | – | 62 | 492.7961 | 983.5776 | 983.5764 | 1.20 | 0 | 48 | 9.7e-05 | 1Score **> 27** indicates **identity** Score **> 20** indicates **homology** | U | K.NIVVVEGVR.I |
| 12188 | 54 | – | 62 | 492.7963 | 983.5780 | 983.5764 | 1.63 | 0 | 28 | 0.025 | 1Score **> 29** indicates **identity** Score **> 24** indicates **homology** | U | K.NIVVVEGVR.I |
| 12189 | 54 | – | 62 | 492.7965 | 983.5785 | 983.5764 | 2.14 | 0 | 43 | 0.0015 | 1Score **> 29** indicates **identity** Score **> 27** indicates **homology** | U | K.NIVVVEGVR.I |
| 138640 | 54 | – | 73 | 731.0888 | 2190.2446 | 2190.2412 | 1.52 | 1 | 37 | 0.00033 | 1Score **> 33** indicates **identity** Score **> 15** indicates **homology** | U | K.NIVVVEGVRIPFLLSGTSYK.D |
| 138730 | 54 | – | 73 | 731.4196 | 2191.2370 | 2191.2252 | 5.36 | 1 | 28 | 0.0021 | 1Score **> 33** indicates **identity** Score **> 14** indicates **homology** | U | K.NIVVVEGVRIPFLLSGTSYK.D  + Deamidated (NQ) |
| 32769 | 63 | – | 73 | 613.3454 | 1224.6762 | 1224.6754 | 0.63 | 0 | 50 | 2.1e-05 | 1Score **> 32** indicates **identity** Score **> 16** indicates **homology** | U | R.IPFLLSGTSYK.D |
| 32770 | 63 | – | 73 | 613.3456 | 1224.6766 | 1224.6754 | 0.97 | 0 | 62 | 1.4e-06 | 1Score **> 32** indicates **identity** Score **> 16** indicates **homology** | U | R.IPFLLSGTSYK.D |
| 32771 | 63 | – | 73 | 613.3461 | 1224.6776 | 1224.6754 | 1.77 | 0 | 60 | 2.4e-06 | 1Score **> 32** indicates **identity** Score **> 16** indicates **homology** | U | R.IPFLLSGTSYK.D |
| 32772 | 63 | – | 73 | 613.3461 | 1224.6777 | 1224.6754 | 1.85 | 0 | 36 | 0.00041 | 1Score **> 32** indicates **identity** Score **> 15** indicates **homology** | U | R.IPFLLSGTSYK.D |
| 32773 | 63 | – | 73 | 613.3464 | 1224.6783 | 1224.6754 | 2.32 | 0 | 62 | 1.5e-06 | 1Score **> 32** indicates **identity** Score **> 16** indicates **homology** | U | R.IPFLLSGTSYK.D |
| 144979 | 63 | – | 82 | 569.3038 | 2273.1861 | 2273.1878 | -0.73 | 1 | 44 | 6.9e-05 | 1Score **> 37** indicates **identity** Score **> 15** indicates **homology** | U | R.IPFLLSGTSYKDLMPHDLAR.A |
| 144980 | 63 | – | 82 | 758.7360 | 2273.1862 | 2273.1878 | -0.69 | 1 | 52 | 1.3e-05 | 1Score **> 37** indicates **identity** Score **> 16** indicates **homology** | U | R.IPFLLSGTSYKDLMPHDLAR.A |
| 144982 | 63 | – | 82 | 758.7368 | 2273.1885 | 2273.1878 | 0.29 | 1 | 37 | 0.0016 | 1Score **> 37** indicates **identity** Score **> 22** indicates **homology** | U | R.IPFLLSGTSYKDLMPHDLAR.A |
| 144983 | 63 | – | 82 | 569.3044 | 2273.1886 | 2273.1878 | 0.33 | 1 | 46 | 5.3e-05 | 1Score **> 37** indicates **identity** Score **> 15** indicates **homology** | U | R.IPFLLSGTSYKDLMPHDLAR.A |
| 144984 | 63 | – | 82 | 758.7371 | 2273.1894 | 2273.1878 | 0.69 | 1 | 54 | 9.6e-06 | 1Score **> 37** indicates **identity** Score **> 16** indicates **homology** | U | R.IPFLLSGTSYKDLMPHDLAR.A |
| 144985 | 63 | – | 82 | 569.3049 | 2273.1905 | 2273.1878 | 1.17 | 1 | 46 | 5.2e-05 | 1Score **> 37** indicates **identity** Score **> 15** indicates **homology** | U | R.IPFLLSGTSYKDLMPHDLAR.A |
| 144986 | 63 | – | 82 | 569.3049 | 2273.1907 | 2273.1878 | 1.27 | 1 | 33 | 0.00087 | 1Score **> 37** indicates **identity** Score **> 15** indicates **homology** | U | R.IPFLLSGTSYKDLMPHDLAR.A |
| 144987 | 63 | – | 82 | 758.7398 | 2273.1975 | 2273.1878 | 4.28 | 1 | 18 | 0.022 | 1Score **> 37** indicates **identity** Score **> 14** indicates **homology** | U | R.IPFLLSGTSYKDLMPHDLAR.A |
| 145859 | 63 | – | 82 | 573.3072 | 2289.1999 | 2289.1827 | 7.49 | 1 | 15 | 0.036 | 1Score **> 37** indicates **identity** Score **> 13** indicates **homology** | U | R.IPFLLSGTSYKDLMPHDLAR.A  + Oxidation (M) |
| 17675 | 74 | – | 82 | 356.5148 | 1066.5226 | 1066.5229 | -0.31 | 0 | 18 | 0.021 | 1Score **> 30** indicates **identity** Score **> 14** indicates **homology** | U | K.DLMPHDLAR.A |
| 9547 | 83 | – | 91 | 469.2818 | 936.5490 | 936.5505 | -1.62 | 0 | 46 | 0.00011 | 1Score **> 23** indicates **identity** Score **> 19** indicates **homology** | U | R.AALSGLLHR.T |
| 9548 | 83 | – | 91 | 469.2826 | 936.5507 | 936.5505 | 0.26 | 0 | 68 | 5.7e-07 | 1Score **> 23** indicates **identity** Score **> 18** indicates **homology** | U | R.AALSGLLHR.T |
| 9549 | 83 | – | 91 | 469.2827 | 936.5509 | 936.5505 | 0.41 | 0 | 73 | 1.5e-07 | 1Score **> 23** indicates **identity** Score **> 17** indicates **homology** | U | R.AALSGLLHR.T |
| 9550 | 83 | – | 91 | 469.2828 | 936.5511 | 936.5505 | 0.67 | 0 | 39 | 0.00024 | 1Score **> 23** indicates **identity** Score **> 15** indicates **homology** | U | R.AALSGLLHR.T |
| 9551 | 83 | – | 91 | 469.2829 | 936.5512 | 936.5505 | 0.75 | 0 | 64 | 4.6e-06 | 1Score **> 23** indicates **identity** Score **> 23** indicates **homology** | U | R.AALSGLLHR.T |
| 9552 | 83 | – | 91 | 469.2830 | 936.5515 | 936.5505 | 1.13 | 0 | 64 | 9.4e-07 | 1Score **> 23** indicates **identity** Score **> 17** indicates **homology** | U | R.AALSGLLHR.T |
| 9553 | 83 | – | 91 | 469.2834 | 936.5522 | 936.5505 | 1.84 | 0 | 53 | 2.7e-05 | 1Score **> 23** indicates **identity** Score **> 20** indicates **homology** | U | R.AALSGLLHR.T |
| 9554 | 83 | – | 91 | 469.2834 | 936.5523 | 936.5505 | 1.99 | 0 | 58 | 3.4e-06 | 1Score **> 25** indicates **identity** Score **> 16** indicates **homology** | U | R.AALSGLLHR.T |
| 9555 | 83 | – | 91 | 469.2844 | 936.5542 | 936.5505 | 3.93 | 0 | 42 | 0.0001 | 1Score **> 25** indicates **identity** Score **> 15** indicates **homology** | U | R.AALSGLLHR.T |
| 152469 | 92 | – | 112 | 797.7734 | 2390.2985 | 2390.3097 | -4.71 | 1 | 26 | 0.0035 | 1Score **> 36** indicates **identity** Score **> 14** indicates **homology** | U | R.TNIPKDVVDYIIFGTVIQEVK.T |
| 152470 | 92 | – | 112 | 797.7746 | 2390.3019 | 2390.3097 | -3.28 | 1 | 28 | 0.0024 | 1Score **> 36** indicates **identity** Score **> 14** indicates **homology** | U | R.TNIPKDVVDYIIFGTVIQEVK.T |
| 152474 | 92 | – | 112 | 797.7773 | 2390.3102 | 2390.3097 | 0.19 | 1 | 61 | 1.8e-06 | 1Score **> 35** indicates **identity** Score **> 16** indicates **homology** | U | R.TNIPKDVVDYIIFGTVIQEVK.T |
| 152477 | 92 | – | 112 | 1196.1629 | 2390.3112 | 2390.3097 | 0.60 | 1 | 70 | 2.9e-07 | 1Score **> 35** indicates **identity** Score **> 17** indicates **homology** | U | R.TNIPKDVVDYIIFGTVIQEVK.T |
| 152479 | 92 | – | 112 | 797.7778 | 2390.3116 | 2390.3097 | 0.80 | 1 | 46 | 4.5e-05 | 1Score **> 35** indicates **identity** Score **> 15** indicates **homology** | U | R.TNIPKDVVDYIIFGTVIQEVK.T |
| 152480 | 92 | – | 112 | 1196.1631 | 2390.3117 | 2390.3097 | 0.83 | 1 | 107 | 1e-10 | 1Score **> 35** indicates **identity** Score **> 19** indicates **homology** | U | R.TNIPKDVVDYIIFGTVIQEVK.T |
| 152481 | 92 | – | 112 | 598.5853 | 2390.3119 | 2390.3097 | 0.91 | 1 | 46 | 5e-05 | 1Score **> 35** indicates **identity** Score **> 15** indicates **homology** | U | R.TNIPKDVVDYIIFGTVIQEVK.T |
| 152482 | 92 | – | 112 | 598.5853 | 2390.3120 | 2390.3097 | 0.94 | 1 | 47 | 4e-05 | 1Score **> 35** indicates **identity** Score **> 15** indicates **homology** | U | R.TNIPKDVVDYIIFGTVIQEVK.T |
| 152485 | 92 | – | 112 | 797.7783 | 2390.3130 | 2390.3097 | 1.38 | 1 | 55 | 6.5e-06 | 1Score **> 35** indicates **identity** Score **> 16** indicates **homology** | U | R.TNIPKDVVDYIIFGTVIQEVK.T |
| 152486 | 92 | – | 112 | 797.7784 | 2390.3134 | 2390.3097 | 1.53 | 1 | 61 | 2e-06 | 1Score **> 35** indicates **identity** Score **> 16** indicates **homology** | U | R.TNIPKDVVDYIIFGTVIQEVK.T |
| 152489 | 92 | – | 112 | 797.7793 | 2390.3162 | 2390.3097 | 2.70 | 1 | 31 | 0.0012 | 1Score **> 35** indicates **identity** Score **> 14** indicates **homology** | U | R.TNIPKDVVDYIIFGTVIQEVK.T |
| 152490 | 92 | – | 112 | 797.7794 | 2390.3163 | 2390.3097 | 2.75 | 1 | 42 | 0.00011 | 1Score **> 35** indicates **identity** Score **> 15** indicates **homology** | U | R.TNIPKDVVDYIIFGTVIQEVK.T |
| 152581 | 92 | – | 112 | 1196.6603 | 2391.3060 | 2391.2937 | 5.12 | 1 | 69 | 3.4e-07 | 1Score **> 35** indicates **identity** Score **> 17** indicates **homology** | U | R.TNIPKDVVDYIIFGTVIQEVK.T  + Deamidated (NQ) |
| 179139 | 92 | – | 118 | 755.6676 | 3018.6414 | 3018.6390 | 0.80 | 2 | 20 | 0.013 | 1Score **> 35** indicates **identity** Score **> 14** indicates **homology** | U | R.TNIPKDVVDYIIFGTVIQEVKTSNVAR.E |
| 104607 | 97 | – | 112 | 613.3354 | 1836.9844 | 1836.9873 | -1.58 | 0 | 39 | 0.00022 | 1Score **> 35** indicates **identity** Score **> 15** indicates **homology** | U | K.DVVDYIIFGTVIQEVK.T |
| 104609 | 97 | – | 112 | 919.5016 | 1836.9886 | 1836.9873 | 0.68 | 0 | 36 | 0.0014 | 1Score **> 35** indicates **identity** Score **> 19** indicates **homology** | U | K.DVVDYIIFGTVIQEVK.T |
| 104616 | 97 | – | 112 | 613.3391 | 1836.9953 | 1836.9873 | 4.36 | 0 | 25 | 0.0044 | 1Score **> 35** indicates **identity** Score **> 14** indicates **homology** | U | K.DVVDYIIFGTVIQEVK.T |
| 104617 | 97 | – | 112 | 613.3393 | 1836.9962 | 1836.9873 | 4.81 | 0 | 52 | 1.4e-05 | 1Score **> 35** indicates **identity** Score **> 16** indicates **homology** | U | K.DVVDYIIFGTVIQEVK.T |
| 104622 | 97 | – | 112 | 919.5065 | 1836.9985 | 1836.9873 | 6.10 | 0 | 35 | 0.00054 | 1Score **> 35** indicates **identity** Score **> 15** indicates **homology** | U | K.DVVDYIIFGTVIQEVK.T |
| 104736 | 97 | – | 112 | 613.6700 | 1837.9883 | 1837.9713 | 9.20 | 0 | 19 | 0.018 | 1Score **> 35** indicates **identity** Score **> 14** indicates **homology** | U | K.DVVDYIIFGTVIQEVK.T  + Deamidated (NQ) |
| 17532 | 119 | – | 129 | 533.2647 | 1064.5149 | 1064.5138 | 1.04 | 0 | 49 | 2.4e-05 | 1Score **> 31** indicates **identity** Score **> 16** indicates **homology** | U | R.EAALGAGFSDK.T |
| 198361 | 119 | – | 175 | 989.8194 | 5932.8727 | 5932.9030 | -5.09 | 1 | 20 | 0.013 | 1Score **> 35** indicates **identity** Score **> 14** indicates **homology** | U | R.EAALGAGFSDKTPAHTVTMACISSNQAMTTAVGLIASGQCDVVVAGGVELMSDVPIR.H  + HNE (H); Oxidation (M) |
| 198381 | 119 | – | 175 | 1196.0047 | 5974.9873 | 5974.9751 | 2.05 | 1 | 23 | 0.0077 | 1Score **> 35** indicates **identity** Score **> 14** indicates **homology** | U | R.EAALGAGFSDKTPAHTVTMACISSNQAMTTAVGLIASGQCDVVVAGGVELMSDVPIR.H  + 2 HNE (C); Oxidation (M) |
| 198383 | 119 | – | 175 | 997.0027 | 5975.9725 | 5975.9591 | 2.24 | 1 | 15 | 0.038 | 1Score **> 35** indicates **identity** Score **> 13** indicates **homology** | U | R.EAALGAGFSDKTPAHTVTMACISSNQAMTTAVGLIASGQCDVVVAGGVELMSDVPIR.H  + Deamidated (NQ); 2 HNE (C); Oxidation (M) |
| 198384 | 119 | – | 175 | 1196.2070 | 5975.9986 | 5975.9591 | 6.61 | 1 | 34 | 0.00063 | 1Score **> 35** indicates **identity** Score **> 15** indicates **homology** | U | R.EAALGAGFSDKTPAHTVTMACISSNQAMTTAVGLIASGQCDVVVAGGVELMSDVPIR.H  + Deamidated (NQ); 2 HNE (C); Oxidation (M) |
| 181882 | 203 | – | 230 | 784.8839 | 3135.5066 | 3135.5084 | -0.57 | 1 | 44 | 7.8e-05 | 1Score **> 37** indicates **identity** Score **> 15** indicates **homology** | U | K.FRLNFLSPELPAVAEFSTNETMGHSADR.L |
| 173031 | 205 | – | 230 | 945.4517 | 2833.3333 | 2833.3228 | 3.70 | 0 | 22 | 0.0081 | 1Score **> 35** indicates **identity** Score **> 14** indicates **homology** | U | R.LNFLSPELPAVAEFSTNETMGHSADR.L  + Deamidated (NQ) |
| 7872 | 231 | – | 239 | 453.2638 | 904.5131 | 904.5130 | 0.048 | 0 | 45 | 0.0004 | 1Score **> 29** indicates **identity** Score **> 24** indicates **homology** | U | R.LAAAFAVSR.M |
| 7873 | 231 | – | 239 | 453.2640 | 904.5135 | 904.5130 | 0.52 | 0 | 35 | 0.0026 | 1Score **> 29** indicates **identity** Score **> 22** indicates **homology** | U | R.LAAAFAVSR.M |
| 7874 | 231 | – | 239 | 453.2640 | 904.5135 | 904.5130 | 0.55 | 0 | 57 | 7.3e-05 | 1Score **> 29** indicates **identity** | U | R.LAAAFAVSR.M |
| 7876 | 231 | – | 239 | 453.2642 | 904.5138 | 904.5130 | 0.80 | 0 | 45 | 0.00083 | 1Score **> 29** indicates **identity** Score **> 27** indicates **homology** | U | R.LAAAFAVSR.M |
| 7877 | 231 | – | 239 | 453.2642 | 904.5138 | 904.5130 | 0.81 | 0 | 39 | 0.002 | 1Score **> 29** indicates **identity** Score **> 24** indicates **homology** | U | R.LAAAFAVSR.M |
| 7878 | 231 | – | 239 | 453.2642 | 904.5138 | 904.5130 | 0.86 | 0 | 66 | 1e-05 | 1Score **> 29** indicates **identity** | U | R.LAAAFAVSR.M |
| 7879 | 231 | – | 239 | 453.2642 | 904.5139 | 904.5130 | 0.95 | 0 | 54 | 0.00011 | 1Score **> 29** indicates **identity** Score **> 27** indicates **homology** | U | R.LAAAFAVSR.M |
| 7880 | 231 | – | 239 | 453.2643 | 904.5141 | 904.5130 | 1.13 | 0 | 42 | 0.003 | 1Score **> 29** indicates **identity** | U | R.LAAAFAVSR.M |
| 7881 | 231 | – | 239 | 453.2643 | 904.5141 | 904.5130 | 1.14 | 0 | 28 | 0.0066 | 1Score **> 29** indicates **identity** Score **> 19** indicates **homology** | U | R.LAAAFAVSR.M |
| 7882 | 231 | – | 239 | 453.2643 | 904.5141 | 904.5130 | 1.18 | 0 | 52 | 0.00023 | 1Score **> 29** indicates **identity** Score **> 28** indicates **homology** | U | R.LAAAFAVSR.M |
| 7883 | 231 | – | 239 | 453.2643 | 904.5141 | 904.5130 | 1.19 | 0 | 44 | 0.00076 | 1Score **> 29** indicates **identity** Score **> 26** indicates **homology** | U | R.LAAAFAVSR.M |
| 7884 | 231 | – | 239 | 453.2643 | 904.5141 | 904.5130 | 1.22 | 0 | 46 | 0.00084 | 1Score **> 29** indicates **identity** Score **> 27** indicates **homology** | U | R.LAAAFAVSR.M |
| 7885 | 231 | – | 239 | 453.2646 | 904.5147 | 904.5130 | 1.81 | 0 | 61 | 2.1e-05 | 1Score **> 27** indicates **identity** | U | R.LAAAFAVSR.M |
| 7887 | 231 | – | 239 | 453.2649 | 904.5153 | 904.5130 | 2.47 | 0 | 45 | 0.0011 | 1Score **> 28** indicates **identity** | U | R.LAAAFAVSR.M |
| 7888 | 231 | – | 239 | 453.2649 | 904.5153 | 904.5130 | 2.48 | 0 | 45 | 0.00067 | 1Score **> 28** indicates **identity** Score **> 26** indicates **homology** | U | R.LAAAFAVSR.M |
| 7889 | 231 | – | 239 | 453.2649 | 904.5153 | 904.5130 | 2.54 | 0 | 45 | 0.0004 | 1Score **> 28** indicates **identity** Score **> 24** indicates **homology** | U | R.LAAAFAVSR.M |
| 7890 | 231 | – | 239 | 453.2652 | 904.5159 | 904.5130 | 3.16 | 0 | 39 | 0.0019 | 1Score **> 28** indicates **identity** Score **> 24** indicates **homology** | U | R.LAAAFAVSR.M |
| 27249 | 240 | – | 248 | 585.7575 | 1169.5005 | 1169.5023 | -1.47 | 0 | 21 | 0.011 | 1Score **> 26** indicates **identity** Score **> 14** indicates **homology** | U | R.MEQDEYALR.S  + Oxidation (M) |
| 27250 | 240 | – | 248 | 585.7592 | 1169.5039 | 1169.5023 | 1.44 | 0 | 21 | 0.011 | 1Score **> 26** indicates **identity** Score **> 14** indicates **homology** | U | R.MEQDEYALR.S  + Oxidation (M) |
| 27251 | 240 | – | 248 | 585.7594 | 1169.5042 | 1169.5023 | 1.67 | 0 | 36 | 0.00042 | 1Score **> 26** indicates **identity** Score **> 15** indicates **homology** | U | R.MEQDEYALR.S  + Oxidation (M) |
| 27252 | 240 | – | 248 | 585.7597 | 1169.5048 | 1169.5023 | 2.20 | 0 | 18 | 0.02 | 1Score **> 26** indicates **identity** Score **> 14** indicates **homology** | U | R.MEQDEYALR.S  + Oxidation (M) |
| 126648 | 255 | – | 273 | 517.0364 | 2064.1164 | 2064.1004 | 7.75 | 2 | 20 | 0.013 | 1Score **> 35** indicates **identity** Score **> 14** indicates **homology** | U | K.KAQDEGHLSDIVPFKVPGK.D |
| 114216 | 256 | – | 273 | 485.0083 | 1936.0039 | 1936.0054 | -0.78 | 1 | 15 | 0.039 | 1Score **> 36** indicates **identity** Score **> 13** indicates **homology** | U | K.AQDEGHLSDIVPFKVPGK.D |
| 157796 | 256 | – | 278 | 827.7724 | 2480.2954 | 2480.2911 | 1.73 | 2 | 31 | 0.0012 | 1Score **> 37** indicates **identity** Score **> 14** indicates **homology** | U | K.AQDEGHLSDIVPFKVPGKDTVTK.D |
| 90684 | 336 | – | 349 | 572.2989 | 1713.8749 | 1713.8726 | 1.37 | 1 | 18 | 0.019 | 1Score **> 35** indicates **identity** Score **> 14** indicates **homology** | U | K.AYLRDFIYVSQDPK.D |
| 90689 | 336 | – | 349 | 572.2995 | 1713.8767 | 1713.8726 | 2.36 | 1 | 19 | 0.017 | 1Score **> 35** indicates **identity** Score **> 14** indicates **homology** | U | K.AYLRDFIYVSQDPK.D |
| 90690 | 336 | – | 349 | 857.9458 | 1713.8771 | 1713.8726 | 2.65 | 1 | 41 | 0.00028 | 1Score **> 35** indicates **identity** Score **> 18** indicates **homology** | U | K.AYLRDFIYVSQDPK.D |
| 181432 | 336 | – | 362 | 1038.2102 | 3111.6087 | 3111.6281 | -6.23 | 2 | 49 | 2.8e-05 | 1Score **> 37** indicates **identity** Score **> 16** indicates **homology** | U | K.AYLRDFIYVSQDPKDQLLLGPTYATPK.V |
| 181437 | 336 | – | 362 | 778.9127 | 3111.6216 | 3111.6281 | -2.07 | 2 | 15 | 0.042 | 1Score **> 37** indicates **identity** Score **> 13** indicates **homology** | U | K.AYLRDFIYVSQDPKDQLLLGPTYATPK.V |
| 181438 | 336 | – | 362 | 778.9135 | 3111.6249 | 3111.6281 | -1.00 | 2 | 27 | 0.0031 | 1Score **> 37** indicates **identity** Score **> 14** indicates **homology** | U | K.AYLRDFIYVSQDPKDQLLLGPTYATPK.V |
| 181439 | 336 | – | 362 | 778.9142 | 3111.6279 | 3111.6281 | -0.064 | 2 | 18 | 0.022 | 1Score **> 37** indicates **identity** Score **> 14** indicates **homology** | U | K.AYLRDFIYVSQDPKDQLLLGPTYATPK.V |
| 181440 | 336 | – | 362 | 778.9146 | 3111.6295 | 3111.6281 | 0.46 | 2 | 44 | 7.6e-05 | 1Score **> 37** indicates **identity** Score **> 15** indicates **homology** | U | K.AYLRDFIYVSQDPKDQLLLGPTYATPK.V |
| 181441 | 336 | – | 362 | 778.9147 | 3111.6297 | 3111.6281 | 0.54 | 2 | 42 | 0.00012 | 1Score **> 37** indicates **identity** Score **> 15** indicates **homology** | U | K.AYLRDFIYVSQDPKDQLLLGPTYATPK.V |
| 181442 | 336 | – | 362 | 1038.2173 | 3111.6301 | 3111.6281 | 0.64 | 2 | 79 | 3.6e-08 | 1Score **> 37** indicates **identity** Score **> 17** indicates **homology** | U | K.AYLRDFIYVSQDPKDQLLLGPTYATPK.V |
| 181444 | 336 | – | 362 | 1038.2178 | 3111.6316 | 3111.6281 | 1.13 | 2 | 76 | 7.6e-08 | 1Score **> 37** indicates **identity** Score **> 17** indicates **homology** | U | K.AYLRDFIYVSQDPKDQLLLGPTYATPK.V |
| 181445 | 336 | – | 362 | 778.9152 | 3111.6318 | 3111.6281 | 1.20 | 2 | 41 | 0.00014 | 1Score **> 37** indicates **identity** Score **> 15** indicates **homology** | U | K.AYLRDFIYVSQDPKDQLLLGPTYATPK.V |
| 181446 | 336 | – | 362 | 778.9153 | 3111.6319 | 3111.6281 | 1.24 | 2 | 45 | 6e-05 | 1Score **> 37** indicates **identity** Score **> 15** indicates **homology** | U | K.AYLRDFIYVSQDPKDQLLLGPTYATPK.V |
| 181447 | 336 | – | 362 | 1038.2187 | 3111.6343 | 3111.6281 | 2.00 | 2 | 75 | 1e-07 | 1Score **> 37** indicates **identity** Score **> 17** indicates **homology** | U | K.AYLRDFIYVSQDPKDQLLLGPTYATPK.V |
| 181448 | 336 | – | 362 | 778.9169 | 3111.6384 | 3111.6281 | 3.31 | 2 | 28 | 0.0022 | 1Score **> 37** indicates **identity** Score **> 14** indicates **homology** | U | K.AYLRDFIYVSQDPKDQLLLGPTYATPK.V |
| 181478 | 336 | – | 362 | 1557.3198 | 3112.6250 | 3112.6121 | 4.16 | 2 | 43 | 0.0001 | 1Score **> 37** indicates **identity** Score **> 15** indicates **homology** | U | K.AYLRDFIYVSQDPKDQLLLGPTYATPK.V  + Deamidated (NQ) |
| 181480 | 336 | – | 362 | 1038.5518 | 3112.6335 | 3112.6121 | 6.89 | 2 | 54 | 9e-06 | 1Score **> 37** indicates **identity** Score **> 16** indicates **homology** | U | K.AYLRDFIYVSQDPKDQLLLGPTYATPK.V  + Deamidated (NQ) |
| 181481 | 336 | – | 362 | 779.1661 | 3112.6351 | 3112.6121 | 7.40 | 2 | 20 | 0.012 | 1Score **> 37** indicates **identity** Score **> 14** indicates **homology** | U | K.AYLRDFIYVSQDPKDQLLLGPTYATPK.V  + Deamidated (NQ) |
| 31357 | 340 | – | 349 | 606.3051 | 1210.5957 | 1210.5870 | 7.14 | 0 | 33 | 0.00086 | 1Score **> 31** indicates **identity** Score **> 15** indicates **homology** | U | R.DFIYVSQDPK.D |
| 163935 | 340 | – | 362 | 870.4498 | 2608.3276 | 2608.3425 | -5.72 | 1 | 26 | 0.0035 | 1Score **> 37** indicates **identity** Score **> 14** indicates **homology** | U | R.DFIYVSQDPKDQLLLGPTYATPK.V |
| 163938 | 340 | – | 362 | 870.4512 | 2608.3317 | 2608.3425 | -4.12 | 1 | 21 | 0.012 | 1Score **> 37** indicates **identity** Score **> 14** indicates **homology** | U | R.DFIYVSQDPKDQLLLGPTYATPK.V |
| 163942 | 340 | – | 362 | 870.4537 | 2608.3394 | 2608.3425 | -1.19 | 1 | 32 | 0.0011 | 1Score **> 37** indicates **identity** Score **> 14** indicates **homology** | U | R.DFIYVSQDPKDQLLLGPTYATPK.V |
| 163944 | 340 | – | 362 | 870.4543 | 2608.3412 | 2608.3425 | -0.51 | 1 | 22 | 0.0089 | 1Score **> 37** indicates **identity** Score **> 14** indicates **homology** | U | R.DFIYVSQDPKDQLLLGPTYATPK.V |
| 163945 | 340 | – | 362 | 1305.1796 | 2608.3446 | 2608.3425 | 0.83 | 1 | 87 | 7.6e-09 | 1Score **> 37** indicates **identity** Score **> 18** indicates **homology** | U | R.DFIYVSQDPKDQLLLGPTYATPK.V |
| 163946 | 340 | – | 362 | 870.4555 | 2608.3448 | 2608.3425 | 0.87 | 1 | 52 | 1.4e-05 | 1Score **> 37** indicates **identity** Score **> 16** indicates **homology** | U | R.DFIYVSQDPKDQLLLGPTYATPK.V |
| 163947 | 340 | – | 362 | 1305.1799 | 2608.3453 | 2608.3425 | 1.08 | 1 | 62 | 1.4e-06 | 1Score **> 37** indicates **identity** Score **> 16** indicates **homology** | U | R.DFIYVSQDPKDQLLLGPTYATPK.V |
| 163948 | 340 | – | 362 | 870.4557 | 2608.3454 | 2608.3425 | 1.10 | 1 | 57 | 4.7e-06 | 1Score **> 37** indicates **identity** Score **> 16** indicates **homology** | U | R.DFIYVSQDPKDQLLLGPTYATPK.V |
| 163949 | 340 | – | 362 | 870.4558 | 2608.3456 | 2608.3425 | 1.21 | 1 | 49 | 2.3e-05 | 1Score **> 37** indicates **identity** Score **> 16** indicates **homology** | U | R.DFIYVSQDPKDQLLLGPTYATPK.V |
| 163950 | 340 | – | 362 | 870.4560 | 2608.3462 | 2608.3425 | 1.45 | 1 | 46 | 5.3e-05 | 1Score **> 37** indicates **identity** Score **> 15** indicates **homology** | U | R.DFIYVSQDPKDQLLLGPTYATPK.V |
| 163951 | 340 | – | 362 | 1305.1812 | 2608.3479 | 2608.3425 | 2.07 | 1 | 94 | 1.5e-09 | 1Score **> 37** indicates **identity** Score **> 18** indicates **homology** | U | R.DFIYVSQDPKDQLLLGPTYATPK.V |
| 163952 | 340 | – | 362 | 870.4567 | 2608.3482 | 2608.3425 | 2.21 | 1 | 69 | 3.1e-07 | 1Score **> 37** indicates **identity** Score **> 17** indicates **homology** | U | R.DFIYVSQDPKDQLLLGPTYATPK.V |
| 163953 | 340 | – | 362 | 870.4574 | 2608.3504 | 2608.3425 | 3.06 | 1 | 25 | 0.0047 | 1Score **> 37** indicates **identity** Score **> 14** indicates **homology** | U | R.DFIYVSQDPKDQLLLGPTYATPK.V |
| 163954 | 340 | – | 362 | 870.4580 | 2608.3521 | 2608.3425 | 3.69 | 1 | 55 | 6.9e-06 | 1Score **> 37** indicates **identity** Score **> 16** indicates **homology** | U | R.DFIYVSQDPKDQLLLGPTYATPK.V |
| 163960 | 340 | – | 362 | 870.4596 | 2608.3569 | 2608.3425 | 5.55 | 1 | 38 | 0.00026 | 1Score **> 37** indicates **identity** Score **> 15** indicates **homology** | U | R.DFIYVSQDPKDQLLLGPTYATPK.V |
| 163961 | 340 | – | 362 | 870.4597 | 2608.3572 | 2608.3425 | 5.64 | 1 | 36 | 0.0004 | 1Score **> 37** indicates **identity** Score **> 15** indicates **homology** | U | R.DFIYVSQDPKDQLLLGPTYATPK.V |
| 163966 | 340 | – | 362 | 870.4612 | 2608.3618 | 2608.3425 | 7.40 | 1 | 35 | 0.0005 | 1Score **> 37** indicates **identity** Score **> 15** indicates **homology** | U | R.DFIYVSQDPKDQLLLGPTYATPK.V |
| 164002 | 340 | – | 362 | 870.7863 | 2609.3369 | 2609.3265 | 4.01 | 1 | 31 | 0.0013 | 1Score **> 37** indicates **identity** Score **> 14** indicates **homology** | U | R.DFIYVSQDPKDQLLLGPTYATPK.V  + Deamidated (NQ) |
| 164003 | 340 | – | 362 | 1305.6802 | 2609.3459 | 2609.3265 | 7.45 | 1 | 70 | 2.5e-07 | 1Score **> 37** indicates **identity** Score **> 17** indicates **homology** | U | R.DFIYVSQDPKDQLLLGPTYATPK.V  + Deamidated (NQ) |
| 164004 | 340 | – | 362 | 870.7912 | 2609.3518 | 2609.3265 | 9.69 | 1 | 53 | 1.3e-05 | 1Score **> 37** indicates **identity** Score **> 16** indicates **homology** | U | R.DFIYVSQDPKDQLLLGPTYATPK.V  + Deamidated (NQ) |
| 52278 | 350 | – | 362 | 708.8923 | 1415.7700 | 1415.7660 | 2.81 | 0 | 51 | 3.5e-05 | 1Score **> 34** indicates **identity** Score **> 19** indicates **homology** | U | K.DQLLLGPTYATPK.V |
| 52280 | 350 | – | 362 | 708.8929 | 1415.7713 | 1415.7660 | 3.74 | 0 | 59 | 6.2e-06 | 1Score **> 35** indicates **identity** Score **> 19** indicates **homology** | U | K.DQLLLGPTYATPK.V |
| 52282 | 350 | – | 362 | 708.8935 | 1415.7724 | 1415.7660 | 4.49 | 0 | 50 | 3.5e-05 | 1Score **> 34** indicates **identity** Score **> 18** indicates **homology** | U | K.DQLLLGPTYATPK.V |
| 52284 | 350 | – | 362 | 708.8940 | 1415.7734 | 1415.7660 | 5.21 | 0 | 22 | 0.008 | 1Score **> 35** indicates **identity** Score **> 14** indicates **homology** | U | K.DQLLLGPTYATPK.V |
| 16685 | 408 | – | 417 | 352.5421 | 1054.6044 | 1054.6022 | 2.02 | 1 | 44 | 0.00042 | 1Score **> 29** indicates **identity** Score **> 23** indicates **homology** | U | K.TKVGSPPLEK.F |
| 16686 | 408 | – | 417 | 528.3097 | 1054.6049 | 1054.6022 | 2.51 | 1 | 24 | 0.0071 | 1Score **> 29** indicates **identity** Score **> 15** indicates **homology** | U | K.TKVGSPPLEK.F |
| 16687 | 408 | – | 417 | 352.5426 | 1054.6061 | 1054.6022 | 3.67 | 1 | 37 | 0.0011 | 1Score **> 29** indicates **identity** Score **> 20** indicates **homology** | U | K.TKVGSPPLEK.F |
| 16688 | 408 | – | 417 | 528.3106 | 1054.6065 | 1054.6022 | 4.08 | 1 | 18 | 0.021 | 1Score **> 29** indicates **identity** Score **> 14** indicates **homology** | U | K.TKVGSPPLEK.F |
| 16689 | 408 | – | 417 | 352.5430 | 1054.6072 | 1054.6022 | 4.68 | 1 | 32 | 0.0019 | 1Score **> 29** indicates **identity** Score **> 17** indicates **homology** | U | K.TKVGSPPLEK.F |
| 182548 | 408 | – | 437 | 634.9277 | 3169.6023 | 3169.6131 | -3.43 | 2 | 14 | 0.044 | 1Score **> 38** indicates **identity** Score **> 13** indicates **homology** | U | K.TKVGSPPLEKFNIWGGSLSLGHPFGATGCR.L |
| 182552 | 408 | – | 437 | 634.9304 | 3169.6155 | 3169.6131 | 0.74 | 2 | 30 | 0.0015 | 1Score **> 38** indicates **identity** Score **> 14** indicates **homology** | U | K.TKVGSPPLEKFNIWGGSLSLGHPFGATGCR.L |
| 182592 | 408 | – | 437 | 635.1307 | 3170.6170 | 3170.5972 | 6.24 | 2 | 15 | 0.037 | 1Score **> 37** indicates **identity** Score **> 13** indicates **homology** | U | K.TKVGSPPLEKFNIWGGSLSLGHPFGATGCR.L  + Deamidated (NQ) |
| 182593 | 408 | – | 437 | 635.1317 | 3170.6222 | 3170.5972 | 7.90 | 2 | 38 | 0.00028 | 1Score **> 37** indicates **identity** Score **> 15** indicates **homology** | U | K.TKVGSPPLEKFNIWGGSLSLGHPFGATGCR.L  + Deamidated (NQ) |
| 4336 | 410 | – | 417 | 413.7371 | 825.4597 | 825.4596 | 0.097 | 0 | 34 | 0.0027 | 1Score **> 23** indicates **identity** Score **> 20** indicates **homology** | U | K.VGSPPLEK.F |
| 4337 | 410 | – | 417 | 413.7373 | 825.4601 | 825.4596 | 0.54 | 0 | 40 | 0.0013 | 1Score **> 24** indicates **identity** | U | K.VGSPPLEK.F |
| 4338 | 410 | – | 417 | 413.7375 | 825.4605 | 825.4596 | 1.04 | 0 | 30 | 0.004 | 1Score **> 24** indicates **identity** Score **> 18** indicates **homology** | U | K.VGSPPLEK.F |
| 176476 | 410 | – | 437 | 736.1250 | 2940.4708 | 2940.4705 | 0.096 | 1 | 41 | 0.00015 | 1Score **> 38** indicates **identity** Score **> 15** indicates **homology** | U | K.VGSPPLEKFNIWGGSLSLGHPFGATGCR.L |
| 176515 | 410 | – | 437 | 981.4996 | 2941.4769 | 2941.4545 | 7.60 | 1 | 32 | 0.00091 | 1Score **> 37** indicates **identity** Score **> 15** indicates **homology** | U | K.VGSPPLEKFNIWGGSLSLGHPFGATGCR.L  + Deamidated (NQ) |
| 133117 | 418 | – | 437 | 712.0144 | 2133.0214 | 2133.0215 | -0.0033 | 0 | 24 | 0.0055 | 1Score **> 35** indicates **identity** Score **> 14** indicates **homology** | U | K.FNIWGGSLSLGHPFGATGCR.L |
| 133118 | 418 | – | 437 | 1067.5182 | 2133.0219 | 2133.0215 | 0.21 | 0 | 119 | 6.6e-12 | 1Score **> 35** indicates **identity** Score **> 20** indicates **homology** | U | K.FNIWGGSLSLGHPFGATGCR.L |
| 133119 | 418 | – | 437 | 712.0147 | 2133.0222 | 2133.0215 | 0.34 | 0 | 39 | 0.00023 | 1Score **> 35** indicates **identity** Score **> 15** indicates **homology** | U | K.FNIWGGSLSLGHPFGATGCR.L |
| 133121 | 418 | – | 437 | 1067.5184 | 2133.0223 | 2133.0215 | 0.38 | 0 | 125 | 2e-12 | 1Score **> 35** indicates **identity** Score **> 20** indicates **homology** | U | K.FNIWGGSLSLGHPFGATGCR.L |
| 133122 | 418 | – | 437 | 712.0147 | 2133.0224 | 2133.0215 | 0.44 | 0 | 63 | 1.2e-06 | 1Score **> 35** indicates **identity** Score **> 16** indicates **homology** | U | K.FNIWGGSLSLGHPFGATGCR.L |
| 133123 | 418 | – | 437 | 712.0148 | 2133.0226 | 2133.0215 | 0.55 | 0 | 40 | 0.00017 | 1Score **> 35** indicates **identity** Score **> 15** indicates **homology** | U | K.FNIWGGSLSLGHPFGATGCR.L |
| 133125 | 418 | – | 437 | 712.0149 | 2133.0228 | 2133.0215 | 0.63 | 0 | 62 | 1.7e-06 | 1Score **> 35** indicates **identity** Score **> 16** indicates **homology** | U | K.FNIWGGSLSLGHPFGATGCR.L |
| 133126 | 418 | – | 437 | 712.0149 | 2133.0229 | 2133.0215 | 0.65 | 0 | 54 | 8.5e-06 | 1Score **> 35** indicates **identity** Score **> 16** indicates **homology** | U | K.FNIWGGSLSLGHPFGATGCR.L |
| 133127 | 418 | – | 437 | 1067.5187 | 2133.0229 | 2133.0215 | 0.67 | 0 | 107 | 8.5e-11 | 1Score **> 35** indicates **identity** Score **> 19** indicates **homology** | U | K.FNIWGGSLSLGHPFGATGCR.L |
| 133128 | 418 | – | 437 | 1067.5188 | 2133.0230 | 2133.0215 | 0.72 | 0 | 126 | 1.6e-12 | 1Score **> 35** indicates **identity** Score **> 20** indicates **homology** | U | K.FNIWGGSLSLGHPFGATGCR.L |
| 133129 | 418 | – | 437 | 712.0150 | 2133.0231 | 2133.0215 | 0.77 | 0 | 19 | 0.016 | 1Score **> 35** indicates **identity** Score **> 14** indicates **homology** | U | K.FNIWGGSLSLGHPFGATGCR.L |
| 133130 | 418 | – | 437 | 712.0150 | 2133.0233 | 2133.0215 | 0.88 | 0 | 15 | 0.042 | 1Score **> 35** indicates **identity** Score **> 13** indicates **homology** | U | K.FNIWGGSLSLGHPFGATGCR.L |
| 133131 | 418 | – | 437 | 712.0151 | 2133.0235 | 2133.0215 | 0.94 | 0 | 57 | 4.2e-06 | 1Score **> 35** indicates **identity** Score **> 16** indicates **homology** | U | K.FNIWGGSLSLGHPFGATGCR.L |
| 133135 | 418 | – | 437 | 712.0153 | 2133.0242 | 2133.0215 | 1.30 | 0 | 29 | 0.002 | 1Score **> 35** indicates **identity** Score **> 14** indicates **homology** | U | K.FNIWGGSLSLGHPFGATGCR.L |
| 133136 | 418 | – | 437 | 1067.5194 | 2133.0243 | 2133.0215 | 1.34 | 0 | 79 | 3.6e-08 | 1Score **> 35** indicates **identity** Score **> 17** indicates **homology** | U | K.FNIWGGSLSLGHPFGATGCR.L |
| 133137 | 418 | – | 437 | 712.0155 | 2133.0246 | 2133.0215 | 1.48 | 0 | 19 | 0.016 | 1Score **> 35** indicates **identity** Score **> 14** indicates **homology** | U | K.FNIWGGSLSLGHPFGATGCR.L |
| 133139 | 418 | – | 437 | 1067.5204 | 2133.0262 | 2133.0215 | 2.22 | 0 | 133 | 3.7e-13 | 1Score **> 35** indicates **identity** Score **> 21** indicates **homology** | U | K.FNIWGGSLSLGHPFGATGCR.L |
| 133140 | 418 | – | 437 | 712.0160 | 2133.0263 | 2133.0215 | 2.27 | 0 | 14 | 0.045 | 1Score **> 35** indicates **identity** Score **> 13** indicates **homology** | U | K.FNIWGGSLSLGHPFGATGCR.L |
| 133144 | 418 | – | 437 | 1067.5228 | 2133.0310 | 2133.0215 | 4.48 | 0 | 54 | 8.4e-06 | 1Score **> 36** indicates **identity** Score **> 16** indicates **homology** | U | K.FNIWGGSLSLGHPFGATGCR.L |
| 5108 | 438 | – | 445 | 423.2363 | 844.4581 | 844.4589 | -0.87 | 0 | 39 | 0.00093 | 1Score **> 32** indicates **identity** Score **> 21** indicates **homology** | U | R.LVMAAANR.L |
| 5109 | 438 | – | 445 | 423.2367 | 844.4588 | 844.4589 | -0.12 | 0 | 14 | 0.044 | 1Score **> 32** indicates **identity** Score **> 13** indicates **homology** | U | R.LVMAAANR.L |
| 5942 | 438 | – | 445 | 431.2348 | 860.4551 | 860.4538 | 1.57 | 0 | 26 | 0.0084 | 1Score **> 31** indicates **identity** Score **> 18** indicates **homology** | U | R.LVMAAANR.L  + Oxidation (M) |
| 5944 | 438 | – | 445 | 431.2352 | 860.4559 | 860.4538 | 2.43 | 0 | 26 | 0.0035 | 1Score **> 31** indicates **identity** Score **> 14** indicates **homology** | U | R.LVMAAANR.L  + Oxidation (M) |
| 5945 | 438 | – | 445 | 431.2354 | 860.4563 | 860.4538 | 2.88 | 0 | 21 | 0.012 | 1Score **> 31** indicates **identity** Score **> 15** indicates **homology** | U | R.LVMAAANR.L  + Oxidation (M) |
| 168028 | 449 | – | 475 | 902.4335 | 2704.2785 | 2704.2737 | 1.77 | 0 | 68 | 3.9e-07 | 1Score **> 35** indicates **identity** Score **> 17** indicates **homology** | U | K.DGGQYALVAACAAGGQGHAMIVEAYPK.- |
| 168029 | 449 | – | 475 | 902.4348 | 2704.2827 | 2704.2737 | 3.31 | 0 | 40 | 0.00017 | 1Score **> 36** indicates **identity** Score **> 15** indicates **homology** | U | K.DGGQYALVAACAAGGQGHAMIVEAYPK.- |
| 168030 | 449 | – | 475 | 902.4354 | 2704.2844 | 2704.2737 | 3.93 | 0 | 55 | 7.7e-06 | 1Score **> 36** indicates **identity** Score **> 16** indicates **homology** | U | K.DGGQYALVAACAAGGQGHAMIVEAYPK.- |
| 172569 | 449 | – | 475 | 1411.1706 | 2820.3266 | 2820.3462 | -6.95 | 0 | 16 | 0.03 | 1Score **> 35** indicates **identity** Score **> 14** indicates **homology** | U | K.DGGQYALVAACAAGGQGHAMIVEAYPK.-  + Deamidated (NQ); HNE (C); Oxidation (M) |

---

```
ID   ECHB_MOUSE              Reviewed;         475 AA.
AC   Q99JY0; Q3TEH9; Q8BJI5; Q8BJM0; Q8BK52;
DT   16-AUG-2004, integrated into UniProtKB/Swiss-Prot.
DT   01-JUN-2001, sequence version 1.
DT   28-JUN-2023, entry version 165.
DE   RecName: Full=Trifunctional enzyme subunit beta, mitochondrial;
DE   AltName: Full=TP-beta;
DE   Includes:
DE     RecName: Full=3-ketoacyl-CoA thiolase;
DE              EC=2.3.1.155 {ECO:0000250|UniProtKB:P55084};
DE              EC=2.3.1.16 {ECO:0000250|UniProtKB:P55084};
DE     AltName: Full=Acetyl-CoA acyltransferase;
DE     AltName: Full=Beta-ketothiolase;
DE   Flags: Precursor;
GN   Name=Hadhb;
OS   Mus musculus (Mouse).
OC   Eukaryota; Metazoa; Chordata; Craniata; Vertebrata; Euteleostomi; Mammalia;
OC   Eutheria; Euarchontoglires; Glires; Rodentia; Myomorpha; Muroidea; Muridae;
OC   Murinae; Mus; Mus.
OX   NCBI_TaxID=10090;
RN   [1]
RP   NUCLEOTIDE SEQUENCE [LARGE SCALE MRNA].
RC   STRAIN=C57BL/6J;
RC   TISSUE=Bone marrow, Colon, Hippocampus, Spinal ganglion, Testis, and
RC   Thymus;
RX   PubMed=16141072; DOI=10.1126/science.1112014;
RA   Carninci P., Kasukawa T., Katayama S., Gough J., Frith M.C., Maeda N.,
RA   Oyama R., Ravasi T., Lenhard B., Wells C., Kodzius R., Shimokawa K.,
RA   Bajic V.B., Brenner S.E., Batalov S., Forrest A.R., Zavolan M., Davis M.J.,
RA   Wilming L.G., Aidinis V., Allen J.E., Ambesi-Impiombato A., Apweiler R.,
RA   Aturaliya R.N., Bailey T.L., Bansal M., Baxter L., Beisel K.W., Bersano T.,
RA   Bono H., Chalk A.M., Chiu K.P., Choudhary V., Christoffels A.,
RA   Clutterbuck D.R., Crowe M.L., Dalla E., Dalrymple B.P., de Bono B.,
RA   Della Gatta G., di Bernardo D., Down T., Engstrom P., Fagiolini M.,
RA   Faulkner G., Fletcher C.F., Fukushima T., Furuno M., Futaki S.,
RA   Gariboldi M., Georgii-Hemming P., Gingeras T.R., Gojobori T., Green R.E.,
RA   Gustincich S., Harbers M., Hayashi Y., Hensch T.K., Hirokawa N., Hill D.,
RA   Huminiecki L., Iacono M., Ikeo K., Iwama A., Ishikawa T., Jakt M.,
RA   Kanapin A., Katoh M., Kawasawa Y., Kelso J., Kitamura H., Kitano H.,
RA   Kollias G., Krishnan S.P., Kruger A., Kummerfeld S.K., Kurochkin I.V.,
RA   Lareau L.F., Lazarevic D., Lipovich L., Liu J., Liuni S., McWilliam S.,
RA   Madan Babu M., Madera M., Marchionni L., Matsuda H., Matsuzawa S., Miki H.,
RA   Mignone F., Miyake S., Morris K., Mottagui-Tabar S., Mulder N., Nakano N.,
RA   Nakauchi H., Ng P., Nilsson R., Nishiguchi S., Nishikawa S., Nori F.,
RA   Ohara O., Okazaki Y., Orlando V., Pang K.C., Pavan W.J., Pavesi G.,
RA   Pesole G., Petrovsky N., Piazza S., Reed J., Reid J.F., Ring B.Z.,
RA   Ringwald M., Rost B., Ruan Y., Salzberg S.L., Sandelin A., Schneider C.,
RA   Schoenbach C., Sekiguchi K., Semple C.A., Seno S., Sessa L., Sheng Y.,
RA   Shibata Y., Shimada H., Shimada K., Silva D., Sinclair B., Sperling S.,
RA   Stupka E., Sugiura K., Sultana R., Takenaka Y., Taki K., Tammoja K.,
RA   Tan S.L., Tang S., Taylor M.S., Tegner J., Teichmann S.A., Ueda H.R.,
RA   van Nimwegen E., Verardo R., Wei C.L., Yagi K., Yamanishi H.,
RA   Zabarovsky E., Zhu S., Zimmer A., Hide W., Bult C., Grimmond S.M.,
RA   Teasdale R.D., Liu E.T., Brusic V., Quackenbush J., Wahlestedt C.,
RA   Mattick J.S., Hume D.A., Kai C., Sasaki D., Tomaru Y., Fukuda S.,
RA   Kanamori-Katayama M., Suzuki M., Aoki J., Arakawa T., Iida J., Imamura K.,
RA   Itoh M., Kato T., Kawaji H., Kawagashira N., Kawashima T., Kojima M.,
RA   Kondo S., Konno H., Nakano K., Ninomiya N., Nishio T., Okada M., Plessy C.,
RA   Shibata K., Shiraki T., Suzuki S., Tagami M., Waki K., Watahiki A.,
RA   Okamura-Oho Y., Suzuki H., Kawai J., Hayashizaki Y.;
RT   "The transcriptional landscape of the mammalian genome.";
RL   Science 309:1559-1563(2005).
RN   [2]
RP   NUCLEOTIDE SEQUENCE [LARGE SCALE MRNA].
RC   STRAIN=FVB/N; TISSUE=Mammary tumor;
RX   PubMed=15489334; DOI=10.1101/gr.2596504;
RG   The MGC Project Team;
RT   "The status, quality, and expansion of the NIH full-length cDNA project:
RT   the Mammalian Gene Collection (MGC).";
RL   Genome Res. 14:2121-2127(2004).
RN   [3]
RP   IDENTIFICATION BY MASS SPECTROMETRY [LARGE SCALE ANALYSIS].
RC   TISSUE=Brain, Brown adipose tissue, Heart, Kidney, Liver, Lung,
RC   Pancreas, Spleen, and Testis;
RX   PubMed=21183079; DOI=10.1016/j.cell.2010.12.001;
RA   Huttlin E.L., Jedrychowski M.P., Elias J.E., Goswami T., Rad R.,
RA   Beausoleil S.A., Villen J., Haas W., Sowa M.E., Gygi S.P.;
RT   "A tissue-specific atlas of mouse protein phosphorylation and expression.";
RL   Cell 143:1174-1189(2010).
RN   [4]
RP   SUCCINYLATION [LARGE SCALE ANALYSIS] AT LYS-53; LYS-73; LYS-189; LYS-191;
RP   LYS-273; LYS-292; LYS-294 AND LYS-333, AND IDENTIFICATION BY MASS
RP   SPECTROMETRY [LARGE SCALE ANALYSIS].
RC   TISSUE=Embryonic fibroblast, and Liver;
RX   PubMed=23806337; DOI=10.1016/j.molcel.2013.06.001;
RA   Park J., Chen Y., Tishkoff D.X., Peng C., Tan M., Dai L., Xie Z., Zhang Y.,
RA   Zwaans B.M., Skinner M.E., Lombard D.B., Zhao Y.;
RT   "SIRT5-mediated lysine desuccinylation impacts diverse metabolic
RT   pathways.";
RL   Mol. Cell 50:919-930(2013).
RN   [5]
RP   ACETYLATION [LARGE SCALE ANALYSIS] AT LYS-73; LYS-189; LYS-294; LYS-299;
RP   LYS-333; LYS-349 AND LYS-362, AND IDENTIFICATION BY MASS SPECTROMETRY
RP   [LARGE SCALE ANALYSIS].
RC   TISSUE=Liver;
RX   PubMed=23576753; DOI=10.1073/pnas.1302961110;
RA   Rardin M.J., Newman J.C., Held J.M., Cusack M.P., Sorensen D.J., Li B.,
RA   Schilling B., Mooney S.D., Kahn C.R., Verdin E., Gibson B.W.;
RT   "Label-free quantitative proteomics of the lysine acetylome in mitochondria
RT   identifies substrates of SIRT3 in metabolic pathways.";
RL   Proc. Natl. Acad. Sci. U.S.A. 110:6601-6606(2013).
RN   [6]
RP   INTERACTION WITH MTLN.
RX   PubMed=29949755; DOI=10.1016/j.celrep.2018.05.058;
RA   Makarewich C.A., Baskin K.K., Munir A.Z., Bezprozvannaya S., Sharma G.,
RA   Khemtong C., Shah A.M., McAnally J.R., Malloy C.R., Szweda L.I.,
RA   Bassel-Duby R., Olson E.N.;
RT   "MOXI Is a Mitochondrial Micropeptide That Enhances Fatty Acid beta-
RT   Oxidation.";
RL   Cell Rep. 23:3701-3709(2018).
CC   -!- FUNCTION: Mitochondrial trifunctional enzyme catalyzes the last three
CC       of the four reactions of the mitochondrial beta-oxidation pathway. The
CC       mitochondrial beta-oxidation pathway is the major energy-producing
CC       process in tissues and is performed through four consecutive reactions
CC       breaking down fatty acids into acetyl-CoA. Among the enzymes involved
CC       in this pathway, the trifunctional enzyme exhibits specificity for
CC       long-chain fatty acids. Mitochondrial trifunctional enzyme is a
CC       heterotetrameric complex composed of two proteins, the trifunctional
CC       enzyme subunit alpha/HADHA carries the 2,3-enoyl-CoA hydratase and the
CC       3-hydroxyacyl-CoA dehydrogenase activities, while the trifunctional
CC       enzyme subunit beta/HADHB described here bears the 3-ketoacyl-CoA
CC       thiolase activity. {ECO:0000250|UniProtKB:P55084}.
CC   -!- CATALYTIC ACTIVITY:
CC       Reaction=acetyl-CoA + an acyl-CoA = a 3-oxoacyl-CoA + CoA;
CC         Xref=Rhea:RHEA:21564, ChEBI:CHEBI:57287, ChEBI:CHEBI:57288,
CC         ChEBI:CHEBI:58342, ChEBI:CHEBI:90726; EC=2.3.1.16;
CC         Evidence={ECO:0000250|UniProtKB:P55084};
CC       PhysiologicalDirection=right-to-left; Xref=Rhea:RHEA:21566;
CC         Evidence={ECO:0000250|UniProtKB:P55084};
CC   -!- CATALYTIC ACTIVITY:
CC       Reaction=acetyl-CoA + butanoyl-CoA = 3-oxohexanoyl-CoA + CoA;
CC         Xref=Rhea:RHEA:31111, ChEBI:CHEBI:57287, ChEBI:CHEBI:57288,
CC         ChEBI:CHEBI:57371, ChEBI:CHEBI:62418;
CC         Evidence={ECO:0000250|UniProtKB:P55084};
CC       PhysiologicalDirection=right-to-left; Xref=Rhea:RHEA:31113;
CC         Evidence={ECO:0000250|UniProtKB:P55084};
CC   -!- CATALYTIC ACTIVITY:
CC       Reaction=acetyl-CoA + hexanoyl-CoA = 3-oxooctanoyl-CoA + CoA;
CC         Xref=Rhea:RHEA:31203, ChEBI:CHEBI:57287, ChEBI:CHEBI:57288,
CC         ChEBI:CHEBI:62619, ChEBI:CHEBI:62620;
CC         Evidence={ECO:0000250|UniProtKB:P55084};
CC       PhysiologicalDirection=right-to-left; Xref=Rhea:RHEA:31205;
CC         Evidence={ECO:0000250|UniProtKB:P55084};
CC   -!- CATALYTIC ACTIVITY:
CC       Reaction=acetyl-CoA + octanoyl-CoA = 3-oxodecanoyl-CoA + CoA;
CC         Xref=Rhea:RHEA:31087, ChEBI:CHEBI:57287, ChEBI:CHEBI:57288,
CC         ChEBI:CHEBI:57386, ChEBI:CHEBI:62548;
CC         Evidence={ECO:0000250|UniProtKB:P55084};
CC       PhysiologicalDirection=right-to-left; Xref=Rhea:RHEA:31089;
CC         Evidence={ECO:0000250|UniProtKB:P55084};
CC   -!- CATALYTIC ACTIVITY:
CC       Reaction=acetyl-CoA + decanoyl-CoA = 3-oxododecanoyl-CoA + CoA;
CC         Xref=Rhea:RHEA:31183, ChEBI:CHEBI:57287, ChEBI:CHEBI:57288,
CC         ChEBI:CHEBI:61430, ChEBI:CHEBI:62615;
CC         Evidence={ECO:0000250|UniProtKB:P55084};
CC       PhysiologicalDirection=right-to-left; Xref=Rhea:RHEA:31185;
CC         Evidence={ECO:0000250|UniProtKB:P55084};
CC   -!- CATALYTIC ACTIVITY:
CC       Reaction=acetyl-CoA + dodecanoyl-CoA = 3-oxotetradecanoyl-CoA + CoA;
CC         Xref=Rhea:RHEA:31091, ChEBI:CHEBI:57287, ChEBI:CHEBI:57288,
CC         ChEBI:CHEBI:57375, ChEBI:CHEBI:62543;
CC         Evidence={ECO:0000250|UniProtKB:P55084};
CC       PhysiologicalDirection=right-to-left; Xref=Rhea:RHEA:31093;
CC         Evidence={ECO:0000250|UniProtKB:P55084};
CC   -!- CATALYTIC ACTIVITY:
CC       Reaction=acetyl-CoA + tetradecanoyl-CoA = 3-oxohexadecanoyl-CoA + CoA;
CC         Xref=Rhea:RHEA:18161, ChEBI:CHEBI:57287, ChEBI:CHEBI:57288,
CC         ChEBI:CHEBI:57349, ChEBI:CHEBI:57385; EC=2.3.1.155;
CC         Evidence={ECO:0000250|UniProtKB:P55084};
CC       PhysiologicalDirection=right-to-left; Xref=Rhea:RHEA:18163;
CC         Evidence={ECO:0000250|UniProtKB:P55084};
CC   -!- PATHWAY: Lipid metabolism; fatty acid beta-oxidation.
CC       {ECO:0000250|UniProtKB:P55084}.
CC   -!- SUBUNIT: Heterotetramer of 2 alpha/HADHA and 2 beta/HADHB subunits;
CC       forms the mitochondrial trifunctional enzyme (By similarity). Also
CC       purified as higher order heterooligomers including a 4 alpha/HADHA and
CC       4 beta/HADHB heterooligomer which physiological significance remains
CC       unclear (By similarity). The mitochondrial trifunctional enzyme
CC       interacts with MTLN (PubMed:29949755). Interacts with RSAD2/viperin (By
CC       similarity). {ECO:0000250|UniProtKB:P55084,
CC       ECO:0000269|PubMed:29949755}.
CC   -!- SUBCELLULAR LOCATION: Mitochondrion {ECO:0000250|UniProtKB:P55084}.
CC       Mitochondrion inner membrane {ECO:0000250|UniProtKB:P55084}.
CC       Mitochondrion outer membrane {ECO:0000250|UniProtKB:P55084}.
CC       Endoplasmic reticulum {ECO:0000250|UniProtKB:P55084}. Note=Protein
CC       stability and association with membranes require HADHA.
CC       {ECO:0000250|UniProtKB:P55084}.
CC   -!- PTM: Acetylation of Lys-202 is observed in liver mitochondria from
CC       fasted mice but not from fed mice.
CC   -!- SIMILARITY: Belongs to the thiolase-like superfamily. Thiolase family.
CC       {ECO:0000305}.
CC   ---------------------------------------------------------------------------
CC   Copyrighted by the UniProt Consortium, see https://www.uniprot.org/terms
CC   Distributed under the Creative Commons Attribution (CC BY 4.0) License
CC   ---------------------------------------------------------------------------
DR   EMBL; AK033462; BAC28300.1; -; mRNA.
DR   EMBL; AK076814; BAC36493.1; -; mRNA.
DR   EMBL; AK083164; BAC38790.1; -; mRNA.
DR   EMBL; AK083767; BAC39015.1; -; mRNA.
DR   EMBL; AK150889; BAE29936.1; -; mRNA.
DR   EMBL; AK169637; BAE41269.1; -; mRNA.
DR   EMBL; BC005585; AAH05585.1; -; mRNA.
DR   CCDS; CCDS39045.1; -.
DR   RefSeq; NP_001276727.1; NM_001289798.1.
DR   RefSeq; NP_001276728.1; NM_001289799.1.
DR   RefSeq; NP_663533.1; NM_145558.2.
DR   RefSeq; XP_017176317.1; XM_017320828.1.
DR   RefSeq; XP_017176318.1; XM_017320829.1.
DR   AlphaFoldDB; Q99JY0; -.
DR   SMR; Q99JY0; -.
DR   BioGRID; 231080; 33.
DR   IntAct; Q99JY0; 7.
DR   MINT; Q99JY0; -.
DR   STRING; 10090.ENSMUSP00000110434; -.
DR   iPTMnet; Q99JY0; -.
DR   PhosphoSitePlus; Q99JY0; -.
DR   SwissPalm; Q99JY0; -.
DR   EPD; Q99JY0; -.
DR   jPOST; Q99JY0; -.
DR   MaxQB; Q99JY0; -.
DR   PaxDb; Q99JY0; -.
DR   PeptideAtlas; Q99JY0; -.
DR   ProteomicsDB; 277669; -.
DR   Antibodypedia; 27848; 265 antibodies from 30 providers.
DR   DNASU; 231086; -.
DR   Ensembl; ENSMUST00000026841; ENSMUSP00000026841; ENSMUSG00000059447.
DR   Ensembl; ENSMUST00000114783; ENSMUSP00000110431; ENSMUSG00000059447.
DR   Ensembl; ENSMUST00000114786; ENSMUSP00000110434; ENSMUSG00000059447.
DR   GeneID; 231086; -.
DR   KEGG; mmu:231086; -.
DR   UCSC; uc008wve.2; mouse.
DR   AGR; MGI:2136381; -.
DR   CTD; 3032; -.
DR   MGI; MGI:2136381; Hadhb.
DR   VEuPathDB; HostDB:ENSMUSG00000059447; -.
DR   eggNOG; KOG1392; Eukaryota.
DR   GeneTree; ENSGT01030000234626; -.
DR   HOGENOM; CLU_031026_2_0_1; -.
DR   InParanoid; Q99JY0; -.
DR   OMA; MTAFPEP; -.
DR   OrthoDB; 1826604at2759; -.
DR   PhylomeDB; Q99JY0; -.
DR   TreeFam; TF315243; -.
DR   Reactome; R-MMU-1482798; Acyl chain remodeling of CL.
DR   Reactome; R-MMU-77285; Beta oxidation of myristoyl-CoA to lauroyl-CoA.
DR   Reactome; R-MMU-77305; Beta oxidation of palmitoyl-CoA to myristoyl-CoA.
DR   Reactome; R-MMU-77310; Beta oxidation of lauroyl-CoA to decanoyl-CoA-CoA.
DR   Reactome; R-MMU-77346; Beta oxidation of decanoyl-CoA to octanoyl-CoA-CoA.
DR   Reactome; R-MMU-77348; Beta oxidation of octanoyl-CoA to hexanoyl-CoA.
DR   Reactome; R-MMU-77350; Beta oxidation of hexanoyl-CoA to butanoyl-CoA.
DR   UniPathway; UPA00659; -.
DR   BioGRID-ORCS; 231086; 1 hit in 77 CRISPR screens.
DR   ChiTaRS; Hadhb; mouse.
DR   PRO; PR:Q99JY0; -.
DR   Proteomes; UP000000589; Chromosome 5.
DR   RNAct; Q99JY0; protein.
DR   Bgee; ENSMUSG00000059447; Expressed in spermatocyte and 170 other tissues.
DR   ExpressionAtlas; Q99JY0; baseline and differential.
DR   Genevisible; Q99JY0; MM.
DR   GO; GO:0005783; C:endoplasmic reticulum; ISS:UniProtKB.
DR   GO; GO:0016507; C:mitochondrial fatty acid beta-oxidation multienzyme complex; ISO:MGI.
DR   GO; GO:0005743; C:mitochondrial inner membrane; HDA:MGI.
DR   GO; GO:0042645; C:mitochondrial nucleoid; ISO:MGI.
DR   GO; GO:0005741; C:mitochondrial outer membrane; ISS:UniProtKB.
DR   GO; GO:0005739; C:mitochondrion; IDA:MGI.
DR   GO; GO:0003985; F:acetyl-CoA C-acetyltransferase activity; IDA:MGI.
DR   GO; GO:0003988; F:acetyl-CoA C-acyltransferase activity; IDA:MGI.
DR   GO; GO:0050633; F:acetyl-CoA C-myristoyltransferase activity; IEA:UniProtKB-EC.
DR   GO; GO:0106222; F:lncRNA binding; IDA:MGI.
DR   GO; GO:0044877; F:protein-containing complex binding; ISO:MGI.
DR   GO; GO:0071222; P:cellular response to lipopolysaccharide; IDA:MGI.
DR   GO; GO:0006635; P:fatty acid beta-oxidation; ISO:MGI.
DR   GO; GO:0010467; P:gene expression; IMP:MGI.
DR   CDD; cd00751; thiolase; 1.
DR   Gene3D; 3.40.47.10; -; 1.
DR   InterPro; IPR002155; Thiolase.
DR   InterPro; IPR016039; Thiolase-like.
DR   InterPro; IPR020615; Thiolase_acyl_enz_int_AS.
DR   InterPro; IPR020610; Thiolase_AS.
DR   InterPro; IPR020617; Thiolase_C.
DR   InterPro; IPR020613; Thiolase_CS.
DR   InterPro; IPR020616; Thiolase_N.
DR   PANTHER; PTHR18919; ACETYL-COA C-ACYLTRANSFERASE; 1.
DR   PANTHER; PTHR18919:SF153; TRIFUNCTIONAL ENZYME SUBUNIT BETA, MITOCHONDRIAL; 1.
DR   Pfam; PF02803; Thiolase_C; 1.
DR   Pfam; PF00108; Thiolase_N; 1.
DR   SUPFAM; SSF53901; Thiolase-like; 2.
DR   PROSITE; PS00098; THIOLASE_1; 1.
DR   PROSITE; PS00737; THIOLASE_2; 1.
DR   PROSITE; PS00099; THIOLASE_3; 1.
DR   TIGRFAMs; TIGR01930; AcCoA-C-Actrans; 1.
PE   1: Evidence at protein level;
KW   Acetylation; Acyltransferase; Endoplasmic reticulum; Fatty acid metabolism;
KW   Lipid metabolism; Membrane; Mitochondrion; Mitochondrion inner membrane;
KW   Mitochondrion outer membrane; Reference proteome; Transferase;
KW   Transit peptide.
FT   TRANSIT         1..34
FT                   /note="Mitochondrion"
FT                   /evidence="ECO:0000250"
FT   CHAIN           35..475
FT                   /note="Trifunctional enzyme subunit beta, mitochondrial"
FT                   /id="PRO_0000034082"
FT   INTRAMEM        174..221
FT                   /evidence="ECO:0000250|UniProtKB:P55084"
FT   ACT_SITE        139
FT                   /note="Acyl-thioester intermediate"
FT                   /evidence="ECO:0000250|UniProtKB:P55084"
FT   ACT_SITE        459
FT                   /note="Proton donor/acceptor"
FT                   /evidence="ECO:0000250|UniProtKB:P55084"
FT   SITE            429
FT                   /note="Increases nucleophilicity of active site Cys"
FT                   /evidence="ECO:0000250|UniProtKB:P55084"
FT   MOD_RES         53
FT                   /note="N6-succinyllysine"
FT                   /evidence="ECO:0007744|PubMed:23806337"
FT   MOD_RES         73
FT                   /note="N6-acetyllysine; alternate"
FT                   /evidence="ECO:0007744|PubMed:23576753"
FT   MOD_RES         73
FT                   /note="N6-succinyllysine; alternate"
FT                   /evidence="ECO:0007744|PubMed:23806337"
FT   MOD_RES         189
FT                   /note="N6-acetyllysine; alternate"
FT                   /evidence="ECO:0007744|PubMed:23576753"
FT   MOD_RES         189
FT                   /note="N6-succinyllysine; alternate"
FT                   /evidence="ECO:0007744|PubMed:23806337"
FT   MOD_RES         191
FT                   /note="N6-succinyllysine"
FT                   /evidence="ECO:0007744|PubMed:23806337"
FT   MOD_RES         273
FT                   /note="N6-succinyllysine"
FT                   /evidence="ECO:0007744|PubMed:23806337"
FT   MOD_RES         292
FT                   /note="N6-succinyllysine"
FT                   /evidence="ECO:0007744|PubMed:23806337"
FT   MOD_RES         294
FT                   /note="N6-acetyllysine; alternate"
FT                   /evidence="ECO:0007744|PubMed:23576753"
FT   MOD_RES         294
FT                   /note="N6-succinyllysine; alternate"
FT                   /evidence="ECO:0007744|PubMed:23806337"
FT   MOD_RES         299
FT                   /note="N6-acetyllysine"
FT                   /evidence="ECO:0007744|PubMed:23576753"
FT   MOD_RES         333
FT                   /note="N6-acetyllysine; alternate"
FT                   /evidence="ECO:0007744|PubMed:23576753"
FT   MOD_RES         333
FT                   /note="N6-succinyllysine; alternate"
FT                   /evidence="ECO:0007744|PubMed:23806337"
FT   MOD_RES         349
FT                   /note="N6-acetyllysine"
FT                   /evidence="ECO:0007744|PubMed:23576753"
FT   MOD_RES         362
FT                   /note="N6-acetyllysine"
FT                   /evidence="ECO:0007744|PubMed:23576753"
FT   CONFLICT        24..25
FT                   /note="IR -> HK (in Ref. 1; BAC36493)"
FT                   /evidence="ECO:0000305"
FT   CONFLICT        425
FT                   /note="L -> M (in Ref. 1; BAC38790)"
FT                   /evidence="ECO:0000305"
FT   CONFLICT        450
FT                   /note="G -> R (in Ref. 1; BAC38790)"
FT                   /evidence="ECO:0000305"
FT   CONFLICT        450
FT                   /note="G -> V (in Ref. 1; BAC39015)"
FT                   /evidence="ECO:0000305"
SQ   SEQUENCE   475 AA;  51386 MW;  F131B497C4F5FAF4 CRC64;
     MTTILTSTFR NLSTTSKWAL RSSIRPLSCS SQLHSAPAVQ TKSKKTLAKP NMKNIVVVEG
     VRIPFLLSGT SYKDLMPHDL ARAALSGLLH RTNIPKDVVD YIIFGTVIQE VKTSNVAREA
     ALGAGFSDKT PAHTVTMACI SSNQAMTTAV GLIASGQCDV VVAGGVELMS DVPIRHSRNM
     RKMMLDLNKA KTLGQRLSLL SKFRLNFLSP ELPAVAEFST NETMGHSADR LAAAFAVSRM
     EQDEYALRSH SLAKKAQDEG HLSDIVPFKV PGKDTVTKDN GIRPSSLEQM AKLKPAFIKP
     YGTVTAANSS FLTDGASAML IMSEDRALAM GYKPKAYLRD FIYVSQDPKD QLLLGPTYAT
     PKVLEKAGLT MNDIDAFEFH EAFSGQILAN FKAMDSDWFA QNYMGRKTKV GSPPLEKFNI
     WGGSLSLGHP FGATGCRLVM AAANRLRKDG GQYALVAACA AGGQGHAMIV EAYPK
//
```

**Mascot:** http://www.matrixscience.com/


HNE (C) (+156.1150)
