# Supplementary material for: A common East-Asian ALDH2 mutation causes metabolic disorders and the therapeutic effect of ALDH2 activators
Source: Nat Commun. 2023 Sep 25;14:5971. doi: 10.1038/s41467-023-41570-6 (PMC10520061; doi:10.1038/s41467-023-41570-6)
Supplement: Supplementary file 4 — Supplementary Data 1 [file 41467_2023_41570_MOESM4_ESM.zip › Table S5c/Q99JY0/Q99JY0_KIO-2_K299_K349.html]

Mascot Search Results: Q99JY0
 

# MASCOT Search Results

## Protein View: Q99JY0

### Trifunctional enzyme subunit beta, mitochondrial OS=Mus musculus OX=10090 GN=Hadhb PE=1 SV=1

|  |  |
| --- | --- |
| Database: | Mouse\_UniProt\_proteomes |
| Score: | 6831 |
| Monoisotopic mass (M_r_): | 51639 |
| Calculated pI: | 9.43 |

Sequence similarity is available as an NCBI BLAST search of Q99JY0 against nr.

### Search parameters

|  |  |
| --- | --- |
| MS data file: | `D:\LCMSMS\2023 Users' data\230529-1\230529-1-KIO-2.raw` |
| Enzyme: | Trypsin/P: cuts C-term side of KR. |
| Fixed modifications: | Carbamidomethyl (C) |
| Variable modifications: | Deamidated (NQ), HNE (C), HNE (H), HNE (K), Oxidation (M) |

### Protein sequence coverage: 72%

Matched peptides shown in ***bold red***.

|  |  |  |  |  |  |
| --- | --- | --- | --- | --- | --- |
| `1` | `MTTILTSTFR` | `NLSTTSKWAL` | `RSSIRPLSCS` | `SQLHSAPAVQ` | `TKSKKTLAKP` |
| `51` | `NMKNIVVVEG` | `VRIPFLLSGT` | `SYKDLMPHDL` | `ARAALSGLLH` | `RTNIPKDVVD` |
| `101` | `YIIFGTVIQE` | `VKTSNVAREA` | `ALGAGFSDKT` | `PAHTVTMACI` | `SSNQAMTTAV` |
| `151` | `GLIASGQCDV` | `VVAGGVELMS` | `DVPIRHSRNM` | `RKMMLDLNKA` | `KTLGQRLSLL` |
| `201` | `SKFRLNFLSP` | `ELPAVAEFST` | `NETMGHSADR` | `LAAAFAVSRM` | `EQDEYALRSH` |
| `251` | `SLAKKAQDEG` | `HLSDIVPFKV` | `PGKDTVTKDN` | `GIRPSSLEQM` | `AKLKPAFIKP` |
| `301` | `YGTVTAANSS` | `FLTDGASAML` | `IMSEDRALAM` | `GYKPKAYLRD` | `FIYVSQDPKD` |
| `351` | `QLLLGPTYAT` | `PKVLEKAGLT` | `MNDIDAFEFH` | `EAFSGQILAN` | `FKAMDSDWFA` |
| `401` | `QNYMGRKTKV` | `GSPPLEKFNI` | `WGGSLSLGHP` | `FGATGCRLVM` | `AAANRLRKDG` |
| `451` | `GQYALVAACA` | `AGGQGHAMIV` | `EAYPK` |  |  |

Unformatted sequence string: 475 residues (for pasting into other applications).

|  |  |  |  |
| --- | --- | --- | --- |
| Sort by | residue number | increasing mass | decreasing mass |
| Show | matched peptides only | predicted peptides also |  |

| Query | Start | – | End | Observed | Mr(expt) | Mr(calc) | ppm | M | Score | Expect | Rank | U | Peptide |
| --- | --- | --- | --- | --- | --- | --- | --- | --- | --- | --- | --- | --- | --- |
| 13740 | 54 | – | 62 | 492.7946 | 983.5746 | 983.5764 | -1.82 | 0 | 27 | 0.003 | 1Score **> 29** indicates **identity** Score **> 14** indicates **homology** | U | K.NIVVVEGVR.I |
| 13741 | 54 | – | 62 | 492.7947 | 983.5749 | 983.5764 | -1.55 | 0 | 18 | 0.019 | 1Score **> 29** indicates **identity** Score **> 14** indicates **homology** | U | K.NIVVVEGVR.I |
| 13742 | 54 | – | 62 | 492.7948 | 983.5750 | 983.5764 | -1.44 | 0 | 24 | 0.0058 | 1Score **> 27** indicates **identity** Score **> 14** indicates **homology** | U | K.NIVVVEGVR.I |
| 13743 | 54 | – | 62 | 492.7949 | 983.5753 | 983.5764 | -1.14 | 0 | 19 | 0.027 | 1Score **> 27** indicates **identity** Score **> 16** indicates **homology** | U | K.NIVVVEGVR.I |
| 13744 | 54 | – | 62 | 492.7952 | 983.5759 | 983.5764 | -0.52 | 0 | 25 | 0.0055 | 1Score **> 27** indicates **identity** Score **> 15** indicates **homology** | U | K.NIVVVEGVR.I |
| 13745 | 54 | – | 62 | 492.7953 | 983.5760 | 983.5764 | -0.36 | 0 | 40 | 0.0002 | 1Score **> 27** indicates **identity** Score **> 15** indicates **homology** | U | K.NIVVVEGVR.I |
| 13746 | 54 | – | 62 | 492.7954 | 983.5763 | 983.5764 | -0.064 | 0 | 41 | 0.00014 | 1Score **> 27** indicates **identity** Score **> 15** indicates **homology** | U | K.NIVVVEGVR.I |
| 13747 | 54 | – | 62 | 492.7955 | 983.5764 | 983.5764 | 0.0010 | 0 | 35 | 0.00098 | 1Score **> 27** indicates **identity** Score **> 18** indicates **homology** | U | K.NIVVVEGVR.I |
| 13748 | 54 | – | 62 | 492.7955 | 983.5764 | 983.5764 | 0.029 | 0 | 48 | 0.0001 | 1Score **> 27** indicates **identity** Score **> 21** indicates **homology** | U | K.NIVVVEGVR.I |
| 13749 | 54 | – | 62 | 492.7955 | 983.5764 | 983.5764 | 0.050 | 0 | 30 | 0.003 | 1Score **> 27** indicates **identity** Score **> 17** indicates **homology** | U | K.NIVVVEGVR.I |
| 13750 | 54 | – | 62 | 492.7957 | 983.5768 | 983.5764 | 0.39 | 0 | 30 | 0.0015 | 1Score **> 27** indicates **identity** Score **> 14** indicates **homology** | U | K.NIVVVEGVR.I |
| 13751 | 54 | – | 62 | 492.7957 | 983.5768 | 983.5764 | 0.41 | 0 | 45 | 0.00012 | 1Score **> 27** indicates **identity** Score **> 18** indicates **homology** | U | K.NIVVVEGVR.I |
| 13752 | 54 | – | 62 | 492.7957 | 983.5769 | 983.5764 | 0.49 | 0 | 24 | 0.0056 | 1Score **> 27** indicates **identity** Score **> 14** indicates **homology** | U | K.NIVVVEGVR.I |
| 13753 | 54 | – | 62 | 492.7958 | 983.5770 | 983.5764 | 0.63 | 0 | 43 | 0.00021 | 1Score **> 27** indicates **identity** Score **> 18** indicates **homology** | U | K.NIVVVEGVR.I |
| 13754 | 54 | – | 62 | 492.7958 | 983.5770 | 983.5764 | 0.67 | 0 | 32 | 0.00093 | 1Score **> 27** indicates **identity** Score **> 15** indicates **homology** | U | K.NIVVVEGVR.I |
| 13755 | 54 | – | 62 | 492.7958 | 983.5771 | 983.5764 | 0.76 | 0 | 28 | 0.0023 | 1Score **> 27** indicates **identity** Score **> 14** indicates **homology** | U | K.NIVVVEGVR.I |
| 13756 | 54 | – | 62 | 492.7960 | 983.5774 | 983.5764 | 1.04 | 0 | 27 | 0.0032 | 1Score **> 27** indicates **identity** Score **> 14** indicates **homology** | U | K.NIVVVEGVR.I |
| 13757 | 54 | – | 62 | 492.7963 | 983.5780 | 983.5764 | 1.59 | 0 | 34 | 0.0041 | 1Score **> 29** indicates **identity** Score **> 23** indicates **homology** | U | K.NIVVVEGVR.I |
| 13759 | 54 | – | 62 | 492.7969 | 983.5793 | 983.5764 | 2.97 | 0 | 37 | 0.0025 | 1Score **> 28** indicates **identity** Score **> 24** indicates **homology** | U | K.NIVVVEGVR.I |
| 13763 | 54 | – | 62 | 492.7983 | 983.5821 | 983.5764 | 5.77 | 0 | 37 | 0.0026 | 1Score **> 29** indicates **identity** Score **> 24** indicates **homology** | U | K.NIVVVEGVR.I |
| 143807 | 54 | – | 73 | 731.0857 | 2190.2354 | 2190.2412 | -2.66 | 1 | 19 | 0.018 | 1Score **> 33** indicates **identity** Score **> 14** indicates **homology** | U | K.NIVVVEGVRIPFLLSGTSYK.D |
| 143808 | 54 | – | 73 | 1096.1296 | 2190.2447 | 2190.2412 | 1.56 | 1 | 63 | 1.2e-06 | 1Score **> 33** indicates **identity** Score **> 16** indicates **homology** | U | K.NIVVVEGVRIPFLLSGTSYK.D |
| 143809 | 54 | – | 73 | 731.0889 | 2190.2447 | 2190.2412 | 1.60 | 1 | 17 | 0.024 | 1Score **> 33** indicates **identity** Score **> 14** indicates **homology** | U | K.NIVVVEGVRIPFLLSGTSYK.D |
| 36536 | 63 | – | 73 | 613.3443 | 1224.6740 | 1224.6754 | -1.15 | 0 | 42 | 0.00012 | 1Score **> 32** indicates **identity** Score **> 15** indicates **homology** | U | R.IPFLLSGTSYK.D |
| 36538 | 63 | – | 73 | 613.3454 | 1224.6762 | 1224.6754 | 0.66 | 0 | 56 | 5.6e-06 | 1Score **> 32** indicates **identity** Score **> 16** indicates **homology** | U | R.IPFLLSGTSYK.D |
| 36539 | 63 | – | 73 | 613.3456 | 1224.6766 | 1224.6754 | 0.96 | 0 | 68 | 4.1e-07 | 1Score **> 32** indicates **identity** Score **> 17** indicates **homology** | U | R.IPFLLSGTSYK.D |
| 36540 | 63 | – | 73 | 613.3456 | 1224.6767 | 1224.6754 | 1.06 | 0 | 79 | 3.7e-08 | 1Score **> 32** indicates **identity** Score **> 17** indicates **homology** | U | R.IPFLLSGTSYK.D |
| 36541 | 63 | – | 73 | 613.3457 | 1224.6768 | 1224.6754 | 1.17 | 0 | 77 | 6.1e-08 | 1Score **> 32** indicates **identity** Score **> 17** indicates **homology** | U | R.IPFLLSGTSYK.D |
| 36542 | 63 | – | 73 | 613.3457 | 1224.6769 | 1224.6754 | 1.25 | 0 | 36 | 0.00038 | 1Score **> 32** indicates **identity** Score **> 15** indicates **homology** | U | R.IPFLLSGTSYK.D |
| 36543 | 63 | – | 73 | 613.3459 | 1224.6772 | 1224.6754 | 1.43 | 0 | 50 | 2.2e-05 | 1Score **> 32** indicates **identity** Score **> 16** indicates **homology** | U | R.IPFLLSGTSYK.D |
| 36544 | 63 | – | 73 | 613.3460 | 1224.6775 | 1224.6754 | 1.67 | 0 | 64 | 9.2e-07 | 1Score **> 32** indicates **identity** Score **> 17** indicates **homology** | U | R.IPFLLSGTSYK.D |
| 36546 | 63 | – | 73 | 613.3469 | 1224.6793 | 1224.6754 | 3.16 | 0 | 41 | 0.00014 | 1Score **> 32** indicates **identity** Score **> 15** indicates **homology** | U | R.IPFLLSGTSYK.D |
| 36547 | 63 | – | 73 | 613.3472 | 1224.6798 | 1224.6754 | 3.55 | 0 | 39 | 0.00022 | 1Score **> 32** indicates **identity** Score **> 15** indicates **homology** | U | R.IPFLLSGTSYK.D |
| 149914 | 63 | – | 82 | 569.3037 | 2273.1857 | 2273.1878 | -0.93 | 1 | 24 | 0.0056 | 1Score **> 37** indicates **identity** Score **> 14** indicates **homology** | U | R.IPFLLSGTSYKDLMPHDLAR.A |
| 149915 | 63 | – | 82 | 569.3044 | 2273.1884 | 2273.1878 | 0.28 | 1 | 45 | 5.5e-05 | 1Score **> 37** indicates **identity** Score **> 15** indicates **homology** | U | R.IPFLLSGTSYKDLMPHDLAR.A |
| 149917 | 63 | – | 82 | 758.7368 | 2273.1886 | 2273.1878 | 0.37 | 1 | 67 | 1.5e-06 | 1Score **> 37** indicates **identity** Score **> 21** indicates **homology** | U | R.IPFLLSGTSYKDLMPHDLAR.A |
| 149919 | 63 | – | 82 | 758.7370 | 2273.1891 | 2273.1878 | 0.55 | 1 | 61 | 2e-06 | 1Score **> 37** indicates **identity** Score **> 16** indicates **homology** | U | R.IPFLLSGTSYKDLMPHDLAR.A |
| 149920 | 63 | – | 82 | 569.3046 | 2273.1891 | 2273.1878 | 0.58 | 1 | 47 | 3.6e-05 | 1Score **> 37** indicates **identity** Score **> 15** indicates **homology** | U | R.IPFLLSGTSYKDLMPHDLAR.A |
| 149921 | 63 | – | 82 | 758.7370 | 2273.1891 | 2273.1878 | 0.59 | 1 | 49 | 2.7e-05 | 1Score **> 37** indicates **identity** Score **> 16** indicates **homology** | U | R.IPFLLSGTSYKDLMPHDLAR.A |
| 149922 | 63 | – | 82 | 569.3046 | 2273.1892 | 2273.1878 | 0.60 | 1 | 53 | 1e-05 | 1Score **> 37** indicates **identity** Score **> 16** indicates **homology** | U | R.IPFLLSGTSYKDLMPHDLAR.A |
| 149923 | 63 | – | 82 | 569.3047 | 2273.1898 | 2273.1878 | 0.86 | 1 | 35 | 0.00056 | 1Score **> 37** indicates **identity** Score **> 15** indicates **homology** | U | R.IPFLLSGTSYKDLMPHDLAR.A |
| 149924 | 63 | – | 82 | 569.3048 | 2273.1900 | 2273.1878 | 0.97 | 1 | 15 | 0.039 | 1Score **> 37** indicates **identity** Score **> 13** indicates **homology** | U | R.IPFLLSGTSYKDLMPHDLAR.A |
| 149925 | 63 | – | 82 | 758.7373 | 2273.1901 | 2273.1878 | 1.02 | 1 | 59 | 2.8e-06 | 1Score **> 37** indicates **identity** Score **> 16** indicates **homology** | U | R.IPFLLSGTSYKDLMPHDLAR.A |
| 149926 | 63 | – | 82 | 569.3049 | 2273.1903 | 2273.1878 | 1.10 | 1 | 52 | 1.3e-05 | 1Score **> 37** indicates **identity** Score **> 16** indicates **homology** | U | R.IPFLLSGTSYKDLMPHDLAR.A |
| 150864 | 63 | – | 82 | 573.3060 | 2289.1949 | 2289.1827 | 5.33 | 1 | 29 | 0.002 | 1Score **> 37** indicates **identity** Score **> 14** indicates **homology** | U | R.IPFLLSGTSYKDLMPHDLAR.A  + Oxidation (M) |
| 150868 | 63 | – | 82 | 573.3067 | 2289.1977 | 2289.1827 | 6.52 | 1 | 22 | 0.0079 | 1Score **> 37** indicates **identity** Score **> 14** indicates **homology** | U | R.IPFLLSGTSYKDLMPHDLAR.A  + Oxidation (M) |
| 150869 | 63 | – | 82 | 764.0732 | 2289.1979 | 2289.1827 | 6.64 | 1 | 18 | 0.02 | 1Score **> 37** indicates **identity** Score **> 14** indicates **homology** | U | R.IPFLLSGTSYKDLMPHDLAR.A  + Oxidation (M) |
| 19842 | 74 | – | 82 | 356.5148 | 1066.5225 | 1066.5229 | -0.38 | 0 | 19 | 0.017 | 1Score **> 30** indicates **identity** Score **> 14** indicates **homology** | U | K.DLMPHDLAR.A |
| 19843 | 74 | – | 82 | 356.5150 | 1066.5232 | 1066.5229 | 0.21 | 0 | 26 | 0.0038 | 1Score **> 30** indicates **identity** Score **> 14** indicates **homology** | U | K.DLMPHDLAR.A |
| 19844 | 74 | – | 82 | 356.5151 | 1066.5235 | 1066.5229 | 0.54 | 0 | 25 | 0.0043 | 1Score **> 30** indicates **identity** Score **> 14** indicates **homology** | U | K.DLMPHDLAR.A |
| 10790 | 83 | – | 91 | 469.2821 | 936.5496 | 936.5505 | -0.89 | 0 | 43 | 8.5e-05 | 1Score **> 23** indicates **identity** Score **> 15** indicates **homology** | U | R.AALSGLLHR.T |
| 10791 | 83 | – | 91 | 469.2822 | 936.5499 | 936.5505 | -0.67 | 0 | 76 | 7.8e-08 | 1Score **> 23** indicates **identity** Score **> 17** indicates **homology** | U | R.AALSGLLHR.T |
| 10792 | 83 | – | 91 | 469.2822 | 936.5499 | 936.5505 | -0.64 | 0 | 66 | 6e-07 | 1Score **> 23** indicates **identity** Score **> 17** indicates **homology** | U | R.AALSGLLHR.T |
| 10793 | 83 | – | 91 | 469.2825 | 936.5505 | 936.5505 | 0.017 | 0 | 48 | 7.7e-05 | 1Score **> 23** indicates **identity** Score **> 20** indicates **homology** | U | R.AALSGLLHR.T |
| 10794 | 83 | – | 91 | 469.2833 | 936.5521 | 936.5505 | 1.78 | 0 | 45 | 6.5e-05 | 1Score **> 23** indicates **identity** Score **> 15** indicates **homology** | U | R.AALSGLLHR.T |
| 10795 | 83 | – | 91 | 469.2834 | 936.5523 | 936.5505 | 1.96 | 0 | 22 | 0.0078 | 1Score **> 23** indicates **identity** Score **> 14** indicates **homology** | U | R.AALSGLLHR.T |
| 10796 | 83 | – | 91 | 469.2843 | 936.5540 | 936.5505 | 3.80 | 0 | 47 | 0.00037 | 1Score **> 25** indicates **identity** | U | R.AALSGLLHR.T |
| 157523 | 92 | – | 112 | 797.7769 | 2390.3089 | 2390.3097 | -0.35 | 1 | 61 | 1.8e-06 | 1Score **> 35** indicates **identity** Score **> 16** indicates **homology** | U | R.TNIPKDVVDYIIFGTVIQEVK.T |
| 157525 | 92 | – | 112 | 1196.1621 | 2390.3096 | 2390.3097 | -0.058 | 1 | 92 | 2.3e-09 | 1Score **> 35** indicates **identity** Score **> 18** indicates **homology** | U | R.TNIPKDVVDYIIFGTVIQEVK.T |
| 157526 | 92 | – | 112 | 598.5848 | 2390.3103 | 2390.3097 | 0.23 | 1 | 56 | 5.7e-06 | 1Score **> 35** indicates **identity** Score **> 16** indicates **homology** | U | R.TNIPKDVVDYIIFGTVIQEVK.T |
| 157527 | 92 | – | 112 | 797.7774 | 2390.3105 | 2390.3097 | 0.33 | 1 | 49 | 2.7e-05 | 1Score **> 35** indicates **identity** Score **> 16** indicates **homology** | U | R.TNIPKDVVDYIIFGTVIQEVK.T |
| 157530 | 92 | – | 112 | 797.7779 | 2390.3119 | 2390.3097 | 0.92 | 1 | 36 | 0.00042 | 1Score **> 35** indicates **identity** Score **> 15** indicates **homology** | U | R.TNIPKDVVDYIIFGTVIQEVK.T |
| 157532 | 92 | – | 112 | 598.5855 | 2390.3130 | 2390.3097 | 1.38 | 1 | 43 | 0.0001 | 1Score **> 35** indicates **identity** Score **> 15** indicates **homology** | U | R.TNIPKDVVDYIIFGTVIQEVK.T |
| 157533 | 92 | – | 112 | 1196.1641 | 2390.3136 | 2390.3097 | 1.63 | 1 | 31 | 0.0013 | 1Score **> 35** indicates **identity** Score **> 14** indicates **homology** | U | R.TNIPKDVVDYIIFGTVIQEVK.T |
| 157534 | 92 | – | 112 | 797.7785 | 2390.3136 | 2390.3097 | 1.63 | 1 | 69 | 3.1e-07 | 1Score **> 35** indicates **identity** Score **> 17** indicates **homology** | U | R.TNIPKDVVDYIIFGTVIQEVK.T |
| 157537 | 92 | – | 112 | 797.7786 | 2390.3141 | 2390.3097 | 1.83 | 1 | 61 | 2e-06 | 1Score **> 35** indicates **identity** Score **> 16** indicates **homology** | U | R.TNIPKDVVDYIIFGTVIQEVK.T |
| 157538 | 92 | – | 112 | 797.7787 | 2390.3141 | 2390.3097 | 1.85 | 1 | 61 | 2.1e-06 | 1Score **> 35** indicates **identity** Score **> 16** indicates **homology** | U | R.TNIPKDVVDYIIFGTVIQEVK.T |
| 157540 | 92 | – | 112 | 797.7788 | 2390.3145 | 2390.3097 | 1.99 | 1 | 40 | 0.00018 | 1Score **> 35** indicates **identity** Score **> 15** indicates **homology** | U | R.TNIPKDVVDYIIFGTVIQEVK.T |
| 157542 | 92 | – | 112 | 1196.1651 | 2390.3156 | 2390.3097 | 2.44 | 1 | 98 | 7e-10 | 1Score **> 35** indicates **identity** Score **> 19** indicates **homology** | U | R.TNIPKDVVDYIIFGTVIQEVK.T |
| 157546 | 92 | – | 112 | 797.7801 | 2390.3184 | 2390.3097 | 3.63 | 1 | 33 | 0.00088 | 1Score **> 35** indicates **identity** Score **> 15** indicates **homology** | U | R.TNIPKDVVDYIIFGTVIQEVK.T |
| 157548 | 92 | – | 112 | 797.7808 | 2390.3206 | 2390.3097 | 4.57 | 1 | 40 | 0.00017 | 1Score **> 34** indicates **identity** Score **> 15** indicates **homology** | U | R.TNIPKDVVDYIIFGTVIQEVK.T |
| 157549 | 92 | – | 112 | 797.7811 | 2390.3215 | 2390.3097 | 4.91 | 1 | 44 | 7.9e-05 | 1Score **> 35** indicates **identity** Score **> 15** indicates **homology** | U | R.TNIPKDVVDYIIFGTVIQEVK.T |
| 157551 | 92 | – | 112 | 797.7826 | 2390.3258 | 2390.3097 | 6.74 | 1 | 22 | 0.0094 | 1Score **> 34** indicates **identity** Score **> 14** indicates **homology** | U | R.TNIPKDVVDYIIFGTVIQEVK.T |
| 157552 | 92 | – | 112 | 797.7830 | 2390.3273 | 2390.3097 | 7.34 | 1 | 30 | 0.0017 | 1Score **> 34** indicates **identity** Score **> 14** indicates **homology** | U | R.TNIPKDVVDYIIFGTVIQEVK.T |
| 184312 | 92 | – | 118 | 1007.5554 | 3019.6442 | 3019.6230 | 7.03 | 2 | 35 | 0.00054 | 1Score **> 34** indicates **identity** Score **> 15** indicates **homology** | U | R.TNIPKDVVDYIIFGTVIQEVKTSNVAR.E  + Deamidated (NQ) |
| 110510 | 97 | – | 112 | 919.4977 | 1836.9808 | 1836.9873 | -3.57 | 0 | 33 | 0.0025 | 1Score **> 35** indicates **identity** Score **> 19** indicates **homology** | U | K.DVVDYIIFGTVIQEVK.T |
| 110513 | 97 | – | 112 | 919.4988 | 1836.9830 | 1836.9873 | -2.36 | 0 | 54 | 6.6e-05 | 1Score **> 35** indicates **identity** Score **> 24** indicates **homology** | U | K.DVVDYIIFGTVIQEVK.T |
| 110523 | 97 | – | 112 | 613.3360 | 1836.9863 | 1836.9873 | -0.57 | 0 | 47 | 4e-05 | 1Score **> 35** indicates **identity** Score **> 15** indicates **homology** | U | K.DVVDYIIFGTVIQEVK.T |
| 110533 | 97 | – | 112 | 613.3367 | 1836.9883 | 1836.9873 | 0.52 | 0 | 43 | 9.9e-05 | 1Score **> 35** indicates **identity** Score **> 15** indicates **homology** | U | K.DVVDYIIFGTVIQEVK.T |
| 110534 | 97 | – | 112 | 613.3369 | 1836.9889 | 1836.9873 | 0.83 | 0 | 36 | 0.00043 | 1Score **> 35** indicates **identity** Score **> 15** indicates **homology** | U | K.DVVDYIIFGTVIQEVK.T |
| 110535 | 97 | – | 112 | 919.5018 | 1836.9891 | 1836.9873 | 0.98 | 0 | 69 | 1.8e-06 | 1Score **> 35** indicates **identity** Score **> 24** indicates **homology** | U | K.DVVDYIIFGTVIQEVK.T |
| 110536 | 97 | – | 112 | 613.3370 | 1836.9891 | 1836.9873 | 0.98 | 0 | 53 | 1.1e-05 | 1Score **> 35** indicates **identity** Score **> 16** indicates **homology** | U | K.DVVDYIIFGTVIQEVK.T |
| 110542 | 97 | – | 112 | 613.3380 | 1836.9920 | 1836.9873 | 2.55 | 0 | 35 | 0.00055 | 1Score **> 35** indicates **identity** Score **> 15** indicates **homology** | U | K.DVVDYIIFGTVIQEVK.T |
| 110543 | 97 | – | 112 | 919.5033 | 1836.9921 | 1836.9873 | 2.61 | 0 | 17 | 0.024 | 1Score **> 35** indicates **identity** Score **> 14** indicates **homology** | U | K.DVVDYIIFGTVIQEVK.T |
| 110544 | 97 | – | 112 | 613.3382 | 1836.9927 | 1836.9873 | 2.94 | 0 | 52 | 1.4e-05 | 1Score **> 35** indicates **identity** Score **> 16** indicates **homology** | U | K.DVVDYIIFGTVIQEVK.T |
| 110545 | 97 | – | 112 | 613.3389 | 1836.9949 | 1836.9873 | 4.11 | 0 | 37 | 0.00032 | 1Score **> 35** indicates **identity** Score **> 15** indicates **homology** | U | K.DVVDYIIFGTVIQEVK.T |
| 19685 | 119 | – | 129 | 533.2632 | 1064.5119 | 1064.5138 | -1.83 | 0 | 27 | 0.0031 | 1Score **> 31** indicates **identity** Score **> 14** indicates **homology** | U | R.EAALGAGFSDK.T |
| 19687 | 119 | – | 129 | 533.2643 | 1064.5140 | 1064.5138 | 0.18 | 0 | 51 | 1.8e-05 | 1Score **> 31** indicates **identity** Score **> 16** indicates **homology** | U | R.EAALGAGFSDK.T |
| 19688 | 119 | – | 129 | 533.2646 | 1064.5147 | 1064.5138 | 0.84 | 0 | 61 | 3.5e-06 | 1Score **> 31** indicates **identity** Score **> 19** indicates **homology** | U | R.EAALGAGFSDK.T |
| 201650 | 119 | – | 175 | 989.8242 | 5932.9013 | 5932.9030 | -0.27 | 1 | 23 | 0.0075 | 1Score **> 35** indicates **identity** Score **> 14** indicates **homology** | U | R.EAALGAGFSDKTPAHTVTMACISSNQAMTTAVGLIASGQCDVVVAGGVELMSDVPIR.H  + HNE (H); Oxidation (M) |
| 201655 | 119 | – | 175 | 989.9866 | 5933.8757 | 5933.8870 | -1.89 | 1 | 14 | 0.045 | 1Score **> 35** indicates **identity** Score **> 13** indicates **homology** | U | R.EAALGAGFSDKTPAHTVTMACISSNQAMTTAVGLIASGQCDVVVAGGVELMSDVPIR.H  + Deamidated (NQ); HNE (H); Oxidation (M) |
| 201657 | 119 | – | 175 | 989.9885 | 5933.8871 | 5933.8870 | 0.028 | 1 | 20 | 0.014 | 1Score **> 35** indicates **identity** Score **> 14** indicates **homology** | U | R.EAALGAGFSDKTPAHTVTMACISSNQAMTTAVGLIASGQCDVVVAGGVELMSDVPIR.H  + Deamidated (NQ); HNE (H); Oxidation (M) |
| 201658 | 119 | – | 175 | 989.9899 | 5933.8958 | 5933.8870 | 1.48 | 1 | 14 | 0.047 | 1Score **> 35** indicates **identity** Score **> 13** indicates **homology** | U | R.EAALGAGFSDKTPAHTVTMACISSNQAMTTAVGLIASGQCDVVVAGGVELMSDVPIR.H  + Deamidated (NQ); HNE (H); Oxidation (M) |
| 201659 | 119 | – | 175 | 989.9901 | 5933.8970 | 5933.8870 | 1.69 | 1 | 15 | 0.038 | 1Score **> 35** indicates **identity** Score **> 13** indicates **homology** | U | R.EAALGAGFSDKTPAHTVTMACISSNQAMTTAVGLIASGQCDVVVAGGVELMSDVPIR.H  + Deamidated (NQ); HNE (H); Oxidation (M) |
| 201660 | 119 | – | 175 | 1187.7868 | 5933.8975 | 5933.8870 | 1.78 | 1 | 17 | 0.025 | 1Score **> 35** indicates **identity** Score **> 14** indicates **homology** | U | R.EAALGAGFSDKTPAHTVTMACISSNQAMTTAVGLIASGQCDVVVAGGVELMSDVPIR.H  + Deamidated (NQ); HNE (H); Oxidation (M) |
| 201661 | 119 | – | 175 | 989.9910 | 5933.9025 | 5933.8870 | 2.62 | 1 | 26 | 0.0033 | 1Score **> 35** indicates **identity** Score **> 14** indicates **homology** | U | R.EAALGAGFSDKTPAHTVTMACISSNQAMTTAVGLIASGQCDVVVAGGVELMSDVPIR.H  + Deamidated (NQ); HNE (H); Oxidation (M) |
| 201723 | 119 | – | 175 | 997.1559 | 5976.8920 | 5976.9431 | -8.54 | 1 | 27 | 0.0032 | 1Score **> 35** indicates **identity** Score **> 14** indicates **homology** | U | R.EAALGAGFSDKTPAHTVTMACISSNQAMTTAVGLIASGQCDVVVAGGVELMSDVPIR.H  + 2 Deamidated (NQ); 2 HNE (C); Oxidation (M) |
| 186791 | 203 | – | 230 | 1046.5125 | 3136.5156 | 3136.4924 | 7.42 | 1 | 63 | 1.1e-06 | 1Score **> 37** indicates **identity** Score **> 16** indicates **homology** | U | K.FRLNFLSPELPAVAEFSTNETMGHSADR.L  + Deamidated (NQ) |
| 186799 | 203 | – | 230 | 785.3811 | 3137.4953 | 3137.4764 | 6.03 | 1 | 16 | 0.034 | 1Score **> 36** indicates **identity** Score **> 13** indicates **homology** | U | K.FRLNFLSPELPAVAEFSTNETMGHSADR.L  + 2 Deamidated (NQ) |
| 178102 | 205 | – | 230 | 945.1176 | 2832.3310 | 2832.3388 | -2.77 | 0 | 30 | 0.0014 | 1Score **> 35** indicates **identity** Score **> 14** indicates **homology** | U | R.LNFLSPELPAVAEFSTNETMGHSADR.L |
| 178106 | 205 | – | 230 | 945.1229 | 2832.3468 | 2832.3388 | 2.82 | 0 | 60 | 2.4e-06 | 1Score **> 36** indicates **identity** Score **> 16** indicates **homology** | U | R.LNFLSPELPAVAEFSTNETMGHSADR.L |
| 178661 | 205 | – | 230 | 950.4484 | 2848.3233 | 2848.3338 | -3.67 | 0 | 32 | 0.001 | 1Score **> 35** indicates **identity** Score **> 14** indicates **homology** | U | R.LNFLSPELPAVAEFSTNETMGHSADR.L  + Oxidation (M) |
| 8863 | 231 | – | 239 | 453.2633 | 904.5121 | 904.5130 | -1.02 | 0 | 46 | 0.0003 | 1Score **> 29** indicates **identity** Score **> 23** indicates **homology** | U | R.LAAAFAVSR.M |
| 8864 | 231 | – | 239 | 453.2634 | 904.5123 | 904.5130 | -0.81 | 0 | 46 | 0.0011 | 1Score **> 29** indicates **identity** | U | R.LAAAFAVSR.M |
| 8865 | 231 | – | 239 | 453.2636 | 904.5126 | 904.5130 | -0.42 | 0 | 59 | 5.2e-05 | 1Score **> 29** indicates **identity** | U | R.LAAAFAVSR.M |
| 8866 | 231 | – | 239 | 453.2636 | 904.5126 | 904.5130 | -0.42 | 0 | 59 | 4.4e-05 | 1Score **> 29** indicates **identity** Score **> 28** indicates **homology** | U | R.LAAAFAVSR.M |
| 8867 | 231 | – | 239 | 453.2637 | 904.5128 | 904.5130 | -0.23 | 0 | 45 | 0.00061 | 1Score **> 29** indicates **identity** Score **> 25** indicates **homology** | U | R.LAAAFAVSR.M |
| 8868 | 231 | – | 239 | 453.2637 | 904.5128 | 904.5130 | -0.22 | 0 | 46 | 0.001 | 1Score **> 29** indicates **identity** | U | R.LAAAFAVSR.M |
| 8869 | 231 | – | 239 | 453.2637 | 904.5129 | 904.5130 | -0.13 | 0 | 34 | 0.0053 | 1Score **> 29** indicates **identity** Score **> 24** indicates **homology** | U | R.LAAAFAVSR.M |
| 8870 | 231 | – | 239 | 453.2639 | 904.5132 | 904.5130 | 0.13 | 0 | 40 | 0.0029 | 1Score **> 29** indicates **identity** Score **> 27** indicates **homology** | U | R.LAAAFAVSR.M |
| 8871 | 231 | – | 239 | 453.2640 | 904.5134 | 904.5130 | 0.46 | 0 | 61 | 3.4e-05 | 1Score **> 29** indicates **identity** | U | R.LAAAFAVSR.M |
| 8872 | 231 | – | 239 | 453.2640 | 904.5134 | 904.5130 | 0.46 | 0 | 53 | 0.00012 | 1Score **> 29** indicates **identity** Score **> 26** indicates **homology** | U | R.LAAAFAVSR.M |
| 8875 | 231 | – | 239 | 453.2641 | 904.5136 | 904.5130 | 0.58 | 0 | 41 | 0.0016 | 1Score **> 29** indicates **identity** Score **> 26** indicates **homology** | U | R.LAAAFAVSR.M |
| 8876 | 231 | – | 239 | 453.2641 | 904.5136 | 904.5130 | 0.59 | 0 | 59 | 5.3e-05 | 1Score **> 29** indicates **identity** | U | R.LAAAFAVSR.M |
| 8877 | 231 | – | 239 | 453.2641 | 904.5136 | 904.5130 | 0.65 | 0 | 46 | 0.00085 | 1Score **> 29** indicates **identity** Score **> 28** indicates **homology** | U | R.LAAAFAVSR.M |
| 8879 | 231 | – | 239 | 453.2643 | 904.5140 | 904.5130 | 1.07 | 0 | 40 | 0.0014 | 1Score **> 29** indicates **identity** Score **> 24** indicates **homology** | U | R.LAAAFAVSR.M |
| 8880 | 231 | – | 239 | 453.2643 | 904.5141 | 904.5130 | 1.17 | 0 | 53 | 0.00015 | 1Score **> 29** indicates **identity** Score **> 27** indicates **homology** | U | R.LAAAFAVSR.M |
| 8882 | 231 | – | 239 | 453.2645 | 904.5145 | 904.5130 | 1.60 | 0 | 40 | 0.0014 | 1Score **> 27** indicates **identity** Score **> 24** indicates **homology** | U | R.LAAAFAVSR.M |
| 28584 | 240 | – | 248 | 577.7604 | 1153.5062 | 1153.5073 | -1.02 | 0 | 47 | 4.1e-05 | 1Score **> 28** indicates **identity** Score **> 15** indicates **homology** | U | R.MEQDEYALR.S |
| 30318 | 240 | – | 248 | 585.7585 | 1169.5025 | 1169.5023 | 0.24 | 0 | 35 | 0.00056 | 1Score **> 26** indicates **identity** Score **> 15** indicates **homology** | U | R.MEQDEYALR.S  + Oxidation (M) |
| 30319 | 240 | – | 248 | 585.7588 | 1169.5030 | 1169.5023 | 0.64 | 0 | 18 | 0.019 | 1Score **> 26** indicates **identity** Score **> 14** indicates **homology** | U | R.MEQDEYALR.S  + Oxidation (M) |
| 30320 | 240 | – | 248 | 585.7588 | 1169.5031 | 1169.5023 | 0.74 | 0 | 21 | 0.012 | 1Score **> 26** indicates **identity** Score **> 14** indicates **homology** | U | R.MEQDEYALR.S  + Oxidation (M) |
| 30321 | 240 | – | 248 | 585.7590 | 1169.5035 | 1169.5023 | 1.03 | 0 | 18 | 0.02 | 1Score **> 26** indicates **identity** Score **> 14** indicates **homology** | U | R.MEQDEYALR.S  + Oxidation (M) |
| 30322 | 240 | – | 248 | 585.7593 | 1169.5040 | 1169.5023 | 1.47 | 0 | 31 | 0.0012 | 1Score **> 26** indicates **identity** Score **> 14** indicates **homology** | U | R.MEQDEYALR.S  + Oxidation (M) |
| 132074 | 255 | – | 273 | 413.8281 | 2064.1042 | 2064.1004 | 1.83 | 2 | 27 | 0.0031 | 1Score **> 36** indicates **identity** Score **> 14** indicates **homology** | U | K.KAQDEGHLSDIVPFKVPGK.D |
| 132076 | 255 | – | 273 | 517.0335 | 2064.1048 | 2064.1004 | 2.12 | 2 | 18 | 0.021 | 1Score **> 36** indicates **identity** Score **> 14** indicates **homology** | U | K.KAQDEGHLSDIVPFKVPGK.D |
| 132077 | 255 | – | 273 | 517.0341 | 2064.1071 | 2064.1004 | 3.25 | 2 | 38 | 0.00028 | 1Score **> 36** indicates **identity** Score **> 15** indicates **homology** | U | K.KAQDEGHLSDIVPFKVPGK.D |
| 132078 | 255 | – | 273 | 517.0341 | 2064.1072 | 2064.1004 | 3.32 | 2 | 34 | 0.00068 | 1Score **> 36** indicates **identity** Score **> 15** indicates **homology** | U | K.KAQDEGHLSDIVPFKVPGK.D |
| 120150 | 256 | – | 273 | 485.0088 | 1936.0063 | 1936.0054 | 0.45 | 1 | 14 | 0.049 | 1Score **> 36** indicates **identity** Score **> 13** indicates **homology** | U | K.AQDEGHLSDIVPFKVPGK.D |
| 120153 | 256 | – | 273 | 485.0097 | 1936.0095 | 1936.0054 | 2.11 | 1 | 14 | 0.044 | 1Score **> 36** indicates **identity** Score **> 13** indicates **homology** | U | K.AQDEGHLSDIVPFKVPGK.D |
| 120158 | 256 | – | 273 | 646.3464 | 1936.0174 | 1936.0054 | 6.21 | 1 | 15 | 0.036 | 1Score **> 36** indicates **identity** Score **> 13** indicates **homology** | U | K.AQDEGHLSDIVPFKVPGK.D |
| 162749 | 256 | – | 278 | 621.0802 | 2480.2917 | 2480.2911 | 0.22 | 2 | 24 | 0.0052 | 1Score **> 37** indicates **identity** Score **> 14** indicates **homology** | U | K.AQDEGHLSDIVPFKVPGKDTVTK.D |
| 162750 | 256 | – | 278 | 621.0803 | 2480.2920 | 2480.2911 | 0.36 | 2 | 29 | 0.0018 | 1Score **> 37** indicates **identity** Score **> 14** indicates **homology** | U | K.AQDEGHLSDIVPFKVPGKDTVTK.D |
| 162752 | 256 | – | 278 | 827.7714 | 2480.2923 | 2480.2911 | 0.48 | 2 | 27 | 0.0028 | 1Score **> 37** indicates **identity** Score **> 14** indicates **homology** | U | K.AQDEGHLSDIVPFKVPGKDTVTK.D |
| 162754 | 256 | – | 278 | 827.7715 | 2480.2927 | 2480.2911 | 0.64 | 2 | 24 | 0.0062 | 1Score **> 37** indicates **identity** Score **> 14** indicates **homology** | U | K.AQDEGHLSDIVPFKVPGKDTVTK.D |
| 73782 | 279 | – | 292 | 773.3863 | 1544.7581 | 1544.7617 | -2.31 | 1 | 34 | 0.0058 | 1Score **> 34** indicates **identity** Score **> 24** indicates **homology** | U | K.DNGIRPSSLEQMAK.L |
| 73783 | 279 | – | 292 | 515.9268 | 1544.7586 | 1544.7617 | -1.97 | 1 | 15 | 0.036 | 1Score **> 34** indicates **identity** Score **> 13** indicates **homology** | U | K.DNGIRPSSLEQMAK.L |
| 73784 | 279 | – | 292 | 773.3867 | 1544.7588 | 1544.7617 | -1.88 | 1 | 27 | 0.025 | 1Score **> 34** indicates **identity** Score **> 24** indicates **homology** | U | K.DNGIRPSSLEQMAK.L |
| 75815 | 279 | – | 292 | 781.3847 | 1560.7548 | 1560.7566 | -1.12 | 1 | 66 | 2.4e-05 | 1Score **> 33** indicates **identity** Score **> 32** indicates **homology** | U | K.DNGIRPSSLEQMAK.L  + Oxidation (M) |
| 75816 | 279 | – | 292 | 781.3858 | 1560.7570 | 1560.7566 | 0.29 | 1 | 45 | 0.00049 | 1Score **> 33** indicates **identity** Score **> 24** indicates **homology** | U | K.DNGIRPSSLEQMAK.L  + Oxidation (M) |
| 196067 | 293 | – | 326 | 944.7288 | 3774.8862 | 3774.9100 | -6.30 | 2 | 24 | 0.0053 | 1Score **> 38** indicates **identity** Score **> 14** indicates **homology** | U | K.LKPAFIKPYGTVTAANSSFLTDGASAMLIMSEDR.A  + Deamidated (NQ); HNE (K); Oxidation (M) |
| 177683 | 300 | – | 326 | 941.1031 | 2820.2876 | 2820.2946 | -2.48 | 0 | 25 | 0.0047 | 1Score **> 33** indicates **identity** Score **> 14** indicates **homology** | U | K.PYGTVTAANSSFLTDGASAMLIMSEDR.A  + Oxidation (M) |
| 13431 | 327 | – | 335 | 489.7757 | 977.5368 | 977.5368 | 0.044 | 1 | 32 | 0.00098 | 1Score **> 30** indicates **identity** Score **> 15** indicates **homology** | U | R.ALAMGYKPK.A |
| 96579 | 336 | – | 349 | 572.2961 | 1713.8664 | 1713.8726 | -3.63 | 1 | 17 | 0.023 | 1Score **> 35** indicates **identity** Score **> 14** indicates **homology** | U | K.AYLRDFIYVSQDPK.D |
| 96592 | 336 | – | 349 | 572.2986 | 1713.8740 | 1713.8726 | 0.84 | 1 | 15 | 0.036 | 1Score **> 35** indicates **identity** Score **> 13** indicates **homology** | U | K.AYLRDFIYVSQDPK.D |
| 96596 | 336 | – | 349 | 572.2990 | 1713.8752 | 1713.8726 | 1.50 | 1 | 21 | 0.012 | 1Score **> 35** indicates **identity** Score **> 14** indicates **homology** | U | K.AYLRDFIYVSQDPK.D |
| 96597 | 336 | – | 349 | 857.9449 | 1713.8752 | 1713.8726 | 1.50 | 1 | 32 | 0.0017 | 1Score **> 35** indicates **identity** Score **> 17** indicates **homology** | U | K.AYLRDFIYVSQDPK.D |
| 96598 | 336 | – | 349 | 572.2990 | 1713.8753 | 1713.8726 | 1.58 | 1 | 25 | 0.0048 | 1Score **> 35** indicates **identity** Score **> 14** indicates **homology** | U | K.AYLRDFIYVSQDPK.D |
| 96599 | 336 | – | 349 | 857.9450 | 1713.8754 | 1713.8726 | 1.65 | 1 | 42 | 0.00023 | 1Score **> 35** indicates **identity** Score **> 19** indicates **homology** | U | K.AYLRDFIYVSQDPK.D |
| 96602 | 336 | – | 349 | 857.9463 | 1713.8781 | 1713.8726 | 3.20 | 1 | 47 | 3.8e-05 | 1Score **> 35** indicates **identity** Score **> 16** indicates **homology** | U | K.AYLRDFIYVSQDPK.D |
| 186384 | 336 | – | 362 | 778.9131 | 3111.6234 | 3111.6281 | -1.48 | 2 | 29 | 0.0018 | 1Score **> 37** indicates **identity** Score **> 14** indicates **homology** | U | K.AYLRDFIYVSQDPKDQLLLGPTYATPK.V |
| 186385 | 336 | – | 362 | 778.9133 | 3111.6242 | 3111.6281 | -1.23 | 2 | 39 | 0.00024 | 1Score **> 37** indicates **identity** Score **> 15** indicates **homology** | U | K.AYLRDFIYVSQDPKDQLLLGPTYATPK.V |
| 186386 | 336 | – | 362 | 778.9138 | 3111.6261 | 3111.6281 | -0.63 | 2 | 31 | 0.0013 | 1Score **> 37** indicates **identity** Score **> 14** indicates **homology** | U | K.AYLRDFIYVSQDPKDQLLLGPTYATPK.V |
| 186388 | 336 | – | 362 | 1038.2167 | 3111.6282 | 3111.6281 | 0.055 | 2 | 63 | 1.2e-06 | 1Score **> 37** indicates **identity** Score **> 16** indicates **homology** | U | K.AYLRDFIYVSQDPKDQLLLGPTYATPK.V |
| 186389 | 336 | – | 362 | 778.9148 | 3111.6301 | 3111.6281 | 0.65 | 2 | 51 | 1.8e-05 | 1Score **> 37** indicates **identity** Score **> 16** indicates **homology** | U | K.AYLRDFIYVSQDPKDQLLLGPTYATPK.V |
| 186390 | 336 | – | 362 | 778.9149 | 3111.6305 | 3111.6281 | 0.78 | 2 | 39 | 0.00024 | 1Score **> 37** indicates **identity** Score **> 15** indicates **homology** | U | K.AYLRDFIYVSQDPKDQLLLGPTYATPK.V |
| 186391 | 336 | – | 362 | 778.9149 | 3111.6305 | 3111.6281 | 0.79 | 2 | 47 | 4.1e-05 | 1Score **> 37** indicates **identity** Score **> 15** indicates **homology** | U | K.AYLRDFIYVSQDPKDQLLLGPTYATPK.V |
| 186392 | 336 | – | 362 | 1038.2175 | 3111.6306 | 3111.6281 | 0.82 | 2 | 85 | 9.9e-09 | 1Score **> 37** indicates **identity** Score **> 18** indicates **homology** | U | K.AYLRDFIYVSQDPKDQLLLGPTYATPK.V |
| 186393 | 336 | – | 362 | 1038.2175 | 3111.6306 | 3111.6281 | 0.82 | 2 | 48 | 3e-05 | 1Score **> 37** indicates **identity** Score **> 16** indicates **homology** | U | K.AYLRDFIYVSQDPKDQLLLGPTYATPK.V |
| 186394 | 336 | – | 362 | 778.9152 | 3111.6319 | 3111.6281 | 1.22 | 2 | 40 | 0.00016 | 1Score **> 37** indicates **identity** Score **> 15** indicates **homology** | U | K.AYLRDFIYVSQDPKDQLLLGPTYATPK.V |
| 186395 | 336 | – | 362 | 778.9153 | 3111.6319 | 3111.6281 | 1.24 | 2 | 21 | 0.011 | 1Score **> 37** indicates **identity** Score **> 14** indicates **homology** | U | K.AYLRDFIYVSQDPKDQLLLGPTYATPK.V |
| 186396 | 336 | – | 362 | 778.9157 | 3111.6335 | 3111.6281 | 1.76 | 2 | 33 | 0.00084 | 1Score **> 37** indicates **identity** Score **> 15** indicates **homology** | U | K.AYLRDFIYVSQDPKDQLLLGPTYATPK.V |
| 186397 | 336 | – | 362 | 1038.2185 | 3111.6336 | 3111.6281 | 1.77 | 2 | 51 | 1.8e-05 | 1Score **> 37** indicates **identity** Score **> 16** indicates **homology** | U | K.AYLRDFIYVSQDPKDQLLLGPTYATPK.V |
| 186398 | 336 | – | 362 | 1038.2190 | 3111.6352 | 3111.6281 | 2.31 | 2 | 55 | 6.9e-06 | 1Score **> 37** indicates **identity** Score **> 16** indicates **homology** | U | K.AYLRDFIYVSQDPKDQLLLGPTYATPK.V |
| 186399 | 336 | – | 362 | 1038.2195 | 3111.6366 | 3111.6281 | 2.76 | 2 | 75 | 9.9e-08 | 1Score **> 37** indicates **identity** Score **> 17** indicates **homology** | U | K.AYLRDFIYVSQDPKDQLLLGPTYATPK.V |
| 186422 | 336 | – | 362 | 779.1632 | 3112.6237 | 3112.6121 | 3.74 | 2 | 31 | 0.0014 | 1Score **> 37** indicates **identity** Score **> 14** indicates **homology** | U | K.AYLRDFIYVSQDPKDQLLLGPTYATPK.V  + Deamidated (NQ) |
| 186423 | 336 | – | 362 | 1557.3233 | 3112.6321 | 3112.6121 | 6.45 | 2 | 40 | 0.00017 | 1Score **> 37** indicates **identity** Score **> 15** indicates **homology** | U | K.AYLRDFIYVSQDPKDQLLLGPTYATPK.V  + Deamidated (NQ) |
| 186426 | 336 | – | 362 | 1038.5527 | 3112.6362 | 3112.6121 | 7.75 | 2 | 41 | 0.00013 | 1Score **> 37** indicates **identity** Score **> 15** indicates **homology** | U | K.AYLRDFIYVSQDPKDQLLLGPTYATPK.V  + Deamidated (NQ) |
| 186430 | 336 | – | 362 | 1038.5545 | 3112.6418 | 3112.6121 | 9.55 | 2 | 37 | 0.00054 | 1Score **> 37** indicates **identity** Score **> 16** indicates **homology** | U | K.AYLRDFIYVSQDPKDQLLLGPTYATPK.V  + Deamidated (NQ) |
| 186431 | 336 | – | 362 | 1038.5546 | 3112.6420 | 3112.6121 | 9.62 | 2 | 21 | 0.0099 | 1Score **> 37** indicates **identity** Score **> 14** indicates **homology** | U | K.AYLRDFIYVSQDPKDQLLLGPTYATPK.V  + Deamidated (NQ) |
| 189472 | 336 | – | 362 | 1090.9010 | 3269.6811 | 3269.7111 | -9.18 | 2 | 21 | 0.012 | 1Score **> 37** indicates **identity** Score **> 14** indicates **homology** | U | K.AYLRDFIYVSQDPKDQLLLGPTYATPK.V  + 2 Deamidated (NQ); HNE (K) |
| 34913 | 340 | – | 349 | 606.3022 | 1210.5898 | 1210.5870 | 2.30 | 0 | 48 | 2.9e-05 | 1Score **> 32** indicates **identity** Score **> 16** indicates **homology** | U | R.DFIYVSQDPK.D |
| 34914 | 340 | – | 349 | 606.3022 | 1210.5899 | 1210.5870 | 2.38 | 0 | 47 | 3.6e-05 | 1Score **> 32** indicates **identity** Score **> 15** indicates **homology** | U | R.DFIYVSQDPK.D |
| 34915 | 340 | – | 349 | 606.3028 | 1210.5910 | 1210.5870 | 3.29 | 0 | 19 | 0.017 | 1Score **> 32** indicates **identity** Score **> 14** indicates **homology** | U | R.DFIYVSQDPK.D |
| 34917 | 340 | – | 349 | 606.3047 | 1210.5948 | 1210.5870 | 6.45 | 0 | 33 | 0.00085 | 1Score **> 31** indicates **identity** Score **> 15** indicates **homology** | U | R.DFIYVSQDPK.D |
| 34919 | 340 | – | 349 | 606.3052 | 1210.5958 | 1210.5870 | 7.23 | 0 | 33 | 0.00079 | 1Score **> 31** indicates **identity** Score **> 15** indicates **homology** | U | R.DFIYVSQDPK.D |
| 168886 | 340 | – | 362 | 870.4530 | 2608.3373 | 2608.3425 | -2.00 | 1 | 26 | 0.0035 | 1Score **> 37** indicates **identity** Score **> 14** indicates **homology** | U | R.DFIYVSQDPKDQLLLGPTYATPK.V |
| 168887 | 340 | – | 362 | 870.4533 | 2608.3380 | 2608.3425 | -1.73 | 1 | 47 | 3.8e-05 | 1Score **> 37** indicates **identity** Score **> 15** indicates **homology** | U | R.DFIYVSQDPKDQLLLGPTYATPK.V |
| 168888 | 340 | – | 362 | 870.4548 | 2608.3424 | 2608.3425 | -0.0096 | 1 | 27 | 0.0028 | 1Score **> 37** indicates **identity** Score **> 14** indicates **homology** | U | R.DFIYVSQDPKDQLLLGPTYATPK.V |
| 168889 | 340 | – | 362 | 870.4549 | 2608.3428 | 2608.3425 | 0.12 | 1 | 23 | 0.0071 | 1Score **> 37** indicates **identity** Score **> 14** indicates **homology** | U | R.DFIYVSQDPKDQLLLGPTYATPK.V |
| 168890 | 340 | – | 362 | 1305.1789 | 2608.3433 | 2608.3425 | 0.33 | 1 | 26 | 0.0033 | 1Score **> 37** indicates **identity** Score **> 14** indicates **homology** | U | R.DFIYVSQDPKDQLLLGPTYATPK.V |
| 168891 | 340 | – | 362 | 870.4551 | 2608.3435 | 2608.3425 | 0.41 | 1 | 63 | 1.2e-06 | 1Score **> 37** indicates **identity** Score **> 16** indicates **homology** | U | R.DFIYVSQDPKDQLLLGPTYATPK.V |
| 168892 | 340 | – | 362 | 870.4552 | 2608.3436 | 2608.3425 | 0.45 | 1 | 64 | 9.8e-07 | 1Score **> 37** indicates **identity** Score **> 17** indicates **homology** | U | R.DFIYVSQDPKDQLLLGPTYATPK.V |
| 168893 | 340 | – | 362 | 1305.1791 | 2608.3437 | 2608.3425 | 0.48 | 1 | 80 | 3e-08 | 1Score **> 37** indicates **identity** Score **> 18** indicates **homology** | U | R.DFIYVSQDPKDQLLLGPTYATPK.V |
| 168894 | 340 | – | 362 | 870.4553 | 2608.3440 | 2608.3425 | 0.58 | 1 | 60 | 2.6e-06 | 1Score **> 37** indicates **identity** Score **> 16** indicates **homology** | U | R.DFIYVSQDPKDQLLLGPTYATPK.V |
| 168895 | 340 | – | 362 | 1305.1793 | 2608.3440 | 2608.3425 | 0.60 | 1 | 66 | 7.1e-07 | 1Score **> 37** indicates **identity** Score **> 17** indicates **homology** | U | R.DFIYVSQDPKDQLLLGPTYATPK.V |
| 168896 | 340 | – | 362 | 870.4553 | 2608.3441 | 2608.3425 | 0.63 | 1 | 58 | 3.9e-06 | 1Score **> 37** indicates **identity** Score **> 16** indicates **homology** | U | R.DFIYVSQDPKDQLLLGPTYATPK.V |
| 168897 | 340 | – | 362 | 1305.1795 | 2608.3444 | 2608.3425 | 0.73 | 1 | 64 | 9.8e-07 | 1Score **> 37** indicates **identity** Score **> 17** indicates **homology** | U | R.DFIYVSQDPKDQLLLGPTYATPK.V |
| 168898 | 340 | – | 362 | 870.4555 | 2608.3448 | 2608.3425 | 0.88 | 1 | 60 | 2.5e-06 | 1Score **> 37** indicates **identity** Score **> 16** indicates **homology** | U | R.DFIYVSQDPKDQLLLGPTYATPK.V |
| 168899 | 340 | – | 362 | 1305.1797 | 2608.3449 | 2608.3425 | 0.92 | 1 | 101 | 3.5e-10 | 1Score **> 37** indicates **identity** Score **> 19** indicates **homology** | U | R.DFIYVSQDPKDQLLLGPTYATPK.V |
| 168901 | 340 | – | 362 | 870.4558 | 2608.3455 | 2608.3425 | 1.14 | 1 | 59 | 3.2e-06 | 1Score **> 37** indicates **identity** Score **> 16** indicates **homology** | U | R.DFIYVSQDPKDQLLLGPTYATPK.V |
| 168902 | 340 | – | 362 | 870.4559 | 2608.3459 | 2608.3425 | 1.32 | 1 | 44 | 7e-05 | 1Score **> 37** indicates **identity** Score **> 15** indicates **homology** | U | R.DFIYVSQDPKDQLLLGPTYATPK.V |
| 168903 | 340 | – | 362 | 870.4559 | 2608.3460 | 2608.3425 | 1.35 | 1 | 53 | 1e-05 | 1Score **> 37** indicates **identity** Score **> 16** indicates **homology** | U | R.DFIYVSQDPKDQLLLGPTYATPK.V |
| 168904 | 340 | – | 362 | 870.4560 | 2608.3463 | 2608.3425 | 1.47 | 1 | 27 | 0.0031 | 1Score **> 37** indicates **identity** Score **> 14** indicates **homology** | U | R.DFIYVSQDPKDQLLLGPTYATPK.V |
| 168905 | 340 | – | 362 | 870.4561 | 2608.3465 | 2608.3425 | 1.53 | 1 | 52 | 1.4e-05 | 1Score **> 37** indicates **identity** Score **> 16** indicates **homology** | U | R.DFIYVSQDPKDQLLLGPTYATPK.V |
| 168906 | 340 | – | 362 | 870.4561 | 2608.3466 | 2608.3425 | 1.57 | 1 | 65 | 8.8e-07 | 1Score **> 37** indicates **identity** Score **> 17** indicates **homology** | U | R.DFIYVSQDPKDQLLLGPTYATPK.V |
| 168908 | 340 | – | 362 | 870.4565 | 2608.3477 | 2608.3425 | 2.01 | 1 | 33 | 0.0008 | 1Score **> 37** indicates **identity** Score **> 15** indicates **homology** | U | R.DFIYVSQDPKDQLLLGPTYATPK.V |
| 168909 | 340 | – | 362 | 870.4566 | 2608.3479 | 2608.3425 | 2.08 | 1 | 48 | 2.8e-05 | 1Score **> 37** indicates **identity** Score **> 16** indicates **homology** | U | R.DFIYVSQDPKDQLLLGPTYATPK.V |
| 168910 | 340 | – | 362 | 870.4567 | 2608.3481 | 2608.3425 | 2.17 | 1 | 61 | 1.8e-06 | 1Score **> 37** indicates **identity** Score **> 16** indicates **homology** | U | R.DFIYVSQDPKDQLLLGPTYATPK.V |
| 168911 | 340 | – | 362 | 870.4572 | 2608.3498 | 2608.3425 | 2.80 | 1 | 48 | 3e-05 | 1Score **> 37** indicates **identity** Score **> 16** indicates **homology** | U | R.DFIYVSQDPKDQLLLGPTYATPK.V |
| 168912 | 340 | – | 362 | 870.4582 | 2608.3528 | 2608.3425 | 3.95 | 1 | 43 | 9.8e-05 | 1Score **> 37** indicates **identity** Score **> 15** indicates **homology** | U | R.DFIYVSQDPKDQLLLGPTYATPK.V |
| 168914 | 340 | – | 362 | 870.4614 | 2608.3622 | 2608.3425 | 7.58 | 1 | 42 | 0.00011 | 1Score **> 37** indicates **identity** Score **> 15** indicates **homology** | U | R.DFIYVSQDPKDQLLLGPTYATPK.V |
| 168915 | 340 | – | 362 | 870.4617 | 2608.3634 | 2608.3425 | 8.01 | 1 | 25 | 0.0043 | 1Score **> 37** indicates **identity** Score **> 14** indicates **homology** | U | R.DFIYVSQDPKDQLLLGPTYATPK.V |
| 168946 | 340 | – | 362 | 870.7885 | 2609.3437 | 2609.3265 | 6.60 | 1 | 20 | 0.013 | 1Score **> 37** indicates **identity** Score **> 14** indicates **homology** | U | R.DFIYVSQDPKDQLLLGPTYATPK.V  + Deamidated (NQ) |
| 168947 | 340 | – | 362 | 870.7887 | 2609.3442 | 2609.3265 | 6.78 | 1 | 15 | 0.041 | 1Score **> 37** indicates **identity** Score **> 13** indicates **homology** | U | R.DFIYVSQDPKDQLLLGPTYATPK.V  + Deamidated (NQ) |
| 168948 | 340 | – | 362 | 1305.6798 | 2609.3450 | 2609.3265 | 7.10 | 1 | 33 | 0.00077 | 1Score **> 37** indicates **identity** Score **> 15** indicates **homology** | U | R.DFIYVSQDPKDQLLLGPTYATPK.V  + Deamidated (NQ) |
| 168949 | 340 | – | 362 | 870.7897 | 2609.3474 | 2609.3265 | 8.01 | 1 | 16 | 0.034 | 1Score **> 37** indicates **identity** Score **> 14** indicates **homology** | U | R.DFIYVSQDPKDQLLLGPTYATPK.V  + Deamidated (NQ) |
| 168951 | 340 | – | 362 | 870.7904 | 2609.3494 | 2609.3265 | 8.77 | 1 | 22 | 0.0096 | 1Score **> 37** indicates **identity** Score **> 14** indicates **homology** | U | R.DFIYVSQDPKDQLLLGPTYATPK.V  + Deamidated (NQ) |
| 168952 | 340 | – | 362 | 1305.6830 | 2609.3514 | 2609.3265 | 9.55 | 1 | 58 | 3.8e-06 | 1Score **> 37** indicates **identity** Score **> 16** indicates **homology** | U | R.DFIYVSQDPKDQLLLGPTYATPK.V  + Deamidated (NQ) |
| 57617 | 350 | – | 362 | 708.8902 | 1415.7658 | 1415.7660 | -0.13 | 0 | 33 | 0.0008 | 1Score **> 34** indicates **identity** Score **> 15** indicates **homology** | U | K.DQLLLGPTYATPK.V |
| 57622 | 350 | – | 362 | 708.8914 | 1415.7682 | 1415.7660 | 1.56 | 0 | 51 | 2.7e-05 | 1Score **> 35** indicates **identity** Score **> 18** indicates **homology** | U | K.DQLLLGPTYATPK.V |
| 57623 | 350 | – | 362 | 708.8914 | 1415.7683 | 1415.7660 | 1.58 | 0 | 63 | 2.7e-06 | 1Score **> 35** indicates **identity** Score **> 20** indicates **homology** | U | K.DQLLLGPTYATPK.V |
| 57625 | 350 | – | 362 | 708.8915 | 1415.7684 | 1415.7660 | 1.64 | 0 | 65 | 1.7e-06 | 1Score **> 35** indicates **identity** Score **> 20** indicates **homology** | U | K.DQLLLGPTYATPK.V |
| 57626 | 350 | – | 362 | 708.8917 | 1415.7688 | 1415.7660 | 1.94 | 0 | 34 | 0.00079 | 1Score **> 35** indicates **identity** Score **> 15** indicates **homology** | U | K.DQLLLGPTYATPK.V |
| 57629 | 350 | – | 362 | 708.8922 | 1415.7698 | 1415.7660 | 2.69 | 0 | 61 | 4e-06 | 1Score **> 34** indicates **identity** Score **> 20** indicates **homology** | U | K.DQLLLGPTYATPK.V |
| 57633 | 350 | – | 362 | 708.8942 | 1415.7739 | 1415.7660 | 5.55 | 0 | 32 | 0.0011 | 1Score **> 35** indicates **identity** Score **> 14** indicates **homology** | U | K.DQLLLGPTYATPK.V |
| 18786 | 408 | – | 417 | 352.5416 | 1054.6030 | 1054.6022 | 0.73 | 1 | 26 | 0.0037 | 1Score **> 29** indicates **identity** Score **> 14** indicates **homology** | U | K.TKVGSPPLEK.F |
| 18787 | 408 | – | 417 | 352.5418 | 1054.6035 | 1054.6022 | 1.14 | 1 | 49 | 0.0001 | 1Score **> 29** indicates **identity** Score **> 22** indicates **homology** | U | K.TKVGSPPLEK.F |
| 18788 | 408 | – | 417 | 528.3093 | 1054.6040 | 1054.6022 | 1.65 | 1 | 34 | 0.00091 | 1Score **> 29** indicates **identity** Score **> 16** indicates **homology** | U | K.TKVGSPPLEK.F |
| 18789 | 408 | – | 417 | 352.5421 | 1054.6044 | 1054.6022 | 2.07 | 1 | 35 | 0.0018 | 1Score **> 29** indicates **identity** Score **> 20** indicates **homology** | U | K.TKVGSPPLEK.F |
| 18790 | 408 | – | 417 | 528.3097 | 1054.6048 | 1054.6022 | 2.37 | 1 | 17 | 0.025 | 1Score **> 29** indicates **identity** Score **> 14** indicates **homology** | U | K.TKVGSPPLEK.F |
| 18791 | 408 | – | 417 | 528.3099 | 1054.6052 | 1054.6022 | 2.75 | 1 | 46 | 0.00013 | 1Score **> 29** indicates **identity** Score **> 20** indicates **homology** | U | K.TKVGSPPLEK.F |
| 18792 | 408 | – | 417 | 352.5432 | 1054.6078 | 1054.6022 | 5.27 | 1 | 34 | 0.00067 | 1Score **> 29** indicates **identity** Score **> 15** indicates **homology** | U | K.TKVGSPPLEK.F |
| 18793 | 408 | – | 417 | 528.3112 | 1054.6079 | 1054.6022 | 5.33 | 1 | 16 | 0.035 | 1Score **> 29** indicates **identity** Score **> 13** indicates **homology** | U | K.TKVGSPPLEK.F |
| 4908 | 410 | – | 417 | 413.7371 | 825.4596 | 825.4596 | 0.0024 | 0 | 39 | 0.0015 | 1Score **> 23** indicates **identity** | U | K.VGSPPLEK.F |
| 4909 | 410 | – | 417 | 413.7372 | 825.4597 | 825.4596 | 0.17 | 0 | 49 | 0.00016 | 1Score **> 23** indicates **identity** | U | K.VGSPPLEK.F |
| 4910 | 410 | – | 417 | 413.7376 | 825.4606 | 825.4596 | 1.18 | 0 | 43 | 0.00061 | 1Score **> 24** indicates **identity** Score **> 23** indicates **homology** | U | K.VGSPPLEK.F |
| 4911 | 410 | – | 417 | 413.7386 | 825.4627 | 825.4596 | 3.78 | 0 | 18 | 0.022 | 1Score **> 26** indicates **identity** Score **> 14** indicates **homology** | U | K.VGSPPLEK.F |
| 181725 | 410 | – | 437 | 736.1254 | 2940.4726 | 2940.4705 | 0.71 | 1 | 20 | 0.014 | 1Score **> 38** indicates **identity** Score **> 14** indicates **homology** | U | K.VGSPPLEKFNIWGGSLSLGHPFGATGCR.L |
| 138392 | 418 | – | 437 | 712.0143 | 2133.0211 | 2133.0215 | -0.16 | 0 | 22 | 0.0093 | 1Score **> 35** indicates **identity** Score **> 14** indicates **homology** | U | K.FNIWGGSLSLGHPFGATGCR.L |
| 138394 | 418 | – | 437 | 712.0144 | 2133.0215 | 2133.0215 | 0.0094 | 0 | 77 | 8.4e-08 | 1Score **> 35** indicates **identity** Score **> 18** indicates **homology** | U | K.FNIWGGSLSLGHPFGATGCR.L |
| 138397 | 418 | – | 437 | 1067.5181 | 2133.0217 | 2133.0215 | 0.11 | 0 | 109 | 6.6e-11 | 1Score **> 35** indicates **identity** Score **> 19** indicates **homology** | U | K.FNIWGGSLSLGHPFGATGCR.L |
| 138398 | 418 | – | 437 | 712.0146 | 2133.0220 | 2133.0215 | 0.28 | 0 | 16 | 0.03 | 1Score **> 35** indicates **identity** Score **> 14** indicates **homology** | U | K.FNIWGGSLSLGHPFGATGCR.L |
| 138400 | 418 | – | 437 | 1067.5184 | 2133.0222 | 2133.0215 | 0.35 | 0 | 119 | 6.6e-12 | 1Score **> 35** indicates **identity** Score **> 20** indicates **homology** | U | K.FNIWGGSLSLGHPFGATGCR.L |
| 138401 | 418 | – | 437 | 712.0147 | 2133.0223 | 2133.0215 | 0.38 | 0 | 54 | 9.3e-06 | 1Score **> 35** indicates **identity** Score **> 16** indicates **homology** | U | K.FNIWGGSLSLGHPFGATGCR.L |
| 138402 | 418 | – | 437 | 1067.5184 | 2133.0223 | 2133.0215 | 0.41 | 0 | 128 | 1e-12 | 1Score **> 35** indicates **identity** Score **> 20** indicates **homology** | U | K.FNIWGGSLSLGHPFGATGCR.L |
| 138404 | 418 | – | 437 | 712.0148 | 2133.0226 | 2133.0215 | 0.56 | 0 | 78 | 6.2e-08 | 1Score **> 35** indicates **identity** Score **> 19** indicates **homology** | U | K.FNIWGGSLSLGHPFGATGCR.L |
| 138405 | 418 | – | 437 | 712.0149 | 2133.0229 | 2133.0215 | 0.66 | 0 | 84 | 1.3e-08 | 1Score **> 35** indicates **identity** Score **> 18** indicates **homology** | U | K.FNIWGGSLSLGHPFGATGCR.L |
| 138406 | 418 | – | 437 | 1067.5187 | 2133.0229 | 2133.0215 | 0.67 | 0 | 60 | 2.4e-06 | 1Score **> 35** indicates **identity** Score **> 16** indicates **homology** | U | K.FNIWGGSLSLGHPFGATGCR.L |
| 138407 | 418 | – | 437 | 1067.5190 | 2133.0234 | 2133.0215 | 0.91 | 0 | 83 | 1.5e-08 | 1Score **> 35** indicates **identity** Score **> 18** indicates **homology** | U | K.FNIWGGSLSLGHPFGATGCR.L |
| 138408 | 418 | – | 437 | 712.0152 | 2133.0237 | 2133.0215 | 1.04 | 0 | 27 | 0.003 | 1Score **> 35** indicates **identity** Score **> 14** indicates **homology** | U | K.FNIWGGSLSLGHPFGATGCR.L |
| 138409 | 418 | – | 437 | 1067.5193 | 2133.0240 | 2133.0215 | 1.17 | 0 | 64 | 1.1e-06 | 1Score **> 35** indicates **identity** Score **> 16** indicates **homology** | U | K.FNIWGGSLSLGHPFGATGCR.L |
| 138411 | 418 | – | 437 | 712.0154 | 2133.0243 | 2133.0215 | 1.33 | 0 | 24 | 0.0061 | 1Score **> 35** indicates **identity** Score **> 14** indicates **homology** | U | K.FNIWGGSLSLGHPFGATGCR.L |
| 138412 | 418 | – | 437 | 712.0154 | 2133.0245 | 2133.0215 | 1.41 | 0 | 23 | 0.0076 | 1Score **> 35** indicates **identity** Score **> 14** indicates **homology** | U | K.FNIWGGSLSLGHPFGATGCR.L |
| 138413 | 418 | – | 437 | 712.0154 | 2133.0245 | 2133.0215 | 1.41 | 0 | 41 | 0.00016 | 1Score **> 35** indicates **identity** Score **> 15** indicates **homology** | U | K.FNIWGGSLSLGHPFGATGCR.L |
| 138414 | 418 | – | 437 | 712.0155 | 2133.0246 | 2133.0215 | 1.46 | 0 | 26 | 0.0039 | 1Score **> 35** indicates **identity** Score **> 14** indicates **homology** | U | K.FNIWGGSLSLGHPFGATGCR.L |
| 138417 | 418 | – | 437 | 1067.5198 | 2133.0250 | 2133.0215 | 1.68 | 0 | 130 | 6.7e-13 | 1Score **> 35** indicates **identity** Score **> 21** indicates **homology** | U | K.FNIWGGSLSLGHPFGATGCR.L |
| 138418 | 418 | – | 437 | 712.0156 | 2133.0250 | 2133.0215 | 1.69 | 0 | 54 | 8.3e-06 | 1Score **> 35** indicates **identity** Score **> 16** indicates **homology** | U | K.FNIWGGSLSLGHPFGATGCR.L |
| 138419 | 418 | – | 437 | 712.0156 | 2133.0251 | 2133.0215 | 1.71 | 0 | 41 | 0.00016 | 1Score **> 35** indicates **identity** Score **> 15** indicates **homology** | U | K.FNIWGGSLSLGHPFGATGCR.L |
| 138420 | 418 | – | 437 | 712.0159 | 2133.0259 | 2133.0215 | 2.09 | 0 | 23 | 0.0065 | 1Score **> 35** indicates **identity** Score **> 14** indicates **homology** | U | K.FNIWGGSLSLGHPFGATGCR.L |
| 138422 | 418 | – | 437 | 1067.5204 | 2133.0263 | 2133.0215 | 2.27 | 0 | 56 | 5.6e-06 | 1Score **> 35** indicates **identity** Score **> 16** indicates **homology** | U | K.FNIWGGSLSLGHPFGATGCR.L |
| 138424 | 418 | – | 437 | 1067.5213 | 2133.0281 | 2133.0215 | 3.13 | 0 | 80 | 3e-08 | 1Score **> 36** indicates **identity** Score **> 18** indicates **homology** | U | K.FNIWGGSLSLGHPFGATGCR.L |
| 138431 | 418 | – | 437 | 1067.5231 | 2133.0316 | 2133.0215 | 4.73 | 0 | 108 | 6.9e-11 | 1Score **> 36** indicates **identity** Score **> 19** indicates **homology** | U | K.FNIWGGSLSLGHPFGATGCR.L |
| 138675 | 418 | – | 437 | 1068.0197 | 2134.0249 | 2134.0055 | 9.11 | 0 | 14 | 0.046 | 1Score **> 36** indicates **identity** Score **> 13** indicates **homology** | U | K.FNIWGGSLSLGHPFGATGCR.L  + Deamidated (NQ) |
| 5783 | 438 | – | 445 | 423.2364 | 844.4583 | 844.4589 | -0.72 | 0 | 50 | 0.00022 | 1Score **> 32** indicates **identity** Score **> 26** indicates **homology** | U | R.LVMAAANR.L |
| 5784 | 438 | – | 445 | 423.2365 | 844.4584 | 844.4589 | -0.51 | 0 | 30 | 0.0084 | 1Score **> 32** indicates **identity** Score **> 22** indicates **homology** | U | R.LVMAAANR.L |
| 6631 | 438 | – | 445 | 431.2330 | 860.4514 | 860.4538 | -2.73 | 0 | 27 | 0.0041 | 1Score **> 29** indicates **identity** Score **> 15** indicates **homology** | U | R.LVMAAANR.L  + Oxidation (M) |
| 6632 | 438 | – | 445 | 431.2345 | 860.4544 | 860.4538 | 0.65 | 0 | 25 | 0.007 | 1Score **> 31** indicates **identity** Score **> 16** indicates **homology** | U | R.LVMAAANR.L  + Oxidation (M) |
| 6633 | 438 | – | 445 | 431.2347 | 860.4548 | 860.4538 | 1.15 | 0 | 23 | 0.011 | 1Score **> 31** indicates **identity** Score **> 16** indicates **homology** | U | R.LVMAAANR.L  + Oxidation (M) |
| 6634 | 438 | – | 445 | 431.2352 | 860.4559 | 860.4538 | 2.42 | 0 | 16 | 0.044 | 1Score **> 31** indicates **identity** Score **> 14** indicates **homology** | U | R.LVMAAANR.L  + Oxidation (M) |
| 178108 | 448 | – | 475 | 709.1014 | 2832.3765 | 2832.3687 | 2.75 | 1 | 31 | 0.0013 | 1Score **> 37** indicates **identity** Score **> 14** indicates **homology** | U | R.KDGGQYALVAACAAGGQGHAMIVEAYPK.- |
| 172964 | 449 | – | 475 | 902.4311 | 2704.2714 | 2704.2737 | -0.85 | 0 | 39 | 0.00021 | 1Score **> 35** indicates **identity** Score **> 15** indicates **homology** | U | K.DGGQYALVAACAAGGQGHAMIVEAYPK.- |
| 172965 | 449 | – | 475 | 902.4324 | 2704.2755 | 2704.2737 | 0.64 | 0 | 77 | 6.4e-08 | 1Score **> 35** indicates **identity** Score **> 17** indicates **homology** | U | K.DGGQYALVAACAAGGQGHAMIVEAYPK.- |
| 172966 | 449 | – | 475 | 902.4327 | 2704.2763 | 2704.2737 | 0.95 | 0 | 33 | 0.00077 | 1Score **> 35** indicates **identity** Score **> 15** indicates **homology** | U | K.DGGQYALVAACAAGGQGHAMIVEAYPK.- |
| 172967 | 449 | – | 475 | 902.4331 | 2704.2775 | 2704.2737 | 1.37 | 0 | 66 | 5.9e-07 | 1Score **> 35** indicates **identity** Score **> 17** indicates **homology** | U | K.DGGQYALVAACAAGGQGHAMIVEAYPK.- |
| 172969 | 449 | – | 475 | 902.4341 | 2704.2804 | 2704.2737 | 2.46 | 0 | 19 | 0.016 | 1Score **> 36** indicates **identity** Score **> 14** indicates **homology** | U | K.DGGQYALVAACAAGGQGHAMIVEAYPK.- |
| 172970 | 449 | – | 475 | 902.4342 | 2704.2809 | 2704.2737 | 2.63 | 0 | 59 | 3e-06 | 1Score **> 36** indicates **identity** Score **> 16** indicates **homology** | U | K.DGGQYALVAACAAGGQGHAMIVEAYPK.- |
| 172989 | 449 | – | 475 | 902.7685 | 2705.2836 | 2705.2578 | 9.55 | 0 | 37 | 0.00033 | 1Score **> 36** indicates **identity** Score **> 15** indicates **homology** | U | K.DGGQYALVAACAAGGQGHAMIVEAYPK.-  + Deamidated (NQ) |

---

```
ID   ECHB_MOUSE              Reviewed;         475 AA.
AC   Q99JY0; Q3TEH9; Q8BJI5; Q8BJM0; Q8BK52;
DT   16-AUG-2004, integrated into UniProtKB/Swiss-Prot.
DT   01-JUN-2001, sequence version 1.
DT   28-JUN-2023, entry version 165.
DE   RecName: Full=Trifunctional enzyme subunit beta, mitochondrial;
DE   AltName: Full=TP-beta;
DE   Includes:
DE     RecName: Full=3-ketoacyl-CoA thiolase;
DE              EC=2.3.1.155 {ECO:0000250|UniProtKB:P55084};
DE              EC=2.3.1.16 {ECO:0000250|UniProtKB:P55084};
DE     AltName: Full=Acetyl-CoA acyltransferase;
DE     AltName: Full=Beta-ketothiolase;
DE   Flags: Precursor;
GN   Name=Hadhb;
OS   Mus musculus (Mouse).
OC   Eukaryota; Metazoa; Chordata; Craniata; Vertebrata; Euteleostomi; Mammalia;
OC   Eutheria; Euarchontoglires; Glires; Rodentia; Myomorpha; Muroidea; Muridae;
OC   Murinae; Mus; Mus.
OX   NCBI_TaxID=10090;
RN   [1]
RP   NUCLEOTIDE SEQUENCE [LARGE SCALE MRNA].
RC   STRAIN=C57BL/6J;
RC   TISSUE=Bone marrow, Colon, Hippocampus, Spinal ganglion, Testis, and
RC   Thymus;
RX   PubMed=16141072; DOI=10.1126/science.1112014;
RA   Carninci P., Kasukawa T., Katayama S., Gough J., Frith M.C., Maeda N.,
RA   Oyama R., Ravasi T., Lenhard B., Wells C., Kodzius R., Shimokawa K.,
RA   Bajic V.B., Brenner S.E., Batalov S., Forrest A.R., Zavolan M., Davis M.J.,
RA   Wilming L.G., Aidinis V., Allen J.E., Ambesi-Impiombato A., Apweiler R.,
RA   Aturaliya R.N., Bailey T.L., Bansal M., Baxter L., Beisel K.W., Bersano T.,
RA   Bono H., Chalk A.M., Chiu K.P., Choudhary V., Christoffels A.,
RA   Clutterbuck D.R., Crowe M.L., Dalla E., Dalrymple B.P., de Bono B.,
RA   Della Gatta G., di Bernardo D., Down T., Engstrom P., Fagiolini M.,
RA   Faulkner G., Fletcher C.F., Fukushima T., Furuno M., Futaki S.,
RA   Gariboldi M., Georgii-Hemming P., Gingeras T.R., Gojobori T., Green R.E.,
RA   Gustincich S., Harbers M., Hayashi Y., Hensch T.K., Hirokawa N., Hill D.,
RA   Huminiecki L., Iacono M., Ikeo K., Iwama A., Ishikawa T., Jakt M.,
RA   Kanapin A., Katoh M., Kawasawa Y., Kelso J., Kitamura H., Kitano H.,
RA   Kollias G., Krishnan S.P., Kruger A., Kummerfeld S.K., Kurochkin I.V.,
RA   Lareau L.F., Lazarevic D., Lipovich L., Liu J., Liuni S., McWilliam S.,
RA   Madan Babu M., Madera M., Marchionni L., Matsuda H., Matsuzawa S., Miki H.,
RA   Mignone F., Miyake S., Morris K., Mottagui-Tabar S., Mulder N., Nakano N.,
RA   Nakauchi H., Ng P., Nilsson R., Nishiguchi S., Nishikawa S., Nori F.,
RA   Ohara O., Okazaki Y., Orlando V., Pang K.C., Pavan W.J., Pavesi G.,
RA   Pesole G., Petrovsky N., Piazza S., Reed J., Reid J.F., Ring B.Z.,
RA   Ringwald M., Rost B., Ruan Y., Salzberg S.L., Sandelin A., Schneider C.,
RA   Schoenbach C., Sekiguchi K., Semple C.A., Seno S., Sessa L., Sheng Y.,
RA   Shibata Y., Shimada H., Shimada K., Silva D., Sinclair B., Sperling S.,
RA   Stupka E., Sugiura K., Sultana R., Takenaka Y., Taki K., Tammoja K.,
RA   Tan S.L., Tang S., Taylor M.S., Tegner J., Teichmann S.A., Ueda H.R.,
RA   van Nimwegen E., Verardo R., Wei C.L., Yagi K., Yamanishi H.,
RA   Zabarovsky E., Zhu S., Zimmer A., Hide W., Bult C., Grimmond S.M.,
RA   Teasdale R.D., Liu E.T., Brusic V., Quackenbush J., Wahlestedt C.,
RA   Mattick J.S., Hume D.A., Kai C., Sasaki D., Tomaru Y., Fukuda S.,
RA   Kanamori-Katayama M., Suzuki M., Aoki J., Arakawa T., Iida J., Imamura K.,
RA   Itoh M., Kato T., Kawaji H., Kawagashira N., Kawashima T., Kojima M.,
RA   Kondo S., Konno H., Nakano K., Ninomiya N., Nishio T., Okada M., Plessy C.,
RA   Shibata K., Shiraki T., Suzuki S., Tagami M., Waki K., Watahiki A.,
RA   Okamura-Oho Y., Suzuki H., Kawai J., Hayashizaki Y.;
RT   "The transcriptional landscape of the mammalian genome.";
RL   Science 309:1559-1563(2005).
RN   [2]
RP   NUCLEOTIDE SEQUENCE [LARGE SCALE MRNA].
RC   STRAIN=FVB/N; TISSUE=Mammary tumor;
RX   PubMed=15489334; DOI=10.1101/gr.2596504;
RG   The MGC Project Team;
RT   "The status, quality, and expansion of the NIH full-length cDNA project:
RT   the Mammalian Gene Collection (MGC).";
RL   Genome Res. 14:2121-2127(2004).
RN   [3]
RP   IDENTIFICATION BY MASS SPECTROMETRY [LARGE SCALE ANALYSIS].
RC   TISSUE=Brain, Brown adipose tissue, Heart, Kidney, Liver, Lung,
RC   Pancreas, Spleen, and Testis;
RX   PubMed=21183079; DOI=10.1016/j.cell.2010.12.001;
RA   Huttlin E.L., Jedrychowski M.P., Elias J.E., Goswami T., Rad R.,
RA   Beausoleil S.A., Villen J., Haas W., Sowa M.E., Gygi S.P.;
RT   "A tissue-specific atlas of mouse protein phosphorylation and expression.";
RL   Cell 143:1174-1189(2010).
RN   [4]
RP   SUCCINYLATION [LARGE SCALE ANALYSIS] AT LYS-53; LYS-73; LYS-189; LYS-191;
RP   LYS-273; LYS-292; LYS-294 AND LYS-333, AND IDENTIFICATION BY MASS
RP   SPECTROMETRY [LARGE SCALE ANALYSIS].
RC   TISSUE=Embryonic fibroblast, and Liver;
RX   PubMed=23806337; DOI=10.1016/j.molcel.2013.06.001;
RA   Park J., Chen Y., Tishkoff D.X., Peng C., Tan M., Dai L., Xie Z., Zhang Y.,
RA   Zwaans B.M., Skinner M.E., Lombard D.B., Zhao Y.;
RT   "SIRT5-mediated lysine desuccinylation impacts diverse metabolic
RT   pathways.";
RL   Mol. Cell 50:919-930(2013).
RN   [5]
RP   ACETYLATION [LARGE SCALE ANALYSIS] AT LYS-73; LYS-189; LYS-294; LYS-299;
RP   LYS-333; LYS-349 AND LYS-362, AND IDENTIFICATION BY MASS SPECTROMETRY
RP   [LARGE SCALE ANALYSIS].
RC   TISSUE=Liver;
RX   PubMed=23576753; DOI=10.1073/pnas.1302961110;
RA   Rardin M.J., Newman J.C., Held J.M., Cusack M.P., Sorensen D.J., Li B.,
RA   Schilling B., Mooney S.D., Kahn C.R., Verdin E., Gibson B.W.;
RT   "Label-free quantitative proteomics of the lysine acetylome in mitochondria
RT   identifies substrates of SIRT3 in metabolic pathways.";
RL   Proc. Natl. Acad. Sci. U.S.A. 110:6601-6606(2013).
RN   [6]
RP   INTERACTION WITH MTLN.
RX   PubMed=29949755; DOI=10.1016/j.celrep.2018.05.058;
RA   Makarewich C.A., Baskin K.K., Munir A.Z., Bezprozvannaya S., Sharma G.,
RA   Khemtong C., Shah A.M., McAnally J.R., Malloy C.R., Szweda L.I.,
RA   Bassel-Duby R., Olson E.N.;
RT   "MOXI Is a Mitochondrial Micropeptide That Enhances Fatty Acid beta-
RT   Oxidation.";
RL   Cell Rep. 23:3701-3709(2018).
CC   -!- FUNCTION: Mitochondrial trifunctional enzyme catalyzes the last three
CC       of the four reactions of the mitochondrial beta-oxidation pathway. The
CC       mitochondrial beta-oxidation pathway is the major energy-producing
CC       process in tissues and is performed through four consecutive reactions
CC       breaking down fatty acids into acetyl-CoA. Among the enzymes involved
CC       in this pathway, the trifunctional enzyme exhibits specificity for
CC       long-chain fatty acids. Mitochondrial trifunctional enzyme is a
CC       heterotetrameric complex composed of two proteins, the trifunctional
CC       enzyme subunit alpha/HADHA carries the 2,3-enoyl-CoA hydratase and the
CC       3-hydroxyacyl-CoA dehydrogenase activities, while the trifunctional
CC       enzyme subunit beta/HADHB described here bears the 3-ketoacyl-CoA
CC       thiolase activity. {ECO:0000250|UniProtKB:P55084}.
CC   -!- CATALYTIC ACTIVITY:
CC       Reaction=acetyl-CoA + an acyl-CoA = a 3-oxoacyl-CoA + CoA;
CC         Xref=Rhea:RHEA:21564, ChEBI:CHEBI:57287, ChEBI:CHEBI:57288,
CC         ChEBI:CHEBI:58342, ChEBI:CHEBI:90726; EC=2.3.1.16;
CC         Evidence={ECO:0000250|UniProtKB:P55084};
CC       PhysiologicalDirection=right-to-left; Xref=Rhea:RHEA:21566;
CC         Evidence={ECO:0000250|UniProtKB:P55084};
CC   -!- CATALYTIC ACTIVITY:
CC       Reaction=acetyl-CoA + butanoyl-CoA = 3-oxohexanoyl-CoA + CoA;
CC         Xref=Rhea:RHEA:31111, ChEBI:CHEBI:57287, ChEBI:CHEBI:57288,
CC         ChEBI:CHEBI:57371, ChEBI:CHEBI:62418;
CC         Evidence={ECO:0000250|UniProtKB:P55084};
CC       PhysiologicalDirection=right-to-left; Xref=Rhea:RHEA:31113;
CC         Evidence={ECO:0000250|UniProtKB:P55084};
CC   -!- CATALYTIC ACTIVITY:
CC       Reaction=acetyl-CoA + hexanoyl-CoA = 3-oxooctanoyl-CoA + CoA;
CC         Xref=Rhea:RHEA:31203, ChEBI:CHEBI:57287, ChEBI:CHEBI:57288,
CC         ChEBI:CHEBI:62619, ChEBI:CHEBI:62620;
CC         Evidence={ECO:0000250|UniProtKB:P55084};
CC       PhysiologicalDirection=right-to-left; Xref=Rhea:RHEA:31205;
CC         Evidence={ECO:0000250|UniProtKB:P55084};
CC   -!- CATALYTIC ACTIVITY:
CC       Reaction=acetyl-CoA + octanoyl-CoA = 3-oxodecanoyl-CoA + CoA;
CC         Xref=Rhea:RHEA:31087, ChEBI:CHEBI:57287, ChEBI:CHEBI:57288,
CC         ChEBI:CHEBI:57386, ChEBI:CHEBI:62548;
CC         Evidence={ECO:0000250|UniProtKB:P55084};
CC       PhysiologicalDirection=right-to-left; Xref=Rhea:RHEA:31089;
CC         Evidence={ECO:0000250|UniProtKB:P55084};
CC   -!- CATALYTIC ACTIVITY:
CC       Reaction=acetyl-CoA + decanoyl-CoA = 3-oxododecanoyl-CoA + CoA;
CC         Xref=Rhea:RHEA:31183, ChEBI:CHEBI:57287, ChEBI:CHEBI:57288,
CC         ChEBI:CHEBI:61430, ChEBI:CHEBI:62615;
CC         Evidence={ECO:0000250|UniProtKB:P55084};
CC       PhysiologicalDirection=right-to-left; Xref=Rhea:RHEA:31185;
CC         Evidence={ECO:0000250|UniProtKB:P55084};
CC   -!- CATALYTIC ACTIVITY:
CC       Reaction=acetyl-CoA + dodecanoyl-CoA = 3-oxotetradecanoyl-CoA + CoA;
CC         Xref=Rhea:RHEA:31091, ChEBI:CHEBI:57287, ChEBI:CHEBI:57288,
CC         ChEBI:CHEBI:57375, ChEBI:CHEBI:62543;
CC         Evidence={ECO:0000250|UniProtKB:P55084};
CC       PhysiologicalDirection=right-to-left; Xref=Rhea:RHEA:31093;
CC         Evidence={ECO:0000250|UniProtKB:P55084};
CC   -!- CATALYTIC ACTIVITY:
CC       Reaction=acetyl-CoA + tetradecanoyl-CoA = 3-oxohexadecanoyl-CoA + CoA;
CC         Xref=Rhea:RHEA:18161, ChEBI:CHEBI:57287, ChEBI:CHEBI:57288,
CC         ChEBI:CHEBI:57349, ChEBI:CHEBI:57385; EC=2.3.1.155;
CC         Evidence={ECO:0000250|UniProtKB:P55084};
CC       PhysiologicalDirection=right-to-left; Xref=Rhea:RHEA:18163;
CC         Evidence={ECO:0000250|UniProtKB:P55084};
CC   -!- PATHWAY: Lipid metabolism; fatty acid beta-oxidation.
CC       {ECO:0000250|UniProtKB:P55084}.
CC   -!- SUBUNIT: Heterotetramer of 2 alpha/HADHA and 2 beta/HADHB subunits;
CC       forms the mitochondrial trifunctional enzyme (By similarity). Also
CC       purified as higher order heterooligomers including a 4 alpha/HADHA and
CC       4 beta/HADHB heterooligomer which physiological significance remains
CC       unclear (By similarity). The mitochondrial trifunctional enzyme
CC       interacts with MTLN (PubMed:29949755). Interacts with RSAD2/viperin (By
CC       similarity). {ECO:0000250|UniProtKB:P55084,
CC       ECO:0000269|PubMed:29949755}.
CC   -!- SUBCELLULAR LOCATION: Mitochondrion {ECO:0000250|UniProtKB:P55084}.
CC       Mitochondrion inner membrane {ECO:0000250|UniProtKB:P55084}.
CC       Mitochondrion outer membrane {ECO:0000250|UniProtKB:P55084}.
CC       Endoplasmic reticulum {ECO:0000250|UniProtKB:P55084}. Note=Protein
CC       stability and association with membranes require HADHA.
CC       {ECO:0000250|UniProtKB:P55084}.
CC   -!- PTM: Acetylation of Lys-202 is observed in liver mitochondria from
CC       fasted mice but not from fed mice.
CC   -!- SIMILARITY: Belongs to the thiolase-like superfamily. Thiolase family.
CC       {ECO:0000305}.
CC   ---------------------------------------------------------------------------
CC   Copyrighted by the UniProt Consortium, see https://www.uniprot.org/terms
CC   Distributed under the Creative Commons Attribution (CC BY 4.0) License
CC   ---------------------------------------------------------------------------
DR   EMBL; AK033462; BAC28300.1; -; mRNA.
DR   EMBL; AK076814; BAC36493.1; -; mRNA.
DR   EMBL; AK083164; BAC38790.1; -; mRNA.
DR   EMBL; AK083767; BAC39015.1; -; mRNA.
DR   EMBL; AK150889; BAE29936.1; -; mRNA.
DR   EMBL; AK169637; BAE41269.1; -; mRNA.
DR   EMBL; BC005585; AAH05585.1; -; mRNA.
DR   CCDS; CCDS39045.1; -.
DR   RefSeq; NP_001276727.1; NM_001289798.1.
DR   RefSeq; NP_001276728.1; NM_001289799.1.
DR   RefSeq; NP_663533.1; NM_145558.2.
DR   RefSeq; XP_017176317.1; XM_017320828.1.
DR   RefSeq; XP_017176318.1; XM_017320829.1.
DR   AlphaFoldDB; Q99JY0; -.
DR   SMR; Q99JY0; -.
DR   BioGRID; 231080; 33.
DR   IntAct; Q99JY0; 7.
DR   MINT; Q99JY0; -.
DR   STRING; 10090.ENSMUSP00000110434; -.
DR   iPTMnet; Q99JY0; -.
DR   PhosphoSitePlus; Q99JY0; -.
DR   SwissPalm; Q99JY0; -.
DR   EPD; Q99JY0; -.
DR   jPOST; Q99JY0; -.
DR   MaxQB; Q99JY0; -.
DR   PaxDb; Q99JY0; -.
DR   PeptideAtlas; Q99JY0; -.
DR   ProteomicsDB; 277669; -.
DR   Antibodypedia; 27848; 265 antibodies from 30 providers.
DR   DNASU; 231086; -.
DR   Ensembl; ENSMUST00000026841; ENSMUSP00000026841; ENSMUSG00000059447.
DR   Ensembl; ENSMUST00000114783; ENSMUSP00000110431; ENSMUSG00000059447.
DR   Ensembl; ENSMUST00000114786; ENSMUSP00000110434; ENSMUSG00000059447.
DR   GeneID; 231086; -.
DR   KEGG; mmu:231086; -.
DR   UCSC; uc008wve.2; mouse.
DR   AGR; MGI:2136381; -.
DR   CTD; 3032; -.
DR   MGI; MGI:2136381; Hadhb.
DR   VEuPathDB; HostDB:ENSMUSG00000059447; -.
DR   eggNOG; KOG1392; Eukaryota.
DR   GeneTree; ENSGT01030000234626; -.
DR   HOGENOM; CLU_031026_2_0_1; -.
DR   InParanoid; Q99JY0; -.
DR   OMA; MTAFPEP; -.
DR   OrthoDB; 1826604at2759; -.
DR   PhylomeDB; Q99JY0; -.
DR   TreeFam; TF315243; -.
DR   Reactome; R-MMU-1482798; Acyl chain remodeling of CL.
DR   Reactome; R-MMU-77285; Beta oxidation of myristoyl-CoA to lauroyl-CoA.
DR   Reactome; R-MMU-77305; Beta oxidation of palmitoyl-CoA to myristoyl-CoA.
DR   Reactome; R-MMU-77310; Beta oxidation of lauroyl-CoA to decanoyl-CoA-CoA.
DR   Reactome; R-MMU-77346; Beta oxidation of decanoyl-CoA to octanoyl-CoA-CoA.
DR   Reactome; R-MMU-77348; Beta oxidation of octanoyl-CoA to hexanoyl-CoA.
DR   Reactome; R-MMU-77350; Beta oxidation of hexanoyl-CoA to butanoyl-CoA.
DR   UniPathway; UPA00659; -.
DR   BioGRID-ORCS; 231086; 1 hit in 77 CRISPR screens.
DR   ChiTaRS; Hadhb; mouse.
DR   PRO; PR:Q99JY0; -.
DR   Proteomes; UP000000589; Chromosome 5.
DR   RNAct; Q99JY0; protein.
DR   Bgee; ENSMUSG00000059447; Expressed in spermatocyte and 170 other tissues.
DR   ExpressionAtlas; Q99JY0; baseline and differential.
DR   Genevisible; Q99JY0; MM.
DR   GO; GO:0005783; C:endoplasmic reticulum; ISS:UniProtKB.
DR   GO; GO:0016507; C:mitochondrial fatty acid beta-oxidation multienzyme complex; ISO:MGI.
DR   GO; GO:0005743; C:mitochondrial inner membrane; HDA:MGI.
DR   GO; GO:0042645; C:mitochondrial nucleoid; ISO:MGI.
DR   GO; GO:0005741; C:mitochondrial outer membrane; ISS:UniProtKB.
DR   GO; GO:0005739; C:mitochondrion; IDA:MGI.
DR   GO; GO:0003985; F:acetyl-CoA C-acetyltransferase activity; IDA:MGI.
DR   GO; GO:0003988; F:acetyl-CoA C-acyltransferase activity; IDA:MGI.
DR   GO; GO:0050633; F:acetyl-CoA C-myristoyltransferase activity; IEA:UniProtKB-EC.
DR   GO; GO:0106222; F:lncRNA binding; IDA:MGI.
DR   GO; GO:0044877; F:protein-containing complex binding; ISO:MGI.
DR   GO; GO:0071222; P:cellular response to lipopolysaccharide; IDA:MGI.
DR   GO; GO:0006635; P:fatty acid beta-oxidation; ISO:MGI.
DR   GO; GO:0010467; P:gene expression; IMP:MGI.
DR   CDD; cd00751; thiolase; 1.
DR   Gene3D; 3.40.47.10; -; 1.
DR   InterPro; IPR002155; Thiolase.
DR   InterPro; IPR016039; Thiolase-like.
DR   InterPro; IPR020615; Thiolase_acyl_enz_int_AS.
DR   InterPro; IPR020610; Thiolase_AS.
DR   InterPro; IPR020617; Thiolase_C.
DR   InterPro; IPR020613; Thiolase_CS.
DR   InterPro; IPR020616; Thiolase_N.
DR   PANTHER; PTHR18919; ACETYL-COA C-ACYLTRANSFERASE; 1.
DR   PANTHER; PTHR18919:SF153; TRIFUNCTIONAL ENZYME SUBUNIT BETA, MITOCHONDRIAL; 1.
DR   Pfam; PF02803; Thiolase_C; 1.
DR   Pfam; PF00108; Thiolase_N; 1.
DR   SUPFAM; SSF53901; Thiolase-like; 2.
DR   PROSITE; PS00098; THIOLASE_1; 1.
DR   PROSITE; PS00737; THIOLASE_2; 1.
DR   PROSITE; PS00099; THIOLASE_3; 1.
DR   TIGRFAMs; TIGR01930; AcCoA-C-Actrans; 1.
PE   1: Evidence at protein level;
KW   Acetylation; Acyltransferase; Endoplasmic reticulum; Fatty acid metabolism;
KW   Lipid metabolism; Membrane; Mitochondrion; Mitochondrion inner membrane;
KW   Mitochondrion outer membrane; Reference proteome; Transferase;
KW   Transit peptide.
FT   TRANSIT         1..34
FT                   /note="Mitochondrion"
FT                   /evidence="ECO:0000250"
FT   CHAIN           35..475
FT                   /note="Trifunctional enzyme subunit beta, mitochondrial"
FT                   /id="PRO_0000034082"
FT   INTRAMEM        174..221
FT                   /evidence="ECO:0000250|UniProtKB:P55084"
FT   ACT_SITE        139
FT                   /note="Acyl-thioester intermediate"
FT                   /evidence="ECO:0000250|UniProtKB:P55084"
FT   ACT_SITE        459
FT                   /note="Proton donor/acceptor"
FT                   /evidence="ECO:0000250|UniProtKB:P55084"
FT   SITE            429
FT                   /note="Increases nucleophilicity of active site Cys"
FT                   /evidence="ECO:0000250|UniProtKB:P55084"
FT   MOD_RES         53
FT                   /note="N6-succinyllysine"
FT                   /evidence="ECO:0007744|PubMed:23806337"
FT   MOD_RES         73
FT                   /note="N6-acetyllysine; alternate"
FT                   /evidence="ECO:0007744|PubMed:23576753"
FT   MOD_RES         73
FT                   /note="N6-succinyllysine; alternate"
FT                   /evidence="ECO:0007744|PubMed:23806337"
FT   MOD_RES         189
FT                   /note="N6-acetyllysine; alternate"
FT                   /evidence="ECO:0007744|PubMed:23576753"
FT   MOD_RES         189
FT                   /note="N6-succinyllysine; alternate"
FT                   /evidence="ECO:0007744|PubMed:23806337"
FT   MOD_RES         191
FT                   /note="N6-succinyllysine"
FT                   /evidence="ECO:0007744|PubMed:23806337"
FT   MOD_RES         273
FT                   /note="N6-succinyllysine"
FT                   /evidence="ECO:0007744|PubMed:23806337"
FT   MOD_RES         292
FT                   /note="N6-succinyllysine"
FT                   /evidence="ECO:0007744|PubMed:23806337"
FT   MOD_RES         294
FT                   /note="N6-acetyllysine; alternate"
FT                   /evidence="ECO:0007744|PubMed:23576753"
FT   MOD_RES         294
FT                   /note="N6-succinyllysine; alternate"
FT                   /evidence="ECO:0007744|PubMed:23806337"
FT   MOD_RES         299
FT                   /note="N6-acetyllysine"
FT                   /evidence="ECO:0007744|PubMed:23576753"
FT   MOD_RES         333
FT                   /note="N6-acetyllysine; alternate"
FT                   /evidence="ECO:0007744|PubMed:23576753"
FT   MOD_RES         333
FT                   /note="N6-succinyllysine; alternate"
FT                   /evidence="ECO:0007744|PubMed:23806337"
FT   MOD_RES         349
FT                   /note="N6-acetyllysine"
FT                   /evidence="ECO:0007744|PubMed:23576753"
FT   MOD_RES         362
FT                   /note="N6-acetyllysine"
FT                   /evidence="ECO:0007744|PubMed:23576753"
FT   CONFLICT        24..25
FT                   /note="IR -> HK (in Ref. 1; BAC36493)"
FT                   /evidence="ECO:0000305"
FT   CONFLICT        425
FT                   /note="L -> M (in Ref. 1; BAC38790)"
FT                   /evidence="ECO:0000305"
FT   CONFLICT        450
FT                   /note="G -> R (in Ref. 1; BAC38790)"
FT                   /evidence="ECO:0000305"
FT   CONFLICT        450
FT                   /note="G -> V (in Ref. 1; BAC39015)"
FT                   /evidence="ECO:0000305"
SQ   SEQUENCE   475 AA;  51386 MW;  F131B497C4F5FAF4 CRC64;
     MTTILTSTFR NLSTTSKWAL RSSIRPLSCS SQLHSAPAVQ TKSKKTLAKP NMKNIVVVEG
     VRIPFLLSGT SYKDLMPHDL ARAALSGLLH RTNIPKDVVD YIIFGTVIQE VKTSNVAREA
     ALGAGFSDKT PAHTVTMACI SSNQAMTTAV GLIASGQCDV VVAGGVELMS DVPIRHSRNM
     RKMMLDLNKA KTLGQRLSLL SKFRLNFLSP ELPAVAEFST NETMGHSADR LAAAFAVSRM
     EQDEYALRSH SLAKKAQDEG HLSDIVPFKV PGKDTVTKDN GIRPSSLEQM AKLKPAFIKP
     YGTVTAANSS FLTDGASAML IMSEDRALAM GYKPKAYLRD FIYVSQDPKD QLLLGPTYAT
     PKVLEKAGLT MNDIDAFEFH EAFSGQILAN FKAMDSDWFA QNYMGRKTKV GSPPLEKFNI
     WGGSLSLGHP FGATGCRLVM AAANRLRKDG GQYALVAACA AGGQGHAMIV EAYPK
//
```

**Mascot:** http://www.matrixscience.com/


Score **> 37** indicates **identity**  
Score **> 16** indicates **homology**
