# Supplementary material for: A common East-Asian ALDH2 mutation causes metabolic disorders and the therapeutic effect of ALDH2 activators
Source: Nat Commun. 2023 Sep 25;14:5971. doi: 10.1038/s41467-023-41570-6 (PMC10520061; doi:10.1038/s41467-023-41570-6)
Supplement: Supplementary file 4 — Supplementary Data 1 [file 41467_2023_41570_MOESM4_ESM.zip › Table S5c/Q99LC3/Q99LC3_JIO-1-_H202.html]

Mascot Search Results: Q99LC3
 

# MASCOT Search Results

## Protein View: Q99LC3

### NADH dehydrogenase [ubiquinone] 1 alpha subcomplex subunit 10, mitochondrial OS=Mus musculus OX=10090 GN=Ndufa10 PE=1 SV=1

|  |  |
| --- | --- |
| Database: | Mouse\_UniProt\_proteomes |
| Score: | 2935 |
| Monoisotopic mass (M_r_): | 40863 |
| Calculated pI: | 7.63 |

Sequence similarity is available as an NCBI BLAST search of Q99LC3 against nr.

### Search parameters

|  |  |
| --- | --- |
| MS data file: | `D:\LCMSMS\2023 Users' data\230529-1\230529-1-KIO-1.raw` |
| Enzyme: | Trypsin/P: cuts C-term side of KR. |
| Fixed modifications: | Carbamidomethyl (C) |
| Variable modifications: | Deamidated (NQ), HNE (C), HNE (H), HNE (K), Oxidation (M) |

### Protein sequence coverage: 58%

Matched peptides shown in ***bold red***.

|  |  |  |  |  |  |
| --- | --- | --- | --- | --- | --- |
| `1` | `MALRLLRLVP` | `ASAPARGLAA` | `GAQRVGRIHT` | `SVHCKLRYGL` | `LAAILGDKTT` |
| `51` | `KKLHEYSRVI` | `TVDGNICSGK` | `NKLAKEIAQQ` | `LGMKHYPEAG` | `IQYSSTTTGD` |
| `101` | `GRPLDIEFSG` | `SCSLEKFYDD` | `PKSNDGNSYR` | `LQSWLYASRL` | `LQYADALEHL` |
| `151` | `LSTGQGVVLE` | `RSIYSDFVFL` | `EAMYNQGYIR` | `KQCVDHYNEI` | `KRLTLPEYLP` |
| `201` | `PHAVIYIDVP` | `VPEVQSRIQK` | `KGDPHEMKVT` | `SAYLQDIENA` | `YKKTFLPKMS` |
| `251` | `EMCEVLVYDS` | `WEAEDPTKVV` | `EDIEYLKYNK` | `GPWLKQDDWT` | `FHYLRMLVQD` |
| `301` | `KTEVLNYTTI` | `PVYLPEITIG` | `AHQGSRIYNS` | `FRELPGRKYA` | `PGYNAEVGDK` |
| `351` | `WIWLK` |  |  |  |  |

Unformatted sequence string: 355 residues (for pasting into other applications).

|  |  |  |  |
| --- | --- | --- | --- |
| Sort by | residue number | increasing mass | decreasing mass |
| Show | matched peptides only | predicted peptides also |  |

| Query | Start | – | End | Observed | Mr(expt) | Mr(calc) | ppm | M | Score | Expect | Rank | U | Peptide |
| --- | --- | --- | --- | --- | --- | --- | --- | --- | --- | --- | --- | --- | --- |
| 50607 | 36 | – | 48 | 468.2854 | 1401.8345 | 1401.8344 | 0.12 | 1 | 40 | 0.00019 | 1Score **> 31** indicates **identity** Score **> 15** indicates **homology** | U | K.LRYGLLAAILGDK.T |
| 50608 | 36 | – | 48 | 468.2858 | 1401.8354 | 1401.8344 | 0.78 | 1 | 52 | 0.00012 | 1Score **> 31** indicates **identity** Score **> 26** indicates **homology** | U | K.LRYGLLAAILGDK.T |
| 93062 | 36 | – | 51 | 434.0132 | 1732.0238 | 1732.0247 | -0.48 | 2 | 44 | 7.2e-05 | 1Score **> 30** indicates **identity** Score **> 15** indicates **homology** | U | K.LRYGLLAAILGDKTTK.K |
| 93063 | 36 | – | 51 | 434.0134 | 1732.0245 | 1732.0247 | -0.11 | 2 | 38 | 0.0003 | 1Score **> 30** indicates **identity** Score **> 15** indicates **homology** | U | K.LRYGLLAAILGDKTTK.K |
| 93066 | 36 | – | 51 | 434.0137 | 1732.0255 | 1732.0247 | 0.48 | 2 | 38 | 0.00026 | 1Score **> 30** indicates **identity** Score **> 15** indicates **homology** | U | K.LRYGLLAAILGDKTTK.K |
| 93067 | 36 | – | 51 | 578.3492 | 1732.0256 | 1732.0247 | 0.56 | 2 | 32 | 0.001 | 1Score **> 30** indicates **identity** Score **> 14** indicates **homology** | U | K.LRYGLLAAILGDKTTK.K |
| 93068 | 36 | – | 51 | 434.0138 | 1732.0259 | 1732.0247 | 0.72 | 2 | 14 | 0.045 | 1Score **> 30** indicates **identity** Score **> 13** indicates **homology** | U | K.LRYGLLAAILGDKTTK.K |
| 93069 | 36 | – | 51 | 578.3494 | 1732.0262 | 1732.0247 | 0.91 | 2 | 36 | 0.00046 | 1Score **> 30** indicates **identity** Score **> 15** indicates **homology** | U | K.LRYGLLAAILGDKTTK.K |
| 93072 | 36 | – | 51 | 578.3510 | 1732.0312 | 1732.0247 | 3.76 | 2 | 25 | 0.0043 | 1Score **> 29** indicates **identity** Score **> 14** indicates **homology** | U | K.LRYGLLAAILGDKTTK.K |
| 23826 | 38 | – | 48 | 567.3313 | 1132.6481 | 1132.6492 | -0.94 | 0 | 58 | 3.4e-06 | 1Score **> 32** indicates **identity** Score **> 16** indicates **homology** | U | R.YGLLAAILGDK.T |
| 23827 | 38 | – | 48 | 567.3321 | 1132.6496 | 1132.6492 | 0.35 | 0 | 46 | 4.4e-05 | 1Score **> 32** indicates **identity** Score **> 15** indicates **homology** | U | R.YGLLAAILGDK.T |
| 57670 | 38 | – | 51 | 488.6201 | 1462.8385 | 1462.8395 | -0.68 | 1 | 43 | 0.0001 | 1Score **> 32** indicates **identity** Score **> 15** indicates **homology** | U | R.YGLLAAILGDKTTK.K |
| 57671 | 38 | – | 51 | 488.6201 | 1462.8386 | 1462.8395 | -0.62 | 1 | 29 | 0.0021 | 1Score **> 32** indicates **identity** Score **> 14** indicates **homology** | U | R.YGLLAAILGDKTTK.K |
| 57675 | 38 | – | 51 | 488.6207 | 1462.8402 | 1462.8395 | 0.50 | 1 | 24 | 0.0051 | 1Score **> 32** indicates **identity** Score **> 14** indicates **homology** | U | R.YGLLAAILGDKTTK.K |
| 57676 | 38 | – | 51 | 488.6207 | 1462.8403 | 1462.8395 | 0.53 | 1 | 59 | 2.7e-06 | 1Score **> 32** indicates **identity** Score **> 16** indicates **homology** | U | R.YGLLAAILGDKTTK.K |
| 57677 | 38 | – | 51 | 488.6208 | 1462.8405 | 1462.8395 | 0.68 | 1 | 26 | 0.0037 | 1Score **> 32** indicates **identity** Score **> 14** indicates **homology** | U | R.YGLLAAILGDKTTK.K |
| 57678 | 38 | – | 51 | 732.4280 | 1462.8414 | 1462.8395 | 1.27 | 1 | 59 | 2.9e-06 | 1Score **> 32** indicates **identity** Score **> 16** indicates **homology** | U | R.YGLLAAILGDKTTK.K |
| 57679 | 38 | – | 51 | 488.6211 | 1462.8416 | 1462.8395 | 1.43 | 1 | 42 | 0.00011 | 1Score **> 32** indicates **identity** Score **> 15** indicates **homology** | U | R.YGLLAAILGDKTTK.K |
| 57680 | 38 | – | 51 | 488.6212 | 1462.8418 | 1462.8395 | 1.60 | 1 | 53 | 1.2e-05 | 1Score **> 32** indicates **identity** Score **> 16** indicates **homology** | U | R.YGLLAAILGDKTTK.K |
| 57682 | 38 | – | 51 | 488.6213 | 1462.8422 | 1462.8395 | 1.81 | 1 | 52 | 1.4e-05 | 1Score **> 32** indicates **identity** Score **> 16** indicates **homology** | U | R.YGLLAAILGDKTTK.K |
| 57683 | 38 | – | 51 | 488.6215 | 1462.8425 | 1462.8395 | 2.07 | 1 | 46 | 4.9e-05 | 1Score **> 32** indicates **identity** Score **> 15** indicates **homology** | U | R.YGLLAAILGDKTTK.K |
| 57684 | 38 | – | 51 | 732.4287 | 1462.8428 | 1462.8395 | 2.28 | 1 | 42 | 0.00011 | 1Score **> 32** indicates **identity** Score **> 15** indicates **homology** | U | R.YGLLAAILGDKTTK.K |
| 57685 | 38 | – | 51 | 732.4288 | 1462.8430 | 1462.8395 | 2.35 | 1 | 78 | 5.1e-08 | 1Score **> 31** indicates **identity** Score **> 17** indicates **homology** | U | R.YGLLAAILGDKTTK.K |
| 57686 | 38 | – | 51 | 488.6216 | 1462.8431 | 1462.8395 | 2.43 | 1 | 52 | 1.4e-05 | 1Score **> 31** indicates **identity** Score **> 16** indicates **homology** | U | R.YGLLAAILGDKTTK.K |
| 57688 | 38 | – | 51 | 732.4288 | 1462.8431 | 1462.8395 | 2.46 | 1 | 78 | 4.5e-08 | 1Score **> 31** indicates **identity** Score **> 17** indicates **homology** | U | R.YGLLAAILGDKTTK.K |
| 57689 | 38 | – | 51 | 488.6220 | 1462.8443 | 1462.8395 | 3.24 | 1 | 28 | 0.0026 | 1Score **> 32** indicates **identity** Score **> 14** indicates **homology** | U | R.YGLLAAILGDKTTK.K |
| 57691 | 38 | – | 51 | 488.6227 | 1462.8464 | 1462.8395 | 4.71 | 1 | 33 | 0.00079 | 1Score **> 31** indicates **identity** Score **> 15** indicates **homology** | U | R.YGLLAAILGDKTTK.K |
| 36538 | 59 | – | 70 | 631.8235 | 1261.6324 | 1261.6337 | -1.01 | 0 | 30 | 0.0016 | 1Score **> 33** indicates **identity** Score **> 14** indicates **homology** | U | R.VITVDGNICSGK.N |
| 36540 | 59 | – | 70 | 631.8237 | 1261.6328 | 1261.6337 | -0.71 | 0 | 55 | 7.1e-06 | 1Score **> 33** indicates **identity** Score **> 16** indicates **homology** | U | R.VITVDGNICSGK.N |
| 36542 | 59 | – | 70 | 631.8240 | 1261.6334 | 1261.6337 | -0.23 | 0 | 45 | 6.7e-05 | 1Score **> 33** indicates **identity** Score **> 15** indicates **homology** | U | R.VITVDGNICSGK.N |
| 36544 | 59 | – | 70 | 631.8241 | 1261.6336 | 1261.6337 | -0.049 | 0 | 49 | 2.4e-05 | 1Score **> 33** indicates **identity** Score **> 16** indicates **homology** | U | R.VITVDGNICSGK.N |
| 36545 | 59 | – | 70 | 631.8244 | 1261.6343 | 1261.6337 | 0.53 | 0 | 63 | 1.3e-06 | 1Score **> 33** indicates **identity** Score **> 16** indicates **homology** | U | R.VITVDGNICSGK.N |
| 36546 | 59 | – | 70 | 631.8245 | 1261.6345 | 1261.6337 | 0.64 | 0 | 29 | 0.0018 | 1Score **> 33** indicates **identity** Score **> 14** indicates **homology** | U | R.VITVDGNICSGK.N |
| 36547 | 59 | – | 70 | 631.8246 | 1261.6346 | 1261.6337 | 0.72 | 0 | 58 | 3.9e-06 | 1Score **> 33** indicates **identity** Score **> 16** indicates **homology** | U | R.VITVDGNICSGK.N |
| 36548 | 59 | – | 70 | 631.8247 | 1261.6349 | 1261.6337 | 1.02 | 0 | 61 | 1.8e-06 | 1Score **> 34** indicates **identity** Score **> 16** indicates **homology** | U | R.VITVDGNICSGK.N |
| 36549 | 59 | – | 70 | 631.8249 | 1261.6352 | 1261.6337 | 1.25 | 0 | 26 | 0.0036 | 1Score **> 33** indicates **identity** Score **> 14** indicates **homology** | U | R.VITVDGNICSGK.N |
| 36551 | 59 | – | 70 | 631.8256 | 1261.6366 | 1261.6337 | 2.34 | 0 | 38 | 0.00026 | 1Score **> 33** indicates **identity** Score **> 15** indicates **homology** | U | R.VITVDGNICSGK.N |
| 36553 | 59 | – | 70 | 631.8264 | 1261.6383 | 1261.6337 | 3.67 | 0 | 24 | 0.006 | 1Score **> 33** indicates **identity** Score **> 14** indicates **homology** | U | R.VITVDGNICSGK.N |
| 36554 | 59 | – | 70 | 631.8269 | 1261.6392 | 1261.6337 | 4.41 | 0 | 40 | 0.00019 | 1Score **> 33** indicates **identity** Score **> 15** indicates **homology** | U | R.VITVDGNICSGK.N |
| 85926 | 117 | – | 130 | 839.3605 | 1676.7065 | 1676.7067 | -0.11 | 1 | 70 | 2.8e-07 | 1Score **> 25** indicates **identity** Score **> 17** indicates **homology** | U | K.FYDDPKSNDGNSYR.L |
| 85927 | 117 | – | 130 | 839.3606 | 1676.7067 | 1676.7067 | 0.046 | 1 | 61 | 1.9e-06 | 1Score **> 25** indicates **identity** Score **> 16** indicates **homology** | U | K.FYDDPKSNDGNSYR.L |
| 85928 | 117 | – | 130 | 559.9096 | 1676.7071 | 1676.7067 | 0.26 | 1 | 26 | 0.0038 | 1Score **> 25** indicates **identity** Score **> 14** indicates **homology** | U | K.FYDDPKSNDGNSYR.L |
| 85930 | 117 | – | 130 | 559.9101 | 1676.7086 | 1676.7067 | 1.15 | 1 | 53 | 1e-05 | 1Score **> 26** indicates **identity** Score **> 16** indicates **homology** | U | K.FYDDPKSNDGNSYR.L |
| 85931 | 117 | – | 130 | 559.9104 | 1676.7092 | 1676.7067 | 1.54 | 1 | 48 | 3.3e-05 | 1Score **> 25** indicates **identity** Score **> 15** indicates **homology** | U | K.FYDDPKSNDGNSYR.L |
| 22798 | 131 | – | 139 | 562.2979 | 1122.5812 | 1122.5822 | -0.82 | 0 | 48 | 0.00046 | 1Score **> 32** indicates **identity** Score **> 28** indicates **homology** | U | R.LQSWLYASR.L |
| 22799 | 131 | – | 139 | 562.2981 | 1122.5816 | 1122.5822 | -0.52 | 0 | 34 | 0.0013 | 1Score **> 32** indicates **identity** Score **> 17** indicates **homology** | U | R.LQSWLYASR.L |
| 22801 | 131 | – | 139 | 562.2982 | 1122.5818 | 1122.5822 | -0.29 | 0 | 56 | 8.7e-05 | 1Score **> 32** indicates **identity** Score **> 28** indicates **homology** | U | R.LQSWLYASR.L |
| 22802 | 131 | – | 139 | 562.2985 | 1122.5824 | 1122.5822 | 0.18 | 0 | 47 | 0.00064 | 1Score **> 32** indicates **identity** Score **> 28** indicates **homology** | U | R.LQSWLYASR.L |
| 22803 | 131 | – | 139 | 562.2987 | 1122.5828 | 1122.5822 | 0.58 | 0 | 48 | 0.00069 | 1Score **> 32** indicates **identity** Score **> 29** indicates **homology** | U | R.LQSWLYASR.L |
| 22804 | 131 | – | 139 | 562.2988 | 1122.5830 | 1122.5822 | 0.72 | 0 | 26 | 0.0069 | 1Score **> 32** indicates **identity** Score **> 17** indicates **homology** | U | R.LQSWLYASR.L |
| 22806 | 131 | – | 139 | 562.2988 | 1122.5830 | 1122.5822 | 0.78 | 0 | 57 | 8.6e-05 | 1Score **> 32** indicates **identity** Score **> 29** indicates **homology** | U | R.LQSWLYASR.L |
| 22807 | 131 | – | 139 | 562.2990 | 1122.5834 | 1122.5822 | 1.11 | 0 | 20 | 0.041 | 1Score **> 32** indicates **identity** Score **> 18** indicates **homology** | U | R.LQSWLYASR.L |
| 22809 | 131 | – | 139 | 562.2993 | 1122.5840 | 1122.5822 | 1.59 | 0 | 60 | 4.3e-05 | 1Score **> 32** indicates **identity** Score **> 29** indicates **homology** | U | R.LQSWLYASR.L |
| 22810 | 131 | – | 139 | 562.2993 | 1122.5840 | 1122.5822 | 1.64 | 0 | 58 | 9.6e-05 | 1Score **> 32** indicates **identity** Score **> 30** indicates **homology** | U | R.LQSWLYASR.L |
| 22812 | 131 | – | 139 | 562.2999 | 1122.5853 | 1122.5822 | 2.79 | 0 | 46 | 0.00033 | 1Score **> 32** indicates **identity** Score **> 24** indicates **homology** | U | R.LQSWLYASR.L |
| 22813 | 131 | – | 139 | 562.3008 | 1122.5871 | 1122.5822 | 4.40 | 0 | 35 | 0.0024 | 1Score **> 32** indicates **identity** Score **> 21** indicates **homology** | U | R.LQSWLYASR.L |
| 154486 | 140 | – | 161 | 1213.1588 | 2424.3030 | 2424.3013 | 0.73 | 0 | 133 | 3.3e-13 | 1Score **> 36** indicates **identity** Score **> 21** indicates **homology** | U | R.LLQYADALEHLLSTGQGVVLER.S |
| 154487 | 140 | – | 161 | 809.1083 | 2424.3032 | 2424.3013 | 0.78 | 0 | 100 | 4.2e-10 | 1Score **> 36** indicates **identity** Score **> 19** indicates **homology** | U | R.LLQYADALEHLLSTGQGVVLER.S |
| 154489 | 140 | – | 161 | 607.0835 | 2424.3050 | 2424.3013 | 1.55 | 0 | 18 | 0.023 | 1Score **> 36** indicates **identity** Score **> 14** indicates **homology** | U | R.LLQYADALEHLLSTGQGVVLER.S |
| 154490 | 140 | – | 161 | 809.1108 | 2424.3106 | 2424.3013 | 3.84 | 0 | 39 | 0.00023 | 1Score **> 36** indicates **identity** Score **> 15** indicates **homology** | U | R.LLQYADALEHLLSTGQGVVLER.S |
| 154491 | 140 | – | 161 | 809.1133 | 2424.3179 | 2424.3013 | 6.88 | 0 | 38 | 0.00029 | 1Score **> 36** indicates **identity** Score **> 15** indicates **homology** | U | R.LLQYADALEHLLSTGQGVVLER.S |
| 154607 | 140 | – | 161 | 809.4385 | 2425.2936 | 2425.2853 | 3.44 | 0 | 47 | 3.9e-05 | 1Score **> 36** indicates **identity** Score **> 15** indicates **homology** | U | R.LLQYADALEHLLSTGQGVVLER.S  + Deamidated (NQ) |
| 73954 | 181 | – | 192 | 795.3963 | 1588.7780 | 1588.7780 | 0.011 | 2 | 47 | 0.0029 | 1Score **> 34** indicates **identity** | U | R.KQCVDHYNEIKR.L |
| 40893 | 182 | – | 191 | 653.2987 | 1304.5829 | 1304.5819 | 0.76 | 0 | 28 | 0.0022 | 1Score **> 28** indicates **identity** Score **> 14** indicates **homology** | U | K.QCVDHYNEIK.R |
| 57387 | 182 | – | 192 | 731.3498 | 1460.6851 | 1460.6830 | 1.42 | 1 | 33 | 0.042 | 1Score **> 32** indicates **identity** | U | K.QCVDHYNEIKR.L |
| 178566 | 192 | – | 217 | 751.1725 | 3000.6609 | 3000.6437 | 5.73 | 1 | 22 | 0.0092 | 1Score **> 34** indicates **identity** Score **> 14** indicates **homology** | U | K.RLTLPEYLPPHAVIYIDVPVPEVQSR.I |
| 173409 | 193 | – | 217 | 949.1879 | 2844.5420 | 2844.5426 | -0.21 | 0 | 33 | 0.00078 | 1Score **> 35** indicates **identity** Score **> 15** indicates **homology** | U | R.LTLPEYLPPHAVIYIDVPVPEVQSR.I |
| 173411 | 193 | – | 217 | 949.1898 | 2844.5476 | 2844.5426 | 1.76 | 0 | 63 | 1.3e-06 | 1Score **> 36** indicates **identity** Score **> 16** indicates **homology** | U | R.LTLPEYLPPHAVIYIDVPVPEVQSR.I |
| 173412 | 193 | – | 217 | 1423.2818 | 2844.5490 | 2844.5426 | 2.27 | 0 | 48 | 3.5e-05 | 1Score **> 35** indicates **identity** Score **> 15** indicates **homology** | U | R.LTLPEYLPPHAVIYIDVPVPEVQSR.I |
| 173413 | 193 | – | 217 | 949.1905 | 2844.5497 | 2844.5426 | 2.50 | 0 | 45 | 6.1e-05 | 1Score **> 35** indicates **identity** Score **> 15** indicates **homology** | U | R.LTLPEYLPPHAVIYIDVPVPEVQSR.I |
| 173414 | 193 | – | 217 | 949.1911 | 2844.5515 | 2844.5426 | 3.13 | 0 | 40 | 0.00017 | 1Score **> 35** indicates **identity** Score **> 15** indicates **homology** | U | R.LTLPEYLPPHAVIYIDVPVPEVQSR.I |
| 178565 | 193 | – | 217 | 1001.2236 | 3000.6490 | 3000.6576 | -2.86 | 0 | 31 | 0.0012 | 1Score **> 34** indicates **identity** Score **> 14** indicates **homology** | U | R.LTLPEYLPPHAVIYIDVPVPEVQSR.I  + HNE (H) |
| 77359 | 229 | – | 242 | 807.8980 | 1613.7815 | 1613.7937 | -7.51 | 0 | 38 | 0.00025 | 1Score **> 33** indicates **identity** Score **> 15** indicates **homology** | U | K.VTSAYLQDIENAYK.K |
| 94097 | 229 | – | 243 | 581.6364 | 1741.8875 | 1741.8886 | -0.66 | 1 | 22 | 0.009 | 1Score **> 35** indicates **identity** Score **> 14** indicates **homology** | U | K.VTSAYLQDIENAYKK.T |
| 94100 | 229 | – | 243 | 581.6376 | 1741.8910 | 1741.8886 | 1.36 | 1 | 40 | 0.00016 | 1Score **> 35** indicates **identity** Score **> 15** indicates **homology** | U | K.VTSAYLQDIENAYKK.T |
| 21367 | 269 | – | 277 | 554.2999 | 1106.5853 | 1106.5859 | -0.58 | 0 | 30 | 0.0016 | 1Score **> 32** indicates **identity** Score **> 14** indicates **homology** | U | K.VVEDIEYLK.Y |
| 21372 | 269 | – | 277 | 554.3022 | 1106.5899 | 1106.5859 | 3.58 | 0 | 28 | 0.0022 | 1Score **> 33** indicates **identity** Score **> 14** indicates **homology** | U | K.VVEDIEYLK.Y |
| 13492 | 278 | – | 285 | 503.2795 | 1004.5444 | 1004.5443 | 0.081 | 1 | 28 | 0.0033 | 1Score **> 32** indicates **identity** Score **> 16** indicates **homology** | U | K.YNKGPWLK.Q |
| 150808 | 278 | – | 295 | 592.5464 | 2366.1563 | 2366.1596 | -1.39 | 2 | 31 | 0.0013 | 1Score **> 36** indicates **identity** Score **> 14** indicates **homology** | U | K.YNKGPWLKQDDWTFHYLR.M |
| 150813 | 278 | – | 295 | 789.7278 | 2366.1614 | 2366.1596 | 0.76 | 2 | 19 | 0.017 | 1Score **> 36** indicates **identity** Score **> 14** indicates **homology** | U | K.YNKGPWLKQDDWTFHYLR.M |
| 150814 | 278 | – | 295 | 592.5477 | 2366.1617 | 2366.1596 | 0.90 | 2 | 74 | 1.2e-07 | 1Score **> 36** indicates **identity** Score **> 17** indicates **homology** | U | K.YNKGPWLKQDDWTFHYLR.M |
| 150815 | 278 | – | 295 | 592.5478 | 2366.1622 | 2366.1596 | 1.08 | 2 | 74 | 1.3e-07 | 1Score **> 36** indicates **identity** Score **> 17** indicates **homology** | U | K.YNKGPWLKQDDWTFHYLR.M |
| 150816 | 278 | – | 295 | 474.2397 | 2366.1622 | 2366.1596 | 1.09 | 2 | 27 | 0.0031 | 1Score **> 36** indicates **identity** Score **> 14** indicates **homology** | U | K.YNKGPWLKQDDWTFHYLR.M |
| 150818 | 278 | – | 295 | 474.2398 | 2366.1624 | 2366.1596 | 1.18 | 2 | 44 | 7.8e-05 | 1Score **> 36** indicates **identity** Score **> 15** indicates **homology** | U | K.YNKGPWLKQDDWTFHYLR.M |
| 150819 | 278 | – | 295 | 592.5479 | 2366.1626 | 2366.1596 | 1.27 | 2 | 59 | 3.2e-06 | 1Score **> 36** indicates **identity** Score **> 16** indicates **homology** | U | K.YNKGPWLKQDDWTFHYLR.M |
| 150820 | 278 | – | 295 | 474.2398 | 2366.1626 | 2366.1596 | 1.28 | 2 | 23 | 0.0075 | 1Score **> 36** indicates **identity** Score **> 14** indicates **homology** | U | K.YNKGPWLKQDDWTFHYLR.M |
| 150821 | 278 | – | 295 | 474.2399 | 2366.1629 | 2366.1596 | 1.37 | 2 | 48 | 3.4e-05 | 1Score **> 36** indicates **identity** Score **> 15** indicates **homology** | U | K.YNKGPWLKQDDWTFHYLR.M |
| 150822 | 278 | – | 295 | 592.5480 | 2366.1631 | 2366.1596 | 1.46 | 2 | 58 | 3.3e-06 | 1Score **> 36** indicates **identity** Score **> 16** indicates **homology** | U | K.YNKGPWLKQDDWTFHYLR.M |
| 150909 | 278 | – | 295 | 592.7971 | 2367.1592 | 2367.1436 | 6.56 | 2 | 30 | 0.0014 | 1Score **> 36** indicates **identity** Score **> 14** indicates **homology** | U | K.YNKGPWLKQDDWTFHYLR.M  + Deamidated (NQ) |
| 116638 | 281 | – | 295 | 654.6635 | 1960.9687 | 1960.9584 | 5.22 | 1 | 29 | 0.0019 | 1Score **> 35** indicates **identity** Score **> 14** indicates **homology** | U | K.GPWLKQDDWTFHYLR.M |
| 116641 | 281 | – | 295 | 654.6641 | 1960.9705 | 1960.9584 | 6.15 | 1 | 19 | 0.017 | 1Score **> 35** indicates **identity** Score **> 14** indicates **homology** | U | K.GPWLKQDDWTFHYLR.M |
| 116644 | 281 | – | 295 | 491.2503 | 1960.9722 | 1960.9584 | 7.03 | 1 | 60 | 2.3e-06 | 1Score **> 35** indicates **identity** Score **> 16** indicates **homology** | U | K.GPWLKQDDWTFHYLR.M |
| 116647 | 281 | – | 295 | 491.2509 | 1960.9744 | 1960.9584 | 8.17 | 1 | 59 | 2.8e-06 | 1Score **> 36** indicates **identity** Score **> 16** indicates **homology** | U | K.GPWLKQDDWTFHYLR.M |
| 48310 | 286 | – | 295 | 460.8825 | 1379.6255 | 1379.6259 | -0.23 | 0 | 30 | 0.0015 | 1Score **> 30** indicates **identity** Score **> 14** indicates **homology** | U | K.QDDWTFHYLR.M |
| 48311 | 286 | – | 295 | 460.8825 | 1379.6256 | 1379.6259 | -0.21 | 0 | 20 | 0.013 | 1Score **> 30** indicates **identity** Score **> 14** indicates **homology** | U | K.QDDWTFHYLR.M |
| 48312 | 286 | – | 295 | 690.8204 | 1379.6262 | 1379.6259 | 0.26 | 0 | 51 | 1.6e-05 | 1Score **> 30** indicates **identity** Score **> 16** indicates **homology** | U | K.QDDWTFHYLR.M |
| 48313 | 286 | – | 295 | 460.8827 | 1379.6264 | 1379.6259 | 0.40 | 0 | 28 | 0.0023 | 1Score **> 30** indicates **identity** Score **> 14** indicates **homology** | U | K.QDDWTFHYLR.M |
| 48316 | 286 | – | 295 | 690.8210 | 1379.6274 | 1379.6259 | 1.11 | 0 | 69 | 3.5e-07 | 1Score **> 30** indicates **identity** Score **> 17** indicates **homology** | U | K.QDDWTFHYLR.M |
| 48317 | 286 | – | 295 | 460.8832 | 1379.6277 | 1379.6259 | 1.30 | 0 | 20 | 0.013 | 1Score **> 30** indicates **identity** Score **> 14** indicates **homology** | U | K.QDDWTFHYLR.M |
| 48318 | 286 | – | 295 | 690.8215 | 1379.6285 | 1379.6259 | 1.92 | 0 | 80 | 2.9e-08 | 1Score **> 30** indicates **identity** Score **> 18** indicates **homology** | U | K.QDDWTFHYLR.M |
| 170653 | 302 | – | 326 | 925.1608 | 2772.4605 | 2772.4334 | 9.78 | 0 | 28 | 0.0022 | 1Score **> 37** indicates **identity** Score **> 14** indicates **homology** | U | K.TEVLNYTTIPVYLPEITIGAHQGSR.I  + Deamidated (NQ) |
| 45699 | 327 | – | 337 | 451.2418 | 1350.7037 | 1350.7044 | -0.51 | 1 | 24 | 0.0069 | 1Score **> 33** indicates **identity** Score **> 15** indicates **homology** | U | R.IYNSFRELPGR.K |
| 133659 | 338 | – | 355 | 713.3755 | 2137.1048 | 2137.0996 | 2.41 | 2 | 29 | 0.002 | 1Score **> 37** indicates **identity** Score **> 14** indicates **homology** | U | R.KYAPGYNAEVGDKWIWLK.- |
| 133661 | 338 | – | 355 | 535.2839 | 2137.1065 | 2137.0996 | 3.22 | 2 | 27 | 0.0031 | 1Score **> 36** indicates **identity** Score **> 14** indicates **homology** | U | R.KYAPGYNAEVGDKWIWLK.- |
| 133662 | 338 | – | 355 | 713.3764 | 2137.1075 | 2137.0996 | 3.69 | 2 | 25 | 0.0042 | 1Score **> 37** indicates **identity** Score **> 14** indicates **homology** | U | R.KYAPGYNAEVGDKWIWLK.- |
| 133664 | 338 | – | 355 | 713.3781 | 2137.1126 | 2137.0996 | 6.06 | 2 | 41 | 0.00015 | 1Score **> 36** indicates **identity** Score **> 15** indicates **homology** | U | R.KYAPGYNAEVGDKWIWLK.- |
| 121293 | 339 | – | 355 | 670.6811 | 2009.0214 | 2009.0047 | 8.35 | 1 | 37 | 0.00031 | 1Score **> 36** indicates **identity** Score **> 15** indicates **homology** | U | K.YAPGYNAEVGDKWIWLK.- |
| 121294 | 339 | – | 355 | 670.6813 | 2009.0221 | 2009.0047 | 8.68 | 1 | 37 | 0.00031 | 1Score **> 36** indicates **identity** Score **> 15** indicates **homology** | U | K.YAPGYNAEVGDKWIWLK.- |
| 121296 | 339 | – | 355 | 1005.5184 | 2009.0222 | 2009.0047 | 8.74 | 1 | 52 | 1.4e-05 | 1Score **> 36** indicates **identity** Score **> 16** indicates **homology** | U | K.YAPGYNAEVGDKWIWLK.- |
| 121297 | 339 | – | 355 | 670.6816 | 2009.0228 | 2009.0047 | 9.04 | 1 | 44 | 8.1e-05 | 1Score **> 36** indicates **identity** Score **> 15** indicates **homology** | U | K.YAPGYNAEVGDKWIWLK.- |
| 121298 | 339 | – | 355 | 1005.5192 | 2009.0238 | 2009.0047 | 9.53 | 1 | 38 | 0.0003 | 1Score **> 36** indicates **identity** Score **> 15** indicates **homology** | U | K.YAPGYNAEVGDKWIWLK.- |

---

```
ID   NDUAA_MOUSE             Reviewed;         355 AA.
AC   Q99LC3; Q3UKK0; Q8BL57; Q9CW21;
DT   27-JUN-2003, integrated into UniProtKB/Swiss-Prot.
DT   01-JUN-2001, sequence version 1.
DT   28-JUN-2023, entry version 166.
DE   RecName: Full=NADH dehydrogenase [ubiquinone] 1 alpha subcomplex subunit 10, mitochondrial;
DE   AltName: Full=Complex I-42kD;
DE            Short=CI-42kD;
DE   AltName: Full=NADH-ubiquinone oxidoreductase 42 kDa subunit;
DE   Flags: Precursor;
GN   Name=Ndufa10;
OS   Mus musculus (Mouse).
OC   Eukaryota; Metazoa; Chordata; Craniata; Vertebrata; Euteleostomi; Mammalia;
OC   Eutheria; Euarchontoglires; Glires; Rodentia; Myomorpha; Muroidea; Muridae;
OC   Murinae; Mus; Mus.
OX   NCBI_TaxID=10090;
RN   [1]
RP   NUCLEOTIDE SEQUENCE [LARGE SCALE MRNA].
RX   PubMed=15489334; DOI=10.1101/gr.2596504;
RG   The MGC Project Team;
RT   "The status, quality, and expansion of the NIH full-length cDNA project:
RT   the Mammalian Gene Collection (MGC).";
RL   Genome Res. 14:2121-2127(2004).
RN   [2]
RP   NUCLEOTIDE SEQUENCE [LARGE SCALE MRNA] OF 1-330.
RC   STRAIN=C57BL/6J; TISSUE=Brain, and Placenta;
RX   PubMed=16141072; DOI=10.1126/science.1112014;
RA   Carninci P., Kasukawa T., Katayama S., Gough J., Frith M.C., Maeda N.,
RA   Oyama R., Ravasi T., Lenhard B., Wells C., Kodzius R., Shimokawa K.,
RA   Bajic V.B., Brenner S.E., Batalov S., Forrest A.R., Zavolan M., Davis M.J.,
RA   Wilming L.G., Aidinis V., Allen J.E., Ambesi-Impiombato A., Apweiler R.,
RA   Aturaliya R.N., Bailey T.L., Bansal M., Baxter L., Beisel K.W., Bersano T.,
RA   Bono H., Chalk A.M., Chiu K.P., Choudhary V., Christoffels A.,
RA   Clutterbuck D.R., Crowe M.L., Dalla E., Dalrymple B.P., de Bono B.,
RA   Della Gatta G., di Bernardo D., Down T., Engstrom P., Fagiolini M.,
RA   Faulkner G., Fletcher C.F., Fukushima T., Furuno M., Futaki S.,
RA   Gariboldi M., Georgii-Hemming P., Gingeras T.R., Gojobori T., Green R.E.,
RA   Gustincich S., Harbers M., Hayashi Y., Hensch T.K., Hirokawa N., Hill D.,
RA   Huminiecki L., Iacono M., Ikeo K., Iwama A., Ishikawa T., Jakt M.,
RA   Kanapin A., Katoh M., Kawasawa Y., Kelso J., Kitamura H., Kitano H.,
RA   Kollias G., Krishnan S.P., Kruger A., Kummerfeld S.K., Kurochkin I.V.,
RA   Lareau L.F., Lazarevic D., Lipovich L., Liu J., Liuni S., McWilliam S.,
RA   Madan Babu M., Madera M., Marchionni L., Matsuda H., Matsuzawa S., Miki H.,
RA   Mignone F., Miyake S., Morris K., Mottagui-Tabar S., Mulder N., Nakano N.,
RA   Nakauchi H., Ng P., Nilsson R., Nishiguchi S., Nishikawa S., Nori F.,
RA   Ohara O., Okazaki Y., Orlando V., Pang K.C., Pavan W.J., Pavesi G.,
RA   Pesole G., Petrovsky N., Piazza S., Reed J., Reid J.F., Ring B.Z.,
RA   Ringwald M., Rost B., Ruan Y., Salzberg S.L., Sandelin A., Schneider C.,
RA   Schoenbach C., Sekiguchi K., Semple C.A., Seno S., Sessa L., Sheng Y.,
RA   Shibata Y., Shimada H., Shimada K., Silva D., Sinclair B., Sperling S.,
RA   Stupka E., Sugiura K., Sultana R., Takenaka Y., Taki K., Tammoja K.,
RA   Tan S.L., Tang S., Taylor M.S., Tegner J., Teichmann S.A., Ueda H.R.,
RA   van Nimwegen E., Verardo R., Wei C.L., Yagi K., Yamanishi H.,
RA   Zabarovsky E., Zhu S., Zimmer A., Hide W., Bult C., Grimmond S.M.,
RA   Teasdale R.D., Liu E.T., Brusic V., Quackenbush J., Wahlestedt C.,
RA   Mattick J.S., Hume D.A., Kai C., Sasaki D., Tomaru Y., Fukuda S.,
RA   Kanamori-Katayama M., Suzuki M., Aoki J., Arakawa T., Iida J., Imamura K.,
RA   Itoh M., Kato T., Kawaji H., Kawagashira N., Kawashima T., Kojima M.,
RA   Kondo S., Konno H., Nakano K., Ninomiya N., Nishio T., Okada M., Plessy C.,
RA   Shibata K., Shiraki T., Suzuki S., Tagami M., Waki K., Watahiki A.,
RA   Okamura-Oho Y., Suzuki H., Kawai J., Hayashizaki Y.;
RT   "The transcriptional landscape of the mammalian genome.";
RL   Science 309:1559-1563(2005).
RN   [3]
RP   PROTEIN SEQUENCE OF 38-48; 59-70; 76-84; 131-161; 193-217; 229-243;
RP   269-277; 286-295; 302-332 AND 339-350, AND IDENTIFICATION BY MASS
RP   SPECTROMETRY.
RC   STRAIN=C57BL/6J; TISSUE=Brain, and Hippocampus;
RA   Lubec G., Klug S., Kang S.U.;
RL   Submitted (APR-2007) to UniProtKB.
RN   [4]
RP   IDENTIFICATION BY MASS SPECTROMETRY [LARGE SCALE ANALYSIS].
RC   TISSUE=Brain, Brown adipose tissue, Heart, Kidney, Liver, Lung,
RC   Pancreas, Spleen, and Testis;
RX   PubMed=21183079; DOI=10.1016/j.cell.2010.12.001;
RA   Huttlin E.L., Jedrychowski M.P., Elias J.E., Goswami T., Rad R.,
RA   Beausoleil S.A., Villen J., Haas W., Sowa M.E., Gygi S.P.;
RT   "A tissue-specific atlas of mouse protein phosphorylation and expression.";
RL   Cell 143:1174-1189(2010).
RN   [5]
RP   SUCCINYLATION [LARGE SCALE ANALYSIS] AT LYS-122 AND LYS-285, AND
RP   IDENTIFICATION BY MASS SPECTROMETRY [LARGE SCALE ANALYSIS].
RC   TISSUE=Liver;
RX   PubMed=23806337; DOI=10.1016/j.molcel.2013.06.001;
RA   Park J., Chen Y., Tishkoff D.X., Peng C., Tan M., Dai L., Xie Z., Zhang Y.,
RA   Zwaans B.M., Skinner M.E., Lombard D.B., Zhao Y.;
RT   "SIRT5-mediated lysine desuccinylation impacts diverse metabolic
RT   pathways.";
RL   Mol. Cell 50:919-930(2013).
RN   [6]
RP   ACETYLATION [LARGE SCALE ANALYSIS] AT LYS-122, AND IDENTIFICATION BY MASS
RP   SPECTROMETRY [LARGE SCALE ANALYSIS].
RC   TISSUE=Liver;
RX   PubMed=23576753; DOI=10.1073/pnas.1302961110;
RA   Rardin M.J., Newman J.C., Held J.M., Cusack M.P., Sorensen D.J., Li B.,
RA   Schilling B., Mooney S.D., Kahn C.R., Verdin E., Gibson B.W.;
RT   "Label-free quantitative proteomics of the lysine acetylome in mitochondria
RT   identifies substrates of SIRT3 in metabolic pathways.";
RL   Proc. Natl. Acad. Sci. U.S.A. 110:6601-6606(2013).
RN   [7]
RP   FUNCTION, PHOSPHORYLATION AT SER-250, AND MUTAGENESIS OF SER-250.
RX   PubMed=24652937; DOI=10.1126/science.1249161;
RA   Morais V.A., Haddad D., Craessaerts K., De Bock P.J., Swerts J., Vilain S.,
RA   Aerts L., Overbergh L., Gruenewald A., Seibler P., Klein C., Gevaert K.,
RA   Verstreken P., De Strooper B.;
RT   "PINK1 loss-of-function mutations affect mitochondrial complex I activity
RT   via NdufA10 ubiquinone uncoupling.";
RL   Science 344:203-207(2014).
CC   -!- FUNCTION: Accessory subunit of the mitochondrial membrane respiratory
CC       chain NADH dehydrogenase (Complex I), that is believed not to be
CC       involved in catalysis. Complex I functions in the transfer of electrons
CC       from NADH to the respiratory chain. The immediate electron acceptor for
CC       the enzyme is believed to be ubiquinone. {ECO:0000269|PubMed:24652937}.
CC   -!- COFACTOR:
CC       Name=FAD; Xref=ChEBI:CHEBI:57692;
CC       Note=Binds 1 FAD per subunit.;
CC   -!- SUBUNIT: Complex I is composed of 45 different subunits. This a
CC       component of the hydrophobic protein fraction.
CC       {ECO:0000250|UniProtKB:O95299}.
CC   -!- SUBCELLULAR LOCATION: Mitochondrion matrix
CC       {ECO:0000250|UniProtKB:O95299}.
CC   -!- PTM: Phosphorylation at Ser-250 by PINK1 is required for the binding
CC       and/or reduction of the complex I substrate ubiquinone.
CC       {ECO:0000269|PubMed:24652937}.
CC   -!- PTM: Acetylation of Lys-242 is observed in liver mitochondria from
CC       fasted mice but not from fed mice.
CC   -!- SIMILARITY: Belongs to the complex I NDUFA10 subunit family.
CC       {ECO:0000305}.
CC   -!- SEQUENCE CAUTION:
CC       Sequence=BAB23961.2; Type=Erroneous initiation; Evidence={ECO:0000305};
CC   ---------------------------------------------------------------------------
CC   Copyrighted by the UniProt Consortium, see https://www.uniprot.org/terms
CC   Distributed under the Creative Commons Attribution (CC BY 4.0) License
CC   ---------------------------------------------------------------------------
DR   EMBL; BC003439; AAH03439.1; -; mRNA.
DR   EMBL; AK005339; BAB23961.2; ALT_INIT; mRNA.
DR   EMBL; AK145979; BAE26801.1; -; mRNA.
DR   EMBL; AK046292; BAC32674.1; -; mRNA.
DR   CCDS; CCDS15167.1; -.
DR   RefSeq; NP_077159.1; NM_024197.1.
DR   PDB; 6G2J; EM; 3.30 A; O=1-355.
DR   PDB; 6G72; EM; 3.90 A; O=1-355.
DR   PDB; 6ZR2; EM; 3.10 A; O=1-355.
DR   PDB; 6ZTQ; EM; 3.00 A; O=1-355.
DR   PDB; 7AK5; EM; 3.17 A; O=1-355.
DR   PDB; 7AK6; EM; 3.82 A; O=1-355.
DR   PDB; 7B93; EM; 3.04 A; O=1-355.
DR   PDB; 7PSA; EM; 3.40 A; O=1-355.
DR   PDBsum; 6G2J; -.
DR   PDBsum; 6G72; -.
DR   PDBsum; 6ZR2; -.
DR   PDBsum; 6ZTQ; -.
DR   PDBsum; 7AK5; -.
DR   PDBsum; 7AK6; -.
DR   PDBsum; 7B93; -.
DR   PDBsum; 7PSA; -.
DR   AlphaFoldDB; Q99LC3; -.
DR   SMR; Q99LC3; -.
DR   BioGRID; 212064; 76.
DR   ComplexPortal; CPX-266; Mitochondrial respiratory chain complex I.
DR   CORUM; Q99LC3; -.
DR   IntAct; Q99LC3; 7.
DR   STRING; 10090.ENSMUSP00000027478; -.
DR   GlyGen; Q99LC3; 1 site, 1 O-linked glycan (1 site).
DR   iPTMnet; Q99LC3; -.
DR   PhosphoSitePlus; Q99LC3; -.
DR   SwissPalm; Q99LC3; -.
DR   REPRODUCTION-2DPAGE; Q99LC3; -.
DR   EPD; Q99LC3; -.
DR   jPOST; Q99LC3; -.
DR   MaxQB; Q99LC3; -.
DR   PaxDb; Q99LC3; -.
DR   PeptideAtlas; Q99LC3; -.
DR   ProteomicsDB; 253046; -.
DR   Antibodypedia; 34509; 210 antibodies from 30 providers.
DR   DNASU; 67273; -.
DR   Ensembl; ENSMUST00000027478; ENSMUSP00000027478; ENSMUSG00000026260.
DR   GeneID; 67273; -.
DR   KEGG; mmu:67273; -.
DR   UCSC; uc007cbh.1; mouse.
DR   AGR; MGI:1914523; -.
DR   CTD; 4705; -.
DR   MGI; MGI:1914523; Ndufa10.
DR   VEuPathDB; HostDB:ENSMUSG00000026260; -.
DR   eggNOG; KOG3877; Eukaryota.
DR   GeneTree; ENSGT00390000016151; -.
DR   HOGENOM; CLU_050591_0_0_1; -.
DR   InParanoid; Q99LC3; -.
DR   OMA; MFRQGYI; -.
DR   OrthoDB; 6357at2759; -.
DR   PhylomeDB; Q99LC3; -.
DR   TreeFam; TF314616; -.
DR   Reactome; R-MMU-611105; Respiratory electron transport.
DR   Reactome; R-MMU-6799198; Complex I biogenesis.
DR   BioGRID-ORCS; 67273; 21 hits in 78 CRISPR screens.
DR   ChiTaRS; Ndufa10; mouse.
DR   PRO; PR:Q99LC3; -.
DR   Proteomes; UP000000589; Chromosome 1.
DR   RNAct; Q99LC3; protein.
DR   Bgee; ENSMUSG00000026260; Expressed in heart right ventricle and 258 other tissues.
DR   ExpressionAtlas; Q99LC3; baseline and differential.
DR   Genevisible; Q99LC3; MM.
DR   GO; GO:0005737; C:cytoplasm; IBA:GO_Central.
DR   GO; GO:0005759; C:mitochondrial matrix; IEA:UniProtKB-SubCell.
DR   GO; GO:0005747; C:mitochondrial respiratory chain complex I; ISS:UniProtKB.
DR   GO; GO:0005739; C:mitochondrion; HDA:MGI.
DR   GO; GO:0043209; C:myelin sheath; HDA:UniProtKB.
DR   GO; GO:0008137; F:NADH dehydrogenase (ubiquinone) activity; IDA:MGI.
DR   GO; GO:0009060; P:aerobic respiration; NAS:ComplexPortal.
DR   GO; GO:0006120; P:mitochondrial electron transport, NADH to ubiquinone; IDA:MGI.
DR   GO; GO:0032981; P:mitochondrial respiratory chain complex I assembly; ISS:UniProtKB.
DR   GO; GO:0042776; P:proton motive force-driven mitochondrial ATP synthesis; NAS:ComplexPortal.
DR   CDD; cd02030; NDUO42; 1.
DR   Gene3D; 3.40.50.300; P-loop containing nucleotide triphosphate hydrolases; 1.
DR   InterPro; IPR031314; DNK_dom.
DR   InterPro; IPR015828; NDUFA10.
DR   InterPro; IPR027417; P-loop_NTPase.
DR   PANTHER; PTHR10513; DEOXYNUCLEOSIDE KINASE; 1.
DR   PANTHER; PTHR10513:SF15; NADH DEHYDROGENASE [UBIQUINONE] 1 ALPHA SUBCOMPLEX SUBUNIT 10, MITOCHONDRIAL; 1.
DR   Pfam; PF01712; dNK; 1.
DR   PIRSF; PIRSF000543; NADH_UQ_42KD; 1.
DR   SUPFAM; SSF52540; P-loop containing nucleoside triphosphate hydrolases; 1.
PE   1: Evidence at protein level;
KW   3D-structure; Acetylation; Direct protein sequencing; Electron transport;
KW   FAD; Flavoprotein; Mitochondrion; Phosphoprotein; Reference proteome;
KW   Respiratory chain; Transit peptide; Transport.
FT   TRANSIT         1..35
FT                   /note="Mitochondrion"
FT                   /evidence="ECO:0000250|UniProtKB:P34942"
FT   CHAIN           36..355
FT                   /note="NADH dehydrogenase [ubiquinone] 1 alpha subcomplex
FT                   subunit 10, mitochondrial"
FT                   /id="PRO_0000019989"
FT   MOD_RES         122
FT                   /note="N6-acetyllysine; alternate"
FT                   /evidence="ECO:0007744|PubMed:23576753"
FT   MOD_RES         122
FT                   /note="N6-succinyllysine; alternate"
FT                   /evidence="ECO:0007744|PubMed:23806337"
FT   MOD_RES         250
FT                   /note="Phosphoserine; by PINK1"
FT                   /evidence="ECO:0000269|PubMed:24652937"
FT   MOD_RES         285
FT                   /note="N6-succinyllysine"
FT                   /evidence="ECO:0007744|PubMed:23806337"
FT   MUTAGEN         250
FT                   /note="S->A: Leads to mitochondrial depolarization."
FT                   /evidence="ECO:0000269|PubMed:24652937"
FT   MUTAGEN         250
FT                   /note="S->D: Phosphomimetic mutant; able to rescue
FT                   mitochondrial depolarization in a Pink1 mutant background."
FT                   /evidence="ECO:0000269|PubMed:24652937"
FT   CONFLICT        191
FT                   /note="K -> E (in Ref. 2; BAB23961)"
FT                   /evidence="ECO:0000305"
FT   CONFLICT        266
FT                   /note="P -> T (in Ref. 2; BAC32674)"
FT                   /evidence="ECO:0000305"
FT   CONFLICT        294
FT                   /note="L -> S (in Ref. 2; BAB23961)"
FT                   /evidence="ECO:0000305"
FT   HELIX           40..45
FT                   /evidence="ECO:0007829|PDB:6ZTQ"
FT   TURN            50..52
FT                   /evidence="ECO:0007829|PDB:6ZTQ"
FT   STRAND          59..63
FT                   /evidence="ECO:0007829|PDB:6ZTQ"
FT   STRAND          65..69
FT                   /evidence="ECO:0007829|PDB:6ZTQ"
FT   HELIX           70..81
FT                   /evidence="ECO:0007829|PDB:6ZTQ"
FT   STRAND          84..86
FT                   /evidence="ECO:0007829|PDB:7B93"
FT   TURN            94..97
FT                   /evidence="ECO:0007829|PDB:6ZTQ"
FT   TURN            106..108
FT                   /evidence="ECO:0007829|PDB:6ZTQ"
FT   HELIX           114..119
FT                   /evidence="ECO:0007829|PDB:6ZTQ"
FT   HELIX           127..153
FT                   /evidence="ECO:0007829|PDB:6ZTQ"
FT   STRAND          157..161
FT                   /evidence="ECO:0007829|PDB:6ZTQ"
FT   HELIX           163..165
FT                   /evidence="ECO:0007829|PDB:7B93"
FT   HELIX           167..175
FT                   /evidence="ECO:0007829|PDB:6ZTQ"
FT   HELIX           181..194
FT                   /evidence="ECO:0007829|PDB:6ZTQ"
FT   HELIX           195..197
FT                   /evidence="ECO:0007829|PDB:7B93"
FT   STRAND          202..207
FT                   /evidence="ECO:0007829|PDB:6ZTQ"
FT   HELIX           211..219
FT                   /evidence="ECO:0007829|PDB:6ZTQ"
FT   TURN            220..222
FT                   /evidence="ECO:0007829|PDB:6G2J"
FT   HELIX           226..228
FT                   /evidence="ECO:0007829|PDB:6ZTQ"
FT   HELIX           232..250
FT                   /evidence="ECO:0007829|PDB:6ZTQ"
FT   STRAND          253..255
FT                   /evidence="ECO:0007829|PDB:6ZTQ"
FT   HELIX           260..263
FT                   /evidence="ECO:0007829|PDB:6ZTQ"
FT   HELIX           266..273
FT                   /evidence="ECO:0007829|PDB:6ZTQ"
FT   TURN            282..285
FT                   /evidence="ECO:0007829|PDB:6ZTQ"
FT   HELIX           288..299
FT                   /evidence="ECO:0007829|PDB:6ZTQ"
FT   HELIX           301..305
FT                   /evidence="ECO:0007829|PDB:6ZTQ"
FT   HELIX           306..308
FT                   /evidence="ECO:0007829|PDB:6ZTQ"
FT   TURN            315..317
FT                   /evidence="ECO:0007829|PDB:6ZTQ"
FT   HELIX           321..331
FT                   /evidence="ECO:0007829|PDB:6ZTQ"
FT   HELIX           335..337
FT                   /evidence="ECO:0007829|PDB:7B93"
FT   STRAND          338..340
FT                   /evidence="ECO:0007829|PDB:6G2J"
FT   HELIX           341..343
FT                   /evidence="ECO:0007829|PDB:7AK5"
FT   TURN            345..348
FT                   /evidence="ECO:0007829|PDB:6ZTQ"
FT   HELIX           352..354
FT                   /evidence="ECO:0007829|PDB:6ZTQ"
SQ   SEQUENCE   355 AA;  40603 MW;  75364533B079A688 CRC64;
     MALRLLRLVP ASAPARGLAA GAQRVGRIHT SVHCKLRYGL LAAILGDKTT KKLHEYSRVI
     TVDGNICSGK NKLAKEIAQQ LGMKHYPEAG IQYSSTTTGD GRPLDIEFSG SCSLEKFYDD
     PKSNDGNSYR LQSWLYASRL LQYADALEHL LSTGQGVVLE RSIYSDFVFL EAMYNQGYIR
     KQCVDHYNEI KRLTLPEYLP PHAVIYIDVP VPEVQSRIQK KGDPHEMKVT SAYLQDIENA
     YKKTFLPKMS EMCEVLVYDS WEAEDPTKVV EDIEYLKYNK GPWLKQDDWT FHYLRMLVQD
     KTEVLNYTTI PVYLPEITIG AHQGSRIYNS FRELPGRKYA PGYNAEVGDK WIWLK
//
```

**Mascot:** http://www.matrixscience.com/


HNE (H) (+156.1150)
