# Supplementary material for: A common East-Asian ALDH2 mutation causes metabolic disorders and the therapeutic effect of ALDH2 activators
Source: Nat Commun. 2023 Sep 25;14:5971. doi: 10.1038/s41467-023-41570-6 (PMC10520061; doi:10.1038/s41467-023-41570-6)
Supplement: Supplementary file 4 — Supplementary Data 1 [file 41467_2023_41570_MOESM4_ESM.zip › Table S5c/Q9CQB4/Q9CQB4_KIO-4.html]

Mascot Search Results: Q9CQB4
 

# MASCOT Search Results

## Protein View: Q9CQB4

### Cytochrome b-c1 complex subunit 7 OS=Mus musculus OX=10090 GN=Uqcrb PE=1 SV=1

|  |  |
| --- | --- |
| Database: | Mouse\_UniProt\_proteomes |
| Score: | 2024 |
| Monoisotopic mass (M_r_): | 13553 |
| Calculated pI: | 9.10 |

Sequence similarity is available as an NCBI BLAST search of Q9CQB4 against nr.

### Search parameters

|  |  |
| --- | --- |
| MS data file: | `D:\LCMSMS\2023 Users' data\230529-1\230529-1-KIO-4.raw` |
| Enzyme: | Trypsin/P: cuts C-term side of KR. |
| Fixed modifications: | Carbamidomethyl (C) |
| Variable modifications: | Deamidated (NQ), HNE (C), HNE (H), HNE (K), Oxidation (M) |

### Protein sequence coverage: 63%

Matched peptides shown in ***bold red***.

|  |  |  |  |  |  |
| --- | --- | --- | --- | --- | --- |
| `1` | `MAGRSAVSAS` | `SKWLDGFRKW` | `YYNAAGFNKL` | `GLMRDDTLHE` | `TEDVKEAIRR` |
| `51` | `LPEDLYNDRM` | `FRIKRALDLT` | `MRHQILPKDQ` | `WTKYEEDKFY` | `LEPYLKEVIR` |
| `101` | `ERKEREEWAK` | `K` |  |  |  |

Unformatted sequence string: 111 residues (for pasting into other applications).

|  |  |  |  |
| --- | --- | --- | --- |
| Sort by | residue number | increasing mass | decreasing mass |
| Show | matched peptides only | predicted peptides also |  |

| Query | Start | – | End | Observed | Mr(expt) | Mr(calc) | ppm | M | Score | Expect | Rank | U | Peptide |
| --- | --- | --- | --- | --- | --- | --- | --- | --- | --- | --- | --- | --- | --- |
| 51320 | 19 | – | 29 | 681.3373 | 1360.6601 | 1360.6564 | 2.76 | 1 | 47 | 6.2e-05 | 1Score **> 33** indicates **identity** Score **> 18** indicates **homology** | U | R.KWYYNAAGFNK.L |
| 51322 | 19 | – | 29 | 681.3378 | 1360.6610 | 1360.6564 | 3.36 | 1 | 36 | 0.00078 | 1Score **> 33** indicates **identity** Score **> 17** indicates **homology** | U | R.KWYYNAAGFNK.L |
| 37067 | 20 | – | 29 | 617.2844 | 1232.5543 | 1232.5614 | -5.81 | 0 | 17 | 0.025 | 1Score **> 28** indicates **identity** Score **> 14** indicates **homology** | U | K.WYYNAAGFNK.L |
| 37068 | 20 | – | 29 | 617.2874 | 1232.5603 | 1232.5614 | -0.96 | 0 | 30 | 0.0015 | 1Score **> 29** indicates **identity** Score **> 14** indicates **homology** | U | K.WYYNAAGFNK.L |
| 37069 | 20 | – | 29 | 617.2878 | 1232.5610 | 1232.5614 | -0.37 | 0 | 22 | 0.0096 | 1Score **> 29** indicates **identity** Score **> 14** indicates **homology** | U | K.WYYNAAGFNK.L |
| 37070 | 20 | – | 29 | 617.2881 | 1232.5617 | 1232.5614 | 0.22 | 0 | 42 | 0.00011 | 1Score **> 29** indicates **identity** Score **> 15** indicates **homology** | U | K.WYYNAAGFNK.L |
| 37071 | 20 | – | 29 | 617.2884 | 1232.5622 | 1232.5614 | 0.60 | 0 | 40 | 0.00016 | 1Score **> 29** indicates **identity** Score **> 15** indicates **homology** | U | K.WYYNAAGFNK.L |
| 37072 | 20 | – | 29 | 617.2886 | 1232.5627 | 1232.5614 | 1.03 | 0 | 45 | 6.3e-05 | 1Score **> 30** indicates **identity** Score **> 15** indicates **homology** | U | K.WYYNAAGFNK.L |
| 37073 | 20 | – | 29 | 617.2891 | 1232.5637 | 1232.5614 | 1.83 | 0 | 31 | 0.0014 | 1Score **> 30** indicates **identity** Score **> 14** indicates **homology** | U | K.WYYNAAGFNK.L |
| 37214 | 20 | – | 29 | 617.7814 | 1233.5483 | 1233.5454 | 2.28 | 0 | 29 | 0.002 | 1Score **> 29** indicates **identity** Score **> 14** indicates **homology** | U | K.WYYNAAGFNK.L  + Deamidated (NQ) |
| 37215 | 20 | – | 29 | 617.7818 | 1233.5490 | 1233.5454 | 2.91 | 0 | 32 | 0.00098 | 1Score **> 28** indicates **identity** Score **> 15** indicates **homology** | U | K.WYYNAAGFNK.L  + Deamidated (NQ) |
| 152868 | 30 | – | 49 | 781.0645 | 2340.1717 | 2340.1743 | -1.13 | 2 | 18 | 0.019 | 1Score **> 37** indicates **identity** Score **> 14** indicates **homology** | U | K.LGLMRDDTLHETEDVKEAIR.R |
| 152869 | 30 | – | 49 | 586.0508 | 2340.1739 | 2340.1743 | -0.19 | 2 | 29 | 0.0021 | 1Score **> 37** indicates **identity** Score **> 14** indicates **homology** | U | K.LGLMRDDTLHETEDVKEAIR.R |
| 152871 | 30 | – | 49 | 781.0653 | 2340.1741 | 2340.1743 | -0.11 | 2 | 27 | 0.0031 | 1Score **> 37** indicates **identity** Score **> 14** indicates **homology** | U | K.LGLMRDDTLHETEDVKEAIR.R |
| 152872 | 30 | – | 49 | 586.0510 | 2340.1750 | 2340.1743 | 0.30 | 2 | 31 | 0.0012 | 1Score **> 37** indicates **identity** Score **> 14** indicates **homology** | U | K.LGLMRDDTLHETEDVKEAIR.R |
| 152873 | 30 | – | 49 | 469.0423 | 2340.1751 | 2340.1743 | 0.32 | 2 | 16 | 0.034 | 1Score **> 37** indicates **identity** Score **> 13** indicates **homology** | U | K.LGLMRDDTLHETEDVKEAIR.R |
| 152876 | 30 | – | 49 | 781.0659 | 2340.1759 | 2340.1743 | 0.67 | 2 | 26 | 0.0037 | 1Score **> 37** indicates **identity** Score **> 14** indicates **homology** | U | K.LGLMRDDTLHETEDVKEAIR.R |
| 44691 | 35 | – | 45 | 651.2953 | 1300.5760 | 1300.5783 | -1.77 | 0 | 42 | 0.00012 | 1Score **> 27** indicates **identity** Score **> 15** indicates **homology** | U | R.DDTLHETEDVK.E |
| 44697 | 35 | – | 45 | 651.3023 | 1300.5901 | 1300.5783 | 9.11 | 0 | 38 | 0.00029 | 1Score **> 28** indicates **identity** Score **> 15** indicates **homology** | U | R.DDTLHETEDVK.E |
| 102812 | 35 | – | 49 | 885.9302 | 1769.8458 | 1769.8432 | 1.49 | 1 | 63 | 2.2e-06 | 1Score **> 33** indicates **identity** Score **> 19** indicates **homology** | U | R.DDTLHETEDVKEAIR.R |
| 43482 | 50 | – | 59 | 430.8850 | 1289.6332 | 1289.6364 | -2.49 | 1 | 16 | 0.029 | 1Score **> 33** indicates **identity** Score **> 14** indicates **homology** | U | R.RLPEDLYNDR.M |
| 43485 | 50 | – | 59 | 645.8242 | 1289.6338 | 1289.6364 | -2.02 | 1 | 37 | 0.00037 | 1Score **> 33** indicates **identity** Score **> 15** indicates **homology** | U | R.RLPEDLYNDR.M |
| 43486 | 50 | – | 59 | 430.8854 | 1289.6343 | 1289.6364 | -1.62 | 1 | 15 | 0.039 | 1Score **> 33** indicates **identity** Score **> 13** indicates **homology** | U | R.RLPEDLYNDR.M |
| 43487 | 50 | – | 59 | 645.8247 | 1289.6349 | 1289.6364 | -1.12 | 1 | 35 | 0.00059 | 1Score **> 33** indicates **identity** Score **> 15** indicates **homology** | U | R.RLPEDLYNDR.M |
| 43488 | 50 | – | 59 | 430.8857 | 1289.6353 | 1289.6364 | -0.87 | 1 | 25 | 0.0046 | 1Score **> 33** indicates **identity** Score **> 14** indicates **homology** | U | R.RLPEDLYNDR.M |
| 43489 | 50 | – | 59 | 430.8857 | 1289.6354 | 1289.6364 | -0.79 | 1 | 18 | 0.02 | 1Score **> 33** indicates **identity** Score **> 14** indicates **homology** | U | R.RLPEDLYNDR.M |
| 43493 | 50 | – | 59 | 430.8860 | 1289.6362 | 1289.6364 | -0.12 | 1 | 28 | 0.0024 | 1Score **> 33** indicates **identity** Score **> 14** indicates **homology** | U | R.RLPEDLYNDR.M |
| 43494 | 50 | – | 59 | 645.8255 | 1289.6364 | 1289.6364 | 0.0023 | 1 | 54 | 1.3e-05 | 1Score **> 33** indicates **identity** Score **> 18** indicates **homology** | U | R.RLPEDLYNDR.M |
| 43495 | 50 | – | 59 | 645.8257 | 1289.6368 | 1289.6364 | 0.35 | 1 | 65 | 5.8e-06 | 1Score **> 33** indicates **identity** Score **> 25** indicates **homology** | U | R.RLPEDLYNDR.M |
| 43496 | 50 | – | 59 | 645.8257 | 1289.6369 | 1289.6364 | 0.38 | 1 | 67 | 3.5e-06 | 1Score **> 33** indicates **identity** Score **> 25** indicates **homology** | U | R.RLPEDLYNDR.M |
| 43497 | 50 | – | 59 | 645.8258 | 1289.6370 | 1289.6364 | 0.45 | 1 | 67 | 3.5e-06 | 1Score **> 33** indicates **identity** Score **> 25** indicates **homology** | U | R.RLPEDLYNDR.M |
| 43498 | 50 | – | 59 | 645.8258 | 1289.6370 | 1289.6364 | 0.46 | 1 | 67 | 3.5e-06 | 1Score **> 33** indicates **identity** Score **> 25** indicates **homology** | U | R.RLPEDLYNDR.M |
| 43499 | 50 | – | 59 | 645.8258 | 1289.6370 | 1289.6364 | 0.47 | 1 | 49 | 2.9e-05 | 1Score **> 33** indicates **identity** Score **> 16** indicates **homology** | U | R.RLPEDLYNDR.M |
| 43500 | 50 | – | 59 | 430.8863 | 1289.6370 | 1289.6364 | 0.50 | 1 | 19 | 0.016 | 1Score **> 33** indicates **identity** Score **> 14** indicates **homology** | U | R.RLPEDLYNDR.M |
| 43501 | 50 | – | 59 | 645.8258 | 1289.6371 | 1289.6364 | 0.58 | 1 | 67 | 3.4e-06 | 1Score **> 33** indicates **identity** Score **> 25** indicates **homology** | U | R.RLPEDLYNDR.M |
| 43502 | 50 | – | 59 | 430.8863 | 1289.6372 | 1289.6364 | 0.60 | 1 | 35 | 0.00056 | 1Score **> 33** indicates **identity** Score **> 15** indicates **homology** | U | R.RLPEDLYNDR.M |
| 43505 | 50 | – | 59 | 645.8260 | 1289.6374 | 1289.6364 | 0.76 | 1 | 18 | 0.028 | 1Score **> 33** indicates **identity** Score **> 15** indicates **homology** | U | R.RLPEDLYNDR.M |
| 43506 | 50 | – | 59 | 645.8261 | 1289.6376 | 1289.6364 | 0.93 | 1 | 67 | 3.9e-06 | 1Score **> 33** indicates **identity** Score **> 25** indicates **homology** | U | R.RLPEDLYNDR.M |
| 43507 | 50 | – | 59 | 645.8261 | 1289.6376 | 1289.6364 | 0.94 | 1 | 67 | 3.5e-06 | 1Score **> 33** indicates **identity** Score **> 25** indicates **homology** | U | R.RLPEDLYNDR.M |
| 43508 | 50 | – | 59 | 430.8865 | 1289.6377 | 1289.6364 | 1.01 | 1 | 43 | 9.1e-05 | 1Score **> 33** indicates **identity** Score **> 15** indicates **homology** | U | R.RLPEDLYNDR.M |
| 43509 | 50 | – | 59 | 645.8262 | 1289.6378 | 1289.6364 | 1.06 | 1 | 64 | 5.9e-06 | 1Score **> 33** indicates **identity** Score **> 24** indicates **homology** | U | R.RLPEDLYNDR.M |
| 43511 | 50 | – | 59 | 430.8865 | 1289.6378 | 1289.6364 | 1.09 | 1 | 35 | 0.0005 | 1Score **> 33** indicates **identity** Score **> 15** indicates **homology** | U | R.RLPEDLYNDR.M |
| 43513 | 50 | – | 59 | 430.8866 | 1289.6379 | 1289.6364 | 1.16 | 1 | 35 | 0.00054 | 1Score **> 33** indicates **identity** Score **> 15** indicates **homology** | U | R.RLPEDLYNDR.M |
| 43514 | 50 | – | 59 | 645.8262 | 1289.6379 | 1289.6364 | 1.16 | 1 | 34 | 0.00087 | 1Score **> 33** indicates **identity** Score **> 16** indicates **homology** | U | R.RLPEDLYNDR.M |
| 43515 | 50 | – | 59 | 645.8262 | 1289.6379 | 1289.6364 | 1.18 | 1 | 54 | 3.3e-05 | 1Score **> 33** indicates **identity** Score **> 21** indicates **homology** | U | R.RLPEDLYNDR.M |
| 43516 | 50 | – | 59 | 430.8866 | 1289.6380 | 1289.6364 | 1.22 | 1 | 39 | 0.00021 | 1Score **> 33** indicates **identity** Score **> 15** indicates **homology** | U | R.RLPEDLYNDR.M |
| 43518 | 50 | – | 59 | 645.8263 | 1289.6380 | 1289.6364 | 1.29 | 1 | 46 | 5.3e-05 | 1Score **> 33** indicates **identity** Score **> 15** indicates **homology** | U | R.RLPEDLYNDR.M |
| 43519 | 50 | – | 59 | 430.8866 | 1289.6381 | 1289.6364 | 1.29 | 1 | 39 | 0.00021 | 1Score **> 33** indicates **identity** Score **> 15** indicates **homology** | U | R.RLPEDLYNDR.M |
| 43520 | 50 | – | 59 | 645.8263 | 1289.6381 | 1289.6364 | 1.34 | 1 | 39 | 0.00022 | 1Score **> 33** indicates **identity** Score **> 15** indicates **homology** | U | R.RLPEDLYNDR.M |
| 43521 | 50 | – | 59 | 430.8867 | 1289.6382 | 1289.6364 | 1.41 | 1 | 27 | 0.0031 | 1Score **> 33** indicates **identity** Score **> 14** indicates **homology** | U | R.RLPEDLYNDR.M |
| 43522 | 50 | – | 59 | 645.8265 | 1289.6385 | 1289.6364 | 1.63 | 1 | 38 | 0.001 | 1Score **> 33** indicates **identity** Score **> 20** indicates **homology** | U | R.RLPEDLYNDR.M |
| 43523 | 50 | – | 59 | 645.8266 | 1289.6386 | 1289.6364 | 1.71 | 1 | 64 | 4.8e-06 | 1Score **> 33** indicates **identity** Score **> 23** indicates **homology** | U | R.RLPEDLYNDR.M |
| 43524 | 50 | – | 59 | 430.8868 | 1289.6386 | 1289.6364 | 1.74 | 1 | 32 | 0.0011 | 1Score **> 33** indicates **identity** Score **> 14** indicates **homology** | U | R.RLPEDLYNDR.M |
| 43525 | 50 | – | 59 | 645.8266 | 1289.6386 | 1289.6364 | 1.76 | 1 | 67 | 2.5e-06 | 1Score **> 33** indicates **identity** Score **> 23** indicates **homology** | U | R.RLPEDLYNDR.M |
| 43526 | 50 | – | 59 | 645.8267 | 1289.6389 | 1289.6364 | 1.96 | 1 | 27 | 0.003 | 1Score **> 33** indicates **identity** Score **> 14** indicates **homology** | U | R.RLPEDLYNDR.M |
| 26380 | 51 | – | 59 | 567.7744 | 1133.5343 | 1133.5353 | -0.89 | 0 | 44 | 7.6e-05 | 1Score **> 31** indicates **identity** Score **> 15** indicates **homology** | U | R.LPEDLYNDR.M |
| 26385 | 51 | – | 59 | 567.7758 | 1133.5370 | 1133.5353 | 1.55 | 0 | 47 | 3.8e-05 | 1Score **> 31** indicates **identity** Score **> 15** indicates **homology** | U | R.LPEDLYNDR.M |
| 4585 | 66 | – | 72 | 410.2236 | 818.4327 | 818.4320 | 0.88 | 0 | 37 | 0.0086 | 1Score **> 29** indicates **identity** | U | R.ALDLTMR.H |
| 4586 | 66 | – | 72 | 410.2240 | 818.4335 | 818.4320 | 1.87 | 0 | 32 | 0.024 | 1Score **> 28** indicates **identity** | U | R.ALDLTMR.H |
| 156249 | 79 | – | 96 | 1198.0770 | 2394.1394 | 2394.1419 | -1.06 | 2 | 60 | 2.1e-06 | 1Score **> 35** indicates **identity** Score **> 16** indicates **homology** | U | K.DQWTKYEEDKFYLEPYLK.E |
| 156250 | 79 | – | 96 | 799.0539 | 2394.1399 | 2394.1419 | -0.84 | 2 | 24 | 0.0062 | 1Score **> 35** indicates **identity** Score **> 14** indicates **homology** | U | K.DQWTKYEEDKFYLEPYLK.E |
| 156252 | 79 | – | 96 | 799.0539 | 2394.1400 | 2394.1419 | -0.82 | 2 | 22 | 0.0095 | 1Score **> 35** indicates **identity** Score **> 14** indicates **homology** | U | K.DQWTKYEEDKFYLEPYLK.E |
| 156255 | 79 | – | 96 | 799.0550 | 2394.1433 | 2394.1419 | 0.57 | 2 | 55 | 6.5e-06 | 1Score **> 35** indicates **identity** Score **> 16** indicates **homology** | U | K.DQWTKYEEDKFYLEPYLK.E |
| 156256 | 79 | – | 96 | 799.0551 | 2394.1434 | 2394.1419 | 0.62 | 2 | 33 | 0.00084 | 1Score **> 35** indicates **identity** Score **> 15** indicates **homology** | U | K.DQWTKYEEDKFYLEPYLK.E |
| 156257 | 79 | – | 96 | 799.0551 | 2394.1436 | 2394.1419 | 0.69 | 2 | 60 | 2.2e-06 | 1Score **> 35** indicates **identity** Score **> 16** indicates **homology** | U | K.DQWTKYEEDKFYLEPYLK.E |
| 156259 | 79 | – | 96 | 599.5434 | 2394.1445 | 2394.1419 | 1.09 | 2 | 27 | 0.0032 | 1Score **> 35** indicates **identity** Score **> 14** indicates **homology** | U | K.DQWTKYEEDKFYLEPYLK.E |
| 156260 | 79 | – | 96 | 1198.0796 | 2394.1447 | 2394.1419 | 1.14 | 2 | 95 | 1.3e-09 | 1Score **> 35** indicates **identity** Score **> 18** indicates **homology** | U | K.DQWTKYEEDKFYLEPYLK.E |
| 156261 | 79 | – | 96 | 799.0555 | 2394.1447 | 2394.1419 | 1.15 | 2 | 49 | 2.6e-05 | 1Score **> 35** indicates **identity** Score **> 16** indicates **homology** | U | K.DQWTKYEEDKFYLEPYLK.E |
| 156263 | 79 | – | 96 | 799.0557 | 2394.1453 | 2394.1419 | 1.40 | 2 | 46 | 5e-05 | 1Score **> 35** indicates **identity** Score **> 15** indicates **homology** | U | K.DQWTKYEEDKFYLEPYLK.E |
| 156269 | 79 | – | 96 | 599.5448 | 2394.1499 | 2394.1419 | 3.33 | 2 | 29 | 0.0017 | 1Score **> 36** indicates **identity** Score **> 14** indicates **homology** | U | K.DQWTKYEEDKFYLEPYLK.E |
| 156271 | 79 | – | 96 | 799.0577 | 2394.1512 | 2394.1419 | 3.86 | 2 | 60 | 2.6e-06 | 1Score **> 36** indicates **identity** Score **> 16** indicates **homology** | U | K.DQWTKYEEDKFYLEPYLK.E |
| 156272 | 79 | – | 96 | 1198.0829 | 2394.1512 | 2394.1419 | 3.86 | 2 | 75 | 9.4e-08 | 1Score **> 36** indicates **identity** Score **> 17** indicates **homology** | U | K.DQWTKYEEDKFYLEPYLK.E |
| 98883 | 84 | – | 96 | 579.6192 | 1735.8357 | 1735.8345 | 0.74 | 1 | 30 | 0.0016 | 1Score **> 35** indicates **identity** Score **> 14** indicates **homology** | U | K.YEEDKFYLEPYLK.E |
| 98885 | 84 | – | 96 | 868.9264 | 1735.8383 | 1735.8345 | 2.22 | 1 | 72 | 4.1e-07 | 1Score **> 34** indicates **identity** Score **> 20** indicates **homology** | U | K.YEEDKFYLEPYLK.E |
| 98886 | 84 | – | 96 | 868.9266 | 1735.8386 | 1735.8345 | 2.36 | 1 | 40 | 0.00017 | 1Score **> 34** indicates **identity** Score **> 15** indicates **homology** | U | K.YEEDKFYLEPYLK.E |
| 98887 | 84 | – | 96 | 579.6202 | 1735.8387 | 1735.8345 | 2.44 | 1 | 39 | 0.00023 | 1Score **> 34** indicates **identity** Score **> 15** indicates **homology** | U | K.YEEDKFYLEPYLK.E |
| 98900 | 84 | – | 96 | 579.6245 | 1735.8516 | 1735.8345 | 9.89 | 1 | 16 | 0.028 | 1Score **> 35** indicates **identity** Score **> 14** indicates **homology** | U | K.YEEDKFYLEPYLK.E |
| 145422 | 84 | – | 100 | 559.2899 | 2233.1305 | 2233.1306 | -0.046 | 2 | 60 | 2.4e-06 | 1Score **> 37** indicates **identity** Score **> 16** indicates **homology** | U | K.YEEDKFYLEPYLKEVIR.E |
| 145423 | 84 | – | 100 | 559.2899 | 2233.1306 | 2233.1306 | -0.019 | 2 | 53 | 1e-05 | 1Score **> 37** indicates **identity** Score **> 16** indicates **homology** | U | K.YEEDKFYLEPYLKEVIR.E |
| 145424 | 84 | – | 100 | 745.3843 | 2233.1311 | 2233.1306 | 0.22 | 2 | 30 | 0.0016 | 1Score **> 37** indicates **identity** Score **> 14** indicates **homology** | U | K.YEEDKFYLEPYLKEVIR.E |
| 145426 | 84 | – | 100 | 559.2902 | 2233.1318 | 2233.1306 | 0.51 | 2 | 30 | 0.0016 | 1Score **> 37** indicates **identity** Score **> 14** indicates **homology** | U | K.YEEDKFYLEPYLKEVIR.E |
| 145427 | 84 | – | 100 | 745.3846 | 2233.1319 | 2233.1306 | 0.59 | 2 | 25 | 0.0047 | 1Score **> 37** indicates **identity** Score **> 14** indicates **homology** | U | K.YEEDKFYLEPYLKEVIR.E |
| 145431 | 84 | – | 100 | 559.2916 | 2233.1371 | 2233.1306 | 2.90 | 2 | 39 | 0.00022 | 1Score **> 37** indicates **identity** Score **> 15** indicates **homology** | U | K.YEEDKFYLEPYLKEVIR.E |

---

```
ID   Q9CQB4_MOUSE            Unreviewed;       111 AA.
AC   Q9CQB4;
DT   01-JUN-2001, integrated into UniProtKB/TrEMBL.
DT   01-JUN-2001, sequence version 1.
DT   28-JUN-2023, entry version 161.
DE   RecName: Full=Cytochrome b-c1 complex subunit 7 {ECO:0000256|ARBA:ARBA00016323, ECO:0000256|PIRNR:PIRNR000022};
GN   Name=Uqcrb {ECO:0000313|EMBL:AAH86921.1,
GN   ECO:0000313|Ensembl:ENSMUSP00000021993.5,
GN   ECO:0000313|MGI:MGI:1914780};
OS   Mus musculus (Mouse).
OC   Eukaryota; Metazoa; Chordata; Craniata; Vertebrata; Euteleostomi; Mammalia;
OC   Eutheria; Euarchontoglires; Glires; Rodentia; Myomorpha; Muroidea; Muridae;
OC   Murinae; Mus; Mus.
OX   NCBI_TaxID=10090 {ECO:0000313|EMBL:BAB31397.1};
RN   [1] {ECO:0000313|EMBL:BAB31397.1}
RP   NUCLEOTIDE SEQUENCE.
RC   STRAIN=BALB/C {ECO:0000313|EMBL:BAE40296.1}, and C57BL/6J
RC   {ECO:0000313|EMBL:BAB31397.1};
RC   TISSUE=Cerebellum {ECO:0000313|EMBL:BAB31397.1}, Heart
RC   {ECO:0000313|EMBL:BAE40676.1}, and Stomach
RC   {ECO:0000313|EMBL:BAB25983.1};
RX   PubMed=10349636; DOI=10.1016/S0076-6879(99)03004-9;
RA   Carninci P., Hayashizaki Y.;
RT   "High-efficiency full-length cDNA cloning.";
RL   Methods Enzymol. 303:19-44(1999).
RN   [2] {ECO:0000313|EMBL:BAB31397.1}
RP   NUCLEOTIDE SEQUENCE.
RC   STRAIN=BALB/C {ECO:0000313|EMBL:BAE40296.1}, and C57BL/6J
RC   {ECO:0000313|EMBL:BAB31397.1};
RC   TISSUE=Cerebellum {ECO:0000313|EMBL:BAB31397.1}, Heart
RC   {ECO:0000313|EMBL:BAE40676.1}, and Stomach
RC   {ECO:0000313|EMBL:BAB25983.1};
RX   PubMed=11042159; DOI=10.1101/gr.145100;
RA   Carninci P., Shibata Y., Hayatsu N., Sugahara Y., Shibata K., Itoh M.,
RA   Konno H., Okazaki Y., Muramatsu M., Hayashizaki Y.;
RT   "Normalization and subtraction of cap-trapper-selected cDNAs to prepare
RT   full-length cDNA libraries for rapid discovery of new genes.";
RL   Genome Res. 10:1617-1630(2000).
RN   [3] {ECO:0000313|EMBL:BAB31397.1}
RP   NUCLEOTIDE SEQUENCE.
RC   STRAIN=BALB/C {ECO:0000313|EMBL:BAE40296.1}, and C57BL/6J
RC   {ECO:0000313|EMBL:BAB31397.1};
RC   TISSUE=Cerebellum {ECO:0000313|EMBL:BAB31397.1}, Heart
RC   {ECO:0000313|EMBL:BAE40676.1}, and Stomach
RC   {ECO:0000313|EMBL:BAB25983.1};
RX   PubMed=11076861; DOI=10.1101/gr.152600;
RA   Shibata K., Itoh M., Aizawa K., Nagaoka S., Sasaki N., Carninci P.,
RA   Konno H., Akiyama J., Nishi K., Kitsunai T., Tashiro H., Itoh M., Sumi N.,
RA   Ishii Y., Nakamura S., Hazama M., Nishine T., Harada A., Yamamoto R.,
RA   Matsumoto H., Sakaguchi S., Ikegami T., Kashiwagi K., Fujiwake S.,
RA   Inoue K., Togawa Y., Izawa M., Ohara E., Watahiki M., Yoneda Y.,
RA   Ishikawa T., Ozawa K., Tanaka T., Matsuura S., Kawai J., Okazaki Y.,
RA   Muramatsu M., Inoue Y., Kira A., Hayashizaki Y.;
RT   "RIKEN integrated sequence analysis (RISA) system--384-format sequencing
RT   pipeline with 384 multicapillary sequencer.";
RL   Genome Res. 10:1757-1771(2000).
RN   [4] {ECO:0000313|EMBL:BAB31397.1}
RP   NUCLEOTIDE SEQUENCE.
RC   STRAIN=C57BL/6J {ECO:0000313|EMBL:BAB31397.1};
RC   TISSUE=Cerebellum {ECO:0000313|EMBL:BAB31397.1}, and Stomach
RC   {ECO:0000313|EMBL:BAB25983.1};
RA   Adachi J., Aizawa K., Akahira S., Akimura T., Arai A., Aono H., Arakawa T.,
RA   Bono H., Carninci P., Fukuda S., Fukunishi Y., Furuno M., Hanagaki T.,
RA   Hara A., Hayatsu N., Hiramoto K., Hiraoka T., Hori F., Imotani K.,
RA   Ishii Y., Itoh M., Izawa M., Kasukawa T., Kato H., Kawai J., Kojima Y.,
RA   Konno H., Kouda M., Koya S., Kurihara C., Matsuyama T., Miyazaki A.,
RA   Nishi K., Nomura K., Numazaki R., Ohno M., Okazaki Y., Okido T., Owa C.,
RA   Saito H., Saito R., Sakai C., Sakai K., Sano H., Sasaki D., Shibata K.,
RA   Shibata Y., Shinagawa A., Shiraki T., Sogabe Y., Suzuki H., Tagami M.,
RA   Tagawa A., Takahashi F., Tanaka T., Tejima Y., Toya T., Yamamura T.,
RA   Yasunishi A., Yoshida K., Yoshino M., Muramatsu M., Hayashizaki Y.;
RL   Submitted (AUG-2000) to the EMBL/GenBank/DDBJ databases.
RN   [5] {ECO:0000313|EMBL:BAB31397.1}
RP   NUCLEOTIDE SEQUENCE.
RC   STRAIN=BALB/C {ECO:0000313|EMBL:BAE40296.1}, and C57BL/6J
RC   {ECO:0000313|EMBL:BAB31397.1};
RC   TISSUE=Cerebellum {ECO:0000313|EMBL:BAB31397.1}, Heart
RC   {ECO:0000313|EMBL:BAE40676.1}, and Stomach
RC   {ECO:0000313|EMBL:BAB25983.1};
RX   PubMed=11217851; DOI=10.1038/35055500;
RG   The RIKEN Genome Exploration Research Group Phase II Team and the FANTOM Consortium;
RT   "Functional annotation of a full-length mouse cDNA collection.";
RL   Nature 409:685-690(2001).
RN   [6] {ECO:0000313|EMBL:BAB31397.1}
RP   NUCLEOTIDE SEQUENCE.
RC   STRAIN=BALB/C {ECO:0000313|EMBL:BAE40296.1}, and C57BL/6J
RC   {ECO:0000313|EMBL:BAB31397.1};
RC   TISSUE=Cerebellum {ECO:0000313|EMBL:BAB31397.1}, Heart
RC   {ECO:0000313|EMBL:BAE40676.1}, and Stomach
RC   {ECO:0000313|EMBL:BAB25983.1};
RX   PubMed=12466851; DOI=10.1038/nature01266;
RG   The FANTOM Consortium and the RIKEN Genome Exploration Research Group Phase I and II Team;
RT   "Analysis of the mouse transcriptome based on functional annotation of
RT   60,770 full-length cDNAs.";
RL   Nature 420:563-573(2002).
RN   [7] {ECO:0000313|EMBL:AAH86921.1}
RP   NUCLEOTIDE SEQUENCE [LARGE SCALE MRNA].
RC   TISSUE=Kidney {ECO:0000313|EMBL:AAH86921.1};
RX   PubMed=15489334; DOI=10.1101/gr.2596504;
RG   The MGC Project Team;
RA   Gerhard D.S., Wagner L., Feingold E.A., Shenmen C.M., Grouse L.H.,
RA   Schuler G., Klein S.L., Old S., Rasooly R., Good P., Guyer M., Peck A.M.,
RA   Derge J.G., Lipman D., Collins F.S., Jang W., Sherry S., Feolo M.,
RA   Misquitta L., Lee E., Rotmistrovsky K., Greenhut S.F., Schaefer C.F.,
RA   Buetow K., Bonner T.I., Haussler D., Kent J., Kiekhaus M., Furey T.,
RA   Brent M., Prange C., Schreiber K., Shapiro N., Bhat N.K., Hopkins R.F.,
RA   Hsie F., Driscoll T., Soares M.B., Casavant T.L., Scheetz T.E.,
RA   Brown-stein M.J., Usdin T.B., Toshiyuki S., Carninci P., Piao Y.,
RA   Dudekula D.B., Ko M.S., Kawakami K., Suzuki Y., Sugano S., Gruber C.E.,
RA   Smith M.R., Simmons B., Moore T., Waterman R., Johnson S.L., Ruan Y.,
RA   Wei C.L., Mathavan S., Gunaratne P.H., Wu J., Garcia A.M., Hulyk S.W.,
RA   Fuh E., Yuan Y., Sneed A., Kowis C., Hodgson A., Muzny D.M., McPherson J.,
RA   Gibbs R.A., Fahey J., Helton E., Ketteman M., Madan A., Rodrigues S.,
RA   Sanchez A., Whiting M., Madari A., Young A.C., Wetherby K.D., Granite S.J.,
RA   Kwong P.N., Brinkley C.P., Pearson R.L., Bouffard G.G., Blakesly R.W.,
RA   Green E.D., Dickson M.C., Rodriguez A.C., Grimwood J., Schmutz J.,
RA   Myers R.M., Butterfield Y.S., Griffith M., Griffith O.L., Krzywinski M.I.,
RA   Liao N., Morin R., Morrin R., Palmquist D., Petrescu A.S., Skalska U.,
RA   Smailus D.E., Stott J.M., Schnerch A., Schein J.E., Jones S.J., Holt R.A.,
RA   Baross A., Marra M.A., Clifton S., Makowski K.A., Bosak S., Malek J.;
RT   "The status, quality, and expansion of the NIH full-length cDNA project:
RT   the Mammalian Gene Collection (MGC).";
RL   Genome Res. 14:2121-2127(2004).
RN   [8] {ECO:0000313|EMBL:BAE40296.1}
RP   NUCLEOTIDE SEQUENCE.
RC   STRAIN=BALB/C {ECO:0000313|EMBL:BAE40296.1}, and C57BL/6J
RC   {ECO:0000313|EMBL:BAE40676.1};
RC   TISSUE=Heart {ECO:0000313|EMBL:BAE40676.1};
RA   Arakawa T., Carninci P., Fukuda S., Hashizume W., Hayashida K., Hori F.,
RA   Iida J., Imamura K., Imotani K., Itoh M., Kanagawa S., Kawai J., Kojima M.,
RA   Konno H., Murata M., Nakamura M., Ninomiya N., Nishiyori H., Nomura K.,
RA   Ohno M., Sakazume N., Sano H., Sasaki D., Shibata K., Shiraki T.,
RA   Tagami M., Tagami Y., Waki K., Watahiki A., Muramatsu M., Hayashizaki Y.;
RL   Submitted (APR-2004) to the EMBL/GenBank/DDBJ databases.
RN   [9] {ECO:0000313|EMBL:BAB31397.1}
RP   NUCLEOTIDE SEQUENCE.
RC   STRAIN=BALB/C {ECO:0000313|EMBL:BAE40296.1}, and C57BL/6J
RC   {ECO:0000313|EMBL:BAB31397.1};
RC   TISSUE=Cerebellum {ECO:0000313|EMBL:BAB31397.1}, Heart
RC   {ECO:0000313|EMBL:BAE40676.1}, and Stomach
RC   {ECO:0000313|EMBL:BAB25983.1};
RG   The FANTOM Consortium;
RG   Riken Genome Exploration Research Group and Genome Science Group (Genome Network Project Core Group);
RT   "The Transcriptional Landscape of the Mammalian Genome.";
RL   Science 309:1559-1563(2005).
RN   [10] {ECO:0000313|EMBL:BAB31397.1}
RP   NUCLEOTIDE SEQUENCE.
RC   STRAIN=BALB/C {ECO:0000313|EMBL:BAE40296.1}, and C57BL/6J
RC   {ECO:0000313|EMBL:BAB31397.1};
RC   TISSUE=Cerebellum {ECO:0000313|EMBL:BAB31397.1}, Heart
RC   {ECO:0000313|EMBL:BAE40676.1}, and Stomach
RC   {ECO:0000313|EMBL:BAB25983.1};
RX   PubMed=16141073; DOI=10.1126/science.1112009;
RG   RIKEN Genome Exploration Research Group and Genome Science Group (Genome Network Project Core Group) and the FANTOM Consortium;
RT   "Antisense Transcription in the Mammalian Transcriptome.";
RL   Science 309:1564-1566(2005).
RN   [11] {ECO:0000313|Ensembl:ENSMUSP00000021993.5, ECO:0000313|Proteomes:UP000000589}
RP   NUCLEOTIDE SEQUENCE [LARGE SCALE GENOMIC DNA].
RC   STRAIN=C57BL/6J {ECO:0000313|Ensembl:ENSMUSP00000021993.5,
RC   ECO:0000313|Proteomes:UP000000589};
RX   PubMed=19468303; DOI=10.1371/journal.pbio.1000112;
RA   Church D.M., Goodstadt L., Hillier L.W., Zody M.C., Goldstein S., She X.,
RA   Bult C.J., Agarwala R., Cherry J.L., DiCuccio M., Hlavina W., Kapustin Y.,
RA   Meric P., Maglott D., Birtle Z., Marques A.C., Graves T., Zhou S.,
RA   Teague B., Potamousis K., Churas C., Place M., Herschleb J., Runnheim R.,
RA   Forrest D., Amos-Landgraf J., Schwartz D.C., Cheng Z., Lindblad-Toh K.,
RA   Eichler E.E., Ponting C.P.;
RT   "Lineage-specific biology revealed by a finished genome assembly of the
RT   mouse.";
RL   PLoS Biol. 7:E1000112-E1000112(2009).
RN   [12] {ECO:0007829|PubMed:21183079}
RP   IDENTIFICATION BY MASS SPECTROMETRY [LARGE SCALE ANALYSIS].
RX   PubMed=21183079; DOI=10.1016/j.cell.2010.12.001;
RA   Huttlin E.L., Jedrychowski M.P., Elias J.E., Goswami T., Rad R.,
RA   Beausoleil S.A., Villen J., Haas W., Sowa M.E., Gygi S.P.;
RT   "A tissue-specific atlas of mouse protein phosphorylation and expression.";
RL   Cell 143:1174-1189(2010).
RN   [13] {ECO:0007829|PubMed:23806337}
RP   IDENTIFICATION BY MASS SPECTROMETRY [LARGE SCALE ANALYSIS].
RX   PubMed=23806337; DOI=10.1016/j.molcel.2013.06.001;
RA   Park J., Chen Y., Tishkoff D.X., Peng C., Tan M., Dai L., Xie Z., Zhang Y.,
RA   Zwaans B.M., Skinner M.E., Lombard D.B., Zhao Y.;
RT   "SIRT5-mediated lysine desuccinylation impacts diverse metabolic
RT   pathways.";
RL   Mol. Cell 50:919-930(2013).
RN   [14] {ECO:0007829|PubMed:23576753}
RP   IDENTIFICATION BY MASS SPECTROMETRY [LARGE SCALE ANALYSIS].
RX   PubMed=23576753; DOI=10.1073/pnas.1302961110;
RA   Rardin M.J., Newman J.C., Held J.M., Cusack M.P., Sorensen D.J., Li B.,
RA   Schilling B., Mooney S.D., Kahn C.R., Verdin E., Gibson B.W.;
RT   "Label-free quantitative proteomics of the lysine acetylome in mitochondria
RT   identifies substrates of SIRT3 in metabolic pathways.";
RL   Proc. Natl. Acad. Sci. U.S.A. 110:6601-6606(2013).
RN   [15] {ECO:0007829|PDB:7O37}
RP   STRUCTURE BY ELECTRON MICROSCOPY (2.60 ANGSTROMS) OF 2-111.
RX   PubMed=34616041; DOI=10.1038/s41586-021-03927-z;
RA   Vercellino I., Sazanov L.A.;
RT   "Structure and assembly of the mammalian mitochondrial supercomplex
RT   CIII<sub>2</sub>CIV.";
RL   Nature 598:364-367(2021).
RN   [16] {ECO:0000313|Ensembl:ENSMUSP00000021993.5}
RP   IDENTIFICATION.
RC   STRAIN=C57BL/6J {ECO:0000313|Ensembl:ENSMUSP00000021993.5};
RG   Ensembl;
RL   Submitted (MAR-2023) to UniProtKB.
CC   -!- FUNCTION: Component of the ubiquinol-cytochrome c oxidoreductase, a
CC       multisubunit transmembrane complex that is part of the mitochondrial
CC       electron transport chain which drives oxidative phosphorylation.
CC       {ECO:0000256|PIRNR:PIRNR000022}.
CC   -!- SUBCELLULAR LOCATION: Mitochondrion inner membrane
CC       {ECO:0000256|PIRNR:PIRNR000022}.
CC   -!- SIMILARITY: Belongs to the UQCRB/QCR7 family.
CC       {ECO:0000256|ARBA:ARBA00008554, ECO:0000256|PIRNR:PIRNR000022}.
CC   ---------------------------------------------------------------------------
CC   Copyrighted by the UniProt Consortium, see https://www.uniprot.org/terms
CC   Distributed under the Creative Commons Attribution (CC BY 4.0) License
CC   ---------------------------------------------------------------------------
DR   EMBL; BC086921; AAH86921.1; -; mRNA.
DR   EMBL; BC100601; AAI00602.1; -; mRNA.
DR   EMBL; AK008945; BAB25983.1; -; mRNA.
DR   EMBL; AK018769; BAB31397.1; -; mRNA.
DR   EMBL; AK168361; BAE40296.1; -; mRNA.
DR   EMBL; AK168855; BAE40676.1; -; mRNA.
DR   RefSeq; NP_080495.1; NM_026219.1.
DR   PDB; 7O37; EM; 3.20 A; F/Q=2-111.
DR   ProteomicsDB; 338744; -.
DR   TopDownProteomics; Q9CQB4; -.
DR   Antibodypedia; 42750; 182 antibodies from 27 providers.
DR   DNASU; 67530; -.
DR   Ensembl; ENSMUST00000021993.5; ENSMUSP00000021993.5; ENSMUSG00000021520.5.
DR   GeneID; 67530; -.
DR   KEGG; mmu:67530; -.
DR   UCSC; uc007qzu.1; mouse.
DR   AGR; MGI:1914780; -.
DR   CTD; 7381; -.
DR   MGI; MGI:1914780; Uqcrb.
DR   VEuPathDB; HostDB:ENSMUSG00000021520; -.
DR   GeneTree; ENSGT00390000012916; -.
DR   HOGENOM; CLU_115154_2_0_1; -.
DR   OMA; PLAQWYT; -.
DR   OrthoDB; 5477553at2759; -.
DR   TreeFam; TF105035; -.
DR   BioGRID-ORCS; 67530; 24 hits in 79 CRISPR screens.
DR   ChiTaRS; Uqcrb; mouse.
DR   Proteomes; UP000000589; Chromosome 13.
DR   Bgee; ENSMUSG00000021520; Expressed in right kidney and 81 other tissues.
DR   GO; GO:0005750; C:mitochondrial respiratory chain complex III; IEA:InterPro.
DR   GO; GO:0006122; P:mitochondrial electron transport, ubiquinol to cytochrome c; IEA:InterPro.
DR   Gene3D; 1.10.1090.10; Cytochrome b-c1 complex subunit 7; 1.
DR   InterPro; IPR003197; QCR7.
DR   InterPro; IPR036544; QCR7_sf.
DR   PANTHER; PTHR12022:SF12; CYTOCHROME B-C1 COMPLEX SUBUNIT 7; 1.
DR   PANTHER; PTHR12022; UBIQUINOL-CYTOCHROME C REDUCTASE COMPLEX 14 KD PROTEIN; 1.
DR   Pfam; PF02271; UCR_14kD; 1.
DR   PIRSF; PIRSF000022; Bc1_14K; 1.
DR   SUPFAM; SSF81524; 14 kDa protein of cytochrome bc1 complex (Ubiquinol-cytochrome c reductase); 1.
PE   1: Evidence at protein level;
KW   3D-structure {ECO:0007829|PDB:7O37};
KW   Electron transport {ECO:0000256|PIRNR:PIRNR000022};
KW   Membrane {ECO:0000256|ARBA:ARBA00023136, ECO:0000256|PIRNR:PIRNR000022};
KW   Mitochondrion {ECO:0000256|ARBA:ARBA00023128,
KW   ECO:0000256|PIRNR:PIRNR000022};
KW   Mitochondrion inner membrane {ECO:0000256|ARBA:ARBA00022792,
KW   ECO:0000256|PIRNR:PIRNR000022};
KW   Proteomics identification {ECO:0007829|EPD:Q9CQB4,
KW   ECO:0007829|MaxQB:Q9CQB4};
KW   Reference proteome {ECO:0000313|Proteomes:UP000000589};
KW   Respiratory chain {ECO:0000256|PIRNR:PIRNR000022};
KW   Transport {ECO:0000256|ARBA:ARBA00022448, ECO:0000256|PIRNR:PIRNR000022}.
SQ   SEQUENCE   111 AA;  13561 MW;  A4C9B3415470894A CRC64;
     MAGRSAVSAS SKWLDGFRKW YYNAAGFNKL GLMRDDTLHE TEDVKEAIRR LPEDLYNDRM
     FRIKRALDLT MRHQILPKDQ WTKYEEDKFY LEPYLKEVIR ERKEREEWAK K
//
```

**Mascot:** http://www.matrixscience.com/
