# Supplementary material for: A common East-Asian ALDH2 mutation causes metabolic disorders and the therapeutic effect of ALDH2 activators
Source: Nat Commun. 2023 Sep 25;14:5971. doi: 10.1038/s41467-023-41570-6 (PMC10520061; doi:10.1038/s41467-023-41570-6)
Supplement: Supplementary file 4 — Supplementary Data 1 [file 41467_2023_41570_MOESM4_ESM.zip › Table S5c/Q9CR68/Q9CR68_KIO60-2_K130.html]

Mascot Search Results: Q9CR68
 

# MASCOT Search Results

## Protein View: Q9CR68

### Cytochrome b-c1 complex subunit Rieske, mitochondrial OS=Mus musculus OX=10090 GN=Uqcrfs1 PE=1 SV=1

|  |  |
| --- | --- |
| Database: | Mouse\_UniProt\_proteomes |
| Score: | 3304 |
| Monoisotopic mass (M_r_): | 29634 |
| Calculated pI: | 8.91 |

Sequence similarity is available as an NCBI BLAST search of Q9CR68 against nr.

### Search parameters

|  |  |
| --- | --- |
| MS data file: | `D:\LCMSMS\2023 Users' data\230529-1\230529-1-KI60-2.raw` |
| Enzyme: | Trypsin/P: cuts C-term side of KR. |
| Fixed modifications: | Carbamidomethyl (C) |
| Variable modifications: | Deamidated (NQ), HNE (C), HNE (H), HNE (K), Oxidation (M) |

### Protein sequence coverage: 83%

Matched peptides shown in ***bold red***.

|  |  |  |  |  |  |
| --- | --- | --- | --- | --- | --- |
| `1` | `MLSVAARSGP` | `FAPVLSATSR` | `GVAGALRPLL` | `QGAVPAASEP` | `PVLDVKRPFL` |
| `51` | `CRESLSGQAA` | `ARPLVATVGL` | `NVPASVRFSH` | `TDVKVPDFSD` | `YRRAEVLDST` |
| `101` | `KSSKESSEAR` | `KGFSYLVTAT` | `TTVGVAYAAK` | `NVVSQFVSSM` | `SASADVLAMS` |
| `151` | `KIEIKLSDIP` | `EGKNMAFKWR` | `GKPLFVRHRT` | `KKEIDQEAAV` | `EVSQLRDPQH` |
| `201` | `DLDRVKKPEW` | `VILIGVCTHL` | `GCVPIANAGD` | `FGGYYCPCHG` | `SHYDASGRIR` |
| `251` | `KGPAPLNLEV` | `PAYEFTSDDV` | `VVVG` |  |  |

Unformatted sequence string: 274 residues (for pasting into other applications).

|  |  |  |  |
| --- | --- | --- | --- |
| Sort by | residue number | increasing mass | decreasing mass |
| Show | matched peptides only | predicted peptides also |  |

| Query | Start | – | End | Observed | Mr(expt) | Mr(calc) | ppm | M | Score | Expect | Rank | U | Peptide |
| --- | --- | --- | --- | --- | --- | --- | --- | --- | --- | --- | --- | --- | --- |
| 42819 | 8 | – | 20 | 645.3442 | 1288.6738 | 1288.6776 | -2.95 | 0 | 25 | 0.0041 | 1Score **> 34** indicates **identity** Score **> 14** indicates **homology** | U | R.SGPFAPVLSATSR.G |
| 42821 | 8 | – | 20 | 645.3454 | 1288.6763 | 1288.6776 | -0.94 | 0 | 28 | 0.0024 | 1Score **> 34** indicates **identity** Score **> 14** indicates **homology** | U | R.SGPFAPVLSATSR.G |
| 42823 | 8 | – | 20 | 645.3458 | 1288.6770 | 1288.6776 | -0.45 | 0 | 40 | 0.00019 | 1Score **> 34** indicates **identity** Score **> 15** indicates **homology** | U | R.SGPFAPVLSATSR.G |
| 42826 | 8 | – | 20 | 645.3460 | 1288.6775 | 1288.6776 | -0.075 | 0 | 46 | 4.7e-05 | 1Score **> 34** indicates **identity** Score **> 15** indicates **homology** | U | R.SGPFAPVLSATSR.G |
| 42829 | 8 | – | 20 | 645.3462 | 1288.6778 | 1288.6776 | 0.18 | 0 | 56 | 5.1e-06 | 1Score **> 34** indicates **identity** Score **> 16** indicates **homology** | U | R.SGPFAPVLSATSR.G |
| 42830 | 8 | – | 20 | 645.3462 | 1288.6778 | 1288.6776 | 0.20 | 0 | 52 | 1.4e-05 | 1Score **> 34** indicates **identity** Score **> 16** indicates **homology** | U | R.SGPFAPVLSATSR.G |
| 42832 | 8 | – | 20 | 645.3463 | 1288.6780 | 1288.6776 | 0.37 | 0 | 63 | 1.2e-06 | 1Score **> 34** indicates **identity** Score **> 16** indicates **homology** | U | R.SGPFAPVLSATSR.G |
| 42833 | 8 | – | 20 | 645.3463 | 1288.6780 | 1288.6776 | 0.38 | 0 | 17 | 0.024 | 1Score **> 34** indicates **identity** Score **> 14** indicates **homology** | U | R.SGPFAPVLSATSR.G |
| 42834 | 8 | – | 20 | 645.3463 | 1288.6781 | 1288.6776 | 0.41 | 0 | 45 | 6e-05 | 1Score **> 34** indicates **identity** Score **> 15** indicates **homology** | U | R.SGPFAPVLSATSR.G |
| 42837 | 8 | – | 20 | 645.3464 | 1288.6782 | 1288.6776 | 0.50 | 0 | 45 | 6.1e-05 | 1Score **> 34** indicates **identity** Score **> 15** indicates **homology** | U | R.SGPFAPVLSATSR.G |
| 42838 | 8 | – | 20 | 645.3464 | 1288.6783 | 1288.6776 | 0.58 | 0 | 57 | 4.5e-06 | 1Score **> 34** indicates **identity** Score **> 16** indicates **homology** | U | R.SGPFAPVLSATSR.G |
| 42842 | 8 | – | 20 | 645.3469 | 1288.6792 | 1288.6776 | 1.26 | 0 | 44 | 7.4e-05 | 1Score **> 34** indicates **identity** Score **> 15** indicates **homology** | U | R.SGPFAPVLSATSR.G |
| 42846 | 8 | – | 20 | 645.3475 | 1288.6805 | 1288.6776 | 2.29 | 0 | 41 | 0.00014 | 1Score **> 34** indicates **identity** Score **> 15** indicates **homology** | U | R.SGPFAPVLSATSR.G |
| 166205 | 21 | – | 46 | 842.4832 | 2524.4277 | 2524.4377 | -3.95 | 1 | 22 | 0.0091 | 1Score **> 33** indicates **identity** Score **> 14** indicates **homology** | U | R.GVAGALRPLLQGAVPAASEPPVLDVK.R |
| 166206 | 21 | – | 46 | 842.4857 | 2524.4352 | 2524.4377 | -0.98 | 1 | 17 | 0.027 | 1Score **> 32** indicates **identity** Score **> 14** indicates **homology** | U | R.GVAGALRPLLQGAVPAASEPPVLDVK.R |
| 166208 | 21 | – | 46 | 842.4874 | 2524.4404 | 2524.4377 | 1.07 | 1 | 69 | 3.1e-07 | 1Score **> 32** indicates **identity** Score **> 17** indicates **homology** | U | R.GVAGALRPLLQGAVPAASEPPVLDVK.R |
| 166209 | 21 | – | 46 | 842.4875 | 2524.4407 | 2524.4377 | 1.18 | 1 | 74 | 1.2e-07 | 1Score **> 32** indicates **identity** Score **> 17** indicates **homology** | U | R.GVAGALRPLLQGAVPAASEPPVLDVK.R |
| 166210 | 21 | – | 46 | 632.1175 | 2524.4409 | 2524.4377 | 1.28 | 1 | 25 | 0.0045 | 1Score **> 32** indicates **identity** Score **> 14** indicates **homology** | U | R.GVAGALRPLLQGAVPAASEPPVLDVK.R |
| 166211 | 21 | – | 46 | 842.4877 | 2524.4413 | 2524.4377 | 1.44 | 1 | 71 | 2.1e-07 | 1Score **> 32** indicates **identity** Score **> 17** indicates **homology** | U | R.GVAGALRPLLQGAVPAASEPPVLDVK.R |
| 166212 | 21 | – | 46 | 1263.2283 | 2524.4420 | 2524.4377 | 1.70 | 1 | 54 | 9.1e-06 | 1Score **> 32** indicates **identity** Score **> 16** indicates **homology** | U | R.GVAGALRPLLQGAVPAASEPPVLDVK.R |
| 166213 | 21 | – | 46 | 632.1178 | 2524.4422 | 2524.4377 | 1.79 | 1 | 33 | 0.00082 | 1Score **> 32** indicates **identity** Score **> 15** indicates **homology** | U | R.GVAGALRPLLQGAVPAASEPPVLDVK.R |
| 166238 | 21 | – | 46 | 842.8216 | 2525.4429 | 2525.4217 | 8.38 | 1 | 17 | 0.026 | 1Score **> 31** indicates **identity** Score **> 14** indicates **homology** | U | R.GVAGALRPLLQGAVPAASEPPVLDVK.R  + Deamidated (NQ) |
| 166239 | 21 | – | 46 | 1263.7303 | 2525.4460 | 2525.4217 | 9.63 | 1 | 42 | 0.00014 | 1Score **> 31** indicates **identity** Score **> 16** indicates **homology** | U | R.GVAGALRPLLQGAVPAASEPPVLDVK.R  + Deamidated (NQ) |
| 13717 | 53 | – | 62 | 495.2548 | 988.4950 | 988.4937 | 1.29 | 0 | 62 | 3.2e-06 | 1Score **> 31** indicates **identity** Score **> 20** indicates **homology** | U | R.ESLSGQAAAR.P |
| 162771 | 53 | – | 77 | 821.7941 | 2462.3604 | 2462.3605 | -0.061 | 1 | 40 | 0.00018 | 1Score **> 34** indicates **identity** Score **> 15** indicates **homology** | U | R.ESLSGQAAARPLVATVGLNVPASVR.F |
| 162772 | 53 | – | 77 | 821.7945 | 2462.3618 | 2462.3605 | 0.51 | 1 | 66 | 6.3e-07 | 1Score **> 34** indicates **identity** Score **> 17** indicates **homology** | U | R.ESLSGQAAARPLVATVGLNVPASVR.F |
| 162774 | 53 | – | 77 | 821.7949 | 2462.3628 | 2462.3605 | 0.94 | 1 | 64 | 1e-06 | 1Score **> 34** indicates **identity** Score **> 16** indicates **homology** | U | R.ESLSGQAAARPLVATVGLNVPASVR.F |
| 162777 | 53 | – | 77 | 821.7952 | 2462.3638 | 2462.3605 | 1.32 | 1 | 53 | 1e-05 | 1Score **> 34** indicates **identity** Score **> 16** indicates **homology** | U | R.ESLSGQAAARPLVATVGLNVPASVR.F |
| 162778 | 53 | – | 77 | 1232.1893 | 2462.3641 | 2462.3605 | 1.46 | 1 | 19 | 0.017 | 1Score **> 34** indicates **identity** Score **> 14** indicates **homology** | U | R.ESLSGQAAARPLVATVGLNVPASVR.F |
| 162779 | 53 | – | 77 | 821.7953 | 2462.3641 | 2462.3605 | 1.48 | 1 | 26 | 0.0039 | 1Score **> 34** indicates **identity** Score **> 14** indicates **homology** | U | R.ESLSGQAAARPLVATVGLNVPASVR.F |
| 162783 | 53 | – | 77 | 821.7976 | 2462.3709 | 2462.3605 | 4.24 | 1 | 27 | 0.003 | 1Score **> 34** indicates **identity** Score **> 14** indicates **homology** | U | R.ESLSGQAAARPLVATVGLNVPASVR.F |
| 66019 | 63 | – | 77 | 746.9465 | 1491.8785 | 1491.8773 | 0.77 | 0 | 46 | 0.00015 | 1Score **> 31** indicates **identity** Score **> 20** indicates **homology** | U | R.PLVATVGLNVPASVR.F |
| 66020 | 63 | – | 77 | 746.9493 | 1491.8840 | 1491.8773 | 4.45 | 0 | 62 | 7.3e-06 | 1Score **> 31** indicates **identity** Score **> 23** indicates **homology** | U | R.PLVATVGLNVPASVR.F |
| 14269 | 85 | – | 92 | 499.7327 | 997.4508 | 997.4505 | 0.24 | 0 | 22 | 0.0095 | 1Score **> 27** indicates **identity** Score **> 14** indicates **homology** | U | K.VPDFSDYR.R |
| 14270 | 85 | – | 92 | 499.7328 | 997.4510 | 997.4505 | 0.50 | 0 | 42 | 0.00014 | 1Score **> 27** indicates **identity** Score **> 16** indicates **homology** | U | K.VPDFSDYR.R |
| 14271 | 85 | – | 92 | 499.7328 | 997.4511 | 997.4505 | 0.56 | 0 | 43 | 0.0001 | 1Score **> 27** indicates **identity** Score **> 15** indicates **homology** | U | K.VPDFSDYR.R |
| 14272 | 85 | – | 92 | 499.7328 | 997.4511 | 997.4505 | 0.61 | 0 | 42 | 0.00012 | 1Score **> 27** indicates **identity** Score **> 15** indicates **homology** | U | K.VPDFSDYR.R |
| 14273 | 85 | – | 92 | 499.7331 | 997.4516 | 997.4505 | 1.10 | 0 | 32 | 0.00097 | 1Score **> 27** indicates **identity** Score **> 15** indicates **homology** | U | K.VPDFSDYR.R |
| 27909 | 85 | – | 93 | 385.5244 | 1153.5513 | 1153.5516 | -0.28 | 1 | 18 | 0.019 | 1Score **> 31** indicates **identity** Score **> 14** indicates **homology** | U | K.VPDFSDYRR.A |
| 27911 | 85 | – | 93 | 577.7830 | 1153.5514 | 1153.5516 | -0.21 | 1 | 29 | 0.0018 | 1Score **> 31** indicates **identity** Score **> 14** indicates **homology** | U | K.VPDFSDYRR.A |
| 27912 | 85 | – | 93 | 577.7830 | 1153.5514 | 1153.5516 | -0.20 | 1 | 20 | 0.014 | 1Score **> 31** indicates **identity** Score **> 14** indicates **homology** | U | K.VPDFSDYRR.A |
| 27913 | 85 | – | 93 | 385.5245 | 1153.5517 | 1153.5516 | 0.029 | 1 | 20 | 0.014 | 1Score **> 31** indicates **identity** Score **> 14** indicates **homology** | U | K.VPDFSDYRR.A |
| 27914 | 85 | – | 93 | 385.5246 | 1153.5521 | 1153.5516 | 0.40 | 1 | 20 | 0.014 | 1Score **> 30** indicates **identity** Score **> 14** indicates **homology** | U | K.VPDFSDYRR.A |
| 27916 | 85 | – | 93 | 385.5247 | 1153.5524 | 1153.5516 | 0.67 | 1 | 14 | 0.049 | 1Score **> 30** indicates **identity** Score **> 13** indicates **homology** | U | K.VPDFSDYRR.A |
| 15620 | 93 | – | 101 | 509.7804 | 1017.5463 | 1017.5455 | 0.81 | 1 | 35 | 0.0087 | 1Score **> 33** indicates **identity** Score **> 27** indicates **homology** | U | R.RAEVLDSTK.S |
| 130922 | 111 | – | 130 | 1024.5475 | 2047.0805 | 2047.0990 | -9.03 | 1 | 36 | 0.00045 | 1Score **> 36** indicates **identity** Score **> 15** indicates **homology** | U | R.KGFSYLVTATTTVGVAYAAK.N |
| 130935 | 111 | – | 130 | 683.3703 | 2047.0892 | 2047.0990 | -4.78 | 1 | 29 | 0.0021 | 1Score **> 36** indicates **identity** Score **> 14** indicates **homology** | U | R.KGFSYLVTATTTVGVAYAAK.N |
| 130942 | 111 | – | 130 | 683.3714 | 2047.0923 | 2047.0990 | -3.27 | 1 | 26 | 0.0035 | 1Score **> 36** indicates **identity** Score **> 14** indicates **homology** | U | R.KGFSYLVTATTTVGVAYAAK.N |
| 130943 | 111 | – | 130 | 683.3716 | 2047.0929 | 2047.0990 | -2.97 | 1 | 50 | 2e-05 | 1Score **> 36** indicates **identity** Score **> 16** indicates **homology** | U | R.KGFSYLVTATTTVGVAYAAK.N |
| 130944 | 111 | – | 130 | 683.3719 | 2047.0940 | 2047.0990 | -2.45 | 1 | 39 | 0.00023 | 1Score **> 36** indicates **identity** Score **> 15** indicates **homology** | U | R.KGFSYLVTATTTVGVAYAAK.N |
| 130945 | 111 | – | 130 | 683.3720 | 2047.0941 | 2047.0990 | -2.38 | 1 | 35 | 0.00049 | 1Score **> 36** indicates **identity** Score **> 15** indicates **homology** | U | R.KGFSYLVTATTTVGVAYAAK.N |
| 130946 | 111 | – | 130 | 683.3723 | 2047.0951 | 2047.0990 | -1.91 | 1 | 40 | 0.00016 | 1Score **> 36** indicates **identity** Score **> 15** indicates **homology** | U | R.KGFSYLVTATTTVGVAYAAK.N |
| 130947 | 111 | – | 130 | 683.3724 | 2047.0955 | 2047.0990 | -1.72 | 1 | 35 | 0.00053 | 1Score **> 36** indicates **identity** Score **> 15** indicates **homology** | U | R.KGFSYLVTATTTVGVAYAAK.N |
| 130948 | 111 | – | 130 | 683.3725 | 2047.0956 | 2047.0990 | -1.67 | 1 | 31 | 0.0012 | 1Score **> 36** indicates **identity** Score **> 14** indicates **homology** | U | R.KGFSYLVTATTTVGVAYAAK.N |
| 130949 | 111 | – | 130 | 683.3726 | 2047.0960 | 2047.0990 | -1.47 | 1 | 16 | 0.033 | 1Score **> 36** indicates **identity** Score **> 13** indicates **homology** | U | R.KGFSYLVTATTTVGVAYAAK.N |
| 130951 | 111 | – | 130 | 683.3728 | 2047.0966 | 2047.0990 | -1.16 | 1 | 23 | 0.0072 | 1Score **> 36** indicates **identity** Score **> 14** indicates **homology** | U | R.KGFSYLVTATTTVGVAYAAK.N |
| 130952 | 111 | – | 130 | 683.3729 | 2047.0968 | 2047.0990 | -1.09 | 1 | 43 | 9.5e-05 | 1Score **> 36** indicates **identity** Score **> 15** indicates **homology** | U | R.KGFSYLVTATTTVGVAYAAK.N |
| 130953 | 111 | – | 130 | 683.3729 | 2047.0969 | 2047.0990 | -1.03 | 1 | 26 | 0.004 | 1Score **> 36** indicates **identity** Score **> 14** indicates **homology** | U | R.KGFSYLVTATTTVGVAYAAK.N |
| 130954 | 111 | – | 130 | 683.3729 | 2047.0969 | 2047.0990 | -1.01 | 1 | 59 | 2.7e-06 | 1Score **> 36** indicates **identity** Score **> 16** indicates **homology** | U | R.KGFSYLVTATTTVGVAYAAK.N |
| 130957 | 111 | – | 130 | 683.3731 | 2047.0974 | 2047.0990 | -0.76 | 1 | 37 | 0.00037 | 1Score **> 36** indicates **identity** Score **> 15** indicates **homology** | U | R.KGFSYLVTATTTVGVAYAAK.N |
| 130958 | 111 | – | 130 | 683.3731 | 2047.0975 | 2047.0990 | -0.74 | 1 | 40 | 0.00017 | 1Score **> 36** indicates **identity** Score **> 15** indicates **homology** | U | R.KGFSYLVTATTTVGVAYAAK.N |
| 130961 | 111 | – | 130 | 683.3734 | 2047.0983 | 2047.0990 | -0.34 | 1 | 47 | 4.3e-05 | 1Score **> 36** indicates **identity** Score **> 15** indicates **homology** | U | R.KGFSYLVTATTTVGVAYAAK.N |
| 130962 | 111 | – | 130 | 683.3734 | 2047.0983 | 2047.0990 | -0.33 | 1 | 45 | 5.7e-05 | 1Score **> 36** indicates **identity** Score **> 15** indicates **homology** | U | R.KGFSYLVTATTTVGVAYAAK.N |
| 130964 | 111 | – | 130 | 683.3734 | 2047.0985 | 2047.0990 | -0.25 | 1 | 23 | 0.0076 | 1Score **> 36** indicates **identity** Score **> 14** indicates **homology** | U | R.KGFSYLVTATTTVGVAYAAK.N |
| 130965 | 111 | – | 130 | 683.3736 | 2047.0988 | 2047.0990 | -0.075 | 1 | 41 | 0.00014 | 1Score **> 36** indicates **identity** Score **> 15** indicates **homology** | U | R.KGFSYLVTATTTVGVAYAAK.N |
| 130968 | 111 | – | 130 | 683.3737 | 2047.0993 | 2047.0990 | 0.15 | 1 | 37 | 0.00035 | 1Score **> 36** indicates **identity** Score **> 15** indicates **homology** | U | R.KGFSYLVTATTTVGVAYAAK.N |
| 130969 | 111 | – | 130 | 683.3737 | 2047.0993 | 2047.0990 | 0.17 | 1 | 31 | 0.0013 | 1Score **> 36** indicates **identity** Score **> 14** indicates **homology** | U | R.KGFSYLVTATTTVGVAYAAK.N |
| 130970 | 111 | – | 130 | 683.3738 | 2047.0995 | 2047.0990 | 0.25 | 1 | 56 | 5.9e-06 | 1Score **> 36** indicates **identity** Score **> 16** indicates **homology** | U | R.KGFSYLVTATTTVGVAYAAK.N |
| 130972 | 111 | – | 130 | 683.3739 | 2047.0998 | 2047.0990 | 0.38 | 1 | 48 | 2.9e-05 | 1Score **> 36** indicates **identity** Score **> 16** indicates **homology** | U | R.KGFSYLVTATTTVGVAYAAK.N |
| 130973 | 111 | – | 130 | 683.3740 | 2047.1000 | 2047.0990 | 0.50 | 1 | 34 | 0.00069 | 1Score **> 36** indicates **identity** Score **> 15** indicates **homology** | U | R.KGFSYLVTATTTVGVAYAAK.N |
| 130974 | 111 | – | 130 | 683.3741 | 2047.1004 | 2047.0990 | 0.67 | 1 | 24 | 0.0058 | 1Score **> 36** indicates **identity** Score **> 14** indicates **homology** | U | R.KGFSYLVTATTTVGVAYAAK.N |
| 130975 | 111 | – | 130 | 683.3741 | 2047.1005 | 2047.0990 | 0.76 | 1 | 62 | 1.5e-06 | 1Score **> 36** indicates **identity** Score **> 16** indicates **homology** | U | R.KGFSYLVTATTTVGVAYAAK.N |
| 130978 | 111 | – | 130 | 683.3743 | 2047.1010 | 2047.0990 | 0.96 | 1 | 20 | 0.013 | 1Score **> 36** indicates **identity** Score **> 14** indicates **homology** | U | R.KGFSYLVTATTTVGVAYAAK.N |
| 130979 | 111 | – | 130 | 683.3743 | 2047.1011 | 2047.0990 | 1.01 | 1 | 29 | 0.0021 | 1Score **> 36** indicates **identity** Score **> 14** indicates **homology** | U | R.KGFSYLVTATTTVGVAYAAK.N |
| 130981 | 111 | – | 130 | 683.3744 | 2047.1013 | 2047.0990 | 1.11 | 1 | 42 | 0.0001 | 1Score **> 36** indicates **identity** Score **> 15** indicates **homology** | U | R.KGFSYLVTATTTVGVAYAAK.N |
| 130982 | 111 | – | 130 | 683.3744 | 2047.1015 | 2047.0990 | 1.20 | 1 | 19 | 0.017 | 1Score **> 36** indicates **identity** Score **> 14** indicates **homology** | U | R.KGFSYLVTATTTVGVAYAAK.N |
| 130983 | 111 | – | 130 | 683.3745 | 2047.1015 | 2047.0990 | 1.23 | 1 | 37 | 0.00032 | 1Score **> 36** indicates **identity** Score **> 15** indicates **homology** | U | R.KGFSYLVTATTTVGVAYAAK.N |
| 130986 | 111 | – | 130 | 683.3747 | 2047.1022 | 2047.0990 | 1.55 | 1 | 49 | 2.8e-05 | 1Score **> 36** indicates **identity** Score **> 16** indicates **homology** | U | R.KGFSYLVTATTTVGVAYAAK.N |
| 130989 | 111 | – | 130 | 683.3748 | 2047.1027 | 2047.0990 | 1.80 | 1 | 48 | 3.2e-05 | 1Score **> 36** indicates **identity** Score **> 15** indicates **homology** | U | R.KGFSYLVTATTTVGVAYAAK.N |
| 130990 | 111 | – | 130 | 683.3750 | 2047.1033 | 2047.0990 | 2.08 | 1 | 35 | 0.00053 | 1Score **> 36** indicates **identity** Score **> 15** indicates **homology** | U | R.KGFSYLVTATTTVGVAYAAK.N |
| 130991 | 111 | – | 130 | 683.3750 | 2047.1033 | 2047.0990 | 2.09 | 1 | 67 | 5.1e-07 | 1Score **> 36** indicates **identity** Score **> 17** indicates **homology** | U | R.KGFSYLVTATTTVGVAYAAK.N |
| 130992 | 111 | – | 130 | 683.3750 | 2047.1033 | 2047.0990 | 2.10 | 1 | 21 | 0.01 | 1Score **> 36** indicates **identity** Score **> 14** indicates **homology** | U | R.KGFSYLVTATTTVGVAYAAK.N |
| 130997 | 111 | – | 130 | 683.3752 | 2047.1039 | 2047.0990 | 2.38 | 1 | 49 | 2.8e-05 | 1Score **> 36** indicates **identity** Score **> 16** indicates **homology** | U | R.KGFSYLVTATTTVGVAYAAK.N |
| 130999 | 111 | – | 130 | 683.3754 | 2047.1043 | 2047.0990 | 2.58 | 1 | 47 | 4e-05 | 1Score **> 35** indicates **identity** Score **> 15** indicates **homology** | U | R.KGFSYLVTATTTVGVAYAAK.N |
| 131000 | 111 | – | 130 | 683.3755 | 2047.1046 | 2047.0990 | 2.72 | 1 | 32 | 0.0011 | 1Score **> 36** indicates **identity** Score **> 14** indicates **homology** | U | R.KGFSYLVTATTTVGVAYAAK.N |
| 131001 | 111 | – | 130 | 683.3755 | 2047.1048 | 2047.0990 | 2.82 | 1 | 16 | 0.035 | 1Score **> 36** indicates **identity** Score **> 13** indicates **homology** | U | R.KGFSYLVTATTTVGVAYAAK.N |
| 131002 | 111 | – | 130 | 683.3757 | 2047.1051 | 2047.0990 | 2.99 | 1 | 35 | 0.00052 | 1Score **> 35** indicates **identity** Score **> 15** indicates **homology** | U | R.KGFSYLVTATTTVGVAYAAK.N |
| 131003 | 111 | – | 130 | 683.3758 | 2047.1057 | 2047.0990 | 3.25 | 1 | 21 | 0.0099 | 1Score **> 35** indicates **identity** Score **> 14** indicates **homology** | U | R.KGFSYLVTATTTVGVAYAAK.N |
| 131004 | 111 | – | 130 | 683.3759 | 2047.1059 | 2047.0990 | 3.36 | 1 | 42 | 0.00011 | 1Score **> 35** indicates **identity** Score **> 15** indicates **homology** | U | R.KGFSYLVTATTTVGVAYAAK.N |
| 131005 | 111 | – | 130 | 683.3761 | 2047.1064 | 2047.0990 | 3.61 | 1 | 29 | 0.003 | 1Score **> 35** indicates **identity** Score **> 16** indicates **homology** | U | R.KGFSYLVTATTTVGVAYAAK.N |
| 131007 | 111 | – | 130 | 683.3761 | 2047.1066 | 2047.0990 | 3.70 | 1 | 44 | 6.8e-05 | 1Score **> 35** indicates **identity** Score **> 15** indicates **homology** | U | R.KGFSYLVTATTTVGVAYAAK.N |
| 131008 | 111 | – | 130 | 683.3762 | 2047.1068 | 2047.0990 | 3.81 | 1 | 41 | 0.00015 | 1Score **> 35** indicates **identity** Score **> 15** indicates **homology** | U | R.KGFSYLVTATTTVGVAYAAK.N |
| 131010 | 111 | – | 130 | 683.3774 | 2047.1103 | 2047.0990 | 5.50 | 1 | 49 | 2.5e-05 | 1Score **> 35** indicates **identity** Score **> 16** indicates **homology** | U | R.KGFSYLVTATTTVGVAYAAK.N |
| 131012 | 111 | – | 130 | 683.3775 | 2047.1106 | 2047.0990 | 5.67 | 1 | 50 | 2.1e-05 | 1Score **> 35** indicates **identity** Score **> 16** indicates **homology** | U | R.KGFSYLVTATTTVGVAYAAK.N |
| 131013 | 111 | – | 130 | 683.3775 | 2047.1108 | 2047.0990 | 5.74 | 1 | 27 | 0.0028 | 1Score **> 35** indicates **identity** Score **> 14** indicates **homology** | U | R.KGFSYLVTATTTVGVAYAAK.N |
| 131015 | 111 | – | 130 | 683.3776 | 2047.1109 | 2047.0990 | 5.83 | 1 | 63 | 1.3e-06 | 1Score **> 35** indicates **identity** Score **> 16** indicates **homology** | U | R.KGFSYLVTATTTVGVAYAAK.N |
| 131016 | 111 | – | 130 | 683.3777 | 2047.1112 | 2047.0990 | 5.95 | 1 | 49 | 2.7e-05 | 1Score **> 35** indicates **identity** Score **> 16** indicates **homology** | U | R.KGFSYLVTATTTVGVAYAAK.N |
| 131017 | 111 | – | 130 | 683.3777 | 2047.1113 | 2047.0990 | 6.03 | 1 | 70 | 2.7e-07 | 1Score **> 35** indicates **identity** Score **> 17** indicates **homology** | U | R.KGFSYLVTATTTVGVAYAAK.N |
| 131018 | 111 | – | 130 | 683.3782 | 2047.1128 | 2047.0990 | 6.72 | 1 | 38 | 0.00027 | 1Score **> 35** indicates **identity** Score **> 15** indicates **homology** | U | R.KGFSYLVTATTTVGVAYAAK.N |
| 131020 | 111 | – | 130 | 683.3785 | 2047.1137 | 2047.0990 | 7.20 | 1 | 17 | 0.028 | 1Score **> 35** indicates **identity** Score **> 14** indicates **homology** | U | R.KGFSYLVTATTTVGVAYAAK.N |
| 131021 | 111 | – | 130 | 683.3787 | 2047.1143 | 2047.0990 | 7.47 | 1 | 46 | 5.2e-05 | 1Score **> 35** indicates **identity** Score **> 15** indicates **homology** | U | R.KGFSYLVTATTTVGVAYAAK.N |
| 131023 | 111 | – | 130 | 683.3796 | 2047.1168 | 2047.0990 | 8.71 | 1 | 35 | 0.00055 | 1Score **> 35** indicates **identity** Score **> 15** indicates **homology** | U | R.KGFSYLVTATTTVGVAYAAK.N |
| 131024 | 111 | – | 130 | 683.3800 | 2047.1182 | 2047.0990 | 9.36 | 1 | 20 | 0.014 | 1Score **> 35** indicates **identity** Score **> 14** indicates **homology** | U | R.KGFSYLVTATTTVGVAYAAK.N |
| 131025 | 111 | – | 130 | 683.3801 | 2047.1185 | 2047.0990 | 9.53 | 1 | 67 | 4.9e-07 | 1Score **> 35** indicates **identity** Score **> 17** indicates **homology** | U | R.KGFSYLVTATTTVGVAYAAK.N |
| 131026 | 111 | – | 130 | 683.3802 | 2047.1189 | 2047.0990 | 9.71 | 1 | 35 | 0.00048 | 1Score **> 35** indicates **identity** Score **> 15** indicates **homology** | U | R.KGFSYLVTATTTVGVAYAAK.N |
| 200333 | 111 | – | 151 | 869.6455 | 4343.1911 | 4343.2321 | -9.44 | 2 | 15 | 0.042 | 1Score **> 37** indicates **identity** Score **> 13** indicates **homology** | U | R.KGFSYLVTATTTVGVAYAAKNVVSQFVSSMSASADVLAMSK.I  + Deamidated (NQ); HNE (K) |
| 141200 | 131 | – | 151 | 720.0233 | 2157.0482 | 2157.0446 | 1.67 | 0 | 45 | 5.8e-05 | 1Score **> 35** indicates **identity** Score **> 15** indicates **homology** | U | K.NVVSQFVSSMSASADVLAMSK.I |
| 141201 | 131 | – | 151 | 1079.5322 | 2157.0499 | 2157.0446 | 2.47 | 0 | 47 | 3.9e-05 | 1Score **> 35** indicates **identity** Score **> 15** indicates **homology** | U | K.NVVSQFVSSMSASADVLAMSK.I |
| 6180 | 156 | – | 163 | 429.7321 | 857.4497 | 857.4494 | 0.35 | 0 | 15 | 0.043 | 1Score **> 27** indicates **identity** Score **> 13** indicates **homology** | U | K.LSDIPEGK.N |
| 6181 | 156 | – | 163 | 429.7323 | 857.4500 | 857.4494 | 0.65 | 0 | 19 | 0.017 | 1Score **> 27** indicates **identity** Score **> 14** indicates **homology** | U | K.LSDIPEGK.N |
| 4240 | 171 | – | 177 | 408.7580 | 815.5014 | 815.5018 | -0.42 | 1 | 24 | 0.023 | 1Score **> 22** indicates **identity** Score **> 21** indicates **homology** | U | R.GKPLFVR.H |
| 4241 | 171 | – | 177 | 408.7583 | 815.5020 | 815.5018 | 0.33 | 1 | 30 | 0.0068 | 1Score **> 22** indicates **identity** Score **> 21** indicates **homology** | U | R.GKPLFVR.H |
| 4243 | 171 | – | 177 | 408.7583 | 815.5021 | 815.5018 | 0.40 | 1 | 22 | 0.035 | 1Score **> 22** indicates **identity** Score **> 20** indicates **homology** | U | R.GKPLFVR.H |
| 4244 | 171 | – | 177 | 408.7586 | 815.5027 | 815.5018 | 1.10 | 1 | 22 | 0.0098 | 1Score **> 22** indicates **identity** Score **> 15** indicates **homology** | U | R.GKPLFVR.H |
| 173854 | 182 | – | 204 | 673.5886 | 2690.3252 | 2690.3260 | -0.30 | 2 | 57 | 4.1e-06 | 1Score **> 37** indicates **identity** Score **> 16** indicates **homology** | U | K.KEIDQEAAVEVSQLRDPQHDLDR.V |
| 173856 | 182 | – | 204 | 673.5893 | 2690.3282 | 2690.3260 | 0.83 | 2 | 53 | 1e-05 | 1Score **> 37** indicates **identity** Score **> 16** indicates **homology** | U | K.KEIDQEAAVEVSQLRDPQHDLDR.V |
| 173859 | 182 | – | 204 | 897.7841 | 2690.3305 | 2690.3260 | 1.69 | 2 | 17 | 0.027 | 1Score **> 37** indicates **identity** Score **> 14** indicates **homology** | U | K.KEIDQEAAVEVSQLRDPQHDLDR.V |
| 173889 | 182 | – | 204 | 673.8379 | 2691.3225 | 2691.3100 | 4.64 | 2 | 23 | 0.0077 | 1Score **> 37** indicates **identity** Score **> 14** indicates **homology** | U | K.KEIDQEAAVEVSQLRDPQHDLDR.V  + Deamidated (NQ) |
| 168119 | 183 | – | 204 | 641.5652 | 2562.2319 | 2562.2310 | 0.34 | 1 | 20 | 0.014 | 1Score **> 36** indicates **identity** Score **> 14** indicates **homology** | U | K.EIDQEAAVEVSQLRDPQHDLDR.V |
| 168121 | 183 | – | 204 | 855.0846 | 2562.2321 | 2562.2310 | 0.43 | 1 | 20 | 0.012 | 1Score **> 36** indicates **identity** Score **> 14** indicates **homology** | U | K.EIDQEAAVEVSQLRDPQHDLDR.V |
| 168122 | 183 | – | 204 | 641.5654 | 2562.2324 | 2562.2310 | 0.53 | 1 | 18 | 0.019 | 1Score **> 36** indicates **identity** Score **> 14** indicates **homology** | U | K.EIDQEAAVEVSQLRDPQHDLDR.V |
| 168125 | 183 | – | 204 | 855.0850 | 2562.2332 | 2562.2310 | 0.87 | 1 | 37 | 0.00034 | 1Score **> 36** indicates **identity** Score **> 15** indicates **homology** | U | K.EIDQEAAVEVSQLRDPQHDLDR.V |
| 168126 | 183 | – | 204 | 641.5656 | 2562.2332 | 2562.2310 | 0.87 | 1 | 22 | 0.0081 | 1Score **> 36** indicates **identity** Score **> 14** indicates **homology** | U | K.EIDQEAAVEVSQLRDPQHDLDR.V |
| 168129 | 183 | – | 204 | 1282.1240 | 2562.2334 | 2562.2310 | 0.92 | 1 | 24 | 0.0066 | 1Score **> 36** indicates **identity** Score **> 15** indicates **homology** | U | K.EIDQEAAVEVSQLRDPQHDLDR.V |
| 168130 | 183 | – | 204 | 1282.1240 | 2562.2335 | 2562.2310 | 0.99 | 1 | 20 | 0.016 | 1Score **> 36** indicates **identity** Score **> 14** indicates **homology** | U | K.EIDQEAAVEVSQLRDPQHDLDR.V |
| 168131 | 183 | – | 204 | 641.5657 | 2562.2337 | 2562.2310 | 1.06 | 1 | 30 | 0.0014 | 1Score **> 36** indicates **identity** Score **> 14** indicates **homology** | U | K.EIDQEAAVEVSQLRDPQHDLDR.V |
| 168132 | 183 | – | 204 | 855.0852 | 2562.2337 | 2562.2310 | 1.06 | 1 | 31 | 0.0012 | 1Score **> 36** indicates **identity** Score **> 14** indicates **homology** | U | K.EIDQEAAVEVSQLRDPQHDLDR.V |
| 168133 | 183 | – | 204 | 641.5658 | 2562.2341 | 2562.2310 | 1.22 | 1 | 19 | 0.015 | 1Score **> 36** indicates **identity** Score **> 14** indicates **homology** | U | K.EIDQEAAVEVSQLRDPQHDLDR.V |
| 168134 | 183 | – | 204 | 1282.1244 | 2562.2343 | 2562.2310 | 1.29 | 1 | 33 | 0.001 | 1Score **> 36** indicates **identity** Score **> 16** indicates **homology** | U | K.EIDQEAAVEVSQLRDPQHDLDR.V |
| 168135 | 183 | – | 204 | 855.0854 | 2562.2344 | 2562.2310 | 1.34 | 1 | 35 | 0.00051 | 1Score **> 36** indicates **identity** Score **> 15** indicates **homology** | U | K.EIDQEAAVEVSQLRDPQHDLDR.V |
| 168136 | 183 | – | 204 | 641.5659 | 2562.2344 | 2562.2310 | 1.34 | 1 | 32 | 0.00093 | 1Score **> 36** indicates **identity** Score **> 15** indicates **homology** | U | K.EIDQEAAVEVSQLRDPQHDLDR.V |
| 168138 | 183 | – | 204 | 641.5664 | 2562.2366 | 2562.2310 | 2.19 | 1 | 30 | 0.0015 | 1Score **> 36** indicates **identity** Score **> 14** indicates **homology** | U | K.EIDQEAAVEVSQLRDPQHDLDR.V |
| 168139 | 183 | – | 204 | 855.0861 | 2562.2366 | 2562.2310 | 2.19 | 1 | 27 | 0.0029 | 1Score **> 36** indicates **identity** Score **> 14** indicates **homology** | U | K.EIDQEAAVEVSQLRDPQHDLDR.V |
| 168163 | 183 | – | 204 | 855.4131 | 2563.2174 | 2563.2150 | 0.92 | 1 | 17 | 0.026 | 1Score **> 35** indicates **identity** Score **> 14** indicates **homology** | U | K.EIDQEAAVEVSQLRDPQHDLDR.V  + Deamidated (NQ) |
| 168164 | 183 | – | 204 | 641.8146 | 2563.2294 | 2563.2150 | 5.63 | 1 | 22 | 0.0086 | 1Score **> 36** indicates **identity** Score **> 14** indicates **homology** | U | K.EIDQEAAVEVSQLRDPQHDLDR.V  + Deamidated (NQ) |
| 168168 | 183 | – | 204 | 641.8156 | 2563.2331 | 2563.2150 | 7.06 | 1 | 14 | 0.05 | 1Score **> 36** indicates **identity** Score **> 13** indicates **homology** | U | K.EIDQEAAVEVSQLRDPQHDLDR.V  + Deamidated (NQ) |
| 168185 | 183 | – | 204 | 855.4199 | 2563.2379 | 2563.2150 | 8.94 | 1 | 17 | 0.024 | 1Score **> 36** indicates **identity** Score **> 14** indicates **homology** | U | K.EIDQEAAVEVSQLRDPQHDLDR.V  + Deamidated (NQ) |
| 177714 | 183 | – | 206 | 930.8063 | 2789.3972 | 2789.3944 | 1.00 | 2 | 33 | 0.00075 | 1Score **> 37** indicates **identity** Score **> 15** indicates **homology** | U | K.EIDQEAAVEVSQLRDPQHDLDRVK.K |
| 177716 | 183 | – | 206 | 930.8068 | 2789.3985 | 2789.3944 | 1.48 | 2 | 34 | 0.00061 | 1Score **> 37** indicates **identity** Score **> 15** indicates **homology** | U | K.EIDQEAAVEVSQLRDPQHDLDRVK.K |
| 177717 | 183 | – | 206 | 930.8068 | 2789.3986 | 2789.3944 | 1.53 | 2 | 39 | 0.0002 | 1Score **> 37** indicates **identity** Score **> 15** indicates **homology** | U | K.EIDQEAAVEVSQLRDPQHDLDRVK.K |
| 177719 | 183 | – | 206 | 930.8070 | 2789.3992 | 2789.3944 | 1.73 | 2 | 27 | 0.0028 | 1Score **> 37** indicates **identity** Score **> 14** indicates **homology** | U | K.EIDQEAAVEVSQLRDPQHDLDRVK.K |
| 177757 | 183 | – | 206 | 931.1424 | 2790.4053 | 2790.3784 | 9.63 | 2 | 32 | 0.00097 | 1Score **> 37** indicates **identity** Score **> 15** indicates **homology** | U | K.EIDQEAAVEVSQLRDPQHDLDRVK.K  + Deamidated (NQ) |
| 35291 | 197 | – | 206 | 408.2105 | 1221.6097 | 1221.6102 | -0.43 | 1 | 14 | 0.048 | 1Score **> 33** indicates **identity** Score **> 13** indicates **homology** | U | R.DPQHDLDRVK.K |
| 202496 | 205 | – | 248 | 984.4675 | 4917.3009 | 4917.3037 | -0.57 | 2 | 25 | 0.0047 | 1Score **> 35** indicates **identity** Score **> 14** indicates **homology** | U | R.VKKPEWVILIGVCTHLGCVPIANAGDFGGYYCPCHGSHYDASGR.I |
| 202498 | 205 | – | 248 | 820.5577 | 4917.3027 | 4917.3037 | -0.19 | 2 | 31 | 0.0011 | 1Score **> 35** indicates **identity** Score **> 14** indicates **homology** | U | R.VKKPEWVILIGVCTHLGCVPIANAGDFGGYYCPCHGSHYDASGR.I |
| 202499 | 205 | – | 248 | 820.5578 | 4917.3034 | 4917.3037 | -0.063 | 2 | 34 | 0.00059 | 1Score **> 35** indicates **identity** Score **> 15** indicates **homology** | U | R.VKKPEWVILIGVCTHLGCVPIANAGDFGGYYCPCHGSHYDASGR.I |
| 177515 | 249 | – | 274 | 929.1666 | 2784.4780 | 2784.4698 | 2.93 | 2 | 41 | 0.00015 | 1Score **> 36** indicates **identity** Score **> 15** indicates **homology** | U | R.IRKGPAPLNLEVPAYEFTSDDVVVVG.- |
| 177517 | 249 | – | 274 | 929.1695 | 2784.4866 | 2784.4698 | 6.02 | 2 | 38 | 0.00025 | 1Score **> 36** indicates **identity** Score **> 15** indicates **homology** | U | R.IRKGPAPLNLEVPAYEFTSDDVVVVG.- |
| 177558 | 249 | – | 274 | 929.4987 | 2785.4743 | 2785.4538 | 7.35 | 2 | 33 | 0.00074 | 1Score **> 36** indicates **identity** Score **> 15** indicates **homology** | U | R.IRKGPAPLNLEVPAYEFTSDDVVVVG.-  + Deamidated (NQ) |
| 165781 | 251 | – | 274 | 1258.6469 | 2515.2792 | 2515.2846 | -2.18 | 1 | 20 | 0.012 | 1Score **> 37** indicates **identity** Score **> 14** indicates **homology** | U | R.KGPAPLNLEVPAYEFTSDDVVVVG.- |
| 165782 | 251 | – | 274 | 1258.6470 | 2515.2794 | 2515.2846 | -2.10 | 1 | 27 | 0.003 | 1Score **> 37** indicates **identity** Score **> 14** indicates **homology** | U | R.KGPAPLNLEVPAYEFTSDDVVVVG.- |
| 165801 | 251 | – | 274 | 1258.6513 | 2515.2881 | 2515.2846 | 1.36 | 1 | 57 | 4.3e-06 | 1Score **> 37** indicates **identity** Score **> 16** indicates **homology** | U | R.KGPAPLNLEVPAYEFTSDDVVVVG.- |
| 165802 | 251 | – | 274 | 839.4367 | 2515.2882 | 2515.2846 | 1.43 | 1 | 28 | 0.0022 | 1Score **> 37** indicates **identity** Score **> 14** indicates **homology** | U | R.KGPAPLNLEVPAYEFTSDDVVVVG.- |
| 165803 | 251 | – | 274 | 1258.6516 | 2515.2886 | 2515.2846 | 1.58 | 1 | 63 | 1.3e-06 | 1Score **> 37** indicates **identity** Score **> 16** indicates **homology** | U | R.KGPAPLNLEVPAYEFTSDDVVVVG.- |
| 165805 | 251 | – | 274 | 839.4370 | 2515.2892 | 2515.2846 | 1.80 | 1 | 27 | 0.0031 | 1Score **> 37** indicates **identity** Score **> 14** indicates **homology** | U | R.KGPAPLNLEVPAYEFTSDDVVVVG.- |
| 165807 | 251 | – | 274 | 1258.6520 | 2515.2894 | 2515.2846 | 1.88 | 1 | 41 | 0.00014 | 1Score **> 37** indicates **identity** Score **> 15** indicates **homology** | U | R.KGPAPLNLEVPAYEFTSDDVVVVG.- |
| 165812 | 251 | – | 274 | 839.4379 | 2515.2919 | 2515.2846 | 2.88 | 1 | 34 | 0.00065 | 1Score **> 37** indicates **identity** Score **> 15** indicates **homology** | U | R.KGPAPLNLEVPAYEFTSDDVVVVG.- |
| 165813 | 251 | – | 274 | 1258.6532 | 2515.2919 | 2515.2846 | 2.90 | 1 | 33 | 0.00089 | 1Score **> 37** indicates **identity** Score **> 15** indicates **homology** | U | R.KGPAPLNLEVPAYEFTSDDVVVVG.- |
| 165823 | 251 | – | 274 | 1258.6619 | 2515.3093 | 2515.2846 | 9.82 | 1 | 36 | 0.00047 | 1Score **> 37** indicates **identity** Score **> 15** indicates **homology** | U | R.KGPAPLNLEVPAYEFTSDDVVVVG.- |

---

```
ID   UCRI_MOUSE              Reviewed;         274 AA.
AC   Q9CR68; Q5SVV1;
DT   13-SEP-2004, integrated into UniProtKB/Swiss-Prot.
DT   01-JUN-2001, sequence version 1.
DT   28-JUN-2023, entry version 161.
DE   RecName: Full=Cytochrome b-c1 complex subunit Rieske, mitochondrial;
DE            EC=7.1.1.8;
DE   AltName: Full=Complex III subunit 5;
DE   AltName: Full=Cytochrome b-c1 complex subunit 5;
DE   AltName: Full=Rieske iron-sulfur protein;
DE            Short=RISP;
DE   AltName: Full=Rieske protein UQCRFS1 {ECO:0000305};
DE   AltName: Full=Ubiquinol-cytochrome c reductase iron-sulfur subunit;
DE   Contains:
DE     RecName: Full=Cytochrome b-c1 complex subunit 9 {ECO:0000250|UniProtKB:P13272};
DE              Short=Su9 {ECO:0000250|UniProtKB:P13272};
DE              Short=Subunit 9 {ECO:0000250|UniProtKB:P13272};
DE     AltName: Full=8 kDa subunit 9 {ECO:0000250|UniProtKB:P13272};
DE     AltName: Full=Complex III subunit IX;
DE     AltName: Full=Cytochrome b-c1 complex subunit 11;
DE     AltName: Full=UQCRFS1 mitochondrial targeting sequence;
DE              Short=UQCRFS1 MTS;
DE     AltName: Full=Ubiquinol-cytochrome c reductase 8 kDa protein {ECO:0000250|UniProtKB:P13272};
DE   Flags: Precursor;
GN   Name=Uqcrfs1 {ECO:0000312|MGI:MGI:1913944};
OS   Mus musculus (Mouse).
OC   Eukaryota; Metazoa; Chordata; Craniata; Vertebrata; Euteleostomi; Mammalia;
OC   Eutheria; Euarchontoglires; Glires; Rodentia; Myomorpha; Muroidea; Muridae;
OC   Murinae; Mus; Mus.
OX   NCBI_TaxID=10090;
RN   [1]
RP   NUCLEOTIDE SEQUENCE [LARGE SCALE MRNA].
RC   STRAIN=C57BL/6J; TISSUE=Bone marrow, and Liver;
RX   PubMed=16141072; DOI=10.1126/science.1112014;
RA   Carninci P., Kasukawa T., Katayama S., Gough J., Frith M.C., Maeda N.,
RA   Oyama R., Ravasi T., Lenhard B., Wells C., Kodzius R., Shimokawa K.,
RA   Bajic V.B., Brenner S.E., Batalov S., Forrest A.R., Zavolan M., Davis M.J.,
RA   Wilming L.G., Aidinis V., Allen J.E., Ambesi-Impiombato A., Apweiler R.,
RA   Aturaliya R.N., Bailey T.L., Bansal M., Baxter L., Beisel K.W., Bersano T.,
RA   Bono H., Chalk A.M., Chiu K.P., Choudhary V., Christoffels A.,
RA   Clutterbuck D.R., Crowe M.L., Dalla E., Dalrymple B.P., de Bono B.,
RA   Della Gatta G., di Bernardo D., Down T., Engstrom P., Fagiolini M.,
RA   Faulkner G., Fletcher C.F., Fukushima T., Furuno M., Futaki S.,
RA   Gariboldi M., Georgii-Hemming P., Gingeras T.R., Gojobori T., Green R.E.,
RA   Gustincich S., Harbers M., Hayashi Y., Hensch T.K., Hirokawa N., Hill D.,
RA   Huminiecki L., Iacono M., Ikeo K., Iwama A., Ishikawa T., Jakt M.,
RA   Kanapin A., Katoh M., Kawasawa Y., Kelso J., Kitamura H., Kitano H.,
RA   Kollias G., Krishnan S.P., Kruger A., Kummerfeld S.K., Kurochkin I.V.,
RA   Lareau L.F., Lazarevic D., Lipovich L., Liu J., Liuni S., McWilliam S.,
RA   Madan Babu M., Madera M., Marchionni L., Matsuda H., Matsuzawa S., Miki H.,
RA   Mignone F., Miyake S., Morris K., Mottagui-Tabar S., Mulder N., Nakano N.,
RA   Nakauchi H., Ng P., Nilsson R., Nishiguchi S., Nishikawa S., Nori F.,
RA   Ohara O., Okazaki Y., Orlando V., Pang K.C., Pavan W.J., Pavesi G.,
RA   Pesole G., Petrovsky N., Piazza S., Reed J., Reid J.F., Ring B.Z.,
RA   Ringwald M., Rost B., Ruan Y., Salzberg S.L., Sandelin A., Schneider C.,
RA   Schoenbach C., Sekiguchi K., Semple C.A., Seno S., Sessa L., Sheng Y.,
RA   Shibata Y., Shimada H., Shimada K., Silva D., Sinclair B., Sperling S.,
RA   Stupka E., Sugiura K., Sultana R., Takenaka Y., Taki K., Tammoja K.,
RA   Tan S.L., Tang S., Taylor M.S., Tegner J., Teichmann S.A., Ueda H.R.,
RA   van Nimwegen E., Verardo R., Wei C.L., Yagi K., Yamanishi H.,
RA   Zabarovsky E., Zhu S., Zimmer A., Hide W., Bult C., Grimmond S.M.,
RA   Teasdale R.D., Liu E.T., Brusic V., Quackenbush J., Wahlestedt C.,
RA   Mattick J.S., Hume D.A., Kai C., Sasaki D., Tomaru Y., Fukuda S.,
RA   Kanamori-Katayama M., Suzuki M., Aoki J., Arakawa T., Iida J., Imamura K.,
RA   Itoh M., Kato T., Kawaji H., Kawagashira N., Kawashima T., Kojima M.,
RA   Kondo S., Konno H., Nakano K., Ninomiya N., Nishio T., Okada M., Plessy C.,
RA   Shibata K., Shiraki T., Suzuki S., Tagami M., Waki K., Watahiki A.,
RA   Okamura-Oho Y., Suzuki H., Kawai J., Hayashizaki Y.;
RT   "The transcriptional landscape of the mammalian genome.";
RL   Science 309:1559-1563(2005).
RN   [2]
RP   NUCLEOTIDE SEQUENCE [LARGE SCALE GENOMIC DNA].
RC   STRAIN=C57BL/6J;
RX   PubMed=19468303; DOI=10.1371/journal.pbio.1000112;
RA   Church D.M., Goodstadt L., Hillier L.W., Zody M.C., Goldstein S., She X.,
RA   Bult C.J., Agarwala R., Cherry J.L., DiCuccio M., Hlavina W., Kapustin Y.,
RA   Meric P., Maglott D., Birtle Z., Marques A.C., Graves T., Zhou S.,
RA   Teague B., Potamousis K., Churas C., Place M., Herschleb J., Runnheim R.,
RA   Forrest D., Amos-Landgraf J., Schwartz D.C., Cheng Z., Lindblad-Toh K.,
RA   Eichler E.E., Ponting C.P.;
RT   "Lineage-specific biology revealed by a finished genome assembly of the
RT   mouse.";
RL   PLoS Biol. 7:E1000112-E1000112(2009).
RN   [3]
RP   NUCLEOTIDE SEQUENCE [LARGE SCALE MRNA].
RC   STRAIN=FVB/N; TISSUE=Mammary tumor;
RX   PubMed=15489334; DOI=10.1101/gr.2596504;
RG   The MGC Project Team;
RT   "The status, quality, and expansion of the NIH full-length cDNA project:
RT   the Mammalian Gene Collection (MGC).";
RL   Genome Res. 14:2121-2127(2004).
RN   [4]
RP   PROTEIN SEQUENCE OF 8-46; 85-92; 94-101; 131-151; 156-163; 171-177 AND
RP   183-204, AND IDENTIFICATION BY MASS SPECTROMETRY.
RC   STRAIN=C57BL/6J; TISSUE=Brain;
RA   Lubec G., Kang S.U.;
RL   Submitted (APR-2007) to UniProtKB.
RN   [5]
RP   SUBUNIT.
RX   PubMed=19026783; DOI=10.1016/j.molcel.2008.10.021;
RA   Acin-Perez R., Fernandez-Silva P., Peleato M.L., Perez-Martos A.,
RA   Enriquez J.A.;
RT   "Respiratory active mitochondrial supercomplexes.";
RL   Mol. Cell 32:529-539(2008).
RN   [6]
RP   IDENTIFICATION BY MASS SPECTROMETRY [LARGE SCALE ANALYSIS].
RC   TISSUE=Brain, Brown adipose tissue, Heart, Kidney, Liver, Lung,
RC   Pancreas, Spleen, and Testis;
RX   PubMed=21183079; DOI=10.1016/j.cell.2010.12.001;
RA   Huttlin E.L., Jedrychowski M.P., Elias J.E., Goswami T., Rad R.,
RA   Beausoleil S.A., Villen J., Haas W., Sowa M.E., Gygi S.P.;
RT   "A tissue-specific atlas of mouse protein phosphorylation and expression.";
RL   Cell 143:1174-1189(2010).
RN   [7]
RP   PTM, IDENTIFICATION BY MASS SPECTROMETRY, SUBUNIT, INTERACTION WITH TTC19,
RP   AND FUNCTION.
RX   PubMed=28673544; DOI=10.1016/j.molcel.2017.06.001;
RA   Bottani E., Cerutti R., Harbour M.E., Ravaglia S., Dogan S.A., Giordano C.,
RA   Fearnley I.M., D'Amati G., Viscomi C., Fernandez-Vizarra E., Zeviani M.;
RT   "TTC19 plays a husbandry role on UQCRFS1 turnover in the biogenesis of
RT   mitochondrial respiratory complex III.";
RL   Mol. Cell 67:96-105(2017).
CC   -!- FUNCTION: [Cytochrome b-c1 complex subunit Rieske, mitochondrial]:
CC       Component of the ubiquinol-cytochrome c oxidoreductase, a multisubunit
CC       transmembrane complex that is part of the mitochondrial electron
CC       transport chain which drives oxidative phosphorylation. The respiratory
CC       chain contains 3 multisubunit complexes succinate dehydrogenase
CC       (complex II, CII), ubiquinol-cytochrome c oxidoreductase (cytochrome b-
CC       c1 complex, complex III, CIII) and cytochrome c oxidase (complex IV,
CC       CIV), that cooperate to transfer electrons derived from NADH and
CC       succinate to molecular oxygen, creating an electrochemical gradient
CC       over the inner membrane that drives transmembrane transport and the ATP
CC       synthase. The cytochrome b-c1 complex catalyzes electron transfer from
CC       ubiquinol to cytochrome c, linking this redox reaction to translocation
CC       of protons across the mitochondrial inner membrane, with protons being
CC       carried across the membrane as hydrogens on the quinol. In the process
CC       called Q cycle, 2 protons are consumed from the matrix, 4 protons are
CC       released into the intermembrane space and 2 electrons are passed to
CC       cytochrome c. The Rieske protein is a catalytic core subunit containing
CC       a [2Fe-2S] iron-sulfur cluster. It cycles between 2 conformational
CC       states during catalysis to transfer electrons from the quinol bound in
CC       the Q(0) site in cytochrome b to cytochrome c1 (By similarity).
CC       Incorporation of UQCRFS1 is the penultimate step in complex III
CC       assembly (By similarity). {ECO:0000250|UniProtKB:P08067,
CC       ECO:0000250|UniProtKB:P47985}.
CC   -!- FUNCTION: [Cytochrome b-c1 complex subunit 9]: Component of the
CC       ubiquinol-cytochrome c oxidoreductase (cytochrome b-c1 complex, complex
CC       III, CIII). UQCRFS1 undergoes proteolytic processing once it is
CC       incorporated in the complex III dimer. One of the fragments, called
CC       subunit 9, corresponds to its mitochondrial targeting sequence (MTS).
CC       The proteolytic processing is necessary for the correct insertion of
CC       UQCRFS1 in the complex III dimer, but the persistence of UQCRFS1-
CC       derived fragments may prevent newly imported UQCRFS1 to be processed
CC       and assembled into complex III and is detrimental for the complex III
CC       structure and function. {ECO:0000269|PubMed:28673544}.
CC   -!- CATALYTIC ACTIVITY:
CC       Reaction=a quinol + 2 Fe(III)-[cytochrome c](out) = a quinone + 2
CC         Fe(II)-[cytochrome c](out) + 2 H(+)(out); Xref=Rhea:RHEA:11484,
CC         Rhea:RHEA-COMP:10350, Rhea:RHEA-COMP:14399, ChEBI:CHEBI:15378,
CC         ChEBI:CHEBI:24646, ChEBI:CHEBI:29033, ChEBI:CHEBI:29034,
CC         ChEBI:CHEBI:132124; EC=7.1.1.8;
CC         Evidence={ECO:0000250|UniProtKB:P08067};
CC   -!- COFACTOR:
CC       Name=[2Fe-2S] cluster; Xref=ChEBI:CHEBI:190135;
CC         Evidence={ECO:0000255|PROSITE-ProRule:PRU00628};
CC       Note=Binds 1 [2Fe-2S] cluster per subunit. Fe-S cluster delivery to the
CC       Rieske protein is mediated by components of the iron sulfur (Fe-S)
CC       cluster assembly machinery that reside in the mitochondrial matrix
CC       (including HSC20 and LYRM7) (By similarity).
CC       {ECO:0000250|UniProtKB:P47985, ECO:0000255|PROSITE-ProRule:PRU00628};
CC   -!- SUBUNIT: [Cytochrome b-c1 complex subunit Rieske, mitochondrial]:
CC       Component of the ubiquinol-cytochrome c oxidoreductase (cytochrome b-c1
CC       complex, complex III, CIII), a multisubunit enzyme composed of 11
CC       subunits. The complex is composed of 3 respiratory subunits cytochrome
CC       b, cytochrome c1 and Rieske protein UQCRFS1, 2 core protein subunits
CC       UQCRC1/QCR1 and UQCRC2/QCR2, and 6 low-molecular weight protein
CC       subunits UQCRH/QCR6, UQCRB/QCR7, UQCRQ/QCR8, UQCR10/QCR9, UQCR11/QCR10
CC       and subunit 9, the cleavage product of Rieske protein UQCRFS1 (By
CC       similarity). The complex exists as an obligatory dimer and forms
CC       supercomplexes (SCs) in the inner mitochondrial membrane with NADH-
CC       ubiquinone oxidoreductase (complex I, CI) and cytochrome c oxidase
CC       (complex IV, CIV), resulting in different assemblies (supercomplex
CC       SCI(1)III(2)IV(1) and megacomplex MCI(2)III(2)IV(2)) (PubMed:19026783).
CC       Incorporation of the Rieske protein UQCRFS1 is the penultimate step in
CC       complex III assembly (By similarity). Interacts with TTC19, which is
CC       involved in the clearance of UQCRFS1 fragments (PubMed:28673544).
CC       {ECO:0000250|UniProtKB:P13272, ECO:0000250|UniProtKB:P47985,
CC       ECO:0000269|PubMed:19026783, ECO:0000269|PubMed:28673544}.
CC   -!- SUBUNIT: [Cytochrome b-c1 complex subunit 9]: Component of the
CC       ubiquinol-cytochrome c oxidoreductase (cytochrome b-c1 complex, complex
CC       III, CIII). Subunit 9 corresponds to the mitochondrial targeting
CC       sequence (MTS) of Rieske protein UQCRFS1. It is retained after
CC       processing and incorporated inside complex III, where it remains bound
CC       to the complex and localizes between the 2 core subunits UQCRC1/QCR1
CC       and UQCRC2/QCR2. {ECO:0000250|UniProtKB:P13272}.
CC   -!- SUBCELLULAR LOCATION: Mitochondrion inner membrane
CC       {ECO:0000250|UniProtKB:Q5ZLR5}; Single-pass membrane protein
CC       {ECO:0000250|UniProtKB:Q5ZLR5}.
CC   -!- PTM: Proteolytic processing is necessary for the correct insertion of
CC       UQCRFS1 in the complex III dimer. Several fragments are generated
CC       during UQCRFS1 insertion, most probably due to the endogenous matrix-
CC       processing peptidase (MPP) activity of the 2 core protein subunits
CC       UQCRC1/QCR1 and UQCRC2/QCR2, which are homologous to the 2
CC       mitochondrial-processing peptidase (MPP) subunits beta-MPP and alpha-
CC       MPP respectively. The action of the protease is also necessary for the
CC       clearance of the UQCRFS1 fragments. {ECO:0000269|PubMed:28673544}.
CC   -!- MISCELLANEOUS: The Rieske protein is a high potential 2Fe-2S protein.
CC   -!- SIMILARITY: Belongs to the Rieske iron-sulfur protein family.
CC       {ECO:0000305}.
CC   -!- CAUTION: Several peptides are generated during UQCRFS1 insertion
CC       (PubMed:28673544). According to some authors, the identification of the
CC       transit peptide as the subunit 9, does not necessary imply that it must
CC       be considered as a structural subunit of the complex III dimer as
CC       additional fragments from UQCRFS1 are also present (PubMed:28673544).
CC       {ECO:0000269|PubMed:28673544}.
CC   ---------------------------------------------------------------------------
CC   Copyrighted by the UniProt Consortium, see https://www.uniprot.org/terms
CC   Distributed under the Creative Commons Attribution (CC BY 4.0) License
CC   ---------------------------------------------------------------------------
DR   EMBL; AK003966; BAB23097.1; -; mRNA.
DR   EMBL; AK012180; BAB28081.1; -; mRNA.
DR   EMBL; AK014470; BAB29374.1; -; mRNA.
DR   EMBL; AK153139; BAE31751.1; -; mRNA.
DR   EMBL; AK152391; BAE31179.1; -; mRNA.
DR   EMBL; AL611944; CAI24872.1; -; Genomic_DNA.
DR   EMBL; BC019934; AAH19934.1; -; mRNA.
DR   CCDS; CCDS26416.1; -.
DR   RefSeq; NP_079986.1; NM_025710.2.
DR   AlphaFoldDB; Q9CR68; -.
DR   SMR; Q9CR68; -.
DR   BioGRID; 211651; 72.
DR   ComplexPortal; CPX-563; Mitochondrial respiratory chain complex III.
DR   CORUM; Q9CR68; -.
DR   IntAct; Q9CR68; 7.
DR   MINT; Q9CR68; -.
DR   STRING; 10090.ENSMUSP00000045284; -.
DR   GlyGen; Q9CR68; 1 site, 1 O-linked glycan (1 site).
DR   iPTMnet; Q9CR68; -.
DR   PhosphoSitePlus; Q9CR68; -.
DR   SwissPalm; Q9CR68; -.
DR   EPD; Q9CR68; -.
DR   jPOST; Q9CR68; -.
DR   MaxQB; Q9CR68; -.
DR   PaxDb; Q9CR68; -.
DR   PeptideAtlas; Q9CR68; -.
DR   ProteomicsDB; 297800; -.
DR   Antibodypedia; 28808; 333 antibodies from 34 providers.
DR   DNASU; 66694; -.
DR   Ensembl; ENSMUST00000042834; ENSMUSP00000045284; ENSMUSG00000038462.
DR   GeneID; 66694; -.
DR   KEGG; mmu:66694; -.
DR   UCSC; uc007pyv.2; mouse.
DR   AGR; MGI:1913944; -.
DR   CTD; 7386; -.
DR   MGI; MGI:1913944; Uqcrfs1.
DR   VEuPathDB; HostDB:ENSMUSG00000038462; -.
DR   eggNOG; KOG1671; Eukaryota.
DR   GeneTree; ENSGT00390000001014; -.
DR   HOGENOM; CLU_055690_0_1_1; -.
DR   InParanoid; Q9CR68; -.
DR   OMA; KATVHDF; -.
DR   OrthoDB; 166674at2759; -.
DR   PhylomeDB; Q9CR68; -.
DR   TreeFam; TF105037; -.
DR   Reactome; R-MMU-611105; Respiratory electron transport.
DR   BioGRID-ORCS; 66694; 26 hits in 77 CRISPR screens.
DR   ChiTaRS; Uqcrfs1; mouse.
DR   PRO; PR:Q9CR68; -.
DR   Proteomes; UP000000589; Chromosome 13.
DR   RNAct; Q9CR68; protein.
DR   Bgee; ENSMUSG00000038462; Expressed in myocardium of ventricle and 245 other tissues.
DR   Genevisible; Q9CR68; MM.
DR   GO; GO:0005743; C:mitochondrial inner membrane; HDA:MGI.
DR   GO; GO:0031966; C:mitochondrial membrane; ISO:MGI.
DR   GO; GO:0005750; C:mitochondrial respiratory chain complex III; ISO:ComplexPortal.
DR   GO; GO:0005751; C:mitochondrial respiratory chain complex IV; ISO:MGI.
DR   GO; GO:0005739; C:mitochondrion; IDA:MGI.
DR   GO; GO:0043209; C:myelin sheath; HDA:UniProtKB.
DR   GO; GO:0051537; F:2 iron, 2 sulfur cluster binding; IEA:UniProtKB-KW.
DR   GO; GO:0046872; F:metal ion binding; IEA:UniProtKB-KW.
DR   GO; GO:0016491; F:oxidoreductase activity; IBA:GO_Central.
DR   GO; GO:0044877; F:protein-containing complex binding; ISO:MGI.
DR   GO; GO:0008121; F:ubiquinol-cytochrome-c reductase activity; IEA:UniProtKB-EC.
DR   GO; GO:0045333; P:cellular respiration; NAS:ComplexPortal.
DR   GO; GO:0006122; P:mitochondrial electron transport, ubiquinol to cytochrome c; IBA:GO_Central.
DR   GO; GO:0022904; P:respiratory electron transport chain; ISS:UniProtKB.
DR   GO; GO:0046677; P:response to antibiotic; IEA:Ensembl.
DR   GO; GO:0009725; P:response to hormone; IEA:Ensembl.
DR   GO; GO:0009410; P:response to xenobiotic stimulus; IEA:Ensembl.
DR   CDD; cd03470; Rieske_cytochrome_bc1; 1.
DR   Gene3D; 2.102.10.10; Rieske [2Fe-2S] iron-sulphur domain; 1.
DR   Gene3D; 1.20.5.270; Ubiquinol cytochrome reductase, transmembrane domain; 1.
DR   InterPro; IPR037008; bc1_Rieske_TM_sf.
DR   InterPro; IPR011070; Globular_prot_asu/bsu.
DR   InterPro; IPR017941; Rieske_2Fe-2S.
DR   InterPro; IPR036922; Rieske_2Fe-2S_sf.
DR   InterPro; IPR014349; Rieske_Fe-S_prot.
DR   InterPro; IPR005805; Rieske_Fe-S_prot_C.
DR   InterPro; IPR004192; Rieske_TM.
DR   InterPro; IPR015248; Ubiqinol_cyt_c_Rdtase_N.
DR   InterPro; IPR006317; Ubiquinol_cyt_c_Rdtase_Fe-S-su.
DR   PANTHER; PTHR10134; CYTOCHROME B-C1 COMPLEX SUBUNIT RIESKE, MITOCHONDRIAL; 1.
DR   PANTHER; PTHR10134:SF20; CYTOCHROME B-C1 COMPLEX SUBUNIT RIESKE, MITOCHONDRIAL-RELATED; 1.
DR   Pfam; PF00355; Rieske; 1.
DR   Pfam; PF09165; Ubiq-Cytc-red_N; 1.
DR   Pfam; PF02921; UCR_TM; 1.
DR   PRINTS; PR00162; RIESKE.
DR   SUPFAM; SSF50022; ISP domain; 1.
DR   SUPFAM; SSF81502; ISP transmembrane anchor; 1.
DR   SUPFAM; SSF56568; Non-globular alpha+beta subunits of globular proteins; 1.
DR   PROSITE; PS51296; RIESKE; 1.
DR   TIGRFAMs; TIGR01416; Rieske_proteo; 1.
PE   1: Evidence at protein level;
KW   2Fe-2S; Direct protein sequencing; Disulfide bond; Electron transport;
KW   Iron; Iron-sulfur; Membrane; Metal-binding; Mitochondrion;
KW   Mitochondrion inner membrane; Reference proteome; Respiratory chain;
KW   Transit peptide; Translocase; Transmembrane; Transmembrane helix;
KW   Transport.
FT   CHAIN           1..78
FT                   /note="Cytochrome b-c1 complex subunit 9"
FT                   /evidence="ECO:0000269|PubMed:28673544"
FT                   /id="PRO_0000307244"
FT   CHAIN           79..274
FT                   /note="Cytochrome b-c1 complex subunit Rieske,
FT                   mitochondrial"
FT                   /id="PRO_0000030667"
FT   TOPO_DOM        79..103
FT                   /note="Mitochondrial matrix"
FT                   /evidence="ECO:0000250|UniProtKB:P13272"
FT   TRANSMEM        104..140
FT                   /note="Helical"
FT                   /evidence="ECO:0000250|UniProtKB:P13272"
FT   TOPO_DOM        141..274
FT                   /note="Mitochondrial intermembrane"
FT                   /evidence="ECO:0000250|UniProtKB:P13272"
FT   DOMAIN          187..272
FT                   /note="Rieske"
FT                   /evidence="ECO:0000255|PROSITE-ProRule:PRU00628"
FT   BINDING         217
FT                   /ligand="[2Fe-2S] cluster"
FT                   /ligand_id="ChEBI:CHEBI:190135"
FT                   /evidence="ECO:0000250|UniProtKB:P13272"
FT   BINDING         219
FT                   /ligand="[2Fe-2S] cluster"
FT                   /ligand_id="ChEBI:CHEBI:190135"
FT                   /evidence="ECO:0000250|UniProtKB:P13272"
FT   BINDING         236
FT                   /ligand="[2Fe-2S] cluster"
FT                   /ligand_id="ChEBI:CHEBI:190135"
FT                   /evidence="ECO:0000250|UniProtKB:P13272"
FT   BINDING         239
FT                   /ligand="[2Fe-2S] cluster"
FT                   /ligand_id="ChEBI:CHEBI:190135"
FT                   /evidence="ECO:0000250|UniProtKB:P13272"
FT   BINDING         241
FT                   /ligand="[2Fe-2S] cluster"
FT                   /ligand_id="ChEBI:CHEBI:190135"
FT                   /evidence="ECO:0000250|UniProtKB:P13272"
FT   DISULFID        222..238
FT                   /evidence="ECO:0000250|UniProtKB:P13272"
SQ   SEQUENCE   274 AA;  29368 MW;  17E55DE0BBD09961 CRC64;
     MLSVAARSGP FAPVLSATSR GVAGALRPLL QGAVPAASEP PVLDVKRPFL CRESLSGQAA
     ARPLVATVGL NVPASVRFSH TDVKVPDFSD YRRAEVLDST KSSKESSEAR KGFSYLVTAT
     TTVGVAYAAK NVVSQFVSSM SASADVLAMS KIEIKLSDIP EGKNMAFKWR GKPLFVRHRT
     KKEIDQEAAV EVSQLRDPQH DLDRVKKPEW VILIGVCTHL GCVPIANAGD FGGYYCPCHG
     SHYDASGRIR KGPAPLNLEV PAYEFTSDDV VVVG
//
```

**Mascot:** http://www.matrixscience.com/


Deamidated (NQ) (+0.9840)
