# Supplementary material for: A common East-Asian ALDH2 mutation causes metabolic disorders and the therapeutic effect of ALDH2 activators
Source: Nat Commun. 2023 Sep 25;14:5971. doi: 10.1038/s41467-023-41570-6 (PMC10520061; doi:10.1038/s41467-023-41570-6)
Supplement: Supplementary file 4 — Supplementary Data 1 [file 41467_2023_41570_MOESM4_ESM.zip › Table S5c/Q9CZ13/Q9CZ13_KIO-1_H188_K447_C453.html]

Mascot Search Results: Q9CZ13
 

# MASCOT Search Results

## Protein View: Q9CZ13

### Cytochrome b-c1 complex subunit 1, mitochondrial OS=Mus musculus OX=10090 GN=Uqcrc1 PE=1 SV=2

|  |  |
| --- | --- |
| Database: | Mouse\_UniProt\_proteomes |
| Score: | 10563 |
| Monoisotopic mass (M_r_): | 53446 |
| Calculated pI: | 5.81 |

Sequence similarity is available as an NCBI BLAST search of Q9CZ13 against nr.

### Search parameters

|  |  |
| --- | --- |
| MS data file: | `D:\LCMSMS\2023 Users' data\230529-1\230529-1-KIO-1.raw` |
| Enzyme: | Trypsin/P: cuts C-term side of KR. |
| Fixed modifications: | Carbamidomethyl (C) |
| Variable modifications: | Deamidated (NQ), HNE (C), HNE (H), HNE (K), Oxidation (M) |

### Protein sequence coverage: 83%

Matched peptides shown in ***bold red***.

|  |  |  |  |  |  |
| --- | --- | --- | --- | --- | --- |
| `1` | `MAASAVCRAA` | `CSGTQVLLRT` | `RRSPALLRLP` | `ALRGTATFAQ` | `ALQSVPETQV` |
| `51` | `SILDNGLRVA` | `SEQSSHATCT` | `VGVWIDAGSR` | `YETEKNNGAG` | `YFLEHLAFKG` |
| `101` | `TKNRPGNALE` | `KEVESIGAHL` | `NAYSTREHTA` | `YLIKALSKDL` | `PKVVELLADI` |
| `151` | `VQNSSLEDSQ` | `IEKERDVILR` | `EMQENDASMQ` | `NVVFDYLHAT` | `AFQGTPLAQA` |
| `201` | `VEGPSENVRR` | `LSRTDLTDYL` | `NRHYKAPRMV` | `LAAAGGVEHQ` | `QLLDLAQKHL` |
| `251` | `SSVSRVYEED` | `AVPGLTPCRF` | `TGSEIRHRDD` | `ALPLAHVAIA` | `VEGPGWANPD` |
| `301` | `NVTLQVANAI` | `IGHYDCTYGG` | `GVHLSSPLAS` | `VAVANKLCQS` | `FQTFNISYSD` |
| `351` | `TGLLGAHFVC` | `DAMSIDDMVF` | `FLQGQWMRLC` | `TSATESEVTR` | `GKNILRNALV` |
| `401` | `SHLDGTTPVC` | `EDIGRSLLTY` | `GRRIPLAEWE` | `SRIQEVDAQM` | `LRDICSKYFY` |
| `451` | `DQCPAVAGYG` | `PIEQLPDYNR` | `IRSGMFWLRF` |  |  |

Unformatted sequence string: 480 residues (for pasting into other applications).

|  |  |  |  |
| --- | --- | --- | --- |
| Sort by | residue number | increasing mass | decreasing mass |
| Show | matched peptides only | predicted peptides also |  |

| Query | Start | – | End | Observed | Mr(expt) | Mr(calc) | ppm | M | Score | Expect | Rank | U | Peptide |
| --- | --- | --- | --- | --- | --- | --- | --- | --- | --- | --- | --- | --- | --- |
| 164345 | 34 | – | 58 | 1308.6847 | 2615.3548 | 2615.3555 | -0.27 | 0 | 124 | 2.4e-12 | 1Score **> 37** indicates **identity** Score **> 20** indicates **homology** | U | R.GTATFAQALQSVPETQVSILDNGLR.V |
| 164346 | 34 | – | 58 | 1308.6849 | 2615.3553 | 2615.3555 | -0.062 | 0 | 112 | 3.4e-11 | 1Score **> 37** indicates **identity** Score **> 19** indicates **homology** | U | R.GTATFAQALQSVPETQVSILDNGLR.V |
| 164347 | 34 | – | 58 | 872.7924 | 2615.3554 | 2615.3555 | -0.054 | 0 | 76 | 7.4e-08 | 1Score **> 37** indicates **identity** Score **> 17** indicates **homology** | U | R.GTATFAQALQSVPETQVSILDNGLR.V |
| 164348 | 34 | – | 58 | 872.7937 | 2615.3592 | 2615.3555 | 1.41 | 0 | 82 | 1.9e-08 | 1Score **> 37** indicates **identity** Score **> 18** indicates **homology** | U | R.GTATFAQALQSVPETQVSILDNGLR.V |
| 164350 | 34 | – | 58 | 1308.6869 | 2615.3592 | 2615.3555 | 1.43 | 0 | 60 | 2.4e-06 | 1Score **> 37** indicates **identity** Score **> 16** indicates **homology** | U | R.GTATFAQALQSVPETQVSILDNGLR.V |
| 164354 | 34 | – | 58 | 872.7950 | 2615.3631 | 2615.3555 | 2.91 | 0 | 48 | 2.9e-05 | 1Score **> 37** indicates **identity** Score **> 16** indicates **homology** | U | R.GTATFAQALQSVPETQVSILDNGLR.V |
| 164412 | 34 | – | 58 | 873.1210 | 2616.3412 | 2616.3395 | 0.65 | 0 | 21 | 0.011 | 1Score **> 37** indicates **identity** Score **> 14** indicates **homology** | U | R.GTATFAQALQSVPETQVSILDNGLR.V  + Deamidated (NQ) |
| 164414 | 34 | – | 58 | 1309.1789 | 2616.3432 | 2616.3395 | 1.42 | 0 | 116 | 1.2e-11 | 1Score **> 37** indicates **identity** Score **> 20** indicates **homology** | U | R.GTATFAQALQSVPETQVSILDNGLR.V  + Deamidated (NQ) |
| 164415 | 34 | – | 58 | 1309.1794 | 2616.3442 | 2616.3395 | 1.81 | 0 | 112 | 3.3e-11 | 1Score **> 37** indicates **identity** Score **> 19** indicates **homology** | U | R.GTATFAQALQSVPETQVSILDNGLR.V  + Deamidated (NQ) |
| 164417 | 34 | – | 58 | 873.1237 | 2616.3493 | 2616.3395 | 3.76 | 0 | 74 | 1.1e-07 | 1Score **> 37** indicates **identity** Score **> 17** indicates **homology** | U | R.GTATFAQALQSVPETQVSILDNGLR.V  + Deamidated (NQ) |
| 164421 | 34 | – | 58 | 873.1258 | 2616.3555 | 2616.3395 | 6.12 | 0 | 48 | 3.1e-05 | 1Score **> 37** indicates **identity** Score **> 16** indicates **homology** | U | R.GTATFAQALQSVPETQVSILDNGLR.V  + Deamidated (NQ) |
| 164495 | 34 | – | 58 | 873.4510 | 2617.3313 | 2617.3235 | 2.96 | 0 | 42 | 0.00011 | 1Score **> 37** indicates **identity** Score **> 15** indicates **homology** | U | R.GTATFAQALQSVPETQVSILDNGLR.V  + 2 Deamidated (NQ) |
| 164497 | 34 | – | 58 | 873.4529 | 2617.3369 | 2617.3235 | 5.11 | 0 | 56 | 6.2e-06 | 1Score **> 37** indicates **identity** Score **> 16** indicates **homology** | U | R.GTATFAQALQSVPETQVSILDNGLR.V  + 2 Deamidated (NQ) |
| 147912 | 59 | – | 80 | 773.3663 | 2317.0769 | 2317.0757 | 0.52 | 0 | 15 | 0.039 | 1Score **> 34** indicates **identity** Score **> 13** indicates **homology** | U | R.VASEQSSHATCTVGVWIDAGSR.Y |
| 177293 | 59 | – | 85 | 990.1327 | 2967.3763 | 2967.3669 | 3.17 | 1 | 22 | 0.0079 | 1Score **> 35** indicates **identity** Score **> 14** indicates **homology** | U | R.VASEQSSHATCTVGVWIDAGSRYETEK.N |
| 177294 | 59 | – | 85 | 742.8513 | 2967.3763 | 2967.3669 | 3.17 | 1 | 27 | 0.0031 | 1Score **> 35** indicates **identity** Score **> 14** indicates **homology** | U | R.VASEQSSHATCTVGVWIDAGSRYETEK.N |
| 196076 | 59 | – | 99 | 906.8366 | 4529.1467 | 4529.1346 | 2.67 | 2 | 15 | 0.039 | 1Score **> 35** indicates **identity** Score **> 13** indicates **homology** | U | R.VASEQSSHATCTVGVWIDAGSRYETEKNNGAGYFLEHLAFK.G |
| 196078 | 59 | – | 99 | 907.0318 | 4530.1224 | 4530.1186 | 0.83 | 2 | 22 | 0.0091 | 1Score **> 35** indicates **identity** Score **> 14** indicates **homology** | U | R.VASEQSSHATCTVGVWIDAGSRYETEKNNGAGYFLEHLAFK.G  + Deamidated (NQ) |
| 141579 | 81 | – | 99 | 558.5262 | 2230.0757 | 2230.0694 | 2.79 | 1 | 22 | 0.0091 | 1Score **> 35** indicates **identity** Score **> 14** indicates **homology** | U | R.YETEKNNGAGYFLEHLAFK.G |
| 141581 | 81 | – | 99 | 558.5276 | 2230.0812 | 2230.0694 | 5.26 | 1 | 41 | 0.00014 | 1Score **> 35** indicates **identity** Score **> 15** indicates **homology** | U | R.YETEKNNGAGYFLEHLAFK.G |
| 141585 | 81 | – | 99 | 558.5295 | 2230.0890 | 2230.0694 | 8.75 | 1 | 39 | 0.00023 | 1Score **> 35** indicates **identity** Score **> 15** indicates **homology** | U | R.YETEKNNGAGYFLEHLAFK.G |
| 141586 | 81 | – | 99 | 558.5302 | 2230.0915 | 2230.0694 | 9.91 | 1 | 27 | 0.0031 | 1Score **> 36** indicates **identity** Score **> 14** indicates **homology** | U | R.YETEKNNGAGYFLEHLAFK.G |
| 141650 | 81 | – | 99 | 558.7717 | 2231.0578 | 2231.0535 | 1.94 | 1 | 14 | 0.047 | 1Score **> 34** indicates **identity** Score **> 13** indicates **homology** | U | R.YETEKNNGAGYFLEHLAFK.G  + Deamidated (NQ) |
| 159706 | 81 | – | 102 | 630.0665 | 2516.2371 | 2516.2336 | 1.40 | 2 | 20 | 0.013 | 1Score **> 36** indicates **identity** Score **> 14** indicates **homology** | U | R.YETEKNNGAGYFLEHLAFKGTK.N |
| 159707 | 81 | – | 102 | 630.0668 | 2516.2383 | 2516.2336 | 1.88 | 2 | 34 | 0.00059 | 1Score **> 37** indicates **identity** Score **> 15** indicates **homology** | U | R.YETEKNNGAGYFLEHLAFKGTK.N |
| 159758 | 81 | – | 102 | 630.3104 | 2517.2124 | 2517.2176 | -2.05 | 2 | 49 | 2.6e-05 | 1Score **> 36** indicates **identity** Score **> 16** indicates **homology** | U | R.YETEKNNGAGYFLEHLAFKGTK.N  + Deamidated (NQ) |
| 159760 | 81 | – | 102 | 630.3117 | 2517.2175 | 2517.2176 | -0.017 | 2 | 16 | 0.032 | 1Score **> 36** indicates **identity** Score **> 13** indicates **homology** | U | R.YETEKNNGAGYFLEHLAFKGTK.N  + Deamidated (NQ) |
| 72564 | 86 | – | 99 | 527.6002 | 1579.7787 | 1579.7783 | 0.26 | 0 | 47 | 3.6e-05 | 1Score **> 34** indicates **identity** Score **> 15** indicates **homology** | U | K.NNGAGYFLEHLAFK.G |
| 72567 | 86 | – | 99 | 790.8972 | 1579.7798 | 1579.7783 | 0.93 | 0 | 52 | 1.5e-05 | 1Score **> 34** indicates **identity** Score **> 16** indicates **homology** | U | K.NNGAGYFLEHLAFK.G |
| 72569 | 86 | – | 99 | 527.6010 | 1579.7811 | 1579.7783 | 1.78 | 0 | 15 | 0.043 | 1Score **> 34** indicates **identity** Score **> 13** indicates **homology** | U | K.NNGAGYFLEHLAFK.G |
| 72688 | 86 | – | 99 | 527.9268 | 1580.7586 | 1580.7623 | -2.38 | 0 | 42 | 0.00013 | 1Score **> 33** indicates **identity** Score **> 15** indicates **homology** | U | K.NNGAGYFLEHLAFK.G  + Deamidated (NQ) |
| 72701 | 86 | – | 99 | 527.9285 | 1580.7637 | 1580.7623 | 0.88 | 0 | 40 | 0.00017 | 1Score **> 34** indicates **identity** Score **> 15** indicates **homology** | U | K.NNGAGYFLEHLAFK.G  + Deamidated (NQ) |
| 107432 | 86 | – | 102 | 622.9866 | 1865.9380 | 1865.9424 | -2.34 | 1 | 29 | 0.0021 | 1Score **> 35** indicates **identity** Score **> 14** indicates **homology** | U | K.NNGAGYFLEHLAFKGTK.N |
| 107436 | 86 | – | 102 | 622.9898 | 1865.9477 | 1865.9424 | 2.84 | 1 | 36 | 0.00047 | 1Score **> 36** indicates **identity** Score **> 15** indicates **homology** | U | K.NNGAGYFLEHLAFKGTK.N |
| 107509 | 86 | – | 102 | 623.3183 | 1866.9331 | 1866.9264 | 3.56 | 1 | 20 | 0.014 | 1Score **> 36** indicates **identity** Score **> 14** indicates **homology** | U | K.NNGAGYFLEHLAFKGTK.N  + Deamidated (NQ) |
| 164856 | 103 | – | 126 | 526.0697 | 2625.3121 | 2625.3259 | -5.23 | 2 | 20 | 0.013 | 1Score **> 37** indicates **identity** Score **> 14** indicates **homology** | U | K.NRPGNALEKEVESIGAHLNAYSTR.E |
| 164858 | 103 | – | 126 | 657.3358 | 2625.3140 | 2625.3259 | -4.51 | 2 | 18 | 0.022 | 1Score **> 37** indicates **identity** Score **> 14** indicates **homology** | U | K.NRPGNALEKEVESIGAHLNAYSTR.E |
| 164861 | 103 | – | 126 | 657.3363 | 2625.3162 | 2625.3259 | -3.70 | 2 | 26 | 0.0039 | 1Score **> 37** indicates **identity** Score **> 14** indicates **homology** | U | K.NRPGNALEKEVESIGAHLNAYSTR.E |
| 164864 | 103 | – | 126 | 657.3374 | 2625.3207 | 2625.3259 | -1.98 | 2 | 39 | 0.00022 | 1Score **> 37** indicates **identity** Score **> 15** indicates **homology** | U | K.NRPGNALEKEVESIGAHLNAYSTR.E |
| 164865 | 103 | – | 126 | 657.3377 | 2625.3217 | 2625.3259 | -1.58 | 2 | 21 | 0.01 | 1Score **> 37** indicates **identity** Score **> 14** indicates **homology** | U | K.NRPGNALEKEVESIGAHLNAYSTR.E |
| 164867 | 103 | – | 126 | 876.1161 | 2625.3264 | 2625.3259 | 0.19 | 2 | 68 | 3.9e-07 | 1Score **> 37** indicates **identity** Score **> 17** indicates **homology** | U | K.NRPGNALEKEVESIGAHLNAYSTR.E |
| 164868 | 103 | – | 126 | 657.3389 | 2625.3265 | 2625.3259 | 0.24 | 2 | 59 | 2.9e-06 | 1Score **> 37** indicates **identity** Score **> 16** indicates **homology** | U | K.NRPGNALEKEVESIGAHLNAYSTR.E |
| 164869 | 103 | – | 126 | 657.3389 | 2625.3265 | 2625.3259 | 0.25 | 2 | 37 | 0.00035 | 1Score **> 37** indicates **identity** Score **> 15** indicates **homology** | U | K.NRPGNALEKEVESIGAHLNAYSTR.E |
| 164870 | 103 | – | 126 | 526.0726 | 2625.3268 | 2625.3259 | 0.34 | 2 | 29 | 0.0019 | 1Score **> 37** indicates **identity** Score **> 14** indicates **homology** | U | K.NRPGNALEKEVESIGAHLNAYSTR.E |
| 164871 | 103 | – | 126 | 657.3392 | 2625.3276 | 2625.3259 | 0.64 | 2 | 78 | 5.1e-08 | 1Score **> 37** indicates **identity** Score **> 17** indicates **homology** | U | K.NRPGNALEKEVESIGAHLNAYSTR.E |
| 164872 | 103 | – | 126 | 526.0728 | 2625.3277 | 2625.3259 | 0.68 | 2 | 55 | 6.7e-06 | 1Score **> 37** indicates **identity** Score **> 16** indicates **homology** | U | K.NRPGNALEKEVESIGAHLNAYSTR.E |
| 164874 | 103 | – | 126 | 526.0729 | 2625.3281 | 2625.3259 | 0.86 | 2 | 57 | 4.7e-06 | 1Score **> 37** indicates **identity** Score **> 16** indicates **homology** | U | K.NRPGNALEKEVESIGAHLNAYSTR.E |
| 164875 | 103 | – | 126 | 876.1167 | 2625.3282 | 2625.3259 | 0.89 | 2 | 76 | 6.7e-08 | 1Score **> 37** indicates **identity** Score **> 17** indicates **homology** | U | K.NRPGNALEKEVESIGAHLNAYSTR.E |
| 164876 | 103 | – | 126 | 657.3393 | 2625.3283 | 2625.3259 | 0.91 | 2 | 34 | 0.00062 | 1Score **> 37** indicates **identity** Score **> 15** indicates **homology** | U | K.NRPGNALEKEVESIGAHLNAYSTR.E |
| 164877 | 103 | – | 126 | 657.3394 | 2625.3284 | 2625.3259 | 0.98 | 2 | 60 | 2.2e-06 | 1Score **> 37** indicates **identity** Score **> 16** indicates **homology** | U | K.NRPGNALEKEVESIGAHLNAYSTR.E |
| 164878 | 103 | – | 126 | 526.0731 | 2625.3290 | 2625.3259 | 1.18 | 2 | 53 | 9.8e-06 | 1Score **> 37** indicates **identity** Score **> 16** indicates **homology** | U | K.NRPGNALEKEVESIGAHLNAYSTR.E |
| 164879 | 103 | – | 126 | 657.3396 | 2625.3291 | 2625.3259 | 1.23 | 2 | 59 | 2.9e-06 | 1Score **> 37** indicates **identity** Score **> 16** indicates **homology** | U | K.NRPGNALEKEVESIGAHLNAYSTR.E |
| 164881 | 103 | – | 126 | 657.3396 | 2625.3292 | 2625.3259 | 1.28 | 2 | 61 | 2e-06 | 1Score **> 37** indicates **identity** Score **> 16** indicates **homology** | U | K.NRPGNALEKEVESIGAHLNAYSTR.E |
| 164883 | 103 | – | 126 | 526.0732 | 2625.3294 | 2625.3259 | 1.35 | 2 | 15 | 0.037 | 1Score **> 37** indicates **identity** Score **> 13** indicates **homology** | U | K.NRPGNALEKEVESIGAHLNAYSTR.E |
| 164884 | 103 | – | 126 | 526.0732 | 2625.3295 | 2625.3259 | 1.37 | 2 | 58 | 3.6e-06 | 1Score **> 37** indicates **identity** Score **> 16** indicates **homology** | U | K.NRPGNALEKEVESIGAHLNAYSTR.E |
| 164885 | 103 | – | 126 | 526.0732 | 2625.3296 | 2625.3259 | 1.43 | 2 | 60 | 2.3e-06 | 1Score **> 37** indicates **identity** Score **> 16** indicates **homology** | U | K.NRPGNALEKEVESIGAHLNAYSTR.E |
| 164886 | 103 | – | 126 | 657.3397 | 2625.3297 | 2625.3259 | 1.45 | 2 | 49 | 2.3e-05 | 1Score **> 37** indicates **identity** Score **> 16** indicates **homology** | U | K.NRPGNALEKEVESIGAHLNAYSTR.E |
| 164887 | 103 | – | 126 | 526.0732 | 2625.3298 | 2625.3259 | 1.51 | 2 | 17 | 0.026 | 1Score **> 37** indicates **identity** Score **> 14** indicates **homology** | U | K.NRPGNALEKEVESIGAHLNAYSTR.E |
| 164888 | 103 | – | 126 | 657.3397 | 2625.3299 | 2625.3259 | 1.53 | 2 | 57 | 4.3e-06 | 1Score **> 37** indicates **identity** Score **> 16** indicates **homology** | U | K.NRPGNALEKEVESIGAHLNAYSTR.E |
| 164889 | 103 | – | 126 | 526.0733 | 2625.3302 | 2625.3259 | 1.64 | 2 | 62 | 1.4e-06 | 1Score **> 37** indicates **identity** Score **> 16** indicates **homology** | U | K.NRPGNALEKEVESIGAHLNAYSTR.E |
| 164890 | 103 | – | 126 | 876.1174 | 2625.3303 | 2625.3259 | 1.68 | 2 | 72 | 1.9e-07 | 1Score **> 37** indicates **identity** Score **> 17** indicates **homology** | U | K.NRPGNALEKEVESIGAHLNAYSTR.E |
| 164891 | 103 | – | 126 | 876.1174 | 2625.3303 | 2625.3259 | 1.70 | 2 | 26 | 0.0033 | 1Score **> 37** indicates **identity** Score **> 14** indicates **homology** | U | K.NRPGNALEKEVESIGAHLNAYSTR.E |
| 164892 | 103 | – | 126 | 526.0734 | 2625.3304 | 2625.3259 | 1.72 | 2 | 44 | 7e-05 | 1Score **> 37** indicates **identity** Score **> 15** indicates **homology** | U | K.NRPGNALEKEVESIGAHLNAYSTR.E |
| 164893 | 103 | – | 126 | 657.3399 | 2625.3304 | 2625.3259 | 1.73 | 2 | 73 | 1.6e-07 | 1Score **> 37** indicates **identity** Score **> 18** indicates **homology** | U | K.NRPGNALEKEVESIGAHLNAYSTR.E |
| 164895 | 103 | – | 126 | 526.0734 | 2625.3307 | 2625.3259 | 1.84 | 2 | 51 | 1.6e-05 | 1Score **> 37** indicates **identity** Score **> 16** indicates **homology** | U | K.NRPGNALEKEVESIGAHLNAYSTR.E |
| 164896 | 103 | – | 126 | 876.1175 | 2625.3307 | 2625.3259 | 1.84 | 2 | 72 | 6.3e-07 | 1Score **> 37** indicates **identity** Score **> 22** indicates **homology** | U | K.NRPGNALEKEVESIGAHLNAYSTR.E |
| 164897 | 103 | – | 126 | 526.0735 | 2625.3309 | 2625.3259 | 1.92 | 2 | 40 | 0.00018 | 1Score **> 37** indicates **identity** Score **> 15** indicates **homology** | U | K.NRPGNALEKEVESIGAHLNAYSTR.E |
| 164898 | 103 | – | 126 | 657.3400 | 2625.3310 | 2625.3259 | 1.96 | 2 | 50 | 2e-05 | 1Score **> 37** indicates **identity** Score **> 16** indicates **homology** | U | K.NRPGNALEKEVESIGAHLNAYSTR.E |
| 164899 | 103 | – | 126 | 526.0736 | 2625.3314 | 2625.3259 | 2.10 | 2 | 33 | 0.00089 | 1Score **> 37** indicates **identity** Score **> 15** indicates **homology** | U | K.NRPGNALEKEVESIGAHLNAYSTR.E |
| 164900 | 103 | – | 126 | 657.3401 | 2625.3314 | 2625.3259 | 2.12 | 2 | 70 | 2.5e-07 | 1Score **> 37** indicates **identity** Score **> 17** indicates **homology** | U | K.NRPGNALEKEVESIGAHLNAYSTR.E |
| 164901 | 103 | – | 126 | 876.1178 | 2625.3316 | 2625.3259 | 2.19 | 2 | 77 | 6.7e-08 | 1Score **> 37** indicates **identity** Score **> 17** indicates **homology** | U | K.NRPGNALEKEVESIGAHLNAYSTR.E |
| 164902 | 103 | – | 126 | 876.1178 | 2625.3317 | 2625.3259 | 2.23 | 2 | 76 | 6.8e-08 | 1Score **> 37** indicates **identity** Score **> 17** indicates **homology** | U | K.NRPGNALEKEVESIGAHLNAYSTR.E |
| 164903 | 103 | – | 126 | 657.3405 | 2625.3329 | 2625.3259 | 2.66 | 2 | 60 | 5.5e-06 | 1Score **> 37** indicates **identity** Score **> 20** indicates **homology** | U | K.NRPGNALEKEVESIGAHLNAYSTR.E |
| 164904 | 103 | – | 126 | 526.0739 | 2625.3329 | 2625.3259 | 2.69 | 2 | 31 | 0.0013 | 1Score **> 37** indicates **identity** Score **> 14** indicates **homology** | U | K.NRPGNALEKEVESIGAHLNAYSTR.E |
| 164905 | 103 | – | 126 | 526.0739 | 2625.3332 | 2625.3259 | 2.77 | 2 | 32 | 0.00091 | 1Score **> 37** indicates **identity** Score **> 15** indicates **homology** | U | K.NRPGNALEKEVESIGAHLNAYSTR.E |
| 164906 | 103 | – | 126 | 876.1185 | 2625.3337 | 2625.3259 | 3.00 | 2 | 56 | 1.3e-05 | 1Score **> 37** indicates **identity** Score **> 20** indicates **homology** | U | K.NRPGNALEKEVESIGAHLNAYSTR.E |
| 164907 | 103 | – | 126 | 657.3409 | 2625.3344 | 2625.3259 | 3.25 | 2 | 31 | 0.0014 | 1Score **> 37** indicates **identity** Score **> 14** indicates **homology** | U | K.NRPGNALEKEVESIGAHLNAYSTR.E |
| 164908 | 103 | – | 126 | 657.3411 | 2625.3352 | 2625.3259 | 3.55 | 2 | 38 | 0.00026 | 1Score **> 37** indicates **identity** Score **> 15** indicates **homology** | U | K.NRPGNALEKEVESIGAHLNAYSTR.E |
| 164910 | 103 | – | 126 | 657.3424 | 2625.3403 | 2625.3259 | 5.52 | 2 | 48 | 3e-05 | 1Score **> 37** indicates **identity** Score **> 16** indicates **homology** | U | K.NRPGNALEKEVESIGAHLNAYSTR.E |
| 164938 | 103 | – | 126 | 657.5845 | 2626.3089 | 2626.3099 | -0.36 | 2 | 53 | 1.2e-05 | 1Score **> 37** indicates **identity** Score **> 16** indicates **homology** | U | K.NRPGNALEKEVESIGAHLNAYSTR.E  + Deamidated (NQ) |
| 164939 | 103 | – | 126 | 526.2692 | 2626.3097 | 2626.3099 | -0.053 | 2 | 33 | 0.00089 | 1Score **> 37** indicates **identity** Score **> 15** indicates **homology** | U | K.NRPGNALEKEVESIGAHLNAYSTR.E  + Deamidated (NQ) |
| 164941 | 103 | – | 126 | 526.2695 | 2626.3109 | 2626.3099 | 0.39 | 2 | 33 | 0.00076 | 1Score **> 37** indicates **identity** Score **> 15** indicates **homology** | U | K.NRPGNALEKEVESIGAHLNAYSTR.E  + Deamidated (NQ) |
| 164943 | 103 | – | 126 | 657.5853 | 2626.3123 | 2626.3099 | 0.91 | 2 | 35 | 0.00053 | 1Score **> 37** indicates **identity** Score **> 15** indicates **homology** | U | K.NRPGNALEKEVESIGAHLNAYSTR.E  + Deamidated (NQ) |
| 164944 | 103 | – | 126 | 526.2698 | 2626.3126 | 2626.3099 | 1.05 | 2 | 43 | 9.1e-05 | 1Score **> 37** indicates **identity** Score **> 15** indicates **homology** | U | K.NRPGNALEKEVESIGAHLNAYSTR.E  + Deamidated (NQ) |
| 164946 | 103 | – | 126 | 526.2700 | 2626.3136 | 2626.3099 | 1.43 | 2 | 36 | 0.00042 | 1Score **> 37** indicates **identity** Score **> 15** indicates **homology** | U | K.NRPGNALEKEVESIGAHLNAYSTR.E  + Deamidated (NQ) |
| 164949 | 103 | – | 126 | 876.4476 | 2626.3209 | 2626.3099 | 4.19 | 2 | 52 | 1.2e-05 | 1Score **> 37** indicates **identity** Score **> 16** indicates **homology** | U | K.NRPGNALEKEVESIGAHLNAYSTR.E  + Deamidated (NQ) |
| 164950 | 103 | – | 126 | 657.5881 | 2626.3234 | 2626.3099 | 5.16 | 2 | 47 | 4.4e-05 | 1Score **> 37** indicates **identity** Score **> 15** indicates **homology** | U | K.NRPGNALEKEVESIGAHLNAYSTR.E  + Deamidated (NQ) |
| 164951 | 103 | – | 126 | 876.4495 | 2626.3267 | 2626.3099 | 6.41 | 2 | 18 | 0.023 | 1Score **> 37** indicates **identity** Score **> 14** indicates **homology** | U | K.NRPGNALEKEVESIGAHLNAYSTR.E  + Deamidated (NQ) |
| 164953 | 103 | – | 126 | 657.5895 | 2626.3288 | 2626.3099 | 7.21 | 2 | 16 | 0.028 | 1Score **> 37** indicates **identity** Score **> 14** indicates **homology** | U | K.NRPGNALEKEVESIGAHLNAYSTR.E  + Deamidated (NQ) |
| 164954 | 103 | – | 126 | 657.5900 | 2626.3309 | 2626.3099 | 8.00 | 2 | 25 | 0.0048 | 1Score **> 37** indicates **identity** Score **> 14** indicates **homology** | U | K.NRPGNALEKEVESIGAHLNAYSTR.E  + Deamidated (NQ) |
| 164955 | 103 | – | 126 | 657.5903 | 2626.3320 | 2626.3099 | 8.42 | 2 | 29 | 0.0018 | 1Score **> 37** indicates **identity** Score **> 14** indicates **homology** | U | K.NRPGNALEKEVESIGAHLNAYSTR.E  + Deamidated (NQ) |
| 164987 | 103 | – | 126 | 657.8334 | 2627.3044 | 2627.2939 | 4.00 | 2 | 28 | 0.0023 | 1Score **> 37** indicates **identity** Score **> 14** indicates **homology** | U | K.NRPGNALEKEVESIGAHLNAYSTR.E  + 2 Deamidated (NQ) |
| 164989 | 103 | – | 126 | 657.8348 | 2627.3100 | 2627.2939 | 6.14 | 2 | 22 | 0.0091 | 1Score **> 37** indicates **identity** Score **> 14** indicates **homology** | U | K.NRPGNALEKEVESIGAHLNAYSTR.E  + 2 Deamidated (NQ) |
| 164991 | 103 | – | 126 | 657.8356 | 2627.3134 | 2627.2939 | 7.44 | 2 | 47 | 4.1e-05 | 1Score **> 37** indicates **identity** Score **> 15** indicates **homology** | U | K.NRPGNALEKEVESIGAHLNAYSTR.E  + 2 Deamidated (NQ) |
| 81785 | 112 | – | 126 | 823.9100 | 1645.8055 | 1645.8060 | -0.31 | 0 | 72 | 1.2e-06 | 1Score **> 34** indicates **identity** Score **> 25** indicates **homology** | U | K.EVESIGAHLNAYSTR.E |
| 81786 | 112 | – | 126 | 823.9101 | 1645.8057 | 1645.8060 | -0.18 | 0 | 77 | 4.1e-07 | 1Score **> 34** indicates **identity** Score **> 26** indicates **homology** | U | K.EVESIGAHLNAYSTR.E |
| 81788 | 112 | – | 126 | 823.9109 | 1645.8073 | 1645.8060 | 0.82 | 0 | 72 | 6.3e-07 | 1Score **> 34** indicates **identity** Score **> 23** indicates **homology** | U | K.EVESIGAHLNAYSTR.E |
| 81789 | 112 | – | 126 | 549.6098 | 1645.8077 | 1645.8060 | 1.06 | 0 | 45 | 6.3e-05 | 1Score **> 34** indicates **identity** Score **> 15** indicates **homology** | U | K.EVESIGAHLNAYSTR.E |
| 81790 | 112 | – | 126 | 549.6099 | 1645.8079 | 1645.8060 | 1.15 | 0 | 42 | 0.00011 | 1Score **> 34** indicates **identity** Score **> 15** indicates **homology** | U | K.EVESIGAHLNAYSTR.E |
| 81791 | 112 | – | 126 | 549.6102 | 1645.8088 | 1645.8060 | 1.71 | 0 | 25 | 0.005 | 1Score **> 34** indicates **identity** Score **> 14** indicates **homology** | U | K.EVESIGAHLNAYSTR.E |
| 11636 | 127 | – | 134 | 487.7687 | 973.5228 | 973.5233 | -0.48 | 0 | 22 | 0.013 | 1Score **> 31** indicates **identity** Score **> 15** indicates **homology** | U | R.EHTAYLIK.A |
| 11637 | 127 | – | 134 | 487.7687 | 973.5229 | 973.5233 | -0.41 | 0 | 20 | 0.028 | 1Score **> 31** indicates **identity** Score **> 17** indicates **homology** | U | R.EHTAYLIK.A |
| 11639 | 127 | – | 134 | 487.7692 | 973.5238 | 973.5233 | 0.54 | 0 | 42 | 0.00044 | 1Score **> 33** indicates **identity** Score **> 21** indicates **homology** | U | R.EHTAYLIK.A |
| 11640 | 127 | – | 134 | 487.7693 | 973.5240 | 973.5233 | 0.78 | 0 | 43 | 0.0013 | 1Score **> 33** indicates **identity** Score **> 27** indicates **homology** | U | R.EHTAYLIK.A |
| 11642 | 127 | – | 134 | 487.7696 | 973.5247 | 973.5233 | 1.52 | 0 | 25 | 0.027 | 1Score **> 33** indicates **identity** Score **> 22** indicates **homology** | U | R.EHTAYLIK.A |
| 6426 | 135 | – | 142 | 436.2663 | 870.5181 | 870.5174 | 0.80 | 1 | 44 | 0.00023 | 1Score **> 27** indicates **identity** Score **> 20** indicates **homology** | U | K.ALSKDLPK.V |
| 6428 | 135 | – | 142 | 436.2667 | 870.5189 | 870.5174 | 1.66 | 1 | 42 | 0.0013 | 1Score **> 27** indicates **identity** Score **> 26** indicates **homology** | U | K.ALSKDLPK.V |
| 164210 | 143 | – | 165 | 872.1230 | 2613.3473 | 2613.3497 | -0.93 | 1 | 48 | 3.5e-05 | 1Score **> 37** indicates **identity** Score **> 15** indicates **homology** | U | K.VVELLADIVQNSSLEDSQIEKER.D |
| 164214 | 143 | – | 165 | 872.1249 | 2613.3527 | 2613.3497 | 1.16 | 1 | 69 | 3.3e-07 | 1Score **> 37** indicates **identity** Score **> 17** indicates **homology** | U | K.VVELLADIVQNSSLEDSQIEKER.D |
| 164215 | 143 | – | 165 | 872.1249 | 2613.3528 | 2613.3497 | 1.17 | 1 | 24 | 0.0061 | 1Score **> 37** indicates **identity** Score **> 14** indicates **homology** | U | K.VVELLADIVQNSSLEDSQIEKER.D |
| 164217 | 143 | – | 165 | 654.3457 | 2613.3537 | 2613.3497 | 1.54 | 1 | 20 | 0.015 | 1Score **> 37** indicates **identity** Score **> 14** indicates **homology** | U | K.VVELLADIVQNSSLEDSQIEKER.D |
| 164224 | 143 | – | 165 | 872.1262 | 2613.3566 | 2613.3497 | 2.66 | 1 | 45 | 6.5e-05 | 1Score **> 37** indicates **identity** Score **> 15** indicates **homology** | U | K.VVELLADIVQNSSLEDSQIEKER.D |
| 164229 | 143 | – | 165 | 872.1273 | 2613.3601 | 2613.3497 | 3.96 | 1 | 42 | 0.00011 | 1Score **> 37** indicates **identity** Score **> 15** indicates **homology** | U | K.VVELLADIVQNSSLEDSQIEKER.D |
| 164230 | 143 | – | 165 | 872.1274 | 2613.3604 | 2613.3497 | 4.09 | 1 | 73 | 1.4e-07 | 1Score **> 37** indicates **identity** Score **> 17** indicates **homology** | U | K.VVELLADIVQNSSLEDSQIEKER.D |
| 164279 | 143 | – | 165 | 872.4579 | 2614.3520 | 2614.3337 | 6.98 | 1 | 43 | 9.4e-05 | 1Score **> 37** indicates **identity** Score **> 15** indicates **homology** | U | K.VVELLADIVQNSSLEDSQIEKER.D  + Deamidated (NQ) |
| 164286 | 143 | – | 165 | 872.4597 | 2614.3574 | 2614.3337 | 9.06 | 1 | 29 | 0.0017 | 1Score **> 37** indicates **identity** Score **> 14** indicates **homology** | U | K.VVELLADIVQNSSLEDSQIEKER.D  + Deamidated (NQ) |
| 164288 | 143 | – | 165 | 872.4606 | 2614.3599 | 2614.3337 | 10.00 | 1 | 32 | 0.001 | 1Score **> 37** indicates **identity** Score **> 15** indicates **homology** | U | K.VVELLADIVQNSSLEDSQIEKER.D  + Deamidated (NQ) |
| 183487 | 143 | – | 170 | 803.4349 | 3209.7106 | 3209.7143 | -1.14 | 2 | 14 | 0.045 | 1Score **> 36** indicates **identity** Score **> 13** indicates **homology** | U | K.VVELLADIVQNSSLEDSQIEKERDVILR.E |
| 183488 | 143 | – | 170 | 803.4356 | 3209.7133 | 3209.7143 | -0.32 | 2 | 42 | 0.00012 | 1Score **> 36** indicates **identity** Score **> 15** indicates **homology** | U | K.VVELLADIVQNSSLEDSQIEKERDVILR.E |
| 183489 | 143 | – | 170 | 642.9510 | 3209.7185 | 3209.7143 | 1.30 | 2 | 15 | 0.038 | 1Score **> 36** indicates **identity** Score **> 13** indicates **homology** | U | K.VVELLADIVQNSSLEDSQIEKERDVILR.E |
| 183492 | 143 | – | 170 | 1070.9153 | 3209.7241 | 3209.7143 | 3.06 | 2 | 46 | 4.7e-05 | 1Score **> 36** indicates **identity** Score **> 15** indicates **homology** | U | K.VVELLADIVQNSSLEDSQIEKERDVILR.E |
| 183494 | 143 | – | 170 | 1070.9155 | 3209.7248 | 3209.7143 | 3.27 | 2 | 22 | 0.0082 | 1Score **> 36** indicates **identity** Score **> 14** indicates **homology** | U | K.VVELLADIVQNSSLEDSQIEKERDVILR.E |
| 195741 | 171 | – | 209 | 1489.3556 | 4465.0450 | 4465.0842 | -8.78 | 0 | 35 | 0.00047 | 1Score **> 34** indicates **identity** Score **> 15** indicates **homology** | U | R.EMQENDASMQNVVFDYLHATAFQGTPLAQAVEGPSENVR.R  + HNE (H); Oxidation (M) |
| 195744 | 171 | – | 209 | 1490.3614 | 4468.0624 | 4468.0363 | 5.85 | 0 | 29 | 0.0021 | 1Score **> 34** indicates **identity** Score **> 14** indicates **homology** | U | R.EMQENDASMQNVVFDYLHATAFQGTPLAQAVEGPSENVR.R  + 3 Deamidated (NQ); HNE (H); Oxidation (M) |
| 57975 | 211 | – | 222 | 489.5918 | 1465.7537 | 1465.7525 | 0.83 | 1 | 26 | 0.0034 | 1Score **> 34** indicates **identity** Score **> 14** indicates **homology** | U | R.LSRTDLTDYLNR.H |
| 57976 | 211 | – | 222 | 733.8842 | 1465.7538 | 1465.7525 | 0.86 | 1 | 42 | 0.00083 | 1Score **> 34** indicates **identity** Score **> 23** indicates **homology** | U | R.LSRTDLTDYLNR.H |
| 57977 | 211 | – | 222 | 489.5919 | 1465.7538 | 1465.7525 | 0.91 | 1 | 35 | 0.0012 | 1Score **> 34** indicates **identity** Score **> 18** indicates **homology** | U | R.LSRTDLTDYLNR.H |
| 57978 | 211 | – | 222 | 489.5921 | 1465.7544 | 1465.7525 | 1.29 | 1 | 33 | 0.0014 | 1Score **> 34** indicates **identity** Score **> 17** indicates **homology** | U | R.LSRTDLTDYLNR.H |
| 57979 | 211 | – | 222 | 489.5921 | 1465.7546 | 1465.7525 | 1.42 | 1 | 33 | 0.0016 | 1Score **> 34** indicates **identity** Score **> 18** indicates **homology** | U | R.LSRTDLTDYLNR.H |
| 57980 | 211 | – | 222 | 733.8849 | 1465.7552 | 1465.7525 | 1.82 | 1 | 41 | 0.0011 | 1Score **> 34** indicates **identity** Score **> 24** indicates **homology** | U | R.LSRTDLTDYLNR.H |
| 21578 | 214 | – | 222 | 555.7742 | 1109.5338 | 1109.5353 | -1.37 | 0 | 30 | 0.0067 | 1Score **> 30** indicates **identity** Score **> 21** indicates **homology** | U | R.TDLTDYLNR.H |
| 21580 | 214 | – | 222 | 555.7748 | 1109.5351 | 1109.5353 | -0.15 | 0 | 49 | 0.00027 | 1Score **> 30** indicates **identity** Score **> 26** indicates **homology** | U | R.TDLTDYLNR.H |
| 21582 | 214 | – | 222 | 555.7750 | 1109.5355 | 1109.5353 | 0.18 | 0 | 31 | 0.0064 | 1Score **> 31** indicates **identity** Score **> 21** indicates **homology** | U | R.TDLTDYLNR.H |
| 21587 | 214 | – | 222 | 555.7755 | 1109.5364 | 1109.5353 | 0.96 | 0 | 62 | 2.4e-05 | 1Score **> 31** indicates **identity** Score **> 28** indicates **homology** | U | R.TDLTDYLNR.H |
| 21588 | 214 | – | 222 | 555.7756 | 1109.5367 | 1109.5353 | 1.23 | 0 | 68 | 7.7e-06 | 1Score **> 31** indicates **identity** Score **> 30** indicates **homology** | U | R.TDLTDYLNR.H |
| 21589 | 214 | – | 222 | 555.7756 | 1109.5367 | 1109.5353 | 1.27 | 0 | 35 | 0.006 | 1Score **> 31** indicates **identity** Score **> 25** indicates **homology** | U | R.TDLTDYLNR.H |
| 21590 | 214 | – | 222 | 555.7756 | 1109.5367 | 1109.5353 | 1.31 | 0 | 69 | 8.3e-06 | 1Score **> 31** indicates **identity** Score **> 30** indicates **homology** | U | R.TDLTDYLNR.H |
| 21591 | 214 | – | 222 | 555.7757 | 1109.5368 | 1109.5353 | 1.37 | 0 | 66 | 8.7e-06 | 1Score **> 31** indicates **identity** Score **> 28** indicates **homology** | U | R.TDLTDYLNR.H |
| 21593 | 214 | – | 222 | 555.7757 | 1109.5368 | 1109.5353 | 1.38 | 0 | 48 | 0.00027 | 1Score **> 31** indicates **identity** Score **> 25** indicates **homology** | U | R.TDLTDYLNR.H |
| 66840 | 214 | – | 225 | 513.5920 | 1537.7541 | 1537.7525 | 1.05 | 1 | 29 | 0.002 | 1Score **> 33** indicates **identity** Score **> 14** indicates **homology** | U | R.TDLTDYLNRHYK.A |
| 66841 | 214 | – | 225 | 385.4459 | 1537.7545 | 1537.7525 | 1.32 | 1 | 14 | 0.044 | 1Score **> 34** indicates **identity** Score **> 13** indicates **homology** | U | R.TDLTDYLNRHYK.A |
| 66842 | 214 | – | 225 | 385.4463 | 1537.7562 | 1537.7525 | 2.42 | 1 | 23 | 0.0075 | 1Score **> 34** indicates **identity** Score **> 14** indicates **homology** | U | R.TDLTDYLNRHYK.A |
| 66843 | 214 | – | 225 | 385.4464 | 1537.7564 | 1537.7525 | 2.55 | 1 | 22 | 0.0095 | 1Score **> 34** indicates **identity** Score **> 14** indicates **homology** | U | R.TDLTDYLNRHYK.A |
| 66844 | 214 | – | 225 | 513.5929 | 1537.7568 | 1537.7525 | 2.82 | 1 | 41 | 0.00014 | 1Score **> 33** indicates **identity** Score **> 15** indicates **homology** | U | R.TDLTDYLNRHYK.A |
| 66845 | 214 | – | 225 | 513.5931 | 1537.7575 | 1537.7525 | 3.25 | 1 | 43 | 9.7e-05 | 1Score **> 33** indicates **identity** Score **> 15** indicates **homology** | U | R.TDLTDYLNRHYK.A |
| 66846 | 214 | – | 225 | 769.8867 | 1537.7589 | 1537.7525 | 4.18 | 1 | 20 | 0.013 | 1Score **> 34** indicates **identity** Score **> 14** indicates **homology** | U | R.TDLTDYLNRHYK.A |
| 106967 | 214 | – | 228 | 466.4933 | 1861.9439 | 1861.9435 | 0.24 | 2 | 21 | 0.011 | 1Score **> 36** indicates **identity** Score **> 14** indicates **homology** | U | R.TDLTDYLNRHYKAPR.M |
| 106968 | 214 | – | 228 | 466.4934 | 1861.9445 | 1861.9435 | 0.55 | 2 | 28 | 0.0024 | 1Score **> 36** indicates **identity** Score **> 14** indicates **homology** | U | R.TDLTDYLNRHYKAPR.M |
| 106970 | 214 | – | 228 | 466.4940 | 1861.9471 | 1861.9435 | 1.95 | 2 | 27 | 0.0026 | 1Score **> 36** indicates **identity** Score **> 14** indicates **homology** | U | R.TDLTDYLNRHYKAPR.M |
| 106971 | 214 | – | 228 | 621.6566 | 1861.9479 | 1861.9435 | 2.40 | 2 | 15 | 0.037 | 1Score **> 36** indicates **identity** Score **> 13** indicates **homology** | U | R.TDLTDYLNRHYKAPR.M |
| 129119 | 229 | – | 248 | 698.0472 | 2091.1197 | 2091.1146 | 2.43 | 0 | 17 | 0.024 | 1Score **> 36** indicates **identity** Score **> 14** indicates **homology** | U | R.MVLAAAGGVEHQQLLDLAQK.H |
| 2673 | 249 | – | 255 | 393.2164 | 784.4183 | 784.4191 | -1.07 | 0 | 25 | 0.029 | 1Score **> 23** indicates **identity** Score **> 22** indicates **homology** | U | K.HLSSVSR.V |
| 76196 | 256 | – | 269 | 803.3797 | 1604.7448 | 1604.7505 | -3.50 | 0 | 48 | 3.2e-05 | 1Score **> 32** indicates **identity** Score **> 15** indicates **homology** | U | R.VYEEDAVPGLTPCR.F |
| 76197 | 256 | – | 269 | 803.3798 | 1604.7451 | 1604.7505 | -3.32 | 0 | 22 | 0.0079 | 1Score **> 32** indicates **identity** Score **> 14** indicates **homology** | U | R.VYEEDAVPGLTPCR.F |
| 76198 | 256 | – | 269 | 803.3800 | 1604.7455 | 1604.7505 | -3.07 | 0 | 67 | 5.7e-07 | 1Score **> 32** indicates **identity** Score **> 17** indicates **homology** | U | R.VYEEDAVPGLTPCR.F |
| 76200 | 256 | – | 269 | 803.3806 | 1604.7466 | 1604.7505 | -2.37 | 0 | 53 | 1e-05 | 1Score **> 32** indicates **identity** Score **> 16** indicates **homology** | U | R.VYEEDAVPGLTPCR.F |
| 76201 | 256 | – | 269 | 803.3811 | 1604.7476 | 1604.7505 | -1.77 | 0 | 66 | 7.1e-07 | 1Score **> 32** indicates **identity** Score **> 17** indicates **homology** | U | R.VYEEDAVPGLTPCR.F |
| 76202 | 256 | – | 269 | 803.3816 | 1604.7486 | 1604.7505 | -1.14 | 0 | 54 | 9.3e-06 | 1Score **> 32** indicates **identity** Score **> 16** indicates **homology** | U | R.VYEEDAVPGLTPCR.F |
| 76203 | 256 | – | 269 | 803.3816 | 1604.7487 | 1604.7505 | -1.09 | 0 | 67 | 5.4e-07 | 1Score **> 32** indicates **identity** Score **> 17** indicates **homology** | U | R.VYEEDAVPGLTPCR.F |
| 76204 | 256 | – | 269 | 803.3817 | 1604.7488 | 1604.7505 | -1.01 | 0 | 71 | 2.2e-07 | 1Score **> 32** indicates **identity** Score **> 17** indicates **homology** | U | R.VYEEDAVPGLTPCR.F |
| 76205 | 256 | – | 269 | 803.3818 | 1604.7490 | 1604.7505 | -0.93 | 0 | 40 | 0.00017 | 1Score **> 32** indicates **identity** Score **> 15** indicates **homology** | U | R.VYEEDAVPGLTPCR.F |
| 76207 | 256 | – | 269 | 803.3823 | 1604.7500 | 1604.7505 | -0.31 | 0 | 56 | 5.5e-06 | 1Score **> 32** indicates **identity** Score **> 16** indicates **homology** | U | R.VYEEDAVPGLTPCR.F |
| 76208 | 256 | – | 269 | 803.3823 | 1604.7500 | 1604.7505 | -0.31 | 0 | 66 | 6.8e-07 | 1Score **> 32** indicates **identity** Score **> 17** indicates **homology** | U | R.VYEEDAVPGLTPCR.F |
| 76210 | 256 | – | 269 | 803.3824 | 1604.7503 | 1604.7505 | -0.11 | 0 | 51 | 1.5e-05 | 1Score **> 32** indicates **identity** Score **> 16** indicates **homology** | U | R.VYEEDAVPGLTPCR.F |
| 76211 | 256 | – | 269 | 803.3825 | 1604.7504 | 1604.7505 | -0.0087 | 0 | 47 | 4.1e-05 | 1Score **> 32** indicates **identity** Score **> 15** indicates **homology** | U | R.VYEEDAVPGLTPCR.F |
| 76212 | 256 | – | 269 | 803.3826 | 1604.7507 | 1604.7505 | 0.14 | 0 | 85 | 1.2e-08 | 1Score **> 32** indicates **identity** Score **> 18** indicates **homology** | U | R.VYEEDAVPGLTPCR.F |
| 76213 | 256 | – | 269 | 803.3826 | 1604.7507 | 1604.7505 | 0.15 | 0 | 54 | 9.2e-06 | 1Score **> 32** indicates **identity** Score **> 16** indicates **homology** | U | R.VYEEDAVPGLTPCR.F |
| 76214 | 256 | – | 269 | 803.3826 | 1604.7507 | 1604.7505 | 0.15 | 0 | 68 | 4.9e-07 | 1Score **> 32** indicates **identity** Score **> 17** indicates **homology** | U | R.VYEEDAVPGLTPCR.F |
| 76215 | 256 | – | 269 | 803.3827 | 1604.7508 | 1604.7505 | 0.23 | 0 | 72 | 1.7e-07 | 1Score **> 32** indicates **identity** Score **> 17** indicates **homology** | U | R.VYEEDAVPGLTPCR.F |
| 76216 | 256 | – | 269 | 803.3829 | 1604.7512 | 1604.7505 | 0.45 | 0 | 66 | 6.6e-07 | 1Score **> 32** indicates **identity** Score **> 17** indicates **homology** | U | R.VYEEDAVPGLTPCR.F |
| 76217 | 256 | – | 269 | 803.3830 | 1604.7514 | 1604.7505 | 0.60 | 0 | 74 | 1.1e-07 | 1Score **> 32** indicates **identity** Score **> 17** indicates **homology** | U | R.VYEEDAVPGLTPCR.F |
| 76218 | 256 | – | 269 | 535.9244 | 1604.7515 | 1604.7505 | 0.63 | 0 | 21 | 0.011 | 1Score **> 32** indicates **identity** Score **> 14** indicates **homology** | U | R.VYEEDAVPGLTPCR.F |
| 76219 | 256 | – | 269 | 535.9247 | 1604.7523 | 1604.7505 | 1.17 | 0 | 23 | 0.0077 | 1Score **> 32** indicates **identity** Score **> 14** indicates **homology** | U | R.VYEEDAVPGLTPCR.F |
| 76220 | 256 | – | 269 | 803.3835 | 1604.7525 | 1604.7505 | 1.28 | 0 | 62 | 1.6e-06 | 1Score **> 32** indicates **identity** Score **> 16** indicates **homology** | U | R.VYEEDAVPGLTPCR.F |
| 76221 | 256 | – | 269 | 535.9248 | 1604.7525 | 1604.7505 | 1.28 | 0 | 26 | 0.0045 | 1Score **> 32** indicates **identity** Score **> 15** indicates **homology** | U | R.VYEEDAVPGLTPCR.F |
| 76222 | 256 | – | 269 | 803.3835 | 1604.7525 | 1604.7505 | 1.30 | 0 | 38 | 0.0003 | 1Score **> 32** indicates **identity** Score **> 15** indicates **homology** | U | R.VYEEDAVPGLTPCR.F |
| 76223 | 256 | – | 269 | 803.3838 | 1604.7531 | 1604.7505 | 1.66 | 0 | 84 | 1.3e-08 | 1Score **> 32** indicates **identity** Score **> 18** indicates **homology** | U | R.VYEEDAVPGLTPCR.F |
| 76224 | 256 | – | 269 | 803.3839 | 1604.7532 | 1604.7505 | 1.72 | 0 | 59 | 3.2e-06 | 1Score **> 32** indicates **identity** Score **> 16** indicates **homology** | U | R.VYEEDAVPGLTPCR.F |
| 76225 | 256 | – | 269 | 803.3839 | 1604.7532 | 1604.7505 | 1.72 | 0 | 38 | 0.00029 | 1Score **> 32** indicates **identity** Score **> 15** indicates **homology** | U | R.VYEEDAVPGLTPCR.F |
| 76226 | 256 | – | 269 | 803.3841 | 1604.7536 | 1604.7505 | 1.94 | 0 | 19 | 0.015 | 1Score **> 32** indicates **identity** Score **> 14** indicates **homology** | U | R.VYEEDAVPGLTPCR.F |
| 76227 | 256 | – | 269 | 803.3843 | 1604.7540 | 1604.7505 | 2.22 | 0 | 53 | 9.8e-06 | 1Score **> 32** indicates **identity** Score **> 16** indicates **homology** | U | R.VYEEDAVPGLTPCR.F |
| 76228 | 256 | – | 269 | 803.3843 | 1604.7541 | 1604.7505 | 2.28 | 0 | 56 | 5.3e-06 | 1Score **> 32** indicates **identity** Score **> 16** indicates **homology** | U | R.VYEEDAVPGLTPCR.F |
| 76229 | 256 | – | 269 | 803.3847 | 1604.7548 | 1604.7505 | 2.71 | 0 | 84 | 1.3e-08 | 1Score **> 32** indicates **identity** Score **> 18** indicates **homology** | U | R.VYEEDAVPGLTPCR.F |
| 76230 | 256 | – | 269 | 803.3847 | 1604.7549 | 1604.7505 | 2.78 | 0 | 34 | 0.00062 | 1Score **> 32** indicates **identity** Score **> 15** indicates **homology** | U | R.VYEEDAVPGLTPCR.F |
| 76232 | 256 | – | 269 | 803.3852 | 1604.7558 | 1604.7505 | 3.31 | 0 | 69 | 3.3e-07 | 1Score **> 32** indicates **identity** Score **> 17** indicates **homology** | U | R.VYEEDAVPGLTPCR.F |
| 76234 | 256 | – | 269 | 803.3861 | 1604.7577 | 1604.7505 | 4.54 | 0 | 30 | 0.0014 | 1Score **> 33** indicates **identity** Score **> 14** indicates **homology** | U | R.VYEEDAVPGLTPCR.F |
| 152731 | 256 | – | 276 | 799.3910 | 2395.1512 | 2395.1478 | 1.41 | 1 | 17 | 0.026 | 1Score **> 35** indicates **identity** Score **> 14** indicates **homology** | U | R.VYEEDAVPGLTPCRFTGSEIR.H |
| 152733 | 256 | – | 276 | 1198.5834 | 2395.1522 | 2395.1478 | 1.82 | 1 | 17 | 0.027 | 1Score **> 36** indicates **identity** Score **> 14** indicates **homology** | U | R.VYEEDAVPGLTPCRFTGSEIR.H |
| 152734 | 256 | – | 276 | 799.3913 | 2395.1522 | 2395.1478 | 1.83 | 1 | 33 | 0.00078 | 1Score **> 36** indicates **identity** Score **> 15** indicates **homology** | U | R.VYEEDAVPGLTPCRFTGSEIR.H |
| 152737 | 256 | – | 276 | 799.3919 | 2395.1540 | 2395.1478 | 2.58 | 1 | 27 | 0.0031 | 1Score **> 36** indicates **identity** Score **> 14** indicates **homology** | U | R.VYEEDAVPGLTPCRFTGSEIR.H |
| 152739 | 256 | – | 276 | 799.3926 | 2395.1559 | 2395.1478 | 3.37 | 1 | 32 | 0.001 | 1Score **> 36** indicates **identity** Score **> 14** indicates **homology** | U | R.VYEEDAVPGLTPCRFTGSEIR.H |
| 3613 | 270 | – | 276 | 405.2111 | 808.4076 | 808.4079 | -0.34 | 0 | 32 | 0.0015 | 1Score **> 25** indicates **identity** Score **> 17** indicates **homology** | U | R.FTGSEIR.H |
| 3614 | 270 | – | 276 | 405.2111 | 808.4077 | 808.4079 | -0.22 | 0 | 17 | 0.023 | 1Score **> 25** indicates **identity** Score **> 14** indicates **homology** | U | R.FTGSEIR.H |
| 3615 | 270 | – | 276 | 405.2114 | 808.4082 | 808.4079 | 0.31 | 0 | 31 | 0.0012 | 1Score **> 24** indicates **identity** Score **> 14** indicates **homology** | U | R.FTGSEIR.H |
| 3616 | 270 | – | 276 | 405.2114 | 808.4082 | 808.4079 | 0.33 | 0 | 27 | 0.0047 | 1Score **> 24** indicates **identity** Score **> 17** indicates **homology** | U | R.FTGSEIR.H |
| 3617 | 270 | – | 276 | 405.2115 | 808.4084 | 808.4079 | 0.55 | 0 | 46 | 0.00011 | 1Score **> 24** indicates **identity** Score **> 19** indicates **homology** | U | R.FTGSEIR.H |
| 3618 | 270 | – | 276 | 405.2119 | 808.4093 | 808.4079 | 1.73 | 0 | 36 | 0.00046 | 1Score **> 24** indicates **identity** Score **> 15** indicates **homology** | U | R.FTGSEIR.H |
| 3620 | 270 | – | 276 | 405.2128 | 808.4110 | 808.4079 | 3.84 | 0 | 22 | 0.009 | 1Score **> 24** indicates **identity** Score **> 14** indicates **homology** | U | R.FTGSEIR.H |
| 198424 | 277 | – | 336 | 1240.6372 | 6198.1498 | 6198.1134 | 5.87 | 1 | 33 | 0.00074 | 1Score **> 34** indicates **identity** Score **> 15** indicates **homology** | U | R.HRDDALPLAHVAIAVEGPGWANPDNVTLQVANAIIGHYDCTYGGGVHLSSPLASVAVANK.L |
| 45788 | 379 | – | 390 | 677.3158 | 1352.6170 | 1352.6242 | -5.29 | 0 | 33 | 0.0008 | 1Score **> 30** indicates **identity** Score **> 15** indicates **homology** | U | R.LCTSATESEVTR.G |
| 45789 | 379 | – | 390 | 677.3174 | 1352.6202 | 1352.6242 | -2.98 | 0 | 29 | 0.0018 | 1Score **> 30** indicates **identity** Score **> 14** indicates **homology** | U | R.LCTSATESEVTR.G |
| 45790 | 379 | – | 390 | 677.3182 | 1352.6219 | 1352.6242 | -1.70 | 0 | 78 | 4.5e-08 | 1Score **> 30** indicates **identity** Score **> 17** indicates **homology** | U | R.LCTSATESEVTR.G |
| 45791 | 379 | – | 390 | 677.3184 | 1352.6223 | 1352.6242 | -1.43 | 0 | 30 | 0.011 | 1Score **> 32** indicates **identity** Score **> 23** indicates **homology** | U | R.LCTSATESEVTR.G |
| 45792 | 379 | – | 390 | 677.3187 | 1352.6228 | 1352.6242 | -1.05 | 0 | 84 | 1.4e-08 | 1Score **> 32** indicates **identity** Score **> 18** indicates **homology** | U | R.LCTSATESEVTR.G |
| 45794 | 379 | – | 390 | 677.3190 | 1352.6235 | 1352.6242 | -0.54 | 0 | 70 | 2.8e-07 | 1Score **> 32** indicates **identity** Score **> 17** indicates **homology** | U | R.LCTSATESEVTR.G |
| 45795 | 379 | – | 390 | 677.3190 | 1352.6235 | 1352.6242 | -0.53 | 0 | 78 | 4.8e-08 | 1Score **> 32** indicates **identity** Score **> 17** indicates **homology** | U | R.LCTSATESEVTR.G |
| 45796 | 379 | – | 390 | 677.3191 | 1352.6236 | 1352.6242 | -0.43 | 0 | 27 | 0.0029 | 1Score **> 32** indicates **identity** Score **> 14** indicates **homology** | U | R.LCTSATESEVTR.G |
| 45797 | 379 | – | 390 | 677.3192 | 1352.6239 | 1352.6242 | -0.20 | 0 | 75 | 1e-07 | 1Score **> 32** indicates **identity** Score **> 17** indicates **homology** | U | R.LCTSATESEVTR.G |
| 45798 | 379 | – | 390 | 677.3193 | 1352.6241 | 1352.6242 | -0.072 | 0 | 70 | 2.7e-07 | 1Score **> 31** indicates **identity** Score **> 17** indicates **homology** | U | R.LCTSATESEVTR.G |
| 45799 | 379 | – | 390 | 677.3196 | 1352.6247 | 1352.6242 | 0.36 | 0 | 16 | 0.034 | 1Score **> 31** indicates **identity** Score **> 13** indicates **homology** | U | R.LCTSATESEVTR.G |
| 45800 | 379 | – | 390 | 677.3198 | 1352.6250 | 1352.6242 | 0.64 | 0 | 78 | 4.7e-08 | 1Score **> 31** indicates **identity** Score **> 17** indicates **homology** | U | R.LCTSATESEVTR.G |
| 45802 | 379 | – | 390 | 677.3199 | 1352.6252 | 1352.6242 | 0.76 | 0 | 70 | 2.8e-07 | 1Score **> 31** indicates **identity** Score **> 17** indicates **homology** | U | R.LCTSATESEVTR.G |
| 45803 | 379 | – | 390 | 677.3199 | 1352.6253 | 1352.6242 | 0.83 | 0 | 54 | 8.8e-06 | 1Score **> 31** indicates **identity** Score **> 16** indicates **homology** | U | R.LCTSATESEVTR.G |
| 45804 | 379 | – | 390 | 677.3200 | 1352.6255 | 1352.6242 | 0.93 | 0 | 55 | 6.8e-06 | 1Score **> 32** indicates **identity** Score **> 16** indicates **homology** | U | R.LCTSATESEVTR.G |
| 45805 | 379 | – | 390 | 677.3202 | 1352.6259 | 1352.6242 | 1.26 | 0 | 42 | 0.0001 | 1Score **> 32** indicates **identity** Score **> 15** indicates **homology** | U | R.LCTSATESEVTR.G |
| 45806 | 379 | – | 390 | 677.3219 | 1352.6293 | 1352.6242 | 3.79 | 0 | 14 | 0.047 | 1Score **> 32** indicates **identity** Score **> 14** indicates **homology** | U | R.LCTSATESEVTR.G |
| 66833 | 379 | – | 392 | 513.5881 | 1537.7426 | 1537.7406 | 1.26 | 1 | 28 | 0.0024 | 1Score **> 33** indicates **identity** Score **> 14** indicates **homology** | U | R.LCTSATESEVTRGK.N |
| 66834 | 379 | – | 392 | 769.8793 | 1537.7440 | 1537.7406 | 2.23 | 1 | 34 | 0.00075 | 1Score **> 33** indicates **identity** Score **> 15** indicates **homology** | U | R.LCTSATESEVTRGK.N |
| 66835 | 379 | – | 392 | 513.5890 | 1537.7453 | 1537.7406 | 3.03 | 1 | 28 | 0.0033 | 1Score **> 33** indicates **identity** Score **> 16** indicates **homology** | U | R.LCTSATESEVTRGK.N |
| 125677 | 397 | – | 415 | 685.3368 | 2052.9886 | 2052.9899 | -0.60 | 0 | 42 | 0.00012 | 1Score **> 35** indicates **identity** Score **> 15** indicates **homology** | U | R.NALVSHLDGTTPVCEDIGR.S |
| 125678 | 397 | – | 415 | 685.3373 | 2052.9900 | 2052.9899 | 0.042 | 0 | 53 | 9.8e-06 | 1Score **> 35** indicates **identity** Score **> 16** indicates **homology** | U | R.NALVSHLDGTTPVCEDIGR.S |
| 125679 | 397 | – | 415 | 1027.5023 | 2052.9901 | 2052.9899 | 0.10 | 0 | 93 | 1.9e-09 | 1Score **> 35** indicates **identity** Score **> 18** indicates **homology** | U | R.NALVSHLDGTTPVCEDIGR.S |
| 125680 | 397 | – | 415 | 685.3373 | 2052.9901 | 2052.9899 | 0.13 | 0 | 70 | 3.4e-07 | 1Score **> 35** indicates **identity** Score **> 18** indicates **homology** | U | R.NALVSHLDGTTPVCEDIGR.S |
| 125681 | 397 | – | 415 | 1027.5026 | 2052.9906 | 2052.9899 | 0.34 | 0 | 25 | 0.0059 | 1Score **> 35** indicates **identity** Score **> 16** indicates **homology** | U | R.NALVSHLDGTTPVCEDIGR.S |
| 125682 | 397 | – | 415 | 685.3375 | 2052.9907 | 2052.9899 | 0.39 | 0 | 71 | 3e-07 | 1Score **> 35** indicates **identity** Score **> 18** indicates **homology** | U | R.NALVSHLDGTTPVCEDIGR.S |
| 125683 | 397 | – | 415 | 685.3376 | 2052.9908 | 2052.9899 | 0.48 | 0 | 70 | 4.3e-07 | 1Score **> 35** indicates **identity** Score **> 19** indicates **homology** | U | R.NALVSHLDGTTPVCEDIGR.S |
| 125684 | 397 | – | 415 | 685.3376 | 2052.9910 | 2052.9899 | 0.56 | 0 | 70 | 3.6e-07 | 1Score **> 35** indicates **identity** Score **> 18** indicates **homology** | U | R.NALVSHLDGTTPVCEDIGR.S |
| 125685 | 397 | – | 415 | 685.3378 | 2052.9915 | 2052.9899 | 0.80 | 0 | 66 | 9.9e-07 | 1Score **> 35** indicates **identity** Score **> 19** indicates **homology** | U | R.NALVSHLDGTTPVCEDIGR.S |
| 125686 | 397 | – | 415 | 685.3378 | 2052.9916 | 2052.9899 | 0.86 | 0 | 71 | 3.6e-07 | 1Score **> 35** indicates **identity** Score **> 19** indicates **homology** | U | R.NALVSHLDGTTPVCEDIGR.S |
| 125687 | 397 | – | 415 | 685.3378 | 2052.9917 | 2052.9899 | 0.90 | 0 | 66 | 9.4e-07 | 1Score **> 35** indicates **identity** Score **> 18** indicates **homology** | U | R.NALVSHLDGTTPVCEDIGR.S |
| 125689 | 397 | – | 415 | 1027.5034 | 2052.9922 | 2052.9899 | 1.14 | 0 | 101 | 3.3e-10 | 1Score **> 35** indicates **identity** Score **> 19** indicates **homology** | U | R.NALVSHLDGTTPVCEDIGR.S |
| 125690 | 397 | – | 415 | 1027.5034 | 2052.9922 | 2052.9899 | 1.15 | 0 | 89 | 4.3e-09 | 1Score **> 35** indicates **identity** Score **> 18** indicates **homology** | U | R.NALVSHLDGTTPVCEDIGR.S |
| 125691 | 397 | – | 415 | 685.3381 | 2052.9925 | 2052.9899 | 1.26 | 0 | 77 | 1e-07 | 1Score **> 35** indicates **identity** Score **> 20** indicates **homology** | U | R.NALVSHLDGTTPVCEDIGR.S |
| 125692 | 397 | – | 415 | 685.3382 | 2052.9927 | 2052.9899 | 1.37 | 0 | 71 | 3.6e-07 | 1Score **> 35** indicates **identity** Score **> 19** indicates **homology** | U | R.NALVSHLDGTTPVCEDIGR.S |
| 125693 | 397 | – | 415 | 1027.5037 | 2052.9929 | 2052.9899 | 1.46 | 0 | 119 | 6.6e-12 | 1Score **> 35** indicates **identity** Score **> 20** indicates **homology** | U | R.NALVSHLDGTTPVCEDIGR.S |
| 125694 | 397 | – | 415 | 685.3384 | 2052.9933 | 2052.9899 | 1.66 | 0 | 50 | 2.3e-05 | 1Score **> 35** indicates **identity** Score **> 16** indicates **homology** | U | R.NALVSHLDGTTPVCEDIGR.S |
| 125695 | 397 | – | 415 | 685.3384 | 2052.9934 | 2052.9899 | 1.74 | 0 | 56 | 5.6e-06 | 1Score **> 35** indicates **identity** Score **> 16** indicates **homology** | U | R.NALVSHLDGTTPVCEDIGR.S |
| 125696 | 397 | – | 415 | 1027.5041 | 2052.9937 | 2052.9899 | 1.85 | 0 | 97 | 8.8e-10 | 1Score **> 35** indicates **identity** Score **> 19** indicates **homology** | U | R.NALVSHLDGTTPVCEDIGR.S |
| 125697 | 397 | – | 415 | 685.3385 | 2052.9938 | 2052.9899 | 1.92 | 0 | 71 | 3.6e-07 | 1Score **> 35** indicates **identity** Score **> 19** indicates **homology** | U | R.NALVSHLDGTTPVCEDIGR.S |
| 125698 | 397 | – | 415 | 685.3386 | 2052.9941 | 2052.9899 | 2.06 | 0 | 66 | 9.9e-07 | 1Score **> 35** indicates **identity** Score **> 19** indicates **homology** | U | R.NALVSHLDGTTPVCEDIGR.S |
| 125699 | 397 | – | 415 | 685.3387 | 2052.9942 | 2052.9899 | 2.10 | 0 | 71 | 3.7e-07 | 1Score **> 35** indicates **identity** Score **> 19** indicates **homology** | U | R.NALVSHLDGTTPVCEDIGR.S |
| 125700 | 397 | – | 415 | 685.3389 | 2052.9947 | 2052.9899 | 2.37 | 0 | 70 | 4.1e-07 | 1Score **> 35** indicates **identity** Score **> 19** indicates **homology** | U | R.NALVSHLDGTTPVCEDIGR.S |
| 125701 | 397 | – | 415 | 1027.5047 | 2052.9948 | 2052.9899 | 2.38 | 0 | 94 | 1.7e-09 | 1Score **> 35** indicates **identity** Score **> 18** indicates **homology** | U | R.NALVSHLDGTTPVCEDIGR.S |
| 125702 | 397 | – | 415 | 685.3389 | 2052.9948 | 2052.9899 | 2.38 | 0 | 70 | 3.5e-07 | 1Score **> 35** indicates **identity** Score **> 18** indicates **homology** | U | R.NALVSHLDGTTPVCEDIGR.S |
| 125703 | 397 | – | 415 | 1027.5049 | 2052.9952 | 2052.9899 | 2.60 | 0 | 84 | 1.4e-08 | 1Score **> 35** indicates **identity** Score **> 18** indicates **homology** | U | R.NALVSHLDGTTPVCEDIGR.S |
| 125704 | 397 | – | 415 | 1027.5051 | 2052.9956 | 2052.9899 | 2.77 | 0 | 85 | 1.2e-08 | 1Score **> 35** indicates **identity** Score **> 18** indicates **homology** | U | R.NALVSHLDGTTPVCEDIGR.S |
| 125705 | 397 | – | 415 | 1027.5052 | 2052.9958 | 2052.9899 | 2.89 | 0 | 73 | 1.3e-07 | 1Score **> 35** indicates **identity** Score **> 17** indicates **homology** | U | R.NALVSHLDGTTPVCEDIGR.S |
| 125706 | 397 | – | 415 | 1027.5059 | 2052.9973 | 2052.9899 | 3.64 | 0 | 106 | 1.2e-10 | 1Score **> 35** indicates **identity** Score **> 19** indicates **homology** | U | R.NALVSHLDGTTPVCEDIGR.S |
| 125709 | 397 | – | 415 | 1027.5083 | 2053.0020 | 2052.9899 | 5.92 | 0 | 91 | 2.9e-09 | 1Score **> 35** indicates **identity** Score **> 18** indicates **homology** | U | R.NALVSHLDGTTPVCEDIGR.S |
| 125714 | 397 | – | 415 | 685.3426 | 2053.0059 | 2052.9899 | 7.83 | 0 | 39 | 0.00022 | 1Score **> 36** indicates **identity** Score **> 15** indicates **homology** | U | R.NALVSHLDGTTPVCEDIGR.S |
| 125715 | 397 | – | 415 | 1027.5103 | 2053.0061 | 2052.9899 | 7.90 | 0 | 98 | 6.5e-10 | 1Score **> 36** indicates **identity** Score **> 19** indicates **homology** | U | R.NALVSHLDGTTPVCEDIGR.S |
| 125787 | 397 | – | 415 | 1027.9942 | 2053.9739 | 2053.9739 | 0.013 | 0 | 103 | 2.3e-10 | 1Score **> 34** indicates **identity** Score **> 19** indicates **homology** | U | R.NALVSHLDGTTPVCEDIGR.S  + Deamidated (NQ) |
| 125788 | 397 | – | 415 | 1027.9943 | 2053.9741 | 2053.9739 | 0.13 | 0 | 75 | 8.9e-08 | 1Score **> 34** indicates **identity** Score **> 17** indicates **homology** | U | R.NALVSHLDGTTPVCEDIGR.S  + Deamidated (NQ) |
| 125789 | 397 | – | 415 | 685.6659 | 2053.9760 | 2053.9739 | 1.02 | 0 | 46 | 4.4e-05 | 1Score **> 34** indicates **identity** Score **> 15** indicates **homology** | U | R.NALVSHLDGTTPVCEDIGR.S  + Deamidated (NQ) |
| 125790 | 397 | – | 415 | 1027.9955 | 2053.9765 | 2053.9739 | 1.30 | 0 | 63 | 1.4e-06 | 1Score **> 34** indicates **identity** Score **> 16** indicates **homology** | U | R.NALVSHLDGTTPVCEDIGR.S  + Deamidated (NQ) |
| 125791 | 397 | – | 415 | 685.6670 | 2053.9793 | 2053.9739 | 2.64 | 0 | 31 | 0.0013 | 1Score **> 35** indicates **identity** Score **> 14** indicates **homology** | U | R.NALVSHLDGTTPVCEDIGR.S  + Deamidated (NQ) |
| 125792 | 397 | – | 415 | 1027.9985 | 2053.9825 | 2053.9739 | 4.20 | 0 | 66 | 6.5e-07 | 1Score **> 35** indicates **identity** Score **> 17** indicates **homology** | U | R.NALVSHLDGTTPVCEDIGR.S  + Deamidated (NQ) |
| 125793 | 397 | – | 415 | 685.6691 | 2053.9856 | 2053.9739 | 5.70 | 0 | 14 | 0.045 | 1Score **> 35** indicates **identity** Score **> 13** indicates **homology** | U | R.NALVSHLDGTTPVCEDIGR.S  + Deamidated (NQ) |
| 3627 | 416 | – | 422 | 405.2281 | 808.4417 | 808.4443 | -3.18 | 0 | 15 | 0.043 | 1Score **> 22** indicates **identity** Score **> 13** indicates **homology** | U | R.SLLTYGR.R |
| 3628 | 416 | – | 422 | 405.2283 | 808.4421 | 808.4443 | -2.70 | 0 | 28 | 0.0023 | 1Score **> 22** indicates **identity** Score **> 14** indicates **homology** | U | R.SLLTYGR.R |
| 3630 | 416 | – | 422 | 405.2293 | 808.4440 | 808.4443 | -0.30 | 0 | 37 | 0.00038 | 1Score **> 22** indicates **identity** Score **> 15** indicates **homology** | U | R.SLLTYGR.R |
| 3631 | 416 | – | 422 | 405.2294 | 808.4443 | 808.4443 | 0.020 | 0 | 51 | 1.8e-05 | 1Score **> 22** indicates **identity** Score **> 16** indicates **homology** | U | R.SLLTYGR.R |
| 3632 | 416 | – | 422 | 405.2295 | 808.4445 | 808.4443 | 0.20 | 0 | 47 | 4.4e-05 | 1Score **> 22** indicates **identity** Score **> 15** indicates **homology** | U | R.SLLTYGR.R |
| 3633 | 416 | – | 422 | 405.2296 | 808.4446 | 808.4443 | 0.40 | 0 | 51 | 1.7e-05 | 1Score **> 22** indicates **identity** Score **> 16** indicates **homology** | U | R.SLLTYGR.R |
| 3634 | 416 | – | 422 | 405.2296 | 808.4446 | 808.4443 | 0.44 | 0 | 39 | 0.00024 | 1Score **> 22** indicates **identity** Score **> 15** indicates **homology** | U | R.SLLTYGR.R |
| 3635 | 416 | – | 422 | 405.2296 | 808.4447 | 808.4443 | 0.50 | 0 | 39 | 0.00022 | 1Score **> 22** indicates **identity** Score **> 15** indicates **homology** | U | R.SLLTYGR.R |
| 3636 | 416 | – | 422 | 405.2298 | 808.4450 | 808.4443 | 0.82 | 0 | 25 | 0.0046 | 1Score **> 22** indicates **identity** Score **> 14** indicates **homology** | U | R.SLLTYGR.R |
| 3637 | 416 | – | 422 | 405.2298 | 808.4450 | 808.4443 | 0.90 | 0 | 25 | 0.0047 | 1Score **> 22** indicates **identity** Score **> 14** indicates **homology** | U | R.SLLTYGR.R |
| 3638 | 416 | – | 422 | 405.2300 | 808.4454 | 808.4443 | 1.36 | 0 | 36 | 0.00046 | 1Score **> 22** indicates **identity** Score **> 15** indicates **homology** | U | R.SLLTYGR.R |
| 3639 | 416 | – | 422 | 405.2302 | 808.4458 | 808.4443 | 1.85 | 0 | 23 | 0.0067 | 1Score **> 22** indicates **identity** Score **> 14** indicates **homology** | U | R.SLLTYGR.R |
| 35895 | 423 | – | 432 | 419.5620 | 1255.6642 | 1255.6673 | -2.46 | 1 | 24 | 0.0062 | 1Score **> 32** indicates **identity** Score **> 14** indicates **homology** | U | R.RIPLAEWESR.I |
| 35897 | 423 | – | 432 | 419.5622 | 1255.6649 | 1255.6673 | -1.93 | 1 | 23 | 0.0071 | 1Score **> 33** indicates **identity** Score **> 14** indicates **homology** | U | R.RIPLAEWESR.I |
| 35898 | 423 | – | 432 | 419.5624 | 1255.6652 | 1255.6673 | -1.62 | 1 | 26 | 0.0038 | 1Score **> 33** indicates **identity** Score **> 14** indicates **homology** | U | R.RIPLAEWESR.I |
| 35899 | 423 | – | 432 | 419.5624 | 1255.6653 | 1255.6673 | -1.61 | 1 | 25 | 0.0041 | 1Score **> 33** indicates **identity** Score **> 14** indicates **homology** | U | R.RIPLAEWESR.I |
| 35900 | 423 | – | 432 | 419.5624 | 1255.6655 | 1255.6673 | -1.45 | 1 | 19 | 0.021 | 1Score **> 33** indicates **identity** Score **> 15** indicates **homology** | U | R.RIPLAEWESR.I |
| 35901 | 423 | – | 432 | 419.5627 | 1255.6661 | 1255.6673 | -0.92 | 1 | 14 | 0.045 | 1Score **> 33** indicates **identity** Score **> 14** indicates **homology** | U | R.RIPLAEWESR.I |
| 35902 | 423 | – | 432 | 419.5628 | 1255.6665 | 1255.6673 | -0.65 | 1 | 42 | 0.00011 | 1Score **> 33** indicates **identity** Score **> 15** indicates **homology** | U | R.RIPLAEWESR.I |
| 35903 | 423 | – | 432 | 419.5628 | 1255.6665 | 1255.6673 | -0.63 | 1 | 20 | 0.013 | 1Score **> 33** indicates **identity** Score **> 14** indicates **homology** | U | R.RIPLAEWESR.I |
| 35904 | 423 | – | 432 | 419.5628 | 1255.6666 | 1255.6673 | -0.56 | 1 | 24 | 0.0051 | 1Score **> 33** indicates **identity** Score **> 14** indicates **homology** | U | R.RIPLAEWESR.I |
| 35907 | 423 | – | 432 | 419.5630 | 1255.6672 | 1255.6673 | -0.073 | 1 | 31 | 0.0012 | 1Score **> 33** indicates **identity** Score **> 14** indicates **homology** | U | R.RIPLAEWESR.I |
| 35908 | 423 | – | 432 | 419.5631 | 1255.6675 | 1255.6673 | 0.18 | 1 | 30 | 0.0016 | 1Score **> 33** indicates **identity** Score **> 14** indicates **homology** | U | R.RIPLAEWESR.I |
| 35909 | 423 | – | 432 | 419.5631 | 1255.6675 | 1255.6673 | 0.18 | 1 | 23 | 0.0066 | 1Score **> 33** indicates **identity** Score **> 14** indicates **homology** | U | R.RIPLAEWESR.I |
| 35910 | 423 | – | 432 | 628.8410 | 1255.6675 | 1255.6673 | 0.18 | 1 | 23 | 0.039 | 1Score **> 33** indicates **identity** Score **> 21** indicates **homology** | U | R.RIPLAEWESR.I |
| 35912 | 423 | – | 432 | 419.5632 | 1255.6679 | 1255.6673 | 0.46 | 1 | 36 | 0.00046 | 1Score **> 33** indicates **identity** Score **> 15** indicates **homology** | U | R.RIPLAEWESR.I |
| 35914 | 423 | – | 432 | 628.8413 | 1255.6680 | 1255.6673 | 0.57 | 1 | 66 | 2.5e-05 | 1Score **> 33** indicates **identity** | U | R.RIPLAEWESR.I |
| 35915 | 423 | – | 432 | 628.8413 | 1255.6680 | 1255.6673 | 0.58 | 1 | 52 | 0.00024 | 1Score **> 33** indicates **identity** Score **> 28** indicates **homology** | U | R.RIPLAEWESR.I |
| 35916 | 423 | – | 432 | 419.5633 | 1255.6681 | 1255.6673 | 0.61 | 1 | 30 | 0.0015 | 1Score **> 33** indicates **identity** Score **> 14** indicates **homology** | U | R.RIPLAEWESR.I |
| 35917 | 423 | – | 432 | 628.8413 | 1255.6681 | 1255.6673 | 0.62 | 1 | 30 | 0.011 | 1Score **> 33** indicates **identity** Score **> 23** indicates **homology** | U | R.RIPLAEWESR.I |
| 35918 | 423 | – | 432 | 419.5633 | 1255.6681 | 1255.6673 | 0.62 | 1 | 37 | 0.00031 | 1Score **> 33** indicates **identity** Score **> 15** indicates **homology** | U | R.RIPLAEWESR.I |
| 35919 | 423 | – | 432 | 419.5633 | 1255.6681 | 1255.6673 | 0.66 | 1 | 29 | 0.002 | 1Score **> 33** indicates **identity** Score **> 14** indicates **homology** | U | R.RIPLAEWESR.I |
| 35920 | 423 | – | 432 | 419.5633 | 1255.6681 | 1255.6673 | 0.68 | 1 | 23 | 0.0063 | 1Score **> 33** indicates **identity** Score **> 14** indicates **homology** | U | R.RIPLAEWESR.I |
| 35921 | 423 | – | 432 | 419.5633 | 1255.6681 | 1255.6673 | 0.69 | 1 | 29 | 0.0019 | 1Score **> 33** indicates **identity** Score **> 14** indicates **homology** | U | R.RIPLAEWESR.I |
| 35923 | 423 | – | 432 | 419.5633 | 1255.6682 | 1255.6673 | 0.74 | 1 | 41 | 0.00013 | 1Score **> 33** indicates **identity** Score **> 15** indicates **homology** | U | R.RIPLAEWESR.I |
| 35924 | 423 | – | 432 | 419.5634 | 1255.6683 | 1255.6673 | 0.77 | 1 | 26 | 0.0039 | 1Score **> 33** indicates **identity** Score **> 14** indicates **homology** | U | R.RIPLAEWESR.I |
| 35925 | 423 | – | 432 | 419.5634 | 1255.6683 | 1255.6673 | 0.82 | 1 | 21 | 0.011 | 1Score **> 33** indicates **identity** Score **> 14** indicates **homology** | U | R.RIPLAEWESR.I |
| 35927 | 423 | – | 432 | 628.8416 | 1255.6687 | 1255.6673 | 1.10 | 1 | 23 | 0.023 | 1Score **> 32** indicates **identity** Score **> 19** indicates **homology** | U | R.RIPLAEWESR.I |
| 35928 | 423 | – | 432 | 628.8417 | 1255.6688 | 1255.6673 | 1.17 | 1 | 28 | 0.021 | 1Score **> 33** indicates **identity** Score **> 24** indicates **homology** | U | R.RIPLAEWESR.I |
| 35929 | 423 | – | 432 | 419.5635 | 1255.6688 | 1255.6673 | 1.19 | 1 | 38 | 0.00028 | 1Score **> 33** indicates **identity** Score **> 15** indicates **homology** | U | R.RIPLAEWESR.I |
| 35930 | 423 | – | 432 | 419.5635 | 1255.6688 | 1255.6673 | 1.20 | 1 | 31 | 0.0013 | 1Score **> 33** indicates **identity** Score **> 14** indicates **homology** | U | R.RIPLAEWESR.I |
| 35933 | 423 | – | 432 | 628.8418 | 1255.6690 | 1255.6673 | 1.36 | 1 | 53 | 0.00025 | 1Score **> 33** indicates **identity** Score **> 29** indicates **homology** | U | R.RIPLAEWESR.I |
| 35934 | 423 | – | 432 | 419.5636 | 1255.6690 | 1255.6673 | 1.36 | 1 | 38 | 0.00025 | 1Score **> 33** indicates **identity** Score **> 15** indicates **homology** | U | R.RIPLAEWESR.I |
| 35935 | 423 | – | 432 | 628.8418 | 1255.6690 | 1255.6673 | 1.39 | 1 | 32 | 0.01 | 1Score **> 33** indicates **identity** Score **> 24** indicates **homology** | U | R.RIPLAEWESR.I |
| 35938 | 423 | – | 432 | 419.5638 | 1255.6696 | 1255.6673 | 1.84 | 1 | 15 | 0.049 | 1Score **> 33** indicates **identity** Score **> 14** indicates **homology** | U | R.RIPLAEWESR.I |
| 35940 | 423 | – | 432 | 419.5640 | 1255.6700 | 1255.6673 | 2.19 | 1 | 23 | 0.0063 | 1Score **> 33** indicates **identity** Score **> 14** indicates **homology** | U | R.RIPLAEWESR.I |
| 35943 | 423 | – | 432 | 628.8424 | 1255.6703 | 1255.6673 | 2.42 | 1 | 45 | 0.0037 | 1Score **> 33** indicates **identity** | U | R.RIPLAEWESR.I |
| 35944 | 423 | – | 432 | 419.5641 | 1255.6704 | 1255.6673 | 2.51 | 1 | 31 | 0.0011 | 1Score **> 33** indicates **identity** Score **> 14** indicates **homology** | U | R.RIPLAEWESR.I |
| 35946 | 423 | – | 432 | 419.5641 | 1255.6705 | 1255.6673 | 2.55 | 1 | 29 | 0.0017 | 1Score **> 33** indicates **identity** Score **> 14** indicates **homology** | U | R.RIPLAEWESR.I |
| 35947 | 423 | – | 432 | 628.8428 | 1255.6710 | 1255.6673 | 2.99 | 1 | 35 | 0.0049 | 1Score **> 33** indicates **identity** Score **> 24** indicates **homology** | U | R.RIPLAEWESR.I |
| 35948 | 423 | – | 432 | 419.5643 | 1255.6711 | 1255.6673 | 3.02 | 1 | 42 | 0.00012 | 1Score **> 33** indicates **identity** Score **> 15** indicates **homology** | U | R.RIPLAEWESR.I |
| 35949 | 423 | – | 432 | 419.5644 | 1255.6715 | 1255.6673 | 3.35 | 1 | 16 | 0.034 | 1Score **> 33** indicates **identity** Score **> 13** indicates **homology** | U | R.RIPLAEWESR.I |
| 20706 | 424 | – | 432 | 550.7902 | 1099.5659 | 1099.5662 | -0.27 | 0 | 27 | 0.0065 | 1Score **> 32** indicates **identity** Score **> 18** indicates **homology** | U | R.IPLAEWESR.I |
| 20707 | 424 | – | 432 | 550.7909 | 1099.5672 | 1099.5662 | 0.96 | 0 | 44 | 0.00094 | 1Score **> 32** indicates **identity** Score **> 26** indicates **homology** | U | R.IPLAEWESR.I |
| 20708 | 424 | – | 432 | 550.7912 | 1099.5678 | 1099.5662 | 1.50 | 0 | 58 | 7.9e-05 | 1Score **> 32** indicates **identity** Score **> 30** indicates **homology** | U | R.IPLAEWESR.I |
| 30590 | 433 | – | 442 | 601.8143 | 1201.6140 | 1201.6125 | 1.22 | 0 | 55 | 0.00032 | 1Score **> 33** indicates **identity** Score **> 32** indicates **homology** | U | R.IQEVDAQMLR.D |
| 30592 | 433 | – | 442 | 601.8149 | 1201.6152 | 1201.6125 | 2.22 | 0 | 48 | 0.00087 | 1Score **> 33** indicates **identity** Score **> 30** indicates **homology** | U | R.IQEVDAQMLR.D |
| 101092 | 433 | – | 447 | 602.6348 | 1804.8826 | 1804.8811 | 0.82 | 1 | 26 | 0.0036 | 1Score **> 35** indicates **identity** Score **> 14** indicates **homology** | U | R.IQEVDAQMLRDICSK.Y |
| 101096 | 433 | – | 447 | 602.6364 | 1804.8874 | 1804.8811 | 3.45 | 1 | 14 | 0.049 | 1Score **> 35** indicates **identity** Score **> 13** indicates **homology** | U | R.IQEVDAQMLRDICSK.Y |
| 101098 | 433 | – | 447 | 903.4515 | 1804.8885 | 1804.8811 | 4.09 | 1 | 25 | 0.0045 | 1Score **> 35** indicates **identity** Score **> 14** indicates **homology** | U | R.IQEVDAQMLRDICSK.Y |
| 196573 | 433 | – | 470 | 1157.0437 | 4624.1457 | 4624.1488 | -0.66 | 2 | 22 | 0.0088 | 1Score **> 34** indicates **identity** Score **> 14** indicates **homology** | U | R.IQEVDAQMLRDICSKYFYDQCPAVAGYGPIEQLPDYNR.I  + 3 Deamidated (NQ); HNE (C) |
| 196616 | 433 | – | 470 | 1160.5468 | 4638.1579 | 4638.1757 | -3.83 | 2 | 25 | 0.0045 | 1Score **> 34** indicates **identity** Score **> 14** indicates **homology** | U | R.IQEVDAQMLRDICSKYFYDQCPAVAGYGPIEQLPDYNR.I  + Deamidated (NQ); HNE (C); Oxidation (M) |
| 196795 | 433 | – | 470 | 1170.7983 | 4679.1639 | 4679.2022 | -8.19 | 2 | 26 | 0.0037 | 1Score **> 34** indicates **identity** Score **> 14** indicates **homology** | U | R.IQEVDAQMLRDICSKYFYDQCPAVAGYGPIEQLPDYNR.I  + Deamidated (NQ); HNE (K) |
| 186321 | 443 | – | 470 | 1114.1760 | 3339.5063 | 3339.4852 | 6.30 | 1 | 33 | 0.0008 | 1Score **> 32** indicates **identity** Score **> 15** indicates **homology** | U | R.DICSKYFYDQCPAVAGYGPIEQLPDYNR.I  + Deamidated (NQ) |
| 169187 | 448 | – | 470 | 1368.6225 | 2735.2305 | 2735.2326 | -0.76 | 0 | 75 | 9e-08 | 1Score **> 32** indicates **identity** Score **> 17** indicates **homology** | U | K.YFYDQCPAVAGYGPIEQLPDYNR.I |
| 169188 | 448 | – | 470 | 1368.6236 | 2735.2326 | 2735.2326 | 0.0022 | 0 | 81 | 2.6e-08 | 1Score **> 32** indicates **identity** Score **> 18** indicates **homology** | U | K.YFYDQCPAVAGYGPIEQLPDYNR.I |
| 169189 | 448 | – | 470 | 912.7518 | 2735.2336 | 2735.2326 | 0.38 | 0 | 62 | 1.6e-06 | 1Score **> 33** indicates **identity** Score **> 16** indicates **homology** | U | K.YFYDQCPAVAGYGPIEQLPDYNR.I |
| 169190 | 448 | – | 470 | 912.7523 | 2735.2351 | 2735.2326 | 0.90 | 0 | 78 | 5.4e-08 | 1Score **> 33** indicates **identity** Score **> 17** indicates **homology** | U | K.YFYDQCPAVAGYGPIEQLPDYNR.I |
| 169191 | 448 | – | 470 | 1368.6253 | 2735.2360 | 2735.2326 | 1.24 | 0 | 68 | 4.2e-07 | 1Score **> 33** indicates **identity** Score **> 17** indicates **homology** | U | K.YFYDQCPAVAGYGPIEQLPDYNR.I |
| 169192 | 448 | – | 470 | 912.7528 | 2735.2366 | 2735.2326 | 1.45 | 0 | 51 | 1.6e-05 | 1Score **> 33** indicates **identity** Score **> 16** indicates **homology** | U | K.YFYDQCPAVAGYGPIEQLPDYNR.I |
| 169193 | 448 | – | 470 | 912.7529 | 2735.2368 | 2735.2326 | 1.55 | 0 | 67 | 5.8e-07 | 1Score **> 33** indicates **identity** Score **> 17** indicates **homology** | U | K.YFYDQCPAVAGYGPIEQLPDYNR.I |
| 169194 | 448 | – | 470 | 1368.6259 | 2735.2373 | 2735.2326 | 1.73 | 0 | 80 | 3.2e-08 | 1Score **> 33** indicates **identity** Score **> 17** indicates **homology** | U | K.YFYDQCPAVAGYGPIEQLPDYNR.I |
| 169195 | 448 | – | 470 | 912.7531 | 2735.2374 | 2735.2326 | 1.75 | 0 | 76 | 1.8e-07 | 1Score **> 33** indicates **identity** Score **> 21** indicates **homology** | U | K.YFYDQCPAVAGYGPIEQLPDYNR.I |
| 169196 | 448 | – | 470 | 1368.6273 | 2735.2401 | 2735.2326 | 2.74 | 0 | 83 | 2.5e-08 | 1Score **> 33** indicates **identity** Score **> 19** indicates **homology** | U | K.YFYDQCPAVAGYGPIEQLPDYNR.I |
| 169197 | 448 | – | 470 | 912.7544 | 2735.2414 | 2735.2326 | 3.21 | 0 | 31 | 0.0016 | 1Score **> 33** indicates **identity** Score **> 16** indicates **homology** | U | K.YFYDQCPAVAGYGPIEQLPDYNR.I |
| 169198 | 448 | – | 470 | 1368.6282 | 2735.2418 | 2735.2326 | 3.36 | 0 | 92 | 2.6e-09 | 1Score **> 33** indicates **identity** Score **> 18** indicates **homology** | U | K.YFYDQCPAVAGYGPIEQLPDYNR.I |
| 169199 | 448 | – | 470 | 1368.6292 | 2735.2438 | 2735.2326 | 4.11 | 0 | 98 | 6.4e-10 | 1Score **> 33** indicates **identity** Score **> 19** indicates **homology** | U | K.YFYDQCPAVAGYGPIEQLPDYNR.I |
| 169200 | 448 | – | 470 | 912.7558 | 2735.2457 | 2735.2326 | 4.78 | 0 | 76 | 2.2e-07 | 1Score **> 33** indicates **identity** Score **> 22** indicates **homology** | U | K.YFYDQCPAVAGYGPIEQLPDYNR.I |
| 169201 | 448 | – | 470 | 912.7577 | 2735.2514 | 2735.2326 | 6.87 | 0 | 38 | 0.00081 | 1Score **> 34** indicates **identity** Score **> 19** indicates **homology** | U | K.YFYDQCPAVAGYGPIEQLPDYNR.I |
| 169203 | 448 | – | 470 | 912.7602 | 2735.2589 | 2735.2326 | 9.60 | 0 | 24 | 0.012 | 1Score **> 34** indicates **identity** Score **> 17** indicates **homology** | U | K.YFYDQCPAVAGYGPIEQLPDYNR.I |
| 169267 | 448 | – | 470 | 913.0842 | 2736.2306 | 2736.2166 | 5.13 | 0 | 21 | 0.01 | 1Score **> 32** indicates **identity** Score **> 14** indicates **homology** | U | K.YFYDQCPAVAGYGPIEQLPDYNR.I  + Deamidated (NQ) |
| 169268 | 448 | – | 470 | 1369.1288 | 2736.2430 | 2736.2166 | 9.64 | 0 | 80 | 3.4e-08 | 1Score **> 33** indicates **identity** Score **> 17** indicates **homology** | U | K.YFYDQCPAVAGYGPIEQLPDYNR.I  + Deamidated (NQ) |
| 169269 | 448 | – | 470 | 913.0885 | 2736.2435 | 2736.2166 | 9.84 | 0 | 22 | 0.0096 | 1Score **> 33** indicates **identity** Score **> 14** indicates **homology** | U | K.YFYDQCPAVAGYGPIEQLPDYNR.I  + Deamidated (NQ) |
| 178695 | 448 | – | 472 | 1002.4815 | 3004.4226 | 3004.4178 | 1.61 | 1 | 78 | 4.8e-08 | 1Score **> 36** indicates **identity** Score **> 17** indicates **homology** | U | K.YFYDQCPAVAGYGPIEQLPDYNRIR.S |
| 178696 | 448 | – | 472 | 1002.4818 | 3004.4236 | 3004.4178 | 1.93 | 1 | 71 | 2.1e-07 | 1Score **> 36** indicates **identity** Score **> 17** indicates **homology** | U | K.YFYDQCPAVAGYGPIEQLPDYNRIR.S |
| 178698 | 448 | – | 472 | 1002.4818 | 3004.4236 | 3004.4178 | 1.95 | 1 | 75 | 1e-07 | 1Score **> 36** indicates **identity** Score **> 17** indicates **homology** | U | K.YFYDQCPAVAGYGPIEQLPDYNRIR.S |
| 178699 | 448 | – | 472 | 1002.4821 | 3004.4246 | 3004.4178 | 2.28 | 1 | 67 | 5.3e-07 | 1Score **> 36** indicates **identity** Score **> 17** indicates **homology** | U | K.YFYDQCPAVAGYGPIEQLPDYNRIR.S |
| 178701 | 448 | – | 472 | 1002.4826 | 3004.4258 | 3004.4178 | 2.69 | 1 | 77 | 5.6e-08 | 1Score **> 36** indicates **identity** Score **> 17** indicates **homology** | U | K.YFYDQCPAVAGYGPIEQLPDYNRIR.S |
| 178703 | 448 | – | 472 | 1002.4837 | 3004.4292 | 3004.4178 | 3.82 | 1 | 17 | 0.026 | 1Score **> 36** indicates **identity** Score **> 14** indicates **homology** | U | K.YFYDQCPAVAGYGPIEQLPDYNRIR.S |
| 178707 | 448 | – | 472 | 1002.4892 | 3004.4457 | 3004.4178 | 9.29 | 1 | 50 | 2e-05 | 1Score **> 37** indicates **identity** Score **> 16** indicates **homology** | U | K.YFYDQCPAVAGYGPIEQLPDYNRIR.S |
| 178722 | 448 | – | 472 | 1503.7192 | 3005.4238 | 3005.4018 | 7.34 | 1 | 17 | 0.027 | 1Score **> 36** indicates **identity** Score **> 14** indicates **homology** | U | K.YFYDQCPAVAGYGPIEQLPDYNRIR.S  + Deamidated (NQ) |
| 178723 | 448 | – | 472 | 1002.8174 | 3005.4304 | 3005.4018 | 9.53 | 1 | 20 | 0.013 | 1Score **> 36** indicates **identity** Score **> 14** indicates **homology** | U | K.YFYDQCPAVAGYGPIEQLPDYNRIR.S  + Deamidated (NQ) |
| 178791 | 448 | – | 472 | 1003.1405 | 3006.3997 | 3006.3858 | 4.63 | 1 | 35 | 0.00055 | 1Score **> 35** indicates **identity** Score **> 15** indicates **homology** | U | K.YFYDQCPAVAGYGPIEQLPDYNRIR.S  + 2 Deamidated (NQ) |
| 178792 | 448 | – | 472 | 1003.1434 | 3006.4083 | 3006.3858 | 7.49 | 1 | 15 | 0.036 | 1Score **> 35** indicates **identity** Score **> 13** indicates **homology** | U | K.YFYDQCPAVAGYGPIEQLPDYNRIR.S  + 2 Deamidated (NQ) |
| 7355 | 473 | – | 479 | 448.7259 | 895.4372 | 895.4374 | -0.24 | 0 | 42 | 0.0016 | 1Score **> 27** indicates **identity** | U | R.SGMFWLR.F |
| 7356 | 473 | – | 479 | 448.7259 | 895.4373 | 895.4374 | -0.11 | 0 | 42 | 0.0018 | 1Score **> 27** indicates **identity** | U | R.SGMFWLR.F |
| 7357 | 473 | – | 479 | 448.7261 | 895.4377 | 895.4374 | 0.25 | 0 | 43 | 0.0011 | 1Score **> 25** indicates **identity** | U | R.SGMFWLR.F |
| 7358 | 473 | – | 479 | 448.7269 | 895.4392 | 895.4374 | 1.92 | 0 | 33 | 0.00079 | 1Score **> 27** indicates **identity** Score **> 15** indicates **homology** | U | R.SGMFWLR.F |
| 7359 | 473 | – | 479 | 448.7282 | 895.4418 | 895.4374 | 4.88 | 0 | 28 | 0.0025 | 1Score **> 27** indicates **identity** Score **> 14** indicates **homology** | U | R.SGMFWLR.F |
| 15613 | 473 | – | 480 | 522.2609 | 1042.5073 | 1042.5059 | 1.36 | 1 | 54 | 9e-06 | 1Score **> 28** indicates **identity** Score **> 16** indicates **homology** | U | R.SGMFWLRF.- |
| 15614 | 473 | – | 480 | 522.2611 | 1042.5077 | 1042.5059 | 1.80 | 1 | 53 | 1.1e-05 | 1Score **> 28** indicates **identity** Score **> 16** indicates **homology** | U | R.SGMFWLRF.- |
| 15615 | 473 | – | 480 | 522.2613 | 1042.5080 | 1042.5059 | 2.05 | 1 | 48 | 3.4e-05 | 1Score **> 29** indicates **identity** Score **> 15** indicates **homology** | U | R.SGMFWLRF.- |
| 16999 | 473 | – | 480 | 530.2574 | 1058.5003 | 1058.5008 | -0.47 | 1 | 17 | 0.024 | 1Score **> 28** indicates **identity** Score **> 14** indicates **homology** | U | R.SGMFWLRF.-  + Oxidation (M) |
| 17000 | 473 | – | 480 | 530.2576 | 1058.5006 | 1058.5008 | -0.12 | 1 | 20 | 0.013 | 1Score **> 28** indicates **identity** Score **> 14** indicates **homology** | U | R.SGMFWLRF.-  + Oxidation (M) |
| 17001 | 473 | – | 480 | 530.2581 | 1058.5017 | 1058.5008 | 0.84 | 1 | 21 | 0.0099 | 1Score **> 28** indicates **identity** Score **> 14** indicates **homology** | U | R.SGMFWLRF.-  + Oxidation (M) |

---

```
ID   QCR1_MOUSE              Reviewed;         480 AA.
AC   Q9CZ13; Q3TV75; Q9CWL6;
DT   20-JUN-2001, integrated into UniProtKB/Swiss-Prot.
DT   27-JUL-2011, sequence version 2.
DT   28-JUN-2023, entry version 182.
DE   RecName: Full=Cytochrome b-c1 complex subunit 1, mitochondrial;
DE   AltName: Full=Complex III subunit 1;
DE   AltName: Full=Core protein I;
DE   AltName: Full=Ubiquinol-cytochrome-c reductase complex core protein 1;
DE   Flags: Precursor;
GN   Name=Uqcrc1;
OS   Mus musculus (Mouse).
OC   Eukaryota; Metazoa; Chordata; Craniata; Vertebrata; Euteleostomi; Mammalia;
OC   Eutheria; Euarchontoglires; Glires; Rodentia; Myomorpha; Muroidea; Muridae;
OC   Murinae; Mus; Mus.
OX   NCBI_TaxID=10090;
RN   [1]
RP   NUCLEOTIDE SEQUENCE [LARGE SCALE MRNA].
RC   STRAIN=C57BL/6J; TISSUE=Bone marrow, and Embryo;
RX   PubMed=16141072; DOI=10.1126/science.1112014;
RA   Carninci P., Kasukawa T., Katayama S., Gough J., Frith M.C., Maeda N.,
RA   Oyama R., Ravasi T., Lenhard B., Wells C., Kodzius R., Shimokawa K.,
RA   Bajic V.B., Brenner S.E., Batalov S., Forrest A.R., Zavolan M., Davis M.J.,
RA   Wilming L.G., Aidinis V., Allen J.E., Ambesi-Impiombato A., Apweiler R.,
RA   Aturaliya R.N., Bailey T.L., Bansal M., Baxter L., Beisel K.W., Bersano T.,
RA   Bono H., Chalk A.M., Chiu K.P., Choudhary V., Christoffels A.,
RA   Clutterbuck D.R., Crowe M.L., Dalla E., Dalrymple B.P., de Bono B.,
RA   Della Gatta G., di Bernardo D., Down T., Engstrom P., Fagiolini M.,
RA   Faulkner G., Fletcher C.F., Fukushima T., Furuno M., Futaki S.,
RA   Gariboldi M., Georgii-Hemming P., Gingeras T.R., Gojobori T., Green R.E.,
RA   Gustincich S., Harbers M., Hayashi Y., Hensch T.K., Hirokawa N., Hill D.,
RA   Huminiecki L., Iacono M., Ikeo K., Iwama A., Ishikawa T., Jakt M.,
RA   Kanapin A., Katoh M., Kawasawa Y., Kelso J., Kitamura H., Kitano H.,
RA   Kollias G., Krishnan S.P., Kruger A., Kummerfeld S.K., Kurochkin I.V.,
RA   Lareau L.F., Lazarevic D., Lipovich L., Liu J., Liuni S., McWilliam S.,
RA   Madan Babu M., Madera M., Marchionni L., Matsuda H., Matsuzawa S., Miki H.,
RA   Mignone F., Miyake S., Morris K., Mottagui-Tabar S., Mulder N., Nakano N.,
RA   Nakauchi H., Ng P., Nilsson R., Nishiguchi S., Nishikawa S., Nori F.,
RA   Ohara O., Okazaki Y., Orlando V., Pang K.C., Pavan W.J., Pavesi G.,
RA   Pesole G., Petrovsky N., Piazza S., Reed J., Reid J.F., Ring B.Z.,
RA   Ringwald M., Rost B., Ruan Y., Salzberg S.L., Sandelin A., Schneider C.,
RA   Schoenbach C., Sekiguchi K., Semple C.A., Seno S., Sessa L., Sheng Y.,
RA   Shibata Y., Shimada H., Shimada K., Silva D., Sinclair B., Sperling S.,
RA   Stupka E., Sugiura K., Sultana R., Takenaka Y., Taki K., Tammoja K.,
RA   Tan S.L., Tang S., Taylor M.S., Tegner J., Teichmann S.A., Ueda H.R.,
RA   van Nimwegen E., Verardo R., Wei C.L., Yagi K., Yamanishi H.,
RA   Zabarovsky E., Zhu S., Zimmer A., Hide W., Bult C., Grimmond S.M.,
RA   Teasdale R.D., Liu E.T., Brusic V., Quackenbush J., Wahlestedt C.,
RA   Mattick J.S., Hume D.A., Kai C., Sasaki D., Tomaru Y., Fukuda S.,
RA   Kanamori-Katayama M., Suzuki M., Aoki J., Arakawa T., Iida J., Imamura K.,
RA   Itoh M., Kato T., Kawaji H., Kawagashira N., Kawashima T., Kojima M.,
RA   Kondo S., Konno H., Nakano K., Ninomiya N., Nishio T., Okada M., Plessy C.,
RA   Shibata K., Shiraki T., Suzuki S., Tagami M., Waki K., Watahiki A.,
RA   Okamura-Oho Y., Suzuki H., Kawai J., Hayashizaki Y.;
RT   "The transcriptional landscape of the mammalian genome.";
RL   Science 309:1559-1563(2005).
RN   [2]
RP   NUCLEOTIDE SEQUENCE [LARGE SCALE GENOMIC DNA].
RA   Mural R.J., Adams M.D., Myers E.W., Smith H.O., Venter J.C.;
RL   Submitted (JUL-2005) to the EMBL/GenBank/DDBJ databases.
RN   [3]
RP   PROTEIN SEQUENCE OF 59-80; 86-99; 112-134; 143-163; 214-222; 229-248;
RP   256-276; 379-390; 397-442; 448-470 AND 473-479, AND IDENTIFICATION BY MASS
RP   SPECTROMETRY.
RC   STRAIN=C57BL/6J; TISSUE=Brain, and Hippocampus;
RA   Lubec G., Kang S.U., Klug S., Yang J.W., Zigmond M.;
RL   Submitted (JUL-2007) to UniProtKB.
RN   [4]
RP   SUBUNIT.
RX   PubMed=19026783; DOI=10.1016/j.molcel.2008.10.021;
RA   Acin-Perez R., Fernandez-Silva P., Peleato M.L., Perez-Martos A.,
RA   Enriquez J.A.;
RT   "Respiratory active mitochondrial supercomplexes.";
RL   Mol. Cell 32:529-539(2008).
RN   [5]
RP   IDENTIFICATION BY MASS SPECTROMETRY [LARGE SCALE ANALYSIS].
RC   TISSUE=Brain, Brown adipose tissue, Heart, Kidney, Liver, Lung,
RC   Pancreas, Spleen, and Testis;
RX   PubMed=21183079; DOI=10.1016/j.cell.2010.12.001;
RA   Huttlin E.L., Jedrychowski M.P., Elias J.E., Goswami T., Rad R.,
RA   Beausoleil S.A., Villen J., Haas W., Sowa M.E., Gygi S.P.;
RT   "A tissue-specific atlas of mouse protein phosphorylation and expression.";
RL   Cell 143:1174-1189(2010).
RN   [6]
RP   SUCCINYLATION [LARGE SCALE ANALYSIS] AT LYS-163, AND IDENTIFICATION BY MASS
RP   SPECTROMETRY [LARGE SCALE ANALYSIS].
RC   TISSUE=Liver;
RX   PubMed=23806337; DOI=10.1016/j.molcel.2013.06.001;
RA   Park J., Chen Y., Tishkoff D.X., Peng C., Tan M., Dai L., Xie Z., Zhang Y.,
RA   Zwaans B.M., Skinner M.E., Lombard D.B., Zhao Y.;
RT   "SIRT5-mediated lysine desuccinylation impacts diverse metabolic
RT   pathways.";
RL   Mol. Cell 50:919-930(2013).
RN   [7]
RP   ACETYLATION [LARGE SCALE ANALYSIS] AT LYS-111; LYS-138; LYS-163 AND
RP   LYS-248, AND IDENTIFICATION BY MASS SPECTROMETRY [LARGE SCALE ANALYSIS].
RC   TISSUE=Liver;
RX   PubMed=23576753; DOI=10.1073/pnas.1302961110;
RA   Rardin M.J., Newman J.C., Held J.M., Cusack M.P., Sorensen D.J., Li B.,
RA   Schilling B., Mooney S.D., Kahn C.R., Verdin E., Gibson B.W.;
RT   "Label-free quantitative proteomics of the lysine acetylome in mitochondria
RT   identifies substrates of SIRT3 in metabolic pathways.";
RL   Proc. Natl. Acad. Sci. U.S.A. 110:6601-6606(2013).
RN   [8]
RP   TISSUE SPECIFICITY, AND DISRUPTION PHENOTYPE.
RX   PubMed=30666338; DOI=10.1007/s00018-019-03007-6;
RA   Shan W., Li J., Xu W., Li H., Zuo Z.;
RT   "Critical role of UQCRC1 in embryo survival, brain ischemic tolerance and
RT   normal cognition in mice.";
RL   Cell. Mol. Life Sci. 76:1381-1396(2019).
RN   [9]
RP   INTERACTION WITH UQCC6.
RX   PubMed=32161263; DOI=10.1038/s41467-020-14999-2;
RA   Zhang S., Reljic B., Liang C., Kerouanton B., Francisco J.C., Peh J.H.,
RA   Mary C., Jagannathan N.S., Olexiouk V., Tang C., Fidelito G., Nama S.,
RA   Cheng R.K., Wee C.L., Wang L.C., Duek Roggli P., Sampath P., Lane L.,
RA   Petretto E., Sobota R.M., Jesuthasan S., Tucker-Kellogg L., Reversade B.,
RA   Menschaert G., Sun L., Stroud D.A., Ho L.;
RT   "Mitochondrial peptide BRAWNIN is essential for vertebrate respiratory
RT   complex III assembly.";
RL   Nat. Commun. 11:1312-1312(2020).
RN   [10]
RP   INTERACTION WITH STMP1.
RX   PubMed=35101990; DOI=10.1073/pnas.2120476119;
RA   Makarewich C.A., Munir A.Z., Bezprozvannaya S., Gibson A.M., Young Kim S.,
RA   Martin-Sandoval M.S., Mathews T.P., Szweda L.I., Bassel-Duby R.,
RA   Olson E.N.;
RT   "The cardiac-enriched microprotein mitolamban regulates mitochondrial
RT   respiratory complex assembly and function in mice.";
RL   Proc. Natl. Acad. Sci. U.S.A. 119:0-0(2022).
CC   -!- FUNCTION: Component of the ubiquinol-cytochrome c oxidoreductase, a
CC       multisubunit transmembrane complex that is part of the mitochondrial
CC       electron transport chain which drives oxidative phosphorylation. The
CC       respiratory chain contains 3 multisubunit complexes succinate
CC       dehydrogenase (complex II, CII), ubiquinol-cytochrome c oxidoreductase
CC       (cytochrome b-c1 complex, complex III, CIII) and cytochrome c oxidase
CC       (complex IV, CIV), that cooperate to transfer electrons derived from
CC       NADH and succinate to molecular oxygen, creating an electrochemical
CC       gradient over the inner membrane that drives transmembrane transport
CC       and the ATP synthase. The cytochrome b-c1 complex catalyzes electron
CC       transfer from ubiquinol to cytochrome c, linking this redox reaction to
CC       translocation of protons across the mitochondrial inner membrane, with
CC       protons being carried across the membrane as hydrogens on the quinol.
CC       In the process called Q cycle, 2 protons are consumed from the matrix,
CC       4 protons are released into the intermembrane space and 2 electrons are
CC       passed to cytochrome c (By similarity). The 2 core subunits UQCRC1/QCR1
CC       and UQCRC2/QCR2 are homologous to the 2 mitochondrial-processing
CC       peptidase (MPP) subunits beta-MPP and alpha-MPP respectively, and they
CC       seem to have preserved their MPP processing properties. May be involved
CC       in the in situ processing of UQCRFS1 into the mature Rieske protein and
CC       its mitochondrial targeting sequence (MTS)/subunit 9 when incorporated
CC       into complex III (By similarity). Seems to play an important role in
CC       the maintenance of proper mitochondrial function in nigral dopaminergic
CC       neurons (By similarity). {ECO:0000250|UniProtKB:P07256,
CC       ECO:0000250|UniProtKB:P31800, ECO:0000250|UniProtKB:P31930}.
CC   -!- SUBUNIT: Component of the ubiquinol-cytochrome c oxidoreductase
CC       (cytochrome b-c1 complex, complex III, CIII), a multisubunit enzyme
CC       composed of 11 subunits. The complex is composed of 3 respiratory
CC       subunits cytochrome b, cytochrome c1 and Rieske protein UQCRFS1, 2 core
CC       protein subunits UQCRC1/QCR1 and UQCRC2/QCR2, and 6 low-molecular
CC       weight protein subunits UQCRH/QCR6, UQCRB/QCR7, UQCRQ/QCR8,
CC       UQCR10/QCR9, UQCR11/QCR10 and subunit 9, the cleavage product of Rieske
CC       protein UQCRFS1 (By similarity). The complex exists as an obligatory
CC       dimer and forms supercomplexes (SCs) in the inner mitochondrial
CC       membrane with NADH-ubiquinone oxidoreductase (complex I, CI) and
CC       cytochrome c oxidase (complex IV, CIV), resulting in different
CC       assemblies (supercomplex SCI(1)III(2)IV(1) and megacomplex
CC       MCI(2)III(2)IV(2)) (PubMed:19026783). Interacts with UQCC6
CC       (PubMed:32161263). Interacts with STMP1 (PubMed:35101990).
CC       {ECO:0000250|UniProtKB:P31800, ECO:0000269|PubMed:19026783,
CC       ECO:0000269|PubMed:32161263, ECO:0000269|PubMed:35101990}.
CC   -!- SUBCELLULAR LOCATION: Mitochondrion inner membrane
CC       {ECO:0000250|UniProtKB:P07256}; Peripheral membrane protein
CC       {ECO:0000250|UniProtKB:P07256}; Matrix side
CC       {ECO:0000250|UniProtKB:P07256}.
CC   -!- TISSUE SPECIFICITY: Expressed in neurons and astrocytes of the cerebral
CC       cortex and hippocampus (at protein level).
CC       {ECO:0000269|PubMed:30666338}.
CC   -!- PTM: Acetylation of Lys-138 is observed in liver mitochondria from
CC       fasted mice but not from fed mice.
CC   -!- DISRUPTION PHENOTYPE: Results in early embryonic lethality.
CC       {ECO:0000269|PubMed:30666338}.
CC   -!- SIMILARITY: Belongs to the peptidase M16 family. UQCRC1/QCR1 subfamily.
CC       {ECO:0000305}.
CC   ---------------------------------------------------------------------------
CC   Copyrighted by the UniProt Consortium, see https://www.uniprot.org/terms
CC   Distributed under the Creative Commons Attribution (CC BY 4.0) License
CC   ---------------------------------------------------------------------------
DR   EMBL; AK013128; BAB28666.1; -; mRNA.
DR   EMBL; AK010553; BAB27022.1; -; mRNA.
DR   EMBL; AK151764; BAE30670.1; -; mRNA.
DR   EMBL; AK160337; BAE35744.1; -; mRNA.
DR   EMBL; CH466560; EDL21324.1; -; Genomic_DNA.
DR   CCDS; CCDS23540.1; -.
DR   RefSeq; NP_079683.2; NM_025407.2.
DR   PDB; 7O37; EM; 3.20 A; A/L=35-480.
DR   PDB; 7O3C; EM; 3.30 A; A/L=35-480.
DR   PDB; 7O3E; EM; 3.60 A; A/L=35-480.
DR   PDB; 7O3H; EM; 2.60 A; A/L=35-480.
DR   PDBsum; 7O37; -.
DR   PDBsum; 7O3C; -.
DR   PDBsum; 7O3E; -.
DR   PDBsum; 7O3H; -.
DR   AlphaFoldDB; Q9CZ13; -.
DR   SMR; Q9CZ13; -.
DR   BioGRID; 204459; 58.
DR   ComplexPortal; CPX-563; Mitochondrial respiratory chain complex III.
DR   CORUM; Q9CZ13; -.
DR   IntAct; Q9CZ13; 11.
DR   MINT; Q9CZ13; -.
DR   STRING; 10090.ENSMUSP00000026743; -.
DR   MEROPS; M16.975; -.
DR   MEROPS; M16.981; -.
DR   GlyGen; Q9CZ13; 2 sites, 1 O-linked glycan (2 sites).
DR   iPTMnet; Q9CZ13; -.
DR   PhosphoSitePlus; Q9CZ13; -.
DR   SwissPalm; Q9CZ13; -.
DR   REPRODUCTION-2DPAGE; IPI00111885; -.
DR   REPRODUCTION-2DPAGE; Q9CZ13; -.
DR   UCD-2DPAGE; Q9CZ13; -.
DR   EPD; Q9CZ13; -.
DR   jPOST; Q9CZ13; -.
DR   MaxQB; Q9CZ13; -.
DR   PaxDb; Q9CZ13; -.
DR   PeptideAtlas; Q9CZ13; -.
DR   ProteomicsDB; 301902; -.
DR   TopDownProteomics; Q9CZ13; -.
DR   Antibodypedia; 1257; 399 antibodies from 29 providers.
DR   DNASU; 22273; -.
DR   Ensembl; ENSMUST00000026743; ENSMUSP00000026743; ENSMUSG00000025651.
DR   GeneID; 22273; -.
DR   KEGG; mmu:22273; -.
DR   UCSC; uc009rrg.1; mouse.
DR   AGR; MGI:107876; -.
DR   CTD; 7384; -.
DR   MGI; MGI:107876; Uqcrc1.
DR   VEuPathDB; HostDB:ENSMUSG00000025651; -.
DR   eggNOG; KOG0960; Eukaryota.
DR   GeneTree; ENSGT00940000158931; -.
DR   HOGENOM; CLU_009902_4_2_1; -.
DR   InParanoid; Q9CZ13; -.
DR   OMA; WSNPDNV; -.
DR   OrthoDB; 167798at2759; -.
DR   PhylomeDB; Q9CZ13; -.
DR   TreeFam; TF105032; -.
DR   Reactome; R-MMU-611105; Respiratory electron transport.
DR   BioGRID-ORCS; 22273; 24 hits in 79 CRISPR screens.
DR   ChiTaRS; Uqcrc1; mouse.
DR   PRO; PR:Q9CZ13; -.
DR   Proteomes; UP000000589; Chromosome 9.
DR   RNAct; Q9CZ13; protein.
DR   Bgee; ENSMUSG00000025651; Expressed in proximal tubule and 143 other tissues.
DR   ExpressionAtlas; Q9CZ13; baseline and differential.
DR   Genevisible; Q9CZ13; MM.
DR   GO; GO:0005743; C:mitochondrial inner membrane; HDA:MGI.
DR   GO; GO:0005750; C:mitochondrial respiratory chain complex III; ISO:MGI.
DR   GO; GO:0005739; C:mitochondrion; IDA:MGI.
DR   GO; GO:0043209; C:myelin sheath; HDA:UniProtKB.
DR   GO; GO:0046872; F:metal ion binding; IEA:InterPro.
DR   GO; GO:0044877; F:protein-containing complex binding; ISO:MGI.
DR   GO; GO:0031625; F:ubiquitin protein ligase binding; ISO:MGI.
DR   GO; GO:0045333; P:cellular respiration; NAS:ComplexPortal.
DR   GO; GO:0006122; P:mitochondrial electron transport, ubiquinol to cytochrome c; IDA:MGI.
DR   GO; GO:0034551; P:mitochondrial respiratory chain complex III assembly; ISS:UniProtKB.
DR   GO; GO:0014823; P:response to activity; IEA:Ensembl.
DR   GO; GO:0043279; P:response to alkaloid; IEA:Ensembl.
DR   Gene3D; 3.30.830.10; Metalloenzyme, LuxS/M16 peptidase-like; 2.
DR   InterPro; IPR011249; Metalloenz_LuxS/M16.
DR   InterPro; IPR011765; Pept_M16_N.
DR   InterPro; IPR007863; Peptidase_M16_C.
DR   PANTHER; PTHR11851:SF116; CYTOCHROME B-C1 COMPLEX SUBUNIT 1, MITOCHONDRIAL; 1.
DR   PANTHER; PTHR11851; METALLOPROTEASE; 1.
DR   Pfam; PF00675; Peptidase_M16; 1.
DR   Pfam; PF05193; Peptidase_M16_C; 1.
DR   SUPFAM; SSF63411; LuxS/MPP-like metallohydrolase; 2.
PE   1: Evidence at protein level;
KW   3D-structure; Acetylation; Direct protein sequencing; Electron transport;
KW   Membrane; Mitochondrion; Mitochondrion inner membrane; Phosphoprotein;
KW   Reference proteome; Respiratory chain; Transit peptide; Transport.
FT   TRANSIT         1..34
FT                   /note="Mitochondrion"
FT                   /evidence="ECO:0000250"
FT   CHAIN           35..480
FT                   /note="Cytochrome b-c1 complex subunit 1, mitochondrial"
FT                   /id="PRO_0000026787"
FT   MOD_RES         111
FT                   /note="N6-acetyllysine"
FT                   /evidence="ECO:0007744|PubMed:23576753"
FT   MOD_RES         138
FT                   /note="N6-acetyllysine"
FT                   /evidence="ECO:0007744|PubMed:23576753"
FT   MOD_RES         163
FT                   /note="N6-acetyllysine; alternate"
FT                   /evidence="ECO:0007744|PubMed:23576753"
FT   MOD_RES         163
FT                   /note="N6-succinyllysine; alternate"
FT                   /evidence="ECO:0007744|PubMed:23806337"
FT   MOD_RES         212
FT                   /note="Phosphoserine"
FT                   /evidence="ECO:0000250|UniProtKB:Q68FY0"
FT   MOD_RES         214
FT                   /note="Phosphothreonine"
FT                   /evidence="ECO:0000250|UniProtKB:Q68FY0"
FT   MOD_RES         248
FT                   /note="N6-acetyllysine"
FT                   /evidence="ECO:0007744|PubMed:23576753"
FT   CONFLICT        223
FT                   /note="H -> N (in Ref. 1; BAB28666)"
FT                   /evidence="ECO:0000305"
FT   CONFLICT        318
FT                   /note="Y -> C (in Ref. 1; BAB28666)"
FT                   /evidence="ECO:0000305"
FT   HELIX           38..43
FT                   /evidence="ECO:0007829|PDB:7O3H"
FT   STRAND          49..52
FT                   /evidence="ECO:0007829|PDB:7O3H"
FT   STRAND          58..63
FT                   /evidence="ECO:0007829|PDB:7O3H"
FT   STRAND          67..76
FT                   /evidence="ECO:0007829|PDB:7O3H"
FT   HELIX           79..81
FT                   /evidence="ECO:0007829|PDB:7O3H"
FT   STRAND          84..86
FT                   /evidence="ECO:0007829|PDB:7O3H"
FT   HELIX           89..96
FT                   /evidence="ECO:0007829|PDB:7O3H"
FT   STRAND          101..104
FT                   /evidence="ECO:0007829|PDB:7O3H"
FT   HELIX           107..115
FT                   /evidence="ECO:0007829|PDB:7O3H"
FT   STRAND          119..124
FT                   /evidence="ECO:0007829|PDB:7O3H"
FT   STRAND          129..137
FT                   /evidence="ECO:0007829|PDB:7O3H"
FT   HELIX           140..152
FT                   /evidence="ECO:0007829|PDB:7O3H"
FT   HELIX           158..175
FT                   /evidence="ECO:0007829|PDB:7O3H"
FT   HELIX           179..191
FT                   /evidence="ECO:0007829|PDB:7O3H"
FT   TURN            196..198
FT                   /evidence="ECO:0007829|PDB:7O3H"
FT   HELIX           205..210
FT                   /evidence="ECO:0007829|PDB:7O3H"
FT   HELIX           213..223
FT                   /evidence="ECO:0007829|PDB:7O3H"
FT   HELIX           226..228
FT                   /evidence="ECO:0007829|PDB:7O3H"
FT   STRAND          229..236
FT                   /evidence="ECO:0007829|PDB:7O3H"
FT   HELIX           239..249
FT                   /evidence="ECO:0007829|PDB:7O3H"
FT   STRAND          250..253
FT                   /evidence="ECO:0007829|PDB:7O37"
FT   STRAND          258..260
FT                   /evidence="ECO:0007829|PDB:7O3H"
FT   STRAND          273..278
FT                   /evidence="ECO:0007829|PDB:7O3H"
FT   STRAND          282..292
FT                   /evidence="ECO:0007829|PDB:7O3H"
FT   HELIX           300..311
FT                   /evidence="ECO:0007829|PDB:7O3H"
FT   STRAND          313..315
FT                   /evidence="ECO:0007829|PDB:7O3C"
FT   HELIX           321..323
FT                   /evidence="ECO:0007829|PDB:7O3H"
FT   HELIX           327..335
FT                   /evidence="ECO:0007829|PDB:7O3H"
FT   STRAND          339..348
FT                   /evidence="ECO:0007829|PDB:7O3H"
FT   STRAND          351..360
FT                   /evidence="ECO:0007829|PDB:7O3H"
FT   HELIX           362..364
FT                   /evidence="ECO:0007829|PDB:7O3H"
FT   HELIX           365..381
FT                   /evidence="ECO:0007829|PDB:7O3H"
FT   HELIX           385..402
FT                   /evidence="ECO:0007829|PDB:7O3H"
FT   HELIX           406..418
FT                   /evidence="ECO:0007829|PDB:7O3H"
FT   HELIX           426..434
FT                   /evidence="ECO:0007829|PDB:7O3H"
FT   HELIX           438..448
FT                   /evidence="ECO:0007829|PDB:7O3H"
FT   TURN            449..451
FT                   /evidence="ECO:0007829|PDB:7O3H"
FT   STRAND          455..461
FT                   /evidence="ECO:0007829|PDB:7O3H"
FT   STRAND          463..465
FT                   /evidence="ECO:0007829|PDB:7O3H"
FT   HELIX           468..474
FT                   /evidence="ECO:0007829|PDB:7O3H"
SQ   SEQUENCE   480 AA;  52852 MW;  8150C97C655A91C9 CRC64;
     MAASAVCRAA CSGTQVLLRT RRSPALLRLP ALRGTATFAQ ALQSVPETQV SILDNGLRVA
     SEQSSHATCT VGVWIDAGSR YETEKNNGAG YFLEHLAFKG TKNRPGNALE KEVESIGAHL
     NAYSTREHTA YLIKALSKDL PKVVELLADI VQNSSLEDSQ IEKERDVILR EMQENDASMQ
     NVVFDYLHAT AFQGTPLAQA VEGPSENVRR LSRTDLTDYL NRHYKAPRMV LAAAGGVEHQ
     QLLDLAQKHL SSVSRVYEED AVPGLTPCRF TGSEIRHRDD ALPLAHVAIA VEGPGWANPD
     NVTLQVANAI IGHYDCTYGG GVHLSSPLAS VAVANKLCQS FQTFNISYSD TGLLGAHFVC
     DAMSIDDMVF FLQGQWMRLC TSATESEVTR GKNILRNALV SHLDGTTPVC EDIGRSLLTY
     GRRIPLAEWE SRIQEVDAQM LRDICSKYFY DQCPAVAGYG PIEQLPDYNR IRSGMFWLRF
//
```

**Mascot:** http://www.matrixscience.com/


HNE (K) (+156.1150)
