# Supplementary material for: A common East-Asian ALDH2 mutation causes metabolic disorders and the therapeutic effect of ALDH2 activators
Source: Nat Commun. 2023 Sep 25;14:5971. doi: 10.1038/s41467-023-41570-6 (PMC10520061; doi:10.1038/s41467-023-41570-6)
Supplement: Supplementary file 4 — Supplementary Data 1 [file 41467_2023_41570_MOESM4_ESM.zip › Table S5c/Q9CZ13/Q9CZ13_KIO-2_K111.html]

Mascot Search Results: Q9CZ13
 

# MASCOT Search Results

## Protein View: Q9CZ13

### Cytochrome b-c1 complex subunit 1, mitochondrial OS=Mus musculus OX=10090 GN=Uqcrc1 PE=1 SV=2

|  |  |
| --- | --- |
| Database: | Mouse\_UniProt\_proteomes |
| Score: | 11859 |
| Monoisotopic mass (M_r_): | 53446 |
| Calculated pI: | 5.81 |

Sequence similarity is available as an NCBI BLAST search of Q9CZ13 against nr.

### Search parameters

|  |  |
| --- | --- |
| MS data file: | `D:\LCMSMS\2023 Users' data\230529-1\230529-1-KIO-2.raw` |
| Enzyme: | Trypsin/P: cuts C-term side of KR. |
| Fixed modifications: | Carbamidomethyl (C) |
| Variable modifications: | Deamidated (NQ), HNE (C), HNE (H), HNE (K), Oxidation (M) |

### Protein sequence coverage: 80%

Matched peptides shown in ***bold red***.

|  |  |  |  |  |  |
| --- | --- | --- | --- | --- | --- |
| `1` | `MAASAVCRAA` | `CSGTQVLLRT` | `RRSPALLRLP` | `ALRGTATFAQ` | `ALQSVPETQV` |
| `51` | `SILDNGLRVA` | `SEQSSHATCT` | `VGVWIDAGSR` | `YETEKNNGAG` | `YFLEHLAFKG` |
| `101` | `TKNRPGNALE` | `KEVESIGAHL` | `NAYSTREHTA` | `YLIKALSKDL` | `PKVVELLADI` |
| `151` | `VQNSSLEDSQ` | `IEKERDVILR` | `EMQENDASMQ` | `NVVFDYLHAT` | `AFQGTPLAQA` |
| `201` | `VEGPSENVRR` | `LSRTDLTDYL` | `NRHYKAPRMV` | `LAAAGGVEHQ` | `QLLDLAQKHL` |
| `251` | `SSVSRVYEED` | `AVPGLTPCRF` | `TGSEIRHRDD` | `ALPLAHVAIA` | `VEGPGWANPD` |
| `301` | `NVTLQVANAI` | `IGHYDCTYGG` | `GVHLSSPLAS` | `VAVANKLCQS` | `FQTFNISYSD` |
| `351` | `TGLLGAHFVC` | `DAMSIDDMVF` | `FLQGQWMRLC` | `TSATESEVTR` | `GKNILRNALV` |
| `401` | `SHLDGTTPVC` | `EDIGRSLLTY` | `GRRIPLAEWE` | `SRIQEVDAQM` | `LRDICSKYFY` |
| `451` | `DQCPAVAGYG` | `PIEQLPDYNR` | `IRSGMFWLRF` |  |  |

Unformatted sequence string: 480 residues (for pasting into other applications).

|  |  |  |  |
| --- | --- | --- | --- |
| Sort by | residue number | increasing mass | decreasing mass |
| Show | matched peptides only | predicted peptides also |  |

| Query | Start | – | End | Observed | Mr(expt) | Mr(calc) | ppm | M | Score | Expect | Rank | U | Peptide |
| --- | --- | --- | --- | --- | --- | --- | --- | --- | --- | --- | --- | --- | --- |
| 169321 | 34 | – | 58 | 1308.6774 | 2615.3403 | 2615.3555 | -5.80 | 0 | 81 | 2.5e-08 | 1Score **> 37** indicates **identity** Score **> 18** indicates **homology** | U | R.GTATFAQALQSVPETQVSILDNGLR.V |
| 169324 | 34 | – | 58 | 1308.6827 | 2615.3509 | 2615.3555 | -1.78 | 0 | 57 | 5.6e-06 | 1Score **> 37** indicates **identity** Score **> 17** indicates **homology** | U | R.GTATFAQALQSVPETQVSILDNGLR.V |
| 169325 | 34 | – | 58 | 872.7920 | 2615.3541 | 2615.3555 | -0.53 | 0 | 43 | 9.5e-05 | 1Score **> 37** indicates **identity** Score **> 15** indicates **homology** | U | R.GTATFAQALQSVPETQVSILDNGLR.V |
| 169327 | 34 | – | 58 | 872.7926 | 2615.3561 | 2615.3555 | 0.23 | 0 | 89 | 4.9e-09 | 1Score **> 37** indicates **identity** Score **> 18** indicates **homology** | U | R.GTATFAQALQSVPETQVSILDNGLR.V |
| 169328 | 34 | – | 58 | 1308.6855 | 2615.3564 | 2615.3555 | 0.34 | 0 | 114 | 1.9e-11 | 1Score **> 37** indicates **identity** Score **> 20** indicates **homology** | U | R.GTATFAQALQSVPETQVSILDNGLR.V |
| 169329 | 34 | – | 58 | 872.7928 | 2615.3567 | 2615.3555 | 0.47 | 0 | 71 | 2.2e-07 | 1Score **> 37** indicates **identity** Score **> 17** indicates **homology** | U | R.GTATFAQALQSVPETQVSILDNGLR.V |
| 169330 | 34 | – | 58 | 872.7930 | 2615.3571 | 2615.3555 | 0.62 | 0 | 73 | 1.5e-07 | 1Score **> 37** indicates **identity** Score **> 17** indicates **homology** | U | R.GTATFAQALQSVPETQVSILDNGLR.V |
| 169332 | 34 | – | 58 | 872.7935 | 2615.3587 | 2615.3555 | 1.23 | 0 | 22 | 0.0094 | 1Score **> 37** indicates **identity** Score **> 14** indicates **homology** | U | R.GTATFAQALQSVPETQVSILDNGLR.V |
| 169333 | 34 | – | 58 | 1308.6867 | 2615.3589 | 2615.3555 | 1.31 | 0 | 108 | 8.3e-11 | 1Score **> 37** indicates **identity** Score **> 19** indicates **homology** | U | R.GTATFAQALQSVPETQVSILDNGLR.V |
| 169335 | 34 | – | 58 | 872.7952 | 2615.3638 | 2615.3555 | 3.17 | 0 | 79 | 4.2e-08 | 1Score **> 37** indicates **identity** Score **> 17** indicates **homology** | U | R.GTATFAQALQSVPETQVSILDNGLR.V |
| 169382 | 34 | – | 58 | 873.1201 | 2616.3384 | 2616.3395 | -0.43 | 0 | 48 | 3.3e-05 | 1Score **> 37** indicates **identity** Score **> 15** indicates **homology** | U | R.GTATFAQALQSVPETQVSILDNGLR.V  + Deamidated (NQ) |
| 169387 | 34 | – | 58 | 873.1208 | 2616.3405 | 2616.3395 | 0.39 | 0 | 78 | 5.1e-08 | 1Score **> 37** indicates **identity** Score **> 17** indicates **homology** | U | R.GTATFAQALQSVPETQVSILDNGLR.V  + Deamidated (NQ) |
| 169390 | 34 | – | 58 | 873.1212 | 2616.3419 | 2616.3395 | 0.92 | 0 | 16 | 0.028 | 1Score **> 37** indicates **identity** Score **> 14** indicates **homology** | U | R.GTATFAQALQSVPETQVSILDNGLR.V  + Deamidated (NQ) |
| 169394 | 34 | – | 58 | 1309.1816 | 2616.3487 | 2616.3395 | 3.53 | 0 | 98 | 6.5e-10 | 1Score **> 37** indicates **identity** Score **> 19** indicates **homology** | U | R.GTATFAQALQSVPETQVSILDNGLR.V  + Deamidated (NQ) |
| 169395 | 34 | – | 58 | 873.1246 | 2616.3520 | 2616.3395 | 4.78 | 0 | 46 | 4.6e-05 | 1Score **> 37** indicates **identity** Score **> 15** indicates **homology** | U | R.GTATFAQALQSVPETQVSILDNGLR.V  + Deamidated (NQ) |
| 169436 | 34 | – | 58 | 873.4540 | 2617.3403 | 2617.3235 | 6.41 | 0 | 39 | 0.00021 | 1Score **> 37** indicates **identity** Score **> 15** indicates **homology** | U | R.GTATFAQALQSVPETQVSILDNGLR.V  + 2 Deamidated (NQ) |
| 152829 | 59 | – | 80 | 773.3634 | 2317.0683 | 2317.0757 | -3.19 | 0 | 32 | 0.00098 | 1Score **> 33** indicates **identity** Score **> 15** indicates **homology** | U | R.VASEQSSHATCTVGVWIDAGSR.Y |
| 152830 | 59 | – | 80 | 773.3638 | 2317.0696 | 2317.0757 | -2.65 | 0 | 23 | 0.0071 | 1Score **> 33** indicates **identity** Score **> 14** indicates **homology** | U | R.VASEQSSHATCTVGVWIDAGSR.Y |
| 152832 | 59 | – | 80 | 773.3643 | 2317.0711 | 2317.0757 | -2.01 | 0 | 54 | 8.4e-06 | 1Score **> 33** indicates **identity** Score **> 16** indicates **homology** | U | R.VASEQSSHATCTVGVWIDAGSR.Y |
| 152833 | 59 | – | 80 | 773.3645 | 2317.0716 | 2317.0757 | -1.77 | 0 | 32 | 0.0011 | 1Score **> 33** indicates **identity** Score **> 14** indicates **homology** | U | R.VASEQSSHATCTVGVWIDAGSR.Y |
| 152834 | 59 | – | 80 | 1159.5432 | 2317.0719 | 2317.0757 | -1.66 | 0 | 74 | 3.1e-07 | 1Score **> 33** indicates **identity** Score **> 21** indicates **homology** | U | R.VASEQSSHATCTVGVWIDAGSR.Y |
| 152835 | 59 | – | 80 | 773.3646 | 2317.0720 | 2317.0757 | -1.59 | 0 | 29 | 0.0021 | 1Score **> 33** indicates **identity** Score **> 14** indicates **homology** | U | R.VASEQSSHATCTVGVWIDAGSR.Y |
| 152837 | 59 | – | 80 | 773.3654 | 2317.0744 | 2317.0757 | -0.58 | 0 | 15 | 0.035 | 1Score **> 34** indicates **identity** Score **> 13** indicates **homology** | U | R.VASEQSSHATCTVGVWIDAGSR.Y |
| 152838 | 59 | – | 80 | 1159.5450 | 2317.0755 | 2317.0757 | -0.094 | 0 | 66 | 6.5e-07 | 1Score **> 34** indicates **identity** Score **> 17** indicates **homology** | U | R.VASEQSSHATCTVGVWIDAGSR.Y |
| 152839 | 59 | – | 80 | 773.3658 | 2317.0756 | 2317.0757 | -0.038 | 0 | 21 | 0.01 | 1Score **> 34** indicates **identity** Score **> 14** indicates **homology** | U | R.VASEQSSHATCTVGVWIDAGSR.Y |
| 152842 | 59 | – | 80 | 773.3666 | 2317.0780 | 2317.0757 | 0.98 | 0 | 49 | 2.6e-05 | 1Score **> 34** indicates **identity** Score **> 16** indicates **homology** | U | R.VASEQSSHATCTVGVWIDAGSR.Y |
| 152849 | 59 | – | 80 | 773.3678 | 2317.0815 | 2317.0757 | 2.48 | 0 | 19 | 0.017 | 1Score **> 34** indicates **identity** Score **> 14** indicates **homology** | U | R.VASEQSSHATCTVGVWIDAGSR.Y |
| 152851 | 59 | – | 80 | 773.3687 | 2317.0842 | 2317.0757 | 3.64 | 0 | 17 | 0.026 | 1Score **> 34** indicates **identity** Score **> 14** indicates **homology** | U | R.VASEQSSHATCTVGVWIDAGSR.Y |
| 152857 | 59 | – | 80 | 773.3714 | 2317.0923 | 2317.0757 | 7.17 | 0 | 33 | 0.00076 | 1Score **> 34** indicates **identity** Score **> 15** indicates **homology** | U | R.VASEQSSHATCTVGVWIDAGSR.Y |
| 152887 | 59 | – | 80 | 773.6972 | 2318.0697 | 2318.0597 | 4.30 | 0 | 19 | 0.017 | 1Score **> 33** indicates **identity** Score **> 14** indicates **homology** | U | R.VASEQSSHATCTVGVWIDAGSR.Y  + Deamidated (NQ) |
| 152889 | 59 | – | 80 | 773.6985 | 2318.0738 | 2318.0597 | 6.07 | 0 | 14 | 0.049 | 1Score **> 34** indicates **identity** Score **> 13** indicates **homology** | U | R.VASEQSSHATCTVGVWIDAGSR.Y  + Deamidated (NQ) |
| 152890 | 59 | – | 80 | 773.6995 | 2318.0768 | 2318.0597 | 7.36 | 0 | 19 | 0.017 | 1Score **> 34** indicates **identity** Score **> 14** indicates **homology** | U | R.VASEQSSHATCTVGVWIDAGSR.Y  + Deamidated (NQ) |
| 182497 | 59 | – | 85 | 990.1282 | 2967.3627 | 2967.3669 | -1.41 | 1 | 54 | 8.6e-06 | 1Score **> 34** indicates **identity** Score **> 16** indicates **homology** | U | R.VASEQSSHATCTVGVWIDAGSRYETEK.N |
| 182499 | 59 | – | 85 | 742.8495 | 2967.3688 | 2967.3669 | 0.64 | 1 | 20 | 0.013 | 1Score **> 34** indicates **identity** Score **> 14** indicates **homology** | U | R.VASEQSSHATCTVGVWIDAGSRYETEK.N |
| 182500 | 59 | – | 85 | 742.8496 | 2967.3693 | 2967.3669 | 0.83 | 1 | 32 | 0.00097 | 1Score **> 34** indicates **identity** Score **> 15** indicates **homology** | U | R.VASEQSSHATCTVGVWIDAGSRYETEK.N |
| 182501 | 59 | – | 85 | 990.1310 | 2967.3712 | 2967.3669 | 1.45 | 1 | 64 | 9e-07 | 1Score **> 35** indicates **identity** Score **> 17** indicates **homology** | U | R.VASEQSSHATCTVGVWIDAGSRYETEK.N |
| 182536 | 59 | – | 85 | 990.4648 | 2968.3727 | 2968.3509 | 7.34 | 1 | 19 | 0.017 | 1Score **> 34** indicates **identity** Score **> 14** indicates **homology** | U | R.VASEQSSHATCTVGVWIDAGSRYETEK.N  + Deamidated (NQ) |
| 199613 | 59 | – | 99 | 906.8331 | 4529.1293 | 4529.1346 | -1.17 | 2 | 16 | 0.033 | 1Score **> 35** indicates **identity** Score **> 13** indicates **homology** | U | R.VASEQSSHATCTVGVWIDAGSRYETEKNNGAGYFLEHLAFK.G |
| 199614 | 59 | – | 99 | 906.8345 | 4529.1362 | 4529.1346 | 0.35 | 2 | 16 | 0.033 | 1Score **> 35** indicates **identity** Score **> 13** indicates **homology** | U | R.VASEQSSHATCTVGVWIDAGSRYETEKNNGAGYFLEHLAFK.G |
| 199621 | 59 | – | 99 | 907.0321 | 4530.1240 | 4530.1186 | 1.20 | 2 | 17 | 0.024 | 1Score **> 35** indicates **identity** Score **> 14** indicates **homology** | U | R.VASEQSSHATCTVGVWIDAGSRYETEKNNGAGYFLEHLAFK.G  + Deamidated (NQ) |
| 146575 | 81 | – | 99 | 558.5254 | 2230.0725 | 2230.0694 | 1.38 | 1 | 34 | 0.00059 | 1Score **> 35** indicates **identity** Score **> 15** indicates **homology** | U | R.YETEKNNGAGYFLEHLAFK.G |
| 146577 | 81 | – | 99 | 744.3656 | 2230.0749 | 2230.0694 | 2.43 | 1 | 35 | 0.00054 | 1Score **> 35** indicates **identity** Score **> 15** indicates **homology** | U | R.YETEKNNGAGYFLEHLAFK.G |
| 146578 | 81 | – | 99 | 558.5262 | 2230.0756 | 2230.0694 | 2.76 | 1 | 41 | 0.00013 | 1Score **> 35** indicates **identity** Score **> 15** indicates **homology** | U | R.YETEKNNGAGYFLEHLAFK.G |
| 146579 | 81 | – | 99 | 744.3662 | 2230.0767 | 2230.0694 | 3.27 | 1 | 46 | 5.2e-05 | 1Score **> 35** indicates **identity** Score **> 15** indicates **homology** | U | R.YETEKNNGAGYFLEHLAFK.G |
| 146581 | 81 | – | 99 | 558.5269 | 2230.0786 | 2230.0694 | 4.12 | 1 | 28 | 0.0025 | 1Score **> 35** indicates **identity** Score **> 14** indicates **homology** | U | R.YETEKNNGAGYFLEHLAFK.G |
| 146582 | 81 | – | 99 | 558.5270 | 2230.0787 | 2230.0694 | 4.17 | 1 | 55 | 6.4e-06 | 1Score **> 35** indicates **identity** Score **> 16** indicates **homology** | U | R.YETEKNNGAGYFLEHLAFK.G |
| 146583 | 81 | – | 99 | 744.3672 | 2230.0796 | 2230.0694 | 4.57 | 1 | 56 | 5.3e-06 | 1Score **> 35** indicates **identity** Score **> 16** indicates **homology** | U | R.YETEKNNGAGYFLEHLAFK.G |
| 146584 | 81 | – | 99 | 558.5274 | 2230.0805 | 2230.0694 | 4.96 | 1 | 44 | 6.7e-05 | 1Score **> 35** indicates **identity** Score **> 15** indicates **homology** | U | R.YETEKNNGAGYFLEHLAFK.G |
| 146662 | 81 | – | 99 | 558.7703 | 2231.0519 | 2231.0535 | -0.68 | 1 | 15 | 0.037 | 1Score **> 34** indicates **identity** Score **> 13** indicates **homology** | U | R.YETEKNNGAGYFLEHLAFK.G  + Deamidated (NQ) |
| 146663 | 81 | – | 99 | 558.7707 | 2231.0539 | 2231.0535 | 0.19 | 1 | 15 | 0.036 | 1Score **> 34** indicates **identity** Score **> 13** indicates **homology** | U | R.YETEKNNGAGYFLEHLAFK.G  + Deamidated (NQ) |
| 146664 | 81 | – | 99 | 558.7712 | 2231.0556 | 2231.0535 | 0.96 | 1 | 27 | 0.0026 | 1Score **> 34** indicates **identity** Score **> 14** indicates **homology** | U | R.YETEKNNGAGYFLEHLAFK.G  + Deamidated (NQ) |
| 146669 | 81 | – | 99 | 744.6935 | 2231.0586 | 2231.0535 | 2.31 | 1 | 45 | 5.6e-05 | 1Score **> 34** indicates **identity** Score **> 15** indicates **homology** | U | R.YETEKNNGAGYFLEHLAFK.G  + Deamidated (NQ) |
| 146670 | 81 | – | 99 | 744.6935 | 2231.0587 | 2231.0535 | 2.34 | 1 | 27 | 0.0032 | 1Score **> 34** indicates **identity** Score **> 14** indicates **homology** | U | R.YETEKNNGAGYFLEHLAFK.G  + Deamidated (NQ) |
| 146671 | 81 | – | 99 | 558.7723 | 2231.0602 | 2231.0535 | 3.01 | 1 | 37 | 0.00031 | 1Score **> 35** indicates **identity** Score **> 15** indicates **homology** | U | R.YETEKNNGAGYFLEHLAFK.G  + Deamidated (NQ) |
| 146672 | 81 | – | 99 | 558.7726 | 2231.0613 | 2231.0535 | 3.53 | 1 | 15 | 0.043 | 1Score **> 35** indicates **identity** Score **> 13** indicates **homology** | U | R.YETEKNNGAGYFLEHLAFK.G  + Deamidated (NQ) |
| 164777 | 81 | – | 102 | 630.0656 | 2516.2333 | 2516.2336 | -0.11 | 2 | 34 | 0.0007 | 1Score **> 36** indicates **identity** Score **> 15** indicates **homology** | U | R.YETEKNNGAGYFLEHLAFKGTK.N |
| 164779 | 81 | – | 102 | 630.0663 | 2516.2361 | 2516.2336 | 1.03 | 2 | 36 | 0.00045 | 1Score **> 36** indicates **identity** Score **> 15** indicates **homology** | U | R.YETEKNNGAGYFLEHLAFKGTK.N |
| 164835 | 81 | – | 102 | 630.3111 | 2517.2153 | 2517.2176 | -0.89 | 2 | 35 | 0.00057 | 1Score **> 36** indicates **identity** Score **> 15** indicates **homology** | U | R.YETEKNNGAGYFLEHLAFKGTK.N  + Deamidated (NQ) |
| 164840 | 81 | – | 102 | 630.3125 | 2517.2210 | 2517.2176 | 1.35 | 2 | 36 | 0.0004 | 1Score **> 36** indicates **identity** Score **> 15** indicates **homology** | U | R.YETEKNNGAGYFLEHLAFKGTK.N  + Deamidated (NQ) |
| 78378 | 86 | – | 99 | 790.8943 | 1579.7741 | 1579.7783 | -2.67 | 0 | 52 | 1.4e-05 | 1Score **> 34** indicates **identity** Score **> 16** indicates **homology** | U | K.NNGAGYFLEHLAFK.G |
| 78380 | 86 | – | 99 | 790.8952 | 1579.7759 | 1579.7783 | -1.50 | 0 | 60 | 2.6e-06 | 1Score **> 34** indicates **identity** Score **> 16** indicates **homology** | U | K.NNGAGYFLEHLAFK.G |
| 78382 | 86 | – | 99 | 790.8958 | 1579.7770 | 1579.7783 | -0.86 | 0 | 61 | 2.1e-06 | 1Score **> 34** indicates **identity** Score **> 16** indicates **homology** | U | K.NNGAGYFLEHLAFK.G |
| 78383 | 86 | – | 99 | 527.6001 | 1579.7784 | 1579.7783 | 0.049 | 0 | 35 | 0.00048 | 1Score **> 34** indicates **identity** Score **> 15** indicates **homology** | U | K.NNGAGYFLEHLAFK.G |
| 78385 | 86 | – | 99 | 527.6003 | 1579.7790 | 1579.7783 | 0.45 | 0 | 38 | 0.00026 | 1Score **> 34** indicates **identity** Score **> 15** indicates **homology** | U | K.NNGAGYFLEHLAFK.G |
| 78386 | 86 | – | 99 | 527.6003 | 1579.7792 | 1579.7783 | 0.57 | 0 | 47 | 3.9e-05 | 1Score **> 34** indicates **identity** Score **> 15** indicates **homology** | U | K.NNGAGYFLEHLAFK.G |
| 78389 | 86 | – | 99 | 527.6008 | 1579.7805 | 1579.7783 | 1.41 | 0 | 23 | 0.0075 | 1Score **> 34** indicates **identity** Score **> 14** indicates **homology** | U | K.NNGAGYFLEHLAFK.G |
| 78542 | 86 | – | 99 | 791.3876 | 1580.7607 | 1580.7623 | -1.04 | 0 | 60 | 2.6e-06 | 1Score **> 33** indicates **identity** Score **> 16** indicates **homology** | U | K.NNGAGYFLEHLAFK.G  + Deamidated (NQ) |
| 78543 | 86 | – | 99 | 527.9278 | 1580.7617 | 1580.7623 | -0.42 | 0 | 57 | 4.9e-06 | 1Score **> 33** indicates **identity** Score **> 16** indicates **homology** | U | K.NNGAGYFLEHLAFK.G  + Deamidated (NQ) |
| 78556 | 86 | – | 99 | 527.9287 | 1580.7642 | 1580.7623 | 1.22 | 0 | 57 | 4.5e-06 | 1Score **> 34** indicates **identity** Score **> 16** indicates **homology** | U | K.NNGAGYFLEHLAFK.G  + Deamidated (NQ) |
| 78562 | 86 | – | 99 | 527.9294 | 1580.7665 | 1580.7623 | 2.65 | 0 | 45 | 6.3e-05 | 1Score **> 33** indicates **identity** Score **> 15** indicates **homology** | U | K.NNGAGYFLEHLAFK.G  + Deamidated (NQ) |
| 78577 | 86 | – | 99 | 791.3946 | 1580.7747 | 1580.7623 | 7.84 | 0 | 55 | 7.1e-06 | 1Score **> 34** indicates **identity** Score **> 16** indicates **homology** | U | K.NNGAGYFLEHLAFK.G  + Deamidated (NQ) |
| 113408 | 86 | – | 102 | 622.9861 | 1865.9366 | 1865.9424 | -3.13 | 1 | 34 | 0.0007 | 1Score **> 35** indicates **identity** Score **> 15** indicates **homology** | U | K.NNGAGYFLEHLAFKGTK.N |
| 113409 | 86 | – | 102 | 622.9883 | 1865.9432 | 1865.9424 | 0.40 | 1 | 36 | 0.00038 | 1Score **> 36** indicates **identity** Score **> 15** indicates **homology** | U | K.NNGAGYFLEHLAFKGTK.N |
| 113410 | 86 | – | 102 | 622.9890 | 1865.9451 | 1865.9424 | 1.46 | 1 | 36 | 0.00043 | 1Score **> 36** indicates **identity** Score **> 15** indicates **homology** | U | K.NNGAGYFLEHLAFKGTK.N |
| 113411 | 86 | – | 102 | 622.9890 | 1865.9453 | 1865.9424 | 1.56 | 1 | 42 | 0.00013 | 1Score **> 36** indicates **identity** Score **> 15** indicates **homology** | U | K.NNGAGYFLEHLAFKGTK.N |
| 113497 | 86 | – | 102 | 623.3168 | 1866.9285 | 1866.9264 | 1.09 | 1 | 47 | 3.6e-05 | 1Score **> 35** indicates **identity** Score **> 15** indicates **homology** | U | K.NNGAGYFLEHLAFKGTK.N  + Deamidated (NQ) |
| 113501 | 86 | – | 102 | 623.3187 | 1866.9343 | 1866.9264 | 4.24 | 1 | 39 | 0.00023 | 1Score **> 35** indicates **identity** Score **> 15** indicates **homology** | U | K.NNGAGYFLEHLAFKGTK.N  + Deamidated (NQ) |
| 113510 | 86 | – | 102 | 623.3214 | 1866.9423 | 1866.9264 | 8.53 | 1 | 38 | 0.00026 | 1Score **> 36** indicates **identity** Score **> 15** indicates **homology** | U | K.NNGAGYFLEHLAFKGTK.N  + Deamidated (NQ) |
| 169774 | 103 | – | 126 | 657.3346 | 2625.3094 | 2625.3259 | -6.27 | 2 | 27 | 0.0029 | 1Score **> 37** indicates **identity** Score **> 14** indicates **homology** | U | K.NRPGNALEKEVESIGAHLNAYSTR.E |
| 169776 | 103 | – | 126 | 657.3361 | 2625.3152 | 2625.3259 | -4.08 | 2 | 18 | 0.019 | 1Score **> 37** indicates **identity** Score **> 14** indicates **homology** | U | K.NRPGNALEKEVESIGAHLNAYSTR.E |
| 169777 | 103 | – | 126 | 526.0705 | 2625.3159 | 2625.3259 | -3.80 | 2 | 27 | 0.0029 | 1Score **> 37** indicates **identity** Score **> 14** indicates **homology** | U | K.NRPGNALEKEVESIGAHLNAYSTR.E |
| 169778 | 103 | – | 126 | 657.3365 | 2625.3171 | 2625.3259 | -3.35 | 2 | 45 | 5.5e-05 | 1Score **> 37** indicates **identity** Score **> 15** indicates **homology** | U | K.NRPGNALEKEVESIGAHLNAYSTR.E |
| 169779 | 103 | – | 126 | 657.3367 | 2625.3177 | 2625.3259 | -3.11 | 2 | 39 | 0.00021 | 1Score **> 37** indicates **identity** Score **> 15** indicates **homology** | U | K.NRPGNALEKEVESIGAHLNAYSTR.E |
| 169780 | 103 | – | 126 | 526.0711 | 2625.3193 | 2625.3259 | -2.52 | 2 | 20 | 0.014 | 1Score **> 37** indicates **identity** Score **> 14** indicates **homology** | U | K.NRPGNALEKEVESIGAHLNAYSTR.E |
| 169781 | 103 | – | 126 | 657.3371 | 2625.3194 | 2625.3259 | -2.46 | 2 | 58 | 3.5e-06 | 1Score **> 37** indicates **identity** Score **> 16** indicates **homology** | U | K.NRPGNALEKEVESIGAHLNAYSTR.E |
| 169782 | 103 | – | 126 | 876.1141 | 2625.3203 | 2625.3259 | -2.10 | 2 | 41 | 0.00013 | 1Score **> 37** indicates **identity** Score **> 15** indicates **homology** | U | K.NRPGNALEKEVESIGAHLNAYSTR.E |
| 169783 | 103 | – | 126 | 526.0715 | 2625.3211 | 2625.3259 | -1.81 | 2 | 21 | 0.0098 | 1Score **> 37** indicates **identity** Score **> 14** indicates **homology** | U | K.NRPGNALEKEVESIGAHLNAYSTR.E |
| 169784 | 103 | – | 126 | 526.0715 | 2625.3213 | 2625.3259 | -1.74 | 2 | 21 | 0.013 | 1Score **> 37** indicates **identity** Score **> 14** indicates **homology** | U | K.NRPGNALEKEVESIGAHLNAYSTR.E |
| 169785 | 103 | – | 126 | 657.3376 | 2625.3214 | 2625.3259 | -1.69 | 2 | 53 | 1e-05 | 1Score **> 37** indicates **identity** Score **> 16** indicates **homology** | U | K.NRPGNALEKEVESIGAHLNAYSTR.E |
| 169787 | 103 | – | 126 | 657.3378 | 2625.3222 | 2625.3259 | -1.38 | 2 | 38 | 0.00029 | 1Score **> 37** indicates **identity** Score **> 15** indicates **homology** | U | K.NRPGNALEKEVESIGAHLNAYSTR.E |
| 169788 | 103 | – | 126 | 526.0721 | 2625.3239 | 2625.3259 | -0.73 | 2 | 30 | 0.0015 | 1Score **> 37** indicates **identity** Score **> 14** indicates **homology** | U | K.NRPGNALEKEVESIGAHLNAYSTR.E |
| 169789 | 103 | – | 126 | 526.0722 | 2625.3245 | 2625.3259 | -0.52 | 2 | 18 | 0.021 | 1Score **> 37** indicates **identity** Score **> 14** indicates **homology** | U | K.NRPGNALEKEVESIGAHLNAYSTR.E |
| 169790 | 103 | – | 126 | 657.3386 | 2625.3251 | 2625.3259 | -0.28 | 2 | 30 | 0.0016 | 1Score **> 37** indicates **identity** Score **> 14** indicates **homology** | U | K.NRPGNALEKEVESIGAHLNAYSTR.E |
| 169791 | 103 | – | 126 | 526.0723 | 2625.3254 | 2625.3259 | -0.19 | 2 | 51 | 1.6e-05 | 1Score **> 37** indicates **identity** Score **> 16** indicates **homology** | U | K.NRPGNALEKEVESIGAHLNAYSTR.E |
| 169792 | 103 | – | 126 | 526.0724 | 2625.3257 | 2625.3259 | -0.069 | 2 | 39 | 0.00022 | 1Score **> 37** indicates **identity** Score **> 15** indicates **homology** | U | K.NRPGNALEKEVESIGAHLNAYSTR.E |
| 169793 | 103 | – | 126 | 657.3387 | 2625.3258 | 2625.3259 | -0.032 | 2 | 64 | 1.1e-06 | 1Score **> 37** indicates **identity** Score **> 16** indicates **homology** | U | K.NRPGNALEKEVESIGAHLNAYSTR.E |
| 169794 | 103 | – | 126 | 526.0724 | 2625.3258 | 2625.3259 | -0.029 | 2 | 54 | 9e-06 | 1Score **> 37** indicates **identity** Score **> 16** indicates **homology** | U | K.NRPGNALEKEVESIGAHLNAYSTR.E |
| 169795 | 103 | – | 126 | 526.0725 | 2625.3261 | 2625.3259 | 0.11 | 2 | 41 | 0.00015 | 1Score **> 37** indicates **identity** Score **> 15** indicates **homology** | U | K.NRPGNALEKEVESIGAHLNAYSTR.E |
| 169796 | 103 | – | 126 | 876.1160 | 2625.3263 | 2625.3259 | 0.16 | 2 | 77 | 6.6e-08 | 1Score **> 37** indicates **identity** Score **> 17** indicates **homology** | U | K.NRPGNALEKEVESIGAHLNAYSTR.E |
| 169797 | 103 | – | 126 | 657.3388 | 2625.3263 | 2625.3259 | 0.16 | 2 | 59 | 2.9e-06 | 1Score **> 37** indicates **identity** Score **> 16** indicates **homology** | U | K.NRPGNALEKEVESIGAHLNAYSTR.E |
| 169798 | 103 | – | 126 | 526.0726 | 2625.3264 | 2625.3259 | 0.19 | 2 | 44 | 6.9e-05 | 1Score **> 37** indicates **identity** Score **> 15** indicates **homology** | U | K.NRPGNALEKEVESIGAHLNAYSTR.E |
| 169799 | 103 | – | 126 | 526.0727 | 2625.3270 | 2625.3259 | 0.43 | 2 | 45 | 5.5e-05 | 1Score **> 37** indicates **identity** Score **> 15** indicates **homology** | U | K.NRPGNALEKEVESIGAHLNAYSTR.E |
| 169800 | 103 | – | 126 | 657.3390 | 2625.3270 | 2625.3259 | 0.45 | 2 | 37 | 0.00036 | 1Score **> 37** indicates **identity** Score **> 15** indicates **homology** | U | K.NRPGNALEKEVESIGAHLNAYSTR.E |
| 169801 | 103 | – | 126 | 657.3390 | 2625.3271 | 2625.3259 | 0.45 | 2 | 53 | 1e-05 | 1Score **> 37** indicates **identity** Score **> 16** indicates **homology** | U | K.NRPGNALEKEVESIGAHLNAYSTR.E |
| 169803 | 103 | – | 126 | 657.3391 | 2625.3272 | 2625.3259 | 0.49 | 2 | 59 | 3.1e-06 | 1Score **> 37** indicates **identity** Score **> 16** indicates **homology** | U | K.NRPGNALEKEVESIGAHLNAYSTR.E |
| 169804 | 103 | – | 126 | 526.0727 | 2625.3272 | 2625.3259 | 0.52 | 2 | 30 | 0.0019 | 1Score **> 37** indicates **identity** Score **> 15** indicates **homology** | U | K.NRPGNALEKEVESIGAHLNAYSTR.E |
| 169805 | 103 | – | 126 | 657.3391 | 2625.3273 | 2625.3259 | 0.56 | 2 | 55 | 6.9e-06 | 1Score **> 37** indicates **identity** Score **> 16** indicates **homology** | U | K.NRPGNALEKEVESIGAHLNAYSTR.E |
| 169806 | 103 | – | 126 | 876.1164 | 2625.3273 | 2625.3259 | 0.56 | 2 | 77 | 6.5e-08 | 1Score **> 37** indicates **identity** Score **> 17** indicates **homology** | U | K.NRPGNALEKEVESIGAHLNAYSTR.E |
| 169807 | 103 | – | 126 | 526.0728 | 2625.3274 | 2625.3259 | 0.59 | 2 | 60 | 2.3e-06 | 1Score **> 37** indicates **identity** Score **> 16** indicates **homology** | U | K.NRPGNALEKEVESIGAHLNAYSTR.E |
| 169808 | 103 | – | 126 | 876.1165 | 2625.3277 | 2625.3259 | 0.70 | 2 | 17 | 0.027 | 1Score **> 37** indicates **identity** Score **> 14** indicates **homology** | U | K.NRPGNALEKEVESIGAHLNAYSTR.E |
| 169809 | 103 | – | 126 | 657.3393 | 2625.3281 | 2625.3259 | 0.84 | 2 | 62 | 1.6e-06 | 1Score **> 37** indicates **identity** Score **> 16** indicates **homology** | U | K.NRPGNALEKEVESIGAHLNAYSTR.E |
| 169810 | 103 | – | 126 | 876.1166 | 2625.3281 | 2625.3259 | 0.84 | 2 | 77 | 6.4e-08 | 1Score **> 37** indicates **identity** Score **> 17** indicates **homology** | U | K.NRPGNALEKEVESIGAHLNAYSTR.E |
| 169811 | 103 | – | 126 | 657.3393 | 2625.3281 | 2625.3259 | 0.87 | 2 | 63 | 1.2e-06 | 1Score **> 37** indicates **identity** Score **> 16** indicates **homology** | U | K.NRPGNALEKEVESIGAHLNAYSTR.E |
| 169812 | 103 | – | 126 | 876.1167 | 2625.3283 | 2625.3259 | 0.93 | 2 | 40 | 0.00019 | 1Score **> 37** indicates **identity** Score **> 15** indicates **homology** | U | K.NRPGNALEKEVESIGAHLNAYSTR.E |
| 169813 | 103 | – | 126 | 657.3394 | 2625.3283 | 2625.3259 | 0.93 | 2 | 56 | 5.2e-06 | 1Score **> 37** indicates **identity** Score **> 16** indicates **homology** | U | K.NRPGNALEKEVESIGAHLNAYSTR.E |
| 169814 | 103 | – | 126 | 526.0729 | 2625.3284 | 2625.3259 | 0.95 | 2 | 61 | 2.1e-06 | 1Score **> 37** indicates **identity** Score **> 16** indicates **homology** | U | K.NRPGNALEKEVESIGAHLNAYSTR.E |
| 169815 | 103 | – | 126 | 876.1169 | 2625.3287 | 2625.3259 | 1.10 | 2 | 76 | 6.8e-08 | 1Score **> 37** indicates **identity** Score **> 17** indicates **homology** | U | K.NRPGNALEKEVESIGAHLNAYSTR.E |
| 169816 | 103 | – | 126 | 526.0731 | 2625.3289 | 2625.3259 | 1.16 | 2 | 55 | 7.2e-06 | 1Score **> 37** indicates **identity** Score **> 16** indicates **homology** | U | K.NRPGNALEKEVESIGAHLNAYSTR.E |
| 169817 | 103 | – | 126 | 657.3395 | 2625.3289 | 2625.3259 | 1.17 | 2 | 55 | 6.7e-06 | 1Score **> 37** indicates **identity** Score **> 16** indicates **homology** | U | K.NRPGNALEKEVESIGAHLNAYSTR.E |
| 169818 | 103 | – | 126 | 876.1170 | 2625.3291 | 2625.3259 | 1.24 | 2 | 77 | 6.5e-08 | 1Score **> 37** indicates **identity** Score **> 17** indicates **homology** | U | K.NRPGNALEKEVESIGAHLNAYSTR.E |
| 169819 | 103 | – | 126 | 526.0732 | 2625.3295 | 2625.3259 | 1.40 | 2 | 54 | 8.9e-06 | 1Score **> 37** indicates **identity** Score **> 16** indicates **homology** | U | K.NRPGNALEKEVESIGAHLNAYSTR.E |
| 169820 | 103 | – | 126 | 657.3398 | 2625.3300 | 2625.3259 | 1.57 | 2 | 49 | 2.7e-05 | 1Score **> 37** indicates **identity** Score **> 16** indicates **homology** | U | K.NRPGNALEKEVESIGAHLNAYSTR.E |
| 169821 | 103 | – | 126 | 526.0733 | 2625.3302 | 2625.3259 | 1.64 | 2 | 50 | 2.1e-05 | 1Score **> 37** indicates **identity** Score **> 16** indicates **homology** | U | K.NRPGNALEKEVESIGAHLNAYSTR.E |
| 169823 | 103 | – | 126 | 657.3402 | 2625.3317 | 2625.3259 | 2.23 | 2 | 42 | 0.0001 | 1Score **> 37** indicates **identity** Score **> 15** indicates **homology** | U | K.NRPGNALEKEVESIGAHLNAYSTR.E |
| 169824 | 103 | – | 126 | 657.3406 | 2625.3333 | 2625.3259 | 2.82 | 2 | 37 | 0.00031 | 1Score **> 37** indicates **identity** Score **> 15** indicates **homology** | U | K.NRPGNALEKEVESIGAHLNAYSTR.E |
| 169826 | 103 | – | 126 | 657.3410 | 2625.3347 | 2625.3259 | 3.36 | 2 | 39 | 0.00021 | 1Score **> 37** indicates **identity** Score **> 15** indicates **homology** | U | K.NRPGNALEKEVESIGAHLNAYSTR.E |
| 169827 | 103 | – | 126 | 657.3411 | 2625.3351 | 2625.3259 | 3.54 | 2 | 26 | 0.0038 | 1Score **> 37** indicates **identity** Score **> 14** indicates **homology** | U | K.NRPGNALEKEVESIGAHLNAYSTR.E |
| 169851 | 103 | – | 126 | 526.2683 | 2626.3052 | 2626.3099 | -1.78 | 2 | 31 | 0.0013 | 1Score **> 37** indicates **identity** Score **> 14** indicates **homology** | U | K.NRPGNALEKEVESIGAHLNAYSTR.E  + Deamidated (NQ) |
| 169852 | 103 | – | 126 | 657.5839 | 2626.3064 | 2626.3099 | -1.33 | 2 | 33 | 0.00081 | 1Score **> 37** indicates **identity** Score **> 15** indicates **homology** | U | K.NRPGNALEKEVESIGAHLNAYSTR.E  + Deamidated (NQ) |
| 169855 | 103 | – | 126 | 876.4435 | 2626.3088 | 2626.3099 | -0.42 | 2 | 22 | 0.0095 | 1Score **> 37** indicates **identity** Score **> 14** indicates **homology** | U | K.NRPGNALEKEVESIGAHLNAYSTR.E  + Deamidated (NQ) |
| 169856 | 103 | – | 126 | 526.2691 | 2626.3089 | 2626.3099 | -0.37 | 2 | 15 | 0.038 | 1Score **> 37** indicates **identity** Score **> 13** indicates **homology** | U | K.NRPGNALEKEVESIGAHLNAYSTR.E  + Deamidated (NQ) |
| 169858 | 103 | – | 126 | 526.2694 | 2626.3105 | 2626.3099 | 0.22 | 2 | 24 | 0.0057 | 1Score **> 37** indicates **identity** Score **> 14** indicates **homology** | U | K.NRPGNALEKEVESIGAHLNAYSTR.E  + Deamidated (NQ) |
| 169859 | 103 | – | 126 | 657.5851 | 2626.3114 | 2626.3099 | 0.58 | 2 | 29 | 0.0019 | 1Score **> 37** indicates **identity** Score **> 14** indicates **homology** | U | K.NRPGNALEKEVESIGAHLNAYSTR.E  + Deamidated (NQ) |
| 169860 | 103 | – | 126 | 657.5852 | 2626.3115 | 2626.3099 | 0.64 | 2 | 39 | 0.00024 | 1Score **> 37** indicates **identity** Score **> 15** indicates **homology** | U | K.NRPGNALEKEVESIGAHLNAYSTR.E  + Deamidated (NQ) |
| 169861 | 103 | – | 126 | 657.5857 | 2626.3138 | 2626.3099 | 1.48 | 2 | 52 | 1.2e-05 | 1Score **> 37** indicates **identity** Score **> 16** indicates **homology** | U | K.NRPGNALEKEVESIGAHLNAYSTR.E  + Deamidated (NQ) |
| 169862 | 103 | – | 126 | 526.2703 | 2626.3152 | 2626.3099 | 2.03 | 2 | 34 | 0.00061 | 1Score **> 37** indicates **identity** Score **> 15** indicates **homology** | U | K.NRPGNALEKEVESIGAHLNAYSTR.E  + Deamidated (NQ) |
| 169863 | 103 | – | 126 | 526.2705 | 2626.3162 | 2626.3099 | 2.41 | 2 | 35 | 0.0032 | 1Score **> 37** indicates **identity** Score **> 23** indicates **homology** | U | K.NRPGNALEKEVESIGAHLNAYSTR.E  + Deamidated (NQ) |
| 169864 | 103 | – | 126 | 657.5874 | 2626.3203 | 2626.3099 | 3.97 | 2 | 64 | 9.7e-07 | 1Score **> 37** indicates **identity** Score **> 17** indicates **homology** | U | K.NRPGNALEKEVESIGAHLNAYSTR.E  + Deamidated (NQ) |
| 169867 | 103 | – | 126 | 526.2725 | 2626.3259 | 2626.3099 | 6.11 | 2 | 34 | 0.00071 | 1Score **> 37** indicates **identity** Score **> 15** indicates **homology** | U | K.NRPGNALEKEVESIGAHLNAYSTR.E  + Deamidated (NQ) |
| 169869 | 103 | – | 126 | 657.5891 | 2626.3274 | 2626.3099 | 6.67 | 2 | 37 | 0.00032 | 1Score **> 37** indicates **identity** Score **> 15** indicates **homology** | U | K.NRPGNALEKEVESIGAHLNAYSTR.E  + Deamidated (NQ) |
| 176175 | 103 | – | 126 | 928.4733 | 2782.3982 | 2782.4249 | -9.61 | 2 | 19 | 0.018 | 1Score **> 37** indicates **identity** Score **> 14** indicates **homology** | U | K.NRPGNALEKEVESIGAHLNAYSTR.E  + Deamidated (NQ); HNE (K) |
| 87587 | 112 | – | 126 | 549.6093 | 1645.8059 | 1645.8060 | -0.025 | 0 | 46 | 5.3e-05 | 1Score **> 34** indicates **identity** Score **> 15** indicates **homology** | U | K.EVESIGAHLNAYSTR.E |
| 87588 | 112 | – | 126 | 549.6093 | 1645.8061 | 1645.8060 | 0.086 | 0 | 16 | 0.031 | 1Score **> 34** indicates **identity** Score **> 14** indicates **homology** | U | K.EVESIGAHLNAYSTR.E |
| 87589 | 112 | – | 126 | 823.9103 | 1645.8061 | 1645.8060 | 0.093 | 0 | 92 | 2.6e-08 | 1Score **> 34** indicates **identity** Score **> 29** indicates **homology** | U | K.EVESIGAHLNAYSTR.E |
| 87591 | 112 | – | 126 | 823.9106 | 1645.8067 | 1645.8060 | 0.43 | 0 | 81 | 2.1e-07 | 1Score **> 34** indicates **identity** Score **> 27** indicates **homology** | U | K.EVESIGAHLNAYSTR.E |
| 87593 | 112 | – | 126 | 549.6097 | 1645.8072 | 1645.8060 | 0.73 | 0 | 53 | 1.1e-05 | 1Score **> 34** indicates **identity** Score **> 16** indicates **homology** | U | K.EVESIGAHLNAYSTR.E |
| 87594 | 112 | – | 126 | 823.9110 | 1645.8075 | 1645.8060 | 0.91 | 0 | 60 | 8e-06 | 1Score **> 34** indicates **identity** Score **> 21** indicates **homology** | U | K.EVESIGAHLNAYSTR.E |
| 87595 | 112 | – | 126 | 549.6098 | 1645.8076 | 1645.8060 | 0.96 | 0 | 46 | 4.6e-05 | 1Score **> 34** indicates **identity** Score **> 15** indicates **homology** | U | K.EVESIGAHLNAYSTR.E |
| 13106 | 127 | – | 134 | 487.7685 | 973.5225 | 973.5233 | -0.75 | 0 | 23 | 0.016 | 1Score **> 31** indicates **identity** Score **> 17** indicates **homology** | U | R.EHTAYLIK.A |
| 13108 | 127 | – | 134 | 487.7689 | 973.5232 | 973.5233 | -0.021 | 0 | 43 | 0.00092 | 1Score **> 31** indicates **identity** Score **> 25** indicates **homology** | U | R.EHTAYLIK.A |
| 13109 | 127 | – | 134 | 487.7690 | 973.5234 | 973.5233 | 0.19 | 0 | 33 | 0.011 | 1Score **> 33** indicates **identity** Score **> 26** indicates **homology** | U | R.EHTAYLIK.A |
| 7155 | 135 | – | 142 | 436.2665 | 870.5184 | 870.5174 | 1.09 | 1 | 47 | 0.00016 | 1Score **> 27** indicates **identity** Score **> 22** indicates **homology** | U | K.ALSKDLPK.V |
| 185463 | 139 | – | 165 | 767.6647 | 3066.6298 | 3066.6084 | 6.97 | 2 | 23 | 0.0073 | 1Score **> 36** indicates **identity** Score **> 14** indicates **homology** | U | K.DLPKVVELLADIVQNSSLEDSQIEKER.D |
| 153557 | 143 | – | 163 | 1165.1113 | 2328.2081 | 2328.2060 | 0.89 | 0 | 103 | 2.1e-10 | 1Score **> 37** indicates **identity** Score **> 19** indicates **homology** | U | K.VVELLADIVQNSSLEDSQIEK.E |
| 169135 | 143 | – | 165 | 872.1210 | 2613.3411 | 2613.3497 | -3.31 | 1 | 38 | 0.00026 | 1Score **> 37** indicates **identity** Score **> 15** indicates **homology** | U | K.VVELLADIVQNSSLEDSQIEKER.D |
| 169136 | 143 | – | 165 | 872.1211 | 2613.3416 | 2613.3497 | -3.11 | 1 | 14 | 0.049 | 1Score **> 37** indicates **identity** Score **> 13** indicates **homology** | U | K.VVELLADIVQNSSLEDSQIEKER.D |
| 169145 | 143 | – | 165 | 872.1240 | 2613.3502 | 2613.3497 | 0.18 | 1 | 74 | 1.2e-07 | 1Score **> 37** indicates **identity** Score **> 17** indicates **homology** | U | K.VVELLADIVQNSSLEDSQIEKER.D |
| 169149 | 143 | – | 165 | 872.1242 | 2613.3509 | 2613.3497 | 0.45 | 1 | 71 | 2.4e-07 | 1Score **> 37** indicates **identity** Score **> 17** indicates **homology** | U | K.VVELLADIVQNSSLEDSQIEKER.D |
| 169151 | 143 | – | 165 | 872.1243 | 2613.3512 | 2613.3497 | 0.57 | 1 | 20 | 0.014 | 1Score **> 37** indicates **identity** Score **> 14** indicates **homology** | U | K.VVELLADIVQNSSLEDSQIEKER.D |
| 169152 | 143 | – | 165 | 654.3452 | 2613.3516 | 2613.3497 | 0.73 | 1 | 63 | 1.3e-06 | 1Score **> 37** indicates **identity** Score **> 16** indicates **homology** | U | K.VVELLADIVQNSSLEDSQIEKER.D |
| 169153 | 143 | – | 165 | 1307.6831 | 2613.3517 | 2613.3497 | 0.77 | 1 | 101 | 3e-10 | 1Score **> 37** indicates **identity** Score **> 19** indicates **homology** | U | K.VVELLADIVQNSSLEDSQIEKER.D |
| 169156 | 143 | – | 165 | 654.3453 | 2613.3522 | 2613.3497 | 0.97 | 1 | 30 | 0.0016 | 1Score **> 37** indicates **identity** Score **> 14** indicates **homology** | U | K.VVELLADIVQNSSLEDSQIEKER.D |
| 169157 | 143 | – | 165 | 654.3454 | 2613.3525 | 2613.3497 | 1.09 | 1 | 29 | 0.0018 | 1Score **> 37** indicates **identity** Score **> 14** indicates **homology** | U | K.VVELLADIVQNSSLEDSQIEKER.D |
| 169160 | 143 | – | 165 | 872.1249 | 2613.3527 | 2613.3497 | 1.17 | 1 | 87 | 6.7e-09 | 1Score **> 37** indicates **identity** Score **> 18** indicates **homology** | U | K.VVELLADIVQNSSLEDSQIEKER.D |
| 169161 | 143 | – | 165 | 872.1249 | 2613.3529 | 2613.3497 | 1.24 | 1 | 54 | 8.3e-06 | 1Score **> 37** indicates **identity** Score **> 16** indicates **homology** | U | K.VVELLADIVQNSSLEDSQIEKER.D |
| 169162 | 143 | – | 165 | 1307.6839 | 2613.3532 | 2613.3497 | 1.33 | 1 | 135 | 2.2e-13 | 1Score **> 37** indicates **identity** Score **> 21** indicates **homology** | U | K.VVELLADIVQNSSLEDSQIEKER.D |
| 169163 | 143 | – | 165 | 872.1251 | 2613.3534 | 2613.3497 | 1.42 | 1 | 88 | 6e-09 | 1Score **> 37** indicates **identity** Score **> 18** indicates **homology** | U | K.VVELLADIVQNSSLEDSQIEKER.D |
| 169166 | 143 | – | 165 | 872.1254 | 2613.3544 | 2613.3497 | 1.80 | 1 | 76 | 8.1e-08 | 1Score **> 37** indicates **identity** Score **> 17** indicates **homology** | U | K.VVELLADIVQNSSLEDSQIEKER.D |
| 169167 | 143 | – | 165 | 872.1254 | 2613.3544 | 2613.3497 | 1.80 | 1 | 66 | 6.6e-07 | 1Score **> 37** indicates **identity** Score **> 17** indicates **homology** | U | K.VVELLADIVQNSSLEDSQIEKER.D |
| 169170 | 143 | – | 165 | 872.1258 | 2613.3557 | 2613.3497 | 2.29 | 1 | 35 | 0.00048 | 1Score **> 37** indicates **identity** Score **> 15** indicates **homology** | U | K.VVELLADIVQNSSLEDSQIEKER.D |
| 169173 | 143 | – | 165 | 872.1264 | 2613.3573 | 2613.3497 | 2.91 | 1 | 58 | 3.9e-06 | 1Score **> 37** indicates **identity** Score **> 16** indicates **homology** | U | K.VVELLADIVQNSSLEDSQIEKER.D |
| 169175 | 143 | – | 165 | 872.1264 | 2613.3575 | 2613.3497 | 2.99 | 1 | 18 | 0.019 | 1Score **> 37** indicates **identity** Score **> 14** indicates **homology** | U | K.VVELLADIVQNSSLEDSQIEKER.D |
| 169177 | 143 | – | 165 | 872.1267 | 2613.3582 | 2613.3497 | 3.24 | 1 | 34 | 0.00064 | 1Score **> 37** indicates **identity** Score **> 15** indicates **homology** | U | K.VVELLADIVQNSSLEDSQIEKER.D |
| 169181 | 143 | – | 165 | 872.1271 | 2613.3594 | 2613.3497 | 3.71 | 1 | 49 | 2.8e-05 | 1Score **> 37** indicates **identity** Score **> 16** indicates **homology** | U | K.VVELLADIVQNSSLEDSQIEKER.D |
| 169183 | 143 | – | 165 | 872.1272 | 2613.3598 | 2613.3497 | 3.85 | 1 | 21 | 0.012 | 1Score **> 37** indicates **identity** Score **> 14** indicates **homology** | U | K.VVELLADIVQNSSLEDSQIEKER.D |
| 169189 | 143 | – | 165 | 872.1286 | 2613.3639 | 2613.3497 | 5.45 | 1 | 48 | 3.1e-05 | 1Score **> 37** indicates **identity** Score **> 16** indicates **homology** | U | K.VVELLADIVQNSSLEDSQIEKER.D |
| 169190 | 143 | – | 165 | 872.1286 | 2613.3641 | 2613.3497 | 5.51 | 1 | 16 | 0.032 | 1Score **> 37** indicates **identity** Score **> 13** indicates **homology** | U | K.VVELLADIVQNSSLEDSQIEKER.D |
| 169245 | 143 | – | 165 | 872.4522 | 2614.3347 | 2614.3337 | 0.39 | 1 | 30 | 0.0017 | 1Score **> 37** indicates **identity** Score **> 15** indicates **homology** | U | K.VVELLADIVQNSSLEDSQIEKER.D  + Deamidated (NQ) |
| 169247 | 143 | – | 165 | 872.4534 | 2614.3383 | 2614.3337 | 1.75 | 1 | 71 | 2.2e-07 | 1Score **> 37** indicates **identity** Score **> 17** indicates **homology** | U | K.VVELLADIVQNSSLEDSQIEKER.D  + Deamidated (NQ) |
| 188296 | 143 | – | 170 | 803.4325 | 3209.7010 | 3209.7143 | -4.15 | 2 | 15 | 0.043 | 1Score **> 36** indicates **identity** Score **> 13** indicates **homology** | U | K.VVELLADIVQNSSLEDSQIEKERDVILR.E |
| 188299 | 143 | – | 170 | 803.4350 | 3209.7110 | 3209.7143 | -1.01 | 2 | 34 | 0.00069 | 1Score **> 36** indicates **identity** Score **> 15** indicates **homology** | U | K.VVELLADIVQNSSLEDSQIEKERDVILR.E |
| 188300 | 143 | – | 170 | 1070.9121 | 3209.7144 | 3209.7143 | 0.018 | 2 | 15 | 0.042 | 1Score **> 36** indicates **identity** Score **> 13** indicates **homology** | U | K.VVELLADIVQNSSLEDSQIEKERDVILR.E |
| 188303 | 143 | – | 170 | 803.4365 | 3209.7168 | 3209.7143 | 0.78 | 2 | 41 | 0.00016 | 1Score **> 36** indicates **identity** Score **> 15** indicates **homology** | U | K.VVELLADIVQNSSLEDSQIEKERDVILR.E |
| 188304 | 143 | – | 170 | 1070.9130 | 3209.7170 | 3209.7143 | 0.86 | 2 | 30 | 0.0017 | 1Score **> 36** indicates **identity** Score **> 14** indicates **homology** | U | K.VVELLADIVQNSSLEDSQIEKERDVILR.E |
| 188308 | 143 | – | 170 | 642.9518 | 3209.7225 | 3209.7143 | 2.54 | 2 | 23 | 0.0063 | 1Score **> 35** indicates **identity** Score **> 14** indicates **homology** | U | K.VVELLADIVQNSSLEDSQIEKERDVILR.E |
| 188309 | 143 | – | 170 | 1070.9148 | 3209.7226 | 3209.7143 | 2.60 | 2 | 53 | 1.1e-05 | 1Score **> 36** indicates **identity** Score **> 16** indicates **homology** | U | K.VVELLADIVQNSSLEDSQIEKERDVILR.E |
| 188310 | 143 | – | 170 | 1070.9149 | 3209.7230 | 3209.7143 | 2.71 | 2 | 18 | 0.023 | 1Score **> 36** indicates **identity** Score **> 14** indicates **homology** | U | K.VVELLADIVQNSSLEDSQIEKERDVILR.E |
| 188316 | 143 | – | 170 | 803.4389 | 3209.7264 | 3209.7143 | 3.77 | 2 | 36 | 0.00047 | 1Score **> 35** indicates **identity** Score **> 15** indicates **homology** | U | K.VVELLADIVQNSSLEDSQIEKERDVILR.E |
| 188333 | 143 | – | 170 | 803.6837 | 3210.7056 | 3210.6983 | 2.28 | 2 | 17 | 0.027 | 1Score **> 36** indicates **identity** Score **> 14** indicates **homology** | U | K.VVELLADIVQNSSLEDSQIEKERDVILR.E  + Deamidated (NQ) |
| 199170 | 171 | – | 209 | 1484.6885 | 4451.0436 | 4451.0573 | -3.08 | 0 | 62 | 1.6e-06 | 1Score **> 34** indicates **identity** Score **> 16** indicates **homology** | U | R.EMQENDASMQNVVFDYLHATAFQGTPLAQAVEGPSENVR.R  + 2 Deamidated (NQ); HNE (H) |
| 199186 | 171 | – | 209 | 1485.3535 | 4453.0387 | 4453.0253 | 3.00 | 0 | 51 | 1.5e-05 | 1Score **> 33** indicates **identity** Score **> 16** indicates **homology** | U | R.EMQENDASMQNVVFDYLHATAFQGTPLAQAVEGPSENVR.R  + 4 Deamidated (NQ); HNE (H) |
| 199262 | 171 | – | 209 | 1117.2694 | 4465.0486 | 4465.0842 | -7.98 | 0 | 25 | 0.005 | 1Score **> 34** indicates **identity** Score **> 14** indicates **homology** | U | R.EMQENDASMQNVVFDYLHATAFQGTPLAQAVEGPSENVR.R  + HNE (H); Oxidation (M) |
| 63585 | 211 | – | 222 | 489.5916 | 1465.7531 | 1465.7525 | 0.40 | 1 | 25 | 0.0076 | 1Score **> 34** indicates **identity** Score **> 17** indicates **homology** | U | R.LSRTDLTDYLNR.H |
| 63587 | 211 | – | 222 | 489.5920 | 1465.7542 | 1465.7525 | 1.15 | 1 | 28 | 0.0059 | 1Score **> 34** indicates **identity** Score **> 18** indicates **homology** | U | R.LSRTDLTDYLNR.H |
| 63589 | 211 | – | 222 | 489.5928 | 1465.7565 | 1465.7525 | 2.73 | 1 | 23 | 0.0071 | 1Score **> 34** indicates **identity** Score **> 14** indicates **homology** | U | R.LSRTDLTDYLNR.H |
| 23975 | 214 | – | 222 | 555.7743 | 1109.5341 | 1109.5353 | -1.05 | 0 | 30 | 0.016 | 1Score **> 30** indicates **identity** Score **> 24** indicates **homology** | U | R.TDLTDYLNR.H |
| 23980 | 214 | – | 222 | 555.7748 | 1109.5351 | 1109.5353 | -0.14 | 0 | 61 | 3e-05 | 1Score **> 30** indicates **identity** Score **> 28** indicates **homology** | U | R.TDLTDYLNR.H |
| 23984 | 214 | – | 222 | 555.7752 | 1109.5358 | 1109.5353 | 0.41 | 0 | 66 | 8.6e-06 | 1Score **> 31** indicates **identity** Score **> 28** indicates **homology** | U | R.TDLTDYLNR.H |
| 23985 | 214 | – | 222 | 555.7752 | 1109.5358 | 1109.5353 | 0.47 | 0 | 16 | 0.029 | 1Score **> 31** indicates **identity** Score **> 14** indicates **homology** | U | R.TDLTDYLNR.H |
| 23986 | 214 | – | 222 | 555.7753 | 1109.5361 | 1109.5353 | 0.71 | 0 | 63 | 2e-05 | 1Score **> 30** indicates **identity** Score **> 28** indicates **homology** | U | R.TDLTDYLNR.H |
| 23987 | 214 | – | 222 | 555.7754 | 1109.5362 | 1109.5353 | 0.78 | 0 | 62 | 2e-05 | 1Score **> 30** indicates **identity** Score **> 28** indicates **homology** | U | R.TDLTDYLNR.H |
| 23989 | 214 | – | 222 | 555.7755 | 1109.5365 | 1109.5353 | 1.05 | 0 | 48 | 0.00027 | 1Score **> 31** indicates **identity** Score **> 25** indicates **homology** | U | R.TDLTDYLNR.H |
| 23990 | 214 | – | 222 | 555.7756 | 1109.5367 | 1109.5353 | 1.30 | 0 | 66 | 1.2e-05 | 1Score **> 31** indicates **identity** Score **> 30** indicates **homology** | U | R.TDLTDYLNR.H |
| 24052 | 214 | – | 222 | 556.2685 | 1110.5224 | 1110.5193 | 2.78 | 0 | 31 | 0.0023 | 1Score **> 29** indicates **identity** Score **> 17** indicates **homology** | U | R.TDLTDYLNR.H  + Deamidated (NQ) |
| 134446 | 229 | – | 248 | 698.0403 | 2091.0989 | 2091.1146 | -7.51 | 0 | 16 | 0.032 | 1Score **> 36** indicates **identity** Score **> 13** indicates **homology** | U | R.MVLAAAGGVEHQQLLDLAQK.H |
| 134481 | 229 | – | 248 | 698.0467 | 2091.1182 | 2091.1146 | 1.70 | 0 | 21 | 0.01 | 1Score **> 36** indicates **identity** Score **> 14** indicates **homology** | U | R.MVLAAAGGVEHQQLLDLAQK.H |
| 134485 | 229 | – | 248 | 698.0470 | 2091.1193 | 2091.1146 | 2.22 | 0 | 31 | 0.0011 | 1Score **> 36** indicates **identity** Score **> 14** indicates **homology** | U | R.MVLAAAGGVEHQQLLDLAQK.H |
| 134491 | 229 | – | 248 | 698.0474 | 2091.1204 | 2091.1146 | 2.76 | 0 | 44 | 8e-05 | 1Score **> 36** indicates **identity** Score **> 15** indicates **homology** | U | R.MVLAAAGGVEHQQLLDLAQK.H |
| 134493 | 229 | – | 248 | 698.0475 | 2091.1206 | 2091.1146 | 2.83 | 0 | 48 | 3e-05 | 1Score **> 36** indicates **identity** Score **> 16** indicates **homology** | U | R.MVLAAAGGVEHQQLLDLAQK.H |
| 134496 | 229 | – | 248 | 698.0478 | 2091.1216 | 2091.1146 | 3.33 | 0 | 39 | 0.00024 | 1Score **> 36** indicates **identity** Score **> 15** indicates **homology** | U | R.MVLAAAGGVEHQQLLDLAQK.H |
| 134500 | 229 | – | 248 | 698.0491 | 2091.1253 | 2091.1146 | 5.12 | 0 | 43 | 8.8e-05 | 1Score **> 35** indicates **identity** Score **> 15** indicates **homology** | U | R.MVLAAAGGVEHQQLLDLAQK.H |
| 136113 | 229 | – | 248 | 703.3747 | 2107.1022 | 2107.1096 | -3.49 | 0 | 18 | 0.022 | 1Score **> 36** indicates **identity** Score **> 14** indicates **homology** | U | R.MVLAAAGGVEHQQLLDLAQK.H  + Oxidation (M) |
| 3072 | 249 | – | 255 | 393.2164 | 784.4183 | 784.4191 | -1.07 | 0 | 18 | 0.022 | 1Score **> 23** indicates **identity** Score **> 14** indicates **homology** | U | K.HLSSVSR.V |
| 3073 | 249 | – | 255 | 393.2169 | 784.4193 | 784.4191 | 0.25 | 0 | 28 | 0.019 | 1Score **> 23** indicates **identity** Score **> 23** indicates **homology** | U | K.HLSSVSR.V |
| 82059 | 256 | – | 269 | 803.3804 | 1604.7462 | 1604.7505 | -2.64 | 0 | 42 | 0.00012 | 1Score **> 32** indicates **identity** Score **> 15** indicates **homology** | U | R.VYEEDAVPGLTPCR.F |
| 82060 | 256 | – | 269 | 803.3806 | 1604.7467 | 1604.7505 | -2.36 | 0 | 36 | 0.00045 | 1Score **> 32** indicates **identity** Score **> 15** indicates **homology** | U | R.VYEEDAVPGLTPCR.F |
| 82061 | 256 | – | 269 | 803.3807 | 1604.7469 | 1604.7505 | -2.22 | 0 | 30 | 0.0014 | 1Score **> 32** indicates **identity** Score **> 14** indicates **homology** | U | R.VYEEDAVPGLTPCR.F |
| 82062 | 256 | – | 269 | 803.3809 | 1604.7472 | 1604.7505 | -2.06 | 0 | 41 | 0.00014 | 1Score **> 32** indicates **identity** Score **> 15** indicates **homology** | U | R.VYEEDAVPGLTPCR.F |
| 82063 | 256 | – | 269 | 803.3809 | 1604.7473 | 1604.7505 | -1.99 | 0 | 51 | 1.7e-05 | 1Score **> 32** indicates **identity** Score **> 16** indicates **homology** | U | R.VYEEDAVPGLTPCR.F |
| 82064 | 256 | – | 269 | 803.3810 | 1604.7474 | 1604.7505 | -1.91 | 0 | 80 | 3.4e-08 | 1Score **> 32** indicates **identity** Score **> 17** indicates **homology** | U | R.VYEEDAVPGLTPCR.F |
| 82065 | 256 | – | 269 | 535.9232 | 1604.7477 | 1604.7505 | -1.70 | 0 | 14 | 0.044 | 1Score **> 32** indicates **identity** Score **> 13** indicates **homology** | U | R.VYEEDAVPGLTPCR.F |
| 82066 | 256 | – | 269 | 803.3815 | 1604.7485 | 1604.7505 | -1.20 | 0 | 55 | 7.1e-06 | 1Score **> 32** indicates **identity** Score **> 16** indicates **homology** | U | R.VYEEDAVPGLTPCR.F |
| 82067 | 256 | – | 269 | 803.3822 | 1604.7498 | 1604.7505 | -0.39 | 0 | 68 | 4.3e-07 | 1Score **> 32** indicates **identity** Score **> 17** indicates **homology** | U | R.VYEEDAVPGLTPCR.F |
| 82068 | 256 | – | 269 | 803.3823 | 1604.7501 | 1604.7505 | -0.25 | 0 | 62 | 1.6e-06 | 1Score **> 32** indicates **identity** Score **> 16** indicates **homology** | U | R.VYEEDAVPGLTPCR.F |
| 82069 | 256 | – | 269 | 803.3824 | 1604.7502 | 1604.7505 | -0.18 | 0 | 67 | 4.7e-07 | 1Score **> 32** indicates **identity** Score **> 17** indicates **homology** | U | R.VYEEDAVPGLTPCR.F |
| 82070 | 256 | – | 269 | 803.3824 | 1604.7503 | 1604.7505 | -0.096 | 0 | 52 | 1.3e-05 | 1Score **> 32** indicates **identity** Score **> 16** indicates **homology** | U | R.VYEEDAVPGLTPCR.F |
| 82071 | 256 | – | 269 | 803.3826 | 1604.7506 | 1604.7505 | 0.075 | 0 | 72 | 2.1e-07 | 1Score **> 32** indicates **identity** Score **> 17** indicates **homology** | U | R.VYEEDAVPGLTPCR.F |
| 82072 | 256 | – | 269 | 803.3826 | 1604.7507 | 1604.7505 | 0.15 | 0 | 33 | 0.0024 | 1Score **> 32** indicates **identity** Score **> 19** indicates **homology** | U | R.VYEEDAVPGLTPCR.F |
| 82073 | 256 | – | 269 | 803.3827 | 1604.7509 | 1604.7505 | 0.30 | 0 | 45 | 6.1e-05 | 1Score **> 32** indicates **identity** Score **> 15** indicates **homology** | U | R.VYEEDAVPGLTPCR.F |
| 82074 | 256 | – | 269 | 803.3828 | 1604.7511 | 1604.7505 | 0.42 | 0 | 67 | 5.3e-07 | 1Score **> 32** indicates **identity** Score **> 17** indicates **homology** | U | R.VYEEDAVPGLTPCR.F |
| 82076 | 256 | – | 269 | 535.9244 | 1604.7515 | 1604.7505 | 0.62 | 0 | 26 | 0.0043 | 1Score **> 32** indicates **identity** Score **> 15** indicates **homology** | U | R.VYEEDAVPGLTPCR.F |
| 82077 | 256 | – | 269 | 803.3830 | 1604.7515 | 1604.7505 | 0.66 | 0 | 63 | 1.1e-06 | 1Score **> 32** indicates **identity** Score **> 16** indicates **homology** | U | R.VYEEDAVPGLTPCR.F |
| 82078 | 256 | – | 269 | 535.9245 | 1604.7518 | 1604.7505 | 0.81 | 0 | 22 | 0.0084 | 1Score **> 32** indicates **identity** Score **> 14** indicates **homology** | U | R.VYEEDAVPGLTPCR.F |
| 82079 | 256 | – | 269 | 803.3832 | 1604.7519 | 1604.7505 | 0.91 | 0 | 54 | 9e-06 | 1Score **> 32** indicates **identity** Score **> 16** indicates **homology** | U | R.VYEEDAVPGLTPCR.F |
| 82080 | 256 | – | 269 | 535.9246 | 1604.7519 | 1604.7505 | 0.93 | 0 | 21 | 0.01 | 1Score **> 32** indicates **identity** Score **> 14** indicates **homology** | U | R.VYEEDAVPGLTPCR.F |
| 82081 | 256 | – | 269 | 803.3835 | 1604.7524 | 1604.7505 | 1.21 | 0 | 67 | 5.6e-07 | 1Score **> 32** indicates **identity** Score **> 17** indicates **homology** | U | R.VYEEDAVPGLTPCR.F |
| 82082 | 256 | – | 269 | 803.3835 | 1604.7524 | 1604.7505 | 1.22 | 0 | 79 | 3.7e-08 | 1Score **> 32** indicates **identity** Score **> 17** indicates **homology** | U | R.VYEEDAVPGLTPCR.F |
| 82084 | 256 | – | 269 | 803.3839 | 1604.7533 | 1604.7505 | 1.74 | 0 | 51 | 1.5e-05 | 1Score **> 32** indicates **identity** Score **> 16** indicates **homology** | U | R.VYEEDAVPGLTPCR.F |
| 82086 | 256 | – | 269 | 803.3846 | 1604.7546 | 1604.7505 | 2.57 | 0 | 55 | 7.5e-06 | 1Score **> 32** indicates **identity** Score **> 16** indicates **homology** | U | R.VYEEDAVPGLTPCR.F |
| 82087 | 256 | – | 269 | 803.3851 | 1604.7557 | 1604.7505 | 3.27 | 0 | 46 | 4.6e-05 | 1Score **> 32** indicates **identity** Score **> 15** indicates **homology** | U | R.VYEEDAVPGLTPCR.F |
| 82088 | 256 | – | 269 | 803.3854 | 1604.7562 | 1604.7505 | 3.58 | 0 | 64 | 9.1e-07 | 1Score **> 32** indicates **identity** Score **> 17** indicates **homology** | U | R.VYEEDAVPGLTPCR.F |
| 82090 | 256 | – | 269 | 803.3856 | 1604.7566 | 1604.7505 | 3.80 | 0 | 29 | 0.0021 | 1Score **> 32** indicates **identity** Score **> 14** indicates **homology** | U | R.VYEEDAVPGLTPCR.F |
| 157782 | 256 | – | 276 | 799.3883 | 2395.1430 | 2395.1478 | -1.99 | 1 | 23 | 0.0063 | 1Score **> 35** indicates **identity** Score **> 14** indicates **homology** | U | R.VYEEDAVPGLTPCRFTGSEIR.H |
| 4085 | 270 | – | 276 | 405.2104 | 808.4062 | 808.4079 | -2.17 | 0 | 31 | 0.0013 | 1Score **> 24** indicates **identity** Score **> 14** indicates **homology** | U | R.FTGSEIR.H |
| 4086 | 270 | – | 276 | 405.2107 | 808.4068 | 808.4079 | -1.36 | 0 | 23 | 0.0069 | 1Score **> 24** indicates **identity** Score **> 14** indicates **homology** | U | R.FTGSEIR.H |
| 4087 | 270 | – | 276 | 405.2108 | 808.4071 | 808.4079 | -0.99 | 0 | 33 | 0.0025 | 1Score **> 24** indicates **identity** Score **> 19** indicates **homology** | U | R.FTGSEIR.H |
| 4089 | 270 | – | 276 | 405.2109 | 808.4073 | 808.4079 | -0.73 | 0 | 24 | 0.015 | 1Score **> 25** indicates **identity** Score **> 18** indicates **homology** | U | R.FTGSEIR.H |
| 4090 | 270 | – | 276 | 405.2113 | 808.4081 | 808.4079 | 0.24 | 0 | 38 | 0.00051 | 1Score **> 25** indicates **identity** Score **> 18** indicates **homology** | U | R.FTGSEIR.H |
| 4091 | 270 | – | 276 | 405.2114 | 808.4082 | 808.4079 | 0.36 | 0 | 43 | 0.00014 | 1Score **> 24** indicates **identity** Score **> 17** indicates **homology** | U | R.FTGSEIR.H |
| 4092 | 270 | – | 276 | 405.2119 | 808.4093 | 808.4079 | 1.66 | 0 | 28 | 0.0024 | 1Score **> 24** indicates **identity** Score **> 14** indicates **homology** | U | R.FTGSEIR.H |
| 201765 | 277 | – | 336 | 1034.0282 | 6198.1253 | 6198.1134 | 1.92 | 1 | 21 | 0.01 | 1Score **> 34** indicates **identity** Score **> 14** indicates **homology** | U | R.HRDDALPLAHVAIAVEGPGWANPDNVTLQVANAIIGHYDCTYGGGVHLSSPLASVAVANK.L |
| 201766 | 277 | – | 336 | 1240.6334 | 6198.1306 | 6198.1134 | 2.77 | 1 | 26 | 0.0039 | 1Score **> 34** indicates **identity** Score **> 14** indicates **homology** | U | R.HRDDALPLAHVAIAVEGPGWANPDNVTLQVANAIIGHYDCTYGGGVHLSSPLASVAVANK.L |
| 201767 | 277 | – | 336 | 1240.6340 | 6198.1334 | 6198.1134 | 3.22 | 1 | 25 | 0.005 | 1Score **> 34** indicates **identity** Score **> 14** indicates **homology** | U | R.HRDDALPLAHVAIAVEGPGWANPDNVTLQVANAIIGHYDCTYGGGVHLSSPLASVAVANK.L |
| 50810 | 379 | – | 390 | 677.3147 | 1352.6148 | 1352.6242 | -6.95 | 0 | 56 | 5.7e-06 | 1Score **> 30** indicates **identity** Score **> 16** indicates **homology** | U | R.LCTSATESEVTR.G |
| 50811 | 379 | – | 390 | 677.3153 | 1352.6161 | 1352.6242 | -5.96 | 0 | 24 | 0.0054 | 1Score **> 30** indicates **identity** Score **> 14** indicates **homology** | U | R.LCTSATESEVTR.G |
| 50812 | 379 | – | 390 | 677.3157 | 1352.6169 | 1352.6242 | -5.38 | 0 | 14 | 0.047 | 1Score **> 30** indicates **identity** Score **> 13** indicates **homology** | U | R.LCTSATESEVTR.G |
| 50813 | 379 | – | 390 | 677.3168 | 1352.6191 | 1352.6242 | -3.79 | 0 | 31 | 0.0035 | 1Score **> 31** indicates **identity** Score **> 19** indicates **homology** | U | R.LCTSATESEVTR.G |
| 50814 | 379 | – | 390 | 677.3180 | 1352.6214 | 1352.6242 | -2.07 | 0 | 48 | 3e-05 | 1Score **> 30** indicates **identity** Score **> 16** indicates **homology** | U | R.LCTSATESEVTR.G |
| 50815 | 379 | – | 390 | 677.3182 | 1352.6218 | 1352.6242 | -1.79 | 0 | 24 | 0.0061 | 1Score **> 30** indicates **identity** Score **> 14** indicates **homology** | U | R.LCTSATESEVTR.G |
| 50816 | 379 | – | 390 | 677.3185 | 1352.6225 | 1352.6242 | -1.26 | 0 | 79 | 4e-08 | 1Score **> 32** indicates **identity** Score **> 17** indicates **homology** | U | R.LCTSATESEVTR.G |
| 50817 | 379 | – | 390 | 677.3185 | 1352.6225 | 1352.6242 | -1.24 | 0 | 78 | 4.7e-08 | 1Score **> 32** indicates **identity** Score **> 17** indicates **homology** | U | R.LCTSATESEVTR.G |
| 50818 | 379 | – | 390 | 677.3186 | 1352.6226 | 1352.6242 | -1.15 | 0 | 81 | 2.5e-08 | 1Score **> 32** indicates **identity** Score **> 18** indicates **homology** | U | R.LCTSATESEVTR.G |
| 50819 | 379 | – | 390 | 677.3188 | 1352.6231 | 1352.6242 | -0.80 | 0 | 74 | 1e-07 | 1Score **> 32** indicates **identity** Score **> 17** indicates **homology** | U | R.LCTSATESEVTR.G |
| 50820 | 379 | – | 390 | 677.3191 | 1352.6236 | 1352.6242 | -0.45 | 0 | 69 | 3.3e-07 | 1Score **> 32** indicates **identity** Score **> 17** indicates **homology** | U | R.LCTSATESEVTR.G |
| 50821 | 379 | – | 390 | 677.3192 | 1352.6238 | 1352.6242 | -0.25 | 0 | 75 | 9.8e-08 | 1Score **> 32** indicates **identity** Score **> 17** indicates **homology** | U | R.LCTSATESEVTR.G |
| 50822 | 379 | – | 390 | 677.3194 | 1352.6243 | 1352.6242 | 0.086 | 0 | 55 | 7.4e-06 | 1Score **> 31** indicates **identity** Score **> 16** indicates **homology** | U | R.LCTSATESEVTR.G |
| 50823 | 379 | – | 390 | 677.3195 | 1352.6244 | 1352.6242 | 0.18 | 0 | 81 | 2.4e-08 | 1Score **> 31** indicates **identity** Score **> 18** indicates **homology** | U | R.LCTSATESEVTR.G |
| 50824 | 379 | – | 390 | 677.3195 | 1352.6245 | 1352.6242 | 0.20 | 0 | 81 | 2.3e-08 | 1Score **> 31** indicates **identity** Score **> 18** indicates **homology** | U | R.LCTSATESEVTR.G |
| 50825 | 379 | – | 390 | 677.3198 | 1352.6250 | 1352.6242 | 0.57 | 0 | 38 | 0.0024 | 1Score **> 31** indicates **identity** Score **> 24** indicates **homology** | U | R.LCTSATESEVTR.G |
| 50827 | 379 | – | 390 | 677.3200 | 1352.6254 | 1352.6242 | 0.90 | 0 | 18 | 0.027 | 1Score **> 31** indicates **identity** Score **> 14** indicates **homology** | U | R.LCTSATESEVTR.G |
| 50828 | 379 | – | 390 | 677.3204 | 1352.6262 | 1352.6242 | 1.46 | 0 | 60 | 2.2e-06 | 1Score **> 32** indicates **identity** Score **> 16** indicates **homology** | U | R.LCTSATESEVTR.G |
| 131224 | 397 | – | 415 | 1027.4987 | 2052.9829 | 2052.9899 | -3.38 | 0 | 57 | 4.3e-06 | 1Score **> 34** indicates **identity** Score **> 16** indicates **homology** | U | R.NALVSHLDGTTPVCEDIGR.S |
| 131226 | 397 | – | 415 | 685.3370 | 2052.9890 | 2052.9899 | -0.40 | 0 | 60 | 3.1e-06 | 1Score **> 35** indicates **identity** Score **> 18** indicates **homology** | U | R.NALVSHLDGTTPVCEDIGR.S |
| 131227 | 397 | – | 415 | 1027.5020 | 2052.9894 | 2052.9899 | -0.25 | 0 | 44 | 8.3e-05 | 1Score **> 35** indicates **identity** Score **> 15** indicates **homology** | U | R.NALVSHLDGTTPVCEDIGR.S |
| 131228 | 397 | – | 415 | 685.3374 | 2052.9903 | 2052.9899 | 0.20 | 0 | 66 | 8.2e-07 | 1Score **> 35** indicates **identity** Score **> 17** indicates **homology** | U | R.NALVSHLDGTTPVCEDIGR.S |
| 131229 | 397 | – | 415 | 685.3374 | 2052.9905 | 2052.9899 | 0.31 | 0 | 70 | 4e-07 | 1Score **> 35** indicates **identity** Score **> 18** indicates **homology** | U | R.NALVSHLDGTTPVCEDIGR.S |
| 131230 | 397 | – | 415 | 1027.5026 | 2052.9906 | 2052.9899 | 0.37 | 0 | 99 | 5.3e-10 | 1Score **> 35** indicates **identity** Score **> 19** indicates **homology** | U | R.NALVSHLDGTTPVCEDIGR.S |
| 131231 | 397 | – | 415 | 685.3376 | 2052.9908 | 2052.9899 | 0.47 | 0 | 65 | 8.4e-07 | 1Score **> 35** indicates **identity** Score **> 17** indicates **homology** | U | R.NALVSHLDGTTPVCEDIGR.S |
| 131232 | 397 | – | 415 | 685.3376 | 2052.9909 | 2052.9899 | 0.52 | 0 | 70 | 3.4e-07 | 1Score **> 35** indicates **identity** Score **> 18** indicates **homology** | U | R.NALVSHLDGTTPVCEDIGR.S |
| 131233 | 397 | – | 415 | 1027.5028 | 2052.9910 | 2052.9899 | 0.57 | 0 | 99 | 5e-10 | 1Score **> 35** indicates **identity** Score **> 19** indicates **homology** | U | R.NALVSHLDGTTPVCEDIGR.S |
| 131234 | 397 | – | 415 | 1027.5028 | 2052.9911 | 2052.9899 | 0.59 | 0 | 95 | 3.7e-09 | 1Score **> 35** indicates **identity** Score **> 23** indicates **homology** | U | R.NALVSHLDGTTPVCEDIGR.S |
| 131235 | 397 | – | 415 | 685.3376 | 2052.9911 | 2052.9899 | 0.60 | 0 | 71 | 3.7e-07 | 1Score **> 35** indicates **identity** Score **> 19** indicates **homology** | U | R.NALVSHLDGTTPVCEDIGR.S |
| 131236 | 397 | – | 415 | 1027.5029 | 2052.9912 | 2052.9899 | 0.67 | 0 | 96 | 1.1e-09 | 1Score **> 35** indicates **identity** Score **> 18** indicates **homology** | U | R.NALVSHLDGTTPVCEDIGR.S |
| 131237 | 397 | – | 415 | 1027.5030 | 2052.9914 | 2052.9899 | 0.74 | 0 | 87 | 7.1e-09 | 1Score **> 35** indicates **identity** Score **> 18** indicates **homology** | U | R.NALVSHLDGTTPVCEDIGR.S |
| 131238 | 397 | – | 415 | 685.3378 | 2052.9916 | 2052.9899 | 0.87 | 0 | 65 | 1e-06 | 1Score **> 35** indicates **identity** Score **> 18** indicates **homology** | U | R.NALVSHLDGTTPVCEDIGR.S |
| 131239 | 397 | – | 415 | 685.3378 | 2052.9917 | 2052.9899 | 0.87 | 0 | 75 | 1.5e-07 | 1Score **> 35** indicates **identity** Score **> 19** indicates **homology** | U | R.NALVSHLDGTTPVCEDIGR.S |
| 131240 | 397 | – | 415 | 685.3379 | 2052.9918 | 2052.9899 | 0.93 | 0 | 67 | 8e-07 | 1Score **> 35** indicates **identity** Score **> 18** indicates **homology** | U | R.NALVSHLDGTTPVCEDIGR.S |
| 131241 | 397 | – | 415 | 685.3379 | 2052.9919 | 2052.9899 | 1.00 | 0 | 66 | 7.1e-07 | 1Score **> 35** indicates **identity** Score **> 17** indicates **homology** | U | R.NALVSHLDGTTPVCEDIGR.S |
| 131242 | 397 | – | 415 | 685.3379 | 2052.9920 | 2052.9899 | 1.02 | 0 | 66 | 8.6e-07 | 1Score **> 35** indicates **identity** Score **> 18** indicates **homology** | U | R.NALVSHLDGTTPVCEDIGR.S |
| 131243 | 397 | – | 415 | 1027.5033 | 2052.9920 | 2052.9899 | 1.03 | 0 | 106 | 1.2e-10 | 1Score **> 35** indicates **identity** Score **> 19** indicates **homology** | U | R.NALVSHLDGTTPVCEDIGR.S |
| 131244 | 397 | – | 415 | 685.3380 | 2052.9920 | 2052.9899 | 1.06 | 0 | 71 | 3.9e-07 | 1Score **> 35** indicates **identity** Score **> 20** indicates **homology** | U | R.NALVSHLDGTTPVCEDIGR.S |
| 131245 | 397 | – | 415 | 685.3380 | 2052.9923 | 2052.9899 | 1.17 | 0 | 70 | 4.2e-07 | 1Score **> 35** indicates **identity** Score **> 19** indicates **homology** | U | R.NALVSHLDGTTPVCEDIGR.S |
| 131246 | 397 | – | 415 | 1027.5035 | 2052.9924 | 2052.9899 | 1.22 | 0 | 97 | 7.8e-10 | 1Score **> 35** indicates **identity** Score **> 19** indicates **homology** | U | R.NALVSHLDGTTPVCEDIGR.S |
| 131247 | 397 | – | 415 | 685.3382 | 2052.9929 | 2052.9899 | 1.46 | 0 | 57 | 2.8e-05 | 1Score **> 35** indicates **identity** Score **> 24** indicates **homology** | U | R.NALVSHLDGTTPVCEDIGR.S |
| 131248 | 397 | – | 415 | 685.3382 | 2052.9929 | 2052.9899 | 1.47 | 0 | 65 | 9.3e-07 | 1Score **> 35** indicates **identity** Score **> 17** indicates **homology** | U | R.NALVSHLDGTTPVCEDIGR.S |
| 131249 | 397 | – | 415 | 1027.5041 | 2052.9937 | 2052.9899 | 1.87 | 0 | 101 | 3.3e-10 | 1Score **> 35** indicates **identity** Score **> 19** indicates **homology** | U | R.NALVSHLDGTTPVCEDIGR.S |
| 131250 | 397 | – | 415 | 1027.5042 | 2052.9938 | 2052.9899 | 1.94 | 0 | 79 | 3.9e-08 | 1Score **> 35** indicates **identity** Score **> 17** indicates **homology** | U | R.NALVSHLDGTTPVCEDIGR.S |
| 131251 | 397 | – | 415 | 685.3391 | 2052.9955 | 2052.9899 | 2.73 | 0 | 36 | 0.00039 | 1Score **> 35** indicates **identity** Score **> 15** indicates **homology** | U | R.NALVSHLDGTTPVCEDIGR.S |
| 131252 | 397 | – | 415 | 685.3392 | 2052.9958 | 2052.9899 | 2.88 | 0 | 71 | 2.7e-07 | 1Score **> 35** indicates **identity** Score **> 18** indicates **homology** | U | R.NALVSHLDGTTPVCEDIGR.S |
| 131253 | 397 | – | 415 | 685.3393 | 2052.9960 | 2052.9899 | 2.98 | 0 | 35 | 0.0036 | 1Score **> 35** indicates **identity** Score **> 23** indicates **homology** | U | R.NALVSHLDGTTPVCEDIGR.S |
| 131254 | 397 | – | 415 | 685.3397 | 2052.9972 | 2052.9899 | 3.58 | 0 | 60 | 2.5e-06 | 1Score **> 35** indicates **identity** Score **> 16** indicates **homology** | U | R.NALVSHLDGTTPVCEDIGR.S |
| 131255 | 397 | – | 415 | 685.3400 | 2052.9982 | 2052.9899 | 4.07 | 0 | 49 | 2.5e-05 | 1Score **> 35** indicates **identity** Score **> 16** indicates **homology** | U | R.NALVSHLDGTTPVCEDIGR.S |
| 131256 | 397 | – | 415 | 1027.5066 | 2052.9987 | 2052.9899 | 4.28 | 0 | 83 | 1.5e-08 | 1Score **> 35** indicates **identity** Score **> 18** indicates **homology** | U | R.NALVSHLDGTTPVCEDIGR.S |
| 131317 | 397 | – | 415 | 685.6645 | 2053.9718 | 2053.9739 | -1.03 | 0 | 41 | 0.00014 | 1Score **> 34** indicates **identity** Score **> 15** indicates **homology** | U | R.NALVSHLDGTTPVCEDIGR.S  + Deamidated (NQ) |
| 131318 | 397 | – | 415 | 1027.9934 | 2053.9722 | 2053.9739 | -0.83 | 0 | 101 | 3.4e-10 | 1Score **> 34** indicates **identity** Score **> 19** indicates **homology** | U | R.NALVSHLDGTTPVCEDIGR.S  + Deamidated (NQ) |
| 131319 | 397 | – | 415 | 1027.9936 | 2053.9727 | 2053.9739 | -0.56 | 0 | 73 | 1.4e-07 | 1Score **> 34** indicates **identity** Score **> 17** indicates **homology** | U | R.NALVSHLDGTTPVCEDIGR.S  + Deamidated (NQ) |
| 131320 | 397 | – | 415 | 1027.9943 | 2053.9740 | 2053.9739 | 0.036 | 0 | 25 | 0.0046 | 1Score **> 34** indicates **identity** Score **> 14** indicates **homology** | U | R.NALVSHLDGTTPVCEDIGR.S  + Deamidated (NQ) |
| 131321 | 397 | – | 415 | 1027.9993 | 2053.9840 | 2053.9739 | 4.94 | 0 | 56 | 5.6e-06 | 1Score **> 35** indicates **identity** Score **> 16** indicates **homology** | U | R.NALVSHLDGTTPVCEDIGR.S  + Deamidated (NQ) |
| 131322 | 397 | – | 415 | 1028.0024 | 2053.9902 | 2053.9739 | 7.95 | 0 | 82 | 2.2e-08 | 1Score **> 35** indicates **identity** Score **> 18** indicates **homology** | U | R.NALVSHLDGTTPVCEDIGR.S  + Deamidated (NQ) |
| 131324 | 397 | – | 415 | 1028.0030 | 2053.9914 | 2053.9739 | 8.54 | 0 | 23 | 0.0069 | 1Score **> 35** indicates **identity** Score **> 14** indicates **homology** | U | R.NALVSHLDGTTPVCEDIGR.S  + Deamidated (NQ) |
| 131325 | 397 | – | 415 | 1028.0035 | 2053.9925 | 2053.9739 | 9.08 | 0 | 16 | 0.032 | 1Score **> 35** indicates **identity** Score **> 13** indicates **homology** | U | R.NALVSHLDGTTPVCEDIGR.S  + Deamidated (NQ) |
| 131326 | 397 | – | 415 | 685.6718 | 2053.9936 | 2053.9739 | 9.61 | 0 | 35 | 0.00048 | 1Score **> 35** indicates **identity** Score **> 15** indicates **homology** | U | R.NALVSHLDGTTPVCEDIGR.S  + Deamidated (NQ) |
| 4103 | 416 | – | 422 | 405.2288 | 808.4430 | 808.4443 | -1.64 | 0 | 42 | 0.00012 | 1Score **> 22** indicates **identity** Score **> 15** indicates **homology** | U | R.SLLTYGR.R |
| 4104 | 416 | – | 422 | 405.2288 | 808.4430 | 808.4443 | -1.64 | 0 | 15 | 0.038 | 1Score **> 22** indicates **identity** Score **> 13** indicates **homology** | U | R.SLLTYGR.R |
| 4105 | 416 | – | 422 | 405.2292 | 808.4438 | 808.4443 | -0.63 | 0 | 25 | 0.005 | 1Score **> 22** indicates **identity** Score **> 14** indicates **homology** | U | R.SLLTYGR.R |
| 4106 | 416 | – | 422 | 405.2293 | 808.4440 | 808.4443 | -0.42 | 0 | 47 | 3.9e-05 | 1Score **> 22** indicates **identity** Score **> 16** indicates **homology** | U | R.SLLTYGR.R |
| 4107 | 416 | – | 422 | 405.2293 | 808.4440 | 808.4443 | -0.33 | 0 | 51 | 2e-05 | 1Score **> 22** indicates **identity** Score **> 16** indicates **homology** | U | R.SLLTYGR.R |
| 4108 | 416 | – | 422 | 405.2293 | 808.4441 | 808.4443 | -0.29 | 0 | 27 | 0.0032 | 1Score **> 22** indicates **identity** Score **> 14** indicates **homology** | U | R.SLLTYGR.R |
| 4109 | 416 | – | 422 | 405.2294 | 808.4443 | 808.4443 | 0.020 | 0 | 31 | 0.0013 | 1Score **> 22** indicates **identity** Score **> 14** indicates **homology** | U | R.SLLTYGR.R |
| 4110 | 416 | – | 422 | 405.2296 | 808.4446 | 808.4443 | 0.40 | 0 | 34 | 0.0007 | 1Score **> 22** indicates **identity** Score **> 15** indicates **homology** | U | R.SLLTYGR.R |
| 4111 | 416 | – | 422 | 405.2296 | 808.4447 | 808.4443 | 0.51 | 0 | 51 | 2e-05 | 1Score **> 22** indicates **identity** Score **> 16** indicates **homology** | U | R.SLLTYGR.R |
| 4112 | 416 | – | 422 | 405.2297 | 808.4448 | 808.4443 | 0.61 | 0 | 31 | 0.0013 | 1Score **> 22** indicates **identity** Score **> 14** indicates **homology** | U | R.SLLTYGR.R |
| 4113 | 416 | – | 422 | 405.2299 | 808.4452 | 808.4443 | 1.06 | 0 | 38 | 0.00025 | 1Score **> 22** indicates **identity** Score **> 15** indicates **homology** | U | R.SLLTYGR.R |
| 4114 | 416 | – | 422 | 405.2302 | 808.4458 | 808.4443 | 1.81 | 0 | 38 | 0.00029 | 1Score **> 22** indicates **identity** Score **> 15** indicates **homology** | U | R.SLLTYGR.R |
| 39967 | 423 | – | 432 | 419.5611 | 1255.6615 | 1255.6673 | -4.58 | 1 | 27 | 0.003 | 1Score **> 32** indicates **identity** Score **> 14** indicates **homology** | U | R.RIPLAEWESR.I |
| 39968 | 423 | – | 432 | 419.5615 | 1255.6627 | 1255.6673 | -3.68 | 1 | 24 | 0.0057 | 1Score **> 33** indicates **identity** Score **> 14** indicates **homology** | U | R.RIPLAEWESR.I |
| 39969 | 423 | – | 432 | 419.5617 | 1255.6632 | 1255.6673 | -3.21 | 1 | 20 | 0.014 | 1Score **> 32** indicates **identity** Score **> 14** indicates **homology** | U | R.RIPLAEWESR.I |
| 39970 | 423 | – | 432 | 419.5622 | 1255.6649 | 1255.6673 | -1.88 | 1 | 30 | 0.0016 | 1Score **> 33** indicates **identity** Score **> 14** indicates **homology** | U | R.RIPLAEWESR.I |
| 39971 | 423 | – | 432 | 628.8401 | 1255.6656 | 1255.6673 | -1.35 | 1 | 30 | 0.014 | 1Score **> 33** indicates **identity** Score **> 24** indicates **homology** | U | R.RIPLAEWESR.I |
| 39973 | 423 | – | 432 | 419.5626 | 1255.6661 | 1255.6673 | -0.93 | 1 | 28 | 0.0025 | 1Score **> 33** indicates **identity** Score **> 14** indicates **homology** | U | R.RIPLAEWESR.I |
| 39974 | 423 | – | 432 | 419.5627 | 1255.6662 | 1255.6673 | -0.82 | 1 | 23 | 0.0077 | 1Score **> 33** indicates **identity** Score **> 14** indicates **homology** | U | R.RIPLAEWESR.I |
| 39975 | 423 | – | 432 | 419.5628 | 1255.6665 | 1255.6673 | -0.63 | 1 | 31 | 0.0014 | 1Score **> 33** indicates **identity** Score **> 14** indicates **homology** | U | R.RIPLAEWESR.I |
| 39976 | 423 | – | 432 | 419.5628 | 1255.6665 | 1255.6673 | -0.61 | 1 | 35 | 0.00051 | 1Score **> 33** indicates **identity** Score **> 15** indicates **homology** | U | R.RIPLAEWESR.I |
| 39977 | 423 | – | 432 | 419.5628 | 1255.6666 | 1255.6673 | -0.55 | 1 | 25 | 0.0041 | 1Score **> 33** indicates **identity** Score **> 14** indicates **homology** | U | R.RIPLAEWESR.I |
| 39979 | 423 | – | 432 | 419.5629 | 1255.6668 | 1255.6673 | -0.42 | 1 | 24 | 0.0058 | 1Score **> 33** indicates **identity** Score **> 14** indicates **homology** | U | R.RIPLAEWESR.I |
| 39980 | 423 | – | 432 | 419.5629 | 1255.6668 | 1255.6673 | -0.41 | 1 | 23 | 0.0066 | 1Score **> 33** indicates **identity** Score **> 14** indicates **homology** | U | R.RIPLAEWESR.I |
| 39981 | 423 | – | 432 | 419.5629 | 1255.6670 | 1255.6673 | -0.23 | 1 | 29 | 0.0019 | 1Score **> 33** indicates **identity** Score **> 14** indicates **homology** | U | R.RIPLAEWESR.I |
| 39983 | 423 | – | 432 | 628.8408 | 1255.6670 | 1255.6673 | -0.21 | 1 | 29 | 0.011 | 1Score **> 33** indicates **identity** Score **> 23** indicates **homology** | U | R.RIPLAEWESR.I |
| 39984 | 423 | – | 432 | 628.8408 | 1255.6671 | 1255.6673 | -0.18 | 1 | 41 | 0.0059 | 1Score **> 33** indicates **identity** Score **> 31** indicates **homology** | U | R.RIPLAEWESR.I |
| 39987 | 423 | – | 432 | 419.5630 | 1255.6672 | 1255.6673 | -0.049 | 1 | 41 | 0.00013 | 1Score **> 33** indicates **identity** Score **> 15** indicates **homology** | U | R.RIPLAEWESR.I |
| 39988 | 423 | – | 432 | 628.8409 | 1255.6673 | 1255.6673 | 0.0032 | 1 | 63 | 4.8e-05 | 1Score **> 33** indicates **identity** | U | R.RIPLAEWESR.I |
| 39989 | 423 | – | 432 | 419.5631 | 1255.6674 | 1255.6673 | 0.065 | 1 | 36 | 0.00043 | 1Score **> 33** indicates **identity** Score **> 15** indicates **homology** | U | R.RIPLAEWESR.I |
| 39990 | 423 | – | 432 | 419.5631 | 1255.6674 | 1255.6673 | 0.065 | 1 | 22 | 0.0084 | 1Score **> 33** indicates **identity** Score **> 14** indicates **homology** | U | R.RIPLAEWESR.I |
| 39991 | 423 | – | 432 | 628.8410 | 1255.6674 | 1255.6673 | 0.11 | 1 | 33 | 0.0096 | 1Score **> 33** indicates **identity** Score **> 26** indicates **homology** | U | R.RIPLAEWESR.I |
| 39993 | 423 | – | 432 | 628.8410 | 1255.6674 | 1255.6673 | 0.12 | 1 | 44 | 0.0039 | 1Score **> 33** indicates **identity** | U | R.RIPLAEWESR.I |
| 39994 | 423 | – | 432 | 419.5631 | 1255.6674 | 1255.6673 | 0.13 | 1 | 17 | 0.023 | 1Score **> 33** indicates **identity** Score **> 14** indicates **homology** | U | R.RIPLAEWESR.I |
| 39995 | 423 | – | 432 | 628.8410 | 1255.6674 | 1255.6673 | 0.13 | 1 | 21 | 0.046 | 1Score **> 33** indicates **identity** Score **> 20** indicates **homology** | U | R.RIPLAEWESR.I |
| 39997 | 423 | – | 432 | 419.5632 | 1255.6679 | 1255.6673 | 0.48 | 1 | 25 | 0.0043 | 1Score **> 33** indicates **identity** Score **> 14** indicates **homology** | U | R.RIPLAEWESR.I |
| 39998 | 423 | – | 432 | 419.5632 | 1255.6679 | 1255.6673 | 0.50 | 1 | 40 | 0.00018 | 1Score **> 33** indicates **identity** Score **> 15** indicates **homology** | U | R.RIPLAEWESR.I |
| 39999 | 423 | – | 432 | 419.5633 | 1255.6679 | 1255.6673 | 0.52 | 1 | 31 | 0.0012 | 1Score **> 33** indicates **identity** Score **> 14** indicates **homology** | U | R.RIPLAEWESR.I |
| 40000 | 423 | – | 432 | 628.8412 | 1255.6679 | 1255.6673 | 0.52 | 1 | 61 | 8.1e-05 | 1Score **> 33** indicates **identity** | U | R.RIPLAEWESR.I |
| 40002 | 423 | – | 432 | 419.5633 | 1255.6680 | 1255.6673 | 0.61 | 1 | 23 | 0.0071 | 1Score **> 33** indicates **identity** Score **> 14** indicates **homology** | U | R.RIPLAEWESR.I |
| 40003 | 423 | – | 432 | 419.5633 | 1255.6682 | 1255.6673 | 0.75 | 1 | 27 | 0.0033 | 1Score **> 33** indicates **identity** Score **> 15** indicates **homology** | U | R.RIPLAEWESR.I |
| 40005 | 423 | – | 432 | 628.8415 | 1255.6684 | 1255.6673 | 0.87 | 1 | 26 | 0.02 | 1Score **> 33** indicates **identity** Score **> 21** indicates **homology** | U | R.RIPLAEWESR.I |
| 40006 | 423 | – | 432 | 628.8416 | 1255.6686 | 1255.6673 | 1.03 | 1 | 53 | 0.00024 | 1Score **> 33** indicates **identity** Score **> 29** indicates **homology** | U | R.RIPLAEWESR.I |
| 40008 | 423 | – | 432 | 419.5635 | 1255.6687 | 1255.6673 | 1.09 | 1 | 20 | 0.013 | 1Score **> 32** indicates **identity** Score **> 14** indicates **homology** | U | R.RIPLAEWESR.I |
| 40009 | 423 | – | 432 | 628.8417 | 1255.6688 | 1255.6673 | 1.25 | 1 | 17 | 0.047 | 1Score **> 33** indicates **identity** Score **> 17** indicates **homology** | U | R.RIPLAEWESR.I |
| 40011 | 423 | – | 432 | 419.5638 | 1255.6696 | 1255.6673 | 1.82 | 1 | 39 | 0.00021 | 1Score **> 33** indicates **identity** Score **> 15** indicates **homology** | U | R.RIPLAEWESR.I |
| 23012 | 424 | – | 432 | 550.7906 | 1099.5666 | 1099.5662 | 0.37 | 0 | 53 | 0.00014 | 1Score **> 32** indicates **identity** Score **> 27** indicates **homology** | U | R.IPLAEWESR.I |
| 23013 | 424 | – | 432 | 550.7908 | 1099.5670 | 1099.5662 | 0.71 | 0 | 67 | 1.3e-05 | 1Score **> 32** indicates **identity** Score **> 31** indicates **homology** | U | R.IPLAEWESR.I |
| 34032 | 433 | – | 442 | 601.8094 | 1201.6043 | 1201.6125 | -6.78 | 0 | 34 | 0.0024 | 1Score **> 32** indicates **identity** Score **> 21** indicates **homology** | U | R.IQEVDAQMLR.D |
| 34044 | 433 | – | 442 | 601.8136 | 1201.6126 | 1201.6125 | 0.12 | 0 | 49 | 0.00088 | 1Score **> 33** indicates **identity** Score **> 31** indicates **homology** | U | R.IQEVDAQMLR.D |
| 34045 | 433 | – | 442 | 601.8139 | 1201.6133 | 1201.6125 | 0.70 | 0 | 35 | 0.0065 | 1Score **> 33** indicates **identity** Score **> 26** indicates **homology** | U | R.IQEVDAQMLR.D |
| 34046 | 433 | – | 442 | 601.8139 | 1201.6133 | 1201.6125 | 0.71 | 0 | 63 | 4.1e-05 | 1Score **> 33** indicates **identity** Score **> 32** indicates **homology** | U | R.IQEVDAQMLR.D |
| 35668 | 433 | – | 442 | 609.8108 | 1217.6070 | 1217.6074 | -0.34 | 0 | 27 | 0.0031 | 1Score **> 33** indicates **identity** Score **> 14** indicates **homology** | U | R.IQEVDAQMLR.D  + Oxidation (M) |
| 35672 | 433 | – | 442 | 609.8141 | 1217.6137 | 1217.6074 | 5.20 | 0 | 19 | 0.018 | 1Score **> 33** indicates **identity** Score **> 14** indicates **homology** | U | R.IQEVDAQMLR.D  + Oxidation (M) |
| 174143 | 448 | – | 470 | 912.7496 | 2735.2269 | 2735.2326 | -2.09 | 0 | 21 | 0.011 | 1Score **> 32** indicates **identity** Score **> 14** indicates **homology** | U | K.YFYDQCPAVAGYGPIEQLPDYNR.I |
| 174144 | 448 | – | 470 | 912.7496 | 2735.2269 | 2735.2326 | -2.09 | 0 | 29 | 0.002 | 1Score **> 32** indicates **identity** Score **> 14** indicates **homology** | U | K.YFYDQCPAVAGYGPIEQLPDYNR.I |
| 174145 | 448 | – | 470 | 912.7500 | 2735.2282 | 2735.2326 | -1.62 | 0 | 30 | 0.0015 | 1Score **> 32** indicates **identity** Score **> 14** indicates **homology** | U | K.YFYDQCPAVAGYGPIEQLPDYNR.I |
| 174146 | 448 | – | 470 | 912.7503 | 2735.2290 | 2735.2326 | -1.30 | 0 | 61 | 1.8e-06 | 1Score **> 32** indicates **identity** Score **> 16** indicates **homology** | U | K.YFYDQCPAVAGYGPIEQLPDYNR.I |
| 174147 | 448 | – | 470 | 912.7512 | 2735.2318 | 2735.2326 | -0.27 | 0 | 63 | 1.3e-06 | 1Score **> 32** indicates **identity** Score **> 16** indicates **homology** | U | K.YFYDQCPAVAGYGPIEQLPDYNR.I |
| 174148 | 448 | – | 470 | 1368.6235 | 2735.2324 | 2735.2326 | -0.056 | 0 | 93 | 2e-09 | 1Score **> 32** indicates **identity** Score **> 18** indicates **homology** | U | K.YFYDQCPAVAGYGPIEQLPDYNR.I |
| 174149 | 448 | – | 470 | 912.7518 | 2735.2336 | 2735.2326 | 0.37 | 0 | 16 | 0.032 | 1Score **> 33** indicates **identity** Score **> 13** indicates **homology** | U | K.YFYDQCPAVAGYGPIEQLPDYNR.I |
| 174151 | 448 | – | 470 | 912.7519 | 2735.2339 | 2735.2326 | 0.48 | 0 | 74 | 1.2e-07 | 1Score **> 33** indicates **identity** Score **> 17** indicates **homology** | U | K.YFYDQCPAVAGYGPIEQLPDYNR.I |
| 174152 | 448 | – | 470 | 912.7521 | 2735.2346 | 2735.2326 | 0.72 | 0 | 69 | 3.5e-07 | 1Score **> 33** indicates **identity** Score **> 17** indicates **homology** | U | K.YFYDQCPAVAGYGPIEQLPDYNR.I |
| 174153 | 448 | – | 470 | 1368.6249 | 2735.2352 | 2735.2326 | 0.96 | 0 | 55 | 7e-06 | 1Score **> 33** indicates **identity** Score **> 16** indicates **homology** | U | K.YFYDQCPAVAGYGPIEQLPDYNR.I |
| 174154 | 448 | – | 470 | 1368.6249 | 2735.2353 | 2735.2326 | 1.00 | 0 | 90 | 3.3e-09 | 1Score **> 33** indicates **identity** Score **> 18** indicates **homology** | U | K.YFYDQCPAVAGYGPIEQLPDYNR.I |
| 174155 | 448 | – | 470 | 912.7525 | 2735.2355 | 2735.2326 | 1.08 | 0 | 66 | 6.5e-07 | 1Score **> 33** indicates **identity** Score **> 17** indicates **homology** | U | K.YFYDQCPAVAGYGPIEQLPDYNR.I |
| 174156 | 448 | – | 470 | 1368.6252 | 2735.2358 | 2735.2326 | 1.18 | 0 | 80 | 3.2e-08 | 1Score **> 33** indicates **identity** Score **> 17** indicates **homology** | U | K.YFYDQCPAVAGYGPIEQLPDYNR.I |
| 174157 | 448 | – | 470 | 1368.6252 | 2735.2359 | 2735.2326 | 1.23 | 0 | 80 | 3.2e-08 | 1Score **> 33** indicates **identity** Score **> 17** indicates **homology** | U | K.YFYDQCPAVAGYGPIEQLPDYNR.I |
| 174158 | 448 | – | 470 | 912.7527 | 2735.2362 | 2735.2326 | 1.32 | 0 | 78 | 4.4e-08 | 1Score **> 33** indicates **identity** Score **> 17** indicates **homology** | U | K.YFYDQCPAVAGYGPIEQLPDYNR.I |
| 174159 | 448 | – | 470 | 912.7529 | 2735.2367 | 2735.2326 | 1.52 | 0 | 60 | 2.5e-06 | 1Score **> 33** indicates **identity** Score **> 16** indicates **homology** | U | K.YFYDQCPAVAGYGPIEQLPDYNR.I |
| 174226 | 448 | – | 470 | 913.0824 | 2736.2255 | 2736.2166 | 3.24 | 0 | 21 | 0.0099 | 1Score **> 32** indicates **identity** Score **> 14** indicates **homology** | U | K.YFYDQCPAVAGYGPIEQLPDYNR.I  + Deamidated (NQ) |
| 174227 | 448 | – | 470 | 1369.1225 | 2736.2304 | 2736.2166 | 5.06 | 0 | 57 | 4.7e-06 | 1Score **> 32** indicates **identity** Score **> 16** indicates **homology** | U | K.YFYDQCPAVAGYGPIEQLPDYNR.I  + Deamidated (NQ) |
| 174228 | 448 | – | 470 | 1369.1242 | 2736.2339 | 2736.2166 | 6.32 | 0 | 71 | 2.1e-07 | 1Score **> 33** indicates **identity** Score **> 17** indicates **homology** | U | K.YFYDQCPAVAGYGPIEQLPDYNR.I  + Deamidated (NQ) |
| 174229 | 448 | – | 470 | 1369.1244 | 2736.2342 | 2736.2166 | 6.42 | 0 | 53 | 1.1e-05 | 1Score **> 33** indicates **identity** Score **> 16** indicates **homology** | U | K.YFYDQCPAVAGYGPIEQLPDYNR.I  + Deamidated (NQ) |
| 174230 | 448 | – | 470 | 913.0855 | 2736.2346 | 2736.2166 | 6.56 | 0 | 22 | 0.0094 | 1Score **> 33** indicates **identity** Score **> 14** indicates **homology** | U | K.YFYDQCPAVAGYGPIEQLPDYNR.I  + Deamidated (NQ) |
| 174231 | 448 | – | 470 | 1369.1257 | 2736.2368 | 2736.2166 | 7.39 | 0 | 65 | 8.8e-07 | 1Score **> 33** indicates **identity** Score **> 17** indicates **homology** | U | K.YFYDQCPAVAGYGPIEQLPDYNR.I  + Deamidated (NQ) |
| 174232 | 448 | – | 470 | 913.0864 | 2736.2373 | 2736.2166 | 7.55 | 0 | 20 | 0.014 | 1Score **> 33** indicates **identity** Score **> 14** indicates **homology** | U | K.YFYDQCPAVAGYGPIEQLPDYNR.I  + Deamidated (NQ) |
| 174233 | 448 | – | 470 | 913.0865 | 2736.2376 | 2736.2166 | 7.67 | 0 | 19 | 0.015 | 1Score **> 33** indicates **identity** Score **> 14** indicates **homology** | U | K.YFYDQCPAVAGYGPIEQLPDYNR.I  + Deamidated (NQ) |
| 174234 | 448 | – | 470 | 913.0865 | 2736.2376 | 2736.2166 | 7.67 | 0 | 28 | 0.0021 | 1Score **> 33** indicates **identity** Score **> 14** indicates **homology** | U | K.YFYDQCPAVAGYGPIEQLPDYNR.I  + Deamidated (NQ) |
| 174235 | 448 | – | 470 | 913.0870 | 2736.2391 | 2736.2166 | 8.21 | 0 | 20 | 0.014 | 1Score **> 33** indicates **identity** Score **> 14** indicates **homology** | U | K.YFYDQCPAVAGYGPIEQLPDYNR.I  + Deamidated (NQ) |
| 174236 | 448 | – | 470 | 1369.1274 | 2736.2403 | 2736.2166 | 8.67 | 0 | 32 | 0.0011 | 1Score **> 33** indicates **identity** Score **> 14** indicates **homology** | U | K.YFYDQCPAVAGYGPIEQLPDYNR.I  + Deamidated (NQ) |
| 174237 | 448 | – | 470 | 913.0874 | 2736.2404 | 2736.2166 | 8.68 | 0 | 20 | 0.014 | 1Score **> 33** indicates **identity** Score **> 14** indicates **homology** | U | K.YFYDQCPAVAGYGPIEQLPDYNR.I  + Deamidated (NQ) |
| 183912 | 448 | – | 472 | 1002.4816 | 3004.4231 | 3004.4178 | 1.77 | 1 | 57 | 4.3e-06 | 1Score **> 36** indicates **identity** Score **> 16** indicates **homology** | U | K.YFYDQCPAVAGYGPIEQLPDYNRIR.S |
| 183913 | 448 | – | 472 | 1002.4825 | 3004.4256 | 3004.4178 | 2.62 | 1 | 56 | 5.1e-06 | 1Score **> 36** indicates **identity** Score **> 16** indicates **homology** | U | K.YFYDQCPAVAGYGPIEQLPDYNRIR.S |
| 8249 | 473 | – | 479 | 448.7253 | 895.4360 | 895.4374 | -1.62 | 0 | 37 | 0.0011 | 1Score **> 27** indicates **identity** Score **> 20** indicates **homology** | U | R.SGMFWLR.F |
| 8250 | 473 | – | 479 | 448.7258 | 895.4370 | 895.4374 | -0.50 | 0 | 47 | 0.00062 | 1Score **> 27** indicates **identity** | U | R.SGMFWLR.F |
| 8251 | 473 | – | 479 | 448.7259 | 895.4373 | 895.4374 | -0.17 | 0 | 47 | 0.00059 | 1Score **> 27** indicates **identity** | U | R.SGMFWLR.F |
| 8253 | 473 | – | 479 | 448.7260 | 895.4375 | 895.4374 | 0.075 | 0 | 34 | 0.00076 | 1Score **> 27** indicates **identity** Score **> 15** indicates **homology** | U | R.SGMFWLR.F |
| 8254 | 473 | – | 479 | 448.7263 | 895.4380 | 895.4374 | 0.66 | 0 | 42 | 0.0011 | 1Score **> 25** indicates **identity** | U | R.SGMFWLR.F |
| 8255 | 473 | – | 479 | 448.7263 | 895.4381 | 895.4374 | 0.69 | 0 | 37 | 0.00042 | 1Score **> 25** indicates **identity** Score **> 16** indicates **homology** | U | R.SGMFWLR.F |
| 8257 | 473 | – | 479 | 448.7268 | 895.4391 | 895.4374 | 1.85 | 0 | 38 | 0.00087 | 1Score **> 27** indicates **identity** Score **> 20** indicates **homology** | U | R.SGMFWLR.F |
| 8258 | 473 | – | 479 | 448.7282 | 895.4418 | 895.4374 | 4.87 | 0 | 38 | 0.00072 | 1Score **> 27** indicates **identity** Score **> 19** indicates **homology** | U | R.SGMFWLR.F |
| 17632 | 473 | – | 480 | 522.2598 | 1042.5051 | 1042.5059 | -0.75 | 1 | 52 | 1.4e-05 | 1Score **> 28** indicates **identity** Score **> 16** indicates **homology** | U | R.SGMFWLRF.- |
| 17633 | 473 | – | 480 | 522.2604 | 1042.5062 | 1042.5059 | 0.30 | 1 | 54 | 9e-06 | 1Score **> 28** indicates **identity** Score **> 16** indicates **homology** | U | R.SGMFWLRF.- |
| 17634 | 473 | – | 480 | 522.2609 | 1042.5073 | 1042.5059 | 1.38 | 1 | 31 | 0.0012 | 1Score **> 28** indicates **identity** Score **> 14** indicates **homology** | U | R.SGMFWLRF.- |
| 19102 | 473 | – | 480 | 530.2563 | 1058.4981 | 1058.5008 | -2.53 | 1 | 31 | 0.0013 | 1Score **> 28** indicates **identity** Score **> 14** indicates **homology** | U | R.SGMFWLRF.-  + Oxidation (M) |
| 19103 | 473 | – | 480 | 530.2572 | 1058.4998 | 1058.5008 | -0.90 | 1 | 21 | 0.01 | 1Score **> 27** indicates **identity** Score **> 14** indicates **homology** | U | R.SGMFWLRF.-  + Oxidation (M) |
| 19105 | 473 | – | 480 | 530.2578 | 1058.5011 | 1058.5008 | 0.26 | 1 | 21 | 0.01 | 1Score **> 28** indicates **identity** Score **> 14** indicates **homology** | U | R.SGMFWLRF.-  + Oxidation (M) |

---

```
ID   QCR1_MOUSE              Reviewed;         480 AA.
AC   Q9CZ13; Q3TV75; Q9CWL6;
DT   20-JUN-2001, integrated into UniProtKB/Swiss-Prot.
DT   27-JUL-2011, sequence version 2.
DT   28-JUN-2023, entry version 182.
DE   RecName: Full=Cytochrome b-c1 complex subunit 1, mitochondrial;
DE   AltName: Full=Complex III subunit 1;
DE   AltName: Full=Core protein I;
DE   AltName: Full=Ubiquinol-cytochrome-c reductase complex core protein 1;
DE   Flags: Precursor;
GN   Name=Uqcrc1;
OS   Mus musculus (Mouse).
OC   Eukaryota; Metazoa; Chordata; Craniata; Vertebrata; Euteleostomi; Mammalia;
OC   Eutheria; Euarchontoglires; Glires; Rodentia; Myomorpha; Muroidea; Muridae;
OC   Murinae; Mus; Mus.
OX   NCBI_TaxID=10090;
RN   [1]
RP   NUCLEOTIDE SEQUENCE [LARGE SCALE MRNA].
RC   STRAIN=C57BL/6J; TISSUE=Bone marrow, and Embryo;
RX   PubMed=16141072; DOI=10.1126/science.1112014;
RA   Carninci P., Kasukawa T., Katayama S., Gough J., Frith M.C., Maeda N.,
RA   Oyama R., Ravasi T., Lenhard B., Wells C., Kodzius R., Shimokawa K.,
RA   Bajic V.B., Brenner S.E., Batalov S., Forrest A.R., Zavolan M., Davis M.J.,
RA   Wilming L.G., Aidinis V., Allen J.E., Ambesi-Impiombato A., Apweiler R.,
RA   Aturaliya R.N., Bailey T.L., Bansal M., Baxter L., Beisel K.W., Bersano T.,
RA   Bono H., Chalk A.M., Chiu K.P., Choudhary V., Christoffels A.,
RA   Clutterbuck D.R., Crowe M.L., Dalla E., Dalrymple B.P., de Bono B.,
RA   Della Gatta G., di Bernardo D., Down T., Engstrom P., Fagiolini M.,
RA   Faulkner G., Fletcher C.F., Fukushima T., Furuno M., Futaki S.,
RA   Gariboldi M., Georgii-Hemming P., Gingeras T.R., Gojobori T., Green R.E.,
RA   Gustincich S., Harbers M., Hayashi Y., Hensch T.K., Hirokawa N., Hill D.,
RA   Huminiecki L., Iacono M., Ikeo K., Iwama A., Ishikawa T., Jakt M.,
RA   Kanapin A., Katoh M., Kawasawa Y., Kelso J., Kitamura H., Kitano H.,
RA   Kollias G., Krishnan S.P., Kruger A., Kummerfeld S.K., Kurochkin I.V.,
RA   Lareau L.F., Lazarevic D., Lipovich L., Liu J., Liuni S., McWilliam S.,
RA   Madan Babu M., Madera M., Marchionni L., Matsuda H., Matsuzawa S., Miki H.,
RA   Mignone F., Miyake S., Morris K., Mottagui-Tabar S., Mulder N., Nakano N.,
RA   Nakauchi H., Ng P., Nilsson R., Nishiguchi S., Nishikawa S., Nori F.,
RA   Ohara O., Okazaki Y., Orlando V., Pang K.C., Pavan W.J., Pavesi G.,
RA   Pesole G., Petrovsky N., Piazza S., Reed J., Reid J.F., Ring B.Z.,
RA   Ringwald M., Rost B., Ruan Y., Salzberg S.L., Sandelin A., Schneider C.,
RA   Schoenbach C., Sekiguchi K., Semple C.A., Seno S., Sessa L., Sheng Y.,
RA   Shibata Y., Shimada H., Shimada K., Silva D., Sinclair B., Sperling S.,
RA   Stupka E., Sugiura K., Sultana R., Takenaka Y., Taki K., Tammoja K.,
RA   Tan S.L., Tang S., Taylor M.S., Tegner J., Teichmann S.A., Ueda H.R.,
RA   van Nimwegen E., Verardo R., Wei C.L., Yagi K., Yamanishi H.,
RA   Zabarovsky E., Zhu S., Zimmer A., Hide W., Bult C., Grimmond S.M.,
RA   Teasdale R.D., Liu E.T., Brusic V., Quackenbush J., Wahlestedt C.,
RA   Mattick J.S., Hume D.A., Kai C., Sasaki D., Tomaru Y., Fukuda S.,
RA   Kanamori-Katayama M., Suzuki M., Aoki J., Arakawa T., Iida J., Imamura K.,
RA   Itoh M., Kato T., Kawaji H., Kawagashira N., Kawashima T., Kojima M.,
RA   Kondo S., Konno H., Nakano K., Ninomiya N., Nishio T., Okada M., Plessy C.,
RA   Shibata K., Shiraki T., Suzuki S., Tagami M., Waki K., Watahiki A.,
RA   Okamura-Oho Y., Suzuki H., Kawai J., Hayashizaki Y.;
RT   "The transcriptional landscape of the mammalian genome.";
RL   Science 309:1559-1563(2005).
RN   [2]
RP   NUCLEOTIDE SEQUENCE [LARGE SCALE GENOMIC DNA].
RA   Mural R.J., Adams M.D., Myers E.W., Smith H.O., Venter J.C.;
RL   Submitted (JUL-2005) to the EMBL/GenBank/DDBJ databases.
RN   [3]
RP   PROTEIN SEQUENCE OF 59-80; 86-99; 112-134; 143-163; 214-222; 229-248;
RP   256-276; 379-390; 397-442; 448-470 AND 473-479, AND IDENTIFICATION BY MASS
RP   SPECTROMETRY.
RC   STRAIN=C57BL/6J; TISSUE=Brain, and Hippocampus;
RA   Lubec G., Kang S.U., Klug S., Yang J.W., Zigmond M.;
RL   Submitted (JUL-2007) to UniProtKB.
RN   [4]
RP   SUBUNIT.
RX   PubMed=19026783; DOI=10.1016/j.molcel.2008.10.021;
RA   Acin-Perez R., Fernandez-Silva P., Peleato M.L., Perez-Martos A.,
RA   Enriquez J.A.;
RT   "Respiratory active mitochondrial supercomplexes.";
RL   Mol. Cell 32:529-539(2008).
RN   [5]
RP   IDENTIFICATION BY MASS SPECTROMETRY [LARGE SCALE ANALYSIS].
RC   TISSUE=Brain, Brown adipose tissue, Heart, Kidney, Liver, Lung,
RC   Pancreas, Spleen, and Testis;
RX   PubMed=21183079; DOI=10.1016/j.cell.2010.12.001;
RA   Huttlin E.L., Jedrychowski M.P., Elias J.E., Goswami T., Rad R.,
RA   Beausoleil S.A., Villen J., Haas W., Sowa M.E., Gygi S.P.;
RT   "A tissue-specific atlas of mouse protein phosphorylation and expression.";
RL   Cell 143:1174-1189(2010).
RN   [6]
RP   SUCCINYLATION [LARGE SCALE ANALYSIS] AT LYS-163, AND IDENTIFICATION BY MASS
RP   SPECTROMETRY [LARGE SCALE ANALYSIS].
RC   TISSUE=Liver;
RX   PubMed=23806337; DOI=10.1016/j.molcel.2013.06.001;
RA   Park J., Chen Y., Tishkoff D.X., Peng C., Tan M., Dai L., Xie Z., Zhang Y.,
RA   Zwaans B.M., Skinner M.E., Lombard D.B., Zhao Y.;
RT   "SIRT5-mediated lysine desuccinylation impacts diverse metabolic
RT   pathways.";
RL   Mol. Cell 50:919-930(2013).
RN   [7]
RP   ACETYLATION [LARGE SCALE ANALYSIS] AT LYS-111; LYS-138; LYS-163 AND
RP   LYS-248, AND IDENTIFICATION BY MASS SPECTROMETRY [LARGE SCALE ANALYSIS].
RC   TISSUE=Liver;
RX   PubMed=23576753; DOI=10.1073/pnas.1302961110;
RA   Rardin M.J., Newman J.C., Held J.M., Cusack M.P., Sorensen D.J., Li B.,
RA   Schilling B., Mooney S.D., Kahn C.R., Verdin E., Gibson B.W.;
RT   "Label-free quantitative proteomics of the lysine acetylome in mitochondria
RT   identifies substrates of SIRT3 in metabolic pathways.";
RL   Proc. Natl. Acad. Sci. U.S.A. 110:6601-6606(2013).
RN   [8]
RP   TISSUE SPECIFICITY, AND DISRUPTION PHENOTYPE.
RX   PubMed=30666338; DOI=10.1007/s00018-019-03007-6;
RA   Shan W., Li J., Xu W., Li H., Zuo Z.;
RT   "Critical role of UQCRC1 in embryo survival, brain ischemic tolerance and
RT   normal cognition in mice.";
RL   Cell. Mol. Life Sci. 76:1381-1396(2019).
RN   [9]
RP   INTERACTION WITH UQCC6.
RX   PubMed=32161263; DOI=10.1038/s41467-020-14999-2;
RA   Zhang S., Reljic B., Liang C., Kerouanton B., Francisco J.C., Peh J.H.,
RA   Mary C., Jagannathan N.S., Olexiouk V., Tang C., Fidelito G., Nama S.,
RA   Cheng R.K., Wee C.L., Wang L.C., Duek Roggli P., Sampath P., Lane L.,
RA   Petretto E., Sobota R.M., Jesuthasan S., Tucker-Kellogg L., Reversade B.,
RA   Menschaert G., Sun L., Stroud D.A., Ho L.;
RT   "Mitochondrial peptide BRAWNIN is essential for vertebrate respiratory
RT   complex III assembly.";
RL   Nat. Commun. 11:1312-1312(2020).
RN   [10]
RP   INTERACTION WITH STMP1.
RX   PubMed=35101990; DOI=10.1073/pnas.2120476119;
RA   Makarewich C.A., Munir A.Z., Bezprozvannaya S., Gibson A.M., Young Kim S.,
RA   Martin-Sandoval M.S., Mathews T.P., Szweda L.I., Bassel-Duby R.,
RA   Olson E.N.;
RT   "The cardiac-enriched microprotein mitolamban regulates mitochondrial
RT   respiratory complex assembly and function in mice.";
RL   Proc. Natl. Acad. Sci. U.S.A. 119:0-0(2022).
CC   -!- FUNCTION: Component of the ubiquinol-cytochrome c oxidoreductase, a
CC       multisubunit transmembrane complex that is part of the mitochondrial
CC       electron transport chain which drives oxidative phosphorylation. The
CC       respiratory chain contains 3 multisubunit complexes succinate
CC       dehydrogenase (complex II, CII), ubiquinol-cytochrome c oxidoreductase
CC       (cytochrome b-c1 complex, complex III, CIII) and cytochrome c oxidase
CC       (complex IV, CIV), that cooperate to transfer electrons derived from
CC       NADH and succinate to molecular oxygen, creating an electrochemical
CC       gradient over the inner membrane that drives transmembrane transport
CC       and the ATP synthase. The cytochrome b-c1 complex catalyzes electron
CC       transfer from ubiquinol to cytochrome c, linking this redox reaction to
CC       translocation of protons across the mitochondrial inner membrane, with
CC       protons being carried across the membrane as hydrogens on the quinol.
CC       In the process called Q cycle, 2 protons are consumed from the matrix,
CC       4 protons are released into the intermembrane space and 2 electrons are
CC       passed to cytochrome c (By similarity). The 2 core subunits UQCRC1/QCR1
CC       and UQCRC2/QCR2 are homologous to the 2 mitochondrial-processing
CC       peptidase (MPP) subunits beta-MPP and alpha-MPP respectively, and they
CC       seem to have preserved their MPP processing properties. May be involved
CC       in the in situ processing of UQCRFS1 into the mature Rieske protein and
CC       its mitochondrial targeting sequence (MTS)/subunit 9 when incorporated
CC       into complex III (By similarity). Seems to play an important role in
CC       the maintenance of proper mitochondrial function in nigral dopaminergic
CC       neurons (By similarity). {ECO:0000250|UniProtKB:P07256,
CC       ECO:0000250|UniProtKB:P31800, ECO:0000250|UniProtKB:P31930}.
CC   -!- SUBUNIT: Component of the ubiquinol-cytochrome c oxidoreductase
CC       (cytochrome b-c1 complex, complex III, CIII), a multisubunit enzyme
CC       composed of 11 subunits. The complex is composed of 3 respiratory
CC       subunits cytochrome b, cytochrome c1 and Rieske protein UQCRFS1, 2 core
CC       protein subunits UQCRC1/QCR1 and UQCRC2/QCR2, and 6 low-molecular
CC       weight protein subunits UQCRH/QCR6, UQCRB/QCR7, UQCRQ/QCR8,
CC       UQCR10/QCR9, UQCR11/QCR10 and subunit 9, the cleavage product of Rieske
CC       protein UQCRFS1 (By similarity). The complex exists as an obligatory
CC       dimer and forms supercomplexes (SCs) in the inner mitochondrial
CC       membrane with NADH-ubiquinone oxidoreductase (complex I, CI) and
CC       cytochrome c oxidase (complex IV, CIV), resulting in different
CC       assemblies (supercomplex SCI(1)III(2)IV(1) and megacomplex
CC       MCI(2)III(2)IV(2)) (PubMed:19026783). Interacts with UQCC6
CC       (PubMed:32161263). Interacts with STMP1 (PubMed:35101990).
CC       {ECO:0000250|UniProtKB:P31800, ECO:0000269|PubMed:19026783,
CC       ECO:0000269|PubMed:32161263, ECO:0000269|PubMed:35101990}.
CC   -!- SUBCELLULAR LOCATION: Mitochondrion inner membrane
CC       {ECO:0000250|UniProtKB:P07256}; Peripheral membrane protein
CC       {ECO:0000250|UniProtKB:P07256}; Matrix side
CC       {ECO:0000250|UniProtKB:P07256}.
CC   -!- TISSUE SPECIFICITY: Expressed in neurons and astrocytes of the cerebral
CC       cortex and hippocampus (at protein level).
CC       {ECO:0000269|PubMed:30666338}.
CC   -!- PTM: Acetylation of Lys-138 is observed in liver mitochondria from
CC       fasted mice but not from fed mice.
CC   -!- DISRUPTION PHENOTYPE: Results in early embryonic lethality.
CC       {ECO:0000269|PubMed:30666338}.
CC   -!- SIMILARITY: Belongs to the peptidase M16 family. UQCRC1/QCR1 subfamily.
CC       {ECO:0000305}.
CC   ---------------------------------------------------------------------------
CC   Copyrighted by the UniProt Consortium, see https://www.uniprot.org/terms
CC   Distributed under the Creative Commons Attribution (CC BY 4.0) License
CC   ---------------------------------------------------------------------------
DR   EMBL; AK013128; BAB28666.1; -; mRNA.
DR   EMBL; AK010553; BAB27022.1; -; mRNA.
DR   EMBL; AK151764; BAE30670.1; -; mRNA.
DR   EMBL; AK160337; BAE35744.1; -; mRNA.
DR   EMBL; CH466560; EDL21324.1; -; Genomic_DNA.
DR   CCDS; CCDS23540.1; -.
DR   RefSeq; NP_079683.2; NM_025407.2.
DR   PDB; 7O37; EM; 3.20 A; A/L=35-480.
DR   PDB; 7O3C; EM; 3.30 A; A/L=35-480.
DR   PDB; 7O3E; EM; 3.60 A; A/L=35-480.
DR   PDB; 7O3H; EM; 2.60 A; A/L=35-480.
DR   PDBsum; 7O37; -.
DR   PDBsum; 7O3C; -.
DR   PDBsum; 7O3E; -.
DR   PDBsum; 7O3H; -.
DR   AlphaFoldDB; Q9CZ13; -.
DR   SMR; Q9CZ13; -.
DR   BioGRID; 204459; 58.
DR   ComplexPortal; CPX-563; Mitochondrial respiratory chain complex III.
DR   CORUM; Q9CZ13; -.
DR   IntAct; Q9CZ13; 11.
DR   MINT; Q9CZ13; -.
DR   STRING; 10090.ENSMUSP00000026743; -.
DR   MEROPS; M16.975; -.
DR   MEROPS; M16.981; -.
DR   GlyGen; Q9CZ13; 2 sites, 1 O-linked glycan (2 sites).
DR   iPTMnet; Q9CZ13; -.
DR   PhosphoSitePlus; Q9CZ13; -.
DR   SwissPalm; Q9CZ13; -.
DR   REPRODUCTION-2DPAGE; IPI00111885; -.
DR   REPRODUCTION-2DPAGE; Q9CZ13; -.
DR   UCD-2DPAGE; Q9CZ13; -.
DR   EPD; Q9CZ13; -.
DR   jPOST; Q9CZ13; -.
DR   MaxQB; Q9CZ13; -.
DR   PaxDb; Q9CZ13; -.
DR   PeptideAtlas; Q9CZ13; -.
DR   ProteomicsDB; 301902; -.
DR   TopDownProteomics; Q9CZ13; -.
DR   Antibodypedia; 1257; 399 antibodies from 29 providers.
DR   DNASU; 22273; -.
DR   Ensembl; ENSMUST00000026743; ENSMUSP00000026743; ENSMUSG00000025651.
DR   GeneID; 22273; -.
DR   KEGG; mmu:22273; -.
DR   UCSC; uc009rrg.1; mouse.
DR   AGR; MGI:107876; -.
DR   CTD; 7384; -.
DR   MGI; MGI:107876; Uqcrc1.
DR   VEuPathDB; HostDB:ENSMUSG00000025651; -.
DR   eggNOG; KOG0960; Eukaryota.
DR   GeneTree; ENSGT00940000158931; -.
DR   HOGENOM; CLU_009902_4_2_1; -.
DR   InParanoid; Q9CZ13; -.
DR   OMA; WSNPDNV; -.
DR   OrthoDB; 167798at2759; -.
DR   PhylomeDB; Q9CZ13; -.
DR   TreeFam; TF105032; -.
DR   Reactome; R-MMU-611105; Respiratory electron transport.
DR   BioGRID-ORCS; 22273; 24 hits in 79 CRISPR screens.
DR   ChiTaRS; Uqcrc1; mouse.
DR   PRO; PR:Q9CZ13; -.
DR   Proteomes; UP000000589; Chromosome 9.
DR   RNAct; Q9CZ13; protein.
DR   Bgee; ENSMUSG00000025651; Expressed in proximal tubule and 143 other tissues.
DR   ExpressionAtlas; Q9CZ13; baseline and differential.
DR   Genevisible; Q9CZ13; MM.
DR   GO; GO:0005743; C:mitochondrial inner membrane; HDA:MGI.
DR   GO; GO:0005750; C:mitochondrial respiratory chain complex III; ISO:MGI.
DR   GO; GO:0005739; C:mitochondrion; IDA:MGI.
DR   GO; GO:0043209; C:myelin sheath; HDA:UniProtKB.
DR   GO; GO:0046872; F:metal ion binding; IEA:InterPro.
DR   GO; GO:0044877; F:protein-containing complex binding; ISO:MGI.
DR   GO; GO:0031625; F:ubiquitin protein ligase binding; ISO:MGI.
DR   GO; GO:0045333; P:cellular respiration; NAS:ComplexPortal.
DR   GO; GO:0006122; P:mitochondrial electron transport, ubiquinol to cytochrome c; IDA:MGI.
DR   GO; GO:0034551; P:mitochondrial respiratory chain complex III assembly; ISS:UniProtKB.
DR   GO; GO:0014823; P:response to activity; IEA:Ensembl.
DR   GO; GO:0043279; P:response to alkaloid; IEA:Ensembl.
DR   Gene3D; 3.30.830.10; Metalloenzyme, LuxS/M16 peptidase-like; 2.
DR   InterPro; IPR011249; Metalloenz_LuxS/M16.
DR   InterPro; IPR011765; Pept_M16_N.
DR   InterPro; IPR007863; Peptidase_M16_C.
DR   PANTHER; PTHR11851:SF116; CYTOCHROME B-C1 COMPLEX SUBUNIT 1, MITOCHONDRIAL; 1.
DR   PANTHER; PTHR11851; METALLOPROTEASE; 1.
DR   Pfam; PF00675; Peptidase_M16; 1.
DR   Pfam; PF05193; Peptidase_M16_C; 1.
DR   SUPFAM; SSF63411; LuxS/MPP-like metallohydrolase; 2.
PE   1: Evidence at protein level;
KW   3D-structure; Acetylation; Direct protein sequencing; Electron transport;
KW   Membrane; Mitochondrion; Mitochondrion inner membrane; Phosphoprotein;
KW   Reference proteome; Respiratory chain; Transit peptide; Transport.
FT   TRANSIT         1..34
FT                   /note="Mitochondrion"
FT                   /evidence="ECO:0000250"
FT   CHAIN           35..480
FT                   /note="Cytochrome b-c1 complex subunit 1, mitochondrial"
FT                   /id="PRO_0000026787"
FT   MOD_RES         111
FT                   /note="N6-acetyllysine"
FT                   /evidence="ECO:0007744|PubMed:23576753"
FT   MOD_RES         138
FT                   /note="N6-acetyllysine"
FT                   /evidence="ECO:0007744|PubMed:23576753"
FT   MOD_RES         163
FT                   /note="N6-acetyllysine; alternate"
FT                   /evidence="ECO:0007744|PubMed:23576753"
FT   MOD_RES         163
FT                   /note="N6-succinyllysine; alternate"
FT                   /evidence="ECO:0007744|PubMed:23806337"
FT   MOD_RES         212
FT                   /note="Phosphoserine"
FT                   /evidence="ECO:0000250|UniProtKB:Q68FY0"
FT   MOD_RES         214
FT                   /note="Phosphothreonine"
FT                   /evidence="ECO:0000250|UniProtKB:Q68FY0"
FT   MOD_RES         248
FT                   /note="N6-acetyllysine"
FT                   /evidence="ECO:0007744|PubMed:23576753"
FT   CONFLICT        223
FT                   /note="H -> N (in Ref. 1; BAB28666)"
FT                   /evidence="ECO:0000305"
FT   CONFLICT        318
FT                   /note="Y -> C (in Ref. 1; BAB28666)"
FT                   /evidence="ECO:0000305"
FT   HELIX           38..43
FT                   /evidence="ECO:0007829|PDB:7O3H"
FT   STRAND          49..52
FT                   /evidence="ECO:0007829|PDB:7O3H"
FT   STRAND          58..63
FT                   /evidence="ECO:0007829|PDB:7O3H"
FT   STRAND          67..76
FT                   /evidence="ECO:0007829|PDB:7O3H"
FT   HELIX           79..81
FT                   /evidence="ECO:0007829|PDB:7O3H"
FT   STRAND          84..86
FT                   /evidence="ECO:0007829|PDB:7O3H"
FT   HELIX           89..96
FT                   /evidence="ECO:0007829|PDB:7O3H"
FT   STRAND          101..104
FT                   /evidence="ECO:0007829|PDB:7O3H"
FT   HELIX           107..115
FT                   /evidence="ECO:0007829|PDB:7O3H"
FT   STRAND          119..124
FT                   /evidence="ECO:0007829|PDB:7O3H"
FT   STRAND          129..137
FT                   /evidence="ECO:0007829|PDB:7O3H"
FT   HELIX           140..152
FT                   /evidence="ECO:0007829|PDB:7O3H"
FT   HELIX           158..175
FT                   /evidence="ECO:0007829|PDB:7O3H"
FT   HELIX           179..191
FT                   /evidence="ECO:0007829|PDB:7O3H"
FT   TURN            196..198
FT                   /evidence="ECO:0007829|PDB:7O3H"
FT   HELIX           205..210
FT                   /evidence="ECO:0007829|PDB:7O3H"
FT   HELIX           213..223
FT                   /evidence="ECO:0007829|PDB:7O3H"
FT   HELIX           226..228
FT                   /evidence="ECO:0007829|PDB:7O3H"
FT   STRAND          229..236
FT                   /evidence="ECO:0007829|PDB:7O3H"
FT   HELIX           239..249
FT                   /evidence="ECO:0007829|PDB:7O3H"
FT   STRAND          250..253
FT                   /evidence="ECO:0007829|PDB:7O37"
FT   STRAND          258..260
FT                   /evidence="ECO:0007829|PDB:7O3H"
FT   STRAND          273..278
FT                   /evidence="ECO:0007829|PDB:7O3H"
FT   STRAND          282..292
FT                   /evidence="ECO:0007829|PDB:7O3H"
FT   HELIX           300..311
FT                   /evidence="ECO:0007829|PDB:7O3H"
FT   STRAND          313..315
FT                   /evidence="ECO:0007829|PDB:7O3C"
FT   HELIX           321..323
FT                   /evidence="ECO:0007829|PDB:7O3H"
FT   HELIX           327..335
FT                   /evidence="ECO:0007829|PDB:7O3H"
FT   STRAND          339..348
FT                   /evidence="ECO:0007829|PDB:7O3H"
FT   STRAND          351..360
FT                   /evidence="ECO:0007829|PDB:7O3H"
FT   HELIX           362..364
FT                   /evidence="ECO:0007829|PDB:7O3H"
FT   HELIX           365..381
FT                   /evidence="ECO:0007829|PDB:7O3H"
FT   HELIX           385..402
FT                   /evidence="ECO:0007829|PDB:7O3H"
FT   HELIX           406..418
FT                   /evidence="ECO:0007829|PDB:7O3H"
FT   HELIX           426..434
FT                   /evidence="ECO:0007829|PDB:7O3H"
FT   HELIX           438..448
FT                   /evidence="ECO:0007829|PDB:7O3H"
FT   TURN            449..451
FT                   /evidence="ECO:0007829|PDB:7O3H"
FT   STRAND          455..461
FT                   /evidence="ECO:0007829|PDB:7O3H"
FT   STRAND          463..465
FT                   /evidence="ECO:0007829|PDB:7O3H"
FT   HELIX           468..474
FT                   /evidence="ECO:0007829|PDB:7O3H"
SQ   SEQUENCE   480 AA;  52852 MW;  8150C97C655A91C9 CRC64;
     MAASAVCRAA CSGTQVLLRT RRSPALLRLP ALRGTATFAQ ALQSVPETQV SILDNGLRVA
     SEQSSHATCT VGVWIDAGSR YETEKNNGAG YFLEHLAFKG TKNRPGNALE KEVESIGAHL
     NAYSTREHTA YLIKALSKDL PKVVELLADI VQNSSLEDSQ IEKERDVILR EMQENDASMQ
     NVVFDYLHAT AFQGTPLAQA VEGPSENVRR LSRTDLTDYL NRHYKAPRMV LAAAGGVEHQ
     QLLDLAQKHL SSVSRVYEED AVPGLTPCRF TGSEIRHRDD ALPLAHVAIA VEGPGWANPD
     NVTLQVANAI IGHYDCTYGG GVHLSSPLAS VAVANKLCQS FQTFNISYSD TGLLGAHFVC
     DAMSIDDMVF FLQGQWMRLC TSATESEVTR GKNILRNALV SHLDGTTPVC EDIGRSLLTY
     GRRIPLAEWE SRIQEVDAQM LRDICSKYFY DQCPAVAGYG PIEQLPDYNR IRSGMFWLRF
//
```

**Mascot:** http://www.matrixscience.com/


Deamidated (NQ) (+0.9840)
