# Supplementary material for: A common East-Asian ALDH2 mutation causes metabolic disorders and the therapeutic effect of ALDH2 activators
Source: Nat Commun. 2023 Sep 25;14:5971. doi: 10.1038/s41467-023-41570-6 (PMC10520061; doi:10.1038/s41467-023-41570-6)
Supplement: Supplementary file 4 — Supplementary Data 1 [file 41467_2023_41570_MOESM4_ESM.zip › Table S5c/Q9D0M3/Q9D0M3_KIO60-1-H107.html]

Mascot Search Results: Q9D0M3
 

# MASCOT Search Results

## Protein View: Q9D0M3

### Cytochrome c1, heme protein, mitochondrial OS=Mus musculus OX=10090 GN=Cyc1 PE=1 SV=1

|  |  |
| --- | --- |
| Database: | Mouse\_UniProt\_proteomes |
| Score: | 3837 |
| Monoisotopic mass (M_r_): | 35533 |
| Calculated pI: | 9.24 |

Sequence similarity is available as an NCBI BLAST search of Q9D0M3 against nr.

### Search parameters

|  |  |
| --- | --- |
| MS data file: | `D:\LCMSMS\2023 Users' data\230529-1\230529-1-KI60-1.raw` |
| Enzyme: | Trypsin/P: cuts C-term side of KR. |
| Fixed modifications: | Carbamidomethyl (C) |
| Variable modifications: | Deamidated (NQ), HNE (C), HNE (H), HNE (K), Oxidation (M) |

### Protein sequence coverage: 40%

Matched peptides shown in ***bold red***.

|  |  |  |  |  |  |
| --- | --- | --- | --- | --- | --- |
| `1` | `MAAAAASLRR` | `TVLGPRGVGL` | `PGASAPGLLG` | `GARSRQLPLR` | `TPQAVSLSSK` |
| `51` | `SGPSRGRKVM` | `LSALGMLAAG` | `GAGLAVALHS` | `AVSASDLELH` | `PPSYPWSHRG` |
| `101` | `LLSSLDHTSI` | `RRGFQVYKQV` | `CSSCHSMDYV` | `AYRHLVGVCY` | `TEEEAKALAE` |
| `151` | `EVEVQDGPND` | `DGEMFMRPGK` | `LSDYFPKPYP` | `NPEAARAANN` | `GALPPDLSYI` |
| `201` | `VRARHGGEDY` | `VFSLLTGYCE` | `PPTGVSLREG` | `LYFNPYFPGQ` | `AIGMAPPIYT` |
| `251` | `EVLEYDDGTP` | `ATMSQVAKDV` | `ATFLRWASEP` | `EHDHRKRMGL` | `KMLLMMGLLL` |
| `301` | `PLTYAMKRHK` | `WSVLKSRKLA` | `YRPPK` |  |  |

Unformatted sequence string: 325 residues (for pasting into other applications).

|  |  |  |  |
| --- | --- | --- | --- |
| Sort by | residue number | increasing mass | decreasing mass |
| Show | matched peptides only | predicted peptides also |  |

| Query | Start | – | End | Observed | Mr(expt) | Mr(calc) | ppm | M | Score | Expect | Rank | U | Peptide |
| --- | --- | --- | --- | --- | --- | --- | --- | --- | --- | --- | --- | --- | --- |
| 41828 | 100 | – | 111 | 433.5731 | 1297.6974 | 1297.6990 | -1.22 | 0 | 45 | 5.9e-05 | 1Score **> 33** indicates **identity** Score **> 15** indicates **homology** | U | R.GLLSSLDHTSIR.R |
| 41829 | 100 | – | 111 | 433.5733 | 1297.6980 | 1297.6990 | -0.80 | 0 | 48 | 3.8e-05 | 1Score **> 33** indicates **identity** Score **> 17** indicates **homology** | U | R.GLLSSLDHTSIR.R |
| 41830 | 100 | – | 111 | 649.8565 | 1297.6985 | 1297.6990 | -0.39 | 0 | 51 | 1.7e-05 | 1Score **> 33** indicates **identity** Score **> 16** indicates **homology** | U | R.GLLSSLDHTSIR.R |
| 41831 | 100 | – | 111 | 433.5735 | 1297.6985 | 1297.6990 | -0.35 | 0 | 47 | 5.3e-05 | 1Score **> 33** indicates **identity** Score **> 17** indicates **homology** | U | R.GLLSSLDHTSIR.R |
| 41832 | 100 | – | 111 | 433.5735 | 1297.6986 | 1297.6990 | -0.32 | 0 | 44 | 7.4e-05 | 1Score **> 33** indicates **identity** Score **> 15** indicates **homology** | U | R.GLLSSLDHTSIR.R |
| 41833 | 100 | – | 111 | 433.5735 | 1297.6986 | 1297.6990 | -0.31 | 0 | 51 | 1.8e-05 | 1Score **> 33** indicates **identity** Score **> 16** indicates **homology** | U | R.GLLSSLDHTSIR.R |
| 41834 | 100 | – | 111 | 433.5735 | 1297.6987 | 1297.6990 | -0.22 | 0 | 47 | 9.3e-05 | 1Score **> 33** indicates **identity** Score **> 19** indicates **homology** | U | R.GLLSSLDHTSIR.R |
| 41835 | 100 | – | 111 | 433.5735 | 1297.6987 | 1297.6990 | -0.21 | 0 | 49 | 2.9e-05 | 1Score **> 33** indicates **identity** Score **> 16** indicates **homology** | U | R.GLLSSLDHTSIR.R |
| 41836 | 100 | – | 111 | 433.5735 | 1297.6987 | 1297.6990 | -0.20 | 0 | 16 | 0.03 | 1Score **> 33** indicates **identity** Score **> 14** indicates **homology** | U | R.GLLSSLDHTSIR.R |
| 41837 | 100 | – | 111 | 649.8567 | 1297.6988 | 1297.6990 | -0.18 | 0 | 50 | 2.2e-05 | 1Score **> 33** indicates **identity** Score **> 16** indicates **homology** | U | R.GLLSSLDHTSIR.R |
| 41838 | 100 | – | 111 | 433.5736 | 1297.6991 | 1297.6990 | 0.044 | 0 | 46 | 5.8e-05 | 1Score **> 33** indicates **identity** Score **> 16** indicates **homology** | U | R.GLLSSLDHTSIR.R |
| 41839 | 100 | – | 111 | 433.5736 | 1297.6991 | 1297.6990 | 0.046 | 0 | 47 | 3.5e-05 | 1Score **> 33** indicates **identity** Score **> 15** indicates **homology** | U | R.GLLSSLDHTSIR.R |
| 41840 | 100 | – | 111 | 649.8568 | 1297.6991 | 1297.6990 | 0.10 | 0 | 67 | 4.9e-07 | 1Score **> 33** indicates **identity** Score **> 17** indicates **homology** | U | R.GLLSSLDHTSIR.R |
| 41841 | 100 | – | 111 | 433.5737 | 1297.6992 | 1297.6990 | 0.17 | 0 | 51 | 1.9e-05 | 1Score **> 33** indicates **identity** Score **> 16** indicates **homology** | U | R.GLLSSLDHTSIR.R |
| 41842 | 100 | – | 111 | 433.5737 | 1297.6993 | 1297.6990 | 0.26 | 0 | 51 | 1.8e-05 | 1Score **> 33** indicates **identity** Score **> 16** indicates **homology** | U | R.GLLSSLDHTSIR.R |
| 41843 | 100 | – | 111 | 433.5738 | 1297.6995 | 1297.6990 | 0.42 | 0 | 38 | 0.00027 | 1Score **> 33** indicates **identity** Score **> 15** indicates **homology** | U | R.GLLSSLDHTSIR.R |
| 41845 | 100 | – | 111 | 433.5738 | 1297.6997 | 1297.6990 | 0.52 | 0 | 48 | 3.7e-05 | 1Score **> 33** indicates **identity** Score **> 17** indicates **homology** | U | R.GLLSSLDHTSIR.R |
| 41846 | 100 | – | 111 | 433.5739 | 1297.6998 | 1297.6990 | 0.60 | 0 | 40 | 0.00017 | 1Score **> 33** indicates **identity** Score **> 15** indicates **homology** | U | R.GLLSSLDHTSIR.R |
| 41847 | 100 | – | 111 | 649.8572 | 1297.6998 | 1297.6990 | 0.61 | 0 | 67 | 5e-07 | 1Score **> 33** indicates **identity** Score **> 17** indicates **homology** | U | R.GLLSSLDHTSIR.R |
| 41850 | 100 | – | 111 | 433.5748 | 1297.7025 | 1297.6990 | 2.69 | 0 | 51 | 1.8e-05 | 1Score **> 33** indicates **identity** Score **> 16** indicates **homology** | U | R.GLLSSLDHTSIR.R |
| 41851 | 100 | – | 111 | 433.5757 | 1297.7052 | 1297.6990 | 4.75 | 0 | 33 | 0.00078 | 1Score **> 33** indicates **identity** Score **> 15** indicates **homology** | U | R.GLLSSLDHTSIR.R |
| 58689 | 100 | – | 111 | 485.6072 | 1453.7998 | 1453.8140 | -9.82 | 0 | 27 | 0.0027 | 1Score **> 33** indicates **identity** Score **> 14** indicates **homology** | U | R.GLLSSLDHTSIR.R  + HNE (H) |
| 58691 | 100 | – | 111 | 485.6072 | 1453.7999 | 1453.8140 | -9.75 | 0 | 29 | 0.0033 | 1Score **> 33** indicates **identity** Score **> 16** indicates **homology** | U | R.GLLSSLDHTSIR.R  + HNE (H) |
| 58699 | 100 | – | 111 | 485.6076 | 1453.8009 | 1453.8140 | -9.06 | 0 | 23 | 0.0073 | 1Score **> 33** indicates **identity** Score **> 14** indicates **homology** | U | R.GLLSSLDHTSIR.R  + HNE (H) |
| 58702 | 100 | – | 111 | 364.4576 | 1453.8012 | 1453.8140 | -8.86 | 0 | 17 | 0.024 | 1Score **> 33** indicates **identity** Score **> 14** indicates **homology** | U | R.GLLSSLDHTSIR.R  + HNE (H) |
| 58703 | 100 | – | 111 | 485.6077 | 1453.8012 | 1453.8140 | -8.86 | 0 | 21 | 0.021 | 1Score **> 33** indicates **identity** Score **> 17** indicates **homology** | U | R.GLLSSLDHTSIR.R  + HNE (H) |
| 58706 | 100 | – | 111 | 485.6078 | 1453.8014 | 1453.8140 | -8.68 | 0 | 19 | 0.017 | 1Score **> 33** indicates **identity** Score **> 14** indicates **homology** | U | R.GLLSSLDHTSIR.R  + HNE (H) |
| 58681 | 100 | – | 112 | 485.6068 | 1453.7985 | 1453.8001 | -1.12 | 1 | 17 | 0.024 | 1Score **> 33** indicates **identity** Score **> 14** indicates **homology** | U | R.GLLSSLDHTSIRR.G |
| 58684 | 100 | – | 112 | 485.6070 | 1453.7990 | 1453.8001 | -0.74 | 1 | 30 | 0.0019 | 1Score **> 33** indicates **identity** Score **> 16** indicates **homology** | U | R.GLLSSLDHTSIRR.G |
| 58685 | 100 | – | 112 | 485.6070 | 1453.7991 | 1453.8001 | -0.71 | 1 | 27 | 0.018 | 1Score **> 33** indicates **identity** Score **> 22** indicates **homology** | U | R.GLLSSLDHTSIRR.G |
| 58692 | 100 | – | 112 | 485.6072 | 1453.7999 | 1453.8001 | -0.17 | 1 | 24 | 0.01 | 1Score **> 33** indicates **identity** Score **> 17** indicates **homology** | U | R.GLLSSLDHTSIRR.G |
| 68593 | 134 | – | 146 | 767.8617 | 1533.7088 | 1533.7133 | -2.94 | 0 | 64 | 1.1e-06 | 1Score **> 31** indicates **identity** Score **> 16** indicates **homology** | U | R.HLVGVCYTEEEAK.A |
| 68594 | 134 | – | 146 | 767.8630 | 1533.7115 | 1533.7133 | -1.19 | 0 | 67 | 4.9e-07 | 1Score **> 31** indicates **identity** Score **> 17** indicates **homology** | U | R.HLVGVCYTEEEAK.A |
| 68595 | 134 | – | 146 | 512.2445 | 1533.7115 | 1533.7133 | -1.17 | 0 | 36 | 0.00042 | 1Score **> 31** indicates **identity** Score **> 15** indicates **homology** | U | R.HLVGVCYTEEEAK.A |
| 68597 | 134 | – | 146 | 512.2447 | 1533.7122 | 1533.7133 | -0.74 | 0 | 27 | 0.0029 | 1Score **> 31** indicates **identity** Score **> 14** indicates **homology** | U | R.HLVGVCYTEEEAK.A |
| 68598 | 134 | – | 146 | 767.8636 | 1533.7126 | 1533.7133 | -0.47 | 0 | 82 | 2e-08 | 1Score **> 32** indicates **identity** Score **> 18** indicates **homology** | U | R.HLVGVCYTEEEAK.A |
| 68599 | 134 | – | 146 | 512.2449 | 1533.7127 | 1533.7133 | -0.39 | 0 | 26 | 0.0037 | 1Score **> 32** indicates **identity** Score **> 14** indicates **homology** | U | R.HLVGVCYTEEEAK.A |
| 68600 | 134 | – | 146 | 767.8636 | 1533.7127 | 1533.7133 | -0.39 | 0 | 78 | 5e-08 | 1Score **> 32** indicates **identity** Score **> 17** indicates **homology** | U | R.HLVGVCYTEEEAK.A |
| 68601 | 134 | – | 146 | 767.8637 | 1533.7128 | 1533.7133 | -0.32 | 0 | 43 | 9.5e-05 | 1Score **> 32** indicates **identity** Score **> 15** indicates **homology** | U | R.HLVGVCYTEEEAK.A |
| 68602 | 134 | – | 146 | 767.8640 | 1533.7135 | 1533.7133 | 0.14 | 0 | 46 | 5.2e-05 | 1Score **> 32** indicates **identity** Score **> 15** indicates **homology** | U | R.HLVGVCYTEEEAK.A |
| 68603 | 134 | – | 146 | 512.2452 | 1533.7137 | 1533.7133 | 0.26 | 0 | 45 | 6e-05 | 1Score **> 32** indicates **identity** Score **> 15** indicates **homology** | U | R.HLVGVCYTEEEAK.A |
| 68604 | 134 | – | 146 | 767.8642 | 1533.7139 | 1533.7133 | 0.40 | 0 | 76 | 7.1e-08 | 1Score **> 32** indicates **identity** Score **> 17** indicates **homology** | U | R.HLVGVCYTEEEAK.A |
| 68605 | 134 | – | 146 | 767.8644 | 1533.7142 | 1533.7133 | 0.54 | 0 | 19 | 0.018 | 1Score **> 32** indicates **identity** Score **> 14** indicates **homology** | U | R.HLVGVCYTEEEAK.A |
| 68606 | 134 | – | 146 | 767.8647 | 1533.7149 | 1533.7133 | 1.01 | 0 | 65 | 7.4e-07 | 1Score **> 31** indicates **identity** Score **> 17** indicates **homology** | U | R.HLVGVCYTEEEAK.A |
| 68607 | 134 | – | 146 | 767.8655 | 1533.7164 | 1533.7133 | 1.98 | 0 | 74 | 1.3e-07 | 1Score **> 32** indicates **identity** Score **> 17** indicates **homology** | U | R.HLVGVCYTEEEAK.A |
| 167271 | 147 | – | 170 | 878.7320 | 2633.1743 | 2633.1738 | 0.19 | 1 | 46 | 4.6e-05 | 1Score **> 32** indicates **identity** Score **> 15** indicates **homology** | U | K.ALAEEVEVQDGPNDDGEMFMRPGK.L |
| 109750 | 171 | – | 186 | 622.3081 | 1863.9025 | 1863.9155 | -6.98 | 1 | 14 | 0.049 | 1Score **> 35** indicates **identity** Score **> 13** indicates **homology** | U | K.LSDYFPKPYPNPEAAR.A |
| 109760 | 171 | – | 186 | 622.3123 | 1863.9151 | 1863.9155 | -0.24 | 1 | 52 | 1.4e-05 | 1Score **> 35** indicates **identity** Score **> 16** indicates **homology** | U | K.LSDYFPKPYPNPEAAR.A |
| 109765 | 171 | – | 186 | 622.3127 | 1863.9163 | 1863.9155 | 0.43 | 1 | 58 | 3.5e-06 | 1Score **> 35** indicates **identity** Score **> 16** indicates **homology** | U | K.LSDYFPKPYPNPEAAR.A |
| 109766 | 171 | – | 186 | 932.9655 | 1863.9164 | 1863.9155 | 0.49 | 1 | 56 | 5.7e-06 | 1Score **> 35** indicates **identity** Score **> 16** indicates **homology** | U | K.LSDYFPKPYPNPEAAR.A |
| 109767 | 171 | – | 186 | 622.3128 | 1863.9166 | 1863.9155 | 0.59 | 1 | 54 | 8.4e-06 | 1Score **> 35** indicates **identity** Score **> 16** indicates **homology** | U | K.LSDYFPKPYPNPEAAR.A |
| 109769 | 171 | – | 186 | 622.3129 | 1863.9168 | 1863.9155 | 0.71 | 1 | 39 | 0.00024 | 1Score **> 35** indicates **identity** Score **> 15** indicates **homology** | U | K.LSDYFPKPYPNPEAAR.A |
| 109770 | 171 | – | 186 | 622.3129 | 1863.9169 | 1863.9155 | 0.74 | 1 | 44 | 7e-05 | 1Score **> 35** indicates **identity** Score **> 15** indicates **homology** | U | K.LSDYFPKPYPNPEAAR.A |
| 109771 | 171 | – | 186 | 932.9658 | 1863.9171 | 1863.9155 | 0.85 | 1 | 46 | 4.5e-05 | 1Score **> 35** indicates **identity** Score **> 15** indicates **homology** | U | K.LSDYFPKPYPNPEAAR.A |
| 109772 | 171 | – | 186 | 622.3130 | 1863.9172 | 1863.9155 | 0.91 | 1 | 54 | 7.8e-06 | 1Score **> 35** indicates **identity** Score **> 16** indicates **homology** | U | K.LSDYFPKPYPNPEAAR.A |
| 109773 | 171 | – | 186 | 932.9659 | 1863.9173 | 1863.9155 | 0.94 | 1 | 46 | 4.9e-05 | 1Score **> 35** indicates **identity** Score **> 15** indicates **homology** | U | K.LSDYFPKPYPNPEAAR.A |
| 109774 | 171 | – | 186 | 932.9660 | 1863.9175 | 1863.9155 | 1.05 | 1 | 59 | 2.9e-06 | 1Score **> 35** indicates **identity** Score **> 16** indicates **homology** | U | K.LSDYFPKPYPNPEAAR.A |
| 109775 | 171 | – | 186 | 622.3131 | 1863.9175 | 1863.9155 | 1.05 | 1 | 57 | 4.5e-06 | 1Score **> 35** indicates **identity** Score **> 16** indicates **homology** | U | K.LSDYFPKPYPNPEAAR.A |
| 109776 | 171 | – | 186 | 932.9661 | 1863.9176 | 1863.9155 | 1.10 | 1 | 43 | 9.3e-05 | 1Score **> 35** indicates **identity** Score **> 15** indicates **homology** | U | K.LSDYFPKPYPNPEAAR.A |
| 109777 | 171 | – | 186 | 622.3132 | 1863.9177 | 1863.9155 | 1.16 | 1 | 51 | 1.5e-05 | 1Score **> 35** indicates **identity** Score **> 16** indicates **homology** | U | K.LSDYFPKPYPNPEAAR.A |
| 109779 | 171 | – | 186 | 622.3132 | 1863.9178 | 1863.9155 | 1.23 | 1 | 20 | 0.014 | 1Score **> 35** indicates **identity** Score **> 14** indicates **homology** | U | K.LSDYFPKPYPNPEAAR.A |
| 109781 | 171 | – | 186 | 622.3139 | 1863.9199 | 1863.9155 | 2.36 | 1 | 47 | 4.3e-05 | 1Score **> 35** indicates **identity** Score **> 15** indicates **homology** | U | K.LSDYFPKPYPNPEAAR.A |
| 109783 | 171 | – | 186 | 622.3146 | 1863.9220 | 1863.9155 | 3.49 | 1 | 29 | 0.0018 | 1Score **> 35** indicates **identity** Score **> 14** indicates **homology** | U | K.LSDYFPKPYPNPEAAR.A |
| 109797 | 171 | – | 186 | 932.9707 | 1863.9269 | 1863.9155 | 6.11 | 1 | 37 | 0.00034 | 1Score **> 35** indicates **identity** Score **> 15** indicates **homology** | U | K.LSDYFPKPYPNPEAAR.A |
| 87451 | 187 | – | 202 | 835.9462 | 1669.8779 | 1669.8787 | -0.51 | 0 | 60 | 2.7e-06 | 1Score **> 35** indicates **identity** Score **> 17** indicates **homology** | U | R.AANNGALPPDLSYIVR.A |
| 87452 | 187 | – | 202 | 835.9462 | 1669.8779 | 1669.8787 | -0.47 | 0 | 45 | 0.00012 | 1Score **> 35** indicates **identity** Score **> 18** indicates **homology** | U | R.AANNGALPPDLSYIVR.A |
| 87453 | 187 | – | 202 | 835.9463 | 1669.8780 | 1669.8787 | -0.46 | 0 | 58 | 6.8e-06 | 1Score **> 35** indicates **identity** Score **> 19** indicates **homology** | U | R.AANNGALPPDLSYIVR.A |
| 87454 | 187 | – | 202 | 835.9465 | 1669.8784 | 1669.8787 | -0.23 | 0 | 47 | 8.8e-05 | 1Score **> 35** indicates **identity** Score **> 19** indicates **homology** | U | R.AANNGALPPDLSYIVR.A |
| 87455 | 187 | – | 202 | 835.9465 | 1669.8784 | 1669.8787 | -0.18 | 0 | 40 | 0.00017 | 1Score **> 35** indicates **identity** Score **> 15** indicates **homology** | U | R.AANNGALPPDLSYIVR.A |
| 87456 | 187 | – | 202 | 835.9466 | 1669.8787 | 1669.8787 | -0.0048 | 0 | 63 | 3.8e-06 | 1Score **> 35** indicates **identity** Score **> 21** indicates **homology** | U | R.AANNGALPPDLSYIVR.A |
| 87457 | 187 | – | 202 | 835.9467 | 1669.8789 | 1669.8787 | 0.074 | 0 | 60 | 6.2e-06 | 1Score **> 35** indicates **identity** Score **> 20** indicates **homology** | U | R.AANNGALPPDLSYIVR.A |
| 87458 | 187 | – | 202 | 835.9472 | 1669.8798 | 1669.8787 | 0.65 | 0 | 68 | 1.3e-06 | 1Score **> 36** indicates **identity** Score **> 21** indicates **homology** | U | R.AANNGALPPDLSYIVR.A |
| 87459 | 187 | – | 202 | 835.9472 | 1669.8798 | 1669.8787 | 0.66 | 0 | 52 | 1.4e-05 | 1Score **> 36** indicates **identity** Score **> 16** indicates **homology** | U | R.AANNGALPPDLSYIVR.A |
| 87461 | 187 | – | 202 | 835.9477 | 1669.8808 | 1669.8787 | 1.24 | 0 | 55 | 7.7e-06 | 1Score **> 36** indicates **identity** Score **> 16** indicates **homology** | U | R.AANNGALPPDLSYIVR.A |
| 87463 | 187 | – | 202 | 835.9480 | 1669.8814 | 1669.8787 | 1.57 | 0 | 51 | 1.8e-05 | 1Score **> 35** indicates **identity** Score **> 16** indicates **homology** | U | R.AANNGALPPDLSYIVR.A |
| 87464 | 187 | – | 202 | 557.6344 | 1669.8814 | 1669.8787 | 1.58 | 0 | 24 | 0.0058 | 1Score **> 35** indicates **identity** Score **> 14** indicates **homology** | U | R.AANNGALPPDLSYIVR.A |
| 87469 | 187 | – | 202 | 835.9495 | 1669.8844 | 1669.8787 | 3.41 | 0 | 16 | 0.032 | 1Score **> 35** indicates **identity** Score **> 13** indicates **homology** | U | R.AANNGALPPDLSYIVR.A |
| 87470 | 187 | – | 202 | 835.9496 | 1669.8847 | 1669.8787 | 3.54 | 0 | 52 | 2.2e-05 | 1Score **> 35** indicates **identity** Score **> 18** indicates **homology** | U | R.AANNGALPPDLSYIVR.A |
| 87471 | 187 | – | 202 | 557.6357 | 1669.8852 | 1669.8787 | 3.85 | 0 | 28 | 0.0023 | 1Score **> 35** indicates **identity** Score **> 14** indicates **homology** | U | R.AANNGALPPDLSYIVR.A |
| 87596 | 187 | – | 202 | 557.9613 | 1670.8620 | 1670.8627 | -0.42 | 0 | 27 | 0.003 | 1Score **> 35** indicates **identity** Score **> 14** indicates **homology** | U | R.AANNGALPPDLSYIVR.A  + Deamidated (NQ) |
| 87597 | 187 | – | 202 | 836.4384 | 1670.8623 | 1670.8627 | -0.29 | 0 | 62 | 1.8e-06 | 1Score **> 35** indicates **identity** Score **> 17** indicates **homology** | U | R.AANNGALPPDLSYIVR.A  + Deamidated (NQ) |
| 87598 | 187 | – | 202 | 836.4388 | 1670.8631 | 1670.8627 | 0.21 | 0 | 65 | 7.6e-07 | 1Score **> 35** indicates **identity** Score **> 17** indicates **homology** | U | R.AANNGALPPDLSYIVR.A  + Deamidated (NQ) |
| 87599 | 187 | – | 202 | 836.4389 | 1670.8632 | 1670.8627 | 0.27 | 0 | 54 | 8.6e-06 | 1Score **> 35** indicates **identity** Score **> 16** indicates **homology** | U | R.AANNGALPPDLSYIVR.A  + Deamidated (NQ) |
| 87600 | 187 | – | 202 | 557.9617 | 1670.8632 | 1670.8627 | 0.30 | 0 | 26 | 0.0033 | 1Score **> 35** indicates **identity** Score **> 14** indicates **homology** | U | R.AANNGALPPDLSYIVR.A  + Deamidated (NQ) |
| 87601 | 187 | – | 202 | 836.4389 | 1670.8633 | 1670.8627 | 0.35 | 0 | 59 | 3.2e-06 | 1Score **> 35** indicates **identity** Score **> 17** indicates **homology** | U | R.AANNGALPPDLSYIVR.A  + Deamidated (NQ) |
| 87602 | 187 | – | 202 | 836.4390 | 1670.8634 | 1670.8627 | 0.42 | 0 | 68 | 5.1e-07 | 1Score **> 35** indicates **identity** Score **> 17** indicates **homology** | U | R.AANNGALPPDLSYIVR.A  + Deamidated (NQ) |
| 87603 | 187 | – | 202 | 836.4392 | 1670.8638 | 1670.8627 | 0.65 | 0 | 59 | 3.2e-06 | 1Score **> 35** indicates **identity** Score **> 16** indicates **homology** | U | R.AANNGALPPDLSYIVR.A  + Deamidated (NQ) |
| 87604 | 187 | – | 202 | 836.4393 | 1670.8641 | 1670.8627 | 0.78 | 0 | 62 | 1.7e-06 | 1Score **> 35** indicates **identity** Score **> 17** indicates **homology** | U | R.AANNGALPPDLSYIVR.A  + Deamidated (NQ) |
| 87605 | 187 | – | 202 | 836.4396 | 1670.8646 | 1670.8627 | 1.10 | 0 | 60 | 3.1e-06 | 1Score **> 35** indicates **identity** Score **> 17** indicates **homology** | U | R.AANNGALPPDLSYIVR.A  + Deamidated (NQ) |
| 87606 | 187 | – | 202 | 557.9622 | 1670.8648 | 1670.8627 | 1.20 | 0 | 35 | 0.00049 | 1Score **> 35** indicates **identity** Score **> 15** indicates **homology** | U | R.AANNGALPPDLSYIVR.A  + Deamidated (NQ) |
| 87609 | 187 | – | 202 | 836.4398 | 1670.8650 | 1670.8627 | 1.33 | 0 | 71 | 2.4e-07 | 1Score **> 35** indicates **identity** Score **> 17** indicates **homology** | U | R.AANNGALPPDLSYIVR.A  + Deamidated (NQ) |
| 87610 | 187 | – | 202 | 836.4399 | 1670.8653 | 1670.8627 | 1.52 | 0 | 59 | 3.2e-06 | 1Score **> 35** indicates **identity** Score **> 17** indicates **homology** | U | R.AANNGALPPDLSYIVR.A  + Deamidated (NQ) |
| 87611 | 187 | – | 202 | 836.4399 | 1670.8653 | 1670.8627 | 1.52 | 0 | 59 | 3.3e-06 | 1Score **> 35** indicates **identity** Score **> 17** indicates **homology** | U | R.AANNGALPPDLSYIVR.A  + Deamidated (NQ) |
| 87612 | 187 | – | 202 | 836.4399 | 1670.8653 | 1670.8627 | 1.55 | 0 | 70 | 2.9e-07 | 1Score **> 35** indicates **identity** Score **> 18** indicates **homology** | U | R.AANNGALPPDLSYIVR.A  + Deamidated (NQ) |
| 87613 | 187 | – | 202 | 836.4403 | 1670.8661 | 1670.8627 | 1.98 | 0 | 62 | 1.7e-06 | 1Score **> 35** indicates **identity** Score **> 16** indicates **homology** | U | R.AANNGALPPDLSYIVR.A  + Deamidated (NQ) |
| 87614 | 187 | – | 202 | 836.4404 | 1670.8663 | 1670.8627 | 2.13 | 0 | 56 | 5.9e-06 | 1Score **> 35** indicates **identity** Score **> 16** indicates **homology** | U | R.AANNGALPPDLSYIVR.A  + Deamidated (NQ) |
| 87615 | 187 | – | 202 | 836.4405 | 1670.8665 | 1670.8627 | 2.25 | 0 | 57 | 4.5e-06 | 1Score **> 35** indicates **identity** Score **> 16** indicates **homology** | U | R.AANNGALPPDLSYIVR.A  + Deamidated (NQ) |
| 87618 | 187 | – | 202 | 836.4413 | 1670.8681 | 1670.8627 | 3.21 | 0 | 58 | 3.7e-06 | 1Score **> 36** indicates **identity** Score **> 16** indicates **homology** | U | R.AANNGALPPDLSYIVR.A  + Deamidated (NQ) |
| 87632 | 187 | – | 202 | 557.9667 | 1670.8783 | 1670.8627 | 9.32 | 0 | 26 | 0.004 | 1Score **> 35** indicates **identity** Score **> 14** indicates **homology** | U | R.AANNGALPPDLSYIVR.A  + Deamidated (NQ) |
| 87747 | 187 | – | 202 | 836.9350 | 1671.8555 | 1671.8468 | 5.24 | 0 | 48 | 3e-05 | 1Score **> 35** indicates **identity** Score **> 16** indicates **homology** | U | R.AANNGALPPDLSYIVR.A  + 2 Deamidated (NQ) |
| 87749 | 187 | – | 202 | 836.9359 | 1671.8573 | 1671.8468 | 6.32 | 0 | 27 | 0.003 | 1Score **> 35** indicates **identity** Score **> 14** indicates **homology** | U | R.AANNGALPPDLSYIVR.A  + 2 Deamidated (NQ) |
| 176425 | 203 | – | 228 | 721.1021 | 2880.3794 | 2880.3865 | -2.47 | 1 | 73 | 1.6e-07 | 1Score **> 36** indicates **identity** Score **> 17** indicates **homology** | U | R.ARHGGEDYVFSLLTGYCEPPTGVSLR.E |
| 176426 | 203 | – | 228 | 721.1026 | 2880.3812 | 2880.3865 | -1.84 | 1 | 28 | 0.0025 | 1Score **> 36** indicates **identity** Score **> 14** indicates **homology** | U | R.ARHGGEDYVFSLLTGYCEPPTGVSLR.E |
| 176427 | 203 | – | 228 | 961.1357 | 2880.3852 | 2880.3865 | -0.43 | 1 | 56 | 6.1e-06 | 1Score **> 36** indicates **identity** Score **> 16** indicates **homology** | U | R.ARHGGEDYVFSLLTGYCEPPTGVSLR.E |
| 176428 | 203 | – | 228 | 961.1359 | 2880.3857 | 2880.3865 | -0.26 | 1 | 80 | 2.9e-08 | 1Score **> 36** indicates **identity** Score **> 18** indicates **homology** | U | R.ARHGGEDYVFSLLTGYCEPPTGVSLR.E |
| 176429 | 203 | – | 228 | 721.1041 | 2880.3872 | 2880.3865 | 0.24 | 1 | 75 | 9.9e-08 | 1Score **> 36** indicates **identity** Score **> 17** indicates **homology** | U | R.ARHGGEDYVFSLLTGYCEPPTGVSLR.E |
| 176430 | 203 | – | 228 | 1441.2013 | 2880.3880 | 2880.3865 | 0.51 | 1 | 22 | 0.0082 | 1Score **> 36** indicates **identity** Score **> 14** indicates **homology** | U | R.ARHGGEDYVFSLLTGYCEPPTGVSLR.E |
| 176431 | 203 | – | 228 | 961.1425 | 2880.4058 | 2880.3865 | 6.70 | 1 | 49 | 2.3e-05 | 1Score **> 37** indicates **identity** Score **> 16** indicates **homology** | U | R.ARHGGEDYVFSLLTGYCEPPTGVSLR.E |
| 167976 | 205 | – | 228 | 885.4222 | 2653.2447 | 2653.2483 | -1.36 | 0 | 19 | 0.017 | 1Score **> 35** indicates **identity** Score **> 14** indicates **homology** | U | R.HGGEDYVFSLLTGYCEPPTGVSLR.E |
| 167977 | 205 | – | 228 | 885.4236 | 2653.2489 | 2653.2483 | 0.22 | 0 | 57 | 4.9e-06 | 1Score **> 35** indicates **identity** Score **> 16** indicates **homology** | U | R.HGGEDYVFSLLTGYCEPPTGVSLR.E |
| 167978 | 205 | – | 228 | 1327.6330 | 2653.2514 | 2653.2483 | 1.16 | 0 | 130 | 6.3e-13 | 1Score **> 35** indicates **identity** Score **> 21** indicates **homology** | U | R.HGGEDYVFSLLTGYCEPPTGVSLR.E |
| 167979 | 205 | – | 228 | 1327.6331 | 2653.2516 | 2653.2483 | 1.23 | 0 | 98 | 6.7e-10 | 1Score **> 35** indicates **identity** Score **> 19** indicates **homology** | U | R.HGGEDYVFSLLTGYCEPPTGVSLR.E |
| 167980 | 205 | – | 228 | 885.4248 | 2653.2525 | 2653.2483 | 1.59 | 0 | 71 | 2.2e-07 | 1Score **> 35** indicates **identity** Score **> 17** indicates **homology** | U | R.HGGEDYVFSLLTGYCEPPTGVSLR.E |
| 167981 | 205 | – | 228 | 885.4250 | 2653.2532 | 2653.2483 | 1.85 | 0 | 49 | 2.5e-05 | 1Score **> 35** indicates **identity** Score **> 16** indicates **homology** | U | R.HGGEDYVFSLLTGYCEPPTGVSLR.E |
| 4116 | 269 | – | 275 | 411.2291 | 820.4436 | 820.4443 | -0.83 | 0 | 24 | 0.017 | 1Score **> 27** indicates **identity** Score **> 19** indicates **homology** | U | K.DVATFLR.W |
| 4117 | 269 | – | 275 | 411.2291 | 820.4436 | 820.4443 | -0.82 | 0 | 21 | 0.037 | 1Score **> 27** indicates **identity** Score **> 19** indicates **homology** | U | K.DVATFLR.W |
| 4118 | 269 | – | 275 | 411.2291 | 820.4437 | 820.4443 | -0.76 | 0 | 21 | 0.02 | 1Score **> 27** indicates **identity** Score **> 17** indicates **homology** | U | K.DVATFLR.W |
| 4119 | 269 | – | 275 | 411.2291 | 820.4437 | 820.4443 | -0.74 | 0 | 26 | 0.012 | 1Score **> 27** indicates **identity** Score **> 19** indicates **homology** | U | K.DVATFLR.W |
| 4120 | 269 | – | 275 | 411.2292 | 820.4439 | 820.4443 | -0.44 | 0 | 18 | 0.02 | 1Score **> 27** indicates **identity** Score **> 14** indicates **homology** | U | K.DVATFLR.W |
| 4121 | 269 | – | 275 | 411.2293 | 820.4440 | 820.4443 | -0.36 | 0 | 23 | 0.033 | 1Score **> 27** indicates **identity** Score **> 21** indicates **homology** | U | K.DVATFLR.W |
| 4122 | 269 | – | 275 | 411.2293 | 820.4440 | 820.4443 | -0.32 | 0 | 23 | 0.025 | 1Score **> 27** indicates **identity** Score **> 19** indicates **homology** | U | K.DVATFLR.W |
| 4125 | 269 | – | 275 | 411.2294 | 820.4442 | 820.4443 | -0.082 | 0 | 21 | 0.024 | 1Score **> 27** indicates **identity** Score **> 18** indicates **homology** | U | K.DVATFLR.W |
| 4126 | 269 | – | 275 | 411.2294 | 820.4443 | 820.4443 | 0.0012 | 0 | 22 | 0.026 | 1Score **> 27** indicates **identity** Score **> 19** indicates **homology** | U | K.DVATFLR.W |
| 4127 | 269 | – | 275 | 411.2295 | 820.4444 | 820.4443 | 0.067 | 0 | 18 | 0.021 | 1Score **> 27** indicates **identity** Score **> 14** indicates **homology** | U | K.DVATFLR.W |
| 4129 | 269 | – | 275 | 411.2295 | 820.4444 | 820.4443 | 0.084 | 0 | 32 | 0.0061 | 1Score **> 27** indicates **identity** Score **> 22** indicates **homology** | U | K.DVATFLR.W |
| 4132 | 269 | – | 275 | 411.2295 | 820.4445 | 820.4443 | 0.27 | 0 | 30 | 0.011 | 1Score **> 27** indicates **identity** Score **> 23** indicates **homology** | U | K.DVATFLR.W |
| 4134 | 269 | – | 275 | 411.2296 | 820.4446 | 820.4443 | 0.36 | 0 | 22 | 0.0083 | 1Score **> 27** indicates **identity** Score **> 14** indicates **homology** | U | K.DVATFLR.W |
| 4137 | 269 | – | 275 | 411.2297 | 820.4448 | 820.4443 | 0.63 | 0 | 28 | 0.015 | 1Score **> 26** indicates **identity** Score **> 22** indicates **homology** | U | K.DVATFLR.W |
| 4139 | 269 | – | 275 | 411.2297 | 820.4449 | 820.4443 | 0.71 | 0 | 28 | 0.015 | 1Score **> 26** indicates **identity** Score **> 22** indicates **homology** | U | K.DVATFLR.W |
| 4140 | 269 | – | 275 | 411.2297 | 820.4449 | 820.4443 | 0.75 | 0 | 26 | 0.018 | 1Score **> 26** indicates **identity** Score **> 21** indicates **homology** | U | K.DVATFLR.W |
| 4141 | 269 | – | 275 | 411.2298 | 820.4450 | 820.4443 | 0.88 | 0 | 25 | 0.016 | 1Score **> 26** indicates **identity** Score **> 19** indicates **homology** | U | K.DVATFLR.W |
| 4144 | 269 | – | 275 | 411.2300 | 820.4454 | 820.4443 | 1.32 | 0 | 22 | 0.0095 | 1Score **> 26** indicates **identity** Score **> 14** indicates **homology** | U | K.DVATFLR.W |
| 4153 | 269 | – | 275 | 411.2308 | 820.4470 | 820.4443 | 3.34 | 0 | 21 | 0.02 | 1Score **> 26** indicates **identity** Score **> 16** indicates **homology** | U | K.DVATFLR.W |
| 4158 | 269 | – | 275 | 411.2315 | 820.4485 | 820.4443 | 5.13 | 0 | 22 | 0.023 | 1Score **> 27** indicates **identity** Score **> 19** indicates **homology** | U | K.DVATFLR.W |
| 38048 | 276 | – | 285 | 421.8550 | 1262.5431 | 1262.5428 | 0.25 | 0 | 35 | 0.00056 | 1Score **> 26** indicates **identity** Score **> 15** indicates **homology** | U | R.WASEPEHDHR.K |
| 5109 | 319 | – | 325 | 422.7552 | 843.4959 | 843.4966 | -0.90 | 1 | 26 | 0.024 | 1Score **> 28** indicates **identity** Score **> 23** indicates **homology** | U | K.LAYRPPK.- |
| 5111 | 319 | – | 325 | 422.7554 | 843.4963 | 843.4966 | -0.42 | 1 | 24 | 0.042 | 1Score **> 28** indicates **identity** Score **> 23** indicates **homology** | U | K.LAYRPPK.- |
| 5118 | 319 | – | 325 | 422.7558 | 843.4970 | 843.4966 | 0.39 | 1 | 31 | 0.02 | 1Score **> 28** indicates **identity** Score **> 26** indicates **homology** | U | K.LAYRPPK.- |

---

```
ID   CY1_MOUSE               Reviewed;         325 AA.
AC   Q9D0M3; Q3TDC5; Q3UAN2; Q63ZW4; Q9DCG0;
DT   11-JUL-2002, integrated into UniProtKB/Swiss-Prot.
DT   01-JUN-2001, sequence version 1.
DT   28-JUN-2023, entry version 175.
DE   RecName: Full=Cytochrome c1, heme protein, mitochondrial;
DE            EC=7.1.1.8;
DE   AltName: Full=Complex III subunit 4;
DE   AltName: Full=Complex III subunit IV;
DE   AltName: Full=Cytochrome b-c1 complex subunit 4;
DE   AltName: Full=Ubiquinol-cytochrome-c reductase complex cytochrome c1 subunit;
DE            Short=Cytochrome c-1;
DE   Flags: Precursor;
GN   Name=Cyc1;
OS   Mus musculus (Mouse).
OC   Eukaryota; Metazoa; Chordata; Craniata; Vertebrata; Euteleostomi; Mammalia;
OC   Eutheria; Euarchontoglires; Glires; Rodentia; Myomorpha; Muroidea; Muridae;
OC   Murinae; Mus; Mus.
OX   NCBI_TaxID=10090;
RN   [1]
RP   NUCLEOTIDE SEQUENCE [LARGE SCALE MRNA] (ISOFORMS 1 AND 2).
RC   STRAIN=C57BL/6J, and NOD; TISSUE=Bone marrow, Embryo, and Kidney;
RX   PubMed=16141072; DOI=10.1126/science.1112014;
RA   Carninci P., Kasukawa T., Katayama S., Gough J., Frith M.C., Maeda N.,
RA   Oyama R., Ravasi T., Lenhard B., Wells C., Kodzius R., Shimokawa K.,
RA   Bajic V.B., Brenner S.E., Batalov S., Forrest A.R., Zavolan M., Davis M.J.,
RA   Wilming L.G., Aidinis V., Allen J.E., Ambesi-Impiombato A., Apweiler R.,
RA   Aturaliya R.N., Bailey T.L., Bansal M., Baxter L., Beisel K.W., Bersano T.,
RA   Bono H., Chalk A.M., Chiu K.P., Choudhary V., Christoffels A.,
RA   Clutterbuck D.R., Crowe M.L., Dalla E., Dalrymple B.P., de Bono B.,
RA   Della Gatta G., di Bernardo D., Down T., Engstrom P., Fagiolini M.,
RA   Faulkner G., Fletcher C.F., Fukushima T., Furuno M., Futaki S.,
RA   Gariboldi M., Georgii-Hemming P., Gingeras T.R., Gojobori T., Green R.E.,
RA   Gustincich S., Harbers M., Hayashi Y., Hensch T.K., Hirokawa N., Hill D.,
RA   Huminiecki L., Iacono M., Ikeo K., Iwama A., Ishikawa T., Jakt M.,
RA   Kanapin A., Katoh M., Kawasawa Y., Kelso J., Kitamura H., Kitano H.,
RA   Kollias G., Krishnan S.P., Kruger A., Kummerfeld S.K., Kurochkin I.V.,
RA   Lareau L.F., Lazarevic D., Lipovich L., Liu J., Liuni S., McWilliam S.,
RA   Madan Babu M., Madera M., Marchionni L., Matsuda H., Matsuzawa S., Miki H.,
RA   Mignone F., Miyake S., Morris K., Mottagui-Tabar S., Mulder N., Nakano N.,
RA   Nakauchi H., Ng P., Nilsson R., Nishiguchi S., Nishikawa S., Nori F.,
RA   Ohara O., Okazaki Y., Orlando V., Pang K.C., Pavan W.J., Pavesi G.,
RA   Pesole G., Petrovsky N., Piazza S., Reed J., Reid J.F., Ring B.Z.,
RA   Ringwald M., Rost B., Ruan Y., Salzberg S.L., Sandelin A., Schneider C.,
RA   Schoenbach C., Sekiguchi K., Semple C.A., Seno S., Sessa L., Sheng Y.,
RA   Shibata Y., Shimada H., Shimada K., Silva D., Sinclair B., Sperling S.,
RA   Stupka E., Sugiura K., Sultana R., Takenaka Y., Taki K., Tammoja K.,
RA   Tan S.L., Tang S., Taylor M.S., Tegner J., Teichmann S.A., Ueda H.R.,
RA   van Nimwegen E., Verardo R., Wei C.L., Yagi K., Yamanishi H.,
RA   Zabarovsky E., Zhu S., Zimmer A., Hide W., Bult C., Grimmond S.M.,
RA   Teasdale R.D., Liu E.T., Brusic V., Quackenbush J., Wahlestedt C.,
RA   Mattick J.S., Hume D.A., Kai C., Sasaki D., Tomaru Y., Fukuda S.,
RA   Kanamori-Katayama M., Suzuki M., Aoki J., Arakawa T., Iida J., Imamura K.,
RA   Itoh M., Kato T., Kawaji H., Kawagashira N., Kawashima T., Kojima M.,
RA   Kondo S., Konno H., Nakano K., Ninomiya N., Nishio T., Okada M., Plessy C.,
RA   Shibata K., Shiraki T., Suzuki S., Tagami M., Waki K., Watahiki A.,
RA   Okamura-Oho Y., Suzuki H., Kawai J., Hayashizaki Y.;
RT   "The transcriptional landscape of the mammalian genome.";
RL   Science 309:1559-1563(2005).
RN   [2]
RP   NUCLEOTIDE SEQUENCE [LARGE SCALE MRNA] (ISOFORMS 1 AND 2).
RC   TISSUE=Jaw;
RX   PubMed=15489334; DOI=10.1101/gr.2596504;
RG   The MGC Project Team;
RT   "The status, quality, and expansion of the NIH full-length cDNA project:
RT   the Mammalian Gene Collection (MGC).";
RL   Genome Res. 14:2121-2127(2004).
RN   [3]
RP   PROTEIN SEQUENCE OF 100-111; 134-202; 269-285 AND 292-307, AND
RP   IDENTIFICATION BY MASS SPECTROMETRY.
RC   STRAIN=C57BL/6J; TISSUE=Brain, and Hippocampus;
RA   Lubec G., Klug S., Kang S.U.;
RL   Submitted (APR-2007) to UniProtKB.
RN   [4]
RP   SUBUNIT.
RX   PubMed=19026783; DOI=10.1016/j.molcel.2008.10.021;
RA   Acin-Perez R., Fernandez-Silva P., Peleato M.L., Perez-Martos A.,
RA   Enriquez J.A.;
RT   "Respiratory active mitochondrial supercomplexes.";
RL   Mol. Cell 32:529-539(2008).
RN   [5]
RP   IDENTIFICATION BY MASS SPECTROMETRY [LARGE SCALE ANALYSIS].
RC   TISSUE=Brain, Brown adipose tissue, Heart, Kidney, Liver, Lung,
RC   Pancreas, Spleen, and Testis;
RX   PubMed=21183079; DOI=10.1016/j.cell.2010.12.001;
RA   Huttlin E.L., Jedrychowski M.P., Elias J.E., Goswami T., Rad R.,
RA   Beausoleil S.A., Villen J., Haas W., Sowa M.E., Gygi S.P.;
RT   "A tissue-specific atlas of mouse protein phosphorylation and expression.";
RL   Cell 143:1174-1189(2010).
RN   [6]
RP   INTERACTION WITH FLVCR2.
RX   PubMed=32973183; DOI=10.1038/s41467-020-18607-1;
RA   Li Y., Ivica N.A., Dong T., Papageorgiou D.P., He Y., Brown D.R.,
RA   Kleyman M., Hu G., Chen W.W., Sullivan L.B., Del Rosario A., Hammond P.T.,
RA   Vander Heiden M.G., Chen J.;
RT   "MFSD7C switches mitochondrial ATP synthesis to thermogenesis in response
RT   to heme.";
RL   Nat. Commun. 11:4837-4837(2020).
CC   -!- FUNCTION: Component of the ubiquinol-cytochrome c oxidoreductase, a
CC       multisubunit transmembrane complex that is part of the mitochondrial
CC       electron transport chain which drives oxidative phosphorylation. The
CC       respiratory chain contains 3 multisubunit complexes succinate
CC       dehydrogenase (complex II, CII), ubiquinol-cytochrome c oxidoreductase
CC       (cytochrome b-c1 complex, complex III, CIII) and cytochrome c oxidase
CC       (complex IV, CIV), that cooperate to transfer electrons derived from
CC       NADH and succinate to molecular oxygen, creating an electrochemical
CC       gradient over the inner membrane that drives transmembrane transport
CC       and the ATP synthase. The cytochrome b-c1 complex catalyzes electron
CC       transfer from ubiquinol to cytochrome c, linking this redox reaction to
CC       translocation of protons across the mitochondrial inner membrane, with
CC       protons being carried across the membrane as hydrogens on the quinol.
CC       In the process called Q cycle, 2 protons are consumed from the matrix,
CC       4 protons are released into the intermembrane space and 2 electrons are
CC       passed to cytochrome c. Cytochrome c1 is a catalytic core subunit
CC       containing a c-type heme. It transfers electrons from the [2Fe-2S]
CC       iron-sulfur cluster of the Rieske protein to cytochrome c.
CC       {ECO:0000250|UniProtKB:P07143}.
CC   -!- CATALYTIC ACTIVITY:
CC       Reaction=a quinol + 2 Fe(III)-[cytochrome c](out) = a quinone + 2
CC         Fe(II)-[cytochrome c](out) + 2 H(+)(out); Xref=Rhea:RHEA:11484,
CC         Rhea:RHEA-COMP:10350, Rhea:RHEA-COMP:14399, ChEBI:CHEBI:15378,
CC         ChEBI:CHEBI:24646, ChEBI:CHEBI:29033, ChEBI:CHEBI:29034,
CC         ChEBI:CHEBI:132124; EC=7.1.1.8;
CC         Evidence={ECO:0000250|UniProtKB:P07143};
CC   -!- COFACTOR:
CC       Name=heme c; Xref=ChEBI:CHEBI:61717;
CC         Evidence={ECO:0000250|UniProtKB:P00125};
CC       Note=Binds 1 heme c group covalently per subunit.
CC       {ECO:0000250|UniProtKB:P00125};
CC   -!- SUBUNIT: Component of the ubiquinol-cytochrome c oxidoreductase
CC       (cytochrome b-c1 complex, complex III, CIII), a multisubunit enzyme
CC       composed of 11 subunits. The complex is composed of 3 respiratory
CC       subunits cytochrome b, cytochrome c1 and Rieske protein UQCRFS1, 2 core
CC       protein subunits UQCRC1/QCR1 and UQCRC2/QCR2, and 6 low-molecular
CC       weight protein subunits UQCRH/QCR6, UQCRB/QCR7, UQCRQ/QCR8,
CC       UQCR10/QCR9, UQCR11/QCR10 and subunit 9, the cleavage product of Rieske
CC       protein UQCRFS1 (By similarity). The complex exists as an obligatory
CC       dimer and forms supercomplexes (SCs) in the inner mitochondrial
CC       membrane with NADH-ubiquinone oxidoreductase (complex I, CI) and
CC       cytochrome c oxidase (complex IV, CIV), resulting in different
CC       assemblies (supercomplex SCI(1)III(2)IV(1) and megacomplex
CC       MCI(2)III(2)IV(2)) (PubMed:19026783). Interacts with FLVCR2; this
CC       interaction occurs in the absence of heme and is disrupted upon heme
CC       binding. {ECO:0000250|UniProtKB:P00125, ECO:0000269|PubMed:19026783,
CC       ECO:0000269|PubMed:32973183}.
CC   -!- SUBCELLULAR LOCATION: Mitochondrion inner membrane
CC       {ECO:0000250|UniProtKB:P07143}; Single-pass membrane protein
CC       {ECO:0000250|UniProtKB:P07143}.
CC   -!- ALTERNATIVE PRODUCTS:
CC       Event=Alternative splicing; Named isoforms=2;
CC       Name=1;
CC         IsoId=Q9D0M3-1; Sequence=Displayed;
CC       Name=2;
CC         IsoId=Q9D0M3-2; Sequence=VSP_025056;
CC   -!- SIMILARITY: Belongs to the cytochrome c family. {ECO:0000305}.
CC   -!- SEQUENCE CAUTION:
CC       Sequence=AAH82790.1; Type=Erroneous initiation; Evidence={ECO:0000305};
CC   ---------------------------------------------------------------------------
CC   Copyrighted by the UniProt Consortium, see https://www.uniprot.org/terms
CC   Distributed under the Creative Commons Attribution (CC BY 4.0) License
CC   ---------------------------------------------------------------------------
DR   EMBL; AK002815; BAB22380.1; -; mRNA.
DR   EMBL; AK011288; BAB27518.1; -; mRNA.
DR   EMBL; AK151299; BAE30282.1; -; mRNA.
DR   EMBL; AK170272; BAE41677.1; -; mRNA.
DR   EMBL; BC005620; AAH05620.1; -; mRNA.
DR   EMBL; BC082790; AAH82790.1; ALT_INIT; mRNA.
DR   CCDS; CCDS27567.1; -. [Q9D0M3-1]
DR   RefSeq; NP_079843.1; NM_025567.2. [Q9D0M3-1]
DR   PDB; 7O37; EM; 3.20 A; D/O=85-325.
DR   PDB; 7O3C; EM; 3.30 A; D/O=85-325.
DR   PDB; 7O3E; EM; 3.60 A; D/O=85-325.
DR   PDB; 7O3H; EM; 2.60 A; D/O=85-325.
DR   PDBsum; 7O37; -.
DR   PDBsum; 7O3C; -.
DR   PDBsum; 7O3E; -.
DR   PDBsum; 7O3H; -.
DR   AlphaFoldDB; Q9D0M3; -.
DR   SMR; Q9D0M3; -.
DR   BioGRID; 211479; 82.
DR   ComplexPortal; CPX-563; Mitochondrial respiratory chain complex III.
DR   CORUM; Q9D0M3; -.
DR   IntAct; Q9D0M3; 16.
DR   MINT; Q9D0M3; -.
DR   STRING; 10090.ENSMUSP00000023210; -.
DR   iPTMnet; Q9D0M3; -.
DR   PhosphoSitePlus; Q9D0M3; -.
DR   SwissPalm; Q9D0M3; -.
DR   EPD; Q9D0M3; -.
DR   jPOST; Q9D0M3; -.
DR   MaxQB; Q9D0M3; -.
DR   PaxDb; Q9D0M3; -.
DR   PeptideAtlas; Q9D0M3; -.
DR   ProteomicsDB; 283990; -. [Q9D0M3-1]
DR   ProteomicsDB; 283991; -. [Q9D0M3-2]
DR   TopDownProteomics; Q9D0M3-1; -. [Q9D0M3-1]
DR   Antibodypedia; 810; 436 antibodies from 33 providers.
DR   DNASU; 66445; -.
DR   Ensembl; ENSMUST00000023210; ENSMUSP00000023210; ENSMUSG00000022551. [Q9D0M3-1]
DR   GeneID; 66445; -.
DR   KEGG; mmu:66445; -.
DR   UCSC; uc007wjs.1; mouse. [Q9D0M3-1]
DR   AGR; MGI:1913695; -.
DR   CTD; 1537; -.
DR   MGI; MGI:1913695; Cyc1.
DR   VEuPathDB; HostDB:ENSMUSG00000022551; -.
DR   eggNOG; KOG3052; Eukaryota.
DR   GeneTree; ENSGT00390000012445; -.
DR   HOGENOM; CLU_040334_1_0_1; -.
DR   InParanoid; Q9D0M3; -.
DR   OMA; WVKKFKW; -.
DR   OrthoDB; 275461at2759; -.
DR   PhylomeDB; Q9D0M3; -.
DR   TreeFam; TF314799; -.
DR   Reactome; R-MMU-611105; Respiratory electron transport.
DR   BioGRID-ORCS; 66445; 28 hits in 83 CRISPR screens.
DR   ChiTaRS; Cyc1; mouse.
DR   PRO; PR:Q9D0M3; -.
DR   Proteomes; UP000000589; Chromosome 15.
DR   RNAct; Q9D0M3; protein.
DR   Bgee; ENSMUSG00000022551; Expressed in ileal epithelium and 266 other tissues.
DR   ExpressionAtlas; Q9D0M3; baseline and differential.
DR   Genevisible; Q9D0M3; MM.
DR   GO; GO:0005743; C:mitochondrial inner membrane; HDA:MGI.
DR   GO; GO:0005750; C:mitochondrial respiratory chain complex III; ISO:MGI.
DR   GO; GO:0005739; C:mitochondrion; IDA:MGI.
DR   GO; GO:0020037; F:heme binding; IEA:InterPro.
DR   GO; GO:0046872; F:metal ion binding; IEA:UniProtKB-KW.
DR   GO; GO:0008121; F:ubiquinol-cytochrome-c reductase activity; IEA:UniProtKB-EC.
DR   GO; GO:0045333; P:cellular respiration; NAS:ComplexPortal.
DR   GO; GO:0006122; P:mitochondrial electron transport, ubiquinol to cytochrome c; IBA:GO_Central.
DR   GO; GO:0033762; P:response to glucagon; ISO:MGI.
DR   Gene3D; 1.10.760.10; Cytochrome c-like domain; 1.
DR   Gene3D; 1.20.5.100; Cytochrome c1, transmembrane anchor, C-terminal; 1.
DR   InterPro; IPR009056; Cyt_c-like_dom.
DR   InterPro; IPR036909; Cyt_c-like_dom_sf.
DR   InterPro; IPR002326; Cyt_c1.
DR   InterPro; IPR021157; Cyt_c1_TM_anchor_C.
DR   PANTHER; PTHR10266; CYTOCHROME C1; 1.
DR   PANTHER; PTHR10266:SF3; CYTOCHROME C1, HEME PROTEIN, MITOCHONDRIAL; 1.
DR   Pfam; PF02167; Cytochrom_C1; 1.
DR   PRINTS; PR00603; CYTOCHROMEC1.
DR   SUPFAM; SSF46626; Cytochrome c; 1.
DR   SUPFAM; SSF81496; Cytochrome c1 subunit of cytochrome bc1 complex (Ubiquinol-cytochrome c reductase), transmembrane anchor; 1.
DR   PROSITE; PS51007; CYTC; 1.
PE   1: Evidence at protein level;
KW   3D-structure; Alternative splicing; Direct protein sequencing;
KW   Electron transport; Heme; Iron; Membrane; Metal-binding; Mitochondrion;
KW   Mitochondrion inner membrane; Reference proteome; Respiratory chain;
KW   Transit peptide; Translocase; Transmembrane; Transmembrane helix;
KW   Transport.
FT   TRANSIT         1..84
FT                   /note="Mitochondrion"
FT                   /evidence="ECO:0000250"
FT   CHAIN           85..325
FT                   /note="Cytochrome c1, heme protein, mitochondrial"
FT                   /id="PRO_0000006555"
FT   TOPO_DOM        85..281
FT                   /note="Mitochondrial intermembrane"
FT                   /evidence="ECO:0000250|UniProtKB:P00125"
FT   TRANSMEM        282..315
FT                   /note="Helical"
FT                   /evidence="ECO:0000250|UniProtKB:P00125"
FT   TOPO_DOM        316..325
FT                   /note="Mitochondrial matrix"
FT                   /evidence="ECO:0000250|UniProtKB:P00125"
FT   DOMAIN          108..209
FT                   /note="Cytochrome c"
FT                   /evidence="ECO:0000255|PROSITE-ProRule:PRU00433"
FT   BINDING         121
FT                   /ligand="heme c"
FT                   /ligand_id="ChEBI:CHEBI:61717"
FT                   /note="covalent"
FT                   /evidence="ECO:0000250|UniProtKB:P00125"
FT   BINDING         124
FT                   /ligand="heme c"
FT                   /ligand_id="ChEBI:CHEBI:61717"
FT                   /note="covalent"
FT                   /evidence="ECO:0000250|UniProtKB:P00125"
FT   BINDING         125
FT                   /ligand="heme c"
FT                   /ligand_id="ChEBI:CHEBI:61717"
FT                   /ligand_part="Fe"
FT                   /ligand_part_id="ChEBI:CHEBI:18248"
FT                   /note="axial binding residue"
FT                   /evidence="ECO:0000250|UniProtKB:P00125"
FT   BINDING         244
FT                   /ligand="heme c"
FT                   /ligand_id="ChEBI:CHEBI:61717"
FT                   /ligand_part="Fe"
FT                   /ligand_part_id="ChEBI:CHEBI:18248"
FT                   /note="axial binding residue"
FT                   /evidence="ECO:0000250|UniProtKB:P00125"
FT   VAR_SEQ         1..59
FT                   /note="Missing (in isoform 2)"
FT                   /evidence="ECO:0000303|PubMed:15489334,
FT                   ECO:0000303|PubMed:16141072"
FT                   /id="VSP_025056"
FT   CONFLICT        44
FT                   /note="A -> R (in Ref. 1; BAB22380)"
FT                   /evidence="ECO:0000305"
FT   CONFLICT        318
FT                   /note="K -> R (in Ref. 1; BAB22380)"
FT                   /evidence="ECO:0000305"
FT   STRAND          100..102
FT                   /evidence="ECO:0007829|PDB:7O3H"
FT   HELIX           107..119
FT                   /evidence="ECO:0007829|PDB:7O3H"
FT   HELIX           121..123
FT                   /evidence="ECO:0007829|PDB:7O3H"
FT   HELIX           132..135
FT                   /evidence="ECO:0007829|PDB:7O3H"
FT   TURN            137..139
FT                   /evidence="ECO:0007829|PDB:7O3H"
FT   HELIX           142..150
FT                   /evidence="ECO:0007829|PDB:7O3H"
FT   STRAND          152..156
FT                   /evidence="ECO:0007829|PDB:7O3H"
FT   STRAND          165..168
FT                   /evidence="ECO:0007829|PDB:7O3H"
FT   STRAND          178..181
FT                   /evidence="ECO:0007829|PDB:7O3H"
FT   HELIX           182..188
FT                   /evidence="ECO:0007829|PDB:7O3H"
FT   TURN            189..191
FT                   /evidence="ECO:0007829|PDB:7O3H"
FT   TURN            200..202
FT                   /evidence="ECO:0007829|PDB:7O3H"
FT   TURN            205..207
FT                   /evidence="ECO:0007829|PDB:7O3H"
FT   HELIX           208..215
FT                   /evidence="ECO:0007829|PDB:7O3H"
FT   STRAND          240..244
FT                   /evidence="ECO:0007829|PDB:7O3H"
FT   HELIX           263..278
FT                   /evidence="ECO:0007829|PDB:7O3H"
FT   HELIX           282..312
FT                   /evidence="ECO:0007829|PDB:7O3H"
FT   TURN            313..316
FT                   /evidence="ECO:0007829|PDB:7O3H"
FT   STRAND          318..321
FT                   /evidence="ECO:0007829|PDB:7O3H"
SQ   SEQUENCE   325 AA;  35328 MW;  5F7DD8B78677E9BF CRC64;
     MAAAAASLRR TVLGPRGVGL PGASAPGLLG GARSRQLPLR TPQAVSLSSK SGPSRGRKVM
     LSALGMLAAG GAGLAVALHS AVSASDLELH PPSYPWSHRG LLSSLDHTSI RRGFQVYKQV
     CSSCHSMDYV AYRHLVGVCY TEEEAKALAE EVEVQDGPND DGEMFMRPGK LSDYFPKPYP
     NPEAARAANN GALPPDLSYI VRARHGGEDY VFSLLTGYCE PPTGVSLREG LYFNPYFPGQ
     AIGMAPPIYT EVLEYDDGTP ATMSQVAKDV ATFLRWASEP EHDHRKRMGL KMLLMMGLLL
     PLTYAMKRHK WSVLKSRKLA YRPPK
//
```

**Mascot:** http://www.matrixscience.com/


HNE (H) (+156.1150)
