# Supplementary material for: A common East-Asian ALDH2 mutation causes metabolic disorders and the therapeutic effect of ALDH2 activators
Source: Nat Commun. 2023 Sep 25;14:5971. doi: 10.1038/s41467-023-41570-6 (PMC10520061; doi:10.1038/s41467-023-41570-6)
Supplement: Supplementary file 4 — Supplementary Data 1 [file 41467_2023_41570_MOESM4_ESM.zip › Table S5c/Q9DB77/Q9DB77_KIO-1-k183.html]

Mascot Search Results: Q9DB77
 

# MASCOT Search Results

## Protein View: Q9DB77

### Cytochrome b-c1 complex subunit 2, mitochondrial OS=Mus musculus OX=10090 GN=Uqcrc2 PE=1 SV=1

|  |  |
| --- | --- |
| Database: | Mouse\_UniProt\_proteomes |
| Score: | 7792 |
| Monoisotopic mass (M_r_): | 48262 |
| Calculated pI: | 9.26 |

Sequence similarity is available as an NCBI BLAST search of Q9DB77 against nr.

### Search parameters

|  |  |
| --- | --- |
| MS data file: | `D:\LCMSMS\2023 Users' data\230529-1\230529-1-KIO-1.raw` |
| Enzyme: | Trypsin/P: cuts C-term side of KR. |
| Fixed modifications: | Carbamidomethyl (C) |
| Variable modifications: | Deamidated (NQ), HNE (C), HNE (H), HNE (K), Oxidation (M) |

### Protein sequence coverage: 72%

Matched peptides shown in ***bold red***.

|  |  |  |  |  |  |
| --- | --- | --- | --- | --- | --- |
| `1` | `MKLLSRAGSF` | `SRFYSLKVAP` | `KVKTSAAPGG` | `VPLQPQDLEF` | `TKLPNGLVIA` |
| `51` | `SLENYAPLSR` | `IGLFVKAGSR` | `YEDSNNLGTS` | `HLLRLASSLT` | `TKGASSFKIT` |
| `101` | `RGIEAVGGKL` | `SVTATRENMA` | `YTVEGIRSDI` | `EILMEFLLNV` | `TTAPEFRRWE` |
| `151` | `VAALRSQLKI` | `DKAVAFQNSQ` | `TRIIENLHDV` | `AYKNALANPL` | `YCPDYRMGKI` |
| `201` | `TSEELHYFVQ` | `NHFTSARMAL` | `VGLGVSHSVL` | `KQVAEQFLNM` | `RGGLGLAGAK` |
| `251` | `AKYRGGEIRE` | `QNGDNLVHAA` | `IVAESAAIGN` | `AEANAFSVLQ` | `HLLGAGPHIK` |
| `301` | `RGNNTTSLLS` | `QSVAKGSHQP` | `FDVSAFNASY` | `SDSGLFGIYT` | `ISQAAAAGEV` |
| `351` | `INAAYNQVKA` | `VAQGNLSSAD` | `VQAAKNKLKA` | `GYLMSVETSE` | `GFLSEIGSQA` |
| `401` | `LAAGSYMPPS` | `TVLQQIDSVA` | `DADVVKAAKK` | `FVSGKKSMAA` | `SGNLGHTPFL` |
| `451` | `DEL` |  |  |  |  |

Unformatted sequence string: 453 residues (for pasting into other applications).

|  |  |  |  |
| --- | --- | --- | --- |
| Sort by | residue number | increasing mass | decreasing mass |
| Show | matched peptides only | predicted peptides also |  |

| Query | Start | – | End | Observed | Mr(expt) | Mr(calc) | ppm | M | Score | Expect | Rank | U | Peptide |
| --- | --- | --- | --- | --- | --- | --- | --- | --- | --- | --- | --- | --- | --- |
| 194145 | 22 | – | 60 | 1023.5599 | 4090.2106 | 4090.2103 | 0.095 | 2 | 36 | 0.00047 | 1Score **> 33** indicates **identity** Score **> 15** indicates **homology** | U | K.VKTSAAPGGVPLQPQDLEFTKLPNGLVIASLENYAPLSR.I |
| 194149 | 22 | – | 60 | 1023.8056 | 4091.1932 | 4091.1943 | -0.26 | 2 | 35 | 0.00057 | 1Score **> 34** indicates **identity** Score **> 15** indicates **homology** | U | K.VKTSAAPGGVPLQPQDLEFTKLPNGLVIASLENYAPLSR.I  + Deamidated (NQ) |
| 194150 | 22 | – | 60 | 1364.7388 | 4091.1945 | 4091.1943 | 0.053 | 2 | 26 | 0.0039 | 1Score **> 34** indicates **identity** Score **> 14** indicates **homology** | U | K.VKTSAAPGGVPLQPQDLEFTKLPNGLVIASLENYAPLSR.I  + Deamidated (NQ) |
| 115865 | 24 | – | 42 | 978.5103 | 1955.0060 | 1955.0000 | 3.05 | 0 | 28 | 0.0022 | 1Score **> 36** indicates **identity** Score **> 14** indicates **homology** | U | K.TSAAPGGVPLQPQDLEFTK.L |
| 115866 | 24 | – | 42 | 978.5110 | 1955.0074 | 1955.0000 | 3.78 | 0 | 50 | 1.9e-05 | 1Score **> 36** indicates **identity** Score **> 16** indicates **homology** | U | K.TSAAPGGVPLQPQDLEFTK.L |
| 192895 | 24 | – | 60 | 966.7688 | 3863.0460 | 3863.0469 | -0.21 | 1 | 36 | 0.0004 | 1Score **> 36** indicates **identity** Score **> 15** indicates **homology** | U | K.TSAAPGGVPLQPQDLEFTKLPNGLVIASLENYAPLSR.I |
| 192896 | 24 | – | 60 | 966.7705 | 3863.0528 | 3863.0469 | 1.53 | 1 | 29 | 0.0021 | 1Score **> 35** indicates **identity** Score **> 14** indicates **homology** | U | K.TSAAPGGVPLQPQDLEFTKLPNGLVIASLENYAPLSR.I |
| 192897 | 24 | – | 60 | 1288.6916 | 3863.0529 | 3863.0469 | 1.57 | 1 | 34 | 0.0006 | 1Score **> 35** indicates **identity** Score **> 15** indicates **homology** | U | K.TSAAPGGVPLQPQDLEFTKLPNGLVIASLENYAPLSR.I |
| 192900 | 24 | – | 60 | 1289.0245 | 3864.0517 | 3864.0309 | 5.40 | 1 | 35 | 0.00047 | 1Score **> 36** indicates **identity** Score **> 15** indicates **homology** | U | K.TSAAPGGVPLQPQDLEFTKLPNGLVIASLENYAPLSR.I  + Deamidated (NQ) |
| 192902 | 24 | – | 60 | 967.0226 | 3864.0613 | 3864.0309 | 7.87 | 1 | 34 | 0.00067 | 1Score **> 35** indicates **identity** Score **> 15** indicates **homology** | U | K.TSAAPGGVPLQPQDLEFTKLPNGLVIASLENYAPLSR.I  + Deamidated (NQ) |
| 192905 | 24 | – | 60 | 1289.3540 | 3865.0401 | 3865.0149 | 6.52 | 1 | 24 | 0.0054 | 1Score **> 36** indicates **identity** Score **> 14** indicates **homology** | U | K.TSAAPGGVPLQPQDLEFTKLPNGLVIASLENYAPLSR.I  + 2 Deamidated (NQ) |
| 192906 | 24 | – | 60 | 1289.3578 | 3865.0515 | 3865.0149 | 9.46 | 1 | 25 | 0.0045 | 1Score **> 35** indicates **identity** Score **> 14** indicates **homology** | U | K.TSAAPGGVPLQPQDLEFTKLPNGLVIASLENYAPLSR.I  + 2 Deamidated (NQ) |
| 113225 | 43 | – | 60 | 964.0370 | 1926.0595 | 1926.0574 | 1.09 | 0 | 58 | 3.6e-06 | 1Score **> 35** indicates **identity** Score **> 16** indicates **homology** | U | K.LPNGLVIASLENYAPLSR.I |
| 113226 | 43 | – | 60 | 643.0271 | 1926.0595 | 1926.0574 | 1.09 | 0 | 34 | 0.00069 | 1Score **> 35** indicates **identity** Score **> 15** indicates **homology** | U | K.LPNGLVIASLENYAPLSR.I |
| 113326 | 43 | – | 60 | 964.5229 | 1927.0313 | 1927.0414 | -5.25 | 0 | 58 | 1.3e-05 | 1Score **> 36** indicates **identity** Score **> 21** indicates **homology** | U | K.LPNGLVIASLENYAPLSR.I  + Deamidated (NQ) |
| 119503 | 67 | – | 84 | 995.4907 | 1988.9668 | 1988.9664 | 0.23 | 1 | 60 | 3.6e-06 | 1Score **> 35** indicates **identity** Score **> 18** indicates **homology** | U | K.AGSRYEDSNNLGTSHLLR.L |
| 119504 | 67 | – | 84 | 663.9965 | 1988.9675 | 1988.9664 | 0.58 | 1 | 30 | 0.0016 | 1Score **> 35** indicates **identity** Score **> 14** indicates **homology** | U | K.AGSRYEDSNNLGTSHLLR.L |
| 119505 | 67 | – | 84 | 498.2492 | 1988.9675 | 1988.9664 | 0.58 | 1 | 35 | 0.00057 | 1Score **> 35** indicates **identity** Score **> 15** indicates **homology** | U | K.AGSRYEDSNNLGTSHLLR.L |
| 119506 | 67 | – | 84 | 663.9966 | 1988.9681 | 1988.9664 | 0.86 | 1 | 57 | 4.1e-06 | 1Score **> 35** indicates **identity** Score **> 16** indicates **homology** | U | K.AGSRYEDSNNLGTSHLLR.L |
| 119507 | 67 | – | 84 | 995.4913 | 1988.9681 | 1988.9664 | 0.86 | 1 | 77 | 1.4e-07 | 1Score **> 35** indicates **identity** Score **> 21** indicates **homology** | U | K.AGSRYEDSNNLGTSHLLR.L |
| 119508 | 67 | – | 84 | 663.9967 | 1988.9682 | 1988.9664 | 0.92 | 1 | 65 | 8.2e-07 | 1Score **> 35** indicates **identity** Score **> 17** indicates **homology** | U | K.AGSRYEDSNNLGTSHLLR.L |
| 119509 | 67 | – | 84 | 498.2494 | 1988.9685 | 1988.9664 | 1.08 | 1 | 44 | 7.2e-05 | 1Score **> 35** indicates **identity** Score **> 15** indicates **homology** | U | K.AGSRYEDSNNLGTSHLLR.L |
| 119510 | 67 | – | 84 | 498.2494 | 1988.9687 | 1988.9664 | 1.14 | 1 | 57 | 4.3e-06 | 1Score **> 35** indicates **identity** Score **> 16** indicates **homology** | U | K.AGSRYEDSNNLGTSHLLR.L |
| 119511 | 67 | – | 84 | 995.4917 | 1988.9689 | 1988.9664 | 1.25 | 1 | 49 | 0.00011 | 1Score **> 35** indicates **identity** Score **> 22** indicates **homology** | U | K.AGSRYEDSNNLGTSHLLR.L |
| 119512 | 67 | – | 84 | 498.2496 | 1988.9694 | 1988.9664 | 1.51 | 1 | 46 | 5.1e-05 | 1Score **> 35** indicates **identity** Score **> 16** indicates **homology** | U | K.AGSRYEDSNNLGTSHLLR.L |
| 119513 | 67 | – | 84 | 498.2497 | 1988.9697 | 1988.9664 | 1.65 | 1 | 60 | 2.8e-06 | 1Score **> 35** indicates **identity** Score **> 17** indicates **homology** | U | K.AGSRYEDSNNLGTSHLLR.L |
| 119514 | 67 | – | 84 | 663.9972 | 1988.9698 | 1988.9664 | 1.71 | 1 | 60 | 2.5e-06 | 1Score **> 35** indicates **identity** Score **> 16** indicates **homology** | U | K.AGSRYEDSNNLGTSHLLR.L |
| 119515 | 67 | – | 84 | 498.2498 | 1988.9700 | 1988.9664 | 1.80 | 1 | 16 | 0.031 | 1Score **> 35** indicates **identity** Score **> 14** indicates **homology** | U | K.AGSRYEDSNNLGTSHLLR.L |
| 119516 | 67 | – | 84 | 663.9973 | 1988.9700 | 1988.9664 | 1.84 | 1 | 54 | 8.1e-06 | 1Score **> 35** indicates **identity** Score **> 16** indicates **homology** | U | K.AGSRYEDSNNLGTSHLLR.L |
| 119517 | 67 | – | 84 | 498.2498 | 1988.9701 | 1988.9664 | 1.85 | 1 | 49 | 2.3e-05 | 1Score **> 35** indicates **identity** Score **> 16** indicates **homology** | U | K.AGSRYEDSNNLGTSHLLR.L |
| 119518 | 67 | – | 84 | 663.9974 | 1988.9702 | 1988.9664 | 1.94 | 1 | 59 | 3.5e-06 | 1Score **> 35** indicates **identity** Score **> 17** indicates **homology** | U | K.AGSRYEDSNNLGTSHLLR.L |
| 119519 | 67 | – | 84 | 498.2499 | 1988.9703 | 1988.9664 | 1.99 | 1 | 24 | 0.0052 | 1Score **> 35** indicates **identity** Score **> 14** indicates **homology** | U | K.AGSRYEDSNNLGTSHLLR.L |
| 119520 | 67 | – | 84 | 498.2499 | 1988.9705 | 1988.9664 | 2.05 | 1 | 58 | 3.5e-06 | 1Score **> 35** indicates **identity** Score **> 16** indicates **homology** | U | K.AGSRYEDSNNLGTSHLLR.L |
| 119521 | 67 | – | 84 | 498.2499 | 1988.9706 | 1988.9664 | 2.10 | 1 | 49 | 2.3e-05 | 1Score **> 35** indicates **identity** Score **> 16** indicates **homology** | U | K.AGSRYEDSNNLGTSHLLR.L |
| 119522 | 67 | – | 84 | 663.9978 | 1988.9715 | 1988.9664 | 2.57 | 1 | 26 | 0.0033 | 1Score **> 35** indicates **identity** Score **> 14** indicates **homology** | U | K.AGSRYEDSNNLGTSHLLR.L |
| 119525 | 67 | – | 84 | 663.9991 | 1988.9755 | 1988.9664 | 4.60 | 1 | 30 | 0.0014 | 1Score **> 35** indicates **identity** Score **> 14** indicates **homology** | U | K.AGSRYEDSNNLGTSHLLR.L |
| 119629 | 67 | – | 84 | 664.3243 | 1989.9511 | 1989.9504 | 0.36 | 1 | 51 | 1.7e-05 | 1Score **> 35** indicates **identity** Score **> 16** indicates **homology** | U | K.AGSRYEDSNNLGTSHLLR.L  + Deamidated (NQ) |
| 119630 | 67 | – | 84 | 498.4952 | 1989.9517 | 1989.9504 | 0.64 | 1 | 43 | 8.9e-05 | 1Score **> 35** indicates **identity** Score **> 15** indicates **homology** | U | K.AGSRYEDSNNLGTSHLLR.L  + Deamidated (NQ) |
| 119632 | 67 | – | 84 | 664.3248 | 1989.9524 | 1989.9504 | 1.03 | 1 | 47 | 3.7e-05 | 1Score **> 35** indicates **identity** Score **> 15** indicates **homology** | U | K.AGSRYEDSNNLGTSHLLR.L  + Deamidated (NQ) |
| 77862 | 71 | – | 84 | 540.2648 | 1617.7726 | 1617.7747 | -1.27 | 0 | 69 | 3.7e-07 | 1Score **> 33** indicates **identity** Score **> 17** indicates **homology** | U | R.YEDSNNLGTSHLLR.L |
| 77864 | 71 | – | 84 | 540.2652 | 1617.7739 | 1617.7747 | -0.50 | 0 | 22 | 0.0082 | 1Score **> 33** indicates **identity** Score **> 14** indicates **homology** | U | R.YEDSNNLGTSHLLR.L |
| 77866 | 71 | – | 84 | 540.2653 | 1617.7740 | 1617.7747 | -0.39 | 0 | 73 | 2e-07 | 1Score **> 33** indicates **identity** Score **> 19** indicates **homology** | U | R.YEDSNNLGTSHLLR.L |
| 77867 | 71 | – | 84 | 540.2654 | 1617.7745 | 1617.7747 | -0.11 | 0 | 65 | 1.1e-06 | 1Score **> 33** indicates **identity** Score **> 18** indicates **homology** | U | R.YEDSNNLGTSHLLR.L |
| 77868 | 71 | – | 84 | 540.2661 | 1617.7764 | 1617.7747 | 1.06 | 0 | 23 | 0.024 | 1Score **> 33** indicates **identity** Score **> 19** indicates **homology** | U | R.YEDSNNLGTSHLLR.L |
| 77870 | 71 | – | 84 | 540.2663 | 1617.7770 | 1617.7747 | 1.45 | 0 | 73 | 2.2e-07 | 1Score **> 33** indicates **identity** Score **> 19** indicates **homology** | U | R.YEDSNNLGTSHLLR.L |
| 77871 | 71 | – | 84 | 809.8959 | 1617.7773 | 1617.7747 | 1.62 | 0 | 99 | 4.8e-10 | 1Score **> 34** indicates **identity** Score **> 19** indicates **homology** | U | R.YEDSNNLGTSHLLR.L |
| 77872 | 71 | – | 84 | 809.8960 | 1617.7774 | 1617.7747 | 1.68 | 0 | 99 | 5.3e-10 | 1Score **> 34** indicates **identity** Score **> 19** indicates **homology** | U | R.YEDSNNLGTSHLLR.L |
| 77873 | 71 | – | 84 | 540.2664 | 1617.7774 | 1617.7747 | 1.69 | 0 | 75 | 5.9e-07 | 1Score **> 34** indicates **identity** Score **> 25** indicates **homology** | U | R.YEDSNNLGTSHLLR.L |
| 77874 | 71 | – | 84 | 540.2665 | 1617.7778 | 1617.7747 | 1.92 | 0 | 21 | 0.011 | 1Score **> 34** indicates **identity** Score **> 14** indicates **homology** | U | R.YEDSNNLGTSHLLR.L |
| 77877 | 71 | – | 84 | 540.2676 | 1617.7810 | 1617.7747 | 3.93 | 0 | 42 | 0.00016 | 1Score **> 34** indicates **identity** Score **> 16** indicates **homology** | U | R.YEDSNNLGTSHLLR.L |
| 77878 | 71 | – | 84 | 540.2682 | 1617.7827 | 1617.7747 | 4.98 | 0 | 24 | 0.0065 | 1Score **> 34** indicates **identity** Score **> 14** indicates **homology** | U | R.YEDSNNLGTSHLLR.L |
| 77879 | 71 | – | 84 | 540.2683 | 1617.7830 | 1617.7747 | 5.18 | 0 | 27 | 0.0031 | 1Score **> 34** indicates **identity** Score **> 15** indicates **homology** | U | R.YEDSNNLGTSHLLR.L |
| 78102 | 71 | – | 84 | 540.5940 | 1618.7603 | 1618.7587 | 0.97 | 0 | 50 | 2.1e-05 | 1Score **> 33** indicates **identity** Score **> 16** indicates **homology** | U | R.YEDSNNLGTSHLLR.L  + Deamidated (NQ) |
| 78103 | 71 | – | 84 | 540.5950 | 1618.7630 | 1618.7587 | 2.69 | 0 | 16 | 0.032 | 1Score **> 33** indicates **identity** Score **> 13** indicates **homology** | U | R.YEDSNNLGTSHLLR.L  + Deamidated (NQ) |
| 4084 | 85 | – | 92 | 410.7424 | 819.4703 | 819.4702 | 0.17 | 0 | 15 | 0.035 | 1Score **> 22** indicates **identity** Score **> 13** indicates **homology** | U | R.LASSLTTK.G |
| 4085 | 85 | – | 92 | 410.7425 | 819.4704 | 819.4702 | 0.28 | 0 | 30 | 0.0019 | 1Score **> 22** indicates **identity** Score **> 15** indicates **homology** | U | R.LASSLTTK.G |
| 4087 | 85 | – | 92 | 410.7430 | 819.4714 | 819.4702 | 1.56 | 0 | 37 | 0.00077 | 1Score **> 22** indicates **identity** Score **> 18** indicates **homology** | U | R.LASSLTTK.G |
| 57137 | 102 | – | 116 | 486.9479 | 1457.8220 | 1457.8202 | 1.21 | 1 | 54 | 9.1e-06 | 1Score **> 34** indicates **identity** Score **> 16** indicates **homology** | U | R.GIEAVGGKLSVTATR.E |
| 57158 | 102 | – | 116 | 486.9492 | 1457.8257 | 1457.8202 | 3.79 | 1 | 29 | 0.0018 | 1Score **> 34** indicates **identity** Score **> 14** indicates **homology** | U | R.GIEAVGGKLSVTATR.E |
| 1401 | 110 | – | 116 | 374.2213 | 746.4281 | 746.4286 | -0.79 | 0 | 26 | 0.0076 | 1Score **> 23** indicates **identity** Score **> 18** indicates **homology** | U | K.LSVTATR.E |
| 1403 | 110 | – | 116 | 374.2220 | 746.4294 | 746.4286 | 1.07 | 0 | 55 | 3.7e-05 | 1Score **> 24** indicates **identity** | U | K.LSVTATR.E |
| 121368 | 110 | – | 127 | 671.0091 | 2010.0055 | 2010.0204 | -7.44 | 1 | 24 | 0.0059 | 1Score **> 36** indicates **identity** Score **> 14** indicates **homology** | U | K.LSVTATRENMAYTVEGIR.S |
| 38606 | 117 | – | 127 | 641.8110 | 1281.6075 | 1281.6023 | 4.04 | 0 | 58 | 4e-06 | 1Score **> 31** indicates **identity** Score **> 16** indicates **homology** | U | R.ENMAYTVEGIR.S |
| 38609 | 117 | – | 127 | 641.8132 | 1281.6119 | 1281.6023 | 7.49 | 0 | 53 | 0.00039 | 1Score **> 31** indicates **identity** | U | R.ENMAYTVEGIR.S |
| 13130 | 148 | – | 155 | 500.7885 | 999.5625 | 999.5614 | 1.10 | 1 | 42 | 0.0056 | 1Score **> 32** indicates **identity** | U | R.RWEVAALR.S |
| 13131 | 148 | – | 155 | 500.7885 | 999.5625 | 999.5614 | 1.14 | 1 | 28 | 0.044 | 1Score **> 32** indicates **identity** Score **> 27** indicates **homology** | U | R.RWEVAALR.S |
| 13132 | 148 | – | 155 | 500.7885 | 999.5625 | 999.5614 | 1.16 | 1 | 34 | 0.036 | 1Score **> 32** indicates **identity** | U | R.RWEVAALR.S |
| 13133 | 148 | – | 155 | 500.7886 | 999.5626 | 999.5614 | 1.26 | 1 | 45 | 0.0033 | 1Score **> 32** indicates **identity** | U | R.RWEVAALR.S |
| 13134 | 148 | – | 155 | 500.7887 | 999.5629 | 999.5614 | 1.57 | 1 | 31 | 0.021 | 1Score **> 32** indicates **identity** Score **> 27** indicates **homology** | U | R.RWEVAALR.S |
| 13136 | 148 | – | 155 | 500.7890 | 999.5634 | 999.5614 | 2.07 | 1 | 53 | 0.00047 | 1Score **> 32** indicates **identity** | U | R.RWEVAALR.S |
| 13138 | 148 | – | 155 | 500.7895 | 999.5645 | 999.5614 | 3.16 | 1 | 46 | 0.0025 | 1Score **> 32** indicates **identity** | U | R.RWEVAALR.S |
| 113910 | 156 | – | 172 | 484.2675 | 1933.0409 | 1933.0381 | 1.44 | 2 | 45 | 6.6e-05 | 1Score **> 35** indicates **identity** Score **> 15** indicates **homology** | U | R.SQLKIDKAVAFQNSQTR.I |
| 113911 | 156 | – | 172 | 645.3547 | 1933.0424 | 1933.0381 | 2.20 | 2 | 66 | 6.8e-07 | 1Score **> 35** indicates **identity** Score **> 17** indicates **homology** | U | R.SQLKIDKAVAFQNSQTR.I |
| 113913 | 156 | – | 172 | 967.5287 | 1933.0429 | 1933.0381 | 2.47 | 2 | 67 | 7.8e-07 | 1Score **> 35** indicates **identity** Score **> 18** indicates **homology** | U | R.SQLKIDKAVAFQNSQTR.I |
| 113914 | 156 | – | 172 | 484.2681 | 1933.0434 | 1933.0381 | 2.75 | 2 | 31 | 0.0012 | 1Score **> 35** indicates **identity** Score **> 14** indicates **homology** | U | R.SQLKIDKAVAFQNSQTR.I |
| 113915 | 156 | – | 172 | 967.5293 | 1933.0440 | 1933.0381 | 3.05 | 2 | 58 | 5.6e-06 | 1Score **> 35** indicates **identity** Score **> 18** indicates **homology** | U | R.SQLKIDKAVAFQNSQTR.I |
| 113916 | 156 | – | 172 | 645.3553 | 1933.0441 | 1933.0381 | 3.13 | 2 | 51 | 2.4e-05 | 1Score **> 35** indicates **identity** Score **> 17** indicates **homology** | U | R.SQLKIDKAVAFQNSQTR.I |
| 59205 | 160 | – | 172 | 739.3902 | 1476.7659 | 1476.7685 | -1.72 | 1 | 18 | 0.021 | 1Score **> 35** indicates **identity** Score **> 14** indicates **homology** | U | K.IDKAVAFQNSQTR.I |
| 59208 | 160 | – | 172 | 493.2632 | 1476.7679 | 1476.7685 | -0.39 | 1 | 48 | 2.9e-05 | 1Score **> 35** indicates **identity** Score **> 16** indicates **homology** | U | K.IDKAVAFQNSQTR.I |
| 59209 | 160 | – | 172 | 739.3914 | 1476.7682 | 1476.7685 | -0.18 | 1 | 53 | 7.7e-05 | 1Score **> 35** indicates **identity** Score **> 24** indicates **homology** | U | K.IDKAVAFQNSQTR.I |
| 59210 | 160 | – | 172 | 493.2634 | 1476.7684 | 1476.7685 | -0.025 | 1 | 59 | 2.9e-06 | 1Score **> 35** indicates **identity** Score **> 16** indicates **homology** | U | K.IDKAVAFQNSQTR.I |
| 59211 | 160 | – | 172 | 493.2634 | 1476.7685 | 1476.7685 | 0.018 | 1 | 54 | 7.9e-06 | 1Score **> 35** indicates **identity** Score **> 16** indicates **homology** | U | K.IDKAVAFQNSQTR.I |
| 22602 | 163 | – | 172 | 561.2887 | 1120.5628 | 1120.5625 | 0.27 | 0 | 59 | 3e-06 | 1Score **> 32** indicates **identity** Score **> 16** indicates **homology** | U | K.AVAFQNSQTR.I |
| 22603 | 163 | – | 172 | 561.2890 | 1120.5634 | 1120.5625 | 0.78 | 0 | 45 | 5.6e-05 | 1Score **> 32** indicates **identity** Score **> 15** indicates **homology** | U | K.AVAFQNSQTR.I |
| 22681 | 163 | – | 172 | 561.7808 | 1121.5471 | 1121.5465 | 0.49 | 0 | 22 | 0.0079 | 1Score **> 31** indicates **identity** Score **> 14** indicates **homology** | U | K.AVAFQNSQTR.I  + Deamidated (NQ) |
| 42004 | 173 | – | 183 | 438.9062 | 1313.6967 | 1313.6979 | -0.91 | 0 | 27 | 0.0033 | 1Score **> 34** indicates **identity** Score **> 15** indicates **homology** | U | R.IIENLHDVAYK.N |
| 42012 | 173 | – | 183 | 438.9068 | 1313.6984 | 1313.6979 | 0.39 | 0 | 15 | 0.038 | 1Score **> 34** indicates **identity** Score **> 13** indicates **homology** | U | R.IIENLHDVAYK.N |
| 42013 | 173 | – | 183 | 438.9069 | 1313.6987 | 1313.6979 | 0.63 | 0 | 37 | 0.0004 | 1Score **> 34** indicates **identity** Score **> 16** indicates **homology** | U | R.IIENLHDVAYK.N |
| 42015 | 173 | – | 183 | 438.9070 | 1313.6992 | 1313.6979 | 1.01 | 0 | 41 | 0.00029 | 1Score **> 34** indicates **identity** Score **> 18** indicates **homology** | U | R.IIENLHDVAYK.N |
| 42017 | 173 | – | 183 | 657.8570 | 1313.6994 | 1313.6979 | 1.14 | 0 | 56 | 2.3e-05 | 1Score **> 34** indicates **identity** Score **> 22** indicates **homology** | U | R.IIENLHDVAYK.N |
| 42018 | 173 | – | 183 | 657.8571 | 1313.6997 | 1313.6979 | 1.37 | 0 | 72 | 2.1e-06 | 1Score **> 34** indicates **identity** Score **> 28** indicates **homology** | U | R.IIENLHDVAYK.N |
| 42019 | 173 | – | 183 | 438.9073 | 1313.7001 | 1313.6979 | 1.65 | 0 | 44 | 0.0002 | 1Score **> 34** indicates **identity** Score **> 19** indicates **homology** | U | R.IIENLHDVAYK.N |
| 42020 | 173 | – | 183 | 438.9073 | 1313.7002 | 1313.6979 | 1.73 | 0 | 42 | 0.0002 | 1Score **> 34** indicates **identity** Score **> 17** indicates **homology** | U | R.IIENLHDVAYK.N |
| 42022 | 173 | – | 183 | 438.9076 | 1313.7009 | 1313.6979 | 2.30 | 0 | 21 | 0.011 | 1Score **> 33** indicates **identity** Score **> 14** indicates **homology** | U | R.IIENLHDVAYK.N |
| 173934 | 173 | – | 196 | 954.8125 | 2861.4156 | 2861.4170 | -0.50 | 1 | 52 | 1.4e-05 | 1Score **> 37** indicates **identity** Score **> 16** indicates **homology** | U | R.IIENLHDVAYKNALANPLYCPDYR.M |
| 173936 | 173 | – | 196 | 716.3622 | 2861.4196 | 2861.4170 | 0.91 | 1 | 45 | 6.6e-05 | 1Score **> 37** indicates **identity** Score **> 15** indicates **homology** | U | R.IIENLHDVAYKNALANPLYCPDYR.M |
| 173937 | 173 | – | 196 | 716.3622 | 2861.4198 | 2861.4170 | 0.99 | 1 | 42 | 0.00012 | 1Score **> 37** indicates **identity** Score **> 15** indicates **homology** | U | R.IIENLHDVAYKNALANPLYCPDYR.M |
| 173938 | 173 | – | 196 | 954.8141 | 2861.4205 | 2861.4170 | 1.22 | 1 | 66 | 7.3e-07 | 1Score **> 37** indicates **identity** Score **> 17** indicates **homology** | U | R.IIENLHDVAYKNALANPLYCPDYR.M |
| 173939 | 173 | – | 196 | 716.3624 | 2861.4206 | 2861.4170 | 1.26 | 1 | 48 | 3.3e-05 | 1Score **> 37** indicates **identity** Score **> 15** indicates **homology** | U | R.IIENLHDVAYKNALANPLYCPDYR.M |
| 173940 | 173 | – | 196 | 954.8144 | 2861.4215 | 2861.4170 | 1.57 | 1 | 84 | 2.4e-08 | 1Score **> 37** indicates **identity** Score **> 20** indicates **homology** | U | R.IIENLHDVAYKNALANPLYCPDYR.M |
| 173941 | 173 | – | 196 | 954.8145 | 2861.4216 | 2861.4170 | 1.61 | 1 | 89 | 1.3e-08 | 1Score **> 37** indicates **identity** Score **> 23** indicates **homology** | U | R.IIENLHDVAYKNALANPLYCPDYR.M |
| 179158 | 173 | – | 196 | 1007.5014 | 3019.4825 | 3019.5000 | -5.81 | 1 | 31 | 0.0013 | 1Score **> 37** indicates **identity** Score **> 14** indicates **homology** | U | R.IIENLHDVAYKNALANPLYCPDYR.M  + 2 Deamidated (NQ); HNE (K) |
| 70657 | 184 | – | 196 | 783.8697 | 1565.7248 | 1565.7296 | -3.11 | 0 | 67 | 5.5e-07 | 1Score **> 32** indicates **identity** Score **> 17** indicates **homology** | U | K.NALANPLYCPDYR.M |
| 70659 | 184 | – | 196 | 783.8719 | 1565.7292 | 1565.7296 | -0.30 | 0 | 55 | 6.7e-06 | 1Score **> 32** indicates **identity** Score **> 16** indicates **homology** | U | K.NALANPLYCPDYR.M |
| 70660 | 184 | – | 196 | 783.8720 | 1565.7295 | 1565.7296 | -0.11 | 0 | 76 | 6.9e-08 | 1Score **> 32** indicates **identity** Score **> 17** indicates **homology** | U | K.NALANPLYCPDYR.M |
| 70661 | 184 | – | 196 | 783.8721 | 1565.7297 | 1565.7296 | 0.037 | 0 | 90 | 3.3e-09 | 1Score **> 32** indicates **identity** Score **> 18** indicates **homology** | U | K.NALANPLYCPDYR.M |
| 70663 | 184 | – | 196 | 783.8722 | 1565.7299 | 1565.7296 | 0.19 | 0 | 67 | 4.8e-07 | 1Score **> 32** indicates **identity** Score **> 17** indicates **homology** | U | K.NALANPLYCPDYR.M |
| 70664 | 184 | – | 196 | 783.8728 | 1565.7311 | 1565.7296 | 0.96 | 0 | 67 | 5.1e-07 | 1Score **> 32** indicates **identity** Score **> 17** indicates **homology** | U | K.NALANPLYCPDYR.M |
| 70666 | 184 | – | 196 | 783.8733 | 1565.7321 | 1565.7296 | 1.58 | 0 | 32 | 0.001 | 1Score **> 32** indicates **identity** Score **> 14** indicates **homology** | U | K.NALANPLYCPDYR.M |
| 70667 | 184 | – | 196 | 783.8733 | 1565.7321 | 1565.7296 | 1.60 | 0 | 85 | 1e-08 | 1Score **> 32** indicates **identity** Score **> 18** indicates **homology** | U | K.NALANPLYCPDYR.M |
| 70668 | 184 | – | 196 | 783.8734 | 1565.7322 | 1565.7296 | 1.67 | 0 | 35 | 0.0005 | 1Score **> 32** indicates **identity** Score **> 15** indicates **homology** | U | K.NALANPLYCPDYR.M |
| 70669 | 184 | – | 196 | 783.8735 | 1565.7325 | 1565.7296 | 1.81 | 0 | 48 | 3.4e-05 | 1Score **> 32** indicates **identity** Score **> 15** indicates **homology** | U | K.NALANPLYCPDYR.M |
| 137311 | 200 | – | 217 | 727.0194 | 2178.0365 | 2178.0494 | -5.92 | 0 | 22 | 0.0087 | 1Score **> 35** indicates **identity** Score **> 14** indicates **homology** | U | K.ITSEELHYFVQNHFTSAR.M |
| 137312 | 200 | – | 217 | 727.0197 | 2178.0373 | 2178.0494 | -5.55 | 0 | 19 | 0.016 | 1Score **> 35** indicates **identity** Score **> 14** indicates **homology** | U | K.ITSEELHYFVQNHFTSAR.M |
| 137315 | 200 | – | 217 | 545.5181 | 2178.0434 | 2178.0494 | -2.78 | 0 | 35 | 0.00052 | 1Score **> 36** indicates **identity** Score **> 15** indicates **homology** | U | K.ITSEELHYFVQNHFTSAR.M |
| 137316 | 200 | – | 217 | 727.0219 | 2178.0438 | 2178.0494 | -2.57 | 0 | 36 | 0.00044 | 1Score **> 36** indicates **identity** Score **> 15** indicates **homology** | U | K.ITSEELHYFVQNHFTSAR.M |
| 137318 | 200 | – | 217 | 727.0228 | 2178.0464 | 2178.0494 | -1.36 | 0 | 26 | 0.0036 | 1Score **> 36** indicates **identity** Score **> 14** indicates **homology** | U | K.ITSEELHYFVQNHFTSAR.M |
| 137319 | 200 | – | 217 | 545.5192 | 2178.0475 | 2178.0494 | -0.86 | 0 | 28 | 0.0024 | 1Score **> 36** indicates **identity** Score **> 14** indicates **homology** | U | K.ITSEELHYFVQNHFTSAR.M |
| 137320 | 200 | – | 217 | 727.0232 | 2178.0478 | 2178.0494 | -0.75 | 0 | 37 | 0.00033 | 1Score **> 36** indicates **identity** Score **> 15** indicates **homology** | U | K.ITSEELHYFVQNHFTSAR.M |
| 137321 | 200 | – | 217 | 727.0233 | 2178.0479 | 2178.0494 | -0.68 | 0 | 33 | 0.00076 | 1Score **> 36** indicates **identity** Score **> 15** indicates **homology** | U | K.ITSEELHYFVQNHFTSAR.M |
| 137323 | 200 | – | 217 | 545.5193 | 2178.0482 | 2178.0494 | -0.54 | 0 | 37 | 0.00034 | 1Score **> 36** indicates **identity** Score **> 15** indicates **homology** | U | K.ITSEELHYFVQNHFTSAR.M |
| 137324 | 200 | – | 217 | 545.5196 | 2178.0493 | 2178.0494 | -0.037 | 0 | 34 | 0.0006 | 1Score **> 36** indicates **identity** Score **> 15** indicates **homology** | U | K.ITSEELHYFVQNHFTSAR.M |
| 137325 | 200 | – | 217 | 545.5196 | 2178.0494 | 2178.0494 | -0.013 | 0 | 28 | 0.0023 | 1Score **> 36** indicates **identity** Score **> 14** indicates **homology** | U | K.ITSEELHYFVQNHFTSAR.M |
| 137326 | 200 | – | 217 | 727.0237 | 2178.0494 | 2178.0494 | 0.0037 | 0 | 21 | 0.0099 | 1Score **> 36** indicates **identity** Score **> 14** indicates **homology** | U | K.ITSEELHYFVQNHFTSAR.M |
| 137327 | 200 | – | 217 | 727.0239 | 2178.0497 | 2178.0494 | 0.15 | 0 | 31 | 0.0012 | 1Score **> 36** indicates **identity** Score **> 14** indicates **homology** | U | K.ITSEELHYFVQNHFTSAR.M |
| 137328 | 200 | – | 217 | 545.5197 | 2178.0498 | 2178.0494 | 0.20 | 0 | 23 | 0.0077 | 1Score **> 36** indicates **identity** Score **> 14** indicates **homology** | U | K.ITSEELHYFVQNHFTSAR.M |
| 137329 | 200 | – | 217 | 727.0239 | 2178.0499 | 2178.0494 | 0.24 | 0 | 27 | 0.0028 | 1Score **> 36** indicates **identity** Score **> 14** indicates **homology** | U | K.ITSEELHYFVQNHFTSAR.M |
| 137330 | 200 | – | 217 | 727.0241 | 2178.0504 | 2178.0494 | 0.45 | 0 | 33 | 0.00074 | 1Score **> 36** indicates **identity** Score **> 15** indicates **homology** | U | K.ITSEELHYFVQNHFTSAR.M |
| 137331 | 200 | – | 217 | 727.0241 | 2178.0504 | 2178.0494 | 0.45 | 0 | 50 | 2e-05 | 1Score **> 36** indicates **identity** Score **> 16** indicates **homology** | U | K.ITSEELHYFVQNHFTSAR.M |
| 137332 | 200 | – | 217 | 545.5199 | 2178.0505 | 2178.0494 | 0.49 | 0 | 47 | 4e-05 | 1Score **> 36** indicates **identity** Score **> 15** indicates **homology** | U | K.ITSEELHYFVQNHFTSAR.M |
| 137333 | 200 | – | 217 | 545.5200 | 2178.0507 | 2178.0494 | 0.61 | 0 | 33 | 0.00073 | 1Score **> 36** indicates **identity** Score **> 15** indicates **homology** | U | K.ITSEELHYFVQNHFTSAR.M |
| 137334 | 200 | – | 217 | 727.0243 | 2178.0511 | 2178.0494 | 0.76 | 0 | 18 | 0.02 | 1Score **> 36** indicates **identity** Score **> 14** indicates **homology** | U | K.ITSEELHYFVQNHFTSAR.M |
| 137335 | 200 | – | 217 | 727.0244 | 2178.0513 | 2178.0494 | 0.88 | 0 | 18 | 0.021 | 1Score **> 36** indicates **identity** Score **> 14** indicates **homology** | U | K.ITSEELHYFVQNHFTSAR.M |
| 137336 | 200 | – | 217 | 545.5201 | 2178.0515 | 2178.0494 | 0.94 | 0 | 39 | 0.00024 | 1Score **> 36** indicates **identity** Score **> 15** indicates **homology** | U | K.ITSEELHYFVQNHFTSAR.M |
| 137338 | 200 | – | 217 | 727.0245 | 2178.0517 | 2178.0494 | 1.07 | 0 | 31 | 0.0013 | 1Score **> 36** indicates **identity** Score **> 14** indicates **homology** | U | K.ITSEELHYFVQNHFTSAR.M |
| 137339 | 200 | – | 217 | 1090.0331 | 2178.0517 | 2178.0494 | 1.07 | 0 | 72 | 1.9e-07 | 1Score **> 36** indicates **identity** Score **> 17** indicates **homology** | U | K.ITSEELHYFVQNHFTSAR.M |
| 137340 | 200 | – | 217 | 545.5203 | 2178.0521 | 2178.0494 | 1.22 | 0 | 43 | 8.6e-05 | 1Score **> 36** indicates **identity** Score **> 15** indicates **homology** | U | K.ITSEELHYFVQNHFTSAR.M |
| 137341 | 200 | – | 217 | 727.0247 | 2178.0522 | 2178.0494 | 1.26 | 0 | 51 | 1.8e-05 | 1Score **> 36** indicates **identity** Score **> 16** indicates **homology** | U | K.ITSEELHYFVQNHFTSAR.M |
| 137343 | 200 | – | 217 | 727.0247 | 2178.0523 | 2178.0494 | 1.33 | 0 | 47 | 4.1e-05 | 1Score **> 36** indicates **identity** Score **> 15** indicates **homology** | U | K.ITSEELHYFVQNHFTSAR.M |
| 137344 | 200 | – | 217 | 727.0247 | 2178.0523 | 2178.0494 | 1.33 | 0 | 49 | 2.4e-05 | 1Score **> 36** indicates **identity** Score **> 16** indicates **homology** | U | K.ITSEELHYFVQNHFTSAR.M |
| 137345 | 200 | – | 217 | 545.5204 | 2178.0524 | 2178.0494 | 1.37 | 0 | 25 | 0.0042 | 1Score **> 36** indicates **identity** Score **> 14** indicates **homology** | U | K.ITSEELHYFVQNHFTSAR.M |
| 137346 | 200 | – | 217 | 545.5204 | 2178.0524 | 2178.0494 | 1.39 | 0 | 39 | 0.0002 | 1Score **> 36** indicates **identity** Score **> 15** indicates **homology** | U | K.ITSEELHYFVQNHFTSAR.M |
| 137348 | 200 | – | 217 | 727.0248 | 2178.0526 | 2178.0494 | 1.44 | 0 | 25 | 0.0041 | 1Score **> 36** indicates **identity** Score **> 14** indicates **homology** | U | K.ITSEELHYFVQNHFTSAR.M |
| 137349 | 200 | – | 217 | 727.0248 | 2178.0526 | 2178.0494 | 1.49 | 0 | 23 | 0.007 | 1Score **> 36** indicates **identity** Score **> 14** indicates **homology** | U | K.ITSEELHYFVQNHFTSAR.M |
| 137350 | 200 | – | 217 | 545.5205 | 2178.0528 | 2178.0494 | 1.54 | 0 | 34 | 0.00065 | 1Score **> 36** indicates **identity** Score **> 15** indicates **homology** | U | K.ITSEELHYFVQNHFTSAR.M |
| 137351 | 200 | – | 217 | 727.0249 | 2178.0528 | 2178.0494 | 1.55 | 0 | 59 | 2.8e-06 | 1Score **> 36** indicates **identity** Score **> 16** indicates **homology** | U | K.ITSEELHYFVQNHFTSAR.M |
| 137352 | 200 | – | 217 | 545.5206 | 2178.0532 | 2178.0494 | 1.72 | 0 | 29 | 0.002 | 1Score **> 36** indicates **identity** Score **> 14** indicates **homology** | U | K.ITSEELHYFVQNHFTSAR.M |
| 137353 | 200 | – | 217 | 545.5206 | 2178.0533 | 2178.0494 | 1.76 | 0 | 43 | 8.5e-05 | 1Score **> 36** indicates **identity** Score **> 15** indicates **homology** | U | K.ITSEELHYFVQNHFTSAR.M |
| 137354 | 200 | – | 217 | 727.0251 | 2178.0534 | 2178.0494 | 1.83 | 0 | 48 | 2.9e-05 | 1Score **> 36** indicates **identity** Score **> 16** indicates **homology** | U | K.ITSEELHYFVQNHFTSAR.M |
| 137355 | 200 | – | 217 | 727.0251 | 2178.0534 | 2178.0494 | 1.85 | 0 | 49 | 2.4e-05 | 1Score **> 36** indicates **identity** Score **> 16** indicates **homology** | U | K.ITSEELHYFVQNHFTSAR.M |
| 137357 | 200 | – | 217 | 545.5207 | 2178.0536 | 2178.0494 | 1.93 | 0 | 43 | 8.8e-05 | 1Score **> 36** indicates **identity** Score **> 15** indicates **homology** | U | K.ITSEELHYFVQNHFTSAR.M |
| 137358 | 200 | – | 217 | 545.5207 | 2178.0537 | 2178.0494 | 1.98 | 0 | 30 | 0.0016 | 1Score **> 36** indicates **identity** Score **> 14** indicates **homology** | U | K.ITSEELHYFVQNHFTSAR.M |
| 137359 | 200 | – | 217 | 545.5207 | 2178.0539 | 2178.0494 | 2.04 | 0 | 47 | 3.7e-05 | 1Score **> 36** indicates **identity** Score **> 15** indicates **homology** | U | K.ITSEELHYFVQNHFTSAR.M |
| 137362 | 200 | – | 217 | 545.5210 | 2178.0548 | 2178.0494 | 2.46 | 0 | 21 | 0.01 | 1Score **> 36** indicates **identity** Score **> 14** indicates **homology** | U | K.ITSEELHYFVQNHFTSAR.M |
| 137364 | 200 | – | 217 | 545.5211 | 2178.0553 | 2178.0494 | 2.69 | 0 | 21 | 0.011 | 1Score **> 36** indicates **identity** Score **> 14** indicates **homology** | U | K.ITSEELHYFVQNHFTSAR.M |
| 137366 | 200 | – | 217 | 545.5213 | 2178.0560 | 2178.0494 | 3.04 | 0 | 43 | 8.9e-05 | 1Score **> 36** indicates **identity** Score **> 15** indicates **homology** | U | K.ITSEELHYFVQNHFTSAR.M |
| 137367 | 200 | – | 217 | 545.5213 | 2178.0561 | 2178.0494 | 3.06 | 0 | 14 | 0.044 | 1Score **> 36** indicates **identity** Score **> 13** indicates **homology** | U | K.ITSEELHYFVQNHFTSAR.M |
| 137368 | 200 | – | 217 | 727.0260 | 2178.0562 | 2178.0494 | 3.13 | 0 | 46 | 4.7e-05 | 1Score **> 36** indicates **identity** Score **> 15** indicates **homology** | U | K.ITSEELHYFVQNHFTSAR.M |
| 137369 | 200 | – | 217 | 545.5214 | 2178.0566 | 2178.0494 | 3.29 | 0 | 22 | 0.0085 | 1Score **> 36** indicates **identity** Score **> 14** indicates **homology** | U | K.ITSEELHYFVQNHFTSAR.M |
| 137370 | 200 | – | 217 | 727.0261 | 2178.0566 | 2178.0494 | 3.31 | 0 | 16 | 0.034 | 1Score **> 36** indicates **identity** Score **> 13** indicates **homology** | U | K.ITSEELHYFVQNHFTSAR.M |
| 137372 | 200 | – | 217 | 545.5215 | 2178.0570 | 2178.0494 | 3.47 | 0 | 23 | 0.0071 | 1Score **> 36** indicates **identity** Score **> 14** indicates **homology** | U | K.ITSEELHYFVQNHFTSAR.M |
| 137375 | 200 | – | 217 | 727.0268 | 2178.0585 | 2178.0494 | 4.19 | 0 | 26 | 0.0041 | 1Score **> 36** indicates **identity** Score **> 14** indicates **homology** | U | K.ITSEELHYFVQNHFTSAR.M |
| 137376 | 200 | – | 217 | 545.5220 | 2178.0587 | 2178.0494 | 4.29 | 0 | 44 | 7.8e-05 | 1Score **> 36** indicates **identity** Score **> 15** indicates **homology** | U | K.ITSEELHYFVQNHFTSAR.M |
| 137377 | 200 | – | 217 | 727.0274 | 2178.0603 | 2178.0494 | 5.02 | 0 | 51 | 1.6e-05 | 1Score **> 36** indicates **identity** Score **> 16** indicates **homology** | U | K.ITSEELHYFVQNHFTSAR.M |
| 137378 | 200 | – | 217 | 727.0277 | 2178.0612 | 2178.0494 | 5.41 | 0 | 18 | 0.02 | 1Score **> 36** indicates **identity** Score **> 14** indicates **homology** | U | K.ITSEELHYFVQNHFTSAR.M |
| 137445 | 200 | – | 217 | 545.7672 | 2179.0399 | 2179.0334 | 2.95 | 0 | 36 | 0.00038 | 1Score **> 35** indicates **identity** Score **> 15** indicates **homology** | U | K.ITSEELHYFVQNHFTSAR.M  + Deamidated (NQ) |
| 137446 | 200 | – | 217 | 545.7673 | 2179.0401 | 2179.0334 | 3.08 | 0 | 40 | 0.00017 | 1Score **> 35** indicates **identity** Score **> 15** indicates **homology** | U | K.ITSEELHYFVQNHFTSAR.M  + Deamidated (NQ) |
| 137449 | 200 | – | 217 | 727.3542 | 2179.0408 | 2179.0334 | 3.38 | 0 | 29 | 0.0017 | 1Score **> 35** indicates **identity** Score **> 14** indicates **homology** | U | K.ITSEELHYFVQNHFTSAR.M  + Deamidated (NQ) |
| 137452 | 200 | – | 217 | 545.7684 | 2179.0444 | 2179.0334 | 5.03 | 0 | 18 | 0.022 | 1Score **> 35** indicates **identity** Score **> 14** indicates **homology** | U | K.ITSEELHYFVQNHFTSAR.M  + Deamidated (NQ) |
| 137459 | 200 | – | 217 | 727.3566 | 2179.0480 | 2179.0334 | 6.68 | 0 | 18 | 0.019 | 1Score **> 35** indicates **identity** Score **> 14** indicates **homology** | U | K.ITSEELHYFVQNHFTSAR.M  + Deamidated (NQ) |
| 137473 | 200 | – | 217 | 727.3584 | 2179.0535 | 2179.0334 | 9.22 | 0 | 25 | 0.0047 | 1Score **> 35** indicates **identity** Score **> 14** indicates **homology** | U | K.ITSEELHYFVQNHFTSAR.M  + Deamidated (NQ) |
| 51514 | 218 | – | 231 | 470.9430 | 1409.8070 | 1409.8065 | 0.40 | 0 | 46 | 4.5e-05 | 1Score **> 31** indicates **identity** Score **> 15** indicates **homology** | U | R.MALVGLGVSHSVLK.Q |
| 51515 | 218 | – | 231 | 705.9109 | 1409.8072 | 1409.8065 | 0.53 | 0 | 49 | 3.5e-05 | 1Score **> 31** indicates **identity** Score **> 17** indicates **homology** | U | R.MALVGLGVSHSVLK.Q |
| 51517 | 218 | – | 231 | 470.9431 | 1409.8074 | 1409.8065 | 0.66 | 0 | 62 | 1.7e-06 | 1Score **> 31** indicates **identity** Score **> 16** indicates **homology** | U | R.MALVGLGVSHSVLK.Q |
| 51518 | 218 | – | 231 | 705.9110 | 1409.8074 | 1409.8065 | 0.70 | 0 | 80 | 1.2e-07 | 1Score **> 32** indicates **identity** Score **> 24** indicates **homology** | U | R.MALVGLGVSHSVLK.Q |
| 51519 | 218 | – | 231 | 470.9431 | 1409.8075 | 1409.8065 | 0.73 | 0 | 25 | 0.005 | 1Score **> 32** indicates **identity** Score **> 14** indicates **homology** | U | R.MALVGLGVSHSVLK.Q |
| 51520 | 218 | – | 231 | 705.9111 | 1409.8077 | 1409.8065 | 0.86 | 0 | 52 | 1.2e-05 | 1Score **> 32** indicates **identity** Score **> 16** indicates **homology** | U | R.MALVGLGVSHSVLK.Q |
| 51521 | 218 | – | 231 | 470.9434 | 1409.8083 | 1409.8065 | 1.31 | 0 | 44 | 7.2e-05 | 1Score **> 32** indicates **identity** Score **> 15** indicates **homology** | U | R.MALVGLGVSHSVLK.Q |
| 51522 | 218 | – | 231 | 705.9116 | 1409.8086 | 1409.8065 | 1.54 | 0 | 79 | 6.1e-08 | 1Score **> 32** indicates **identity** Score **> 19** indicates **homology** | U | R.MALVGLGVSHSVLK.Q |
| 51523 | 218 | – | 231 | 470.9436 | 1409.8090 | 1409.8065 | 1.83 | 0 | 22 | 0.0095 | 1Score **> 32** indicates **identity** Score **> 14** indicates **homology** | U | R.MALVGLGVSHSVLK.Q |
| 51524 | 218 | – | 231 | 470.9438 | 1409.8095 | 1409.8065 | 2.15 | 0 | 17 | 0.026 | 1Score **> 31** indicates **identity** Score **> 14** indicates **homology** | U | R.MALVGLGVSHSVLK.Q |
| 51525 | 218 | – | 231 | 470.9438 | 1409.8095 | 1409.8065 | 2.19 | 0 | 49 | 2.5e-05 | 1Score **> 31** indicates **identity** Score **> 16** indicates **homology** | U | R.MALVGLGVSHSVLK.Q |
| 51526 | 218 | – | 231 | 470.9438 | 1409.8096 | 1409.8065 | 2.22 | 0 | 43 | 9.6e-05 | 1Score **> 31** indicates **identity** Score **> 15** indicates **homology** | U | R.MALVGLGVSHSVLK.Q |
| 51527 | 218 | – | 231 | 470.9441 | 1409.8106 | 1409.8065 | 2.95 | 0 | 36 | 0.00045 | 1Score **> 32** indicates **identity** Score **> 15** indicates **homology** | U | R.MALVGLGVSHSVLK.Q |
| 53378 | 218 | – | 231 | 476.2762 | 1425.8067 | 1425.8014 | 3.75 | 0 | 17 | 0.027 | 1Score **> 33** indicates **identity** Score **> 14** indicates **homology** | U | R.MALVGLGVSHSVLK.Q  + Oxidation (M) |
| 33791 | 232 | – | 241 | 618.3146 | 1234.6147 | 1234.6128 | 1.53 | 0 | 29 | 0.0018 | 1Score **> 33** indicates **identity** Score **> 14** indicates **homology** | U | K.QVAEQFLNMR.G |
| 33792 | 232 | – | 241 | 618.3148 | 1234.6150 | 1234.6128 | 1.79 | 0 | 44 | 8.1e-05 | 1Score **> 33** indicates **identity** Score **> 15** indicates **homology** | U | K.QVAEQFLNMR.G |
| 33793 | 232 | – | 241 | 618.3148 | 1234.6151 | 1234.6128 | 1.82 | 0 | 43 | 8.9e-05 | 1Score **> 33** indicates **identity** Score **> 15** indicates **homology** | U | K.QVAEQFLNMR.G |
| 33794 | 232 | – | 241 | 618.3150 | 1234.6155 | 1234.6128 | 2.13 | 0 | 46 | 4.8e-05 | 1Score **> 33** indicates **identity** Score **> 15** indicates **homology** | U | K.QVAEQFLNMR.G |
| 35332 | 232 | – | 241 | 626.3112 | 1250.6079 | 1250.6077 | 0.16 | 0 | 35 | 0.00048 | 1Score **> 32** indicates **identity** Score **> 15** indicates **homology** | U | K.QVAEQFLNMR.G  + Oxidation (M) |
| 35348 | 232 | – | 241 | 626.3146 | 1250.6147 | 1250.6077 | 5.53 | 0 | 28 | 0.0023 | 1Score **> 33** indicates **identity** Score **> 14** indicates **homology** | U | K.QVAEQFLNMR.G  + Oxidation (M) |
| 1318 | 242 | – | 250 | 372.2227 | 742.4309 | 742.4337 | -3.87 | 0 | 38 | 0.0024 | 1Score **> 24** indicates **identity** | U | R.GGLGLAGAK.A |
| 1320 | 242 | – | 250 | 372.2249 | 742.4352 | 742.4337 | 1.99 | 0 | 47 | 0.00028 | 1Score **> 24** indicates **identity** | U | R.GGLGLAGAK.A |
| 197150 | 255 | – | 301 | 803.4262 | 4814.5135 | 4814.4964 | 3.56 | 2 | 42 | 0.01 | 1Score **> 35** indicates **identity** | U | R.GGEIREQNGDNLVHAAIVAESAAIGNAEANAFSVLQHLLGAGPHIKR.G |
| 194324 | 260 | – | 300 | 1037.5412 | 4146.1358 | 4146.1246 | 2.70 | 0 | 39 | 0.00022 | 1Score **> 37** indicates **identity** Score **> 15** indicates **homology** | U | R.EQNGDNLVHAAIVAESAAIGNAEANAFSVLQHLLGAGPHIK.R |
| 194325 | 260 | – | 300 | 830.2353 | 4146.1403 | 4146.1246 | 3.79 | 0 | 46 | 4.6e-05 | 1Score **> 37** indicates **identity** Score **> 15** indicates **homology** | U | R.EQNGDNLVHAAIVAESAAIGNAEANAFSVLQHLLGAGPHIK.R |
| 194916 | 260 | – | 300 | 718.0479 | 4302.2435 | 4302.2396 | 0.90 | 0 | 26 | 0.0034 | 1Score **> 36** indicates **identity** Score **> 14** indicates **homology** | U | R.EQNGDNLVHAAIVAESAAIGNAEANAFSVLQHLLGAGPHIK.R  + HNE (K) |
| 194917 | 260 | – | 301 | 861.4560 | 4302.2435 | 4302.2257 | 4.14 | 1 | 41 | 0.018 | 1Score **> 36** indicates **identity** | U | R.EQNGDNLVHAAIVAESAAIGNAEANAFSVLQHLLGAGPHIKR.G |
| 71947 | 301 | – | 315 | 788.4260 | 1574.8375 | 1574.8376 | -0.099 | 1 | 38 | 0.00029 | 1Score **> 35** indicates **identity** Score **> 15** indicates **homology** | U | K.RGNNTTSLLSQSVAK.G |
| 71950 | 301 | – | 315 | 788.4263 | 1574.8381 | 1574.8376 | 0.28 | 1 | 63 | 6.8e-06 | 1Score **> 35** indicates **identity** Score **> 24** indicates **homology** | U | K.RGNNTTSLLSQSVAK.G |
| 71951 | 301 | – | 315 | 788.4263 | 1574.8381 | 1574.8376 | 0.30 | 1 | 68 | 2.4e-06 | 1Score **> 35** indicates **identity** Score **> 24** indicates **homology** | U | K.RGNNTTSLLSQSVAK.G |
| 71952 | 301 | – | 315 | 525.9535 | 1574.8386 | 1574.8376 | 0.64 | 1 | 56 | 6.3e-06 | 1Score **> 35** indicates **identity** Score **> 17** indicates **homology** | U | K.RGNNTTSLLSQSVAK.G |
| 71955 | 301 | – | 315 | 525.9536 | 1574.8388 | 1574.8376 | 0.77 | 1 | 58 | 1.2e-05 | 1Score **> 35** indicates **identity** Score **> 22** indicates **homology** | U | K.RGNNTTSLLSQSVAK.G |
| 71956 | 301 | – | 315 | 788.4272 | 1574.8398 | 1574.8376 | 1.37 | 1 | 20 | 0.012 | 1Score **> 35** indicates **identity** Score **> 14** indicates **homology** | U | K.RGNNTTSLLSQSVAK.G |
| 71957 | 301 | – | 315 | 525.9547 | 1574.8422 | 1574.8376 | 2.91 | 1 | 19 | 0.017 | 1Score **> 35** indicates **identity** Score **> 14** indicates **homology** | U | K.RGNNTTSLLSQSVAK.G |
| 72057 | 301 | – | 315 | 788.9148 | 1575.8151 | 1575.8216 | -4.12 | 1 | 44 | 0.00019 | 1Score **> 35** indicates **identity** Score **> 19** indicates **homology** | U | K.RGNNTTSLLSQSVAK.G  + Deamidated (NQ) |
| 72061 | 301 | – | 315 | 788.9178 | 1575.8211 | 1575.8216 | -0.32 | 1 | 58 | 1.7e-05 | 1Score **> 35** indicates **identity** Score **> 23** indicates **homology** | U | K.RGNNTTSLLSQSVAK.G  + Deamidated (NQ) |
| 72062 | 301 | – | 315 | 526.2817 | 1575.8232 | 1575.8216 | 0.97 | 1 | 29 | 0.0019 | 1Score **> 35** indicates **identity** Score **> 14** indicates **homology** | U | K.RGNNTTSLLSQSVAK.G  + Deamidated (NQ) |
| 72063 | 301 | – | 315 | 788.9190 | 1575.8235 | 1575.8216 | 1.21 | 1 | 34 | 0.0038 | 1Score **> 35** indicates **identity** Score **> 22** indicates **homology** | U | K.RGNNTTSLLSQSVAK.G  + Deamidated (NQ) |
| 72064 | 301 | – | 315 | 788.9190 | 1575.8235 | 1575.8216 | 1.22 | 1 | 35 | 0.002 | 1Score **> 35** indicates **identity** Score **> 21** indicates **homology** | U | K.RGNNTTSLLSQSVAK.G  + Deamidated (NQ) |
| 72066 | 301 | – | 315 | 526.2826 | 1575.8259 | 1575.8216 | 2.74 | 1 | 25 | 0.005 | 1Score **> 35** indicates **identity** Score **> 14** indicates **homology** | U | K.RGNNTTSLLSQSVAK.G  + Deamidated (NQ) |
| 72067 | 301 | – | 315 | 526.2827 | 1575.8261 | 1575.8216 | 2.86 | 1 | 45 | 0.00016 | 1Score **> 35** indicates **identity** Score **> 19** indicates **homology** | U | K.RGNNTTSLLSQSVAK.G  + Deamidated (NQ) |
| 52616 | 302 | – | 315 | 710.3755 | 1418.7364 | 1418.7365 | -0.091 | 0 | 55 | 6.3e-06 | 1Score **> 34** indicates **identity** Score **> 16** indicates **homology** | U | R.GNNTTSLLSQSVAK.G |
| 52618 | 302 | – | 315 | 710.3761 | 1418.7377 | 1418.7365 | 0.87 | 0 | 112 | 3.7e-11 | 1Score **> 34** indicates **identity** Score **> 21** indicates **homology** | U | R.GNNTTSLLSQSVAK.G |
| 52619 | 302 | – | 315 | 710.3762 | 1418.7379 | 1418.7365 | 0.97 | 0 | 112 | 3.6e-11 | 1Score **> 34** indicates **identity** Score **> 20** indicates **homology** | U | R.GNNTTSLLSQSVAK.G |
| 52620 | 302 | – | 315 | 710.3763 | 1418.7380 | 1418.7365 | 1.02 | 0 | 98 | 6.2e-10 | 1Score **> 34** indicates **identity** Score **> 19** indicates **homology** | U | R.GNNTTSLLSQSVAK.G |
| 52621 | 302 | – | 315 | 710.3766 | 1418.7386 | 1418.7365 | 1.45 | 0 | 112 | 3.7e-11 | 1Score **> 34** indicates **identity** Score **> 21** indicates **homology** | U | R.GNNTTSLLSQSVAK.G |
| 52622 | 302 | – | 315 | 710.3767 | 1418.7389 | 1418.7365 | 1.65 | 0 | 84 | 1.5e-08 | 1Score **> 34** indicates **identity** Score **> 18** indicates **homology** | U | R.GNNTTSLLSQSVAK.G |
| 52624 | 302 | – | 315 | 710.3769 | 1418.7393 | 1418.7365 | 1.97 | 0 | 80 | 3.5e-08 | 1Score **> 35** indicates **identity** Score **> 17** indicates **homology** | U | R.GNNTTSLLSQSVAK.G |
| 52625 | 302 | – | 315 | 710.3774 | 1418.7403 | 1418.7365 | 2.68 | 0 | 93 | 1.8e-09 | 1Score **> 34** indicates **identity** Score **> 18** indicates **homology** | U | R.GNNTTSLLSQSVAK.G |
| 52628 | 302 | – | 315 | 710.3780 | 1418.7415 | 1418.7365 | 3.51 | 0 | 59 | 3.2e-06 | 1Score **> 34** indicates **identity** Score **> 16** indicates **homology** | U | R.GNNTTSLLSQSVAK.G |
| 52630 | 302 | – | 315 | 710.3785 | 1418.7425 | 1418.7365 | 4.23 | 0 | 65 | 8.1e-07 | 1Score **> 35** indicates **identity** Score **> 17** indicates **homology** | U | R.GNNTTSLLSQSVAK.G |
| 52725 | 302 | – | 315 | 710.8671 | 1419.7196 | 1419.7205 | -0.67 | 0 | 60 | 2.6e-06 | 1Score **> 34** indicates **identity** Score **> 16** indicates **homology** | U | R.GNNTTSLLSQSVAK.G  + Deamidated (NQ) |
| 52727 | 302 | – | 315 | 710.8678 | 1419.7210 | 1419.7205 | 0.34 | 0 | 85 | 1e-08 | 1Score **> 34** indicates **identity** Score **> 18** indicates **homology** | U | R.GNNTTSLLSQSVAK.G  + Deamidated (NQ) |
| 52729 | 302 | – | 315 | 710.8682 | 1419.7218 | 1419.7205 | 0.88 | 0 | 80 | 3.4e-08 | 1Score **> 34** indicates **identity** Score **> 18** indicates **homology** | U | R.GNNTTSLLSQSVAK.G  + Deamidated (NQ) |
| 52730 | 302 | – | 315 | 710.8686 | 1419.7226 | 1419.7205 | 1.48 | 0 | 67 | 6.4e-07 | 1Score **> 34** indicates **identity** Score **> 17** indicates **homology** | U | R.GNNTTSLLSQSVAK.G  + Deamidated (NQ) |
| 52732 | 302 | – | 315 | 710.8688 | 1419.7231 | 1419.7205 | 1.80 | 0 | 79 | 3.7e-08 | 1Score **> 34** indicates **identity** Score **> 17** indicates **homology** | U | R.GNNTTSLLSQSVAK.G  + Deamidated (NQ) |
| 52734 | 302 | – | 315 | 710.8702 | 1419.7258 | 1419.7205 | 3.70 | 0 | 61 | 1.8e-06 | 1Score **> 34** indicates **identity** Score **> 16** indicates **homology** | U | R.GNNTTSLLSQSVAK.G  + Deamidated (NQ) |
| 52736 | 302 | – | 315 | 710.8714 | 1419.7283 | 1419.7205 | 5.50 | 0 | 27 | 0.015 | 1Score **> 34** indicates **identity** Score **> 21** indicates **homology** | U | R.GNNTTSLLSQSVAK.G  + Deamidated (NQ) |
| 196293 | 316 | – | 359 | 1522.7355 | 4565.1845 | 4565.1775 | 1.54 | 0 | 33 | 0.00079 | 1Score **> 36** indicates **identity** Score **> 15** indicates **homology** | U | K.GSHQPFDVSAFNASYSDSGLFGIYTISQAAAAGEVINAAYNQVK.A |
| 196318 | 316 | – | 359 | 1523.4039 | 4567.1899 | 4567.1455 | 9.72 | 0 | 49 | 2.4e-05 | 1Score **> 36** indicates **identity** Score **> 16** indicates **homology** | U | K.GSHQPFDVSAFNASYSDSGLFGIYTISQAAAAGEVINAAYNQVK.A  + 2 Deamidated (NQ) |
| 65795 | 360 | – | 375 | 765.3990 | 1528.7834 | 1528.7845 | -0.74 | 0 | 83 | 1.7e-08 | 1Score **> 34** indicates **identity** Score **> 18** indicates **homology** | U | K.AVAQGNLSSADVQAAK.N |
| 65796 | 360 | – | 375 | 765.3992 | 1528.7838 | 1528.7845 | -0.44 | 0 | 59 | 2.7e-06 | 1Score **> 34** indicates **identity** Score **> 16** indicates **homology** | U | K.AVAQGNLSSADVQAAK.N |
| 65797 | 360 | – | 375 | 765.3993 | 1528.7840 | 1528.7845 | -0.33 | 0 | 101 | 3.5e-10 | 1Score **> 34** indicates **identity** Score **> 19** indicates **homology** | U | K.AVAQGNLSSADVQAAK.N |
| 65799 | 360 | – | 375 | 765.3994 | 1528.7842 | 1528.7845 | -0.19 | 0 | 76 | 6.7e-08 | 1Score **> 34** indicates **identity** Score **> 17** indicates **homology** | U | K.AVAQGNLSSADVQAAK.N |
| 65802 | 360 | – | 375 | 765.3995 | 1528.7844 | 1528.7845 | -0.045 | 0 | 84 | 1.3e-08 | 1Score **> 34** indicates **identity** Score **> 18** indicates **homology** | U | K.AVAQGNLSSADVQAAK.N |
| 65804 | 360 | – | 375 | 765.3997 | 1528.7848 | 1528.7845 | 0.21 | 0 | 101 | 3.6e-10 | 1Score **> 34** indicates **identity** Score **> 19** indicates **homology** | U | K.AVAQGNLSSADVQAAK.N |
| 65805 | 360 | – | 375 | 765.3999 | 1528.7852 | 1528.7845 | 0.45 | 0 | 96 | 9e-10 | 1Score **> 34** indicates **identity** Score **> 19** indicates **homology** | U | K.AVAQGNLSSADVQAAK.N |
| 65806 | 360 | – | 375 | 765.3999 | 1528.7852 | 1528.7845 | 0.46 | 0 | 101 | 3.1e-10 | 1Score **> 34** indicates **identity** Score **> 19** indicates **homology** | U | K.AVAQGNLSSADVQAAK.N |
| 65807 | 360 | – | 375 | 765.3999 | 1528.7853 | 1528.7845 | 0.53 | 0 | 100 | 3.8e-10 | 1Score **> 34** indicates **identity** Score **> 19** indicates **homology** | U | K.AVAQGNLSSADVQAAK.N |
| 65808 | 360 | – | 375 | 765.4002 | 1528.7858 | 1528.7845 | 0.85 | 0 | 108 | 8.1e-11 | 1Score **> 34** indicates **identity** Score **> 19** indicates **homology** | U | K.AVAQGNLSSADVQAAK.N |
| 65809 | 360 | – | 375 | 765.4003 | 1528.7861 | 1528.7845 | 1.01 | 0 | 107 | 9.6e-11 | 1Score **> 34** indicates **identity** Score **> 19** indicates **homology** | U | K.AVAQGNLSSADVQAAK.N |
| 65810 | 360 | – | 375 | 765.4004 | 1528.7863 | 1528.7845 | 1.15 | 0 | 81 | 2.7e-08 | 1Score **> 34** indicates **identity** Score **> 18** indicates **homology** | U | K.AVAQGNLSSADVQAAK.N |
| 65813 | 360 | – | 375 | 765.4013 | 1528.7880 | 1528.7845 | 2.31 | 0 | 96 | 9.4e-10 | 1Score **> 34** indicates **identity** Score **> 19** indicates **homology** | U | K.AVAQGNLSSADVQAAK.N |
| 65820 | 360 | – | 375 | 765.4043 | 1528.7940 | 1528.7845 | 6.20 | 0 | 78 | 5.3e-08 | 1Score **> 34** indicates **identity** Score **> 17** indicates **homology** | U | K.AVAQGNLSSADVQAAK.N |
| 65894 | 360 | – | 375 | 765.8926 | 1529.7707 | 1529.7685 | 1.41 | 0 | 72 | 2e-07 | 1Score **> 34** indicates **identity** Score **> 18** indicates **homology** | U | K.AVAQGNLSSADVQAAK.N  + Deamidated (NQ) |
| 65898 | 360 | – | 375 | 765.8940 | 1529.7735 | 1529.7685 | 3.27 | 0 | 85 | 1.2e-08 | 1Score **> 34** indicates **identity** Score **> 18** indicates **homology** | U | K.AVAQGNLSSADVQAAK.N  + Deamidated (NQ) |
| 65905 | 360 | – | 375 | 765.8954 | 1529.7763 | 1529.7685 | 5.10 | 0 | 94 | 1.7e-09 | 1Score **> 34** indicates **identity** Score **> 18** indicates **homology** | U | K.AVAQGNLSSADVQAAK.N  + Deamidated (NQ) |
| 65908 | 360 | – | 375 | 765.8962 | 1529.7779 | 1529.7685 | 6.14 | 0 | 49 | 2.6e-05 | 1Score **> 34** indicates **identity** Score **> 16** indicates **homology** | U | K.AVAQGNLSSADVQAAK.N  + Deamidated (NQ) |
| 96158 | 437 | – | 453 | 880.4208 | 1758.8270 | 1758.8247 | 1.31 | 0 | 67 | 4.7e-07 | 1Score **> 32** indicates **identity** Score **> 17** indicates **homology** | U | K.SMAASGNLGHTPFLDEL.- |
| 96160 | 437 | – | 453 | 880.4210 | 1758.8274 | 1758.8247 | 1.56 | 0 | 19 | 0.017 | 1Score **> 32** indicates **identity** Score **> 14** indicates **homology** | U | K.SMAASGNLGHTPFLDEL.- |
| 96161 | 437 | – | 453 | 880.4222 | 1758.8298 | 1758.8247 | 2.89 | 0 | 39 | 0.00022 | 1Score **> 32** indicates **identity** Score **> 15** indicates **homology** | U | K.SMAASGNLGHTPFLDEL.- |

---

```
ID   QCR2_MOUSE              Reviewed;         453 AA.
AC   Q9DB77; Q8BK11; Q9CVK7;
DT   20-JUN-2001, integrated into UniProtKB/Swiss-Prot.
DT   01-JUN-2001, sequence version 1.
DT   28-JUN-2023, entry version 183.
DE   RecName: Full=Cytochrome b-c1 complex subunit 2, mitochondrial;
DE   AltName: Full=Complex III subunit 2;
DE   AltName: Full=Core protein II;
DE   AltName: Full=Ubiquinol-cytochrome-c reductase complex core protein 2;
DE   Flags: Precursor;
GN   Name=Uqcrc2;
OS   Mus musculus (Mouse).
OC   Eukaryota; Metazoa; Chordata; Craniata; Vertebrata; Euteleostomi; Mammalia;
OC   Eutheria; Euarchontoglires; Glires; Rodentia; Myomorpha; Muroidea; Muridae;
OC   Murinae; Mus; Mus.
OX   NCBI_TaxID=10090;
RN   [1]
RP   NUCLEOTIDE SEQUENCE [LARGE SCALE MRNA].
RC   STRAIN=C57BL/6J; TISSUE=Cerebellum, Pancreas, and Thymus;
RX   PubMed=16141072; DOI=10.1126/science.1112014;
RA   Carninci P., Kasukawa T., Katayama S., Gough J., Frith M.C., Maeda N.,
RA   Oyama R., Ravasi T., Lenhard B., Wells C., Kodzius R., Shimokawa K.,
RA   Bajic V.B., Brenner S.E., Batalov S., Forrest A.R., Zavolan M., Davis M.J.,
RA   Wilming L.G., Aidinis V., Allen J.E., Ambesi-Impiombato A., Apweiler R.,
RA   Aturaliya R.N., Bailey T.L., Bansal M., Baxter L., Beisel K.W., Bersano T.,
RA   Bono H., Chalk A.M., Chiu K.P., Choudhary V., Christoffels A.,
RA   Clutterbuck D.R., Crowe M.L., Dalla E., Dalrymple B.P., de Bono B.,
RA   Della Gatta G., di Bernardo D., Down T., Engstrom P., Fagiolini M.,
RA   Faulkner G., Fletcher C.F., Fukushima T., Furuno M., Futaki S.,
RA   Gariboldi M., Georgii-Hemming P., Gingeras T.R., Gojobori T., Green R.E.,
RA   Gustincich S., Harbers M., Hayashi Y., Hensch T.K., Hirokawa N., Hill D.,
RA   Huminiecki L., Iacono M., Ikeo K., Iwama A., Ishikawa T., Jakt M.,
RA   Kanapin A., Katoh M., Kawasawa Y., Kelso J., Kitamura H., Kitano H.,
RA   Kollias G., Krishnan S.P., Kruger A., Kummerfeld S.K., Kurochkin I.V.,
RA   Lareau L.F., Lazarevic D., Lipovich L., Liu J., Liuni S., McWilliam S.,
RA   Madan Babu M., Madera M., Marchionni L., Matsuda H., Matsuzawa S., Miki H.,
RA   Mignone F., Miyake S., Morris K., Mottagui-Tabar S., Mulder N., Nakano N.,
RA   Nakauchi H., Ng P., Nilsson R., Nishiguchi S., Nishikawa S., Nori F.,
RA   Ohara O., Okazaki Y., Orlando V., Pang K.C., Pavan W.J., Pavesi G.,
RA   Pesole G., Petrovsky N., Piazza S., Reed J., Reid J.F., Ring B.Z.,
RA   Ringwald M., Rost B., Ruan Y., Salzberg S.L., Sandelin A., Schneider C.,
RA   Schoenbach C., Sekiguchi K., Semple C.A., Seno S., Sessa L., Sheng Y.,
RA   Shibata Y., Shimada H., Shimada K., Silva D., Sinclair B., Sperling S.,
RA   Stupka E., Sugiura K., Sultana R., Takenaka Y., Taki K., Tammoja K.,
RA   Tan S.L., Tang S., Taylor M.S., Tegner J., Teichmann S.A., Ueda H.R.,
RA   van Nimwegen E., Verardo R., Wei C.L., Yagi K., Yamanishi H.,
RA   Zabarovsky E., Zhu S., Zimmer A., Hide W., Bult C., Grimmond S.M.,
RA   Teasdale R.D., Liu E.T., Brusic V., Quackenbush J., Wahlestedt C.,
RA   Mattick J.S., Hume D.A., Kai C., Sasaki D., Tomaru Y., Fukuda S.,
RA   Kanamori-Katayama M., Suzuki M., Aoki J., Arakawa T., Iida J., Imamura K.,
RA   Itoh M., Kato T., Kawaji H., Kawagashira N., Kawashima T., Kojima M.,
RA   Kondo S., Konno H., Nakano K., Ninomiya N., Nishio T., Okada M., Plessy C.,
RA   Shibata K., Shiraki T., Suzuki S., Tagami M., Waki K., Watahiki A.,
RA   Okamura-Oho Y., Suzuki H., Kawai J., Hayashizaki Y.;
RT   "The transcriptional landscape of the mammalian genome.";
RL   Science 309:1559-1563(2005).
RN   [2]
RP   NUCLEOTIDE SEQUENCE [LARGE SCALE MRNA].
RX   PubMed=15489334; DOI=10.1101/gr.2596504;
RG   The MGC Project Team;
RT   "The status, quality, and expansion of the NIH full-length cDNA project:
RT   the Mammalian Gene Collection (MGC).";
RL   Genome Res. 14:2121-2127(2004).
RN   [3]
RP   PROTEIN SEQUENCE OF 24-60; 71-85; 117-147; 163-196; 200-241; 301-315;
RP   360-375 AND 436-453, AND IDENTIFICATION BY MASS SPECTROMETRY.
RC   STRAIN=C57BL/6J; TISSUE=Brain, and Hippocampus;
RA   Lubec G., Kang S.U., Klug S., Yang J.W., Zigmond M.;
RL   Submitted (JUL-2007) to UniProtKB.
RN   [4]
RP   SUBUNIT.
RX   PubMed=19026783; DOI=10.1016/j.molcel.2008.10.021;
RA   Acin-Perez R., Fernandez-Silva P., Peleato M.L., Perez-Martos A.,
RA   Enriquez J.A.;
RT   "Respiratory active mitochondrial supercomplexes.";
RL   Mol. Cell 32:529-539(2008).
RN   [5]
RP   IDENTIFICATION BY MASS SPECTROMETRY [LARGE SCALE ANALYSIS].
RC   TISSUE=Brain, Brown adipose tissue, Heart, Kidney, Liver, Lung,
RC   Pancreas, Spleen, and Testis;
RX   PubMed=21183079; DOI=10.1016/j.cell.2010.12.001;
RA   Huttlin E.L., Jedrychowski M.P., Elias J.E., Goswami T., Rad R.,
RA   Beausoleil S.A., Villen J., Haas W., Sowa M.E., Gygi S.P.;
RT   "A tissue-specific atlas of mouse protein phosphorylation and expression.";
RL   Cell 143:1174-1189(2010).
RN   [6]
RP   ACETYLATION [LARGE SCALE ANALYSIS] AT LYS-66; LYS-199 AND LYS-250, AND
RP   IDENTIFICATION BY MASS SPECTROMETRY [LARGE SCALE ANALYSIS].
RC   TISSUE=Liver;
RX   PubMed=23576753; DOI=10.1073/pnas.1302961110;
RA   Rardin M.J., Newman J.C., Held J.M., Cusack M.P., Sorensen D.J., Li B.,
RA   Schilling B., Mooney S.D., Kahn C.R., Verdin E., Gibson B.W.;
RT   "Label-free quantitative proteomics of the lysine acetylome in mitochondria
RT   identifies substrates of SIRT3 in metabolic pathways.";
RL   Proc. Natl. Acad. Sci. U.S.A. 110:6601-6606(2013).
RN   [7]
RP   SUBUNIT, AND TISSUE SPECIFICITY.
RX   PubMed=30666338; DOI=10.1007/s00018-019-03007-6;
RA   Shan W., Li J., Xu W., Li H., Zuo Z.;
RT   "Critical role of UQCRC1 in embryo survival, brain ischemic tolerance and
RT   normal cognition in mice.";
RL   Cell. Mol. Life Sci. 76:1381-1396(2019).
RN   [8]
RP   INTERACTION WITH RAB5IF.
RX   PubMed=31536960; DOI=10.1016/j.isci.2019.08.057;
RA   Moutaoufik M.T., Malty R., Amin S., Zhang Q., Phanse S., Gagarinova A.,
RA   Zilocchi M., Hoell L., Minic Z., Gagarinova M., Aoki H., Stockwell J.,
RA   Jessulat M., Goebels F., Broderick K., Scott N.E., Vlasblom J., Musso G.,
RA   Prasad B., Lamantea E., Garavaglia B., Rajput A., Murayama K., Okazaki Y.,
RA   Foster L.J., Bader G.D., Cayabyab F.S., Babu M.;
RT   "Rewiring of the Human Mitochondrial Interactome during Neuronal
RT   Reprogramming Reveals Regulators of the Respirasome and Neurogenesis.";
RL   IScience 19:1114-1132(2019).
RN   [9]
RP   INTERACTION WITH STMP1.
RX   PubMed=35101990; DOI=10.1073/pnas.2120476119;
RA   Makarewich C.A., Munir A.Z., Bezprozvannaya S., Gibson A.M., Young Kim S.,
RA   Martin-Sandoval M.S., Mathews T.P., Szweda L.I., Bassel-Duby R.,
RA   Olson E.N.;
RT   "The cardiac-enriched microprotein mitolamban regulates mitochondrial
RT   respiratory complex assembly and function in mice.";
RL   Proc. Natl. Acad. Sci. U.S.A. 119:0-0(2022).
CC   -!- FUNCTION: Component of the ubiquinol-cytochrome c oxidoreductase, a
CC       multisubunit transmembrane complex that is part of the mitochondrial
CC       electron transport chain which drives oxidative phosphorylation. The
CC       respiratory chain contains 3 multisubunit complexes succinate
CC       dehydrogenase (complex II, CII), ubiquinol-cytochrome c oxidoreductase
CC       (cytochrome b-c1 complex, complex III, CIII) and cytochrome c oxidase
CC       (complex IV, CIV), that cooperate to transfer electrons derived from
CC       NADH and succinate to molecular oxygen, creating an electrochemical
CC       gradient over the inner membrane that drives transmembrane transport
CC       and the ATP synthase. The cytochrome b-c1 complex catalyzes electron
CC       transfer from ubiquinol to cytochrome c, linking this redox reaction to
CC       translocation of protons across the mitochondrial inner membrane, with
CC       protons being carried across the membrane as hydrogens on the quinol.
CC       In the process called Q cycle, 2 protons are consumed from the matrix,
CC       4 protons are released into the intermembrane space and 2 electrons are
CC       passed to cytochrome c (By similarity). The 2 core subunits UQCRC1/QCR1
CC       and UQCRC2/QCR2 are homologous to the 2 mitochondrial-processing
CC       peptidase (MPP) subunits beta-MPP and alpha-MPP respectively, and they
CC       seem to have preserved their MPP processing properties. May be involved
CC       in the in situ processing of UQCRFS1 into the mature Rieske protein and
CC       its mitochondrial targeting sequence (MTS)/subunit 9 when incorporated
CC       into complex III (By similarity). {ECO:0000250|UniProtKB:P07257,
CC       ECO:0000250|UniProtKB:P23004}.
CC   -!- SUBUNIT: Component of the ubiquinol-cytochrome c oxidoreductase
CC       (cytochrome b-c1 complex, complex III, CIII), a multisubunit enzyme
CC       composed of 11 subunits. The complex is composed of 3 respiratory
CC       subunits cytochrome b, cytochrome c1 and Rieske protein UQCRFS1, 2 core
CC       protein subunits UQCRC1/QCR1 and UQCRC2/QCR2, and 6 low-molecular
CC       weight protein subunits UQCRH/QCR6, UQCRB/QCR7, UQCRQ/QCR8,
CC       UQCR10/QCR9, UQCR11/QCR10 and subunit 9, the cleavage product of Rieske
CC       protein UQCRFS1 (By similarity). The complex exists as an obligatory
CC       dimer and forms supercomplexes (SCs) in the inner mitochondrial
CC       membrane with NADH-ubiquinone oxidoreductase (complex I, CI) and
CC       cytochrome c oxidase (complex IV, CIV), resulting in different
CC       assemblies (supercomplex SCI(1)III(2)IV(1) and megacomplex
CC       MCI(2)III(2)IV(2)) (PubMed:19026783, PubMed:30666338). Interacts with
CC       RAB5IF (PubMed:31536960). Interacts with STMP1 (PubMed:35101990).
CC       {ECO:0000250|UniProtKB:P23004, ECO:0000269|PubMed:19026783,
CC       ECO:0000269|PubMed:30666338, ECO:0000269|PubMed:31536960,
CC       ECO:0000269|PubMed:35101990}.
CC   -!- SUBCELLULAR LOCATION: Mitochondrion inner membrane
CC       {ECO:0000250|UniProtKB:P07257}; Peripheral membrane protein
CC       {ECO:0000250|UniProtKB:P07257}; Matrix side
CC       {ECO:0000250|UniProtKB:P07257}.
CC   -!- TISSUE SPECIFICITY: Expressed in neurons and astrocytes of the cerebral
CC       cortex and hippocampus (at protein level).
CC       {ECO:0000269|PubMed:30666338}.
CC   -!- PTM: Acetylation of Lys-159 and Lys-250 is observed in liver
CC       mitochondria from fasted mice but not from fed mice.
CC   -!- SIMILARITY: Belongs to the peptidase M16 family. UQCRC2/QCR2 subfamily.
CC       {ECO:0000305}.
CC   ---------------------------------------------------------------------------
CC   Copyrighted by the UniProt Consortium, see https://www.uniprot.org/terms
CC   Distributed under the Creative Commons Attribution (CC BY 4.0) License
CC   ---------------------------------------------------------------------------
DR   EMBL; AK005151; BAB23845.1; -; mRNA.
DR   EMBL; AK007669; BAB25176.1; -; mRNA.
DR   EMBL; AK077583; BAC36876.1; -; mRNA.
DR   EMBL; AK088103; BAC40146.1; -; mRNA.
DR   EMBL; BC003423; AAH03423.1; -; mRNA.
DR   CCDS; CCDS21795.1; -.
DR   PIR; PC7073; PC7073.
DR   RefSeq; NP_080175.1; NM_025899.2.
DR   PDB; 7O37; EM; 3.20 A; B/M=15-453.
DR   PDB; 7O3C; EM; 3.30 A; B/M=15-453.
DR   PDB; 7O3E; EM; 3.60 A; B/M=15-453.
DR   PDB; 7O3H; EM; 2.60 A; B/M=15-453.
DR   PDBsum; 7O37; -.
DR   PDBsum; 7O3C; -.
DR   PDBsum; 7O3E; -.
DR   PDBsum; 7O3H; -.
DR   AlphaFoldDB; Q9DB77; -.
DR   SMR; Q9DB77; -.
DR   BioGRID; 211868; 76.
DR   ComplexPortal; CPX-563; Mitochondrial respiratory chain complex III.
DR   CORUM; Q9DB77; -.
DR   IntAct; Q9DB77; 16.
DR   MINT; Q9DB77; -.
DR   STRING; 10090.ENSMUSP00000033176; -.
DR   MEROPS; M16.974; -.
DR   GlyGen; Q9DB77; 1 site, 1 O-linked glycan (1 site).
DR   iPTMnet; Q9DB77; -.
DR   PhosphoSitePlus; Q9DB77; -.
DR   SwissPalm; Q9DB77; -.
DR   EPD; Q9DB77; -.
DR   jPOST; Q9DB77; -.
DR   MaxQB; Q9DB77; -.
DR   PaxDb; Q9DB77; -.
DR   PeptideAtlas; Q9DB77; -.
DR   ProteomicsDB; 300330; -.
DR   TopDownProteomics; Q9DB77; -.
DR   Antibodypedia; 1267; 265 antibodies from 30 providers.
DR   DNASU; 67003; -.
DR   Ensembl; ENSMUST00000033176; ENSMUSP00000033176; ENSMUSG00000030884.
DR   GeneID; 67003; -.
DR   KEGG; mmu:67003; -.
DR   UCSC; uc009jms.1; mouse.
DR   AGR; MGI:1914253; -.
DR   CTD; 7385; -.
DR   MGI; MGI:1914253; Uqcrc2.
DR   VEuPathDB; HostDB:ENSMUSG00000030884; -.
DR   eggNOG; KOG2583; Eukaryota.
DR   GeneTree; ENSGT00940000154915; -.
DR   HOGENOM; CLU_009902_0_0_1; -.
DR   InParanoid; Q9DB77; -.
DR   OMA; WVGEFFT; -.
DR   OrthoDB; 314823at2759; -.
DR   PhylomeDB; Q9DB77; -.
DR   TreeFam; TF105033; -.
DR   Reactome; R-MMU-611105; Respiratory electron transport.
DR   BioGRID-ORCS; 67003; 24 hits in 79 CRISPR screens.
DR   ChiTaRS; Uqcrc2; mouse.
DR   PRO; PR:Q9DB77; -.
DR   Proteomes; UP000000589; Chromosome 7.
DR   RNAct; Q9DB77; protein.
DR   Bgee; ENSMUSG00000030884; Expressed in paneth cell and 267 other tissues.
DR   ExpressionAtlas; Q9DB77; baseline and differential.
DR   Genevisible; Q9DB77; MM.
DR   GO; GO:0005743; C:mitochondrial inner membrane; HDA:MGI.
DR   GO; GO:0005750; C:mitochondrial respiratory chain complex III; IDA:UniProtKB.
DR   GO; GO:0005751; C:mitochondrial respiratory chain complex IV; ISO:MGI.
DR   GO; GO:0005739; C:mitochondrion; IDA:MGI.
DR   GO; GO:0043209; C:myelin sheath; HDA:UniProtKB.
DR   GO; GO:0005654; C:nucleoplasm; ISO:MGI.
DR   GO; GO:0046872; F:metal ion binding; IEA:InterPro.
DR   GO; GO:0004222; F:metalloendopeptidase activity; IEA:InterPro.
DR   GO; GO:0044877; F:protein-containing complex binding; ISO:MGI.
DR   GO; GO:0045333; P:cellular respiration; NAS:ComplexPortal.
DR   GO; GO:0006122; P:mitochondrial electron transport, ubiquinol to cytochrome c; NAS:ComplexPortal.
DR   GO; GO:0006508; P:proteolysis; IEA:InterPro.
DR   GO; GO:0009410; P:response to xenobiotic stimulus; IEA:Ensembl.
DR   Gene3D; 3.30.830.10; Metalloenzyme, LuxS/M16 peptidase-like; 2.
DR   InterPro; IPR011249; Metalloenz_LuxS/M16.
DR   InterPro; IPR011765; Pept_M16_N.
DR   InterPro; IPR001431; Pept_M16_Zn_BS.
DR   InterPro; IPR007863; Peptidase_M16_C.
DR   PANTHER; PTHR11851:SF226; CYTOCHROME B-C1 COMPLEX SUBUNIT 2, MITOCHONDRIAL; 1.
DR   PANTHER; PTHR11851; METALLOPROTEASE; 1.
DR   Pfam; PF00675; Peptidase_M16; 1.
DR   Pfam; PF05193; Peptidase_M16_C; 1.
DR   SUPFAM; SSF63411; LuxS/MPP-like metallohydrolase; 2.
DR   PROSITE; PS00143; INSULINASE; 1.
PE   1: Evidence at protein level;
KW   3D-structure; Acetylation; Direct protein sequencing; Electron transport;
KW   Membrane; Mitochondrion; Mitochondrion inner membrane; Phosphoprotein;
KW   Reference proteome; Respiratory chain; Transit peptide; Transport.
FT   TRANSIT         1..14
FT                   /note="Mitochondrion"
FT                   /evidence="ECO:0000250"
FT   CHAIN           15..453
FT                   /note="Cytochrome b-c1 complex subunit 2, mitochondrial"
FT                   /id="PRO_0000026792"
FT   MOD_RES         66
FT                   /note="N6-acetyllysine"
FT                   /evidence="ECO:0007744|PubMed:23576753"
FT   MOD_RES         199
FT                   /note="N6-acetyllysine"
FT                   /evidence="ECO:0007744|PubMed:23576753"
FT   MOD_RES         250
FT                   /note="N6-acetyllysine"
FT                   /evidence="ECO:0007744|PubMed:23576753"
FT   MOD_RES         368
FT                   /note="Phosphoserine"
FT                   /evidence="ECO:0000250|UniProtKB:P32551"
FT   CONFLICT        36
FT                   /note="Q -> L (in Ref. 1; BAB25176)"
FT                   /evidence="ECO:0000305"
FT   CONFLICT        319
FT                   /note="Q -> R (in Ref. 1; BAC36876)"
FT                   /evidence="ECO:0000305"
FT   STRAND          39..42
FT                   /evidence="ECO:0007829|PDB:7O3H"
FT   STRAND          48..52
FT                   /evidence="ECO:0007829|PDB:7O3H"
FT   STRAND          57..66
FT                   /evidence="ECO:0007829|PDB:7O3H"
FT   HELIX           69..71
FT                   /evidence="ECO:0007829|PDB:7O3H"
FT   STRAND          74..76
FT                   /evidence="ECO:0007829|PDB:7O3C"
FT   HELIX           79..85
FT                   /evidence="ECO:0007829|PDB:7O3H"
FT   TURN            86..88
FT                   /evidence="ECO:0007829|PDB:7O3H"
FT   STRAND          91..93
FT                   /evidence="ECO:0007829|PDB:7O3C"
FT   HELIX           96..105
FT                   /evidence="ECO:0007829|PDB:7O3H"
FT   STRAND          109..114
FT                   /evidence="ECO:0007829|PDB:7O3H"
FT   STRAND          119..126
FT                   /evidence="ECO:0007829|PDB:7O3H"
FT   TURN            127..129
FT                   /evidence="ECO:0007829|PDB:7O3H"
FT   HELIX           130..142
FT                   /evidence="ECO:0007829|PDB:7O3H"
FT   HELIX           148..165
FT                   /evidence="ECO:0007829|PDB:7O3H"
FT   HELIX           169..181
FT                   /evidence="ECO:0007829|PDB:7O3H"
FT   STRAND          182..185
FT                   /evidence="ECO:0007829|PDB:7O3H"
FT   TURN            194..199
FT                   /evidence="ECO:0007829|PDB:7O3H"
FT   HELIX           202..212
FT                   /evidence="ECO:0007829|PDB:7O3H"
FT   HELIX           215..217
FT                   /evidence="ECO:0007829|PDB:7O3H"
FT   STRAND          218..225
FT                   /evidence="ECO:0007829|PDB:7O3H"
FT   HELIX           227..237
FT                   /evidence="ECO:0007829|PDB:7O3H"
FT   STRAND          256..261
FT                   /evidence="ECO:0007829|PDB:7O3H"
FT   STRAND          265..275
FT                   /evidence="ECO:0007829|PDB:7O3H"
FT   STRAND          278..280
FT                   /evidence="ECO:0007829|PDB:7O37"
FT   HELIX           281..293
FT                   /evidence="ECO:0007829|PDB:7O3H"
FT   HELIX           308..314
FT                   /evidence="ECO:0007829|PDB:7O3H"
FT   STRAND          322..330
FT                   /evidence="ECO:0007829|PDB:7O3H"
FT   STRAND          333..344
FT                   /evidence="ECO:0007829|PDB:7O3H"
FT   HELIX           347..362
FT                   /evidence="ECO:0007829|PDB:7O3H"
FT   HELIX           368..386
FT                   /evidence="ECO:0007829|PDB:7O3H"
FT   HELIX           389..402
FT                   /evidence="ECO:0007829|PDB:7O3H"
FT   HELIX           409..417
FT                   /evidence="ECO:0007829|PDB:7O3H"
FT   HELIX           421..433
FT                   /evidence="ECO:0007829|PDB:7O3H"
FT   STRAND          436..442
FT                   /evidence="ECO:0007829|PDB:7O3H"
FT   HELIX           450..452
FT                   /evidence="ECO:0007829|PDB:7O37"
SQ   SEQUENCE   453 AA;  48235 MW;  24A2BDDDA657867E CRC64;
     MKLLSRAGSF SRFYSLKVAP KVKTSAAPGG VPLQPQDLEF TKLPNGLVIA SLENYAPLSR
     IGLFVKAGSR YEDSNNLGTS HLLRLASSLT TKGASSFKIT RGIEAVGGKL SVTATRENMA
     YTVEGIRSDI EILMEFLLNV TTAPEFRRWE VAALRSQLKI DKAVAFQNSQ TRIIENLHDV
     AYKNALANPL YCPDYRMGKI TSEELHYFVQ NHFTSARMAL VGLGVSHSVL KQVAEQFLNM
     RGGLGLAGAK AKYRGGEIRE QNGDNLVHAA IVAESAAIGN AEANAFSVLQ HLLGAGPHIK
     RGNNTTSLLS QSVAKGSHQP FDVSAFNASY SDSGLFGIYT ISQAAAAGEV INAAYNQVKA
     VAQGNLSSAD VQAAKNKLKA GYLMSVETSE GFLSEIGSQA LAAGSYMPPS TVLQQIDSVA
     DADVVKAAKK FVSGKKSMAA SGNLGHTPFL DEL
//
```

**Mascot:** http://www.matrixscience.com/


Score **> 34** indicates **identity**  
Score **> 16** indicates **homology**
