# Supplementary material for: A common East-Asian ALDH2 mutation causes metabolic disorders and the therapeutic effect of ALDH2 activators
Source: Nat Commun. 2023 Sep 25;14:5971. doi: 10.1038/s41467-023-41570-6 (PMC10520061; doi:10.1038/s41467-023-41570-6)
Supplement: Supplementary file 4 — Supplementary Data 1 [file 41467_2023_41570_MOESM4_ESM.zip › Table S5c/Q9Z2Z6/Q9Z2Z6_KIO-2_K12_C136.html]

Mascot Search Results: Q9Z2Z6
 

# MASCOT Search Results

## Protein View: Q9Z2Z6

### Mitochondrial carnitine/acylcarnitine carrier protein OS=Mus musculus OX=10090 GN=Slc25a20 PE=1 SV=1

|  |  |
| --- | --- |
| Database: | Mouse\_UniProt\_proteomes |
| Score: | 2677 |
| Monoisotopic mass (M_r_): | 33347 |
| Calculated pI: | 9.24 |

Sequence similarity is available as an NCBI BLAST search of Q9Z2Z6 against nr.

### Search parameters

|  |  |
| --- | --- |
| MS data file: | `D:\LCMSMS\2023 Users' data\230529-1\230529-1-KIO-2.raw` |
| Enzyme: | Trypsin/P: cuts C-term side of KR. |
| Fixed modifications: | Carbamidomethyl (C) |
| Variable modifications: | Deamidated (NQ), HNE (C), HNE (H), HNE (K), Oxidation (M) |

### Protein sequence coverage: 69%

Matched peptides shown in ***bold red***.

|  |  |  |  |  |  |
| --- | --- | --- | --- | --- | --- |
| `1` | `MADEPKPISP` | `FKNLLAGGFG` | `GMCLVFVGHP` | `LDTVKVRLQT` | `QPPSLSGQPP` |
| `51` | `MYSGTLDCFR` | `KTLMREGITG` | `LYRGMAAPII` | `GVTPMFAVCF` | `FGFGLGKKLQ` |
| `101` | `QKSPEDELSY` | `PQLFTAGMLS` | `GVFTTGIMTP` | `GERIKCLLQI` | `QASSGENKYS` |
| `151` | `GTLDCAKKLY` | `QEFGIRGFYK` | `GTVLTLMRDV` | `PASGMYFMTY` | `EWLKNLFTPE` |
| `201` | `GKSVSDLSVP` | `RILVAGGFAG` | `IFNWAVAIPP` | `DVLKSRFQTA` | `PPGKYPNGFR` |
| `251` | `DVLRELIREE` | `GVTSLYKGFN` | `AVMIRAFPAN` | `AACFLGFEIA` | `MKFLNWIAPN` |
| `301` | `L` |  |  |  |  |

Unformatted sequence string: 301 residues (for pasting into other applications).

|  |  |  |  |
| --- | --- | --- | --- |
| Sort by | residue number | increasing mass | decreasing mass |
| Show | matched peptides only | predicted peptides also |  |

| Query | Start | – | End | Observed | Mr(expt) | Mr(calc) | ppm | M | Score | Expect | Rank | U | Peptide |
| --- | --- | --- | --- | --- | --- | --- | --- | --- | --- | --- | --- | --- | --- |
| 195968 | 2 | – | 35 | 942.9928 | 3767.9420 | 3767.9671 | -6.66 | 2 | 15 | 0.036 | 1Score **> 37** indicates **identity** Score **> 13** indicates **homology** | U | M.ADEPKPISPFKNLLAGGFGGMCLVFVGHPLDTVK.V  + Deamidated (NQ); HNE (K) |
| 158239 | 13 | – | 35 | 801.4162 | 2401.2267 | 2401.2287 | -0.84 | 0 | 51 | 1.7e-05 | 1Score **> 37** indicates **identity** Score **> 16** indicates **homology** | U | K.NLLAGGFGGMCLVFVGHPLDTVK.V |
| 158240 | 13 | – | 35 | 1201.6220 | 2401.2295 | 2401.2287 | 0.32 | 0 | 54 | 9.6e-06 | 1Score **> 37** indicates **identity** Score **> 16** indicates **homology** | U | K.NLLAGGFGGMCLVFVGHPLDTVK.V |
| 158242 | 13 | – | 35 | 1201.6251 | 2401.2356 | 2401.2287 | 2.89 | 0 | 94 | 1.4e-09 | 1Score **> 37** indicates **identity** Score **> 18** indicates **homology** | U | K.NLLAGGFGGMCLVFVGHPLDTVK.V |
| 158244 | 13 | – | 35 | 801.4197 | 2401.2372 | 2401.2287 | 3.55 | 0 | 40 | 0.00019 | 1Score **> 37** indicates **identity** Score **> 15** indicates **homology** | U | K.NLLAGGFGGMCLVFVGHPLDTVK.V |
| 167720 | 38 | – | 60 | 1291.1195 | 2580.2244 | 2580.1989 | 9.91 | 0 | 21 | 0.011 | 1Score **> 35** indicates **identity** Score **> 14** indicates **homology** | U | R.LQTQPPSLSGQPPMYSGTLDCFR.K  + Deamidated (NQ) |
| 173042 | 38 | – | 61 | 903.4441 | 2707.3106 | 2707.3098 | 0.28 | 1 | 42 | 0.00011 | 1Score **> 36** indicates **identity** Score **> 15** indicates **homology** | U | R.LQTQPPSLSGQPPMYSGTLDCFRK.T |
| 173043 | 38 | – | 61 | 903.4443 | 2707.3112 | 2707.3098 | 0.51 | 1 | 34 | 0.00067 | 1Score **> 36** indicates **identity** Score **> 15** indicates **homology** | U | R.LQTQPPSLSGQPPMYSGTLDCFRK.T |
| 173044 | 38 | – | 61 | 903.4446 | 2707.3119 | 2707.3098 | 0.75 | 1 | 33 | 0.00088 | 1Score **> 37** indicates **identity** Score **> 15** indicates **homology** | U | R.LQTQPPSLSGQPPMYSGTLDCFRK.T |
| 173046 | 38 | – | 61 | 903.4451 | 2707.3134 | 2707.3098 | 1.31 | 1 | 41 | 0.00016 | 1Score **> 37** indicates **identity** Score **> 15** indicates **homology** | U | R.LQTQPPSLSGQPPMYSGTLDCFRK.T |
| 173680 | 38 | – | 61 | 908.7738 | 2723.2996 | 2723.3047 | -1.90 | 1 | 15 | 0.041 | 1Score **> 36** indicates **identity** Score **> 13** indicates **homology** | U | R.LQTQPPSLSGQPPMYSGTLDCFRK.T  + Oxidation (M) |
| 173681 | 38 | – | 61 | 908.7748 | 2723.3026 | 2723.3047 | -0.78 | 1 | 28 | 0.0026 | 1Score **> 36** indicates **identity** Score **> 14** indicates **homology** | U | R.LQTQPPSLSGQPPMYSGTLDCFRK.T  + Oxidation (M) |
| 9061 | 66 | – | 73 | 454.7454 | 907.4763 | 907.4763 | 0.0066 | 0 | 48 | 0.00069 | 1Score **> 29** indicates **identity** | U | R.EGITGLYR.G |
| 9063 | 66 | – | 73 | 454.7457 | 907.4769 | 907.4763 | 0.61 | 0 | 56 | 0.00011 | 1Score **> 29** indicates **identity** | U | R.EGITGLYR.G |
| 9064 | 66 | – | 73 | 454.7460 | 907.4774 | 907.4763 | 1.22 | 0 | 53 | 0.00022 | 1Score **> 29** indicates **identity** | U | R.EGITGLYR.G |
| 172218 | 134 | – | 157 | 671.8396 | 2683.3292 | 2683.3309 | -0.62 | 2 | 20 | 0.013 | 1Score **> 37** indicates **identity** Score **> 14** indicates **homology** | U | R.IKCLLQIQASSGENKYSGTLDCAK.K |
| 172266 | 134 | – | 157 | 672.0877 | 2684.3217 | 2684.3149 | 2.53 | 2 | 31 | 0.0013 | 1Score **> 37** indicates **identity** Score **> 14** indicates **homology** | U | R.IKCLLQIQASSGENKYSGTLDCAK.K  + Deamidated (NQ) |
| 176203 | 134 | – | 157 | 929.1299 | 2784.3679 | 2784.3925 | -8.82 | 2 | 17 | 0.026 | 1Score **> 37** indicates **identity** Score **> 14** indicates **homology** | U | R.IKCLLQIQASSGENKYSGTLDCAK.K  + 2 Deamidated (NQ); HNE (C) |
| 61316 | 136 | – | 148 | 724.3647 | 1446.7148 | 1446.7137 | 0.78 | 0 | 54 | 9.3e-06 | 1Score **> 34** indicates **identity** Score **> 16** indicates **homology** | U | K.CLLQIQASSGENK.Y |
| 61318 | 136 | – | 148 | 724.3651 | 1446.7157 | 1446.7137 | 1.44 | 0 | 44 | 6.7e-05 | 1Score **> 34** indicates **identity** Score **> 15** indicates **homology** | U | K.CLLQIQASSGENK.Y |
| 61319 | 136 | – | 148 | 724.3653 | 1446.7161 | 1446.7137 | 1.72 | 0 | 43 | 9.5e-05 | 1Score **> 34** indicates **identity** Score **> 15** indicates **homology** | U | K.CLLQIQASSGENK.Y |
| 160524 | 136 | – | 157 | 815.0575 | 2442.1507 | 2442.1519 | -0.50 | 1 | 57 | 4.6e-06 | 1Score **> 35** indicates **identity** Score **> 16** indicates **homology** | U | K.CLLQIQASSGENKYSGTLDCAK.K |
| 160525 | 136 | – | 157 | 1222.0828 | 2442.1510 | 2442.1519 | -0.35 | 1 | 98 | 5.9e-10 | 1Score **> 35** indicates **identity** Score **> 19** indicates **homology** | U | K.CLLQIQASSGENKYSGTLDCAK.K |
| 160526 | 136 | – | 157 | 815.0581 | 2442.1525 | 2442.1519 | 0.27 | 1 | 98 | 7e-10 | 1Score **> 35** indicates **identity** Score **> 19** indicates **homology** | U | K.CLLQIQASSGENKYSGTLDCAK.K |
| 160527 | 136 | – | 157 | 1222.0837 | 2442.1529 | 2442.1519 | 0.41 | 1 | 125 | 1.8e-12 | 1Score **> 35** indicates **identity** Score **> 20** indicates **homology** | U | K.CLLQIQASSGENKYSGTLDCAK.K |
| 160529 | 136 | – | 157 | 815.0586 | 2442.1539 | 2442.1519 | 0.83 | 1 | 58 | 3.7e-06 | 1Score **> 35** indicates **identity** Score **> 16** indicates **homology** | U | K.CLLQIQASSGENKYSGTLDCAK.K |
| 160530 | 136 | – | 157 | 815.0586 | 2442.1541 | 2442.1519 | 0.90 | 1 | 93 | 1.7e-09 | 1Score **> 35** indicates **identity** Score **> 18** indicates **homology** | U | K.CLLQIQASSGENKYSGTLDCAK.K |
| 160531 | 136 | – | 157 | 1222.0844 | 2442.1543 | 2442.1519 | 0.98 | 1 | 61 | 2e-06 | 1Score **> 35** indicates **identity** Score **> 16** indicates **homology** | U | K.CLLQIQASSGENKYSGTLDCAK.K |
| 160532 | 136 | – | 157 | 815.0587 | 2442.1544 | 2442.1519 | 1.03 | 1 | 36 | 0.0004 | 1Score **> 35** indicates **identity** Score **> 15** indicates **homology** | U | K.CLLQIQASSGENKYSGTLDCAK.K |
| 160534 | 136 | – | 157 | 815.0596 | 2442.1571 | 2442.1519 | 2.12 | 1 | 25 | 0.0045 | 1Score **> 35** indicates **identity** Score **> 14** indicates **homology** | U | K.CLLQIQASSGENKYSGTLDCAK.K |
| 160582 | 136 | – | 157 | 815.3903 | 2443.1491 | 2443.1359 | 5.42 | 1 | 39 | 0.00021 | 1Score **> 35** indicates **identity** Score **> 15** indicates **homology** | U | K.CLLQIQASSGENKYSGTLDCAK.K  + Deamidated (NQ) |
| 160586 | 136 | – | 157 | 815.3917 | 2443.1533 | 2443.1359 | 7.14 | 1 | 36 | 0.00044 | 1Score **> 35** indicates **identity** Score **> 15** indicates **homology** | U | K.CLLQIQASSGENKYSGTLDCAK.K  + Deamidated (NQ) |
| 167270 | 136 | – | 158 | 857.7548 | 2570.2427 | 2570.2468 | -1.62 | 2 | 47 | 3.9e-05 | 1Score **> 36** indicates **identity** Score **> 15** indicates **homology** | U | K.CLLQIQASSGENKYSGTLDCAKK.L |
| 167271 | 136 | – | 158 | 643.5684 | 2570.2447 | 2570.2468 | -0.85 | 2 | 16 | 0.034 | 1Score **> 37** indicates **identity** Score **> 13** indicates **homology** | U | K.CLLQIQASSGENKYSGTLDCAKK.L |
| 167272 | 136 | – | 158 | 643.5685 | 2570.2450 | 2570.2468 | -0.73 | 2 | 37 | 0.00031 | 1Score **> 36** indicates **identity** Score **> 15** indicates **homology** | U | K.CLLQIQASSGENKYSGTLDCAKK.L |
| 167326 | 136 | – | 158 | 858.0913 | 2571.2521 | 2571.2308 | 8.26 | 2 | 32 | 0.0011 | 1Score **> 37** indicates **identity** Score **> 14** indicates **homology** | U | K.CLLQIQASSGENKYSGTLDCAKK.L  + Deamidated (NQ) |
| 15720 | 149 | – | 157 | 507.7320 | 1013.4495 | 1013.4488 | 0.68 | 0 | 28 | 0.0022 | 1Score **> 25** indicates **identity** Score **> 14** indicates **homology** | U | K.YSGTLDCAK.K |
| 27284 | 149 | – | 158 | 571.7800 | 1141.5455 | 1141.5437 | 1.50 | 1 | 50 | 4.9e-05 | 1Score **> 30** indicates **identity** Score **> 20** indicates **homology** | U | K.YSGTLDCAKK.L |
| 28533 | 158 | – | 166 | 385.2163 | 1152.6270 | 1152.6291 | -1.86 | 1 | 20 | 0.012 | 1Score **> 31** indicates **identity** Score **> 14** indicates **homology** | U | K.KLYQEFGIR.G |
| 28535 | 158 | – | 166 | 385.2168 | 1152.6286 | 1152.6291 | -0.44 | 1 | 30 | 0.0045 | 1Score **> 32** indicates **identity** Score **> 20** indicates **homology** | U | K.KLYQEFGIR.G |
| 28536 | 158 | – | 166 | 577.3217 | 1152.6288 | 1152.6291 | -0.28 | 1 | 36 | 0.00098 | 1Score **> 32** indicates **identity** Score **> 18** indicates **homology** | U | K.KLYQEFGIR.G |
| 28537 | 158 | – | 166 | 577.3218 | 1152.6290 | 1152.6291 | -0.087 | 1 | 63 | 1.5e-05 | 1Score **> 32** indicates **identity** Score **> 27** indicates **homology** | U | K.KLYQEFGIR.G |
| 28538 | 158 | – | 166 | 385.2170 | 1152.6291 | 1152.6291 | 0.022 | 1 | 24 | 0.017 | 1Score **> 32** indicates **identity** Score **> 19** indicates **homology** | U | K.KLYQEFGIR.G |
| 28539 | 158 | – | 166 | 577.3219 | 1152.6292 | 1152.6291 | 0.052 | 1 | 53 | 6.1e-05 | 1Score **> 32** indicates **identity** Score **> 23** indicates **homology** | U | K.KLYQEFGIR.G |
| 28540 | 158 | – | 166 | 577.3219 | 1152.6293 | 1152.6291 | 0.15 | 1 | 63 | 1.5e-05 | 1Score **> 32** indicates **identity** Score **> 27** indicates **homology** | U | K.KLYQEFGIR.G |
| 28543 | 158 | – | 166 | 385.2172 | 1152.6298 | 1152.6291 | 0.57 | 1 | 21 | 0.031 | 1Score **> 32** indicates **identity** Score **> 18** indicates **homology** | U | K.KLYQEFGIR.G |
| 28547 | 158 | – | 166 | 577.3234 | 1152.6323 | 1152.6291 | 2.76 | 1 | 36 | 0.0016 | 1Score **> 32** indicates **identity** Score **> 21** indicates **homology** | U | K.KLYQEFGIR.G |
| 16499 | 159 | – | 166 | 513.2731 | 1024.5317 | 1024.5342 | -2.41 | 0 | 22 | 0.0085 | 1Score **> 30** indicates **identity** Score **> 14** indicates **homology** | U | K.LYQEFGIR.G |
| 16500 | 159 | – | 166 | 513.2734 | 1024.5323 | 1024.5342 | -1.82 | 0 | 18 | 0.022 | 1Score **> 30** indicates **identity** Score **> 14** indicates **homology** | U | K.LYQEFGIR.G |
| 16501 | 159 | – | 166 | 513.2737 | 1024.5328 | 1024.5342 | -1.35 | 0 | 17 | 0.024 | 1Score **> 30** indicates **identity** Score **> 14** indicates **homology** | U | K.LYQEFGIR.G |
| 16503 | 159 | – | 166 | 513.2748 | 1024.5350 | 1024.5342 | 0.78 | 0 | 43 | 0.0001 | 1Score **> 30** indicates **identity** Score **> 15** indicates **homology** | U | K.LYQEFGIR.G |
| 54085 | 167 | – | 178 | 462.5922 | 1384.7547 | 1384.7537 | 0.69 | 1 | 42 | 0.00013 | 1Score **> 34** indicates **identity** Score **> 15** indicates **homology** | U | R.GFYKGTVLTLMR.D |
| 54087 | 167 | – | 178 | 462.5923 | 1384.7551 | 1384.7537 | 1.00 | 1 | 35 | 0.0005 | 1Score **> 34** indicates **identity** Score **> 15** indicates **homology** | U | R.GFYKGTVLTLMR.D |
| 54090 | 167 | – | 178 | 462.5926 | 1384.7560 | 1384.7537 | 1.67 | 1 | 21 | 0.0097 | 1Score **> 34** indicates **identity** Score **> 14** indicates **homology** | U | R.GFYKGTVLTLMR.D |
| 54092 | 167 | – | 178 | 462.5928 | 1384.7566 | 1384.7537 | 2.09 | 1 | 20 | 0.014 | 1Score **> 34** indicates **identity** Score **> 14** indicates **homology** | U | R.GFYKGTVLTLMR.D |
| 8039 | 171 | – | 178 | 445.7599 | 889.5053 | 889.5055 | -0.27 | 0 | 26 | 0.0059 | 1Score **> 29** indicates **identity** Score **> 16** indicates **homology** | U | K.GTVLTLMR.D |
| 8040 | 171 | – | 178 | 445.7600 | 889.5055 | 889.5055 | -0.011 | 0 | 44 | 0.00029 | 1Score **> 29** indicates **identity** Score **> 21** indicates **homology** | U | K.GTVLTLMR.D |
| 120218 | 179 | – | 194 | 969.4458 | 1936.8771 | 1936.8739 | 1.65 | 0 | 48 | 2.8e-05 | 1Score **> 31** indicates **identity** Score **> 16** indicates **homology** | U | R.DVPASGMYFMTYEWLK.N |
| 8833 | 195 | – | 202 | 453.2396 | 904.4646 | 904.4654 | -0.95 | 0 | 24 | 0.0053 | 1Score **> 30** indicates **identity** Score **> 14** indicates **homology** | U | K.NLFTPEGK.S |
| 8834 | 195 | – | 202 | 453.2396 | 904.4647 | 904.4654 | -0.84 | 0 | 35 | 0.00053 | 1Score **> 30** indicates **identity** Score **> 15** indicates **homology** | U | K.NLFTPEGK.S |
| 8836 | 195 | – | 202 | 453.2399 | 904.4652 | 904.4654 | -0.29 | 0 | 28 | 0.0025 | 1Score **> 30** indicates **identity** Score **> 15** indicates **homology** | U | K.NLFTPEGK.S |
| 8837 | 195 | – | 202 | 453.2399 | 904.4652 | 904.4654 | -0.28 | 0 | 28 | 0.0031 | 1Score **> 30** indicates **identity** Score **> 15** indicates **homology** | U | K.NLFTPEGK.S |
| 8838 | 195 | – | 202 | 453.2405 | 904.4664 | 904.4654 | 1.12 | 0 | 35 | 0.00054 | 1Score **> 31** indicates **identity** Score **> 15** indicates **homology** | U | K.NLFTPEGK.S |
| 12179 | 203 | – | 211 | 480.2610 | 958.5074 | 958.5084 | -1.05 | 0 | 53 | 1e-05 | 1Score **> 31** indicates **identity** Score **> 16** indicates **homology** | U | K.SVSDLSVPR.I |
| 12181 | 203 | – | 211 | 480.2611 | 958.5077 | 958.5084 | -0.67 | 0 | 58 | 3.6e-06 | 1Score **> 31** indicates **identity** Score **> 16** indicates **homology** | U | K.SVSDLSVPR.I |
| 12183 | 203 | – | 211 | 480.2614 | 958.5083 | 958.5084 | -0.021 | 0 | 35 | 0.0021 | 1Score **> 30** indicates **identity** Score **> 20** indicates **homology** | U | K.SVSDLSVPR.I |
| 12184 | 203 | – | 211 | 480.2616 | 958.5086 | 958.5084 | 0.26 | 0 | 54 | 8.3e-06 | 1Score **> 30** indicates **identity** Score **> 16** indicates **homology** | U | K.SVSDLSVPR.I |
| 12185 | 203 | – | 211 | 480.2620 | 958.5095 | 958.5084 | 1.18 | 0 | 49 | 2.4e-05 | 1Score **> 30** indicates **identity** Score **> 16** indicates **homology** | U | K.SVSDLSVPR.I |
| 78268 | 237 | – | 250 | 527.2702 | 1578.7888 | 1578.7943 | -3.51 | 1 | 24 | 0.0054 | 1Score **> 34** indicates **identity** Score **> 14** indicates **homology** | U | R.FQTAPPGKYPNGFR.D |
| 78274 | 237 | – | 250 | 527.2722 | 1578.7948 | 1578.7943 | 0.30 | 1 | 34 | 0.0007 | 1Score **> 34** indicates **identity** Score **> 15** indicates **homology** | U | R.FQTAPPGKYPNGFR.D |
| 78276 | 237 | – | 250 | 527.2723 | 1578.7952 | 1578.7943 | 0.56 | 1 | 34 | 0.00067 | 1Score **> 34** indicates **identity** Score **> 15** indicates **homology** | U | R.FQTAPPGKYPNGFR.D |
| 78277 | 237 | – | 250 | 527.2726 | 1578.7960 | 1578.7943 | 1.08 | 1 | 16 | 0.035 | 1Score **> 34** indicates **identity** Score **> 13** indicates **homology** | U | R.FQTAPPGKYPNGFR.D |
| 78281 | 237 | – | 250 | 790.4074 | 1578.8002 | 1578.7943 | 3.74 | 1 | 39 | 0.00024 | 1Score **> 34** indicates **identity** Score **> 15** indicates **homology** | U | R.FQTAPPGKYPNGFR.D |
| 78286 | 237 | – | 250 | 790.4103 | 1578.8060 | 1578.7943 | 7.39 | 1 | 40 | 0.0002 | 1Score **> 34** indicates **identity** Score **> 15** indicates **homology** | U | R.FQTAPPGKYPNGFR.D |
| 78384 | 237 | – | 250 | 527.6003 | 1579.7789 | 1579.7783 | 0.38 | 1 | 21 | 0.011 | 1Score **> 34** indicates **identity** Score **> 14** indicates **homology** | U | R.FQTAPPGKYPNGFR.D  + Deamidated (NQ) |
| 78388 | 237 | – | 250 | 527.6007 | 1579.7802 | 1579.7783 | 1.21 | 1 | 38 | 0.0003 | 1Score **> 34** indicates **identity** Score **> 15** indicates **homology** | U | R.FQTAPPGKYPNGFR.D  + Deamidated (NQ) |
| 78391 | 237 | – | 250 | 527.6009 | 1579.7810 | 1579.7783 | 1.71 | 1 | 22 | 0.0079 | 1Score **> 34** indicates **identity** Score **> 14** indicates **homology** | U | R.FQTAPPGKYPNGFR.D  + Deamidated (NQ) |
| 78392 | 237 | – | 250 | 527.6010 | 1579.7811 | 1579.7783 | 1.78 | 1 | 24 | 0.018 | 1Score **> 34** indicates **identity** Score **> 20** indicates **homology** | U | R.FQTAPPGKYPNGFR.D  + Deamidated (NQ) |
| 78393 | 237 | – | 250 | 527.6010 | 1579.7812 | 1579.7783 | 1.81 | 1 | 37 | 0.00037 | 1Score **> 34** indicates **identity** Score **> 15** indicates **homology** | U | R.FQTAPPGKYPNGFR.D  + Deamidated (NQ) |
| 78394 | 237 | – | 250 | 527.6015 | 1579.7828 | 1579.7783 | 2.83 | 1 | 40 | 0.00016 | 1Score **> 34** indicates **identity** Score **> 15** indicates **homology** | U | R.FQTAPPGKYPNGFR.D  + Deamidated (NQ) |
| 78395 | 237 | – | 250 | 790.8989 | 1579.7832 | 1579.7783 | 3.07 | 1 | 24 | 0.0052 | 1Score **> 34** indicates **identity** Score **> 14** indicates **homology** | U | R.FQTAPPGKYPNGFR.D  + Deamidated (NQ) |
| 131868 | 237 | – | 254 | 688.3660 | 2062.0762 | 2062.0748 | 0.64 | 2 | 18 | 0.02 | 1Score **> 36** indicates **identity** Score **> 14** indicates **homology** | U | R.FQTAPPGKYPNGFRDVLR.E |
| 131869 | 237 | – | 254 | 516.5264 | 2062.0764 | 2062.0748 | 0.75 | 2 | 15 | 0.037 | 1Score **> 36** indicates **identity** Score **> 13** indicates **homology** | U | R.FQTAPPGKYPNGFRDVLR.E |
| 131872 | 237 | – | 254 | 688.3661 | 2062.0766 | 2062.0748 | 0.83 | 2 | 21 | 0.011 | 1Score **> 36** indicates **identity** Score **> 14** indicates **homology** | U | R.FQTAPPGKYPNGFRDVLR.E |
| 131874 | 237 | – | 254 | 516.5266 | 2062.0772 | 2062.0748 | 1.14 | 2 | 16 | 0.032 | 1Score **> 36** indicates **identity** Score **> 13** indicates **homology** | U | R.FQTAPPGKYPNGFRDVLR.E |
| 131982 | 237 | – | 254 | 516.7715 | 2063.0571 | 2063.0589 | -0.87 | 2 | 22 | 0.0082 | 1Score **> 36** indicates **identity** Score **> 14** indicates **homology** | U | R.FQTAPPGKYPNGFRDVLR.E  + Deamidated (NQ) |
| 131984 | 237 | – | 254 | 516.7721 | 2063.0595 | 2063.0589 | 0.30 | 2 | 22 | 0.0092 | 1Score **> 36** indicates **identity** Score **> 14** indicates **homology** | U | R.FQTAPPGKYPNGFRDVLR.E  + Deamidated (NQ) |
| 131985 | 237 | – | 254 | 516.7724 | 2063.0604 | 2063.0589 | 0.73 | 2 | 18 | 0.021 | 1Score **> 36** indicates **identity** Score **> 14** indicates **homology** | U | R.FQTAPPGKYPNGFRDVLR.E  + Deamidated (NQ) |
| 131987 | 237 | – | 254 | 688.6943 | 2063.0610 | 2063.0589 | 1.04 | 2 | 24 | 0.0054 | 1Score **> 36** indicates **identity** Score **> 14** indicates **homology** | U | R.FQTAPPGKYPNGFRDVLR.E  + Deamidated (NQ) |
| 131988 | 237 | – | 254 | 516.7725 | 2063.0611 | 2063.0589 | 1.08 | 2 | 31 | 0.0012 | 1Score **> 36** indicates **identity** Score **> 14** indicates **homology** | U | R.FQTAPPGKYPNGFRDVLR.E  + Deamidated (NQ) |
| 131990 | 237 | – | 254 | 516.7728 | 2063.0620 | 2063.0589 | 1.53 | 2 | 26 | 0.0036 | 1Score **> 36** indicates **identity** Score **> 14** indicates **homology** | U | R.FQTAPPGKYPNGFRDVLR.E  + Deamidated (NQ) |
| 131991 | 237 | – | 254 | 688.6947 | 2063.0622 | 2063.0589 | 1.60 | 2 | 32 | 0.001 | 1Score **> 36** indicates **identity** Score **> 15** indicates **homology** | U | R.FQTAPPGKYPNGFRDVLR.E  + Deamidated (NQ) |
| 131993 | 237 | – | 254 | 688.6948 | 2063.0627 | 2063.0589 | 1.84 | 2 | 33 | 0.00074 | 1Score **> 36** indicates **identity** Score **> 15** indicates **homology** | U | R.FQTAPPGKYPNGFRDVLR.E  + Deamidated (NQ) |
| 131994 | 237 | – | 254 | 516.7730 | 2063.0629 | 2063.0589 | 1.95 | 2 | 23 | 0.0075 | 1Score **> 36** indicates **identity** Score **> 14** indicates **homology** | U | R.FQTAPPGKYPNGFRDVLR.E  + Deamidated (NQ) |
| 131995 | 237 | – | 254 | 516.7730 | 2063.0629 | 2063.0589 | 1.97 | 2 | 18 | 0.019 | 1Score **> 36** indicates **identity** Score **> 14** indicates **homology** | U | R.FQTAPPGKYPNGFRDVLR.E  + Deamidated (NQ) |
| 131996 | 237 | – | 254 | 516.7730 | 2063.0629 | 2063.0589 | 1.98 | 2 | 33 | 0.00085 | 1Score **> 36** indicates **identity** Score **> 15** indicates **homology** | U | R.FQTAPPGKYPNGFRDVLR.E  + Deamidated (NQ) |
| 131997 | 237 | – | 254 | 688.6950 | 2063.0632 | 2063.0589 | 2.08 | 2 | 25 | 0.0048 | 1Score **> 36** indicates **identity** Score **> 15** indicates **homology** | U | R.FQTAPPGKYPNGFRDVLR.E  + Deamidated (NQ) |
| 131998 | 237 | – | 254 | 516.7731 | 2063.0632 | 2063.0589 | 2.11 | 2 | 30 | 0.0015 | 1Score **> 36** indicates **identity** Score **> 14** indicates **homology** | U | R.FQTAPPGKYPNGFRDVLR.E  + Deamidated (NQ) |
| 132001 | 237 | – | 254 | 516.7733 | 2063.0640 | 2063.0589 | 2.48 | 2 | 37 | 0.00031 | 1Score **> 36** indicates **identity** Score **> 15** indicates **homology** | U | R.FQTAPPGKYPNGFRDVLR.E  + Deamidated (NQ) |
| 132002 | 237 | – | 254 | 516.7733 | 2063.0642 | 2063.0589 | 2.57 | 2 | 15 | 0.037 | 1Score **> 36** indicates **identity** Score **> 13** indicates **homology** | U | R.FQTAPPGKYPNGFRDVLR.E  + Deamidated (NQ) |
| 132004 | 237 | – | 254 | 516.7739 | 2063.0666 | 2063.0589 | 3.76 | 2 | 24 | 0.0061 | 1Score **> 36** indicates **identity** Score **> 14** indicates **homology** | U | R.FQTAPPGKYPNGFRDVLR.E  + Deamidated (NQ) |
| 72512 | 255 | – | 267 | 512.9464 | 1535.8173 | 1535.8195 | -1.42 | 1 | 29 | 0.002 | 1Score **> 34** indicates **identity** Score **> 14** indicates **homology** | U | R.ELIREEGVTSLYK.G |
| 72516 | 255 | – | 267 | 768.9161 | 1535.8177 | 1535.8195 | -1.18 | 1 | 30 | 0.0083 | 1Score **> 34** indicates **identity** Score **> 22** indicates **homology** | U | R.ELIREEGVTSLYK.G |
| 72523 | 255 | – | 267 | 768.9170 | 1535.8195 | 1535.8195 | 0.035 | 1 | 51 | 0.00022 | 1Score **> 34** indicates **identity** Score **> 27** indicates **homology** | U | R.ELIREEGVTSLYK.G |
| 72524 | 255 | – | 267 | 768.9172 | 1535.8198 | 1535.8195 | 0.19 | 1 | 51 | 0.00055 | 1Score **> 34** indicates **identity** Score **> 31** indicates **homology** | U | R.ELIREEGVTSLYK.G |
| 72525 | 255 | – | 267 | 512.9473 | 1535.8201 | 1535.8195 | 0.38 | 1 | 27 | 0.0028 | 1Score **> 34** indicates **identity** Score **> 14** indicates **homology** | U | R.ELIREEGVTSLYK.G |
| 72526 | 255 | – | 267 | 512.9473 | 1535.8202 | 1535.8195 | 0.44 | 1 | 28 | 0.0026 | 1Score **> 34** indicates **identity** Score **> 14** indicates **homology** | U | R.ELIREEGVTSLYK.G |
| 72527 | 255 | – | 267 | 512.9474 | 1535.8204 | 1535.8195 | 0.56 | 1 | 38 | 0.0003 | 1Score **> 34** indicates **identity** Score **> 15** indicates **homology** | U | R.ELIREEGVTSLYK.G |
| 72528 | 255 | – | 267 | 512.9474 | 1535.8204 | 1535.8195 | 0.60 | 1 | 19 | 0.016 | 1Score **> 34** indicates **identity** Score **> 14** indicates **homology** | U | R.ELIREEGVTSLYK.G |
| 72529 | 255 | – | 267 | 768.9177 | 1535.8209 | 1535.8195 | 0.91 | 1 | 41 | 0.0036 | 1Score **> 34** indicates **identity** Score **> 29** indicates **homology** | U | R.ELIREEGVTSLYK.G |
| 72530 | 255 | – | 267 | 768.9178 | 1535.8210 | 1535.8195 | 0.99 | 1 | 33 | 0.0037 | 1Score **> 34** indicates **identity** Score **> 22** indicates **homology** | U | R.ELIREEGVTSLYK.G |
| 72532 | 255 | – | 267 | 768.9178 | 1535.8211 | 1535.8195 | 1.03 | 1 | 31 | 0.0064 | 1Score **> 34** indicates **identity** Score **> 21** indicates **homology** | U | R.ELIREEGVTSLYK.G |
| 72534 | 255 | – | 267 | 768.9179 | 1535.8213 | 1535.8195 | 1.20 | 1 | 33 | 0.012 | 1Score **> 34** indicates **identity** Score **> 26** indicates **homology** | U | R.ELIREEGVTSLYK.G |
| 72536 | 255 | – | 267 | 512.9478 | 1535.8216 | 1535.8195 | 1.37 | 1 | 26 | 0.006 | 1Score **> 34** indicates **identity** Score **> 16** indicates **homology** | U | R.ELIREEGVTSLYK.G |
| 72537 | 255 | – | 267 | 512.9479 | 1535.8218 | 1535.8195 | 1.48 | 1 | 31 | 0.0019 | 1Score **> 34** indicates **identity** Score **> 16** indicates **homology** | U | R.ELIREEGVTSLYK.G |
| 72539 | 255 | – | 267 | 512.9480 | 1535.8223 | 1535.8195 | 1.83 | 1 | 25 | 0.0041 | 1Score **> 34** indicates **identity** Score **> 14** indicates **homology** | U | R.ELIREEGVTSLYK.G |
| 72540 | 255 | – | 267 | 768.9186 | 1535.8226 | 1535.8195 | 2.05 | 1 | 22 | 0.024 | 1Score **> 34** indicates **identity** Score **> 18** indicates **homology** | U | R.ELIREEGVTSLYK.G |
| 72541 | 255 | – | 267 | 768.9186 | 1535.8227 | 1535.8195 | 2.06 | 1 | 24 | 0.033 | 1Score **> 34** indicates **identity** Score **> 22** indicates **homology** | U | R.ELIREEGVTSLYK.G |
| 72542 | 255 | – | 267 | 512.9483 | 1535.8230 | 1535.8195 | 2.30 | 1 | 25 | 0.0048 | 1Score **> 34** indicates **identity** Score **> 14** indicates **homology** | U | R.ELIREEGVTSLYK.G |
| 72543 | 255 | – | 267 | 512.9484 | 1535.8235 | 1535.8195 | 2.59 | 1 | 26 | 0.0034 | 1Score **> 34** indicates **identity** Score **> 14** indicates **homology** | U | R.ELIREEGVTSLYK.G |
| 72544 | 255 | – | 267 | 512.9485 | 1535.8236 | 1535.8195 | 2.68 | 1 | 34 | 0.0007 | 1Score **> 34** indicates **identity** Score **> 15** indicates **homology** | U | R.ELIREEGVTSLYK.G |
| 159573 | 255 | – | 275 | 607.0790 | 2424.2868 | 2424.2835 | 1.35 | 2 | 40 | 0.00017 | 1Score **> 36** indicates **identity** Score **> 15** indicates **homology** | U | R.ELIREEGVTSLYKGFNAVMIR.A |
| 159574 | 255 | – | 275 | 607.0796 | 2424.2893 | 2424.2835 | 2.40 | 2 | 37 | 0.00036 | 1Score **> 36** indicates **identity** Score **> 15** indicates **homology** | U | R.ELIREEGVTSLYKGFNAVMIR.A |
| 8997 | 268 | – | 275 | 454.2446 | 906.4747 | 906.4745 | 0.17 | 0 | 28 | 0.0031 | 1Score **> 30** indicates **identity** Score **> 16** indicates **homology** | U | K.GFNAVMIR.A |
| 8998 | 268 | – | 275 | 454.2447 | 906.4749 | 906.4745 | 0.42 | 0 | 29 | 0.0017 | 1Score **> 29** indicates **identity** Score **> 14** indicates **homology** | U | K.GFNAVMIR.A |
| 8999 | 268 | – | 275 | 454.2448 | 906.4749 | 906.4745 | 0.44 | 0 | 36 | 0.0005 | 1Score **> 29** indicates **identity** Score **> 16** indicates **homology** | U | K.GFNAVMIR.A |
| 9000 | 268 | – | 275 | 454.2448 | 906.4750 | 906.4745 | 0.51 | 0 | 36 | 0.00045 | 1Score **> 29** indicates **identity** Score **> 15** indicates **homology** | U | K.GFNAVMIR.A |
| 9001 | 268 | – | 275 | 454.2448 | 906.4751 | 906.4745 | 0.57 | 0 | 30 | 0.0014 | 1Score **> 29** indicates **identity** Score **> 14** indicates **homology** | U | K.GFNAVMIR.A |
| 10030 | 268 | – | 275 | 462.2414 | 922.4682 | 922.4695 | -1.42 | 0 | 19 | 0.016 | 1Score **> 29** indicates **identity** Score **> 14** indicates **homology** | U | K.GFNAVMIR.A  + Oxidation (M) |
| 112428 | 276 | – | 292 | 929.4522 | 1856.8898 | 1856.8953 | -2.95 | 0 | 64 | 1.1e-06 | 1Score **> 34** indicates **identity** Score **> 16** indicates **homology** | U | R.AFPANAACFLGFEIAMK.F |
| 112429 | 276 | – | 292 | 929.4544 | 1856.8943 | 1856.8953 | -0.53 | 0 | 49 | 2.5e-05 | 1Score **> 34** indicates **identity** Score **> 16** indicates **homology** | U | R.AFPANAACFLGFEIAMK.F |
| 112430 | 276 | – | 292 | 619.9726 | 1856.8959 | 1856.8953 | 0.29 | 0 | 19 | 0.015 | 1Score **> 34** indicates **identity** Score **> 14** indicates **homology** | U | R.AFPANAACFLGFEIAMK.F |
| 112440 | 276 | – | 292 | 929.4589 | 1856.9033 | 1856.8953 | 4.28 | 0 | 65 | 7.5e-07 | 1Score **> 34** indicates **identity** Score **> 17** indicates **homology** | U | R.AFPANAACFLGFEIAMK.F |
| 21734 | 293 | – | 301 | 544.3000 | 1086.5855 | 1086.5862 | -0.64 | 0 | 24 | 0.0058 | 1Score **> 34** indicates **identity** Score **> 14** indicates **homology** | U | K.FLNWIAPNL.- |
| 21735 | 293 | – | 301 | 544.3002 | 1086.5858 | 1086.5862 | -0.40 | 0 | 18 | 0.021 | 1Score **> 34** indicates **identity** Score **> 14** indicates **homology** | U | K.FLNWIAPNL.- |
| 21736 | 293 | – | 301 | 544.3002 | 1086.5858 | 1086.5862 | -0.40 | 0 | 23 | 0.0064 | 1Score **> 34** indicates **identity** Score **> 14** indicates **homology** | U | K.FLNWIAPNL.- |
| 21740 | 293 | – | 301 | 544.3010 | 1086.5874 | 1086.5862 | 1.09 | 0 | 16 | 0.031 | 1Score **> 34** indicates **identity** Score **> 14** indicates **homology** | U | K.FLNWIAPNL.- |
| 21741 | 293 | – | 301 | 544.3010 | 1086.5875 | 1086.5862 | 1.17 | 0 | 19 | 0.016 | 1Score **> 34** indicates **identity** Score **> 14** indicates **homology** | U | K.FLNWIAPNL.- |

---

```
ID   MCAT_MOUSE              Reviewed;         301 AA.
AC   Q9Z2Z6; Q3U801;
DT   11-JUL-2002, integrated into UniProtKB/Swiss-Prot.
DT   01-MAY-1999, sequence version 1.
DT   28-JUN-2023, entry version 160.
DE   RecName: Full=Mitochondrial carnitine/acylcarnitine carrier protein {ECO:0000305};
DE   AltName: Full=Carnitine/acylcarnitine translocase;
DE            Short=CAC;
DE            Short=CACT;
DE            Short=mCAC;
DE   AltName: Full=Solute carrier family 25 member 20;
GN   Name=Slc25a20 {ECO:0000312|EMBL:BAA74768.1}; Synonyms=Cac, Cact;
OS   Mus musculus (Mouse).
OC   Eukaryota; Metazoa; Chordata; Craniata; Vertebrata; Euteleostomi; Mammalia;
OC   Eutheria; Euarchontoglires; Glires; Rodentia; Myomorpha; Muroidea; Muridae;
OC   Murinae; Mus; Mus.
OX   NCBI_TaxID=10090;
RN   [1]
RP   NUCLEOTIDE SEQUENCE [MRNA].
RA   Lu K., Kuwajima M., Shima K., Nakamura Y., Nishimori H.;
RT   "Cloning, genomic structure and chromosomal localization of murine
RT   mitochondrial carnitine-acylcarnitine translocase gene and mutation
RT   analysis in the juvenile visceral steatosis (JVS) mouse.";
RL   Submitted (AUG-1998) to the EMBL/GenBank/DDBJ databases.
RN   [2]
RP   NUCLEOTIDE SEQUENCE [LARGE SCALE MRNA].
RC   STRAIN=C57BL/6J; TISSUE=Bone marrow;
RX   PubMed=16141072; DOI=10.1126/science.1112014;
RA   Carninci P., Kasukawa T., Katayama S., Gough J., Frith M.C., Maeda N.,
RA   Oyama R., Ravasi T., Lenhard B., Wells C., Kodzius R., Shimokawa K.,
RA   Bajic V.B., Brenner S.E., Batalov S., Forrest A.R., Zavolan M., Davis M.J.,
RA   Wilming L.G., Aidinis V., Allen J.E., Ambesi-Impiombato A., Apweiler R.,
RA   Aturaliya R.N., Bailey T.L., Bansal M., Baxter L., Beisel K.W., Bersano T.,
RA   Bono H., Chalk A.M., Chiu K.P., Choudhary V., Christoffels A.,
RA   Clutterbuck D.R., Crowe M.L., Dalla E., Dalrymple B.P., de Bono B.,
RA   Della Gatta G., di Bernardo D., Down T., Engstrom P., Fagiolini M.,
RA   Faulkner G., Fletcher C.F., Fukushima T., Furuno M., Futaki S.,
RA   Gariboldi M., Georgii-Hemming P., Gingeras T.R., Gojobori T., Green R.E.,
RA   Gustincich S., Harbers M., Hayashi Y., Hensch T.K., Hirokawa N., Hill D.,
RA   Huminiecki L., Iacono M., Ikeo K., Iwama A., Ishikawa T., Jakt M.,
RA   Kanapin A., Katoh M., Kawasawa Y., Kelso J., Kitamura H., Kitano H.,
RA   Kollias G., Krishnan S.P., Kruger A., Kummerfeld S.K., Kurochkin I.V.,
RA   Lareau L.F., Lazarevic D., Lipovich L., Liu J., Liuni S., McWilliam S.,
RA   Madan Babu M., Madera M., Marchionni L., Matsuda H., Matsuzawa S., Miki H.,
RA   Mignone F., Miyake S., Morris K., Mottagui-Tabar S., Mulder N., Nakano N.,
RA   Nakauchi H., Ng P., Nilsson R., Nishiguchi S., Nishikawa S., Nori F.,
RA   Ohara O., Okazaki Y., Orlando V., Pang K.C., Pavan W.J., Pavesi G.,
RA   Pesole G., Petrovsky N., Piazza S., Reed J., Reid J.F., Ring B.Z.,
RA   Ringwald M., Rost B., Ruan Y., Salzberg S.L., Sandelin A., Schneider C.,
RA   Schoenbach C., Sekiguchi K., Semple C.A., Seno S., Sessa L., Sheng Y.,
RA   Shibata Y., Shimada H., Shimada K., Silva D., Sinclair B., Sperling S.,
RA   Stupka E., Sugiura K., Sultana R., Takenaka Y., Taki K., Tammoja K.,
RA   Tan S.L., Tang S., Taylor M.S., Tegner J., Teichmann S.A., Ueda H.R.,
RA   van Nimwegen E., Verardo R., Wei C.L., Yagi K., Yamanishi H.,
RA   Zabarovsky E., Zhu S., Zimmer A., Hide W., Bult C., Grimmond S.M.,
RA   Teasdale R.D., Liu E.T., Brusic V., Quackenbush J., Wahlestedt C.,
RA   Mattick J.S., Hume D.A., Kai C., Sasaki D., Tomaru Y., Fukuda S.,
RA   Kanamori-Katayama M., Suzuki M., Aoki J., Arakawa T., Iida J., Imamura K.,
RA   Itoh M., Kato T., Kawaji H., Kawagashira N., Kawashima T., Kojima M.,
RA   Kondo S., Konno H., Nakano K., Ninomiya N., Nishio T., Okada M., Plessy C.,
RA   Shibata K., Shiraki T., Suzuki S., Tagami M., Waki K., Watahiki A.,
RA   Okamura-Oho Y., Suzuki H., Kawai J., Hayashizaki Y.;
RT   "The transcriptional landscape of the mammalian genome.";
RL   Science 309:1559-1563(2005).
RN   [3]
RP   NUCLEOTIDE SEQUENCE [LARGE SCALE MRNA].
RC   STRAIN=Czech II; TISSUE=Mammary gland;
RX   PubMed=15489334; DOI=10.1101/gr.2596504;
RG   The MGC Project Team;
RT   "The status, quality, and expansion of the NIH full-length cDNA project:
RT   the Mammalian Gene Collection (MGC).";
RL   Genome Res. 14:2121-2127(2004).
RN   [4]
RP   TISSUE SPECIFICITY.
RX   PubMed=19287344; DOI=10.1203/pdr.0b013e3181a283c1;
RA   Camacho J.A., Rioseco-Camacho N.;
RT   "The human and mouse SLC25A29 mitochondrial transporters rescue the
RT   deficient ornithine metabolism in fibroblasts of patients with the
RT   hyperornithinemia-hyperammonemia-homocitrullinuria (HHH) syndrome.";
RL   Pediatr. Res. 66:35-41(2009).
RN   [5]
RP   IDENTIFICATION BY MASS SPECTROMETRY [LARGE SCALE ANALYSIS].
RC   TISSUE=Brown adipose tissue, Heart, Kidney, Liver, Lung, Spleen, and
RC   Testis;
RX   PubMed=21183079; DOI=10.1016/j.cell.2010.12.001;
RA   Huttlin E.L., Jedrychowski M.P., Elias J.E., Goswami T., Rad R.,
RA   Beausoleil S.A., Villen J., Haas W., Sowa M.E., Gygi S.P.;
RT   "A tissue-specific atlas of mouse protein phosphorylation and expression.";
RL   Cell 143:1174-1189(2010).
RN   [6]
RP   SUCCINYLATION [LARGE SCALE ANALYSIS] AT LYS-170, AND IDENTIFICATION BY MASS
RP   SPECTROMETRY [LARGE SCALE ANALYSIS].
RC   TISSUE=Liver;
RX   PubMed=23806337; DOI=10.1016/j.molcel.2013.06.001;
RA   Park J., Chen Y., Tishkoff D.X., Peng C., Tan M., Dai L., Xie Z., Zhang Y.,
RA   Zwaans B.M., Skinner M.E., Lombard D.B., Zhao Y.;
RT   "SIRT5-mediated lysine desuccinylation impacts diverse metabolic
RT   pathways.";
RL   Mol. Cell 50:919-930(2013).
RN   [7]
RP   ACETYLATION [LARGE SCALE ANALYSIS] AT LYS-148; LYS-157 AND LYS-170, AND
RP   IDENTIFICATION BY MASS SPECTROMETRY [LARGE SCALE ANALYSIS].
RC   TISSUE=Liver;
RX   PubMed=23576753; DOI=10.1073/pnas.1302961110;
RA   Rardin M.J., Newman J.C., Held J.M., Cusack M.P., Sorensen D.J., Li B.,
RA   Schilling B., Mooney S.D., Kahn C.R., Verdin E., Gibson B.W.;
RT   "Label-free quantitative proteomics of the lysine acetylome in mitochondria
RT   identifies substrates of SIRT3 in metabolic pathways.";
RL   Proc. Natl. Acad. Sci. U.S.A. 110:6601-6606(2013).
CC   -!- FUNCTION: Mediates the electroneutral exchange of acylcarnitines (O-
CC       acyl-(R)-carnitine or L-acylcarnitine) of different acyl chain lengths
CC       (ranging from O-acetyl-(R)-carnitine to long-chain O-acyl-(R)-
CC       carnitines) with free carnitine ((R)-carnitine or L-carnitine) across
CC       the mitochondrial inner membrane, via a ping-pong mechanism. Key player
CC       in the mitochondrial oxidation pathway, it translocates the fatty acids
CC       in the form of acylcarnitines into the mitochondrial matrix, where the
CC       carnitine palmitoyltransferase 2 (CPT-2) activates them to undergo
CC       fatty acid beta-oxidation. Catalyzes the unidirectional transport
CC       (uniport) of carnitine at lower rates than the antiport (exchange).
CC       {ECO:0000250|UniProtKB:O43772}.
CC   -!- CATALYTIC ACTIVITY:
CC       Reaction=(R)-carnitine(out) + O-acetyl-(R)-carnitine(in) = (R)-
CC         carnitine(in) + O-acetyl-(R)-carnitine(out); Xref=Rhea:RHEA:49908,
CC         ChEBI:CHEBI:16347, ChEBI:CHEBI:57589;
CC         Evidence={ECO:0000250|UniProtKB:P97521};
CC   -!- CATALYTIC ACTIVITY:
CC       Reaction=(R)-carnitine(out) + O-acyl-(R)-carnitine(in) = (R)-
CC         carnitine(in) + O-acyl-(R)-carnitine(out); Xref=Rhea:RHEA:49924,
CC         ChEBI:CHEBI:16347, ChEBI:CHEBI:75659;
CC         Evidence={ECO:0000250|UniProtKB:O43772};
CC   -!- CATALYTIC ACTIVITY:
CC       Reaction=(R)-carnitine(out) + O-propanoyl-(R)-carnitine(in) = (R)-
CC         carnitine(in) + O-propanoyl-(R)-carnitine(out); Xref=Rhea:RHEA:49912,
CC         ChEBI:CHEBI:16347, ChEBI:CHEBI:53210;
CC         Evidence={ECO:0000250|UniProtKB:P97521};
CC   -!- CATALYTIC ACTIVITY:
CC       Reaction=(R)-carnitine(out) + O-hexadecanoyl-(R)-carnitine(in) = (R)-
CC         carnitine(in) + O-hexadecanoyl-(R)-carnitine(out);
CC         Xref=Rhea:RHEA:49916, ChEBI:CHEBI:16347, ChEBI:CHEBI:17490;
CC         Evidence={ECO:0000250|UniProtKB:P97521};
CC   -!- CATALYTIC ACTIVITY:
CC       Reaction=(R)-carnitine(out) + O-octanoyl-(R)-carnitine(in) = (R)-
CC         carnitine(in) + O-octanoyl-(R)-carnitine(out); Xref=Rhea:RHEA:49920,
CC         ChEBI:CHEBI:16347, ChEBI:CHEBI:18102;
CC         Evidence={ECO:0000250|UniProtKB:P97521};
CC   -!- CATALYTIC ACTIVITY:
CC       Reaction=(R)-carnitine(in) = (R)-carnitine(out); Xref=Rhea:RHEA:34959,
CC         ChEBI:CHEBI:16347; Evidence={ECO:0000250|UniProtKB:O43772};
CC   -!- SUBCELLULAR LOCATION: Mitochondrion inner membrane {ECO:0000250};
CC       Multi-pass membrane protein {ECO:0000250}.
CC   -!- TISSUE SPECIFICITY: Widely expressed, with highest levels in the liver,
CC       intermediate levels in heart, testis and kidney and low levels in
CC       brain, including cortex, cerebellum, hippocampus and hypothalamus.
CC       {ECO:0000269|PubMed:19287344}.
CC   -!- SIMILARITY: Belongs to the mitochondrial carrier (TC 2.A.29) family.
CC       {ECO:0000305}.
CC   ---------------------------------------------------------------------------
CC   Copyrighted by the UniProt Consortium, see https://www.uniprot.org/terms
CC   Distributed under the Creative Commons Attribution (CC BY 4.0) License
CC   ---------------------------------------------------------------------------
DR   EMBL; AB017112; BAA74768.1; -; mRNA.
DR   EMBL; AK075624; BAC35865.1; -; mRNA.
DR   EMBL; AK152436; BAE31218.1; -; mRNA.
DR   EMBL; BC029733; AAH29733.1; -; mRNA.
DR   CCDS; CCDS23535.1; -.
DR   RefSeq; NP_065266.1; NM_020520.4.
DR   AlphaFoldDB; Q9Z2Z6; -.
DR   SMR; Q9Z2Z6; -.
DR   BioGRID; 208247; 5.
DR   IntAct; Q9Z2Z6; 3.
DR   STRING; 10090.ENSMUSP00000035222; -.
DR   iPTMnet; Q9Z2Z6; -.
DR   PhosphoSitePlus; Q9Z2Z6; -.
DR   SwissPalm; Q9Z2Z6; -.
DR   EPD; Q9Z2Z6; -.
DR   jPOST; Q9Z2Z6; -.
DR   MaxQB; Q9Z2Z6; -.
DR   PaxDb; Q9Z2Z6; -.
DR   ProteomicsDB; 252741; -.
DR   Antibodypedia; 3111; 252 antibodies from 27 providers.
DR   DNASU; 57279; -.
DR   Ensembl; ENSMUST00000035222; ENSMUSP00000035222; ENSMUSG00000032602.
DR   GeneID; 57279; -.
DR   KEGG; mmu:57279; -.
DR   UCSC; uc009rqs.1; mouse.
DR   AGR; MGI:1928738; -.
DR   CTD; 788; -.
DR   MGI; MGI:1928738; Slc25a20.
DR   VEuPathDB; HostDB:ENSMUSG00000032602; -.
DR   eggNOG; KOG0758; Eukaryota.
DR   GeneTree; ENSGT00940000157863; -.
DR   HOGENOM; CLU_015166_16_0_1; -.
DR   InParanoid; Q9Z2Z6; -.
DR   OMA; MAGIANW; -.
DR   OrthoDB; 4064096at2759; -.
DR   PhylomeDB; Q9Z2Z6; -.
DR   TreeFam; TF300894; -.
DR   Reactome; R-MMU-200425; Carnitine metabolism.
DR   BioGRID-ORCS; 57279; 3 hits in 80 CRISPR screens.
DR   ChiTaRS; Slc25a20; mouse.
DR   PRO; PR:Q9Z2Z6; -.
DR   Proteomes; UP000000589; Chromosome 9.
DR   RNAct; Q9Z2Z6; protein.
DR   Bgee; ENSMUSG00000032602; Expressed in brown adipose tissue and 255 other tissues.
DR   Genevisible; Q9Z2Z6; MM.
DR   GO; GO:0005829; C:cytosol; ISO:MGI.
DR   GO; GO:0005740; C:mitochondrial envelope; IBA:GO_Central.
DR   GO; GO:0005743; C:mitochondrial inner membrane; HDA:MGI.
DR   GO; GO:0005739; C:mitochondrion; HDA:MGI.
DR   GO; GO:0015227; F:acyl carnitine transmembrane transporter activity; ISS:UniProtKB.
DR   GO; GO:1902603; P:carnitine transmembrane transport; ISS:UniProtKB.
DR   GO; GO:0001701; P:in utero embryonic development; IMP:MGI.
DR   GO; GO:0006869; P:lipid transport; IEA:UniProtKB-KW.
DR   GO; GO:0006839; P:mitochondrial transport; IBA:GO_Central.
DR   Gene3D; 1.50.40.10; Mitochondrial carrier domain; 2.
DR   InterPro; IPR018108; Mitochondrial_sb/sol_carrier.
DR   InterPro; IPR023395; Mt_carrier_dom_sf.
DR   PANTHER; PTHR45624; MITOCHONDRIAL BASIC AMINO ACIDS TRANSPORTER-RELATED; 1.
DR   PANTHER; PTHR45624:SF56; MITOCHONDRIAL CARNITINE/ACYLCARNITINE CARRIER PROTEIN; 1.
DR   Pfam; PF00153; Mito_carr; 3.
DR   SUPFAM; SSF103506; Mitochondrial carrier; 1.
DR   PROSITE; PS50920; SOLCAR; 3.
PE   1: Evidence at protein level;
KW   Acetylation; Lipid transport; Membrane; Mitochondrion;
KW   Mitochondrion inner membrane; Reference proteome; Repeat; Transmembrane;
KW   Transmembrane helix; Transport.
FT   INIT_MET        1
FT                   /note="Removed"
FT                   /evidence="ECO:0000250|UniProtKB:O43772"
FT   CHAIN           2..301
FT                   /note="Mitochondrial carnitine/acylcarnitine carrier
FT                   protein"
FT                   /id="PRO_0000090630"
FT   TOPO_DOM        2..12
FT                   /note="Cytoplasmic"
FT                   /evidence="ECO:0000255"
FT   TRANSMEM        13..31
FT                   /note="Helical; Name=1"
FT                   /evidence="ECO:0000255"
FT   TOPO_DOM        32..73
FT                   /note="Mitochondrial matrix"
FT                   /evidence="ECO:0000255"
FT   TRANSMEM        74..93
FT                   /note="Helical; Name=2"
FT                   /evidence="ECO:0000255"
FT   TOPO_DOM        94..112
FT                   /note="Cytoplasmic"
FT                   /evidence="ECO:0000255"
FT   TRANSMEM        113..131
FT                   /note="Helical; Name=3"
FT                   /evidence="ECO:0000255"
FT   TOPO_DOM        132..170
FT                   /note="Mitochondrial matrix"
FT                   /evidence="ECO:0000255"
FT   TRANSMEM        171..190
FT                   /note="Helical; Name=4"
FT                   /evidence="ECO:0000255"
FT   TOPO_DOM        191..211
FT                   /note="Cytoplasmic"
FT                   /evidence="ECO:0000255"
FT   TRANSMEM        212..230
FT                   /note="Helical; Name=5"
FT                   /evidence="ECO:0000255"
FT   TOPO_DOM        231..267
FT                   /note="Mitochondrial matrix"
FT                   /evidence="ECO:0000255"
FT   TRANSMEM        268..287
FT                   /note="Helical; Name=6"
FT                   /evidence="ECO:0000255"
FT   TOPO_DOM        288..301
FT                   /note="Cytoplasmic"
FT                   /evidence="ECO:0000255"
FT   REPEAT          8..99
FT                   /note="Solcar 1"
FT   REPEAT          108..196
FT                   /note="Solcar 2"
FT   REPEAT          207..293
FT                   /note="Solcar 3"
FT   MOD_RES         2
FT                   /note="N-acetylalanine"
FT                   /evidence="ECO:0000250|UniProtKB:O43772"
FT   MOD_RES         148
FT                   /note="N6-acetyllysine"
FT                   /evidence="ECO:0007744|PubMed:23576753"
FT   MOD_RES         157
FT                   /note="N6-acetyllysine"
FT                   /evidence="ECO:0007744|PubMed:23576753"
FT   MOD_RES         170
FT                   /note="N6-acetyllysine; alternate"
FT                   /evidence="ECO:0007744|PubMed:23576753"
FT   MOD_RES         170
FT                   /note="N6-succinyllysine; alternate"
FT                   /evidence="ECO:0007744|PubMed:23806337"
SQ   SEQUENCE   301 AA;  33027 MW;  0E332BF4D7584490 CRC64;
     MADEPKPISP FKNLLAGGFG GMCLVFVGHP LDTVKVRLQT QPPSLSGQPP MYSGTLDCFR
     KTLMREGITG LYRGMAAPII GVTPMFAVCF FGFGLGKKLQ QKSPEDELSY PQLFTAGMLS
     GVFTTGIMTP GERIKCLLQI QASSGENKYS GTLDCAKKLY QEFGIRGFYK GTVLTLMRDV
     PASGMYFMTY EWLKNLFTPE GKSVSDLSVP RILVAGGFAG IFNWAVAIPP DVLKSRFQTA
     PPGKYPNGFR DVLRELIREE GVTSLYKGFN AVMIRAFPAN AACFLGFEIA MKFLNWIAPN
     L
//
```

**Mascot:** http://www.matrixscience.com/


HNE (C) (+156.1150)
