# Supplementary material for: A common East-Asian ALDH2 mutation causes metabolic disorders and the therapeutic effect of ALDH2 activators
Source: Nat Commun. 2023 Sep 25;14:5971. doi: 10.1038/s41467-023-41570-6 (PMC10520061; doi:10.1038/s41467-023-41570-6)
Supplement: Supplementary file 4 — Supplementary Data 1 [file 41467_2023_41570_MOESM4_ESM.zip › Table S5a/T1683_Acaa2_K375.pdf]

MATRIX SCIENCE Mascot Search Results

Peptide View

MS/MS Fragmentation of **GGKYAVGSACIGGGQGIALIIQNTA**
Found in **Q8BWT1** in **Mouse_UniProt_proteomes**, 3-ketoacyl-CoA thiolase, mitochondrial OS=Mus musculus OX=10090 GN=Acaa2 PE=1 SV=3

Match to Query 88533: 2531.327592 from(844.783140,3+) intensity(5977617.5000) rtinseconds(5157) scans(95783) index(85872)
Title: File: "D:\Data\2019 User's data\190516-5\190517-1 raw data\190516-5-T1683_1_10.raw"; SpectrumID: "95783"; scans: "95783"
Data file D:\Data\2019 User's data\190516-5\190516-5\190516-5-T1683_1_10.mgf

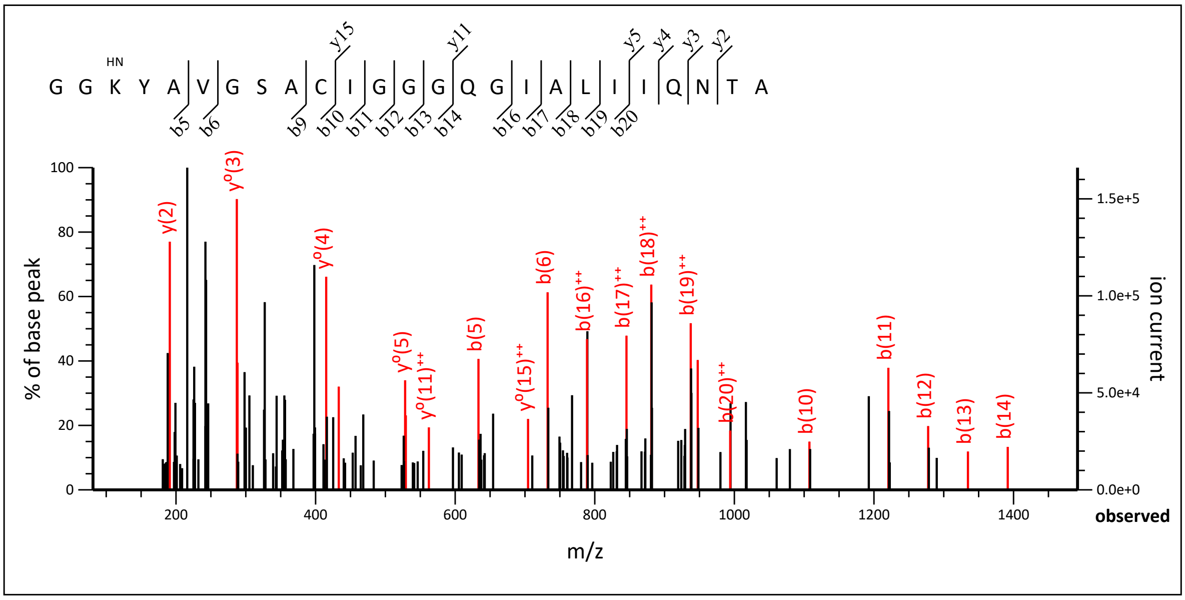

81.06 to 1491.69

Label all possible matches ○ Label matches used for scoring ◉

Monoisotopic mass of neutral peptide Mr(calc): 2531.3418
Fixed modifications: Carbamidomethyl (C) (apply to specified residues or termini only)
Variable modifications:
K3 : HNE (K)
Ions Score: 44 **Expect:** 0.027
Matches : 23/264 fragment ions using 45 most intense peaks (help)

#	b	b++	b*	b*++	b0	b0++	Seq.	y	y++	y*	y*++	y0	y0++	#
1	58.0287	29.5180					G							25
2	115.0502	58.0287					G	2475.3276	1238.1674	2458.3010	1229.6542	2457.3170	1229.1621	24
3	399.2602	200.1337	382.2336	191.6205			K	2418.3061	1209.6567	2401.2796	1201.1434	2400.2955	1200.6514	23
4	562.3235	281.6654	545.2970	273.1521			Y	2134.0961	1067.5517	2117.0696	1059.0384	2116.0856	1058.5464	22
5	633.3606	317.1840	616.3341	308.6707			A	1971.0328	986.0200	1954.0062	977.5068	1953.0222	977.0148	21
6	732.4291	366.7182	715.4025	358.2049			V	1899.9957	950.5015	1882.9691	941.9882	1881.9851	941.4962	20
7	789.4505	395.2289	772.4240	386.7156			G	1800.9273	900.9673	1783.9007	892.4540	1782.9167	891.9620	19
8	876.4825	438.7449	859.4560	430.2316	858.4720	429.7396	S	1743.9058	872.4565	1726.8792	863.9433	1725.8952	863.4513	18
9	947.5197	474.2635	930.4931	465.7502	929.5091	465.2582	A	1656.8738	828.9405	1639.8472	820.4272	1638.8632	819.9352	17
10	1107.5503	554.2788	1090.5238	545.7655	1089.5397	545.2735	C	1585.8367	793.4220	1568.8101	784.9087	1567.8261	784.4167	16
11	1220.6344	610.8208	1203.6078	602.3075	1202.6238	601.8155	I	1425.8060	713.4066	1408.7795	704.8934	1407.7954	704.4014	15
12	1277.6558	639.3316	1260.6293	630.8183	1259.6453	630.3263	G	1312.7219	656.8646	1295.6954	648.3513	1294.7114	647.8593	14
13	1334.6773	667.8423	1317.6508	659.3290	1316.6667	658.8370	G	1255.7005	628.3539	1238.6739	619.8406	1237.6899	619.3486	13
14	1391.6988	696.3530	1374.6722	687.8397	1373.6882	687.3477	G	1198.6790	599.8431	1181.6525	591.3299	1180.6684	590.8379	12
15	1519.7573	760.3823	1502.7308	751.8690	1501.7468	751.3770	Q	1141.6576	571.3324	1124.6310	562.8191	1123.6470	562.3271	11
16	1576.7788	788.8930	1559.7523	780.3798	1558.7682	779.8878	G	1013.5990	507.3031	996.5724	498.7898	995.5884	498.2978	10
17	1689.8629	845.4351	1672.8363	836.9218	1671.8523	836.4298	I	956.5775	478.7924	939.5510	470.2791	938.5669	469.7871	9
18	1760.9000	880.9536	1743.8734	872.4404	1742.8894	871.9483	A	843.4934	422.2504	826.4669	413.7371	825.4829	413.2451	8
19	1873.9840	937.4957	1856.9575	928.9824	1855.9735	928.4904	L	772.4563	386.7318	755.4298	378.2185	754.4458	377.7265	7
20	1987.0681	994.0377	1970.0416	985.5244	1969.0575	985.0324	I	659.3723	330.1898	642.3457	321.6765	641.3617	321.1845	6
21	2100.1522	1050.5797	2083.1256	1042.0665	2082.1416	1041.5744	I	546.2882	273.6477	529.2617	265.1345	528.2776	264.6425	5
22	2228.2108	1114.6090	2211.1842	1106.0957	2210.2002	1105.6037	Q	433.2041	217.1057	416.1776	208.5924	415.1936	208.1004	4

23	2342.2537	1171.6305	2325.2271	1163.1172	2324.2431	1162.6252	N	305.1456	153.0764	288.1190	144.5631	287.1350	144.0711	3
24	2443.3014	1222.1543	2426.2748	1213.6410	2425.2908	1213.1490	T	**191.1026**	96.0550			173.0921	87.0497	2
25							A	90.0550	45.5311					1

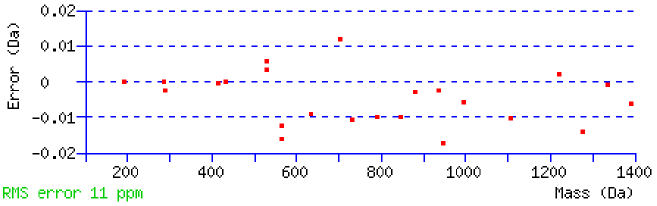

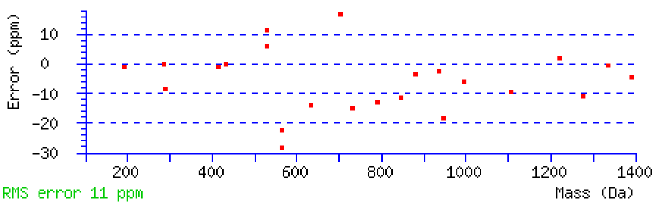

NCBI **BLAST** search of GGKYAVGSACIGGGQGIALIIQNTA
(Parameters: blastp, nr protein database, expect=20000, no filter, PAM30)
Other BLAST web gateways

All matches to this query

Score	Mr(calc)	Delta	Sequence	Site Analysis
43.6	2531.3418	-0.0142	GGKYAVGSACIGGGQGIALIIQNTA	HNE K3 100.00%
42.6	2531.3278	-0.0002	RGGKYAVGSACIGGGQGIALIIQNTA	

Mascot: http://www.matrixscience.com/
