# Supplementary material for: A common East-Asian ALDH2 mutation causes metabolic disorders and the therapeutic effect of ALDH2 activators
Source: Nat Commun. 2023 Sep 25;14:5971. doi: 10.1038/s41467-023-41570-6 (PMC10520061; doi:10.1038/s41467-023-41570-6)
Supplement: Supplementary file 4 — Supplementary Data 1 [file 41467_2023_41570_MOESM4_ESM.zip › Table S5a/T1683_Atp5fla_K175.pdf]

MATRIX SCIENCE Mascot Search Results

Peptide View

MS/MS Fragmentation of **RVGLKAPGIIPR**
Found in **Q03265** in **Mouse_UniProt_proteomes**, ATP synthase subunit alpha, mitochondrial OS=Mus musculus OX=10090 GN=Atp5f1a PE=1 SV=1

Match to Query 43190: 1431.914736 from(358.985960,4+) intensity(5436234.0000) rtinseconds(2574) scans(36291) index(29324)
Title: File: "D:\Data\2019 User's data\190516-5\190517-1 raw data\190516-5-T1683_1_10.raw"; SpectrumID: "36291"; scans: "36291"
Data file D:\Data\2019 User's data\190516-5\190516-5\190516-5-T1683_1_10.mgf

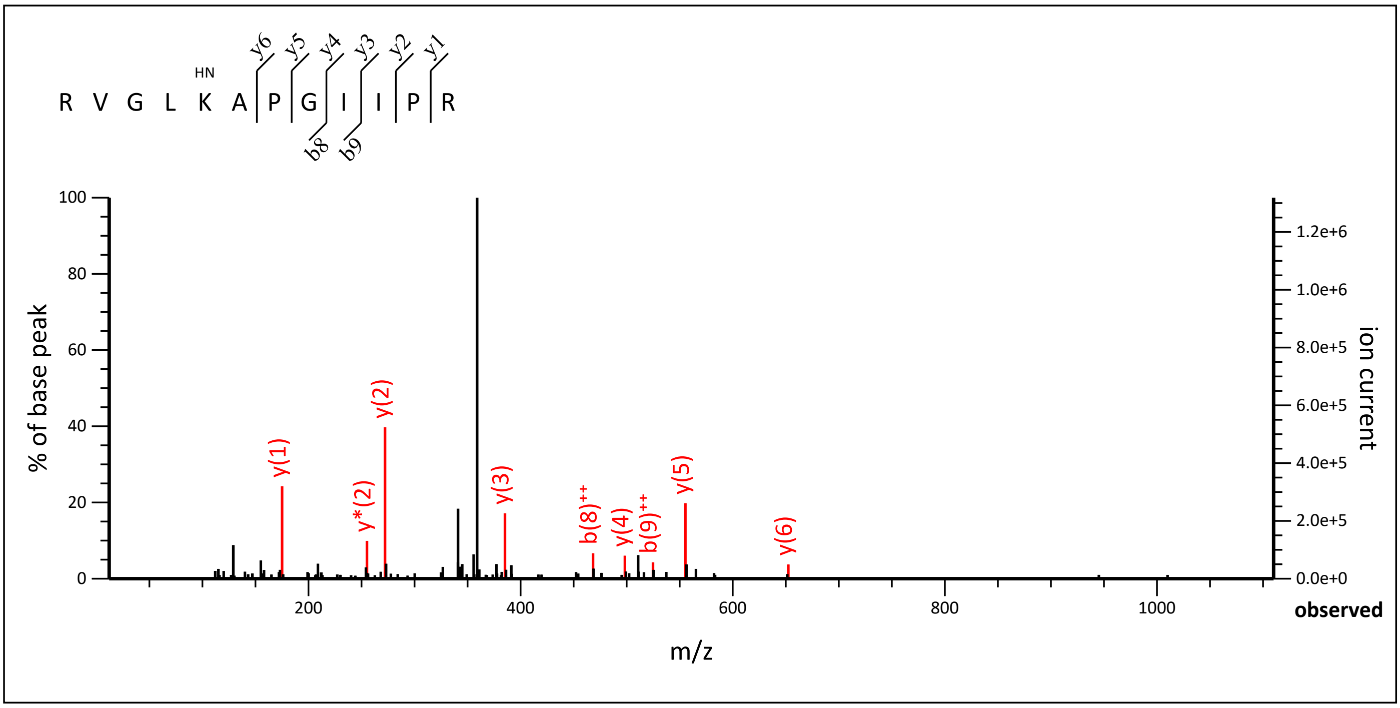

12.09 to 1110

Label all possible matches ○ Label matches used for scoring ◉

Monoisotopic mass of neutral peptide Mr(calc): 1431.9289
Fixed modifications: Carbamidomethyl (C) (apply to specified residues or termini only)
Variable modifications:
K5 : HNE (K)
Ions Score: 31 **Expect:** 0.018
Matches : 9/88 fragment ions using 19 most intense peaks (help)

#	b	b^{++}	b*	b^{*++}	Seq.	y	y^{++}	y*	y^{*++}	#
1	157.1084	79.0578	140.0818	70.5446	R					12
2	256.1768	128.5920	239.1503	120.0788	V	1276.8351	638.9212	1259.8086	630.4079	11
3	313.1983	157.1028	296.1717	148.5895	G	1177.7667	589.3870	1160.7402	580.8737	10
4	426.2823	213.6448	409.2558	205.1315	L	1120.7452	560.8763	1103.7187	552.3630	9
5	710.4923	355.7498	693.4658	347.2365	K	1007.6612	504.3342	990.6346	495.8210	8
6	781.5294	391.2684	764.5029	382.7551	A	723.4512	362.2292	706.4246	353.7160	7
7	878.5822	439.7947	861.5557	431.2815	P	***652.4141***	326.7107	635.3875	318.1974	6
8	935.6037	**468.3055**	918.5771	459.7922	G	***555.3613***	278.1843	538.3348	269.6710	5
9	1048.6877	**524.8475**	1031.6612	516.3342	I	***498.3398***	249.6736	481.3133	241.1603	4
10	1161.7718	581.3895	1144.7452	572.8763	I	***385.2558***	193.1315	368.2292	184.6183	3
11	1258.8246	629.9159	1241.7980	621.4026	P	***272.1717***	136.5895	255.1452	128.0762	2

**12**					**R**	*175.1190*	88.0631	158.0924	79.5498	**1**

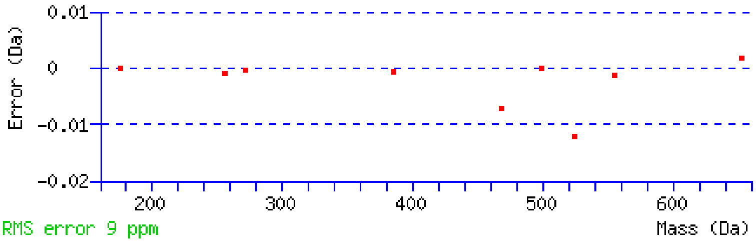

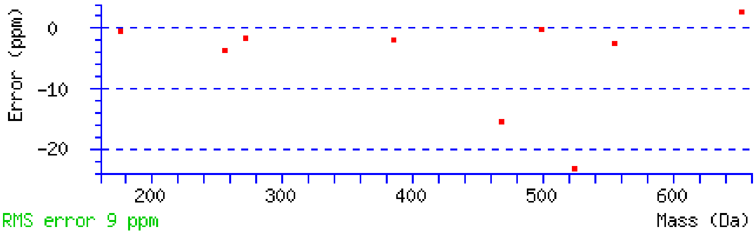

NCBI **BLAST** search of RVGLKAPGIIPR
(Parameters: blastp, nr protein database, expect=20000, no filter, PAM30)
Other BLAST web gateways

All matches to this query

Score	Mr(calc)	Delta	Sequence
31.1	1431.9289	-0.0142	RVGLKAPGIIPR
14.4	1431.8887	0.0260	MLKHPLLPR

Mascot: http://www.matrixscience.com/
