# Supplementary material for: A common East-Asian ALDH2 mutation causes metabolic disorders and the therapeutic effect of ALDH2 activators
Source: Nat Commun. 2023 Sep 25;14:5971. doi: 10.1038/s41467-023-41570-6 (PMC10520061; doi:10.1038/s41467-023-41570-6)
Supplement: Supplementary file 4 — Supplementary Data 1 [file 41467_2023_41570_MOESM4_ESM.zip › Table S5a/T1683_chchd3_k24.pdf]

MATRIX SCIENCE Mascot Search Results

Peptide View

MS/MS Fragmentation of **VTFEADENENITVVK**
Found in **D3Z0L4** in **Mouse_UniProt_proteomes**, MICOS complex subunit (Fragment) OS=Mus musculus OX=10090 GN=Chchd3 PE=1 SV=1

Match to Query 67650: 1862.935872 from(621.985900,3+) intensity(3045310.5000) rtinseconds(3204) scans(51019) index(43339)
Title: File: "D:\Data\2019 User's data\190516-5\190517-1 raw data\190516-5-T1683_1_10.raw"; SpectrumID: "51019"; scans: "51019"
Data file D:\Data\2019 User's data\190516-5\190516-5\190516-5-T1683_1_10.mgf

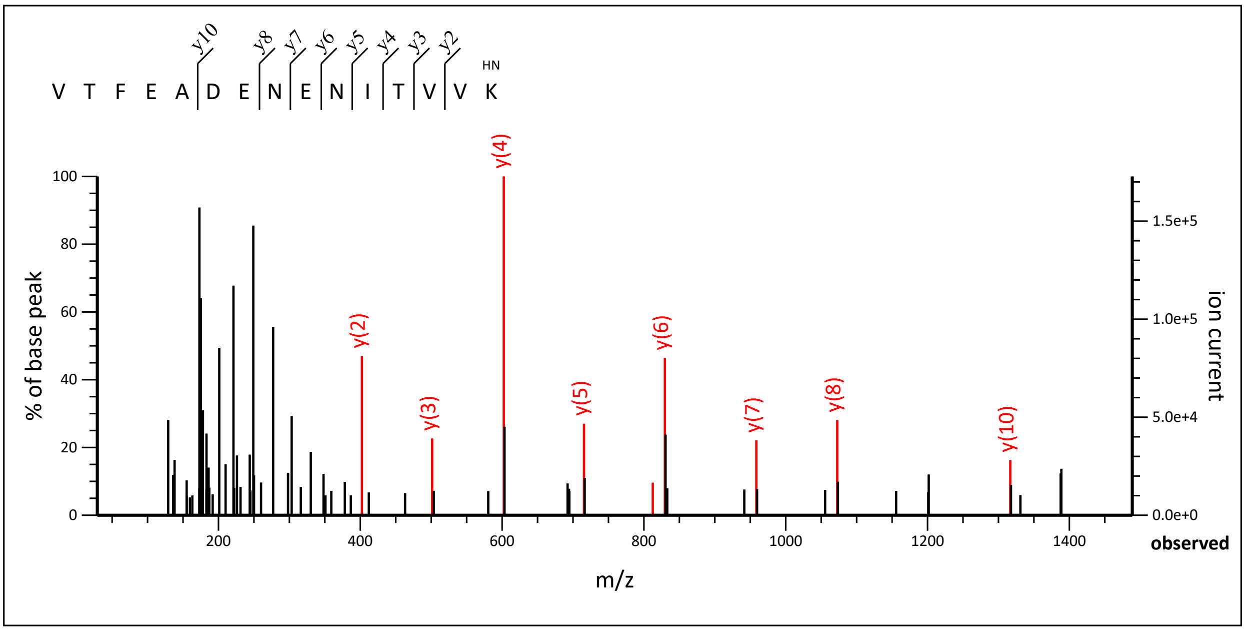

29.1 to 1488.73

Label all possible matches ○ Label matches used for scoring ◉

Monoisotopic mass of neutral peptide Mr(calc): 1862.9513
Fixed modifications: Carbamidomethyl (C) (apply to specified residues or termini only)
Variable modifications:
K15 : HNE (K)
Ions Score: 58 **Expect:** 0.00059
Matches : 9/146 fragment ions using 13 most intense peaks (help)

#	b	b^{++}	b*	b^{*++}	b^{0}	b^{0++}	Seq.	y	y^{++}	y*	y^{*++}	y^{0}	y^{0++}	#
1	100.0757	50.5415					V							15
2	201.1234	101.0653			183.1128	92.0600	T	1764.8902	882.9487	1747.8636	874.4355	1746.8796	873.9434	14
3	348.1918	174.5995			330.1812	165.5942	F	1663.8425	832.4249	1646.8160	823.9116	1645.8319	823.4196	13
4	477.2344	239.1208			459.2238	230.1155	E	1516.7741	758.8907	1499.7475	750.3774	1498.7635	749.8854	12
5	548.2715	274.6394			530.2609	265.6341	A	1387.7315	694.3694	1370.7050	685.8561	1369.7209	685.3641	11
6	663.2984	332.1529			645.2879	323.1476	D	***1316.6944***	658.8508	1299.6678	650.3376	1298.6838	649.8455	10
7	792.3410	396.6742			774.3305	387.6689	E	1201.6674	601.3374	1184.6409	592.8241	1183.6569	592.3321	9
8	906.3840	453.6956	889.3574	445.1823	888.3734	444.6903	N	***1072.6249***	536.8161	1055.5983	528.3028	1054.6143	527.8108	8
9	1035.4265	518.2169	1018.4000	509.7036	1017.4160	509.2116	E	***958.5819***	479.7946	941.5554	471.2813	940.5714	470.7893	7
10	1149.4695	575.2384	1132.4429	566.7251	1131.4589	566.2331	N	***829.5393***	415.2733	812.5128	406.7600	811.5288	406.2680	6
11	1262.5535	631.7804	1245.5270	623.2671	1244.5430	622.7751	I	***715.4964***	358.2518	698.4699	349.7386	697.4858	349.2466	5
12	1363.6012	682.3042	1346.5747	673.7910	1345.5907	673.2990	T	***602.4123***	301.7098	585.3858	293.1965	584.4018	292.7045	4
13	1462.6696	731.8385	1445.6431	723.3252	1444.6591	722.8332	V	***501.3647***	251.1860	484.3381	242.6727			3
14	1561.7380	781.3727	1544.7115	772.8594	1543.7275	772.3674	V	***402.2962***	201.6518	385.2697	193.1385			2
15							K	303.2278	152.1176	286.2013	143.6043			1

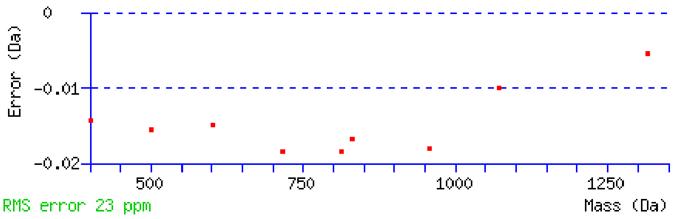

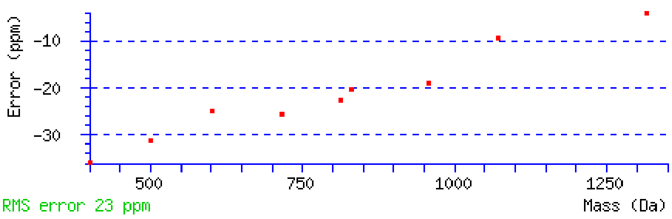

NCBI **BLAST** search of VTFEADENENITVVK
(Parameters: blastp, nr protein database, expect=20000, no filter, PAM30)
Other BLAST web gateways

All matches to this query

Score	Mr(calc)	Delta	Sequence
58.3	1862.9513	-0.0155	VTFEADENENITVVK

**Mascot:** http://www.matrixscience.com/
