# Supplementary material for: A common East-Asian ALDH2 mutation causes metabolic disorders and the therapeutic effect of ALDH2 activators
Source: Nat Commun. 2023 Sep 25;14:5971. doi: 10.1038/s41467-023-41570-6 (PMC10520061; doi:10.1038/s41467-023-41570-6)
Supplement: Supplementary file 4 — Supplementary Data 1 [file 41467_2023_41570_MOESM4_ESM.zip › Table S5a/T1683_Immt_K218.pdf]

MATRIX SCIENCE Mascot Search Results

Peptide View

MS/MS Fragmentation of **KAVDEAADALLK**
Found in **E9Q800** in **Mouse_UniProt_proteomes**, MICOS complex subunit MIC60 OS=Mus musculus OX=10090 GN=Immt PE=1 SV=1

Match to Query 40725: 1398.782202 from(467.268010,3+) intensity(1239045.8750) rtinseconds(2893) scans(43767) index(36438)
Title: File: "D:\Data\2019 User's data\190516-5\190517-1 raw data\190516-5-T1683_1_10.raw"; SpectrumID: "43767"; scans: "43767"
Data file D:\Data\2019 User's data\190516-5\190516-5\190516-5-T1683_1_10.mgf

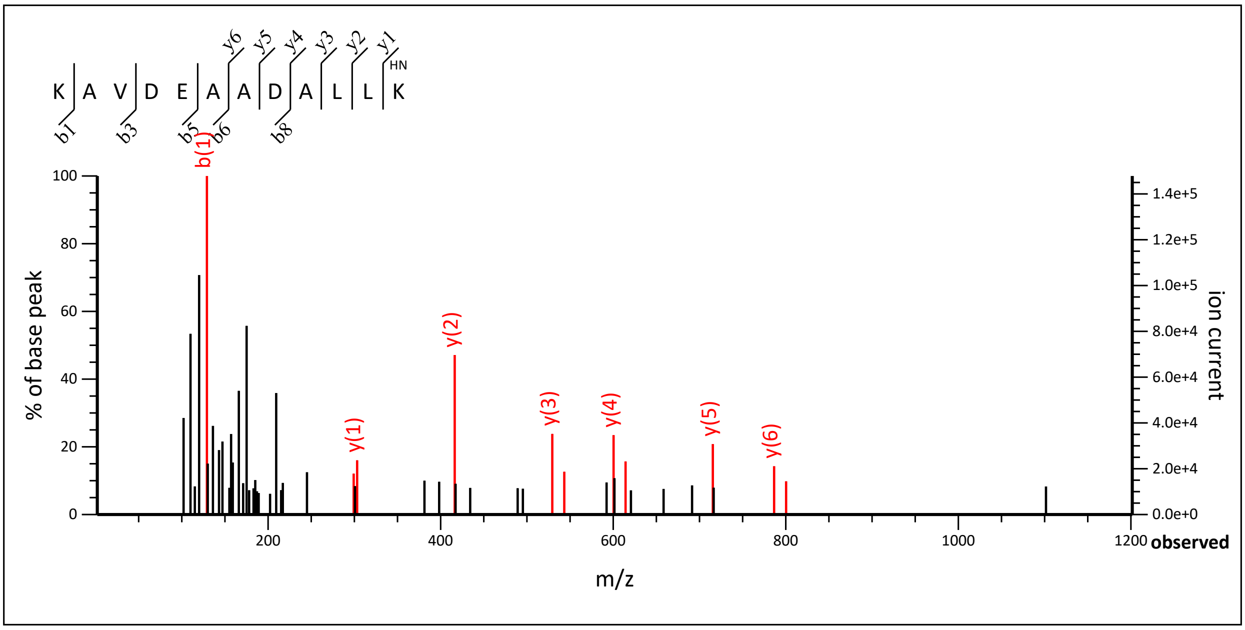

2.06 to 1201.58

Label all possible matches ○ Label matches used for scoring ◉

```
Monoisotopic mass of neutral peptide Mr(calc): 1398.7970
Fixed modifications: Carbamidomethyl (C) (apply to specified residues or termini only)
Variable modifications:
K12    : HNE (K)
Ions Score: 42  Expect: 0.015
Matches : 11/118 fragment ions using 22 most intense peaks   (help)
```

#	b	b^{++}	b*	b^{*++}	b^0	b^{0++}	Seq.	y	y^{++}	y*	y^{*++}	y^0	y^{0++}	#
1	***129.1022***	65.0548	112.0757	56.5415			K							12
2	200.1394	100.5733	183.1128	92.0600			A	1271.7093	636.3583	1254.6828	627.8450	1253.6987	627.3530	11
3	***299.2078***	150.1075	282.1812	141.5942			V	1200.6722	600.8397	1183.6456	592.3265	1182.6616	591.8345	10
4	414.2347	207.6210	397.2082	199.1077	396.2241	198.6157	D	1101.6038	551.3055	1084.5772	542.7923	1083.5932	542.3002	9
5	***543.2773***	272.1423	526.2508	263.6290	525.2667	263.1370	E	986.5768	493.7921	969.5503	485.2788	968.5663	484.7868	8
6	***614.3144***	307.6608	597.2879	299.1476	596.3039	298.6556	A	857.5342	429.2708	840.5077	420.7575	839.5237	420.2655	7
7	685.3515	343.1794	668.3250	334.6661	667.3410	334.1741	A	***786.4971***	393.7522	769.4706	385.2389	768.4866	384.7469	6
8	***800.3785***	400.6929	783.3519	392.1796	782.3679	391.6876	D	***715.4600***	358.2336	698.4335	349.7204	697.4495	349.2284	5
9	871.4156	436.2114	854.3890	427.6982	853.4050	427.2061	A	***600.4331***	300.7202	583.4065	292.2069			4
10	984.4997	492.7535	967.4731	484.2402	966.4891	483.7482	L	***529.3960***	265.2016	512.3694	256.6883			3
11	1097.5837	549.2955	1080.5572	540.7822	1079.5732	540.2902	L	***416.3119***	208.6596	399.2853	200.1463			2
12							K	***303.2278***	152.1176	286.2013	143.6043			1

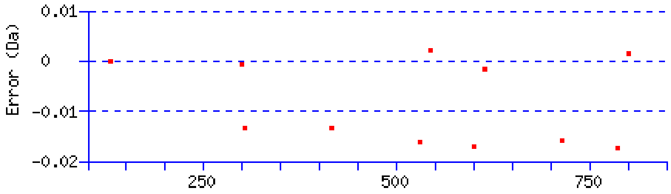

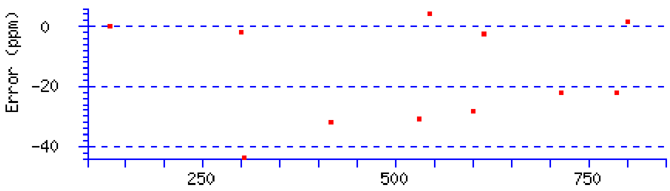

NCBI **BLAST** search of KAVDEAADALLK
(Parameters: blastp, nr protein database, expect=20000, no filter, PAM30)
Other BLAST web gateways

All matches to this query

Score	Mr(calc)	Delta	Sequence	Site Analysis
42.4	1398.7970	-0.0148	KAVDEAADALLK	HNE K12 99.98%
18.7	1398.7970	-0.0148	DLQSQIDALIK	
16.2	1398.8023	-0.0201	LHNYWLLKR	
11.0	1398.7792	0.0030	MPKEQQELLK	
9.1	1398.7830	-0.0008	NRDRQQAILK	
9.1	1398.7830	-0.0008	NRDRQQAILK	
9.1	1398.7830	-0.0008	NRDRQQAILK	
5.8	1398.7943	-0.0121	QIDTRAALVEKR	
5.8	1398.8082	-0.0260	GKVNEADIKLR	
5.8	1398.7983	-0.0161	NGNGKGWLRLK	

Mascot: http://www.matrixscience.com/
