# Supplementary material for: A common East-Asian ALDH2 mutation causes metabolic disorders and the therapeutic effect of ALDH2 activators
Source: Nat Commun. 2023 Sep 25;14:5971. doi: 10.1038/s41467-023-41570-6 (PMC10520061; doi:10.1038/s41467-023-41570-6)
Supplement: Supplementary file 4 — Supplementary Data 1 [file 41467_2023_41570_MOESM4_ESM.zip › Table S5a/T1683_Uqcrb_H39.pdf]

MATRIX SCIENCE Mascot Search Results

Peptide View

MS/MS Fragmentation of **DDTLHETEDVKEAIR**
Found in **Q9CQB4** in **Mouse_UniProt_proteomes**, Cytochrome b-c1 complex subunit 7 OS=Mus musculus OX=10090 GN=Uqcrb PE=1 SV=1

Match to Query 70392: 1925.943372 from(642.988400,3+) intensity(5762060.5000) rtinseconds(2415) scans(32575) index(25785)
Title: File: "D:\Data\2019 User's data\190516-5\190517-1 raw data\190516-5-T1683_1_10.raw"; SpectrumID: "32575"; scans: "32575"
Data file D:\Data\2019 User's data\190516-5\190516-5\190516-5-T1683_1_10.mgf

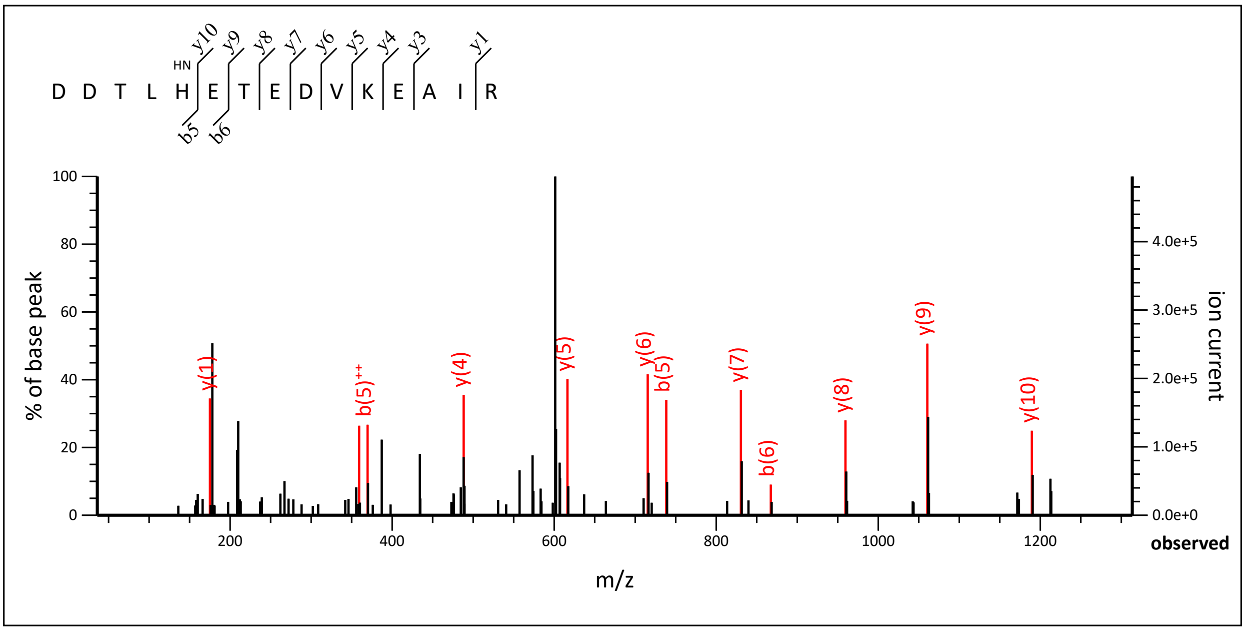

36.07 to 1313.52

Label all possible matches ○ Label matches used for scoring ◉

Monoisotopic mass of neutral peptide Mr(calc): 1925.9582
Fixed modifications: Carbamidomethyl (C) (apply to specified residues or termini only)
Variable modifications:
H5 : HNE (H)
Ions Score: 56 **Expect:** 0.00097
Matches : 12/142 fragment ions using 22 most intense peaks (help)

#	b	b^{++}	b^{*}	b^{*++}	b^{0}	b^{0++}	Seq.	y	y^{++}	y^{*}	y^{*++}	y^{0}	y^{0++}	#
1	116.0342	58.5207			98.0237	49.5155	D							15
2	231.0612	116.0342			213.0506	107.0289	D	1811.9385	906.4729	1794.9120	897.9596	1793.9280	897.4676	14
3	332.1088	166.5581			314.0983	157.5528	T	1696.9116	848.9594	1679.8850	840.4462	1678.9010	839.9542	13
4	445.1929	223.1001			427.1823	214.0948	L	1595.8639	798.4356	1578.8374	789.9223	1577.8533	789.4303	12
5	**738.3668**	**369.6871**			720.3563	360.6818	H	1482.7799	741.8936	1465.7533	733.3803	1464.7693	732.8883	11
6	**867.4094**	434.2084			849.3989	425.2031	E	***1189.6059***	595.3066	1172.5794	586.7933	1171.5953	586.3013	10
7	968.4571	484.7322			950.4466	475.7269	T	***1060.5633***	530.7853	1043.5368	522.2720	1042.5528	521.7800	9
8	1097.4997	549.2535			1079.4891	540.2482	E	***959.5156***	480.2615	942.4891	471.7482	941.5051	471.2562	8
9	1212.5267	606.7670			1194.5161	597.7617	D	***830.4730***	415.7402	813.4465	407.2269	812.4625	406.7349	7
10	1311.5951	656.3012			1293.5845	647.2959	V	***715.4461***	358.2267	698.4196	349.7134	697.4355	349.2214	6
11	1439.6900	720.3487	1422.6635	711.8354	1421.6795	711.3434	K	***616.3777***	308.6925	599.3511	300.1792	598.3671	299.6872	5
12	1568.7326	784.8700	1551.7061	776.3567	1550.7221	775.8647	E	***488.2827***	244.6450	471.2562	236.1317	470.2722	235.6397	4
13	1639.7697	820.3885	1622.7432	811.8752	1621.7592	811.3832	A	***359.2401***	180.1237	342.2136	171.6104			3
14	1752.8538	876.9305	1735.8273	868.4173	1734.8432	867.9253	I	288.2030	144.6051	271.1765	136.0919			2
15							R	***175.1190***	88.0631	158.0924	79.5498			1

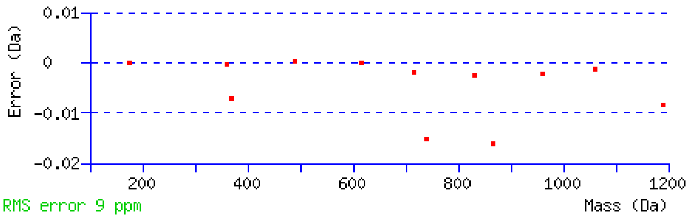

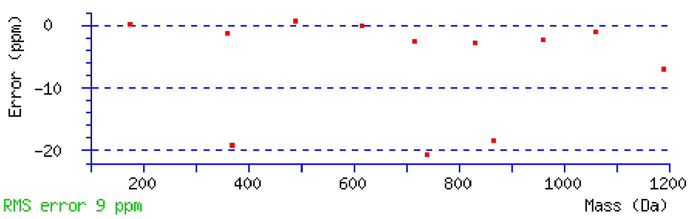

NCBI **BLAST** search of DDTLHETEDVKEAIR
(Parameters: blastp, nr protein database, expect=20000, no filter, PAM30)
Other BLAST web gateways

All matches to this query

Score	Mr(calc)	Delta	Sequence	Site Analysis
55.6	1925.9582	-0.0148	DDTLHETEDVKEAIR	HNE H5 100.00%
3.1	1925.9292	0.0142	MKGLIDEVDQDFTSR	

Mascot: http://www.matrixscience.com/
