# Supplementary material for: A common East-Asian ALDH2 mutation causes metabolic disorders and the therapeutic effect of ALDH2 activators
Source: Nat Commun. 2023 Sep 25;14:5971. doi: 10.1038/s41467-023-41570-6 (PMC10520061; doi:10.1038/s41467-023-41570-6)
Supplement: Supplementary file 4 — Supplementary Data 1 [file 41467_2023_41570_MOESM4_ESM.zip › Table S5a/T1683_Uqcrc1_K3.pdf]

MATRIX SCIENCE Mascot Search Results

Peptide View

MS/MS Fragmentation of **DEKNNGAGYFLEHLAFK**
Found in **A0A0A6YVZ0** in **Mouse_UniProt_proteomes**, Cytochrome b-c1 complex subunit 1, mitochondrial (Fragment) OS=Mus musculus OX=10090 GN=Uqcrc1 PE=1 SV=1

Match to Query 77428: 2108.064176 from(528.023320,4+) intensity(634213.1250) rtinseconds(5327) scans(99673) index(89577)
Title: File: "D:\Data\2019 User's data\190516-5\190517-1 raw data\190516-5-T1683_1_10.raw"; SpectrumID: "99673"; scans: "99673"
Data file D:\Data\2019 User's data\190516-5\190516-5\190516-5-T1683_1_10.mgf

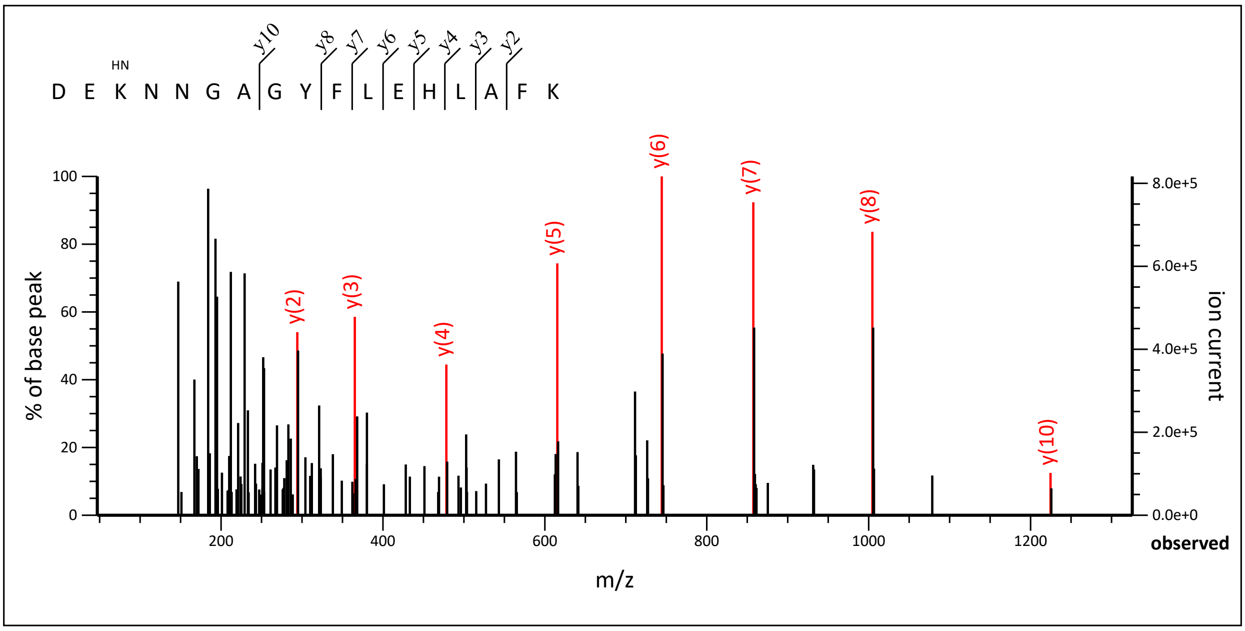

47.11 to 1325.62

Label all possible matches ○ Label matches used for scoring ◉

Monoisotopic mass of neutral peptide Mr(calc): 2108.0578
Fixed modifications: Carbamidomethyl (C) (apply to specified residues or termini only)
Variable modifications:
K3 : HNE (K)
Ions Score: 63 **Expect:** 0.00022
Matches : 8/178 fragment ions using 10 most intense peaks (help)

#	b	b^{++}	b*	b^{*++}	b^{0}	b^{0++}	Seq.	y	y^{++}	y*	y^{*++}	y^{0}	y^{0++}	#
1	116.0342	58.5207			98.0237	49.5155	D							17
2	245.0768	123.0420			227.0662	114.0368	E	1994.0382	997.5227	1977.0116	989.0095	1976.0276	988.5175	16
3	529.2868	265.1470	512.2603	256.6338	511.2762	256.1418	K	1864.9956	933.0014	1847.9690	924.4882	1846.9850	923.9962	15
4	643.3297	322.1685	626.3032	313.6552	625.3192	313.1632	N	1580.7856	790.8964	1563.7591	782.3832	1562.7750	781.8912	14
5	757.3727	379.1900	740.3461	370.6767	739.3621	370.1847	N	1466.7427	733.8750	1449.7161	725.3617	1448.7321	724.8697	13
6	814.3941	407.7007	797.3676	399.1874	796.3836	398.6954	G	1352.6998	676.8535	1335.6732	668.3402	1334.6892	667.8482	12
7	885.4312	443.2193	868.4047	434.7060	867.4207	434.2140	A	1295.6783	648.3428	1278.6517	639.8295	1277.6677	639.3375	11
8	942.4527	471.7300	925.4262	463.2167	924.4421	462.7247	G	***1224.6412***	612.8242	1207.6146	604.3109	1206.6306	603.8189	10
9	1105.5160	553.2617	1088.4895	544.7484	1087.5055	544.2564	Y	1167.6197	584.3135	1150.5932	575.8002	1149.6091	575.3082	9
10	1252.5844	626.7959	1235.5579	618.2826	1234.5739	617.7906	F	***1004.5564***	502.7818	987.5298	494.2686	986.5458	493.7765	8
11	1365.6685	683.3379	1348.6420	674.8246	1347.6579	674.3326	L	***857.4880***	429.2476	840.4614	420.7343	839.4774	420.2423	7
12	1494.7111	747.8592	1477.6846	739.3459	1476.7005	738.8539	E	***744.4039***	372.7056	727.3774	364.1923	726.3933	363.7003	6
13	1631.7700	816.3886	1614.7435	807.8754	1613.7594	807.3834	H	***615.3613***	308.1843	598.3348	299.6710			5
14	1744.8541	872.9307	1727.8275	864.4174	1726.8435	863.9254	L	***478.3024***	239.6548	461.2758	231.1416			4
15	1815.8912	908.4492	1798.8646	899.9360	1797.8806	899.4440	A	***365.2183***	183.1128	348.1918	174.5995			3
16	1962.9596	981.9834	1945.9331	973.4702	1944.9490	972.9782	F	***294.1812***	147.5942	277.1547	139.0810			2
17							K	147.1128	74.0600	130.0863	65.5468			1

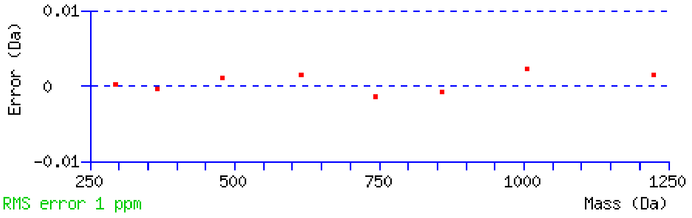

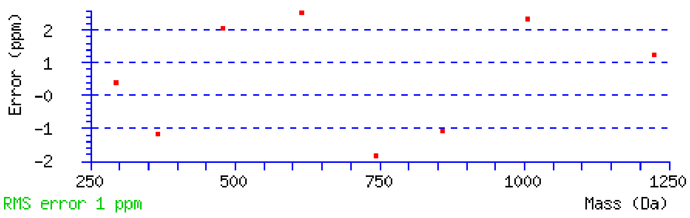

NCBI **BLAST** search of DEKNNGAGYFLEHLAFK
(Parameters: blastp, nr protein database, expect=20000, no filter, PAM30)
Other BLAST web gateways

All matches to this query

Score	Mr(calc)	Delta	Sequence	Site Analysis
63.2	2108.0578	0.0063	DEKNNGAGYFLEHLAFK	HNE K3 100.00%
63.2	2108.0830	-0.0188	IEKNNGAGYFLEHLAFK	
63.2	2108.0830	-0.0188	LEKNNGAGYFLEHLAFK	
63.2	2108.0578	0.0064	NEKNNGAGYFLEHLAFK	
63.2	2108.0578	0.0064	NEKNNGAGYFLEHLAFK	
63.2	2108.0578	0.0064	NEKNNGAGYFLEHLAFK	
0.9	2108.0534	0.0108	AADIWSLGCTIIEMATGK	
0.9	2108.0632	0.0010	ALDLLMESNAIIQSMAAK	
0.1	2108.0300	0.0342	SYQEHIRQPPEEHFRR	

Mascot: http://www.matrixscience.com/
