# Supplementary material for: A common East-Asian ALDH2 mutation causes metabolic disorders and the therapeutic effect of ALDH2 activators
Source: Nat Commun. 2023 Sep 25;14:5971. doi: 10.1038/s41467-023-41570-6 (PMC10520061; doi:10.1038/s41467-023-41570-6)
Supplement: Supplementary file 4 — Supplementary Data 1 [file 41467_2023_41570_MOESM4_ESM.zip › Table S5a/T1683_Uqcrfs1_K101.pdf]

MATRIX SCIENCE Mascot Search Results

Peptide View

MS/MS Fragmentation of **AEVLDSTKSSKESSEAR**
Found in **Q9CR68** in **Mouse_UniProt_proteomes**, Cytochrome b-c1 complex subunit Rieske, mitochondrial OS=Mus musculus OX=10090 GN=Uqcrfs1 PE=1 SV=1

Match to Query 72449: 1978.991536 from(495.755160,4+) intensity(1501178.7500) rtinseconds(1307) scans(6930) index(1471)
Title: File: "D:\Data\2019 User's data\190516-5\190517-1 raw data\190516-5-T1683_1_10.raw"; SpectrumID: "6930"; scans: "6930"
Data file D:\Data\2019 User's data\190516-5\190516-5\190516-5-T1683_1_10.mgf

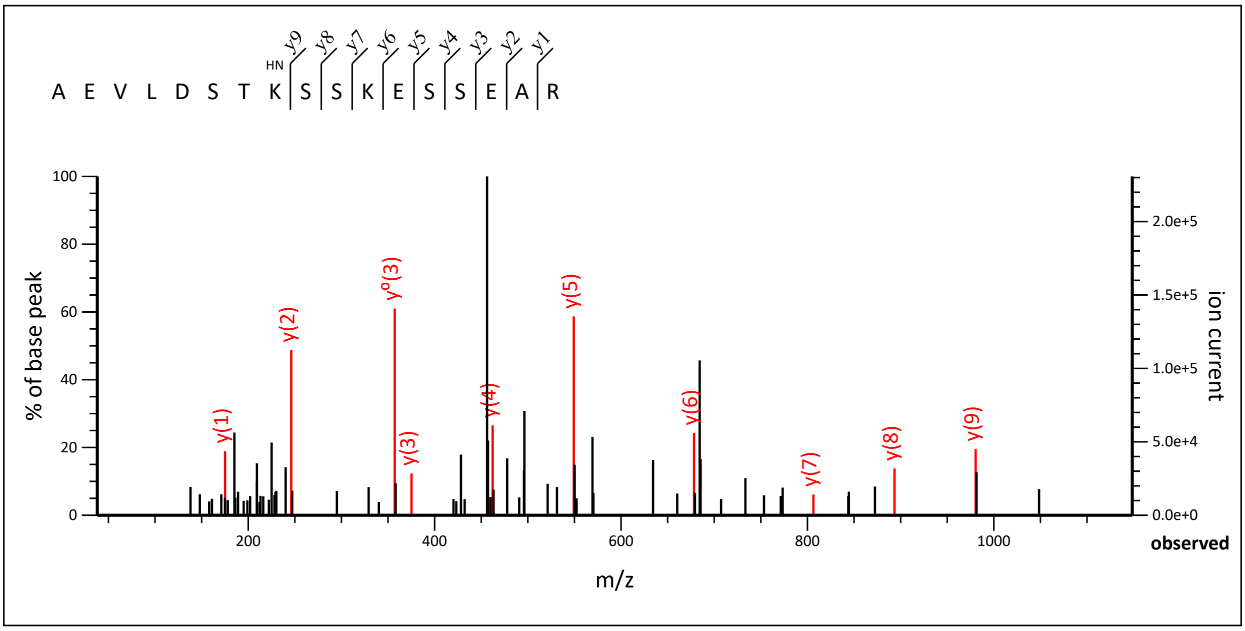

38.06 to 1148.53

Label all possible matches ○ Label matches used for scoring ◉

Monoisotopic mass of neutral peptide Mr(calc): 1979.0058
Fixed modifications: Carbamidomethyl (C) (apply to specified residues or termini only)
Variable modifications:
K8 : HNE (K)
Ions Score: 42 **Expect:** 0.028
Matches : 10/172 fragment ions using 27 most intense peaks (help)

#	b	b^{++}	b*	b^{*++}	b^0	b^{0++}	Seq.	y	y^{++}	y*	y^{*++}	y^0	y^{0++}	#
1	72.0444	36.5258					A							17
2	201.0870	101.0471			183.0764	92.0418	E	1908.9760	954.9917	1891.9495	946.4784	1890.9655	945.9864	16
3	300.1554	150.5813			282.1448	141.5761	V	1779.9334	890.4704	1762.9069	881.9571	1761.9229	881.4651	15
4	413.2395	207.1234			395.2289	198.1181	L	1680.8650	840.9362	1663.8385	832.4229	1662.8545	831.9309	14
5	528.2664	264.6368			510.2558	255.6316	D	1567.7810	784.3941	1550.7544	775.8808	1549.7704	775.3888	13
6	615.2984	308.1529			597.2879	299.1476	S	1452.7540	726.8807	1435.7275	718.3674	1434.7435	717.8754	12
7	716.3461	358.6767			698.3355	349.6714	T	1365.7220	683.3646	1348.6955	674.8514	1347.7114	674.3594	11
8	1000.5561	500.7817	983.5296	492.2684	982.5455	491.7764	K	1264.6743	632.8408	1247.6478	624.3275	1246.6638	623.8355	10
9	1087.5881	544.2977	1070.5616	535.7844	1069.5776	535.2924	S	***980.4643***	490.7358	963.4378	482.2225	962.4538	481.7305	9
10	1174.6202	587.8137	1157.5936	579.3004	1156.6096	578.8084	S	***893.4323***	447.2198	876.4058	438.7065	875.4217	438.2145	8
11	1302.7151	651.8612	1285.6886	643.3479	1284.7046	642.8559	K	***806.4003***	403.7038	789.3737	395.1905	788.3897	394.6985	7
12	1431.7577	716.3825	1414.7312	707.8692	1413.7472	707.3772	E	***678.3053***	339.6563	661.2788	331.1430	660.2947	330.6510	6
13	1518.7897	759.8985	1501.7632	751.3852	1500.7792	750.8932	S	***549.2627***	275.1350	532.2362	266.6217	531.2522	266.1297	5
14	1605.8218	803.4145	1588.7952	794.9012	1587.8112	794.4092	S	***462.2307***	231.6190	445.2041	223.1057	444.2201	222.6137	4
15	1734.8644	867.9358	1717.8378	859.4225	1716.8538	858.9305	E	***375.1987***	188.1030	358.1721	179.5897	357.1881	179.0977	3
16	1805.9015	903.4544	1788.8749	894.9411	1787.8909	894.4491	A	***246.1561***	123.5817	229.1295	115.0684			2
17							R	***175.1190***	88.0631	158.0924	79.5498			1

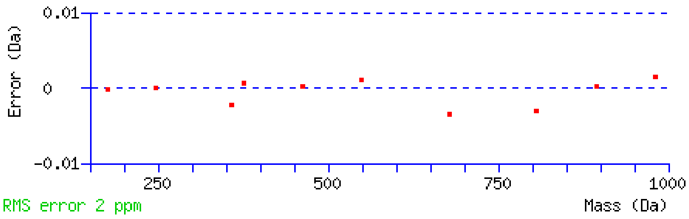

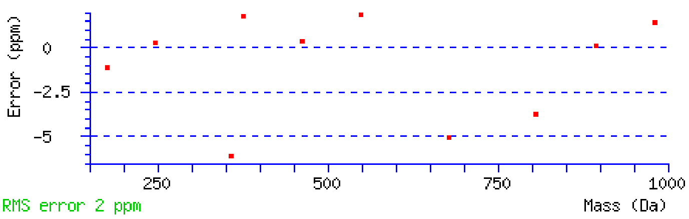

NCBI **BLAST** search of AEVLDSTKSSKESSEAR
(Parameters: blastp, nr protein database, expect=20000, no filter, PAM30)
Other BLAST web gateways

All matches to this query

Score	Mr(calc)	Delta	Sequence	Site Analysis
41.5	1979.0058	-0.0143	AEVLDSTKSSKESSEAR	HNE K8 99.72%
16.1	1979.0058	-0.0143	AEVLDSTKSSKESSEAR	HNE K11 0.28%

Mascot: http://www.matrixscience.com/
