# Supplementary material for: A common East-Asian ALDH2 mutation causes metabolic disorders and the therapeutic effect of ALDH2 activators
Source: Nat Commun. 2023 Sep 25;14:5971. doi: 10.1038/s41467-023-41570-6 (PMC10520061; doi:10.1038/s41467-023-41570-6)
Supplement: Supplementary file 4 — Supplementary Data 1 [file 41467_2023_41570_MOESM4_ESM.zip › Table S5a/T1690_ACO2_K144.pdf]

MATRIX SCIENCE Mascot Search Results

Peptide View

MS/MS Fragmentation of **AKDINQEVYNFLATAGAK**
Found in **Q99KI0** in **Mouse_UniProt_proteomes**, Aconitate hydratase, mitochondrial OS=Mus musculus OX=10090 GN=Aco2 PE=1 SV=1

Match to Query 78049: 2108.102256 from(528.032840,4+) intensity(3018207.7500) rtinseconds(5188) scans(96197) index(86321)
Title: File: "D:\Data\2019 User's data\190516-5\190516-5_T1690_1_10.raw"; SpectrumID: "96197"; scans: "96197"
Data file D:\Data\2019 User's data\190516-5\190516-5\190516-5_T1690_1_10.mgf

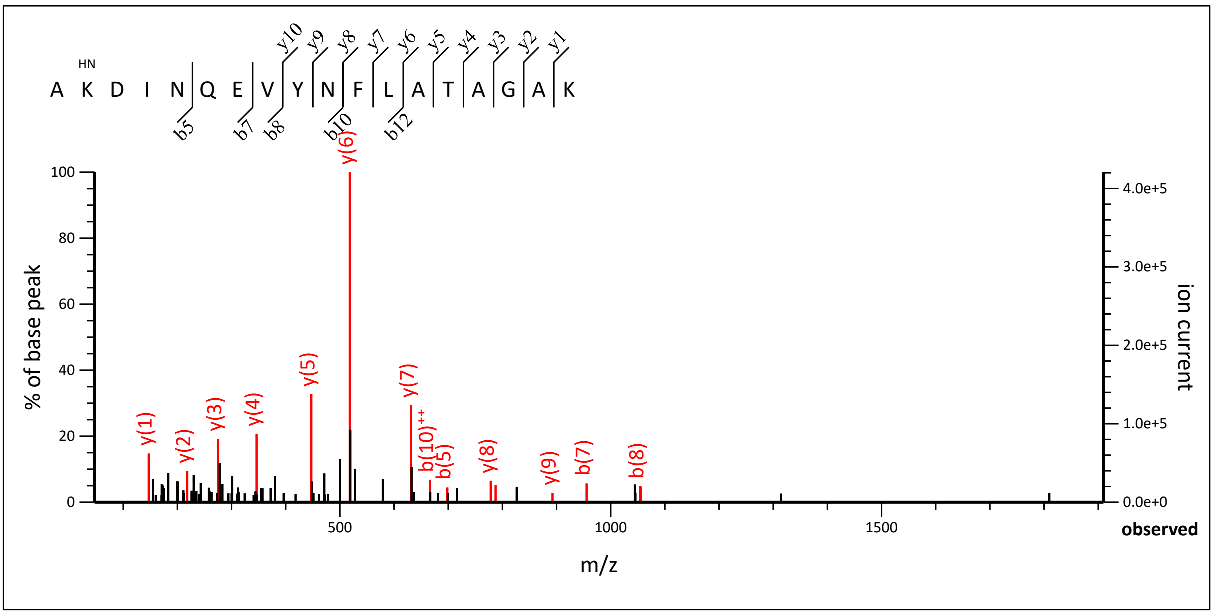

47.11 to 1909.7

Label all possible matches ○ Label matches used for scoring ◉

Monoisotopic mass of neutral peptide Mr(calc): 2108.1153
Fixed modifications: Carbamidomethyl (C) (apply to specified residues or termini only)
Variable modifications:
K2 : HNE (K)
Ions Score: 78 **Expect:** 7.8e-006
Matches : 15/190 fragment ions using 21 most intense peaks (help)

#	b	b^{++}	b*	b^{*++}	b^0	b^{0++}	Seq.	y	y^{++}	y*	y^{*++}	y^0	y^{0++}	#
1	72.0444	36.5258					A							18
2	356.2544	178.6308	339.2278	170.1176			K	2038.0855	1019.5464	2021.0590	1011.0331	2020.0750	1010.5411	17
3	471.2813	236.1443	454.2548	227.6310	453.2708	227.1390	D	1753.8755	877.4414	1736.8490	868.9281	1735.8650	868.4361	16
4	584.3654	292.6863	567.3388	284.1731	566.3548	283.6811	I	1638.8486	819.9279	1621.8221	811.4147	1620.8380	810.9227	15
5	**698.4083**	349.7078	681.3818	341.1945	680.3978	340.7025	N	1525.7645	763.3859	1508.7380	754.8726	1507.7540	754.3806	14
6	826.4669	413.7371	809.4403	405.2238	808.4563	404.7318	Q	1411.7216	706.3644	1394.6951	697.8512	1393.7110	697.3592	13
7	**955.5095**	478.2584	938.4829	469.7451	937.4989	469.2531	E	1283.6630	642.3352	1266.6365	633.8219	1265.6525	633.3299	12
8	**1054.5779**	527.7926	1037.5514	519.2793	1036.5673	518.7873	V	1154.6204	577.8139	1137.5939	569.3006	1136.6099	568.8086	11
9	1217.6412	609.3243	1200.6147	600.8110	1199.6307	600.3190	Y	***1055.5520***	528.2796	1038.5255	519.7664	1037.5415	519.2744	10
10	1331.6842	**666.3457**	1314.6576	657.8324	1313.6736	657.3404	N	***892.4887***	446.7480	875.4621	438.2347	874.4781	437.7427	9
11	1478.7526	739.8799	1461.7260	731.3666	1460.7420	730.8746	F	***778.4458***	389.7265	761.4192	381.2132	760.4352	380.7212	8
12	1591.8366	796.4220	1574.8101	787.9087	1573.8261	787.4167	L	***631.3774***	316.1923	614.3508	307.6790	613.3668	307.1870	7
13	1662.8738	831.9405	1645.8472	823.4272	1644.8632	822.9352	A	***518.2933***	259.6503	501.2667	251.1370	500.2827	250.6450	6
14	1763.9214	882.4644	1746.8949	873.9511	1745.9109	873.4591	T	***447.2562***	224.1317	430.2296	215.6185	429.2456	215.1264	5
15	1834.9585	917.9829	1817.9320	909.4696	1816.9480	908.9776	A	***346.2085***	173.6079	329.1819	165.0946			4
16	1891.9800	946.4936	1874.9535	937.9804	1873.9694	937.4884	G	***275.1714***	138.0893	258.1448	129.5761			3
17	1963.0171	982.0122	1945.9906	973.4989	1945.0066	973.0069	A	***218.1499***	109.5786	201.1234	101.0653			2
18							K	***147.1128***	74.0600	130.0863	65.5468			1

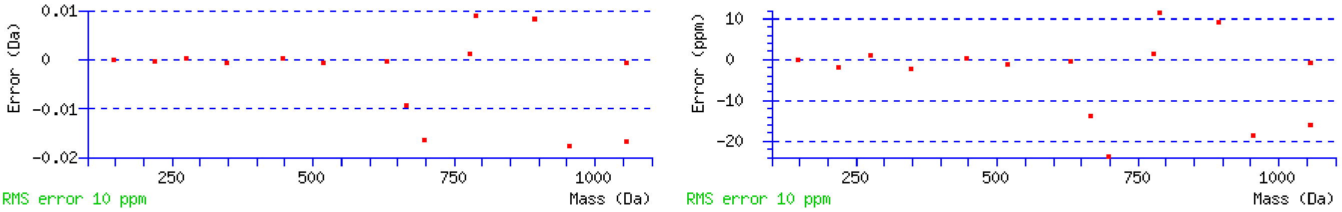

NCBI **BLAST** search of AKDINQEVYNFLATAGAK
(Parameters: blastp, nr protein database, expect=20000, no filter, PAM30)
Other BLAST web gateways

All matches to this query

Score	Mr(calc)	Delta	Sequence	Site Analysis
77.7	2108.1153	-0.0131	AKDINQEVYNFLATAGAK	HNE K2 100.00%
77.7	2108.1014	0.0008	RAKDINQEVYNFLATAGAK	
3.9	2108.0718	0.0305	IKACTGSLNMTLQRASAAAK	
3.9	2108.0718	0.0305	IKACTGSLNMTLQRASAAAK	
3.3	2108.0888	0.0135	ELTSKNSALEYQLLEIQK	
3.3	2108.0888	0.0135	ELTSKNSALEYQLLEIQK	
3.3	2108.1153	-0.0131	KQFNRNLSEFGLIQEK	
3.3	2108.1153	-0.0131	KQFNRNLSEFGLIQEK	
3.3	2108.1153	-0.0131	KQFNRNLSEFGLIQEK	
3.3	2108.0864	0.0159	LQGKFNFHTMTIQIEK	

Mascot: http://www.matrixscience.com/
