# Supplementary material for: A common East-Asian ALDH2 mutation causes metabolic disorders and the therapeutic effect of ALDH2 activators
Source: Nat Commun. 2023 Sep 25;14:5971. doi: 10.1038/s41467-023-41570-6 (PMC10520061; doi:10.1038/s41467-023-41570-6)
Supplement: Supplementary file 4 — Supplementary Data 1 [file 41467_2023_41570_MOESM4_ESM.zip › Table S5a/T1690_HSPA8_K168.pdf]

MATRIX SCIENCE Mascot Search Results

Peptide View

MS/MS Fragmentation of **IINEPTAAAIAYGLDK**
Found in **Q504P4** in **Mouse_UniProt_proteomes**, Heat shock cognate 71 kDa protein OS=Mus musculus OX=10090 GN=Hspa8 PE=1 SV=1

Match to Query 66109: 1814.988612 from(606.003480,3+) intensity(3148655.7500) rtinseconds(4378) scans(77842) index(68919)
Title: File: "D:\Data\2019 User's data\190516-5\190516-5_T1690_1_10.raw"; SpectrumID: "77842"; scans: "77842"
Data file D:\Data\2019 User's data\190516-5\190516-5\190516-5_T1690_1_10.mgf

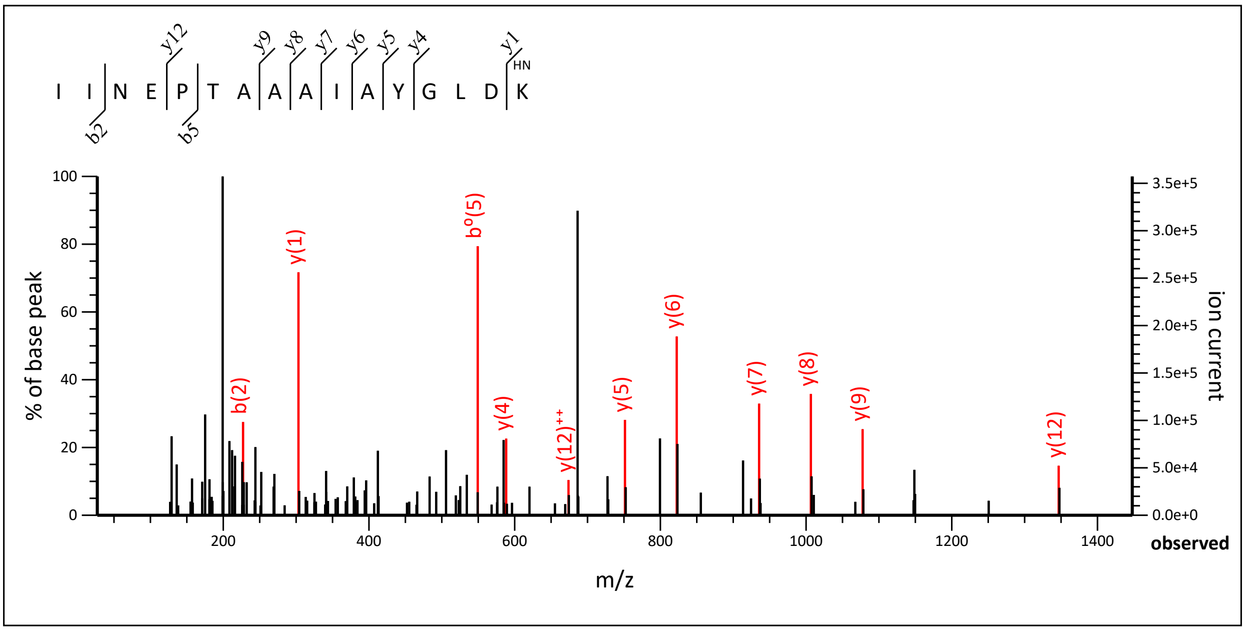

27.09 to 1447.75

Label all possible matches ○ Label matches used for scoring ◉

Monoisotopic mass of neutral peptide Mr(calc): 1815.0029
Fixed modifications: Carbamidomethyl (C) (apply to specified residues or termini only)
Variable modifications:
K16 : HNE (K)
Ions Score: 39 **Expect:** 0.043
Matches : 11/168 fragment ions using 25 most intense peaks (help)

#	b	b^{++}	b^*	b^{*++}	b^0	b^{0++}	Seq.	y	y^{++}	y^*	y^{*++}	y^0	y^{0++}	#
1	114.0913	57.5493					I							16
2	**227.1754**	114.0913					I	1702.9262	851.9667	1685.8996	843.4535	1684.9156	842.9614	15
3	341.2183	171.1128	324.1918	162.5995			N	1589.8421	795.4247	1572.8156	786.9114	1571.8316	786.4194	14
4	470.2609	235.6341	453.2344	227.1208	452.2504	226.6288	E	1475.7992	738.4032	1458.7726	729.8900	1457.7886	729.3980	13
5	567.3137	284.1605	550.2871	275.6472	**549.3031**	275.1552	P	***1346.7566***	**673.8819**	1329.7300	665.3687	1328.7460	664.8767	12
6	668.3614	334.6843	651.3348	326.1710	650.3508	325.6790	T	1249.7038	625.3556	1232.6773	616.8423	1231.6933	616.3503	11
7	739.3985	370.2029	722.3719	361.6896	721.3879	361.1976	A	1148.6562	574.8317	1131.6296	566.3184	1130.6456	565.8264	10
8	810.4356	405.7214	793.4090	397.2082	792.4250	396.7162	A	***1077.6190***	539.3132	1060.5925	530.7999	1059.6085	530.3079	9
9	881.4727	441.2400	864.4462	432.7267	863.4621	432.2347	A	***1006.5819***	503.7946	989.5554	495.2813	988.5714	494.7893	8
10	994.5568	497.7820	977.5302	489.2688	976.5462	488.7767	I	***935.5448***	468.2760	918.5183	459.7628	917.5342	459.2708	7
11	1065.5939	533.3006	1048.5673	524.7873	1047.5833	524.2953	A	***822.4607***	411.7340	805.4342	403.2207	804.4502	402.7287	6
12	1228.6572	614.8322	1211.6307	606.3190	1210.6467	605.8270	Y	***751.4236***	376.2155	734.3971	367.7022	733.4131	367.2102	5
13	1285.6787	643.3430	1268.6521	634.8297	1267.6681	634.3377	G	***588.3603***	294.6838	571.3338	286.1705	570.3497	285.6785	4
14	1398.7627	699.8850	1381.7362	691.3717	1380.7522	690.8797	L	531.3388	266.1731	514.3123	257.6598	513.3283	257.1678	3
15	1513.7897	757.3985	1496.7631	748.8852	1495.7791	748.3932	D	418.2548	209.6310	401.2282	201.1178	400.2442	200.6257	2
16							K	***303.2278***	152.1176	286.2013	143.6043			1

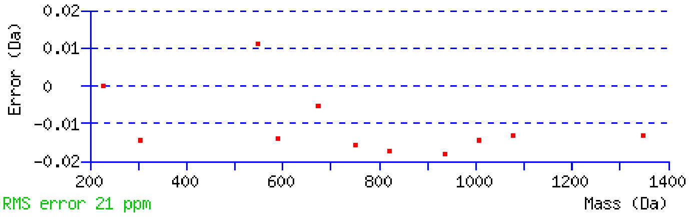

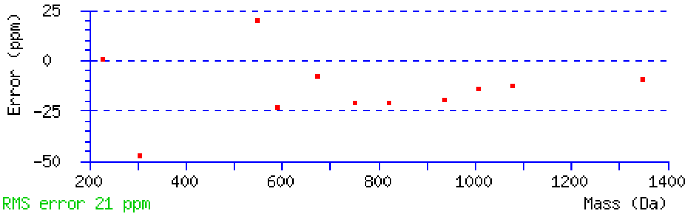

NCBI **BLAST** search of IINEPTAAAIAYGLDK
(Parameters: blastp, nr protein database, expect=20000, no filter, PAM30)
Other BLAST web gateways

All matches to this query

Score	Mr(calc)	Delta	Sequence
63.7	1814.9890	-0.0004	IINEPTAAAIAYGLDKR
39.3	1815.0029	-0.0143	IINEPTAAAIAYGLDK

Mascot: http://www.matrixscience.com/
