# Supplementary material for: A common East-Asian ALDH2 mutation causes metabolic disorders and the therapeutic effect of ALDH2 activators
Source: Nat Commun. 2023 Sep 25;14:5971. doi: 10.1038/s41467-023-41570-6 (PMC10520061; doi:10.1038/s41467-023-41570-6)
Supplement: Supplementary file 4 — Supplementary Data 1 [file 41467_2023_41570_MOESM4_ESM.zip › Table S5a/T1690_Myh4_K1525.pdf]

MATRIX SCIENCE Mascot Search Results

Peptide View

MS/MS Fragmentation of **NLQQEISDLTEQIAEGGK**
Found in **Q5SX39** in **Mouse_UniProt_proteomes**, Myosin-4 OS=Mus musculus OX=10090 GN=Myh4 PE=2 SV=1

Match to Query 78723: 2128.081392 from(710.367740,3+) intensity(842973.6250) rtinseconds(5609) scans(105806) index(95456)
Title: File: "D:\Data\2019 User's data\190516-5\190516-5_T1690_1_10.raw"; SpectrumID: "105806"; scans: "105806"
Data file D:\Data\2019 User's data\190516-5\190516-5\190516-5_T1690_1_10.mgf

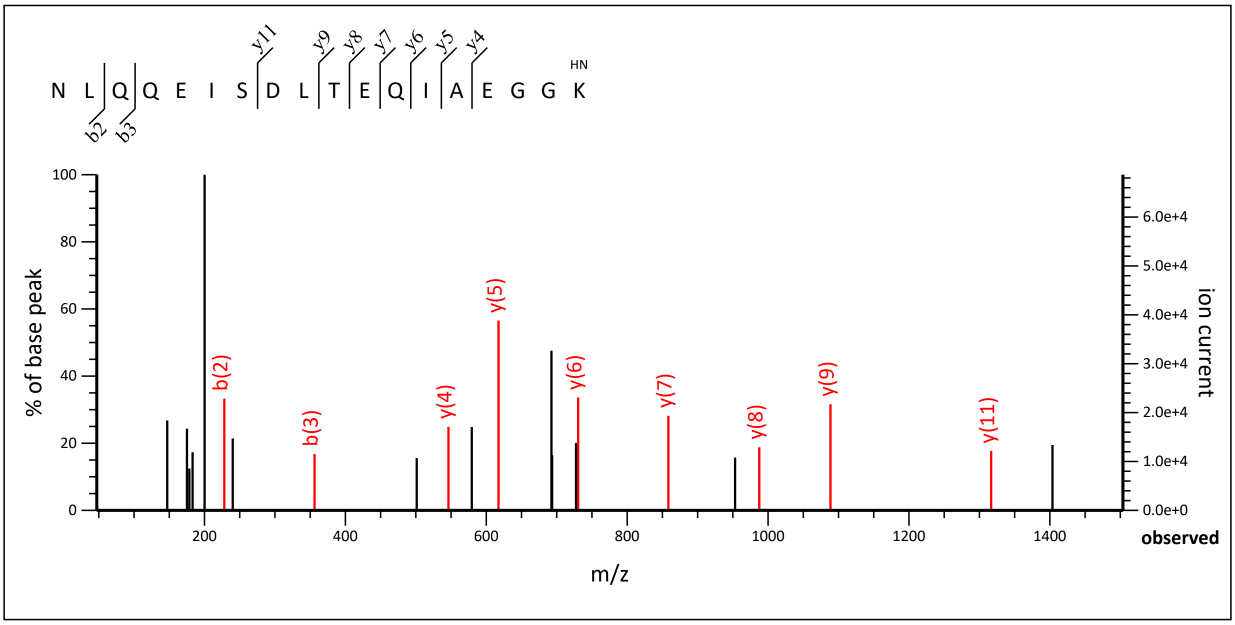

47.1 to 1503.7

Label all possible matches ○ Label matches used for scoring ◉

Monoisotopic mass of neutral peptide Mr(calc): 2128.0899
Fixed modifications: Carbamidomethyl (C) (apply to specified residues or termini only)
Variable modifications:
K18 : HNE (K)
Ions Score: 42 **Expect:** 0.031
Matches : 9/190 fragment ions using 15 most intense peaks (help)

#	b	b^{++}	b*	b^{*++}	b^0	b^{0++}	Seq.	y	y^{++}	y*	y^{*++}	y^0	y^{0++}	#
1	115.0502	58.0287	98.0237	49.5155			N							18
2	**228.1343**	114.5708	211.1077	106.0575			L	2015.0543	1008.0308	1998.0277	999.5175	1997.0437	999.0255	17
3	**356.1928**	178.6001	339.1663	170.0868			Q	1901.9702	951.4888	1884.9437	942.9755	1883.9597	942.4835	16
4	484.2514	242.6293	467.2249	234.1161			Q	1773.9117	887.4595	1756.8851	878.9462	1755.9011	878.4542	15
5	613.2940	307.1506	596.2675	298.6374	595.2835	298.1454	E	1645.8531	823.4302	1628.8265	814.9169	1627.8425	814.4249	14
6	726.3781	363.6927	709.3515	355.1794	708.3675	354.6874	I	1516.8105	758.9089	1499.7839	750.3956	1498.7999	749.9036	13
7	813.4101	407.2087	796.3836	398.6954	795.3995	398.2034	S	1403.7264	702.3668	1386.6999	693.8536	1385.7159	693.3616	12
8	928.4371	464.7222	911.4105	456.2089	910.4265	455.7169	D	***1316.6944***	658.8508	1299.6678	650.3376	1298.6838	649.8456	11
9	1041.5211	521.2642	1024.4946	512.7509	1023.5106	512.2589	L	1201.6674	601.3374	1184.6409	592.8241	1183.6569	592.3321	10
10	1142.5688	571.7880	1125.5422	563.2748	1124.5582	562.7828	T	***1088.5834***	544.7953	1071.5568	536.2821	1070.5728	535.7900	9
11	1271.6114	636.3093	1254.5848	627.7961	1253.6008	627.3040	E	***987.5357***	494.2715	970.5092	485.7582	969.5251	485.2662	8
12	1399.6700	700.3386	1382.6434	691.8253	1381.6594	691.3333	Q	***858.4931***	429.7502	841.4666	421.2369	840.4825	420.7449	7
13	1512.7540	756.8807	1495.7275	748.3674	1494.7435	747.8754	I	***730.4345***	365.7209	713.4080	357.2076	712.4240	356.7156	6
14	1583.7911	792.3992	1566.7646	783.8859	1565.7806	783.3939	A	***617.3505***	309.1789	600.3239	300.6656	599.3399	300.1736	5
15	1712.8337	856.9205	1695.8072	848.4072	1694.8232	847.9152	E	***546.3134***	273.6603	529.2868	265.1470	528.3028	264.6550	4
16	1769.8552	885.4312	1752.8287	876.9180	1751.8446	876.4260	G	417.2708	209.1390	400.2442	200.6257			3
17	1826.8767	913.9420	1809.8501	905.4287	1808.8661	904.9367	G	360.2493	180.6283	343.2227	172.1150			2
18							K	303.2278	152.1176	286.2013	143.6043			1

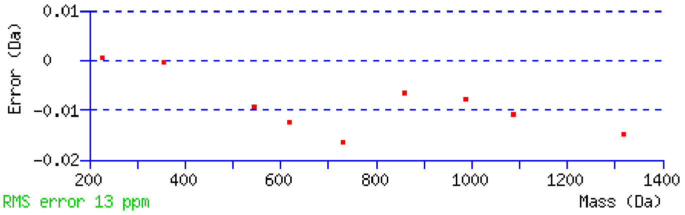

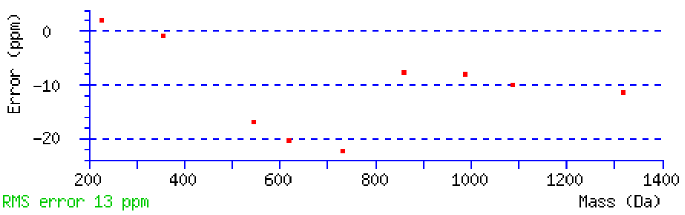

NCBI **BLAST** search of NLQQEISDLTEQIAEGGK
(Parameters: blastp, nr protein database, expect=20000, no filter, PAM30)
Other BLAST web gateways

All matches to this query

Score	Mr(calc)	Delta	Sequence
58.3	2128.0760	0.0054	NLQQEISDLTEQIAEGGKR
42.0	2128.0899	-0.0085	NLQQEISDLTEQIAEGGK
1.9	2128.1011	-0.0197	QIEAKDNKITELLNDVER
1.9	2128.1011	-0.0197	QIEAKDNKITELLNDVER
0.7	2128.0721	0.0092	KNMHSANQKIQGAQDK

Mascot: http://www.matrixscience.com/
