# Supplementary material for: A common East-Asian ALDH2 mutation causes metabolic disorders and the therapeutic effect of ALDH2 activators
Source: Nat Commun. 2023 Sep 25;14:5971. doi: 10.1038/s41467-023-41570-6 (PMC10520061; doi:10.1038/s41467-023-41570-6)
Supplement: Supplementary file 4 — Supplementary Data 1 [file 41467_2023_41570_MOESM4_ESM.zip › Table S5a/T1690_pdha1_H121.pdf]

MATRIX SCIENCE Mascot Search Results

Peptide View

MS/MS Fragmentation of **AHGFTFTR**
Found in **P35486** in **Mouse_UniProt_proteomes**, Pyruvate dehydrogenase E1 component subunit alpha, somatic form, mitochondrial OS=Mus musculus OX=10090 GN=Pdha1 PE=1 SV=1

Match to Query 19203: 1091.562702 from(364.861510,3+) intensity(15776231.0000) rtinseconds(2077) scans(24430) index(18124)
Title: File: "D:\Data\2019 User's data\190516-5\190516-5_T1690_1_10.raw"; SpectrumID: "24430"; scans: "24430"
Data file D:\Data\2019 User's data\190516-5\190516-5\190516-5_T1690_1_10.mgf

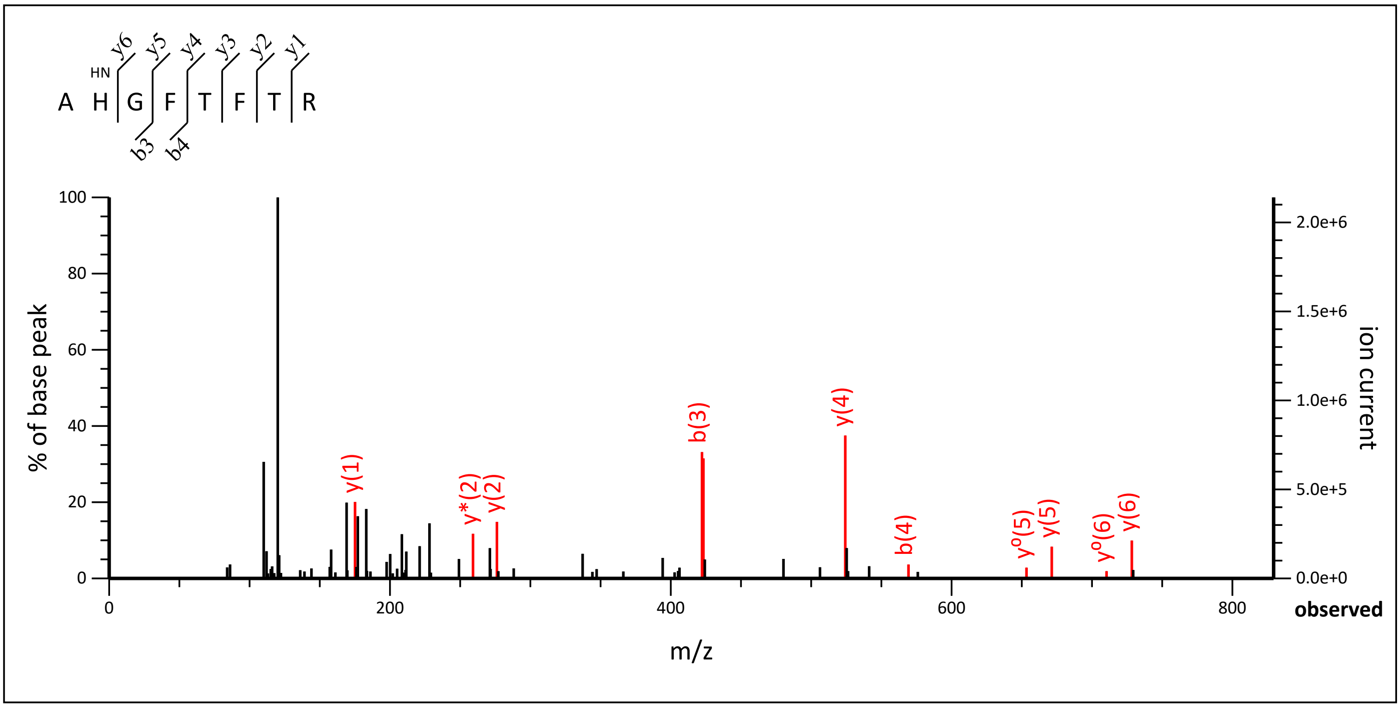

0 to 829.36

Label all possible matches ○ Label matches used for scoring ◉

Monoisotopic mass of neutral peptide Mr(calc): 1091.5764
Fixed modifications: Carbamidomethyl (C) (apply to specified residues or termini only)
Variable modifications:
H2 : HNE (H)
Ions Score: 37 **Expect:** 0.037
Matches : 11/60 fragment ions using 20 most intense peaks (help)

#	b	b^{++}	b^{0}	b^{0++}	Seq.	y	y^{++}	y^{*}	y^{*++}	y^{0}	y^{0++}	#
1	72.0444	36.5258			A							8
2	365.2183	183.1128			H	1021.5465	511.2769	1004.5200	502.7636	1003.5360	502.2716	7
3	**422.2398**	211.6235			G	***728.3726***	364.6899	711.3461	356.1767	710.3620	355.6847	6
4	**569.3082**	285.1577			F	***671.3511***	336.1792	654.3246	327.6659	653.3406	327.1739	5
5	670.3559	335.6816	652.3453	326.6763	T	***524.2827***	262.6450	507.2562	254.1317	506.2722	253.6397	4
6	817.4243	409.2158	799.4137	400.2105	F	***423.2350***	212.1212	406.2085	203.6079	405.2245	203.1159	3
7	918.4720	459.7396	900.4614	450.7343	T	***276.1666***	138.5870	259.1401	130.0737	258.1561	129.5817	2
8					R	***175.1190***	88.0631	158.0924	79.5498			1

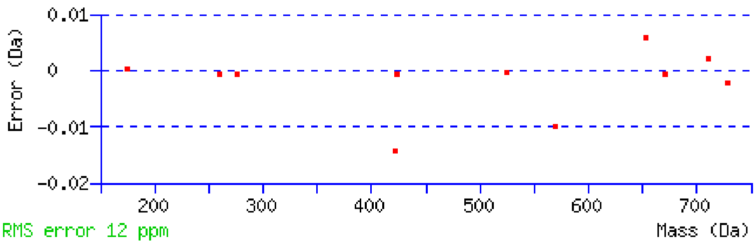

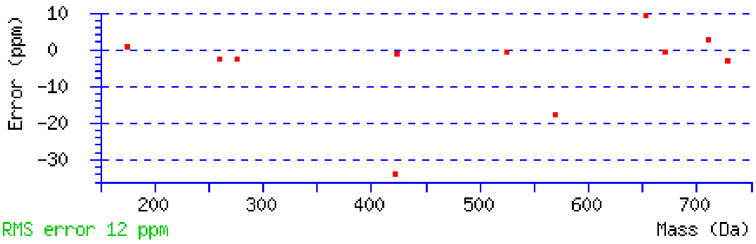

NCBI **BLAST** search of AHGFTFTR
(Parameters: blastp, nr protein database, expect=20000, no filter, PAM30)
Other BLAST web gateways

All matches to this query

Score	Mr(calc)	Delta	Sequence
37.4	1091.5764	-0.0137	AHGFTFTR
2.4	1091.5611	0.0016	IEAVQEFTR

Mascot: http://www.matrixscience.com/
