# Supplementary material for: A common East-Asian ALDH2 mutation causes metabolic disorders and the therapeutic effect of ALDH2 activators
Source: Nat Commun. 2023 Sep 25;14:5971. doi: 10.1038/s41467-023-41570-6 (PMC10520061; doi:10.1038/s41467-023-41570-6)
Supplement: Supplementary file 4 — Supplementary Data 1 [file 41467_2023_41570_MOESM4_ESM.zip › Table S5a/T1718_Acaa2_k25.pdf]

Mascot Search Results

Peptide View

MS/MS Fragmentation of **TPFGAYGGLLK**
Found in **Q8BWT1** in **Mouse_UniProt_proteomes**, 3-ketoacyl-CoA thiolase, mitochondrial OS=Mus musculus OX=10090 GN=Acaa2 PE=1 SV=3

Match to Query 21220: 1122.607768 from(562.311160,2+) intensity(10956909.0000) rtinseconds(4588) scans(82661) index(73521)
Title: File: "D:\Data\2019 User's data\190516-5\190516-5-T1718_1_10.raw"; SpectrumID: "82661"; scans: "82661"
Data file D:\Data\2019 User's data\190516-5\190516-5\190516-5-T1718_1_10.mgf

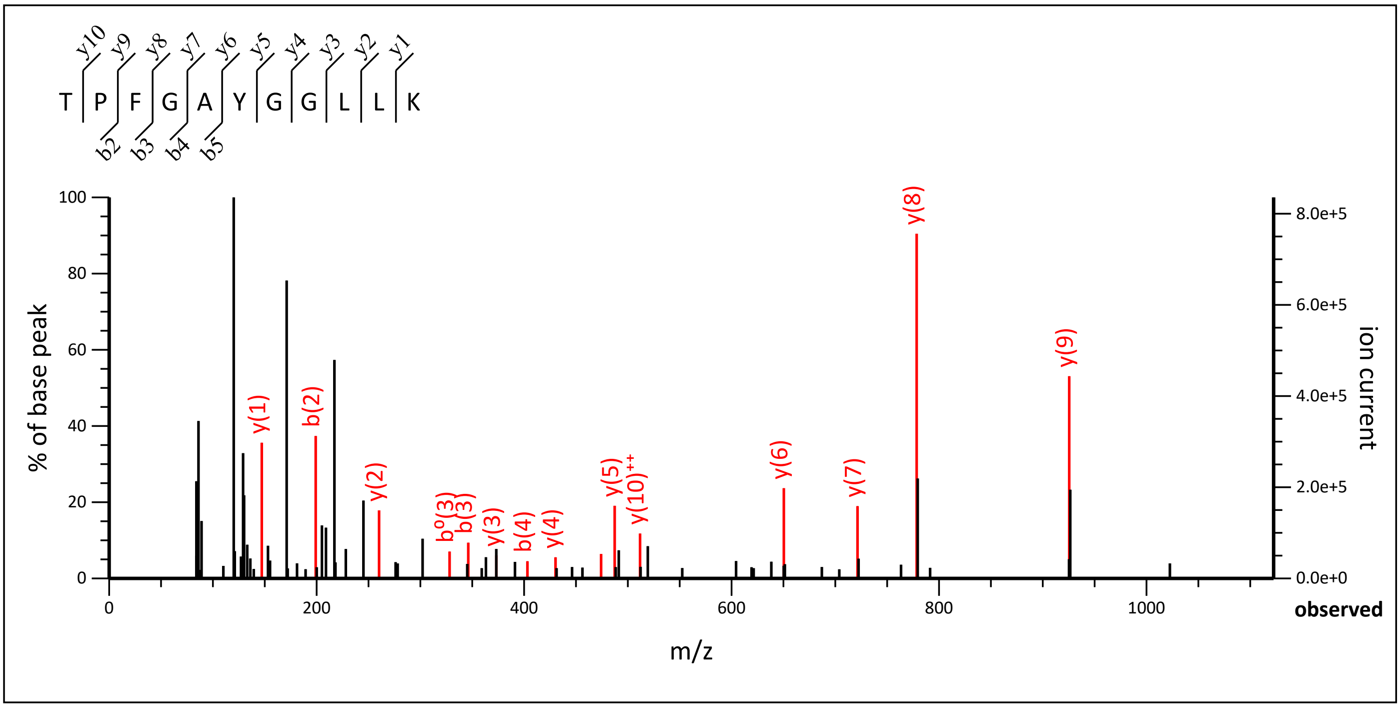

0 to 1122.55

Label all possible matches ○ Label matches used for scoring ◉

Monoisotopic mass of neutral peptide Mr(calc): 1122.6073
Fixed modifications: Carbamidomethyl (C) (apply to specified residues or termini only)
Ions Score: 50 **Expect:** 0.0018
Matches : 15/80 fragment ions using 40 most intense peaks (help)

#	b	b^{++}	b^0	b^{0++}	Seq.	y	y^{++}	y*	y^{*++}	#
1	102.0550	51.5311	84.0444	42.5258	T					11
2	**199.1077**	100.0575	181.0972	91.0522	P	1022.5669	**511.7871**	1005.5404	503.2738	10
3	**346.1761**	173.5917	328.1656	164.5864	F	***925.5142***	463.2607	908.4876	454.7475	9
4	**403.1976**	202.1024	385.1870	193.0972	G	***778.4458***	389.7265	761.4192	381.2132	8
5	**474.2347**	237.6210	456.2241	228.6157	A	***721.4243***	361.2158	704.3978	352.7025	7
6	637.2980	319.1527	619.2875	310.1474	Y	***650.3872***	325.6972	633.3606	317.1840	6
7	694.3195	347.6634	676.3089	338.6581	G	***487.3239***	244.1656	470.2973	235.6523	5
8	751.3410	376.1741	733.3304	367.1688	G	***430.3024***	215.6548	413.2758	207.1416	4
9	864.4250	432.7162	846.4145	423.7109	L	***373.2809***	187.1441	356.2544	178.6308	3
10	977.5091	489.2582	959.4985	480.2529	L	***260.1969***	130.6021	243.1703	122.0888	2
11					K	***147.1128***	74.0600	130.0863	65.5468	1

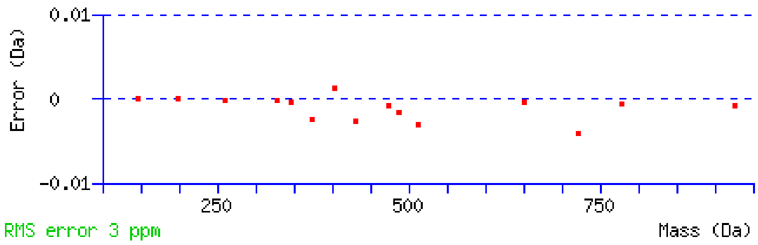

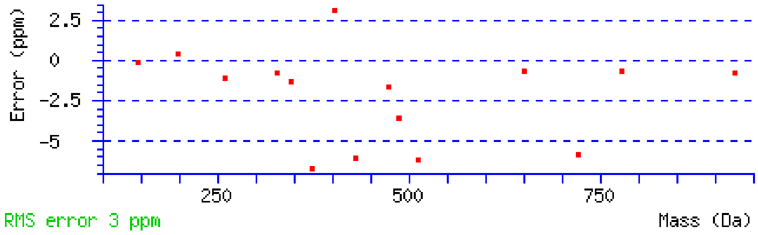

NCBI **BLAST** search of TPFGAYGGLLK
(Parameters: blastp, nr protein database, expect=20000, no filter, PAM30)
Other BLAST web gateways

All matches to this query

Score	Mr(calc)	Delta	Sequence
49.6	1122.6073	0.0004	TPFGAYGGLLK

**Mascot:** http://www.matrixscience.com/
