# Supplementary material for: A common East-Asian ALDH2 mutation causes metabolic disorders and the therapeutic effect of ALDH2 activators
Source: Nat Commun. 2023 Sep 25;14:5971. doi: 10.1038/s41467-023-41570-6 (PMC10520061; doi:10.1038/s41467-023-41570-6)
Supplement: Supplementary file 4 — Supplementary Data 1 [file 41467_2023_41570_MOESM4_ESM.zip › Table S5a/T1718_Gdp2_K652.pdf]

MATRIX SCIENCE Mascot Search Results

Peptide View

MS/MS Fragmentation of **FHKFDEDEKGFITIVDVQR**
Found in **A2AQR0** in **Mouse_UniProt_proteomes**, Glycerol-3-phosphate dehydrogenase OS=Mus musculus OX=10090 GN=Gpd2 PE=1 SV=1

Match to Query 86699: 2478.272896 from(620.575500,4+) intensity(871579.8750) rtinseconds(4241) scans(74687) index(65929)
Title: File: "D:\Data\2019 User's data\190516-5\190516-5-T1718_1_10.raw"; SpectrumID: "74687"; scans: "74687"
Data file D:\Data\2019 User's data\190516-5\190516-5\190516-5-T1718_1_10.mgf

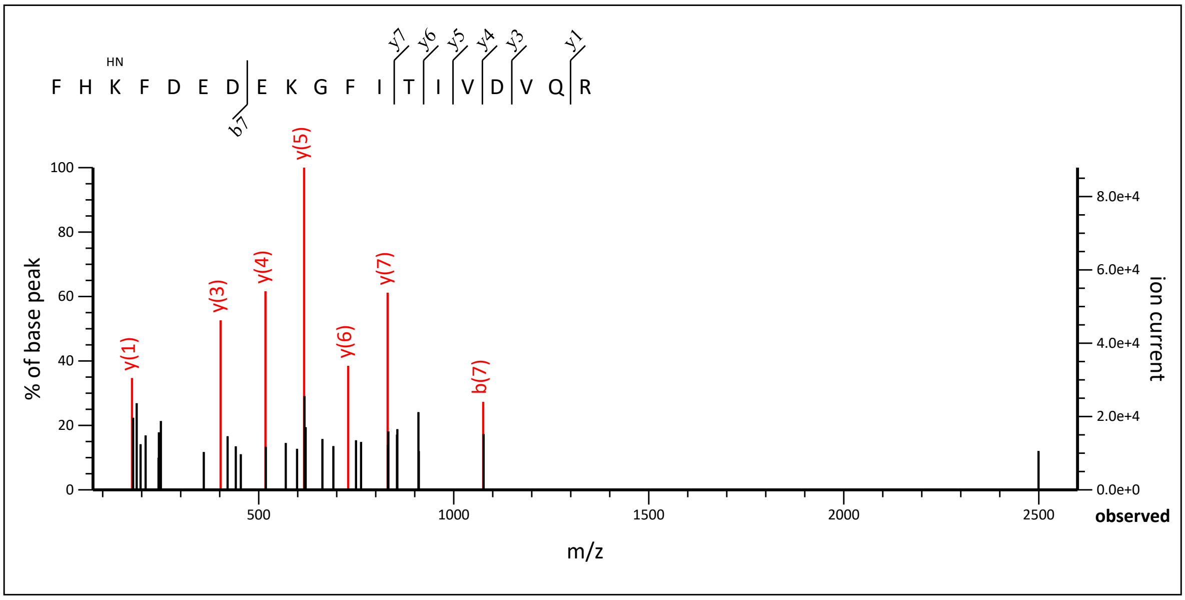

75.12 to 2599.32

Label all possible matches ○ Label matches used for scoring ◉

Monoisotopic mass of neutral peptide Mr(calc): 2478.2795
Fixed modifications: Carbamidomethyl (C) (apply to specified residues or termini only)
Variable modifications:
K3 : HNE (K)
Ions Score: 42 **Expect:** 0.039
Matches : 7/198 fragment ions using 9 most intense peaks (help)

#	b	b++	b*	b*++	b0	b0++	Seq.	y	y++	y*	y*++	y0	y0++	#
1	148.0757	74.5415					F							19
2	285.1346	143.0709					H	2332.2183	1166.6128	2315.1918	1158.0995	2314.2078	1157.6075	18
3	569.3446	285.1759	552.3180	276.6627			K	2195.1594	1098.0834	2178.1329	1089.5701	2177.1489	1089.0781	17
4	716.4130	358.7101	699.3865	350.1969			F	1910.9494	955.9784	1893.9229	947.4651	1892.9389	946.9731	16
5	831.4400	416.2236	814.4134	407.7103	813.4294	407.2183	D	1763.8810	882.4441	1746.8545	873.9309	1745.8705	873.4389	15
6	960.4825	480.7449	943.4560	472.2316	942.4720	471.7396	E	1648.8541	824.9307	1631.8275	816.4174	1630.8435	815.9254	14
7	**1075.5095**	538.2584	1058.4829	529.7451	1057.4989	529.2531	D	1519.8115	760.4094	1502.7849	751.8961	1501.8009	751.4041	13
8	1204.5521	602.7797	1187.5255	594.2664	1186.5415	593.7744	E	1404.7845	702.8959	1387.7580	694.3826	1386.7740	693.8906	12
9	1332.6470	666.8272	1315.6205	658.3139	1314.6365	657.8219	K	1275.7419	638.3746	1258.7154	629.8613	1257.7314	629.3693	11
10	1389.6685	695.3379	1372.6420	686.8246	1371.6579	686.3326	G	1147.6470	574.3271	1130.6204	565.8139	1129.6364	565.3218	10
11	1536.7369	768.8721	1519.7104	760.3588	1518.7264	759.8668	F	1090.6255	545.8164	1073.5990	537.3031	1072.6150	536.8111	9
12	1649.8210	825.4141	1632.7944	816.9009	1631.8104	816.4088	I	943.5571	472.2822	926.5306	463.7689	925.5465	463.2769	8
13	1750.8687	875.9380	1733.8421	867.4247	1732.8581	866.9327	T	***830.4730***	415.7402	813.4465	407.2269	812.4625	406.7349	7
14	1863.9527	932.4800	1846.9262	923.9667	1845.9422	923.4747	I	***729.4254***	365.2163	712.3988	356.7030	711.4148	356.2110	6
15	1963.0211	982.0142	1945.9946	973.5009	1945.0106	973.0089	V	***616.3413***	308.6743	599.3148	300.1610	598.3307	299.6690	5
16	2078.0481	1039.5277	2061.0215	1031.0144	2060.0375	1030.5224	D	***517.2729***	259.1401	500.2463	250.6268	499.2623	250.1348	4
17	2177.1165	1089.0619	2160.0900	1080.5486	2159.1059	1080.0566	V	***402.2459***	201.6266	385.2194	193.1133			3
18	2305.1751	1153.0912	2288.1485	1144.5779	2287.1645	1144.0859	Q	303.1775	152.0924	286.1510	143.5791			2
19							R	***175.1190***	88.0631	158.0924	79.5498			1

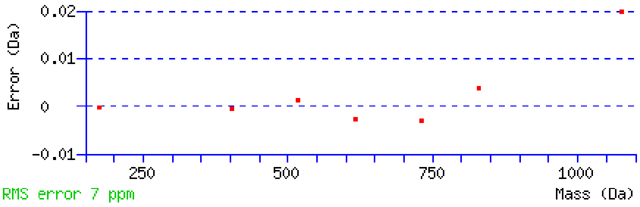

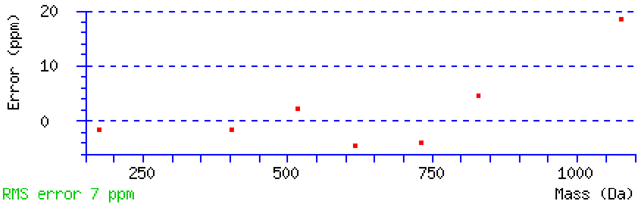

NCBI **BLAST** search of FHKFDEDEKGFITIVDVQR
(Parameters: blastp, nr protein database, expect=20000, no filter, PAM30)
Other BLAST web gateways

All matches to this query

Score	Mr(calc)	Delta	Sequence	Site Analysis
41.8	2478.2795	-0.0066	FHKFDEDEKGFITIVDVQR	HNE K3 72.36%
41.8	2478.2795	-0.0066	FHKFDEDEKGFITIVDVQR	
37.6	2478.2795	-0.0066	FHKFDEDEKGFITIVDVQR	HNE K9 27.64%
4.4	2478.2940	-0.0211	CSLRLTHYIEEANVGRGYIK	
4.3	2478.2703	0.0026	PHTFVGVHVRRGDYLEVMPNR	
4.1	2478.2754	-0.0025	FIEQNKEKFTQLDLEDVQVR	
3.1	2478.3212	-0.0483	ASSLQKLKGLMEDVMQNIK	
3.1	2478.3212	-0.0483	ASSLQKLKGLMEDVMQNIK	
3.1	2478.2272	0.0457	DLETEIKGKGMQVSTDTLDVQR	
3.1	2478.2325	0.0404	KRYISQNDMIAILDYHNQVR	

Mascot: http://www.matrixscience.com/
