# Supplementary material for: A common East-Asian ALDH2 mutation causes metabolic disorders and the therapeutic effect of ALDH2 activators
Source: Nat Commun. 2023 Sep 25;14:5971. doi: 10.1038/s41467-023-41570-6 (PMC10520061; doi:10.1038/s41467-023-41570-6)
Supplement: Supplementary file 4 — Supplementary Data 1 [file 41467_2023_41570_MOESM4_ESM.zip › Table S5a/T1718_Gdp2_K658.pdf]

MATRIX SCIENCE Mascot Search Results

Peptide View

MS/MS Fragmentation of **FHKFDEDEKGFITIVDVQR**
Found in **A2AQR0** in **Mouse_UniProt_proteomes**, Glycerol-3-phosphate dehydrogenase OS=Mus musculus OX=10090 GN=Gpd2 PE=1 SV=1

Match to Query 86695: 2478.267296 from(620.574100,4+) intensity(1203251.0000) rtinseconds(4262) scans(75166) index(66385)
Title: File: "D:\Data\2019 User's data\190516-5\190516-5-T1718_1_10.raw"; SpectrumID: "75166"; scans: "75166"
Data file D:\Data\2019 User's data\190516-5\190516-5\190516-5-T1718_1_10.mgf

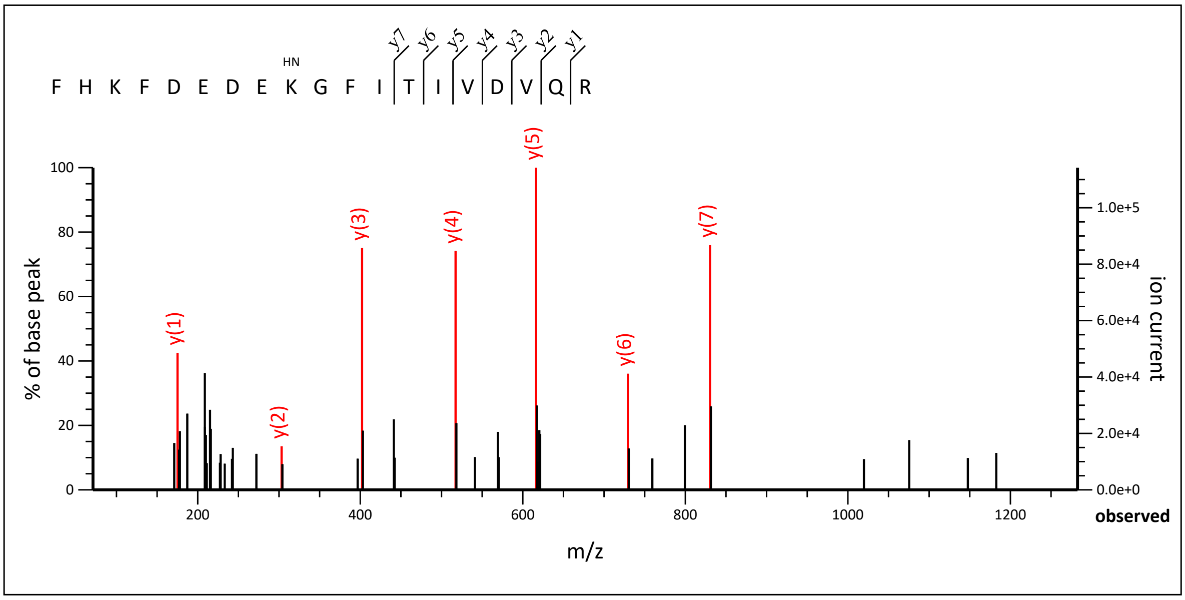

71.11 to 1282.61

Label all possible matches ○ Label matches used for scoring ◉

Monoisotopic mass of neutral peptide Mr(calc): 2478.2795
Fixed modifications: Carbamidomethyl (C) (apply to specified residues or termini only)
Variable modifications:
K9 : HNE (K)
Ions Score: 48 **Expect:** 0.0099
Matches : 7/198 fragment ions using 10 most intense peaks (help)

#	b	b^{++}	b*	b*$^{++}$	b^{0}	b^{0++}	Seq.	y	y^{++}	y*	y*$^{++}$	y^{0}	y^{0++}	#
1	148.0757	74.5415					F							19
2	285.1346	143.0709					H	2332.2183	1166.6128	2315.1918	1158.0995	2314.2078	1157.6075	18
3	413.2296	207.1184	396.2030	198.6051			K	2195.1594	1098.0834	2178.1329	1089.5701	2177.1489	1089.0781	17
4	560.2980	280.6526	543.2714	272.1394			F	2067.0645	1034.0359	2050.0379	1025.5226	2049.0539	1025.0306	16
5	675.3249	338.1661	658.2984	329.6528	657.3144	329.1608	D	1919.9961	960.5017	1902.9695	951.9884	1901.9855	951.4964	15
6	804.3675	402.6874	787.3410	394.1741	786.3569	393.6821	E	1804.9691	902.9882	1787.9426	894.4749	1786.9585	893.9829	14
7	919.3945	460.2009	902.3679	451.6876	901.3839	451.1956	D	1675.9265	838.4669	1658.9000	829.9536	1657.9159	829.4616	13
8	1048.4371	524.7222	1031.4105	516.2089	1030.4265	515.7169	E	1560.8996	780.9534	1543.8730	772.4401	1542.8890	771.9481	12
9	1332.6470	666.8272	1315.6205	658.3139	1314.6365	657.8219	K	1431.8570	716.4321	1414.8304	707.9189	1413.8464	707.4268	11
10	1389.6685	695.3379	1372.6420	686.8246	1371.6579	686.3326	G	1147.6470	574.3271	1130.6204	565.8139	1129.6364	565.3218	10
11	1536.7369	768.8721	1519.7104	760.3588	1518.7264	759.8668	F	1090.6255	545.8164	1073.5990	537.3031	1072.6150	536.8111	9
12	1649.8210	825.4141	1632.7944	816.9009	1631.8104	816.4088	I	943.5571	472.2822	926.5306	463.7689	925.5465	463.2769	8
13	1750.8687	875.9380	1733.8421	867.4247	1732.8581	866.9327	T	*830.4730*	415.7402	813.4465	407.2269	812.4625	406.7349	7
14	1863.9527	932.4800	1846.9262	923.9667	1845.9422	923.4747	I	*729.4254*	365.2163	712.3988	356.7030	711.4148	356.2110	6
15	1963.0211	982.0142	1945.9946	973.5009	1945.0106	973.0089	V	*616.3413*	308.6743	599.3148	300.1610	598.3307	299.6690	5
16	2078.0481	1039.5277	2061.0215	1031.0144	2060.0375	1030.5224	D	*517.2729*	259.1401	500.2463	250.6268	499.2623	250.1348	4
17	2177.1165	1089.0619	2160.0900	1080.5486	2159.1059	1080.0566	V	*402.2459*	201.6266	385.2194	193.1133			3
18	2305.1751	1153.0912	2288.1485	1144.5779	2287.1645	1144.0859	Q	*303.1775*	152.0924	286.1510	143.5791			2
19							R	*175.1190*	88.0631	158.0924	79.5498			1

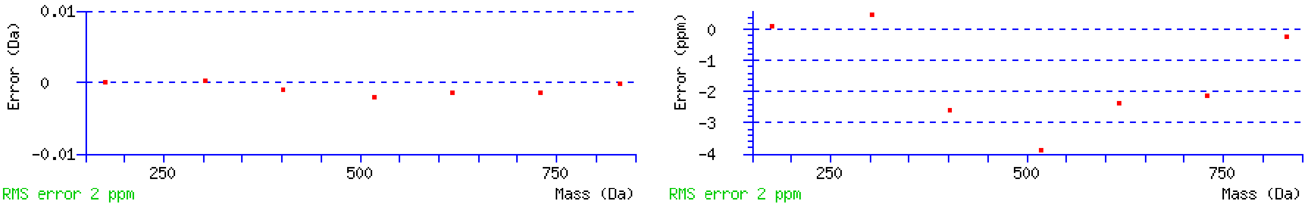

NCBI **BLAST** search of FHKFDEDEKGFITIVDVQR
(Parameters: blastp, nr protein database, expect=20000, no filter, PAM30)
Other BLAST web gateways

All matches to this query

Score	Mr(calc)	Delta	Sequence	Site Analysis
47.8	2478.2795	-0.0122	FHKFDEDEKGFITIVDVQR	HNE K9 50.00%
47.8	2478.2795	-0.0122	FHKFDEDEKGFITIVDVQR	HNE K3 50.00%
47.8	2478.2795	-0.0122	FHKFDEDEKGFITIVDVQR	
14.4	2478.2272	0.0401	DLETEIKGKGMQVSTDTLDVQR	
4.5	2478.2703	-0.0030	PHTFVGVHVRRGDYLEVMPNR	
3.6	2478.2754	-0.0081	FIEQNKEKFTQLDLEDVQVR	
3.5	2478.2940	-0.0267	CSLRLTHYIEEANVGRGYIK	
3.1	2478.2914	-0.0241	EVGEARGWLSSLRAHIMPAGIGR	
3.0	2478.2325	0.0348	KRYISQNDMIAILDYHNQVR	
3.0	2478.2325	0.0348	KRYISQNDMIAILDYHNQVR	

Mascot: http://www.matrixscience.com/
