# Supplementary material for: A common East-Asian ALDH2 mutation causes metabolic disorders and the therapeutic effect of ALDH2 activators
Source: Nat Commun. 2023 Sep 25;14:5971. doi: 10.1038/s41467-023-41570-6 (PMC10520061; doi:10.1038/s41467-023-41570-6)
Supplement: Supplementary file 4 — Supplementary Data 1 [file 41467_2023_41570_MOESM4_ESM.zip › Table S5a/T1718_Ndufb4_H59.pdf]

MATRIX SCIENCE Mascot Search Results

Peptide View

MS/MS Fragmentation of **VSHIEDPALIR**
Found in **Q9CQC7** in **Mouse_UniProt_proteomes**, NADH dehydrogenase [ubiquinone] 1 beta subcomplex subunit 4 OS=Mus musculus OX=10090 GN=Ndufb4 PE=1 SV=3

Match to Query 41105: 1404.784032 from(469.268620,3+) intensity(16048582.0000) rtinseconds(2520) scans(34869) index(28086)
Title: File: "D:\Data\2019 User's data\190516-5\190516-5-T1718_1_10.raw"; SpectrumID: "34869"; scans: "34869"
Data file D:\Data\2019 User's data\190516-5\190516-5\190516-5-T1718_1_10.mgf

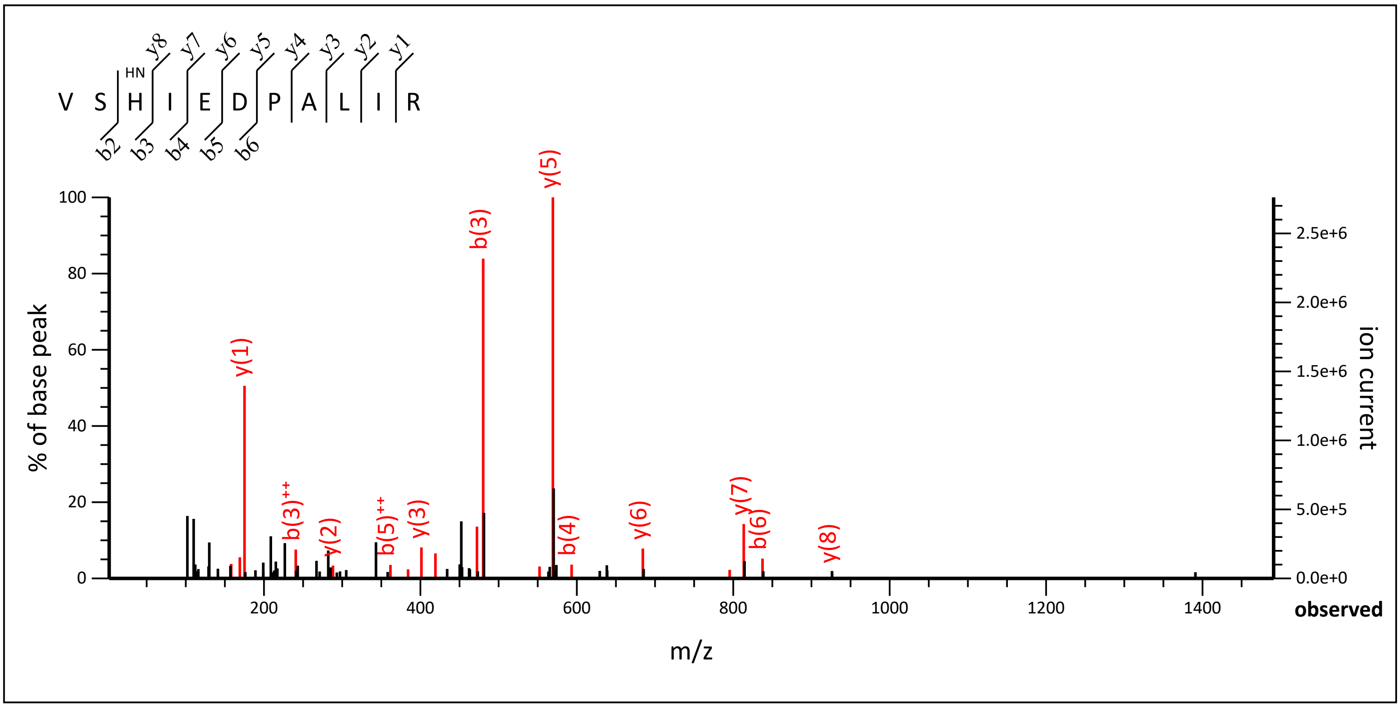

2.06 to 1490.92

Label all possible matches ○ Label matches used for scoring ◉

Monoisotopic mass of neutral peptide Mr(calc): 1404.7977
Fixed modifications: Carbamidomethyl (C) (apply to specified residues or termini only)
Variable modifications:
H3 : HNE (H)
Ions Score: 39 **Expect:** 0.031
Matches : 19/88 fragment ions using 47 most intense peaks (help)

#	b	b++	b0	b0++	Seq.	y	y++	y*	y*++	y0	y0++	#
1	100.0757	50.5415			V							11
2	187.1077	94.0575	169.0972	85.0522	S	1306.7365	653.8719	1289.7100	645.3586	1288.7260	644.8666	10
3	**480.2817**	**240.6445**	462.2711	231.6392	H	1219.7045	610.3559	1202.6780	601.8426	1201.6939	601.3506	9
4	**593.3657**	297.1865	575.3552	288.1812	I	***926.5306***	463.7689	909.5040	455.2556	908.5200	454.7636	8
5	722.4083	**361.7078**	704.3978	352.7025	E	***813.4465***	407.2269	796.4199	398.7136	795.4359	398.2216	7
6	**837.4353**	**419.2213**	819.4247	410.2160	D	***684.4039***	342.7056	667.3774	334.1923	666.3933	333.7003	6
7	934.4880	467.7477	916.4775	458.7424	P	***569.3770***	285.1921	552.3504	276.6788			5
8	1005.5251	503.2662	987.5146	494.2609	A	***472.3242***	236.6657	455.2976	228.1525			4
9	1118.6092	559.8082	1100.5986	550.8030	L	***401.2871***	201.1472	384.2605	192.6339			3
10	1231.6933	616.3503	1213.6827	607.3450	I	***288.2030***	144.6051	271.1765	136.0919			2
11					R	***175.1190***	88.0631	158.0924	79.5498			1

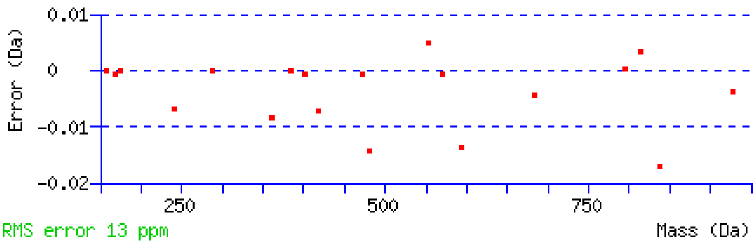

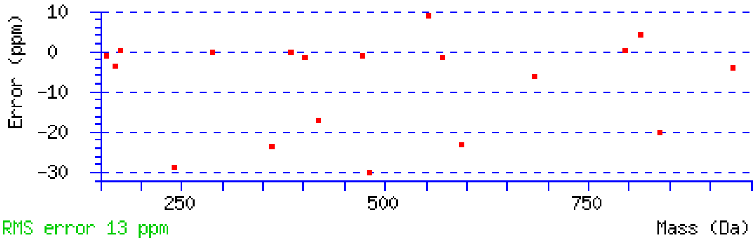

NCBI **BLAST** search of VSHIEDPALIR
(Parameters: blastp, nr protein database, expect=20000, no filter, PAM30)
Other BLAST web gateways

All matches to this query

Score	Mr(calc)	Delta	Sequence
39.0	1404.7977	-0.0136	VSHIEDPALIR
37.8	1404.7837	0.0003	RVSHIEDPALIR
0.1	1404.7837	0.0003	ETALHERPLLAR

Mascot: http://www.matrixscience.com/
