# Supplementary material for: A common East-Asian ALDH2 mutation causes metabolic disorders and the therapeutic effect of ALDH2 activators
Source: Nat Commun. 2023 Sep 25;14:5971. doi: 10.1038/s41467-023-41570-6 (PMC10520061; doi:10.1038/s41467-023-41570-6)
Supplement: Supplementary file 4 — Supplementary Data 1 [file 41467_2023_41570_MOESM4_ESM.zip › Table S5a/T1718_Ndufc2_H8.pdf]

MATRIX SCIENCE Mascot Search Results

Peptide View

MS/MS Fragmentation of **MNGRPGHEPLKFLPDEAR**
Found in **Q9CQ54** in **Mouse_UniProt_proteomes**, NADH dehydrogenase [ubiquinone] 1 subunit C2 OS=Mus musculus OX=10090 GN=Ndufc2 PE=1 SV=1

Match to Query 80737: 2236.088336 from(560.029360,4+) intensity(1645829.5000) rtinseconds(4219) scans(74180) index(65446)
Title: File: "D:\Data\2019 User's data\190516-5\190516-5-T1718_1_10.raw"; SpectrumID: "74180"; scans: "74180"
Data file D:\Data\2019 User's data\190516-5\190516-5\190516-5-T1718_1_10.mgf

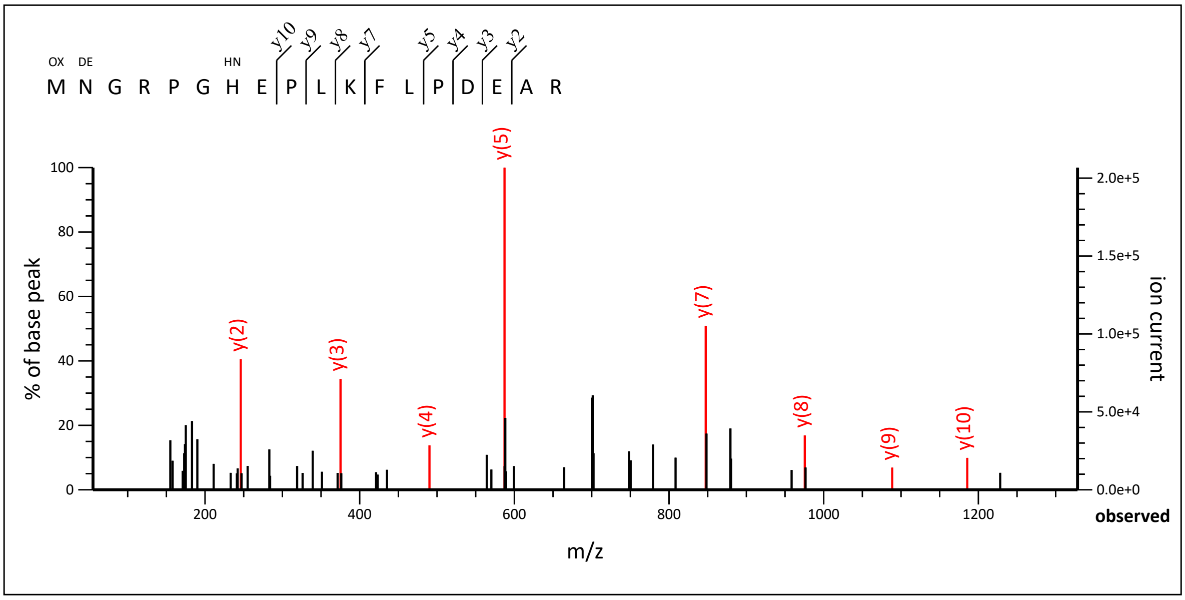

55.12 to 1328.24

Label all possible matches ○ Label matches used for scoring ◉

Monoisotopic mass of neutral peptide Mr(calc): 2236.1310
Fixed modifications: Carbamidomethyl (C) (apply to specified residues or termini only)
Variable modifications:
M1 : Oxidation (M), with neutral losses 0.0000(shown in table), 63.9983
N2 : Deamidated (NQ)
H7 : HNE (H)
Ions Score: 59 **Expect:** 0.00054
Matches : 8/270 fragment ions using 11 most intense peaks (help)

#	b	b^{++}	b*	b^{*++}	b^{0}	b^{0++}	Seq.	y	y^{++}	y*	y^{*++}	y^{0}	y^{0++}	#
1	148.0427	74.5250					M							18
2	263.0696	132.0384	246.0431	123.5252			N	2090.1029	1045.5551	2073.0764	1037.0418	2072.0924	1036.5498	17
3	320.0911	160.5492	303.0645	152.0359			G	1975.0760	988.0416	1958.0494	979.5284	1957.0654	979.0363	16
4	476.1922	238.5997	459.1656	230.0865			R	1918.0545	959.5309	1901.0280	951.0176	1900.0439	950.5256	15
5	573.2450	287.1261	556.2184	278.6128			P	1761.9534	881.4803	1744.9268	872.9671	1743.9428	872.4751	14
6	630.2664	315.6368	613.2399	307.1236			G	1664.9006	832.9540	1647.8741	824.4407	1646.8901	823.9487	13
7	923.4404	462.2238	906.4138	453.7105			H	1607.8792	804.4432	1590.8526	795.9299	1589.8686	795.4379	12
8	1052.4830	526.7451	1035.4564	518.2318	1034.4724	517.7398	E	1314.7052	657.8563	1297.6787	649.3430	1296.6947	648.8510	11
9	1149.5357	575.2715	1132.5092	566.7582	1131.5252	566.2662	P	***1185.6626***	593.3350	1168.6361	584.8217	1167.6521	584.3297	10
10	1262.6198	631.8135	1245.5932	623.3003	1244.6092	622.8082	L	***1088.6099***	544.8086	1071.5833	536.2953	1070.5993	535.8033	9
11	1390.7147	695.8610	1373.6882	687.3477	1372.7042	686.8557	K	***975.5258***	488.2665	958.4993	479.7533	957.5152	479.2613	8
12	1537.7832	769.3952	1520.7566	760.8819	1519.7726	760.3899	F	***847.4308***	424.2191	830.4043	415.7058	829.4203	415.2138	7
13	1650.8672	825.9373	1633.8407	817.4240	1632.8567	816.9320	L	700.3624	350.6849	683.3359	342.1716	682.3519	341.6796	6
14	1747.9200	874.4636	1730.8934	865.9504	1729.9094	865.4584	P	***587.2784***	294.1428	570.2518	285.6295	569.2678	285.1375	5
15	1862.9469	931.9771	1845.9204	923.4638	1844.9364	922.9718	D	***490.2256***	245.6164	473.1991	237.1032	472.2150	236.6112	4
16	1991.9895	996.4984	1974.9630	987.9851	1973.9790	987.4931	E	***375.1987***	188.1030	358.1721	179.5897	357.1881	179.0977	3
17	2063.0266	1032.0170	2046.0001	1023.5037	2045.0161	1023.0117	A	***246.1561***	123.5817	229.1295	115.0684			2
18							R	175.1190	88.0631	158.0924	79.5498			1

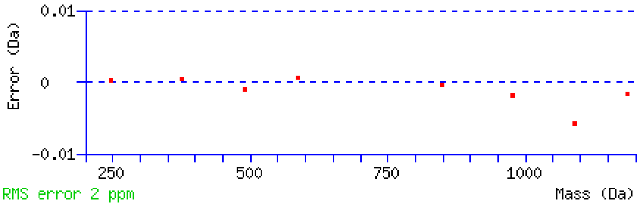

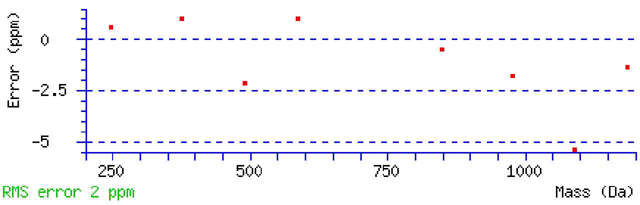

NCBI **BLAST** search of MNGRPGHEPLKFLPDEAR
(Parameters: blastp, nr protein database, expect=20000, no filter, PAM30)
Other BLAST web gateways

All matches to this query

Score	Mr(calc)	Delta	Sequence	Site Analysis
59.0	2236.1310	-0.0427	MNGRPGHEPLKFLPDEAR	HNE H7 100.00%
21.6	2236.1310	-0.0427	MNGRPGHEPLKFLPDEAR	

Mascot: http://www.matrixscience.com/
