# Supplementary material for: A common East-Asian ALDH2 mutation causes metabolic disorders and the therapeutic effect of ALDH2 activators
Source: Nat Commun. 2023 Sep 25;14:5971. doi: 10.1038/s41467-023-41570-6 (PMC10520061; doi:10.1038/s41467-023-41570-6)
Supplement: Supplementary file 4 — Supplementary Data 1 [file 41467_2023_41570_MOESM4_ESM.zip › Table S5a/T1718_Pcca_H275.pdf]

MATRIX SCIENCE Mascot Search Results

Peptide View

MS/MS Fragmentation of **HIEIQVLGDKHGNALWLNERECSIQR**
Found in **Q91ZA3** in **Mouse_UniProt_proteomes**, Propionyl-CoA carboxylase alpha chain, mitochondrial OS=Mus musculus OX=10090 GN=Pcca PE=1 SV=2

Match to Query 97249: 3270.677456 from(818.676640,4+) intensity(736254.4375) rtinseconds(4095) scans(71322) index(62731)
Title: File: "D:\Data\2019 User's data\190516-5\190516-5-T1718_1_10.raw"; SpectrumID: "71322"; scans: "71322"
Data file D:\Data\2019 User's data\190516-5\190516-5\190516-5-T1718_1_10.mgf

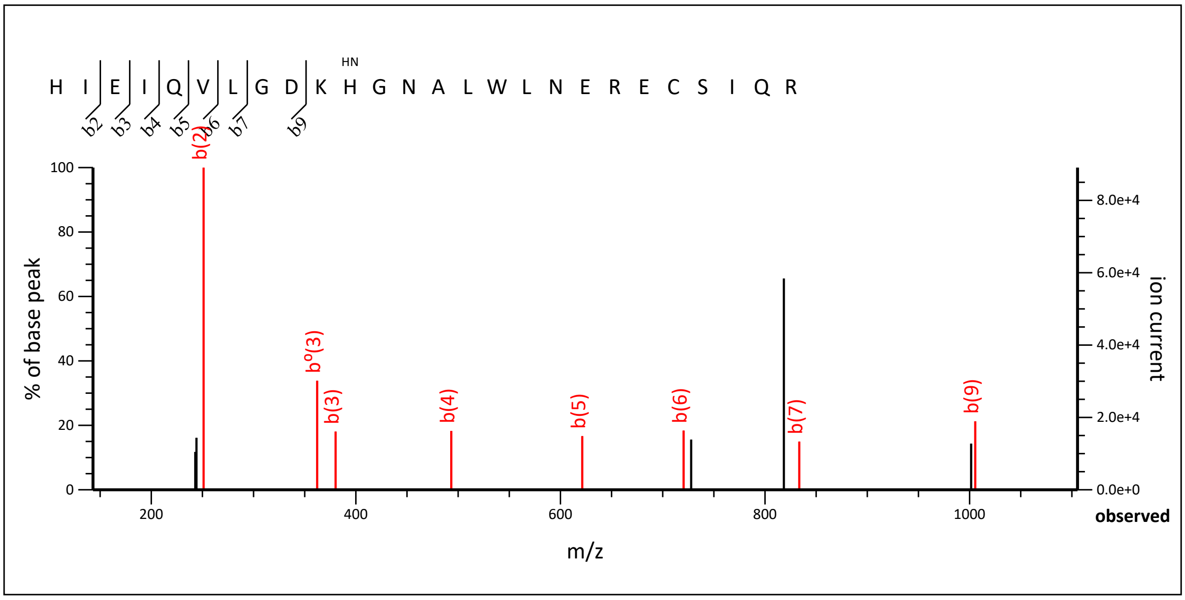

143.02 to 1105.52

Label all possible matches ○ Label matches used for scoring ◉

Monoisotopic mass of neutral peptide Mr(calc): 3270.6931
Fixed modifications: Carbamidomethyl (C) (apply to specified residues or termini only)
Variable modifications:
H11 : HNE (H)
Ions Score: 43 **Expect:** 0.036
Matches : 8/282 fragment ions using 12 most intense peaks (help)

#	b	b^{++}	b*	b^{*++}	b^0	b^{0++}	Seq.	y	y^{++}	y*	y^{*++}	y^0	y^{0++}	#
1	138.0662	69.5367					H							26
2	***251.1503***	126.0788					I	3134.6415	1567.8244	3117.6150	1559.3111	3116.6310	1558.8191	25
3	***380.1928***	190.6001			362.1823	181.5948	E	3021.5575	1511.2824	3004.5309	1502.7691	3003.5469	1502.2771	24
4	***493.2769***	247.1421			475.2663	238.1368	I	2892.5149	1446.7611	2875.4883	1438.2478	2874.5043	1437.7558	23
5	***621.3355***	311.1714	604.3089	302.6581	603.3249	302.1661	Q	2779.4308	1390.2190	2762.4043	1381.7058	2761.4202	1381.2138	22
6	***720.4039***	360.7056	703.3774	352.1923	702.3933	351.7003	V	2651.3722	1326.1898	2634.3457	1317.6765	2633.3617	1317.1845	21
7	***833.4880***	417.2476	816.4614	408.7343	815.4774	408.2423	L	2552.3038	1276.6555	2535.2773	1268.1423	2534.2933	1267.6503	20
8	890.5094	445.7584	873.4829	437.2451	872.4989	436.7531	G	2439.2198	1220.1135	2422.1932	1211.6002	2421.2092	1211.1082	19
9	***1005.5364***	503.2718	988.5098	494.7585	987.5258	494.2665	D	2382.1983	1191.6028	2365.1717	1183.0895	2364.1877	1182.5975	18
10	1133.6313	567.3193	1116.6048	558.8060	1115.6208	558.3140	K	2267.1713	1134.0893	2250.1448	1125.5760	2249.1608	1125.0840	17
11	1426.8053	713.9063	1409.7787	705.3930	1408.7947	704.9010	H	2139.0764	1070.0418	2122.0498	1061.5286	2121.0658	1061.0365	16
12	1483.8267	742.4170	1466.8002	733.9037	1465.8162	733.4117	G	1845.9024	923.4549	1828.8759	914.9416	1827.8919	914.4496	15
13	1597.8697	799.4385	1580.8431	790.9252	1579.8591	790.4332	N	1788.8810	894.9441	1771.8544	886.4309	1770.8704	885.9388	14
14	1668.9068	834.9570	1651.8802	826.4438	1650.8962	825.9517	A	1674.8381	837.9227	1657.8115	829.4094	1656.8275	828.9174	13
15	1781.9908	891.4991	1764.9643	882.9858	1763.9803	882.4938	L	1603.8009	802.4041	1586.7744	793.8908	1585.7904	793.3988	12
16	1968.0702	984.5387	1951.0436	976.0254	1950.0596	975.5334	W	1490.7169	745.8621	1473.6903	737.3488	1472.7063	736.8568	11
17	2081.1542	1041.0807	2064.1277	1032.5675	2063.1437	1032.0755	L	1304.6376	652.8224	1287.6110	644.3091	1286.6270	643.8171	10
18	2195.1971	1098.1022	2178.1706	1089.5889	2177.1866	1089.0969	N	1191.5535	596.2804	1174.5269	587.7671	1173.5429	587.2751	9
19	2324.2397	1162.6235	2307.2132	1154.1102	2306.2292	1153.6182	E	1077.5106	539.2589	1060.4840	530.7456	1059.5000	530.2536	8
20	2480.3409	1240.6741	2463.3143	1232.1608	2462.3303	1231.6688	R	948.4680	474.7376	931.4414	466.2244	930.4574	465.7323	7
21	2609.3834	1305.1954	2592.3569	1296.6821	2591.3729	1296.1901	E	792.3669	396.6871	775.3403	388.1738	774.3563	387.6818	6
22	2769.4141	1385.2107	2752.3875	1376.6974	2751.4035	1376.2054	C	663.3243	332.1658	646.2977	323.6525	645.3137	323.1605	5

23	2856.4461	1428.7267	2839.4196	1420.2134	2838.4356	1419.7214	S	503.2936	252.1504	486.2671	243.6372	485.2831	243.1452	4
24	2969.5302	1485.2687	2952.5036	1476.7555	2951.5196	1476.2634	I	416.2616	208.6344	399.2350	200.1212			3
25	3097.5888	1549.2980	3080.5622	1540.7847	3079.5782	1540.2927	Q	303.1775	152.0924	286.1510	143.5791			2
26							R	175.1190	88.0631	158.0924	79.5498			1

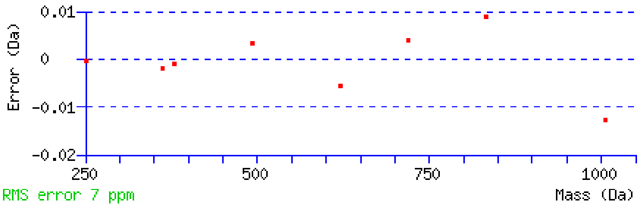

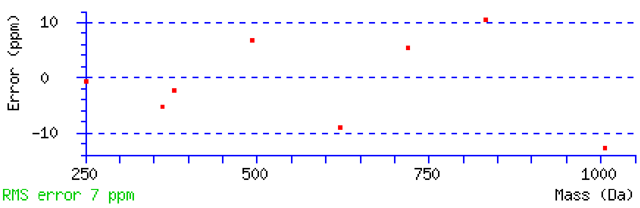

NCBI **BLAST** search of HIEIQVLGDKHGNALWLNERECSIQR
(Parameters: blastp, nr protein database, expect=20000, no filter, PAM30)
Other BLAST web gateways

All matches to this query

Score	Mr(calc)	Delta	Sequence	Site Analysis
43.3	3270.6931	-0.0157	HIEIQVLGDKHGNALWLNERECSIQR	HNE H11 99.97%
43.3	3270.6931	-0.0157	HIEIQVLGDKHGNALWLNERECSIQR	
22.4	3270.6230	0.0544	HIEVQILGDQYGNILHLYERDCSIQR	
6.0	3270.6443	0.0331	HLELPGQPLNNYHMKTLLLYECEK	
6.0	3270.6443	0.0331	HLELPGQPLNNYHMKTLLLYECEK	
6.0	3270.6443	0.0331	HLELPGQPLNNYHMKTLLLYECEK	
4.6	3270.6435	0.0340	LHKNLEGNHDQHGRNNLLASYIYYVFR	
3.7	3270.6905	-0.0130	HIQELMETKETVTTEVVNLSNRNEK	
3.7	3270.6905	-0.0130	HIQELMETKETVTTEVVNLSNRNEK	
3.7	3270.6905	-0.0130	HIQELMETKETVTTEVVNLSNRNEK	

Mascot: http://www.matrixscience.com/
