# Supplementary material for: A common East-Asian ALDH2 mutation causes metabolic disorders and the therapeutic effect of ALDH2 activators
Source: Nat Commun. 2023 Sep 25;14:5971. doi: 10.1038/s41467-023-41570-6 (PMC10520061; doi:10.1038/s41467-023-41570-6)
Supplement: Supplementary file 4 — Supplementary Data 1 [file 41467_2023_41570_MOESM4_ESM.zip › Table S5a/T1718_pcx_H574.pdf]

MATRIX SCIENCE Mascot Search Results

Peptide View

MS/MS Fragmentation of **DAHQSLLATR**
Found in **G5E8R3** in **Mouse_UniProt_proteomes**, Pyruvate carboxylase OS=Mus musculus OX=10090 GN=Pcx PE=1 SV=1

Match to Query 32026: 1266.682122 from(423.234650,3+) intensity(1652817.1250) rtinseconds(1733) scans(16623) index(10775)
Title: File: "D:\Data\2019 User's data\190516-5\190516-5-T1718_1_10.raw"; SpectrumID: "16623"; scans: "16623"
Data file D:\Data\2019 User's data\190516-5\190516-5\190516-5-T1718_1_10.mgf

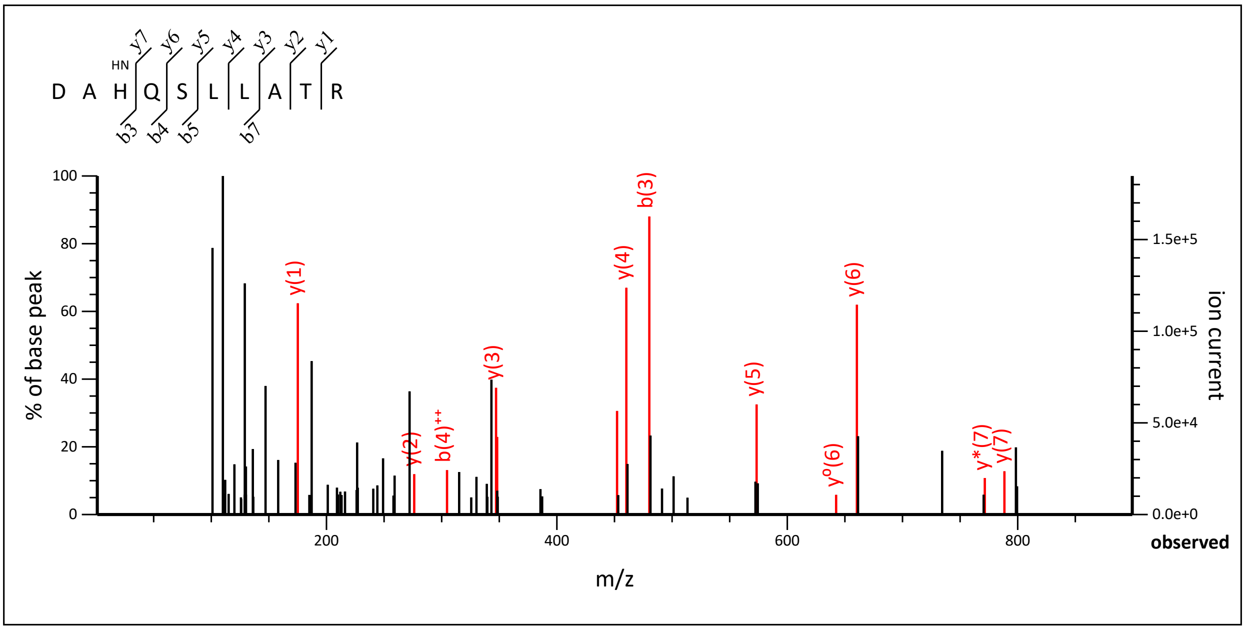

1.07 to 899.45

Label all possible matches ○ Label matches used for scoring ◉

Monoisotopic mass of neutral peptide Mr(calc): 1266.6932
Fixed modifications: Carbamidomethyl (C) (apply to specified residues or termini only)
Variable modifications:
H3 : HNE (H)
Ions Score: 39 **Expect:** 0.029
Matches : 13/100 fragment ions using 27 most intense peaks (help)

#	b	b^{++}	b*	b^{*++}	b^0	b^{0++}	Seq.	y	y^{++}	y*	y^{*++}	y^0	y^{0++}	#
1	116.0342	58.5207			98.0237	49.5155	D							10
2	187.0713	94.0393			169.0608	85.0340	A	1152.6735	576.8404	1135.6470	568.3271	1134.6630	567.8351	9
3	**480.2453**	240.6263			462.2347	231.6210	H	1081.6364	541.3218	1064.6099	532.8086	1063.6259	532.3166	8
4	608.3039	**304.6556**	591.2773	296.1423	590.2933	295.6503	Q	***788.4625***	394.7349	771.4359	386.2216	770.4519	385.7296	7
5	695.3359	**348.1716**	678.3093	339.6583	677.3253	339.1663	S	***660.4039***	330.7056	643.3774	322.1923	642.3933	321.7003	6
6	808.4199	404.7136	791.3934	396.2003	790.4094	395.7083	L	***573.3719***	287.1896	556.3453	278.6763	555.3613	278.1843	5
7	921.5040	461.2556	904.4775	452.7424	903.4934	452.2504	L	***460.2878***	230.6475	443.2613	222.1343	442.2772	221.6423	4
8	992.5411	496.7742	975.5146	488.2609	974.5306	487.7689	A	***347.2037***	174.1055	330.1772	165.5922	329.1932	165.1002	3
9	1093.5888	547.2980	1076.5623	538.7848	1075.5782	538.2928	T	***276.1666***	138.5870	259.1401	130.0737	258.1561	129.5817	2
10							R	***175.1190***	88.0631	158.0924	79.5498			1

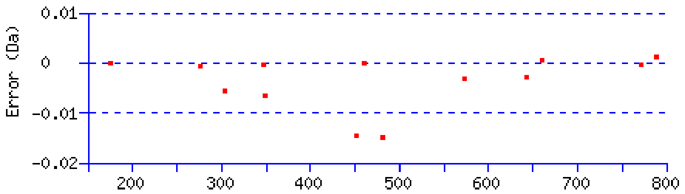

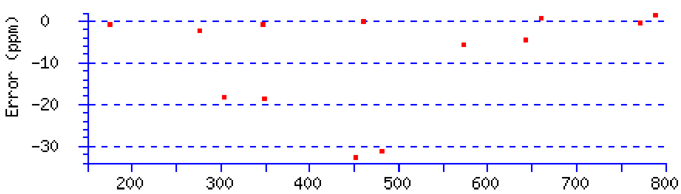

NCBI **BLAST** search of DAHQSLLATR
(Parameters: blastp, nr protein database, expect=20000, no filter, PAM30)

Other BLAST web gateways

All matches to this query

Score	Mr(calc)	Delta	Sequence
38.6	1266.6932	-0.0111	DAHQSLLATR
2.3	1266.6819	0.0002	HNAEISALEK

**Mascot:** http://www.matrixscience.com/
