# Supplementary material for: A common East-Asian ALDH2 mutation causes metabolic disorders and the therapeutic effect of ALDH2 activators
Source: Nat Commun. 2023 Sep 25;14:5971. doi: 10.1038/s41467-023-41570-6 (PMC10520061; doi:10.1038/s41467-023-41570-6)
Supplement: Supplementary file 4 — Supplementary Data 1 [file 41467_2023_41570_MOESM4_ESM.zip › Table S5a/T1718_Sdhb_H246.pdf]

# MATRIX SCIENCE Mascot Search Results

## Peptide View

MS/MS Fragmentation of **CHTIMNCTQTCPK**
Found in **Q9CQA3** in **Mouse_UniProt_proteomes**, Succinate dehydrogenase [ubiquinone] iron-sulfur subunit, mitochondrial OS=Mus musculus OX=10090 GN=Sdhb PE=1 SV=1

Match to Query 64585: 1805.778582 from(602.933470,3+) intensity(5096597.0000) rtinseconds(1532) scans(11962) index(6356)
Title: File: "D:\Data\2019 User's data\190516-5\190516-5-T1718_1_10.raw"; SpectrumID: "11962"; scans: "11962"
Data file D:\Data\2019 User's data\190516-5\190516-5\190516-5-T1718_1_10.mgf

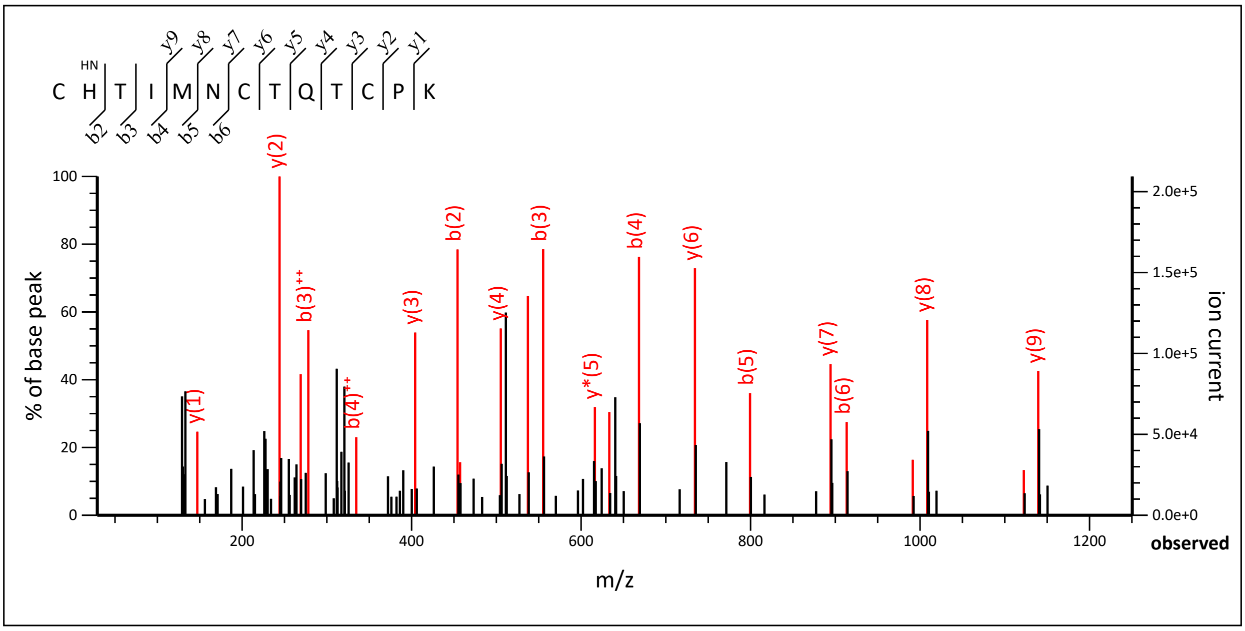


29.1 to 1250.43

Label all possible matches ○ Label matches used for scoring ◉

**Monoisotopic mass of neutral peptide Mr(calc):** 1805.7933
**Fixed modifications:** Carbamidomethyl (C) (apply to specified residues or termini only)
**Variable modifications:**
**H2** : HNE (H)
**Ions Score:** 50 **Expect:** 0.0006
**Matches :** 23/124 fragment ions using 42 most intense peaks (help)

| # | b | b++ | b* | b*++ | b0 | b0++ | Seq. | y | y++ | y* | y*++ | y0 | y0++ | # |
|---|---|---|---|---|---|---|---|---|---|---|---|---|---|---|
| 1 | 161.0379 | 81.0226 | | | | | C | | | | | | | 13 |
| 2 | **454.2119** | 227.6096 | | | | | H | 1646.7699 | 823.8886 | 1629.7434 | 815.3753 | 1628.7593 | 814.8833 | 12 |
| 3 | **555.2595** | **278.1334** | | | 537.2490 | 269.1281 | T | 1353.5960 | 677.3016 | 1336.5694 | 668.7883 | 1335.5854 | 668.2963 | 11 |
| 4 | **668.3436** | **334.6754** | | | 650.3330 | 325.6702 | I | 1252.5483 | 626.7778 | 1235.5217 | 618.2645 | 1234.5377 | 617.7725 | 10 |
| 5 | **799.3841** | 400.1957 | | | 781.3735 | 391.1904 | M | ***1139.4642*** | 570.2357 | 1122.4377 | 561.7225 | 1121.4536 | 561.2305 | 9 |
| 6 | **913.4270** | **457.2171** | 896.4005 | 448.7039 | 895.4165 | 448.2119 | N | ***1008.4237*** | 504.7155 | 991.3972 | 496.2022 | 990.4132 | 495.7102 | 8 |
| 7 | 1073.4577 | **537.2325** | 1056.4311 | 528.7192 | 1055.4471 | 528.2272 | C | ***894.3808*** | 447.6940 | 877.3543 | 439.1808 | 876.3702 | 438.6888 | 7 |
| 8 | 1174.5053 | 587.7563 | 1157.4788 | 579.2430 | 1156.4948 | 578.7510 | T | ***734.3502*** | 367.6787 | 717.3236 | 359.1654 | 716.3396 | 358.6734 | 6 |
| 9 | 1302.5639 | 651.7856 | 1285.5374 | 643.2723 | 1284.5534 | 642.7803 | Q | ***633.3025*** | 317.1549 | 616.2759 | 308.6416 | 615.2919 | 308.1496 | 5 |
| 10 | 1403.6116 | 702.3094 | 1386.5851 | 693.7962 | 1385.6010 | 693.3042 | T | ***505.2439*** | 253.1256 | 488.2173 | 244.6123 | 487.2333 | 244.1203 | 4 |
| 11 | 1563.6423 | 782.3248 | 1546.6157 | 773.8115 | 1545.6317 | 773.3195 | C | ***404.1962*** | 202.6017 | 387.1697 | 194.0885 | | | 3 |
| 12 | 1660.6950 | 830.8511 | 1643.6685 | 822.3379 | 1642.6845 | 821.8459 | P | ***244.1656*** | 122.5864 | 227.1390 | 114.0731 | | | 2 |
| 13 | | | | | | | K | ***147.1128*** | 74.0600 | 130.0863 | 65.5468 | | | 1 |

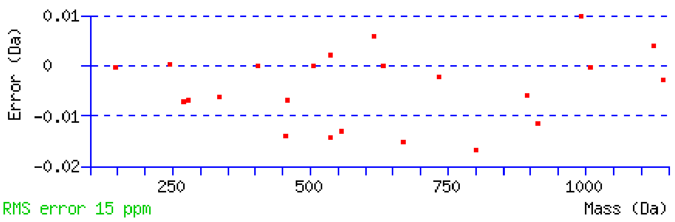


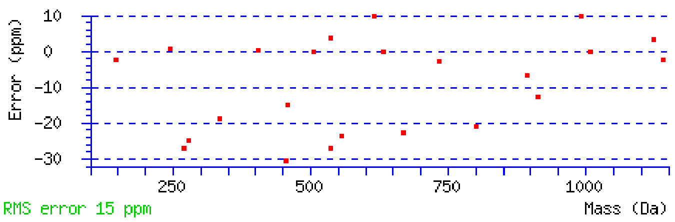


NCBI **BLAST** search of CHTIMNCTQTCPK
(Parameters: blastp, nr protein database, expect=20000, no filter, PAM30)
Other BLAST web gateways

**All matches to this query**

| Score | Mr(calc) | Delta | Sequence | Site Analysis |
|---|---|---|---|---|
| 50.3 | 1805.7933 | -0.0147 | CHTIMNCTQTCPK | HNE H2 100.00% |

**Mascot:** http://www.matrixscience.com/
